# Transcriptome analysis reveals a potential regulatory mechanism of the lnc-5423.6/IGFBP5 axis in the early stages of mouse thymic involution: lnc-5423.6/IGFBP5 axis regulates thymic involution

**DOI:** 10.3724/abbs.2023042

**Published:** 2023-04-19

**Authors:** Bingxin Li, Yaqiong Ye, Longsheng Hong, Wanyan Li, Qingru Wu, Wenjun Liu, Yongjiang Ma, Danning Xu, Yugu Li

**Affiliations:** 1 College of Veterinary Medicine South China Agricultural University Guangzhou 510642 China; 2 College of Animal Science & Technology Guangdong Province Key Laboratory of Waterfowl Healthy Breeding Zhongkai University of Agriculture and Engineering Guangzhou 510225 China; 3 School of Life Science and Engineering Foshan University Foshan 528000 China

**Keywords:** thymic involution, ceRNA, lnc-5423.6, miR-193b-3p, IGFBP5

## Abstract

Age-related thymic involution is one of the significant reasons for induced immunity decline. Recent evidence has indicated that lncRNAs are widely involved in regulating organ development. However, the lncRNA expression profiles in mouse thymic involution have not been reported. In this study, we collect mouse thymus at the ages of 1 month, 3 months, and 6 months for sequencing to observe the lncRNA and gene expression profiles in the early stages of thymic involution. Through bioinformatics analysis, a triple regulatory network of lncRNA-miRNA-mRNA that contains 29 lncRNAs, 145 miRNAs and 12 mRNAs that may be related to thymic involution is identified. Among them, IGFBP5 can reduce the viability, inhibit proliferation and promote apoptosis of mouse medullary thymic epithelial cell line 1 (MTEC1) cells through the p53 signaling pathway. In addition, miR-193b-3p can alleviate MTEC1 cell apoptosis by targeting *IGFBP5*. Notably, lnc-5423.6 can act as a molecular sponge of miR-193b-3p to regulate the expression of IGFBP5. In summary, lnc-5423.6 enhances the expression of IGFBP5 by adsorption of miR-193b-3p, thereby promoting MTEC1 cell apoptosis.

## Introduction

The thymus plays a vital role in the immune system by producing antigen-specific T lymphocytes [1]. Therefore, thymic involution is considered one of the important indexes of immune senescence [2]. Interestingly, in most vertebrates, the volume and mass of the thymus decrease with age after puberty, which shows a conservative evolutionary process named age-related thymic involution [3]. Previous studies reported that the mouse thymus reached a maximum at 1 month of age, and the number of thymocytes in both females and males was dramatically decreased at 3 months of age [ 4, 5] . The decrease in thymus size and the deposition of adipocytes are the major features of age-related thymic involution [ 6, 7] . Adipocytes gradually replace thymic epithelial cells (TECs), resulting in a decrease in the output of naive T cells, which ultimately reduces immunity and increases the risk of immune-related diseases [8]. Therefore, exploring the regulatory mechanisms of age-related thymic involution is essential for understanding thymic involution and regeneration.

Long noncoding RNAs (lncRNAs) are a new type of single-stranded regulatory RNA with multiple exons and endogenous coding [9]. lncRNAs play critical roles in regulating gene expression during the biological process of organ development [10]. With strong homology to the coding sequence, lncRNAs have rich microRNA (miRNA) binding sites [11]. Salmena *et al*. [12] proposed a competing endogenous RNA (ceRNA) hypothesis on the regulatory mechanism of lncRNAs and mRNAs, which was later confirmed by many studies. In this theory, lncRNAs can act as molecular sponges by adsorbing miRNAs to reduce their abundances, therefore regulating the expression of target mRNAs [13]. Nevertheless, only a few lncRNAs have functional annotations [14]. In a previous study, we identified 17498 lncRNAs in the mouse thymus and found that 64 lncRNAs might participate in castration-regulated thymic involution through ceRNA [15]. However, the understanding of lncRNAs regulating thymic involution is still not clear.

In this study, we identified the expression profiles of lncRNAs and genes in the thymus during the thymus development stage (1 month old) and involution stages (3 months old and 6 months old) by high-throughput sequencing and analysed the functions of key lncRNAs and genes. This study provides valuable data support for further research on the mechanism by which lncRNAs regulate thymic involution and regeneration.

## Materials and Methods

### Animals and treatment

Ninety BALB/c mice (50% male and 50% female) were purchased from Huafukang (Beijing, China). Mice were kept in a specific pathogen-free environment (12/12 h light/dark cycle, 22–24°C, 40%–60% humidity) and subdivided into six groups with15 mice per group: 1-month-old females (F1), 1-month-old males (M1), 3-month-old females (F3), 3-month-old males (M3), 6-month-old females (F6), and 6-month-old males (M6). The thymuses were collected in a sterile environment. Among them, 5 thymuses per group were weighed and immersed in 4% paraformaldehyde, and the rest were snap frozen in liquid nitrogen and stored at ‒80°C. The study was conducted under the guidance of the Animal Protection Committee of South China Agricultural University (Guangzhou, China).

### Thymus index analysis

Mice were weighed and euthanized. Five thymuses per group were washed three times with saline. Then, the surface moisture was removed with filter paper and weighed. The thymus index is the ratio of the thymus weight (mg)/body weight (g).

### RNA sequencing

Total RNA was extracted from the thymus using TRIzol reagent (Invitrogen, Carlsbad, USA). Bioanalyzer 2100 (Agilent, Santa Clara, USA), NanoDrop 2000 (Thermo Fisher Scientific, Waltham, USA), and agarose gel electrophoresis were used to detect the purity, content, and integrity of total RNA. The A260/A280 cut-off value was set to greater than 1.8; the A260/A230 cut-off value was set to greater than 2.0, and the RNA integrity number cut-off value was set to greater than 7.0. cDNA libraries were constructed for all groups using equal amounts of total RNA pooled from 5 thymuses in each group as described in previous studies [ 16– 18] . The Epicenter Ribo-Zero Gold kit (Illumina, San Diego, USA) was used to remove ribosomal RNA (rRNA) from 10 μg of total RNA. After removal of rRNAs, the remaining RNA was fragmented into short fragments using divalent cations under high temperature with the Next® Magnesium RNA Fragmentation Module (E6150S; NEB, Ipswich, USA). Then, the cleaved RNA fragments were reverse-transcribed to generate cDNA using SuperScript™ II Reverse Transcriptase (1896649; Invitrogen, Carlsbad, USA), which were subsequently used to synthesize U-labelled second-stranded DNA with *E*. *coli* DNA polymerase I (m0209; NEB), RNase H (m0297; NEB) and dUTP Solution (R0133; Thermo Fisher Scientific). The average insert size for the final cDNA library was 300±50 bp. Finally, these cDNAs were subject to 2×150 bp paired-end sequencing (PE150) on an Illumina HiSeq 4000 (Illumina) by LC-BIO-tech (Hangzhou, China) following the manufacturer’s protocol.

### Transcript and lncRNA identification

First, Cutadapt (cutadapt-1.9) was used to remove the raw data containing adapter contamination, low-quality bases, and undefined base reads. Second, FastQC ( http://www.bioinformatics.babraham.ac.uk/projects/fastqc/) was used to verify the sequence quality to obtain clean data. Next, TopHat2 was used to map clean data to the mouse genome. Finally, StringTie ( http://ccb.jhu.edu/software/stringtie/, version: stringtie-1.3.4d) and Ballgown were used to estimate the expression levels in this study.

For lncRNA identification, transcripts overlapping with known mRNAs or shorter than 200 nt were discarded. Then, CNCI, CPC, and Pfam were used to predict transcripts with coding potential, the CNCI cut-off value was set to less than 0, and the CPC cut-off value was set to less than –1 to obtain potential lncRNAs.

### RNA expression and function analysis

The differentially expressed lncRNAs (DELs) and genes (DEGs) were screened according to the cut-off values of |log _2_(fold change)| >1 with *P*<0.05 by the R package Ballgown. The functions of DEGs in the thymus between development and degenerative stages (F3 vs F1, F6 vs F1, M3 vs M1 and M6 vs M1) were obtained via Gene Ontology (GO) and Kyoto Encyclopedia of Genes and Genomes (KEGG) of the DAVID online tool ( https://david.ncifcrf.gov/summary.jsp).

### Transcription factor-binding site analysis of DEGs

First, the putative promoter sequence was obtained from Browser1 of the UCSC genome, which was defined as 1 kb upstream of the transcription start site. Next, TESS v6.0 was used to scan the position-weight matrices in the TRANSFAC database. The cut-off value of the relative fraction line was 0.9. Finally, hypergeometric tests were performed using internal Perl scripts. *P*<0.05 was defined as a rich transcription factor.

### Protein-protein interaction (PPI) network identification and analysis

The Search Tool for the Retrieval of Interacting Genes (STRING) database ( https://string-db.org/) was used to build two interaction networks of DEGs in the male and female comparison groups. Then, nodes with a combined confidence score of less than 0.4 and unconnected nodes in the PPI network were removed. Cytoscape software (version 3.7.1) was used to visualize PPI network diagrams.

### ceRNA network identification

According to the PPI network analysis, the overlapping genes in the two networks were selected for further analysis. miRWalk 3.0 was used to predict the targeting relationship between related miRNAs and these genes. The selection criteria were set at *P*<0.05, the target gene binding region was the 3′UTR, and the seed sequence length was at least 7 mer. TargetScan and miRanda were used to predict miRNAs related to the intersection of male and female DELs according to target score >80 and miRanda MEF≤‒30 kcal/mol. Finally, Cytoscape was used to visualize the ceRNA network to investigate lncRNA regulation of mRNA through miRNA.

### Quantitative real time-PCR (qRT-PCR) verification

TRIzol reagent (Invitrogen) was used to extract the total RNA from thymuses. Ten micrograms of total RNA from each sample was reverse transcribed using the ReverTra Ace qRT-PCR RT kit (Toyobo, Osaka, Japan). Next, 10 DELs and 10 DEGs were randomly screened for sequencing data verification in all comparison groups. Finally, qRT-PCR was performed using SYBR Green Real-Time PCR Master Mix (Toyobo) on a Bio-Rad CFX96 real-time PCR system (Bio-Rad, Hercules, USA). The primers used in the study are listed in Supplementary Table S1. *Actb* and *U6* were used as internal controls for mRNA and miRNA to normalize the data, respectively. All experiments were repeated three times, and the relative expression levels of genes were measured according to the cycle threshold (Ct) and calculated using the 2 ^–∆∆Ct^ method.

### Plasmid construction and RNA oligonucleotide synthesis

According to the sequencing results, IGFBP5 was first selected for functional verification, then the ORF region of IGFBP5 was amplified by PCR and cloned it into the pEGFP-N1 plasmid between the *Xho*I and *Eco*RI restriction sites. The small interfering RNA of *IGFBP5* (si- *IGFBP5*; siG2101120228246499, 5′-CCCAAGCACACTCGCATTT-3′) and the negative control (siNC; siN0000001) used in this study were synthesized by RioBio (Guangzhou, China).

The miR-193b-3p mimic (miR10004859; 5′-AACUGGCCCACAAAGUCCCGCU-3′), mimic negative control (miR1N0000001), miR-193b-3p inhibitor (miR20004859; 5′-AGCGGGACUUUGUGGGCCAGUU-3′), and inhibitor negative control (miR2N0000001) were designed and synthesized by RiboBio. The 3′UTR fragment sequence of *IGFBP5* containing the miR-193b-3p binding site was synthesized and subcloned into *Xho*I/ *Not*I restriction sites in the psiCHECK 2 double luciferase reporter vector. Meanwhile, the IGFBP5 double fluorescence mutant carrier was obtained by converting the miR-193b-3p binding site from GCCAG to CAAGA.

The sequence fragment of lnc-5423.6 containing the miR-193b-3p binding site was amplified and subcloned into pcDNA3.1 *Bam*HI and *Xho*I restriction sites in the overexpression vector. In addition, the amplified sequence fragment containing the miR-193b-3p binding site was subcloned into the *Xho*I/ *Not*I restriction site in the psiCHECK 2 double luciferase reporter vector. Meanwhile, the lnc-5423.6 double fluorescence mutant carrier was obtained by converting the miR-193b-3p binding site from GCGGGAC to CGCCCTG.

### Cell culture and transfection

Mouse medullary thymic epithelial cell line 1 (MTEC1) cells were obtained from Peking University Health Science Center and kept in our laboratory. MTEC1 cells were cultured in DMEM (Gibco, Grand Island, USA) supplemented with 10% fetal bovine serum (Gibco) at 37°C and 5% CO _2_.

All transient transfections were performed using Lipofectamine 2000 reagent (Invitrogen) according to the manufacturer’s instructions. The transfection doses of DNA in 96-well plates, 12-well plates, and 6-well plates were 0.1 μg/well, 1 μg/well, and 2.5 μg/well, respectively. The final concentration of siRNA was 0.1 μM.

### Dual‐luciferase reporter assay

The 293T cells (ATCC, Manassas, USA) were inoculated into 96-well culture plates. When the cells fusion degree reached 60%, miR-193b-3p mimic or mimic NC was cotransfected into cells with IGFBP5 and lnc-5423.6 wild-type and mutant double luciferase reporter vectors using Lipofectamine 2000 reagent (Invitrogen). After 48 h, the firefly luciferase and Renilla luminescence luciferase activities were detected by a dual luciferase reporter gene detection kit (Beyotime, Shanghai, China) and a multifunctional enzyme standard (Thermo Fisher Scientific).

### Cell counting kit-8 (CCK-8) assay

MTEC1 cells were inoculated in 96-well plates. The transfection experiment was carried out when the cell fusion degree reached 60%. According to the manufacturer’s instructions, 48 h after transfection, 10 μL of CCK-8 reagent (Beyotime) was added to each well and incubated for 1.5 h. Finally, the OD value of each group of cells was detected at 450 nm with a microplate reader (1410101; Thermo Fisher Scientific).

### 5-Ethynyl-2′-deoxyuridine (EdU) assay

MTEC1 cells were inoculated in 12-well plates. After transient transfection for 48 h, 300 μL of culture medium containing 50 μM EdU reagent (RioBio) was added to each well and incubated for 2 h. Subsequently, the cells were fixed for Apollo and DNA staining. Finally, three fields were randomly selected under a fluorescence microscope (DM4000 B; Leica, Weztlar, Germany) to observe the number of EdU-positive cells in each group.

### Flow cytometry assays

MTEC1 cells were inoculated in 6-well plates. After transient transfection for 48 h, the cells were collected for V-FITC and PI staining, and a flow cytometer (C09753; Beckman Coulter, Brea, USA) was used for apoptosis detection. FlowJo V10 (Beckman Coulter) was used to analyse and visualize the results of flow cytometry.

### Western blot analysis

MTEC1 cells were inoculated into 6-well culture plates. When the cell fusion degree reached 60%, the overexpression vector, siRNA, miRNA, mimic and negative control were transfected into the cells. After 48 h, cell proteins were extracted using RIPA buffer and protease inhibitor mixture and then separated by 10% SDS-PAGE and immunoblotted using various antibodies according to standard western blot procedures. Rabbit anti-IGFBP5 (ab254324; Abcam, Cambridge, UK), rabbit anti-p53 (5395S; Cell Signaling Technology, Danvers, USA), rabbit anti-p21 (2947S; Cell Signaling Technology), rabbit anti-Bax (5023P; Cell Signaling Technology), rabbit anti-Cyclin B1 (ab55184; Abcam), rabbit anti-Cyclin D1 (ab134175; Abcam) and rabbit anti-β-actin (ab179467; Abcam) of 1:1000 dilution were incubated with protein blots at 4°C overnight. Subsequently, the secondary antibody (goat anti-rabbit) (A0208; Beyotime) of 1:1000 dilution was use to incubate with the blots at 37°C for 1 h. Finally, chemiluminescence detection was carried out using the ECL Plus (Solarbio, Beijing, China).

### Statistical analysis

Data are expressed as the mean±standard deviation (SD). Student’s *t* test was used to assess the difference between the two groups. One-way ANOVA followed by Tukey’s test was used to measure differences among more than two groups. *P*<0.05 was considered statistically significant.

## Results

### Morphological and histological changes in the thymus during involution

**Figure 1:**
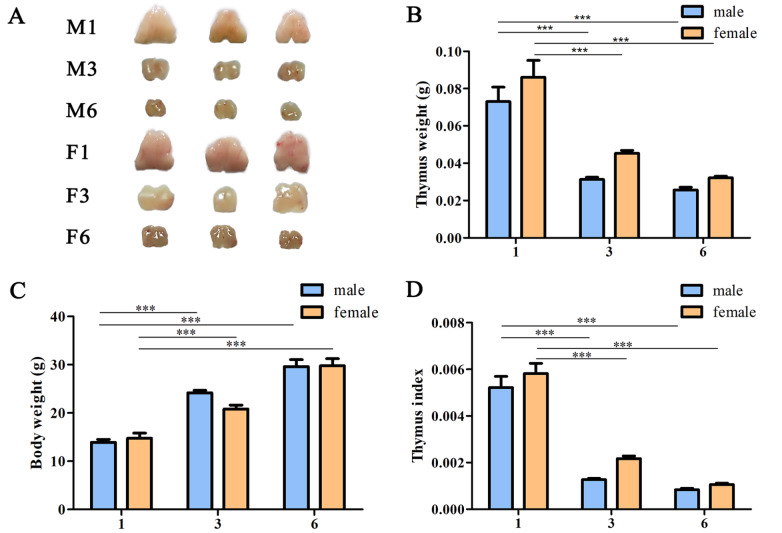
Figure 1 Morphological and histological observation of age-related thymic involution (A) Thymus tissues in 1-month-old, 3-month-old, and 6-month-old mice. (B) Thymus weight. (C) Body weight. (D) Thymus index. Data are expressed as the mean±standard deviation (SD). n=5. *** P<0.001.

To investigate morphological changes during thymic development and involution, we analysed thymus indexes of 1-, 3-, and 6-month-old mice. Morphological and weight changes showed that the thymus at 3 and 6 months of age was significantly degenerated compared with that at 1 month of age ( Figure 1A,B and Supplementary Figure S1). The bodyweight gradually increased with age ( Figure 1C). Therefore, the thymus index results showed that it is inversely proportional to age ( Figure 1D).

### Identification of DEGs associated with thymic involution

**Figure 2:**
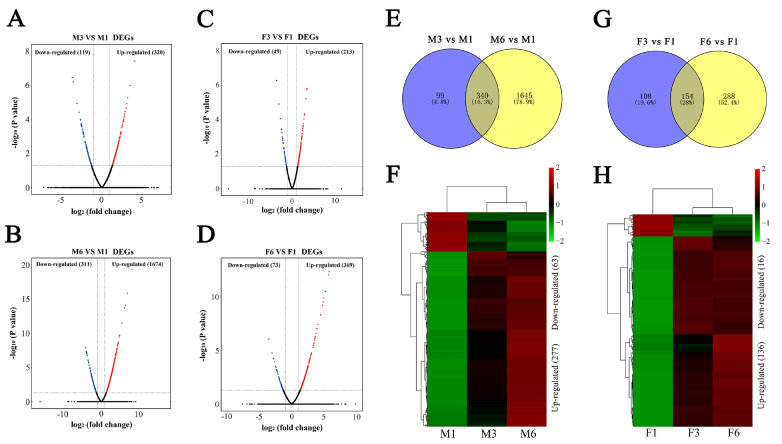
Figure 2 Identification of DEGs between different ages during thymic involution (A–D) Volcano plots of DEGs in males and females. Venn and heatmaps of overlapping DEGs in males (E,F) and females (G,H), respectively. |log 2(fold change)|>1 and P<0.05 represent a significant difference.

In this study, we identified 46699 genes in the thymus development and involution stages of male and female mice by RNA-seq ( Supplementary Table S2). Among these genes, we identified 439 and 1985 DEGs in the M3 group and M6 group respectively when compared with those in the M1 group ( Figure 2A,B and Supplementary Tables S3 and S4). Compared with the F1group, F3 and F6 groups had 262 and 442 DEGs, respectively ( Figure 2C,D and Supplementary Tables S5 and S6). Further analysis showed that 340 DEGs (277 upregulated and 63 downregulated) overlapped in the M3 and M6 groups compared with those in the M1 group ( Figure 2E,F).In addition, 154 DEGs (136 upregulated and 16 downregulated) overlapped in the F3 and F6 groups compared with those in the F1 group ( Figure 2G), among which 152 DEGs (136 upregulated and 16 downregulated) had the same expression trend ( Figure 2H). These results indicate that age can significantly affect the gene expression profile of thymus.

### GO and KEGG pathway analysis of DEGs during thymic involution

**Figure 3:**
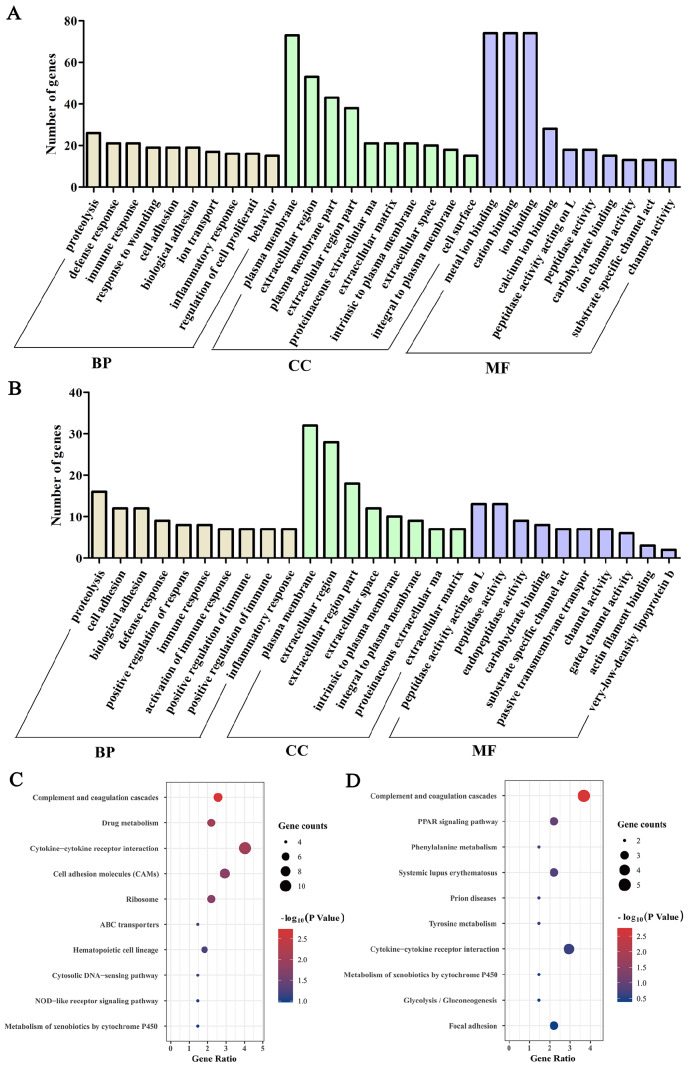
Figure 3 GO and KEGG pathway analysis of DEGs during age-related thymic involution GO analysis of DEGs in males (A) and females (B). KEGG pathway analysis of DEGs in males (C) and females (D). P<0.05 represents a significant difference.

The DAVID tool was used for GO term and KEGG pathway (KP) analysis of DEGs. The enriched GO terms included biological process (BP), cellular component (CC), and molecular function (MF). The top ten GO terms were displayed with *P*<0.05 in 3 categories based on the number of genes. The BP category in males mainly included proteolysis, immune response, cell adhesion, ion transport, regulation of cell proliferation, fatty acid metabolic process, and complement activation ( Figure 3A and Supplementary Table S7). The CC category in males mainly included plasma membrane, extracellular region, plasma membrane part, and extracellular region part ( Figure 3A). The MF category in males mainly included metal ion binding, cation binding, and ion binding ( Figure 3A). The enrichment analysis of DEGs in females showed similar results. BP categories in females mainly included activation of proteolysis, cell adhesion, immune response, and lipid transport ( Figure 3B and Supplementary Table S8). CC categories in females mainly included plasma membrane, extracellular region, and extracellular region part ( Figure 3B). MF categories in females mainly included peptidase activity, very-low-density lipoprotein binding, and very-low-density lipoprotein receptor activity ( Figure 3B).

KP analysis results showed that male DEGs were mainly involved in complement and coagulation cascades, drug metabolism, cytokine-cytokine receptor interactions, cell adhesion molecules (CAMs), ribosomes, and ABC transporters ( Figure 3C). The cell adhesion molecule pathway, including *H2-K1*, *Siglec1*, *Cldn1*, *Cd22*, *Cd4*, *Sdc4*, *Sdc2*, and *Itgam*, is essential for lymphocyte migration [19]. ABC transporters are one of the lipid transport pathways involved in fatty acid metabolism [20]. These results indicate that CAMs and ABC transporters are closely related to male thymic involution. In addition, female KP analysis results showed that there was only one significant enrichment pathway for female DEGs: complement and coagulation cascades, which mainly involves *C1ra*, *C4b*, *Serping1*, *Cfd*, and *C1qc* ( Figure 3D). The complement system can produce proinflammatory mediators through a proteolytic cascade and bind to thymocytes to inhibit their development [ 21, 22] . These results indicated that DEGs are closely related to adipogenesis and cell development in the thymus.

### Potential regulatory mechanism of DEGs during thymic involution

Gene expression is largely controlled by transcription factors. To determine the transcription factors of DEGs, TESS software was used to enrich the transcription factor-binding sites for up- and downregulated DEGs in males and females. The results showed that the binding sites of AP-1, Arnt, CAC-binding, Ncx, Nkx2-5, PU.1, and SREBP-1 were significantly enriched in the upregulated DEGs in males with increasing age ( Supplementary Figure S2A). The binding sites of AP-2γ, MyoD, NF-κB, and POU3F2 were significantly enriched in downregulated DEGs in males (Supplementary Figure S2B). Additionally, the binding sites of AP-1, GR, and SREBP-1 were significantly enriched in upregulated DEGs in females ( Supplementary Figure S2C). The binding sites of SREBP-1, TBP, and Tel-2 were significantly enriched in downregulated DEGs in females ( Supplementary Figure S2D). These findings provide insight into the regulatory mechanism of thymic involution in mice.

### PPI network identification and analysis of DEGs in males and females

Based on the STRING database analysis, Cytoscape was used to construct DEG networks to obtain key genes in male and female mouse thymus. The PPI network in males included 156 nodes and 353 edges with interaction score>0.4 ( Supplementary Figure S3A), and the PPI network in females included 35 nodes and 46 edges ( Supplementary Figure S3B). Next, 16 overlapping DEGs were found in the PPI networks in males and females using Venny software. Considering their key positions in the two PPI networks, these 16 DEGs may be more critical than other genes. Therefore, we chose these 16 key DEGs for further analysis.

### Identification of DELs associated with thymic involution

**Figure 4:**
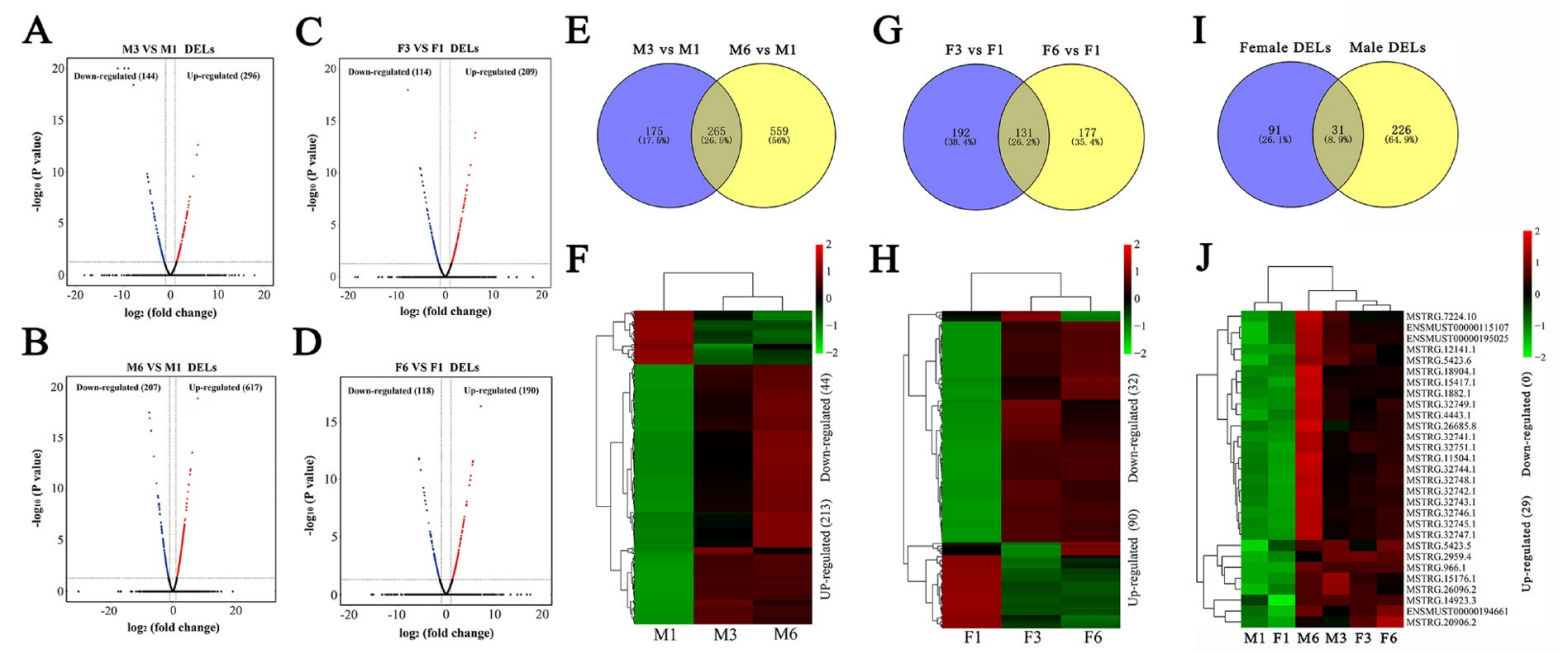
Figure 4 Identification of DELs between different ages during thymic involution (A–D) Volcano plots of DELs in males and females. Venn diagram and heatmap of overlapping DELs in males (E,F) and females (G,H), respectively. Venn diagram and heatmap of DELs overlapping in the thymic development and involution stages of males and females (I,J).

In this study, we identified 21521 lncRNAs in the thymus by RNA-seq ( Supplementary Table S9). Among these lncRNAs, 440 and 824 DELs were identified in the M3 and M6 groups respectively when compared with those in the M1 group ( Figure 4A,B and Supplementary Tables S10 and S11). Compared with the F1 group, the F3 and F6 groups had 323 and 308 DELs respectively ( Figure 4C,D and Supplementary Tables S12 and S13). Further analysis showed that 265 DELs overlapped in the M3 and M6 groups compared with those in the M1 group ( Figure 4E), among which 257 (213 upregulated and 44 downregulated) had the same expression trend ( Figure 4F). In addition, 131 DELs overlapped in the F3 and F6 groups compared with those in the F1 group ( Figure 4G), among which 122 (90 upregulated and 32 downregulated) had the same expression trend ( Figure 4H). Next, 31 overlapping DELs were identified in both the male and female groups at 3 months and 6 months ( Figure 4I), among which 29 (29 upregulated and 0 downregulated) had the same expression trend ( Figure 4J). Therefore, these 29 DELs are expected to be more critical than other lncRNAs for age-related thymic involution.

**Figure 5:**
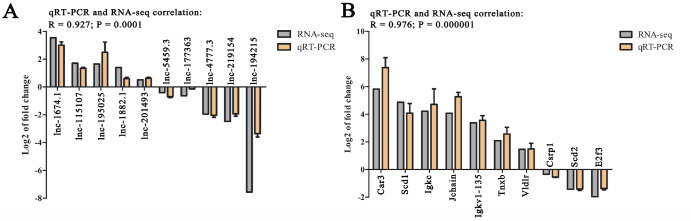
Figure 5 Verification of DELs (A) and DEGs (B) by qRT-PCR The thymus of F3 and F1 mice was used for validation of lnc-1882.1 and lnc-177363. The thymus of M3 and M1 mice was used for validation of lnc-115107, lnc-195025, lnc-201493, lnc-5459.3, and lnc-4777.3. The thymus of F6 and F1 mice was used for validation of Car3, Jchain, Tnxb, Vldlr, Casp1, Scd2, and E2f3. The thymus of M6 and M1 mice was used for validation of lnc-1674.1, lnc-219154, lnc-194215, Scd1, Igkc, and Igkv1-135. Log 2 of fold change was expressed as the mean±SD, n=3.

To verify the reliability of the RNA-seq data, 10 DELs and 10 DEGs were randomly selected from all groups for qRT-PCR verification. The results showed that the correlation between RNA-seq and qRT-PCR was 0.927 and 0.976, respectively ( Figure 5). Correlation results indicated the reliability of the RNA-seq data.

### Identification of the lncRNA-miRNA-mRNA network related to thymic involution

**Figure 6:**
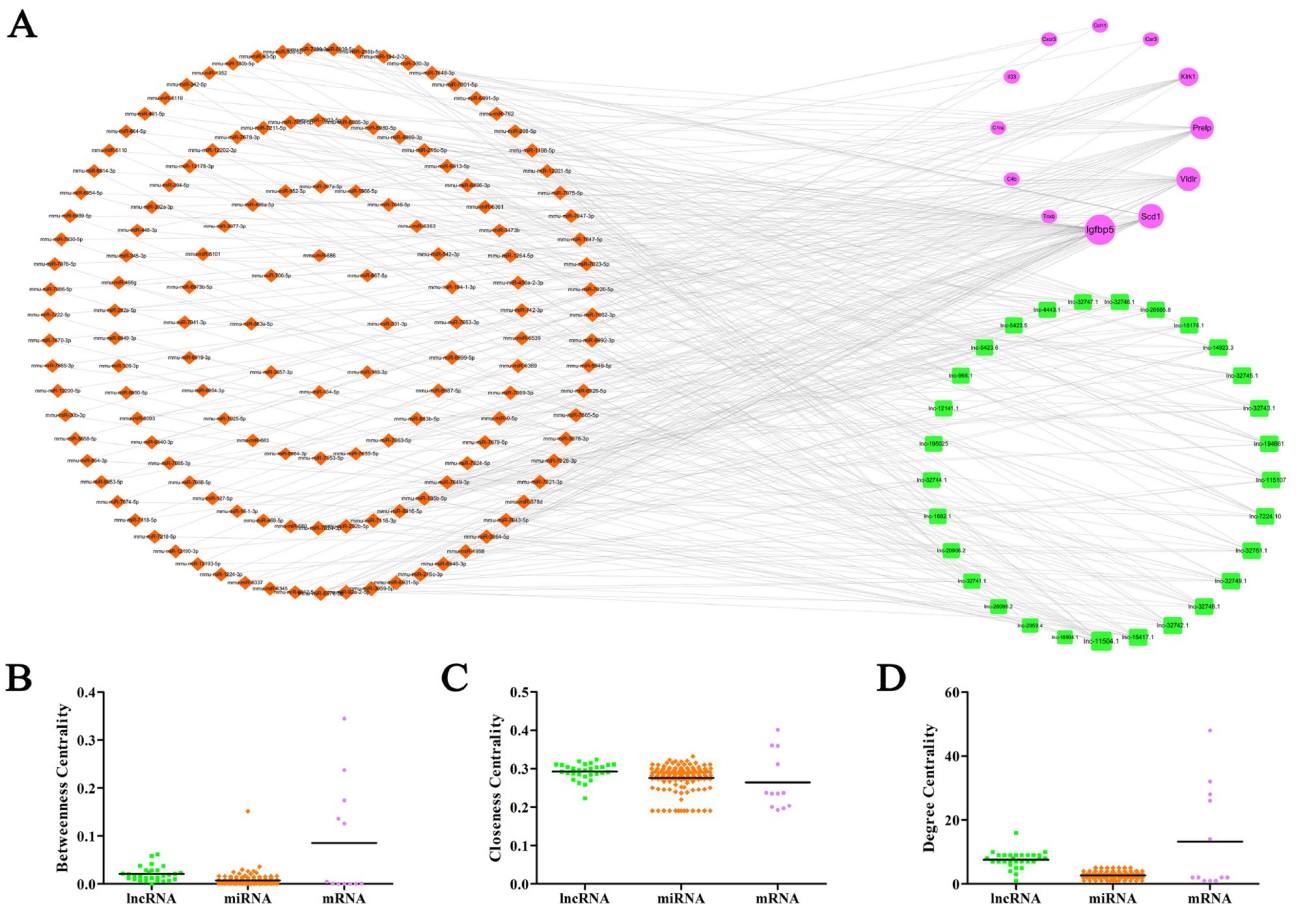
Figure 6 The novel lncRNA-miRNA-mRNA triple regulatory network associated with mouse age-related thymic involution (A) lncRNA-miRNA-mRNA network. (B) The betweenness centrality of lncRNAs, miRNAs and mRNAs. (C) Closeness centrality. (D) The degree centrality. The green rectangle represents lncRNA; the brown diamond represents miRNA; the purple circle represents mRNA.

The ceRNA theory is a vital way of regulating gene expression. In this theory, lncRNAs can interact with mRNAs through competitive adsorption of miRNAs with shared binding sites. In this study, we used miRwalk 3.0 to predict the upstream target miRNAs of the aforementioned 16 key overlapping DEGs. Finally, we predicted that 195 miRNAs regulated the expressions of 13 DEGs ( *Igfbp5*, *Scd1*, *Vldlr*, *Prelp*, *Klrk1*, *Cxcr3*, *Il33*, *Car3*, *Ccl11*, *Tnxb*, *C4b*, *Cyp2f2*, and *C1ra*). However, no potential miRNA was observed upstream of *Cfd*, *Dcn*, and *Serping1*. Meanwhile, TargetScan and Miranda were used to predict the downstream miRNAs of the 29 key DELs mentioned above. Finally, based on mRNA-miRNA and lncRNA-miRNA prediction, we constructed a lncRNA-miRNA-mRNA triple regulatory network containing 29 lncRNAs, 145 miRNAs, and 12 mRNAs ( Figure 6A). Centrality indicators in network analysis, including betweenness centrality (BC), closeness centrality (CC), and degree centrality (DC), were used to determine the importance of individual nodes with functions in the network ( Figure 6B–D). The Kruskal–Wallis test showed that lncRNAs were significantly different from miRNAs and mRNAs in the BC, CC, and DC modules, indicating that lncRNAs play critical roles in this ceRNA network.

Damage to the three-dimensional reticular structure of TECs and the deposition of adipocytes are typical characteristics of thymic involution [ 8, 23] . IGFBP5 is the most conserved member of the IGFBP5 family in vertebrates and plays key roles in the regulation of cell apoptosis, differentiation and survival [24]. This study revealed that the expression of *IGFBP5* was significantly increased during thymic involution, so it might play key roles in thymic epithelial cell apoptosis and adipocyte deposition. Meanwhile, we found that 28 lncRNAs might participate in the regulation of *IGFBP5* through ceRNA ( Supplementary Figure S4A). SCD1 and VLDLR are vital regulators of fat metabolism and production [ 25, 26] . The increased expression of *SCD1* and *VLDLR* during thymic involution suggests that they may be involved in the process of adipocyte deposition. In addition, we found that 26 lncRNAs might be involved in regulating *SCD1*, and 23 lncRNAs might be involved in the regulation of *VLDLR* ( Supplementary Figure S4B,C). These results suggest that lncRNAs may play important roles in thymic involution as ceRNAs.

### IGFBP5 promotes MTEC1 cell apoptosis

**Figure 7:**
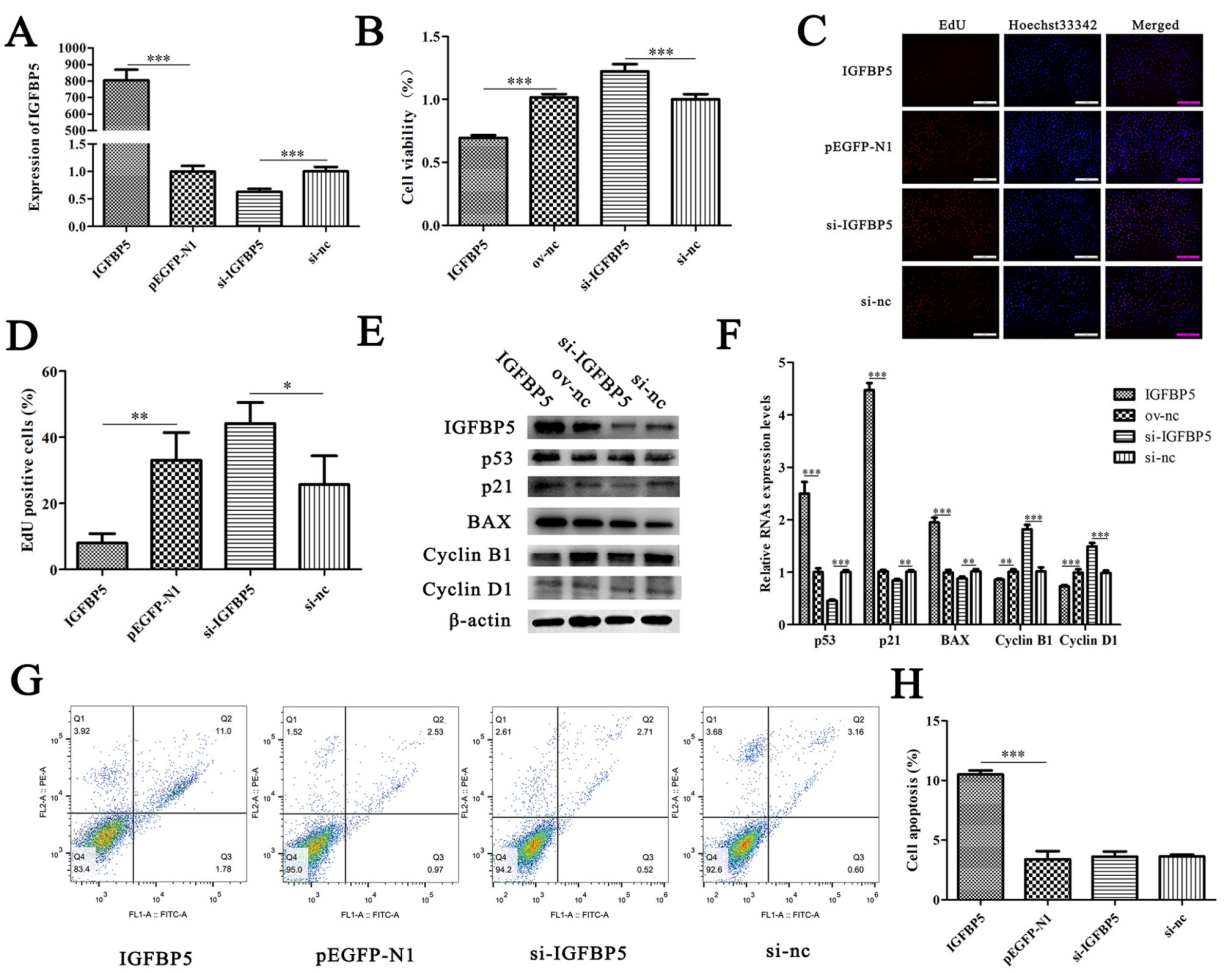
Figure 7 IGFBP5 inhibits the proliferation and promotes the apoptosis of MTEC1 cells (A) qRT-PCR was used to verify the transfection efficiency of the IGFBP5 overexpression plasmid and si- IGFBP5 in MTEC1 cells ( n=3). (B) CCK-8 assay was used to detect the effect of IGFBP5 on the viability of MTEC1 cells ( n=6). (C,D) The effect of IGFBP5 on the proliferation of MTEC1 cells was evaluated by EdU incorporation assay ( n=3). (E) Western blot analysis was used to verify the effect of IGFBP5 on apoptosis-related genes in MTEC1 cells ( n=3). (F) qRT-PCR was used to verify the effect of IGFBP5 on apoptosis-related genes. (G,H) The effect of IGFBP5 on MTEC1 cell apoptosis ( n=3). Data are shown as the mean±SD. * P<0.05,** P<0.01, *** P<0.001.

To explore the effects of IGFBP5 on TECs, we constructed IGFBP5 overexpression plasmids and specific siRNAs. The transfection efficiency of plasmid and siRNA in MTEC1 cells was verified at the mRNA level ( Figure 7A). CCK-8 and EdU assay results showed that IGFBP5 inhibited the viability and proliferation of MTEC1 cells, and siRNA showed the opposite results ( Figure 7B–D). qRT‒PCR and western blot analysis showed that the expressions of IGFBP5, p53, p21, and BAX were significantly increased in the IGFBP5 group, and si- *IGFBP5* significantly decreased the expressions of IGFBP5 and p21 ( Figure 7E,F). In addition, IGFBP5 promoted the apoptosis of MTEC1 cells, but the protective effect of si- *IGFBP5* on apoptosis was not significant, which might be associated with the small number of apoptotic cells in the control group ( Figure 7G,H). These results indicated that IGFBP5 plays a key role in the proliferation and apoptosis of MTEC1 cells.

### miR-193b-3p alleviates MTEC1 cell apoptosis by targeting *IGFBP5*

**Figure 8:**
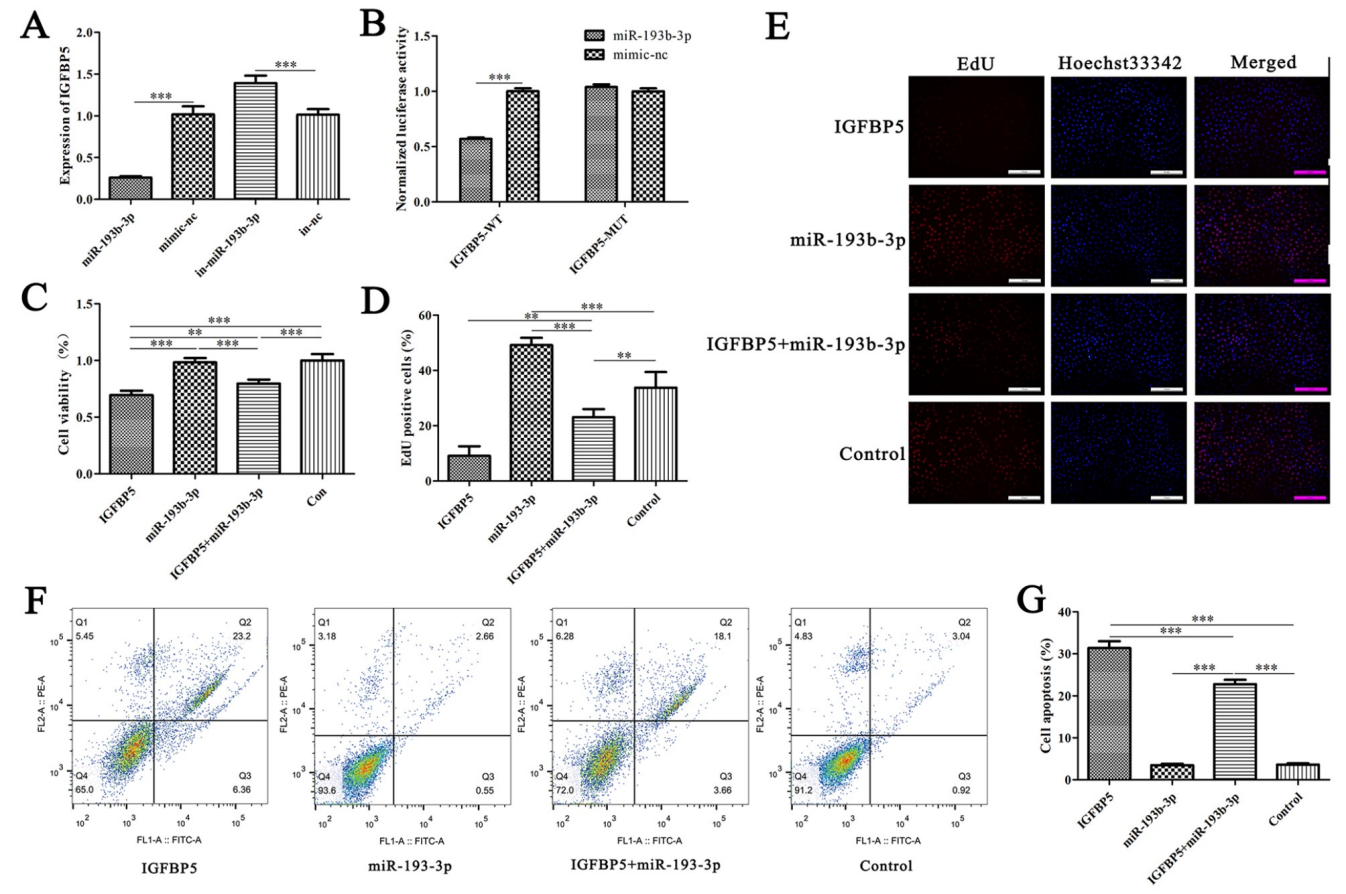
Figure 8 miR-193b-3p alleviates MTEC1 cell apoptosis by targeting *IGFBP5* (A) qRT-PCR was used to verify the transfection efficiency of miR-193b-3p in MTEC1 cells ( n=3). (B) The results of the dual-luciferase reporter assay. (C) CCK-8 assay was used to detect the effect of miR-193b-3p on the viability of MTEC1 cells ( n=6). (D, E) The effect of miR-193b-3p on the proliferation of MTEC1 cells was evaluated by EdU incorporation assay ( n=3). (F, G) The effect of miR-193b-3p on MTEC1 cell apoptosis ( n=3). Data are shown as the mean±SD. ** P<0.01, *** P<0.001.

Our previous data revealed that miR-193b-3p could target *IGFBP5*. To verify the targeting relationship between miR-193b-3p and IGFBP5, we transfected miR-193b-3p mimic, inhibitor and negative control into MTEC1 cells. qRT-PCR results showed that miR-193b-3p could significantly reduce the expression of *IGFBP5* ( Figure 8A). The results of the dual-luciferase reporter assay showed that miR-193b-3p could target the 3′UTR of *IGFBP5* ( Figure 8B). Subsequently, we analysed the effect of miR-193b-3p on the proliferation and apoptosis of MTEC1 cells by CCK-8 assay, EdU assay and flow cytometry. The results showed that miR-193b-3p significantly alleviated the decrease in cell viability, the inhibition of cell proliferation and the increase in apoptosis of MTEC1 cells induced by IGFBP5 ( Figure 8C–G). These results suggest that miR-193b-3p can regulate the proliferation and apoptosis of MTEC1 cells by targeting *IGFBP5*.

### lnc-5423.6 regulates IGFBP5 expression as a molecular sponge of miR-193b-3p to promote apoptosis of MTEC1 cells

**Figure 9:**
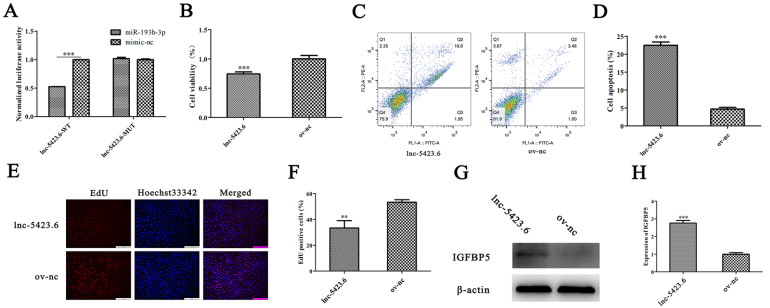
Figure 9 lnc-5423.6 promotes the apoptosis of MTEC1 cells by upregulating IGFBP5 (A) The results of the dual-luciferase reporter assay. (B) CCK-8 assay was used to detect the effect of lnc-5423.6 on the viability of MTEC1 cells ( n=6). (C,D) The effect of lnc-5423.6 on MTEC1 cell apoptosis ( n=3). (E,F) The effect of lnc-5423.6 on the proliferation of MTEC1 cells was evaluated by EdU incorporation assay ( n=3). (G,H) Western blot analysis was used to verify the effect of lnc-5423.6 on IGFBP5 in MTEC1 cells ( n=3). ** P<0.01, *** P<0.001.

Bioinformatics analysis revealed that lnc-5423.6 is a molecular sponge of miR-193b-3p. Through dual-luciferase reporter gene assays, we verified that lnc-5423.6 could bind to miR-193b-3p ( Figure 9A). Subsequently, we analysed the effects of lnc-5423.6 on MTEC1 cell apoptosis and IGFBP5 expression by CCK-8 assay, EdU assay, flow cytometry and western blot analysis. The results showed that lnc-5423.6 could significantly reduce cell viability, promote apoptosis and inhibit cell proliferation ( Figure 9B–F). Moreover, lnc-5423.6 significantly increased the expression of IGFBP5 in MTEC1 cells ( Figure 9G,H). These results indicated that lnc-5423.6 can promote the expression of IGFBP5 through targeted adsorption of miR-193b-3p, thereby regulating the proliferation and apoptosis of MTEC1 cells.

## Discussion

The thymus serves as a central immune organ, and its age-related degradation has a strong inhibitory effect on the production of thymic hormones and naive T lymphocytes [ 27, 28] . However, the mechanism of age-related thymic involution is not clear. As a new type of RNA, the function of lncRNAs in the thymus was initially revealed by Wei *et al*. [29]. In this study, we identified a total of 21521 lncRNAs and 46699 genes in the early stages of thymic involution. The results of qRT-PCR of randomly selected DELs and DEGs showed that the expression trends of these RNAs in RNA-seq and qRT-PCR were consistent, which partly proved the reliability of the experimental data.

To further explore the roles of DEGs in the process of thymic involution, GO and KEGG in DAVID were used to perform functional enrichment analysis. BP analysis results showed that DEGs were significantly enriched in GO terms related to thymus development and immunity, including cell adhesion, extracellular matrix organization, potassium ion transport, protein processing, activation of the immune response, complement activation, inflammatory response, and humoral immune response. Cell adhesion molecules mediate a series of biological processes between lymphocytes and other cell types during the “T-cell homing” process to trigger the dynamic rearrangement of the cytoskeleton and further lead to lymphocyte migration [ 19, 30] . The thymus extracellular matrix increases the output of thymocytes *in vivo* and promotes the differentiation of TECs *in vitro* [31]. Potassium ion transport and protein processing are necessary to maintain regular thymic physiological activity. K ^+^ maintains the cation homeostasis of cells by compensating for negative charges and activates the protein translation process [32]. The immune response gradually weakens as the thymus degenerates. This phenomenon is related to the decreased output of naive T lymphocytes in the thymus and old lymphocytes’ insensitivity to new pathogenic microorganisms [ 33, 34] . In addition, as part of innate immunity, dendritic cells can trigger naive T lymphocytes and secrete cytokines to recruit and activate macrophages to resist pathogens [35]. With increasing age, the number of antigen-presenting cells, such as macrophages and dendritic cells, gradually decreases [ 36, 37] . Additionally, aging can affect humoral immunity by reducing the number of B-cell pools and antibody production [38]. Complement activation is closely related to the inflammatory response and thymocyte development. The complement system can generate proinflammatory mediators through interaction with proteolytic cascades of classical, lectin, and alternative pathways to activate the inflammatory response to target lysis of target cells [21]. Meanwhile, thymocytes easily bind with complement proteins, and the amount of complement binding is inversely proportional to cell development, which partially explains why thymic involution induces immune decline [ 22, 39] . It is well known that thymic involution in male mammals is faster than that in females, but the regulatory mechanism remains unknown. In this study, KP analysis results showed that DEGs in males were mainly concentrated in complement and coagulation cascades, drug metabolism, cytokine-cytokine receptor interaction, cell adhesion molecules (CAMs), ribosome, and ABC transporters, but only one pathway was enriched in DEGs in females. Studies have shown that vascular endothelial growth factor can induce thymic involution, which may be related to its enhanced adhesion of thymocytes to endothelial cells [ 19, 40] . Adipocyte deposition is one of the signs of thymic involution. ABC transporters, as one of the fatty acid metabolic pathways, may be closely related to adipocyte deposition in the thymus [20]. However, further research is needed to reveal the mechanism of thymic involution in mice of different sexes.

lncRNAs are widely involved in aging by absorbing miRNAs through the ceRNA mechanism [ 41, 42] . However, the specific expression of lncRNAs in the thymus needs further investigation. In this study, we hypothesize that lncRNAs may be involved in age-related thymic involution through the ceRNA mechanism. Based on the ceRNA theory, we constructed a novel ceRNA network related to thymic involution. The scale-free distribution of the ceRNA network indicated that lncRNAs play essential roles in thymic involution regulation.

Adipocyte deposition is one of the models to explain age-related thymic involution [43]. In addition, adipocytes produce thymus-suppressing factors, such as leukemia inhibitory factor, tumor suppressor M and interleukin-6, which are associated with age-related thymic involution [44]. The roles of SCD1 and VLDLR in thymic involution have not been reported, but studies have found that SCD1 is a lipogenic protein required for producing new fat, which is regulated by the transcription factor SREBP-1 [45]. VLDLR is a member of the low-density lipoprotein receptor superfamily [46]. ER stress stimulates liver steatosis by increasing the expression of VLDLR [25]. Inhibiting the upregulation of VLDLR can protect mice from hepatic steatosis induced by a high-fat diet [47]. These studies indicate that lncRNAs may act as ceRNAs to regulate the expressions of SCD1 and VLDLR to participate in adipocyte deposition during age-related thymic involution.

In recent years, the function of IGFBP5 in mediating cell proliferation, differentiation and apoptosis was gradually revealed [ 24, 48] . However, there are many controversies regarding the function of IGFBP5. Studies have found that IGFBP5 can increase the invasion of glioblastoma pleomorphic cells and inhibit cell proliferation through the EMT and Akt signaling pathways [49]. In addition, IGFBP5 plays an important role in methamphetamine-induced dopaminergic neuron apoptosis [50]. However, IGFBP5 overexpression can promote the proliferation and inhibit the apoptosis of rat nucleus pulposus cells derived from intervertebral disc degeneration by inducing inactivation of the ERK/MAPK axis [51]. This is mainly due to the different roles played by IGFBP5 in different species, different tissues, different developmental stages, or different cells. In this study, we found that the expression level of IGFBP5 was significantly increased in all stages of thymic involution, which means that IGFBP5 might play a key role in the process of thymic involution. TECs provide a unique microenvironment for the differentiation, development and maturity of thymocytes [8]. In this study, IGFBP5 was found to inhibit the proliferation and promote the apoptosis of MTEC1 cells. Injury of the three-dimensional mesh structure composed of TECs is considered to be one of the signs of thymic involution. Therefore, IGFBP5 may play key roles in thymic involution. In addition, given the role of lncRNAs in the ceRNA network, they might play a key role in IGFBP5 regulation. The results of this study provide new insight into thymic involution.

In recent years, we showed that miRNAs are widely involved in the regulation of thymic epithelial cell development [ 52– 54] . Through comprehensive analysis, we selected and verified the regulatory relationship between miR-193b-3p and IGFBP5. The results showed that miR-193b-3p could directly bind to the 3’UTR of *IGFBP5*. miR-193b-3p could alleviate the apoptosis and proliferation inhibition of MTEC1 cells caused by IGFBP5. These results suggest that miR-193b-3p specifically regulates the expression of IGFBP5 in MTEC1 cells. In addition, the action pathways of miR-193b-3p *in vivo* are diverse. For example, miR-193b-3p can alleviate focal cerebral ischemia and reperfusion injury in rats [55], regulate cartilage formation and chondrocyte metabolism by targeting HDAC3 [56], and regulate hepatocyte apoptosis in selenium-deficient broilers by targeting MAML1 [57].

lncRNAs can participate in the proliferation and aging physiological processes in different ways [ 58, 59] . In this study, we hypothesized that lnc-5423.6 may adjust the expression of IGFBP5 by adsorbing miR-193b-3p. A dual-luciferase reporter assay showed that lnc-5423.6 could target miR-193b-3p. The results of CCK-8 assay, EdU assay and flow cytometric analysis showed that overexpression of lnc-5423.6 could promote apoptosis and inhibit cell viability and cell proliferation. Moreover, overexpression of lnc-5423.6 significantly increased the expression of IGFBP5 at the mRNA and protein levels. This result indicates that lnc-5423.6 may act as a ceRNA to increase the expression of IGFBP5 by adsorbing miR-193b-3p to promote MTEC1 cell apoptosis.

In summary, we comprehensively analysed the expression profiles of lncRNAs and genes during the early stages of thymic involution in mice and identified a triple regulatory ceRNA network of lncRNA-miRNA-mRNA related to thymic involution. Among them, IGFBP5 plays a key role in thymic epithelial cell apoptosis. In addition, lnc-5423.6 could promote the expression of IGFBP5 by adsorbing miR-193b-3p to induce apoptosis of MTEC1 cells, which provides a valuable reference for further exploring the role of lncRNAs in age-related thymic involution.

## Data Availability Statement

The raw data in this study have been deposited in the Gene Expression Omnibus of the National Center for Biotechnology Information database (NCBI-GEO) under accession number GSE162271.

## Supporting information

Table_S8

Table_S11

Table_S1

Table_S13

Table_S3

Table_S6

356FigS1-S4

Table_S2

Table_S12

Table_S9

Table_S4

Table_S7

Table_S5

Table_S10

## Supplementary Data

Supplementary data is available at *Acta Biochimica et Biophysica Sinica* online.
