# Supplementary material for: Transcriptome analysis reveals a potential regulatory mechanism of the lnc-5423.6/IGFBP5 axis in the early stages of mouse thymic involution: lnc-5423.6/IGFBP5 axis regulates thymic involution
Source: Acta Biochim Biophys Sin (Shanghai). 2023 Apr 19;55(4):548–60. doi: 10.3724/abbs.2023042 (PMC10195152; doi:10.3724/abbs.2023042)
Supplement: Table_S8 [file Table_S8.pdf]

Category	Term	Count	%	PValue	Genes	List Total	Pop Hits	Pop Total
GOTERM_	activation	6	4.411765	3.65E-06	IGHG1, C1	91	36	13588
GOTERM_	compleme	6	4.411765	3.65E-06	IGHG1, C1	91	36	13588
GOTERM_	activation	7	5.147059	2.19E-05	IGHG1, C1	91	86	13588
GOTERM_	humoral ir	6	4.411765	2.79E-05	IGHG1, C1	91	54	13588
GOTERM_	compleme	5	3.676471	4.32E-05	IGHG1, C1	91	30	13588
GOTERM_	protein ma	6	4.411765	6.88E-05	IGHG1, C1	91	65	13588
GOTERM_	humoral ir	5	3.676471	8.05E-05	IGHG1, C1	91	35	13588
GOTERM_	acute infla	6	4.411765	1.96E-04	IGHG1, C1	91	81	13588
GOTERM_	positive re	8	5.882353	2.31E-04	IGHG1, C1	91	186	13588
GOTERM_	positive re	7	5.147059	2.81E-04	IGHG1, C1	91	136	13588
GOTERM_	protein pr	6	4.411765	3.22E-04	IGHG1, C1	91	90	13588
GOTERM_	protein ma	6	4.411765	4.34E-04	IGHG1, C1	91	96	13588
GOTERM_	immunogl	5	3.676471	7.96E-04	IGHG1, C1	91	63	13588
GOTERM_	B cell med	5	3.676471	8.95E-04	IGHG1, C1	91	65	13588
GOTERM_	cell adhes	12	8.823529	0.001165	SIGLEC1, A	91	561	13588
GOTERM_	biological	12	8.823529	0.001182	SIGLEC1, A	91	562	13588
GOTERM_	immune e	6	4.411765	0.001483	IGHG1, C1	91	126	13588
GOTERM_	lymphocyt	5	3.676471	0.001605	IGHG1, C1	91	76	13588
GOTERM_	adaptive ir	5	3.676471	0.002319	IGHG1, C1	91	84	13588
GOTERM_	adaptive ir	5	3.676471	0.002319	IGHG1, C1	91	84	13588
GOTERM_	positive re	7	5.147059	0.002443	IGHG1, C1	91	206	13588
GOTERM_	leukocyte	5	3.676471	0.002862	IGHG1, C1	91	89	13588
GOTERM_	proteolysis	16	11.76471	0.003094	IGHG1, AD	91	1034	13588
GOTERM_	inflammat	7	5.147059	0.003778	CCL11, IGI	91	225	13588
GOTERM_	innate imr	5	3.676471	0.005529	C1RA, C4E	91	107	13588
GOTERM_	defense re	9	6.617647	0.009609	CCL11, IGI	91	448	13588
GOTERM_	cell morph	7	5.147059	0.016651	SEMA5A, S	91	309	13588
GOTERM_	peptidyl-s	3	2.205882	0.017811	SPOCK2, D	91	31	13588
GOTERM_	response t	7	5.147059	0.027563	CCL11, IGI	91	347	13588
GOTERM_	cellular co	7	5.147059	0.028934	SEMA5A, S	91	351	13588
GOTERM_	extracellul	4	2.941176	0.029403	TNXB, SPC	91	101	13588
GOTERM_	chemical h	7	5.147059	0.034087	SCN1A, LD	91	365	13588
GOTERM_	immune re	8	5.882353	0.036536	CCL11, IGI	91	471	13588
GOTERM_	lipid trans	4	2.941176	0.044418	SPNS2, LD	91	119	13588
GOTERM_	respiratory	4	2.941176	0.049151	PDPN, ALI	91	124	13588
GOTERM_	peptide cr	2	1.470588	0.051789	SPOCK2, D	91	8	13588
GOTERM_	lipid locali	4	2.941176	0.053109	SPNS2, LD	91	128	13588
GOTERM_	regulation	3	2.205882	0.054753	IGHG1, FA	91	57	13588
GOTERM_	positive re	3	2.205882	0.054753	IGHG1, KL	91	57	13588
GOTERM_	sex differe	4	2.941176	0.055144	PGR, RXFP	91	130	13588
GOTERM_	cellular di-	4	2.941176	0.059324	RYR3, MT1	91	134	13588
GOTERM_	white fat c	2	1.470588	0.064316	SCD1, FAE	91	10	13588
GOTERM_	di-, tri-val	4	2.941176	0.072728	RYR3, MT1	91	146	13588
GOTERM_	extracellul	4	2.941176	0.076274	TNXB, SPC	91	149	13588
GOTERM_	cellular ca	4	2.941176	0.079895	RYR3, MT1	91	152	13588
GOTERM_	chondroiti	2	1.470588	0.088881	SPOCK2, D	91	14	13588
GOTERM_	cellular ior	5	3.676471	0.094987	SCN1A, RY	91	261	13588
GOTERM_	tube deve	5	3.676471	0.098026	CCL11, PG	91	264	13588
GOTERM_	reproduct	5	3.676471	0.098026	PLAG1, PG	91	264	13588
GOTERM_	chondroiti	2	1.470588	0.100924	SPOCK2, D	91	16	13588
GOTERM_	cellular ch	5	3.676471	0.10215	SCN1A, RY	91	268	13588
GOTERM_	tube morp	4	2.941176	0.104492	CCL11, PG	91	171	13588
GOTERM_	gland mor	3	2.205882	0.106835	CCL11, PL	91	84	13588
GOTERM_	peptide cr	2	1.470588	0.106886	SPOCK2, D	91	17	13588
GOTERM_	face devel	2	1.470588	0.106886	ALDH1A3,	91	17	13588
GOTERM_	neuron pr	4	2.941176	0.111412	SEMA5A, I	91	176	13588
GOTERM_	regulation	2	1.470588	0.112809	IGHG1, SE	91	18	13588

GOTERM_ regulation	3	2.205882	0.115388	IGHG1, KL	91	88	13588
GOTERM_ branching	2	1.470588	0.118693	CCL11, PG	91	19	13588
GOTERM_ cation hor	4	2.941176	0.122839	RYR3, MT1	91	184	13588
GOTERM_ fatty acid r	4	2.941176	0.122839	SCD1, TNX	91	184	13588
GOTERM_ branching	3	2.205882	0.126316	CCL11, PG	91	93	13588
GOTERM_ endocytos	4	2.941176	0.128709	IGHG1, LD	91	188	13588
GOTERM_ membrane	4	2.941176	0.128709	IGHG1, LD	91	188	13588
GOTERM_ ion homeo	5	3.676471	0.129669	SCN1A, RY	91	293	13588
GOTERM_ collagen fi	2	1.470588	0.130345	TNXB, DPT	91	21	13588
GOTERM_ head deve	2	1.470588	0.130345	ALDH1A3,	91	21	13588
GOTERM_ cellular me	3	2.205882	0.142005	RYR3, MT1	91	100	13588
GOTERM_ positive re	2	1.470588	0.147539	IGHG1, FA	91	24	13588
GOTERM_ regulation	3	2.205882	0.148853	IGHG1, FA	91	103	13588
GOTERM_ cell projec	4	2.941176	0.150008	SEMA5A, I	91	202	13588
GOTERM_ developm	3	2.205882	0.15115	PGR, RXFP	91	104	13588
GOTERM_ metal ion	3	2.205882	0.155766	RYR3, MT1	91	106	13588
GOTERM_ cholestero	2	1.470588	0.158815	LDLR, FAB	91	26	13588
GOTERM_ sterol hom	2	1.470588	0.158815	LDLR, FAB	91	26	13588
GOTERM_ mammary	2	1.470588	0.158815	CCL11, PG	91	26	13588
GOTERM_ cell part m	4	2.941176	0.165873	SEMA5A, I	91	212	13588
GOTERM_ response t	2	1.470588	0.175452	CYP2F2, M	91	29	13588
GOTERM_ brown fat	2	1.470588	0.175452	SCD1, FAB	91	29	13588
GOTERM_ neuron pr	4	2.941176	0.175625	SEMA5A, I	91	218	13588
GOTERM_ transmem	6	4.411765	0.189256	KCNH1, SL	91	460	13588
GOTERM_ homeosta	7	5.147059	0.190205	SCN1A, LD	91	584	13588
GOTERM_ ion transp	8	5.882353	0.192139	KCNH1, KC	91	712	13588
GOTERM_ cellular ho	5	3.676471	0.192546	SCN1A, RY	91	343	13588
GOTERM_ mammary	2	1.470588	0.197129	CCL11, PG	91	33	13588
GOTERM_ proteoglyc	2	1.470588	0.197129	SPOCK2, D	91	33	13588
GOTERM_ morphoge	3	2.205882	0.200771	CCL11, PG	91	125	13588
GOTERM_ lipid home	2	1.470588	0.20246	LDLR, FAB	91	34	13588
GOTERM_ reproduct	3	2.205882	0.212873	PLAG1, PG	91	130	13588
GOTERM_ triglycerid	2	1.470588	0.213017	TNXB, PCK	91	36	13588
GOTERM_ cell motion	5	3.676471	0.225653	SEMA5A, I	91	367	13588
GOTERM_ positive re	2	1.470588	0.228593	IGHG1, FA	91	39	13588
GOTERM_ glycosami	2	1.470588	0.233718	SPOCK2, D	91	40	13588
GOTERM_ positive re	2	1.470588	0.233718	IGHG1, KL	91	40	13588
GOTERM_ positive re	2	1.470588	0.233718	IGHG1, KL	91	40	13588
GOTERM_ cytokine p	2	1.470588	0.248889	AVPR2, FA	91	43	13588
GOTERM_ acylglycer	2	1.470588	0.248889	TNXB, PCK	91	43	13588
GOTERM_ positive re	2	1.470588	0.248889	TNXB, SPO	91	43	13588
GOTERM_ cation tran	6	4.411765	0.255323	KCNH1, KC	91	515	13588
GOTERM_ neutral lip	2	1.470588	0.258838	TNXB, PCK	91	45	13588
GOTERM_ glycerol et	2	1.470588	0.258838	TNXB, PCK	91	45	13588
GOTERM_ regulation	2	1.470588	0.263763	KLRK1, SEI	91	46	13588
GOTERM_ positive re	2	1.470588	0.263763	IGHG1, KL	91	46	13588
GOTERM_ regulation	2	1.470588	0.268656	PDPN, GA	91	47	13588
GOTERM_ membrane	4	2.941176	0.269057	IGHG1, LD	91	272	13588
GOTERM_ organic et	2	1.470588	0.273517	TNXB, PCK	91	48	13588
GOTERM_ potassium	3	2.205882	0.286396	KCNH1, KC	91	160	13588
GOTERM_ steroid me	3	2.205882	0.288852	LDLR, SDR	91	161	13588
GOTERM_ lipid biosy	4	2.941176	0.292503	CDS2, SCD	91	285	13588
GOTERM_ aminoglyc	2	1.470588	0.292643	SPOCK2, D	91	52	13588
GOTERM_ axonogen	3	2.205882	0.29376	SEMA5A, I	91	163	13588
GOTERM_ developm	2	1.470588	0.297346	RXFP2, PD	91	53	13588
GOTERM_ actin cytos	3	2.205882	0.298664	TNXB, SHR	91	165	13588
GOTERM_ neuron de	4	2.941176	0.305196	SEMA5A, I	91	292	13588
GOTERM_ actin filam	2	1.470588	0.311271	SHROOM4	91	56	13588

GOTERM_ male sex c	2	1.470588	0.315851	RXFP2, PD	91	57	13588
GOTERM_ morphoge	3	2.205882	0.318223	CCL11, PG	91	173	13588
GOTERM_ monovale	4	2.941176	0.325191	KCNH1, K(	91	303	13588
GOTERM_ actin filam	3	2.205882	0.325529	TNXB, SHI	91	176	13588
GOTERM_ fat cell dif	2	1.470588	0.333875	SCD1, FAE	91	61	13588
GOTERM_ metal ion	5	3.676471	0.336177	KCNH1, K(	91	442	13588
GOTERM_ cell morph	3	2.205882	0.34008	SEMA5A, I	91	182	13588
GOTERM_ regulation	3	2.205882	0.349729	TNXB, FAE	91	186	13588
GOTERM_ regulation	2	1.470588	0.351428	IGHG1, KL	91	65	13588
GOTERM_ cell projec	4	2.941176	0.354281	SEMA5A, I	91	319	13588
GOTERM_ lymphocyt	3	2.205882	0.361726	KLRK1, ITC	91	191	13588
GOTERM_ transmem	3	2.205882	0.364116	SS18, PDG	91	192	13588
GOTERM_ regulation	3	2.205882	0.364116	TNXB, FAE	91	192	13588
GOTERM_ growth	3	2.205882	0.366503	CCL11, PL	91	193	13588
GOTERM_ cytoskelet	4	2.941176	0.366975	SS18, TNX	91	326	13588
GOTERM_ vesicle-me	5	3.676471	0.372508	IGHG1, LD	91	466	13588
GOTERM_ neuron mi	2	1.470588	0.372728	MDGA1, N	91	70	13588
GOTERM_ regulation	2	1.470588	0.372728	IGHG1, KL	91	70	13588
GOTERM_ cholesterc	2	1.470588	0.372728	LDLR, VLD	91	70	13588
GOTERM_ gland dev	3	2.205882	0.376016	CCL11, PL	91	197	13588
GOTERM_ regulation	3	2.205882	0.380751	TNXB, FAE	91	199	13588
GOTERM_ integrin-m	2	1.470588	0.397377	ADAM28,	91	76	13588
GOTERM_ sterol met	2	1.470588	0.401391	LDLR, VLD	91	77	13588
GOTERM_ cell morph	3	2.205882	0.411153	SEMA5A, I	91	212	13588
GOTERM_ leukocyte	3	2.205882	0.427227	KLRK1, ITC	91	219	13588
GOTERM_ negative r	5	3.676471	0.432697	INHBB, FA	91	506	13588
GOTERM_ mammary	2	1.470588	0.436347	CCL11, PG	91	86	13588
GOTERM_ response t	2	1.470588	0.440104	CYGB, SRX	91	87	13588
GOTERM_ response t	2	1.470588	0.443836	SS18, FAB	91	88	13588
GOTERM_ gonad dev	2	1.470588	0.443836	PGR, RXFF	91	88	13588
GOTERM_ cellular ca	2	1.470588	0.454886	RYR3, BAN	91	91	13588
GOTERM_ regulation	2	1.470588	0.458521	HTRA3, IG	91	92	13588
GOTERM_ polysacch	2	1.470588	0.458521	SPOCK2, D	91	92	13588
GOTERM_ oxidation	6	4.411765	0.461001	SCD1, CYF	91	672	13588
GOTERM_ cell-cell a	3	2.205882	0.465296	TNXB, PDI	91	236	13588
GOTERM_ sulfur met	2	1.470588	0.465719	SPOCK2, D	91	94	13588
GOTERM_ regulation	2	1.470588	0.465719	TNXB, SPC	91	94	13588
GOTERM_ calcium io	2	1.470588	0.469283	RYR3, BAN	91	95	13588
GOTERM_ tissue mor	3	2.205882	0.469679	CCL11, PG	91	238	13588
GOTERM_ locomotor	3	2.205882	0.471863	CCL11, SC	91	239	13588
GOTERM_ regulation	2	1.470588	0.476339	PDPN, GA	91	97	13588
GOTERM_ regulation	5	3.676471	0.479802	PGR, MYC	91	538	13588
GOTERM_ axon guid	2	1.470588	0.479833	SEMA5A, S	91	98	13588
GOTERM_ rhythmic p	2	1.470588	0.479833	PGR, PER3	91	98	13588
GOTERM_ carboxylic	2	1.470588	0.483303	PDPN, SLC	91	99	13588
GOTERM_ developm	2	1.470588	0.486751	CCL11, PL	91	100	13588
GOTERM_ organic ac	2	1.470588	0.486751	PDPN, SLC	91	100	13588
GOTERM_ cell activat	3	2.205882	0.486999	KLRK1, ITC	91	246	13588
GOTERM_ positive re	3	2.205882	0.491275	IGHG1, AL	91	248	13588
GOTERM_ neuron dif	4	2.941176	0.495149	SEMA5A, I	91	399	13588
GOTERM_ vasculatur	3	2.205882	0.495529	SEMA5A, I	91	250	13588
GOTERM_ positive re	3	2.205882	0.495529	IGHG1, AL	91	250	13588
GOTERM_ positive re	3	2.205882	0.49976	IGHG1, AL	91	252	13588
GOTERM_ behavior	4	2.941176	0.505166	CCL11, SC	91	405	13588
GOTERM_ chemotaxi	2	1.470588	0.516772	CCL11, EN	91	109	13588
GOTERM_ taxis	2	1.470588	0.516772	CCL11, EN	91	109	13588
GOTERM_ epithelial t	2	1.470588	0.523203	CCL11, PG	91	111	13588
GOTERM_ lung deve	2	1.470588	0.523203	PDPN, PD	91	111	13588

GOTERM_ negative r	4	2.941176	0.526522	INHBB, FA	91	418	13588
GOTERM_ respiratory	2	1.470588	0.529551	PDPN, PD	91	113	13588
GOTERM_ epithelium	3	2.205882	0.538828	CCL11, PG	91	271	13588
GOTERM_ enzyme lir	3	2.205882	0.542819	SS18, PDG	91	273	13588
GOTERM_ negative r	4	2.941176	0.545789	INHBB, FA	91	430	13588
GOTERM_ negative r	4	2.941176	0.552113	INHBB, FA	91	434	13588
GOTERM_ regulation	3	2.205882	0.556601	TNXB, SER	91	280	13588
GOTERM_ sodium io	2	1.470588	0.565916	SCN1A, SC	91	125	13588
GOTERM_ regulation	3	2.205882	0.575782	TNXB, FAE	91	290	13588
GOTERM_ glycerolipi	2	1.470588	0.577409	TNXB, PCK	91	129	13588
GOTERM_ positive re	2	1.470588	0.58583	IGHG1, IN	91	132	13588
GOTERM_ regulation	3	2.205882	0.596187	TNXB, FAE	91	301	13588
GOTERM_ regulation	3	2.205882	0.596187	TNXB, FAE	91	301	13588
GOTERM_ regulation	2	1.470588	0.604841	INHBB, KL	91	139	13588
GOTERM_ protein an	5	3.676471	0.618009	BLK, PDGF	91	640	13588
GOTERM_ glycoprote	2	1.470588	0.625512	SPOCK2, D	91	147	13588
GOTERM_ regulation	2	1.470588	0.659146	INHBB, UH	91	161	13588
GOTERM_ phospholi	2	1.470588	0.6637	CDS2, TNX	91	163	13588
GOTERM_ forebrain	2	1.470588	0.672629	ALDH1A3,	91	167	13588
GOTERM_ induction	2	1.470588	0.672629	IGHG1, CA	91	167	13588
GOTERM_ induction	2	1.470588	0.672629	IGHG1, CA	91	167	13588
GOTERM_ phosphoru	6	4.411765	0.687212	PTPRZ1, B	91	866	13588
GOTERM_ phosphate	6	4.411765	0.687212	PTPRZ1, B	91	866	13588
GOTERM_ organoph	2	1.470588	0.69187	CDS2, TNX	91	176	13588
GOTERM_ phosphory	5	3.676471	0.707226	BLK, PDGF	91	718	13588
GOTERM_ negative r	3	2.205882	0.710485	FABP4, PE	91	372	13588
GOTERM_ regulation	4	2.941176	0.715095	IGHG1, AL	91	553	13588
GOTERM_ regulation	4	2.941176	0.723111	IGHG1, AL	91	560	13588
GOTERM_ regulation	4	2.941176	0.726493	IGHG1, AL	91	563	13588
GOTERM_ intracellula	6	4.411765	0.733261	PGR, SS18	91	915	13588
GOTERM_ blood ves	2	1.470588	0.734324	SEMA5A, I	91	198	13588
GOTERM_ negative r	3	2.205882	0.74385	FABP4, PE	91	397	13588
GOTERM_ negative r	3	2.205882	0.748877	FABP4, PE	91	401	13588
GOTERM_ negative r	3	2.205882	0.759882	FABP4, PE	91	410	13588
GOTERM_ regulation	9	6.617647	0.767857	KCNH1, P	91	1465	13588
GOTERM_ negative r	2	1.470588	0.777091	MYO16, D	91	224	13588
GOTERM_ protein co	2	1.470588	0.781564	GAS7, SCA	91	227	13588
GOTERM_ protein co	2	1.470588	0.781564	GAS7, SCA	91	227	13588
GOTERM_ regulation	9	6.617647	0.782899	KCNH1, P	91	1488	13588
GOTERM_ negative r	2	1.470588	0.787391	PER3, NR2	91	231	13588
GOTERM_ protein kir	2	1.470588	0.794459	SS18, AVP	91	236	13588
GOTERM_ cell migrat	2	1.470588	0.799946	MDGA1, N	91	240	13588
GOTERM_ blood ves	2	1.470588	0.805288	SEMA5A, I	91	244	13588
GOTERM_ cell prolife	2	1.470588	0.809201	PDPN, ITG	91	247	13588
GOTERM_ apoptosis	3	2.205882	0.818534	TNS4, CAS	91	465	13588
GOTERM_ regulation	2	1.470588	0.820481	HTRA3, IG	91	256	13588
GOTERM_ programm	3	2.205882	0.825922	TNS4, CAS	91	473	13588
GOTERM_ positive re	2	1.470588	0.82646	RXFP2, VL	91	261	13588
GOTERM_ pattern sp	2	1.470588	0.851523	SEMA5A, I	91	284	13588
GOTERM_ localizatio	2	1.470588	0.851523	MDGA1, N	91	284	13588
GOTERM_ positive re	2	1.470588	0.851523	FABP4, DD	91	284	13588
GOTERM_ cell motilit	2	1.470588	0.851523	MDGA1, N	91	284	13588
GOTERM_ skeletal sy	2	1.470588	0.852528	PDGFRA, I	91	285	13588
GOTERM_ response t	3	2.205882	0.852872	AVPR2, CA	91	505	13588
GOTERM_ cell death	3	2.205882	0.854425	TNS4, CAS	91	507	13588
GOTERM_ death	3	2.205882	0.863437	TNS4, CAS	91	519	13588
GOTERM_ positive re	2	1.470588	0.872135	RXFP2, VL	91	306	13588
GOTERM_ negative r	2	1.470588	0.873862	PER3, NR2	91	308	13588

GOTERM_ negative r	2	1.470588	0.875566	PER3, NR2	91	310	13588
GOTERM_ macromol	2	1.470588	0.897163	GAS7, SCA	91	338	13588
GOTERM_ macromol	2	1.470588	0.915624	GAS7, SCA	91	367	13588
GOTERM_ regulation	3	2.205882	0.919634	PER3, YAP	91	616	13588
GOTERM_ cell surface	13	9.558824	0.919845	ADAM28,	91	2495	13588
GOTERM_ cellular res	2	1.470588	0.93449	CASP12, S	91	404	13588
GOTERM_ regulation	11	8.088235	0.939445	PLAG1, KC	91	2227	13588
GOTERM_ transcripti	8	5.882353	0.958315	PLAG1, PG	91	1772	13588
GOTERM_ positive re	2	1.470588	0.963219	AVPR2, YA	91	488	13588
GOTERM_ positive re	2	1.470588	0.971706	KLRK1, YA	91	526	13588
GOTERM_ positive re	2	1.470588	0.976365	KLRK1, YA	91	552	13588
GOTERM_ positive re	2	1.470588	0.97717	KLRK1, YA	91	557	13588
GOTERM_ cell cycle	2	1.470588	0.98431	PDPN, UH	91	611	13588
GOTERM_ positive re	2	1.470588	0.986539	AVPR2, YA	91	633	13588
GOTERM_ G-protein	6	4.411765	0.996539	AVPR2, OI	91	1877	13588
GOTERM_ neurologic	4	2.941176	0.999374	OLFR56, S	91	1681	13588
GOTERM_ sensory pe	3	2.205882	0.999388	OLFR56, P	91	1402	13588
GOTERM_ sensory pe	2	1.470588	0.999568	OLFR56, C	91	1117	13588
GOTERM_ cognition	3	2.205882	0.999638	OLFR56, P	91	1480	13588
GOTERM_ sensory pe	2	1.470588	0.999749	OLFR56, C	91	1192	13588
GOTERM_ developm	1	0.735294	1	PGR	91	101	13588
GOTERM_ negative r	1	0.735294	1	INHBB	91	14	13588
GOTERM_ regulation	1	0.735294	1	PDPN	91	107	13588
GOTERM_ vitamin tra	1	0.735294	1	PDPN	91	17	13588
GOTERM_ gluconeog	1	0.735294	1	PCK1	91	20	13588
GOTERM_ regulation	1	0.735294	1	INHBB	91	125	13588
GOTERM_ positive re	1	0.735294	1	YAP1	91	510	13588
GOTERM_ monocarb	1	0.735294	1	SLCO2A1	91	19	13588
GOTERM_ collagen m	1	0.735294	1	TNXB	91	23	13588
GOTERM_ response t	1	0.735294	1	MT1	91	53	13588
GOTERM_ negative r	1	0.735294	1	SERPING1	91	5	13588
GOTERM_ odontoge	1	0.735294	1	PDGFRA	91	41	13588
GOTERM_ defense re	1	0.735294	1	IGHG1	91	108	13588
GOTERM_ female go	1	0.735294	1	PGR	91	53	13588
GOTERM_ lymphang	1	0.735294	1	PDPN	91	3	13588
GOTERM_ positive re	1	0.735294	1	IGHG1	91	7	13588
GOTERM_ generation	1	0.735294	1	PCK1	91	261	13588
GOTERM_ detoxificat	1	0.735294	1	MT1	91	3	13588
GOTERM_ limbic syst	1	0.735294	1	ALDH1A3	91	33	13588
GOTERM_ polyol me	1	0.735294	1	PCK1	91	43	13588
GOTERM_ endoplasm	1	0.735294	1	CASP12	91	17	13588
GOTERM_ negative r	1	0.735294	1	SERPING1	91	78	13588
GOTERM_ male geni	1	0.735294	1	PDGFRA	91	15	13588
GOTERM_ nitric oxid	1	0.735294	1	MT1	91	2	13588
GOTERM_ regulation	1	0.735294	1	IGHG1	91	83	13588
GOTERM_ small GTPa	1	0.735294	1	ARF3	91	258	13588
GOTERM_ mononucl	1	0.735294	1	ITGAD	91	43	13588
GOTERM_ regulation	1	0.735294	1	IGHG1	91	8	13588
GOTERM_ response t	1	0.735294	1	MT1	91	92	13588
GOTERM_ developm	1	0.735294	1	CCL11	91	16	13588
GOTERM_ microtubu	1	0.735294	1	SS18	91	111	13588
GOTERM_ actin poly	1	0.735294	1	GAS7	91	13	13588
GOTERM_ regulation	1	0.735294	1	MYO16	91	73	13588
GOTERM_ female sex	1	0.735294	1	PGR	91	64	13588
GOTERM_ blood coa	1	0.735294	1	SERPING1	91	70	13588
GOTERM_ lung alveo	1	0.735294	1	PDPN	91	22	13588
GOTERM_ immune re	1	0.735294	1	KLRK1	91	48	13588
GOTERM_ positive re	1	0.735294	1	IGHG1	91	34	13588

GOTERM_ second-m	1	0.735294	1	RXFP2	91	118	13588
GOTERM_ negative r	1	0.735294	1	MYO16	91	7	13588
GOTERM_ neuromus	1	0.735294	1	SCN1A	91	12	13588
GOTERM_ zinc ion hc	1	0.735294	1	MT1	91	8	13588
GOTERM_ progester	1	0.735294	1	PGR	91	3	13588
GOTERM_ pyruvate r	1	0.735294	1	PCK1	91	25	13588
GOTERM_ regulation	1	0.735294	1	INHBB	91	1	13588
GOTERM_ regulation	1	0.735294	1	SERPING1	91	4	13588
GOTERM_ embryonic	1	0.735294	1	ALDH1A3	91	359	13588
GOTERM_ I-kappaB I	1	0.735294	1	AVPR2	91	28	13588
GOTERM_ regulation	1	0.735294	1	RXFP2	91	32	13588
GOTERM_ osteoblast	1	0.735294	1	IGFBP5	91	47	13588
GOTERM_ multicellul	1	0.735294	1	TNXB	91	26	13588
GOTERM_ cell cycle a	1	0.735294	1	UHMK1	91	57	13588
GOTERM_ camera-ty	1	0.735294	1	ALDH1A3	91	130	13588
GOTERM_ ovulation	1	0.735294	1	PGR	91	48	13588
GOTERM_ transition	1	0.735294	1	SCARA5	91	70	13588
GOTERM_ negative r	1	0.735294	1	FABP4	91	132	13588
GOTERM_ positive re	1	0.735294	1	PDPN	91	44	13588
GOTERM_ fatty acid I	1	0.735294	1	SCD1	91	81	13588
GOTERM_ positive re	1	0.735294	1	IGHG1	91	34	13588
GOTERM_ stimulator	1	0.735294	1	KLRK1	91	2	13588
GOTERM_ retinoic ac	1	0.735294	1	ALDH1A3	91	2	13588
GOTERM_ ovulation	1	0.735294	1	PGR	91	13	13588
GOTERM_ muscle sys	1	0.735294	1	RYR3	91	67	13588
GOTERM_ metencep	1	0.735294	1	MYO16	91	41	13588
GOTERM_ regulation	1	0.735294	1	TNXB	91	155	13588
GOTERM_ regulation	1	0.735294	1	RXFP2	91	73	13588
GOTERM_ regulation	1	0.735294	1	SCN1A	91	54	13588
GOTERM_ positive re	1	0.735294	1	INHBB	91	53	13588
GOTERM_ compleme	1	0.735294	1	CFD	91	10	13588
GOTERM_ apoptosis	1	0.735294	1	CASP12	91	4	13588
GOTERM_ tertiary bra	1	0.735294	1	PGR	91	3	13588
GOTERM_ lipid catab	1	0.735294	1	ENPP2	91	134	13588
GOTERM_ regulation	1	0.735294	1	SPOCK2	91	40	13588
GOTERM_ retinoic ac	1	0.735294	1	ALDH1A3	91	17	13588
GOTERM_ lipoprotein	1	0.735294	1	LDLR	91	76	13588
GOTERM_ positive re	1	0.735294	1	PDPN	91	41	13588
GOTERM_ adult loco	1	0.735294	1	SCN1A	91	62	13588
GOTERM_ prostaglar	1	0.735294	1	PDPN	91	14	13588
GOTERM_ lymphocyt	1	0.735294	1	ITGAD	91	42	13588
GOTERM_ embryonic	1	0.735294	1	ALDH1A3	91	241	13588
GOTERM_ positive re	1	0.735294	1	YAP1	91	358	13588
GOTERM_ positive re	1	0.735294	1	RXFP2	91	42	13588
GOTERM_ ossificatio	1	0.735294	1	IGFBP5	91	106	13588
GOTERM_ interferon	1	0.735294	1	AVPR2	91	5	13588
GOTERM_ cellular ma	1	0.735294	1	GAS7	91	245	13588
GOTERM_ one-carbo	1	0.735294	1	CAR3	91	117	13588
GOTERM_ fatty acid t	1	0.735294	1	SLCO2A1	91	13	13588
GOTERM_ cellular ma	1	0.735294	1	LDLR	91	609	13588
GOTERM_ positive re	1	0.735294	1	IGHG1	91	8	13588
GOTERM_ optic cup	1	0.735294	1	ALDH1A3	91	2	13588
GOTERM_ genitalia c	1	0.735294	1	PDGFRA	91	23	13588
GOTERM_ retinoid m	1	0.735294	1	ALDH1A3	91	28	13588
GOTERM_ ER-nuclea	1	0.735294	1	CASP12	91	25	13588
GOTERM_ negative r	1	0.735294	1	FABP4	91	51	13588
GOTERM_ ovulation	1	0.735294	1	PGR	91	47	13588
GOTERM_ regulation	1	0.735294	1	TNXB	91	93	13588

GOTERM_ morphoge	1	0.735294	1	ALDH1A3	91	78	13588
GOTERM_ negative r	1	0.735294	1	FABP4	91	53	13588
GOTERM_ regulation	1	0.735294	1	TNXB	91	45	13588
GOTERM_ very-low-	1	0.735294	1	VLDLR	91	1	13588
GOTERM_ diterpenoi	1	0.735294	1	ALDH1A3	91	28	13588
GOTERM_ G-protein	1	0.735294	1	RXFP2	91	60	13588
GOTERM_ vascular e	1	0.735294	1	PDGFRA	91	14	13588
GOTERM_ cell-matrix	1	0.735294	1	TNXB	91	50	13588
GOTERM_ positive re	1	0.735294	1	VLDLR	91	130	13588
GOTERM_ positive re	1	0.735294	1	ALDH1A3	91	172	13588
GOTERM_ response t	1	0.735294	1	FABP4	91	184	13588
GOTERM_ fat-soluble	1	0.735294	1	ALDH1A3	91	34	13588
GOTERM_ regulation	1	0.735294	1	TNXB	91	46	13588
GOTERM_ polyol bio	1	0.735294	1	PCK1	91	5	13588
GOTERM_ positive re	1	0.735294	1	YAP1	91	475	13588
GOTERM_ positive re	1	0.735294	1	RXFP2	91	32	13588
GOTERM_ regulation	1	0.735294	1	RXFP2	91	75	13588
GOTERM_ negative r	1	0.735294	1	SERPING1	91	12	13588
GOTERM_ odontoge	1	0.735294	1	PDGFRA	91	45	13588
GOTERM_ positive re	1	0.735294	1	IGHG1	91	13	13588
GOTERM_ muscle co	1	0.735294	1	RYR3	91	60	13588
GOTERM_ striatum d	1	0.735294	1	ALDH1A3	91	10	13588
GOTERM_ prostate g	1	0.735294	1	PLAG1	91	13	13588
GOTERM_ positive re	1	0.735294	1	INHBB	91	14	13588
GOTERM_ protein an	1	0.735294	1	UHMK1	91	66	13588
GOTERM_ positive re	1	0.735294	1	VLDLR	91	135	13588
GOTERM_ fear respo	1	0.735294	1	PENK	91	19	13588
GOTERM_ monosacc	1	0.735294	1	PCK1	91	31	13588
GOTERM_ posttransc	1	0.735294	1	IGF2BP3	91	148	13588
GOTERM_ regulation	1	0.735294	1	PER3	91	67	13588
GOTERM_ two-comp	1	0.735294	1	KCNH1	91	8	13588
GOTERM_ cell aging	1	0.735294	1	PRELP	91	29	13588
GOTERM_ in utero e	1	0.735294	1	PDGFRA	91	267	13588
GOTERM_ olfactory p	1	0.735294	1	ALDH1A3	91	2	13588
GOTERM_ regulation	1	0.735294	1	RXFP2	91	62	13588
GOTERM_ cellular alk	1	0.735294	1	CYP2F2	91	21	13588
GOTERM_ regulation	1	0.735294	1	PDPN	91	92	13588
GOTERM_ eye develo	1	0.735294	1	ALDH1A3	91	157	13588
GOTERM_ negative r	1	0.735294	1	SERPING1	91	26	13588
GOTERM_ regulation	1	0.735294	1	MYO16	91	12	13588
GOTERM_ natural kill	1	0.735294	1	KLRK1	91	16	13588
GOTERM_ response t	1	0.735294	1	CYP2F2	91	16	13588
GOTERM_ positive re	1	0.735294	1	IGHG1	91	122	13588
GOTERM_ regulation	1	0.735294	1	UHMK1	91	71	13588
GOTERM_ positive re	1	0.735294	1	KLRK1	91	23	13588
GOTERM_ carbohydr	1	0.735294	1	SLC35C1	91	60	13588
GOTERM_ regulation	1	0.735294	1	SCN1A	91	45	13588
GOTERM_ open track	1	0.735294	1	ANO1	91	1	13588
GOTERM_ transmissi	1	0.735294	1	SCN1A	91	226	13588
GOTERM_ energy de	1	0.735294	1	PCK1	91	98	13588
GOTERM_ regionaliza	1	0.735294	1	NR2F2	91	214	13588
GOTERM_ skeletal m	1	0.735294	1	NR2F2	91	72	13588
GOTERM_ sexual rep	1	0.735294	1	PGR	91	386	13588
GOTERM_ response t	1	0.735294	1	CASP12	91	54	13588
GOTERM_ regulation	1	0.735294	1	PGR	91	63	13588
GOTERM_ negative r	1	0.735294	1	FABP4	91	101	13588
GOTERM_ negative r	1	0.735294	1	MYO16	91	18	13588
GOTERM_ multicellul	1	0.735294	1	PGR	91	409	13588

GOTERM_	regulation	1	0.735294	1	IGHG1	91	8	13588
GOTERM_	negative r	1	0.735294	1	SERPING1	91	65	13588
GOTERM_	di-, tri-val	1	0.735294	1	SCARA5	91	161	13588
GOTERM_	regulation	1	0.735294	1	IGF2BP3	91	100	13588
GOTERM_	antibacter	1	0.735294	1	IGHG1	91	3	13588
GOTERM_	positive re	1	0.735294	1	RXFP2	91	42	13588
GOTERM_	cellular ca	1	0.735294	1	PCK1	91	56	13588
GOTERM_	hindbrain	1	0.735294	1	MYO16	91	67	13588
GOTERM_	striated m	1	0.735294	1	NR2F2	91	127	13588
GOTERM_	telenceph	1	0.735294	1	ALDH1A3	91	72	13588
GOTERM_	regulation	1	0.735294	1	INHBB	91	26	13588
GOTERM_	T cell activ	1	0.735294	1	ITGAD	91	116	13588
GOTERM_	gamete ge	1	0.735294	1	PGR	91	331	13588
GOTERM_	positive re	1	0.735294	1	PDPN	91	37	13588
GOTERM_	negative r	1	0.735294	1	SERPING1	91	3	13588
GOTERM_	activation	1	0.735294	1	RXFP2	91	32	13588
GOTERM_	embryonic	1	0.735294	1	PDGFRA	91	425	13588
GOTERM_	lipid glyco	1	0.735294	1	SLC35C1	91	6	13588
GOTERM_	regulation	1	0.735294	1	IGHG1	91	30	13588
GOTERM_	sensory or	1	0.735294	1	ALDH1A3	91	257	13588
GOTERM_	phagocyto	1	0.735294	1	IGHG1	91	13	13588
GOTERM_	cholestero	1	0.735294	1	LDLR	91	23	13588
GOTERM_	glycerol m	1	0.735294	1	PCK1	91	23	13588
GOTERM_	protein oli	1	0.735294	1	SCARA5	91	72	13588
GOTERM_	positive re	1	0.735294	1	YAP1	91	530	13588
GOTERM_	negative r	1	0.735294	1	INHBB	91	1	13588
GOTERM_	negative r	1	0.735294	1	SERPING1	91	1	13588
GOTERM_	positive re	1	0.735294	1	IGHG1	91	34	13588
GOTERM_	female pre	1	0.735294	1	PGR	91	68	13588
GOTERM_	sensory pe	1	0.735294	1	PENK	91	33	13588
GOTERM_	vitamin A	1	0.735294	1	ALDH1A3	91	26	13588
GOTERM_	naphthale	1	0.735294	1	CYP2F2	91	1	13588
GOTERM_	neural nuc	1	0.735294	1	ALDH1A3	91	8	13588
GOTERM_	G-protein	1	0.735294	1	RXFP2	91	55	13588
GOTERM_	positive re	1	0.735294	1	ALDH1A3	91	189	13588
GOTERM_	acylglycer	1	0.735294	1	PCK1	91	12	13588
GOTERM_	positive re	1	0.735294	1	KLRK1	91	163	13588
GOTERM_	B cell activ	1	0.735294	1	BANK1	91	78	13588
GOTERM_	negative r	1	0.735294	1	SERPING1	91	72	13588
GOTERM_	negative r	1	0.735294	1	INHBB	91	17	13588
GOTERM_	immune re	1	0.735294	1	KLRK1	91	41	13588
GOTERM_	skeletal m	1	0.735294	1	NR2F2	91	74	13588
GOTERM_	palate dev	1	0.735294	1	PDGFRA	91	35	13588
GOTERM_	head morp	1	0.735294	1	PDGFRA	91	14	13588
GOTERM_	urogenital	1	0.735294	1	PLAG1	91	146	13588
GOTERM_	mammary	1	0.735294	1	PGR	91	6	13588
GOTERM_	regulation	1	0.735294	1	RXFP2	91	60	13588
GOTERM_	negative r	1	0.735294	1	SERPING1	91	7	13588
GOTERM_	cofactor tr	1	0.735294	1	PDPN	91	11	13588
GOTERM_	developm	1	0.735294	1	PGR	91	57	13588
GOTERM_	adult beha	1	0.735294	1	SCN1A	91	93	13588
GOTERM_	radial patt	1	0.735294	1	NR2F2	91	1	13588
GOTERM_	positive re	1	0.735294	1	IGHG1	91	14	13588
GOTERM_	positive re	1	0.735294	1	IGHG1	91	10	13588
GOTERM_	iron ion ho	1	0.735294	1	SCARA5	91	35	13588
GOTERM_	hypersens	1	0.735294	1	IGHG1	91	5	13588
GOTERM_	multicellul	1	0.735294	1	TNXB	91	24	13588
GOTERM_	regulation	1	0.735294	1	UHMK1	91	7	13588

GOTERM_	fibril orgar	1	0.735294	1	TNXB	91	3	13588
GOTERM_	wound he	1	0.735294	1	SERPING1	91	112	13588
GOTERM_	chloride tr	1	0.735294	1	ANO1	91	61	13588
GOTERM_	neuromus	1	0.735294	1	SCN1A	91	60	13588
GOTERM_	oxygen tra	1	0.735294	1	CYGB	91	11	13588
GOTERM_	positive re	1	0.735294	1	IGHG1	91	11	13588
GOTERM_	glycerolipi	1	0.735294	1	PCK1	91	58	13588
GOTERM_	epithelial (	1	0.735294	1	PGR	91	123	13588
GOTERM_	positive re	1	0.735294	1	KLRK1	91	23	13588
GOTERM_	negative r	1	0.735294	1	INHBB	91	1	13588
GOTERM_	alcohol bi	1	0.735294	1	PCK1	91	37	13588
GOTERM_	type II hyp	1	0.735294	1	IGHG1	91	4	13588
GOTERM_	lipid modi	1	0.735294	1	SLC35C1	91	47	13588
GOTERM_	positive re	1	0.735294	1	IGHG1	91	8	13588
GOTERM_	regulation	1	0.735294	1	KLRK1	91	19	13588
GOTERM_	intracellula	1	0.735294	1	PGR	91	32	13588
GOTERM_	response t	1	0.735294	1	MT1	91	7	13588
GOTERM_	cellular res	1	0.735294	1	SCARA5	91	11	13588
GOTERM_	response t	1	0.735294	1	FABP4	91	19	13588
GOTERM_	isoprenoic	1	0.735294	1	ALDH1A3	91	49	13588
GOTERM_	acute infla	1	0.735294	1	IGHG1	91	5	13588
GOTERM_	regulation	1	0.735294	1	MYO16	91	96	13588
GOTERM_	T cell proli	1	0.735294	1	ITGAD	91	28	13588
GOTERM_	benzene a	1	0.735294	1	CYP2F2	91	6	13588
GOTERM_	regulation	1	0.735294	1	SCN1A	91	119	13588
GOTERM_	regulation	1	0.735294	1	RXFP2	91	70	13588
GOTERM_	positive re	1	0.735294	1	IGHG1	91	14	13588
GOTERM_	regulation	1	0.735294	1	IGHG1	91	8	13588
GOTERM_	male gona	1	0.735294	1	RXFP2	91	39	13588
GOTERM_	regulation	1	0.735294	1	INHBB	91	64	13588
GOTERM_	negative r	1	0.735294	1	INHBB	91	1	13588
GOTERM_	positive re	1	0.735294	1	KLRK1	91	62	13588
GOTERM_	alditol me	1	0.735294	1	PCK1	91	25	13588
GOTERM_	peptidyl-s	1	0.735294	1	UHMK1	91	22	13588
GOTERM_	regulation	1	0.735294	1	IGHG1	91	13	13588
GOTERM_	epithelial (	1	0.735294	1	PGR	91	10	13588
GOTERM_	regulation	1	0.735294	1	UHMK1	91	90	13588
GOTERM_	myeloid le	1	0.735294	1	IGHG1	91	17	13588
GOTERM_	neuronal a	1	0.735294	1	SCN1A	91	6	13588
GOTERM_	glycerol bi	1	0.735294	1	PCK1	91	2	13588
GOTERM_	hemostasi	1	0.735294	1	SERPING1	91	71	13588
GOTERM_	activation	1	0.735294	1	RXFP2	91	41	13588
GOTERM_	protein tri	1	0.735294	1	SCARA5	91	3	13588
GOTERM_	regulation	1	0.735294	1	ALDH1A3	91	3	13588
GOTERM_	regulation	1	0.735294	1	PDPN	91	110	13588
GOTERM_	positive re	1	0.735294	1	ALDH1A3	91	1	13588
GOTERM_	negative r	1	0.735294	1	INHBB	91	27	13588
GOTERM_	elastic fibe	1	0.735294	1	TNXB	91	4	13588
GOTERM_	actin filam	1	0.735294	1	GAS7	91	15	13588
GOTERM_	cellular res	1	0.735294	1	CASP12	91	17	13588
GOTERM_	folic acid t	1	0.735294	1	PDPN	91	3	13588
GOTERM_	unsaturate	1	0.735294	1	PDPN	91	36	13588
GOTERM_	negative r	1	0.735294	1	INHBB	91	72	13588
GOTERM_	positive re	1	0.735294	1	IGHG1	91	8	13588
GOTERM_	regulation	1	0.735294	1	IGHG1	91	15	13588
GOTERM_	regulation	1	0.735294	1	AVPR2	91	65	13588
GOTERM_	steroid bic	1	0.735294	1	SDR42E1	91	71	13588
GOTERM_	hexose me	1	0.735294	1	PCK1	91	169	13588

GOTERM_	cell recogı	1	0.735294	1	IGHG1	91	47	13588
GOTERM_	regulation	1	0.735294	1	INHBB	91	1	13588
GOTERM_	behaviora	1	0.735294	1	PENK	91	14	13588
GOTERM_	protein hc	1	0.735294	1	SCARA5	91	53	13588
GOTERM_	fermentati	1	0.735294	1	PCK1	91	2	13588
GOTERM_	organ gro	1	0.735294	1	PLAG1	91	22	13588
GOTERM_	epithelial (	1	0.735294	1	PGR	91	25	13588
GOTERM_	response t	1	0.735294	1	CASP12	91	87	13588
GOTERM_	regulation	1	0.735294	1	INHBB	91	36	13588
GOTERM_	positive re	1	0.735294	1	AVPR2	91	20	13588
GOTERM_	virus-infec	1	0.735294	1	CASP12	91	3	13588
GOTERM_	xenobiotic	1	0.735294	1	CYP2F2	91	13	13588
GOTERM_	behaviora	1	0.735294	1	PENK	91	14	13588
GOTERM_	regulation	1	0.735294	1	RXFP2	91	62	13588
GOTERM_	regulation	1	0.735294	1	IGHG1	91	56	13588
GOTERM_	antimicrot	1	0.735294	1	IGHG1	91	4	13588
GOTERM_	regulation	1	0.735294	1	IGHG1	91	25	13588
GOTERM_	hexose bic	1	0.735294	1	PCK1	91	25	13588
GOTERM_	negative r	1	0.735294	1	SERPING1	91	30	13588
GOTERM_	alditol bio	1	0.735294	1	PCK1	91	2	13588
GOTERM_	cell-substı	1	0.735294	1	TNXB	91	57	13588
GOTERM_	secondary	1	0.735294	1	ALDH1A3	91	69	13588
GOTERM_	glycerol et	1	0.735294	1	PCK1	91	13	13588
GOTERM_	regulation	1	0.735294	1	UHMK1	91	42	13588
GOTERM_	cell cycle p	1	0.735294	1	UHMK1	91	393	13588
GOTERM_	cellular ma	1	0.735294	1	GAS7	91	217	13588
GOTERM_	amine trar	1	0.735294	1	PDPN	91	92	13588
GOTERM_	eye morph	1	0.735294	1	ALDH1A3	91	73	13588
GOTERM_	cyclic-nuc	1	0.735294	1	RXFP2	91	66	13588
GOTERM_	ephrin rec	1	0.735294	1	SS18	91	6	13588
GOTERM_	regulation	1	0.735294	1	TNXB	91	165	13588
GOTERM_	regulation	1	0.735294	1	SERPING1	91	1	13588
GOTERM_	blood circ	1	0.735294	1	AVPR2	91	111	13588
GOTERM_	neuropept	1	0.735294	1	PENK	91	78	13588
GOTERM_	neutral lip	1	0.735294	1	PCK1	91	12	13588
GOTERM_	homophili	1	0.735294	1	PCDH17	91	117	13588
GOTERM_	regulation	1	0.735294	1	TNXB	91	84	13588
GOTERM_	positive re	1	0.735294	1	SPOCK2	91	27	13588
GOTERM_	striated m	1	0.735294	1	RYR3	91	24	13588
GOTERM_	response t	1	0.735294	1	IGHG1	91	157	13588
GOTERM_	positive re	1	0.735294	1	KLRK1	91	15	13588
GOTERM_	positive re	1	0.735294	1	YAP1	91	416	13588
GOTERM_	regulation	1	0.735294	1	UHMK1	91	30	13588
GOTERM_	autophagy	1	0.735294	1	WIPI1	91	31	13588
GOTERM_	adult walk	1	0.735294	1	SCN1A	91	27	13588
GOTERM_	microtubu	1	0.735294	1	SS18	91	211	13588
GOTERM_	embryonic	1	0.735294	1	ALDH1A3	91	161	13588
GOTERM_	regulation	1	0.735294	1	KLRK1	91	20	13588
GOTERM_	negative r	1	0.735294	1	MYO16	91	14	13588
GOTERM_	regulation	1	0.735294	1	IGHG1	91	13	13588
GOTERM_	cellular hc	1	0.735294	1	ALDH1A3	91	52	13588
GOTERM_	iron ion tr	1	0.735294	1	SCARA5	91	2	13588
GOTERM_	positive re	1	0.735294	1	KLRK1	91	15	13588
GOTERM_	multicellul	1	0.735294	1	PLAG1	91	39	13588
GOTERM_	lymph ves	1	0.735294	1	PDPN	91	6	13588
GOTERM_	limb devel	1	0.735294	1	NR2F2	91	119	13588
GOTERM_	ion transm	1	0.735294	1	SCARA5	91	42	13588
GOTERM_	prostate g	1	0.735294	1	PLAG1	91	39	13588

GOTERM_	type IIa hy	1	0.735294	1	IGHG1	91	4	13588
GOTERM_	protein loc	1	0.735294	1	ARF3	91	753	13588
GOTERM_	cerebellum	1	0.735294	1	MYO16	91	35	13588
GOTERM_	regulation	1	0.735294	1	SERPING1	91	89	13588
GOTERM_	negative r	1	0.735294	1	SERPING1	91	20	13588
GOTERM_	muscle tiss	1	0.735294	1	NR2F2	91	136	13588
GOTERM_	regulation	1	0.735294	1	KLRK1	91	30	13588
GOTERM_	dephosph	1	0.735294	1	PTPRZ1	91	141	13588
GOTERM_	maternal p	1	0.735294	1	PGR	91	12	13588
GOTERM_	regulation	1	0.735294	1	MYO16	91	214	13588
GOTERM_	cAMP-me	1	0.735294	1	RXFP2	91	61	13588
GOTERM_	terpenoid	1	0.735294	1	ALDH1A3	91	29	13588
GOTERM_	establishm	1	0.735294	1	ARF3	91	656	13588
GOTERM_	lipoprotei	1	0.735294	1	VLDLR	91	5	13588
GOTERM_	regulation	1	0.735294	1	TNXB	91	121	13588
GOTERM_	response t	1	0.735294	1	CASP12	91	26	13588
GOTERM_	angiogene	1	0.735294	1	SEMA5A	91	133	13588
GOTERM_	circadian r	1	0.735294	1	PER3	91	37	13588
GOTERM_	chordate e	1	0.735294	1	PDGFRA	91	421	13588
GOTERM_	camera-ty	1	0.735294	1	ALDH1A3	91	48	13588
GOTERM_	regulation	1	0.735294	1	MYO16	91	11	13588
GOTERM_	regulation	1	0.735294	1	SERPING1	91	26	13588
GOTERM_	leukocyte	1	0.735294	1	ITGAD	91	43	13588
GOTERM_	regulation	1	0.735294	1	RXFP2	91	67	13588
GOTERM_	patterning	1	0.735294	1	SEMA5A	91	24	13588
GOTERM_	regulation	1	0.735294	1	SERPING1	91	4	13588
GOTERM_	activation	1	0.735294	1	KLRK1	91	14	13588
GOTERM_	triglycerid	1	0.735294	1	PCK1	91	8	13588
GOTERM_	prostanoic	1	0.735294	1	PDPN	91	14	13588
GOTERM_	negative r	1	0.735294	1	PER3	91	21	13588
GOTERM_	positive re	1	0.735294	1	RXFP2	91	42	13588
GOTERM_	positive re	1	0.735294	1	INHBB	91	1	13588
GOTERM_	ovulation	1	0.735294	1	PGR	91	11	13588
GOTERM_	response t	1	0.735294	1	FABP4	91	23	13588
GOTERM_	embryonic	1	0.735294	1	ALDH1A3	91	15	13588
GOTERM_	cellular zir	1	0.735294	1	MT1	91	7	13588
GOTERM_	prostaglar	1	0.735294	1	SLCO2A1	91	1	13588
GOTERM_	regulation	1	0.735294	1	UHMK1	91	35	13588
GOTERM_	carboxylic	1	0.735294	1	SCD1	91	141	13588
GOTERM_	vitamin m	1	0.735294	1	ALDH1A3	91	69	13588
GOTERM_	response t	1	0.735294	1	SCARA5	91	51	13588
GOTERM_	protein pc	1	0.735294	1	GAS7	91	40	13588
GOTERM_	regulation	1	0.735294	1	KLRK1	91	32	13588
GOTERM_	response t	1	0.735294	1	FABP4	91	165	13588
GOTERM_	immune re	1	0.735294	1	KLRK1	91	52	13588
GOTERM_	positive re	1	0.735294	1	INHBB	91	1	13588
GOTERM_	nucleus ac	1	0.735294	1	ALDH1A3	91	1	13588
GOTERM_	inflammat	1	0.735294	1	IGHG1	91	7	13588
GOTERM_	subpallium	1	0.735294	1	ALDH1A3	91	13	13588
GOTERM_	protein hc	1	0.735294	1	SCARA5	91	3	13588
GOTERM_	positive re	1	0.735294	1	KLRK1	91	17	13588
GOTERM_	negative r	1	0.735294	1	INHBB	91	9	13588
GOTERM_	appendag	1	0.735294	1	NR2F2	91	119	13588
GOTERM_	cellular pr	1	0.735294	1	GAS7	91	108	13588
GOTERM_	regulation	1	0.735294	1	INHBB	91	1	13588
GOTERM_	multicellul	1	0.735294	1	PENK	91	37	13588
GOTERM_	regulation	1	0.735294	1	SERPING1	91	12	13588
GOTERM_	anterior/p	1	0.735294	1	NR2F2	91	153	13588

GOTERM_	organic ac	1	0.735294	1	SCD1	91	141	13588
GOTERM_	response t	1	0.735294	1	SCARA5	91	32	13588
GOTERM_	response t	1	0.735294	1	SCARA5	91	251	13588
GOTERM_	gas transp	1	0.735294	1	CYGB	91	13	13588
GOTERM_	nose deve	1	0.735294	1	ALDH1A3	91	9	13588
GOTERM_	face morp	1	0.735294	1	PDGFRA	91	12	13588
GOTERM_	negative r	1	0.735294	1	SERPING1	91	3	13588
GOTERM_	embryonic	1	0.735294	1	ALDH1A3	91	14	13588
GOTERM_	body mor	1	0.735294	1	PDGFRA	91	20	13588
GOTERM_	iron ion tr	1	0.735294	1	SCARA5	91	32	13588
GOTERM_	icosanoid	1	0.735294	1	PDPN	91	34	13588
GOTERM_	response t	1	0.735294	1	FABP4	91	60	13588
GOTERM_	glucose m	1	0.735294	1	PCK1	91	140	13588
GOTERM_	positive re	1	0.735294	1	VLDLR	91	124	13588
GOTERM_	negative r	1	0.735294	1	INHBB	91	9	13588
GOTERM_	immune re	1	0.735294	1	KLRK1	91	44	13588
GOTERM_	phospholi	1	0.735294	1	CDS2	91	83	13588
GOTERM_	leukocyte	1	0.735294	1	IGHG1	91	12	13588
GOTERM_	aging	1	0.735294	1	PRELP	91	61	13588
GOTERM_	amino aci	1	0.735294	1	PDPN	91	71	13588
GOTERM_	response t	1	0.735294	1	AVPR2	91	32	13588
GOTERM_	regulation	1	0.735294	1	IGHG1	91	52	13588
GOTERM_	activated T	1	0.735294	1	ITGAD	91	7	13588
GOTERM_	carbohydr	1	0.735294	1	PCK1	91	83	13588
GOTERM_	female ga	1	0.735294	1	PGR	91	56	13588
GOTERM_	positive re	1	0.735294	1	YAP1	91	419	13588
GOTERM_	innate imr	1	0.735294	1	KLRK1	91	12	13588
GOTERM_	regulation	1	0.735294	1	IGHG1	91	56	13588
GOTERM_	negative r	1	0.735294	1	SERPING1	91	76	13588
GOTERM_	positive re	1	0.735294	1	IGHG1	91	10	13588
GOTERM_	regulation	1	0.735294	1	UHMK1	91	65	13588
GOTERM_	sterol tran	1	0.735294	1	LDLR	91	23	13588
GOTERM_	phagocyto	1	0.735294	1	IGHG1	91	15	13588
GOTERM_	regulation	1	0.735294	1	RXFP2	91	65	13588
GOTERM_	regulation	1	0.735294	1	RXFP2	91	70	13588
GOTERM_	bone deve	1	0.735294	1	IGFBP5	91	118	13588
GOTERM_	regulation	1	0.735294	1	INHBB	91	1	13588
GOTERM_	innate imr	1	0.735294	1	KLRK1	91	4	13588
GOTERM_	platelet-d	1	0.735294	1	PDGFRA	91	23	13588
GOTERM_	muscle or	1	0.735294	1	NR2F2	91	176	13588
GOTERM_	protein tra	1	0.735294	1	ARF3	91	651	13588
GOTERM_	inorganic	1	0.735294	1	ANO1	91	83	13588
GOTERM_	skeletal sy	1	0.735294	1	PDGFRA	91	130	13588
GOTERM_	regulation	1	0.735294	1	IGHG1	91	30	13588
GOTERM_	positive re	1	0.735294	1	KLRK1	91	36	13588
GOTERM_	mammary	1	0.735294	1	CCL11	91	2	13588
GOTERM_	glycerol bi	1	0.735294	1	PCK1	91	1	13588
GOTERM_	negative r	1	0.735294	1	MYO16	91	43	13588
GOTERM_	cellular irc	1	0.735294	1	SCARA5	91	29	13588
GOTERM_	reproduct	1	0.735294	1	PGR	91	409	13588
GOTERM_	hormone i	1	0.735294	1	ALDH1A3	91	90	13588
GOTERM_	regulation	1	0.735294	1	TNXB	91	21	13588
GOTERM_	negative r	1	0.735294	1	PER3	91	23	13588
GOTERM_	circulatory	1	0.735294	1	AVPR2	91	111	13588
GOTERM_	macromol	1	0.735294	1	LDLR	91	654	13588
GOTERM_	lipoprotei	1	0.735294	1	LDLR	91	6	13588
GOTERM_	phagocyto	1	0.735294	1	IGHG1	91	49	13588
GOTERM_	regulation	1	0.735294	1	KLRK1	91	20	13588

GOTERM_	embryonic	1	0.735294	1	ALDH1A3	91	10	13588
GOTERM_	positive re	1	0.735294	1	KLRK1	91	15	13588
GOTERM_	coagulatic	1	0.735294	1	SERPING1	91	70	13588
GOTERM_	regulation	1	0.735294	1	INHBB	91	22	13588
GOTERM_	regulation	1	0.735294	1	SERPING1	91	5	13588
GOTERM_	regulation	1	0.735294	1	ALDH1A3	91	128	13588
GOTERM_	regulation	1	0.735294	1	KLRK1	91	30	13588
GOTERM_	steroid ho	1	0.735294	1	PGR	91	21	13588
GOTERM_	regulation	1	0.735294	1	TNXB	91	75	13588
GOTERM_	cell matur	1	0.735294	1	PGR	91	75	13588
GOTERM_	antibody-	1	0.735294	1	IGHG1	91	4	13588
GOTERM_	anion tran	1	0.735294	1	ANO1	91	130	13588
GOTERM_	actin filam	1	0.735294	1	GAS7	91	8	13588
GOTERM_	regulation	1	0.735294	1	PER3	91	51	13588
GOTERM_	regulation	1	0.735294	1	IGHG1	91	18	13588
GOTERM_	protein an	1	0.735294	1	PTPRZ1	91	114	13588
GOTERM_	negative r	1	0.735294	1	FABP4	91	51	13588
GOTERM_	positive re	1	0.735294	1	IGHG1	91	23	13588
GOTERM_	cell killing	1	0.735294	1	IGHG1	91	16	13588
GOTERM_	monosacc	1	0.735294	1	PCK1	91	191	13588
GOTERM_	regulation	1	0.735294	1	INHBB	91	201	13588

Fold Enricl	Bonferron	Benjamini	FDR
24.88645	0.002612	0.002612	0.005526
24.88645	0.002612	0.002612	0.005526
12.15385	0.015579	0.00782	0.033167
16.59096	0.019782	0.006638	0.042202
24.88645	0.030481	0.007709	0.065374
13.78326	0.048089	0.009808	0.104062
21.33124	0.056036	0.009565	0.121754
11.06064	0.13124	0.019898	0.296777
6.422309	0.152295	0.020441	0.348442
7.68552	0.182525	0.022144	0.424859
9.954579	0.205728	0.02277	0.485413
9.332418	0.266953	0.027837	0.653911
11.85069	0.434402	0.046379	1.196695
11.48605	0.473378	0.048132	1.345618
3.193982	0.565877	0.057861	1.747368
3.188299	0.571089	0.054871	1.772435
7.110413	0.654561	0.064275	2.220546
9.823597	0.68345	0.065425	2.400783
8.888017	0.810333	0.088223	3.451211
8.888017	0.810333	0.088223	3.451211
5.073936	0.8265	0.088067	3.632763
8.38869	0.871555	0.097524	4.242962
2.310541	0.89125	0.100263	4.57909
4.64547	0.933494	0.115916	5.565287
6.977508	0.98112	0.158521	8.044307
2.999706	0.999005	0.250272	13.58819
3.382624	0.999994	0.381775	22.42998
14.45019	0.999997	0.390367	23.80209
3.012192	1	0.523458	34.47789
2.977865	1	0.528016	35.86152
5.913611	1	0.521375	36.32811
2.863646	1	0.562958	40.82064
2.536198	1	0.576693	43.04992
5.019115	1	0.63818	49.70484
4.816732	1	0.664971	53.34403
37.32967	1	0.673676	55.26357
4.666209	1	0.672534	56.1965
7.858878	1	0.673697	57.33312
7.858878	1	0.673697	57.33312
4.594421	1	0.666348	57.59901
4.457274	1	0.684099	60.34983
29.86374	1	0.704904	63.41573
4.090923	1	0.741172	68.08684
4.008555	1	0.749811	69.88386
3.929439	1	0.75817	71.62125
21.33124	1	0.787736	75.53632
2.860511	1	0.802913	77.90199
2.828005	1	0.80632	78.99838
2.828005	1	0.80632	78.99838
18.66484	1	0.809088	79.99603
2.785796	1	0.80631	80.40467
3.492835	1	0.807233	81.16401
5.33281	1	0.808132	81.8957
17.5669	1	0.801852	81.91125
17.5669	1	0.801852	81.91125
3.393606	1	0.809545	83.24944
16.59096	1	0.807585	83.64323

5.09041	1	0.809165	84.34805
15.71776	1	0.812747	85.20948
3.246058	1	0.818451	86.22787
3.246058	1	0.818451	86.22787
4.816732	1	0.822101	87.03081
3.176993	1	0.82284	87.55789
3.176993	1	0.82284	87.55789
2.5481	1	0.81994	87.76358
14.22083	1	0.816373	87.90668
14.22083	1	0.816373	87.90668
4.47956	1	0.839213	90.14006
12.44322	1	0.846441	91.05945
4.349088	1	0.844522	91.26553
2.956806	1	0.842313	91.44316
4.30727	1	0.840119	91.61544
4.226	1	0.845133	92.27928
11.48605	1	0.846825	92.69039
11.48605	1	0.846825	92.69039
11.48605	1	0.846825	92.69039
2.817334	1	0.85604	93.56515
10.29784	1	0.868839	94.59664
10.29784	1	0.868839	94.59664
2.739792	1	0.865214	94.61373
1.947635	1	0.883047	95.81442
1.789779	1	0.880874	95.88794
1.677738	1	0.880184	96.03399
2.176657	1	0.877259	96.06415
9.049617	1	0.880496	96.38885
9.049617	1	0.880496	96.38885
3.583648	1	0.882284	96.62882
8.783452	1	0.881313	96.735
3.445816	1	0.892018	97.32361
8.295482	1	0.889079	97.331
2.034314	1	0.901511	97.91065
7.657368	1	0.902009	98.02748
7.465934	1	0.904928	98.21664
7.465934	1	0.904928	98.21664
7.465934	1	0.904928	98.21664
6.945055	1	0.917835	98.68213
6.945055	1	0.917835	98.68213
6.945055	1	0.917835	98.68213
1.739635	1	0.92138	98.84294
6.636386	1	0.922166	98.92284
6.636386	1	0.922166	98.92284
6.492117	1	0.924174	99.02618
6.492117	1	0.924174	99.02618
6.353986	1	0.926085	99.11961
2.195863	1	0.924181	99.12689
6.221612	1	0.925718	99.20409
2.799725	1	0.933768	99.39277
2.782336	1	0.933586	99.42361
2.095701	1	0.934293	99.46679
5.743026	1	0.932423	99.46838
2.748197	1	0.931278	99.48094
5.634667	1	0.931982	99.51941
2.714885	1	0.931012	99.53286
2.045461	1	0.933846	99.59452
5.33281	1	0.936238	99.64495

5.239252	1	0.937542	99.67904
2.589341	1	0.937361	99.69547
1.971204	1	0.940168	99.73929
2.545205	1	0.938694	99.74126
4.895694	1	0.942266	99.78568
1.689125	1	0.942057	99.79661
2.461297	1	0.942817	99.81397
2.408366	1	0.946853	99.85113
4.594421	1	0.946318	99.8569
1.872335	1	0.946434	99.86613
2.34532	1	0.949035	99.88767
2.333104	1	0.948899	99.89387
2.333104	1	0.948899	99.89387
2.321016	1	0.948768	99.89974
1.832131	1	0.947631	99.90086
1.602132	1	0.949168	99.91319
4.266248	1	0.947926	99.91365
4.266248	1	0.947926	99.91365
4.266248	1	0.947926	99.91365
2.273889	1	0.948293	99.92025
2.251035	1	0.949402	99.92893
3.929439	1	0.956111	99.95291
3.878407	1	0.956729	99.95744
2.113	1	0.959782	99.96681
2.045461	1	0.965019	99.97816
1.475481	1	0.96603	99.98111
3.472527	1	0.966376	99.98287
3.432613	1	0.966759	99.98452
3.393606	1	0.967131	99.98601
3.393606	1	0.967131	99.98601
3.281729	1	0.96991	99.98967
3.246058	1	0.970218	99.99067
3.246058	1	0.970218	99.99067
1.333203	1	0.970164	99.99129
1.898119	1	0.97068	99.99228
3.176993	1	0.969994	99.99238
3.176993	1	0.969994	99.99238
3.143551	1	0.970291	99.99311
1.882168	1	0.969602	99.99319
1.874293	1	0.969477	99.9936
3.078736	1	0.970073	99.99437
1.38772	1	0.970351	99.99491
3.04732	1	0.969572	99.99491
3.04732	1	0.969572	99.99491
3.016539	1	0.969864	99.9954
2.986374	1	0.970149	99.99585
2.986374	1	0.970149	99.99585
1.82096	1	0.969451	99.99588
1.806274	1	0.97	99.99637
1.496929	1	0.970422	99.99676
1.791824	1	0.969785	99.9968
1.791824	1	0.969785	99.9968
1.777603	1	0.970323	99.99718
1.474752	1	0.9712	99.99761
2.739792	1	0.973785	99.99833
2.739792	1	0.973785	99.99833
2.690427	1	0.974846	99.99864
2.690427	1	0.974846	99.99864

1.428887	1	0.975074	99.99877
2.642809	1	0.975228	99.99889
1.652974	1	0.976945	99.99918
1.640865	1	0.977324	99.99928
1.389011	1	0.977458	99.99935
1.376209	1	0.978377	99.99947
1.599843	1	0.978855	99.99955
2.389099	1	0.980376	99.99967
1.544676	1	0.981919	99.99977
2.315018	1	0.981771	99.99978
2.262404	1	0.982954	99.99984
1.488226	1	0.984424	99.99989
1.488226	1	0.984424	99.99989
2.14847	1	0.98551	99.99992
1.166552	1	0.987235	99.99995
2.031547	1	0.988001	99.99996
1.85489	1	0.991904	99.99999
1.832131	1	0.992143	99.99999
1.788248	1	0.992812	100
1.788248	1	0.992812	100
1.788248	1	0.992812	100
1.034541	1	0.993935	100
1.034541	1	0.993935	100
1.696803	1	0.99414	100
1.039824	1	0.995157	100
1.204183	1	0.995235	100
1.080063	1	0.995407	100
1.066562	1	0.995801	100
1.060879	1	0.995883	100
0.979139	1	0.996173	100
1.50827	1	0.996112	100
1.128353	1	0.996551	100
1.117097	1	0.996717	100
1.092576	1	0.997178	100
0.917316	1	0.997459	100
1.333203	1	0.997771	100
1.315583	1	0.997875	100
1.315583	1	0.997875	100
0.903137	1	0.997853	100
1.292802	1	0.997957	100
1.265413	1	0.998151	100
1.244322	1	0.99828	100
1.223924	1	0.998399	100
1.209058	1	0.998468	100
0.963346	1	0.998695	100
1.166552	1	0.998702	100
0.947053	1	0.998805	100
1.144204	1	0.998776	100
1.05154	1	0.9993	100
1.05154	1	0.9993	100
1.05154	1	0.9993	100
1.05154	1	0.9993	100
1.04785	1	0.999291	100
0.887042	1	0.99927	100
0.883542	1	0.999271	100
0.863114	1	0.999404	100
0.975939	1	0.999515	100
0.969602	1	0.99952	100

0.963346	1	0.999525	100
0.883542	1	0.999754	100
0.813726	1	0.999875	100
0.727201	1	0.99989	100
0.778013	1	0.999886	100
0.739201	1	0.999942	100
0.737542	1	0.999954	100
0.674125	1	0.999987	100
0.611962	1	0.999991	100
0.567752	1	0.999996	100
0.54101	1	0.999998	100
0.536153	1	0.999998	100
0.488768	1	0.999999	100
0.471781	1	1	100
0.477311	1	1	100
0.355309	1	1	100
0.319512	1	1	100
0.267357	1	1	100
0.302673	1	1	100
0.250535	1	1	100
1.478403	1	1	100
10.66562	1	1	100
1.395502	1	1	100
8.783452	1	1	100
7.465934	1	1	100
1.194549	1	1	100
0.292782	1	1	100
7.858878	1	1	100
6.492117	1	1	100
2.817334	1	1	100
29.86374	1	1	100
3.641919	1	1	100
1.38258	1	1	100
2.817334	1	1	100
49.77289	1	1	100
21.33124	1	1	100
0.572102	1	1	100
49.77289	1	1	100
4.524809	1	1	100
3.472527	1	1	100
8.783452	1	1	100
1.914342	1	1	100
9.954579	1	1	100
74.65934	1	1	100
1.79902	1	1	100
0.578755	1	1	100
3.472527	1	1	100
18.66484	1	1	100
1.623029	1	1	100
9.332418	1	1	100
1.345213	1	1	100
11.48605	1	1	100
2.045461	1	1	100
2.333104	1	1	100
2.133124	1	1	100
6.787213	1	1	100
3.110806	1	1	100
4.391726	1	1	100

1.265413	1	1	100
21.33124	1	1	100
12.44322	1	1	100
18.66484	1	1	100
49.77289	1	1	100
5.972747	1	1	100
149.3187	1	1	100
37.32967	1	1	100
0.415929	1	1	100
5.33281	1	1	100
4.666209	1	1	100
3.176993	1	1	100
5.743026	1	1	100
2.619626	1	1	100
1.148605	1	1	100
3.110806	1	1	100
2.133124	1	1	100
1.131202	1	1	100
3.393606	1	1	100
1.843441	1	1	100
4.391726	1	1	100
74.65934	1	1	100
74.65934	1	1	100
11.48605	1	1	100
2.228637	1	1	100
3.641919	1	1	100
0.963346	1	1	100
2.045461	1	1	100
2.765161	1	1	100
2.817334	1	1	100
14.93187	1	1	100
37.32967	1	1	100
49.77289	1	1	100
1.114319	1	1	100
3.732967	1	1	100
8.783452	1	1	100
1.964719	1	1	100
3.641919	1	1	100
2.408366	1	1	100
10.66562	1	1	100
3.555207	1	1	100
0.61958	1	1	100
0.417091	1	1	100
3.555207	1	1	100
1.408667	1	1	100
29.86374	1	1	100
0.609464	1	1	100
1.276228	1	1	100
11.48605	1	1	100
0.245187	1	1	100
18.66484	1	1	100
74.65934	1	1	100
6.492117	1	1	100
5.33281	1	1	100
5.972747	1	1	100
2.927817	1	1	100
3.176993	1	1	100
1.605577	1	1	100

1.914342	1	1	100
2.817334	1	1	100
3.318193	1	1	100
149.3187	1	1	100
5.33281	1	1	100
2.488645	1	1	100
10.66562	1	1	100
2.986374	1	1	100
1.148605	1	1	100
0.868132	1	1	100
0.811515	1	1	100
4.391726	1	1	100
3.246058	1	1	100
29.86374	1	1	100
0.314355	1	1	100
4.666209	1	1	100
1.990916	1	1	100
12.44322	1	1	100
3.318193	1	1	100
11.48605	1	1	100
2.488645	1	1	100
14.93187	1	1	100
11.48605	1	1	100
10.66562	1	1	100
2.262404	1	1	100
1.106064	1	1	100
7.858878	1	1	100
4.816732	1	1	100
1.00891	1	1	100
2.228637	1	1	100
18.66484	1	1	100
5.14892	1	1	100
0.559246	1	1	100
74.65934	1	1	100
2.408366	1	1	100
7.110413	1	1	100
1.623029	1	1	100
0.951074	1	1	100
5.743026	1	1	100
12.44322	1	1	100
9.332418	1	1	100
9.332418	1	1	100
1.223924	1	1	100
2.10308	1	1	100
6.492117	1	1	100
2.488645	1	1	100
3.318193	1	1	100
149.3187	1	1	100
0.660702	1	1	100
1.52366	1	1	100
0.697751	1	1	100
2.073871	1	1	100
0.386836	1	1	100
2.765161	1	1	100
2.370138	1	1	100
1.478403	1	1	100
8.295482	1	1	100
0.365082	1	1	100

18.66484	1	1	100
2.29721	1	1	100
0.927445	1	1	100
1.493187	1	1	100
49.77289	1	1	100
3.555207	1	1	100
2.666405	1	1	100
2.228637	1	1	100
1.175738	1	1	100
2.073871	1	1	100
5.743026	1	1	100
1.28723	1	1	100
0.451114	1	1	100
4.03564	1	1	100
49.77289	1	1	100
4.666209	1	1	100
0.351338	1	1	100
24.88645	1	1	100
4.977289	1	1	100
0.581007	1	1	100
11.48605	1	1	100
6.492117	1	1	100
6.492117	1	1	100
2.073871	1	1	100
0.281733	1	1	100
149.3187	1	1	100
149.3187	1	1	100
4.391726	1	1	100
2.195863	1	1	100
4.524809	1	1	100
5.743026	1	1	100
149.3187	1	1	100
18.66484	1	1	100
2.714885	1	1	100
0.790046	1	1	100
12.44322	1	1	100
0.916066	1	1	100
1.914342	1	1	100
2.073871	1	1	100
8.783452	1	1	100
3.641919	1	1	100
2.01782	1	1	100
4.266248	1	1	100
10.66562	1	1	100
1.022731	1	1	100
24.88645	1	1	100
2.488645	1	1	100
21.33124	1	1	100
13.57443	1	1	100
2.619626	1	1	100
1.605577	1	1	100
149.3187	1	1	100
10.66562	1	1	100
14.93187	1	1	100
4.266248	1	1	100
29.86374	1	1	100
6.221612	1	1	100
21.33124	1	1	100

49.77289	1	1	100
1.333203	1	1	100
2.447847	1	1	100
2.488645	1	1	100
13.57443	1	1	100
13.57443	1	1	100
2.57446	1	1	100
1.213973	1	1	100
6.492117	1	1	100
149.3187	1	1	100
4.03564	1	1	100
37.32967	1	1	100
3.176993	1	1	100
18.66484	1	1	100
7.858878	1	1	100
4.666209	1	1	100
21.33124	1	1	100
13.57443	1	1	100
7.858878	1	1	100
3.04732	1	1	100
29.86374	1	1	100
1.555403	1	1	100
5.33281	1	1	100
24.88645	1	1	100
1.254779	1	1	100
2.133124	1	1	100
10.66562	1	1	100
18.66484	1	1	100
3.828684	1	1	100
2.333104	1	1	100
149.3187	1	1	100
2.408366	1	1	100
5.972747	1	1	100
6.787213	1	1	100
11.48605	1	1	100
14.93187	1	1	100
1.659096	1	1	100
8.783452	1	1	100
24.88645	1	1	100
74.65934	1	1	100
2.10308	1	1	100
3.641919	1	1	100
49.77289	1	1	100
49.77289	1	1	100
1.357443	1	1	100
149.3187	1	1	100
5.530322	1	1	100
37.32967	1	1	100
9.954579	1	1	100
8.783452	1	1	100
49.77289	1	1	100
4.147741	1	1	100
2.073871	1	1	100
18.66484	1	1	100
9.954579	1	1	100
2.29721	1	1	100
2.10308	1	1	100
0.883542	1	1	100

3.176993	1	1	100
149.3187	1	1	100
10.66562	1	1	100
2.817334	1	1	100
74.65934	1	1	100
6.787213	1	1	100
5.972747	1	1	100
1.716307	1	1	100
4.147741	1	1	100
7.465934	1	1	100
49.77289	1	1	100
11.48605	1	1	100
10.66562	1	1	100
2.408366	1	1	100
2.666405	1	1	100
37.32967	1	1	100
5.972747	1	1	100
5.972747	1	1	100
4.977289	1	1	100
74.65934	1	1	100
2.619626	1	1	100
2.164039	1	1	100
11.48605	1	1	100
3.555207	1	1	100
0.379946	1	1	100
0.688105	1	1	100
1.623029	1	1	100
2.045461	1	1	100
2.262404	1	1	100
24.88645	1	1	100
0.904962	1	1	100
149.3187	1	1	100
1.345213	1	1	100
1.914342	1	1	100
12.44322	1	1	100
1.276228	1	1	100
1.777603	1	1	100
5.530322	1	1	100
6.221612	1	1	100
0.951074	1	1	100
9.954579	1	1	100
0.358939	1	1	100
4.977289	1	1	100
4.816732	1	1	100
5.530322	1	1	100
0.707671	1	1	100
0.927445	1	1	100
7.465934	1	1	100
10.66562	1	1	100
11.48605	1	1	100
2.871513	1	1	100
74.65934	1	1	100
9.954579	1	1	100
3.828684	1	1	100
24.88645	1	1	100
1.254779	1	1	100
3.555207	1	1	100
3.828684	1	1	100

37.32967	1	1	100
0.198298	1	1	100
4.266248	1	1	100
1.677738	1	1	100
7.465934	1	1	100
1.097931	1	1	100
4.977289	1	1	100
1.058998	1	1	100
12.44322	1	1	100
0.697751	1	1	100
2.447847	1	1	100
5.14892	1	1	100
0.22762	1	1	100
29.86374	1	1	100
1.234039	1	1	100
5.743026	1	1	100
1.122697	1	1	100
4.03564	1	1	100
0.354676	1	1	100
3.110806	1	1	100
13.57443	1	1	100
5.743026	1	1	100
3.472527	1	1	100
2.228637	1	1	100
6.221612	1	1	100
37.32967	1	1	100
10.66562	1	1	100
18.66484	1	1	100
10.66562	1	1	100
7.110413	1	1	100
3.555207	1	1	100
149.3187	1	1	100
13.57443	1	1	100
6.492117	1	1	100
9.954579	1	1	100
21.33124	1	1	100
149.3187	1	1	100
4.266248	1	1	100
1.058998	1	1	100
2.164039	1	1	100
2.927817	1	1	100
3.732967	1	1	100
4.666209	1	1	100
0.904962	1	1	100
2.871513	1	1	100
149.3187	1	1	100
149.3187	1	1	100
21.33124	1	1	100
11.48605	1	1	100
49.77289	1	1	100
8.783452	1	1	100
16.59096	1	1	100
1.254779	1	1	100
1.38258	1	1	100
149.3187	1	1	100
4.03564	1	1	100
12.44322	1	1	100
0.975939	1	1	100

1.058998	1	1	100
4.666209	1	1	100
0.594895	1	1	100
11.48605	1	1	100
16.59096	1	1	100
12.44322	1	1	100
49.77289	1	1	100
10.66562	1	1	100
7.465934	1	1	100
4.666209	1	1	100
4.391726	1	1	100
2.488645	1	1	100
1.066562	1	1	100
1.204183	1	1	100
16.59096	1	1	100
3.393606	1	1	100
1.79902	1	1	100
12.44322	1	1	100
2.447847	1	1	100
2.10308	1	1	100
4.666209	1	1	100
2.871513	1	1	100
21.33124	1	1	100
1.79902	1	1	100
2.666405	1	1	100
0.356369	1	1	100
12.44322	1	1	100
2.666405	1	1	100
1.964719	1	1	100
14.93187	1	1	100
2.29721	1	1	100
6.492117	1	1	100
9.954579	1	1	100
2.29721	1	1	100
2.133124	1	1	100
1.265413	1	1	100
149.3187	1	1	100
37.32967	1	1	100
6.492117	1	1	100
0.848402	1	1	100
0.229368	1	1	100
1.79902	1	1	100
1.148605	1	1	100
4.977289	1	1	100
4.147741	1	1	100
74.65934	1	1	100
149.3187	1	1	100
3.472527	1	1	100
5.14892	1	1	100
0.365082	1	1	100
1.659096	1	1	100
7.110413	1	1	100
6.492117	1	1	100
1.345213	1	1	100
0.228316	1	1	100
24.88645	1	1	100
3.04732	1	1	100
7.465934	1	1	100

14.93187	1	1	100
9.954579	1	1	100
2.133124	1	1	100
6.787213	1	1	100
29.86374	1	1	100
1.166552	1	1	100
4.977289	1	1	100
7.110413	1	1	100
1.990916	1	1	100
1.990916	1	1	100
37.32967	1	1	100
1.148605	1	1	100
18.66484	1	1	100
2.927817	1	1	100
8.295482	1	1	100
1.309813	1	1	100
2.927817	1	1	100
6.492117	1	1	100
9.332418	1	1	100
0.781773	1	1	100
0.742879	1	1	100
