# Supplementary material for: Transcriptome analysis reveals a potential regulatory mechanism of the lnc-5423.6/IGFBP5 axis in the early stages of mouse thymic involution: lnc-5423.6/IGFBP5 axis regulates thymic involution
Source: Acta Biochim Biophys Sin (Shanghai). 2023 Apr 19;55(4):548–60. doi: 10.3724/abbs.2023042 (PMC10195152; doi:10.3724/abbs.2023042)
Supplement: Table_S11 [file Table_S11.pdf]

t_name	fc	log2(fc	pval	regulat	significant
ENSMUST	0.0053	-7.558	3E-18	down	yes
ENSMUST	0.0395	-4.663	8E-10	down	yes
ENSMUST	0.0465	-4.428	4E-09	down	yes
ENSMUST	0.0453	-4.464	4E-09	down	yes
ENSMUST	10.534	3.397	3E-06	up	yes
ENSMUST	10.541	3.398	3E-06	up	yes
ENSMUST	8.7537	3.1299	1E-05	up	yes
ENSMUST	8.1329	3.0238	2E-05	up	yes
ENSMUST	8.0127	3.0023	3E-05	up	yes
ENSMUST	7.5101	2.9088	4E-05	up	yes
ENSMUST	7.1544	2.8388	6E-05	up	yes
ENSMUST	6.9655	2.8002	8E-05	up	yes
ENSMUST	6.9644	2.8	8E-05	up	yes
ENSMUST	6.841	2.7742	9E-05	up	yes
ENSMUST	6.7396	2.7527	0.0001	up	yes
ENSMUST	0.1529	-2.71	0.0001	down	yes
ENSMUST	6.6298	2.729	0.0001	up	yes
ENSMUST	6.2973	2.6547	0.0002	up	yes
ENSMUST	6.1636	2.6238	0.0002	up	yes
ENSMUST	6.1544	2.6216	0.0002	up	yes
ENSMUST	6.1229	2.6142	0.0002	up	yes
ENSMUST	6.1266	2.6151	0.0002	up	yes
ENSMUST	5.9658	2.5767	0.0003	up	yes
ENSMUST	5.9037	2.5616	0.0003	up	yes
ENSMUST	0.1799	-2.475	0.0003	down	yes
ENSMUST	5.7077	2.5129	0.0004	up	yes
ENSMUST	5.5074	2.4614	0.0004	up	yes
ENSMUST	5.5488	2.4722	0.0004	up	yes
ENSMUST	5.4319	2.4415	0.0005	up	yes
ENSMUST	5.4685	2.4512	0.0005	up	yes
ENSMUST	5.4046	2.4342	0.0005	up	yes
ENSMUST	0.1904	-2.393	0.0006	down	yes
ENSMUST	0.1998	-2.323	0.0007	down	yes
ENSMUST	5.1031	2.3514	0.0008	up	yes
ENSMUST	5.1537	2.3656	0.0008	up	yes
ENSMUST	0.2075	-2.268	0.001	down	yes
ENSMUST	4.8297	2.2719	0.0011	up	yes
ENSMUST	4.7841	2.2583	0.0012	up	yes
ENSMUST	4.729	2.2415	0.0012	up	yes
ENSMUST	4.6639	2.2215	0.0013	up	yes
ENSMUST	4.6473	2.2164	0.0016	up	yes
ENSMUST	4.5092	2.1729	0.0016	up	yes
ENSMUST	4.5558	2.1877	0.0017	up	yes
ENSMUST	4.4878	2.166	0.0017	up	yes
ENSMUST	4.5604	2.1892	0.0018	up	yes
ENSMUST	0.2343	-2.094	0.0019	down	yes
ENSMUST	4.552	2.1865	0.0019	up	yes
ENSMUST	4.4207	2.1443	0.002	up	yes
ENSMUST	4.4158	2.1427	0.002	up	yes
ENSMUST	4.4651	2.1587	0.0021	up	yes
ENSMUST	4.3508	2.1213	0.0022	up	yes
ENSMUST	4.3791	2.1306	0.0022	up	yes
ENSMUST	4.3426	2.1186	0.0023	up	yes
ENSMUST	4.2173	2.0763	0.0027	up	yes
ENSMUST	4.1984	2.0698	0.0028	up	yes
ENSMUST	0.246	-2.023	0.0029	down	yes

ENSMUST	4.1525	2.054	0.0031	up	yes
ENSMUST	0.249	-2.006	0.0032	down	yes
ENSMUST	0.2486	-2.008	0.0033	down	yes
ENSMUST	4.0232	2.0083	0.0037	up	yes
ENSMUST	3.9363	1.9769	0.004	up	yes
ENSMUST	0.2635	-1.924	0.0044	down	yes
ENSMUST	3.9011	1.9639	0.0048	up	yes
ENSMUST	3.8829	1.9571	0.0049	up	yes
ENSMUST	3.829	1.937	0.005	up	yes
ENSMUST	3.8419	1.9418	0.0052	up	yes
ENSMUST	3.8564	1.9473	0.0053	up	yes
ENSMUST	3.8005	1.9262	0.0056	up	yes
ENSMUST	3.7573	1.9097	0.0058	up	yes
ENSMUST	3.6535	1.8693	0.007	up	yes
ENSMUST	3.6168	1.8547	0.0073	up	yes
ENSMUST	3.5929	1.8451	0.0075	up	yes
ENSMUST	3.5578	1.831	0.0076	up	yes
ENSMUST	0.283	-1.821	0.0077	down	yes
ENSMUST	3.6057	1.8503	0.0077	up	yes
ENSMUST	3.5884	1.8433	0.0079	up	yes
ENSMUST	3.5447	1.8256	0.0081	up	yes
ENSMUST	3.5793	1.8397	0.0082	up	yes
ENSMUST	3.56	1.8319	0.0082	up	yes
ENSMUST	3.5026	1.8084	0.0083	up	yes
ENSMUST	3.547	1.8266	0.0084	up	yes
ENSMUST	3.5448	1.8257	0.0087	up	yes
ENSMUST	3.4732	1.7963	0.0093	up	yes
ENSMUST	3.4681	1.7941	0.0094	up	yes
ENSMUST	3.3543	1.746	0.0114	up	yes
ENSMUST	0.3055	-1.711	0.0118	down	yes
ENSMUST	3.3406	1.7401	0.0119	up	yes
ENSMUST	3.3519	1.745	0.0121	up	yes
ENSMUST	0.317	-1.657	0.0129	down	yes
ENSMUST	0.3115	-1.683	0.0133	down	yes
ENSMUST	0.3207	-1.641	0.0149	down	yes
ENSMUST	3.1477	1.6543	0.0151	up	yes
ENSMUST	3.1482	1.6545	0.0157	up	yes
ENSMUST	0.3313	-1.594	0.0164	down	yes
ENSMUST	3.1461	1.6536	0.0165	up	yes
ENSMUST	3.1601	1.66	0.0166	up	yes
ENSMUST	0.3362	-1.573	0.0176	down	yes
ENSMUST	0.3322	-1.59	0.0179	down	yes
ENSMUST	3.1184	1.6408	0.018	up	yes
ENSMUST	3.0463	1.6071	0.0182	up	yes
ENSMUST	3.0498	1.6087	0.019	up	yes
ENSMUST	3.0768	1.6214	0.0192	up	yes
ENSMUST	3.053	1.6102	0.0196	up	yes
ENSMUST	0.3434	-1.542	0.0198	down	yes
ENSMUST	3.0664	1.6166	0.0201	up	yes
ENSMUST	3.0279	1.5983	0.0203	up	yes
ENSMUST	3.0364	1.6024	0.0203	up	yes
ENSMUST	0.3397	-1.558	0.0204	down	yes
ENSMUST	0.3386	-1.562	0.0205	down	yes
ENSMUST	3.0308	1.5997	0.0205	up	yes
ENSMUST	0.3408	-1.553	0.0212	down	yes
ENSMUST	2.9675	1.5693	0.0223	up	yes
ENSMUST	2.9866	1.5785	0.0225	up	yes

ENSMUST	2.944	1.5578	0.0228	up	yes
ENSMUST	2.9602	1.5657	0.023	up	yes
ENSMUST	2.9633	1.5672	0.0244	up	yes
ENSMUST	2.9513	1.5613	0.0246	up	yes
ENSMUST	2.9273	1.5496	0.0249	up	yes
ENSMUST	2.9025	1.5373	0.0256	up	yes
ENSMUST	0.3547	-1.495	0.0262	down	yes
ENSMUST	0.3574	-1.484	0.0267	down	yes
ENSMUST	2.8481	1.51	0.0269	up	yes
ENSMUST	0.3584	-1.48	0.0273	down	yes
ENSMUST	0.3621	-1.466	0.0273	down	yes
ENSMUST	2.7621	1.4658	0.0318	up	yes
ENSMUST	2.7011	1.4335	0.0349	up	yes
ENSMUST	0.3783	-1.402	0.035	down	yes
ENSMUST	0.3858	-1.374	0.0372	down	yes
ENSMUST	0.388	-1.366	0.0383	down	yes
ENSMUST	2.6362	1.3985	0.0406	up	yes
ENSMUST	2.6481	1.405	0.0426	up	yes
ENSMUST	2.6102	1.3842	0.0429	up	yes
ENSMUST	2.5917	1.3739	0.0432	up	yes
ENSMUST	2.6156	1.3871	0.0436	up	yes
ENSMUST	2.5988	1.3778	0.0438	up	yes
ENSMUST	2.6145	1.3865	0.0454	up	yes
ENSMUST	2.6203	1.3897	0.0455	up	yes
ENSMUST	0.4	-1.322	0.0467	down	yes
ENSMUST	0.4	-1.322	0.0467	down	yes
ENSMUST	2.5717	1.3627	0.0474	up	yes
ENSMUST	0.3953	-1.339	0.0476	down	yes
ENSMUST	0.4074	-1.295	0.0494	down	yes
ENSMUST	2.5577	1.3548	0.0497	up	yes
ENSMUST	2.5443	1.3472	0.0498	up	yes
MSTRG.6	271.19	8.0831	1E-19	up	yes
MSTRG.1	0.0059	-7.397	1E-17	down	yes
MSTRG.1	0.0081	-6.945	2E-16	down	yes
MSTRG.3	76.564	6.2586	3E-14	up	yes
MSTRG.1	74.935	6.2276	3E-14	up	yes
MSTRG.6	0.0151	-6.054	7E-14	down	yes
MSTRG.2	52.815	5.7229	1E-12	up	yes
MSTRG.1	49.733	5.6361	2E-12	up	yes
MSTRG.1	46.893	5.5513	4E-12	up	yes
MSTRG.2	0.0271	-5.206	3E-11	down	yes
MSTRG.9	36.058	5.1722	4E-11	up	yes
MSTRG.1	31.278	4.9671	2E-10	up	yes
MSTRG.1	0.0366	-4.772	5E-10	down	yes
MSTRG.8	0.0378	-4.724	5E-10	down	yes
MSTRG.2	25.866	4.693	1E-09	up	yes
MSTRG.1	22.764	4.5087	3E-09	up	yes
MSTRG.2	0.0456	-4.453	3E-09	down	yes
MSTRG.5	22.541	4.4945	3E-09	up	yes
MSTRG.4	21.553	4.4298	6E-09	up	yes
MSTRG.1	0.0491	-4.347	6E-09	down	yes
MSTRG.6	0.0521	-4.264	1E-08	down	yes
MSTRG.1	19.613	4.2938	1E-08	up	yes
MSTRG.7	0.0577	-4.115	3E-08	down	yes
MSTRG.1	0.0586	-4.093	3E-08	down	yes
MSTRG.2	15.841	3.9856	8E-08	up	yes
MSTRG.3	15.243	3.93	1E-07	up	yes

MSTRG.5	0.0727	-3.782	2E-07	down	yes
MSTRG.1	0.0714	-3.808	2E-07	down	yes
MSTRG.4	0.0738	-3.761	2E-07	down	yes
MSTRG.7	13.616	3.7672	3E-07	up	yes
MSTRG.3	13.704	3.7765	3E-07	up	yes
MSTRG.2	13.624	3.768	4E-07	up	yes
MSTRG.6	13.313	3.7347	4E-07	up	yes
MSTRG.8	0.0792	-3.658	4E-07	down	yes
MSTRG.5	12.85	3.6837	5E-07	up	yes
MSTRG.8	12.664	3.6626	5E-07	up	yes
MSTRG.6	0.0824	-3.602	6E-07	down	yes
MSTRG.8	0.0802	-3.641	6E-07	down	yes
MSTRG.1	12.555	3.6502	6E-07	up	yes
MSTRG.3	12.692	3.6659	7E-07	up	yes
MSTRG.6	12.327	3.6238	7E-07	up	yes
MSTRG.2	11.935	3.5771	9E-07	up	yes
MSTRG.2	0.086	-3.539	9E-07	down	yes
MSTRG.1	11.73	3.5522	1E-06	up	yes
MSTRG.8	0.0873	-3.519	1E-06	down	yes
MSTRG.3	11.639	3.5409	1E-06	up	yes
MSTRG.3	11.538	3.5284	1E-06	up	yes
MSTRG.5	0.0879	-3.508	1E-06	down	yes
MSTRG.1	0.0883	-3.502	1E-06	down	yes
MSTRG.3	11.478	3.5208	1E-06	up	yes
MSTRG.1	11.648	3.542	1E-06	up	yes
MSTRG.3	11.282	3.4959	1E-06	up	yes
MSTRG.3	11.255	3.4925	2E-06	up	yes
MSTRG.3	11.029	3.4633	2E-06	up	yes
MSTRG.9	0.0936	-3.418	2E-06	down	yes
MSTRG.3	0.0944	-3.406	2E-06	down	yes
MSTRG.9	0.0944	-3.405	2E-06	down	yes
MSTRG.3	10.683	3.4172	2E-06	up	yes
MSTRG.1	10.706	3.4204	2E-06	up	yes
MSTRG.6	10.578	3.403	3E-06	up	yes
MSTRG.3	10.752	3.4266	3E-06	up	yes
MSTRG.2	10.408	3.3796	3E-06	up	yes
MSTRG.6	10.212	3.3522	3E-06	up	yes
MSTRG.6	9.9559	3.3155	5E-06	up	yes
MSTRG.1	9.9671	3.3172	5E-06	up	yes
MSTRG.2	9.6631	3.2725	5E-06	up	yes
MSTRG.4	9.9161	3.3098	5E-06	up	yes
MSTRG.6	9.5813	3.2602	5E-06	up	yes
MSTRG.1	9.623	3.2665	6E-06	up	yes
MSTRG.3	9.3275	3.2215	7E-06	up	yes
MSTRG.1	0.112	-3.158	8E-06	down	yes
MSTRG.3	9.4178	3.2354	8E-06	up	yes
MSTRG.4	9.3026	3.2176	8E-06	up	yes
MSTRG.3	9.3205	3.2204	9E-06	up	yes
MSTRG.8	0.1138	-3.136	1E-05	down	yes
MSTRG.1	8.8083	3.1389	1E-05	up	yes
MSTRG.1	9.0143	3.1722	1E-05	up	yes
MSTRG.3	8.6993	3.1209	1E-05	up	yes
MSTRG.1	0.1158	-3.11	1E-05	down	yes
MSTRG.1	8.762	3.1313	1E-05	up	yes
MSTRG.1	8.7165	3.1237	1E-05	up	yes
MSTRG.2	0.1219	-3.036	1E-05	down	yes
MSTRG.2	8.4931	3.0863	2E-05	up	yes

MSTRG.3	8.6029	3.1048	2E-05	up	yes
MSTRG.6	8.3583	3.0632	2E-05	up	yes
MSTRG.3	8.3259	3.0576	2E-05	up	yes
MSTRG.1	0.1243	-3.009	2E-05	down	yes
MSTRG.1	8.4501	3.079	2E-05	up	yes
MSTRG.4	8.2685	3.0476	2E-05	up	yes
MSTRG.1	8.3433	3.0606	2E-05	up	yes
MSTRG.8	0.1225	-3.03	2E-05	down	yes
MSTRG.2	8.3264	3.0577	2E-05	up	yes
MSTRG.2	8.4012	3.0706	2E-05	up	yes
MSTRG.1	8.1958	3.0349	2E-05	up	yes
MSTRG.1	8.1444	3.0258	2E-05	up	yes
MSTRG.3	8.1534	3.0274	2E-05	up	yes
MSTRG.2	8.3168	3.056	2E-05	up	yes
MSTRG.1	0.127	-2.978	2E-05	down	yes
MSTRG.4	8.0235	3.0042	2E-05	up	yes
MSTRG.2	7.9805	2.9965	2E-05	up	yes
MSTRG.1	8.2294	3.0408	2E-05	up	yes
MSTRG.1	7.8063	2.9646	3E-05	up	yes
MSTRG.2	7.949	2.9908	3E-05	up	yes
MSTRG.9	7.784	2.9605	3E-05	up	yes
MSTRG.2	7.9904	2.9983	3E-05	up	yes
MSTRG.3	7.7322	2.9509	3E-05	up	yes
MSTRG.1	0.1319	-2.923	3E-05	down	yes
MSTRG.2	7.8349	2.9699	3E-05	up	yes
MSTRG.2	7.6981	2.9445	4E-05	up	yes
MSTRG.5	7.6655	2.9384	4E-05	up	yes
MSTRG.3	7.6378	2.9332	4E-05	up	yes
MSTRG.2	7.5648	2.9193	4E-05	up	yes
MSTRG.1	0.1367	-2.871	4E-05	down	yes
MSTRG.6	7.5245	2.9116	4E-05	up	yes
MSTRG.1	0.1377	-2.86	4E-05	down	yes
MSTRG.2	7.5159	2.9099	4E-05	up	yes
MSTRG.9	7.455	2.8982	4E-05	up	yes
MSTRG.1	7.4431	2.8959	5E-05	up	yes
MSTRG.1	7.3349	2.8748	5E-05	up	yes
MSTRG.1	7.3961	2.8868	5E-05	up	yes
MSTRG.3	7.4564	2.8985	5E-05	up	yes
MSTRG.1	7.318	2.8714	5E-05	up	yes
MSTRG.3	7.3822	2.8841	5E-05	up	yes
MSTRG.1	0.142	-2.817	5E-05	down	yes
MSTRG.1	7.3597	2.8796	5E-05	up	yes
MSTRG.6	7.2579	2.8596	5E-05	up	yes
MSTRG.7	7.3557	2.8789	5E-05	up	yes
MSTRG.1	7.2861	2.8652	6E-05	up	yes
MSTRG.9	7.1539	2.8387	6E-05	up	yes
MSTRG.3	7.2377	2.8555	6E-05	up	yes
MSTRG.4	7.2466	2.8573	6E-05	up	yes
MSTRG.3	7.1202	2.8319	7E-05	up	yes
MSTRG.2	7.0736	2.8225	7E-05	up	yes
MSTRG.1	7.1356	2.835	7E-05	up	yes
MSTRG.9	6.9449	2.796	7E-05	up	yes
MSTRG.1	6.9885	2.805	7E-05	up	yes
MSTRG.1	6.9143	2.7896	8E-05	up	yes
MSTRG.1	7.0612	2.8199	8E-05	up	yes
MSTRG.8	0.1484	-2.752	9E-05	down	yes
MSTRG.4	6.8897	2.7844	9E-05	up	yes

MSTRG.1	6.779	2.7611	9E-05	up	yes
MSTRG.9	6.7565	2.7563	9E-05	up	yes
MSTRG.2	6.9537	2.7978	9E-05	up	yes
MSTRG.3	6.7246	2.7494	9E-05	up	yes
MSTRG.7	6.7628	2.7576	9E-05	up	yes
MSTRG.2	6.8965	2.7859	1E-04	up	yes
MSTRG.9	6.6587	2.7352	1E-04	up	yes
MSTRG.1	6.6507	2.7335	1E-04	up	yes
MSTRG.2	0.1511	-2.726	0.0001	down	yes
MSTRG.3	6.6399	2.7312	0.0001	up	yes
MSTRG.3	6.7331	2.7513	0.0001	up	yes
MSTRG.1	6.6002	2.7225	0.0001	up	yes
MSTRG.4	6.547	2.7108	0.0001	up	yes
MSTRG.1	6.6962	2.7433	0.0001	up	yes
MSTRG.3	6.6241	2.7277	0.0001	up	yes
MSTRG.1	6.4821	2.6965	0.0001	up	yes
MSTRG.4	6.5299	2.7071	0.0001	up	yes
MSTRG.2	6.6457	2.7324	0.0001	up	yes
MSTRG.3	6.457	2.6909	0.0001	up	yes
MSTRG.2	6.4496	2.6892	0.0001	up	yes
MSTRG.6	6.5043	2.7014	0.0001	up	yes
MSTRG.1	6.5171	2.7042	0.0001	up	yes
MSTRG.1	6.454	2.6902	0.0001	up	yes
MSTRG.1	6.5488	2.7112	0.0001	up	yes
MSTRG.5	6.3232	2.6606	0.0001	up	yes
MSTRG.2	6.3505	2.6669	0.0001	up	yes
MSTRG.3	6.3568	2.6683	0.0002	up	yes
MSTRG.1	6.4934	2.699	0.0002	up	yes
MSTRG.2	0.1593	-2.65	0.0002	down	yes
MSTRG.3	6.2902	2.6531	0.0002	up	yes
MSTRG.4	6.2184	2.6365	0.0002	up	yes
MSTRG.7	0.1637	-2.611	0.0002	down	yes
MSTRG.3	6.3582	2.6686	0.0002	up	yes
MSTRG.9	6.2225	2.6375	0.0002	up	yes
MSTRG.1	6.187	2.6292	0.0002	up	yes
MSTRG.1	6.1248	2.6147	0.0002	up	yes
MSTRG.2	6.1534	2.6214	0.0002	up	yes
MSTRG.2	6.1651	2.6241	0.0002	up	yes
MSTRG.1	6.1675	2.6247	0.0002	up	yes
MSTRG.1	6.0286	2.5918	0.0002	up	yes
MSTRG.1	6.0905	2.6066	0.0002	up	yes
MSTRG.9	6.0368	2.5938	0.0002	up	yes
MSTRG.4	0.1688	-2.567	0.0002	down	yes
MSTRG.7	0.1724	-2.536	0.0002	down	yes
MSTRG.1	0.1718	-2.541	0.0002	down	yes
MSTRG.4	6.089	2.6062	0.0002	up	yes
MSTRG.4	5.9371	2.5698	0.0002	up	yes
MSTRG.4	5.9752	2.579	0.0002	up	yes
MSTRG.3	5.9546	2.574	0.0002	up	yes
MSTRG.1	5.9676	2.5771	0.0002	up	yes
MSTRG.2	0.1743	-2.521	0.0003	down	yes
MSTRG.3	0.1722	-2.538	0.0003	down	yes
MSTRG.3	5.9168	2.5648	0.0003	up	yes
MSTRG.9	5.8362	2.545	0.0003	up	yes
MSTRG.3	5.8598	2.5508	0.0003	up	yes
MSTRG.2	5.8535	2.5493	0.0003	up	yes
MSTRG.3	5.8497	2.5484	0.0003	up	yes

MSTRG.1	5.8553	2.5497	0.0003	up	yes
MSTRG.2	5.773	2.5293	0.0003	up	yes
MSTRG.9	5.8223	2.5416	0.0003	up	yes
MSTRG.2	5.8804	2.5559	0.0003	up	yes
MSTRG.1	5.7733	2.5294	0.0003	up	yes
MSTRG.1	0.1764	-2.503	0.0003	down	yes
MSTRG.9	5.8947	2.5594	0.0003	up	yes
MSTRG.3	5.8032	2.5368	0.0003	up	yes
MSTRG.1	5.7409	2.5213	0.0003	up	yes
MSTRG.1	5.7124	2.5141	0.0003	up	yes
MSTRG.1	5.8366	2.5451	0.0003	up	yes
MSTRG.3	5.7734	2.5294	0.0003	up	yes
MSTRG.4	5.6637	2.5018	0.0003	up	yes
MSTRG.3	5.8348	2.5447	0.0003	up	yes
MSTRG.1	5.6523	2.4988	0.0003	up	yes
MSTRG.2	0.1793	-2.48	0.0004	down	yes
MSTRG.3	5.6671	2.5026	0.0004	up	yes
MSTRG.3	0.184	-2.442	0.0004	down	yes
MSTRG.9	5.714	2.5145	0.0004	up	yes
MSTRG.1	0.1838	-2.444	0.0004	down	yes
MSTRG.3	5.5973	2.4847	0.0004	up	yes
MSTRG.1	5.5638	2.4761	0.0004	up	yes
MSTRG.2	5.5503	2.4726	0.0004	up	yes
MSTRG.1	5.7026	2.5116	0.0004	up	yes
MSTRG.3	5.6414	2.4961	0.0004	up	yes
MSTRG.6	5.5068	2.4612	0.0004	up	yes
MSTRG.1	5.4779	2.4536	0.0004	up	yes
MSTRG.2	0.1872	-2.417	0.0004	down	yes
MSTRG.1	5.4269	2.4401	0.0005	up	yes
MSTRG.3	5.4295	2.4408	0.0005	up	yes
MSTRG.4	5.5154	2.4635	0.0005	up	yes
MSTRG.2	5.471	2.4518	0.0005	up	yes
MSTRG.2	5.4323	2.4416	0.0005	up	yes
MSTRG.1	5.4512	2.4466	0.0005	up	yes
MSTRG.3	5.442	2.4441	0.0005	up	yes
MSTRG.2	5.3847	2.4289	0.0005	up	yes
MSTRG.3	0.1929	-2.374	0.0005	down	yes
MSTRG.1	0.1883	-2.409	0.0005	down	yes
MSTRG.9	5.3543	2.4207	0.0005	up	yes
MSTRG.1	5.3376	2.4162	0.0006	up	yes
MSTRG.8	0.1944	-2.363	0.0006	down	yes
MSTRG.5	5.2553	2.3938	0.0006	up	yes
MSTRG.7	0.1975	-2.34	0.0006	down	yes
MSTRG.1	0.1973	-2.341	0.0006	down	yes
MSTRG.2	0.1937	-2.368	0.0006	down	yes
MSTRG.1	5.1872	2.375	0.0006	up	yes
MSTRG.2	5.182	2.3735	0.0006	up	yes
MSTRG.2	5.1637	2.3684	0.0006	up	yes
MSTRG.1	5.2177	2.3834	0.0006	up	yes
MSTRG.9	5.1743	2.3714	0.0006	up	yes
MSTRG.1	5.293	2.4041	0.0006	up	yes
MSTRG.6	5.1633	2.3683	0.0007	up	yes
MSTRG.2	0.2013	-2.313	0.0007	down	yes
MSTRG.3	5.1514	2.365	0.0007	up	yes
MSTRG.3	5.0941	2.3488	0.0007	up	yes
MSTRG.3	0.2032	-2.299	0.0007	down	yes
MSTRG.3	5.1716	2.3706	0.0007	up	yes

MSTRG.9	5.0906	2.3478	0.0007	up	yes
MSTRG.5	5.0865	2.3467	0.0007	up	yes
MSTRG.8	5.1173	2.3554	0.0007	up	yes
MSTRG.2	5.0505	2.3364	0.0007	up	yes
MSTRG.1	5.0916	2.3481	0.0007	up	yes
MSTRG.5	5.0404	2.3335	0.0008	up	yes
MSTRG.3	0.2035	-2.297	0.0008	down	yes
MSTRG.9	5.0597	2.339	0.0008	up	yes
MSTRG.2	5.0733	2.3429	0.0008	up	yes
MSTRG.1	5.0908	2.3479	0.0008	up	yes
MSTRG.1	5.0368	2.3325	0.0008	up	yes
MSTRG.1	4.9878	2.3184	0.0008	up	yes
MSTRG.2	0.2039	-2.294	0.0008	down	yes
MSTRG.1	5.0536	2.3373	0.0008	up	yes
MSTRG.2	5.1372	2.361	0.0008	up	yes
MSTRG.2	5.0239	2.3288	0.0008	up	yes
MSTRG.3	4.9716	2.3137	0.0009	up	yes
MSTRG.5	4.9452	2.306	0.0009	up	yes
MSTRG.1	5.0373	2.3326	0.0009	up	yes
MSTRG.2	4.9558	2.3091	0.0009	up	yes
MSTRG.2	4.9444	2.3058	0.0009	up	yes
MSTRG.1	4.9349	2.303	0.0009	up	yes
MSTRG.2	4.9464	2.3064	0.0009	up	yes
MSTRG.1	4.8596	2.2808	0.001	up	yes
MSTRG.3	4.9512	2.3078	0.001	up	yes
MSTRG.2	0.2087	-2.261	0.001	down	yes
MSTRG.7	4.9477	2.3068	0.001	up	yes
MSTRG.3	0.2096	-2.254	0.001	down	yes
MSTRG.1	4.863	2.2818	0.0011	up	yes
MSTRG.2	4.8261	2.2709	0.0011	up	yes
MSTRG.1	0.2138	-2.226	0.0011	down	yes
MSTRG.1	4.8961	2.2916	0.0011	up	yes
MSTRG.3	4.7741	2.2552	0.0012	up	yes
MSTRG.2	0.218	-2.198	0.0012	down	yes
MSTRG.2	4.7611	2.2513	0.0012	up	yes
MSTRG.1	4.8311	2.2724	0.0012	up	yes
MSTRG.7	0.2182	-2.197	0.0013	down	yes
MSTRG.3	4.7889	2.2597	0.0013	up	yes
MSTRG.1	4.7647	2.2524	0.0013	up	yes
MSTRG.1	4.7234	2.2398	0.0014	up	yes
MSTRG.5	0.2221	-2.171	0.0014	down	yes
MSTRG.2	4.6321	2.2117	0.0014	up	yes
MSTRG.4	4.7056	2.2344	0.0014	up	yes
MSTRG.3	4.7449	2.2464	0.0014	up	yes
MSTRG.5	4.6121	2.2054	0.0014	up	yes
MSTRG.2	4.6092	2.2045	0.0014	up	yes
MSTRG.1	0.2203	-2.182	0.0014	down	yes
MSTRG.9	4.6395	2.214	0.0014	up	yes
MSTRG.3	0.2192	-2.19	0.0014	down	yes
MSTRG.6	4.6969	2.2317	0.0015	up	yes
MSTRG.1	4.6976	2.2319	0.0015	up	yes
MSTRG.1	4.6691	2.2231	0.0015	up	yes
MSTRG.1	4.6142	2.2061	0.0015	up	yes
MSTRG.9	0.2262	-2.144	0.0015	down	yes
MSTRG.1	4.5394	2.1825	0.0017	up	yes
MSTRG.1	4.5572	2.1881	0.0017	up	yes
MSTRG.3	4.5045	2.1714	0.0017	up	yes

MSTRG.3	4.5763	2.1942	0.0018	up	yes
MSTRG.5	4.5314	2.1799	0.0018	up	yes
MSTRG.3	4.4646	2.1585	0.0018	up	yes
MSTRG.1	0.2334	-2.099	0.002	down	yes
MSTRG.3	4.4127	2.1417	0.002	up	yes
MSTRG.3	4.3749	2.1293	0.002	up	yes
MSTRG.1	4.4321	2.148	0.0021	up	yes
MSTRG.3	4.3906	2.1344	0.0022	up	yes
MSTRG.3	0.2349	-2.09	0.0022	down	yes
MSTRG.3	4.3096	2.1076	0.0022	up	yes
MSTRG.1	4.43	2.1473	0.0023	up	yes
MSTRG.3	4.2989	2.104	0.0023	up	yes
MSTRG.1	4.2771	2.0966	0.0023	up	yes
MSTRG.3	4.2959	2.1029	0.0024	up	yes
MSTRG.1	4.3603	2.1244	0.0024	up	yes
MSTRG.1	4.2785	2.0971	0.0025	up	yes
MSTRG.1	0.2419	-2.047	0.0026	down	yes
MSTRG.2	4.2964	2.1031	0.0026	up	yes
MSTRG.2	4.2879	2.1003	0.0026	up	yes
MSTRG.5	4.2024	2.0712	0.0026	up	yes
MSTRG.3	0.247	-2.018	0.0027	down	yes
MSTRG.3	4.1933	2.0681	0.0027	up	yes
MSTRG.2	4.215	2.0755	0.0027	up	yes
MSTRG.2	4.1889	2.0666	0.0027	up	yes
MSTRG.1	4.2257	2.0792	0.0027	up	yes
MSTRG.1	4.1794	2.0633	0.0027	up	yes
MSTRG.3	4.2182	2.0766	0.0028	up	yes
MSTRG.2	4.2429	2.0851	0.0028	up	yes
MSTRG.2	0.2423	-2.045	0.0028	down	yes
MSTRG.6	4.1713	2.0605	0.0028	up	yes
MSTRG.2	4.1245	2.0442	0.0029	up	yes
MSTRG.2	4.1893	2.0667	0.0029	up	yes
MSTRG.1	4.1749	2.0618	0.0029	up	yes
MSTRG.2	4.2309	2.081	0.0029	up	yes
MSTRG.3	0.2504	-1.998	0.003	down	yes
MSTRG.2	4.1447	2.0513	0.003	up	yes
MSTRG.1	4.2069	2.0727	0.003	up	yes
MSTRG.3	0.2512	-1.993	0.003	down	yes
MSTRG.1	4.076	2.0272	0.0032	up	yes
MSTRG.3	0.2512	-1.993	0.0032	down	yes
MSTRG.1	0.2497	-2.002	0.0033	down	yes
MSTRG.3	4.0453	2.0162	0.0033	up	yes
MSTRG.2	4.0467	2.0167	0.0034	up	yes
MSTRG.1	4.0176	2.0063	0.0035	up	yes
MSTRG.3	4.0045	2.0016	0.0036	up	yes
MSTRG.1	4.0453	2.0162	0.0036	up	yes
MSTRG.3	4.0115	2.0041	0.0037	up	yes
MSTRG.3	4.0174	2.0063	0.0038	up	yes
MSTRG.4	0.2578	-1.956	0.0038	down	yes
MSTRG.2	0.2607	-1.94	0.0038	down	yes
MSTRG.6	3.9671	1.9881	0.0039	up	yes
MSTRG.3	3.9396	1.9781	0.0039	up	yes
MSTRG.2	3.9488	1.9814	0.0039	up	yes
MSTRG.5	4.0203	2.0073	0.004	up	yes
MSTRG.1	3.9304	1.9747	0.004	up	yes
MSTRG.2	3.9538	1.9832	0.0041	up	yes
MSTRG.1	3.9355	1.9766	0.0042	up	yes

MSTRG.5	3.912	1.9679	0.0043	up	yes
MSTRG.1	3.9482	1.9812	0.0043	up	yes
MSTRG.3	3.9045	1.9651	0.0044	up	yes
MSTRG.3	3.9201	1.9709	0.0044	up	yes
MSTRG.1	3.8668	1.9511	0.0045	up	yes
MSTRG.2	3.9346	1.9762	0.0047	up	yes
MSTRG.1	3.8088	1.9293	0.0048	up	yes
MSTRG.2	3.8503	1.945	0.0049	up	yes
MSTRG.3	3.848	1.9441	0.0049	up	yes
MSTRG.4	3.7917	1.9228	0.005	up	yes
MSTRG.1	3.8143	1.9314	0.005	up	yes
MSTRG.3	3.7821	1.9192	0.005	up	yes
MSTRG.1	3.8284	1.9367	0.0051	up	yes
MSTRG.2	3.8621	1.9494	0.0052	up	yes
MSTRG.1	3.7705	1.9147	0.0053	up	yes
MSTRG.1	3.8177	1.9327	0.0053	up	yes
MSTRG.1	3.8278	1.9365	0.0053	up	yes
MSTRG.7	3.7967	1.9247	0.0054	up	yes
MSTRG.1	3.7821	1.9192	0.0054	up	yes
MSTRG.1	3.7608	1.9111	0.0054	up	yes
MSTRG.2	3.7283	1.8985	0.0055	up	yes
MSTRG.2	3.7189	1.8949	0.0055	up	yes
MSTRG.3	3.7143	1.8931	0.0056	up	yes
MSTRG.3	3.7511	1.9073	0.0057	up	yes
MSTRG.6	3.7804	1.9185	0.0058	up	yes
MSTRG.9	0.2719	-1.879	0.0058	down	yes
MSTRG.1	3.7178	1.8944	0.0059	up	yes
MSTRG.3	3.7631	1.9119	0.006	up	yes
MSTRG.2	0.2764	-1.855	0.0061	down	yes
MSTRG.1	3.7521	1.9077	0.0061	up	yes
MSTRG.3	3.6412	1.8644	0.0064	up	yes
MSTRG.2	3.7002	1.8876	0.0065	up	yes
MSTRG.2	3.6693	1.8755	0.0067	up	yes
MSTRG.1	3.6586	1.8713	0.0067	up	yes
MSTRG.2	3.6103	1.8521	0.0067	up	yes
MSTRG.1	3.5923	1.8449	0.0069	up	yes
MSTRG.2	3.6132	1.8533	0.0069	up	yes
MSTRG.2	3.6398	1.8639	0.0071	up	yes
MSTRG.1	3.5784	1.8393	0.0071	up	yes
MSTRG.1	0.2862	-1.805	0.0072	down	yes
MSTRG.3	3.6064	1.8505	0.0073	up	yes
MSTRG.2	3.656	1.8703	0.0073	up	yes
MSTRG.6	3.5851	1.842	0.0073	up	yes
MSTRG.2	3.629	1.8596	0.0073	up	yes
MSTRG.3	3.5707	1.8362	0.0074	up	yes
MSTRG.1	3.539	1.8233	0.0079	up	yes
MSTRG.1	3.5752	1.838	0.0079	up	yes
MSTRG.2	3.5408	1.8241	0.0081	up	yes
MSTRG.2	3.5415	1.8243	0.0081	up	yes
MSTRG.1	3.5243	1.8173	0.0082	up	yes
MSTRG.1	3.5116	1.8121	0.0082	up	yes
MSTRG.1	3.4885	1.8026	0.0082	up	yes
MSTRG.2	0.2889	-1.791	0.0083	down	yes
MSTRG.1	3.5043	1.8091	0.0083	up	yes
MSTRG.3	3.4932	1.8046	0.0084	up	yes
MSTRG.2	3.4664	1.7934	0.0085	up	yes
MSTRG.8	0.2885	-1.793	0.0085	down	yes

MSTRG.1	3.4673	1.7938	0.0085	up	yes
MSTRG.3	0.2936	-1.768	0.0087	down	yes
MSTRG.2	3.4908	1.8036	0.0087	up	yes
MSTRG.2	0.2988	-1.743	0.0088	down	yes
MSTRG.1	0.2997	-1.739	0.0089	down	yes
MSTRG.1	3.4466	1.7852	0.0089	up	yes
MSTRG.1	0.3003	-1.736	0.009	down	yes
MSTRG.1	3.5233	1.8169	0.0091	up	yes
MSTRG.2	3.4409	1.7828	0.0091	up	yes
MSTRG.1	3.4222	1.7749	0.0092	up	yes
MSTRG.9	3.462	1.7916	0.0094	up	yes
MSTRG.9	3.4133	1.7712	0.0094	up	yes
MSTRG.2	0.3009	-1.733	0.0097	down	yes
MSTRG.1	3.4277	1.7772	0.0097	up	yes
MSTRG.2	3.3845	1.7589	0.0099	up	yes
MSTRG.1	3.3911	1.7618	0.0099	up	yes
MSTRG.1	3.3929	1.7625	0.0101	up	yes
MSTRG.1	3.4174	1.7729	0.0101	up	yes
MSTRG.1	3.4505	1.7868	0.0102	up	yes
MSTRG.1	3.3652	1.7507	0.0102	up	yes
MSTRG.4	3.4498	1.7865	0.0104	up	yes
MSTRG.3	3.3807	1.7573	0.0106	up	yes
MSTRG.2	3.3575	1.7474	0.0107	up	yes
MSTRG.3	3.4141	1.7715	0.0107	up	yes
MSTRG.2	3.3677	1.7518	0.0109	up	yes
MSTRG.1	3.4085	1.7691	0.011	up	yes
MSTRG.2	3.3203	1.7313	0.0111	up	yes
MSTRG.1	0.3102	-1.689	0.0111	down	yes
MSTRG.1	3.366	1.7511	0.0112	up	yes
MSTRG.2	3.301	1.7229	0.0115	up	yes
MSTRG.2	3.2962	1.7208	0.0115	up	yes
MSTRG.1	3.3097	1.7267	0.0117	up	yes
MSTRG.4	3.3624	1.7495	0.0118	up	yes
MSTRG.2	3.322	1.7321	0.0119	up	yes
MSTRG.1	3.2634	1.7064	0.0123	up	yes
MSTRG.7	3.2747	1.7113	0.0123	up	yes
MSTRG.1	3.2659	1.7075	0.0125	up	yes
MSTRG.2	3.336	1.7381	0.0125	up	yes
MSTRG.1	0.3087	-1.696	0.0126	down	yes
MSTRG.1	0.3177	-1.654	0.0126	down	yes
MSTRG.2	3.2776	1.7126	0.0127	up	yes
MSTRG.2	3.2583	1.7041	0.0128	up	yes
MSTRG.1	3.2487	1.6998	0.0128	up	yes
MSTRG.1	3.276	1.7119	0.0129	up	yes
MSTRG.2	0.32	-1.644	0.013	down	yes
MSTRG.2	3.3011	1.723	0.0131	up	yes
MSTRG.9	3.2686	1.7087	0.0133	up	yes
MSTRG.3	0.3199	-1.644	0.0133	down	yes
MSTRG.2	0.3156	-1.664	0.0136	down	yes
MSTRG.1	3.2386	1.6954	0.0136	up	yes
MSTRG.3	3.2747	1.7113	0.0136	up	yes
MSTRG.1	3.2597	1.7047	0.0137	up	yes
MSTRG.1	3.2597	1.7047	0.0137	up	yes
MSTRG.1	0.3234	-1.629	0.0139	down	yes
MSTRG.4	3.1851	1.6714	0.0141	up	yes
MSTRG.2	3.2094	1.6823	0.0141	up	yes
MSTRG.1	3.1732	1.6659	0.0143	up	yes

MSTRG.6	3.1786	1.6684	0.0143	up	yes
MSTRG.1	3.1919	1.6744	0.0146	up	yes
MSTRG.6	3.1482	1.6545	0.015	up	yes
MSTRG.1	3.1433	1.6523	0.0153	up	yes
MSTRG.1	0.3223	-1.633	0.0154	down	yes
MSTRG.2	3.1935	1.6751	0.0155	up	yes
MSTRG.1	0.3295	-1.602	0.0155	down	yes
MSTRG.1	3.1426	1.652	0.0156	up	yes
MSTRG.3	0.3266	-1.614	0.0156	down	yes
MSTRG.3	3.1571	1.6586	0.0157	up	yes
MSTRG.1	3.1922	1.6745	0.0157	up	yes
MSTRG.1	3.1465	1.6538	0.0158	up	yes
MSTRG.2	3.1686	1.6638	0.0159	up	yes
MSTRG.6	3.1136	1.6386	0.0159	up	yes
MSTRG.2	3.1375	1.6496	0.0159	up	yes
MSTRG.2	3.1408	1.6511	0.016	up	yes
MSTRG.1	0.3313	-1.594	0.016	down	yes
MSTRG.3	3.1302	1.6462	0.0161	up	yes
MSTRG.3	3.1224	1.6426	0.0161	up	yes
MSTRG.2	0.332	-1.591	0.0161	down	yes
MSTRG.8	3.1129	1.6383	0.0163	up	yes
MSTRG.1	0.3328	-1.587	0.0163	down	yes
MSTRG.5	3.1539	1.6572	0.0164	up	yes
MSTRG.1	3.1086	1.6363	0.0164	up	yes
MSTRG.2	3.1002	1.6324	0.0164	up	yes
MSTRG.1	3.1202	1.6416	0.0164	up	yes
MSTRG.2	3.1803	1.6692	0.0164	up	yes
MSTRG.8	3.1046	1.6344	0.0166	up	yes
MSTRG.1	3.117	1.6402	0.0166	up	yes
MSTRG.2	3.1207	1.6419	0.0173	up	yes
MSTRG.6	0.3365	-1.571	0.0173	down	yes
MSTRG.1	3.0752	1.6207	0.0174	up	yes
MSTRG.1	3.0898	1.6275	0.0175	up	yes
MSTRG.3	3.1192	1.6412	0.0176	up	yes
MSTRG.2	3.0947	1.6298	0.0177	up	yes
MSTRG.2	3.0765	1.6213	0.0178	up	yes
MSTRG.2	3.046	1.6069	0.018	up	yes
MSTRG.2	3.0485	1.6081	0.0182	up	yes
MSTRG.2	3.0691	1.6178	0.0183	up	yes
MSTRG.1	3.041	1.6046	0.0183	up	yes
MSTRG.9	0.3399	-1.557	0.0184	down	yes
MSTRG.3	0.332	-1.591	0.0186	down	yes
MSTRG.2	3.1132	1.6384	0.0186	up	yes
MSTRG.1	3.0449	1.6064	0.0186	up	yes
MSTRG.3	3.0167	1.593	0.0191	up	yes
MSTRG.1	3.0166	1.5929	0.0192	up	yes
MSTRG.1	0.3438	-1.54	0.0195	down	yes
MSTRG.2	0.3443	-1.538	0.0198	down	yes
MSTRG.1	3.0467	1.6073	0.02	up	yes
MSTRG.3	3.008	1.5888	0.0203	up	yes
MSTRG.2	2.977	1.5739	0.0204	up	yes
MSTRG.2	3.0085	1.589	0.0207	up	yes
MSTRG.1	2.9679	1.5694	0.0207	up	yes
MSTRG.2	2.9838	1.5772	0.0208	up	yes
MSTRG.1	2.9801	1.5754	0.0208	up	yes
MSTRG.2	0.3405	-1.554	0.0209	down	yes
MSTRG.1	0.3454	-1.534	0.021	down	yes

MSTRG.2	2.9643	1.5677	0.0212	up	yes
MSTRG.1	2.9879	1.5791	0.0214	up	yes
MSTRG.1	0.3435	-1.541	0.0216	down	yes
MSTRG.1	0.3483	-1.522	0.0216	down	yes
MSTRG.3	2.9628	1.567	0.0216	up	yes
MSTRG.1	0.3452	-1.535	0.0217	down	yes
MSTRG.1	2.9856	1.578	0.0217	up	yes
MSTRG.1	0.3431	-1.543	0.0219	down	yes
MSTRG.7	0.3477	-1.524	0.022	down	yes
MSTRG.6	2.9556	1.5635	0.022	up	yes
MSTRG.1	0.3486	-1.52	0.0221	down	yes
MSTRG.3	2.9714	1.5711	0.0227	up	yes
MSTRG.1	2.928	1.5499	0.0233	up	yes
MSTRG.1	0.3462	-1.53	0.0236	down	yes
MSTRG.1	0.3547	-1.495	0.0236	down	yes
MSTRG.3	0.3567	-1.487	0.0238	down	yes
MSTRG.1	2.9584	1.5648	0.0238	up	yes
MSTRG.4	0.3508	-1.511	0.0238	down	yes
MSTRG.1	0.3516	-1.508	0.0239	down	yes
MSTRG.1	2.9035	1.5378	0.024	up	yes
MSTRG.1	0.3567	-1.487	0.0243	down	yes
MSTRG.2	2.8753	1.5237	0.025	up	yes
MSTRG.1	2.8795	1.5258	0.0251	up	yes
MSTRG.1	2.8763	1.5242	0.0252	up	yes
MSTRG.1	0.3574	-1.484	0.0254	down	yes
MSTRG.3	0.3548	-1.495	0.0255	down	yes
MSTRG.3	0.3548	-1.495	0.0255	down	yes
MSTRG.1	2.8553	1.5136	0.0256	up	yes
MSTRG.1	2.8848	1.5285	0.0257	up	yes
MSTRG.2	2.9158	1.5439	0.026	up	yes
MSTRG.1	2.9233	1.5476	0.0263	up	yes
MSTRG.1	0.3616	-1.468	0.0263	down	yes
MSTRG.1	0.3542	-1.497	0.0264	down	yes
MSTRG.1	2.8455	1.5087	0.0268	up	yes
MSTRG.5	2.8335	1.5026	0.0268	up	yes
MSTRG.2	2.8316	1.5016	0.0269	up	yes
MSTRG.3	0.3575	-1.484	0.0272	down	yes
MSTRG.2	0.3647	-1.455	0.0273	down	yes
MSTRG.3	2.8322	1.5019	0.0273	up	yes
MSTRG.2	2.8417	1.5067	0.0274	up	yes
MSTRG.1	2.8925	1.5323	0.0275	up	yes
MSTRG.1	2.8856	1.5289	0.0277	up	yes
MSTRG.3	2.811	1.4911	0.0279	up	yes
MSTRG.3	2.881	1.5266	0.0279	up	yes
MSTRG.1	0.3675	-1.444	0.0279	down	yes
MSTRG.1	0.3638	-1.459	0.0282	down	yes
MSTRG.1	2.8061	1.4886	0.0282	up	yes
MSTRG.1	0.3681	-1.442	0.0289	down	yes
MSTRG.2	0.3652	-1.453	0.029	down	yes
MSTRG.2	0.3673	-1.445	0.0293	down	yes
MSTRG.3	0.3625	-1.464	0.0293	down	yes
MSTRG.1	2.8063	1.4887	0.0295	up	yes
MSTRG.3	0.3706	-1.432	0.0295	down	yes
MSTRG.3	2.8146	1.4929	0.0295	up	yes
MSTRG.2	2.8032	1.4871	0.0297	up	yes
MSTRG.2	2.7977	1.4843	0.03	up	yes
MSTRG.1	2.7809	1.4755	0.0305	up	yes

MSTRG.2	2.7692	1.4694	0.0306	up	yes
MSTRG.1	2.7914	1.481	0.0306	up	yes
MSTRG.1	2.7564	1.4628	0.031	up	yes
MSTRG.1	2.7585	1.4639	0.0311	up	yes
MSTRG.5	0.3703	-1.433	0.0314	down	yes
MSTRG.6	0.3713	-1.43	0.0316	down	yes
MSTRG.4	0.3731	-1.422	0.0317	down	yes
MSTRG.6	2.7345	1.4513	0.032	up	yes
MSTRG.1	2.7786	1.4744	0.0322	up	yes
MSTRG.1	0.3744	-1.417	0.0323	down	yes
MSTRG.1	2.7555	1.4623	0.0327	up	yes
MSTRG.7	0.3801	-1.395	0.0335	down	yes
MSTRG.1	2.7109	1.4388	0.0337	up	yes
MSTRG.1	0.3811	-1.392	0.0339	down	yes
MSTRG.2	2.6949	1.4302	0.0346	up	yes
MSTRG.2	0.3791	-1.399	0.0349	down	yes
MSTRG.1	2.7272	1.4474	0.035	up	yes
MSTRG.2	2.6882	1.4266	0.0354	up	yes
MSTRG.3	2.7439	1.4562	0.0354	up	yes
MSTRG.2	2.6828	1.4238	0.0359	up	yes
MSTRG.3	2.7057	1.436	0.0361	up	yes
MSTRG.1	2.7008	1.4334	0.0364	up	yes
MSTRG.1	0.3826	-1.386	0.0366	down	yes
MSTRG.2	2.72	1.4436	0.0367	up	yes
MSTRG.2	2.7118	1.4393	0.0367	up	yes
MSTRG.5	2.6611	1.412	0.0368	up	yes
MSTRG.5	0.3806	-1.394	0.0369	down	yes
MSTRG.2	2.697	1.4313	0.0372	up	yes
MSTRG.9	2.7092	1.4379	0.0373	up	yes
MSTRG.1	0.3799	-1.396	0.0375	down	yes
MSTRG.2	2.6658	1.4146	0.0375	up	yes
MSTRG.2	2.6842	1.4245	0.0377	up	yes
MSTRG.1	2.6872	1.4261	0.0379	up	yes
MSTRG.3	0.3857	-1.374	0.038	down	yes
MSTRG.3	2.7065	1.4364	0.038	up	yes
MSTRG.6	2.6732	1.4186	0.0381	up	yes
MSTRG.2	2.6813	1.4229	0.0381	up	yes
MSTRG.3	2.6425	1.4019	0.0386	up	yes
MSTRG.2	2.6332	1.3968	0.0389	up	yes
MSTRG.3	2.6504	1.4062	0.039	up	yes
MSTRG.9	2.6891	1.4271	0.039	up	yes
MSTRG.2	0.3929	-1.348	0.0396	down	yes
MSTRG.2	2.6326	1.3965	0.0398	up	yes
MSTRG.7	0.3859	-1.374	0.0399	down	yes
MSTRG.1	0.3924	-1.35	0.0402	down	yes
MSTRG.2	2.6542	1.4083	0.0403	up	yes
MSTRG.2	2.6314	1.3958	0.0405	up	yes
MSTRG.2	0.3904	-1.357	0.0406	down	yes
MSTRG.3	2.6779	1.4211	0.0411	up	yes
MSTRG.2	0.3961	-1.336	0.0412	down	yes
MSTRG.3	0.3899	-1.359	0.0416	down	yes
MSTRG.2	2.6091	1.3836	0.042	up	yes
MSTRG.3	0.396	-1.336	0.042	down	yes
MSTRG.1	0.3976	-1.331	0.0422	down	yes
MSTRG.1	0.392	-1.351	0.0423	down	yes
MSTRG.2	2.5879	1.3718	0.0424	up	yes
MSTRG.2	0.3981	-1.329	0.043	down	yes

MSTRG.1	2.5846	1.3699	0.0431	up	yes
MSTRG.3	0.3983	-1.328	0.0433	down	yes
MSTRG.3	2.6456	1.4036	0.0434	up	yes
MSTRG.1	0.393	-1.347	0.0439	down	yes
MSTRG.3	0.3898	-1.359	0.0442	down	yes
MSTRG.2	2.5692	1.3613	0.0444	up	yes
MSTRG.2	0.3995	-1.324	0.0445	down	yes
MSTRG.9	0.3946	-1.342	0.0448	down	yes
MSTRG.2	2.6151	1.3869	0.045	up	yes
MSTRG.1	2.5686	1.361	0.045	up	yes
MSTRG.2	2.5578	1.3549	0.0454	up	yes
MSTRG.3	0.4036	-1.309	0.0455	down	yes
MSTRG.1	0.4028	-1.312	0.0456	down	yes
MSTRG.1	2.5525	1.3519	0.046	up	yes
MSTRG.2	0.4043	-1.307	0.0461	down	yes
MSTRG.8	0.3992	-1.325	0.0463	down	yes
MSTRG.2	2.5526	1.352	0.0465	up	yes
MSTRG.6	2.5612	1.3568	0.0466	up	yes
MSTRG.1	0.4043	-1.307	0.0475	down	yes
MSTRG.8	2.5362	1.3427	0.0478	up	yes
MSTRG.1	0.404	-1.307	0.0478	down	yes
MSTRG.3	0.4072	-1.296	0.0478	down	yes
MSTRG.2	2.5455	1.3479	0.048	up	yes
MSTRG.1	0.4091	-1.29	0.0483	down	yes
MSTRG.1	0.4072	-1.296	0.0484	down	yes
MSTRG.2	2.5654	1.3592	0.0498	up	yes
MSTRG.8	2.5449	1.3476	0.0499	up	yes
