# Supplementary material for: Transcriptome analysis reveals a potential regulatory mechanism of the lnc-5423.6/IGFBP5 axis in the early stages of mouse thymic involution: lnc-5423.6/IGFBP5 axis regulates thymic involution
Source: Acta Biochim Biophys Sin (Shanghai). 2023 Apr 19;55(4):548–60. doi: 10.3724/abbs.2023042 (PMC10195152; doi:10.3724/abbs.2023042)
Supplement: Table_S1 [file Table_S1.pdf]

Supplementary Table S1. Specific primer sequences for qPCR

Gene	Forward primer (5'-3')	Reverse primer (5'-3')
Lnc-1674.1	CTAGTCCAGTACAGCCACAAAG	GCACCTGCTCCACCTATTT
Lnc-115107	AAGAGAGCAAAGCGGTGTAG	GCAGTTAAGAGACTCCCGAAAG
Lnc-195025	AGGTCTGTTCTGTGGGTAGA	GTAGTTCGTGGGAAGCCTATAC
Lnc-1882.1	CGTGGTGAAGTCTCTCTTTCTC	ACCAGCTGACAAACCTCTTC
Lnc-201493	GAGGGTAATAAGGGAAGACAAGG	GCCATTGAGGAACCCATACA
Lnc-5459.3	ATCCGGAGTGCAATGGATAAG	CGGGAAACTCGACTGCATAA
Lnc-177363	CTTGCAGTCTGATTGGGTAGAA	ACCCTCTGCGGTTCATAAATAC
Lnc-4777.3	TGGATGTTGTAATCAGAGAGAGTG	GAAGGCCAAGATCCAGGATAAA
Lnc-219154	AGCGTTTGGAGGCTGATTAT	CTACACATGGTCTGGTGAGATG
Lnc-194215	CTGCACTGCTTTCTTGCTTAC	CATCTGCCACCAAGGAGATATT
Car3	GACAACCAGTCACCCATTGA	GTTCAGGATGGTCTTAGCAGAG
Scd1	AGAGAGAGAGAGAGAGAGAGAGA	GGTTCAGAGGATGGACAGAAAG
Igkc	AGGACGAGTATGAACGACATAAC	GTCTCAGGACCTTTGTCTCTAAC
Jchain	ACCCTTTCCTTCCTTCCTTTG	TCCTGTGTTCATTGGCTGTT
Igkv1-135	CTCCAAAGCGCCTAATCTATCT	GCCTCCACTCTGCTGATTT
Tnxb	CCTGGAATCGAACCACAGATAC	GACTCCTCTTCCTCCTCTTCTT
Vldlr	GCCGAGTCTGATCTTCACTAATC	CCGTGTTCCTTAGTTGCTCTAC
Csrp1	ACCTTACCCTCCTCTGTTTCTA	GGTCTCCTGCTTTGCTCTATT
Scd2	CACCTGTCTCTTCGCGTATTT	CCGTGCCTTGTATGTTCTGT
E2f3	CAGAAGACACCGGCACTTTA	AGGCTGAAGAGAGAGGAAGATA
IGFBP5	TGAAGAAGGACCGCAGAAAG	GTTCGGATTCCTGTCTCATCTC
p53	GCCATGGCCATCTACAAGAA	AATTTCCTTCCACCCGGATAAG
p21	AAGTGTGCCGTTGTCTCTTC	AGTCAAAGTTCCACCGTTCTC
Bax	GTGGTTGCCCTCTTCTACTTT	CAGCCCATGATGGTTCTGAT
Cyclin B1	GGTGTAACGGCCATGTTTATTG	CTGTCTGATCTGGTGCTTAGTG
Cyclin D1	CAGAGGCGGATGAGAACAAG	GAGGGTGGGTTGGAAATGAA
β-actin	CATCCGTAAAGACCTCTATGCCAC	ATGGAGCCACCGATCCACA
