# Supplementary material for: Transcriptome analysis reveals a potential regulatory mechanism of the lnc-5423.6/IGFBP5 axis in the early stages of mouse thymic involution: lnc-5423.6/IGFBP5 axis regulates thymic involution
Source: Acta Biochim Biophys Sin (Shanghai). 2023 Apr 19;55(4):548–60. doi: 10.3724/abbs.2023042 (PMC10195152; doi:10.3724/abbs.2023042)
Supplement: Table_S13 [file Table_S13.pdf]

t_name	fc	log2(fc	pval	regulat	significant
ENSMUST	34.522	5.1095	5E-11	up	yes
ENSMUST	18.494	4.209	2E-08	up	yes
ENSMUST	0.0801	-3.641	6E-07	down	yes
ENSMUST	0.0979	-3.352	4E-06	down	yes
ENSMUST	0.1389	-2.848	6E-05	down	yes
ENSMUST	6.9221	2.7912	7E-05	up	yes
ENSMUST	0.148	-2.757	9E-05	down	yes
ENSMUST	0.1779	-2.491	0.0003	down	yes
ENSMUST	0.1773	-2.495	0.0004	down	yes
ENSMUST	5.5135	2.463	0.0004	up	yes
ENSMUST	0.1823	-2.456	0.0005	down	yes
ENSMUST	5.3316	2.4146	0.0005	up	yes
ENSMUST	5.0102	2.3249	0.0008	up	yes
ENSMUST	0.2097	-2.253	0.0013	down	yes
ENSMUST	0.2255	-2.149	0.0019	down	yes
ENSMUST	0.2342	-2.094	0.0022	down	yes
ENSMUST	4.3018	2.1049	0.0022	up	yes
ENSMUST	4.2705	2.0944	0.0023	up	yes
ENSMUST	0.2331	-2.101	0.0024	down	yes
ENSMUST	0.2348	-2.091	0.0028	down	yes
ENSMUST	0.2408	-2.054	0.0029	down	yes
ENSMUST	4.024	2.0086	0.0033	up	yes
ENSMUST	0.2808	-1.832	0.0072	down	yes
ENSMUST	3.5492	1.8275	0.0075	up	yes
ENSMUST	3.3225	1.7323	0.0103	up	yes
ENSMUST	3.2747	1.7114	0.0123	up	yes
ENSMUST	3.1224	1.6427	0.0157	up	yes
ENSMUST	3.0864	1.6259	0.0163	up	yes
ENSMUST	0.3255	-1.619	0.0164	down	yes
ENSMUST	3.0502	1.6089	0.0172	up	yes
ENSMUST	2.9498	1.5606	0.0195	up	yes
ENSMUST	2.9091	1.5406	0.0212	up	yes
ENSMUST	2.9286	1.5502	0.022	up	yes
ENSMUST	2.8759	1.524	0.0226	up	yes
ENSMUST	0.3589	-1.479	0.0292	down	yes
ENSMUST	2.7589	1.4641	0.0302	up	yes
ENSMUST	2.72	1.4436	0.0306	up	yes
ENSMUST	2.6702	1.4169	0.0336	up	yes
ENSMUST	2.7043	1.4352	0.0353	up	yes
ENSMUST	0.3708	-1.431	0.0373	down	yes
ENSMUST	0.3711	-1.43	0.0374	down	yes
ENSMUST	0.3921	-1.351	0.046	down	yes
ENSMUST	2.5018	1.323	0.0495	up	yes
MSTRG.1	146.95	7.1992	4E-17	up	yes
MSTRG.8	0.0201	-5.638	2E-12	down	yes
MSTRG.1	0.0199	-5.65	2E-12	down	yes
MSTRG.6	47.516	5.5703	3E-12	up	yes
MSTRG.7	46.167	5.5288	3E-12	up	yes
MSTRG.7	43.635	5.4474	6E-12	up	yes
MSTRG.1	0.025	-5.323	2E-11	down	yes
MSTRG.5	32.01	5.0004	1E-10	up	yes
MSTRG.1	28.347	4.8251	3E-10	up	yes
MSTRG.1	0.0367	-4.766	6E-10	down	yes
MSTRG.1	0.0376	-4.733	6E-10	down	yes
MSTRG.1	0.0414	-4.595	1E-09	down	yes
MSTRG.3	0.0436	-4.521	3E-09	down	yes

MSTRG.8	0.0477	-4.391	7E-09	down	yes
MSTRG.1	19.639	4.2957	9E-09	up	yes
MSTRG.3	0.053	-4.238	1E-08	down	yes
MSTRG.2	18.969	4.2456	2E-08	up	yes
MSTRG.5	0.0613	-4.027	5E-08	down	yes
MSTRG.1	14.005	3.8079	2E-07	up	yes
MSTRG.2	13.66	3.7719	3E-07	up	yes
MSTRG.3	12.941	3.6938	4E-07	up	yes
MSTRG.4	12.99	3.6993	5E-07	up	yes
MSTRG.1	12.197	3.6084	6E-07	up	yes
MSTRG.1	12.342	3.6255	6E-07	up	yes
MSTRG.1	12.026	3.5881	7E-07	up	yes
MSTRG.5	11.756	3.5554	9E-07	up	yes
MSTRG.2	11.524	3.5266	1E-06	up	yes
MSTRG.2	11.085	3.4705	1E-06	up	yes
MSTRG.7	10.201	3.3506	3E-06	up	yes
MSTRG.2	10.123	3.3396	3E-06	up	yes
MSTRG.8	0.0979	-3.352	4E-06	down	yes
MSTRG.1	9.6597	3.272	5E-06	up	yes
MSTRG.1	0.1039	-3.267	6E-06	down	yes
MSTRG.1	0.1065	-3.23	6E-06	down	yes
MSTRG.1	0.104	-3.265	7E-06	down	yes
MSTRG.1	9.0151	3.1723	8E-06	up	yes
MSTRG.6	0.1108	-3.173	8E-06	down	yes
MSTRG.3	0.1117	-3.162	1E-05	down	yes
MSTRG.3	8.4866	3.0852	1E-05	up	yes
MSTRG.4	8.3415	3.0603	2E-05	up	yes
MSTRG.2	8.0577	3.0104	2E-05	up	yes
MSTRG.8	0.1246	-3.004	2E-05	down	yes
MSTRG.1	0.1235	-3.018	2E-05	down	yes
MSTRG.3	0.1271	-2.976	3E-05	down	yes
MSTRG.1	7.5995	2.9259	3E-05	up	yes
MSTRG.2	0.1306	-2.937	4E-05	down	yes
MSTRG.2	7.5552	2.9175	4E-05	up	yes
MSTRG.5	7.1046	2.8288	5E-05	up	yes
MSTRG.4	7.0405	2.8157	5E-05	up	yes
MSTRG.8	0.1494	-2.743	0.0001	down	yes
MSTRG.9	0.1546	-2.693	0.0001	down	yes
MSTRG.1	0.1563	-2.678	0.0001	down	yes
MSTRG.3	6.29	2.6531	0.0001	up	yes
MSTRG.2	6.2683	2.6481	0.0001	up	yes
MSTRG.2	5.9606	2.5754	0.0002	up	yes
MSTRG.2	5.9118	2.5636	0.0002	up	yes
MSTRG.2	5.669	2.5031	0.0003	up	yes
MSTRG.3	0.1741	-2.522	0.0003	down	yes
MSTRG.8	5.7123	2.5141	0.0003	up	yes
MSTRG.1	5.5665	2.4768	0.0003	up	yes
MSTRG.6	0.1777	-2.493	0.0003	down	yes
MSTRG.1	0.1733	-2.529	0.0003	down	yes
MSTRG.1	0.1819	-2.459	0.0004	down	yes
MSTRG.2	5.3941	2.4314	0.0004	up	yes
MSTRG.1	5.4807	2.4544	0.0004	up	yes
MSTRG.2	0.1839	-2.443	0.0004	down	yes
MSTRG.4	0.1819	-2.459	0.0004	down	yes
MSTRG.2	0.186	-2.427	0.0005	down	yes
MSTRG.1	5.1987	2.3781	0.0005	up	yes
MSTRG.1	0.1866	-2.422	0.0005	down	yes

MSTRG.3	0.1898	-2.398	0.0005	down	yes
MSTRG.2	5.1395	2.3616	0.0006	up	yes
MSTRG.6	5.1928	2.3765	0.0007	up	yes
MSTRG.1	5.023	2.3285	0.0007	up	yes
MSTRG.2	0.1956	-2.354	0.0007	down	yes
MSTRG.9	0.1939	-2.366	0.0007	down	yes
MSTRG.5	0.1982	-2.335	0.0007	down	yes
MSTRG.6	0.1979	-2.337	0.0007	down	yes
MSTRG.5	5.0841	2.346	0.0008	up	yes
MSTRG.1	0.1948	-2.36	0.0008	down	yes
MSTRG.4	4.8309	2.2723	0.0009	up	yes
MSTRG.1	0.2067	-2.274	0.001	down	yes
MSTRG.2	0.2	-2.322	0.001	down	yes
MSTRG.9	4.908	2.2951	0.001	up	yes
MSTRG.2	4.6939	2.2308	0.0011	up	yes
MSTRG.5	4.5615	2.1895	0.0013	up	yes
MSTRG.1	0.2181	-2.197	0.0013	down	yes
MSTRG.2	4.5478	2.1852	0.0014	up	yes
MSTRG.4	4.4872	2.1658	0.0015	up	yes
MSTRG.3	4.3807	2.1312	0.0017	up	yes
MSTRG.3	4.2785	2.0971	0.002	up	yes
MSTRG.3	4.3737	2.1289	0.002	up	yes
MSTRG.1	4.2283	2.0801	0.0021	up	yes
MSTRG.2	4.3261	2.1131	0.0021	up	yes
MSTRG.2	4.2219	2.0779	0.0022	up	yes
MSTRG.5	4.232	2.0814	0.0022	up	yes
MSTRG.2	0.2312	-2.113	0.0024	down	yes
MSTRG.5	4.1201	2.0427	0.0026	up	yes
MSTRG.3	4.1956	2.0689	0.0027	up	yes
MSTRG.1	4.0697	2.0249	0.003	up	yes
MSTRG.1	4.0027	2.001	0.003	up	yes
MSTRG.7	4.0496	2.0178	0.0031	up	yes
MSTRG.2	4.0571	2.0205	0.0032	up	yes
MSTRG.3	3.9749	1.9909	0.0033	up	yes
MSTRG.1	3.9891	1.9961	0.0033	up	yes
MSTRG.3	3.9208	1.9712	0.0035	up	yes
MSTRG.3	3.9273	1.9735	0.0035	up	yes
MSTRG.3	3.9024	1.9644	0.0036	up	yes
MSTRG.3	0.2534	-1.981	0.0037	down	yes
MSTRG.1	3.8273	1.9363	0.0041	up	yes
MSTRG.1	0.2581	-1.954	0.0041	down	yes
MSTRG.3	0.2549	-1.972	0.0042	down	yes
MSTRG.3	3.8261	1.9359	0.0044	up	yes
MSTRG.2	3.8246	1.9353	0.0046	up	yes
MSTRG.3	3.7343	1.9008	0.0048	up	yes
MSTRG.4	3.7037	1.889	0.0049	up	yes
MSTRG.3	3.7848	1.9202	0.005	up	yes
MSTRG.1	3.7632	1.9119	0.0052	up	yes
MSTRG.2	0.2663	-1.909	0.0052	down	yes
MSTRG.3	0.2703	-1.887	0.0055	down	yes
MSTRG.7	0.2723	-1.877	0.0057	down	yes
MSTRG.9	3.6653	1.8739	0.0058	up	yes
MSTRG.1	0.2739	-1.868	0.0059	down	yes
MSTRG.3	3.5746	1.8378	0.0063	up	yes
MSTRG.4	3.6508	1.8682	0.0063	up	yes
MSTRG.1	0.2783	-1.845	0.0068	down	yes
MSTRG.2	0.2827	-1.823	0.0072	down	yes

MSTRG.5	3.4622	1.7917	0.0076	up	yes
MSTRG.3	0.2879	-1.796	0.0081	down	yes
MSTRG.1	0.286	-1.806	0.0081	down	yes
MSTRG.2	0.291	-1.781	0.0091	down	yes
MSTRG.3	3.3388	1.7393	0.0098	up	yes
MSTRG.3	3.2407	1.6963	0.0111	up	yes
MSTRG.1	3.2654	1.7072	0.0112	up	yes
MSTRG.1	3.2263	1.6899	0.0113	up	yes
MSTRG.1	3.2802	1.7138	0.0113	up	yes
MSTRG.1	0.306	-1.708	0.0115	down	yes
MSTRG.1	3.2026	1.6792	0.012	up	yes
MSTRG.1	0.3053	-1.712	0.012	down	yes
MSTRG.1	0.3006	-1.734	0.0123	down	yes
MSTRG.1	0.3101	-1.689	0.0124	down	yes
MSTRG.1	3.2025	1.6792	0.0126	up	yes
MSTRG.4	0.3128	-1.677	0.0131	down	yes
MSTRG.3	3.2286	1.6909	0.0132	up	yes
MSTRG.2	0.3117	-1.682	0.0133	down	yes
MSTRG.6	0.3077	-1.701	0.0134	down	yes
MSTRG.3	3.1377	1.6497	0.0138	up	yes
MSTRG.2	3.1131	1.6384	0.0142	up	yes
MSTRG.6	0.3091	-1.694	0.0143	down	yes
MSTRG.2	3.1191	1.6411	0.0146	up	yes
MSTRG.3	0.3205	-1.642	0.0153	down	yes
MSTRG.2	0.321	-1.639	0.0153	down	yes
MSTRG.1	3.0368	1.6025	0.0161	up	yes
MSTRG.1	3.056	1.6116	0.0162	up	yes
MSTRG.3	3.0713	1.6188	0.0164	up	yes
MSTRG.2	3.0539	1.6107	0.018	up	yes
MSTRG.1	0.3333	-1.585	0.0186	down	yes
MSTRG.3	2.9972	1.5836	0.0188	up	yes
MSTRG.5	2.953	1.5622	0.0191	up	yes
MSTRG.1	2.977	1.5738	0.0196	up	yes
MSTRG.1	0.335	-1.578	0.0196	down	yes
MSTRG.1	0.3347	-1.579	0.0198	down	yes
MSTRG.6	2.9708	1.5708	0.0201	up	yes
MSTRG.1	2.9445	1.558	0.0202	up	yes
MSTRG.2	2.9869	1.5787	0.0203	up	yes
MSTRG.1	2.9311	1.5515	0.0205	up	yes
MSTRG.9	2.9027	1.5374	0.0206	up	yes
MSTRG.6	2.9227	1.5473	0.0208	up	yes
MSTRG.2	2.9071	1.5396	0.0208	up	yes
MSTRG.9	2.9105	1.5413	0.0209	up	yes
MSTRG.1	2.896	1.534	0.021	up	yes
MSTRG.9	2.9334	1.5526	0.0214	up	yes
MSTRG.4	0.3422	-1.547	0.0217	down	yes
MSTRG.1	0.3447	-1.536	0.0226	down	yes
MSTRG.1	2.858	1.515	0.0227	up	yes
MSTRG.1	2.9045	1.5383	0.0229	up	yes
MSTRG.1	2.912	1.542	0.0231	up	yes
MSTRG.4	2.8495	1.5107	0.0232	up	yes
MSTRG.5	2.846	1.5089	0.0238	up	yes
MSTRG.6	0.3504	-1.513	0.0245	down	yes
MSTRG.1	2.8325	1.5021	0.0245	up	yes
MSTRG.9	2.8284	1.5	0.0245	up	yes
MSTRG.1	0.3435	-1.542	0.0247	down	yes
MSTRG.7	0.3501	-1.514	0.0247	down	yes

MSTRG.6	0.3407	-1.553	0.0249	down	yes
MSTRG.4	2.8187	1.495	0.025	up	yes
MSTRG.9	2.8279	1.4997	0.025	up	yes
MSTRG.2	0.3519	-1.507	0.0258	down	yes
MSTRG.2	2.7989	1.4849	0.0258	up	yes
MSTRG.6	0.3495	-1.517	0.026	down	yes
MSTRG.5	2.786	1.4782	0.0262	up	yes
MSTRG.3	2.806	1.4885	0.0266	up	yes
MSTRG.1	2.773	1.4715	0.0268	up	yes
MSTRG.4	2.8171	1.4942	0.0269	up	yes
MSTRG.2	0.3567	-1.487	0.0269	down	yes
MSTRG.2	0.357	-1.486	0.027	down	yes
MSTRG.2	0.3586	-1.48	0.0277	down	yes
MSTRG.9	0.3588	-1.479	0.0278	down	yes
MSTRG.1	2.7548	1.4619	0.0278	up	yes
MSTRG.1	2.7746	1.4723	0.0279	up	yes
MSTRG.3	0.3597	-1.475	0.0281	down	yes
MSTRG.1	0.3555	-1.492	0.0281	down	yes
MSTRG.1	0.3599	-1.474	0.0282	down	yes
MSTRG.1	0.3573	-1.485	0.0286	down	yes
MSTRG.9	2.7293	1.4486	0.0291	up	yes
MSTRG.1	0.3558	-1.491	0.0295	down	yes
MSTRG.3	0.3527	-1.504	0.0296	down	yes
MSTRG.3	2.7335	1.4508	0.0298	up	yes
MSTRG.4	2.6984	1.4321	0.0318	up	yes
MSTRG.3	2.7483	1.4586	0.032	up	yes
MSTRG.1	2.7061	1.4362	0.0323	up	yes
MSTRG.6	2.7125	1.4396	0.0325	up	yes
MSTRG.2	0.3662	-1.449	0.0325	down	yes
MSTRG.3	2.7382	1.4532	0.0328	up	yes
MSTRG.3	2.6703	1.417	0.0328	up	yes
MSTRG.3	2.6637	1.4134	0.033	up	yes
MSTRG.2	2.6888	1.427	0.0333	up	yes
MSTRG.3	0.3627	-1.463	0.0336	down	yes
MSTRG.1	2.6791	1.4217	0.0339	up	yes
MSTRG.2	0.3669	-1.446	0.0339	down	yes
MSTRG.2	2.6923	1.4288	0.035	up	yes
MSTRG.7	2.6912	1.4282	0.0353	up	yes
MSTRG.9	2.6393	1.4002	0.0356	up	yes
MSTRG.9	2.6193	1.3892	0.0356	up	yes
MSTRG.1	2.6547	1.4085	0.0361	up	yes
MSTRG.8	0.3776	-1.405	0.0362	down	yes
MSTRG.4	0.3783	-1.402	0.0367	down	yes
MSTRG.1	2.6079	1.3829	0.0369	up	yes
MSTRG.5	2.6481	1.4049	0.0379	up	yes
MSTRG.3	2.605	1.3813	0.0385	up	yes
MSTRG.1	0.3783	-1.402	0.0386	down	yes
MSTRG.1	2.5787	1.3667	0.0387	up	yes
MSTRG.6	2.5804	1.3676	0.0387	up	yes
MSTRG.3	2.5804	1.3676	0.0388	up	yes
MSTRG.3	2.5759	1.3651	0.039	up	yes
MSTRG.1	2.5624	1.3575	0.0398	up	yes
MSTRG.2	0.3798	-1.397	0.0401	down	yes
MSTRG.3	2.5619	1.3572	0.0402	up	yes
MSTRG.1	2.6071	1.3824	0.0404	up	yes
MSTRG.7	2.5799	1.3673	0.0408	up	yes
MSTRG.1	2.5629	1.3578	0.0411	up	yes

MSTRG.2	2.5761	1.3652	0.0412	up	yes
MSTRG.3	0.3873	-1.368	0.0417	down	yes
MSTRG.1	0.3847	-1.378	0.0419	down	yes
MSTRG.4	2.54	1.3448	0.042	up	yes
MSTRG.9	2.5369	1.3431	0.0424	up	yes
MSTRG.9	2.5316	1.3401	0.0431	up	yes
MSTRG.2	2.5209	1.334	0.0432	up	yes
MSTRG.1	2.5257	1.3367	0.0433	up	yes
MSTRG.1	2.5145	1.3303	0.0439	up	yes
MSTRG.2	0.3927	-1.349	0.044	down	yes
MSTRG.3	0.3859	-1.374	0.044	down	yes
MSTRG.1	2.5607	1.3565	0.0448	up	yes
MSTRG.3	0.39	-1.358	0.0452	down	yes
MSTRG.9	2.5121	1.3289	0.0453	up	yes
MSTRG.1	2.4963	1.3198	0.0455	up	yes
MSTRG.2	0.3896	-1.36	0.046	down	yes
MSTRG.2	0.3961	-1.336	0.0463	down	yes
MSTRG.3	2.5422	1.3461	0.047	up	yes
MSTRG.3	2.4794	1.31	0.0473	up	yes
MSTRG.1	0.3959	-1.337	0.0474	down	yes
MSTRG.7	2.4805	1.3107	0.0475	up	yes
MSTRG.1	2.5021	1.3231	0.0478	up	yes
MSTRG.6	2.4644	1.3013	0.0487	up	yes
MSTRG.1	2.4783	1.3094	0.0492	up	yes
