# Supplementary material for: Transcriptome analysis reveals a potential regulatory mechanism of the lnc-5423.6/IGFBP5 axis in the early stages of mouse thymic involution: lnc-5423.6/IGFBP5 axis regulates thymic involution
Source: Acta Biochim Biophys Sin (Shanghai). 2023 Apr 19;55(4):548–60. doi: 10.3724/abbs.2023042 (PMC10195152; doi:10.3724/abbs.2023042)
Supplement: Table_S3 [file Table_S3.pdf]

gene_na	fc	log2(fc	pval	regulat	significant
Igkv15-	18.10	4.18	0.00	up	yes
Inafm2	0.08	-3.66	0.00	down	yes
Gm24447	0.09	-3.54	0.00	down	yes
Etohd2	12.20	3.61	0.00	up	yes
Nek6	9.84	3.30	0.00	up	yes
Glp1r	0.12	-3.07	0.00	down	yes
Cyp2e1	9.24	3.21	0.00	up	yes
Bend4	8.55	3.10	0.00	up	yes
Vldlr	8.58	3.10	0.00	up	yes
Car3	7.88	2.98	0.00	up	yes
Zfp422	0.14	-2.83	0.00	down	yes
Kcnj13	7.52	2.91	0.00	up	yes
Iglj2;I	7.40	2.89	0.00	up	yes
Pkd2l2	7.11	2.83	0.00	up	yes
Cd33	6.99	2.80	0.00	up	yes
Gm24265	0.16	-2.65	0.00	down	yes
Kirrel	6.97	2.80	0.00	up	yes
Gm24407	0.16	-2.64	0.00	down	yes
Gm22042	0.16	-2.64	0.00	down	yes
Ighg3	6.95	2.80	0.00	up	yes
Cyp2f2	6.80	2.76	0.00	up	yes
Jchain	6.52	2.70	0.00	up	yes
Gm22614	0.17	-2.53	0.00	down	yes
Igkc	6.23	2.64	0.00	up	yes
Pdp1	0.17	-2.54	0.00	down	yes
Gm21905	0.18	-2.49	0.00	down	yes
C4b	6.12	2.61	0.00	up	yes
Igfbp5	6.06	2.60	0.00	up	yes
Avpr2	0.17	-2.52	0.00	down	yes
Samd3	6.16	2.62	0.00	up	yes
Dnah14	0.18	-2.48	0.00	down	yes
Oas2	5.89	2.56	0.00	up	yes
Rnu1b6	0.19	-2.39	0.00	down	yes
Gm24830	0.19	-2.38	0.00	down	yes
Mettl21	0.19	-2.36	0.00	down	yes
Snord15	0.20	-2.35	0.00	down	yes
1810062	5.53	2.47	0.00	up	yes
Scn7a	5.54	2.47	0.00	up	yes
Igkv1-1	5.42	2.44	0.00	up	yes
Klrk1	5.38	2.43	0.00	up	yes
Gm24305	0.20	-2.30	0.00	down	yes
Tns4	5.39	2.43	0.00	up	yes
Scd1	5.31	2.41	0.00	up	yes
Rnu1b2	0.20	-2.29	0.00	down	yes
Zfp458	0.20	-2.29	0.00	down	yes
Prom2	5.38	2.43	0.00	up	yes
Rnf24	5.28	2.40	0.00	up	yes
Six2	5.30	2.41	0.00	up	yes
Gm26232	0.21	-2.26	0.00	down	yes
Rnu1b1	0.21	-2.26	0.00	down	yes
A730017	5.32	2.41	0.00	up	yes
Bfsp2	0.22	-2.21	0.00	down	yes
Tlr1	5.07	2.34	0.00	up	yes
Gm13443	0.22	-2.19	0.00	down	yes
Rbfox1	4.97	2.31	0.00	up	yes
Apod	4.98	2.32	0.00	up	yes

Rnf26	4.84	2.28	0.00	up	yes
Slc16a2	4.84	2.28	0.00	up	yes
Fcgbp	4.61	2.21	0.00	up	yes
Aldh1a3	4.67	2.22	0.00	up	yes
Kcnc1	4.64	2.21	0.00	up	yes
Fmo2	4.62	2.21	0.00	up	yes
Eif4h	0.24	-2.05	0.00	down	yes
Gm23804	0.24	-2.05	0.00	down	yes
Adamdec	4.52	2.18	0.00	up	yes
Kcnj15	4.50	2.17	0.00	up	yes
Deptor	4.50	2.17	0.00	up	yes
Six1	4.47	2.16	0.00	up	yes
Gm22513	0.24	-2.06	0.00	down	yes
Tmem245	0.25	-2.01	0.00	down	yes
Mid2	4.41	2.14	0.00	up	yes
Trpm6	4.44	2.15	0.00	up	yes
Gxylt2	4.40	2.14	0.00	up	yes
Gm8113	4.28	2.10	0.00	up	yes
Il1b	4.40	2.14	0.00	up	yes
Ccl11	4.31	2.11	0.00	up	yes
Prelp	4.28	2.10	0.00	up	yes
Igkv1-1	4.31	2.11	0.00	up	yes
Aebp1	4.30	2.11	0.00	up	yes
Gm24497	0.26	-1.96	0.00	down	yes
Mir466i	0.26	-1.95	0.00	down	yes
Crtam	4.29	2.10	0.00	up	yes
Fmod	4.30	2.10	0.00	up	yes
Nfam1	4.20	2.07	0.00	up	yes
Gm25939	0.26	-1.92	0.00	down	yes
Gm25628	0.26	-1.92	0.00	down	yes
Scn1a	4.15	2.05	0.00	up	yes
Degs1	4.11	2.04	0.00	up	yes
Gm23472	0.27	-1.91	0.00	down	yes
Slc25a5	4.09	2.03	0.00	up	yes
Gm23238	0.27	-1.90	0.00	down	yes
Il33	4.13	2.04	0.00	up	yes
Lrig3	4.14	2.05	0.00	up	yes
Ighg1	4.07	2.02	0.00	up	yes
Sv2b	4.11	2.04	0.00	up	yes
Samd10	0.27	-1.90	0.00	down	yes
Gm25890	0.27	-1.87	0.00	down	yes
Drap1;E	0.27	-1.87	0.00	down	yes
Cyp27a1	4.09	2.03	0.00	up	yes
Setbp1	3.98	1.99	0.00	up	yes
Plcxd1	0.27	-1.88	0.00	down	yes
Gm13237	0.27	-1.87	0.00	down	yes
Cxcr3	3.99	2.00	0.00	up	yes
1700019	3.97	1.99	0.00	up	yes
Gm8797	0.27	-1.89	0.00	down	yes
Tuba3a	0.28	-1.84	0.01	down	yes
Vaultrc	0.28	-1.85	0.01	down	yes
Gas1	3.91	1.97	0.01	up	yes
Zbtb42	3.96	1.99	0.01	up	yes
Rbpj	0.28	-1.83	0.01	down	yes
Gm18194	3.91	1.97	0.01	up	yes
Gm22317	0.28	-1.82	0.01	down	yes
Blk	3.87	1.95	0.01	up	yes

Amotl2	3.90	1.96	0.01	up	yes
Gm7609	3.94	1.98	0.01	up	yes
Rbm3-ps	0.28	-1.82	0.01	down	yes
Tmem9b	3.82	1.93	0.01	up	yes
Tnfsf8	3.82	1.93	0.01	up	yes
Bpifa1	3.82	1.93	0.01	up	yes
Bank1	3.84	1.94	0.01	up	yes
Gm23444	0.29	-1.78	0.01	down	yes
Eomes	3.74	1.90	0.01	up	yes
Gm26316	0.29	-1.77	0.01	down	yes
Dnase1l	3.72	1.90	0.01	up	yes
Gm18537	0.29	-1.78	0.01	down	yes
Slc27a3	3.73	1.90	0.01	up	yes
Trabd2b	3.77	1.91	0.01	up	yes
Sidt1	3.69	1.88	0.01	up	yes
Spock2	3.68	1.88	0.01	up	yes
Actg-ps	0.29	-1.79	0.01	down	yes
Fam46a	3.70	1.89	0.01	up	yes
Dcn	3.67	1.87	0.01	up	yes
Dnah5	3.69	1.88	0.01	up	yes
CT00954	0.29	-1.77	0.01	down	yes
Rnu12	0.30	-1.74	0.01	down	yes
Abcb1a	3.63	1.86	0.01	up	yes
Phxr2	0.30	-1.72	0.01	down	yes
Svep1	3.62	1.85	0.01	up	yes
Sdk2	3.58	1.84	0.01	up	yes
Hspa1a	3.58	1.84	0.01	up	yes
Orm3;Or	3.53	1.82	0.01	up	yes
AC15782	0.31	-1.69	0.01	down	yes
Antxr1	3.51	1.81	0.01	up	yes
Fcgr2b	3.52	1.81	0.01	up	yes
Elovl7	3.56	1.83	0.01	up	yes
Pgr	3.56	1.83	0.01	up	yes
Il23r	3.54	1.82	0.01	up	yes
Rxfp1	0.31	-1.70	0.01	down	yes
Kcnip3	3.53	1.82	0.01	up	yes
Fer1l5	0.31	-1.70	0.01	down	yes
Gm4070;	3.45	1.79	0.01	up	yes
Mmp28	3.47	1.79	0.01	up	yes
Shisa2	3.43	1.78	0.01	up	yes
Plch2	3.46	1.79	0.01	up	yes
Ophn1	3.52	1.81	0.01	up	yes
Iglc1;I	3.42	1.78	0.01	up	yes
Gm11427	0.32	-1.65	0.01	down	yes
Ace	3.42	1.77	0.01	up	yes
Rhobtb1	3.42	1.77	0.01	up	yes
Mgl2	3.48	1.80	0.01	up	yes
Stag3	0.32	-1.65	0.01	down	yes
Cd300lf	3.41	1.77	0.01	up	yes
Csmd1	3.36	1.75	0.01	up	yes
Gm13680	0.32	-1.63	0.01	down	yes
Cyp2d22	3.39	1.76	0.01	up	yes
Gprin3	3.32	1.73	0.01	up	yes
Zfp964	3.38	1.76	0.01	up	yes
March8	3.34	1.74	0.01	up	yes
Kcnh1	3.34	1.74	0.01	up	yes
Kctd12	3.28	1.72	0.01	up	yes

Siglec1	3.30	1.72	0.01	up	yes
Gm12286	0.33	-1.62	0.01	down	yes
Pla2g2d	3.28	1.71	0.01	up	yes
Gab1	3.29	1.72	0.01	up	yes
Tfap2a	3.39	1.76	0.01	up	yes
Klhl32	3.30	1.72	0.01	up	yes
Hspa1b	3.27	1.71	0.01	up	yes
Snx21	0.33	-1.59	0.01	down	yes
Adh1	3.31	1.73	0.02	up	yes
Gm22146	3.22	1.69	0.02	up	yes
Clca2	3.30	1.72	0.02	up	yes
Hspb1	3.26	1.71	0.02	up	yes
Rnu2-10	0.34	-1.56	0.02	down	yes
Rpsa;Gm	3.18	1.67	0.02	up	yes
Plppr4	3.22	1.69	0.02	up	yes
Gm13167	0.34	-1.55	0.02	down	yes
Slc39a3	0.34	-1.55	0.02	down	yes
Rtp4	3.20	1.68	0.02	up	yes
Fzd1	3.21	1.68	0.02	up	yes
Itgam	3.22	1.69	0.02	up	yes
BC00553	3.14	1.65	0.02	up	yes
Cbll1	3.15	1.65	0.02	up	yes
Arl4c	3.14	1.65	0.02	up	yes
Gm11605	0.34	-1.56	0.02	down	yes
Slc1a3	3.14	1.65	0.02	up	yes
Ms4a6c	3.13	1.65	0.02	up	yes
Golm1	3.15	1.66	0.02	up	yes
AC15417	0.34	-1.54	0.02	down	yes
Cped1	3.19	1.67	0.02	up	yes
Tube1	0.35	-1.52	0.02	down	yes
Gm24950	0.35	-1.52	0.02	down	yes
Ighv2-2	3.20	1.68	0.02	up	yes
Gm22009	0.35	-1.52	0.02	down	yes
Abca5	3.18	1.67	0.02	up	yes
Gpc4	3.13	1.65	0.02	up	yes
Mill1	3.13	1.64	0.02	up	yes
Smoc2	3.12	1.64	0.02	up	yes
Fat2	3.10	1.63	0.02	up	yes
Ikzf3;M	0.35	-1.51	0.02	down	yes
Kif1a	3.15	1.65	0.02	up	yes
Cxcr5	3.15	1.66	0.02	up	yes
Chst1	3.15	1.65	0.02	up	yes
F830016	3.12	1.64	0.02	up	yes
Thrsp	3.12	1.64	0.02	up	yes
Osmr	3.07	1.62	0.02	up	yes
Rn7s6	0.36	-1.49	0.02	down	yes
Nkg7	3.06	1.61	0.02	up	yes
Frmd5	0.34	-1.54	0.02	down	yes
Gm23849	0.36	-1.49	0.02	down	yes
Gm6745	0.35	-1.50	0.02	down	yes
Trim32	3.05	1.61	0.02	up	yes
Cd4	0.36	-1.48	0.02	down	yes
Sema5a	3.05	1.61	0.02	up	yes
Jag1	3.04	1.60	0.02	up	yes
Mdga1	3.04	1.61	0.02	up	yes
Gm21188	3.05	1.61	0.02	up	yes
Bbs12;F	0.36	-1.49	0.02	down	yes

Atp6v0c	3.01	1.59	0.02	up	yes
Mrgprf	3.08	1.62	0.02	up	yes
Cebpa	3.01	1.59	0.02	up	yes
Lsm12	3.00	1.59	0.02	up	yes
Cygb	3.02	1.59	0.02	up	yes
Pttg1ip	0.36	-1.46	0.02	down	yes
P3h2	3.03	1.60	0.02	up	yes
Casp1	3.03	1.60	0.02	up	yes
Tmbim1	2.99	1.58	0.02	up	yes
B4galt6	3.01	1.59	0.02	up	yes
Cd300ld	3.01	1.59	0.02	up	yes
Oas3	2.97	1.57	0.02	up	yes
Gm15737	3.04	1.60	0.02	up	yes
Gbp10;Gl	2.96	1.57	0.02	up	yes
Cd300ld	2.97	1.57	0.02	up	yes
Gm8623	0.36	-1.46	0.02	down	yes
Slamf8	3.05	1.61	0.02	up	yes
Gm10036	0.37	-1.45	0.02	down	yes
Nphs2	0.37	-1.44	0.02	down	yes
H2-K1	2.94	1.56	0.02	up	yes
AI66145	2.98	1.57	0.03	up	yes
C1s1;C1	2.94	1.56	0.03	up	yes
Abca9	2.95	1.56	0.03	up	yes
Ino80c	0.37	-1.43	0.03	down	yes
Nup62;I	2.92	1.55	0.03	up	yes
Gna15	2.97	1.57	0.03	up	yes
Pcdh19	2.99	1.58	0.03	up	yes
Clcf1	2.92	1.55	0.03	up	yes
Gm13232	0.37	-1.43	0.03	down	yes
Arhgap6	2.98	1.57	0.03	up	yes
Spata2	2.91	1.54	0.03	up	yes
Htra3	2.96	1.56	0.03	up	yes
Fgf1	2.92	1.55	0.03	up	yes
Gm8979	2.96	1.56	0.03	up	yes
Ebf1	2.93	1.55	0.03	up	yes
Gm10076	0.37	-1.43	0.03	down	yes
Snora23	0.37	-1.43	0.03	down	yes
Rmi2	0.37	-1.42	0.03	down	yes
Dpp6	2.92	1.55	0.03	up	yes
Xpnpep3	0.37	-1.42	0.03	down	yes
Aox2	2.93	1.55	0.03	up	yes
Ddx60	2.90	1.53	0.03	up	yes
Mpdz	2.93	1.55	0.03	up	yes
Clec4a3	2.96	1.56	0.03	up	yes
Btk	2.92	1.55	0.03	up	yes
Gm13192	0.37	-1.43	0.03	down	yes
Tnfaip2	2.93	1.55	0.03	up	yes
Abca8a	2.91	1.54	0.03	up	yes
Igf2bp3	0.38	-1.41	0.03	down	yes
Dll1	2.95	1.56	0.03	up	yes
Nudt16	0.38	-1.41	0.03	down	yes
Irs1	2.89	1.53	0.03	up	yes
Ttll10	2.94	1.55	0.03	up	yes
Vmn2r84	0.38	-1.40	0.03	down	yes
Zbtb16	2.87	1.52	0.03	up	yes
Fam107a	2.93	1.55	0.03	up	yes
Kbtbd11	2.86	1.51	0.03	up	yes

Sesn3	2.85	1.51	0.03	up	yes
Tnxb	2.86	1.52	0.03	up	yes
Grtp1	0.38	-1.41	0.03	down	yes
Gm5869	0.38	-1.40	0.03	down	yes
Arhgap3.	2.85	1.51	0.03	up	yes
Cnga2	2.91	1.54	0.03	up	yes
Hmgb1-p.	0.37	-1.42	0.03	down	yes
Gm5586	0.38	-1.41	0.03	down	yes
Mylip	2.84	1.50	0.03	up	yes
C3	2.83	1.50	0.03	up	yes
Ltbp1	2.84	1.50	0.03	up	yes
Arhgef2	2.85	1.51	0.03	up	yes
Ccr5	2.84	1.51	0.03	up	yes
Nrbp2	2.83	1.50	0.03	up	yes
Gm10157	0.37	-1.42	0.03	down	yes
Thap12	2.83	1.50	0.03	up	yes
Ifi202b	2.85	1.51	0.03	up	yes
Cyp2g1	2.86	1.52	0.03	up	yes
Cyp2a5	2.82	1.50	0.03	up	yes
Bche	2.88	1.53	0.03	up	yes
Srl	2.83	1.50	0.03	up	yes
Klf9	2.82	1.49	0.03	up	yes
Smad7	2.81	1.49	0.03	up	yes
Igkv14-	2.88	1.53	0.03	up	yes
Dgat2	2.83	1.50	0.03	up	yes
Tmtc1	2.82	1.50	0.03	up	yes
Slc24a4	2.81	1.49	0.03	up	yes
Sox12	0.38	-1.38	0.03	down	yes
Prr18	2.84	1.51	0.03	up	yes
Arid5a	2.79	1.48	0.03	up	yes
Cldn1	2.80	1.48	0.03	up	yes
C1rb	2.85	1.51	0.03	up	yes
Nod1	2.79	1.48	0.03	up	yes
Gm26448	0.39	-1.36	0.03	down	yes
Trp53rk.	2.80	1.48	0.03	up	yes
Igdcc4	2.78	1.48	0.03	up	yes
Limch1	2.78	1.47	0.03	up	yes
Galnt15	2.78	1.48	0.03	up	yes
Cxcl9	2.78	1.48	0.03	up	yes
Prrx1	2.79	1.48	0.03	up	yes
Htra1	2.78	1.48	0.03	up	yes
Prrg3	2.77	1.47	0.03	up	yes
Fen1	0.39	-1.35	0.03	down	yes
Ptprz1	2.78	1.48	0.03	up	yes
Serping	2.77	1.47	0.04	up	yes
Tlr7	2.79	1.48	0.04	up	yes
Ntrk2	2.81	1.49	0.04	up	yes
Dcdc5	2.81	1.49	0.04	up	yes
Serpinb.	2.82	1.50	0.04	up	yes
Plekha6	2.77	1.47	0.04	up	yes
Steap4	2.76	1.47	0.04	up	yes
Cwc25	0.39	-1.34	0.04	down	yes
Il18r1	2.75	1.46	0.04	up	yes
Ifit3	2.76	1.47	0.04	up	yes
Rasip1	2.80	1.49	0.04	up	yes
Mmp19	2.80	1.49	0.04	up	yes
Abi3bp	2.78	1.47	0.04	up	yes

Tmem119	2.80	1.48	0.04	up	yes
Gm8494	0.39	-1.37	0.04	down	yes
Apba2	2.76	1.46	0.04	up	yes
Cpxm1	2.80	1.49	0.04	up	yes
Ifi44l	2.74	1.46	0.04	up	yes
Proser2	2.77	1.47	0.04	up	yes
Snx3	2.73	1.45	0.04	up	yes
Adamts5	2.78	1.47	0.04	up	yes
Myo16	2.74	1.45	0.04	up	yes
Rnu3b2	0.40	-1.33	0.04	down	yes
Zfp451	0.40	-1.33	0.04	down	yes
Nr2c2	0.40	-1.32	0.04	down	yes
Iigp1	2.72	1.45	0.04	up	yes
Rnu3b4	0.40	-1.32	0.04	down	yes
Ptafr	2.75	1.46	0.04	up	yes
Slfn8	2.71	1.44	0.04	up	yes
Klrd1	2.75	1.46	0.04	up	yes
Sdc2	2.74	1.45	0.04	up	yes
Hepacam	2.78	1.47	0.04	up	yes
Ifi27l2	2.71	1.44	0.04	up	yes
Pdgfra;I	2.73	1.45	0.04	up	yes
Slc26a1	0.40	-1.32	0.04	down	yes
Cgnl1	2.72	1.44	0.04	up	yes
Ctsf	2.75	1.46	0.04	up	yes
Rpl7-ps	0.40	-1.33	0.04	down	yes
C1qc	0.40	-1.32	0.04	down	yes
Pm20d1	2.74	1.46	0.04	up	yes
Sdc4	2.69	1.43	0.04	up	yes
Zfp618	2.75	1.46	0.04	up	yes
9130208	2.70	1.43	0.04	up	yes
Edar	2.73	1.45	0.04	up	yes
Tmprss2	2.74	1.45	0.04	up	yes
Rpsa-ps	0.40	-1.33	0.04	down	yes
Ahnak2	2.69	1.43	0.04	up	yes
Enpp2	2.68	1.42	0.04	up	yes
Gm10156	0.40	-1.31	0.04	down	yes
Kctd15	2.71	1.44	0.04	up	yes
Gm4759;	2.67	1.42	0.04	up	yes
Fosl2	2.67	1.42	0.04	up	yes
Plekha8	2.69	1.43	0.04	up	yes
Fkrp	2.67	1.42	0.04	up	yes
Txndc5	2.67	1.42	0.04	up	yes
Kcnq3	2.67	1.42	0.04	up	yes
Art2a-p	2.72	1.45	0.04	up	yes
Slc6a20	0.41	-1.30	0.04	down	yes
Alox5	2.71	1.44	0.04	up	yes
Gm25360	0.41	-1.30	0.04	down	yes
Zc2hc1a	2.71	1.44	0.04	up	yes
Gm35857	0.41	-1.29	0.04	down	yes
Cfd	2.71	1.44	0.04	up	yes
Slit2	2.71	1.44	0.04	up	yes
Dsel	2.68	1.42	0.04	up	yes
Ifi44	2.65	1.41	0.04	up	yes
AC23881	2.68	1.42	0.04	up	yes
Ccdc80	2.66	1.41	0.04	up	yes
Prf1	2.66	1.41	0.04	up	yes
Sema3a	2.71	1.44	0.04	up	yes

Mul1	2.66	1.41	0.04	up	yes
Foxj1	2.66	1.41	0.04	up	yes
Mmp25	2.65	1.41	0.04	up	yes
Plag1	0.41	-1.28	0.04	down	yes
Zfp329;	0.41	-1.28	0.04	down	yes
Pigc	2.65	1.40	0.04	up	yes
Fndc1	2.68	1.42	0.04	up	yes
Icos	2.63	1.40	0.04	up	yes
F3	2.67	1.42	0.04	up	yes
Anxa1	2.64	1.40	0.04	up	yes
Rec114	0.41	-1.28	0.05	down	yes
Erich3	2.70	1.43	0.05	up	yes
Havcr2	2.65	1.41	0.05	up	yes
Ptges	2.65	1.41	0.05	up	yes
C1ra	2.63	1.39	0.05	up	yes
AL59158	0.42	-1.26	0.05	down	yes
Trim26	2.60	1.38	0.05	up	yes
Pgm2l1	0.42	-1.26	0.05	down	yes
Boc	2.60	1.38	0.05	up	yes
Cd22	2.63	1.39	0.05	up	yes
Timp3	2.60	1.38	0.05	up	yes
Ntng2	2.61	1.38	0.05	up	yes
Unc79	2.63	1.39	0.05	up	yes
Zfp462	2.60	1.38	0.05	up	yes
Ltbp4	2.60	1.38	0.05	up	yes
Lzts3	2.61	1.39	0.05	up	yes
C3ar1	2.60	1.38	0.05	up	yes
Calml3	2.65	1.40	0.05	up	yes
Fam13c	2.64	1.40	0.05	up	yes
Cldn12	2.61	1.39	0.05	up	yes
Mx2	2.59	1.37	0.05	up	yes
Clp1	0.42	-1.26	0.05	down	yes
Atp1a2	2.61	1.38	0.05	up	yes
Parva	2.59	1.37	0.05	up	yes
Gm14303	0.42	-1.26	0.05	down	yes
Pde7b	2.62	1.39	0.05	up	yes
Akap5	2.59	1.37	0.05	up	yes
Arl2bp	2.58	1.37	0.05	up	yes
Rflnb	2.58	1.37	0.05	up	yes
AC15315	0.42	-1.26	0.05	down	yes
Rnu3b3	0.42	-1.24	0.05	down	yes
