# Supplementary material for: Transcriptome analysis reveals a potential regulatory mechanism of the lnc-5423.6/IGFBP5 axis in the early stages of mouse thymic involution: lnc-5423.6/IGFBP5 axis regulates thymic involution
Source: Acta Biochim Biophys Sin (Shanghai). 2023 Apr 19;55(4):548–60. doi: 10.3724/abbs.2023042 (PMC10195152; doi:10.3724/abbs.2023042)
Supplement: Table_S6 [file Table_S6.pdf]

gene_na	fc	log2(fc	pval	regulat	significant
Car3	56.64	5.82	0.00	up	yes
Scd1	52.33	5.71	0.00	up	yes
Ighg3	37.04	5.21	0.00	up	yes
Ighg1	31.75	4.99	0.00	up	yes
Cfd	29.26	4.87	0.00	up	yes
Rec114	27.64	4.79	0.00	up	yes
Ces1d	22.04	4.46	0.00	up	yes
Pck1	21.95	4.46	0.00	up	yes
Jchain	16.72	4.06	0.00	up	yes
Igkc	15.10	3.92	0.00	up	yes
Igkv1-1	13.61	3.77	0.00	up	yes
Igfbp5	12.47	3.64	0.00	up	yes
Iglc2;I	12.34	3.63	0.00	up	yes
Thrsp	12.03	3.59	0.00	up	yes
D630045	0.08	-3.59	0.00	down	yes
Igkv10-	11.72	3.55	0.00	up	yes
Scn7a	10.98	3.46	0.00	up	yes
Scn1a	10.71	3.42	0.00	up	yes
Car5b	10.65	3.41	0.00	up	yes
Fabp4	9.97	3.32	0.00	up	yes
Itgad	9.77	3.29	0.00	up	yes
Aoc3	8.31	3.05	0.00	up	yes
Eif3j2	0.12	-3.08	0.00	down	yes
Ighg2b	8.13	3.02	0.00	up	yes
Ighv3-6	8.20	3.04	0.00	up	yes
Pcx	7.79	2.96	0.00	up	yes
Deptor	7.59	2.92	0.00	up	yes
Dgat2	7.56	2.92	0.00	up	yes
C4b	7.50	2.91	0.00	up	yes
Iglc1;I	7.23	2.85	0.00	up	yes
Mid1-ps	0.14	-2.84	0.00	down	yes
Tusc5	7.27	2.86	0.00	up	yes
Gm21742	0.14	-2.85	0.00	down	yes
Mgll	6.97	2.80	0.00	up	yes
Dcn	6.70	2.75	0.00	up	yes
Ifi47;O	6.74	2.75	0.00	up	yes
Ryr3	6.73	2.75	0.00	up	yes
Gm21860	0.15	-2.71	0.00	down	yes
Tns4	6.52	2.70	0.00	up	yes
Abca8a	6.49	2.70	0.00	up	yes
Mgst1	6.47	2.69	0.00	up	yes
Fndc1	6.41	2.68	0.00	up	yes
Chst1	6.34	2.66	0.00	up	yes
Igkv1-1	6.26	2.65	0.00	up	yes
Plin4	5.80	2.54	0.00	up	yes
Pgr	5.85	2.55	0.00	up	yes
Gm23472	0.17	-2.52	0.00	down	yes
Mir29b-	5.74	2.52	0.00	up	yes
Megf6	5.64	2.50	0.00	up	yes
Myo16	5.63	2.49	0.00	up	yes
Gas7	5.66	2.50	0.00	up	yes
Slit3	5.55	2.47	0.00	up	yes
A130051	0.18	-2.46	0.00	down	yes
Aebp1	5.50	2.46	0.00	up	yes
Gm7609	5.63	2.49	0.00	up	yes
Pvrig	5.51	2.46	0.00	up	yes

Prelp	5.36	2.42	0.00	up	yes
Inafm2	5.29	2.40	0.00	up	yes
Slc16a7	5.34	2.42	0.00	up	yes
Il33	5.29	2.40	0.00	up	yes
Uhmk1	0.19	-2.37	0.00	down	yes
Htra3	5.14	2.36	0.00	up	yes
Prom2	5.07	2.34	0.00	up	yes
Gm24924	0.20	-2.33	0.00	down	yes
Blk	4.99	2.32	0.00	up	yes
Ephx2	5.05	2.33	0.00	up	yes
C1qc	4.93	2.30	0.00	up	yes
Scara5	5.03	2.33	0.00	up	yes
Papln	5.03	2.33	0.00	up	yes
Arl2bp	0.20	-2.32	0.00	down	yes
Kcnh1	4.93	2.30	0.00	up	yes
Tril	4.99	2.32	0.00	up	yes
Fam13a	4.86	2.28	0.00	up	yes
Acsl1	4.73	2.24	0.00	up	yes
Cttnbp2	0.21	-2.27	0.00	down	yes
Cd163	4.77	2.25	0.00	up	yes
Igha;Ig	4.67	2.22	0.00	up	yes
Ghr	4.71	2.24	0.00	up	yes
Agpat2	4.68	2.23	0.00	up	yes
Abca8b	4.69	2.23	0.00	up	yes
Pygl	4.63	2.21	0.00	up	yes
Dnal1	4.68	2.23	0.00	up	yes
Me1	4.49	2.17	0.00	up	yes
Mdga1	4.48	2.16	0.00	up	yes
Per3	4.48	2.16	0.00	up	yes
Thbd	4.50	2.17	0.00	up	yes
Rn7s6	0.22	-2.16	0.00	down	yes
Luzp1	4.43	2.15	0.00	up	yes
Il18rap	4.42	2.14	0.00	up	yes
Parva	4.38	2.13	0.00	up	yes
Dpp6	4.34	2.12	0.00	up	yes
Mfap3	4.28	2.10	0.00	up	yes
Lpl	4.26	2.09	0.00	up	yes
Adam28;	4.29	2.10	0.00	up	yes
Pdgfra;	4.28	2.10	0.00	up	yes
Tnxb	4.25	2.09	0.00	up	yes
Zan	4.33	2.11	0.00	up	yes
Ano1	4.32	2.11	0.00	up	yes
Dkk3	4.29	2.10	0.00	up	yes
Nrbp2	4.17	2.06	0.00	up	yes
Atp1a2	4.15	2.05	0.00	up	yes
Ptprd	4.12	2.04	0.00	up	yes
Aldh1a3	4.12	2.04	0.00	up	yes
Ddr2	4.07	2.03	0.00	up	yes
Kcnq5	0.24	-2.03	0.00	down	yes
Ptprq	0.24	-2.04	0.00	down	yes
Mettl21	0.24	-2.05	0.00	down	yes
Glp1r	4.06	2.02	0.00	up	yes
Chpt1	4.07	2.02	0.00	up	yes
Fam110b	4.12	2.04	0.00	up	yes
Plcxd1	4.06	2.02	0.00	up	yes
Cyp2f2	4.07	2.03	0.00	up	yes
Lgals12	4.08	2.03	0.00	up	yes

Cd36	4.00	2.00	0.00	up	yes
Cped1	4.07	2.03	0.00	up	yes
Cavin2	4.01	2.00	0.00	up	yes
Ubtd1	4.04	2.01	0.00	up	yes
Klf11	3.97	1.99	0.00	up	yes
Plekha6	3.97	1.99	0.00	up	yes
Fat3	3.97	1.99	0.00	up	yes
Cspg4	4.02	2.01	0.00	up	yes
Cgnl1	3.90	1.96	0.00	up	yes
E2f3	0.26	-1.97	0.00	down	yes
Apcdd1	3.99	2.00	0.00	up	yes
Cenpb;S	3.86	1.95	0.00	up	yes
Svep1	3.88	1.96	0.00	up	yes
Bank1	3.91	1.97	0.00	up	yes
Adgrd1	3.88	1.96	0.00	up	yes
Cygb	3.86	1.95	0.00	up	yes
C1ra	3.87	1.95	0.00	up	yes
Ngp	3.83	1.94	0.00	up	yes
Slc16a2	3.92	1.97	0.00	up	yes
Mt1	3.92	1.97	0.00	up	yes
Adh1	3.87	1.95	0.00	up	yes
Slc41a2	3.91	1.97	0.00	up	yes
Vldlr	3.79	1.92	0.00	up	yes
Gm27875	0.26	-1.94	0.00	down	yes
Mlxipl	3.86	1.95	0.00	up	yes
Hrnr	0.26	-1.96	0.00	down	yes
Aifm2	3.81	1.93	0.00	up	yes
Angptl4	3.77	1.92	0.00	up	yes
Ldlr	0.27	-1.91	0.00	down	yes
Inhbb	3.82	1.94	0.00	up	yes
Klhl32	3.78	1.92	0.00	up	yes
Ccr8	0.26	-1.92	0.01	down	yes
Btnl9	3.76	1.91	0.01	up	yes
Cxcr3	3.72	1.90	0.01	up	yes
Zfp703	3.63	1.86	0.01	up	yes
AI46413	3.69	1.88	0.01	up	yes
Klrd1	3.70	1.89	0.01	up	yes
Rnu3b4	3.63	1.86	0.01	up	yes
Cpxm1	3.70	1.89	0.01	up	yes
Spock2	3.60	1.85	0.01	up	yes
Adamts5	3.65	1.87	0.01	up	yes
Adamts1	3.68	1.88	0.01	up	yes
Bpifa1	3.62	1.86	0.01	up	yes
Capn12	0.27	-1.88	0.01	down	yes
Nr2f2	3.63	1.86	0.01	up	yes
Adgra2	3.54	1.83	0.01	up	yes
Rxfp2	3.60	1.85	0.01	up	yes
Fcna	3.56	1.83	0.01	up	yes
Zfp532	3.52	1.81	0.01	up	yes
Fcmr	3.56	1.83	0.01	up	yes
Oas1a	3.56	1.83	0.01	up	yes
Pm20d1	3.51	1.81	0.01	up	yes
Npr1	3.50	1.81	0.01	up	yes
Ptprz1	3.49	1.80	0.01	up	yes
Acacb	3.43	1.78	0.01	up	yes
Abca9	3.45	1.79	0.01	up	yes
Pdpn	3.48	1.80	0.01	up	yes

Cebpa	3.42	1.77	0.01	up	yes
Lcn2	3.48	1.80	0.01	up	yes
Tlr4	0.28	-1.81	0.01	down	yes
Reln	3.47	1.79	0.01	up	yes
Ntrk2	3.48	1.80	0.01	up	yes
Abcd2	3.43	1.78	0.01	up	yes
Snhg11;	3.39	1.76	0.01	up	yes
Ptger3	3.44	1.78	0.01	up	yes
Hp	3.47	1.79	0.01	up	yes
Hydin	3.41	1.77	0.01	up	yes
Gm13502	0.29	-1.80	0.01	down	yes
Avpr2	0.29	-1.77	0.01	down	yes
Adamtsl	3.50	1.81	0.01	up	yes
Steap4	3.36	1.75	0.01	up	yes
Aox2	3.36	1.75	0.01	up	yes
Rnase4;	3.36	1.75	0.01	up	yes
Ptges	3.36	1.75	0.01	up	yes
Gm22042	0.30	-1.75	0.01	down	yes
Epb41l1	3.32	1.73	0.01	up	yes
Klrk1	3.31	1.73	0.01	up	yes
Cd5l	3.35	1.75	0.01	up	yes
Tns2	3.26	1.71	0.01	up	yes
Nt5e	3.28	1.72	0.01	up	yes
2900026	3.25	1.70	0.01	up	yes
Gpr83	0.30	-1.72	0.01	down	yes
Tmem8b	3.30	1.72	0.01	up	yes
Rhou	3.26	1.71	0.01	up	yes
Bicc1	3.28	1.71	0.01	up	yes
Pla2r1	3.31	1.72	0.01	up	yes
Gm11427	3.21	1.68	0.01	up	yes
Cpt1a	3.20	1.68	0.01	up	yes
Arl4c	0.31	-1.69	0.01	down	yes
Irs1	3.20	1.68	0.01	up	yes
Mrvi1	3.24	1.70	0.01	up	yes
Gm7694	3.23	1.69	0.01	up	yes
Sorbs3	3.17	1.67	0.01	up	yes
Ipmk	3.15	1.66	0.01	up	yes
Fam213a	3.21	1.68	0.01	up	yes
Fgf1	3.15	1.66	0.01	up	yes
Tshz2	3.18	1.67	0.01	up	yes
Gda	3.16	1.66	0.01	up	yes
Gstm2	3.17	1.66	0.01	up	yes
Paqr9	3.15	1.66	0.01	up	yes
Vcp-rs	3.10	1.63	0.01	up	yes
Rsl1	0.31	-1.69	0.01	down	yes
Siglec1	3.12	1.64	0.01	up	yes
Adhfe1	3.13	1.65	0.01	up	yes
Mylip	3.08	1.62	0.01	up	yes
C1s1;C1	3.10	1.63	0.02	up	yes
Ahnak2	3.10	1.63	0.02	up	yes
Penk	3.13	1.65	0.02	up	yes
Casp12	3.13	1.64	0.02	up	yes
Pnpla3	3.10	1.63	0.02	up	yes
Plag1	0.32	-1.64	0.02	down	yes
Fyb2	3.08	1.62	0.02	up	yes
Cyp27a1	3.12	1.64	0.02	up	yes
Scn2b	0.32	-1.63	0.02	down	yes

Fasn	3.04	1.60	0.02	up	yes
Fhl1	3.13	1.64	0.02	up	yes
Xkr5	0.32	-1.65	0.02	down	yes
Arxes2	3.08	1.62	0.02	up	yes
Serping	3.04	1.61	0.02	up	yes
Atp1a3	3.07	1.62	0.02	up	yes
Shroom4	3.07	1.62	0.02	up	yes
Evc	3.13	1.65	0.02	up	yes
Cdhr1	0.33	-1.62	0.02	down	yes
Proz	0.32	-1.64	0.02	down	yes
Gm24830	0.33	-1.60	0.02	down	yes
S100a9	3.03	1.60	0.02	up	yes
Cd248	3.08	1.62	0.02	up	yes
Ltbp4	2.98	1.58	0.02	up	yes
Tfap2a	3.02	1.60	0.02	up	yes
Tef	2.97	1.57	0.02	up	yes
Clec3b	3.03	1.60	0.02	up	yes
Cmtr2	0.33	-1.61	0.02	down	yes
Rarres2	3.00	1.58	0.02	up	yes
Acot1	3.04	1.60	0.02	up	yes
Asprv1	0.32	-1.62	0.02	down	yes
Ltbp3	2.97	1.57	0.02	up	yes
Shisa6	2.97	1.57	0.02	up	yes
Ttc39a	2.98	1.58	0.02	up	yes
Eomes	0.32	-1.63	0.02	down	yes
Ly6c2;L	2.95	1.56	0.02	up	yes
Crhbp	0.33	-1.61	0.02	down	yes
Cfh	2.94	1.56	0.02	up	yes
Ighd	2.96	1.57	0.02	up	yes
Arf3	0.34	-1.56	0.02	down	yes
Dchs2	2.98	1.58	0.02	up	yes
Slc35c1	2.92	1.55	0.02	up	yes
Rnf185	0.34	-1.56	0.02	down	yes
Rabl3	0.34	-1.57	0.02	down	yes
Krt10	0.33	-1.60	0.02	down	yes
Ccl11	2.95	1.56	0.02	up	yes
Map3k6	2.98	1.57	0.02	up	yes
Rnu1b2	0.34	-1.57	0.02	down	yes
Sox18	2.98	1.57	0.02	up	yes
Cacna2d	3.00	1.58	0.02	up	yes
Mmp2	2.89	1.53	0.02	up	yes
Fzd4	2.91	1.54	0.02	up	yes
Calcb	0.34	-1.57	0.02	down	yes
Tmtc1	2.91	1.54	0.02	up	yes
Dennd5b	2.89	1.53	0.02	up	yes
Smim8	2.97	1.57	0.02	up	yes
Fras1	2.88	1.53	0.02	up	yes
Fam107b	2.87	1.52	0.02	up	yes
Sell	0.34	-1.54	0.02	down	yes
Fgfr1	2.91	1.54	0.02	up	yes
Sema5a	2.87	1.52	0.02	up	yes
Stab1	2.88	1.53	0.02	up	yes
Gm16026	2.87	1.52	0.02	up	yes
Cpa3	2.90	1.53	0.02	up	yes
Pygo1	2.90	1.54	0.02	up	yes
Slco2a1	2.85	1.51	0.02	up	yes
Sdr42e1	2.87	1.52	0.02	up	yes

Prickle[illegible]	2.92	1.54	0.02	up	yes
Yap1	2.84	1.51	0.02	up	yes
Thsd7a	2.91	1.54	0.02	up	yes
Zfp612	2.91	1.54	0.02	up	yes
Rnu3b2	2.84	1.50	0.02	up	yes
Cradd	0.35	-1.52	0.02	down	yes
Arhgap1	0.35	-1.51	0.03	down	yes
Fam184a	0.34	-1.56	0.03	down	yes
Lrig3	2.85	1.51	0.03	up	yes
Gnao1	2.85	1.51	0.03	up	yes
Vwf	2.81	1.49	0.03	up	yes
Mgat5b	2.83	1.50	0.03	up	yes
Prdm1	2.82	1.50	0.03	up	yes
Padi4	0.35	-1.53	0.03	down	yes
Pde7b	2.81	1.49	0.03	up	yes
Rnu1b1	0.35	-1.50	0.03	down	yes
Mok	2.81	1.49	0.03	up	yes
Mpdz	2.81	1.49	0.03	up	yes
Mid1	0.36	-1.48	0.03	down	yes
Naaladl[illegible]	2.80	1.48	0.03	up	yes
Enpp1	2.78	1.47	0.03	up	yes
Zfp185	2.83	1.50	0.03	up	yes
Klk1	2.84	1.50	0.03	up	yes
Pnpla2	2.74	1.46	0.03	up	yes
Il18r1	2.74	1.45	0.03	up	yes
Srxn1	2.76	1.47	0.03	up	yes
Arl5b	2.73	1.45	0.03	up	yes
Gpc4	2.75	1.46	0.03	up	yes
Fam46a	2.75	1.46	0.03	up	yes
Wipi1	2.77	1.47	0.03	up	yes
Rusc2	2.81	1.49	0.03	up	yes
Fah	2.77	1.47	0.03	up	yes
Srgap3	2.73	1.45	0.03	up	yes
Evc2	2.78	1.48	0.03	up	yes
Tbkbp1	2.77	1.47	0.03	up	yes
Rhpn2	2.78	1.48	0.03	up	yes
Plpp3	2.72	1.44	0.03	up	yes
Lrp1	2.71	1.44	0.03	up	yes
Rnu3b3	2.71	1.44	0.03	up	yes
Fignl2	2.76	1.47	0.03	up	yes
Slc39a9	0.37	-1.45	0.03	down	yes
Sdk2	2.71	1.44	0.03	up	yes
Aatk	2.73	1.45	0.03	up	yes
Mgl2	2.76	1.47	0.03	up	yes
Cd300lg	2.71	1.44	0.03	up	yes
Slc9a3r[illegible]	2.73	1.45	0.03	up	yes
Tbx21	2.73	1.45	0.03	up	yes
Lrrc18	2.76	1.47	0.03	up	yes
Dmrta1	2.76	1.46	0.03	up	yes
Mmp8	2.76	1.46	0.03	up	yes
Apod	2.73	1.45	0.03	up	yes
Frmd5	2.76	1.46	0.03	up	yes
Gpm6b	2.70	1.43	0.03	up	yes
Arl4d	2.75	1.46	0.03	up	yes
Xdh	2.68	1.42	0.03	up	yes
Pcdh17	2.70	1.43	0.03	up	yes
Nid1	2.68	1.42	0.03	up	yes

Spns2	2.70	1.43	0.03	up	yes
Aldh5a1	2.73	1.45	0.03	up	yes
Slc11a1	2.70	1.43	0.03	up	yes
Myo6	2.68	1.42	0.03	up	yes
Dpt	2.68	1.42	0.03	up	yes
Gm18537	0.37	-1.43	0.03	down	yes
Scd2;Mi	0.37	-1.42	0.03	down	yes
Ftl1;Gm	2.64	1.40	0.03	up	yes
Slc25a5	2.65	1.40	0.03	up	yes
Cds2	2.64	1.40	0.03	up	yes
Cyp51	0.37	-1.42	0.03	down	yes
Acsbg1	2.68	1.42	0.03	up	yes
1600014	2.64	1.40	0.03	up	yes
Hist1h2	0.38	-1.41	0.03	down	yes
Klf9	2.65	1.41	0.03	up	yes
Rnu3b1	2.65	1.40	0.03	up	yes
Apba2	2.66	1.41	0.04	up	yes
Ar	2.63	1.40	0.04	up	yes
Apob	0.37	-1.45	0.04	down	yes
Palmd	2.68	1.42	0.04	up	yes
Pirb	2.66	1.41	0.04	up	yes
Flt1	2.64	1.40	0.04	up	yes
3110082	0.37	-1.44	0.04	down	yes
Xpnpep3	2.63	1.40	0.04	up	yes
Phactr4	2.62	1.39	0.04	up	yes
Dsg1a	0.37	-1.42	0.04	down	yes
Sspn	2.65	1.41	0.04	up	yes
Lrg1	2.65	1.40	0.04	up	yes
Kcnj8	2.69	1.43	0.04	up	yes
1810062	0.38	-1.40	0.04	down	yes
Olfr920	2.66	1.41	0.04	up	yes
Piezo2	2.66	1.41	0.04	up	yes
Dok3	2.63	1.40	0.04	up	yes
Il23r	2.62	1.39	0.04	up	yes
Trim39	2.59	1.38	0.04	up	yes
Pparg	2.69	1.43	0.04	up	yes
Trpm2;G	2.60	1.38	0.04	up	yes
Nfib	2.59	1.38	0.04	up	yes
Oasl1	0.37	-1.42	0.04	down	yes
Gjb2	0.37	-1.42	0.04	down	yes
Gm35857	2.58	1.36	0.04	up	yes
Sdc2	2.60	1.38	0.04	up	yes
Blvrb	2.63	1.39	0.04	up	yes
Gpx3	2.57	1.36	0.04	up	yes
Ctsf	2.62	1.39	0.04	up	yes
Flnc	2.62	1.39	0.04	up	yes
Plcd4	0.38	-1.41	0.04	down	yes
Trp63	2.56	1.35	0.04	up	yes
Muc3a	2.58	1.37	0.04	up	yes
Phldb2	2.58	1.36	0.04	up	yes
Ebf1	2.56	1.36	0.04	up	yes
Gprc5b	2.57	1.36	0.04	up	yes
Abcb1a	2.59	1.37	0.04	up	yes
Map2	2.59	1.37	0.04	up	yes
Heph	2.59	1.37	0.04	up	yes
Plcd3	2.59	1.38	0.04	up	yes
Aox1	2.55	1.35	0.04	up	yes

Scara3	2.56	1.36	0.04	up	yes
Abl2	0.39	-1.36	0.04	down	yes
Cd22	2.56	1.36	0.04	up	yes
Plxna4	2.56	1.36	0.04	up	yes
Sod3	2.54	1.34	0.04	up	yes
Greb1	2.53	1.34	0.04	up	yes
Mcmdc2	0.38	-1.39	0.04	down	yes
Hist2h3	0.39	-1.35	0.04	down	yes
Pter	2.59	1.37	0.04	up	yes
Dlc1	2.53	1.34	0.04	up	yes
Pdgfc	2.55	1.35	0.04	up	yes
Mrc2	2.56	1.35	0.04	up	yes
Pon3	2.53	1.34	0.04	up	yes
Ace	2.53	1.34	0.04	up	yes
Ikzf4	0.39	-1.36	0.04	down	yes
Ppp1r9a	2.57	1.36	0.04	up	yes
Bmp4	2.56	1.36	0.04	up	yes
Chl1	0.39	-1.35	0.04	down	yes
Zfp976	2.58	1.37	0.05	up	yes
Gucy1a3	2.54	1.34	0.05	up	yes
Dmd	2.54	1.34	0.05	up	yes
Slc2a4	2.52	1.33	0.05	up	yes
Gm26992	2.52	1.33	0.05	up	yes
Paqr8	2.52	1.33	0.05	up	yes
Idi1	0.40	-1.34	0.05	down	yes
Pamr1	2.55	1.35	0.05	up	yes
Prss23	2.52	1.33	0.05	up	yes
Jdp2	2.54	1.34	0.05	up	yes
Ptgs2	0.40	-1.33	0.05	down	yes
Fgl2	2.49	1.32	0.05	up	yes
Rnf26	0.40	-1.33	0.05	down	yes
Spaca6;	2.49	1.32	0.05	up	yes
Slc39a3	0.40	-1.32	0.05	down	yes
Camp	2.54	1.34	0.05	up	yes
Zfp462	2.48	1.31	0.05	up	yes
Tspan12	2.51	1.33	0.05	up	yes
Igf2bp3	0.40	-1.32	0.05	down	yes
Tfcp2l1	2.48	1.31	0.05	up	yes
Gxylt2	2.51	1.33	0.05	up	yes
Pkd2l2	2.46	1.30	0.05	up	yes
Mtus1	2.48	1.31	0.05	up	yes
Fat2	2.46	1.30	0.05	up	yes
Enpp2	2.46	1.30	0.05	up	yes
Ushbp1	2.50	1.32	0.05	up	yes
