# Supplementary material for: Transcriptome analysis reveals a potential regulatory mechanism of the lnc-5423.6/IGFBP5 axis in the early stages of mouse thymic involution: lnc-5423.6/IGFBP5 axis regulates thymic involution
Source: Acta Biochim Biophys Sin (Shanghai). 2023 Apr 19;55(4):548–60. doi: 10.3724/abbs.2023042 (PMC10195152; doi:10.3724/abbs.2023042)
Supplement: 356FigS1-S4 [file 356FigS1-S4.pdf]

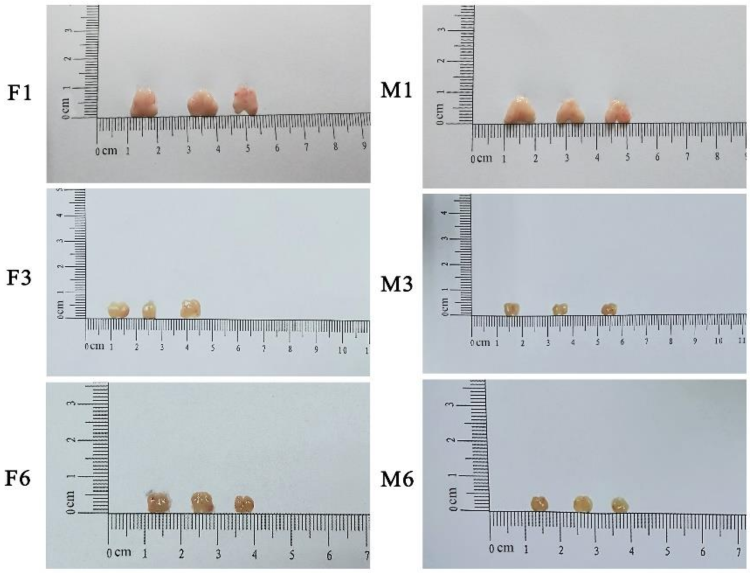

Figure. S1. Morphological observation of age-related thymic involution.

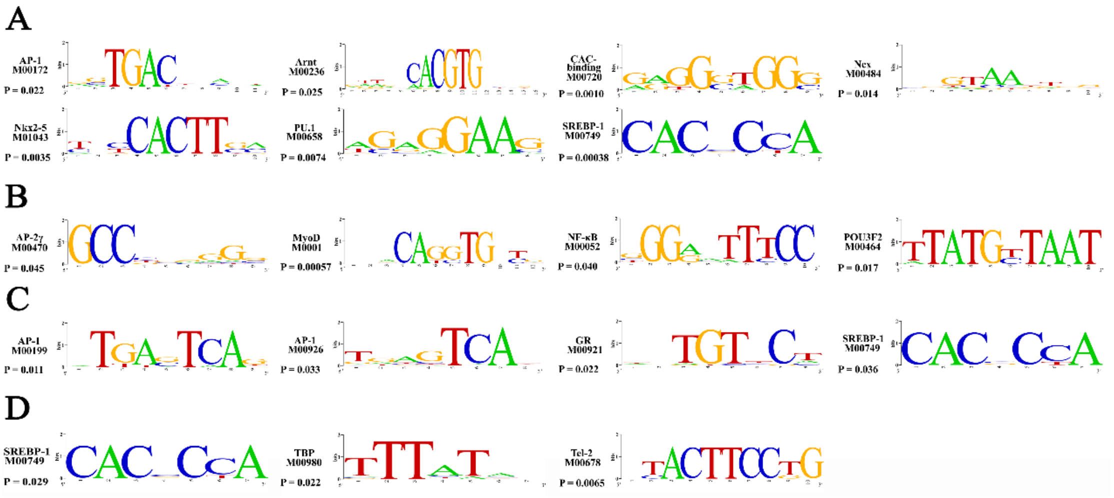

Figure S2. Transcription factor-binding sites of DEGs during age-related thymic involution (A) Sequence logos of transcription factor-binding sites in upregulated DEGs in males. (B) Sequence logos of transcription factor-binding sites in downregulated DEGs in males. (C) Sequence logos of transcription factor-binding sites in upregulated DEGs in females. (D) Sequence logos of transcription factor-binding sites in downregulated DEGs in females. $P < 0.05$ represents a significant difference.

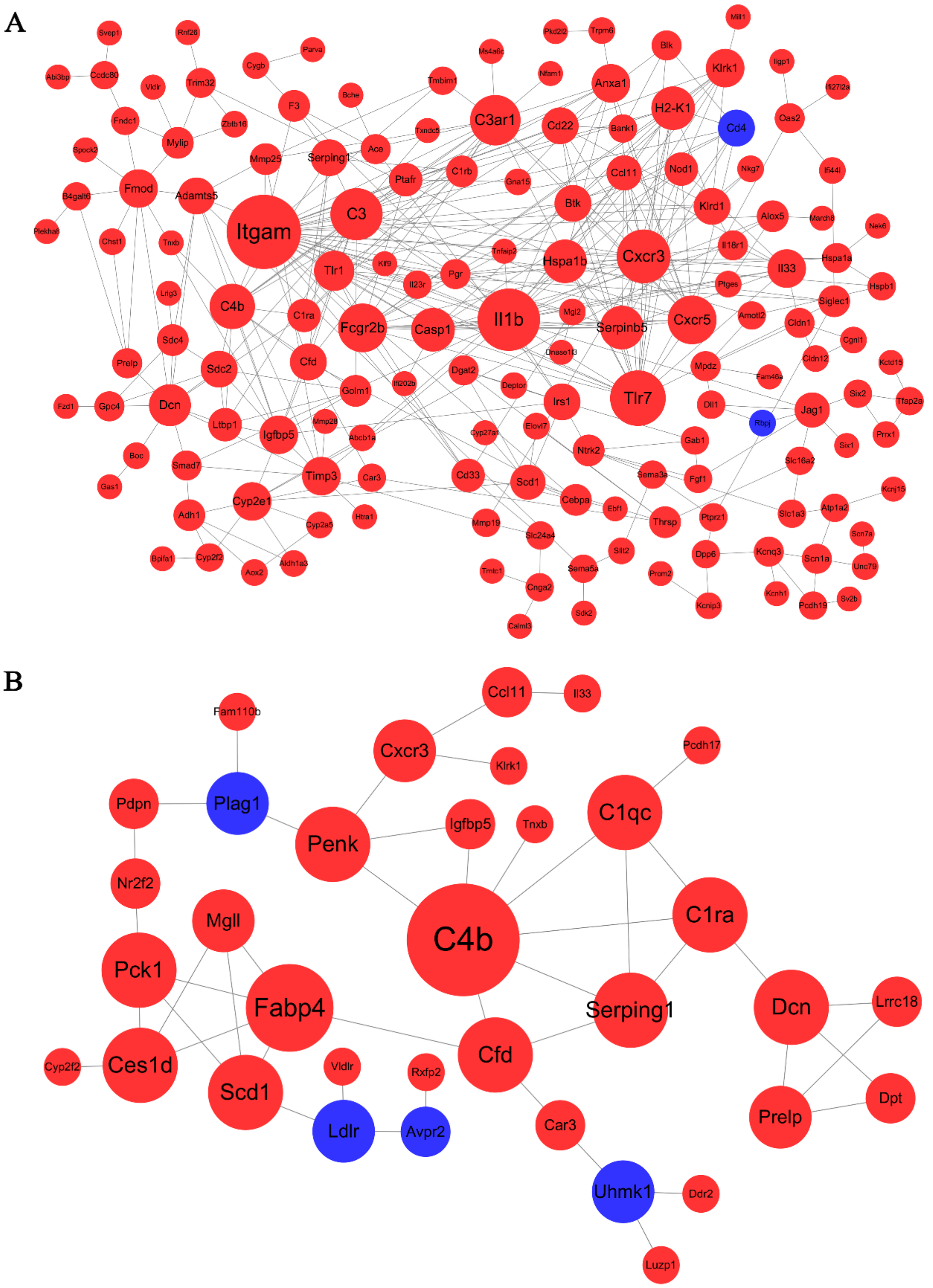

Figure S3. Protein-protein interaction (PPI) networks of DEGs during age-related thymic involution PPI network of DEGs in males (A) and females (B). Red represents the upregulated genes, and blue represents the downregulated genes. The node size is positively correlated with the node degree.

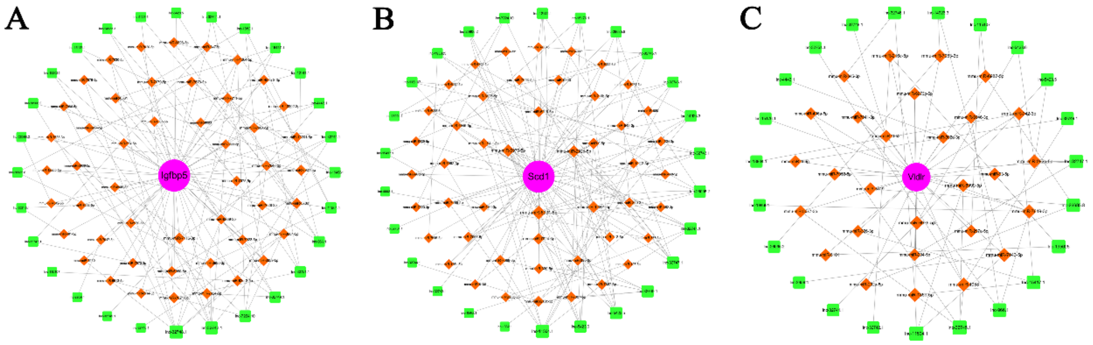

Figure S4. ceRNA networks related to apoptosis and adipocyte deposition during thymus involution (A) *IGFBP5* ceRNA network. (B) *SCD1*. (C) *VLDLR*. The green rectangle represents lncRNA; the brown diamond represents miRNA; the purple circle represents mRNA.
