# Supplementary material for: Transcriptome analysis reveals a potential regulatory mechanism of the lnc-5423.6/IGFBP5 axis in the early stages of mouse thymic involution: lnc-5423.6/IGFBP5 axis regulates thymic involution
Source: Acta Biochim Biophys Sin (Shanghai). 2023 Apr 19;55(4):548–60. doi: 10.3724/abbs.2023042 (PMC10195152; doi:10.3724/abbs.2023042)
Supplement: Table_S12 [file Table_S12.pdf]

t_name	fc	log2(fc	pval	regulat	significant
ENSMUST	39.172	5.2918	2E-11	up	yes
ENSMUST	24.011	4.5856	1E-09	up	yes
ENSMUST	21.756	4.4433	5E-09	up	yes
ENSMUST	0.0961	-3.379	2E-06	down	yes
ENSMUST	0.107	-3.224	7E-06	down	yes
ENSMUST	6.3279	2.6617	0.0002	up	yes
ENSMUST	0.1598	-2.646	0.0002	down	yes
ENSMUST	6.1723	2.6258	0.0002	up	yes
ENSMUST	0.2203	-2.182	0.0013	down	yes
ENSMUST	0.2305	-2.117	0.0018	down	yes
ENSMUST	0.2302	-2.119	0.0019	down	yes
ENSMUST	0.2251	-2.152	0.002	down	yes
ENSMUST	4.3794	2.1307	0.0022	up	yes
ENSMUST	4.0644	2.023	0.0033	up	yes
ENSMUST	0.2565	-1.963	0.0042	down	yes
ENSMUST	3.9279	1.9738	0.0043	up	yes
ENSMUST	3.8784	1.9555	0.0047	up	yes
ENSMUST	3.6695	1.8756	0.0066	up	yes
ENSMUST	3.6195	1.8558	0.0069	up	yes
ENSMUST	3.6087	1.8515	0.0071	up	yes
ENSMUST	0.296	-1.756	0.009	down	yes
ENSMUST	0.3137	-1.673	0.0141	down	yes
ENSMUST	3.0349	1.6016	0.0189	up	yes
ENSMUST	0.3382	-1.564	0.0209	down	yes
ENSMUST	2.9224	1.5472	0.0221	up	yes
ENSMUST	2.9423	1.557	0.0227	up	yes
ENSMUST	0.3472	-1.526	0.0231	down	yes
ENSMUST	0.3459	-1.531	0.0234	down	yes
ENSMUST	2.9432	1.5574	0.024	up	yes
ENSMUST	2.8995	1.5358	0.0244	up	yes
ENSMUST	2.8884	1.5303	0.0246	up	yes
ENSMUST	2.6315	1.3959	0.0377	up	yes
ENSMUST	0.3792	-1.399	0.0384	down	yes
ENSMUST	0.388	-1.366	0.0395	down	yes
ENSMUST	2.6266	1.3932	0.0402	up	yes
ENSMUST	2.5893	1.3726	0.0409	up	yes
ENSMUST	0.3885	-1.364	0.0414	down	yes
ENSMUST	2.5575	1.3547	0.043	up	yes
ENSMUST	0.3923	-1.35	0.0448	down	yes
ENSMUST	2.4872	1.3145	0.0496	up	yes
MSTRG.3	0.0047	-7.743	1E-18	down	yes
MSTRG.2	80.648	6.3336	1E-14	up	yes
MSTRG.1	74.192	6.2132	4E-14	up	yes
MSTRG.6	0.0273	-5.195	3E-11	down	yes
MSTRG.8	0.0286	-5.127	4E-11	down	yes
MSTRG.6	0.0324	-4.948	1E-10	down	yes
MSTRG.4	31.359	4.9708	2E-10	up	yes
MSTRG.1	0.033	-4.921	2E-10	down	yes
MSTRG.1	0.0381	-4.715	7E-10	down	yes
MSTRG.7	0.0425	-4.558	2E-09	down	yes
MSTRG.1	21.758	4.4435	4E-09	up	yes
MSTRG.2	0.0492	-4.344	7E-09	down	yes
MSTRG.1	18.97	4.2457	1E-08	up	yes
MSTRG.5	0.0565	-4.145	2E-08	down	yes
MSTRG.1	18.032	4.1725	2E-08	up	yes
MSTRG.2	17.698	4.1455	3E-08	up	yes

MSTRG.2	16.775	4.0682	5E-08	up	yes
MSTRG.1	16.789	4.0694	5E-08	up	yes
MSTRG.3	0.0638	-3.97	7E-08	down	yes
MSTRG.3	15.44	3.9486	9E-08	up	yes
MSTRG.9	14.809	3.8884	1E-07	up	yes
MSTRG.3	14.199	3.8277	2E-07	up	yes
MSTRG.2	14.032	3.8106	2E-07	up	yes
MSTRG.3	13.676	3.7736	2E-07	up	yes
MSTRG.1	0.0729	-3.778	3E-07	down	yes
MSTRG.2	0.0784	-3.672	4E-07	down	yes
MSTRG.5	12.686	3.6651	5E-07	up	yes
MSTRG.2	0.0833	-3.585	9E-07	down	yes
MSTRG.6	0.0903	-3.469	1E-06	down	yes
MSTRG.3	0.0952	-3.393	2E-06	down	yes
MSTRG.9	10.7	3.4196	2E-06	up	yes
MSTRG.7	10.054	3.3297	3E-06	up	yes
MSTRG.1	9.8121	3.2946	5E-06	up	yes
MSTRG.3	9.1703	3.197	8E-06	up	yes
MSTRG.2	8.7959	3.1368	1E-05	up	yes
MSTRG.3	0.1149	-3.122	1E-05	down	yes
MSTRG.3	8.5436	3.0948	1E-05	up	yes
MSTRG.2	8.4309	3.0757	1E-05	up	yes
MSTRG.5	8.6124	3.1064	2E-05	up	yes
MSTRG.4	0.1224	-3.03	2E-05	down	yes
MSTRG.2	0.1277	-2.969	2E-05	down	yes
MSTRG.4	8.0909	3.0163	3E-05	up	yes
MSTRG.2	7.5507	2.9166	3E-05	up	yes
MSTRG.2	0.1397	-2.84	5E-05	down	yes
MSTRG.2	0.1407	-2.829	5E-05	down	yes
MSTRG.2	7.1458	2.8371	6E-05	up	yes
MSTRG.2	0.1434	-2.802	6E-05	down	yes
MSTRG.1	6.7719	2.7596	8E-05	up	yes
MSTRG.4	6.8444	2.7749	8E-05	up	yes
MSTRG.3	6.6775	2.7393	9E-05	up	yes
MSTRG.3	0.1514	-2.723	1E-04	down	yes
MSTRG.2	6.4796	2.6959	0.0001	up	yes
MSTRG.2	6.4562	2.6907	0.0001	up	yes
MSTRG.1	6.4589	2.6913	0.0001	up	yes
MSTRG.2	6.3229	2.6606	0.0001	up	yes
MSTRG.1	0.1612	-2.633	0.0001	down	yes
MSTRG.2	0.1613	-2.632	0.0002	down	yes
MSTRG.1	0.16	-2.644	0.0002	down	yes
MSTRG.5	6.1663	2.6244	0.0002	up	yes
MSTRG.1	0.1652	-2.598	0.0002	down	yes
MSTRG.2	6.1927	2.6306	0.0002	up	yes
MSTRG.1	6.2239	2.6378	0.0002	up	yes
MSTRG.5	6.0024	2.5855	0.0002	up	yes
MSTRG.2	6.0407	2.5947	0.0002	up	yes
MSTRG.1	0.1664	-2.587	0.0002	down	yes
MSTRG.4	5.9113	2.5635	0.0002	up	yes
MSTRG.2	5.8917	2.5587	0.0002	up	yes
MSTRG.3	0.1741	-2.522	0.0003	down	yes
MSTRG.1	5.6899	2.5084	0.0003	up	yes
MSTRG.1	0.1802	-2.473	0.0003	down	yes
MSTRG.2	5.6707	2.5035	0.0004	up	yes
MSTRG.3	0.1813	-2.463	0.0004	down	yes
MSTRG.1	0.1855	-2.431	0.0004	down	yes

MSTRG.1	0.1857	-2.429	0.0004	down	yes
MSTRG.1	5.3591	2.422	0.0005	up	yes
MSTRG.2	0.1905	-2.392	0.0005	down	yes
MSTRG.6	0.1934	-2.37	0.0005	down	yes
MSTRG.1	0.1936	-2.369	0.0005	down	yes
MSTRG.3	5.262	2.3956	0.0006	up	yes
MSTRG.1	0.1971	-2.343	0.0006	down	yes
MSTRG.2	5.1317	2.3594	0.0007	up	yes
MSTRG.2	5.0883	2.3472	0.0007	up	yes
MSTRG.3	0.2026	-2.304	0.0008	down	yes
MSTRG.2	0.202	-2.308	0.0008	down	yes
MSTRG.1	5.0541	2.3375	0.0008	up	yes
MSTRG.1	4.9429	2.3054	0.0009	up	yes
MSTRG.2	0.2102	-2.25	0.001	down	yes
MSTRG.1	4.825	2.2705	0.0011	up	yes
MSTRG.1	4.7902	2.2601	0.0011	up	yes
MSTRG.5	4.7361	2.2437	0.0011	up	yes
MSTRG.2	0.2136	-2.227	0.0012	down	yes
MSTRG.2	4.6828	2.2274	0.0012	up	yes
MSTRG.5	4.6682	2.2229	0.0012	up	yes
MSTRG.3	4.6571	2.2194	0.0012	up	yes
MSTRG.3	4.6283	2.2105	0.0013	up	yes
MSTRG.9	4.7067	2.2347	0.0014	up	yes
MSTRG.1	4.5647	2.1905	0.0014	up	yes
MSTRG.3	4.5407	2.1829	0.0015	up	yes
MSTRG.1	0.2182	-2.197	0.0015	down	yes
MSTRG.5	4.5151	2.1748	0.0015	up	yes
MSTRG.1	0.2185	-2.194	0.0015	down	yes
MSTRG.1	4.4524	2.1546	0.0016	up	yes
MSTRG.5	4.5597	2.1889	0.0016	up	yes
MSTRG.2	4.4155	2.1426	0.0018	up	yes
MSTRG.1	0.2306	-2.117	0.0019	down	yes
MSTRG.3	4.4464	2.1526	0.002	up	yes
MSTRG.2	4.3297	2.1143	0.002	up	yes
MSTRG.2	0.2336	-2.098	0.002	down	yes
MSTRG.5	4.3174	2.1102	0.002	up	yes
MSTRG.3	4.2945	2.1025	0.0023	up	yes
MSTRG.2	4.2789	2.0973	0.0025	up	yes
MSTRG.2	0.2459	-2.024	0.0028	down	yes
MSTRG.4	4.0916	2.0327	0.0028	up	yes
MSTRG.3	4.1421	2.0504	0.003	up	yes
MSTRG.2	0.2498	-2.001	0.0031	down	yes
MSTRG.1	4.0586	2.021	0.0031	up	yes
MSTRG.3	0.253	-1.983	0.0033	down	yes
MSTRG.2	4.0109	2.0039	0.0035	up	yes
MSTRG.2	3.9547	1.9836	0.0036	up	yes
MSTRG.1	0.266	-1.911	0.0046	down	yes
MSTRG.2	0.2664	-1.908	0.0046	down	yes
MSTRG.6	3.8176	1.9327	0.0049	up	yes
MSTRG.1	0.2627	-1.929	0.005	down	yes
MSTRG.5	3.7456	1.9052	0.0052	up	yes
MSTRG.3	3.7545	1.9086	0.0053	up	yes
MSTRG.8	0.265	-1.916	0.0054	down	yes
MSTRG.2	3.719	1.8949	0.0056	up	yes
MSTRG.3	3.7769	1.9172	0.0057	up	yes
MSTRG.8	0.2772	-1.851	0.0059	down	yes
MSTRG.1	0.2785	-1.844	0.0061	down	yes

MSTRG.2	3.6255	1.8582	0.0066	up	yes
MSTRG.1	3.637	1.8627	0.0066	up	yes
MSTRG.3	0.2766	-1.854	0.0066	down	yes
MSTRG.1	3.6634	1.8732	0.0067	up	yes
MSTRG.2	0.2777	-1.848	0.0067	down	yes
MSTRG.2	3.6124	1.853	0.0069	up	yes
MSTRG.1	3.5673	1.8348	0.0071	up	yes
MSTRG.1	0.2872	-1.8	0.0074	down	yes
MSTRG.3	3.5158	1.8138	0.0075	up	yes
MSTRG.7	3.544	1.8254	0.0075	up	yes
MSTRG.6	0.2834	-1.819	0.0077	down	yes
MSTRG.5	3.5147	1.8134	0.0078	up	yes
MSTRG.1	3.5361	1.8221	0.0079	up	yes
MSTRG.1	0.29	-1.786	0.0079	down	yes
MSTRG.2	0.2922	-1.775	0.0081	down	yes
MSTRG.3	0.2897	-1.787	0.0082	down	yes
MSTRG.1	3.4647	1.7927	0.0085	up	yes
MSTRG.9	0.2951	-1.761	0.0086	down	yes
MSTRG.1	3.4113	1.7703	0.0089	up	yes
MSTRG.2	3.4566	1.7894	0.0091	up	yes
MSTRG.3	3.3942	1.7631	0.0091	up	yes
MSTRG.3	3.3664	1.7512	0.0095	up	yes
MSTRG.3	3.3702	1.7528	0.0096	up	yes
MSTRG.3	3.3749	1.7548	0.0097	up	yes
MSTRG.3	3.4276	1.7772	0.0098	up	yes
MSTRG.1	3.3641	1.7502	0.0101	up	yes
MSTRG.1	0.3015	-1.73	0.0102	down	yes
MSTRG.7	3.4202	1.7741	0.0102	up	yes
MSTRG.1	3.3587	1.7479	0.0103	up	yes
MSTRG.2	3.3868	1.7599	0.0109	up	yes
MSTRG.2	3.3296	1.7354	0.0112	up	yes
MSTRG.2	0.308	-1.699	0.0114	down	yes
MSTRG.1	3.2609	1.7053	0.0115	up	yes
MSTRG.2	0.31	-1.69	0.0115	down	yes
MSTRG.2	0.3106	-1.687	0.0118	down	yes
MSTRG.5	0.313	-1.676	0.0121	down	yes
MSTRG.2	0.3137	-1.673	0.0123	down	yes
MSTRG.2	0.3188	-1.649	0.0137	down	yes
MSTRG.1	3.2362	1.6943	0.0137	up	yes
MSTRG.2	0.3224	-1.633	0.0149	down	yes
MSTRG.1	3.1674	1.6633	0.0151	up	yes
MSTRG.1	3.1531	1.6568	0.0153	up	yes
MSTRG.2	0.3269	-1.613	0.0155	down	yes
MSTRG.3	3.0876	1.6265	0.016	up	yes
MSTRG.3	0.3255	-1.619	0.0162	down	yes
MSTRG.3	3.1399	1.6507	0.0167	up	yes
MSTRG.3	3.0717	1.6191	0.0168	up	yes
MSTRG.2	0.3313	-1.594	0.0168	down	yes
MSTRG.2	0.3237	-1.627	0.0168	down	yes
MSTRG.2	0.3294	-1.602	0.018	down	yes
MSTRG.8	0.3296	-1.601	0.0181	down	yes
MSTRG.4	3.0853	1.6254	0.0182	up	yes
MSTRG.1	3.0062	1.5879	0.0185	up	yes
MSTRG.1	0.3361	-1.573	0.0187	down	yes
MSTRG.2	0.3379	-1.565	0.0188	down	yes
MSTRG.3	2.9919	1.5811	0.0188	up	yes
MSTRG.1	2.9909	1.5806	0.019	up	yes

MSTRG.1	3.0016	1.5857	0.0192	up	yes
MSTRG.1	0.3376	-1.567	0.0193	down	yes
MSTRG.9	3.0022	1.586	0.0195	up	yes
MSTRG.3	3.0445	1.6062	0.0196	up	yes
MSTRG.3	3.0256	1.5972	0.0206	up	yes
MSTRG.3	2.9625	1.5668	0.0207	up	yes
MSTRG.1	0.3429	-1.544	0.0209	down	yes
MSTRG.2	2.9493	1.5604	0.0212	up	yes
MSTRG.1	3.0025	1.5862	0.0213	up	yes
MSTRG.1	0.3408	-1.553	0.0213	down	yes
MSTRG.1	2.9546	1.563	0.0213	up	yes
MSTRG.5	0.3461	-1.531	0.0215	down	yes
MSTRG.3	2.9976	1.5838	0.0216	up	yes
MSTRG.1	2.9282	1.55	0.0219	up	yes
MSTRG.1	0.3482	-1.522	0.0221	down	yes
MSTRG.1	2.8972	1.5347	0.0228	up	yes
MSTRG.2	0.3505	-1.512	0.0229	down	yes
MSTRG.3	2.8858	1.529	0.0232	up	yes
MSTRG.3	0.3495	-1.517	0.0233	down	yes
MSTRG.2	0.345	-1.535	0.0233	down	yes
MSTRG.1	2.9358	1.5538	0.0239	up	yes
MSTRG.2	2.8532	1.5126	0.0245	up	yes
MSTRG.2	2.8578	1.5149	0.0247	up	yes
MSTRG.1	2.8573	1.5147	0.025	up	yes
MSTRG.1	2.8665	1.5193	0.025	up	yes
MSTRG.3	2.912	1.542	0.0251	up	yes
MSTRG.6	2.9172	1.5446	0.0251	up	yes
MSTRG.9	2.8779	1.525	0.0252	up	yes
MSTRG.1	2.8596	1.5158	0.0252	up	yes
MSTRG.1	2.8368	1.5043	0.0253	up	yes
MSTRG.9	2.8533	1.5126	0.0255	up	yes
MSTRG.1	0.3514	-1.509	0.0257	down	yes
MSTRG.1	2.8364	1.5041	0.0257	up	yes
MSTRG.1	2.8314	1.5015	0.0257	up	yes
MSTRG.3	2.8613	1.5167	0.0259	up	yes
MSTRG.9	2.8177	1.4945	0.0264	up	yes
MSTRG.1	2.8343	1.503	0.0266	up	yes
MSTRG.3	2.7969	1.4838	0.0276	up	yes
MSTRG.1	2.837	1.5044	0.0276	up	yes
MSTRG.1	2.7945	1.4826	0.0279	up	yes
MSTRG.1	2.847	1.5095	0.0279	up	yes
MSTRG.5	0.3642	-1.457	0.0281	down	yes
MSTRG.1	2.7864	1.4784	0.0281	up	yes
MSTRG.1	2.8152	1.4932	0.0283	up	yes
MSTRG.2	2.7781	1.4741	0.0283	up	yes
MSTRG.1	0.3634	-1.46	0.0284	down	yes
MSTRG.2	2.8034	1.4872	0.0286	up	yes
MSTRG.2	2.7736	1.4718	0.0287	up	yes
MSTRG.1	0.3661	-1.45	0.0288	down	yes
MSTRG.3	2.7609	1.4651	0.0298	up	yes
MSTRG.1	0.3638	-1.459	0.0299	down	yes
MSTRG.1	2.7458	1.4572	0.0301	up	yes
MSTRG.3	0.3654	-1.453	0.0302	down	yes
MSTRG.1	2.7383	1.4533	0.0304	up	yes
MSTRG.2	2.7316	1.4498	0.0306	up	yes
MSTRG.1	2.7508	1.4599	0.0308	up	yes
MSTRG.1	2.7825	1.4764	0.0308	up	yes

MSTRG.2	2.7187	1.4429	0.0318	up	yes
MSTRG.2	2.7263	1.4469	0.0329	up	yes
MSTRG.3	2.7355	1.4518	0.0331	up	yes
MSTRG.1	2.6951	1.4303	0.0331	up	yes
MSTRG.3	2.7164	1.4417	0.0335	up	yes
MSTRG.1	0.378	-1.404	0.0343	down	yes
MSTRG.1	2.6575	1.41	0.0354	up	yes
MSTRG.1	2.6596	1.4112	0.0356	up	yes
MSTRG.1	2.6448	1.4032	0.0365	up	yes
MSTRG.3	2.6699	1.4168	0.0375	up	yes
MSTRG.1	2.6405	1.4008	0.0382	up	yes
MSTRG.2	2.6353	1.398	0.0391	up	yes
MSTRG.9	2.6514	1.4068	0.0397	up	yes
MSTRG.1	2.5888	1.3723	0.0404	up	yes
MSTRG.2	0.3884	-1.364	0.041	down	yes
MSTRG.3	2.5854	1.3704	0.0421	up	yes
MSTRG.4	2.5736	1.3638	0.0428	up	yes
MSTRG.2	2.5613	1.3569	0.0428	up	yes
MSTRG.1	2.5736	1.3638	0.043	up	yes
MSTRG.3	2.595	1.3757	0.0432	up	yes
MSTRG.3	2.6164	1.3876	0.0434	up	yes
MSTRG.1	2.5701	1.3618	0.0435	up	yes
MSTRG.1	2.5512	1.3512	0.0441	up	yes
MSTRG.3	2.5655	1.3592	0.0444	up	yes
MSTRG.3	2.5723	1.363	0.0444	up	yes
MSTRG.4	2.597	1.3769	0.0451	up	yes
MSTRG.3	0.3974	-1.331	0.0458	down	yes
MSTRG.7	2.5445	1.3474	0.0461	up	yes
MSTRG.3	2.5732	1.3636	0.0472	up	yes
MSTRG.1	2.5466	1.3485	0.0478	up	yes
MSTRG.6	0.4026	-1.313	0.0479	down	yes
MSTRG.3	2.5108	1.3282	0.048	up	yes
MSTRG.4	2.5472	1.3489	0.0486	up	yes
MSTRG.4	0.4061	-1.3	0.0491	down	yes
MSTRG.9	2.4907	1.3166	0.0492	up	yes
MSTRG.8	0.4039	-1.308	0.0492	down	yes
MSTRG.1	2.4885	1.3153	0.0493	up	yes
MSTRG.1	2.5226	1.3349	0.0497	up	yes
MSTRG.2	2.5056	1.3252	0.0498	up	yes
