# Supplementary material for: Transcriptome analysis reveals a potential regulatory mechanism of the lnc-5423.6/IGFBP5 axis in the early stages of mouse thymic involution: lnc-5423.6/IGFBP5 axis regulates thymic involution
Source: Acta Biochim Biophys Sin (Shanghai). 2023 Apr 19;55(4):548–60. doi: 10.3724/abbs.2023042 (PMC10195152; doi:10.3724/abbs.2023042)
Supplement: Table_S9 [file Table_S9.pdf]

t_name	gene_id	gene_name
ENSMUST00000006814	MSTRG.23376	Abhd1
ENSMUST00000011196	MSTRG.13495	1700049J03Rik
ENSMUST00000020222	ENSMUSG00000020033	4930463O16Rik
ENSMUST00000021378	MSTRG.7092	4930512B01Rik
ENSMUST00000022049	ENSMUSG00000021566	Slc6a19os
ENSMUST00000022427	ENSMUSG00000021874	4933413J09Rik
ENSMUST00000022711	ENSMUSG00000022116	4930449E01Rik
ENSMUST00000028061	MSTRG.1715	4930562F07Rik
ENSMUST00000029642	ENSMUSG00000028009	1700061I17Rik
ENSMUST00000030352	MSTRG.21823	Lrp8os1
ENSMUST00000031274	MSTRG.24134	4930522N08Rik
ENSMUST00000031305	MSTRG.24610	Gm9754
ENSMUST00000031402	MSTRG.24886	Cct6a
ENSMUST00000031863	MSTRG.25760	1700111E14Rik
ENSMUST00000032872	ENSMUSG00000030636	1700010L04Rik
ENSMUST00000032900	MSTRG.29218	Sox6os
ENSMUST00000033651	MSTRG.3727	D630029K05Rik
ENSMUST00000034183	ENSMUSG00000031736	Crnde
ENSMUST00000034550	ENSMUSG00000032048	4930510E17Rik
ENSMUST00000035534	MSTRG.26928	4933440N22Rik
ENSMUST00000035860	ENSMUSG00000037884	1700017G19Rik
ENSMUST00000035939	MSTRG.28618	9330171B17Rik
ENSMUST00000036304	ENSMUSG00000037247	Pldi
ENSMUST00000037953	MSTRG.6417	2810032G03Rik
ENSMUST00000038032	MSTRG.8633	5033430I15Rik
ENSMUST00000038250	ENSMUSG00000089887	4930428N03Rik
ENSMUST00000038450	MSTRG.19891	4632404H12Rik
ENSMUST00000039080	MSTRG.386	8430432A02Rik
ENSMUST00000040608	ENSMUSG00000038917	3930402G23Rik
ENSMUST00000041178	ENSMUSG00000034764	1700006J14Rik
ENSMUST00000042671	ENSMUSG00000037535	1700021A07Rik
ENSMUST00000043553	ENSMUSG00000040657	1700063H04Rik
ENSMUST00000044500	MSTRG.9202	Gm4117
ENSMUST00000044964	MSTRG.23512	2210406O10Rik
ENSMUST00000046994	MSTRG.7541	3300002A11Rik
ENSMUST00000047020	ENSMUSG00000040705	A930016O22Rik
ENSMUST00000047607	ENSMUSG00000039798	2600006K01Rik
ENSMUST00000047876	MSTRG.19718	Gm10710
ENSMUST00000047953	MSTRG.27472	3010003L21Rik
ENSMUST00000048606	MSTRG.29893	4921522P10Rik
ENSMUST00000048965	MSTRG.9584	Gm9752
ENSMUST00000049518	MSTRG.9055	Zfp85os
ENSMUST00000049684	MSTRG.6347	Rab10os
ENSMUST00000050571	ENSMUSG00000044522	A730020M07Rik
ENSMUST00000050829	MSTRG.32435	2010007H06Rik
ENSMUST00000050921	ENSMUSG00000020887	A230052G05Rik
ENSMUST00000051089	MSTRG.20088	Gm42743
ENSMUST00000052189	MSTRG.30439	B230317F23Rik
ENSMUST00000053559	ENSMUSG00000044574	5031434C07Rik
ENSMUST00000054653	ENSMUSG00000047383	C2cd6b
ENSMUST00000054837	MSTRG.3029	1700120B22Rik
ENSMUST00000054947	MSTRG.31679	Fbxl12os
ENSMUST00000056145	ENSMUSG00000022187	Gm5546
ENSMUST00000056711	MSTRG.11367	4933427E11Rik
ENSMUST00000056774	MSTRG.14008	2410017I17Rik
ENSMUST00000056994	MSTRG.3781	4921513I03Rik

ENSMUST00000057134	MSTRG.29631	Etos1
ENSMUST00000057889	ENSMUSG00000045238	A730035I17Rik
ENSMUST00000058665	MSTRG.28273	Mtag2
ENSMUST00000058918	ENSMUSG00000048484	Gm7461
ENSMUST00000058942	MSTRG.3080	4930533K18Rik
ENSMUST00000059648	ENSMUSG00000021874	4933413J09Rik
ENSMUST00000059704	MSTRG.19222	4632415L05Rik
ENSMUST00000060147	ENSMUSG00000046764	A530053G22Rik
ENSMUST00000060657	ENSMUSG00000044633	B530045E10Rik
ENSMUST00000060946	MSTRG.20344	A930002I21Rik
ENSMUST00000061823	ENSMUSG00000045928	4933440M02Rik
ENSMUST00000062159	MSTRG.1695	1600012P17Rik
ENSMUST00000062252	MSTRG.9336	Gm9828
ENSMUST00000063103	ENSMUSG00000043773	1700048O20Rik
ENSMUST00000063891	ENSMUSG00000052368	Gm9873
ENSMUST00000064097	ENSMUSG00000052295	8030423F21Rik
ENSMUST00000064101	MSTRG.3720	5330438D12Rik
ENSMUST00000064349	ENSMUSG00000052479	A330008L17Rik
ENSMUST00000064591	MSTRG.28597	Gm9885
ENSMUST00000064646	ENSMUSG00000052658	5830454E08Rik
ENSMUST00000064809	MSTRG.20415	Gm9889
ENSMUST00000065310	MSTRG.29334	1700069B07Rik
ENSMUST00000065383	ENSMUSG00000053117	E330013P04Rik
ENSMUST00000065469	ENSMUSG00000053185	Gm9898
ENSMUST00000065519	MSTRG.23362	Gm9899
ENSMUST00000065709	MSTRG.1755	Gas5
ENSMUST00000065731	MSTRG.18765	2310001K24Rik
ENSMUST00000065740	MSTRG.29327	Gm9905
ENSMUST00000065741	MSTRG.28933	F730035P03Rik
ENSMUST00000065878	MSTRG.26180	4930597O21Rik
ENSMUST00000066038	MSTRG.31649	Gm16568
ENSMUST00000066087	MSTRG.5004	4930563E22Rik
ENSMUST00000066220	MSTRG.23927	Dancr
ENSMUST00000066316	MSTRG.27825	4732471J01Rik
ENSMUST00000066604	MSTRG.31866	Kirrel3os
ENSMUST00000066742	ENSMUSG00000053980	Gm9930
ENSMUST00000066988	MSTRG.2145	A430110L20Rik
ENSMUST00000067136	MSTRG.31149	1700082M22Rik
ENSMUST00000067161	MSTRG.30241	Gm9939
ENSMUST00000067450	ENSMUSG00000041789	2700046A07Rik
ENSMUST00000067468	MSTRG.1447	Gm4793
ENSMUST00000067500	MSTRG.20418	A930005H10Rik
ENSMUST00000067599	MSTRG.111	Gm9947
ENSMUST00000067618	ENSMUSG00000054510	Gm14461
ENSMUST00000067628	MSTRG.23005	Atad3aos
ENSMUST00000067770	MSTRG.25427	D730045B01Rik
ENSMUST00000068068	MSTRG.30055	1700041G16Rik
ENSMUST00000068194	MSTRG.29930	4931415C17Rik
ENSMUST00000068507	MSTRG.33976	A630012P03Rik
ENSMUST00000068526	ENSMUSG00000055125	M5C1000I18Rik
ENSMUST00000068548	ENSMUSG00000055134	9130017K11Rik
ENSMUST00000068704	MSTRG.12887	Mir99ahg
ENSMUST00000068730	ENSMUSG00000079564	Gm11149
ENSMUST00000068836	MSTRG.29423	Gm9967
ENSMUST00000068968	ENSMUSG00000055403	4933427D06Rik
ENSMUST00000069035	MSTRG.28879	A630091E08Rik
ENSMUST00000069431	ENSMUSG00000055704	Gm9978

ENSMUST00000069553	MSTRG.27074	A230083G16Rik
ENSMUST00000069573	MSTRG.2278	1700034H15Rik
ENSMUST00000069741	ENSMUSG00000055944	E130018O15Rik
ENSMUST00000069809	ENSMUSG00000055972	2810407A14Rik
ENSMUST00000069880	ENSMUSG00000056023	Gm9989
ENSMUST00000069943	ENSMUSG00000056061	Gata5os
ENSMUST00000070048	ENSMUSG00000056128	Gm9991
ENSMUST00000070085	MSTRG.20341	AI504432
ENSMUST00000070502	MSTRG.20359	Gm12500
ENSMUST00000070942	MSTRG.9012	A530095I07Rik
ENSMUST00000070956	MSTRG.6029	Gm11696
ENSMUST00000070987	ENSMUSG00000056699	Gm5533
ENSMUST00000071067	MSTRG.30873	C330011M18Rik
ENSMUST00000071101	ENSMUSG00000056771	Gm10010
ENSMUST00000071254	MSTRG.31591	Phxr4
ENSMUST00000071328	ENSMUSG00000075408	6030408B16Rik
ENSMUST00000071374	MSTRG.13285	BC002059
ENSMUST00000072180	ENSMUSG00000059244	Gm10062
ENSMUST00000072769	MSTRG.20496	Gm17494
ENSMUST00000074808	ENSMUSG00000062704	9430002A10Rik
ENSMUST00000074862	ENSMUSG00000061510	Gm10101
ENSMUST00000075081	MSTRG.23285	1500035N22Rik
ENSMUST00000076071	MSTRG.16518	Gm10115
ENSMUST00000076667	MSTRG.18771	0610039K10Rik
ENSMUST00000077142	MSTRG.28000	C230062I16Rik
ENSMUST00000077153	ENSMUSG00000062036	4932415M13Rik
ENSMUST00000077874	MSTRG.17662	Ptpmt1
ENSMUST00000078844	ENSMUSG00000060424	Pantr1
ENSMUST00000079186	ENSMUSG00000060416	Gm839
ENSMUST00000079529	MSTRG.13206	Airn
ENSMUST00000080024	MSTRG.28538	B130024G19Rik
ENSMUST00000080911	MSTRG.32300	C030014I23Rik
ENSMUST00000081331	ENSMUSG00000072723	Gm10044
ENSMUST00000081739	MSTRG.15766	Gm10143
ENSMUST00000081929	MSTRG.26374	Gm15401
ENSMUST00000084298	MSTRG.14645	Gm1976
ENSMUST00000084713	MSTRG.23183	Gm10475
ENSMUST00000084725	MSTRG.13396	D330041H03Rik
ENSMUST00000086432	MSTRG.1434	Ptprv
ENSMUST00000086914	ENSMUSG00000067103	AY702103
ENSMUST00000087588	MSTRG.14090	Gm17080
ENSMUST00000087947	MSTRG.15787	A430093F15Rik
ENSMUST00000088880	MSTRG.10708	Gm6994
ENSMUST00000089083	MSTRG.18528	Gm14164
ENSMUST00000089534	ENSMUSG00000078308	Gm7293
ENSMUST00000090237	MSTRG.25792	Gm10244
ENSMUST00000090409	MSTRG.9823	Gm10248
ENSMUST00000090537	MSTRG.14008	2410017I17Rik
ENSMUST00000090779	MSTRG.20080	Gm20634
ENSMUST00000091270	ENSMUSG00000069074	Gm10258
ENSMUST00000092265	ENSMUSG00000069554	I830134H01Rik
ENSMUST00000092680	MSTRG.2527	4933406P04Rik
ENSMUST00000093501	MSTRG.953	A530040E14Rik
ENSMUST00000093603	ENSMUSG00000070111	Gm10286
ENSMUST00000093873	MSTRG.32130	4930581F22Rik
ENSMUST00000093950	ENSMUSG00000070342	Gm10287
ENSMUST00000094769	MSTRG.22167	9930104L06Rik

ENSMUST00000095448	MSTRG.13603	E230001N04Rik
ENSMUST00000095903	MSTRG.6352	1110002L01Rik
ENSMUST00000096366	MSTRG.34264	1700010D01Rik
ENSMUST00000097320	MSTRG.14065	Runx2os1
ENSMUST00000097448	MSTRG.2128	H3f3aos
ENSMUST00000097514	ENSMUSG00000073528	Gm10530
ENSMUST00000097531	ENSMUSG00000073535	Gm5532
ENSMUST00000097535	MSTRG.1601	E330020D12Rik
ENSMUST00000097612	MSTRG.15014	Gm10545
ENSMUST00000097699	MSTRG.750	Apol7d
ENSMUST00000097740	ENSMUSG00000073679	Mxra8os
ENSMUST00000097750	ENSMUSG00000073686	Gm10564
ENSMUST00000097827	MSTRG.22557	Gm16287
ENSMUST00000097928	MSTRG.29819	Faddos
ENSMUST00000097947	MSTRG.29757	Gm10575
ENSMUST00000097982	ENSMUSG00000073803	4930544L04Rik
ENSMUST00000098075	ENSMUSG00000086712	AI427809
ENSMUST00000098110	MSTRG.29143	AA474408
ENSMUST00000098239	MSTRG.28981	Gm10602
ENSMUST00000098303	ENSMUSG00000054061	Gm9934
ENSMUST00000098305	MSTRG.28807	4632427E13Rik
ENSMUST00000098472	MSTRG.31124	4930513N10Rik
ENSMUST00000098653	ENSMUSG00000074252	Gm10654
ENSMUST00000098658	MSTRG.32602	Gm10655
ENSMUST00000098678	MSTRG.27808	D930028M14Rik
ENSMUST00000098778	MSTRG.27702	Gm10676
ENSMUST00000098781	MSTRG.30358	AA386476
ENSMUST00000098827	MSTRG.32345	Gm10684
ENSMUST00000098828	MSTRG.20127	Gm5544
ENSMUST00000098839	MSTRG.20133	Gm15441
ENSMUST00000098867	MSTRG.20035	6330562C20Rik
ENSMUST00000098916	MSTRG.29972	Gm10699
ENSMUST00000098926	MSTRG.31791	Gm10701
ENSMUST00000099104	MSTRG.19476	Gm10729
ENSMUST00000099292	MSTRG.18410	Gm5535
ENSMUST00000099331	MSTRG.9228	4833422C13Rik
ENSMUST00000099446	MSTRG.9094	CT009718.1
ENSMUST00000099647	ENSMUSG00000074987	Wt1os
ENSMUST00000099661	MSTRG.3050	Gm10797
ENSMUST00000099676	MSTRG.15797	AW112010
ENSMUST00000099693	MSTRG.17740	Gm10804
ENSMUST00000099699	MSTRG.17717	4631405J19Rik
ENSMUST00000099718	ENSMUSG00000085500	Gm16976
ENSMUST00000099888	ENSMUSG00000075184	F930017D23Rik
ENSMUST00000100000	ENSMUSG00000075277	Haglr
ENSMUST00000100291	MSTRG.10888	4930594M22Rik
ENSMUST00000100370	MSTRG.11685	1700001L05Rik
ENSMUST00000100426	MSTRG.16516	Gm10855
ENSMUST00000100448	MSTRG.10584	Gm6878
ENSMUST00000100526	MSTRG.25142	Gm10874
ENSMUST00000100641	MSTRG.24983	Gm10369
ENSMUST00000100683	MSTRG.11169	Gm10373
ENSMUST00000100778	MSTRG.27444	Gm6288
ENSMUST00000100817	ENSMUSG00000072679	D6Ertd474e
ENSMUST00000100821	MSTRG.27354	Gm7457
ENSMUST00000100944	ENSMUSG00000072769	Gm10419
ENSMUST00000101007	MSTRG.4874	9330160F10Rik

ENSMUST00000101077	MSTRG.4772	A530017D24Rik
ENSMUST00000101090	MSTRG.23978	2310040G07Rik
ENSMUST00000101121	ENSMUSG00000072874	Gm6116
ENSMUST00000101278	MSTRG.26374	Gm15401
ENSMUST00000101281	ENSMUSG00000073000	Gm10451
ENSMUST00000101450	ENSMUSG00000073103	Gm10466
ENSMUST00000101522	MSTRG.23235	5031425E22Rik
ENSMUST00000101556	ENSMUSG00000073174	Gm29254
ENSMUST00000105109	ENSMUSG00000078314	Gm14762
ENSMUST00000105311	MSTRG.13289	Gm6712
ENSMUST00000105408	MSTRG.3167	Gm10941
ENSMUST00000105610	MSTRG.12549	Gm17106
ENSMUST00000105760	MSTRG.3296	Gm17151
ENSMUST00000107095	MSTRG.20133	Gm15441
ENSMUST00000107628	MSTRG.5613	D030028A08Rik
ENSMUST00000107991	ENSMUSG00000095348	Gm3892
ENSMUST00000108464	MSTRG.19838	Gm17146
ENSMUST00000108741	ENSMUSG00000078838	Gm17382
ENSMUST00000109032	MSTRG.19018	Gm11008
ENSMUST00000109431	ENSMUSG00000078952	Lncenc1
ENSMUST00000109473	ENSMUSG00000078956	Gm14221
ENSMUST00000109506	ENSMUSG00000078957	1700060C20Rik
ENSMUST00000110279	ENSMUSG00000079045	Prox1os
ENSMUST00000110292	ENSMUSG00000079048	4933413L06Rik
ENSMUST00000110507	MSTRG.25385	8430423G03Rik
ENSMUST00000110860	MSTRG.29945	Gm15353
ENSMUST00000111075	ENSMUSG00000079174	Gm3054
ENSMUST00000111270	MSTRG.17717	4631405J19Rik
ENSMUST00000111461	MSTRG.17662	Ptpmt1
ENSMUST00000111588	ENSMUSG00000079048	4933413L06Rik
ENSMUST00000112103	MSTRG.1443	Gm38399
ENSMUST00000112652	MSTRG.9692	Gm11100
ENSMUST00000112775	ENSMUSG00000079407	1700110I01Rik
ENSMUST00000113396	MSTRG.982	Gm10552
ENSMUST00000113496	MSTRG.15674	Gm14966
ENSMUST00000113671	MSTRG.34311	Gm21986
ENSMUST00000113886	MSTRG.13899	Gm11131
ENSMUST00000114051	MSTRG.26378	Gm15402
ENSMUST00000114080	ENSMUSG00000079528	Clnkos
ENSMUST00000114185	MSTRG.26322	Gm1070
ENSMUST00000114854	MSTRG.33976	A630012P03Rik
ENSMUST00000115107	MSTRG.25671	Lncpint
ENSMUST00000115480	MSTRG.61	2610203C22Rik
ENSMUST00000116172	MSTRG.30600	Gm11175
ENSMUST00000116345	MSTRG.7404	Gm17193
ENSMUST00000117191	MSTRG.4169	Gm16140
ENSMUST00000117249	MSTRG.23927	Dancr
ENSMUST00000118575	MSTRG.15512	Gm16066
ENSMUST00000120155	ENSMUSG00000083545	Gm13320
ENSMUST00000120364	MSTRG.23927	Dancr
ENSMUST00000120398	MSTRG.20110	C920021L13Rik
ENSMUST00000122270	MSTRG.9234	1700119I11Rik
ENSMUST00000122365	MSTRG.12795	Gm28037
ENSMUST00000122806	MSTRG.18786	Gm14302
ENSMUST00000122813	MSTRG.692	2810408I11Rik
ENSMUST00000122831	MSTRG.19123	Uckl1os
ENSMUST00000122837	MSTRG.5571	Gm11520

ENSMUST00000122839	MSTRG.21201	Gm12404
ENSMUST00000122851	ENSMUSG00000085450	Gm13686
ENSMUST00000122854	MSTRG.5688	Gm12356
ENSMUST00000122858	MSTRG.19353	Gm15952
ENSMUST00000122881	MSTRG.8388	2610307P16Rik
ENSMUST00000122882	MSTRG.23512	2210406O10Rik
ENSMUST00000122898	MSTRG.20178	Gm12474
ENSMUST00000122911	MSTRG.4821	Gm12292
ENSMUST00000122923	ENSMUSG00000084910	C630043F03Rik
ENSMUST00000122926	MSTRG.8261	4930470G03Rik
ENSMUST00000122948	ENSMUSG00000087187	Gm13431
ENSMUST00000122953	ENSMUSG00000078314	Gm14762
ENSMUST00000122969	MSTRG.22190	Gm12946
ENSMUST00000122972	MSTRG.10082	Gm15601
ENSMUST00000122987	MSTRG.2723	Gm16365
ENSMUST00000122988	MSTRG.7703	Gm2800
ENSMUST00000122990	MSTRG.17470	Dlx1as
ENSMUST00000123009	MSTRG.13162	4930506C21Rik
ENSMUST00000123014	ENSMUSG00000087586	Gm6938
ENSMUST00000123016	ENSMUSG00000086496	Gm14204
ENSMUST00000123040	MSTRG.34412	2810403D21Rik
ENSMUST00000123044	ENSMUSG00000086712	AI427809
ENSMUST00000123048	MSTRG.18504	Gm14149
ENSMUST00000123051	ENSMUSG00000085840	Gm11261
ENSMUST00000123087	MSTRG.17505	Sp3os
ENSMUST00000123094	MSTRG.5517	Dgkeos
ENSMUST00000123107	MSTRG.21742	0610043K17Rik
ENSMUST00000123110	MSTRG.19123	Uckl1os
ENSMUST00000123117	MSTRG.29439	Gm21984
ENSMUST00000123120	ENSMUSG00000086404	Gm6787
ENSMUST00000123127	ENSMUSG00000086712	AI427809
ENSMUST00000123129	ENSMUSG00000086560	Gm13372
ENSMUST00000123149	MSTRG.25679	2210408F21Rik
ENSMUST00000123155	MSTRG.20251	Gm15886
ENSMUST00000123160	ENSMUSG00000086749	Gm12037
ENSMUST00000123210	ENSMUSG00000086396	4930555B11Rik
ENSMUST00000123212	MSTRG.8673	Phf2os1
ENSMUST00000123224	ENSMUSG00000085141	Gm13429
ENSMUST00000123231	MSTRG.1549	Gm15584
ENSMUST00000123263	MSTRG.17712	Gm13791
ENSMUST00000123272	ENSMUSG00000085399	Foxd2os
ENSMUST00000123278	ENSMUSG00000086375	Gm12127
ENSMUST00000123318	MSTRG.12965	Gm15965
ENSMUST00000123341	MSTRG.13162	4930506C21Rik
ENSMUST00000123348	MSTRG.3714	Kcnmb4os2
ENSMUST00000123363	MSTRG.23572	Gm16014
ENSMUST00000123377	MSTRG.17906	4930533B01Rik
ENSMUST00000123388	MSTRG.4693	4933439C10Rik
ENSMUST00000123390	ENSMUSG00000085725	Gm15873
ENSMUST00000123402	ENSMUSG00000085794	Vax2os
ENSMUST00000123403	MSTRG.18133	4930417H01Rik
ENSMUST00000123420	ENSMUSG00000085532	B430319H21Rik
ENSMUST00000123423	MSTRG.4784	Lrrc75aos2
ENSMUST00000123441	MSTRG.17853	Gm13954
ENSMUST00000123455	ENSMUSG00000086074	Gm13274
ENSMUST00000123457	MSTRG.2993	Hk1os
ENSMUST00000123459	MSTRG.3987	Gm11946

ENSMUST00000123462	MSTRG.33823	A230072C01Rik
ENSMUST00000123483	MSTRG.16853	Gm13387
ENSMUST00000123509	MSTRG.28534	Gm16158
ENSMUST00000123517	MSTRG.28250	Gm15545
ENSMUST00000123535	ENSMUSG00000054944	5330416C01Rik
ENSMUST00000123544	MSTRG.4756	Map2k3os
ENSMUST00000123548	MSTRG.22831	Gm13205
ENSMUST00000123574	ENSMUSG00000086416	Gm14002
ENSMUST00000123581	MSTRG.25498	A430035B10Rik
ENSMUST00000123610	MSTRG.23282	Prkag2os2
ENSMUST00000123613	ENSMUSG00000085838	Chn1os1
ENSMUST00000123620	ENSMUSG00000085375	Gm12506
ENSMUST00000123623	ENSMUSG00000085218	BB218582
ENSMUST00000123633	MSTRG.17884	Ccdc34os
ENSMUST00000123644	MSTRG.27558	Gm15510
ENSMUST00000123663	MSTRG.17515	Gm13707
ENSMUST00000123668	MSTRG.29774	Nctc1
ENSMUST00000123699	MSTRG.17296	Gm13483
ENSMUST00000123700	MSTRG.5478	Mir142hg
ENSMUST00000123703	MSTRG.30276	B430010I23Rik
ENSMUST00000123707	MSTRG.34071	Gm16189
ENSMUST00000123734	MSTRG.4147	Eldr
ENSMUST00000123757	MSTRG.25671	Gm13834
ENSMUST00000123764	ENSMUSG00000086226	Gm12660
ENSMUST00000123777	MSTRG.11121	Gm15941
ENSMUST00000123784	MSTRG.5670	Gm11629
ENSMUST00000123812	MSTRG.5883	Arhgap27os2
ENSMUST00000123841	MSTRG.34350	Ftx
ENSMUST00000123850	MSTRG.34376	5530601H04Rik
ENSMUST00000123882	MSTRG.6195	Gm16045
ENSMUST00000123891	MSTRG.17786	Gm13919
ENSMUST00000123894	MSTRG.32899	4930562D21Rik
ENSMUST00000123899	ENSMUSG00000086205	Gm12679
ENSMUST00000123905	MSTRG.12475	Gm15742
ENSMUST00000123911	ENSMUSG00000086644	Gm13470
ENSMUST00000123920	MSTRG.29946	Gm15351
ENSMUST00000123933	ENSMUSG00000087383	Gm12446
ENSMUST00000123944	ENSMUSG00000086496	Gm14204
ENSMUST00000123949	MSTRG.4698	Gm12264
ENSMUST00000123998	ENSMUSG00000087143	A830082K12Rik
ENSMUST00000124015	MSTRG.25289	2900089D17Rik
ENSMUST00000124018	MSTRG.33737	Rbm3os
ENSMUST00000124028	MSTRG.6051	BC006965
ENSMUST00000124104	MSTRG.25498	A430035B10Rik
ENSMUST00000124106	ENSMUSG00000021268	Meg3
ENSMUST00000124108	MSTRG.4901	Dnah2os
ENSMUST00000124122	ENSMUSG00000085558	4930412C18Rik
ENSMUST00000124166	MSTRG.21674	Junos
ENSMUST00000124182	MSTRG.3882	Gm16217
ENSMUST00000124198	MSTRG.25390	Gm15408
ENSMUST00000124210	MSTRG.17307	A430018G15Rik
ENSMUST00000124238	ENSMUSG00000086137	Gm16248
ENSMUST00000124246	MSTRG.26827	Gt(ROSA)26Sor
ENSMUST00000124274	MSTRG.4093	Gm11973
ENSMUST00000124276	MSTRG.28039	Arhgap33os
ENSMUST00000124298	MSTRG.25150	6330418K02Rik
ENSMUST00000124319	ENSMUSG00000086071	Gm12354

ENSMUST00000124322	MSTRG.16804	Gm13380
ENSMUST00000124326	MSTRG.7076	Gm15561
ENSMUST00000124329	MSTRG.34412	2810403D21Rik
ENSMUST00000124336	MSTRG.18312	AV099323
ENSMUST00000124366	ENSMUSG00000085541	Gm16010
ENSMUST00000124374	MSTRG.11850	Rapgef3os2
ENSMUST00000124376	MSTRG.12985	Gm16310
ENSMUST00000124378	MSTRG.24547	Gm13830
ENSMUST00000124392	MSTRG.23322	Gm16058
ENSMUST00000124394	MSTRG.24280	Gm28050
ENSMUST00000124435	MSTRG.18792	Gm11457
ENSMUST00000124439	MSTRG.17991	Gm14207
ENSMUST00000124462	MSTRG.5882	Arhgap27os3
ENSMUST00000124469	MSTRG.19357	Mccc1os
ENSMUST00000124471	MSTRG.22355	Gm12999
ENSMUST00000124489	ENSMUSG00000084768	Gm13605
ENSMUST00000124503	MSTRG.4390	Gm12122
ENSMUST00000124513	MSTRG.34716	Gm15247
ENSMUST00000124522	ENSMUSG00000045709	Smkr-ps
ENSMUST00000124572	ENSMUSG00000086381	AV064505
ENSMUST00000124589	ENSMUSG00000086952	Gm12596
ENSMUST00000124606	ENSMUSG00000085007	Gm11549
ENSMUST00000124622	MSTRG.17324	Gm13522
ENSMUST00000124643	MSTRG.23504	4931431C16Rik
ENSMUST00000124654	MSTRG.17356	Gm13571
ENSMUST00000124664	MSTRG.33465	4930520O04Rik
ENSMUST00000124673	MSTRG.29203	4933406I18Rik
ENSMUST00000124728	ENSMUSG00000086630	Gm14397
ENSMUST00000124738	MSTRG.22322	Gm12992
ENSMUST00000124746	ENSMUSG00000087416	Gm15906
ENSMUST00000124754	MSTRG.21881	Dmrta2os
ENSMUST00000124762	MSTRG.5004	4930563E22Rik
ENSMUST00000124774	MSTRG.18403	Kif16bos
ENSMUST00000124786	MSTRG.21744	Gm12798
ENSMUST00000124792	MSTRG.21069	Gm11906
ENSMUST00000124795	MSTRG.18060	AV039307
ENSMUST00000124811	MSTRG.25679	2210408F21Rik
ENSMUST00000124813	MSTRG.28529	Gm16157
ENSMUST00000124818	MSTRG.4095	Snhg15
ENSMUST00000124829	ENSMUSG00000085252	8430437L04Rik
ENSMUST00000124833	MSTRG.8295	Gm11290
ENSMUST00000124842	MSTRG.33960	Firre
ENSMUST00000124848	ENSMUSG00000085836	Gm13074
ENSMUST00000124850	MSTRG.18870	9230111E07Rik
ENSMUST00000124853	MSTRG.26997	Gm15856
ENSMUST00000124855	ENSMUSG00000086752	Gm11674
ENSMUST00000124865	MSTRG.5592	Gm11537
ENSMUST00000124897	MSTRG.27704	Mypopos
ENSMUST00000124901	ENSMUSG00000086588	Gm11729
ENSMUST00000124929	MSTRG.30501	1700125H03Rik
ENSMUST00000124940	MSTRG.9758	1810062O18Rik
ENSMUST00000124953	MSTRG.32735	Gm15563
ENSMUST00000124975	MSTRG.22742	Gm13166
ENSMUST00000124976	MSTRG.2474	Gm16234
ENSMUST00000124988	ENSMUSG00000079000	Dnmt3bos
ENSMUST00000124989	ENSMUSG00000085305	Gm12410
ENSMUST00000125001	MSTRG.17662	Ptpmt1

ENSMUST00000125022	MSTRG.34495	BC065397
ENSMUST00000125056	ENSMUSG00000086706	Gm15848
ENSMUST00000125068	MSTRG.4379	Gm12120
ENSMUST00000125071	MSTRG.33108	Gm16185
ENSMUST00000125095	MSTRG.17290	Gm13480
ENSMUST00000125117	MSTRG.14816	1700001G01Rik
ENSMUST00000125121	ENSMUSG00000086658	Gm14260
ENSMUST00000125131	MSTRG.28721	2310044K18Rik
ENSMUST00000125158	ENSMUSG00000087400	Gm15270
ENSMUST00000125161	ENSMUSG00000085451	Gm15137
ENSMUST00000125173	MSTRG.16764	1810059C17Rik
ENSMUST00000125186	MSTRG.20010	B230398E01Rik
ENSMUST00000125191	MSTRG.24831	Tctn2
ENSMUST00000125194	ENSMUSG00000084850	Gm12092
ENSMUST00000125198	MSTRG.16907	Gm13562
ENSMUST00000125204	MSTRG.4246	Gm12060
ENSMUST00000125244	MSTRG.30947	A230103J11Rik
ENSMUST00000125250	MSTRG.24398	Gm15787
ENSMUST00000125269	MSTRG.6164	Gm11739
ENSMUST00000125277	ENSMUSG00000087051	Gm12730
ENSMUST00000125308	MSTRG.7188	Gm15283
ENSMUST00000125317	ENSMUSG00000085333	1700030A11Rik
ENSMUST00000125318	MSTRG.28211	Gm28496
ENSMUST00000125333	MSTRG.32523	Man2c1os
ENSMUST00000125345	MSTRG.18558	2500004C02Rik
ENSMUST00000125354	MSTRG.18206	Gm14005
ENSMUST00000125355	MSTRG.29816	Gm14372
ENSMUST00000125358	MSTRG.8388	2610307P16Rik
ENSMUST00000125365	ENSMUSG00000087306	A230004M16Rik
ENSMUST00000125374	ENSMUSG00000085412	Halr1
ENSMUST00000125396	MSTRG.15406	F830208F22Rik
ENSMUST00000125401	ENSMUSG00000086541	Has2os
ENSMUST00000125406	ENSMUSG00000086003	B230206L02Rik
ENSMUST00000125412	ENSMUSG00000078601	Gm12525
ENSMUST00000125413	ENSMUSG00000086382	Chrna1os
ENSMUST00000125419	MSTRG.34350	Ftx
ENSMUST00000125438	MSTRG.21674	Junos
ENSMUST00000125464	MSTRG.21820	Lrp8os3
ENSMUST00000125494	ENSMUSG00000087625	4930419G24Rik
ENSMUST00000125518	MSTRG.16912	Gm13563
ENSMUST00000125538	ENSMUSG00000087410	2310065F04Rik
ENSMUST00000125558	MSTRG.9751	7330404K18Rik
ENSMUST00000125569	ENSMUSG00000087358	4930453H23Rik
ENSMUST00000125577	MSTRG.6209	Gm11724
ENSMUST00000125582	MSTRG.22661	Gm13052
ENSMUST00000125600	ENSMUSG00000069796	Gm11426
ENSMUST00000125607	MSTRG.6198	Gm11725
ENSMUST00000125609	ENSMUSG00000085146	Eif2c5
ENSMUST00000125611	ENSMUSG00000085702	Mecomos
ENSMUST00000125626	MSTRG.5344	E230016K23Rik
ENSMUST00000125653	MSTRG.6099	Ict1os
ENSMUST00000125657	MSTRG.26433	Ccdc142os
ENSMUST00000125662	MSTRG.25150	6330418K02Rik
ENSMUST00000125681	ENSMUSG00000085526	Gm16083
ENSMUST00000125700	ENSMUSG00000087505	Gm15241
ENSMUST00000125820	ENSMUSG00000085824	Platr9
ENSMUST00000125829	ENSMUSG00000086146	Gm15729

ENSMUST00000125841	MSTRG.18859	Zfas1
ENSMUST00000125852	MSTRG.20367	1700010K24Rik
ENSMUST00000125853	MSTRG.4960	Gm12316
ENSMUST00000125854	ENSMUSG00000086454	Platr14
ENSMUST00000125869	MSTRG.17091	Gm13524
ENSMUST00000125873	MSTRG.6051	BC006965
ENSMUST00000125884	ENSMUSG00000085217	6030471H07Rik
ENSMUST00000125902	MSTRG.12966	4930404I05Rik
ENSMUST00000125903	MSTRG.30278	Gm16193
ENSMUST00000125908	MSTRG.32246	Gm16322
ENSMUST00000125917	MSTRG.21674	Junos
ENSMUST00000125934	MSTRG.750	Apol7d
ENSMUST00000125956	ENSMUSG00000086255	Gm11534
ENSMUST00000125967	MSTRG.5503	C030037D09Rik
ENSMUST00000126002	MSTRG.17731	D930015M05Rik
ENSMUST00000126009	MSTRG.23034	Gm16008
ENSMUST00000126011	ENSMUSG00000087160	Gm16336
ENSMUST00000126016	MSTRG.5363	4930502E09Rik
ENSMUST00000126025	ENSMUSG00000086585	Gm16126
ENSMUST00000126080	MSTRG.18240	9830144P21Rik
ENSMUST00000126086	MSTRG.18915	Gm16796
ENSMUST00000126095	ENSMUSG00000086188	Gm15169
ENSMUST00000126099	MSTRG.19392	Gm12531
ENSMUST00000126122	MSTRG.30116	Gm16159
ENSMUST00000126170	MSTRG.33711	Gm36995
ENSMUST00000126205	ENSMUSG00000087530	Gm15533
ENSMUST00000126219	MSTRG.17653	4933423P22Rik
ENSMUST00000126225	MSTRG.21086	Bach2os
ENSMUST00000126231	MSTRG.22650	4930455G09Rik
ENSMUST00000126238	MSTRG.32634	Gm16759
ENSMUST00000126252	ENSMUSG00000087084	Gm13016
ENSMUST00000126265	ENSMUSG00000085772	D630024D03Rik
ENSMUST00000126270	ENSMUSG00000085931	Gm12648
ENSMUST00000126285	ENSMUSG00000031559	4930555F03Rik
ENSMUST00000126286	MSTRG.33729	Gm14820
ENSMUST00000126289	ENSMUSG00000021268	Meg3
ENSMUST00000126294	ENSMUSG00000087127	Gm12756
ENSMUST00000126326	MSTRG.24580	Gm13837
ENSMUST00000126339	ENSMUSG00000086807	Platr21
ENSMUST00000126361	ENSMUSG00000086578	Gm13583
ENSMUST00000126380	MSTRG.22338	Snhg12
ENSMUST00000126385	MSTRG.922	Gm16341
ENSMUST00000126401	ENSMUSG00000086905	Gm13716
ENSMUST00000126427	MSTRG.12243	Gm15869
ENSMUST00000126447	MSTRG.27441	Gm15762
ENSMUST00000126462	MSTRG.17490	Rapgef4os1
ENSMUST00000126467	ENSMUSG00000085316	D330050G23Rik
ENSMUST00000126470	ENSMUSG00000084890	A830036E02Rik
ENSMUST00000126472	ENSMUSG00000072591	5930412G12Rik
ENSMUST00000126476	MSTRG.7541	3300002A11Rik
ENSMUST00000126480	MSTRG.5555	Gm11542
ENSMUST00000126498	MSTRG.25719	Gm13856
ENSMUST00000126516	MSTRG.750	Apol7d
ENSMUST00000126537	MSTRG.26031	5430402O13Rik
ENSMUST00000126549	ENSMUSG00000084938	BB557941
ENSMUST00000126572	ENSMUSG00000085845	Gm13944
ENSMUST00000126596	MSTRG.1548	A230059L01Rik

ENSMUST00000126622	MSTRG.9336	Gm9828
ENSMUST00000126642	ENSMUSG00000084822	Myadml2os
ENSMUST00000126646	MSTRG.21543	A230083N12Rik
ENSMUST00000126647	ENSMUSG00000085840	Gm11261
ENSMUST00000126659	MSTRG.16509	Gm13388
ENSMUST00000126668	MSTRG.21854	Gm12743
ENSMUST00000126672	MSTRG.19123	Uckl1os
ENSMUST00000126677	MSTRG.12046	Gm15537
ENSMUST00000126679	MSTRG.17614	Gm13710
ENSMUST00000126693	MSTRG.25099	Gm15498
ENSMUST00000126735	MSTRG.18404	Pcsk2os1
ENSMUST00000126769	ENSMUSG00000086008	Gm8817
ENSMUST00000126849	MSTRG.12175	Gm15558
ENSMUST00000126902	MSTRG.28861	Rsf1os1
ENSMUST00000126910	MSTRG.18316	1700026D11Rik
ENSMUST00000126922	ENSMUSG00000087277	2010013B24Rik
ENSMUST00000126926	MSTRG.19879	Gm15417
ENSMUST00000126936	MSTRG.15931	Pip5k1bos
ENSMUST00000126953	MSTRG.17653	4933423P22Rik
ENSMUST00000126959	ENSMUSG00000086219	Srrm4os
ENSMUST00000126965	MSTRG.3778	Grip1os1
ENSMUST00000126966	ENSMUSG00000086077	Gm14396
ENSMUST00000126967	MSTRG.16805	Gm13375
ENSMUST00000127001	MSTRG.6679	2410018L13Rik
ENSMUST00000127006	MSTRG.18874	Gm11476
ENSMUST00000127010	MSTRG.28089	Gm12758
ENSMUST00000127015	MSTRG.21142	Gm12367
ENSMUST00000127017	MSTRG.5211	Gm11192
ENSMUST00000127041	ENSMUSG00000104861	3110039M20Rik
ENSMUST00000127049	MSTRG.24585	Gm14508
ENSMUST00000127060	ENSMUSG00000086153	Gm12923
ENSMUST00000127070	MSTRG.3714	Kcnmb4os2
ENSMUST00000127074	MSTRG.5314	Slfn5os
ENSMUST00000127107	ENSMUSG00000085888	Gm12224
ENSMUST00000127115	MSTRG.12767	Gm15518
ENSMUST00000127129	MSTRG.7128	3110056K07Rik
ENSMUST00000127150	MSTRG.17278	Zeb2os
ENSMUST00000127230	ENSMUSG00000087033	Gm14155
ENSMUST00000127237	ENSMUSG00000086454	Platr14
ENSMUST00000127257	MSTRG.22167	9930104L06Rik
ENSMUST00000127263	MSTRG.6053	2610035D17Rik
ENSMUST00000127284	MSTRG.912	C130036L24Rik
ENSMUST00000127301	MSTRG.31319	9430091E24Rik
ENSMUST00000127307	ENSMUSG00000087167	Gm15891
ENSMUST00000127328	ENSMUSG00000085125	Gm16070
ENSMUST00000127333	ENSMUSG00000085502	Gm12320
ENSMUST00000127337	ENSMUSG00000075437	Gm11681
ENSMUST00000127350	ENSMUSG00000086454	Platr14
ENSMUST00000127359	ENSMUSG00000086746	Gm15222
ENSMUST00000127391	MSTRG.21875	9630013D21Rik
ENSMUST00000127408	ENSMUSG00000085500	Gm16976
ENSMUST00000127413	MSTRG.13038	1600002D24Rik
ENSMUST00000127424	ENSMUSG00000087059	Gm12339
ENSMUST00000127429	ENSMUSG00000072589	Gm10371
ENSMUST00000127441	MSTRG.18870	Gm14321
ENSMUST00000127450	MSTRG.6256	Rptoros
ENSMUST00000127462	ENSMUSG00000086631	Gm12784

ENSMUST00000127488	MSTRG.4093	Gm11973
ENSMUST00000127498	MSTRG.642	Pard3bos2
ENSMUST00000127521	MSTRG.33773	5730405O15Rik
ENSMUST00000127525	MSTRG.18240	9830144P21Rik
ENSMUST00000127527	MSTRG.30900	Gm16183
ENSMUST00000127533	MSTRG.34376	5530601H04Rik
ENSMUST00000127555	MSTRG.15531	Tmem134
ENSMUST00000127563	MSTRG.24527	Gm13822
ENSMUST00000127564	MSTRG.3785	Gm15910
ENSMUST00000127576	MSTRG.3161	4930483K19Rik
ENSMUST00000127600	MSTRG.17477	Gm13663
ENSMUST00000127620	MSTRG.335	Gm16150
ENSMUST00000127631	MSTRG.30498	Gm15991
ENSMUST00000127642	MSTRG.16890	Fcnaos
ENSMUST00000127668	ENSMUSG00000086720	Plxna4os3
ENSMUST00000127672	ENSMUSG00000086353	Gm13481
ENSMUST00000127673	ENSMUSG00000085982	9530051G07Rik
ENSMUST00000127678	MSTRG.21651	Gm12655
ENSMUST00000127688	ENSMUSG00000084774	Gm14110
ENSMUST00000127697	MSTRG.12063	Gm15835
ENSMUST00000127708	MSTRG.15315	B430212C06Rik
ENSMUST00000127711	MSTRG.22462	Gm16224
ENSMUST00000127752	MSTRG.22930	Gm13096
ENSMUST00000127757	MSTRG.30720	Gm11033
ENSMUST00000127763	MSTRG.25384	Gm15410
ENSMUST00000127764	MSTRG.4626	Gm12238
ENSMUST00000127786	MSTRG.34338	Xist
ENSMUST00000127792	MSTRG.24576	C330018A13Rik
ENSMUST00000127817	ENSMUSG00000086111	Gm15326
ENSMUST00000127849	MSTRG.17401	Gm13630
ENSMUST00000127875	ENSMUSG00000085774	Gm13055
ENSMUST00000127920	ENSMUSG00000085129	5031425F14Rik
ENSMUST00000127936	ENSMUSG00000085987	Gm13403
ENSMUST00000127989	MSTRG.24801	Gm16001
ENSMUST00000127990	MSTRG.25197	Gm16120
ENSMUST00000128000	MSTRG.22338	Snhg12
ENSMUST00000128017	ENSMUSG00000084861	1700095J07Rik
ENSMUST00000128026	MSTRG.18558	2500004C02Rik
ENSMUST00000128078	MSTRG.5512	Gm11496
ENSMUST00000128094	MSTRG.21674	Junos
ENSMUST00000128111	ENSMUSG00000087067	Gm11532
ENSMUST00000128125	MSTRG.32520	Gm10658
ENSMUST00000128131	MSTRG.22338	Snhg12
ENSMUST00000128159	MSTRG.12747	Gm15638
ENSMUST00000128160	MSTRG.20015	4930481B07Rik
ENSMUST00000128165	MSTRG.17497	Rapgef4os2
ENSMUST00000128176	MSTRG.18278	A730017L22Rik
ENSMUST00000128178	ENSMUSG00000021268	Meg3
ENSMUST00000128180	ENSMUSG00000079603	Gm13218
ENSMUST00000128181	MSTRG.27760	Gm16175
ENSMUST00000128183	ENSMUSG00000085844	Gm11690
ENSMUST00000128198	ENSMUSG00000086111	Gm15326
ENSMUST00000128213	MSTRG.5400	2610027K06Rik
ENSMUST00000128214	ENSMUSG00000085378	Gm11415
ENSMUST00000128218	MSTRG.25812	Gm16272
ENSMUST00000128240	MSTRG.15187	Gm15345
ENSMUST00000128242	ENSMUSG00000085141	Gm13429

ENSMUST00000128263	MSTRG.22013	Gm12841
ENSMUST00000128292	MSTRG.4801	Zfp286os
ENSMUST00000128298	MSTRG.17058	Gm13610
ENSMUST00000128307	ENSMUSG00000087040	Gm14033
ENSMUST00000128327	ENSMUSG00000074508	Gm10706
ENSMUST00000128342	MSTRG.11572	Gm16576
ENSMUST00000128385	MSTRG.22652	Gm13053
ENSMUST00000128415	MSTRG.33234	Gm15619
ENSMUST00000128443	MSTRG.20992	1700123M08Rik
ENSMUST00000128458	ENSMUSG00000021268	Meg3
ENSMUST00000128476	MSTRG.31260	Gm16208
ENSMUST00000128520	MSTRG.25196	Gm16121
ENSMUST00000128521	ENSMUSG00000086028	Gm12243
ENSMUST00000128542	MSTRG.15627	4930481A15Rik
ENSMUST00000128545	MSTRG.2136	2210411M09Rik
ENSMUST00000128562	ENSMUSG00000086559	Gm14426
ENSMUST00000128563	ENSMUSG00000087628	Gm13028
ENSMUST00000128569	MSTRG.23286	2900005J15Rik
ENSMUST00000128571	ENSMUSG00000063018	2010204K13Rik
ENSMUST00000128577	MSTRG.18528	Gm14164
ENSMUST00000128588	ENSMUSG00000086009	4930587A21Rik
ENSMUST00000128589	MSTRG.16510	Gm13391
ENSMUST00000128591	ENSMUSG00000085020	2310081O03Rik
ENSMUST00000128634	MSTRG.27444	Gm6288
ENSMUST00000128647	MSTRG.4784	Lrrc75aos2
ENSMUST00000128652	ENSMUSG00000086501	4930597A21Rik
ENSMUST00000128654	ENSMUSG00000085743	8430419K02Rik
ENSMUST00000128662	MSTRG.29838	Gm16180
ENSMUST00000128681	ENSMUSG00000078955	Gm14222
ENSMUST00000128728	MSTRG.24628	Gm15690
ENSMUST00000128800	MSTRG.22905	Gm13091
ENSMUST00000128804	MSTRG.11121	Gm15941
ENSMUST00000128806	MSTRG.3987	Gm11946
ENSMUST00000128807	MSTRG.3034	Gm16145
ENSMUST00000128841	MSTRG.22977	Gm16024
ENSMUST00000128848	MSTRG.9758	1810062O18Rik
ENSMUST00000128858	ENSMUSG00000087252	Gm14379
ENSMUST00000128859	MSTRG.17397	Gm13618
ENSMUST00000128872	MSTRG.19757	Gm15535
ENSMUST00000128888	ENSMUSG00000086425	F730016J06Rik
ENSMUST00000128894	MSTRG.15066	Gm15336
ENSMUST00000128904	ENSMUSG00000084890	A830036E02Rik
ENSMUST00000128914	MSTRG.9075	Gm15912
ENSMUST00000128919	ENSMUSG00000085058	8030453O22Rik
ENSMUST00000128943	MSTRG.15315	B430212C06Rik
ENSMUST00000128960	MSTRG.5406	Brip1os
ENSMUST00000128982	MSTRG.33553	Gm2415
ENSMUST00000128996	ENSMUSG00000085665	Gm12059
ENSMUST00000128998	ENSMUSG00000066060	Gm12866
ENSMUST00000129000	MSTRG.18075	Gm14978
ENSMUST00000129007	MSTRG.24411	A630023P12Rik
ENSMUST00000129058	ENSMUSG00000109460	Gm45591
ENSMUST00000129059	MSTRG.28730	2610206C17Rik
ENSMUST00000129067	ENSMUSG00000087678	Gm14120
ENSMUST00000129089	MSTRG.17213	Nr6a1os
ENSMUST00000129097	MSTRG.22923	4930589P08Rik
ENSMUST00000129102	MSTRG.21424	Gm12536

ENSMUST00000129108	ENSMUSG00000087516	Tbx3os1
ENSMUST00000129114	MSTRG.17440	Mettl5os
ENSMUST00000129126	MSTRG.4652	Gm12246
ENSMUST00000129139	MSTRG.5989	Gm11712
ENSMUST00000129158	MSTRG.29987	Gm16346
ENSMUST00000129164	MSTRG.15144	C030005K06Rik
ENSMUST00000129191	MSTRG.2726	E130307A14Rik
ENSMUST00000129207	MSTRG.21012	Gm11844
ENSMUST00000129237	MSTRG.9758	1810062O18Rik
ENSMUST00000129245	ENSMUSG00000021268	Meg3
ENSMUST00000129275	MSTRG.23922	Usp46os1
ENSMUST00000129329	ENSMUSG00000087146	Gm15205
ENSMUST00000129337	MSTRG.5453	Gm11508
ENSMUST00000129343	MSTRG.26514	A430078I02Rik
ENSMUST00000129353	MSTRG.23983	1700112J05Rik
ENSMUST00000129364	MSTRG.18812	Zfp335os
ENSMUST00000129373	MSTRG.29750	B230206H07Rik
ENSMUST00000129379	MSTRG.6260	Gm11767
ENSMUST00000129408	MSTRG.30446	Gm16178
ENSMUST00000129409	MSTRG.28831	C230038L03Rik
ENSMUST00000129425	ENSMUSG00000085772	D630024D03Rik
ENSMUST00000129441	MSTRG.5434	Gm11491
ENSMUST00000129460	MSTRG.21708	Gm12705
ENSMUST00000129470	MSTRG.3024	Gm16135
ENSMUST00000129501	MSTRG.5973	Gm11715
ENSMUST00000129513	ENSMUSG00000087374	Gm15457
ENSMUST00000129546	MSTRG.26049	Hotairm1
ENSMUST00000129551	MSTRG.28374	Gm2788
ENSMUST00000129559	MSTRG.25041	Gm15701
ENSMUST00000129569	MSTRG.13046	Gm15317
ENSMUST00000129570	MSTRG.4095	Snhg15
ENSMUST00000129609	MSTRG.24534	Hnf1aos1
ENSMUST00000129617	MSTRG.21795	Gm12786
ENSMUST00000129661	MSTRG.17751	Mir670hg
ENSMUST00000129675	MSTRG.15674	Gm14966
ENSMUST00000129681	MSTRG.13250	Gm16052
ENSMUST00000129699	ENSMUSG00000087187	Gm13431
ENSMUST00000129701	MSTRG.12997	Gm15976
ENSMUST00000129713	ENSMUSG00000087648	E130018N17Rik
ENSMUST00000129723	ENSMUSG00000085830	Grin1os
ENSMUST00000129735	MSTRG.4147	Eldr
ENSMUST00000129740	MSTRG.552	Gm15834
ENSMUST00000129764	ENSMUSG00000087365	C430049B03Rik
ENSMUST00000129780	MSTRG.5887	C130046K22Rik
ENSMUST00000129791	MSTRG.8425	Gm11373
ENSMUST00000129834	MSTRG.3323	Atcayos
ENSMUST00000129868	MSTRG.18342	9630028H03Rik
ENSMUST00000129875	ENSMUSG00000084789	Gm12974
ENSMUST00000129876	MSTRG.4281	A630052C17Rik
ENSMUST00000129907	ENSMUSG00000078706	Gm53
ENSMUST00000129908	ENSMUSG00000085147	Gm12609
ENSMUST00000129924	MSTRG.4899	Kdm6bos
ENSMUST00000129932	MSTRG.7492	5430427M07Rik
ENSMUST00000129950	MSTRG.3323	Atcayos
ENSMUST00000129953	MSTRG.16065	Hectd2os
ENSMUST00000129965	MSTRG.28534	Gm16158
ENSMUST00000129971	MSTRG.27513	Gm15927

ENSMUST00000129980	MSTRG.34176	4933407K13Rik
ENSMUST00000129988	ENSMUSG00000086070	8430436N08Rik
ENSMUST00000129994	MSTRG.16795	Gm13335
ENSMUST00000130003	MSTRG.8473	Gm16984
ENSMUST00000130004	ENSMUSG00000085416	1700019G24Rik
ENSMUST00000130021	MSTRG.18545	Gm14199
ENSMUST00000130022	MSTRG.18839	Platr29
ENSMUST00000130054	MSTRG.7649	B430119L08Rik
ENSMUST00000130063	MSTRG.34350	Ftx
ENSMUST00000130082	MSTRG.623	2310016D23Rik
ENSMUST00000130083	ENSMUSG00000085668	Gm35202
ENSMUST00000130085	MSTRG.21068	C230012O17Rik
ENSMUST00000130086	MSTRG.32444	Arhgap20os
ENSMUST00000130088	ENSMUSG00000085790	Gm12729
ENSMUST00000130089	MSTRG.18685	4930405A21Rik
ENSMUST00000130099	MSTRG.33773	5730405O15Rik
ENSMUST00000130109	ENSMUSG00000086363	A330102I10Rik
ENSMUST00000130117	MSTRG.20996	Gm11842
ENSMUST00000130123	MSTRG.24405	Gm15788
ENSMUST00000130153	ENSMUSG00000086441	Gm15046
ENSMUST00000130177	ENSMUSG00000087604	Cdrt4os1
ENSMUST00000130184	MSTRG.16253	Gm16726
ENSMUST00000130189	MSTRG.28399	A230056P14Rik
ENSMUST00000130206	MSTRG.21495	Gm11209
ENSMUST00000130251	ENSMUSG00000087390	Gm7598
ENSMUST00000130256	MSTRG.10858	B930095G15Rik
ENSMUST00000130296	MSTRG.14824	Gm16090
ENSMUST00000130361	MSTRG.6679	2410018L13Rik
ENSMUST00000130373	ENSMUSG00000085421	4732490B19Rik
ENSMUST00000130392	MSTRG.4147	Eldr
ENSMUST00000130442	MSTRG.5446	Gm11505
ENSMUST00000130486	ENSMUSG00000086825	Gm15675
ENSMUST00000130493	MSTRG.4437	Gm12158
ENSMUST00000130556	MSTRG.5884	Arhgap27os1
ENSMUST00000130564	ENSMUSG00000085641	4930465M20Rik
ENSMUST00000130570	MSTRG.4693	4933439C10Rik
ENSMUST00000130576	MSTRG.21605	Gm12631
ENSMUST00000130584	MSTRG.33498	Gm16142
ENSMUST00000130607	MSTRG.13622	Gm16195
ENSMUST00000130619	MSTRG.15531	Tmem134
ENSMUST00000130625	ENSMUSG00000084885	3010001F23Rik
ENSMUST00000130630	MSTRG.24398	Gm15787
ENSMUST00000130634	ENSMUSG00000087226	Gm14015
ENSMUST00000130639	MSTRG.13162	4930506C21Rik
ENSMUST00000130657	MSTRG.26190	E230016M11Rik
ENSMUST00000130659	MSTRG.22233	Gm12940
ENSMUST00000130673	MSTRG.5914	Gm11651
ENSMUST00000130679	MSTRG.18881	A530013C23Rik
ENSMUST00000130683	MSTRG.20318	Kcnd3os
ENSMUST00000130684	MSTRG.21674	Junos
ENSMUST00000130696	MSTRG.15195	4930511M06Rik
ENSMUST00000130699	MSTRG.636	Pard3bos1
ENSMUST00000130733	ENSMUSG00000075589	Gm11536
ENSMUST00000130744	MSTRG.34176	4933407K13Rik
ENSMUST00000130746	MSTRG.4708	4930412M03Rik
ENSMUST00000130771	MSTRG.29707	Gm2044
ENSMUST00000130773	ENSMUSG00000085591	Gm13479

ENSMUST00000130827	MSTRG.17278	Zeb2os
ENSMUST00000130840	MSTRG.8795	4930451E10Rik
ENSMUST00000130859	MSTRG.18630	Gssos2
ENSMUST00000130860	MSTRG.4813	Gm12289
ENSMUST00000130874	MSTRG.4422	Gm12150
ENSMUST00000130875	MSTRG.25751	1810058I24Rik
ENSMUST00000130882	ENSMUSG00000086262	A930031H19Rik
ENSMUST00000130884	MSTRG.18316	1700026D11Rik
ENSMUST00000130892	MSTRG.24398	Gm15787
ENSMUST00000130912	MSTRG.24831	Tctn2
ENSMUST00000130943	ENSMUSG00000086190	4930579M01Rik
ENSMUST00000130952	MSTRG.14424	Trmt61b
ENSMUST00000130963	ENSMUSG00000075431	Gm11691
ENSMUST00000131007	MSTRG.32557	Gm16131
ENSMUST00000131019	ENSMUSG00000086209	4933405E24Rik
ENSMUST00000131021	MSTRG.3990	Gm11399
ENSMUST00000131029	MSTRG.24280	Gm28050
ENSMUST00000131044	MSTRG.16442	Gm13185
ENSMUST00000131057	MSTRG.6051	BC006965
ENSMUST00000131064	MSTRG.29930	4931415C17Rik
ENSMUST00000131080	MSTRG.28828	Gm15416
ENSMUST00000131085	MSTRG.29695	Gm15718
ENSMUST00000131093	MSTRG.32759	Gm16144
ENSMUST00000131099	ENSMUSG00000084787	Gm12436
ENSMUST00000131110	ENSMUSG00000087343	1700021N21Rik
ENSMUST00000131129	MSTRG.29781	R74862
ENSMUST00000131146	MSTRG.19861	Gm16069
ENSMUST00000131147	MSTRG.16853	Gm13387
ENSMUST00000131160	MSTRG.28925	Gm15635
ENSMUST00000131188	ENSMUSG00000086006	Gm13293
ENSMUST00000131189	ENSMUSG00000086995	Gm13544
ENSMUST00000131190	ENSMUSG00000087485	Gm13383
ENSMUST00000131198	MSTRG.17365	Gm13620
ENSMUST00000131207	ENSMUSG00000075416	Gm14488
ENSMUST00000131215	MSTRG.9437	2610204G07Rik
ENSMUST00000131216	MSTRG.21875	9630013D21Rik
ENSMUST00000131221	MSTRG.4684	2310058D17Rik
ENSMUST00000131222	MSTRG.10688	Gm4285
ENSMUST00000131274	ENSMUSG00000086273	Rbm46os
ENSMUST00000131275	ENSMUSG00000084844	Hoxb3os
ENSMUST00000131297	MSTRG.16527	Gm13256
ENSMUST00000131299	ENSMUSG00000092201	A530058N18Rik
ENSMUST00000131314	MSTRG.15670	Gm14965
ENSMUST00000131324	ENSMUSG00000046683	0610025J13Rik
ENSMUST00000131342	ENSMUSG00000085831	1700003G13Rik
ENSMUST00000131345	ENSMUSG00000086968	4933431E20Rik
ENSMUST00000131346	MSTRG.18961	Gm10714
ENSMUST00000131357	ENSMUSG00000073976	1700026J12Rik
ENSMUST00000131365	ENSMUSG00000087547	Platr27
ENSMUST00000131381	MSTRG.22150	Gm12915
ENSMUST00000131383	MSTRG.4261	4930538E20Rik
ENSMUST00000131387	ENSMUSG00000086891	Gm15963
ENSMUST00000131416	MSTRG.10879	A330035P11Rik
ENSMUST00000131441	ENSMUSG00000086474	9130204K15Rik
ENSMUST00000131457	ENSMUSG00000087283	Gm13580
ENSMUST00000131498	MSTRG.20918	A830012C17Rik
ENSMUST00000131501	MSTRG.5613	D030028A08Rik

ENSMUST00000131502	MSTRG.26054	Hoxaas3
ENSMUST00000131514	ENSMUSG00000086913	Gm14019
ENSMUST00000131539	ENSMUSG00000087220	Gm11377
ENSMUST00000131564	ENSMUSG00000087143	A830082K12Rik
ENSMUST00000131570	MSTRG.4212	Gm12043
ENSMUST00000131581	ENSMUSG00000085717	Hmgb4os
ENSMUST00000131588	MSTRG.17117	C79798
ENSMUST00000131603	MSTRG.18308	Gm14051
ENSMUST00000131630	ENSMUSG00000086243	Gm12121
ENSMUST00000131634	MSTRG.17685	Gm13783
ENSMUST00000131640	MSTRG.34412	2810403D21Rik
ENSMUST00000131642	MSTRG.4967	Gm12319
ENSMUST00000131663	MSTRG.341	Gm16152
ENSMUST00000131675	ENSMUSG00000087099	Gm11782
ENSMUST00000131687	ENSMUSG00000085860	2410003L11Rik
ENSMUST00000131707	MSTRG.29988	Gm16347
ENSMUST00000131710	ENSMUSG00000075555	Gm10863
ENSMUST00000131733	MSTRG.22351	Gm12981
ENSMUST00000131740	MSTRG.29778	Tspan32os
ENSMUST00000131787	MSTRG.4781	2410006H16Rik
ENSMUST00000131801	MSTRG.18666	Gm14252
ENSMUST00000131841	ENSMUSG00000086775	Snhg7os
ENSMUST00000131845	MSTRG.24534	Hnf1aos1
ENSMUST00000131846	ENSMUSG00000085517	Gm12963
ENSMUST00000131851	MSTRG.4371	Gm12116
ENSMUST00000131864	ENSMUSG00000085721	Gm12796
ENSMUST00000131874	MSTRG.3149	Gm16220
ENSMUST00000131886	ENSMUSG00000085057	Gm13415
ENSMUST00000131896	ENSMUSG00000085026	Srrm3os
ENSMUST00000131898	MSTRG.33465	4930520O04Rik
ENSMUST00000131907	ENSMUSG00000050334	C130071C03Rik
ENSMUST00000131908	ENSMUSG00000087147	Gm14228
ENSMUST00000131931	ENSMUSG00000086999	Bcas1os2
ENSMUST00000131947	MSTRG.34495	BC065397
ENSMUST00000131961	MSTRG.18209	Gm14010
ENSMUST00000131985	MSTRG.12215	Gm15738
ENSMUST00000132007	MSTRG.4128	Gm12000
ENSMUST00000132019	MSTRG.30483	Gm15354
ENSMUST00000132023	ENSMUSG00000085470	1700012C14Rik
ENSMUST00000132069	MSTRG.12059	Gm15859
ENSMUST00000132076	MSTRG.30503	Gm15656
ENSMUST00000132077	MSTRG.3998	Tug1
ENSMUST00000132090	MSTRG.24831	Tctn2
ENSMUST00000132097	ENSMUSG00000085449	Gm15520
ENSMUST00000132100	ENSMUSG00000086053	Gm15178
ENSMUST00000132101	ENSMUSG00000079070	Gm3985
ENSMUST00000132106	ENSMUSG00000084987	Gm13134
ENSMUST00000132111	MSTRG.22322	Gm12992
ENSMUST00000132130	ENSMUSG00000084921	Gm13838
ENSMUST00000132149	MSTRG.18206	Gm14005
ENSMUST00000132167	MSTRG.29774	Nctc1
ENSMUST00000132184	MSTRG.2353	Gm16153
ENSMUST00000132210	MSTRG.24398	Gm15787
ENSMUST00000132211	MSTRG.16455	Gm13187
ENSMUST00000132219	ENSMUSG00000085478	Gm11851
ENSMUST00000132223	MSTRG.3460	Gm15990
ENSMUST00000132226	MSTRG.4693	4933439C10Rik

ENSMUST00000132247	MSTRG.22328	Gm13063
ENSMUST00000132261	ENSMUSG00000085419	Gm11734
ENSMUST00000132267	MSTRG.24429	Gm15559
ENSMUST00000132294	ENSMUSG00000000031	H19
ENSMUST00000132296	MSTRG.5629	Gm11583
ENSMUST00000132326	ENSMUSG00000052371	Hoxd3os1
ENSMUST00000132337	ENSMUSG00000086498	Gm12681
ENSMUST00000132370	MSTRG.1993	4933439K11Rik
ENSMUST00000132378	MSTRG.4643	2010001A14Rik
ENSMUST00000132389	MSTRG.23927	Dancr
ENSMUST00000132394	MSTRG.4666	Gm12256
ENSMUST00000132432	MSTRG.21331	Gm16731
ENSMUST00000132470	ENSMUSG00000085840	Gm11261
ENSMUST00000132485	MSTRG.19526	Gm16206
ENSMUST00000132487	ENSMUSG00000055045	Gm11190
ENSMUST00000132488	MSTRG.9336	Gm9828
ENSMUST00000132553	MSTRG.21114	Gm11934
ENSMUST00000132559	MSTRG.26049	Hotairm1
ENSMUST00000132564	ENSMUSG00000086480	Gm15287
ENSMUST00000132571	MSTRG.14834	Gm15972
ENSMUST00000132584	ENSMUSG00000086003	B230206L02Rik
ENSMUST00000132609	ENSMUSG00000087289	4933424M12Rik
ENSMUST00000132616	MSTRG.31124	4930513N10Rik
ENSMUST00000132690	MSTRG.22001	Gm12827
ENSMUST00000132692	MSTRG.17199	Gm13556
ENSMUST00000132762	MSTRG.20549	Gm15551
ENSMUST00000132792	MSTRG.17499	Rapgef4os3
ENSMUST00000132811	MSTRG.29347	Gm15489
ENSMUST00000132826	MSTRG.28558	Gm7580
ENSMUST00000132834	ENSMUSG00000087003	Cntrobos
ENSMUST00000132840	ENSMUSG00000086850	Gm13257
ENSMUST00000132849	MSTRG.29684	Gm15582
ENSMUST00000132857	ENSMUSG00000086335	Gm12107
ENSMUST00000132861	MSTRG.12987	1700048M11Rik
ENSMUST00000132871	ENSMUSG00000086467	4930571N24Rik
ENSMUST00000132898	ENSMUSG00000085929	Gm13421
ENSMUST00000132913	MSTRG.17109	Gm13536
ENSMUST00000132921	MSTRG.5251	Rab11fip4os2
ENSMUST00000132973	ENSMUSG00000086163	Gm14206
ENSMUST00000133015	MSTRG.21478	Gm12542
ENSMUST00000133025	ENSMUSG00000085545	Gm13553
ENSMUST00000133125	ENSMUSG00000078122	F630028O10Rik
ENSMUST00000133160	MSTRG.5640	Gm11592
ENSMUST00000133179	MSTRG.28067	Gm17077
ENSMUST00000133204	MSTRG.8295	Gm11290
ENSMUST00000133214	MSTRG.33984	Kis2
ENSMUST00000133221	ENSMUSG00000085912	Trp53cor1
ENSMUST00000133231	MSTRG.6051	BC006965
ENSMUST00000133243	MSTRG.25382	Gm15411
ENSMUST00000133244	ENSMUSG00000044689	Gm13749
ENSMUST00000133270	MSTRG.3888	Gm16229
ENSMUST00000133273	MSTRG.11612	Gm17025
ENSMUST00000133274	MSTRG.13566	Gm15420
ENSMUST00000133299	MSTRG.335	Gm16150
ENSMUST00000133302	MSTRG.2726	E130307A14Rik
ENSMUST00000133334	ENSMUSG00000028590	Pramef12os
ENSMUST00000133337	ENSMUSG00000046413	Irx3os

ENSMUST00000133341	MSTRG.1804	Gm16548
ENSMUST00000133350	ENSMUSG00000085757	1700019B21Rik
ENSMUST00000133400	MSTRG.5291	C030013C21Rik
ENSMUST00000133431	MSTRG.21068	C230012O17Rik
ENSMUST00000133449	MSTRG.18723	Snhg17
ENSMUST00000133451	MSTRG.21080	Bach2it1
ENSMUST00000133455	ENSMUSG00000085843	Kank4os
ENSMUST00000133463	MSTRG.16909	0610009E02Rik
ENSMUST00000133467	MSTRG.26827	Gt(ROSA)26Sor
ENSMUST00000133476	MSTRG.24734	Gm15857
ENSMUST00000133489	MSTRG.4245	1700030C12Rik
ENSMUST00000133494	MSTRG.20013	Gm15265
ENSMUST00000133496	ENSMUSG00000087568	Maats1os
ENSMUST00000133499	ENSMUSG00000073759	4933407E24Rik
ENSMUST00000133510	MSTRG.25679	2210408F21Rik
ENSMUST00000133523	ENSMUSG00000084847	Gm15507
ENSMUST00000133528	ENSMUSG00000046413	Irx3os
ENSMUST00000133534	MSTRG.17974	Gm14091
ENSMUST00000133550	MSTRG.17037	D330023K18Rik
ENSMUST00000133570	ENSMUSG00000086968	4933431E20Rik
ENSMUST00000133596	MSTRG.17386	Gm13565
ENSMUST00000133616	MSTRG.33737	Rbm3os
ENSMUST00000133630	MSTRG.29781	R74862
ENSMUST00000133643	MSTRG.18060	AV039307
ENSMUST00000133644	MSTRG.21215	Gm13299
ENSMUST00000133648	MSTRG.17928	Gm13977
ENSMUST00000133653	ENSMUSG00000086189	Gm15462
ENSMUST00000133675	MSTRG.11734	Gm15569
ENSMUST00000133694	MSTRG.25382	Gm15406
ENSMUST00000133698	ENSMUSG00000086910	Gm15755
ENSMUST00000133723	MSTRG.26053	Gm15050
ENSMUST00000133742	MSTRG.22426	Gm12977
ENSMUST00000133751	MSTRG.18669	Gm14170
ENSMUST00000133752	MSTRG.30459	4930512H18Rik
ENSMUST00000133772	MSTRG.21674	Junos
ENSMUST00000133808	MSTRG.16899	Tmem250-ps
ENSMUST00000133815	MSTRG.17904	Gm13964
ENSMUST00000133819	MSTRG.4259	Gm12064
ENSMUST00000133867	ENSMUSG00000086008	Gm8817
ENSMUST00000133871	ENSMUSG00000087132	A930001C03Rik
ENSMUST00000133873	MSTRG.16455	Gm13187
ENSMUST00000133884	MSTRG.21651	Gm12655
ENSMUST00000133940	MSTRG.4557	Gm12205
ENSMUST00000133945	ENSMUSG00000086475	Platr13
ENSMUST00000133948	ENSMUSG00000085743	8430419K02Rik
ENSMUST00000133960	ENSMUSG00000085180	AI838599
ENSMUST00000133963	MSTRG.16227	Gm15491
ENSMUST00000133999	MSTRG.25197	Gm16120
ENSMUST00000134006	ENSMUSG00000087531	Gm15606
ENSMUST00000134059	MSTRG.22872	Gm13073
ENSMUST00000134066	MSTRG.34495	BC065397
ENSMUST00000134069	MSTRG.6221	Gm11738
ENSMUST00000134140	MSTRG.10825	Mir17hg
ENSMUST00000134206	MSTRG.21726	Gm12701
ENSMUST00000134236	MSTRG.22883	Gm13067
ENSMUST00000134250	MSTRG.19932	Gm16048
ENSMUST00000134252	MSTRG.5064	1700016P03Rik

ENSMUST00000134264	MSTRG.18315	Gm14095
ENSMUST00000134275	ENSMUSG00000085991	Gm11479
ENSMUST00000134284	ENSMUSG00000087319	Gm15907
ENSMUST00000134285	ENSMUSG00000086020	Gm12239
ENSMUST00000134289	ENSMUSG00000087042	Gm11611
ENSMUST00000134305	MSTRG.5397	Bcas3os2
ENSMUST00000134312	MSTRG.29705	Gm16201
ENSMUST00000134345	MSTRG.5099	Mir22hg
ENSMUST00000134391	MSTRG.6132	Recql5os1
ENSMUST00000134411	MSTRG.18133	4930417H01Rik
ENSMUST00000134422	ENSMUSG00000086800	4930556L07Rik
ENSMUST00000134427	MSTRG.5642	Gm11613
ENSMUST00000134436	MSTRG.5614	Gm11525
ENSMUST00000134448	ENSMUSG00000086975	Gm15208
ENSMUST00000134467	MSTRG.23488	Gm15522
ENSMUST00000134493	ENSMUSG00000087516	Tbx3os1
ENSMUST00000134494	MSTRG.21912	Gm12823
ENSMUST00000134512	ENSMUSG00000086427	Hoxa11os
ENSMUST00000134527	MSTRG.4095	Snhg15
ENSMUST00000134541	MSTRG.25686	Gm13844
ENSMUST00000134552	MSTRG.24771	Gm15751
ENSMUST00000134563	MSTRG.17751	Mir670hg
ENSMUST00000134580	ENSMUSG00000085582	3110099E03Rik
ENSMUST00000134586	MSTRG.3709	Kcnmb4os1
ENSMUST00000134600	MSTRG.26579	Gm5577
ENSMUST00000134620	ENSMUSG00000075314	Gm1322
ENSMUST00000134624	MSTRG.8388	2610307P16Rik
ENSMUST00000134627	ENSMUSG00000086148	Gm15271
ENSMUST00000134633	MSTRG.23284	Prkag2os1
ENSMUST00000134640	ENSMUSG00000086284	Frmpd1os
ENSMUST00000134649	MSTRG.31363	Gm15655
ENSMUST00000134672	MSTRG.34495	BC065397
ENSMUST00000134673	ENSMUSG00000072591	5930412G12Rik
ENSMUST00000134688	MSTRG.33553	Gm2415
ENSMUST00000134690	MSTRG.33390	Gm7628
ENSMUST00000134692	ENSMUSG00000087199	Gm15818
ENSMUST00000134705	ENSMUSG00000087143	A830082K12Rik
ENSMUST00000134755	ENSMUSG00000075416	Gm14488
ENSMUST00000134787	MSTRG.8388	2610307P16Rik
ENSMUST00000134795	ENSMUSG00000086214	Gm14062
ENSMUST00000134799	MSTRG.22426	Gm12977
ENSMUST00000134801	ENSMUSG00000085071	Gm14066
ENSMUST00000134838	MSTRG.18686	Gm14230
ENSMUST00000134845	MSTRG.29750	B230206H07Rik
ENSMUST00000134911	MSTRG.22307	Gm12970
ENSMUST00000134920	MSTRG.17014	Gm16323
ENSMUST00000134971	ENSMUSG00000085292	Gm12853
ENSMUST00000134992	MSTRG.12375	Gm16618
ENSMUST00000134998	MSTRG.1456	Gm15850
ENSMUST00000135037	MSTRG.4816	2810001G20Rik
ENSMUST00000135042	MSTRG.21514	8030451A03Rik
ENSMUST00000135048	ENSMUSG00000086750	Gm11770
ENSMUST00000135068	MSTRG.10398	Gm15932
ENSMUST00000135086	ENSMUSG00000086860	Gm1720
ENSMUST00000135110	MSTRG.18278	A730017L22Rik
ENSMUST00000135114	MSTRG.34376	5530601H04Rik
ENSMUST00000135116	ENSMUSG00000085440	Sorbs2os

ENSMUST00000135138	MSTRG.29942	Gm15350
ENSMUST00000135144	ENSMUSG00000086389	Gm15998
ENSMUST00000135153	ENSMUSG00000074987	Wt1os
ENSMUST00000135180	ENSMUSG00000085218	BB218582
ENSMUST00000135186	MSTRG.18221	Gm14027
ENSMUST00000135203	MSTRG.13949	Gm4577
ENSMUST00000135240	MSTRG.25386	Gm15409
ENSMUST00000135244	MSTRG.17542	E030042O20Rik
ENSMUST00000135257	MSTRG.15531	Tmem134
ENSMUST00000135268	ENSMUSG00000085235	Gm12576
ENSMUST00000135297	MSTRG.17505	Sp3os
ENSMUST00000135313	ENSMUSG00000086443	4933421A08Rik
ENSMUST00000135347	MSTRG.15658	Frmd8os
ENSMUST00000135360	MSTRG.18454	A930019D19Rik
ENSMUST00000135363	ENSMUSG00000085058	8030453O22Rik
ENSMUST00000135366	MSTRG.24398	Gm15787
ENSMUST00000135378	MSTRG.15070	Gm15337
ENSMUST00000135401	MSTRG.11121	Gm15941
ENSMUST00000135421	MSTRG.6679	2410018L13Rik
ENSMUST00000135433	MSTRG.18206	Gm14005
ENSMUST00000135434	MSTRG.3272	Gm15122
ENSMUST00000135451	ENSMUSG00000087703	Gm15650
ENSMUST00000135468	ENSMUSG00000085585	Gm12223
ENSMUST00000135483	MSTRG.20013	A730011C13Rik
ENSMUST00000135527	MSTRG.32520	Gm10658
ENSMUST00000135564	MSTRG.28529	Gm16157
ENSMUST00000135572	MSTRG.9336	Gm9828
ENSMUST00000135583	MSTRG.11558	Gm16059
ENSMUST00000135612	MSTRG.26103	9130019P16Rik
ENSMUST00000135630	ENSMUSG00000087675	Gm11762
ENSMUST00000135643	ENSMUSG00000049160	Tex50
ENSMUST00000135659	MSTRG.17638	4930443O20Rik
ENSMUST00000135670	ENSMUSG00000055134	9130017K11Rik
ENSMUST00000135674	MSTRG.31699	1700084C06Rik
ENSMUST00000135682	ENSMUSG00000086813	Gm13657
ENSMUST00000135735	MSTRG.17337	Gm13546
ENSMUST00000135749	ENSMUSG00000085737	Gm13442
ENSMUST00000135777	MSTRG.21333	Gm568
ENSMUST00000135792	ENSMUSG00000085353	1700092C17Rik
ENSMUST00000135825	MSTRG.5613	D030028A08Rik
ENSMUST00000135857	MSTRG.5920	Gm11646
ENSMUST00000135858	ENSMUSG00000085968	Gm13027
ENSMUST00000135873	ENSMUSG00000087322	Gm16075
ENSMUST00000135883	MSTRG.6840	Arl4aos
ENSMUST00000135940	MSTRG.3778	Grip1os1
ENSMUST00000135958	MSTRG.24273	Lrrc8dos
ENSMUST00000135987	ENSMUSG00000066176	Gm12511
ENSMUST00000135990	MSTRG.18329	Gm14104
ENSMUST00000136015	ENSMUSG00000086706	Gm15848
ENSMUST00000136022	MSTRG.24996	Abhd11os
ENSMUST00000136025	MSTRG.18206	Gm14012
ENSMUST00000136027	ENSMUSG00000086239	Gm14329
ENSMUST00000136038	MSTRG.3975	8430429K09Rik
ENSMUST00000136051	MSTRG.5303	Gm11423
ENSMUST00000136052	ENSMUSG00000086561	Gm15540
ENSMUST00000136053	MSTRG.4147	Eldr
ENSMUST00000136083	MSTRG.4378	Gm12119

ENSMUST00000136110	MSTRG.25751	1810058I24Rik
ENSMUST00000136115	MSTRG.22000	Gm12828
ENSMUST00000136117	MSTRG.5400	2610027K06Rik
ENSMUST00000136127	MSTRG.22343	Snhg3
ENSMUST00000136149	MSTRG.21086	Bach2os
ENSMUST00000136170	ENSMUSG00000085773	Gm16233
ENSMUST00000136187	MSTRG.10890	Gm15735
ENSMUST00000136190	MSTRG.3058	Gm16212
ENSMUST00000136196	MSTRG.4886	Kcnab3os
ENSMUST00000136201	MSTRG.5503	C030037D09Rik
ENSMUST00000136211	MSTRG.34376	5530601H04Rik
ENSMUST00000136217	ENSMUSG00000085069	Gm13111
ENSMUST00000136218	MSTRG.11782	C230037L18Rik
ENSMUST00000136276	MSTRG.22167	9930104L06Rik
ENSMUST00000136288	ENSMUSG00000086170	Gm12144
ENSMUST00000136294	MSTRG.28831	C230038L03Rik
ENSMUST00000136305	MSTRG.11128	Gm15942
ENSMUST00000136306	ENSMUSG00000085180	AI838599
ENSMUST00000136322	MSTRG.32300	C030014I23Rik
ENSMUST00000136359	ENSMUSG00000000031	H19
ENSMUST00000136376	MSTRG.31679	Fbxl12os
ENSMUST00000136378	MSTRG.18859	Zfas1
ENSMUST00000136400	ENSMUSG00000085319	Gm13330
ENSMUST00000136401	MSTRG.17717	4631405J19Rik
ENSMUST00000136406	ENSMUSG00000087620	5330434G04Rik
ENSMUST00000136438	MSTRG.6053	2610035D17Rik
ENSMUST00000136447	MSTRG.33823	A230072C01Rik
ENSMUST00000136454	MSTRG.5382	Appbp2os
ENSMUST00000136472	ENSMUSG00000086034	Gm15201
ENSMUST00000136489	ENSMUSG00000086445	Gm13191
ENSMUST00000136500	MSTRG.6315	Gm11775
ENSMUST00000136503	MSTRG.25536	Gm4876
ENSMUST00000136518	MSTRG.33960	Firre
ENSMUST00000136525	ENSMUSG00000086096	Gm12688
ENSMUST00000136531	MSTRG.14192	Frs3os
ENSMUST00000136538	MSTRG.30739	Gm10649
ENSMUST00000136542	ENSMUSG00000085501	Gm11772
ENSMUST00000136557	MSTRG.16292	Gm16068
ENSMUST00000136571	MSTRG.25679	2210408F21Rik
ENSMUST00000136578	ENSMUSG00000085025	Gm13715
ENSMUST00000136632	ENSMUSG00000087132	A930001C03Rik
ENSMUST00000136672	MSTRG.28368	Nell1os
ENSMUST00000136690	MSTRG.7128	3110056K07Rik
ENSMUST00000136693	MSTRG.9642	Gm3848
ENSMUST00000136697	MSTRG.15830	A330040F15Rik
ENSMUST00000136744	MSTRG.13038	1600002D24Rik
ENSMUST00000136749	MSTRG.30167	Gm1698
ENSMUST00000136759	MSTRG.19123	Uckl1os
ENSMUST00000136779	MSTRG.34706	Gm15261
ENSMUST00000136793	MSTRG.18262	4930473A02Rik
ENSMUST00000136794	MSTRG.18437	Gm14092
ENSMUST00000136806	MSTRG.26054	Hoxaas3
ENSMUST00000136807	ENSMUSG00000085427	6430710C18Rik
ENSMUST00000136862	MSTRG.2726	E130307A14Rik
ENSMUST00000136879	MSTRG.17385	Gm13561
ENSMUST00000136886	MSTRG.17749	Alkbh3os1
ENSMUST00000136906	MSTRG.8377	1700016G14Rik

ENSMUST00000136908	ENSMUSG00000087400	Gm15270
ENSMUST00000136923	ENSMUSG00000108703	Gm44793
ENSMUST00000136927	MSTRG.5991	Gm11714
ENSMUST00000136941	ENSMUSG00000084918	Gm12708
ENSMUST00000136990	ENSMUSG00000087095	Emx2os
ENSMUST00000136992	MSTRG.21009	Gm11837
ENSMUST00000136998	ENSMUSG00000086509	Nkx2-2os
ENSMUST00000137000	MSTRG.27513	Gm15927
ENSMUST00000137002	ENSMUSG00000086686	F630206G17Rik
ENSMUST00000137010	ENSMUSG00000060808	B9d1os
ENSMUST00000137020	ENSMUSG00000084935	Gm14161
ENSMUST00000137073	MSTRG.17734	Gm13807
ENSMUST00000137077	MSTRG.5476	1110028F11Rik
ENSMUST00000137081	MSTRG.6051	BC006965
ENSMUST00000137096	ENSMUSG00000087668	Gm11186
ENSMUST00000137099	MSTRG.24410	Gm15792
ENSMUST00000137134	ENSMUSG00000087539	Gm13584
ENSMUST00000137136	MSTRG.25729	Gm13861
ENSMUST00000137153	ENSMUSG00000071753	C230004F18Rik
ENSMUST00000137154	MSTRG.5759	Dhx58os
ENSMUST00000137168	MSTRG.20326	Gm42890
ENSMUST00000137175	MSTRG.6001	Gm11657
ENSMUST00000137176	MSTRG.18293	Gm14232
ENSMUST00000137180	MSTRG.18439	BC039771
ENSMUST00000137186	MSTRG.16798	Gm13362
ENSMUST00000137193	MSTRG.28824	Gm15412
ENSMUST00000137198	MSTRG.19357	Mccc1os
ENSMUST00000137199	ENSMUSG00000087143	A830082K12Rik
ENSMUST00000137236	MSTRG.22233	Gm12940
ENSMUST00000137239	MSTRG.21681	9530080O11Rik
ENSMUST00000137241	MSTRG.306	4930439A04Rik
ENSMUST00000137257	MSTRG.24735	Gm15860
ENSMUST00000137259	MSTRG.10527	Gm15918
ENSMUST00000137275	MSTRG.18879	Gm14320
ENSMUST00000137283	ENSMUSG00000086164	Gm13029
ENSMUST00000137295	ENSMUSG00000072591	5930412G12Rik
ENSMUST00000137315	MSTRG.13054	Gm15341
ENSMUST00000137343	MSTRG.22338	Snhg12
ENSMUST00000137351	MSTRG.16568	Gm13261
ENSMUST00000137352	MSTRG.10784	4930517O19Rik
ENSMUST00000137353	ENSMUSG00000087238	Gm12865
ENSMUST00000137359	MSTRG.10006	Gm15512
ENSMUST00000137364	ENSMUSG00000086707	Gm12088
ENSMUST00000137373	MSTRG.27761	Gm16174
ENSMUST00000137397	ENSMUSG00000087450	Gm13994
ENSMUST00000137398	ENSMUSG00000085271	E130006D01Rik
ENSMUST00000137405	ENSMUSG00000084825	2410152P15Rik
ENSMUST00000137411	ENSMUSG00000086703	4930507D10Rik
ENSMUST00000137422	ENSMUSG00000087035	Tmem74bos
ENSMUST00000137442	MSTRG.4693	4933439C10Rik
ENSMUST00000137457	MSTRG.22233	Gm12940
ENSMUST00000137460	ENSMUSG00000084885	3010001F23Rik
ENSMUST00000137463	MSTRG.18686	Gm14230
ENSMUST00000137478	MSTRG.26804	0610040F04Rik
ENSMUST00000137535	ENSMUSG00000086043	Gm12473
ENSMUST00000137539	MSTRG.14612	4833418N02Rik
ENSMUST00000137546	ENSMUSG00000086275	1700121C08Rik

ENSMUST00000137561	MSTRG.5896	Gm11659
ENSMUST00000137567	ENSMUSG00000087080	Gm12199
ENSMUST00000137579	MSTRG.15531	Tmem134
ENSMUST00000137582	MSTRG.17393	Gm13594
ENSMUST00000137598	MSTRG.5651	Gm11614
ENSMUST00000137603	MSTRG.15195	4930511M06Rik
ENSMUST00000137607	MSTRG.21999	Gm12843
ENSMUST00000137629	ENSMUSG00000086166	Gm14342
ENSMUST00000137643	MSTRG.912	C130036L24Rik
ENSMUST00000137661	MSTRG.16886	A230005M16Rik
ENSMUST00000137662	ENSMUSG00000085348	Myhas
ENSMUST00000137673	ENSMUSG00000087001	Gm15475
ENSMUST00000137706	MSTRG.31249	Gm10629
ENSMUST00000137728	MSTRG.18228	AI847159
ENSMUST00000137730	MSTRG.13621	Gm16196
ENSMUST00000137731	ENSMUSG00000085497	Gm15985
ENSMUST00000137738	MSTRG.5254	Gm11205
ENSMUST00000137742	MSTRG.28211	Gm28496
ENSMUST00000137752	MSTRG.22978	Gm16023
ENSMUST00000137765	ENSMUSG00000084963	Gm14936
ENSMUST00000137776	MSTRG.20183	Spag17os
ENSMUST00000137810	ENSMUSG00000085022	Gm5860
ENSMUST00000137830	MSTRG.14424	Trmt61b
ENSMUST00000137869	MSTRG.16907	Gm13562
ENSMUST00000137882	ENSMUSG00000086172	2700068H02Rik
ENSMUST00000137883	MSTRG.13386	BC028777
ENSMUST00000137907	ENSMUSG00000086761	Cep112os1
ENSMUST00000137941	MSTRG.17505	Sp3os
ENSMUST00000137958	ENSMUSG00000084947	Gm15594
ENSMUST00000137995	MSTRG.31235	1810019D21Rik
ENSMUST00000138007	MSTRG.5503	C030037D09Rik
ENSMUST00000138082	ENSMUSG00000085808	Gm14718
ENSMUST00000138087	MSTRG.27870	Gm44924
ENSMUST00000138108	MSTRG.24534	Hnf1aos2
ENSMUST00000138136	ENSMUSG00000085459	Gm12272
ENSMUST00000138137	MSTRG.34376	5530601H04Rik
ENSMUST00000138156	MSTRG.33984	Kis2
ENSMUST00000138170	MSTRG.25536	Gm4876
ENSMUST00000138180	MSTRG.29266	4930583K01Rik
ENSMUST00000138207	MSTRG.12980	Gm15964
ENSMUST00000138236	MSTRG.15315	B430212C06Rik
ENSMUST00000138253	MSTRG.29191	Far1os
ENSMUST00000138256	MSTRG.6314	Gm11773
ENSMUST00000138259	MSTRG.26102	Gm15527
ENSMUST00000138275	MSTRG.25721	Gm14546
ENSMUST00000138288	MSTRG.18922	Gm14455
ENSMUST00000138291	ENSMUSG00000085701	Gm12968
ENSMUST00000138295	ENSMUSG00000086919	Gm11497
ENSMUST00000138298	MSTRG.25705	Gm14540
ENSMUST00000138303	MSTRG.18341	4930545L23Rik
ENSMUST00000138341	MSTRG.25731	Gm14547
ENSMUST00000138361	MSTRG.23039	Gm13648
ENSMUST00000138372	MSTRG.17949	Gm13982
ENSMUST00000138374	ENSMUSG00000087357	Gm12498
ENSMUST00000138377	MSTRG.21382	1700060J05Rik
ENSMUST00000138379	ENSMUSG00000086124	A530076I17Rik
ENSMUST00000138390	ENSMUSG00000085558	4930412C18Rik

ENSMUST00000138391	MSTRG.4668	Gm12259
ENSMUST00000138402	MSTRG.4291	Gm12089
ENSMUST00000138406	ENSMUSG00000085565	Gm15721
ENSMUST00000138411	MSTRG.21514	8030451A03Rik
ENSMUST00000138416	ENSMUSG00000085257	Gm13264
ENSMUST00000138422	MSTRG.26433	Ccdc142os
ENSMUST00000138424	MSTRG.5863	2410004I01Rik
ENSMUST00000138426	MSTRG.21960	Gm12953
ENSMUST00000138430	MSTRG.20251	Gm15886
ENSMUST00000138431	MSTRG.18228	AI847159
ENSMUST00000138442	MSTRG.32098	2810455O05Rik
ENSMUST00000138444	MSTRG.17337	Gm13546
ENSMUST00000138447	MSTRG.8354	4932702P03Rik
ENSMUST00000138460	MSTRG.34376	5530601H04Rik
ENSMUST00000138462	MSTRG.16450	Gm13189
ENSMUST00000138472	ENSMUSG00000086968	4933431E20Rik
ENSMUST00000138486	MSTRG.18206	Gm14005
ENSMUST00000138492	MSTRG.22821	Gm13209
ENSMUST00000138528	MSTRG.17296	Gm13483
ENSMUST00000138535	MSTRG.636	Pard3bos1
ENSMUST00000138539	MSTRG.12743	Gm15591
ENSMUST00000138573	ENSMUSG00000086546	Gm13709
ENSMUST00000138592	MSTRG.18723	Snhg17
ENSMUST00000138593	MSTRG.15531	Tmem134
ENSMUST00000138606	MSTRG.25679	2210408F21Rik
ENSMUST00000138611	MSTRG.672	Ino80dos
ENSMUST00000138614	MSTRG.34320	Gm14858
ENSMUST00000138617	MSTRG.6817	Atxn7l1os2
ENSMUST00000138626	ENSMUSG00000085472	Gm15691
ENSMUST00000138653	MSTRG.25679	2210408F21Rik
ENSMUST00000138655	ENSMUSG00000085569	Gm12602
ENSMUST00000138672	ENSMUSG00000085928	4933427I22Rik
ENSMUST00000138678	ENSMUSG00000072884	Gm10433
ENSMUST00000138702	MSTRG.5979	Gm11722
ENSMUST00000138710	MSTRG.19372	Gm43439
ENSMUST00000138715	MSTRG.11629	4930483J18Rik
ENSMUST00000138729	MSTRG.21973	1700021J08Rik
ENSMUST00000138744	MSTRG.30017	Gm16725
ENSMUST00000138751	ENSMUSG00000074783	AU019990
ENSMUST00000138755	ENSMUSG00000085141	Gm13429
ENSMUST00000138763	MSTRG.4693	4933439C10Rik
ENSMUST00000138767	MSTRG.26100	9530036M11Rik
ENSMUST00000138795	MSTRG.22959	Gm13112
ENSMUST00000138816	MSTRG.13624	Gm16194
ENSMUST00000138817	MSTRG.5150	Gm12343
ENSMUST00000138832	MSTRG.34159	Gm15384
ENSMUST00000138855	MSTRG.5560	Gm11513
ENSMUST00000138861	MSTRG.22148	Gm12925
ENSMUST00000138915	MSTRG.5740	Gm12348
ENSMUST00000138926	MSTRG.27516	Gm15929
ENSMUST00000138928	MSTRG.18861	1110018N20Rik
ENSMUST00000138943	MSTRG.34176	4933407K13Rik
ENSMUST00000138992	MSTRG.8783	Gm15911
ENSMUST00000139005	ENSMUSG00000052371	Hoxd3os1
ENSMUST00000139006	MSTRG.25669	4930412F09Rik
ENSMUST00000139017	MSTRG.5704	Gm12359
ENSMUST00000139025	MSTRG.4713	Gm12265

ENSMUST00000139027	MSTRG.6051	BC006965
ENSMUST00000139042	MSTRG.18685	4930405A21Rik
ENSMUST00000139055	MSTRG.26054	Hoxaas3
ENSMUST00000139056	MSTRG.2257	D730003I15Rik
ENSMUST00000139058	ENSMUSG00000086637	Gm14040
ENSMUST00000139065	ENSMUSG00000087278	A930006I01Rik
ENSMUST00000139089	MSTRG.27389	Sspnos
ENSMUST00000139095	ENSMUSG00000084890	A830036E02Rik
ENSMUST00000139119	MSTRG.21265	Gm12454
ENSMUST00000139140	MSTRG.30762	4933431K23Rik
ENSMUST00000139144	MSTRG.16909	0610009E02Rik
ENSMUST00000139163	MSTRG.33765	Gm14634
ENSMUST00000139185	MSTRG.25685	Mkln1os
ENSMUST00000139194	MSTRG.5517	Dgkeos
ENSMUST00000139214	ENSMUSG00000085295	4930430E12Rik
ENSMUST00000139218	MSTRG.29705	Gm16201
ENSMUST00000139222	ENSMUSG00000087192	4930412L05Rik
ENSMUST00000139265	MSTRG.30501	1700125H03Rik
ENSMUST00000139271	MSTRG.4147	Eldr
ENSMUST00000139272	ENSMUSG00000086035	Gm12610
ENSMUST00000139288	MSTRG.17103	Gm13528
ENSMUST00000139309	MSTRG.1583	Gm15479
ENSMUST00000139327	ENSMUSG00000084853	Gm11791
ENSMUST00000139334	MSTRG.12048	Gm15879
ENSMUST00000139338	ENSMUSG00000085428	Gm14344
ENSMUST00000139351	MSTRG.27372	Sox5os3
ENSMUST00000139356	MSTRG.2726	E130307A14Rik
ENSMUST00000139361	ENSMUSG00000087059	Gm12339
ENSMUST00000139363	MSTRG.20862	4930570G19Rik
ENSMUST00000139377	ENSMUSG00000085135	Gm13713
ENSMUST00000139423	ENSMUSG00000086455	Gm11815
ENSMUST00000139424	ENSMUSG00000086617	Gm11635
ENSMUST00000139429	MSTRG.2726	E130307A14Rik
ENSMUST00000139455	ENSMUSG00000086449	9030204H09Rik
ENSMUST00000139471	ENSMUSG00000087029	Gm14133
ENSMUST00000139479	MSTRG.16458	Gm13175
ENSMUST00000139488	ENSMUSG00000084948	1700061H18Rik
ENSMUST00000139492	MSTRG.17614	Gm13710
ENSMUST00000139493	MSTRG.4147	Eldr
ENSMUST00000139500	MSTRG.24557	4930401G09Rik
ENSMUST00000139512	MSTRG.33554	Gm16295
ENSMUST00000139514	ENSMUSG00000085218	BB218582
ENSMUST00000139529	ENSMUSG00000054618	Gm9951
ENSMUST00000139530	MSTRG.4697	1700007J10Rik
ENSMUST00000139552	MSTRG.33496	2900079G21Rik
ENSMUST00000139588	MSTRG.21068	C230012O17Rik
ENSMUST00000139592	MSTRG.5507	Scpep1os
ENSMUST00000139600	ENSMUSG00000085394	2210414B05Rik
ENSMUST00000139612	ENSMUSG00000086013	Gm15706
ENSMUST00000139618	MSTRG.21999	Gm12843
ENSMUST00000139621	MSTRG.5880	1700028N14Rik
ENSMUST00000139636	MSTRG.18558	2500004C02Rik
ENSMUST00000139653	MSTRG.22653	Tmem51os1
ENSMUST00000139662	MSTRG.1768	Dnm3os
ENSMUST00000139667	MSTRG.19392	Gm12531
ENSMUST00000139677	MSTRG.18932	Gm14453
ENSMUST00000139695	MSTRG.34234	Gm14798

ENSMUST00000139701	MSTRG.25536	Gm4876
ENSMUST00000139718	MSTRG.15531	Tmem134
ENSMUST00000139751	ENSMUSG00000066158	AY512931
ENSMUST00000139752	MSTRG.5624	Gm11574
ENSMUST00000139769	ENSMUSG00000086808	Gm11981
ENSMUST00000139771	MSTRG.19454	4930577N17Rik
ENSMUST00000139791	MSTRG.28326	Gm15700
ENSMUST00000139804	ENSMUSG00000043168	4930426D05Rik
ENSMUST00000139808	ENSMUSG00000078925	D030018L15Rik
ENSMUST00000139822	MSTRG.8341	4930558J22Rik
ENSMUST00000139823	MSTRG.17534	A330043C09Rik
ENSMUST00000139825	MSTRG.552	Gm15834
ENSMUST00000139827	MSTRG.2278	1700034H15Rik
ENSMUST00000139835	MSTRG.21564	Gm11266
ENSMUST00000139864	ENSMUSG00000084848	4933408N05Rik
ENSMUST00000139884	ENSMUSG00000085389	1700003M07Rik
ENSMUST00000139891	MSTRG.2726	E130307A14Rik
ENSMUST00000139895	ENSMUSG00000087273	Gm13203
ENSMUST00000139927	MSTRG.18870	Gm14321
ENSMUST00000139941	MSTRG.5990	Gm11713
ENSMUST00000139948	MSTRG.17263	Arhgap15os
ENSMUST00000139987	MSTRG.15531	Tmem134
ENSMUST00000140000	MSTRG.12095	Gm15983
ENSMUST00000140003	MSTRG.10909	BC037032
ENSMUST00000140028	ENSMUSG00000072591	5930412G12Rik
ENSMUST00000140044	MSTRG.11121	Gm15941
ENSMUST00000140069	MSTRG.4643	2010001A14Rik
ENSMUST00000140080	MSTRG.22198	Gm12945
ENSMUST00000140087	MSTRG.24405	Gm15788
ENSMUST00000140101	ENSMUSG00000087516	Tbx3os1
ENSMUST00000140104	ENSMUSG00000086356	Gm13441
ENSMUST00000140105	MSTRG.6808	F730043M19Rik
ENSMUST00000140161	MSTRG.4164	Etaa1os
ENSMUST00000140185	MSTRG.17936	2700033N17Rik
ENSMUST00000140196	ENSMUSG00000086249	Gm12724
ENSMUST00000140203	ENSMUSG00000085320	Gm11548
ENSMUST00000140253	ENSMUSG00000078925	D030018L15Rik
ENSMUST00000140263	ENSMUSG00000084847	Gm15507
ENSMUST00000140267	MSTRG.15531	Tmem134
ENSMUST00000140271	MSTRG.17470	Dlx1as
ENSMUST00000140273	MSTRG.6269	Gm11769
ENSMUST00000140298	ENSMUSG00000085124	Gm12766
ENSMUST00000140307	MSTRG.1993	4933439K11Rik
ENSMUST00000140320	MSTRG.10784	4930517O19Rik
ENSMUST00000140371	MSTRG.4912	Tnfsf13os
ENSMUST00000140380	MSTRG.5925	Gm11672
ENSMUST00000140405	MSTRG.15531	Tmem134
ENSMUST00000140409	MSTRG.34176	4933407K13Rik
ENSMUST00000140412	MSTRG.17112	Nron
ENSMUST00000140415	ENSMUSG00000086363	A330102I10Rik
ENSMUST00000140443	ENSMUSG00000086822	5330413P13Rik
ENSMUST00000140451	MSTRG.30116	Gm16159
ENSMUST00000140464	MSTRG.17342	Gm13558
ENSMUST00000140465	ENSMUSG00000085616	Gm13850
ENSMUST00000140488	MSTRG.10842	Gm16835
ENSMUST00000140501	MSTRG.22608	Gm13031
ENSMUST00000140509	MSTRG.31235	1810019D21Rik

ENSMUST00000140510	MSTRG.22148	Gm12925
ENSMUST00000140556	MSTRG.5274	Adap2os
ENSMUST00000140569	MSTRG.6809	Gm16267
ENSMUST00000140578	MSTRG.23864	Gm15477
ENSMUST00000140588	ENSMUSG00000087045	4930515B02Rik
ENSMUST00000140646	MSTRG.23460	Gm15513
ENSMUST00000140650	MSTRG.23564	Gm7854
ENSMUST00000140669	MSTRG.30485	Gm15356
ENSMUST00000140673	MSTRG.5400	2610027K06Rik
ENSMUST00000140689	MSTRG.29220	1700003G18Rik
ENSMUST00000140709	MSTRG.25536	Gm4876
ENSMUST00000140716	ENSMUSG00000000031	H19
ENSMUST00000140736	MSTRG.5921	Taco1os
ENSMUST00000140746	MSTRG.25535	Gm15473
ENSMUST00000140807	MSTRG.32200	1700110K17Rik
ENSMUST00000140826	ENSMUSG00000086211	Gm12462
ENSMUST00000140833	MSTRG.24605	Gm15728
ENSMUST00000140881	MSTRG.28088	Gm12762
ENSMUST00000140883	MSTRG.27087	Gm44597
ENSMUST00000140892	MSTRG.12215	Gm15738
ENSMUST00000140928	MSTRG.31938	BC048644
ENSMUST00000140941	MSTRG.20862	4930570G19Rik
ENSMUST00000140949	MSTRG.18351	Gm14210
ENSMUST00000140953	MSTRG.24831	Tctn2
ENSMUST00000140954	MSTRG.34376	5530601H04Rik
ENSMUST00000140978	MSTRG.17447	Gm13631
ENSMUST00000140987	MSTRG.18466	AI646519
ENSMUST00000141008	MSTRG.24398	Gm15787
ENSMUST00000141011	MSTRG.28250	Gm15545
ENSMUST00000141023	MSTRG.17050	Gm13609
ENSMUST00000141027	MSTRG.683	Platr12
ENSMUST00000141032	ENSMUSG00000085707	Gm12212
ENSMUST00000141035	MSTRG.17006	Gm14471
ENSMUST00000141076	ENSMUSG00000087400	Gm15270
ENSMUST00000141082	MSTRG.5344	E230016K23Rik
ENSMUST00000141090	MSTRG.22343	Snhg3
ENSMUST00000141120	ENSMUSG00000087054	Gm12405
ENSMUST00000141133	MSTRG.17227	Gm13496
ENSMUST00000141136	MSTRG.34376	5530601H04Rik
ENSMUST00000141149	MSTRG.17402	Gm13629
ENSMUST00000141162	ENSMUSG00000085032	Gm11217
ENSMUST00000141168	MSTRG.31214	Gm16156
ENSMUST00000141172	MSTRG.12227	Pla2g10os
ENSMUST00000141174	MSTRG.33753	Gm14493
ENSMUST00000141199	ENSMUSG00000085810	Gm16325
ENSMUST00000141216	MSTRG.24738	Gm15747
ENSMUST00000141239	MSTRG.13624	Gm16194
ENSMUST00000141256	MSTRG.12533	Tnk2os
ENSMUST00000141259	MSTRG.22850	Ube4bos1
ENSMUST00000141265	MSTRG.9758	1810062O18Rik
ENSMUST00000141271	ENSMUSG00000087184	Gm11650
ENSMUST00000141275	MSTRG.32246	Gm16322
ENSMUST00000141289	ENSMUSG00000087674	4930447M23Rik
ENSMUST00000141299	ENSMUSG00000086828	Gm15579
ENSMUST00000141300	ENSMUSG00000055408	Hottip
ENSMUST00000141307	MSTRG.27394	Gm15543
ENSMUST00000141341	ENSMUSG00000085261	Gm13814

ENSMUST00000141344	MSTRG.12998	D430001F17Rik
ENSMUST00000141347	ENSMUSG00000087685	1700122E12Rik
ENSMUST00000141375	MSTRG.21202	Gm13307
ENSMUST00000141390	ENSMUSG00000086722	Gm15499
ENSMUST00000141399	MSTRG.6051	BC006965
ENSMUST00000141405	ENSMUSG00000085180	AI838599
ENSMUST00000141420	MSTRG.24411	A630023P12Rik
ENSMUST00000141428	MSTRG.21514	8030451A03Rik
ENSMUST00000141440	MSTRG.27379	Gm15687
ENSMUST00000141450	MSTRG.4382	4930469K13Rik
ENSMUST00000141452	ENSMUSG00000085515	C630028M04Rik
ENSMUST00000141454	MSTRG.25150	6330418K02Rik
ENSMUST00000141455	MSTRG.19225	Gm16093
ENSMUST00000141473	ENSMUSG00000085763	Smc2os
ENSMUST00000141485	MSTRG.19995	Gm15234
ENSMUST00000141488	MSTRG.16449	1700080N15Rik
ENSMUST00000141511	MSTRG.18028	Gm13999
ENSMUST00000141532	MSTRG.24411	A630023P12Rik
ENSMUST00000141538	MSTRG.20983	Gm11831
ENSMUST00000141541	MSTRG.29529	Gm15503
ENSMUST00000141543	ENSMUSG00000085024	C230035I16Rik
ENSMUST00000141559	ENSMUSG00000086733	Gm11685
ENSMUST00000141580	ENSMUSG00000085660	Gm12434
ENSMUST00000141582	ENSMUSG00000086272	BC039966
ENSMUST00000141639	MSTRG.26433	Ccdc142os
ENSMUST00000141656	MSTRG.9786	Gm15935
ENSMUST00000141679	ENSMUSG00000045709	Smkr-ps
ENSMUST00000141681	ENSMUSG00000086266	Igf2os
ENSMUST00000141696	MSTRG.5704	Gm12359
ENSMUST00000141697	MSTRG.33973	Gm14582
ENSMUST00000141700	MSTRG.27194	Gm15987
ENSMUST00000141710	MSTRG.24475	Gm15736
ENSMUST00000141731	ENSMUSG00000086369	E330017L17Rik
ENSMUST00000141741	MSTRG.30104	Gm16933
ENSMUST00000141752	ENSMUSG00000085638	Gm15521
ENSMUST00000141758	ENSMUSG00000086408	Gm11588
ENSMUST00000141769	MSTRG.22653	Tmem51os1
ENSMUST00000141786	MSTRG.18228	AI847159
ENSMUST00000141787	MSTRG.20013	A730011C13Rik
ENSMUST00000141797	MSTRG.13624	Gm16194
ENSMUST00000141809	MSTRG.18723	Snhg17
ENSMUST00000141810	MSTRG.29320	Gm15774
ENSMUST00000141843	MSTRG.18792	Gm11457
ENSMUST00000141846	MSTRG.2082	B230369F24Rik
ENSMUST00000141875	MSTRG.12274	1700056N10Rik
ENSMUST00000141893	MSTRG.21881	Dmrta2os
ENSMUST00000141923	MSTRG.18881	A530013C23Rik
ENSMUST00000141926	MSTRG.18636	Gm16098
ENSMUST00000141929	MSTRG.5682	1700003D09Rik
ENSMUST00000141930	ENSMUSG00000086741	Gm15816
ENSMUST00000141935	MSTRG.16966	Gm13393
ENSMUST00000142008	ENSMUSG00000084954	Gm16351
ENSMUST00000142019	MSTRG.22871	Gm16188
ENSMUST00000142025	MSTRG.15755	Gm9750
ENSMUST00000142044	ENSMUSG00000087340	Gm15228
ENSMUST00000142047	MSTRG.21540	Gm11240
ENSMUST00000142053	ENSMUSG00000085712	Gm15124

ENSMUST00000142067	MSTRG.16065	Hectd2os
ENSMUST00000142068	MSTRG.5108	4931413K12Rik
ENSMUST00000142076	MSTRG.9786	Gm15935
ENSMUST00000142077	MSTRG.32523	Man2c1os
ENSMUST00000142099	MSTRG.9758	1810062O18Rik
ENSMUST00000142107	MSTRG.5808	Gm11626
ENSMUST00000142137	MSTRG.21538	Gm11250
ENSMUST00000142151	ENSMUSG00000086256	Gm12052
ENSMUST00000142165	MSTRG.18936	Ctcflos
ENSMUST00000142207	MSTRG.18936	Ctcflos
ENSMUST00000142212	MSTRG.18757	Gm16751
ENSMUST00000142249	ENSMUSG00000085293	Gm13817
ENSMUST00000142282	MSTRG.6051	BC006965
ENSMUST00000142286	ENSMUSG00000087202	Gm15813
ENSMUST00000142289	MSTRG.22483	Gm13008
ENSMUST00000142292	MSTRG.21674	Junos
ENSMUST00000142297	MSTRG.20967	Gm11827
ENSMUST00000142310	MSTRG.18672	Gm14225
ENSMUST00000142322	MSTRG.3984	Gm11948
ENSMUST00000142330	MSTRG.17717	4631405J19Rik
ENSMUST00000142338	MSTRG.18812	Zfp335os
ENSMUST00000142353	ENSMUSG00000087702	Gm13575
ENSMUST00000142373	MSTRG.21067	Gm11899
ENSMUST00000142374	MSTRG.18884	Gm14236
ENSMUST00000142397	MSTRG.3975	8430429K09Rik
ENSMUST00000142425	MSTRG.21823	Lrp8os1
ENSMUST00000142429	ENSMUSG00000085395	Gm13056
ENSMUST00000142433	MSTRG.20110	C920021L13Rik
ENSMUST00000142445	ENSMUSG00000085267	Gm14235
ENSMUST00000142458	ENSMUSG00000084939	Gm830
ENSMUST00000142492	MSTRG.4785	Lrrc75aos1
ENSMUST00000142509	MSTRG.18630	Gssos1
ENSMUST00000142511	ENSMUSG00000085929	Gm13421
ENSMUST00000142520	MSTRG.6292	Gm11788
ENSMUST00000142533	MSTRG.4775	Gm12279
ENSMUST00000142579	MSTRG.32444	Arhgap20os
ENSMUST00000142581	MSTRG.21742	0610043K17Rik
ENSMUST00000142621	ENSMUSG00000086402	Gm15322
ENSMUST00000142625	MSTRG.25195	Gm16122
ENSMUST00000142634	ENSMUSG00000085871	Ube2uos
ENSMUST00000142642	MSTRG.18520	Gm14154
ENSMUST00000142709	MSTRG.5332	AI662270
ENSMUST00000142712	ENSMUSG00000044387	2410080I02Rik
ENSMUST00000142723	MSTRG.2723	Gm16365
ENSMUST00000142744	MSTRG.20960	Gm11816
ENSMUST00000142751	MSTRG.6225	Gm11747
ENSMUST00000142774	MSTRG.16515	Gm13389
ENSMUST00000142777	MSTRG.4147	Eldr
ENSMUST00000142815	MSTRG.21962	C530005A16Rik
ENSMUST00000142828	ENSMUSG00000086576	Gm13660
ENSMUST00000142833	MSTRG.20373	Gm12522
ENSMUST00000142835	MSTRG.21848	Gm12737
ENSMUST00000142871	ENSMUSG00000087611	4930458D05Rik
ENSMUST00000142879	MSTRG.24411	A630023P12Rik
ENSMUST00000142895	MSTRG.5476	1110028F11Rik
ENSMUST00000142907	MSTRG.34473	3632454L22Rik
ENSMUST00000142937	ENSMUSG00000085411	Gm14319

ENSMUST00000142968	MSTRG.2993	Hk1os
ENSMUST00000142990	MSTRG.21202	Gm13307
ENSMUST00000143016	MSTRG.4566	Gm12207
ENSMUST00000143025	ENSMUSG00000085834	Gm15622
ENSMUST00000143062	MSTRG.24801	Gm16001
ENSMUST00000143065	MSTRG.18206	Gm14005
ENSMUST00000143070	ENSMUSG00000086138	4930471C06Rik
ENSMUST00000143099	MSTRG.25593	6530409C15Rik
ENSMUST00000143103	MSTRG.2738	Cdk19os
ENSMUST00000143109	MSTRG.20363	Gm12524
ENSMUST00000143112	ENSMUSG00000084913	Gm7616
ENSMUST00000143122	MSTRG.2993	Hk1os
ENSMUST00000143129	ENSMUSG00000073274	Gm14636
ENSMUST00000143135	ENSMUSG00000087389	Gm15592
ENSMUST00000143202	ENSMUSG00000085649	A730032A03Rik
ENSMUST00000143208	MSTRG.5487	Gm15893
ENSMUST00000143227	MSTRG.18390	Macrod2os2
ENSMUST00000143242	MSTRG.21674	Junos
ENSMUST00000143245	MSTRG.24146	Tmem150cos
ENSMUST00000143260	MSTRG.8895	Platr25
ENSMUST00000143261	MSTRG.341	Gm16152
ENSMUST00000143266	ENSMUSG00000087691	Gm15674
ENSMUST00000143272	ENSMUSG00000021268	Meg3
ENSMUST00000143287	MSTRG.20013	Gm15265
ENSMUST00000143291	MSTRG.6239	Gm11752
ENSMUST00000143302	MSTRG.5573	Gm11521
ENSMUST00000143306	MSTRG.20110	C920021L13Rik
ENSMUST00000143311	ENSMUSG00000085675	Gm12022
ENSMUST00000143315	ENSMUSG00000046005	D830044D21Rik
ENSMUST00000143324	MSTRG.30739	Gm10649
ENSMUST00000143346	MSTRG.18241	A730036I17Rik
ENSMUST00000143378	ENSMUSG00000043168	4930426D05Rik
ENSMUST00000143382	ENSMUSG00000086636	Gm12949
ENSMUST00000143391	ENSMUSG00000084766	Gm12714
ENSMUST00000143392	MSTRG.10566	1700001G11Rik
ENSMUST00000143428	MSTRG.6259	Gm11766
ENSMUST00000143429	ENSMUSG00000087071	Trp73os
ENSMUST00000143435	MSTRG.6027	Gm11670
ENSMUST00000143453	ENSMUSG00000086822	5330413P13Rik
ENSMUST00000143458	MSTRG.16736	Gm13269
ENSMUST00000143469	MSTRG.17132	Gm13448
ENSMUST00000143473	MSTRG.5240	Gm11201
ENSMUST00000143488	MSTRG.23368	Gm15461
ENSMUST00000143525	MSTRG.17901	Gm13963
ENSMUST00000143551	MSTRG.25593	6530409C15Rik
ENSMUST00000143559	MSTRG.17806	4930547E08Rik
ENSMUST00000143564	MSTRG.33202	5830418P13Rik
ENSMUST00000143587	MSTRG.18859	Zfas1
ENSMUST00000143593	MSTRG.4133	Gm12002
ENSMUST00000143605	MSTRG.13546	Itpr3os
ENSMUST00000143618	MSTRG.21497	Gm11211
ENSMUST00000143638	MSTRG.30057	Gm15346
ENSMUST00000143645	MSTRG.18863	Gm14291
ENSMUST00000143649	ENSMUSG00000087684	1200007C13Rik
ENSMUST00000143651	MSTRG.2726	E130307A14Rik
ENSMUST00000143652	MSTRG.7694	Gm16086
ENSMUST00000143671	MSTRG.13053	1700093J21Rik

ENSMUST00000143673	MSTRG.5332	AI662270
ENSMUST00000143705	MSTRG.22177	Gm12932
ENSMUST00000143706	MSTRG.4169	Gm16140
ENSMUST00000143710	MSTRG.32242	Gm16214
ENSMUST00000143715	ENSMUSG00000085407	1700095J03Rik
ENSMUST00000143717	MSTRG.10909	BC037032
ENSMUST00000143719	MSTRG.31319	9430091E24Rik
ENSMUST00000143732	MSTRG.15406	F830208F22Rik
ENSMUST00000143738	MSTRG.18969	Nespas
ENSMUST00000143755	ENSMUSG00000087110	Gm11264
ENSMUST00000143760	MSTRG.6051	BC006965
ENSMUST00000143765	MSTRG.27063	Gm15884
ENSMUST00000143781	ENSMUSG00000085811	Cep112it
ENSMUST00000143787	MSTRG.2209	9630028B13Rik
ENSMUST00000143796	ENSMUSG00000084937	Gm11228
ENSMUST00000143801	MSTRG.22042	Gm12868
ENSMUST00000143812	MSTRG.9055	Zfp85os
ENSMUST00000143819	MSTRG.5406	Brip1os
ENSMUST00000143821	MSTRG.17307	A430018G15Rik
ENSMUST00000143828	MSTRG.14424	Trmt61b
ENSMUST00000143836	ENSMUSG00000021268	Meg3
ENSMUST00000143857	MSTRG.18225	Gm14022
ENSMUST00000143871	MSTRG.26579	Gm5577
ENSMUST00000143877	MSTRG.2723	Gm16365
ENSMUST00000143887	ENSMUSG00000078706	Gm53
ENSMUST00000143888	ENSMUSG00000086565	Gm13133
ENSMUST00000143893	MSTRG.20318	Kcnd3os
ENSMUST00000143898	ENSMUSG00000087333	Gm13652
ENSMUST00000143945	MSTRG.23864	Gm15477
ENSMUST00000143949	MSTRG.7007	4921518K17Rik
ENSMUST00000143956	ENSMUSG00000086296	D030055H07Rik
ENSMUST00000143964	ENSMUSG00000074783	AU019990
ENSMUST00000143995	ENSMUSG00000087185	Gm13872
ENSMUST00000143999	MSTRG.33960	Firre
ENSMUST00000144002	ENSMUSG00000085399	Foxd2os
ENSMUST00000144006	ENSMUSG00000086095	Gm15328
ENSMUST00000144020	MSTRG.3714	Kcnmb4os2
ENSMUST00000144026	MSTRG.27371	Sox5it
ENSMUST00000144031	MSTRG.22803	Gm13201
ENSMUST00000144033	MSTRG.4912	Tnfsf13os
ENSMUST00000144036	MSTRG.6051	BC006965
ENSMUST00000144078	MSTRG.15315	B430212C06Rik
ENSMUST00000144085	ENSMUSG00000096299	Gm21814
ENSMUST00000144092	ENSMUSG00000086152	Gm11454
ENSMUST00000144095	MSTRG.21505	Aknaos
ENSMUST00000144096	MSTRG.6234	Gm11755
ENSMUST00000144105	MSTRG.26670	4930466I24Rik
ENSMUST00000144109	MSTRG.6808	F730043M19Rik
ENSMUST00000144118	MSTRG.19357	Mccc1os
ENSMUST00000144134	MSTRG.12598	Gm15564
ENSMUST00000144166	MSTRG.22742	Gm13166
ENSMUST00000144178	MSTRG.18235	Gm14023
ENSMUST00000144193	MSTRG.23261	Gm15589
ENSMUST00000144202	MSTRG.24547	Gm13830
ENSMUST00000144230	MSTRG.6104	Gm11695
ENSMUST00000144232	MSTRG.25685	Mkln1os
ENSMUST00000144256	MSTRG.18936	Ctcflos

ENSMUST00000144260	MSTRG.1186	D630008O14Rik
ENSMUST00000144291	MSTRG.12242	Gm15868
ENSMUST00000144294	ENSMUSG00000087369	Gm14696
ENSMUST00000144339	MSTRG.19	Gm16041
ENSMUST00000144350	MSTRG.20869	Gm15577
ENSMUST00000144366	MSTRG.34376	5530601H04Rik
ENSMUST00000144368	ENSMUSG00000085222	Gm13974
ENSMUST00000144407	MSTRG.22046	Gm12898
ENSMUST00000144411	ENSMUSG00000053545	6430503K07Rik
ENSMUST00000144412	MSTRG.33881	Gm14643
ENSMUST00000144419	MSTRG.21135	Gm12364
ENSMUST00000144420	MSTRG.33206	Gm16252
ENSMUST00000144421	MSTRG.34412	2810403D21Rik
ENSMUST00000144424	MSTRG.33496	2900079G21Rik
ENSMUST00000144460	MSTRG.25150	6330418K02Rik
ENSMUST00000144487	ENSMUSG00000043168	4930426D05Rik
ENSMUST00000144542	MSTRG.19353	Gm15952
ENSMUST00000144545	ENSMUSG00000087143	A830082K12Rik
ENSMUST00000144556	ENSMUSG00000085641	4930465M20Rik
ENSMUST00000144568	MSTRG.25498	A430035B10Rik
ENSMUST00000144575	MSTRG.7700	Gm16085
ENSMUST00000144594	ENSMUSG00000085689	Gm16838
ENSMUST00000144604	MSTRG.21741	E130102H24Rik
ENSMUST00000144612	MSTRG.25679	2210408F21Rik
ENSMUST00000144634	ENSMUSG00000087196	Gm13373
ENSMUST00000144637	ENSMUSG00000086592	Gm12682
ENSMUST00000144649	MSTRG.18631	Gm14257
ENSMUST00000144661	MSTRG.18439	BC039771
ENSMUST00000144680	ENSMUSG00000086709	Gm16263
ENSMUST00000144689	ENSMUSG00000085985	Gm11682
ENSMUST00000144703	ENSMUSG00000085558	4930412C18Rik
ENSMUST00000144704	ENSMUSG00000087268	Gm14486
ENSMUST00000144705	MSTRG.22338	Snhg12
ENSMUST00000144719	MSTRG.33713	Flicr
ENSMUST00000144735	ENSMUSG00000087176	D230022J07Rik
ENSMUST00000144738	MSTRG.34715	Gm15726
ENSMUST00000144745	MSTRG.27166	Gm10069
ENSMUST00000144750	MSTRG.30145	1700047A11Rik
ENSMUST00000144757	ENSMUSG00000085658	Gm15704
ENSMUST00000144761	MSTRG.34320	Gm14858
ENSMUST00000144765	MSTRG.21241	Gm12395
ENSMUST00000144774	MSTRG.34376	5530601H04Rik
ENSMUST00000144796	MSTRG.29477	B130055M24Rik
ENSMUST00000144818	ENSMUSG00000055494	Gm14168
ENSMUST00000144822	MSTRG.17672	Gm13778
ENSMUST00000144828	MSTRG.28708	4933406J10Rik
ENSMUST00000144844	MSTRG.3975	8430429K09Rik
ENSMUST00000144849	MSTRG.34376	5530601H04Rik
ENSMUST00000144852	MSTRG.3998	Tug1
ENSMUST00000144853	ENSMUSG00000071753	C230004F18Rik
ENSMUST00000144854	ENSMUSG00000086025	4933406G16Rik
ENSMUST00000144870	ENSMUSG00000079604	Gm13219
ENSMUST00000144875	ENSMUSG00000087540	Gm11412
ENSMUST00000144876	ENSMUSG00000085902	Gm12200
ENSMUST00000144896	MSTRG.6367	Dnmt3aos
ENSMUST00000144915	MSTRG.4456	Gm16033
ENSMUST00000144922	MSTRG.4834	Gm12296

ENSMUST00000144926	MSTRG.10879	A330035P11Rik
ENSMUST00000144932	MSTRG.24588	Gm13842
ENSMUST00000144933	ENSMUSG00000085180	AI838599
ENSMUST00000144975	ENSMUSG00000085734	Gm11684
ENSMUST00000144979	MSTRG.14192	Frs3os
ENSMUST00000144981	ENSMUSG00000085677	4930594O21Rik
ENSMUST00000144984	MSTRG.13040	Gm15340
ENSMUST00000144990	MSTRG.4511	Gm12195
ENSMUST00000144993	ENSMUSG00000054944	5330416C01Rik
ENSMUST00000145018	MSTRG.10784	4930517O19Rik
ENSMUST00000145021	ENSMUSG00000085348	Myhas
ENSMUST00000145030	MSTRG.13249	Gm16046
ENSMUST00000145042	MSTRG.25382	5930430L01Rik
ENSMUST00000145046	MSTRG.22167	9930104L06Rik
ENSMUST00000145050	MSTRG.2278	1700034H15Rik
ENSMUST00000145064	ENSMUSG00000087104	Tmem132cos
ENSMUST00000145068	MSTRG.4662	1700047K16Rik
ENSMUST00000145081	MSTRG.15315	B430212C06Rik
ENSMUST00000145085	ENSMUSG00000084775	Gm16741
ENSMUST00000145095	ENSMUSG00000087622	Gm12290
ENSMUST00000145111	MSTRG.26103	9130019P16Rik
ENSMUST00000145143	MSTRG.22832	Gm17029
ENSMUST00000145163	MSTRG.22939	Gm13645
ENSMUST00000145170	MSTRG.17517	Gm13708
ENSMUST00000145178	MSTRG.18212	Gm14011
ENSMUST00000145182	ENSMUSG00000085806	Gm12023
ENSMUST00000145185	MSTRG.33202	5830418P13Rik
ENSMUST00000145196	ENSMUSG00000086369	E330017L17Rik
ENSMUST00000145206	MSTRG.6053	2610035D17Rik
ENSMUST00000145240	MSTRG.22233	Gm12940
ENSMUST00000145250	MSTRG.34412	2810403D21Rik
ENSMUST00000145262	ENSMUSG00000085860	2410003L11Rik
ENSMUST00000145263	MSTRG.22862	Gm13066
ENSMUST00000145269	MSTRG.22754	1700095A21Rik
ENSMUST00000145279	MSTRG.20183	Spag17os
ENSMUST00000145291	ENSMUSG00000104861	3110039M20Rik
ENSMUST00000145297	MSTRG.29818	Gm14376
ENSMUST00000145338	ENSMUSG00000086386	Gm12108
ENSMUST00000145343	MSTRG.31235	1810019D21Rik
ENSMUST00000145346	ENSMUSG00000085479	9430073C21Rik
ENSMUST00000145372	MSTRG.29943	Gm15347
ENSMUST00000145389	ENSMUSG00000085224	Gm13425
ENSMUST00000145400	MSTRG.19879	Gm15417
ENSMUST00000145410	ENSMUSG00000085541	Gm16010
ENSMUST00000145411	ENSMUSG00000052767	Gm12703
ENSMUST00000145413	ENSMUSG00000085275	Gm14487
ENSMUST00000145415	MSTRG.12564	Gm15829
ENSMUST00000145420	MSTRG.26443	Gm15624
ENSMUST00000145435	MSTRG.4912	Tnfsf13os
ENSMUST00000145437	ENSMUSG00000075389	2810410L24Rik
ENSMUST00000145438	MSTRG.6184	Snhg20
ENSMUST00000145448	ENSMUSG00000085295	4930430E12Rik
ENSMUST00000145461	MSTRG.5332	AI662270
ENSMUST00000145464	MSTRG.7005	Gm16246
ENSMUST00000145488	MSTRG.22262	Gm12979
ENSMUST00000145489	MSTRG.5671	Gm11632
ENSMUST00000145502	MSTRG.622	Gm11579

ENSMUST00000145510	MSTRG.21799	Gm12802
ENSMUST00000145515	ENSMUSG00000084885	3010001F23Rik
ENSMUST00000145517	ENSMUSG00000086337	Gm11535
ENSMUST00000145520	MSTRG.22984	5830444B04Rik
ENSMUST00000145525	MSTRG.18496	E130215H24Rik
ENSMUST00000145533	MSTRG.4693	4933439C10Rik
ENSMUST00000145549	ENSMUSG00000084843	B230312C02Rik
ENSMUST00000145562	MSTRG.31124	4930513N10Rik
ENSMUST00000145563	MSTRG.17477	Gm13663
ENSMUST00000145577	MSTRG.21762	4930456L15Rik
ENSMUST00000145579	MSTRG.18433	Gm14093
ENSMUST00000145581	ENSMUSG00000085180	AI838599
ENSMUST00000145585	MSTRG.16471	4930551O13Rik
ENSMUST00000145596	MSTRG.9828	Kcnma1
ENSMUST00000145613	ENSMUSG00000087143	A830082K12Rik
ENSMUST00000145620	MSTRG.34495	BC065397
ENSMUST00000145637	MSTRG.10566	1700001G11Rik
ENSMUST00000145644	ENSMUSG00000087613	Gm13855
ENSMUST00000145652	MSTRG.5246	Rab11fip4os1
ENSMUST00000145711	ENSMUSG00000085002	Gm12984
ENSMUST00000145769	MSTRG.6808	F730043M19Rik
ENSMUST00000145789	MSTRG.4125	Gm11999
ENSMUST00000145790	MSTRG.3114	Gm15397
ENSMUST00000145794	MSTRG.3648	Gm15663
ENSMUST00000145801	ENSMUSG00000086363	A330102I10Rik
ENSMUST00000145803	MSTRG.34350	Ftx
ENSMUST00000145804	MSTRG.13950	Ppp1r18os
ENSMUST00000145848	ENSMUSG00000086594	Nudt12os
ENSMUST00000145859	MSTRG.18859	Zfas1
ENSMUST00000145865	MSTRG.20155	4930442L01Rik
ENSMUST00000145871	MSTRG.7113	F730035M05Rik
ENSMUST00000145893	MSTRG.20253	Gm15471
ENSMUST00000145894	MSTRG.33708	Gm14703
ENSMUST00000145897	MSTRG.23978	2310040G07Rik
ENSMUST00000145914	MSTRG.23859	Gm15478
ENSMUST00000145916	ENSMUSG00000086907	Gm15298
ENSMUST00000145924	MSTRG.13581	Gm15597
ENSMUST00000145926	MSTRG.17717	4631405J19Rik
ENSMUST00000145941	MSTRG.33729	Gm14820
ENSMUST00000145950	ENSMUSG00000087136	Gm15864
ENSMUST00000145961	MSTRG.28084	Gm12764
ENSMUST00000145971	MSTRG.2726	E130307A14Rik
ENSMUST00000145985	ENSMUSG00000054457	9430021M05Rik
ENSMUST00000145987	MSTRG.3787	Gm4473
ENSMUST00000145994	ENSMUSG00000086701	Gm13595
ENSMUST00000146010	MSTRG.14911	Epb41l4aos
ENSMUST00000146016	ENSMUSG00000085059	Gm11750
ENSMUST00000146034	ENSMUSG00000075325	Gm13582
ENSMUST00000146041	ENSMUSG00000087070	Gm12505
ENSMUST00000146091	MSTRG.1456	Gm15850
ENSMUST00000146094	ENSMUSG00000085424	Fhad1os1
ENSMUST00000146110	MSTRG.17880	Platr8
ENSMUST00000146121	ENSMUSG00000043145	Gm11292
ENSMUST00000146124	MSTRG.22832	Gm17029
ENSMUST00000146132	MSTRG.3489	Gm16235
ENSMUST00000146134	MSTRG.17786	Gm13920
ENSMUST00000146145	MSTRG.29175	2310014F06Rik

ENSMUST00000146152	MSTRG.22460	Gm16225
ENSMUST00000146154	MSTRG.20992	1700123M08Rik
ENSMUST00000146195	MSTRG.17505	Sp3os
ENSMUST00000146199	MSTRG.27533	Gm15494
ENSMUST00000146208	ENSMUSG00000087400	Gm15270
ENSMUST00000146218	MSTRG.3993	Gm12735
ENSMUST00000146222	MSTRG.13050	Gm15342
ENSMUST00000146227	MSTRG.34376	5530601H04Rik
ENSMUST00000146232	MSTRG.28048	Gm21982
ENSMUST00000146250	MSTRG.22869	Gm13068
ENSMUST00000146269	MSTRG.28096	Gm12781
ENSMUST00000146294	MSTRG.3766	Grip1os3
ENSMUST00000146304	MSTRG.4121	Gm11998
ENSMUST00000146314	MSTRG.358	Gm15832
ENSMUST00000146320	ENSMUSG00000086010	Gm15318
ENSMUST00000146326	ENSMUSG00000086769	Gm15587
ENSMUST00000146333	MSTRG.4816	2810001G20Rik
ENSMUST00000146334	MSTRG.4699	Med9os
ENSMUST00000146336	ENSMUSG00000086363	A330102I10Rik
ENSMUST00000146337	ENSMUSG00000086968	4933431E20Rik
ENSMUST00000146346	ENSMUSG00000085870	Gm12829
ENSMUST00000146349	MSTRG.27163	Gm15862
ENSMUST00000146364	ENSMUSG00000072884	Gm10433
ENSMUST00000146366	MSTRG.29633	Gm5602
ENSMUST00000146398	MSTRG.18316	1700026D11Rik
ENSMUST00000146404	ENSMUSG00000085950	Gm13589
ENSMUST00000146456	MSTRG.3975	8430429K09Rik
ENSMUST00000146481	ENSMUSG00000085558	4930412C18Rik
ENSMUST00000146495	ENSMUSG00000085845	Gm13944
ENSMUST00000146501	ENSMUSG00000087192	4930412L05Rik
ENSMUST00000146524	MSTRG.5517	Dgkeos
ENSMUST00000146529	MSTRG.17331	A930012O16Rik
ENSMUST00000146531	ENSMUSG00000086911	Gm12027
ENSMUST00000146532	MSTRG.22877	Gm13070
ENSMUST00000146535	ENSMUSG00000087591	Gm14635
ENSMUST00000146546	MSTRG.24558	Gm13832
ENSMUST00000146560	MSTRG.14612	4833418N02Rik
ENSMUST00000146576	MSTRG.21283	Gm12503
ENSMUST00000146581	MSTRG.3471	Igf1os
ENSMUST00000146587	ENSMUSG00000087470	A630031M04Rik
ENSMUST00000146624	MSTRG.20219	Tspan2os
ENSMUST00000146633	MSTRG.17672	Gm13778
ENSMUST00000146644	ENSMUSG00000084904	Gm14827
ENSMUST00000146657	MSTRG.19709	Gm16000
ENSMUST00000146658	MSTRG.22588	2310026L22Rik
ENSMUST00000146663	ENSMUSG00000091583	Gm17033
ENSMUST00000146671	MSTRG.22742	Gm13166
ENSMUST00000146679	MSTRG.22168	Gm12930
ENSMUST00000146690	ENSMUSG00000085429	Gm13485
ENSMUST00000146700	MSTRG.1438	Gm15445
ENSMUST00000146701	ENSMUSG00000021268	Meg3
ENSMUST00000146702	ENSMUSG00000086405	9330198N18Rik
ENSMUST00000146717	MSTRG.4261	4930538E20Rik
ENSMUST00000146728	MSTRG.23932	1700071G01Rik
ENSMUST00000146744	MSTRG.3581	Tmcc3os
ENSMUST00000146746	ENSMUSG00000085127	4930444E06Rik
ENSMUST00000146751	MSTRG.15613	Gm14963

ENSMUST00000146754	ENSMUSG00000085238	4930479D17Rik
ENSMUST00000146755	ENSMUSG00000085523	Gm15945
ENSMUST00000146766	MSTRG.25674	Gm13833
ENSMUST00000146769	MSTRG.17040	Gm13405
ENSMUST00000146778	MSTRG.25679	2210408F21Rik
ENSMUST00000146779	ENSMUSG00000087615	Pnpla1os
ENSMUST00000146801	MSTRG.34495	BC065397
ENSMUST00000146806	MSTRG.5395	Bcas3os1
ENSMUST00000146845	ENSMUSG00000085797	A530010F05Rik
ENSMUST00000146846	MSTRG.18792	Gm11457
ENSMUST00000146857	MSTRG.8282	Gm11274
ENSMUST00000146868	MSTRG.5578	4833417C18Rik
ENSMUST00000146887	MSTRG.23106	Gm15731
ENSMUST00000146902	MSTRG.26102	Gm15527
ENSMUST00000146909	MSTRG.21142	Gm12367
ENSMUST00000146915	MSTRG.4812	9630013K17Rik
ENSMUST00000146929	ENSMUSG00000084806	Gm15232
ENSMUST00000146938	MSTRG.32461	Gm16124
ENSMUST00000146949	ENSMUSG00000086012	Gm15902
ENSMUST00000146954	ENSMUSG00000087164	Nr5a1os
ENSMUST00000146963	MSTRG.18840	Gm11464
ENSMUST00000146976	MSTRG.21785	Gm12744
ENSMUST00000146980	ENSMUSG00000084762	Platr3
ENSMUST00000146981	MSTRG.25801	4930599N23Rik
ENSMUST00000146982	MSTRG.22019	9530034E10Rik
ENSMUST00000146985	MSTRG.4311	Gm8098
ENSMUST00000147006	ENSMUSG00000087139	Gm11683
ENSMUST00000147019	MSTRG.16736	Gm13269
ENSMUST00000147073	MSTRG.15144	C030005K06Rik
ENSMUST00000147082	ENSMUSG00000087185	Gm13872
ENSMUST00000147130	MSTRG.34220	Gm14764
ENSMUST00000147141	ENSMUSG00000085158	Gm14264
ENSMUST00000147144	ENSMUSG00000086948	Gm14697
ENSMUST00000147173	MSTRG.28374	Gm2788
ENSMUST00000147177	MSTRG.644	Pard3bos3
ENSMUST00000147186	ENSMUSG00000087057	Gm11730
ENSMUST00000147187	MSTRG.18673	Gm14224
ENSMUST00000147198	MSTRG.28807	4632427E13Rik
ENSMUST00000147206	MSTRG.30320	Gm15634
ENSMUST00000147221	ENSMUSG00000084948	1700061H18Rik
ENSMUST00000147230	MSTRG.2393	B430219N15Rik
ENSMUST00000147231	ENSMUSG00000085876	Gm12409
ENSMUST00000147235	ENSMUSG00000086382	Chrna1os
ENSMUST00000147260	MSTRG.26031	5430402O13Rik
ENSMUST00000147294	ENSMUSG00000052951	C130021I20Rik
ENSMUST00000147324	ENSMUSG00000086621	Gm13348
ENSMUST00000147359	MSTRG.4395	4930403D09Rik
ENSMUST00000147378	ENSMUSG00000087695	Gm16291
ENSMUST00000147379	ENSMUSG00000085723	Gm15915
ENSMUST00000147398	MSTRG.29942	Gm15350
ENSMUST00000147410	ENSMUSG00000084844	Hoxb3os
ENSMUST00000147411	ENSMUSG00000087694	A530058N18Rik
ENSMUST00000147412	MSTRG.4555	Gm12204
ENSMUST00000147420	ENSMUSG00000086544	Chn1os3
ENSMUST00000147425	MSTRG.18012	1700020I14Rik
ENSMUST00000147438	ENSMUSG00000085594	Gm11551
ENSMUST00000147445	MSTRG.5993	A830035A12Rik

ENSMUST00000147446	MSTRG.18861	1110018N20Rik
ENSMUST00000147449	ENSMUSG00000087371	Gm15541
ENSMUST00000147463	ENSMUSG00000085946	AA387200
ENSMUST00000147478	MSTRG.34376	5530601H04Rik
ENSMUST00000147487	MSTRG.29477	B130055M24Rik
ENSMUST00000147494	MSTRG.708	Gm10558
ENSMUST00000147538	ENSMUSG00000087685	1700122E12Rik
ENSMUST00000147541	ENSMUSG00000086283	2810433D01Rik
ENSMUST00000147542	MSTRG.26614	Gm15612
ENSMUST00000147548	MSTRG.26190	E230016M11Rik
ENSMUST00000147555	ENSMUSG00000085517	Gm12963
ENSMUST00000147620	ENSMUSG00000075555	Gm10863
ENSMUST00000147633	MSTRG.15531	Tmem134
ENSMUST00000147638	MSTRG.11245	Gm15943
ENSMUST00000147653	MSTRG.3110	Gm15398
ENSMUST00000147678	ENSMUSG00000086478	Gm14102
ENSMUST00000147681	ENSMUSG00000078122	F630028O10Rik
ENSMUST00000147685	MSTRG.16510	Gm13391
ENSMUST00000147689	MSTRG.33465	4930520O04Rik
ENSMUST00000147690	MSTRG.28863	Rsf1os2
ENSMUST00000147704	MSTRG.8361	Gm11342
ENSMUST00000147717	ENSMUSG00000087245	Gm12126
ENSMUST00000147722	MSTRG.22718	Gm16211
ENSMUST00000147754	MSTRG.16065	Hectd2os
ENSMUST00000147762	MSTRG.4095	Snhg15
ENSMUST00000147774	MSTRG.13375	Gm16275
ENSMUST00000147811	MSTRG.8295	Gm11290
ENSMUST00000147847	MSTRG.4601	Gm12227
ENSMUST00000147848	MSTRG.12968	4931406G06Rik
ENSMUST00000147868	MSTRG.18322	Gm14097
ENSMUST00000147870	ENSMUSG00000063018	2010204K13Rik
ENSMUST00000147929	MSTRG.17991	Gm14207
ENSMUST00000147951	ENSMUSG00000086923	4930406D18Rik
ENSMUST00000147959	ENSMUSG00000087268	Gm14486
ENSMUST00000148028	ENSMUSG00000075555	Gm10863
ENSMUST00000148063	MSTRG.25498	A430035B10Rik
ENSMUST00000148064	MSTRG.13518	Gm16278
ENSMUST00000148066	MSTRG.30739	Gm10649
ENSMUST00000148082	MSTRG.25066	Gm16599
ENSMUST00000148087	MSTRG.4257	Gm12063
ENSMUST00000148089	ENSMUSG00000086843	E030013I19Rik
ENSMUST00000148122	MSTRG.7702	Gm16084
ENSMUST00000148132	MSTRG.4468	Gm12167
ENSMUST00000148136	MSTRG.5901	Gm11665
ENSMUST00000148217	ENSMUSG00000084932	Gm15156
ENSMUST00000148230	MSTRG.17037	D330023K18Rik
ENSMUST00000148244	ENSMUSG00000086063	Gm12257
ENSMUST00000148284	ENSMUSG00000085973	Gm14742
ENSMUST00000148293	MSTRG.12560	Gm15658
ENSMUST00000148316	ENSMUSG00000087334	AW495222
ENSMUST00000148318	MSTRG.27528	D030047H15Rik
ENSMUST00000148335	MSTRG.20074	Gm15444
ENSMUST00000148357	MSTRG.30277	Gm16192
ENSMUST00000148364	ENSMUSG00000085803	Gm12056
ENSMUST00000148405	ENSMUSG00000062391	4932435O22Rik
ENSMUST00000148413	MSTRG.4772	A530017D24Rik
ENSMUST00000148420	MSTRG.17901	Gm13966

ENSMUST00000148448	ENSMUSG00000084754	Gm12532
ENSMUST00000148457	ENSMUSG00000086187	Gm12860
ENSMUST00000148464	ENSMUSG00000087693	Gm16191
ENSMUST00000148466	MSTRG.24752	A930024E05Rik
ENSMUST00000148478	MSTRG.19757	Gm15535
ENSMUST00000148496	MSTRG.4164	Etaa1os
ENSMUST00000148522	MSTRG.34104	Gm14817
ENSMUST00000148530	MSTRG.33960	Firre
ENSMUST00000148542	MSTRG.411	Gm29247
ENSMUST00000148548	MSTRG.18241	A730036I17Rik
ENSMUST00000148568	ENSMUSG00000087541	Hopxos
ENSMUST00000148585	MSTRG.61	2610203C22Rik
ENSMUST00000148587	ENSMUSG00000087203	Gm13986
ENSMUST00000148598	ENSMUSG00000086677	Tvp23bos
ENSMUST00000148607	MSTRG.16754	Gm13324
ENSMUST00000148641	MSTRG.27513	Gm15927
ENSMUST00000148651	ENSMUSG00000084885	3010001F23Rik
ENSMUST00000148654	MSTRG.29906	Gm15419
ENSMUST00000148685	MSTRG.18628	Acss2os
ENSMUST00000148687	MSTRG.28716	Gm16638
ENSMUST00000148699	MSTRG.27862	Gm15567
ENSMUST00000148704	MSTRG.13386	BC028777
ENSMUST00000148705	MSTRG.18515	Gm14167
ENSMUST00000148724	MSTRG.6051	BC006965
ENSMUST00000148729	MSTRG.24992	Wbscr25
ENSMUST00000148734	ENSMUSG00000073781	Gm6471
ENSMUST00000148740	MSTRG.6808	F730043M19Rik
ENSMUST00000148743	MSTRG.8310	Gm11335
ENSMUST00000148776	MSTRG.28886	Gm15506
ENSMUST00000148807	MSTRG.15531	Tmem134
ENSMUST00000148844	MSTRG.20369	Gm12523
ENSMUST00000148847	MSTRG.19123	Uckl1os
ENSMUST00000148885	MSTRG.8388	2610307P16Rik
ENSMUST00000148892	MSTRG.21758	BB031773
ENSMUST00000148907	ENSMUSG00000085933	Tmem61
ENSMUST00000148931	MSTRG.29682	Gm16764
ENSMUST00000148972	MSTRG.28925	Gm15635
ENSMUST00000148981	MSTRG.8361	Gm11342
ENSMUST00000148997	MSTRG.18228	AI847159
ENSMUST00000149000	MSTRG.29988	Gm16347
ENSMUST00000149014	MSTRG.16529	Gm13262
ENSMUST00000149025	ENSMUSG00000084756	Gm13881
ENSMUST00000149048	ENSMUSG00000087290	Gm15866
ENSMUST00000149078	MSTRG.21691	Gm12676
ENSMUST00000149084	MSTRG.21086	Bach2os
ENSMUST00000149090	MSTRG.18125	Spata5l1
ENSMUST00000149095	ENSMUSG00000085575	Gm15485
ENSMUST00000149124	MSTRG.33713	Flicr
ENSMUST00000149128	MSTRG.24280	Gm28039
ENSMUST00000149136	ENSMUSG00000020624	4933434M16Rik
ENSMUST00000149137	MSTRG.16702	Gm13270
ENSMUST00000149156	MSTRG.24322	A930041C12Rik
ENSMUST00000149172	MSTRG.12975	A930006K02Rik
ENSMUST00000149224	MSTRG.290	D430040D24Rik
ENSMUST00000149226	ENSMUSG00000085629	Gm11697
ENSMUST00000149231	ENSMUSG00000086544	Chn1os3
ENSMUST00000149239	ENSMUSG00000086221	4930412B13Rik

ENSMUST00000149246	MSTRG.6679	2410018L13Rik
ENSMUST00000149247	MSTRG.22628	Gm13075
ENSMUST00000149252	MSTRG.9500	Gm15327
ENSMUST00000149275	MSTRG.18686	Gm14230
ENSMUST00000149283	MSTRG.17437	Gm13625
ENSMUST00000149285	MSTRG.18783	Gm11455
ENSMUST00000149300	MSTRG.17051	Gm16534
ENSMUST00000149308	MSTRG.33496	2900079G21Rik
ENSMUST00000149319	ENSMUSG00000087035	Tmem74bos
ENSMUST00000149348	MSTRG.21755	Gm12709
ENSMUST00000149367	MSTRG.21672	Gm12694
ENSMUST00000149373	MSTRG.11850	Rapgef3os1
ENSMUST00000149374	MSTRG.20253	Gm15471
ENSMUST00000149378	ENSMUSG00000087364	Gm13398
ENSMUST00000149386	ENSMUSG00000087135	Gm16096
ENSMUST00000149398	MSTRG.16795	Gm13335
ENSMUST00000149413	MSTRG.24595	9530046B11Rik
ENSMUST00000149428	ENSMUSG00000085864	C330019F10Rik
ENSMUST00000149429	ENSMUSG00000085999	Gm13411
ENSMUST00000149438	MSTRG.33729	Gm14820
ENSMUST00000149452	MSTRG.13964	Gm16279
ENSMUST00000149463	MSTRG.28831	C230038L03Rik
ENSMUST00000149472	MSTRG.22242	1700086P04Rik
ENSMUST00000149488	MSTRG.12430	B630019A10Rik
ENSMUST00000149506	MSTRG.28399	A230056P14Rik
ENSMUST00000149515	MSTRG.25660	Gm13782
ENSMUST00000149552	MSTRG.13646	Gm16758
ENSMUST00000149562	MSTRG.3489	Gm16235
ENSMUST00000149574	ENSMUSG00000052188	Gm14964
ENSMUST00000149576	MSTRG.18297	Gm14233
ENSMUST00000149592	ENSMUSG00000086117	Gm12065
ENSMUST00000149601	MSTRG.16065	Hectd2os
ENSMUST00000149608	MSTRG.24571	Gm13840
ENSMUST00000149609	MSTRG.24547	Gm13830
ENSMUST00000149618	ENSMUSG00000054618	Gm9951
ENSMUST00000149619	MSTRG.16853	Gm13387
ENSMUST00000149661	ENSMUSG00000086861	Gm15325
ENSMUST00000149667	MSTRG.18723	Snhg17
ENSMUST00000149677	MSTRG.21142	Gm12367
ENSMUST00000149681	MSTRG.12293	Gm15648
ENSMUST00000149687	MSTRG.23512	2210406O10Rik
ENSMUST00000149696	MSTRG.12285	Gm15585
ENSMUST00000149702	ENSMUSG00000086486	Ift88os
ENSMUST00000149741	MSTRG.22464	Gm15979
ENSMUST00000149815	MSTRG.27452	Far2os2
ENSMUST00000149817	MSTRG.21265	Gm12454
ENSMUST00000149820	MSTRG.665	Gm13748
ENSMUST00000149822	MSTRG.6184	Snhg20
ENSMUST00000149826	ENSMUSG00000085189	Gm11963
ENSMUST00000149830	MSTRG.2726	E130307A14Rik
ENSMUST00000149839	ENSMUSG00000087334	AW495222
ENSMUST00000149848	MSTRG.20960	Gm11816
ENSMUST00000149865	MSTRG.5218	Slc13a2os
ENSMUST00000149873	MSTRG.34709	Gm15246
ENSMUST00000149889	MSTRG.26190	E230016M11Rik
ENSMUST00000149909	MSTRG.1186	D630008O14Rik
ENSMUST00000149914	ENSMUSG00000085125	Gm16070

ENSMUST00000149932	MSTRG.16433	Gm13184
ENSMUST00000149935	MSTRG.15137	Gm16283
ENSMUST00000149938	MSTRG.29945	Gm15353
ENSMUST00000149940	MSTRG.5099	Mir22hg
ENSMUST00000149950	MSTRG.2789	Gm15200
ENSMUST00000149952	MSTRG.33553	Gm2415
ENSMUST00000149958	ENSMUSG00000086824	4930448D08Rik
ENSMUST00000149990	MSTRG.31310	Gm16349
ENSMUST00000149994	ENSMUSG00000086757	Gm2309
ENSMUST00000150005	ENSMUSG00000092397	C130080G10Rik
ENSMUST00000150024	ENSMUSG00000085008	Dbhos
ENSMUST00000150038	MSTRG.15766	Gm10143
ENSMUST00000150073	ENSMUSG00000085402	Gm12111
ENSMUST00000150079	MSTRG.10920	Gm15938
ENSMUST00000150099	ENSMUSG00000085818	Gm13267
ENSMUST00000150111	ENSMUSG00000086000	Gm12493
ENSMUST00000150145	MSTRG.19071	4921531C22Rik
ENSMUST00000150154	MSTRG.18134	Bloc1s6os
ENSMUST00000150171	ENSMUSG00000087575	Gm12976
ENSMUST00000150185	MSTRG.18944	Pmepa1os
ENSMUST00000150202	MSTRG.22653	Tmem51os1
ENSMUST00000150203	MSTRG.21496	Gm11210
ENSMUST00000150239	MSTRG.21869	Ttc39aos1
ENSMUST00000150251	MSTRG.25536	Gm4876
ENSMUST00000150265	ENSMUSG00000086494	2210417A02Rik
ENSMUST00000150276	MSTRG.18969	Nespas
ENSMUST00000150312	ENSMUSG00000086474	9130204K15Rik
ENSMUST00000150314	ENSMUSG00000086763	Plxna4os1
ENSMUST00000150323	MSTRG.21822	Lrp8os2
ENSMUST00000150329	MSTRG.4145	Egfros
ENSMUST00000150364	MSTRG.11753	1810021B22Rik
ENSMUST00000150366	ENSMUSG00000086481	Gm11707
ENSMUST00000150367	MSTRG.32973	A330041J22Rik
ENSMUST00000150375	MSTRG.16912	Gm13563
ENSMUST00000150385	MSTRG.25229	Gm16036
ENSMUST00000150388	MSTRG.13640	Gm28052
ENSMUST00000150389	MSTRG.12334	Gm15764
ENSMUST00000150418	ENSMUSG00000038408	1700018A04Rik
ENSMUST00000150442	MSTRG.5888	Gm11642
ENSMUST00000150450	MSTRG.17815	Gm13883
ENSMUST00000150466	ENSMUSG00000086822	5330413P13Rik
ENSMUST00000150471	MSTRG.5195	Dhrs13os
ENSMUST00000150472	MSTRG.11539	1700027A07Rik
ENSMUST00000150482	MSTRG.17718	Gm13780
ENSMUST00000150490	ENSMUSG00000078706	Gm53
ENSMUST00000150536	ENSMUSG00000085130	Gm11417
ENSMUST00000150544	MSTRG.26648	Gm14573
ENSMUST00000150556	MSTRG.17662	Ptpmt1
ENSMUST00000150581	ENSMUSG00000086454	Platr14
ENSMUST00000150600	MSTRG.2041	Gm15423
ENSMUST00000150613	MSTRG.28250	Gm15545
ENSMUST00000150616	MSTRG.23842	Gm15948
ENSMUST00000150621	ENSMUSG00000085017	Gm13412
ENSMUST00000150627	MSTRG.15531	Tmem134
ENSMUST00000150678	MSTRG.33482	Gm9888
ENSMUST00000150686	MSTRG.29946	Gm15351
ENSMUST00000150690	MSTRG.34694	Gm15239

ENSMUST00000150702	ENSMUSG00000086515	Erich2os
ENSMUST00000150705	ENSMUSG00000085519	Gm13703
ENSMUST00000150712	MSTRG.6053	2610035D17Rik
ENSMUST00000150735	ENSMUSG00000085044	1700109G15Rik
ENSMUST00000150749	MSTRG.692	2810408I11Rik
ENSMUST00000150772	ENSMUSG00000084996	Gm11419
ENSMUST00000150777	MSTRG.23005	Atad3aos
ENSMUST00000150782	MSTRG.3323	Atcayos
ENSMUST00000150794	ENSMUSG00000087378	Gm12414
ENSMUST00000150801	ENSMUSG00000073274	Gm14636
ENSMUST00000150818	ENSMUSG00000085992	Gm11515
ENSMUST00000150851	ENSMUSG00000021268	Meg3
ENSMUST00000150889	MSTRG.13249	Gm16046
ENSMUST00000150895	ENSMUSG00000086981	Gm12171
ENSMUST00000150906	MSTRG.12048	Gm15879
ENSMUST00000150953	ENSMUSG00000086296	D030055H07Rik
ENSMUST00000150973	MSTRG.4567	A630014C17Rik
ENSMUST00000150994	MSTRG.15195	1700066O22Rik
ENSMUST00000151014	MSTRG.29902	Gm15418
ENSMUST00000151028	ENSMUSG00000087032	Gm13874
ENSMUST00000151032	ENSMUSG00000086391	1700042O10Rik
ENSMUST00000151038	ENSMUSG00000085022	Gm5860
ENSMUST00000151040	ENSMUSG00000085833	Gm13003
ENSMUST00000151051	MSTRG.18231	Gm14029
ENSMUST00000151069	MSTRG.16886	A230005M16Rik
ENSMUST00000151091	MSTRG.6238	Gm11753
ENSMUST00000151096	MSTRG.24738	Gm15747
ENSMUST00000151099	ENSMUSG00000087365	C430049B03Rik
ENSMUST00000151101	MSTRG.34643	Gm15243
ENSMUST00000151112	MSTRG.33765	Gm14634
ENSMUST00000151122	ENSMUSG00000085112	A530072M11Rik
ENSMUST00000151136	MSTRG.13905	Gm10501
ENSMUST00000151138	MSTRG.20967	Gm11827
ENSMUST00000151159	MSTRG.24558	Gm13832
ENSMUST00000151164	MSTRG.21199	Fam219aos
ENSMUST00000151166	MSTRG.34412	2810403D21Rik
ENSMUST00000151207	MSTRG.20015	4930481B07Rik
ENSMUST00000151216	MSTRG.22041	Gm12867
ENSMUST00000151220	MSTRG.8354	4932702P03Rik
ENSMUST00000151229	MSTRG.17213	Nr6a1os
ENSMUST00000151267	MSTRG.34176	4933407K13Rik
ENSMUST00000151282	MSTRG.5966	Gm11706
ENSMUST00000151310	MSTRG.17815	Gm13883
ENSMUST00000151331	MSTRG.18723	Snhg17
ENSMUST00000151337	MSTRG.22655	Gm13062
ENSMUST00000151369	ENSMUSG00000086940	Gm12746
ENSMUST00000151374	MSTRG.22343	Snhg3
ENSMUST00000151381	ENSMUSG00000085336	Gm11732
ENSMUST00000151398	ENSMUSG00000073430	Gm10505
ENSMUST00000151401	MSTRG.15531	Tmem134
ENSMUST00000151417	MSTRG.18316	1700026D11Rik
ENSMUST00000151427	MSTRG.18206	Gm14005
ENSMUST00000151434	MSTRG.23512	2210406O10Rik
ENSMUST00000151472	MSTRG.18969	Nespas
ENSMUST00000151512	MSTRG.25161	Gm15672
ENSMUST00000151521	MSTRG.16909	0610009E02Rik
ENSMUST00000151538	MSTRG.18390	Macrod2os1

ENSMUST00000151550	ENSMUSG00000084895	AA672651
ENSMUST00000151564	MSTRG.9642	Gm3848
ENSMUST00000151580	MSTRG.29776	Prr33
ENSMUST00000151637	MSTRG.6808	F730043M19Rik
ENSMUST00000151639	ENSMUSG00000087124	Gm13839
ENSMUST00000151650	MSTRG.6051	BC006965
ENSMUST00000151651	ENSMUSG00000087646	Gm1667
ENSMUST00000151670	ENSMUSG00000086417	Gm12996
ENSMUST00000151676	MSTRG.32930	4930429F24Rik
ENSMUST00000151688	ENSMUSG00000084919	Ptprtos
ENSMUST00000151719	MSTRG.15144	C030005K06Rik
ENSMUST00000151761	MSTRG.17112	Nron
ENSMUST00000151765	ENSMUSG00000085113	B130011K05Rik
ENSMUST00000151778	ENSMUSG00000086286	Gm15138
ENSMUST00000151782	ENSMUSG00000087125	A230108P19Rik
ENSMUST00000151800	MSTRG.25679	2210408F21Rik
ENSMUST00000151816	ENSMUSG00000042976	9930038B18Rik
ENSMUST00000151817	MSTRG.4845	Gm12305
ENSMUST00000151820	MSTRG.17092	Gm13523
ENSMUST00000151825	MSTRG.4704	Gm16062
ENSMUST00000151828	ENSMUSG00000085123	Rubie
ENSMUST00000151836	MSTRG.23034	Gm16008
ENSMUST00000151841	ENSMUSG00000072753	9230020A06Rik
ENSMUST00000151848	ENSMUSG00000085918	Gm13032
ENSMUST00000151884	ENSMUSG00000085899	Gm15338
ENSMUST00000151896	MSTRG.22850	Ube4bos1
ENSMUST00000151901	MSTRG.26590	1700031F10Rik
ENSMUST00000151921	MSTRG.2726	E130307A14Rik
ENSMUST00000151931	ENSMUSG00000086578	Gm13583
ENSMUST00000151945	MSTRG.7509	Gm16876
ENSMUST00000151949	ENSMUSG00000086903	Hotair
ENSMUST00000151967	MSTRG.22830	Gm15969
ENSMUST00000151984	MSTRG.17356	Gm13571
ENSMUST00000151994	MSTRG.16460	Gm13179
ENSMUST00000151998	ENSMUSG00000053613	Notumos
ENSMUST00000152001	ENSMUSG00000087044	1700042G15Rik
ENSMUST00000152014	MSTRG.24398	Gm15787
ENSMUST00000152025	ENSMUSG00000085053	Gm14809
ENSMUST00000152070	MSTRG.12746	Gm15640
ENSMUST00000152073	MSTRG.4170	Gm16141
ENSMUST00000152074	ENSMUSG00000085967	Gm12530
ENSMUST00000152089	MSTRG.34412	2810403D21Rik
ENSMUST00000152109	ENSMUSG00000087365	C430049B03Rik
ENSMUST00000152112	ENSMUSG00000085949	Gm14275
ENSMUST00000152116	ENSMUSG00000087290	Gm15866
ENSMUST00000152118	ENSMUSG00000052951	C130021I20Rik
ENSMUST00000152125	MSTRG.18723	Snhg17
ENSMUST00000152147	MSTRG.25751	1810058I24Rik
ENSMUST00000152166	MSTRG.28368	Nell1os
ENSMUST00000152172	ENSMUSG00000086105	Gm11636
ENSMUST00000152188	MSTRG.30276	B430010I23Rik
ENSMUST00000152192	MSTRG.27374	Sox5os5
ENSMUST00000152203	MSTRG.32444	Arhgap20os
ENSMUST00000152229	ENSMUSG00000086620	Rspo4os
ENSMUST00000152230	ENSMUSG00000085976	Gm13816
ENSMUST00000152250	MSTRG.18347	Gm14211
ENSMUST00000152264	MSTRG.29926	Gm15875

ENSMUST00000152292	MSTRG.8795	4930451E10Rik
ENSMUST00000152302	MSTRG.29669	Gm15677
ENSMUST00000152313	MSTRG.17764	2810002D19Rik
ENSMUST00000152342	MSTRG.27861	Gm44641
ENSMUST00000152351	ENSMUSG00000085922	Fhad1os2
ENSMUST00000152365	MSTRG.19123	Uckl1os
ENSMUST00000152375	MSTRG.16912	Gm13563
ENSMUST00000152379	MSTRG.34495	BC065397
ENSMUST00000152412	MSTRG.17937	G630016G05Rik
ENSMUST00000152439	MSTRG.18207	Gm14009
ENSMUST00000152462	ENSMUSG00000075277	Haglr
ENSMUST00000152524	MSTRG.16710	Stamos
ENSMUST00000152538	ENSMUSG00000086675	Plxna4os2
ENSMUST00000152569	MSTRG.17606	Gm13684
ENSMUST00000152570	MSTRG.32755	Gm15511
ENSMUST00000152575	MSTRG.25382	5930430L01Rik
ENSMUST00000152576	MSTRG.3981	Gm11944
ENSMUST00000152595	MSTRG.28250	Gm15545
ENSMUST00000152600	MSTRG.3463	Gm15344
ENSMUST00000152625	MSTRG.28037	Nphs1os
ENSMUST00000152627	MSTRG.5456	Gm15892
ENSMUST00000152648	ENSMUSG00000086937	Gm15063
ENSMUST00000152663	ENSMUSG00000086968	4933431E20Rik
ENSMUST00000152709	MSTRG.29559	A130023I24Rik
ENSMUST00000152740	MSTRG.17402	Gm13629
ENSMUST00000152750	ENSMUSG00000086594	Nudt12os
ENSMUST00000152780	MSTRG.22984	5830444B04Rik
ENSMUST00000152781	MSTRG.4857	C78197
ENSMUST00000152784	MSTRG.5303	Gm11423
ENSMUST00000152787	MSTRG.23923	Usp46os2
ENSMUST00000152790	MSTRG.4643	2010001A14Rik
ENSMUST00000152799	MSTRG.805	Gm15179
ENSMUST00000152813	MSTRG.18125	Spata5l1
ENSMUST00000152815	ENSMUSG00000087309	4930528P14Rik
ENSMUST00000152822	MSTRG.24831	Tctn2
ENSMUST00000152825	ENSMUSG00000086917	Gm11630
ENSMUST00000152827	ENSMUSG00000086923	4930406D18Rik
ENSMUST00000152862	ENSMUSG00000087487	Ppp2r2cos
ENSMUST00000152863	MSTRG.1258	Gm15392
ENSMUST00000152867	MSTRG.20251	Gm15886
ENSMUST00000152916	MSTRG.34337	Tsix
ENSMUST00000152923	MSTRG.22738	Gm13165
ENSMUST00000152943	MSTRG.22343	Snhg3
ENSMUST00000152951	ENSMUSG00000086333	1700120E14Rik
ENSMUST00000152963	MSTRG.4842	Gm12301
ENSMUST00000152968	ENSMUSG00000086460	Gm12236
ENSMUST00000152986	ENSMUSG00000086548	Gm12972
ENSMUST00000153012	MSTRG.5596	Gm11529
ENSMUST00000153022	MSTRG.14816	1700001G01Rik
ENSMUST00000153023	MSTRG.22653	Tmem51os1
ENSMUST00000153028	ENSMUSG00000074783	AU019990
ENSMUST00000153030	ENSMUSG00000087265	Gm12349
ENSMUST00000153057	MSTRG.25679	2210408F21Rik
ENSMUST00000153087	MSTRG.5902	Gm22000
ENSMUST00000153089	MSTRG.23728	Gm15819
ENSMUST00000153095	MSTRG.29946	Gm15351
ENSMUST00000153106	MSTRG.32555	Gm16130

ENSMUST00000153113	MSTRG.2726	E130307A14Rik
ENSMUST00000153130	MSTRG.25289	2900089D17Rik
ENSMUST00000153180	ENSMUSG00000087604	Cdrt4os1
ENSMUST00000153181	MSTRG.5704	Gm12359
ENSMUST00000153188	MSTRG.19071	4921531C22Rik
ENSMUST00000153208	MSTRG.18589	Gm14198
ENSMUST00000153212	MSTRG.34412	2810403D21Rik
ENSMUST00000153213	MSTRG.5456	Gm15892
ENSMUST00000153228	ENSMUSG00000085863	Gm12801
ENSMUST00000153239	MSTRG.61	2610203C22Rik
ENSMUST00000153253	ENSMUSG00000087690	Gm16031
ENSMUST00000153261	MSTRG.2726	E130307A14Rik
ENSMUST00000153283	ENSMUSG00000087544	Gm13923
ENSMUST00000153297	MSTRG.7298	Plekhd1os
ENSMUST00000153313	MSTRG.3998	Tug1
ENSMUST00000153328	MSTRG.25692	Gm13849
ENSMUST00000153332	MSTRG.17884	Ccdc34os
ENSMUST00000153344	ENSMUSG00000092483	Gm20421
ENSMUST00000153352	MSTRG.14774	Gm15956
ENSMUST00000153354	ENSMUSG00000085182	Gm13596
ENSMUST00000153366	ENSMUSG00000052479	A330008L17Rik
ENSMUST00000153372	MSTRG.26579	Gm5577
ENSMUST00000153402	MSTRG.18342	9630028H03Rik
ENSMUST00000153429	ENSMUSG00000020624	4933434M16Rik
ENSMUST00000153431	MSTRG.4697	1700007J10Rik
ENSMUST00000153443	MSTRG.22832	Gm17029
ENSMUST00000153461	MSTRG.4576	A430108G06Rik
ENSMUST00000153474	MSTRG.22338	Snhg12
ENSMUST00000153497	ENSMUSG00000087410	2310065F04Rik
ENSMUST00000153522	ENSMUSG00000085969	Gm15600
ENSMUST00000153523	MSTRG.18936	Ctcflos
ENSMUST00000153552	MSTRG.30145	1700047A11Rik
ENSMUST00000153559	ENSMUSG00000085599	Gm13449
ENSMUST00000153562	MSTRG.27854	Gm15883
ENSMUST00000153572	MSTRG.12612	4930565N06Rik
ENSMUST00000153581	MSTRG.18012	1700020I14Rik
ENSMUST00000153599	MSTRG.5728	Gm11940
ENSMUST00000153600	MSTRG.16971	Gm13402
ENSMUST00000153610	MSTRG.34473	3632454L22Rik
ENSMUST00000153616	MSTRG.18688	5430405H02Rik
ENSMUST00000153646	MSTRG.10566	1700001G11Rik
ENSMUST00000153654	MSTRG.15531	Tmem134
ENSMUST00000153659	MSTRG.19388	C230034O21Rik
ENSMUST00000153685	MSTRG.8564	A730081D07Rik
ENSMUST00000153701	ENSMUSG00000084920	Gm15230
ENSMUST00000153718	MSTRG.4796	Gm12278
ENSMUST00000153722	MSTRG.2722	Gm16364
ENSMUST00000153726	MSTRG.17927	Gm13972
ENSMUST00000153753	ENSMUSG00000085024	C230035I16Rik
ENSMUST00000153774	ENSMUSG00000086161	Gm15389
ENSMUST00000153785	MSTRG.19375	Fgf2os
ENSMUST00000153786	ENSMUSG00000086021	Gm15767
ENSMUST00000153791	MSTRG.4417	Gm12148
ENSMUST00000153794	MSTRG.4133	Gm12002
ENSMUST00000153795	MSTRG.5577	Gm11527
ENSMUST00000153810	ENSMUSG00000086262	A930031H19Rik
ENSMUST00000153814	MSTRG.22130	Gm12905

ENSMUST00000153817	ENSMUSG00000087575	Gm12976
ENSMUST00000153825	MSTRG.6228	Gm11748
ENSMUST00000153878	MSTRG.18406	Pcsk2os2
ENSMUST00000153879	MSTRG.25227	Gm16035
ENSMUST00000153883	MSTRG.34338	Xist
ENSMUST00000153884	MSTRG.15195	4930511M06Rik
ENSMUST00000153898	MSTRG.3776	Grip1os2
ENSMUST00000153899	MSTRG.6013	Gm11655
ENSMUST00000153921	ENSMUSG00000085928	4933427I22Rik
ENSMUST00000153937	MSTRG.30762	4933431K23Rik
ENSMUST00000153958	MSTRG.9500	Gm15327
ENSMUST00000153969	MSTRG.19879	Gm15417
ENSMUST00000154016	MSTRG.28399	Gm15888
ENSMUST00000154030	MSTRG.18723	Snhg17
ENSMUST00000154044	ENSMUSG00000086789	Lyrm7os
ENSMUST00000154060	MSTRG.24996	Abhd11os
ENSMUST00000154065	MSTRG.18774	Gm16316
ENSMUST00000154066	MSTRG.26514	A430078I02Rik
ENSMUST00000154067	MSTRG.18316	1700026D11Rik
ENSMUST00000154068	ENSMUSG00000085564	Gm12198
ENSMUST00000154072	ENSMUSG00000087024	B230119M05Rik
ENSMUST00000154109	MSTRG.34059	Gm14705
ENSMUST00000154116	MSTRG.2278	1700034H15Rik
ENSMUST00000154159	ENSMUSG00000085759	1700061E18Rik
ENSMUST00000154167	MSTRG.27372	Sox5os3
ENSMUST00000154177	ENSMUSG00000084854	Gm12678
ENSMUST00000154188	MSTRG.34651	Gm15202
ENSMUST00000154194	MSTRG.20992	1700123M08Rik
ENSMUST00000154223	ENSMUSG00000079604	Gm13219
ENSMUST00000154242	MSTRG.18723	Snhg17
ENSMUST00000154246	ENSMUSG00000085766	2810430I11Rik
ENSMUST00000154296	MSTRG.17042	Gm13427
ENSMUST00000154298	MSTRG.8388	2610307P16Rik
ENSMUST00000154305	ENSMUSG00000072591	5930412G12Rik
ENSMUST00000154329	ENSMUSG00000086968	4933431E20Rik
ENSMUST00000154334	MSTRG.34376	5530601H04Rik
ENSMUST00000154346	ENSMUSG00000063018	2010204K13Rik
ENSMUST00000154352	ENSMUSG00000085569	Gm12602
ENSMUST00000154354	MSTRG.4675	2610507I01Rik
ENSMUST00000154360	MSTRG.16449	1700080N15Rik
ENSMUST00000154361	ENSMUSG00000087104	Tmem132cos
ENSMUST00000154379	MSTRG.6051	BC006965
ENSMUST00000154384	MSTRG.8795	4930451E10Rik
ENSMUST00000154407	ENSMUSG00000087132	A930001C03Rik
ENSMUST00000154414	MSTRG.5986	Gm11716
ENSMUST00000154452	MSTRG.5693	Gm12352
ENSMUST00000154457	MSTRG.26433	Ccdc142os
ENSMUST00000154462	MSTRG.21429	Gm12526
ENSMUST00000154471	MSTRG.5887	C130046K22Rik
ENSMUST00000154480	MSTRG.17564	Gm13727
ENSMUST00000154488	MSTRG.33984	Kis2
ENSMUST00000154515	ENSMUSG00000085961	Gm15904
ENSMUST00000154539	MSTRG.24146	Tmem150cos
ENSMUST00000154561	MSTRG.21869	Ttc39aos1
ENSMUST00000154566	MSTRG.3766	Grip1os3
ENSMUST00000154592	MSTRG.22742	Gm13166
ENSMUST00000154615	ENSMUSG00000085896	5330429C05Rik

ENSMUST00000154616	MSTRG.19104	Gm14341
ENSMUST00000154632	MSTRG.28477	BC046251
ENSMUST00000154654	MSTRG.17475	Gm13647
ENSMUST00000154673	MSTRG.27472	3010003L21Rik
ENSMUST00000154678	ENSMUSG00000086144	Gm11379
ENSMUST00000154682	MSTRG.16969	6530402F18Rik
ENSMUST00000154720	MSTRG.22315	Gm12971
ENSMUST00000154775	MSTRG.12747	Gm15638
ENSMUST00000154798	ENSMUSG00000086283	2810433D01Rik
ENSMUST00000154810	MSTRG.34714	G530011O06Rik
ENSMUST00000154820	MSTRG.25685	Mkln1os
ENSMUST00000154825	MSTRG.27525	9430041J12Rik
ENSMUST00000154834	MSTRG.17902	Gm13965
ENSMUST00000154848	MSTRG.29174	Parvaos
ENSMUST00000154854	MSTRG.12966	4930404I05Rik
ENSMUST00000154868	MSTRG.20251	Gm15886
ENSMUST00000154897	MSTRG.23573	Gm16015
ENSMUST00000154919	MSTRG.8356	9330162012Rik
ENSMUST00000154931	MSTRG.22978	Gm16023
ENSMUST00000154946	MSTRG.18346	Gm14209
ENSMUST00000154953	MSTRG.26443	Gm15624
ENSMUST00000154993	MSTRG.33859	Gm14549
ENSMUST00000154997	ENSMUSG00000085345	Gm16064
ENSMUST00000155010	MSTRG.23005	Atad3aos
ENSMUST00000155069	ENSMUSG00000073765	Gm12863
ENSMUST00000155074	MSTRG.28863	Rsf1os2
ENSMUST00000155091	MSTRG.16449	1700080N15Rik
ENSMUST00000155101	MSTRG.28827	Gm15413
ENSMUST00000155103	MSTRG.12334	Gm15764
ENSMUST00000155106	ENSMUSG00000087104	Tmem132cos
ENSMUST00000155136	MSTRG.34473	3632454L22Rik
ENSMUST00000155159	ENSMUSG00000085794	Vax2os
ENSMUST00000155165	MSTRG.4576	A430108G06Rik
ENSMUST00000155166	MSTRG.13250	Gm16049
ENSMUST00000155178	ENSMUSG00000086947	4930522O17Rik
ENSMUST00000155180	MSTRG.3577	Gm16155
ENSMUST00000155184	MSTRG.3767	Gm16321
ENSMUST00000155191	MSTRG.10909	BC037032
ENSMUST00000155211	ENSMUSG00000084945	C030037F17Rik
ENSMUST00000155217	MSTRG.28826	Gm15414
ENSMUST00000155224	ENSMUSG00000086833	Gm12440
ENSMUST00000155292	MSTRG.8388	2610307P16Rik
ENSMUST00000155323	ENSMUSG00000085316	D330050G23Rik
ENSMUST00000155332	MSTRG.3982	Gm12592
ENSMUST00000155345	MSTRG.25679	2210408F21Rik
ENSMUST00000155350	ENSMUSG00000085160	Gm13617
ENSMUST00000155382	ENSMUSG00000084761	Gm12406
ENSMUST00000155384	ENSMUSG00000054418	2900041M22Rik
ENSMUST00000155432	ENSMUSG00000085488	4930557F10Rik
ENSMUST00000155472	ENSMUSG00000085135	Gm13713
ENSMUST00000155482	MSTRG.2726	E130307A14Rik
ENSMUST00000155496	ENSMUSG00000087441	Gm13853
ENSMUST00000155498	MSTRG.4093	Gm11973
ENSMUST00000155505	MSTRG.6208	Gm11723
ENSMUST00000155508	MSTRG.5081	Gm12333
ENSMUST00000155531	MSTRG.5580	Zfp652os
ENSMUST00000155550	MSTRG.3890	Gm16230

ENSMUST00000155562	MSTRG.27373	Sox5os4
ENSMUST00000155578	MSTRG.20961	Gm11817
ENSMUST00000155588	ENSMUSG00000085125	Gm16070
ENSMUST00000155610	MSTRG.11784	Gm15609
ENSMUST00000155621	MSTRG.17662	Ptpmt1
ENSMUST00000155622	ENSMUSG00000087365	C430049B03Rik
ENSMUST00000155674	MSTRG.18706	Gm14286
ENSMUST00000155681	ENSMUSG00000075389	2810410L24Rik
ENSMUST00000155684	MSTRG.16890	Fcnaos
ENSMUST00000155688	ENSMUSG00000087162	Gm14244
ENSMUST00000155691	MSTRG.21098	4933421O10Rik
ENSMUST00000155758	ENSMUSG00000085412	Halr1
ENSMUST00000155796	MSTRG.24877	Gm15903
ENSMUST00000155799	ENSMUSG00000011350	Gm5893
ENSMUST00000155812	MSTRG.14191	Tomm6os
ENSMUST00000155816	MSTRG.22343	Snhg3
ENSMUST00000155822	ENSMUSG00000086321	Gm11413
ENSMUST00000155836	ENSMUSG00000085748	Gm12280
ENSMUST00000155845	MSTRG.22848	Ube4bos3
ENSMUST00000155855	MSTRG.10486	Mipepos
ENSMUST00000155864	MSTRG.23957	Gm15984
ENSMUST00000155877	MSTRG.6813	Atxn7l1os1
ENSMUST00000155883	ENSMUSG00000085531	Slc36a3os
ENSMUST00000155884	MSTRG.18630	Gssos2
ENSMUST00000155906	MSTRG.25999	Gimap1os
ENSMUST00000155922	MSTRG.26052	Hoxaas2
ENSMUST00000155949	MSTRG.16969	6530402F18Rik
ENSMUST00000155952	MSTRG.29781	R74862
ENSMUST00000155976	MSTRG.23422	Gm15614
ENSMUST00000155982	MSTRG.3028	Gm16143
ENSMUST00000155992	ENSMUSG00000086633	Gm16081
ENSMUST00000156011	MSTRG.11131	Gm15940
ENSMUST00000156020	MSTRG.16938	AA645442
ENSMUST00000156068	MSTRG.4980	6330403K07Rik
ENSMUST00000156081	MSTRG.22009	Gm12840
ENSMUST00000156084	MSTRG.30947	A230103J11Rik
ENSMUST00000156086	MSTRG.21675	4930551L18Rik
ENSMUST00000156090	MSTRG.3783	Gm15961
ENSMUST00000156095	MSTRG.17937	G630016G05Rik
ENSMUST00000156099	MSTRG.16909	Gm13568
ENSMUST00000156109	ENSMUSG00000086092	Gm15323
ENSMUST00000156115	MSTRG.25684	Gm14532
ENSMUST00000156144	ENSMUSG00000086136	Gm12718
ENSMUST00000156149	MSTRG.5456	Gm15892
ENSMUST00000156169	MSTRG.24579	Gm14507
ENSMUST00000156185	MSTRG.30720	Gm11033
ENSMUST00000156205	MSTRG.5796	Gm11615
ENSMUST00000156209	ENSMUSG00000086302	Gm13790
ENSMUST00000156210	MSTRG.34709	Gm15246
ENSMUST00000156211	MSTRG.34350	Ftx
ENSMUST00000156219	MSTRG.21933	Gm12847
ENSMUST00000156224	MSTRG.14093	Gm16172
ENSMUST00000156235	ENSMUSG00000085708	Gm16063
ENSMUST00000156236	MSTRG.9491	Gm15286
ENSMUST00000156240	MSTRG.34350	Ftx
ENSMUST00000156242	MSTRG.12991	Gm10785
ENSMUST00000156260	MSTRG.3345	Gm16104

ENSMUST00000156265	MSTRG.21815	Gm12869
ENSMUST00000156271	ENSMUSG00000085517	Gm12963
ENSMUST00000156275	ENSMUSG00000087267	4933427J07Rik
ENSMUST00000156292	ENSMUSG00000086438	Asb17os
ENSMUST00000156297	MSTRG.17112	Nron
ENSMUST00000156311	ENSMUSG00000086987	Gm12484
ENSMUST00000156331	MSTRG.24588	Gm13842
ENSMUST00000156342	ENSMUSG00000052371	Hoxd3os1
ENSMUST00000156345	MSTRG.18491	Gm14123
ENSMUST00000156350	ENSMUSG00000085562	2610028E06Rik
ENSMUST00000156364	MSTRG.33607	Lyzl4os
ENSMUST00000156387	MSTRG.17051	Gm16534
ENSMUST00000156391	MSTRG.24605	Gm15728
ENSMUST00000156395	MSTRG.5313	Unc45bos
ENSMUST00000156401	ENSMUSG00000086771	1700080G11Rik
ENSMUST00000156403	ENSMUSG00000086769	Gm15587
ENSMUST00000156418	MSTRG.17331	A930012O16Rik
ENSMUST00000156443	MSTRG.27370	Sox5os2
ENSMUST00000156446	MSTRG.4456	Gm16034
ENSMUST00000156466	MSTRG.23957	Gm15984
ENSMUST00000156467	MSTRG.25293	Gm15708
ENSMUST00000156477	ENSMUSG00000086552	Dlx4os
ENSMUST00000156494	ENSMUSG00000073051	Gm14812
ENSMUST00000156497	MSTRG.26374	Gm15401
ENSMUST00000156508	ENSMUSG00000086425	F730016J06Rik
ENSMUST00000156511	MSTRG.32200	1700110K17Rik
ENSMUST00000156538	MSTRG.17991	Gm14207
ENSMUST00000156576	ENSMUSG00000086306	Gm11754
ENSMUST00000156579	MSTRG.24398	Gm15787
ENSMUST00000156601	MSTRG.18243	Il1bos
ENSMUST00000156615	MSTRG.13037	2810404F17Rik
ENSMUST00000156633	ENSMUSG00000086904	Gm13404
ENSMUST00000156664	MSTRG.30643	Gm16091
ENSMUST00000156666	ENSMUSG00000086694	Gm16237
ENSMUST00000156682	MSTRG.9846	Zmiz1os1
ENSMUST00000156718	ENSMUSG00000087225	Gm14014
ENSMUST00000156734	MSTRG.26830	Gm15492
ENSMUST00000156790	MSTRG.21875	9630013D21Rik
ENSMUST00000156803	ENSMUSG00000112110	AC158605.1
ENSMUST00000156822	ENSMUSG00000084856	Gm11528
ENSMUST00000156831	ENSMUSG00000085743	8430419K02Rik
ENSMUST00000156875	MSTRG.29191	Far1os
ENSMUST00000156882	MSTRG.4485	Gm16170
ENSMUST00000156905	MSTRG.25792	Gm10244
ENSMUST00000156926	ENSMUSG00000084849	Gm16105
ENSMUST00000156947	ENSMUSG00000085027	Gm11840
ENSMUST00000156950	MSTRG.13567	Gm15458
ENSMUST00000156965	MSTRG.13583	Gm15598
ENSMUST00000156974	MSTRG.34376	5530601H04Rik
ENSMUST00000156994	MSTRG.27367	Sox5os1
ENSMUST00000157010	ENSMUSG00000084980	Slc36a1os
ENSMUST00000157011	MSTRG.21567	Gm11269
ENSMUST00000157018	MSTRG.31316	Gm15895
ENSMUST00000157022	ENSMUSG00000084885	3010001F23Rik
ENSMUST00000157059	MSTRG.17477	Gm13662
ENSMUST00000159006	MSTRG.26049	Hotairm1
ENSMUST00000159031	MSTRG.14615	0610012D04Rik

ENSMUST00000159037	MSTRG.1755	Gas5
ENSMUST00000159040	ENSMUSG00000090203	AU015336
ENSMUST00000159053	MSTRG.24807	Pitpnm2os2
ENSMUST00000159072	MSTRG.24762	AI480526
ENSMUST00000159087	MSTRG.953	A530040E14Rik
ENSMUST00000159119	MSTRG.1755	Gas5
ENSMUST00000159138	MSTRG.1854	Gm16565
ENSMUST00000159142	MSTRG.31064	Gm15889
ENSMUST00000159153	MSTRG.1755	Gas5
ENSMUST00000159157	MSTRG.1755	Gas5
ENSMUST00000159213	ENSMUSG00000089866	Gm15699
ENSMUST00000159244	MSTRG.24131	4930405H06Rik
ENSMUST00000159257	ENSMUSG00000090079	Gm15849
ENSMUST00000159260	ENSMUSG00000090263	D730045A05Rik
ENSMUST00000159268	MSTRG.31622	Gm16302
ENSMUST00000159271	MSTRG.11730	Gm15722
ENSMUST00000159273	MSTRG.2306	4930570N18Rik
ENSMUST00000159296	MSTRG.8925	Gm16132
ENSMUST00000159309	MSTRG.12416	9230117E06Rik
ENSMUST00000159313	MSTRG.16171	Gm16244
ENSMUST00000159324	MSTRG.1152	B230216N24Rik
ENSMUST00000159361	ENSMUSG00000090135	Gm15809
ENSMUST00000159363	MSTRG.32130	4930581F22Rik
ENSMUST00000159399	MSTRG.1755	Gas5
ENSMUST00000159404	MSTRG.1755	Gas5
ENSMUST00000159407	MSTRG.2296	Gm15867
ENSMUST00000159412	MSTRG.11753	1810021B22Rik
ENSMUST00000159438	MSTRG.1755	Gas5
ENSMUST00000159445	ENSMUSG00000089959	Gm16268
ENSMUST00000159471	ENSMUSG00000090227	Gm16554
ENSMUST00000159472	MSTRG.31367	Gm16116
ENSMUST00000159488	MSTRG.22940	Gm16334
ENSMUST00000159495	ENSMUSG00000086474	9130204K15Rik
ENSMUST00000159501	MSTRG.12560	Gm15657
ENSMUST00000159539	MSTRG.13842	C4a
ENSMUST00000159544	MSTRG.26827	Gt(ROSA)26Sor
ENSMUST00000159562	ENSMUSG00000089887	4930428N03Rik
ENSMUST00000159568	ENSMUSG00000090063	Dlx6os1
ENSMUST00000159595	ENSMUSG00000089815	Gm5083
ENSMUST00000159599	MSTRG.27354	Gm7457
ENSMUST00000159602	MSTRG.13177	A230009B12Rik
ENSMUST00000159618	MSTRG.27	4732440D04Rik
ENSMUST00000159621	ENSMUSG00000034764	1700006J14Rik
ENSMUST00000159637	MSTRG.24762	AI480526
ENSMUST00000159663	MSTRG.1755	Gas5
ENSMUST00000159669	MSTRG.13842	C4a
ENSMUST00000159700	ENSMUSG00000021874	4933413J09Rik
ENSMUST00000159701	ENSMUSG00000089990	Gm15852
ENSMUST00000159706	MSTRG.1755	Gas5
ENSMUST00000159731	MSTRG.13206	Airn
ENSMUST00000159738	MSTRG.11907	Nckap5los
ENSMUST00000159740	MSTRG.2128	H3f3aos
ENSMUST00000159791	MSTRG.13206	Airn
ENSMUST00000159793	ENSMUSG00000072679	D6Ertd474e
ENSMUST00000159796	MSTRG.32125	Gm15775
ENSMUST00000159827	ENSMUSG00000090063	Dlx6os1
ENSMUST00000159838	ENSMUSG00000089879	4930448H16Rik

ENSMUST00000159874	MSTRG.6998	Gm15524
ENSMUST00000159884	MSTRG.26561	E230015B07Rik
ENSMUST00000159890	MSTRG.1755	Gas5
ENSMUST00000159897	MSTRG.7792	Gm15995
ENSMUST00000159933	MSTRG.32130	4930581F22Rik
ENSMUST00000159950	MSTRG.24133	Gm16226
ENSMUST00000159953	MSTRG.12419	Gm15651
ENSMUST00000159965	ENSMUSG00000073154	9330158H04Rik
ENSMUST00000159971	ENSMUSG00000090012	Gm16555
ENSMUST00000159981	MSTRG.27724	1700058P15Rik
ENSMUST00000160001	MSTRG.16171	Gm16244
ENSMUST00000160030	MSTRG.25496	Gm16043
ENSMUST00000160065	MSTRG.8923	Gm16133
ENSMUST00000160076	MSTRG.20807	Gm16213
ENSMUST00000160080	MSTRG.20700	Gm16559
ENSMUST00000160089	ENSMUSG00000090104	Slmapos2
ENSMUST00000160091	ENSMUSG00000034764	1700006J14Rik
ENSMUST00000160099	MSTRG.24762	AI480526
ENSMUST00000160105	MSTRG.20217	Gm16160
ENSMUST00000160110	MSTRG.20276	Phtf1os
ENSMUST00000160126	ENSMUSG00000089783	Gm454
ENSMUST00000160137	MSTRG.19609	A730090N16Rik
ENSMUST00000160149	MSTRG.9670	Fhitos
ENSMUST00000160152	MSTRG.1755	Gas5
ENSMUST00000160156	ENSMUSG00000073154	9330158H04Rik
ENSMUST00000160177	MSTRG.13152	E430024P14Rik
ENSMUST00000160178	MSTRG.3086	Gm15647
ENSMUST00000160182	MSTRG.3626	Gm16239
ENSMUST00000160188	ENSMUSG00000072679	D6Ertd474e
ENSMUST00000160203	MSTRG.14062	Runx2os3
ENSMUST00000160227	MSTRG.24762	AI480526
ENSMUST00000160231	MSTRG.25265	0610040B10Rik
ENSMUST00000160242	ENSMUSG00000044021	Muc19
ENSMUST00000160255	MSTRG.24762	AI480526
ENSMUST00000160258	MSTRG.7327	Gm16572
ENSMUST00000160263	MSTRG.19918	Gm16540
ENSMUST00000160264	MSTRG.11116	Gm16136
ENSMUST00000160268	MSTRG.1843	Gm15853
ENSMUST00000160278	MSTRG.8923	Gm16133
ENSMUST00000160306	MSTRG.15476	Gm16146
ENSMUST00000160309	MSTRG.13842	C4a
ENSMUST00000160321	MSTRG.24762	AI480526
ENSMUST00000160334	MSTRG.10431	Gm16573
ENSMUST00000160351	MSTRG.9291	Gm16243
ENSMUST00000160361	ENSMUSG00000090254	Gm1965
ENSMUST00000160368	MSTRG.19174	Gm16337
ENSMUST00000160380	MSTRG.2217	1700007P06Rik
ENSMUST00000160385	MSTRG.9228	4833422C13Rik
ENSMUST00000160420	MSTRG.22167	9930104L06Rik
ENSMUST00000160429	MSTRG.1755	Gas5
ENSMUST00000160432	MSTRG.20808	Gm16231
ENSMUST00000160445	MSTRG.10652	Gm16549
ENSMUST00000160446	MSTRG.130	Gm7568
ENSMUST00000160463	MSTRG.7630	Gm15523
ENSMUST00000160490	MSTRG.29861	Gm16553
ENSMUST00000160494	ENSMUSG00000051297	2410124H12Rik
ENSMUST00000160497	MSTRG.1755	Gas5

ENSMUST00000160516	MSTRG.1755	Gas5
ENSMUST00000160528	MSTRG.13842	C4a
ENSMUST00000160540	ENSMUSG00000089653	Gm15841
ENSMUST00000160544	MSTRG.31369	Gm16117
ENSMUST00000160545	MSTRG.13152	E430024P14Rik
ENSMUST00000160551	MSTRG.1755	Gas5
ENSMUST00000160562	ENSMUSG00000090168	D630014O11Rik
ENSMUST00000160564	MSTRG.1399	Gm15851
ENSMUST00000160568	ENSMUSG00000089780	Gm16306
ENSMUST00000160577	MSTRG.26561	E230015B07Rik
ENSMUST00000160588	MSTRG.1152	B230216N24Rik
ENSMUST00000160604	MSTRG.20733	Gm15689
ENSMUST00000160605	MSTRG.31879	Gm15684
ENSMUST00000160626	MSTRG.13242	Gm16050
ENSMUST00000160657	MSTRG.13842	C4a
ENSMUST00000160659	MSTRG.314	4930556I23Rik
ENSMUST00000160661	ENSMUSG00000021874	4933413J09Rik
ENSMUST00000160679	MSTRG.13842	C4a
ENSMUST00000160698	MSTRG.10933	Gm2245
ENSMUST00000160746	MSTRG.12315	Gm15954
ENSMUST00000160763	MSTRG.13177	A230009B12Rik
ENSMUST00000160797	ENSMUSG00000090016	Gm16578
ENSMUST00000160917	MSTRG.14612	4833418N02Rik
ENSMUST00000160932	MSTRG.13206	Airn
ENSMUST00000160935	MSTRG.14999	2010320O07Rik
ENSMUST00000160938	MSTRG.11229	Gm16006
ENSMUST00000160947	MSTRG.552	Gm15834
ENSMUST00000160954	MSTRG.7602	Gm16339
ENSMUST00000160958	ENSMUSG00000021566	Slc6a19os
ENSMUST00000160960	MSTRG.11962	Galnt6os
ENSMUST00000160992	MSTRG.16168	Gm16541
ENSMUST00000160997	MSTRG.3414	1700025N21Rik
ENSMUST00000161005	MSTRG.1755	Gas5
ENSMUST00000161006	MSTRG.9157	1700023H06Rik
ENSMUST00000161024	MSTRG.31065	Gm15890
ENSMUST00000161091	MSTRG.13178	Gm16168
ENSMUST00000161096	MSTRG.14674	Gm10125
ENSMUST00000161121	MSTRG.13842	C4a
ENSMUST00000161126	MSTRG.540	Gm15759
ENSMUST00000161136	MSTRG.32130	4930581F22Rik
ENSMUST00000161150	MSTRG.953	A530040E14Rik
ENSMUST00000161174	ENSMUSG00000072679	D6Ertd474e
ENSMUST00000161216	MSTRG.33494	2310075C17Rik
ENSMUST00000161229	MSTRG.1755	Gas5
ENSMUST00000161244	ENSMUSG00000089819	Gm15679
ENSMUST00000161274	MSTRG.13178	Gm16168
ENSMUST00000161282	MSTRG.967	Gm16025
ENSMUST00000161291	ENSMUSG00000090191	9230105E05Rik
ENSMUST00000161307	MSTRG.25498	A430035B10Rik
ENSMUST00000161319	MSTRG.19695	Gm3513
ENSMUST00000161362	ENSMUSG00000090254	Gm1965
ENSMUST00000161364	MSTRG.3648	Gm15663
ENSMUST00000161366	MSTRG.25494	Gm16055
ENSMUST00000161380	MSTRG.1755	Gas5
ENSMUST00000161382	MSTRG.10431	Gm16573
ENSMUST00000161383	MSTRG.14674	Gm10125
ENSMUST00000161394	MSTRG.20728	Gm15688

ENSMUST00000161395	ENSMUSG00000089697	Gm15947
ENSMUST00000161397	ENSMUSG00000089870	Gm15562
ENSMUST00000161416	MSTRG.22941	Gm16333
ENSMUST00000161447	MSTRG.26636	Gm15756
ENSMUST00000161449	MSTRG.973	Gm16092
ENSMUST00000161461	MSTRG.1755	Gas5
ENSMUST00000161473	MSTRG.23190	Gm16110
ENSMUST00000161480	MSTRG.24306	A430072P03Rik
ENSMUST00000161483	MSTRG.11753	1810021B22Rik
ENSMUST00000161491	MSTRG.9228	4833422C13Rik
ENSMUST00000161509	MSTRG.155	4930486I03Rik
ENSMUST00000161529	MSTRG.11918	Gm16537
ENSMUST00000161540	MSTRG.972	Gm17017
ENSMUST00000161571	MSTRG.11118	Gm16137
ENSMUST00000161579	MSTRG.2431	Gm16577
ENSMUST00000161583	MSTRG.1766	2810442N19Rik
ENSMUST00000161597	ENSMUSG00000090116	Gm15680
ENSMUST00000161599	MSTRG.10888	4930594M22Rik
ENSMUST00000161614	ENSMUSG00000090257	Gm4524
ENSMUST00000161623	MSTRG.1755	Gas5
ENSMUST00000161626	MSTRG.130	Gm7568
ENSMUST00000161643	MSTRG.10940	Gm16029
ENSMUST00000161661	MSTRG.1152	B230216N24Rik
ENSMUST00000161681	MSTRG.30400	Gm15758
ENSMUST00000161706	MSTRG.13423	Snhg9
ENSMUST00000161735	MSTRG.11753	1810021B22Rik
ENSMUST00000161759	MSTRG.14612	4833418N02Rik
ENSMUST00000161763	MSTRG.982	Gm10552
ENSMUST00000161797	MSTRG.13842	C4a
ENSMUST00000161819	MSTRG.10681	Gm15629
ENSMUST00000161822	MSTRG.33285	4930524O07Rik
ENSMUST00000161823	MSTRG.32130	4930581F22Rik
ENSMUST00000161826	MSTRG.1764	Gm38304
ENSMUST00000161841	ENSMUSG00000089835	Gm7097
ENSMUST00000161848	MSTRG.24630	Gm15749
ENSMUST00000161868	MSTRG.31456	Gm16163
ENSMUST00000161890	MSTRG.13206	Airn
ENSMUST00000161914	MSTRG.31370	Gm16118
ENSMUST00000161917	MSTRG.9202	Gm4117
ENSMUST00000161921	MSTRG.14674	Gm10125
ENSMUST00000161923	MSTRG.967	Gm16025
ENSMUST00000161924	MSTRG.3154	Gm16240
ENSMUST00000161925	MSTRG.7794	Gm15996
ENSMUST00000161943	MSTRG.1803	Gm16587
ENSMUST00000161975	ENSMUSG00000089934	4930473D10Rik
ENSMUST00000161985	MSTRG.24021	Gm16579
ENSMUST00000162027	MSTRG.33305	Gm16343
ENSMUST00000162051	MSTRG.10680	Gm15628
ENSMUST00000162059	MSTRG.13668	4833413E03Rik
ENSMUST00000162121	ENSMUSG00000090160	4930480K15Rik
ENSMUST00000162133	MSTRG.13842	C4a
ENSMUST00000162148	MSTRG.24306	A430072P03Rik
ENSMUST00000162163	MSTRG.1755	Gas5
ENSMUST00000162186	MSTRG.11571	Gm16575
ENSMUST00000162198	MSTRG.31983	Gm10612
ENSMUST00000162209	MSTRG.32372	Gm16536
ENSMUST00000162218	MSTRG.10888	4930594M22Rik

ENSMUST00000162239	MSTRG.26824	Gm16161
ENSMUST00000162289	MSTRG.1755	Gas5
ENSMUST00000162306	MSTRG.33058	Gm16262
ENSMUST00000162318	ENSMUSG00000089760	D030046N08Rik
ENSMUST00000162325	MSTRG.32122	1810008B01Rik
ENSMUST00000162347	MSTRG.963	Gm16028
ENSMUST00000162348	MSTRG.24690	Gm16552
ENSMUST00000162353	MSTRG.953	A530040E14Rik
ENSMUST00000162370	ENSMUSG00000090255	4921534H16Rik
ENSMUST00000162380	ENSMUSG00000089952	4933413C19Rik
ENSMUST00000162391	MSTRG.27454	Far2os1
ENSMUST00000162407	MSTRG.15651	Gm16538
ENSMUST00000162432	MSTRG.13179	Gm16169
ENSMUST00000162450	MSTRG.8830	Etohd2
ENSMUST00000162454	MSTRG.2303	Gm15872
ENSMUST00000162457	MSTRG.30515	Gm15716
ENSMUST00000162458	ENSMUSG00000089881	Gm16280
ENSMUST00000162520	MSTRG.11116	Gm16136
ENSMUST00000162526	MSTRG.4687	Gm10435
ENSMUST00000162545	ENSMUSG00000089713	Gm16564
ENSMUST00000162547	MSTRG.26562	1810020O05Rik
ENSMUST00000162558	MSTRG.1755	Gas5
ENSMUST00000162566	MSTRG.32130	4930581F22Rik
ENSMUST00000162577	MSTRG.24807	Pitpnm2os2
ENSMUST00000162579	MSTRG.11753	1810021B22Rik
ENSMUST00000162608	MSTRG.27020	Gm16556
ENSMUST00000162615	MSTRG.2216	Gm15509
ENSMUST00000162639	MSTRG.24133	Gm16226
ENSMUST00000162641	MSTRG.27970	Gm16282
ENSMUST00000162649	MSTRG.27767	Gm16251
ENSMUST00000162651	MSTRG.16168	Gm16541
ENSMUST00000162677	MSTRG.12560	Gm15657
ENSMUST00000162697	MSTRG.24762	AI480526
ENSMUST00000162724	ENSMUSG00000089679	Gm16299
ENSMUST00000162738	MSTRG.32523	Man2c1os
ENSMUST00000162770	MSTRG.30391	5330439A09Rik
ENSMUST00000162771	MSTRG.3355	Gm15917
ENSMUST00000162822	MSTRG.30400	Gm15758
ENSMUST00000162828	MSTRG.31315	Gm15894
ENSMUST00000162829	MSTRG.16168	Gm16541
ENSMUST00000162837	ENSMUSG00000089656	Gm16271
ENSMUST00000162868	ENSMUSG00000089957	A830011K09Rik
ENSMUST00000162890	MSTRG.25265	0610040B10Rik
ENSMUST00000162911	ENSMUSG00000090255	4921534H16Rik
ENSMUST00000162958	MSTRG.29980	Gm16350
ENSMUST00000163009	MSTRG.3326	Gm16315
ENSMUST00000163052	MSTRG.17740	Gm10804
ENSMUST00000163053	MSTRG.8830	Etohd2
ENSMUST00000163056	ENSMUSG00000089815	Gm5083
ENSMUST00000163069	MSTRG.13641	Tbc1d22bos
ENSMUST00000163077	MSTRG.12560	Gm15657
ENSMUST00000163081	MSTRG.1755	Gas5
ENSMUST00000163160	ENSMUSG00000090990	Gm17197
ENSMUST00000163186	MSTRG.32537	Gm17231
ENSMUST00000163194	MSTRG.25098	Gm17112
ENSMUST00000163196	MSTRG.33277	Gm17041
ENSMUST00000163208	MSTRG.22130	Gm12905

ENSMUST00000163245	MSTRG.13761	Gm20507
ENSMUST00000163273	MSTRG.6302	Gm17178
ENSMUST00000163302	MSTRG.33496	2900079G21Rik
ENSMUST00000163314	ENSMUSG00000086541	Has2os
ENSMUST00000163493	ENSMUSG00000091475	2810468N07Rik
ENSMUST00000163578	MSTRG.13161	T2
ENSMUST00000163627	MSTRG.6417	2810032G03Rik
ENSMUST00000163635	ENSMUSG00000072723	Gm10044
ENSMUST00000163674	MSTRG.29757	Gm10575
ENSMUST00000163681	ENSMUSG00000090249	Gm3287
ENSMUST00000163707	ENSMUSG00000091709	Gm17189
ENSMUST00000163715	MSTRG.9827	Gm17105
ENSMUST00000163731	MSTRG.30154	Proscos
ENSMUST00000163781	MSTRG.11866	9330020H09Rik
ENSMUST00000163802	MSTRG.7402	Gm17139
ENSMUST00000163836	MSTRG.16899	Tmem250-ps
ENSMUST00000163996	MSTRG.29203	4933406I18Rik
ENSMUST00000164057	ENSMUSG00000011350	Gm5893
ENSMUST00000164074	MSTRG.2760	Gm17196
ENSMUST00000164075	MSTRG.13724	Gm17115
ENSMUST00000164104	ENSMUSG00000092090	Gm3294
ENSMUST00000164249	MSTRG.1715	4930562F07Rik
ENSMUST00000164299	ENSMUSG00000090358	Gm2822
ENSMUST00000164319	MSTRG.10808	Gm17066
ENSMUST00000164330	MSTRG.20331	2010016I18Rik
ENSMUST00000164367	MSTRG.7691	Gm17032
ENSMUST00000164379	MSTRG.16388	Gm17203
ENSMUST00000164605	MSTRG.14367	4930405O22Rik
ENSMUST00000164716	MSTRG.22012	Gm17114
ENSMUST00000164734	MSTRG.33675	Gm17120
ENSMUST00000164755	ENSMUSG00000090534	Gm4675
ENSMUST00000164804	MSTRG.6845	Gm17056
ENSMUST00000165070	MSTRG.7508	Gm8378
ENSMUST00000165145	MSTRG.9028	Gm17039
ENSMUST00000165200	MSTRG.7322	Gm17191
ENSMUST00000165350	MSTRG.10808	Gm17066
ENSMUST00000165444	MSTRG.29778	Tspan32os
ENSMUST00000165458	MSTRG.32940	Gm17477
ENSMUST00000165464	MSTRG.1456	Gm15850
ENSMUST00000165610	MSTRG.5260	AU040972
ENSMUST00000165647	MSTRG.2762	5730435O14Rik
ENSMUST00000165676	MSTRG.6847	Gm17024
ENSMUST00000165760	ENSMUSG00000087360	Gm15104
ENSMUST00000165858	MSTRG.32537	Gm17231
ENSMUST00000165880	ENSMUSG00000086541	Has2os
ENSMUST00000165911	MSTRG.13498	Gm17218
ENSMUST00000166003	MSTRG.21514	8030451A03Rik
ENSMUST00000166038	ENSMUSG00000091686	4930471C04Rik
ENSMUST00000166047	MSTRG.24489	Gm17122
ENSMUST00000166070	MSTRG.25536	Gm4876
ENSMUST00000166072	ENSMUSG00000075042	4930431P03Rik
ENSMUST00000166107	ENSMUSG00000066158	AY512931
ENSMUST00000166127	ENSMUSG00000075042	4930431P03Rik
ENSMUST00000166229	MSTRG.30800	Gm9725
ENSMUST00000166290	ENSMUSG00000086541	Has2os
ENSMUST00000166328	ENSMUSG00000091272	Gm17641
ENSMUST00000166507	MSTRG.32724	Gm17098

ENSMUST00000166593	MSTRG.3245	Gm17134
ENSMUST00000166606	MSTRG.24140	Gm17092
ENSMUST00000166636	ENSMUSG00000021268	Meg3
ENSMUST00000166637	ENSMUSG00000090270	Gm17226
ENSMUST00000166653	ENSMUSG00000054510	Gm14461
ENSMUST00000166813	ENSMUSG00000072723	Gm10044
ENSMUST00000166858	MSTRG.22984	5830444B04Rik
ENSMUST00000166902	MSTRG.9892	Arf4os
ENSMUST00000166921	MSTRG.22984	5830444B04Rik
ENSMUST00000166953	MSTRG.11782	C230037L18Rik
ENSMUST00000166971	MSTRG.7323	6530401F13Rik
ENSMUST00000167009	MSTRG.25280	Gm17135
ENSMUST00000167226	MSTRG.3923	Gm17201
ENSMUST00000167359	ENSMUSG00000090925	1810064F22Rik
ENSMUST00000167415	MSTRG.26827	Gt(ROSA)26Sor
ENSMUST00000167536	ENSMUSG00000091526	BB019430
ENSMUST00000167578	ENSMUSG00000091816	Gm17141
ENSMUST00000167642	MSTRG.20326	Gm42890
ENSMUST00000167654	MSTRG.17740	Gm10804
ENSMUST00000167704	MSTRG.19285	1700112D23Rik
ENSMUST00000167763	MSTRG.10379	4930579G18Rik
ENSMUST00000167777	MSTRG.7508	Gm8378
ENSMUST00000167834	MSTRG.9405	1700099I09Rik
ENSMUST00000167886	MSTRG.26450	B230319C09Rik
ENSMUST00000167899	MSTRG.30133	Gm17484
ENSMUST00000167900	MSTRG.29757	Gm10575
ENSMUST00000168000	ENSMUSG00000090709	Gm17173
ENSMUST00000168012	MSTRG.6352	1110002L01Rik
ENSMUST00000168048	MSTRG.16794	Gm17171
ENSMUST00000168123	MSTRG.11681	Gm17206
ENSMUST00000168137	MSTRG.33417	5830462I19Rik
ENSMUST00000168140	MSTRG.16390	Gm4219
ENSMUST00000168187	MSTRG.14696	Gm17036
ENSMUST00000168243	MSTRG.30811	4930579O11Rik
ENSMUST00000168284	MSTRG.2949	Gm17059
ENSMUST00000168347	ENSMUSG00000091393	5330438I03Rik
ENSMUST00000168422	MSTRG.23058	Gm17590
ENSMUST00000168442	MSTRG.31039	Gm3235
ENSMUST00000168470	MSTRG.10667	Gm17233
ENSMUST00000168475	ENSMUSG00000092131	BC050972
ENSMUST00000168496	MSTRG.28730	2610206C17Rik
ENSMUST00000168521	MSTRG.3241	E130317F20Rik
ENSMUST00000168625	MSTRG.9013	Gm17108
ENSMUST00000168682	MSTRG.32634	Gm16759
ENSMUST00000168686	MSTRG.23934	Gm17207
ENSMUST00000168775	MSTRG.18243	Il1bos
ENSMUST00000168789	ENSMUSG00000086578	Gm13583
ENSMUST00000168835	MSTRG.11782	C230037L18Rik
ENSMUST00000168837	ENSMUSG00000055403	4933427D06Rik
ENSMUST00000168897	MSTRG.12650	Gm17103
ENSMUST00000168951	MSTRG.11920	Gm17058
ENSMUST00000168978	MSTRG.13800	Gm20427
ENSMUST00000168990	MSTRG.26417	Gm17034
ENSMUST00000169054	MSTRG.9796	A430057M04Rik
ENSMUST00000169055	MSTRG.15531	Tmem134
ENSMUST00000169111	MSTRG.1993	4933439K11Rik
ENSMUST00000169153	ENSMUSG00000092131	BC050972

ENSMUST00000169163	MSTRG.11316	Gm17140
ENSMUST00000169175	ENSMUSG00000090785	Gm17116
ENSMUST00000169209	ENSMUSG00000085353	1700092C17Rik
ENSMUST00000169242	MSTRG.21220	Gm17167
ENSMUST00000169417	ENSMUSG00000091849	Gm17188
ENSMUST00000169420	ENSMUSG00000092131	BC050972
ENSMUST00000169600	MSTRG.1715	4930562F07Rik
ENSMUST00000169733	MSTRG.14053	1700071M16Rik
ENSMUST00000169791	MSTRG.12776	Dubr
ENSMUST00000169881	ENSMUSG00000090839	Gm17094
ENSMUST00000169887	MSTRG.11928	Gm17057
ENSMUST00000169914	MSTRG.27815	9130221H12Rik
ENSMUST00000169975	MSTRG.10001	Gm17210
ENSMUST00000169981	MSTRG.7749	Gm17111
ENSMUST00000170047	MSTRG.2762	5730435O14Rik
ENSMUST00000170101	ENSMUSG00000101854	1700026F02Rik
ENSMUST00000170103	MSTRG.13759	Gm17251
ENSMUST00000170150	MSTRG.9796	A430057M04Rik
ENSMUST00000170177	ENSMUSG00000072723	Gm10044
ENSMUST00000170214	MSTRG.33678	Gm17200
ENSMUST00000170286	MSTRG.15710	Gm17227
ENSMUST00000170435	ENSMUSG00000091192	Sardhos
ENSMUST00000170636	MSTRG.14380	Gm17133
ENSMUST00000170645	MSTRG.9406	Gm17160
ENSMUST00000170849	MSTRG.12887	Mir99ahg
ENSMUST00000170929	ENSMUSG00000091864	Gm17102
ENSMUST00000170933	MSTRG.21857	Gm17354
ENSMUST00000170938	MSTRG.31679	Fbxl12os
ENSMUST00000170986	MSTRG.26450	B230319C09Rik
ENSMUST00000171079	MSTRG.8473	Gm16984
ENSMUST00000171096	MSTRG.32920	4930542C12Rik
ENSMUST00000171173	MSTRG.31308	Gm17344
ENSMUST00000171190	MSTRG.23286	2900005J15Rik
ENSMUST00000171248	MSTRG.10025	Gm17110
ENSMUST00000171379	MSTRG.14367	4930405O22Rik
ENSMUST00000171408	MSTRG.5344	E230016K23Rik
ENSMUST00000171429	MSTRG.1984	Gm17224
ENSMUST00000171483	MSTRG.18060	AV039307
ENSMUST00000171591	MSTRG.11321	Gm2895
ENSMUST00000171665	MSTRG.27533	Gm15494
ENSMUST00000171722	MSTRG.9405	1700099I09Rik
ENSMUST00000171723	MSTRG.24303	Gm17202
ENSMUST00000171743	MSTRG.15531	Tmem134
ENSMUST00000171747	MSTRG.1905	Gm9929
ENSMUST00000171768	MSTRG.1054	Gm17090
ENSMUST00000172037	MSTRG.18857	Gm17096
ENSMUST00000172047	MSTRG.7508	Gm8378
ENSMUST00000172051	ENSMUSG00000090427	Gm17225
ENSMUST00000172055	ENSMUSG00000091849	Gm17188
ENSMUST00000172072	MSTRG.14053	1700071M16Rik
ENSMUST00000172075	MSTRG.33534	Gm17396
ENSMUST00000172093	MSTRG.33683	Gm17021
ENSMUST00000172128	ENSMUSG00000091283	Gm17234
ENSMUST00000172202	MSTRG.22344	Gm17300
ENSMUST00000172211	ENSMUSG00000090589	Gm17180
ENSMUST00000172285	MSTRG.13932	Gm17705
ENSMUST00000172460	ENSMUSG00000072723	Gm10044

ENSMUST00000172477	MSTRG.14019	Gm20429
ENSMUST00000172480	MSTRG.13989	Gm20508
ENSMUST00000172483	MSTRG.27754	Gm20512
ENSMUST00000172501	MSTRG.13854	1110038B12Rik
ENSMUST00000172522	MSTRG.12461	Gm20319
ENSMUST00000172526	MSTRG.13778	BC051226
ENSMUST00000172531	MSTRG.13854	1110038B12Rik
ENSMUST00000172547	ENSMUSG00000092499	1700092C10Rik
ENSMUST00000172588	MSTRG.26372	Gm20536
ENSMUST00000172591	MSTRG.3864	Gm20492
ENSMUST00000172644	MSTRG.28994	Gm20476
ENSMUST00000172664	ENSMUSG00000092539	Gm20468
ENSMUST00000172700	MSTRG.23625	4930518C09Rik
ENSMUST00000172701	ENSMUSG00000074987	Wt1os
ENSMUST00000172721	MSTRG.32933	Gm3211
ENSMUST00000172729	MSTRG.24745	Gm44574
ENSMUST00000172793	MSTRG.1783	Gm20471
ENSMUST00000172796	MSTRG.13819	Gm20496
ENSMUST00000172812	MSTRG.15629	Malat1
ENSMUST00000172817	MSTRG.13776	Platr17
ENSMUST00000172838	MSTRG.6581	Gm4425
ENSMUST00000172874	ENSMUSG00000092171	4833427F10Rik
ENSMUST00000172891	MSTRG.15971	Gm20407
ENSMUST00000172910	MSTRG.12838	Crybg3
ENSMUST00000172952	ENSMUSG00000092222	Gm20506
ENSMUST00000172972	MSTRG.14204	Gm20540
ENSMUST00000173025	MSTRG.14008	2410017I17Rik
ENSMUST00000173070	MSTRG.13854	1110038B12Rik
ENSMUST00000173141	ENSMUSG00000092392	Gm20546
ENSMUST00000173157	MSTRG.6645	Gm20535
ENSMUST00000173162	MSTRG.13941	Gm20483
ENSMUST00000173165	ENSMUSG00000092220	Gm20528
ENSMUST00000173178	MSTRG.11555	Gm20420
ENSMUST00000173208	MSTRG.24515	4930515G01Rik
ENSMUST00000173249	MSTRG.18728	Gm20412
ENSMUST00000173293	ENSMUSG00000092404	Gm9921
ENSMUST00000173304	ENSMUSG00000092509	Gm20394
ENSMUST00000173312	MSTRG.24745	Gm2479
ENSMUST00000173314	MSTRG.15629	Malat1
ENSMUST00000173374	MSTRG.28460	Gm20457
ENSMUST00000173437	ENSMUSG00000092346	Tlx1os
ENSMUST00000173499	MSTRG.15629	Malat1
ENSMUST00000173523	MSTRG.15629	Malat1
ENSMUST00000173528	MSTRG.24178	Gm20548
ENSMUST00000173553	ENSMUSG00000092515	C87198
ENSMUST00000173576	MSTRG.13928	Gm20522
ENSMUST00000173605	MSTRG.12598	Gm15564
ENSMUST00000173622	MSTRG.18931	Gm20490
ENSMUST00000173637	ENSMUSG00000092602	4931413I07Rik
ENSMUST00000173648	MSTRG.14011	Gm20478
ENSMUST00000173670	MSTRG.24745	Gm2479
ENSMUST00000173672	MSTRG.15657	Neat1
ENSMUST00000173677	MSTRG.5483	4930556N13Rik
ENSMUST00000173695	MSTRG.27343	Gm20400
ENSMUST00000173770	MSTRG.13819	Gm20496
ENSMUST00000173811	MSTRG.13854	1110038B12Rik
ENSMUST00000173827	ENSMUSG00000085702	Mecomos

ENSMUST00000173840	ENSMUSG00000092219	Gm20443
ENSMUST00000173847	MSTRG.6363	Dtnbos
ENSMUST00000173856	ENSMUSG00000092627	D130058E05Rik
ENSMUST00000173900	MSTRG.13990	A930015D03Rik
ENSMUST00000173965	MSTRG.24178	Gm20548
ENSMUST00000173978	MSTRG.26150	BB365896
ENSMUST00000173980	MSTRG.31308	Gm17344
ENSMUST00000173993	MSTRG.31928	Gm20406
ENSMUST00000174002	MSTRG.11294	Gm20405
ENSMUST00000174005	ENSMUSG00000092196	Gm38505
ENSMUST00000174014	MSTRG.32270	Gm20444
ENSMUST00000174019	MSTRG.23519	Gm42936
ENSMUST00000174032	MSTRG.26901	Gm20404
ENSMUST00000174039	MSTRG.13854	1110038B12Rik
ENSMUST00000174074	MSTRG.13824	Gm20513
ENSMUST00000174079	MSTRG.5877	Gm20511
ENSMUST00000174085	MSTRG.12598	Gm15564
ENSMUST00000174141	MSTRG.3865	Gm4189
ENSMUST00000174167	MSTRG.3865	Gm4189
ENSMUST00000174168	MSTRG.14019	Gm20429
ENSMUST00000174173	MSTRG.13854	1110038B12Rik
ENSMUST00000174185	MSTRG.23005	Atad3aos
ENSMUST00000174217	ENSMUSG00000092522	Gm20389
ENSMUST00000174283	ENSMUSG00000092272	Gm19301
ENSMUST00000174285	ENSMUSG00000092570	Plut
ENSMUST00000174287	MSTRG.15657	Neat1
ENSMUST00000174312	MSTRG.24178	Gm20548
ENSMUST00000174321	MSTRG.13778	BC051226
ENSMUST00000174338	MSTRG.15379	Gm20544
ENSMUST00000174380	ENSMUSG00000092239	1700031A10Rik
ENSMUST00000174385	MSTRG.12461	Gm20319
ENSMUST00000174440	ENSMUSG00000092242	Gm20515
ENSMUST00000174452	ENSMUSG00000092402	Gm20485
ENSMUST00000174464	MSTRG.13854	1110038B12Rik
ENSMUST00000174467	MSTRG.13778	BC051226
ENSMUST00000174502	ENSMUSG00000092599	1700010K23Rik
ENSMUST00000174528	MSTRG.24178	Gm20548
ENSMUST00000174530	MSTRG.6641	Gm21989
ENSMUST00000174550	MSTRG.4133	Gm12002
ENSMUST00000174568	MSTRG.4227	9130230N09Rik
ENSMUST00000174600	ENSMUSG00000092525	Gm20461
ENSMUST00000174630	ENSMUSG00000092600	Gm20442
ENSMUST00000174654	MSTRG.2387	Gm20470
ENSMUST00000174714	MSTRG.13854	1110038B12Rik
ENSMUST00000174738	MSTRG.19472	Gm20402
ENSMUST00000174756	MSTRG.13832	Gm20463
ENSMUST00000174760	MSTRG.31556	Gm20416
ENSMUST00000174768	ENSMUSG00000092275	Gm20465
ENSMUST00000174784	MSTRG.15655	Gm20417
ENSMUST00000174808	MSTRG.15629	Malat1
ENSMUST00000174811	ENSMUSG00000092353	Gm20539
ENSMUST00000174821	ENSMUSG00000092220	Gm20528
ENSMUST00000174829	MSTRG.15657	Neat1
ENSMUST00000174851	MSTRG.13795	Gm26940
ENSMUST00000174861	MSTRG.27052	Gm20531
ENSMUST00000174864	MSTRG.24178	Gm20548
ENSMUST00000174870	ENSMUSG00000074987	Wt1os

ENSMUST00000174884	MSTRG.23626	Gm20475
ENSMUST00000175668	ENSMUSG00000093622	Gm20703
ENSMUST00000175674	ENSMUSG00000093726	Gm20667
ENSMUST00000175690	MSTRG.29436	Gm20650
ENSMUST00000175699	MSTRG.11333	Gm20732
ENSMUST00000175717	MSTRG.12942	B130034C11Rik
ENSMUST00000175723	ENSMUSG00000093516	Gm19553
ENSMUST00000175729	MSTRG.33352	4921523L03Rik
ENSMUST00000175753	MSTRG.4797	Gm27194
ENSMUST00000175825	MSTRG.34580	A230072E10Rik
ENSMUST00000175846	MSTRG.31293	Gm20686
ENSMUST00000175854	ENSMUSG00000093540	4930505M18Rik
ENSMUST00000175861	MSTRG.10580	Gm20675
ENSMUST00000175871	ENSMUSG00000093765	Gm20658
ENSMUST00000175891	MSTRG.33353	4930535L15Rik
ENSMUST00000175942	MSTRG.34580	A230072E10Rik
ENSMUST00000176016	MSTRG.5335	AA465934
ENSMUST00000176020	MSTRG.32166	Gm3896
ENSMUST00000176061	MSTRG.31955	Gm20681
ENSMUST00000176106	MSTRG.7653	Gm28577
ENSMUST00000176132	ENSMUSG00000093672	Gm20655
ENSMUST00000176134	MSTRG.18688	5430405H02Rik
ENSMUST00000176143	MSTRG.20084	Gm20627
ENSMUST00000176157	ENSMUSG00000093479	Gm20629
ENSMUST00000176201	MSTRG.22724	Gm20707
ENSMUST00000176215	MSTRG.13407	Rab26os
ENSMUST00000176275	MSTRG.20084	Gm20627
ENSMUST00000176283	ENSMUSG00000093482	Gm20619
ENSMUST00000176290	MSTRG.26132	4930533I22Rik
ENSMUST00000176296	MSTRG.12942	B130034C11Rik
ENSMUST00000176338	ENSMUSG00000093394	Gm20621
ENSMUST00000176344	ENSMUSG00000093497	Gm20713
ENSMUST00000176387	MSTRG.11250	Gm20712
ENSMUST00000176455	MSTRG.3727	D630029K05Rik
ENSMUST00000176473	MSTRG.25258	Gm20635
ENSMUST00000176545	MSTRG.5335	AA465934
ENSMUST00000176549	MSTRG.14430	Gm28727
ENSMUST00000176552	MSTRG.7540	Gm20682
ENSMUST00000176556	ENSMUSG00000093460	Six3os1
ENSMUST00000176569	MSTRG.20035	6330562C20Rik
ENSMUST00000176599	ENSMUSG00000093467	Gm20659
ENSMUST00000176618	ENSMUSG00000093394	Gm20621
ENSMUST00000176654	ENSMUSG00000093380	Gm20685
ENSMUST00000176660	MSTRG.24461	Gm20636
ENSMUST00000176703	MSTRG.11231	9330154K18Rik
ENSMUST00000176721	ENSMUSG00000093673	Gm20644
ENSMUST00000176742	MSTRG.20083	Gm20633
ENSMUST00000176744	MSTRG.28485	Gm20684
ENSMUST00000176749	MSTRG.23640	Gm20700
ENSMUST00000176760	MSTRG.20081	Gm20632
ENSMUST00000176762	MSTRG.11287	Gm20717
ENSMUST00000176841	MSTRG.10586	4930438E09Rik
ENSMUST00000176872	MSTRG.10391	Gm20687
ENSMUST00000176921	MSTRG.13407	Rab26os
ENSMUST00000176934	MSTRG.10587	Gm10860
ENSMUST00000176972	ENSMUSG00000093771	AU023070
ENSMUST00000177012	MSTRG.16950	Gm13381

ENSMUST00000177015	MSTRG.33623	A730085K08Rik
ENSMUST00000177043	MSTRG.7408	Prox2os
ENSMUST00000177048	MSTRG.15981	4931403E22Rik
ENSMUST00000177059	ENSMUSG00000093765	Gm20658
ENSMUST00000177065	ENSMUSG00000093489	Gm20625
ENSMUST00000177127	MSTRG.10585	1700120O09Rik
ENSMUST00000177130	MSTRG.29865	4932443L11Rik
ENSMUST00000177136	ENSMUSG00000060424	Pantr1
ENSMUST00000177150	MSTRG.5335	AA465934
ENSMUST00000177190	MSTRG.14674	Gm10125
ENSMUST00000177201	ENSMUSG00000093738	AI606473
ENSMUST00000177220	ENSMUSG00000093460	Six3os1
ENSMUST00000177239	ENSMUSG00000093650	Gm20631
ENSMUST00000177248	MSTRG.5335	AA465934
ENSMUST00000177251	MSTRG.26980	Gm20692
ENSMUST00000177262	MSTRG.15977	Gm20616
ENSMUST00000177268	ENSMUSG00000093650	Gm20631
ENSMUST00000177278	MSTRG.26768	Gm20705
ENSMUST00000177314	MSTRG.19829	Gm20652
ENSMUST00000177319	ENSMUSG00000087601	Uchl1os
ENSMUST00000177351	MSTRG.1876	Gm20711
ENSMUST00000177363	MSTRG.20073	Gm20628
ENSMUST00000177421	ENSMUSG00000093587	Gm20554
ENSMUST00000177440	ENSMUSG00000086224	2700069I18Rik
ENSMUST00000177453	MSTRG.15977	Gm20616
ENSMUST00000177482	ENSMUSG00000093675	Gm20618
ENSMUST00000177492	ENSMUSG00000093568	Gm20611
ENSMUST00000177547	MSTRG.7598	Gm20036
ENSMUST00000177629	MSTRG.22803	Gm13201
ENSMUST00000177702	ENSMUSG00000085933	Tmem61
ENSMUST00000177890	MSTRG.11685	1700001L05Rik
ENSMUST00000177896	MSTRG.6347	Rab10os
ENSMUST00000178038	MSTRG.33086	1700065D16Rik
ENSMUST00000178043	ENSMUSG00000095348	Gm3892
ENSMUST00000178064	ENSMUSG00000031736	Crnde
ENSMUST00000178074	MSTRG.6347	Rab10os
ENSMUST00000178129	MSTRG.7614	Gm28373
ENSMUST00000178206	ENSMUSG00000094868	Dlx6os2
ENSMUST00000178227	ENSMUSG00000094230	Gm21847
ENSMUST00000178331	MSTRG.6347	Rab10os
ENSMUST00000178424	ENSMUSG00000095369	Gm21859
ENSMUST00000178432	MSTRG.19018	Gm11008
ENSMUST00000178464	MSTRG.14674	Gm10125
ENSMUST00000178493	MSTRG.5517	Dgkeos
ENSMUST00000178516	ENSMUSG00000087358	4930453H23Rik
ENSMUST00000178539	ENSMUSG00000093812	Gm5627
ENSMUST00000178540	ENSMUSG00000056821	1700028I16Rik
ENSMUST00000178584	ENSMUSG00000095651	Gm21817
ENSMUST00000178628	MSTRG.11685	1700001L05Rik
ENSMUST00000178699	MSTRG.15569	Gm21844
ENSMUST00000178769	MSTRG.18390	Macrod2os2
ENSMUST00000178848	ENSMUSG00000030636	1700010L04Rik
ENSMUST00000178904	MSTRG.16044	A830019P07Rik
ENSMUST00000178906	MSTRG.21233	Gm10593
ENSMUST00000178920	MSTRG.2350	Gm21781
ENSMUST00000178938	MSTRG.7616	Gm20069
ENSMUST00000178978	MSTRG.1768	Dnm3os

ENSMUST00000178987	MSTRG.23002	1500002C15Rik
ENSMUST00000178990	MSTRG.30154	Proscos
ENSMUST00000178996	ENSMUSG00000096573	1700009J07Rik
ENSMUST00000179029	ENSMUSG00000031736	Crnde
ENSMUST00000179138	MSTRG.14911	Epb41l4aos
ENSMUST00000179222	ENSMUSG00000031736	Crnde
ENSMUST00000179257	MSTRG.23204	Gm21846
ENSMUST00000179324	ENSMUSG00000078952	Lncenc1
ENSMUST00000179361	ENSMUSG00000030623	Prss23os
ENSMUST00000179400	ENSMUSG00000031736	Crnde
ENSMUST00000179421	ENSMUSG00000031736	Crnde
ENSMUST00000179637	MSTRG.6352	1110002L01Rik
ENSMUST00000179643	MSTRG.6347	Rab10os
ENSMUST00000179662	ENSMUSG00000030636	1700010L04Rik
ENSMUST00000179676	MSTRG.4987	Gm19967
ENSMUST00000179705	MSTRG.11685	1700001L05Rik
ENSMUST00000179771	MSTRG.14751	Mir133a-1hg
ENSMUST00000179908	MSTRG.6347	Rab10os
ENSMUST00000179924	MSTRG.6347	Rab10os
ENSMUST00000180031	MSTRG.9896	9930004E17Rik
ENSMUST00000180102	ENSMUSG00000031736	Crnde
ENSMUST00000180149	MSTRG.6352	1110002L01Rik
ENSMUST00000180221	ENSMUSG00000096322	Gm3898
ENSMUST00000180377	MSTRG.10499	Dleu2
ENSMUST00000180379	MSTRG.27174	2310001H17Rik
ENSMUST00000180383	MSTRG.20107	Gm26594
ENSMUST00000180385	MSTRG.28687	2900076A07Rik
ENSMUST00000180386	MSTRG.33539	Gm26614
ENSMUST00000180389	MSTRG.5568	Gm26830
ENSMUST00000180390	MSTRG.13396	D330041H03Rik
ENSMUST00000180391	MSTRG.6798	4933406C10Rik
ENSMUST00000180395	ENSMUSG00000097364	Gm26719
ENSMUST00000180396	MSTRG.9328	Gm26619
ENSMUST00000180397	MSTRG.10445	1700039M10Rik
ENSMUST00000180399	ENSMUSG00000097218	Gm26552
ENSMUST00000180401	MSTRG.12405	2610020F03Rik
ENSMUST00000180404	MSTRG.19469	5031434O11Rik
ENSMUST00000180406	ENSMUSG00000097548	Gm26748
ENSMUST00000180407	MSTRG.3907	Gm26847
ENSMUST00000180408	MSTRG.29912	E230013L22Rik
ENSMUST00000180409	MSTRG.9168	9330111N05Rik
ENSMUST00000180410	MSTRG.31247	Gm26786
ENSMUST00000180411	MSTRG.30046	Gm17491
ENSMUST00000180412	ENSMUSG00000097310	A930038B10Rik
ENSMUST00000180413	MSTRG.11332	Gm26621
ENSMUST00000180416	MSTRG.9273	Gm26527
ENSMUST00000180418	MSTRG.11163	Gm26854
ENSMUST00000180419	MSTRG.31756	Gm16845
ENSMUST00000180420	MSTRG.2305	4631405K08Rik
ENSMUST00000180421	ENSMUSG00000097924	A730020E08Rik
ENSMUST00000180423	ENSMUSG00000097339	Gm26671
ENSMUST00000180425	ENSMUSG00000097464	Gm26736
ENSMUST00000180426	MSTRG.21256	Gm26881
ENSMUST00000180427	MSTRG.10741	Gm6999
ENSMUST00000180428	MSTRG.24324	Gm26692
ENSMUST00000180429	ENSMUSG00000097416	Gm26670
ENSMUST00000180432	MSTRG.11275	Pvt1

ENSMUST00000180441	ENSMUSG00000097209	AU022754
ENSMUST00000180442	MSTRG.16314	4833407H14Rik
ENSMUST00000180443	MSTRG.4519	Gm26542
ENSMUST00000180445	MSTRG.31068	9330175E14Rik
ENSMUST00000180450	MSTRG.15324	Gm26910
ENSMUST00000180455	ENSMUSG00000097299	Gm26654
ENSMUST00000180456	MSTRG.2569	Gm26740
ENSMUST00000180460	ENSMUSG00000097287	D130017N08Rik
ENSMUST00000180464	MSTRG.3695	Gm26596
ENSMUST00000180465	MSTRG.10595	4930480K23Rik
ENSMUST00000180466	MSTRG.9855	Gm26772
ENSMUST00000180467	MSTRG.28647	6330403N20Rik
ENSMUST00000180468	MSTRG.14432	Gm26510
ENSMUST00000180470	MSTRG.23753	Gm20033
ENSMUST00000180471	ENSMUSG00000097911	Gm26691
ENSMUST00000180472	ENSMUSG00000097250	Gm26771
ENSMUST00000180474	MSTRG.32162	Gm26787
ENSMUST00000180477	MSTRG.3937	A430046D13Rik
ENSMUST00000180478	ENSMUSG00000097156	Gm3764
ENSMUST00000180482	MSTRG.3362	Gm26896
ENSMUST00000180486	MSTRG.33648	A530083I20Rik
ENSMUST00000180488	MSTRG.32762	B230323A14Rik
ENSMUST00000180489	MSTRG.16311	Mirt1
ENSMUST00000180490	ENSMUSG00000097587	4930578M01Rik
ENSMUST00000180491	MSTRG.9344	6430562O15Rik
ENSMUST00000180492	MSTRG.5606	2010300F17Rik
ENSMUST00000180494	MSTRG.17478	Gm17250
ENSMUST00000180495	MSTRG.1472	Gm19705
ENSMUST00000180496	ENSMUSG00000097315	Gm26568
ENSMUST00000180505	MSTRG.15595	Gm10814
ENSMUST00000180506	MSTRG.2733	Gm26824
ENSMUST00000180508	ENSMUSG00000090585	4933406F09Rik
ENSMUST00000180509	ENSMUSG00000097694	G730013B05Rik
ENSMUST00000180511	MSTRG.33751	Gm10489
ENSMUST00000180512	MSTRG.14942	Gm26538
ENSMUST00000180515	ENSMUSG00000097254	C430042M11Rik
ENSMUST00000180516	MSTRG.15016	Gm10544
ENSMUST00000180517	MSTRG.28871	Gm16938
ENSMUST00000180518	ENSMUSG00000097662	Gm2093
ENSMUST00000180524	ENSMUSG00000097393	D030068K23Rik
ENSMUST00000180525	MSTRG.32522	2700012I20Rik
ENSMUST00000180527	MSTRG.26961	4930540M05Rik
ENSMUST00000180529	ENSMUSG00000097378	B230208H11Rik
ENSMUST00000180530	ENSMUSG00000097666	A330094K24Rik
ENSMUST00000180533	MSTRG.32879	4933433G15Rik
ENSMUST00000180534	MSTRG.10458	3110083C13Rik
ENSMUST00000180537	MSTRG.2578	Gm10825
ENSMUST00000180538	MSTRG.16258	Gm26792
ENSMUST00000180546	MSTRG.28603	Gm26633
ENSMUST00000180547	MSTRG.13373	9530082P21Rik
ENSMUST00000180550	MSTRG.14874	Gm26533
ENSMUST00000180551	ENSMUSG00000097474	Gm26584
ENSMUST00000180557	MSTRG.20510	4933405D12Rik
ENSMUST00000180558	ENSMUSG00000097727	F630040K05Rik
ENSMUST00000180561	ENSMUSG00000097720	Gm26849
ENSMUST00000180562	ENSMUSG00000052368	Gm9873
ENSMUST00000180563	MSTRG.32992	Snhg5

ENSMUST00000180569	MSTRG.27122	9330179D12Rik
ENSMUST00000180574	MSTRG.32879	4933433G15Rik
ENSMUST00000180576	MSTRG.7716	Gm16596
ENSMUST00000180581	ENSMUSG00000097173	Gm26829
ENSMUST00000180584	MSTRG.33932	9530027J09Rik
ENSMUST00000180585	MSTRG.11167	Gm17473
ENSMUST00000180586	MSTRG.27159	Gm26770
ENSMUST00000180589	ENSMUSG00000097063	Pantr2
ENSMUST00000180590	ENSMUSG00000097220	Gm26599
ENSMUST00000180591	MSTRG.2061	Gm26801
ENSMUST00000180592	ENSMUSG00000097497	Gm26652
ENSMUST00000180594	ENSMUSG00000097626	4921504A21Rik
ENSMUST00000180597	MSTRG.31914	Gm26747
ENSMUST00000180598	MSTRG.2291	Gm10516
ENSMUST00000180601	MSTRG.23672	9230114K14Rik
ENSMUST00000180602	ENSMUSG00000097450	Gm26893
ENSMUST00000180603	ENSMUSG00000097456	Gm16958
ENSMUST00000180604	MSTRG.10982	4930556M19Rik
ENSMUST00000180608	MSTRG.13713	Gm26858
ENSMUST00000180609	MSTRG.16072	A330032B11Rik
ENSMUST00000180610	ENSMUSG00000097545	Mir124a-1hg
ENSMUST00000180612	MSTRG.31068	9330175E14Rik
ENSMUST00000180613	MSTRG.6797	Gdap10
ENSMUST00000180616	MSTRG.19469	5031434O11Rik
ENSMUST00000180623	ENSMUSG00000097615	Gm2061
ENSMUST00000180624	MSTRG.12186	Gm4262
ENSMUST00000180625	ENSMUSG00000097514	Gm17619
ENSMUST00000180630	MSTRG.30887	Gm26532
ENSMUST00000180631	ENSMUSG00000097738	4930445N18Rik
ENSMUST00000180632	MSTRG.2206	4930532G15Rik
ENSMUST00000180635	ENSMUSG00000097156	Gm3764
ENSMUST00000180638	MSTRG.1895	3110045C21Rik
ENSMUST00000180639	MSTRG.11973	A330009N23Rik
ENSMUST00000180643	MSTRG.7284	2310015A10Rik
ENSMUST00000180645	ENSMUSG00000097396	Gm26556
ENSMUST00000180650	ENSMUSG00000097668	Gm26761
ENSMUST00000180652	MSTRG.23816	Gm26725
ENSMUST00000180653	ENSMUSG00000097525	Platr31
ENSMUST00000180656	MSTRG.9007	Gm26819
ENSMUST00000180666	MSTRG.21629	Gm26525
ENSMUST00000180667	MSTRG.16311	Mirt1
ENSMUST00000180668	ENSMUSG00000097002	Gm2670
ENSMUST00000180670	MSTRG.32274	Gm10687
ENSMUST00000180671	MSTRG.6564	Gm4419
ENSMUST00000180676	MSTRG.33580	Gm2449
ENSMUST00000180679	MSTRG.31328	Gm26816
ENSMUST00000180680	MSTRG.2540	Gm26577
ENSMUST00000180681	MSTRG.27746	Gm26890
ENSMUST00000180682	MSTRG.10434	Gm16973
ENSMUST00000180684	MSTRG.34212	Gm26775
ENSMUST00000180685	MSTRG.33631	Gm26797
ENSMUST00000180690	MSTRG.30417	2500002B13Rik
ENSMUST00000180691	MSTRG.27730	Gm26852
ENSMUST00000180693	MSTRG.29577	5830432E09Rik
ENSMUST00000180695	MSTRG.5032	4732414G09Rik
ENSMUST00000180698	MSTRG.7779	2810029C07Rik
ENSMUST00000180699	MSTRG.27801	Gm26550

ENSMUST00000180700	ENSMUSG00000097248	Gm2694
ENSMUST00000180701	MSTRG.33255	4930500F10Rik
ENSMUST00000180705	MSTRG.28687	2900076A07Rik
ENSMUST00000180708	MSTRG.23698	4932441J04Rik
ENSMUST00000180712	MSTRG.33016	9330159M07Rik
ENSMUST00000180713	MSTRG.1785	Gm26523
ENSMUST00000180715	MSTRG.28625	Gm26646
ENSMUST00000180718	MSTRG.1391	Gm26706
ENSMUST00000180719	ENSMUSG00000097429	Gm26520
ENSMUST00000180721	MSTRG.13396	D330041H03Rik
ENSMUST00000180722	MSTRG.751	D230017M19Rik
ENSMUST00000180723	MSTRG.32997	9430037G07Rik
ENSMUST00000180727	MSTRG.33088	Gm16794
ENSMUST00000180729	ENSMUSG00000097884	Gm26543
ENSMUST00000180730	MSTRG.11269	9930014A18Rik
ENSMUST00000180731	ENSMUSG00000097107	Platr6
ENSMUST00000180732	MSTRG.17690	Gm17281
ENSMUST00000180733	MSTRG.25389	5730422E09Rik
ENSMUST00000180734	MSTRG.8920	Gm16907
ENSMUST00000180735	MSTRG.12640	D930030I03Rik
ENSMUST00000180737	MSTRG.16003	A930007I19Rik
ENSMUST00000180738	MSTRG.7016	Gm17529
ENSMUST00000180740	MSTRG.23312	Gm26608
ENSMUST00000180741	ENSMUSG00000097434	Gm16630
ENSMUST00000180742	ENSMUSG00000097005	4930598A11Rik
ENSMUST00000180743	MSTRG.14433	Gm26561
ENSMUST00000180748	MSTRG.5138	Gm26780
ENSMUST00000180749	ENSMUSG00000097836	Gm26903
ENSMUST00000180750	MSTRG.29900	9530052E02Rik
ENSMUST00000180751	ENSMUSG00000097762	4732463B04Rik
ENSMUST00000180752	MSTRG.10434	Gm16973
ENSMUST00000180753	MSTRG.30298	Gm2366
ENSMUST00000180757	MSTRG.30063	Gm26853
ENSMUST00000180759	MSTRG.6474	Gm5432
ENSMUST00000180763	MSTRG.27378	Gm26666
ENSMUST00000180764	ENSMUSG00000097031	Gm26820
ENSMUST00000180770	MSTRG.2406	Gm26674
ENSMUST00000180772	MSTRG.10826	Gm26773
ENSMUST00000180775	MSTRG.8676	Fam120aos
ENSMUST00000180777	ENSMUSG00000097326	A330048O09Rik
ENSMUST00000180778	ENSMUSG00000097819	Gm26813
ENSMUST00000180779	MSTRG.24149	5430416N02Rik
ENSMUST00000180780	MSTRG.2327	Gm16897
ENSMUST00000180782	MSTRG.6137	B230344G16Rik
ENSMUST00000180783	MSTRG.33016	9330159M07Rik
ENSMUST00000180784	MSTRG.23008	Gm26840
ENSMUST00000180785	ENSMUSG00000097676	Gm26612
ENSMUST00000180789	ENSMUSG00000097222	1010001N08Rik
ENSMUST00000180790	MSTRG.3173	2810425M01Rik
ENSMUST00000180791	MSTRG.19873	4731419I09Rik
ENSMUST00000180796	MSTRG.31912	5033426O07Rik
ENSMUST00000180797	MSTRG.1470	Platr23
ENSMUST00000180798	ENSMUSG00000097695	Gm26905
ENSMUST00000180799	MSTRG.23753	Gm20033
ENSMUST00000180800	MSTRG.7591	D130020L05Rik
ENSMUST00000180801	MSTRG.27986	Gm26554
ENSMUST00000180802	MSTRG.13602	Gm20109

ENSMUST00000180804	ENSMUSG00000097124	A530020G20Rik
ENSMUST00000180805	ENSMUSG00000097165	2210008F06Rik
ENSMUST00000180806	ENSMUSG00000097248	Gm2694
ENSMUST00000180807	MSTRG.8128	Gm26861
ENSMUST00000180808	MSTRG.9344	6430562O15Rik
ENSMUST00000180809	MSTRG.26385	Gm26640
ENSMUST00000180810	ENSMUSG00000097837	Gm26794
ENSMUST00000180811	MSTRG.27122	9330179D12Rik
ENSMUST00000180812	MSTRG.24624	Gm10390
ENSMUST00000180815	ENSMUSG00000097689	4930471D02Rik
ENSMUST00000180816	MSTRG.23528	Gm26647
ENSMUST00000180820	MSTRG.28052	Tmem147os
ENSMUST00000180823	ENSMUSG00000097376	Gm8705
ENSMUST00000180825	MSTRG.25785	Gm26699
ENSMUST00000180830	MSTRG.12399	1300002E11Rik
ENSMUST00000180832	MSTRG.4560	9430098F02Rik
ENSMUST00000180835	MSTRG.1376	6030442K20Rik
ENSMUST00000180836	ENSMUSG00000097709	2810429I04Rik
ENSMUST00000180837	ENSMUSG00000097648	9330185C12Rik
ENSMUST00000180838	MSTRG.19195	Gm2464
ENSMUST00000180839	MSTRG.13701	Gm17276
ENSMUST00000180841	MSTRG.16867	AA543186
ENSMUST00000180842	MSTRG.13326	9330136K24Rik
ENSMUST00000180844	MSTRG.3528	Gm26765
ENSMUST00000180846	MSTRG.31665	Gm26733
ENSMUST00000180850	ENSMUSG00000097888	Gm26682
ENSMUST00000180851	MSTRG.18493	Gm26681
ENSMUST00000180852	MSTRG.11825	2610037D02Rik
ENSMUST00000180853	MSTRG.2393	B430219N15Rik
ENSMUST00000180854	MSTRG.21254	Gm26643
ENSMUST00000180857	MSTRG.10184	Gm26590
ENSMUST00000180860	MSTRG.14758	Gm6277
ENSMUST00000180861	MSTRG.7096	4931403G20Rik
ENSMUST00000180863	MSTRG.25566	Gm26809
ENSMUST00000180864	MSTRG.28087	Gm26762
ENSMUST00000180865	MSTRG.33302	Gm9917
ENSMUST00000180866	MSTRG.24413	Gm26711
ENSMUST00000180868	MSTRG.13507	Gm26694
ENSMUST00000180869	MSTRG.2211	A430105J06Rik
ENSMUST00000180870	MSTRG.31697	Gm26592
ENSMUST00000180874	MSTRG.19156	C030034L19Rik
ENSMUST00000180875	ENSMUSG00000097372	Gm26876
ENSMUST00000180876	ENSMUSG00000097451	Rian
ENSMUST00000180878	ENSMUSG00000097726	9530036O11Rik
ENSMUST00000180879	MSTRG.28687	2900076A07Rik
ENSMUST00000180880	MSTRG.14497	Gm26637
ENSMUST00000180881	ENSMUSG00000097023	Mir9-3hg
ENSMUST00000180882	ENSMUSG00000097399	Gm26555
ENSMUST00000180894	MSTRG.9181	A230107N01Rik
ENSMUST00000180895	MSTRG.18911	A630075F10Rik
ENSMUST00000180896	MSTRG.26517	1600020E01Rik
ENSMUST00000180899	ENSMUSG00000097406	Gm26833
ENSMUST00000180902	ENSMUSG00000096965	3300005D01Rik
ENSMUST00000180904	ENSMUSG00000097043	Gm10556
ENSMUST00000180905	MSTRG.29692	1500002F19Rik
ENSMUST00000180906	MSTRG.8446	1110046J04Rik
ENSMUST00000180908	MSTRG.9042	9430065F17Rik

ENSMUST00000180911	MSTRG.7349	Gm26623
ENSMUST00000180912	MSTRG.23753	Gm20033
ENSMUST00000180913	ENSMUSG00000097067	Gm26743
ENSMUST00000180914	MSTRG.32762	B230323A14Rik
ENSMUST00000180917	MSTRG.10499	Dleu2
ENSMUST00000180919	MSTRG.6112	Gm26613
ENSMUST00000180921	MSTRG.8662	A330033J07Rik
ENSMUST00000180923	MSTRG.12556	1700007L15Rik
ENSMUST00000180926	MSTRG.27957	Gm26604
ENSMUST00000180928	MSTRG.30365	E030037K01Rik
ENSMUST00000180931	MSTRG.15051	Gm26672
ENSMUST00000180932	MSTRG.13526	1700022N22Rik
ENSMUST00000180933	MSTRG.29313	9030407P20Rik
ENSMUST00000180935	MSTRG.23923	Usp46os2
ENSMUST00000180936	MSTRG.33105	Gm26767
ENSMUST00000180937	ENSMUSG00000097716	1700026J14Rik
ENSMUST00000180941	MSTRG.14900	2310026I22Rik
ENSMUST00000180943	MSTRG.22316	Gm26516
ENSMUST00000180944	MSTRG.9610	Gm26680
ENSMUST00000180948	ENSMUSG00000097387	4930563E18Rik
ENSMUST00000180951	ENSMUSG00000097287	D130017N08Rik
ENSMUST00000180953	MSTRG.11946	5330439K02Rik
ENSMUST00000180958	MSTRG.20069	Gm17690
ENSMUST00000180959	MSTRG.26819	Gm26799
ENSMUST00000180962	MSTRG.1472	Gm19705
ENSMUST00000180963	MSTRG.21634	Gm26867
ENSMUST00000180964	MSTRG.26848	1700015O11Rik
ENSMUST00000180965	ENSMUSG00000097450	Gm26893
ENSMUST00000180967	MSTRG.13015	2310043M15Rik
ENSMUST00000180968	MSTRG.22701	Gm26763
ENSMUST00000180969	MSTRG.20440	Gm26530
ENSMUST00000180970	MSTRG.14666	Gm26865
ENSMUST00000180974	MSTRG.32905	C920006O11Rik
ENSMUST00000180975	MSTRG.13378	D930048G16Rik
ENSMUST00000180977	MSTRG.29313	9030407P20Rik
ENSMUST00000180980	ENSMUSG00000097218	Gm26552
ENSMUST00000180981	MSTRG.1431	Gm26642
ENSMUST00000180982	MSTRG.32997	9430037G07Rik
ENSMUST00000180983	MSTRG.28687	2900076A07Rik
ENSMUST00000180984	ENSMUSG00000097156	Gm3764
ENSMUST00000180986	MSTRG.16003	A930007I19Rik
ENSMUST00000180987	MSTRG.9762	6230400D17Rik
ENSMUST00000180988	ENSMUSG00000097832	Gm26912
ENSMUST00000180989	MSTRG.13001	Gm26626
ENSMUST00000180991	MSTRG.12918	Mir155hg
ENSMUST00000180992	MSTRG.8382	Gm26735
ENSMUST00000180995	ENSMUSG00000102548	Gm16701
ENSMUST00000180996	ENSMUSG00000067103	AY702103
ENSMUST00000180997	MSTRG.32845	BC065403
ENSMUST00000180999	MSTRG.12808	Gm16892
ENSMUST00000181000	MSTRG.3488	Gm26546
ENSMUST00000181002	MSTRG.175	Gm26580
ENSMUST00000181003	MSTRG.5267	Gm26563
ENSMUST00000181005	MSTRG.23332	4632411P08Rik
ENSMUST00000181007	ENSMUSG00000097127	Gm26886
ENSMUST00000181008	MSTRG.10163	Gm16617
ENSMUST00000181014	MSTRG.13396	D330041H03Rik

ENSMUST00000181018	ENSMUSG00000097921	Gm26576
ENSMUST00000181020	MSTRG.34342	Jpx
ENSMUST00000181021	ENSMUSG00000097280	AI849053
ENSMUST00000181028	MSTRG.26353	Gm26628
ENSMUST00000181029	MSTRG.13603	E230001N04Rik
ENSMUST00000181030	MSTRG.30777	Gm4890
ENSMUST00000181032	MSTRG.14415	A930029G22Rik
ENSMUST00000181033	ENSMUSG00000097218	Gm26552
ENSMUST00000181034	ENSMUSG00000097093	C330013E15Rik
ENSMUST00000181041	MSTRG.31895	Gm26739
ENSMUST00000181043	MSTRG.9168	9330111N05Rik
ENSMUST00000181044	ENSMUSG00000097310	A930038B10Rik
ENSMUST00000181045	MSTRG.25060	4933404O12Rik
ENSMUST00000181046	ENSMUSG00000097734	B930082K07Rik
ENSMUST00000181047	ENSMUSG00000097040	2610316D01Rik
ENSMUST00000181048	MSTRG.24404	Gm26718
ENSMUST00000181049	MSTRG.11675	AW121686
ENSMUST00000181050	MSTRG.2252	A230020J21Rik
ENSMUST00000181052	MSTRG.13145	Gm26848
ENSMUST00000181053	MSTRG.8662	AC123857.1
ENSMUST00000181054	ENSMUSG00000097182	A830009L08Rik
ENSMUST00000181055	MSTRG.7773	A230087F16Rik
ENSMUST00000181056	MSTRG.5118	Gm26836
ENSMUST00000181058	MSTRG.23117	Gm5106
ENSMUST00000181060	ENSMUSG00000097311	Gm26871
ENSMUST00000181064	MSTRG.26604	Gm26588
ENSMUST00000181068	MSTRG.12039	Gm26675
ENSMUST00000181070	ENSMUSG00000097124	A530020G20Rik
ENSMUST00000181072	MSTRG.10514	Gm26536
ENSMUST00000181073	ENSMUSG00000097378	B230208H11Rik
ENSMUST00000181075	MSTRG.28143	Gm26526
ENSMUST00000181076	MSTRG.8172	Gm26645
ENSMUST00000181077	MSTRG.12288	Gm26635
ENSMUST00000181078	MSTRG.28123	Gm26790
ENSMUST00000181082	ENSMUSG00000097369	Gm26545
ENSMUST00000181084	MSTRG.22768	C230088H06Rik
ENSMUST00000181085	MSTRG.7776	Gm10425
ENSMUST00000181086	ENSMUSG00000097541	Gm26752
ENSMUST00000181087	MSTRG.8554	Gm26514
ENSMUST00000181089	ENSMUSG00000097822	Gm26655
ENSMUST00000181090	MSTRG.25523	1110019D14Rik
ENSMUST00000181094	MSTRG.15094	A930012L18Rik
ENSMUST00000181097	MSTRG.30217	Gm26768
ENSMUST00000181100	ENSMUSG00000097907	Raxos1
ENSMUST00000181101	MSTRG.24378	Gm26779
ENSMUST00000181103	MSTRG.9167	Gm4211
ENSMUST00000181105	MSTRG.20980	Gm26663
ENSMUST00000181106	MSTRG.32273	Gm26737
ENSMUST00000181107	MSTRG.33633	9530059O14Rik
ENSMUST00000181109	MSTRG.16119	Gm27042
ENSMUST00000181112	MSTRG.13705	Gm26549
ENSMUST00000181113	ENSMUSG00000097621	Gm26562
ENSMUST00000181114	MSTRG.25430	1700028E10Rik
ENSMUST00000181118	MSTRG.8171	Gm6556
ENSMUST00000181119	MSTRG.6236	Gm26888
ENSMUST00000181120	MSTRG.30206	Gm26632
ENSMUST00000181121	MSTRG.26685	9530026P05Rik

ENSMUST00000181122	MSTRG.11826	4833422M21Rik
ENSMUST00000181124	ENSMUSG00000097584	1700125G22Rik
ENSMUST00000181125	MSTRG.5858	Gm26668
ENSMUST00000181126	ENSMUSG00000097596	Gm26673
ENSMUST00000181127	MSTRG.2285	Gm26879
ENSMUST00000181128	MSTRG.27602	1810019N24Rik
ENSMUST00000181129	MSTRG.31864	Gm26662
ENSMUST00000181132	ENSMUSG00000097484	Gm26807
ENSMUST00000181133	MSTRG.31918	1700030M09Rik
ENSMUST00000181134	ENSMUSG00000097156	Gm3764
ENSMUST00000181137	MSTRG.15801	4930526L06Rik
ENSMUST00000181139	MSTRG.33088	Gm16794
ENSMUST00000181140	MSTRG.30902	Gm26664
ENSMUST00000181141	MSTRG.24431	Gm26515
ENSMUST00000181144	ENSMUSG00000097645	Gm26863
ENSMUST00000181145	MSTRG.26685	9530026P05Rik
ENSMUST00000181146	ENSMUSG00000097085	Gm26634
ENSMUST00000181147	MSTRG.14573	1110020A21Rik
ENSMUST00000181149	MSTRG.14074	B230354K17Rik
ENSMUST00000181151	ENSMUSG00000097036	1110036E04Rik
ENSMUST00000181152	MSTRG.5267	Gm26563
ENSMUST00000181153	MSTRG.28019	Gm26810
ENSMUST00000181155	MSTRG.15256	Carmn
ENSMUST00000181157	ENSMUSG00000097278	Gm26650
ENSMUST00000181158	MSTRG.9055	Zfp85os
ENSMUST00000181160	ENSMUSG00000096950	Gm9530
ENSMUST00000181164	MSTRG.28687	2900076A07Rik
ENSMUST00000181167	MSTRG.30328	Gm16675
ENSMUST00000181170	MSTRG.1929	B930036N10Rik
ENSMUST00000181175	MSTRG.7470	Gm26764
ENSMUST00000181176	ENSMUSG00000097752	Gm26688
ENSMUST00000181178	ENSMUSG00000097385	Gm26814
ENSMUST00000181180	MSTRG.13165	Gm16702
ENSMUST00000181184	ENSMUSG00000097071	4930544I03Rik
ENSMUST00000181185	MSTRG.2688	Gm26564
ENSMUST00000181186	ENSMUSG00000097095	Gm26547
ENSMUST00000181189	MSTRG.28764	2310010J17Rik
ENSMUST00000181190	MSTRG.25060	4933404O12Rik
ENSMUST00000181191	MSTRG.17689	A330069E16Rik
ENSMUST00000181195	ENSMUSG00000097740	E030044B06Rik
ENSMUST00000181197	ENSMUSG00000097435	Gm26783
ENSMUST00000181198	ENSMUSG00000097632	4930552P12Rik
ENSMUST00000181199	MSTRG.22736	Gm26573
ENSMUST00000181200	MSTRG.31913	Gm26815
ENSMUST00000181202	ENSMUSG00000097726	9530036O11Rik
ENSMUST00000181206	MSTRG.12200	4930509G22Rik
ENSMUST00000181207	MSTRG.11566	Gm26884
ENSMUST00000181209	MSTRG.23193	6030443J06Rik
ENSMUST00000181211	MSTRG.15536	Gm17552
ENSMUST00000181212	MSTRG.4459	2310031A07Rik
ENSMUST00000181214	MSTRG.32223	2610203C20Rik
ENSMUST00000181215	MSTRG.31824	Gm17508
ENSMUST00000181218	ENSMUSG00000097542	Gm26751
ENSMUST00000181220	MSTRG.11638	Gm17597
ENSMUST00000181222	MSTRG.31703	Gm16754
ENSMUST00000181224	MSTRG.28569	AU020206
ENSMUST00000181225	MSTRG.25523	1110019D14Rik

ENSMUST00000181226	MSTRG.2328	A330023F24Rik
ENSMUST00000181230	ENSMUSG00000097074	4833428L15Rik
ENSMUST00000181231	MSTRG.31905	Fendrr
ENSMUST00000181232	MSTRG.12957	Gm17518
ENSMUST00000181235	ENSMUSG00000097697	4833412C05Rik
ENSMUST00000181237	MSTRG.22254	Gm26722
ENSMUST00000181238	MSTRG.27174	2310001H17Rik
ENSMUST00000181241	MSTRG.19058	Gm26869
ENSMUST00000181242	MSTRG.31511	Gm26870
ENSMUST00000181247	ENSMUSG00000097183	Gm17501
ENSMUST00000181248	MSTRG.30084	Gm26804
ENSMUST00000181251	MSTRG.13260	A930024N18Rik
ENSMUST00000181252	MSTRG.11826	4833422M21Rik
ENSMUST00000181253	ENSMUSG00000097451	Rian
ENSMUST00000181254	ENSMUSG00000097540	1700037F24Rik
ENSMUST00000181255	MSTRG.11515	Gm17638
ENSMUST00000181258	MSTRG.26618	Gm26811
ENSMUST00000181259	MSTRG.13396	D330041H03Rik
ENSMUST00000181260	MSTRG.1068	Gm26720
ENSMUST00000181262	MSTRG.4551	Gm26551
ENSMUST00000181264	MSTRG.28687	2900076A07Rik
ENSMUST00000181265	MSTRG.3282	Gm26710
ENSMUST00000181269	ENSMUSG00000096982	Redrum
ENSMUST00000181270	MSTRG.6157	Gm26730
ENSMUST00000181272	ENSMUSG00000097171	Gm17644
ENSMUST00000181273	MSTRG.2328	A330023F24Rik
ENSMUST00000181274	ENSMUSG00000097448	Platr22
ENSMUST00000181277	MSTRG.3428	Gm17249
ENSMUST00000181279	MSTRG.6910	Gm26517
ENSMUST00000181280	ENSMUSG00000097278	Gm26650
ENSMUST00000181282	MSTRG.30883	Gm26887
ENSMUST00000181285	MSTRG.6233	Gm26508
ENSMUST00000181286	MSTRG.19149	Gm16685
ENSMUST00000181289	MSTRG.32540	Gm17322
ENSMUST00000181290	MSTRG.7450	Gm26698
ENSMUST00000181291	MSTRG.13373	9530082P21Rik
ENSMUST00000181292	MSTRG.22126	Gm26606
ENSMUST00000181295	MSTRG.20994	Gm26895
ENSMUST00000181296	MSTRG.32223	2610203C20Rik
ENSMUST00000181299	MSTRG.28550	A730056A06Rik
ENSMUST00000181301	MSTRG.14128	Gm26904
ENSMUST00000181302	MSTRG.32618	Gm26609
ENSMUST00000181303	MSTRG.19469	5031434O11Rik
ENSMUST00000181304	ENSMUSG00000097603	A430010J10Rik
ENSMUST00000181305	MSTRG.19967	1700040D17Rik
ENSMUST00000181306	ENSMUSG00000097003	D930007P13Rik
ENSMUST00000181307	MSTRG.16036	Gm26902
ENSMUST00000181308	ENSMUSG00000097754	Ptgs2os2
ENSMUST00000181309	ENSMUSG00000097823	Gm16701
ENSMUST00000181311	ENSMUSG00000061510	Gm10101
ENSMUST00000181312	MSTRG.25561	Gm26738
ENSMUST00000181315	MSTRG.29900	9530052E02Rik
ENSMUST00000181316	ENSMUSG00000097222	1010001N08Rik
ENSMUST00000181317	MSTRG.27029	Gm26826
ENSMUST00000181318	MSTRG.13728	Gm26693
ENSMUST00000181322	MSTRG.30248	Gm16793
ENSMUST00000181323	MSTRG.7282	Gm26669

ENSMUST00000181325	MSTRG.33616	E530011L22Rik
ENSMUST00000181328	MSTRG.12001	Gm26518
ENSMUST00000181330	MSTRG.2432	AC167234.2
ENSMUST00000181331	MSTRG.19156	C030034L19Rik
ENSMUST00000181335	MSTRG.23602	9630001P10Rik
ENSMUST00000181337	ENSMUSG00000097308	Gm6410
ENSMUST00000181338	MSTRG.30098	Gm26714
ENSMUST00000181342	MSTRG.3032	Gm26789
ENSMUST00000181343	ENSMUSG00000093482	Gm20619
ENSMUST00000181344	MSTRG.10512	4931440J10Rik
ENSMUST00000181345	ENSMUSG00000097738	4930445N18Rik
ENSMUST00000181348	ENSMUSG00000097650	4921507G05Rik
ENSMUST00000181355	MSTRG.26604	Gm26588
ENSMUST00000181356	ENSMUSG00000097156	Gm3764
ENSMUST00000181361	ENSMUSG00000097156	Gm3764
ENSMUST00000181362	MSTRG.11140	Gm26704
ENSMUST00000181370	MSTRG.24210	Gm26703
ENSMUST00000181371	ENSMUSG00000097744	D030040B21Rik
ENSMUST00000181373	MSTRG.8374	C530050E15Rik
ENSMUST00000181374	MSTRG.23193	6030443J06Rik
ENSMUST00000181376	MSTRG.8556	5033403F01Rik
ENSMUST00000181378	ENSMUSG00000097196	Gm26665
ENSMUST00000181380	MSTRG.17313	Gm26603
ENSMUST00000181382	MSTRG.751	D230017M19Rik
ENSMUST00000181383	ENSMUSG00000097482	Gm17634
ENSMUST00000181384	MSTRG.11766	Gm26798
ENSMUST00000181385	MSTRG.1458	Gm26781
ENSMUST00000181387	MSTRG.14483	Gm26749
ENSMUST00000181388	ENSMUSG00000097547	B230110C06Rik
ENSMUST00000181389	MSTRG.23034	Gm16008
ENSMUST00000181390	MSTRG.13284	Gm26873
ENSMUST00000181392	ENSMUSG00000096965	3300005D01Rik
ENSMUST00000181393	ENSMUSG00000097735	D930032P07Rik
ENSMUST00000181395	MSTRG.28067	Gm4673
ENSMUST00000181397	ENSMUSG00000096975	Gm16386
ENSMUST00000181398	ENSMUSG00000106144	Gm43972
ENSMUST00000181400	MSTRG.31713	Gm26511
ENSMUST00000181403	MSTRG.8940	1810034E14Rik
ENSMUST00000181404	ENSMUSG00000097362	Gm26544
ENSMUST00000181405	MSTRG.32857	4930509E16Rik
ENSMUST00000181406	ENSMUSG00000097428	AW047730
ENSMUST00000181408	MSTRG.14501	Gm17315
ENSMUST00000181409	ENSMUSG00000090585	4933406F09Rik
ENSMUST00000181411	MSTRG.11110	Gm26766
ENSMUST00000181414	MSTRG.31893	Gm10614
ENSMUST00000181416	MSTRG.11275	Pvt1
ENSMUST00000181418	MSTRG.24214	D930016D06Rik
ENSMUST00000181420	MSTRG.30753	0610038B21Rik
ENSMUST00000181424	MSTRG.30161	4933416M07Rik
ENSMUST00000181425	MSTRG.14881	Gm26823
ENSMUST00000181427	MSTRG.8108	Gm26601
ENSMUST00000181428	ENSMUSG00000093672	Gm20655
ENSMUST00000181430	MSTRG.10595	4930480K23Rik
ENSMUST00000181432	MSTRG.32021	4933417D19Rik
ENSMUST00000181436	MSTRG.7826	Gm26583
ENSMUST00000181440	ENSMUSG00000097163	BC051077
ENSMUST00000181444	MSTRG.32496	Peak1os

ENSMUST00000181446	MSTRG.18901	Gm26883
ENSMUST00000181447	ENSMUSG00000097466	D430036J16Rik
ENSMUST00000181448	MSTRG.33016	9330159M07Rik
ENSMUST00000181449	MSTRG.33018	Gm26611
ENSMUST00000181452	MSTRG.30661	Gm17435
ENSMUST00000181453	MSTRG.14939	2010110K18Rik
ENSMUST00000181454	MSTRG.28185	2310002F09Rik
ENSMUST00000181457	MSTRG.29518	Gm26690
ENSMUST00000181458	ENSMUSG00000097408	Gm26831
ENSMUST00000181460	ENSMUSG00000097028	Ptgs2os
ENSMUST00000181462	MSTRG.32516	Gm26631
ENSMUST00000181465	MSTRG.13071	C030010L15Rik
ENSMUST00000181466	ENSMUSG00000097076	Platr7
ENSMUST00000181474	MSTRG.31897	Gm26537
ENSMUST00000181475	MSTRG.21159	Gm6297
ENSMUST00000181476	ENSMUSG00000096918	Gm16863
ENSMUST00000181479	MSTRG.2480	B230364G03Rik
ENSMUST00000181480	ENSMUSG00000097201	Gm26700
ENSMUST00000181481	MSTRG.24059	Gm26582
ENSMUST00000181484	ENSMUSG00000096965	3300005D01Rik
ENSMUST00000181485	ENSMUSG00000097184	4632428C04Rik
ENSMUST00000181486	MSTRG.22778	Gm26624
ENSMUST00000181488	MSTRG.24387	1010001B22Rik
ENSMUST00000181489	MSTRG.1131	Panct2
ENSMUST00000181490	MSTRG.8667	6720427I07Rik
ENSMUST00000181491	MSTRG.1472	Gm19705
ENSMUST00000181492	MSTRG.28687	2900076A07Rik
ENSMUST00000181495	MSTRG.7447	4732487G21Rik
ENSMUST00000181496	ENSMUSG00000097391	Mirg
ENSMUST00000181497	ENSMUSG00000097127	Gm26886
ENSMUST00000181498	MSTRG.12075	Gm16861
ENSMUST00000181499	MSTRG.1945	Gm26641
ENSMUST00000181501	MSTRG.27679	9330104G04Rik
ENSMUST00000181502	MSTRG.6300	Gm17586
ENSMUST00000181503	MSTRG.23312	Gm26608
ENSMUST00000181506	MSTRG.24210	Gm26703
ENSMUST00000181508	MSTRG.9168	9330111N05Rik
ENSMUST00000181510	MSTRG.11882	B130046B21Rik
ENSMUST00000181511	ENSMUSG00000097023	Mir9-3hg
ENSMUST00000181515	ENSMUSG00000097638	Carlr
ENSMUST00000181517	MSTRG.27174	2310001H17Rik
ENSMUST00000181519	MSTRG.25962	Gm26625
ENSMUST00000181520	MSTRG.9177	Gm17259
ENSMUST00000181522	MSTRG.22385	Gm26615
ENSMUST00000181523	ENSMUSG00000097249	Gm26889
ENSMUST00000181524	MSTRG.1472	Gm19705
ENSMUST00000181526	MSTRG.12204	2610020C07Rik
ENSMUST00000181527	ENSMUSG00000097451	Rian
ENSMUST00000181528	ENSMUSG00000097502	4930528D03Rik
ENSMUST00000181529	MSTRG.28049	Gm26610
ENSMUST00000181530	MSTRG.31905	Fendrr
ENSMUST00000181532	MSTRG.2569	Gm26740
ENSMUST00000181533	MSTRG.34054	Fmr1os
ENSMUST00000181534	ENSMUSG00000097356	Gm26774
ENSMUST00000181536	MSTRG.11060	Snhg18
ENSMUST00000181538	MSTRG.15163	Gm4221
ENSMUST00000181539	MSTRG.16311	Mirt1

ENSMUST00000181540	ENSMUSG00000097137	Gm26627
ENSMUST00000181546	ENSMUSG00000097282	5031415H12Rik
ENSMUST00000181547	ENSMUSG00000097082	4933440J02Rik
ENSMUST00000181550	ENSMUSG00000097156	Gm3764
ENSMUST00000181551	MSTRG.28185	2310002F09Rik
ENSMUST00000181552	MSTRG.27845	Gm26707
ENSMUST00000181553	ENSMUSG00000051758	4930544M13Rik
ENSMUST00000181554	ENSMUSG00000097797	Gm26901
ENSMUST00000181555	MSTRG.25430	1700028E10Rik
ENSMUST00000181556	MSTRG.22600	2700016F22Rik
ENSMUST00000181558	MSTRG.14662	Gm6225
ENSMUST00000181561	MSTRG.7773	A230087F16Rik
ENSMUST00000181563	MSTRG.505	Gm10561
ENSMUST00000181565	MSTRG.28734	Gm26522
ENSMUST00000181569	MSTRG.24384	Gm26808
ENSMUST00000181570	MSTRG.8145	Gpr137b-ps
ENSMUST00000181572	MSTRG.31511	Gm26870
ENSMUST00000181574	MSTRG.23214	2700038G22Rik
ENSMUST00000181575	MSTRG.1080	5033417F24Rik
ENSMUST00000181576	MSTRG.31680	Gm26521
ENSMUST00000181578	MSTRG.3858	F420014N23Rik
ENSMUST00000181580	MSTRG.20906	Gm26857
ENSMUST00000181581	ENSMUSG00000097288	Gm2155
ENSMUST00000181582	MSTRG.9846	Gm26660
ENSMUST00000181590	MSTRG.2709	4930547M16Rik
ENSMUST00000181593	ENSMUSG00000060424	Pantr1
ENSMUST00000181595	MSTRG.663	Gm26649
ENSMUST00000181596	MSTRG.17521	Gm10822
ENSMUST00000181598	ENSMUSG00000097383	1500026H17Rik
ENSMUST00000181600	MSTRG.33574	4930516B21Rik
ENSMUST00000181602	ENSMUSG00000097742	Gm26535
ENSMUST00000181605	MSTRG.13373	9530082P21Rik
ENSMUST00000181610	MSTRG.22753	Gm26880
ENSMUST00000181611	MSTRG.33038	Gm26882
ENSMUST00000181612	MSTRG.16021	2700046G09Rik
ENSMUST00000181617	MSTRG.32208	Gm17540
ENSMUST00000181618	ENSMUSG00000097049	6530411M01Rik
ENSMUST00000181619	MSTRG.20658	4930539J05Rik
ENSMUST00000181620	MSTRG.24214	D930016D06Rik
ENSMUST00000181622	ENSMUSG00000097156	Gm3764
ENSMUST00000181623	ENSMUSG00000097930	C330002G04Rik
ENSMUST00000181629	ENSMUSG00000097361	4930550C17Rik
ENSMUST00000181631	MSTRG.28506	1700112J16Rik
ENSMUST00000181636	ENSMUSG00000097156	Gm3764
ENSMUST00000181637	MSTRG.27758	Gm26600
ENSMUST00000181638	ENSMUSG00000097910	5033428I22Rik
ENSMUST00000181640	MSTRG.12442	Lppos
ENSMUST00000181641	MSTRG.14939	2010110K18Rik
ENSMUST00000181642	MSTRG.16520	Gm26565
ENSMUST00000181644	ENSMUSG00000097455	Gm26891
ENSMUST00000181647	MSTRG.31935	Gm26812
ENSMUST00000181648	ENSMUSG00000097228	Gm26597
ENSMUST00000181653	MSTRG.19601	Gm26850
ENSMUST00000181654	ENSMUSG00000097934	6720483E21Rik
ENSMUST00000181655	ENSMUSG00000096960	A230028O05Rik
ENSMUST00000181656	ENSMUSG00000097104	Gm26579
ENSMUST00000181657	MSTRG.11275	Pvt1

ENSMUST00000181658	ENSMUSG00000097624	Gm5091
ENSMUST00000181664	MSTRG.14946	Snhg4
ENSMUST00000181668	MSTRG.30053	Gm26909
ENSMUST00000181670	MSTRG.6798	4933406C10Rik
ENSMUST00000181672	ENSMUSG00000097497	Gm26652
ENSMUST00000181674	MSTRG.2791	Gm26860
ENSMUST00000181679	MSTRG.31918	1700030M09Rik
ENSMUST00000181680	MSTRG.2145	A430110L20Rik
ENSMUST00000181685	MSTRG.10852	Gm26679
ENSMUST00000181687	MSTRG.4754	Gm26534
ENSMUST00000181688	MSTRG.2432	4930432B10Rik
ENSMUST00000181689	ENSMUSG00000097840	Gm26756
ENSMUST00000181692	MSTRG.263	Gm26788
ENSMUST00000181694	MSTRG.19566	4930593A02Rik
ENSMUST00000181695	MSTRG.1373	Gm26892
ENSMUST00000181696	MSTRG.26575	Gm26636
ENSMUST00000181697	MSTRG.10146	Gm26782
ENSMUST00000181701	MSTRG.9610	Gm26680
ENSMUST00000181702	ENSMUSG00000097928	Gm26578
ENSMUST00000181704	ENSMUSG00000097286	Gm26684
ENSMUST00000181705	ENSMUSG00000097265	Gm26803
ENSMUST00000181706	ENSMUSG00000097072	Foxl2os
ENSMUST00000181708	ENSMUSG00000097709	2810429I04Rik
ENSMUST00000181710	ENSMUSG00000097134	1110002J07Rik
ENSMUST00000181713	ENSMUSG00000097363	Gm26717
ENSMUST00000181714	MSTRG.14674	Gm26575
ENSMUST00000181717	MSTRG.28626	5430400D12Rik
ENSMUST00000181718	MSTRG.29864	Gm26750
ENSMUST00000181719	MSTRG.33633	9530059O14Rik
ENSMUST00000181720	MSTRG.880	C430014B12Rik
ENSMUST00000181723	ENSMUSG00000096943	Gm26721
ENSMUST00000181725	ENSMUSG00000060424	Pantr1
ENSMUST00000181726	MSTRG.16003	A930007I19Rik
ENSMUST00000181727	MSTRG.7441	Gm26531
ENSMUST00000181729	MSTRG.14889	A830052D11Rik
ENSMUST00000181731	MSTRG.15848	C130060C02Rik
ENSMUST00000181732	MSTRG.12181	Gm26822
ENSMUST00000181737	MSTRG.1052	Gm26683
ENSMUST00000181738	MSTRG.27007	Gm4651
ENSMUST00000181739	MSTRG.10983	4930556M19Rik
ENSMUST00000181743	ENSMUSG00000097862	Gm26859
ENSMUST00000181746	MSTRG.1463	9230116N13Rik
ENSMUST00000181747	MSTRG.11826	4833422M21Rik
ENSMUST00000181751	MSTRG.23090	Gm26825
ENSMUST00000181752	MSTRG.22768	C230088H06Rik
ENSMUST00000181756	ENSMUSG00000097899	Gm16894
ENSMUST00000181757	MSTRG.15000	1700086O06Rik
ENSMUST00000181758	MSTRG.5606	2010300F17Rik
ENSMUST00000181759	ENSMUSG00000097447	Gm26630
ENSMUST00000181760	MSTRG.13396	D330041H03Rik
ENSMUST00000181762	ENSMUSG00000097563	Gm26638
ENSMUST00000181764	MSTRG.23193	6030443J06Rik
ENSMUST00000181765	MSTRG.15163	Gm4221
ENSMUST00000181769	MSTRG.14649	Gm26734
ENSMUST00000181770	MSTRG.26763	Gm26911
ENSMUST00000181773	MSTRG.31483	Gm26759
ENSMUST00000181774	MSTRG.13202	4732491K20Rik

ENSMUST00000181775	MSTRG.14111	Gm26785
ENSMUST00000181776	MSTRG.31935	Gm26812
ENSMUST00000181777	MSTRG.16036	Gm26902
ENSMUST00000181778	MSTRG.31903	Gm26878
ENSMUST00000181780	MSTRG.12399	1300002E11Rik
ENSMUST00000181781	MSTRG.3600	5730420D15Rik
ENSMUST00000181784	MSTRG.28761	E230029C05Rik
ENSMUST00000181794	ENSMUSG00000097241	Gm26907
ENSMUST00000181800	MSTRG.8446	1110046J04Rik
ENSMUST00000181802	MSTRG.11861	Gm26513
ENSMUST00000181803	MSTRG.6439	Gm4755
ENSMUST00000181805	MSTRG.32095	4732419C18Rik
ENSMUST00000181807	MSTRG.12709	Gm26732
ENSMUST00000181809	ENSMUSG00000097532	Gm4349
ENSMUST00000181811	MSTRG.2127	Gm17275
ENSMUST00000181812	MSTRG.31735	Gm16853
ENSMUST00000181813	MSTRG.19416	6430590A07Rik
ENSMUST00000181814	MSTRG.32223	2610203C20Rik
ENSMUST00000181815	ENSMUSG00000097307	Gm26834
ENSMUST00000181816	ENSMUSG00000097609	Gm26659
ENSMUST00000181817	MSTRG.12768	Gm16619
ENSMUST00000181819	MSTRG.20025	Gm16740
ENSMUST00000181820	MSTRG.16231	4930505N22Rik
ENSMUST00000181821	MSTRG.68	1700034P13Rik
ENSMUST00000181824	MSTRG.33546	Gm17399
ENSMUST00000181825	MSTRG.27182	Gm26656
ENSMUST00000181827	ENSMUSG00000097655	Gm26713
ENSMUST00000181829	MSTRG.15162	Gm26742
ENSMUST00000181831	ENSMUSG00000097281	Gm26685
ENSMUST00000181834	MSTRG.12402	Gm26744
ENSMUST00000181835	MSTRG.3557	4933408J17Rik
ENSMUST00000181837	MSTRG.19802	1700113A16Rik
ENSMUST00000181838	ENSMUSG00000097618	Gm26507
ENSMUST00000181839	ENSMUSG00000097102	2310069G16Rik
ENSMUST00000181840	MSTRG.26685	9530026P05Rik
ENSMUST00000181841	ENSMUSG00000097083	D930019O06Rik
ENSMUST00000181842	MSTRG.25236	D430018E03Rik
ENSMUST00000181851	ENSMUSG00000097495	Gm26651
ENSMUST00000181852	ENSMUSG00000097801	Gm26777
ENSMUST00000181854	ENSMUSG00000097293	D630002J18Rik
ENSMUST00000181856	MSTRG.10630	Gm26908
ENSMUST00000181864	ENSMUSG00000097520	4930488L21Rik
ENSMUST00000181865	ENSMUSG00000097524	Gm26846
ENSMUST00000181866	MSTRG.3291	Gm26541
ENSMUST00000181870	MSTRG.11499	1700109K24Rik
ENSMUST00000181871	MSTRG.15000	1700086O06Rik
ENSMUST00000181873	MSTRG.24149	5430416N02Rik
ENSMUST00000181874	ENSMUSG00000097157	Gm26512
ENSMUST00000181875	ENSMUSG00000097847	4930478K11Rik
ENSMUST00000181876	MSTRG.2239	Gm26574
ENSMUST00000181877	MSTRG.15433	Gm26676
ENSMUST00000181881	ENSMUSG00000097278	Gm26650
ENSMUST00000181882	MSTRG.20751	9530052C20Rik
ENSMUST00000181883	ENSMUSG00000097486	Gm17733
ENSMUST00000181884	ENSMUSG00000097694	G730013B05Rik
ENSMUST00000181885	MSTRG.321	Gm26805
ENSMUST00000181886	ENSMUSG00000097644	Gm26862

ENSMUST00000181887	MSTRG.15016	Gm10544
ENSMUST00000181888	MSTRG.2461	Gm26835
ENSMUST00000181895	MSTRG.13133	Gm2885
ENSMUST00000181897	ENSMUSG00000096977	Gm10827
ENSMUST00000181898	ENSMUSG00000097248	Gm2694
ENSMUST00000181899	MSTRG.2903	Gm16998
ENSMUST00000181900	MSTRG.5455	2010015M23Rik
ENSMUST00000181901	ENSMUSG00000097068	Gm26760
ENSMUST00000181903	MSTRG.28687	2900076A07Rik
ENSMUST00000181905	MSTRG.24624	Gm10390
ENSMUST00000181906	MSTRG.3583	Cep83os
ENSMUST00000181910	MSTRG.11269	9930014A18Rik
ENSMUST00000181911	MSTRG.28480	Gm26827
ENSMUST00000181912	MSTRG.8985	4930525G20Rik
ENSMUST00000181913	MSTRG.15383	Gm10532
ENSMUST00000181915	ENSMUSG00000097028	Ptgs2os
ENSMUST00000181918	MSTRG.30642	Gm26586
ENSMUST00000181919	MSTRG.28365	Gm26856
ENSMUST00000181920	MSTRG.13637	Gm26885
ENSMUST00000181921	MSTRG.16358	B230217O12Rik
ENSMUST00000181925	MSTRG.23278	Gm26648
ENSMUST00000181926	ENSMUSG00000097311	Gm26871
ENSMUST00000181927	ENSMUSG00000097870	Gm26868
ENSMUST00000181928	MSTRG.26517	1600020E01Rik
ENSMUST00000181929	MSTRG.23753	Gm20033
ENSMUST00000181930	MSTRG.19156	C030034L19Rik
ENSMUST00000181931	MSTRG.15458	Gm10524
ENSMUST00000181932	MSTRG.28113	B230322F03Rik
ENSMUST00000181936	ENSMUSG00000097547	B230110C06Rik
ENSMUST00000181938	MSTRG.13322	Gm26753
ENSMUST00000181939	ENSMUSG00000097027	Gm26559
ENSMUST00000181940	MSTRG.29577	5830432E09Rik
ENSMUST00000181941	ENSMUSG00000097513	Gm26696
ENSMUST00000181942	MSTRG.23308	4831440E17Rik
ENSMUST00000181943	MSTRG.25975	Gm44696
ENSMUST00000181944	MSTRG.31938	BC048644
ENSMUST00000181947	MSTRG.2328	A330023F24Rik
ENSMUST00000181949	MSTRG.527	4930558J18Rik
ENSMUST00000181951	ENSMUSG00000097283	Gm26686
ENSMUST00000181953	MSTRG.2570	Gm26581
ENSMUST00000181954	ENSMUSG00000097038	Gm26821
ENSMUST00000181955	MSTRG.32223	2610203C20Rik
ENSMUST00000181957	MSTRG.31511	Gm26870
ENSMUST00000181958	MSTRG.28078	G630030J09Rik
ENSMUST00000181959	MSTRG.16364	Gm26874
ENSMUST00000181960	MSTRG.12399	1300002E11Rik
ENSMUST00000181962	MSTRG.14269	Gm16712
ENSMUST00000181968	ENSMUSG00000097313	Gm26569
ENSMUST00000181973	MSTRG.751	D230017M19Rik
ENSMUST00000181996	MSTRG.34336	Gm26952
ENSMUST00000182043	MSTRG.76	Snhg6
ENSMUST00000182046	MSTRG.31198	Gm5914
ENSMUST00000182074	ENSMUSG00000097391	Mirg
ENSMUST00000182075	MSTRG.11275	Pvt1
ENSMUST00000182077	ENSMUSG00000098128	Gm3693
ENSMUST00000182088	ENSMUSG00000097451	Rian
ENSMUST00000182091	MSTRG.12887	Mir99ahg

ENSMUST00000182100	ENSMUSG00000097451	Rian
ENSMUST00000182109	MSTRG.16285	Gm19557
ENSMUST00000182119	ENSMUSG00000097451	Rian
ENSMUST00000182120	MSTRG.1434	Ptprv
ENSMUST00000182121	MSTRG.31936	9530085P06Rik
ENSMUST00000182124	ENSMUSG00000098097	6530403H02Rik
ENSMUST00000182125	ENSMUSG00000097023	Mir9-3hg
ENSMUST00000182127	MSTRG.1474	Gm4258
ENSMUST00000182132	MSTRG.20344	A930002I21Rik
ENSMUST00000182141	MSTRG.11275	Pvt1
ENSMUST00000182145	MSTRG.31917	Gm27045
ENSMUST00000182158	MSTRG.1481	A430106G13Rik
ENSMUST00000182185	ENSMUSG00000097391	Mirg
ENSMUST00000182216	ENSMUSG00000098072	Gm26995
ENSMUST00000182232	MSTRG.32992	Snhg5
ENSMUST00000182238	ENSMUSG00000097451	Rian
ENSMUST00000182244	MSTRG.15256	Carmn
ENSMUST00000182250	MSTRG.26863	Gm26982
ENSMUST00000182259	MSTRG.10499	Dleu2
ENSMUST00000182264	MSTRG.31905	Fendrr
ENSMUST00000182265	MSTRG.28803	Gm26944
ENSMUST00000182275	MSTRG.1435	Gm10535
ENSMUST00000182286	MSTRG.10499	Dleu2
ENSMUST00000182300	ENSMUSG00000097391	Mirg
ENSMUST00000182303	ENSMUSG00000097970	Gm27028
ENSMUST00000182325	MSTRG.10499	Dleu2
ENSMUST00000182330	MSTRG.3998	Tug1
ENSMUST00000182342	ENSMUSG00000097974	Gm10605
ENSMUST00000182345	ENSMUSG00000098204	Gm26984
ENSMUST00000182349	ENSMUSG00000098230	1700095B10Rik
ENSMUST00000182352	MSTRG.31941	Gm27011
ENSMUST00000182370	ENSMUSG00000097451	Rian
ENSMUST00000182380	MSTRG.11275	Pvt1
ENSMUST00000182387	MSTRG.1434	Ptprv
ENSMUST00000182400	MSTRG.20339	Gm27008
ENSMUST00000182406	ENSMUSG00000097451	Rian
ENSMUST00000182408	MSTRG.28807	4632427E13Rik
ENSMUST00000182414	MSTRG.20339	Gm27008
ENSMUST00000182423	MSTRG.31890	Gm26971
ENSMUST00000182424	MSTRG.1478	Gm26936
ENSMUST00000182447	MSTRG.34342	Jpx
ENSMUST00000182453	MSTRG.28806	Gm26981
ENSMUST00000182486	MSTRG.34342	Jpx
ENSMUST00000182488	MSTRG.28052	AC167978.1
ENSMUST00000182496	ENSMUSG00000097976	Gm26918
ENSMUST00000182497	ENSMUSG00000098055	Gm26947
ENSMUST00000182498	MSTRG.76	Snhg6
ENSMUST00000182499	ENSMUSG00000097391	Mirg
ENSMUST00000182516	MSTRG.4773	Gm26964
ENSMUST00000182517	MSTRG.32992	Snhg5
ENSMUST00000182537	MSTRG.10499	Dleu2
ENSMUST00000182541	MSTRG.1434	Ptprv
ENSMUST00000182545	MSTRG.33512	Gm26962
ENSMUST00000182548	MSTRG.12887	Mir99ahg
ENSMUST00000182551	MSTRG.31923	Gm27030
ENSMUST00000182570	MSTRG.1434	Ptprv
ENSMUST00000182578	MSTRG.28807	4632427E13Rik

ENSMUST00000182580	MSTRG.76	Snhg6
ENSMUST00000182590	MSTRG.18166	Gm27003
ENSMUST00000182601	MSTRG.12887	Mir99ahg
ENSMUST00000182623	ENSMUSG00000112117	RMST_1
ENSMUST00000182639	MSTRG.14645	Gm1976
ENSMUST00000182656	MSTRG.12887	Mir99ahg
ENSMUST00000182680	MSTRG.1478	Gm26936
ENSMUST00000182686	MSTRG.28052	Gm26935
ENSMUST00000182689	ENSMUSG00000097451	Rian
ENSMUST00000182690	MSTRG.12488	Gm15743
ENSMUST00000182718	MSTRG.1434	Ptprv
ENSMUST00000182727	MSTRG.10499	Gm27010
ENSMUST00000182733	ENSMUSG00000098172	Gm26973
ENSMUST00000182737	ENSMUSG00000097451	Rian
ENSMUST00000182742	MSTRG.76	Snhg6
ENSMUST00000182748	MSTRG.12887	Mir99ahg
ENSMUST00000182753	ENSMUSG00000098233	Gm26954
ENSMUST00000182756	MSTRG.10499	Dleu2
ENSMUST00000182768	MSTRG.10499	Dleu2
ENSMUST00000182770	ENSMUSG00000097451	Rian
ENSMUST00000182785	ENSMUSG00000098202	B830012L14Rik
ENSMUST00000182789	MSTRG.33514	Gm27002
ENSMUST00000182811	MSTRG.6184	Snhg20
ENSMUST00000182819	MSTRG.76	Snhg6
ENSMUST00000182820	MSTRG.12887	Mir99ahg
ENSMUST00000182826	MSTRG.22338	Snhg12
ENSMUST00000182843	MSTRG.30211	Gm26978
ENSMUST00000182863	MSTRG.31198	Gm5914
ENSMUST00000182865	MSTRG.20658	4930539J05Rik
ENSMUST00000182870	ENSMUSG00000098040	Gm20757
ENSMUST00000182872	MSTRG.32992	Snhg5
ENSMUST00000182878	ENSMUSG00000098014	Gm26967
ENSMUST00000182885	ENSMUSG00000098161	Platr11
ENSMUST00000182891	ENSMUSG00000098001	Gm27048
ENSMUST00000182894	MSTRG.21579	Gm26968
ENSMUST00000182937	ENSMUSG00000097023	Mir9-3hg
ENSMUST00000182943	ENSMUSG00000097961	Gm27000
ENSMUST00000182953	ENSMUSG00000097767	Miat
ENSMUST00000182956	MSTRG.11275	Pvt1
ENSMUST00000182957	MSTRG.10499	Dleu2
ENSMUST00000182971	MSTRG.1476	Gm26979
ENSMUST00000182981	ENSMUSG00000097451	Rian
ENSMUST00000183019	ENSMUSG00000097974	Gm10605
ENSMUST00000183021	MSTRG.31882	A130014A01Rik
ENSMUST00000183027	MSTRG.34342	Jpx
ENSMUST00000183028	MSTRG.11275	Pvt1
ENSMUST00000183031	MSTRG.10505	Gm27017
ENSMUST00000183043	ENSMUSG00000097023	Mir9-3hg
ENSMUST00000183045	MSTRG.32992	Snhg5
ENSMUST00000183054	MSTRG.10499	Dleu2
ENSMUST00000183056	MSTRG.25673	Gm27019
ENSMUST00000183066	MSTRG.10499	Dleu2
ENSMUST00000183068	ENSMUSG00000097391	Mirg
ENSMUST00000183079	MSTRG.10499	Dleu2
ENSMUST00000183083	MSTRG.15253	Bvht
ENSMUST00000183084	ENSMUSG00000097391	Mirg
ENSMUST00000183087	MSTRG.15253	Bvht

ENSMUST00000183089	MSTRG.1434	Ptprv
ENSMUST00000183099	MSTRG.12887	Mir99ahg
ENSMUST00000183124	ENSMUSG00000098051	Gm27032
ENSMUST00000183144	ENSMUSG00000097391	Mirg
ENSMUST00000183168	ENSMUSG00000098097	6530403H02Rik
ENSMUST00000183171	ENSMUSG00000087143	A830082K12Rik
ENSMUST00000183180	ENSMUSG00000097451	Rian
ENSMUST00000183191	ENSMUSG00000098061	Gm26945
ENSMUST00000183194	MSTRG.28052	Tmem147os
ENSMUST00000183212	MSTRG.1434	Ptprv
ENSMUST00000183215	ENSMUSG00000097451	Rian
ENSMUST00000183220	MSTRG.22484	Gm24362
ENSMUST00000183231	ENSMUSG00000098172	Gm26973
ENSMUST00000183235	MSTRG.31886	A330074K22Rik
ENSMUST00000183244	MSTRG.33510	Gm4665
ENSMUST00000183245	MSTRG.10499	Dleu2
ENSMUST00000183264	ENSMUSG00000098107	Gm27007
ENSMUST00000183266	ENSMUSG00000097451	Rian
ENSMUST00000183290	MSTRG.13797	BC051537
ENSMUST00000183297	MSTRG.11275	Pvt1
ENSMUST00000183304	MSTRG.10501	Gm26916
ENSMUST00000183317	MSTRG.1434	Ptprv
ENSMUST00000183323	MSTRG.7344	Gm29361
ENSMUST00000183327	MSTRG.12887	Mir99ahg
ENSMUST00000183328	MSTRG.1434	Ptprv
ENSMUST00000183333	MSTRG.12887	Mir99ahg
ENSMUST00000183339	MSTRG.10619	Gm27177
ENSMUST00000183344	ENSMUSG00000098257	Gm27169
ENSMUST00000183374	MSTRG.19845	1500004A13Rik
ENSMUST00000183387	ENSMUSG00000098627	4930524O08Rik
ENSMUST00000183398	MSTRG.10599	Gm27222
ENSMUST00000183413	ENSMUSG00000099016	Gm27227
ENSMUST00000183423	ENSMUSG00000099148	Gm3331
ENSMUST00000183438	ENSMUSG00000098552	Gm27217
ENSMUST00000183485	MSTRG.10605	Gm27221
ENSMUST00000183512	MSTRG.28956	Gm10603
ENSMUST00000183514	MSTRG.10623	Gm27177
ENSMUST00000183517	MSTRG.32862	Gm27241
ENSMUST00000183611	ENSMUSG00000098684	Gm27246
ENSMUST00000183612	MSTRG.32155	1700027I24Rik
ENSMUST00000183616	MSTRG.31854	Gm27240
ENSMUST00000183642	MSTRG.10618	Gm27176
ENSMUST00000183645	MSTRG.30971	Hm629797
ENSMUST00000183670	MSTRG.32840	Gm27253
ENSMUST00000183707	MSTRG.19845	1500004A13Rik
ENSMUST00000183750	MSTRG.10391	Zfhx2os
ENSMUST00000183752	ENSMUSG00000098534	Gm27167
ENSMUST00000183782	MSTRG.28450	Gm27252
ENSMUST00000183818	MSTRG.31850	Gm27201
ENSMUST00000183834	MSTRG.11954	Gm27209
ENSMUST00000183838	ENSMUSG00000098424	Gm27202
ENSMUST00000183867	MSTRG.27274	Lockd
ENSMUST00000183878	ENSMUSG00000099170	5730403I07Rik
ENSMUST00000183905	MSTRG.27274	Lockd
ENSMUST00000183916	MSTRG.28554	1810026B05Rik
ENSMUST00000183966	MSTRG.19845	1500004A13Rik
ENSMUST00000184013	MSTRG.19845	1500004A13Rik

ENSMUST00000184014	MSTRG.19845	1500004A13Rik
ENSMUST00000184034	MSTRG.10619	Gm27177
ENSMUST00000184073	MSTRG.27973	1110035H17Rik
ENSMUST00000184083	MSTRG.9861	Anxa11os
ENSMUST00000184100	ENSMUSG00000098747	Gm27216
ENSMUST00000184126	MSTRG.32823	2310009A05Rik
ENSMUST00000184161	ENSMUSG00000098434	2010110E17Rik
ENSMUST00000184166	ENSMUSG00000099146	0610031O16Rik
ENSMUST00000184170	MSTRG.19845	1500004A13Rik
ENSMUST00000184175	ENSMUSG00000112117	RMST_1
ENSMUST00000184181	MSTRG.15258	1500015A07Rik
ENSMUST00000184211	ENSMUSG00000099054	Gm27242
ENSMUST00000184260	MSTRG.20388	Gm27244
ENSMUST00000184288	MSTRG.29975	5830468F06Rik
ENSMUST00000184303	MSTRG.32848	Gm27255
ENSMUST00000184319	MSTRG.32823	2310009A05Rik
ENSMUST00000184355	MSTRG.32848	Gm27230
ENSMUST00000184360	MSTRG.28450	Gm27252
ENSMUST00000184373	MSTRG.32863	Gm27232
ENSMUST00000184383	MSTRG.32837	Gm27188
ENSMUST00000184420	MSTRG.28956	Gm10603
ENSMUST00000184491	MSTRG.10619	Gm27177
ENSMUST00000184492	MSTRG.11297	Gm27153
ENSMUST00000184504	MSTRG.27274	Lockd
ENSMUST00000184530	MSTRG.15258	1500015A07Rik
ENSMUST00000184554	MSTRG.28554	1810026B05Rik
ENSMUST00000184563	ENSMUSG00000099094	Gm19569
ENSMUST00000184587	MSTRG.28554	1810026B05Rik
ENSMUST00000184626	MSTRG.22975	Gm27200
ENSMUST00000184645	ENSMUSG00000062036	4932415M13Rik
ENSMUST00000184655	MSTRG.28554	1810026B05Rik
ENSMUST00000184658	ENSMUSG00000099094	Gm19569
ENSMUST00000184671	MSTRG.32848	Gm27230
ENSMUST00000184678	MSTRG.15258	1500015A07Rik
ENSMUST00000184702	ENSMUSG00000099207	Gm10637
ENSMUST00000184724	MSTRG.18771	0610039K10Rik
ENSMUST00000184762	MSTRG.30753	0610038B21Rik
ENSMUST00000184787	MSTRG.16902	Gm27196
ENSMUST00000184792	MSTRG.10619	Gm27177
ENSMUST00000184796	MSTRG.32845	BC065403
ENSMUST00000184835	MSTRG.32848	Gm27230
ENSMUST00000184855	MSTRG.28554	1810026B05Rik
ENSMUST00000184875	MSTRG.28956	Gm10603
ENSMUST00000184958	MSTRG.19845	1500004A13Rik
ENSMUST00000184991	MSTRG.27274	Lockd
ENSMUST00000184994	ENSMUSG00000098284	A330093E20Rik
ENSMUST00000185013	MSTRG.32862	Gm27241
ENSMUST00000185015	ENSMUSG00000098739	Gm27151
ENSMUST00000185046	ENSMUSG00000099146	0610031O16Rik
ENSMUST00000185067	MSTRG.11825	2610037D02Rik
ENSMUST00000185095	MSTRG.1228	Gm27184
ENSMUST00000185132	ENSMUSG00000099020	Gm27159
ENSMUST00000185152	MSTRG.27274	Lockd
ENSMUST00000185169	ENSMUSG00000099025	Gm27162
ENSMUST00000185173	MSTRG.23967	Gm7467
ENSMUST00000185191	MSTRG.32853	Gm27231
ENSMUST00000185204	ENSMUSG00000087022	9130024F11Rik

ENSMUST00000185211	MSTRG.15951	2610016A17Rik
ENSMUST00000185218	MSTRG.244	Gm28417
ENSMUST00000185221	ENSMUSG00000100157	2310034O05Rik
ENSMUST00000185238	ENSMUSG00000100558	1700025F24Rik
ENSMUST00000185242	ENSMUSG00000100277	1810053B23Rik
ENSMUST00000185268	MSTRG.27109	Gm26728
ENSMUST00000185270	MSTRG.3942	Gm29585
ENSMUST00000185272	ENSMUSG00000100826	Snhg14
ENSMUST00000185283	ENSMUSG00000099945	1700126A01Rik
ENSMUST00000185304	MSTRG.998	Gm28375
ENSMUST00000185309	ENSMUSG00000096573	1700009J07Rik
ENSMUST00000185310	MSTRG.1299	2900009J06Rik
ENSMUST00000185323	ENSMUSG00000102106	2310043O21Rik
ENSMUST00000185324	MSTRG.33088	Gm16794
ENSMUST00000185335	ENSMUSG00000101086	Gm28651
ENSMUST00000185351	MSTRG.29494	1700008J07Rik
ENSMUST00000185364	ENSMUSG00000100277	1810053B23Rik
ENSMUST00000185378	MSTRG.32495	Gm29322
ENSMUST00000185385	ENSMUSG00000100014	Gm29290
ENSMUST00000185388	MSTRG.13781	Gm19412
ENSMUST00000185395	ENSMUSG00000062704	9430002A10Rik
ENSMUST00000185402	MSTRG.18688	5430405H02Rik
ENSMUST00000185409	ENSMUSG00000101514	Gm5524
ENSMUST00000185414	MSTRG.26535	2610306M01Rik
ENSMUST00000185424	MSTRG.20418	A930005H10Rik
ENSMUST00000185439	ENSMUSG00000102060	1700061E17Rik
ENSMUST00000185443	ENSMUSG00000099957	2610027F03Rik
ENSMUST00000185446	MSTRG.15787	A430093F15Rik
ENSMUST00000185458	MSTRG.30382	Gm19744
ENSMUST00000185464	ENSMUSG00000100094	1810008I18Rik
ENSMUST00000185465	ENSMUSG00000100627	A830008E24Rik
ENSMUST00000185480	MSTRG.673	Gm20342
ENSMUST00000185486	MSTRG.7668	Gm28875
ENSMUST00000185494	ENSMUSG00000099906	Gm28653
ENSMUST00000185499	MSTRG.3606	4732465J04Rik
ENSMUST00000185501	MSTRG.33019	Gm2396
ENSMUST00000185512	MSTRG.20418	A930005H10Rik
ENSMUST00000185513	ENSMUSG00000096573	1700009J07Rik
ENSMUST00000185516	MSTRG.457	Gm28177
ENSMUST00000185535	MSTRG.1160	Gm7160
ENSMUST00000185536	MSTRG.883	Gm28941
ENSMUST00000185538	MSTRG.32239	Gm28119
ENSMUST00000185540	ENSMUSG00000099465	Gm3830
ENSMUST00000185541	ENSMUSG00000100400	Gm29326
ENSMUST00000185546	ENSMUSG00000100198	1700030O20Rik
ENSMUST00000185548	ENSMUSG00000099552	9830004L10Rik
ENSMUST00000185555	ENSMUSG00000100277	1810053B23Rik
ENSMUST00000185564	MSTRG.32769	Gm28731
ENSMUST00000185587	MSTRG.645	Gm29084
ENSMUST00000185635	ENSMUSG00000100510	AV026068
ENSMUST00000185637	MSTRG.29788	Gm28821
ENSMUST00000185650	MSTRG.14946	Snhg4
ENSMUST00000185652	MSTRG.8940	1810034E14Rik
ENSMUST00000185663	ENSMUSG00000099683	Gm28214
ENSMUST00000185681	MSTRG.20418	A930005H10Rik
ENSMUST00000185693	ENSMUSG00000100826	Snhg14
ENSMUST00000185694	MSTRG.33078	BC043934

ENSMUST00000185716	ENSMUSG00000099810	Gm29509
ENSMUST00000185722	ENSMUSG00000101969	Gm20125
ENSMUST00000185726	ENSMUSG00000100211	1700064M15Rik
ENSMUST00000185727	MSTRG.808	Gm29253
ENSMUST00000185737	ENSMUSG00000100392	Gm28935
ENSMUST00000185746	ENSMUSG00000099907	Gm10421
ENSMUST00000185751	MSTRG.11367	4933427E11Rik
ENSMUST00000185761	ENSMUSG00000101549	Gm29630
ENSMUST00000185768	ENSMUSG00000101968	1700027A15Rik
ENSMUST00000185789	MSTRG.29789	Kcnq1ot1
ENSMUST00000185794	ENSMUSG00000101370	Gm29139
ENSMUST00000185798	ENSMUSG00000097652	Mhrt
ENSMUST00000185804	ENSMUSG00000100252	Mir124-2hg
ENSMUST00000185808	MSTRG.33258	Gm29123
ENSMUST00000185809	ENSMUSG00000101009	1700108F19Rik
ENSMUST00000185813	ENSMUSG00000101476	Gm29570
ENSMUST00000185814	MSTRG.14946	Snhg4
ENSMUST00000185815	ENSMUSG00000100826	Snhg14
ENSMUST00000185820	MSTRG.24831	Tctn2
ENSMUST00000185842	MSTRG.165	Gm28836
ENSMUST00000185848	MSTRG.1563	Gm10138
ENSMUST00000185857	ENSMUSG00000100827	Gm29069
ENSMUST00000185876	MSTRG.34338	Xist
ENSMUST00000185880	MSTRG.10865	1810041H14Rik
ENSMUST00000185881	MSTRG.244	Gm28417
ENSMUST00000185888	MSTRG.1590	Gm28791
ENSMUST00000185910	MSTRG.392	Gm29155
ENSMUST00000185931	MSTRG.29138	1600010M07Rik
ENSMUST00000185938	ENSMUSG00000101334	1700028M03Rik
ENSMUST00000185939	ENSMUSG00000101603	Gm28730
ENSMUST00000185943	MSTRG.23235	5031425E22Rik
ENSMUST00000185960	ENSMUSG00000056031	9330154J02Rik
ENSMUST00000185962	MSTRG.8700	Gm8739
ENSMUST00000185971	ENSMUSG00000100277	1810053B23Rik
ENSMUST00000185973	MSTRG.9181	A230107N01Rik
ENSMUST00000185979	MSTRG.9389	Gm29502
ENSMUST00000185980	MSTRG.385	Gm28782
ENSMUST00000185982	MSTRG.25671	Lncpint
ENSMUST00000185991	MSTRG.725	Gm29113
ENSMUST00000185995	ENSMUSG00000100096	Gm28760
ENSMUST00000186007	MSTRG.14226	Gm19585
ENSMUST00000186011	MSTRG.28139	Gm37494
ENSMUST00000186025	MSTRG.32435	2010007H06Rik
ENSMUST00000186027	ENSMUSG00000101693	Gm19461
ENSMUST00000186030	MSTRG.21301	1700055D18Rik
ENSMUST00000186042	ENSMUSG00000100367	Gm29336
ENSMUST00000186052	MSTRG.15787	A430093F15Rik
ENSMUST00000186055	ENSMUSG00000101903	Gm29291
ENSMUST00000186073	ENSMUSG00000074067	Gm10619
ENSMUST00000186084	ENSMUSG00000101505	1700109G14Rik
ENSMUST00000186098	MSTRG.1038	4933400F21Rik
ENSMUST00000186106	MSTRG.27603	Gm29638
ENSMUST00000186108	ENSMUSG00000101966	Gm5248
ENSMUST00000186117	MSTRG.245	4930568A12Rik
ENSMUST00000186123	ENSMUSG00000101405	Gm28758
ENSMUST00000186130	MSTRG.13267	Gm28873
ENSMUST00000186134	MSTRG.8581	Gm28707

ENSMUST00000186135	MSTRG.30382	Gm19744
ENSMUST00000186147	ENSMUSG00000100291	2310069B03Rik
ENSMUST00000186159	MSTRG.14704	Gm28529
ENSMUST00000186160	MSTRG.15719	1700105P06Rik
ENSMUST00000186169	MSTRG.6169	Gm29292
ENSMUST00000186170	MSTRG.31797	Gm29642
ENSMUST00000186174	ENSMUSG00000047935	Gm5607
ENSMUST00000186180	ENSMUSG00000099378	1700067G17Rik
ENSMUST00000186183	ENSMUSG00000097709	2810429I04Rik
ENSMUST00000186189	MSTRG.25799	1700025N23Rik
ENSMUST00000186214	ENSMUSG00000100510	AV026068
ENSMUST00000186216	MSTRG.4308	Gm29237
ENSMUST00000186230	MSTRG.1308	Gm28800
ENSMUST00000186234	MSTRG.28597	Gm9885
ENSMUST00000186238	MSTRG.33104	Gm19325
ENSMUST00000186240	MSTRG.1350	Gm28913
ENSMUST00000186267	ENSMUSG00000100053	Gm28154
ENSMUST00000186289	MSTRG.392	Gm29155
ENSMUST00000186291	MSTRG.11673	Gm20324
ENSMUST00000186300	MSTRG.10896	Gm28932
ENSMUST00000186325	MSTRG.692	2810408I11Rik
ENSMUST00000186326	MSTRG.5613	D030028A08Rik
ENSMUST00000186327	MSTRG.12671	Gm28750
ENSMUST00000186334	ENSMUSG00000100170	Gm28942
ENSMUST00000186337	MSTRG.988	Gm29055
ENSMUST00000186343	MSTRG.128	Gm28095
ENSMUST00000186344	MSTRG.30055	1700041G16Rik
ENSMUST00000186347	ENSMUSG00000099349	1700047G03Rik
ENSMUST00000186379	ENSMUSG00000099758	Gm10830
ENSMUST00000186386	ENSMUSG00000100510	AV026068
ENSMUST00000186387	ENSMUSG00000102013	E330023G01Rik
ENSMUST00000186411	MSTRG.17533	2600014E21Rik
ENSMUST00000186424	MSTRG.27808	D930028M14Rik
ENSMUST00000186428	MSTRG.445	Gm29453
ENSMUST00000186431	MSTRG.13289	Gm6712
ENSMUST00000186436	MSTRG.672	Ino80dos
ENSMUST00000186444	ENSMUSG00000100594	2810414N06Rik
ENSMUST00000186450	MSTRG.32732	Gm28379
ENSMUST00000186454	ENSMUSG00000101930	Gm5441
ENSMUST00000186462	ENSMUSG00000100169	Gm29087
ENSMUST00000186471	MSTRG.20359	Gm12500
ENSMUST00000186472	ENSMUSG00000100680	1810044D09Rik
ENSMUST00000186473	ENSMUSG00000100182	1810006J02Rik
ENSMUST00000186474	MSTRG.10436	2410022M11Rik
ENSMUST00000186480	MSTRG.12796	Zbtb11os1
ENSMUST00000186500	MSTRG.15813	BE692007
ENSMUST00000186504	ENSMUSG00000101179	Gm29455
ENSMUST00000186510	MSTRG.779	Gm29358
ENSMUST00000186515	MSTRG.1383	Gm28609
ENSMUST00000186531	MSTRG.32003	2810013P06Rik
ENSMUST00000186535	MSTRG.12776	Dubr
ENSMUST00000186560	ENSMUSG00000100108	Gm28100
ENSMUST00000186562	ENSMUSG00000101402	Gm28673
ENSMUST00000186584	MSTRG.16094	Gm28991
ENSMUST00000186595	ENSMUSG00000055048	Gm9962
ENSMUST00000186596	MSTRG.15787	A430093F15Rik
ENSMUST00000186597	MSTRG.1563	Gm10138

ENSMUST00000186604	MSTRG.144	Gm28376
ENSMUST00000186610	ENSMUSG00000097709	2810429I04Rik
ENSMUST00000186612	ENSMUSG00000099971	Gm28287
ENSMUST00000186618	ENSMUSG00000100664	6030442E23Rik
ENSMUST00000186632	MSTRG.33091	C78334
ENSMUST00000186677	MSTRG.2981	Gm28447
ENSMUST00000186681	MSTRG.837	Gm29187
ENSMUST00000186732	ENSMUSG00000101701	4930521E06Rik
ENSMUST00000186737	ENSMUSG00000086712	AI427809
ENSMUST00000186756	ENSMUSG00000099832	Gm29025
ENSMUST00000186760	MSTRG.18765	2310001K24Rik
ENSMUST00000186765	MSTRG.1694	4930439D14Rik
ENSMUST00000186768	MSTRG.15951	2610016A17Rik
ENSMUST00000186769	MSTRG.442	Gm28055
ENSMUST00000186785	MSTRG.1601	E330020D12Rik
ENSMUST00000186786	MSTRG.672	Ino80dos
ENSMUST00000186806	MSTRG.12776	Dubr
ENSMUST00000186817	MSTRG.446	Gm28323
ENSMUST00000186819	ENSMUSG00000100625	1700016G22Rik
ENSMUST00000186821	MSTRG.33094	A930006L05Rik
ENSMUST00000186823	MSTRG.1003	Gm29371
ENSMUST00000186830	ENSMUSG00000102063	Gm28706
ENSMUST00000186838	MSTRG.25427	D730045B01Rik
ENSMUST00000186843	ENSMUSG00000100625	1700016G22Rik
ENSMUST00000186844	MSTRG.3688	Gm28592
ENSMUST00000186872	ENSMUSG00000100134	1700065O20Rik
ENSMUST00000186875	ENSMUSG00000100457	D830032E09Rik
ENSMUST00000186876	MSTRG.6775	Gm29542
ENSMUST00000186885	MSTRG.1158	Gm28187
ENSMUST00000186895	MSTRG.687	Gm28982
ENSMUST00000186931	MSTRG.33769	2010308F09Rik
ENSMUST00000186944	MSTRG.12776	Dubr
ENSMUST00000186958	ENSMUSG00000097482	Gm17634
ENSMUST00000186977	MSTRG.18726	2010009K17Rik
ENSMUST00000186993	MSTRG.18723	Snhg17
ENSMUST00000187011	MSTRG.1038	4933400F21Rik
ENSMUST00000187014	ENSMUSG00000100129	Gm29064
ENSMUST00000187015	MSTRG.33027	4930579C12Rik
ENSMUST00000187055	MSTRG.20418	A930005H10Rik
ENSMUST00000187079	MSTRG.3156	1700094J05Rik
ENSMUST00000187090	MSTRG.14418	2410021H03Rik
ENSMUST00000187091	ENSMUSG00000101634	1700066B17Rik
ENSMUST00000187099	MSTRG.18825	Gm28163
ENSMUST00000187100	MSTRG.728	Gm29114
ENSMUST00000187121	ENSMUSG00000101722	Gm29125
ENSMUST00000187123	ENSMUSG00000100890	1700085C21Rik
ENSMUST00000187127	ENSMUSG00000100791	Gm28199
ENSMUST00000187142	MSTRG.31949	Zfp469
ENSMUST00000187154	ENSMUSG00000100945	Gm29529
ENSMUST00000187158	ENSMUSG00000101568	1700040F17Rik
ENSMUST00000187172	MSTRG.25890	2010310C07Rik
ENSMUST00000187198	ENSMUSG00000099839	Gm29374
ENSMUST00000187199	MSTRG.13267	Gm28869
ENSMUST00000187218	MSTRG.10865	1810041H14Rik
ENSMUST00000187259	MSTRG.32634	Gm16759
ENSMUST00000187265	ENSMUSG00000100111	A330087D11Rik
ENSMUST00000187267	ENSMUSG00000100717	1700120G07Rik

ENSMUST00000187268	MSTRG.263	Gm26788
ENSMUST00000187270	MSTRG.908	9930111H07Rik
ENSMUST00000187272	ENSMUSG00000101683	1700028D13Rik
ENSMUST00000187295	MSTRG.1038	4933400F21Rik
ENSMUST00000187312	MSTRG.28078	Gm10640
ENSMUST00000187322	MSTRG.14946	Snhg4
ENSMUST00000187327	MSTRG.18688	5430405H02Rik
ENSMUST00000187332	MSTRG.2804	Gm29245
ENSMUST00000187343	MSTRG.17002	1700084E18Rik
ENSMUST00000187345	MSTRG.32223	2610203C20Rik
ENSMUST00000187351	MSTRG.27095	Gm28967
ENSMUST00000187352	ENSMUSG00000100777	4930598F16Rik
ENSMUST00000187361	MSTRG.19306	4933429H19Rik
ENSMUST00000187393	MSTRG.33075	Gm28424
ENSMUST00000187396	ENSMUSG00000102096	1700101O22Rik
ENSMUST00000187397	ENSMUSG00000099384	1700110C19Rik
ENSMUST00000187399	ENSMUSG00000090925	1810064F22Rik
ENSMUST00000187401	MSTRG.111	Gm9947
ENSMUST00000187409	MSTRG.23564	Gm7854
ENSMUST00000187415	MSTRG.1612	Gm28512
ENSMUST00000187421	MSTRG.28554	1810026B05Rik
ENSMUST00000187423	MSTRG.10883	1700108J01Rik
ENSMUST00000187450	MSTRG.1440	Gm28277
ENSMUST00000187453	ENSMUSG00000101843	Gm28746
ENSMUST00000187458	MSTRG.29414	2510046G10Rik
ENSMUST00000187461	ENSMUSG00000100510	AV026068
ENSMUST00000187479	ENSMUSG00000101888	1700025H01Rik
ENSMUST00000187483	MSTRG.3606	4732465J04Rik
ENSMUST00000187484	MSTRG.17870	Gm29053
ENSMUST00000187489	ENSMUSG00000099459	Gm28556
ENSMUST00000187497	MSTRG.863	1700016L21Rik
ENSMUST00000187511	MSTRG.1023	Gm19582
ENSMUST00000187514	MSTRG.6029	Gm11696
ENSMUST00000187517	ENSMUSG00000101189	1700029M03Rik
ENSMUST00000187536	ENSMUSG00000099406	Gm29408
ENSMUST00000187542	MSTRG.27108	Gm28809
ENSMUST00000187553	ENSMUSG00000101211	Gm28818
ENSMUST00000187556	ENSMUSG00000102029	Gm29530
ENSMUST00000187558	MSTRG.12571	1700119H24Rik
ENSMUST00000187561	MSTRG.1474	Gm4258
ENSMUST00000187564	ENSMUSG00000100980	Gm29100
ENSMUST00000187589	MSTRG.32949	Gm29054
ENSMUST00000187592	ENSMUSG00000067101	1700010H22Rik
ENSMUST00000187637	ENSMUSG00000101287	Gm28166
ENSMUST00000187655	ENSMUSG00000100965	Gm28449
ENSMUST00000187666	ENSMUSG00000100826	Snhg14
ENSMUST00000187676	MSTRG.32732	Gm28379
ENSMUST00000187685	ENSMUSG00000100936	Gm28865
ENSMUST00000187692	MSTRG.24282	Gm29464
ENSMUST00000187697	MSTRG.15658	Frmd8os
ENSMUST00000187700	ENSMUSG00000100154	Gm29052
ENSMUST00000187706	MSTRG.11894	1700120C14Rik
ENSMUST00000187716	MSTRG.1575	Gm28610
ENSMUST00000187719	ENSMUSG00000100255	Gm28196
ENSMUST00000187724	MSTRG.27108	Gm28809
ENSMUST00000187729	ENSMUSG00000100010	1700010I02Rik
ENSMUST00000187746	ENSMUSG00000100807	Gm29521

ENSMUST00000187757	MSTRG.20331	2010016I18Rik
ENSMUST00000187763	ENSMUSG00000037535	1700021A07Rik
ENSMUST00000187764	MSTRG.390	Gm29156
ENSMUST00000187773	MSTRG.10865	1810041H14Rik
ENSMUST00000187777	ENSMUSG00000099924	Gm28320
ENSMUST00000187779	ENSMUSG00000100174	Gm28719
ENSMUST00000187815	MSTRG.2633	4930579H20Rik
ENSMUST00000187838	MSTRG.20263	Gm29561
ENSMUST00000187848	ENSMUSG00000100094	1810008I18Rik
ENSMUST00000187850	ENSMUSG00000106107	Gm43190
ENSMUST00000187855	MSTRG.3942	Gm29585
ENSMUST00000187867	MSTRG.9449	Gm28989
ENSMUST00000187869	MSTRG.880	C430014B12Rik
ENSMUST00000187873	MSTRG.28126	E130304I02Rik
ENSMUST00000187876	MSTRG.25671	Lncpint
ENSMUST00000187880	MSTRG.5792	Gm28156
ENSMUST00000187882	ENSMUSG00000099669	Gm29012
ENSMUST00000187891	ENSMUSG00000056031	9330154J02Rik
ENSMUST00000187897	MSTRG.15990	1700018L02Rik
ENSMUST00000187898	MSTRG.7477	Gm29362
ENSMUST00000187902	ENSMUSG00000100151	Gm28382
ENSMUST00000187908	MSTRG.775	Gm28364
ENSMUST00000187918	ENSMUSG00000100247	A530053M12Rik
ENSMUST00000187920	ENSMUSG00000099411	2310015D24Rik
ENSMUST00000187923	MSTRG.25160	6330403L08Rik
ENSMUST00000187924	MSTRG.1152	B230216N24Rik
ENSMUST00000187929	MSTRG.860	Gm28940
ENSMUST00000187941	MSTRG.779	Gm29358
ENSMUST00000187956	ENSMUSG00000101224	Gm28209
ENSMUST00000187961	ENSMUSG00000100336	Gm29601
ENSMUST00000187967	MSTRG.11894	1700120C14Rik
ENSMUST00000188014	ENSMUSG00000044574	5031434C07Rik
ENSMUST00000188031	ENSMUSG00000099472	Gm29539
ENSMUST00000188038	MSTRG.28139	Gm37494
ENSMUST00000188040	ENSMUSG00000099950	9130227L01Rik
ENSMUST00000188056	ENSMUSG00000100277	1810053B23Rik
ENSMUST00000188068	MSTRG.1340	Gm28857
ENSMUST00000188069	MSTRG.1088	Gm28535
ENSMUST00000188089	MSTRG.3942	Gm29585
ENSMUST00000188093	ENSMUSG00000097072	Foxl2os
ENSMUST00000188113	MSTRG.826	Gm28410
ENSMUST00000188115	MSTRG.1349	Gm28914
ENSMUST00000188118	MSTRG.14778	Gm29200
ENSMUST00000188123	MSTRG.1426	Gm28892
ENSMUST00000188135	MSTRG.26515	2310040G24Rik
ENSMUST00000188137	ENSMUSG00000101356	Gm28876
ENSMUST00000188156	MSTRG.33016	9330159M07Rik
ENSMUST00000188160	MSTRG.14968	Gm29417
ENSMUST00000188188	ENSMUSG00000100546	Gm29483
ENSMUST00000188198	MSTRG.33016	9330159M07Rik
ENSMUST00000188201	ENSMUSG00000101588	Gm28265
ENSMUST00000188203	MSTRG.1376	6030442K20Rik
ENSMUST00000188207	ENSMUSG00000099340	Gm29540
ENSMUST00000188213	ENSMUSG00000101848	4933417E11Rik
ENSMUST00000188215	MSTRG.25542	Gm29591
ENSMUST00000188217	MSTRG.32952	Gm2087
ENSMUST00000188232	MSTRG.33019	Gm2396

ENSMUST00000188236	MSTRG.23131	1700003C15Rik
ENSMUST00000188243	MSTRG.489	4930444A19Rik
ENSMUST00000188260	MSTRG.20418	A930005H10Rik
ENSMUST00000188271	MSTRG.11930	2310068J16Rik
ENSMUST00000188277	MSTRG.32223	2610203C20Rik
ENSMUST00000188278	MSTRG.11355	1700010B13Rik
ENSMUST00000188292	MSTRG.245	4930568A12Rik
ENSMUST00000188294	MSTRG.29795	4933417O13Rik
ENSMUST00000188296	MSTRG.6029	Gm11696
ENSMUST00000188305	ENSMUSG00000102095	C730036E19Rik
ENSMUST00000188309	ENSMUSG00000102064	Gm28625
ENSMUST00000188318	ENSMUSG00000100605	Gm29243
ENSMUST00000188320	ENSMUSG00000100146	1700020M21Rik
ENSMUST00000188328	MSTRG.23235	5031425E22Rik
ENSMUST00000188329	ENSMUSG00000101414	Gm29101
ENSMUST00000188349	ENSMUSG00000101968	1700027A15Rik
ENSMUST00000188412	MSTRG.32223	2610203C20Rik
ENSMUST00000188418	ENSMUSG00000100666	1700007F19Rik
ENSMUST00000188427	ENSMUSG00000099802	Gm28493
ENSMUST00000188465	MSTRG.23293	1700096K18Rik
ENSMUST00000188474	ENSMUSG00000101061	Platr1
ENSMUST00000188480	MSTRG.15787	A430093F15Rik
ENSMUST00000188481	MSTRG.14416	C030034I22Rik
ENSMUST00000188485	MSTRG.34727	Gm29650
ENSMUST00000188504	ENSMUSG00000100987	Gm1627
ENSMUST00000188519	MSTRG.1160	Gm7160
ENSMUST00000188535	MSTRG.16382	Gm29261
ENSMUST00000188538	ENSMUSG00000101505	1700109G14Rik
ENSMUST00000188544	ENSMUSG00000099878	Gm28178
ENSMUST00000188551	MSTRG.33056	Gm29395
ENSMUST00000188574	MSTRG.26091	Gm28402
ENSMUST00000188577	ENSMUSG00000042360	4930433N12Rik
ENSMUST00000188579	ENSMUSG00000100600	A230077H06Rik
ENSMUST00000188589	MSTRG.1076	Gm29480
ENSMUST00000188595	ENSMUSG00000090925	1810064F22Rik
ENSMUST00000188600	MSTRG.766	Gm29183
ENSMUST00000188602	MSTRG.672	Ino80dos
ENSMUST00000188605	ENSMUSG00000100371	Gm29669
ENSMUST00000188608	MSTRG.7092	4930512B01Rik
ENSMUST00000188611	MSTRG.25671	Lncpint
ENSMUST00000188623	MSTRG.11672	Gm29019
ENSMUST00000188631	MSTRG.1131	Panct2
ENSMUST00000188640	ENSMUSG00000101028	Gm28723
ENSMUST00000188642	ENSMUSG00000097709	2810429I04Rik
ENSMUST00000188647	ENSMUSG00000099390	Gm29225
ENSMUST00000188648	ENSMUSG00000100872	1700065J18Rik
ENSMUST00000188654	MSTRG.30452	1700001D01Rik
ENSMUST00000188662	MSTRG.29138	1600010M07Rik
ENSMUST00000188663	ENSMUSG00000100511	1700111N16Rik
ENSMUST00000188678	ENSMUSG00000099436	Gm28977
ENSMUST00000188698	ENSMUSG00000022116	4930449E01Rik
ENSMUST00000188703	MSTRG.32419	Plet1os
ENSMUST00000188704	ENSMUSG00000100502	Gm28286
ENSMUST00000188711	MSTRG.15951	2610016A17Rik
ENSMUST00000188715	ENSMUSG00000099384	1700110C19Rik
ENSMUST00000188717	MSTRG.1601	E330020D12Rik
ENSMUST00000188725	MSTRG.27675	Gm29442

ENSMUST00000188729	MSTRG.1486	Gm28501
ENSMUST00000188730	MSTRG.14118	Trerf1
ENSMUST00000188737	MSTRG.21822	Lrp8os2
ENSMUST00000188743	MSTRG.1163	Gm28403
ENSMUST00000188747	MSTRG.33004	Gm28229
ENSMUST00000188753	MSTRG.393	Gm29157
ENSMUST00000188763	ENSMUSG00000099470	Gm29340
ENSMUST00000188774	MSTRG.32223	2610203C20Rik
ENSMUST00000188776	MSTRG.3673	1700020G17Rik
ENSMUST00000188786	ENSMUSG00000099996	Gm29065
ENSMUST00000188810	MSTRG.1128	Gm28901
ENSMUST00000188813	ENSMUSG00000046463	5930403N24Rik
ENSMUST00000188819	ENSMUSG00000101581	C430002N11Rik
ENSMUST00000188824	MSTRG.29138	1600010M07Rik
ENSMUST00000188846	MSTRG.1116	Gm10550
ENSMUST00000188852	MSTRG.13688	1700097N02Rik
ENSMUST00000188854	MSTRG.33088	Gm16794
ENSMUST00000188856	ENSMUSG00000099449	Gm28401
ENSMUST00000188874	ENSMUSG00000100943	Gm29229
ENSMUST00000188891	MSTRG.33008	Gm10634
ENSMUST00000188902	ENSMUSG00000100157	2310034O05Rik
ENSMUST00000188909	MSTRG.1231	3830432H09Rik
ENSMUST00000188927	ENSMUSG00000101903	Gm29291
ENSMUST00000188936	MSTRG.30382	Gm19744
ENSMUST00000188945	MSTRG.32223	2610203C20Rik
ENSMUST00000188947	MSTRG.14118	Trerf1
ENSMUST00000188950	ENSMUSG00000099735	1700122H20Rik
ENSMUST00000188956	ENSMUSG00000100253	1700020N18Rik
ENSMUST00000188962	MSTRG.730	Gm29112
ENSMUST00000188966	ENSMUSG00000101776	Gm28268
ENSMUST00000188976	ENSMUSG00000100826	Snhg14
ENSMUST00000188983	ENSMUSG00000100252	Mir124-2hg
ENSMUST00000188984	MSTRG.33238	Gm19667
ENSMUST00000189028	MSTRG.9058	1700100L14Rik
ENSMUST00000189033	MSTRG.28539	Gm28258
ENSMUST00000189045	MSTRG.23235	5031425E22Rik
ENSMUST00000189067	ENSMUSG00000101581	C430002N11Rik
ENSMUST00000189071	ENSMUSG00000085412	Halr1
ENSMUST00000189074	MSTRG.33181	Gm29387
ENSMUST00000189080	MSTRG.1072	Gm29481
ENSMUST00000189096	MSTRG.571	G730003C15Rik
ENSMUST00000189116	MSTRG.3692	1700010J16Rik
ENSMUST00000189129	MSTRG.26461	1700124L16Rik
ENSMUST00000189132	ENSMUSG00000100553	Gm17751
ENSMUST00000189139	MSTRG.863	1700016L21Rik
ENSMUST00000189143	ENSMUSG00000100510	AV026068
ENSMUST00000189148	ENSMUSG00000097072	Foxl2os
ENSMUST00000189150	ENSMUSG00000100106	Gm28856
ENSMUST00000189156	MSTRG.13781	Gm19412
ENSMUST00000189164	MSTRG.14946	Snhg4
ENSMUST00000189171	MSTRG.11721	D130051D11Rik
ENSMUST00000189196	ENSMUSG00000100417	Gm28578
ENSMUST00000189204	MSTRG.31572	Gm16833
ENSMUST00000189205	MSTRG.651	Gm29083
ENSMUST00000189230	MSTRG.26052	Hoxaas2
ENSMUST00000189232	ENSMUSG00000100711	Gm29461
ENSMUST00000189245	ENSMUSG00000102052	Gm28387

ENSMUST00000189247	ENSMUSG00000100444	C530043A13Rik
ENSMUST00000189251	ENSMUSG00000100998	Gm28864
ENSMUST00000189275	MSTRG.25730	Npn2
ENSMUST00000189281	MSTRG.33092	Gm28085
ENSMUST00000189284	ENSMUSG00000100437	Gm29152
ENSMUST00000189288	ENSMUSG00000100658	F730311O21Rik
ENSMUST00000189291	ENSMUSG00000100603	1700129L04Rik
ENSMUST00000189293	ENSMUSG00000099950	9130227L01Rik
ENSMUST00000189296	MSTRG.835	Gm29536
ENSMUST00000189302	ENSMUSG00000073174	Gm29254
ENSMUST00000189306	MSTRG.15813	BE692007
ENSMUST00000189308	MSTRG.26461	1700124L16Rik
ENSMUST00000189322	MSTRG.68	1700034P13Rik
ENSMUST00000189326	ENSMUSG00000100155	Gm2109
ENSMUST00000189331	MSTRG.2469	Gm28289
ENSMUST00000189334	MSTRG.22518	2810405F17Rik
ENSMUST00000189375	ENSMUSG00000101009	1700108F19Rik
ENSMUST00000189381	MSTRG.6321	Gm28192
ENSMUST00000189386	MSTRG.26515	2310040G24Rik
ENSMUST00000189387	MSTRG.27064	Gm29008
ENSMUST00000189394	ENSMUSG00000100798	Gm19589
ENSMUST00000189404	MSTRG.428	Gm28151
ENSMUST00000189408	ENSMUSG00000100826	Snhg14
ENSMUST00000189410	ENSMUSG00000099413	Gm17767
ENSMUST00000189419	MSTRG.8940	1810034E14Rik
ENSMUST00000189431	MSTRG.68	1700034P13Rik
ENSMUST00000189435	MSTRG.11355	1700010B13Rik
ENSMUST00000189441	MSTRG.19105	9230112E08Rik
ENSMUST00000189445	ENSMUSG00000100760	Gm4035
ENSMUST00000189460	ENSMUSG00000101702	1700072G22Rik
ENSMUST00000189464	ENSMUSG00000097709	2810429I04Rik
ENSMUST00000189466	ENSMUSG00000074067	Gm10619
ENSMUST00000189467	ENSMUSG00000100252	Mir124-2hg
ENSMUST00000189481	ENSMUSG00000100393	Gm29491
ENSMUST00000189493	MSTRG.25366	2310047D07Rik
ENSMUST00000189499	ENSMUSG00000101126	Gm10538
ENSMUST00000189507	ENSMUSG00000101746	2310043L19Rik
ENSMUST00000189514	MSTRG.764	6030407O03Rik
ENSMUST00000189527	ENSMUSG00000100120	Gm553
ENSMUST00000189565	MSTRG.15787	A430093F15Rik
ENSMUST00000189574	ENSMUSG00000100600	A230077H06Rik
ENSMUST00000189576	MSTRG.442	Gm28055
ENSMUST00000189581	ENSMUSG00000100826	Snhg14
ENSMUST00000189590	ENSMUSG00000101693	Gm19461
ENSMUST00000189594	MSTRG.2164	1700047M11Rik
ENSMUST00000189625	MSTRG.6637	1700030C10Rik
ENSMUST00000189628	MSTRG.15815	Gm19261
ENSMUST00000189639	MSTRG.25366	2310047D07Rik
ENSMUST00000189646	MSTRG.723	Gm28497
ENSMUST00000189653	MSTRG.28442	Snhg14
ENSMUST00000189675	MSTRG.14704	Gm28529
ENSMUST00000189683	MSTRG.20331	2010016I18Rik
ENSMUST00000189687	ENSMUSG00000102067	Gm29230
ENSMUST00000189722	MSTRG.683	Platr12
ENSMUST00000189748	ENSMUSG00000067101	1700010H22Rik
ENSMUST00000189763	ENSMUSG00000092627	D130058E05Rik
ENSMUST00000189767	ENSMUSG00000100911	1700027F09Rik

ENSMUST00000189781	MSTRG.11393	Gm28502
ENSMUST00000189802	MSTRG.653	Gm4208
ENSMUST00000189815	ENSMUSG00000101581	C430002N11Rik
ENSMUST00000189828	ENSMUSG00000099696	2900052N01Rik
ENSMUST00000189831	ENSMUSG00000099872	1700074H08Rik
ENSMUST00000189843	MSTRG.8633	5033430I15Rik
ENSMUST00000189853	ENSMUSG00000099895	Gm28153
ENSMUST00000189857	MSTRG.33149	Gm28586
ENSMUST00000189860	MSTRG.5575	Gm29477
ENSMUST00000189864	MSTRG.578	Gm28411
ENSMUST00000189867	MSTRG.438	9330175M20Rik
ENSMUST00000189880	ENSMUSG00000099404	Gm28172
ENSMUST00000189899	ENSMUSG00000099576	Gm29040
ENSMUST00000189905	MSTRG.8477	1700011B04Rik
ENSMUST00000189909	MSTRG.18859	Zfas1
ENSMUST00000189912	MSTRG.30382	Gm19744
ENSMUST00000189920	MSTRG.21741	E130102H24Rik
ENSMUST00000189929	ENSMUSG00000055972	2810407A14Rik
ENSMUST00000189938	ENSMUSG00000097482	Gm17634
ENSMUST00000189942	MSTRG.27270	2810454H06Rik
ENSMUST00000189953	ENSMUSG00000099798	Gm29168
ENSMUST00000189960	MSTRG.28554	1810026B05Rik
ENSMUST00000189966	MSTRG.28538	B130024G19Rik
ENSMUST00000189971	ENSMUSG00000100789	Gm29508
ENSMUST00000189974	MSTRG.14432	Gm26510
ENSMUST00000189996	ENSMUSG00000101029	Gm29439
ENSMUST00000190000	ENSMUSG00000099349	1700047G03Rik
ENSMUST00000190006	ENSMUSG00000101447	Gm28826
ENSMUST00000190007	MSTRG.489	4930444A19Rik
ENSMUST00000190020	MSTRG.14118	Trerf1
ENSMUST00000190028	MSTRG.28554	1810026B05Rik
ENSMUST00000190033	MSTRG.32992	Snhg5
ENSMUST00000190040	MSTRG.27336	Gm28523
ENSMUST00000190043	MSTRG.6028	1700096J18Rik
ENSMUST00000190053	ENSMUSG00000095956	1700036A12Rik
ENSMUST00000190074	ENSMUSG00000101565	1700003L19Rik
ENSMUST00000190077	MSTRG.26461	1700124L16Rik
ENSMUST00000190080	MSTRG.14118	Trerf1
ENSMUST00000190088	MSTRG.10898	1700024B18Rik
ENSMUST00000190105	MSTRG.1179	Gm20753
ENSMUST00000190109	MSTRG.32223	2610203C20Rik
ENSMUST00000190113	MSTRG.33014	Gm29562
ENSMUST00000190118	ENSMUSG00000100782	Gm28231
ENSMUST00000190120	MSTRG.5909	1700052K11Rik
ENSMUST00000190132	ENSMUSG00000101930	Gm5441
ENSMUST00000190144	ENSMUSG00000099628	Gm28427
ENSMUST00000190169	ENSMUSG00000099413	Gm17767
ENSMUST00000190179	ENSMUSG00000099774	Gm29341
ENSMUST00000190194	MSTRG.1241	Gm28867
ENSMUST00000190204	ENSMUSG00000100119	2700089I24Rik
ENSMUST00000190206	ENSMUSG00000102098	2310016D03Rik
ENSMUST00000190224	MSTRG.1538	Gm29170
ENSMUST00000190228	MSTRG.452	Gm29670
ENSMUST00000190235	MSTRG.33553	Gm2415
ENSMUST00000190242	MSTRG.1167	Gm28404
ENSMUST00000190305	ENSMUSG00000100466	Gm29572
ENSMUST00000190320	MSTRG.28538	B130024G19Rik

ENSMUST00000190327	MSTRG.2278	1700034H15Rik
ENSMUST00000190332	MSTRG.33211	4932413F04Rik
ENSMUST00000190356	MSTRG.20331	2010016I18Rik
ENSMUST00000190362	ENSMUSG00000100553	Gm17751
ENSMUST00000190364	ENSMUSG00000051198	4930548G14Rik
ENSMUST00000190367	ENSMUSG00000099767	Gm28884
ENSMUST00000190392	ENSMUSG00000101797	Gm29266
ENSMUST00000190396	ENSMUSG00000100022	Gm29590
ENSMUST00000190409	ENSMUSG00000053117	E330013P04Rik
ENSMUST00000190415	MSTRG.13285	BC002059
ENSMUST00000190461	MSTRG.29940	2810030D12Rik
ENSMUST00000190466	MSTRG.13184	Gm28505
ENSMUST00000190485	ENSMUSG00000100627	A830008E24Rik
ENSMUST00000190491	MSTRG.3156	1700094J05Rik
ENSMUST00000190493	ENSMUSG00000100733	4932411K12Rik
ENSMUST00000190504	ENSMUSG00000099364	5730419F03Rik
ENSMUST00000190515	ENSMUSG00000101481	1700123O21Rik
ENSMUST00000190525	ENSMUSG00000101968	1700027A15Rik
ENSMUST00000190547	ENSMUSG00000097709	2810429I04Rik
ENSMUST00000190550	ENSMUSG00000100573	1700081H04Rik
ENSMUST00000190567	MSTRG.23151	A630072M18Rik
ENSMUST00000190575	MSTRG.15489	1700030N03Rik
ENSMUST00000190576	ENSMUSG00000100872	1700065J18Rik
ENSMUST00000190581	MSTRG.29723	C330022C24Rik
ENSMUST00000190598	MSTRG.573	Gm29017
ENSMUST00000190602	ENSMUSG00000099370	Platr10
ENSMUST00000190628	ENSMUSG00000098144	1700029N11Rik
ENSMUST00000190633	ENSMUSG00000101067	Gm29007
ENSMUST00000190663	ENSMUSG00000100334	C230024C17Rik
ENSMUST00000190666	MSTRG.28442	Snhg14
ENSMUST00000190668	MSTRG.15489	1700030N03Rik
ENSMUST00000190673	MSTRG.29940	2810030D12Rik
ENSMUST00000190683	MSTRG.11894	1700120C14Rik
ENSMUST00000190684	ENSMUSG00000100760	Gm4035
ENSMUST00000190685	ENSMUSG00000101643	Gm28307
ENSMUST00000190690	ENSMUSG00000101791	2210011K15Rik
ENSMUST00000190693	MSTRG.27815	9130221H12Rik
ENSMUST00000190698	ENSMUSG00000053117	E330013P04Rik
ENSMUST00000190699	MSTRG.9760	2810402E24Rik
ENSMUST00000190705	ENSMUSG00000099625	Gm29325
ENSMUST00000190718	ENSMUSG00000101648	Gm29388
ENSMUST00000190720	ENSMUSG00000101880	Gm29282
ENSMUST00000190728	ENSMUSG00000101210	Gm28720
ENSMUST00000190732	MSTRG.13093	Gm29050
ENSMUST00000190739	MSTRG.3453	A230060F14Rik
ENSMUST00000190769	ENSMUSG00000099449	Gm28401
ENSMUST00000190783	ENSMUSG00000099553	Gm29538
ENSMUST00000190789	MSTRG.28139	Gm37494
ENSMUST00000190793	MSTRG.2767	BC048559
ENSMUST00000190804	MSTRG.912	C130036L24Rik
ENSMUST00000190818	ENSMUSG00000101009	1700108F19Rik
ENSMUST00000190830	ENSMUSG00000099825	1810012K16Rik
ENSMUST00000190845	ENSMUSG00000100209	Gm28793
ENSMUST00000190861	MSTRG.11389	Gm28068
ENSMUST00000190863	ENSMUSG00000101895	Gm28981
ENSMUST00000190864	ENSMUSG00000099672	Gm28564
ENSMUST00000190865	MSTRG.13093	Gm29050

ENSMUST00000190881	ENSMUSG00000100717	1700120G07Rik
ENSMUST00000190889	MSTRG.25890	2010310C07Rik
ENSMUST00000190898	MSTRG.732	Gm28112
ENSMUST00000190908	ENSMUSG00000100857	1700041M19Rik
ENSMUST00000190910	ENSMUSG00000100775	Gm29107
ENSMUST00000190911	ENSMUSG00000100396	Gm29367
ENSMUST00000190917	MSTRG.22337	Gm28874
ENSMUST00000190918	ENSMUSG00000099502	Gm28640
ENSMUST00000190927	ENSMUSG00000100830	Gm28441
ENSMUST00000190930	MSTRG.18767	2900093K20Rik
ENSMUST00000190938	MSTRG.29496	Gm28198
ENSMUST00000190941	MSTRG.32419	Plet1os
ENSMUST00000190947	MSTRG.28078	Gm10640
ENSMUST00000190967	MSTRG.30382	Gm19744
ENSMUST00000190970	MSTRG.33091	C78334
ENSMUST00000190971	MSTRG.1101	Gm28536
ENSMUST00000190974	MSTRG.16346	Ppnr
ENSMUST00000190991	MSTRG.1140	Gm7967
ENSMUST00000191034	MSTRG.1371	Gm29103
ENSMUST00000191038	MSTRG.29501	1700120K04Rik
ENSMUST00000191042	MSTRG.1140	Gm7967
ENSMUST00000191060	ENSMUSG00000100182	1810006J02Rik
ENSMUST00000191067	ENSMUSG00000046463	5930403N24Rik
ENSMUST00000191072	ENSMUSG00000101603	Gm28730
ENSMUST00000191079	MSTRG.12776	Dubr
ENSMUST00000191084	MSTRG.28538	B130024G19Rik
ENSMUST00000191114	MSTRG.764	6030407O03Rik
ENSMUST00000191115	ENSMUSG00000045075	Gm9796
ENSMUST00000191132	ENSMUSG00000101693	Gm19461
ENSMUST00000191153	MSTRG.14118	Trerf1
ENSMUST00000191154	MSTRG.803	Gm28294
ENSMUST00000191155	MSTRG.29334	1700069B07Rik
ENSMUST00000191157	MSTRG.29138	1600010M07Rik
ENSMUST00000191182	MSTRG.20418	A930005H10Rik
ENSMUST00000191186	MSTRG.23235	5031425E22Rik
ENSMUST00000191207	ENSMUSG00000101693	Gm19461
ENSMUST00000191222	MSTRG.14594	2010106C02Rik
ENSMUST00000191240	MSTRG.25367	Gm29264
ENSMUST00000191246	ENSMUSG00000054061	Gm9934
ENSMUST00000191278	ENSMUSG00000099349	1700047G03Rik
ENSMUST00000191287	MSTRG.68	1700034P13Rik
ENSMUST00000191298	ENSMUSG00000101823	Gm29438
ENSMUST00000191302	MSTRG.28139	Gm37494
ENSMUST00000191315	MSTRG.240	Gm28306
ENSMUST00000191318	MSTRG.578	Gm28411
ENSMUST00000191346	MSTRG.27602	1810019N24Rik
ENSMUST00000191351	ENSMUSG00000101007	Gm29541
ENSMUST00000191357	MSTRG.244	Gm28417
ENSMUST00000191359	ENSMUSG00000100932	Gm29459
ENSMUST00000191362	ENSMUSG00000099639	1700084F23Rik
ENSMUST00000191363	MSTRG.9451	Gm28988
ENSMUST00000191369	ENSMUSG00000099468	Gm28271
ENSMUST00000191384	ENSMUSG00000101009	1700108F19Rik
ENSMUST00000191391	MSTRG.21676	Gm28096
ENSMUST00000191393	ENSMUSG00000097022	BC001981
ENSMUST00000191396	MSTRG.30605	2010320M18Rik
ENSMUST00000191402	MSTRG.472	Gm28777

ENSMUST00000191405	MSTRG.15489	1700030N03Rik
ENSMUST00000191409	MSTRG.1140	Gm7967
ENSMUST00000191426	ENSMUSG00000075408	6030408B16Rik
ENSMUST00000191427	MSTRG.1356	Gm29488
ENSMUST00000191429	MSTRG.3673	1700020G17Rik
ENSMUST00000191447	ENSMUSG00000101009	1700108F19Rik
ENSMUST00000191452	ENSMUSG00000101257	2310015K22Rik
ENSMUST00000191453	MSTRG.26056	Gm29430
ENSMUST00000191455	MSTRG.32634	Gm16759
ENSMUST00000191460	ENSMUSG00000100357	Gm28181
ENSMUST00000191474	MSTRG.1611	Gm28513
ENSMUST00000191479	ENSMUSG00000099370	Platr10
ENSMUST00000191482	MSTRG.1585	Gm28792
ENSMUST00000191492	MSTRG.33136	4930422M22Rik
ENSMUST00000191517	MSTRG.9833	Gm29626
ENSMUST00000191518	MSTRG.22330	Gm28872
ENSMUST00000191519	ENSMUSG00000100496	Gm28639
ENSMUST00000191524	ENSMUSG00000100175	1700025M24Rik
ENSMUST00000191526	ENSMUSG00000101641	Gm29560
ENSMUST00000191539	MSTRG.14226	Gm19585
ENSMUST00000191563	MSTRG.1082	9430060I03Rik
ENSMUST00000191570	MSTRG.20331	2010016I18Rik
ENSMUST00000191576	MSTRG.1037	Gm28499
ENSMUST00000191585	ENSMUSG00000097709	2810429I04Rik
ENSMUST00000191603	ENSMUSG00000099696	2900052N01Rik
ENSMUST00000191612	MSTRG.1449	Gm36938
ENSMUST00000191614	MSTRG.31289	Gm38042
ENSMUST00000191616	MSTRG.1986	Gm37950
ENSMUST00000191619	MSTRG.1331	Gm37278
ENSMUST00000191623	MSTRG.14447	Gm37639
ENSMUST00000191628	MSTRG.597	Gm37531
ENSMUST00000191630	ENSMUSG00000104104	Gm37877
ENSMUST00000191632	MSTRG.15434	D330025C20Rik
ENSMUST00000191634	MSTRG.1850	Gm37856
ENSMUST00000191635	MSTRG.1274	Gm37772
ENSMUST00000191636	MSTRG.1963	Gm37065
ENSMUST00000191637	ENSMUSG00000103228	Gm37727
ENSMUST00000191641	ENSMUSG00000104210	Gm37984
ENSMUST00000191644	MSTRG.32999	Gm37226
ENSMUST00000191648	ENSMUSG00000102633	Gm38224
ENSMUST00000191650	MSTRG.10499	Dleu2
ENSMUST00000191652	ENSMUSG00000103234	Gm37158
ENSMUST00000191654	ENSMUSG00000102778	Gm38165
ENSMUST00000191655	MSTRG.1514	Gm37718
ENSMUST00000191656	MSTRG.1490	Gm37101
ENSMUST00000191658	ENSMUSG00000104112	Gm37293
ENSMUST00000191660	ENSMUSG00000102190	Gm38166
ENSMUST00000191663	ENSMUSG00000103961	Gm36388
ENSMUST00000191669	MSTRG.14230	Gm37593
ENSMUST00000191676	MSTRG.1728	Gm38302
ENSMUST00000191685	ENSMUSG00000104451	Gm37831
ENSMUST00000191687	MSTRG.2043	Gm37519
ENSMUST00000191688	MSTRG.1343	Gm37084
ENSMUST00000191694	MSTRG.33129	1700008A23Rik
ENSMUST00000191696	MSTRG.12909	Gm37694
ENSMUST00000191698	MSTRG.353	Gm37265
ENSMUST00000191701	MSTRG.11001	F830212C03Rik

ENSMUST00000191702	MSTRG.2243	Gm38245
ENSMUST00000191704	MSTRG.1024	4833421G17Rik
ENSMUST00000191707	ENSMUSG00000104155	Gm38103
ENSMUST00000191712	MSTRG.10544	Gm37183
ENSMUST00000191714	ENSMUSG00000102632	Gm37786
ENSMUST00000191718	MSTRG.16454	Gm38348
ENSMUST00000191720	MSTRG.33051	Gm38391
ENSMUST00000191729	MSTRG.10393	Gm37034
ENSMUST00000191734	MSTRG.2158	Gm37258
ENSMUST00000191735	MSTRG.1819	Gm20743
ENSMUST00000191736	MSTRG.12895	Gm37241
ENSMUST00000191737	MSTRG.20330	Gm38244
ENSMUST00000191743	MSTRG.32943	Gm37837
ENSMUST00000191750	MSTRG.1648	Gm37383
ENSMUST00000191751	ENSMUSG00000102384	Gm38143
ENSMUST00000191753	MSTRG.28138	Gm44573
ENSMUST00000191756	MSTRG.19463	Gm37646
ENSMUST00000191759	ENSMUSG00000103694	Gm37530
ENSMUST00000191764	ENSMUSG00000103822	6030460B20Rik
ENSMUST00000191765	MSTRG.4434	Gm37695
ENSMUST00000191767	ENSMUSG00000102936	Gm35584
ENSMUST00000191770	ENSMUSG00000104419	2900092O11Rik
ENSMUST00000191771	ENSMUSG00000102508	Gm37367
ENSMUST00000191772	MSTRG.213	Gm38198
ENSMUST00000191778	MSTRG.31911	Gm37609
ENSMUST00000191779	MSTRG.234	1700001G17Rik
ENSMUST00000191780	ENSMUSG00000104299	Gm9924
ENSMUST00000191784	ENSMUSG00000103765	Gm37857
ENSMUST00000191791	MSTRG.33308	Lsmem2
ENSMUST00000191792	ENSMUSG00000102256	Gm38258
ENSMUST00000191796	ENSMUSG00000104282	Gm37460
ENSMUST00000191800	ENSMUSG00000103438	Gm36991
ENSMUST00000191809	MSTRG.2231	A430027H14Rik
ENSMUST00000191811	MSTRG.1482	Gm37632
ENSMUST00000191816	MSTRG.12897	Gm37606
ENSMUST00000191819	ENSMUSG00000103991	Gm38031
ENSMUST00000191822	MSTRG.2106	Gm38169
ENSMUST00000191824	ENSMUSG00000102705	4632432E15Rik
ENSMUST00000191825	MSTRG.27	4732440D04Rik
ENSMUST00000191827	ENSMUSG00000104205	Gm37396
ENSMUST00000191831	ENSMUSG00000104199	4930420N18Rik
ENSMUST00000191834	MSTRG.1974	Gm37756
ENSMUST00000191839	MSTRG.1816	Gm32391
ENSMUST00000191840	ENSMUSG00000102610	Gm37846
ENSMUST00000191850	MSTRG.362	Gm38070
ENSMUST00000191857	MSTRG.827	Gm37884
ENSMUST00000191858	MSTRG.33355	Gm38134
ENSMUST00000191863	ENSMUSG00000104462	Gm37661
ENSMUST00000191867	MSTRG.25836	Gm37610
ENSMUST00000191873	ENSMUSG00000103630	Gm37242
ENSMUST00000191875	MSTRG.2314	Gm37942
ENSMUST00000191877	MSTRG.26376	Gm38114
ENSMUST00000191880	MSTRG.15042	4930517L18Rik
ENSMUST00000191884	MSTRG.2156	Gm38359
ENSMUST00000191885	MSTRG.2330	Gm32250
ENSMUST00000191887	MSTRG.32799	Gm37663
ENSMUST00000191892	MSTRG.3756	4932442E05Rik

ENSMUST00000191894	MSTRG.16708	D930036K23Rik
ENSMUST00000191895	ENSMUSG00000104312	Gm37912
ENSMUST00000191902	ENSMUSG00000103728	Gm37099
ENSMUST00000191905	ENSMUSG00000102432	Gm29856
ENSMUST00000191912	ENSMUSG00000103662	Gm34294
ENSMUST00000191913	ENSMUSG00000102389	D630023O14Rik
ENSMUST00000191917	ENSMUSG00000104076	Gm37026
ENSMUST00000191922	ENSMUSG00000102480	Gm37926
ENSMUST00000191930	ENSMUSG00000102694	Gm37496
ENSMUST00000191937	MSTRG.1548	A230059L01Rik
ENSMUST00000191945	MSTRG.716	Gm38387
ENSMUST00000191950	ENSMUSG00000104276	Gm37866
ENSMUST00000191953	MSTRG.19750	Gm38313
ENSMUST00000191954	ENSMUSG00000102785	Gm2447
ENSMUST00000191961	ENSMUSG00000102825	Gm36990
ENSMUST00000191963	MSTRG.1474	A130050O07Rik
ENSMUST00000191965	MSTRG.234	1700001G17Rik
ENSMUST00000191979	MSTRG.638	Gm38075
ENSMUST00000191984	ENSMUSG00000107745	Gm43917
ENSMUST00000191994	ENSMUSG00000104519	Gm37161
ENSMUST00000191996	MSTRG.488	Gm37370
ENSMUST00000192000	MSTRG.1963	Gm37065
ENSMUST00000192003	MSTRG.15053	BC037039
ENSMUST00000192004	MSTRG.31891	1700016A09Rik
ENSMUST00000192005	MSTRG.315	4930470B04Rik
ENSMUST00000192006	MSTRG.33080	Gm37195
ENSMUST00000192008	MSTRG.301	Gm37506
ENSMUST00000192010	ENSMUSG00000102699	4930433B08Rik
ENSMUST00000192012	MSTRG.1408	Gm37935
ENSMUST00000192016	ENSMUSG00000102365	Gm37045
ENSMUST00000192022	ENSMUSG00000104015	Gm10745
ENSMUST00000192025	MSTRG.2316	Gm37783
ENSMUST00000192030	ENSMUSG00000103299	Gm38231
ENSMUST00000192036	ENSMUSG00000102687	Gm37219
ENSMUST00000192040	ENSMUSG00000102163	Gm36945
ENSMUST00000192050	MSTRG.224	Gm37906
ENSMUST00000192051	MSTRG.33302	Gm9917
ENSMUST00000192052	MSTRG.2309	Gm38118
ENSMUST00000192053	MSTRG.33346	Gm37401
ENSMUST00000192059	MSTRG.20039	Gm37500
ENSMUST00000192060	MSTRG.19425	Gm31266
ENSMUST00000192062	MSTRG.28074	Gm37292
ENSMUST00000192063	MSTRG.16815	Gm38309
ENSMUST00000192064	MSTRG.12689	Gm37946
ENSMUST00000192072	MSTRG.1667	Gm37648
ENSMUST00000192073	MSTRG.9399	Gm36638
ENSMUST00000192074	MSTRG.14675	Gm2238
ENSMUST00000192078	ENSMUSG00000102676	Gm37435
ENSMUST00000192079	MSTRG.33210	Gm37649
ENSMUST00000192081	ENSMUSG00000102544	Gm5103
ENSMUST00000192082	MSTRG.11613	1110025M09Rik
ENSMUST00000192083	MSTRG.19468	Gm38200
ENSMUST00000192087	MSTRG.16547	Gm10851
ENSMUST00000192088	MSTRG.777	Gm37503
ENSMUST00000192105	ENSMUSG00000103292	Gm35048
ENSMUST00000192111	MSTRG.81	Gm37569
ENSMUST00000192112	MSTRG.16726	Gm38105

ENSMUST00000192113	MSTRG.2025	3110062G12Rik
ENSMUST00000192114	MSTRG.2635	Gm36962
ENSMUST00000192122	ENSMUSG00000103070	Gm37903
ENSMUST00000192127	ENSMUSG00000103917	Gm38072
ENSMUST00000192131	ENSMUSG00000102180	Gm37189
ENSMUST00000192133	MSTRG.7615	Gm37019
ENSMUST00000192134	MSTRG.16837	Gm37139
ENSMUST00000192137	MSTRG.15046	3222401L13Rik
ENSMUST00000192140	MSTRG.1474	Gm37124
ENSMUST00000192147	MSTRG.2311	Gm37691
ENSMUST00000192153	ENSMUSG00000103277	Gm37916
ENSMUST00000192154	MSTRG.19297	Gm37537
ENSMUST00000192162	MSTRG.1631	Gm37622
ENSMUST00000192163	MSTRG.10614	Gm37847
ENSMUST00000192164	MSTRG.2112	Gm31728
ENSMUST00000192167	MSTRG.18678	Gm37274
ENSMUST00000192170	MSTRG.32979	Gm37941
ENSMUST00000192173	ENSMUSG00000102336	Gm37233
ENSMUST00000192176	MSTRG.15626	Gm37376
ENSMUST00000192182	ENSMUSG00000104395	Gm37957
ENSMUST00000192184	MSTRG.1606	Gm37669
ENSMUST00000192190	MSTRG.1786	Gm37644
ENSMUST00000192201	MSTRG.15811	Gm37387
ENSMUST00000192206	MSTRG.7443	Gm37804
ENSMUST00000192212	ENSMUSG00000103107	Gm9867
ENSMUST00000192213	ENSMUSG00000103824	Gm38177
ENSMUST00000192216	MSTRG.202	Gm37880
ENSMUST00000192219	MSTRG.652	Gm37121
ENSMUST00000192224	ENSMUSG00000104079	Gm37024
ENSMUST00000192228	MSTRG.23753	Gm20033
ENSMUST00000192232	ENSMUSG00000102771	Gm33320
ENSMUST00000192234	MSTRG.272	4930403P22Rik
ENSMUST00000192244	MSTRG.16542	Gm37766
ENSMUST00000192251	MSTRG.32860	Gm37105
ENSMUST00000192253	ENSMUSG00000102373	9530018H14Rik
ENSMUST00000192254	MSTRG.19433	Gm37268
ENSMUST00000192256	MSTRG.1684	Gm37578
ENSMUST00000192257	MSTRG.1199	Gm36944
ENSMUST00000192258	MSTRG.844	Gm37932
ENSMUST00000192259	ENSMUSG00000104283	Gm37459
ENSMUST00000192265	MSTRG.24818	Gm37415
ENSMUST00000192266	MSTRG.1939	Gm37154
ENSMUST00000192267	MSTRG.19596	9330121J05Rik
ENSMUST00000192272	MSTRG.1883	Gm37524
ENSMUST00000192285	MSTRG.2220	Gm38155
ENSMUST00000192296	ENSMUSG00000103053	Gm38271
ENSMUST00000192297	ENSMUSG00000103640	Gm31406
ENSMUST00000192299	ENSMUSG00000104025	E330040D14Rik
ENSMUST00000192303	MSTRG.19490	Gm37675
ENSMUST00000192304	ENSMUSG00000098705	Gm3428
ENSMUST00000192309	MSTRG.24792	Gm37939
ENSMUST00000192316	MSTRG.311	4930594C11Rik
ENSMUST00000192320	ENSMUSG00000102460	Gm38197
ENSMUST00000192321	MSTRG.32813	Gm38215
ENSMUST00000192325	MSTRG.2331	Gm37334
ENSMUST00000192326	MSTRG.989	Gm37485
ENSMUST00000192330	MSTRG.32497	Gm37842

ENSMUST00000192336	ENSMUSG00000104017	Gm37363
ENSMUST00000192337	ENSMUSG00000102793	Gm37708
ENSMUST00000192338	MSTRG.42	Gm38024
ENSMUST00000192339	ENSMUSG00000102495	Gm26524
ENSMUST00000192340	ENSMUSG00000103261	Gm36981
ENSMUST00000192342	MSTRG.10499	Gm37472
ENSMUST00000192346	MSTRG.1368	Gm38067
ENSMUST00000192348	MSTRG.2332	9630010A21Rik
ENSMUST00000192350	MSTRG.1824	Gm38190
ENSMUST00000192353	ENSMUSG00000103547	Gm37665
ENSMUST00000192356	ENSMUSG00000104438	Gm37427
ENSMUST00000192361	MSTRG.16732	Gm10848
ENSMUST00000192363	MSTRG.1175	Gm37566
ENSMUST00000192367	MSTRG.198	Gm37331
ENSMUST00000192371	MSTRG.32841	Gm37581
ENSMUST00000192373	MSTRG.24818	Gm37415
ENSMUST00000192375	MSTRG.2202	2010103J01Rik
ENSMUST00000192379	MSTRG.31852	Gm38346
ENSMUST00000192381	ENSMUSG00000103684	Gm38180
ENSMUST00000192385	ENSMUSG00000104167	A030012G06Rik
ENSMUST00000192386	ENSMUSG00000103625	Gm37357
ENSMUST00000192388	ENSMUSG00000102204	Gm36958
ENSMUST00000192389	MSTRG.33161	Gm37204
ENSMUST00000192390	MSTRG.19489	Gm38000
ENSMUST00000192391	MSTRG.19497	Gm20089
ENSMUST00000192396	MSTRG.899	Gm37738
ENSMUST00000192397	MSTRG.16837	Gm38147
ENSMUST00000192399	MSTRG.1189	Gm37053
ENSMUST00000192402	MSTRG.2047	Gm37960
ENSMUST00000192406	ENSMUSG00000103788	Gm37527
ENSMUST00000192407	MSTRG.2252	A230020J21Rik
ENSMUST00000192408	ENSMUSG00000102398	Gm37580
ENSMUST00000192413	MSTRG.18238	Gm4430
ENSMUST00000192416	ENSMUSG00000102900	Gm37811
ENSMUST00000192418	MSTRG.33032	Gm38091
ENSMUST00000192421	ENSMUSG00000103816	Gm38006
ENSMUST00000192423	ENSMUSG00000102629	Gm29999
ENSMUST00000192431	ENSMUSG00000102420	Gm37464
ENSMUST00000192432	ENSMUSG00000103223	Gm37730
ENSMUST00000192440	MSTRG.15010	Gm37751
ENSMUST00000192441	MSTRG.2157	Gm37018
ENSMUST00000192446	MSTRG.25822	Gm38276
ENSMUST00000192448	MSTRG.215	Gm37895
ENSMUST00000192452	MSTRG.2114	Gm37390
ENSMUST00000192459	ENSMUSG00000104398	Gm37964
ENSMUST00000192464	MSTRG.992	Gm37541
ENSMUST00000192469	ENSMUSG00000103686	Gm37073
ENSMUST00000192473	MSTRG.29976	Gm37844
ENSMUST00000192479	MSTRG.15016	Gm10544
ENSMUST00000192480	MSTRG.316	Gm19863
ENSMUST00000192482	MSTRG.22116	Gm17244
ENSMUST00000192484	ENSMUSG00000104029	Gm37210
ENSMUST00000192487	MSTRG.171	Gm36949
ENSMUST00000192490	ENSMUSG00000103635	Gm38061
ENSMUST00000192498	MSTRG.1963	Gm37065
ENSMUST00000192500	ENSMUSG00000104336	Gm34240
ENSMUST00000192501	MSTRG.15051	Gm26672

ENSMUST00000192502	ENSMUSG00000103440	Gm37131
ENSMUST00000192504	ENSMUSG00000103368	Gm36927
ENSMUST00000192513	ENSMUSG00000103375	Gm37181
ENSMUST00000192514	MSTRG.7411	Gm38355
ENSMUST00000192517	ENSMUSG00000103609	Gm37022
ENSMUST00000192524	ENSMUSG00000103219	Gm37787
ENSMUST00000192526	ENSMUSG00000102410	Gm36935
ENSMUST00000192528	ENSMUSG00000102578	Gm10576
ENSMUST00000192533	MSTRG.29794	Gm37364
ENSMUST00000192536	ENSMUSG00000102963	Gm37945
ENSMUST00000192537	ENSMUSG00000103010	Gm37504
ENSMUST00000192541	MSTRG.872	Gm38062
ENSMUST00000192543	ENSMUSG00000102895	5830415G21Rik
ENSMUST00000192549	MSTRG.33330	Gm37247
ENSMUST00000192556	MSTRG.2270	9430037O13Rik
ENSMUST00000192560	ENSMUSG00000104165	Gm38249
ENSMUST00000192563	ENSMUSG00000102291	Gm37542
ENSMUST00000192568	MSTRG.2170	Gm37885
ENSMUST00000192571	MSTRG.19781	Gm37855
ENSMUST00000192574	MSTRG.19583	Gm38186
ENSMUST00000192580	MSTRG.33223	Gm37563
ENSMUST00000192581	ENSMUSG00000103967	Gm38214
ENSMUST00000192582	MSTRG.1726	Gm37212
ENSMUST00000192586	ENSMUSG00000104382	4930402C01Rik
ENSMUST00000192592	MSTRG.991	Gm38010
ENSMUST00000192600	MSTRG.2276	Gm38037
ENSMUST00000192605	ENSMUSG00000103479	Gm37698
ENSMUST00000192612	ENSMUSG00000103755	Gm37805
ENSMUST00000192613	MSTRG.19683	Gm37897
ENSMUST00000192614	MSTRG.212	8030445P17Rik
ENSMUST00000192618	ENSMUSG00000104189	Gm37822
ENSMUST00000192625	ENSMUSG00000103166	Gm10537
ENSMUST00000192626	ENSMUSG00000102674	8030442B05Rik
ENSMUST00000192629	MSTRG.31847	Gm37082
ENSMUST00000192632	ENSMUSG00000104303	Gm36976
ENSMUST00000192635	ENSMUSG00000102353	Gm38345
ENSMUST00000192636	MSTRG.1136	Gm37171
ENSMUST00000192644	MSTRG.1643	Gm37539
ENSMUST00000192646	MSTRG.31848	Gm38292
ENSMUST00000192647	MSTRG.1264	Gm37717
ENSMUST00000192648	MSTRG.19703	6430573P05Rik
ENSMUST00000192652	MSTRG.307	Gm37135
ENSMUST00000192654	MSTRG.12893	9430053O09Rik
ENSMUST00000192658	MSTRG.1924	Gm20045
ENSMUST00000192660	ENSMUSG00000103710	D030062O11Rik
ENSMUST00000192661	MSTRG.17419	Gm38236
ENSMUST00000192663	MSTRG.12908	9530003O04Rik
ENSMUST00000192669	ENSMUSG00000103992	Gm38029
ENSMUST00000192676	MSTRG.10115	Gm37874
ENSMUST00000192679	ENSMUSG00000103087	Gm38122
ENSMUST00000192680	ENSMUSG00000104363	Gm38081
ENSMUST00000192682	MSTRG.19681	Gm37672
ENSMUST00000192689	MSTRG.22319	A930004J17Rik
ENSMUST00000192691	MSTRG.31200	Gm38250
ENSMUST00000192692	ENSMUSG00000102331	Gm19938
ENSMUST00000192693	MSTRG.1850	Gm32999
ENSMUST00000192701	MSTRG.19491	Gm38252

ENSMUST00000192703	ENSMUSG00000104263	9430062P05Rik
ENSMUST00000192705	ENSMUSG00000103448	Gm37133
ENSMUST00000192713	ENSMUSG00000104410	Gm37066
ENSMUST00000192720	MSTRG.17418	Gm37159
ENSMUST00000192728	MSTRG.11333	Gm37296
ENSMUST00000192733	ENSMUSG00000104297	Gm38046
ENSMUST00000192735	MSTRG.32869	Gm7265
ENSMUST00000192739	MSTRG.10394	Gm38129
ENSMUST00000192740	ENSMUSG00000103610	Gm37947
ENSMUST00000192741	MSTRG.19319	Gm37592
ENSMUST00000192742	MSTRG.2171	Gm37069
ENSMUST00000192743	MSTRG.1325	Gm38330
ENSMUST00000192748	MSTRG.2121	Gm37768
ENSMUST00000192751	ENSMUSG00000104334	Gm38282
ENSMUST00000192752	ENSMUSG00000102236	Gm37887
ENSMUST00000192753	MSTRG.25826	5830405F06Rik
ENSMUST00000192754	MSTRG.32787	Gm37660
ENSMUST00000192756	ENSMUSG00000102280	Gm36999
ENSMUST00000192759	ENSMUSG00000103767	Gm37858
ENSMUST00000192763	MSTRG.15432	Gm37015
ENSMUST00000192767	MSTRG.26377	Gm38320
ENSMUST00000192772	MSTRG.28803	6430511E19Rik
ENSMUST00000192778	ENSMUSG00000103114	Gm32200
ENSMUST00000192781	MSTRG.11331	Gm37320
ENSMUST00000192790	ENSMUSG00000102385	Gm38144
ENSMUST00000192791	MSTRG.24763	Gm38102
ENSMUST00000192794	ENSMUSG00000104520	Gm37336
ENSMUST00000192797	MSTRG.2231	A430027H14Rik
ENSMUST00000192800	MSTRG.25071	Gm38082
ENSMUST00000192805	MSTRG.15051	Gm26672
ENSMUST00000192808	MSTRG.1423	Gm37949
ENSMUST00000192813	MSTRG.3109	Gm37438
ENSMUST00000192814	MSTRG.417	Gm38211
ENSMUST00000192816	MSTRG.853	Gm37262
ENSMUST00000192826	MSTRG.1571	Gm37893
ENSMUST00000192828	MSTRG.32829	Gm37326
ENSMUST00000192833	ENSMUSG00000097971	Gm26917
ENSMUST00000192836	MSTRG.1069	Gm37036
ENSMUST00000192839	MSTRG.852	Gm37902
ENSMUST00000192843	ENSMUSG00000102971	A730062M13Rik
ENSMUST00000192845	MSTRG.19502	Gm37465
ENSMUST00000192846	ENSMUSG00000103612	Gm37948
ENSMUST00000192858	ENSMUSG00000102868	Gm37633
ENSMUST00000192860	MSTRG.2190	Gm37214
ENSMUST00000192863	MSTRG.18654	2900097C17Rik
ENSMUST00000192864	ENSMUSG00000097451	Rian
ENSMUST00000192865	MSTRG.11269	9930014A18Rik
ENSMUST00000192868	ENSMUSG00000103971	Gm37679
ENSMUST00000192879	MSTRG.2256	Gm38188
ENSMUST00000192885	ENSMUSG00000103785	Gm35025
ENSMUST00000192889	ENSMUSG00000103995	5730488B01Rik
ENSMUST00000192891	MSTRG.232	Gm37618
ENSMUST00000192894	ENSMUSG00000097063	Pantr2
ENSMUST00000192895	MSTRG.32801	Gm37023
ENSMUST00000192896	MSTRG.29796	Gm38095
ENSMUST00000192898	MSTRG.19601	Gm26850
ENSMUST00000192900	MSTRG.19161	Gm38001

ENSMUST00000192902	ENSMUSG00000102728	Gm37934
ENSMUST00000192904	MSTRG.1446	Gm37333
ENSMUST00000192908	MSTRG.33358	Gm37678
ENSMUST00000192914	MSTRG.1149	Gm37140
ENSMUST00000192921	MSTRG.24926	B230377A18Rik
ENSMUST00000192922	MSTRG.584	D430013B06Rik
ENSMUST00000192924	MSTRG.254	Gm37127
ENSMUST00000192925	ENSMUSG00000103273	Gm37913
ENSMUST00000192928	ENSMUSG00000104330	Gm38285
ENSMUST00000192930	ENSMUSG00000103531	2610300A13Rik
ENSMUST00000192936	MSTRG.15017	Gm38097
ENSMUST00000192940	MSTRG.19291	Gm33051
ENSMUST00000192941	ENSMUSG00000103437	Gm36994
ENSMUST00000192943	ENSMUSG00000104178	Gm9916
ENSMUST00000192947	ENSMUSG00000104174	Gm37701
ENSMUST00000192952	ENSMUSG00000102488	Gm37920
ENSMUST00000192953	MSTRG.33232	Gm37582
ENSMUST00000192957	MSTRG.1990	Gm36937
ENSMUST00000192958	ENSMUSG00000102227	Gm37302
ENSMUST00000192965	ENSMUSG00000103174	Gm37168
ENSMUST00000192966	ENSMUSG00000103187	Gm37087
ENSMUST00000192968	ENSMUSG00000102784	Gm38251
ENSMUST00000192972	ENSMUSG00000103811	Gm38004
ENSMUST00000192973	ENSMUSG00000103201	Gm37329
ENSMUST00000192974	MSTRG.1035	Gm37297
ENSMUST00000192978	ENSMUSG00000103387	1700030I03Rik
ENSMUST00000192979	MSTRG.30092	B230112G18Rik
ENSMUST00000192981	MSTRG.1899	Gm37502
ENSMUST00000192993	ENSMUSG00000104498	Gm37110
ENSMUST00000192994	MSTRG.19321	Gm37123
ENSMUST00000192996	MSTRG.33309	Gm38150
ENSMUST00000192997	MSTRG.14229	Gm36931
ENSMUST00000192999	ENSMUSG00000103870	Gm38362
ENSMUST00000193001	MSTRG.16558	Gm37881
ENSMUST00000193009	ENSMUSG00000104470	Gm38207
ENSMUST00000193014	ENSMUSG00000104190	Gm10472
ENSMUST00000193015	MSTRG.15030	Gm37446
ENSMUST00000193021	ENSMUSG00000102286	Gm37004
ENSMUST00000193022	MSTRG.634	Gm38012
ENSMUST00000193032	MSTRG.521	Gm37382
ENSMUST00000193034	MSTRG.19690	Gm37256
ENSMUST00000193035	ENSMUSG00000103968	2610509F24Rik
ENSMUST00000193036	MSTRG.20086	Gm38227
ENSMUST00000193037	MSTRG.10600	Gm20236
ENSMUST00000193040	MSTRG.252	Gm38336
ENSMUST00000193041	MSTRG.1253	Gm38283
ENSMUST00000193052	MSTRG.495	A130048G24Rik
ENSMUST00000193054	ENSMUSG00000102985	4930456G14Rik
ENSMUST00000193065	MSTRG.19217	Gm37979
ENSMUST00000193066	MSTRG.34350	Gm38020
ENSMUST00000193067	ENSMUSG00000102740	Gm37599
ENSMUST00000193076	MSTRG.1866	Gm37982
ENSMUST00000193079	MSTRG.200	Gm37862
ENSMUST00000193081	MSTRG.16701	Gm37160
ENSMUST00000193082	ENSMUSG00000102858	Gm37086
ENSMUST00000193086	MSTRG.1854	Gm16565
ENSMUST00000193090	MSTRG.19459	Gm38160

ENSMUST00000193092	ENSMUSG00000102189	Gm37194
ENSMUST00000193095	ENSMUSG00000102880	4930517J16Rik
ENSMUST00000193096	MSTRG.591	Gm37977
ENSMUST00000193099	ENSMUSG00000104313	Gm37911
ENSMUST00000193100	MSTRG.8533	Gm10129
ENSMUST00000193106	ENSMUSG00000097797	Gm26901
ENSMUST00000193107	ENSMUSG00000102152	Gm37475
ENSMUST00000193111	ENSMUSG00000103751	Gm37283
ENSMUST00000193112	MSTRG.32797	Gm37879
ENSMUST00000193114	ENSMUSG00000103785	Gm35025
ENSMUST00000193116	ENSMUSG00000102657	Gm37899
ENSMUST00000193120	ENSMUSG00000102886	Gm30667
ENSMUST00000193122	ENSMUSG00000102572	Gm37966
ENSMUST00000193124	MSTRG.16517	Gm37340
ENSMUST00000193126	ENSMUSG00000102198	Gm38173
ENSMUST00000193127	MSTRG.33359	Gm38366
ENSMUST00000193135	MSTRG.33285	4930524O07Rik
ENSMUST00000193136	MSTRG.18689	Gm37790
ENSMUST00000193139	MSTRG.20934	Gm37689
ENSMUST00000193147	MSTRG.29797	Gm38065
ENSMUST00000193148	ENSMUSG00000104209	Gm37400
ENSMUST00000193156	MSTRG.23698	4932441J04Rik
ENSMUST00000193157	MSTRG.278	Gm37909
ENSMUST00000193163	ENSMUSG00000103608	4930442P19Rik
ENSMUST00000193166	MSTRG.1126	Gm37642
ENSMUST00000193169	MSTRG.394	Gm38228
ENSMUST00000193173	MSTRG.1396	Gm37368
ENSMUST00000193176	MSTRG.742	Gm37456
ENSMUST00000193177	MSTRG.1041	Gm37521
ENSMUST00000193178	MSTRG.848	Gm10555
ENSMUST00000193181	MSTRG.1828	Gm38368
ENSMUST00000193187	MSTRG.2061	Gm26801
ENSMUST00000193191	MSTRG.1732	Gm37328
ENSMUST00000193192	ENSMUSG00000103726	Gm30074
ENSMUST00000193193	MSTRG.33362	4833445I07Rik
ENSMUST00000193194	ENSMUSG00000103149	AA914427
ENSMUST00000193198	ENSMUSG00000102628	Gm37671
ENSMUST00000193199	MSTRG.2115	Gm37033
ENSMUST00000193202	ENSMUSG00000022116	4930449E01Rik
ENSMUST00000193207	MSTRG.32844	Gm37983
ENSMUST00000193208	MSTRG.10499	Dleu2
ENSMUST00000193209	MSTRG.2123	Gm38293
ENSMUST00000193214	ENSMUSG00000104277	Gm38299
ENSMUST00000193223	ENSMUSG00000104486	Gm38066
ENSMUST00000193225	MSTRG.14948	Gm36989
ENSMUST00000193239	MSTRG.1767	Gm37767
ENSMUST00000193241	ENSMUSG00000104046	Gm37567
ENSMUST00000193245	MSTRG.2199	Gm34882
ENSMUST00000193246	MSTRG.19747	Gm37726
ENSMUST00000193253	MSTRG.17424	Gm38377
ENSMUST00000193256	MSTRG.8171	Gm6556
ENSMUST00000193259	ENSMUSG00000102330	Gm37234
ENSMUST00000193261	MSTRG.1474	D130019J16Rik
ENSMUST00000193263	ENSMUSG00000103143	Gm37742
ENSMUST00000193264	ENSMUSG00000102715	Gm6209
ENSMUST00000193265	ENSMUSG00000104362	Gm37928
ENSMUST00000193280	MSTRG.13665	Gm38099

ENSMUST00000193281	MSTRG.46	Gm37005
ENSMUST00000193283	ENSMUSG00000104230	Gm37591
ENSMUST00000193284	MSTRG.1316	Gm38026
ENSMUST00000193285	MSTRG.2188	Gm37986
ENSMUST00000193293	ENSMUSG00000104088	Gm38275
ENSMUST00000193295	MSTRG.9400	Gm36638
ENSMUST00000193300	MSTRG.14230	Gm37176
ENSMUST00000193302	MSTRG.16566	Gm37255
ENSMUST00000193304	MSTRG.1238	Gm37745
ENSMUST00000193312	ENSMUSG00000104403	Gm38011
ENSMUST00000193314	ENSMUSG00000102652	Gm37078
ENSMUST00000193315	MSTRG.4168	2610001A08Rik
ENSMUST00000193317	ENSMUSG00000102408	4930568G15Rik
ENSMUST00000193333	MSTRG.258	Gm37354
ENSMUST00000193336	MSTRG.19289	Gm32950
ENSMUST00000193345	ENSMUSG00000103595	Gm37355
ENSMUST00000193347	MSTRG.1650	Gm9694
ENSMUST00000193349	MSTRG.16565	Gm38014
ENSMUST00000193350	ENSMUSG00000103966	Gm37120
ENSMUST00000193352	MSTRG.10612	Gm37094
ENSMUST00000193357	MSTRG.25826	5830405F06Rik
ENSMUST00000193362	ENSMUSG00000103599	Gm37356
ENSMUST00000193369	MSTRG.930	Gm37152
ENSMUST00000193378	MSTRG.1507	Gm37470
ENSMUST00000193380	ENSMUSG00000103973	BC055308
ENSMUST00000193381	ENSMUSG00000103189	Gm37092
ENSMUST00000193385	ENSMUSG00000102178	Gm37869
ENSMUST00000193387	MSTRG.19285	1700112D23Rik
ENSMUST00000193392	MSTRG.199	Gm37211
ENSMUST00000193393	MSTRG.894	Gm37058
ENSMUST00000193395	ENSMUSG00000103692	4930503O07Rik
ENSMUST00000193407	ENSMUSG00000102473	Gm37243
ENSMUST00000193409	ENSMUSG00000104327	Gm37322
ENSMUST00000193413	MSTRG.33170	Gm3081
ENSMUST00000193416	ENSMUSG00000104220	4930578I07Rik
ENSMUST00000193420	MSTRG.31268	C630050I24Rik
ENSMUST00000193428	ENSMUSG00000097797	Gm26901
ENSMUST00000193429	MSTRG.31703	Gm16754
ENSMUST00000193430	ENSMUSG00000104459	Gm37824
ENSMUST00000193434	MSTRG.1445	2610012C04Rik
ENSMUST00000193444	MSTRG.2260	Gm37074
ENSMUST00000193447	ENSMUSG00000103043	Gm37306
ENSMUST00000193450	ENSMUSG00000104238	Gm37587
ENSMUST00000193457	ENSMUSG00000102297	5730585A16Rik
ENSMUST00000193458	ENSMUSG00000102581	Gm37443
ENSMUST00000193461	MSTRG.1712	Gm37294
ENSMUST00000193462	MSTRG.21856	Gm20731
ENSMUST00000193467	ENSMUSG00000103911	Gm38074
ENSMUST00000193469	MSTRG.19486	3110080O07Rik
ENSMUST00000193471	ENSMUSG00000104037	Gm37776
ENSMUST00000193473	MSTRG.1733	Gm38329
ENSMUST00000193475	ENSMUSG00000103394	Gm10152
ENSMUST00000193481	ENSMUSG00000102756	Gm37054
ENSMUST00000193492	ENSMUSG00000103545	1700012J22Rik
ENSMUST00000193494	ENSMUSG00000103385	Gm37752
ENSMUST00000193498	ENSMUSG00000102207	Gm10344
ENSMUST00000193499	ENSMUSG00000103786	Gm37525

ENSMUST00000193501	MSTRG.32816	Gm37710
ENSMUST00000193506	MSTRG.12896	Gm36963
ENSMUST00000193507	MSTRG.19689	Gm37685
ENSMUST00000193511	ENSMUSG00000102404	5530400K19Rik
ENSMUST00000193513	MSTRG.12043	1700016D08Rik
ENSMUST00000193520	MSTRG.6281	Gm3807
ENSMUST00000193524	MSTRG.19444	Gm37498
ENSMUST00000193527	ENSMUSG00000103325	Gm10531
ENSMUST00000193528	ENSMUSG00000103486	Gm10657
ENSMUST00000193530	MSTRG.12887	Mir99ahg
ENSMUST00000193532	MSTRG.1651	9430034N14Rik
ENSMUST00000193534	ENSMUSG00000103762	Gm37861
ENSMUST00000193537	MSTRG.12907	C130023A14Rik
ENSMUST00000193540	ENSMUSG00000102278	Gm37145
ENSMUST00000193542	MSTRG.2219	Gm37254
ENSMUST00000193544	ENSMUSG00000102574	Gm33206
ENSMUST00000193545	MSTRG.33179	Gm37314
ENSMUST00000193546	MSTRG.12890	Gm21816
ENSMUST00000193553	MSTRG.28957	Gm37716
ENSMUST00000193555	MSTRG.721	Gm38218
ENSMUST00000193561	ENSMUSG00000103158	Gm31925
ENSMUST00000193563	MSTRG.33216	Gm37750
ENSMUST00000193564	MSTRG.26379	Gm37736
ENSMUST00000193570	MSTRG.2312	Gm37650
ENSMUST00000193572	MSTRG.32972	Gm38230
ENSMUST00000193573	MSTRG.210	Gm38260
ENSMUST00000193574	MSTRG.20645	Gm37228
ENSMUST00000193576	MSTRG.14229	Gm37248
ENSMUST00000193577	MSTRG.1177	Gm37372
ENSMUST00000193580	ENSMUSG00000102181	Gm37190
ENSMUST00000193581	ENSMUSG00000102595	Gm30097
ENSMUST00000193592	ENSMUSG00000102682	Gm37223
ENSMUST00000193594	MSTRG.1886	Gm38381
ENSMUST00000193595	MSTRG.19817	Gm37584
ENSMUST00000193596	MSTRG.16723	Gm37416
ENSMUST00000193601	MSTRG.16729	2900092N22Rik
ENSMUST00000193604	ENSMUSG00000104183	1700054O19Rik
ENSMUST00000193605	MSTRG.110	Gm37444
ENSMUST00000193607	ENSMUSG00000103754	Gm37284
ENSMUST00000193609	MSTRG.19594	Gm37589
ENSMUST00000193614	MSTRG.484	E330011M16Rik
ENSMUST00000193616	ENSMUSG00000103266	Gm36979
ENSMUST00000193617	ENSMUSG00000102380	Gm38140
ENSMUST00000193623	ENSMUSG00000103494	Gm37410
ENSMUST00000193624	ENSMUSG00000102801	Gm37478
ENSMUST00000193626	MSTRG.33283	Gm37612
ENSMUST00000193635	MSTRG.53	Gm37629
ENSMUST00000193649	ENSMUSG00000102498	Gm19445
ENSMUST00000193657	MSTRG.33224	Gm38077
ENSMUST00000193663	MSTRG.32955	Gm37307
ENSMUST00000193668	MSTRG.33316	Gm37974
ENSMUST00000193672	ENSMUSG00000103695	A830029E22Rik
ENSMUST00000193674	ENSMUSG00000104366	4933409D19Rik
ENSMUST00000193684	MSTRG.19640	Gm37558
ENSMUST00000193685	MSTRG.16702	Gm13270
ENSMUST00000193688	MSTRG.1044	Gm36970
ENSMUST00000193689	MSTRG.33214	Gm37200

ENSMUST00000193690	MSTRG.19429	Gm38047
ENSMUST00000193691	MSTRG.378	Gm37623
ENSMUST00000193705	ENSMUSG00000104520	Gm37336
ENSMUST00000193707	ENSMUSG00000103259	2610105M22Rik
ENSMUST00000193717	MSTRG.17422	Gm37747
ENSMUST00000193724	ENSMUSG00000104509	Gm33994
ENSMUST00000193728	MSTRG.1850	Gm37469
ENSMUST00000193732	ENSMUSG00000097797	Gm26901
ENSMUST00000193733	MSTRG.631	Gm37039
ENSMUST00000193735	ENSMUSG00000102549	Gm38137
ENSMUST00000193744	MSTRG.1309	Gm38248
ENSMUST00000193746	MSTRG.28374	Gm38059
ENSMUST00000193748	ENSMUSG00000102702	Gm37260
ENSMUST00000193750	MSTRG.1699	Gm37115
ENSMUST00000193753	ENSMUSG00000103642	Gm37769
ENSMUST00000193760	MSTRG.2048	Gm37106
ENSMUST00000193762	ENSMUSG00000104423	A030005K14Rik
ENSMUST00000193764	ENSMUSG00000102745	Gm37600
ENSMUST00000193770	MSTRG.1474	Gm37298
ENSMUST00000193775	MSTRG.1820	Gm37403
ENSMUST00000193778	MSTRG.19606	Gm38048
ENSMUST00000193782	MSTRG.33300	Gm34106
ENSMUST00000193794	MSTRG.2240	Gm37422
ENSMUST00000193802	MSTRG.14230	Gm37266
ENSMUST00000193809	MSTRG.3998	Tug1
ENSMUST00000193813	ENSMUSG00000102785	Gm2447
ENSMUST00000193814	ENSMUSG00000103066	Gm36967
ENSMUST00000193815	MSTRG.15049	Gm37165
ENSMUST00000193816	MSTRG.11387	Gm37042
ENSMUST00000193817	MSTRG.15469	Gm37216
ENSMUST00000193819	MSTRG.2058	Gm37463
ENSMUST00000193833	ENSMUSG00000103040	Gm37304
ENSMUST00000193837	ENSMUSG00000103196	Gm37641
ENSMUST00000193840	MSTRG.1326	Gm36951
ENSMUST00000193842	MSTRG.33324	Gm37436
ENSMUST00000193851	MSTRG.1330	Gm37064
ENSMUST00000193853	MSTRG.296	Gm33533
ENSMUST00000193857	ENSMUSG00000103321	4933403L11Rik
ENSMUST00000193863	ENSMUSG00000102249	4930423C22Rik
ENSMUST00000193864	MSTRG.28688	Gm37829
ENSMUST00000193871	ENSMUSG00000102894	Gm37851
ENSMUST00000193873	MSTRG.116	Gm38116
ENSMUST00000193875	MSTRG.31703	Gm16754
ENSMUST00000193881	ENSMUSG00000104068	Gm37199
ENSMUST00000193885	ENSMUSG00000102766	Gm6420
ENSMUST00000193887	MSTRG.1706	Gm37088
ENSMUST00000193896	ENSMUSG00000103513	Gm34780
ENSMUST00000193900	MSTRG.32846	Gm37150
ENSMUST00000193902	MSTRG.18237	Gm4430
ENSMUST00000193903	MSTRG.13034	Gm37259
ENSMUST00000193908	MSTRG.30884	Gm37352
ENSMUST00000193913	MSTRG.31899	Gm33142
ENSMUST00000193915	MSTRG.19471	Gm38034
ENSMUST00000193926	ENSMUSG00000103998	Gm38025
ENSMUST00000193928	MSTRG.513	Gm38056
ENSMUST00000193929	ENSMUSG00000104281	Gm37461
ENSMUST00000193939	MSTRG.1455	Gm37552

ENSMUST00000193945	ENSMUSG00000103713	Gm2136
ENSMUST00000193946	MSTRG.1729	6720464F23Rik
ENSMUST00000193951	ENSMUSG00000102229	Gm37299
ENSMUST00000193955	MSTRG.1818	Gm32569
ENSMUST00000193956	MSTRG.16447	Gm10862
ENSMUST00000193958	MSTRG.1683	Gm37788
ENSMUST00000193962	MSTRG.1711	4933417C20Rik
ENSMUST00000193964	MSTRG.19731	Gm37933
ENSMUST00000193968	MSTRG.1510	Gm37655
ENSMUST00000193969	ENSMUSG00000102975	Gm37347
ENSMUST00000193978	ENSMUSG00000104075	5430433H01Rik
ENSMUST00000193983	MSTRG.269	Gm33280
ENSMUST00000193986	ENSMUSG00000104009	Gm38337
ENSMUST00000193993	MSTRG.19260	Gm37573
ENSMUST00000193999	ENSMUSG00000103794	4930590H14Rik
ENSMUST00000194000	MSTRG.1853	Gm36972
ENSMUST00000194005	ENSMUSG00000102685	Gm37373
ENSMUST00000194008	MSTRG.19208	Gm33819
ENSMUST00000194009	MSTRG.14229	5830444F18Rik
ENSMUST00000194017	ENSMUSG00000102689	Gm37217
ENSMUST00000194018	MSTRG.1469	Gm37799
ENSMUST00000194021	ENSMUSG00000104187	Gm37820
ENSMUST00000194040	ENSMUSG00000102834	1700029B24Rik
ENSMUST00000194052	MSTRG.15	Gm37277
ENSMUST00000194053	MSTRG.1688	Gm37943
ENSMUST00000194058	MSTRG.20329	Gm38253
ENSMUST00000194067	ENSMUSG00000104080	Gm38279
ENSMUST00000194074	ENSMUSG00000102981	Gm38322
ENSMUST00000194090	MSTRG.16456	Gm36988
ENSMUST00000194093	MSTRG.990	Gm38142
ENSMUST00000194096	ENSMUSG00000103015	Gm37549
ENSMUST00000194099	ENSMUSG00000103025	Gm37686
ENSMUST00000194104	MSTRG.33362	4833445I07Rik
ENSMUST00000194105	MSTRG.1719	Gm36975
ENSMUST00000194107	ENSMUSG00000102266	Gm38106
ENSMUST00000194109	ENSMUSG00000103652	Gm37209
ENSMUST00000194110	MSTRG.17423	Gm37666
ENSMUST00000194112	MSTRG.33370	Gm38163
ENSMUST00000194113	MSTRG.10254	Gm37271
ENSMUST00000194114	MSTRG.18	Gm37079
ENSMUST00000194117	MSTRG.16699	Gm37780
ENSMUST00000194121	MSTRG.2050	Gm38146
ENSMUST00000194123	MSTRG.18187	Gm10766
ENSMUST00000194131	ENSMUSG00000102591	Gm38383
ENSMUST00000194133	ENSMUSG00000073535	Gm5532
ENSMUST00000194135	MSTRG.33173	Gm37553
ENSMUST00000194136	MSTRG.590	Gm38125
ENSMUST00000194137	MSTRG.21239	Gm21955
ENSMUST00000194138	ENSMUSG00000097451	Rian
ENSMUST00000194140	MSTRG.33327	4930447F24Rik
ENSMUST00000194141	ENSMUSG00000104033	Gm37773
ENSMUST00000194143	MSTRG.1948	A630035G10Rik
ENSMUST00000194146	MSTRG.33294	A930036K24Rik
ENSMUST00000194148	MSTRG.32949	Gm29054
ENSMUST00000194151	MSTRG.1274	Gm37510
ENSMUST00000194154	ENSMUSG00000103927	Gm9932
ENSMUST00000194155	ENSMUSG00000102717	Gm37759

ENSMUST00000194169	MSTRG.1329	Gm37479
ENSMUST00000194177	MSTRG.2317	Gm37674
ENSMUST00000194178	MSTRG.261	Gm38157
ENSMUST00000194180	MSTRG.1814	Gm37411
ENSMUST00000194198	ENSMUSG00000104109	Gm30292
ENSMUST00000194200	ENSMUSG00000104304	Gm36974
ENSMUST00000194202	MSTRG.32841	Gm37303
ENSMUST00000194203	MSTRG.13190	Gm10513
ENSMUST00000194205	MSTRG.2272	Gm37349
ENSMUST00000194215	MSTRG.14229	E430014B02Rik
ENSMUST00000194216	MSTRG.22885	Gm37795
ENSMUST00000194221	ENSMUSG00000102690	Gm37779
ENSMUST00000194222	ENSMUSG00000102608	Gm37267
ENSMUST00000194223	MSTRG.19589	Gm37696
ENSMUST00000194227	MSTRG.1463	9230116N13Rik
ENSMUST00000194243	MSTRG.25608	Gm37940
ENSMUST00000194245	MSTRG.2076	Gm38300
ENSMUST00000194246	MSTRG.1500	Gm37423
ENSMUST00000194249	MSTRG.303	F830112A20Rik
ENSMUST00000194255	MSTRG.23608	Gm36401
ENSMUST00000194261	ENSMUSG00000102531	1110002O04Rik
ENSMUST00000194270	MSTRG.19841	Gm10253
ENSMUST00000194274	ENSMUSG00000104401	Gm20750
ENSMUST00000194275	MSTRG.31857	Gm37758
ENSMUST00000194281	MSTRG.32499	Gm36940
ENSMUST00000194283	MSTRG.33327	4930447F24Rik
ENSMUST00000194285	MSTRG.869	Gm37645
ENSMUST00000194286	ENSMUSG00000103732	Gm38315
ENSMUST00000194287	MSTRG.9793	C130012C08Rik
ENSMUST00000194289	MSTRG.33010	Gm37560
ENSMUST00000194292	MSTRG.19493	Gm10728
ENSMUST00000194293	MSTRG.16511	Gm38386
ENSMUST00000194295	ENSMUSG00000102794	Gm37712
ENSMUST00000194308	ENSMUSG00000103402	Gm37930
ENSMUST00000194311	ENSMUSG00000104057	Gm38130
ENSMUST00000194316	ENSMUSG00000104136	Gm36955
ENSMUST00000194317	MSTRG.302	Gm38115
ENSMUST00000194318	MSTRG.19448	Gm38246
ENSMUST00000194322	MSTRG.1819	Gm20743
ENSMUST00000194323	ENSMUSG00000102140	Gm38058
ENSMUST00000194331	MSTRG.2155	2700078F05Rik
ENSMUST00000194332	ENSMUSG00000102319	Gm37626
ENSMUST00000194334	ENSMUSG00000103744	Gm38238
ENSMUST00000194336	MSTRG.32982	Gm17530
ENSMUST00000194337	ENSMUSG00000102860	Gm37638
ENSMUST00000194339	MSTRG.1453	A430034D21Rik
ENSMUST00000194340	MSTRG.19313	9530022L04Rik
ENSMUST00000194342	MSTRG.33022	Gm37833
ENSMUST00000194351	MSTRG.31883	Gm38379
ENSMUST00000194356	ENSMUSG00000103102	Gm37049
ENSMUST00000194357	MSTRG.103	Gm37702
ENSMUST00000194358	MSTRG.31862	Gm38217
ENSMUST00000194360	MSTRG.19474	Gm37261
ENSMUST00000194364	MSTRG.32820	Gm38192
ENSMUST00000194368	MSTRG.19254	Gm37136
ENSMUST00000194370	ENSMUSG00000102536	1700039I01Rik
ENSMUST00000194375	MSTRG.2257	D730003I15Rik

ENSMUST00000194378	MSTRG.33202	5830418P13Rik
ENSMUST00000194379	MSTRG.1898	Gm37748
ENSMUST00000194396	ENSMUSG00000103881	Gm10748
ENSMUST00000194403	ENSMUSG00000104487	Gm31373
ENSMUST00000194406	ENSMUSG00000103383	Gm37754
ENSMUST00000194416	ENSMUSG00000103882	Gm37452
ENSMUST00000194417	MSTRG.898	Gm38374
ENSMUST00000194420	ENSMUSG00000103136	Gm37980
ENSMUST00000194422	ENSMUSG00000102168	Gm36943
ENSMUST00000194425	ENSMUSG00000104236	4930533L02Rik
ENSMUST00000194426	MSTRG.290	D430040D24Rik
ENSMUST00000194431	MSTRG.740	Gm38272
ENSMUST00000194442	ENSMUSG00000103424	Gm37535
ENSMUST00000194444	MSTRG.2229	2900042K21Rik
ENSMUST00000194451	MSTRG.16738	Gm37437
ENSMUST00000194459	MSTRG.1615	Gm38352
ENSMUST00000194465	MSTRG.714	Gm37285
ENSMUST00000194467	MSTRG.1498	A130071D04Rik
ENSMUST00000194468	MSTRG.1948	A630035G10Rik
ENSMUST00000194472	ENSMUSG00000102681	Gm37221
ENSMUST00000194474	MSTRG.84	1700047N06Rik
ENSMUST00000194479	ENSMUSG00000102184	Gm37192
ENSMUST00000194483	MSTRG.1474	Gm37534
ENSMUST00000194484	MSTRG.102	Gm38223
ENSMUST00000194486	ENSMUSG00000104454	Gm37827
ENSMUST00000194487	MSTRG.2335	Gm37366
ENSMUST00000194488	ENSMUSG00000103170	Gm37170
ENSMUST00000194490	MSTRG.19771	Gm37876
ENSMUST00000194491	MSTRG.33331	Gm37230
ENSMUST00000194497	MSTRG.19328	Gm37353
ENSMUST00000194500	ENSMUSG00000104329	Gm37324
ENSMUST00000194502	MSTRG.88	Gm38178
ENSMUST00000194503	ENSMUSG00000104340	Gm10522
ENSMUST00000194506	MSTRG.2049	Gm37598
ENSMUST00000194507	ENSMUSG00000103583	Gm38325
ENSMUST00000194508	MSTRG.32871	Gm36936
ENSMUST00000194512	MSTRG.3211	Gm37514
ENSMUST00000194514	ENSMUSG00000102362	4930509J09Rik
ENSMUST00000194515	ENSMUSG00000102999	Gm37762
ENSMUST00000194517	ENSMUSG00000103539	Gm37834
ENSMUST00000194519	ENSMUSG00000103012	Gm37548
ENSMUST00000194520	MSTRG.1425	Gm37677
ENSMUST00000194525	MSTRG.13187	Gm37402
ENSMUST00000194526	MSTRG.23973	A730089K16Rik
ENSMUST00000194529	ENSMUSG00000102307	Gm38194
ENSMUST00000194530	ENSMUSG00000102144	4930429P21Rik
ENSMUST00000194532	MSTRG.12888	Gm31258
ENSMUST00000194534	MSTRG.19506	C130089K02Rik
ENSMUST00000194535	MSTRG.209	Gm37051
ENSMUST00000194537	MSTRG.98	Gm38319
ENSMUST00000194539	MSTRG.1953	Gm37010
ENSMUST00000194541	MSTRG.25650	Rncr4
ENSMUST00000194547	ENSMUSG00000102144	4930429P21Rik
ENSMUST00000194551	ENSMUSG00000103060	Gm36969
ENSMUST00000194553	MSTRG.12887	Mir99ahg
ENSMUST00000194564	MSTRG.118	Gm37138
ENSMUST00000194567	MSTRG.1754	Gm37362

ENSMUST00000194570	MSTRG.1752	Gm37072
ENSMUST00000194571	MSTRG.1731	Gm37873
ENSMUST00000194572	MSTRG.19181	A930014E01Rik
ENSMUST00000194573	MSTRG.33322	Gm37850
ENSMUST00000194579	MSTRG.26131	Gm37397
ENSMUST00000194584	MSTRG.2328	A330023F24Rik
ENSMUST00000194586	MSTRG.2318	Gm38036
ENSMUST00000194588	MSTRG.19477	Gm37342
ENSMUST00000194590	ENSMUSG00000103140	Gm37495
ENSMUST00000194594	ENSMUSG00000103071	Gm38110
ENSMUST00000194599	MSTRG.32802	A430027C01Rik
ENSMUST00000194600	MSTRG.1182	Gm38235
ENSMUST00000194601	MSTRG.16697	Gm38156
ENSMUST00000194607	MSTRG.2150	Gm37664
ENSMUST00000194609	ENSMUSG00000102360	Gm37043
ENSMUST00000194610	MSTRG.33090	Gm38375
ENSMUST00000194616	ENSMUSG00000103145	Gm37744
ENSMUST00000194628	MSTRG.33302	Gm9917
ENSMUST00000194629	ENSMUSG00000102715	Gm6209
ENSMUST00000194631	ENSMUSG00000102926	Gm37151
ENSMUST00000194637	MSTRG.10909	BC037032
ENSMUST00000194640	MSTRG.19499	Gm37526
ENSMUST00000194642	MSTRG.2333	Gm37027
ENSMUST00000194644	MSTRG.211	Gm37628
ENSMUST00000194645	ENSMUSG00000103142	Gm37743
ENSMUST00000194646	ENSMUSG00000104420	E230020A03Rik
ENSMUST00000194647	ENSMUSG00000102723	Gm37936
ENSMUST00000194648	MSTRG.719	6820402A03Rik
ENSMUST00000194651	MSTRG.18910	AY702102
ENSMUST00000194653	ENSMUSG00000102934	Gm38117
ENSMUST00000194659	MSTRG.1818	Gm32569
ENSMUST00000194661	MSTRG.11332	Gm26621
ENSMUST00000194665	MSTRG.19493	Gm10728
ENSMUST00000194667	ENSMUSG00000102995	A330074H02Rik
ENSMUST00000194678	MSTRG.12894	Gm37063
ENSMUST00000194679	MSTRG.17420	Gm37651
ENSMUST00000194683	MSTRG.16714	Gm37499
ENSMUST00000194686	MSTRG.780	Gm37931
ENSMUST00000194697	MSTRG.32948	Gm2065
ENSMUST00000194699	MSTRG.1255	Gm37174
ENSMUST00000194702	ENSMUSG00000102287	Gm37003
ENSMUST00000194706	MSTRG.26924	Gm7292
ENSMUST00000194708	MSTRG.32852	Gm36948
ENSMUST00000194709	ENSMUSG00000102166	Gm36947
ENSMUST00000194710	MSTRG.395	Gm37536
ENSMUST00000194715	MSTRG.635	Gm38278
ENSMUST00000194716	MSTRG.33176	Gm37621
ENSMUST00000194720	ENSMUSG00000102761	Gm37184
ENSMUST00000194722	ENSMUSG00000102619	Gm37849
ENSMUST00000194726	MSTRG.562	Gm37760
ENSMUST00000194728	ENSMUSG00000104211	Gm37985
ENSMUST00000194736	MSTRG.25605	Gm37978
ENSMUST00000194737	MSTRG.173	Gm38243
ENSMUST00000194744	MSTRG.858	Gm19552
ENSMUST00000194752	MSTRG.21219	1700045I11Rik
ENSMUST00000194753	MSTRG.11722	Gm37699
ENSMUST00000194758	ENSMUSG00000103354	Gm38083

ENSMUST00000194760	MSTRG.1354	Gm38262
ENSMUST00000194761	MSTRG.1751	Gm37729
ENSMUST00000194763	ENSMUSG00000104041	Gm37562
ENSMUST00000194765	MSTRG.16534	C630004M23Rik
ENSMUST00000194771	MSTRG.16710	Stamos
ENSMUST00000194773	ENSMUSG00000102521	Gm37781
ENSMUST00000194780	MSTRG.14230	Gm6934
ENSMUST00000194790	MSTRG.145	4921511E07Rik
ENSMUST00000194792	ENSMUSG00000103116	4930539M17Rik
ENSMUST00000194793	MSTRG.1321	Gm37407
ENSMUST00000194803	ENSMUSG00000104331	Gm38284
ENSMUST00000194804	MSTRG.1489	Gm37080
ENSMUST00000194809	ENSMUSG00000102564	Gm37035
ENSMUST00000194811	MSTRG.27352	5330439B14Rik
ENSMUST00000194812	MSTRG.31847	Gm37474
ENSMUST00000194817	MSTRG.32792	Gm38111
ENSMUST00000194820	MSTRG.633	Gm37205
ENSMUST00000194827	MSTRG.1067	Gm37048
ENSMUST00000194830	MSTRG.10622	Gm37513
ENSMUST00000194835	MSTRG.1910	Gm38204
ENSMUST00000194837	ENSMUSG00000102500	Gm37371
ENSMUST00000194838	MSTRG.10158	Gm38316
ENSMUST00000194845	ENSMUSG00000103810	Gm38005
ENSMUST00000194854	MSTRG.30	Gm38372
ENSMUST00000194864	MSTRG.30938	1700051O22Rik
ENSMUST00000194866	ENSMUSG00000103492	Gm37412
ENSMUST00000194867	MSTRG.121	Gm37509
ENSMUST00000194874	MSTRG.1474	Gm26930
ENSMUST00000194880	ENSMUSG00000102623	Gm37673
ENSMUST00000194883	MSTRG.16544	Gm37520
ENSMUST00000194884	MSTRG.1598	Gm38009
ENSMUST00000194886	MSTRG.33	Gm30414
ENSMUST00000194889	MSTRG.12665	Gm36939
ENSMUST00000194891	MSTRG.5356	Gm37391
ENSMUST00000194892	ENSMUSG00000102716	Gm5099
ENSMUST00000194893	MSTRG.14229	C230085N15Rik
ENSMUST00000194899	MSTRG.20861	Gm37482
ENSMUST00000194907	ENSMUSG00000102796	Gm37711
ENSMUST00000194910	MSTRG.1198	Gm38255
ENSMUST00000194911	ENSMUSG00000102405	Gm37863
ENSMUST00000194916	MSTRG.818	Gm37264
ENSMUST00000194919	MSTRG.1099	Gm37250
ENSMUST00000194920	MSTRG.264	A930005N03Rik
ENSMUST00000194923	MSTRG.216	Gm36952
ENSMUST00000194925	MSTRG.543	Gm37607
ENSMUST00000194927	MSTRG.1727	Gm37215
ENSMUST00000194929	MSTRG.1746	Gm37052
ENSMUST00000194930	ENSMUSG00000103258	Gm37518
ENSMUST00000194934	ENSMUSG00000102709	Gm38308
ENSMUST00000194937	MSTRG.12891	9430092D12Rik
ENSMUST00000194938	MSTRG.10601	Gm31748
ENSMUST00000194944	ENSMUSG00000103346	C230057A21Rik
ENSMUST00000194945	MSTRG.2114	Gm36933
ENSMUST00000194946	ENSMUSG00000102467	Gm37167
ENSMUST00000194952	MSTRG.4165	Gm37818
ENSMUST00000194954	ENSMUSG00000102684	Gm37374
ENSMUST00000194955	ENSMUSG00000104343	5730408A14Rik

ENSMUST00000194956	MSTRG.19508	5430420F09Rik
ENSMUST00000194957	MSTRG.13684	Gm9874
ENSMUST00000194958	ENSMUSG00000103331	Gm37995
ENSMUST00000194966	MSTRG.2319	2900035J10Rik
ENSMUST00000194969	MSTRG.1314	Gm37625
ENSMUST00000194970	ENSMUSG00000102946	Gm37883
ENSMUST00000194973	ENSMUSG00000102176	Gm37871
ENSMUST00000194987	MSTRG.16735	Gm38273
ENSMUST00000194988	MSTRG.1901	4930500M09Rik
ENSMUST00000194989	ENSMUSG00000102718	Gm37761
ENSMUST00000194990	ENSMUSG00000103080	Gm38123
ENSMUST00000194991	MSTRG.32986	Gm37012
ENSMUST00000194994	MSTRG.333	Gm37309
ENSMUST00000194997	MSTRG.1686	Gm37060
ENSMUST00000194998	MSTRG.1688	Gm37802
ENSMUST00000195000	MSTRG.33901	Gm37564
ENSMUST00000195003	ENSMUSG00000102815	Gm37813
ENSMUST00000195007	ENSMUSG00000104283	Gm37459
ENSMUST00000195013	ENSMUSG00000104502	Gm37737
ENSMUST00000195024	MSTRG.1319	Gm37588
ENSMUST00000195025	MSTRG.25677	Gm37728
ENSMUST00000195028	MSTRG.330	Gm37062
ENSMUST00000195035	ENSMUSG00000103965	Gm30173
ENSMUST00000195039	ENSMUSG00000102516	Gm38340
ENSMUST00000195048	MSTRG.1644	Gm37571
ENSMUST00000195050	MSTRG.1747	Gm37809
ENSMUST00000195052	MSTRG.33212	Gm38032
ENSMUST00000195053	MSTRG.29786	Gm38321
ENSMUST00000195064	MSTRG.1659	4930518J20Rik
ENSMUST00000195065	ENSMUSG00000103571	Gm37114
ENSMUST00000195066	ENSMUSG00000103809	Gm37061
ENSMUST00000195070	ENSMUSG00000102682	Gm37223
ENSMUST00000195083	MSTRG.2291	Gm10516
ENSMUST00000195090	ENSMUSG00000103775	Gm36997
ENSMUST00000195097	ENSMUSG00000103172	Gm37148
ENSMUST00000195098	MSTRG.32870	Gm37955
ENSMUST00000195108	MSTRG.31200	Gm38250
ENSMUST00000195109	ENSMUSG00000102542	Gm38133
ENSMUST00000195110	MSTRG.1324	Gm38240
ENSMUST00000195116	ENSMUSG00000103401	Gm37929
ENSMUST00000195118	MSTRG.430	Gm37393
ENSMUST00000195121	MSTRG.2026	Gm38104
ENSMUST00000195122	MSTRG.33164	Gm38297
ENSMUST00000195128	MSTRG.201	Gm38286
ENSMUST00000195131	MSTRG.19461	Gm37968
ENSMUST00000195134	MSTRG.379	Gm37915
ENSMUST00000195138	MSTRG.32805	Gm37137
ENSMUST00000195141	ENSMUSG00000103914	Gm38073
ENSMUST00000195143	ENSMUSG00000102495	Gm26524
ENSMUST00000195146	ENSMUSG00000103559	Gm38221
ENSMUST00000195148	ENSMUSG00000103451	Gm33973
ENSMUST00000195153	MSTRG.10609	Gm38378
ENSMUST00000195166	ENSMUSG00000103161	Gm38148
ENSMUST00000195168	ENSMUSG00000104417	Gm37068
ENSMUST00000195172	ENSMUSG00000104087	Gm38277
ENSMUST00000195173	MSTRG.20874	4930566N20Rik
ENSMUST00000195178	ENSMUSG00000103482	Gm37999

ENSMUST00000195179	ENSMUSG00000102686	Gm37220
ENSMUST00000195180	MSTRG.57	Gm37143
ENSMUST00000195185	MSTRG.33126	Gm37249
ENSMUST00000195187	MSTRG.217	Gm37602
ENSMUST00000195193	MSTRG.16855	Gm38287
ENSMUST00000195204	MSTRG.11012	Gm38234
ENSMUST00000195208	MSTRG.34362	Gm37956
ENSMUST00000195213	ENSMUSG00000102614	Gm37843
ENSMUST00000195224	MSTRG.19458	Gm37399
ENSMUST00000195226	MSTRG.16543	Gm36932
ENSMUST00000195237	MSTRG.10965	Gm37310
ENSMUST00000195240	MSTRG.225	Gm37724
ENSMUST00000195241	ENSMUSG00000104433	Gm37426
ENSMUST00000195243	ENSMUSG00000103620	Gm37359
ENSMUST00000195250	MSTRG.32825	Gm37611
ENSMUST00000195252	ENSMUSG00000102865	Gm9839
ENSMUST00000195253	MSTRG.16733	Gm37006
ENSMUST00000195266	MSTRG.999	Gm37017
ENSMUST00000195276	MSTRG.22053	Gm37817
ENSMUST00000195282	MSTRG.9390	Gm37830
ENSMUST00000195284	MSTRG.33311	Gm19721
ENSMUST00000195287	ENSMUSG00000104310	Gm4081
ENSMUST00000195288	ENSMUSG00000102618	Gm37848
ENSMUST00000195289	MSTRG.16705	Gm37126
ENSMUST00000195290	ENSMUSG00000103738	Gm37652
ENSMUST00000195293	MSTRG.33177	Gm37953
ENSMUST00000195294	MSTRG.2267	Gm37432
ENSMUST00000195297	MSTRG.1734	Gm38126
ENSMUST00000195301	MSTRG.33023	Gm37993
ENSMUST00000195303	ENSMUSG00000103603	Gm37020
ENSMUST00000195304	ENSMUSG00000102579	Gm37965
ENSMUST00000195306	MSTRG.33178	Gm38344
ENSMUST00000195308	MSTRG.1831	Gm37864
ENSMUST00000195309	ENSMUSG00000103647	Gm37707
ENSMUST00000195310	MSTRG.236	Gm37905
ENSMUST00000195314	MSTRG.2206	4930532G15Rik
ENSMUST00000195317	MSTRG.917	Gm37914
ENSMUST00000195320	ENSMUSG00000102491	Gm36986
ENSMUST00000195326	MSTRG.16452	Gm37107
ENSMUST00000195331	MSTRG.21704	Gm10305
ENSMUST00000195332	MSTRG.10499	Gm37420
ENSMUST00000195335	ENSMUSG00000103377	Gm37180
ENSMUST00000195338	MSTRG.32868	A530064N14Rik
ENSMUST00000195343	ENSMUSG00000102887	Gm10857
ENSMUST00000195348	MSTRG.1657	A430050A11Rik
ENSMUST00000195349	MSTRG.841	Gm37886
ENSMUST00000195352	ENSMUSG00000102481	Gm37925
ENSMUST00000195353	MSTRG.19498	Gm38055
ENSMUST00000195360	MSTRG.91	Gm38069
ENSMUST00000195366	ENSMUSG00000103345	Gm10723
ENSMUST00000195368	MSTRG.17939	2810405F15Rik
ENSMUST00000195369	MSTRG.15297	Gm37643
ENSMUST00000195377	ENSMUSG00000104086	1700039M15Rik
ENSMUST00000195378	MSTRG.32976	Gm37484
ENSMUST00000195379	MSTRG.2052	Gm37706
ENSMUST00000195380	MSTRG.1834	Gm37860
ENSMUST00000195382	MSTRG.19450	Gm38357

ENSMUST00000195385	ENSMUSG00000103469	Gm9910
ENSMUST00000195386	MSTRG.14633	Gm38109
ENSMUST00000195397	ENSMUSG00000103577	9330162B11Rik
ENSMUST00000195400	MSTRG.1355	Gm37954
ENSMUST00000195401	MSTRG.1748	Gm37653
ENSMUST00000195404	ENSMUSG00000102197	Gm38170
ENSMUST00000195406	ENSMUSG00000094763	Gm1647
ENSMUST00000195407	MSTRG.19326	Gm37640
ENSMUST00000195409	MSTRG.16565	Gm37289
ENSMUST00000195411	MSTRG.714	Gm37570
ENSMUST00000195413	MSTRG.31203	Gm38384
ENSMUST00000195414	MSTRG.1718	9430087J23Rik
ENSMUST00000195416	MSTRG.16709	Gm37894
ENSMUST00000195426	MSTRG.1032	Gm38312
ENSMUST00000195431	MSTRG.16744	Gm36930
ENSMUST00000195434	MSTRG.19595	Gm37488
ENSMUST00000195437	MSTRG.223	Gm37522
ENSMUST00000195439	MSTRG.1338	Gm37163
ENSMUST00000195445	MSTRG.7	Gm37144
ENSMUST00000195446	MSTRG.19271	A830092H15Rik
ENSMUST00000195451	MSTRG.30938	1700051O22Rik
ENSMUST00000195455	ENSMUSG00000103182	Gm37091
ENSMUST00000195460	ENSMUSG00000103119	Gm37583
ENSMUST00000195462	ENSMUSG00000087022	9130024F11Rik
ENSMUST00000195463	MSTRG.2027	Gm36992
ENSMUST00000195466	MSTRG.643	Gm38351
ENSMUST00000195470	ENSMUSG00000102482	Gm37924
ENSMUST00000195473	ENSMUSG00000102461	Gm37166
ENSMUST00000195475	ENSMUSG00000102139	Gm37109
ENSMUST00000195476	ENSMUSG00000104188	Gm37821
ENSMUST00000195478	MSTRG.786	Gm37733
ENSMUST00000195479	ENSMUSG00000102518	Gm38339
ENSMUST00000195489	MSTRG.19608	A330015K06Rik
ENSMUST00000195493	MSTRG.100	Gm38376
ENSMUST00000195494	MSTRG.16550	Gm37975
ENSMUST00000195495	MSTRG.15054	Gm29994
ENSMUST00000195497	MSTRG.23715	Gm37720
ENSMUST00000195500	MSTRG.1572	Gm36527
ENSMUST00000195501	MSTRG.1673	Gm38043
ENSMUST00000195506	MSTRG.99	Gm38120
ENSMUST00000195507	ENSMUSG00000102958	Gm37016
ENSMUST00000195508	MSTRG.1869	Gm2453
ENSMUST00000195509	MSTRG.831	4833412K13Rik
ENSMUST00000195510	MSTRG.21053	Gm37900
ENSMUST00000195512	MSTRG.15567	Gm37206
ENSMUST00000195518	MSTRG.1843	Gm15853
ENSMUST00000195528	ENSMUSG00000103898	Gm30238
ENSMUST00000195529	ENSMUSG00000103761	Gm37859
ENSMUST00000195532	ENSMUSG00000102458	Pisrt1
ENSMUST00000195535	MSTRG.33175	Gm37962
ENSMUST00000195536	MSTRG.33327	4930447F24Rik
ENSMUST00000195540	MSTRG.33119	Gm37637
ENSMUST00000195541	MSTRG.12904	Gm37311
ENSMUST00000195545	ENSMUSG00000102544	Gm5103
ENSMUST00000195546	ENSMUSG00000102698	Gm37777
ENSMUST00000195547	MSTRG.19703	6430573P05Rik
ENSMUST00000195551	MSTRG.32791	Gm38057

ENSMUST00000195552	MSTRG.8670	Gm37238
ENSMUST00000195553	MSTRG.2336	Gm37132
ENSMUST00000195556	ENSMUSG00000102940	Gm37551
ENSMUST00000195562	MSTRG.717	Gm38162
ENSMUST00000195566	MSTRG.2039	Gm37490
ENSMUST00000195569	ENSMUSG00000103215	Gm38388
ENSMUST00000195573	MSTRG.1284	Gm38301
ENSMUST00000195578	MSTRG.1320	Gm37868
ENSMUST00000195583	ENSMUSG00000102969	Gm33366
ENSMUST00000195585	MSTRG.16739	Gm37891
ENSMUST00000195587	ENSMUSG00000103616	1700018A14Rik
ENSMUST00000195593	MSTRG.624	Gm37198
ENSMUST00000195599	ENSMUSG00000103175	Gm37169
ENSMUST00000195603	ENSMUSG00000104367	Gm38079
ENSMUST00000195609	MSTRG.1545	Gm38187
ENSMUST00000195611	ENSMUSG00000103923	Gm37896
ENSMUST00000195618	MSTRG.1721	Gm37529
ENSMUST00000195625	MSTRG.1000	Gm37447
ENSMUST00000195629	MSTRG.12056	Gm37186
ENSMUST00000195630	MSTRG.9033	Gm38307
ENSMUST00000195634	MSTRG.995	Gm37967
ENSMUST00000195635	ENSMUSG00000102408	4930568G15Rik
ENSMUST00000195639	MSTRG.19630	Gm42568
ENSMUST00000195644	ENSMUSG00000104314	Gm37907
ENSMUST00000195645	MSTRG.33012	Gm37614
ENSMUST00000195666	MSTRG.25065	A430110C17Rik
ENSMUST00000195672	MSTRG.25675	5330406M23Rik
ENSMUST00000195673	ENSMUSG00000103366	C630004L07Rik
ENSMUST00000195674	ENSMUSG00000104465	BC002189
ENSMUST00000195675	ENSMUSG00000103579	Gm37113
ENSMUST00000195676	MSTRG.1388	Gm26616
ENSMUST00000195677	MSTRG.28380	Gm37613
ENSMUST00000195679	ENSMUSG00000104192	Gm37253
ENSMUST00000195680	MSTRG.16717	Gm37697
ENSMUST00000195683	MSTRG.7981	Gm30948
ENSMUST00000195685	MSTRG.19608	A330015K06Rik
ENSMUST00000195687	MSTRG.993	Gm38021
ENSMUST00000195692	MSTRG.12889	Gm37466
ENSMUST00000195694	MSTRG.23034	Gm37090
ENSMUST00000195696	ENSMUSG00000103831	Gm37608
ENSMUST00000195700	MSTRG.1740	Gm37634
ENSMUST00000195709	MSTRG.33245	Gm38314
ENSMUST00000195710	MSTRG.137	Gm38342
ENSMUST00000195712	MSTRG.28803	Gm37008
ENSMUST00000195714	MSTRG.19618	Gm37305
ENSMUST00000195716	ENSMUSG00000102196	Gm38171
ENSMUST00000195718	MSTRG.99	Gm38380
ENSMUST00000195719	MSTRG.2107	Gm38331
ENSMUST00000195720	MSTRG.1022	Gm38365
ENSMUST00000195723	MSTRG.214	Gm37077
ENSMUST00000195726	ENSMUSG00000103322	Gm37404
ENSMUST00000195727	ENSMUSG00000104000	Gm38335
ENSMUST00000195730	MSTRG.256	Gm37067
ENSMUST00000195742	MSTRG.25604	Gm37321
ENSMUST00000195761	ENSMUSG00000102321	Gm37792
ENSMUST00000195767	MSTRG.19706	Gm37973
ENSMUST00000195769	MSTRG.31290	Gm38318

ENSMUST00000195771	ENSMUSG00000104328	Gm37323
ENSMUST00000195772	ENSMUSG00000102759	Gm10463
ENSMUST00000195774	MSTRG.14445	Gm38220
ENSMUST00000195777	MSTRG.26375	Gm37969
ENSMUST00000195780	ENSMUSG00000103329	Gm42492
ENSMUST00000195783	MSTRG.2204	Gm37785
ENSMUST00000195784	MSTRG.1737	Gm37083
ENSMUST00000195785	MSTRG.16448	Gm38085
ENSMUST00000195792	MSTRG.12892	Gm38071
ENSMUST00000195799	MSTRG.32969	4933431K14Rik
ENSMUST00000195805	MSTRG.4166	2900018N21Rik
ENSMUST00000195806	ENSMUSG00000103204	Gm38139
ENSMUST00000195807	MSTRG.12043	1700016D08Rik
ENSMUST00000195811	MSTRG.99	Gm37409
ENSMUST00000195813	ENSMUSG00000103056	Gm38268
ENSMUST00000195820	MSTRG.16451	Gm37814
ENSMUST00000195830	MSTRG.31940	Gm37497
ENSMUST00000195831	ENSMUSG00000103079	Gm36307
ENSMUST00000195835	ENSMUSG00000103607	Gm37021
ENSMUST00000195842	MSTRG.875	A630081D01Rik
ENSMUST00000195845	MSTRG.8903	A930032L01Rik
ENSMUST00000195852	MSTRG.33131	Gm38327
ENSMUST00000195853	MSTRG.13188	Gm37332
ENSMUST00000195877	ENSMUSG00000104351	Gm37125
ENSMUST00000195879	ENSMUSG00000102902	Gm38263
ENSMUST00000195880	MSTRG.720	Gm37755
ENSMUST00000195881	MSTRG.16746	Gm38257
ENSMUST00000195887	MSTRG.19364	Gm42922
ENSMUST00000195890	MSTRG.33439	Gm43127
ENSMUST00000195892	ENSMUSG00000105006	Gm9484
ENSMUST00000195895	ENSMUSG00000105735	4933415J04Rik
ENSMUST00000195898	MSTRG.23193	6030443J06Rik
ENSMUST00000195908	ENSMUSG00000105278	Gm42630
ENSMUST00000195914	ENSMUSG00000106292	Gm43174
ENSMUST00000195915	MSTRG.24124	Gm42759
ENSMUST00000195919	MSTRG.24692	Gm42866
ENSMUST00000195921	MSTRG.24768	Gm43409
ENSMUST00000195925	MSTRG.23611	Gm43201
ENSMUST00000195931	MSTRG.24943	Gm42439
ENSMUST00000195932	ENSMUSG00000104961	Gm43835
ENSMUST00000195935	ENSMUSG00000105283	Gm33370
ENSMUST00000195938	ENSMUSG00000105663	Gm43606
ENSMUST00000195940	MSTRG.19891	4632404H12Rik
ENSMUST00000195951	MSTRG.20693	Gm43689
ENSMUST00000195964	MSTRG.20494	Gm42994
ENSMUST00000195967	MSTRG.20193	Gm43463
ENSMUST00000195987	MSTRG.20517	Gm43590
ENSMUST00000195990	MSTRG.23592	Gm42984
ENSMUST00000195991	MSTRG.19760	4933425M03Rik
ENSMUST00000195992	MSTRG.24764	4932422M17Rik
ENSMUST00000195993	MSTRG.20541	Gm43010
ENSMUST00000195996	ENSMUSG00000105550	Gm35585
ENSMUST00000196002	ENSMUSG00000104835	Gm5547
ENSMUST00000196003	ENSMUSG00000105264	Gm42716
ENSMUST00000196013	ENSMUSG00000105183	Gm42564
ENSMUST00000196016	ENSMUSG00000105224	Gm3364
ENSMUST00000196018	ENSMUSG00000105469	A930036I15Rik

ENSMUST00000196026	MSTRG.20486	Gm42777
ENSMUST00000196037	MSTRG.24128	Gm35172
ENSMUST00000196040	MSTRG.20164	Gm43189
ENSMUST00000196044	MSTRG.24938	C230071H17Rik
ENSMUST00000196046	MSTRG.24361	Gm43364
ENSMUST00000196047	MSTRG.23648	Gm43320
ENSMUST00000196052	ENSMUSG00000106157	4930555A03Rik
ENSMUST00000196056	ENSMUSG00000104990	Gm43504
ENSMUST00000196058	ENSMUSG00000105265	Sox2ot
ENSMUST00000196063	MSTRG.23631	Gm42534
ENSMUST00000196065	ENSMUSG00000106123	Gm42638
ENSMUST00000196072	MSTRG.23436	Gm43693
ENSMUST00000196075	MSTRG.23714	Gm42484
ENSMUST00000196080	MSTRG.23256	Gm43389
ENSMUST00000196083	MSTRG.23297	Gm43143
ENSMUST00000196086	MSTRG.20865	Gm42942
ENSMUST00000196087	ENSMUSG00000105917	Gm43612
ENSMUST00000196088	MSTRG.20269	4930509H03Rik
ENSMUST00000196091	ENSMUSG00000105003	Gm40055
ENSMUST00000196101	MSTRG.23663	Gm43048
ENSMUST00000196108	MSTRG.20864	Gm42946
ENSMUST00000196110	MSTRG.20639	Gm42873
ENSMUST00000196113	ENSMUSG00000106380	Gm3519
ENSMUST00000196116	MSTRG.24950	Gm42996
ENSMUST00000196117	MSTRG.20290	Gm42701
ENSMUST00000196120	MSTRG.20064	Gm10685
ENSMUST00000196123	ENSMUSG00000105260	Gm40040
ENSMUST00000196136	MSTRG.8232	Gm42683
ENSMUST00000196142	ENSMUSG00000105297	1700052H01Rik
ENSMUST00000196146	MSTRG.20764	Gm34866
ENSMUST00000196150	ENSMUSG00000104531	Gm43731
ENSMUST00000196186	MSTRG.25224	Gm43556
ENSMUST00000196190	MSTRG.19390	Gm43539
ENSMUST00000196191	MSTRG.23643	Gm43829
ENSMUST00000196200	MSTRG.20493	Gm43569
ENSMUST00000196216	MSTRG.23595	Gm42413
ENSMUST00000196218	MSTRG.23200	A930003O13Rik
ENSMUST00000196219	ENSMUSG00000105008	Gm43652
ENSMUST00000196227	ENSMUSG00000106073	Gm42892
ENSMUST00000196228	ENSMUSG00000105689	Gm43120
ENSMUST00000196232	ENSMUSG00000105939	Gm43322
ENSMUST00000196233	MSTRG.20209	Gm42538
ENSMUST00000196235	MSTRG.24747	Gm43411
ENSMUST00000196238	ENSMUSG00000105334	Gm42680
ENSMUST00000196242	MSTRG.23605	1600023N17Rik
ENSMUST00000196253	MSTRG.20658	Gm43379
ENSMUST00000196262	MSTRG.24374	A430073D23Rik
ENSMUST00000196277	ENSMUSG00000106304	Gm42518
ENSMUST00000196278	MSTRG.24470	Gm42161
ENSMUST00000196283	MSTRG.23751	Gm3716
ENSMUST00000196286	MSTRG.20841	4930597L12Rik
ENSMUST00000196287	MSTRG.24873	Gm42979
ENSMUST00000196290	MSTRG.20507	4930447N08Rik
ENSMUST00000196294	ENSMUSG00000104660	Gm43601
ENSMUST00000196295	MSTRG.23156	Gm43216
ENSMUST00000196302	MSTRG.20229	Gm43062
ENSMUST00000196303	ENSMUSG00000105119	Gm43765

ENSMUST00000196305	MSTRG.24418	Gm42596
ENSMUST00000196307	MSTRG.23709	Gm43715
ENSMUST00000196310	ENSMUSG00000104982	Gm32554
ENSMUST00000196313	ENSMUSG00000106321	Gm43674
ENSMUST00000196325	ENSMUSG00000106492	Gm29707
ENSMUST00000196327	MSTRG.19980	Gm43774
ENSMUST00000196328	ENSMUSG00000105293	Gm42843
ENSMUST00000196332	ENSMUSG00000104865	Gm42708
ENSMUST00000196337	ENSMUSG00000105260	Gm40040
ENSMUST00000196357	ENSMUSG00000105730	Gm43838
ENSMUST00000196362	ENSMUSG00000105975	Gm42609
ENSMUST00000196372	ENSMUSG00000105864	Gm10484
ENSMUST00000196377	ENSMUSG00000104981	Gm43736
ENSMUST00000196382	MSTRG.20606	Gm43350
ENSMUST00000196390	MSTRG.23561	Gm42553
ENSMUST00000196396	ENSMUSG00000105335	Gm42423
ENSMUST00000196399	MSTRG.24149	5430416N02Rik
ENSMUST00000196407	ENSMUSG00000106446	Gm42970
ENSMUST00000196411	ENSMUSG00000104894	Gm43507
ENSMUST00000196412	ENSMUSG00000105146	Gm35409
ENSMUST00000196415	MSTRG.24861	Gm43474
ENSMUST00000196419	MSTRG.20615	Gm43352
ENSMUST00000196422	ENSMUSG00000104875	Gm43397
ENSMUST00000196427	MSTRG.20292	C030032O16Rik
ENSMUST00000196434	MSTRG.24127	Gm35394
ENSMUST00000196438	MSTRG.20601	2010110G14Rik
ENSMUST00000196439	MSTRG.25306	Gm43562
ENSMUST00000196442	ENSMUSG00000106070	Gm43135
ENSMUST00000196445	ENSMUSG00000104786	Gm43573
ENSMUST00000196451	MSTRG.20756	Gm43707
ENSMUST00000196459	MSTRG.24275	Gm43817
ENSMUST00000196466	MSTRG.20519	Snhg8
ENSMUST00000196468	MSTRG.24945	Gm42991
ENSMUST00000196470	ENSMUSG00000104860	Gm42510
ENSMUST00000196473	ENSMUSG00000059244	Gm10062
ENSMUST00000196474	MSTRG.1508	Gm43544
ENSMUST00000196484	MSTRG.24678	4930477O15Rik
ENSMUST00000196486	MSTRG.24797	Gm34086
ENSMUST00000196492	ENSMUSG00000106044	Gm42860
ENSMUST00000196494	ENSMUSG00000105876	Gm43572
ENSMUST00000196503	MSTRG.24850	Gm42838
ENSMUST00000196513	MSTRG.20669	Gm43430
ENSMUST00000196517	ENSMUSG00000105560	Gm42744
ENSMUST00000196522	MSTRG.24358	Gm43365
ENSMUST00000196524	ENSMUSG00000106157	4930555A03Rik
ENSMUST00000196531	MSTRG.19958	Gm36070
ENSMUST00000196532	ENSMUSG00000106566	Gm43836
ENSMUST00000196534	MSTRG.24249	Gm42141
ENSMUST00000196539	MSTRG.23722	Gm43753
ENSMUST00000196543	MSTRG.23582	Gm42462
ENSMUST00000196546	ENSMUSG00000105516	Gm36823
ENSMUST00000196548	ENSMUSG00000104971	9430087B13Rik
ENSMUST00000196549	MSTRG.24472	Gm43094
ENSMUST00000196551	ENSMUSG00000106603	Gm43509
ENSMUST00000196555	ENSMUSG00000105264	Gm42716
ENSMUST00000196562	MSTRG.24719	Gm43361
ENSMUST00000196563	ENSMUSG00000105478	Gm43285

ENSMUST00000196567	MSTRG.23668	2900064K03Rik
ENSMUST00000196582	ENSMUSG00000104617	Gm43356
ENSMUST00000196584	MSTRG.25260	Gm43398
ENSMUST00000196589	ENSMUSG00000106157	4930555A03Rik
ENSMUST00000196593	MSTRG.24214	D930016D06Rik
ENSMUST00000196615	MSTRG.10217	Gm43434
ENSMUST00000196616	ENSMUSG00000106008	Gm42920
ENSMUST00000196620	ENSMUSG00000105811	Gm42707
ENSMUST00000196621	ENSMUSG00000044522	A730020M07Rik
ENSMUST00000196625	ENSMUSG00000104776	Gm43691
ENSMUST00000196629	ENSMUSG00000097287	D130017N08Rik
ENSMUST00000196630	MSTRG.23060	Gm42435
ENSMUST00000196636	MSTRG.20555	Gm42514
ENSMUST00000196642	MSTRG.24709	Gm42830
ENSMUST00000196653	ENSMUSG00000105139	Gm19391
ENSMUST00000196656	MSTRG.23313	Gm43611
ENSMUST00000196660	MSTRG.25109	Gm36266
ENSMUST00000196662	MSTRG.19353	Gm15952
ENSMUST00000196663	MSTRG.23097	Gm42597
ENSMUST00000196665	ENSMUSG00000106212	Gm43112
ENSMUST00000196666	MSTRG.20872	Gm43526
ENSMUST00000196668	ENSMUSG00000105811	Gm42707
ENSMUST00000196669	MSTRG.20433	Gm29151
ENSMUST00000196670	ENSMUSG00000106441	Gm42921
ENSMUST00000196681	MSTRG.24411	A630023P12Rik
ENSMUST00000196693	ENSMUSG00000072769	Gm10419
ENSMUST00000196697	MSTRG.20600	Gm43522
ENSMUST00000196705	MSTRG.20049	Gm43375
ENSMUST00000196714	ENSMUSG00000105729	5330425B07Rik
ENSMUST00000196718	ENSMUSG00000105252	Gm42753
ENSMUST00000196720	ENSMUSG00000105891	A230001M10Rik
ENSMUST00000196721	ENSMUSG00000105018	Gm43546
ENSMUST00000196724	MSTRG.24933	Gm43483
ENSMUST00000196732	MSTRG.19520	Gm43803
ENSMUST00000196743	ENSMUSG00000105011	Gm43210
ENSMUST00000196744	ENSMUSG00000104528	Gm43314
ENSMUST00000196754	ENSMUSG00000105087	Gm42552
ENSMUST00000196765	MSTRG.24205	Gm43545
ENSMUST00000196766	MSTRG.20262	Gm43581
ENSMUST00000196770	MSTRG.20168	Gm43024
ENSMUST00000196771	MSTRG.24425	Gm42595
ENSMUST00000196779	ENSMUSG00000105284	Gm42693
ENSMUST00000196787	ENSMUSG00000106478	Gm36551
ENSMUST00000196791	MSTRG.20878	4930592C13Rik
ENSMUST00000196806	MSTRG.33402	Gm42433
ENSMUST00000196810	ENSMUSG00000106339	Gm43489
ENSMUST00000196822	MSTRG.23705	Gm42481
ENSMUST00000196829	ENSMUSG00000105352	C030018K13Rik
ENSMUST00000196835	MSTRG.23723	Gm43237
ENSMUST00000196840	MSTRG.25085	Gm42456
ENSMUST00000196841	ENSMUSG00000105662	Gm6639
ENSMUST00000196844	ENSMUSG00000055125	M5C1000I18Rik
ENSMUST00000196846	ENSMUSG00000104904	9330198I05Rik
ENSMUST00000196851	MSTRG.23252	Gm43566
ENSMUST00000196856	MSTRG.24948	Gm42993
ENSMUST00000196877	ENSMUSG00000105975	Gm42609
ENSMUST00000196893	ENSMUSG00000106461	Gm20755

ENSMUST00000196896	MSTRG.20250	Gm43387
ENSMUST00000196901	ENSMUSG00000105760	Gm43211
ENSMUST00000196920	MSTRG.24179	Gm42934
ENSMUST00000196922	MSTRG.24312	Gm43592
ENSMUST00000196925	MSTRG.24459	Gm43759
ENSMUST00000196937	ENSMUSG00000105445	Gm42972
ENSMUST00000196944	ENSMUSG00000105871	Gm43147
ENSMUST00000196948	MSTRG.24953	Gm43019
ENSMUST00000196949	MSTRG.20565	Gm43729
ENSMUST00000196955	MSTRG.20311	Gm42659
ENSMUST00000196957	ENSMUSG00000106560	Gm43119
ENSMUST00000196982	ENSMUSG00000104632	Gm42909
ENSMUST00000196987	ENSMUSG00000105265	Sox2ot
ENSMUST00000196989	MSTRG.20631	Gm42876
ENSMUST00000196993	ENSMUSG00000105888	BC037156
ENSMUST00000196994	ENSMUSG00000105342	Gm43244
ENSMUST00000196996	ENSMUSG00000106411	Gm42445
ENSMUST00000197000	MSTRG.20456	4930512P04Rik
ENSMUST00000197001	MSTRG.23585	Gm43181
ENSMUST00000197004	MSTRG.24245	Gm43856
ENSMUST00000197011	MSTRG.28618	9330171B17Rik
ENSMUST00000197014	MSTRG.19342	Gm43668
ENSMUST00000197016	ENSMUSG00000029092	D5Ertd615e
ENSMUST00000197025	ENSMUSG00000106319	Gm42601
ENSMUST00000197032	ENSMUSG00000104847	Gm43177
ENSMUST00000197034	MSTRG.19517	Gm42899
ENSMUST00000197043	MSTRG.24946	Gm42992
ENSMUST00000197047	MSTRG.24949	Gm42995
ENSMUST00000197051	MSTRG.23081	Gm43679
ENSMUST00000197053	MSTRG.20596	Gm42998
ENSMUST00000197054	ENSMUSG00000105401	Gm42949
ENSMUST00000197061	MSTRG.23712	Gm42639
ENSMUST00000197066	ENSMUSG00000106000	Gm16508
ENSMUST00000197067	ENSMUSG00000104547	Gm43022
ENSMUST00000197071	ENSMUSG00000105520	Gm42837
ENSMUST00000197075	MSTRG.20149	Gm42783
ENSMUST00000197083	MSTRG.24445	Gm42780
ENSMUST00000197084	MSTRG.24169	Gm43511
ENSMUST00000197086	ENSMUSG00000106121	Gm42679
ENSMUST00000197090	ENSMUSG00000105287	Gm43577
ENSMUST00000197095	MSTRG.25060	4933404O12Rik
ENSMUST00000197097	ENSMUSG00000104801	Gm43834
ENSMUST00000197100	MSTRG.25225	Gm43557
ENSMUST00000197107	ENSMUSG00000105444	Gm10727
ENSMUST00000197115	ENSMUSG00000106178	Gm42987
ENSMUST00000197116	ENSMUSG00000104588	Gm43666
ENSMUST00000197119	MSTRG.23759	Gm43627
ENSMUST00000197120	MSTRG.23730	Gm42566
ENSMUST00000197122	ENSMUSG00000104625	Gm42448
ENSMUST00000197127	ENSMUSG00000104610	Gm567
ENSMUST00000197132	ENSMUSG00000106398	Gm43357
ENSMUST00000197139	ENSMUSG00000105627	Gm43548
ENSMUST00000197140	ENSMUSG00000105264	Gm42716
ENSMUST00000197148	ENSMUSG00000097183	Gm17501
ENSMUST00000197153	MSTRG.23211	AI506816
ENSMUST00000197165	ENSMUSG00000104592	Gm42721
ENSMUST00000197171	MSTRG.23673	C130083M11Rik

ENSMUST00000197172	MSTRG.19802	1700113A16Rik
ENSMUST00000197174	MSTRG.25326	Gm42636
ENSMUST00000197178	ENSMUSG00000104846	Gm43020
ENSMUST00000197184	MSTRG.24295	Gm42900
ENSMUST00000197198	MSTRG.20502	Gm42836
ENSMUST00000197199	MSTRG.20310	Gm42658
ENSMUST00000197200	ENSMUSG00000104586	4921539H07Rik
ENSMUST00000197208	MSTRG.24721	Gm43541
ENSMUST00000197216	ENSMUSG00000105867	Gm42517
ENSMUST00000197219	MSTRG.19567	A930028O11Rik
ENSMUST00000197222	ENSMUSG00000106512	Gm43078
ENSMUST00000197227	MSTRG.26304	Gm30211
ENSMUST00000197231	MSTRG.20829	1700012D16Rik
ENSMUST00000197233	MSTRG.20338	Gm43071
ENSMUST00000197239	ENSMUSG00000105528	Gm43519
ENSMUST00000197247	MSTRG.23579	Gm43184
ENSMUST00000197249	MSTRG.24932	Gm42591
ENSMUST00000197251	ENSMUSG00000105861	Gm43508
ENSMUST00000197252	MSTRG.23655	Gm43316
ENSMUST00000197257	MSTRG.23671	Gm43178
ENSMUST00000197262	MSTRG.24735	Gm15860
ENSMUST00000197263	MSTRG.19403	Gm42437
ENSMUST00000197267	ENSMUSG00000104659	Gm43619
ENSMUST00000197277	ENSMUSG00000105300	Gm30613
ENSMUST00000197279	ENSMUSG00000057802	Gm10030
ENSMUST00000197286	MSTRG.20151	Gm42782
ENSMUST00000197287	MSTRG.23627	Gm43426
ENSMUST00000197294	MSTRG.20853	Gm43400
ENSMUST00000197297	MSTRG.20840	1700015C17Rik
ENSMUST00000197309	MSTRG.20176	Gm42717
ENSMUST00000197317	MSTRG.19335	Gm43140
ENSMUST00000197321	ENSMUSG00000057802	Gm10030
ENSMUST00000197326	ENSMUSG00000106296	4632404M16Rik
ENSMUST00000197337	MSTRG.10325	Gm43650
ENSMUST00000197338	MSTRG.20480	Gm42928
ENSMUST00000197351	ENSMUSG00000104974	Gm43686
ENSMUST00000197360	MSTRG.20211	Gm42937
ENSMUST00000197370	MSTRG.20579	Gm35986
ENSMUST00000197377	MSTRG.23237	4930580E04Rik
ENSMUST00000197378	MSTRG.20295	Gm43260
ENSMUST00000197379	MSTRG.24376	Gm10416
ENSMUST00000197382	ENSMUSG00000045238	A730035I17Rik
ENSMUST00000197386	MSTRG.20632	Gm29811
ENSMUST00000197390	MSTRG.23239	Gm42509
ENSMUST00000197391	MSTRG.20484	Gm43823
ENSMUST00000197392	ENSMUSG00000105403	Gm43618
ENSMUST00000197397	ENSMUSG00000106310	Gm43523
ENSMUST00000197413	ENSMUSG00000105816	D030025E07Rik
ENSMUST00000197414	ENSMUSG00000106232	Gm43236
ENSMUST00000197424	ENSMUSG00000105912	Gm10440
ENSMUST00000197430	ENSMUSG00000106139	Gm30648
ENSMUST00000197445	ENSMUSG00000106472	Gm43111
ENSMUST00000197451	MSTRG.23002	1500002C15Rik
ENSMUST00000197455	MSTRG.24757	Gm43412
ENSMUST00000197463	ENSMUSG00000072962	Gm16401
ENSMUST00000197472	MSTRG.24780	Gm43133
ENSMUST00000197473	MSTRG.23051	Gm36548

ENSMUST00000197486	MSTRG.23202	A930003O13Rik
ENSMUST00000197492	ENSMUSG00000106519	Gm38509
ENSMUST00000197494	ENSMUSG00000105158	Gm42862
ENSMUST00000197498	MSTRG.20671	Gm31243
ENSMUST00000197499	MSTRG.23120	7330423F06Rik
ENSMUST00000197507	MSTRG.20802	Gm43185
ENSMUST00000197508	MSTRG.24741	Gm42908
ENSMUST00000197509	ENSMUSG00000106223	2400006E01Rik
ENSMUST00000197510	MSTRG.20464	Gm43410
ENSMUST00000197514	MSTRG.20207	Gm42939
ENSMUST00000197524	ENSMUSG00000104905	Gm43718
ENSMUST00000197530	MSTRG.20691	Gm43088
ENSMUST00000197532	MSTRG.24448	Gm43676
ENSMUST00000197536	MSTRG.25335	1700041I07Rik
ENSMUST00000197541	MSTRG.23674	Gm43180
ENSMUST00000197551	MSTRG.23214	2700038G22Rik
ENSMUST00000197552	ENSMUSG00000104626	Gm42675
ENSMUST00000197556	MSTRG.23716	Gm42483
ENSMUST00000197559	ENSMUSG00000106134	Gm42493
ENSMUST00000197562	MSTRG.23628	Gm42536
ENSMUST00000197563	ENSMUSG00000106651	Gm42608
ENSMUST00000197564	ENSMUSG00000104808	Gm42619
ENSMUST00000197569	MSTRG.20405	C130013H08Rik
ENSMUST00000197572	MSTRG.20454	Gm42457
ENSMUST00000197583	ENSMUSG00000104912	Gm43023
ENSMUST00000197593	ENSMUSG00000105526	Gm43490
ENSMUST00000197594	MSTRG.20523	Gm43288
ENSMUST00000197597	MSTRG.24467	Gm43246
ENSMUST00000197606	MSTRG.24214	D930016D06Rik
ENSMUST00000197609	ENSMUSG00000105041	Gm42676
ENSMUST00000197612	MSTRG.20393	Gm43221
ENSMUST00000197620	MSTRG.10211	Gm30275
ENSMUST00000197638	MSTRG.24468	F830115B05Rik
ENSMUST00000197639	ENSMUSG00000105602	Gm10636
ENSMUST00000197640	ENSMUSG00000104919	Gm42617
ENSMUST00000197648	ENSMUSG00000106060	Gm42488
ENSMUST00000197656	MSTRG.24718	Gm43360
ENSMUST00000197661	MSTRG.8222	Gm31887
ENSMUST00000197663	ENSMUSG00000105392	Gm42684
ENSMUST00000197674	ENSMUSG00000105271	Gm42875
ENSMUST00000197676	MSTRG.23884	Gm43815
ENSMUST00000197683	ENSMUSG00000105652	4930519L02Rik
ENSMUST00000197685	MSTRG.20066	Gm43534
ENSMUST00000197695	ENSMUSG00000105337	1700094M23Rik
ENSMUST00000197697	ENSMUSG00000105265	Sox2ot
ENSMUST00000197701	MSTRG.23567	Gm42551
ENSMUST00000197702	MSTRG.20546	Gm43006
ENSMUST00000197703	ENSMUSG00000106036	Gm43608
ENSMUST00000197704	ENSMUSG00000105622	Gm42615
ENSMUST00000197709	MSTRG.24181	1700013M08Rik
ENSMUST00000197710	ENSMUSG00000104935	Gm43414
ENSMUST00000197715	MSTRG.24196	Gm42620
ENSMUST00000197721	ENSMUSG00000105750	Gm43130
ENSMUST00000197729	MSTRG.24198	Gm43547
ENSMUST00000197733	ENSMUSG00000106104	Gm42660
ENSMUST00000197734	MSTRG.23660	Gm42768
ENSMUST00000197735	MSTRG.19514	Gm42901

ENSMUST00000197736	ENSMUSG00000106211	Gm42842
ENSMUST00000197747	MSTRG.20522	Gm43287
ENSMUST00000197753	MSTRG.23646	Gm42919
ENSMUST00000197762	MSTRG.25097	Gm8066
ENSMUST00000197765	MSTRG.19520	Gm43803
ENSMUST00000197775	MSTRG.23641	Gm43648
ENSMUST00000197792	MSTRG.24180	Gm43787
ENSMUST00000197810	ENSMUSG00000106157	4930555A03Rik
ENSMUST00000197818	MSTRG.12356	Gm43388
ENSMUST00000197819	MSTRG.20282	9530097N15Rik
ENSMUST00000197831	ENSMUSG00000105065	Gm42513
ENSMUST00000197832	MSTRG.24944	Gm42438
ENSMUST00000197835	ENSMUSG00000106173	Gm42764
ENSMUST00000197836	MSTRG.20190	Gm43466
ENSMUST00000197838	ENSMUSG00000106511	Gm43521
ENSMUST00000197839	ENSMUSG00000104928	Gm42565
ENSMUST00000197848	MSTRG.20613	A430072C10Rik
ENSMUST00000197851	MSTRG.33441	Gm43814
ENSMUST00000197854	ENSMUSG00000072769	Gm10419
ENSMUST00000197856	ENSMUSG00000105202	C230031I18Rik
ENSMUST00000197865	ENSMUSG00000106507	Gm43056
ENSMUST00000197868	ENSMUSG00000104869	Gm42544
ENSMUST00000197871	MSTRG.23770	Gm43628
ENSMUST00000197875	ENSMUSG00000105270	Gm42863
ENSMUST00000197878	ENSMUSG00000105258	Gm40038
ENSMUST00000197881	MSTRG.23058	Gm17590
ENSMUST00000197883	ENSMUSG00000105048	Gm42966
ENSMUST00000197889	ENSMUSG00000106290	Gm43429
ENSMUST00000197897	MSTRG.20539	Gm43011
ENSMUST00000197902	MSTRG.33403	Gm42432
ENSMUST00000197904	ENSMUSG00000105952	Gm40153
ENSMUST00000197905	MSTRG.24246	Gm32051
ENSMUST00000197906	MSTRG.24942	Gm42440
ENSMUST00000197910	ENSMUSG00000104719	4933437G19Rik
ENSMUST00000197913	ENSMUSG00000105733	Gm42973
ENSMUST00000197918	MSTRG.25259	9530056K15Rik
ENSMUST00000197919	ENSMUSG00000105619	9530034A14Rik
ENSMUST00000197921	MSTRG.24154	Gm42690
ENSMUST00000197924	MSTRG.23654	Gm43317
ENSMUST00000197932	ENSMUSG00000105102	Gm35507
ENSMUST00000197936	MSTRG.24496	Gm42913
ENSMUST00000197944	MSTRG.24717	Gm43359
ENSMUST00000197951	MSTRG.24936	Gm42442
ENSMUST00000197965	MSTRG.25096	9130604C24Rik
ENSMUST00000197967	ENSMUSG00000106498	Gm43032
ENSMUST00000197971	MSTRG.19960	Gm38411
ENSMUST00000197976	ENSMUSG00000105772	Gm42496
ENSMUST00000197977	ENSMUSG00000105265	Sox2ot
ENSMUST00000197982	ENSMUSG00000104667	Gm4961
ENSMUST00000197991	MSTRG.20323	Gm43331
ENSMUST00000197996	ENSMUSG00000106128	Gm43520
ENSMUST00000197999	MSTRG.20223	Gm42681
ENSMUST00000198001	ENSMUSG00000105795	Gm3970
ENSMUST00000198002	ENSMUSG00000104556	Gm43192
ENSMUST00000198003	MSTRG.20195	Gm43464
ENSMUST00000198011	MSTRG.20687	Gm43403
ENSMUST00000198012	MSTRG.24672	Gm43420

ENSMUST00000198013	MSTRG.20043	Gm42671
ENSMUST00000198021	ENSMUSG00000106202	Gm43727
ENSMUST00000198032	ENSMUSG00000105265	Sox2ot
ENSMUST00000198036	MSTRG.23645	Gm43319
ENSMUST00000198040	ENSMUSG00000106365	Gm43568
ENSMUST00000198041	MSTRG.19969	Gm42463
ENSMUST00000198047	ENSMUSG00000105337	1700094M23Rik
ENSMUST00000198052	MSTRG.24401	Gm43137
ENSMUST00000198056	ENSMUSG00000105923	A830019L24Rik
ENSMUST00000198060	MSTRG.20322	Gm43848
ENSMUST00000198074	MSTRG.23755	C230096K16Rik
ENSMUST00000198075	MSTRG.24954	Gm43018
ENSMUST00000198084	MSTRG.24935	Gm42443
ENSMUST00000198085	ENSMUSG00000105456	Gm43745
ENSMUST00000198086	MSTRG.33430	Gm42470
ENSMUST00000198098	MSTRG.24447	Gm43677
ENSMUST00000198100	MSTRG.20758	Gm43560
ENSMUST00000198104	MSTRG.20285	Gm42699
ENSMUST00000198123	ENSMUSG00000105243	Gm43444
ENSMUST00000198124	MSTRG.10211	Gm30275
ENSMUST00000198130	MSTRG.24959	Gm43186
ENSMUST00000198132	MSTRG.24113	Gm33050
ENSMUST00000198136	MSTRG.20575	Gm43653
ENSMUST00000198142	MSTRG.10206	Gm43647
ENSMUST00000198145	MSTRG.24696	1700008B11Rik
ENSMUST00000198148	MSTRG.23653	C030015E24Rik
ENSMUST00000198157	MSTRG.25022	Gm43091
ENSMUST00000198159	ENSMUSG00000104955	1700016F12Rik
ENSMUST00000198162	MSTRG.20250	Gm43149
ENSMUST00000198169	MSTRG.20490	4633401B06Rik
ENSMUST00000198170	ENSMUSG00000105265	Sox2ot
ENSMUST00000198171	ENSMUSG00000106426	Gm36211
ENSMUST00000198175	ENSMUSG00000105961	Gm40123
ENSMUST00000198176	MSTRG.23185	Gm19666
ENSMUST00000198178	MSTRG.19518	C130075A20Rik
ENSMUST00000198191	MSTRG.23621	4930449I04Rik
ENSMUST00000198192	MSTRG.20894	Gm43362
ENSMUST00000198193	ENSMUSG00000104956	4930429D17Rik
ENSMUST00000198195	MSTRG.19794	Gm19439
ENSMUST00000198198	MSTRG.20232	Gm43063
ENSMUST00000198200	MSTRG.20117	Gm43073
ENSMUST00000198215	ENSMUSG00000106140	Gm42208
ENSMUST00000198221	MSTRG.24789	Gm43797
ENSMUST00000198222	MSTRG.20501	Gm4610
ENSMUST00000198232	MSTRG.32410	1700042D02Rik
ENSMUST00000198236	MSTRG.23433	Gm43692
ENSMUST00000198237	ENSMUSG00000105377	Gm43148
ENSMUST00000198244	MSTRG.23367	Gm19409
ENSMUST00000198248	ENSMUSG00000106352	5033403H07Rik
ENSMUST00000198251	MSTRG.19847	Gm43713
ENSMUST00000198256	ENSMUSG00000105257	Gm43144
ENSMUST00000198260	MSTRG.23629	Gm42537
ENSMUST00000198269	MSTRG.20417	Gm43109
ENSMUST00000198276	MSTRG.23271	2310074N15Rik
ENSMUST00000198280	MSTRG.19519	Gm43804
ENSMUST00000198286	ENSMUSG00000105481	6430500D05Rik
ENSMUST00000198291	MSTRG.20690	Gm40155

ENSMUST00000198292	MSTRG.20713	Gm43162
ENSMUST00000198296	MSTRG.23070	Gm43017
ENSMUST00000198299	MSTRG.24490	Gm43335
ENSMUST00000198306	ENSMUSG00000104835	Gm5547
ENSMUST00000198307	ENSMUSG00000105651	1700017M07Rik
ENSMUST00000198310	ENSMUSG00000105571	Gm43662
ENSMUST00000198312	MSTRG.25259	Gm42504
ENSMUST00000198331	MSTRG.23211	AI506816
ENSMUST00000198333	MSTRG.19379	Gm43484
ENSMUST00000198336	MSTRG.20658	2810428J06Rik
ENSMUST00000198339	ENSMUSG00000105579	Gm43251
ENSMUST00000198340	ENSMUSG00000105261	Gm43333
ENSMUST00000198342	MSTRG.23664	Gm42771
ENSMUST00000198349	MSTRG.20218	Gm42682
ENSMUST00000198351	MSTRG.19824	Gm43714
ENSMUST00000198354	MSTRG.20671	Gm31243
ENSMUST00000198374	MSTRG.20482	Gm42929
ENSMUST00000198380	MSTRG.19982	Gm43768
ENSMUST00000198383	ENSMUSG00000104918	Gm42944
ENSMUST00000198390	MSTRG.24289	Gm43423
ENSMUST00000198394	ENSMUSG00000106621	Gm42752
ENSMUST00000198395	ENSMUSG00000105376	Gm36535
ENSMUST00000198402	ENSMUSG00000044522	A730020M07Rik
ENSMUST00000198407	ENSMUSG00000105484	Gm42775
ENSMUST00000198414	MSTRG.20208	Gm42938
ENSMUST00000198417	MSTRG.24712	Gm42829
ENSMUST00000198427	ENSMUSG00000106365	Gm43568
ENSMUST00000198429	MSTRG.19898	Gm42809
ENSMUST00000198434	MSTRG.23706	Gm42480
ENSMUST00000198438	MSTRG.19377	Gm42923
ENSMUST00000198446	MSTRG.20294	Gm43259
ENSMUST00000198450	ENSMUSG00000105942	Gm43175
ENSMUST00000198455	MSTRG.25265	0610040B10Rik
ENSMUST00000198458	ENSMUSG00000052848	C130026L21Rik
ENSMUST00000198459	ENSMUSG00000104782	Gm43326
ENSMUST00000198461	MSTRG.23211	AI506816
ENSMUST00000198462	ENSMUSG00000105701	Gm42587
ENSMUST00000198466	MSTRG.24807	Pitpnm2os2
ENSMUST00000198482	ENSMUSG00000104886	Gm43000
ENSMUST00000198488	MSTRG.19521	Gm43805
ENSMUST00000198493	ENSMUSG00000029092	D5Ertd615e
ENSMUST00000198497	ENSMUSG00000104941	Gm8953
ENSMUST00000198500	MSTRG.23067	Gm43031
ENSMUST00000198509	MSTRG.20519	Snhg8
ENSMUST00000198512	ENSMUSG00000105139	Gm19391
ENSMUST00000198522	MSTRG.24149	5430416N02Rik
ENSMUST00000198527	MSTRG.19222	4632415L05Rik
ENSMUST00000198533	ENSMUSG00000106495	Gm42755
ENSMUST00000198535	MSTRG.19547	Gm43437
ENSMUST00000198537	MSTRG.24327	4930428O21Rik
ENSMUST00000198540	MSTRG.20468	Gm43300
ENSMUST00000198543	MSTRG.20767	Gm43240
ENSMUST00000198544	MSTRG.20323	Gm43328
ENSMUST00000198548	MSTRG.19979	Gm43773
ENSMUST00000198550	MSTRG.25268	Gm42502
ENSMUST00000198557	MSTRG.20129	Gm31305
ENSMUST00000198559	ENSMUSG00000104736	Gm33609

ENSMUST00000198564	MSTRG.20753	Gm43445
ENSMUST00000198569	MSTRG.25093	Gm43012
ENSMUST00000198576	MSTRG.23661	Gm42769
ENSMUST00000198579	MSTRG.23588	Gm42429
ENSMUST00000198582	MSTRG.23751	Gm3716
ENSMUST00000198583	MSTRG.23659	Gm42767
ENSMUST00000198585	ENSMUSG00000072769	Gm10419
ENSMUST00000198593	ENSMUSG00000097639	Platr4
ENSMUST00000198595	MSTRG.23113	Gm42490
ENSMUST00000198600	MSTRG.24737	5830487J09Rik
ENSMUST00000198605	MSTRG.24309	Gm43593
ENSMUST00000198613	MSTRG.20122	Gm43075
ENSMUST00000198616	MSTRG.19340	Gm43667
ENSMUST00000198617	MSTRG.25199	Gm43338
ENSMUST00000198619	ENSMUSG00000105389	BB187690
ENSMUST00000198622	ENSMUSG00000105419	Gm43205
ENSMUST00000198623	ENSMUSG00000028177	1810013D15Rik
ENSMUST00000198627	ENSMUSG00000104634	Gm42461
ENSMUST00000198632	MSTRG.24931	2810432F15Rik
ENSMUST00000198636	MSTRG.20623	Gm42881
ENSMUST00000198640	MSTRG.24937	4930563F08Rik
ENSMUST00000198643	ENSMUSG00000105578	Gm43663
ENSMUST00000198647	MSTRG.20812	Gm42967
ENSMUST00000198654	MSTRG.23710	Gm42479
ENSMUST00000198656	MSTRG.24669	Gm42879
ENSMUST00000198672	ENSMUSG00000104672	Gm43665
ENSMUST00000198676	MSTRG.23696	Gm43042
ENSMUST00000198677	ENSMUSG00000105353	Gm42428
ENSMUST00000198678	MSTRG.24821	Gm43034
ENSMUST00000198681	ENSMUSG00000094230	Gm21847
ENSMUST00000198686	ENSMUSG00000104868	Gm9954
ENSMUST00000198687	ENSMUSG00000106219	5830416I19Rik
ENSMUST00000198691	ENSMUSG00000105639	Gm42516
ENSMUST00000198696	ENSMUSG00000105387	Gm29681
ENSMUST00000198712	ENSMUSG00000105891	A230001M10Rik
ENSMUST00000198715	MSTRG.20507	4930447N08Rik
ENSMUST00000198718	MSTRG.23073	2510017J16Rik
ENSMUST00000198720	MSTRG.23672	9230114K14Rik
ENSMUST00000198723	MSTRG.19281	Gm43344
ENSMUST00000198724	ENSMUSG00000105442	Gm42614
ENSMUST00000198725	ENSMUSG00000106146	Gm43045
ENSMUST00000198731	MSTRG.23301	Gm42585
ENSMUST00000198737	MSTRG.20605	Gm43351
ENSMUST00000198744	ENSMUSG00000106492	Gm29707
ENSMUST00000198749	MSTRG.20870	Gm43527
ENSMUST00000198751	ENSMUSG00000105662	Gm6639
ENSMUST00000198760	MSTRG.23644	Gm43830
ENSMUST00000198771	MSTRG.23657	Gm43046
ENSMUST00000198772	MSTRG.25190	Gm43702
ENSMUST00000198787	MSTRG.25157	Gm42815
ENSMUST00000198804	MSTRG.19402	Gm42436
ENSMUST00000198813	ENSMUSG00000104545	E030032P16Rik
ENSMUST00000198817	ENSMUSG00000104867	Gm43728
ENSMUST00000198822	MSTRG.20681	4930599N24Rik
ENSMUST00000198825	MSTRG.23100	Gm30835
ENSMUST00000198827	MSTRG.20304	Gm40117
ENSMUST00000198829	MSTRG.19964	Gm43401

ENSMUST00000198850	MSTRG.20246	Gm43061
ENSMUST00000198852	MSTRG.24171	Gm43514
ENSMUST00000198853	MSTRG.24840	Gm42633
ENSMUST00000198855	ENSMUSG00000106093	Gm42722
ENSMUST00000198856	MSTRG.19544	Gm43549
ENSMUST00000198857	MSTRG.25223	Gm43708
ENSMUST00000198863	MSTRG.23623	Gm42460
ENSMUST00000198873	MSTRG.25293	Gm15708
ENSMUST00000198874	MSTRG.19376	Gm43488
ENSMUST00000198875	MSTRG.23590	E430021H15Rik
ENSMUST00000198876	ENSMUSG00000104829	Gm43685
ENSMUST00000198879	MSTRG.20507	4930447N08Rik
ENSMUST00000198885	ENSMUSG00000104568	Gm43255
ENSMUST00000198888	MSTRG.20638	Gm42874
ENSMUST00000198897	MSTRG.23647	Gm43321
ENSMUST00000198898	ENSMUSG00000106361	Gm35066
ENSMUST00000198904	MSTRG.23707	Gm42482
ENSMUST00000198917	ENSMUSG00000105155	Gm42910
ENSMUST00000198918	MSTRG.24722	Gm43301
ENSMUST00000198924	ENSMUSG00000105577	Gm34599
ENSMUST00000198927	MSTRG.12349	2010309G21Rik
ENSMUST00000198939	ENSMUSG00000104677	Gm43376
ENSMUST00000198943	MSTRG.20371	Gm43099
ENSMUST00000198947	MSTRG.20035	6330562C20Rik
ENSMUST00000198949	ENSMUSG00000104567	Gm43209
ENSMUST00000198961	ENSMUSG00000105698	Gm31881
ENSMUST00000198962	ENSMUSG00000105857	Gm42817
ENSMUST00000198963	MSTRG.24119	Gm43145
ENSMUST00000198964	ENSMUSG00000104833	Gm42489
ENSMUST00000198971	MSTRG.20255	Gm43065
ENSMUST00000198978	MSTRG.24851	Gm42839
ENSMUST00000198989	ENSMUSG00000106010	Gm42616
ENSMUST00000198992	ENSMUSG00000106120	Gm42697
ENSMUST00000199005	ENSMUSG00000104800	Gm36017
ENSMUST00000199006	MSTRG.19355	Gm43080
ENSMUST00000199007	ENSMUSG00000106515	Gm30382
ENSMUST00000199015	MSTRG.20547	Gm43005
ENSMUST00000199027	ENSMUSG00000106612	Gm43750
ENSMUST00000199037	MSTRG.23887	4933408A14Rik
ENSMUST00000199047	MSTRG.23667	Gm43670
ENSMUST00000199056	MSTRG.23200	A930003O13Rik
ENSMUST00000199058	ENSMUSG00000106306	4933401H06Rik
ENSMUST00000199060	MSTRG.20496	Gm17494
ENSMUST00000199064	ENSMUSG00000105022	Gm43537
ENSMUST00000199065	ENSMUSG00000106639	Gm43121
ENSMUST00000199071	MSTRG.20284	Gm42700
ENSMUST00000199072	MSTRG.19242	Gm43672
ENSMUST00000199075	MSTRG.20421	Gm42687
ENSMUST00000199080	ENSMUSG00000104708	Gm42503
ENSMUST00000199082	ENSMUSG00000106103	Gm43081
ENSMUST00000199084	MSTRG.20489	Gm43609
ENSMUST00000199085	MSTRG.23210	Gm42948
ENSMUST00000199097	MSTRG.24929	Gm42589
ENSMUST00000199099	MSTRG.24798	Gm43661
ENSMUST00000199116	ENSMUSG00000106365	Gm43568
ENSMUST00000199119	ENSMUSG00000106547	B230303O12Rik
ENSMUST00000199123	MSTRG.24399	Gm43138

ENSMUST00000199138	MSTRG.20524	Gm43283
ENSMUST00000199139	ENSMUSG00000105954	Gm42793
ENSMUST00000199155	ENSMUSG00000097639	Platr4
ENSMUST00000199168	MSTRG.20179	Gm42868
ENSMUST00000199172	ENSMUSG00000104535	Gm42686
ENSMUST00000199177	MSTRG.25327	Gm43625
ENSMUST00000199187	MSTRG.20643	Gm43254
ENSMUST00000199190	MSTRG.23600	Gm42521
ENSMUST00000199198	MSTRG.19369	Gm43821
ENSMUST00000199205	MSTRG.20265	Gm38412
ENSMUST00000199206	MSTRG.20515	Gm35065
ENSMUST00000199211	ENSMUSG00000106255	Gm36793
ENSMUST00000199219	MSTRG.20608	5830437K03Rik
ENSMUST00000199221	MSTRG.24434	Gm27680
ENSMUST00000199224	ENSMUSG00000105174	Gm43353
ENSMUST00000199228	ENSMUSG00000105568	Gm42971
ENSMUST00000199230	ENSMUSG00000105511	Gm33758
ENSMUST00000199249	MSTRG.24664	Gm42918
ENSMUST00000199252	MSTRG.23711	Gm42478
ENSMUST00000199255	ENSMUSG00000105959	D530037P16Rik
ENSMUST00000199264	MSTRG.25060	4933404O12Rik
ENSMUST00000199268	MSTRG.20651	Gm42567
ENSMUST00000199271	MSTRG.10287	Gm30214
ENSMUST00000199275	MSTRG.24194	Gm43789
ENSMUST00000199279	MSTRG.24729	Gm43684
ENSMUST00000199282	ENSMUSG00000105247	Gm42519
ENSMUST00000199285	ENSMUSG00000105695	Gm43327
ENSMUST00000199286	ENSMUSG00000105437	Gm42450
ENSMUST00000199289	ENSMUSG00000106054	Gm43623
ENSMUST00000199292	MSTRG.20887	Gm43485
ENSMUST00000199296	MSTRG.23793	Gm42725
ENSMUST00000199304	MSTRG.23586	Gm42555
ENSMUST00000199309	ENSMUSG00000062496	4930431F12Rik
ENSMUST00000199326	MSTRG.23362	Gm9899
ENSMUST00000199336	MSTRG.25248	Gm43378
ENSMUST00000199347	ENSMUSG00000105000	Gm43616
ENSMUST00000199354	MSTRG.24687	Gm42748
ENSMUST00000199355	MSTRG.12357	Gm42870
ENSMUST00000199356	MSTRG.24834	4930404A12Rik
ENSMUST00000199363	ENSMUSG00000105316	Gm42953
ENSMUST00000199366	ENSMUSG00000106048	Gm42444
ENSMUST00000199375	MSTRG.20261	Gm43585
ENSMUST00000199378	ENSMUSG00000106647	Gm42523
ENSMUST00000199380	ENSMUSG00000106210	1700001N15Rik
ENSMUST00000199385	MSTRG.20264	Gm43696
ENSMUST00000199386	ENSMUSG00000105296	Gm19708
ENSMUST00000199400	MSTRG.19581	Gm43589
ENSMUST00000199408	ENSMUSG00000105516	Gm36823
ENSMUST00000199414	MSTRG.19366	Gm43820
ENSMUST00000199417	MSTRG.20469	Gm42664
ENSMUST00000199418	MSTRG.20814	Gm43858
ENSMUST00000199419	ENSMUSG00000097339	Gm26671
ENSMUST00000199431	MSTRG.20209	Gm42538
ENSMUST00000199434	ENSMUSG00000105642	Gm43134
ENSMUST00000199438	ENSMUSG00000105186	Gm43778
ENSMUST00000199441	MSTRG.23786	1110003F10Rik
ENSMUST00000199446	MSTRG.20880	Gm42611

ENSMUST00000199465	MSTRG.20899	Gm43307
ENSMUST00000199474	ENSMUSG00000106538	Gm30301
ENSMUST00000199476	MSTRG.23584	Gm43182
ENSMUST00000199477	ENSMUSG00000055961	BC051076
ENSMUST00000199480	MSTRG.26192	Gm42889
ENSMUST00000199482	ENSMUSG00000104901	Gm42692
ENSMUST00000199486	ENSMUSG00000106554	Gm33474
ENSMUST00000199488	ENSMUSG00000105168	Gm30735
ENSMUST00000199492	MSTRG.20867	Gm43528
ENSMUST00000199495	MSTRG.23220	Gm43054
ENSMUST00000199500	MSTRG.24460	Gm43758
ENSMUST00000199503	ENSMUSG00000106515	Gm30382
ENSMUST00000199508	MSTRG.24299	Gm42902
ENSMUST00000199515	MSTRG.20289	Gm42702
ENSMUST00000199517	MSTRG.24123	C430019N01Rik
ENSMUST00000199524	ENSMUSG00000105767	Gm43607
ENSMUST00000199544	MSTRG.23205	Gm43169
ENSMUST00000199547	MSTRG.23672	9230114K14Rik
ENSMUST00000199551	MSTRG.12359	A530030E21Rik
ENSMUST00000199552	MSTRG.19559	6720482G16Rik
ENSMUST00000199556	ENSMUSG00000104963	Gm42974
ENSMUST00000199565	MSTRG.24114	Gm42605
ENSMUST00000199580	MSTRG.26316	Gm43292
ENSMUST00000199589	ENSMUSG00000104764	Gm43066
ENSMUST00000199595	MSTRG.33424	Gm42763
ENSMUST00000199604	MSTRG.20734	Gm43826
ENSMUST00000199606	MSTRG.25256	BC030343
ENSMUST00000199608	MSTRG.19354	8430422M14Rik
ENSMUST00000199610	MSTRG.23327	C79130
ENSMUST00000199616	MSTRG.3003	4933428P19Rik
ENSMUST00000199630	MSTRG.20166	Gm42819
ENSMUST00000199631	MSTRG.24877	Gm15903
ENSMUST00000199632	MSTRG.23077	4833413G10Rik
ENSMUST00000199642	MSTRG.23601	Gm42520
ENSMUST00000199644	MSTRG.24440	Gm42778
ENSMUST00000199645	MSTRG.20519	Snhg8
ENSMUST00000199648	ENSMUSG00000106466	Gm40348
ENSMUST00000199649	MSTRG.24174	Gm43273
ENSMUST00000199650	MSTRG.23075	Gm43825
ENSMUST00000199653	MSTRG.23670	Gm43176
ENSMUST00000199654	ENSMUSG00000105926	Gm43386
ENSMUST00000199657	MSTRG.23662	Gm42770
ENSMUST00000199660	MSTRG.23193	6030443J06Rik
ENSMUST00000199663	ENSMUSG00000105762	Gm43605
ENSMUST00000199664	MSTRG.25287	Gm43267
ENSMUST00000199676	MSTRG.20866	Gm42943
ENSMUST00000199677	MSTRG.23740	Gm43721
ENSMUST00000199688	MSTRG.23234	Gm42951
ENSMUST00000199690	MSTRG.20268	A530041M06Rik
ENSMUST00000199706	MSTRG.20323	Gm43330
ENSMUST00000199713	ENSMUSG00000105062	Gm43113
ENSMUST00000199721	MSTRG.20069	Gm17690
ENSMUST00000199724	ENSMUSG00000072769	Gm10419
ENSMUST00000199729	MSTRG.23319	Gm43776
ENSMUST00000199733	ENSMUSG00000106524	Gm42706
ENSMUST00000199734	MSTRG.23568	6030400A10Rik
ENSMUST00000199747	ENSMUSG00000104677	Gm43376

ENSMUST00000199759	ENSMUSG00000097339	Gm26671
ENSMUST00000199762	MSTRG.20271	Gm43584
ENSMUST00000199764	ENSMUSG00000105302	Gm19817
ENSMUST00000199778	MSTRG.24939	Gm42441
ENSMUST00000199779	MSTRG.24170	Gm43513
ENSMUST00000199780	MSTRG.24288	Gm43422
ENSMUST00000199783	ENSMUSG00000106491	Gm42446
ENSMUST00000199788	ENSMUSG00000104793	Gm43756
ENSMUST00000199790	ENSMUSG00000106329	Gm10652
ENSMUST00000199792	MSTRG.23713	Gm42640
ENSMUST00000199793	MSTRG.24740	Gm42907
ENSMUST00000199794	MSTRG.19348	Gm43079
ENSMUST00000199796	MSTRG.20445	Gm43651
ENSMUST00000199799	MSTRG.25046	Gm43604
ENSMUST00000199805	MSTRG.23672	9230114K14Rik
ENSMUST00000199806	ENSMUSG00000106528	Gm42841
ENSMUST00000199820	MSTRG.19922	Gm45354
ENSMUST00000199829	ENSMUSG00000104665	Gm43366
ENSMUST00000199836	ENSMUSG00000105354	D130017N08Rik
ENSMUST00000199844	MSTRG.19756	Gm42812
ENSMUST00000199847	MSTRG.24872	Gm40332
ENSMUST00000199851	MSTRG.24788	Gm42651
ENSMUST00000199858	ENSMUSG00000105822	Gm42969
ENSMUST00000199859	MSTRG.20280	Gm42696
ENSMUST00000199867	ENSMUSG00000104888	1500005C15Rik
ENSMUST00000199871	ENSMUSG00000104622	Gm42541
ENSMUST00000199877	MSTRG.20830	A530083M17Rik
ENSMUST00000199879	MSTRG.23591	Gm42983
ENSMUST00000199885	ENSMUSG00000105850	Gm2861
ENSMUST00000199889	ENSMUSG00000097124	A530020G20Rik
ENSMUST00000199893	ENSMUSG00000105356	Gm42603
ENSMUST00000199898	ENSMUSG00000104727	Gm42500
ENSMUST00000199915	MSTRG.19866	Gm43737
ENSMUST00000199936	MSTRG.20554	Gm42515
ENSMUST00000199939	MSTRG.23651	Gm43318
ENSMUST00000199941	MSTRG.23211	AI506816
ENSMUST00000199945	MSTRG.23665	Gm42772
ENSMUST00000199950	ENSMUSG00000104705	4930405N21Rik
ENSMUST00000199951	MSTRG.19573	Gm43570
ENSMUST00000199953	MSTRG.20191	Gm42869
ENSMUST00000199958	ENSMUSG00000105703	Gm43305
ENSMUST00000199962	MSTRG.20750	Gm43646
ENSMUST00000199971	MSTRG.24834	4930404A12Rik
ENSMUST00000199972	MSTRG.20116	Gm42508
ENSMUST00000199975	ENSMUSG00000106575	Gm42613
ENSMUST00000199979	MSTRG.20544	Gm43009
ENSMUST00000199980	ENSMUSG00000105797	Gm5149
ENSMUST00000199981	ENSMUSG00000105059	Gm42498
ENSMUST00000199995	MSTRG.20296	Gm43256
ENSMUST00000199996	MSTRG.20379	Gm43435
ENSMUST00000200000	MSTRG.24326	5830411K02Rik
ENSMUST00000200001	ENSMUSG00000106311	Gm43755
ENSMUST00000200006	MSTRG.19802	1700113A16Rik
ENSMUST00000200007	ENSMUSG00000104684	5430427N15Rik
ENSMUST00000200008	ENSMUSG00000105501	5330426L24Rik
ENSMUST00000200016	MSTRG.23787	Gm43311
ENSMUST00000200017	ENSMUSG00000106300	Gm42765

ENSMUST00000200022	ENSMUSG00000104563	Gm43041
ENSMUST00000200026	MSTRG.20629	Gm42872
ENSMUST00000200032	MSTRG.24806	Gm43339
ENSMUST00000200033	ENSMUSG00000105347	Gm43503
ENSMUST00000200034	MSTRG.24214	D930016D06Rik
ENSMUST00000200044	MSTRG.24280	Gm43421
ENSMUST00000200047	MSTRG.20609	Gm43349
ENSMUST00000200051	ENSMUSG00000105476	Gm35439
ENSMUST00000200055	MSTRG.23345	A230098N10Rik
ENSMUST00000200060	ENSMUSG00000106155	Gm43495
ENSMUST00000200067	MSTRG.24689	Gm43571
ENSMUST00000200070	MSTRG.20320	Gm43847
ENSMUST00000200072	ENSMUSG00000104586	4921539H07Rik
ENSMUST00000200075	MSTRG.20813	Gm43859
ENSMUST00000200077	MSTRG.24116	Gm42604
ENSMUST00000200084	MSTRG.20165	Gm42820
ENSMUST00000200088	ENSMUSG00000105366	Gm43719
ENSMUST00000200089	MSTRG.23658	Gm43047
ENSMUST00000200102	ENSMUSG00000105485	Gm42447
ENSMUST00000200104	MSTRG.23652	Gm43341
ENSMUST00000200108	ENSMUSG00000105681	Gm43428
ENSMUST00000200110	MSTRG.23096	Gm42598
ENSMUST00000200118	MSTRG.24737	5830487J09Rik
ENSMUST00000200120	MSTRG.20323	Gm43329
ENSMUST00000200128	MSTRG.24947	2610011E03Rik
ENSMUST00000200129	ENSMUSG00000106338	Gm43645
ENSMUST00000200130	MSTRG.19345	Gm43203
ENSMUST00000200133	ENSMUSG00000104781	Gm43303
ENSMUST00000200134	MSTRG.24742	Gm43813
ENSMUST00000200139	ENSMUSG00000106357	Gm8013
ENSMUST00000200140	ENSMUSG00000104988	Gm43622
ENSMUST00000200143	MSTRG.23753	Gm20033
ENSMUST00000200150	ENSMUSG00000105440	Gm31693
ENSMUST00000200155	ENSMUSG00000106628	Gm43558
ENSMUST00000200176	ENSMUSG00000105324	Gm42705
ENSMUST00000200179	ENSMUSG00000097311	Gm26871
ENSMUST00000200180	MSTRG.24957	Gm43188
ENSMUST00000200182	ENSMUSG00000104835	Gm5547
ENSMUST00000200184	ENSMUSG00000105282	Gm42981
ENSMUST00000200193	MSTRG.24469	4930557B06Rik
ENSMUST00000200199	ENSMUSG00000106485	3830422I06Rik
ENSMUST00000200213	MSTRG.19921	Gm45477
ENSMUST00000200220	MSTRG.25263	Gm42501
ENSMUST00000200221	ENSMUSG00000106354	Gm42607
ENSMUST00000200222	MSTRG.23051	Gm36548
ENSMUST00000200244	ENSMUSG00000105136	8030487O14Rik
ENSMUST00000200254	ENSMUSG00000105652	4930519L02Rik
ENSMUST00000200255	MSTRG.20897	B230334C09Rik
ENSMUST00000200257	MSTRG.19912	Gm19710
ENSMUST00000200260	MSTRG.33400	Gm43732
ENSMUST00000200263	MSTRG.23320	Gm43502
ENSMUST00000200265	MSTRG.20192	Gm43462
ENSMUST00000200266	MSTRG.23704	Gm42635
ENSMUST00000200275	ENSMUSG00000105322	Gm43751
ENSMUST00000200281	ENSMUSG00000104894	Gm43507
ENSMUST00000200282	ENSMUSG00000106584	4930502C17Rik
ENSMUST00000200283	MSTRG.20581	Gm43072

ENSMUST00000200285	ENSMUSG00000104998	Gm43822
ENSMUST00000200288	ENSMUSG00000104698	Gm42602
ENSMUST00000200303	ENSMUSG00000104938	Gm42840
ENSMUST00000200306	ENSMUSG00000105443	Gm43837
ENSMUST00000200312	MSTRG.20291	Gm43258
ENSMUST00000200319	ENSMUSG00000106500	Gm42540
ENSMUST00000200320	MSTRG.19912	Gm19710
ENSMUST00000200321	MSTRG.20598	Gm42997
ENSMUST00000200324	ENSMUSG00000106275	Gm42495
ENSMUST00000200329	ENSMUSG00000086438	Asb17os
ENSMUST00000200336	ENSMUSG00000105106	Gm43657
ENSMUST00000200343	MSTRG.20815	Gm43857
ENSMUST00000200347	MSTRG.20212	Gm42941
ENSMUST00000200349	MSTRG.20616	Gm42449
ENSMUST00000200359	MSTRG.23791	Gm43290
ENSMUST00000200361	MSTRG.20447	Gm42940
ENSMUST00000200367	MSTRG.20199	Gm43465
ENSMUST00000200369	MSTRG.23717	Gm42486
ENSMUST00000200370	MSTRG.19516	Gm42898
ENSMUST00000200378	ENSMUSG00000105740	Gm42685
ENSMUST00000200385	MSTRG.23614	Gm43200
ENSMUST00000200395	ENSMUSG00000104851	E030026E10Rik
ENSMUST00000200403	MSTRG.24397	Gm43136
ENSMUST00000200405	ENSMUSG00000104939	Gm33651
ENSMUST00000200411	MSTRG.24760	Gm43413
ENSMUST00000200412	MSTRG.19701	4921511C10Rik
ENSMUST00000200416	ENSMUSG00000106168	Gm43538
ENSMUST00000200427	ENSMUSG00000106483	Gm32736
ENSMUST00000200435	MSTRG.20129	Gm31305
ENSMUST00000200437	MSTRG.19919	4930537H20Rik
ENSMUST00000200442	ENSMUSG00000104816	Gm43473
ENSMUST00000200448	MSTRG.24812	Gm43340
ENSMUST00000200449	MSTRG.20603	Gm36520
ENSMUST00000200465	MSTRG.23650	Gm10048
ENSMUST00000200473	MSTRG.23589	Gm42427
ENSMUST00000200487	MSTRG.24491	Gm43336
ENSMUST00000200494	MSTRG.1500	4930596I21Rik
ENSMUST00000200502	MSTRG.20317	Gm43846
ENSMUST00000200507	MSTRG.20677	Gm42822
ENSMUST00000200523	ENSMUSG00000105699	Gm43703
ENSMUST00000200525	MSTRG.20424	Gm43108
ENSMUST00000200529	ENSMUSG00000106306	4933401H06Rik
ENSMUST00000200537	MSTRG.23789	Gm43289
ENSMUST00000200538	MSTRG.24930	Gm42588
ENSMUST00000200563	MSTRG.23362	Gm9899
ENSMUST00000200579	ENSMUSG00000105648	Gm43505
ENSMUST00000200582	ENSMUSG00000106015	Gm42980
ENSMUST00000200590	MSTRG.10187	Gm43766
ENSMUST00000200599	MSTRG.24175	Gm43272
ENSMUST00000200602	MSTRG.19337	Gm43077
ENSMUST00000200608	MSTRG.20809	Gm42710
ENSMUST00000200612	ENSMUSG00000105660	Gm42975
ENSMUST00000200614	MSTRG.25190	Gm43195
ENSMUST00000200632	MSTRG.23578	Gm43183
ENSMUST00000200640	MSTRG.20090	E330034L11Rik
ENSMUST00000200641	ENSMUSG00000105376	Gm36535
ENSMUST00000200644	ENSMUSG00000106549	Gm42653

ENSMUST00000200646	MSTRG.24952	Gm42989
ENSMUST00000200648	ENSMUSG00000106139	Gm30648
ENSMUST00000200652	MSTRG.24955	Gm43187
ENSMUST00000200664	MSTRG.20574	Gm43654
ENSMUST00000200668	MSTRG.24663	Gm42917
ENSMUST00000200675	ENSMUSG00000106332	Gm43506
ENSMUST00000200678	MSTRG.25423	Gm43807
ENSMUST00000200694	MSTRG.23408	Gm43809
ENSMUST00000200696	MSTRG.24004	Gm43084
ENSMUST00000200697	ENSMUSG00000107134	Gm42528
ENSMUST00000200700	ENSMUSG00000107318	Gm30003
ENSMUST00000200707	ENSMUSG00000107198	Gm19619
ENSMUST00000200718	MSTRG.25389	5730422E09Rik
ENSMUST00000200723	MSTRG.23865	Gm42736
ENSMUST00000200729	MSTRG.24969	Gm42884
ENSMUST00000200738	MSTRG.23397	AI839979
ENSMUST00000200747	ENSMUSG00000107373	Gm42668
ENSMUST00000200749	ENSMUSG00000106766	4933424N20Rik
ENSMUST00000200753	MSTRG.23853	1700025A08Rik
ENSMUST00000200757	ENSMUSG00000106735	A330058E17Rik
ENSMUST00000200761	MSTRG.25697	Gm42895
ENSMUST00000200763	ENSMUSG00000106802	Gm42885
ENSMUST00000200768	MSTRG.23792	4930589O11Rik
ENSMUST00000200773	MSTRG.23428	Gm43694
ENSMUST00000200774	MSTRG.23776	Gm42648
ENSMUST00000200783	ENSMUSG00000106863	Gm42109
ENSMUST00000200788	ENSMUSG00000106694	Gm42894
ENSMUST00000200791	ENSMUSG00000107287	Gm43458
ENSMUST00000200801	MSTRG.25804	Gm43479
ENSMUST00000200819	ENSMUSG00000106871	Gm3289
ENSMUST00000200820	ENSMUSG00000106733	Gm36186
ENSMUST00000200842	MSTRG.24916	Gm43165
ENSMUST00000200848	MSTRG.25582	Gm42856
ENSMUST00000200851	ENSMUSG00000087516	Tbx3os1
ENSMUST00000200853	MSTRG.23384	Gm42798
ENSMUST00000200855	ENSMUSG00000107214	4930500F04Rik
ENSMUST00000200866	ENSMUSG00000107322	4933439J24Rik
ENSMUST00000200867	MSTRG.23470	Gm43457
ENSMUST00000200872	MSTRG.24660	Gm42930
ENSMUST00000200879	MSTRG.25394	Gm42787
ENSMUST00000200887	MSTRG.25422	Gm43298
ENSMUST00000200893	ENSMUSG00000106863	Gm42109
ENSMUST00000200900	MSTRG.25360	Gm43553
ENSMUST00000200904	ENSMUSG00000106808	Gm42745
ENSMUST00000200913	MSTRG.23806	4930480C01Rik
ENSMUST00000200924	ENSMUSG00000107172	4933430H06Rik
ENSMUST00000200925	ENSMUSG00000107070	Gm35191
ENSMUST00000200931	MSTRG.25377	Gm43150
ENSMUST00000200934	MSTRG.25372	Gm19719
ENSMUST00000200937	MSTRG.25373	BC028471
ENSMUST00000200940	MSTRG.23682	Gm45495
ENSMUST00000200950	MSTRG.23684	Gm10441
ENSMUST00000200951	ENSMUSG00000085058	8030453O22Rik
ENSMUST00000200956	MSTRG.23396	Gm43660
ENSMUST00000200971	MSTRG.23827	Gm43794
ENSMUST00000200980	ENSMUSG00000106753	Gm43230
ENSMUST00000200982	ENSMUSG00000107360	Gm42558

ENSMUST00000201004	ENSMUSG00000107251	Gm43102
ENSMUST00000201006	MSTRG.24651	Gm43069
ENSMUST00000201018	MSTRG.23414	Gm43810
ENSMUST00000201019	MSTRG.23813	Gm43775
ENSMUST00000201027	ENSMUSG00000106745	Gm43342
ENSMUST00000201032	MSTRG.25442	Gm20559
ENSMUST00000201035	ENSMUSG00000107164	4933425D22Rik
ENSMUST00000201036	MSTRG.24619	Gm43785
ENSMUST00000201039	MSTRG.25708	Gm43168
ENSMUST00000201049	ENSMUSG00000107173	Gm43266
ENSMUST00000201051	MSTRG.23960	Gm42803
ENSMUST00000201056	MSTRG.24583	Gm42473
ENSMUST00000201062	ENSMUSG00000106795	Gm43050
ENSMUST00000201074	MSTRG.23861	Gm42737
ENSMUST00000201080	ENSMUSG00000106799	Gm20756
ENSMUST00000201093	MSTRG.25376	Gm43151
ENSMUST00000201095	MSTRG.24648	Gm43068
ENSMUST00000201096	MSTRG.24618	Gm43051
ENSMUST00000201101	MSTRG.25421	Gm36447
ENSMUST00000201115	MSTRG.23869	3110031N09Rik
ENSMUST00000201125	MSTRG.23376	Abhd1
ENSMUST00000201126	ENSMUSG00000106712	Gm19649
ENSMUST00000201128	MSTRG.25345	D5Ertd605e
ENSMUST00000201137	MSTRG.2214	Gm43497
ENSMUST00000201142	MSTRG.25430	1700028E10Rik
ENSMUST00000201152	ENSMUSG00000106795	Gm43050
ENSMUST00000201153	MSTRG.24002	Gm43594
ENSMUST00000201159	MSTRG.25592	Gm42547
ENSMUST00000201161	MSTRG.23890	Gm9870
ENSMUST00000201164	ENSMUSG00000106791	4930553P18Rik
ENSMUST00000201179	ENSMUSG00000106812	Gm28563
ENSMUST00000201187	MSTRG.23522	Gm42507
ENSMUST00000201190	MSTRG.23850	4930425K10Rik
ENSMUST00000201195	MSTRG.24547	Gm13830
ENSMUST00000201197	MSTRG.23933	Gm42571
ENSMUST00000201201	MSTRG.24965	Gm43480
ENSMUST00000201213	ENSMUSG00000106980	Gm43690
ENSMUST00000201214	MSTRG.23376	Abhd1
ENSMUST00000201216	MSTRG.23490	Gm42560
ENSMUST00000201249	MSTRG.24914	Gm42896
ENSMUST00000201268	MSTRG.24889	Gm42790
ENSMUST00000201273	ENSMUSG00000107021	Gm42853
ENSMUST00000201281	MSTRG.24883	2210412B16Rik
ENSMUST00000201293	MSTRG.24561	A930005G22Rik
ENSMUST00000201308	MSTRG.23937	Gm42599
ENSMUST00000201317	ENSMUSG00000106786	Gm42781
ENSMUST00000201318	ENSMUSG00000106855	5330437M03Rik
ENSMUST00000201324	ENSMUSG00000106799	Gm20756
ENSMUST00000201325	MSTRG.23458	Gm42851
ENSMUST00000201339	MSTRG.23376	Abhd1
ENSMUST00000201340	ENSMUSG00000106905	Gm42741
ENSMUST00000201350	MSTRG.27112	1700018A23Rik
ENSMUST00000201366	MSTRG.23394	4930478M09Rik
ENSMUST00000201367	MSTRG.23950	Gm42577
ENSMUST00000201375	MSTRG.25679	2210408F21Rik
ENSMUST00000201397	MSTRG.25411	Gm42556
ENSMUST00000201398	MSTRG.23420	Gm43313

ENSMUST00000201402	ENSMUSG00000106756	Gm32780
ENSMUST00000201403	ENSMUSG00000107042	4921513H07Rik
ENSMUST00000201412	MSTRG.23955	Gm43747
ENSMUST00000201414	MSTRG.24886	Cct6a
ENSMUST00000201444	MSTRG.25373	BC028471
ENSMUST00000201451	MSTRG.24618	Gm43052
ENSMUST00000201457	MSTRG.24584	Gm42471
ENSMUST00000201470	ENSMUSG00000107126	Gm40289
ENSMUST00000201477	MSTRG.23461	Gm42848
ENSMUST00000201486	MSTRG.24913	Gm42897
ENSMUST00000201492	ENSMUSG00000107208	D130004A15Rik
ENSMUST00000201493	MSTRG.25442	Gm43197
ENSMUST00000201501	ENSMUSG00000107045	Gm43636
ENSMUST00000201506	MSTRG.24657	Gm42656
ENSMUST00000201517	ENSMUSG00000107008	Gm2762
ENSMUST00000201536	ENSMUSG00000107182	Gm43268
ENSMUST00000201539	MSTRG.24621	Gm43788
ENSMUST00000201546	MSTRG.27120	Gm43126
ENSMUST00000201551	ENSMUSG00000107061	Gm19590
ENSMUST00000201553	ENSMUSG00000107199	Gm42550
ENSMUST00000201573	MSTRG.23780	Gm42645
ENSMUST00000201581	MSTRG.24096	Gm43681
ENSMUST00000201584	ENSMUSG00000106714	Gm42546
ENSMUST00000201586	MSTRG.23924	Gm43417
ENSMUST00000201590	ENSMUSG00000106725	Gm43363
ENSMUST00000201604	MSTRG.25395	Gm42788
ENSMUST00000201618	MSTRG.24896	Gm42986
ENSMUST00000201619	ENSMUSG00000106683	Gm43263
ENSMUST00000201640	ENSMUSG00000106970	Gm43377
ENSMUST00000201641	MSTRG.25373	Gm20488
ENSMUST00000201645	MSTRG.23824	Gm43793
ENSMUST00000201648	MSTRG.25417	Gm42531
ENSMUST00000201650	MSTRG.25419	Gm43597
ENSMUST00000201653	MSTRG.24886	Cct6a
ENSMUST00000201655	MSTRG.23419	Gm43312
ENSMUST00000201656	ENSMUSG00000106999	Gm42734
ENSMUST00000201682	MSTRG.2215	Gm43496
ENSMUST00000201689	MSTRG.25635	4930528J11Rik
ENSMUST00000201707	ENSMUSG00000106832	Gm42632
ENSMUST00000201715	MSTRG.24966	Gm43481
ENSMUST00000201718	MSTRG.23857	Gm42735
ENSMUST00000201728	MSTRG.24563	1110006O24Rik
ENSMUST00000201732	ENSMUSG00000106743	Gm42847
ENSMUST00000201733	ENSMUSG00000106754	Gm43101
ENSMUST00000201742	MSTRG.23496	Gm43860
ENSMUST00000201755	MSTRG.23943	6720475M21Rik
ENSMUST00000201762	MSTRG.25439	Gm43196
ENSMUST00000201764	ENSMUSG00000106747	Gm43025
ENSMUST00000201770	MSTRG.23803	Gm42729
ENSMUST00000201771	MSTRG.23469	Gm42846
ENSMUST00000201775	MSTRG.25567	Gm20186
ENSMUST00000201788	MSTRG.24981	Gm42882
ENSMUST00000201790	MSTRG.23397	AI839979
ENSMUST00000201793	MSTRG.15099	Gm43425
ENSMUST00000201811	MSTRG.23479	Gm42559
ENSMUST00000201812	ENSMUSG00000107312	Gm43229
ENSMUST00000201816	MSTRG.23834	Gm42670

ENSMUST00000201818	MSTRG.26506	Gm19265
ENSMUST00000201829	MSTRG.25627	Gm43029
ENSMUST00000201831	MSTRG.25442	Gm20559
ENSMUST00000201839	MSTRG.25013	Gm42731
ENSMUST00000201843	MSTRG.25416	Gm42529
ENSMUST00000201845	MSTRG.23913	Gm43799
ENSMUST00000201848	MSTRG.23841	Gm43282
ENSMUST00000201862	MSTRG.26489	Gm43588
ENSMUST00000201865	MSTRG.23832	C530043K16Rik
ENSMUST00000201868	MSTRG.25682	Gm43800
ENSMUST00000201871	MSTRG.23772	Gm43769
ENSMUST00000201872	MSTRG.23783	Gm43004
ENSMUST00000201893	ENSMUSG00000107029	Gm43123
ENSMUST00000201903	MSTRG.23818	1700126H18Rik
ENSMUST00000201914	ENSMUSG00000106843	Gm42458
ENSMUST00000201916	MSTRG.23926	Gm43416
ENSMUST00000201927	MSTRG.24886	Cct6a
ENSMUST00000201933	ENSMUSG00000106995	Gm33167
ENSMUST00000201934	MSTRG.23925	Gm43415
ENSMUST00000201941	ENSMUSG00000107125	Gm3822
ENSMUST00000201943	MSTRG.25436	Gm8579
ENSMUST00000201944	MSTRG.24539	C730045M19Rik
ENSMUST00000201947	ENSMUSG00000106913	9430007M09Rik
ENSMUST00000201951	ENSMUSG00000106862	Gm42811
ENSMUST00000201966	MSTRG.25797	Gm42962
ENSMUST00000201967	MSTRG.23376	Abhd1
ENSMUST00000201971	MSTRG.24637	2510016D11Rik
ENSMUST00000201973	ENSMUSG00000107385	C330024D21Rik
ENSMUST00000201983	MSTRG.23548	4930487D11Rik
ENSMUST00000201992	MSTRG.25419	Gm43597
ENSMUST00000202001	MSTRG.23947	Gm43167
ENSMUST00000202005	ENSMUSG00000107153	Gm38404
ENSMUST00000202009	ENSMUSG00000106934	Gm42506
ENSMUST00000202013	MSTRG.23376	Abhd1
ENSMUST00000202022	MSTRG.25373	BC028471
ENSMUST00000202026	ENSMUSG00000106762	4930478P22Rik
ENSMUST00000202029	ENSMUSG00000107256	Gm13821
ENSMUST00000202033	MSTRG.23408	Gm43809
ENSMUST00000202037	ENSMUSG00000107384	Gm42557
ENSMUST00000202051	MSTRG.23830	Gm43795
ENSMUST00000202053	MSTRG.24622	Gm43275
ENSMUST00000202056	ENSMUSG00000106675	Gm43798
ENSMUST00000202066	ENSMUSG00000106738	Gm5
ENSMUST00000202069	MSTRG.23775	N4bp2os
ENSMUST00000202073	MSTRG.23425	Gm10461
ENSMUST00000202081	MSTRG.26490	Gm42600
ENSMUST00000202084	ENSMUSG00000106994	Gm10829
ENSMUST00000202091	MSTRG.23900	Gm43499
ENSMUST00000202098	ENSMUSG00000106680	Gm43852
ENSMUST00000202099	MSTRG.23855	Gm42466
ENSMUST00000202120	ENSMUSG00000107106	Gm43760
ENSMUST00000202128	MSTRG.25382	Gm15411
ENSMUST00000202132	MSTRG.17152	Gm43761
ENSMUST00000202139	MSTRG.24089	Gm43172
ENSMUST00000202148	MSTRG.25567	Gm20186
ENSMUST00000202150	MSTRG.24094	Gm43682
ENSMUST00000202154	MSTRG.23685	Gm40304

ENSMUST00000202166	ENSMUSG00000106746	2900064F13Rik
ENSMUST00000202188	ENSMUSG00000107132	Gm15997
ENSMUST00000202206	MSTRG.24599	Gm43637
ENSMUST00000202212	MSTRG.17146	Gm43128
ENSMUST00000202216	MSTRG.25430	1700028E10Rik
ENSMUST00000202228	MSTRG.24988	9030607J07Rik
ENSMUST00000202229	MSTRG.25382	Gm15406
ENSMUST00000202233	MSTRG.23782	Gm42646
ENSMUST00000202246	MSTRG.24090	Gm43173
ENSMUST00000202249	MSTRG.24049	Gm43688
ENSMUST00000202251	ENSMUSG00000107353	4930430O22Rik
ENSMUST00000202262	MSTRG.24886	Cct6a
ENSMUST00000202274	MSTRG.23800	Gm42726
ENSMUST00000202276	MSTRG.24511	Gm42789
ENSMUST00000202292	MSTRG.23930	Gm42572
ENSMUST00000202297	MSTRG.23398	2900076G11Rik
ENSMUST00000202307	ENSMUSG00000087516	Tbx3os1
ENSMUST00000202325	MSTRG.23465	4930557J02Rik
ENSMUST00000202328	MSTRG.23811	Gm43323
ENSMUST00000202329	MSTRG.25736	Gm43748
ENSMUST00000202336	MSTRG.23952	Gm42576
ENSMUST00000202337	ENSMUSG00000107089	Gm43443
ENSMUST00000202338	ENSMUSG00000106845	Gm43156
ENSMUST00000202340	MSTRG.25373	BC028471
ENSMUST00000202348	MSTRG.23966	Gm43658
ENSMUST00000202351	MSTRG.25346	Gm43461
ENSMUST00000202366	ENSMUSG00000107008	Gm2762
ENSMUST00000202376	ENSMUSG00000107300	Gm43279
ENSMUST00000202386	ENSMUSG00000106738	Gm5
ENSMUST00000202387	MSTRG.24645	Gm43579
ENSMUST00000202390	MSTRG.23848	Gm43698
ENSMUST00000202392	MSTRG.25594	Gm42549
ENSMUST00000202396	ENSMUSG00000106944	Gm43843
ENSMUST00000202399	MSTRG.24066	Gm43040
ENSMUST00000202401	MSTRG.23376	Abhd1
ENSMUST00000202425	ENSMUSG00000107148	Gm42888
ENSMUST00000202427	MSTRG.12080	Gm42477
ENSMUST00000202437	MSTRG.23395	Gm43059
ENSMUST00000202446	MSTRG.23802	Gm42727
ENSMUST00000202454	MSTRG.23829	Gm43343
ENSMUST00000202455	MSTRG.27129	Gm43633
ENSMUST00000202460	MSTRG.23991	Gm34648
ENSMUST00000202462	ENSMUSG00000107177	Gm43845
ENSMUST00000202466	MSTRG.24886	Cct6a
ENSMUST00000202469	ENSMUSG00000106986	Gm42887
ENSMUST00000202482	MSTRG.25418	Gm43598
ENSMUST00000202485	MSTRG.25373	BC028471
ENSMUST00000202489	ENSMUSG00000106775	C130093G08Rik
ENSMUST00000202491	MSTRG.25347	Gm43460
ENSMUST00000202492	MSTRG.23826	Gm43792
ENSMUST00000202494	ENSMUSG00000107386	Gm42800
ENSMUST00000202495	MSTRG.25375	Gm43152
ENSMUST00000202498	ENSMUSG00000106673	Gm43374
ENSMUST00000202502	ENSMUSG00000107008	Gm2762
ENSMUST00000202511	MSTRG.25403	Gm43332
ENSMUST00000202512	MSTRG.23541	Gm42858
ENSMUST00000202514	MSTRG.25442	Gm20559

ENSMUST00000202522	MSTRG.23840	Gm43281
ENSMUST00000202529	MSTRG.25716	Gm43164
ENSMUST00000202533	MSTRG.27118	Gm43635
ENSMUST00000202534	MSTRG.23372	Gm43808
ENSMUST00000202542	MSTRG.25567	Gm20186
ENSMUST00000202544	MSTRG.25442	Gm20559
ENSMUST00000202545	MSTRG.24618	Gm43274
ENSMUST00000202546	ENSMUSG00000106991	Gm43399
ENSMUST00000202548	ENSMUSG00000107353	4930430O22Rik
ENSMUST00000202552	ENSMUSG00000107363	Gm43373
ENSMUST00000202553	MSTRG.17157	Gm43764
ENSMUST00000202562	MSTRG.25589	Gm42801
ENSMUST00000202583	ENSMUSG00000106674	Gm20005
ENSMUST00000202592	MSTRG.24535	1810017P11Rik
ENSMUST00000202607	MSTRG.24553	Gm42903
ENSMUST00000202626	ENSMUSG00000106873	BC049739
ENSMUST00000202634	ENSMUSG00000107385	C330024D21Rik
ENSMUST00000202644	MSTRG.23801	Gm42728
ENSMUST00000202649	MSTRG.25558	Gm42852
ENSMUST00000202654	MSTRG.25373	BC028471
ENSMUST00000202662	MSTRG.24994	Gm43372
ENSMUST00000202665	ENSMUSG00000107053	1700021F13Rik
ENSMUST00000202669	MSTRG.25595	Gm42548
ENSMUST00000202671	MSTRG.27130	Gm43634
ENSMUST00000202673	MSTRG.23775	N4bp2os
ENSMUST00000202675	ENSMUSG00000106709	Gm30270
ENSMUST00000202678	ENSMUSG00000106826	Gm42583
ENSMUST00000202680	MSTRG.24073	Gm43599
ENSMUST00000202688	ENSMUSG00000107078	Gm36840
ENSMUST00000202694	MSTRG.23822	Gm43790
ENSMUST00000202696	MSTRG.25442	Gm20559
ENSMUST00000202699	MSTRG.23795	Gm40309
ENSMUST00000202708	MSTRG.23450	Gm10459
ENSMUST00000202719	MSTRG.24906	Gm6598
ENSMUST00000202733	MSTRG.17166	Gm43621
ENSMUST00000202739	ENSMUSG00000107008	Gm2762
ENSMUST00000202742	ENSMUSG00000106741	4930513D17Rik
ENSMUST00000202771	MSTRG.15094	A930012L18Rik
ENSMUST00000202780	MSTRG.25691	Gm43293
ENSMUST00000202782	ENSMUSG00000106915	Gm42655
ENSMUST00000202793	MSTRG.23923	Usp46os2
ENSMUST00000202797	MSTRG.24897	Gm43482
ENSMUST00000202800	ENSMUSG00000106741	4930513D17Rik
ENSMUST00000202826	MSTRG.11339	Peg13
ENSMUST00000202834	MSTRG.23457	Gm42849
ENSMUST00000202838	ENSMUSG00000107234	Gm43371
ENSMUST00000202850	MSTRG.25455	Gm43294
ENSMUST00000202854	MSTRG.24886	Cct6a
ENSMUST00000202859	ENSMUSG00000106957	Gm43085
ENSMUST00000202860	MSTRG.25419	Gm43597
ENSMUST00000202862	MSTRG.23417	Gm43811
ENSMUST00000202871	MSTRG.24918	Gm43166
ENSMUST00000202883	MSTRG.24053	Gm43039
ENSMUST00000202887	MSTRG.23907	Gm42732
ENSMUST00000202890	ENSMUSG00000107117	Gm43842
ENSMUST00000202893	MSTRG.23374	5930420M18Rik
ENSMUST00000202895	MSTRG.24968	2700029L08Rik

ENSMUST00000202906	ENSMUSG00000106897	Gm43027
ENSMUST00000202914	MSTRG.23501	Gm43791
ENSMUST00000202923	ENSMUSG00000106896	G630022F23Rik
ENSMUST00000202926	MSTRG.25564	Gm43844
ENSMUST00000202931	MSTRG.23807	Gm43771
ENSMUST00000202933	MSTRG.25554	Gm43533
ENSMUST00000202943	MSTRG.25802	Gm42963
ENSMUST00000202954	MSTRG.24905	Gm42467
ENSMUST00000202959	MSTRG.23805	C030017G13Rik
ENSMUST00000202960	ENSMUSG00000107240	Gm43231
ENSMUST00000202983	MSTRG.23863	D630030B08Rik
ENSMUST00000202995	MSTRG.23484	Gm42867
ENSMUST00000203013	MSTRG.27004	Gm43915
ENSMUST00000203019	MSTRG.26766	Gm43947
ENSMUST00000203022	ENSMUSG00000108290	Gm44120
ENSMUST00000203027	MSTRG.26568	9930120I10Rik
ENSMUST00000203044	MSTRG.26893	Gm44206
ENSMUST00000203047	ENSMUSG00000108060	4921529L05Rik
ENSMUST00000203049	MSTRG.26598	Gm44264
ENSMUST00000203051	MSTRG.25933	Gm43881
ENSMUST00000203055	MSTRG.27464	Gm44021
ENSMUST00000203063	MSTRG.26558	Gm44198
ENSMUST00000203069	MSTRG.26437	Gm44287
ENSMUST00000203084	MSTRG.27419	Gm44270
ENSMUST00000203086	MSTRG.25899	Gm44731
ENSMUST00000203087	MSTRG.25920	6330419E04Rik
ENSMUST00000203092	MSTRG.25463	Gm44250
ENSMUST00000203093	ENSMUSG00000107480	Gm44165
ENSMUST00000203102	MSTRG.17193	Gm44291
ENSMUST00000203115	ENSMUSG00000107397	4930402H05Rik
ENSMUST00000203125	ENSMUSG00000108014	Gm44380
ENSMUST00000203139	MSTRG.26793	Gm44440
ENSMUST00000203145	ENSMUSG00000107517	Gm44046
ENSMUST00000203153	ENSMUSG00000107878	Gm44106
ENSMUST00000203158	MSTRG.26609	Gm44001
ENSMUST00000203171	ENSMUSG00000107552	Gm44096
ENSMUST00000203175	ENSMUSG00000107734	Gm30055
ENSMUST00000203181	MSTRG.26600	Gm44170
ENSMUST00000203184	MSTRG.26717	Gm44065
ENSMUST00000203186	MSTRG.26606	Gm44005
ENSMUST00000203191	MSTRG.1246	2900060B14Rik
ENSMUST00000203211	ENSMUSG00000108088	Gm44229
ENSMUST00000203218	MSTRG.25915	Gm43872
ENSMUST00000203231	MSTRG.27249	Gm44017
ENSMUST00000203245	ENSMUSG00000107714	Gm34933
ENSMUST00000203249	MSTRG.25382	1810059H22Rik
ENSMUST00000203251	MSTRG.27402	Gm44086
ENSMUST00000203259	ENSMUSG00000107839	4930434O05Rik
ENSMUST00000203260	MSTRG.25908	9430018G01Rik
ENSMUST00000203263	MSTRG.27180	Gm44066
ENSMUST00000203270	ENSMUSG00000107431	Gm44114
ENSMUST00000203277	ENSMUSG00000096299	Gm21814
ENSMUST00000203285	MSTRG.26424	Gm43981
ENSMUST00000203287	MSTRG.27087	Gm44597
ENSMUST00000203289	MSTRG.25484	Gm43961
ENSMUST00000203293	MSTRG.26149	Gm45060
ENSMUST00000203298	MSTRG.26550	Gm44417

ENSMUST00000203300	MSTRG.27273	Gm44238
ENSMUST00000203301	MSTRG.26068	Gm43966
ENSMUST00000203304	ENSMUSG00000107620	Gm44256
ENSMUST00000203306	ENSMUSG00000107488	Gm31579
ENSMUST00000203312	ENSMUSG00000107883	Gm44062
ENSMUST00000203319	MSTRG.25935	Gm43875
ENSMUST00000203332	MSTRG.26912	9530062K07Rik
ENSMUST00000203342	MSTRG.25461	Gm44432
ENSMUST00000203350	MSTRG.27181	Gm44067
ENSMUST00000203360	MSTRG.26815	Gm44040
ENSMUST00000203361	MSTRG.26919	Gm44957
ENSMUST00000203364	MSTRG.26856	Gm44053
ENSMUST00000203367	ENSMUSG00000046764	A530053G22Rik
ENSMUST00000203368	MSTRG.26630	C030015A19Rik
ENSMUST00000203376	MSTRG.26601	Gm44430
ENSMUST00000203378	ENSMUSG00000090254	Gm1965
ENSMUST00000203379	MSTRG.26788	Gm44439
ENSMUST00000203383	MSTRG.26180	4930597O21Rik
ENSMUST00000203387	MSTRG.25901	Gm44253
ENSMUST00000203389	ENSMUSG00000107632	Gm44070
ENSMUST00000203393	MSTRG.26553	9530013L04Rik
ENSMUST00000203397	MSTRG.26123	Gm10209
ENSMUST00000203407	MSTRG.19416	6430590A07Rik
ENSMUST00000203417	ENSMUSG00000108179	Gm44274
ENSMUST00000203419	MSTRG.17245	Gm44027
ENSMUST00000203423	MSTRG.27109	Gm26728
ENSMUST00000203426	ENSMUSG00000107976	Gm44043
ENSMUST00000203437	MSTRG.26446	Gm43890
ENSMUST00000203438	MSTRG.25469	Gm44424
ENSMUST00000203440	ENSMUSG00000108246	Gm43896
ENSMUST00000203442	ENSMUSG00000107768	Gm44275
ENSMUST00000203443	MSTRG.27385	Gm43909
ENSMUST00000203451	ENSMUSG00000087289	4933424M12Rik
ENSMUST00000203453	MSTRG.26046	Gm44077
ENSMUST00000203466	MSTRG.26860	Gm43963
ENSMUST00000203474	MSTRG.26758	Gm43948
ENSMUST00000203503	MSTRG.26764	9530086O07Rik
ENSMUST00000203508	MSTRG.17209	Gm44146
ENSMUST00000203511	ENSMUSG00000107422	Gm44129
ENSMUST00000203540	MSTRG.26403	Gm44433
ENSMUST00000203549	ENSMUSG00000108288	Gm44202
ENSMUST00000203567	MSTRG.27449	4933406L23Rik
ENSMUST00000203569	ENSMUSG00000107812	Gm43935
ENSMUST00000203570	MSTRG.26859	Gm43964
ENSMUST00000203592	ENSMUSG00000107792	Gm43914
ENSMUST00000203605	MSTRG.27396	Gm44164
ENSMUST00000203620	MSTRG.26893	Gm44206
ENSMUST00000203631	MSTRG.26594	Gm44265
ENSMUST00000203635	MSTRG.25923	Gm43880
ENSMUST00000203642	MSTRG.26790	Gm44441
ENSMUST00000203645	MSTRG.25972	Gm45738
ENSMUST00000203660	MSTRG.26554	Gm44414
ENSMUST00000203662	MSTRG.25382	1810059H22Rik
ENSMUST00000203666	MSTRG.26165	Gm44072
ENSMUST00000203667	MSTRG.26161	9330118I20Rik
ENSMUST00000203672	MSTRG.33960	Firre
ENSMUST00000203679	ENSMUSG00000107567	4930480K02Rik

ENSMUST00000203683	MSTRG.17218	Gm44144
ENSMUST00000203686	ENSMUSG00000108276	Gm36640
ENSMUST00000203691	MSTRG.25382	1810059H22Rik
ENSMUST00000203697	MSTRG.27007	Gm4651
ENSMUST00000203699	MSTRG.26865	Gm44033
ENSMUST00000203701	ENSMUSG00000107696	Gm44171
ENSMUST00000203702	MSTRG.26581	4933412L11Rik
ENSMUST00000203703	MSTRG.27207	1700101I11Rik
ENSMUST00000203704	ENSMUSG00000107622	4930512J16Rik
ENSMUST00000203708	MSTRG.27465	Gm44022
ENSMUST00000203709	MSTRG.26765	Gm43953
ENSMUST00000203718	MSTRG.27000	Gm44148
ENSMUST00000203729	ENSMUSG00000096299	Gm21814
ENSMUST00000203736	MSTRG.25382	1810059H22Rik
ENSMUST00000203746	MSTRG.26567	Gm44423
ENSMUST00000203752	ENSMUSG00000107704	Gm36355
ENSMUST00000203765	MSTRG.27244	Gm43952
ENSMUST00000203778	ENSMUSG00000108187	4930511E03Rik
ENSMUST00000203779	MSTRG.27162	Gm43965
ENSMUST00000203780	MSTRG.27174	2310001H17Rik
ENSMUST00000203781	ENSMUSG00000108145	Gm38811
ENSMUST00000203784	MSTRG.26696	Gm44220
ENSMUST00000203790	ENSMUSG00000108048	Gm43990
ENSMUST00000203807	MSTRG.26634	Gm45901
ENSMUST00000203809	ENSMUSG00000107521	Gm43994
ENSMUST00000203811	MSTRG.25914	Gm43879
ENSMUST00000203815	ENSMUSG00000108161	Gm32914
ENSMUST00000203819	MSTRG.19413	Gm44136
ENSMUST00000203822	MSTRG.26928	Gm43863
ENSMUST00000203831	MSTRG.25924	Gm43876
ENSMUST00000203832	MSTRG.26409	Gm44437
ENSMUST00000203836	MSTRG.25886	Gm44284
ENSMUST00000203841	MSTRG.27100	4930417O13Rik
ENSMUST00000203845	ENSMUSG00000108026	Gm44007
ENSMUST00000203852	ENSMUSG00000107614	Gm44196
ENSMUST00000203855	ENSMUSG00000108010	Gm38708
ENSMUST00000203856	ENSMUSG00000107989	Gm19692
ENSMUST00000203858	MSTRG.26888	D830050J10Rik
ENSMUST00000203859	ENSMUSG00000108229	1700102F20Rik
ENSMUST00000203868	ENSMUSG00000107903	Gm43930
ENSMUST00000203871	MSTRG.26318	Gm44110
ENSMUST00000203879	MSTRG.19566	4930593A02Rik
ENSMUST00000203894	ENSMUSG00000107548	Gm43866
ENSMUST00000203905	ENSMUSG00000107496	4933431M02Rik
ENSMUST00000203917	ENSMUSG00000107405	Gm44115
ENSMUST00000203918	MSTRG.26066	Gm44434
ENSMUST00000203920	MSTRG.27339	Gm10400
ENSMUST00000203924	ENSMUSG00000108258	Gm43913
ENSMUST00000203927	ENSMUSG00000107434	Gm4640
ENSMUST00000203932	MSTRG.27457	4732416N19Rik
ENSMUST00000203933	MSTRG.33960	Firre
ENSMUST00000203950	MSTRG.25917	Gm43889
ENSMUST00000203951	MSTRG.26811	Gm44101
ENSMUST00000203953	MSTRG.25913	B230112I24Rik
ENSMUST00000203956	MSTRG.27078	2010008C14Rik
ENSMUST00000203963	MSTRG.29952	9530085L11Rik
ENSMUST00000203964	MSTRG.17202	Gm44183

ENSMUST00000203974	ENSMUSG00000097603	A430010J10Rik
ENSMUST00000203985	MSTRG.26106	Gm44026
ENSMUST00000203987	ENSMUSG00000107876	Gm43936
ENSMUST00000203992	MSTRG.26693	Gm43923
ENSMUST00000203998	MSTRG.25988	Gm38804
ENSMUST00000204002	MSTRG.25940	Gm43883
ENSMUST00000204008	MSTRG.26517	1600020E01Rik
ENSMUST00000204020	ENSMUSG00000107585	3300002P13Rik
ENSMUST00000204021	MSTRG.26005	Gm44764
ENSMUST00000204022	MSTRG.26197	Gm38825
ENSMUST00000204031	MSTRG.26736	Gm44102
ENSMUST00000204033	ENSMUSG00000107933	Gm30498
ENSMUST00000204037	MSTRG.27166	Gm10069
ENSMUST00000204047	MSTRG.26517	1600020E01Rik
ENSMUST00000204049	MSTRG.27366	Gm44068
ENSMUST00000204055	MSTRG.26548	Gm44416
ENSMUST00000204058	MSTRG.26767	Gm43946
ENSMUST00000204068	MSTRG.26517	1600020E01Rik
ENSMUST00000204070	ENSMUSG00000107651	Gm44166
ENSMUST00000204075	MSTRG.26063	Gm44445
ENSMUST00000204079	ENSMUSG00000108194	1700097M23Rik
ENSMUST00000204081	MSTRG.27166	Gm10069
ENSMUST00000204082	ENSMUSG00000107882	Gm44278
ENSMUST00000204098	MSTRG.26406	Gm44286
ENSMUST00000204101	ENSMUSG00000108094	Gm31108
ENSMUST00000204106	ENSMUSG00000108173	Gm44231
ENSMUST00000204112	MSTRG.26560	Gm44097
ENSMUST00000204118	ENSMUSG00000107460	Gm44186
ENSMUST00000204123	ENSMUSG00000108206	Gm44427
ENSMUST00000204130	MSTRG.27109	Gm26728
ENSMUST00000204131	MSTRG.26689	Gm44224
ENSMUST00000204151	MSTRG.26547	Gm44415
ENSMUST00000204153	MSTRG.26529	Gm44214
ENSMUST00000204154	MSTRG.26476	Gm44012
ENSMUST00000204155	MSTRG.27477	AC132412.1
ENSMUST00000204157	MSTRG.27301	Gm44140
ENSMUST00000204159	ENSMUSG00000107580	Gm44124
ENSMUST00000204162	MSTRG.26537	Gm44153
ENSMUST00000204173	ENSMUSG00000107766	Gm44073
ENSMUST00000204195	ENSMUSG00000107893	Gm44192
ENSMUST00000204200	MSTRG.26794	Gm44436
ENSMUST00000204215	MSTRG.26401	6330415B21Rik
ENSMUST00000204216	MSTRG.26510	Gm44941
ENSMUST00000204219	MSTRG.26551	Gm44418
ENSMUST00000204220	ENSMUSG00000108038	D030044L04Rik
ENSMUST00000204222	MSTRG.26341	Gm44194
ENSMUST00000204226	ENSMUSG00000107619	Gm32479
ENSMUST00000204231	MSTRG.25945	Gm44141
ENSMUST00000204237	MSTRG.27263	Gm36328
ENSMUST00000204240	ENSMUSG00000107637	1700069P05Rik
ENSMUST00000204244	MSTRG.26763	Gm26911
ENSMUST00000204246	MSTRG.26400	Gm44234
ENSMUST00000204247	MSTRG.27405	Gm44087
ENSMUST00000204251	MSTRG.25922	Gm43884
ENSMUST00000204262	MSTRG.26321	Gm44321
ENSMUST00000204269	MSTRG.26755	Gm44116
ENSMUST00000204271	MSTRG.27333	Gm30524

ENSMUST00000204274	ENSMUSG00000108020	6820426E19Rik
ENSMUST00000204275	ENSMUSG00000107667	C530044C16Rik
ENSMUST00000204286	MSTRG.26607	Gm43999
ENSMUST00000204288	ENSMUSG00000107689	Gm44386
ENSMUST00000204295	MSTRG.29949	E330037G11Rik
ENSMUST00000204296	MSTRG.25459	Gm43921
ENSMUST00000204297	MSTRG.17221	5730507A11Rik
ENSMUST00000204298	ENSMUSG00000107528	Gm44442
ENSMUST00000204312	MSTRG.26404	Gm44235
ENSMUST00000204314	MSTRG.26959	Gm44317
ENSMUST00000204323	MSTRG.27209	Gm44243
ENSMUST00000204328	MSTRG.27174	2310001H17Rik
ENSMUST00000204330	MSTRG.27285	Gm19434
ENSMUST00000204345	MSTRG.26093	Gm44008
ENSMUST00000204346	ENSMUSG00000107699	Gm43970
ENSMUST00000204352	MSTRG.26861	Gm43912
ENSMUST00000204358	MSTRG.27201	Gm43992
ENSMUST00000204360	MSTRG.27340	Gm43958
ENSMUST00000204364	MSTRG.26914	Gm44103
ENSMUST00000204379	MSTRG.26402	Gm44435
ENSMUST00000204381	MSTRG.26536	Gm44093
ENSMUST00000204387	ENSMUSG00000108092	Gm44189
ENSMUST00000204388	MSTRG.26405	Gm44438
ENSMUST00000204392	MSTRG.27325	Gm44013
ENSMUST00000204395	MSTRG.26151	Gm45061
ENSMUST00000204396	MSTRG.17189	Gm44443
ENSMUST00000204397	MSTRG.26806	Gm17055
ENSMUST00000204401	MSTRG.26642	Gm44105
ENSMUST00000204405	MSTRG.25930	Gm43888
ENSMUST00000204406	MSTRG.26822	Gm44081
ENSMUST00000204407	MSTRG.27028	Gm5112
ENSMUST00000204412	ENSMUSG00000108228	6430584L05Rik
ENSMUST00000204426	MSTRG.27260	1700051K13Rik
ENSMUST00000204442	ENSMUSG00000107605	Gm44117
ENSMUST00000204444	MSTRG.26928	4933440N22Rik
ENSMUST00000204449	MSTRG.26685	9530026P05Rik
ENSMUST00000204451	MSTRG.26087	Gm44080
ENSMUST00000204452	ENSMUSG00000108168	Gm43864
ENSMUST00000204461	MSTRG.17188	Gm44283
ENSMUST00000204462	MSTRG.27305	Gm44401
ENSMUST00000204469	ENSMUSG00000107815	Gm44257
ENSMUST00000204474	ENSMUSG00000108234	Gm44428
ENSMUST00000204490	MSTRG.26001	4833403J16Rik
ENSMUST00000204491	ENSMUSG00000107516	Gm30784
ENSMUST00000204492	MSTRG.26635	Gm44236
ENSMUST00000204498	MSTRG.26839	Gm44280
ENSMUST00000204503	ENSMUSG00000107964	4930447C11Rik
ENSMUST00000204505	MSTRG.25928	Gm43873
ENSMUST00000204522	MSTRG.26760	Gm43942
ENSMUST00000204538	MSTRG.26673	Gm44249
ENSMUST00000204539	ENSMUSG00000107841	Gm44185
ENSMUST00000204543	MSTRG.26523	Gm44369
ENSMUST00000204549	MSTRG.27324	Gm43940
ENSMUST00000204551	MSTRG.26531	Gm44036
ENSMUST00000204556	ENSMUSG00000107809	Gm44209
ENSMUST00000204559	MSTRG.26906	Gm44154
ENSMUST00000204561	MSTRG.27392	Gm43910

ENSMUST00000204568	MSTRG.26620	Gm44207
ENSMUST00000204570	MSTRG.25382	1810059H22Rik
ENSMUST00000204572	MSTRG.27252	Gm43984
ENSMUST00000204577	ENSMUSG00000107546	Gm20560
ENSMUST00000204579	ENSMUSG00000046764	A530053G22Rik
ENSMUST00000204588	MSTRG.27240	Gm45769
ENSMUST00000204589	MSTRG.25382	1810059H22Rik
ENSMUST00000204597	MSTRG.27100	4930417O13Rik
ENSMUST00000204604	ENSMUSG00000107440	Gm40377
ENSMUST00000204605	ENSMUSG00000107558	2010109P13Rik
ENSMUST00000204608	ENSMUSG00000097603	A430010J10Rik
ENSMUST00000204618	MSTRG.27463	Gm44020
ENSMUST00000204623	ENSMUSG00000108220	Gm44056
ENSMUST00000204630	MSTRG.27242	Gm38910
ENSMUST00000204631	MSTRG.26669	Gm44095
ENSMUST00000204633	ENSMUSG00000108255	Gm16499
ENSMUST00000204640	MSTRG.26608	Gm43937
ENSMUST00000204642	ENSMUSG00000107879	9330102E08Rik
ENSMUST00000204652	MSTRG.27183	Gm44078
ENSMUST00000204662	MSTRG.26641	Gm44123
ENSMUST00000204663	MSTRG.26517	1600020E01Rik
ENSMUST00000204669	ENSMUSG00000107927	Gm44090
ENSMUST00000204670	MSTRG.27468	2610017A05Rik
ENSMUST00000204678	ENSMUSG00000107660	Gm6559
ENSMUST00000204686	MSTRG.26132	Gm45193
ENSMUST00000204689	MSTRG.25663	Gm44296
ENSMUST00000204703	ENSMUSG00000107967	Gm44094
ENSMUST00000204720	MSTRG.26885	Gm44157
ENSMUST00000204722	ENSMUSG00000107732	Gm44204
ENSMUST00000204727	MSTRG.25931	Gm43887
ENSMUST00000204729	MSTRG.33960	Firre
ENSMUST00000204735	MSTRG.26585	Gm44187
ENSMUST00000204736	MSTRG.26768	Gm44104
ENSMUST00000204738	MSTRG.26517	1600020E01Rik
ENSMUST00000204742	MSTRG.25919	Gm43869
ENSMUST00000204759	MSTRG.26804	0610040F04Rik
ENSMUST00000204769	MSTRG.17201	Gm35808
ENSMUST00000204781	MSTRG.26763	Gm26911
ENSMUST00000204791	ENSMUSG00000108151	Gm33201
ENSMUST00000204809	MSTRG.25382	1810059H22Rik
ENSMUST00000204811	MSTRG.26443	Gm15624
ENSMUST00000204812	ENSMUSG00000108071	Gm3455
ENSMUST00000204816	MSTRG.21761	Gm44037
ENSMUST00000204817	MSTRG.27388	Gm43931
ENSMUST00000204822	ENSMUSG00000107559	Gm44193
ENSMUST00000204824	MSTRG.26185	Gm36816
ENSMUST00000204833	MSTRG.27289	Gm43969
ENSMUST00000204835	MSTRG.26580	Gm44168
ENSMUST00000204844	ENSMUSG00000107412	Gm20383
ENSMUST00000204847	MSTRG.26190	E230016M11Rik
ENSMUST00000204848	ENSMUSG00000097924	A730020E08Rik
ENSMUST00000204851	ENSMUSG00000108046	Gm43924
ENSMUST00000204853	MSTRG.25918	Gm43874
ENSMUST00000204863	MSTRG.26330	Gm44175
ENSMUST00000204871	MSTRG.25921	Gm43882
ENSMUST00000204883	MSTRG.26845	Gm44200
ENSMUST00000204889	MSTRG.26879	Gm44079

ENSMUST00000204898	MSTRG.27306	Gm44069
ENSMUST00000204899	MSTRG.26018	Gm43980
ENSMUST00000204905	MSTRG.26130	Gm44049
ENSMUST00000204906	MSTRG.26749	Gm44045
ENSMUST00000204910	MSTRG.27150	Gm44215
ENSMUST00000204911	MSTRG.27100	4930417O13Rik
ENSMUST00000204912	MSTRG.26005	Gm44764
ENSMUST00000204928	MSTRG.25916	Gm43885
ENSMUST00000204931	MSTRG.25927	Gm43871
ENSMUST00000204933	MSTRG.27269	Gm44366
ENSMUST00000204941	MSTRG.25382	1810059H22Rik
ENSMUST00000204945	MSTRG.26564	Gm43904
ENSMUST00000204946	MSTRG.26993	Gm44260
ENSMUST00000204953	MSTRG.26469	Gm43920
ENSMUST00000204957	ENSMUSG00000108297	Gm44167
ENSMUST00000204964	MSTRG.26571	Gm44064
ENSMUST00000204973	MSTRG.26128	Gm3793
ENSMUST00000204974	MSTRG.33960	Firre
ENSMUST00000204991	MSTRG.33960	Firre
ENSMUST00000204995	MSTRG.27250	Gm43982
ENSMUST00000205000	MSTRG.27100	4930417O13Rik
ENSMUST00000205001	MSTRG.27484	Gm10388
ENSMUST00000205007	MSTRG.26538	Gm44152
ENSMUST00000205011	MSTRG.26342	1700040L08Rik
ENSMUST00000205022	MSTRG.26331	Gm44174
ENSMUST00000205028	MSTRG.26517	1600020E01Rik
ENSMUST00000205029	MSTRG.25929	Gm43877
ENSMUST00000205038	MSTRG.27202	Gm44000
ENSMUST00000205039	MSTRG.25925	Gm43886
ENSMUST00000205047	MSTRG.26006	Gm7932
ENSMUST00000205051	MSTRG.27174	2310001H17Rik
ENSMUST00000205056	MSTRG.27430	Gm44085
ENSMUST00000205059	MSTRG.33960	Firre
ENSMUST00000205061	MSTRG.26407	Gm44288
ENSMUST00000205062	MSTRG.27003	Gm43916
ENSMUST00000205064	MSTRG.26633	Gm44421
ENSMUST00000205073	ENSMUSG00000107396	4930425L21Rik
ENSMUST00000205074	ENSMUSG00000108254	Gm43908
ENSMUST00000205083	MSTRG.27157	Gm44190
ENSMUST00000205093	MSTRG.26685	9530026P05Rik
ENSMUST00000205102	MSTRG.25932	Gm43870
ENSMUST00000205104	ENSMUSG00000107767	Gm44448
ENSMUST00000205107	MSTRG.26384	D530018E20Rik
ENSMUST00000205110	MSTRG.27084	4930557K07Rik
ENSMUST00000205126	MSTRG.26622	Gm43903
ENSMUST00000205132	ENSMUSG00000108119	Gm43891
ENSMUST00000205137	MSTRG.26919	Gm44957
ENSMUST00000205146	ENSMUSG00000107902	Gm32592
ENSMUST00000205150	MSTRG.26995	Gm44014
ENSMUST00000205153	ENSMUSG00000046764	A530053G22Rik
ENSMUST00000205157	MSTRG.26334	Gm44130
ENSMUST00000205169	ENSMUSG00000107977	Gm44121
ENSMUST00000205172	MSTRG.25999	Gimap1os
ENSMUST00000205178	MSTRG.26750	Gm44044
ENSMUST00000205183	MSTRG.26922	Gm44237
ENSMUST00000205184	ENSMUSG00000107622	4930512J16Rik
ENSMUST00000205187	MSTRG.26825	Gm43868

ENSMUST00000205194	MSTRG.26526	Gm44292
ENSMUST00000205197	MSTRG.26185	Gm36816
ENSMUST00000205198	ENSMUSG00000107526	1700003I16Rik
ENSMUST00000205203	MSTRG.26000	Gm44226
ENSMUST00000205205	ENSMUSG00000108062	Gm44028
ENSMUST00000205212	MSTRG.26541	Gm44091
ENSMUST00000205216	ENSMUSG00000107785	Gm45083
ENSMUST00000205219	MSTRG.26846	Gm44199
ENSMUST00000205221	ENSMUSG00000107715	Gm44135
ENSMUST00000205226	MSTRG.26569	B130021K23Rik
ENSMUST00000205229	MSTRG.26616	Gm44178
ENSMUST00000205236	ENSMUSG00000108236	0610033M10Rik
ENSMUST00000205253	ENSMUSG00000108079	Gm44210
ENSMUST00000205260	MSTRG.28549	4930429H19Rik
ENSMUST00000205272	MSTRG.27780	Gm44796
ENSMUST00000205274	MSTRG.15723	Snhg1
ENSMUST00000205280	ENSMUSG00000108756	Gm44894
ENSMUST00000205284	MSTRG.29487	Gm39090
ENSMUST00000205285	MSTRG.28615	Gm10616
ENSMUST00000205294	ENSMUSG00000108473	Gm44739
ENSMUST00000205314	ENSMUSG00000108671	2700080J24Rik
ENSMUST00000205317	MSTRG.28579	Gm44694
ENSMUST00000205323	ENSMUSG00000108657	Gm35611
ENSMUST00000205334	ENSMUSG00000108569	Gm4593
ENSMUST00000205370	MSTRG.26366	Particl
ENSMUST00000205388	MSTRG.27885	Gm44684
ENSMUST00000205389	MSTRG.15723	Snhg1
ENSMUST00000205394	MSTRG.29378	Gm44876
ENSMUST00000205395	MSTRG.26339	Gm44770
ENSMUST00000205396	MSTRG.29537	Gm45121
ENSMUST00000205400	MSTRG.28400	Gm44509
ENSMUST00000205403	MSTRG.28085	2310043P16Rik
ENSMUST00000205409	MSTRG.28203	Gm44780
ENSMUST00000205412	MSTRG.28131	D530033B14Rik
ENSMUST00000205414	MSTRG.26667	Gm44711
ENSMUST00000205418	MSTRG.28544	Gm36696
ENSMUST00000205445	MSTRG.29231	Gm45151
ENSMUST00000205458	MSTRG.28554	Gm44552
ENSMUST00000205469	ENSMUSG00000108722	Gm34225
ENSMUST00000205476	ENSMUSG00000108553	Gm38569
ENSMUST00000205492	MSTRG.28553	Gm44645
ENSMUST00000205493	ENSMUSG00000108583	Gm44854
ENSMUST00000205504	ENSMUSG00000108711	Gm38991
ENSMUST00000205509	ENSMUSG00000108513	Gm30075
ENSMUST00000205522	ENSMUSG00000108354	4931431B13Rik
ENSMUST00000205527	MSTRG.28185	2310002F09Rik
ENSMUST00000205528	ENSMUSG00000108600	Gm32884
ENSMUST00000205529	ENSMUSG00000108383	Gm6567
ENSMUST00000205532	MSTRG.26460	Gm21284
ENSMUST00000205533	ENSMUSG00000108308	Gm45218
ENSMUST00000205539	MSTRG.15723	Snhg1
ENSMUST00000205544	MSTRG.28579	Gm44835
ENSMUST00000205547	MSTRG.26656	Gm45216
ENSMUST00000205549	MSTRG.26661	Gm44981
ENSMUST00000205557	MSTRG.28610	Gm39041
ENSMUST00000205558	MSTRG.28585	Gm45059
ENSMUST00000205565	MSTRG.29353	Gm44746

ENSMUST00000205567	MSTRG.28581	Gm44695
ENSMUST00000205571	ENSMUSG00000108709	4933431G14Rik
ENSMUST00000205574	ENSMUSG00000108790	Gm44806
ENSMUST00000205576	MSTRG.15723	Snhg1
ENSMUST00000205580	ENSMUSG00000108597	Gm44708
ENSMUST00000205586	ENSMUSG00000108409	1700025J12Rik
ENSMUST00000205591	ENSMUSG00000108373	Gm45168
ENSMUST00000205603	ENSMUSG00000108410	Gm44740
ENSMUST00000205608	MSTRG.28669	Gm44649
ENSMUST00000205611	MSTRG.28591	Gm44607
ENSMUST00000205621	ENSMUSG00000108604	Gm44850
ENSMUST00000205627	ENSMUSG00000108627	Gm45079
ENSMUST00000205637	ENSMUSG00000108545	Gm44613
ENSMUST00000205645	MSTRG.28082	Gm44567
ENSMUST00000205647	MSTRG.27679	9330104G04Rik
ENSMUST00000205660	MSTRG.29423	Gm9967
ENSMUST00000205662	ENSMUSG00000085725	Gm15873
ENSMUST00000205674	ENSMUSG00000108371	Gm38832
ENSMUST00000205675	MSTRG.29225	Gm44867
ENSMUST00000205683	ENSMUSG00000108334	Gm30771
ENSMUST00000205692	MSTRG.29542	Gm44673
ENSMUST00000205693	ENSMUSG00000091890	A830073O21Rik
ENSMUST00000205702	ENSMUSG00000108330	Gm44882
ENSMUST00000205704	MSTRG.29478	Gm45110
ENSMUST00000205705	MSTRG.28625	Gm26646
ENSMUST00000205707	MSTRG.28554	C130083A15Rik
ENSMUST00000205717	ENSMUSG00000108776	Gm45169
ENSMUST00000205726	MSTRG.28455	Gm32633
ENSMUST00000205735	MSTRG.28655	Gm44851
ENSMUST00000205745	MSTRG.29520	Gm45205
ENSMUST00000205759	MSTRG.28587	5330411O13Rik
ENSMUST00000205762	MSTRG.28589	1700011D18Rik
ENSMUST00000205775	ENSMUSG00000108446	Gm44997
ENSMUST00000205787	MSTRG.27825	4732471J01Rik
ENSMUST00000205794	ENSMUSG00000045928	4933440M02Rik
ENSMUST00000205799	MSTRG.28097	Gm45091
ENSMUST00000205810	ENSMUSG00000108428	Gm45058
ENSMUST00000205812	MSTRG.27540	Gm44878
ENSMUST00000205825	MSTRG.15723	Snhg1
ENSMUST00000205831	MSTRG.28458	Gm45212
ENSMUST00000205833	MSTRG.28104	Gm45096
ENSMUST00000205846	MSTRG.29353	Gm45191
ENSMUST00000205847	MSTRG.28226	Gm44646
ENSMUST00000205849	ENSMUSG00000108778	Gm20083
ENSMUST00000205852	MSTRG.15723	Snhg1
ENSMUST00000205858	ENSMUSG00000108417	Gm30928
ENSMUST00000205859	MSTRG.28609	Gm44639
ENSMUST00000205869	MSTRG.28101	Gm44836
ENSMUST00000205875	ENSMUSG00000108375	Gm45003
ENSMUST00000205892	ENSMUSG00000108345	4933435G04Rik
ENSMUST00000205893	MSTRG.28871	Gm16938
ENSMUST00000205909	MSTRG.15723	Snhg1
ENSMUST00000205924	MSTRG.29467	Gm31897
ENSMUST00000205925	MSTRG.28829	Gm44633
ENSMUST00000205934	ENSMUSG00000108325	Gm44898
ENSMUST00000205940	MSTRG.28089	Gm12758
ENSMUST00000205941	MSTRG.29234	Gm44777

ENSMUST00000205955	ENSMUSG00000108757	Gm45202
ENSMUST00000205957	MSTRG.29244	Gm45153
ENSMUST00000205970	ENSMUSG00000108446	Gm44997
ENSMUST00000205973	MSTRG.29238	C430039J01Rik
ENSMUST00000205975	MSTRG.27705	Mypopos
ENSMUST00000205978	ENSMUSG00000108722	Gm34225
ENSMUST00000205988	ENSMUSG00000108382	4930448A20Rik
ENSMUST00000205989	ENSMUSG00000097247	1500012K07Rik
ENSMUST00000205992	MSTRG.29487	Gm39090
ENSMUST00000205998	MSTRG.29365	4930413G21Rik
ENSMUST00000206001	ENSMUSG00000086631	Gm12784
ENSMUST00000206007	MSTRG.22910	Gm9768
ENSMUST00000206013	ENSMUSG00000090457	4930571K23Rik
ENSMUST00000206022	MSTRG.28630	Gm21057
ENSMUST00000206025	ENSMUSG00000108724	Gm44734
ENSMUST00000206029	MSTRG.15723	Snhg1
ENSMUST00000206035	ENSMUSG00000108676	1700123J17Rik
ENSMUST00000206040	ENSMUSG00000108754	Gm44748
ENSMUST00000206043	MSTRG.29217	Gm45025
ENSMUST00000206054	ENSMUSG00000108661	Gm45172
ENSMUST00000206056	MSTRG.28662	Gm44931
ENSMUST00000206057	MSTRG.29500	Gm44759
ENSMUST00000206060	ENSMUSG00000108351	Gm36736
ENSMUST00000206063	ENSMUSG00000108572	Gm44883
ENSMUST00000206075	MSTRG.27513	Gm15927
ENSMUST00000206078	ENSMUSG00000108548	Gm45006
ENSMUST00000206080	MSTRG.26449	5430434F05Rik
ENSMUST00000206082	MSTRG.28468	Gm45082
ENSMUST00000206096	MSTRG.28552	Gm44758
ENSMUST00000206111	MSTRG.28630	Gm21057
ENSMUST00000206112	MSTRG.26370	Gm45053
ENSMUST00000206113	MSTRG.15723	Snhg1
ENSMUST00000206120	MSTRG.26338	Gm44769
ENSMUST00000206130	MSTRG.28110	Gm44636
ENSMUST00000206135	MSTRG.15723	Snhg1
ENSMUST00000206141	ENSMUSG00000108658	Gm45138
ENSMUST00000206146	ENSMUSG00000108519	4833421K07Rik
ENSMUST00000206152	MSTRG.26658	4930517G19Rik
ENSMUST00000206155	MSTRG.15723	Snhg1
ENSMUST00000206172	ENSMUSG00000108432	Gm44756
ENSMUST00000206185	MSTRG.26466	Gm44750
ENSMUST00000206187	ENSMUSG00000108665	Gm44897
ENSMUST00000206188	MSTRG.28408	Gm34121
ENSMUST00000206189	ENSMUSG00000108531	Gm39091
ENSMUST00000206190	ENSMUSG00000108581	Gm44896
ENSMUST00000206197	MSTRG.29440	Gm44735
ENSMUST00000206201	MSTRG.29241	AV356131
ENSMUST00000206202	MSTRG.29350	Gm44985
ENSMUST00000206208	ENSMUSG00000108467	Gm45211
ENSMUST00000206216	ENSMUSG00000087530	Gm15533
ENSMUST00000206225	ENSMUSG00000108717	Gm44940
ENSMUST00000206226	ENSMUSG00000108431	Gm42375
ENSMUST00000206227	ENSMUSG00000108593	Gm44581
ENSMUST00000206231	MSTRG.29467	Gm31897
ENSMUST00000206239	MSTRG.29529	Gm15503
ENSMUST00000206249	ENSMUSG00000108449	Gm44507
ENSMUST00000206250	MSTRG.26663	Gm44712

ENSMUST00000206255	MSTRG.15723	Snhg1
ENSMUST00000206276	ENSMUSG00000108811	4933432K03Rik
ENSMUST00000206281	MSTRG.28487	Gm45081
ENSMUST00000206282	MSTRG.28131	D530033B14Rik
ENSMUST00000206288	MSTRG.28403	Gm44510
ENSMUST00000206290	MSTRG.27694	4833404L02Rik
ENSMUST00000206292	MSTRG.29343	Gm44986
ENSMUST00000206297	MSTRG.29508	Gm45204
ENSMUST00000206300	MSTRG.27825	4732471J01Rik
ENSMUST00000206304	MSTRG.27704	Mypopos
ENSMUST00000206316	MSTRG.28554	Gm44553
ENSMUST00000206318	ENSMUSG00000108675	Gm44970
ENSMUST00000206322	MSTRG.29225	Gm44867
ENSMUST00000206342	MSTRG.28223	5430431A17Rik
ENSMUST00000206345	MSTRG.28067	Gm4673
ENSMUST00000206354	ENSMUSG00000108483	Gm45184
ENSMUST00000206358	MSTRG.28569	AU020206
ENSMUST00000206367	ENSMUSG00000108787	2200007N16Rik
ENSMUST00000206372	MSTRG.28181	Gm36546
ENSMUST00000206373	MSTRG.28200	Gm44757
ENSMUST00000206375	MSTRG.29485	Gm44760
ENSMUST00000206381	MSTRG.27704	Mypopos
ENSMUST00000206383	MSTRG.15723	Snhg1
ENSMUST00000206384	MSTRG.28635	Gm45203
ENSMUST00000206387	MSTRG.28621	Gm44974
ENSMUST00000206401	MSTRG.28578	Gm45210
ENSMUST00000206403	MSTRG.28627	Gm44950
ENSMUST00000206404	ENSMUSG00000108770	Gm44719
ENSMUST00000206417	MSTRG.28395	Gm44616
ENSMUST00000206423	MSTRG.28632	Gm44949
ENSMUST00000206436	MSTRG.27825	4732471J01Rik
ENSMUST00000206440	MSTRG.27513	Gm15927
ENSMUST00000206449	MSTRG.28629	Gm44951
ENSMUST00000206457	ENSMUSG00000108313	Gm45052
ENSMUST00000206459	ENSMUSG00000108685	Gm45149
ENSMUST00000206461	ENSMUSG00000108373	Gm45168
ENSMUST00000206462	MSTRG.15723	Snhg1
ENSMUST00000206468	ENSMUSG00000108620	Gm20670
ENSMUST00000206473	ENSMUSG00000108645	Gm44722
ENSMUST00000206475	ENSMUSG00000097693	4930471M09Rik
ENSMUST00000206485	MSTRG.27537	Gm44877
ENSMUST00000206492	MSTRG.29234	Gm44777
ENSMUST00000206494	MSTRG.28651	Gm18310
ENSMUST00000206497	MSTRG.29533	Gm32816
ENSMUST00000206502	MSTRG.29399	Gm45141
ENSMUST00000206503	ENSMUSG00000108644	Gm44920
ENSMUST00000206512	MSTRG.29531	2610306O10Rik
ENSMUST00000206516	MSTRG.29506	9430064I24Rik
ENSMUST00000206519	MSTRG.15723	Snhg1
ENSMUST00000206521	ENSMUSG00000108448	Gm21269
ENSMUST00000206525	ENSMUSG00000108469	Gm45054
ENSMUST00000206533	ENSMUSG00000108456	4732496C06Rik
ENSMUST00000206534	MSTRG.29220	1700003G18Rik
ENSMUST00000206544	MSTRG.28408	Gm34121
ENSMUST00000206549	ENSMUSG00000108814	Gm30717
ENSMUST00000206550	MSTRG.28472	Gm44794
ENSMUST00000206555	ENSMUSG00000108413	BC026762

ENSMUST00000206557	MSTRG.28455	Gm32633
ENSMUST00000206563	MSTRG.29353	Gm44745
ENSMUST00000206576	ENSMUSG00000108751	Gm45217
ENSMUST00000206579	ENSMUSG00000108532	Gm32647
ENSMUST00000206580	ENSMUSG00000108395	Gm34811
ENSMUST00000206586	MSTRG.29388	Gm44874
ENSMUST00000206600	MSTRG.28577	2310001K20Rik
ENSMUST00000206605	ENSMUSG00000108695	Gm2511
ENSMUST00000206633	ENSMUSG00000108479	2200002A13Rik
ENSMUST00000206634	ENSMUSG00000108749	Gm44767
ENSMUST00000206640	ENSMUSG00000108412	Gm45005
ENSMUST00000206663	ENSMUSG00000108778	Gm20083
ENSMUST00000206666	MSTRG.28180	Gm44816
ENSMUST00000206668	ENSMUSG00000108697	4930486N12Rik
ENSMUST00000206670	ENSMUSG00000108474	Gm44895
ENSMUST00000206671	MSTRG.28580	Gm44834
ENSMUST00000206678	ENSMUSG00000108315	Gm44601
ENSMUST00000206697	MSTRG.29559	A130023I24Rik
ENSMUST00000206701	ENSMUSG00000108504	Gm4265
ENSMUST00000206710	MSTRG.29237	Gm45033
ENSMUST00000206723	MSTRG.28205	Gm45124
ENSMUST00000206736	MSTRG.28103	Gm45095
ENSMUST00000206737	ENSMUSG00000108360	Gm36159
ENSMUST00000206739	MSTRG.28452	Gm44627
ENSMUST00000206742	ENSMUSG00000108575	Gm35665
ENSMUST00000206746	ENSMUSG00000108318	Gm45170
ENSMUST00000206747	MSTRG.28185	2310002F09Rik
ENSMUST00000206756	ENSMUSG00000108470	Gm4598
ENSMUST00000206759	MSTRG.29463	Gm44729
ENSMUST00000206763	ENSMUSG00000108434	Gm45768
ENSMUST00000206774	MSTRG.28595	Gm39038
ENSMUST00000206776	MSTRG.28555	Gm44686
ENSMUST00000206785	ENSMUSG00000108507	6720469O03Rik
ENSMUST00000206786	MSTRG.15723	Snhg1
ENSMUST00000206788	MSTRG.27812	4933430L12Rik
ENSMUST00000206795	MSTRG.28607	Gm31510
ENSMUST00000206810	ENSMUSG00000108592	Gm38973
ENSMUST00000206812	ENSMUSG00000108670	Gm44815
ENSMUST00000206822	ENSMUSG00000108425	Gm44706
ENSMUST00000206829	ENSMUSG00000108616	Gm35040
ENSMUST00000206838	MSTRG.26357	Gm45051
ENSMUST00000206841	MSTRG.29356	Gm44664
ENSMUST00000206856	MSTRG.29562	Gm19514
ENSMUST00000206863	ENSMUSG00000108723	Gm44837
ENSMUST00000206867	ENSMUSG00000108356	9130221F21Rik
ENSMUST00000206873	MSTRG.29447	Gm31749
ENSMUST00000206881	ENSMUSG00000108417	Gm30928
ENSMUST00000206885	MSTRG.28637	Gm45206
ENSMUST00000206896	MSTRG.28669	Gm44649
ENSMUST00000206898	MSTRG.29358	Gm44663
ENSMUST00000206903	MSTRG.29423	Gm9967
ENSMUST00000206905	ENSMUSG00000108779	Gm45691
ENSMUST00000206921	ENSMUSG00000108722	Gm34225
ENSMUST00000206926	MSTRG.15723	Snhg1
ENSMUST00000206927	ENSMUSG00000108422	Gm44765
ENSMUST00000206930	MSTRG.29234	Gm44777
ENSMUST00000206938	MSTRG.26660	Gm45828

ENSMUST00000206939	MSTRG.29449	Gm44939
ENSMUST00000206943	MSTRG.28402	Gm44508
ENSMUST00000206944	MSTRG.29534	Gm44672
ENSMUST00000206950	ENSMUSG00000108311	Gm44737
ENSMUST00000206954	ENSMUSG00000108481	Gm33248
ENSMUST00000206961	MSTRG.15723	Snhg1
ENSMUST00000206967	MSTRG.29364	Gm20274
ENSMUST00000206970	MSTRG.28061	Gm44662
ENSMUST00000206972	MSTRG.5382	Appbp2os
ENSMUST00000206975	MSTRG.26345	Gm44771
ENSMUST00000206978	MSTRG.28469	Gm44721
ENSMUST00000206991	MSTRG.28546	Gm45715
ENSMUST00000206993	MSTRG.28592	4921513I08Rik
ENSMUST00000207005	MSTRG.28091	BC060293
ENSMUST00000207008	MSTRG.28474	Gm44792
ENSMUST00000207013	MSTRG.28102	Gm45094
ENSMUST00000207018	MSTRG.28187	Gm44814
ENSMUST00000207029	MSTRG.28765	Gm45222
ENSMUST00000207035	ENSMUSG00000109371	Gm44626
ENSMUST00000207036	MSTRG.29674	Gm10584
ENSMUST00000207037	MSTRG.28765	Gm45223
ENSMUST00000207045	MSTRG.28352	Gm14377
ENSMUST00000207047	MSTRG.28683	Gm44967
ENSMUST00000207051	MSTRG.29292	4930560O18Rik
ENSMUST00000207056	MSTRG.28743	Gm44995
ENSMUST00000207057	ENSMUSG00000108828	Gm34549
ENSMUST00000207061	ENSMUSG00000109394	A230057D06Rik
ENSMUST00000207066	MSTRG.28700	Gm45088
ENSMUST00000207074	ENSMUSG00000109291	Gm2814
ENSMUST00000207081	MSTRG.5160	Gm45027
ENSMUST00000207091	ENSMUSG00000109421	Gm44998
ENSMUST00000207095	MSTRG.28769	Dlg2
ENSMUST00000207096	MSTRG.29264	Gm45084
ENSMUST00000207099	MSTRG.27954	Gm38979
ENSMUST00000207119	MSTRG.28710	Gm44724
ENSMUST00000207120	ENSMUSG00000099853	Gm29328
ENSMUST00000207127	ENSMUSG00000108993	Gm44846
ENSMUST00000207133	MSTRG.6251	A930037H05Rik
ENSMUST00000207134	ENSMUSG00000108942	Gm44660
ENSMUST00000207140	MSTRG.28490	Gm44752
ENSMUST00000207156	ENSMUSG00000109401	Cpeb1os1
ENSMUST00000207166	ENSMUSG00000109082	Gm44586
ENSMUST00000207174	ENSMUSG00000108961	Gm32540
ENSMUST00000207177	MSTRG.28437	Gm44559
ENSMUST00000207179	ENSMUSG00000109133	Gm45098
ENSMUST00000207184	ENSMUSG00000094841	Gm10610
ENSMUST00000207187	MSTRG.28800	Gm45201
ENSMUST00000207189	MSTRG.29804	E230032D23Rik
ENSMUST00000207190	MSTRG.28433	Gm44742
ENSMUST00000207191	MSTRG.28917	Gm44914
ENSMUST00000207194	MSTRG.28436	Gm44562
ENSMUST00000207195	MSTRG.29898	Gm44789
ENSMUST00000207198	ENSMUSG00000109122	A230103L15Rik
ENSMUST00000207202	MSTRG.28787	Gm44679
ENSMUST00000207222	MSTRG.29676	Gm34908
ENSMUST00000207224	ENSMUSG00000109321	A330076H08Rik
ENSMUST00000207234	ENSMUSG00000109101	Gm45132

ENSMUST00000207245	ENSMUSG00000109351	Gm44632
ENSMUST00000207250	MSTRG.29875	Gm45117
ENSMUST00000207266	MSTRG.29842	Gm44775
ENSMUST00000207269	ENSMUSG00000109214	Gm44659
ENSMUST00000207273	MSTRG.6251	A930037H05Rik
ENSMUST00000207290	MSTRG.28226	Gm44646
ENSMUST00000207292	MSTRG.29123	Gm44864
ENSMUST00000207308	ENSMUSG00000108866	Gm35082
ENSMUST00000207311	MSTRG.29284	Gm44550
ENSMUST00000207313	MSTRG.28730	2610206C17Rik
ENSMUST00000207324	MSTRG.28426	Gm44936
ENSMUST00000207326	MSTRG.28693	Gm45845
ENSMUST00000207355	MSTRG.28718	Gm45838
ENSMUST00000207356	ENSMUSG00000108828	Gm34549
ENSMUST00000207365	MSTRG.29271	Gm45155
ENSMUST00000207366	MSTRG.28357	Gm45041
ENSMUST00000207369	ENSMUSG00000109013	Gm44723
ENSMUST00000207374	MSTRG.29112	Gm44773
ENSMUST00000207382	ENSMUSG00000109366	Gm44698
ENSMUST00000207402	MSTRG.29623	Gm44647
ENSMUST00000207408	ENSMUSG00000108865	Gm44617
ENSMUST00000207411	ENSMUSG00000109162	2900027M19Rik
ENSMUST00000207413	ENSMUSG00000109232	Gm44577
ENSMUST00000207423	MSTRG.28776	Gm44907
ENSMUST00000207427	MSTRG.28520	Gm45102
ENSMUST00000207434	MSTRG.28809	Gm45200
ENSMUST00000207435	MSTRG.28895	Gm44975
ENSMUST00000207445	MSTRG.68	1700034P13Rik
ENSMUST00000207446	MSTRG.6251	A930037H05Rik
ENSMUST00000207451	MSTRG.28681	Gm44899
ENSMUST00000207456	MSTRG.29815	Gm44999
ENSMUST00000207458	MSTRG.28761	E230029C05Rik
ENSMUST00000207466	MSTRG.29629	4930513N20Rik
ENSMUST00000207479	MSTRG.28799	B230206I08Rik
ENSMUST00000207499	ENSMUSG00000109089	4833411C07Rik
ENSMUST00000207503	ENSMUSG00000109057	Gm44741
ENSMUST00000207510	ENSMUSG00000109418	Gm44800
ENSMUST00000207512	ENSMUSG00000109341	Gm30873
ENSMUST00000207515	ENSMUSG00000109111	6530437J22Rik
ENSMUST00000207517	ENSMUSG00000109134	Gm45076
ENSMUST00000207526	ENSMUSG00000109194	Gm44570
ENSMUST00000207528	ENSMUSG00000109548	Gm45066
ENSMUST00000207548	MSTRG.27561	Gm45133
ENSMUST00000207552	ENSMUSG00000108930	Gm10648
ENSMUST00000207572	ENSMUSG00000108861	5930435M05Rik
ENSMUST00000207573	ENSMUSG00000108893	Gm44927
ENSMUST00000207574	MSTRG.28795	Gm45130
ENSMUST00000207575	ENSMUSG00000109338	Gm45030
ENSMUST00000207577	MSTRG.28678	Gm45718
ENSMUST00000207579	MSTRG.28782	Gm45182
ENSMUST00000207592	MSTRG.28896	Gm45188
ENSMUST00000207593	MSTRG.28798	4930567K12Rik
ENSMUST00000207600	MSTRG.27903	Gm31024
ENSMUST00000207605	ENSMUSG00000109545	Gm44788
ENSMUST00000207606	ENSMUSG00000109419	Gm45163
ENSMUST00000207610	ENSMUSG00000109284	B230311B06Rik
ENSMUST00000207620	ENSMUSG00000108968	Gm45011

ENSMUST00000207623	MSTRG.29115	Gm44772
ENSMUST00000207625	MSTRG.28941	A930030B08Rik
ENSMUST00000207627	ENSMUSG00000109021	Gm44812
ENSMUST00000207632	MSTRG.28508	Gm44667
ENSMUST00000207639	ENSMUSG00000109095	Gm44799
ENSMUST00000207640	ENSMUSG00000109395	Gm45199
ENSMUST00000207643	ENSMUSG00000109190	Gm44621
ENSMUST00000207644	ENSMUSG00000109529	Gm44801
ENSMUST00000207648	ENSMUSG00000109381	Gm44826
ENSMUST00000207649	MSTRG.27911	Gm44709
ENSMUST00000207651	MSTRG.29321	Gm45047
ENSMUST00000207657	MSTRG.28695	Gm45014
ENSMUST00000207661	ENSMUSG00000109407	Gm34664
ENSMUST00000207667	MSTRG.28441	Gm44830
ENSMUST00000207674	ENSMUSG00000109458	Gm44889
ENSMUST00000207688	MSTRG.29892	Gm45073
ENSMUST00000207694	ENSMUSG00000109321	A330076H08Rik
ENSMUST00000207696	MSTRG.28780	Gm45176
ENSMUST00000207697	MSTRG.28897	Gm45187
ENSMUST00000207700	MSTRG.27563	Gm45841
ENSMUST00000207705	MSTRG.28430	Gm44937
ENSMUST00000207718	ENSMUSG00000109473	B930025P03Rik
ENSMUST00000207723	MSTRG.28540	Gm44532
ENSMUST00000207739	ENSMUSG00000109114	Gm44753
ENSMUST00000207742	ENSMUSG00000109402	Gm45040
ENSMUST00000207744	ENSMUSG00000109562	Gm45178
ENSMUST00000207746	MSTRG.28000	C230062I16Rik
ENSMUST00000207756	MSTRG.27577	Gm44697
ENSMUST00000207760	MSTRG.28940	Gm38405
ENSMUST00000207763	ENSMUSG00000109413	Gm45165
ENSMUST00000207774	MSTRG.28518	Gm44669
ENSMUST00000207795	ENSMUSG00000108884	Gm45792
ENSMUST00000207806	MSTRG.29331	2210406H18Rik
ENSMUST00000207808	ENSMUSG00000109461	Gm44848
ENSMUST00000207816	MSTRG.28440	Gm44560
ENSMUST00000207822	MSTRG.28765	Gm45221
ENSMUST00000207829	ENSMUSG00000109200	Gm44969
ENSMUST00000207830	MSTRG.28501	Gm44755
ENSMUST00000207837	ENSMUSG00000109286	Gm44541
ENSMUST00000207838	MSTRG.29651	Gm44625
ENSMUST00000207840	MSTRG.28794	C030038I04Rik
ENSMUST00000207844	MSTRG.28797	4931412I15Rik
ENSMUST00000207847	MSTRG.28767	Gm45220
ENSMUST00000207859	ENSMUSG00000109028	Gm35842
ENSMUST00000207861	MSTRG.29313	9030407P20Rik
ENSMUST00000207878	ENSMUSG00000108967	Gm45181
ENSMUST00000207898	ENSMUSG00000108983	Gm44926
ENSMUST00000207900	MSTRG.28751	Gm45793
ENSMUST00000207903	ENSMUSG00000109113	Gm32916
ENSMUST00000207911	MSTRG.28510	Gm44668
ENSMUST00000207912	MSTRG.28532	4930405G09Rik
ENSMUST00000207914	ENSMUSG00000109089	4833411C07Rik
ENSMUST00000207920	ENSMUSG00000109452	Gm44530
ENSMUST00000207934	MSTRG.29648	Gm44623
ENSMUST00000207952	MSTRG.28538	B130024G19Rik
ENSMUST00000207954	MSTRG.29621	Gm45719
ENSMUST00000207959	MSTRG.29804	E230032D23Rik

ENSMUST00000207963	ENSMUSG00000109091	Gm44827
ENSMUST00000207965	ENSMUSG00000109424	Gm44658
ENSMUST00000207966	MSTRG.34412	2810403D21Rik
ENSMUST00000208005	ENSMUSG00000101969	Gm20125
ENSMUST00000208008	MSTRG.28443	Gm44829
ENSMUST00000208015	MSTRG.28000	C230062I16Rik
ENSMUST00000208016	MSTRG.28386	Gm45235
ENSMUST00000208024	MSTRG.28925	Gm15635
ENSMUST00000208026	ENSMUSG00000108918	Gm44802
ENSMUST00000208028	MSTRG.28761	E230029C05Rik
ENSMUST00000208034	MSTRG.6251	A930037H05Rik
ENSMUST00000208041	MSTRG.28538	B130024G19Rik
ENSMUST00000208046	MSTRG.28341	Gm32031
ENSMUST00000208051	MSTRG.28777	Gm45131
ENSMUST00000208055	ENSMUSG00000108934	Gm44732
ENSMUST00000208060	MSTRG.28383	Gm44913
ENSMUST00000208065	ENSMUSG00000109551	4930435N07Rik
ENSMUST00000208075	MSTRG.29312	4930588G17Rik
ENSMUST00000208078	ENSMUSG00000108969	Gm45044
ENSMUST00000208080	ENSMUSG00000109385	Gm44518
ENSMUST00000208097	MSTRG.29890	E230020D15Rik
ENSMUST00000208101	MSTRG.28724	Gm26708
ENSMUST00000208123	ENSMUSG00000109139	Gm44798
ENSMUST00000208140	ENSMUSG00000108987	Gm45056
ENSMUST00000208141	ENSMUSG00000109313	Gm44693
ENSMUST00000208150	ENSMUSG00000108924	4933436H12Rik
ENSMUST00000208152	MSTRG.6251	A930037H05Rik
ENSMUST00000208157	MSTRG.29330	Gm44901
ENSMUST00000208165	ENSMUSG00000109503	Gm44786
ENSMUST00000208167	ENSMUSG00000109093	Gm19950
ENSMUST00000208188	MSTRG.28788	Gm44675
ENSMUST00000208201	MSTRG.28697	Gm45867
ENSMUST00000208218	ENSMUSG00000109080	Gm38944
ENSMUST00000208228	MSTRG.27946	Gm44700
ENSMUST00000208229	MSTRG.27948	Gm44699
ENSMUST00000208234	ENSMUSG00000109262	Gm44744
ENSMUST00000208236	MSTRG.28724	Gm26708
ENSMUST00000208250	MSTRG.28927	Gm34280
ENSMUST00000208257	ENSMUSG00000097697	4833412C05Rik
ENSMUST00000208262	MSTRG.28975	Gm44545
ENSMUST00000208263	ENSMUSG00000108854	D830036C21Rik
ENSMUST00000208266	MSTRG.28761	E230029C05Rik
ENSMUST00000208277	ENSMUSG00000109508	Gm44956
ENSMUST00000208279	ENSMUSG00000108842	Gm45001
ENSMUST00000208280	MSTRG.27757	Gm45114
ENSMUST00000208287	MSTRG.29290	Gm44763
ENSMUST00000208295	ENSMUSG00000109347	Gm45175
ENSMUST00000208302	ENSMUSG00000109244	Gm44751
ENSMUST00000208305	ENSMUSG00000109018	Gm44809
ENSMUST00000208321	MSTRG.28940	Gm38405
ENSMUST00000208323	ENSMUSG00000109052	Gm45012
ENSMUST00000208325	MSTRG.28763	Gm44861
ENSMUST00000208333	ENSMUSG00000108847	B830042I05Rik
ENSMUST00000208342	MSTRG.28000	C230062I16Rik
ENSMUST00000208344	MSTRG.28348	Gm45737
ENSMUST00000208346	ENSMUSG00000109458	Gm44889
ENSMUST00000208351	MSTRG.29280	Gm44549

ENSMUST00000208353	MSTRG.29664	Gm44546
ENSMUST00000208358	ENSMUSG00000109473	B930025P03Rik
ENSMUST00000208362	MSTRG.28761	E230029C05Rik
ENSMUST00000208364	ENSMUSG00000109552	Gm44531
ENSMUST00000208375	MSTRG.28692	Gm45016
ENSMUST00000208383	MSTRG.29678	Gm35147
ENSMUST00000208403	MSTRG.28714	AC154141.1
ENSMUST00000208414	MSTRG.28791	Gm44680
ENSMUST00000208425	ENSMUSG00000109361	Gm44946
ENSMUST00000208426	ENSMUSG00000109436	Gm45075
ENSMUST00000208431	ENSMUSG00000108905	Gm44618
ENSMUST00000208444	MSTRG.28796	Gm45129
ENSMUST00000208447	MSTRG.28792	Gm44681
ENSMUST00000208448	ENSMUSG00000109402	Gm45040
ENSMUST00000208452	ENSMUSG00000108956	Gm45179
ENSMUST00000208460	ENSMUSG00000109127	Gm31135
ENSMUST00000208473	MSTRG.28762	Gm44860
ENSMUST00000208476	MSTRG.6251	A930037H05Rik
ENSMUST00000208480	ENSMUSG00000109107	Gm44781
ENSMUST00000208484	MSTRG.28242	D7Bwg0826e
ENSMUST00000208485	ENSMUSG00000109056	A630009H07Rik
ENSMUST00000208490	MSTRG.27997	Gm44873
ENSMUST00000208498	MSTRG.28434	9330162G02Rik
ENSMUST00000208508	MSTRG.30328	Gm16675
ENSMUST00000208510	MSTRG.5543	Gm21885
ENSMUST00000208511	ENSMUSG00000109097	Gm29683
ENSMUST00000208520	MSTRG.28789	Gm44676
ENSMUST00000208527	ENSMUSG00000109333	Gm30790
ENSMUST00000208537	MSTRG.28689	Gm45698
ENSMUST00000208543	MSTRG.28890	Gm19656
ENSMUST00000208547	MSTRG.28761	E230029C05Rik
ENSMUST00000208548	MSTRG.28940	Gm38405
ENSMUST00000208559	MSTRG.28362	Gm44822
ENSMUST00000208560	ENSMUSG00000109193	Gm39027
ENSMUST00000208576	ENSMUSG00000109304	Gm45112
ENSMUST00000208578	MSTRG.27567	Gm45148
ENSMUST00000208587	ENSMUSG00000109479	Gm44919
ENSMUST00000208595	MSTRG.29675	Gm44892
ENSMUST00000208604	ENSMUSG00000108949	Gm45069
ENSMUST00000208615	ENSMUSG00000038917	3930402G23Rik
ENSMUST00000208621	MSTRG.28379	9130015G15Rik
ENSMUST00000208629	MSTRG.29856	Gm45231
ENSMUST00000208630	ENSMUSG00000109125	Gm45159
ENSMUST00000208636	MSTRG.28379	9130015G15Rik
ENSMUST00000208646	MSTRG.28886	Gm15506
ENSMUST00000208648	ENSMUSG00000109167	Gm44652
ENSMUST00000208652	MSTRG.29328	Gm45137
ENSMUST00000208664	MSTRG.28706	Gm44916
ENSMUST00000208679	ENSMUSG00000108015	Gm32591
ENSMUST00000208685	ENSMUSG00000040705	A930016O22Rik
ENSMUST00000208693	MSTRG.2424	4930598N05Rik
ENSMUST00000208696	MSTRG.28378	Gm32849
ENSMUST00000208699	ENSMUSG00000053528	A530021J07Rik
ENSMUST00000208700	MSTRG.29650	Gm44624
ENSMUST00000208702	ENSMUSG00000108903	Gm45029
ENSMUST00000208727	ENSMUSG00000109237	9130214F15Rik
ENSMUST00000208733	MSTRG.29823	Gm44930

ENSMUST00000208745	MSTRG.28971	Gm44953
ENSMUST00000208748	ENSMUSG00000109209	Gm45104
ENSMUST00000208755	ENSMUSG00000109329	Gm33926
ENSMUST00000208774	MSTRG.29275	Gm44866
ENSMUST00000208780	ENSMUSG00000109359	Gm44797
ENSMUST00000208786	ENSMUSG00000108978	Gm44689
ENSMUST00000208787	MSTRG.28744	Gm44996
ENSMUST00000208790	MSTRG.28374	Gm2788
ENSMUST00000208794	ENSMUSG00000109124	Gm44516
ENSMUST00000208795	MSTRG.29653	Gm44982
ENSMUST00000208797	MSTRG.29804	E230032D23Rik
ENSMUST00000208799	ENSMUSG00000108932	Gm31463
ENSMUST00000208802	MSTRG.28506	1700112J16Rik
ENSMUST00000208803	ENSMUSG00000109083	Gm44992
ENSMUST00000208814	ENSMUSG00000030623	Prss23os
ENSMUST00000208817	MSTRG.29672	Gm44891
ENSMUST00000208822	ENSMUSG00000109321	A330076H08Rik
ENSMUST00000208830	MSTRG.29317	Gm44661
ENSMUST00000208842	MSTRG.28042	1700019A23Rik
ENSMUST00000208847	ENSMUSG00000109277	Gm39121
ENSMUST00000208849	ENSMUSG00000108832	Gm44832
ENSMUST00000208858	ENSMUSG00000109194	Gm44570
ENSMUST00000208859	ENSMUSG00000109284	B230311B06Rik
ENSMUST00000208880	MSTRG.28758	Gm45120
ENSMUST00000208883	MSTRG.28813	9530078K11Rik
ENSMUST00000208884	ENSMUSG00000109422	Gm45174
ENSMUST00000208885	MSTRG.27916	Gm44710
ENSMUST00000208887	ENSMUSG00000109294	Gm44808
ENSMUST00000208892	MSTRG.28716	Gm16638
ENSMUST00000208893	MSTRG.29795	4933417O13Rik
ENSMUST00000208911	ENSMUSG00000108970	Gm45906
ENSMUST00000208925	MSTRG.34246	Gm39526
ENSMUST00000208927	MSTRG.28341	Gm32031
ENSMUST00000208935	MSTRG.2424	4930598N05Rik
ENSMUST00000208953	ENSMUSG00000109554	A230057D06Rik
ENSMUST00000208961	ENSMUSG00000108824	A230057D06Rik
ENSMUST00000208963	MSTRG.28738	Gm44704
ENSMUST00000208965	ENSMUSG00000045813	Gm9801
ENSMUST00000208973	MSTRG.27564	4933431C10Rik
ENSMUST00000208983	ENSMUSG00000109071	Gm44840
ENSMUST00000208988	ENSMUSG00000099853	Gm29328
ENSMUST00000208993	MSTRG.28341	Gm32031
ENSMUST00000208997	MSTRG.2424	4930598N05Rik
ENSMUST00000209007	MSTRG.29904	Gm44716
ENSMUST00000209012	ENSMUSG00000109249	Gm44610
ENSMUST00000209020	MSTRG.68	1700034P13Rik
ENSMUST00000209027	MSTRG.29278	1700016B15Rik
ENSMUST00000209043	MSTRG.27566	Gm36371
ENSMUST00000209045	ENSMUSG00000109346	Gm44515
ENSMUST00000209046	ENSMUSG00000109383	Gm36584
ENSMUST00000209050	ENSMUSG00000109364	Gm44948
ENSMUST00000209052	ENSMUSG00000109170	Gm45086
ENSMUST00000209053	ENSMUSG00000109215	4930453L07Rik
ENSMUST00000209064	ENSMUSG00000030623	Prss23os
ENSMUST00000209071	ENSMUSG00000109097	Gm29683
ENSMUST00000209073	ENSMUSG00000109158	Gm45166
ENSMUST00000209078	ENSMUSG00000109106	4933430H16Rik

ENSMUST00000209081	ENSMUSG00000108846	Gm10623
ENSMUST00000209089	ENSMUSG00000109111	6530437J22Rik
ENSMUST00000209090	MSTRG.28897	Gm45187
ENSMUST00000209091	MSTRG.29891	Gm45074
ENSMUST00000209101	MSTRG.28229	Gm15396
ENSMUST00000209104	MSTRG.28781	Gm45177
ENSMUST00000209109	MSTRG.27957	Gm26604
ENSMUST00000209111	ENSMUSG00000070574	2310016G11Rik
ENSMUST00000209115	ENSMUSG00000109006	B230209E15Rik
ENSMUST00000209119	MSTRG.28530	Gm44888
ENSMUST00000209120	ENSMUSG00000108899	Gm44811
ENSMUST00000209122	ENSMUSG00000108865	Gm44617
ENSMUST00000209128	ENSMUSG00000109062	Gm44622
ENSMUST00000209134	ENSMUSG00000109009	Gm44929
ENSMUST00000209142	MSTRG.28703	Gm45807
ENSMUST00000209149	ENSMUSG00000108868	Gm32850
ENSMUST00000209151	MSTRG.28790	Gm44677
ENSMUST00000209160	MSTRG.28947	Gm39059
ENSMUST00000209164	MSTRG.28764	2310010J17Rik
ENSMUST00000209166	MSTRG.28764	2310010J17Rik
ENSMUST00000209170	MSTRG.28769	Dlg2
ENSMUST00000209183	ENSMUSG00000108820	Gm44620
ENSMUST00000209189	ENSMUSG00000109556	Gm38843
ENSMUST00000209194	MSTRG.27943	Gm44702
ENSMUST00000209210	ENSMUSG00000110231	Gm6329
ENSMUST00000209213	MSTRG.12215	Gm15738
ENSMUST00000209224	ENSMUSG00000109598	Gm36356
ENSMUST00000209232	ENSMUSG00000109996	Gm31898
ENSMUST00000209235	ENSMUSG00000110176	Gm35520
ENSMUST00000209240	MSTRG.29992	Gm45394
ENSMUST00000209267	MSTRG.30759	Gm45286
ENSMUST00000209271	MSTRG.30398	Gm45540
ENSMUST00000209272	MSTRG.30800	Gm9725
ENSMUST00000209273	MSTRG.30762	4933431K23Rik
ENSMUST00000209288	MSTRG.28374	Gm2788
ENSMUST00000209298	MSTRG.27661	Gm45508
ENSMUST00000209301	MSTRG.28308	Gm45441
ENSMUST00000209302	MSTRG.30748	4933421D24Rik
ENSMUST00000209312	ENSMUSG00000109603	Gm32389
ENSMUST00000209330	MSTRG.29002	Gm35363
ENSMUST00000209336	ENSMUSG00000101299	Gm28175
ENSMUST00000209340	MSTRG.28265	Gm45552
ENSMUST00000209344	MSTRG.8956	Gm36445
ENSMUST00000209358	ENSMUSG00000109643	Gm31545
ENSMUST00000209367	ENSMUSG00000110389	Gm45579
ENSMUST00000209370	MSTRG.30175	Svet1
ENSMUST00000209393	ENSMUSG00000109792	Gm45426
ENSMUST00000209399	MSTRG.18591	Gm45609
ENSMUST00000209402	MSTRG.29366	Gm45847
ENSMUST00000209405	ENSMUSG00000109887	Gm28756
ENSMUST00000209406	ENSMUSG00000109628	BC024386
ENSMUST00000209414	MSTRG.30149	Gm45250
ENSMUST00000209418	MSTRG.29910	Gm20100
ENSMUST00000209427	MSTRG.30157	5430430B14Rik
ENSMUST00000209429	MSTRG.12399	1300002E11Rik
ENSMUST00000209433	MSTRG.28681	Gm44899
ENSMUST00000209439	MSTRG.28375	Gm45282

ENSMUST00000209449	MSTRG.12399	1300002E11Rik
ENSMUST00000209455	ENSMUSG00000110264	E330018M18Rik
ENSMUST00000209468	MSTRG.12910	E330011O21Rik
ENSMUST00000209471	ENSMUSG00000109634	Gm45474
ENSMUST00000209486	MSTRG.30147	Gm45251
ENSMUST00000209492	MSTRG.30427	Gm45358
ENSMUST00000209493	MSTRG.31009	Gm6658
ENSMUST00000209494	MSTRG.30012	Gm45493
ENSMUST00000209497	MSTRG.28338	C86187
ENSMUST00000209499	MSTRG.30752	Gm45407
ENSMUST00000209502	ENSMUSG00000110205	Gm33594
ENSMUST00000209503	ENSMUSG00000110037	4930543N07Rik
ENSMUST00000209512	MSTRG.30242	Gm34368
ENSMUST00000209524	ENSMUSG00000109915	7420700N18Rik
ENSMUST00000209529	MSTRG.29696	B930086L07Rik
ENSMUST00000209535	ENSMUSG00000109936	Gm45889
ENSMUST00000209540	MSTRG.14969	Gm45884
ENSMUST00000209541	MSTRG.28281	Gm10252
ENSMUST00000209549	ENSMUSG00000109871	Gm45276
ENSMUST00000209552	ENSMUSG00000110157	Gm32507
ENSMUST00000209554	ENSMUSG00000109999	Gm35368
ENSMUST00000209560	ENSMUSG00000109633	C230079O03Rik
ENSMUST00000209561	MSTRG.29693	Gm45502
ENSMUST00000209572	MSTRG.31026	Gm45427
ENSMUST00000209584	ENSMUSG00000086095	Gm15328
ENSMUST00000209585	ENSMUSG00000110335	1700015I17Rik
ENSMUST00000209595	ENSMUSG00000086175	Gm15802
ENSMUST00000209597	MSTRG.29165	1700012D14Rik
ENSMUST00000209603	ENSMUSG00000078956	Gm14221
ENSMUST00000209605	MSTRG.29915	Gm45592
ENSMUST00000209615	ENSMUSG00000109083	Gm44992
ENSMUST00000209618	ENSMUSG00000109716	Gm42196
ENSMUST00000209619	ENSMUSG00000109956	Gm45428
ENSMUST00000209624	MSTRG.27662	Gm45509
ENSMUST00000209629	MSTRG.28331	Gm45629
ENSMUST00000209642	MSTRG.15663	Gm42067
ENSMUST00000209644	ENSMUSG00000109766	Gm45415
ENSMUST00000209647	MSTRG.30141	Gm45411
ENSMUST00000209648	ENSMUSG00000110151	Gm38416
ENSMUST00000209650	MSTRG.29589	Gm45278
ENSMUST00000209658	MSTRG.25689	Gm43154
ENSMUST00000209660	ENSMUSG00000084954	Gm16351
ENSMUST00000209663	MSTRG.31285	Gm39244
ENSMUST00000209665	ENSMUSG00000109803	Gm45604
ENSMUST00000209672	ENSMUSG00000109931	Gm39929
ENSMUST00000209681	ENSMUSG00000110276	Gm45330
ENSMUST00000209682	MSTRG.17324	Gm13522
ENSMUST00000209685	ENSMUSG00000109674	Gm45470
ENSMUST00000209694	ENSMUSG00000109770	Gm30085
ENSMUST00000209697	MSTRG.29920	4930465I24Rik
ENSMUST00000209713	MSTRG.29763	Gm45416
ENSMUST00000209717	MSTRG.30339	Gm45481
ENSMUST00000209728	MSTRG.12399	1300002E11Rik
ENSMUST00000209731	MSTRG.29154	B430319F04Rik
ENSMUST00000209736	ENSMUSG00000110227	Gm45634
ENSMUST00000209738	MSTRG.27593	1700047O18Rik
ENSMUST00000209741	ENSMUSG00000109921	Gm33326

ENSMUST00000209747	MSTRG.29590	Gm10578
ENSMUST00000209754	MSTRG.31040	Gm45293
ENSMUST00000209762	ENSMUSG00000110260	Gm30504
ENSMUST00000209767	MSTRG.29692	1500002F19Rik
ENSMUST00000209774	ENSMUSG00000110140	5430421F17Rik
ENSMUST00000209775	MSTRG.31001	Gm45641
ENSMUST00000209781	MSTRG.30408	A230085B16Rik
ENSMUST00000209784	ENSMUSG00000110058	Gm5907
ENSMUST00000209785	MSTRG.21256	Gm26881
ENSMUST00000209793	ENSMUSG00000110351	Gm32050
ENSMUST00000209799	ENSMUSG00000110075	Gm45305
ENSMUST00000209801	MSTRG.505	Gm10561
ENSMUST00000209804	MSTRG.30793	Gm45872
ENSMUST00000209808	MSTRG.28258	Gm45669
ENSMUST00000209810	ENSMUSG00000110115	Gm32486
ENSMUST00000209814	ENSMUSG00000110242	G630064G18Rik
ENSMUST00000209816	ENSMUSG00000109973	Gm45397
ENSMUST00000209819	ENSMUSG00000109704	Gm38414
ENSMUST00000209823	MSTRG.30925	Gm42031
ENSMUST00000209836	MSTRG.5104	Gm45606
ENSMUST00000209837	MSTRG.18591	Gm45609
ENSMUST00000209845	MSTRG.30444	Gm45483
ENSMUST00000209849	MSTRG.28374	Gm2788
ENSMUST00000209850	ENSMUSG00000109816	4930483O08Rik
ENSMUST00000209854	ENSMUSG00000110225	Gm45528
ENSMUST00000209855	MSTRG.8956	Gm36445
ENSMUST00000209874	ENSMUSG00000109780	Gm45447
ENSMUST00000209884	ENSMUSG00000110135	Gm45839
ENSMUST00000209893	ENSMUSG00000109775	Gm45297
ENSMUST00000209896	ENSMUSG00000097910	5033428I22Rik
ENSMUST00000209897	ENSMUSG00000109890	AU023762
ENSMUST00000209900	ENSMUSG00000109665	Gm45458
ENSMUST00000209917	MSTRG.30407	Gm45262
ENSMUST00000209918	ENSMUSG00000110187	Gm45496
ENSMUST00000209925	ENSMUSG00000109604	Gm45346
ENSMUST00000209929	MSTRG.30387	Gm45553
ENSMUST00000209931	ENSMUSG00000110411	Gm45457
ENSMUST00000209933	ENSMUSG00000110382	Gm29895
ENSMUST00000209934	ENSMUSG00000109748	Gm35392
ENSMUST00000209936	ENSMUSG00000109632	Gm31479
ENSMUST00000209940	ENSMUSG00000110365	Gm38947
ENSMUST00000209943	ENSMUSG00000109743	Gm45255
ENSMUST00000209947	MSTRG.17751	Mir670hg
ENSMUST00000209951	MSTRG.8956	Gm36445
ENSMUST00000209955	MSTRG.29016	Gm45399
ENSMUST00000209959	MSTRG.29165	1700012D14Rik
ENSMUST00000209960	MSTRG.21553	Gm42303
ENSMUST00000209966	ENSMUSG00000109827	Gm6213
ENSMUST00000209973	ENSMUSG00000109622	Gm45517
ENSMUST00000209975	ENSMUSG00000109839	Gm45677
ENSMUST00000209977	ENSMUSG00000109995	B020031H02Rik
ENSMUST00000209980	MSTRG.28996	Gm45620
ENSMUST00000209998	ENSMUSG00000109998	Gm45437
ENSMUST00000209999	ENSMUSG00000109961	Gm31983
ENSMUST00000210004	ENSMUSG00000110293	Gm45557
ENSMUST00000210007	MSTRG.12220	Gm32817
ENSMUST00000210010	ENSMUSG00000110290	Gm45336

ENSMUST00000210022	MSTRG.9321	Gm5086
ENSMUST00000210028	MSTRG.30060	Gm45555
ENSMUST00000210033	MSTRG.12437	Gm45338
ENSMUST00000210035	MSTRG.27646	Gm38948
ENSMUST00000210038	MSTRG.28315	Gm45442
ENSMUST00000210042	ENSMUSG00000110269	Gm45258
ENSMUST00000210049	ENSMUSG00000109853	Gm33045
ENSMUST00000210054	ENSMUSG00000109828	Gm45662
ENSMUST00000210059	MSTRG.27663	Gm45510
ENSMUST00000210072	MSTRG.30109	5830408C22Rik
ENSMUST00000210076	MSTRG.28998	Gm45548
ENSMUST00000210090	MSTRG.30406	Gm45264
ENSMUST00000210097	MSTRG.30109	5830408C22Rik
ENSMUST00000210102	ENSMUSG00000110417	Gm31727
ENSMUST00000210105	ENSMUSG00000109714	Gm35021
ENSMUST00000210109	MSTRG.29971	Gm45520
ENSMUST00000210111	MSTRG.30163	Gm45266
ENSMUST00000210130	ENSMUSG00000109846	Gm45627
ENSMUST00000210131	MSTRG.5505	Gm45883
ENSMUST00000210140	ENSMUSG00000110007	Gm45352
ENSMUST00000210144	MSTRG.18591	Gm45609
ENSMUST00000210161	MSTRG.30746	Gm45406
ENSMUST00000210176	MSTRG.18591	Gm45609
ENSMUST00000210178	ENSMUSG00000109869	Gm39158
ENSMUST00000210196	ENSMUSG00000110377	Gm45363
ENSMUST00000210200	MSTRG.18410	Gm5535
ENSMUST00000210201	ENSMUSG00000086095	Gm15328
ENSMUST00000210206	ENSMUSG00000110137	Gm45577
ENSMUST00000210217	MSTRG.30095	Gm45412
ENSMUST00000210223	ENSMUSG00000110236	Gm40493
ENSMUST00000210243	MSTRG.29772	Gm45630
ENSMUST00000210244	ENSMUSG00000110141	Gm45684
ENSMUST00000210253	ENSMUSG00000110278	Gm5608
ENSMUST00000210255	MSTRG.30433	Gm45644
ENSMUST00000210258	ENSMUSG00000110222	Gm39149
ENSMUST00000210260	ENSMUSG00000110016	Gm20751
ENSMUST00000210263	ENSMUSG00000100335	2310008N11Rik
ENSMUST00000210265	ENSMUSG00000109829	Gm45605
ENSMUST00000210285	MSTRG.30231	Gm45349
ENSMUST00000210294	MSTRG.30469	Gm10997
ENSMUST00000210301	ENSMUSG00000110084	Gm45257
ENSMUST00000210303	ENSMUSG00000110329	Gm45304
ENSMUST00000210310	MSTRG.30442	Gm45484
ENSMUST00000210318	MSTRG.27581	Gm45631
ENSMUST00000210319	ENSMUSG00000110320	Gm31152
ENSMUST00000210323	MSTRG.463	Gm31812
ENSMUST00000210335	ENSMUSG00000110390	Gm45869
ENSMUST00000210339	ENSMUSG00000110304	Gm36380
ENSMUST00000210340	MSTRG.29165	1700012D14Rik
ENSMUST00000210342	MSTRG.29571	Gm45532
ENSMUST00000210356	ENSMUSG00000110027	C030029H02Rik
ENSMUST00000210360	MSTRG.28275	Gm45619
ENSMUST00000210371	MSTRG.12437	Gm45338
ENSMUST00000210376	MSTRG.30460	Gm45418
ENSMUST00000210377	MSTRG.30404	Gm45263
ENSMUST00000210378	MSTRG.12215	Gm15738
ENSMUST00000210380	MSTRG.29072	Gm45667

ENSMUST00000210383	MSTRG.28294	0610005C13Rik
ENSMUST00000210389	MSTRG.30144	Gm45413
ENSMUST00000210393	MSTRG.31041	Gm45295
ENSMUST00000210409	ENSMUSG00000109997	9430099M06Rik
ENSMUST00000210415	MSTRG.29705	Gm16201
ENSMUST00000210418	MSTRG.30749	Gm45285
ENSMUST00000210429	ENSMUSG00000109909	Gm45663
ENSMUST00000210430	ENSMUSG00000110383	Gm45580
ENSMUST00000210432	ENSMUSG00000110071	Gm45512
ENSMUST00000210436	MSTRG.28327	Gm45310
ENSMUST00000210446	ENSMUSG00000109772	Gm39132
ENSMUST00000210452	MSTRG.30122	Gm45343
ENSMUST00000210456	ENSMUSG00000109785	Gm45542
ENSMUST00000210460	MSTRG.28292	Gm45564
ENSMUST00000210463	MSTRG.28374	Gm2788
ENSMUST00000210468	MSTRG.30243	Gm45469
ENSMUST00000210484	MSTRG.29583	Gm45280
ENSMUST00000210487	ENSMUSG00000110216	Gm36325
ENSMUST00000210502	MSTRG.29919	Gm7562
ENSMUST00000210553	ENSMUSG00000109961	Gm31983
ENSMUST00000210567	ENSMUSG00000109628	BC024386
ENSMUST00000210569	MSTRG.28294	0610005C13Rik
ENSMUST00000210575	ENSMUSG00000110341	Gm45424
ENSMUST00000210577	MSTRG.18410	Gm5535
ENSMUST00000210581	MSTRG.31953	Gm45353
ENSMUST00000210582	MSTRG.30120	D830025C05Rik
ENSMUST00000210596	MSTRG.30109	5830408C22Rik
ENSMUST00000210603	MSTRG.30241	Gm9939
ENSMUST00000210606	ENSMUSG00000086822	5330413P13Rik
ENSMUST00000210613	ENSMUSG00000109759	1700011L03Rik
ENSMUST00000210614	ENSMUSG00000097910	5033428I22Rik
ENSMUST00000210623	MSTRG.28294	0610005C13Rik
ENSMUST00000210633	ENSMUSG00000097910	5033428I22Rik
ENSMUST00000210635	MSTRG.30432	Gm45359
ENSMUST00000210646	MSTRG.29079	Gm45912
ENSMUST00000210653	MSTRG.8956	Gm36445
ENSMUST00000210658	MSTRG.12437	Gm45338
ENSMUST00000210665	MSTRG.31003	Gm45640
ENSMUST00000210666	MSTRG.30472	Gm45242
ENSMUST00000210672	ENSMUSG00000110329	Gm45304
ENSMUST00000210674	ENSMUSG00000109930	Gm34730
ENSMUST00000210683	ENSMUSG00000092178	Gm45351
ENSMUST00000210685	MSTRG.31035	Gm45294
ENSMUST00000210693	MSTRG.30460	Gm45418
ENSMUST00000210694	ENSMUSG00000109612	Gm45253
ENSMUST00000210698	MSTRG.30424	Gm45360
ENSMUST00000210717	ENSMUSG00000109761	5430403N17Rik
ENSMUST00000210723	MSTRG.30152	Gm45371
ENSMUST00000210728	MSTRG.31017	Gm45464
ENSMUST00000210732	ENSMUSG00000100335	2310008N11Rik
ENSMUST00000210736	MSTRG.29996	3110080E11Rik
ENSMUST00000210738	MSTRG.12220	Gm32817
ENSMUST00000210752	MSTRG.30417	2500002B13Rik
ENSMUST00000210768	ENSMUSG00000110207	Gm33586
ENSMUST00000210771	ENSMUSG00000109980	Gm45538
ENSMUST00000210776	ENSMUSG00000110368	Gm45518
ENSMUST00000210787	ENSMUSG00000109083	Gm44992

ENSMUST00000210788	MSTRG.29165	1700012D14Rik
ENSMUST00000210795	MSTRG.12437	Gm45338
ENSMUST00000210796	MSTRG.29137	Gm45515
ENSMUST00000210802	ENSMUSG00000095385	D630033O11Rik
ENSMUST00000210822	ENSMUSG00000109700	Gm21123
ENSMUST00000210827	MSTRG.9321	Gm5086
ENSMUST00000210835	ENSMUSG00000110125	Gm33148
ENSMUST00000210839	MSTRG.12910	E330011O21Rik
ENSMUST00000210843	MSTRG.30132	6430710M23Rik
ENSMUST00000210851	MSTRG.17751	Mir670hg
ENSMUST00000210852	MSTRG.30405	Gm45265
ENSMUST00000210866	MSTRG.28294	0610005C13Rik
ENSMUST00000210896	MSTRG.31126	Gm29682
ENSMUST00000210897	MSTRG.9321	Gm5086
ENSMUST00000210903	MSTRG.30122	Gm45342
ENSMUST00000210912	MSTRG.30965	Gm39214
ENSMUST00000210917	MSTRG.15663	Gm42067
ENSMUST00000210926	MSTRG.30174	Gm45652
ENSMUST00000210938	MSTRG.12220	Gm32817
ENSMUST00000210940	MSTRG.29146	Gm45819
ENSMUST00000210942	MSTRG.29607	Gm45328
ENSMUST00000210943	ENSMUSG00000110116	4933430A20Rik
ENSMUST00000210951	MSTRG.30241	Gm9939
ENSMUST00000210953	MSTRG.29577	5830432E09Rik
ENSMUST00000210956	ENSMUSG00000109870	Gm35850
ENSMUST00000210961	ENSMUSG00000086822	5330413P13Rik
ENSMUST00000210965	MSTRG.28294	0610005C13Rik
ENSMUST00000210969	ENSMUSG00000110058	Gm5907
ENSMUST00000210975	MSTRG.30430	AW046200
ENSMUST00000210981	ENSMUSG00000110363	Gm45576
ENSMUST00000211015	ENSMUSG00000110015	Gm33968
ENSMUST00000211017	ENSMUSG00000109886	Gm45271
ENSMUST00000211025	MSTRG.18410	Gm5535
ENSMUST00000211028	MSTRG.30162	Gm45267
ENSMUST00000211039	ENSMUSG00000110146	Gm45487
ENSMUST00000211048	MSTRG.31031	Gm45289
ENSMUST00000211052	MSTRG.29019	Gm45527
ENSMUST00000211053	ENSMUSG00000110030	Gm45546
ENSMUST00000211055	MSTRG.28374	Gm2788
ENSMUST00000211063	MSTRG.29166	Gm45378
ENSMUST00000211070	MSTRG.29590	Gm10578
ENSMUST00000211086	MSTRG.28321	Gm45311
ENSMUST00000211111	ENSMUSG00000109734	Gm45272
ENSMUST00000211137	MSTRG.30119	Gm45570
ENSMUST00000211142	ENSMUSG00000110101	Gm6249
ENSMUST00000211143	MSTRG.30326	Gm45607
ENSMUST00000211147	MSTRG.30785	Gm45435
ENSMUST00000211157	ENSMUSG00000110017	Gm45237
ENSMUST00000211167	MSTRG.31557	Gm45494
ENSMUST00000211170	MSTRG.29366	Gm45846
ENSMUST00000211176	MSTRG.28302	Gm31597
ENSMUST00000211177	MSTRG.31033	Gm45290
ENSMUST00000211184	MSTRG.29692	1500002F19Rik
ENSMUST00000211189	ENSMUSG00000109688	Gm34597
ENSMUST00000211191	ENSMUSG00000100510	AV026068
ENSMUST00000211194	MSTRG.18591	Gm45609
ENSMUST00000211202	MSTRG.29983	Gm45675

ENSMUST00000211208	MSTRG.21256	Gm26881
ENSMUST00000211232	MSTRG.31030	Gm45292
ENSMUST00000211237	ENSMUSG00000110078	Gm45241
ENSMUST00000211251	ENSMUSG00000110080	Gm6145
ENSMUST00000211253	ENSMUSG00000109704	Gm38414
ENSMUST00000211255	ENSMUSG00000109936	Gm45889
ENSMUST00000211262	MSTRG.31285	Gm39244
ENSMUST00000211264	ENSMUSG00000110052	1700020G03Rik
ENSMUST00000211266	ENSMUSG00000110063	Gm31045
ENSMUST00000211274	ENSMUSG00000109621	Gm45370
ENSMUST00000211284	MSTRG.30430	AW046200
ENSMUST00000211287	MSTRG.31029	4831440D22Rik
ENSMUST00000211291	MSTRG.34337	Tsix
ENSMUST00000211295	MSTRG.31043	Gm45472
ENSMUST00000211296	ENSMUSG00000109880	Gm34096
ENSMUST00000211302	MSTRG.29175	2310014F06Rik
ENSMUST00000211303	ENSMUSG00000092536	Gm20501
ENSMUST00000211305	MSTRG.30138	Gm32098
ENSMUST00000211313	ENSMUSG00000110246	C130073E24Rik
ENSMUST00000211328	MSTRG.30306	Gm45244
ENSMUST00000211331	ENSMUSG00000110311	Gm45410
ENSMUST00000211335	MSTRG.12910	E330011O21Rik
ENSMUST00000211342	MSTRG.29189	Gm45355
ENSMUST00000211351	ENSMUSG00000109958	Gm32786
ENSMUST00000211358	ENSMUSG00000110210	Gm33756
ENSMUST00000211359	ENSMUSG00000110384	Gm45301
ENSMUST00000211364	MSTRG.31007	Gm45643
ENSMUST00000211371	ENSMUSG00000109898	6330420H09Rik
ENSMUST00000211380	MSTRG.30600	Gm11175
ENSMUST00000211381	ENSMUSG00000109819	B930018H19Rik
ENSMUST00000211382	MSTRG.29555	Gm45670
ENSMUST00000211391	MSTRG.30321	Gm45693
ENSMUST00000211394	MSTRG.28305	Gm45444
ENSMUST00000211401	MSTRG.30426	Gm19269
ENSMUST00000211415	MSTRG.27590	Gm45593
ENSMUST00000211418	ENSMUSG00000109998	Gm45437
ENSMUST00000211421	MSTRG.30798	Gm31105
ENSMUST00000211426	ENSMUSG00000110060	Gm9860
ENSMUST00000211427	MSTRG.30471	Gm45345
ENSMUST00000211428	MSTRG.28981	Gm10602
ENSMUST00000211430	MSTRG.17751	Mir670hg
ENSMUST00000211437	MSTRG.29580	Gm45240
ENSMUST00000211443	MSTRG.12399	1300002E11Rik
ENSMUST00000211447	MSTRG.30110	Gm45745
ENSMUST00000211449	MSTRG.30423	Gm45534
ENSMUST00000211460	MSTRG.12437	Gm45338
ENSMUST00000211464	ENSMUSG00000110249	9330121K16Rik
ENSMUST00000211466	MSTRG.30762	Gm45714
ENSMUST00000211467	MSTRG.30173	Gm9911
ENSMUST00000211468	MSTRG.29688	Gm45501
ENSMUST00000211474	MSTRG.31020	Gm45666
ENSMUST00000211475	MSTRG.30771	Gm45728
ENSMUST00000211481	MSTRG.30358	AA386476
ENSMUST00000211483	ENSMUSG00000109945	Gm45601
ENSMUST00000211485	MSTRG.30834	Gm45449
ENSMUST00000211499	MSTRG.12437	Gm45338
ENSMUST00000211504	MSTRG.29366	Gm45847

ENSMUST00000211520	ENSMUSG00000110366	Gm45885
ENSMUST00000211521	MSTRG.28294	0610005C13Rik
ENSMUST00000211524	MSTRG.29572	Gm45597
ENSMUST00000211528	MSTRG.28334	Gm45309
ENSMUST00000211532	MSTRG.29083	Gm45632
ENSMUST00000211533	ENSMUSG00000110357	A030001D20Rik
ENSMUST00000211534	ENSMUSG00000110409	Gm9908
ENSMUST00000211539	MSTRG.30342	Gm45537
ENSMUST00000211546	ENSMUSG00000110089	Gm36243
ENSMUST00000211548	ENSMUSG00000110214	Gm45587
ENSMUST00000211564	MSTRG.29757	Gm10575
ENSMUST00000211572	ENSMUSG00000110388	Gm30329
ENSMUST00000211577	ENSMUSG00000085980	Gm12408
ENSMUST00000211586	ENSMUSG00000110338	Gm45584
ENSMUST00000211588	ENSMUSG00000110308	Gm45516
ENSMUST00000211613	MSTRG.30334	Gm45479
ENSMUST00000211634	MSTRG.29989	Gm45393
ENSMUST00000211642	ENSMUSG00000110051	Gm45322
ENSMUST00000211653	MSTRG.30109	5830408C22Rik
ENSMUST00000211661	MSTRG.30440	Gm45809
ENSMUST00000211662	ENSMUSG00000110367	Gm34853
ENSMUST00000211673	ENSMUSG00000110017	Gm45237
ENSMUST00000211692	ENSMUSG00000092536	Gm20501
ENSMUST00000211696	MSTRG.30226	Gm33831
ENSMUST00000211700	MSTRG.30429	4930412F12Rik
ENSMUST00000211705	ENSMUSG00000109870	Gm35850
ENSMUST00000211726	ENSMUSG00000109744	Gm45461
ENSMUST00000211727	ENSMUSG00000109731	Gm45425
ENSMUST00000211729	MSTRG.8956	Gm36445
ENSMUST00000211750	ENSMUSG00000110018	5430437J10Rik
ENSMUST00000211758	MSTRG.30040	Gm45453
ENSMUST00000211767	ENSMUSG00000110240	Gm45486
ENSMUST00000211774	MSTRG.30150	Gm45572
ENSMUST00000211787	MSTRG.30430	AW046200
ENSMUST00000211790	MSTRG.30459	4930512H18Rik
ENSMUST00000211793	MSTRG.28981	Gm10602
ENSMUST00000211799	ENSMUSG00000109808	5430402P08Rik
ENSMUST00000211831	MSTRG.30724	Gm45705
ENSMUST00000211835	ENSMUSG00000110426	Gm45757
ENSMUST00000211857	MSTRG.31109	Gm45854
ENSMUST00000211865	MSTRG.31057	Gm45873
ENSMUST00000211876	MSTRG.32021	Gm45842
ENSMUST00000211881	MSTRG.31413	Gm45720
ENSMUST00000211921	ENSMUSG00000110661	Gm31805
ENSMUST00000211931	MSTRG.31925	Gm45833
ENSMUST00000211940	MSTRG.31994	Gm45894
ENSMUST00000211950	ENSMUSG00000101854	1700026F02Rik
ENSMUST00000211951	ENSMUSG00000110582	Gm30052
ENSMUST00000211953	MSTRG.31286	Lncbate1
ENSMUST00000211969	MSTRG.31143	K230015D01Rik
ENSMUST00000211986	MSTRG.19754	Gm45790
ENSMUST00000211988	MSTRG.31142	Gm45731
ENSMUST00000211989	MSTRG.31125	Gm31659
ENSMUST00000211992	MSTRG.31051	Gm45774
ENSMUST00000212007	MSTRG.31986	A530010L16Rik
ENSMUST00000212022	MSTRG.30626	Gm45694
ENSMUST00000212043	MSTRG.32105	Gm45890

ENSMUST00000212057	ENSMUSG00000110712	Gm39662
ENSMUST00000212068	MSTRG.30733	Gm45820
ENSMUST00000212077	MSTRG.31961	Gm20735
ENSMUST00000212079	MSTRG.32223	2610203C20Rik
ENSMUST00000212110	MSTRG.31135	Gm45877
ENSMUST00000212119	ENSMUSG00000110633	Gm32122
ENSMUST00000212123	MSTRG.30498	Gm15991
ENSMUST00000212132	MSTRG.30846	D830024N08Rik
ENSMUST00000212137	MSTRG.31062	Gm45804
ENSMUST00000212145	MSTRG.31508	Gm32856
ENSMUST00000212158	MSTRG.31932	Gm42047
ENSMUST00000212178	ENSMUSG00000110698	Gm45875
ENSMUST00000212180	MSTRG.31508	Gm32856
ENSMUST00000212186	ENSMUSG00000110647	Gm17745
ENSMUST00000212188	MSTRG.32105	Gm45890
ENSMUST00000212189	MSTRG.30652	Gm45779
ENSMUST00000212190	MSTRG.31115	Gm45760
ENSMUST00000212196	ENSMUSG00000110693	Gm45899
ENSMUST00000212199	MSTRG.31102	Gm45812
ENSMUST00000212203	MSTRG.31424	Gm45747
ENSMUST00000212231	MSTRG.30731	Gm45822
ENSMUST00000212232	MSTRG.31173	Gm20163
ENSMUST00000212247	MSTRG.31986	A530010L16Rik
ENSMUST00000212248	ENSMUSG00000110634	Gm45895
ENSMUST00000212250	MSTRG.32105	Gm45890
ENSMUST00000212253	MSTRG.4542	Gm39822
ENSMUST00000212260	ENSMUSG00000110629	4930567H12Rik
ENSMUST00000212266	ENSMUSG00000101854	1700026F02Rik
ENSMUST00000212267	MSTRG.31412	Gm45721
ENSMUST00000212286	MSTRG.33538	Gm33460
ENSMUST00000212288	MSTRG.31173	Gm20163
ENSMUST00000212289	MSTRG.31125	Gm31659
ENSMUST00000212305	ENSMUSG00000110665	Gm31786
ENSMUST00000212309	ENSMUSG00000097986	Gm26953
ENSMUST00000212310	MSTRG.31111	Gm45853
ENSMUST00000212313	ENSMUSG00000110616	Gm36879
ENSMUST00000212328	MSTRG.31146	Gm33023
ENSMUST00000212331	ENSMUSG00000110636	Gm39397
ENSMUST00000212334	MSTRG.31985	C230057M02Rik
ENSMUST00000212339	MSTRG.32094	6030466F02Rik
ENSMUST00000212356	MSTRG.32105	Gm45890
ENSMUST00000212366	ENSMUSG00000110520	Gm45776
ENSMUST00000212373	MSTRG.32223	2610203C20Rik
ENSMUST00000212384	MSTRG.30604	6330537M06Rik
ENSMUST00000212387	MSTRG.31961	Gm20735
ENSMUST00000212406	MSTRG.31486	Gm31718
ENSMUST00000212411	MSTRG.32014	Gm4316
ENSMUST00000212413	MSTRG.31048	Gm45909
ENSMUST00000212427	MSTRG.30707	Gm35572
ENSMUST00000212444	MSTRG.31985	C230057M02Rik
ENSMUST00000212448	MSTRG.31098	Gm45758
ENSMUST00000212450	MSTRG.31146	Gm33023
ENSMUST00000212456	ENSMUSG00000110524	Gm39228
ENSMUST00000212460	MSTRG.32112	Gm45732
ENSMUST00000212490	MSTRG.32014	Gm4316
ENSMUST00000212602	ENSMUSG00000110427	4933406B17Rik
ENSMUST00000212619	ENSMUSG00000110542	Gm39139

ENSMUST00000212621	ENSMUSG00000110542	Gm39139
ENSMUST00000212623	ENSMUSG00000110654	Gm45746
ENSMUST00000212635	MSTRG.31912	5033426O07Rik
ENSMUST00000212638	ENSMUSG00000097520	4930488L21Rik
ENSMUST00000212639	MSTRG.31486	Gm31718
ENSMUST00000212647	MSTRG.31124	4930513N10Rik
ENSMUST00000212669	MSTRG.31064	Gm15889
ENSMUST00000212672	MSTRG.31128	Gm45887
ENSMUST00000212679	MSTRG.33549	Gm45897
ENSMUST00000212682	MSTRG.31100	Gm31224
ENSMUST00000212695	ENSMUSG00000110626	Gm45805
ENSMUST00000212698	ENSMUSG00000110437	Gm45876
ENSMUST00000212699	MSTRG.30661	Gm17435
ENSMUST00000212701	MSTRG.31990	Gm45743
ENSMUST00000212728	MSTRG.31995	Gm45894
ENSMUST00000212738	ENSMUSG00000110614	Gm45817
ENSMUST00000212751	MSTRG.32103	Gm29773
ENSMUST00000212759	MSTRG.31081	Gm45797
ENSMUST00000212778	MSTRG.31075	Gm45767
ENSMUST00000212780	ENSMUSG00000110554	Gm35256
ENSMUST00000212782	ENSMUSG00000110500	Gm32568
ENSMUST00000212785	ENSMUSG00000110534	Gm45708
ENSMUST00000212800	ENSMUSG00000110710	C78859
ENSMUST00000212816	ENSMUSG00000110670	Gm30132
ENSMUST00000212830	ENSMUSG00000110592	4930488N15Rik
ENSMUST00000212850	MSTRG.31195	4930578M07Rik
ENSMUST00000212860	MSTRG.32093	Gm45781
ENSMUST00000212863	MSTRG.31122	Gm45762
ENSMUST00000212881	MSTRG.31988	Gm39271
ENSMUST00000212891	ENSMUSG00000110640	Gm45903
ENSMUST00000212899	MSTRG.31932	Gm42047
ENSMUST00000212914	MSTRG.31487	A630001O12Rik
ENSMUST00000212917	MSTRG.31221	Gm45752
ENSMUST00000212925	MSTRG.31103	Gm31036
ENSMUST00000212930	ENSMUSG00000110670	Gm30132
ENSMUST00000212946	MSTRG.31195	4930578M07Rik
ENSMUST00000212957	MSTRG.31125	Gm31659
ENSMUST00000212988	ENSMUSG00000110620	Gm45733
ENSMUST00000213002	MSTRG.30013	Gm35934
ENSMUST00000213014	ENSMUSG00000110507	8030455M16Rik
ENSMUST00000213021	ENSMUSG00000110672	Gm45786
ENSMUST00000213047	ENSMUSG00000110687	Gm45764
ENSMUST00000213052	MSTRG.31460	Gm45709
ENSMUST00000213063	MSTRG.30725	Gm45706
ENSMUST00000213082	ENSMUSG00000110455	Gm45904
ENSMUST00000213085	ENSMUSG00000110629	4930567H12Rik
ENSMUST00000213107	ENSMUSG00000111308	AC156031.3
ENSMUST00000213112	ENSMUSG00000111629	AC126257.4
ENSMUST00000213122	ENSMUSG00000111494	AC153955.4
ENSMUST00000213132	MSTRG.33631	Gm26797
ENSMUST00000213137	MSTRG.31591	CAAA01194877.1
ENSMUST00000213140	MSTRG.32252	AC162938.2
ENSMUST00000213173	MSTRG.32433	AC160052.1
ENSMUST00000213180	MSTRG.32333	AC061963.1
ENSMUST00000213191	ENSMUSG00000111520	AC166902.2
ENSMUST00000213195	ENSMUSG00000110888	AC126257.1
ENSMUST00000213227	ENSMUSG00000111825	AC166370.1

ENSMUST00000213234	MSTRG.2508	AC161825.1
ENSMUST00000213244	MSTRG.32201	AC132474.3
ENSMUST00000213295	ENSMUSG00000110827	AC159895.1
ENSMUST00000213311	ENSMUSG00000111836	AC140363.2
ENSMUST00000213319	ENSMUSG00000111256	AC153729.4
ENSMUST00000213330	ENSMUSG00000111422	AC153536.3
ENSMUST00000213336	ENSMUSG00000110824	AC133947.1
ENSMUST00000213341	ENSMUSG00000111074	AC160051.1
ENSMUST00000213342	ENSMUSG00000075184	F930017D23Rik
ENSMUST00000213353	MSTRG.2812	AC152922.2
ENSMUST00000213359	ENSMUSG00000111363	AC117195.1
ENSMUST00000213372	MSTRG.2525	AC153845.3
ENSMUST00000213385	MSTRG.33665	AC164123.1
ENSMUST00000213406	ENSMUSG00000111498	AC110091.4
ENSMUST00000213429	ENSMUSG00000095385	D630033O11Rik
ENSMUST00000213434	ENSMUSG00000111796	AC120386.1
ENSMUST00000213443	ENSMUSG00000110749	AC160123.1
ENSMUST00000213446	MSTRG.33463	4921528I07Rik
ENSMUST00000213455	ENSMUSG00000111364	CT025751.1
ENSMUST00000213462	MSTRG.2687	AC113059.1
ENSMUST00000213469	MSTRG.2803	AC112265.1
ENSMUST00000213473	MSTRG.32333	AC061963.1
ENSMUST00000213487	MSTRG.32238	CT009696.4
ENSMUST00000213492	MSTRG.33638	AC124778.1
ENSMUST00000213494	MSTRG.32298	AC122428.5
ENSMUST00000213497	MSTRG.2709	4930547M16Rik
ENSMUST00000213522	MSTRG.2803	AC112265.1
ENSMUST00000213541	MSTRG.32330	AC061963.2
ENSMUST00000213560	MSTRG.32238	CT009696.4
ENSMUST00000213571	ENSMUSG00000110874	AC121988.1
ENSMUST00000213573	MSTRG.33647	AC125374.3
ENSMUST00000213583	ENSMUSG00000111489	AC154826.2
ENSMUST00000213590	ENSMUSG00000111156	CT009721.1
ENSMUST00000213591	MSTRG.33620	AC165080.4
ENSMUST00000213592	MSTRG.31766	AC159308.5
ENSMUST00000213594	ENSMUSG00000111811	AC122305.5
ENSMUST00000213617	MSTRG.2684	AC153959.1
ENSMUST00000213634	MSTRG.32381	AC122273.2
ENSMUST00000213646	MSTRG.32273	Gm26737
ENSMUST00000213677	MSTRG.33595	AC123708.2
ENSMUST00000213692	MSTRG.2541	AC153556.1
ENSMUST00000213695	MSTRG.33605	AC163350.2
ENSMUST00000213703	MSTRG.32957	AC125106.1
ENSMUST00000213706	ENSMUSG00000111746	AC160116.2
ENSMUST00000213709	MSTRG.32282	AC122428.1
ENSMUST00000213726	MSTRG.32251	AC162938.3
ENSMUST00000213741	MSTRG.31591	AC241616.1
ENSMUST00000213746	MSTRG.32300	C030014I23Rik
ENSMUST00000213761	ENSMUSG00000110958	AC153891.1
ENSMUST00000213810	ENSMUSG00000111389	AC164092.4
ENSMUST00000213811	MSTRG.2856	AC153954.3
ENSMUST00000213816	MSTRG.32894	AC160334.3
ENSMUST00000213841	ENSMUSG00000111674	AC153566.2
ENSMUST00000213846	ENSMUSG00000111550	AC123035.1
ENSMUST00000213849	MSTRG.3550	AC161054.2
ENSMUST00000213881	ENSMUSG00000111752	AC100386.1
ENSMUST00000213884	MSTRG.32381	AC122273.2

ENSMUST00000213901	MSTRG.3530	AC122335.3
ENSMUST00000213905	MSTRG.33627	AC120394.2
ENSMUST00000213907	ENSMUSG00000111189	AC140409.2
ENSMUST00000213914	ENSMUSG00000100837	1700063D05Rik
ENSMUST00000213934	ENSMUSG00000111215	AC091522.1
ENSMUST00000213948	MSTRG.31835	AC167244.1
ENSMUST00000213973	ENSMUSG00000111008	AC129016.1
ENSMUST00000213974	ENSMUSG00000052658	5830454E08Rik
ENSMUST00000213976	MSTRG.31663	AC166098.1
ENSMUST00000213982	ENSMUSG00000111439	AC138284.2
ENSMUST00000213985	MSTRG.2798	AC125206.2
ENSMUST00000214010	MSTRG.31614	CT030247.2
ENSMUST00000214014	ENSMUSG00000111871	AC113304.1
ENSMUST00000214016	MSTRG.2405	AC153569.1
ENSMUST00000214034	ENSMUSG00000111045	AC164625.1
ENSMUST00000214037	MSTRG.32570	AC160637.1
ENSMUST00000214038	MSTRG.31869	AC160116.1
ENSMUST00000214047	MSTRG.2673	AC153969.1
ENSMUST00000214055	ENSMUSG00000110758	AC153729.1
ENSMUST00000214060	ENSMUSG00000111097	AC154264.1
ENSMUST00000214070	MSTRG.2799	AC125206.1
ENSMUST00000214073	ENSMUSG00000111840	AC159809.2
ENSMUST00000214076	MSTRG.33188	AC164161.1
ENSMUST00000214084	ENSMUSG00000110844	AC127581.1
ENSMUST00000214088	ENSMUSG00000111002	CT010514.1
ENSMUST00000214091	ENSMUSG00000111785	AC120394.4
ENSMUST00000214092	MSTRG.2658	AC153899.2
ENSMUST00000214108	ENSMUSG00000111354	AC160394.4
ENSMUST00000214113	ENSMUSG00000110823	AC167973.1
ENSMUST00000214120	ENSMUSG00000110895	AC123832.1
ENSMUST00000214129	ENSMUSG00000111377	AC153536.2
ENSMUST00000214134	MSTRG.5329	AL603745.2
ENSMUST00000214135	MSTRG.3420	AC150314.4
ENSMUST00000214165	MSTRG.31760	AC163623.3
ENSMUST00000214172	ENSMUSG00000111103	AC165442.1
ENSMUST00000214178	ENSMUSG00000110902	AC153862.2
ENSMUST00000214196	MSTRG.33208	AC165256.1
ENSMUST00000214199	ENSMUSG00000111013	AC122326.1
ENSMUST00000214203	MSTRG.3564	AC122901.1
ENSMUST00000214235	MSTRG.32589	AC158238.2
ENSMUST00000214237	MSTRG.33461	AC166112.1
ENSMUST00000214246	ENSMUSG00000111285	AC172194.1
ENSMUST00000214251	MSTRG.32678	AC114645.3
ENSMUST00000214258	MSTRG.32178	AC138284.1
ENSMUST00000214265	MSTRG.33463	4921528I07Rik
ENSMUST00000214276	ENSMUSG00000111840	AC159809.2
ENSMUST00000214286	ENSMUSG00000111532	AC161595.2
ENSMUST00000214288	ENSMUSG00000111013	AC122326.1
ENSMUST00000214294	ENSMUSG00000110727	CT025619.1
ENSMUST00000214307	MSTRG.33580	Gm2449
ENSMUST00000214308	MSTRG.32686	AC114645.4
ENSMUST00000214323	MSTRG.2523	AC158620.1
ENSMUST00000214327	ENSMUSG00000111357	AC140363.1
ENSMUST00000214355	ENSMUSG00000110843	AC153829.1
ENSMUST00000214387	ENSMUSG00000111318	AC158630.2
ENSMUST00000214389	MSTRG.32174	AC138284.7
ENSMUST00000214403	MSTRG.33586	AC117245.4

ENSMUST00000214407	MSTRG.32203	AC132474.1
ENSMUST00000214413	MSTRG.33683	Gm17021
ENSMUST00000214422	MSTRG.32307	AC151971.9
ENSMUST00000214426	MSTRG.33648	A530083I20Rik
ENSMUST00000214440	MSTRG.32257	AC126459.3
ENSMUST00000214447	ENSMUSG00000110862	CT025619.2
ENSMUST00000214449	MSTRG.2688	Gm26564
ENSMUST00000214451	MSTRG.33611	AC163350.1
ENSMUST00000214468	MSTRG.31778	AC093926.2
ENSMUST00000214496	ENSMUSG00000111752	AC100386.1
ENSMUST00000214497	MSTRG.32651	AC160118.1
ENSMUST00000214500	ENSMUSG00000110988	AC163719.1
ENSMUST00000214503	MSTRG.33040	AC162946.1
ENSMUST00000214553	MSTRG.2668	AC153971.2
ENSMUST00000214556	MSTRG.2726	AC160403.1
ENSMUST00000214559	MSTRG.32672	AC122463.1
ENSMUST00000214560	MSTRG.29065	AC139579.1
ENSMUST00000214568	ENSMUSG00000100837	1700063D05Rik
ENSMUST00000214570	ENSMUSG00000111868	AC156273.3
ENSMUST00000214575	ENSMUSG00000111873	AC164092.5
ENSMUST00000214586	ENSMUSG00000111417	AC158987.1
ENSMUST00000214597	MSTRG.32130	4930581F22Rik
ENSMUST00000214603	MSTRG.32278	AC124577.2
ENSMUST00000214606	MSTRG.32134	AC140448.1
ENSMUST00000214634	MSTRG.2660	AC153899.1
ENSMUST00000214646	ENSMUSG00000111836	AC140363.2
ENSMUST00000214651	MSTRG.2822	AC153536.1
ENSMUST00000214657	ENSMUSG00000110794	AC151843.1
ENSMUST00000214661	MSTRG.33218	AC145736.1
ENSMUST00000214665	MSTRG.2698	AC131667.1
ENSMUST00000214671	ENSMUSG00000111533	AC137127.3
ENSMUST00000214683	MSTRG.33676	AC132852.1
ENSMUST00000214686	ENSMUSG00000111182	AC158799.1
ENSMUST00000214688	MSTRG.31821	CT025653.1
ENSMUST00000214691	MSTRG.2536	AC117639.2
ENSMUST00000214714	ENSMUSG00000110730	AC118476.2
ENSMUST00000214717	MSTRG.32317	AC151971.6
ENSMUST00000214720	ENSMUSG00000111056	AC132114.1
ENSMUST00000214721	MSTRG.3569	AC127596.2
ENSMUST00000214727	ENSMUSG00000110767	AC134859.1
ENSMUST00000214736	MSTRG.32368	AC122426.1
ENSMUST00000214737	ENSMUSG00000111250	AC159886.2
ENSMUST00000214742	ENSMUSG00000111243	AC131777.1
ENSMUST00000214749	ENSMUSG00000111729	AC131777.3
ENSMUST00000214762	MSTRG.32321	AC122305.3
ENSMUST00000214766	ENSMUSG00000111487	AC150314.2
ENSMUST00000214777	ENSMUSG00000043773	1700048O20Rik
ENSMUST00000214783	MSTRG.32183	AC105958.1
ENSMUST00000214789	MSTRG.2655	AC153912.3
ENSMUST00000214791	MSTRG.32258	AC126459.1
ENSMUST00000214795	MSTRG.31829	CT025678.1
ENSMUST00000214803	MSTRG.32341	AC122305.1
ENSMUST00000214808	ENSMUSG00000111053	AC164092.2
ENSMUST00000214812	ENSMUSG00000111449	AC122220.1
ENSMUST00000214815	MSTRG.33611	AC115863.1
ENSMUST00000214817	ENSMUSG00000111146	AC108846.3
ENSMUST00000214824	ENSMUSG00000111535	AC153955.5

ENSMUST00000214838	MSTRG.33608	AC159810.1
ENSMUST00000214845	MSTRG.33688	CAAA01180111.2
ENSMUST00000214867	ENSMUSG00000111627	AC133948.1
ENSMUST00000214881	ENSMUSG00000111489	AC154826.2
ENSMUST00000214890	ENSMUSG00000111100	CT010488.2
ENSMUST00000214902	MSTRG.2772	AC115297.1
ENSMUST00000214913	ENSMUSG00000110864	AC164105.2
ENSMUST00000214920	MSTRG.31615	CT030247.1
ENSMUST00000214931	MSTRG.32328	AC142113.1
ENSMUST00000214959	ENSMUSG00000111397	AC153380.1
ENSMUST00000214960	ENSMUSG00000111429	AC098716.1
ENSMUST00000214963	MSTRG.33594	AC110091.1
ENSMUST00000214968	ENSMUSG00000111565	AC159462.2
ENSMUST00000214985	MSTRG.32254	AC126459.4
ENSMUST00000214999	ENSMUSG00000052143	Gm9869
ENSMUST00000215028	ENSMUSG00000110779	AC116726.1
ENSMUST00000215070	ENSMUSG00000079564	Gm11149
ENSMUST00000215085	ENSMUSG00000110874	AC121988.1
ENSMUST00000215086	ENSMUSG00000111533	AC137127.3
ENSMUST00000215097	MSTRG.2664	AC153971.1
ENSMUST00000215107	ENSMUSG00000111360	AC110091.3
ENSMUST00000215131	MSTRG.32314	AC151971.5
ENSMUST00000215132	ENSMUSG00000111290	AC160562.2
ENSMUST00000215135	ENSMUSG00000111102	AC153556.3
ENSMUST00000215148	MSTRG.32346	AC122305.2
ENSMUST00000215154	ENSMUSG00000110988	AC163719.1
ENSMUST00000215162	ENSMUSG00000111474	AC158630.3
ENSMUST00000215174	MSTRG.32168	AC164087.1
ENSMUST00000215184	MSTRG.31730	AC122525.2
ENSMUST00000215190	ENSMUSG00000111631	AC137843.1
ENSMUST00000215206	ENSMUSG00000111233	AC118476.4
ENSMUST00000215213	ENSMUSG00000110937	AC156631.1
ENSMUST00000215216	MSTRG.32358	AC126804.1
ENSMUST00000215220	ENSMUSG00000111652	AC154406.1
ENSMUST00000215223	MSTRG.32963	CT009769.1
ENSMUST00000215241	ENSMUSG00000111110	AC163342.1
ENSMUST00000215242	MSTRG.32366	4930448E22Rik
ENSMUST00000215257	ENSMUSG00000111000	AC165080.1
ENSMUST00000215259	MSTRG.33605	AC163350.2
ENSMUST00000215297	MSTRG.32593	AC112680.4
ENSMUST00000215316	ENSMUSG00000111043	AC157950.1
ENSMUST00000215328	MSTRG.31565	AC156791.1
ENSMUST00000215330	MSTRG.32198	AC135353.1
ENSMUST00000215342	MSTRG.32225	CT009696.3
ENSMUST00000215353	ENSMUSG00000111646	AC153912.5
ENSMUST00000215356	ENSMUSG00000043773	1700048O20Rik
ENSMUST00000215366	MSTRG.2535	AC117639.1
ENSMUST00000215389	MSTRG.32264	AC124577.4
ENSMUST00000215400	ENSMUSG00000111078	AC027700.1
ENSMUST00000215411	MSTRG.31760	AC163623.3
ENSMUST00000215429	ENSMUSG00000110737	AC158630.1
ENSMUST00000215430	MSTRG.32257	AC126459.3
ENSMUST00000215445	ENSMUSG00000111282	AC116503.1
ENSMUST00000215448	MSTRG.2648	AC153912.2
ENSMUST00000215460	ENSMUSG00000111169	AC160394.2
ENSMUST00000215481	MSTRG.33588	AC117245.1
ENSMUST00000215483	ENSMUSG00000110741	AC166352.1

ENSMUST00000215490	ENSMUSG00000111232	AC113291.3
ENSMUST00000215491	MSTRG.31706	AC163637.1
ENSMUST00000215497	MSTRG.32251	AC162938.3
ENSMUST00000215507	ENSMUSG00000111037	AC126257.3
ENSMUST00000215520	MSTRG.2648	AC153912.2
ENSMUST00000215539	ENSMUSG00000111014	AC160124.1
ENSMUST00000215551	MSTRG.32303	AC122428.4
ENSMUST00000215560	ENSMUSG00000110831	AC158592.1
ENSMUST00000215570	ENSMUSG00000091272	Gm17641
ENSMUST00000215582	MSTRG.31679	Fbxl12os
ENSMUST00000215586	ENSMUSG00000111169	AC160394.2
ENSMUST00000215598	MSTRG.31814	AC156163.1
ENSMUST00000215599	ENSMUSG00000075184	F930017D23Rik
ENSMUST00000215601	ENSMUSG00000111794	AC140409.4
ENSMUST00000215604	MSTRG.33206	Gm16252
ENSMUST00000215630	ENSMUSG00000111598	AC131660.2
ENSMUST00000215633	MSTRG.2542	AC153556.2
ENSMUST00000215635	ENSMUSG00000110729	AC164092.1
ENSMUST00000215636	MSTRG.2846	AC135669.2
ENSMUST00000215645	ENSMUSG00000111556	AC107755.1
ENSMUST00000215648	ENSMUSG00000110784	AC164881.1
ENSMUST00000215652	ENSMUSG00000097074	4833428L15Rik
ENSMUST00000215662	ENSMUSG00000111720	AC118476.6
ENSMUST00000215667	MSTRG.2767	BC048559
ENSMUST00000215680	ENSMUSG00000110817	AC079245.1
ENSMUST00000215700	ENSMUSG00000110835	AC153862.1
ENSMUST00000215724	ENSMUSG00000111556	AC107755.1
ENSMUST00000215747	ENSMUSG00000111422	AC153536.3
ENSMUST00000215759	MSTRG.33633	9530059O14Rik
ENSMUST00000215762	MSTRG.2504	AC158622.3
ENSMUST00000215774	MSTRG.32254	AC126459.5
ENSMUST00000215803	MSTRG.32336	AC061963.3
ENSMUST00000215824	ENSMUSG00000111752	AC100386.1
ENSMUST00000215827	ENSMUSG00000111398	AC107851.2
ENSMUST00000215835	ENSMUSG00000111055	AC153958.1
ENSMUST00000215837	MSTRG.33639	AC124778.3
ENSMUST00000215840	MSTRG.2654	AC153912.4
ENSMUST00000215849	MSTRG.32365	AC164105.3
ENSMUST00000215869	MSTRG.32220	AC156031.1
ENSMUST00000215873	MSTRG.2407	AC153569.2
ENSMUST00000215882	ENSMUSG00000110795	CAAA01216754.1
ENSMUST00000215889	MSTRG.2525	AC153845.3
ENSMUST00000215895	ENSMUSG00000110982	AC158355.1
ENSMUST00000215913	MSTRG.32259	AC126459.2
ENSMUST00000215920	MSTRG.3423	AC150314.3
ENSMUST00000215937	MSTRG.5309	AL603745.3
ENSMUST00000215946	MSTRG.32307	AC151971.9
ENSMUST00000215963	MSTRG.32578	AC156795.1
ENSMUST00000215971	MSTRG.33195	AC156633.1
ENSMUST00000216012	ENSMUSG00000110998	AC160394.1
ENSMUST00000216017	MSTRG.32894	AC160334.3
ENSMUST00000216041	MSTRG.2504	AC158622.3
ENSMUST00000216043	MSTRG.33594	AC110091.1
ENSMUST00000216052	MSTRG.33677	AC132852.2
ENSMUST00000216055	MSTRG.31805	AC141438.1
ENSMUST00000216064	ENSMUSG00000111695	AC154264.2
ENSMUST00000216096	MSTRG.3529	AC122335.2

ENSMUST00000216103	ENSMUSG00000111489	AC154826.2
ENSMUST00000216113	ENSMUSG00000111490	AC134248.1
ENSMUST00000216122	ENSMUSG00000110986	AC153970.1
ENSMUST00000216140	MSTRG.33202	5830418P13Rik
ENSMUST00000216147	MSTRG.32382	AC122273.1
ENSMUST00000216155	ENSMUSG00000110794	AC151843.1
ENSMUST00000216159	MSTRG.32323	AC122305.4
ENSMUST00000216170	MSTRG.32595	AC160562.1
ENSMUST00000216190	ENSMUSG00000110823	AC167973.1
ENSMUST00000216193	MSTRG.32309	AC151971.10
ENSMUST00000216204	ENSMUSG00000110992	AC140409.1
ENSMUST00000216206	MSTRG.32169	AC164087.2
ENSMUST00000216217	ENSMUSG00000111157	AC156832.3
ENSMUST00000216236	ENSMUSG00000111365	AC102554.1
ENSMUST00000216269	ENSMUSG00000111094	AC145731.1
ENSMUST00000216270	MSTRG.31756	Gm16845
ENSMUST00000216282	ENSMUSG00000111818	AC114645.5
ENSMUST00000216299	MSTRG.32236	CT025619.4
ENSMUST00000216305	MSTRG.33219	AC145736.2
ENSMUST00000216311	ENSMUSG00000111544	AC122188.2
ENSMUST00000216321	MSTRG.32302	AC122428.2
ENSMUST00000216324	ENSMUSG00000110929	AC134606.1
ENSMUST00000216374	ENSMUSG00000111444	AC160562.3
ENSMUST00000216389	ENSMUSG00000111199	AC161595.1
ENSMUST00000216441	ENSMUSG00000111077	AC153566.1
ENSMUST00000216448	MSTRG.32267	AC148328.1
ENSMUST00000216452	MSTRG.33631	Gm26797
ENSMUST00000216455	MSTRG.32251	AC162938.3
ENSMUST00000216467	MSTRG.2798	AC125206.2
ENSMUST00000216469	MSTRG.5314	Slfn5os
ENSMUST00000216477	ENSMUSG00000111049	AC131801.1
ENSMUST00000216483	ENSMUSG00000079564	Gm11149
ENSMUST00000216487	MSTRG.32155	CT025617.1
ENSMUST00000216490	MSTRG.32356	AC162176.1
ENSMUST00000216518	MSTRG.2808	AC152922.1
ENSMUST00000216522	MSTRG.32369	AC122426.2
ENSMUST00000216531	ENSMUSG00000111489	AC154826.2
ENSMUST00000216533	ENSMUSG00000111829	AC163342.2
ENSMUST00000216546	MSTRG.2818	AC163661.1
ENSMUST00000216555	ENSMUSG00000110814	AC131660.1
ENSMUST00000216565	MSTRG.32606	AC113291.2
ENSMUST00000216569	ENSMUSG00000111803	AC154264.3
ENSMUST00000216588	ENSMUSG00000111035	AC138739.1
ENSMUST00000216590	MSTRG.31591	Phxr4
ENSMUST00000216597	ENSMUSG00000111167	AC160051.2
ENSMUST00000216598	MSTRG.2542	AC153556.2
ENSMUST00000216604	MSTRG.32251	AC162938.3
ENSMUST00000216609	MSTRG.32373	AC126804.2
ENSMUST00000216615	MSTRG.31773	AC159308.4
ENSMUST00000216646	MSTRG.31613	CT030247.5
ENSMUST00000216660	ENSMUSG00000111110	AC163342.1
ENSMUST00000216664	ENSMUSG00000110902	AC153862.2
ENSMUST00000216683	MSTRG.2504	AC158622.3
ENSMUST00000216687	MSTRG.33201	AC165256.2
ENSMUST00000216695	ENSMUSG00000111853	AC161596.2
ENSMUST00000216696	ENSMUSG00000111780	AC166902.3
ENSMUST00000216699	MSTRG.33634	AC164612.1

ENSMUST00000216704	MSTRG.32164	AC138284.4
ENSMUST00000216712	MSTRG.33206	Gm16252
ENSMUST00000216718	ENSMUSG00000111360	AC110091.3
ENSMUST00000216728	ENSMUSG00000111555	CT485606.2
ENSMUST00000216737	ENSMUSG00000111348	AC133947.2
ENSMUST00000216739	ENSMUSG00000111116	AC153955.2
ENSMUST00000216740	MSTRG.2822	AC153536.1
ENSMUST00000216749	MSTRG.32592	AC112680.3
ENSMUST00000216778	MSTRG.33195	AC156633.1
ENSMUST00000216780	MSTRG.31769	AC159308.3
ENSMUST00000216787	ENSMUSG00000111362	AC153955.3
ENSMUST00000216791	MSTRG.33616	E530011L22Rik
ENSMUST00000216792	ENSMUSG00000111189	AC140409.2
ENSMUST00000216803	MSTRG.31821	CT025653.1
ENSMUST00000216827	ENSMUSG00000111841	AC140460.1
ENSMUST00000216842	MSTRG.5332	AI662270
ENSMUST00000216854	ENSMUSG00000111290	AC160562.2
ENSMUST00000216860	ENSMUSG00000111511	AC160966.2
ENSMUST00000216861	MSTRG.32596	AC160394.3
ENSMUST00000216894	ENSMUSG00000111839	AC166078.2
ENSMUST00000216896	ENSMUSG00000111570	CT009741.1
ENSMUST00000216899	MSTRG.32590	AC158238.1
ENSMUST00000216902	MSTRG.2512	AC171277.1
ENSMUST00000216917	MSTRG.31694	AC159314.1
ENSMUST00000216918	MSTRG.33590	AC117245.5
ENSMUST00000216924	MSTRG.33648	A530083I20Rik
ENSMUST00000216940	ENSMUSG00000111343	AC159819.3
ENSMUST00000216941	MSTRG.32564	CT033754.1
ENSMUST00000216952	MSTRG.31637	AC155249.2
ENSMUST00000216959	MSTRG.2855	AC153954.2
ENSMUST00000216960	MSTRG.32272	AC148328.2
ENSMUST00000216972	ENSMUSG00000111412	AC107740.1
ENSMUST00000216975	MSTRG.32240	CT025619.3
ENSMUST00000216981	ENSMUSG00000110813	CT030724.1
ENSMUST00000217008	ENSMUSG00000111535	AC153955.5
ENSMUST00000217021	ENSMUSG00000111429	AC098716.1
ENSMUST00000217028	MSTRG.33572	AC166052.1
ENSMUST00000217030	ENSMUSG00000111769	AC124011.1
ENSMUST00000217044	ENSMUSG00000110799	AC153356.1
ENSMUST00000217047	ENSMUSG00000110938	AC158622.2
ENSMUST00000217068	ENSMUSG00000111335	AC160334.1
ENSMUST00000217069	ENSMUSG00000111641	AC153955.6
ENSMUST00000217076	ENSMUSG00000111167	AC160051.2
ENSMUST00000217077	MSTRG.33658	AC133650.1
ENSMUST00000217090	ENSMUSG00000111167	AC160051.2
ENSMUST00000217104	ENSMUSG00000111498	AC110091.4
ENSMUST00000217109	ENSMUSG00000111852	AC113527.1
ENSMUST00000217125	MSTRG.2822	AC153536.1
ENSMUST00000217127	ENSMUSG00000111806	AC121793.2
ENSMUST00000217151	MSTRG.32345	Gm10684
ENSMUST00000217153	MSTRG.33625	AC120394.1
ENSMUST00000217156	MSTRG.29023	AC123830.1
ENSMUST00000217162	ENSMUSG00000111341	AC165080.3
ENSMUST00000217183	ENSMUSG00000111733	CAAA01216754.6
ENSMUST00000217201	ENSMUSG00000111765	AC157516.1
ENSMUST00000217206	ENSMUSG00000111665	AC131684.1
ENSMUST00000217207	ENSMUSG00000110853	AC161245.1

ENSMUST00000217210	ENSMUSG00000110766	AC154295.1
ENSMUST00000217211	ENSMUSG00000111774	AC166078.1
ENSMUST00000217220	ENSMUSG00000110827	AC159895.1
ENSMUST00000217222	ENSMUSG00000111523	CAAA01125382.4
ENSMUST00000217239	MSTRG.32596	AC160394.3
ENSMUST00000217243	MSTRG.33654	AC133650.2
ENSMUST00000217258	ENSMUSG00000111726	CT009721.2
ENSMUST00000217260	MSTRG.32963	CT009769.1
ENSMUST00000217285	MSTRG.2544	AC153556.4
ENSMUST00000217287	MSTRG.33626	AC120394.3
ENSMUST00000217288	MSTRG.32215	AC156031.2
ENSMUST00000217302	ENSMUSG00000111324	AC131777.2
ENSMUST00000217329	MSTRG.32308	AC151971.1
ENSMUST00000217338	ENSMUSG00000046463	5930403N24Rik
ENSMUST00000217345	MSTRG.32181	AC138284.3
ENSMUST00000217349	ENSMUSG00000111649	AC126806.1
ENSMUST00000217357	MSTRG.32257	AC126459.3
ENSMUST00000217363	ENSMUSG00000111821	AC158622.5
ENSMUST00000217369	ENSMUSG00000111479	AC154826.1
ENSMUST00000217378	ENSMUSG00000111646	AC153912.5
ENSMUST00000217385	MSTRG.32366	4930448E22Rik
ENSMUST00000217388	MSTRG.33586	AC117245.4
ENSMUST00000217392	ENSMUSG00000111807	AC160394.5
ENSMUST00000217400	ENSMUSG00000111398	AC107851.2
ENSMUST00000217413	MSTRG.32286	AC124577.3
ENSMUST00000217414	MSTRG.32202	AC132474.2
ENSMUST00000217419	MSTRG.31855	CT030644.1
ENSMUST00000217423	ENSMUSG00000111563	AC113059.3
ENSMUST00000217425	MSTRG.32325	AC142113.2
ENSMUST00000217430	MSTRG.32592	AC112680.3
ENSMUST00000217440	MSTRG.32179	AC138284.6
ENSMUST00000217441	ENSMUSG00000111182	AC158799.1
ENSMUST00000217446	ENSMUSG00000110884	AC164161.2
ENSMUST00000217447	ENSMUSG00000111443	AC116180.1
ENSMUST00000217456	ENSMUSG00000110752	AC133192.1
ENSMUST00000217458	ENSMUSG00000111034	AC153955.1
ENSMUST00000217460	MSTRG.32290	AC122428.3
ENSMUST00000217463	MSTRG.32300	C030014I23Rik
ENSMUST00000217474	ENSMUSG00000110755	AC151971.2
ENSMUST00000217490	MSTRG.31733	AC122525.1
ENSMUST00000217491	MSTRG.33619	AC165080.2
ENSMUST00000217495	ENSMUSG00000111546	AC117245.3
ENSMUST00000217501	ENSMUSG00000111422	AC153536.3
ENSMUST00000217505	MSTRG.2798	AC125206.2
ENSMUST00000217507	MSTRG.32254	AC126459.5
ENSMUST00000217520	MSTRG.31678	AC164565.1
ENSMUST00000217529	ENSMUSG00000111657	AC126796.1
ENSMUST00000217535	MSTRG.2525	AC153845.3
ENSMUST00000217540	MSTRG.32306	AC151971.3
ENSMUST00000217543	ENSMUSG00000111874	AC156793.1
ENSMUST00000217551	MSTRG.32251	AC162938.3
ENSMUST00000217561	ENSMUSG00000111176	AC157950.2
ENSMUST00000217562	ENSMUSG00000111836	AC140363.2
ENSMUST00000217574	ENSMUSG00000111379	AC157476.2
ENSMUST00000217582	ENSMUSG00000111865	AC153729.5
ENSMUST00000217585	MSTRG.32333	AC061963.1
ENSMUST00000217609	ENSMUSG00000111050	AC156832.2

ENSMUST00000217619	MSTRG.19658	AC122876.1
ENSMUST00000217622	ENSMUSG00000111435	AC123842.1
ENSMUST00000217623	ENSMUSG00000111022	AC164881.2
ENSMUST00000217629	ENSMUSG00000110896	AC116726.2
ENSMUST00000217654	MSTRG.31817	CT025660.2
ENSMUST00000217666	ENSMUSG00000112258	AC154313.1
ENSMUST00000217669	MSTRG.6424	AC122860.1
ENSMUST00000217680	ENSMUSG00000112647	AC115805.1
ENSMUST00000217700	ENSMUSG00000112161	AC160063.1
ENSMUST00000217715	ENSMUSG00000112702	AC111046.1
ENSMUST00000217728	ENSMUSG00000111947	AC131120.1
ENSMUST00000217740	MSTRG.2974	D830039M14Rik
ENSMUST00000217743	ENSMUSG00000112841	AC164567.5
ENSMUST00000217744	ENSMUSG00000112604	AC161247.1
ENSMUST00000217762	MSTRG.6992	AC159624.1
ENSMUST00000217769	ENSMUSG00000112035	AC122018.1
ENSMUST00000217783	ENSMUSG00000112514	AC153512.4
ENSMUST00000217786	ENSMUSG00000112635	CT025625.1
ENSMUST00000217788	MSTRG.3042	AC153382.2
ENSMUST00000217795	ENSMUSG00000112805	AC153504.2
ENSMUST00000217797	ENSMUSG00000097323	4930426I24Rik
ENSMUST00000217798	ENSMUSG00000112501	AC153940.1
ENSMUST00000217799	ENSMUSG00000112242	AL589661.2
ENSMUST00000217800	MSTRG.23050	CAAA01066804.1
ENSMUST00000217802	MSTRG.3241	E130317F20Rik
ENSMUST00000217817	ENSMUSG00000112365	AC117232.4
ENSMUST00000217818	MSTRG.2861	AC153529.2
ENSMUST00000217821	MSTRG.6897	AC164550.1
ENSMUST00000217825	ENSMUSG00000111972	AC162467.1
ENSMUST00000217839	MSTRG.7217	AC163033.2
ENSMUST00000217840	ENSMUSG00000112540	AC160060.1
ENSMUST00000217849	ENSMUSG00000112711	AC135019.3
ENSMUST00000217853	MSTRG.3725	AC123720.1
ENSMUST00000217855	MSTRG.3647	AC140299.1
ENSMUST00000217857	ENSMUSG00000112532	AC147634.1
ENSMUST00000217859	MSTRG.2978	AC165164.1
ENSMUST00000217860	ENSMUSG00000112110	AC158605.1
ENSMUST00000217866	ENSMUSG00000112117	RMST_1
ENSMUST00000217867	MSTRG.3081	AC087891.1
ENSMUST00000217871	ENSMUSG00000112117	RMST_1
ENSMUST00000217874	MSTRG.3849	AC134329.1
ENSMUST00000217882	MSTRG.6391	AC158232.1
ENSMUST00000217888	MSTRG.7517	AC125487.1
ENSMUST00000217893	MSTRG.3632	AC153821.3
ENSMUST00000217897	ENSMUSG00000112761	AC134329.2
ENSMUST00000217919	ENSMUSG00000112497	AC099715.1
ENSMUST00000217920	MSTRG.2483	AC153370.2
ENSMUST00000217927	MSTRG.6425	AC139752.1
ENSMUST00000217932	ENSMUSG00000112819	AC241534.4
ENSMUST00000217933	ENSMUSG00000112803	AC159326.2
ENSMUST00000217947	MSTRG.3222	AC164623.1
ENSMUST00000217951	ENSMUSG00000112174	CT009504.2
ENSMUST00000217953	MSTRG.3040	AC153382.1
ENSMUST00000217955	MSTRG.3031	AC153516.1
ENSMUST00000217956	MSTRG.3849	AC134329.1
ENSMUST00000217963	ENSMUSG00000112303	AC155929.3
ENSMUST00000217965	MSTRG.6352	AC160138.1

ENSMUST00000217967	MSTRG.6421	AC122860.2
ENSMUST00000217986	MSTRG.7242	AC124346.3
ENSMUST00000217990	MSTRG.7258	AC165249.1
ENSMUST00000217997	ENSMUSG00000112705	AC119892.1
ENSMUST00000218015	ENSMUSG00000091526	BB019430
ENSMUST00000218017	ENSMUSG00000112792	AC157570.4
ENSMUST00000218027	MSTRG.3736	AC139638.1
ENSMUST00000218033	MSTRG.7200	AC124414.1
ENSMUST00000218037	ENSMUSG00000112235	AC139754.3
ENSMUST00000218041	MSTRG.7245	AC124572.2
ENSMUST00000218053	ENSMUSG00000112556	AC129336.2
ENSMUST00000218056	ENSMUSG00000112539	AC124413.3
ENSMUST00000218060	ENSMUSG00000112117	RMST_1
ENSMUST00000218063	MSTRG.3478	AC168315.1
ENSMUST00000218068	MSTRG.3775	AC158686.1
ENSMUST00000218069	ENSMUSG00000112783	AC121286.2
ENSMUST00000218071	MSTRG.3413	AC102114.1
ENSMUST00000218077	MSTRG.3459	AC150899.1
ENSMUST00000218078	MSTRG.7284	2310015A10Rik
ENSMUST00000218080	ENSMUSG00000112630	AC153547.1
ENSMUST00000218097	ENSMUSG00000112139	AC114002.2
ENSMUST00000218099	ENSMUSG00000112771	AC160863.4
ENSMUST00000218100	MSTRG.3030	AC155909.1
ENSMUST00000218103	ENSMUSG00000112701	AC163282.2
ENSMUST00000218105	MSTRG.3632	AC153821.3
ENSMUST00000218108	MSTRG.2547	AC153557.3
ENSMUST00000218113	ENSMUSG00000112458	AC135019.1
ENSMUST00000218132	MSTRG.3598	AC153366.1
ENSMUST00000218143	MSTRG.3191	AC160405.2
ENSMUST00000218145	ENSMUSG00000112071	CT010463.1
ENSMUST00000218155	MSTRG.7244	AC124572.1
ENSMUST00000218156	ENSMUSG00000112000	AC153501.1
ENSMUST00000218164	ENSMUSG00000112619	AC122829.3
ENSMUST00000218174	MSTRG.3051	AC153379.3
ENSMUST00000218180	MSTRG.2963	AC153517.1
ENSMUST00000218182	ENSMUSG00000112280	AC153962.3
ENSMUST00000218188	ENSMUSG00000112845	AC153361.2
ENSMUST00000218189	MSTRG.33202	5830418P13Rik
ENSMUST00000218193	ENSMUSG00000112924	AC132304.3
ENSMUST00000218201	MSTRG.3588	AC159542.1
ENSMUST00000218205	MSTRG.3042	AC153382.2
ENSMUST00000218206	MSTRG.2878	AC153526.2
ENSMUST00000218207	ENSMUSG00000112300	AC160405.1
ENSMUST00000218233	ENSMUSG00000112598	CT573364.1
ENSMUST00000218234	ENSMUSG00000111885	AC122405.1
ENSMUST00000218235	MSTRG.7287	CT030161.3
ENSMUST00000218247	MSTRG.3198	AC160411.1
ENSMUST00000218248	MSTRG.6898	AC130661.1
ENSMUST00000218252	ENSMUSG00000112169	CT009504.1
ENSMUST00000218255	ENSMUSG00000112117	RMST_1
ENSMUST00000218256	MSTRG.7228	AC124556.1
ENSMUST00000218261	MSTRG.3524	AC151984.1
ENSMUST00000218277	ENSMUSG00000112041	AC104880.1
ENSMUST00000218285	MSTRG.3803	AC160029.3
ENSMUST00000218300	ENSMUSG00000112444	AC153548.3
ENSMUST00000218306	MSTRG.3049	AC153379.2
ENSMUST00000218314	ENSMUSG00000112117	RMST_1

ENSMUST00000218320	ENSMUSG00000111935	AC152981.1
ENSMUST00000218323	ENSMUSG00000111933	AC108401.1
ENSMUST00000218326	ENSMUSG00000112367	AC155832.1
ENSMUST00000218335	ENSMUSG00000112392	AC159297.4
ENSMUST00000218341	MSTRG.7209	AC120002.3
ENSMUST00000218344	MSTRG.7009	AC123067.2
ENSMUST00000218348	MSTRG.3700	AC153497.1
ENSMUST00000218349	ENSMUSG00000112410	CT009504.3
ENSMUST00000218350	ENSMUSG00000112028	AC079680.1
ENSMUST00000218351	MSTRG.2885	AC153524.4
ENSMUST00000218352	MSTRG.6391	AC158232.1
ENSMUST00000218354	ENSMUSG00000112897	AC157017.3
ENSMUST00000218355	MSTRG.3013	AC122539.3
ENSMUST00000218356	ENSMUSG00000112348	AC158398.1
ENSMUST00000218368	ENSMUSG00000112041	AC104880.1
ENSMUST00000218374	MSTRG.6387	AC241534.1
ENSMUST00000218379	MSTRG.3623	AC153504.1
ENSMUST00000218388	ENSMUSG00000112749	AC153938.3
ENSMUST00000218392	ENSMUSG00000112400	AC160863.2
ENSMUST00000218396	ENSMUSG00000112289	AC153937.3
ENSMUST00000218411	MSTRG.7290	CT030161.1
ENSMUST00000218415	ENSMUSG00000112158	AC102369.2
ENSMUST00000218416	MSTRG.27178	AC142191.1
ENSMUST00000218418	ENSMUSG00000112532	AC147634.1
ENSMUST00000218424	ENSMUSG00000112573	AC153567.2
ENSMUST00000218446	ENSMUSG00000111912	AC152946.1
ENSMUST00000218453	MSTRG.7217	AC163033.2
ENSMUST00000218454	MSTRG.3352	AC159474.1
ENSMUST00000218472	ENSMUSG00000112721	AC168276.2
ENSMUST00000218473	ENSMUSG00000095953	6030469F06Rik
ENSMUST00000218476	MSTRG.2892	AC155941.3
ENSMUST00000218485	ENSMUSG00000112188	AC153937.2
ENSMUST00000218493	ENSMUSG00000112700	AC155929.4
ENSMUST00000218495	ENSMUSG00000112722	AC156268.1
ENSMUST00000218497	MSTRG.6995	AC154734.1
ENSMUST00000218501	ENSMUSG00000112206	AC153544.1
ENSMUST00000218509	ENSMUSG00000112257	AC121805.1
ENSMUST00000218518	ENSMUSG00000112290	AC153938.2
ENSMUST00000218522	ENSMUSG00000112818	AC153830.1
ENSMUST00000218529	MSTRG.3100	AC153509.2
ENSMUST00000218530	MSTRG.2682	AC153962.1
ENSMUST00000218537	ENSMUSG00000112505	AC241534.3
ENSMUST00000218544	MSTRG.3202	AC160411.2
ENSMUST00000218555	MSTRG.7292	AC163282.1
ENSMUST00000218557	MSTRG.3969	AC226737.1
ENSMUST00000218567	ENSMUSG00000112452	AC166361.1
ENSMUST00000218588	ENSMUSG00000112543	AC121986.1
ENSMUST00000218594	MSTRG.3000	AC121961.1
ENSMUST00000218599	MSTRG.3774	AC158686.2
ENSMUST00000218600	ENSMUSG00000112137	AC102312.1
ENSMUST00000218616	MSTRG.3187	AC164573.1
ENSMUST00000218634	ENSMUSG00000112211	AC114575.2
ENSMUST00000218639	ENSMUSG00000112420	AC156636.1
ENSMUST00000218641	MSTRG.3209	AC153887.2
ENSMUST00000218657	ENSMUSG00000112131	AC168274.1
ENSMUST00000218661	ENSMUSG00000112563	AC153526.5
ENSMUST00000218662	MSTRG.3191	AC160405.2

ENSMUST00000218664	ENSMUSG00000112596	AC153494.1
ENSMUST00000218666	MSTRG.7289	CT030161.2
ENSMUST00000218673	MSTRG.3520	AC132465.3
ENSMUST00000218674	MSTRG.3704	AC126943.1
ENSMUST00000218706	MSTRG.3640	AC121844.1
ENSMUST00000218707	ENSMUSG00000112733	AC155941.5
ENSMUST00000218708	MSTRG.7522	AC122317.2
ENSMUST00000218733	MSTRG.2622	AC101677.2
ENSMUST00000218746	ENSMUSG00000112014	AC140264.3
ENSMUST00000218751	ENSMUSG00000112865	AC155255.2
ENSMUST00000218757	MSTRG.3080	4930533K18Rik
ENSMUST00000218768	MSTRG.3584	AC153506.1
ENSMUST00000218774	ENSMUSG00000112739	AC155941.6
ENSMUST00000218783	ENSMUSG00000112681	AC164576.1
ENSMUST00000218784	ENSMUSG00000112319	AC152414.1
ENSMUST00000218795	MSTRG.2889	AC155941.2
ENSMUST00000218800	MSTRG.2414	AC122390.1
ENSMUST00000218808	ENSMUSG00000112576	AC121805.2
ENSMUST00000218818	ENSMUSG00000112765	AC122832.3
ENSMUST00000218833	ENSMUSG00000112227	AC157019.2
ENSMUST00000218836	ENSMUSG00000112071	CT010463.1
ENSMUST00000218838	ENSMUSG00000112082	AC101882.1
ENSMUST00000218840	ENSMUSG00000112352	AC153550.1
ENSMUST00000218845	ENSMUSG00000112483	AC156636.2
ENSMUST00000218852	ENSMUSG00000112527	AC124413.2
ENSMUST00000218870	ENSMUSG00000112832	AC129304.2
ENSMUST00000218886	MSTRG.3191	AC160405.2
ENSMUST00000218917	MSTRG.7262	AC154585.1
ENSMUST00000218925	ENSMUSG00000112733	AC155941.5
ENSMUST00000218927	ENSMUSG00000112763	AC162376.3
ENSMUST00000218937	ENSMUSG00000112616	AC160028.4
ENSMUST00000218957	ENSMUSG00000112307	AC112274.1
ENSMUST00000218961	MSTRG.3038	AC153512.1
ENSMUST00000218963	ENSMUSG00000112291	AC168276.1
ENSMUST00000218971	MSTRG.3802	AC160029.1
ENSMUST00000218986	MSTRG.3652	AC125108.1
ENSMUST00000218987	MSTRG.3815	AC110381.1
ENSMUST00000218993	MSTRG.3479	AC168315.3
ENSMUST00000218999	MSTRG.3394	AC158605.2
ENSMUST00000219006	ENSMUSG00000112537	AC127568.2
ENSMUST00000219008	MSTRG.3195	AC160405.3
ENSMUST00000219013	MSTRG.3393	AC158605.3
ENSMUST00000219014	MSTRG.3198	AC160411.1
ENSMUST00000219015	MSTRG.7283	AC108401.3
ENSMUST00000219016	MSTRG.3611	AC153365.1
ENSMUST00000219020	MSTRG.2419	AC122301.2
ENSMUST00000219021	ENSMUSG00000097134	1110002J07Rik
ENSMUST00000219022	ENSMUSG00000112716	AC134528.2
ENSMUST00000219025	ENSMUSG00000112256	AC151895.1
ENSMUST00000219031	MSTRG.6408	CT009738.1
ENSMUST00000219032	ENSMUSG00000112854	AC154039.2
ENSMUST00000219034	MSTRG.7238	AC148324.1
ENSMUST00000219046	MSTRG.3752	AC158804.2
ENSMUST00000219062	MSTRG.7304	AC127337.2
ENSMUST00000219067	MSTRG.3639	AC121844.2
ENSMUST00000219077	ENSMUSG00000112409	AC155832.2
ENSMUST00000219079	MSTRG.3858	F420014N23Rik

ENSMUST00000219081	ENSMUSG00000112888	AC166256.1
ENSMUST00000219095	MSTRG.3814	AC110381.3
ENSMUST00000219113	ENSMUSG00000112073	AC131720.3
ENSMUST00000219114	MSTRG.2980	AC124421.1
ENSMUST00000219123	ENSMUSG00000112246	AC121871.1
ENSMUST00000219124	MSTRG.3641	AC121844.3
ENSMUST00000219127	MSTRG.6888	AC164550.2
ENSMUST00000219142	MSTRG.2984	AC124421.2
ENSMUST00000219148	MSTRG.2901	AC164624.1
ENSMUST00000219154	MSTRG.3217	AC153887.3
ENSMUST00000219161	ENSMUSG00000112142	AC115891.3
ENSMUST00000219164	ENSMUSG00000111910	AC162908.1
ENSMUST00000219170	ENSMUSG00000112874	AC115805.2
ENSMUST00000219184	ENSMUSG00000112121	AC163903.1
ENSMUST00000219192	MSTRG.3080	4930533K18Rik
ENSMUST00000219196	ENSMUSG00000112875	AC122197.2
ENSMUST00000219198	MSTRG.6419	AC124134.1
ENSMUST00000219210	MSTRG.3720	5330438D12Rik
ENSMUST00000219217	MSTRG.27178	AC142191.1
ENSMUST00000219219	MSTRG.3781	4921513I03Rik
ENSMUST00000219221	ENSMUSG00000112792	AC157570.4
ENSMUST00000219233	ENSMUSG00000111977	AC132265.1
ENSMUST00000219242	MSTRG.2422	AC138290.1
ENSMUST00000219259	MSTRG.2483	AC153370.2
ENSMUST00000219269	ENSMUSG00000112664	AC166361.3
ENSMUST00000219273	MSTRG.3720	5330438D12Rik
ENSMUST00000219283	ENSMUSG00000112324	AC153548.2
ENSMUST00000219287	ENSMUSG00000112477	AC153506.3
ENSMUST00000219298	ENSMUSG00000112660	AC160410.1
ENSMUST00000219313	MSTRG.3612	AC153365.2
ENSMUST00000219319	ENSMUSG00000112030	AC133174.1
ENSMUST00000219320	MSTRG.7525	AC122317.3
ENSMUST00000219321	MSTRG.3084	AC087890.1
ENSMUST00000219324	MSTRG.2978	AC165164.1
ENSMUST00000219328	MSTRG.2415	Gm28905
ENSMUST00000219333	ENSMUSG00000112866	AC153967.1
ENSMUST00000219336	ENSMUSG00000112054	CT030030.1
ENSMUST00000219349	MSTRG.7284	2310015A10Rik
ENSMUST00000219353	MSTRG.7217	AC163033.2
ENSMUST00000219355	ENSMUSG00000112587	AC100590.3
ENSMUST00000219367	ENSMUSG00000073000	Gm10451
ENSMUST00000219371	ENSMUSG00000112041	AC104880.1
ENSMUST00000219379	ENSMUSG00000112261	AC167222.1
ENSMUST00000219395	ENSMUSG00000112212	AC153530.1
ENSMUST00000219396	ENSMUSG00000112097	AC122829.1
ENSMUST00000219397	MSTRG.3770	AC162467.3
ENSMUST00000219411	ENSMUSG00000112054	CT030030.1
ENSMUST00000219416	ENSMUSG00000112089	AC153977.2
ENSMUST00000219418	ENSMUSG00000112183	AC139754.1
ENSMUST00000219423	MSTRG.6995	AC154734.1
ENSMUST00000219429	ENSMUSG00000112095	AC132332.1
ENSMUST00000219433	ENSMUSG00000112805	AC153504.2
ENSMUST00000219440	MSTRG.3632	AC153821.3
ENSMUST00000219444	ENSMUSG00000112117	RMST_1
ENSMUST00000219456	ENSMUSG00000112120	AC153379.1
ENSMUST00000219458	MSTRG.2894	AC153949.1
ENSMUST00000219461	MSTRG.3597	AC153366.2

ENSMUST00000219466	ENSMUSG00000111924	AC153935.1
ENSMUST00000219468	ENSMUSG00000112164	AC114575.1
ENSMUST00000219472	ENSMUSG00000112404	AC159282.1
ENSMUST00000219480	ENSMUSG00000112549	AC115785.1
ENSMUST00000219484	ENSMUSG00000112265	AC101882.2
ENSMUST00000219485	ENSMUSG00000112586	AC132384.14
ENSMUST00000219492	ENSMUSG00000100550	2310039L15Rik
ENSMUST00000219494	ENSMUSG00000112338	AC116557.1
ENSMUST00000219499	MSTRG.6441	AC140354.1
ENSMUST00000219501	ENSMUSG00000112358	AC108802.1
ENSMUST00000219513	MSTRG.7230	AC159296.1
ENSMUST00000219521	ENSMUSG00000112342	AC168058.1
ENSMUST00000219525	ENSMUSG00000112728	AC123694.2
ENSMUST00000219528	MSTRG.3631	AC153821.2
ENSMUST00000219530	ENSMUSG00000112137	AC102312.1
ENSMUST00000219535	MSTRG.3953	AC131081.1
ENSMUST00000219536	ENSMUSG00000111994	AC159477.1
ENSMUST00000219546	MSTRG.3645	AC140299.2
ENSMUST00000219576	ENSMUSG00000111952	AC159709.1
ENSMUST00000219578	ENSMUSG00000112109	AC159466.1
ENSMUST00000219581	ENSMUSG00000112857	AC124556.2
ENSMUST00000219583	ENSMUSG00000112188	AC153937.2
ENSMUST00000219594	ENSMUSG00000112029	AC160028.1
ENSMUST00000219595	MSTRG.6429	AC117236.1
ENSMUST00000219596	MSTRG.3443	AC122408.3
ENSMUST00000219606	ENSMUSG00000112117	RMST_1
ENSMUST00000219608	ENSMUSG00000112666	AC132384.16
ENSMUST00000219610	ENSMUSG00000112054	CT030030.1
ENSMUST00000219611	ENSMUSG00000112757	AC134528.3
ENSMUST00000219618	MSTRG.2882	AC105169.2
ENSMUST00000219623	ENSMUSG00000086012	Gm15902
ENSMUST00000219628	MSTRG.3805	AC160029.2
ENSMUST00000219638	ENSMUSG00000112762	AC131596.1
ENSMUST00000219640	MSTRG.3871	AC114678.1
ENSMUST00000219645	ENSMUSG00000112535	AC160028.3
ENSMUST00000219668	ENSMUSG00000112014	AC140264.3
ENSMUST00000219678	MSTRG.6882	AC154864.1
ENSMUST00000219680	MSTRG.6995	AC154734.1
ENSMUST00000219688	MSTRG.3628	AC153821.1
ENSMUST00000219695	MSTRG.3198	AC160411.1
ENSMUST00000219700	ENSMUSG00000112108	AC121788.2
ENSMUST00000219716	MSTRG.3769	AC162467.4
ENSMUST00000219726	ENSMUSG00000112117	RMST_1
ENSMUST00000219731	MSTRG.2890	AC155941.4
ENSMUST00000219735	ENSMUSG00000112112	AC154300.1
ENSMUST00000219739	MSTRG.3042	AC153382.2
ENSMUST00000219740	ENSMUSG00000112386	AC108401.2
ENSMUST00000219743	ENSMUSG00000112396	AC122829.2
ENSMUST00000219775	ENSMUSG00000111913	AC153498.1
ENSMUST00000219777	ENSMUSG00000112075	AC117648.1
ENSMUST00000219785	ENSMUSG00000112753	AC139638.2
ENSMUST00000219794	ENSMUSG00000112564	AC153500.1
ENSMUST00000219800	ENSMUSG00000111926	AC124413.1
ENSMUST00000219810	MSTRG.7516	AC125487.2
ENSMUST00000219828	MSTRG.2867	AC153530.2
ENSMUST00000219831	MSTRG.3386	AC171183.3
ENSMUST00000219852	MSTRG.2604	AC159502.1

ENSMUST00000219875	ENSMUSG00000112295	AC153801.2
ENSMUST00000219876	ENSMUSG00000112548	AC147615.2
ENSMUST00000219913	ENSMUSG00000112677	AC155710.2
ENSMUST00000219920	ENSMUSG00000111994	AC159477.1
ENSMUST00000219933	MSTRG.3706	AC166836.1
ENSMUST00000219938	MSTRG.3523	AC132465.2
ENSMUST00000219940	ENSMUSG00000112905	AC117199.1
ENSMUST00000219952	ENSMUSG00000111903	AC093467.1
ENSMUST00000219954	ENSMUSG00000112569	CT010463.2
ENSMUST00000219957	MSTRG.6996	AC154734.2
ENSMUST00000219965	ENSMUSG00000112498	AL691505.1
ENSMUST00000219975	ENSMUSG00000112679	AC155644.2
ENSMUST00000219984	ENSMUSG00000112805	AC153504.2
ENSMUST00000219998	MSTRG.3110	Gm15398
ENSMUST00000220000	ENSMUSG00000112397	AC153369.1
ENSMUST00000220010	ENSMUSG00000111994	AC159477.1
ENSMUST00000220011	ENSMUSG00000112876	AC155293.3
ENSMUST00000220013	MSTRG.3817	AC110381.2
ENSMUST00000220021	ENSMUSG00000111943	AC153890.1
ENSMUST00000220023	MSTRG.3796	AC140264.2
ENSMUST00000220031	MSTRG.7258	AC165249.1
ENSMUST00000220034	MSTRG.3753	AC153495.1
ENSMUST00000220039	ENSMUSG00000112430	AC102312.2
ENSMUST00000220040	ENSMUSG00000112796	AC123694.3
ENSMUST00000220043	ENSMUSG00000073000	Gm10451
ENSMUST00000220050	ENSMUSG00000111918	AC155830.1
ENSMUST00000220056	MSTRG.3721	AC139376.1
ENSMUST00000220057	ENSMUSG00000112633	AC167229.1
ENSMUST00000220058	ENSMUSG00000100550	2310039L15Rik
ENSMUST00000220065	ENSMUSG00000112421	AC125361.1
ENSMUST00000220066	MSTRG.3725	AC123720.1
ENSMUST00000220067	ENSMUSG00000112121	AC163903.1
ENSMUST00000220074	ENSMUSG00000112778	AC160060.4
ENSMUST00000220077	MSTRG.7213	AC124453.1
ENSMUST00000220081	ENSMUSG00000112513	AC153364.1
ENSMUST00000220083	MSTRG.3840	AC158802.1
ENSMUST00000220086	ENSMUSG00000112523	AC122024.3
ENSMUST00000220094	MSTRG.2978	AC165164.1
ENSMUST00000220118	MSTRG.3408	AC158636.2
ENSMUST00000220125	MSTRG.7284	2310015A10Rik
ENSMUST00000220129	ENSMUSG00000112599	AC160863.3
ENSMUST00000220132	ENSMUSG00000112821	AC119892.2
ENSMUST00000220145	ENSMUSG00000112361	AC153526.3
ENSMUST00000220146	ENSMUSG00000044633	B530045E10Rik
ENSMUST00000220149	ENSMUSG00000112682	AC153940.2
ENSMUST00000220154	MSTRG.7295	AC134537.3
ENSMUST00000220157	MSTRG.7217	AC163033.2
ENSMUST00000220160	MSTRG.3831	AC152944.1
ENSMUST00000220168	ENSMUSG00000112848	CAAA01115014.1
ENSMUST00000220169	MSTRG.3407	AC158636.1
ENSMUST00000220177	ENSMUSG00000112785	AC163296.3
ENSMUST00000220181	ENSMUSG00000112517	AC111092.1
ENSMUST00000220184	MSTRG.3757	AC154039.1
ENSMUST00000220192	ENSMUSG00000112117	RMST_1
ENSMUST00000220196	ENSMUSG00000112914	AC118230.1
ENSMUST00000220205	MSTRG.7206	AC120002.2
ENSMUST00000220211	ENSMUSG00000112532	AC147634.1

ENSMUST00000220217	ENSMUSG00000111951	AC155929.1
ENSMUST00000220221	ENSMUSG00000112843	AC152979.5
ENSMUST00000220224	ENSMUSG00000112197	AC129336.1
ENSMUST00000220232	ENSMUSG00000112404	AC159282.1
ENSMUST00000220233	ENSMUSG00000074776	Gm10754
ENSMUST00000220243	MSTRG.3015	AC166359.1
ENSMUST00000220244	MSTRG.3589	AC159379.2
ENSMUST00000220248	ENSMUSG00000112481	AC155710.1
ENSMUST00000220251	MSTRG.3725	AC123720.1
ENSMUST00000220253	MSTRG.3627	AC153821.4
ENSMUST00000220254	MSTRG.3772	AC162467.2
ENSMUST00000220255	MSTRG.6353	AC160138.2
ENSMUST00000220256	MSTRG.2472	AC118202.1
ENSMUST00000220270	ENSMUSG00000112730	AC155834.1
ENSMUST00000220278	ENSMUSG00000112340	AC110043.1
ENSMUST00000220288	ENSMUSG00000112117	RMST_1
ENSMUST00000220293	ENSMUSG00000112765	AC122832.3
ENSMUST00000220296	MSTRG.2481	AC153370.1
ENSMUST00000220305	MSTRG.3720	5330438D12Rik
ENSMUST00000220313	MSTRG.2977	AC165164.2
ENSMUST00000220315	MSTRG.6374	CT572999.2
ENSMUST00000220316	MSTRG.7285	AC108401.4
ENSMUST00000220318	ENSMUSG00000111927	AC100590.1
ENSMUST00000220320	ENSMUSG00000112819	AC241534.4
ENSMUST00000220322	ENSMUSG00000112121	AC163903.1
ENSMUST00000220337	MSTRG.6376	AC162932.1
ENSMUST00000220339	ENSMUSG00000112533	AC069074.2
ENSMUST00000220354	ENSMUSG00000112234	AC153938.1
ENSMUST00000220360	ENSMUSG00000112787	AC110816.2
ENSMUST00000220361	MSTRG.2437	AC156952.1
ENSMUST00000220363	ENSMUSG00000112383	AC153512.2
ENSMUST00000220364	ENSMUSG00000112527	AC124413.2
ENSMUST00000220380	ENSMUSG00000112622	AC122850.1
ENSMUST00000220387	ENSMUSG00000112580	AC164629.8
ENSMUST00000220398	ENSMUSG00000112314	AC155712.2
ENSMUST00000220401	MSTRG.2885	AC153524.4
ENSMUST00000220423	MSTRG.7300	AC127337.1
ENSMUST00000220425	MSTRG.3761	AC144942.1
ENSMUST00000220426	ENSMUSG00000112425	AC153512.3
ENSMUST00000220432	ENSMUSG00000112265	AC101882.2
ENSMUST00000220435	ENSMUSG00000112019	AC152819.1
ENSMUST00000220436	MSTRG.2886	AC153524.1
ENSMUST00000220444	MSTRG.3473	AC139754.4
MSTRG.10.1	MSTRG.10	Lypla1
MSTRG.10000.1	MSTRG.10000	Parg
MSTRG.10002.1	MSTRG.10002	.
MSTRG.10003.1	MSTRG.10003	.
MSTRG.10010.9	MSTRG.10010	Mapk8
MSTRG.10011.1	MSTRG.10011	Mapk8
MSTRG.10012.1	MSTRG.10012	Mapk8
MSTRG.10013.1	MSTRG.10013	.
MSTRG.1002.1	MSTRG.1002	Gm29371
MSTRG.10022.1	MSTRG.10022	Fam35a
MSTRG.10026.1	MSTRG.10026	.
MSTRG.10027.1	MSTRG.10027	.
MSTRG.10029.1	MSTRG.10029	Wapl
MSTRG.10033.1	MSTRG.10033	Ccser2

MSTRG.10034.1	MSTRG.10034	Ccser2
MSTRG.10035.3	MSTRG.10035	Ghitm
MSTRG.10038.1	MSTRG.10038	.
MSTRG.10039.1	MSTRG.10039	.
MSTRG.10041.1	MSTRG.10041	Nrg3
MSTRG.10045.1	MSTRG.10045	Nrg3
MSTRG.1005.1	MSTRG.1005	.
MSTRG.10052.1	MSTRG.10052	.
MSTRG.10053.1	MSTRG.10053	.
MSTRG.10054.1	MSTRG.10054	.
MSTRG.10055.1	MSTRG.10055	.
MSTRG.10056.1	MSTRG.10056	.
MSTRG.10057.1	MSTRG.10057	.
MSTRG.1006.1	MSTRG.1006	.
MSTRG.10067.1	MSTRG.10067	.
MSTRG.10069.9	MSTRG.10069	Txndc16
MSTRG.10073.1	MSTRG.10073	Styx
MSTRG.10073.3	MSTRG.10073	Styx
MSTRG.10074.1	MSTRG.10074	Styx
MSTRG.10081.1	MSTRG.10081	Gm1821
MSTRG.10084.1	MSTRG.10084	Ddhd1
MSTRG.10085.1	MSTRG.10085	Ddhd1
MSTRG.10086.1	MSTRG.10086	Ddhd1
MSTRG.10087.1	MSTRG.10087	Ddhd1
MSTRG.10089.1	MSTRG.10089	Cdkn3
MSTRG.1009.1	MSTRG.1009	Gigyf2
MSTRG.10090.1	MSTRG.10090	Cdkn3
MSTRG.10093.1	MSTRG.10093	Gmfb
MSTRG.10093.4	MSTRG.10093	Gmfb
MSTRG.10093.6	MSTRG.10093	Gmfb
MSTRG.10095.1	MSTRG.10095	Gch1
MSTRG.10096.1	MSTRG.10096	Gch1
MSTRG.10097.1	MSTRG.10097	Gch1
MSTRG.1010.1	MSTRG.1010	Gigyf2
MSTRG.10100.1	MSTRG.10100	Mapk1ip1l
MSTRG.10102.1	MSTRG.10102	Socs4
MSTRG.10103.1	MSTRG.10103	Socs4
MSTRG.10105.1	MSTRG.10105	Mapk1ip1l
MSTRG.10108.1	MSTRG.10108	Fbxo34
MSTRG.10109.1	MSTRG.10109	Fbxo34
MSTRG.10111.1	MSTRG.10111	Atg14
MSTRG.10113.1	MSTRG.10113	Dlgap5
MSTRG.10114.1	MSTRG.10114	Dlgap5
MSTRG.10116.1	MSTRG.10116	.
MSTRG.10119.1	MSTRG.10119	Peli2
MSTRG.10120.1	MSTRG.10120	Peli2
MSTRG.10122.1	MSTRG.10122	Peli2
MSTRG.10125.1	MSTRG.10125	Ktn1
MSTRG.10126.1	MSTRG.10126	Ktn1
MSTRG.10128.1	MSTRG.10128	Ktn1
MSTRG.10130.1	MSTRG.10130	Tmem260
MSTRG.10131.1	MSTRG.10131	Tmem260
MSTRG.10132.1	MSTRG.10132	Tmem260
MSTRG.10135.1	MSTRG.10135	Exoc5
MSTRG.10138.1	MSTRG.10138	Naa30
MSTRG.10142.1	MSTRG.10142	Rpph1
MSTRG.10147.1	MSTRG.10147	.

MSTRG.1015.1	MSTRG.1015	Atg16l1
MSTRG.10151.1	MSTRG.10151	Osgep
MSTRG.10152.1	MSTRG.10152	Osgep
MSTRG.10154.1	MSTRG.10154	Osgep
MSTRG.1016.1	MSTRG.1016	Atg16l1
MSTRG.10161.1	MSTRG.10161	Mettl17
MSTRG.10164.1	MSTRG.10164	Arhgef40
MSTRG.10165.1	MSTRG.10165	Hnrnpc
MSTRG.10172.1	MSTRG.10172	Hnrnpc
MSTRG.10173.1	MSTRG.10173	Hnrnpc
MSTRG.10174.1	MSTRG.10174	Hnrnpc
MSTRG.10186.1	MSTRG.10186	Chd8
MSTRG.10189.1	MSTRG.10189	Tox4
MSTRG.1019.1	MSTRG.1019	.
MSTRG.10190.1	MSTRG.10190	Rab2b
MSTRG.10198.1	MSTRG.10198	Slc7a7
MSTRG.10206.1	MSTRG.10206	Gm43647
MSTRG.10206.3	MSTRG.10206	Gm43647
MSTRG.10206.4	MSTRG.10206	Gm43647
MSTRG.10206.5	MSTRG.10206	Gm43647
MSTRG.1021.1	MSTRG.1021	Sag
MSTRG.1023.2	MSTRG.1023	Gm19582
MSTRG.10253.1	MSTRG.10253	Ppp1r3e
MSTRG.1026.1	MSTRG.1026	Usp40
MSTRG.10266.1	MSTRG.10266	Trav15d-1-dv6d-1
MSTRG.10273.1	MSTRG.10273	Trav9d-3
MSTRG.10279.1	MSTRG.10279	.
MSTRG.1029.1	MSTRG.1029	Hjurp
MSTRG.10298.1	MSTRG.10298	Trav9n-4
MSTRG.10300.1	MSTRG.10300	Trav15n-1
MSTRG.10301.1	MSTRG.10301	Gm43650
MSTRG.10302.1	MSTRG.10302	Trav9n-4
MSTRG.10312.1	MSTRG.10312	Trav14n-2
MSTRG.10314.1	MSTRG.10314	Gm43650
MSTRG.10315.1	MSTRG.10315	Trav9n-4
MSTRG.10318.1	MSTRG.10318	Gm43650
MSTRG.10319.1	MSTRG.10319	Gm43650
MSTRG.10322.1	MSTRG.10322	Gm43650
MSTRG.10329.1	MSTRG.10329	Trav7-4
MSTRG.1034.1	MSTRG.1034	Agap1
MSTRG.10340.1	MSTRG.10340	.
MSTRG.10344.1	MSTRG.10344	Trav12-1
MSTRG.10346.1	MSTRG.10346	Trav14-1
MSTRG.10347.1	MSTRG.10347	Trav15-1-dv6-1
MSTRG.10372.1	MSTRG.10372	.
MSTRG.10374.1	MSTRG.10374	.
MSTRG.10380.1	MSTRG.10380	Acin1
MSTRG.10381.1	MSTRG.10381	Acin1
MSTRG.10386.1	MSTRG.10386	Ngdn
MSTRG.10388.1	MSTRG.10388	Dhrs2
MSTRG.1040.1	MSTRG.1040	Agap1
MSTRG.10416.1	MSTRG.10416	.
MSTRG.10416.2	MSTRG.10416	.
MSTRG.10420.1	MSTRG.10420	.
MSTRG.10430.1	MSTRG.10430	Parp4
MSTRG.10438.1	MSTRG.10438	.
MSTRG.10441.1	MSTRG.10441	Cryl1

MSTRG.10447.1	MSTRG.10447	Xpo4
MSTRG.1045.1	MSTRG.1045	.
MSTRG.10450.1	MSTRG.10450	Lats2
MSTRG.10453.1	MSTRG.10453	Ska3
MSTRG.10456.1	MSTRG.10456	Zdhhc20
MSTRG.10460.1	MSTRG.10460	Gm5142
MSTRG.10466.6	MSTRG.10466	Setdb2
MSTRG.10467.1	MSTRG.10467	Setdb2
MSTRG.10470.1	MSTRG.10470	Cab39l
MSTRG.10472.1	MSTRG.10472	Cab39l
MSTRG.10473.1	MSTRG.10473	.
MSTRG.10474.1	MSTRG.10474	.
MSTRG.10475.1	MSTRG.10475	.
MSTRG.1048.1	MSTRG.1048	Ramp1
MSTRG.10484.1	MSTRG.10484	Nupl1
MSTRG.10488.1	MSTRG.10488	Mipep
MSTRG.10495.1	MSTRG.10495	Ebpl
MSTRG.1050.1	MSTRG.1050	Lrrfip1
MSTRG.10516.1	MSTRG.10516	Wdfy2
MSTRG.10519.1	MSTRG.10519	.
MSTRG.10523.1	MSTRG.10523	Fdft1
MSTRG.10529.1	MSTRG.10529	Xkr6
MSTRG.1053.11	MSTRG.1053	Scly
MSTRG.10530.1	MSTRG.10530	Xkr6
MSTRG.10533.1	MSTRG.10533	.
MSTRG.10536.1	MSTRG.10536	Msra
MSTRG.10536.2	MSTRG.10536	Msra
MSTRG.10537.1	MSTRG.10537	Msra
MSTRG.10539.1	MSTRG.10539	Kif13b
MSTRG.10548.1	MSTRG.10548	Hmbox1
MSTRG.10551.1	MSTRG.10551	Extl3
MSTRG.10554.1	MSTRG.10554	Zfp395
MSTRG.10559.3	MSTRG.10559	Ccdc25
MSTRG.10572.1	MSTRG.10572	Ptk2b
MSTRG.10578.1	MSTRG.10578	Bnip3l
MSTRG.10582.1	MSTRG.10582	Ebf2
MSTRG.1059.1	MSTRG.1059	Traf3ip1
MSTRG.10607.1	MSTRG.10607	Gm16867
MSTRG.10610.1	MSTRG.10610	Gm16867
MSTRG.10611.1	MSTRG.10611	Gm16867
MSTRG.10613.1	MSTRG.10613	Gm16867
MSTRG.10620.1	MSTRG.10620	Gm27177
MSTRG.10632.1	MSTRG.10632	Ppp3cc
MSTRG.10633.1	MSTRG.10633	Ppp3cc
MSTRG.10640.1	MSTRG.10640	Fam160b2
MSTRG.10644.1	MSTRG.10644	Xpo7
MSTRG.10646.1	MSTRG.10646	Xpo7
MSTRG.10648.1	MSTRG.10648	.
MSTRG.10649.1	MSTRG.10649	.
MSTRG.10654.1	MSTRG.10654	Fndc3a
MSTRG.10655.1	MSTRG.10655	Fndc3a
MSTRG.10656.1	MSTRG.10656	Fndc3a
MSTRG.10657.1	MSTRG.10657	.
MSTRG.10660.1	MSTRG.10660	Med4
MSTRG.10663.1	MSTRG.10663	Sucla2
MSTRG.10671.1	MSTRG.10671	Lpar6
MSTRG.10686.1	MSTRG.10686	.

MSTRG.10691.2	MSTRG.10691	Gtf2f2
MSTRG.10692.1	MSTRG.10692	Gtf2f2
MSTRG.10695.1	MSTRG.10695	Gtf2f2
MSTRG.10696.1	MSTRG.10696	Gtf2f2
MSTRG.10699.1	MSTRG.10699	Nufip1
MSTRG.1070.1	MSTRG.1070	Hdac4
MSTRG.10702.1	MSTRG.10702	Tsc22d1
MSTRG.10703.1	MSTRG.10703	Tsc22d1
MSTRG.1071.1	MSTRG.1071	Gm29481
MSTRG.10710.1	MSTRG.10710	Enox1
MSTRG.10715.1	MSTRG.10715	Akap11
MSTRG.10716.1	MSTRG.10716	.
MSTRG.10717.1	MSTRG.10717	.
MSTRG.10720.1	MSTRG.10720	Dgkh
MSTRG.10721.1	MSTRG.10721	Dgkh
MSTRG.10722.1	MSTRG.10722	Dgkh
MSTRG.10724.1	MSTRG.10724	.
MSTRG.10729.1	MSTRG.10729	Naa16
MSTRG.1073.1	MSTRG.1073	Gm29481
MSTRG.10730.1	MSTRG.10730	Naa16
MSTRG.10733.1	MSTRG.10733	Elf1
MSTRG.10734.1	MSTRG.10734	Elf1
MSTRG.10735.1	MSTRG.10735	Elf1
MSTRG.10738.1	MSTRG.10738	Sugt1
MSTRG.10739.1	MSTRG.10739	Sugt1
MSTRG.10743.1	MSTRG.10743	Gm6999
MSTRG.10745.1	MSTRG.10745	.
MSTRG.10747.1	MSTRG.10747	Diaph3
MSTRG.1075.1	MSTRG.1075	Gpc1
MSTRG.10750.1	MSTRG.10750	Tdrd3
MSTRG.10751.1	MSTRG.10751	Tdrd3
MSTRG.10752.1	MSTRG.10752	Tdrd3
MSTRG.10753.1	MSTRG.10753	Tdrd3
MSTRG.10754.1	MSTRG.10754	Tdrd3
MSTRG.10755.1	MSTRG.10755	Tdrd3
MSTRG.10756.1	MSTRG.10756	Tdrd3
MSTRG.10757.1	MSTRG.10757	Tdrd3
MSTRG.10760.1	MSTRG.10760	.
MSTRG.10761.1	MSTRG.10761	.
MSTRG.10762.1	MSTRG.10762	.
MSTRG.10763.1	MSTRG.10763	.
MSTRG.10764.1	MSTRG.10764	.
MSTRG.10767.1	MSTRG.10767	Pcdh9
MSTRG.10768.1	MSTRG.10768	.
MSTRG.10773.1	MSTRG.10773	Bora
MSTRG.10773.2	MSTRG.10773	Bora
MSTRG.10776.1	MSTRG.10776	Pibf1
MSTRG.10778.1	MSTRG.10778	Pibf1
MSTRG.10779.1	MSTRG.10779	Pibf1
MSTRG.10780.1	MSTRG.10780	Pibf1
MSTRG.10781.1	MSTRG.10781	Pibf1
MSTRG.10783.1	MSTRG.10783	Klf12
MSTRG.10786.1	MSTRG.10786	4930517O19Rik
MSTRG.10787.1	MSTRG.10787	.
MSTRG.10791.1	MSTRG.10791	Uchl3
MSTRG.10792.1	MSTRG.10792	Uchl3
MSTRG.10793.1	MSTRG.10793	.

MSTRG.108.1	MSTRG.108	Eya1
MSTRG.10801.1	MSTRG.10801	Mycbp2
MSTRG.10805.1	MSTRG.10805	Slain1
MSTRG.10806.1	MSTRG.10806	Slain1os
MSTRG.10809.1	MSTRG.10809	Rbm26
MSTRG.10812.1	MSTRG.10812	Ndfip2
MSTRG.10814.1	MSTRG.10814	.
MSTRG.10815.1	MSTRG.10815	.
MSTRG.10818.1	MSTRG.10818	.
MSTRG.10819.1	MSTRG.10819	.
MSTRG.10820.1	MSTRG.10820	.
MSTRG.10821.1	MSTRG.10821	.
MSTRG.10822.1	MSTRG.10822	.
MSTRG.10824.1	MSTRG.10824	.
MSTRG.10830.1	MSTRG.10830	Abcc4
MSTRG.10832.1	MSTRG.10832	Abcc4
MSTRG.10834.1	MSTRG.10834	Abcc4
MSTRG.10835.1	MSTRG.10835	Abcc4
MSTRG.10839.1	MSTRG.10839	.
MSTRG.10843.1	MSTRG.10843	.
MSTRG.10845.1	MSTRG.10845	Rap2a
MSTRG.10846.1	MSTRG.10846	Rap2a
MSTRG.10848.1	MSTRG.10848	Mbnl2
MSTRG.10849.1	MSTRG.10849	Mbnl2
MSTRG.10850.1	MSTRG.10850	Mbnl2
MSTRG.10851.1	MSTRG.10851	Mbnl2
MSTRG.10853.1	MSTRG.10853	Mbnl2
MSTRG.10855.1	MSTRG.10855	.
MSTRG.10859.2	MSTRG.10859	Stk24
MSTRG.10859.4	MSTRG.10859	Stk24
MSTRG.1086.1	MSTRG.1086	Sned1
MSTRG.10860.1	MSTRG.10860	Stk24
MSTRG.10861.1	MSTRG.10861	Stk24
MSTRG.10862.1	MSTRG.10862	.
MSTRG.10864.1	MSTRG.10864	1810041H14Rik
MSTRG.10867.1	MSTRG.10867	Ubac2
MSTRG.10867.2	MSTRG.10867	Ubac2
MSTRG.10869.1	MSTRG.10869	Ubac2
MSTRG.10872.1	MSTRG.10872	Dock9
MSTRG.10874.1	MSTRG.10874	Dock9
MSTRG.10875.1	MSTRG.10875	Dock9
MSTRG.10876.1	MSTRG.10876	Dock9
MSTRG.10877.1	MSTRG.10877	Dock9
MSTRG.10886.1	MSTRG.10886	Pcca
MSTRG.10903.6	MSTRG.10903	Selenop
MSTRG.10905.1	MSTRG.10905	Ccdc152
MSTRG.10906.1	MSTRG.10906	.
MSTRG.10925.1	MSTRG.10925	.
MSTRG.10926.1	MSTRG.10926	.
MSTRG.10931.1	MSTRG.10931	Fyb
MSTRG.10931.2	MSTRG.10931	Fyb
MSTRG.10932.1	MSTRG.10932	Fyb
MSTRG.10933.1	MSTRG.10933	Gm2245
MSTRG.10933.2	MSTRG.10933	Gm2245
MSTRG.10936.1	MSTRG.10936	.
MSTRG.10943.1	MSTRG.10943	Wdr70
MSTRG.10944.1	MSTRG.10944	Wdr70

MSTRG.10945.1	MSTRG.10945	Wdr70
MSTRG.10947.1	MSTRG.10947	Gm2310
MSTRG.10950.1	MSTRG.10950	.
MSTRG.10951.1	MSTRG.10951	.
MSTRG.10953.1	MSTRG.10953	2410089E03Rik
MSTRG.10954.1	MSTRG.10954	2410089E03Rik
MSTRG.10955.1	MSTRG.10955	2410089E03Rik
MSTRG.10957.1	MSTRG.10957	2410089E03Rik
MSTRG.10958.1	MSTRG.10958	.
MSTRG.10960.1	MSTRG.10960	Nipbl
MSTRG.10961.1	MSTRG.10961	Nipbl
MSTRG.10962.1	MSTRG.10962	.
MSTRG.10963.1	MSTRG.10963	.
MSTRG.10966.1	MSTRG.10966	Slc1a3
MSTRG.10967.1	MSTRG.10967	Slc1a3
MSTRG.10968.1	MSTRG.10968	Slc1a3
MSTRG.10970.1	MSTRG.10970	Nadk2
MSTRG.10975.1	MSTRG.10975	Il7r
MSTRG.10979.1	MSTRG.10979	Rad1
MSTRG.10980.1	MSTRG.10980	Rad1
MSTRG.10985.5	MSTRG.10985	Sub1
MSTRG.10986.1	MSTRG.10986	.
MSTRG.10988.1	MSTRG.10988	.
MSTRG.10992.1	MSTRG.10992	Gm25713
MSTRG.10995.1	MSTRG.10995	Golph3
MSTRG.10997.1	MSTRG.10997	Pdzd2
MSTRG.10998.1	MSTRG.10998	Pdzd2
MSTRG.11.1	MSTRG.11	Tcea1
MSTRG.11003.1	MSTRG.11003	.
MSTRG.11006.1	MSTRG.11006	Drosha
MSTRG.11007.1	MSTRG.11007	Drosha
MSTRG.11008.1	MSTRG.11008	Drosha
MSTRG.11009.1	MSTRG.11009	.
MSTRG.11013.1	MSTRG.11013	Cdh12
MSTRG.11019.1	MSTRG.11019	Cdh18
MSTRG.11022.1	MSTRG.11022	Myo10
MSTRG.11024.1	MSTRG.11024	Myo10
MSTRG.11025.1	MSTRG.11025	Myo10
MSTRG.11027.1	MSTRG.11027	Retreg1
MSTRG.11029.1	MSTRG.11029	Retreg1
MSTRG.11033.1	MSTRG.11033	Fbxl7
MSTRG.11036.1	MSTRG.11036	Ank
MSTRG.11037.1	MSTRG.11037	Ank
MSTRG.11039.1	MSTRG.11039	Otulin
MSTRG.11040.1	MSTRG.11040	Otulin
MSTRG.11043.1	MSTRG.11043	Trio
MSTRG.11044.1	MSTRG.11044	Trio
MSTRG.11045.1	MSTRG.11045	Trio
MSTRG.11047.1	MSTRG.11047	Ctnnd2
MSTRG.1105.1	MSTRG.1105	D2hgdh
MSTRG.11052.1	MSTRG.11052	Ankrd33b
MSTRG.11055.1	MSTRG.11055	.
MSTRG.11059.1	MSTRG.11059	.
MSTRG.1106.1	MSTRG.1106	D2hgdh
MSTRG.11065.1	MSTRG.11065	Cpq
MSTRG.11066.2	MSTRG.11066	Cpq
MSTRG.1107.1	MSTRG.1107	D2hgdh

MSTRG.11071.1	MSTRG.11071	Mtdh
MSTRG.11072.1	MSTRG.11072	Mtdh
MSTRG.11079.1	MSTRG.11079	Pop1
MSTRG.11081.1	MSTRG.11081	Nipal2
MSTRG.11082.1	MSTRG.11082	Stk3
MSTRG.11083.1	MSTRG.11083	Stk3
MSTRG.11084.1	MSTRG.11084	Stk3
MSTRG.11086.1	MSTRG.11086	Stk3
MSTRG.11087.1	MSTRG.11087	Stk3
MSTRG.11090.1	MSTRG.11090	Vps13b
MSTRG.11093.1	MSTRG.11093	Vps13b
MSTRG.11095.1	MSTRG.11095	Vps13b
MSTRG.11096.1	MSTRG.11096	Vps13b
MSTRG.11097.1	MSTRG.11097	Vps13b
MSTRG.11098.1	MSTRG.11098	Cox6c
MSTRG.11111.1	MSTRG.11111	.
MSTRG.11112.1	MSTRG.11112	.
MSTRG.11119.1	MSTRG.11119	Grhl2
MSTRG.11129.1	MSTRG.11129	Ncald
MSTRG.11130.1	MSTRG.11130	Ncald
MSTRG.11135.1	MSTRG.11135	Ubr5
MSTRG.11136.1	MSTRG.11136	.
MSTRG.11145.1	MSTRG.11145	Slc25a32
MSTRG.11146.1	MSTRG.11146	Slc25a32
MSTRG.1115.1	MSTRG.1115	Thap4
MSTRG.11151.1	MSTRG.11151	Rims2
MSTRG.11153.1	MSTRG.11153	Rims2
MSTRG.11155.1	MSTRG.11155	Lrp12
MSTRG.11156.1	MSTRG.11156	Lrp12
MSTRG.11157.1	MSTRG.11157	Lrp12
MSTRG.11158.1	MSTRG.11158	Lrp12
MSTRG.11159.1	MSTRG.11159	Lrp12
MSTRG.11161.1	MSTRG.11161	Oxr1
MSTRG.11162.1	MSTRG.11162	Oxr1
MSTRG.11164.1	MSTRG.11164	Oxr1
MSTRG.11165.1	MSTRG.11165	.
MSTRG.1117.1	MSTRG.1117	Atg4b
MSTRG.11171.1	MSTRG.11171	Emc2
MSTRG.11172.1	MSTRG.11172	.
MSTRG.11178.1	MSTRG.11178	.
MSTRG.11180.1	MSTRG.11180	1700022A22Rik
MSTRG.11181.1	MSTRG.11181	.
MSTRG.11182.1	MSTRG.11182	.
MSTRG.11187.1	MSTRG.11187	Csmd3
MSTRG.11188.1	MSTRG.11188	.
MSTRG.11190.1	MSTRG.11190	Trps1
MSTRG.11191.1	MSTRG.11191	Trps1
MSTRG.11192.1	MSTRG.11192	Trps1
MSTRG.11193.1	MSTRG.11193	Trps1
MSTRG.11194.1	MSTRG.11194	Trps1
MSTRG.11195.1	MSTRG.11195	Trps1
MSTRG.11196.1	MSTRG.11196	Trps1
MSTRG.11199.1	MSTRG.11199	Utp23
MSTRG.11200.1	MSTRG.11200	.
MSTRG.11205.2	MSTRG.11205	Ext1
MSTRG.11205.3	MSTRG.11205	Ext1
MSTRG.11205.4	MSTRG.11205	Ext1

MSTRG.11206.1	MSTRG.11206	Ext1
MSTRG.11208.1	MSTRG.11208	Samd12
MSTRG.11213.1	MSTRG.11213	.
MSTRG.11219.1	MSTRG.11219	Mtbp
MSTRG.1122.1	MSTRG.1122	Ing5
MSTRG.11220.1	MSTRG.11220	Mtbp
MSTRG.11221.1	MSTRG.11221	Mtbp
MSTRG.11222.1	MSTRG.11222	Mtbp
MSTRG.11224.1	MSTRG.11224	Sntb1
MSTRG.11225.1	MSTRG.11225	Sntb1
MSTRG.11226.1	MSTRG.11226	Sntb1
MSTRG.11230.1	MSTRG.11230	Zhx2
MSTRG.11232.1	MSTRG.11232	Zhx2
MSTRG.11233.1	MSTRG.11233	.
MSTRG.11234.1	MSTRG.11234	Derl1
MSTRG.11234.3	MSTRG.11234	Derl1
MSTRG.11236.1	MSTRG.11236	Tbc1d31
MSTRG.11239.1	MSTRG.11239	Zhx1
MSTRG.11241.1	MSTRG.11241	Wdyhv1
MSTRG.11248.1	MSTRG.11248	Fam91a1
MSTRG.1125.1	MSTRG.1125	St8sia4
MSTRG.11251.1	MSTRG.11251	Tmem65
MSTRG.11252.1	MSTRG.11252	Tmem65
MSTRG.11253.1	MSTRG.11253	Tmem65
MSTRG.11259.1	MSTRG.11259	Mtss1
MSTRG.11259.2	MSTRG.11259	Mtss1
MSTRG.11264.1	MSTRG.11264	Nsmce2
MSTRG.11264.2	MSTRG.11264	Nsmce2
MSTRG.11265.1	MSTRG.11265	Nsmce2
MSTRG.11266.1	MSTRG.11266	.
MSTRG.1127.1	MSTRG.1127	.
MSTRG.11271.1	MSTRG.11271	.
MSTRG.11272.2	MSTRG.11272	.
MSTRG.11272.3	MSTRG.11272	.
MSTRG.11273.1	MSTRG.11273	.
MSTRG.11275.1	MSTRG.11275	Pvt1
MSTRG.11275.11	MSTRG.11275	Pvt1
MSTRG.11275.12	MSTRG.11275	Pvt1
MSTRG.11275.9	MSTRG.11275	Pvt1
MSTRG.11276.1	MSTRG.11276	Pvt1
MSTRG.11277.1	MSTRG.11277	Pvt1
MSTRG.11278.1	MSTRG.11278	Pvt1
MSTRG.11280.1	MSTRG.11280	Pvt1
MSTRG.11281.1	MSTRG.11281	Pvt1
MSTRG.11285.1	MSTRG.11285	Gm25628
MSTRG.11285.3	MSTRG.11285	Fam49b
MSTRG.11288.1	MSTRG.11288	Asap1
MSTRG.11289.1	MSTRG.11289	Asap1
MSTRG.11290.1	MSTRG.11290	Asap1
MSTRG.11291.1	MSTRG.11291	Asap1
MSTRG.11298.1	MSTRG.11298	Kcnq3
MSTRG.11299.1	MSTRG.11299	Kcnq3
MSTRG.11300.1	MSTRG.11300	Kcnq3
MSTRG.11301.1	MSTRG.11301	Kcnq3
MSTRG.11302.1	MSTRG.11302	Kcnq3
MSTRG.11304.1	MSTRG.11304	Kcnq3
MSTRG.11305.1	MSTRG.11305	Kcnq3

MSTRG.11307.1	MSTRG.11307	Kcnq3
MSTRG.11308.1	MSTRG.11308	Kcnq3
MSTRG.11310.1	MSTRG.11310	.
MSTRG.11312.1	MSTRG.11312	Tmem71
MSTRG.11317.1	MSTRG.11317	Tg
MSTRG.11318.4	MSTRG.11318	Sla
MSTRG.11323.1	MSTRG.11323	.
MSTRG.11325.1	MSTRG.11325	1700012I11Rik
MSTRG.11326.1	MSTRG.11326	1700012I11Rik
MSTRG.11328.1	MSTRG.11328	Zfat
MSTRG.11329.1	MSTRG.11329	Zfat
MSTRG.1133.1	MSTRG.1133	Slco6b1
MSTRG.11333.1	MSTRG.11333	Gm20732
MSTRG.11333.5	MSTRG.11333	Gm20732
MSTRG.11335.1	MSTRG.11335	.
MSTRG.11336.1	MSTRG.11336	.
MSTRG.11341.1	MSTRG.11341	Trappc9
MSTRG.11345.1	MSTRG.11345	Ago2
MSTRG.11346.3	MSTRG.11346	Ptk2
MSTRG.11347.1	MSTRG.11347	Ptk2
MSTRG.11349.1	MSTRG.11349	Ptk2
MSTRG.1135.1	MSTRG.1135	Gm7135
MSTRG.11352.1	MSTRG.11352	Dennd3
MSTRG.11354.1	MSTRG.11354	Slc45a4
MSTRG.11358.1	MSTRG.11358	.
MSTRG.11364.1	MSTRG.11364	.
MSTRG.11370.1	MSTRG.11370	Slurp1
MSTRG.11372.1	MSTRG.11372	Ly6d
MSTRG.11375.1	MSTRG.11375	.
MSTRG.11376.1	MSTRG.11376	.
MSTRG.11377.1	MSTRG.11377	.
MSTRG.11378.1	MSTRG.11378	.
MSTRG.1138.1	MSTRG.1138	D1Ertd622e
MSTRG.11380.1	MSTRG.11380	.
MSTRG.1139.1	MSTRG.1139	D1Ertd622e
MSTRG.11391.1	MSTRG.11391	Gm5960
MSTRG.11410.1	MSTRG.11410	Zc3h3
MSTRG.11411.1	MSTRG.11411	Zc3h3
MSTRG.11413.1	MSTRG.11413	.
MSTRG.11416.1	MSTRG.11416	.
MSTRG.1142.1	MSTRG.1142	Gm7967
MSTRG.11438.1	MSTRG.11438	Parp10
MSTRG.11444.1	MSTRG.11444	Oplah
MSTRG.1145.1	MSTRG.1145	Gin1
MSTRG.11458.1	MSTRG.11458	.
MSTRG.11459.1	MSTRG.11459	.
MSTRG.1146.15	MSTRG.1146	Pam
MSTRG.11462.1	MSTRG.11462	Mroh1
MSTRG.1147.1	MSTRG.1147	Pam
MSTRG.11474.1	MSTRG.11474	.
MSTRG.11475.1	MSTRG.11475	.
MSTRG.1148.1	MSTRG.1148	Pam
MSTRG.11483.1	MSTRG.11483	Arhgap39
MSTRG.11484.1	MSTRG.11484	Arhgap39
MSTRG.11486.1	MSTRG.11486	Arhgap39
MSTRG.11487.1	MSTRG.11487	Arhgap39
MSTRG.11488.1	MSTRG.11488	Arhgap39

MSTRG.11490.1	MSTRG.11490	Zfp251
MSTRG.11493.1	MSTRG.11493	Zfp7
MSTRG.11494.1	MSTRG.11494	Zfp7
MSTRG.11495.1	MSTRG.11495	.
MSTRG.11496.1	MSTRG.11496	.
MSTRG.11500.1	MSTRG.11500	Rbfox2
MSTRG.11501.1	MSTRG.11501	Rbfox2
MSTRG.11502.1	MSTRG.11502	Rbfox2
MSTRG.11504.1	MSTRG.11504	Rbfox2
MSTRG.11508.1	MSTRG.11508	.
MSTRG.11509.1	MSTRG.11509	.
MSTRG.11510.1	MSTRG.11510	.
MSTRG.11521.1	MSTRG.11521	.
MSTRG.11526.1	MSTRG.11526	.
MSTRG.11530.1	MSTRG.11530	.
MSTRG.11536.1	MSTRG.11536	Lgals1
MSTRG.11538.7	MSTRG.11538	Sh3bp1
MSTRG.1154.1	MSTRG.1154	Pam
MSTRG.1155.1	MSTRG.1155	Pam
MSTRG.11551.1	MSTRG.11551	Tmem184b
MSTRG.11553.1	MSTRG.11553	Pla2g6
MSTRG.11554.1	MSTRG.11554	Pla2g6
MSTRG.11556.1	MSTRG.11556	Pla2g6
MSTRG.11559.1	MSTRG.11559	Csnk1e
MSTRG.1156.1	MSTRG.1156	Pam
MSTRG.11566.2	MSTRG.11566	Gm26884
MSTRG.11568.1	MSTRG.11568	Gtpbp1
MSTRG.1157.1	MSTRG.1157	.
MSTRG.11570.1	MSTRG.11570	Sun2
MSTRG.11572.1	MSTRG.11572	Gm16576
MSTRG.11573.1	MSTRG.11573	Gm16576
MSTRG.11576.1	MSTRG.11576	Dnal4
MSTRG.11581.18	MSTRG.11581	Apobec3
MSTRG.11582.1	MSTRG.11582	Apobec3
MSTRG.11586.1	MSTRG.11586	Mgat3
MSTRG.11591.2	MSTRG.11591	Rps19bp1
MSTRG.11594.1	MSTRG.11594	Fam83f
MSTRG.11598.1	MSTRG.11598	.
MSTRG.11600.1	MSTRG.11600	Tnrc6b
MSTRG.11601.1	MSTRG.11601	Tnrc6b
MSTRG.11602.1	MSTRG.11602	Tnrc6b
MSTRG.11603.1	MSTRG.11603	Tnrc6b
MSTRG.11607.1	MSTRG.11607	Slc25a17
MSTRG.11608.2	MSTRG.11608	Rbx1
MSTRG.11608.3	MSTRG.11608	Rbx1
MSTRG.1161.1	MSTRG.1161	Gm7160
MSTRG.11615.1	MSTRG.11615	Mkl1
MSTRG.11617.1	MSTRG.11617	Gm25131
MSTRG.11618.1	MSTRG.11618	Mkl1
MSTRG.11620.1	MSTRG.11620	Mkl1
MSTRG.11620.2	MSTRG.11620	Mkl1
MSTRG.11625.1	MSTRG.11625	Mkl1
MSTRG.11626.1	MSTRG.11626	Mkl1
MSTRG.11628.1	MSTRG.11628	Mkl1
MSTRG.11636.1	MSTRG.11636	Zc3h7b
MSTRG.1164.1	MSTRG.1164	Gm28403
MSTRG.11640.1	MSTRG.11640	Tef

MSTRG.11643.1	MSTRG.11643	Aco2
MSTRG.11647.1	MSTRG.11647	.
MSTRG.11649.2	MSTRG.11649	Ccdc134
MSTRG.11649.3	MSTRG.11649	Ccdc134
MSTRG.11653.1	MSTRG.11653	Xrcc6
MSTRG.11658.1	MSTRG.11658	Xrcc6
MSTRG.1166.1	MSTRG.1166	Pign
MSTRG.11662.9	MSTRG.11662	Smdt1
MSTRG.11664.1	MSTRG.11664	Ndufa6
MSTRG.11666.1	MSTRG.11666	.
MSTRG.11667.1	MSTRG.11667	.
MSTRG.11668.1	MSTRG.11668	.
MSTRG.11669.1	MSTRG.11669	.
MSTRG.11679.1	MSTRG.11679	.
MSTRG.11684.1	MSTRG.11684	Arfgap3
MSTRG.1169.1	MSTRG.1169	Pign
MSTRG.117.1	MSTRG.117	Kcnb2
MSTRG.11703.1	MSTRG.11703	Arhgap8
MSTRG.11704.1	MSTRG.11704	Arhgap8
MSTRG.11705.1	MSTRG.11705	Arhgap8
MSTRG.11711.1	MSTRG.11711	Fam118a
MSTRG.11717.1	MSTRG.11717	Atxn10
MSTRG.11718.1	MSTRG.11718	Atxn10
MSTRG.1172.1	MSTRG.1172	Pign
MSTRG.11721.1	MSTRG.11721	D130051D11Rik
MSTRG.11721.4	MSTRG.11721	Mirlet7c-2
MSTRG.11721.5	MSTRG.11721	.
MSTRG.11721.6	MSTRG.11721	.
MSTRG.11721.7	MSTRG.11721	.
MSTRG.11721.8	MSTRG.11721	.
MSTRG.11722.1	MSTRG.11722	Gm37699
MSTRG.11728.1	MSTRG.11728	Celsr1
MSTRG.11737.1	MSTRG.11737	Tbc1d22a
MSTRG.11737.3	MSTRG.11737	Tbc1d22a
MSTRG.11738.1	MSTRG.11738	Tbc1d22a
MSTRG.1174.1	MSTRG.1174	2310035C23Rik
MSTRG.11742.1	MSTRG.11742	Brd1
MSTRG.11749.1	MSTRG.11749	Mov10l1
MSTRG.11750.1	MSTRG.11750	Mov10l1
MSTRG.11751.1	MSTRG.11751	Mov10l1
MSTRG.11756.1	MSTRG.11756	Mapk11
MSTRG.1176.1	MSTRG.1176	.
MSTRG.11760.1	MSTRG.11760	Selenoo
MSTRG.11764.1	MSTRG.11764	Mapk12
MSTRG.11768.1	MSTRG.11768	.
MSTRG.11769.1	MSTRG.11769	.
MSTRG.11770.1	MSTRG.11770	.
MSTRG.11780.1	MSTRG.11780	.
MSTRG.11785.1	MSTRG.11785	.
MSTRG.11786.1	MSTRG.11786	.
MSTRG.11787.1	MSTRG.11787	.
MSTRG.11791.1	MSTRG.11791	Cpne8
MSTRG.11794.1	MSTRG.11794	Gm4335
MSTRG.11795.1	MSTRG.11795	Gm4335
MSTRG.11799.1	MSTRG.11799	Yaf2
MSTRG.11800.1	MSTRG.11800	Yaf2
MSTRG.11801.1	MSTRG.11801	Yaf2

MSTRG.11805.1	MSTRG.11805	Prickle1
MSTRG.11806.1	MSTRG.11806	Prickle1
MSTRG.11807.1	MSTRG.11807	Prickle1
MSTRG.11807.2	MSTRG.11807	Prickle1
MSTRG.11807.3	MSTRG.11807	Prickle1
MSTRG.1181.1	MSTRG.1181	Phlpp1
MSTRG.11812.1	MSTRG.11812	Nell2
MSTRG.11813.1	MSTRG.11813	.
MSTRG.11816.2	MSTRG.11816	.
MSTRG.11816.3	MSTRG.11816	.
MSTRG.11816.5	MSTRG.11816	A130051J06Rik
MSTRG.11819.1	MSTRG.11819	Gm17546
MSTRG.11820.1	MSTRG.11820	Ano6
MSTRG.11822.1	MSTRG.11822	Ano6
MSTRG.11823.1	MSTRG.11823	Ano6
MSTRG.11823.2	MSTRG.11823	Ano6
MSTRG.11825.2	MSTRG.11825	2610037D02Rik
MSTRG.11827.1	MSTRG.11827	2610037D02Rik
MSTRG.11829.1	MSTRG.11829	Slc38a1
MSTRG.1183.1	MSTRG.1183	Phlpp1
MSTRG.11831.1	MSTRG.11831	Slc38a1
MSTRG.11833.1	MSTRG.11833	.
MSTRG.11836.1	MSTRG.11836	Pced1b
MSTRG.11837.1	MSTRG.11837	Pced1b
MSTRG.11838.1	MSTRG.11838	Pced1b
MSTRG.11839.1	MSTRG.11839	Pced1b
MSTRG.11841.1	MSTRG.11841	Pced1b
MSTRG.11842.1	MSTRG.11842	Pced1b
MSTRG.11843.1	MSTRG.11843	Pced1b
MSTRG.11844.1	MSTRG.11844	Pced1b
MSTRG.11845.1	MSTRG.11845	Pced1b
MSTRG.11847.1	MSTRG.11847	Rpap3
MSTRG.11853.1	MSTRG.11853	.
MSTRG.11856.1	MSTRG.11856	Hdac7
MSTRG.11857.1	MSTRG.11857	Hdac7
MSTRG.11863.1	MSTRG.11863	Asb8
MSTRG.11867.2	MSTRG.11867	4930415O20Rik
MSTRG.11872.1	MSTRG.11872	Arf3
MSTRG.11873.1	MSTRG.11873	.
MSTRG.11874.1	MSTRG.11874	Lmbr1l
MSTRG.11877.1	MSTRG.11877	.
MSTRG.11879.1	MSTRG.11879	Tuba1b
MSTRG.1188.1	MSTRG.1188	D630008O14Rik
MSTRG.1188.2	MSTRG.1188	Bcl2
MSTRG.1188.3	MSTRG.1188	Bcl2
MSTRG.11882.1	MSTRG.11882	B130046B21Rik
MSTRG.11884.1	MSTRG.11884	Prkag1
MSTRG.11885.1	MSTRG.11885	.
MSTRG.1189.1	MSTRG.1189	Gm37053
MSTRG.1189.3	MSTRG.1189	Bcl2
MSTRG.11892.1	MSTRG.11892	Kcnh3
MSTRG.11899.1	MSTRG.11899	Fmnl3
MSTRG.11900.1	MSTRG.11900	Fmnl3
MSTRG.11901.1	MSTRG.11901	Fmnl3
MSTRG.11903.1	MSTRG.11903	Fmnl3
MSTRG.11905.1	MSTRG.11905	.
MSTRG.11908.1	MSTRG.11908	Nckap5l

MSTRG.11908.2	MSTRG.11908	Nckap5l
MSTRG.11909.1	MSTRG.11909	.
MSTRG.11910.1	MSTRG.11910	.
MSTRG.1192.1	MSTRG.1192	Vps4b
MSTRG.11920.1	MSTRG.11920	Cox14
MSTRG.11920.2	MSTRG.11920	Cox14
MSTRG.11923.1	MSTRG.11923	Cers5
MSTRG.11925.1	MSTRG.11925	Lima1
MSTRG.1193.1	MSTRG.1193	.
MSTRG.11933.1	MSTRG.11933	Mettl7a1
MSTRG.11936.1	MSTRG.11936	Dip2b
MSTRG.11937.1	MSTRG.11937	Dip2b
MSTRG.11938.1	MSTRG.11938	Dip2b
MSTRG.1194.1	MSTRG.1194	.
MSTRG.11940.1	MSTRG.11940	Atf1
MSTRG.11944.1	MSTRG.11944	Slc11a2
MSTRG.11947.1	MSTRG.11947	5330439K02Rik
MSTRG.1195.1	MSTRG.1195	.
MSTRG.11951.1	MSTRG.11951	Tfcp2
MSTRG.11955.1	MSTRG.11955	Pou6f1
MSTRG.11956.1	MSTRG.11956	Dazap2
MSTRG.11959.1	MSTRG.11959	Bin2
MSTRG.11959.2	MSTRG.11959	Bin2
MSTRG.11964.1	MSTRG.11964	Galnt6
MSTRG.11966.1	MSTRG.11966	Fignl2
MSTRG.11967.1	MSTRG.11967	Fignl2
MSTRG.11968.1	MSTRG.11968	.
MSTRG.11969.1	MSTRG.11969	.
MSTRG.1197.1	MSTRG.1197	Cdh19
MSTRG.11971.1	MSTRG.11971	Acvr1b
MSTRG.11986.1	MSTRG.11986	Spryd3
MSTRG.11989.1	MSTRG.11989	.
MSTRG.12.1	MSTRG.12	Tcea1
MSTRG.12009.1	MSTRG.12009	Atf7
MSTRG.12010.1	MSTRG.12010	Atf7
MSTRG.12011.1	MSTRG.12011	Atf7
MSTRG.12013.1	MSTRG.12013	.
MSTRG.12014.1	MSTRG.12014	.
MSTRG.12017.4	MSTRG.12017	Cbx5
MSTRG.12018.1	MSTRG.12018	Cbx5
MSTRG.12019.1	MSTRG.12019	Cbx5
MSTRG.12019.2	MSTRG.12019	Cbx5
MSTRG.1202.1	MSTRG.1202	Gm29088
MSTRG.12022.1	MSTRG.12022	Zfp385a
MSTRG.12025.1	MSTRG.12025	Itga5
MSTRG.12027.1	MSTRG.12027	Nckap1l
MSTRG.12031.1	MSTRG.12031	.
MSTRG.12032.1	MSTRG.12032	.
MSTRG.12033.1	MSTRG.12033	.
MSTRG.12034.1	MSTRG.12034	.
MSTRG.12041.1	MSTRG.12041	Zfp597
MSTRG.1205.1	MSTRG.1205	.
MSTRG.12054.1	MSTRG.12054	Adcy9
MSTRG.12055.1	MSTRG.12055	Adcy9
MSTRG.12057.1	MSTRG.12057	Crebbp
MSTRG.12060.1	MSTRG.12060	Gm15859
MSTRG.12061.1	MSTRG.12061	Hmox2

MSTRG.12068.1	MSTRG.12068	Pam16
MSTRG.1207.1	MSTRG.1207	Gm7195
MSTRG.1209.1	MSTRG.1209	Gm28358
MSTRG.12091.1	MSTRG.12091	Ubn1
MSTRG.12096.1	MSTRG.12096	.
MSTRG.12097.1	MSTRG.12097	.
MSTRG.12099.1	MSTRG.12099	Mettl22
MSTRG.12103.1	MSTRG.12103	.
MSTRG.12104.1	MSTRG.12104	.
MSTRG.12105.1	MSTRG.12105	.
MSTRG.12106.1	MSTRG.12106	.
MSTRG.12107.1	MSTRG.12107	.
MSTRG.12108.1	MSTRG.12108	.
MSTRG.12109.1	MSTRG.12109	.
MSTRG.12110.1	MSTRG.12110	.
MSTRG.12111.1	MSTRG.12111	.
MSTRG.12112.1	MSTRG.12112	.
MSTRG.12113.1	MSTRG.12113	.
MSTRG.12114.1	MSTRG.12114	.
MSTRG.12115.1	MSTRG.12115	.
MSTRG.12116.1	MSTRG.12116	.
MSTRG.12117.1	MSTRG.12117	.
MSTRG.12118.1	MSTRG.12118	.
MSTRG.12119.1	MSTRG.12119	.
MSTRG.12120.1	MSTRG.12120	.
MSTRG.12121.1	MSTRG.12121	.
MSTRG.12122.1	MSTRG.12122	.
MSTRG.12123.1	MSTRG.12123	.
MSTRG.12124.1	MSTRG.12124	.
MSTRG.12125.1	MSTRG.12125	.
MSTRG.12126.1	MSTRG.12126	.
MSTRG.12127.1	MSTRG.12127	.
MSTRG.12128.1	MSTRG.12128	.
MSTRG.12130.1	MSTRG.12130	Rbfox1
MSTRG.12131.1	MSTRG.12131	Rbfox1
MSTRG.12132.1	MSTRG.12132	Rbfox1
MSTRG.12134.1	MSTRG.12134	Rbfox1
MSTRG.12135.1	MSTRG.12135	Rbfox1
MSTRG.12136.1	MSTRG.12136	Rbfox1
MSTRG.12137.1	MSTRG.12137	Rbfox1
MSTRG.12139.1	MSTRG.12139	Rbfox1
MSTRG.12140.1	MSTRG.12140	Rbfox1
MSTRG.12141.1	MSTRG.12141	Rbfox1
MSTRG.12142.1	MSTRG.12142	Rbfox1
MSTRG.12143.1	MSTRG.12143	Rbfox1
MSTRG.12144.1	MSTRG.12144	Rbfox1
MSTRG.12145.1	MSTRG.12145	Rbfox1
MSTRG.12146.1	MSTRG.12146	Rbfox1
MSTRG.12147.1	MSTRG.12147	Rbfox1
MSTRG.12148.1	MSTRG.12148	Rbfox1
MSTRG.12150.1	MSTRG.12150	Rbfox1
MSTRG.12152.1	MSTRG.12152	Rbfox1
MSTRG.12153.1	MSTRG.12153	Rbfox1
MSTRG.12154.1	MSTRG.12154	Rbfox1
MSTRG.12160.1	MSTRG.12160	.
MSTRG.12161.1	MSTRG.12161	.
MSTRG.12163.1	MSTRG.12163	.

MSTRG.1217.1	MSTRG.1217	Gli2
MSTRG.12170.1	MSTRG.12170	.
MSTRG.12172.1	MSTRG.12172	Ciita
MSTRG.12175.1	MSTRG.12175	Gm15558
MSTRG.12175.2	MSTRG.12175	Gm15558
MSTRG.12175.4	MSTRG.12175	Gm15558
MSTRG.12176.1	MSTRG.12176	Clec16a
MSTRG.12177.1	MSTRG.12177	.
MSTRG.12182.1	MSTRG.12182	Rmi2
MSTRG.12187.1	MSTRG.12187	Litaf
MSTRG.12192.1	MSTRG.12192	Snx29
MSTRG.12198.1	MSTRG.12198	Zc3h7a
MSTRG.12199.1	MSTRG.12199	Zc3h7a
MSTRG.1220.1	MSTRG.1220	Gli2
MSTRG.12204.2	MSTRG.12204	2610020C07Rik
MSTRG.12208.1	MSTRG.12208	Cpped1
MSTRG.1221.1	MSTRG.1221	Gli2
MSTRG.12210.1	MSTRG.12210	.
MSTRG.12211.1	MSTRG.12211	.
MSTRG.12214.1	MSTRG.12214	Mkl2
MSTRG.12216.1	MSTRG.12216	Mkl2
MSTRG.12217.1	MSTRG.12217	Mkl2
MSTRG.1222.1	MSTRG.1222	Gli2
MSTRG.12221.1	MSTRG.12221	.
MSTRG.12223.1	MSTRG.12223	Parn
MSTRG.12225.1	MSTRG.12225	.
MSTRG.1223.1	MSTRG.1223	Gli2
MSTRG.12231.1	MSTRG.12231	Pdxdc1
MSTRG.12232.1	MSTRG.12232	Pdxdc1
MSTRG.12233.2	MSTRG.12233	Mpv17l
MSTRG.12234.1	MSTRG.12234	Mpv17l
MSTRG.12238.1	MSTRG.12238	.
MSTRG.1224.1	MSTRG.1224	Gli2
MSTRG.12244.1	MSTRG.12244	.
MSTRG.12245.1	MSTRG.12245	.
MSTRG.12247.1	MSTRG.12247	Abcc1
MSTRG.12249.1	MSTRG.12249	Abcc1
MSTRG.12250.1	MSTRG.12250	Abcc1
MSTRG.12252.5	MSTRG.12252	Ube2v2
MSTRG.12253.1	MSTRG.12253	Ube2v2
MSTRG.12257.1	MSTRG.12257	Spidr
MSTRG.12258.1	MSTRG.12258	Spidr
MSTRG.12259.1	MSTRG.12259	Spidr
MSTRG.1226.1	MSTRG.1226	Ralb
MSTRG.12262.1	MSTRG.12262	Prkdc
MSTRG.12263.2	MSTRG.12263	Prkdc
MSTRG.12264.1	MSTRG.12264	Prkdc
MSTRG.12269.1	MSTRG.12269	.
MSTRG.12273.1	MSTRG.12273	Gm22558
MSTRG.12275.1	MSTRG.12275	.
MSTRG.12276.1	MSTRG.12276	.
MSTRG.12281.7	MSTRG.12281	Ypel1
MSTRG.12281.9	MSTRG.12281	Ypel1
MSTRG.12282.1	MSTRG.12282	Ypel1
MSTRG.12285.1	MSTRG.12285	Gm15585
MSTRG.12287.1	MSTRG.12287	Hic2
MSTRG.1229.1	MSTRG.1229	Gm27184

MSTRG.12292.2	MSTRG.12292	Ube2l3
MSTRG.12292.8	MSTRG.12292	Ube2l3
MSTRG.12294.1	MSTRG.12294	Ube2l3
MSTRG.123.1	MSTRG.123	Sbspon
MSTRG.12310.1	MSTRG.12310	Med15
MSTRG.12317.1	MSTRG.12317	.
MSTRG.12328.1	MSTRG.12328	Tango2
MSTRG.12335.1	MSTRG.12335	Comt
MSTRG.12337.1	MSTRG.12337	.
MSTRG.12339.1	MSTRG.12339	Gnb1l
MSTRG.12341.1	MSTRG.12341	Gm28539
MSTRG.12343.1	MSTRG.12343	Hira
MSTRG.12344.1	MSTRG.12344	Hira
MSTRG.12347.1	MSTRG.12347	Hira
MSTRG.1236.1	MSTRG.1236	Ptpn4
MSTRG.12363.1	MSTRG.12363	A930003A15Rik
MSTRG.12392.1	MSTRG.12392	.
MSTRG.12396.1	MSTRG.12396	Vps8
MSTRG.12397.1	MSTRG.12397	Vps8
MSTRG.12399.10	MSTRG.12399	1300002E11Rik
MSTRG.12399.11	MSTRG.12399	1300002E11Rik
MSTRG.12399.12	MSTRG.12399	1300002E11Rik
MSTRG.12399.13	MSTRG.12399	1300002E11Rik
MSTRG.12399.3	MSTRG.12399	1300002E11Rik
MSTRG.12399.8	MSTRG.12399	1300002E11Rik
MSTRG.12399.9	MSTRG.12399	1300002E11Rik
MSTRG.12401.1	MSTRG.12401	.
MSTRG.12407.1	MSTRG.12407	Tmem41a
MSTRG.12411.1	MSTRG.12411	Tra2b
MSTRG.12414.1	MSTRG.12414	Tbccd1
MSTRG.12415.3	MSTRG.12415	Dgkg
MSTRG.12415.5	MSTRG.12415	Dgkg
MSTRG.12417.1	MSTRG.12417	Dgkg
MSTRG.1242.1	MSTRG.1242	Clasp1
MSTRG.12420.1	MSTRG.12420	Dgkg
MSTRG.12426.1	MSTRG.12426	.
MSTRG.12430.1	MSTRG.12430	B630019A10Rik
MSTRG.12430.7	MSTRG.12430	B630019A10Rik
MSTRG.12434.1	MSTRG.12434	St6gal1
MSTRG.12435.1	MSTRG.12435	St6gal1
MSTRG.12439.1	MSTRG.12439	Bcl6
MSTRG.1244.1	MSTRG.1244	Clasp1
MSTRG.12445.1	MSTRG.12445	Lpp
MSTRG.12446.1	MSTRG.12446	Lpp
MSTRG.12447.1	MSTRG.12447	Lpp
MSTRG.12448.1	MSTRG.12448	Lpp
MSTRG.12449.1	MSTRG.12449	Lpp
MSTRG.12450.1	MSTRG.12450	Lpp
MSTRG.12453.1	MSTRG.12453	Lpp
MSTRG.12454.1	MSTRG.12454	Lpp
MSTRG.12459.1	MSTRG.12459	.
MSTRG.12463.1	MSTRG.12463	.
MSTRG.12465.1	MSTRG.12465	Opa1
MSTRG.12469.1	MSTRG.12469	Atp13a3
MSTRG.12469.2	MSTRG.12469	Atp13a3
MSTRG.12470.1	MSTRG.12470	Atp13a3
MSTRG.12471.1	MSTRG.12471	Atp13a3

MSTRG.12476.1	MSTRG.12476	Xxylt1
MSTRG.12477.1	MSTRG.12477	Xxylt1
MSTRG.12479.1	MSTRG.12479	Acap2
MSTRG.12480.1	MSTRG.12480	Acap2
MSTRG.12481.1	MSTRG.12481	Acap2
MSTRG.12484.1	MSTRG.12484	.
MSTRG.12485.1	MSTRG.12485	.
MSTRG.12489.1	MSTRG.12489	Bdh1
MSTRG.12491.1	MSTRG.12491	Dlg1
MSTRG.12492.1	MSTRG.12492	Dlg1
MSTRG.12494.1	MSTRG.12494	Dlg1
MSTRG.12500.1	MSTRG.12500	Senp5
MSTRG.12501.1	MSTRG.12501	Senp5
MSTRG.12504.1	MSTRG.12504	Senp5
MSTRG.12506.1	MSTRG.12506	Senp5
MSTRG.12508.1	MSTRG.12508	.
MSTRG.1251.1	MSTRG.1251	3110009E18Rik
MSTRG.12511.1	MSTRG.12511	Pak2
MSTRG.12514.1	MSTRG.12514	Nrros
MSTRG.1252.1	MSTRG.1252	3110009E18Rik
MSTRG.12524.1	MSTRG.12524	Ubxn7
MSTRG.12525.1	MSTRG.12525	Ubxn7
MSTRG.12530.1	MSTRG.12530	Tfrc
MSTRG.12532.1	MSTRG.12532	Tnk2
MSTRG.12534.1	MSTRG.12534	Tnk2
MSTRG.12535.1	MSTRG.12535	Fyttd1
MSTRG.12538.1	MSTRG.12538	Fyttd1
MSTRG.12539.1	MSTRG.12539	Fyttd1
MSTRG.1254.6	MSTRG.1254	Insig2
MSTRG.12542.1	MSTRG.12542	Osbpl11
MSTRG.12543.1	MSTRG.12543	Osbpl11
MSTRG.12544.1	MSTRG.12544	.
MSTRG.12546.1	MSTRG.12546	Snx4
MSTRG.12558.1	MSTRG.12558	Zfp148
MSTRG.1256.1	MSTRG.1256	.
MSTRG.12567.1	MSTRG.12567	Kalrn
MSTRG.12568.1	MSTRG.12568	Kalrn
MSTRG.12573.1	MSTRG.12573	Hacd2
MSTRG.12574.1	MSTRG.12574	Hacd2
MSTRG.12575.1	MSTRG.12575	Hacd2
MSTRG.12576.1	MSTRG.12576	Hacd2
MSTRG.12580.1	MSTRG.12580	Sec22a
MSTRG.12581.1	MSTRG.12581	Sec22a
MSTRG.12582.1	MSTRG.12582	Sec22a
MSTRG.12584.1	MSTRG.12584	Dirc2
MSTRG.12585.1	MSTRG.12585	Dirc2
MSTRG.12587.1	MSTRG.12587	Hspbap1
MSTRG.12588.1	MSTRG.12588	Hspbap1
MSTRG.12593.1	MSTRG.12593	Fam162a
MSTRG.12594.1	MSTRG.12594	Ccdc58
MSTRG.12594.2	MSTRG.12594	Ccdc58
MSTRG.12595.1	MSTRG.12595	Ccdc58
MSTRG.12603.1	MSTRG.12603	Slc15a2
MSTRG.12609.1	MSTRG.12609	Iqcb1
MSTRG.12610.1	MSTRG.12610	Eaf2
MSTRG.12614.1	MSTRG.12614	Polq
MSTRG.12615.1	MSTRG.12615	Polq

MSTRG.12616.1	MSTRG.12616	Polq
MSTRG.12619.1	MSTRG.12619	Stxbp5l
MSTRG.12620.1	MSTRG.12620	Stxbp5l
MSTRG.12627.1	MSTRG.12627	.
MSTRG.12628.1	MSTRG.12628	.
MSTRG.1263.1	MSTRG.1263	Gm28928
MSTRG.12630.1	MSTRG.12630	Gsk3b
MSTRG.12631.1	MSTRG.12631	Gsk3b
MSTRG.12632.1	MSTRG.12632	Gsk3b
MSTRG.12634.1	MSTRG.12634	Gsk3b
MSTRG.12642.1	MSTRG.12642	Cd80
MSTRG.12645.1	MSTRG.12645	Timmdc1
MSTRG.12647.1	MSTRG.12647	.
MSTRG.12649.1	MSTRG.12649	Tmem39a
MSTRG.1265.1	MSTRG.1265	.
MSTRG.12653.1	MSTRG.12653	Arhgap31
MSTRG.12654.1	MSTRG.12654	Arhgap31
MSTRG.12655.1	MSTRG.12655	Arhgap31
MSTRG.12656.1	MSTRG.12656	Arhgap31
MSTRG.12658.1	MSTRG.12658	B4galt4
MSTRG.12660.1	MSTRG.12660	.
MSTRG.12662.1	MSTRG.12662	Lsamp
MSTRG.12667.1	MSTRG.12667	Lsamp
MSTRG.12678.1	MSTRG.12678	Gramd1c
MSTRG.12681.1	MSTRG.12681	Zbtb20
MSTRG.12684.1	MSTRG.12684	Zbtb20
MSTRG.12685.1	MSTRG.12685	Zbtb20
MSTRG.12686.1	MSTRG.12686	Zbtb20
MSTRG.12688.1	MSTRG.12688	Zbtb20
MSTRG.1269.1	MSTRG.1269	Lypd1
MSTRG.12690.1	MSTRG.12690	Zbtb20
MSTRG.12695.1	MSTRG.12695	Zbtb20
MSTRG.12699.1	MSTRG.12699	Naa50
MSTRG.1270.1	MSTRG.1270	Lypd1
MSTRG.12701.1	MSTRG.12701	Usf3
MSTRG.12703.1	MSTRG.12703	Sidt1
MSTRG.12704.1	MSTRG.12704	Sidt1
MSTRG.12705.1	MSTRG.12705	Sidt1
MSTRG.12706.1	MSTRG.12706	Sidt1
MSTRG.12707.1	MSTRG.12707	Sidt1
MSTRG.12708.1	MSTRG.12708	Sidt1
MSTRG.1271.1	MSTRG.1271	Lypd1
MSTRG.12711.1	MSTRG.12711	Spice1
MSTRG.12712.1	MSTRG.12712	Spice1
MSTRG.12713.1	MSTRG.12713	.
MSTRG.12716.1	MSTRG.12716	Boc
MSTRG.12718.1	MSTRG.12718	.
MSTRG.1272.1	MSTRG.1272	Lypd1
MSTRG.12721.1	MSTRG.12721	.
MSTRG.12723.1	MSTRG.12723	Cd200r1
MSTRG.12724.1	MSTRG.12724	Cd200r1
MSTRG.12728.1	MSTRG.12728	Atg3
MSTRG.12729.1	MSTRG.12729	.
MSTRG.12731.1	MSTRG.12731	Btla
MSTRG.12732.1	MSTRG.12732	Btla
MSTRG.12733.1	MSTRG.12733	Btla
MSTRG.12735.1	MSTRG.12735	Cd200

MSTRG.12742.1	MSTRG.12742	Abhd10
MSTRG.12744.1	MSTRG.12744	Abhd10
MSTRG.12747.4	MSTRG.12747	Gm15638
MSTRG.12748.1	MSTRG.12748	Gm15638
MSTRG.1275.1	MSTRG.1275	Mgat5
MSTRG.12751.1	MSTRG.12751	Nectin3
MSTRG.12754.1	MSTRG.12754	.
MSTRG.12758.1	MSTRG.12758	Myh15
MSTRG.12759.1	MSTRG.12759	Myh15
MSTRG.1276.1	MSTRG.1276	Mgat5
MSTRG.12761.1	MSTRG.12761	Dzip3
MSTRG.12763.1	MSTRG.12763	Dzip3
MSTRG.12764.1	MSTRG.12764	Dzip3
MSTRG.12769.1	MSTRG.12769	.
MSTRG.1277.1	MSTRG.1277	Mgat5
MSTRG.1277.2	MSTRG.1277	Mgat5
MSTRG.1277.3	MSTRG.1277	Mgat5
MSTRG.12770.1	MSTRG.12770	.
MSTRG.12771.1	MSTRG.12771	.
MSTRG.12773.1	MSTRG.12773	.
MSTRG.12775.1	MSTRG.12775	Bbx
MSTRG.12779.1	MSTRG.12779	Cblb
MSTRG.1278.1	MSTRG.1278	Mgat5
MSTRG.12780.1	MSTRG.12780	Cblb
MSTRG.12780.2	MSTRG.12780	Cblb
MSTRG.12783.1	MSTRG.12783	.
MSTRG.12785.1	MSTRG.12785	.
MSTRG.12786.1	MSTRG.12786	.
MSTRG.1279.1	MSTRG.1279	Mgat5
MSTRG.12790.1	MSTRG.12790	Nxpe3
MSTRG.12791.1	MSTRG.12791	.
MSTRG.12800.1	MSTRG.12800	Senp7
MSTRG.12802.1	MSTRG.12802	Impg2
MSTRG.12803.1	MSTRG.12803	.
MSTRG.12808.1	MSTRG.12808	Gm16892
MSTRG.1281.1	MSTRG.1281	Mgat5
MSTRG.12810.1	MSTRG.12810	Tbc1d23
MSTRG.12812.1	MSTRG.12812	.
MSTRG.12817.1	MSTRG.12817	Cmss1
MSTRG.12818.1	MSTRG.12818	Cmss1
MSTRG.12819.1	MSTRG.12819	Cmss1
MSTRG.12820.1	MSTRG.12820	Cmss1
MSTRG.12821.1	MSTRG.12821	Cmss1
MSTRG.12822.1	MSTRG.12822	.
MSTRG.12823.1	MSTRG.12823	.
MSTRG.12826.8	MSTRG.12826	St3gal6
MSTRG.12828.1	MSTRG.12828	St3gal6
MSTRG.12830.1	MSTRG.12830	.
MSTRG.12831.1	MSTRG.12831	.
MSTRG.12840.1	MSTRG.12840	.
MSTRG.12841.1	MSTRG.12841	.
MSTRG.12844.1	MSTRG.12844	Nsun3
MSTRG.12845.1	MSTRG.12845	Nsun3
MSTRG.12847.1	MSTRG.12847	Arl13b
MSTRG.1285.1	MSTRG.1285	Mgat5
MSTRG.1285.2	MSTRG.1285	Mgat5
MSTRG.1285.3	MSTRG.1285	Mgat5

MSTRG.12850.1	MSTRG.12850	Arl13b
MSTRG.12855.1	MSTRG.12855	Zfp654
MSTRG.12856.1	MSTRG.12856	Zfp654
MSTRG.12857.1	MSTRG.12857	Zfp654
MSTRG.1286.1	MSTRG.1286	Mgat5
MSTRG.12860.1	MSTRG.12860	Htr1f
MSTRG.12862.1	MSTRG.12862	.
MSTRG.12863.1	MSTRG.12863	.
MSTRG.12864.1	MSTRG.12864	.
MSTRG.12865.1	MSTRG.12865	.
MSTRG.12866.1	MSTRG.12866	.
MSTRG.12869.1	MSTRG.12869	Robo1
MSTRG.1287.1	MSTRG.1287	Mgat5
MSTRG.12870.1	MSTRG.12870	Robo1
MSTRG.12871.1	MSTRG.12871	Robo1
MSTRG.12872.1	MSTRG.12872	Robo1
MSTRG.12873.1	MSTRG.12873	Robo1
MSTRG.12874.1	MSTRG.12874	Robo1
MSTRG.12875.1	MSTRG.12875	.
MSTRG.12876.1	MSTRG.12876	.
MSTRG.12877.1	MSTRG.12877	.
MSTRG.1290.1	MSTRG.1290	.
MSTRG.12901.1	MSTRG.12901	Mir99ahg
MSTRG.12902.1	MSTRG.12902	Mir99ahg
MSTRG.12903.1	MSTRG.12903	Mir99ahg
MSTRG.12905.1	MSTRG.12905	Mir99ahg
MSTRG.12906.1	MSTRG.12906	Mir99ahg
MSTRG.12914.1	MSTRG.12914	D16Ertd472e
MSTRG.12915.1	MSTRG.12915	.
MSTRG.12916.1	MSTRG.12916	.
MSTRG.12917.1	MSTRG.12917	.
MSTRG.12917.2	MSTRG.12917	.
MSTRG.12919.1	MSTRG.12919	Mir155hg
MSTRG.12929.1	MSTRG.12929	Cyyr1
MSTRG.12930.1	MSTRG.12930	.
MSTRG.12931.1	MSTRG.12931	.
MSTRG.12935.1	MSTRG.12935	Ltn1
MSTRG.12938.1	MSTRG.12938	.
MSTRG.1294.1	MSTRG.1294	Mcm6
MSTRG.12941.1	MSTRG.12941	Cct8
MSTRG.12945.1	MSTRG.12945	.
MSTRG.12947.1	MSTRG.12947	.
MSTRG.12949.1	MSTRG.12949	.
MSTRG.12950.1	MSTRG.12950	.
MSTRG.12954.1	MSTRG.12954	Scaf4
MSTRG.12955.1	MSTRG.12955	Scaf4
MSTRG.1296.1	MSTRG.1296	Dars
MSTRG.1297.1	MSTRG.1297	Dars
MSTRG.12971.1	MSTRG.12971	Ifnar2
MSTRG.12972.1	MSTRG.12972	Gm21970
MSTRG.12976.1	MSTRG.12976	A930006K02Rik
MSTRG.12984.1	MSTRG.12984	Mrps6
MSTRG.12993.1	MSTRG.12993	Gm10785
MSTRG.12995.1	MSTRG.12995	Itsn1
MSTRG.1300.1	MSTRG.1300	2900009J06Rik
MSTRG.13002.2	MSTRG.13002	Runx1
MSTRG.13002.20	MSTRG.13002	Runx1

MSTRG.13002.23	MSTRG.13002	Runx1
MSTRG.13002.25	MSTRG.13002	Runx1
MSTRG.13002.26	MSTRG.13002	Runx1
MSTRG.13002.27	MSTRG.13002	Runx1
MSTRG.13002.28	MSTRG.13002	Runx1
MSTRG.13002.29	MSTRG.13002	Runx1
MSTRG.13002.3	MSTRG.13002	Runx1
MSTRG.13002.4	MSTRG.13002	Runx1
MSTRG.13002.5	MSTRG.13002	Runx1
MSTRG.13004.1	MSTRG.13004	Runx1
MSTRG.13006.1	MSTRG.13006	Cbr1
MSTRG.13008.1	MSTRG.13008	Setd4
MSTRG.13009.1	MSTRG.13009	.
MSTRG.13010.1	MSTRG.13010	Setd4
MSTRG.13012.1	MSTRG.13012	.
MSTRG.13019.1	MSTRG.13019	Hlcs
MSTRG.1302.1	MSTRG.1302	Map3k19
MSTRG.13021.1	MSTRG.13021	Hlcs
MSTRG.13023.1	MSTRG.13023	Hlcs
MSTRG.13024.1	MSTRG.13024	Hlcs
MSTRG.13026.1	MSTRG.13026	.
MSTRG.13031.1	MSTRG.13031	.
MSTRG.13035.1	MSTRG.13035	.
MSTRG.13035.2	MSTRG.13035	.
MSTRG.13036.1	MSTRG.13036	.
MSTRG.13041.1	MSTRG.13041	1600002D24Rik
MSTRG.13043.1	MSTRG.13043	1600002D24Rik
MSTRG.1305.1	MSTRG.1305	Rab3gap1
MSTRG.13052.1	MSTRG.13052	Brwd1
MSTRG.1306.1	MSTRG.1306	Rab3gap1
MSTRG.13060.1	MSTRG.13060	Mx2
MSTRG.13065.1	MSTRG.13065	Prdm15
MSTRG.13067.1	MSTRG.13067	C2cd2
MSTRG.13073.1	MSTRG.13073	.
MSTRG.13074.1	MSTRG.13074	.
MSTRG.13075.1	MSTRG.13075	.
MSTRG.13077.1	MSTRG.13077	.
MSTRG.13081.1	MSTRG.13081	.
MSTRG.13086.1	MSTRG.13086	Scaf8
MSTRG.13086.2	MSTRG.13086	.
MSTRG.13088.1	MSTRG.13088	Scaf8
MSTRG.13089.1	MSTRG.13089	.
MSTRG.13090.1	MSTRG.13090	.
MSTRG.13091.1	MSTRG.13091	.
MSTRG.13092.1	MSTRG.13092	.
MSTRG.13094.1	MSTRG.13094	Tmem242
MSTRG.13097.1	MSTRG.13097	Tmem242
MSTRG.131.1	MSTRG.131	Stau2
MSTRG.1310.1	MSTRG.1310	Zranb3
MSTRG.13102.1	MSTRG.13102	Zdhhc14
MSTRG.13103.1	MSTRG.13103	Zdhhc14
MSTRG.13103.2	MSTRG.13103	Zdhhc14
MSTRG.13103.3	MSTRG.13103	Zdhhc14
MSTRG.13104.1	MSTRG.13104	Zdhhc14
MSTRG.13104.2	MSTRG.13104	Zdhhc14
MSTRG.13104.3	MSTRG.13104	Zdhhc14
MSTRG.13105.1	MSTRG.13105	Zdhhc14

MSTRG.13108.1	MSTRG.13108	Zdhhc14
MSTRG.13109.1	MSTRG.13109	Zdhhc14
MSTRG.1311.1	MSTRG.1311	Zranb3
MSTRG.13110.1	MSTRG.13110	Zdhhc14
MSTRG.13113.1	MSTRG.13113	Arid1b
MSTRG.13115.1	MSTRG.13115	Arid1b
MSTRG.13116.1	MSTRG.13116	Arid1b
MSTRG.13117.1	MSTRG.13117	Arid1b
MSTRG.13117.2	MSTRG.13117	Arid1b
MSTRG.13118.1	MSTRG.13118	Arid1b
MSTRG.13120.1	MSTRG.13120	Synj2
MSTRG.13122.1	MSTRG.13122	Serac1
MSTRG.13123.1	MSTRG.13123	Serac1
MSTRG.13126.1	MSTRG.13126	.
MSTRG.13127.1	MSTRG.13127	.
MSTRG.1313.1	MSTRG.1313	Zranb3
MSTRG.13133.1	MSTRG.13133	Gm2885
MSTRG.13134.1	MSTRG.13134	Gm2885
MSTRG.13141.1	MSTRG.13141	Tmem181a
MSTRG.13142.1	MSTRG.13142	Dynlt1c
MSTRG.13144.1	MSTRG.13144	Dynlt1a
MSTRG.13145.2	MSTRG.13145	Dynlt1c
MSTRG.1315.1	MSTRG.1315	Zranb3
MSTRG.13161.17	MSTRG.13161	Sft2d1
MSTRG.13161.5	MSTRG.13161	Sft2d1
MSTRG.13161.8	MSTRG.13161	Mpc1
MSTRG.13174.1	MSTRG.13174	Qk
MSTRG.13175.1	MSTRG.13175	Qk
MSTRG.13181.1	MSTRG.13181	Park2
MSTRG.13183.1	MSTRG.13183	Park2
MSTRG.13185.1	MSTRG.13185	Park2
MSTRG.13186.1	MSTRG.13186	Park2
MSTRG.13192.1	MSTRG.13192	Agpat4
MSTRG.13193.1	MSTRG.13193	Agpat4
MSTRG.13194.1	MSTRG.13194	Agpat4
MSTRG.13195.1	MSTRG.13195	Agpat4
MSTRG.13197.1	MSTRG.13197	.
MSTRG.13199.1	MSTRG.13199	Map3k4
MSTRG.132.1	MSTRG.132	Stau2
MSTRG.13201.1	MSTRG.13201	Map3k4
MSTRG.13204.1	MSTRG.13204	Pnldc1
MSTRG.13206.1	MSTRG.13206	Airn
MSTRG.13206.2	MSTRG.13206	Airn
MSTRG.13207.1	MSTRG.13207	Airn
MSTRG.13208.1	MSTRG.13208	Gm23833
MSTRG.13211.1	MSTRG.13211	Airn
MSTRG.13213.1	MSTRG.13213	Airn
MSTRG.13223.1	MSTRG.13223	.
MSTRG.13239.1	MSTRG.13239	.
MSTRG.13246.1	MSTRG.13246	Tcte2
MSTRG.13247.1	MSTRG.13247	Tcte2
MSTRG.13252.1	MSTRG.13252	Afdn
MSTRG.13253.1	MSTRG.13253	Afdn
MSTRG.13256.1	MSTRG.13256	Wdr27
MSTRG.13257.1	MSTRG.13257	Wdr27
MSTRG.13258.1	MSTRG.13258	Wdr27
MSTRG.13264.2	MSTRG.13264	Gm3448

MSTRG.13274.1	MSTRG.13274	Rgmb
MSTRG.13275.1	MSTRG.13275	.
MSTRG.13276.1	MSTRG.13276	.
MSTRG.13278.1	MSTRG.13278	Chd1
MSTRG.13279.1	MSTRG.13279	Chd1
MSTRG.13280.1	MSTRG.13280	Chd1
MSTRG.13280.2	MSTRG.13280	Chd1
MSTRG.13280.3	MSTRG.13280	Chd1
MSTRG.13283.1	MSTRG.13283	Chd1
MSTRG.13286.1	MSTRG.13286	Zfp97
MSTRG.13286.2	MSTRG.13286	.
MSTRG.13287.1	MSTRG.13287	BC002059
MSTRG.13288.1	MSTRG.13288	Zfp97
MSTRG.13288.2	MSTRG.13288	.
MSTRG.13290.1	MSTRG.13290	Zfp960
MSTRG.13290.2	MSTRG.13290	Zfp97
MSTRG.13292.1	MSTRG.13292	Zfp97
MSTRG.13293.1	MSTRG.13293	Zfp97
MSTRG.13297.1	MSTRG.13297	Zfp97
MSTRG.13298.1	MSTRG.13298	.
MSTRG.13299.1	MSTRG.13299	Gm6712
MSTRG.133.2	MSTRG.133	Ube2w
MSTRG.133.5	MSTRG.133	Ube2w
MSTRG.13300.1	MSTRG.13300	.
MSTRG.13302.1	MSTRG.13302	Lnpep
MSTRG.13311.1	MSTRG.13311	.
MSTRG.13315.1	MSTRG.13315	Zfp760
MSTRG.13316.1	MSTRG.13316	Zfp760
MSTRG.13318.1	MSTRG.13318	Zfp677
MSTRG.13321.1	MSTRG.13321	.
MSTRG.13324.1	MSTRG.13324	Zfp53
MSTRG.13325.1	MSTRG.13325	Zfp53
MSTRG.13331.1	MSTRG.13331	.
MSTRG.13333.1	MSTRG.13333	.
MSTRG.13335.1	MSTRG.13335	.
MSTRG.13339.1	MSTRG.13339	Zfp995
MSTRG.13341.1	MSTRG.13341	Zfp942
MSTRG.13342.1	MSTRG.13342	Zfp942
MSTRG.13348.1	MSTRG.13348	Zfp943
MSTRG.13351.1	MSTRG.13351	Zfp994
MSTRG.13352.1	MSTRG.13352	.
MSTRG.13353.1	MSTRG.13353	.
MSTRG.13354.2	MSTRG.13354	Zfp944
MSTRG.13356.1	MSTRG.13356	Zfp758
MSTRG.13358.1	MSTRG.13358	.
MSTRG.13359.1	MSTRG.13359	.
MSTRG.1336.1	MSTRG.1336	Gm29427
MSTRG.13360.1	MSTRG.13360	.
MSTRG.13368.1	MSTRG.13368	.
MSTRG.13376.1	MSTRG.13376	Flywch2
MSTRG.13379.1	MSTRG.13379	Srrm2
MSTRG.13379.2	MSTRG.13379	Srrm2
MSTRG.13379.3	MSTRG.13379	Srrm2
MSTRG.13384.1	MSTRG.13384	Amdhd2
MSTRG.13394.1	MSTRG.13394	Abca3
MSTRG.13395.1	MSTRG.13395	Abca3
MSTRG.13396.1	MSTRG.13396	D330041H03Rik

MSTRG.13398.1	MSTRG.13398	Eci1
MSTRG.134.1	MSTRG.134	Ube2w
MSTRG.13401.1	MSTRG.13401	.
MSTRG.13410.1	MSTRG.13410	Pkd1
MSTRG.13426.1	MSTRG.13426	Ndufb10
MSTRG.13430.1	MSTRG.13430	Hagh
MSTRG.13431.1	MSTRG.13431	.
MSTRG.13439.1	MSTRG.13439	Mapk8ip3
MSTRG.13441.1	MSTRG.13441	Ift140
MSTRG.13442.1	MSTRG.13442	Ift140
MSTRG.13444.1	MSTRG.13444	Ift140
MSTRG.13445.1	MSTRG.13445	Ift140
MSTRG.13446.1	MSTRG.13446	Ift140
MSTRG.13448.1	MSTRG.13448	Telo2
MSTRG.13451.1	MSTRG.13451	Clcn7
MSTRG.13452.1	MSTRG.13452	Clcn7
MSTRG.13455.1	MSTRG.13455	.
MSTRG.13457.1	MSTRG.13457	Unkl
MSTRG.13458.1	MSTRG.13458	Unkl
MSTRG.1346.1	MSTRG.1346	Rassf5
MSTRG.13465.1	MSTRG.13465	Lmf1
MSTRG.13466.1	MSTRG.13466	Lmf1
MSTRG.13467.1	MSTRG.13467	Lmf1
MSTRG.13468.1	MSTRG.13468	Lmf1
MSTRG.13469.1	MSTRG.13469	Lmf1
MSTRG.13479.1	MSTRG.13479	Axin1
MSTRG.13481.1	MSTRG.13481	Arhgdig
MSTRG.13489.4	MSTRG.13489	Neurl1b
MSTRG.13490.1	MSTRG.13490	Neurl1b
MSTRG.13491.1	MSTRG.13491	Neurl1b
MSTRG.13492.1	MSTRG.13492	Neurl1b
MSTRG.13516.1	MSTRG.13516	Gm26694
MSTRG.1352.1	MSTRG.1352	Fam72a
MSTRG.13521.1	MSTRG.13521	D630044L22Rik
MSTRG.13524.1	MSTRG.13524	Capn15
MSTRG.13525.1	MSTRG.13525	Capn15
MSTRG.13528.1	MSTRG.13528	Rab11fip3
MSTRG.13529.1	MSTRG.13529	Rab11fip3
MSTRG.13530.1	MSTRG.13530	Rab11fip3
MSTRG.13531.1	MSTRG.13531	Rab11fip3
MSTRG.13532.2	MSTRG.13532	Kifc5b
MSTRG.13540.1	MSTRG.13540	Ggnbp1
MSTRG.13541.1	MSTRG.13541	Ggnbp1
MSTRG.13542.1	MSTRG.13542	Ggnbp1
MSTRG.13551.1	MSTRG.13551	Pacsin1
MSTRG.13553.1	MSTRG.13553	Pacsin1
MSTRG.13554.1	MSTRG.13554	Pacsin1
MSTRG.13556.1	MSTRG.13556	Nudt3
MSTRG.13557.5	MSTRG.13557	Hmga1
MSTRG.13557.6	MSTRG.13557	Hmga1
MSTRG.13557.7	MSTRG.13557	Hmga1
MSTRG.13558.3	MSTRG.13558	AI413582
MSTRG.13558.4	MSTRG.13558	AI413582
MSTRG.13559.1	MSTRG.13559	.
MSTRG.13560.1	MSTRG.13560	Nudt3
MSTRG.13561.1	MSTRG.13561	Nudt3
MSTRG.13563.1	MSTRG.13563	Nudt3

MSTRG.13563.2	MSTRG.13563	Nudt3
MSTRG.13564.1	MSTRG.13564	Nudt3
MSTRG.13568.1	MSTRG.13568	D17Wsu92e
MSTRG.1357.1	MSTRG.1357	Srgap2
MSTRG.13571.1	MSTRG.13571	D17Wsu92e
MSTRG.13572.1	MSTRG.13572	D17Wsu92e
MSTRG.13576.1	MSTRG.13576	Taf11
MSTRG.13578.1	MSTRG.13578	Anks1
MSTRG.13579.1	MSTRG.13579	Anks1
MSTRG.13579.2	MSTRG.13579	Anks1
MSTRG.13580.1	MSTRG.13580	Anks1
MSTRG.13582.1	MSTRG.13582	Anks1
MSTRG.1359.1	MSTRG.1359	Srgap2
MSTRG.13590.1	MSTRG.13590	Def6
MSTRG.13590.2	MSTRG.13590	Def6
MSTRG.13594.1	MSTRG.13594	Ppard
MSTRG.13599.1	MSTRG.13599	Tead3
MSTRG.136.1	MSTRG.136	Ube2w
MSTRG.1360.1	MSTRG.1360	Gm8532
MSTRG.13600.1	MSTRG.13600	Tead3
MSTRG.13605.1	MSTRG.13605	Gm22146
MSTRG.13605.2	MSTRG.13605	Gm22146
MSTRG.13610.1	MSTRG.13610	Srpk1
MSTRG.13613.1	MSTRG.13613	Mapk14
MSTRG.13614.1	MSTRG.13614	Mapk14
MSTRG.13616.1	MSTRG.13616	Brpf3
MSTRG.13629.1	MSTRG.13629	Ppil1
MSTRG.1363.1	MSTRG.1363	.
MSTRG.13632.1	MSTRG.13632	BC004004
MSTRG.13636.1	MSTRG.13636	Fgd2
MSTRG.13643.1	MSTRG.13643	Gm28043
MSTRG.13650.1	MSTRG.13650	Abcg1
MSTRG.13650.2	MSTRG.13650	Abcg1
MSTRG.13652.1	MSTRG.13652	Abcg1
MSTRG.13654.1	MSTRG.13654	.
MSTRG.13657.1	MSTRG.13657	.
MSTRG.1366.1	MSTRG.1366	Rab29
MSTRG.13662.1	MSTRG.13662	Wdr4
MSTRG.13663.1	MSTRG.13663	Wdr4
MSTRG.13666.1	MSTRG.13666	Gm24970
MSTRG.13672.2	MSTRG.13672	Zfand3
MSTRG.13672.3	MSTRG.13672	Zfand3
MSTRG.13673.1	MSTRG.13673	Zfand3
MSTRG.13673.2	MSTRG.13673	Zfand3
MSTRG.13674.1	MSTRG.13674	Zfand3
MSTRG.13675.1	MSTRG.13675	Zfand3
MSTRG.13676.1	MSTRG.13676	Zfand3
MSTRG.13677.1	MSTRG.13677	Zfand3
MSTRG.13679.1	MSTRG.13679	Btbd9
MSTRG.13681.1	MSTRG.13681	Btbd9
MSTRG.13685.1	MSTRG.13685	Btbd9
MSTRG.13686.1	MSTRG.13686	Btbd9
MSTRG.13687.1	MSTRG.13687	.
MSTRG.13689.1	MSTRG.13689	.
MSTRG.13691.1	MSTRG.13691	1700097N02Rik
MSTRG.13691.2	MSTRG.13691	1700097N02Rik
MSTRG.13692.1	MSTRG.13692	Dnah8

MSTRG.13694.1	MSTRG.13694	Dnah8
MSTRG.13694.2	MSTRG.13694	Dnah8
MSTRG.13694.3	MSTRG.13694	Dnah8
MSTRG.13694.4	MSTRG.13694	Dnah8
MSTRG.13695.1	MSTRG.13695	Dnah8
MSTRG.13696.1	MSTRG.13696	.
MSTRG.13698.1	MSTRG.13698	Pdxk-ps
MSTRG.13700.1	MSTRG.13700	Rrp1b
MSTRG.13704.1	MSTRG.13704	Brd4
MSTRG.13704.2	MSTRG.13704	Brd4
MSTRG.13706.1	MSTRG.13706	Gm26549
MSTRG.13710.1	MSTRG.13710	Cyp4f16
MSTRG.13711.1	MSTRG.13711	Cyp4f16
MSTRG.13714.1	MSTRG.13714	Wiz
MSTRG.13715.1	MSTRG.13715	Wiz
MSTRG.13718.1	MSTRG.13718	.
MSTRG.13720.1	MSTRG.13720	.
MSTRG.13723.1	MSTRG.13723	Zfp871
MSTRG.13729.1	MSTRG.13729	Gm26693
MSTRG.13734.1	MSTRG.13734	.
MSTRG.13739.1	MSTRG.13739	Zfp763
MSTRG.13744.1	MSTRG.13744	Zfp955b
MSTRG.13745.1	MSTRG.13745	Zfp955b
MSTRG.13747.1	MSTRG.13747	Zfp101
MSTRG.13748.1	MSTRG.13748	Zfp101
MSTRG.13748.2	MSTRG.13748	Zfp101
MSTRG.13749.2	MSTRG.13749	Zfp81
MSTRG.1375.1	MSTRG.1375	Dstyk
MSTRG.13751.1	MSTRG.13751	Adamts10
MSTRG.13752.1	MSTRG.13752	Adamts10
MSTRG.13754.1	MSTRG.13754	Myo1f
MSTRG.13755.1	MSTRG.13755	Myo1f
MSTRG.13756.1	MSTRG.13756	Rab11b
MSTRG.13758.1	MSTRG.13758	Rab11b
MSTRG.13764.1	MSTRG.13764	March2
MSTRG.13769.4	MSTRG.13769	Ndufa7
MSTRG.1377.1	MSTRG.1377	6030442K20Rik
MSTRG.13770.1	MSTRG.13770	Ndufa7
MSTRG.13771.1	MSTRG.13771	Ndufa7
MSTRG.13773.1	MSTRG.13773	Kifc1
MSTRG.13775.1	MSTRG.13775	Kifc1
MSTRG.13777.1	MSTRG.13777	BC051226
MSTRG.13790.1	MSTRG.13790	H2-K1
MSTRG.13791.1	MSTRG.13791	H2-K2
MSTRG.13804.1	MSTRG.13804	Col11a2
MSTRG.13806.1	MSTRG.13806	.
MSTRG.13808.1	MSTRG.13808	H2-DMb2
MSTRG.13811.1	MSTRG.13811	.
MSTRG.13813.1	MSTRG.13813	.
MSTRG.13815.1	MSTRG.13815	.
MSTRG.13821.1	MSTRG.13821	Gm15821
MSTRG.13821.2	MSTRG.13821	Gm15821
MSTRG.13828.1	MSTRG.13828	.
MSTRG.13831.2	MSTRG.13831	Rnf5
MSTRG.13838.1	MSTRG.13838	Prrt1
MSTRG.1384.1	MSTRG.1384	Pik3c2b
MSTRG.13854.6	MSTRG.13854	1110038B12Rik

MSTRG.1386.1	MSTRG.1386	Mdm4
MSTRG.13865.3	MSTRG.13865	Nfkbil1
MSTRG.13867.1	MSTRG.13867	Nfkbil1
MSTRG.13872.1	MSTRG.13872	Ddx39b
MSTRG.13875.1	MSTRG.13875	.
MSTRG.13875.2	MSTRG.13875	.
MSTRG.13876.1	MSTRG.13876	.
MSTRG.13880.1	MSTRG.13880	Gm18734
MSTRG.13881.1	MSTRG.13881	Gm18734
MSTRG.13882.1	MSTRG.13882	Gm18734
MSTRG.13884.1	MSTRG.13884	.
MSTRG.13885.1	MSTRG.13885	Gm18734
MSTRG.13885.2	MSTRG.13885	.
MSTRG.13886.1	MSTRG.13886	H2-Q1
MSTRG.13886.2	MSTRG.13886	H2-Q1
MSTRG.13888.1	MSTRG.13888	H2-Q1
MSTRG.13889.1	MSTRG.13889	H2-Q1
MSTRG.13890.1	MSTRG.13890	H2-Q1
MSTRG.13891.1	MSTRG.13891	H2-Q1
MSTRG.13892.1	MSTRG.13892	H2-Q2
MSTRG.13893.1	MSTRG.13893	H2-Q1
MSTRG.13893.2	MSTRG.13893	H2-Q1
MSTRG.13894.1	MSTRG.13894	H2-Q2
MSTRG.13895.1	MSTRG.13895	H2-Q1
MSTRG.13897.1	MSTRG.13897	H2-Q2
MSTRG.13899.2	MSTRG.13899	Gm11131
MSTRG.13900.1	MSTRG.13900	H2-Q6
MSTRG.13900.2	MSTRG.13900	H2-Q6
MSTRG.13900.3	MSTRG.13900	H2-Q6
MSTRG.13906.1	MSTRG.13906	Gm10501
MSTRG.13913.1	MSTRG.13913	Msh5
MSTRG.13914.1	MSTRG.13914	Msh5
MSTRG.13914.2	MSTRG.13914	Apom
MSTRG.1394.1	MSTRG.1394	Zc3h11a
MSTRG.13942.1	MSTRG.13942	Gm20483
MSTRG.13947.1	MSTRG.13947	Ddr1
MSTRG.13948.1	MSTRG.13948	Gm23864
MSTRG.13952.1	MSTRG.13952	Nrm
MSTRG.13955.1	MSTRG.13955	Ppp1r18
MSTRG.13959.1	MSTRG.13959	Flot1
MSTRG.13960.1	MSTRG.13960	Tubb5
MSTRG.1397.1	MSTRG.1397	Atp2b4
MSTRG.13972.1	MSTRG.13972	Ppp1r11
MSTRG.13975.1	MSTRG.13975	Trim26
MSTRG.13985.1	MSTRG.13985	Abcf1
MSTRG.13992.1	MSTRG.13992	A930015D03Rik
MSTRG.13993.1	MSTRG.13993	A930015D03Rik
MSTRG.13994.1	MSTRG.13994	H2-T24
MSTRG.13995.1	MSTRG.13995	Gm6034
MSTRG.13996.1	MSTRG.13996	Gm6034
MSTRG.13998.1	MSTRG.13998	Gm6034
MSTRG.13998.2	MSTRG.13998	Gm6034
MSTRG.14.1	MSTRG.14	.
MSTRG.140.1	MSTRG.140	Eloc
MSTRG.14002.1	MSTRG.14002	2410017I17Rik
MSTRG.14022.1	MSTRG.14022	.
MSTRG.14025.1	MSTRG.14025	Olfr755-ps1

MSTRG.14027.1	MSTRG.14027	Olfr103
MSTRG.14038.1	MSTRG.14038	.
MSTRG.14039.1	MSTRG.14039	.
MSTRG.14042.1	MSTRG.14042	.
MSTRG.14043.1	MSTRG.14043	.
MSTRG.14044.1	MSTRG.14044	.
MSTRG.14045.1	MSTRG.14045	Cd2ap
MSTRG.14047.1	MSTRG.14047	Cd2ap
MSTRG.14049.1	MSTRG.14049	.
MSTRG.1406.1	MSTRG.1406	Adipor1
MSTRG.14060.1	MSTRG.14060	Clic5
MSTRG.14067.1	MSTRG.14067	Runx2
MSTRG.14068.1	MSTRG.14068	Supt3
MSTRG.14069.1	MSTRG.14069	Supt3
MSTRG.14070.1	MSTRG.14070	Supt3
MSTRG.14071.1	MSTRG.14071	Supt3
MSTRG.14072.1	MSTRG.14072	.
MSTRG.14079.1	MSTRG.14079	.
MSTRG.14080.1	MSTRG.14080	.
MSTRG.14080.2	MSTRG.14080	Aars2
MSTRG.14080.3	MSTRG.14080	.
MSTRG.14080.4	MSTRG.14080	.
MSTRG.14085.1	MSTRG.14085	.
MSTRG.14091.1	MSTRG.14091	.
MSTRG.14095.1	MSTRG.14095	Mrpl14
MSTRG.14098.1	MSTRG.14098	Mrps18a
MSTRG.141.1	MSTRG.141	.
MSTRG.1410.1	MSTRG.1410	Klhl12
MSTRG.1411.1	MSTRG.1411	Klhl12
MSTRG.14110.12	MSTRG.14110	Tjap1
MSTRG.14114.5	MSTRG.14114	Crip3
MSTRG.14114.6	MSTRG.14114	Crip3
MSTRG.14114.7	MSTRG.14114	Crip3
MSTRG.14114.8	MSTRG.14114	Crip3
MSTRG.14114.9	MSTRG.14114	Crip3
MSTRG.14119.1	MSTRG.14119	Trerf1
MSTRG.14120.1	MSTRG.14120	Trerf1
MSTRG.14122.1	MSTRG.14122	Trerf1
MSTRG.14125.1	MSTRG.14125	Trerf1
MSTRG.14126.1	MSTRG.14126	Trerf1
MSTRG.1413.1	MSTRG.1413	Rabif
MSTRG.14132.1	MSTRG.14132	Gm26904
MSTRG.14136.1	MSTRG.14136	Gm26904
MSTRG.14137.1	MSTRG.14137	Ptk7
MSTRG.14139.1	MSTRG.14139	Gm26904
MSTRG.1414.1	MSTRG.1414	Rabif
MSTRG.14142.1	MSTRG.14142	Rrp36
MSTRG.14143.1	MSTRG.14143	Rrp36
MSTRG.14150.1	MSTRG.14150	Cnpy3
MSTRG.14155.1	MSTRG.14155	Gm26904
MSTRG.14156.1	MSTRG.14156	.
MSTRG.14158.5	MSTRG.14158	Mrps10
MSTRG.14159.1	MSTRG.14159	Tfeb
MSTRG.14162.1	MSTRG.14162	Tfeb
MSTRG.14164.1	MSTRG.14164	Tfeb
MSTRG.14165.1	MSTRG.14165	.
MSTRG.14167.1	MSTRG.14167	Foxp4

MSTRG.14168.1	MSTRG.14168	Foxp4
MSTRG.14172.1	MSTRG.14172	.
MSTRG.14178.1	MSTRG.14178	Ccnd3
MSTRG.14179.6	MSTRG.14179	Ccnd3
MSTRG.14179.7	MSTRG.14179	Ccnd3
MSTRG.1418.1	MSTRG.1418	Kdm5b
MSTRG.14180.1	MSTRG.14180	Ccnd3
MSTRG.14180.2	MSTRG.14180	Ccnd3
MSTRG.14181.1	MSTRG.14181	Ccnd3
MSTRG.14183.1	MSTRG.14183	Bysl
MSTRG.14185.1	MSTRG.14185	Med20
MSTRG.14187.1	MSTRG.14187	Gm20517
MSTRG.14188.1	MSTRG.14188	Gm20517
MSTRG.14189.1	MSTRG.14189	Gm20517
MSTRG.14195.1	MSTRG.14195	Nfya
MSTRG.14196.1	MSTRG.14196	Nfya
MSTRG.14197.2	MSTRG.14197	Oard1
MSTRG.14197.3	MSTRG.14197	Oard1
MSTRG.142.1	MSTRG.142	Ly96
MSTRG.1420.2	MSTRG.1420	Gm4204
MSTRG.14200.1	MSTRG.14200	Unc5cl
MSTRG.14201.1	MSTRG.14201	Unc5cl
MSTRG.14202.1	MSTRG.14202	Unc5cl
MSTRG.14207.1	MSTRG.14207	.
MSTRG.14208.1	MSTRG.14208	.
MSTRG.14210.1	MSTRG.14210	Rftn1
MSTRG.14211.1	MSTRG.14211	Rftn1
MSTRG.14212.1	MSTRG.14212	Rftn1
MSTRG.14216.1	MSTRG.14216	Plcl2
MSTRG.14216.2	MSTRG.14216	Plcl2
MSTRG.14217.1	MSTRG.14217	Plcl2
MSTRG.14219.1	MSTRG.14219	Tbc1d5
MSTRG.14221.1	MSTRG.14221	Tbc1d5
MSTRG.14222.1	MSTRG.14222	Tbc1d5
MSTRG.14223.1	MSTRG.14223	Tbc1d5
MSTRG.14224.1	MSTRG.14224	Tbc1d5
MSTRG.14227.1	MSTRG.14227	Gm19585
MSTRG.14228.1	MSTRG.14228	.
MSTRG.14230.1	MSTRG.14230	Gm37593
MSTRG.14230.10	MSTRG.14230	Gm37176
MSTRG.14230.2	MSTRG.14230	Gm37176
MSTRG.14230.3	MSTRG.14230	Gm37176
MSTRG.14230.4	MSTRG.14230	Gm37176
MSTRG.14230.5	MSTRG.14230	Gm37176
MSTRG.14230.6	MSTRG.14230	Gm37176
MSTRG.14231.1	MSTRG.14231	Satb1
MSTRG.14232.1	MSTRG.14232	.
MSTRG.14233.1	MSTRG.14233	.
MSTRG.14234.1	MSTRG.14234	Gm37176
MSTRG.14235.1	MSTRG.14235	.
MSTRG.14235.2	MSTRG.14235	.
MSTRG.14235.3	MSTRG.14235	.
MSTRG.14236.1	MSTRG.14236	.
MSTRG.14237.1	MSTRG.14237	.
MSTRG.14238.1	MSTRG.14238	.
MSTRG.14240.1	MSTRG.14240	.
MSTRG.14240.2	MSTRG.14240	.

MSTRG.14240.3	MSTRG.14240	.
MSTRG.14240.4	MSTRG.14240	.
MSTRG.14241.1	MSTRG.14241	.
MSTRG.14242.1	MSTRG.14242	.
MSTRG.14243.1	MSTRG.14243	.
MSTRG.14244.1	MSTRG.14244	.
MSTRG.14245.1	MSTRG.14245	.
MSTRG.14246.1	MSTRG.14246	Rab5a
MSTRG.14248.1	MSTRG.14248	Pp2d1
MSTRG.14250.1	MSTRG.14250	Kat2b
MSTRG.14251.1	MSTRG.14251	Kat2b
MSTRG.14252.1	MSTRG.14252	Kat2b
MSTRG.14254.1	MSTRG.14254	Kat2b
MSTRG.14255.1	MSTRG.14255	Kat2b
MSTRG.14256.1	MSTRG.14256	Kat2b
MSTRG.14258.1	MSTRG.14258	Sgo1
MSTRG.14259.1	MSTRG.14259	.
MSTRG.14261.1	MSTRG.14261	Pot1b
MSTRG.14262.1	MSTRG.14262	Pot1b
MSTRG.14264.1	MSTRG.14264	Pot1b
MSTRG.1427.1	MSTRG.1427	Ppp1r12b
MSTRG.14271.1	MSTRG.14271	Ccdc94
MSTRG.1428.1	MSTRG.1428	Ppp1r12b
MSTRG.14296.1	MSTRG.14296	Kdm4b
MSTRG.143.1	MSTRG.143	.
MSTRG.14306.1	MSTRG.14306	Rfx2
MSTRG.14307.1	MSTRG.14307	Rfx2
MSTRG.14308.1	MSTRG.14308	Rfx2
MSTRG.14309.1	MSTRG.14309	Rfx2
MSTRG.14310.1	MSTRG.14310	Rfx2
MSTRG.14320.1	MSTRG.14320	Mllt1
MSTRG.14330.1	MSTRG.14330	.
MSTRG.14331.1	MSTRG.14331	Gm11110
MSTRG.14336.1	MSTRG.14336	.
MSTRG.14341.1	MSTRG.14341	.
MSTRG.14342.1	MSTRG.14342	.
MSTRG.14343.1	MSTRG.14343	.
MSTRG.14344.1	MSTRG.14344	.
MSTRG.14347.1	MSTRG.14347	Efna5
MSTRG.14349.1	MSTRG.14349	Efna5
MSTRG.14350.1	MSTRG.14350	Efna5
MSTRG.14351.1	MSTRG.14351	Efna5
MSTRG.14352.1	MSTRG.14352	Efna5
MSTRG.14353.1	MSTRG.14353	Efna5
MSTRG.14354.1	MSTRG.14354	Efna5
MSTRG.14355.1	MSTRG.14355	.
MSTRG.14356.1	MSTRG.14356	.
MSTRG.14359.1	MSTRG.14359	Fbxl17
MSTRG.14360.2	MSTRG.14360	Fbxl17
MSTRG.14360.3	MSTRG.14360	Fbxl17
MSTRG.14363.1	MSTRG.14363	Fbxl17
MSTRG.14364.1	MSTRG.14364	Fbxl17
MSTRG.14365.1	MSTRG.14365	Fbxl17
MSTRG.14367.1	MSTRG.14367	4930405O22Rik
MSTRG.14367.3	MSTRG.14367	4930405O22Rik
MSTRG.14368.1	MSTRG.14368	Fbxl17
MSTRG.14371.1	MSTRG.14371	Fbxl17

MSTRG.14372.1	MSTRG.14372	Fbxl17
MSTRG.14373.1	MSTRG.14373	Fbxl17
MSTRG.14375.1	MSTRG.14375	Pja2
MSTRG.14377.1	MSTRG.14377	Pja2
MSTRG.14378.1	MSTRG.14378	.
MSTRG.14382.1	MSTRG.14382	Tmem232
MSTRG.14385.1	MSTRG.14385	Vapa
MSTRG.14389.1	MSTRG.14389	Ppp4r1
MSTRG.14392.1	MSTRG.14392	.
MSTRG.14394.1	MSTRG.14394	Ankrd12
MSTRG.14398.1	MSTRG.14398	.
MSTRG.14400.1	MSTRG.14400	Ddx11
MSTRG.14405.1	MSTRG.14405	.
MSTRG.14411.1	MSTRG.14411	Ptprm
MSTRG.14414.1	MSTRG.14414	.
MSTRG.14420.1	MSTRG.14420	Ndc80
MSTRG.14422.1	MSTRG.14422	Ndc80
MSTRG.14424.4	MSTRG.14424	Trmt61b
MSTRG.14432.3	MSTRG.14432	Gm26510
MSTRG.14434.1	MSTRG.14434	Gm26561
MSTRG.14435.1	MSTRG.14435	Gm26510
MSTRG.14436.1	MSTRG.14436	Gm26510
MSTRG.14437.1	MSTRG.14437	Gm26510
MSTRG.14439.1	MSTRG.14439	Gm26561
MSTRG.14444.1	MSTRG.14444	Gm26561
MSTRG.14446.1	MSTRG.14446	Gm26561
MSTRG.14449.1	MSTRG.14449	Ypel5
MSTRG.14450.2	MSTRG.14450	Lbh
MSTRG.14451.4	MSTRG.14451	Lclat1
MSTRG.14452.1	MSTRG.14452	Lclat1
MSTRG.14456.1	MSTRG.14456	Lclat1
MSTRG.14460.1	MSTRG.14460	Memo1
MSTRG.14460.2	MSTRG.14460	Memo1
MSTRG.14461.1	MSTRG.14461	Memo1
MSTRG.14463.1	MSTRG.14463	Spast
MSTRG.14465.1	MSTRG.14465	Spast
MSTRG.14466.1	MSTRG.14466	Spast
MSTRG.14470.1	MSTRG.14470	Yipf4
MSTRG.14475.1	MSTRG.14475	.
MSTRG.14480.1	MSTRG.14480	Birc6
MSTRG.14482.1	MSTRG.14482	Birc6
MSTRG.14486.1	MSTRG.14486	Ttc27
MSTRG.14488.1	MSTRG.14488	Strn
MSTRG.14495.1	MSTRG.14495	Eif2ak2
MSTRG.14496.1	MSTRG.14496	Eif2ak2
MSTRG.14499.1	MSTRG.14499	.
MSTRG.14507.1	MSTRG.14507	.
MSTRG.14513.1	MSTRG.14513	Atl2
MSTRG.14515.1	MSTRG.14515	.
MSTRG.14522.1	MSTRG.14522	.
MSTRG.14529.2	MSTRG.14529	Sos1
MSTRG.14531.1	MSTRG.14531	Sos1
MSTRG.14532.1	MSTRG.14532	.
MSTRG.14534.1	MSTRG.14534	Map4k3
MSTRG.14537.1	MSTRG.14537	Tmem178
MSTRG.14537.2	MSTRG.14537	Tmem178
MSTRG.14538.1	MSTRG.14538	.

MSTRG.14540.1	MSTRG.14540	Thumpd2
MSTRG.14541.1	MSTRG.14541	.
MSTRG.14544.1	MSTRG.14544	Slc8a1
MSTRG.14545.1	MSTRG.14545	Slc8a1
MSTRG.14546.1	MSTRG.14546	Slc8a1
MSTRG.14551.1	MSTRG.14551	Eml4
MSTRG.14552.1	MSTRG.14552	.
MSTRG.14554.1	MSTRG.14554	.
MSTRG.14556.1	MSTRG.14556	.
MSTRG.14557.1	MSTRG.14557	.
MSTRG.14558.1	MSTRG.14558	.
MSTRG.14559.1	MSTRG.14559	.
MSTRG.14560.1	MSTRG.14560	.
MSTRG.14562.1	MSTRG.14562	Thada
MSTRG.14562.2	MSTRG.14562	Thada
MSTRG.14562.3	MSTRG.14562	Thada
MSTRG.14562.4	MSTRG.14562	Thada
MSTRG.14562.5	MSTRG.14562	Thada
MSTRG.14563.1	MSTRG.14563	Thada
MSTRG.14564.1	MSTRG.14564	Thada
MSTRG.14566.1	MSTRG.14566	Thada
MSTRG.14571.1	MSTRG.14571	Lrpprc
MSTRG.14575.1	MSTRG.14575	Ppm1b
MSTRG.14576.1	MSTRG.14576	Ppm1b
MSTRG.1458.1	MSTRG.1458	Gm26781
MSTRG.14580.1	MSTRG.14580	Camkmt
MSTRG.14581.1	MSTRG.14581	.
MSTRG.14581.2	MSTRG.14581	.
MSTRG.14583.1	MSTRG.14583	Srbd1
MSTRG.14584.1	MSTRG.14584	Srbd1
MSTRG.14587.1	MSTRG.14587	Prkce
MSTRG.14588.1	MSTRG.14588	Prkce
MSTRG.14589.1	MSTRG.14589	Prkce
MSTRG.14590.1	MSTRG.14590	Prkce
MSTRG.14592.1	MSTRG.14592	Prkce
MSTRG.14593.1	MSTRG.14593	Prkce
MSTRG.14595.1	MSTRG.14595	Prkce
MSTRG.14596.1	MSTRG.14596	Prkce
MSTRG.14597.1	MSTRG.14597	Prkce
MSTRG.14598.1	MSTRG.14598	Prkce
MSTRG.1460.1	MSTRG.1460	Gm26781
MSTRG.14603.1	MSTRG.14603	Pigf
MSTRG.14604.3	MSTRG.14604	Cript
MSTRG.14609.1	MSTRG.14609	Mcfd2
MSTRG.14617.1	MSTRG.14617	Msh2
MSTRG.1462.1	MSTRG.1462	Camsap2
MSTRG.14620.1	MSTRG.14620	Msh2
MSTRG.14622.1	MSTRG.14622	Kcnk12
MSTRG.14623.1	MSTRG.14623	.
MSTRG.14626.1	MSTRG.14626	Fbxo11
MSTRG.14626.2	MSTRG.14626	Fbxo11
MSTRG.14626.3	MSTRG.14626	Fbxo11
MSTRG.14627.1	MSTRG.14627	Fbxo11
MSTRG.14628.1	MSTRG.14628	Fbxo11
MSTRG.14631.1	MSTRG.14631	Foxn2
MSTRG.14635.1	MSTRG.14635	Ston1
MSTRG.14637.1	MSTRG.14637	Fshr

MSTRG.14638.1	MSTRG.14638	.
MSTRG.14640.1	MSTRG.14640	.
MSTRG.14643.5	MSTRG.14643	2700099C18Rik
MSTRG.14645.1	MSTRG.14645	Gm1976
MSTRG.14645.4	MSTRG.14645	Gm1976
MSTRG.14647.1	MSTRG.14647	Gm1976
MSTRG.1465.1	MSTRG.1465	Ddx59
MSTRG.14653.1	MSTRG.14653	.
MSTRG.14657.1	MSTRG.14657	Crem
MSTRG.14658.1	MSTRG.14658	Crem
MSTRG.14659.1	MSTRG.14659	Crem
MSTRG.14660.1	MSTRG.14660	Crem
MSTRG.14661.1	MSTRG.14661	Crem
MSTRG.14664.1	MSTRG.14664	Map3k8
MSTRG.14669.1	MSTRG.14669	Svil
MSTRG.14670.1	MSTRG.14670	Svil
MSTRG.14672.1	MSTRG.14672	Zfp438
MSTRG.14673.1	MSTRG.14673	Zfp438
MSTRG.14679.2	MSTRG.14679	Zeb1
MSTRG.14679.3	MSTRG.14679	Zeb1
MSTRG.14680.1	MSTRG.14680	Zeb1
MSTRG.14681.1	MSTRG.14681	Zeb1
MSTRG.14683.1	MSTRG.14683	Arhgap12
MSTRG.14684.1	MSTRG.14684	Arhgap12
MSTRG.14685.1	MSTRG.14685	Arhgap12
MSTRG.14686.1	MSTRG.14686	Arhgap12
MSTRG.14688.1	MSTRG.14688	Arhgap12
MSTRG.14689.1	MSTRG.14689	Arhgap12
MSTRG.14690.1	MSTRG.14690	Arhgap12
MSTRG.14697.1	MSTRG.14697	Kif5b
MSTRG.14698.1	MSTRG.14698	.
MSTRG.14699.4	MSTRG.14699	.
MSTRG.147.1	MSTRG.147	Jph1
MSTRG.14700.1	MSTRG.14700	.
MSTRG.14703.1	MSTRG.14703	Epc1
MSTRG.14704.1	MSTRG.14704	Gm28529
MSTRG.14707.1	MSTRG.14707	Mpp7
MSTRG.14708.1	MSTRG.14708	Mpp7
MSTRG.14709.1	MSTRG.14709	Mpp7
MSTRG.14710.1	MSTRG.14710	Mpp7
MSTRG.14712.1	MSTRG.14712	.
MSTRG.14713.1	MSTRG.14713	.
MSTRG.14714.1	MSTRG.14714	.
MSTRG.14718.1	MSTRG.14718	.
MSTRG.14722.1	MSTRG.14722	Ccny
MSTRG.14723.1	MSTRG.14723	.
MSTRG.14724.1	MSTRG.14724	.
MSTRG.14725.17	MSTRG.14725	Wac
MSTRG.14725.19	MSTRG.14725	Wac
MSTRG.14726.1	MSTRG.14726	Wac
MSTRG.14726.2	MSTRG.14726	Wac
MSTRG.1473.1	MSTRG.1473	Gm4258
MSTRG.14733.1	MSTRG.14733	Thoc1
MSTRG.14734.1	MSTRG.14734	Thoc1
MSTRG.14735.1	MSTRG.14735	Thoc1
MSTRG.14736.1	MSTRG.14736	Thoc1
MSTRG.14738.1	MSTRG.14738	Rock1

MSTRG.14738.10	MSTRG.14738	Rock1
MSTRG.14738.11	MSTRG.14738	Rock1
MSTRG.14738.12	MSTRG.14738	Rock1
MSTRG.14738.13	MSTRG.14738	Rock1
MSTRG.14738.14	MSTRG.14738	Rock1
MSTRG.14738.15	MSTRG.14738	Rock1
MSTRG.14738.16	MSTRG.14738	Rock1
MSTRG.14738.17	MSTRG.14738	Rock1
MSTRG.14738.18	MSTRG.14738	Rock1
MSTRG.14738.19	MSTRG.14738	Rock1
MSTRG.14738.2	MSTRG.14738	Rock1
MSTRG.14738.20	MSTRG.14738	Rock1
MSTRG.14738.3	MSTRG.14738	Rock1
MSTRG.14738.4	MSTRG.14738	Rock1
MSTRG.14738.5	MSTRG.14738	Rock1
MSTRG.14738.6	MSTRG.14738	Rock1
MSTRG.14738.7	MSTRG.14738	Rock1
MSTRG.14738.8	MSTRG.14738	Rock1
MSTRG.14738.9	MSTRG.14738	Rock1
MSTRG.1474.1	MSTRG.1474	Gm4258
MSTRG.1474.2	MSTRG.1474	Gm4258
MSTRG.14746.1	MSTRG.14746	Esco1
MSTRG.14747.1	MSTRG.14747	Esco1
MSTRG.14748.1	MSTRG.14748	Esco1
MSTRG.1475.1	MSTRG.1475	Gm37298
MSTRG.14757.1	MSTRG.14757	Rbbp8
MSTRG.14759.1	MSTRG.14759	Gm6277
MSTRG.14763.1	MSTRG.14763	Tmem241
MSTRG.14764.1	MSTRG.14764	Tmem241
MSTRG.14765.1	MSTRG.14765	Tmem241
MSTRG.14766.1	MSTRG.14766	Tmem241
MSTRG.14767.1	MSTRG.14767	Tmem241
MSTRG.14768.1	MSTRG.14768	Tmem241
MSTRG.14769.1	MSTRG.14769	Tmem241
MSTRG.1477.1	MSTRG.1477	Gm4258
MSTRG.14770.1	MSTRG.14770	Tmem241
MSTRG.14773.1	MSTRG.14773	Npc1
MSTRG.14781.1	MSTRG.14781	Ttc39c
MSTRG.14784.1	MSTRG.14784	Impact
MSTRG.14786.1	MSTRG.14786	Zfp521
MSTRG.14787.1	MSTRG.14787	Zfp521
MSTRG.1479.1	MSTRG.1479	Gm4258
MSTRG.14790.1	MSTRG.14790	.
MSTRG.14790.2	MSTRG.14790	.
MSTRG.14790.3	MSTRG.14790	.
MSTRG.14791.1	MSTRG.14791	.
MSTRG.14792.1	MSTRG.14792	Ss18
MSTRG.14792.2	MSTRG.14792	Ss18
MSTRG.14792.3	MSTRG.14792	Ss18
MSTRG.14792.4	MSTRG.14792	Ss18
MSTRG.14792.5	MSTRG.14792	Taf4b
MSTRG.14792.6	MSTRG.14792	Ss18
MSTRG.14792.7	MSTRG.14792	Ss18
MSTRG.14794.1	MSTRG.14794	Ss18
MSTRG.14798.1	MSTRG.14798	Kctd1
MSTRG.1480.1	MSTRG.1480	Gm4258
MSTRG.1480.2	MSTRG.1480	Gm4258

MSTRG.14800.1	MSTRG.14800	Kctd1
MSTRG.14801.1	MSTRG.14801	Kctd1
MSTRG.14802.1	MSTRG.14802	Kctd1
MSTRG.14803.1	MSTRG.14803	Kctd1
MSTRG.14803.2	MSTRG.14803	Kctd1
MSTRG.14804.1	MSTRG.14804	Kctd1
MSTRG.14805.1	MSTRG.14805	Kctd1
MSTRG.14806.1	MSTRG.14806	Kctd1
MSTRG.14807.1	MSTRG.14807	Kctd1
MSTRG.14808.1	MSTRG.14808	Kctd1
MSTRG.14809.1	MSTRG.14809	Kctd1
MSTRG.14810.1	MSTRG.14810	.
MSTRG.14811.1	MSTRG.14811	.
MSTRG.14811.2	MSTRG.14811	.
MSTRG.14811.3	MSTRG.14811	.
MSTRG.14811.4	MSTRG.14811	.
MSTRG.14812.1	MSTRG.14812	.
MSTRG.14814.1	MSTRG.14814	.
MSTRG.14815.1	MSTRG.14815	.
MSTRG.14818.1	MSTRG.14818	1700001G01Rik
MSTRG.14828.1	MSTRG.14828	Trappc8
MSTRG.14831.1	MSTRG.14831	Rnf138
MSTRG.14832.1	MSTRG.14832	Rnf138
MSTRG.14833.1	MSTRG.14833	Rnf138
MSTRG.1484.1	MSTRG.1484	Gm4258
MSTRG.14843.1	MSTRG.14843	.
MSTRG.14845.1	MSTRG.14845	Galnt1
MSTRG.14846.1	MSTRG.14846	Galnt1
MSTRG.14854.1	MSTRG.14854	.
MSTRG.14855.1	MSTRG.14855	.
MSTRG.14860.1	MSTRG.14860	AW554918
MSTRG.14861.1	MSTRG.14861	AW554918
MSTRG.14863.1	MSTRG.14863	AW554918
MSTRG.14864.1	MSTRG.14864	AW554918
MSTRG.14866.1	MSTRG.14866	.
MSTRG.14869.1	MSTRG.14869	Pik3c3
MSTRG.1487.1	MSTRG.1487	Nek7
MSTRG.14875.1	MSTRG.14875	Gm26533
MSTRG.1488.1	MSTRG.1488	Nek7
MSTRG.14880.1	MSTRG.14880	Wdr33
MSTRG.14881.1	MSTRG.14881	Gm26823
MSTRG.14882.1	MSTRG.14882	Wdr33
MSTRG.14885.1	MSTRG.14885	Iws1
MSTRG.14886.2	MSTRG.14886	Map3k2
MSTRG.14887.1	MSTRG.14887	Map3k2
MSTRG.14892.1	MSTRG.14892	Gypc
MSTRG.14893.1	MSTRG.14893	Gypc
MSTRG.14899.1	MSTRG.14899	.
MSTRG.14902.1	MSTRG.14902	Gm22814
MSTRG.14903.1	MSTRG.14903	Camk4
MSTRG.14904.1	MSTRG.14904	Camk4
MSTRG.14905.1	MSTRG.14905	Camk4
MSTRG.14905.2	MSTRG.14905	Camk4
MSTRG.14905.3	MSTRG.14905	Camk4
MSTRG.14907.1	MSTRG.14907	Camk4
MSTRG.14908.1	MSTRG.14908	Camk4
MSTRG.14909.1	MSTRG.14909	Camk4

MSTRG.1491.1	MSTRG.1491	Nek7
MSTRG.14915.3	MSTRG.14915	Gm10548
MSTRG.14917.1	MSTRG.14917	Apc
MSTRG.14918.1	MSTRG.14918	Apc
MSTRG.14919.1	MSTRG.14919	Apc
MSTRG.1492.1	MSTRG.1492	Nek7
MSTRG.14923.3	MSTRG.14923	Pkd2l2
MSTRG.14929.1	MSTRG.14929	Brd8
MSTRG.1493.1	MSTRG.1493	Nek7
MSTRG.14937.1	MSTRG.14937	Ctnna1
MSTRG.14940.2	MSTRG.14940	Fam53c
MSTRG.14945.1	MSTRG.14945	Sil1
MSTRG.14947.1	MSTRG.14947	Matr3
MSTRG.14951.3	MSTRG.14951	Paip2
MSTRG.14952.1	MSTRG.14952	Slc23a1
MSTRG.14954.1	MSTRG.14954	.
MSTRG.14958.1	MSTRG.14958	Dnajc18
MSTRG.14959.1	MSTRG.14959	Dnajc18
MSTRG.1496.1	MSTRG.1496	Ptprc
MSTRG.14960.1	MSTRG.14960	Dnajc18
MSTRG.14966.4	MSTRG.14966	Ube2d2a
MSTRG.14966.5	MSTRG.14966	Ube2d2a
MSTRG.14967.1	MSTRG.14967	.
MSTRG.1497.1	MSTRG.1497	Ptprc
MSTRG.14970.1	MSTRG.14970	Pura
MSTRG.14972.1	MSTRG.14972	.
MSTRG.14993.1	MSTRG.14993	.
MSTRG.14994.1	MSTRG.14994	.
MSTRG.14995.1	MSTRG.14995	.
MSTRG.14996.1	MSTRG.14996	.
MSTRG.1500.1	MSTRG.1500	4930596I21Rik
MSTRG.15005.1	MSTRG.15005	.
MSTRG.15006.1	MSTRG.15006	.
MSTRG.15008.1	MSTRG.15008	Gnpda1
MSTRG.1501.1	MSTRG.1501	Dennd1b
MSTRG.1503.1	MSTRG.1503	Dennd1b
MSTRG.1505.1	MSTRG.1505	Dennd1b
MSTRG.15050.1	MSTRG.15050	Pcdhga6
MSTRG.15052.1	MSTRG.15052	Pcdhga12
MSTRG.15057.1	MSTRG.15057	.
MSTRG.1506.1	MSTRG.1506	Dennd1b
MSTRG.15064.1	MSTRG.15064	Arhgap26
MSTRG.15065.1	MSTRG.15065	Arhgap26
MSTRG.15067.1	MSTRG.15067	Arhgap26
MSTRG.15072.1	MSTRG.15072	Nr3c1
MSTRG.15075.1	MSTRG.15075	Prelid2
MSTRG.15076.1	MSTRG.15076	Prelid2
MSTRG.15080.1	MSTRG.15080	Rbm27
MSTRG.15086.1	MSTRG.15086	Gm3650
MSTRG.15089.1	MSTRG.15089	Dcp2
MSTRG.1509.1	MSTRG.1509	Dennd1b
MSTRG.15092.1	MSTRG.15092	Mcc
MSTRG.15093.1	MSTRG.15093	Mcc
MSTRG.15095.1	MSTRG.15095	A930012L18Rik
MSTRG.15096.1	MSTRG.15096	Mcc
MSTRG.15097.1	MSTRG.15097	Mcc
MSTRG.15098.1	MSTRG.15098	Mcc

MSTRG.15102.1	MSTRG.15102	Ythdc2
MSTRG.15103.1	MSTRG.15103	Ythdc2
MSTRG.15104.1	MSTRG.15104	.
MSTRG.15106.1	MSTRG.15106	Pggt1b
MSTRG.15109.1	MSTRG.15109	.
MSTRG.15114.1	MSTRG.15114	Fem1c
MSTRG.15115.1	MSTRG.15115	Fem1c
MSTRG.15116.1	MSTRG.15116	Fem1c
MSTRG.15118.1	MSTRG.15118	Eif1a
MSTRG.1512.1	MSTRG.1512	Dennd1b
MSTRG.15121.1	MSTRG.15121	Ap3s1
MSTRG.15122.1	MSTRG.15122	Ap3s1
MSTRG.15125.1	MSTRG.15125	Commd10
MSTRG.15127.1	MSTRG.15127	Commd10
MSTRG.15129.1	MSTRG.15129	.
MSTRG.1513.1	MSTRG.1513	Dennd1b
MSTRG.15130.1	MSTRG.15130	.
MSTRG.15132.1	MSTRG.15132	.
MSTRG.15134.1	MSTRG.15134	Dtwd2
MSTRG.15135.1	MSTRG.15135	Dtwd2
MSTRG.15136.1	MSTRG.15136	Dtwd2
MSTRG.15138.1	MSTRG.15138	.
MSTRG.15141.1	MSTRG.15141	.
MSTRG.15142.5	MSTRG.15142	Dmxl1
MSTRG.15143.1	MSTRG.15143	Dmxl1
MSTRG.15144.1	MSTRG.15144	C030005K06Rik
MSTRG.15144.2	MSTRG.15144	C030005K06Rik
MSTRG.15145.1	MSTRG.15145	Tnfaip8
MSTRG.15147.1	MSTRG.15147	Tnfaip8
MSTRG.15148.1	MSTRG.15148	Tnfaip8
MSTRG.15149.2	MSTRG.15149	Srfbp1
MSTRG.15154.1	MSTRG.15154	Snx2
MSTRG.15158.1	MSTRG.15158	Csnk1g3
MSTRG.15159.1	MSTRG.15159	Csnk1g3
MSTRG.1516.1	MSTRG.1516	Gm4788
MSTRG.15164.1	MSTRG.15164	.
MSTRG.15166.1	MSTRG.15166	.
MSTRG.15168.1	MSTRG.15168	.
MSTRG.15169.1	MSTRG.15169	.
MSTRG.15170.1	MSTRG.15170	.
MSTRG.15171.1	MSTRG.15171	.
MSTRG.15172.1	MSTRG.15172	.
MSTRG.15173.1	MSTRG.15173	.
MSTRG.15175.1	MSTRG.15175	Gramd3
MSTRG.15176.1	MSTRG.15176	Gramd3
MSTRG.15178.1	MSTRG.15178	Aldh7a1
MSTRG.15180.1	MSTRG.15180	Aldh7a1
MSTRG.15184.1	MSTRG.15184	Lmnb1
MSTRG.15188.1	MSTRG.15188	March3
MSTRG.15189.1	MSTRG.15189	March3
MSTRG.15190.1	MSTRG.15190	March3
MSTRG.15191.1	MSTRG.15191	March3
MSTRG.15192.1	MSTRG.15192	March3
MSTRG.15198.1	MSTRG.15198	1700011I03Rik
MSTRG.1520.1	MSTRG.1520	Gm38019
MSTRG.15203.1	MSTRG.15203	Isoc1
MSTRG.15206.1	MSTRG.15206	Gm4951

MSTRG.15218.1	MSTRG.15218	.
MSTRG.15219.1	MSTRG.15219	.
MSTRG.1522.1	MSTRG.1522	Cfh
MSTRG.15220.1	MSTRG.15220	.
MSTRG.15221.1	MSTRG.15221	.
MSTRG.15222.1	MSTRG.15222	.
MSTRG.15224.1	MSTRG.15224	.
MSTRG.15225.1	MSTRG.15225	.
MSTRG.1523.1	MSTRG.1523	.
MSTRG.1524.1	MSTRG.1524	.
MSTRG.15241.1	MSTRG.15241	Hmgxb3
MSTRG.15244.1	MSTRG.15244	Ppargc1b
MSTRG.15245.1	MSTRG.15245	Ppargc1b
MSTRG.15246.1	MSTRG.15246	Ppargc1b
MSTRG.15248.1	MSTRG.15248	Ppargc1b
MSTRG.15249.1	MSTRG.15249	Ppargc1b
MSTRG.1525.1	MSTRG.1525	.
MSTRG.15253.3	MSTRG.15253	Bvht
MSTRG.15256.1	MSTRG.15256	Carmn
MSTRG.1526.1	MSTRG.1526	.
MSTRG.15261.1	MSTRG.15261	Ablim3
MSTRG.15264.1	MSTRG.15264	.
MSTRG.15267.1	MSTRG.15267	.
MSTRG.1528.1	MSTRG.1528	Cdc73
MSTRG.15286.1	MSTRG.15286	Nedd4l
MSTRG.15287.1	MSTRG.15287	Nedd4l
MSTRG.15288.1	MSTRG.15288	Nedd4l
MSTRG.15289.1	MSTRG.15289	Nedd4l
MSTRG.1529.1	MSTRG.1529	Cdc73
MSTRG.15290.1	MSTRG.15290	Nedd4l
MSTRG.15291.1	MSTRG.15291	Nedd4l
MSTRG.15292.1	MSTRG.15292	Nedd4l
MSTRG.15293.1	MSTRG.15293	Nedd4l
MSTRG.15294.1	MSTRG.15294	Nedd4l
MSTRG.15296.1	MSTRG.15296	Zfp532
MSTRG.15298.1	MSTRG.15298	Zfp532
MSTRG.15299.1	MSTRG.15299	Zfp532
MSTRG.15301.6	MSTRG.15301	Sec11c
MSTRG.15304.1	MSTRG.15304	.
MSTRG.15304.2	MSTRG.15304	.
MSTRG.15305.1	MSTRG.15305	.
MSTRG.15306.1	MSTRG.15306	.
MSTRG.1531.1	MSTRG.1531	B3galt2
MSTRG.15313.1	MSTRG.15313	Impa2
MSTRG.15314.1	MSTRG.15314	Impa2
MSTRG.15318.1	MSTRG.15318	Afg3l2
MSTRG.1532.1	MSTRG.1532	Cdc73
MSTRG.15321.5	MSTRG.15321	Ptpn2
MSTRG.15322.1	MSTRG.15322	Ptpn2
MSTRG.15323.1	MSTRG.15323	Ptpn2
MSTRG.15327.1	MSTRG.15327	Cep192
MSTRG.15329.1	MSTRG.15329	Mc2r
MSTRG.15330.1	MSTRG.15330	Mc2r
MSTRG.15332.1	MSTRG.15332	Ldlrad4
MSTRG.15334.1	MSTRG.15334	Ldlrad4
MSTRG.15336.1	MSTRG.15336	Fam210a
MSTRG.15339.1	MSTRG.15339	4930503L19Rik

MSTRG.15341.1	MSTRG.15341	Stard6
MSTRG.15349.1	MSTRG.15349	Tcf4
MSTRG.15350.1	MSTRG.15350	Tcf4
MSTRG.15351.1	MSTRG.15351	Tcf4
MSTRG.15352.1	MSTRG.15352	Tcf4
MSTRG.15354.1	MSTRG.15354	Tcf4
MSTRG.15356.1	MSTRG.15356	Smad4
MSTRG.15356.2	MSTRG.15356	Smad4
MSTRG.15357.1	MSTRG.15357	.
MSTRG.15359.1	MSTRG.15359	.
MSTRG.15360.3	MSTRG.15360	Ska1
MSTRG.15365.1	MSTRG.15365	Gm23119
MSTRG.15367.1	MSTRG.15367	.
MSTRG.15374.1	MSTRG.15374	Dym
MSTRG.15375.1	MSTRG.15375	Dym
MSTRG.15377.1	MSTRG.15377	Dym
MSTRG.15378.1	MSTRG.15378	Dym
MSTRG.15382.1	MSTRG.15382	Ctif
MSTRG.15384.1	MSTRG.15384	Ctif
MSTRG.15386.1	MSTRG.15386	Zbtb7c
MSTRG.15387.1	MSTRG.15387	.
MSTRG.15388.1	MSTRG.15388	.
MSTRG.1539.1	MSTRG.1539	Uchl5
MSTRG.15390.1	MSTRG.15390	Smad2
MSTRG.15391.1	MSTRG.15391	Smad2
MSTRG.15393.1	MSTRG.15393	Pias2
MSTRG.15394.1	MSTRG.15394	Pias2
MSTRG.15398.1	MSTRG.15398	8030462N17Rik
MSTRG.15399.1	MSTRG.15399	4930465K10Rik
MSTRG.154.1	MSTRG.154	Pkhd1
MSTRG.1540.1	MSTRG.1540	Uchl5
MSTRG.15400.1	MSTRG.15400	8030462N17Rik
MSTRG.1541.1	MSTRG.1541	Uchl5
MSTRG.15414.1	MSTRG.15414	Setbp1
MSTRG.15415.1	MSTRG.15415	Setbp1
MSTRG.15416.1	MSTRG.15416	Setbp1
MSTRG.15417.1	MSTRG.15417	Setbp1
MSTRG.1542.1	MSTRG.1542	.
MSTRG.15422.2	MSTRG.15422	Adnp2
MSTRG.15424.1	MSTRG.15424	.
MSTRG.15427.1	MSTRG.15427	Txnl4a
MSTRG.15428.1	MSTRG.15428	Txnl4a
MSTRG.15429.1	MSTRG.15429	Txnl4a
MSTRG.15437.1	MSTRG.15437	.
MSTRG.15438.1	MSTRG.15438	.
MSTRG.15441.1	MSTRG.15441	Atp9b
MSTRG.15442.1	MSTRG.15442	Atp9b
MSTRG.15443.1	MSTRG.15443	AC117949.2
MSTRG.15445.1	MSTRG.15445	Atp9b
MSTRG.15446.1	MSTRG.15446	Atp9b
MSTRG.15447.1	MSTRG.15447	Atp9b
MSTRG.15450.1	MSTRG.15450	Mbp
MSTRG.15453.1	MSTRG.15453	Zfp516
MSTRG.15454.1	MSTRG.15454	Zfp516
MSTRG.15456.1	MSTRG.15456	.
MSTRG.15459.1	MSTRG.15459	.
MSTRG.15460.1	MSTRG.15460	.

MSTRG.15462.1	MSTRG.15462	Tshz1
MSTRG.15463.1	MSTRG.15463	Tshz1
MSTRG.15464.1	MSTRG.15464	Tshz1
MSTRG.15467.1	MSTRG.15467	Zfp407
MSTRG.15468.1	MSTRG.15468	Zfp407
MSTRG.15470.1	MSTRG.15470	Zfp407
MSTRG.15471.1	MSTRG.15471	Zfp407
MSTRG.15471.2	MSTRG.15471	Zfp407
MSTRG.15472.1	MSTRG.15472	Zfp407
MSTRG.15475.3	MSTRG.15475	Cyb5a
MSTRG.15478.1	MSTRG.15478	Timm21
MSTRG.15480.1	MSTRG.15480	Socs6
MSTRG.15483.1	MSTRG.15483	Cd226
MSTRG.15484.1	MSTRG.15484	Cd226
MSTRG.15485.1	MSTRG.15485	.
MSTRG.15488.1	MSTRG.15488	.
MSTRG.15490.1	MSTRG.15490	1700030N03Rik
MSTRG.15493.1	MSTRG.15493	Ighmbp2
MSTRG.15499.1	MSTRG.15499	Lrp5
MSTRG.1550.1	MSTRG.1550	.
MSTRG.15500.1	MSTRG.15500	Lrp5
MSTRG.15502.1	MSTRG.15502	Ppp6r3
MSTRG.15506.1	MSTRG.15506	Ppp6r3
MSTRG.15506.2	MSTRG.15506	Ppp6r3
MSTRG.15513.1	MSTRG.15513	Kmt5b
MSTRG.15523.1	MSTRG.15523	.
MSTRG.15523.2	MSTRG.15523	.
MSTRG.15523.3	MSTRG.15523	.
MSTRG.15523.4	MSTRG.15523	.
MSTRG.15523.5	MSTRG.15523	.
MSTRG.15523.6	MSTRG.15523	.
MSTRG.15529.1	MSTRG.15529	Pitpnm1
MSTRG.1553.1	MSTRG.1553	.
MSTRG.15534.1	MSTRG.15534	Pold4
MSTRG.15539.5	MSTRG.15539	Clcf1
MSTRG.15546.1	MSTRG.15546	Kdm2a
MSTRG.15547.1	MSTRG.15547	Kdm2a
MSTRG.15555.1	MSTRG.15555	4930533O14Rik
MSTRG.1556.1	MSTRG.1556	.
MSTRG.15563.1	MSTRG.15563	Gm960
MSTRG.15568.1	MSTRG.15568	Gm21992
MSTRG.15569.2	MSTRG.15569	Gm21844
MSTRG.15582.1	MSTRG.15582	Pacs1
MSTRG.15583.1	MSTRG.15583	Pacs1
MSTRG.15585.1	MSTRG.15585	Pacs1
MSTRG.15586.1	MSTRG.15586	Pacs1
MSTRG.15587.1	MSTRG.15587	Pacs1
MSTRG.15589.1	MSTRG.15589	Pacs1
MSTRG.1559.1	MSTRG.1559	Tpr
MSTRG.15602.1	MSTRG.15602	Mrpl49
MSTRG.15612.1	MSTRG.15612	Atg2a
MSTRG.15618.1	MSTRG.15618	.
MSTRG.15626.1	MSTRG.15626	Ltbp3
MSTRG.15626.2	MSTRG.15626	Ltbp3
MSTRG.1565.10	MSTRG.1565	Swt1
MSTRG.1565.6	MSTRG.1565	Swt1
MSTRG.1566.1	MSTRG.1566	Swt1

MSTRG.1567.1	MSTRG.1567	Swt1
MSTRG.15675.1	MSTRG.15675	Gm14966
MSTRG.15678.1	MSTRG.15678	.
MSTRG.15679.4	MSTRG.15679	Trmt112
MSTRG.1568.1	MSTRG.1568	Swt1
MSTRG.15687.1	MSTRG.15687	Bad
MSTRG.15693.1	MSTRG.15693	Fermt3
MSTRG.15702.1	MSTRG.15702	.
MSTRG.15706.1	MSTRG.15706	Macrod1
MSTRG.15710.1	MSTRG.15710	Rcor2
MSTRG.15715.14	MSTRG.15715	Mark2
MSTRG.15716.1	MSTRG.15716	Mark2
MSTRG.15716.2	MSTRG.15716	1700105P06Rik
MSTRG.15716.3	MSTRG.15716	Mark2
MSTRG.15717.1	MSTRG.15717	Mark2
MSTRG.15723.10	MSTRG.15723	Snhg1
MSTRG.15726.1	MSTRG.15726	Stx5a
MSTRG.15728.1	MSTRG.15728	Polr2g
MSTRG.1573.5	MSTRG.1573	1700025G04Rik
MSTRG.15748.1	MSTRG.15748	Ahnak
MSTRG.15749.1	MSTRG.15749	Ahnak
MSTRG.15750.1	MSTRG.15750	Ahnak
MSTRG.15751.1	MSTRG.15751	Ahnak
MSTRG.1576.1	MSTRG.1576	1700025G04Rik
MSTRG.1577.1	MSTRG.1577	1700025G04Rik
MSTRG.15773.1	MSTRG.15773	Sdhaf2
MSTRG.15777.1	MSTRG.15777	.
MSTRG.15787.1	MSTRG.15787	A430093F15Rik
MSTRG.15787.5	MSTRG.15787	A430093F15Rik
MSTRG.15787.7	MSTRG.15787	A430093F15Rik
MSTRG.15788.1	MSTRG.15788	A430093F15Rik
MSTRG.15789.2	MSTRG.15789	A430093F15Rik
MSTRG.15789.3	MSTRG.15789	A430093F15Rik
MSTRG.158.1	MSTRG.158	Pkhd1
MSTRG.1580.1	MSTRG.1580	Tsen15
MSTRG.15812.2	MSTRG.15812	Ms4a6b
MSTRG.15812.4	MSTRG.15812	Ms4a6c
MSTRG.15813.1	MSTRG.15813	BE692007
MSTRG.15824.1	MSTRG.15824	Patl1
MSTRG.15826.1	MSTRG.15826	.
MSTRG.15832.1	MSTRG.15832	A330040F15Rik
MSTRG.15834.1	MSTRG.15834	A330040F15Rik
MSTRG.15838.1	MSTRG.15838	A330040F15Rik
MSTRG.15843.1	MSTRG.15843	.
MSTRG.15850.1	MSTRG.15850	Gnaq
MSTRG.15852.1	MSTRG.15852	Gnaq
MSTRG.15853.1	MSTRG.15853	Gnaq
MSTRG.15854.1	MSTRG.15854	Gnaq
MSTRG.15856.1	MSTRG.15856	Gnaq
MSTRG.15858.1	MSTRG.15858	Gnaq
MSTRG.15859.1	MSTRG.15859	Gnaq
MSTRG.15861.1	MSTRG.15861	Gnaq
MSTRG.15862.1	MSTRG.15862	Gnaq
MSTRG.15863.1	MSTRG.15863	Gnaq
MSTRG.15864.1	MSTRG.15864	Gnaq
MSTRG.15866.1	MSTRG.15866	Gna14
MSTRG.15869.1	MSTRG.15869	Prune2

MSTRG.1587.3	MSTRG.1587	Arpc5
MSTRG.1587.4	MSTRG.1587	Arpc5
MSTRG.15873.1	MSTRG.15873	Gcnt1
MSTRG.15875.1	MSTRG.15875	Vps13a
MSTRG.15876.1	MSTRG.15876	Vps13a
MSTRG.15878.1	MSTRG.15878	Vps13a
MSTRG.15879.1	MSTRG.15879	Vps13a
MSTRG.15880.1	MSTRG.15880	Vps13a
MSTRG.15881.1	MSTRG.15881	Vps13a
MSTRG.15882.1	MSTRG.15882	Vps13a
MSTRG.15883.1	MSTRG.15883	Vps13a
MSTRG.15885.1	MSTRG.15885	Rfk
MSTRG.15887.1	MSTRG.15887	.
MSTRG.15888.1	MSTRG.15888	.
MSTRG.15891.1	MSTRG.15891	Ostf1
MSTRG.15892.1	MSTRG.15892	Ostf1
MSTRG.15893.1	MSTRG.15893	Nmrk1
MSTRG.15896.1	MSTRG.15896	Carnmt1
MSTRG.15897.1	MSTRG.15897	Carnmt1
MSTRG.15898.1	MSTRG.15898	Carnmt1
MSTRG.15899.1	MSTRG.15899	Carnmt1
MSTRG.15907.1	MSTRG.15907	.
MSTRG.15909.1	MSTRG.15909	.
MSTRG.1591.1	MSTRG.1591	Smg7
MSTRG.15911.1	MSTRG.15911	1110059E24Rik
MSTRG.15912.1	MSTRG.15912	1110059E24Rik
MSTRG.15913.1	MSTRG.15913	Abhd17b
MSTRG.1592.1	MSTRG.1592	Smg7
MSTRG.15926.1	MSTRG.15926	Fxn
MSTRG.15929.1	MSTRG.15929	Pip5k1b
MSTRG.1593.1	MSTRG.1593	Smg7
MSTRG.15930.1	MSTRG.15930	Pip5k1b
MSTRG.15932.1	MSTRG.15932	Pip5k1b
MSTRG.15933.1	MSTRG.15933	Pip5k1b
MSTRG.15935.1	MSTRG.15935	Pip5k1b
MSTRG.15936.1	MSTRG.15936	Pip5k1b
MSTRG.15937.1	MSTRG.15937	Pip5k1b
MSTRG.15938.1	MSTRG.15938	Pip5k1b
MSTRG.15939.1	MSTRG.15939	Pip5k1b
MSTRG.1594.1	MSTRG.1594	.
MSTRG.15941.1	MSTRG.15941	Pip5k1b
MSTRG.15946.1	MSTRG.15946	Pgm5
MSTRG.15950.1	MSTRG.15950	Kank1
MSTRG.15954.1	MSTRG.15954	.
MSTRG.15955.1	MSTRG.15955	.
MSTRG.15957.1	MSTRG.15957	Dock8
MSTRG.15958.1	MSTRG.15958	Dock8
MSTRG.15959.1	MSTRG.15959	Dock8
MSTRG.1596.1	MSTRG.1596	.
MSTRG.15960.1	MSTRG.15960	Dock8
MSTRG.15966.1	MSTRG.15966	Rfx3
MSTRG.15967.1	MSTRG.15967	Rfx3
MSTRG.15968.1	MSTRG.15968	Rfx3
MSTRG.15970.1	MSTRG.15970	Rfx3
MSTRG.15973.1	MSTRG.15973	Rfx3
MSTRG.15975.1	MSTRG.15975	Smarca2
MSTRG.15978.1	MSTRG.15978	Smarca2

MSTRG.15979.1	MSTRG.15979	Smarca2
MSTRG.15980.1	MSTRG.15980	Smarca2
MSTRG.15988.1	MSTRG.15988	Cdc37l1
MSTRG.1600.1	MSTRG.1600	Lamc1
MSTRG.16002.1	MSTRG.16002	.
MSTRG.16014.1	MSTRG.16014	Minpp1
MSTRG.16016.1	MSTRG.16016	Sgms1
MSTRG.16017.1	MSTRG.16017	Sgms1
MSTRG.16018.1	MSTRG.16018	Sgms1
MSTRG.16019.1	MSTRG.16019	Sgms1
MSTRG.1602.1	MSTRG.1602	E330020D12Rik
MSTRG.16020.1	MSTRG.16020	Sgms1
MSTRG.16025.1	MSTRG.16025	Pten
MSTRG.16026.1	MSTRG.16026	Pten
MSTRG.16027.1	MSTRG.16027	.
MSTRG.16030.1	MSTRG.16030	Stambpl1
MSTRG.16031.1	MSTRG.16031	Stambpl1
MSTRG.16032.1	MSTRG.16032	Stambpl1
MSTRG.16038.2	MSTRG.16038	Ifit2
MSTRG.16039.1	MSTRG.16039	Ifit2
MSTRG.16045.1	MSTRG.16045	A830019P07Rik
MSTRG.16049.1	MSTRG.16049	Kif20b
MSTRG.16050.1	MSTRG.16050	Kif20b
MSTRG.16052.1	MSTRG.16052	.
MSTRG.16053.1	MSTRG.16053	.
MSTRG.16055.1	MSTRG.16055	Rpp30
MSTRG.16056.1	MSTRG.16056	Rpp30
MSTRG.16060.1	MSTRG.16060	Pcgf5
MSTRG.16064.1	MSTRG.16064	Hectd2
MSTRG.16067.1	MSTRG.16067	Tnks2
MSTRG.1607.1	MSTRG.1607	Shcbp1l
MSTRG.16074.3	MSTRG.16074	March5
MSTRG.16077.1	MSTRG.16077	.
MSTRG.16080.1	MSTRG.16080	Exoc6
MSTRG.16081.1	MSTRG.16081	Exoc6
MSTRG.16087.1	MSTRG.16087	Fra10ac1
MSTRG.16088.1	MSTRG.16088	.
MSTRG.16088.2	MSTRG.16088	.
MSTRG.16089.1	MSTRG.16089	.
MSTRG.16090.1	MSTRG.16090	.
MSTRG.16097.1	MSTRG.16097	Tbc1d12
MSTRG.16098.1	MSTRG.16098	Tbc1d12
MSTRG.16099.1	MSTRG.16099	Tbc1d12
MSTRG.16101.1	MSTRG.16101	Tbc1d12
MSTRG.16103.1	MSTRG.16103	.
MSTRG.16105.1	MSTRG.16105	.
MSTRG.16108.1	MSTRG.16108	Cyp2c54
MSTRG.16112.1	MSTRG.16112	Sorbs1
MSTRG.16113.1	MSTRG.16113	Sorbs1
MSTRG.16116.1	MSTRG.16116	.
MSTRG.16126.1	MSTRG.16126	.
MSTRG.16126.2	MSTRG.16126	.
MSTRG.16126.3	MSTRG.16126	.
MSTRG.16126.4	MSTRG.16126	.
MSTRG.16126.5	MSTRG.16126	.
MSTRG.16126.6	MSTRG.16126	.
MSTRG.16128.1	MSTRG.16128	.

MSTRG.16130.1	MSTRG.16130	.
MSTRG.16131.1	MSTRG.16131	Dntt
MSTRG.16132.1	MSTRG.16132	Dntt
MSTRG.16132.2	MSTRG.16132	Dntt
MSTRG.16135.1	MSTRG.16135	Tll2
MSTRG.16137.1	MSTRG.16137	Tm9sf3
MSTRG.16137.2	MSTRG.16137	Tm9sf3
MSTRG.16143.1	MSTRG.16143	Lcor
MSTRG.16144.1	MSTRG.16144	Lcor
MSTRG.16147.1	MSTRG.16147	Arhgap19
MSTRG.16148.1	MSTRG.16148	Arhgap19
MSTRG.16149.1	MSTRG.16149	Arhgap19
MSTRG.16163.1	MSTRG.16163	Ubtd1
MSTRG.16164.1	MSTRG.16164	Ubtd1
MSTRG.16165.1	MSTRG.16165	Ubtd1
MSTRG.16167.1	MSTRG.16167	R3hcc1l
MSTRG.16183.1	MSTRG.16183	Dnmbp
MSTRG.16188.1	MSTRG.16188	Pkd2l1
MSTRG.16190.1	MSTRG.16190	Erlin1
MSTRG.16191.1	MSTRG.16191	Erlin1
MSTRG.16194.1	MSTRG.16194	Scd3
MSTRG.16196.1	MSTRG.16196	.
MSTRG.16197.1	MSTRG.16197	Scd4
MSTRG.16198.1	MSTRG.16198	.
MSTRG.16199.1	MSTRG.16199	.
MSTRG.162.1	MSTRG.162	Tram2
MSTRG.1620.1	MSTRG.1620	Cacna1e
MSTRG.16203.1	MSTRG.16203	Hif1an
MSTRG.16206.1	MSTRG.16206	Gm20538
MSTRG.1621.1	MSTRG.1621	Cacna1e
MSTRG.16213.1	MSTRG.16213	Lzts2
MSTRG.16217.1	MSTRG.16217	Btrc
MSTRG.16218.1	MSTRG.16218	Btrc
MSTRG.16219.1	MSTRG.16219	Btrc
MSTRG.16225.1	MSTRG.16225	Fbxw4
MSTRG.16230.1	MSTRG.16230	9130011E15Rik
MSTRG.16241.1	MSTRG.16241	.
MSTRG.16244.2	MSTRG.16244	Borcs7
MSTRG.16245.1	MSTRG.16245	Borcs7
MSTRG.16246.1	MSTRG.16246	Borcs7
MSTRG.16247.1	MSTRG.16247	Borcs7
MSTRG.16251.1	MSTRG.16251	Gbf1
MSTRG.16252.1	MSTRG.16252	Gbf1
MSTRG.16255.7	MSTRG.16255	Psd
MSTRG.1626.1	MSTRG.1626	Xpr1
MSTRG.16260.1	MSTRG.16260	Arl3
MSTRG.16263.1	MSTRG.16263	Wbp1l
MSTRG.16264.1	MSTRG.16264	Wbp1l
MSTRG.16265.1	MSTRG.16265	Wbp1l
MSTRG.16268.1	MSTRG.16268	Pcgf6
MSTRG.16270.1	MSTRG.16270	Cnnm2
MSTRG.16271.1	MSTRG.16271	Cnnm2
MSTRG.16274.1	MSTRG.16274	.
MSTRG.1628.1	MSTRG.1628	Stx6
MSTRG.16281.1	MSTRG.16281	Sh3pxd2a
MSTRG.16283.1	MSTRG.16283	Sh3pxd2a
MSTRG.16287.1	MSTRG.16287	Sfr1

MSTRG.16287.2	MSTRG.16287	Sfr1
MSTRG.16287.3	MSTRG.16287	Sfr1
MSTRG.16287.4	MSTRG.16287	Sfr1
MSTRG.16289.1	MSTRG.16289	Slk
MSTRG.1629.1	MSTRG.1629	Stx6
MSTRG.16291.1	MSTRG.16291	Slk
MSTRG.16298.1	MSTRG.16298	Cfap43
MSTRG.163.1	MSTRG.163	Tram2
MSTRG.16304.1	MSTRG.16304	Add3
MSTRG.16307.1	MSTRG.16307	Mxi1
MSTRG.16310.1	MSTRG.16310	.
MSTRG.16311.1	MSTRG.16311	Mirt1
MSTRG.16311.4	MSTRG.16311	Mirt1
MSTRG.16311.5	MSTRG.16311	Mirt1
MSTRG.16313.1	MSTRG.16313	Mirt1
MSTRG.16313.2	MSTRG.16313	4833407H14Rik
MSTRG.1632.1	MSTRG.1632	BC034090
MSTRG.16323.1	MSTRG.16323	Bbip1
MSTRG.16323.2	MSTRG.16323	Bbip1
MSTRG.16323.3	MSTRG.16323	Bbip1
MSTRG.16327.1	MSTRG.16327	Zdhhc6
MSTRG.16329.1	MSTRG.16329	Vti1a
MSTRG.16330.1	MSTRG.16330	Vti1a
MSTRG.16331.2	MSTRG.16331	Vti1a
MSTRG.16333.1	MSTRG.16333	Vti1a
MSTRG.16335.1	MSTRG.16335	Vti1a
MSTRG.16336.1	MSTRG.16336	Vti1a
MSTRG.16337.1	MSTRG.16337	Vti1a
MSTRG.16338.1	MSTRG.16338	Vti1a
MSTRG.1634.1	MSTRG.1634	Acbd6
MSTRG.16340.17	MSTRG.16340	Tcf7l2
MSTRG.16341.1	MSTRG.16341	Tcf7l2
MSTRG.16342.1	MSTRG.16342	Tcf7l2
MSTRG.16343.1	MSTRG.16343	Tcf7l2
MSTRG.16344.1	MSTRG.16344	Tcf7l2
MSTRG.16345.1	MSTRG.16345	Tcf7l2
MSTRG.16348.1	MSTRG.16348	Tcf7l2
MSTRG.16349.1	MSTRG.16349	.
MSTRG.1635.1	MSTRG.1635	Acbd6
MSTRG.16352.1	MSTRG.16352	Nhlrc2
MSTRG.16353.1	MSTRG.16353	Nhlrc2
MSTRG.16357.1	MSTRG.16357	Afap1l2
MSTRG.16359.1	MSTRG.16359	B230217O12Rik
MSTRG.1636.1	MSTRG.1636	Acbd6
MSTRG.16360.1	MSTRG.16360	B230217O12Rik
MSTRG.16366.1	MSTRG.16366	Ablim1
MSTRG.16367.1	MSTRG.16367	Ablim1
MSTRG.16367.2	MSTRG.16367	Ablim1
MSTRG.16368.1	MSTRG.16368	Ablim1
MSTRG.16369.1	MSTRG.16369	Ablim1
MSTRG.16369.2	MSTRG.16369	Ablim1
MSTRG.1637.1	MSTRG.1637	Acbd6
MSTRG.16370.1	MSTRG.16370	Ablim1
MSTRG.16372.1	MSTRG.16372	Atrnl1
MSTRG.16373.1	MSTRG.16373	Atrnl1
MSTRG.16374.1	MSTRG.16374	Atrnl1
MSTRG.16375.1	MSTRG.16375	Atrnl1

MSTRG.16376.1	MSTRG.16376	Atrnl1
MSTRG.16378.1	MSTRG.16378	Atrnl1
MSTRG.16379.1	MSTRG.16379	Atrnl1
MSTRG.1638.1	MSTRG.1638	Acbd6
MSTRG.16386.1	MSTRG.16386	Pdzd8
MSTRG.16387.1	MSTRG.16387	.
MSTRG.16389.1	MSTRG.16389	Gm17203
MSTRG.1639.1	MSTRG.1639	Acbd6
MSTRG.16391.1	MSTRG.16391	.
MSTRG.16393.1	MSTRG.16393	Fam204a
MSTRG.16396.1	MSTRG.16396	Cacul1
MSTRG.16398.1	MSTRG.16398	.
MSTRG.1640.1	MSTRG.1640	Acbd6
MSTRG.16403.1	MSTRG.16403	Fam45a
MSTRG.16405.1	MSTRG.16405	Fam45a
MSTRG.16405.2	MSTRG.16405	Fam45a
MSTRG.16408.1	MSTRG.16408	Prdx3
MSTRG.1641.1	MSTRG.1641	Acbd6
MSTRG.16410.1	MSTRG.16410	.
MSTRG.16412.1	MSTRG.16412	Grk5
MSTRG.16413.1	MSTRG.16413	Grk5
MSTRG.16414.1	MSTRG.16414	Grk5
MSTRG.16415.1	MSTRG.16415	Grk5
MSTRG.16416.1	MSTRG.16416	Grk5
MSTRG.16417.1	MSTRG.16417	Grk5
MSTRG.16419.1	MSTRG.16419	Grk5
MSTRG.16421.1	MSTRG.16421	Grk5
MSTRG.16422.1	MSTRG.16422	Grk5
MSTRG.16424.1	MSTRG.16424	Zfp950
MSTRG.16426.1	MSTRG.16426	Nmt2
MSTRG.16428.1	MSTRG.16428	Nmt2
MSTRG.16434.7	MSTRG.16434	Fam107b
MSTRG.16437.1	MSTRG.16437	Fam107b
MSTRG.16438.1	MSTRG.16438	Fam107b
MSTRG.16439.1	MSTRG.16439	Fam107b
MSTRG.16440.1	MSTRG.16440	Gm13185
MSTRG.16443.3	MSTRG.16443	Prpf18
MSTRG.16453.1	MSTRG.16453	Frmd4a
MSTRG.16459.1	MSTRG.16459	Frmd4a
MSTRG.16463.1	MSTRG.16463	Sephs1
MSTRG.16464.1	MSTRG.16464	Sephs1
MSTRG.16469.1	MSTRG.16469	.
MSTRG.16475.1	MSTRG.16475	.
MSTRG.16481.1	MSTRG.16481	Camk1d
MSTRG.16483.1	MSTRG.16483	Camk1d
MSTRG.16484.1	MSTRG.16484	Camk1d
MSTRG.16485.1	MSTRG.16485	Camk1d
MSTRG.16486.1	MSTRG.16486	Camk1d
MSTRG.16487.1	MSTRG.16487	Camk1d
MSTRG.16488.1	MSTRG.16488	Camk1d
MSTRG.16489.1	MSTRG.16489	Camk1d
MSTRG.16490.1	MSTRG.16490	Camk1d
MSTRG.16491.1	MSTRG.16491	Camk1d
MSTRG.16492.1	MSTRG.16492	Camk1d
MSTRG.16493.1	MSTRG.16493	Camk1d
MSTRG.16494.1	MSTRG.16494	Camk1d
MSTRG.16495.1	MSTRG.16495	Camk1d

MSTRG.16497.1	MSTRG.16497	Camk1d
MSTRG.16497.2	MSTRG.16497	Camk1d
MSTRG.16497.3	MSTRG.16497	Camk1d
MSTRG.16497.4	MSTRG.16497	Camk1d
MSTRG.16497.5	MSTRG.16497	Camk1d
MSTRG.16497.6	MSTRG.16497	Camk1d
MSTRG.16497.7	MSTRG.16497	Camk1d
MSTRG.16498.1	MSTRG.16498	Camk1d
MSTRG.16498.2	MSTRG.16498	Camk1d
MSTRG.16500.1	MSTRG.16500	Upf2
MSTRG.16501.1	MSTRG.16501	Upf2
MSTRG.16502.1	MSTRG.16502	Upf2
MSTRG.16504.1	MSTRG.16504	Usp6nl
MSTRG.16505.1	MSTRG.16505	Usp6nl
MSTRG.16506.1	MSTRG.16506	Usp6nl
MSTRG.16507.1	MSTRG.16507	Usp6nl
MSTRG.16508.1	MSTRG.16508	Usp6nl
MSTRG.16512.1	MSTRG.16512	.
MSTRG.16514.1	MSTRG.16514	Celf2
MSTRG.1652.1	MSTRG.1652	Tor1aip1
MSTRG.16523.1	MSTRG.16523	.
MSTRG.16524.1	MSTRG.16524	.
MSTRG.16526.1	MSTRG.16526	Gata3
MSTRG.16527.1	MSTRG.16527	Gm13256
MSTRG.16531.1	MSTRG.16531	Taf3
MSTRG.16533.1	MSTRG.16533	Taf3
MSTRG.16536.1	MSTRG.16536	Itih5
MSTRG.1654.1	MSTRG.1654	Tor1aip2
MSTRG.16540.1	MSTRG.16540	.
MSTRG.16544.1	MSTRG.16544	Gm37520
MSTRG.16544.3	MSTRG.16544	Prkcq
MSTRG.16546.1	MSTRG.16546	Pfkfb3
MSTRG.16547.1	MSTRG.16547	Gm10851
MSTRG.16548.1	MSTRG.16548	Pfkfb3
MSTRG.16549.1	MSTRG.16549	Pfkfb3
MSTRG.16550.1	MSTRG.16550	Gm37975
MSTRG.16550.3	MSTRG.16550	Gm37975
MSTRG.16550.4	MSTRG.16550	Pfkfb3
MSTRG.16556.1	MSTRG.16556	Il2ra
MSTRG.16560.1	MSTRG.16560	Il15ra
MSTRG.16564.1	MSTRG.16564	.
MSTRG.16567.1	MSTRG.16567	Mindy3
MSTRG.16570.1	MSTRG.16570	Sfmbt2
MSTRG.16571.1	MSTRG.16571	Sfmbt2
MSTRG.16572.1	MSTRG.16572	Sfmbt2
MSTRG.1658.1	MSTRG.1658	Nphs2
MSTRG.166.1	MSTRG.166	.
MSTRG.1663.1	MSTRG.1663	Axdnd1
MSTRG.1664.1	MSTRG.1664	Axdnd1
MSTRG.1666.1	MSTRG.1666	Soat1
MSTRG.1666.2	MSTRG.1666	Soat1
MSTRG.1668.1	MSTRG.1668	Soat1
MSTRG.16693.1	MSTRG.16693	Trdmt1
MSTRG.16694.1	MSTRG.16694	.
MSTRG.16698.1	MSTRG.16698	Rsu1
MSTRG.1670.1	MSTRG.1670	Abl2
MSTRG.16706.1	MSTRG.16706	St8sia6

MSTRG.1671.1	MSTRG.1671	Abl2
MSTRG.16712.1	MSTRG.16712	Stam
MSTRG.16713.1	MSTRG.16713	Stam
MSTRG.16719.1	MSTRG.16719	.
MSTRG.1673.1	MSTRG.1673	Gm38043
MSTRG.1674.1	MSTRG.1674	Abl2
MSTRG.1674.2	MSTRG.1674	Abl2
MSTRG.16741.1	MSTRG.16741	Nsun6
MSTRG.16742.1	MSTRG.16742	Nsun6
MSTRG.16743.1	MSTRG.16743	Nsun6
MSTRG.16748.1	MSTRG.16748	Plxdc2
MSTRG.16749.1	MSTRG.16749	Plxdc2
MSTRG.16749.2	MSTRG.16749	Plxdc2
MSTRG.16751.1	MSTRG.16751	Plxdc2
MSTRG.16752.1	MSTRG.16752	.
MSTRG.16756.6	MSTRG.16756	Commd3
MSTRG.16759.1	MSTRG.16759	Pip4k2a
MSTRG.16760.1	MSTRG.16760	Pip4k2a
MSTRG.16763.1	MSTRG.16763	.
MSTRG.16767.1	MSTRG.16767	Mllt10
MSTRG.1677.1	MSTRG.1677	Tor3a
MSTRG.16770.1	MSTRG.16770	Mllt10
MSTRG.16772.1	MSTRG.16772	Mllt10
MSTRG.16774.1	MSTRG.16774	Dnajc1
MSTRG.16775.1	MSTRG.16775	Dnajc1
MSTRG.16776.1	MSTRG.16776	Dnajc1
MSTRG.16778.1	MSTRG.16778	Dnajc1
MSTRG.16779.1	MSTRG.16779	Dnajc1
MSTRG.16779.2	MSTRG.16779	Dnajc1
MSTRG.16780.1	MSTRG.16780	Dnajc1
MSTRG.16782.1	MSTRG.16782	Msrb2
MSTRG.16783.1	MSTRG.16783	Msrb2
MSTRG.16784.1	MSTRG.16784	.
MSTRG.16796.1	MSTRG.16796	Etl4
MSTRG.168.1	MSTRG.168	.
MSTRG.16800.1	MSTRG.16800	.
MSTRG.16801.1	MSTRG.16801	.
MSTRG.16803.1	MSTRG.16803	Arhgap21
MSTRG.16806.1	MSTRG.16806	.
MSTRG.16810.1	MSTRG.16810	Acbd5
MSTRG.16812.1	MSTRG.16812	Mastl
MSTRG.16813.1	MSTRG.16813	Mastl
MSTRG.1682.1	MSTRG.1682	Rasal2
MSTRG.16821.1	MSTRG.16821	Abi1
MSTRG.16822.1	MSTRG.16822	Abi1
MSTRG.16822.2	MSTRG.16822	Abi1
MSTRG.16823.1	MSTRG.16823	Abi1
MSTRG.16824.1	MSTRG.16824	.
MSTRG.16825.1	MSTRG.16825	.
MSTRG.16832.1	MSTRG.16832	Zmynd19
MSTRG.16834.1	MSTRG.16834	Arrdc1
MSTRG.16835.1	MSTRG.16835	Arrdc1
MSTRG.16838.1	MSTRG.16838	Ehmt1
MSTRG.16839.1	MSTRG.16839	Ehmt1
MSTRG.16840.1	MSTRG.16840	Ehmt1
MSTRG.16841.1	MSTRG.16841	Ehmt1
MSTRG.16841.2	MSTRG.16841	Ehmt1

MSTRG.16848.1	MSTRG.16848	Pnpla7
MSTRG.16850.1	MSTRG.16850	Pnpla7
MSTRG.16865.3	MSTRG.16865	Ssna1
MSTRG.16880.1	MSTRG.16880	Traf2
MSTRG.16889.1	MSTRG.16889	Rabl6
MSTRG.1690.1	MSTRG.1690	Angptl1
MSTRG.1691.1	MSTRG.1691	Ralgps2
MSTRG.16912.2	MSTRG.16912	Gm13563
MSTRG.1692.1	MSTRG.1692	Ralgps2
MSTRG.16922.1	MSTRG.16922	Snhg7
MSTRG.16925.3	MSTRG.16925	Adamts13
MSTRG.1693.1	MSTRG.1693	Ralgps2
MSTRG.16942.1	MSTRG.16942	Rxra
MSTRG.16953.1	MSTRG.16953	Tsc1
MSTRG.1696.1	MSTRG.1696	Pappa2
MSTRG.16961.1	MSTRG.16961	Ddx31
MSTRG.16962.1	MSTRG.16962	Ddx31
MSTRG.1697.1	MSTRG.1697	Pappa2
MSTRG.16971.1	MSTRG.16971	Gm13402
MSTRG.16971.2	MSTRG.16971	Gm13402
MSTRG.16972.1	MSTRG.16972	Gm13402
MSTRG.16973.1	MSTRG.16973	Med27
MSTRG.16977.1	MSTRG.16977	Rapgef1
MSTRG.16989.1	MSTRG.16989	Sptan1
MSTRG.16995.1	MSTRG.16995	Pkn3
MSTRG.16996.3	MSTRG.16996	Zdhhc12
MSTRG.1700.1	MSTRG.1700	Rfwd2
MSTRG.17001.1	MSTRG.17001	Kyat1
MSTRG.17004.1	MSTRG.17004	Gm28035
MSTRG.17009.1	MSTRG.17009	.
MSTRG.1701.1	MSTRG.1701	Rfwd2
MSTRG.1701.2	MSTRG.1701	Rfwd2
MSTRG.17010.1	MSTRG.17010	.
MSTRG.17016.1	MSTRG.17016	.
MSTRG.17018.1	MSTRG.17018	Cstad
MSTRG.1702.1	MSTRG.1702	Rfwd2
MSTRG.17020.1	MSTRG.17020	Ntmt1
MSTRG.1703.1	MSTRG.1703	Rfwd2
MSTRG.17030.1	MSTRG.17030	Fubp3
MSTRG.17035.1	MSTRG.17035	Fnbp1
MSTRG.17036.1	MSTRG.17036	D330023K18Rik
MSTRG.1704.1	MSTRG.1704	Rfwd2
MSTRG.17044.1	MSTRG.17044	Abl1
MSTRG.17045.1	MSTRG.17045	Abl1
MSTRG.17049.1	MSTRG.17049	Nup214
MSTRG.1705.1	MSTRG.1705	Rfwd2
MSTRG.17052.1	MSTRG.17052	Fam78a
MSTRG.17056.1	MSTRG.17056	Prrc2b
MSTRG.17056.2	MSTRG.17056	Prrc2b
MSTRG.17056.3	MSTRG.17056	Prrc2b
MSTRG.17065.6	MSTRG.17065	Swi5
MSTRG.17067.1	MSTRG.17067	Golga2
MSTRG.1708.1	MSTRG.1708	Rfwd2
MSTRG.17081.1	MSTRG.17081	Eng
MSTRG.17090.1	MSTRG.17090	Stxbp1
MSTRG.17097.1	MSTRG.17097	Zbtb34
MSTRG.17098.1	MSTRG.17098	Zbtb34

MSTRG.17099.1	MSTRG.17099	Zbtb34
MSTRG.17100.1	MSTRG.17100	Zbtb34
MSTRG.17101.1	MSTRG.17101	Zbtb34
MSTRG.17115.1	MSTRG.17115	Pbx3
MSTRG.17118.1	MSTRG.17118	Pbx3
MSTRG.17119.1	MSTRG.17119	Pbx3
MSTRG.17123.1	MSTRG.17123	Mapkap1
MSTRG.1713.5	MSTRG.1713	4930523C07Rik
MSTRG.17131.1	MSTRG.17131	Phf19
MSTRG.17139.1	MSTRG.17139	Rab14
MSTRG.17139.2	MSTRG.17139	Rab14
MSTRG.1714.1	MSTRG.1714	4930523C07Rik
MSTRG.17145.1	MSTRG.17145	Gsn
MSTRG.17149.1	MSTRG.17149	Ggta1
MSTRG.17150.1	MSTRG.17150	Ggta1
MSTRG.17151.1	MSTRG.17151	Ggta1
MSTRG.17153.1	MSTRG.17153	Ggta1
MSTRG.17154.1	MSTRG.17154	Ggta1
MSTRG.1717.1	MSTRG.1717	.
MSTRG.17174.1	MSTRG.17174	Rabgap1
MSTRG.17175.1	MSTRG.17175	Strbp
MSTRG.17177.1	MSTRG.17177	Strbp
MSTRG.17178.1	MSTRG.17178	Strbp
MSTRG.17180.1	MSTRG.17180	Strbp
MSTRG.17181.1	MSTRG.17181	Mir5128
MSTRG.17181.3	MSTRG.17181	Strbp
MSTRG.17182.1	MSTRG.17182	Strbp
MSTRG.17185.1	MSTRG.17185	Dennd1a
MSTRG.1719.2	MSTRG.1719	Gm36975
MSTRG.17190.1	MSTRG.17190	Dennd1a
MSTRG.17191.1	MSTRG.17191	Dennd1a
MSTRG.17192.1	MSTRG.17192	Dennd1a
MSTRG.17194.1	MSTRG.17194	Dennd1a
MSTRG.17195.1	MSTRG.17195	Dennd1a
MSTRG.17196.1	MSTRG.17196	Dennd1a
MSTRG.17198.1	MSTRG.17198	Dennd1a
MSTRG.17203.3	MSTRG.17203	Psmb7
MSTRG.17211.1	MSTRG.17211	Nr6a1
MSTRG.17212.1	MSTRG.17212	Nr6a1
MSTRG.17214.1	MSTRG.17214	Nr6a1
MSTRG.17215.1	MSTRG.17215	Nr6a1
MSTRG.17216.1	MSTRG.17216	Nr6a1
MSTRG.1722.1	MSTRG.1722	Gm36975
MSTRG.17220.1	MSTRG.17220	Nr6a1
MSTRG.17222.1	MSTRG.17222	Nr6a1
MSTRG.17223.1	MSTRG.17223	Nr6a1
MSTRG.17224.1	MSTRG.17224	Nr6a1
MSTRG.17225.1	MSTRG.17225	Nr6a1
MSTRG.17226.1	MSTRG.17226	Nr6a1
MSTRG.1723.1	MSTRG.1723	Rabgap1l
MSTRG.17232.1	MSTRG.17232	.
MSTRG.17233.1	MSTRG.17233	.
MSTRG.17235.1	MSTRG.17235	.
MSTRG.17237.1	MSTRG.17237	Scai
MSTRG.17238.1	MSTRG.17238	Scai
MSTRG.1724.1	MSTRG.1724	Rabgap1l
MSTRG.17240.1	MSTRG.17240	Scai

MSTRG.17242.1	MSTRG.17242	Scai
MSTRG.17246.1	MSTRG.17246	Scai
MSTRG.1725.1	MSTRG.1725	Rabgap1l
MSTRG.17251.1	MSTRG.17251	Kynu
MSTRG.17259.1	MSTRG.17259	Arhgap15
MSTRG.17260.1	MSTRG.17260	Arhgap15
MSTRG.17261.1	MSTRG.17261	Arhgap15
MSTRG.17262.1	MSTRG.17262	Arhgap15
MSTRG.17264.1	MSTRG.17264	Arhgap15
MSTRG.17265.1	MSTRG.17265	Arhgap15
MSTRG.17267.1	MSTRG.17267	Gtdc1
MSTRG.17268.1	MSTRG.17268	Gtdc1
MSTRG.17270.1	MSTRG.17270	Gtdc1
MSTRG.17271.1	MSTRG.17271	Gtdc1
MSTRG.17272.1	MSTRG.17272	.
MSTRG.17273.1	MSTRG.17273	.
MSTRG.17274.1	MSTRG.17274	.
MSTRG.17275.1	MSTRG.17275	.
MSTRG.17277.1	MSTRG.17277	Zeb2
MSTRG.17282.1	MSTRG.17282	Epc2
MSTRG.17287.1	MSTRG.17287	Mbd5
MSTRG.17289.5	MSTRG.17289	Lypd6b
MSTRG.17292.1	MSTRG.17292	Lypd6b
MSTRG.17293.1	MSTRG.17293	Lypd6b
MSTRG.17295.1	MSTRG.17295	Mmadhc
MSTRG.17302.1	MSTRG.17302	.
MSTRG.17304.1	MSTRG.17304	Nmi
MSTRG.17305.1	MSTRG.17305	Nmi
MSTRG.17314.1	MSTRG.17314	Fmnl2
MSTRG.17315.1	MSTRG.17315	Fmnl2
MSTRG.17316.1	MSTRG.17316	Fmnl2
MSTRG.17318.1	MSTRG.17318	Fmnl2
MSTRG.17327.1	MSTRG.17327	.
MSTRG.17330.1	MSTRG.17330	.
MSTRG.17333.1	MSTRG.17333	Gpd2
MSTRG.17340.1	MSTRG.17340	Acvr1
MSTRG.1735.1	MSTRG.1735	Rabgap1l
MSTRG.17352.1	MSTRG.17352	Tanc1
MSTRG.17354.1	MSTRG.17354	Wdsub1
MSTRG.17359.1	MSTRG.17359	.
MSTRG.1736.1	MSTRG.1736	Rabgap1l
MSTRG.17363.1	MSTRG.17363	Baz2b
MSTRG.17367.1	MSTRG.17367	Baz2b
MSTRG.17368.1	MSTRG.17368	Baz2b
MSTRG.17370.1	MSTRG.17370	March7
MSTRG.17374.1	MSTRG.17374	.
MSTRG.17376.1	MSTRG.17376	Psmd14
MSTRG.17378.1	MSTRG.17378	Psmd14
MSTRG.17382.1	MSTRG.17382	Ifih1
MSTRG.17390.1	MSTRG.17390	Cobll1
MSTRG.17391.1	MSTRG.17391	Cobll1
MSTRG.17392.1	MSTRG.17392	Cobll1
MSTRG.17395.2	MSTRG.17395	Scn2a
MSTRG.17398.1	MSTRG.17398	Ttc21b
MSTRG.174.1	MSTRG.174	Kcnq5
MSTRG.17409.1	MSTRG.17409	Stk39
MSTRG.1741.11	MSTRG.1741	Cenpl

MSTRG.17410.1	MSTRG.17410	Stk39
MSTRG.17411.1	MSTRG.17411	Stk39
MSTRG.17412.1	MSTRG.17412	Stk39
MSTRG.17413.1	MSTRG.17413	Stk39
MSTRG.17414.1	MSTRG.17414	Stk39
MSTRG.17415.1	MSTRG.17415	Stk39
MSTRG.1742.1	MSTRG.1742	Cenpl
MSTRG.17421.1	MSTRG.17421	Cers6
MSTRG.17421.2	MSTRG.17421	Cers6
MSTRG.17429.1	MSTRG.17429	Ppig
MSTRG.17430.1	MSTRG.17430	Ppig
MSTRG.17432.1	MSTRG.17432	Ppig
MSTRG.17437.1	MSTRG.17437	Mettl5
MSTRG.17439.1	MSTRG.17439	Mettl5
MSTRG.17442.1	MSTRG.17442	Ubr3
MSTRG.17444.1	MSTRG.17444	Ubr3
MSTRG.17445.1	MSTRG.17445	Ubr3
MSTRG.17448.1	MSTRG.17448	Myo3b
MSTRG.17451.1	MSTRG.17451	Gorasp2
MSTRG.17453.1	MSTRG.17453	Tlk1
MSTRG.17453.2	MSTRG.17453	Tlk1
MSTRG.17456.1	MSTRG.17456	Mettl8
MSTRG.17457.1	MSTRG.17457	Mettl8
MSTRG.1746.1	MSTRG.1746	Gm37052
MSTRG.17460.1	MSTRG.17460	Dcaf17
MSTRG.17465.1	MSTRG.17465	Slc25a12
MSTRG.17473.1	MSTRG.17473	.
MSTRG.17474.1	MSTRG.17474	.
MSTRG.17480.1	MSTRG.17480	Itga6
MSTRG.17481.1	MSTRG.17481	Itga6
MSTRG.17482.1	MSTRG.17482	Itga6
MSTRG.17486.1	MSTRG.17486	Map3k20
MSTRG.17487.1	MSTRG.17487	Map3k20
MSTRG.17491.1	MSTRG.17491	Rapgef4
MSTRG.17492.1	MSTRG.17492	Rapgef4
MSTRG.17493.1	MSTRG.17493	Rapgef4
MSTRG.17494.1	MSTRG.17494	Rapgef4
MSTRG.17495.1	MSTRG.17495	Rapgef4
MSTRG.17496.1	MSTRG.17496	Rapgef4
MSTRG.17498.1	MSTRG.17498	Rapgef4
MSTRG.17500.1	MSTRG.17500	Rapgef4
MSTRG.17504.1	MSTRG.17504	Sp3
MSTRG.17508.1	MSTRG.17508	Gm13666
MSTRG.17511.1	MSTRG.17511	Ola1
MSTRG.17516.12	MSTRG.17516	Wipf1
MSTRG.17516.8	MSTRG.17516	Wipf1
MSTRG.17517.2	MSTRG.17517	Gm13708
MSTRG.17517.3	MSTRG.17517	Gm13708
MSTRG.17522.1	MSTRG.17522	Gm10822
MSTRG.17525.1	MSTRG.17525	.
MSTRG.17527.1	MSTRG.17527	Lnpk
MSTRG.17528.1	MSTRG.17528	Lnpk
MSTRG.17529.1	MSTRG.17529	Lnpk
MSTRG.17537.1	MSTRG.17537	.
MSTRG.17538.1	MSTRG.17538	.
MSTRG.17541.1	MSTRG.17541	Nfe2l2
MSTRG.17547.1	MSTRG.17547	Pde11a

MSTRG.17548.1	MSTRG.17548	Pde11a
MSTRG.17549.1	MSTRG.17549	Pde11a
MSTRG.1755.18	MSTRG.1755	Gas5
MSTRG.17553.1	MSTRG.17553	.
MSTRG.17555.1	MSTRG.17555	Osbpl6
MSTRG.17563.1	MSTRG.17563	Sestd1
MSTRG.17565.1	MSTRG.17565	Cwc22
MSTRG.17566.1	MSTRG.17566	Cwc22
MSTRG.17567.1	MSTRG.17567	.
MSTRG.17568.5	MSTRG.17568	Ube2e3
MSTRG.17569.1	MSTRG.17569	.
MSTRG.17572.1	MSTRG.17572	Ssfa2
MSTRG.17573.1	MSTRG.17573	.
MSTRG.17574.1	MSTRG.17574	.
MSTRG.17575.1	MSTRG.17575	.
MSTRG.17576.1	MSTRG.17576	.
MSTRG.17577.1	MSTRG.17577	.
MSTRG.17578.1	MSTRG.17578	.
MSTRG.1758.1	MSTRG.1758	.
MSTRG.17581.1	MSTRG.17581	Itga4
MSTRG.17590.1	MSTRG.17590	Nckap1
MSTRG.17594.1	MSTRG.17594	Nup35
MSTRG.17597.1	MSTRG.17597	.
MSTRG.17598.1	MSTRG.17598	.
MSTRG.17600.1	MSTRG.17600	.
MSTRG.17602.1	MSTRG.17602	Zc3h15
MSTRG.17603.1	MSTRG.17603	.
MSTRG.17604.1	MSTRG.17604	.
MSTRG.17610.1	MSTRG.17610	Calcrl
MSTRG.17611.1	MSTRG.17611	Calcrl
MSTRG.17612.1	MSTRG.17612	Calcrl
MSTRG.17613.1	MSTRG.17613	.
MSTRG.17615.1	MSTRG.17615	Gm13710
MSTRG.17616.1	MSTRG.17616	.
MSTRG.17617.1	MSTRG.17617	.
MSTRG.17637.1	MSTRG.17637	Tnks1bp1
MSTRG.17639.1	MSTRG.17639	.
MSTRG.17640.1	MSTRG.17640	.
MSTRG.17641.1	MSTRG.17641	Olfr1033
MSTRG.17642.1	MSTRG.17642	.
MSTRG.17645.1	MSTRG.17645	.
MSTRG.17646.1	MSTRG.17646	.
MSTRG.17647.1	MSTRG.17647	.
MSTRG.17648.1	MSTRG.17648	.
MSTRG.17649.1	MSTRG.17649	.
MSTRG.17650.1	MSTRG.17650	.
MSTRG.17651.1	MSTRG.17651	.
MSTRG.17654.1	MSTRG.17654	Ptprj
MSTRG.17658.6	MSTRG.17658	Mtch2
MSTRG.17666.1	MSTRG.17666	Celf1
MSTRG.17667.1	MSTRG.17667	Celf1
MSTRG.17673.1	MSTRG.17673	Gm13778
MSTRG.17674.1	MSTRG.17674	Gm13778
MSTRG.1769.1	MSTRG.1769	Dnm3
MSTRG.17698.1	MSTRG.17698	Ambra1
MSTRG.17700.1	MSTRG.17700	Ambra1
MSTRG.17701.1	MSTRG.17701	Ambra1

MSTRG.17702.1	MSTRG.17702	Ambra1
MSTRG.17703.1	MSTRG.17703	Ambra1
MSTRG.17703.2	MSTRG.17703	Ambra1
MSTRG.17704.1	MSTRG.17704	Ambra1
MSTRG.17705.1	MSTRG.17705	Ambra1
MSTRG.17709.1	MSTRG.17709	Dgkz
MSTRG.17716.1	MSTRG.17716	Prdm11
MSTRG.17721.1	MSTRG.17721	Phf21a
MSTRG.1773.1	MSTRG.1773	Vamp4
MSTRG.17732.1	MSTRG.17732	D930015M05Rik
MSTRG.17733.1	MSTRG.17733	Cry2
MSTRG.17741.1	MSTRG.17741	.
MSTRG.17746.1	MSTRG.17746	Hsd17b12
MSTRG.17747.1	MSTRG.17747	Hsd17b12
MSTRG.17748.9	MSTRG.17748	Alkbh3
MSTRG.1775.1	MSTRG.1775	Fmo1
MSTRG.17750.1	MSTRG.17750	Alkbh3
MSTRG.17755.1	MSTRG.17755	.
MSTRG.17756.1	MSTRG.17756	.
MSTRG.17757.1	MSTRG.17757	.
MSTRG.17761.1	MSTRG.17761	Ttc17
MSTRG.17763.1	MSTRG.17763	Ttc17
MSTRG.17765.3	MSTRG.17765	Api5
MSTRG.17766.1	MSTRG.17766	.
MSTRG.17767.1	MSTRG.17767	.
MSTRG.17769.1	MSTRG.17769	Gm10800
MSTRG.17769.2	MSTRG.17769	Gm10800
MSTRG.17770.1	MSTRG.17770	.
MSTRG.17771.1	MSTRG.17771	.
MSTRG.17775.1	MSTRG.17775	B230118H07Rik
MSTRG.17776.1	MSTRG.17776	B230118H07Rik
MSTRG.17778.1	MSTRG.17778	.
MSTRG.17782.1	MSTRG.17782	Commd9
MSTRG.17786.1	MSTRG.17786	Ldlrad3
MSTRG.17786.2	MSTRG.17786	Gm13919
MSTRG.17786.5	MSTRG.17786	Gm13919
MSTRG.17788.1	MSTRG.17788	Ldlrad3
MSTRG.17789.1	MSTRG.17789	Ldlrad3
MSTRG.17790.1	MSTRG.17790	Ldlrad3
MSTRG.17792.1	MSTRG.17792	Ldlrad3
MSTRG.178.1	MSTRG.178	Smap1
MSTRG.17801.2	MSTRG.17801	Abtb2
MSTRG.17802.1	MSTRG.17802	Abtb2
MSTRG.17803.1	MSTRG.17803	Abtb2
MSTRG.17804.1	MSTRG.17804	Abtb2
MSTRG.17804.2	MSTRG.17804	Abtb2
MSTRG.17805.1	MSTRG.17805	.
MSTRG.17812.1	MSTRG.17812	Caprin1
MSTRG.17816.1	MSTRG.17816	D430041D05Rik
MSTRG.17817.1	MSTRG.17817	D430041D05Rik
MSTRG.17820.1	MSTRG.17820	D430041D05Rik
MSTRG.17821.1	MSTRG.17821	D430041D05Rik
MSTRG.17822.1	MSTRG.17822	D430041D05Rik
MSTRG.17824.1	MSTRG.17824	D430041D05Rik
MSTRG.17825.1	MSTRG.17825	D430041D05Rik
MSTRG.17826.1	MSTRG.17826	D430041D05Rik
MSTRG.17827.1	MSTRG.17827	D430041D05Rik

MSTRG.17832.1	MSTRG.17832	.
MSTRG.17834.1	MSTRG.17834	Cstf3
MSTRG.17836.1	MSTRG.17836	Cstf3
MSTRG.17838.1	MSTRG.17838	Tcp11l1
MSTRG.1784.1	MSTRG.1784	Prrx1
MSTRG.17846.1	MSTRG.17846	.
MSTRG.17849.1	MSTRG.17849	Pax6os1
MSTRG.17854.1	MSTRG.17854	Elp4
MSTRG.17855.1	MSTRG.17855	Elp4
MSTRG.17856.1	MSTRG.17856	Elp4
MSTRG.17857.1	MSTRG.17857	Elp4
MSTRG.17858.1	MSTRG.17858	Elp4
MSTRG.17859.1	MSTRG.17859	Elp4
MSTRG.17862.1	MSTRG.17862	Dnajc24
MSTRG.17864.1	MSTRG.17864	Dnajc24
MSTRG.17866.1	MSTRG.17866	Dnajc24
MSTRG.17867.1	MSTRG.17867	Dnajc24
MSTRG.17874.1	MSTRG.17874	Mettl15
MSTRG.17875.1	MSTRG.17875	Mettl15
MSTRG.17876.1	MSTRG.17876	Mettl15
MSTRG.17878.1	MSTRG.17878	Kif18a
MSTRG.17882.6	MSTRG.17882	Ccdc34
MSTRG.17885.1	MSTRG.17885	.
MSTRG.17891.1	MSTRG.17891	Gm21985
MSTRG.17892.1	MSTRG.17892	Gm21985
MSTRG.17894.1	MSTRG.17894	Gm21985
MSTRG.17895.1	MSTRG.17895	Gm21985
MSTRG.17896.1	MSTRG.17896	Gm13940
MSTRG.17897.1	MSTRG.17897	Gm21985
MSTRG.17898.1	MSTRG.17898	Gm21985
MSTRG.179.1	MSTRG.179	Smap1
MSTRG.1790.1	MSTRG.1790	.
MSTRG.17905.1	MSTRG.17905	Fmn1
MSTRG.17908.1	MSTRG.17908	Aven
MSTRG.17910.1	MSTRG.17910	Aven
MSTRG.17911.1	MSTRG.17911	Aven
MSTRG.17912.1	MSTRG.17912	Aven
MSTRG.17913.1	MSTRG.17913	Aven
MSTRG.17914.1	MSTRG.17914	Aven
MSTRG.17915.1	MSTRG.17915	Aven
MSTRG.17919.1	MSTRG.17919	Scg5
MSTRG.17926.1	MSTRG.17926	Dph6
MSTRG.17930.1	MSTRG.17930	BC052040
MSTRG.17932.1	MSTRG.17932	BC052040
MSTRG.17933.1	MSTRG.17933	BC052040
MSTRG.17934.1	MSTRG.17934	BC052040
MSTRG.17935.1	MSTRG.17935	BC052040
MSTRG.17938.1	MSTRG.17938	G630016G05Rik
MSTRG.1794.1	MSTRG.1794	Scyl3
MSTRG.17941.1	MSTRG.17941	Spred1
MSTRG.17942.1	MSTRG.17942	Spred1
MSTRG.17946.1	MSTRG.17946	Meis2
MSTRG.17947.1	MSTRG.17947	Meis2
MSTRG.17950.1	MSTRG.17950	.
MSTRG.17951.1	MSTRG.17951	.
MSTRG.17951.2	MSTRG.17951	.
MSTRG.17951.3	MSTRG.17951	.

MSTRG.17952.1	MSTRG.17952	.
MSTRG.17957.1	MSTRG.17957	.
MSTRG.17959.1	MSTRG.17959	Eif2ak4
MSTRG.1796.1	MSTRG.1796	BC055324
MSTRG.17960.1	MSTRG.17960	Eif2ak4
MSTRG.17961.1	MSTRG.17961	Eif2ak4
MSTRG.17962.1	MSTRG.17962	Eif2ak4
MSTRG.17963.1	MSTRG.17963	Eif2ak4
MSTRG.17964.1	MSTRG.17964	Eif2ak4
MSTRG.17967.1	MSTRG.17967	Bmf
MSTRG.17969.1	MSTRG.17969	Bub1b
MSTRG.1797.1	MSTRG.1797	BC055324
MSTRG.17970.1	MSTRG.17970	Inafm2
MSTRG.17972.4	MSTRG.17972	Knstrn
MSTRG.17973.1	MSTRG.17973	Knstrn
MSTRG.17978.1	MSTRG.17978	.
MSTRG.17979.1	MSTRG.17979	.
MSTRG.17983.1	MSTRG.17983	Knl1
MSTRG.17984.1	MSTRG.17984	Knl1
MSTRG.17986.1	MSTRG.17986	Rad51
MSTRG.17997.1	MSTRG.17997	Dnajc17
MSTRG.180.1	MSTRG.180	.
MSTRG.18000.1	MSTRG.18000	Ino80
MSTRG.18001.1	MSTRG.18001	Ino80
MSTRG.18002.1	MSTRG.18002	Ino80
MSTRG.18003.1	MSTRG.18003	Ino80
MSTRG.18009.1	MSTRG.18009	Chp1
MSTRG.18010.1	MSTRG.18010	Chp1
MSTRG.18015.1	MSTRG.18015	Nusap1
MSTRG.18021.1	MSTRG.18021	Mga
MSTRG.18021.2	MSTRG.18021	Mga
MSTRG.18022.1	MSTRG.18022	Mga
MSTRG.18025.1	MSTRG.18025	Mapkbp1
MSTRG.18026.1	MSTRG.18026	Mapkbp1
MSTRG.18035.1	MSTRG.18035	Snap23
MSTRG.18036.1	MSTRG.18036	Lrrc57
MSTRG.18038.1	MSTRG.18038	Haus2
MSTRG.18041.1	MSTRG.18041	Tmem62
MSTRG.18044.1	MSTRG.18044	Tmem87a
MSTRG.18046.1	MSTRG.18046	Ganc
MSTRG.18047.1	MSTRG.18047	Ganc
MSTRG.18048.1	MSTRG.18048	Capn3
MSTRG.18051.1	MSTRG.18051	Zfp106
MSTRG.18052.1	MSTRG.18052	Zfp106
MSTRG.18053.4	MSTRG.18053	Ccndbp1
MSTRG.18057.1	MSTRG.18057	Cdan1
MSTRG.18058.1	MSTRG.18058	Cdan1
MSTRG.18059.1	MSTRG.18059	Cdan1
MSTRG.18062.1	MSTRG.18062	Ubr1
MSTRG.18063.1	MSTRG.18063	Ubr1
MSTRG.18064.1	MSTRG.18064	Ubr1
MSTRG.18065.1	MSTRG.18065	Ubr1
MSTRG.18069.1	MSTRG.18069	Adal
MSTRG.1807.1	MSTRG.1807	.
MSTRG.1807.2	MSTRG.1807	.
MSTRG.18077.1	MSTRG.18077	Ppip5k1
MSTRG.1808.1	MSTRG.1808	.

MSTRG.18086.5	MSTRG.18086	Serf2
MSTRG.18089.1	MSTRG.18089	Mfap1b
MSTRG.181.1	MSTRG.181	.
MSTRG.18100.1	MSTRG.18100	Casc4
MSTRG.18101.1	MSTRG.18101	Casc4
MSTRG.18102.1	MSTRG.18102	Casc4
MSTRG.18103.1	MSTRG.18103	Mageb3
MSTRG.18105.1	MSTRG.18105	Casc4
MSTRG.18106.1	MSTRG.18106	Casc4
MSTRG.18107.1	MSTRG.18107	Mageb3
MSTRG.18107.2	MSTRG.18107	Ctdspl2
MSTRG.18108.1	MSTRG.18108	Casc4
MSTRG.1811.1	MSTRG.1811	Nme7
MSTRG.18111.1	MSTRG.18111	Ctdspl2
MSTRG.18112.1	MSTRG.18112	Ctdspl2
MSTRG.18113.1	MSTRG.18113	Ctdspl2
MSTRG.18114.1	MSTRG.18114	.
MSTRG.18116.1	MSTRG.18116	Eif3j1
MSTRG.18118.1	MSTRG.18118	Spg11
MSTRG.18119.1	MSTRG.18119	.
MSTRG.1812.1	MSTRG.1812	Nme7
MSTRG.18129.1	MSTRG.18129	Bambi-ps1
MSTRG.1813.1	MSTRG.1813	Nme7
MSTRG.18130.1	MSTRG.18130	Bambi-ps1
MSTRG.18139.1	MSTRG.18139	Sema6d
MSTRG.18140.2	MSTRG.18140	Dut
MSTRG.18147.1	MSTRG.18147	Cep152
MSTRG.18151.1	MSTRG.18151	Cops2
MSTRG.18153.1	MSTRG.18153	Cops2
MSTRG.18156.1	MSTRG.18156	Fam227b
MSTRG.18161.1	MSTRG.18161	Atp8b4
MSTRG.18162.1	MSTRG.18162	Atp8b4
MSTRG.18166.1	MSTRG.18166	Gm27003
MSTRG.18167.1	MSTRG.18167	Gabpb1
MSTRG.18170.1	MSTRG.18170	Usp50
MSTRG.18175.1	MSTRG.18175	Trpm7
MSTRG.18177.1	MSTRG.18177	Ap4e1
MSTRG.18179.1	MSTRG.18179	Ap4e1
MSTRG.18189.1	MSTRG.18189	.
MSTRG.18190.1	MSTRG.18190	.
MSTRG.18194.1	MSTRG.18194	Kcnip3
MSTRG.18203.1	MSTRG.18203	Acoxl
MSTRG.18205.7	MSTRG.18205	Bcl2l11
MSTRG.18205.8	MSTRG.18205	Bcl2l11
MSTRG.18208.1	MSTRG.18208	Gm14005
MSTRG.18215.1	MSTRG.18215	Anapc1
MSTRG.18216.1	MSTRG.18216	Anapc1
MSTRG.18219.1	MSTRG.18219	Zc3h8
MSTRG.18222.1	MSTRG.18222	Zc3h6
MSTRG.18223.1	MSTRG.18223	Zc3h6
MSTRG.18230.1	MSTRG.18230	AI847159
MSTRG.18232.1	MSTRG.18232	.
MSTRG.18255.1	MSTRG.18255	Vps16
MSTRG.18258.1	MSTRG.18258	Ptpra
MSTRG.18259.1	MSTRG.18259	Ptpra
MSTRG.18259.2	MSTRG.18259	Ptpra
MSTRG.18260.1	MSTRG.18260	Ptpra

MSTRG.18263.1	MSTRG.18263	4930473A02Rik
MSTRG.18264.1	MSTRG.18264	4930473A02Rik
MSTRG.18265.1	MSTRG.18265	4930473A02Rik
MSTRG.18268.1	MSTRG.18268	Ubox5
MSTRG.18269.1	MSTRG.18269	Ubox5
MSTRG.1827.1	MSTRG.1827	Dcaf6
MSTRG.18270.1	MSTRG.18270	Ubox5
MSTRG.18271.1	MSTRG.18271	Ubox5
MSTRG.18275.1	MSTRG.18275	Itpa
MSTRG.18280.1	MSTRG.18280	4930402H24Rik
MSTRG.18281.1	MSTRG.18281	4930402H24Rik
MSTRG.18282.1	MSTRG.18282	4930402H24Rik
MSTRG.18283.1	MSTRG.18283	4930402H24Rik
MSTRG.18285.4	MSTRG.18285	Atrn
MSTRG.18286.1	MSTRG.18286	Atrn
MSTRG.18288.1	MSTRG.18288	Atrn
MSTRG.18295.4	MSTRG.18295	Cdc25b
MSTRG.18298.1	MSTRG.18298	Pank2
MSTRG.18299.1	MSTRG.18299	Pank2
MSTRG.183.1	MSTRG.183	Lmbrd1
MSTRG.1830.1	MSTRG.1830	Dcaf6
MSTRG.18307.1	MSTRG.18307	Slc23a2
MSTRG.18309.4	MSTRG.18309	Tmem230
MSTRG.18309.5	MSTRG.18309	Tmem230
MSTRG.18313.1	MSTRG.18313	AV099323
MSTRG.18319.1	MSTRG.18319	Gpcpd1
MSTRG.1832.1	MSTRG.1832	Dcaf6
MSTRG.18321.1	MSTRG.18321	1110034G24Rik
MSTRG.18324.1	MSTRG.18324	1110034G24Rik
MSTRG.18327.1	MSTRG.18327	.
MSTRG.18331.1	MSTRG.18331	Crls1
MSTRG.18334.1	MSTRG.18334	Fermt1
MSTRG.18335.1	MSTRG.18335	.
MSTRG.18336.1	MSTRG.18336	.
MSTRG.18337.1	MSTRG.18337	.
MSTRG.18344.1	MSTRG.18344	Plcb4
MSTRG.18345.1	MSTRG.18345	Plcb4
MSTRG.18348.1	MSTRG.18348	Plcb4
MSTRG.18349.1	MSTRG.18349	Plcb4
MSTRG.18352.1	MSTRG.18352	Plcb4
MSTRG.18354.1	MSTRG.18354	.
MSTRG.18355.1	MSTRG.18355	.
MSTRG.18356.4	MSTRG.18356	Mkks
MSTRG.18358.1	MSTRG.18358	Slx4ip
MSTRG.18359.1	MSTRG.18359	Slx4ip
MSTRG.18360.1	MSTRG.18360	Slx4ip
MSTRG.18361.1	MSTRG.18361	Slx4ip
MSTRG.18362.1	MSTRG.18362	Slx4ip
MSTRG.18363.1	MSTRG.18363	Slx4ip
MSTRG.18364.1	MSTRG.18364	Slx4ip
MSTRG.18365.1	MSTRG.18365	.
MSTRG.18366.1	MSTRG.18366	.
MSTRG.18368.1	MSTRG.18368	Btbd3
MSTRG.18372.1	MSTRG.18372	Tasp1
MSTRG.18373.1	MSTRG.18373	Tasp1
MSTRG.18374.1	MSTRG.18374	Tasp1
MSTRG.18375.1	MSTRG.18375	Tasp1

MSTRG.18380.1	MSTRG.18380	Esf1
MSTRG.18382.1	MSTRG.18382	Ndufaf5
MSTRG.18383.1	MSTRG.18383	Ndufaf5
MSTRG.18386.1	MSTRG.18386	Macrod2
MSTRG.18393.1	MSTRG.18393	Macrod2
MSTRG.184.1	MSTRG.184	Lmbrd1
MSTRG.1840.1	MSTRG.1840	.
MSTRG.18400.1	MSTRG.18400	Kif16b
MSTRG.18401.1	MSTRG.18401	Kif16b
MSTRG.18402.1	MSTRG.18402	Kif16b
MSTRG.18407.1	MSTRG.18407	Pcsk2
MSTRG.1841.1	MSTRG.1841	.
MSTRG.18416.1	MSTRG.18416	Mgme1
MSTRG.18417.1	MSTRG.18417	.
MSTRG.18418.1	MSTRG.18418	.
MSTRG.18427.1	MSTRG.18427	Dtd1
MSTRG.18428.1	MSTRG.18428	Dtd1
MSTRG.18429.1	MSTRG.18429	Dtd1
MSTRG.18431.1	MSTRG.18431	.
MSTRG.18438.1	MSTRG.18438	Slc24a3
MSTRG.18446.1	MSTRG.18446	Kiz
MSTRG.18447.1	MSTRG.18447	Kiz
MSTRG.18448.1	MSTRG.18448	Kiz
MSTRG.18449.1	MSTRG.18449	Kiz
MSTRG.18450.1	MSTRG.18450	Kiz
MSTRG.18451.1	MSTRG.18451	Kiz
MSTRG.18453.1	MSTRG.18453	Ralgapa2
MSTRG.18455.1	MSTRG.18455	Ralgapa2
MSTRG.18456.1	MSTRG.18456	Ralgapa2
MSTRG.18457.1	MSTRG.18457	Ralgapa2
MSTRG.18458.1	MSTRG.18458	Ralgapa2
MSTRG.18459.1	MSTRG.18459	Ralgapa2
MSTRG.18461.1	MSTRG.18461	Ralgapa2
MSTRG.18462.1	MSTRG.18462	Ralgapa2
MSTRG.18464.1	MSTRG.18464	.
MSTRG.18468.1	MSTRG.18468	.
MSTRG.1847.1	MSTRG.1847	Fam78b
MSTRG.18478.1	MSTRG.18478	Zfp120
MSTRG.18481.1	MSTRG.18481	Zfp120
MSTRG.18482.1	MSTRG.18482	Zfp120
MSTRG.18485.1	MSTRG.18485	.
MSTRG.18486.3	MSTRG.18486	3300002I08Rik
MSTRG.18488.1	MSTRG.18488	3300002I08Rik
MSTRG.18497.1	MSTRG.18497	Pygb
MSTRG.18499.8	MSTRG.18499	Abhd12
MSTRG.185.1	MSTRG.185	Lmbrd1
MSTRG.1850.1	MSTRG.1850	Gm36972
MSTRG.1850.10	MSTRG.1850	Gm37856
MSTRG.1850.11	MSTRG.1850	Gm37856
MSTRG.1850.3	MSTRG.1850	Gm37856
MSTRG.1850.4	MSTRG.1850	Gm23402
MSTRG.1850.5	MSTRG.1850	Gm37856
MSTRG.1850.7	MSTRG.1850	Cd247
MSTRG.1850.8	MSTRG.1850	Cd247
MSTRG.1850.9	MSTRG.1850	Cd247
MSTRG.18500.1	MSTRG.18500	Abhd12
MSTRG.18505.1	MSTRG.18505	Gm14149

MSTRG.18506.1	MSTRG.18506	.
MSTRG.18509.12	MSTRG.18509	Ninl
MSTRG.18521.1	MSTRG.18521	.
MSTRG.18526.1	MSTRG.18526	Csnk2a1
MSTRG.1854.12	MSTRG.1854	Cd247
MSTRG.1854.8	MSTRG.1854	Cd247
MSTRG.18540.1	MSTRG.18540	Gm26841
MSTRG.18541.1	MSTRG.18541	H13
MSTRG.18543.1	MSTRG.18543	Tpx2
MSTRG.18550.1	MSTRG.18550	Tm9sf4
MSTRG.18553.1	MSTRG.18553	Plagl2
MSTRG.18556.1	MSTRG.18556	Kif3b
MSTRG.18557.1	MSTRG.18557	Kif3b
MSTRG.18563.1	MSTRG.18563	Nol4l
MSTRG.18565.1	MSTRG.18565	Commd7
MSTRG.18573.1	MSTRG.18573	Cdk5rap1
MSTRG.18574.1	MSTRG.18574	Cdk5rap1
MSTRG.18575.1	MSTRG.18575	Cdk5rap1
MSTRG.18576.1	MSTRG.18576	Cbfa2t2
MSTRG.18578.1	MSTRG.18578	Cbfa2t2
MSTRG.18579.1	MSTRG.18579	Cbfa2t2
MSTRG.1858.1	MSTRG.1858	Pou2f1
MSTRG.18584.1	MSTRG.18584	Chmp4b
MSTRG.18585.1	MSTRG.18585	Chmp4b
MSTRG.1859.1	MSTRG.1859	Pou2f1
MSTRG.18593.1	MSTRG.18593	.
MSTRG.18595.1	MSTRG.18595	Raly
MSTRG.18595.2	MSTRG.18595	Raly
MSTRG.18597.1	MSTRG.18597	Raly
MSTRG.18597.2	MSTRG.18597	a
MSTRG.18599.1	MSTRG.18599	Raly
MSTRG.186.1	MSTRG.186	Lmbrd1
MSTRG.1860.1	MSTRG.1860	Pou2f1
MSTRG.18600.1	MSTRG.18600	Raly
MSTRG.18603.1	MSTRG.18603	Eif2s2
MSTRG.18604.1	MSTRG.18604	a
MSTRG.18606.1	MSTRG.18606	.
MSTRG.18609.1	MSTRG.18609	Pigu
MSTRG.1861.1	MSTRG.1861	Pou2f1
MSTRG.18612.1	MSTRG.18612	Itch
MSTRG.18613.1	MSTRG.18613	Itch
MSTRG.18615.2	MSTRG.18615	Dynlrb1
MSTRG.18618.1	MSTRG.18618	Ncoa6
MSTRG.18619.1	MSTRG.18619	Ncoa6
MSTRG.1862.1	MSTRG.1862	Pou2f1
MSTRG.18620.1	MSTRG.18620	Ncoa6
MSTRG.18620.2	MSTRG.18620	Ncoa6
MSTRG.18622.1	MSTRG.18622	Ncoa6
MSTRG.18623.1	MSTRG.18623	Ncoa6
MSTRG.1863.1	MSTRG.1863	.
MSTRG.18633.1	MSTRG.18633	Edem2
MSTRG.18641.1	MSTRG.18641	Trpc4ap
MSTRG.18641.2	MSTRG.18641	Trpc4ap
MSTRG.18641.3	MSTRG.18641	Trpc4ap
MSTRG.18641.4	MSTRG.18641	Trpc4ap
MSTRG.18642.1	MSTRG.18642	Trpc4ap
MSTRG.18642.2	MSTRG.18642	Trpc4ap

MSTRG.18643.1	MSTRG.18643	Trpc4ap
MSTRG.18645.1	MSTRG.18645	Uqcc1
MSTRG.18646.1	MSTRG.18646	Uqcc1
MSTRG.18647.1	MSTRG.18647	Uqcc1
MSTRG.18648.1	MSTRG.18648	Uqcc1
MSTRG.1865.3	MSTRG.1865	Uck2
MSTRG.1865.6	MSTRG.1865	Uck2
MSTRG.18651.1	MSTRG.18651	6430550D23Rik
MSTRG.18654.1	MSTRG.18654	2900097C17Rik
MSTRG.18654.2	MSTRG.18654	2900097C17Rik
MSTRG.18657.1	MSTRG.18657	Phf20
MSTRG.18659.1	MSTRG.18659	Phf20
MSTRG.1866.1	MSTRG.1866	Gm37982
MSTRG.1866.3	MSTRG.1866	Gm37982
MSTRG.18660.1	MSTRG.18660	Phf20
MSTRG.18661.1	MSTRG.18661	Phf20
MSTRG.18662.1	MSTRG.18662	Phf20
MSTRG.18674.1	MSTRG.18674	Epb41l1
MSTRG.18679.1	MSTRG.18679	Cpne1
MSTRG.1868.1	MSTRG.1868	Uck2
MSTRG.18681.10	MSTRG.18681	Rbm39
MSTRG.18681.14	MSTRG.18681	Rbm39
MSTRG.18681.17	MSTRG.18681	Rbm39
MSTRG.18681.18	MSTRG.18681	Rbm39
MSTRG.18681.26	MSTRG.18681	Rbm39
MSTRG.18691.1	MSTRG.18691	5430405H02Rik
MSTRG.18692.1	MSTRG.18692	5430405H02Rik
MSTRG.18694.1	MSTRG.18694	5430405H02Rik
MSTRG.18696.2	MSTRG.18696	Sla2
MSTRG.18696.5	MSTRG.18696	Sla2
MSTRG.187.1	MSTRG.187	Lmbrd1
MSTRG.18701.4	MSTRG.18701	Dsn1
MSTRG.18705.1	MSTRG.18705	Manbal
MSTRG.18709.1	MSTRG.18709	Rbl1
MSTRG.18711.1	MSTRG.18711	Rbl1
MSTRG.18712.1	MSTRG.18712	Rbl1
MSTRG.1872.1	MSTRG.1872	.
MSTRG.18722.1	MSTRG.18722	Ctnnbl1
MSTRG.18723.1	MSTRG.18723	Snhg17
MSTRG.18723.7	MSTRG.18723	Snhg17
MSTRG.18729.10	MSTRG.18729	Snhg11
MSTRG.18729.2	MSTRG.18729	Snhg11
MSTRG.18729.4	MSTRG.18729	Snhg11
MSTRG.18729.6	MSTRG.18729	Snhg11
MSTRG.18733.1	MSTRG.18733	Ralgapb
MSTRG.18737.1	MSTRG.18737	Ppp1r16b
MSTRG.18739.1	MSTRG.18739	.
MSTRG.18742.1	MSTRG.18742	Dhx35
MSTRG.18744.1	MSTRG.18744	.
MSTRG.18745.1	MSTRG.18745	.
MSTRG.18749.1	MSTRG.18749	Chd6
MSTRG.1875.1	MSTRG.1875	Lmx1a
MSTRG.18750.1	MSTRG.18750	Chd6
MSTRG.18751.1	MSTRG.18751	Chd6
MSTRG.18754.1	MSTRG.18754	Zhx3
MSTRG.18754.2	MSTRG.18754	Gm27206
MSTRG.18755.1	MSTRG.18755	Zhx3

MSTRG.18756.1	MSTRG.18756	Zhx3
MSTRG.18759.1	MSTRG.18759	Zhx3
MSTRG.18766.5	MSTRG.18766	Oser1
MSTRG.18768.1	MSTRG.18768	Oser1
MSTRG.18772.4	MSTRG.18772	Pkig
MSTRG.18773.1	MSTRG.18773	Pkig
MSTRG.18775.1	MSTRG.18775	Pkig
MSTRG.18776.1	MSTRG.18776	Pkig
MSTRG.18779.1	MSTRG.18779	Slpi
MSTRG.18779.3	MSTRG.18779	Slpi
MSTRG.1878.1	MSTRG.1878	Pbx1
MSTRG.18784.1	MSTRG.18784	Stk4
MSTRG.18784.2	MSTRG.18784	Stk4
MSTRG.18784.3	MSTRG.18784	Stk4
MSTRG.18784.4	MSTRG.18784	Stk4
MSTRG.18784.5	MSTRG.18784	Stk4
MSTRG.18784.6	MSTRG.18784	Stk4
MSTRG.18784.7	MSTRG.18784	Stk4
MSTRG.1879.1	MSTRG.1879	Pbx1
MSTRG.18792.4	MSTRG.18792	Gm11457
MSTRG.188.1	MSTRG.188	Lmbrd1
MSTRG.1880.1	MSTRG.1880	Pbx1
MSTRG.18801.1	MSTRG.18801	Neurl2
MSTRG.18804.1	MSTRG.18804	Pltp
MSTRG.18805.1	MSTRG.18805	.
MSTRG.18808.1	MSTRG.18808	Cd40
MSTRG.18809.1	MSTRG.18809	.
MSTRG.1881.1	MSTRG.1881	Pbx1
MSTRG.18814.1	MSTRG.18814	Slc12a5
MSTRG.18819.1	MSTRG.18819	Elmo2
MSTRG.1882.1	MSTRG.1882	Pbx1
MSTRG.18820.1	MSTRG.18820	Elmo2
MSTRG.18822.1	MSTRG.18822	.
MSTRG.18824.1	MSTRG.18824	Eya2
MSTRG.18828.1	MSTRG.18828	Eya2
MSTRG.18829.1	MSTRG.18829	Eya2
MSTRG.18830.1	MSTRG.18830	Eya2
MSTRG.18831.1	MSTRG.18831	Eya2
MSTRG.18832.1	MSTRG.18832	Eya2
MSTRG.18833.1	MSTRG.18833	Eya2
MSTRG.18834.1	MSTRG.18834	Eya2
MSTRG.18836.1	MSTRG.18836	Zmynd8
MSTRG.1884.1	MSTRG.1884	Pbx1
MSTRG.18842.2	MSTRG.18842	Ncoa3
MSTRG.18843.1	MSTRG.18843	Ncoa3
MSTRG.18846.1	MSTRG.18846	Arfgef2
MSTRG.1885.1	MSTRG.1885	Pbx1
MSTRG.18853.1	MSTRG.18853	Stau1
MSTRG.18854.1	MSTRG.18854	Stau1
MSTRG.18855.1	MSTRG.18855	Stau1
MSTRG.18867.1	MSTRG.18867	Slc9a8
MSTRG.18871.1	MSTRG.18871	Gm11476
MSTRG.18871.2	MSTRG.18871	Gm11476
MSTRG.18875.1	MSTRG.18875	Gm11476
MSTRG.1888.1	MSTRG.1888	Pbx1
MSTRG.18885.1	MSTRG.18885	.
MSTRG.18886.1	MSTRG.18886	.

MSTRG.1889.1	MSTRG.1889	Pbx1
MSTRG.18890.1	MSTRG.18890	Nfatc2
MSTRG.18893.1	MSTRG.18893	Atp9a
MSTRG.18894.1	MSTRG.18894	Atp9a
MSTRG.18895.1	MSTRG.18895	Zfp64
MSTRG.18898.1	MSTRG.18898	Zfp64
MSTRG.18899.1	MSTRG.18899	Zfp64
MSTRG.189.1	MSTRG.189	.
MSTRG.18902.1	MSTRG.18902	Gm26883
MSTRG.18903.1	MSTRG.18903	Gm26883
MSTRG.18904.1	MSTRG.18904	Gm26883
MSTRG.18905.1	MSTRG.18905	Gm26883
MSTRG.18907.1	MSTRG.18907	Gm26883
MSTRG.18908.1	MSTRG.18908	Gm26883
MSTRG.18909.1	MSTRG.18909	Gm26883
MSTRG.1892.1	MSTRG.1892	Nuf2
MSTRG.18920.1	MSTRG.18920	.
MSTRG.18926.1	MSTRG.18926	.
MSTRG.18928.1	MSTRG.18928	.
MSTRG.18928.2	MSTRG.18928	.
MSTRG.18933.1	MSTRG.18933	Gm14453
MSTRG.18934.1	MSTRG.18934	.
MSTRG.18938.1	MSTRG.18938	.
MSTRG.18942.1	MSTRG.18942	Pmepa1
MSTRG.18943.1	MSTRG.18943	Pmepa1
MSTRG.18951.1	MSTRG.18951	Ppp4r1l-ps
MSTRG.18952.1	MSTRG.18952	Ppp4r1l-ps
MSTRG.18954.1	MSTRG.18954	Ppp4r1l-ps
MSTRG.18955.1	MSTRG.18955	Ppp4r1l-ps
MSTRG.18957.1	MSTRG.18957	Rab22a
MSTRG.18968.1	MSTRG.18968	.
MSTRG.18972.1	MSTRG.18972	Gnas
MSTRG.18973.1	MSTRG.18973	Nespas
MSTRG.18978.1	MSTRG.18978	.
MSTRG.18980.1	MSTRG.18980	.
MSTRG.18982.1	MSTRG.18982	.
MSTRG.18984.1	MSTRG.18984	Zfp831
MSTRG.18985.1	MSTRG.18985	Zfp831
MSTRG.18988.1	MSTRG.18988	Gm14401
MSTRG.19014.1	MSTRG.19014	Gm6710
MSTRG.19024.1	MSTRG.19024	.
MSTRG.19027.1	MSTRG.19027	.
MSTRG.19030.1	MSTRG.19030	.
MSTRG.19042.1	MSTRG.19042	.
MSTRG.19043.1	MSTRG.19043	.
MSTRG.19044.1	MSTRG.19044	.
MSTRG.19053.1	MSTRG.19053	.
MSTRG.19058.1	MSTRG.19058	Gm14326
MSTRG.19063.1	MSTRG.19063	.
MSTRG.19066.2	MSTRG.19066	Cdh4
MSTRG.19068.1	MSTRG.19068	Cdh4
MSTRG.1907.1	MSTRG.1907	Gm9929
MSTRG.19070.1	MSTRG.19070	Taf4
MSTRG.19074.1	MSTRG.19074	Osbpl2
MSTRG.19077.1	MSTRG.19077	Ss18l1
MSTRG.19078.2	MSTRG.19078	Mtg2
MSTRG.19092.4	MSTRG.19092	Ppdpf

MSTRG.19094.1	MSTRG.19094	Helz2
MSTRG.19099.1	MSTRG.19099	Gmeb2
MSTRG.191.1	MSTRG.191	.
MSTRG.19106.1	MSTRG.19106	Slc2a4rg-ps
MSTRG.19110.1	MSTRG.19110	Tpd52l2
MSTRG.19114.1	MSTRG.19114	Rtel1
MSTRG.19123.2	MSTRG.19123	Uckl1os
MSTRG.19128.1	MSTRG.19128	.
MSTRG.1913.1	MSTRG.1913	Gm27552
MSTRG.1913.2	MSTRG.1913	Gm27552
MSTRG.1913.3	MSTRG.1913	Gm27552
MSTRG.19130.1	MSTRG.19130	.
MSTRG.19132.1	MSTRG.19132	.
MSTRG.19133.1	MSTRG.19133	.
MSTRG.19134.1	MSTRG.19134	.
MSTRG.19135.1	MSTRG.19135	.
MSTRG.19137.1	MSTRG.19137	.
MSTRG.19138.1	MSTRG.19138	.
MSTRG.19140.1	MSTRG.19140	.
MSTRG.19143.1	MSTRG.19143	Pkia
MSTRG.19148.1	MSTRG.19148	Il7
MSTRG.19150.1	MSTRG.19150	Gm16685
MSTRG.19151.1	MSTRG.19151	Gm16685
MSTRG.19152.1	MSTRG.19152	.
MSTRG.19153.1	MSTRG.19153	.
MSTRG.19154.1	MSTRG.19154	.
MSTRG.19157.1	MSTRG.19157	C030034L19Rik
MSTRG.19158.1	MSTRG.19158	C030034L19Rik
MSTRG.19160.1	MSTRG.19160	Gm38001
MSTRG.19162.1	MSTRG.19162	Zfp704
MSTRG.19163.1	MSTRG.19163	Zfp704
MSTRG.19165.1	MSTRG.19165	Mrps28
MSTRG.19166.1	MSTRG.19166	Mrps28
MSTRG.19169.1	MSTRG.19169	Tpd52
MSTRG.19170.1	MSTRG.19170	Tpd52
MSTRG.19171.1	MSTRG.19171	Tpd52
MSTRG.19175.1	MSTRG.19175	Pag1
MSTRG.19176.1	MSTRG.19176	Pag1
MSTRG.19187.1	MSTRG.19187	Snx16
MSTRG.19188.1	MSTRG.19188	Snx16
MSTRG.19189.1	MSTRG.19189	.
MSTRG.19191.1	MSTRG.19191	Slc7a12
MSTRG.192.2	MSTRG.192	Gm6473
MSTRG.19207.1	MSTRG.19207	.
MSTRG.19209.1	MSTRG.19209	.
MSTRG.19212.1	MSTRG.19212	.
MSTRG.19215.1	MSTRG.19215	Cyp7b1
MSTRG.19216.1	MSTRG.19216	Armc1
MSTRG.19223.6	MSTRG.19223	Mtfr1
MSTRG.19223.9	MSTRG.19223	Mtfr1
MSTRG.19226.1	MSTRG.19226	Pde7a
MSTRG.19227.1	MSTRG.19227	Pde7a
MSTRG.19231.1	MSTRG.19231	Cp
MSTRG.19232.1	MSTRG.19232	Hps3
MSTRG.19233.1	MSTRG.19233	Hps3
MSTRG.19236.1	MSTRG.19236	.
MSTRG.19239.1	MSTRG.19239	.

MSTRG.19243.1	MSTRG.19243	.
MSTRG.19246.1	MSTRG.19246	Tbl1xr1
MSTRG.19247.1	MSTRG.19247	Tbl1xr1
MSTRG.19257.1	MSTRG.19257	Spata16
MSTRG.1926.1	MSTRG.1926	Ufc1
MSTRG.19261.1	MSTRG.19261	Nlgn1
MSTRG.19263.1	MSTRG.19263	Nlgn1
MSTRG.19264.1	MSTRG.19264	Nlgn1
MSTRG.19265.1	MSTRG.19265	Nlgn1
MSTRG.19266.1	MSTRG.19266	Nlgn1
MSTRG.19267.1	MSTRG.19267	Nlgn1
MSTRG.19268.1	MSTRG.19268	Nlgn1
MSTRG.19269.1	MSTRG.19269	Nlgn1
MSTRG.1927.1	MSTRG.1927	Ufc1
MSTRG.19270.1	MSTRG.19270	Nlgn1
MSTRG.19275.1	MSTRG.19275	Tnfsf10
MSTRG.19277.1	MSTRG.19277	Gm25152
MSTRG.19292.1	MSTRG.19292	.
MSTRG.19295.1	MSTRG.19295	Tnik
MSTRG.19295.2	MSTRG.19295	Tnik
MSTRG.19298.1	MSTRG.19298	Tnik
MSTRG.19298.2	MSTRG.19298	Tnik
MSTRG.19298.3	MSTRG.19298	Tnik
MSTRG.19300.1	MSTRG.19300	Tnik
MSTRG.19301.1	MSTRG.19301	Tnik
MSTRG.19303.1	MSTRG.19303	Tnik
MSTRG.19304.1	MSTRG.19304	Tnik
MSTRG.19305.1	MSTRG.19305	Tnik
MSTRG.19311.1	MSTRG.19311	.
MSTRG.19324.1	MSTRG.19324	Mfn1
MSTRG.19325.7	MSTRG.19325	Gnb4
MSTRG.19331.1	MSTRG.19331	.
MSTRG.19332.1	MSTRG.19332	.
MSTRG.19334.3	MSTRG.19334	Ccdc39
MSTRG.19336.1	MSTRG.19336	.
MSTRG.19339.1	MSTRG.19339	Fxr1
MSTRG.1934.1	MSTRG.1934	Dedd
MSTRG.19346.1	MSTRG.19346	Atp11b
MSTRG.19349.1	MSTRG.19349	Acad9
MSTRG.19350.1	MSTRG.19350	Acad9
MSTRG.19352.1	MSTRG.19352	Dcun1d1
MSTRG.1937.1	MSTRG.1937	Pfdn2
MSTRG.19373.1	MSTRG.19373	Bbs12
MSTRG.19379.9	MSTRG.19379	Spata5
MSTRG.1938.1	MSTRG.1938	Pfdn2
MSTRG.19380.1	MSTRG.19380	Spata5
MSTRG.19381.1	MSTRG.19381	Spata5
MSTRG.19382.1	MSTRG.19382	Spata5
MSTRG.19383.1	MSTRG.19383	Spata5
MSTRG.19385.1	MSTRG.19385	Spata5
MSTRG.19395.1	MSTRG.19395	Pgrmc2
MSTRG.19396.1	MSTRG.19396	Pgrmc2
MSTRG.19398.1	MSTRG.19398	Larp1b
MSTRG.19399.1	MSTRG.19399	Larp1b
MSTRG.19400.1	MSTRG.19400	Larp1b
MSTRG.19404.1	MSTRG.19404	Larp1b
MSTRG.19405.1	MSTRG.19405	Larp1b

MSTRG.19407.1	MSTRG.19407	Larp1b
MSTRG.19410.1	MSTRG.19410	Mfsd8
MSTRG.19415.1	MSTRG.19415	Abhd18
MSTRG.19418.1	MSTRG.19418	Abhd18
MSTRG.19419.1	MSTRG.19419	Abhd18
MSTRG.19420.1	MSTRG.19420	Abhd18
MSTRG.19421.1	MSTRG.19421	.
MSTRG.19422.1	MSTRG.19422	.
MSTRG.19424.1	MSTRG.19424	Gm31266
MSTRG.19427.1	MSTRG.19427	Sclt1
MSTRG.19428.1	MSTRG.19428	Sclt1
MSTRG.19430.1	MSTRG.19430	Sclt1
MSTRG.19432.1	MSTRG.19432	D3Ertd751e
MSTRG.19434.1	MSTRG.19434	.
MSTRG.19435.1	MSTRG.19435	.
MSTRG.19436.1	MSTRG.19436	.
MSTRG.19437.1	MSTRG.19437	.
MSTRG.19443.1	MSTRG.19443	Slc7a11
MSTRG.19445.1	MSTRG.19445	Slc7a11
MSTRG.19446.1	MSTRG.19446	Slc7a11
MSTRG.19454.1	MSTRG.19454	4930577N17Rik
MSTRG.19455.1	MSTRG.19455	Elf2
MSTRG.19456.1	MSTRG.19456	Elf2
MSTRG.19464.1	MSTRG.19464	Naa15
MSTRG.19465.1	MSTRG.19465	Naa15
MSTRG.19467.1	MSTRG.19467	Setd7
MSTRG.19470.1	MSTRG.19470	.
MSTRG.19473.2	MSTRG.19473	Foxo1
MSTRG.19478.1	MSTRG.19478	Maml3
MSTRG.19479.1	MSTRG.19479	Maml3
MSTRG.19481.1	MSTRG.19481	Maml3
MSTRG.19482.1	MSTRG.19482	Maml3
MSTRG.19483.1	MSTRG.19483	Maml3
MSTRG.19487.1	MSTRG.19487	Maml3
MSTRG.19488.1	MSTRG.19488	Maml3
MSTRG.1949.1	MSTRG.1949	A630035G10Rik
MSTRG.19494.1	MSTRG.19494	Maml3
MSTRG.19495.1	MSTRG.19495	Maml3
MSTRG.1950.1	MSTRG.1950	A630035G10Rik
MSTRG.19500.1	MSTRG.19500	Maml3
MSTRG.19501.1	MSTRG.19501	Maml3
MSTRG.19504.1	MSTRG.19504	Maml3
MSTRG.19506.1	MSTRG.19506	C130089K02Rik
MSTRG.19507.1	MSTRG.19507	Maml3
MSTRG.19509.1	MSTRG.19509	Maml3
MSTRG.19513.1	MSTRG.19513	.
MSTRG.19522.1	MSTRG.19522	Lhfp
MSTRG.19529.1	MSTRG.19529	.
MSTRG.19535.1	MSTRG.19535	.
MSTRG.19540.1	MSTRG.19540	Alg5
MSTRG.1955.1	MSTRG.1955	Vangl2
MSTRG.19550.8	MSTRG.19550	Rnf13
MSTRG.19556.1	MSTRG.19556	Tsc22d2
MSTRG.19557.2	MSTRG.19557	Serp1
MSTRG.19557.3	MSTRG.19557	Serp1
MSTRG.19558.4	MSTRG.19558	Eif2a
MSTRG.1956.1	MSTRG.1956	Vangl2

MSTRG.19564.1	MSTRG.19564	Siah2
MSTRG.19565.1	MSTRG.19565	Siah2
MSTRG.1957.1	MSTRG.1957	Vangl2
MSTRG.19572.1	MSTRG.19572	4930449A18Rik
MSTRG.19577.1	MSTRG.19577	Med12l
MSTRG.19587.1	MSTRG.19587	.
MSTRG.19588.1	MSTRG.19588	.
MSTRG.1959.1	MSTRG.1959	Slamf1
MSTRG.19593.1	MSTRG.19593	Mbnl1
MSTRG.1960.1	MSTRG.1960	Slamf1
MSTRG.19602.2	MSTRG.19602	Gmps
MSTRG.19604.1	MSTRG.19604	Gm26939
MSTRG.1961.1	MSTRG.1961	.
MSTRG.19610.1	MSTRG.19610	Kcnab1
MSTRG.19615.1	MSTRG.19615	Tiparp
MSTRG.19617.13	MSTRG.19617	Ccnl1
MSTRG.19617.14	MSTRG.19617	Ccnl1
MSTRG.19617.16	MSTRG.19617	Ccnl1
MSTRG.19617.7	MSTRG.19617	Ccnl1
MSTRG.19617.9	MSTRG.19617	Ccnl1
MSTRG.19623.1	MSTRG.19623	Rsrc1
MSTRG.19624.1	MSTRG.19624	Rsrc1
MSTRG.19625.1	MSTRG.19625	Rsrc1
MSTRG.19626.1	MSTRG.19626	Rsrc1
MSTRG.19627.1	MSTRG.19627	Rsrc1
MSTRG.1963.1	MSTRG.1963	Cd84
MSTRG.1963.10	MSTRG.1963	Cd84
MSTRG.1963.11	MSTRG.1963	Gm37065
MSTRG.1963.12	MSTRG.1963	Cd84
MSTRG.1963.17	MSTRG.1963	Gm37065
MSTRG.1963.2	MSTRG.1963	Cd84
MSTRG.1963.3	MSTRG.1963	Cd84
MSTRG.1963.4	MSTRG.1963	Gm37065
MSTRG.1963.5	MSTRG.1963	Cd84
MSTRG.1963.6	MSTRG.1963	Cd84
MSTRG.1963.7	MSTRG.1963	Cd84
MSTRG.1963.8	MSTRG.1963	Gm37065
MSTRG.1963.9	MSTRG.1963	Cd84
MSTRG.19633.1	MSTRG.19633	.
MSTRG.19636.1	MSTRG.19636	.
MSTRG.19637.4	MSTRG.19637	Kpna4
MSTRG.19639.1	MSTRG.19639	Kpna4
MSTRG.19643.1	MSTRG.19643	Ppm1l
MSTRG.19645.1	MSTRG.19645	Ppm1l
MSTRG.19646.1	MSTRG.19646	Ppm1l
MSTRG.19647.1	MSTRG.19647	Ppm1l
MSTRG.19648.1	MSTRG.19648	Ppm1l
MSTRG.19649.1	MSTRG.19649	Ppm1l
MSTRG.1965.1	MSTRG.1965	Gm10521
MSTRG.19651.1	MSTRG.19651	Ppm1l
MSTRG.19653.1	MSTRG.19653	Ppm1l
MSTRG.19657.1	MSTRG.19657	Sptssb
MSTRG.1966.1	MSTRG.1966	.
MSTRG.19663.1	MSTRG.19663	Ift80
MSTRG.1967.1	MSTRG.1967	Gm37065
MSTRG.19671.1	MSTRG.19671	.
MSTRG.19672.1	MSTRG.19672	.

MSTRG.19673.1	MSTRG.19673	Gm6098
MSTRG.19676.1	MSTRG.19676	Wdr49
MSTRG.19684.1	MSTRG.19684	Fstl5
MSTRG.19685.1	MSTRG.19685	Fstl5
MSTRG.19687.1	MSTRG.19687	Golim4
MSTRG.19688.1	MSTRG.19688	Golim4
MSTRG.19692.1	MSTRG.19692	Gm9762
MSTRG.19696.1	MSTRG.19696	Fnip2
MSTRG.19697.1	MSTRG.19697	Rapgef2
MSTRG.19699.1	MSTRG.19699	Rapgef2
MSTRG.19701.2	MSTRG.19701	4921511C10Rik
MSTRG.19701.3	MSTRG.19701	4921511C10Rik
MSTRG.19702.1	MSTRG.19702	Rapgef2
MSTRG.19710.1	MSTRG.19710	Pdgfc
MSTRG.19714.1	MSTRG.19714	.
MSTRG.19716.1	MSTRG.19716	.
MSTRG.19720.1	MSTRG.19720	Dchs2
MSTRG.19721.1	MSTRG.19721	Dchs2
MSTRG.19723.3	MSTRG.19723	Tmem131l
MSTRG.19723.4	MSTRG.19723	Tmem131l
MSTRG.19726.1	MSTRG.19726	Sh3d19
MSTRG.19727.1	MSTRG.19727	Sh3d19
MSTRG.19734.1	MSTRG.19734	Gm37240
MSTRG.19735.1	MSTRG.19735	Gm37240
MSTRG.19738.1	MSTRG.19738	Gm37240
MSTRG.19739.1	MSTRG.19739	Gm37240
MSTRG.19744.1	MSTRG.19744	Gm37240
MSTRG.19745.1	MSTRG.19745	Fbxw7
MSTRG.19746.1	MSTRG.19746	Gm37240
MSTRG.19748.1	MSTRG.19748	Gm37240
MSTRG.1975.1	MSTRG.1975	Copa
MSTRG.19751.1	MSTRG.19751	Gm37240
MSTRG.19759.1	MSTRG.19759	Gm37240
MSTRG.19766.1	MSTRG.19766	Lrba
MSTRG.19768.1	MSTRG.19768	Lrba
MSTRG.19770.1	MSTRG.19770	Lrba
MSTRG.19776.1	MSTRG.19776	Dclk2
MSTRG.19777.1	MSTRG.19777	Dclk2
MSTRG.19778.1	MSTRG.19778	Dclk2
MSTRG.19798.1	MSTRG.19798	Iqgap3
MSTRG.19799.1	MSTRG.19799	Iqgap3
MSTRG.1980.1	MSTRG.1980	Dcaf8
MSTRG.19800.1	MSTRG.19800	.
MSTRG.19802.1	MSTRG.19802	1700113A16Rik
MSTRG.19802.2	MSTRG.19802	1700113A16Rik
MSTRG.19803.1	MSTRG.19803	1700113A16Rik
MSTRG.19805.1	MSTRG.19805	Gm38392
MSTRG.19807.1	MSTRG.19807	Tsacc
MSTRG.1981.1	MSTRG.1981	Dcaf8
MSTRG.19818.1	MSTRG.19818	.
MSTRG.19818.2	MSTRG.19818	.
MSTRG.19835.8	MSTRG.19835	Slc50a1
MSTRG.19839.1	MSTRG.19839	Rit1
MSTRG.19840.1	MSTRG.19840	Rit1
MSTRG.19844.1	MSTRG.19844	5830417I10Rik
MSTRG.19845.2	MSTRG.19845	1500004A13Rik
MSTRG.19845.8	MSTRG.19845	1500004A13Rik

MSTRG.19846.1	MSTRG.19846	Gon4l
MSTRG.19852.1	MSTRG.19852	Ash1l
MSTRG.19853.1	MSTRG.19853	Gm26465
MSTRG.19854.1	MSTRG.19854	Ash1l
MSTRG.19856.1	MSTRG.19856	Ash1l
MSTRG.19883.1	MSTRG.19883	Kcnn3
MSTRG.19891.1	MSTRG.19891	4632404H12Rik
MSTRG.19893.1	MSTRG.19893	Il6ra
MSTRG.19894.1	MSTRG.19894	Il6ra
MSTRG.19895.1	MSTRG.19895	Il6ra
MSTRG.19896.1	MSTRG.19896	Il6ra
MSTRG.19897.1	MSTRG.19897	Il6ra
MSTRG.19900.1	MSTRG.19900	Jtb
MSTRG.19900.2	MSTRG.19900	Jtb
MSTRG.19902.3	MSTRG.19902	Nup210l
MSTRG.19928.1	MSTRG.19928	.
MSTRG.1993.2	MSTRG.1993	4933439K11Rik
MSTRG.19935.1	MSTRG.19935	Snapin
MSTRG.19947.1	MSTRG.19947	.
MSTRG.19948.1	MSTRG.19948	.
MSTRG.19949.1	MSTRG.19949	.
MSTRG.19954.1	MSTRG.19954	.
MSTRG.19958.1	MSTRG.19958	Gm36070
MSTRG.19960.1	MSTRG.19960	Gm38411
MSTRG.19960.2	MSTRG.19960	Gm38411
MSTRG.19960.3	MSTRG.19960	Gm38411
MSTRG.19960.4	MSTRG.19960	Gm38411
MSTRG.19960.6	MSTRG.19960	Gm38411
MSTRG.19960.7	MSTRG.19960	Gm38411
MSTRG.19960.8	MSTRG.19960	Gm38411
MSTRG.19962.1	MSTRG.19962	Gm38411
MSTRG.19963.1	MSTRG.19963	Rorc
MSTRG.1997.1	MSTRG.1997	Aim2
MSTRG.19975.1	MSTRG.19975	Snx27
MSTRG.1998.1	MSTRG.1998	Aim2
MSTRG.19981.1	MSTRG.19981	Snx27
MSTRG.19984.1	MSTRG.19984	Psmd4
MSTRG.19986.1	MSTRG.19986	Vps72
MSTRG.19990.1	MSTRG.19990	Pip5k1a
MSTRG.19994.1	MSTRG.19994	Tuft1
MSTRG.20000.1	MSTRG.20000	Gm15264
MSTRG.20002.1	MSTRG.20002	Pogz
MSTRG.20002.2	MSTRG.20002	Pogz
MSTRG.20008.1	MSTRG.20008	.
MSTRG.20011.1	MSTRG.20011	.
MSTRG.20013.1	MSTRG.20013	Gm15265
MSTRG.20013.3	MSTRG.20013	Gm15265
MSTRG.20013.4	MSTRG.20013	Gm15265
MSTRG.20013.5	MSTRG.20013	Gm15265
MSTRG.20017.2	MSTRG.20017	Scnm1
MSTRG.20024.1	MSTRG.20024	Gabpb2
MSTRG.20027.2	MSTRG.20027	Cdc42se1
MSTRG.20031.1	MSTRG.20031	Ctss
MSTRG.20033.1	MSTRG.20033	Prune1
MSTRG.20045.1	MSTRG.20045	.
MSTRG.20052.1	MSTRG.20052	Vps45
MSTRG.20053.1	MSTRG.20053	Vps45

MSTRG.20054.8	MSTRG.20054	Otud7b
MSTRG.20055.1	MSTRG.20055	Otud7b
MSTRG.20063.1	MSTRG.20063	BC107364
MSTRG.20073.1	MSTRG.20073	Bola1
MSTRG.20079.1	MSTRG.20079	Hist2h3c2
MSTRG.20080.1	MSTRG.20080	Hist2h3c1
MSTRG.2009.1	MSTRG.2009	Opn3
MSTRG.20093.1	MSTRG.20093	.
MSTRG.20093.2	MSTRG.20093	.
MSTRG.20094.1	MSTRG.20094	.
MSTRG.20094.2	MSTRG.20094	.
MSTRG.20095.1	MSTRG.20095	.
MSTRG.20096.4	MSTRG.20096	Adamtsl4
MSTRG.20097.1	MSTRG.20097	.
MSTRG.20098.1	MSTRG.20098	.
MSTRG.20104.1	MSTRG.20104	Rprd2
MSTRG.20109.1	MSTRG.20109	Mrps21
MSTRG.2011.1	MSTRG.2011	Ifi203
MSTRG.2011.2	MSTRG.2011	Ifi203
MSTRG.20111.1	MSTRG.20111	C920021L13Rik
MSTRG.20118.1	MSTRG.20118	Bcl9
MSTRG.2012.1	MSTRG.2012	Mndal
MSTRG.20120.1	MSTRG.20120	Chd1l
MSTRG.2013.1	MSTRG.2013	Ifi203
MSTRG.20131.1	MSTRG.20131	Polr3c
MSTRG.20137.1	MSTRG.20137	Polr3gl
MSTRG.2014.1	MSTRG.2014	Ifi203
MSTRG.2014.2	MSTRG.2014	Ifi203
MSTRG.2014.3	MSTRG.2014	Ifi203
MSTRG.20148.1	MSTRG.20148	Polr3c
MSTRG.20150.2	MSTRG.20150	Rnf115
MSTRG.20152.1	MSTRG.20152	.
MSTRG.2016.1	MSTRG.2016	Ifi203
MSTRG.20162.1	MSTRG.20162	Phgdh
MSTRG.20169.1	MSTRG.20169	Wars2
MSTRG.2017.1	MSTRG.2017	Mndal
MSTRG.20170.1	MSTRG.20170	Wars2
MSTRG.20172.1	MSTRG.20172	Wars2
MSTRG.20174.1	MSTRG.20174	Wars2
MSTRG.20175.1	MSTRG.20175	Wars2
MSTRG.20186.1	MSTRG.20186	Gdap2
MSTRG.20187.1	MSTRG.20187	Gdap2
MSTRG.20188.1	MSTRG.20188	.
MSTRG.2020.1	MSTRG.2020	Exo1
MSTRG.20200.1	MSTRG.20200	.
MSTRG.20204.1	MSTRG.20204	.
MSTRG.20206.1	MSTRG.20206	Igsf3
MSTRG.2021.1	MSTRG.2021	Exo1
MSTRG.20214.1	MSTRG.20214	Slc22a15
MSTRG.20216.1	MSTRG.20216	Vangl1
MSTRG.20221.1	MSTRG.20221	Tspan2
MSTRG.20238.1	MSTRG.20238	.
MSTRG.2024.1	MSTRG.2024	Cep170
MSTRG.20241.1	MSTRG.20241	Trim33
MSTRG.20242.1	MSTRG.20242	Trim33
MSTRG.20243.1	MSTRG.20243	Trim33
MSTRG.20244.1	MSTRG.20244	Trim33

MSTRG.20245.1	MSTRG.20245	Trim33
MSTRG.20247.1	MSTRG.20247	Trim33
MSTRG.20251.1	MSTRG.20251	Gm15886
MSTRG.20251.2	MSTRG.20251	Gm15886
MSTRG.20260.1	MSTRG.20260	.
MSTRG.20267.1	MSTRG.20267	Lrig2
MSTRG.20274.1	MSTRG.20274	Rsbn1
MSTRG.20275.1	MSTRG.20275	Rsbn1
MSTRG.20278.1	MSTRG.20278	Phtf1
MSTRG.20281.1	MSTRG.20281	Magi3
MSTRG.20286.1	MSTRG.20286	Magi3
MSTRG.20288.1	MSTRG.20288	Magi3
MSTRG.20293.1	MSTRG.20293	Magi3
MSTRG.20297.1	MSTRG.20297	Magi3
MSTRG.20298.1	MSTRG.20298	Magi3
MSTRG.203.1	MSTRG.203	Adgrb3
MSTRG.2030.1	MSTRG.2030	Cep170
MSTRG.20302.1	MSTRG.20302	Mov10
MSTRG.20303.1	MSTRG.20303	Mov10
MSTRG.20306.1	MSTRG.20306	Cttnbp2nl
MSTRG.20307.1	MSTRG.20307	Cttnbp2nl
MSTRG.2031.1	MSTRG.2031	Cep170
MSTRG.20313.1	MSTRG.20313	St7l
MSTRG.20315.1	MSTRG.20315	.
MSTRG.2032.1	MSTRG.2032	Cep170
MSTRG.20331.1	MSTRG.20331	2010016I18Rik
MSTRG.20331.4	MSTRG.20331	2010016I18Rik
MSTRG.20333.4	MSTRG.20333	Cept1
MSTRG.20334.1	MSTRG.20334	Cept1
MSTRG.20337.1	MSTRG.20337	Dram2
MSTRG.20342.1	MSTRG.20342	Gm27008
MSTRG.20345.1	MSTRG.20345	Gm27008
MSTRG.20348.1	MSTRG.20348	Slc16a4
MSTRG.20350.1	MSTRG.20350	.
MSTRG.20354.1	MSTRG.20354	Ahcyl1
MSTRG.20361.1	MSTRG.20361	Gnai3
MSTRG.20365.2	MSTRG.20365	Cyb561d1
MSTRG.2038.1	MSTRG.2038	Sdccag8
MSTRG.20382.1	MSTRG.20382	Taf13
MSTRG.20385.1	MSTRG.20385	Wdr47
MSTRG.20396.1	MSTRG.20396	Vav3
MSTRG.20397.1	MSTRG.20397	Vav3
MSTRG.20398.1	MSTRG.20398	Vav3
MSTRG.20399.1	MSTRG.20399	Vav3
MSTRG.2040.1	MSTRG.2040	Sdccag8
MSTRG.20400.1	MSTRG.20400	Vav3
MSTRG.20401.1	MSTRG.20401	Vav3
MSTRG.20402.1	MSTRG.20402	Vav3
MSTRG.20403.1	MSTRG.20403	Vav3
MSTRG.20407.1	MSTRG.20407	Amy2-ps1
MSTRG.20408.1	MSTRG.20408	.
MSTRG.20410.1	MSTRG.20410	Amy1
MSTRG.20412.1	MSTRG.20412	.
MSTRG.20413.1	MSTRG.20413	.
MSTRG.20420.1	MSTRG.20420	Dph5
MSTRG.20428.1	MSTRG.20428	Slc30a7
MSTRG.20431.1	MSTRG.20431	Cdc14a

MSTRG.20432.1	MSTRG.20432	Cdc14a
MSTRG.20434.1	MSTRG.20434	Cdc14a
MSTRG.20435.1	MSTRG.20435	Cdc14a
MSTRG.20436.1	MSTRG.20436	Cdc14a
MSTRG.20437.1	MSTRG.20437	Cdc14a
MSTRG.20438.1	MSTRG.20438	Cdc14a
MSTRG.20444.1	MSTRG.20444	Dbt
MSTRG.20448.1	MSTRG.20448	Lrrc39
MSTRG.20449.1	MSTRG.20449	Lrrc39
MSTRG.20455.1	MSTRG.20455	Mfsd14a
MSTRG.20458.1	MSTRG.20458	Agl
MSTRG.2046.4	MSTRG.2046	Akt3
MSTRG.2046.5	MSTRG.2046	Akt3
MSTRG.20460.1	MSTRG.20460	Frrs1
MSTRG.20461.1	MSTRG.20461	Frrs1
MSTRG.20462.1	MSTRG.20462	Frrs1
MSTRG.20465.1	MSTRG.20465	Dpyd
MSTRG.20466.1	MSTRG.20466	Dpyd
MSTRG.20471.1	MSTRG.20471	.
MSTRG.20473.1	MSTRG.20473	Alg14
MSTRG.20474.1	MSTRG.20474	Alg14
MSTRG.20475.1	MSTRG.20475	Alg14
MSTRG.20476.1	MSTRG.20476	Alg14
MSTRG.20477.1	MSTRG.20477	Alg14
MSTRG.20483.1	MSTRG.20483	Cnn3
MSTRG.20488.1	MSTRG.20488	Abcd3
MSTRG.205.1	MSTRG.205	Adgrb3
MSTRG.20500.1	MSTRG.20500	Bcar3
MSTRG.20503.1	MSTRG.20503	Bcar3
MSTRG.20504.1	MSTRG.20504	Bcar3
MSTRG.20509.1	MSTRG.20509	Pde5a
MSTRG.2051.1	MSTRG.2051	Akt3
MSTRG.20518.1	MSTRG.20518	Prss12
MSTRG.20524.1	MSTRG.20524	Gm43283
MSTRG.20525.1	MSTRG.20525	Synpo2
MSTRG.20528.1	MSTRG.20528	Mettl14
MSTRG.2053.1	MSTRG.2053	Akt3
MSTRG.20530.1	MSTRG.20530	Gm4617
MSTRG.20532.1	MSTRG.20532	Ugt8a
MSTRG.20533.1	MSTRG.20533	Ugt8a
MSTRG.20534.1	MSTRG.20534	Ugt8a
MSTRG.20535.1	MSTRG.20535	Ugt8a
MSTRG.20536.1	MSTRG.20536	Ugt8a
MSTRG.20537.1	MSTRG.20537	Ugt8a
MSTRG.20538.15	MSTRG.20538	Camk2d
MSTRG.2054.1	MSTRG.2054	Akt3
MSTRG.20540.1	MSTRG.20540	Camk2d
MSTRG.20542.1	MSTRG.20542	Camk2d
MSTRG.20543.1	MSTRG.20543	Camk2d
MSTRG.20545.1	MSTRG.20545	Camk2d
MSTRG.20548.1	MSTRG.20548	Camk2d
MSTRG.2055.1	MSTRG.2055	Akt3
MSTRG.20550.1	MSTRG.20550	Camk2d
MSTRG.20551.1	MSTRG.20551	Camk2d
MSTRG.20552.1	MSTRG.20552	Camk2d
MSTRG.20553.1	MSTRG.20553	Camk2d
MSTRG.20556.1	MSTRG.20556	Camk2d

MSTRG.20557.1	MSTRG.20557	Camk2d
MSTRG.2056.1	MSTRG.2056	Akt3
MSTRG.20566.1	MSTRG.20566	Zgrf1
MSTRG.20568.1	MSTRG.20568	5730508B09Rik
MSTRG.20569.1	MSTRG.20569	5730508B09Rik
MSTRG.2057.1	MSTRG.2057	Akt3
MSTRG.20570.1	MSTRG.20570	5730508B09Rik
MSTRG.20573.1	MSTRG.20573	Tifa
MSTRG.20578.1	MSTRG.20578	.
MSTRG.20582.1	MSTRG.20582	Elovl6
MSTRG.20583.1	MSTRG.20583	Elovl6
MSTRG.20584.1	MSTRG.20584	Elovl6
MSTRG.20588.1	MSTRG.20588	Pla2g12a
MSTRG.2059.1	MSTRG.2059	Akt3
MSTRG.20593.1	MSTRG.20593	Sec24b
MSTRG.20594.1	MSTRG.20594	Sec24b
MSTRG.206.1	MSTRG.206	Adgrb3
MSTRG.20607.1	MSTRG.20607	2010110G14Rik
MSTRG.20611.1	MSTRG.20611	.
MSTRG.20617.1	MSTRG.20617	Lef1
MSTRG.20618.1	MSTRG.20618	Lef1
MSTRG.20619.1	MSTRG.20619	Lef1
MSTRG.20619.2	MSTRG.20619	Lef1
MSTRG.20619.3	MSTRG.20619	Lef1
MSTRG.20620.1	MSTRG.20620	Lef1
MSTRG.20621.1	MSTRG.20621	Lef1
MSTRG.20624.1	MSTRG.20624	Papss1
MSTRG.20634.1	MSTRG.20634	.
MSTRG.20637.1	MSTRG.20637	Gstcd
MSTRG.2064.1	MSTRG.2064	1700016C15Rik
MSTRG.20642.1	MSTRG.20642	Arhgef38
MSTRG.20646.1	MSTRG.20646	Gm37228
MSTRG.20648.1	MSTRG.20648	Tet2
MSTRG.20649.1	MSTRG.20649	Tet2
MSTRG.20650.1	MSTRG.20650	Tet2
MSTRG.20654.1	MSTRG.20654	Cenpe
MSTRG.20657.1	MSTRG.20657	.
MSTRG.20658.1	MSTRG.20658	Cisd2
MSTRG.20664.1	MSTRG.20664	Manba
MSTRG.20665.1	MSTRG.20665	Manba
MSTRG.20666.1	MSTRG.20666	Manba
MSTRG.20667.1	MSTRG.20667	.
MSTRG.2067.1	MSTRG.2067	Adss
MSTRG.20670.1	MSTRG.20670	Nfkb1
MSTRG.20675.1	MSTRG.20675	Bank1
MSTRG.20677.3	MSTRG.20677	Ppp3ca
MSTRG.20678.1	MSTRG.20678	Ppp3ca
MSTRG.20679.1	MSTRG.20679	Ppp3ca
MSTRG.20680.1	MSTRG.20680	4930599N24Rik
MSTRG.20682.1	MSTRG.20682	Ppp3ca
MSTRG.20683.1	MSTRG.20683	Ppp3ca
MSTRG.20685.1	MSTRG.20685	Ppp3ca
MSTRG.20686.1	MSTRG.20686	.
MSTRG.2069.1	MSTRG.2069	Gm16432
MSTRG.20696.1	MSTRG.20696	Dapp1
MSTRG.20697.1	MSTRG.20697	Dapp1
MSTRG.20698.1	MSTRG.20698	Dapp1

MSTRG.20703.1	MSTRG.20703	Metap1
MSTRG.20705.1	MSTRG.20705	Eif4e
MSTRG.20707.1	MSTRG.20707	.
MSTRG.20709.1	MSTRG.20709	Tspan5
MSTRG.20710.1	MSTRG.20710	Tspan5
MSTRG.20714.1	MSTRG.20714	Rap1gds1
MSTRG.20715.1	MSTRG.20715	Rap1gds1
MSTRG.20717.1	MSTRG.20717	Stpg2
MSTRG.20718.1	MSTRG.20718	Stpg2
MSTRG.20722.1	MSTRG.20722	.
MSTRG.20723.1	MSTRG.20723	.
MSTRG.20724.1	MSTRG.20724	.
MSTRG.20726.1	MSTRG.20726	.
MSTRG.20727.1	MSTRG.20727	.
MSTRG.2073.1	MSTRG.2073	Gm16432
MSTRG.20730.1	MSTRG.20730	Unc5c
MSTRG.20731.1	MSTRG.20731	Unc5c
MSTRG.20732.1	MSTRG.20732	Unc5c
MSTRG.20736.1	MSTRG.20736	Pdlim5
MSTRG.20748.1	MSTRG.20748	Pkn2
MSTRG.20749.1	MSTRG.20749	Pkn2
MSTRG.2075.1	MSTRG.2075	.
MSTRG.20754.1	MSTRG.20754	.
MSTRG.20762.1	MSTRG.20762	.
MSTRG.20766.1	MSTRG.20766	.
MSTRG.20769.1	MSTRG.20769	Odf2l
MSTRG.20771.1	MSTRG.20771	Znhit6
MSTRG.20778.1	MSTRG.20778	Ssx2ip
MSTRG.20780.8	MSTRG.20780	Spata1
MSTRG.20781.1	MSTRG.20781	Ctbs
MSTRG.20782.1	MSTRG.20782	Spata1
MSTRG.20787.1	MSTRG.20787	Prkacb
MSTRG.20789.1	MSTRG.20789	.
MSTRG.20790.1	MSTRG.20790	.
MSTRG.20791.1	MSTRG.20791	.
MSTRG.20794.1	MSTRG.20794	.
MSTRG.20798.1	MSTRG.20798	Miga1
MSTRG.20799.1	MSTRG.20799	Miga1
MSTRG.20811.1	MSTRG.20811	Usp33
MSTRG.20816.1	MSTRG.20816	Zzz3
MSTRG.20818.1	MSTRG.20818	Zzz3
MSTRG.20819.1	MSTRG.20819	Zzz3
MSTRG.20820.1	MSTRG.20820	Zzz3
MSTRG.20821.1	MSTRG.20821	Zzz3
MSTRG.20822.1	MSTRG.20822	Zzz3
MSTRG.20827.1	MSTRG.20827	St6galnac3
MSTRG.20828.1	MSTRG.20828	St6galnac3
MSTRG.20831.1	MSTRG.20831	St6galnac3
MSTRG.20834.1	MSTRG.20834	St6galnac3
MSTRG.20838.1	MSTRG.20838	St6galnac3
MSTRG.20839.1	MSTRG.20839	St6galnac3
MSTRG.20842.1	MSTRG.20842	St6galnac3
MSTRG.20843.1	MSTRG.20843	St6galnac3
MSTRG.20845.1	MSTRG.20845	St6galnac3
MSTRG.20847.1	MSTRG.20847	St6galnac3
MSTRG.20849.1	MSTRG.20849	St6galnac3
MSTRG.2085.1	MSTRG.2085	Efcab2

MSTRG.20850.1	MSTRG.20850	St6galnac3
MSTRG.20856.1	MSTRG.20856	Tyw3
MSTRG.2087.1	MSTRG.2087	Smyd3
MSTRG.20871.1	MSTRG.20871	Negr1
MSTRG.2088.1	MSTRG.2088	Smyd3
MSTRG.20881.1	MSTRG.20881	Ankrd13c
MSTRG.20882.1	MSTRG.20882	Ankrd13c
MSTRG.20883.1	MSTRG.20883	Ankrd13c
MSTRG.20884.1	MSTRG.20884	Ankrd13c
MSTRG.20889.1	MSTRG.20889	Lrrc7
MSTRG.20892.1	MSTRG.20892	Depdc1a
MSTRG.20893.1	MSTRG.20893	Depdc1a
MSTRG.20896.1	MSTRG.20896	Lrrc40
MSTRG.2090.1	MSTRG.2090	Smyd3
MSTRG.20901.1	MSTRG.20901	.
MSTRG.20902.1	MSTRG.20902	.
MSTRG.20906.2	MSTRG.20906	Gm26857
MSTRG.20906.3	MSTRG.20906	Gm26857
MSTRG.20908.1	MSTRG.20908	Lyn
MSTRG.2091.1	MSTRG.2091	Smyd3
MSTRG.20913.1	MSTRG.20913	Plag1
MSTRG.20914.1	MSTRG.20914	Plag1
MSTRG.2092.1	MSTRG.2092	Smyd3
MSTRG.20920.1	MSTRG.20920	.
MSTRG.20921.1	MSTRG.20921	.
MSTRG.20927.1	MSTRG.20927	Fam110b
MSTRG.2093.1	MSTRG.2093	Smyd3
MSTRG.20931.1	MSTRG.20931	.
MSTRG.20938.1	MSTRG.20938	Tox
MSTRG.2094.1	MSTRG.2094	Smyd3
MSTRG.20941.1	MSTRG.20941	Tox
MSTRG.20941.2	MSTRG.20941	Tox
MSTRG.20941.3	MSTRG.20941	Tox
MSTRG.20941.4	MSTRG.20941	Tox
MSTRG.20942.2	MSTRG.20942	.
MSTRG.20945.1	MSTRG.20945	.
MSTRG.20945.2	MSTRG.20945	.
MSTRG.20946.1	MSTRG.20946	.
MSTRG.20947.1	MSTRG.20947	.
MSTRG.20947.2	MSTRG.20947	.
MSTRG.20947.3	MSTRG.20947	.
MSTRG.20948.1	MSTRG.20948	.
MSTRG.2095.3	MSTRG.2095	Smyd3
MSTRG.20950.1	MSTRG.20950	Rab2a
MSTRG.20951.1	MSTRG.20951	Rab2a
MSTRG.20952.1	MSTRG.20952	Rab2a
MSTRG.20953.1	MSTRG.20953	Rab2a
MSTRG.20953.2	MSTRG.20953	Rab2a
MSTRG.20954.1	MSTRG.20954	Rab2a
MSTRG.20965.1	MSTRG.20965	Plekhf2
MSTRG.20966.1	MSTRG.20966	Plekhf2
MSTRG.2097.1	MSTRG.2097	Smyd3
MSTRG.20971.1	MSTRG.20971	.
MSTRG.20972.3	MSTRG.20972	Trp53inp1
MSTRG.20972.4	MSTRG.20972	Trp53inp1
MSTRG.20976.1	MSTRG.20976	Dpy19l4
MSTRG.20978.1	MSTRG.20978	Dpy19l4

MSTRG.20979.1	MSTRG.20979	Dpy19l4
MSTRG.2098.1	MSTRG.2098	Smyd3
MSTRG.20986.1	MSTRG.20986	Rad54b
MSTRG.20987.1	MSTRG.20987	Rad54b
MSTRG.20988.1	MSTRG.20988	Rad54b
MSTRG.20989.1	MSTRG.20989	Gm11832
MSTRG.20990.1	MSTRG.20990	.
MSTRG.21001.1	MSTRG.21001	.
MSTRG.21005.1	MSTRG.21005	.
MSTRG.21008.1	MSTRG.21008	Tmem55a
MSTRG.21011.1	MSTRG.21011	Necab1
MSTRG.21014.1	MSTRG.21014	.
MSTRG.21014.2	MSTRG.21014	.
MSTRG.21014.3	MSTRG.21014	.
MSTRG.21030.1	MSTRG.21030	Gm11867
MSTRG.21031.1	MSTRG.21031	Gm11867
MSTRG.21040.3	MSTRG.21040	Wwp1
MSTRG.21041.1	MSTRG.21041	.
MSTRG.21048.1	MSTRG.21048	Coq3
MSTRG.21050.1	MSTRG.21050	Fbxl4
MSTRG.21052.1	MSTRG.21052	Fbxl4
MSTRG.21057.1	MSTRG.21057	Usp45
MSTRG.21057.2	MSTRG.21057	Usp45
MSTRG.21058.1	MSTRG.21058	Usp45
MSTRG.21060.1	MSTRG.21060	Pnisr
MSTRG.21063.1	MSTRG.21063	Mms22l
MSTRG.21064.1	MSTRG.21064	.
MSTRG.21066.1	MSTRG.21066	Klhl32
MSTRG.21073.1	MSTRG.21073	Manea
MSTRG.21074.1	MSTRG.21074	.
MSTRG.21078.1	MSTRG.21078	Map3k7
MSTRG.21082.1	MSTRG.21082	Bach2
MSTRG.21083.1	MSTRG.21083	Bach2
MSTRG.21084.1	MSTRG.21084	Bach2
MSTRG.21084.2	MSTRG.21084	Bach2
MSTRG.2109.1	MSTRG.2109	Coq8a
MSTRG.21092.1	MSTRG.21092	Ankrd6
MSTRG.21093.1	MSTRG.21093	Ankrd6
MSTRG.21094.1	MSTRG.21094	Ankrd6
MSTRG.21095.1	MSTRG.21095	Ankrd6
MSTRG.21096.1	MSTRG.21096	Ankrd6
MSTRG.21097.1	MSTRG.21097	Ankrd6
MSTRG.21101.1	MSTRG.21101	Casp8ap2
MSTRG.21102.1	MSTRG.21102	Casp8ap2
MSTRG.21106.1	MSTRG.21106	.
MSTRG.21107.1	MSTRG.21107	.
MSTRG.21107.2	MSTRG.21107	.
MSTRG.21108.1	MSTRG.21108	.
MSTRG.2111.1	MSTRG.2111	Psen2
MSTRG.21111.1	MSTRG.21111	Gabrr2
MSTRG.21113.1	MSTRG.21113	.
MSTRG.21118.1	MSTRG.21118	Rngtt
MSTRG.21119.1	MSTRG.21119	Rngtt
MSTRG.21120.1	MSTRG.21120	Rngtt
MSTRG.21121.1	MSTRG.21121	.
MSTRG.21127.1	MSTRG.21127	Rars2
MSTRG.21130.1	MSTRG.21130	.

MSTRG.21133.1	MSTRG.21133	Zfp292
MSTRG.21136.1	MSTRG.21136	Mob3b
MSTRG.21137.1	MSTRG.21137	Mob3b
MSTRG.21138.1	MSTRG.21138	Mob3b
MSTRG.21143.1	MSTRG.21143	.
MSTRG.21145.1	MSTRG.21145	Gm12381
MSTRG.21147.1	MSTRG.21147	.
MSTRG.21148.1	MSTRG.21148	.
MSTRG.21155.1	MSTRG.21155	Aptx
MSTRG.2116.1	MSTRG.2116	Itpkb
MSTRG.21164.1	MSTRG.21164	B4galt1
MSTRG.21173.1	MSTRG.21173	Ube2r2
MSTRG.21177.1	MSTRG.21177	Ube2r2
MSTRG.21182.1	MSTRG.21182	Ubap2
MSTRG.21184.1	MSTRG.21184	Ubap2
MSTRG.21185.1	MSTRG.21185	Ubap2
MSTRG.21186.1	MSTRG.21186	Ubap2
MSTRG.21187.1	MSTRG.21187	Ubap2
MSTRG.2119.1	MSTRG.2119	Lin9
MSTRG.21194.1	MSTRG.21194	Ubap1
MSTRG.21196.1	MSTRG.21196	Kif24
MSTRG.21197.1	MSTRG.21197	Kif24
MSTRG.21198.1	MSTRG.21198	Kif24
MSTRG.21232.1	MSTRG.21232	Gm2163
MSTRG.21238.1	MSTRG.21238	.
MSTRG.2124.1	MSTRG.2124	Gm38293
MSTRG.21245.1	MSTRG.21245	4930578G10Rik
MSTRG.2125.1	MSTRG.2125	Gm38293
MSTRG.21251.1	MSTRG.21251	Fancg
MSTRG.21261.1	MSTRG.21261	Sit1
MSTRG.21265.2	MSTRG.21265	Gm12454
MSTRG.21273.1	MSTRG.21273	Spaar
MSTRG.21280.1	MSTRG.21280	Glipr2
MSTRG.21281.1	MSTRG.21281	Glipr2
MSTRG.21284.1	MSTRG.21284	.
MSTRG.21288.1	MSTRG.21288	Gne
MSTRG.2129.1	MSTRG.2129	.
MSTRG.21292.15	MSTRG.21292	Rnf38
MSTRG.21292.20	MSTRG.21292	Rnf38
MSTRG.21292.22	MSTRG.21292	Rnf38
MSTRG.21292.23	MSTRG.21292	Rnf38
MSTRG.21295.1	MSTRG.21295	Zcchc7
MSTRG.21295.2	MSTRG.21295	Zcchc7
MSTRG.21296.1	MSTRG.21296	Gm22639
MSTRG.21300.1	MSTRG.21300	Zbtb5
MSTRG.21306.1	MSTRG.21306	Trmt10b
MSTRG.21307.1	MSTRG.21307	Trmt10b
MSTRG.21311.1	MSTRG.21311	Slc25a51
MSTRG.21320.1	MSTRG.21320	Tstd2
MSTRG.21321.1	MSTRG.21321	Tstd2
MSTRG.21323.1	MSTRG.21323	Ncbp1
MSTRG.21330.1	MSTRG.21330	Trim14
MSTRG.21341.1	MSTRG.21341	.
MSTRG.21344.1	MSTRG.21344	Tgfbr1
MSTRG.21348.1	MSTRG.21348	.
MSTRG.21350.1	MSTRG.21350	.
MSTRG.21356.1	MSTRG.21356	Invs

MSTRG.21358.1	MSTRG.21358	Invs
MSTRG.21360.1	MSTRG.21360	Tex10
MSTRG.21363.1	MSTRG.21363	Acnat2
MSTRG.21371.1	MSTRG.21371	.
MSTRG.21376.1	MSTRG.21376	.
MSTRG.21385.1	MSTRG.21385	Fsd1l
MSTRG.21389.1	MSTRG.21389	Tmem38b
MSTRG.21390.1	MSTRG.21390	Tmem38b
MSTRG.21392.1	MSTRG.21392	Zfp462
MSTRG.21393.1	MSTRG.21393	Zfp462
MSTRG.21394.1	MSTRG.21394	.
MSTRG.21395.1	MSTRG.21395	Rad23b
MSTRG.21397.1	MSTRG.21397	Rad23b
MSTRG.21401.1	MSTRG.21401	.
MSTRG.2141.1	MSTRG.2141	Cnih4
MSTRG.21414.1	MSTRG.21414	Tmem245
MSTRG.21414.2	MSTRG.21414	Tmem245
MSTRG.21415.1	MSTRG.21415	Tmem245
MSTRG.21416.1	MSTRG.21416	Tmem245
MSTRG.21418.1	MSTRG.21418	Ptpn3
MSTRG.2142.1	MSTRG.2142	Cnih4
MSTRG.21420.1	MSTRG.21420	Ptpn3
MSTRG.21421.1	MSTRG.21421	Ptpn3
MSTRG.21422.1	MSTRG.21422	Ptpn3
MSTRG.21423.1	MSTRG.21423	Ptpn3
MSTRG.21425.1	MSTRG.21425	.
MSTRG.2143.1	MSTRG.2143	.
MSTRG.21431.1	MSTRG.21431	Pakap
MSTRG.21433.1	MSTRG.21433	Pakap
MSTRG.2144.1	MSTRG.2144	.
MSTRG.21440.1	MSTRG.21440	AI314180
MSTRG.21442.1	MSTRG.21442	Dnajc25
MSTRG.21443.1	MSTRG.21443	Dnajc25
MSTRG.21444.1	MSTRG.21444	Dnajc25
MSTRG.21445.1	MSTRG.21445	Gm20503
MSTRG.21446.1	MSTRG.21446	Gm20503
MSTRG.21447.1	MSTRG.21447	.
MSTRG.21449.1	MSTRG.21449	Ugcg
MSTRG.21450.1	MSTRG.21450	.
MSTRG.21451.1	MSTRG.21451	.
MSTRG.21455.1	MSTRG.21455	Susd1
MSTRG.21456.1	MSTRG.21456	Susd1
MSTRG.21457.1	MSTRG.21457	Susd1
MSTRG.21460.1	MSTRG.21460	Ptbp3
MSTRG.21465.1	MSTRG.21465	E130308A19Rik
MSTRG.21466.1	MSTRG.21466	E130308A19Rik
MSTRG.21467.1	MSTRG.21467	E130308A19Rik
MSTRG.21468.1	MSTRG.21468	E130308A19Rik
MSTRG.21469.1	MSTRG.21469	E130308A19Rik
MSTRG.21470.1	MSTRG.21470	E130308A19Rik
MSTRG.21471.1	MSTRG.21471	E130308A19Rik
MSTRG.21472.1	MSTRG.21472	E130308A19Rik
MSTRG.21473.1	MSTRG.21473	E130308A19Rik
MSTRG.21474.1	MSTRG.21474	Inip
MSTRG.21474.2	MSTRG.21474	Inip
MSTRG.21476.1	MSTRG.21476	Snx30
MSTRG.21477.1	MSTRG.21477	Snx30

MSTRG.21479.1	MSTRG.21479	.
MSTRG.2148.1	MSTRG.2148	.
MSTRG.21481.1	MSTRG.21481	Bspry
MSTRG.21488.1	MSTRG.21488	Cdc26
MSTRG.21492.3	MSTRG.21492	Pole3
MSTRG.21498.1	MSTRG.21498	.
MSTRG.21503.1	MSTRG.21503	Orm2
MSTRG.21508.1	MSTRG.21508	.
MSTRG.21515.1	MSTRG.21515	Tnc
MSTRG.21517.1	MSTRG.21517	.
MSTRG.2152.1	MSTRG.2152	Dnah14
MSTRG.21524.1	MSTRG.21524	Gm11751
MSTRG.21527.1	MSTRG.21527	Megf9
MSTRG.21529.1	MSTRG.21529	Cdk5rap2
MSTRG.21530.1	MSTRG.21530	Cdk5rap2
MSTRG.21531.1	MSTRG.21531	Cdk5rap2
MSTRG.21532.1	MSTRG.21532	Cdk5rap2
MSTRG.21533.1	MSTRG.21533	Cdk5rap2
MSTRG.21534.1	MSTRG.21534	.
MSTRG.21535.1	MSTRG.21535	.
MSTRG.21536.1	MSTRG.21536	.
MSTRG.21544.1	MSTRG.21544	Kdm4c
MSTRG.21547.1	MSTRG.21547	.
MSTRG.21548.1	MSTRG.21548	.
MSTRG.21550.1	MSTRG.21550	Ptprd
MSTRG.21552.1	MSTRG.21552	Ptprd
MSTRG.21563.1	MSTRG.21563	Nfib
MSTRG.21569.1	MSTRG.21569	Zdhhc21
MSTRG.21576.1	MSTRG.21576	Ccdc171
MSTRG.21582.1	MSTRG.21582	Bnc2
MSTRG.21585.1	MSTRG.21585	Cntln
MSTRG.21593.1	MSTRG.21593	Dennd4c
MSTRG.21597.1	MSTRG.21597	.
MSTRG.21598.9	MSTRG.21598	Mllt3
MSTRG.21599.1	MSTRG.21599	Mllt3
MSTRG.21600.1	MSTRG.21600	Mllt3
MSTRG.21600.2	MSTRG.21600	Mllt3
MSTRG.21603.1	MSTRG.21603	Mllt3
MSTRG.21603.2	MSTRG.21603	Mllt3
MSTRG.21603.3	MSTRG.21603	Mllt3
MSTRG.21604.1	MSTRG.21604	Mllt3
MSTRG.21608.1	MSTRG.21608	.
MSTRG.21612.1	MSTRG.21612	Focad
MSTRG.21613.1	MSTRG.21613	Focad
MSTRG.21615.1	MSTRG.21615	Gm10583
MSTRG.21625.1	MSTRG.21625	.
MSTRG.21628.1	MSTRG.21628	.
MSTRG.21629.2	MSTRG.21629	Gm26525
MSTRG.2163.1	MSTRG.2163	1700047M11Rik
MSTRG.21644.1	MSTRG.21644	.
MSTRG.21645.1	MSTRG.21645	.
MSTRG.21650.1	MSTRG.21650	Gm12649
MSTRG.21652.1	MSTRG.21652	Gm12655
MSTRG.21653.1	MSTRG.21653	.
MSTRG.21655.1	MSTRG.21655	.
MSTRG.21658.1	MSTRG.21658	Caap1
MSTRG.21659.1	MSTRG.21659	Caap1

MSTRG.21660.1	MSTRG.21660	Caap1
MSTRG.21662.1	MSTRG.21662	.
MSTRG.21665.1	MSTRG.21665	Plaa
MSTRG.21668.1	MSTRG.21668	Ift74
MSTRG.21678.1	MSTRG.21678	Cyp2j13
MSTRG.21679.1	MSTRG.21679	Cyp2j13
MSTRG.2168.1	MSTRG.2168	Trp53bp2
MSTRG.21683.1	MSTRG.21683	Hook1
MSTRG.21684.1	MSTRG.21684	Hook1
MSTRG.21685.1	MSTRG.21685	Hook1
MSTRG.21686.1	MSTRG.21686	.
MSTRG.21688.1	MSTRG.21688	.
MSTRG.21690.1	MSTRG.21690	E130114P18Rik
MSTRG.21693.1	MSTRG.21693	Nfia
MSTRG.21694.1	MSTRG.21694	Nfia
MSTRG.21694.2	MSTRG.21694	Nfia
MSTRG.21696.1	MSTRG.21696	Tm2d1
MSTRG.21703.1	MSTRG.21703	Atg4c
MSTRG.21711.1	MSTRG.21711	Itgb3bp
MSTRG.21712.1	MSTRG.21712	Itgb3bp
MSTRG.21713.1	MSTRG.21713	Itgb3bp
MSTRG.21714.1	MSTRG.21714	Itgb3bp
MSTRG.21715.1	MSTRG.21715	Itgb3bp
MSTRG.21716.1	MSTRG.21716	Itgb3bp
MSTRG.21717.1	MSTRG.21717	Itgb3bp
MSTRG.21719.1	MSTRG.21719	Efcab7
MSTRG.21720.1	MSTRG.21720	Efcab7
MSTRG.21724.1	MSTRG.21724	Ror1
MSTRG.21727.1	MSTRG.21727	Ror1
MSTRG.21728.1	MSTRG.21728	Ror1
MSTRG.21729.1	MSTRG.21729	Ror1
MSTRG.21733.1	MSTRG.21733	Cachd1
MSTRG.2174.1	MSTRG.2174	Disp1
MSTRG.21746.1	MSTRG.21746	Leprot
MSTRG.21748.1	MSTRG.21748	Gm22533
MSTRG.21749.1	MSTRG.21749	Dnajc6
MSTRG.2175.1	MSTRG.2175	Disp1
MSTRG.21750.1	MSTRG.21750	.
MSTRG.21751.1	MSTRG.21751	.
MSTRG.21754.1	MSTRG.21754	Sgip1
MSTRG.21757.1	MSTRG.21757	Tctex1d1
MSTRG.21758.1	MSTRG.21758	BB031773
MSTRG.2176.1	MSTRG.2176	Disp1
MSTRG.21760.1	MSTRG.21760	.
MSTRG.21764.1	MSTRG.21764	Oma1
MSTRG.21765.1	MSTRG.21765	Oma1
MSTRG.21767.1	MSTRG.21767	.
MSTRG.21769.1	MSTRG.21769	Wdr78
MSTRG.2177.1	MSTRG.2177	Disp1
MSTRG.21771.1	MSTRG.21771	Wdr78
MSTRG.21778.1	MSTRG.21778	Fyb2
MSTRG.21779.1	MSTRG.21779	Fyb2
MSTRG.2178.1	MSTRG.2178	Disp1
MSTRG.21782.1	MSTRG.21782	Plpp3
MSTRG.21783.1	MSTRG.21783	Plpp3
MSTRG.21789.1	MSTRG.21789	Usp24
MSTRG.2179.1	MSTRG.2179	Disp1

MSTRG.21792.7	MSTRG.21792	Ssbp3
MSTRG.21793.1	MSTRG.21793	Ssbp3
MSTRG.21795.1	MSTRG.21795	Gm12786
MSTRG.21797.1	MSTRG.21797	.
MSTRG.21801.1	MSTRG.21801	Tceanc2
MSTRG.21808.1	MSTRG.21808	Hspb11
MSTRG.21809.1	MSTRG.21809	Hspb11
MSTRG.21813.1	MSTRG.21813	Yipf1
MSTRG.21814.1	MSTRG.21814	Yipf1
MSTRG.21817.1	MSTRG.21817	Ndc1
MSTRG.21818.1	MSTRG.21818	Ndc1
MSTRG.21824.1	MSTRG.21824	Lrp8
MSTRG.21825.1	MSTRG.21825	Lrp8
MSTRG.21826.1	MSTRG.21826	Lrp8
MSTRG.2183.1	MSTRG.2183	Aida
MSTRG.21830.1	MSTRG.21830	.
MSTRG.21839.1	MSTRG.21839	Btf3l4
MSTRG.21840.1	MSTRG.21840	Btf3l4
MSTRG.21846.1	MSTRG.21846	Gm12739
MSTRG.21851.1	MSTRG.21851	Prpf38a
MSTRG.21851.5	MSTRG.21851	Prpf38a
MSTRG.21853.1	MSTRG.21853	Orc1
MSTRG.2186.1	MSTRG.2186	Mia3
MSTRG.21862.1	MSTRG.21862	Eps15
MSTRG.21867.1	MSTRG.21867	Osbpl9
MSTRG.21873.1	MSTRG.21873	Gm12811
MSTRG.21878.1	MSTRG.21878	Gm12808
MSTRG.21888.1	MSTRG.21888	Agbl4
MSTRG.21891.1	MSTRG.21891	Agbl4
MSTRG.21892.1	MSTRG.21892	Agbl4
MSTRG.21894.1	MSTRG.21894	.
MSTRG.21895.1	MSTRG.21895	.
MSTRG.21897.1	MSTRG.21897	Spata6
MSTRG.21899.1	MSTRG.21899	Spata6
MSTRG.21906.1	MSTRG.21906	Skint2
MSTRG.21908.1	MSTRG.21908	Skint2
MSTRG.21910.1	MSTRG.21910	Skint10
MSTRG.21916.1	MSTRG.21916	Trabd2b
MSTRG.21917.1	MSTRG.21917	Trabd2b
MSTRG.21918.1	MSTRG.21918	Trabd2b
MSTRG.21921.1	MSTRG.21921	Cmpk1
MSTRG.21922.1	MSTRG.21922	Cmpk1
MSTRG.21923.1	MSTRG.21923	Cmpk1
MSTRG.21928.1	MSTRG.21928	Faah
MSTRG.21932.1	MSTRG.21932	Atpaf1
MSTRG.21935.1	MSTRG.21935	Mob3c
MSTRG.21939.1	MSTRG.21939	Nsun4
MSTRG.21943.1	MSTRG.21943	AL670603.1
MSTRG.21944.1	MSTRG.21944	Pik3r3
MSTRG.21945.1	MSTRG.21945	Pik3r3
MSTRG.21947.1	MSTRG.21947	Mast2
MSTRG.21976.1	MSTRG.21976	Tesk2
MSTRG.21977.1	MSTRG.21977	Tesk2
MSTRG.21981.1	MSTRG.21981	Mutyh
MSTRG.21989.1	MSTRG.21989	.
MSTRG.21995.1	MSTRG.21995	.
MSTRG.22.1	MSTRG.22	Atp6v1h

MSTRG.2200.1	MSTRG.2200	Rab3gap2
MSTRG.22002.1	MSTRG.22002	.
MSTRG.22003.1	MSTRG.22003	.
MSTRG.22005.1	MSTRG.22005	Eri3
MSTRG.22006.1	MSTRG.22006	Eri3
MSTRG.22016.1	MSTRG.22016	.
MSTRG.22018.1	MSTRG.22018	St3gal3
MSTRG.22020.1	MSTRG.22020	St3gal3
MSTRG.22021.1	MSTRG.22021	St3gal3
MSTRG.22024.1	MSTRG.22024	.
MSTRG.22025.1	MSTRG.22025	.
MSTRG.22028.1	MSTRG.22028	Ptprf
MSTRG.22037.1	MSTRG.22037	Olfr1340
MSTRG.22037.2	MSTRG.22037	Olfr1340
MSTRG.22044.1	MSTRG.22044	Svbp
MSTRG.22048.1	MSTRG.22048	P3h1
MSTRG.22061.1	MSTRG.22061	.
MSTRG.22067.1	MSTRG.22067	Hivep3
MSTRG.22068.1	MSTRG.22068	Hivep3
MSTRG.22069.1	MSTRG.22069	Hivep3
MSTRG.22070.1	MSTRG.22070	Hivep3
MSTRG.22071.1	MSTRG.22071	Hivep3
MSTRG.22072.1	MSTRG.22072	Hivep3
MSTRG.22074.1	MSTRG.22074	Hivep3
MSTRG.2208.1	MSTRG.2208	Eprs
MSTRG.22087.1	MSTRG.22087	Zfp69
MSTRG.22088.1	MSTRG.22088	Zfp69
MSTRG.22089.1	MSTRG.22089	Zfp69
MSTRG.22090.1	MSTRG.22090	Zfp69
MSTRG.22091.1	MSTRG.22091	.
MSTRG.22098.1	MSTRG.22098	Gm12877
MSTRG.22101.1	MSTRG.22101	Ppt1
MSTRG.22103.1	MSTRG.22103	Cap1
MSTRG.22105.1	MSTRG.22105	Gm12891
MSTRG.22105.2	MSTRG.22105	.
MSTRG.22110.1	MSTRG.22110	Trit1
MSTRG.22111.1	MSTRG.22111	Trit1
MSTRG.22112.1	MSTRG.22112	Trit1
MSTRG.22125.1	MSTRG.22125	Akirin1
MSTRG.22129.3	MSTRG.22129	Mycbp
MSTRG.22143.1	MSTRG.22143	Macf1
MSTRG.22144.1	MSTRG.22144	Macf1
MSTRG.22146.1	MSTRG.22146	Macf1
MSTRG.22149.1	MSTRG.22149	Macf1
MSTRG.22153.1	MSTRG.22153	Inpp5b
MSTRG.22154.1	MSTRG.22154	Inpp5b
MSTRG.22155.1	MSTRG.22155	Inpp5b
MSTRG.22157.1	MSTRG.22157	Mtf1
MSTRG.22159.1	MSTRG.22159	Mtf1
MSTRG.22160.1	MSTRG.22160	Mtf1
MSTRG.22175.1	MSTRG.22175	.
MSTRG.2218.10	MSTRG.2218	Gpatch2
MSTRG.22186.3	MSTRG.22186	Stk40
MSTRG.22189.1	MSTRG.22189	Map7d1
MSTRG.22194.1	MSTRG.22194	Thrap3
MSTRG.22200.1	MSTRG.22200	Ago3
MSTRG.22201.1	MSTRG.22201	Ago3

MSTRG.22202.1	MSTRG.22202	Ago3
MSTRG.2222.1	MSTRG.2222	Ush2a
MSTRG.22223.1	MSTRG.22223	AU040320
MSTRG.22226.1	MSTRG.22226	Zmym4
MSTRG.22227.1	MSTRG.22227	Zmym4
MSTRG.22228.1	MSTRG.22228	Zmym4
MSTRG.22229.1	MSTRG.22229	Zmym4
MSTRG.22232.1	MSTRG.22232	.
MSTRG.22235.1	MSTRG.22235	Zmym1
MSTRG.22244.1	MSTRG.22244	Rnf19b
MSTRG.22246.1	MSTRG.22246	S100pbp
MSTRG.22248.1	MSTRG.22248	Yars
MSTRG.2225.11	MSTRG.2225	Kctd3
MSTRG.22250.1	MSTRG.22250	C77080
MSTRG.22253.1	MSTRG.22253	Sync
MSTRG.22257.1	MSTRG.22257	Zbtb8a
MSTRG.22259.1	MSTRG.22259	Bsdc1
MSTRG.2226.1	MSTRG.2226	Kctd3
MSTRG.22263.1	MSTRG.22263	Marcksl1
MSTRG.22266.1	MSTRG.22266	Hdac1
MSTRG.22267.1	MSTRG.22267	Hdac1
MSTRG.22268.1	MSTRG.22268	Hdac1
MSTRG.22269.1	MSTRG.22269	Hdac1
MSTRG.2227.1	MSTRG.2227	Kctd3
MSTRG.22270.1	MSTRG.22270	Hdac1
MSTRG.22272.1	MSTRG.22272	Lck
MSTRG.22273.1	MSTRG.22273	Lck
MSTRG.22274.1	MSTRG.22274	.
MSTRG.22274.2	MSTRG.22274	.
MSTRG.22274.3	MSTRG.22274	.
MSTRG.22275.1	MSTRG.22275	.
MSTRG.22277.1	MSTRG.22277	Tmem39b
MSTRG.22277.2	MSTRG.22277	Tmem39b
MSTRG.22280.1	MSTRG.22280	Khdrbs1
MSTRG.22281.1	MSTRG.22281	Khdrbs1
MSTRG.22288.2	MSTRG.22288	Dcdc2b
MSTRG.22293.1	MSTRG.22293	Kpna6
MSTRG.22296.1	MSTRG.22296	Pef1
MSTRG.22301.1	MSTRG.22301	Fabp3
MSTRG.22303.1	MSTRG.22303	Zcchc17
MSTRG.22304.1	MSTRG.22304	Zcchc17
MSTRG.22310.1	MSTRG.22310	Matn1
MSTRG.22314.1	MSTRG.22314	Pum1
MSTRG.22321.1	MSTRG.22321	Srsf4
MSTRG.22322.1	MSTRG.22322	Gm12992
MSTRG.22325.1	MSTRG.22325	Ythdf2
MSTRG.22328.1	MSTRG.22328	Gm13063
MSTRG.22333.1	MSTRG.22333	Taf12
MSTRG.22335.1	MSTRG.22335	.
MSTRG.22336.1	MSTRG.22336	.
MSTRG.22341.1	MSTRG.22341	Trnau1ap
MSTRG.22347.1	MSTRG.22347	Phactr4
MSTRG.22348.1	MSTRG.22348	Phactr4
MSTRG.22352.1	MSTRG.22352	Sesn2
MSTRG.22358.1	MSTRG.22358	.
MSTRG.2236.1	MSTRG.2236	Ptpn14
MSTRG.22360.1	MSTRG.22360	Eya3

MSTRG.22361.1	MSTRG.22361	Eya3
MSTRG.22362.1	MSTRG.22362	Eya3
MSTRG.22363.1	MSTRG.22363	Eya3
MSTRG.2237.1	MSTRG.2237	Ptpn14
MSTRG.22372.1	MSTRG.22372	Stx12
MSTRG.22375.1	MSTRG.22375	.
MSTRG.22376.1	MSTRG.22376	.
MSTRG.22377.1	MSTRG.22377	.
MSTRG.22378.1	MSTRG.22378	Ahdc1
MSTRG.22380.1	MSTRG.22380	Ahdc1
MSTRG.22383.1	MSTRG.22383	Ahdc1
MSTRG.22387.1	MSTRG.22387	Wasf2
MSTRG.22389.1	MSTRG.22389	Wasf2
MSTRG.22390.1	MSTRG.22390	Wasf2
MSTRG.22391.1	MSTRG.22391	Wasf2
MSTRG.22392.1	MSTRG.22392	Wasf2
MSTRG.22393.1	MSTRG.22393	Wasf2
MSTRG.22394.1	MSTRG.22394	Wasf2
MSTRG.22399.1	MSTRG.22399	Wdtc1
MSTRG.22401.1	MSTRG.22401	Wdtc1
MSTRG.22402.1	MSTRG.22402	Wdtc1
MSTRG.22404.1	MSTRG.22404	Slc9a1
MSTRG.22405.1	MSTRG.22405	Slc9a1
MSTRG.22414.1	MSTRG.22414	Zdhhc18
MSTRG.22416.1	MSTRG.22416	.
MSTRG.22418.1	MSTRG.22418	Gm22662
MSTRG.22418.3	MSTRG.22418	Arid1a
MSTRG.22418.4	MSTRG.22418	Arid1a
MSTRG.22418.5	MSTRG.22418	Arid1a
MSTRG.22418.6	MSTRG.22418	Arid1a
MSTRG.22419.1	MSTRG.22419	Pigv
MSTRG.22419.4	MSTRG.22419	Pigv
MSTRG.2242.1	MSTRG.2242	Smyd2
MSTRG.22422.1	MSTRG.22422	Arid1a
MSTRG.22424.1	MSTRG.22424	Arid1a
MSTRG.22431.1	MSTRG.22431	Dhdds
MSTRG.22432.1	MSTRG.22432	Dhdds
MSTRG.22435.2	MSTRG.22435	Cd52
MSTRG.22441.1	MSTRG.22441	Cep85
MSTRG.22442.1	MSTRG.22442	Cep85
MSTRG.22443.1	MSTRG.22443	Cep85
MSTRG.22446.2	MSTRG.22446	Stmn1
MSTRG.2245.1	MSTRG.2245	Rps6kc1
MSTRG.22454.1	MSTRG.22454	Ldlrap1
MSTRG.22466.1	MSTRG.22466	Srrm1
MSTRG.22477.1	MSTRG.22477	Hmgcl
MSTRG.2248.1	MSTRG.2248	Vash2
MSTRG.2249.1	MSTRG.2249	.
MSTRG.22500.1	MSTRG.22500	Ephb2
MSTRG.22501.1	MSTRG.22501	Ephb2
MSTRG.22502.1	MSTRG.22502	Ephb2
MSTRG.22503.1	MSTRG.22503	Ephb2
MSTRG.22504.1	MSTRG.22504	Ephb2
MSTRG.22505.1	MSTRG.22505	Ephb2
MSTRG.22506.1	MSTRG.22506	Ephb2
MSTRG.22507.1	MSTRG.22507	Ephb2
MSTRG.22508.1	MSTRG.22508	Ephb2

MSTRG.22509.1	MSTRG.22509	Ephb2
MSTRG.2251.1	MSTRG.2251	Mfsd7b
MSTRG.22510.1	MSTRG.22510	Ephb2
MSTRG.22512.1	MSTRG.22512	Zbtb40
MSTRG.22513.1	MSTRG.22513	Zbtb40
MSTRG.22515.1	MSTRG.22515	Zbtb40
MSTRG.22516.1	MSTRG.22516	.
MSTRG.22517.1	MSTRG.22517	.
MSTRG.22519.1	MSTRG.22519	Gm13005
MSTRG.22528.1	MSTRG.22528	Usp48
MSTRG.22530.1	MSTRG.22530	Usp48
MSTRG.22531.1	MSTRG.22531	Usp48
MSTRG.22532.1	MSTRG.22532	Usp48
MSTRG.22542.1	MSTRG.22542	Pla2g2e
MSTRG.22546.1	MSTRG.22546	Otud3
MSTRG.22547.1	MSTRG.22547	Otud3
MSTRG.22548.1	MSTRG.22548	Otud3
MSTRG.22552.1	MSTRG.22552	Gm45533
MSTRG.22555.2	MSTRG.22555	Minos1
MSTRG.22556.1	MSTRG.22556	Minos1
MSTRG.22558.1	MSTRG.22558	.
MSTRG.22561.1	MSTRG.22561	Capzb
MSTRG.22563.1	MSTRG.22563	Pqlc2
MSTRG.22564.1	MSTRG.22564	Pqlc2
MSTRG.22567.1	MSTRG.22567	Alpl
MSTRG.22567.2	MSTRG.22567	Alpl
MSTRG.22567.3	MSTRG.22567	Ece1
MSTRG.22567.4	MSTRG.22567	Ece1
MSTRG.22568.1	MSTRG.22568	.
MSTRG.22571.1	MSTRG.22571	Ece1
MSTRG.22572.1	MSTRG.22572	Eif4g3
MSTRG.22574.1	MSTRG.22574	Eif4g3
MSTRG.22575.1	MSTRG.22575	Eif4g3
MSTRG.22576.1	MSTRG.22576	Eif4g3
MSTRG.22576.2	MSTRG.22576	Eif4g3
MSTRG.22577.1	MSTRG.22577	Eif4g3
MSTRG.22578.1	MSTRG.22578	Eif4g3
MSTRG.22579.1	MSTRG.22579	Eif4g3
MSTRG.2258.1	MSTRG.2258	.
MSTRG.22580.1	MSTRG.22580	Eif4g3
MSTRG.22581.1	MSTRG.22581	Eif4g3
MSTRG.22582.1	MSTRG.22582	Eif4g3
MSTRG.22582.2	MSTRG.22582	Eif4g3
MSTRG.22582.3	MSTRG.22582	Eif4g3
MSTRG.22582.4	MSTRG.22582	Eif4g3
MSTRG.22582.5	MSTRG.22582	Eif4g3
MSTRG.22583.1	MSTRG.22583	Eif4g3
MSTRG.22584.1	MSTRG.22584	Eif4g3
MSTRG.22586.1	MSTRG.22586	Eif4g3
MSTRG.22587.1	MSTRG.22587	Eif4g3
MSTRG.2259.1	MSTRG.2259	.
MSTRG.22594.1	MSTRG.22594	Gm21969
MSTRG.226.1	MSTRG.226	.
MSTRG.22602.1	MSTRG.22602	Arhgef10l
MSTRG.22605.1	MSTRG.22605	Rcc2
MSTRG.22617.1	MSTRG.22617	Spata21
MSTRG.22618.2	MSTRG.22618	4921514A10Rik

MSTRG.22619.1	MSTRG.22619	Szrd1
MSTRG.22624.1	MSTRG.22624	Plekhm2
MSTRG.22625.1	MSTRG.22625	Plekhm2
MSTRG.22627.1	MSTRG.22627	Clcnka
MSTRG.2263.1	MSTRG.2263	Tmem206
MSTRG.22635.1	MSTRG.22635	Spen
MSTRG.22636.1	MSTRG.22636	Spen
MSTRG.22639.1	MSTRG.22639	.
MSTRG.22641.1	MSTRG.22641	Ddi2
MSTRG.22642.1	MSTRG.22642	Ddi2
MSTRG.22646.1	MSTRG.22646	Efhd2
MSTRG.22657.1	MSTRG.22657	Kazn
MSTRG.22658.1	MSTRG.22658	.
MSTRG.22659.1	MSTRG.22659	.
MSTRG.2266.1	MSTRG.2266	Gm37432
MSTRG.2266.2	MSTRG.2266	.
MSTRG.22660.1	MSTRG.22660	.
MSTRG.22662.1	MSTRG.22662	.
MSTRG.22663.1	MSTRG.22663	.
MSTRG.22665.1	MSTRG.22665	.
MSTRG.22670.1	MSTRG.22670	Prdm2
MSTRG.22673.1	MSTRG.22673	Prdm2
MSTRG.22675.1	MSTRG.22675	.
MSTRG.22676.1	MSTRG.22676	.
MSTRG.22677.1	MSTRG.22677	.
MSTRG.22678.1	MSTRG.22678	.
MSTRG.2268.1	MSTRG.2268	.
MSTRG.22682.1	MSTRG.22682	Dhrs3
MSTRG.22688.1	MSTRG.22688	Vps13d
MSTRG.2269.1	MSTRG.2269	.
MSTRG.22690.1	MSTRG.22690	Zfp987
MSTRG.22693.1	MSTRG.22693	.
MSTRG.22698.1	MSTRG.22698	Gm13212
MSTRG.22699.1	MSTRG.22699	Gm13212
MSTRG.22700.1	MSTRG.22700	Gm13212
MSTRG.22717.1	MSTRG.22717	Gm13137
MSTRG.22722.1	MSTRG.22722	Zfp984
MSTRG.22724.4	MSTRG.22724	Zfp984
MSTRG.22724.5	MSTRG.22724	Zfp984
MSTRG.22745.1	MSTRG.22745	Gm26573
MSTRG.22746.1	MSTRG.22746	Gm26573
MSTRG.22751.1	MSTRG.22751	Zfp981
MSTRG.22759.2	MSTRG.22759	Zfp993
MSTRG.22760.1	MSTRG.22760	Gm26573
MSTRG.22764.1	MSTRG.22764	Gm26573
MSTRG.22771.1	MSTRG.22771	C230088H06Rik
MSTRG.22781.1	MSTRG.22781	C230088H06Rik
MSTRG.22800.1	MSTRG.22800	Clcn6
MSTRG.22818.1	MSTRG.22818	Exosc10
MSTRG.22820.1	MSTRG.22820	Exosc10
MSTRG.22823.1	MSTRG.22823	Gm572
MSTRG.22825.1	MSTRG.22825	.
MSTRG.2283.1	MSTRG.2283	Traf5
MSTRG.22833.1	MSTRG.22833	Pex14
MSTRG.22834.1	MSTRG.22834	Pex14
MSTRG.22835.1	MSTRG.22835	Pex14
MSTRG.22836.1	MSTRG.22836	Pex14

MSTRG.22837.1	MSTRG.22837	Pex14
MSTRG.22838.1	MSTRG.22838	Pex14
MSTRG.22844.1	MSTRG.22844	Kif1b
MSTRG.22845.1	MSTRG.22845	Kif1b
MSTRG.22851.1	MSTRG.22851	Ube4b
MSTRG.22852.1	MSTRG.22852	Ube4b
MSTRG.22853.1	MSTRG.22853	.
MSTRG.22854.1	MSTRG.22854	.
MSTRG.22861.1	MSTRG.22861	Ctnnbip1
MSTRG.22867.1	MSTRG.22867	Pik3cd
MSTRG.2287.1	MSTRG.2287	Rcor3
MSTRG.22870.1	MSTRG.22870	Slc25a33
MSTRG.22878.1	MSTRG.22878	Spsb1
MSTRG.22879.1	MSTRG.22879	Spsb1
MSTRG.2288.1	MSTRG.2288	Rcor3
MSTRG.22880.1	MSTRG.22880	Spsb1
MSTRG.22881.1	MSTRG.22881	Spsb1
MSTRG.22882.1	MSTRG.22882	.
MSTRG.22887.1	MSTRG.22887	.
MSTRG.22889.1	MSTRG.22889	H6pd
MSTRG.2289.1	MSTRG.2289	Rcor3
MSTRG.22895.1	MSTRG.22895	Rere
MSTRG.22895.2	MSTRG.22895	Rere
MSTRG.22895.3	MSTRG.22895	Rere
MSTRG.22897.1	MSTRG.22897	Rere
MSTRG.22898.1	MSTRG.22898	Gm13092
MSTRG.229.1	MSTRG.229	Prim2
MSTRG.22901.1	MSTRG.22901	Rere
MSTRG.22901.2	MSTRG.22901	Rere
MSTRG.22902.1	MSTRG.22902	Rere
MSTRG.22903.1	MSTRG.22903	Rere
MSTRG.22904.1	MSTRG.22904	Gm13091
MSTRG.22905.1	MSTRG.22905	Gm13091
MSTRG.22905.2	MSTRG.22905	Gm13091
MSTRG.22906.1	MSTRG.22906	Rere
MSTRG.22918.1	MSTRG.22918	Camta1
MSTRG.22925.1	MSTRG.22925	Camta1
MSTRG.22928.1	MSTRG.22928	Acot7
MSTRG.22931.1	MSTRG.22931	Acot7
MSTRG.22942.1	MSTRG.22942	Kcnab2
MSTRG.22945.1	MSTRG.22945	Dffb
MSTRG.22952.1	MSTRG.22952	Wrap73
MSTRG.22955.1	MSTRG.22955	Megf6
MSTRG.22966.1	MSTRG.22966	Faap20
MSTRG.22967.1	MSTRG.22967	Rer1
MSTRG.22967.4	MSTRG.22967	Rer1
MSTRG.22970.1	MSTRG.22970	Ski
MSTRG.22971.1	MSTRG.22971	Ski
MSTRG.22972.1	MSTRG.22972	Ski
MSTRG.22974.1	MSTRG.22974	Prkcz
MSTRG.22976.1	MSTRG.22976	.
MSTRG.22978.1	MSTRG.22978	Gm16023
MSTRG.22986.1	MSTRG.22986	Cfap74
MSTRG.22988.1	MSTRG.22988	Cfap74
MSTRG.22991.1	MSTRG.22991	Gnb1
MSTRG.22993.1	MSTRG.22993	Gnb1
MSTRG.22994.1	MSTRG.22994	Gnb1

MSTRG.22995.1	MSTRG.22995	Gnb1
MSTRG.22996.1	MSTRG.22996	Nadk
MSTRG.22998.1	MSTRG.22998	.
MSTRG.23.1	MSTRG.23	Atp6v1h
MSTRG.230.1	MSTRG.230	Prim2
MSTRG.2300.1	MSTRG.2300	Diexf
MSTRG.23000.2	MSTRG.23000	Ssu72
MSTRG.23000.5	MSTRG.23000	Ssu72
MSTRG.23001.1	MSTRG.23001	Atad3a
MSTRG.23006.1	MSTRG.23006	Atad3aos
MSTRG.23013.1	MSTRG.23013	Mrpl20
MSTRG.23019.1	MSTRG.23019	Ints11
MSTRG.23020.1	MSTRG.23020	Ints11
MSTRG.23021.1	MSTRG.23021	Ints11
MSTRG.23025.1	MSTRG.23025	Ube2j2
MSTRG.23026.1	MSTRG.23026	Ube2j2
MSTRG.23027.1	MSTRG.23027	.
MSTRG.23029.10	MSTRG.23029	9430015G10Rik
MSTRG.23029.12	MSTRG.23029	9430015G10Rik
MSTRG.23029.5	MSTRG.23029	9430015G10Rik
MSTRG.23032.1	MSTRG.23032	Sdf4
MSTRG.23032.2	MSTRG.23032	Sdf4
MSTRG.23033.4	MSTRG.23033	Tnfrsf4
MSTRG.23037.1	MSTRG.23037	Gm16008
MSTRG.2304.1	MSTRG.2304	.
MSTRG.23044.1	MSTRG.23044	AW011738
MSTRG.23044.2	MSTRG.23044	AW011738
MSTRG.23044.3	MSTRG.23044	AW011738
MSTRG.23052.1	MSTRG.23052	.
MSTRG.23053.1	MSTRG.23053	.
MSTRG.23055.1	MSTRG.23055	Cdk6
MSTRG.23056.1	MSTRG.23056	Cdk6
MSTRG.23057.1	MSTRG.23057	Cdk6
MSTRG.23062.1	MSTRG.23062	Mterf1b
MSTRG.23065.1	MSTRG.23065	Akap9
MSTRG.2307.4	MSTRG.2307	Lamb3
MSTRG.23076.1	MSTRG.23076	Pex1
MSTRG.23083.1	MSTRG.23083	Ankib1
MSTRG.23084.1	MSTRG.23084	Ankib1
MSTRG.23092.1	MSTRG.23092	.
MSTRG.231.1	MSTRG.231	Prim2
MSTRG.231.2	MSTRG.231	Prim2
MSTRG.23105.1	MSTRG.23105	.
MSTRG.23116.1	MSTRG.23116	Slc25a40
MSTRG.23119.1	MSTRG.23119	Abcb1b
MSTRG.23125.1	MSTRG.23125	Crot
MSTRG.23126.1	MSTRG.23126	Crot
MSTRG.23129.1	MSTRG.23129	.
MSTRG.23130.1	MSTRG.23130	.
MSTRG.23133.1	MSTRG.23133	1700003C15Rik
MSTRG.23134.3	MSTRG.23134	Tmem243
MSTRG.23139.1	MSTRG.23139	.
MSTRG.23145.1	MSTRG.23145	.
MSTRG.23153.1	MSTRG.23153	.
MSTRG.23155.1	MSTRG.23155	Ptpn12
MSTRG.23157.1	MSTRG.23157	Ptpn12
MSTRG.23160.1	MSTRG.23160	Gsap

MSTRG.23161.1	MSTRG.23161	Gsap
MSTRG.23162.1	MSTRG.23162	Gsap
MSTRG.23167.1	MSTRG.23167	Fam185a
MSTRG.23187.1	MSTRG.23187	Reln
MSTRG.23198.1	MSTRG.23198	6030443J06Rik
MSTRG.23207.1	MSTRG.23207	Lhfpl3
MSTRG.23208.1	MSTRG.23208	Lhfpl3
MSTRG.23209.1	MSTRG.23209	Lhfpl3
MSTRG.2321.1	MSTRG.2321	Plekhg1
MSTRG.23211.2	MSTRG.23211	AI506816
MSTRG.23217.9	MSTRG.23217	Pus7
MSTRG.23219.7	MSTRG.23219	Rint1
MSTRG.2322.1	MSTRG.2322	Plekhg1
MSTRG.23221.1	MSTRG.23221	Rint1
MSTRG.23223.1	MSTRG.23223	4933427G23Rik
MSTRG.23224.1	MSTRG.23224	4933427G23Rik
MSTRG.23225.1	MSTRG.23225	4933427G23Rik
MSTRG.23226.8	MSTRG.23226	Fam126a
MSTRG.23227.1	MSTRG.23227	Fam126a
MSTRG.23228.1	MSTRG.23228	Fam126a
MSTRG.23229.1	MSTRG.23229	Fam126a
MSTRG.23231.1	MSTRG.23231	Fam126a
MSTRG.23232.1	MSTRG.23232	Fam126a
MSTRG.23235.1	MSTRG.23235	5031425E22Rik
MSTRG.23235.4	MSTRG.23235	5031425E22Rik
MSTRG.23235.5	MSTRG.23235	5031425E22Rik
MSTRG.23235.7	MSTRG.23235	5031425E22Rik
MSTRG.23242.1	MSTRG.23242	Kmt2e
MSTRG.23244.1	MSTRG.23244	Srpk2
MSTRG.23245.1	MSTRG.23245	Srpk2
MSTRG.23246.1	MSTRG.23246	Srpk2
MSTRG.23247.1	MSTRG.23247	Srpk2
MSTRG.23248.1	MSTRG.23248	Srpk2
MSTRG.23249.1	MSTRG.23249	Srpk2
MSTRG.23250.1	MSTRG.23250	Srpk2
MSTRG.23254.1	MSTRG.23254	Klhl7
MSTRG.23258.1	MSTRG.23258	Nupl2
MSTRG.23259.1	MSTRG.23259	.
MSTRG.23266.1	MSTRG.23266	Agap3
MSTRG.23276.1	MSTRG.23276	Smarcd3
MSTRG.23277.3	MSTRG.23277	Nub1
MSTRG.23278.1	MSTRG.23278	Gm26648
MSTRG.2328.3	MSTRG.2328	A330023F24Rik
MSTRG.2328.4	MSTRG.2328	A330023F24Rik
MSTRG.23283.1	MSTRG.23283	Prkag2
MSTRG.23292.1	MSTRG.23292	.
MSTRG.23294.1	MSTRG.23294	.
MSTRG.23295.1	MSTRG.23295	.
MSTRG.23296.1	MSTRG.23296	.
MSTRG.23299.1	MSTRG.23299	Xrcc2
MSTRG.23305.1	MSTRG.23305	Kmt2c
MSTRG.23306.1	MSTRG.23306	Kmt2c
MSTRG.23307.1	MSTRG.23307	Kmt2c
MSTRG.23311.1	MSTRG.23311	Paxip1
MSTRG.23324.1	MSTRG.23324	Rbm33
MSTRG.23328.1	MSTRG.23328	Lmbr1
MSTRG.23329.1	MSTRG.23329	Lmbr1

MSTRG.23331.1	MSTRG.23331	Lmbr1
MSTRG.23334.1	MSTRG.23334	Ube3c
MSTRG.23336.1	MSTRG.23336	.
MSTRG.23340.1	MSTRG.23340	Dnajb6
MSTRG.23341.1	MSTRG.23341	Dnajb6
MSTRG.23343.1	MSTRG.23343	Dnajb6
MSTRG.23349.1	MSTRG.23349	.
MSTRG.23350.1	MSTRG.23350	.
MSTRG.23355.1	MSTRG.23355	Hadhb
MSTRG.23360.7	MSTRG.23360	Selenoi
MSTRG.23373.1	MSTRG.23373	Ost4
MSTRG.23373.2	MSTRG.23373	Ost4
MSTRG.2338.1	MSTRG.2338	Akap12
MSTRG.23380.1	MSTRG.23380	Atraid
MSTRG.23382.1	MSTRG.23382	Mpv17
MSTRG.2340.1	MSTRG.2340	Akap12
MSTRG.23404.1	MSTRG.23404	Slc4a1ap
MSTRG.23408.6	MSTRG.23408	Mrpl33
MSTRG.23409.1	MSTRG.23409	Mrpl33
MSTRG.23412.1	MSTRG.23412	Gm43809
MSTRG.23413.1	MSTRG.23413	Gm43809
MSTRG.23416.1	MSTRG.23416	Babam2
MSTRG.23419.9	MSTRG.23419	Ppp1cb
MSTRG.2342.1	MSTRG.2342	Akap12
MSTRG.23427.1	MSTRG.23427	Yes1
MSTRG.23432.1	MSTRG.23432	Gm20671
MSTRG.23438.1	MSTRG.23438	Depdc5
MSTRG.2344.1	MSTRG.2344	Zbtb2
MSTRG.23440.1	MSTRG.23440	Gm43850
MSTRG.23443.1	MSTRG.23443	Uvssa
MSTRG.23454.1	MSTRG.23454	Nsd2
MSTRG.23459.1	MSTRG.23459	.
MSTRG.23466.1	MSTRG.23466	Poln
MSTRG.23467.1	MSTRG.23467	Poln
MSTRG.2347.1	MSTRG.2347	Mthfd1l
MSTRG.23473.1	MSTRG.23473	Fam193a
MSTRG.23473.2	MSTRG.23473	Fam193a
MSTRG.23473.3	MSTRG.23473	Fam193a
MSTRG.23473.4	MSTRG.23473	Fam193a
MSTRG.23474.1	MSTRG.23474	.
MSTRG.23476.1	MSTRG.23476	Fam193a
MSTRG.23476.2	MSTRG.23476	Fam193a
MSTRG.23477.1	MSTRG.23477	Fam193a
MSTRG.23478.1	MSTRG.23478	Fam193a
MSTRG.23485.1	MSTRG.23485	Add1
MSTRG.23493.1	MSTRG.23493	Htt
MSTRG.23508.1	MSTRG.23508	Afap1
MSTRG.23521.1	MSTRG.23521	D5Ertd579e
MSTRG.23523.1	MSTRG.23523	D5Ertd579e
MSTRG.23525.1	MSTRG.23525	D5Ertd579e
MSTRG.23529.1	MSTRG.23529	Jakmip1
MSTRG.23530.1	MSTRG.23530	Jakmip1
MSTRG.23532.1	MSTRG.23532	Jakmip1
MSTRG.23533.1	MSTRG.23533	Jakmip1
MSTRG.23534.1	MSTRG.23534	Jakmip1
MSTRG.23537.1	MSTRG.23537	Stx18
MSTRG.23538.1	MSTRG.23538	Stx18

MSTRG.23539.1	MSTRG.23539	Stx18
MSTRG.23540.1	MSTRG.23540	Stx18
MSTRG.23545.1	MSTRG.23545	Zbtb49
MSTRG.23547.1	MSTRG.23547	Lyar
MSTRG.23550.1	MSTRG.23550	Slc2a9
MSTRG.23555.1	MSTRG.23555	.
MSTRG.23557.1	MSTRG.23557	Rab28
MSTRG.23558.1	MSTRG.23558	Rab28
MSTRG.2356.1	MSTRG.2356	Esr1
MSTRG.23562.1	MSTRG.23562	.
MSTRG.23569.1	MSTRG.23569	Cpeb2
MSTRG.2357.1	MSTRG.2357	.
MSTRG.23570.1	MSTRG.23570	.
MSTRG.23574.1	MSTRG.23574	.
MSTRG.23583.1	MSTRG.23583	Fbxl5
MSTRG.23591.1	MSTRG.23591	Gm42982
MSTRG.23593.1	MSTRG.23593	Gm42984
MSTRG.23597.1	MSTRG.23597	.
MSTRG.23598.5	MSTRG.23598	Med28
MSTRG.2360.1	MSTRG.2360	Mtrf1l
MSTRG.23604.1	MSTRG.23604	.
MSTRG.23609.1	MSTRG.23609	Lcorl
MSTRG.23610.1	MSTRG.23610	Lcorl
MSTRG.23612.1	MSTRG.23612	Lcorl
MSTRG.23615.1	MSTRG.23615	Lcorl
MSTRG.23616.1	MSTRG.23616	Lcorl
MSTRG.23617.1	MSTRG.23617	Lcorl
MSTRG.23619.1	MSTRG.23619	Lcorl
MSTRG.23620.1	MSTRG.23620	Lcorl
MSTRG.23634.1	MSTRG.23634	5730480H06Rik
MSTRG.23635.1	MSTRG.23635	5730480H06Rik
MSTRG.23636.1	MSTRG.23636	5730480H06Rik
MSTRG.23638.1	MSTRG.23638	5730480H06Rik
MSTRG.2366.3	MSTRG.2366	Cnksr3
MSTRG.2367.1	MSTRG.2367	Cnksr3
MSTRG.23679.1	MSTRG.23679	Anapc4
MSTRG.2368.1	MSTRG.2368	Cnksr3
MSTRG.23681.1	MSTRG.23681	Smim20
MSTRG.23686.1	MSTRG.23686	Rbpj
MSTRG.23687.1	MSTRG.23687	Rbpj
MSTRG.23689.1	MSTRG.23689	Tbc1d19
MSTRG.23691.1	MSTRG.23691	Stim2
MSTRG.23692.1	MSTRG.23692	Stim2
MSTRG.23695.1	MSTRG.23695	.
MSTRG.2370.1	MSTRG.2370	AC147512.1
MSTRG.23701.1	MSTRG.23701	4932441J04Rik
MSTRG.2371.1	MSTRG.2371	.
MSTRG.23718.1	MSTRG.23718	.
MSTRG.23719.1	MSTRG.23719	.
MSTRG.23720.1	MSTRG.23720	.
MSTRG.23721.1	MSTRG.23721	.
MSTRG.23727.1	MSTRG.23727	Rell1
MSTRG.23729.1	MSTRG.23729	.
MSTRG.23736.1	MSTRG.23736	Tbc1d1
MSTRG.23738.1	MSTRG.23738	Tbc1d1
MSTRG.23739.1	MSTRG.23739	Tbc1d1
MSTRG.23741.1	MSTRG.23741	Tbc1d1

MSTRG.23743.1	MSTRG.23743	Tbc1d1
MSTRG.23744.1	MSTRG.23744	Tbc1d1
MSTRG.23745.1	MSTRG.23745	Tbc1d1
MSTRG.23747.1	MSTRG.23747	Tbc1d1
MSTRG.23748.1	MSTRG.23748	Tbc1d1
MSTRG.23749.1	MSTRG.23749	Tbc1d1
MSTRG.2375.1	MSTRG.2375	.
MSTRG.23750.1	MSTRG.23750	Tbc1d1
MSTRG.23752.1	MSTRG.23752	Gm3716
MSTRG.23760.1	MSTRG.23760	Fam114a1
MSTRG.23762.1	MSTRG.23762	Tmem156
MSTRG.23766.1	MSTRG.23766	Wdr19
MSTRG.23768.1	MSTRG.23768	Rfc1
MSTRG.23769.1	MSTRG.23769	Rfc1
MSTRG.23771.1	MSTRG.23771	Rfc1
MSTRG.23774.1	MSTRG.23774	.
MSTRG.23777.1	MSTRG.23777	N4bp2
MSTRG.23778.1	MSTRG.23778	.
MSTRG.23779.1	MSTRG.23779	.
MSTRG.2379.1	MSTRG.2379	Ipcef1
MSTRG.23790.1	MSTRG.23790	Smim14
MSTRG.23795.1	MSTRG.23795	Gm40309
MSTRG.23796.1	MSTRG.23796	Ube2k
MSTRG.23796.2	MSTRG.23796	Ube2k
MSTRG.23797.1	MSTRG.23797	Ube2k
MSTRG.23798.1	MSTRG.23798	Ube2k
MSTRG.23799.1	MSTRG.23799	Ube2k
MSTRG.238.1	MSTRG.238	.
MSTRG.2380.1	MSTRG.2380	Ipcef1
MSTRG.23811.13	MSTRG.23811	Rbm47
MSTRG.23812.1	MSTRG.23812	Rbm47
MSTRG.23814.1	MSTRG.23814	Rbm47
MSTRG.23815.1	MSTRG.23815	Rbm47
MSTRG.2383.1	MSTRG.2383	Pcmt1
MSTRG.23831.1	MSTRG.23831	Apbb2
MSTRG.23833.1	MSTRG.23833	Apbb2
MSTRG.23836.1	MSTRG.23836	Tmem33
MSTRG.23838.1	MSTRG.23838	Limch1
MSTRG.23843.1	MSTRG.23843	Limch1
MSTRG.23846.1	MSTRG.23846	.
MSTRG.23849.1	MSTRG.23849	Gm21905
MSTRG.23849.3	MSTRG.23849	Gm5108
MSTRG.23852.1	MSTRG.23852	.
MSTRG.23858.1	MSTRG.23858	Atp8a1
MSTRG.23859.2	MSTRG.23859	Gm15478
MSTRG.23860.1	MSTRG.23860	Atp8a1
MSTRG.23866.1	MSTRG.23866	.
MSTRG.23868.1	MSTRG.23868	.
MSTRG.23879.1	MSTRG.23879	Atp10d
MSTRG.23880.1	MSTRG.23880	Atp10d
MSTRG.23881.1	MSTRG.23881	Atp10d
MSTRG.23883.1	MSTRG.23883	Nfxl1
MSTRG.23891.5	MSTRG.23891	Slain2
MSTRG.23891.7	MSTRG.23891	Slain2
MSTRG.23892.1	MSTRG.23892	Slain2
MSTRG.23897.4	MSTRG.23897	Tec
MSTRG.23898.1	MSTRG.23898	Tec

MSTRG.23899.1	MSTRG.23899	Tec
MSTRG.23901.1	MSTRG.23901	Tec
MSTRG.23904.1	MSTRG.23904	Cwh43
MSTRG.23908.1	MSTRG.23908	Gm42732
MSTRG.23909.1	MSTRG.23909	.
MSTRG.23910.1	MSTRG.23910	Ociad2
MSTRG.23917.1	MSTRG.23917	Dcun1d4
MSTRG.23918.1	MSTRG.23918	Dcun1d4
MSTRG.23919.1	MSTRG.23919	Dcun1d4
MSTRG.23927.5	MSTRG.23927	Dancr
MSTRG.23931.1	MSTRG.23931	Fryl
MSTRG.23932.1	MSTRG.23932	1700071G01Rik
MSTRG.23934.1	MSTRG.23934	Gm17207
MSTRG.23934.2	MSTRG.23934	Gm17207
MSTRG.23935.1	MSTRG.23935	Fryl
MSTRG.23938.1	MSTRG.23938	Scfd2
MSTRG.23940.1	MSTRG.23940	Scfd2
MSTRG.23941.1	MSTRG.23941	Scfd2
MSTRG.23944.1	MSTRG.23944	Scfd2
MSTRG.23945.1	MSTRG.23945	Scfd2
MSTRG.23946.1	MSTRG.23946	Scfd2
MSTRG.23948.1	MSTRG.23948	Scfd2
MSTRG.23949.1	MSTRG.23949	Scfd2
MSTRG.23953.1	MSTRG.23953	Chic2
MSTRG.23954.1	MSTRG.23954	Chic2
MSTRG.23970.1	MSTRG.23970	Cep135
MSTRG.23971.1	MSTRG.23971	Cep135
MSTRG.23972.1	MSTRG.23972	Cep135
MSTRG.2398.1	MSTRG.2398	Ust
MSTRG.23985.1	MSTRG.23985	.
MSTRG.2399.1	MSTRG.2399	Ust
MSTRG.23993.1	MSTRG.23993	.
MSTRG.23994.1	MSTRG.23994	.
MSTRG.23995.1	MSTRG.23995	.
MSTRG.23996.1	MSTRG.23996	.
MSTRG.23999.1	MSTRG.23999	Epha5
MSTRG.2400.1	MSTRG.2400	.
MSTRG.2400.2	MSTRG.2400	.
MSTRG.24003.1	MSTRG.24003	Adgrl3
MSTRG.24005.1	MSTRG.24005	Adgrl3
MSTRG.24009.1	MSTRG.24009	Cenpc1
MSTRG.2401.1	MSTRG.2401	.
MSTRG.2401.2	MSTRG.2401	.
MSTRG.24010.1	MSTRG.24010	Cenpc1
MSTRG.24013.1	MSTRG.24013	Tmprss11d
MSTRG.24016.1	MSTRG.24016	Uba6
MSTRG.24017.1	MSTRG.24017	Uba6
MSTRG.24019.1	MSTRG.24019	.
MSTRG.2402.1	MSTRG.2402	.
MSTRG.24024.1	MSTRG.24024	.
MSTRG.24025.1	MSTRG.24025	.
MSTRG.24032.1	MSTRG.24032	Rufy3
MSTRG.24035.1	MSTRG.24035	Slc4a4
MSTRG.24036.1	MSTRG.24036	Slc4a4
MSTRG.24040.1	MSTRG.24040	.
MSTRG.24042.1	MSTRG.24042	Ankrd17
MSTRG.24043.1	MSTRG.24043	Ankrd17

MSTRG.24044.1	MSTRG.24044	Ankrd17
MSTRG.24045.1	MSTRG.24045	Ankrd17
MSTRG.24047.1	MSTRG.24047	Parm1
MSTRG.24051.1	MSTRG.24051	.
MSTRG.24057.1	MSTRG.24057	.
MSTRG.24062.1	MSTRG.24062	G3bp2
MSTRG.24065.1	MSTRG.24065	Uso1
MSTRG.24077.1	MSTRG.24077	.
MSTRG.2408.1	MSTRG.2408	AC153569.2
MSTRG.24084.1	MSTRG.24084	Scarb2
MSTRG.24087.1	MSTRG.24087	.
MSTRG.24092.1	MSTRG.24092	Sept11
MSTRG.24093.1	MSTRG.24093	Sept11
MSTRG.2410.1	MSTRG.2410	Stxbp5
MSTRG.24103.1	MSTRG.24103	.
MSTRG.24108.1	MSTRG.24108	Mrpl1
MSTRG.24111.1	MSTRG.24111	Cnot6l
MSTRG.24115.1	MSTRG.24115	Fras1
MSTRG.24117.3	MSTRG.24117	Bmp2k
MSTRG.24118.1	MSTRG.24118	Bmp2k
MSTRG.2412.1	MSTRG.2412	Stxbp5
MSTRG.24122.1	MSTRG.24122	Antxr2
MSTRG.24126.1	MSTRG.24126	Rasgef1b
MSTRG.2413.1	MSTRG.2413	Stxbp5
MSTRG.24138.1	MSTRG.24138	Gm35911
MSTRG.24140.1	MSTRG.24140	Gm17092
MSTRG.24148.1	MSTRG.24148	Sec31a
MSTRG.24149.1	MSTRG.24149	5430416N02Rik
MSTRG.24157.1	MSTRG.24157	Lin54
MSTRG.24158.1	MSTRG.24158	Lin54
MSTRG.24159.1	MSTRG.24159	Lin54
MSTRG.2416.1	MSTRG.2416	Gm28905
MSTRG.24160.1	MSTRG.24160	Lin54
MSTRG.24161.1	MSTRG.24161	Lin54
MSTRG.24164.1	MSTRG.24164	Cops4
MSTRG.24168.1	MSTRG.24168	Helq
MSTRG.24172.1	MSTRG.24172	.
MSTRG.24176.1	MSTRG.24176	.
MSTRG.2418.1	MSTRG.2418	Gm28905
MSTRG.24183.3	MSTRG.24183	Arhgap24
MSTRG.24185.1	MSTRG.24185	Arhgap24
MSTRG.24187.1	MSTRG.24187	Arhgap24
MSTRG.24189.1	MSTRG.24189	Arhgap24
MSTRG.24190.1	MSTRG.24190	Arhgap24
MSTRG.24203.1	MSTRG.24203	Aff1
MSTRG.24203.2	MSTRG.24203	Aff1
MSTRG.24204.1	MSTRG.24204	Aff1
MSTRG.24204.2	MSTRG.24204	Aff1
MSTRG.24208.1	MSTRG.24208	.
MSTRG.24213.1	MSTRG.24213	.
MSTRG.24215.1	MSTRG.24215	.
MSTRG.24216.1	MSTRG.24216	.
MSTRG.24217.1	MSTRG.24217	.
MSTRG.24218.1	MSTRG.24218	.
MSTRG.24219.1	MSTRG.24219	.
MSTRG.24220.1	MSTRG.24220	.
MSTRG.24221.1	MSTRG.24221	.

MSTRG.24222.1	MSTRG.24222	.
MSTRG.24223.1	MSTRG.24223	.
MSTRG.24224.1	MSTRG.24224	.
MSTRG.24225.1	MSTRG.24225	.
MSTRG.24226.1	MSTRG.24226	.
MSTRG.24226.2	MSTRG.24226	.
MSTRG.24227.1	MSTRG.24227	.
MSTRG.24228.1	MSTRG.24228	Zfp951
MSTRG.24229.3	MSTRG.24229	Zfp951
MSTRG.24229.4	MSTRG.24229	Zfp951
MSTRG.24230.1	MSTRG.24230	Zfp951
MSTRG.24231.1	MSTRG.24231	Zfp951
MSTRG.24231.2	MSTRG.24231	Zfp951
MSTRG.24232.1	MSTRG.24232	Zfp951
MSTRG.24233.1	MSTRG.24233	Zfp951
MSTRG.24234.1	MSTRG.24234	Zfp951
MSTRG.24235.1	MSTRG.24235	Zfp951
MSTRG.24236.1	MSTRG.24236	Zfp951
MSTRG.24237.1	MSTRG.24237	Zfp951
MSTRG.24238.1	MSTRG.24238	Zfp951
MSTRG.24239.1	MSTRG.24239	Zfp951
MSTRG.24240.1	MSTRG.24240	Zfp951
MSTRG.24241.1	MSTRG.24241	Zfp951
MSTRG.24244.1	MSTRG.24244	Abcg3
MSTRG.24249.1	MSTRG.24249	Gm42141
MSTRG.24252.1	MSTRG.24252	Lrrc8c
MSTRG.24253.1	MSTRG.24253	Lrrc8c
MSTRG.24256.1	MSTRG.24256	Gbp8
MSTRG.24258.1	MSTRG.24258	Gbp8
MSTRG.24259.1	MSTRG.24259	Gbp8
MSTRG.24260.1	MSTRG.24260	Gbp9
MSTRG.24262.1	MSTRG.24262	Gm43302
MSTRG.24263.1	MSTRG.24263	Gm43302
MSTRG.24267.1	MSTRG.24267	Lrrc8d
MSTRG.24268.1	MSTRG.24268	Lrrc8d
MSTRG.24269.1	MSTRG.24269	Lrrc8d
MSTRG.24270.1	MSTRG.24270	Lrrc8d
MSTRG.24271.1	MSTRG.24271	Lrrc8d
MSTRG.24276.1	MSTRG.24276	Zfp326
MSTRG.24277.1	MSTRG.24277	.
MSTRG.24278.1	MSTRG.24278	.
MSTRG.24279.8	MSTRG.24279	Cdc7
MSTRG.24281.1	MSTRG.24281	Gm28050
MSTRG.24285.1	MSTRG.24285	Zfp644
MSTRG.24286.1	MSTRG.24286	Gm24191
MSTRG.24286.2	MSTRG.24286	Gm24191
MSTRG.24287.1	MSTRG.24287	Zfp644
MSTRG.2429.1	MSTRG.2429	Shprh
MSTRG.24290.1	MSTRG.24290	Tgfbr3
MSTRG.24293.1	MSTRG.24293	Tgfbr3
MSTRG.24294.1	MSTRG.24294	Tgfbr3
MSTRG.24296.4	MSTRG.24296	Brdt
MSTRG.2430.1	MSTRG.2430	Shprh
MSTRG.24300.1	MSTRG.24300	Gm42902
MSTRG.24315.1	MSTRG.24315	Btbd8
MSTRG.24317.1	MSTRG.24317	Glmn
MSTRG.24318.1	MSTRG.24318	Gm42669

MSTRG.24319.1	MSTRG.24319	Glmn
MSTRG.24321.1	MSTRG.24321	Rpap2
MSTRG.24323.1	MSTRG.24323	Rpap2
MSTRG.24324.1	MSTRG.24324	Gm26692
MSTRG.24324.2	MSTRG.24324	Gm26692
MSTRG.24325.1	MSTRG.24325	Gm26692
MSTRG.24330.1	MSTRG.24330	Fam69a
MSTRG.24332.1	MSTRG.24332	Fam69a
MSTRG.24333.1	MSTRG.24333	Fam69a
MSTRG.24338.1	MSTRG.24338	Ccdc18
MSTRG.24342.1	MSTRG.24342	Mtf2
MSTRG.24343.1	MSTRG.24343	Mtf2
MSTRG.24345.1	MSTRG.24345	Tmed5
MSTRG.24348.1	MSTRG.24348	.
MSTRG.24349.1	MSTRG.24349	.
MSTRG.24355.1	MSTRG.24355	.
MSTRG.2436.1	MSTRG.2436	.
MSTRG.24369.1	MSTRG.24369	5430403G16Rik
MSTRG.24370.1	MSTRG.24370	Zfp932
MSTRG.24372.1	MSTRG.24372	4930522L14Rik
MSTRG.24375.1	MSTRG.24375	A430073D23Rik
MSTRG.24377.1	MSTRG.24377	.
MSTRG.24378.3	MSTRG.24378	.
MSTRG.24379.1	MSTRG.24379	Gm26808
MSTRG.2438.1	MSTRG.2438	Utrn
MSTRG.2438.2	MSTRG.2438	Utrn
MSTRG.24380.1	MSTRG.24380	.
MSTRG.24380.2	MSTRG.24380	.
MSTRG.24380.3	MSTRG.24380	.
MSTRG.24380.4	MSTRG.24380	.
MSTRG.24381.1	MSTRG.24381	.
MSTRG.24382.1	MSTRG.24382	.
MSTRG.24389.1	MSTRG.24389	Zfp932
MSTRG.2439.1	MSTRG.2439	Utrn
MSTRG.24390.1	MSTRG.24390	.
MSTRG.24398.3	MSTRG.24398	Gm15787
MSTRG.2440.1	MSTRG.2440	Utrn
MSTRG.24400.1	MSTRG.24400	Golga3
MSTRG.24411.2	MSTRG.24411	A630023P12Rik
MSTRG.24414.2	MSTRG.24414	Fbrsl1
MSTRG.2442.1	MSTRG.2442	Utrn
MSTRG.24420.1	MSTRG.24420	Chek2
MSTRG.24422.1	MSTRG.24422	Chek2
MSTRG.24439.1	MSTRG.24439	Ttc28
MSTRG.24441.1	MSTRG.24441	Ttc28
MSTRG.24444.1	MSTRG.24444	Ttc28
MSTRG.24450.1	MSTRG.24450	.
MSTRG.24452.1	MSTRG.24452	Tpst2
MSTRG.24454.1	MSTRG.24454	.
MSTRG.24463.1	MSTRG.24463	Grk3
MSTRG.24464.1	MSTRG.24464	Grk3
MSTRG.24471.1	MSTRG.24471	2900026A02Rik
MSTRG.24477.1	MSTRG.24477	.
MSTRG.2448.1	MSTRG.2448	Phactr2
MSTRG.24480.1	MSTRG.24480	Selplg
MSTRG.24480.2	MSTRG.24480	Selplg
MSTRG.24484.1	MSTRG.24484	Sart3

MSTRG.24486.1	MSTRG.24486	Sart3
MSTRG.2449.1	MSTRG.2449	Phactr2
MSTRG.2450.1	MSTRG.2450	Phactr2
MSTRG.24507.1	MSTRG.24507	Fam222a
MSTRG.24509.1	MSTRG.24509	.
MSTRG.24512.1	MSTRG.24512	Tchp
MSTRG.24517.1	MSTRG.24517	Ankrd13a
MSTRG.24522.1	MSTRG.24522	Rpl37rt
MSTRG.24523.1	MSTRG.24523	Rpl37rt
MSTRG.24529.3	MSTRG.24529	2210016L21Rik
MSTRG.24530.1	MSTRG.24530	2210016L21Rik
MSTRG.24532.1	MSTRG.24532	Sppl3
MSTRG.24532.2	MSTRG.24532	Sppl3
MSTRG.24533.1	MSTRG.24533	Sppl3
MSTRG.24538.1	MSTRG.24538	Sppl3
MSTRG.2454.1	MSTRG.2454	Pex3
MSTRG.24540.1	MSTRG.24540	Sppl3
MSTRG.24542.1	MSTRG.24542	Sppl3
MSTRG.24551.1	MSTRG.24551	Rnf10
MSTRG.24559.1	MSTRG.24559	Gm13832
MSTRG.2456.1	MSTRG.2456	.
MSTRG.24560.1	MSTRG.24560	Gm13832
MSTRG.24572.1	MSTRG.24572	Pxn
MSTRG.24578.1	MSTRG.24578	Ccdc60
MSTRG.2458.1	MSTRG.2458	Aig1
MSTRG.24587.1	MSTRG.24587	Prkab1
MSTRG.2459.1	MSTRG.2459	Aig1
MSTRG.24593.1	MSTRG.24593	.
MSTRG.24594.3	MSTRG.24594	Suds3
MSTRG.24597.1	MSTRG.24597	Taok3
MSTRG.24598.1	MSTRG.24598	Taok3
MSTRG.246.1	MSTRG.246	.
MSTRG.2460.1	MSTRG.2460	Aig1
MSTRG.24600.1	MSTRG.24600	Taok3
MSTRG.24600.2	MSTRG.24600	Taok3
MSTRG.24600.3	MSTRG.24600	Taok3
MSTRG.24601.2	MSTRG.24601	Taok3
MSTRG.2461.1	MSTRG.2461	Gm26835
MSTRG.2461.2	MSTRG.2461	Gm26835
MSTRG.2461.3	MSTRG.2461	Gm26835
MSTRG.24615.1	MSTRG.24615	2410131K14Rik
MSTRG.24616.1	MSTRG.24616	.
MSTRG.24617.1	MSTRG.24617	.
MSTRG.24620.1	MSTRG.24620	Med13l
MSTRG.24622.1	MSTRG.24622	Gm43275
MSTRG.24625.1	MSTRG.24625	Rbm19
MSTRG.24626.1	MSTRG.24626	Rbm19
MSTRG.24632.1	MSTRG.24632	Rasal1
MSTRG.24633.1	MSTRG.24633	Rasal1
MSTRG.24635.1	MSTRG.24635	Dtx1
MSTRG.24638.1	MSTRG.24638	Tpcn1
MSTRG.24639.1	MSTRG.24639	Tpcn1
MSTRG.24640.1	MSTRG.24640	Tpcn1
MSTRG.24641.1	MSTRG.24641	Tpcn1
MSTRG.2465.1	MSTRG.2465	Hivep2
MSTRG.24650.1	MSTRG.24650	Oas3
MSTRG.24654.1	MSTRG.24654	Oas1c

MSTRG.24658.1	MSTRG.24658	Rph3a
MSTRG.2466.1	MSTRG.2466	Hivep2
MSTRG.24666.1	MSTRG.24666	Hectd4
MSTRG.2467.1	MSTRG.2467	Hivep2
MSTRG.24674.1	MSTRG.24674	Erp29
MSTRG.2468.1	MSTRG.2468	Hivep2
MSTRG.24682.1	MSTRG.24682	Acad12
MSTRG.24683.1	MSTRG.24683	Acad10
MSTRG.24685.1	MSTRG.24685	Acad10
MSTRG.24691.1	MSTRG.24691	Atxn2
MSTRG.24693.1	MSTRG.24693	Atxn2
MSTRG.24694.1	MSTRG.24694	Atxn2
MSTRG.24697.1	MSTRG.24697	.
MSTRG.24703.1	MSTRG.24703	Tctn1
MSTRG.24710.1	MSTRG.24710	.
MSTRG.24717.1	MSTRG.24717	Gm43359
MSTRG.24727.1	MSTRG.24727	Camkk2
MSTRG.24728.1	MSTRG.24728	Camkk2
MSTRG.24731.1	MSTRG.24731	Anapc5
MSTRG.24742.1	MSTRG.24742	Gm43813
MSTRG.24743.1	MSTRG.24743	Mlxip
MSTRG.24746.1	MSTRG.24746	Kdm2b
MSTRG.24748.1	MSTRG.24748	Kdm2b
MSTRG.24749.1	MSTRG.24749	Kdm2b
MSTRG.2475.1	MSTRG.2475	Vta1
MSTRG.24750.1	MSTRG.24750	Kdm2b
MSTRG.24751.1	MSTRG.24751	Kdm2b
MSTRG.24752.2	MSTRG.24752	A930024E05Rik
MSTRG.24753.1	MSTRG.24753	Gm44574
MSTRG.24755.1	MSTRG.24755	.
MSTRG.2476.1	MSTRG.2476	Vta1
MSTRG.24761.1	MSTRG.24761	Tmem120b
MSTRG.24772.2	MSTRG.24772	Diablo
MSTRG.24779.1	MSTRG.24779	Vps33a
MSTRG.24784.1	MSTRG.24784	Denr
MSTRG.24785.1	MSTRG.24785	Ccdc62
MSTRG.24793.1	MSTRG.24793	Hip1r
MSTRG.24796.1	MSTRG.24796	Vps37b
MSTRG.248.1	MSTRG.248	.
MSTRG.24800.1	MSTRG.24800	.
MSTRG.24804.1	MSTRG.24804	.
MSTRG.24809.1	MSTRG.24809	Pitpnm2
MSTRG.24810.1	MSTRG.24810	Pitpnm2os1
MSTRG.24810.2	MSTRG.24810	Pitpnm2os1
MSTRG.2482.1	MSTRG.2482	AC153370.2
MSTRG.2482.2	MSTRG.2482	AC153370.2
MSTRG.24827.1	MSTRG.24827	Zfp664
MSTRG.24828.1	MSTRG.24828	Zfp664
MSTRG.24838.1	MSTRG.24838	Ccdc92
MSTRG.2484.1	MSTRG.2484	AC153370.1
MSTRG.24841.1	MSTRG.24841	.
MSTRG.24845.1	MSTRG.24845	.
MSTRG.24848.1	MSTRG.24848	Dhx37
MSTRG.24852.1	MSTRG.24852	Ncor2
MSTRG.24853.1	MSTRG.24853	Ncor2
MSTRG.24854.1	MSTRG.24854	Ncor2
MSTRG.24854.2	MSTRG.24854	Ncor2

MSTRG.24857.1	MSTRG.24857	.
MSTRG.24859.1	MSTRG.24859	.
MSTRG.24864.1	MSTRG.24864	Stx2
MSTRG.24868.1	MSTRG.24868	Adgrd1
MSTRG.24871.1	MSTRG.24871	.
MSTRG.24875.1	MSTRG.24875	Nipsnap2
MSTRG.24876.4	MSTRG.24876	Nipsnap2
MSTRG.24880.6	MSTRG.24880	Crcp
MSTRG.24881.1	MSTRG.24881	Crcp
MSTRG.24885.1	MSTRG.24885	Psph
MSTRG.2489.1	MSTRG.2489	Abracl
MSTRG.24891.1	MSTRG.24891	Phkg1
MSTRG.24892.1	MSTRG.24892	Phkg1
MSTRG.24899.1	MSTRG.24899	Rabgef1
MSTRG.2490.1	MSTRG.2490	Abracl
MSTRG.24901.1	MSTRG.24901	Rabgef1
MSTRG.24902.1	MSTRG.24902	Rabgef1
MSTRG.24903.1	MSTRG.24903	Rabgef1
MSTRG.24907.1	MSTRG.24907	Tmem248
MSTRG.24910.1	MSTRG.24910	Tyw1
MSTRG.24911.1	MSTRG.24911	Tyw1
MSTRG.24915.1	MSTRG.24915	Wbscr17
MSTRG.24917.1	MSTRG.24917	Wbscr17
MSTRG.24919.1	MSTRG.24919	Wbscr17
MSTRG.2492.1	MSTRG.2492	Ccdc28a
MSTRG.24920.1	MSTRG.24920	.
MSTRG.24923.1	MSTRG.24923	Gatsl2
MSTRG.24925.1	MSTRG.24925	Rcc1l
MSTRG.24934.1	MSTRG.24934	Auts2
MSTRG.2494.1	MSTRG.2494	Reps1
MSTRG.24940.1	MSTRG.24940	Auts2
MSTRG.24941.1	MSTRG.24941	Auts2
MSTRG.2495.1	MSTRG.2495	Reps1
MSTRG.24951.1	MSTRG.24951	Auts2
MSTRG.24956.1	MSTRG.24956	Auts2
MSTRG.24958.1	MSTRG.24958	Auts2
MSTRG.24960.1	MSTRG.24960	Auts2
MSTRG.24973.1	MSTRG.24973	Gtf2ird1
MSTRG.24975.1	MSTRG.24975	Gtf2ird1
MSTRG.24977.1	MSTRG.24977	Gtf2ird1
MSTRG.24977.2	MSTRG.24977	Gtf2ird1
MSTRG.2498.1	MSTRG.2498	Nhsl1
MSTRG.24980.1	MSTRG.24980	Gtf2ird1
MSTRG.24986.1	MSTRG.24986	.
MSTRG.24990.1	MSTRG.24990	Mettl27
MSTRG.24998.1	MSTRG.24998	Stx1a
MSTRG.24999.1	MSTRG.24999	Stx1a
MSTRG.25.1	MSTRG.25	Gm19026
MSTRG.2500.1	MSTRG.2500	Nhsl1
MSTRG.25000.1	MSTRG.25000	Stx1a
MSTRG.25004.1	MSTRG.25004	Tbl2
MSTRG.2501.1	MSTRG.2501	Nhsl1
MSTRG.25014.1	MSTRG.25014	Hip1
MSTRG.25015.1	MSTRG.25015	.
MSTRG.25016.1	MSTRG.25016	.
MSTRG.25023.1	MSTRG.25023	Gm43091
MSTRG.25026.1	MSTRG.25026	Por

MSTRG.25029.1	MSTRG.25029	Tmem120a
MSTRG.25034.3	MSTRG.25034	Ywhag
MSTRG.25034.4	MSTRG.25034	Ywhag
MSTRG.25038.1	MSTRG.25038	Dtx2
MSTRG.2504.4	MSTRG.2504	AC158622.3
MSTRG.25042.1	MSTRG.25042	Rasa4
MSTRG.25044.1	MSTRG.25044	Orai2
MSTRG.25049.1	MSTRG.25049	.
MSTRG.2505.1	MSTRG.2505	AC158622.3
MSTRG.25052.1	MSTRG.25052	Prkrip1
MSTRG.25053.1	MSTRG.25053	.
MSTRG.25054.1	MSTRG.25054	.
MSTRG.25055.1	MSTRG.25055	.
MSTRG.25056.1	MSTRG.25056	.
MSTRG.25059.1	MSTRG.25059	Ift22
MSTRG.25062.1	MSTRG.25062	Fis1
MSTRG.25066.2	MSTRG.25066	Gm16599
MSTRG.25066.3	MSTRG.25066	Gm16599
MSTRG.25066.4	MSTRG.25066	Cux1
MSTRG.25066.5	MSTRG.25066	Gm16599
MSTRG.25066.6	MSTRG.25066	Gm16599
MSTRG.25066.7	MSTRG.25066	Gm16599
MSTRG.25067.1	MSTRG.25067	Cux1
MSTRG.25069.1	MSTRG.25069	Cux1
MSTRG.25070.1	MSTRG.25070	Cux1
MSTRG.25070.2	MSTRG.25070	Cux1
MSTRG.25072.1	MSTRG.25072	Cux1
MSTRG.25073.1	MSTRG.25073	Cux1
MSTRG.25073.2	MSTRG.25073	Cux1
MSTRG.25088.1	MSTRG.25088	Srrt
MSTRG.25090.4	MSTRG.25090	Trip6
MSTRG.25092.1	MSTRG.25092	Slc12a9
MSTRG.25101.1	MSTRG.25101	.
MSTRG.25106.1	MSTRG.25106	Mospd3
MSTRG.25109.1	MSTRG.25109	Gm36266
MSTRG.25109.2	MSTRG.25109	Gm36266
MSTRG.2511.1	MSTRG.2511	Pex7
MSTRG.25115.1	MSTRG.25115	Agfg2
MSTRG.25116.1	MSTRG.25116	Agfg2
MSTRG.25117.1	MSTRG.25117	Agfg2
MSTRG.25118.1	MSTRG.25118	.
MSTRG.25123.1	MSTRG.25123	.
MSTRG.25125.1	MSTRG.25125	Zkscan1
MSTRG.25126.1	MSTRG.25126	Zkscan1
MSTRG.25129.1	MSTRG.25129	Zscan21
MSTRG.2513.1	MSTRG.2513	Pex7
MSTRG.25140.1	MSTRG.25140	.
MSTRG.25148.1	MSTRG.25148	Zfp157
MSTRG.2515.1	MSTRG.2515	Map3k5
MSTRG.25159.1	MSTRG.25159	Pdgfa
MSTRG.2516.1	MSTRG.2516	Map3k5
MSTRG.2517.1	MSTRG.2517	Map3k5
MSTRG.25170.1	MSTRG.25170	3110082I17Rik
MSTRG.25172.1	MSTRG.25172	Gpr146
MSTRG.2518.1	MSTRG.2518	Map3k5
MSTRG.25181.1	MSTRG.25181	.
MSTRG.25183.1	MSTRG.25183	Snx8

MSTRG.25184.1	MSTRG.25184	Snx8
MSTRG.25185.1	MSTRG.25185	Snx8
MSTRG.25189.1	MSTRG.25189	.
MSTRG.2519.1	MSTRG.2519	Map3k5
MSTRG.25191.1	MSTRG.25191	.
MSTRG.25192.1	MSTRG.25192	Mad1l1
MSTRG.25193.1	MSTRG.25193	Gm43702
MSTRG.25194.1	MSTRG.25194	Mad1l1
MSTRG.25194.2	MSTRG.25194	Mad1l1
MSTRG.25194.3	MSTRG.25194	Mad1l1
MSTRG.25194.4	MSTRG.25194	Mad1l1
MSTRG.25194.5	MSTRG.25194	Mad1l1
MSTRG.25197.3	MSTRG.25197	Gm16120
MSTRG.25197.4	MSTRG.25197	Gm16120
MSTRG.25197.5	MSTRG.25197	Gm16120
MSTRG.25198.1	MSTRG.25198	Mad1l1
MSTRG.25201.1	MSTRG.25201	Nudt1
MSTRG.25204.1	MSTRG.25204	Lfng
MSTRG.25205.1	MSTRG.25205	Lfng
MSTRG.25207.1	MSTRG.25207	.
MSTRG.2521.1	MSTRG.2521	Map7
MSTRG.25211.1	MSTRG.25211	Amz1
MSTRG.25212.1	MSTRG.25212	Amz1
MSTRG.25214.1	MSTRG.25214	Gna12
MSTRG.25216.1	MSTRG.25216	Card11
MSTRG.25219.1	MSTRG.25219	Card11
MSTRG.2522.1	MSTRG.2522	Map7
MSTRG.25220.3	MSTRG.25220	Card11
MSTRG.25226.1	MSTRG.25226	Sdk1
MSTRG.25233.1	MSTRG.25233	Wipi2
MSTRG.25237.1	MSTRG.25237	Fbxl18
MSTRG.25240.1	MSTRG.25240	Actb
MSTRG.25247.1	MSTRG.25247	Rnf216
MSTRG.25249.1	MSTRG.25249	Rnf216
MSTRG.25250.1	MSTRG.25250	Rnf216
MSTRG.25252.1	MSTRG.25252	Rnf216
MSTRG.25253.1	MSTRG.25253	Rnf216
MSTRG.25261.2	MSTRG.25261	Kdelr2
MSTRG.25264.10	MSTRG.25264	Zdhhc4
MSTRG.25264.11	MSTRG.25264	Zdhhc4
MSTRG.25272.1	MSTRG.25272	Usp42
MSTRG.25284.1	MSTRG.25284	Lmtk2
MSTRG.25288.1	MSTRG.25288	.
MSTRG.25291.1	MSTRG.25291	Bri3
MSTRG.25298.1	MSTRG.25298	Smurf1
MSTRG.25299.1	MSTRG.25299	Smurf1
MSTRG.25300.1	MSTRG.25300	Smurf1
MSTRG.25310.1	MSTRG.25310	Arpc1a
MSTRG.25310.2	MSTRG.25310	Arpc1a
MSTRG.25313.1	MSTRG.25313	Zkscan5
MSTRG.25315.1	MSTRG.25315	.
MSTRG.2532.1	MSTRG.2532	Pde7b
MSTRG.25325.1	MSTRG.25325	Cyp3a16
MSTRG.2533.1	MSTRG.2533	Pde7b
MSTRG.25330.1	MSTRG.25330	Cdk8
MSTRG.25333.1	MSTRG.25333	Rpl21
MSTRG.25334.1	MSTRG.25334	Usp12

MSTRG.25339.3	MSTRG.25339	Lnx2
MSTRG.25340.1	MSTRG.25340	Lnx2
MSTRG.25342.1	MSTRG.25342	Lnx2
MSTRG.25348.1	MSTRG.25348	.
MSTRG.25355.1	MSTRG.25355	Pan3
MSTRG.25357.1	MSTRG.25357	Pan3
MSTRG.25361.1	MSTRG.25361	Mtus2
MSTRG.25363.1	MSTRG.25363	Slc7a1
MSTRG.25364.1	MSTRG.25364	Slc7a1
MSTRG.25365.3	MSTRG.25365	Ubl3
MSTRG.25365.7	MSTRG.25365	Ubl3
MSTRG.25368.1	MSTRG.25368	Gm29264
MSTRG.2537.1	MSTRG.2537	Ahi1
MSTRG.25374.1	MSTRG.25374	.
MSTRG.25374.2	MSTRG.25374	.
MSTRG.25374.3	MSTRG.25374	BC028471
MSTRG.25374.4	MSTRG.25374	.
MSTRG.25379.1	MSTRG.25379	B3glct
MSTRG.25380.1	MSTRG.25380	B3glct
MSTRG.25381.10	MSTRG.25381	Hsph1
MSTRG.25382.10	MSTRG.25382	1810059H22Rik
MSTRG.25382.12	MSTRG.25382	1810059H22Rik
MSTRG.25382.13	MSTRG.25382	1810059H22Rik
MSTRG.25382.15	MSTRG.25382	1810059H22Rik
MSTRG.25382.23	MSTRG.25382	Gm42791
MSTRG.25386.2	MSTRG.25386	Gm15409
MSTRG.25387.1	MSTRG.25387	Gm42791
MSTRG.25388.1	MSTRG.25388	Gm42791
MSTRG.25390.1	MSTRG.25390	Gm15408
MSTRG.25392.1	MSTRG.25392	Hmgb1
MSTRG.25393.1	MSTRG.25393	Hmgb1
MSTRG.25397.1	MSTRG.25397	5730422E09Rik
MSTRG.25398.1	MSTRG.25398	5730422E09Rik
MSTRG.25399.1	MSTRG.25399	5730422E09Rik
MSTRG.25402.1	MSTRG.25402	Hmgb1
MSTRG.25406.1	MSTRG.25406	Fry
MSTRG.25408.1	MSTRG.25408	Fry
MSTRG.25409.1	MSTRG.25409	Fry
MSTRG.25426.1	MSTRG.25426	Pds5b
MSTRG.2543.1	MSTRG.2543	.
MSTRG.25431.1	MSTRG.25431	Gm42906
MSTRG.25432.1	MSTRG.25432	D730045B01Rik
MSTRG.25437.1	MSTRG.25437	Gm8579
MSTRG.25440.1	MSTRG.25440	.
MSTRG.25441.1	MSTRG.25441	.
MSTRG.25444.1	MSTRG.25444	Gm20559
MSTRG.25446.1	MSTRG.25446	Samd9l
MSTRG.25447.1	MSTRG.25447	Samd9l
MSTRG.25447.2	MSTRG.25447	Samd9l
MSTRG.25447.3	MSTRG.25447	Samd9l
MSTRG.25448.1	MSTRG.25448	Samd9l
MSTRG.25449.1	MSTRG.25449	Samd9l
MSTRG.2545.1	MSTRG.2545	.
MSTRG.25452.1	MSTRG.25452	Vps50
MSTRG.25453.1	MSTRG.25453	Vps50
MSTRG.25454.1	MSTRG.25454	Vps50
MSTRG.25457.1	MSTRG.25457	.

MSTRG.25466.1	MSTRG.25466	Gm44424
MSTRG.2547.1	MSTRG.2547	AC153557.3
MSTRG.2547.2	MSTRG.2547	AC153557.3
MSTRG.25470.1	MSTRG.25470	Pon2
MSTRG.25473.2	MSTRG.25473	Sem1
MSTRG.25474.1	MSTRG.25474	Sem1
MSTRG.25475.1	MSTRG.25475	.
MSTRG.25477.1	MSTRG.25477	Sdhaf3
MSTRG.25478.1	MSTRG.25478	Sdhaf3
MSTRG.25479.1	MSTRG.25479	Sdhaf3
MSTRG.25480.1	MSTRG.25480	Sdhaf3
MSTRG.25481.1	MSTRG.25481	Sdhaf3
MSTRG.25489.1	MSTRG.25489	.
MSTRG.25492.1	MSTRG.25492	Umad1
MSTRG.25493.1	MSTRG.25493	Umad1
MSTRG.25498.4	MSTRG.25498	A430035B10Rik
MSTRG.25499.1	MSTRG.25499	Glcci1
MSTRG.2550.1	MSTRG.2550	Gm2539
MSTRG.25501.1	MSTRG.25501	.
MSTRG.25502.1	MSTRG.25502	.
MSTRG.25507.1	MSTRG.25507	Phf14
MSTRG.25508.1	MSTRG.25508	Phf14
MSTRG.2551.1	MSTRG.2551	Gm2539
MSTRG.25510.1	MSTRG.25510	Phf14
MSTRG.25511.1	MSTRG.25511	Phf14
MSTRG.25514.1	MSTRG.25514	.
MSTRG.25518.1	MSTRG.25518	Bmt2
MSTRG.25519.1	MSTRG.25519	Bmt2
MSTRG.2552.1	MSTRG.2552	E030030I06Rik
MSTRG.25520.1	MSTRG.25520	Bmt2
MSTRG.25521.1	MSTRG.25521	.
MSTRG.25527.1	MSTRG.25527	Foxp2
MSTRG.2553.1	MSTRG.2553	Gm19791
MSTRG.25530.1	MSTRG.25530	Mdfic
MSTRG.25531.1	MSTRG.25531	Mdfic
MSTRG.25533.1	MSTRG.25533	Mdfic
MSTRG.25534.1	MSTRG.25534	Mdfic
MSTRG.2555.1	MSTRG.2555	E030030I06Rik
MSTRG.25550.1	MSTRG.25550	St7
MSTRG.25553.1	MSTRG.25553	St7
MSTRG.25555.1	MSTRG.25555	St7
MSTRG.25556.1	MSTRG.25556	St7
MSTRG.25557.1	MSTRG.25557	St7
MSTRG.25559.1	MSTRG.25559	St7
MSTRG.25560.1	MSTRG.25560	St7
MSTRG.25565.1	MSTRG.25565	.
MSTRG.25566.1	MSTRG.25566	Lsm8
MSTRG.25566.2	MSTRG.25566	Lsm8
MSTRG.25567.1	MSTRG.25567	Gm20186
MSTRG.25567.2	MSTRG.25567	Gm20186
MSTRG.25567.5	MSTRG.25567	Gm20186
MSTRG.25568.1	MSTRG.25568	.
MSTRG.2557.1	MSTRG.2557	E030030I06Rik
MSTRG.25570.1	MSTRG.25570	Ing3
MSTRG.25570.2	MSTRG.25570	Ing3
MSTRG.25572.1	MSTRG.25572	Fam3c
MSTRG.2558.1	MSTRG.2558	E030030I06Rik

MSTRG.25580.1	MSTRG.25580	Cadps2
MSTRG.25583.1	MSTRG.25583	Wasl
MSTRG.25584.1	MSTRG.25584	.
MSTRG.25585.1	MSTRG.25585	.
MSTRG.25587.1	MSTRG.25587	Pot1a
MSTRG.2559.1	MSTRG.2559	.
MSTRG.25591.1	MSTRG.25591	Grm8
MSTRG.25596.1	MSTRG.25596	6530409C15Rik
MSTRG.25599.1	MSTRG.25599	Gcc1
MSTRG.25602.1	MSTRG.25602	.
MSTRG.25606.1	MSTRG.25606	Snd1
MSTRG.25609.1	MSTRG.25609	Snd1
MSTRG.2561.1	MSTRG.2561	Slc2a12
MSTRG.25610.1	MSTRG.25610	Snd1
MSTRG.25611.1	MSTRG.25611	Snd1
MSTRG.25612.1	MSTRG.25612	Snd1
MSTRG.25613.1	MSTRG.25613	Snd1
MSTRG.25613.2	MSTRG.25613	Snd1
MSTRG.25615.1	MSTRG.25615	Rbm28
MSTRG.25622.1	MSTRG.25622	.
MSTRG.25625.1	MSTRG.25625	Irf5
MSTRG.25631.1	MSTRG.25631	Ahcyl2
MSTRG.25632.1	MSTRG.25632	Ahcyl2
MSTRG.25633.1	MSTRG.25633	Ahcyl2
MSTRG.25637.1	MSTRG.25637	Ube2h
MSTRG.25637.10	MSTRG.25637	Ube2h
MSTRG.25637.11	MSTRG.25637	Ube2h
MSTRG.25637.5	MSTRG.25637	Ube2h
MSTRG.25637.6	MSTRG.25637	Ube2h
MSTRG.25637.7	MSTRG.25637	Ube2h
MSTRG.25637.9	MSTRG.25637	Ube2h
MSTRG.25638.1	MSTRG.25638	Ube2h
MSTRG.25639.1	MSTRG.25639	Ube2h
MSTRG.2564.1	MSTRG.2564	Gm4895
MSTRG.25640.1	MSTRG.25640	Ube2h
MSTRG.25642.1	MSTRG.25642	Nrf1
MSTRG.25643.1	MSTRG.25643	Nrf1
MSTRG.25643.2	MSTRG.25643	Nrf1
MSTRG.25658.1	MSTRG.25658	Cep41
MSTRG.25659.1	MSTRG.25659	Cep41
MSTRG.2566.1	MSTRG.2566	Raet1e
MSTRG.2566.10	MSTRG.2566	H60b
MSTRG.2566.11	MSTRG.2566	Gm26740
MSTRG.2566.8	MSTRG.2566	Raet1d
MSTRG.2566.9	MSTRG.2566	H60b
MSTRG.2567.1	MSTRG.2567	Gm26740
MSTRG.2567.2	MSTRG.2567	Gm26740
MSTRG.2567.3	MSTRG.2567	Gm26740
MSTRG.2568.1	MSTRG.2568	Raet1e
MSTRG.2568.2	MSTRG.2568	Raet1e
MSTRG.25680.1	MSTRG.25680	2210408F21Rik
MSTRG.25681.1	MSTRG.25681	2210408F21Rik
MSTRG.25688.1	MSTRG.25688	Mkln1
MSTRG.25693.1	MSTRG.25693	Gm13849
MSTRG.25694.1	MSTRG.25694	Gm13849
MSTRG.25695.1	MSTRG.25695	Gm13849
MSTRG.25696.1	MSTRG.25696	.

MSTRG.25698.1	MSTRG.25698	Chchd3
MSTRG.25699.1	MSTRG.25699	Chchd3
MSTRG.25700.1	MSTRG.25700	Chchd3
MSTRG.25701.1	MSTRG.25701	Chchd3
MSTRG.25702.1	MSTRG.25702	Chchd3
MSTRG.25703.1	MSTRG.25703	Chchd3
MSTRG.25704.1	MSTRG.25704	Chchd3
MSTRG.25709.1	MSTRG.25709	Exoc4
MSTRG.25710.1	MSTRG.25710	Exoc4
MSTRG.25711.1	MSTRG.25711	Exoc4
MSTRG.25712.1	MSTRG.25712	Exoc4
MSTRG.25712.2	MSTRG.25712	Exoc4
MSTRG.25715.1	MSTRG.25715	Exoc4
MSTRG.25717.1	MSTRG.25717	Exoc4
MSTRG.25718.1	MSTRG.25718	Exoc4
MSTRG.25724.1	MSTRG.25724	.
MSTRG.25727.1	MSTRG.25727	Cald1
MSTRG.25728.1	MSTRG.25728	Cald1
MSTRG.2573.1	MSTRG.2573	Gm26740
MSTRG.25732.11	MSTRG.25732	Agbl3
MSTRG.25738.1	MSTRG.25738	Tmem140
MSTRG.25740.1	MSTRG.25740	Wdr91
MSTRG.25745.1	MSTRG.25745	Cnot4
MSTRG.25746.1	MSTRG.25746	Cnot4
MSTRG.25748.1	MSTRG.25748	Cnot4
MSTRG.25749.1	MSTRG.25749	Cnot4
MSTRG.2575.1	MSTRG.2575	H60b
MSTRG.25750.1	MSTRG.25750	Cnot4
MSTRG.25754.1	MSTRG.25754	.
MSTRG.25755.2	MSTRG.25755	Mtpn
MSTRG.25758.3	MSTRG.25758	Creb3l2
MSTRG.2576.1	MSTRG.2576	Gm26740
MSTRG.25761.1	MSTRG.25761	Dgki
MSTRG.25767.1	MSTRG.25767	Trim24
MSTRG.25768.1	MSTRG.25768	.
MSTRG.25776.1	MSTRG.25776	Ubn2
MSTRG.25778.1	MSTRG.25778	Ubn2
MSTRG.25779.1	MSTRG.25779	Ubn2
MSTRG.25780.1	MSTRG.25780	Ubn2
MSTRG.25781.1	MSTRG.25781	Ubn2
MSTRG.25789.1	MSTRG.25789	Rab19
MSTRG.25790.1	MSTRG.25790	Rab19
MSTRG.25793.1	MSTRG.25793	.
MSTRG.25794.1	MSTRG.25794	.
MSTRG.25807.1	MSTRG.25807	.
MSTRG.25809.1	MSTRG.25809	Slc37a3
MSTRG.2581.1	MSTRG.2581	.
MSTRG.25816.1	MSTRG.25816	Agk
MSTRG.25817.1	MSTRG.25817	Agk
MSTRG.25818.1	MSTRG.25818	Agk
MSTRG.2582.1	MSTRG.2582	.
MSTRG.25821.1	MSTRG.25821	Ssbp1
MSTRG.25823.1	MSTRG.25823	Trbv1
MSTRG.25824.1	MSTRG.25824	.
MSTRG.25828.2	MSTRG.25828	Trbv31
MSTRG.25828.3	MSTRG.25828	Trbv31
MSTRG.25833.1	MSTRG.25833	.

MSTRG.25834.1	MSTRG.25834	.
MSTRG.25835.1	MSTRG.25835	.
MSTRG.2584.1	MSTRG.2584	Eya4
MSTRG.25849.1	MSTRG.25849	Trbv20
MSTRG.2585.1	MSTRG.2585	Eya4
MSTRG.25850.1	MSTRG.25850	.
MSTRG.25850.2	MSTRG.25850	.
MSTRG.25851.1	MSTRG.25851	.
MSTRG.2587.1	MSTRG.2587	Eya4
MSTRG.2588.1	MSTRG.2588	Eya4
MSTRG.25880.1	MSTRG.25880	Trbc2
MSTRG.2589.1	MSTRG.2589	Eya4
MSTRG.2590.1	MSTRG.2590	Eya4
MSTRG.25905.1	MSTRG.25905	Tpk1
MSTRG.25906.1	MSTRG.25906	Tpk1
MSTRG.25907.1	MSTRG.25907	Tpk1
MSTRG.25909.1	MSTRG.25909	Tpk1
MSTRG.2591.1	MSTRG.2591	Eya4
MSTRG.25910.1	MSTRG.25910	Tpk1
MSTRG.25911.1	MSTRG.25911	Tpk1
MSTRG.25936.1	MSTRG.25936	Cntnap2
MSTRG.25943.1	MSTRG.25943	.
MSTRG.25944.1	MSTRG.25944	Gm44141
MSTRG.25947.1	MSTRG.25947	.
MSTRG.25948.1	MSTRG.25948	.
MSTRG.25949.1	MSTRG.25949	.
MSTRG.2595.1	MSTRG.2595	.
MSTRG.25950.1	MSTRG.25950	.
MSTRG.25951.1	MSTRG.25951	.
MSTRG.25952.1	MSTRG.25952	.
MSTRG.25952.2	MSTRG.25952	.
MSTRG.25953.1	MSTRG.25953	.
MSTRG.25960.1	MSTRG.25960	Cul1
MSTRG.25965.1	MSTRG.25965	Ezh2
MSTRG.25966.1	MSTRG.25966	Ezh2
MSTRG.25968.1	MSTRG.25968	.
MSTRG.25969.1	MSTRG.25969	.
MSTRG.25969.2	MSTRG.25969	.
MSTRG.25969.3	MSTRG.25969	.
MSTRG.2597.1	MSTRG.2597	Enpp1
MSTRG.25987.9	MSTRG.25987	Gm44965
MSTRG.25989.1	MSTRG.25989	Gm38804
MSTRG.2599.1	MSTRG.2599	Med23
MSTRG.25998.1	MSTRG.25998	Gm28053
MSTRG.25999.1	MSTRG.25999	Gimap1os
MSTRG.2600.1	MSTRG.2600	Med23
MSTRG.26007.4	MSTRG.26007	Malsu1
MSTRG.2601.1	MSTRG.2601	Med23
MSTRG.26010.1	MSTRG.26010	Igf2bp3
MSTRG.26011.1	MSTRG.26011	Igf2bp3
MSTRG.26013.1	MSTRG.26013	Igf2bp3
MSTRG.26016.1	MSTRG.26016	Ccdc126
MSTRG.26020.1	MSTRG.26020	Fam221a
MSTRG.26021.1	MSTRG.26021	.
MSTRG.26023.1	MSTRG.26023	Mpp6
MSTRG.26024.1	MSTRG.26024	Mpp6
MSTRG.26026.1	MSTRG.26026	Dfna5

MSTRG.26028.1	MSTRG.26028	Osbpl3
MSTRG.26029.1	MSTRG.26029	Osbpl3
MSTRG.26034.1	MSTRG.26034	5430402O13Rik
MSTRG.26037.1	MSTRG.26037	Nfe2l3
MSTRG.26039.1	MSTRG.26039	Hnrnpa2b1
MSTRG.26040.1	MSTRG.26040	.
MSTRG.26042.1	MSTRG.26042	.
MSTRG.26044.1	MSTRG.26044	Skap2
MSTRG.26045.1	MSTRG.26045	Skap2
MSTRG.2605.1	MSTRG.2605	.
MSTRG.26062.1	MSTRG.26062	Hibadh
MSTRG.26064.1	MSTRG.26064	Hibadh
MSTRG.26065.1	MSTRG.26065	Gm44434
MSTRG.26067.1	MSTRG.26067	.
MSTRG.26073.1	MSTRG.26073	Jazf1
MSTRG.26074.1	MSTRG.26074	Jazf1
MSTRG.26075.1	MSTRG.26075	Jazf1
MSTRG.26076.1	MSTRG.26076	Jazf1
MSTRG.26079.1	MSTRG.26079	Jazf1
MSTRG.2608.1	MSTRG.2608	Epb41l2
MSTRG.26080.1	MSTRG.26080	Jazf1
MSTRG.26081.1	MSTRG.26081	Jazf1
MSTRG.26083.1	MSTRG.26083	Jazf1
MSTRG.26086.1	MSTRG.26086	Creb5
MSTRG.26089.1	MSTRG.26089	Fkbp14
MSTRG.2609.1	MSTRG.2609	Epb41l2
MSTRG.26094.4	MSTRG.26094	Chn2
MSTRG.26095.1	MSTRG.26095	Chn2
MSTRG.26096.1	MSTRG.26096	Chn2
MSTRG.26096.2	MSTRG.26096	Chn2
MSTRG.26097.1	MSTRG.26097	Chn2
MSTRG.26098.1	MSTRG.26098	Chn2
MSTRG.26099.1	MSTRG.26099	Chn2
MSTRG.26110.1	MSTRG.26110	Znrf2
MSTRG.26112.1	MSTRG.26112	Nod1
MSTRG.26113.1	MSTRG.26113	Nod1
MSTRG.2612.1	MSTRG.2612	.
MSTRG.26120.1	MSTRG.26120	Pde1c
MSTRG.26126.1	MSTRG.26126	Nt5c3
MSTRG.2613.1	MSTRG.2613	.
MSTRG.26134.1	MSTRG.26134	Gm45193
MSTRG.26138.1	MSTRG.26138	Herc6
MSTRG.26139.1	MSTRG.26139	Herc6
MSTRG.26140.1	MSTRG.26140	Herc6
MSTRG.26141.1	MSTRG.26141	Herc6
MSTRG.26142.1	MSTRG.26142	Herc6
MSTRG.26145.1	MSTRG.26145	.
MSTRG.26146.1	MSTRG.26146	.
MSTRG.2615.1	MSTRG.2615	Samd3
MSTRG.26152.1	MSTRG.26152	Fam13a
MSTRG.26154.1	MSTRG.26154	Herc3
MSTRG.26157.1	MSTRG.26157	.
MSTRG.26159.1	MSTRG.26159	.
MSTRG.2616.1	MSTRG.2616	Samd3
MSTRG.26160.1	MSTRG.26160	.
MSTRG.26168.1	MSTRG.26168	Grid2
MSTRG.26169.1	MSTRG.26169	Grid2

MSTRG.26171.1	MSTRG.26171	Hpgds
MSTRG.26173.1	MSTRG.26173	Hpgds
MSTRG.26175.1	MSTRG.26175	Smarcad1
MSTRG.26176.1	MSTRG.26176	.
MSTRG.26182.1	MSTRG.26182	Gng12
MSTRG.26183.1	MSTRG.26183	Gng12
MSTRG.26184.1	MSTRG.26184	Gng12
MSTRG.2620.1	MSTRG.2620	L3mbtl3
MSTRG.2621.1	MSTRG.2621	L3mbtl3
MSTRG.2621.2	MSTRG.2621	L3mbtl3
MSTRG.26210.1	MSTRG.26210	Igkv1-117
MSTRG.26236.1	MSTRG.26236	Rn7s6
MSTRG.26236.2	MSTRG.26236	Rn7s6
MSTRG.26236.3	MSTRG.26236	Rn7s6
MSTRG.26236.4	MSTRG.26236	Rn7s6
MSTRG.26236.5	MSTRG.26236	Rn7s6
MSTRG.2626.1	MSTRG.2626	Arhgap18
MSTRG.26264.1	MSTRG.26264	.
MSTRG.26282.3	MSTRG.26282	Igkv18-36
MSTRG.26315.1	MSTRG.26315	Eif2ak3
MSTRG.26323.1	MSTRG.26323	Smyd1
MSTRG.26324.1	MSTRG.26324	Smyd1
MSTRG.26325.1	MSTRG.26325	Smyd1
MSTRG.26326.1	MSTRG.26326	Smyd1
MSTRG.26330.1	MSTRG.26330	Gm44175
MSTRG.26330.2	MSTRG.26330	Gm44175
MSTRG.26330.3	MSTRG.26330	Gm44175
MSTRG.26330.4	MSTRG.26330	Gm44175
MSTRG.26332.1	MSTRG.26332	Gm44174
MSTRG.26347.1	MSTRG.26347	Immt
MSTRG.26358.4	MSTRG.26358	0610030E20Rik
MSTRG.26358.5	MSTRG.26358	0610030E20Rik
MSTRG.26358.6	MSTRG.26358	0610030E20Rik
MSTRG.26363.1	MSTRG.26363	Vamp5
MSTRG.26365.6	MSTRG.26365	Mat2a
MSTRG.2638.1	MSTRG.2638	.
MSTRG.26380.1	MSTRG.26380	Kcmf1
MSTRG.26382.6	MSTRG.26382	Kcmf1
MSTRG.26383.1	MSTRG.26383	Kcmf1
MSTRG.26387.1	MSTRG.26387	Gm18402
MSTRG.26390.1	MSTRG.26390	.
MSTRG.26391.1	MSTRG.26391	.
MSTRG.26391.2	MSTRG.26391	.
MSTRG.26391.3	MSTRG.26391	.
MSTRG.26391.4	MSTRG.26391	.
MSTRG.26391.5	MSTRG.26391	.
MSTRG.26392.1	MSTRG.26392	.
MSTRG.26392.2	MSTRG.26392	.
MSTRG.26394.1	MSTRG.26394	.
MSTRG.26397.1	MSTRG.26397	.
MSTRG.26408.1	MSTRG.26408	Ctnna2
MSTRG.26411.1	MSTRG.26411	Gcfc2
MSTRG.26412.1	MSTRG.26412	Gcfc2
MSTRG.26414.4	MSTRG.26414	Pole4
MSTRG.2642.1	MSTRG.2642	Themis
MSTRG.2643.1	MSTRG.2643	AC159472.1
MSTRG.2643.2	MSTRG.2643	AC159472.1

MSTRG.2643.3	MSTRG.2643	AC159472.1
MSTRG.2643.5	MSTRG.2643	Themis
MSTRG.26435.11	MSTRG.26435	Wbp1
MSTRG.26435.4	MSTRG.26435	Wbp1
MSTRG.26439.1	MSTRG.26439	Rtkn
MSTRG.26444.1	MSTRG.26444	Dctn1
MSTRG.26447.2	MSTRG.26447	Mob1a
MSTRG.26452.1	MSTRG.26452	Dguok
MSTRG.26455.1	MSTRG.26455	Stambp
MSTRG.26456.1	MSTRG.26456	Gm18537
MSTRG.26458.1	MSTRG.26458	Stambp
MSTRG.26459.1	MSTRG.26459	Stambp
MSTRG.2646.1	MSTRG.2646	4930519F09Rik
MSTRG.26474.1	MSTRG.26474	Exoc6b
MSTRG.26479.1	MSTRG.26479	Exoc6b
MSTRG.26483.1	MSTRG.26483	Sfxn5
MSTRG.26491.1	MSTRG.26491	Alms1
MSTRG.26500.1	MSTRG.26500	.
MSTRG.2651.1	MSTRG.2651	Echdc1
MSTRG.26517.3	MSTRG.26517	1600020E01Rik
MSTRG.26518.1	MSTRG.26518	1600020E01Rik
MSTRG.26519.1	MSTRG.26519	1600020E01Rik
MSTRG.26520.1	MSTRG.26520	1600020E01Rik
MSTRG.26533.1	MSTRG.26533	.
MSTRG.26537.1	MSTRG.26537	Gm44153
MSTRG.26537.2	MSTRG.26537	Gm44153
MSTRG.26539.1	MSTRG.26539	Aak1
MSTRG.26539.2	MSTRG.26539	Aak1
MSTRG.26540.1	MSTRG.26540	Aak1
MSTRG.26543.1	MSTRG.26543	Nfu1
MSTRG.26544.1	MSTRG.26544	.
MSTRG.26545.1	MSTRG.26545	.
MSTRG.26549.1	MSTRG.26549	Antxr1
MSTRG.26557.1	MSTRG.26557	Arhgap25
MSTRG.26557.2	MSTRG.26557	Arhgap25
MSTRG.26572.1	MSTRG.26572	.
MSTRG.26577.1	MSTRG.26577	Hmces
MSTRG.26583.1	MSTRG.26583	Rab7
MSTRG.26584.1	MSTRG.26584	Rab7
MSTRG.26584.2	MSTRG.26584	Rab7
MSTRG.26591.1	MSTRG.26591	Dnajb8
MSTRG.26592.1	MSTRG.26592	.
MSTRG.26596.1	MSTRG.26596	Eefsec
MSTRG.26597.1	MSTRG.26597	Eefsec
MSTRG.26603.1	MSTRG.26603	Kbtbd12
MSTRG.26615.1	MSTRG.26615	.
MSTRG.26617.15	MSTRG.26617	Tpra1
MSTRG.26617.3	MSTRG.26617	Tpra1
MSTRG.2662.1	MSTRG.2662	Hint3
MSTRG.26628.1	MSTRG.26628	Gm20426
MSTRG.26638.1	MSTRG.26638	Slc41a3
MSTRG.2665.1	MSTRG.2665	Ncoa7
MSTRG.26653.1	MSTRG.26653	Tmem43
MSTRG.26655.1	MSTRG.26655	Xpc
MSTRG.26659.1	MSTRG.26659	Slc6a6
MSTRG.2666.1	MSTRG.2666	Ncoa7
MSTRG.26662.1	MSTRG.26662	Slc6a6

MSTRG.26665.1	MSTRG.26665	Ccdc174
MSTRG.2667.1	MSTRG.2667	Ncoa7
MSTRG.26674.1	MSTRG.26674	Mrps25
MSTRG.26677.1	MSTRG.26677	.
MSTRG.26680.1	MSTRG.26680	Adamts9
MSTRG.26681.1	MSTRG.26681	Adamts9
MSTRG.26682.1	MSTRG.26682	Adamts9
MSTRG.26684.1	MSTRG.26684	Adamts9
MSTRG.26685.4	MSTRG.26685	9530026P05Rik
MSTRG.26685.8	MSTRG.26685	9530026P05Rik
MSTRG.26687.1	MSTRG.26687	9530026P05Rik
MSTRG.26688.1	MSTRG.26688	9530026P05Rik
MSTRG.26692.1	MSTRG.26692	9530026P05Rik
MSTRG.26694.1	MSTRG.26694	9530026P05Rik
MSTRG.26695.1	MSTRG.26695	9530026P05Rik
MSTRG.2670.1	MSTRG.2670	Ncoa7
MSTRG.26700.1	MSTRG.26700	Magi1
MSTRG.26701.1	MSTRG.26701	.
MSTRG.26705.1	MSTRG.26705	Slc25a26
MSTRG.26706.1	MSTRG.26706	Slc25a26
MSTRG.26708.1	MSTRG.26708	Lrig1
MSTRG.26709.1	MSTRG.26709	Lrig1
MSTRG.26713.1	MSTRG.26713	Fam19a1
MSTRG.26723.1	MSTRG.26723	Frmd4b
MSTRG.26724.1	MSTRG.26724	Frmd4b
MSTRG.26725.1	MSTRG.26725	Frmd4b
MSTRG.26726.1	MSTRG.26726	Frmd4b
MSTRG.26727.1	MSTRG.26727	Frmd4b
MSTRG.26729.1	MSTRG.26729	Frmd4b
MSTRG.26730.1	MSTRG.26730	Frmd4b
MSTRG.26731.1	MSTRG.26731	Frmd4b
MSTRG.26734.1	MSTRG.26734	Frmd4b
MSTRG.26738.1	MSTRG.26738	Mitf
MSTRG.26739.1	MSTRG.26739	Mitf
MSTRG.26740.1	MSTRG.26740	Mitf
MSTRG.26741.1	MSTRG.26741	Mitf
MSTRG.26742.1	MSTRG.26742	Mitf
MSTRG.26743.1	MSTRG.26743	Mitf
MSTRG.26745.1	MSTRG.26745	Mitf
MSTRG.26752.1	MSTRG.26752	Shq1
MSTRG.26759.1	MSTRG.26759	Gm43948
MSTRG.26759.2	MSTRG.26759	Gm43948
MSTRG.2676.1	MSTRG.2676	.
MSTRG.26769.1	MSTRG.26769	Foxp1
MSTRG.26770.1	MSTRG.26770	Foxp1
MSTRG.26771.2	MSTRG.26771	Foxp1
MSTRG.26772.1	MSTRG.26772	Foxp1
MSTRG.26774.1	MSTRG.26774	Foxp1
MSTRG.26774.2	MSTRG.26774	Foxp1
MSTRG.26775.1	MSTRG.26775	Foxp1
MSTRG.26780.1	MSTRG.26780	Gm20696
MSTRG.26781.1	MSTRG.26781	.
MSTRG.26782.1	MSTRG.26782	.
MSTRG.26785.1	MSTRG.26785	.
MSTRG.26787.1	MSTRG.26787	Chl1
MSTRG.26792.1	MSTRG.26792	Chl1
MSTRG.26795.1	MSTRG.26795	.

MSTRG.26799.1	MSTRG.26799	.
MSTRG.268.1	MSTRG.268	Neurl3
MSTRG.2680.1	MSTRG.2680	Zufsp
MSTRG.26800.1	MSTRG.26800	.
MSTRG.26801.1	MSTRG.26801	.
MSTRG.26802.6	MSTRG.26802	Sumf1
MSTRG.26809.1	MSTRG.26809	Itpr1
MSTRG.26812.1	MSTRG.26812	Itpr1
MSTRG.26814.1	MSTRG.26814	Itpr1
MSTRG.26820.1	MSTRG.26820	Gm26799
MSTRG.26846.1	MSTRG.26846	Gm44199
MSTRG.26849.1	MSTRG.26849	1700015O11Rik
MSTRG.26854.1	MSTRG.26854	Fancd2
MSTRG.26862.1	MSTRG.26862	Irak2
MSTRG.26867.1	MSTRG.26867	Tamm41
MSTRG.26868.1	MSTRG.26868	Tamm41
MSTRG.26874.1	MSTRG.26874	Vgll4
MSTRG.26876.1	MSTRG.26876	.
MSTRG.26878.1	MSTRG.26878	Pparg
MSTRG.26882.1	MSTRG.26882	Raf1
MSTRG.26883.1	MSTRG.26883	Mkrn2
MSTRG.26887.1	MSTRG.26887	Raf1
MSTRG.26894.1	MSTRG.26894	Efcab12
MSTRG.26899.1	MSTRG.26899	Tmcc1
MSTRG.2690.1	MSTRG.2690	Nt5dc1
MSTRG.26900.1	MSTRG.26900	Tmcc1
MSTRG.26902.1	MSTRG.26902	Tmcc1
MSTRG.26904.1	MSTRG.26904	Tmcc1
MSTRG.26916.1	MSTRG.26916	Zfand4
MSTRG.26917.1	MSTRG.26917	Zfand4
MSTRG.26918.1	MSTRG.26918	Zfand4
MSTRG.2692.1	MSTRG.2692	Nt5dc1
MSTRG.26927.1	MSTRG.26927	Hnrnpf
MSTRG.26928.1	MSTRG.26928	4933440N22Rik
MSTRG.2693.1	MSTRG.2693	Nt5dc1
MSTRG.26934.1	MSTRG.26934	Csgalnact2
MSTRG.26941.1	MSTRG.26941	Wnt5b
MSTRG.26942.1	MSTRG.26942	Wnt5b
MSTRG.26943.1	MSTRG.26943	Wnt5b
MSTRG.26944.1	MSTRG.26944	Wnt5b
MSTRG.26946.1	MSTRG.26946	Wnt5b
MSTRG.26947.1	MSTRG.26947	Wnt5b
MSTRG.26948.1	MSTRG.26948	Wnt5b
MSTRG.2695.1	MSTRG.2695	.
MSTRG.26950.1	MSTRG.26950	Wnt5b
MSTRG.26951.1	MSTRG.26951	Wnt5b
MSTRG.26952.1	MSTRG.26952	Wnt5b
MSTRG.26956.4	MSTRG.26956	Dcp1b
MSTRG.26960.1	MSTRG.26960	Erc1
MSTRG.26962.1	MSTRG.26962	Rad52
MSTRG.26963.1	MSTRG.26963	Rad52
MSTRG.26965.1	MSTRG.26965	Ninj2
MSTRG.26969.1	MSTRG.26969	Wnk1
MSTRG.26973.1	MSTRG.26973	Ccdc77
MSTRG.26974.1	MSTRG.26974	Ccdc77
MSTRG.26976.1	MSTRG.26976	Kdm5a
MSTRG.26978.1	MSTRG.26978	.

MSTRG.26982.1	MSTRG.26982	Hdhd5
MSTRG.26986.1	MSTRG.26986	Atp6v1e1
MSTRG.26987.1	MSTRG.26987	Atp6v1e1
MSTRG.26989.1	MSTRG.26989	Bcl2l13
MSTRG.2699.1	MSTRG.2699	Hs3st5
MSTRG.26991.1	MSTRG.26991	Bid
MSTRG.26998.1	MSTRG.26998	.
MSTRG.27001.1	MSTRG.27001	Usp18
MSTRG.27005.1	MSTRG.27005	Mical3
MSTRG.27006.1	MSTRG.27006	Mical3
MSTRG.27008.1	MSTRG.27008	Mical3
MSTRG.27012.1	MSTRG.27012	Mug2
MSTRG.27026.1	MSTRG.27026	Slc2a3
MSTRG.27030.1	MSTRG.27030	Gm26826
MSTRG.27037.1	MSTRG.27037	.
MSTRG.27038.1	MSTRG.27038	.
MSTRG.27040.1	MSTRG.27040	Vmn2r27
MSTRG.27042.1	MSTRG.27042	.
MSTRG.27044.1	MSTRG.27044	Pex5
MSTRG.27053.1	MSTRG.27053	.
MSTRG.27054.1	MSTRG.27054	.
MSTRG.27075.1	MSTRG.27075	A230083G16Rik
MSTRG.27085.1	MSTRG.27085	4930557K07Rik
MSTRG.27086.1	MSTRG.27086	4930557K07Rik
MSTRG.27091.1	MSTRG.27091	Nop2
MSTRG.27093.1	MSTRG.27093	Iffo1
MSTRG.27099.1	MSTRG.27099	Vamp1
MSTRG.27100.2	MSTRG.27100	4930417O13Rik
MSTRG.27100.4	MSTRG.27100	4930417O13Rik
MSTRG.27100.6	MSTRG.27100	4930417O13Rik
MSTRG.27105.1	MSTRG.27105	Tnfrsf1a
MSTRG.2711.1	MSTRG.2711	Slc16a10
MSTRG.27116.1	MSTRG.27116	D6Wsu163e
MSTRG.27117.1	MSTRG.27117	.
MSTRG.27119.1	MSTRG.27119	Gm43635
MSTRG.2713.1	MSTRG.2713	Slc16a10
MSTRG.27132.1	MSTRG.27132	Cracr2a
MSTRG.27133.1	MSTRG.27133	Cracr2a
MSTRG.27136.1	MSTRG.27136	Tspan9
MSTRG.27139.1	MSTRG.27139	.
MSTRG.2714.1	MSTRG.2714	Slc16a10
MSTRG.27141.1	MSTRG.27141	Klrb1a
MSTRG.27142.1	MSTRG.27142	Klrb1c
MSTRG.27149.1	MSTRG.27149	.
MSTRG.27153.1	MSTRG.27153	Klrb1c
MSTRG.27153.2	MSTRG.27153	Klrb1c
MSTRG.27155.1	MSTRG.27155	Gm44511
MSTRG.27158.1	MSTRG.27158	Tulp3
MSTRG.27167.1	MSTRG.27167	Gm10069
MSTRG.2717.1	MSTRG.2717	Rpf2
MSTRG.27170.1	MSTRG.27170	Gm10069
MSTRG.27174.3	MSTRG.27174	2310001H17Rik
MSTRG.27175.1	MSTRG.27175	2310001H17Rik
MSTRG.27176.1	MSTRG.27176	2310001H17Rik
MSTRG.27177.1	MSTRG.27177	2310001H17Rik
MSTRG.27179.1	MSTRG.27179	Cd69
MSTRG.27179.2	MSTRG.27179	Cd69

MSTRG.27179.3	MSTRG.27179	Cd69
MSTRG.27179.4	MSTRG.27179	Cd69
MSTRG.27183.1	MSTRG.27183	BC035044
MSTRG.27183.3	MSTRG.27183	BC035044
MSTRG.27183.6	MSTRG.27183	BC035044
MSTRG.27184.1	MSTRG.27184	BC035044
MSTRG.27185.1	MSTRG.27185	Clec2g
MSTRG.27185.2	MSTRG.27185	Clec2g
MSTRG.27188.1	MSTRG.27188	BC035044
MSTRG.27189.1	MSTRG.27189	Gm15987
MSTRG.27193.1	MSTRG.27193	Clec2g
MSTRG.27195.1	MSTRG.27195	Clec2g
MSTRG.2720.1	MSTRG.2720	Fyn
MSTRG.2721.1	MSTRG.2721	Gm6963
MSTRG.2721.3	MSTRG.2721	Fyn
MSTRG.27211.1	MSTRG.27211	Klrk1
MSTRG.27212.1	MSTRG.27212	.
MSTRG.27216.1	MSTRG.27216	Klrc2
MSTRG.27220.1	MSTRG.27220	Gm16242
MSTRG.27223.1	MSTRG.27223	Klra17
MSTRG.27226.1	MSTRG.27226	Gm33962
MSTRG.27232.1	MSTRG.27232	5430401F13Rik
MSTRG.27233.1	MSTRG.27233	Gm16571
MSTRG.27239.1	MSTRG.27239	.
MSTRG.27241.1	MSTRG.27241	.
MSTRG.27246.1	MSTRG.27246	Lrp6
MSTRG.27247.1	MSTRG.27247	Lrp6
MSTRG.2725.1	MSTRG.2725	Traf3ip2
MSTRG.27251.1	MSTRG.27251	Etv6
MSTRG.27253.1	MSTRG.27253	Etv6
MSTRG.27254.1	MSTRG.27254	Etv6
MSTRG.27256.1	MSTRG.27256	Etv6
MSTRG.2726.10	MSTRG.2726	E130307A14Rik
MSTRG.2726.12	MSTRG.2726	E130307A14Rik
MSTRG.2726.13	MSTRG.2726	E130307A14Rik
MSTRG.2726.15	MSTRG.2726	E130307A14Rik
MSTRG.2726.2	MSTRG.2726	E130307A14Rik
MSTRG.2726.9	MSTRG.2726	E130307A14Rik
MSTRG.27262.1	MSTRG.27262	Dusp16
MSTRG.27264.1	MSTRG.27264	Dusp16
MSTRG.27267.1	MSTRG.27267	Crebl2
MSTRG.27274.10	MSTRG.27274	Lockd
MSTRG.27274.5	MSTRG.27274	Lockd
MSTRG.27274.7	MSTRG.27274	Lockd
MSTRG.27274.8	MSTRG.27274	Lockd
MSTRG.27274.9	MSTRG.27274	Lockd
MSTRG.27275.1	MSTRG.27275	Gm44238
MSTRG.27275.2	MSTRG.27275	Lockd
MSTRG.27275.3	MSTRG.27275	Lockd
MSTRG.27275.4	MSTRG.27275	Lockd
MSTRG.27276.1	MSTRG.27276	Lockd
MSTRG.2728.1	MSTRG.2728	E130307A14Rik
MSTRG.27283.1	MSTRG.27283	Hebp1
MSTRG.2729.1	MSTRG.2729	E130307A14Rik
MSTRG.27292.1	MSTRG.27292	Gucy2c
MSTRG.27295.1	MSTRG.27295	Gm44140
MSTRG.27302.1	MSTRG.27302	Atf7ip

MSTRG.27303.1	MSTRG.27303	Atf7ip
MSTRG.27315.1	MSTRG.27315	Ptpro
MSTRG.27319.1	MSTRG.27319	Dera
MSTRG.27320.1	MSTRG.27320	Dera
MSTRG.27327.1	MSTRG.27327	Aebp2
MSTRG.27329.1	MSTRG.27329	Plekha5
MSTRG.27334.1	MSTRG.27334	Gm30524
MSTRG.27338.1	MSTRG.27338	Pde3a
MSTRG.27344.1	MSTRG.27344	Gm20400
MSTRG.27347.1	MSTRG.27347	Recql
MSTRG.2735.1	MSTRG.2735	Gtf3c6
MSTRG.27357.1	MSTRG.27357	St8sia1
MSTRG.27358.1	MSTRG.27358	St8sia1
MSTRG.27359.1	MSTRG.27359	St8sia1
MSTRG.27361.1	MSTRG.27361	Etnk1
MSTRG.27362.1	MSTRG.27362	1700126G02Rik
MSTRG.27363.1	MSTRG.27363	1700126G02Rik
MSTRG.27368.1	MSTRG.27368	Sox5
MSTRG.27369.1	MSTRG.27369	Sox5
MSTRG.2737.6	MSTRG.2737	Cdk19
MSTRG.27375.1	MSTRG.27375	Sox5
MSTRG.27383.1	MSTRG.27383	Rassf8
MSTRG.27386.1	MSTRG.27386	.
MSTRG.2739.1	MSTRG.2739	Cdk19
MSTRG.27391.1	MSTRG.27391	Lrmp
MSTRG.27395.2	MSTRG.27395	Etfrf1
MSTRG.27399.1	MSTRG.27399	Itpr2
MSTRG.2740.1	MSTRG.2740	Cdk19
MSTRG.27400.1	MSTRG.27400	Itpr2
MSTRG.27401.1	MSTRG.27401	Itpr2
MSTRG.27401.2	MSTRG.27401	Itpr2
MSTRG.27403.1	MSTRG.27403	Itpr2
MSTRG.27404.1	MSTRG.27404	Itpr2
MSTRG.27406.1	MSTRG.27406	Itpr2
MSTRG.27407.1	MSTRG.27407	Itpr2
MSTRG.27408.1	MSTRG.27408	Itpr2
MSTRG.27409.1	MSTRG.27409	Itpr2
MSTRG.27410.1	MSTRG.27410	Itpr2
MSTRG.27411.1	MSTRG.27411	Itpr2
MSTRG.27412.1	MSTRG.27412	Itpr2
MSTRG.27413.1	MSTRG.27413	Itpr2
MSTRG.27414.1	MSTRG.27414	Itpr2
MSTRG.27415.1	MSTRG.27415	.
MSTRG.27415.2	MSTRG.27415	.
MSTRG.27416.1	MSTRG.27416	.
MSTRG.27417.1	MSTRG.27417	.
MSTRG.27426.1	MSTRG.27426	Arntl2
MSTRG.27428.1	MSTRG.27428	Ppfibp1
MSTRG.2743.1	MSTRG.2743	.
MSTRG.27434.1	MSTRG.27434	Mrps35
MSTRG.27437.1	MSTRG.27437	Mansc4
MSTRG.27439.1	MSTRG.27439	Klhl42
MSTRG.27439.2	MSTRG.27439	Klhl42
MSTRG.2744.2	MSTRG.2744	Fig4
MSTRG.27442.1	MSTRG.27442	Gm15762
MSTRG.27445.1	MSTRG.27445	Gm6288
MSTRG.27447.1	MSTRG.27447	Ccdc91

MSTRG.27448.1	MSTRG.27448	Ccdc91
MSTRG.27450.1	MSTRG.27450	.
MSTRG.27451.1	MSTRG.27451	.
MSTRG.27456.1	MSTRG.27456	Ergic2
MSTRG.27466.1	MSTRG.27466	Tmtc1
MSTRG.2747.1	MSTRG.2747	Fig4
MSTRG.27481.1	MSTRG.27481	Etfbkmt
MSTRG.27482.1	MSTRG.27482	Etfbkmt
MSTRG.27483.6	MSTRG.27483	Amn1
MSTRG.27485.1	MSTRG.27485	.
MSTRG.27486.1	MSTRG.27486	.
MSTRG.27491.1	MSTRG.27491	.
MSTRG.27494.1	MSTRG.27494	Bicd1
MSTRG.27501.1	MSTRG.27501	Gm15922
MSTRG.2751.1	MSTRG.2751	Zbtb24
MSTRG.2752.1	MSTRG.2752	.
MSTRG.27520.1	MSTRG.27520	Lair1
MSTRG.27521.1	MSTRG.27521	Lair1
MSTRG.27524.1	MSTRG.27524	Gm15931
MSTRG.27534.1	MSTRG.27534	Gm15494
MSTRG.27555.5	MSTRG.27555	Fiz1
MSTRG.27565.1	MSTRG.27565	Zfp787
MSTRG.27568.1	MSTRG.27568	Zfp444
MSTRG.27569.1	MSTRG.27569	Galp
MSTRG.27572.1	MSTRG.27572	Zfp667
MSTRG.27574.1	MSTRG.27574	.
MSTRG.27575.1	MSTRG.27575	.
MSTRG.27578.1	MSTRG.27578	.
MSTRG.27584.1	MSTRG.27584	Vmn2r38
MSTRG.27586.1	MSTRG.27586	.
MSTRG.27587.1	MSTRG.27587	.
MSTRG.27588.1	MSTRG.27588	.
MSTRG.27595.2	MSTRG.27595	Gm18194
MSTRG.27599.1	MSTRG.27599	Zscan22
MSTRG.276.1	MSTRG.276	.
MSTRG.27600.1	MSTRG.27600	.
MSTRG.27607.1	MSTRG.27607	Slc27a5
MSTRG.2761.1	MSTRG.2761	Ccdc162
MSTRG.27611.1	MSTRG.27611	.
MSTRG.27612.1	MSTRG.27612	.
MSTRG.27615.1	MSTRG.27615	.
MSTRG.27616.1	MSTRG.27616	.
MSTRG.27618.1	MSTRG.27618	6330408A02Rik
MSTRG.27620.1	MSTRG.27620	.
MSTRG.27647.1	MSTRG.27647	Bicra
MSTRG.27653.1	MSTRG.27653	Dhx34
MSTRG.27656.1	MSTRG.27656	Bbc3
MSTRG.27658.1	MSTRG.27658	Sae1
MSTRG.27659.1	MSTRG.27659	Sae1
MSTRG.27666.1	MSTRG.27666	Npas1
MSTRG.27671.1	MSTRG.27671	Gm29443
MSTRG.27673.1	MSTRG.27673	Arhgap35
MSTRG.2768.1	MSTRG.2768	BC048559
MSTRG.27684.1	MSTRG.27684	Gm32772
MSTRG.27685.1	MSTRG.27685	.
MSTRG.27686.1	MSTRG.27686	.
MSTRG.27687.1	MSTRG.27687	.

MSTRG.27689.1	MSTRG.27689	Slc1a5
MSTRG.2769.1	MSTRG.2769	Cep57l1
MSTRG.27696.1	MSTRG.27696	.
MSTRG.27706.1	MSTRG.27706	Mypop
MSTRG.27709.1	MSTRG.27709	Sympk
MSTRG.2771.1	MSTRG.2771	Sesn1
MSTRG.2773.1	MSTRG.2773	Sesn1
MSTRG.27732.1	MSTRG.27732	Klc3
MSTRG.27735.1	MSTRG.27735	Mark4
MSTRG.27736.1	MSTRG.27736	Exoc3l2
MSTRG.2775.1	MSTRG.2775	Sesn1
MSTRG.27751.1	MSTRG.27751	Clptm1
MSTRG.27762.1	MSTRG.27762	.
MSTRG.27763.1	MSTRG.27763	.
MSTRG.27764.1	MSTRG.27764	.
MSTRG.2777.1	MSTRG.2777	Foxo3
MSTRG.27776.1	MSTRG.27776	Zfp180
MSTRG.27784.1	MSTRG.27784	Kcnn4
MSTRG.27784.2	MSTRG.27784	Kcnn4
MSTRG.27784.3	MSTRG.27784	Kcnn4
MSTRG.27786.1	MSTRG.27786	Kcnn4
MSTRG.27787.5	MSTRG.27787	Zfp61
MSTRG.2780.1	MSTRG.2780	Afg1l
MSTRG.27800.1	MSTRG.27800	Phldb3
MSTRG.2781.1	MSTRG.2781	Afg1l
MSTRG.27811.1	MSTRG.27811	Dedd2
MSTRG.27819.1	MSTRG.27819	Arhgef1
MSTRG.2782.1	MSTRG.2782	Afg1l
MSTRG.2783.1	MSTRG.2783	Afg1l
MSTRG.2784.1	MSTRG.2784	Afg1l
MSTRG.27849.1	MSTRG.27849	.
MSTRG.27850.1	MSTRG.27850	.
MSTRG.27852.1	MSTRG.27852	.
MSTRG.27875.1	MSTRG.27875	2310022A10Rik
MSTRG.27876.1	MSTRG.27876	2310022A10Rik
MSTRG.27879.1	MSTRG.27879	.
MSTRG.2788.1	MSTRG.2788	Ostm1
MSTRG.27880.1	MSTRG.27880	.
MSTRG.27881.1	MSTRG.27881	.
MSTRG.27884.1	MSTRG.27884	Map3k10
MSTRG.27894.1	MSTRG.27894	Zfp850
MSTRG.27895.1	MSTRG.27895	Zfp850
MSTRG.27898.1	MSTRG.27898	.
MSTRG.27899.1	MSTRG.27899	.
MSTRG.279.3	MSTRG.279	Fer1l5
MSTRG.27912.1	MSTRG.27912	Supt5
MSTRG.27914.1	MSTRG.27914	.
MSTRG.2792.1	MSTRG.2792	Sec63
MSTRG.2793.1	MSTRG.2793	Sec63
MSTRG.27930.4	MSTRG.27930	Fbxo17
MSTRG.27931.1	MSTRG.27931	Fbxo17
MSTRG.27932.1	MSTRG.27932	Fbxo17
MSTRG.27933.1	MSTRG.27933	Fbxo17
MSTRG.27936.1	MSTRG.27936	Sars2
MSTRG.2794.1	MSTRG.2794	Sec63
MSTRG.27941.1	MSTRG.27941	Rinl
MSTRG.27949.1	MSTRG.27949	Actn4

MSTRG.27950.1	MSTRG.27950	Actn4
MSTRG.27955.1	MSTRG.27955	Ryr1
MSTRG.2796.1	MSTRG.2796	Sec63
MSTRG.27961.1	MSTRG.27961	.
MSTRG.27964.1	MSTRG.27964	Catsperg1
MSTRG.27967.1	MSTRG.27967	Spint2
MSTRG.27971.1	MSTRG.27971	Rasgrp4
MSTRG.27977.1	MSTRG.27977	.
MSTRG.27981.1	MSTRG.27981	Sipa1l3
MSTRG.27984.2	MSTRG.27984	Zfp790
MSTRG.27990.1	MSTRG.27990	Zfp260
MSTRG.27991.1	MSTRG.27991	Zfp260
MSTRG.27994.1	MSTRG.27994	Zfp27
MSTRG.27999.1	MSTRG.27999	Zfp74
MSTRG.28.1	MSTRG.28	Rb1cc1
MSTRG.28001.1	MSTRG.28001	C230062I16Rik
MSTRG.28003.1	MSTRG.28003	C230062I16Rik
MSTRG.28006.1	MSTRG.28006	Zfp568
MSTRG.28007.1	MSTRG.28007	Zfp568
MSTRG.28009.1	MSTRG.28009	Zfp14
MSTRG.2801.1	MSTRG.2801	Scml4
MSTRG.28024.1	MSTRG.28024	Wdr62
MSTRG.28029.1	MSTRG.28029	.
MSTRG.28046.1	MSTRG.28046	.
MSTRG.28052.3	MSTRG.28052	Tmem147os
MSTRG.28053.5	MSTRG.28053	Gapdhs
MSTRG.28053.6	MSTRG.28053	Gapdhs
MSTRG.28054.1	MSTRG.28054	Gapdhs
MSTRG.28064.1	MSTRG.28064	Gm44662
MSTRG.28067.1	MSTRG.28067	Gm17077
MSTRG.2807.1	MSTRG.2807	.
MSTRG.28084.1	MSTRG.28084	Gm12764
MSTRG.2809.1	MSTRG.2809	Pdss2
MSTRG.28093.1	MSTRG.28093	4931406P16Rik
MSTRG.28094.1	MSTRG.28094	4931406P16Rik
MSTRG.2810.1	MSTRG.2810	Pdss2
MSTRG.28105.1	MSTRG.28105	.
MSTRG.28109.1	MSTRG.28109	Gpatch1
MSTRG.28111.1	MSTRG.28111	Gpatch1
MSTRG.28120.1	MSTRG.28120	Dpy19l3
MSTRG.28124.1	MSTRG.28124	Gm26790
MSTRG.28128.1	MSTRG.28128	Ccne1
MSTRG.28129.1	MSTRG.28129	Uri1
MSTRG.28129.2	MSTRG.28129	Uri1
MSTRG.28133.1	MSTRG.28133	D530033B14Rik
MSTRG.28134.1	MSTRG.28134	D530033B14Rik
MSTRG.28135.1	MSTRG.28135	D530033B14Rik
MSTRG.28136.1	MSTRG.28136	.
MSTRG.2814.1	MSTRG.2814	Pdss2
MSTRG.28140.1	MSTRG.28140	Gm37494
MSTRG.28141.1	MSTRG.28141	Gm37494
MSTRG.28142.1	MSTRG.28142	Gm37494
MSTRG.28150.1	MSTRG.28150	Gm20449
MSTRG.28156.1	MSTRG.28156	2610021A01Rik
MSTRG.28158.1	MSTRG.28158	Zfp788
MSTRG.28161.1	MSTRG.28161	Zfp141
MSTRG.28162.1	MSTRG.28162	Zfp141

MSTRG.28169.1	MSTRG.28169	Gm17768
MSTRG.2817.1	MSTRG.2817	Bend3
MSTRG.28171.1	MSTRG.28171	Zfp715
MSTRG.28176.1	MSTRG.28176	Zfp719
MSTRG.28192.1	MSTRG.28192	2310002F09Rik
MSTRG.28196.1	MSTRG.28196	Gm45122
MSTRG.28201.1	MSTRG.28201	Shank1
MSTRG.28218.1	MSTRG.28218	Pold1
MSTRG.28231.1	MSTRG.28231	.
MSTRG.28234.1	MSTRG.28234	Vrk3
MSTRG.2824.1	MSTRG.2824	Qrsl1
MSTRG.28246.1	MSTRG.28246	.
MSTRG.28250.3	MSTRG.28250	Gm15545
MSTRG.28250.5	MSTRG.28250	Gm15545
MSTRG.28255.1	MSTRG.28255	Nosip
MSTRG.28256.1	MSTRG.28256	Nosip
MSTRG.28265.1	MSTRG.28265	Gm45552
MSTRG.28265.2	MSTRG.28265	Gm45552
MSTRG.28276.1	MSTRG.28276	.
MSTRG.2828.1	MSTRG.2828	Crybg1
MSTRG.28285.1	MSTRG.28285	Gys1
MSTRG.283.1	MSTRG.283	Cnnm3
MSTRG.2831.1	MSTRG.2831	Atg5
MSTRG.28313.1	MSTRG.28313	Tmem143
MSTRG.28314.5	MSTRG.28314	Emp3
MSTRG.28319.1	MSTRG.28319	Nomo1
MSTRG.2832.1	MSTRG.2832	Atg5
MSTRG.28320.1	MSTRG.28320	.
MSTRG.28323.1	MSTRG.28323	Sergef
MSTRG.28324.1	MSTRG.28324	Sergef
MSTRG.28325.1	MSTRG.28325	Sergef
MSTRG.28328.1	MSTRG.28328	Sergef
MSTRG.28329.1	MSTRG.28329	Sergef
MSTRG.28330.1	MSTRG.28330	Sergef
MSTRG.28344.1	MSTRG.28344	.
MSTRG.28349.1	MSTRG.28349	Uevld
MSTRG.2835.1	MSTRG.2835	Prep
MSTRG.28354.1	MSTRG.28354	Mrgprx2
MSTRG.28356.1	MSTRG.28356	Zdhhc13
MSTRG.28358.1	MSTRG.28358	Zdhhc13
MSTRG.28359.1	MSTRG.28359	Zdhhc13
MSTRG.2836.1	MSTRG.2836	Prep
MSTRG.28361.1	MSTRG.28361	Htatip2
MSTRG.28363.1	MSTRG.28363	Prmt3
MSTRG.28364.1	MSTRG.28364	Prmt3
MSTRG.28366.1	MSTRG.28366	.
MSTRG.28369.1	MSTRG.28369	Nell1
MSTRG.2837.1	MSTRG.2837	Prep
MSTRG.28371.1	MSTRG.28371	Nell1
MSTRG.28381.1	MSTRG.28381	Nav2
MSTRG.28382.1	MSTRG.28382	Nav2
MSTRG.28390.1	MSTRG.28390	.
MSTRG.28391.1	MSTRG.28391	.
MSTRG.28397.1	MSTRG.28397	Nipa2
MSTRG.28398.1	MSTRG.28398	Nipa2
MSTRG.28407.1	MSTRG.28407	Oca2
MSTRG.2841.1	MSTRG.2841	Hace1

MSTRG.28411.1	MSTRG.28411	Herc2
MSTRG.2842.1	MSTRG.2842	Hace1
MSTRG.28420.1	MSTRG.28420	Gabrg3
MSTRG.28422.1	MSTRG.28422	.
MSTRG.28424.1	MSTRG.28424	Atp10a
MSTRG.28425.1	MSTRG.28425	Atp10a
MSTRG.28427.1	MSTRG.28427	Atp10a
MSTRG.28428.1	MSTRG.28428	Atp10a
MSTRG.28429.1	MSTRG.28429	Atp10a
MSTRG.2843.1	MSTRG.2843	Hace1
MSTRG.28431.1	MSTRG.28431	Atp10a
MSTRG.28438.1	MSTRG.28438	Ube3a
MSTRG.28439.1	MSTRG.28439	Ube3a
MSTRG.2844.1	MSTRG.2844	Hace1
MSTRG.2845.1	MSTRG.2845	Hace1
MSTRG.28450.2	MSTRG.28450	Gm27252
MSTRG.28451.1	MSTRG.28451	.
MSTRG.28453.1	MSTRG.28453	.
MSTRG.28455.1	MSTRG.28455	Gm32633
MSTRG.28456.1	MSTRG.28456	Gm32633
MSTRG.28457.1	MSTRG.28457	Gm32633
MSTRG.28464.1	MSTRG.28464	Mcee
MSTRG.28465.1	MSTRG.28465	Mcee
MSTRG.28466.1	MSTRG.28466	Mcee
MSTRG.2847.1	MSTRG.2847	Hace1
MSTRG.28470.1	MSTRG.28470	Apba2
MSTRG.28481.1	MSTRG.28481	Gm26827
MSTRG.28484.1	MSTRG.28484	Chsy1
MSTRG.28491.1	MSTRG.28491	Lrrk1
MSTRG.28497.1	MSTRG.28497	Asb7
MSTRG.28498.1	MSTRG.28498	Asb7
MSTRG.28499.1	MSTRG.28499	Asb7
MSTRG.2850.1	MSTRG.2850	.
MSTRG.28503.1	MSTRG.28503	Adamts17
MSTRG.28505.1	MSTRG.28505	.
MSTRG.28509.1	MSTRG.28509	Mef2a
MSTRG.2851.1	MSTRG.2851	.
MSTRG.28512.1	MSTRG.28512	Lrrc28
MSTRG.28516.1	MSTRG.28516	.
MSTRG.2852.1	MSTRG.2852	.
MSTRG.28522.1	MSTRG.28522	.
MSTRG.28525.1	MSTRG.28525	Igf1r
MSTRG.28527.1	MSTRG.28527	Pgpep1l
MSTRG.28528.1	MSTRG.28528	Pgpep1l
MSTRG.2853.1	MSTRG.2853	AC153954.2
MSTRG.28531.1	MSTRG.28531	Gm16157
MSTRG.28532.1	MSTRG.28532	4930405G09Rik
MSTRG.28533.1	MSTRG.28533	4930405G09Rik
MSTRG.28535.1	MSTRG.28535	.
MSTRG.28541.1	MSTRG.28541	.
MSTRG.28545.1	MSTRG.28545	Mctp2
MSTRG.28548.1	MSTRG.28548	.
MSTRG.28557.1	MSTRG.28557	Slco3a1
MSTRG.28559.1	MSTRG.28559	Slco3a1
MSTRG.28559.2	MSTRG.28559	Slco3a1
MSTRG.28559.3	MSTRG.28559	Slco3a1
MSTRG.28560.1	MSTRG.28560	Slco3a1

MSTRG.28561.1	MSTRG.28561	Slco3a1
MSTRG.28561.2	MSTRG.28561	Slco3a1
MSTRG.28562.1	MSTRG.28562	Slco3a1
MSTRG.28563.1	MSTRG.28563	Slco3a1
MSTRG.28564.1	MSTRG.28564	Slco3a1
MSTRG.28565.1	MSTRG.28565	.
MSTRG.28567.1	MSTRG.28567	.
MSTRG.28568.1	MSTRG.28568	.
MSTRG.28569.1	MSTRG.28569	AU020206
MSTRG.28569.2	MSTRG.28569	AU020206
MSTRG.28570.1	MSTRG.28570	AU020206
MSTRG.28571.1	MSTRG.28571	AU020206
MSTRG.28571.2	MSTRG.28571	AU020206
MSTRG.28571.3	MSTRG.28571	AU020206
MSTRG.28571.4	MSTRG.28571	AU020206
MSTRG.28573.1	MSTRG.28573	Klhl25
MSTRG.28582.1	MSTRG.28582	Akap13
MSTRG.28593.1	MSTRG.28593	Ntrk3
MSTRG.28594.1	MSTRG.28594	Ntrk3
MSTRG.28596.1	MSTRG.28596	Ntrk3
MSTRG.28598.1	MSTRG.28598	Ntrk3
MSTRG.28598.2	MSTRG.28598	Ntrk3
MSTRG.28598.3	MSTRG.28598	Ntrk3
MSTRG.28599.1	MSTRG.28599	Ntrk3
MSTRG.286.1	MSTRG.286	Gm42417
MSTRG.2860.1	MSTRG.2860	Ascc3
MSTRG.28600.1	MSTRG.28600	Ntrk3
MSTRG.2862.1	MSTRG.2862	Ascc3
MSTRG.28624.1	MSTRG.28624	Ap3s2
MSTRG.28629.1	MSTRG.28629	Gm44951
MSTRG.28631.1	MSTRG.28631	Zfp710
MSTRG.28633.1	MSTRG.28633	.
MSTRG.2864.1	MSTRG.2864	Ascc3
MSTRG.28643.1	MSTRG.28643	Unc45a
MSTRG.28646.1	MSTRG.28646	Vps33b
MSTRG.28649.1	MSTRG.28649	Prc1
MSTRG.28651.1	MSTRG.28651	Gm18310
MSTRG.28657.1	MSTRG.28657	Blm
MSTRG.28659.1	MSTRG.28659	Blm
MSTRG.28663.1	MSTRG.28663	Crtc3
MSTRG.28664.1	MSTRG.28664	Crtc3
MSTRG.28666.1	MSTRG.28666	Crtc3
MSTRG.28670.1	MSTRG.28670	Gm44649
MSTRG.28671.1	MSTRG.28671	Iqgap1
MSTRG.28672.1	MSTRG.28672	Iqgap1
MSTRG.28673.1	MSTRG.28673	Iqgap1
MSTRG.28677.1	MSTRG.28677	Sec11a
MSTRG.28679.1	MSTRG.28679	Gm45718
MSTRG.28690.2	MSTRG.28690	Fam103a1
MSTRG.28695.1	MSTRG.28695	Gm45014
MSTRG.28699.1	MSTRG.28699	Hdgfl3
MSTRG.287.1	MSTRG.287	Ankrd39
MSTRG.2870.1	MSTRG.2870	Ros1
MSTRG.28705.1	MSTRG.28705	Efl1
MSTRG.28706.1	MSTRG.28706	Gm44916
MSTRG.28707.1	MSTRG.28707	Efl1
MSTRG.28711.1	MSTRG.28711	Gm44724

MSTRG.28717.1	MSTRG.28717	Gm16638
MSTRG.28720.1	MSTRG.28720	Il16
MSTRG.28727.1	MSTRG.28727	.
MSTRG.28729.1	MSTRG.28729	Zfand6
MSTRG.28731.1	MSTRG.28731	.
MSTRG.28732.1	MSTRG.28732	.
MSTRG.28737.1	MSTRG.28737	Gm44704
MSTRG.28739.1	MSTRG.28739	Tmem135
MSTRG.28742.1	MSTRG.28742	Tmem135
MSTRG.28753.1	MSTRG.28753	Hikeshi
MSTRG.28754.1	MSTRG.28754	Hikeshi
MSTRG.28755.1	MSTRG.28755	Hikeshi
MSTRG.28756.5	MSTRG.28756	Eed
MSTRG.28757.1	MSTRG.28757	Eed
MSTRG.28759.1	MSTRG.28759	Eed
MSTRG.2876.1	MSTRG.2876	Gopc
MSTRG.28760.1	MSTRG.28760	Eed
MSTRG.28766.1	MSTRG.28766	Picalm
MSTRG.2877.1	MSTRG.2877	Gopc
MSTRG.28771.4	MSTRG.28771	Tmem126a
MSTRG.28772.1	MSTRG.28772	.
MSTRG.28773.1	MSTRG.28773	.
MSTRG.2880.1	MSTRG.2880	.
MSTRG.28803.1	MSTRG.28803	Gm26944
MSTRG.28803.3	MSTRG.28803	Gm26944
MSTRG.28805.1	MSTRG.28805	Pcf11
MSTRG.28807.1	MSTRG.28807	4632427E13Rik
MSTRG.28814.1	MSTRG.28814	.
MSTRG.28816.1	MSTRG.28816	.
MSTRG.28817.1	MSTRG.28817	.
MSTRG.28818.1	MSTRG.28818	.
MSTRG.28834.1	MSTRG.28834	Nars2
MSTRG.28835.1	MSTRG.28835	Nars2
MSTRG.28836.1	MSTRG.28836	Nars2
MSTRG.28837.1	MSTRG.28837	.
MSTRG.28839.1	MSTRG.28839	Gab2
MSTRG.28840.1	MSTRG.28840	Gab2
MSTRG.28841.1	MSTRG.28841	Gab2
MSTRG.28842.1	MSTRG.28842	Gab2
MSTRG.28843.1	MSTRG.28843	Gab2
MSTRG.28844.1	MSTRG.28844	Gab2
MSTRG.28854.1	MSTRG.28854	Aamdc
MSTRG.28855.1	MSTRG.28855	Aamdc
MSTRG.28858.1	MSTRG.28858	Rsf1
MSTRG.28862.1	MSTRG.28862	Rsf1
MSTRG.28866.1	MSTRG.28866	Rsf1os2
MSTRG.28874.1	MSTRG.28874	Acer3
MSTRG.28875.1	MSTRG.28875	Acer3
MSTRG.28880.1	MSTRG.28880	A630091E08Rik
MSTRG.28881.1	MSTRG.28881	.
MSTRG.28883.1	MSTRG.28883	Emsy
MSTRG.28884.1	MSTRG.28884	Emsy
MSTRG.28885.1	MSTRG.28885	.
MSTRG.28887.1	MSTRG.28887	Gm15506
MSTRG.28892.1	MSTRG.28892	Wnt11
MSTRG.28898.1	MSTRG.28898	Uvrag
MSTRG.28900.1	MSTRG.28900	Uvrag

MSTRG.28901.1	MSTRG.28901	Uvrag
MSTRG.28902.1	MSTRG.28902	Uvrag
MSTRG.28907.1	MSTRG.28907	Gdpd5
MSTRG.2891.1	MSTRG.2891	Mcm9
MSTRG.28912.1	MSTRG.28912	Arrb1
MSTRG.28914.1	MSTRG.28914	.
MSTRG.28926.1	MSTRG.28926	Xrra1
MSTRG.28927.2	MSTRG.28927	Gm34280
MSTRG.28934.1	MSTRG.28934	.
MSTRG.28934.2	MSTRG.28934	.
MSTRG.28939.1	MSTRG.28939	Xrra1
MSTRG.28940.1	MSTRG.28940	Gm38405
MSTRG.28940.2	MSTRG.28940	Gm38405
MSTRG.28940.3	MSTRG.28940	Gm38405
MSTRG.28940.4	MSTRG.28940	Gm38405
MSTRG.28940.8	MSTRG.28940	Gm38405
MSTRG.28944.1	MSTRG.28944	Rnf169
MSTRG.28945.1	MSTRG.28945	Rnf169
MSTRG.28949.1	MSTRG.28949	Ucp2
MSTRG.2895.1	MSTRG.2895	.
MSTRG.28951.1	MSTRG.28951	Dnajb13
MSTRG.2896.1	MSTRG.2896	.
MSTRG.28960.1	MSTRG.28960	Mrpl48
MSTRG.28963.3	MSTRG.28963	Rab6a
MSTRG.28966.1	MSTRG.28966	Gm45209
MSTRG.28968.1	MSTRG.28968	Gm45209
MSTRG.28970.1	MSTRG.28970	Fam168a
MSTRG.28972.5	MSTRG.28972	Relt
MSTRG.28972.7	MSTRG.28972	Relt
MSTRG.28977.1	MSTRG.28977	Clpb
MSTRG.2898.1	MSTRG.2898	Man1a
MSTRG.28985.1	MSTRG.28985	Fchsd2
MSTRG.28987.1	MSTRG.28987	Fchsd2
MSTRG.28988.1	MSTRG.28988	Fchsd2
MSTRG.2899.2	MSTRG.2899	Man1a
MSTRG.2899.3	MSTRG.2899	Man1a
MSTRG.28990.1	MSTRG.28990	Fchsd2
MSTRG.28992.1	MSTRG.28992	Atg16l2
MSTRG.28999.1	MSTRG.28999	Gm45837
MSTRG.29.1	MSTRG.29	Rb1cc1
MSTRG.2900.1	MSTRG.2900	Man1a
MSTRG.29000.1	MSTRG.29000	Gm45837
MSTRG.29010.1	MSTRG.29010	Stim1
MSTRG.29012.1	MSTRG.29012	Stim1
MSTRG.29013.1	MSTRG.29013	Gm18255
MSTRG.2902.1	MSTRG.2902	Man1a
MSTRG.29025.1	MSTRG.29025	Trim5
MSTRG.29026.1	MSTRG.29026	Trim5
MSTRG.29029.3	MSTRG.29029	Trim34b
MSTRG.29030.1	MSTRG.29030	Trim34b
MSTRG.29034.1	MSTRG.29034	Trim30a
MSTRG.29036.1	MSTRG.29036	.
MSTRG.2904.1	MSTRG.2904	.
MSTRG.29042.1	MSTRG.29042	Lrrc51
MSTRG.29045.1	MSTRG.29045	Lrrc51
MSTRG.29047.1	MSTRG.29047	Numa1
MSTRG.29049.1	MSTRG.29049	Numa1

MSTRG.2905.1	MSTRG.2905	.
MSTRG.29050.1	MSTRG.29050	Numa1
MSTRG.29051.1	MSTRG.29051	Numa1
MSTRG.29052.1	MSTRG.29052	Numa1
MSTRG.29053.1	MSTRG.29053	Gm45313
MSTRG.29054.1	MSTRG.29054	Numa1
MSTRG.29055.1	MSTRG.29055	Rnf121
MSTRG.2906.1	MSTRG.2906	.
MSTRG.29061.1	MSTRG.29061	Art5
MSTRG.29064.1	MSTRG.29064	Nup98
MSTRG.29066.1	MSTRG.29066	.
MSTRG.29069.1	MSTRG.29069	Fam160a2
MSTRG.29081.1	MSTRG.29081	.
MSTRG.29093.1	MSTRG.29093	Gm20663
MSTRG.29094.1	MSTRG.29094	Gm8982
MSTRG.29094.2	MSTRG.29094	Gm4070
MSTRG.29095.1	MSTRG.29095	Gm4070
MSTRG.29095.2	MSTRG.29095	Gvin1
MSTRG.29095.3	MSTRG.29095	Gvin1
MSTRG.29101.1	MSTRG.29101	Gm22372
MSTRG.29103.1	MSTRG.29103	Gm22372
MSTRG.29105.1	MSTRG.29105	.
MSTRG.29106.1	MSTRG.29106	Gm1966
MSTRG.29107.1	MSTRG.29107	Gm1966
MSTRG.29109.1	MSTRG.29109	.
MSTRG.29118.6	MSTRG.29118	Rpl27a
MSTRG.29121.1	MSTRG.29121	St5
MSTRG.29122.1	MSTRG.29122	St5
MSTRG.29131.1	MSTRG.29131	Dennd5a
MSTRG.29133.1	MSTRG.29133	Tmem41b
MSTRG.29134.1	MSTRG.29134	Tmem41b
MSTRG.29145.1	MSTRG.29145	1600010M07Rik
MSTRG.29157.1	MSTRG.29157	Rnf141
MSTRG.29158.1	MSTRG.29158	Rnf141
MSTRG.2916.1	MSTRG.2916	Gcc2
MSTRG.29161.1	MSTRG.29161	Galnt18
MSTRG.29162.1	MSTRG.29162	Galnt18
MSTRG.29163.1	MSTRG.29163	Galnt18
MSTRG.29167.1	MSTRG.29167	1700012D14Rik
MSTRG.2917.1	MSTRG.2917	Gcc2
MSTRG.29171.1	MSTRG.29171	Usp47
MSTRG.29173.1	MSTRG.29173	Parva
MSTRG.29179.1	MSTRG.29179	Tead1
MSTRG.29183.1	MSTRG.29183	Btbd10
MSTRG.29185.1	MSTRG.29185	Btbd10
MSTRG.29186.1	MSTRG.29186	Btbd10
MSTRG.29188.1	MSTRG.29188	Arntl
MSTRG.29193.1	MSTRG.29193	Spon1
MSTRG.29195.1	MSTRG.29195	Rras2
MSTRG.29196.1	MSTRG.29196	Rras2
MSTRG.2920.1	MSTRG.2920	Gm23058
MSTRG.29200.1	MSTRG.29200	.
MSTRG.29202.1	MSTRG.29202	.
MSTRG.29204.1	MSTRG.29204	4933406I18Rik
MSTRG.29206.1	MSTRG.29206	Pde3b
MSTRG.29207.1	MSTRG.29207	Pde3b
MSTRG.29209.1	MSTRG.29209	.

MSTRG.2921.1	MSTRG.2921	Lims1
MSTRG.29213.1	MSTRG.29213	Insc
MSTRG.29216.1	MSTRG.29216	Sox6
MSTRG.29224.1	MSTRG.29224	Plekha7
MSTRG.29226.1	MSTRG.29226	Plekha7
MSTRG.29227.1	MSTRG.29227	Plekha7
MSTRG.29228.1	MSTRG.29228	Plekha7
MSTRG.2923.1	MSTRG.2923	Lims1
MSTRG.29230.1	MSTRG.29230	Nucb2
MSTRG.29236.1	MSTRG.29236	Pik3c2a
MSTRG.2924.1	MSTRG.2924	Lims1
MSTRG.29240.1	MSTRG.29240	Pik3c2a
MSTRG.29242.1	MSTRG.29242	.
MSTRG.29245.1	MSTRG.29245	Xylt1
MSTRG.29247.1	MSTRG.29247	Xylt1
MSTRG.29248.1	MSTRG.29248	Xylt1
MSTRG.29249.1	MSTRG.29249	Xylt1
MSTRG.29250.1	MSTRG.29250	Xylt1
MSTRG.29253.1	MSTRG.29253	Arl6ip1
MSTRG.29256.1	MSTRG.29256	Syt17
MSTRG.29264.1	MSTRG.29264	Gm45084
MSTRG.29265.1	MSTRG.29265	Smg1
MSTRG.29267.1	MSTRG.29267	.
MSTRG.29268.1	MSTRG.29268	.
MSTRG.29269.1	MSTRG.29269	.
MSTRG.29271.1	MSTRG.29271	Gm45155
MSTRG.29271.2	MSTRG.29271	Gm45155
MSTRG.29279.1	MSTRG.29279	9030624J02Rik
MSTRG.29281.1	MSTRG.29281	9030624J02Rik
MSTRG.29282.1	MSTRG.29282	9030624J02Rik
MSTRG.29283.1	MSTRG.29283	9030624J02Rik
MSTRG.29285.1	MSTRG.29285	9030624J02Rik
MSTRG.29289.1	MSTRG.29289	Iqck
MSTRG.29296.1	MSTRG.29296	Eri2
MSTRG.29298.1	MSTRG.29298	Rexo5
MSTRG.29299.1	MSTRG.29299	Rexo5
MSTRG.29300.1	MSTRG.29300	Rexo5
MSTRG.29302.1	MSTRG.29302	Dcun1d3
MSTRG.29303.1	MSTRG.29303	Dcun1d3
MSTRG.29305.1	MSTRG.29305	Dcun1d3
MSTRG.29307.1	MSTRG.29307	Lyrm1
MSTRG.29314.5	MSTRG.29314	BC030336
MSTRG.29314.6	MSTRG.29314	BC030336
MSTRG.29315.1	MSTRG.29315	BC030336
MSTRG.29342.1	MSTRG.29342	Ndufab1
MSTRG.29349.1	MSTRG.29349	Plk1
MSTRG.29354.1	MSTRG.29354	Prkcb
MSTRG.29355.1	MSTRG.29355	Prkcb
MSTRG.29357.4	MSTRG.29357	Prkcb
MSTRG.2936.1	MSTRG.2936	P4ha1
MSTRG.29363.1	MSTRG.29363	Prkcb
MSTRG.29369.1	MSTRG.29369	Arhgap17
MSTRG.29372.1	MSTRG.29372	.
MSTRG.29374.1	MSTRG.29374	Kdm8
MSTRG.29375.1	MSTRG.29375	.
MSTRG.29381.1	MSTRG.29381	Il21r
MSTRG.29382.1	MSTRG.29382	.

MSTRG.29385.1	MSTRG.29385	Gtf3c1
MSTRG.29385.2	MSTRG.29385	Gtf3c1
MSTRG.29392.1	MSTRG.29392	Xpo6
MSTRG.29394.1	MSTRG.29394	Xpo6
MSTRG.29396.1	MSTRG.29396	Xpo6
MSTRG.2940.1	MSTRG.2940	Mcu
MSTRG.29408.1	MSTRG.29408	.
MSTRG.2941.1	MSTRG.2941	Mcu
MSTRG.29417.1	MSTRG.29417	.
MSTRG.29419.1	MSTRG.29419	Slx1b
MSTRG.2942.1	MSTRG.2942	Mcu
MSTRG.29423.2	MSTRG.29423	Gm9967
MSTRG.29423.4	MSTRG.29423	Gm9967
MSTRG.29424.1	MSTRG.29424	Gm9967
MSTRG.29425.7	MSTRG.29425	Ypel3
MSTRG.29425.8	MSTRG.29425	Ypel3
MSTRG.29431.9	MSTRG.29431	Ino80e
MSTRG.29435.1	MSTRG.29435	Tmem219
MSTRG.29437.1	MSTRG.29437	Gm20650
MSTRG.2944.1	MSTRG.2944	Mcu
MSTRG.29447.1	MSTRG.29447	Gm31749
MSTRG.29451.1	MSTRG.29451	.
MSTRG.29464.1	MSTRG.29464	.
MSTRG.29467.1	MSTRG.29467	Gm31897
MSTRG.29467.2	MSTRG.29467	Gm31897
MSTRG.29470.1	MSTRG.29470	Itgal
MSTRG.29479.1	MSTRG.29479	B130055M24Rik
MSTRG.2948.1	MSTRG.2948	Micu1
MSTRG.29480.1	MSTRG.29480	Prr14
MSTRG.29482.1	MSTRG.29482	.
MSTRG.29493.1	MSTRG.29493	Fbxl19
MSTRG.29502.1	MSTRG.29502	.
MSTRG.29503.1	MSTRG.29503	.
MSTRG.29515.1	MSTRG.29515	Kat8
MSTRG.29519.1	MSTRG.29519	.
MSTRG.29525.1	MSTRG.29525	Gm6916
MSTRG.29532.19	MSTRG.29532	Tial1
MSTRG.29532.3	MSTRG.29532	Tial1
MSTRG.2954.1	MSTRG.2954	Dnajb12
MSTRG.29540.1	MSTRG.29540	Inpp5f
MSTRG.29543.1	MSTRG.29543	Gm44674
MSTRG.29544.1	MSTRG.29544	.
MSTRG.29546.1	MSTRG.29546	.
MSTRG.29548.1	MSTRG.29548	Edrf1
MSTRG.29552.1	MSTRG.29552	Bccip
MSTRG.29560.1	MSTRG.29560	Adam12
MSTRG.29563.1	MSTRG.29563	Adam12
MSTRG.29564.1	MSTRG.29564	Adam12
MSTRG.29565.1	MSTRG.29565	Adam12
MSTRG.29566.1	MSTRG.29566	Adam12
MSTRG.29567.1	MSTRG.29567	Adam12
MSTRG.29568.1	MSTRG.29568	Adam12
MSTRG.29577.1	MSTRG.29577	5830432E09Rik
MSTRG.29577.2	MSTRG.29577	5830432E09Rik
MSTRG.29577.3	MSTRG.29577	5830432E09Rik
MSTRG.29577.4	MSTRG.29577	5830432E09Rik
MSTRG.29577.5	MSTRG.29577	5830432E09Rik

MSTRG.29579.1	MSTRG.29579	Mki67
MSTRG.29580.1	MSTRG.29580	Gm45240
MSTRG.29581.1	MSTRG.29581	Mki67
MSTRG.29584.1	MSTRG.29584	Mgmt
MSTRG.29585.1	MSTRG.29585	Mgmt
MSTRG.29586.1	MSTRG.29586	Mgmt
MSTRG.29587.1	MSTRG.29587	Mgmt
MSTRG.2959.4	MSTRG.2959	Spock2
MSTRG.29591.1	MSTRG.29591	Ebf3
MSTRG.29593.1	MSTRG.29593	.
MSTRG.29594.1	MSTRG.29594	.
MSTRG.29597.1	MSTRG.29597	Tcerg1l
MSTRG.29605.1	MSTRG.29605	Stk32c
MSTRG.29611.1	MSTRG.29611	Inpp5a
MSTRG.29612.1	MSTRG.29612	Inpp5a
MSTRG.29613.1	MSTRG.29613	Inpp5a
MSTRG.29614.1	MSTRG.29614	Inpp5a
MSTRG.29617.1	MSTRG.29617	Fgfr2
MSTRG.29622.1	MSTRG.29622	Fgfr2
MSTRG.29626.1	MSTRG.29626	Fgfr2
MSTRG.29645.1	MSTRG.29645	Fgfr2
MSTRG.29646.1	MSTRG.29646	Fgfr2
MSTRG.29670.3	MSTRG.29670	Chst15
MSTRG.29671.1	MSTRG.29671	Chst15
MSTRG.29687.1	MSTRG.29687	Fgfr2
MSTRG.29689.1	MSTRG.29689	Fgfr2
MSTRG.29691.1	MSTRG.29691	Fgfr2
MSTRG.29706.1	MSTRG.29706	Gm16201
MSTRG.29713.1	MSTRG.29713	Zfp511
MSTRG.2973.1	MSTRG.2973	Eif4ebp2
MSTRG.2973.4	MSTRG.2973	AC122197.1
MSTRG.29735.1	MSTRG.29735	Ptdss2
MSTRG.29736.1	MSTRG.29736	Ptdss2
MSTRG.2976.1	MSTRG.2976	Lrrc20
MSTRG.29762.1	MSTRG.29762	Chid1
MSTRG.29780.1	MSTRG.29780	Tspan32
MSTRG.29784.6	MSTRG.29784	Tssc4
MSTRG.29801.1	MSTRG.29801	Cars
MSTRG.29803.3	MSTRG.29803	Tnfrsf26
MSTRG.29805.1	MSTRG.29805	E230032D23Rik
MSTRG.29829.1	MSTRG.29829	Tpcn2
MSTRG.29833.1	MSTRG.29833	.
MSTRG.29847.1	MSTRG.29847	Pnpla6
MSTRG.29859.1	MSTRG.29859	Fcer2a
MSTRG.2987.1	MSTRG.2987	.
MSTRG.29881.1	MSTRG.29881	.
MSTRG.29883.1	MSTRG.29883	Zfp958
MSTRG.29884.1	MSTRG.29884	Zfp958
MSTRG.29888.1	MSTRG.29888	.
MSTRG.29894.5	MSTRG.29894	Arglu1
MSTRG.299.1	MSTRG.299	.
MSTRG.29901.1	MSTRG.29901	9530052E02Rik
MSTRG.29907.1	MSTRG.29907	.
MSTRG.29911.1	MSTRG.29911	Rab20
MSTRG.29933.1	MSTRG.29933	Pcid2
MSTRG.2994.1	MSTRG.2994	Hk1
MSTRG.29951.1	MSTRG.29951	Dcun1d2

MSTRG.29958.1	MSTRG.29958	Rasa3
MSTRG.29959.1	MSTRG.29959	Rasa3
MSTRG.29965.4	MSTRG.29965	Fbxo25
MSTRG.29966.1	MSTRG.29966	.
MSTRG.29968.1	MSTRG.29968	.
MSTRG.29969.1	MSTRG.29969	.
MSTRG.29978.1	MSTRG.29978	Arhgef10
MSTRG.29979.1	MSTRG.29979	Arhgef10
MSTRG.29982.1	MSTRG.29982	Gm45408
MSTRG.29990.1	MSTRG.29990	Csmd1
MSTRG.29991.1	MSTRG.29991	Csmd1
MSTRG.29993.1	MSTRG.29993	Csmd1
MSTRG.29995.1	MSTRG.29995	Csmd1
MSTRG.29997.1	MSTRG.29997	Csmd1
MSTRG.29998.1	MSTRG.29998	Csmd1
MSTRG.29999.1	MSTRG.29999	Csmd1
MSTRG.30000.1	MSTRG.30000	Csmd1
MSTRG.30001.1	MSTRG.30001	Csmd1
MSTRG.30002.1	MSTRG.30002	Csmd1
MSTRG.30003.1	MSTRG.30003	Csmd1
MSTRG.30004.1	MSTRG.30004	Csmd1
MSTRG.30005.1	MSTRG.30005	Csmd1
MSTRG.30006.1	MSTRG.30006	Csmd1
MSTRG.30007.1	MSTRG.30007	Csmd1
MSTRG.30008.1	MSTRG.30008	Csmd1
MSTRG.30009.1	MSTRG.30009	Csmd1
MSTRG.30011.1	MSTRG.30011	Agpat5
MSTRG.30016.1	MSTRG.30016	Mcph1
MSTRG.30021.1	MSTRG.30021	.
MSTRG.30023.1	MSTRG.30023	Gm35998
MSTRG.30024.1	MSTRG.30024	.
MSTRG.30025.1	MSTRG.30025	Gm21119
MSTRG.30027.1	MSTRG.30027	.
MSTRG.30031.1	MSTRG.30031	Alg11
MSTRG.30036.1	MSTRG.30036	Vps36
MSTRG.30042.1	MSTRG.30042	Mrps31
MSTRG.30044.3	MSTRG.30044	Smim19
MSTRG.30047.1	MSTRG.30047	Slc20a2
MSTRG.30048.1	MSTRG.30048	Slc20a2
MSTRG.30049.1	MSTRG.30049	Slc20a2
MSTRG.30050.1	MSTRG.30050	Slc20a2
MSTRG.30058.1	MSTRG.30058	.
MSTRG.3006.1	MSTRG.3006	.
MSTRG.30061.1	MSTRG.30061	Gm7760
MSTRG.30062.1	MSTRG.30062	4930467E23Rik
MSTRG.30064.1	MSTRG.30064	Gm7760
MSTRG.30064.2	MSTRG.30064	Gm45754
MSTRG.30064.3	MSTRG.30064	Gm45754
MSTRG.30065.1	MSTRG.30065	Gm7760
MSTRG.30066.1	MSTRG.30066	4930467E23Rik
MSTRG.30067.1	MSTRG.30067	4930467E23Rik
MSTRG.30070.1	MSTRG.30070	Gm20786
MSTRG.30076.1	MSTRG.30076	Gm15319
MSTRG.30077.1	MSTRG.30077	Gm15319
MSTRG.30077.2	MSTRG.30077	.
MSTRG.30078.1	MSTRG.30078	Gm15319
MSTRG.30078.2	MSTRG.30078	Gm15319

MSTRG.30079.1	MSTRG.30079	Gm26804
MSTRG.3008.1	MSTRG.3008	Ccar1
MSTRG.30081.1	MSTRG.30081	Gm26804
MSTRG.30082.1	MSTRG.30082	Gm26804
MSTRG.30083.1	MSTRG.30083	Gm26804
MSTRG.30084.1	MSTRG.30084	Gm26804
MSTRG.30084.2	MSTRG.30084	Gm26804
MSTRG.30084.4	MSTRG.30084	Gm26804
MSTRG.30087.1	MSTRG.30087	.
MSTRG.30088.1	MSTRG.30088	Gm21092
MSTRG.30089.1	MSTRG.30089	.
MSTRG.3009.1	MSTRG.3009	Ccar1
MSTRG.30097.1	MSTRG.30097	.
MSTRG.30100.1	MSTRG.30100	.
MSTRG.30106.1	MSTRG.30106	Plekha2
MSTRG.30107.1	MSTRG.30107	Plekha2
MSTRG.30108.1	MSTRG.30108	Plekha2
MSTRG.30111.1	MSTRG.30111	5830408C22Rik
MSTRG.30112.1	MSTRG.30112	.
MSTRG.30114.1	MSTRG.30114	Tacc1
MSTRG.30118.1	MSTRG.30118	Letm2
MSTRG.3012.1	MSTRG.3012	Ccar1
MSTRG.30124.1	MSTRG.30124	Nsd3
MSTRG.30127.1	MSTRG.30127	Kcnu1
MSTRG.30134.1	MSTRG.30134	Gm17484
MSTRG.30142.1	MSTRG.30142	Hook3
MSTRG.30143.1	MSTRG.30143	Hook3
MSTRG.30147.1	MSTRG.30147	Gm45251
MSTRG.30148.1	MSTRG.30148	Rnf170
MSTRG.30164.1	MSTRG.30164	.
MSTRG.30165.1	MSTRG.30165	.
MSTRG.30166.1	MSTRG.30166	.
MSTRG.30172.1	MSTRG.30172	Unc5d
MSTRG.30176.1	MSTRG.30176	.
MSTRG.30177.1	MSTRG.30177	.
MSTRG.30183.1	MSTRG.30183	Fut10
MSTRG.30185.1	MSTRG.30185	Fut10
MSTRG.30186.1	MSTRG.30186	Fut10
MSTRG.30188.1	MSTRG.30188	Purg
MSTRG.30190.1	MSTRG.30190	Wrn
MSTRG.30191.1	MSTRG.30191	Wrn
MSTRG.30192.1	MSTRG.30192	Wrn
MSTRG.30193.1	MSTRG.30193	Wrn
MSTRG.30197.1	MSTRG.30197	.
MSTRG.30201.1	MSTRG.30201	Gtf2e2
MSTRG.30203.1	MSTRG.30203	Gtf2e2
MSTRG.30204.1	MSTRG.30204	Gtf2e2
MSTRG.30208.1	MSTRG.30208	Rbpms
MSTRG.30209.1	MSTRG.30209	Rbpms
MSTRG.30210.1	MSTRG.30210	Rbpms
MSTRG.30212.1	MSTRG.30212	Rbpms
MSTRG.30213.1	MSTRG.30213	Rbpms
MSTRG.30214.1	MSTRG.30214	Rbpms
MSTRG.30222.1	MSTRG.30222	Saraf
MSTRG.30224.1	MSTRG.30224	.
MSTRG.30227.1	MSTRG.30227	.
MSTRG.30228.1	MSTRG.30228	.

MSTRG.30229.1	MSTRG.30229	.
MSTRG.3023.1	MSTRG.3023	Rufy2
MSTRG.30232.1	MSTRG.30232	Gm45349
MSTRG.30233.1	MSTRG.30233	Gm45349
MSTRG.30236.1	MSTRG.30236	Tnks
MSTRG.30237.1	MSTRG.30237	Tnks
MSTRG.30238.1	MSTRG.30238	Tnks
MSTRG.30239.1	MSTRG.30239	Tnks
MSTRG.30240.1	MSTRG.30240	Tnks
MSTRG.30245.1	MSTRG.30245	Ppp1r3b
MSTRG.30246.1	MSTRG.30246	.
MSTRG.30250.1	MSTRG.30250	Mfhas1
MSTRG.30253.1	MSTRG.30253	Prag1
MSTRG.30255.1	MSTRG.30255	Lonrf1
MSTRG.30256.1	MSTRG.30256	Lonrf1
MSTRG.30257.1	MSTRG.30257	Lonrf1
MSTRG.3026.1	MSTRG.3026	Hnrnph3
MSTRG.30260.1	MSTRG.30260	Dlc1
MSTRG.30261.1	MSTRG.30261	Dlc1
MSTRG.30262.1	MSTRG.30262	Dlc1
MSTRG.30263.1	MSTRG.30263	.
MSTRG.30266.1	MSTRG.30266	Msr1
MSTRG.30270.1	MSTRG.30270	Vps37a
MSTRG.30271.1	MSTRG.30271	Vps37a
MSTRG.30275.1	MSTRG.30275	Mtus1
MSTRG.30281.1	MSTRG.30281	Frg1
MSTRG.30282.1	MSTRG.30282	Frg1
MSTRG.30283.1	MSTRG.30283	.
MSTRG.30284.1	MSTRG.30284	.
MSTRG.30285.1	MSTRG.30285	.
MSTRG.30288.1	MSTRG.30288	Adam34
MSTRG.30294.1	MSTRG.30294	.
MSTRG.30301.1	MSTRG.30301	Pdlim3
MSTRG.30302.1	MSTRG.30302	.
MSTRG.30310.1	MSTRG.30310	Ufsp2
MSTRG.30314.1	MSTRG.30314	Snx25
MSTRG.30315.1	MSTRG.30315	Snx25
MSTRG.30317.1	MSTRG.30317	Snx25
MSTRG.30318.1	MSTRG.30318	Snx25
MSTRG.30324.1	MSTRG.30324	Primpol
MSTRG.30325.1	MSTRG.30325	Primpol
MSTRG.30330.1	MSTRG.30330	Gm16675
MSTRG.30332.1	MSTRG.30332	Irf2
MSTRG.30333.1	MSTRG.30333	Irf2
MSTRG.30338.1	MSTRG.30338	Irf2
MSTRG.30341.1	MSTRG.30341	Stox2
MSTRG.30343.1	MSTRG.30343	Stox2
MSTRG.30344.1	MSTRG.30344	Stox2
MSTRG.30345.1	MSTRG.30345	Stox2
MSTRG.30346.1	MSTRG.30346	Stox2
MSTRG.30347.1	MSTRG.30347	Stox2
MSTRG.30348.1	MSTRG.30348	Stox2
MSTRG.30349.1	MSTRG.30349	Stox2
MSTRG.30350.1	MSTRG.30350	Stox2
MSTRG.30351.1	MSTRG.30351	Stox2
MSTRG.30352.1	MSTRG.30352	Stox2
MSTRG.30355.1	MSTRG.30355	.

MSTRG.30359.1	MSTRG.30359	AA386476
MSTRG.3036.1	MSTRG.3036	.
MSTRG.30362.1	MSTRG.30362	Rwdd4a
MSTRG.30369.1	MSTRG.30369	Wwc2
MSTRG.3037.1	MSTRG.3037	.
MSTRG.30371.1	MSTRG.30371	Dctd
MSTRG.30372.1	MSTRG.30372	.
MSTRG.30373.1	MSTRG.30373	.
MSTRG.30374.1	MSTRG.30374	.
MSTRG.30375.1	MSTRG.30375	.
MSTRG.30376.1	MSTRG.30376	.
MSTRG.30383.1	MSTRG.30383	.
MSTRG.30385.1	MSTRG.30385	Aga
MSTRG.3039.1	MSTRG.3039	.
MSTRG.30393.1	MSTRG.30393	.
MSTRG.30394.1	MSTRG.30394	.
MSTRG.30395.1	MSTRG.30395	.
MSTRG.30396.1	MSTRG.30396	.
MSTRG.30401.1	MSTRG.30401	Wdr17
MSTRG.30410.1	MSTRG.30410	.
MSTRG.30412.1	MSTRG.30412	Cep44
MSTRG.30415.1	MSTRG.30415	Fbxo8
MSTRG.30421.2	MSTRG.30421	Galnt7
MSTRG.30422.1	MSTRG.30422	Galnt7
MSTRG.30425.1	MSTRG.30425	Galnt7
MSTRG.30428.1	MSTRG.30428	Galnt7
MSTRG.30431.1	MSTRG.30431	AW046200
MSTRG.30437.1	MSTRG.30437	Clcn3
MSTRG.30438.1	MSTRG.30438	Clcn3
MSTRG.30441.1	MSTRG.30441	Nek1
MSTRG.30443.1	MSTRG.30443	Nek1
MSTRG.30448.1	MSTRG.30448	Sh3rf1
MSTRG.30448.2	MSTRG.30448	Sh3rf1
MSTRG.30449.1	MSTRG.30449	Sh3rf1
MSTRG.3045.1	MSTRG.3045	Jmjd1c
MSTRG.30450.1	MSTRG.30450	Sh3rf1
MSTRG.30453.1	MSTRG.30453	Sh3rf1
MSTRG.30456.1	MSTRG.30456	.
MSTRG.30462.1	MSTRG.30462	Ddx60
MSTRG.30463.1	MSTRG.30463	Ddx60
MSTRG.30464.1	MSTRG.30464	Ddx60
MSTRG.30465.1	MSTRG.30465	.
MSTRG.30467.5	MSTRG.30467	Klhl2
MSTRG.30468.1	MSTRG.30468	Klhl2
MSTRG.30470.1	MSTRG.30470	Klhl2
MSTRG.30479.1	MSTRG.30479	March1
MSTRG.30484.1	MSTRG.30484	March1
MSTRG.30489.1	MSTRG.30489	Sh2d4a
MSTRG.30493.1	MSTRG.30493	Psd3
MSTRG.30494.1	MSTRG.30494	Psd3
MSTRG.30495.1	MSTRG.30495	Psd3
MSTRG.30496.1	MSTRG.30496	Psd3
MSTRG.30497.1	MSTRG.30497	Psd3
MSTRG.305.1	MSTRG.305	Inpp4a
MSTRG.30502.1	MSTRG.30502	Csgalnact1
MSTRG.30504.1	MSTRG.30504	Csgalnact1
MSTRG.30505.1	MSTRG.30505	Csgalnact1

MSTRG.30506.1	MSTRG.30506	Csgalnact1
MSTRG.30507.1	MSTRG.30507	Csgalnact1
MSTRG.30509.1	MSTRG.30509	Csgalnact1
MSTRG.30513.1	MSTRG.30513	Lpl
MSTRG.30517.1	MSTRG.30517	Atp6v1b2
MSTRG.3052.1	MSTRG.3052	AC153379.3
MSTRG.30520.1	MSTRG.30520	Lzts1
MSTRG.30521.1	MSTRG.30521	Lzts1
MSTRG.30522.1	MSTRG.30522	Lzts1
MSTRG.30524.1	MSTRG.30524	Zfp930
MSTRG.30525.1	MSTRG.30525	Zfp930
MSTRG.30528.3	MSTRG.30528	D130040H23Rik
MSTRG.30529.1	MSTRG.30529	D130040H23Rik
MSTRG.30530.1	MSTRG.30530	D130040H23Rik
MSTRG.30533.1	MSTRG.30533	Gm10033
MSTRG.30535.1	MSTRG.30535	.
MSTRG.30537.1	MSTRG.30537	AC166750.1
MSTRG.30538.1	MSTRG.30538	AC166750.1
MSTRG.3054.1	MSTRG.3054	Rtkn2
MSTRG.30540.1	MSTRG.30540	Zfp868
MSTRG.30542.1	MSTRG.30542	.
MSTRG.30546.1	MSTRG.30546	Tm6sf2
MSTRG.30547.1	MSTRG.30547	Sugp1
MSTRG.3055.1	MSTRG.3055	Rtkn2
MSTRG.30550.1	MSTRG.30550	Gm20422
MSTRG.30551.1	MSTRG.30551	Gm20422
MSTRG.30555.1	MSTRG.30555	Gatad2a
MSTRG.30555.2	MSTRG.30555	Gatad2a
MSTRG.30558.1	MSTRG.30558	Yjefn3
MSTRG.3056.1	MSTRG.3056	Rtkn2
MSTRG.30561.1	MSTRG.30561	Gatad2a
MSTRG.30562.1	MSTRG.30562	Gatad2a
MSTRG.30563.1	MSTRG.30563	Gatad2a
MSTRG.30564.1	MSTRG.30564	Gatad2a
MSTRG.30565.1	MSTRG.30565	Gatad2a
MSTRG.30565.2	MSTRG.30565	Gatad2a
MSTRG.3057.1	MSTRG.3057	Rtkn2
MSTRG.30570.1	MSTRG.30570	Tmem161a
MSTRG.30572.1	MSTRG.30572	Slc25a42
MSTRG.30576.1	MSTRG.30576	Homer3
MSTRG.30583.1	MSTRG.30583	Crtc1
MSTRG.30586.1	MSTRG.30586	Klhl26
MSTRG.30590.1	MSTRG.30590	Kxd1
MSTRG.30595.1	MSTRG.30595	Ell
MSTRG.30596.1	MSTRG.30596	Ell
MSTRG.3060.1	MSTRG.3060	Arid5b
MSTRG.3061.1	MSTRG.3061	Arid5b
MSTRG.30610.1	MSTRG.30610	Mast3
MSTRG.30613.1	MSTRG.30613	Il12rb1
MSTRG.30617.5	MSTRG.30617	Zfp617
MSTRG.30618.1	MSTRG.30618	Zfp617
MSTRG.3062.1	MSTRG.3062	Arid5b
MSTRG.30620.1	MSTRG.30620	Zfp617
MSTRG.30621.12	MSTRG.30621	Zfp961
MSTRG.30622.1	MSTRG.30622	Zfp961
MSTRG.30623.1	MSTRG.30623	Zfp961
MSTRG.30624.1	MSTRG.30624	Zfp961

MSTRG.30625.1	MSTRG.30625	Zfp961
MSTRG.30629.1	MSTRG.30629	Hsh2d
MSTRG.3063.1	MSTRG.3063	Arid5b
MSTRG.3064.1	MSTRG.3064	Arid5b
MSTRG.30640.1	MSTRG.30640	.
MSTRG.30646.1	MSTRG.30646	Ap1m1
MSTRG.3065.1	MSTRG.3065	Arid5b
MSTRG.30651.1	MSTRG.30651	.
MSTRG.30656.1	MSTRG.30656	1700030K09Rik
MSTRG.30664.1	MSTRG.30664	Med26
MSTRG.30665.1	MSTRG.30665	Gm35857
MSTRG.30665.2	MSTRG.30665	Gm35857
MSTRG.30667.1	MSTRG.30667	.
MSTRG.30667.2	MSTRG.30667	.
MSTRG.30667.3	MSTRG.30667	.
MSTRG.30668.1	MSTRG.30668	.
MSTRG.3067.1	MSTRG.3067	Arid5b
MSTRG.30674.1	MSTRG.30674	Unc13a
MSTRG.3068.1	MSTRG.3068	Arid5b
MSTRG.30680.1	MSTRG.30680	Babam1
MSTRG.30684.11	MSTRG.30684	Dda1
MSTRG.30684.12	MSTRG.30684	Dda1
MSTRG.30684.13	MSTRG.30684	Dda1
MSTRG.30684.8	MSTRG.30684	Dda1
MSTRG.3069.1	MSTRG.3069	Arid5b
MSTRG.30695.1	MSTRG.30695	Slc27a1
MSTRG.30696.1	MSTRG.30696	Pgls
MSTRG.30702.1	MSTRG.30702	Jak3
MSTRG.30708.1	MSTRG.30708	Gm35572
MSTRG.30709.1	MSTRG.30709	Gm35572
MSTRG.30711.2	MSTRG.30711	Crry-ps
MSTRG.30713.1	MSTRG.30713	Large1
MSTRG.30714.1	MSTRG.30714	Large1
MSTRG.30716.1	MSTRG.30716	Large1
MSTRG.30718.1	MSTRG.30718	Large1
MSTRG.30719.1	MSTRG.30719	Large1
MSTRG.3072.1	MSTRG.3072	Rhobtb1
MSTRG.30721.1	MSTRG.30721	Gm11033
MSTRG.30728.7	MSTRG.30728	Hmgxb4
MSTRG.30729.1	MSTRG.30729	Hmgxb4
MSTRG.3073.1	MSTRG.3073	Rhobtb1
MSTRG.30730.1	MSTRG.30730	Hmgxb4
MSTRG.30736.1	MSTRG.30736	Iqcm
MSTRG.30747.1	MSTRG.30747	Arhgap10
MSTRG.30750.1	MSTRG.30750	Arhgap10
MSTRG.30751.1	MSTRG.30751	Arhgap10
MSTRG.30754.1	MSTRG.30754	0610038B21Rik
MSTRG.30756.1	MSTRG.30756	Prmt9
MSTRG.30757.1	MSTRG.30757	Prmt9
MSTRG.3076.1	MSTRG.3076	Ccdc6
MSTRG.30764.1	MSTRG.30764	4933431K23Rik
MSTRG.30768.5	MSTRG.30768	Slc10a7
MSTRG.30768.7	MSTRG.30768	Slc10a7
MSTRG.30768.8	MSTRG.30768	Slc10a7
MSTRG.30769.1	MSTRG.30769	Slc10a7
MSTRG.3077.1	MSTRG.3077	Ccdc6
MSTRG.30770.1	MSTRG.30770	.

MSTRG.30772.1	MSTRG.30772	Zfp827
MSTRG.30773.1	MSTRG.30773	Zfp827
MSTRG.30775.1	MSTRG.30775	Mmaa
MSTRG.30776.1	MSTRG.30776	Mmaa
MSTRG.30779.1	MSTRG.30779	Smad1
MSTRG.3078.1	MSTRG.3078	Ccdc6
MSTRG.30780.1	MSTRG.30780	Smad1
MSTRG.30784.3	MSTRG.30784	Anapc10
MSTRG.30787.1	MSTRG.30787	.
MSTRG.3079.1	MSTRG.3079	.
MSTRG.30790.1	MSTRG.30790	.
MSTRG.30792.1	MSTRG.30792	Frem3
MSTRG.30796.1	MSTRG.30796	Gab1
MSTRG.30797.1	MSTRG.30797	Gab1
MSTRG.30802.1	MSTRG.30802	Inpp4b
MSTRG.30803.1	MSTRG.30803	Inpp4b
MSTRG.30804.1	MSTRG.30804	Inpp4b
MSTRG.30805.1	MSTRG.30805	Inpp4b
MSTRG.30806.1	MSTRG.30806	Inpp4b
MSTRG.30807.1	MSTRG.30807	Inpp4b
MSTRG.30808.1	MSTRG.30808	Inpp4b
MSTRG.30809.1	MSTRG.30809	Inpp4b
MSTRG.30810.1	MSTRG.30810	Inpp4b
MSTRG.30812.1	MSTRG.30812	Inpp4b
MSTRG.30814.1	MSTRG.30814	Inpp4b
MSTRG.30816.1	MSTRG.30816	Inpp4b
MSTRG.30817.1	MSTRG.30817	Inpp4b
MSTRG.30818.1	MSTRG.30818	Inpp4b
MSTRG.30819.1	MSTRG.30819	Inpp4b
MSTRG.30820.1	MSTRG.30820	Inpp4b
MSTRG.30821.1	MSTRG.30821	Inpp4b
MSTRG.30822.1	MSTRG.30822	Inpp4b
MSTRG.30823.1	MSTRG.30823	Inpp4b
MSTRG.30826.1	MSTRG.30826	Il15
MSTRG.30827.1	MSTRG.30827	.
MSTRG.30829.1	MSTRG.30829	Gm9655
MSTRG.30830.1	MSTRG.30830	Gm9655
MSTRG.30837.1	MSTRG.30837	Tbc1d9
MSTRG.30843.1	MSTRG.30843	Scoc
MSTRG.30848.1	MSTRG.30848	Tecr
MSTRG.3085.1	MSTRG.3085	Bicc1
MSTRG.30850.1	MSTRG.30850	Tecr
MSTRG.30852.4	MSTRG.30852	Gipc1
MSTRG.30853.1	MSTRG.30853	Gipc1
MSTRG.30857.1	MSTRG.30857	Ddx39
MSTRG.30861.1	MSTRG.30861	Adgrl1
MSTRG.30868.1	MSTRG.30868	Palm3
MSTRG.3088.4	MSTRG.3088	Tfam
MSTRG.30880.3	MSTRG.30880	Nanos3
MSTRG.30884.3	MSTRG.30884	Gm37352
MSTRG.30890.1	MSTRG.30890	D8Ertd738e
MSTRG.30894.1	MSTRG.30894	.
MSTRG.30899.1	MSTRG.30899	Cacna1a
MSTRG.30903.1	MSTRG.30903	Gm26664
MSTRG.30906.1	MSTRG.30906	Trmt1
MSTRG.30910.1	MSTRG.30910	Nfix
MSTRG.30911.1	MSTRG.30911	Nfix

MSTRG.30917.1	MSTRG.30917	Rtbdn
MSTRG.3092.1	MSTRG.3092	Ank3
MSTRG.30922.1	MSTRG.30922	Rnaseh2a
MSTRG.30927.1	MSTRG.30927	.
MSTRG.30929.1	MSTRG.30929	Man2b1
MSTRG.3093.1	MSTRG.3093	Ank3
MSTRG.30931.1	MSTRG.30931	Vps35
MSTRG.30934.1	MSTRG.30934	4921524J17Rik
MSTRG.30936.1	MSTRG.30936	4921524J17Rik
MSTRG.30937.1	MSTRG.30937	4921524J17Rik
MSTRG.3094.1	MSTRG.3094	Ank3
MSTRG.30940.1	MSTRG.30940	Hook2
MSTRG.30943.1	MSTRG.30943	Asna1
MSTRG.30946.1	MSTRG.30946	Tnpo2
MSTRG.30947.1	MSTRG.30947	A230103J11Rik
MSTRG.3095.1	MSTRG.3095	Ank3
MSTRG.30951.1	MSTRG.30951	.
MSTRG.30955.1	MSTRG.30955	.
MSTRG.30956.1	MSTRG.30956	.
MSTRG.30957.1	MSTRG.30957	.
MSTRG.3096.1	MSTRG.3096	Ank3
MSTRG.30962.1	MSTRG.30962	Neto2
MSTRG.30967.1	MSTRG.30967	Phkb
MSTRG.30969.1	MSTRG.30969	Phkb
MSTRG.3097.1	MSTRG.3097	Ank3
MSTRG.30970.1	MSTRG.30970	Phkb
MSTRG.30976.1	MSTRG.30976	Lonp2
MSTRG.3098.1	MSTRG.3098	Ank3
MSTRG.30980.7	MSTRG.30980	Cnep1r1
MSTRG.30985.1	MSTRG.30985	Papd5
MSTRG.30986.1	MSTRG.30986	Papd5
MSTRG.30987.1	MSTRG.30987	Papd5
MSTRG.30988.1	MSTRG.30988	Papd5
MSTRG.30989.1	MSTRG.30989	Papd5
MSTRG.30992.1	MSTRG.30992	Adcy7
MSTRG.30995.1	MSTRG.30995	Cyld
MSTRG.30998.1	MSTRG.30998	Chd9
MSTRG.31.1	MSTRG.31	Gm26983
MSTRG.31004.1	MSTRG.31004	Chd9
MSTRG.31005.1	MSTRG.31005	Chd9
MSTRG.31008.1	MSTRG.31008	Chd9
MSTRG.31010.1	MSTRG.31010	Chd9
MSTRG.31011.1	MSTRG.31011	Chd9
MSTRG.31012.1	MSTRG.31012	Chd9
MSTRG.31014.1	MSTRG.31014	Rbl2
MSTRG.31015.1	MSTRG.31015	Rbl2
MSTRG.31024.1	MSTRG.31024	Fto
MSTRG.31024.2	MSTRG.31024	Fto
MSTRG.31025.1	MSTRG.31025	Fto
MSTRG.31027.1	MSTRG.31027	Fto
MSTRG.31028.1	MSTRG.31028	Fto
MSTRG.3103.1	MSTRG.3103	.
MSTRG.31032.1	MSTRG.31032	Fto
MSTRG.31034.3	MSTRG.31034	Fto
MSTRG.31034.4	MSTRG.31034	Fto
MSTRG.31035.1	MSTRG.31035	Gm45294
MSTRG.31035.2	MSTRG.31035	Gm45294

MSTRG.31036.1	MSTRG.31036	Fto
MSTRG.31037.1	MSTRG.31037	Fto
MSTRG.31037.2	MSTRG.31037	Fto
MSTRG.31038.1	MSTRG.31038	Fto
MSTRG.31054.1	MSTRG.31054	Nudt21
MSTRG.31061.1	MSTRG.31061	Nup93
MSTRG.31069.1	MSTRG.31069	Nlrc5
MSTRG.31072.1	MSTRG.31072	.
MSTRG.31079.1	MSTRG.31079	Fam192a
MSTRG.31079.11	MSTRG.31079	Fam192a
MSTRG.31082.1	MSTRG.31082	Rspry1
MSTRG.31083.1	MSTRG.31083	Rspry1
MSTRG.31085.1	MSTRG.31085	Ciapin1
MSTRG.31101.1	MSTRG.31101	Gm31224
MSTRG.31110.1	MSTRG.31110	Csnk2a2
MSTRG.31123.1	MSTRG.31123	Cnot1
MSTRG.31131.2	MSTRG.31131	Cklf
MSTRG.31133.1	MSTRG.31133	Gm45711
MSTRG.31136.1	MSTRG.31136	Cmtm4
MSTRG.31137.1	MSTRG.31137	Cmtm4
MSTRG.31138.1	MSTRG.31138	Cmtm4
MSTRG.31140.1	MSTRG.31140	.
MSTRG.31151.1	MSTRG.31151	Terb1
MSTRG.31154.1	MSTRG.31154	Nae1
MSTRG.31160.1	MSTRG.31160	Cbfb
MSTRG.31163.1	MSTRG.31163	D230025D16Rik
MSTRG.31164.1	MSTRG.31164	D230025D16Rik
MSTRG.31167.1	MSTRG.31167	Tradd
MSTRG.31172.1	MSTRG.31172	4931428F04Rik
MSTRG.31176.4	MSTRG.31176	Zdhhc1
MSTRG.31186.1	MSTRG.31186	Gfod2
MSTRG.31187.1	MSTRG.31187	Gfod2
MSTRG.31188.1	MSTRG.31188	Gfod2
MSTRG.3119.1	MSTRG.3119	Specc1l
MSTRG.31190.1	MSTRG.31190	Ranbp10
MSTRG.31192.1	MSTRG.31192	Tsnaxip1
MSTRG.31199.1	MSTRG.31199	Atp6v0d1
MSTRG.312.1	MSTRG.312	Mgat4a
MSTRG.31204.1	MSTRG.31204	Agrp
MSTRG.31220.1	MSTRG.31220	.
MSTRG.31226.1	MSTRG.31226	Dus2
MSTRG.31227.1	MSTRG.31227	Dus2
MSTRG.31228.1	MSTRG.31228	Dus2
MSTRG.31229.13	MSTRG.31229	Nfatc3
MSTRG.31229.5	MSTRG.31229	Nfatc3
MSTRG.31229.6	MSTRG.31229	Nfatc3
MSTRG.31230.1	MSTRG.31230	Nfatc3
MSTRG.31232.1	MSTRG.31232	Nfatc3
MSTRG.31233.1	MSTRG.31233	Nfatc3
MSTRG.31238.1	MSTRG.31238	.
MSTRG.31239.1	MSTRG.31239	.
MSTRG.31241.1	MSTRG.31241	Slc7a6
MSTRG.31242.1	MSTRG.31242	Slc7a6
MSTRG.31245.1	MSTRG.31245	Prmt7
MSTRG.31246.1	MSTRG.31246	Prmt7
MSTRG.31252.1	MSTRG.31252	Tango6
MSTRG.31254.1	MSTRG.31254	Sntb2

MSTRG.31255.1	MSTRG.31255	Sntb2
MSTRG.31258.7	MSTRG.31258	Chtf8
MSTRG.31258.8	MSTRG.31258	Chtf8
MSTRG.31272.1	MSTRG.31272	Nfat5
MSTRG.31274.1	MSTRG.31274	.
MSTRG.31279.1	MSTRG.31279	Wwp2
MSTRG.31280.1	MSTRG.31280	Wwp2
MSTRG.31281.1	MSTRG.31281	Wwp2
MSTRG.31282.1	MSTRG.31282	Wwp2
MSTRG.31287.1	MSTRG.31287	Zfhx3
MSTRG.31291.1	MSTRG.31291	Zfhx3
MSTRG.31292.1	MSTRG.31292	Zfhx3
MSTRG.31297.1	MSTRG.31297	Txnl4b
MSTRG.31303.1	MSTRG.31303	Zfp821
MSTRG.31312.1	MSTRG.31312	Vac14
MSTRG.31318.1	MSTRG.31318	Pdpr
MSTRG.31323.1	MSTRG.31323	Glg1
MSTRG.31323.2	MSTRG.31323	Glg1
MSTRG.31324.1	MSTRG.31324	Glg1
MSTRG.31325.1	MSTRG.31325	Glg1
MSTRG.31333.1	MSTRG.31333	Ddx19b
MSTRG.31340.1	MSTRG.31340	Zfp1
MSTRG.31343.1	MSTRG.31343	Cfdp1
MSTRG.31348.1	MSTRG.31348	.
MSTRG.31358.1	MSTRG.31358	Wdr59
MSTRG.31359.1	MSTRG.31359	Znrf1
MSTRG.31359.2	MSTRG.31359	Znrf1
MSTRG.31364.1	MSTRG.31364	.
MSTRG.31366.1	MSTRG.31366	Wwox
MSTRG.31368.1	MSTRG.31368	Wwox
MSTRG.31371.1	MSTRG.31371	Wwox
MSTRG.31372.1	MSTRG.31372	Wwox
MSTRG.31373.1	MSTRG.31373	Wwox
MSTRG.31374.1	MSTRG.31374	Wwox
MSTRG.31376.1	MSTRG.31376	Wwox
MSTRG.31377.1	MSTRG.31377	Wwox
MSTRG.31378.1	MSTRG.31378	Wwox
MSTRG.31379.1	MSTRG.31379	Wwox
MSTRG.31380.1	MSTRG.31380	Wwox
MSTRG.31382.1	MSTRG.31382	Wwox
MSTRG.31383.2	MSTRG.31383	.
MSTRG.31385.1	MSTRG.31385	Cdyl2
MSTRG.31386.1	MSTRG.31386	Cdyl2
MSTRG.31391.1	MSTRG.31391	Cmc2
MSTRG.31395.1	MSTRG.31395	Gan
MSTRG.31397.1	MSTRG.31397	.
MSTRG.31398.1	MSTRG.31398	.
MSTRG.31402.1	MSTRG.31402	Cmip
MSTRG.31402.2	MSTRG.31402	Cmip
MSTRG.31402.3	MSTRG.31402	Cmip
MSTRG.31402.4	MSTRG.31402	Cmip
MSTRG.31402.5	MSTRG.31402	Cmip
MSTRG.31402.6	MSTRG.31402	Cmip
MSTRG.31405.1	MSTRG.31405	.
MSTRG.31408.1	MSTRG.31408	.
MSTRG.31415.1	MSTRG.31415	Cdh13
MSTRG.31418.1	MSTRG.31418	Mlycd

MSTRG.31419.1	MSTRG.31419	Mlycd
MSTRG.31430.1	MSTRG.31430	Klhl36
MSTRG.31431.1	MSTRG.31431	.
MSTRG.31431.2	MSTRG.31431	.
MSTRG.31432.1	MSTRG.31432	.
MSTRG.31433.1	MSTRG.31433	.
MSTRG.31437.1	MSTRG.31437	.
MSTRG.31438.1	MSTRG.31438	Gm24459
MSTRG.31445.1	MSTRG.31445	2310022B05Rik
MSTRG.31449.1	MSTRG.31449	Ttc13
MSTRG.31449.2	MSTRG.31449	Ttc13
MSTRG.31449.3	MSTRG.31449	Ttc13
MSTRG.31450.1	MSTRG.31450	Ttc13
MSTRG.31451.1	MSTRG.31451	Ttc13
MSTRG.31452.3	MSTRG.31452	Arv1
MSTRG.31452.4	MSTRG.31452	Arv1
MSTRG.31453.1	MSTRG.31453	Arv1
MSTRG.31454.1	MSTRG.31454	Arv1
MSTRG.31455.1	MSTRG.31455	Arv1
MSTRG.31462.1	MSTRG.31462	Egln1
MSTRG.31464.1	MSTRG.31464	Egln1
MSTRG.31465.1	MSTRG.31465	.
MSTRG.31467.1	MSTRG.31467	Tsnax
MSTRG.3147.1	MSTRG.3147	Gm5134
MSTRG.31470.1	MSTRG.31470	.
MSTRG.31471.1	MSTRG.31471	.
MSTRG.31481.1	MSTRG.31481	Tarbp1
MSTRG.31482.1	MSTRG.31482	.
MSTRG.31484.1	MSTRG.31484	Gm26759
MSTRG.31488.1	MSTRG.31488	Gm31718
MSTRG.31491.1	MSTRG.31491	Rbm34
MSTRG.31493.1	MSTRG.31493	Nrp1
MSTRG.31495.1	MSTRG.31495	.
MSTRG.31497.1	MSTRG.31497	Pard3
MSTRG.31501.1	MSTRG.31501	2610044O15Rik8
MSTRG.31502.1	MSTRG.31502	.
MSTRG.31505.1	MSTRG.31505	Ccdc7a
MSTRG.31508.1	MSTRG.31508	Gm32856
MSTRG.31509.1	MSTRG.31509	Gm32856
MSTRG.3151.1	MSTRG.3151	Prmt2
MSTRG.31510.1	MSTRG.31510	.
MSTRG.31511.1	MSTRG.31511	Gm26870
MSTRG.31511.5	MSTRG.31511	Gm26870
MSTRG.31514.1	MSTRG.31514	Gm10718
MSTRG.31525.1	MSTRG.31525	Cwf19l2
MSTRG.31526.1	MSTRG.31526	Cwf19l2
MSTRG.31530.1	MSTRG.31530	.
MSTRG.31533.1	MSTRG.31533	Kbtbd3
MSTRG.31540.1	MSTRG.31540	Casp12
MSTRG.31542.1	MSTRG.31542	Pdgfd
MSTRG.31545.1	MSTRG.31545	.
MSTRG.31546.1	MSTRG.31546	.
MSTRG.31548.1	MSTRG.31548	Dync2h1
MSTRG.31549.2	MSTRG.31549	Dcun1d5
MSTRG.31549.3	MSTRG.31549	Dcun1d5
MSTRG.31549.5	MSTRG.31549	Dcun1d5
MSTRG.31551.1	MSTRG.31551	.

MSTRG.31554.1	MSTRG.31554	.
MSTRG.31558.1	MSTRG.31558	.
MSTRG.31561.1	MSTRG.31561	AC155907.1
MSTRG.31569.1	MSTRG.31569	Arhgap42
MSTRG.31570.1	MSTRG.31570	Arhgap42
MSTRG.31571.1	MSTRG.31571	Arhgap42
MSTRG.31575.1	MSTRG.31575	AC154994.1
MSTRG.31580.1	MSTRG.31580	Cntn5
MSTRG.31589.1	MSTRG.31589	Mtmr2
MSTRG.31590.1	MSTRG.31590	Mtmr2
MSTRG.31593.1	MSTRG.31593	Maml2
MSTRG.31594.1	MSTRG.31594	Maml2
MSTRG.31596.1	MSTRG.31596	Maml2
MSTRG.31598.1	MSTRG.31598	Maml2
MSTRG.31599.1	MSTRG.31599	Maml2
MSTRG.31600.1	MSTRG.31600	Maml2
MSTRG.31601.1	MSTRG.31601	Maml2
MSTRG.31601.2	MSTRG.31601	Maml2
MSTRG.31602.1	MSTRG.31602	Maml2
MSTRG.31603.1	MSTRG.31603	Maml2
MSTRG.31605.4	MSTRG.31605	Fam76b
MSTRG.31606.1	MSTRG.31606	Fam76b
MSTRG.31608.1	MSTRG.31608	Sesn3
MSTRG.31624.1	MSTRG.31624	Izumo1r
MSTRG.31626.1	MSTRG.31626	.
MSTRG.31627.1	MSTRG.31627	.
MSTRG.31629.1	MSTRG.31629	Panx1
MSTRG.31630.1	MSTRG.31630	Panx1
MSTRG.31633.1	MSTRG.31633	Med17
MSTRG.31639.1	MSTRG.31639	Slc36a4
MSTRG.31640.1	MSTRG.31640	Slc36a4
MSTRG.31643.1	MSTRG.31643	Taf1d
MSTRG.31643.2	MSTRG.31643	Taf1d
MSTRG.3165.1	MSTRG.3165	Pcbp3
MSTRG.31651.4	MSTRG.31651	Chordc1
MSTRG.31655.1	MSTRG.31655	.
MSTRG.31659.1	MSTRG.31659	Gm45699
MSTRG.3166.1	MSTRG.3166	Pcbp3
MSTRG.31662.1	MSTRG.31662	Zfp560
MSTRG.31669.1	MSTRG.31669	Gm26733
MSTRG.31672.1	MSTRG.31672	.
MSTRG.31677.1	MSTRG.31677	Zfp846
MSTRG.31682.1	MSTRG.31682	Fbxl12
MSTRG.31686.1	MSTRG.31686	.
MSTRG.31691.1	MSTRG.31691	Dnmt1
MSTRG.317.1	MSTRG.317	2010300C02Rik
MSTRG.31700.1	MSTRG.31700	1700084C06Rik
MSTRG.31702.1	MSTRG.31702	Cdc37
MSTRG.31718.1	MSTRG.31718	AC166992.1
MSTRG.31719.1	MSTRG.31719	AC166992.1
MSTRG.31738.1	MSTRG.31738	Dnm2
MSTRG.31738.2	MSTRG.31738	Dnm2
MSTRG.31739.1	MSTRG.31739	Dnm2
MSTRG.31740.1	MSTRG.31740	Dnm2
MSTRG.31743.1	MSTRG.31743	.
MSTRG.31746.1	MSTRG.31746	AB124611
MSTRG.31748.1	MSTRG.31748	Carm1

MSTRG.31749.1	MSTRG.31749	Carm1
MSTRG.3175.1	MSTRG.3175	Itgb2
MSTRG.31750.1	MSTRG.31750	Carm1
MSTRG.31754.1	MSTRG.31754	.
MSTRG.31767.1	MSTRG.31767	.
MSTRG.3177.4	MSTRG.3177	Sumo3
MSTRG.31770.1	MSTRG.31770	.
MSTRG.31771.3	MSTRG.31771	Zfp810
MSTRG.31772.1	MSTRG.31772	Zfp810
MSTRG.31775.1	MSTRG.31775	Zfp810
MSTRG.31777.1	MSTRG.31777	Anln
MSTRG.31780.2	MSTRG.31780	Rp9
MSTRG.31782.1	MSTRG.31782	Rp9
MSTRG.31788.1	MSTRG.31788	Bbs9
MSTRG.31789.1	MSTRG.31789	Bbs9
MSTRG.3179.1	MSTRG.3179	Trpm2
MSTRG.31794.1	MSTRG.31794	Dpy19l1
MSTRG.318.1	MSTRG.318	2010300C02Rik
MSTRG.31801.1	MSTRG.31801	.
MSTRG.31806.1	MSTRG.31806	.
MSTRG.31809.1	MSTRG.31809	Acad8
MSTRG.31810.1	MSTRG.31810	Acad8
MSTRG.31811.1	MSTRG.31811	Acad8
MSTRG.31823.1	MSTRG.31823	Zbtb44
MSTRG.31828.1	MSTRG.31828	Aplp2
MSTRG.3183.1	MSTRG.3183	Pfkl
MSTRG.31831.1	MSTRG.31831	Prdm10
MSTRG.31832.1	MSTRG.31832	Prdm10
MSTRG.31840.1	MSTRG.31840	.
MSTRG.31841.1	MSTRG.31841	.
MSTRG.31849.1	MSTRG.31849	Fli1
MSTRG.31856.1	MSTRG.31856	CT030644.1
MSTRG.31858.1	MSTRG.31858	CT030644.1
MSTRG.3186.1	MSTRG.3186	.
MSTRG.31863.1	MSTRG.31863	Kirrel3
MSTRG.31865.1	MSTRG.31865	Kirrel3
MSTRG.31870.1	MSTRG.31870	AC160116.1
MSTRG.31872.1	MSTRG.31872	Cdon
MSTRG.31873.1	MSTRG.31873	Cdon
MSTRG.31877.1	MSTRG.31877	Gm20388
MSTRG.31877.2	MSTRG.31877	Gm20388
MSTRG.31889.1	MSTRG.31889	Gse1
MSTRG.31890.1	MSTRG.31890	Gm26971
MSTRG.3192.1	MSTRG.3192	AC160405.2
MSTRG.31929.1	MSTRG.31929	Gm20388
MSTRG.31930.1	MSTRG.31930	Gm20388
MSTRG.31933.1	MSTRG.31933	Gm20388
MSTRG.31934.1	MSTRG.31934	Gm20388
MSTRG.31947.1	MSTRG.31947	Gm20388
MSTRG.31948.1	MSTRG.31948	Gm20388
MSTRG.31951.1	MSTRG.31951	Gm20388
MSTRG.31958.1	MSTRG.31958	Gm20388
MSTRG.3196.1	MSTRG.3196	Agpat3
MSTRG.31963.1	MSTRG.31963	Gm20388
MSTRG.31964.1	MSTRG.31964	Rnf166
MSTRG.31968.1	MSTRG.31968	Piezo1
MSTRG.31973.1	MSTRG.31973	Gm20388

MSTRG.31978.1	MSTRG.31978	Cbfa2t3
MSTRG.31978.2	MSTRG.31978	Cbfa2t3
MSTRG.31978.3	MSTRG.31978	Cbfa2t3
MSTRG.31979.1	MSTRG.31979	Cbfa2t3
MSTRG.31980.1	MSTRG.31980	Gm20388
MSTRG.31982.1	MSTRG.31982	Cbfa2t3
MSTRG.31984.1	MSTRG.31984	Gm20388
MSTRG.31984.2	MSTRG.31984	Gm20388
MSTRG.31987.1	MSTRG.31987	Gm20388
MSTRG.31997.1	MSTRG.31997	Ankrd11
MSTRG.31997.2	MSTRG.31997	Ankrd11
MSTRG.31997.3	MSTRG.31997	Ankrd11
MSTRG.31997.4	MSTRG.31997	Ankrd11
MSTRG.31997.5	MSTRG.31997	Ankrd11
MSTRG.31997.6	MSTRG.31997	Ankrd11
MSTRG.31997.7	MSTRG.31997	Ankrd11
MSTRG.31997.8	MSTRG.31997	Ankrd11
MSTRG.31999.1	MSTRG.31999	Gm20388
MSTRG.320.1	MSTRG.320	Tsga10
MSTRG.32000.1	MSTRG.32000	Ankrd11
MSTRG.32000.2	MSTRG.32000	Ankrd11
MSTRG.32001.1	MSTRG.32001	Ankrd11
MSTRG.32002.1	MSTRG.32002	Ankrd11
MSTRG.32003.1	MSTRG.32003	2810013P06Rik
MSTRG.32004.1	MSTRG.32004	Gm20388
MSTRG.32005.7	MSTRG.32005	Spg7
MSTRG.32006.1	MSTRG.32006	Spg7
MSTRG.32007.1	MSTRG.32007	Spg7
MSTRG.32008.1	MSTRG.32008	Spg7
MSTRG.32009.1	MSTRG.32009	Spg7
MSTRG.32010.1	MSTRG.32010	Spg7
MSTRG.32018.1	MSTRG.32018	Chmp1a
MSTRG.32026.1	MSTRG.32026	Fanca
MSTRG.32027.1	MSTRG.32027	Gm20388
MSTRG.32040.1	MSTRG.32040	Gm20388
MSTRG.3205.1	MSTRG.3205	.
MSTRG.3206.1	MSTRG.3206	.
MSTRG.3210.1	MSTRG.3210	Olfr1357
MSTRG.32100.1	MSTRG.32100	Ccsap
MSTRG.32109.1	MSTRG.32109	Taf5l
MSTRG.32113.1	MSTRG.32113	Gm20388
MSTRG.32116.1	MSTRG.32116	Galnt2
MSTRG.32117.1	MSTRG.32117	Galnt2
MSTRG.32118.1	MSTRG.32118	Galnt2
MSTRG.32118.2	MSTRG.32118	Galnt2
MSTRG.32119.1	MSTRG.32119	Galnt2
MSTRG.3212.1	MSTRG.3212	.
MSTRG.32120.1	MSTRG.32120	Galnt2
MSTRG.32121.1	MSTRG.32121	Galnt2
MSTRG.32123.1	MSTRG.32123	Gm20388
MSTRG.32125.1	MSTRG.32125	Gm15775
MSTRG.32127.1	MSTRG.32127	St3gal4
MSTRG.32128.1	MSTRG.32128	St3gal4
MSTRG.32135.1	MSTRG.32135	Tirap
MSTRG.32136.1	MSTRG.32136	Tirap
MSTRG.3214.1	MSTRG.3214	Olfr1357
MSTRG.32140.1	MSTRG.32140	Fam118b

MSTRG.32141.1	MSTRG.32141	Fam118b
MSTRG.32143.1	MSTRG.32143	Fam118b
MSTRG.32145.1	MSTRG.32145	.
MSTRG.3215.1	MSTRG.3215	Olfr1357
MSTRG.32156.1	MSTRG.32156	CT025617.1
MSTRG.32160.1	MSTRG.32160	Stt3a
MSTRG.32161.1	MSTRG.32161	Stt3a
MSTRG.3217.1	MSTRG.3217	AC153887.3
MSTRG.32175.1	MSTRG.32175	.
MSTRG.32180.1	MSTRG.32180	Ccdc15
MSTRG.32190.1	MSTRG.32190	.
MSTRG.32191.1	MSTRG.32191	.
MSTRG.32192.1	MSTRG.32192	.
MSTRG.32193.1	MSTRG.32193	.
MSTRG.32204.1	MSTRG.32204	Gramd1b
MSTRG.32205.1	MSTRG.32205	Gramd1b
MSTRG.32206.1	MSTRG.32206	Gramd1b
MSTRG.3221.1	MSTRG.3221	2610008E11Rik
MSTRG.32211.1	MSTRG.32211	Ubash3b
MSTRG.32212.1	MSTRG.32212	Ubash3b
MSTRG.32213.1	MSTRG.32213	Ubash3b
MSTRG.32214.1	MSTRG.32214	Ubash3b
MSTRG.32216.1	MSTRG.32216	Ubash3b
MSTRG.32218.1	MSTRG.32218	Ubash3b
MSTRG.32219.1	MSTRG.32219	Ubash3b
MSTRG.32222.1	MSTRG.32222	.
MSTRG.32223.1	MSTRG.32223	2610203C20Rik
MSTRG.32223.6	MSTRG.32223	2610203C20Rik
MSTRG.32226.1	MSTRG.32226	2610203C20Rik
MSTRG.32228.1	MSTRG.32228	2610203C20Rik
MSTRG.32229.1	MSTRG.32229	CT009696.3
MSTRG.32230.1	MSTRG.32230	2610203C20Rik
MSTRG.32232.1	MSTRG.32232	CT009696.3
MSTRG.32234.1	MSTRG.32234	2610203C20Rik
MSTRG.32236.1	MSTRG.32236	CT025619.4
MSTRG.32237.1	MSTRG.32237	2610203C20Rik
MSTRG.32244.4	MSTRG.32244	Tbcel
MSTRG.32244.8	MSTRG.32244	Tbcel
MSTRG.32254.1	MSTRG.32254	AC126459.5
MSTRG.32254.3	MSTRG.32254	AC126459.5
MSTRG.32254.4	MSTRG.32254	AC126459.5
MSTRG.32254.6	MSTRG.32254	AC126459.5
MSTRG.32255.1	MSTRG.32255	AC126459.3
MSTRG.32256.1	MSTRG.32256	.
MSTRG.32265.1	MSTRG.32265	Nlrx1
MSTRG.32271.1	MSTRG.32271	Rnf26
MSTRG.32277.1	MSTRG.32277	Cbl
MSTRG.32280.1	MSTRG.32280	Cbl
MSTRG.32289.1	MSTRG.32289	Vps11
MSTRG.32297.1	MSTRG.32297	Ccdc84
MSTRG.323.7	MSTRG.323	Mitd1
MSTRG.32310.1	MSTRG.32310	.
MSTRG.32311.1	MSTRG.32311	.
MSTRG.32313.1	MSTRG.32313	Ddx6
MSTRG.32322.1	MSTRG.32322	Cd3e
MSTRG.32326.9	MSTRG.32326	Ift46
MSTRG.32333.3	MSTRG.32333	AC061963.1

MSTRG.32335.1	MSTRG.32335	Atp5l
MSTRG.32339.1	MSTRG.32339	.
MSTRG.32353.1	MSTRG.32353	Il10ra
MSTRG.32353.2	MSTRG.32353	Il10ra
MSTRG.32360.1	MSTRG.32360	AC134586.1
MSTRG.32363.1	MSTRG.32363	Bud13
MSTRG.32370.1	MSTRG.32370	Cadm1
MSTRG.32376.1	MSTRG.32376	Rnf214
MSTRG.32378.1	MSTRG.32378	Pcsk7
MSTRG.32379.1	MSTRG.32379	Pcsk7
MSTRG.32385.1	MSTRG.32385	Sik3
MSTRG.32385.2	MSTRG.32385	Sik3
MSTRG.32387.1	MSTRG.32387	Sik3
MSTRG.32389.1	MSTRG.32389	Sik3
MSTRG.32390.1	MSTRG.32390	Sik3
MSTRG.32391.1	MSTRG.32391	Sik3
MSTRG.32396.1	MSTRG.32396	.
MSTRG.32397.1	MSTRG.32397	.
MSTRG.32398.1	MSTRG.32398	.
MSTRG.32401.1	MSTRG.32401	Zbtb16
MSTRG.32403.1	MSTRG.32403	Zbtb16
MSTRG.32404.1	MSTRG.32404	Zbtb16
MSTRG.32405.1	MSTRG.32405	Zbtb16
MSTRG.32406.1	MSTRG.32406	Zbtb16
MSTRG.32407.1	MSTRG.32407	Zbtb16
MSTRG.32414.1	MSTRG.32414	Usp28
MSTRG.32416.1	MSTRG.32416	Ttc12
MSTRG.32417.1	MSTRG.32417	Ttc12
MSTRG.32421.10	MSTRG.32421	Pts
MSTRG.32421.7	MSTRG.32421	Pts
MSTRG.32422.1	MSTRG.32422	Sdhd
MSTRG.32439.1	MSTRG.32439	Sik2
MSTRG.32440.1	MSTRG.32440	Sik2
MSTRG.32441.1	MSTRG.32441	Sik2
MSTRG.32442.1	MSTRG.32442	Sik2
MSTRG.32460.1	MSTRG.32460	Cul5
MSTRG.32462.1	MSTRG.32462	Gm16124
MSTRG.32464.1	MSTRG.32464	Slc35f2
MSTRG.32479.1	MSTRG.32479	Etfa
MSTRG.32480.1	MSTRG.32480	Etfa
MSTRG.32485.1	MSTRG.32485	Scaper
MSTRG.32486.1	MSTRG.32486	Scaper
MSTRG.32487.1	MSTRG.32487	Scaper
MSTRG.32488.2	MSTRG.32488	AC107819.3
MSTRG.32489.1	MSTRG.32489	AC107819.3
MSTRG.32498.1	MSTRG.32498	Peak1
MSTRG.325.7	MSTRG.325	Txndc9
MSTRG.32501.1	MSTRG.32501	Hmg20a
MSTRG.32502.1	MSTRG.32502	Hmg20a
MSTRG.32507.1	MSTRG.32507	Snupn
MSTRG.32508.4	MSTRG.32508	Commd4
MSTRG.32514.1	MSTRG.32514	Ppcdc
MSTRG.3252.1	MSTRG.3252	Arid3a
MSTRG.32527.1	MSTRG.32527	Scamp2
MSTRG.32529.5	MSTRG.32529	Csk
MSTRG.32530.1	MSTRG.32530	Csk
MSTRG.32536.1	MSTRG.32536	.

MSTRG.32537.1	MSTRG.32537	Gm17231
MSTRG.32538.1	MSTRG.32538	Gm17231
MSTRG.32541.1	MSTRG.32541	Cyp11a1
MSTRG.32545.1	MSTRG.32545	Pml
MSTRG.32546.1	MSTRG.32546	Pml
MSTRG.32549.1	MSTRG.32549	Nptn
MSTRG.32550.1	MSTRG.32550	Nptn
MSTRG.32551.1	MSTRG.32551	Nptn
MSTRG.32566.1	MSTRG.32566	Arih1
MSTRG.32572.1	MSTRG.32572	Senp8
MSTRG.32573.1	MSTRG.32573	Senp8
MSTRG.32576.1	MSTRG.32576	Myo9a
MSTRG.32577.1	MSTRG.32577	Myo9a
MSTRG.32579.1	MSTRG.32579	AC156795.1
MSTRG.32580.1	MSTRG.32580	AC156795.1
MSTRG.32582.1	MSTRG.32582	AC156795.1
MSTRG.32583.1	MSTRG.32583	Myo9a
MSTRG.32585.1	MSTRG.32585	AC156795.1
MSTRG.32588.1	MSTRG.32588	Thsd4
MSTRG.32597.1	MSTRG.32597	AC160562.1
MSTRG.32600.1	MSTRG.32600	AC140488.1
MSTRG.32601.1	MSTRG.32601	AC140488.1
MSTRG.32605.1	MSTRG.32605	Glce
MSTRG.32607.1	MSTRG.32607	.
MSTRG.32609.1	MSTRG.32609	.
MSTRG.32613.1	MSTRG.32613	Coro2b
MSTRG.32616.1	MSTRG.32616	.
MSTRG.32620.1	MSTRG.32620	Map2k5
MSTRG.32621.1	MSTRG.32621	Map2k5
MSTRG.32627.1	MSTRG.32627	Pias1
MSTRG.32627.2	MSTRG.32627	Pias1
MSTRG.32630.1	MSTRG.32630	Smad3
MSTRG.32631.1	MSTRG.32631	Smad3
MSTRG.32645.1	MSTRG.32645	Zwilch
MSTRG.32646.1	MSTRG.32646	Zwilch
MSTRG.32653.1	MSTRG.32653	Map2k1
MSTRG.32658.1	MSTRG.32658	Megf11
MSTRG.32660.1	MSTRG.32660	Dennd4a
MSTRG.32667.1	MSTRG.32667	Igdcc4
MSTRG.32673.1	MSTRG.32673	Gm16218
MSTRG.32677.1	MSTRG.32677	Clpx
MSTRG.32685.1	MSTRG.32685	Mtfmt
MSTRG.32690.9	MSTRG.32690	Pif1
MSTRG.32698.1	MSTRG.32698	Dapk2
MSTRG.32699.1	MSTRG.32699	Zfp609
MSTRG.32699.2	MSTRG.32699	Zfp609
MSTRG.327.1	MSTRG.327	Eif5b
MSTRG.32703.1	MSTRG.32703	Zfp609
MSTRG.32704.1	MSTRG.32704	Zfp609
MSTRG.32705.1	MSTRG.32705	Zfp609
MSTRG.32709.1	MSTRG.32709	Trip4
MSTRG.32710.1	MSTRG.32710	Trip4
MSTRG.32712.1	MSTRG.32712	Pclaf
MSTRG.32714.1	MSTRG.32714	Trip4
MSTRG.32716.1	MSTRG.32716	Csnk1g1
MSTRG.32717.1	MSTRG.32717	Csnk1g1
MSTRG.32719.1	MSTRG.32719	Csnk1g1

MSTRG.32720.1	MSTRG.32720	Csnk1g1
MSTRG.32721.1	MSTRG.32721	Csnk1g1
MSTRG.32726.1	MSTRG.32726	Rab8b
MSTRG.32731.1	MSTRG.32731	Herc1
MSTRG.32736.1	MSTRG.32736	Usp3
MSTRG.32738.1	MSTRG.32738	Vps13c
MSTRG.32741.1	MSTRG.32741	Rora
MSTRG.32742.1	MSTRG.32742	Rora
MSTRG.32743.1	MSTRG.32743	Rora
MSTRG.32744.1	MSTRG.32744	Rora
MSTRG.32745.1	MSTRG.32745	Rora
MSTRG.32746.1	MSTRG.32746	Rora
MSTRG.32747.1	MSTRG.32747	Rora
MSTRG.32748.1	MSTRG.32748	Rora
MSTRG.32749.1	MSTRG.32749	Rora
MSTRG.32751.1	MSTRG.32751	Rora
MSTRG.32752.1	MSTRG.32752	Rora
MSTRG.32753.1	MSTRG.32753	Rora
MSTRG.32754.1	MSTRG.32754	Rora
MSTRG.32756.1	MSTRG.32756	Gm15511
MSTRG.32763.1	MSTRG.32763	B230323A14Rik
MSTRG.32764.1	MSTRG.32764	B230323A14Rik
MSTRG.32771.1	MSTRG.32771	Bnip2
MSTRG.32772.10	MSTRG.32772	Gtf2a2
MSTRG.32774.1	MSTRG.32774	Myo1e
MSTRG.32775.1	MSTRG.32775	Myo1e
MSTRG.32777.1	MSTRG.32777	Rnf111
MSTRG.32778.1	MSTRG.32778	Rnf111
MSTRG.32780.1	MSTRG.32780	Rnf111
MSTRG.32790.15	MSTRG.32790	Tcf12
MSTRG.32790.21	MSTRG.32790	Tcf12
MSTRG.32790.23	MSTRG.32790	Tcf12
MSTRG.32794.1	MSTRG.32794	Tcf12
MSTRG.32795.1	MSTRG.32795	Tcf12
MSTRG.32796.1	MSTRG.32796	Gm27152
MSTRG.32798.1	MSTRG.32798	Tcf12
MSTRG.32800.1	MSTRG.32800	Tcf12
MSTRG.32804.1	MSTRG.32804	Gm27450
MSTRG.32806.1	MSTRG.32806	Tcf12
MSTRG.32807.1	MSTRG.32807	Tcf12
MSTRG.32808.1	MSTRG.32808	Tcf12
MSTRG.32811.1	MSTRG.32811	Rab27a
MSTRG.32821.1	MSTRG.32821	Pigb
MSTRG.32822.1	MSTRG.32822	Pigb
MSTRG.32828.1	MSTRG.32828	Unc13c
MSTRG.32832.1	MSTRG.32832	Fam214a
MSTRG.32833.1	MSTRG.32833	Fam214a
MSTRG.32834.1	MSTRG.32834	Fam214a
MSTRG.32835.1	MSTRG.32835	Fam214a
MSTRG.3284.1	MSTRG.3284	Rexo1
MSTRG.32843.1	MSTRG.32843	Zfp280d
MSTRG.32847.1	MSTRG.32847	Gm27230
MSTRG.32848.1	MSTRG.32848	Gm27230
MSTRG.32848.2	MSTRG.32848	Gm27230
MSTRG.32848.4	MSTRG.32848	Gm27230
MSTRG.32848.5	MSTRG.32848	Gm27230
MSTRG.32855.1	MSTRG.32855	Tex9

MSTRG.32859.1	MSTRG.32859	Rfx7
MSTRG.32867.1	MSTRG.32867	Nedd4
MSTRG.32872.1	MSTRG.32872	Nedd4
MSTRG.32885.1	MSTRG.32885	.
MSTRG.32887.1	MSTRG.32887	Lrrc1
MSTRG.32888.1	MSTRG.32888	Lrrc1
MSTRG.3289.1	MSTRG.3289	Btbd2
MSTRG.32890.1	MSTRG.32890	Gclc
MSTRG.32891.1	MSTRG.32891	Gclc
MSTRG.32893.1	MSTRG.32893	Elovl5
MSTRG.32896.1	MSTRG.32896	Slc17a5
MSTRG.329.1	MSTRG.329	Rev1
MSTRG.32902.1	MSTRG.32902	Ick
MSTRG.32902.2	MSTRG.32902	Rn7sk
MSTRG.32902.3	MSTRG.32902	Ddx43
MSTRG.32903.1	MSTRG.32903	Ick
MSTRG.32929.1	MSTRG.32929	Tmem30a
MSTRG.3293.10	MSTRG.3293	Izumo4
MSTRG.32935.1	MSTRG.32935	.
MSTRG.32939.1	MSTRG.32939	Senp6
MSTRG.32942.1	MSTRG.32942	Mei4
MSTRG.32953.1	MSTRG.32953	Lca5
MSTRG.32954.1	MSTRG.32954	Lca5
MSTRG.32961.1	MSTRG.32961	Bckdhb
MSTRG.32981.1	MSTRG.32981	Dopey1
MSTRG.32989.1	MSTRG.32989	Cep162
MSTRG.3299.5	MSTRG.3299	Oaz1
MSTRG.32990.1	MSTRG.32990	Cep162
MSTRG.32992.3	MSTRG.32992	Snhg5
MSTRG.32993.5	MSTRG.32993	Zfp949
MSTRG.32995.1	MSTRG.32995	Zfp949
MSTRG.33001.1	MSTRG.33001	Nt5e
MSTRG.33005.1	MSTRG.33005	.
MSTRG.33013.1	MSTRG.33013	.
MSTRG.33030.1	MSTRG.33030	Mthfs
MSTRG.33031.1	MSTRG.33031	Mthfs
MSTRG.33033.1	MSTRG.33033	Mthfs
MSTRG.33035.1	MSTRG.33035	.
MSTRG.33039.1	MSTRG.33039	AC162946.1
MSTRG.33042.1	MSTRG.33042	.
MSTRG.33047.1	MSTRG.33047	Plscr1
MSTRG.33053.1	MSTRG.33053	Plod2
MSTRG.33055.1	MSTRG.33055	1190002N15Rik
MSTRG.33059.1	MSTRG.33059	Slc9a9
MSTRG.33060.1	MSTRG.33060	Slc9a9
MSTRG.33061.1	MSTRG.33061	Slc9a9
MSTRG.33062.1	MSTRG.33062	Slc9a9
MSTRG.33063.1	MSTRG.33063	Slc9a9
MSTRG.33064.1	MSTRG.33064	Slc9a9
MSTRG.33064.2	MSTRG.33064	Slc9a9
MSTRG.33065.1	MSTRG.33065	Slc9a9
MSTRG.33067.1	MSTRG.33067	Slc9a9
MSTRG.33068.1	MSTRG.33068	Slc9a9
MSTRG.33069.1	MSTRG.33069	Slc9a9
MSTRG.33070.1	MSTRG.33070	Slc9a9
MSTRG.33071.1	MSTRG.33071	Slc9a9
MSTRG.33077.1	MSTRG.33077	Gk5

MSTRG.33082.1	MSTRG.33082	Tfdp2
MSTRG.33083.1	MSTRG.33083	Tfdp2
MSTRG.33084.1	MSTRG.33084	Tfdp2
MSTRG.33085.2	MSTRG.33085	Atp1b3
MSTRG.33085.5	MSTRG.33085	Atp1b3
MSTRG.33089.1	MSTRG.33089	Gm16794
MSTRG.33098.1	MSTRG.33098	Rasa2
MSTRG.33099.1	MSTRG.33099	Rasa2
MSTRG.33102.1	MSTRG.33102	Pxylp1
MSTRG.33105.1	MSTRG.33105	Gm26767
MSTRG.33106.1	MSTRG.33106	Gm26767
MSTRG.3311.1	MSTRG.3311	Pias4
MSTRG.33110.1	MSTRG.33110	Nmnat3
MSTRG.33112.1	MSTRG.33112	Nmnat3
MSTRG.33113.1	MSTRG.33113	Nmnat3
MSTRG.3312.1	MSTRG.3312	Pias4
MSTRG.33123.1	MSTRG.33123	Pik3cb
MSTRG.33124.1	MSTRG.33124	Pik3cb
MSTRG.33125.1	MSTRG.33125	Pik3cb
MSTRG.33128.1	MSTRG.33128	Esyt3
MSTRG.33133.1	MSTRG.33133	Cep70
MSTRG.33137.1	MSTRG.33137	4930422M22Rik
MSTRG.33141.1	MSTRG.33141	Armc8
MSTRG.33144.1	MSTRG.33144	Il20rb
MSTRG.33145.1	MSTRG.33145	Il20rb
MSTRG.33146.1	MSTRG.33146	Il20rb
MSTRG.33147.5	MSTRG.33147	Nck1
MSTRG.33148.1	MSTRG.33148	Nck1
MSTRG.33150.1	MSTRG.33150	Nck1
MSTRG.33154.1	MSTRG.33154	.
MSTRG.33157.1	MSTRG.33157	Gm8661
MSTRG.33159.1	MSTRG.33159	Stag1
MSTRG.33160.1	MSTRG.33160	Stag1
MSTRG.33162.1	MSTRG.33162	Stag1
MSTRG.33163.1	MSTRG.33163	Stag1
MSTRG.33165.1	MSTRG.33165	Stag1
MSTRG.33167.1	MSTRG.33167	Stag1
MSTRG.33169.1	MSTRG.33169	Stag1
MSTRG.33171.1	MSTRG.33171	Stag1
MSTRG.33182.1	MSTRG.33182	Gm29387
MSTRG.33185.1	MSTRG.33185	Cep63
MSTRG.33189.1	MSTRG.33189	Ryk
MSTRG.3319.1	MSTRG.3319	Eef2
MSTRG.33194.1	MSTRG.33194	Slco2a1
MSTRG.33197.1	MSTRG.33197	Gm20425
MSTRG.33198.4	MSTRG.33198	Cdv3
MSTRG.33198.8	MSTRG.33198	Cdv3
MSTRG.33198.9	MSTRG.33198	Cdv3
MSTRG.332.1	MSTRG.332	Pdcl3
MSTRG.33202.10	MSTRG.33202	5830418P13Rik
MSTRG.33202.12	MSTRG.33202	5830418P13Rik
MSTRG.33202.4	MSTRG.33202	5830418P13Rik
MSTRG.33202.5	MSTRG.33202	5830418P13Rik
MSTRG.33202.6	MSTRG.33202	5830418P13Rik
MSTRG.33202.7	MSTRG.33202	5830418P13Rik
MSTRG.33202.9	MSTRG.33202	5830418P13Rik
MSTRG.33204.1	MSTRG.33204	Bfsp2

MSTRG.3322.1	MSTRG.3322	Dapk3
MSTRG.33228.1	MSTRG.33228	.
MSTRG.33237.1	MSTRG.33237	Atp2c1
MSTRG.33239.1	MSTRG.33239	Atp2c1
MSTRG.33241.1	MSTRG.33241	Atp2c1
MSTRG.33248.1	MSTRG.33248	Wdr82
MSTRG.33249.1	MSTRG.33249	.
MSTRG.33259.1	MSTRG.33259	Poc1a
MSTRG.33260.1	MSTRG.33260	Poc1a
MSTRG.33266.1	MSTRG.33266	.
MSTRG.33272.1	MSTRG.33272	Rad54l2
MSTRG.33274.1	MSTRG.33274	Mapkapk3
MSTRG.33279.1	MSTRG.33279	Vprbp
MSTRG.33280.1	MSTRG.33280	Vprbp
MSTRG.33282.1	MSTRG.33282	Vprbp
MSTRG.33284.1	MSTRG.33284	Manf
MSTRG.33292.1	MSTRG.33292	Dock3
MSTRG.33293.1	MSTRG.33293	.
MSTRG.33320.1	MSTRG.33320	Rbm5
MSTRG.33325.1	MSTRG.33325	Rbm6
MSTRG.33328.1	MSTRG.33328	4930447F24Rik
MSTRG.33337.1	MSTRG.33337	Rnf123
MSTRG.33354.1	MSTRG.33354	Ip6k1
MSTRG.33373.1	MSTRG.33373	Arih2
MSTRG.33379.1	MSTRG.33379	Nckipsd
MSTRG.3338.1	MSTRG.3338	Tbxa2r
MSTRG.33384.1	MSTRG.33384	.
MSTRG.33385.4	MSTRG.33385	Shisa5
MSTRG.33389.1	MSTRG.33389	Ccdc51
MSTRG.33397.1	MSTRG.33397	Map4
MSTRG.33399.1	MSTRG.33399	Map4
MSTRG.33401.1	MSTRG.33401	Map4
MSTRG.33404.1	MSTRG.33404	Map4
MSTRG.33405.2	MSTRG.33405	Elp6
MSTRG.33406.1	MSTRG.33406	Elp6
MSTRG.33407.1	MSTRG.33407	Elp6
MSTRG.33408.1	MSTRG.33408	Elp6
MSTRG.33412.1	MSTRG.33412	Smarcc1
MSTRG.33413.1	MSTRG.33413	Smarcc1
MSTRG.33414.1	MSTRG.33414	Smarcc1
MSTRG.33418.1	MSTRG.33418	Scap
MSTRG.33421.1	MSTRG.33421	Klhl18
MSTRG.33422.1	MSTRG.33422	Klhl18
MSTRG.33428.1	MSTRG.33428	Lrrc2
MSTRG.33432.1	MSTRG.33432	Ccdc12
MSTRG.33433.1	MSTRG.33433	Ccdc12
MSTRG.33440.1	MSTRG.33440	.
MSTRG.33446.1	MSTRG.33446	Lrrfip2
MSTRG.33447.1	MSTRG.33447	Lrrfip2
MSTRG.33448.1	MSTRG.33448	Lrrfip2
MSTRG.33450.1	MSTRG.33450	Mlh1
MSTRG.33462.1	MSTRG.33462	.
MSTRG.33468.1	MSTRG.33468	Glb1
MSTRG.3347.1	MSTRG.3347	Nfic
MSTRG.33474.1	MSTRG.33474	Cnot10
MSTRG.33476.1	MSTRG.33476	Dync1li1
MSTRG.33476.2	MSTRG.33476	Dync1li1

MSTRG.33478.1	MSTRG.33478	Cmtm6
MSTRG.33479.1	MSTRG.33479	Cmtm6
MSTRG.33481.1	MSTRG.33481	Cmtm7
MSTRG.33482.2	MSTRG.33482	Gm9888
MSTRG.33486.1	MSTRG.33486	.
MSTRG.33487.1	MSTRG.33487	.
MSTRG.33488.1	MSTRG.33488	.
MSTRG.33490.1	MSTRG.33490	Arpp21
MSTRG.33490.2	MSTRG.33490	Arpp21
MSTRG.33491.1	MSTRG.33491	Arpp21
MSTRG.33492.1	MSTRG.33492	Mir128-2
MSTRG.33493.1	MSTRG.33493	Arpp21
MSTRG.33495.1	MSTRG.33495	Arpp21
MSTRG.33496.4	MSTRG.33496	2900079G21Rik
MSTRG.335.1	MSTRG.335	Gm16150
MSTRG.335.2	MSTRG.335	Gm16150
MSTRG.33503.1	MSTRG.33503	Osbpl10
MSTRG.33504.1	MSTRG.33504	Osbpl10
MSTRG.33505.1	MSTRG.33505	Osbpl10
MSTRG.33506.1	MSTRG.33506	Osbpl10
MSTRG.33507.1	MSTRG.33507	Osbpl10
MSTRG.33508.1	MSTRG.33508	Osbpl10
MSTRG.33509.1	MSTRG.33509	Osbpl10
MSTRG.33511.1	MSTRG.33511	Osbpl10
MSTRG.33513.1	MSTRG.33513	Osbpl10
MSTRG.33515.1	MSTRG.33515	Osbpl10
MSTRG.33517.1	MSTRG.33517	Stt3b
MSTRG.33518.1	MSTRG.33518	Stt3b
MSTRG.33520.1	MSTRG.33520	Stt3b
MSTRG.33520.2	MSTRG.33520	Stt3b
MSTRG.33520.3	MSTRG.33520	Stt3b
MSTRG.33522.1	MSTRG.33522	Rbms3
MSTRG.33523.1	MSTRG.33523	Rbms3
MSTRG.33524.1	MSTRG.33524	Rbms3
MSTRG.33525.1	MSTRG.33525	Rbms3
MSTRG.33526.1	MSTRG.33526	Rbms3
MSTRG.33527.1	MSTRG.33527	Rbms3
MSTRG.33530.1	MSTRG.33530	Rbms3
MSTRG.33531.1	MSTRG.33531	Rbms3
MSTRG.33532.1	MSTRG.33532	Rbms3
MSTRG.33533.1	MSTRG.33533	Rbms3
MSTRG.33544.1	MSTRG.33544	Cmc1
MSTRG.33551.1	MSTRG.33551	Itga9
MSTRG.33556.1	MSTRG.33556	Ctdspl
MSTRG.33560.1	MSTRG.33560	.
MSTRG.33569.1	MSTRG.33569	Slc22a14
MSTRG.33571.7	MSTRG.33571	Acvr2b
MSTRG.33573.1	MSTRG.33573	Acvr2b
MSTRG.33576.1	MSTRG.33576	Exog
MSTRG.33577.1	MSTRG.33577	Exog
MSTRG.3359.1	MSTRG.3359	Gm10778
MSTRG.336.1	MSTRG.336	Aff3
MSTRG.3360.1	MSTRG.3360	Gm10778
MSTRG.33612.1	MSTRG.33612	Trak1
MSTRG.33613.1	MSTRG.33613	Trak1
MSTRG.33614.1	MSTRG.33614	Trak1
MSTRG.33621.1	MSTRG.33621	.

MSTRG.33629.1	MSTRG.33629	Ano10
MSTRG.33635.1	MSTRG.33635	AC124778.3
MSTRG.33636.1	MSTRG.33636	.
MSTRG.33642.1	MSTRG.33642	Tcaim
MSTRG.33644.1	MSTRG.33644	Gm35549
MSTRG.33645.1	MSTRG.33645	Gm35549
MSTRG.33657.1	MSTRG.33657	Cdcp1
MSTRG.3366.1	MSTRG.3366	Zfp433
MSTRG.33660.1	MSTRG.33660	AC133650.3
MSTRG.33663.1	MSTRG.33663	Exosc7
MSTRG.33667.1	MSTRG.33667	Lars2
MSTRG.33668.1	MSTRG.33668	Lars2
MSTRG.33669.1	MSTRG.33669	Lars2
MSTRG.33681.1	MSTRG.33681	Ccr9
MSTRG.33686.2	MSTRG.33686	Ppp2r3d
MSTRG.33687.1	MSTRG.33687	2010315B03Rik
MSTRG.33690.1	MSTRG.33690	2010315B03Rik
MSTRG.33691.1	MSTRG.33691	.
MSTRG.33693.1	MSTRG.33693	Ppp2r3d
MSTRG.33697.1	MSTRG.33697	Gm14359
MSTRG.33698.1	MSTRG.33698	.
MSTRG.33699.1	MSTRG.33699	.
MSTRG.337.1	MSTRG.337	Aff3
MSTRG.33712.1	MSTRG.33712	Gm36995
MSTRG.33718.1	MSTRG.33718	Otud5
MSTRG.33743.1	MSTRG.33743	.
MSTRG.33746.1	MSTRG.33746	.
MSTRG.33750.1	MSTRG.33750	Rpgr
MSTRG.33755.1	MSTRG.33755	Atp6ap2
MSTRG.33756.1	MSTRG.33756	.
MSTRG.33757.1	MSTRG.33757	.
MSTRG.33759.7	MSTRG.33759	Bcor
MSTRG.33759.8	MSTRG.33759	Bcor
MSTRG.33759.9	MSTRG.33759	Bcor
MSTRG.3376.1	MSTRG.3376	Zfp781
MSTRG.33760.1	MSTRG.33760	Bcor
MSTRG.33761.1	MSTRG.33761	Bcor
MSTRG.3377.1	MSTRG.3377	AC172027.3
MSTRG.33771.1	MSTRG.33771	2010308F09Rik
MSTRG.33774.1	MSTRG.33774	.
MSTRG.33775.1	MSTRG.33775	.
MSTRG.33780.1	MSTRG.33780	Cask
MSTRG.33781.1	MSTRG.33781	.
MSTRG.33782.1	MSTRG.33782	.
MSTRG.33783.1	MSTRG.33783	.
MSTRG.33785.1	MSTRG.33785	Fundc1
MSTRG.33787.1	MSTRG.33787	Kdm6a
MSTRG.33792.1	MSTRG.33792	Slc9a7
MSTRG.33793.1	MSTRG.33793	Slc9a7
MSTRG.33794.1	MSTRG.33794	Slc9a7
MSTRG.33798.1	MSTRG.33798	.
MSTRG.33800.1	MSTRG.33800	Jade3
MSTRG.33804.1	MSTRG.33804	Rbm10
MSTRG.33816.1	MSTRG.33816	.
MSTRG.33817.1	MSTRG.33817	.
MSTRG.33818.1	MSTRG.33818	.
MSTRG.33819.1	MSTRG.33819	.

MSTRG.33820.1	MSTRG.33820	.
MSTRG.33823.2	MSTRG.33823	A230072C01Rik
MSTRG.33826.1	MSTRG.33826	Zfp182
MSTRG.33827.1	MSTRG.33827	.
MSTRG.33829.1	MSTRG.33829	E330010L02Rik
MSTRG.33831.1	MSTRG.33831	Wdr44
MSTRG.33832.1	MSTRG.33832	Wdr44
MSTRG.33833.1	MSTRG.33833	Wdr44
MSTRG.33837.1	MSTRG.33837	.
MSTRG.33838.1	MSTRG.33838	.
MSTRG.33839.1	MSTRG.33839	.
MSTRG.33840.1	MSTRG.33840	.
MSTRG.33841.1	MSTRG.33841	.
MSTRG.33843.1	MSTRG.33843	.
MSTRG.33844.1	MSTRG.33844	.
MSTRG.33845.1	MSTRG.33845	.
MSTRG.33848.1	MSTRG.33848	Dock11
MSTRG.33854.1	MSTRG.33854	Sept6
MSTRG.33855.1	MSTRG.33855	Sept6
MSTRG.33861.1	MSTRG.33861	C330007P06Rik
MSTRG.33862.1	MSTRG.33862	C330007P06Rik
MSTRG.33864.1	MSTRG.33864	C330007P06Rik
MSTRG.33867.1	MSTRG.33867	.
MSTRG.33878.1	MSTRG.33878	.
MSTRG.33882.1	MSTRG.33882	Gm14643
MSTRG.33889.1	MSTRG.33889	Gria3
MSTRG.33895.1	MSTRG.33895	.
MSTRG.33896.1	MSTRG.33896	.
MSTRG.33897.1	MSTRG.33897	.
MSTRG.33898.1	MSTRG.33898	.
MSTRG.33899.1	MSTRG.33899	.
MSTRG.339.1	MSTRG.339	Aff3
MSTRG.3390.1	MSTRG.3390	Zfp873
MSTRG.33900.1	MSTRG.33900	.
MSTRG.33904.1	MSTRG.33904	Stag2
MSTRG.33905.1	MSTRG.33905	Stag2
MSTRG.33907.1	MSTRG.33907	.
MSTRG.33908.1	MSTRG.33908	.
MSTRG.33909.1	MSTRG.33909	.
MSTRG.33910.4	MSTRG.33910	Sh2d1a
MSTRG.33911.1	MSTRG.33911	Sh2d1a
MSTRG.33914.1	MSTRG.33914	Tenm1
MSTRG.33917.1	MSTRG.33917	Tenm1
MSTRG.33922.1	MSTRG.33922	Smarca1
MSTRG.33924.1	MSTRG.33924	Ocrl
MSTRG.33927.1	MSTRG.33927	.
MSTRG.33934.1	MSTRG.33934	Bcorl1
MSTRG.33937.1	MSTRG.33937	Aifm1
MSTRG.33940.1	MSTRG.33940	Zfp280c
MSTRG.33943.1	MSTRG.33943	.
MSTRG.33945.1	MSTRG.33945	Enox2
MSTRG.33946.1	MSTRG.33946	Enox2
MSTRG.33948.1	MSTRG.33948	Enox2
MSTRG.33949.1	MSTRG.33949	Enox2
MSTRG.33952.1	MSTRG.33952	Enox2
MSTRG.33953.1	MSTRG.33953	Enox2
MSTRG.33954.1	MSTRG.33954	Enox2

MSTRG.33955.1	MSTRG.33955	Enox2
MSTRG.33956.1	MSTRG.33956	Enox2
MSTRG.33956.2	MSTRG.33956	Enox2
MSTRG.33957.1	MSTRG.33957	Enox2
MSTRG.33960.4	MSTRG.33960	Firre
MSTRG.33960.8	MSTRG.33960	Firre
MSTRG.33961.1	MSTRG.33961	Firre
MSTRG.33962.1	MSTRG.33962	Firre
MSTRG.33965.1	MSTRG.33965	Mbnl3
MSTRG.33966.1	MSTRG.33966	Mbnl3
MSTRG.33967.1	MSTRG.33967	Mbnl3
MSTRG.33968.1	MSTRG.33968	.
MSTRG.3397.1	MSTRG.3397	.
MSTRG.33970.1	MSTRG.33970	Usp26
MSTRG.3399.1	MSTRG.3399	Tdg
MSTRG.33995.1	MSTRG.33995	.
MSTRG.340.1	MSTRG.340	Aff3
MSTRG.3400.1	MSTRG.3400	Tdg
MSTRG.34004.1	MSTRG.34004	Arhgef6
MSTRG.34005.1	MSTRG.34005	Arhgef6
MSTRG.34007.1	MSTRG.34007	Gm44333
MSTRG.34010.1	MSTRG.34010	.
MSTRG.34014.1	MSTRG.34014	Fgf13
MSTRG.34017.1	MSTRG.34017	Fgf13
MSTRG.34018.1	MSTRG.34018	Fgf13
MSTRG.34019.1	MSTRG.34019	Fgf13
MSTRG.34020.1	MSTRG.34020	Fgf13
MSTRG.34021.1	MSTRG.34021	Fgf13
MSTRG.34022.1	MSTRG.34022	Fgf13
MSTRG.34023.1	MSTRG.34023	Fgf13
MSTRG.34024.1	MSTRG.34024	Fgf13
MSTRG.34025.1	MSTRG.34025	Fgf13
MSTRG.34026.1	MSTRG.34026	Fgf13
MSTRG.34027.1	MSTRG.34027	Fgf13
MSTRG.34028.1	MSTRG.34028	Fgf13
MSTRG.34029.1	MSTRG.34029	Fgf13
MSTRG.3403.1	MSTRG.3403	Hcfc2
MSTRG.34030.1	MSTRG.34030	Fgf13
MSTRG.34031.1	MSTRG.34031	Fgf13
MSTRG.34033.1	MSTRG.34033	.
MSTRG.34035.1	MSTRG.34035	Atp11c
MSTRG.34037.1	MSTRG.34037	Atp11c
MSTRG.34038.1	MSTRG.34038	Atp11c
MSTRG.34048.1	MSTRG.34048	.
MSTRG.34049.1	MSTRG.34049	.
MSTRG.34050.1	MSTRG.34050	.
MSTRG.34052.1	MSTRG.34052	.
MSTRG.34058.1	MSTRG.34058	.
MSTRG.34064.1	MSTRG.34064	.
MSTRG.34067.1	MSTRG.34067	Mtm1
MSTRG.34068.1	MSTRG.34068	Mtm1
MSTRG.34072.1	MSTRG.34072	Cd99l2
MSTRG.34073.1	MSTRG.34073	Cd99l2
MSTRG.34074.1	MSTRG.34074	Cd99l2
MSTRG.34081.1	MSTRG.34081	Nsdhl
MSTRG.34098.1	MSTRG.34098	Xlr5a
MSTRG.341.1	MSTRG.341	Gm16152

MSTRG.341.3	MSTRG.341	Gm16152
MSTRG.341.6	MSTRG.341	Aff3
MSTRG.34101.2	MSTRG.34101	Bcap31
MSTRG.34110.1	MSTRG.34110	.
MSTRG.34114.1	MSTRG.34114	.
MSTRG.34116.1	MSTRG.34116	L1cam
MSTRG.34119.1	MSTRG.34119	Gm8545
MSTRG.34119.2	MSTRG.34119	Mecp2
MSTRG.3412.1	MSTRG.3412	Chst11
MSTRG.3412.2	MSTRG.3412	Chst11
MSTRG.34123.1	MSTRG.34123	Mecp2
MSTRG.34125.1	MSTRG.34125	.
MSTRG.34126.7	MSTRG.34126	Emd
MSTRG.34134.1	MSTRG.34134	.
MSTRG.34136.1	MSTRG.34136	.
MSTRG.34147.1	MSTRG.34147	Gab3
MSTRG.34149.1	MSTRG.34149	Gab3
MSTRG.34151.1	MSTRG.34151	Gab3
MSTRG.34152.1	MSTRG.34152	Gab3
MSTRG.34153.1	MSTRG.34153	Gab3
MSTRG.34154.1	MSTRG.34154	Gab3
MSTRG.34165.1	MSTRG.34165	Fundc2
MSTRG.34166.1	MSTRG.34166	Fundc2
MSTRG.34168.1	MSTRG.34168	Cmc4
MSTRG.34170.1	MSTRG.34170	Brcc3
MSTRG.34171.1	MSTRG.34171	Brcc3
MSTRG.34172.1	MSTRG.34172	Brcc3
MSTRG.34174.1	MSTRG.34174	Brcc3
MSTRG.34175.1	MSTRG.34175	.
MSTRG.34176.6	MSTRG.34176	4933407K13Rik
MSTRG.34176.7	MSTRG.34176	4933407K13Rik
MSTRG.34177.1	MSTRG.34177	Gm7153
MSTRG.34178.1	MSTRG.34178	4933407K13Rik
MSTRG.34179.1	MSTRG.34179	4933407K13Rik
MSTRG.34180.1	MSTRG.34180	4933407K13Rik
MSTRG.34181.1	MSTRG.34181	4933407K13Rik
MSTRG.34184.1	MSTRG.34184	Tbl1x
MSTRG.34185.1	MSTRG.34185	Tbl1x
MSTRG.34187.1	MSTRG.34187	Tbl1x
MSTRG.34188.1	MSTRG.34188	Prkx
MSTRG.34190.1	MSTRG.34190	Pbsn
MSTRG.34191.1	MSTRG.34191	.
MSTRG.34192.1	MSTRG.34192	.
MSTRG.34193.1	MSTRG.34193	.
MSTRG.34196.1	MSTRG.34196	Prrg1
MSTRG.34197.1	MSTRG.34197	Prrg1
MSTRG.34198.1	MSTRG.34198	Prrg1
MSTRG.34199.1	MSTRG.34199	Prrg1
MSTRG.34200.1	MSTRG.34200	Prrg1
MSTRG.34202.1	MSTRG.34202	Prrg1
MSTRG.34203.1	MSTRG.34203	Prrg1
MSTRG.34204.1	MSTRG.34204	Prrg1
MSTRG.34207.1	MSTRG.34207	.
MSTRG.34211.1	MSTRG.34211	.
MSTRG.34217.1	MSTRG.34217	Gk
MSTRG.34219.1	MSTRG.34219	Gk
MSTRG.34223.1	MSTRG.34223	.

MSTRG.34225.1	MSTRG.34225	.
MSTRG.34228.1	MSTRG.34228	.
MSTRG.34231.1	MSTRG.34231	Pola1
MSTRG.34231.2	MSTRG.34231	Pola1
MSTRG.34238.1	MSTRG.34238	Klhl15
MSTRG.34239.1	MSTRG.34239	Klhl15
MSTRG.34243.1	MSTRG.34243	.
MSTRG.34247.1	MSTRG.34247	Gm39526
MSTRG.34252.2	MSTRG.34252	.
MSTRG.34253.1	MSTRG.34253	.
MSTRG.34254.1	MSTRG.34254	.
MSTRG.34255.1	MSTRG.34255	.
MSTRG.34255.2	MSTRG.34255	.
MSTRG.34255.3	MSTRG.34255	.
MSTRG.34255.4	MSTRG.34255	.
MSTRG.34256.1	MSTRG.34256	.
MSTRG.34256.2	MSTRG.34256	.
MSTRG.34256.3	MSTRG.34256	.
MSTRG.34256.4	MSTRG.34256	.
MSTRG.34257.1	MSTRG.34257	.
MSTRG.3426.1	MSTRG.3426	Tcp11l2
MSTRG.34260.1	MSTRG.34260	Msn
MSTRG.34261.1	MSTRG.34261	Msn
MSTRG.34263.2	MSTRG.34263	Zc3h12b
MSTRG.34265.1	MSTRG.34265	Zc3h12b
MSTRG.34268.1	MSTRG.34268	Zc3h12b
MSTRG.34273.1	MSTRG.34273	Hsf3
MSTRG.34281.1	MSTRG.34281	.
MSTRG.34293.1	MSTRG.34293	Dlg3
MSTRG.34294.1	MSTRG.34294	Dlg3
MSTRG.34298.1	MSTRG.34298	Kif4
MSTRG.3430.1	MSTRG.3430	Polr3b
MSTRG.34301.1	MSTRG.34301	Snx12
MSTRG.34304.1	MSTRG.34304	.
MSTRG.34305.1	MSTRG.34305	.
MSTRG.3431.1	MSTRG.3431	Polr3b
MSTRG.34315.1	MSTRG.34315	Ogt
MSTRG.34323.1	MSTRG.34323	Gm14858
MSTRG.34324.1	MSTRG.34324	Hdac8
MSTRG.34325.1	MSTRG.34325	Hdac8
MSTRG.34333.1	MSTRG.34333	4930519F16Rik
MSTRG.34335.1	MSTRG.34335	Chic1
MSTRG.34337.4	MSTRG.34337	Tsix
MSTRG.34343.1	MSTRG.34343	Jpx
MSTRG.34350.2	MSTRG.34350	Ftx
MSTRG.34350.6	MSTRG.34350	Ftx
MSTRG.34350.8	MSTRG.34350	Ftx
MSTRG.3436.1	MSTRG.3436	Mterf2
MSTRG.34360.1	MSTRG.34360	Slc16a2
MSTRG.34364.4	MSTRG.34364	Rlim
MSTRG.34368.1	MSTRG.34368	Abcb7
MSTRG.34372.1	MSTRG.34372	Zdhhc15
MSTRG.34373.1	MSTRG.34373	Zdhhc15
MSTRG.34377.1	MSTRG.34377	5530601H04Rik
MSTRG.34378.1	MSTRG.34378	5530601H04Rik
MSTRG.3438.1	MSTRG.3438	Cry1
MSTRG.3439.1	MSTRG.3439	Cry1

MSTRG.34390.1	MSTRG.34390	Atrx
MSTRG.34391.1	MSTRG.34391	Atrx
MSTRG.34391.2	MSTRG.34391	Atrx
MSTRG.34392.1	MSTRG.34392	Atrx
MSTRG.34393.1	MSTRG.34393	Cox7b
MSTRG.34395.1	MSTRG.34395	.
MSTRG.344.1	MSTRG.344	Aff3
MSTRG.344.3	MSTRG.344	Aff3
MSTRG.344.4	MSTRG.344	Aff3
MSTRG.3440.1	MSTRG.3440	.
MSTRG.34402.1	MSTRG.34402	.
MSTRG.34403.1	MSTRG.34403	.
MSTRG.34404.1	MSTRG.34404	.
MSTRG.34410.1	MSTRG.34410	Brwd3
MSTRG.34411.1	MSTRG.34411	Brwd3
MSTRG.34413.1	MSTRG.34413	2810403D21Rik
MSTRG.34414.1	MSTRG.34414	2810403D21Rik
MSTRG.34418.1	MSTRG.34418	Sh3bgrl
MSTRG.3442.1	MSTRG.3442	Btbd11
MSTRG.34422.1	MSTRG.34422	Apool
MSTRG.34423.1	MSTRG.34423	Apool
MSTRG.34424.1	MSTRG.34424	.
MSTRG.34428.1	MSTRG.34428	Chm
MSTRG.34430.1	MSTRG.34430	.
MSTRG.34431.1	MSTRG.34431	.
MSTRG.34432.1	MSTRG.34432	.
MSTRG.34433.1	MSTRG.34433	.
MSTRG.34435.1	MSTRG.34435	.
MSTRG.34436.1	MSTRG.34436	.
MSTRG.34437.1	MSTRG.34437	.
MSTRG.34438.1	MSTRG.34438	.
MSTRG.34439.1	MSTRG.34439	.
MSTRG.34440.1	MSTRG.34440	.
MSTRG.34441.1	MSTRG.34441	.
MSTRG.34442.1	MSTRG.34442	.
MSTRG.34444.1	MSTRG.34444	.
MSTRG.34445.1	MSTRG.34445	.
MSTRG.34446.1	MSTRG.34446	.
MSTRG.34447.1	MSTRG.34447	.
MSTRG.34449.1	MSTRG.34449	Diaph2
MSTRG.34453.1	MSTRG.34453	Cstf2
MSTRG.34457.1	MSTRG.34457	Trmt2b
MSTRG.34459.1	MSTRG.34459	Trmt2b
MSTRG.34461.1	MSTRG.34461	Cenpi
MSTRG.34471.1	MSTRG.34471	Zmat1
MSTRG.34472.1	MSTRG.34472	.
MSTRG.34474.1	MSTRG.34474	.
MSTRG.34475.1	MSTRG.34475	.
MSTRG.34476.1	MSTRG.34476	.
MSTRG.34477.1	MSTRG.34477	.
MSTRG.34478.1	MSTRG.34478	.
MSTRG.34487.1	MSTRG.34487	Gprasp1
MSTRG.34491.1	MSTRG.34491	Kir3dl2
MSTRG.34505.1	MSTRG.34505	Il1rapl2
MSTRG.3451.1	MSTRG.3451	.
MSTRG.34510.1	MSTRG.34510	D330045A20Rik
MSTRG.34516.1	MSTRG.34516	Rbm41

MSTRG.34517.1	MSTRG.34517	Rbm41
MSTRG.3452.1	MSTRG.3452	.
MSTRG.34520.1	MSTRG.34520	Tsc22d3
MSTRG.34526.1	MSTRG.34526	.
MSTRG.34531.1	MSTRG.34531	Acsl4
MSTRG.34531.2	MSTRG.34531	Acsl4
MSTRG.34532.1	MSTRG.34532	Acsl4
MSTRG.34534.1	MSTRG.34534	Ammecr1
MSTRG.34538.1	MSTRG.34538	Mir652
MSTRG.34540.1	MSTRG.34540	Tmem164
MSTRG.34541.1	MSTRG.34541	.
MSTRG.34542.1	MSTRG.34542	.
MSTRG.34543.1	MSTRG.34543	.
MSTRG.34545.1	MSTRG.34545	Lhfpl1
MSTRG.34548.1	MSTRG.34548	Alg13
MSTRG.34550.1	MSTRG.34550	.
MSTRG.34551.1	MSTRG.34551	.
MSTRG.34553.1	MSTRG.34553	Gm15097
MSTRG.34554.1	MSTRG.34554	.
MSTRG.34559.1	MSTRG.34559	Gnl3l
MSTRG.34563.1	MSTRG.34563	Tmem29
MSTRG.34566.1	MSTRG.34566	.
MSTRG.34568.1	MSTRG.34568	.
MSTRG.34570.1	MSTRG.34570	Apex2
MSTRG.34573.1	MSTRG.34573	Apex2
MSTRG.34574.1	MSTRG.34574	Apex2
MSTRG.34582.1	MSTRG.34582	Fam120c
MSTRG.34584.1	MSTRG.34584	Fam120c
MSTRG.34599.1	MSTRG.34599	Kantr
MSTRG.34599.9	MSTRG.34599	Kantr
MSTRG.34613.1	MSTRG.34613	Gm15155
MSTRG.34615.1	MSTRG.34615	.
MSTRG.34619.1	MSTRG.34619	Phex
MSTRG.34622.1	MSTRG.34622	.
MSTRG.34625.1	MSTRG.34625	.
MSTRG.34630.1	MSTRG.34630	A830080D01Rik
MSTRG.34633.1	MSTRG.34633	Sh3kbp1
MSTRG.34634.1	MSTRG.34634	Sh3kbp1
MSTRG.34635.1	MSTRG.34635	Sh3kbp1
MSTRG.34636.1	MSTRG.34636	Sh3kbp1
MSTRG.34637.1	MSTRG.34637	Sh3kbp1
MSTRG.34647.1	MSTRG.34647	Cdkl5
MSTRG.34649.1	MSTRG.34649	Scml2
MSTRG.3465.1	MSTRG.3465	Ttc41
MSTRG.34653.1	MSTRG.34653	Nhs
MSTRG.34655.1	MSTRG.34655	Nhs
MSTRG.34661.1	MSTRG.34661	Txlng
MSTRG.34662.1	MSTRG.34662	Txlng
MSTRG.34663.1	MSTRG.34663	Txlng
MSTRG.34665.1	MSTRG.34665	Ap1s2
MSTRG.3467.1	MSTRG.3467	Nt5dc3
MSTRG.34670.1	MSTRG.34670	Ctps2
MSTRG.34673.4	MSTRG.34673	Zrsr2
MSTRG.34675.1	MSTRG.34675	.
MSTRG.3468.1	MSTRG.3468	Nt5dc3
MSTRG.34685.1	MSTRG.34685	Ofd1
MSTRG.34687.1	MSTRG.34687	.

MSTRG.34689.1	MSTRG.34689	.
MSTRG.3469.1	MSTRG.3469	Nt5dc3
MSTRG.34693.1	MSTRG.34693	Prps2
MSTRG.34699.1	MSTRG.34699	Frmpd4
MSTRG.347.1	MSTRG.347	Npas2
MSTRG.34712.1	MSTRG.34712	Mid1
MSTRG.34716.1	MSTRG.34716	Gm15247
MSTRG.34717.1	MSTRG.34717	Mid1
MSTRG.34718.1	MSTRG.34718	Mid1
MSTRG.34718.2	MSTRG.34718	Mid1
MSTRG.34719.1	MSTRG.34719	Mid1
MSTRG.34719.2	MSTRG.34719	Mid1
MSTRG.34720.1	MSTRG.34720	Mid1
MSTRG.34721.10	MSTRG.34721	Gm21887
MSTRG.34721.11	MSTRG.34721	Gm21887
MSTRG.34721.12	MSTRG.34721	Gm21887
MSTRG.34721.13	MSTRG.34721	Gm21887
MSTRG.34721.15	MSTRG.34721	Gm21887
MSTRG.34721.16	MSTRG.34721	Gm21887
MSTRG.34721.2	MSTRG.34721	Gm21887
MSTRG.34721.3	MSTRG.34721	Gm21887
MSTRG.34721.4	MSTRG.34721	Gm21887
MSTRG.34721.5	MSTRG.34721	Gm21887
MSTRG.34721.6	MSTRG.34721	Gm21887
MSTRG.34721.7	MSTRG.34721	Gm21887
MSTRG.34722.1	MSTRG.34722	Gm21887
MSTRG.34723.1	MSTRG.34723	Gm21887
MSTRG.34723.10	MSTRG.34723	Gm21887
MSTRG.34723.11	MSTRG.34723	Gm21887
MSTRG.34723.5	MSTRG.34723	Gm21887
MSTRG.34723.6	MSTRG.34723	Gm21887
MSTRG.34723.7	MSTRG.34723	Gm21887
MSTRG.34723.9	MSTRG.34723	Gm21887
MSTRG.34724.1	MSTRG.34724	.
MSTRG.34731.1	MSTRG.34731	.
MSTRG.34732.1	MSTRG.34732	.
MSTRG.34736.1	MSTRG.34736	Uty
MSTRG.34737.1	MSTRG.34737	Uty
MSTRG.34738.1	MSTRG.34738	Uty
MSTRG.34740.1	MSTRG.34740	.
MSTRG.34741.1	MSTRG.34741	.
MSTRG.34742.1	MSTRG.34742	.
MSTRG.34743.1	MSTRG.34743	.
MSTRG.34745.1	MSTRG.34745	.
MSTRG.34746.1	MSTRG.34746	.
MSTRG.34747.1	MSTRG.34747	.
MSTRG.34749.1	MSTRG.34749	Gm28930
MSTRG.34750.1	MSTRG.34750	.
MSTRG.34750.2	MSTRG.34750	.
MSTRG.34751.1	MSTRG.34751	.
MSTRG.34751.2	MSTRG.34751	.
MSTRG.34752.1	MSTRG.34752	Mid1-ps1
MSTRG.34752.10	MSTRG.34752	.
MSTRG.34752.12	MSTRG.34752	Erdr1
MSTRG.34752.13	MSTRG.34752	Erdr1
MSTRG.34752.14	MSTRG.34752	Erdr1
MSTRG.34752.15	MSTRG.34752	Erdr1

MSTRG.34752.16	MSTRG.34752	Erdr1
MSTRG.34752.17	MSTRG.34752	Erdr1
MSTRG.34752.5	MSTRG.34752	Mid1-ps1
MSTRG.34752.6	MSTRG.34752	Mid1-ps1
MSTRG.34752.7	MSTRG.34752	Mid1-ps1
MSTRG.34752.8	MSTRG.34752	Mid1-ps1
MSTRG.34752.9	MSTRG.34752	Mid1-ps1
MSTRG.34754.1	MSTRG.34754	.
MSTRG.34755.1	MSTRG.34755	.
MSTRG.34756.1	MSTRG.34756	.
MSTRG.34756.2	MSTRG.34756	.
MSTRG.34757.1	MSTRG.34757	Gm21860
MSTRG.34757.2	MSTRG.34757	Gm21860
MSTRG.34758.1	MSTRG.34758	.
MSTRG.34759.1	MSTRG.34759	.
MSTRG.3476.1	MSTRG.3476	Parpbp
MSTRG.34760.1	MSTRG.34760	Gm21860
MSTRG.34760.2	MSTRG.34760	Gm21860
MSTRG.34760.3	MSTRG.34760	Gm21860
MSTRG.34762.1	MSTRG.34762	Mid1-ps1
MSTRG.34762.2	MSTRG.34762	Mid1-ps1
MSTRG.34762.3	MSTRG.34762	Mid1-ps1
MSTRG.34762.4	MSTRG.34762	.
MSTRG.34763.1	MSTRG.34763	Mid1-ps1
MSTRG.34763.2	MSTRG.34763	Mid1-ps1
MSTRG.34764.3	MSTRG.34764	Erdr1
MSTRG.34764.5	MSTRG.34764	Erdr1
MSTRG.34765.1	MSTRG.34765	Gm21748
MSTRG.3477.1	MSTRG.3477	Parpbp
MSTRG.3480.1	MSTRG.3480	Parpbp
MSTRG.3482.1	MSTRG.3482	Nup37
MSTRG.3485.1	MSTRG.3485	Washc3
MSTRG.3492.1	MSTRG.3492	Chpt1
MSTRG.3497.1	MSTRG.3497	Gas2l3
MSTRG.3498.1	MSTRG.3498	Gas2l3
MSTRG.3499.4	MSTRG.3499	Nr1h4
MSTRG.3500.1	MSTRG.3500	Nr1h4
MSTRG.3501.1	MSTRG.3501	Nr1h4
MSTRG.3505.1	MSTRG.3505	Actr6
MSTRG.3506.1	MSTRG.3506	Actr6
MSTRG.3511.1	MSTRG.3511	Apaf1
MSTRG.3513.1	MSTRG.3513	Apaf1
MSTRG.3514.1	MSTRG.3514	Apaf1
MSTRG.3517.1	MSTRG.3517	Anks1b
MSTRG.352.1	MSTRG.352	Tbc1d8
MSTRG.3532.1	MSTRG.3532	Nedd1
MSTRG.3534.1	MSTRG.3534	Nedd1
MSTRG.3535.1	MSTRG.3535	.
MSTRG.3536.1	MSTRG.3536	.
MSTRG.3539.1	MSTRG.3539	Cdk17
MSTRG.354.1	MSTRG.354	Tbc1d8
MSTRG.3540.1	MSTRG.3540	Cdk17
MSTRG.3540.2	MSTRG.3540	Cdk17
MSTRG.3541.1	MSTRG.3541	Cdk17
MSTRG.3542.1	MSTRG.3542	Cdk17
MSTRG.3544.1	MSTRG.3544	Cdk17
MSTRG.3545.1	MSTRG.3545	Cdk17

MSTRG.3547.1	MSTRG.3547	Elk3
MSTRG.3548.2	MSTRG.3548	Elk3
MSTRG.3549.1	MSTRG.3549	Elk3
MSTRG.355.1	MSTRG.355	Tbc1d8
MSTRG.3553.1	MSTRG.3553	Hal
MSTRG.356.1	MSTRG.356	Tbc1d8
MSTRG.3560.1	MSTRG.3560	Usp44
MSTRG.3561.1	MSTRG.3561	Usp44
MSTRG.3563.1	MSTRG.3563	Metap2
MSTRG.3566.1	MSTRG.3566	Vezt
MSTRG.3571.1	MSTRG.3571	Nr2c1
MSTRG.3572.1	MSTRG.3572	Nr2c1
MSTRG.3574.1	MSTRG.3574	Nr2c1
MSTRG.3578.1	MSTRG.3578	Tmcc3
MSTRG.3579.1	MSTRG.3579	Tmcc3
MSTRG.3586.1	MSTRG.3586	Cep83
MSTRG.3587.1	MSTRG.3587	Cep83
MSTRG.359.1	MSTRG.359	Rnf149
MSTRG.3591.1	MSTRG.3591	Plxnc1
MSTRG.3593.1	MSTRG.3593	Cradd
MSTRG.3594.1	MSTRG.3594	Cradd
MSTRG.3595.1	MSTRG.3595	Cradd
MSTRG.3596.1	MSTRG.3596	Cradd
MSTRG.36.1	MSTRG.36	.
MSTRG.3603.1	MSTRG.3603	Ube2n
MSTRG.3605.1	MSTRG.3605	Mir3058
MSTRG.3607.1	MSTRG.3607	4732465J04Rik
MSTRG.3608.1	MSTRG.3608	4732465J04Rik
MSTRG.3613.1	MSTRG.3613	AC153365.2
MSTRG.3617.1	MSTRG.3617	.
MSTRG.3618.1	MSTRG.3618	.
MSTRG.3620.1	MSTRG.3620	Atp2b1
MSTRG.3621.1	MSTRG.3621	Atp2b1
MSTRG.3622.1	MSTRG.3622	Atp2b1
MSTRG.3629.1	MSTRG.3629	AC153821.1
MSTRG.363.1	MSTRG.363	Il1r1
MSTRG.3636.1	MSTRG.3636	Tmtc3
MSTRG.3637.1	MSTRG.3637	.
MSTRG.364.1	MSTRG.364	Il1r1
MSTRG.3642.1	MSTRG.3642	Tmtc2
MSTRG.3643.1	MSTRG.3643	Tmtc2
MSTRG.3650.1	MSTRG.3650	Mettl25
MSTRG.3653.1	MSTRG.3653	Mettl25
MSTRG.366.1	MSTRG.366	Map4k4
MSTRG.3663.1	MSTRG.3663	Acss3
MSTRG.3665.1	MSTRG.3665	Pawr
MSTRG.3668.1	MSTRG.3668	Ppp1r12a
MSTRG.3670.1	MSTRG.3670	.
MSTRG.3678.1	MSTRG.3678	Csrp2
MSTRG.368.1	MSTRG.368	Il1rl2
MSTRG.3680.1	MSTRG.3680	Zdhhc17
MSTRG.3684.1	MSTRG.3684	AC124399.3
MSTRG.3685.1	MSTRG.3685	Osbpl8
MSTRG.3691.3	MSTRG.3691	Glipr1
MSTRG.3693.1	MSTRG.3693	1700010J16Rik
MSTRG.3702.1	MSTRG.3702	Thap2
MSTRG.3702.3	MSTRG.3702	Thap2

MSTRG.371.1	MSTRG.371	Il1rl1
MSTRG.3711.1	MSTRG.3711	Kcnmb4
MSTRG.3712.1	MSTRG.3712	Kcnmb4
MSTRG.3713.1	MSTRG.3713	Kcnmb4
MSTRG.3715.1	MSTRG.3715	.
MSTRG.3716.1	MSTRG.3716	.
MSTRG.3717.1	MSTRG.3717	.
MSTRG.3719.1	MSTRG.3719	Cnot2
MSTRG.3719.2	MSTRG.3719	Cnot2
MSTRG.3720.1	MSTRG.3720	5330438D12Rik
MSTRG.3722.1	MSTRG.3722	Cnot2
MSTRG.3725.3	MSTRG.3725	AC123720.1
MSTRG.3732.1	MSTRG.3732	Frs2
MSTRG.3733.1	MSTRG.3733	Frs2
MSTRG.3734.1	MSTRG.3734	Frs2
MSTRG.374.1	MSTRG.374	.
MSTRG.3741.1	MSTRG.3741	Cpsf6
MSTRG.3749.1	MSTRG.3749	Mdm1
MSTRG.3750.1	MSTRG.3750	Mdm1
MSTRG.3751.1	MSTRG.3751	Mdm1
MSTRG.3754.1	MSTRG.3754	AC153495.1
MSTRG.376.1	MSTRG.376	Gm28140
MSTRG.3760.1	MSTRG.3760	Irak3
MSTRG.3763.1	MSTRG.3763	Tmbim4
MSTRG.3764.1	MSTRG.3764	Tmbim4
MSTRG.3771.1	MSTRG.3771	Grip1
MSTRG.3773.1	MSTRG.3773	Grip1
MSTRG.3777.1	MSTRG.3777	Grip1
MSTRG.3784.1	MSTRG.3784	Msrb3
MSTRG.3786.1	MSTRG.3786	Lemd3
MSTRG.3790.1	MSTRG.3790	Lemd3
MSTRG.3791.1	MSTRG.3791	Lemd3
MSTRG.3792.1	MSTRG.3792	Lemd3
MSTRG.3797.1	MSTRG.3797	Rassf3
MSTRG.3799.1	MSTRG.3799	Tbk1
MSTRG.38.1	MSTRG.38	.
MSTRG.3806.1	MSTRG.3806	AC160029.3
MSTRG.3807.1	MSTRG.3807	.
MSTRG.381.1	MSTRG.381	.
MSTRG.3810.1	MSTRG.3810	.
MSTRG.3812.1	MSTRG.3812	Ppm1h
MSTRG.3812.2	MSTRG.3812	Ppm1h
MSTRG.3812.3	MSTRG.3812	Ppm1h
MSTRG.3814.1	MSTRG.3814	Ppm1h
MSTRG.3814.2	MSTRG.3814	AC110381.3
MSTRG.3814.3	MSTRG.3814	AC110381.3
MSTRG.3816.1	MSTRG.3816	AC110381.2
MSTRG.3819.1	MSTRG.3819	Ppm1h
MSTRG.3820.1	MSTRG.3820	Ppm1h
MSTRG.3821.1	MSTRG.3821	Ppm1h
MSTRG.3822.1	MSTRG.3822	Ppm1h
MSTRG.3823.1	MSTRG.3823	Ppm1h
MSTRG.3825.5	MSTRG.3825	Ppm1h
MSTRG.3827.1	MSTRG.3827	Mon2
MSTRG.3829.1	MSTRG.3829	Fam19a2
MSTRG.3833.1	MSTRG.3833	.
MSTRG.3836.1	MSTRG.3836	Usp15

MSTRG.3837.1	MSTRG.3837	Usp15
MSTRG.3838.1	MSTRG.3838	Usp15
MSTRG.3841.1	MSTRG.3841	.
MSTRG.3852.1	MSTRG.3852	Os9
MSTRG.3860.1	MSTRG.3860	Pip4k2c
MSTRG.3863.6	MSTRG.3863	Mbd6
MSTRG.3879.1	MSTRG.3879	R3hdm2
MSTRG.3880.1	MSTRG.3880	R3hdm2
MSTRG.3891.1	MSTRG.3891	Nemp1
MSTRG.3892.1	MSTRG.3892	Nemp1
MSTRG.3897.3	MSTRG.3897	Ptges3
MSTRG.3897.5	MSTRG.3897	Ptges3
MSTRG.3907.2	MSTRG.3907	Gm26847
MSTRG.3909.1	MSTRG.3909	Timeless
MSTRG.3912.1	MSTRG.3912	Stat2
MSTRG.3913.1	MSTRG.3913	Stat2
MSTRG.3919.1	MSTRG.3919	.
MSTRG.3923.2	MSTRG.3923	Slc39a5
MSTRG.3927.1	MSTRG.3927	.
MSTRG.3928.1	MSTRG.3928	.
MSTRG.3930.1	MSTRG.3930	Rpl41
MSTRG.3937.1	MSTRG.3937	A430046D13Rik
MSTRG.3938.1	MSTRG.3938	A430046D13Rik
MSTRG.3952.1	MSTRG.3952	Pym1
MSTRG.3961.1	MSTRG.3961	.
MSTRG.3962.1	MSTRG.3962	.
MSTRG.3966.1	MSTRG.3966	Tespa1
MSTRG.3970.1	MSTRG.3970	AC226737.1
MSTRG.3972.1	MSTRG.3972	.
MSTRG.3974.1	MSTRG.3974	Rnf185
MSTRG.3975.3	MSTRG.3975	8430429K09Rik
MSTRG.3976.1	MSTRG.3976	8430429K09Rik
MSTRG.398.1	MSTRG.398	Uxs1
MSTRG.399.1	MSTRG.399	Uxs1
MSTRG.3990.1	MSTRG.3990	Gm11399
MSTRG.3990.2	MSTRG.3990	Gm11399
MSTRG.3991.1	MSTRG.3991	Gm11399
MSTRG.3996.1	MSTRG.3996	Drg1
MSTRG.40.1	MSTRG.40	.
MSTRG.400.1	MSTRG.400	Uxs1
MSTRG.4007.1	MSTRG.4007	Ccdc157
MSTRG.4011.1	MSTRG.4011	Gm11960
MSTRG.4011.2	MSTRG.4011	Mtmr3
MSTRG.4013.1	MSTRG.4013	Mtmr3
MSTRG.4013.2	MSTRG.4013	Mtmr3
MSTRG.4013.3	MSTRG.4013	Mtmr3
MSTRG.402.1	MSTRG.402	Tpp2
MSTRG.4021.1	MSTRG.4021	Nf2
MSTRG.4022.1	MSTRG.4022	Nf2
MSTRG.4023.1	MSTRG.4023	Nf2
MSTRG.4024.1	MSTRG.4024	Nf2
MSTRG.4026.1	MSTRG.4026	Nipsnap1
MSTRG.4036.1	MSTRG.4036	Ewsr1
MSTRG.4039.1	MSTRG.4039	Emid1
MSTRG.4040.1	MSTRG.4040	Emid1
MSTRG.4041.1	MSTRG.4041	.
MSTRG.4043.1	MSTRG.4043	Ccdc117

MSTRG.4045.1	MSTRG.4045	.
MSTRG.4047.1	MSTRG.4047	Gm25142
MSTRG.4048.1	MSTRG.4048	Znrf3
MSTRG.4049.1	MSTRG.4049	Znrf3
MSTRG.405.1	MSTRG.405	.
MSTRG.4050.1	MSTRG.4050	Znrf3
MSTRG.4050.2	MSTRG.4050	Znrf3
MSTRG.4051.1	MSTRG.4051	Znrf3
MSTRG.4053.2	MSTRG.4053	Polm
MSTRG.4058.1	MSTRG.4058	Urgcp
MSTRG.4059.1	MSTRG.4059	Urgcp
MSTRG.406.1	MSTRG.406	.
MSTRG.4061.1	MSTRG.4061	Ube2d-ps
MSTRG.4067.1	MSTRG.4067	Nudcd3
MSTRG.4079.1	MSTRG.4079	Ogdh
MSTRG.408.1	MSTRG.408	Ercc5
MSTRG.4084.1	MSTRG.4084	.
MSTRG.4085.1	MSTRG.4085	.
MSTRG.4087.1	MSTRG.4087	.
MSTRG.4091.1	MSTRG.4091	Pkd1l1
MSTRG.4093.1	MSTRG.4093	Gm11973
MSTRG.4093.3	MSTRG.4093	Gm11973
MSTRG.4095.3	MSTRG.4095	Snhg15
MSTRG.4096.1	MSTRG.4096	Ccm2
MSTRG.4099.1	MSTRG.4099	Gm11975
MSTRG.4099.2	MSTRG.4099	Gm11975
MSTRG.4100.1	MSTRG.4100	Ccm2
MSTRG.4101.1	MSTRG.4101	Ccm2
MSTRG.4108.1	MSTRG.4108	.
MSTRG.4109.1	MSTRG.4109	.
MSTRG.411.9	MSTRG.411	Tex30
MSTRG.4111.1	MSTRG.4111	Abca13
MSTRG.4112.1	MSTRG.4112	Abca13
MSTRG.4117.1	MSTRG.4117	Zpbp
MSTRG.4121.1	MSTRG.4121	Gm11998
MSTRG.4121.2	MSTRG.4121	Gm11998
MSTRG.4122.1	MSTRG.4122	.
MSTRG.4124.1	MSTRG.4124	4930512M02Rik
MSTRG.413.1	MSTRG.413	.
MSTRG.4131.1	MSTRG.4131	.
MSTRG.4134.1	MSTRG.4134	.
MSTRG.4135.1	MSTRG.4135	.
MSTRG.4136.1	MSTRG.4136	.
MSTRG.4142.1	MSTRG.4142	Egfr
MSTRG.4143.1	MSTRG.4143	Egfr
MSTRG.4144.1	MSTRG.4144	Egfr
MSTRG.4146.1	MSTRG.4146	Egfr
MSTRG.4148.1	MSTRG.4148	Eldr
MSTRG.415.1	MSTRG.415	.
MSTRG.4155.1	MSTRG.4155	Ppp3r1
MSTRG.4157.1	MSTRG.4157	Wdr92
MSTRG.4158.1	MSTRG.4158	Wdr92
MSTRG.4160.1	MSTRG.4160	C1d
MSTRG.4161.1	MSTRG.4161	.
MSTRG.4167.1	MSTRG.4167	Meis1
MSTRG.4171.1	MSTRG.4171	.
MSTRG.4173.1	MSTRG.4173	Spred2

MSTRG.4175.1	MSTRG.4175	Actr2
MSTRG.4176.1	MSTRG.4176	Actr2
MSTRG.4177.1	MSTRG.4177	Actr2
MSTRG.4178.1	MSTRG.4178	Actr2
MSTRG.4179.1	MSTRG.4179	Actr2
MSTRG.4185.1	MSTRG.4185	.
MSTRG.4186.1	MSTRG.4186	.
MSTRG.4187.1	MSTRG.4187	.
MSTRG.4190.1	MSTRG.4190	Sertad2
MSTRG.4191.1	MSTRG.4191	Sertad2
MSTRG.4194.1	MSTRG.4194	Aftph
MSTRG.4195.1	MSTRG.4195	Aftph
MSTRG.4197.1	MSTRG.4197	Aftph
MSTRG.4201.1	MSTRG.4201	.
MSTRG.4204.1	MSTRG.4204	Vps54
MSTRG.4206.1	MSTRG.4206	Vps54
MSTRG.4211.1	MSTRG.4211	Ugp2
MSTRG.4216.1	MSTRG.4216	Wdpcp
MSTRG.4217.1	MSTRG.4217	Wdpcp
MSTRG.4218.1	MSTRG.4218	Wdpcp
MSTRG.422.1	MSTRG.422	.
MSTRG.4220.1	MSTRG.4220	Wdpcp
MSTRG.4223.1	MSTRG.4223	Ehbp1
MSTRG.4225.1	MSTRG.4225	Gm12057
MSTRG.4227.1	MSTRG.4227	9130230N09Rik
MSTRG.4228.1	MSTRG.4228	Gm12057
MSTRG.4229.1	MSTRG.4229	Gm20456
MSTRG.423.1	MSTRG.423	.
MSTRG.4232.1	MSTRG.4232	Gm28048
MSTRG.4234.1	MSTRG.4234	.
MSTRG.4237.1	MSTRG.4237	.
MSTRG.424.1	MSTRG.424	Gm37196
MSTRG.4241.1	MSTRG.4241	Usp34
MSTRG.4242.1	MSTRG.4242	Usp34
MSTRG.4249.1	MSTRG.4249	0610010F05Rik
MSTRG.4251.1	MSTRG.4251	0610010F05Rik
MSTRG.4254.1	MSTRG.4254	Pus10
MSTRG.4260.1	MSTRG.4260	Bcl11a
MSTRG.4263.1	MSTRG.4263	.
MSTRG.4264.1	MSTRG.4264	.
MSTRG.4268.1	MSTRG.4268	Fancl
MSTRG.4270.1	MSTRG.4270	Fancl
MSTRG.4272.1	MSTRG.4272	Fancl
MSTRG.4273.1	MSTRG.4273	Fancl
MSTRG.4280.1	MSTRG.4280	A630052C17Rik
MSTRG.4284.1	MSTRG.4284	Cfap36
MSTRG.4285.1	MSTRG.4285	Cfap36
MSTRG.4288.1	MSTRG.4288	Ccdc88a
MSTRG.4289.1	MSTRG.4289	Ccdc88a
MSTRG.429.1	MSTRG.429	Slc39a10
MSTRG.4292.1	MSTRG.4292	Gm12089
MSTRG.4298.1	MSTRG.4298	Gm12093
MSTRG.4301.1	MSTRG.4301	Rtn4
MSTRG.4309.1	MSTRG.4309	Sptbn1
MSTRG.431.1	MSTRG.431	.
MSTRG.4310.1	MSTRG.4310	Sptbn1
MSTRG.4316.1	MSTRG.4316	Acyp2

MSTRG.4317.1	MSTRG.4317	Acyp2
MSTRG.4318.1	MSTRG.4318	Acyp2
MSTRG.4320.1	MSTRG.4320	Psme4
MSTRG.4323.1	MSTRG.4323	Psme4
MSTRG.4324.1	MSTRG.4324	.
MSTRG.4325.1	MSTRG.4325	.
MSTRG.4329.1	MSTRG.4329	Asb3
MSTRG.433.1	MSTRG.433	C230029F24Rik
MSTRG.4331.1	MSTRG.4331	Erlec1
MSTRG.4332.1	MSTRG.4332	Asb3
MSTRG.4333.1	MSTRG.4333	Asb3
MSTRG.4335.1	MSTRG.4335	Asb3
MSTRG.4337.1	MSTRG.4337	Asb3
MSTRG.4338.1	MSTRG.4338	Asb3
MSTRG.4339.1	MSTRG.4339	Asb3
MSTRG.4340.1	MSTRG.4340	Asb3
MSTRG.4341.1	MSTRG.4341	Asb3
MSTRG.4342.1	MSTRG.4342	Asb3
MSTRG.4343.1	MSTRG.4343	Asb3
MSTRG.4347.1	MSTRG.4347	Nsg2
MSTRG.435.1	MSTRG.435	.
MSTRG.4354.1	MSTRG.4354	Nprl3
MSTRG.4357.1	MSTRG.4357	Ubtd2
MSTRG.4359.1	MSTRG.4359	Ubtd2
MSTRG.436.1	MSTRG.436	.
MSTRG.4360.1	MSTRG.4360	Ubtd2
MSTRG.4364.1	MSTRG.4364	Gm12114
MSTRG.4365.1	MSTRG.4365	Fbxw11
MSTRG.4367.1	MSTRG.4367	Fbxw11
MSTRG.4368.1	MSTRG.4368	Fbxw11
MSTRG.4369.1	MSTRG.4369	Fbxw11
MSTRG.437.1	MSTRG.437	.
MSTRG.4376.1	MSTRG.4376	.
MSTRG.4384.1	MSTRG.4384	4930469K13Rik
MSTRG.4394.1	MSTRG.4394	Dock2
MSTRG.4397.1	MSTRG.4397	Dock2
MSTRG.4398.1	MSTRG.4398	Dock2
MSTRG.4400.1	MSTRG.4400	Dock2
MSTRG.4401.1	MSTRG.4401	Dock2
MSTRG.4403.1	MSTRG.4403	Spdl1
MSTRG.4405.1	MSTRG.4405	.
MSTRG.4406.1	MSTRG.4406	.
MSTRG.4409.1	MSTRG.4409	Hmmr
MSTRG.441.10	MSTRG.441	Nabp1
MSTRG.441.3	MSTRG.441	Nabp1
MSTRG.4416.1	MSTRG.4416	.
MSTRG.4421.1	MSTRG.4421	Ccnjl
MSTRG.4427.1	MSTRG.4427	Ublcp1
MSTRG.4428.1	MSTRG.4428	Ublcp1
MSTRG.4430.1	MSTRG.4430	Ublcp1
MSTRG.4431.1	MSTRG.4431	.
MSTRG.4433.1	MSTRG.4433	.
MSTRG.4438.1	MSTRG.4438	Ebf1
MSTRG.4439.1	MSTRG.4439	Ebf1
MSTRG.444.1	MSTRG.444	Myo1b
MSTRG.4440.1	MSTRG.4440	Ebf1
MSTRG.4441.1	MSTRG.4441	Ebf1

MSTRG.4442.1	MSTRG.4442	Ebf1
MSTRG.4443.1	MSTRG.4443	Ebf1
MSTRG.4445.1	MSTRG.4445	Ebf1
MSTRG.4446.1	MSTRG.4446	Ebf1
MSTRG.4447.1	MSTRG.4447	Ebf1
MSTRG.4448.1	MSTRG.4448	Ebf1
MSTRG.4450.1	MSTRG.4450	Clint1
MSTRG.4450.2	MSTRG.4450	Clint1
MSTRG.4452.1	MSTRG.4452	Lsm11
MSTRG.4461.1	MSTRG.4461	.
MSTRG.4465.1	MSTRG.4465	Gm12167
MSTRG.4466.1	MSTRG.4466	.
MSTRG.4473.1	MSTRG.4473	Sgcd
MSTRG.4477.1	MSTRG.4477	.
MSTRG.4478.1	MSTRG.4478	.
MSTRG.4479.1	MSTRG.4479	.
MSTRG.4480.1	MSTRG.4480	.
MSTRG.4498.1	MSTRG.4498	.
MSTRG.4503.1	MSTRG.4503	Cnot6
MSTRG.4504.1	MSTRG.4504	.
MSTRG.4510.1	MSTRG.4510	Tbc1d9b
MSTRG.4513.1	MSTRG.4513	.
MSTRG.4519.1	MSTRG.4519	Gm26542
MSTRG.4533.1	MSTRG.4533	.
MSTRG.4539.1	MSTRG.4539	Jade2
MSTRG.454.1	MSTRG.454	Inpp1
MSTRG.4540.1	MSTRG.4540	.
MSTRG.4540.2	MSTRG.4540	.
MSTRG.4540.3	MSTRG.4540	.
MSTRG.4540.4	MSTRG.4540	.
MSTRG.4540.5	MSTRG.4540	.
MSTRG.4540.6	MSTRG.4540	.
MSTRG.4540.7	MSTRG.4540	.
MSTRG.4541.1	MSTRG.4541	Gm39822
MSTRG.4544.1	MSTRG.4544	.
MSTRG.4545.1	MSTRG.4545	.
MSTRG.4546.1	MSTRG.4546	.
MSTRG.4554.1	MSTRG.4554	Ube2b
MSTRG.4554.2	MSTRG.4554	Ube2b
MSTRG.4565.5	MSTRG.4565	Skp1a
MSTRG.4567.1	MSTRG.4567	A630014C17Rik
MSTRG.4567.2	MSTRG.4567	A630014C17Rik
MSTRG.4570.1	MSTRG.4570	Hspa4
MSTRG.4579.1	MSTRG.4579	Il4
MSTRG.4581.1	MSTRG.4581	.
MSTRG.4585.1	MSTRG.4585	Rad50
MSTRG.4587.1	MSTRG.4587	Slc22a5
MSTRG.4588.1	MSTRG.4588	.
MSTRG.459.1	MSTRG.459	Stat4
MSTRG.4591.8	MSTRG.4591	Gm12216
MSTRG.4592.1	MSTRG.4592	Gm12216
MSTRG.4595.1	MSTRG.4595	Meikin
MSTRG.4598.1	MSTRG.4598	.
MSTRG.4599.1	MSTRG.4599	.
MSTRG.460.1	MSTRG.460	Stat4
MSTRG.4600.1	MSTRG.4600	Cdc42se2
MSTRG.4600.6	MSTRG.4600	Cdc42se2

MSTRG.4602.1	MSTRG.4602	Cdc42se2
MSTRG.4604.5	MSTRG.4604	Lyrm7
MSTRG.4606.1	MSTRG.4606	Lyrm7
MSTRG.4607.1	MSTRG.4607	Lyrm7
MSTRG.4609.1	MSTRG.4609	Fnip1
MSTRG.461.1	MSTRG.461	Stat4
MSTRG.4613.4	MSTRG.4613	Gpx3
MSTRG.4618.1	MSTRG.4618	Ccdc69
MSTRG.4625.1	MSTRG.4625	G3bp1
MSTRG.4629.1	MSTRG.4629	Mfap3
MSTRG.4636.1	MSTRG.4636	Galnt10
MSTRG.4638.1	MSTRG.4638	Galnt10
MSTRG.4639.1	MSTRG.4639	Galnt10
MSTRG.4641.1	MSTRG.4641	Galnt10
MSTRG.4643.3	MSTRG.4643	2010001A14Rik
MSTRG.4643.5	MSTRG.4643	2010001A14Rik
MSTRG.4643.6	MSTRG.4643	2010001A14Rik
MSTRG.4644.1	MSTRG.4644	2010001A14Rik
MSTRG.4646.1	MSTRG.4646	Larp1
MSTRG.4654.1	MSTRG.4654	.
MSTRG.4656.1	MSTRG.4656	Irgm2
MSTRG.4657.1	MSTRG.4657	Irgm2
MSTRG.4667.1	MSTRG.4667	Zfp39
MSTRG.4670.1	MSTRG.4670	Gm12259
MSTRG.4676.5	MSTRG.4676	Mrpl55
MSTRG.4694.1	MSTRG.4694	4933439C10Rik
MSTRG.4696.1	MSTRG.4696	Mprip
MSTRG.470.1	MSTRG.470	Dnah7a
MSTRG.4703.5	MSTRG.4703	Flcn
MSTRG.4708.1	MSTRG.4708	4930412M03Rik
MSTRG.4708.2	MSTRG.4708	4930412M03Rik
MSTRG.4708.3	MSTRG.4708	Gm27511
MSTRG.4715.1	MSTRG.4715	Tom1l2
MSTRG.4716.1	MSTRG.4716	Tom1l2
MSTRG.4725.1	MSTRG.4725	Alkbh5
MSTRG.4729.1	MSTRG.4729	Gm26837
MSTRG.473.1	MSTRG.473	Pms1
MSTRG.4730.1	MSTRG.4730	Gm26837
MSTRG.474.1	MSTRG.474	Pms1
MSTRG.4749.1	MSTRG.4749	Dhrs7b
MSTRG.4750.1	MSTRG.4750	Dhrs7b
MSTRG.4752.1	MSTRG.4752	Tmem11
MSTRG.4753.1	MSTRG.4753	Tmem11
MSTRG.476.1	MSTRG.476	Pms1
MSTRG.4763.1	MSTRG.4763	Aldh3a2
MSTRG.4769.1	MSTRG.4769	.
MSTRG.4777.1	MSTRG.4777	Ubb
MSTRG.4777.2	MSTRG.4777	Ubb
MSTRG.4777.3	MSTRG.4777	Ubb
MSTRG.4777.4	MSTRG.4777	Ubb
MSTRG.4779.1	MSTRG.4779	Zfp287
MSTRG.4781.1	MSTRG.4781	2410006H16Rik
MSTRG.4787.1	MSTRG.4787	Mmgt2
MSTRG.479.1	MSTRG.479	.
MSTRG.4794.1	MSTRG.4794	Ncor1
MSTRG.4803.1	MSTRG.4803	Zfp286
MSTRG.4805.1	MSTRG.4805	Tvp23b

MSTRG.4810.1	MSTRG.4810	Hs3st3b1
MSTRG.4815.1	MSTRG.4815	Cox10
MSTRG.4819.1	MSTRG.4819	Gm12291
MSTRG.482.1	MSTRG.482	Gtf3c3
MSTRG.4827.1	MSTRG.4827	Arhgap44
MSTRG.4829.1	MSTRG.4829	Map2k4
MSTRG.483.1	MSTRG.483	.
MSTRG.4831.1	MSTRG.4831	Zkscan6
MSTRG.4832.1	MSTRG.4832	Zkscan6
MSTRG.4836.1	MSTRG.4836	Shisa6
MSTRG.4837.1	MSTRG.4837	Shisa6
MSTRG.4840.1	MSTRG.4840	.
MSTRG.4847.1	MSTRG.4847	Ntn1
MSTRG.4849.1	MSTRG.4849	Stx8
MSTRG.4852.1	MSTRG.4852	Stx8
MSTRG.4853.1	MSTRG.4853	Stx8
MSTRG.4855.1	MSTRG.4855	Stx8
MSTRG.4856.1	MSTRG.4856	Stx8
MSTRG.4857.1	MSTRG.4857	C78197
MSTRG.4858.1	MSTRG.4858	Stx8
MSTRG.4860.1	MSTRG.4860	Pik3r5
MSTRG.4861.1	MSTRG.4861	Pik3r5
MSTRG.4866.1	MSTRG.4866	Ndel1
MSTRG.487.1	MSTRG.487	.
MSTRG.4871.1	MSTRG.4871	.
MSTRG.4887.1	MSTRG.4887	.
MSTRG.4889.2	MSTRG.4889	Wrap53
MSTRG.489.1	MSTRG.489	4930444A19Rik
MSTRG.4894.1	MSTRG.4894	.
MSTRG.4907.1	MSTRG.4907	Mpdu1
MSTRG.491.1	MSTRG.491	Ankrd44
MSTRG.4918.1	MSTRG.4918	Zbtb4
MSTRG.4928.3	MSTRG.4928	Ybx2
MSTRG.493.1	MSTRG.493	Ankrd44
MSTRG.494.1	MSTRG.494	Ankrd44
MSTRG.4941.1	MSTRG.4941	Dlg4
MSTRG.4954.2	MSTRG.4954	Cxcl16
MSTRG.4958.1	MSTRG.4958	Mink1
MSTRG.496.1	MSTRG.496	Ankrd44
MSTRG.4972.1	MSTRG.4972	Zfp3
MSTRG.4975.1	MSTRG.4975	Kif1c
MSTRG.498.2	MSTRG.498	Hspe1
MSTRG.4981.1	MSTRG.4981	.
MSTRG.4982.2	MSTRG.4982	Derl2
MSTRG.4985.1	MSTRG.4985	Rabep1
MSTRG.4990.1	MSTRG.4990	Rabep1
MSTRG.4991.1	MSTRG.4991	Rabep1
MSTRG.4992.1	MSTRG.4992	Rabep1
MSTRG.500.1	MSTRG.500	Sf3b1
MSTRG.5007.3	MSTRG.5007	Ube2g1
MSTRG.5008.1	MSTRG.5008	Gm24143
MSTRG.5009.1	MSTRG.5009	Ube2g1
MSTRG.5016.1	MSTRG.5016	Spns3
MSTRG.5017.1	MSTRG.5017	Spns3
MSTRG.5018.1	MSTRG.5018	Spns3
MSTRG.5019.1	MSTRG.5019	Spns3
MSTRG.502.1	MSTRG.502	Mob4

MSTRG.5020.1	MSTRG.5020	Spns3
MSTRG.5036.1	MSTRG.5036	Itgae
MSTRG.5037.1	MSTRG.5037	Itgae
MSTRG.5043.1	MSTRG.5043	.
MSTRG.5045.1	MSTRG.5045	Rap1gap2
MSTRG.5048.1	MSTRG.5048	Pafah1b1
MSTRG.5050.1	MSTRG.5050	Pafah1b1
MSTRG.5052.1	MSTRG.5052	Mettl16
MSTRG.5053.1	MSTRG.5053	Mettl16
MSTRG.5054.1	MSTRG.5054	Mettl16
MSTRG.5055.1	MSTRG.5055	Mettl16
MSTRG.5056.1	MSTRG.5056	Mettl16
MSTRG.5057.1	MSTRG.5057	Mettl16
MSTRG.5058.1	MSTRG.5058	Mettl16
MSTRG.5061.1	MSTRG.5061	Rtn4rl1
MSTRG.5062.1	MSTRG.5062	Rtn4rl1
MSTRG.5063.1	MSTRG.5063	Rtn4rl1
MSTRG.5077.1	MSTRG.5077	Smg6
MSTRG.5078.1	MSTRG.5078	Smg6
MSTRG.5080.1	MSTRG.5080	Smg6
MSTRG.5082.1	MSTRG.5082	Gm12333
MSTRG.5083.1	MSTRG.5083	Smg6
MSTRG.5084.1	MSTRG.5084	Smg6
MSTRG.5087.1	MSTRG.5087	Smg6
MSTRG.5088.1	MSTRG.5088	Smg6
MSTRG.5089.1	MSTRG.5089	Smg6
MSTRG.509.1	MSTRG.509	.
MSTRG.5090.1	MSTRG.5090	Smg6
MSTRG.5091.1	MSTRG.5091	Smg6
MSTRG.5095.1	MSTRG.5095	Smyd4
MSTRG.5096.1	MSTRG.5096	Smyd4
MSTRG.5100.1	MSTRG.5100	Tlcd2
MSTRG.5104.1	MSTRG.5104	Gm45606
MSTRG.5106.1	MSTRG.5106	.
MSTRG.511.1	MSTRG.511	Plcl1
MSTRG.5110.1	MSTRG.5110	Pitpna
MSTRG.5114.1	MSTRG.5114	Crk
MSTRG.5115.1	MSTRG.5115	Crk
MSTRG.5116.1	MSTRG.5116	Crk
MSTRG.512.1	MSTRG.512	Plcl1
MSTRG.5122.1	MSTRG.5122	Vps53
MSTRG.5125.1	MSTRG.5125	Fam57a
MSTRG.5126.1	MSTRG.5126	Glod4
MSTRG.513.1	MSTRG.513	Gm38056
MSTRG.5130.1	MSTRG.5130	Nxn
MSTRG.5133.1	MSTRG.5133	Nxn
MSTRG.5135.1	MSTRG.5135	Abr
MSTRG.5139.1	MSTRG.5139	Tusc5
MSTRG.5143.1	MSTRG.5143	Gosr1
MSTRG.5144.1	MSTRG.5144	Gosr1
MSTRG.5145.1	MSTRG.5145	Gosr1
MSTRG.515.1	MSTRG.515	Plcl1
MSTRG.5152.1	MSTRG.5152	Nsrp1
MSTRG.5154.1	MSTRG.5154	Nsrp1
MSTRG.5155.1	MSTRG.5155	Nsrp1
MSTRG.516.1	MSTRG.516	Plcl1
MSTRG.5162.1	MSTRG.5162	Ssh2

MSTRG.5165.1	MSTRG.5165	Ssh2
MSTRG.5167.1	MSTRG.5167	Ssh2
MSTRG.5168.1	MSTRG.5168	Ssh2
MSTRG.5169.1	MSTRG.5169	Ssh2
MSTRG.517.1	MSTRG.517	Plcl1
MSTRG.5174.1	MSTRG.5174	Abhd15
MSTRG.5177.1	MSTRG.5177	Taok1
MSTRG.5182.1	MSTRG.5182	Myo18a
MSTRG.5184.1	MSTRG.5184	Myo18a
MSTRG.5185.1	MSTRG.5185	Myo18a
MSTRG.519.1	MSTRG.519	Plcl1
MSTRG.5191.1	MSTRG.5191	Fam222b
MSTRG.520.1	MSTRG.520	Plcl1
MSTRG.5207.1	MSTRG.5207	Supt6
MSTRG.5209.1	MSTRG.5209	Sdf2
MSTRG.5217.1	MSTRG.5217	Foxn1
MSTRG.523.1	MSTRG.523	Plcl1
MSTRG.5231.1	MSTRG.5231	Nlk
MSTRG.5233.1	MSTRG.5233	Nlk
MSTRG.5234.1	MSTRG.5234	Nlk
MSTRG.5234.3	MSTRG.5234	Nlk
MSTRG.5235.1	MSTRG.5235	Nlk
MSTRG.5238.1	MSTRG.5238	.
MSTRG.524.1	MSTRG.524	Plcl1
MSTRG.5242.1	MSTRG.5242	Ksr1
MSTRG.5247.1	MSTRG.5247	Rab11fip4
MSTRG.525.1	MSTRG.525	Plcl1
MSTRG.5252.1	MSTRG.5252	Rab11fip4
MSTRG.5253.1	MSTRG.5253	.
MSTRG.526.1	MSTRG.526	Plcl1
MSTRG.5260.1	MSTRG.5260	AU040972
MSTRG.5261.1	MSTRG.5261	Nf1
MSTRG.5264.1	MSTRG.5264	.
MSTRG.5271.1	MSTRG.5271	Atad5
MSTRG.5272.1	MSTRG.5272	Atad5
MSTRG.5275.1	MSTRG.5275	.
MSTRG.5279.1	MSTRG.5279	Rhot1
MSTRG.5280.1	MSTRG.5280	Rhot1
MSTRG.5281.1	MSTRG.5281	Rhot1
MSTRG.5283.1	MSTRG.5283	Rhbdl3
MSTRG.5284.1	MSTRG.5284	Rhbdl3
MSTRG.5287.1	MSTRG.5287	.
MSTRG.530.1	MSTRG.530	Tyw5
MSTRG.5306.1	MSTRG.5306	Rffl
MSTRG.5318.1	MSTRG.5318	.
MSTRG.532.12	MSTRG.532	Kctd18
MSTRG.5320.1	MSTRG.5320	.
MSTRG.5325.1	MSTRG.5325	.
MSTRG.5329.1	MSTRG.5329	AL603745.2
MSTRG.5329.2	MSTRG.5329	AL603745.2
MSTRG.5335.2	MSTRG.5335	AA465934
MSTRG.534.1	MSTRG.534	Kctd18
MSTRG.535.1	MSTRG.535	Kctd18
MSTRG.5353.1	MSTRG.5353	Tada2a
MSTRG.5357.2	MSTRG.5357	Aatf
MSTRG.5358.1	MSTRG.5358	Aatf
MSTRG.536.1	MSTRG.536	Kctd18

MSTRG.5362.1	MSTRG.5362	Dhrs11
MSTRG.5372.1	MSTRG.5372	Usp32
MSTRG.5374.1	MSTRG.5374	Usp32
MSTRG.5376.1	MSTRG.5376	Usp32
MSTRG.5377.1	MSTRG.5377	Usp32
MSTRG.5378.1	MSTRG.5378	Usp32
MSTRG.538.1	MSTRG.538	Aox1
MSTRG.5381.1	MSTRG.5381	Appbp2
MSTRG.5384.1	MSTRG.5384	Bcas3
MSTRG.5386.1	MSTRG.5386	Bcas3
MSTRG.5387.1	MSTRG.5387	Bcas3
MSTRG.5388.1	MSTRG.5388	Bcas3
MSTRG.5389.1	MSTRG.5389	Bcas3
MSTRG.5390.1	MSTRG.5390	Bcas3
MSTRG.5391.1	MSTRG.5391	Bcas3
MSTRG.5392.1	MSTRG.5392	Bcas3
MSTRG.5393.1	MSTRG.5393	Bcas3
MSTRG.5394.1	MSTRG.5394	Bcas3
MSTRG.5396.1	MSTRG.5396	Bcas3
MSTRG.54.1	MSTRG.54	Sntg1
MSTRG.54.2	MSTRG.54	Sntg1
MSTRG.5403.1	MSTRG.5403	Brip1
MSTRG.5404.1	MSTRG.5404	Brip1
MSTRG.5410.1	MSTRG.5410	Med13
MSTRG.5412.1	MSTRG.5412	.
MSTRG.5417.1	MSTRG.5417	Vmp1
MSTRG.5418.1	MSTRG.5418	Vmp1
MSTRG.5419.1	MSTRG.5419	Vmp1
MSTRG.542.1	MSTRG.542	Gm15759
MSTRG.5423.1	MSTRG.5423	.
MSTRG.5423.10	MSTRG.5423	.
MSTRG.5423.2	MSTRG.5423	.
MSTRG.5423.3	MSTRG.5423	.
MSTRG.5423.4	MSTRG.5423	.
MSTRG.5423.5	MSTRG.5423	.
MSTRG.5423.6	MSTRG.5423	.
MSTRG.5423.7	MSTRG.5423	.
MSTRG.5423.8	MSTRG.5423	.
MSTRG.5423.9	MSTRG.5423	.
MSTRG.5424.1	MSTRG.5424	.
MSTRG.5425.1	MSTRG.5425	.
MSTRG.5426.3	MSTRG.5426	Ypel2
MSTRG.5428.1	MSTRG.5428	Gdpd1
MSTRG.5431.1	MSTRG.5431	Prr11
MSTRG.5438.1	MSTRG.5438	Ppm1e
MSTRG.5439.1	MSTRG.5439	Ppm1e
MSTRG.544.1	MSTRG.544	.
MSTRG.5441.1	MSTRG.5441	Ppm1e
MSTRG.5443.1	MSTRG.5443	Ppm1e
MSTRG.5447.1	MSTRG.5447	.
MSTRG.5457.1	MSTRG.5457	Cuedc1
MSTRG.5459.1	MSTRG.5459	Rad51c
MSTRG.5459.10	MSTRG.5459	Supt4a
MSTRG.5459.2	MSTRG.5459	Gm23137
MSTRG.5459.3	MSTRG.5459	Gm23137
MSTRG.5459.4	MSTRG.5459	Gm23137
MSTRG.5459.5	MSTRG.5459	Gm23137

MSTRG.5459.6	MSTRG.5459	Gm23137
MSTRG.5459.7	MSTRG.5459	Gm23137
MSTRG.5459.8	MSTRG.5459	Gm23137
MSTRG.5459.9	MSTRG.5459	Gm23137
MSTRG.5462.1	MSTRG.5462	Rnu1a1
MSTRG.5462.2	MSTRG.5462	Rnu1a1
MSTRG.5470.1	MSTRG.5470	Sept4
MSTRG.5474.1	MSTRG.5474	Rnf43
MSTRG.5479.1	MSTRG.5479	Mir142b
MSTRG.5481.1	MSTRG.5481	Mir142hg
MSTRG.5484.1	MSTRG.5484	Akap1
MSTRG.5488.1	MSTRG.5488	Msi2
MSTRG.5489.1	MSTRG.5489	Msi2
MSTRG.549.5	MSTRG.549	Orc2
MSTRG.5490.1	MSTRG.5490	Msi2
MSTRG.5494.1	MSTRG.5494	Msi2
MSTRG.5495.1	MSTRG.5495	Msi2
MSTRG.5496.1	MSTRG.5496	Msi2
MSTRG.5497.1	MSTRG.5497	Msi2
MSTRG.5497.2	MSTRG.5497	Msi2
MSTRG.5498.1	MSTRG.5498	Msi2
MSTRG.5499.1	MSTRG.5499	Msi2
MSTRG.55.1	MSTRG.55	Sntg1
MSTRG.5500.1	MSTRG.5500	Msi2
MSTRG.5501.1	MSTRG.5501	Msi2
MSTRG.5501.2	MSTRG.5501	Msi2
MSTRG.5504.1	MSTRG.5504	.
MSTRG.5505.10	MSTRG.5505	Mmd
MSTRG.5505.2	MSTRG.5505	Gm45883
MSTRG.5505.8	MSTRG.5505	Gm45883
MSTRG.551.1	MSTRG.551	Orc2
MSTRG.5514.1	MSTRG.5514	Dgke
MSTRG.5515.1	MSTRG.5515	Dgke
MSTRG.5516.1	MSTRG.5516	Dgke
MSTRG.5517.1	MSTRG.5517	Dgkeos
MSTRG.5520.1	MSTRG.5520	Gm525
MSTRG.5521.1	MSTRG.5521	Gm525
MSTRG.5522.1	MSTRG.5522	.
MSTRG.5525.1	MSTRG.5525	Stxbp4
MSTRG.5527.1	MSTRG.5527	Stxbp4
MSTRG.5530.1	MSTRG.5530	Tom1l1
MSTRG.5533.1	MSTRG.5533	Utp18
MSTRG.5534.1	MSTRG.5534	Utp18
MSTRG.5540.1	MSTRG.5540	Spag9
MSTRG.5542.1	MSTRG.5542	.
MSTRG.5545.1	MSTRG.5545	Luc7l3
MSTRG.5549.1	MSTRG.5549	Lrrc59
MSTRG.556.1	MSTRG.556	Trak2
MSTRG.5563.1	MSTRG.5563	Spop
MSTRG.5564.1	MSTRG.5564	Spop
MSTRG.557.1	MSTRG.557	Trak2
MSTRG.5572.1	MSTRG.5572	Gm11520
MSTRG.5573.2	MSTRG.5573	Gm11521
MSTRG.5574.2	MSTRG.5574	Fam117a
MSTRG.5574.5	MSTRG.5574	Fam117a
MSTRG.5574.6	MSTRG.5574	Fam117a
MSTRG.5574.8	MSTRG.5574	Fam117a

MSTRG.5579.1	MSTRG.5579	.
MSTRG.5582.1	MSTRG.5582	Zfp652
MSTRG.5584.1	MSTRG.5584	Zfp652
MSTRG.5586.5	MSTRG.5586	Abi3
MSTRG.5587.1	MSTRG.5587	Gngt2
MSTRG.559.1	MSTRG.559	Stradb
MSTRG.5598.1	MSTRG.5598	Skap1
MSTRG.5599.1	MSTRG.5599	Skap1
MSTRG.5600.1	MSTRG.5600	Skap1
MSTRG.5601.1	MSTRG.5601	Skap1
MSTRG.5602.1	MSTRG.5602	Skap1
MSTRG.5603.1	MSTRG.5603	Skap1
MSTRG.5605.1	MSTRG.5605	Skap1
MSTRG.5607.1	MSTRG.5607	Skap1
MSTRG.5608.1	MSTRG.5608	Skap1
MSTRG.5613.2	MSTRG.5613	D030028A08Rik
MSTRG.5626.1	MSTRG.5626	Osbpl7
MSTRG.5635.1	MSTRG.5635	Arhgap23
MSTRG.5638.1	MSTRG.5638	Npepps
MSTRG.5639.1	MSTRG.5639	Npepps
MSTRG.5641.1	MSTRG.5641	.
MSTRG.5642.2	MSTRG.5642	Gm11613
MSTRG.5644.1	MSTRG.5644	Mllt6
MSTRG.5654.1	MSTRG.5654	Cwc25
MSTRG.5658.1	MSTRG.5658	.
MSTRG.5659.1	MSTRG.5659	.
MSTRG.566.1	MSTRG.566	Casp8
MSTRG.566.2	MSTRG.566	Casp8
MSTRG.566.3	MSTRG.566	Casp8
MSTRG.5664.1	MSTRG.5664	Plxdc1
MSTRG.5665.1	MSTRG.5665	Plxdc1
MSTRG.5669.1	MSTRG.5669	Arl5c
MSTRG.567.1	MSTRG.567	Casp8
MSTRG.5671.3	MSTRG.5671	Fbxl20
MSTRG.5671.9	MSTRG.5671	Fbxl20
MSTRG.5679.1	MSTRG.5679	Stard3
MSTRG.5691.1	MSTRG.5691	Erbb2
MSTRG.5693.1	MSTRG.5693	Grb7
MSTRG.5693.3	MSTRG.5693	Grb7
MSTRG.5693.8	MSTRG.5693	Grb7
MSTRG.5696.1	MSTRG.5696	Ikzf3
MSTRG.5697.1	MSTRG.5697	Ikzf3
MSTRG.5704.2	MSTRG.5704	Gm12359
MSTRG.5706.1	MSTRG.5706	Rapgefl1
MSTRG.5710.1	MSTRG.5710	Ccr7
MSTRG.5713.1	MSTRG.5713	Wipf2
MSTRG.5714.1	MSTRG.5714	Gm23640
MSTRG.5716.1	MSTRG.5716	Wipf2
MSTRG.5720.1	MSTRG.5720	Rara
MSTRG.5721.1	MSTRG.5721	Rara
MSTRG.5722.1	MSTRG.5722	Rara
MSTRG.5724.6	MSTRG.5724	Top2a
MSTRG.5744.1	MSTRG.5744	Nt5c3b
MSTRG.575.1	MSTRG.575	Als2
MSTRG.5750.1	MSTRG.5750	Acly
MSTRG.5756.1	MSTRG.5756	Nkiras2
MSTRG.5765.1	MSTRG.5765	Stat5b

MSTRG.5769.1	MSTRG.5769	Stat3
MSTRG.5770.1	MSTRG.5770	Stat3
MSTRG.5787.1	MSTRG.5787	Cntnap1
MSTRG.5804.1	MSTRG.5804	Gm27029
MSTRG.581.1	MSTRG.581	Gm973
MSTRG.5820.1	MSTRG.5820	Nbr1
MSTRG.5827.1	MSTRG.5827	Lsm12
MSTRG.583.1	MSTRG.583	Sumo1
MSTRG.5830.1	MSTRG.5830	Hdac5
MSTRG.5831.1	MSTRG.5831	Hdac5
MSTRG.5845.1	MSTRG.5845	Gpatch8
MSTRG.5847.1	MSTRG.5847	Gpatch8
MSTRG.5849.1	MSTRG.5849	Gpatch8
MSTRG.585.1	MSTRG.585	Sumo1
MSTRG.5852.1	MSTRG.5852	Adam11
MSTRG.5854.1	MSTRG.5854	.
MSTRG.586.1	MSTRG.586	Sumo1
MSTRG.5866.1	MSTRG.5866	Dcakd
MSTRG.5867.1	MSTRG.5867	Gm26668
MSTRG.5869.1	MSTRG.5869	Gm26668
MSTRG.587.1	MSTRG.587	.
MSTRG.5871.1	MSTRG.5871	Plcd3
MSTRG.5874.1	MSTRG.5874	Acbd4
MSTRG.5875.1	MSTRG.5875	Acbd4
MSTRG.5891.1	MSTRG.5891	Nsf
MSTRG.5899.1	MSTRG.5899	Kansl1
MSTRG.5911.1	MSTRG.5911	Tlk2
MSTRG.5912.1	MSTRG.5912	Tlk2
MSTRG.5913.1	MSTRG.5913	Tlk2
MSTRG.5917.1	MSTRG.5917	Kcnh6
MSTRG.5918.1	MSTRG.5918	Kcnh6
MSTRG.5924.1	MSTRG.5924	Map3k3
MSTRG.5931.1	MSTRG.5931	Prr29
MSTRG.5946.1	MSTRG.5946	Ern1
MSTRG.5947.1	MSTRG.5947	Ern1
MSTRG.5949.1	MSTRG.5949	Tex2
MSTRG.5950.1	MSTRG.5950	Tex2
MSTRG.5952.1	MSTRG.5952	Pecam1
MSTRG.5954.1	MSTRG.5954	Pecam1
MSTRG.5954.2	MSTRG.5954	Pecam1
MSTRG.5957.1	MSTRG.5957	Milr1
MSTRG.5961.1	MSTRG.5961	Kpna2
MSTRG.5965.1	MSTRG.5965	Cep95
MSTRG.5968.1	MSTRG.5968	Smurf2
MSTRG.5969.1	MSTRG.5969	Smurf2
MSTRG.5971.1	MSTRG.5971	Smurf2
MSTRG.5972.1	MSTRG.5972	Smurf2
MSTRG.5980.1	MSTRG.5980	Psmd12
MSTRG.5982.1	MSTRG.5982	Pitpnc1
MSTRG.5983.1	MSTRG.5983	Pitpnc1
MSTRG.5984.1	MSTRG.5984	Pitpnc1
MSTRG.5985.1	MSTRG.5985	Pitpnc1
MSTRG.5986.4	MSTRG.5986	Pitpnc1
MSTRG.5987.1	MSTRG.5987	Pitpnc1
MSTRG.5988.1	MSTRG.5988	Pitpnc1
MSTRG.5991.1	MSTRG.5991	Gm11714
MSTRG.5991.2	MSTRG.5991	Gm11714

MSTRG.5991.4	MSTRG.5991	Gm11714
MSTRG.5991.5	MSTRG.5991	Gm11714
MSTRG.5992.1	MSTRG.5992	Pitpnc1
MSTRG.6000.2	MSTRG.6000	Prkca
MSTRG.6003.1	MSTRG.6003	Prkca
MSTRG.6004.1	MSTRG.6004	Prkca
MSTRG.6005.1	MSTRG.6005	Prkca
MSTRG.6006.1	MSTRG.6006	Prkca
MSTRG.6007.1	MSTRG.6007	Prkca
MSTRG.6008.1	MSTRG.6008	Prkca
MSTRG.6009.1	MSTRG.6009	Prkca
MSTRG.6010.1	MSTRG.6010	Prkca
MSTRG.6011.1	MSTRG.6011	Prkca
MSTRG.6012.1	MSTRG.6012	Prkca
MSTRG.6012.2	MSTRG.6012	Prkca
MSTRG.6013.1	MSTRG.6013	Gm11655
MSTRG.6013.2	MSTRG.6013	Gm11655
MSTRG.6013.3	MSTRG.6013	Gm11655
MSTRG.6014.1	MSTRG.6014	Prkca
MSTRG.6015.1	MSTRG.6015	Prkca
MSTRG.6017.1	MSTRG.6017	Prkca
MSTRG.6019.1	MSTRG.6019	Prkca
MSTRG.602.1	MSTRG.602	Wdr12
MSTRG.6020.1	MSTRG.6020	Prkca
MSTRG.6021.1	MSTRG.6021	Prkca
MSTRG.6022.1	MSTRG.6022	Prkca
MSTRG.6025.1	MSTRG.6025	Apoh
MSTRG.6031.1	MSTRG.6031	Gm11696
MSTRG.6036.1	MSTRG.6036	Arsg
MSTRG.604.1	MSTRG.604	Carf
MSTRG.6044.3	MSTRG.6044	Map2k6
MSTRG.6044.4	MSTRG.6044	Map2k6
MSTRG.6048.1	MSTRG.6048	Map2k6
MSTRG.6049.1	MSTRG.6049	Map2k6
MSTRG.605.1	MSTRG.605	Carf
MSTRG.6050.1	MSTRG.6050	Map2k6
MSTRG.6054.1	MSTRG.6054	2610035D17Rik
MSTRG.6056.1	MSTRG.6056	D11Wsu47e
MSTRG.6058.1	MSTRG.6058	Cpsf4l
MSTRG.6061.1	MSTRG.6061	Slc39a11
MSTRG.607.1	MSTRG.607	Carf
MSTRG.6075.1	MSTRG.6075	Cd300ld2
MSTRG.6078.1	MSTRG.6078	Cd300ld5
MSTRG.608.1	MSTRG.608	Carf
MSTRG.6082.1	MSTRG.6082	Fdxr
MSTRG.6089.1	MSTRG.6089	Tmem104
MSTRG.6090.1	MSTRG.6090	Tmem104
MSTRG.6091.1	MSTRG.6091	Tmem104
MSTRG.6092.1	MSTRG.6092	Tmem104
MSTRG.6093.1	MSTRG.6093	Tmem104
MSTRG.6095.1	MSTRG.6095	Cdr2l
MSTRG.6096.1	MSTRG.6096	Cdr2l
MSTRG.6099.1	MSTRG.6099	Ict1os
MSTRG.6102.1	MSTRG.6102	Jpt1
MSTRG.6104.1	MSTRG.6104	Gm11695
MSTRG.6105.1	MSTRG.6105	Gm11695
MSTRG.6107.1	MSTRG.6107	Slc16a5

MSTRG.6117.1	MSTRG.6117	Nup85
MSTRG.6128.8	MSTRG.6128	Recql5
MSTRG.6130.1	MSTRG.6130	Recql5
MSTRG.6134.1	MSTRG.6134	Sap30bpos
MSTRG.6143.1	MSTRG.6143	Trim47
MSTRG.6145.1	MSTRG.6145	Mrpl38
MSTRG.615.1	MSTRG.615	Abi2
MSTRG.6150.1	MSTRG.6150	Ten1
MSTRG.616.1	MSTRG.616	Abi2
MSTRG.6173.1	MSTRG.6173	Ube2o
MSTRG.6174.1	MSTRG.6174	Ube2o
MSTRG.618.1	MSTRG.618	Raph1
MSTRG.6180.1	MSTRG.6180	.
MSTRG.6182.1	MSTRG.6182	Mxra7
MSTRG.6188.1	MSTRG.6188	.
MSTRG.6190.1	MSTRG.6190	Sec14l1
MSTRG.6194.1	MSTRG.6194	Sept9
MSTRG.6196.2	MSTRG.6196	Sept9
MSTRG.6199.1	MSTRG.6199	.
MSTRG.6201.1	MSTRG.6201	Tnrc6c
MSTRG.6203.1	MSTRG.6203	Tnrc6c
MSTRG.6204.1	MSTRG.6204	Tnrc6c
MSTRG.6205.1	MSTRG.6205	Tnrc6c
MSTRG.6213.1	MSTRG.6213	Afmid
MSTRG.6214.3	MSTRG.6214	Birc5
MSTRG.622.2	MSTRG.622	Gm11579
MSTRG.6223.1	MSTRG.6223	Cyth1
MSTRG.6243.1	MSTRG.6243	.
MSTRG.6245.1	MSTRG.6245	Card14
MSTRG.6246.1	MSTRG.6246	Card14
MSTRG.625.1	MSTRG.625	2310016D23Rik
MSTRG.6260.1	MSTRG.6260	Gm11767
MSTRG.627.1	MSTRG.627	2310016D23Rik
MSTRG.6270.1	MSTRG.6270	.
MSTRG.6274.1	MSTRG.6274	Faap100
MSTRG.6282.1	MSTRG.6282	Gcgr
MSTRG.6291.1	MSTRG.6291	Gm16755
MSTRG.6294.1	MSTRG.6294	.
MSTRG.6295.3	MSTRG.6295	Alyref
MSTRG.6299.1	MSTRG.6299	Mafg
MSTRG.6325.1	MSTRG.6325	Hexdc
MSTRG.6329.1	MSTRG.6329	Foxk2
MSTRG.6330.1	MSTRG.6330	.
MSTRG.6336.1	MSTRG.6336	.
MSTRG.6337.1	MSTRG.6337	.
MSTRG.6340.1	MSTRG.6340	Tbcd
MSTRG.6342.1	MSTRG.6342	Tbcd
MSTRG.6344.1	MSTRG.6344	B3gntl1
MSTRG.6347.2	MSTRG.6347	Rab10os
MSTRG.6347.6	MSTRG.6347	Rab10os
MSTRG.6349.1	MSTRG.6349	.
MSTRG.6350.1	MSTRG.6350	.
MSTRG.6352.1	MSTRG.6352	1110002L01Rik
MSTRG.6355.1	MSTRG.6355	Asxl2
MSTRG.6357.1	MSTRG.6357	Dtnb
MSTRG.6358.1	MSTRG.6358	Dtnb
MSTRG.6361.1	MSTRG.6361	Dtnb

MSTRG.6362.1	MSTRG.6362	Dtnb
MSTRG.6365.1	MSTRG.6365	Dnmt3a
MSTRG.6366.1	MSTRG.6366	Dnmt3a
MSTRG.6366.2	MSTRG.6366	Dnmt3a
MSTRG.6369.1	MSTRG.6369	Adcy3
MSTRG.637.1	MSTRG.637	Pard3b
MSTRG.6370.1	MSTRG.6370	Adcy3
MSTRG.6371.1	MSTRG.6371	Adcy3
MSTRG.6372.1	MSTRG.6372	Adcy3
MSTRG.6379.1	MSTRG.6379	Ncoa1
MSTRG.6380.1	MSTRG.6380	Ncoa1
MSTRG.6382.1	MSTRG.6382	Ncoa1
MSTRG.6384.1	MSTRG.6384	Ncoa1
MSTRG.6385.1	MSTRG.6385	Ncoa1
MSTRG.639.1	MSTRG.639	Pard3b
MSTRG.6395.1	MSTRG.6395	Wdcp
MSTRG.6396.1	MSTRG.6396	Wdcp
MSTRG.6401.1	MSTRG.6401	Atad2b
MSTRG.6404.1	MSTRG.6404	Klhl29
MSTRG.6405.1	MSTRG.6405	Klhl29
MSTRG.6406.1	MSTRG.6406	Klhl29
MSTRG.6407.1	MSTRG.6407	Klhl29
MSTRG.6408.1	MSTRG.6408	CT009738.1
MSTRG.6409.1	MSTRG.6409	Klhl29
MSTRG.6410.1	MSTRG.6410	Klhl29
MSTRG.6411.1	MSTRG.6411	Klhl29
MSTRG.6412.1	MSTRG.6412	Klhl29
MSTRG.6413.1	MSTRG.6413	Klhl29
MSTRG.6414.1	MSTRG.6414	Klhl29
MSTRG.6415.1	MSTRG.6415	Klhl29
MSTRG.6415.2	MSTRG.6415	Klhl29
MSTRG.6416.1	MSTRG.6416	Klhl29
MSTRG.6427.1	MSTRG.6427	Ldah
MSTRG.6431.1	MSTRG.6431	Slc7a15
MSTRG.6432.1	MSTRG.6432	Slc7a15
MSTRG.6433.1	MSTRG.6433	Slc7a15
MSTRG.6434.1	MSTRG.6434	Slc7a15
MSTRG.6438.1	MSTRG.6438	.
MSTRG.6442.1	MSTRG.6442	AC140354.1
MSTRG.6443.2	MSTRG.6443	Laptm4a
MSTRG.6445.1	MSTRG.6445	Pum2
MSTRG.6456.1	MSTRG.6456	Smc6
MSTRG.6458.1	MSTRG.6458	.
MSTRG.646.1	MSTRG.646	Pard3b
MSTRG.6460.1	MSTRG.6460	Fam49a
MSTRG.6461.2	MSTRG.6461	CT010452.1
MSTRG.6467.1	MSTRG.6467	AC154517.1
MSTRG.647.1	MSTRG.647	Pard3b
MSTRG.6472.1	MSTRG.6472	Gm4804
MSTRG.6477.1	MSTRG.6477	.
MSTRG.6478.1	MSTRG.6478	.
MSTRG.6480.1	MSTRG.6480	Lpin1
MSTRG.6482.1	MSTRG.6482	E2f6
MSTRG.6485.1	MSTRG.6485	Greb1
MSTRG.6487.1	MSTRG.6487	.
MSTRG.6489.1	MSTRG.6489	Rock2
MSTRG.6498.1	MSTRG.6498	Nol10

MSTRG.65.1	MSTRG.65	Mybl1
MSTRG.6503.1	MSTRG.6503	Hpcal1
MSTRG.6504.1	MSTRG.6504	Hpcal1
MSTRG.6505.1	MSTRG.6505	Hpcal1
MSTRG.6506.1	MSTRG.6506	Hpcal1
MSTRG.6507.1	MSTRG.6507	Hpcal1
MSTRG.6508.1	MSTRG.6508	Hpcal1
MSTRG.6509.1	MSTRG.6509	Hpcal1
MSTRG.6524.1	MSTRG.6524	.
MSTRG.6528.1	MSTRG.6528	.
MSTRG.6531.1	MSTRG.6531	CT033799.2
MSTRG.6538.1	MSTRG.6538	.
MSTRG.6539.1	MSTRG.6539	.
MSTRG.6540.1	MSTRG.6540	.
MSTRG.6543.1	MSTRG.6543	.
MSTRG.6544.1	MSTRG.6544	AC238676.1
MSTRG.6547.1	MSTRG.6547	.
MSTRG.6548.1	MSTRG.6548	AC124601.2
MSTRG.6551.1	MSTRG.6551	AC124601.2
MSTRG.6553.1	MSTRG.6553	.
MSTRG.6558.1	MSTRG.6558	AC124772.5
MSTRG.656.1	MSTRG.656	Adam23
MSTRG.6562.1	MSTRG.6562	Ywhaq
MSTRG.6566.1	MSTRG.6566	Gm19196
MSTRG.6567.1	MSTRG.6567	Gm19196
MSTRG.657.1	MSTRG.657	Adam23
MSTRG.6570.1	MSTRG.6570	.
MSTRG.6571.1	MSTRG.6571	.
MSTRG.6572.1	MSTRG.6572	.
MSTRG.6573.1	MSTRG.6573	.
MSTRG.6575.1	MSTRG.6575	.
MSTRG.6577.1	MSTRG.6577	.
MSTRG.6578.1	MSTRG.6578	.
MSTRG.6578.2	MSTRG.6578	.
MSTRG.6579.1	MSTRG.6579	.
MSTRG.6582.1	MSTRG.6582	AC123808.1
MSTRG.6586.1	MSTRG.6586	.
MSTRG.659.1	MSTRG.659	Adam23
MSTRG.6590.1	MSTRG.6590	Taf1b
MSTRG.6592.1	MSTRG.6592	.
MSTRG.6601.1	MSTRG.6601	AC134254.3
MSTRG.6624.1	MSTRG.6624	.
MSTRG.6635.1	MSTRG.6635	.
MSTRG.6640.1	MSTRG.6640	Zfp125
MSTRG.6642.1	MSTRG.6642	Asap2
MSTRG.6643.1	MSTRG.6643	Asap2
MSTRG.6647.1	MSTRG.6647	Itgb1bp1
MSTRG.6670.1	MSTRG.6670	.
MSTRG.6672.1	MSTRG.6672	2410018L13Rik
MSTRG.6673.1	MSTRG.6673	AC166341.3
MSTRG.6673.2	MSTRG.6673	AC166341.3
MSTRG.6676.1	MSTRG.6676	AC166341.3
MSTRG.6681.1	MSTRG.6681	2410018L13Rik
MSTRG.6685.1	MSTRG.6685	.
MSTRG.6688.1	MSTRG.6688	.
MSTRG.6689.1	MSTRG.6689	.
MSTRG.6694.1	MSTRG.6694	.

MSTRG.67.1	MSTRG.67	Vcpip1
MSTRG.6702.1	MSTRG.6702	.
MSTRG.6706.1	MSTRG.6706	9030624G23Rik
MSTRG.6708.1	MSTRG.6708	.
MSTRG.6722.1	MSTRG.6722	.
MSTRG.6729.1	MSTRG.6729	AC159307.1
MSTRG.6731.1	MSTRG.6731	Kidins220
MSTRG.6732.1	MSTRG.6732	Kidins220
MSTRG.6733.1	MSTRG.6733	Kidins220
MSTRG.6736.1	MSTRG.6736	Rsad2
MSTRG.6739.1	MSTRG.6739	Rnf144a
MSTRG.674.1	MSTRG.674	Ino80dos
MSTRG.6740.1	MSTRG.6740	Rnf144a
MSTRG.6741.1	MSTRG.6741	Rnf144a
MSTRG.6746.3	MSTRG.6746	Adi1
MSTRG.675.1	MSTRG.675	Ino80dos
MSTRG.6751.1	MSTRG.6751	Tssc1
MSTRG.6754.1	MSTRG.6754	AC136986.1
MSTRG.6754.2	MSTRG.6754	AC136986.1
MSTRG.6755.1	MSTRG.6755	Tssc1
MSTRG.6756.1	MSTRG.6756	Tssc1
MSTRG.6757.1	MSTRG.6757	Tssc1
MSTRG.6758.1	MSTRG.6758	Tssc1
MSTRG.6760.1	MSTRG.6760	.
MSTRG.6761.1	MSTRG.6761	.
MSTRG.6763.1	MSTRG.6763	AC162181.1
MSTRG.6766.2	MSTRG.6766	Tmem18
MSTRG.6769.1	MSTRG.6769	Acp1
MSTRG.677.1	MSTRG.677	Ndufs1
MSTRG.6772.1	MSTRG.6772	.
MSTRG.6777.1	MSTRG.6777	Bcap29
MSTRG.6778.1	MSTRG.6778	Bcap29
MSTRG.6780.1	MSTRG.6780	Dus4l
MSTRG.6781.3	MSTRG.6781	Cog5
MSTRG.6786.1	MSTRG.6786	Cog5
MSTRG.6789.1	MSTRG.6789	Cog5
MSTRG.6790.1	MSTRG.6790	Cog5
MSTRG.6793.1	MSTRG.6793	Prkar2b
MSTRG.6798.3	MSTRG.6798	4933406C10Rik
MSTRG.6802.1	MSTRG.6802	4933406C10Rik
MSTRG.6804.1	MSTRG.6804	Sypl
MSTRG.6807.1	MSTRG.6807	.
MSTRG.6812.1	MSTRG.6812	Atxn7l1
MSTRG.6814.1	MSTRG.6814	Atxn7l1
MSTRG.6815.1	MSTRG.6815	Atxn7l1
MSTRG.6816.1	MSTRG.6816	Atxn7l1
MSTRG.6818.1	MSTRG.6818	Atxn7l1os2
MSTRG.6820.1	MSTRG.6820	Snx13
MSTRG.6822.1	MSTRG.6822	Snx13
MSTRG.6825.1	MSTRG.6825	Ahr
MSTRG.6826.1	MSTRG.6826	Ahr
MSTRG.6831.1	MSTRG.6831	.
MSTRG.6833.10	MSTRG.6833	Bzw2
MSTRG.684.1	MSTRG.684	.
MSTRG.6843.1	MSTRG.6843	Lsmem1
MSTRG.6844.1	MSTRG.6844	Lsmem1
MSTRG.6853.1	MSTRG.6853	Zfp277

MSTRG.6854.1	MSTRG.6854	Zfp277
MSTRG.6856.1	MSTRG.6856	Dock4
MSTRG.6857.1	MSTRG.6857	.
MSTRG.686.1	MSTRG.686	Mettl21a
MSTRG.6860.1	MSTRG.6860	Immp2l
MSTRG.6861.1	MSTRG.6861	Immp2l
MSTRG.6863.1	MSTRG.6863	Immp2l
MSTRG.6865.1	MSTRG.6865	Immp2l
MSTRG.6866.1	MSTRG.6866	Immp2l
MSTRG.6869.1	MSTRG.6869	Immp2l
MSTRG.6871.1	MSTRG.6871	Immp2l
MSTRG.6872.1	MSTRG.6872	Immp2l
MSTRG.6873.1	MSTRG.6873	Immp2l
MSTRG.6874.1	MSTRG.6874	Immp2l
MSTRG.6879.1	MSTRG.6879	Immp2l
MSTRG.6883.1	MSTRG.6883	AC154864.1
MSTRG.6887.1	MSTRG.6887	.
MSTRG.689.1	MSTRG.689	Creb1
MSTRG.6891.1	MSTRG.6891	Pnpla8
MSTRG.6892.1	MSTRG.6892	Pnpla8
MSTRG.6893.1	MSTRG.6893	Pnpla8
MSTRG.6894.1	MSTRG.6894	Pnpla8
MSTRG.6895.1	MSTRG.6895	Pnpla8
MSTRG.6899.1	MSTRG.6899	Nrcam
MSTRG.690.1	MSTRG.690	Creb1
MSTRG.6901.1	MSTRG.6901	.
MSTRG.6902.1	MSTRG.6902	.
MSTRG.6903.1	MSTRG.6903	.
MSTRG.6904.1	MSTRG.6904	.
MSTRG.6907.1	MSTRG.6907	Scfd1
MSTRG.6908.1	MSTRG.6908	Scfd1
MSTRG.6914.1	MSTRG.6914	Ap4s1
MSTRG.6915.1	MSTRG.6915	Ap4s1
MSTRG.6918.1	MSTRG.6918	Hectd1
MSTRG.6919.1	MSTRG.6919	.
MSTRG.6921.1	MSTRG.6921	Nubpl
MSTRG.6922.1	MSTRG.6922	Nubpl
MSTRG.6926.1	MSTRG.6926	Akap6
MSTRG.6927.1	MSTRG.6927	Akap6
MSTRG.6932.1	MSTRG.6932	AC155256.2
MSTRG.6932.10	MSTRG.6932	AC165349.1
MSTRG.6932.2	MSTRG.6932	AC165349.1
MSTRG.6932.3	MSTRG.6932	Gm22634
MSTRG.6932.4	MSTRG.6932	Gm22634
MSTRG.6932.6	MSTRG.6932	AC165349.1
MSTRG.6932.8	MSTRG.6932	AC165349.1
MSTRG.6932.9	MSTRG.6932	AC165349.1
MSTRG.6938.1	MSTRG.6938	Rpl31-ps17
MSTRG.6938.2	MSTRG.6938	Gm22634
MSTRG.6938.3	MSTRG.6938	Gm22634
MSTRG.6938.4	MSTRG.6938	Gm22634
MSTRG.694.1	MSTRG.694	Ccnyl1
MSTRG.6941.1	MSTRG.6941	AC165349.2
MSTRG.6945.1	MSTRG.6945	Snx6
MSTRG.6946.1	MSTRG.6946	Snx6
MSTRG.6947.1	MSTRG.6947	Snx6
MSTRG.6948.1	MSTRG.6948	Snx6

MSTRG.695.1	MSTRG.695	.
MSTRG.6951.1	MSTRG.6951	.
MSTRG.6955.1	MSTRG.6955	Baz1a
MSTRG.6956.1	MSTRG.6956	Baz1a
MSTRG.6958.1	MSTRG.6958	Baz1a
MSTRG.696.1	MSTRG.696	.
MSTRG.6964.1	MSTRG.6964	2700097O09Rik
MSTRG.6966.1	MSTRG.6966	Srp54a
MSTRG.6967.1	MSTRG.6967	Srp54a
MSTRG.6968.1	MSTRG.6968	.
MSTRG.6972.1	MSTRG.6972	Srp54a
MSTRG.6973.1	MSTRG.6973	.
MSTRG.6978.1	MSTRG.6978	Srp54b
MSTRG.6979.1	MSTRG.6979	Srp54b
MSTRG.6984.1	MSTRG.6984	Ppp2r3c
MSTRG.6986.1	MSTRG.6986	1110008L16Rik
MSTRG.6990.1	MSTRG.6990	Brms1l
MSTRG.6997.1	MSTRG.6997	AC154734.1
MSTRG.7003.1	MSTRG.7003	Mipol1
MSTRG.7004.1	MSTRG.7004	Mipol1
MSTRG.7011.1	MSTRG.7011	Ttc6
MSTRG.7013.1	MSTRG.7013	.
MSTRG.7021.1	MSTRG.7021	Trappc6b
MSTRG.7022.1	MSTRG.7022	Trappc6b
MSTRG.7022.2	MSTRG.7022	Trappc6b
MSTRG.7025.1	MSTRG.7025	.
MSTRG.7030.1	MSTRG.7030	AC156558.1
MSTRG.7033.1	MSTRG.7033	Ctage5
MSTRG.7034.1	MSTRG.7034	Ctage5
MSTRG.7036.1	MSTRG.7036	Klhl28
MSTRG.7037.1	MSTRG.7037	Klhl28
MSTRG.704.1	MSTRG.704	Pikfyve
MSTRG.7040.1	MSTRG.7040	Togaram1
MSTRG.7047.1	MSTRG.7047	Fancm
MSTRG.7048.1	MSTRG.7048	Fancm
MSTRG.7050.1	MSTRG.7050	Mis18bp1
MSTRG.7051.1	MSTRG.7051	Mis18bp1
MSTRG.7052.1	MSTRG.7052	.
MSTRG.7054.1	MSTRG.7054	.
MSTRG.7056.1	MSTRG.7056	.
MSTRG.7057.1	MSTRG.7057	.
MSTRG.706.1	MSTRG.706	Map2
MSTRG.7061.1	MSTRG.7061	Sos2
MSTRG.7062.1	MSTRG.7062	Rps29
MSTRG.7062.2	MSTRG.7062	Rps29
MSTRG.7062.3	MSTRG.7062	Rn7s1
MSTRG.7064.1	MSTRG.7064	Klhdc1
MSTRG.7077.1	MSTRG.7077	Nemf
MSTRG.7077.2	MSTRG.7077	Nemf
MSTRG.7077.3	MSTRG.7077	Nemf
MSTRG.7083.1	MSTRG.7083	.
MSTRG.7084.2	MSTRG.7084	AC157822.3
MSTRG.7084.3	MSTRG.7084	AC157822.3
MSTRG.7086.1	MSTRG.7086	L2hgdh
MSTRG.7088.1	MSTRG.7088	Atp5s
MSTRG.7089.1	MSTRG.7089	Atp5s
MSTRG.7094.1	MSTRG.7094	Map4k5

MSTRG.7097.1	MSTRG.7097	4931403G20Rik
MSTRG.7098.1	MSTRG.7098	Map4k5
MSTRG.7099.1	MSTRG.7099	Map4k5
MSTRG.71.1	MSTRG.71	Sgk3
MSTRG.7107.1	MSTRG.7107	Nin
MSTRG.7108.1	MSTRG.7108	Nin
MSTRG.7109.1	MSTRG.7109	Nin
MSTRG.7110.1	MSTRG.7110	Nin
MSTRG.7114.2	MSTRG.7114	Tmx1
MSTRG.7116.1	MSTRG.7116	AC159238.2
MSTRG.7122.1	MSTRG.7122	Frmd6
MSTRG.7123.1	MSTRG.7123	.
MSTRG.7125.1	MSTRG.7125	Actr10
MSTRG.7127.1	MSTRG.7127	Psma3
MSTRG.7128.2	MSTRG.7128	3110056K07Rik
MSTRG.7129.1	MSTRG.7129	3110056K07Rik
MSTRG.7131.1	MSTRG.7131	Arid4a
MSTRG.7132.1	MSTRG.7132	Arid4a
MSTRG.7134.1	MSTRG.7134	Tomm20l
MSTRG.7137.1	MSTRG.7137	2700049A03Rik
MSTRG.7138.1	MSTRG.7138	2700049A03Rik
MSTRG.7141.1	MSTRG.7141	2700049A03Rik
MSTRG.7142.1	MSTRG.7142	2700049A03Rik
MSTRG.7143.1	MSTRG.7143	2700049A03Rik
MSTRG.7144.1	MSTRG.7144	2700049A03Rik
MSTRG.715.1	MSTRG.715	Kansl1l
MSTRG.7157.1	MSTRG.7157	Ppm1a
MSTRG.7161.1	MSTRG.7161	Six4
MSTRG.7164.1	MSTRG.7164	Mnat1
MSTRG.7165.1	MSTRG.7165	Mnat1
MSTRG.7166.1	MSTRG.7166	Gm23910
MSTRG.7168.1	MSTRG.7168	Mnat1
MSTRG.7169.1	MSTRG.7169	Mnat1
MSTRG.7170.1	MSTRG.7170	Mnat1
MSTRG.7171.1	MSTRG.7171	Mnat1
MSTRG.7172.1	MSTRG.7172	Mnat1
MSTRG.7173.1	MSTRG.7173	Mnat1
MSTRG.7174.1	MSTRG.7174	Mnat1
MSTRG.7175.1	MSTRG.7175	Mnat1
MSTRG.7178.1	MSTRG.7178	Slc38a6
MSTRG.7179.1	MSTRG.7179	Slc38a6
MSTRG.7180.1	MSTRG.7180	Slc38a6
MSTRG.7181.1	MSTRG.7181	Slc38a6
MSTRG.7184.1	MSTRG.7184	Prkch
MSTRG.7185.1	MSTRG.7185	Prkch
MSTRG.7186.1	MSTRG.7186	Prkch
MSTRG.7187.1	MSTRG.7187	Prkch
MSTRG.7191.1	MSTRG.7191	Snapc1
MSTRG.7192.1	MSTRG.7192	Snapc1
MSTRG.7196.1	MSTRG.7196	Wdr89
MSTRG.7197.1	MSTRG.7197	.
MSTRG.7201.1	MSTRG.7201	Ppp2r5e
MSTRG.7204.1	MSTRG.7204	Tex21
MSTRG.7205.1	MSTRG.7205	Tex21
MSTRG.7212.1	MSTRG.7212	Zbtb25
MSTRG.7218.1	MSTRG.7218	Plekhg3
MSTRG.7220.1	MSTRG.7220	Sptb

MSTRG.7224.10	MSTRG.7224	Rab15
MSTRG.7224.11	MSTRG.7224	Rab15
MSTRG.7225.1	MSTRG.7225	Fntb
MSTRG.7226.1	MSTRG.7226	Fntb
MSTRG.7227.4	MSTRG.7227	Max
MSTRG.7232.1	MSTRG.7232	Fut8
MSTRG.7236.1	MSTRG.7236	Fut8
MSTRG.724.1	MSTRG.724	Gm29113
MSTRG.7240.1	MSTRG.7240	Gphn
MSTRG.7249.1	MSTRG.7249	Mpp5
MSTRG.7251.1	MSTRG.7251	Atp6v1d
MSTRG.7255.1	MSTRG.7255	Tmem229b
MSTRG.7256.1	MSTRG.7256	Tmem229b
MSTRG.7259.1	MSTRG.7259	Pigh
MSTRG.726.1	MSTRG.726	.
MSTRG.7261.1	MSTRG.7261	Arg2
MSTRG.7263.1	MSTRG.7263	Arg2
MSTRG.7267.1	MSTRG.7267	Vti1b
MSTRG.727.1	MSTRG.727	.
MSTRG.7270.1	MSTRG.7270	Zfyve26
MSTRG.7272.1	MSTRG.7272	Rad51b
MSTRG.7273.1	MSTRG.7273	Rad51b
MSTRG.7275.1	MSTRG.7275	Rad51b
MSTRG.7276.1	MSTRG.7276	Rad51b
MSTRG.7277.1	MSTRG.7277	Rad51b
MSTRG.7278.1	MSTRG.7278	Rad51b
MSTRG.7279.1	MSTRG.7279	Rad51b
MSTRG.7280.1	MSTRG.7280	Rad51b
MSTRG.7284.1	MSTRG.7284	2310015A10Rik
MSTRG.7286.1	MSTRG.7286	Actn1
MSTRG.7288.1	MSTRG.7288	Actn1
MSTRG.7289.1	MSTRG.7289	CT030161.2
MSTRG.7291.1	MSTRG.7291	Dcaf5
MSTRG.73.1	MSTRG.73	Sgk3
MSTRG.7302.1	MSTRG.7302	Susd6
MSTRG.7303.1	MSTRG.7303	Susd6
MSTRG.7305.13	MSTRG.7305	Srsf5
MSTRG.7305.7	MSTRG.7305	Srsf5
MSTRG.7305.9	MSTRG.7305	Srsf5
MSTRG.7306.1	MSTRG.7306	Slc10a1
MSTRG.7308.1	MSTRG.7308	Smoc1
MSTRG.7309.1	MSTRG.7309	Smoc1
MSTRG.7310.1	MSTRG.7310	Smoc1
MSTRG.7311.1	MSTRG.7311	Smoc1
MSTRG.7317.1	MSTRG.7317	Gm20498
MSTRG.7321.1	MSTRG.7321	Gm20498
MSTRG.7326.1	MSTRG.7326	Med6
MSTRG.733.1	MSTRG.733	Ikzf2
MSTRG.7330.1	MSTRG.7330	Pcnx
MSTRG.7333.1	MSTRG.7333	Sipa1l1
MSTRG.7334.1	MSTRG.7334	Sipa1l1
MSTRG.7335.1	MSTRG.7335	Sipa1l1
MSTRG.7336.1	MSTRG.7336	Sipa1l1
MSTRG.7337.1	MSTRG.7337	Sipa1l1
MSTRG.734.1	MSTRG.734	Ikzf2
MSTRG.7340.1	MSTRG.7340	Dcaf4
MSTRG.7345.1	MSTRG.7345	Zfyve1

MSTRG.7346.1	MSTRG.7346	Zfyve1
MSTRG.7347.1	MSTRG.7347	Zfyve1
MSTRG.7357.1	MSTRG.7357	Numb
MSTRG.7360.1	MSTRG.7360	Numb
MSTRG.7361.1	MSTRG.7361	Numb
MSTRG.7362.1	MSTRG.7362	Numb
MSTRG.7363.1	MSTRG.7363	Numb
MSTRG.7364.1	MSTRG.7364	Numb
MSTRG.7366.1	MSTRG.7366	Numb
MSTRG.7375.1	MSTRG.7375	Abcd4
MSTRG.7377.2	MSTRG.7377	Npc2
MSTRG.7380.1	MSTRG.7380	Ptgr2
MSTRG.7381.1	MSTRG.7381	Ptgr2
MSTRG.7382.1	MSTRG.7382	Ptgr2
MSTRG.7383.1	MSTRG.7383	Ptgr2
MSTRG.7391.1	MSTRG.7391	Bbof1
MSTRG.7392.1	MSTRG.7392	Bbof1
MSTRG.7395.1	MSTRG.7395	Lin52
MSTRG.7396.1	MSTRG.7396	Lin52
MSTRG.7398.1	MSTRG.7398	Lin52
MSTRG.74.1	MSTRG.74	Sgk3
MSTRG.7409.1	MSTRG.7409	.
MSTRG.741.1	MSTRG.741	Spag16
MSTRG.7414.1	MSTRG.7414	.
MSTRG.7422.1	MSTRG.7422	Mlh3
MSTRG.7428.1	MSTRG.7428	Jdp2
MSTRG.7429.1	MSTRG.7429	Jdp2
MSTRG.7432.1	MSTRG.7432	Batf
MSTRG.7433.1	MSTRG.7433	Batf
MSTRG.7435.1	MSTRG.7435	Ift43
MSTRG.7439.1	MSTRG.7439	Ttll5
MSTRG.7440.1	MSTRG.7440	Ttll5
MSTRG.7445.4	MSTRG.7445	Gpatch2l
MSTRG.7445.7	MSTRG.7445	Gpatch2l
MSTRG.7446.1	MSTRG.7446	Gpatch2l
MSTRG.7453.1	MSTRG.7453	.
MSTRG.7454.1	MSTRG.7454	.
MSTRG.7463.1	MSTRG.7463	Tmed8
MSTRG.7472.1	MSTRG.7472	Sptlc2
MSTRG.7473.1	MSTRG.7473	.
MSTRG.7474.1	MSTRG.7474	.
MSTRG.7482.1	MSTRG.7482	Adck1
MSTRG.7484.1	MSTRG.7484	Adck1
MSTRG.7485.1	MSTRG.7485	Adck1
MSTRG.7486.1	MSTRG.7486	Adck1
MSTRG.7488.4	MSTRG.7488	Gtf2a1
MSTRG.7490.1	MSTRG.7490	.
MSTRG.7495.1	MSTRG.7495	CR974487.2
MSTRG.7496.1	MSTRG.7496	Cep128
MSTRG.7498.1	MSTRG.7498	Cep128
MSTRG.7499.1	MSTRG.7499	Cep128
MSTRG.7501.1	MSTRG.7501	Cep128
MSTRG.7502.1	MSTRG.7502	Cep128
MSTRG.7510.1	MSTRG.7510	Ston2
MSTRG.7514.1	MSTRG.7514	.
MSTRG.7518.1	MSTRG.7518	.
MSTRG.7520.1	MSTRG.7520	.

MSTRG.7529.1	MSTRG.7529	Ttc8
MSTRG.753.1	MSTRG.753	D230017M19Rik
MSTRG.7534.1	MSTRG.7534	Eml5
MSTRG.7539.1	MSTRG.7539	Foxn3
MSTRG.7545.1	MSTRG.7545	Foxn3
MSTRG.7546.1	MSTRG.7546	Foxn3
MSTRG.7546.4	MSTRG.7546	Foxn3
MSTRG.7553.1	MSTRG.7553	Efcab11
MSTRG.7554.1	MSTRG.7554	Efcab11
MSTRG.7562.1	MSTRG.7562	Nrde2
MSTRG.7570.1	MSTRG.7570	Ttc7b
MSTRG.7571.1	MSTRG.7571	Ttc7b
MSTRG.7575.1	MSTRG.7575	Rps6ka5
MSTRG.7576.7	MSTRG.7576	9030617O03Rik
MSTRG.7577.1	MSTRG.7577	9030617O03Rik
MSTRG.7578.1	MSTRG.7578	9030617O03Rik
MSTRG.7584.1	MSTRG.7584	AC126262.2
MSTRG.7584.5	MSTRG.7584	AC126262.2
MSTRG.7584.7	MSTRG.7584	Ccdc88c
MSTRG.7584.8	MSTRG.7584	Mir1190
MSTRG.7588.1	MSTRG.7588	AC136640.1
MSTRG.759.1	MSTRG.759	Xrcc5
MSTRG.7593.1	MSTRG.7593	Trip11
MSTRG.7594.1	MSTRG.7594	Trip11
MSTRG.7596.1	MSTRG.7596	Trip11
MSTRG.7605.1	MSTRG.7605	.
MSTRG.7607.1	MSTRG.7607	Rin3
MSTRG.7608.1	MSTRG.7608	Rin3
MSTRG.7609.1	MSTRG.7609	Rin3
MSTRG.761.1	MSTRG.761	Smarcal1
MSTRG.7612.1	MSTRG.7612	Golga5
MSTRG.7617.1	MSTRG.7617	Itpk1
MSTRG.7624.1	MSTRG.7624	Btbd7
MSTRG.7625.1	MSTRG.7625	Btbd7
MSTRG.7631.1	MSTRG.7631	Asb2
MSTRG.765.1	MSTRG.765	6030407O03Rik
MSTRG.7650.1	MSTRG.7650	.
MSTRG.7654.1	MSTRG.7654	.
MSTRG.7656.1	MSTRG.7656	Gm8918
MSTRG.7657.1	MSTRG.7657	Gm8918
MSTRG.7657.2	MSTRG.7657	Serpina3h
MSTRG.7659.1	MSTRG.7659	Serpina3i
MSTRG.7665.1	MSTRG.7665	Clmn
MSTRG.7667.1	MSTRG.7667	4930408O17Rik
MSTRG.7677.1	MSTRG.7677	Gskip
MSTRG.7678.1	MSTRG.7678	Gskip
MSTRG.7679.1	MSTRG.7679	.
MSTRG.7684.1	MSTRG.7684	Vrk1
MSTRG.7685.1	MSTRG.7685	Vrk1
MSTRG.7686.1	MSTRG.7686	Vrk1
MSTRG.7688.1	MSTRG.7688	Vrk1
MSTRG.7690.1	MSTRG.7690	.
MSTRG.7695.1	MSTRG.7695	Gm16086
MSTRG.7696.1	MSTRG.7696	.
MSTRG.7697.1	MSTRG.7697	.
MSTRG.7698.1	MSTRG.7698	.
MSTRG.7699.1	MSTRG.7699	.

MSTRG.7701.1	MSTRG.7701	.
MSTRG.7704.1	MSTRG.7704	.
MSTRG.7705.1	MSTRG.7705	Bcl11b
MSTRG.7705.2	MSTRG.7705	Bcl11b
MSTRG.7713.1	MSTRG.7713	.
MSTRG.7718.1	MSTRG.7718	Evl
MSTRG.7719.1	MSTRG.7719	Evl
MSTRG.772.1	MSTRG.772	Tns1
MSTRG.7720.1	MSTRG.7720	Evl
MSTRG.7724.1	MSTRG.7724	.
MSTRG.7726.1	MSTRG.7726	.
MSTRG.7729.1	MSTRG.7729	Slc25a29
MSTRG.7731.1	MSTRG.7731	Slc25a47
MSTRG.7736.1	MSTRG.7736	Wdr25
MSTRG.7737.1	MSTRG.7737	Wdr25
MSTRG.7740.1	MSTRG.7740	Wdr25
MSTRG.7744.1	MSTRG.7744	Ppp2r5c
MSTRG.7745.1	MSTRG.7745	AL591582.1
MSTRG.7745.3	MSTRG.7745	AL591582.1
MSTRG.7745.7	MSTRG.7745	AL591582.1
MSTRG.7753.1	MSTRG.7753	Wdr20
MSTRG.7754.1	MSTRG.7754	Wdr20
MSTRG.7756.1	MSTRG.7756	Mok
MSTRG.7766.1	MSTRG.7766	Rcor1
MSTRG.7767.1	MSTRG.7767	Rcor1
MSTRG.7769.1	MSTRG.7769	AC153152.5
MSTRG.7771.1	MSTRG.7771	4930595D18Rik
MSTRG.7771.3	MSTRG.7771	4930595D18Rik
MSTRG.7771.4	MSTRG.7771	4930595D18Rik
MSTRG.7771.5	MSTRG.7771	4930595D18Rik
MSTRG.7771.6	MSTRG.7771	4930595D18Rik
MSTRG.7782.1	MSTRG.7782	Zfyve21
MSTRG.7783.1	MSTRG.7783	Zfyve21
MSTRG.7786.1	MSTRG.7786	Ppp1r13b
MSTRG.7789.1	MSTRG.7789	Trmt61a
MSTRG.7793.1	MSTRG.7793	Apopt1
MSTRG.7795.1	MSTRG.7795	Apopt1
MSTRG.7796.1	MSTRG.7796	Apopt1
MSTRG.7797.1	MSTRG.7797	Apopt1
MSTRG.7799.1	MSTRG.7799	Klc1
MSTRG.7803.1	MSTRG.7803	Tdrd9
MSTRG.7804.1	MSTRG.7804	Tdrd9
MSTRG.7820.1	MSTRG.7820	Gpr132
MSTRG.7822.1	MSTRG.7822	.
MSTRG.7831.1	MSTRG.7831	Pacs2
MSTRG.7837.1	MSTRG.7837	Mta1
MSTRG.7839.3	MSTRG.7839	Crip1
MSTRG.7847.1	MSTRG.7847	Ighv7-1
MSTRG.785.1	MSTRG.785	Usp37
MSTRG.7873.1	MSTRG.7873	.
MSTRG.7913.1	MSTRG.7913	.
MSTRG.7914.1	MSTRG.7914	.
MSTRG.7920.1	MSTRG.7920	Ighv11-2
MSTRG.7927.1	MSTRG.7927	Ighv7-4
MSTRG.7927.2	MSTRG.7927	Ighv7-4
MSTRG.7928.1	MSTRG.7928	Ighv9-1
MSTRG.7967.1	MSTRG.7967	Zfp386

MSTRG.7968.2	MSTRG.7968	Ighv8-5
MSTRG.797.1	MSTRG.797	Nhej1
MSTRG.798.1	MSTRG.798	Nhej1
MSTRG.7997.1	MSTRG.7997	Ighv1-66
MSTRG.80.1	MSTRG.80	Arfgef1
MSTRG.8020.1	MSTRG.8020	Esyt2
MSTRG.8020.2	MSTRG.8020	Esyt2
MSTRG.8021.1	MSTRG.8021	Esyt2
MSTRG.8022.1	MSTRG.8022	Esyt2
MSTRG.8025.1	MSTRG.8025	Ncapg2
MSTRG.8035.1	MSTRG.8035	Sp4
MSTRG.8036.1	MSTRG.8036	Sp4
MSTRG.8037.1	MSTRG.8037	Sp4
MSTRG.8038.1	MSTRG.8038	.
MSTRG.8039.1	MSTRG.8039	.
MSTRG.8041.1	MSTRG.8041	.
MSTRG.8042.1	MSTRG.8042	.
MSTRG.8044.1	MSTRG.8044	.
MSTRG.8045.1	MSTRG.8045	.
MSTRG.8047.1	MSTRG.8047	AC122379.1
MSTRG.8050.1	MSTRG.8050	Gm16505
MSTRG.8051.1	MSTRG.8051	Asb13
MSTRG.8057.1	MSTRG.8057	Fam208b
MSTRG.8058.1	MSTRG.8058	Fam208b
MSTRG.8059.1	MSTRG.8059	Fam208b
MSTRG.8062.1	MSTRG.8062	AC139323.1
MSTRG.8064.1	MSTRG.8064	.
MSTRG.8065.1	MSTRG.8065	.
MSTRG.8067.1	MSTRG.8067	Akr1e1
MSTRG.8068.1	MSTRG.8068	.
MSTRG.8072.1	MSTRG.8072	AC154269.1
MSTRG.8074.1	MSTRG.8074	AC127590.1
MSTRG.8076.1	MSTRG.8076	Pitrm1
MSTRG.8078.7	MSTRG.8078	Adarb2
MSTRG.8087.1	MSTRG.8087	Wdr37
MSTRG.8094.1	MSTRG.8094	.
MSTRG.8096.1	MSTRG.8096	AC127281.1
MSTRG.8103.1	MSTRG.8103	Dip2c
MSTRG.8105.1	MSTRG.8105	Dip2c
MSTRG.8110.1	MSTRG.8110	Dip2c
MSTRG.8112.1	MSTRG.8112	Dip2c
MSTRG.8113.1	MSTRG.8113	Dip2c
MSTRG.8114.1	MSTRG.8114	Dip2c
MSTRG.8121.1	MSTRG.8121	Zmynd11
MSTRG.8129.1	MSTRG.8129	Gm26861
MSTRG.8137.1	MSTRG.8137	Heatr1
MSTRG.8140.1	MSTRG.8140	Edaradd
MSTRG.8143.1	MSTRG.8143	Ero1lb
MSTRG.8157.1	MSTRG.8157	.
MSTRG.8159.1	MSTRG.8159	CT009546.3
MSTRG.8163.1	MSTRG.8163	Lyst
MSTRG.8164.1	MSTRG.8164	.
MSTRG.8165.1	MSTRG.8165	.
MSTRG.8168.2	MSTRG.8168	Psma2
MSTRG.8179.1	MSTRG.8179	B3galnt2
MSTRG.8183.1	MSTRG.8183	Gm26129
MSTRG.8183.2	MSTRG.8183	Gm26129

MSTRG.8183.3	MSTRG.8183	Gm26129
MSTRG.8186.1	MSTRG.8186	Arid4b
MSTRG.8188.1	MSTRG.8188	.
MSTRG.8199.1	MSTRG.8199	Sugct
MSTRG.8204.1	MSTRG.8204	CT009754.2
MSTRG.8209.1	MSTRG.8209	Mir466i
MSTRG.8209.2	MSTRG.8209	Mir466i
MSTRG.8211.1	MSTRG.8211	Cdk13
MSTRG.8212.1	MSTRG.8212	Cdk13
MSTRG.8215.1	MSTRG.8215	Vps41
MSTRG.822.1	MSTRG.822	.
MSTRG.823.1	MSTRG.823	.
MSTRG.8230.1	MSTRG.8230	Stard3nl
MSTRG.8231.1	MSTRG.8231	Stard3nl
MSTRG.8235.1	MSTRG.8235	Elmo1
MSTRG.8236.1	MSTRG.8236	Elmo1
MSTRG.8237.1	MSTRG.8237	Elmo1
MSTRG.8238.1	MSTRG.8238	Elmo1
MSTRG.8239.1	MSTRG.8239	Elmo1
MSTRG.8241.1	MSTRG.8241	Elmo1
MSTRG.8242.2	MSTRG.8242	AC132085.1
MSTRG.8242.3	MSTRG.8242	AC132085.1
MSTRG.8242.4	MSTRG.8242	AC132085.1
MSTRG.8243.1	MSTRG.8243	Elmo1
MSTRG.8244.2	MSTRG.8244	Elmo1
MSTRG.8245.1	MSTRG.8245	Elmo1
MSTRG.8246.1	MSTRG.8246	Elmo1
MSTRG.8247.1	MSTRG.8247	AC132085.1
MSTRG.8249.1	MSTRG.8249	Elmo1
MSTRG.8254.1	MSTRG.8254	.
MSTRG.8268.1	MSTRG.8268	.
MSTRG.8272.1	MSTRG.8272	Hist1h4j
MSTRG.8276.1	MSTRG.8276	Hist1h2bp
MSTRG.8276.2	MSTRG.8276	Hist1h4n
MSTRG.8281.1	MSTRG.8281	.
MSTRG.8287.2	MSTRG.8287	Hist1h2bq
MSTRG.829.1	MSTRG.829	Farsb
MSTRG.8290.1	MSTRG.8290	.
MSTRG.8290.2	MSTRG.8290	.
MSTRG.8290.3	MSTRG.8290	.
MSTRG.8290.4	MSTRG.8290	.
MSTRG.8301.1	MSTRG.8301	.
MSTRG.8306.1	MSTRG.8306	.
MSTRG.8307.1	MSTRG.8307	.
MSTRG.8315.1	MSTRG.8315	Hist1h2bh
MSTRG.8315.2	MSTRG.8315	Hist1h2bh
MSTRG.8320.1	MSTRG.8320	Gm11338
MSTRG.8320.2	MSTRG.8320	Gm11338
MSTRG.8327.1	MSTRG.8327	Hfe
MSTRG.833.1	MSTRG.833	Acsl3
MSTRG.8344.1	MSTRG.8344	Carmil1
MSTRG.8345.1	MSTRG.8345	Carmil1
MSTRG.8349.1	MSTRG.8349	Tdp2
MSTRG.8352.1	MSTRG.8352	.
MSTRG.8362.1	MSTRG.8362	Cmah
MSTRG.8363.1	MSTRG.8363	Cmah
MSTRG.8365.1	MSTRG.8365	Cmah

MSTRG.8366.1	MSTRG.8366	Cmah
MSTRG.8373.1	MSTRG.8373	Ripor2
MSTRG.8375.1	MSTRG.8375	Ripor2
MSTRG.8376.1	MSTRG.8376	.
MSTRG.8384.1	MSTRG.8384	.
MSTRG.8385.1	MSTRG.8385	.
MSTRG.8386.5	MSTRG.8386	AL590503.1
MSTRG.8387.1	MSTRG.8387	AL590503.1
MSTRG.8389.1	MSTRG.8389	2610307P16Rik
MSTRG.8389.2	MSTRG.8389	2610307P16Rik
MSTRG.8389.3	MSTRG.8389	2610307P16Rik
MSTRG.8389.4	MSTRG.8389	2610307P16Rik
MSTRG.8389.5	MSTRG.8389	2610307P16Rik
MSTRG.839.1	MSTRG.839	Gm29536
MSTRG.8391.1	MSTRG.8391	2610307P16Rik
MSTRG.8392.1	MSTRG.8392	2610307P16Rik
MSTRG.8393.1	MSTRG.8393	2610307P16Rik
MSTRG.8394.1	MSTRG.8394	2610307P16Rik
MSTRG.8395.1	MSTRG.8395	2610307P16Rik
MSTRG.8395.2	MSTRG.8395	2610307P16Rik
MSTRG.8397.1	MSTRG.8397	2610307P16Rik
MSTRG.8397.2	MSTRG.8397	2610307P16Rik
MSTRG.8397.3	MSTRG.8397	2610307P16Rik
MSTRG.8398.1	MSTRG.8398	2610307P16Rik
MSTRG.8399.1	MSTRG.8399	2610307P16Rik
MSTRG.840.4	MSTRG.840	Ap1s3
MSTRG.8400.1	MSTRG.8400	2610307P16Rik
MSTRG.8401.1	MSTRG.8401	2610307P16Rik
MSTRG.8409.1	MSTRG.8409	Cdkal1
MSTRG.8410.1	MSTRG.8410	Cdkal1
MSTRG.8411.1	MSTRG.8411	Cdkal1
MSTRG.8412.5	MSTRG.8412	E2f3
MSTRG.8413.1	MSTRG.8413	E2f3
MSTRG.8423.1	MSTRG.8423	Dusp22
MSTRG.8433.1	MSTRG.8433	Gmds
MSTRG.8435.1	MSTRG.8435	Gmds
MSTRG.8440.1	MSTRG.8440	.
MSTRG.8441.1	MSTRG.8441	.
MSTRG.845.1	MSTRG.845	.
MSTRG.8451.7	MSTRG.8451	Bphl
MSTRG.8452.1	MSTRG.8452	Bphl
MSTRG.8459.1	MSTRG.8459	Slc22a23
MSTRG.8462.1	MSTRG.8462	Slc22a23
MSTRG.847.1	MSTRG.847	Wdfy1
MSTRG.8475.1	MSTRG.8475	Eci2
MSTRG.8479.1	MSTRG.8479	AC132604.1
MSTRG.8491.1	MSTRG.8491	AC134860.5
MSTRG.8493.1	MSTRG.8493	Lyrm4
MSTRG.8494.1	MSTRG.8494	Lyrm4
MSTRG.8495.1	MSTRG.8495	Lyrm4
MSTRG.8498.1	MSTRG.8498	Fars2
MSTRG.8499.1	MSTRG.8499	Fars2
MSTRG.85.1	MSTRG.85	Cspp1
MSTRG.8500.1	MSTRG.8500	Fars2
MSTRG.8500.2	MSTRG.8500	Fars2
MSTRG.8502.1	MSTRG.8502	AC140397.2
MSTRG.8504.1	MSTRG.8504	Fars2

MSTRG.8505.1	MSTRG.8505	Fars2
MSTRG.8516.1	MSTRG.8516	F13a1
MSTRG.8518.1	MSTRG.8518	F13a1
MSTRG.8527.1	MSTRG.8527	Rreb1
MSTRG.8528.2	MSTRG.8528	AC022682.1
MSTRG.8529.1	MSTRG.8529	Rreb1
MSTRG.854.1	MSTRG.854	.
MSTRG.8541.1	MSTRG.8541	.
MSTRG.8545.1	MSTRG.8545	AC125223.2
MSTRG.8551.1	MSTRG.8551	AC147639.2
MSTRG.8552.1	MSTRG.8552	AC147639.2
MSTRG.8553.1	MSTRG.8553	.
MSTRG.8555.1	MSTRG.8555	Slc35b3
MSTRG.8557.1	MSTRG.8557	Tmem14c
MSTRG.8565.1	MSTRG.8565	A730081D07Rik
MSTRG.8566.1	MSTRG.8566	A730081D07Rik
MSTRG.8572.1	MSTRG.8572	Nedd9
MSTRG.8573.3	MSTRG.8573	AC167669.4
MSTRG.8574.1	MSTRG.8574	Nedd9
MSTRG.8576.1	MSTRG.8576	Nedd9
MSTRG.8577.1	MSTRG.8577	Nedd9
MSTRG.8583.1	MSTRG.8583	Hivep1
MSTRG.8584.1	MSTRG.8584	Hivep1
MSTRG.8585.2	MSTRG.8585	Hivep1
MSTRG.8587.1	MSTRG.8587	Tbc1d7
MSTRG.8589.1	MSTRG.8589	Gfod1
MSTRG.859.1	MSTRG.859	Rhbdd1
MSTRG.8590.1	MSTRG.8590	Gfod1
MSTRG.8591.1	MSTRG.8591	Gfod1
MSTRG.8592.1	MSTRG.8592	Gfod1
MSTRG.8594.1	MSTRG.8594	Gfod1
MSTRG.8599.1	MSTRG.8599	AC091785.2
MSTRG.86.1	MSTRG.86	Cspp1
MSTRG.8601.1	MSTRG.8601	Sirt5
MSTRG.8608.1	MSTRG.8608	.
MSTRG.8611.1	MSTRG.8611	.
MSTRG.8613.1	MSTRG.8613	Jarid2
MSTRG.8613.2	MSTRG.8613	Jarid2
MSTRG.8615.1	MSTRG.8615	Jarid2
MSTRG.8616.1	MSTRG.8616	Jarid2
MSTRG.862.1	MSTRG.862	Rhbdd1
MSTRG.8623.1	MSTRG.8623	Atxn1
MSTRG.8625.1	MSTRG.8625	Atxn1
MSTRG.8626.2	MSTRG.8626	Atxn1
MSTRG.8627.1	MSTRG.8627	Atxn1
MSTRG.8628.1	MSTRG.8628	Atxn1
MSTRG.8629.1	MSTRG.8629	Atxn1
MSTRG.8631.2	MSTRG.8631	Atxn1
MSTRG.8632.1	MSTRG.8632	Atxn1
MSTRG.8637.1	MSTRG.8637	Cap2
MSTRG.8639.1	MSTRG.8639	Cap2
MSTRG.8640.1	MSTRG.8640	Cap2
MSTRG.8641.1	MSTRG.8641	Cap2
MSTRG.8643.1	MSTRG.8643	Cap2
MSTRG.8647.1	MSTRG.8647	Kif13a
MSTRG.8651.1	MSTRG.8651	Kif13a
MSTRG.8652.1	MSTRG.8652	Kif13a

MSTRG.8656.1	MSTRG.8656	Rnf144b
MSTRG.866.1	MSTRG.866	Dock10
MSTRG.8669.1	MSTRG.8669	Gm37238
MSTRG.867.1	MSTRG.867	Dock10
MSTRG.8672.1	MSTRG.8672	Phf2
MSTRG.8673.1	MSTRG.8673	Phf2os1
MSTRG.8673.3	MSTRG.8673	Phf2os1
MSTRG.8674.1	MSTRG.8674	Phf2
MSTRG.8676.1	MSTRG.8676	Fam120aos
MSTRG.868.1	MSTRG.868	Dock10
MSTRG.8680.1	MSTRG.8680	Susd3
MSTRG.8685.1	MSTRG.8685	Ippk
MSTRG.8689.1	MSTRG.8689	Aspn
MSTRG.8692.1	MSTRG.8692	.
MSTRG.8693.1	MSTRG.8693	.
MSTRG.8697.1	MSTRG.8697	Iars
MSTRG.870.1	MSTRG.870	Dock10
MSTRG.8701.1	MSTRG.8701	Gm8739
MSTRG.8703.1	MSTRG.8703	Fbxw17
MSTRG.8706.1	MSTRG.8706	.
MSTRG.871.1	MSTRG.871	Dock10
MSTRG.8718.1	MSTRG.8718	CT010575.2
MSTRG.8719.1	MSTRG.8719	CT010575.2
MSTRG.872.1	MSTRG.872	Gm38062
MSTRG.8720.1	MSTRG.8720	.
MSTRG.8722.1	MSTRG.8722	Secisbp2
MSTRG.8723.1	MSTRG.8723	.
MSTRG.8723.2	MSTRG.8723	.
MSTRG.8727.1	MSTRG.8727	Secisbp2
MSTRG.8728.1	MSTRG.8728	Gm15440
MSTRG.8729.19	MSTRG.8729	Sema4d
MSTRG.873.1	MSTRG.873	Dock10
MSTRG.873.2	MSTRG.873	Dock10
MSTRG.8731.1	MSTRG.8731	Sema4d
MSTRG.8733.1	MSTRG.8733	Syk
MSTRG.8735.1	MSTRG.8735	Nfil3
MSTRG.874.1	MSTRG.874	Dock10
MSTRG.8742.1	MSTRG.8742	.
MSTRG.8743.1	MSTRG.8743	.
MSTRG.8750.7	MSTRG.8750	Rnf44
MSTRG.8752.1	MSTRG.8752	Uimc1
MSTRG.8753.1	MSTRG.8753	Uimc1
MSTRG.8756.1	MSTRG.8756	Zfp346
MSTRG.8759.1	MSTRG.8759	.
MSTRG.876.1	MSTRG.876	Dock10
MSTRG.8760.1	MSTRG.8760	.
MSTRG.8760.2	MSTRG.8760	.
MSTRG.8760.3	MSTRG.8760	.
MSTRG.8760.4	MSTRG.8760	.
MSTRG.8760.5	MSTRG.8760	.
MSTRG.8760.6	MSTRG.8760	.
MSTRG.8761.1	MSTRG.8761	.
MSTRG.8776.1	MSTRG.8776	Pdlim7
MSTRG.8777.1	MSTRG.8777	Pdlim7
MSTRG.8784.1	MSTRG.8784	B4galt7
MSTRG.8785.1	MSTRG.8785	B4galt7
MSTRG.8790.1	MSTRG.8790	B230219D22Rik

MSTRG.8793.1	MSTRG.8793	Pcbd2
MSTRG.8794.1	MSTRG.8794	Pcbd2
MSTRG.8802.1	MSTRG.8802	H2afy
MSTRG.8808.1	MSTRG.8808	.
MSTRG.8812.1	MSTRG.8812	.
MSTRG.8814.3	MSTRG.8814	Idnk
MSTRG.8814.4	MSTRG.8814	Idnk
MSTRG.882.1	MSTRG.882	Mff
MSTRG.8826.2	MSTRG.8826	AC124392.1
MSTRG.8828.1	MSTRG.8828	Gm5084
MSTRG.8833.1	MSTRG.8833	Dapk1
MSTRG.8834.1	MSTRG.8834	Dapk1
MSTRG.8837.1	MSTRG.8837	Dapk1
MSTRG.8838.1	MSTRG.8838	Dapk1
MSTRG.8839.1	MSTRG.8839	.
MSTRG.8855.1	MSTRG.8855	AC124426.5
MSTRG.8856.1	MSTRG.8856	Zfp808
MSTRG.8858.1	MSTRG.8858	AC124426.5
MSTRG.8859.1	MSTRG.8859	AC124426.5
MSTRG.8863.1	MSTRG.8863	Zfp808
MSTRG.8866.1	MSTRG.8866	Zfp808
MSTRG.8882.1	MSTRG.8882	Gm24020
MSTRG.8884.1	MSTRG.8884	Platr25
MSTRG.8888.1	MSTRG.8888	Zfp934
MSTRG.8889.1	MSTRG.8889	Zfp934
MSTRG.8893.1	MSTRG.8893	Platr25
MSTRG.8895.10	MSTRG.8895	Platr25
MSTRG.8899.1	MSTRG.8899	Gm5141
MSTRG.890.1	MSTRG.890	Slc19a3
MSTRG.8906.1	MSTRG.8906	2010111I01Rik
MSTRG.8909.1	MSTRG.8909	2010111I01Rik
MSTRG.891.1	MSTRG.891	.
MSTRG.8911.1	MSTRG.8911	2010111I01Rik
MSTRG.8912.1	MSTRG.8912	2010111I01Rik
MSTRG.8913.1	MSTRG.8913	2010111I01Rik
MSTRG.8916.1	MSTRG.8916	2010111I01Rik
MSTRG.8924.1	MSTRG.8924	Fancc
MSTRG.893.1	MSTRG.893	Pid1
MSTRG.8930.1	MSTRG.8930	Ercc6l2
MSTRG.8937.1	MSTRG.8937	Cdc14b
MSTRG.8938.1	MSTRG.8938	Cdc14b
MSTRG.8944.1	MSTRG.8944	Aaed1
MSTRG.8945.1	MSTRG.8945	Aaed1
MSTRG.8948.1	MSTRG.8948	.
MSTRG.8953.1	MSTRG.8953	Cntnap3
MSTRG.8954.1	MSTRG.8954	Cntnap3
MSTRG.8958.1	MSTRG.8958	Gm36445
MSTRG.8960.1	MSTRG.8960	.
MSTRG.8963.1	MSTRG.8963	.
MSTRG.8964.1	MSTRG.8964	.
MSTRG.8969.1	MSTRG.8969	Mterf3
MSTRG.8973.1	MSTRG.8973	Zfp759
MSTRG.8988.1	MSTRG.8988	.
MSTRG.8989.1	MSTRG.8989	.
MSTRG.8991.1	MSTRG.8991	CT571266.1
MSTRG.8993.1	MSTRG.8993	.
MSTRG.8994.1	MSTRG.8994	.

MSTRG.8995.1	MSTRG.8995	.
MSTRG.8996.1	MSTRG.8996	.
MSTRG.90.1	MSTRG.90	A830018L16Rik
MSTRG.9001.1	MSTRG.9001	Mtrr
MSTRG.9002.1	MSTRG.9002	Mtrr
MSTRG.901.1	MSTRG.901	Fbxo36
MSTRG.9010.1	MSTRG.9010	Papd7
MSTRG.9015.1	MSTRG.9015	.
MSTRG.9015.2	MSTRG.9015	.
MSTRG.9017.1	MSTRG.9017	.
MSTRG.9021.1	MSTRG.9021	Zfp457
MSTRG.9025.1	MSTRG.9025	Zfp595
MSTRG.9026.1	MSTRG.9026	Gm28044
MSTRG.9027.1	MSTRG.9027	Gm28041
MSTRG.9038.1	MSTRG.9038	Gm26965
MSTRG.9039.1	MSTRG.9039	Gm26965
MSTRG.904.1	MSTRG.904	A630001G21Rik
MSTRG.9041.1	MSTRG.9041	Zfp748
MSTRG.9044.1	MSTRG.9044	Zfp729b
MSTRG.9045.1	MSTRG.9045	Zfp729a
MSTRG.9046.1	MSTRG.9046	CT030194.1
MSTRG.9046.2	MSTRG.9046	CT030194.1
MSTRG.9046.3	MSTRG.9046	CT030194.1
MSTRG.9047.1	MSTRG.9047	CT573016.1
MSTRG.9048.1	MSTRG.9048	CT030194.1
MSTRG.9049.1	MSTRG.9049	Zfp738
MSTRG.905.1	MSTRG.905	A630001G21Rik
MSTRG.9050.1	MSTRG.9050	CT030194.1
MSTRG.9053.1	MSTRG.9053	CT030194.1
MSTRG.9059.1	MSTRG.9059	Ice1
MSTRG.9062.4	MSTRG.9062	Ndufs6
MSTRG.9067.1	MSTRG.9067	Tert
MSTRG.9069.1	MSTRG.9069	Slc12a7
MSTRG.9070.1	MSTRG.9070	Slc12a7
MSTRG.9071.1	MSTRG.9071	Slc12a7
MSTRG.9072.1	MSTRG.9072	Slc12a7
MSTRG.9083.1	MSTRG.9083	Cep72
MSTRG.9092.1	MSTRG.9092	Ccdc127
MSTRG.9096.1	MSTRG.9096	Zfp825
MSTRG.9102.1	MSTRG.9102	CT009718.4
MSTRG.9104.1	MSTRG.9104	.
MSTRG.9112.1	MSTRG.9112	Rfesd
MSTRG.9114.1	MSTRG.9114	Arsk
MSTRG.9116.1	MSTRG.9116	Ttc37
MSTRG.9117.1	MSTRG.9117	Ttc37
MSTRG.9118.1	MSTRG.9118	Ttc37
MSTRG.9122.1	MSTRG.9122	Mctp1
MSTRG.9123.1	MSTRG.9123	Mctp1
MSTRG.9124.1	MSTRG.9124	Mctp1
MSTRG.9130.1	MSTRG.9130	Slf1
MSTRG.9131.1	MSTRG.9131	Slf1
MSTRG.9134.1	MSTRG.9134	2210408I21Rik
MSTRG.9135.1	MSTRG.9135	2210408I21Rik
MSTRG.9137.1	MSTRG.9137	.
MSTRG.9138.1	MSTRG.9138	.
MSTRG.9141.1	MSTRG.9141	Gm28526
MSTRG.9144.1	MSTRG.9144	Fam172a

MSTRG.9146.1	MSTRG.9146	Fam172a
MSTRG.9147.1	MSTRG.9147	Fam172a
MSTRG.9148.1	MSTRG.9148	Fam172a
MSTRG.9150.1	MSTRG.9150	Fam172a
MSTRG.9151.1	MSTRG.9151	Fam172a
MSTRG.9151.2	MSTRG.9151	Fam172a
MSTRG.9152.1	MSTRG.9152	Fam172a
MSTRG.916.1	MSTRG.916	Psmd1
MSTRG.9160.1	MSTRG.9160	Polr3g
MSTRG.9161.1	MSTRG.9161	Polr3g
MSTRG.9163.1	MSTRG.9163	Mblac2
MSTRG.9165.1	MSTRG.9165	Mblac2
MSTRG.9166.1	MSTRG.9166	Mblac2
MSTRG.9179.1	MSTRG.9179	.
MSTRG.918.1	MSTRG.918	Armc9
MSTRG.9180.1	MSTRG.9180	.
MSTRG.9183.1	MSTRG.9183	Tmem161b
MSTRG.9186.1	MSTRG.9186	Tmem161b
MSTRG.9187.1	MSTRG.9187	Tmem161b
MSTRG.9188.3	MSTRG.9188	Cox7c
MSTRG.9189.1	MSTRG.9189	.
MSTRG.919.1	MSTRG.919	Armc9
MSTRG.9190.1	MSTRG.9190	.
MSTRG.9193.1	MSTRG.9193	Rasa1
MSTRG.9193.2	MSTRG.9193	Rasa1
MSTRG.9194.1	MSTRG.9194	.
MSTRG.9195.1	MSTRG.9195	.
MSTRG.920.1	MSTRG.920	Armc9
MSTRG.9203.3	MSTRG.9203	Xrcc4
MSTRG.9204.1	MSTRG.9204	Xrcc4
MSTRG.9205.1	MSTRG.9205	Xrcc4
MSTRG.9206.1	MSTRG.9206	Xrcc4
MSTRG.9208.1	MSTRG.9208	Xrcc4
MSTRG.9209.1	MSTRG.9209	Xrcc4
MSTRG.921.1	MSTRG.921	Armc9
MSTRG.9210.1	MSTRG.9210	Xrcc4
MSTRG.9213.1	MSTRG.9213	Tmem167
MSTRG.9216.1	MSTRG.9216	Atg10
MSTRG.9217.1	MSTRG.9217	Atg10
MSTRG.9218.1	MSTRG.9218	Atg10
MSTRG.9219.1	MSTRG.9219	Atg10
MSTRG.9222.11	MSTRG.9222	Ssbp2
MSTRG.9222.15	MSTRG.9222	Ssbp2
MSTRG.9222.16	MSTRG.9222	Ssbp2
MSTRG.9222.17	MSTRG.9222	Ssbp2
MSTRG.9222.19	MSTRG.9222	Ssbp2
MSTRG.9223.1	MSTRG.9223	Ssbp2
MSTRG.9224.1	MSTRG.9224	Ssbp2
MSTRG.9226.1	MSTRG.9226	Ssbp2
MSTRG.9227.1	MSTRG.9227	Ssbp2
MSTRG.923.1	MSTRG.923	.
MSTRG.9236.1	MSTRG.9236	Rasgrf2
MSTRG.9237.1	MSTRG.9237	.
MSTRG.9240.1	MSTRG.9240	Msh3
MSTRG.9241.1	MSTRG.9241	Msh3
MSTRG.9242.1	MSTRG.9242	Msh3
MSTRG.9246.1	MSTRG.9246	Zfyve16

MSTRG.9247.1	MSTRG.9247	.
MSTRG.9248.1	MSTRG.9248	.
MSTRG.925.1	MSTRG.925	Nmur1
MSTRG.9250.1	MSTRG.9250	Serinc5
MSTRG.9250.2	MSTRG.9250	Serinc5
MSTRG.9250.3	MSTRG.9250	Serinc5
MSTRG.9251.1	MSTRG.9251	Serinc5
MSTRG.9254.2	MSTRG.9254	AC130217.2
MSTRG.9257.1	MSTRG.9257	Papd4
MSTRG.9258.1	MSTRG.9258	Papd4
MSTRG.9260.1	MSTRG.9260	Homer1
MSTRG.9262.1	MSTRG.9262	Homer1
MSTRG.9267.1	MSTRG.9267	Jmy
MSTRG.9268.1	MSTRG.9268	.
MSTRG.9275.1	MSTRG.9275	Scamp1
MSTRG.928.6	MSTRG.928	Pde6d
MSTRG.9281.1	MSTRG.9281	Tbca
MSTRG.9283.1	MSTRG.9283	Zbed3
MSTRG.929.1	MSTRG.929	Pde6d
MSTRG.9293.1	MSTRG.9293	.
MSTRG.9302.1	MSTRG.9302	Iqgap2
MSTRG.9302.2	MSTRG.9302	Iqgap2
MSTRG.9303.1	MSTRG.9303	Iqgap2
MSTRG.9304.1	MSTRG.9304	Iqgap2
MSTRG.9305.1	MSTRG.9305	Iqgap2
MSTRG.9306.1	MSTRG.9306	Iqgap2
MSTRG.9308.1	MSTRG.9308	Poc5
MSTRG.9309.1	MSTRG.9309	Poc5
MSTRG.9310.1	MSTRG.9310	Poc5
MSTRG.932.1	MSTRG.932	.
MSTRG.933.1	MSTRG.933	.
MSTRG.9333.1	MSTRG.9333	.
MSTRG.934.1	MSTRG.934	.
MSTRG.9347.1	MSTRG.9347	Fcho2
MSTRG.9348.1	MSTRG.9348	Fcho2
MSTRG.935.1	MSTRG.935	Gm6264
MSTRG.9350.1	MSTRG.9350	AC129085.1
MSTRG.9353.1	MSTRG.9353	Gm5453
MSTRG.9355.1	MSTRG.9355	Tnpo1
MSTRG.9356.1	MSTRG.9356	Tnpo1
MSTRG.9357.2	MSTRG.9357	Smn1
MSTRG.9357.8	MSTRG.9357	Smn1
MSTRG.9360.1	MSTRG.9360	Bdp1
MSTRG.9361.1	MSTRG.9361	Bdp1
MSTRG.9362.1	MSTRG.9362	Bdp1
MSTRG.937.2	MSTRG.937	C130026I21Rik
MSTRG.9371.1	MSTRG.9371	.
MSTRG.9383.1	MSTRG.9383	Ccdc125
MSTRG.9387.5	MSTRG.9387	Slc30a5
MSTRG.939.1	MSTRG.939	C130026I21Rik
MSTRG.9397.1	MSTRG.9397	Pik3r1
MSTRG.9397.2	MSTRG.9397	Pik3r1
MSTRG.94.1	MSTRG.94	Slco5a1
MSTRG.9401.1	MSTRG.9401	.
MSTRG.9403.1	MSTRG.9403	Cd180
MSTRG.9407.1	MSTRG.9407	Mast4
MSTRG.941.1	MSTRG.941	Gm7582

MSTRG.9413.1	MSTRG.9413	Nln
MSTRG.9416.1	MSTRG.9416	.
MSTRG.9418.3	MSTRG.9418	Ppwd1
MSTRG.942.1	MSTRG.942	Gm7582
MSTRG.9421.1	MSTRG.9421	Cenpk
MSTRG.9423.1	MSTRG.9423	Adamts6
MSTRG.9424.1	MSTRG.9424	Adamts6
MSTRG.9425.1	MSTRG.9425	Adamts6
MSTRG.9426.1	MSTRG.9426	Adamts6
MSTRG.9427.1	MSTRG.9427	Adamts6
MSTRG.9428.1	MSTRG.9428	Adamts6
MSTRG.9429.1	MSTRG.9429	Adamts6
MSTRG.943.1	MSTRG.943	Gm7582
MSTRG.9430.1	MSTRG.9430	Adamts6
MSTRG.9431.1	MSTRG.9431	Adamts6
MSTRG.9432.1	MSTRG.9432	Adamts6
MSTRG.9433.1	MSTRG.9433	Adamts6
MSTRG.9434.1	MSTRG.9434	Adamts6
MSTRG.9436.1	MSTRG.9436	Cwc27
MSTRG.9438.1	MSTRG.9438	Cwc27
MSTRG.9439.1	MSTRG.9439	Cwc27
MSTRG.944.1	MSTRG.944	Gm7582
MSTRG.9440.1	MSTRG.9440	Cwc27
MSTRG.9441.1	MSTRG.9441	Cwc27
MSTRG.9442.2	MSTRG.9442	Srek1ip1
MSTRG.9443.1	MSTRG.9443	Cwc27
MSTRG.9444.1	MSTRG.9444	.
MSTRG.9445.1	MSTRG.9445	.
MSTRG.945.1	MSTRG.945	C130026I21Rik
MSTRG.9455.1	MSTRG.9455	Kif2a
MSTRG.9457.1	MSTRG.9457	Kif2a
MSTRG.9458.5	MSTRG.9458	Zswim6
MSTRG.9458.6	MSTRG.9458	Zswim6
MSTRG.9460.1	MSTRG.9460	Zswim6
MSTRG.9461.1	MSTRG.9461	Zswim6
MSTRG.9461.2	MSTRG.9461	Zswim6
MSTRG.9461.3	MSTRG.9461	Zswim6
MSTRG.9464.1	MSTRG.9464	Zswim6
MSTRG.9464.2	MSTRG.9464	Zswim6
MSTRG.9464.3	MSTRG.9464	Zswim6
MSTRG.9464.4	MSTRG.9464	Zswim6
MSTRG.9465.1	MSTRG.9465	Zswim6
MSTRG.9466.1	MSTRG.9466	Zswim6
MSTRG.9470.1	MSTRG.9470	Ndufaf2
MSTRG.9471.1	MSTRG.9471	Ndufaf2
MSTRG.9473.1	MSTRG.9473	Ercc8
MSTRG.9475.1	MSTRG.9475	Depdc1b
MSTRG.9476.1	MSTRG.9476	Depdc1b
MSTRG.9477.1	MSTRG.9477	Depdc1b
MSTRG.9478.1	MSTRG.9478	Depdc1b
MSTRG.9479.1	MSTRG.9479	.
MSTRG.9481.1	MSTRG.9481	Pde4d
MSTRG.9487.1	MSTRG.9487	Pde4d
MSTRG.9488.1	MSTRG.9488	.
MSTRG.9489.1	MSTRG.9489	.
MSTRG.949.1	MSTRG.949	A530032D15Rik
MSTRG.9494.1	MSTRG.9494	Mier3

MSTRG.9495.1	MSTRG.9495	.
MSTRG.9497.1	MSTRG.9497	Mier3
MSTRG.9499.1	MSTRG.9499	Map3k1
MSTRG.95.1	MSTRG.95	Slco5a1
MSTRG.9501.1	MSTRG.9501	.
MSTRG.9504.1	MSTRG.9504	Ankrd55
MSTRG.951.1	MSTRG.951	C130026I21Rik
MSTRG.9511.1	MSTRG.9511	AC154767.2
MSTRG.9512.1	MSTRG.9512	Slc38a9
MSTRG.9513.1	MSTRG.9513	Slc38a9
MSTRG.9517.1	MSTRG.9517	Skiv2l2
MSTRG.952.1	MSTRG.952	Gm6264
MSTRG.9529.1	MSTRG.9529	BC067074
MSTRG.9530.1	MSTRG.9530	.
MSTRG.9532.1	MSTRG.9532	.
MSTRG.9535.1	MSTRG.9535	Arl15
MSTRG.9535.2	MSTRG.9535	Arl15
MSTRG.9536.1	MSTRG.9536	Arl15
MSTRG.9537.1	MSTRG.9537	Arl15
MSTRG.9537.2	MSTRG.9537	Arl15
MSTRG.9538.1	MSTRG.9538	Arl15
MSTRG.9539.1	MSTRG.9539	Arl15
MSTRG.9541.1	MSTRG.9541	AC126549.1
MSTRG.9543.1	MSTRG.9543	Arl15
MSTRG.9545.1	MSTRG.9545	Ndufs4
MSTRG.9545.2	MSTRG.9545	Ndufs4
MSTRG.9546.1	MSTRG.9546	Ndufs4
MSTRG.9547.1	MSTRG.9547	Ndufs4
MSTRG.955.1	MSTRG.955	Gm6264
MSTRG.9551.1	MSTRG.9551	Mocs2
MSTRG.9557.1	MSTRG.9557	.
MSTRG.9560.1	MSTRG.9560	.
MSTRG.9561.1	MSTRG.9561	.
MSTRG.9563.1	MSTRG.9563	Parp8
MSTRG.9564.1	MSTRG.9564	Parp8
MSTRG.9565.1	MSTRG.9565	Parp8
MSTRG.9566.1	MSTRG.9566	Parp8
MSTRG.9566.2	MSTRG.9566	Parp8
MSTRG.9567.1	MSTRG.9567	.
MSTRG.957.1	MSTRG.957	Gm2619
MSTRG.9571.1	MSTRG.9571	.
MSTRG.9574.1	MSTRG.9574	.
MSTRG.9575.1	MSTRG.9575	.
MSTRG.9579.1	MSTRG.9579	Tmem267
MSTRG.9579.2	MSTRG.9579	Tmem267
MSTRG.9579.3	MSTRG.9579	Tmem267
MSTRG.9579.4	MSTRG.9579	Tmem267
MSTRG.959.1	MSTRG.959	Sp110
MSTRG.9600.1	MSTRG.9600	BC147527
MSTRG.9602.1	MSTRG.9602	.
MSTRG.9605.1	MSTRG.9605	.
MSTRG.961.1	MSTRG.961	C130026I21Rik
MSTRG.9614.1	MSTRG.9614	Gm26680
MSTRG.962.1	MSTRG.962	C130026I21Rik
MSTRG.9620.1	MSTRG.9620	.
MSTRG.9623.1	MSTRG.9623	Pxk
MSTRG.9626.1	MSTRG.9626	Kctd6

MSTRG.9630.1	MSTRG.9630	4930452B06Rik
MSTRG.9634.1	MSTRG.9634	Gm3839
MSTRG.9638.1	MSTRG.9638	Ptprg
MSTRG.964.1	MSTRG.964	Gm16026
MSTRG.9645.1	MSTRG.9645	Fhit
MSTRG.9649.1	MSTRG.9649	Fhit
MSTRG.965.1	MSTRG.965	Gm2619
MSTRG.9650.1	MSTRG.9650	Fhit
MSTRG.9651.1	MSTRG.9651	Fhit
MSTRG.9653.1	MSTRG.9653	Fhit
MSTRG.9654.1	MSTRG.9654	Fhit
MSTRG.9655.1	MSTRG.9655	Fhit
MSTRG.9657.1	MSTRG.9657	Fhit
MSTRG.9658.1	MSTRG.9658	Fhit
MSTRG.966.1	MSTRG.966	Gm2619
MSTRG.9661.1	MSTRG.9661	Fhit
MSTRG.9663.1	MSTRG.9663	Fhit
MSTRG.9663.2	MSTRG.9663	Fhit
MSTRG.9664.1	MSTRG.9664	Fhit
MSTRG.9666.1	MSTRG.9666	Fhit
MSTRG.9667.1	MSTRG.9667	Fhit
MSTRG.9669.1	MSTRG.9669	Fhit
MSTRG.9675.1	MSTRG.9675	AC154682.1
MSTRG.9677.1	MSTRG.9677	AC154682.1
MSTRG.9682.1	MSTRG.9682	Cadps
MSTRG.9694.1	MSTRG.9694	Thoc7
MSTRG.9695.1	MSTRG.9695	Thoc7
MSTRG.9697.1	MSTRG.9697	Atxn7
MSTRG.9698.1	MSTRG.9698	Atxn7
MSTRG.9699.1	MSTRG.9699	Atxn7
MSTRG.9702.1	MSTRG.9702	Slc4a7
MSTRG.9703.1	MSTRG.9703	Slc4a7
MSTRG.9704.1	MSTRG.9704	Slc4a7
MSTRG.9705.1	MSTRG.9705	Slc4a7
MSTRG.9706.1	MSTRG.9706	Slc4a7
MSTRG.9709.1	MSTRG.9709	AC158388.3
MSTRG.971.1	MSTRG.971	Gm2427
MSTRG.9712.1	MSTRG.9712	.
MSTRG.9718.1	MSTRG.9718	Top2b
MSTRG.9719.1	MSTRG.9719	Top2b
MSTRG.972.1	MSTRG.972	Gm17017
MSTRG.9725.3	MSTRG.9725	AC154681.1
MSTRG.9725.4	MSTRG.9725	AC154681.1
MSTRG.9726.1	MSTRG.9726	Thrb
MSTRG.9728.1	MSTRG.9728	.
MSTRG.9731.1	MSTRG.9731	Ube2e1
MSTRG.9732.1	MSTRG.9732	.
MSTRG.9735.1	MSTRG.9735	Ube2e2
MSTRG.9737.1	MSTRG.9737	Ube2e2
MSTRG.9743.1	MSTRG.9743	Gng2
MSTRG.9746.1	MSTRG.9746	Kcnk5
MSTRG.9748.1	MSTRG.9748	Nudt13
MSTRG.975.1	MSTRG.975	Gm7592
MSTRG.9758.1	MSTRG.9758	1810062O18Rik
MSTRG.9758.2	MSTRG.9758	1810062O18Rik
MSTRG.976.1	MSTRG.976	Gm7592
MSTRG.9768.1	MSTRG.9768	.

MSTRG.977.1	MSTRG.977	Gm7592
MSTRG.9775.1	MSTRG.9775	Adk
MSTRG.9776.1	MSTRG.9776	Adk
MSTRG.9777.1	MSTRG.9777	Adk
MSTRG.978.1	MSTRG.978	Gm7592
MSTRG.9782.1	MSTRG.9782	Kat6b
MSTRG.9788.1	MSTRG.9788	Samd8
MSTRG.9789.1	MSTRG.9789	Samd8
MSTRG.9790.1	MSTRG.9790	Samd8
MSTRG.9791.1	MSTRG.9791	Samd8
MSTRG.9800.1	MSTRG.9800	Lrmda
MSTRG.9801.1	MSTRG.9801	Lrmda
MSTRG.9803.1	MSTRG.9803	Lrmda
MSTRG.9804.1	MSTRG.9804	Lrmda
MSTRG.9805.1	MSTRG.9805	Lrmda
MSTRG.9806.1	MSTRG.9806	Lrmda
MSTRG.9807.1	MSTRG.9807	Lrmda
MSTRG.9809.1	MSTRG.9809	Lrmda
MSTRG.981.1	MSTRG.981	Sp110
MSTRG.9810.1	MSTRG.9810	Lrmda
MSTRG.9811.1	MSTRG.9811	Lrmda
MSTRG.9812.1	MSTRG.9812	Lrmda
MSTRG.9813.1	MSTRG.9813	Lrmda
MSTRG.9814.1	MSTRG.9814	Lrmda
MSTRG.9815.1	MSTRG.9815	Lrmda
MSTRG.9816.1	MSTRG.9816	Lrmda
MSTRG.9817.1	MSTRG.9817	Lrmda
MSTRG.9820.1	MSTRG.9820	Lrmda
MSTRG.9830.1	MSTRG.9830	Kcnma1
MSTRG.9832.1	MSTRG.9832	Kcnma1
MSTRG.9834.1	MSTRG.9834	Kcnma1
MSTRG.9835.1	MSTRG.9835	Kcnma1
MSTRG.9836.1	MSTRG.9836	Kcnma1
MSTRG.9837.1	MSTRG.9837	Kcnma1
MSTRG.9838.1	MSTRG.9838	Kcnma1
MSTRG.9845.1	MSTRG.9845	Zcchc24
MSTRG.9846.3	MSTRG.9846	Gm26660
MSTRG.9852.1	MSTRG.9852	Zmiz1
MSTRG.9856.1	MSTRG.9856	Zmiz1
MSTRG.9857.1	MSTRG.9857	Zmiz1
MSTRG.9858.1	MSTRG.9858	Zmiz1
MSTRG.9860.2	MSTRG.9860	Anxa11
MSTRG.9873.1	MSTRG.9873	Cphx3
MSTRG.9879.1	MSTRG.9879	Slmap
MSTRG.9880.1	MSTRG.9880	Gm2178
MSTRG.9882.1	MSTRG.9882	Slmap
MSTRG.9883.1	MSTRG.9883	Slmap
MSTRG.9886.1	MSTRG.9886	Slmap
MSTRG.9887.1	MSTRG.9887	Slmap
MSTRG.9891.1	MSTRG.9891	Dennd6a
MSTRG.9894.1	MSTRG.9894	Arf4
MSTRG.9900.1	MSTRG.9900	Appl1
MSTRG.9904.1	MSTRG.9904	Arhgef3
MSTRG.9905.1	MSTRG.9905	Arhgef3
MSTRG.9906.1	MSTRG.9906	Arhgef3
MSTRG.9907.1	MSTRG.9907	Arhgef3
MSTRG.9908.1	MSTRG.9908	Arhgef3

MSTRG.9909.1	MSTRG.9909	Arhgef3
MSTRG.9910.1	MSTRG.9910	Arhgef3
MSTRG.9911.1	MSTRG.9911	Arhgef3
MSTRG.9911.2	MSTRG.9911	Arhgef3
MSTRG.9912.1	MSTRG.9912	Arhgef3
MSTRG.9914.1	MSTRG.9914	.
MSTRG.9925.1	MSTRG.9925	Cacna2d3
MSTRG.9926.1	MSTRG.9926	Cacna2d3
MSTRG.9935.1	MSTRG.9935	Chdh
MSTRG.9943.3	MSTRG.9943	Tkt
MSTRG.9945.1	MSTRG.9945	Prkcd
MSTRG.9947.1	MSTRG.9947	Rft1
MSTRG.9948.1	MSTRG.9948	Sfmbt1
MSTRG.9952.1	MSTRG.9952	Tmem110
MSTRG.9953.1	MSTRG.9953	Tmem110
MSTRG.9954.1	MSTRG.9954	Tmem110
MSTRG.996.1	MSTRG.996	Dis3l2
MSTRG.9962.1	MSTRG.9962	Glt8d1
MSTRG.9963.1	MSTRG.9963	Glt8d1
MSTRG.997.1	MSTRG.997	Dis3l2
MSTRG.9970.1	MSTRG.9970	Pbrm1
MSTRG.9973.1	MSTRG.9973	Smim4
MSTRG.9979.1	MSTRG.9979	Sh3bp5
MSTRG.9980.1	MSTRG.9980	.
MSTRG.9982.1	MSTRG.9982	Ankrd28
MSTRG.9987.2	MSTRG.9987	Hacl1
MSTRG.9987.3	MSTRG.9987	Hacl1
MSTRG.9987.4	MSTRG.9987	Hacl1
MSTRG.9987.5	MSTRG.9987	Hacl1
MSTRG.9989.1	MSTRG.9989	.
MSTRG.9994.1	MSTRG.9994	.
MSTRG.9995.1	MSTRG.9995	.
MSTRG.9998.1	MSTRG.9998	Parg
