# Supplementary material for: Transcriptome analysis reveals a potential regulatory mechanism of the lnc-5423.6/IGFBP5 axis in the early stages of mouse thymic involution: lnc-5423.6/IGFBP5 axis regulates thymic involution
Source: Acta Biochim Biophys Sin (Shanghai). 2023 Apr 19;55(4):548–60. doi: 10.3724/abbs.2023042 (PMC10195152; doi:10.3724/abbs.2023042)
Supplement: Table_S4 [file Table_S4.pdf]

gene_na	fc	log2(fc	pval	regulat	significant
Cyp2e1	312.50	8.29	0.00	up	yes
Car3	134.54	7.07	0.00	up	yes
Cfd	90.51	6.50	0.00	up	yes
Igfbp5	80.44	6.33	0.00	up	yes
Tg	48.26	5.59	0.00	up	yes
Fmo2	31.81	4.99	0.00	up	yes
Thrsp	31.41	4.97	0.00	up	yes
Scd1	29.06	4.86	0.00	up	yes
Vldlr	25.09	4.65	0.00	up	yes
C4b	24.20	4.60	0.00	up	yes
Ighg3	24.33	4.60	0.00	up	yes
Cyp2f2	24.02	4.59	0.00	up	yes
Kirrel	23.01	4.52	0.00	up	yes
Scn7a	22.01	4.46	0.00	up	yes
Fam107a	21.55	4.43	0.00	up	yes
Gm23472	0.06	-4.18	0.00	down	yes
Bpifa1	19.69	4.30	0.00	up	yes
Pgr	19.68	4.30	0.00	up	yes
Igkc	18.57	4.21	0.00	up	yes
Jchain	18.48	4.21	0.00	up	yes
Scn1a	18.70	4.23	0.00	up	yes
Gpx3	18.05	4.17	0.00	up	yes
Prom2	18.39	4.20	0.00	up	yes
Gm22042	0.06	-4.02	0.00	down	yes
Iglc2;I	17.42	4.12	0.00	up	yes
Igkv15-	17.14	4.10	0.00	up	yes
Rnu1b1	0.06	-3.95	0.00	down	yes
Gm22614	0.07	-3.92	0.00	down	yes
Mgst1	16.71	4.06	0.00	up	yes
Cped1	16.73	4.06	0.00	up	yes
Ighg1	16.09	4.01	0.00	up	yes
Rnu1b2	0.07	-3.89	0.00	down	yes
Prelp	15.66	3.97	0.00	up	yes
Chst1	15.93	3.99	0.00	up	yes
Svep1	15.65	3.97	0.00	up	yes
Tns4	15.53	3.96	0.00	up	yes
Fabp4	14.95	3.90	0.00	up	yes
Aebp1	15.12	3.92	0.00	up	yes
Gm24830	0.07	-3.78	0.00	down	yes
Ephx2	15.08	3.91	0.00	up	yes
Gm26232	0.07	-3.76	0.00	down	yes
Abca8a	13.95	3.80	0.00	up	yes
Dcn	13.58	3.76	0.00	up	yes
Nrbp2	13.56	3.76	0.00	up	yes
Mdga1	13.59	3.76	0.00	up	yes
Htra3	13.56	3.76	0.00	up	yes
Apod	13.58	3.76	0.00	up	yes
Slit3	13.38	3.74	0.00	up	yes
Tnxb	13.02	3.70	0.00	up	yes
Adam28;	13.05	3.71	0.00	up	yes
Abca5	13.29	3.73	0.00	up	yes
Kcnh1	13.08	3.71	0.00	up	yes
Dgat2	13.04	3.70	0.00	up	yes
Gpd1	12.80	3.68	0.00	up	yes
Aoc3	12.77	3.67	0.00	up	yes
Ighv2-2	12.94	3.69	0.00	up	yes

Deptor	12.47	3.64	0.00	up	yes
Rnu1b6	0.09	-3.51	0.00	down	yes
Kcnj15	12.29	3.62	0.00	up	yes
Zfp46	0.09	-3.52	0.00	down	yes
Adh1	12.55	3.65	0.00	up	yes
Ptprd	12.11	3.60	0.00	up	yes
Hspb1	12.09	3.60	0.00	up	yes
Igkv1-1	11.92	3.58	0.00	up	yes
Fndc1	12.01	3.59	0.00	up	yes
Cd36	11.71	3.55	0.00	up	yes
Irs1	11.67	3.54	0.00	up	yes
Gm25890	0.09	-3.40	0.00	down	yes
Hbb-bt	11.51	3.52	0.00	up	yes
Gm24497	0.10	-3.39	0.00	down	yes
Setbp1	11.39	3.51	0.00	up	yes
Ace	11.49	3.52	0.00	up	yes
Steap4	11.26	3.49	0.00	up	yes
Iglc1;I	11.26	3.49	0.00	up	yes
Cd33	11.37	3.51	0.00	up	yes
Atp1a2	11.22	3.49	0.00	up	yes
Gm25939	0.10	-3.34	0.00	down	yes
Adamts1	11.39	3.51	0.00	up	yes
Gm8113	10.84	3.44	0.00	up	yes
Lrig3	11.11	3.47	0.00	up	yes
Hbb-bs	10.74	3.42	0.00	up	yes
1810062	10.71	3.42	0.00	up	yes
Trabd2b	10.46	3.39	0.00	up	yes
Igkv1-1	10.37	3.37	0.00	up	yes
Ghr	10.21	3.35	0.00	up	yes
Pygl	10.23	3.36	0.00	up	yes
Cfh	10.15	3.34	0.00	up	yes
Gpx1	10.15	3.34	0.00	up	yes
Hba-a2;	10.05	3.33	0.00	up	yes
Il33	10.29	3.36	0.00	up	yes
Lrg1	10.26	3.36	0.00	up	yes
4930469	0.11	-3.23	0.00	down	yes
Sdk2	10.02	3.32	0.00	up	yes
Gas1	10.05	3.33	0.00	up	yes
Acacb	9.90	3.31	0.00	up	yes
Cyp27a1	10.22	3.35	0.00	up	yes
Ryr3	9.92	3.31	0.00	up	yes
Shisa2	9.72	3.28	0.00	up	yes
Unc5a	9.96	3.32	0.00	up	yes
Nek6	9.65	3.27	0.00	up	yes
Pcx	9.44	3.24	0.00	up	yes
Gp2	9.44	3.24	0.00	up	yes
Fam13a	9.28	3.21	0.00	up	yes
Cyp2d22	9.46	3.24	0.00	up	yes
Pdgfra;	9.21	3.20	0.00	up	yes
Aox2	9.21	3.20	0.00	up	yes
Gm24265	0.12	-3.05	0.00	down	yes
Gm23444	0.12	-3.05	0.00	down	yes
Adgrd1	9.05	3.18	0.00	up	yes
Ophn1	9.17	3.20	0.00	up	yes
Ltbp3	8.87	3.15	0.00	up	yes
Gm25679	0.12	-3.03	0.00	down	yes
Mir29b-	8.83	3.14	0.00	up	yes

Klhl10	0.12	-3.03	0.00	down	yes
Dpyd	8.89	3.15	0.00	up	yes
Cebpa	8.76	3.13	0.00	up	yes
Evc2	8.95	3.16	0.00	up	yes
Dll1	8.90	3.15	0.00	up	yes
Ctsf	8.95	3.16	0.00	up	yes
Rbfox1	8.81	3.14	0.00	up	yes
Gm24305	0.13	-3.00	0.00	down	yes
Sema5a	8.64	3.11	0.00	up	yes
Vwf	8.65	3.11	0.00	up	yes
Npr1	8.71	3.12	0.00	up	yes
Bend4	8.74	3.13	0.00	up	yes
Mgat5b	8.66	3.11	0.00	up	yes
Cgn	8.56	3.10	0.00	up	yes
Ptges	8.61	3.11	0.00	up	yes
Ptprz1	8.52	3.09	0.00	up	yes
Six1	8.50	3.09	0.00	up	yes
Fat2	8.40	3.07	0.00	up	yes
Ano1	8.54	3.09	0.00	up	yes
Kcnip3	8.54	3.10	0.00	up	yes
Pygo1	8.49	3.09	0.00	up	yes
Plin4	8.30	3.05	0.00	up	yes
Rusc2	8.64	3.11	0.00	up	yes
Ebf1	8.35	3.06	0.00	up	yes
Il18rap	8.37	3.07	0.00	up	yes
Blk	8.33	3.06	0.00	up	yes
Ddr2	8.27	3.05	0.00	up	yes
Ntrk2	8.37	3.06	0.00	up	yes
Chpt1	8.20	3.04	0.00	up	yes
Plch2	8.25	3.04	0.00	up	yes
Six2	8.24	3.04	0.00	up	yes
Ttll10	8.33	3.06	0.00	up	yes
Adhfe1	8.31	3.05	0.00	up	yes
Clca2	8.32	3.06	0.00	up	yes
Plxna4	8.24	3.04	0.00	up	yes
Gm24407	0.13	-2.89	0.00	down	yes
Aldh1a3	8.14	3.03	0.00	up	yes
Stab1	8.04	3.01	0.00	up	yes
Aifm2	8.14	3.02	0.00	up	yes
Dcdc5	8.26	3.05	0.00	up	yes
Muc5b	8.01	3.00	0.00	up	yes
Gda	8.01	3.00	0.00	up	yes
Mrgprf	8.11	3.02	0.00	up	yes
Lepr	8.17	3.03	0.00	up	yes
Dlc1	7.87	2.98	0.00	up	yes
Nos3	8.13	3.02	0.00	up	yes
Tmtc1	7.90	2.98	0.00	up	yes
Sema3f	7.81	2.97	0.00	up	yes
Lpl	7.70	2.94	0.00	up	yes
Papln	7.87	2.98	0.00	up	yes
Unc13b	7.79	2.96	0.00	up	yes
Gm26316	0.14	-2.82	0.00	down	yes
Jag1	7.64	2.93	0.00	up	yes
Smoc2	7.70	2.94	0.00	up	yes
Lcn2	7.81	2.96	0.00	up	yes
Adamts5	7.82	2.97	0.00	up	yes
Serping	7.61	2.93	0.00	up	yes

Tns2	7.56	2.92	0.00	up	yes
Abcc9	7.58	2.92	0.00	up	yes
Gm27980	7.76	2.96	0.00	up	yes
Lrrn4	7.73	2.95	0.00	up	yes
Ifitm1	7.57	2.92	0.00	up	yes
Rhpn2	7.66	2.94	0.00	up	yes
Pnpla3	7.59	2.92	0.00	up	yes
Klf9	7.45	2.90	0.00	up	yes
Asph	7.43	2.89	0.00	up	yes
Rgma	7.40	2.89	0.00	up	yes
Hspa1a	7.42	2.89	0.00	up	yes
Mid2	7.44	2.90	0.00	up	yes
Ahnak2	7.35	2.88	0.00	up	yes
Fzd4	7.36	2.88	0.00	up	yes
Plekhh3	7.49	2.91	0.00	up	yes
Abca9	7.30	2.87	0.00	up	yes
Fmod	7.45	2.90	0.00	up	yes
Mmp28	7.31	2.87	0.00	up	yes
Prex2	7.32	2.87	0.00	up	yes
Il18r1	7.16	2.84	0.00	up	yes
Cavin2	7.24	2.86	0.00	up	yes
Per3	7.17	2.84	0.00	up	yes
Dnah14	0.15	-2.75	0.00	down	yes
Fzd3	7.14	2.84	0.00	up	yes
1700019	7.19	2.85	0.00	up	yes
Ltbp4	7.10	2.83	0.00	up	yes
Sned1;M	7.12	2.83	0.00	up	yes
Ighg2b	7.06	2.82	0.00	up	yes
Rnu2-10	0.15	-2.71	0.00	down	yes
Fcgbp	7.05	2.82	0.00	up	yes
Antxr1	7.07	2.82	0.00	up	yes
Zfp503	7.16	2.84	0.00	up	yes
Abcd2	7.13	2.83	0.00	up	yes
Fgfr1	7.05	2.82	0.00	up	yes
Slc2a13	7.10	2.83	0.00	up	yes
Pkd2l2	7.01	2.81	0.00	up	yes
Pard3	6.98	2.80	0.00	up	yes
Auts2	6.96	2.80	0.00	up	yes
Galnt15	6.96	2.80	0.00	up	yes
Slc16a2	7.04	2.82	0.00	up	yes
Mpdz	7.03	2.81	0.00	up	yes
Fhl1	7.14	2.84	0.00	up	yes
Pparg	7.13	2.83	0.00	up	yes
Fah	6.99	2.81	0.00	up	yes
Zfp462	6.87	2.78	0.00	up	yes
Abhd14b	7.00	2.81	0.00	up	yes
Tfap2a	7.14	2.84	0.00	up	yes
Kcnj13	6.89	2.78	0.00	up	yes
Cgnl1	6.90	2.79	0.00	up	yes
Gm22146	6.81	2.77	0.00	up	yes
March8	6.89	2.78	0.00	up	yes
Bank1	7.01	2.81	0.00	up	yes
Gm24950	0.16	-2.66	0.00	down	yes
Mtus1	6.87	2.78	0.00	up	yes
Tfcp2l1	6.81	2.77	0.00	up	yes
Lama5	6.78	2.76	0.00	up	yes
Gm15737	6.94	2.80	0.00	up	yes

Fignl2	6.92	2.79	0.00	up	yes
Gprc5c	6.78	2.76	0.00	up	yes
Cpa3	6.93	2.79	0.00	up	yes
Gm23849	0.16	-2.62	0.00	down	yes
Anxa1	6.69	2.74	0.00	up	yes
Gm18537	0.16	-2.65	0.00	down	yes
Zfp612	6.82	2.77	0.00	up	yes
Fosl2	6.61	2.73	0.00	up	yes
Mrvi1	6.73	2.75	0.00	up	yes
Nos1ap	7.01	2.81	0.00	up	yes
Epb41l1	6.66	2.73	0.00	up	yes
Enpp2	6.59	2.72	0.00	up	yes
Zfp618	6.76	2.76	0.00	up	yes
Apol7e	0.16	-2.64	0.00	down	yes
Fyb2	6.56	2.71	0.00	up	yes
Bcar1	6.58	2.72	0.00	up	yes
Dnm1	6.76	2.76	0.00	up	yes
Ccl11	6.62	2.73	0.00	up	yes
Gm13237	0.16	-2.61	0.00	down	yes
AI46413	6.60	2.72	0.00	up	yes
Fat3	6.55	2.71	0.00	up	yes
Phka1	6.63	2.73	0.00	up	yes
Sorbs3	6.56	2.71	0.00	up	yes
Phldb2	6.60	2.72	0.00	up	yes
Slc2a4	6.54	2.71	0.00	up	yes
Csmd1	6.54	2.71	0.00	up	yes
Igkv14-	6.74	2.75	0.00	up	yes
G0s2	6.70	2.74	0.00	up	yes
Cygb	6.53	2.71	0.00	up	yes
Edar	6.59	2.72	0.00	up	yes
Slc4a3	6.62	2.73	0.00	up	yes
Arhgap3	6.46	2.69	0.00	up	yes
Atp8b1	6.60	2.72	0.00	up	yes
Lrp1	6.41	2.68	0.00	up	yes
Bche	6.63	2.73	0.00	up	yes
Lama2	6.42	2.68	0.00	up	yes
Amotl2	6.53	2.71	0.00	up	yes
C1s1;C1	6.43	2.69	0.00	up	yes
Fam46a	6.47	2.69	0.00	up	yes
Pdpn	6.48	2.70	0.00	up	yes
Ablim3	6.50	2.70	0.00	up	yes
Gucy1a3	6.50	2.70	0.00	up	yes
Gab1	6.40	2.68	0.00	up	yes
Plekha6	6.39	2.68	0.00	up	yes
C1rb	6.52	2.70	0.00	up	yes
Adamtsl	6.46	2.69	0.00	up	yes
Xdh	6.28	2.65	0.00	up	yes
Osmr	6.28	2.65	0.00	up	yes
Gm13167	0.17	-2.56	0.00	down	yes
Plekhh2	6.45	2.69	0.00	up	yes
C1ra	6.31	2.66	0.00	up	yes
Isl1	6.34	2.66	0.00	up	yes
Prrg3	6.27	2.65	0.00	up	yes
Cacna1h	6.35	2.67	0.00	up	yes
Dmd	6.41	2.68	0.00	up	yes
Mtmr11	6.36	2.67	0.00	up	yes
Bicc1	6.34	2.66	0.00	up	yes

Tnfsf12	6.35	2.67	0.00	up	yes
Slc1a3	6.25	2.64	0.00	up	yes
Eya4	6.27	2.65	0.00	up	yes
Lgals12	6.40	2.68	0.00	up	yes
Podn	6.47	2.69	0.00	up	yes
Sod3	6.24	2.64	0.00	up	yes
Timp3	6.20	2.63	0.00	up	yes
Fcgr2b	6.25	2.64	0.00	up	yes
Sv2b	6.37	2.67	0.00	up	yes
Cyp2j9	6.33	2.66	0.00	up	yes
Aox1	6.17	2.63	0.00	up	yes
Rnu3b2	0.18	-2.51	0.00	down	yes
Megf8	6.13	2.62	0.00	up	yes
Pbx1	6.13	2.62	0.00	up	yes
Pdzd2	6.14	2.62	0.00	up	yes
Spaca6;I	6.18	2.63	0.00	up	yes
Naaladl	6.25	2.64	0.00	up	yes
Rnu3b4	0.18	-2.50	0.00	down	yes
Arhgap4	6.14	2.62	0.00	up	yes
Fras1	6.11	2.61	0.00	up	yes
Gstm2	6.22	2.64	0.00	up	yes
Oas1g	6.27	2.65	0.00	up	yes
Fzd1	6.18	2.63	0.00	up	yes
Ltbp1	6.11	2.61	0.00	up	yes
Cd300lf	6.21	2.64	0.00	up	yes
F3	6.19	2.63	0.00	up	yes
Msln	6.21	2.63	0.00	up	yes
Inha	6.12	2.61	0.00	up	yes
Hist3h2I	0.18	-2.49	0.00	down	yes
Met	6.10	2.61	0.00	up	yes
Mgll	6.03	2.59	0.00	up	yes
Mmp19	6.19	2.63	0.00	up	yes
Gxylt2	6.14	2.62	0.00	up	yes
Rgs4	6.05	2.60	0.00	up	yes
Meis2	6.02	2.59	0.00	up	yes
Map3k6	6.20	2.63	0.00	up	yes
Elf3	6.14	2.62	0.00	up	yes
Rhou	6.07	2.60	0.00	up	yes
Casp12	6.13	2.62	0.00	up	yes
Sspn	6.12	2.61	0.00	up	yes
Neo1	5.99	2.58	0.00	up	yes
Scara3	6.06	2.60	0.00	up	yes
Nid1	5.97	2.58	0.00	up	yes
Cd163	6.05	2.60	0.00	up	yes
Klrk1	5.98	2.58	0.00	up	yes
Lamb1	5.95	2.57	0.00	up	yes
Tmem119	6.07	2.60	0.00	up	yes
Gm23804	0.18	-2.45	0.00	down	yes
Gpc4	5.99	2.58	0.00	up	yes
Apcdd1	6.03	2.59	0.00	up	yes
Slc12a2	5.88	2.56	0.00	up	yes
Tshz2	5.96	2.58	0.00	up	yes
Hspa1b	5.90	2.56	0.00	up	yes
Amotl1	5.86	2.55	0.00	up	yes
Tmem30b	5.90	2.56	0.00	up	yes
Myo1b	5.89	2.56	0.00	up	yes
Dennd5b	5.86	2.55	0.00	up	yes

Plppr4	5.89	2.56	0.00	up	yes
Btnl9	5.95	2.57	0.00	up	yes
Lifr	5.80	2.54	0.00	up	yes
Gprc5a	5.89	2.56	0.00	up	yes
Clu	5.79	2.53	0.00	up	yes
Adcy4	5.89	2.56	0.00	up	yes
Thsd7a	5.93	2.57	0.00	up	yes
Ptger3	5.86	2.55	0.00	up	yes
Tie1	5.84	2.54	0.00	up	yes
Flt1	5.78	2.53	0.00	up	yes
Kcnk2	5.79	2.53	0.00	up	yes
Rnu3b1	0.19	-2.41	0.00	down	yes
Sh3rf2	5.92	2.56	0.00	up	yes
CT00948	5.89	2.56	0.00	up	yes
Penk	5.79	2.53	0.00	up	yes
Lrrc75b	5.90	2.56	0.00	up	yes
Thbd	5.77	2.53	0.00	up	yes
Bace2	5.85	2.55	0.00	up	yes
Cd248	5.82	2.54	0.00	up	yes
Kctd15	5.77	2.53	0.00	up	yes
Six4	5.69	2.51	0.00	up	yes
Rnu3b3	0.19	-2.40	0.00	down	yes
Rab34	5.93	2.57	0.00	up	yes
Ripk4	5.72	2.52	0.00	up	yes
Pkd2	5.68	2.51	0.00	up	yes
Slc27a3	5.73	2.52	0.00	up	yes
Gli2	5.70	2.51	0.00	up	yes
Alox5	5.79	2.53	0.00	up	yes
Chst3	5.76	2.52	0.00	up	yes
Snai2	5.72	2.52	0.00	up	yes
Scn1b	5.71	2.51	0.00	up	yes
Loxl1	5.79	2.53	0.00	up	yes
Ephb3	5.76	2.53	0.00	up	yes
Ar	5.61	2.49	0.00	up	yes
Rbm3-ps	0.19	-2.40	0.00	down	yes
Rhobtb1	5.74	2.52	0.00	up	yes
Acsl1	5.61	2.49	0.00	up	yes
Pdgfc	5.67	2.50	0.00	up	yes
Nr2f2	5.71	2.51	0.00	up	yes
Rhbdf1	5.65	2.50	0.00	up	yes
Npdc1;E	5.64	2.50	0.00	up	yes
Plpp3	5.58	2.48	0.00	up	yes
Hoxa3;H	5.63	2.49	0.00	up	yes
Pm20d1	5.69	2.51	0.00	up	yes
Arhgef2	5.61	2.49	0.00	up	yes
Olfml2a	5.53	2.47	0.00	up	yes
Nipal2	5.67	2.50	0.00	up	yes
Rasip1	5.68	2.50	0.00	up	yes
Smagp	5.65	2.50	0.00	up	yes
Dpp6	5.57	2.48	0.00	up	yes
Gm13232	0.19	-2.39	0.00	down	yes
Paqr7	5.68	2.50	0.00	up	yes
Mir205	5.51	2.46	0.00	up	yes
Zc2hc1a	5.67	2.50	0.00	up	yes
Ccdc85a	5.67	2.50	0.00	up	yes
Tenm3	5.48	2.45	0.00	up	yes
Hydin	5.52	2.46	0.00	up	yes

Aldh5a1	5.57	2.48	0.00	up	yes
Arhgef1:	5.45	2.45	0.00	up	yes
Proser2	5.57	2.48	0.00	up	yes
Per2	5.48	2.45	0.00	up	yes
Fgf1	5.48	2.45	0.00	up	yes
Rnase4;:	5.50	2.46	0.00	up	yes
Adgrg2	5.46	2.45	0.00	up	yes
Itgam	5.52	2.46	0.00	up	yes
Ifi27l2:	5.41	2.44	0.00	up	yes
Plcb1	5.54	2.47	0.00	up	yes
Rin2	5.42	2.44	0.00	up	yes
Fzd8	5.49	2.46	0.00	up	yes
Cenpk	0.19	-2.37	0.00	down	yes
Dchs2	5.51	2.46	0.00	up	yes
Igha;Ig:	5.39	2.43	0.00	up	yes
Adarb1	5.44	2.44	0.00	up	yes
Id4	5.45	2.45	0.00	up	yes
C130074	5.44	2.44	0.00	up	yes
Snhg11;(	5.40	2.43	0.00	up	yes
Zfp532	5.44	2.44	0.00	up	yes
Kcnj2	5.42	2.44	0.00	up	yes
Col7a1	5.40	2.43	0.00	up	yes
Nfam1	5.44	2.44	0.00	up	yes
Me1	5.34	2.42	0.00	up	yes
Eya1	5.35	2.42	0.00	up	yes
Siglec1	5.37	2.42	0.00	up	yes
Irak3	5.45	2.45	0.00	up	yes
2900026:	5.32	2.41	0.00	up	yes
Lum	5.40	2.43	0.00	up	yes
Dab2	5.36	2.42	0.00	up	yes
Boc	5.31	2.41	0.00	up	yes
Pigr	5.39	2.43	0.00	up	yes
Kdr	5.35	2.42	0.00	up	yes
Lama4	5.35	2.42	0.00	up	yes
Hspg2	5.28	2.40	0.00	up	yes
Gm23238	0.20	-2.30	0.00	down	yes
Peg3	5.34	2.42	0.00	up	yes
Il1r1	5.28	2.40	0.00	up	yes
Trim29	5.28	2.40	0.00	up	yes
Clstn1	5.27	2.40	0.00	up	yes
BC06707	5.31	2.41	0.00	up	yes
Igdcc4	5.29	2.40	0.00	up	yes
Cavin3	5.41	2.44	0.00	up	yes
Kcnq3	5.27	2.40	0.00	up	yes
Cldn1	5.28	2.40	0.00	up	yes
Sdc2	5.32	2.41	0.00	up	yes
Tjp1	5.27	2.40	0.00	up	yes
Fam171a	5.29	2.40	0.00	up	yes
Slc46a2	5.23	2.39	0.00	up	yes
Efs	5.24	2.39	0.00	up	yes
Il17rc	5.37	2.42	0.00	up	yes
B4galnt	5.38	2.43	0.00	up	yes
Tlr1	5.29	2.40	0.00	up	yes
Dagla	5.36	2.42	0.00	up	yes
Col18a1	5.21	2.38	0.00	up	yes
Mapk12	5.35	2.42	0.00	up	yes
Gm26448	0.21	-2.27	0.00	down	yes

Sdk1	5.24	2.39	0.00	up	yes
Ccdc115	0.21	-2.28	0.00	down	yes
Klhdc7a	5.22	2.38	0.00	up	yes
Slc9a3r	5.30	2.41	0.00	up	yes
Tnfaip2	5.26	2.40	0.00	up	yes
Gna14	5.24	2.39	0.00	up	yes
Ltf	5.27	2.40	0.00	up	yes
Ephb4	5.18	2.37	0.00	up	yes
Unc79	5.26	2.40	0.00	up	yes
Slit2	5.30	2.41	0.00	up	yes
Serpina	5.17	2.37	0.00	up	yes
P2ry1	5.34	2.42	0.00	up	yes
Six5	5.21	2.38	0.00	up	yes
Itgb5	5.18	2.37	0.00	up	yes
Hmcn2	5.27	2.40	0.00	up	yes
Ccl9	5.23	2.39	0.00	up	yes
Hmgb1-p	0.21	-2.27	0.00	down	yes
Nav1	5.15	2.36	0.00	up	yes
Gsn	5.13	2.36	0.00	up	yes
Dzip1l	5.25	2.39	0.00	up	yes
Sorbs2	5.25	2.39	0.00	up	yes
Pcdh19	5.30	2.41	0.00	up	yes
Spns2	5.22	2.38	0.00	up	yes
Tshz3	5.30	2.41	0.00	up	yes
Fam213a	5.28	2.40	0.00	up	yes
Zan	5.25	2.39	0.00	up	yes
Msrb3	5.16	2.37	0.00	up	yes
Mt2	5.23	2.39	0.00	up	yes
Cx3cl1	5.14	2.36	0.00	up	yes
Erich3	5.30	2.41	0.00	up	yes
Cyp1b1	5.10	2.35	0.00	up	yes
Hmgcs2	5.12	2.36	0.00	up	yes
Sntb2	5.09	2.35	0.00	up	yes
Prrx1	5.14	2.36	0.00	up	yes
Cobl	5.10	2.35	0.00	up	yes
Cxcr3	5.17	2.37	0.00	up	yes
Arhgef1	5.08	2.35	0.00	up	yes
Ngp	5.11	2.35	0.00	up	yes
Trp63	5.08	2.34	0.00	up	yes
Fat1	5.07	2.34	0.00	up	yes
Foxg1	5.14	2.36	0.00	up	yes
Snord15	0.21	-2.25	0.00	down	yes
C3	5.06	2.34	0.00	up	yes
Il23r	5.14	2.36	0.00	up	yes
Fasn	5.05	2.34	0.00	up	yes
Large2	5.15	2.36	0.00	up	yes
Kif5c	5.13	2.36	0.00	up	yes
Sgms2	5.22	2.38	0.00	up	yes
Rnf217	5.11	2.35	0.00	up	yes
Nt5e	5.11	2.35	0.00	up	yes
Plk2	5.15	2.36	0.00	up	yes
AI66145	5.13	2.36	0.00	up	yes
Heyl	5.09	2.35	0.00	up	yes
Oplah	5.06	2.34	0.00	up	yes
Ctnnd2	5.06	2.34	0.00	up	yes
Gm23971	0.22	-2.21	0.00	down	yes
Hykk	5.18	2.37	0.00	up	yes

Dennd2a	5.17	2.37	0.00	up	yes
Nfib	5.00	2.32	0.00	up	yes
Tle1	5.02	2.33	0.00	up	yes
Gm8707	5.00	2.32	0.00	up	yes
Lrp4	4.99	2.32	0.00	up	yes
Pcdh17	5.04	2.33	0.00	up	yes
Obsl1	4.99	2.32	0.00	up	yes
Dcdc2b	5.01	2.32	0.00	up	yes
Stra6	5.14	2.36	0.00	up	yes
B4galt6	5.02	2.33	0.00	up	yes
Dnah5	5.05	2.34	0.00	up	yes
Iqsec2	4.99	2.32	0.00	up	yes
Colec12	4.96	2.31	0.00	up	yes
Epas1	4.93	2.30	0.00	up	yes
Cpe	5.01	2.32	0.00	up	yes
Cdc42ep	5.03	2.33	0.00	up	yes
Tsc22d3	4.92	2.30	0.00	up	yes
Smad6	5.07	2.34	0.00	up	yes
Col6a5	4.95	2.31	0.00	up	yes
Trpm5	4.97	2.31	0.00	up	yes
Mxra8	5.00	2.32	0.00	up	yes
Kif26b	5.04	2.33	0.00	up	yes
Enpp1	4.98	2.32	0.00	up	yes
D630045	0.22	-2.19	0.00	down	yes
Fam109a	5.05	2.34	0.00	up	yes
Pamr1	5.04	2.33	0.00	up	yes
Gm8319	0.22	-2.20	0.00	down	yes
Dok3	4.98	2.32	0.00	up	yes
Enah	4.90	2.29	0.00	up	yes
Mycl	4.96	2.31	0.00	up	yes
Pcgf2	4.94	2.30	0.00	up	yes
Parvb	4.93	2.30	0.00	up	yes
Park2	4.98	2.31	0.00	up	yes
Cadps2	4.97	2.31	0.00	up	yes
Klrd1	4.98	2.32	0.00	up	yes
Htra1	4.90	2.29	0.00	up	yes
Mrc2	4.94	2.31	0.00	up	yes
Adgrl2	4.90	2.29	0.00	up	yes
Pde3a	4.97	2.31	0.00	up	yes
Dnm3	4.89	2.29	0.00	up	yes
Csf1	4.87	2.28	0.00	up	yes
Cyp4v3;	4.92	2.30	0.00	up	yes
Myo16	4.88	2.29	0.00	up	yes
Col12a1	4.84	2.28	0.00	up	yes
Tspan15	4.99	2.32	0.00	up	yes
Txndc5	4.84	2.27	0.00	up	yes
Abi3bp	4.91	2.30	0.00	up	yes
Tbx21	4.92	2.30	0.00	up	yes
Casp1	4.93	2.30	0.00	up	yes
Thbs2	4.85	2.28	0.00	up	yes
Phlda3	4.83	2.27	0.00	up	yes
Slc6a9	4.91	2.30	0.00	up	yes
Sptb	4.81	2.27	0.00	up	yes
Abcb1a	4.90	2.29	0.00	up	yes
Gm8623	0.22	-2.19	0.00	down	yes
Avil	4.83	2.27	0.00	up	yes
Gpt2	4.81	2.27	0.00	up	yes

Fcgrt	4.83	2.27	0.00	up	yes
Plaur	4.93	2.30	0.00	up	yes
Tead1	4.83	2.27	0.00	up	yes
Tril	4.87	2.28	0.00	up	yes
Cachd1	4.76	2.25	0.00	up	yes
Bcam	4.77	2.25	0.00	up	yes
Bmpr1a	4.75	2.25	0.00	up	yes
Cd300ld	4.77	2.25	0.00	up	yes
Usp35	4.73	2.24	0.00	up	yes
Klhl29	4.74	2.24	0.00	up	yes
Ifnlr1	4.78	2.26	0.00	up	yes
Hic1	4.79	2.26	0.00	up	yes
Lamb2	4.74	2.24	0.00	up	yes
Stox2	4.73	2.24	0.00	up	yes
Wwc2	4.74	2.25	0.00	up	yes
Esr1	4.78	2.26	0.00	up	yes
Yap1	4.72	2.24	0.00	up	yes
Fgfr2;C	4.73	2.24	0.00	up	yes
Tsku	4.77	2.25	0.00	up	yes
Glis3	4.74	2.24	0.00	up	yes
Gm7609	4.86	2.28	0.00	up	yes
Dlg5;AC	4.71	2.24	0.00	up	yes
Phldb1	4.72	2.24	0.00	up	yes
Ptpn13	4.68	2.23	0.00	up	yes
Fzd7	4.69	2.23	0.00	up	yes
Cdh3	4.68	2.23	0.00	up	yes
Ahnak	4.66	2.22	0.00	up	yes
Wdfy3	4.67	2.22	0.00	up	yes
Gm12286	0.22	-2.16	0.00	down	yes
Ago4	4.74	2.24	0.00	up	yes
Robo1	4.65	2.22	0.00	up	yes
Adgrf5	4.66	2.22	0.00	up	yes
Cavin1	4.65	2.22	0.00	up	yes
Zfp651	4.68	2.23	0.00	up	yes
Mecp2;G	0.23	-2.10	0.00	down	yes
Ccr6	4.71	2.23	0.00	up	yes
Pax9	4.66	2.22	0.00	up	yes
Errfi1	4.62	2.21	0.00	up	yes
Wnk2	4.67	2.22	0.00	up	yes
Frem2	4.62	2.21	0.00	up	yes
K230010	4.68	2.23	0.00	up	yes
Vwa1	4.64	2.21	0.00	up	yes
Tenm4	4.60	2.20	0.00	up	yes
Eps8l2	4.64	2.21	0.00	up	yes
Nhs	4.66	2.22	0.00	up	yes
Nova2	4.64	2.21	0.00	up	yes
Adgrl4	4.62	2.21	0.00	up	yes
Cdh11	4.67	2.22	0.00	up	yes
Cpd	4.57	2.19	0.00	up	yes
Cd300ld	4.64	2.21	0.00	up	yes
Cd300lg	4.61	2.20	0.00	up	yes
Limch1	4.58	2.20	0.00	up	yes
Cdcp1	4.57	2.19	0.00	up	yes
Mmrn2	4.60	2.20	0.00	up	yes
Sema3c	4.68	2.23	0.00	up	yes
Oas2	4.58	2.20	0.00	up	yes
S100a9	4.62	2.21	0.00	up	yes

Zfp964	4.67	2.22	0.00	up	yes
Tmbim1	4.56	2.19	0.00	up	yes
Ppl	4.56	2.19	0.00	up	yes
App	4.55	2.18	0.00	up	yes
Pde7b	4.63	2.21	0.00	up	yes
Igfbp7	4.58	2.20	0.00	up	yes
Mettl7a	4.56	2.19	0.00	up	yes
Garem1	4.68	2.23	0.00	up	yes
Slc24a4	4.59	2.20	0.00	up	yes
Cpxm1	4.67	2.22	0.00	up	yes
Pirb	4.62	2.21	0.00	up	yes
Tmem9b	4.55	2.19	0.00	up	yes
Csf3r	4.58	2.20	0.00	up	yes
F830016	4.59	2.20	0.00	up	yes
Pnpla2	4.51	2.17	0.00	up	yes
Stard13	4.58	2.19	0.00	up	yes
Sdc4	4.51	2.17	0.00	up	yes
Pros1	4.54	2.18	0.00	up	yes
Map3k9	4.59	2.20	0.00	up	yes
Ptprm	4.52	2.18	0.00	up	yes
Kif13a	4.51	2.17	0.00	up	yes
Elovl7	4.61	2.20	0.00	up	yes
Grk3	4.50	2.17	0.00	up	yes
Mgl2	4.65	2.22	0.00	up	yes
Slco2b1	4.49	2.17	0.00	up	yes
Reps2	4.58	2.20	0.00	up	yes
Foxc1	4.54	2.18	0.00	up	yes
Rarg	4.50	2.17	0.00	up	yes
Exph5	4.54	2.18	0.00	up	yes
Dclk1	4.50	2.17	0.00	up	yes
Rhoj	4.57	2.19	0.00	up	yes
Acsbg1	4.54	2.18	0.00	up	yes
Cables1	4.62	2.21	0.00	up	yes
Fam198b	4.55	2.18	0.00	up	yes
Meis1	4.46	2.16	0.00	up	yes
Gm10182	0.24	-2.06	0.00	down	yes
Gabrp	4.56	2.19	0.00	up	yes
Nfatc4	4.56	2.19	0.00	up	yes
Egfr	4.45	2.15	0.00	up	yes
Mpp2	4.53	2.18	0.00	up	yes
Slc25a5	4.45	2.15	0.00	up	yes
Tspan12	4.58	2.19	0.00	up	yes
Esam	4.49	2.17	0.00	up	yes
Zfhx3	4.44	2.15	0.00	up	yes
Serpinb	4.58	2.20	0.00	up	yes
Col6a6	4.45	2.15	0.00	up	yes
Klf4	4.48	2.16	0.00	up	yes
Pxdn	4.45	2.15	0.00	up	yes
Gdpd5	4.48	2.16	0.00	up	yes
Rarres2	4.47	2.16	0.00	up	yes
Tns3	4.40	2.14	0.00	up	yes
Mfap3l	4.47	2.16	0.00	up	yes
Nid2	4.44	2.15	0.00	up	yes
Zcchc14	4.41	2.14	0.00	up	yes
Slc9a2	4.41	2.14	0.00	up	yes
Smpd3	4.43	2.15	0.00	up	yes
Bcl11a	4.41	2.14	0.00	up	yes

Cebpb	4.49	2.17	0.00	up	yes
Emcn	4.46	2.16	0.00	up	yes
Robo4	4.48	2.16	0.00	up	yes
Cdc42ep	4.44	2.15	0.00	up	yes
Slc22a2	4.38	2.13	0.00	up	yes
Rnf150	4.38	2.13	0.00	up	yes
Parva	4.38	2.13	0.00	up	yes
Agrn	4.35	2.12	0.00	up	yes
Adgrg6	4.41	2.14	0.00	up	yes
Colgalt	4.47	2.16	0.00	up	yes
Tspan7	4.38	2.13	0.00	up	yes
Gm10036	0.25	-2.02	0.00	down	yes
C77080	4.35	2.12	0.00	up	yes
Slc26a1	4.45	2.15	0.00	up	yes
Rbfox2	4.35	2.12	0.00	up	yes
Pald1	4.39	2.13	0.00	up	yes
Arap3	4.36	2.12	0.00	up	yes
Emilin2	4.39	2.13	0.00	up	yes
Afap1l2	4.34	2.12	0.00	up	yes
Adam15	4.36	2.12	0.00	up	yes
Jag2	4.33	2.12	0.00	up	yes
Trpc1	4.40	2.14	0.00	up	yes
Thbs1	4.35	2.12	0.00	up	yes
Lox	4.36	2.12	0.00	up	yes
Grhl2	4.34	2.12	0.00	up	yes
Pde1c	4.37	2.13	0.00	up	yes
Myrf	4.36	2.12	0.00	up	yes
Flrt3	4.39	2.13	0.00	up	yes
Tgm2	4.31	2.11	0.00	up	yes
Trpm6	4.41	2.14	0.00	up	yes
Cyb561	4.37	2.13	0.00	up	yes
Soga1	4.31	2.11	0.00	up	yes
Nbea	4.32	2.11	0.00	up	yes
Cd22	4.35	2.12	0.00	up	yes
Ebf3	4.42	2.14	0.00	up	yes
Emp1	0.25	-2.00	0.00	down	yes
Cdk18	4.36	2.12	0.00	up	yes
Fam196a	4.37	2.13	0.00	up	yes
Slc1a4	4.30	2.10	0.00	up	yes
Clec1a	4.39	2.13	0.00	up	yes
Sh3d19	4.29	2.10	0.00	up	yes
Tek	4.34	2.12	0.00	up	yes
Btk	4.33	2.12	0.00	up	yes
Nav3	4.34	2.12	0.00	up	yes
Sult1a1	4.28	2.10	0.00	up	yes
Gm4951	4.35	2.12	0.00	up	yes
Gm7390	0.25	-2.00	0.00	down	yes
Cpeb3	4.34	2.12	0.00	up	yes
Stk36	4.39	2.14	0.00	up	yes
Cplx2	4.30	2.10	0.00	up	yes
Tst	4.29	2.10	0.00	up	yes
Dsc3	4.26	2.09	0.00	up	yes
Tpbg	4.31	2.11	0.00	up	yes
1810011	4.32	2.11	0.00	up	yes
Mir466i	0.25	-1.98	0.00	down	yes
Fbln1	4.26	2.09	0.00	up	yes
Paqr9	4.29	2.10	0.00	up	yes

Sema4c	4.23	2.08	0.00	up	yes
Snta1	4.36	2.12	0.00	up	yes
Bgn	4.23	2.08	0.00	up	yes
Arhgef1	4.25	2.09	0.00	up	yes
Mill1	4.26	2.09	0.00	up	yes
Plvap	4.21	2.07	0.00	up	yes
Pi16	4.29	2.10	0.00	up	yes
Clstn3	4.34	2.12	0.00	up	yes
Sphk1	4.31	2.11	0.00	up	yes
H2-K1	4.20	2.07	0.00	up	yes
Adap2	4.26	2.09	0.00	up	yes
Lpin3	4.32	2.11	0.00	up	yes
Golm1	4.23	2.08	0.00	up	yes
Tbx3	4.32	2.11	0.00	up	yes
Fermt2	4.22	2.08	0.00	up	yes
Mt1	4.24	2.09	0.00	up	yes
Tcaf1	4.26	2.09	0.00	up	yes
Tmem231	4.28	2.10	0.00	up	yes
5830411	4.21	2.08	0.00	up	yes
Gm44341	0.25	-1.99	0.00	down	yes
Plce1	4.19	2.07	0.00	up	yes
Fam13c	4.31	2.11	0.00	up	yes
Dnah6	4.25	2.09	0.00	up	yes
Card10	4.21	2.07	0.00	up	yes
Nfia	4.17	2.06	0.00	up	yes
Shank1	4.23	2.08	0.00	up	yes
Gm13680	0.26	-1.97	0.00	down	yes
Emp2	4.16	2.06	0.00	up	yes
Pcsk5	4.22	2.08	0.00	up	yes
Il34	4.25	2.09	0.00	up	yes
Acot11	4.22	2.08	0.00	up	yes
Mgat3	4.14	2.05	0.00	up	yes
Kank1	4.15	2.05	0.00	up	yes
Selenon	4.17	2.06	0.00	up	yes
Pclo	4.14	2.05	0.00	up	yes
Tmem91	4.19	2.07	0.00	up	yes
Tmem176	4.14	2.05	0.00	up	yes
Shank3	4.15	2.05	0.00	up	yes
Hepacam	4.24	2.08	0.00	up	yes
Gm24339	0.26	-1.95	0.00	down	yes
Prr5	4.19	2.07	0.00	up	yes
Glul;Mi	4.11	2.04	0.00	up	yes
Adamts9	4.11	2.04	0.00	up	yes
Fcrl1	4.23	2.08	0.00	up	yes
Tbkbp1	4.18	2.06	0.00	up	yes
Cpxm2	4.20	2.07	0.00	up	yes
Wwc1	4.15	2.05	0.00	up	yes
Smim8	0.25	-1.99	0.00	down	yes
Gnao1	4.20	2.07	0.00	up	yes
Inhbb	4.16	2.06	0.00	up	yes
Gm4070;	4.10	2.03	0.00	up	yes
Lyrm1	4.21	2.07	0.00	up	yes
Vaultrc	0.26	-1.93	0.00	down	yes
Pcdhb17	4.20	2.07	0.00	up	yes
Eps8	4.11	2.04	0.00	up	yes
Igfbp4	4.07	2.03	0.00	up	yes
Olfr137	4.14	2.05	0.00	up	yes

Aqp1	4.07	2.03	0.00	up	yes
Rhob	4.07	2.03	0.00	up	yes
Lsr	4.15	2.05	0.00	up	yes
Tmem176l	4.07	2.02	0.00	up	yes
Slc10a1	0.26	-1.92	0.00	down	yes
Kif21a	4.08	2.03	0.00	up	yes
Cdh1	4.06	2.02	0.00	up	yes
Zfp324	0.26	-1.93	0.00	down	yes
Ankrd35	4.19	2.07	0.00	up	yes
Selp	4.16	2.06	0.00	up	yes
Stc1	4.17	2.06	0.00	up	yes
Pdgfb	4.10	2.03	0.00	up	yes
Rpl7-ps	0.26	-1.95	0.00	down	yes
Gm10076	0.26	-1.95	0.00	down	yes
Hpgd	4.06	2.02	0.00	up	yes
Gli3	4.07	2.02	0.00	up	yes
Mtss1l	4.06	2.02	0.00	up	yes
Zfp423	4.11	2.04	0.00	up	yes
Gm4617	0.27	-1.91	0.00	down	yes
Nupr1	4.17	2.06	0.00	up	yes
Sema3a	4.15	2.05	0.00	up	yes
Sel1l3	4.11	2.04	0.00	up	yes
Dtx4	4.05	2.02	0.00	up	yes
Tlr4	4.06	2.02	0.00	up	yes
Pi15	4.14	2.05	0.00	up	yes
Aatk	4.11	2.04	0.00	up	yes
Gfpt2	4.07	2.03	0.00	up	yes
Anpep	4.02	2.01	0.00	up	yes
Hist1h2l	0.27	-1.91	0.00	down	yes
Dchs1	4.06	2.02	0.00	up	yes
Tubb4b-]	0.27	-1.91	0.00	down	yes
Kank2	4.02	2.01	0.00	up	yes
St5	4.01	2.01	0.00	up	yes
Ndufaf4	4.09	2.03	0.00	up	yes
Tmprss2	4.11	2.04	0.00	up	yes
Gpc6	4.05	2.02	0.00	up	yes
Cd19	4.04	2.01	0.00	up	yes
Arhgef2	4.10	2.03	0.00	up	yes
Asns	4.07	2.02	0.00	up	yes
Gm13835	3.99	2.00	0.00	up	yes
Tmem2	4.00	2.00	0.00	up	yes
4833423	4.12	2.04	0.00	up	yes
Mlph	4.07	2.03	0.00	up	yes
Btbd3	4.01	2.00	0.00	up	yes
Cp	4.02	2.01	0.00	up	yes
Sox9	4.00	2.00	0.00	up	yes
Igfbp3	4.01	2.01	0.00	up	yes
Ly6c1;L	4.00	2.00	0.00	up	yes
Plau	4.00	2.00	0.00	up	yes
Ltbp2	3.98	1.99	0.00	up	yes
Gprin3	3.99	2.00	0.00	up	yes
Crispld	4.01	2.00	0.00	up	yes
Tnfrsf1	4.07	2.02	0.00	up	yes
Ryk	4.01	2.00	0.00	up	yes
Cldn15	4.08	2.03	0.00	up	yes
Tm4sf1	4.13	2.05	0.00	up	yes
Lgr4	3.98	1.99	0.00	up	yes

Rcn3	4.06	2.02	0.00	up	yes
Clcf1	3.97	1.99	0.00	up	yes
Tlr7	4.02	2.01	0.00	up	yes
Asap3	3.99	2.00	0.00	up	yes
Fam46c	3.97	1.99	0.00	up	yes
Fermt1	3.95	1.98	0.00	up	yes
Maf	3.95	1.98	0.00	up	yes
Slc46a1	4.09	2.03	0.00	up	yes
Atp7b	3.96	1.98	0.00	up	yes
Nucb2	4.07	2.03	0.00	up	yes
Gpat3	4.03	2.01	0.00	up	yes
Sdc1	3.94	1.98	0.00	up	yes
Itgb4	3.93	1.97	0.00	up	yes
Kctd12	3.93	1.98	0.00	up	yes
Dnase1l	3.93	1.98	0.00	up	yes
Reln	3.98	1.99	0.00	up	yes
Cldn7	4.07	2.02	0.00	up	yes
Klf15	3.96	1.98	0.00	up	yes
Ildr1	3.95	1.98	0.00	up	yes
Pax5	3.92	1.97	0.00	up	yes
Lipg	4.03	2.01	0.00	up	yes
Sox5	4.00	2.00	0.00	up	yes
Gm12816	0.27	-1.87	0.00	down	yes
Cxcr5	4.00	2.00	0.00	up	yes
Naprt	3.99	2.00	0.00	up	yes
AC15417	0.27	-1.89	0.00	down	yes
Acot1	3.96	1.98	0.00	up	yes
Ehf	3.94	1.98	0.00	up	yes
Etv1	4.05	2.02	0.00	up	yes
Pex11a	4.04	2.01	0.00	up	yes
Gm9844	0.27	-1.90	0.00	down	yes
Cyp2g1	3.98	1.99	0.00	up	yes
Hmmr	0.28	-1.85	0.00	down	yes
Lrrk1	3.90	1.96	0.00	up	yes
Slc4a4	3.90	1.96	0.00	up	yes
Gm6745	0.27	-1.88	0.00	down	yes
Efnb1	3.91	1.97	0.00	up	yes
Lilrb4a	3.92	1.97	0.00	up	yes
Crim1	3.89	1.96	0.00	up	yes
Pknox2	3.94	1.98	0.00	up	yes
Gpnmb	3.93	1.97	0.00	up	yes
Myo5b	3.91	1.97	0.00	up	yes
Rnf26	3.89	1.96	0.00	up	yes
Hist1h1	0.28	-1.84	0.00	down	yes
Myo6	3.89	1.96	0.00	up	yes
Ptprb	3.88	1.95	0.00	up	yes
Prss23	3.95	1.98	0.01	up	yes
Clmn	3.88	1.95	0.01	up	yes
Hist1h1	0.28	-1.84	0.01	down	yes
Rab15	3.87	1.95	0.01	up	yes
Aldh6a1	3.87	1.95	0.01	up	yes
Perp	3.86	1.95	0.01	up	yes
C3ar1	3.89	1.96	0.01	up	yes
Slc35e4	3.99	2.00	0.01	up	yes
Tef	3.86	1.95	0.01	up	yes
Thsd4	3.90	1.96	0.01	up	yes
Fcna	3.95	1.98	0.01	up	yes

Ppp1r13	3.88	1.96	0.01	up	yes
Cc2d2a	3.90	1.96	0.01	up	yes
Muc3a	3.88	1.96	0.01	up	yes
Fblim1	3.94	1.98	0.01	up	yes
Rmi2	0.28	-1.83	0.01	down	yes
Nckap5	3.92	1.97	0.01	up	yes
Zfp316	3.86	1.95	0.01	up	yes
Nacc2	3.86	1.95	0.01	up	yes
Plekha8	3.87	1.95	0.01	up	yes
Slc7a2	3.86	1.95	0.01	up	yes
Thbs3	3.96	1.98	0.01	up	yes
Erbb2	3.86	1.95	0.01	up	yes
St14	3.85	1.94	0.01	up	yes
Gm5596	0.28	-1.86	0.01	down	yes
Il7r	3.83	1.94	0.01	up	yes
Ifi202b	3.87	1.95	0.01	up	yes
Grm8;Mi	3.86	1.95	0.01	up	yes
Pon3	3.86	1.95	0.01	up	yes
Src	3.82	1.93	0.01	up	yes
Msc	3.87	1.95	0.01	up	yes
Lamb3	3.82	1.93	0.01	up	yes
Gmnn	0.28	-1.84	0.01	down	yes
Arhgap2	3.82	1.93	0.01	up	yes
Art2a-p	3.93	1.97	0.01	up	yes
Gmfg-ps	0.28	-1.84	0.01	down	yes
Nuak2	3.86	1.95	0.01	up	yes
Gm7879	0.28	-1.84	0.01	down	yes
Ighd	3.83	1.94	0.01	up	yes
Hr	3.85	1.94	0.01	up	yes
Sowahc	3.82	1.93	0.01	up	yes
Hey1	3.82	1.93	0.01	up	yes
Sparcl1	3.79	1.92	0.01	up	yes
Hemgn	0.28	-1.84	0.01	down	yes
Alox5ap	3.85	1.94	0.01	up	yes
Zfp970	3.86	1.95	0.01	up	yes
Asrgl1	0.29	-1.80	0.01	down	yes
Fam20c	3.81	1.93	0.01	up	yes
Gprc5b	3.79	1.92	0.01	up	yes
Ifitm3	3.77	1.91	0.01	up	yes
6430548	3.80	1.93	0.01	up	yes
Gjc1	3.85	1.95	0.01	up	yes
Myo1f	3.77	1.91	0.01	up	yes
Azin2	3.83	1.94	0.01	up	yes
Rhoc	3.80	1.93	0.01	up	yes
Ajuba	3.77	1.91	0.01	up	yes
Gja1	3.78	1.92	0.01	up	yes
Slco2a1	3.75	1.91	0.01	up	yes
Rasef	3.79	1.92	0.01	up	yes
AC15354	0.29	-1.80	0.01	down	yes
Dlec1	3.84	1.94	0.01	up	yes
Rxfp2	3.83	1.94	0.01	up	yes
Osbpl6	3.77	1.91	0.01	up	yes
Padi4	0.28	-1.84	0.01	down	yes
Siglecg	3.81	1.93	0.01	up	yes
Mylk	3.74	1.90	0.01	up	yes
Eln	3.76	1.91	0.01	up	yes
Fndc3b	3.74	1.90	0.01	up	yes

Itga3	3.74	1.90	0.01	up	yes
Zfp768	3.77	1.92	0.01	up	yes
Msr1	3.83	1.94	0.01	up	yes
Zbtb16	3.73	1.90	0.01	up	yes
Tlr3	3.78	1.92	0.01	up	yes
Dapk2	3.80	1.93	0.01	up	yes
Plpp1	3.77	1.91	0.01	up	yes
Lzts2	3.72	1.90	0.01	up	yes
S100a8	3.74	1.90	0.01	up	yes
Chchd10	3.74	1.90	0.01	up	yes
Tcp11l1	3.73	1.90	0.01	up	yes
Igf1r	3.71	1.89	0.01	up	yes
Gins2	0.29	-1.78	0.01	down	yes
Trpm2;G	3.72	1.90	0.01	up	yes
Cd9	3.73	1.90	0.01	up	yes
Lrrtm2	3.75	1.91	0.01	up	yes
Shroom2	3.72	1.90	0.01	up	yes
Hap1	3.74	1.90	0.01	up	yes
Cilp	3.78	1.92	0.01	up	yes
Epcam	3.71	1.89	0.01	up	yes
Orm2;Or	3.70	1.89	0.01	up	yes
Trim7	3.72	1.89	0.01	up	yes
Pld1	3.70	1.89	0.01	up	yes
Mecom	3.76	1.91	0.01	up	yes
Ces2g	3.72	1.89	0.01	up	yes
March3	3.76	1.91	0.01	up	yes
Cd38	3.69	1.89	0.01	up	yes
Aldh1l2	3.77	1.92	0.01	up	yes
Ednra	3.75	1.91	0.01	up	yes
A730017	3.79	1.92	0.01	up	yes
Pacsin3	3.83	1.94	0.01	up	yes
Pcdh1	3.70	1.89	0.01	up	yes
Pear1;L	3.70	1.89	0.01	up	yes
Ptms	0.29	-1.77	0.01	down	yes
Tanc2	3.69	1.88	0.01	up	yes
Dock1	3.68	1.88	0.01	up	yes
Rras	3.69	1.88	0.01	up	yes
P3h2	3.74	1.90	0.01	up	yes
Slc35f6	3.68	1.88	0.01	up	yes
Gprin1	3.74	1.90	0.01	up	yes
Spry2	3.71	1.89	0.01	up	yes
Sapcd2	0.29	-1.80	0.01	down	yes
Cdh2	3.75	1.91	0.01	up	yes
Cacna1c	3.72	1.89	0.01	up	yes
Fut2	3.78	1.92	0.01	up	yes
Cyp2a5	3.68	1.88	0.01	up	yes
Zfp109	3.73	1.90	0.01	up	yes
Dysf	3.67	1.87	0.01	up	yes
Zbtb7c	3.69	1.88	0.01	up	yes
Pck2	3.69	1.88	0.01	up	yes
Plxnb2	3.65	1.87	0.01	up	yes
Itga1;P	3.65	1.87	0.01	up	yes
Fzd2	3.71	1.89	0.01	up	yes
Ifi205	3.72	1.90	0.01	up	yes
Vma21	3.68	1.88	0.01	up	yes
Gca	3.74	1.90	0.01	up	yes
Mcc	3.65	1.87	0.01	up	yes

Fkbp9	3.65	1.87	0.01	up	yes
Megf6	3.65	1.87	0.01	up	yes
Bcar3	3.66	1.87	0.01	up	yes
Hdac11	3.65	1.87	0.01	up	yes
Wfs1	3.69	1.88	0.01	up	yes
Zfp961	0.30	-1.76	0.01	down	yes
Rbms3	3.63	1.86	0.01	up	yes
Slc15a2	3.74	1.90	0.01	up	yes
1700088	3.68	1.88	0.01	up	yes
Rbpj	0.30	-1.75	0.01	down	yes
Dpt	3.65	1.87	0.01	up	yes
Fat4	3.62	1.86	0.01	up	yes
Tgfb3	3.66	1.87	0.01	up	yes
Trpv4	3.72	1.90	0.01	up	yes
Calml3	3.70	1.89	0.01	up	yes
Col14a1	3.64	1.86	0.01	up	yes
Nr1h3	3.66	1.87	0.01	up	yes
Pawr	3.67	1.88	0.01	up	yes
Naip5	3.66	1.87	0.01	up	yes
Kdelc2	3.66	1.87	0.01	up	yes
Cnksr3	3.61	1.85	0.01	up	yes
Pmp22	3.64	1.87	0.01	up	yes
Gm21860	0.30	-1.75	0.01	down	yes
Hist1h2	0.30	-1.74	0.01	down	yes
Sik1	3.61	1.85	0.01	up	yes
Creb3l2	3.61	1.85	0.01	up	yes
Arhgap2	3.73	1.90	0.01	up	yes
Lhfp	3.64	1.86	0.01	up	yes
Sulf1	3.61	1.85	0.01	up	yes
Ngfr	3.70	1.89	0.01	up	yes
Gna15	3.67	1.88	0.01	up	yes
Hist1h3	0.30	-1.73	0.01	down	yes
Gab2	3.60	1.85	0.01	up	yes
Rbpms	3.61	1.85	0.01	up	yes
Myof	3.60	1.85	0.01	up	yes
Gm15452	0.29	-1.76	0.01	down	yes
Hecw2	3.63	1.86	0.01	up	yes
Vegfa	3.63	1.86	0.01	up	yes
Etl4	3.58	1.84	0.01	up	yes
Akap7	3.66	1.87	0.01	up	yes
Prdm1	3.62	1.86	0.01	up	yes
Cited2	3.59	1.84	0.01	up	yes
Decr1	3.59	1.84	0.01	up	yes
Samd5	3.59	1.84	0.01	up	yes
Fes	3.59	1.84	0.01	up	yes
Ushbp1	3.63	1.86	0.01	up	yes
Col5a2	3.58	1.84	0.01	up	yes
Cep55	0.30	-1.73	0.01	down	yes
Myo3b	3.60	1.85	0.01	up	yes
Ppfibp2	3.59	1.84	0.01	up	yes
Yes1	3.60	1.85	0.01	up	yes
Rasal2	3.57	1.83	0.01	up	yes
Phf24	3.67	1.87	0.01	up	yes
Pdgfrb	3.56	1.83	0.01	up	yes
Angptl4	3.59	1.84	0.01	up	yes
Ldb2	3.63	1.86	0.01	up	yes
Gpc2;Ga	3.58	1.84	0.01	up	yes

Ttc39b	3.56	1.83	0.01	up	yes
Rgmb	3.56	1.83	0.01	up	yes
Rubcnl	3.58	1.84	0.01	up	yes
Caskin2	3.56	1.83	0.01	up	yes
Dlk2	3.65	1.87	0.01	up	yes
Cpeb2	3.56	1.83	0.01	up	yes
Alcam	3.54	1.82	0.01	up	yes
Pik3r6	3.62	1.86	0.01	up	yes
Vcan	3.59	1.84	0.01	up	yes
Gm11605	0.30	-1.74	0.01	down	yes
Rbm47	3.53	1.82	0.01	up	yes
Il17re	3.57	1.84	0.01	up	yes
Spock2	3.53	1.82	0.01	up	yes
Cd4	0.31	-1.70	0.01	down	yes
Pcna-ps	0.31	-1.71	0.01	down	yes
Ywhah	0.31	-1.70	0.01	down	yes
Igf2	3.64	1.86	0.01	up	yes
Fap	3.62	1.86	0.01	up	yes
Nectin2	3.56	1.83	0.01	up	yes
Fam129a	3.51	1.81	0.01	up	yes
Sgce	3.61	1.85	0.01	up	yes
Tns1	3.51	1.81	0.01	up	yes
Slc4a11	3.55	1.83	0.01	up	yes
Peli3	3.61	1.85	0.01	up	yes
Pxdc1	3.56	1.83	0.01	up	yes
Gm13192	0.30	-1.73	0.01	down	yes
Pltp	3.50	1.81	0.01	up	yes
Mmp2	3.50	1.81	0.01	up	yes
She	3.57	1.84	0.01	up	yes
Rgs9	3.58	1.84	0.01	up	yes
Dock7	3.50	1.81	0.01	up	yes
Dnaja4	3.53	1.82	0.01	up	yes
Syn3	3.55	1.83	0.01	up	yes
Hist1h2l	0.31	-1.69	0.01	down	yes
Sh3tc1	3.53	1.82	0.01	up	yes
Rpl7a-p	0.30	-1.74	0.01	down	yes
Gm8186	0.30	-1.73	0.01	down	yes
Gm5620	0.31	-1.70	0.01	down	yes
Lca5	0.30	-1.72	0.01	down	yes
Kalrn	3.49	1.80	0.01	up	yes
Fbln5	3.52	1.82	0.01	up	yes
Slc35f5	3.51	1.81	0.01	up	yes
Kcnh6	3.60	1.85	0.01	up	yes
Gm10156	0.31	-1.71	0.01	down	yes
Cd34	3.47	1.80	0.01	up	yes
Nrtn	3.56	1.83	0.01	up	yes
Kitl	3.48	1.80	0.01	up	yes
Pde4a	3.48	1.80	0.01	up	yes
Klrb1b;	3.60	1.85	0.01	up	yes
Fgd4	3.48	1.80	0.01	up	yes
Dbp	3.48	1.80	0.01	up	yes
Mfap3	3.46	1.79	0.01	up	yes
Xrra1	0.31	-1.68	0.01	down	yes
Igsf10	3.47	1.79	0.01	up	yes
Esrp1	3.46	1.79	0.01	up	yes
Adgrg5	3.47	1.80	0.01	up	yes
Rad54b;	0.31	-1.68	0.01	down	yes

Wwtr1	3.46	1.79	0.01	up	yes
H2-Ea-p	3.44	1.78	0.01	up	yes
Itga8	3.49	1.80	0.01	up	yes
Camsap3	3.50	1.81	0.01	up	yes
Lmntd2	3.53	1.82	0.01	up	yes
A4galt	3.50	1.81	0.01	up	yes
Neurl3	3.47	1.79	0.01	up	yes
Hs3st3a	3.52	1.81	0.01	up	yes
Gm8797	0.31	-1.71	0.01	down	yes
S100a1	3.45	1.79	0.01	up	yes
Smarcd3	3.50	1.81	0.01	up	yes
5730409	3.49	1.80	0.01	up	yes
Sema6b	3.52	1.82	0.01	up	yes
Slc11a1	3.50	1.81	0.01	up	yes
Fam217b	3.52	1.82	0.01	up	yes
Tmcc2	3.47	1.80	0.01	up	yes
Unc5c	3.44	1.78	0.01	up	yes
Atp6v0e	3.48	1.80	0.01	up	yes
Ccdc80	3.44	1.78	0.01	up	yes
Cldn5	3.53	1.82	0.01	up	yes
Kcnma1	3.44	1.78	0.01	up	yes
Fgl2	3.44	1.78	0.01	up	yes
Flt4	3.50	1.81	0.01	up	yes
Zfp521	3.45	1.79	0.01	up	yes
Steap3	3.44	1.78	0.01	up	yes
Mras	3.44	1.78	0.01	up	yes
Speg	3.49	1.80	0.01	up	yes
S100a6	3.45	1.79	0.01	up	yes
Mir7676	3.43	1.78	0.01	up	yes
Fam84b	3.43	1.78	0.01	up	yes
Maged1	3.41	1.77	0.01	up	yes
Gpr153	3.47	1.80	0.01	up	yes
Magi1	3.43	1.78	0.01	up	yes
Gm9800	0.32	-1.66	0.01	down	yes
Farp1	3.40	1.77	0.01	up	yes
Pkhd1l1	3.40	1.77	0.01	up	yes
Zfp69	0.31	-1.69	0.01	down	yes
Hck	3.43	1.78	0.01	up	yes
Bhlhe40	3.41	1.77	0.01	up	yes
Gm5619	0.31	-1.70	0.01	down	yes
Plekhg3	3.40	1.77	0.01	up	yes
Ackr4	3.40	1.77	0.01	up	yes
Cyp2b19	3.45	1.79	0.01	up	yes
Bnc1	3.42	1.78	0.01	up	yes
Gm10282	0.32	-1.65	0.01	down	yes
Olfml1	3.43	1.78	0.01	up	yes
Sash1	3.39	1.76	0.01	up	yes
Ttc23	3.43	1.78	0.01	up	yes
Agap1	3.39	1.76	0.01	up	yes
Zfyve9	3.40	1.76	0.01	up	yes
Ror1	3.40	1.77	0.01	up	yes
Frmpd1	3.44	1.78	0.01	up	yes
Susd4	3.41	1.77	0.01	up	yes
Tbx2	3.42	1.77	0.01	up	yes
Rai14	3.37	1.75	0.01	up	yes
Gm5815	0.32	-1.65	0.01	down	yes
Acer2	3.40	1.76	0.01	up	yes

Nhsl2	3.40	1.76	0.01	up	yes
H2-K2	3.42	1.77	0.01	up	yes
Tgfbr3	3.36	1.75	0.01	up	yes
Nfil3	3.40	1.77	0.01	up	yes
Lmo7	3.37	1.75	0.01	up	yes
Ston2	3.36	1.75	0.01	up	yes
Dact2	3.36	1.75	0.01	up	yes
Hdac9	3.42	1.77	0.01	up	yes
Krt14	3.36	1.75	0.01	up	yes
Fam234a	3.37	1.75	0.01	up	yes
Gm23402	0.32	-1.63	0.01	down	yes
Sh3pxd2l	3.35	1.75	0.01	up	yes
Knl1	0.32	-1.63	0.01	down	yes
Ly86	3.41	1.77	0.01	up	yes
Ndnf	3.41	1.77	0.01	up	yes
Pou2af1	3.36	1.75	0.01	up	yes
F2rl1	3.36	1.75	0.01	up	yes
Ptafr	3.40	1.77	0.01	up	yes
Bmp1	3.36	1.75	0.01	up	yes
Nod1	3.36	1.75	0.01	up	yes
Itga9	3.36	1.75	0.01	up	yes
Sox18	3.40	1.77	0.01	up	yes
Ccdc120	3.38	1.76	0.01	up	yes
Hmgb1-p	0.32	-1.63	0.01	down	yes
Stox1	3.39	1.76	0.01	up	yes
Entpd1	3.33	1.74	0.01	up	yes
Map7	3.36	1.75	0.01	up	yes
Phxr2	0.32	-1.63	0.01	down	yes
Mark1	3.36	1.75	0.01	up	yes
Mall	3.36	1.75	0.01	up	yes
Fxyd3	3.38	1.76	0.01	up	yes
Arhgef1	3.34	1.74	0.01	up	yes
Gm2225	0.32	-1.64	0.01	down	yes
Gm9134	0.31	-1.67	0.01	down	yes
Arhgef1	3.37	1.75	0.01	up	yes
Spint1	3.33	1.74	0.01	up	yes
Slc22a1	3.36	1.75	0.01	up	yes
Rgl3	3.41	1.77	0.01	up	yes
AC15919	0.32	-1.66	0.01	down	yes
Osbpl10	3.33	1.73	0.01	up	yes
Vsig10	3.35	1.75	0.01	up	yes
Plekha1	3.32	1.73	0.01	up	yes
Hist1h4	0.33	-1.62	0.01	down	yes
Gm11336	0.32	-1.63	0.01	down	yes
Cdc42bp	3.31	1.73	0.01	up	yes
Lama3	3.31	1.73	0.01	up	yes
Bok	3.41	1.77	0.01	up	yes
Kif26a	3.32	1.73	0.01	up	yes
Samd3	3.39	1.76	0.01	up	yes
Serpine	3.31	1.73	0.01	up	yes
Srgap3	3.31	1.73	0.01	up	yes
Cttnbp2	3.31	1.73	0.01	up	yes
Tln2	3.33	1.73	0.01	up	yes
Mgp	3.34	1.74	0.01	up	yes
Srgap1	3.32	1.73	0.01	up	yes
Gimap3	3.29	1.72	0.01	up	yes
Pld2	3.32	1.73	0.01	up	yes

Il2rb	3.29	1.72	0.01	up	yes
Parm1	3.29	1.72	0.01	up	yes
Cdc42bpl	3.29	1.72	0.01	up	yes
Efnb2	3.29	1.72	0.01	up	yes
AC15294	0.32	-1.64	0.01	down	yes
Mfap5	3.37	1.75	0.01	up	yes
Fads3	3.32	1.73	0.01	up	yes
Scarf2	3.29	1.72	0.01	up	yes
Ntng2	3.29	1.72	0.01	up	yes
Gm44357	0.33	-1.60	0.01	down	yes
Cacna1a	3.30	1.72	0.01	up	yes
Csf1r	3.28	1.71	0.01	up	yes
Plxnb1	3.27	1.71	0.01	up	yes
Gas7	3.28	1.71	0.01	up	yes
Eda	3.33	1.74	0.01	up	yes
Gm9320	0.33	-1.61	0.01	down	yes
Sesn3	3.27	1.71	0.01	up	yes
Rrm2	0.33	-1.60	0.01	down	yes
Lrrk2	3.27	1.71	0.01	up	yes
Ikzf3;M	0.33	-1.59	0.01	down	yes
Gm5431	3.32	1.73	0.01	up	yes
Melk	0.33	-1.60	0.01	down	yes
Kdf1	3.31	1.73	0.01	up	yes
Sorl1	3.26	1.71	0.01	up	yes
Nr2f6	3.32	1.73	0.01	up	yes
Nbl1	3.30	1.72	0.01	up	yes
C1qtnf9	3.33	1.74	0.01	up	yes
Snapc2	3.29	1.72	0.01	up	yes
Gm24119	0.33	-1.61	0.01	down	yes
Trim2	3.28	1.71	0.01	up	yes
Hist1h4	0.33	-1.59	0.01	down	yes
Pbk	0.33	-1.59	0.01	down	yes
Ntn4	3.32	1.73	0.01	up	yes
Rbm20	3.33	1.74	0.01	up	yes
St8sia6	3.25	1.70	0.01	up	yes
2310030	3.29	1.72	0.01	up	yes
Pls1	3.25	1.70	0.01	up	yes
Col6a3	3.25	1.70	0.01	up	yes
Rims3	0.33	-1.62	0.01	down	yes
Napsa	3.27	1.71	0.01	up	yes
Meox2	3.30	1.72	0.01	up	yes
Gm43305	0.33	-1.60	0.01	down	yes
Stard8	3.27	1.71	0.01	up	yes
Clec14a	3.26	1.71	0.01	up	yes
Carmil3	3.24	1.70	0.01	up	yes
Arrdc4	3.23	1.69	0.01	up	yes
Hist1h2	0.33	-1.58	0.01	down	yes
Rpl31-p	0.33	-1.59	0.01	down	yes
Gm10293	0.33	-1.61	0.01	down	yes
Laptm4b	3.24	1.69	0.01	up	yes
Aacs	3.23	1.69	0.01	up	yes
Abca13	3.24	1.70	0.02	up	yes
Anxa3	3.29	1.72	0.02	up	yes
Man1c1	3.23	1.69	0.02	up	yes
Lrrc49	3.30	1.72	0.02	up	yes
Tmtc2	3.25	1.70	0.02	up	yes
Nuf2	0.34	-1.58	0.02	down	yes

Rsad1	3.32	1.73	0.02	up	yes
Itsn1	3.22	1.69	0.02	up	yes
Scn2b	3.25	1.70	0.02	up	yes
Spry4	3.29	1.72	0.02	up	yes
Naip2	3.24	1.70	0.02	up	yes
Cobll1	3.22	1.69	0.02	up	yes
Cdca3	0.34	-1.57	0.02	down	yes
Mapk13	3.28	1.71	0.02	up	yes
Cdk14	3.25	1.70	0.02	up	yes
Chn2	3.22	1.69	0.02	up	yes
Rxra	3.20	1.68	0.02	up	yes
Lgi2	3.23	1.69	0.02	up	yes
Ier5l	3.28	1.71	0.02	up	yes
Cldn12	3.23	1.69	0.02	up	yes
Tdrd5	0.33	-1.59	0.02	down	yes
Rps10-p	0.33	-1.60	0.02	down	yes
Rab11fi	3.20	1.68	0.02	up	yes
Ubash3b	3.19	1.67	0.02	up	yes
Lyrm7	0.34	-1.58	0.02	down	yes
Dhrs7	3.22	1.69	0.02	up	yes
Aph1b	3.22	1.69	0.02	up	yes
Gm17068	3.26	1.71	0.02	up	yes
Fam78b	3.22	1.69	0.02	up	yes
Itpripl	3.19	1.67	0.02	up	yes
Gm27883	0.34	-1.57	0.02	down	yes
Trim32	3.19	1.68	0.02	up	yes
AC16093	0.33	-1.58	0.02	down	yes
Zeb2;Mi	3.17	1.67	0.02	up	yes
Stc2	3.24	1.70	0.02	up	yes
Map3k5	3.18	1.67	0.02	up	yes
Nxpe2	3.25	1.70	0.02	up	yes
Gfra2	3.19	1.67	0.02	up	yes
Sulf2	3.17	1.67	0.02	up	yes
Samd4	3.20	1.68	0.02	up	yes
P2ry6	3.21	1.68	0.02	up	yes
Gm8325	0.33	-1.58	0.02	down	yes
Cep170b	3.16	1.66	0.02	up	yes
B4galt4	3.24	1.70	0.02	up	yes
Hist1h2	0.34	-1.55	0.02	down	yes
Efna5	3.18	1.67	0.02	up	yes
Ptgs1	3.19	1.67	0.02	up	yes
Dock9	3.16	1.66	0.02	up	yes
Clspn	0.34	-1.55	0.02	down	yes
Jun	3.16	1.66	0.02	up	yes
Prdm16	3.26	1.70	0.02	up	yes
AC15382	0.34	-1.55	0.02	down	yes
Zbtb10	3.17	1.67	0.02	up	yes
Gcnt2	3.18	1.67	0.02	up	yes
Bmpr2	3.14	1.65	0.02	up	yes
Cdh13	3.17	1.66	0.02	up	yes
Pla2g7	3.16	1.66	0.02	up	yes
Epn2	3.17	1.67	0.02	up	yes
Fhad1	3.22	1.69	0.02	up	yes
Nr3c2	3.16	1.66	0.02	up	yes
H2-DMb2	3.14	1.65	0.02	up	yes
Tnks1bp	3.14	1.65	0.02	up	yes
Unc93b1	3.14	1.65	0.02	up	yes

Pdzrn3	3.20	1.68	0.02	up	yes
Hspb8	3.14	1.65	0.02	up	yes
Gas6	3.13	1.65	0.02	up	yes
AC12553	0.34	-1.56	0.02	down	yes
Pid1	3.19	1.67	0.02	up	yes
Fam20a	3.21	1.68	0.02	up	yes
Maml3	3.13	1.65	0.02	up	yes
H2-Ab1	3.12	1.64	0.02	up	yes
Rassf8	3.15	1.65	0.02	up	yes
Anxa8	3.16	1.66	0.02	up	yes
Zfp516	3.12	1.64	0.02	up	yes
Kbtbd11	3.12	1.64	0.02	up	yes
Slc5a6	3.13	1.65	0.02	up	yes
Ncapg	0.35	-1.53	0.02	down	yes
Shpk	3.16	1.66	0.02	up	yes
Mylip	3.11	1.64	0.02	up	yes
Gm4799	0.34	-1.56	0.02	down	yes
Sox7	3.17	1.67	0.02	up	yes
Fgd6	3.12	1.64	0.02	up	yes
Hist1h1	0.35	-1.52	0.02	down	yes
Foxq1	3.17	1.67	0.02	up	yes
Gpr174	0.35	-1.53	0.02	down	yes
Galnt18	3.12	1.64	0.02	up	yes
Fcgr3	3.14	1.65	0.02	up	yes
Cks2	0.35	-1.53	0.02	down	yes
Mocos	3.12	1.64	0.02	up	yes
Crybg3	3.11	1.64	0.02	up	yes
Cdc25c	0.35	-1.53	0.02	down	yes
Cstad	0.34	-1.54	0.02	down	yes
Sort1	3.10	1.63	0.02	up	yes
Il13ra1	3.10	1.63	0.02	up	yes
Mb21d2	3.14	1.65	0.02	up	yes
Sema3g	3.13	1.65	0.02	up	yes
Axl	3.09	1.63	0.02	up	yes
Mxd3	0.35	-1.53	0.02	down	yes
Olfr135	0.34	-1.55	0.02	down	yes
Cd300a	3.11	1.64	0.02	up	yes
Sfxn5	3.13	1.65	0.02	up	yes
Apbb2	3.11	1.63	0.02	up	yes
Osbpl5	3.10	1.63	0.02	up	yes
Espn	3.11	1.64	0.02	up	yes
Echdc2	3.17	1.66	0.02	up	yes
Fn1	3.09	1.63	0.02	up	yes
Cmklr1	3.09	1.63	0.02	up	yes
Adra2a	3.14	1.65	0.02	up	yes
Fcer1g	3.13	1.65	0.02	up	yes
Gsap	3.09	1.63	0.02	up	yes
Mapre3	3.11	1.64	0.02	up	yes
Myo1e	3.08	1.62	0.02	up	yes
Nnat	3.18	1.67	0.02	up	yes
Fgd5	3.08	1.62	0.02	up	yes
Ccl21a	3.07	1.62	0.02	up	yes
Dsc2	3.08	1.62	0.02	up	yes
Tnk1	3.10	1.63	0.02	up	yes
Dusp15	3.17	1.67	0.02	up	yes
Ggt5	3.09	1.63	0.02	up	yes
Cmtm8	3.14	1.65	0.02	up	yes

Stap2	3.10	1.63	0.02	up	yes
Rian	3.15	1.66	0.02	up	yes
Ckap2	0.35	-1.51	0.02	down	yes
Trim26	3.06	1.61	0.02	up	yes
Ctnnal1	3.08	1.62	0.02	up	yes
P4ha2	3.11	1.64	0.02	up	yes
Tead3	3.06	1.61	0.02	up	yes
Cenpi	0.35	-1.50	0.02	down	yes
Themis2	3.07	1.62	0.02	up	yes
Socs2	3.08	1.62	0.02	up	yes
Ppic	3.09	1.63	0.02	up	yes
St3gal6	3.05	1.61	0.02	up	yes
Depdc1a	0.35	-1.50	0.02	down	yes
Hunk	3.05	1.61	0.02	up	yes
Samd14	3.07	1.62	0.02	up	yes
Carmil1	3.06	1.61	0.02	up	yes
Gm8494	0.34	-1.54	0.02	down	yes
Acvr1	3.08	1.62	0.02	up	yes
Cd55	3.07	1.62	0.02	up	yes
Mmp23	3.11	1.64	0.02	up	yes
Igsf9b	3.05	1.61	0.02	up	yes
Plek2	3.10	1.63	0.02	up	yes
Mctp1	3.08	1.62	0.02	up	yes
Chic1	3.05	1.61	0.02	up	yes
Gm5586	0.35	-1.52	0.02	down	yes
Fbxo5	0.36	-1.49	0.02	down	yes
Nfe2l1	3.03	1.60	0.02	up	yes
C2;Cfb;	3.04	1.60	0.02	up	yes
Palld	3.03	1.60	0.02	up	yes
Kcnb1	3.07	1.62	0.02	up	yes
Oat	3.03	1.60	0.02	up	yes
Amot	3.04	1.60	0.02	up	yes
Mef2c	3.05	1.61	0.02	up	yes
Rhbdl3	3.04	1.60	0.02	up	yes
Slc36a1	3.02	1.60	0.02	up	yes
Fry	3.02	1.60	0.02	up	yes
Hist1h4	0.36	-1.48	0.02	down	yes
Asf1b	0.36	-1.48	0.02	down	yes
Col6a1	3.02	1.59	0.02	up	yes
Trim47;	3.02	1.60	0.02	up	yes
Alox15	3.03	1.60	0.02	up	yes
Neurl1a	3.10	1.63	0.02	up	yes
Hist1h3	0.36	-1.48	0.02	down	yes
Arhgap2	3.05	1.61	0.02	up	yes
Nectin1	3.01	1.59	0.02	up	yes
Cuedc1	3.03	1.60	0.02	up	yes
Myo1d	3.02	1.59	0.02	up	yes
Afap1l1	3.07	1.62	0.02	up	yes
Ptprg	3.01	1.59	0.02	up	yes
Fam60a	0.36	-1.48	0.02	down	yes
Sgo2a	0.36	-1.48	0.02	down	yes
Nkg7	3.01	1.59	0.02	up	yes
Lmod1	3.05	1.61	0.02	up	yes
Jam2	3.01	1.59	0.02	up	yes
Nudt7	3.03	1.60	0.02	up	yes
Optn	3.03	1.60	0.02	up	yes
Gadd45g	3.07	1.62	0.02	up	yes

Nectin4	3.04	1.60	0.02	up	yes
Sgk1	3.00	1.58	0.02	up	yes
Anxa9	3.08	1.62	0.02	up	yes
Amz1	3.08	1.62	0.02	up	yes
Prr5l	3.02	1.59	0.02	up	yes
Rn7s6	0.36	-1.47	0.02	down	yes
Cdk6	2.98	1.58	0.02	up	yes
Rps4l	3.02	1.59	0.02	up	yes
Iigp1	2.99	1.58	0.02	up	yes
Dsp	2.98	1.58	0.02	up	yes
Hist2h2	0.36	-1.47	0.02	down	yes
Apbb1	3.02	1.59	0.02	up	yes
Pcbp4	3.02	1.60	0.02	up	yes
Syt15	3.05	1.61	0.02	up	yes
Cald1	2.99	1.58	0.02	up	yes
Emc9	3.08	1.62	0.02	up	yes
Stk38l	3.00	1.58	0.02	up	yes
Prss8	3.02	1.60	0.02	up	yes
Etohd2	3.02	1.60	0.02	up	yes
Mmp25	3.00	1.58	0.02	up	yes
Dmtn	3.01	1.59	0.02	up	yes
Sspo	2.98	1.57	0.02	up	yes
Mboat7	2.98	1.57	0.02	up	yes
Gsta4	3.00	1.58	0.02	up	yes
Sh3rf3	2.98	1.58	0.02	up	yes
Top2a;G	0.36	-1.46	0.02	down	yes
Trbv24	0.35	-1.50	0.02	down	yes
Tmem150	2.99	1.58	0.02	up	yes
Pglyrp1	3.00	1.58	0.02	up	yes
Gm3534	0.36	-1.47	0.02	down	yes
Gm14303	0.36	-1.47	0.02	down	yes
Robo2	3.00	1.59	0.02	up	yes
Gm22068	0.36	-1.46	0.02	down	yes
Rflnb	2.97	1.57	0.02	up	yes
Tlr13	2.98	1.58	0.02	up	yes
Arhgap4	2.97	1.57	0.02	up	yes
Cybb	2.97	1.57	0.02	up	yes
Aplp2	2.96	1.57	0.02	up	yes
Lsm5	0.36	-1.49	0.02	down	yes
Gm14494	0.36	-1.49	0.02	down	yes
Epha4	3.01	1.59	0.02	up	yes
Cnksr1	3.01	1.59	0.02	up	yes
Ifi47;O	0.36	-1.46	0.02	down	yes
Dcbld2	2.97	1.57	0.02	up	yes
Fmn1	2.96	1.57	0.02	up	yes
Wfdc18	3.02	1.60	0.02	up	yes
Slc27a1	2.95	1.56	0.02	up	yes
Nup62;I	2.96	1.56	0.02	up	yes
Svbp	0.36	-1.47	0.02	down	yes
Hlx	3.00	1.58	0.02	up	yes
Pecr	3.02	1.60	0.02	up	yes
Gucy1b3	3.02	1.59	0.02	up	yes
AC12474	0.37	-1.45	0.02	down	yes
Pbx3	2.98	1.58	0.02	up	yes
Ccdc71	0.37	-1.45	0.02	down	yes
Hmgb1-p	0.36	-1.45	0.02	down	yes
Mcm10	0.37	-1.45	0.02	down	yes

Rap1gap	3.03	1.60	0.02	up	yes
Fam129b	2.94	1.56	0.02	up	yes
Lpcat2	2.95	1.56	0.02	up	yes
Nradd	2.98	1.58	0.02	up	yes
Ccdc8	2.96	1.57	0.02	up	yes
Epha1	2.96	1.56	0.02	up	yes
Vcl	2.94	1.56	0.02	up	yes
Erg	3.01	1.59	0.02	up	yes
Adgrl3	3.00	1.59	0.02	up	yes
Rpl17-p	0.37	-1.45	0.02	down	yes
Arhgef5	2.94	1.56	0.02	up	yes
Gm12666	0.36	-1.48	0.02	down	yes
H2-Eb1	2.93	1.55	0.02	up	yes
Pik3ap1	2.94	1.56	0.03	up	yes
Gm10075	0.36	-1.45	0.03	down	yes
Agpat2	2.96	1.56	0.03	up	yes
Alpk1	2.94	1.56	0.03	up	yes
Arhgdib	0.37	-1.44	0.03	down	yes
Runx2	2.94	1.56	0.03	up	yes
Bdkrb2	2.97	1.57	0.03	up	yes
Gm2a	2.92	1.55	0.03	up	yes
Arhgef1	2.93	1.55	0.03	up	yes
Csf2rb;	2.93	1.55	0.03	up	yes
Crat	2.93	1.55	0.03	up	yes
Gm7551	0.36	-1.46	0.03	down	yes
Krt222	2.98	1.58	0.03	up	yes
Cnga2	3.00	1.59	0.03	up	yes
Pcna	0.37	-1.43	0.03	down	yes
Diaph3	0.37	-1.43	0.03	down	yes
Gm10291	0.36	-1.46	0.03	down	yes
AC15388	0.37	-1.44	0.03	down	yes
Shcbp1	0.37	-1.44	0.03	down	yes
Kif4	0.37	-1.43	0.03	down	yes
Cep126	2.95	1.56	0.03	up	yes
Coro2a	2.97	1.57	0.03	up	yes
Gm22009	0.37	-1.43	0.03	down	yes
Epm2a	2.94	1.56	0.03	up	yes
Osbpl1a	2.94	1.56	0.03	up	yes
Gpr63	2.99	1.58	0.03	up	yes
Epha2	2.94	1.56	0.03	up	yes
Rhbdf2	2.90	1.54	0.03	up	yes
Smad7	2.90	1.54	0.03	up	yes
Ank3	2.90	1.54	0.03	up	yes
Eif4ebp	2.94	1.56	0.03	up	yes
Fam210b	2.90	1.54	0.03	up	yes
Gm13127	0.36	-1.46	0.03	down	yes
Alox12e	2.90	1.53	0.03	up	yes
Gm16589	0.36	-1.46	0.03	down	yes
Gm5869	0.37	-1.43	0.03	down	yes
Grhl1	2.92	1.54	0.03	up	yes
Gm24336	0.37	-1.44	0.03	down	yes
Kansl1;	0.37	-1.42	0.03	down	yes
Ndc80	0.37	-1.42	0.03	down	yes
Hsd11b1	2.89	1.53	0.03	up	yes
Ckap2l	0.37	-1.42	0.03	down	yes
Slc25a1	2.89	1.53	0.03	up	yes
Mpzl1	2.96	1.56	0.03	up	yes

Gaa	2.88	1.53	0.03	up	yes
Mn1	2.91	1.54	0.03	up	yes
Ephx1;Gr	2.88	1.53	0.03	up	yes
Nrp2	2.88	1.53	0.03	up	yes
Ube2c	0.37	-1.42	0.03	down	yes
Dock6	2.88	1.53	0.03	up	yes
Aldh2	2.88	1.53	0.03	up	yes
Gm17509	0.37	-1.43	0.03	down	yes
Phactr2	2.88	1.53	0.03	up	yes
Rasgrp3	2.89	1.53	0.03	up	yes
Dmpk	2.89	1.53	0.03	up	yes
Cd63	2.88	1.53	0.03	up	yes
Enpp5	2.88	1.53	0.03	up	yes
Icosl	2.88	1.53	0.03	up	yes
Pcdhga4	2.87	1.52	0.03	up	yes
4930595I	0.38	-1.41	0.03	down	yes
Tmem102	2.93	1.55	0.03	up	yes
Abhd5	2.89	1.53	0.03	up	yes
Clip3	2.92	1.54	0.03	up	yes
Hist1h2l	0.38	-1.41	0.03	down	yes
Fzd6	2.87	1.52	0.03	up	yes
Sema6a	2.90	1.53	0.03	up	yes
Cenpm	0.37	-1.42	0.03	down	yes
Cd93	2.86	1.52	0.03	up	yes
Apba2	2.88	1.53	0.03	up	yes
Gm7600	0.37	-1.42	0.03	down	yes
Btbd11	2.86	1.52	0.03	up	yes
Snora23	0.38	-1.41	0.03	down	yes
Rps13-p	0.37	-1.44	0.03	down	yes
Lrfn4	2.88	1.53	0.03	up	yes
Kif11	0.38	-1.40	0.03	down	yes
Cd7	2.87	1.52	0.03	up	yes
Hist2h2l	0.38	-1.40	0.03	down	yes
Tnfsf8	2.89	1.53	0.03	up	yes
Klf11	2.86	1.52	0.03	up	yes
Hs1bp3	2.86	1.52	0.03	up	yes
Olfm1	2.89	1.53	0.03	up	yes
Mgat4b	2.87	1.52	0.03	up	yes
C1qtnf1	2.87	1.52	0.03	up	yes
Kif15	0.38	-1.40	0.03	down	yes
Ciita	2.85	1.51	0.03	up	yes
Il1b	2.92	1.54	0.03	up	yes
Aspm	0.38	-1.40	0.03	down	yes
Rffl	2.84	1.51	0.03	up	yes
Zfp703	2.85	1.51	0.03	up	yes
Marveld	2.93	1.55	0.03	up	yes
Jcad	2.85	1.51	0.03	up	yes
Crb2	2.91	1.54	0.03	up	yes
Shisa6	2.85	1.51	0.03	up	yes
Nusap1	0.38	-1.39	0.03	down	yes
Ecm2;Asj	0.38	-1.39	0.03	down	yes
Snora73l	0.38	-1.40	0.03	down	yes
Gm5564	0.38	-1.40	0.03	down	yes
Sec24d	2.84	1.50	0.03	up	yes
Gstm1	2.85	1.51	0.03	up	yes
Unc5b	2.85	1.51	0.03	up	yes
Arhgap2	2.84	1.51	0.03	up	yes

Esco2	0.38	-1.39	0.03	down	yes
Spata2	2.83	1.50	0.03	up	yes
Mki67	0.38	-1.39	0.03	down	yes
Ust	2.84	1.51	0.03	up	yes
Nlgn1	2.84	1.51	0.03	up	yes
Cttn	2.84	1.50	0.03	up	yes
Gm3756	0.37	-1.43	0.03	down	yes
Gpr68	2.83	1.50	0.03	up	yes
Cish	0.38	-1.40	0.03	down	yes
Tdrd7	2.83	1.50	0.03	up	yes
Tacstd2	2.84	1.51	0.03	up	yes
Rnf125	0.38	-1.38	0.03	down	yes
Hist1h4	0.38	-1.38	0.03	down	yes
Cd101	2.85	1.51	0.03	up	yes
Ptn	2.87	1.52	0.03	up	yes
Capg	2.83	1.50	0.03	up	yes
Copz2	2.82	1.50	0.03	up	yes
Mme	2.87	1.52	0.03	up	yes
Rps3a3	0.38	-1.38	0.03	down	yes
Ccnb2	0.38	-1.38	0.03	down	yes
Gm9833	0.38	-1.39	0.03	down	yes
Tcn2	2.81	1.49	0.03	up	yes
Notch4	2.82	1.50	0.03	up	yes
Lrrn3	2.83	1.50	0.03	up	yes
Zfp2	2.85	1.51	0.03	up	yes
Kctd21	2.83	1.50	0.03	up	yes
Gm18194	2.84	1.51	0.03	up	yes
A430105	2.82	1.50	0.03	up	yes
Rhbdd2	2.81	1.49	0.03	up	yes
Tmem106	2.82	1.49	0.03	up	yes
Slc46a3	2.82	1.50	0.03	up	yes
Gm10263	0.38	-1.38	0.03	down	yes
Pgm2l1	0.39	-1.37	0.03	down	yes
Il7	2.80	1.49	0.03	up	yes
4930539	2.80	1.49	0.03	up	yes
Ncs1	2.82	1.50	0.03	up	yes
Trbv1	0.38	-1.38	0.03	down	yes
Gm9761	0.38	-1.41	0.03	down	yes
Snx9	2.80	1.49	0.03	up	yes
Nckap1	2.80	1.48	0.03	up	yes
Fbxo10	2.80	1.48	0.03	up	yes
Cdk1	0.39	-1.37	0.03	down	yes
Fer1l5	0.38	-1.41	0.03	down	yes
Mrnip	0.38	-1.41	0.03	down	yes
Hist1h3	0.39	-1.37	0.03	down	yes
Cacng4	0.38	-1.39	0.03	down	yes
Gm7332	0.38	-1.38	0.03	down	yes
Tssc1	2.79	1.48	0.03	up	yes
Lrr1	0.38	-1.41	0.03	down	yes
Plac8	2.81	1.49	0.03	up	yes
Nkd2	2.80	1.49	0.03	up	yes
Tpt1-ps	0.38	-1.38	0.03	down	yes
Prr18	2.84	1.51	0.03	up	yes
Bub1	0.39	-1.37	0.03	down	yes
Bbs2	2.80	1.49	0.03	up	yes
Hip1r	2.78	1.48	0.03	up	yes
Zfp1	0.39	-1.37	0.03	down	yes

Ecm1;Mi	2.80	1.48	0.03	up	yes
Cdh5	2.79	1.48	0.03	up	yes
Stil	0.39	-1.36	0.03	down	yes
Tbc1d30	2.81	1.49	0.03	up	yes
Hist1h2	0.39	-1.36	0.03	down	yes
Crip2	2.81	1.49	0.03	up	yes
Rpsa-ps	0.38	-1.41	0.03	down	yes
Fam135a	2.79	1.48	0.03	up	yes
Nr1d2	2.77	1.47	0.03	up	yes
Ifi44l	2.79	1.48	0.03	up	yes
Oas1a	2.80	1.49	0.03	up	yes
Map3k7c	2.83	1.50	0.03	up	yes
Cebpd	2.79	1.48	0.03	up	yes
Trim3	2.79	1.48	0.03	up	yes
BC00553	2.77	1.47	0.03	up	yes
Hist1h2	0.39	-1.36	0.03	down	yes
Rad51ap	0.39	-1.37	0.03	down	yes
Nlrc4	2.82	1.50	0.03	up	yes
Sema6c	2.82	1.49	0.03	up	yes
Sh3bp4	2.77	1.47	0.03	up	yes
Gm11263	0.38	-1.39	0.03	down	yes
Lnx1	2.80	1.48	0.03	up	yes
Gm8822	0.39	-1.36	0.03	down	yes
Fer	2.79	1.48	0.03	up	yes
Nfasc	2.78	1.47	0.03	up	yes
Hist1h1	0.39	-1.35	0.03	down	yes
Col6a2	2.76	1.46	0.03	up	yes
Map3k20	2.76	1.46	0.03	up	yes
Ercc6l	0.39	-1.35	0.03	down	yes
Hs3st3b	2.76	1.47	0.03	up	yes
Gm13436	0.39	-1.35	0.03	down	yes
Mapkapk	2.76	1.46	0.03	up	yes
Uggt2	2.78	1.47	0.04	up	yes
Spink5	2.77	1.47	0.04	up	yes
Dmxl2	2.76	1.46	0.04	up	yes
Tgfb2	2.79	1.48	0.04	up	yes
Plxna2	2.75	1.46	0.04	up	yes
Aurkb	0.39	-1.35	0.04	down	yes
Ms4a6c	2.75	1.46	0.04	up	yes
Itgax	2.75	1.46	0.04	up	yes
F11r	2.75	1.46	0.04	up	yes
Cbx7	2.75	1.46	0.04	up	yes
Pclaf	0.39	-1.34	0.04	down	yes
Muc5ac	2.81	1.49	0.04	up	yes
Phf11d	2.77	1.47	0.04	up	yes
Oasl2	2.74	1.46	0.04	up	yes
Sh3tc2	2.76	1.46	0.04	up	yes
Ska1	0.39	-1.35	0.04	down	yes
Slc16a9	2.78	1.48	0.04	up	yes
Nr1h4	0.39	-1.34	0.04	down	yes
Gm13493	0.39	-1.35	0.04	down	yes
Tgfbi	2.74	1.45	0.04	up	yes
Birc5	0.40	-1.34	0.04	down	yes
Hmgb2	0.40	-1.34	0.04	down	yes
Nol3	0.39	-1.36	0.04	down	yes
Rhbdl2	2.77	1.47	0.04	up	yes
Synpo2	2.73	1.45	0.04	up	yes

Dnah12	2.77	1.47	0.04	up	yes
Apobr	2.75	1.46	0.04	up	yes
Ccsap	0.39	-1.34	0.04	down	yes
Foxn1	2.73	1.45	0.04	up	yes
Cds1	2.73	1.45	0.04	up	yes
Gm12966	0.39	-1.35	0.04	down	yes
CT01057	0.39	-1.35	0.04	down	yes
Ehd2	2.73	1.45	0.04	up	yes
2810408	0.39	-1.34	0.04	down	yes
G6pc3	2.75	1.46	0.04	up	yes
B3galt5	2.74	1.45	0.04	up	yes
Cd276	2.76	1.47	0.04	up	yes
Gm12174	0.39	-1.36	0.04	down	yes
Amph	2.80	1.48	0.04	up	yes
Nudt12	2.76	1.47	0.04	up	yes
Bnc2	2.75	1.46	0.04	up	yes
Col19a1	2.77	1.47	0.04	up	yes
Rpl5-ps	0.39	-1.37	0.04	down	yes
Hist1h3	0.40	-1.33	0.04	down	yes
Aldh1l1	2.72	1.44	0.04	up	yes
Clec12a	2.75	1.46	0.04	up	yes
Lurap1l	2.75	1.46	0.04	up	yes
9130208	2.72	1.45	0.04	up	yes
Cenph	0.39	-1.35	0.04	down	yes
Krt5	2.71	1.44	0.04	up	yes
Cdca2	0.40	-1.33	0.04	down	yes
Grb10	2.72	1.45	0.04	up	yes
Prom1	2.74	1.45	0.04	up	yes
Tbc1d2	2.76	1.47	0.04	up	yes
Olfr920	2.75	1.46	0.04	up	yes
Slamf7	2.73	1.45	0.04	up	yes
Gm21887	2.71	1.44	0.04	up	yes
5330417	2.77	1.47	0.04	up	yes
Fbxl2	0.40	-1.34	0.04	down	yes
Serinc2	2.74	1.45	0.04	up	yes
Skint7	2.75	1.46	0.04	up	yes
Rab3il1	2.73	1.45	0.04	up	yes
Rab40c	2.71	1.44	0.04	up	yes
Rps6ka2	2.72	1.44	0.04	up	yes
Epb41l5	2.72	1.44	0.04	up	yes
Atp6v0a	2.70	1.43	0.04	up	yes
Ttk	0.40	-1.32	0.04	down	yes
Gm5518	0.40	-1.32	0.04	down	yes
Col1a2	2.70	1.43	0.04	up	yes
Fam214b	2.71	1.44	0.04	up	yes
2700099	0.40	-1.33	0.04	down	yes
Tirap	2.72	1.44	0.04	up	yes
Ndrg2	2.70	1.43	0.04	up	yes
Fam43a	2.70	1.44	0.04	up	yes
Hipk2	2.69	1.43	0.04	up	yes
Gucy1a2	2.70	1.43	0.04	up	yes
Serpinf	2.73	1.45	0.04	up	yes
Wdr89	0.40	-1.32	0.04	down	yes
Pyurf;G	2.71	1.44	0.04	up	yes
Ticrr	0.40	-1.32	0.04	down	yes
Acpp	2.69	1.43	0.04	up	yes
Abca1	2.69	1.43	0.04	up	yes

Ssh1	2.69	1.43	0.04	up	yes
Comt	2.69	1.43	0.04	up	yes
Gpc1	2.69	1.43	0.04	up	yes
Fstl1	2.69	1.43	0.04	up	yes
Pou2f2;I	2.70	1.43	0.04	up	yes
Slco5a1	2.69	1.43	0.04	up	yes
Bckdhb	2.70	1.43	0.04	up	yes
Gm10073	0.40	-1.33	0.04	down	yes
Ell2	2.69	1.43	0.04	up	yes
Hip1	2.68	1.42	0.04	up	yes
Ctsk	2.73	1.45	0.04	up	yes
Shisa4	2.72	1.44	0.04	up	yes
Tmem64	2.68	1.42	0.04	up	yes
Noxo1	2.75	1.46	0.04	up	yes
Fnbp1l;I	2.68	1.42	0.04	up	yes
Syne2;M	2.68	1.42	0.04	up	yes
Eya2	2.68	1.42	0.04	up	yes
Morn1	2.73	1.45	0.04	up	yes
Arpin	2.69	1.43	0.04	up	yes
Mis18bp	0.40	-1.31	0.04	down	yes
Cdh17	2.74	1.45	0.04	up	yes
Hal	2.69	1.43	0.04	up	yes
Cd74;Mi	2.67	1.42	0.04	up	yes
Pqlc1	2.68	1.42	0.04	up	yes
Kndc1	2.68	1.42	0.04	up	yes
Dmrt2	2.68	1.42	0.04	up	yes
Ccl21b	2.74	1.45	0.04	up	yes
Gabarap	2.67	1.42	0.04	up	yes
AC15315	0.40	-1.32	0.04	down	yes
Klf5	2.71	1.44	0.04	up	yes
Entpd3	2.71	1.44	0.04	up	yes
Acad11;I	2.67	1.41	0.04	up	yes
Gm5453	0.40	-1.31	0.04	down	yes
Prkaa2	2.66	1.41	0.04	up	yes
Micall1	2.66	1.41	0.04	up	yes
Msi1	2.69	1.43	0.04	up	yes
Cdca5	0.41	-1.30	0.04	down	yes
Hist1h3	0.41	-1.30	0.04	down	yes
Cdc6	0.41	-1.30	0.04	down	yes
Ccdc102	2.68	1.42	0.04	up	yes
Itgb8	2.67	1.42	0.04	up	yes
Gm14165	0.40	-1.31	0.04	down	yes
Gm37164	0.40	-1.31	0.04	down	yes
Sema3b	2.70	1.43	0.04	up	yes
Itgad	2.67	1.42	0.04	up	yes
Derl3	2.78	1.47	0.04	up	yes
Fxyd6	2.72	1.44	0.04	up	yes
Cbx3-ps	0.41	-1.30	0.04	down	yes
Ttc39c	2.68	1.42	0.04	up	yes
Lyn	2.65	1.41	0.04	up	yes
Zfp568	2.65	1.41	0.04	up	yes
Spdl1	0.41	-1.30	0.04	down	yes
Wnt7b	2.68	1.42	0.04	up	yes
Ptpn21	2.66	1.41	0.04	up	yes
Srl	2.67	1.41	0.04	up	yes
Acaca	2.64	1.40	0.04	up	yes
Cbr2	2.64	1.40	0.04	up	yes

Cpq	2.66	1.41	0.04	up	yes
Trip10	2.65	1.41	0.04	up	yes
Aox4	2.68	1.42	0.04	up	yes
Zfp629	0.41	-1.29	0.04	down	yes
4930562	2.68	1.42	0.04	up	yes
Gm10132	0.41	-1.30	0.04	down	yes
Mmp15	2.65	1.40	0.04	up	yes
Bcl3	2.66	1.41	0.04	up	yes
Hhip	2.64	1.40	0.04	up	yes
Podxl	2.64	1.40	0.04	up	yes
Zfp870	2.67	1.42	0.04	up	yes
Ubtd1	2.65	1.40	0.04	up	yes
AC15656	0.41	-1.30	0.04	down	yes
Cdhr5	2.72	1.45	0.04	up	yes
Hist2h4	0.41	-1.28	0.04	down	yes
Cadm1	2.64	1.40	0.04	up	yes
Trbv29	0.41	-1.29	0.04	down	yes
Cdca8	0.41	-1.29	0.04	down	yes
Hist1h4	0.41	-1.28	0.04	down	yes
Eps8l1	2.66	1.41	0.04	up	yes
Chil1	2.67	1.42	0.04	up	yes
Gm4759;	2.63	1.39	0.04	up	yes
Morc4	2.69	1.43	0.04	up	yes
Lypd8	2.68	1.42	0.04	up	yes
Dlg2	2.63	1.40	0.04	up	yes
Rps24-p	0.41	-1.29	0.04	down	yes
Tsc22d1	2.62	1.39	0.04	up	yes
Lvrn	2.68	1.42	0.04	up	yes
Smad9	2.67	1.42	0.04	up	yes
Lgalsl	2.64	1.40	0.04	up	yes
Bard1	0.41	-1.28	0.04	down	yes
Lrrc8c	0.41	-1.28	0.04	down	yes
Sgo1	0.41	-1.28	0.04	down	yes
Dnah10	2.66	1.41	0.05	up	yes
Gm10051	0.41	-1.28	0.05	down	yes
Tjp2	2.62	1.39	0.05	up	yes
Kif1a	2.67	1.42	0.05	up	yes
Ahdc1	0.41	-1.28	0.05	down	yes
Zc3h12c	2.62	1.39	0.05	up	yes
Arsb	2.61	1.39	0.05	up	yes
Nudt1	0.41	-1.30	0.05	down	yes
Fmo1	2.62	1.39	0.05	up	yes
Actn3	2.62	1.39	0.05	up	yes
Nme4	0.41	-1.30	0.05	down	yes
Igsf9	2.62	1.39	0.05	up	yes
Enpep	2.61	1.38	0.05	up	yes
Cenpe	0.41	-1.27	0.05	down	yes
Serpinh	2.62	1.39	0.05	up	yes
Nrg1	2.65	1.41	0.05	up	yes
Kirrel3	2.64	1.40	0.05	up	yes
Uhrf1bp	2.61	1.38	0.05	up	yes
CT03017	0.41	-1.27	0.05	down	yes
Pkia	2.62	1.39	0.05	up	yes
Frmd4a	2.60	1.38	0.05	up	yes
Eea1	2.60	1.38	0.05	up	yes
Stard10	2.62	1.39	0.05	up	yes
Gm5905	0.42	-1.26	0.05	down	yes

Fkbp10	2.62	1.39	0.05	up	yes
Klhl32	2.62	1.39	0.05	up	yes
Bhlhe41	2.60	1.38	0.05	up	yes
F2r	2.59	1.37	0.05	up	yes
Tmod2	2.64	1.40	0.05	up	yes
Irf8	2.59	1.37	0.05	up	yes
Tspan2	0.42	-1.26	0.05	down	yes
Hfe	2.62	1.39	0.05	up	yes
Ppfibp1	2.60	1.38	0.05	up	yes
Nectin3	2.60	1.38	0.05	up	yes
Slc16a7	2.61	1.38	0.05	up	yes
Hist1h2	0.42	-1.26	0.05	down	yes
Sh2b2	2.60	1.38	0.05	up	yes
Cyth4	2.59	1.37	0.05	up	yes
Ncapg2	0.42	-1.26	0.05	down	yes
Gcsam	2.65	1.40	0.05	up	yes
Gm6498	2.66	1.41	0.05	up	yes
Efcab11	0.41	-1.28	0.05	down	yes
Zbtb46	2.59	1.37	0.05	up	yes
Tcf7l2	2.59	1.37	0.05	up	yes
Bmp6	2.63	1.40	0.05	up	yes
Gm10443	0.42	-1.26	0.05	down	yes
Adamts2	2.59	1.37	0.05	up	yes
Tlcd1;M	2.60	1.38	0.05	up	yes
Dse	2.58	1.37	0.05	up	yes
Crtap	2.59	1.37	0.05	up	yes
Wbscr17	2.59	1.38	0.05	up	yes
Ccdc148	2.60	1.38	0.05	up	yes
Gm21541	2.60	1.38	0.05	up	yes
Scin	2.60	1.38	0.05	up	yes
Hexb	2.58	1.37	0.05	up	yes
Rps6-ps	0.41	-1.29	0.05	down	yes
Dhfr	0.42	-1.25	0.05	down	yes
Nek2	0.42	-1.25	0.05	down	yes
Garnl3	2.62	1.39	0.05	up	yes
Ctnnd1;	2.57	1.36	0.05	up	yes
4930415	0.41	-1.30	0.05	down	yes
Kctd12b	2.60	1.38	0.05	up	yes
Scpep1	2.58	1.37	0.05	up	yes
Naip6	2.59	1.37	0.05	up	yes
Tppp	2.57	1.36	0.05	up	yes
Syngr1	2.57	1.36	0.05	up	yes
Dctpp1	0.42	-1.26	0.05	down	yes
Ctf1	2.61	1.38	0.05	up	yes
Hes1	2.60	1.38	0.05	up	yes
Sh2d4b	2.56	1.36	0.05	up	yes
Dixdc1	2.59	1.37	0.05	up	yes
Zgrf1	0.42	-1.25	0.05	down	yes
