# Supplementary material for: Transcriptome analysis reveals a potential regulatory mechanism of the lnc-5423.6/IGFBP5 axis in the early stages of mouse thymic involution: lnc-5423.6/IGFBP5 axis regulates thymic involution
Source: Acta Biochim Biophys Sin (Shanghai). 2023 Apr 19;55(4):548–60. doi: 10.3724/abbs.2023042 (PMC10195152; doi:10.3724/abbs.2023042)
Supplement: Table_S7 [file Table_S7.pdf]

Category	Term	Count	%	PValue	Genes	List Total	Pop Hits	Pop Total
GOTERM_	inflammat	16	5.860806	6.18E-07	IGHG1, IL2	191	225	13588
GOTERM_	response t	19	6.959707	1.80E-06	IGHG1, IL2	191	347	13588
GOTERM_	defense re	21	7.692308	4.69E-06	IGHG1, H2	191	448	13588
GOTERM_	immune re	21	7.692308	9.85E-06	IGHG1, MI	191	471	13588
GOTERM_	acute infla	9	3.296703	1.70E-05	IGHG1, C1	191	81	13588
GOTERM_	positive re	12	4.395604	7.06E-05	SLC1A3, N	191	189	13588
GOTERM_	potassium	11	4.029304	8.36E-05	KCNH1, K(	191	160	13588
GOTERM_	innate imr	9	3.296703	1.26E-04	C1RA, IL18	191	107	13588
GOTERM_	compleme	6	2.197802	1.34E-04	IGHG1, C1	191	36	13588
GOTERM_	activation	6	2.197802	1.34E-04	IGHG1, C1	191	36	13588
GOTERM_	activation	8	2.930403	1.98E-04	IGHG1, C1	191	86	13588
GOTERM_	positive re	11	4.029304	2.87E-04	IGHG1, H2	191	186	13588
GOTERM_	immune e	9	3.296703	3.88E-04	IGHG1, C1	191	126	13588
GOTERM_	positive re	9	3.296703	6.46E-04	IGHG1, H2	191	136	13588
GOTERM_	positive re	10	3.663004	7.02E-04	NOD1, NL	191	172	13588
GOTERM_	compleme	5	1.831502	7.64E-04	IGHG1, C1	191	30	13588
GOTERM_	taxis	8	2.930403	8.39E-04	CCL11, C3	191	109	13588
GOTERM_	chemotaxi	8	2.930403	8.39E-04	CCL11, C3	191	109	13588
GOTERM_	cell adhes	19	6.959707	8.93E-04	AEBP1, TN	191	561	13588
GOTERM_	biological	19	6.959707	9.11E-04	AEBP1, TN	191	562	13588
GOTERM_	humoral ir	6	2.197802	9.20E-04	IGHG1, C1	191	54	13588
GOTERM_	humoral ir	5	1.831502	0.001382	IGHG1, C1	191	35	13588
GOTERM_	leukocyte	7	2.564103	0.001535	IGHG1, C1	191	89	13588
GOTERM_	protein pr	7	2.564103	0.001626	IGHG1, C1	191	90	13588
GOTERM_	behavior	15	5.494505	0.001682	C3AR1, SC	191	405	13588
GOTERM_	immunogl	6	2.197802	0.001849	IGHG1, C1	191	63	13588
GOTERM_	regulation	4	1.465201	0.001881	IGHG1, FC	191	18	13588
GOTERM_	locomotor	11	4.029304	0.001974	CCL11, C3	191	239	13588
GOTERM_	B cell med	6	2.197802	0.002125	IGHG1, C1	191	65	13588
GOTERM_	protein ma	6	2.197802	0.002125	IGHG1, C1	191	65	13588
GOTERM_	protein ma	7	2.564103	0.00226	IGHG1, C1	191	96	13588
GOTERM_	positive re	10	3.663004	0.002457	IGHG1, H2	191	206	13588
GOTERM_	extracellul	7	2.564103	0.002918	SMOC2, T	191	101	13588
GOTERM_	cell morph	10	3.663004	0.002983	SEMA5A, S	191	212	13588
GOTERM_	positive re	5	1.831502	0.002986	SMOC2, T	191	43	13588
GOTERM_	monovale	12	4.395604	0.0036	KCNH1, K(	191	303	13588
GOTERM_	leukocyte	10	3.663004	0.003686	CXCR5, FC	191	219	13588
GOTERM_	lymphocyt	6	2.197802	0.004202	IGHG1, C1	191	76	13588
GOTERM_	proteolysis	26	9.52381	0.005027	IGHG1, AE	191	1034	13588
GOTERM_	positive re	4	1.465201	0.006149	SMOC2, S	191	27	13588
GOTERM_	adaptive ir	6	2.197802	0.006421	IGHG1, C1	191	84	13588
GOTERM_	adaptive ir	6	2.197802	0.006421	IGHG1, C1	191	84	13588
GOTERM_	blood ves	9	3.296703	0.006724	SEMA5A, S	191	198	13588
GOTERM_	cytokine-r	5	1.831502	0.007722	CEBPA, IL2	191	56	13588
GOTERM_	regulation	6	2.197802	0.007793	IGHG1, H2	191	88	13588
GOTERM_	cell activat	10	3.663004	0.007797	CXCR5, FC	191	246	13588
GOTERM_	regulation	16	5.860806	0.00861	CEBPA, FC	191	538	13588
GOTERM_	metal ion	14	5.128205	0.009305	KCNH1, ST	191	442	13588
GOTERM_	regulation	4	1.465201	0.009908	NOD1, TLI	191	32	13588
GOTERM_	positive re	5	1.831502	0.010996	NOD1, KLI	191	62	13588
GOTERM_	regulation	5	1.831502	0.012922	IGHG1, H2	191	65	13588
GOTERM_	regulation	3	1.098901	0.013709	IGHG1, FC	191	13	13588
GOTERM_	regulation	3	1.098901	0.013709	IGHG1, FC	191	13	13588
GOTERM_	cell morph	8	2.930403	0.014105	SEMA5A, S	191	182	13588
GOTERM_	ear develo	6	2.197802	0.015318	SIX1, PRR)	191	104	13588
GOTERM_	middle ea	3	1.098901	0.015848	PRRX1, SI)	191	14	13588
GOTERM_	regulation	5	1.831502	0.016588	IGHG1, H2	191	70	13588

GOTERM_ lymphocyt	8	2.930403	0.017925	CXCR5, CL	191	191	13588
GOTERM_ positive re	4	1.465201	0.018197	IGHG1, H2	191	40	13588
GOTERM_ positive re	4	1.465201	0.018197	IGHG1, H2	191	40	13588
GOTERM_ regulation	4	1.465201	0.018197	SMOC2, S	191	40	13588
GOTERM_ extracellul	7	2.564103	0.018304	SMOC2, T	191	149	13588
GOTERM_ gland dev	8	2.930403	0.020889	CCL11, PG	191	197	13588
GOTERM_ blood ves	9	3.296703	0.021509	SEMA5A,	191	244	13588
GOTERM_ ear morph	5	1.831502	0.021771	SIX1, PRR	191	76	13588
GOTERM_ response t	7	2.564103	0.023014	IGHG1, H2	191	157	13588
GOTERM_ B cell activ	5	1.831502	0.023694	CXCR5, CL	191	78	13588
GOTERM_ vasculatur	9	3.296703	0.024521	SEMA5A,	191	250	13588
GOTERM_ neuron di	12	4.395604	0.024712	SEMA5A,	191	399	13588
GOTERM_ regulation	4	1.465201	0.024847	TNXB, NO	191	45	13588
GOTERM_ regulation	3	1.098901	0.02569	IGHG1, FC	191	18	13588
GOTERM_ positive re	3	1.098901	0.02569	NOD1, IL1	191	18	13588
GOTERM_ regulation	4	1.465201	0.026314	TNXB, NO	191	46	13588
GOTERM_ positive re	4	1.465201	0.026314	IGHG1, H2	191	46	13588
GOTERM_ axonogen	7	2.564103	0.027036	SEMA5A, I	191	163	13588
GOTERM_ positive re	7	2.564103	0.027036	SLC1A3, N	191	163	13588
GOTERM_ cation trar	14	5.128205	0.028967	KCNH1, ST	191	515	13588
GOTERM_ cell morph	10	3.663004	0.029348	SEMA5A,	191	309	13588
GOTERM_ gland mor	5	1.831502	0.03007	CCL11, PG	191	84	13588
GOTERM_ morphoge	7	2.564103	0.034294	CCL11, PG	191	173	13588
GOTERM_ epithelium	9	3.296703	0.03684	CCL11, PG	191	271	13588
GOTERM_ neuron pr	7	2.564103	0.037285	SEMA5A, I	191	176	13588
GOTERM_ positive re	3	1.098901	0.037403	PRRX1, GA	191	22	13588
GOTERM_ negative r	8	2.930403	0.038132	CEBPA, NU	191	224	13588
GOTERM_ angiogene	6	2.197802	0.03891	SEMA5A, I	191	133	13588
GOTERM_ regulation	3	1.098901	0.040596	PRRX1, GA	191	23	13588
GOTERM_ positive re	3	1.098901	0.040596	IGHG1, FC	191	23	13588
GOTERM_ progester	2	0.732601	0.041368	PGR, KLF9	191	3	13588
GOTERM_ ion transp	17	6.227106	0.041548	KCNH1, ST	191	712	13588
GOTERM_ regulation	5	1.831502	0.042749	SMOC2, T	191	94	13588
GOTERM_ regulation	4	1.465201	0.043461	IGHG1, H2	191	56	13588
GOTERM_ regulation	4	1.465201	0.043461	IGHG1, H2	191	56	13588
GOTERM_ hemopoie	9	3.296703	0.044022	CEBPA, CX	191	281	13588
GOTERM_ fatty acid	7	2.564103	0.044923	SCD1, TNX	191	184	13588
GOTERM_ regulation	4	1.465201	0.045419	IGHG1, FC	191	57	13588
GOTERM_ regulation	6	2.197802	0.045587	NOD1, TLI	191	139	13588
GOTERM_ regulation	3	1.098901	0.047275	IGHG1, FC	191	25	13588
GOTERM_ axon guid	5	1.831502	0.048546	SEMA5A,	191	98	13588
GOTERM_ leukocyte	6	2.197802	0.049167	CEBPA, CL	191	142	13588
GOTERM_ tissue mor	8	2.930403	0.049923	CCL11, PG	191	238	13588
GOTERM_ regulation	9	3.296703	0.051209	TNXB, NU	191	290	13588
GOTERM_ cell-cell si	9	3.296703	0.051209	LTBP4, SIX	191	290	13588
GOTERM_ regulation	7	2.564103	0.052932	TNXB, NU	191	192	13588
GOTERM_ embryonic	8	2.930403	0.05306	CEBPA, AL	191	241	13588
GOTERM_ neuron de	9	3.296703	0.053099	SEMA5A,	191	292	13588
GOTERM_ fat cell dif	4	1.465201	0.053679	CEBPA, SC	191	61	13588
GOTERM_ immune sy	9	3.296703	0.055509	CEBPA, CX	191	295	13588
GOTERM_ regulation	4	1.465201	0.058061	PGR, SERP	191	63	13588
GOTERM_ cellular co	10	3.663004	0.058465	SEMA5A,	191	351	13588
GOTERM_ embryonic	4	1.465201	0.060314	SIX1, PRR	191	64	13588
GOTERM_ regulation	9	3.296703	0.060999	TNXB, NU	191	301	13588
GOTERM_ regulation	9	3.296703	0.060999	TNXB, NU	191	301	13588
GOTERM_ regulation	7	2.564103	0.061325	TNXB, NU	191	199	13588
GOTERM_ cell projec	7	2.564103	0.06443	SEMA5A, I	191	202	13588
GOTERM_ regulation	3	1.098901	0.065532	IGHG1, FC	191	30	13588

GOTERM_	regulation	3	1.098901	0.065532	IGHG1, FC	191	30	13588
GOTERM_	acute-pha	3	1.098901	0.065532	IL1B, ORM	191	30	13588
GOTERM_	positive re	9	3.296703	0.065903	GM13232,	191	306	13588
GOTERM_	sensory or	8	2.930403	0.069519	ALDH1A3,	191	257	13588
GOTERM_	cell motio	10	3.663004	0.073152	SEMA5A, I	191	367	13588
GOTERM_	embryonic	6	2.197802	0.075704	ALDH1A3,	191	161	13588
GOTERM_	cell part m	7	2.564103	0.077404	SEMA5A, I	191	212	13588
GOTERM_	positive re	3	1.098901	0.077432	NOD1, NU	191	33	13588
GOTERM_	tube deve	8	2.930403	0.078179	CCL11, CE	191	264	13588
GOTERM_	icosanoid	3	1.098901	0.08154	CYP2D22,	191	34	13588
GOTERM_	positive re	3	1.098901	0.08154	IGHG1, FC	191	34	13588
GOTERM_	positive re	3	1.098901	0.08154	IGHG1, H2	191	34	13588
GOTERM_	positive re	3	1.098901	0.08154	IGHG1, H2	191	34	13588
GOTERM_	palate dev	3	1.098901	0.085713	PRRX1, PD	191	35	13588
GOTERM_	neuron pr	7	2.564103	0.085777	SEMA5A, I	191	218	13588
GOTERM_	regulation	4	1.465201	0.087666	TNXB, NO	191	75	13588
GOTERM_	mechanor	3	1.098901	0.089949	NTRK2, DL	191	36	13588
GOTERM_	unsaturate	3	1.098901	0.089949	CYP2D22,	191	36	13588
GOTERM_	enzyme lir	8	2.930403	0.090123	LTBP1, SM	191	273	13588
GOTERM_	positive re	2	0.732601	0.093889	TLR1, IL1B	191	7	13588
GOTERM_	negative r	2	0.732601	0.093889	MYO16, G	191	7	13588
GOTERM_	detection	2	0.732601	0.093889	NOD1, TLI	191	7	13588
GOTERM_	positive re	2	0.732601	0.093889	PRRX1, GA	191	7	13588
GOTERM_	response t	12	4.395604	0.097365	GM13232,	191	505	13588
GOTERM_	morphoge	5	1.831502	0.098202	CCL11, PG	191	125	13588
GOTERM_	cytosolic c	3	1.098901	0.098602	GNA15, IL	191	38	13588
GOTERM_	regulation	5	1.831502	0.104788	ACE, ADH	191	128	13588
GOTERM_	skeletal sy	8	2.930403	0.1054	GM8797, S	191	285	13588
GOTERM_	regulation	2	0.732601	0.106565	IGHG1, FC	191	8	13588
GOTERM_	regulation	2	0.732601	0.106565	IGHG1, C3	191	8	13588
GOTERM_	positive re	2	0.732601	0.106565	IGHG1, C3	191	8	13588
GOTERM_	positive re	2	0.732601	0.106565	IGHG1, C3	191	8	13588
GOTERM_	regulation	2	0.732601	0.106565	IGHG1, C3	191	8	13588
GOTERM_	response t	2	0.732601	0.106565	IL1B, CASI	191	8	13588
GOTERM_	peptide cr	2	0.732601	0.106565	SPOCK2, D	191	8	13588
GOTERM_	positive re	2	0.732601	0.106565	IGHG1, C3	191	8	13588
GOTERM_	regulation	3	1.098901	0.107476	NOD1, NU	191	40	13588
GOTERM_	skeletal sy	5	1.831502	0.109288	SIX1, PRR)	191	130	13588
GOTERM_	embryonic	4	1.465201	0.110272	SIX1, PRR)	191	83	13588
GOTERM_	odontoge	3	1.098901	0.111991	PDGFRA, I	191	41	13588
GOTERM_	negative r	6	2.197802	0.112167	SIX2, DLL1	191	182	13588
GOTERM_	generation	4	1.465201	0.113242	LTBP4, NT	191	84	13588
GOTERM_	regulation	4	1.465201	0.113242	TNXB, NU	191	84	13588
GOTERM_	cell-cell ad	7	2.564103	0.113254	TNXB, FAT	191	236	13588
GOTERM_	positive re	5	1.831502	0.113873	IGHG1, FC	191	132	13588
GOTERM_	positive re	3	1.098901	0.116555	TLR1, IL1B	191	42	13588
GOTERM_	regulation	6	2.197802	0.119947	TNXB, NU	191	186	13588
GOTERM_	cytokine p	3	1.098901	0.121165	NOD1, CD	191	43	13588
GOTERM_	inner ear c	4	1.465201	0.125421	SIX1, PRR)	191	88	13588
GOTERM_	positive re	4	1.465201	0.125421	NOD1, NU	191	88	13588
GOTERM_	odontoge	3	1.098901	0.130515	PDGFRA, I	191	45	13588
GOTERM_	positive re	2	0.732601	0.131392	IGHG1, C3	191	10	13588
GOTERM_	positive re	2	0.732601	0.131392	IGHG1, C3	191	10	13588
GOTERM_	regulation	2	0.732601	0.131392	SMAD7, A	191	10	13588
GOTERM_	compleme	2	0.732601	0.131392	C3, CFD	191	10	13588
GOTERM_	cellular ca	4	1.465201	0.134852	GNA15, IL	191	91	13588
GOTERM_	B cell diffe	3	1.098901	0.135252	CLCF1, NF	191	46	13588
GOTERM_	regulation	4	1.465201	0.138049	HTRA1, SE	191	92	13588

GOTERM_ positive re	3	1.098901	0.140026	NOD1, GA	191	47	13588
GOTERM_ hemopoie	7	2.564103	0.140354	CEBPA, CL	191	251	13588
GOTERM_ regulation	4	1.465201	0.141272	TNXB, NO	191	93	13588
GOTERM_ branching	4	1.465201	0.141272	CCL11, PG	191	93	13588
GOTERM_ positive re	2	0.732601	0.143546	IGHG1, C3	191	11	13588
GOTERM_ detection	2	0.732601	0.143546	NOD1, TLI	191	11	13588
GOTERM_ regulation	2	0.732601	0.143546	MYO16, G	191	11	13588
GOTERM_ transformi	3	1.098901	0.144837	LTBP1, SM	191	48	13588
GOTERM_ calcium io	4	1.465201	0.147793	GNA15, IL	191	95	13588
GOTERM_ oxidation	14	5.128205	0.147949	SCD1, CYF	191	672	13588
GOTERM_ urogenital	5	1.831502	0.148221	SERPINB5,	191	146	13588
GOTERM_ response t	3	1.098901	0.149681	TLR1, IL1B	191	49	13588
GOTERM_ cell fate cc	5	1.831502	0.150815	SIX1, DLL1	191	147	13588
GOTERM_ regulation	6	2.197802	0.150939	SLC1A3, S	191	201	13588
GOTERM_ regulation	3	1.098901	0.154558	SMAD7, SI	191	50	13588
GOTERM_ regulation	2	0.732601	0.155532	MYO16, G	191	12	13588
GOTERM_ positive re	7	2.564103	0.159682	GNA15, AI	191	261	13588
GOTERM_ translatior	8	2.930403	0.160034	GM10036,	191	319	13588
GOTERM_ regulation	3	1.098901	0.164402	IGHG1, FC	191	52	13588
GOTERM_ cellular me	4	1.465201	0.164505	GNA15, IL	191	100	13588
GOTERM_ regulation	2	0.732601	0.16735	TLR1, IL1B	191	13	13588
GOTERM_ fatty acid t	2	0.732601	0.16735	ANXA1, SI	191	13	13588
GOTERM_ positive re	2	0.732601	0.16735	IGHG1, C3	191	13	13588
GOTERM_ developm	4	1.465201	0.167914	CEBPA, PG	191	101	13588
GOTERM_ regulation	4	1.465201	0.171343	SIX1, NTRI	191	102	13588
GOTERM_ regulation	5	1.831502	0.17222	TNXB, NO	191	155	13588
GOTERM_ Notch sigr	3	1.098901	0.174353	DLL1, JAG	191	54	13588
GOTERM_ regulation	4	1.465201	0.174792	IGHG1, FC	191	103	13588
GOTERM_ positive re	13	4.761905	0.17648	CEBPA, GN	191	633	13588
GOTERM_ artery mor	2	0.732601	0.179004	SMAD7, P	191	14	13588
GOTERM_ positive re	2	0.732601	0.179004	IGHG1, C3	191	14	13588
GOTERM_ positive re	2	0.732601	0.179004	IGHG1, C3	191	14	13588
GOTERM_ chondroiti	2	0.732601	0.179004	SPOCK2, D	191	14	13588
GOTERM_ negative r	2	0.732601	0.179004	MYO16, G	191	14	13588
GOTERM_ regulation	5	1.831502	0.183306	SIX1, NTRI	191	159	13588
GOTERM_ metal ion	4	1.465201	0.185254	GNA15, IL	191	106	13588
GOTERM_ kidney dev	4	1.465201	0.188778	SIX1, SIX2,	191	107	13588
GOTERM_ positive re	3	1.098901	0.189451	IGHG1, C3	191	57	13588
GOTERM_ cell-substr	3	1.098901	0.189451	ARHGAP6	191	57	13588
GOTERM_ regulation	2	0.732601	0.190496	IGHG1, C3	191	15	13588
GOTERM_ phagocyto	2	0.732601	0.190496	IGHG1, FC	191	15	13588
GOTERM_ induction	2	0.732601	0.190496	SIX1, FGF1	191	15	13588
GOTERM_ defense re	4	1.465201	0.192319	IGHG1, H2	191	108	13588
GOTERM_ metaneph	3	1.098901	0.194524	SIX1, SIX2,	191	58	13588
GOTERM_ secretion	6	2.197802	0.197825	KIRREL, LT	191	221	13588
GOTERM_ cell fate sp	3	1.098901	0.199614	SIX1, RBPJ	191	59	13588
GOTERM_ actin cytos	5	1.831502	0.200267	ARHGAP6	191	165	13588
GOTERM_ positive re	2	0.732601	0.201828	NOD1, IL1	191	16	13588
GOTERM_ chondroiti	2	0.732601	0.201828	SPOCK2, D	191	16	13588
GOTERM_ positive re	2	0.732601	0.201828	NOD1, IL1	191	16	13588
GOTERM_ response t	3	1.098901	0.204719	GM13232,	191	60	13588
GOTERM_ positive re	7	2.564103	0.206283	FOSL2, PR	191	284	13588
GOTERM_ aging	3	1.098901	0.209839	NUP62, IFI	191	61	13588
GOTERM_ peptide cr	2	0.732601	0.213002	SPOCK2, D	191	17	13588
GOTERM_ retinoic ac	2	0.732601	0.213002	ADH1, ALI	191	17	13588
GOTERM_ neutrophil	2	0.732601	0.213002	IL1B, ITGA	191	17	13588
GOTERM_ face devel	2	0.732601	0.213002	ALDH1A3,	191	17	13588
GOTERM_ lymphocyt	4	1.465201	0.213899	CLCF1, CD	191	114	13588

GOTERM_	tube morp	5	1.831502	0.217656	CCL11, PG	191	171	13588
GOTERM_	negative r	2	0.732601	0.22402	MYO16, G	191	18	13588
GOTERM_	regulation	3	1.098901	0.225272	TLR1, IL1B	191	64	13588
GOTERM_	negative r	3	1.098901	0.230436	FCGR2B, II	191	65	13588
GOTERM_	lipid trans	4	1.465201	0.232261	PLEKHA8,	191	119	13588
GOTERM_	actin filam	5	1.831502	0.232607	ARHGAP6	191	176	13588
GOTERM_	protein kir	6	2.197802	0.233893	IL23R, CLC	191	236	13588
GOTERM_	monocarb	2	0.732601	0.234885	ANXA1, SI	191	19	13588
GOTERM_	branching	2	0.732601	0.234885	CCL11, PG	191	19	13588
GOTERM_	chordate e	9	3.296703	0.237094	CEBPA, SI)	191	421	13588
GOTERM_	regulation	4	1.465201	0.239686	TNXB, GAI	191	121	13588
GOTERM_	inner ear r	3	1.098901	0.240786	SIX1, PRR)	191	67	13588
GOTERM_	positive re	11	4.029304	0.244788	CEBPA, GI	191	552	13588
GOTERM_	embryonic	9	3.296703	0.244795	CEBPA, SI)	191	425	13588
GOTERM_	negative r	2	0.732601	0.245599	FCGR2B, S	191	20	13588
GOTERM_	negative r	2	0.732601	0.245599	NUP62, IL	191	20	13588
GOTERM_	epithelial c	4	1.465201	0.247151	PGR, SIX1,	191	123	13588
GOTERM_	respiratory	4	1.465201	0.250897	CEBPA, AL	191	124	13588
GOTERM_	positive re	11	4.029304	0.253319	CEBPA, GI	191	557	13588
GOTERM_	sodium io	4	1.465201	0.254652	SCN1A, SL	191	125	13588
GOTERM_	steroid ho	2	0.732601	0.256163	PGR, KLF9	191	21	13588
GOTERM_	head deve	2	0.732601	0.256163	ALDH1A3,	191	21	13588
GOTERM_	positive re	2	0.732601	0.256163	SLC1A3, N	191	21	13588
GOTERM_	regulation	2	0.732601	0.256163	TNXB, GAI	191	21	13588
GOTERM_	cellular alk	2	0.732601	0.256163	CYP2F2, A	191	21	13588
GOTERM_	cell-cell si	2	0.732601	0.256163	SIX1, FGF1	191	21	13588
GOTERM_	developm	2	0.732601	0.256163	SIX1, FGF1	191	21	13588
GOTERM_	response t	5	1.831502	0.258751	GM13232,	191	184	13588
GOTERM_	positive re	6	2.197802	0.263767	IGHG1, NC	191	248	13588
GOTERM_	lipid locali	4	1.465201	0.265962	PLEKHA8,	191	128	13588
GOTERM_	auditory re	2	0.732601	0.26658	DLL1, JAG	191	22	13588
GOTERM_	regulation	2	0.732601	0.26658	PRRX1, GA	191	22	13588
GOTERM_	positive re	2	0.732601	0.26658	SLC1A3, N	191	22	13588
GOTERM_	eye morph	3	1.098901	0.271929	ALDH1A3,	191	73	13588
GOTERM_	regulation	3	1.098901	0.271929	MYO16, G	191	73	13588
GOTERM_	positive re	4	1.465201	0.273537	GAB1, TLR	191	130	13588
GOTERM_	positive re	6	2.197802	0.27374	IGHG1, NC	191	250	13588
GOTERM_	calcium-ir	2	0.732601	0.276852	CLDN1, CI	191	23	13588
GOTERM_	positive re	2	0.732601	0.276852	H2-K1, KL	191	23	13588
GOTERM_	positive re	2	0.732601	0.276852	TRIM32, IL	191	23	13588
GOTERM_	antigen pr	2	0.732601	0.276852	H2-K1, FC	191	23	13588
GOTERM_	collagen n	2	0.732601	0.276852	TNXB, MN	191	23	13588
GOTERM_	positive re	2	0.732601	0.276852	H2-K1, KL	191	23	13588
GOTERM_	platelet-d	2	0.732601	0.276852	GAB1, PD(	191	23	13588
GOTERM_	regulation	3	1.098901	0.277122	GM13232,	191	74	13588
GOTERM_	positive re	6	2.197802	0.277424	IGHG1, NC	191	252	13588
GOTERM_	cell death	10	3.663004	0.279408	TNS4, CLC	191	507	13588
GOTERM_	regulation	4	1.465201	0.281135	SIX1, NTRI	191	132	13588
GOTERM_	negative r	4	1.465201	0.281135	ZFP462, N	191	132	13588
GOTERM_	transmem	5	1.831502	0.281578	NTRK2, GA	191	192	13588
GOTERM_	cell matur	3	1.098901	0.282313	CEBPA, PG	191	75	13588
GOTERM_	positive re	2	0.732601	0.286981	IL1B, IRS1	191	24	13588
GOTERM_	positive re	2	0.732601	0.286981	CEBPA, GI	191	24	13588
GOTERM_	positive re	2	0.732601	0.286981	IGHG1, C3	191	24	13588
GOTERM_	positive re	2	0.732601	0.286981	SLC1A3, N	191	24	13588
GOTERM_	multicellul	2	0.732601	0.286981	TNXB, MN	191	24	13588
GOTERM_	embryonic	2	0.732601	0.286981	PRRX1, GA	191	24	13588
GOTERM_	integrin-n	3	1.098901	0.2875	ADAM28,	191	76	13588

GOTERM_	cell projec	7	2.564103	0.288215	SEMA5A, I	191	319	13588
GOTERM_	cellular di-	4	1.465201	0.288751	GNA15, IL	191	134	13588
GOTERM_	positive re	4	1.465201	0.292566	GAB1, TLR	191	135	13588
GOTERM_	transmem	3	1.098901	0.297862	LTBP1, SM	191	78	13588
GOTERM_	cellular ior	6	2.197802	0.302153	GNA15, S(	191	261	13588
GOTERM_	death	10	3.663004	0.302257	TNS4, CLC	191	519	13588
GOTERM_	mammary	2	0.732601	0.306817	CCL11, PG	191	26	13588
GOTERM_	regulation	2	0.732601	0.306817	LTBP4, SEI	191	26	13588
GOTERM_	multicellul	2	0.732601	0.306817	TNXB, MM	191	26	13588
GOTERM_	vitamin A	2	0.732601	0.306817	ADH1, ALI	191	26	13588
GOTERM_	negative r	2	0.732601	0.306817	FCGR2B, S	191	26	13588
GOTERM_	fatty acid I	3	1.098901	0.313361	SCD1, ELC	191	81	13588
GOTERM_	positive re	3	1.098901	0.313361	GNA15, AI	191	81	13588
GOTERM_	transmem	9	3.296703	0.315267	KCNH1, SI	191	460	13588
GOTERM_	organic ac	4	1.465201	0.315513	SCD1, SLC	191	141	13588
GOTERM_	carboxylic	4	1.465201	0.315513	SCD1, SLC	191	141	13588
GOTERM_	regulation	2	0.732601	0.316528	NOD1, TLI	191	27	13588
GOTERM_	cell chemo	2	0.732601	0.316528	IL1B, ITGA	191	27	13588
GOTERM_	salivary gl	2	0.732601	0.316528	SEMA3A, I	191	27	13588
GOTERM_	leukocyte	2	0.732601	0.316528	IL1B, ITGA	191	27	13588
GOTERM_	positive re	10	3.663004	0.32359	CEBPA, GI	191	530	13588
GOTERM_	regulation	3	1.098901	0.323653	IGHG1, FC	191	83	13588
GOTERM_	cellular ch	6	2.197802	0.32454	GNA15, S(	191	268	13588
GOTERM_	antigen pr	2	0.732601	0.326104	H2-K1, FC	191	28	13588
GOTERM_	retinoid m	2	0.732601	0.326104	ADH1, ALI	191	28	13588
GOTERM_	I-kappaB I	2	0.732601	0.326104	TLR1, BTK	191	28	13588
GOTERM_	diterpenoi	2	0.732601	0.326104	ADH1, ALI	191	28	13588
GOTERM_	JAK-STAT	2	0.732601	0.326104	IL23R, CLC	191	28	13588
GOTERM_	di-, tri-val	4	1.465201	0.33467	GNA15, IL	191	146	13588
GOTERM_	brown fat	2	0.732601	0.335547	CEBPA, SC	191	29	13588
GOTERM_	cell aging	2	0.732601	0.335547	NUP62, PF	191	29	13588
GOTERM_	terpenoid	2	0.732601	0.335547	ADH1, ALI	191	29	13588
GOTERM_	mammary	3	1.098901	0.339017	CCL11, PG	191	86	13588
GOTERM_	regulation	4	1.465201	0.342329	SIX1, NTRI	191	148	13588
GOTERM_	programm	9	3.296703	0.342488	TNS4, CLC	191	473	13588
GOTERM_	antigen pr	3	1.098901	0.344115	MILL1, H2	191	87	13588
GOTERM_	negative r	2	0.732601	0.344858	FCGR2B, S	191	30	13588
GOTERM_	inner ear r	2	0.732601	0.344858	DLL1, JAG	191	30	13588
GOTERM_	regulation	2	0.732601	0.344858	H2-K1, KL	191	30	13588
GOTERM_	regulation	2	0.732601	0.344858	H2-K1, KL	191	30	13588
GOTERM_	regulation	2	0.732601	0.344858	SMAD7, H	191	30	13588
GOTERM_	cellular hc	7	2.564103	0.344992	GNA15, S(	191	343	13588
GOTERM_	positive re	2	0.732601	0.354039	GAS1, FGF	191	31	13588
GOTERM_	salivary gl	2	0.732601	0.354039	SEMA3A, I	191	31	13588
GOTERM_	peptidyl-s	2	0.732601	0.354039	SPOCK2, I	191	31	13588
GOTERM_	cellular ca	4	1.465201	0.35775	GNA15, IL	191	152	13588
GOTERM_	regulation	3	1.098901	0.359333	GM13232,	191	90	13588
GOTERM_	hormone r	3	1.098901	0.359333	ACE, ADH	191	90	13588
GOTERM_	response t	2	0.732601	0.363092	HSPB1, HS	191	32	13588
GOTERM_	intracellula	2	0.732601	0.363092	PGR, KLF9	191	32	13588
GOTERM_	regulation	2	0.732601	0.363092	KLRK1, TLI	191	32	13588
GOTERM_	regulation	10	3.663004	0.369066	IGHG1, N(	191	553	13588
GOTERM_	regulation	3	1.098901	0.369407	GAB1, SEN	191	92	13588
GOTERM_	mammary	2	0.732601	0.372019	CCL11, PG	191	33	13588
GOTERM_	proteogly(	2	0.732601	0.372019	SPOCK2, I	191	33	13588
GOTERM_	adult beha	3	1.098901	0.374421	SCN1A, AI	191	93	13588
GOTERM_	fat-soluble	2	0.732601	0.380821	ADH1, ALI	191	34	13588
GOTERM_	myeloid le	2	0.732601	0.380821	CEBPA, CI	191	34	13588

GOTERM_	regulation	10	3.663004	0.383061	IGHG1, NC	191	560	13588
GOTERM_	regulation	10	3.663004	0.389072	IGHG1, NC	191	563	13588
GOTERM_	regulation	3	1.098901	0.389364	MYO16, G	191	96	13588
GOTERM_	embryonic	2	0.732601	0.389501	DLL1, ZBT	191	35	13588
GOTERM_	antigen pr	2	0.732601	0.389501	H2-K1, FC	191	35	13588
GOTERM_	cerebellun	2	0.732601	0.389501	MYO16, G	191	35	13588
GOTERM_	myeloid le	2	0.732601	0.389501	FCGR2B, T	191	35	13588
GOTERM_	ion homeo	6	2.197802	0.389833	GNA15, SC	191	293	13588
GOTERM_	regulation	4	1.465201	0.392687	NTRK2, IL1	191	161	13588
GOTERM_	embryonic	7	2.564103	0.392778	ALDH1A3,	191	359	13588
GOTERM_	embryonic	3	1.098901	0.39431	PRRX1, GA	191	97	13588
GOTERM_	embryonic	3	1.098901	0.39431	PRRX1, GA	191	97	13588
GOTERM_	elevation c	2	0.732601	0.398059	GNA15, IL	191	36	13588
GOTERM_	triglycerid	2	0.732601	0.398059	TNXB, DGA	191	36	13588
GOTERM_	positive re	2	0.732601	0.398059	EDAR, CAS	191	36	13588
GOTERM_	positive re	2	0.732601	0.398059	IL1B, FGF1	191	36	13588
GOTERM_	intracellula	15	5.494505	0.403295	GNA15, IL	191	915	13588
GOTERM_	carboxylic	3	1.098901	0.404148	SLC1A3, A	191	99	13588
GOTERM_	positive re	2	0.732601	0.406499	CEBPA, GN	191	37	13588
GOTERM_	regulation	4	1.465201	0.407014	TNXB, GAI	191	165	13588
GOTERM_	response t	4	1.465201	0.407014	GM13232,	191	165	13588
GOTERM_	developm	3	1.098901	0.409039	CCL11, GA	191	100	13588
GOTERM_	organic ac	3	1.098901	0.409039	SLC1A3, A	191	100	13588
GOTERM_	induction	4	1.465201	0.412884	IGHG1, NC	191	167	13588
GOTERM_	induction	4	1.465201	0.412884	IGHG1, NC	191	167	13588
GOTERM_	negative r	3	1.098901	0.41391	NUP62, IL1	191	101	13588
GOTERM_	response t	2	0.732601	0.41482	IL1B, PTAF	191	38	13588
GOTERM_	positive re	2	0.732601	0.423026	IGHG1, C3	191	39	13588
GOTERM_	developm	3	1.098901	0.428403	PGR, GM1	191	104	13588
GOTERM_	negative r	4	1.465201	0.429247	NUP62, HT	191	171	13588
GOTERM_	regulation	2	0.732601	0.431117	IL1B, FGF1	191	40	13588
GOTERM_	glycosami	2	0.732601	0.431117	SPOCK2, D	191	40	13588
GOTERM_	cell migrat	5	1.831502	0.433184	NUP62, M	191	240	13588
GOTERM_	exocrine s	2	0.732601	0.439095	SEMA3A, I	191	41	13588
GOTERM_	regulation	2	0.732601	0.439095	SMAD7, A	191	41	13588
GOTERM_	hormone s	2	0.732601	0.439095	LTBP4, IRS	191	41	13588
GOTERM_	metencep	2	0.732601	0.439095	MYO16, G	191	41	13588
GOTERM_	immune re	2	0.732601	0.439095	KLRK1, NF	191	41	13588
GOTERM_	regulation	3	1.098901	0.442707	GAB1, SEN	191	107	13588
GOTERM_	positive re	2	0.732601	0.446962	GM13232,	191	42	13588
GOTERM_	hormone t	2	0.732601	0.446962	LTBP4, IRS	191	42	13588
GOTERM_	positive re	3	1.098901	0.452133	TRIM32, C	191	109	13588
GOTERM_	negative r	2	0.732601	0.454719	MYO16, G	191	43	13588
GOTERM_	acylglycer	2	0.732601	0.454719	TNXB, DGA	191	43	13588
GOTERM_	polyol me	2	0.732601	0.454719	DGAT2, PT	191	43	13588
GOTERM_	leukocyte	2	0.732601	0.454719	IL1B, ITGA	191	43	13588
GOTERM_	regulation	3	1.098901	0.456812	GAB1, SEN	191	110	13588
GOTERM_	epithelial t	3	1.098901	0.461469	CCL11, PG	191	111	13588
GOTERM_	lung deve	3	1.098901	0.461469	CEBPA, PD	191	111	13588
GOTERM_	immune re	2	0.732601	0.462368	KLRK1, NF	191	44	13588
GOTERM_	wound he	3	1.098901	0.466102	F3, SERPIN	191	112	13588
GOTERM_	neutral lip	2	0.732601	0.46991	TNXB, DGA	191	45	13588
GOTERM_	positive re	2	0.732601	0.46991	GM13232,	191	45	13588
GOTERM_	glycerol et	2	0.732601	0.46991	TNXB, DGA	191	45	13588
GOTERM_	respiratory	3	1.098901	0.470711	CEBPA, PD	191	113	13588
GOTERM_	apoptosis	8	2.930403	0.475701	TNS4, CLC	191	465	13588
GOTERM_	nucleus or	2	0.732601	0.477347	GM13232,	191	46	13588
GOTERM_	regulation	2	0.732601	0.477347	KLRK1, SEI	191	46	13588

GOTERM_	appendag	3	1.098901	0.479859	PRRX1, GA	191	115	13588
GOTERM_	limb morp	3	1.098901	0.479859	PRRX1, GA	191	115	13588
GOTERM_	cation hor	4	1.465201	0.481072	GNA15, IL	191	184	13588
GOTERM_	secretion l	4	1.465201	0.484049	LTBP4, NT	191	186	13588
GOTERM_	negative r	4	1.465201	0.484049	NUP62, HT	191	186	13588
GOTERM_	regulation	2	0.732601	0.48468	NTRK2, SE	191	47	13588
GOTERM_	osteoblast	2	0.732601	0.48468	GM8797, I	191	47	13588
GOTERM_	cell recogi	2	0.732601	0.48468	IGHG1, SE	191	47	13588
GOTERM_	membrane	4	1.465201	0.491818	IGHG1, FC	191	188	13588
GOTERM_	endocytos	4	1.465201	0.491818	IGHG1, FC	191	188	13588
GOTERM_	organic et	2	0.732601	0.49191	TNXB, DGA	191	48	13588
GOTERM_	immune re	2	0.732601	0.49191	KLRK1, NF	191	48	13588
GOTERM_	limb devel	3	1.098901	0.497863	PRRX1, GA	191	119	13588
GOTERM_	appendag	3	1.098901	0.497863	PRRX1, GA	191	119	13588
GOTERM_	phagocyto	2	0.732601	0.49904	IGHG1, FC	191	49	13588
GOTERM_	isoprenoic	2	0.732601	0.49904	ADH1, ALI	191	49	13588
GOTERM_	regulation	2	0.732601	0.50607	NOD1, HS	191	50	13588
GOTERM_	molting cy	2	0.732601	0.50607	EDAR, RBF	191	50	13588
GOTERM_	regulation	2	0.732601	0.50607	TRIM32, IL	191	50	13588
GOTERM_	hair cycle	2	0.732601	0.50607	EDAR, RBF	191	50	13588
GOTERM_	cell-matrix	2	0.732601	0.50607	ARHGAP6	191	50	13588
GOTERM_	regulation	2	0.732601	0.50607	NOD1, HS	191	50	13588
GOTERM_	hair follicle	2	0.732601	0.50607	EDAR, RBF	191	50	13588
GOTERM_	growth	4	1.465201	0.507645	CCL11, AP	191	193	13588
GOTERM_	positive re	3	1.098901	0.511104	IGHG1, FC	191	122	13588
GOTERM_	reproduct	5	1.831502	0.511533	PGR, GM1	191	264	13588
GOTERM_	response t	2	0.732601	0.513002	HSPB1, HS	191	51	13588
GOTERM_	regulation	2	0.732601	0.513002	CEBPA, GN	191	51	13588
GOTERM_	regulation	2	0.732601	0.513002	JAG1, ZBT	191	51	13588
GOTERM_	regulation	2	0.732601	0.513002	NOD1, HS	191	51	13588
GOTERM_	negative r	2	0.732601	0.513002	NUP62, IL1	191	51	13588
GOTERM_	negative r	2	0.732601	0.513002	NUP62, IL1	191	51	13588
GOTERM_	in utero er	5	1.831502	0.515438	CEBPA, GA	191	267	13588
GOTERM_	positive re	3	1.098901	0.519804	GAB1, TLR	191	124	13588
GOTERM_	molting cy	2	0.732601	0.519838	EDAR, RBF	191	52	13588
GOTERM_	hair cycle	2	0.732601	0.519838	EDAR, RBF	191	52	13588
GOTERM_	aminoglyc	2	0.732601	0.519838	SPOCK2, D	191	52	13588
GOTERM_	cellular hc	2	0.732601	0.519838	ADH1, ALI	191	52	13588
GOTERM_	immune re	2	0.732601	0.519838	KLRK1, NF	191	52	13588
GOTERM_	regulation	4	1.465201	0.523335	GNA15, AI	191	196	13588
GOTERM_	epidermis	3	1.098901	0.524116	GAB1, EDA	191	125	13588
GOTERM_	regulation	3	1.098901	0.524116	NTRK2, IL1	191	125	13588
GOTERM_	developm	2	0.732601	0.526578	GM13232,	191	53	13588
GOTERM_	negative r	2	0.732601	0.526578	NUP62, IL1	191	53	13588
GOTERM_	regulation	2	0.732601	0.539776	FCGR2B, N	191	55	13588
GOTERM_	glycerolipi	3	1.098901	0.541101	TNXB, DGA	191	129	13588
GOTERM_	sex differe	3	1.098901	0.545282	PGR, GM1	191	130	13588
GOTERM_	reproduct	3	1.098901	0.545282	PGR, GM1	191	130	13588
GOTERM_	positive re	2	0.732601	0.546238	CD4, FGF1	191	56	13588
GOTERM_	male sex c	2	0.732601	0.552609	GM13232,	191	57	13588
GOTERM_	cell cycle a	2	0.732601	0.552609	GAS1, SES	191	57	13588
GOTERM_	regulation	2	0.732601	0.552609	NTRK2, SE	191	57	13588
GOTERM_	regulation	5	1.831502	0.55516	TNXB, GAI	191	280	13588
GOTERM_	ectoderm	3	1.098901	0.557665	GAB1, EDA	191	133	13588
GOTERM_	regulation	2	0.732601	0.558891	IL1B, IRS1	191	58	13588
GOTERM_	pattern sp	5	1.831502	0.564225	SEMA5A, S	191	284	13588
GOTERM_	cell motilit	5	1.831502	0.564225	NUP62, M	191	284	13588
GOTERM_	localizatio	5	1.831502	0.564225	NUP62, M	191	284	13588

GOTERM_ lipid biosy	5	1.831502	0.567016	SCD1, DG/	191	285	13588
GOTERM_ positive re	2	0.732601	0.571193	CD4, FGF1	191	60	13588
GOTERM_ detection	2	0.732601	0.571193	NOD1, TLI	191	60	13588
GOTERM_ neuromus	2	0.732601	0.571193	SCN1A, SL	191	60	13588
GOTERM_ embryonic	2	0.732601	0.577216	CEBPA, G/	191	61	13588
GOTERM_ chemical h	6	2.197802	0.581427	GNA15, S(	191	365	13588
GOTERM_ positive re	2	0.732601	0.583154	CD4, FGF1	191	62	13588
GOTERM_ adult loco	2	0.732601	0.583154	SCN1A, AT	191	62	13588
GOTERM_ positive re	2	0.732601	0.583154	CD4, FGF1	191	62	13588
GOTERM_ regulation	2	0.732601	0.583154	SV2B, ATP	191	62	13588
GOTERM_ response t	2	0.732601	0.594783	ACE, CASF	191	64	13588
GOTERM_ negative r	6	2.197802	0.598364	CEBPA, FZ	191	372	13588
GOTERM_ alcohol ca	2	0.732601	0.600476	ADH1, GM	191	65	13588
GOTERM_ regulation	2	0.732601	0.600476	EDAR, CAS	191	65	13588
GOTERM_ response t	2	0.732601	0.600476	ACE, CASF	191	65	13588
GOTERM_ regulation	2	0.732601	0.600476	ACE, ATP1	191	65	13588
GOTERM_ regulation	3	1.098901	0.601007	FCGR2B, (	191	144	13588
GOTERM_ positive re	8	2.930403	0.606841	CEBPA, GI	191	526	13588
GOTERM_ regulation	2	0.732601	0.611625	CEBPA, GI	191	67	13588
GOTERM_ hindbrain	2	0.732601	0.611625	MYO16, G	191	67	13588
GOTERM_ glycoprote	3	1.098901	0.612262	SPOCK2, (	191	147	13588
GOTERM_ transmissi	4	1.465201	0.615497	SCN1A, AI	191	226	13588
GOTERM_ female pre	2	0.732601	0.617082	PGR, KLF9	191	68	13588
GOTERM_ vitamin m	2	0.732601	0.622463	ADH1, ALI	191	69	13588
GOTERM_ secondary	2	0.732601	0.622463	ADH1, ALI	191	69	13588
GOTERM_ coagulatic	2	0.732601	0.627769	F3, SERPIN	191	70	13588
GOTERM_ blood coa	2	0.732601	0.627769	F3, SERPIN	191	70	13588
GOTERM_ negative r	5	1.831502	0.628387	CEBPA, PF	191	308	13588
GOTERM_ regulation	2	0.732601	0.633001	EDAR, CAS	191	71	13588
GOTERM_ hemostasi	2	0.732601	0.633001	F3, SERPIN	191	71	13588
GOTERM_ negative r	5	1.831502	0.633457	CEBPA, PF	191	310	13588
GOTERM_ regulation	3	1.098901	0.637581	FCGR2B, (	191	154	13588
GOTERM_ endocrine	2	0.732601	0.63816	SIX1, RBPJ	191	72	13588
GOTERM_ regulation	3	1.098901	0.644575	FCGR2B, (	191	156	13588
GOTERM_ eye develc	3	1.098901	0.648031	ALDH1A3,	191	157	13588
GOTERM_ negative r	6	2.197802	0.655613	CEBPA, FZ	191	397	13588
GOTERM_ positive re	7	2.564103	0.656971	CEBPA, GI	191	475	13588
GOTERM_ response t	2	0.732601	0.658083	IFI27L2A, '	191	76	13588
GOTERM_ negative r	2	0.732601	0.658083	FCGR2B, S	191	76	13588
GOTERM_ di-, tri-val	3	1.098901	0.661593	STEAP4, T	191	161	13588
GOTERM_ negative r	6	2.197802	0.664278	CEBPA, FZ	191	401	13588
GOTERM_ positive re	2	0.732601	0.667632	CD4, FGF1	191	78	13588
GOTERM_ cytoskelet	5	1.831502	0.672404	ARHGAP6	191	326	13588
GOTERM_ neurotrans	2	0.732601	0.676915	SV2B, ATP	191	80	13588
GOTERM_ protein an	9	3.296703	0.677307	TRPM6, SI	191	640	13588
GOTERM_ positive re	7	2.564103	0.682398	CEBPA, GI	191	488	13588
GOTERM_ negative r	6	2.197802	0.683258	CEBPA, FZ	191	410	13588
GOTERM_ response t	4	1.465201	0.685485	SLC1A3, H	191	251	13588
GOTERM_ regulation	4	1.465201	0.69829	HTRA1, SE	191	256	13588
GOTERM_ negative r	6	2.197802	0.69952	CEBPA, FZ	191	418	13588
GOTERM_ response t	2	0.732601	0.707421	GAB1, CY(	191	87	13588
GOTERM_ response t	2	0.732601	0.707421	CD4, BOC	191	87	13588
GOTERM_ placenta c	2	0.732601	0.707421	CEBPA, G/	191	87	13588
GOTERM_ response t	2	0.732601	0.711539	SLC1A3, A	191	88	13588
GOTERM_ gonad dev	2	0.732601	0.711539	PGR, GM1	191	88	13588
GOTERM_ homeosta	8	2.930403	0.713831	GNA15, S(	191	584	13588
GOTERM_ synaptic tr	3	1.098901	0.71461	APBA2, SV	191	178	13588
GOTERM_ negative r	7	2.564103	0.715553	CEBPA, FZ	191	506	13588

GOTERM_ regulation	2	0.732601	0.715598	F3, SERPIN	191	89	13588
GOTERM_ detection	2	0.732601	0.719601	NOD1, TLI	191	90	13588
GOTERM_ regulation	2	0.732601	0.719601	EDAR, CAS	191	90	13588
GOTERM_ positive re	7	2.564103	0.722592	CEBPA, GN	191	510	13588
GOTERM_ negative r	6	2.197802	0.722826	CEBPA, FZ	191	430	13588
GOTERM_ polysacch	2	0.732601	0.72744	SPOCK2, D	191	92	13588
GOTERM_ response t	2	0.732601	0.72744	SLC1A3, A	191	92	13588
GOTERM_ negative r	6	2.197802	0.730304	CEBPA, FZ	191	434	13588
GOTERM_ sulfur met	2	0.732601	0.735061	SPOCK2, D	191	94	13588
GOTERM_ membrane	4	1.465201	0.736618	IGHG1, FC	191	272	13588
GOTERM_ positive re	5	1.831502	0.741513	CEBPA, GN	191	358	13588
GOTERM_ myeloid ce	2	0.732601	0.742469	CEBPA, CD	191	96	13588
GOTERM_ response t	2	0.732601	0.749672	SLC1A3, A	191	98	13588
GOTERM_ regulation	2	0.732601	0.756674	SLC1A3, N	191	100	13588
GOTERM_ positive re	2	0.732601	0.756674	CD4, FGF1	191	100	13588
GOTERM_ germ cell	2	0.732601	0.760101	GM13232,	191	101	13588
GOTERM_ regulation	8	2.930403	0.76384	CEBPA, GN	191	616	13588
GOTERM_ ossificatio	2	0.732601	0.776532	GM8797, I	191	106	13588
GOTERM_ regulation	2	0.732601	0.779682	SLC1A3, N	191	107	13588
GOTERM_ blood circ	2	0.732601	0.791843	ACE, ATP1	191	111	13588
GOTERM_ circulatory	2	0.732601	0.791843	ACE, ATP1	191	111	13588
GOTERM_ phosphory	9	3.296703	0.792553	TRPM6, SN	191	718	13588
GOTERM_ regulation	2	0.732601	0.797672	SLC1A3, N	191	113	13588
GOTERM_ regulation	3	1.098901	0.804163	MYO16, G	191	214	13588
GOTERM_ positive re	3	1.098901	0.804163	IL1B, JAG1	191	214	13588
GOTERM_ regionaliz	3	1.098901	0.804163	DLL1, GAS	191	214	13588
GOTERM_ T cell activ	2	0.732601	0.806111	CD4, ITGA	191	116	13588
GOTERM_ homophili	2	0.732601	0.808845	FAT2, PCD	191	117	13588
GOTERM_ bone deve	2	0.732601	0.811541	GM8797, I	191	118	13588
GOTERM_ regulation	2	0.732601	0.8142	SCN1A, SN	191	119	13588
GOTERM_ calcium io	2	0.732601	0.819405	TRPM6, SL	191	121	13588
GOTERM_ heart deve	3	1.098901	0.822273	SMAD7, G	191	223	13588
GOTERM_ striated m	2	0.732601	0.834166	SMAD7, S	191	127	13588
GOTERM_ negative r	3	1.098901	0.8371	CEBPA, PR	191	231	13588
GOTERM_ positive re	5	1.831502	0.837863	CEBPA, GN	191	416	13588
GOTERM_ anion tran	2	0.732601	0.84109	CLCA2, SL	191	130	13588
GOTERM_ camera-ty	2	0.732601	0.84109	ALDH1A3,	191	130	13588
GOTERM_ positive re	5	1.831502	0.841913	CEBPA, GN	191	419	13588
GOTERM_ gamete ge	4	1.465201	0.845642	PGR, GM1	191	331	13588
GOTERM_ lipid catab	2	0.732601	0.849877	ENPP2, PL	191	134	13588
GOTERM_ muscle tis	2	0.732601	0.854087	SMAD7, S	191	136	13588
GOTERM_ glucose m	2	0.732601	0.862158	GM12286,	191	140	13588
GOTERM_ phosphate	10	3.663004	0.862245	TRPM6, SN	191	866	13588
GOTERM_ phosphoru	10	3.663004	0.862245	TRPM6, SN	191	866	13588
GOTERM_ cellular an	2	0.732601	0.864106	SLC1A3, C	191	141	13588
GOTERM_ response t	2	0.732601	0.867919	SLC1A3, A	191	143	13588
GOTERM_ spermatog	3	1.098901	0.875125	GM13232,	191	255	13588
GOTERM_ male gam	3	1.098901	0.875125	GM13232,	191	255	13588
GOTERM_ anterior/p	2	0.732601	0.885447	DLL1, ZBT	191	153	13588
GOTERM_ cell surfac	30	10.98901	0.88793	C3AR1, GN	191	2495	13588
GOTERM_ vesicle-me	5	1.831502	0.895047	IGHG1, FC	191	466	13588
GOTERM_ phospholi	2	0.732601	0.900659	TNXB, PLC	191	163	13588
GOTERM_ forebrain	2	0.732601	0.906165	ALDH1A3,	191	167	13588
GOTERM_ hexose me	2	0.732601	0.908803	GM12286,	191	169	13588
GOTERM_ sexual rep	4	1.465201	0.909812	PGR, GM1	191	386	13588
GOTERM_ reproduct	2	0.732601	0.91386	GM13232,	191	173	13588
GOTERM_ positive re	2	0.732601	0.916283	JAG1, BOC	191	175	13588
GOTERM_ organoph	2	0.732601	0.917469	TNXB, PLC	191	176	13588

GOTERM_ muscle or	2	0.732601	0.917469	SMAD7, S	191	176	13588
GOTERM_ nitrogen c	3	1.098901	0.927044	CEBPA, SL	191	302	13588
GOTERM_ reproduct	4	1.465201	0.928635	PGR, GM1	191	409	13588
GOTERM_ multicellul	4	1.465201	0.928635	PGR, GM1	191	409	13588
GOTERM_ cellular ma	6	2.197802	0.932127	GM8797,	191	609	13588
GOTERM_ monosacc	2	0.732601	0.933377	GM12286,	191	191	13588
GOTERM_ macromol	6	2.197802	0.954646	GM8797,	191	654	13588
GOTERM_ regulation	15	5.494505	0.956233	KCNH1, C	191	1465	13588
GOTERM_ DNA repa	2	0.732601	0.957234	GM13232,	191	222	13588
GOTERM_ regulation	15	5.494505	0.962456	KCNH1, C	191	1488	13588
GOTERM_ negative r	2	0.732601	0.966478	NUP62, HS	191	239	13588
GOTERM_ negative r	2	0.732601	0.968797	NUP62, HS	191	244	13588
GOTERM_ negative r	2	0.732601	0.969241	NUP62, HS	191	245	13588
GOTERM_ cell prolife	2	0.732601	0.970111	IL1B, ITGA	191	247	13588
GOTERM_ small GTPa	2	0.732601	0.974475	ARHGAP3	191	258	13588
GOTERM_ modificati	4	1.465201	0.975255	GM8797,	191	508	13588
GOTERM_ modificati	4	1.465201	0.975255	GM8797,	191	508	13588
GOTERM_ cell cycle p	3	1.098901	0.975469	GAS1, NEK	191	393	13588
GOTERM_ cellular res	3	1.098901	0.978574	GM13232,	191	404	13588
GOTERM_ proteolysis	4	1.465201	0.981486	GM8797,	191	534	13588
GOTERM_ cell divisio	2	0.732601	0.981658	ZBTB16, N	191	281	13588
GOTERM_ cellular pr	4	1.465201	0.982101	GM8797,	191	537	13588
GOTERM_ protein loc	6	2.197802	0.982315	PLEKHA8,	191	753	13588
GOTERM_ DNA meta	3	1.098901	0.98264	GM13232,	191	421	13588
GOTERM_ protein tra	5	1.831502	0.982831	PLEKHA8,	191	651	13588
GOTERM_ response t	2	0.732601	0.983175	GM13232,	191	287	13588
GOTERM_ establishm	5	1.831502	0.98371	PLEKHA8,	191	656	13588
GOTERM_ protein ca	4	1.465201	0.985565	GM8797,	191	556	13588
GOTERM_ regulation	21	7.692308	0.992052	KCNH1, C	191	2227	13588
GOTERM_ cell cycle	4	1.465201	0.992347	ANXA1, G.	191	611	13588
GOTERM_ chromoso	2	0.732601	0.996898	GM8494, I	191	404	13588
GOTERM_ transcripti	11	4.029304	0.999889	CEBPA, PG	191	1772	13588
GOTERM_ neurologi	7	2.564103	0.999998	SCN1A, SL	191	1681	13588
GOTERM_ G-protein	7	2.564103	1	C3AR1, GI	191	1877	13588
GOTERM_ cognition	4	1.465201	1	SLC1A3, A	191	1480	13588
GOTERM_ sensory pe	3	1.098901	1	SLC1A3, C	191	1402	13588
GOTERM_ regulation	1	0.3663	1	H2-K1	191	18	13588
GOTERM_ spermatid	1	0.3663	1	GM13232,	191	63	13588
GOTERM_ regulation	1	0.3663	1	NTRK2	191	20	13588
GOTERM_ developm	1	0.3663	1	CCL11	191	16	13588
GOTERM_ L-amino a	1	0.3663	1	SLC1A3	191	19	13588
GOTERM_ photorece	1	0.3663	1	NTRK2	191	28	13588
GOTERM_ regulation	1	0.3663	1	TLR7	191	4	13588
GOTERM_ protein kir	1	0.3663	1	IRS1	191	8	13588
GOTERM_ RNA trans	1	0.3663	1	NUP62	191	66	13588
GOTERM_ gamma-a	1	0.3663	1	SLC1A3	191	1	13588
GOTERM_ negative r	1	0.3663	1	SEMA3A	191	28	13588
GOTERM_ lipoprotei	1	0.3663	1	VLDLR	191	5	13588
GOTERM_ mechanos	1	0.3663	1	SLC1A3	191	8	13588
GOTERM_ branching	1	0.3663	1	SIX1	191	28	13588
GOTERM_ synaptic v	1	0.3663	1	SV2B	191	29	13588
GOTERM_ regulation	1	0.3663	1	FCGR2B	191	80	13588
GOTERM_ regulation	1	0.3663	1	EDAR	191	8	13588
GOTERM_ axonal fas	1	0.3663	1	SEMA3A	191	7	13588
GOTERM_ smoothen	1	0.3663	1	BOC	191	28	13588
GOTERM_ regulation	1	0.3663	1	IL1B	191	7	13588
GOTERM_ osteoclast	1	0.3663	1	CD300LF	191	9	13588
GOTERM_ heart sept	1	0.3663	1	SMAD7	191	7	13588

GOTERM_ negative r	1	0.3663	1	ZBTB16	191	21	13588
GOTERM_ positive re	1	0.3663	1	IRS1	191	44	13588
GOTERM_ regulation	1	0.3663	1	JAG1	191	8	13588
GOTERM_ neuromus	1	0.3663	1	SLC1A3	191	34	13588
GOTERM_ positive re	1	0.3663	1	CD4	191	94	13588
GOTERM_ organic ar	1	0.3663	1	SLC1A3	191	39	13588
GOTERM_ elastic fibe	1	0.3663	1	TNXB	191	4	13588
GOTERM_ phenol me	1	0.3663	1	CYP2D22	191	30	13588
GOTERM_ eye photo	1	0.3663	1	NTRK2	191	21	13588
GOTERM_ positive re	1	0.3663	1	GAB1	191	16	13588
GOTERM_ cardiac mu	1	0.3663	1	SMAD7	191	57	13588
GOTERM_ mesoneph	1	0.3663	1	ZBTB16	191	5	13588
GOTERM_ multicellul	1	0.3663	1	IL1B	191	71	13588
GOTERM_ DNA catak	1	0.3663	1	DNASE1L3	191	21	13588
GOTERM_ monohydr	1	0.3663	1	ADH1	191	2	13588
GOTERM_ type II hyp	1	0.3663	1	IGHG1	191	4	13588
GOTERM_ sensory pe	1	0.3663	1	SLC1A3	191	84	13588
GOTERM_ negative r	1	0.3663	1	SEMA3A	191	32	13588
GOTERM_ positive re	1	0.3663	1	CASP1	191	7	13588
GOTERM_ positive re	1	0.3663	1	TRIM32	191	14	13588
GOTERM_ response t	1	0.3663	1	TIMP3	191	42	13588
GOTERM_ negative r	1	0.3663	1	FCGR2B	191	35	13588
GOTERM_ skeletal m	1	0.3663	1	SIX1	191	72	13588
GOTERM_ neuronal a	1	0.3663	1	SCN1A	191	6	13588
GOTERM_ nerve deve	1	0.3663	1	SLC1A3	191	24	13588
GOTERM_ phosphoir	1	0.3663	1	PLCH2	191	63	13588
GOTERM_ regulation	1	0.3663	1	EDAR	191	13	13588
GOTERM_ neural nuc	1	0.3663	1	ALDH1A3	191	8	13588
GOTERM_ auditory b	1	0.3663	1	SLC1A3	191	5	13588
GOTERM_ naphthale	1	0.3663	1	CYP2F2	191	1	13588
GOTERM_ regulation	1	0.3663	1	NUP62	191	228	13588
GOTERM_ peptide m	1	0.3663	1	ACE	191	38	13588
GOTERM_ DNA catak	1	0.3663	1	DNASE1L3	191	15	13588
GOTERM_ regulation	1	0.3663	1	SEMA3A	191	97	13588
GOTERM_ xenobiotic	1	0.3663	1	CYP2F2	191	13	13588
GOTERM_ response t	1	0.3663	1	SLC1A3	191	20	13588
GOTERM_ nucleobas	1	0.3663	1	ATP1A2	191	179	13588
GOTERM_ response t	1	0.3663	1	ADH1	191	76	13588
GOTERM_ ovulation	1	0.3663	1	PGR	191	47	13588
GOTERM_ embryo im	1	0.3663	1	KLF9	191	20	13588
GOTERM_ cellular pr	1	0.3663	1	SIX2	191	299	13588
GOTERM_ positive re	1	0.3663	1	EDAR	191	15	13588
GOTERM_ positive re	1	0.3663	1	NFAM1	191	1	13588
GOTERM_ regulation	1	0.3663	1	BOC	191	42	13588
GOTERM_ positive re	1	0.3663	1	NOD1	191	15	13588
GOTERM_ regulation	1	0.3663	1	EDAR	191	30	13588
GOTERM_ inositol ph	1	0.3663	1	PTAFR	191	2	13588
GOTERM_ olfactory p	1	0.3663	1	ALDH1A3	191	2	13588
GOTERM_ ovulation	1	0.3663	1	PGR	191	13	13588
GOTERM_ positive re	1	0.3663	1	TLR1	191	4	13588
GOTERM_ regulation	1	0.3663	1	ATP1A2	191	25	13588
GOTERM_ vasculoge	1	0.3663	1	NTRK2	191	43	13588
GOTERM_ gas transp	1	0.3663	1	CYGB	191	13	13588
GOTERM_ eye photo	1	0.3663	1	NTRK2	191	23	13588
GOTERM_ segment s	1	0.3663	1	DLL1	191	11	13588
GOTERM_ glutamate	1	0.3663	1	NTRK2	191	6	13588
GOTERM_ urea cycle	1	0.3663	1	CEBPA	191	9	13588
GOTERM_ purine nuc	1	0.3663	1	ATP1A2	191	94	13588

GOTERM_ mesenchy	1	0.3663	1	RBPJ	191	49	13588
GOTERM_ inositol m	1	0.3663	1	PTAFR	191	18	13588
GOTERM_ peptide ho	1	0.3663	1	IRS1	191	34	13588
GOTERM_ organelle	1	0.3663	1	NEK6	191	197	13588
GOTERM_ regulation	1	0.3663	1	FCGR2B	191	32	13588
GOTERM_ ATP biosy	1	0.3663	1	ATP1A2	191	82	13588
GOTERM_ head morp	1	0.3663	1	PDGFRA	191	14	13588
GOTERM_ positive re	1	0.3663	1	H2-K1	191	9	13588
GOTERM_ response t	1	0.3663	1	ABCB1A	191	53	13588
GOTERM_ very-long	1	0.3663	1	CYP2D22	191	9	13588
GOTERM_ DNA ligati	1	0.3663	1	GM13232,	191	2	13588
GOTERM_ regulation	1	0.3663	1	BOC	191	43	13588
GOTERM_ Rho prote	1	0.3663	1	ARHGAP3	191	27	13588
GOTERM_ microtubu	1	0.3663	1	KIF1A	191	101	13588
GOTERM_ translatior	1	0.3663	1	GM5869	191	43	13588
GOTERM_ positive re	1	0.3663	1	CASP1	191	7	13588
GOTERM_ nuclear im	1	0.3663	1	SIX2	191	58	13588
GOTERM_ mitotic cel	1	0.3663	1	NEK6	191	244	13588
GOTERM_ positive re	1	0.3663	1	CASP1	191	10	13588
GOTERM_ membrane	1	0.3663	1	B4GALT6	191	23	13588
GOTERM_ determina	1	0.3663	1	DLL1	191	44	13588
GOTERM_ negative r	1	0.3663	1	HSPA1A, H	191	10	13588
GOTERM_ thyroid gla	1	0.3663	1	SIX1	191	14	13588
GOTERM_ striatum d	1	0.3663	1	ALDH1A3	191	10	13588
GOTERM_ amine bio	1	0.3663	1	SLC1A3	191	73	13588
GOTERM_ tertiary bra	1	0.3663	1	PGR	191	3	13588
GOTERM_ positive re	1	0.3663	1	EDAR	191	5	13588
GOTERM_ positive re	1	0.3663	1	KLRK1	191	17	13588
GOTERM_ regulation	1	0.3663	1	TLR1	191	8	13588
GOTERM_ cell-substr	1	0.3663	1	ARHGAP6	191	14	13588
GOTERM_ membrane	1	0.3663	1	B4GALT6	191	64	13588
GOTERM_ protein loc	1	0.3663	1	SIX2	191	61	13588
GOTERM_ actin cytos	1	0.3663	1	ANTXR1	191	12	13588
GOTERM_ negative r	1	0.3663	1	SEMA3A	191	47	13588
GOTERM_ retinal gar	1	0.3663	1	SLIT2	191	14	13588
GOTERM_ peptide tra	1	0.3663	1	IRS1	191	47	13588
GOTERM_ positive re	1	0.3663	1	NOD1	191	36	13588
GOTERM_ negative r	1	0.3663	1	SEMA3A	191	10	13588
GOTERM_ Wnt recep	1	0.3663	1	FZD1	191	130	13588
GOTERM_ activation	1	0.3663	1	TLR1	191	51	13588
GOTERM_ regulation	1	0.3663	1	SERPING1	191	5	13588
GOTERM_ multicellul	1	0.3663	1	MMP19	191	18	13588
GOTERM_ maintenar	1	0.3663	1	CD4	191	21	13588
GOTERM_ regulation	1	0.3663	1	RBPJ	191	8	13588
GOTERM_ activation	1	0.3663	1	KLRK1	191	14	13588
GOTERM_ regulation	1	0.3663	1	RBPJ	191	8	13588
GOTERM_ regulation	1	0.3663	1	NUP62	191	13	13588
GOTERM_ regulation	1	0.3663	1	TLR7	191	4	13588
GOTERM_ regulation	1	0.3663	1	TLR7	191	7	13588
GOTERM_ regenerati	1	0.3663	1	TIMP3	191	23	13588
GOTERM_ dichotomo	1	0.3663	1	SEMA3A	191	6	13588
GOTERM_ compartm	1	0.3663	1	DLL1	191	4	13588
GOTERM_ sphingolip	1	0.3663	1	B4GALT6	191	21	13588
GOTERM_ regulation	1	0.3663	1	SEMA3A	191	161	13588
GOTERM_ long-chair	1	0.3663	1	ANXA1	191	7	13588
GOTERM_ inositol ph	1	0.3663	1	PTAFR	191	4	13588
GOTERM_ DNA fragr	1	0.3663	1	DNASE1L3	191	13	13588
GOTERM_ ribonucleo	1	0.3663	1	ATP1A2	191	93	13588

GOTERM_	positive re	1	0.3663	1	KLRK1	191	15	13588
GOTERM_	glycolipid	1	0.3663	1	PLEKHA8	191	4	13588
GOTERM_	genitalia c	1	0.3663	1	PDGFRA	191	23	13588
GOTERM_	negative r	1	0.3663	1	HSPA1A, H	191	10	13588
GOTERM_	white fat c	1	0.3663	1	SCD1	191	10	13588
GOTERM_	retinol me	1	0.3663	1	ADH1	191	10	13588
GOTERM_	hindbrain	1	0.3663	1	GAS1	191	26	13588
GOTERM_	base-excis	1	0.3663	1	GM13232,	191	19	13588
GOTERM_	gamma-a	1	0.3663	1	SLC1A3	191	4	13588
GOTERM_	ventricular	1	0.3663	1	SMAD7	191	5	13588
GOTERM_	negative r	1	0.3663	1	SERPING1	191	7	13588
GOTERM_	positive re	1	0.3663	1	GAB1	191	59	13588
GOTERM_	chromatin	1	0.3663	1	GM8494	191	315	13588
GOTERM_	visual lear	1	0.3663	1	ATP1A2	191	29	13588
GOTERM_	positive re	1	0.3663	1	IL1B	191	17	13588
GOTERM_	activated T	1	0.3663	1	ITGAM	191	7	13588
GOTERM_	negative r	1	0.3663	1	FCGR2B	191	22	13588
GOTERM_	mesenchy	1	0.3663	1	RBPJ	191	47	13588
GOTERM_	negative r	1	0.3663	1	FCGR2B	191	53	13588
GOTERM_	purine nuc	1	0.3663	1	ATP1A2	191	106	13588
GOTERM_	L-glutama	1	0.3663	1	SLC1A3	191	4	13588
GOTERM_	learning o	1	0.3663	1	ATP1A2	191	89	13588
GOTERM_	inflammat	1	0.3663	1	IGHG1	191	7	13588
GOTERM_	monohydr	1	0.3663	1	ADH1	191	4	13588
GOTERM_	drug meta	1	0.3663	1	CYP2D22	191	8	13588
GOTERM_	positive re	1	0.3663	1	JAG1	191	4	13588
GOTERM_	arachidon	1	0.3663	1	CYP2D22	191	1	13588
GOTERM_	catecholar	1	0.3663	1	CYP2D22	191	30	13588
GOTERM_	regulation	1	0.3663	1	IRS1	191	8	13588
GOTERM_	chromoso	1	0.3663	1	NEK6	191	64	13588
GOTERM_	response t	1	0.3663	1	ADH1	191	21	13588
GOTERM_	regulation	1	0.3663	1	ALDH1A3	191	3	13588
GOTERM_	cerebellun	1	0.3663	1	GAS1	191	22	13588
GOTERM_	positive re	1	0.3663	1	IRS1	191	37	13588
GOTERM_	regulation	1	0.3663	1	ATP1A2	191	13	13588
GOTERM_	regulation	1	0.3663	1	CASP1	191	13	13588
GOTERM_	heat gene	1	0.3663	1	IL1B	191	7	13588
GOTERM_	protein ub	1	0.3663	1	TRIM32	191	66	13588
GOTERM_	actomyosi	1	0.3663	1	LIMCH1	191	25	13588
GOTERM_	membrane	1	0.3663	1	SMAD7	191	36	13588
GOTERM_	regulation	1	0.3663	1	SEMA3A	191	37	13588
GOTERM_	positive re	1	0.3663	1	TLR7	191	7	13588
GOTERM_	purine nuc	1	0.3663	1	ATP1A2	191	160	13588
GOTERM_	prostate g	1	0.3663	1	SERPINB5	191	39	13588
GOTERM_	spermatid	1	0.3663	1	GM13232,	191	7	13588
GOTERM_	negative r	1	0.3663	1	HTRA1	191	16	13588
GOTERM_	antigen pr	1	0.3663	1	H2-K1	191	5	13588
GOTERM_	regulation	1	0.3663	1	SERPING1	191	4	13588
GOTERM_	cartilage c	1	0.3663	1	PRRX1	191	78	13588
GOTERM_	peptidyl-t	1	0.3663	1	BTK	191	37	13588
GOTERM_	outer ear r	1	0.3663	1	GAS1	191	4	13588
GOTERM_	glycosylati	1	0.3663	1	CD4	191	91	13588
GOTERM_	female go	1	0.3663	1	PGR	191	53	13588
GOTERM_	regulation	1	0.3663	1	SERPING1	191	4	13588
GOTERM_	cell junctic	1	0.3663	1	ARHGAP6	191	32	13588
GOTERM_	L-glutama	1	0.3663	1	SLC1A3	191	12	13588
GOTERM_	regulation	1	0.3663	1	EDAR	191	35	13588
GOTERM_	regulation	1	0.3663	1	CASP1	191	15	13588

GOTERM_ negative r	1	0.3663	1	FCGR2B	191	3	13588
GOTERM_ regulation	1	0.3663	1	EDAR	191	42	13588
GOTERM_ positive re	1	0.3663	1	IRS1	191	41	13588
GOTERM_ negative r	1	0.3663	1	SEMA3A	191	53	13588
GOTERM_ fibril orga	1	0.3663	1	TNXB	191	3	13588
GOTERM_ ethanol ca	1	0.3663	1	ADH1	191	2	13588
GOTERM_ negative r	1	0.3663	1	DLL1	191	34	13588
GOTERM_ negative r	1	0.3663	1	SEMA3A	191	16	13588
GOTERM_ nose deve	1	0.3663	1	ALDH1A3	191	9	13588
GOTERM_ negative r	1	0.3663	1	SERPING1	191	72	13588
GOTERM_ cell killing	1	0.3663	1	IGHG1	191	16	13588
GOTERM_ fibroblast	1	0.3663	1	FGF1	191	30	13588
GOTERM_ polyol bio	1	0.3663	1	PTAFR	191	5	13588
GOTERM_ alcohol bi	1	0.3663	1	PTAFR	191	37	13588
GOTERM_ regulation	1	0.3663	1	SEMA3A	191	16	13588
GOTERM_ MAPKKK c	1	0.3663	1	GAB1	191	114	13588
GOTERM_ nucleobas	1	0.3663	1	NUP62	191	74	13588
GOTERM_ synaptic v	1	0.3663	1	SV2B	191	17	13588
GOTERM_ catechol n	1	0.3663	1	CYP2D22	191	30	13588
GOTERM_ negative r	1	0.3663	1	SEMA3A	191	40	13588
GOTERM_ substrate a	1	0.3663	1	ANTXR1	191	1	13588
GOTERM_ negative r	1	0.3663	1	FCGR2B	191	14	13588
GOTERM_ muscle ce	1	0.3663	1	SIX1	191	6	13588
GOTERM_ response t	1	0.3663	1	ADH1	191	30	13588
GOTERM_ positive re	1	0.3663	1	H2-K1	191	13	13588
GOTERM_ negative r	1	0.3663	1	ZFP462	191	26	13588
GOTERM_ regulation	1	0.3663	1	CASP1	191	30	13588
GOTERM_ activation	1	0.3663	1	GAB1	191	50	13588
GOTERM_ macropha	1	0.3663	1	CEBPA	191	7	13588
GOTERM_ benzene a	1	0.3663	1	CYP2F2	191	6	13588
GOTERM_ cytokine b	1	0.3663	1	NOD1	191	13	13588
GOTERM_ epidermal	1	0.3663	1	GAB1	191	11	13588
GOTERM_ detection	1	0.3663	1	TLR1	191	31	13588
GOTERM_ positive re	1	0.3663	1	CASP1	191	17	13588
GOTERM_ leukocyte	1	0.3663	1	IGHG1	191	12	13588
GOTERM_ negative r	1	0.3663	1	IL1B	191	12	13588
GOTERM_ subpalliun	1	0.3663	1	ALDH1A3	191	13	13588
GOTERM_ neurotrans	1	0.3663	1	SV2B	191	36	13588
GOTERM_ negative r	1	0.3663	1	FCGR2B	191	3	13588
GOTERM_ male gern	1	0.3663	1	ZBTB16	191	1	13588
GOTERM_ positive re	1	0.3663	1	EDAR	191	9	13588
GOTERM_ regulation	1	0.3663	1	IL1B	191	7	13588
GOTERM_ germ-line	1	0.3663	1	ZBTB16	191	1	13588
GOTERM_ maternal p	1	0.3663	1	PGR	191	12	13588
GOTERM_ morphoge	1	0.3663	1	ALDH1A3	191	78	13588
GOTERM_ regulation	1	0.3663	1	NOD1	191	6	13588
GOTERM_ ribonucleo	1	0.3663	1	ATP1A2	191	111	13588
GOTERM_ urea meta	1	0.3663	1	CEBPA	191	9	13588
GOTERM_ protein an	1	0.3663	1	CD4	191	48	13588
GOTERM_ dichotomo	1	0.3663	1	SEMA3A	191	5	13588
GOTERM_ neutral lip	1	0.3663	1	DGAT2	191	12	13588
GOTERM_ protein an	1	0.3663	1	CD4	191	50	13588
GOTERM_ pigmentat	1	0.3663	1	EDAR	191	56	13588
GOTERM_ regulation	1	0.3663	1	SCN1A	191	45	13588
GOTERM_ stimulator	1	0.3663	1	KLRK1	191	2	13588
GOTERM_ regulation	1	0.3663	1	GNA15	191	28	13588
GOTERM_ biopolyme	1	0.3663	1	CD4	191	91	13588
GOTERM_ carbohydr	1	0.3663	1	PTAFR	191	83	13588

GOTERM_	negative r	1	0.3663	1	FCGR2B	191	13	13588
GOTERM_	protein im	1	0.3663	1	SIX2	191	56	13588
GOTERM_	acidic ami	1	0.3663	1	SLC1A3	191	12	13588
GOTERM_	sebaceous	1	0.3663	1	RBPJ	191	3	13588
GOTERM_	negative r	1	0.3663	1	ZFP462	191	30	13588
GOTERM_	glycerol m	1	0.3663	1	DGAT2	191	23	13588
GOTERM_	retinoic ac	1	0.3663	1	ALDH1A3	191	2	13588
GOTERM_	regulation	1	0.3663	1	ATP1A2	191	12	13588
GOTERM_	ureteric bu	1	0.3663	1	SIX1	191	28	13588
GOTERM_	nucleus ac	1	0.3663	1	ALDH1A3	191	1	13588
GOTERM_	somitoger	1	0.3663	1	DLL1	191	41	13588
GOTERM_	male geni	1	0.3663	1	PDGFRA	191	15	13588
GOTERM_	positive re	1	0.3663	1	IL1B	191	4	13588
GOTERM_	leukotrien	1	0.3663	1	ALOX5	191	17	13588
GOTERM_	purine nuc	1	0.3663	1	ATP1A2	191	137	13588
GOTERM_	icosanoid	1	0.3663	1	ALOX5	191	27	13588
GOTERM_	epidermal	1	0.3663	1	RBPJ	191	51	13588
GOTERM_	regulation	1	0.3663	1	NEK6	191	16	13588
GOTERM_	multicellul	1	0.3663	1	APBA2	191	39	13588
GOTERM_	regulation	1	0.3663	1	SCN1A	191	54	13588
GOTERM_	glycerolipi	1	0.3663	1	DGAT2	191	58	13588
GOTERM_	female sex	1	0.3663	1	PGR	191	64	13588
GOTERM_	positive re	1	0.3663	1	FOSL2	191	11	13588
GOTERM_	negative r	1	0.3663	1	NUP62	191	3	13588
GOTERM_	purine ribo	1	0.3663	1	ATP1A2	191	93	13588
GOTERM_	positive re	1	0.3663	1	CASP1	191	53	13588
GOTERM_	hair follicle	1	0.3663	1	RBPJ	191	16	13588
GOTERM_	ATP metal	1	0.3663	1	ATP1A2	191	90	13588
GOTERM_	cellular an	1	0.3663	1	SLC1A3	191	48	13588
GOTERM_	negative r	1	0.3663	1	FCGR2B	191	5	13588
GOTERM_	transition	1	0.3663	1	STEAP4	191	70	13588
GOTERM_	reduction	1	0.3663	1	ATP1A2	191	1	13588
GOTERM_	actin filam	1	0.3663	1	ARHGAP6	191	56	13588
GOTERM_	regulation	1	0.3663	1	FCGR2B	191	80	13588
GOTERM_	branching	1	0.3663	1	SEMA3A	191	18	13588
GOTERM_	cellular ca	1	0.3663	1	PTAFR	191	56	13588
GOTERM_	carbohydr	1	0.3663	1	GM12286	191	81	13588
GOTERM_	regulation	1	0.3663	1	ATP1A2	191	16	13588
GOTERM_	phagocyto	1	0.3663	1	IGHG1	191	13	13588
GOTERM_	protein pa	1	0.3663	1	CD4	191	1	13588
GOTERM_	auditory re	1	0.3663	1	DLL1	191	5	13588
GOTERM_	cellular ex	1	0.3663	1	ITGAM	191	6	13588
GOTERM_	neuron mi	1	0.3663	1	MDGA1	191	70	13588
GOTERM_	regulation	1	0.3663	1	SMAD7	191	60	13588
GOTERM_	regulation	1	0.3663	1	NFAM1	191	13	13588
GOTERM_	positive re	1	0.3663	1	JAG1	191	25	13588
GOTERM_	regulation	1	0.3663	1	H2-K1	191	11	13588
GOTERM_	Ras protei	1	0.3663	1	ARHGAP3	191	59	13588
GOTERM_	RNA catab	1	0.3663	1	OAS2	191	48	13588
GOTERM_	glucose ca	1	0.3663	1	GM12286	191	52	13588
GOTERM_	mitosis	1	0.3663	1	NEK6	191	190	13588
GOTERM_	regulation	1	0.3663	1	IL1B	191	11	13588
GOTERM_	regulation	1	0.3663	1	IL1B	191	10	13588
GOTERM_	glycerol et	1	0.3663	1	DGAT2	191	13	13588
GOTERM_	optic cup	1	0.3663	1	ALDH1A3	191	2	13588
GOTERM_	dorsal/ver	1	0.3663	1	GAS1	191	64	13588
GOTERM_	mesenchy	1	0.3663	1	RBPJ	191	50	13588
GOTERM_	negative r	1	0.3663	1	ATP1A2	191	100	13588

GOTERM_	cellular co	1	0.3663	1	DNASE1L3	191	39	13588
GOTERM_	patterning	1	0.3663	1	SEMA5A	191	24	13588
GOTERM_	negative r	1	0.3663	1	SEMA3A	191	2	13588
GOTERM_	natural kill	1	0.3663	1	KLRK1	191	16	13588
GOTERM_	segmenta	1	0.3663	1	DLL1	191	54	13588
GOTERM_	response t	1	0.3663	1	SLC1A3	191	14	13588
GOTERM_	regulation	1	0.3663	1	BOC	191	16	13588
GOTERM_	establishm	1	0.3663	1	NUP62	191	66	13588
GOTERM_	ovulation	1	0.3663	1	PGR	191	48	13588
GOTERM_	response t	1	0.3663	1	KCNIP3	191	19	13588
GOTERM_	anatomica	1	0.3663	1	HSPA1A, H	191	83	13588
GOTERM_	regulation	1	0.3663	1	KLRK1	191	19	13588
GOTERM_	positive re	1	0.3663	1	KLRK1	191	36	13588
GOTERM_	innate imr	1	0.3663	1	KLRK1	191	4	13588
GOTERM_	hindlimb r	1	0.3663	1	ZBTB16	191	34	13588
GOTERM_	regulation	1	0.3663	1	SERPING1	191	1	13588
GOTERM_	regulation	1	0.3663	1	SEMA3A	191	16	13588
GOTERM_	positive re	1	0.3663	1	NFAM1	191	34	13588
GOTERM_	triglycerid	1	0.3663	1	DGAT2	191	8	13588
GOTERM_	regulation	1	0.3663	1	CD4	191	108	13588
GOTERM_	response t	1	0.3663	1	ADH1	191	115	13588
GOTERM_	liver devel	1	0.3663	1	CEBPA	191	43	13588
GOTERM_	negative r	1	0.3663	1	FCGR2B	191	35	13588
GOTERM_	ribonucleo	1	0.3663	1	ATP1A2	191	125	13588
GOTERM_	focal adhe	1	0.3663	1	ARHGAP6	191	8	13588
GOTERM_	prostate g	1	0.3663	1	SERPINB5	191	27	13588
GOTERM_	oxygen ar	1	0.3663	1	FMO2	191	47	13588
GOTERM_	positive re	1	0.3663	1	GNA15	191	27	13588
GOTERM_	protein pc	1	0.3663	1	TTLL10	191	3	13588
GOTERM_	regulation	1	0.3663	1	SERPING1	191	12	13588
GOTERM_	negative r	1	0.3663	1	GAS1	191	7	13588
GOTERM_	negative r	1	0.3663	1	IL1B	191	1	13588
GOTERM_	protein an	1	0.3663	1	NTRK2	191	66	13588
GOTERM_	alditol me	1	0.3663	1	DGAT2	191	25	13588
GOTERM_	pathway-r	1	0.3663	1	SMAD7	191	5	13588
GOTERM_	negative r	1	0.3663	1	FCGR2B	191	14	13588
GOTERM_	peptide se	1	0.3663	1	IRS1	191	36	13588
GOTERM_	oxygen tra	1	0.3663	1	CYGB	191	11	13588
GOTERM_	stress-acti	1	0.3663	1	GAB1	191	35	13588
GOTERM_	response t	1	0.3663	1	ADH1	191	133	13588
GOTERM_	keratinocy	1	0.3663	1	RBPJ	191	48	13588
GOTERM_	protein an	1	0.3663	1	CD4	191	25	13588
GOTERM_	regulation	1	0.3663	1	ATP1A2	191	12	13588
GOTERM_	sensory pe	1	0.3663	1	SLC1A3	191	92	13588
GOTERM_	regulation	1	0.3663	1	EDAR	191	22	13588
GOTERM_	regulation	1	0.3663	1	CD4	191	18	13588
GOTERM_	negative r	1	0.3663	1	DLL1	191	2	13588
GOTERM_	positive re	1	0.3663	1	KLRK1	191	15	13588
GOTERM_	response t	1	0.3663	1	SLC1A3	191	5	13588
GOTERM_	two-comp	1	0.3663	1	KCNH1	191	8	13588
GOTERM_	innate imr	1	0.3663	1	KLRK1	191	12	13588
GOTERM_	inorganic	1	0.3663	1	CLCA2	191	83	13588
GOTERM_	somite spe	1	0.3663	1	DLL1	191	4	13588
GOTERM_	positive re	1	0.3663	1	CD4	191	66	13588
GOTERM_	amino acid	1	0.3663	1	SLC1A3	191	5	13588
GOTERM_	leukotrien	1	0.3663	1	ALOX5	191	19	13588
GOTERM_	M phase	1	0.3663	1	NEK6	191	283	13588
GOTERM_	nucleotide	1	0.3663	1	ATP1A2	191	174	13588

GOTERM_ detection	1	0.3663	1	TLR1	191	1	13588
GOTERM_ detection	1	0.3663	1	TLR1	191	4	13588
GOTERM_ neuron re	1	0.3663	1	SEMA3A	191	12	13588
GOTERM_ negative r	1	0.3663	1	FCGR2B	191	1	13588
GOTERM_ nucleoside	1	0.3663	1	ATP1A2	191	115	13588
GOTERM_ developm	1	0.3663	1	PGR	191	57	13588
GOTERM_ one-carbo	1	0.3663	1	CAR3	191	117	13588
GOTERM_ amine trar	1	0.3663	1	SLC1A3	191	92	13588
GOTERM_ drug trans	1	0.3663	1	ABCB1A	191	14	13588
GOTERM_ epidermal	1	0.3663	1	RBPJ	191	1	13588
GOTERM_ positive re	1	0.3663	1	CASP1	191	7	13588
GOTERM_ protein tar	1	0.3663	1	SIX2	191	133	13588
GOTERM_ regulation	1	0.3663	1	ARHGAP6	191	93	13588
GOTERM_ negative r	1	0.3663	1	NUP62	191	13	13588
GOTERM_ response t	1	0.3663	1	TIMP3	191	33	13588
GOTERM_ cranial ner	1	0.3663	1	SLC1A3	191	18	13588
GOTERM_ regulation	1	0.3663	1	BOC	191	22	13588
GOTERM_ regulation	1	0.3663	1	NFAM1	191	59	13588
GOTERM_ leg morph	1	0.3663	1	ZBTB16	191	3	13588
GOTERM_ monosacc	1	0.3663	1	GM12286	191	54	13588
GOTERM_ regulation	1	0.3663	1	TLR7	191	15	13588
GOTERM_ negative r	1	0.3663	1	SERPING1	191	12	13588
GOTERM_ positive re	1	0.3663	1	CD4	191	99	13588
GOTERM_ protein an	1	0.3663	1	CD4	191	91	13588
GOTERM_ hexose ca	1	0.3663	1	GM12286	191	52	13588
GOTERM_ inner ear r	1	0.3663	1	DLL1	191	5	13588
GOTERM_ regulation	1	0.3663	1	TLR7	191	7	13588
GOTERM_ negative r	1	0.3663	1	FCGR2B	191	13	13588
GOTERM_ visual beh	1	0.3663	1	ATP1A2	191	31	13588
GOTERM_ protein loc	1	0.3663	1	SIX2	191	97	13588
GOTERM_ negative r	1	0.3663	1	IL1B	191	17	13588
GOTERM_ regulation	1	0.3663	1	IL1B	191	20	13588
GOTERM_ negative r	1	0.3663	1	IL1B	191	10	13588
GOTERM_ positive re	1	0.3663	1	CASP1	191	7	13588
GOTERM_ regulation	1	0.3663	1	BOC	191	10	13588
GOTERM_ positive re	1	0.3663	1	EDAR	191	12	13588
GOTERM_ intracellula	1	0.3663	1	SIX2	191	276	13588
GOTERM_ regulation	1	0.3663	1	IL1B	191	4	13588
GOTERM_ diencepha	1	0.3663	1	RBPJ	191	38	13588
GOTERM_ regulation	1	0.3663	1	SMAD7	191	6	13588
GOTERM_ vascular e	1	0.3663	1	PDGFRA	191	14	13588
GOTERM_ response t	1	0.3663	1	SLC1A3	191	39	13588
GOTERM_ response t	1	0.3663	1	IRS1	191	59	13588
GOTERM_ response t	1	0.3663	1	ADH1	191	1	13588
GOTERM_ JNK cascad	1	0.3663	1	GAB1	191	32	13588
GOTERM_ iron ion tr	1	0.3663	1	STEAP4	191	32	13588
GOTERM_ L-amino a	1	0.3663	1	SLC1A3	191	5	13588
GOTERM_ T cell proli	1	0.3663	1	ITGAM	191	28	13588
GOTERM_ regulation	1	0.3663	1	DLL1	191	3	13588
GOTERM_ positive re	1	0.3663	1	TLR7	191	4	13588
GOTERM_ positive re	1	0.3663	1	BOC	191	9	13588
GOTERM_ antigen pr	1	0.3663	1	H2-K1	191	17	13588
GOTERM_ interleukir	1	0.3663	1	GAB1	191	4	13588
GOTERM_ amide bio	1	0.3663	1	CEBPA	191	10	13588
GOTERM_ leukocyte	1	0.3663	1	ITGAM	191	43	13588
GOTERM_ mitochond	1	0.3663	1	CEBPA	191	97	13588
GOTERM_ neurotrans	1	0.3663	1	ATP1A2	191	7	13588
GOTERM_ cellular ma	1	0.3663	1	SIX2	191	301	13588

GOTERM_ dopamine	1	0.3663	1	CYP2D22	191	19	13588
GOTERM_ neuron fat	1	0.3663	1	DLL1	191	47	13588
GOTERM_ type IIa hy	1	0.3663	1	IGHG1	191	4	13588
GOTERM_ cellular ca	1	0.3663	1	GM12286	191	60	13588
GOTERM_ generatior	1	0.3663	1	GM12286	191	261	13588
GOTERM_ icosanoid	1	0.3663	1	ANXA1	191	1	13588
GOTERM_ lymph noc	1	0.3663	1	CXCR5	191	19	13588
GOTERM_ lymphocyt	1	0.3663	1	ITGAM	191	42	13588
GOTERM_ antigen pr	1	0.3663	1	FCGR2B	191	16	13588
GOTERM_ dephosph	1	0.3663	1	PTPRZ1	191	141	13588
GOTERM_ regulation	1	0.3663	1	TLR7	191	14	13588
GOTERM_ regulation	1	0.3663	1	NFAM1	191	16	13588
GOTERM_ ureteric bu	1	0.3663	1	SIX1	191	42	13588
GOTERM_ negative r	1	0.3663	1	FCGR2B	191	3	13588
GOTERM_ nucleocyto	1	0.3663	1	SIX2	191	96	13588
GOTERM_ behaviora	1	0.3663	1	ADH1	191	5	13588
GOTERM_ cell redox	1	0.3663	1	TXNDC5	191	62	13588
GOTERM_ male gona	1	0.3663	1	GM13232,	191	39	13588
GOTERM_ epithelial (	1	0.3663	1	PGR	191	10	13588
GOTERM_ negative r	1	0.3663	1	SEMA3A	191	38	13588
GOTERM_ spermatid	1	0.3663	1	GM13232,	191	59	13588
GOTERM_ regulation	1	0.3663	1	NOD1	191	10	13588
GOTERM_ regulation	1	0.3663	1	KLRK1	191	20	13588
GOTERM_ purine ribo	1	0.3663	1	ATP1A2	191	101	13588
GOTERM_ T cell selec	1	0.3663	1	CD4	191	21	13588
GOTERM_ face morp	1	0.3663	1	PDGFRA	191	12	13588
GOTERM_ negative r	1	0.3663	1	SERPING1	191	5	13588
GOTERM_ negative r	1	0.3663	1	NUP62	191	13	13588
GOTERM_ protein mo	1	0.3663	1	TRIM32	191	104	13588
GOTERM_ M phase c	1	0.3663	1	NEK6	191	194	13588
GOTERM_ unsaturate	1	0.3663	1	ALOX5	191	28	13588
GOTERM_ negative r	1	0.3663	1	SERPING1	191	1	13588
GOTERM_ glutamate	1	0.3663	1	SLC1A3	191	12	13588
GOTERM_ forelimb n	1	0.3663	1	ZBTB16	191	27	13588
GOTERM_ temperatu	1	0.3663	1	IL1B	191	18	13588
GOTERM_ regulation	1	0.3663	1	TLR7	191	4	13588
GOTERM_ negative r	1	0.3663	1	ATP1A2	191	2	13588
GOTERM_ maintenar	1	0.3663	1	CD4	191	28	13588
GOTERM_ cholesterc	1	0.3663	1	VLDLR	191	70	13588
GOTERM_ chromatin	1	0.3663	1	GM8494	191	236	13588
GOTERM_ collagen fi	1	0.3663	1	TNXB	191	21	13588
GOTERM_ positive re	1	0.3663	1	NFAM1	191	4	13588
GOTERM_ glutamine	1	0.3663	1	SLC1A3	191	15	13588
GOTERM_ regulation	1	0.3663	1	SEMA3A	191	4	13588
GOTERM_ regulation	1	0.3663	1	BOC	191	27	13588
GOTERM_ response t	1	0.3663	1	ADH1	191	29	13588
GOTERM_ regulation	1	0.3663	1	CASP1	191	7	13588
GOTERM_ insulin rec	1	0.3663	1	IRS1	191	28	13588
GOTERM_ regulation	1	0.3663	1	NEK6	191	154	13588
GOTERM_ epithelial (	1	0.3663	1	PGR	191	25	13588
GOTERM_ retinal rod	1	0.3663	1	NTRK2	191	5	13588
GOTERM_ regulation	1	0.3663	1	IL1B	191	45	13588
GOTERM_ negative r	1	0.3663	1	FCGR2B	191	35	13588
GOTERM_ lipoprotei	1	0.3663	1	CD4	191	76	13588
GOTERM_ defense re	1	0.3663	1	NOD1	191	23	13588
GOTERM_ antimicrob	1	0.3663	1	IGHG1	191	4	13588
GOTERM_ inositol bi	1	0.3663	1	PTAFR	191	3	13588
GOTERM_ limbic syst	1	0.3663	1	ALDH1A3	191	33	13588

GOTERM_ positive re	1	0.3663	1	IL1B	191	3	13588
GOTERM_ regulation	1	0.3663	1	CD4	191	53	13588
GOTERM_ prostanoic	1	0.3663	1	PTGES	191	14	13588
GOTERM_ maintenar	1	0.3663	1	CD4	191	33	13588
GOTERM_ positive re	1	0.3663	1	NTRK2	191	3	13588
GOTERM_ arachidon	1	0.3663	1	ANXA1	191	1	13588
GOTERM_ sterol met	1	0.3663	1	VLDLR	191	77	13588
GOTERM_ embryonic	1	0.3663	1	ALDH1A3	191	14	13588
GOTERM_ purine ribo	1	0.3663	1	ATP1A2	191	107	13588
GOTERM_ regulation	1	0.3663	1	NTRK2	191	22	13588
GOTERM_ positive re	1	0.3663	1	CASP1	191	3	13588
GOTERM_ glycoprote	1	0.3663	1	CD4	191	117	13588
GOTERM_ cell cycle p	1	0.3663	1	NEK6	191	328	13588
GOTERM_ response t	1	0.3663	1	CYP2F2	191	29	13588
GOTERM_ negative r	1	0.3663	1	SEMA3A	191	71	13588
GOTERM_ lipoprotei	1	0.3663	1	CD4	191	55	13588
GOTERM_ multicellul	1	0.3663	1	KCNIP3	191	37	13588
GOTERM_ sphingolip	1	0.3663	1	B4GALT6	191	62	13588
GOTERM_ activation	1	0.3663	1	TLR1	191	7	13588
GOTERM_ response t	1	0.3663	1	ADH1	191	32	13588
GOTERM_ sensory pe	1	0.3663	1	CNGA2	191	1117	13588
GOTERM_ negative r	1	0.3663	1	FCGR2B	191	53	13588
GOTERM_ negative r	1	0.3663	1	IL1B	191	72	13588
GOTERM_ interleukin	1	0.3663	1	NOD1	191	1	13588
GOTERM_ neural tub	1	0.3663	1	GAS1	191	83	13588
GOTERM_ positive re	1	0.3663	1	KLRK1	191	15	13588
GOTERM_ regulation	1	0.3663	1	SEMA3A	191	3	13588
GOTERM_ mRNA tra	1	0.3663	1	NUP62	191	62	13588
GOTERM_ cytokine n	1	0.3663	1	NOD1	191	14	13588
GOTERM_ negative r	1	0.3663	1	FCGR2B	191	3	13588
GOTERM_ fever	1	0.3663	1	IL1B	191	4	13588
GOTERM_ glycolysis	1	0.3663	1	GM12286	191	44	13588
GOTERM_ regulation	1	0.3663	1	SEMA3A	191	108	13588
GOTERM_ neural tub	1	0.3663	1	GAS1	191	24	13588
GOTERM_ cellular res	1	0.3663	1	IRS1	191	43	13588
GOTERM_ inositol tri	1	0.3663	1	PTAFR	191	2	13588
GOTERM_ response t	1	0.3663	1	SLC1A3	191	19	13588
GOTERM_ brain-deri	1	0.3663	1	NTRK2	191	1	13588
GOTERM_ biogenic a	1	0.3663	1	CYP2D22	191	80	13588
GOTERM_ rhythmic p	1	0.3663	1	PGR	191	98	13588
GOTERM_ cardiac mu	1	0.3663	1	SMAD7	191	18	13588
GOTERM_ positive re	1	0.3663	1	ALDH1A3	191	1	13588
GOTERM_ regulation	1	0.3663	1	TLR7	191	11	13588
GOTERM_ response t	1	0.3663	1	ABCB1A	191	12	13588
GOTERM_ negative r	1	0.3663	1	IL1B	191	7	13588
GOTERM_ microtubu	1	0.3663	1	KIF1A	191	211	13588
GOTERM_ nucleoside	1	0.3663	1	ATP1A2	191	95	13588
GOTERM_ negative r	1	0.3663	1	ATP1A2	191	10	13588
GOTERM_ embryonic	1	0.3663	1	GAS1	191	29	13588
GOTERM_ telomere c	1	0.3663	1	HSPA1A, H	191	24	13588
GOTERM_ cell structu	1	0.3663	1	DNASE1L3	191	15	13588
GOTERM_ determina	1	0.3663	1	DLL1	191	44	13588
GOTERM_ regulation	1	0.3663	1	IL1B	191	2	13588
GOTERM_ negative r	1	0.3663	1	SERPING1	191	78	13588
GOTERM_ heart mor	1	0.3663	1	SMAD7	191	74	13588
GOTERM_ negative r	1	0.3663	1	SEMA3A	191	93	13588
GOTERM_ regulation	1	0.3663	1	FCGR2B	191	82	13588
GOTERM_ purine ribo	1	0.3663	1	ATP1A2	191	119	13588

GOTERM_	anti-apop	1	0.3663	1	HSPA1A, H	191	88	13588
GOTERM_	salivary gl	1	0.3663	1	EDAR	191	5	13588
GOTERM_	negative r	1	0.3663	1	GAS1	191	27	13588
GOTERM_	leukocyte	1	0.3663	1	ITGAM	191	14	13588
GOTERM_	nucleobas	1	0.3663	1	ATP1A2	191	179	13588
GOTERM_	telenceph	1	0.3663	1	ALDH1A3	191	72	13588
GOTERM_	apoptotic	1	0.3663	1	DNASE1L3	191	19	13588
GOTERM_	telomere r	1	0.3663	1	HSPA1A, H	191	23	13588
GOTERM_	antigen pr	1	0.3663	1	FCGR2B	191	16	13588
GOTERM_	body mor	1	0.3663	1	PDGFRA	191	20	13588
GOTERM_	feeding be	1	0.3663	1	NTRK2	191	51	13588
GOTERM_	acid secre	1	0.3663	1	ANXA1	191	3	13588
GOTERM_	mammary	1	0.3663	1	PGR	191	6	13588
GOTERM_	embryonic	1	0.3663	1	ZBTB16	191	22	13588
GOTERM_	response t	1	0.3663	1	CYP2F2	191	16	13588
GOTERM_	regulation	1	0.3663	1	SEMA3A	191	50	13588
GOTERM_	myeloid le	1	0.3663	1	IGHG1	191	17	13588
GOTERM_	negative r	1	0.3663	1	SERPING1	191	3	13588
GOTERM_	photorece	1	0.3663	1	NTRK2	191	26	13588
GOTERM_	aromatic a	1	0.3663	1	IL4I1	191	21	13588
GOTERM_	regulation	1	0.3663	1	CASP1	191	14	13588
GOTERM_	coenzyme	1	0.3663	1	SLC27A3	191	143	13588
GOTERM_	learning	1	0.3663	1	ATP1A2	191	57	13588
GOTERM_	glyceroph	1	0.3663	1	PLCH2	191	88	13588
GOTERM_	response t	1	0.3663	1	TLR1	191	1	13588
GOTERM_	cellular an	1	0.3663	1	CEBPA	191	39	13588
GOTERM_	exocytosis	1	0.3663	1	SV2B	191	110	13588
GOTERM_	hypersens	1	0.3663	1	IGHG1	191	5	13588
GOTERM_	response t	1	0.3663	1	SLC1A3	191	20	13588
GOTERM_	insulin-like	1	0.3663	1	IRS1	191	9	13588
GOTERM_	maintenar	1	0.3663	1	CD4	191	25	13588
GOTERM_	positive re	1	0.3663	1	EDAR	191	4	13588
GOTERM_	camera-ty	1	0.3663	1	ALDH1A3	191	48	13588
GOTERM_	sensory pe	1	0.3663	1	KCNIP3	191	33	13588
GOTERM_	protein an	1	0.3663	1	PTPRZ1	191	114	13588
GOTERM_	cell junctio	1	0.3663	1	ARHGAP6	191	21	13588
GOTERM_	regulation	1	0.3663	1	NEK6	191	36	13588
GOTERM_	positive re	1	0.3663	1	IL1B	191	5	13588
GOTERM_	negative r	1	0.3663	1	SEMA3A	191	8	13588
GOTERM_	regulation	1	0.3663	1	FOSL2	191	20	13588
GOTERM_	negative r	1	0.3663	1	SEMA3A	191	4	13588
GOTERM_	regulation	1	0.3663	1	SEMA3A	191	39	13588
GOTERM_	regulation	1	0.3663	1	KCNIP3	191	80	13588
GOTERM_	peptidyl-t	1	0.3663	1	BTK	191	37	13588
GOTERM_	regulation	1	0.3663	1	BOC	191	20	13588
GOTERM_	base-excis	1	0.3663	1	GM13232,	191	1	13588
GOTERM_	regulation	1	0.3663	1	NTRK2	191	8	13588
GOTERM_	tissue rege	1	0.3663	1	TIMP3	191	12	13588
GOTERM_	thymus de	1	0.3663	1	SIX1	191	22	13588
GOTERM_	RNA locali	1	0.3663	1	NUP62	191	67	13588
GOTERM_	positive re	1	0.3663	1	IRS1	191	6	13588
GOTERM_	activation	1	0.3663	1	GNA15	191	25	13588
GOTERM_	positive re	1	0.3663	1	CD4	191	35	13588
GOTERM_	mononucl	1	0.3663	1	ITGAM	191	43	13588
GOTERM_	neuromus	1	0.3663	1	SCN1A	191	12	13588
GOTERM_	negative r	1	0.3663	1	SEMA3A	191	31	13588
GOTERM_	asymmetri	1	0.3663	1	ZBTB16	191	1	13588
GOTERM_	negative r	1	0.3663	1	FCGR2B	191	10	13588

GOTERM_ protein m	1	0.3663	1	TRIM32	191	79	13588
GOTERM_ interleukir	1	0.3663	1	NOD1	191	1	13588
GOTERM_ regulation	1	0.3663	1	DLL1	191	3	13588
GOTERM_ second-m	1	0.3663	1	GNA15	191	118	13588
GOTERM_ regulation	1	0.3663	1	ATP1A2	191	15	13588
GOTERM_ antibody-	1	0.3663	1	IGHG1	191	4	13588
GOTERM_ embryonic	1	0.3663	1	ALDH1A3	191	10	13588
GOTERM_ defense re	1	0.3663	1	TLR7	191	15	13588
GOTERM_ cofactor m	1	0.3663	1	SLC27A3	191	182	13588
GOTERM_ labyrinthir	1	0.3663	1	GAB1	191	35	13588
GOTERM_ regulation	1	0.3663	1	NFAM1	191	58	13588
GOTERM_ dicarboxyl	1	0.3663	1	SLC1A3	191	11	13588
GOTERM_ mammary	1	0.3663	1	CCL11	191	2	13588
GOTERM_ steroid me	1	0.3663	1	VLDLR	191	161	13588
GOTERM_ amino acid	1	0.3663	1	SLC1A3	191	71	13588
GOTERM_ nucleic aci	1	0.3663	1	NUP62	191	66	13588
GOTERM_ insulin sec	1	0.3663	1	IRS1	191	27	13588
GOTERM_ T cell diffe	1	0.3663	1	CD4	191	76	13588
GOTERM_ negative r	1	0.3663	1	ATP1A2	191	9	13588
GOTERM_ negative r	1	0.3663	1	SERPING1	191	3	13588
GOTERM_ regulation	1	0.3663	1	IL1B	191	36	13588
GOTERM_ prostaglar	1	0.3663	1	PTGES	191	14	13588
GOTERM_ activation	1	0.3663	1	NOD1	191	33	13588
GOTERM_ tube lume	1	0.3663	1	EDAR	191	5	13588
GOTERM_ antigen re	1	0.3663	1	NFAM1	191	36	13588
GOTERM_ regulation	1	0.3663	1	NFAM1	191	2	13588
GOTERM_ mast cell a	1	0.3663	1	FCGR2B	191	15	13588
GOTERM_ regulation	1	0.3663	1	NTRK2	191	12	13588
GOTERM_ regulation	1	0.3663	1	CASP1	191	3	13588
GOTERM_ adult walk	1	0.3663	1	SCN1A	191	27	13588
GOTERM_ positive re	1	0.3663	1	ARHGAP6	191	13	13588
GOTERM_ intracellula	1	0.3663	1	SIX2	191	431	13588
GOTERM_ epithelial t	1	0.3663	1	RBPJ	191	14	13588
GOTERM_ response t	1	0.3663	1	IRS1	191	95	13588
GOTERM_ positive re	1	0.3663	1	CASP1	191	3	13588
GOTERM_ nuclear tra	1	0.3663	1	SIX2	191	98	13588
GOTERM_ negative r	1	0.3663	1	HSPA1A, H	191	22	13588
GOTERM_ alkene bio	1	0.3663	1	ALOX5	191	17	13588
GOTERM_ positive re	1	0.3663	1	GNA15	191	26	13588
GOTERM_ muscle tiss	1	0.3663	1	SMAD7	191	18	13588
GOTERM_ positive re	1	0.3663	1	CD4	191	16	13588
GOTERM_ embryonic	1	0.3663	1	ALDH1A3	191	15	13588
GOTERM_ ethanol m	1	0.3663	1	ADH1	191	4	13588
GOTERM_ negative r	1	0.3663	1	IL1B	191	27	13588
GOTERM_ detection	1	0.3663	1	TLR1	191	1	13588
GOTERM_ long-chai	1	0.3663	1	SLC27A3	191	15	13588
GOTERM_ excretion	1	0.3663	1	KIRREL	191	21	13588
GOTERM_ myoblast r	1	0.3663	1	SIX1	191	2	13588
GOTERM_ inositol tri	1	0.3663	1	PTAFR	191	4	13588
GOTERM_ acyl-CoA	1	0.3663	1	SLC27A3	191	20	13588
GOTERM_ regulation	1	0.3663	1	NEK6	191	36	13588
GOTERM_ positive re	1	0.3663	1	TLR7	191	4	13588
GOTERM_ negative r	1	0.3663	1	DLL1	191	2	13588
GOTERM_ DNA ligati	1	0.3663	1	GM13232,	191	4	13588
GOTERM_ ribonuclec	1	0.3663	1	ATP1A2	191	102	13588
GOTERM_ phosphoir	1	0.3663	1	GNA15	191	40	13588
GOTERM_ glutamine	1	0.3663	1	SLC1A3	191	43	13588
GOTERM_ determina	1	0.3663	1	DLL1	191	43	13588

GOTERM_ glutamate	1	0.3663	1	SLC1A3	191	3	13588
GOTERM_ regulation	1	0.3663	1	CASP1	191	7	13588
GOTERM_ skeletal m	1	0.3663	1	SIX1	191	74	13588
GOTERM_ tube lume	1	0.3663	1	EDAR	191	59	13588
GOTERM_ protein an	1	0.3663	1	CD4	191	1	13588
GOTERM_ negative r	1	0.3663	1	FCGR2B	191	3	13588
GOTERM_ cellular re:	1	0.3663	1	IRS1	191	58	13588
GOTERM_ positive re	1	0.3663	1	NOD1	191	36	13588
GOTERM_ activation	1	0.3663	1	GNA15	191	26	13588
GOTERM_ hormone	1	0.3663	1	ACE	191	5	13588
GOTERM_ regulation	1	0.3663	1	KLRK1	191	20	13588
GOTERM_ very-low-	1	0.3663	1	VLDLR	191	1	13588
GOTERM_ protein an	1	0.3663	1	GNA15	191	18	13588
GOTERM_ antigen pr	1	0.3663	1	FCGR2B	191	19	13588
GOTERM_ dorsal/ver	1	0.3663	1	GAS1	191	16	13588
GOTERM_ negative r	1	0.3663	1	FCGR2B	191	52	13588
GOTERM_ pituitary g	1	0.3663	1	RBPJ	191	29	13588
GOTERM_ acute infla	1	0.3663	1	IGHG1	191	5	13588
GOTERM_ female ga	1	0.3663	1	PGR	191	56	13588
GOTERM_ negative r	1	0.3663	1	FCGR2B	191	36	13588
GOTERM_ regulation	1	0.3663	1	GNA15	191	32	13588
GOTERM_ stem cell	1	0.3663	1	ZBTB16	191	10	13588
GOTERM_ regulation	1	0.3663	1	HTRA1	191	21	13588
GOTERM_ ventricula	1	0.3663	1	SMAD7	191	15	13588
GOTERM_ sensory pe	1	0.3663	1	CNGA2	191	1192	13588
GOTERM_ antibacter	1	0.3663	1	IGHG1	191	3	13588
GOTERM_ regulation	1	0.3663	1	CASP1	191	3	13588
GOTERM_ nuclear di	1	0.3663	1	NEK6	191	190	13588
GOTERM_ protein im	1	0.3663	1	SIX2	191	82	13588
GOTERM_ collagen c	1	0.3663	1	MMP19	191	17	13588
GOTERM_ positive re	1	0.3663	1	NOD1	191	8	13588
GOTERM_ positive re	1	0.3663	1	CD4	191	101	13588
GOTERM_ positive re	1	0.3663	1	BOC	191	4	13588
GOTERM_ diol metal	1	0.3663	1	CYP2D22	191	30	13588
GOTERM_ regulation	1	0.3663	1	SMAD7	191	1	13588
GOTERM_ negative c	1	0.3663	1	SEMA3A	191	4	13588
GOTERM_ positive re	1	0.3663	1	GNA15	191	26	13588
GOTERM_ positive re	1	0.3663	1	TLR7	191	9	13588
GOTERM_ chloride tr	1	0.3663	1	CLCA2	191	61	13588
GOTERM_ morphoge	1	0.3663	1	JAG1	191	7	13588
GOTERM_ positive re	1	0.3663	1	NOD1	191	5	13588
GOTERM_ regulation	1	0.3663	1	TRIM32	191	32	13588
GOTERM_ activation	1	0.3663	1	GAB1	191	13	13588
GOTERM_ ovulation	1	0.3663	1	PGR	191	11	13588
GOTERM_ positive re	1	0.3663	1	IGHG1	191	7	13588
GOTERM_ regulation	1	0.3663	1	IRS1	191	6	13588
GOTERM_ negative r	1	0.3663	1	SEMA3A	191	20	13588
GOTERM_ B cell rece	1	0.3663	1	NFAM1	191	14	13588
GOTERM_ regulation	1	0.3663	1	NUP62	191	181	13588
GOTERM_ cellular an	1	0.3663	1	SLC1A3	191	44	13588
GOTERM_ acylglycer	1	0.3663	1	DGAT2	191	12	13588
GOTERM_ negative r	1	0.3663	1	FCGR2B	191	17	13588
GOTERM_ negative r	1	0.3663	1	HTRA1	191	15	13588
GOTERM_ macropha	1	0.3663	1	TLR1	191	10	13588

Fold Enricl	Bonferron	Benjamini	FDR
5.058941	8.21E-04	8.21E-04	0.001012
3.895348	0.00239	0.001196	0.002948
3.334751	0.006213	0.002075	0.007677
3.171908	0.013008	0.003268	0.016128
7.904596	0.022293	0.004499	0.027769
4.516912	0.089602	0.015524	0.115577
4.890969	0.105218	0.015757	0.136864
5.983853	0.154443	0.020752	0.20645
11.85689	0.163638	0.019659	0.219892
11.85689	0.163638	0.019659	0.219892
6.617801	0.231442	0.02598	0.32376
4.207285	0.317463	0.034126	0.469407
5.081526	0.40319	0.042101	0.633839
4.707884	0.57659	0.063971	1.053133
4.136126	0.606621	0.06447	1.142767
11.85689	0.637689	0.065444	1.242909
5.221384	0.672286	0.067351	1.364934
5.221384	0.672286	0.067351	1.364934
2.409422	0.694874	0.067443	1.451669
2.405135	0.70223	0.065087	1.481295
7.904596	0.70592	0.062385	1.496427
10.16305	0.840795	0.087784	2.238275
5.595388	0.87018	0.092643	2.483702
5.533217	0.884971	0.093622	2.628908
2.634865	0.893213	0.092678	2.718059
6.775368	0.914533	0.097408	2.98459
15.80919	0.918099	0.095244	3.035505
3.274289	0.927668	0.096084	3.183799
6.566895	0.94079	0.099398	3.422259
6.566895	0.94079	0.099398	3.422259
5.187391	0.950551	0.101821	3.636354
3.453464	0.961984	0.106626	3.947971
4.930589	0.979412	0.121408	4.670928
3.355725	0.981141	0.120225	4.773897
8.272251	0.981203	0.116787	4.777747
2.81748	0.991716	0.135198	5.734064
3.248464	0.992615	0.134426	5.867435
5.616423	0.996286	0.14775	6.661066
1.788854	0.998766	0.169761	7.919484
10.53946	0.999724	0.19871	9.604497
5.081526	0.999809	0.201723	10.00968
5.081526	0.999809	0.201723	10.00968
3.233698	0.999872	0.205385	10.45717
6.351907	0.999966	0.227067	11.91936
4.850547	0.99997	0.224005	12.02287
2.891925	0.99997	0.219404	12.02863
2.115728	0.99999	0.234535	13.20135
2.253346	0.999996	0.246011	14.19213
8.89267	0.999998	0.254779	15.04281
5.737207	1	0.273443	16.55803
5.472412	1	0.307733	19.17998
16.41724	1	0.317636	20.22787
16.41724	1	0.317636	20.22787
3.127093	1	0.319753	20.75133
4.104309	1	0.336554	22.33232
15.24458	1	0.34051	23.0137
5.081526	1	0.347869	23.95612

2.979743	1	0.364634	25.63096
7.114136	1	0.363621	25.96692
7.114136	1	0.363621	25.96692
7.114136	1	0.363621	25.96692
3.342212	1	0.360064	26.09936
2.888989	1	0.394074	29.22199
2.624067	1	0.397688	29.95238
4.680353	1	0.396107	30.25799
3.171908	1	0.408131	31.69569
4.560344	1	0.412071	32.46985
2.561089	1	0.41777	33.39953
2.13959	1	0.415136	33.61334
6.323677	1	0.41185	33.76318
11.85689	1	0.417513	34.69498
11.85689	1	0.417513	34.69498
6.186205	1	0.420286	35.3757
6.186205	1	0.420286	35.3757
3.05515	1	0.424149	36.15632
3.05515	1	0.424149	36.15632
1.93394	1	0.441817	38.19902
2.302309	1	0.441315	38.59512
4.234605	1	0.444595	39.33836
2.878552	1	0.484453	43.52202
2.362628	1	0.504703	45.91051
2.829486	1	0.504094	46.31819
9.701095	1	0.50043	46.42649
2.540763	1	0.502537	47.08681
3.209385	1	0.505027	47.7827
9.279308	1	0.515536	49.26279
9.279308	1	0.515536	49.26279
47.42757	1	0.5177	49.92686
1.6986	1	0.514729	50.08099
3.784115	1	0.520484	51.0948
5.081526	1	0.522002	51.68677
5.081526	1	0.522002	51.68677
2.278549	1	0.522252	52.14908
2.706465	1	0.525239	52.88196
4.992376	1	0.524923	53.2807
3.07085	1	0.522031	53.41537
8.536963	1	0.531024	54.74625
3.629661	1	0.536533	55.72416
3.005973	1	0.53706	56.19508
2.391306	1	0.538571	56.76221
2.207835	1	0.543861	57.71022
2.207835	1	0.543861	57.71022
2.593695	1	0.552048	58.94985
2.361539	1	0.548973	59.04109
2.192713	1	0.545318	59.06818
4.665007	1	0.54545	59.47707
2.170414	1	0.553997	60.74132
4.516912	1	0.566896	62.44224
2.026819	1	0.56569	62.70497
4.446335	1	0.573579	63.88603
2.12715	1	0.574087	64.31457
2.12715	1	0.574087	64.31457
2.50246	1	0.572398	64.51697
2.465295	1	0.587329	66.39067
7.114136	1	0.590101	67.03278

7.114136	1	0.590101	67.03278
7.114136	1	0.590101	67.03278
2.092393	1	0.588638	67.24668
2.214517	1	0.60533	69.26197
1.938457	1	0.621203	71.16892
2.651231	1	0.630797	72.44145
2.349007	1	0.635815	73.25978
6.467396	1	0.632499	73.27305
2.155799	1	0.632754	73.62495
6.277179	1	0.645507	75.15597
6.277179	1	0.645507	75.15597
6.277179	1	0.645507	75.15597
6.277179	1	0.645507	75.15597
6.097831	1	0.661307	76.94096
2.284356	1	0.658273	76.96725
3.794206	1	0.663347	77.73424
5.928447	1	0.66996	78.6293
5.928447	1	0.66996	78.6293
2.084729	1	0.667474	78.69585
20.3261	1	0.679987	80.09446
20.3261	1	0.679987	80.09446
20.3261	1	0.679987	80.09446
20.3261	1	0.679987	80.09446
1.690488	1	0.690753	81.30868
2.845654	1	0.690908	81.59023
5.616423	1	0.689372	81.72337
2.778959	1	0.709526	83.67204
1.99695	1	0.708735	83.85409
17.78534	1	0.709932	84.19488
17.78534	1	0.709932	84.19488
17.78534	1	0.709932	84.19488
17.78534	1	0.709932	84.19488
17.78534	1	0.709932	84.19488
17.78534	1	0.709932	84.19488
17.78534	1	0.709932	84.19488
17.78534	1	0.709932	84.19488
5.335602	1	0.710213	84.45664
2.736206	1	0.713635	84.96512
3.428499	1	0.714139	85.23474
5.205465	1	0.717139	85.69507
2.34532	1	0.714888	85.74148
3.387684	1	0.715683	86.02142
3.387684	1	0.715683	86.02142
2.110125	1	0.712916	86.02456
2.694749	1	0.712204	86.18347
5.081526	1	0.718298	86.85227
2.294883	1	0.726447	87.65479
4.963351	1	0.727573	87.93157
3.233698	1	0.737924	88.85373
3.233698	1	0.737924	88.85373
4.742757	1	0.750193	89.87037
14.22827	1	0.750106	90.03625
14.22827	1	0.750106	90.03625
14.22827	1	0.750106	90.03625
14.22827	1	0.750106	90.03625
3.127093	1	0.757203	90.66659
4.639654	1	0.755778	90.73693
3.093103	1	0.760851	91.21551

4.540938	1	0.763624	91.53964
1.984022	1	0.762038	91.59223
3.059843	1	0.762012	91.73807
3.059843	1	0.762012	91.73807
12.93479	1	0.765488	92.08913
12.93479	1	0.765488	92.08913
12.93479	1	0.765488	92.08913
4.446335	1	0.766394	92.28202
2.995426	1	0.771445	92.70737
1.482112	1	0.769494	92.7292
2.436348	1	0.767842	92.76716
4.355594	1	0.769134	92.96753
2.419774	1	0.769612	93.11944
2.123623	1	0.767633	93.13585
4.268482	1	0.774077	93.59956
11.85689	1	0.774142	93.71915
1.908006	1	0.781535	94.20582
1.784109	1	0.780156	94.24552
4.104309	1	0.787749	94.71627
2.845654	1	0.785844	94.72697
10.94482	1	0.789913	95.0134
10.94482	1	0.789913	95.0134
10.94482	1	0.789913	95.0134
2.81748	1	0.789026	95.06832
2.789857	1	0.794212	95.39073
2.294883	1	0.793989	95.47001
3.952298	1	0.796354	95.65732
2.762771	1	0.795232	95.69498
1.461039	1	0.796663	95.83691
10.16305	1	0.799743	96.04101
10.16305	1	0.799743	96.04101
10.16305	1	0.799743	96.04101
10.16305	1	0.799743	96.04101
10.16305	1	0.799743	96.04101
2.23715	1	0.806198	96.36729
2.68458	1	0.80799	96.50656
2.65949	1	0.812686	96.74588
3.744282	1	0.812044	96.78984
3.744282	1	0.812044	96.78984
9.485515	1	0.812092	96.85692
9.485515	1	0.812092	96.85692
9.485515	1	0.812092	96.85692
2.634865	1	0.813558	96.97081
3.679726	1	0.815688	97.1034
1.93144	1	0.819704	97.29175
3.617357	1	0.821013	97.38894
2.155799	1	0.820354	97.4236
8.89267	1	0.821261	97.50472
8.89267	1	0.821261	97.50472
8.89267	1	0.821261	97.50472
3.557068	1	0.824401	97.64866
1.753484	1	0.825273	97.72323
3.498755	1	0.829392	97.88461
8.369572	1	0.832772	98.01903
8.369572	1	0.832772	98.01903
8.369572	1	0.832772	98.01903
8.369572	1	0.832772	98.01903
2.496188	1	0.832517	98.05569

2.080157	1	0.836726	98.20238
7.904596	1	0.844682	98.42737
3.334751	1	0.844924	98.4684
3.283447	1	0.850763	98.62724
2.391306	1	0.851776	98.67957
2.021061	1	0.850724	98.6893
1.808679	1	0.850985	98.7248
7.488564	1	0.85084	98.75156
7.488564	1	0.85084	98.75156
1.520837	1	0.852371	98.80927
2.351781	1	0.854396	98.8738
3.185434	1	0.854394	98.90019
1.417672	1	0.858228	98.99135
1.506523	1	0.856788	98.99151
7.114136	1	0.856395	99.00893
7.114136	1	0.856395	99.00893
2.31354	1	0.856983	99.04179
2.294883	1	0.860378	99.11694
1.404946	1	0.862032	99.16255
2.276524	1	0.862307	99.18668
6.775368	1	0.862803	99.21326
6.775368	1	0.862803	99.21326
6.775368	1	0.862803	99.21326
6.775368	1	0.862803	99.21326
6.775368	1	0.862803	99.21326
6.775368	1	0.862803	99.21326
6.775368	1	0.862803	99.21326
1.933189	1	0.864615	99.2569
1.721162	1	0.869288	99.33508
2.223168	1	0.87054	99.36681
6.467396	1	0.869945	99.37548
6.467396	1	0.869945	99.37548
6.467396	1	0.869945	99.37548
2.923618	1	0.874779	99.44601
2.923618	1	0.874779	99.44601
2.188965	1	0.875306	99.46571
1.707393	1	0.874261	99.46815
6.186205	1	0.876459	99.50425
6.186205	1	0.876459	99.50425
6.186205	1	0.876459	99.50425
6.186205	1	0.876459	99.50425
6.186205	1	0.876459	99.50425
6.186205	1	0.876459	99.50425
6.186205	1	0.876459	99.50425
2.884109	1	0.875504	99.50727
1.693842	1	0.874586	99.51063
1.403183	1	0.875528	99.53217
2.155799	1	0.876182	99.55019
2.155799	1	0.876182	99.55019
1.85264	1	0.875435	99.55471
2.845654	1	0.875009	99.56211
5.928447	1	0.878819	99.60648
5.928447	1	0.878819	99.60648
5.928447	1	0.878819	99.60648
5.928447	1	0.878819	99.60648
5.928447	1	0.878819	99.60648
5.928447	1	0.878819	99.60648
2.808212	1	0.87817	99.61115

1.561096	1	0.877733	99.61749
2.123623	1	0.877109	99.62218
2.107892	1	0.879936	99.65402
2.736206	1	0.884192	99.69407
1.635434	1	0.887316	99.72328
1.370739	1	0.886295	99.72396
5.472412	1	0.889611	99.75206
5.472412	1	0.889611	99.75206
5.472412	1	0.889611	99.75206
5.472412	1	0.889611	99.75206
5.472412	1	0.889611	99.75206
2.634865	1	0.894663	99.78772
2.634865	1	0.894663	99.78772
1.391896	1	0.895344	99.79716
2.018195	1	0.894509	99.79835
2.018195	1	0.894509	99.79835
5.26973	1	0.894379	99.8032
5.26973	1	0.894379	99.8032
5.26973	1	0.894379	99.8032
5.26973	1	0.894379	99.8032
1.34229	1	0.899644	99.83397
2.571375	1	0.898677	99.83422
1.592717	1	0.898434	99.83774
5.081526	1	0.898787	99.84379
5.081526	1	0.898787	99.84379
5.081526	1	0.898787	99.84379
5.081526	1	0.898787	99.84379
5.081526	1	0.898787	99.84379
1.949078	1	0.905057	99.87331
4.906301	1	0.90481	99.87601
4.906301	1	0.90481	99.87601
4.906301	1	0.90481	99.87601
2.481675	1	0.906681	99.8862
1.922739	1	0.908389	99.89518
1.353641	1	0.907576	99.8956
2.45315	1	0.907934	99.89975
4.742757	1	0.90759	99.90159
4.742757	1	0.90759	99.90159
4.742757	1	0.90759	99.90159
4.742757	1	0.90759	99.90159
4.742757	1	0.90759	99.90159
1.451865	1	0.906764	99.90192
4.589765	1	0.912866	99.92189
4.589765	1	0.912866	99.92189
4.589765	1	0.912866	99.92189
1.872141	1	0.914747	99.92892
2.371379	1	0.915037	99.93174
2.371379	1	0.915037	99.93174
4.446335	1	0.916905	99.93801
4.446335	1	0.916905	99.93801
4.446335	1	0.916905	99.93801
1.286462	1	0.920281	99.94687
2.319827	1	0.919684	99.94734
4.311598	1	0.920664	99.9508
4.311598	1	0.920664	99.9508
2.294883	1	0.921488	99.95379
4.184786	1	0.924959	99.96095
4.184786	1	0.924959	99.96095

1.270381	1	0.925632	99.9632
1.263612	1	0.92869	99.96865
2.223168	1	0.928114	99.9689
4.065221	1	0.927439	99.96901
4.065221	1	0.927439	99.96901
4.065221	1	0.927439	99.96901
4.065221	1	0.927439	99.96901
1.45682	1	0.926888	99.96928
1.767487	1	0.927929	99.97155
1.387157	1	0.927234	99.97162
2.200248	1	0.927444	99.97277
2.200248	1	0.927444	99.97277
3.952298	1	0.929028	99.97541
3.952298	1	0.929028	99.97541
3.952298	1	0.929028	99.97541
3.952298	1	0.929028	99.97541
1.166252	1	0.931471	99.97868
2.155799	1	0.931263	99.97918
3.845479	1	0.931945	99.98048
1.724639	1	0.931542	99.98076
1.724639	1	0.931542	99.98076
2.134241	1	0.93203	99.9818
2.134241	1	0.93203	99.9818
1.703985	1	0.933572	99.98365
1.703985	1	0.933572	99.98365
2.11311	1	0.933474	99.98411
3.744282	1	0.93331	99.98451
3.648275	1	0.937246	99.98771
2.052155	1	0.939496	99.98945
1.664125	1	0.939305	99.98971
3.557068	1	0.939658	99.99024
3.557068	1	0.939658	99.99024
1.482112	1	0.940113	99.99081
3.47031	1	0.942538	99.99226
3.47031	1	0.942538	99.99226
3.47031	1	0.942538	99.99226
3.47031	1	0.942538	99.99226
3.47031	1	0.942538	99.99226
1.994618	1	0.943744	99.99304
3.387684	1	0.945241	99.99386
3.387684	1	0.945241	99.99386
1.958019	1	0.947136	99.99473
3.308901	1	0.94778	99.99513
3.308901	1	0.94778	99.99513
3.308901	1	0.94778	99.99513
3.308901	1	0.94778	99.99513
1.940219	1	0.94819	99.99542
1.922739	1	0.949764	99.99602
1.922739	1	0.949764	99.99602
3.233698	1	0.949623	99.99613
1.905572	1	0.950752	99.99655
3.161838	1	0.95189	99.99693
3.161838	1	0.95189	99.99693
3.161838	1	0.95189	99.99693
1.888709	1	0.951714	99.99701
1.223937	1	0.953334	99.99744
3.093103	1	0.953518	99.99756
3.093103	1	0.953518	99.99756

1.855862	1	0.954065	99.99775
1.855862	1	0.954065	99.99775
1.546551	1	0.954068	99.99783
1.529922	1	0.954801	99.99803
1.529922	1	0.954801	99.99803
3.027292	1	0.954567	99.99807
3.027292	1	0.954567	99.99807
3.027292	1	0.954567	99.99807
1.513646	1	0.956956	99.99846
1.513646	1	0.956956	99.99846
2.964223	1	0.956518	99.99847
2.964223	1	0.956518	99.99847
1.79348	1	0.958369	99.99874
1.79348	1	0.958369	99.99874
2.903729	1	0.95836	99.99878
2.903729	1	0.95836	99.99878
2.845654	1	0.960541	99.99903
2.845654	1	0.960541	99.99903
2.845654	1	0.960541	99.99903
2.845654	1	0.960541	99.99903
2.845654	1	0.960541	99.99903
2.845654	1	0.960541	99.99903
2.845654	1	0.960541	99.99903
1.474432	1	0.960678	99.99908
1.749378	1	0.961496	99.99918
1.347374	1	0.96122	99.9992
2.789857	1	0.961319	99.99923
2.789857	1	0.961319	99.99923
2.789857	1	0.961319	99.99923
2.789857	1	0.961319	99.99923
2.789857	1	0.961319	99.99923
2.789857	1	0.961319	99.99923
1.332235	1	0.961764	99.99929
1.721162	1	0.96288	99.99939
2.736206	1	0.962477	99.99939
2.736206	1	0.962477	99.99939
2.736206	1	0.962477	99.99939
2.736206	1	0.962477	99.99939
2.736206	1	0.962477	99.99939
1.451865	1	0.963279	99.99946
1.707393	1	0.96314	99.99948
1.707393	1	0.96314	99.99948
2.68458	1	0.963579	99.99952
2.68458	1	0.963579	99.99952
2.586959	1	0.967498	99.9997
1.65445	1	0.967541	99.99971
1.641724	1	0.968464	99.99975
1.641724	1	0.968464	99.99975
2.540763	1	0.968395	99.99976
2.496188	1	0.969945	99.99981
2.496188	1	0.969945	99.99981
2.496188	1	0.969945	99.99981
1.270381	1	0.970345	99.99983
1.604692	1	0.970727	99.99984
2.45315	1	0.970742	99.99985
1.252489	1	0.971915	99.99988
1.252489	1	0.971915	99.99988
1.252489	1	0.971915	99.99988

1.248094	1	0.972361	99.99989
2.371379	1	0.973175	99.9999
2.371379	1	0.973175	99.9999
2.371379	1	0.973175	99.9999
2.332504	1	0.974449	99.99992
1.169447	1	0.975224	99.99994
2.294883	1	0.975365	99.99994
2.294883	1	0.975365	99.99994
2.294883	1	0.975365	99.99994
2.294883	1	0.975365	99.99994
2.223168	1	0.977879	99.99996
1.147441	1	0.97843	99.99997
2.188965	1	0.978645	99.99997
2.188965	1	0.978645	99.99997
2.188965	1	0.978645	99.99997
2.188965	1	0.978645	99.99997
1.482112	1	0.978505	99.99997
1.081998	1	0.97954	99.99998
2.123623	1	0.980316	99.99998
2.123623	1	0.980316	99.99998
1.451865	1	0.980209	99.99998
1.259139	1	0.980647	99.99998
2.092393	1	0.98074	99.99999
2.062068	1	0.981603	99.99999
2.062068	1	0.981603	99.99999
2.03261	1	0.982423	99.99999
2.03261	1	0.982423	99.99999
1.154892	1	0.982324	99.99999
2.003982	1	0.98299	99.99999
2.003982	1	0.98299	99.99999
1.147441	1	0.982864	99.99999
1.385871	1	0.983426	99.99999
1.976149	1	0.983327	99.99999
1.368103	1	0.984289	100
1.359389	1	0.984701	100
1.075184	1	0.985798	100
1.048399	1	0.98584	100
1.872141	1	0.985843	100
1.872141	1	0.985843	100
1.325615	1	0.986235	100
1.064459	1	0.98649	100
1.824137	1	0.986847	100
1.091125	1	0.987412	100
1.778534	1	0.987922	100
1.000425	1	0.987822	100
1.02047	1	0.988403	100
1.041093	1	0.988374	100
1.133727	1	0.98854	100
1.111584	1	0.990108	100
1.021168	1	0.990131	100
1.635434	1	0.990969	100
1.635434	1	0.990969	100
1.635434	1	0.990969	100
1.616849	1	0.991329	100
1.616849	1	0.991329	100
0.974539	1	0.991473	100
1.199012	1	0.991445	100
0.984169	1	0.991436	100

1.598682	1	0.991325	100
1.580919	1	0.991664	100
1.580919	1	0.991664	100
0.97645	1	0.991884	100
0.99267	1	0.991799	100
1.546551	1	0.992193	100
1.546551	1	0.992193	100
0.983521	1	0.992391	100
1.513646	1	0.99278	100
1.046196	1	0.992838	100
0.993594	1	0.993225	100
1.482112	1	0.993224	100
1.451865	1	0.993809	100
1.422827	1	0.994341	100
1.422827	1	0.994341	100
1.40874	1	0.99455	100
0.923914	1	0.994779	100
1.34229	1	0.995666	100
1.329745	1	0.995822	100
1.281826	1	0.996545	100
1.281826	1	0.996545	100
0.891744	1	0.996535	100
1.259139	1	0.996783	100
0.997309	1	0.997093	100
0.997309	1	0.997093	100
0.997309	1	0.997093	100
1.226575	1	0.997151	100
1.216092	1	0.997248	100
1.205786	1	0.997342	100
1.195653	1	0.997433	100
1.17589	1	0.997641	100
0.957059	1	0.997734	100
1.120336	1	0.998195	100
0.923914	1	0.998276	100
0.855064	1	0.998275	100
1.094482	1	0.998365	100
1.094482	1	0.998365	100
0.848942	1	0.998367	100
0.859714	1	0.998472	100
1.061811	1	0.998588	100
1.046196	1	0.998698	100
1.016305	1	0.998911	100
0.821494	1	0.998894	100
0.821494	1	0.998894	100
1.009097	1	0.998926	100
0.994984	1	0.999008	100
0.836957	1	0.999166	100
0.836957	1	0.999166	100
0.929952	1	0.999367	100
0.855407	1	0.999401	100
0.763319	1	0.999511	100
0.8729	1	0.999586	100
0.851992	1	0.999651	100
0.84191	1	0.999677	100
0.737216	1	0.999682	100
0.822443	1	0.999722	100
0.813044	1	0.999742	100
0.808425	1	0.999749	100

0.808425	1	0.999749	100
0.706702	1	0.999829	100
0.695759	1	0.999838	100
0.695759	1	0.999838	100
0.7009	1	0.99986	100
0.744936	1	0.999865	100
0.652673	1	0.999961	100
0.72841	1	0.999964	100
0.640913	1	0.999966	100
0.717151	1	0.999977	100
0.595325	1	0.999984	100
0.583126	1	0.999987	100
0.580746	1	0.999987	100
0.576043	1	0.999988	100
0.551483	1	0.999993	100
0.560168	1	0.999993	100
0.560168	1	0.999993	100
0.543064	1	0.999993	100
0.528277	1	0.999995	100
0.532894	1	0.999997	100
0.506344	1	0.999997	100
0.529917	1	0.999997	100
0.566863	1	0.999997	100
0.506946	1	0.999997	100
0.546401	1	0.999997	100
0.495759	1	0.999997	100
0.542236	1	0.999998	100
0.511808	1	0.999998	100
0.670844	1	1	100
0.465737	1	1	100
0.352185	1	1	100
0.441622	1	1	100
0.296246	1	1	100
0.265311	1	1	100
0.192274	1	1	100
0.152228	1	1	100
3.952298	1	1	100
1.129228	1	1	100
3.557068	1	1	100
4.446335	1	1	100
3.744282	1	1	100
2.540763	1	1	100
17.78534	1	1	100
8.89267	1	1	100
1.077899	1	1	100
71.14136	1	1	100
2.540763	1	1	100
14.22827	1	1	100
8.89267	1	1	100
2.540763	1	1	100
2.45315	1	1	100
0.889267	1	1	100
8.89267	1	1	100
10.16305	1	1	100
2.540763	1	1	100
10.16305	1	1	100
7.904596	1	1	100
10.16305	1	1	100

3.387684	1	1	100
1.616849	1	1	100
8.89267	1	1	100
2.092393	1	1	100
0.756823	1	1	100
1.824137	1	1	100
17.78534	1	1	100
2.371379	1	1	100
3.387684	1	1	100
4.446335	1	1	100
1.248094	1	1	100
14.22827	1	1	100
1.001991	1	1	100
3.387684	1	1	100
35.57068	1	1	100
17.78534	1	1	100
0.846921	1	1	100
2.223168	1	1	100
10.16305	1	1	100
5.081526	1	1	100
1.693842	1	1	100
2.03261	1	1	100
0.988074	1	1	100
11.85689	1	1	100
2.964223	1	1	100
1.129228	1	1	100
5.472412	1	1	100
8.89267	1	1	100
14.22827	1	1	100
71.14136	1	1	100
0.312024	1	1	100
1.872141	1	1	100
4.742757	1	1	100
0.733416	1	1	100
5.472412	1	1	100
3.557068	1	1	100
0.397438	1	1	100
0.936071	1	1	100
1.513646	1	1	100
3.557068	1	1	100
0.237931	1	1	100
4.742757	1	1	100
71.14136	1	1	100
1.693842	1	1	100
4.742757	1	1	100
2.371379	1	1	100
35.57068	1	1	100
35.57068	1	1	100
5.472412	1	1	100
17.78534	1	1	100
2.845654	1	1	100
1.65445	1	1	100
5.472412	1	1	100
3.093103	1	1	100
6.467396	1	1	100
11.85689	1	1	100
7.904596	1	1	100
0.756823	1	1	100

1.451865	1	1	100
3.952298	1	1	100
2.092393	1	1	100
0.361124	1	1	100
2.223168	1	1	100
0.867578	1	1	100
5.081526	1	1	100
7.904596	1	1	100
1.34229	1	1	100
7.904596	1	1	100
35.57068	1	1	100
1.65445	1	1	100
2.634865	1	1	100
0.70437	1	1	100
1.65445	1	1	100
10.16305	1	1	100
1.226575	1	1	100
0.291563	1	1	100
7.114136	1	1	100
3.093103	1	1	100
1.616849	1	1	100
7.114136	1	1	100
5.081526	1	1	100
7.114136	1	1	100
0.974539	1	1	100
23.71379	1	1	100
14.22827	1	1	100
4.184786	1	1	100
8.89267	1	1	100
5.081526	1	1	100
1.111584	1	1	100
1.166252	1	1	100
5.928447	1	1	100
1.513646	1	1	100
5.081526	1	1	100
1.513646	1	1	100
1.976149	1	1	100
7.114136	1	1	100
0.547241	1	1	100
1.394929	1	1	100
14.22827	1	1	100
3.952298	1	1	100
3.387684	1	1	100
8.89267	1	1	100
5.081526	1	1	100
8.89267	1	1	100
5.472412	1	1	100
17.78534	1	1	100
10.16305	1	1	100
3.093103	1	1	100
11.85689	1	1	100
17.78534	1	1	100
3.387684	1	1	100
0.441872	1	1	100
10.16305	1	1	100
17.78534	1	1	100
5.472412	1	1	100
0.764961	1	1	100

4.742757	1	1	100
17.78534	1	1	100
3.093103	1	1	100
7.114136	1	1	100
7.114136	1	1	100
7.114136	1	1	100
2.736206	1	1	100
3.744282	1	1	100
17.78534	1	1	100
14.22827	1	1	100
10.16305	1	1	100
1.205786	1	1	100
0.225846	1	1	100
2.45315	1	1	100
4.184786	1	1	100
10.16305	1	1	100
3.233698	1	1	100
1.513646	1	1	100
1.34229	1	1	100
0.671145	1	1	100
17.78534	1	1	100
0.799341	1	1	100
10.16305	1	1	100
17.78534	1	1	100
8.89267	1	1	100
17.78534	1	1	100
71.14136	1	1	100
2.371379	1	1	100
8.89267	1	1	100
1.111584	1	1	100
3.387684	1	1	100
23.71379	1	1	100
3.233698	1	1	100
1.922739	1	1	100
5.472412	1	1	100
5.472412	1	1	100
10.16305	1	1	100
1.077899	1	1	100
2.845654	1	1	100
1.976149	1	1	100
1.922739	1	1	100
10.16305	1	1	100
0.444634	1	1	100
1.824137	1	1	100
10.16305	1	1	100
4.446335	1	1	100
14.22827	1	1	100
17.78534	1	1	100
0.912069	1	1	100
1.922739	1	1	100
17.78534	1	1	100
0.781773	1	1	100
1.34229	1	1	100
17.78534	1	1	100
2.223168	1	1	100
5.928447	1	1	100
2.03261	1	1	100
4.742757	1	1	100

23.71379	1	1	100
1.693842	1	1	100
1.735155	1	1	100
1.34229	1	1	100
23.71379	1	1	100
35.57068	1	1	100
2.092393	1	1	100
4.446335	1	1	100
7.904596	1	1	100
0.988074	1	1	100
4.446335	1	1	100
2.371379	1	1	100
14.22827	1	1	100
1.922739	1	1	100
4.446335	1	1	100
0.624047	1	1	100
0.96137	1	1	100
4.184786	1	1	100
2.371379	1	1	100
1.778534	1	1	100
71.14136	1	1	100
5.081526	1	1	100
11.85689	1	1	100
2.371379	1	1	100
5.472412	1	1	100
2.736206	1	1	100
2.371379	1	1	100
1.422827	1	1	100
10.16305	1	1	100
11.85689	1	1	100
5.472412	1	1	100
6.467396	1	1	100
2.294883	1	1	100
4.184786	1	1	100
5.928447	1	1	100
5.928447	1	1	100
5.472412	1	1	100
1.976149	1	1	100
23.71379	1	1	100
71.14136	1	1	100
7.904596	1	1	100
10.16305	1	1	100
71.14136	1	1	100
5.928447	1	1	100
0.912069	1	1	100
11.85689	1	1	100
0.640913	1	1	100
7.904596	1	1	100
1.482112	1	1	100
14.22827	1	1	100
5.928447	1	1	100
1.422827	1	1	100
1.270381	1	1	100
1.580919	1	1	100
35.57068	1	1	100
2.540763	1	1	100
0.781773	1	1	100
0.857125	1	1	100

5.472412	1	1	100
1.270381	1	1	100
5.928447	1	1	100
23.71379	1	1	100
2.371379	1	1	100
3.093103	1	1	100
35.57068	1	1	100
5.928447	1	1	100
2.540763	1	1	100
71.14136	1	1	100
1.735155	1	1	100
4.742757	1	1	100
17.78534	1	1	100
4.184786	1	1	100
0.51928	1	1	100
2.634865	1	1	100
1.394929	1	1	100
4.446335	1	1	100
1.824137	1	1	100
1.317433	1	1	100
1.226575	1	1	100
1.111584	1	1	100
6.467396	1	1	100
23.71379	1	1	100
0.764961	1	1	100
1.34229	1	1	100
4.446335	1	1	100
0.79046	1	1	100
1.482112	1	1	100
14.22827	1	1	100
1.016305	1	1	100
71.14136	1	1	100
1.270381	1	1	100
0.889267	1	1	100
3.952298	1	1	100
1.270381	1	1	100
0.878288	1	1	100
4.446335	1	1	100
5.472412	1	1	100
71.14136	1	1	100
14.22827	1	1	100
11.85689	1	1	100
1.016305	1	1	100
1.185689	1	1	100
5.472412	1	1	100
2.845654	1	1	100
6.467396	1	1	100
1.205786	1	1	100
1.482112	1	1	100
1.368103	1	1	100
0.374428	1	1	100
6.467396	1	1	100
7.114136	1	1	100
5.472412	1	1	100
35.57068	1	1	100
1.111584	1	1	100
1.422827	1	1	100
0.711414	1	1	100

1.824137	1	1	100
2.964223	1	1	100
35.57068	1	1	100
4.446335	1	1	100
1.317433	1	1	100
5.081526	1	1	100
4.446335	1	1	100
1.077899	1	1	100
1.482112	1	1	100
3.744282	1	1	100
0.857125	1	1	100
3.744282	1	1	100
1.976149	1	1	100
17.78534	1	1	100
2.092393	1	1	100
71.14136	1	1	100
4.446335	1	1	100
2.092393	1	1	100
8.89267	1	1	100
0.658716	1	1	100
0.618621	1	1	100
1.65445	1	1	100
2.03261	1	1	100
0.569131	1	1	100
8.89267	1	1	100
2.634865	1	1	100
1.513646	1	1	100
2.634865	1	1	100
23.71379	1	1	100
5.928447	1	1	100
10.16305	1	1	100
71.14136	1	1	100
1.077899	1	1	100
2.845654	1	1	100
14.22827	1	1	100
5.081526	1	1	100
1.976149	1	1	100
6.467396	1	1	100
2.03261	1	1	100
0.534897	1	1	100
1.482112	1	1	100
2.845654	1	1	100
5.928447	1	1	100
0.773276	1	1	100
3.233698	1	1	100
3.952298	1	1	100
35.57068	1	1	100
4.742757	1	1	100
14.22827	1	1	100
8.89267	1	1	100
5.928447	1	1	100
0.857125	1	1	100
17.78534	1	1	100
1.077899	1	1	100
14.22827	1	1	100
3.744282	1	1	100
0.251383	1	1	100
0.408858	1	1	100

71.14136	1	1	100
17.78534	1	1	100
5.928447	1	1	100
71.14136	1	1	100
0.618621	1	1	100
1.248094	1	1	100
0.608046	1	1	100
0.773276	1	1	100
5.081526	1	1	100
71.14136	1	1	100
10.16305	1	1	100
0.534897	1	1	100
0.764961	1	1	100
5.472412	1	1	100
2.155799	1	1	100
3.952298	1	1	100
3.233698	1	1	100
1.205786	1	1	100
23.71379	1	1	100
1.317433	1	1	100
4.742757	1	1	100
5.928447	1	1	100
0.7186	1	1	100
0.781773	1	1	100
1.368103	1	1	100
14.22827	1	1	100
10.16305	1	1	100
5.472412	1	1	100
2.294883	1	1	100
0.733416	1	1	100
4.184786	1	1	100
3.557068	1	1	100
7.114136	1	1	100
10.16305	1	1	100
7.114136	1	1	100
5.928447	1	1	100
0.257759	1	1	100
17.78534	1	1	100
1.872141	1	1	100
11.85689	1	1	100
5.081526	1	1	100
1.824137	1	1	100
1.205786	1	1	100
71.14136	1	1	100
2.223168	1	1	100
2.223168	1	1	100
14.22827	1	1	100
2.540763	1	1	100
23.71379	1	1	100
17.78534	1	1	100
7.904596	1	1	100
4.184786	1	1	100
17.78534	1	1	100
7.114136	1	1	100
1.65445	1	1	100
0.733416	1	1	100
10.16305	1	1	100
0.23635	1	1	100

3.744282	1	1	100
1.513646	1	1	100
17.78534	1	1	100
1.185689	1	1	100
0.272572	1	1	100
71.14136	1	1	100
3.744282	1	1	100
1.693842	1	1	100
4.446335	1	1	100
0.504549	1	1	100
5.081526	1	1	100
4.446335	1	1	100
1.693842	1	1	100
23.71379	1	1	100
0.741056	1	1	100
14.22827	1	1	100
1.147441	1	1	100
1.824137	1	1	100
7.114136	1	1	100
1.872141	1	1	100
1.205786	1	1	100
7.114136	1	1	100
3.557068	1	1	100
0.70437	1	1	100
3.387684	1	1	100
5.928447	1	1	100
14.22827	1	1	100
5.472412	1	1	100
0.684052	1	1	100
0.366708	1	1	100
2.540763	1	1	100
71.14136	1	1	100
5.928447	1	1	100
2.634865	1	1	100
3.952298	1	1	100
17.78534	1	1	100
35.57068	1	1	100
2.540763	1	1	100
1.016305	1	1	100
0.301446	1	1	100
3.387684	1	1	100
17.78534	1	1	100
4.742757	1	1	100
17.78534	1	1	100
2.634865	1	1	100
2.45315	1	1	100
10.16305	1	1	100
2.540763	1	1	100
0.461957	1	1	100
2.845654	1	1	100
14.22827	1	1	100
1.580919	1	1	100
2.03261	1	1	100
0.936071	1	1	100
3.093103	1	1	100
17.78534	1	1	100
23.71379	1	1	100
2.155799	1	1	100

23.71379	1	1	100
1.34229	1	1	100
5.081526	1	1	100
2.155799	1	1	100
23.71379	1	1	100
71.14136	1	1	100
0.923914	1	1	100
5.081526	1	1	100
0.664873	1	1	100
3.233698	1	1	100
23.71379	1	1	100
0.608046	1	1	100
0.216894	1	1	100
2.45315	1	1	100
1.001991	1	1	100
1.293479	1	1	100
1.922739	1	1	100
1.147441	1	1	100
10.16305	1	1	100
2.223168	1	1	100
0.06369	1	1	100
1.34229	1	1	100
0.988074	1	1	100
71.14136	1	1	100
0.857125	1	1	100
4.742757	1	1	100
23.71379	1	1	100
1.147441	1	1	100
5.081526	1	1	100
23.71379	1	1	100
17.78534	1	1	100
1.616849	1	1	100
0.658716	1	1	100
2.964223	1	1	100
1.65445	1	1	100
35.57068	1	1	100
3.744282	1	1	100
71.14136	1	1	100
0.889267	1	1	100
0.725932	1	1	100
3.952298	1	1	100
71.14136	1	1	100
6.467396	1	1	100
5.928447	1	1	100
10.16305	1	1	100
0.337163	1	1	100
0.748856	1	1	100
7.114136	1	1	100
2.45315	1	1	100
2.964223	1	1	100
4.742757	1	1	100
1.616849	1	1	100
35.57068	1	1	100
0.912069	1	1	100
0.96137	1	1	100
0.764961	1	1	100
0.867578	1	1	100
0.597827	1	1	100

0.808425	1	1	100
14.22827	1	1	100
2.634865	1	1	100
5.081526	1	1	100
0.397438	1	1	100
0.988074	1	1	100
3.744282	1	1	100
3.093103	1	1	100
4.446335	1	1	100
3.557068	1	1	100
1.394929	1	1	100
23.71379	1	1	100
11.85689	1	1	100
3.233698	1	1	100
4.446335	1	1	100
1.422827	1	1	100
4.184786	1	1	100
23.71379	1	1	100
2.736206	1	1	100
3.387684	1	1	100
5.081526	1	1	100
0.497492	1	1	100
1.248094	1	1	100
0.808425	1	1	100
71.14136	1	1	100
1.824137	1	1	100
0.64674	1	1	100
14.22827	1	1	100
3.557068	1	1	100
7.904596	1	1	100
2.845654	1	1	100
17.78534	1	1	100
1.482112	1	1	100
2.155799	1	1	100
0.624047	1	1	100
3.387684	1	1	100
1.976149	1	1	100
14.22827	1	1	100
8.89267	1	1	100
3.557068	1	1	100
17.78534	1	1	100
1.824137	1	1	100
0.889267	1	1	100
1.922739	1	1	100
3.557068	1	1	100
71.14136	1	1	100
8.89267	1	1	100
5.928447	1	1	100
3.233698	1	1	100
1.061811	1	1	100
11.85689	1	1	100
2.845654	1	1	100
2.03261	1	1	100
1.65445	1	1	100
5.928447	1	1	100
2.294883	1	1	100
71.14136	1	1	100
7.114136	1	1	100

0.900524	1	1	100
71.14136	1	1	100
23.71379	1	1	100
0.602893	1	1	100
4.742757	1	1	100
17.78534	1	1	100
7.114136	1	1	100
4.742757	1	1	100
0.390887	1	1	100
2.03261	1	1	100
1.226575	1	1	100
6.467396	1	1	100
35.57068	1	1	100
0.441872	1	1	100
1.001991	1	1	100
1.077899	1	1	100
2.634865	1	1	100
0.936071	1	1	100
7.904596	1	1	100
23.71379	1	1	100
1.976149	1	1	100
5.081526	1	1	100
2.155799	1	1	100
14.22827	1	1	100
1.976149	1	1	100
35.57068	1	1	100
4.742757	1	1	100
5.928447	1	1	100
23.71379	1	1	100
2.634865	1	1	100
5.472412	1	1	100
0.165061	1	1	100
5.081526	1	1	100
0.748856	1	1	100
23.71379	1	1	100
0.725932	1	1	100
3.233698	1	1	100
4.184786	1	1	100
2.736206	1	1	100
3.952298	1	1	100
4.446335	1	1	100
4.742757	1	1	100
17.78534	1	1	100
2.634865	1	1	100
71.14136	1	1	100
4.742757	1	1	100
3.387684	1	1	100
35.57068	1	1	100
17.78534	1	1	100
3.557068	1	1	100
1.976149	1	1	100
17.78534	1	1	100
35.57068	1	1	100
17.78534	1	1	100
0.697464	1	1	100
1.778534	1	1	100
1.65445	1	1	100
1.65445	1	1	100

23.71379	1	1	100
10.16305	1	1	100
0.96137	1	1	100
1.205786	1	1	100
71.14136	1	1	100
23.71379	1	1	100
1.226575	1	1	100
1.976149	1	1	100
2.736206	1	1	100
14.22827	1	1	100
3.557068	1	1	100
71.14136	1	1	100
3.952298	1	1	100
3.744282	1	1	100
4.446335	1	1	100
1.368103	1	1	100
2.45315	1	1	100
14.22827	1	1	100
1.270381	1	1	100
1.976149	1	1	100
2.223168	1	1	100
7.114136	1	1	100
3.387684	1	1	100
4.742757	1	1	100
0.059682	1	1	100
23.71379	1	1	100
23.71379	1	1	100
0.374428	1	1	100
0.867578	1	1	100
4.184786	1	1	100
8.89267	1	1	100
0.70437	1	1	100
17.78534	1	1	100
2.371379	1	1	100
71.14136	1	1	100
17.78534	1	1	100
2.736206	1	1	100
7.904596	1	1	100
1.166252	1	1	100
10.16305	1	1	100
14.22827	1	1	100
2.223168	1	1	100
5.472412	1	1	100
6.467396	1	1	100
10.16305	1	1	100
11.85689	1	1	100
3.557068	1	1	100
5.081526	1	1	100
0.393046	1	1	100
1.616849	1	1	100
5.928447	1	1	100
4.184786	1	1	100
4.742757	1	1	100
7.114136	1	1	100
