# Supplementary material for: Transcriptome analysis reveals a potential regulatory mechanism of the lnc-5423.6/IGFBP5 axis in the early stages of mouse thymic involution: lnc-5423.6/IGFBP5 axis regulates thymic involution
Source: Acta Biochim Biophys Sin (Shanghai). 2023 Apr 19;55(4):548–60. doi: 10.3724/abbs.2023042 (PMC10195152; doi:10.3724/abbs.2023042)
Supplement: Table_S5 [file Table_S5.pdf]

gene_na	fc	log2(fc	pval	regulat	significant
Rn7s6	0.08	-3.61	0.00	down	yes
Ighg1	11.31	3.50	0.00	up	yes
Ifi47;O	11.22	3.49	0.00	up	yes
Car3	10.94	3.45	0.00	up	yes
Scd1	9.40	3.23	0.00	up	yes
Zswim6	0.12	-3.07	0.00	down	yes
Igkv1-1	7.41	2.89	0.00	up	yes
Scn7a	7.45	2.90	0.00	up	yes
Zfp422	0.15	-2.72	0.00	down	yes
Igkv10-	6.89	2.78	0.00	up	yes
Gpx1	6.18	2.63	0.00	up	yes
Gas7	6.06	2.60	0.00	up	yes
Igkc	5.87	2.55	0.00	up	yes
C1qc	5.88	2.55	0.00	up	yes
Iglc2;I	5.95	2.57	0.00	up	yes
Jchain	5.64	2.50	0.00	up	yes
Uhmk1	0.18	-2.45	0.00	down	yes
Gm22146	0.18	-2.44	0.00	down	yes
Klrk1	5.51	2.46	0.00	up	yes
A130051	0.20	-2.36	0.00	down	yes
Kdelr3	5.24	2.39	0.00	up	yes
Scn1a	5.11	2.35	0.00	up	yes
Dnah14	4.96	2.31	0.00	up	yes
Ccl11	4.91	2.30	0.00	up	yes
Zfand2a	0.21	-2.25	0.00	down	yes
Ighg3	4.90	2.29	0.00	up	yes
Ighv3-6	4.86	2.28	0.00	up	yes
Cdkn2d	0.22	-2.20	0.00	down	yes
Luzp1	4.70	2.23	0.00	up	yes
Deptor	4.71	2.24	0.00	up	yes
1810062	0.22	-2.18	0.00	down	yes
Myo16	4.69	2.23	0.00	up	yes
Slc16a2	4.74	2.25	0.00	up	yes
Tns4	4.62	2.21	0.00	up	yes
Plag1	0.23	-2.14	0.00	down	yes
Ces1d	4.51	2.17	0.00	up	yes
Gm8189	4.33	2.11	0.00	up	yes
Cnga1	0.23	-2.12	0.00	down	yes
Aebp1	4.36	2.13	0.00	up	yes
Mir29c;I	4.37	2.13	0.00	up	yes
C920009	4.26	2.09	0.00	up	yes
Bfsp2	0.24	-2.05	0.00	down	yes
C1ra	4.29	2.10	0.00	up	yes
C4b	4.26	2.09	0.00	up	yes
Dcn	4.22	2.08	0.00	up	yes
Ryr3	4.29	2.10	0.00	up	yes
Klhl32	4.20	2.07	0.00	up	yes
Kcnh1	4.19	2.07	0.00	up	yes
Siglec1	4.14	2.05	0.00	up	yes
Srxn1	4.14	2.05	0.00	up	yes
Adam28;	4.05	2.02	0.00	up	yes
Parva	4.03	2.01	0.00	up	yes
Cyp2f2	4.06	2.02	0.00	up	yes
Dnal1	4.08	2.03	0.00	up	yes
Gm7609	4.13	2.05	0.00	up	yes
Rsad2	4.04	2.01	0.00	up	yes

Shroom4	4.03	2.01	0.00	up	yes
Map2	4.02	2.01	0.00	up	yes
Abca8a	3.98	1.99	0.00	up	yes
Prelp	3.92	1.97	0.00	up	yes
Avpr2	0.26	-1.92	0.00	down	yes
Eomes	3.90	1.96	0.00	up	yes
Wipi1	3.94	1.98	0.00	up	yes
Nr2f2	3.95	1.98	0.00	up	yes
Samd3	3.93	1.98	0.00	up	yes
Mgll	3.86	1.95	0.00	up	yes
Igf2bp3	0.27	-1.90	0.00	down	yes
Ntn1	0.27	-1.89	0.00	down	yes
Rxfp2	3.88	1.95	0.01	up	yes
Fcmr	3.84	1.94	0.01	up	yes
Cfd	3.82	1.93	0.01	up	yes
Nr1d1	3.67	1.88	0.01	up	yes
Cxcr3	3.71	1.89	0.01	up	yes
Ptprz1	3.65	1.87	0.01	up	yes
Slfn3	0.29	-1.81	0.01	down	yes
Trpm6	3.63	1.86	0.01	up	yes
Degs1	3.55	1.83	0.01	up	yes
Gm24924	0.29	-1.78	0.01	down	yes
Tlr7	3.53	1.82	0.01	up	yes
Capn12	0.29	-1.78	0.01	down	yes
Ifi206	3.51	1.81	0.01	up	yes
Prom2	3.49	1.80	0.01	up	yes
Vcp-rs	3.44	1.78	0.01	up	yes
Rnf185	0.30	-1.74	0.01	down	yes
Coq2	0.30	-1.74	0.01	down	yes
Emp1	0.30	-1.73	0.01	down	yes
Kcnq5	0.30	-1.72	0.01	down	yes
Scara5	3.48	1.80	0.01	up	yes
Htra3	3.44	1.78	0.01	up	yes
Gm26075	0.31	-1.71	0.01	down	yes
Il33	3.45	1.79	0.01	up	yes
Igkv1-1	3.40	1.76	0.01	up	yes
Ndufaf4	3.39	1.76	0.01	up	yes
Cpxm1	3.44	1.78	0.01	up	yes
Xpnpep3	3.37	1.75	0.01	up	yes
Zfp703	3.35	1.74	0.01	up	yes
Per3	3.35	1.75	0.01	up	yes
Gm16026	3.33	1.74	0.01	up	yes
Ifi44	3.32	1.73	0.01	up	yes
Blk	3.33	1.74	0.01	up	yes
Pdpn	3.37	1.75	0.01	up	yes
Mgl2	3.39	1.76	0.01	up	yes
Ifit3	3.32	1.73	0.01	up	yes
Ifi44l	3.32	1.73	0.01	up	yes
Pgr	3.37	1.75	0.01	up	yes
Serping	3.29	1.72	0.01	up	yes
5830411	3.29	1.72	0.01	up	yes
Ikzf3;M	0.32	-1.66	0.01	down	yes
Spock2	3.24	1.70	0.01	up	yes
F830016	3.29	1.72	0.01	up	yes
Rusc2	3.36	1.75	0.01	up	yes
Xrra1	0.32	-1.65	0.01	down	yes
Gxylt2	3.26	1.70	0.01	up	yes

Cradd	0.32	-1.64	0.01	down	yes
Inhbb	3.25	1.70	0.01	up	yes
Serpina	3.26	1.71	0.01	up	yes
Iigp1	3.16	1.66	0.01	up	yes
Slc7a2	3.18	1.67	0.01	up	yes
Ighd	3.18	1.67	0.01	up	yes
Gm18537	0.32	-1.62	0.02	down	yes
Cygb	3.16	1.66	0.02	up	yes
Fam110b	3.20	1.68	0.02	up	yes
Tfap2a	3.17	1.66	0.02	up	yes
Pdgfra;	3.14	1.65	0.02	up	yes
Tnfsf8	3.12	1.64	0.02	up	yes
Pde7b	3.15	1.65	0.02	up	yes
Zfp12	0.33	-1.59	0.02	down	yes
Proz	3.11	1.64	0.02	up	yes
Muc6	0.33	-1.59	0.02	down	yes
Dpp6	3.11	1.63	0.02	up	yes
4930595	0.34	-1.57	0.02	down	yes
Mdga1	3.07	1.62	0.02	up	yes
Mctp1	3.09	1.63	0.02	up	yes
AC16766	0.34	-1.57	0.02	down	yes
Evc	3.16	1.66	0.02	up	yes
Gm4070;	3.04	1.60	0.02	up	yes
Ddr2	3.05	1.61	0.02	up	yes
Pvrig	3.11	1.63	0.02	up	yes
Hbb-bs	0.34	-1.56	0.02	down	yes
Rbfox1	3.05	1.61	0.02	up	yes
AI46413	3.07	1.62	0.02	up	yes
Casp12	3.05	1.61	0.02	up	yes
Tlr11	3.05	1.61	0.02	up	yes
Itga8	3.07	1.62	0.02	up	yes
Naip6	3.03	1.60	0.02	up	yes
Tmbim1	2.98	1.58	0.02	up	yes
Itgad	3.04	1.60	0.02	up	yes
Cd22	3.01	1.59	0.02	up	yes
Aoc3	2.99	1.58	0.02	up	yes
Aldh1a3	2.98	1.58	0.02	up	yes
Igfbp5	2.95	1.56	0.02	up	yes
Svbp	0.34	-1.57	0.02	down	yes
Ap4s1	0.35	-1.52	0.02	down	yes
Asrgl1	0.35	-1.52	0.02	down	yes
Slco2a1	2.93	1.55	0.02	up	yes
Lypd1	0.35	-1.53	0.02	down	yes
Ano1	2.98	1.58	0.02	up	yes
Cdc42ep	2.98	1.58	0.02	up	yes
Lum	2.95	1.56	0.02	up	yes
Plscr2	2.99	1.58	0.02	up	yes
Iglc1;I	2.91	1.54	0.02	up	yes
Tmem240	2.94	1.56	0.02	up	yes
Cped1	2.96	1.57	0.02	up	yes
Cmpk2	2.92	1.55	0.02	up	yes
Tnfaip2	2.90	1.54	0.02	up	yes
Rbpj	0.36	-1.48	0.02	down	yes
Mt1	2.94	1.56	0.02	up	yes
Sdr42e1	2.89	1.53	0.02	up	yes
Hba-a2;	0.36	-1.48	0.02	down	yes
Alpk1	2.88	1.53	0.02	up	yes

Ccr5	2.87	1.52	0.03	up	yes
Fap	2.93	1.55	0.03	up	yes
Kcnc1	2.87	1.52	0.03	up	yes
Dbp	2.85	1.51	0.03	up	yes
Cx3cl1	2.84	1.51	0.03	up	yes
Mx1	2.84	1.51	0.03	up	yes
Pcdh17	2.84	1.51	0.03	up	yes
Fyb2	2.83	1.50	0.03	up	yes
1600014(	2.82	1.50	0.03	up	yes
Mrvi1	2.86	1.51	0.03	up	yes
Arf3	0.37	-1.45	0.03	down	yes
Zfp976	2.89	1.53	0.03	up	yes
Apod	2.85	1.51	0.03	up	yes
Pxdc1	2.85	1.51	0.03	up	yes
Tshz2	2.82	1.49	0.03	up	yes
Gm4951	2.82	1.49	0.03	up	yes
Bank1	2.81	1.49	0.03	up	yes
Setbp1	2.77	1.47	0.03	up	yes
Bend3	2.77	1.47	0.03	up	yes
Clec14a	2.79	1.48	0.03	up	yes
Slc35c1	2.76	1.46	0.03	up	yes
C1s1;C1.	2.76	1.47	0.03	up	yes
Dnah3	2.81	1.49	0.03	up	yes
Vldlr	2.75	1.46	0.03	up	yes
Art2a-p.	2.80	1.49	0.03	up	yes
Klk1	2.81	1.49	0.03	up	yes
Sema5a	2.74	1.45	0.03	up	yes
Slit3	2.77	1.47	0.03	up	yes
Olfr920	2.77	1.47	0.03	up	yes
Gm6630	0.38	-1.41	0.03	down	yes
Gpr35	2.77	1.47	0.03	up	yes
Cgnl1	2.73	1.45	0.03	up	yes
Ifit3b	2.75	1.46	0.03	up	yes
Hydin	2.76	1.46	0.03	up	yes
Plch2	2.74	1.45	0.03	up	yes
Ifi208	2.71	1.44	0.03	up	yes
Apcdd1	2.79	1.48	0.03	up	yes
Pck1	2.79	1.48	0.03	up	yes
Plek2	2.77	1.47	0.03	up	yes
Nrbp2	2.71	1.44	0.03	up	yes
Lrrc18	2.76	1.47	0.03	up	yes
Hr	2.73	1.45	0.03	up	yes
2900026.	2.69	1.43	0.03	up	yes
Mx2	2.69	1.43	0.04	up	yes
Serpinb.	2.77	1.47	0.04	up	yes
Dchs2	2.73	1.45	0.04	up	yes
Fcgbp	2.68	1.42	0.04	up	yes
A730049I	2.75	1.46	0.04	up	yes
Tshz3	2.74	1.46	0.04	up	yes
Dmrta1	2.73	1.45	0.04	up	yes
Evc2	2.72	1.45	0.04	up	yes
Hbb-bt	0.38	-1.39	0.04	down	yes
Ldlr	0.39	-1.37	0.04	down	yes
Dpt	2.66	1.41	0.04	up	yes
Bicc1	2.68	1.42	0.04	up	yes
Arhgef9	0.38	-1.40	0.04	down	yes
Cst7	2.69	1.43	0.04	up	yes

Mycbp	0.39	-1.37	0.04	down	yes
Ephb3	2.67	1.42	0.04	up	yes
Phactr4	2.64	1.40	0.04	up	yes
Xaf1	2.63	1.40	0.04	up	yes
Mok	2.67	1.41	0.04	up	yes
Enpp2	2.63	1.40	0.04	up	yes
Ly6c2;L	2.64	1.40	0.04	up	yes
Amotl2	2.64	1.40	0.04	up	yes
Megf6	2.63	1.39	0.04	up	yes
Penk	2.65	1.41	0.04	up	yes
Dsel	2.61	1.39	0.04	up	yes
Zfp451	2.60	1.38	0.04	up	yes
Yap1	2.60	1.38	0.04	up	yes
Ms4a6c	2.60	1.38	0.04	up	yes
Tmtc1	2.61	1.38	0.04	up	yes
Rxfp1	0.39	-1.35	0.04	down	yes
Tnxb	2.58	1.36	0.04	up	yes
Dpy19l4	0.40	-1.32	0.04	down	yes
Zfp612	2.63	1.39	0.04	up	yes
Kif1a	2.63	1.39	0.04	up	yes
Mllt3	2.56	1.36	0.04	up	yes
AC23881	2.58	1.37	0.04	up	yes
Slc41a2	2.64	1.40	0.04	up	yes
Gcnt2	2.60	1.38	0.05	up	yes
Arhgap2	2.55	1.35	0.05	up	yes
Gm24830	0.40	-1.31	0.05	down	yes
Fabp4	2.54	1.34	0.05	up	yes
Rcn3	2.62	1.39	0.05	up	yes
BC00553	0.41	-1.29	0.05	down	yes
Ptn	2.58	1.37	0.05	up	yes
Tusc5	2.59	1.37	0.05	up	yes
Fbxl12	0.41	-1.29	0.05	down	yes
Spns2	2.54	1.35	0.05	up	yes
Hspa1a	2.52	1.33	0.05	up	yes
Cds2	2.50	1.32	0.05	up	yes
