# Supplementary material for: Transcriptome analysis reveals a potential regulatory mechanism of the lnc-5423.6/IGFBP5 axis in the early stages of mouse thymic involution: lnc-5423.6/IGFBP5 axis regulates thymic involution
Source: Acta Biochim Biophys Sin (Shanghai). 2023 Apr 19;55(4):548–60. doi: 10.3724/abbs.2023042 (PMC10195152; doi:10.3724/abbs.2023042)
Supplement: Table_S10 [file Table_S10.pdf]

t_name	fc	log2(fc	pval	regulat	significant
ENSMUST	0.0043	-7.854	4E-19	down	yes
ENSMUST	18.471	4.2072	3E-08	up	yes
ENSMUST	0.0665	-3.91	9E-08	down	yes
ENSMUST	0.0892	-3.487	1E-06	down	yes
ENSMUST	11.354	3.5051	2E-06	up	yes
ENSMUST	9.4097	3.2341	7E-06	up	yes
ENSMUST	0.1334	-2.906	3E-05	down	yes
ENSMUST	6.7184	2.7481	0.0001	up	yes
ENSMUST	0.1857	-2.429	0.0004	down	yes
ENSMUST	4.3941	2.1356	0.0021	up	yes
ENSMUST	4.4844	2.1649	0.0022	up	yes
ENSMUST	4.1204	2.0428	0.0035	up	yes
ENSMUST	0.2564	-1.964	0.0038	down	yes
ENSMUST	0.2625	-1.929	0.0038	down	yes
ENSMUST	0.2655	-1.913	0.0043	down	yes
ENSMUST	3.92	1.9709	0.0046	up	yes
ENSMUST	0.2829	-1.822	0.0066	down	yes
ENSMUST	3.6727	1.8768	0.0074	up	yes
ENSMUST	0.3014	-1.73	0.0099	down	yes
ENSMUST	3.4397	1.7823	0.0102	up	yes
ENSMUST	0.3059	-1.709	0.0108	down	yes
ENSMUST	3.2731	1.7106	0.0138	up	yes
ENSMUST	3.2056	1.6806	0.0149	up	yes
ENSMUST	0.3285	-1.606	0.0157	down	yes
ENSMUST	0.3318	-1.591	0.0157	down	yes
ENSMUST	3.1374	1.6496	0.0169	up	yes
ENSMUST	3.1315	1.6469	0.0187	up	yes
ENSMUST	3.1064	1.6352	0.0193	up	yes
ENSMUST	3.0408	1.6044	0.0194	up	yes
ENSMUST	3.0617	1.6143	0.0197	up	yes
ENSMUST	3.0866	1.626	0.0201	up	yes
ENSMUST	3.0652	1.616	0.0213	up	yes
ENSMUST	2.9316	1.5517	0.0244	up	yes
ENSMUST	2.9086	1.5403	0.0248	up	yes
ENSMUST	2.886	1.5291	0.0273	up	yes
ENSMUST	0.3736	-1.42	0.0308	down	yes
ENSMUST	2.7939	1.4823	0.0319	up	yes
ENSMUST	0.3844	-1.379	0.0339	down	yes
ENSMUST	2.7009	1.4334	0.0363	up	yes
ENSMUST	0.3856	-1.375	0.0369	down	yes
ENSMUST	2.7348	1.4514	0.0377	up	yes
ENSMUST	0.3921	-1.351	0.0386	down	yes
ENSMUST	0.3965	-1.335	0.0426	down	yes
ENSMUST	2.6028	1.3801	0.0472	up	yes
ENSMUST	2.6186	1.3888	0.0474	up	yes
ENSMUST	2.5965	1.3766	0.0482	up	yes
ENSMUST	0.408	-1.293	0.0493	down	yes
MSTRG.6	0.0004	-11.22	3E-29	down	yes
MSTRG.5	0.0011	-9.81	6E-25	down	yes
MSTRG.1	0.002	-8.941	2E-22	down	yes
MSTRG.1	61.527	5.9431	3E-13	up	yes
MSTRG.1	63.08	5.9791	3E-13	up	yes
MSTRG.1	50.644	5.6623	2E-12	up	yes
MSTRG.1	0.0329	-4.924	1E-10	down	yes
MSTRG.5	30.207	4.9168	3E-10	up	yes
MSTRG.2	0.035	-4.838	3E-10	down	yes

MSTRG.3	0.0363	-4.785	4E-10	down	yes
MSTRG.5	0.0398	-4.652	1E-09	down	yes
MSTRG.1	0.0497	-4.331	6E-09	down	yes
MSTRG.1	0.0505	-4.307	8E-09	down	yes
MSTRG.8	0.054	-4.21	1E-08	down	yes
MSTRG.1	16.107	4.0096	7E-08	up	yes
MSTRG.1	0.0687	-3.863	1E-07	down	yes
MSTRG.8	0.0692	-3.854	1E-07	down	yes
MSTRG.2	15.116	3.918	2E-07	up	yes
MSTRG.4	0.0764	-3.711	3E-07	down	yes
MSTRG.2	14.244	3.8323	3E-07	up	yes
MSTRG.3	0.0758	-3.723	3E-07	down	yes
MSTRG.7	0.0786	-3.67	4E-07	down	yes
MSTRG.1	0.0805	-3.635	4E-07	down	yes
MSTRG.1	12.707	3.6675	6E-07	up	yes
MSTRG.3	12.151	3.603	8E-07	up	yes
MSTRG.5	12.187	3.6073	8E-07	up	yes
MSTRG.2	0.0882	-3.504	1E-06	down	yes
MSTRG.2	0.0899	-3.476	1E-06	down	yes
MSTRG.1	11.919	3.5751	1E-06	up	yes
MSTRG.6	11.885	3.5711	1E-06	up	yes
MSTRG.3	11.769	3.5569	1E-06	up	yes
MSTRG.1	0.0956	-3.386	2E-06	down	yes
MSTRG.1	0.0962	-3.378	2E-06	down	yes
MSTRG.7	0.0963	-3.376	2E-06	down	yes
MSTRG.7	10.616	3.4082	3E-06	up	yes
MSTRG.7	0.0987	-3.341	3E-06	down	yes
MSTRG.1	10.282	3.3621	3E-06	up	yes
MSTRG.7	0.1022	-3.29	4E-06	down	yes
MSTRG.4	0.1022	-3.29	4E-06	down	yes
MSTRG.1	0.1075	-3.218	5E-06	down	yes
MSTRG.9	9.2015	3.2019	9E-06	up	yes
MSTRG.2	9.0501	3.1779	1E-05	up	yes
MSTRG.5	0.1236	-3.016	2E-05	down	yes
MSTRG.2	8.3902	3.0687	2E-05	up	yes
MSTRG.9	8.1424	3.0255	2E-05	up	yes
MSTRG.3	0.1343	-2.897	3E-05	down	yes
MSTRG.1	7.8829	2.9787	3E-05	up	yes
MSTRG.7	0.1388	-2.849	4E-05	down	yes
MSTRG.3	7.6766	2.9405	4E-05	up	yes
MSTRG.4	7.4955	2.906	4E-05	up	yes
MSTRG.9	7.2347	2.8549	6E-05	up	yes
MSTRG.5	7.1987	2.8477	6E-05	up	yes
MSTRG.2	0.1504	-2.734	8E-05	down	yes
MSTRG.3	0.1519	-2.719	8E-05	down	yes
MSTRG.2	6.8768	2.7817	9E-05	up	yes
MSTRG.2	6.7982	2.7651	0.0001	up	yes
MSTRG.5	6.6284	2.7287	0.0001	up	yes
MSTRG.2	6.6018	2.7229	0.0001	up	yes
MSTRG.9	6.5613	2.714	0.0001	up	yes
MSTRG.1	6.5827	2.7187	0.0001	up	yes
MSTRG.2	0.1683	-2.571	0.0002	down	yes
MSTRG.3	6.4233	2.6833	0.0002	up	yes
MSTRG.1	6.11	2.6112	0.0002	up	yes
MSTRG.1	0.1758	-2.508	0.0003	down	yes
MSTRG.2	0.1767	-2.501	0.0003	down	yes
MSTRG.1	0.1795	-2.478	0.0003	down	yes

MSTRG.5	0.183	-2.45	0.0003	down	yes
MSTRG.1	5.6706	2.5035	0.0004	up	yes
MSTRG.2	5.4956	2.4583	0.0005	up	yes
MSTRG.3	0.1996	-2.325	0.0006	down	yes
MSTRG.3	0.2028	-2.302	0.0007	down	yes
MSTRG.1	4.9861	2.3179	0.0009	up	yes
MSTRG.8.	0.2118	-2.239	0.0009	down	yes
MSTRG.1	5.1209	2.3564	0.0009	up	yes
MSTRG.1	0.2085	-2.262	0.0009	down	yes
MSTRG.1	0.2147	-2.22	0.001	down	yes
MSTRG.1	4.9013	2.2932	0.001	up	yes
MSTRG.3.	4.9749	2.3147	0.001	up	yes
MSTRG.2	0.2184	-2.195	0.0011	down	yes
MSTRG.3	4.7919	2.2606	0.0011	up	yes
MSTRG.1	0.2167	-2.206	0.0012	down	yes
MSTRG.4	0.2232	-2.164	0.0013	down	yes
MSTRG.1	4.752	2.2485	0.0013	up	yes
MSTRG.1	0.2239	-2.159	0.0013	down	yes
MSTRG.1	4.7057	2.2344	0.0013	up	yes
MSTRG.3	0.2233	-2.163	0.0013	down	yes
MSTRG.2	0.2233	-2.163	0.0013	down	yes
MSTRG.5	4.6891	2.2293	0.0014	up	yes
MSTRG.3	4.8014	2.2635	0.0014	up	yes
MSTRG.1	4.7374	2.2441	0.0014	up	yes
MSTRG.9	4.7031	2.2336	0.0016	up	yes
MSTRG.8.	4.5708	2.1924	0.0016	up	yes
MSTRG.1	0.2281	-2.132	0.0016	down	yes
MSTRG.4	0.2287	-2.128	0.0016	down	yes
MSTRG.8	0.2303	-2.118	0.0017	down	yes
MSTRG.4	4.6236	2.209	0.0017	up	yes
MSTRG.2	0.2345	-2.092	0.0018	down	yes
MSTRG.1	4.5128	2.174	0.0018	up	yes
MSTRG.1	4.4715	2.1607	0.0018	up	yes
MSTRG.1	4.4915	2.1672	0.0018	up	yes
MSTRG.1	4.5294	2.1793	0.0018	up	yes
MSTRG.2	4.4771	2.1625	0.0019	up	yes
MSTRG.1	4.5741	2.1935	0.0019	up	yes
MSTRG.1	4.5553	2.1875	0.0019	up	yes
MSTRG.1	4.4449	2.1522	0.002	up	yes
MSTRG.5	4.3943	2.1356	0.002	up	yes
MSTRG.3	4.4062	2.1395	0.0021	up	yes
MSTRG.3	0.238	-2.071	0.0023	down	yes
MSTRG.3.	4.3224	2.1118	0.0023	up	yes
MSTRG.2	4.2935	2.1022	0.0024	up	yes
MSTRG.3	4.2318	2.0813	0.0027	up	yes
MSTRG.8.	4.246	2.0861	0.0028	up	yes
MSTRG.1	0.2523	-1.987	0.0029	down	yes
MSTRG.1	0.2531	-1.982	0.0029	down	yes
MSTRG.1	4.147	2.0521	0.003	up	yes
MSTRG.3	4.2468	2.0864	0.003	up	yes
MSTRG.2	0.2536	-1.979	0.003	down	yes
MSTRG.1	0.2544	-1.975	0.0031	down	yes
MSTRG.8	0.2566	-1.962	0.0032	down	yes
MSTRG.3	4.112	2.0399	0.0032	up	yes
MSTRG.3	4.1786	2.063	0.0034	up	yes
MSTRG.3	4.1411	2.05	0.0035	up	yes
MSTRG.1	0.2568	-1.962	0.0035	down	yes

MSTRG.2	4.0509	2.0182	0.0037	up	yes
MSTRG.1	0.2605	-1.941	0.0038	down	yes
MSTRG.6	4.0149	2.0054	0.0038	up	yes
MSTRG.1	3.9803	1.9929	0.0039	up	yes
MSTRG.3	0.2664	-1.908	0.0041	down	yes
MSTRG.2	0.2682	-1.899	0.0042	down	yes
MSTRG.1	3.9228	1.9719	0.0043	up	yes
MSTRG.3	3.9138	1.9686	0.0044	up	yes
MSTRG.3	3.9067	1.9659	0.0045	up	yes
MSTRG.3	4.0101	2.0036	0.0045	up	yes
MSTRG.8	3.9629	1.9866	0.0046	up	yes
MSTRG.4	3.9686	1.9886	0.0047	up	yes
MSTRG.1	3.9101	1.9672	0.0048	up	yes
MSTRG.2	0.2745	-1.865	0.0049	down	yes
MSTRG.2	3.8635	1.9499	0.005	up	yes
MSTRG.3	0.2765	-1.855	0.0051	down	yes
MSTRG.2	3.8484	1.9443	0.0051	up	yes
MSTRG.9	0.2764	-1.855	0.0051	down	yes
MSTRG.2	0.2722	-1.877	0.0052	down	yes
MSTRG.1	3.8204	1.9337	0.0053	up	yes
MSTRG.3	3.7762	1.9169	0.0055	up	yes
MSTRG.2	0.2778	-1.848	0.0055	down	yes
MSTRG.2	0.2818	-1.827	0.0057	down	yes
MSTRG.6	3.7982	1.9253	0.0057	up	yes
MSTRG.2	0.2772	-1.851	0.0059	down	yes
MSTRG.3	3.7805	1.9186	0.006	up	yes
MSTRG.1	3.8097	1.9297	0.0061	up	yes
MSTRG.3	3.785	1.9203	0.0062	up	yes
MSTRG.3	3.7228	1.8964	0.0062	up	yes
MSTRG.2	0.2832	-1.82	0.0062	down	yes
MSTRG.1	0.2796	-1.839	0.0063	down	yes
MSTRG.3	3.721	1.8957	0.0066	up	yes
MSTRG.3	3.6564	1.8704	0.0067	up	yes
MSTRG.2	0.2838	-1.817	0.0067	down	yes
MSTRG.2	0.2857	-1.807	0.0067	down	yes
MSTRG.3	3.6871	1.8825	0.0068	up	yes
MSTRG.2	0.2921	-1.776	0.0072	down	yes
MSTRG.1	3.6105	1.8522	0.0073	up	yes
MSTRG.2	3.6213	1.8565	0.0073	up	yes
MSTRG.6	3.6181	1.8552	0.0074	up	yes
MSTRG.2	0.2874	-1.799	0.0075	down	yes
MSTRG.2	3.582	1.8408	0.0075	up	yes
MSTRG.1	3.5515	1.8284	0.0078	up	yes
MSTRG.2	3.6121	1.8528	0.008	up	yes
MSTRG.7	0.2985	-1.744	0.0081	down	yes
MSTRG.9	0.2991	-1.741	0.0082	down	yes
MSTRG.1	3.5389	1.8233	0.0084	up	yes
MSTRG.2	0.297	-1.752	0.0086	down	yes
MSTRG.3	3.5027	1.8085	0.0092	up	yes
MSTRG.9	3.4554	1.7888	0.0093	up	yes
MSTRG.3	3.5482	1.8271	0.0094	up	yes
MSTRG.3	3.4431	1.7837	0.0096	up	yes
MSTRG.3	3.4416	1.7831	0.0097	up	yes
MSTRG.1	3.4572	1.7896	0.0097	up	yes
MSTRG.1	3.4148	1.7718	0.0099	up	yes
MSTRG.3	3.4209	1.7744	0.01	up	yes
MSTRG.1	3.5155	1.8137	0.01	up	yes

MSTRG.3	3.4012	1.766	0.0102	up	yes
MSTRG.1	0.3046	-1.715	0.0103	down	yes
MSTRG.4	3.4801	1.7991	0.0104	up	yes
MSTRG.3	3.3847	1.759	0.0104	up	yes
MSTRG.1	0.3049	-1.714	0.0106	down	yes
MSTRG.2	3.3985	1.7649	0.0108	up	yes
MSTRG.1	3.4152	1.772	0.0108	up	yes
MSTRG.1	0.3125	-1.678	0.0109	down	yes
MSTRG.2	0.313	-1.676	0.0109	down	yes
MSTRG.1	3.3853	1.7593	0.0111	up	yes
MSTRG.1	3.3797	1.7569	0.0117	up	yes
MSTRG.3	3.3298	1.7354	0.0118	up	yes
MSTRG.8	0.3157	-1.663	0.0118	down	yes
MSTRG.2	0.3149	-1.667	0.0119	down	yes
MSTRG.5	3.3445	1.7418	0.012	up	yes
MSTRG.4	0.3164	-1.66	0.0122	down	yes
MSTRG.2	3.3263	1.7339	0.0124	up	yes
MSTRG.9	3.3056	1.7249	0.0125	up	yes
MSTRG.1	3.2777	1.7127	0.0127	up	yes
MSTRG.1	3.2905	1.7183	0.0127	up	yes
MSTRG.1	3.2886	1.7175	0.0128	up	yes
MSTRG.3	3.2687	1.7087	0.0129	up	yes
MSTRG.1	0.3232	-1.63	0.013	down	yes
MSTRG.7	3.2688	1.7088	0.0133	up	yes
MSTRG.6	3.2445	1.698	0.0134	up	yes
MSTRG.1	0.3232	-1.629	0.0139	down	yes
MSTRG.1	3.2505	1.7007	0.014	up	yes
MSTRG.4	3.2691	1.7089	0.0141	up	yes
MSTRG.5	3.2227	1.6883	0.0141	up	yes
MSTRG.8	0.3286	-1.606	0.0143	down	yes
MSTRG.1	3.2107	1.6829	0.0143	up	yes
MSTRG.2	0.3253	-1.62	0.0145	down	yes
MSTRG.1	3.2058	1.6807	0.0145	up	yes
MSTRG.1	3.1992	1.6777	0.0145	up	yes
MSTRG.5	0.3254	-1.62	0.0146	down	yes
MSTRG.9	3.1916	1.6743	0.0147	up	yes
MSTRG.1	0.3263	-1.616	0.0148	down	yes
MSTRG.3	3.2078	1.6816	0.0148	up	yes
MSTRG.2	3.1844	1.671	0.0148	up	yes
MSTRG.7	0.3314	-1.593	0.015	down	yes
MSTRG.1	3.1801	1.6691	0.0151	up	yes
MSTRG.3	3.1862	1.6718	0.0152	up	yes
MSTRG.1	0.3323	-1.59	0.0153	down	yes
MSTRG.1	3.2042	1.68	0.0153	up	yes
MSTRG.1	3.1889	1.6731	0.0154	up	yes
MSTRG.1	3.1595	1.6597	0.0157	up	yes
MSTRG.7	0.3342	-1.581	0.0158	down	yes
MSTRG.1	3.1552	1.6578	0.0158	up	yes
MSTRG.2	3.1492	1.655	0.0159	up	yes
MSTRG.2	0.3347	-1.579	0.0159	down	yes
MSTRG.5	0.3344	-1.58	0.016	down	yes
MSTRG.1	0.3358	-1.574	0.0162	down	yes
MSTRG.6	3.1381	1.6499	0.0162	up	yes
MSTRG.2	3.1316	1.6469	0.0163	up	yes
MSTRG.3	3.1581	1.6591	0.0164	up	yes
MSTRG.6	3.1352	1.6485	0.0166	up	yes
MSTRG.1	3.1087	1.6363	0.017	up	yes

MSTRG.2	3.1498	1.6553	0.017	up	yes
MSTRG.6	3.1098	1.6368	0.017	up	yes
MSTRG.1	3.1143	1.6389	0.0173	up	yes
MSTRG.1	3.1263	1.6444	0.0173	up	yes
MSTRG.5	3.1086	1.6363	0.0174	up	yes
MSTRG.1	3.1275	1.645	0.0174	up	yes
MSTRG.1	3.0931	1.629	0.0175	up	yes
MSTRG.2	0.3381	-1.564	0.0175	down	yes
MSTRG.1	3.0955	1.6302	0.0178	up	yes
MSTRG.2	3.1378	1.6497	0.0178	up	yes
MSTRG.8	0.3416	-1.55	0.0179	down	yes
MSTRG.1	3.0844	1.625	0.0179	up	yes
MSTRG.1	3.0691	1.6178	0.0182	up	yes
MSTRG.2	3.1481	1.6545	0.0183	up	yes
MSTRG.2	0.3431	-1.543	0.0187	down	yes
MSTRG.2	3.0866	1.626	0.019	up	yes
MSTRG.3	3.0703	1.6184	0.0191	up	yes
MSTRG.4	3.0653	1.616	0.0191	up	yes
MSTRG.1	3.0787	1.6223	0.0192	up	yes
MSTRG.2	0.3413	-1.551	0.0193	down	yes
MSTRG.2	3.056	1.6117	0.0193	up	yes
MSTRG.3	3.0883	1.6268	0.0194	up	yes
MSTRG.1	0.3397	-1.558	0.0195	down	yes
MSTRG.1	3.0217	1.5954	0.0201	up	yes
MSTRG.1	3.041	1.6046	0.0201	up	yes
MSTRG.9	3.0231	1.596	0.0202	up	yes
MSTRG.1	3.0394	1.6038	0.0203	up	yes
MSTRG.1	0.3497	-1.516	0.0203	down	yes
MSTRG.3	3.0282	1.5985	0.0204	up	yes
MSTRG.2	3.084	1.6248	0.0204	up	yes
MSTRG.2	3.0583	1.6127	0.0204	up	yes
MSTRG.3	3.0393	1.6037	0.0211	up	yes
MSTRG.1	3.0299	1.5993	0.0212	up	yes
MSTRG.3	3.0038	1.5868	0.0213	up	yes
MSTRG.1	3.0387	1.6035	0.0217	up	yes
MSTRG.1	3.006	1.5878	0.0229	up	yes
MSTRG.1	0.356	-1.49	0.0234	down	yes
MSTRG.2	2.9669	1.5689	0.0235	up	yes
MSTRG.3	2.9594	1.5653	0.0237	up	yes
MSTRG.2	2.9579	1.5646	0.0238	up	yes
MSTRG.2	0.3587	-1.479	0.0239	down	yes
MSTRG.9	2.9262	1.549	0.024	up	yes
MSTRG.1	2.9525	1.562	0.0242	up	yes
MSTRG.3	2.9873	1.5789	0.0243	up	yes
MSTRG.1	0.3536	-1.5	0.0246	down	yes
MSTRG.1	2.9153	1.5436	0.025	up	yes
MSTRG.1	0.3553	-1.493	0.0251	down	yes
MSTRG.1	0.3647	-1.455	0.0254	down	yes
MSTRG.2	2.9162	1.5441	0.0255	up	yes
MSTRG.1	2.9204	1.5462	0.0256	up	yes
MSTRG.2	2.8817	1.5269	0.0256	up	yes
MSTRG.2	2.9431	1.5573	0.0256	up	yes
MSTRG.3	0.3591	-1.478	0.0257	down	yes
MSTRG.3	0.359	-1.478	0.0257	down	yes
MSTRG.7	2.9124	1.5422	0.0259	up	yes
MSTRG.2	2.9474	1.5595	0.0262	up	yes
MSTRG.2	0.3674	-1.444	0.0263	down	yes

MSTRG.2	2.9081	1.5401	0.0265	up	yes
MSTRG.2	2.8675	1.5198	0.0275	up	yes
MSTRG.1	2.8561	1.5141	0.0276	up	yes
MSTRG.5	0.3634	-1.46	0.0278	down	yes
MSTRG.9	2.8376	1.5047	0.0281	up	yes
MSTRG.2	2.8559	1.5139	0.0287	up	yes
MSTRG.2	2.8985	1.5353	0.0289	up	yes
MSTRG.4	2.8542	1.5131	0.029	up	yes
MSTRG.3	2.8495	1.5107	0.0297	up	yes
MSTRG.6	0.3727	-1.424	0.0299	down	yes
MSTRG.1	2.835	1.5034	0.0299	up	yes
MSTRG.5	2.7979	1.4843	0.0299	up	yes
MSTRG.3	2.8259	1.4987	0.03	up	yes
MSTRG.1	2.8116	1.4914	0.0304	up	yes
MSTRG.1	2.7906	1.4806	0.0308	up	yes
MSTRG.3	2.8288	1.5002	0.0308	up	yes
MSTRG.4	2.8339	1.5028	0.0309	up	yes
MSTRG.3	2.8117	1.4915	0.031	up	yes
MSTRG.1	2.7775	1.4738	0.0312	up	yes
MSTRG.7	0.3802	-1.395	0.0316	down	yes
MSTRG.9	2.7792	1.4747	0.0319	up	yes
MSTRG.6	0.3765	-1.409	0.0319	down	yes
MSTRG.3	0.3813	-1.391	0.032	down	yes
MSTRG.3	2.7594	1.4644	0.0324	up	yes
MSTRG.1	2.8002	1.4855	0.0324	up	yes
MSTRG.2	2.7944	1.4825	0.0325	up	yes
MSTRG.2	0.3811	-1.392	0.0325	down	yes
MSTRG.2	2.7882	1.4793	0.0325	up	yes
MSTRG.2	2.819	1.4952	0.0327	up	yes
MSTRG.2	2.7951	1.4829	0.0333	up	yes
MSTRG.3	2.7436	1.4561	0.0336	up	yes
MSTRG.1	2.7417	1.4551	0.0336	up	yes
MSTRG.1	2.7836	1.4769	0.0338	up	yes
MSTRG.5	2.7321	1.45	0.0344	up	yes
MSTRG.3	2.7649	1.4672	0.0345	up	yes
MSTRG.3	2.787	1.4787	0.0345	up	yes
MSTRG.1	2.7783	1.4742	0.0349	up	yes
MSTRG.2	2.7614	1.4654	0.0349	up	yes
MSTRG.1	2.7622	1.4658	0.035	up	yes
MSTRG.1	2.7833	1.4768	0.035	up	yes
MSTRG.6	2.7292	1.4485	0.035	up	yes
MSTRG.4	2.7683	1.469	0.035	up	yes
MSTRG.1	2.721	1.4441	0.0351	up	yes
MSTRG.1	2.7207	1.444	0.0353	up	yes
MSTRG.2	2.7368	1.4525	0.0354	up	yes
MSTRG.1	2.7105	1.4386	0.0355	up	yes
MSTRG.2	0.3885	-1.364	0.0356	down	yes
MSTRG.2	2.7047	1.4354	0.0363	up	yes
MSTRG.3	2.6979	1.4318	0.0366	up	yes
MSTRG.2	2.7111	1.4389	0.0366	up	yes
MSTRG.1	2.692	1.4287	0.0367	up	yes
MSTRG.2	2.7243	1.4459	0.0368	up	yes
MSTRG.1	2.6981	1.4319	0.0369	up	yes
MSTRG.1	0.3853	-1.376	0.0374	down	yes
MSTRG.8	2.6804	1.4225	0.0374	up	yes
MSTRG.5	2.7232	1.4453	0.0374	up	yes
MSTRG.1	0.3918	-1.352	0.0376	down	yes

MSTRG.2	2.7041	1.4352	0.0379	up	yes
MSTRG.3	0.3896	-1.36	0.039	down	yes
MSTRG.1	2.7101	1.4384	0.0392	up	yes
MSTRG.7	0.3954	-1.339	0.0393	down	yes
MSTRG.1	0.3941	-1.344	0.0394	down	yes
MSTRG.2	2.6562	1.4094	0.0398	up	yes
MSTRG.1	2.6512	1.4066	0.04	up	yes
MSTRG.2	2.6839	1.4244	0.0401	up	yes
MSTRG.3	2.6573	1.41	0.0402	up	yes
MSTRG.3	2.6752	1.4196	0.0403	up	yes
MSTRG.3	2.6786	1.4215	0.0404	up	yes
MSTRG.3	2.66	1.4114	0.0411	up	yes
MSTRG.2	2.6346	1.3976	0.0412	up	yes
MSTRG.8	2.6523	1.4073	0.0412	up	yes
MSTRG.7	2.6573	1.41	0.0413	up	yes
MSTRG.3	2.6436	1.4025	0.0414	up	yes
MSTRG.2	2.6717	1.4177	0.0415	up	yes
MSTRG.1	2.6586	1.4107	0.0423	up	yes
MSTRG.2	2.6355	1.3981	0.0426	up	yes
MSTRG.1	2.6362	1.3985	0.0429	up	yes
MSTRG.3	0.3941	-1.343	0.043	down	yes
MSTRG.5	2.6101	1.3841	0.0432	up	yes
MSTRG.3	2.609	1.3835	0.0433	up	yes
MSTRG.2	2.6374	1.3991	0.0433	up	yes
MSTRG.3	2.6423	1.4018	0.0434	up	yes
MSTRG.2	2.6149	1.3867	0.0434	up	yes
MSTRG.1	0.4024	-1.313	0.0435	down	yes
MSTRG.1	0.402	-1.315	0.044	down	yes
MSTRG.1	2.6304	1.3953	0.0444	up	yes
MSTRG.9	2.5997	1.3783	0.0444	up	yes
MSTRG.9	2.6205	1.3898	0.0447	up	yes
MSTRG.3	2.5944	1.3754	0.0454	up	yes
MSTRG.3	2.6242	1.3919	0.0455	up	yes
MSTRG.2	2.5845	1.3699	0.0463	up	yes
MSTRG.9	2.6255	1.3926	0.0464	up	yes
MSTRG.9	2.5877	1.3717	0.047	up	yes
MSTRG.3	2.578	1.3663	0.0476	up	yes
MSTRG.2	2.6077	1.3828	0.048	up	yes
MSTRG.2	2.5371	1.3432	0.0492	up	yes
MSTRG.3	2.5404	1.3451	0.0494	up	yes
MSTRG.6	2.5501	1.3505	0.0499	up	yes
MSTRG.2	0.4096	-1.288	0.0499	down	yes
