# Supplementary material for: Transcriptome analysis reveals a potential regulatory mechanism of the lnc-5423.6/IGFBP5 axis in the early stages of mouse thymic involution: lnc-5423.6/IGFBP5 axis regulates thymic involution
Source: Acta Biochim Biophys Sin (Shanghai). 2023 Apr 19;55(4):548–60. doi: 10.3724/abbs.2023042 (PMC10195152; doi:10.3724/abbs.2023042)
Supplement: Table_S2 [file Table_S2.pdf]

gene_id	gene_na	transcript_id
ENSMUSG	H19	ENSMUST00000136359;ENSMUST00000140716;ENSMUST00000132
ENSMUSG	Tbx4	ENSMUST00000108047
ENSMUSG	Ngfr	ENSMUST00000000122
ENSMUSG	Wnt3	ENSMUST00000000127
ENSMUSG	Wnt9a	ENSMUST00000000128;ENSMUST00000108783
ENSMUSG	Fer	ENSMUST00000000129;ENSMUST00000038080
ENSMUSG	Slc22a1	ENSMUST00000052348;ENSMUST00000150791;ENSMUST00000145
ENSMUSG	Itgb2l	ENSMUST00000000161;ENSMUST00000113773;ENSMUST00000131
ENSMUSG	Igsf5	ENSMUST00000113794;ENSMUST00000000163;ENSMUST00000136
ENSMUSG	Fgf23	ENSMUST00000000186
ENSMUSG	Fgf6	ENSMUST00000000187
ENSMUSG	Nalcn	ENSMUST00000000201
ENSMUSG	Btbd17	ENSMUST00000156053
ENSMUSG	Slfn4	ENSMUST00000000208;ENSMUST00000167596;ENSMUST00000215
ENSMUSG	Th	ENSMUST00000105929;ENSMUST00000140344
ENSMUSG	Ins2	ENSMUST00000105930;ENSMUST00000105933
ENSMUSG	Scnn1g	ENSMUST00000000221
ENSMUSG	Lhx2	ENSMUST00000143783;ENSMUST00000149664;ENSMUST0000000C
ENSMUSG	Glra1	ENSMUST00000102716
ENSMUSG	Tpd52l1	ENSMUST00000000305;ENSMUST00000214644;ENSMUST00000213
ENSMUSG	Pemt	ENSMUST00000150954;ENSMUST00000000310;ENSMUST00000102
ENSMUSG	Bcl6b	ENSMUST00000000326;ENSMUST00000153449;ENSMUST0000014C
ENSMUSG	Clec10a	ENSMUST00000102571;ENSMUST00000178945;ENSMUST0000000C
ENSMUSG	Rem1	ENSMUST00000000369;ENSMUST00000139370
ENSMUSG	Fap	ENSMUST00000102732;ENSMUST00000128139;ENSMUST0000000C
ENSMUSG	Gcg	ENSMUST00000102733;ENSMUST00000136686;ENSMUST00000155
ENSMUSG	Egfl6	ENSMUST00000000412
ENSMUSG	Cttnbp2	ENSMUST00000152499;ENSMUST00000146775;ENSMUST00000148
ENSMUSG	Acvrl1	ENSMUST00000000542;ENSMUST00000144229;ENSMUST0000013C
ENSMUSG	Gpa33	ENSMUST00000060833;ENSMUST00000027847
ENSMUSG	Gabra2	ENSMUST00000197284;ENSMUST00000197124;ENSMUST00000199
ENSMUSG	Adora3	ENSMUST00000000574;ENSMUST00000197613
ENSMUSG	Vmn2r88	ENSMUST00000159674
ENSMUSG	Grm6	ENSMUST00000156890;ENSMUST00000171427;ENSMUST00000126
ENSMUSG	Sema4f	ENSMUST00000000641;ENSMUST00000125579;ENSMUST00000203
ENSMUSG	Hoxb6	ENSMUST00000173432;ENSMUST00000000704
ENSMUSG	Btn1a1	ENSMUST00000041674;ENSMUST00000110434
ENSMUSG	Icosl	ENSMUST00000105393;ENSMUST00000217675;ENSMUST00000219
ENSMUSG	Polr3d	ENSMUST00000180358;ENSMUST00000000793
ENSMUSG	Car4	ENSMUST00000139416;ENSMUST00000103194;ENSMUST00000138
ENSMUSG	Fasl	ENSMUST00000000834;ENSMUST00000193648
ENSMUSG	Dbh	ENSMUST00000000910
ENSMUSG	Top1mt	ENSMUST00000000958
ENSMUSG	Gm38393	ENSMUST00000179360;ENSMUST00000191456
ENSMUSG	Ccl3	ENSMUST00000001008
ENSMUSG	S100a5	ENSMUST00000107329;ENSMUST00000001049
ENSMUSG	Epn2	ENSMUST00000108711;ENSMUST00000108712;ENSMUST00000001
ENSMUSG	B9d1	ENSMUST00000102657;ENSMUST00000140548;ENSMUST00000138
ENSMUSG	C1ql4	ENSMUST00000064462
ENSMUSG	Oas1h	ENSMUST00000072476
ENSMUSG	Slc26a3	ENSMUST00000171616;ENSMUST00000001254;ENSMUST00000165
ENSMUSG	Sema6b	ENSMUST00000167545;ENSMUST00000001256
ENSMUSG	Gabrg1	ENSMUST00000031119
ENSMUSG	Ckb	ENSMUST00000222705;ENSMUST00000001304;ENSMUST00000223
ENSMUSG	Fndc5	ENSMUST00000102600
ENSMUSG	Meox1	ENSMUST00000057054

ENSMUSG	Sost	ENSMUST00000001534
ENSMUSG	Nkx2-1	ENSMUST00000178477
ENSMUSG	Irx2	ENSMUST00000074372;ENSMUST00000163393
ENSMUSG	Sgca	ENSMUST00000103162;ENSMUST00000152042
ENSMUSG	Dlx3	ENSMUST00000092768
ENSMUSG	Ell2	ENSMUST00000001583;ENSMUST00000220572;ENSMUST00000222
ENSMUSG	Mnx1	ENSMUST00000165512
ENSMUSG	Csn3	ENSMUST00000113267;ENSMUST00000144929;ENSMUST0000014C
ENSMUSG	Hoxc13	ENSMUST00000001700
ENSMUSG	Hoxc11	ENSMUST00000001701
ENSMUSG	Hoxc8	ENSMUST00000001703;ENSMUST00000189484
ENSMUSG	Hoxc6	ENSMUST00000173210;ENSMUST00000173421;ENSMUST00000001
ENSMUSG	Tat	ENSMUST00000001720
ENSMUSG	Marveld	ENSMUST00000001722;ENSMUST00000051430;ENSMUST00000155
ENSMUSG	Tspan33	ENSMUST00000046750;ENSMUST00000115250;ENSMUST00000137
ENSMUSG	Folh1	ENSMUST00000001824
ENSMUSG	Dsg4	ENSMUST00000019426
ENSMUSG	Hoxd13	ENSMUST00000001872
ENSMUSG	Aif1l	ENSMUST00000001920;ENSMUST00000140649;ENSMUST00000138
ENSMUSG	Pnck	ENSMUST00000002087;ENSMUST00000129106;ENSMUST00000144
ENSMUSG	Hsf2bp	ENSMUST00000002145
ENSMUSG	Peg3	ENSMUST00000051209;ENSMUST00000143703;ENSMUST00000155
ENSMUSG	Zim1	ENSMUST00000002336
ENSMUSG	Cd320	ENSMUST00000002379;ENSMUST00000173418;ENSMUST00000087
ENSMUSG	Ncan	ENSMUST00000002412
ENSMUSG	Abhd3	ENSMUST00000117828;ENSMUST00000144150
ENSMUSG	Ikzf4	ENSMUST00000133342;ENSMUST00000221150;ENSMUST00000223
ENSMUSG	Zfp40	ENSMUST00000037057;ENSMUST00000140313;ENSMUST00000172
ENSMUSG	Shh	ENSMUST00000002708
ENSMUSG	Alkbh7	ENSMUST00000074141;ENSMUST00000002737;ENSMUST0000014S
ENSMUSG	Pspn	ENSMUST00000002740
ENSMUSG	Prkd1	ENSMUST00000002765
ENSMUSG	Fkbp7	ENSMUST00000002809;ENSMUST00000154097;ENSMUST00000176
ENSMUSG	Ggct	ENSMUST00000131475;ENSMUST00000002874;ENSMUST00000204
ENSMUSG	Epdr1	ENSMUST00000002885;ENSMUST00000220755
ENSMUSG	Kcnn1	ENSMUST00000110081;ENSMUST00000110078;ENSMUST00000212
ENSMUSG	Arrdc2	ENSMUST00000213095;ENSMUST00000002989;ENSMUST00000212
ENSMUSG	Ppp1r17	ENSMUST00000052827
ENSMUSG	Cyp2c29	ENSMUST00000177087
ENSMUSG	Car11	ENSMUST00000003360;ENSMUST00000210872;ENSMUST00000211
ENSMUSG	Dlgap1	ENSMUST00000133983;ENSMUST00000060072;ENSMUST00000155
ENSMUSG	Atp8b3	ENSMUST00000020383
ENSMUSG	Ccdc65	ENSMUST00000003444
ENSMUSG	Fkbp11	ENSMUST00000003445
ENSMUSG	Rab3b	ENSMUST00000106650;ENSMUST00000003502
ENSMUSG	Dll3	ENSMUST00000108315;ENSMUST00000138721
ENSMUSG	Phyhip	ENSMUST00000003561
ENSMUSG	Crhr2	ENSMUST00000003568;ENSMUST00000212633;ENSMUST0000016S
ENSMUSG	Psg18	ENSMUST00000098783
ENSMUSG	Dusp3	ENSMUST00000151678;ENSMUST00000003612;ENSMUST00000107
ENSMUSG	Fosb	ENSMUST00000003640;ENSMUST00000208326;ENSMUST00000208
ENSMUSG	Sec14l2	ENSMUST00000003681;ENSMUST00000132421;ENSMUST00000133
ENSMUSG	Calb2	ENSMUST00000212297;ENSMUST00000003754
ENSMUSG	Hlf	ENSMUST00000004051;ENSMUST00000043658;ENSMUST00000107
ENSMUSG	Grm3	ENSMUST00000004076
ENSMUSG	Brinp2	ENSMUST00000195271;ENSMUST00000004133;ENSMUST00000194
ENSMUSG	Gstm5	ENSMUST00000004134;ENSMUST00000167523;ENSMUST00000172

ENSMUSG	Gstm7	ENSMUST00000004137;ENSMUST00000106687;ENSMUST00000133
ENSMUSG	Gstm3	ENSMUST00000004136
ENSMUSG	Col5a3	ENSMUST00000004201;ENSMUST00000145974
ENSMUSG	Cacna1b	ENSMUST00000070864;ENSMUST00000102939;ENSMUST0000010C
ENSMUSG	Trmt10a	ENSMUST00000040321;ENSMUST00000161791;ENSMUST00000162
ENSMUSG	Etv1	ENSMUST00000095767;ENSMUST00000161164;ENSMUST00000162
ENSMUSG	Pax2	ENSMUST00000174490;ENSMUST00000004340;ENSMUST00000173
ENSMUSG	Il12b	ENSMUST00000170513;ENSMUST00000102796
ENSMUSG	Gpx6	ENSMUST00000004453
ENSMUSG	Gpx5	ENSMUST00000110491
ENSMUSG	Spic	ENSMUST00000004473;ENSMUST00000133724;ENSMUST00000138
ENSMUSG	9330159	ENSMUST00000092627;ENSMUST00000213490;ENSMUST00000213
ENSMUSG	Sst	ENSMUST00000004480
ENSMUSG	Il11	ENSMUST00000094892;ENSMUST00000163481
ENSMUSG	Col26a1	ENSMUST00000111103;ENSMUST00000141744;ENSMUST00000125
ENSMUSG	Psg17	ENSMUST00000004655
ENSMUSG	Psg19	ENSMUST00000004657;ENSMUST00000182916
ENSMUSG	Sgce	ENSMUST00000115577;ENSMUST00000115579;ENSMUST00000004
ENSMUSG	Tyr	ENSMUST00000004770
ENSMUSG	Ghrhr	ENSMUST00000204988;ENSMUST00000063578
ENSMUSG	Hdac9	ENSMUST00000209902;ENSMUST00000209990;ENSMUST00000211
ENSMUSG	Mtfp1	ENSMUST00000004868;ENSMUST00000123607
ENSMUSG	Rab23	ENSMUST00000195006;ENSMUST00000140354;ENSMUST00000088
ENSMUSG	Ccl24	ENSMUST00000004936;ENSMUST00000201401
ENSMUSG	Pou1f1	ENSMUST00000175882;ENSMUST00000004964;ENSMUST00000184
ENSMUSG	Mapk13	ENSMUST00000004986;ENSMUST00000124099;ENSMUST00000133
ENSMUSG	Pax3	ENSMUST00000004994;ENSMUST00000087086;ENSMUST00000172
ENSMUSG	Crabp2	ENSMUST00000005019;ENSMUST00000163040
ENSMUSG	Nes	ENSMUST00000159830;ENSMUST00000090973;ENSMUST0000016C
ENSMUSG	Bcan	ENSMUST00000090971;ENSMUST00000192274
ENSMUSG	Hapln2	ENSMUST00000160737
ENSMUSG	Syt5	ENSMUST00000065957;ENSMUST00000206023
ENSMUSG	Ccdc130	ENSMUST00000098578;ENSMUST00000005120;ENSMUST00000164
ENSMUSG	Slc1a2	ENSMUST00000080210;ENSMUST00000125085;ENSMUST00000136
ENSMUSG	Wisp1	ENSMUST00000005255;ENSMUST00000133863
ENSMUSG	Shbg	ENSMUST00000005334
ENSMUSG	Corin	ENSMUST00000167460;ENSMUST00000005352;ENSMUST00000176
ENSMUSG	G6pc2	ENSMUST00000005364
ENSMUSG	Prlr	ENSMUST00000005400;ENSMUST00000145428;ENSMUST00000124
ENSMUSG	Fgfr4	ENSMUST00000005452;ENSMUST00000162167
ENSMUSG	Fcer1a	ENSMUST00000049706
ENSMUSG	Casp14	ENSMUST00000005488
ENSMUSG	Slc1a6	ENSMUST00000005490
ENSMUSG	Mlxipl	ENSMUST00000202431;ENSMUST00000141770;ENSMUST00000201
ENSMUSG	Myl10	ENSMUST00000196068;ENSMUST00000005611;ENSMUST00000196
ENSMUSG	Msh4	ENSMUST00000005630;ENSMUST00000189189;ENSMUST00000186
ENSMUSG	Mef2c	ENSMUST00000198199;ENSMUST00000200123;ENSMUST00000005
ENSMUSG	Mrvi1	ENSMUST00000127935;ENSMUST00000005751;ENSMUST00000125
ENSMUSG	Tomm40l	ENSMUST00000005817;ENSMUST00000111327;ENSMUST00000111
ENSMUSG	Nr1i3	ENSMUST00000005820;ENSMUST00000155126;ENSMUST00000152
ENSMUSG	Apoa2	ENSMUST00000111321
ENSMUSG	Pvalb	ENSMUST00000005860
ENSMUSG	Mmp8	ENSMUST00000018765
ENSMUSG	Sqor	ENSMUST00000005953;ENSMUST00000124460;ENSMUST00000147
ENSMUSG	Tnfsf14	ENSMUST00000005976
ENSMUSG	Gata6	ENSMUST00000047762
ENSMUSG	Cnga2	ENSMUST00000006020;ENSMUST00000145642

ENSMUSG	Prl4a1	ENSMUST00000021779
ENSMUSG	Trh	ENSMUST00000006046
ENSMUSG	Otx1	ENSMUST00000006071;ENSMUST00000172395
ENSMUSG	Shpk	ENSMUST00000131927;ENSMUST00000006105
ENSMUSG	Trpv1	ENSMUST00000102526
ENSMUSG	Ephb3	ENSMUST00000006112;ENSMUST00000161063;ENSMUST0000015
ENSMUSG	Tyrp1	ENSMUST00000006151
ENSMUSG	Pdc	ENSMUST00000191228;ENSMUST00000185698
ENSMUSG	Kptn	ENSMUST00000130170;ENSMUST00000006178;ENSMUST00000152
ENSMUSG	Calcoco	ENSMUST00000068686
ENSMUSG	Upk3bl	ENSMUST00000139999;ENSMUST00000111137;ENSMUST0000000
ENSMUSG	Fam131c	ENSMUST00000006380
ENSMUSG	Fblim1	ENSMUST00000006381;ENSMUST00000105784;ENSMUST00000105
ENSMUSG	Ccdc159	ENSMUST00000006403;ENSMUST00000170304;ENSMUST00000217
ENSMUSG	Atp6v1b	ENSMUST00000006431;ENSMUST00000205867;ENSMUST0000020
ENSMUSG	Upk1a	ENSMUST00000006476
ENSMUSG	Ggt1	ENSMUST00000140219;ENSMUST00000152657;ENSMUST0000012
ENSMUSG	Gcat	ENSMUST00000006544;ENSMUST00000171999
ENSMUSG	Tek	ENSMUST00000073939;ENSMUST00000102798;ENSMUST00000071
ENSMUSG	Mpl	ENSMUST00000102671;ENSMUST00000216417
ENSMUSG	Epha2	ENSMUST00000006614;ENSMUST00000131026;ENSMUST00000145
ENSMUSG	Slc34a3	ENSMUST00000006638;ENSMUST00000142095
ENSMUSG	Nsmf	ENSMUST00000100334;ENSMUST00000152122;ENSMUST0000011
ENSMUSG	Itih3	ENSMUST00000006697
ENSMUSG	Itih1	ENSMUST00000163118
ENSMUSG	Ihh	ENSMUST00000164097;ENSMUST00000189364
ENSMUSG	Prkag3	ENSMUST00000188073
ENSMUSG	Cryba2	ENSMUST00000006721;ENSMUST00000133833
ENSMUSG	Slc4a1	ENSMUST00000006749
ENSMUSG	Runx1t1	ENSMUST00000006761;ENSMUST00000150583;ENSMUST00000105
ENSMUSG	Tcf23	ENSMUST00000006818
ENSMUSG	Aplp1	ENSMUST00000006828;ENSMUST00000208792;ENSMUST0000020
ENSMUSG	Zfp184	ENSMUST00000176511;ENSMUST00000176580;ENSMUST00000102
ENSMUSG	Cyp27b1	ENSMUST00000172069
ENSMUSG	Tph2	ENSMUST00000006949
ENSMUSG	Krt23	ENSMUST00000006969
ENSMUSG	Ttc25	ENSMUST00000092684;ENSMUST00000132143;ENSMUST0000000
ENSMUSG	Hap1	ENSMUST00000138603;ENSMUST00000103124;ENSMUST0000017
ENSMUSG	Klk4	ENSMUST00000007161
ENSMUSG	Chrd	ENSMUST00000115423;ENSMUST00000007171;ENSMUST0000015
ENSMUSG	Syngr3	ENSMUST00000007236
ENSMUSG	Ceacam9	ENSMUST00000001984
ENSMUSG	Scube2	ENSMUST00000007423
ENSMUSG	Mc5r	ENSMUST00000172148
ENSMUSG	Hapln4	ENSMUST00000007738;ENSMUST00000213092
ENSMUSG	Gabrb2	ENSMUST00000007797
ENSMUSG	Dio2	ENSMUST00000082432
ENSMUSG	Cpt1c	ENSMUST00000211901;ENSMUST00000212836;ENSMUST0000006
ENSMUSG	Twist2	ENSMUST00000007949;ENSMUST00000186075
ENSMUSG	Tcap	ENSMUST00000008021
ENSMUSG	Hmgcll1	ENSMUST00000008052;ENSMUST00000121592;ENSMUST0000018
ENSMUSG	Cers4	ENSMUST00000177010;ENSMUST00000008350;ENSMUST0000017
ENSMUSG	Fut1	ENSMUST00000210150;ENSMUST00000008605;ENSMUST0000020
ENSMUSG	Elavl2	ENSMUST00000008633;ENSMUST00000107118;ENSMUST00000107
ENSMUSG	Cldn13	ENSMUST00000008987
ENSMUSG	Cd163	ENSMUST00000032234;ENSMUST00000112541;ENSMUST0000020
ENSMUSG	Clec3a	ENSMUST00000009018

ENSMUSG	Slc1a7	ENSMUST00000106713;ENSMUST00000124190
ENSMUSG	Rsph14	ENSMUST00000166088;ENSMUST00000159761
ENSMUSG	Tbx1	ENSMUST00000009241
ENSMUSG	Gnat2	ENSMUST00000058669;ENSMUST00000145101;ENSMUST00000151
ENSMUSG	Spatc1l	ENSMUST00000009259
ENSMUSG	Mymk	ENSMUST00000009358
ENSMUSG	Fam163b	ENSMUST00000151224
ENSMUSG	Ascl2	ENSMUST00000009392;ENSMUST00000121862
ENSMUSG	Lpo	ENSMUST00000103177;ENSMUST00000157004;ENSMUST00000136
ENSMUSG	Slc16a1	ENSMUST00000009522
ENSMUSG	Syn2	ENSMUST00000169345;ENSMUST00000009538;ENSMUST00000203
ENSMUSG	Myod1	ENSMUST00000072514
ENSMUSG	Otog	ENSMUST00000164538;ENSMUST00000210281
ENSMUSG	Odam	ENSMUST00000129757
ENSMUSG	St6galn	ENSMUST00000009732
ENSMUSG	Taf7l	ENSMUST00000009740;ENSMUST00000131311
ENSMUSG	Sardh	ENSMUST00000091224;ENSMUST00000102886;ENSMUST00000142
ENSMUSG	Tex15	ENSMUST00000124496;ENSMUST00000009772
ENSMUSG	Pla2g12	ENSMUST00000009790
ENSMUSG	Oit3	ENSMUST00000009798;ENSMUST00000162493
ENSMUSG	Tex11	ENSMUST00000113718
ENSMUSG	Kcnd1	ENSMUST00000009875;ENSMUST00000125440
ENSMUSG	Pou6f2	ENSMUST00000139064;ENSMUST00000175703;ENSMUST00000175
ENSMUSG	Wnt3a	ENSMUST00000010044
ENSMUSG	Nxf2	ENSMUST00000143059;ENSMUST00000113187;ENSMUST0000010
ENSMUSG	Kif19a	ENSMUST00000126555;ENSMUST00000138804;ENSMUST00000084
ENSMUSG	Slc38a3	ENSMUST00000167868;ENSMUST00000010208;ENSMUST00000194
ENSMUSG	Cacna2d	ENSMUST00000010210;ENSMUST00000168532;ENSMUST00000164
ENSMUSG	Epn3	ENSMUST00000127305;ENSMUST00000010224;ENSMUST00000142
ENSMUSG	Rnf112	ENSMUST00000054927;ENSMUST00000060255;ENSMUST00000102
ENSMUSG	Slc47a1	ENSMUST00000010267;ENSMUST00000131723;ENSMUST00000147
ENSMUSG	Pifo	ENSMUST00000010280;ENSMUST00000066319
ENSMUSG	Prox1	ENSMUST00000175916;ENSMUST00000010319
ENSMUSG	Myt1	ENSMUST00000108756
ENSMUSG	Gm266	ENSMUST00000010673
ENSMUSG	Dazl	ENSMUST00000010736
ENSMUSG	Apol7a	ENSMUST00000176074;ENSMUST00000177135
ENSMUSG	Acaa1b	ENSMUST00000010795;ENSMUST00000127952
ENSMUSG	Phlda2	ENSMUST00000010904;ENSMUST00000207561
ENSMUSG	Asz1	ENSMUST00000136626
ENSMUSG	Wnt2	ENSMUST00000010941
ENSMUSG	Gabra1	ENSMUST00000020707
ENSMUSG	1700006	ENSMUST00000176722
ENSMUSG	Mcoln2	ENSMUST00000011152;ENSMUST00000169533;ENSMUST00000098
ENSMUSG	Slc5a1	ENSMUST00000011178
ENSMUSG	Panx3	ENSMUST00000011262;ENSMUST00000215047
ENSMUSG	Cfap161	ENSMUST00000119134;ENSMUST00000011298
ENSMUSG	Vipr2	ENSMUST00000176078;ENSMUST00000011315;ENSMUST0000010
ENSMUSG	Gm5893	ENSMUST00000164057;ENSMUST00000155799
ENSMUSG	Cpb1	ENSMUST00000011607
ENSMUSG	Slc25a4	ENSMUST00000058661
ENSMUSG	Pinlyp	ENSMUST00000011776
ENSMUSG	4930579	ENSMUST00000012186
ENSMUSG	Mogat1	ENSMUST00000113524;ENSMUST00000152111;ENSMUST00000012
ENSMUSG	Wnt8a	ENSMUST00000012426
ENSMUSG	Nanog	ENSMUST00000112581;ENSMUST00000112580;ENSMUST00000012
ENSMUSG	Mlkl	ENSMUST00000056157;ENSMUST00000120432;ENSMUST00000135

ENSMUSG	Phox2b	ENSMUST00000012664
ENSMUSG	2200002	ENSMUST00000013227
ENSMUSG	Tmem190	ENSMUST00000013235;ENSMUST00000206395
ENSMUSG	4931406	ENSMUST00000013497;ENSMUST00000168213;ENSMUST00000171
ENSMUSG	Iglon5	ENSMUST00000107974
ENSMUSG	Igf2bp1	ENSMUST00000013559
ENSMUSG	B4galnt	ENSMUST00000038343
ENSMUSG	Bcas1	ENSMUST00000109152;ENSMUST00000013667;ENSMUST00000156
ENSMUSG	Aldh1a2	ENSMUST00000034723
ENSMUSG	Snx31	ENSMUST00000013755;ENSMUST00000162888
ENSMUSG	1810065	ENSMUST00000013797
ENSMUSG	Myl2	ENSMUST00000111751
ENSMUSG	Mcemp1	ENSMUST00000014118
ENSMUSG	Pax5	ENSMUST00000014174;ENSMUST00000107825;ENSMUST00000107
ENSMUSG	Trpv4	ENSMUST00000071968;ENSMUST00000112225;ENSMUST00000133
ENSMUSG	Klhl3	ENSMUST00000091583;ENSMUST00000160860
ENSMUSG	Gip	ENSMUST00000103157
ENSMUSG	Clec4f	ENSMUST00000014686
ENSMUSG	Kif1a	ENSMUST00000171796;ENSMUST00000190723;ENSMUST00000188
ENSMUSG	Alx3	ENSMUST00000014747
ENSMUSG	Ceacam1	ENSMUST00000014830
ENSMUSG	Plekhg4	ENSMUST00000160650;ENSMUST00000214056;ENSMUST00000159
ENSMUSG	Stc1	ENSMUST00000014957
ENSMUSG	Gata2	ENSMUST00000170089;ENSMUST00000015197;ENSMUST00000205
ENSMUSG	Ptgds	ENSMUST00000114259;ENSMUST00000015234;ENSMUST00000114
ENSMUSG	Cyp2j9	ENSMUST00000055693;ENSMUST00000126509
ENSMUSG	Xk	ENSMUST00000015486
ENSMUSG	Pcolce2	ENSMUST00000015498;ENSMUST00000151210
ENSMUSG	Dennd6b	ENSMUST00000078953
ENSMUSG	Ace2	ENSMUST00000112271
ENSMUSG	Gzmb	ENSMUST00000015581
ENSMUSG	Notch4	ENSMUST00000015612;ENSMUST00000126950;ENSMUST00000173
ENSMUSG	Fam163a	ENSMUST00000015628
ENSMUSG	2310057	ENSMUST00000140558;ENSMUST00000015663
ENSMUSG	Nkx2-5	ENSMUST00000015723
ENSMUSG	Gata5	ENSMUST00000015771
ENSMUSG	Steap1	ENSMUST00000015796
ENSMUSG	Steap2	ENSMUST00000115426;ENSMUST00000164219;ENSMUST00000115
ENSMUSG	Awat1	ENSMUST00000096361
ENSMUSG	Arnt2	ENSMUST00000085077;ENSMUST00000209133;ENSMUST00000208
ENSMUSG	Nlrp5	ENSMUST00000015866
ENSMUSG	Eps8	ENSMUST00000058210;ENSMUST00000100841;ENSMUST00000147
ENSMUSG	Abo	ENSMUST00000114045
ENSMUSG	Tnr	ENSMUST00000192069;ENSMUST00000195199;ENSMUST00000193
ENSMUSG	Rxrg	ENSMUST00000015987;ENSMUST00000111380
ENSMUSG	Fcrls	ENSMUST00000090986
ENSMUSG	Fcgr1	ENSMUST00000029748
ENSMUSG	Lbp	ENSMUST00000109491;ENSMUST00000152541;ENSMUST00000016
ENSMUSG	Syt14	ENSMUST00000195354;ENSMUST00000215093;ENSMUST00000016
ENSMUSG	Lonrf3	ENSMUST00000135758;ENSMUST00000016383;ENSMUST00000126
ENSMUSG	Tubb1	ENSMUST00000016399
ENSMUSG	Atp1b4	ENSMUST00000016471
ENSMUSG	Kcnq2	ENSMUST00000149964;ENSMUST00000103049;ENSMUST00000152
ENSMUSG	Eef1a2	ENSMUST00000055990
ENSMUSG	Mpped2	ENSMUST00000111063;ENSMUST00000144155;ENSMUST00000125
ENSMUSG	Wt1	ENSMUST00000143043;ENSMUST00000213301;ENSMUST00000133
ENSMUSG	Il19	ENSMUST00000187410

ENSMUSG	Dyrk3	ENSMUST00000016670;ENSMUST00000189756
ENSMUSG	Il10	ENSMUST00000016673
ENSMUSG	Foxred2	ENSMUST00000016696;ENSMUST00000140096;ENSMUST00000117
ENSMUSG	Phf21b	ENSMUST00000016768;ENSMUST00000159939;ENSMUST0000015
ENSMUSG	Nlrp14	ENSMUST00000142623
ENSMUSG	Bik	ENSMUST00000016902
ENSMUSG	Scube1	ENSMUST00000171496;ENSMUST00000144773
ENSMUSG	Pom121l	ENSMUST00000117882;ENSMUST00000017126
ENSMUSG	Matn4	ENSMUST00000103103;ENSMUST00000154940
ENSMUSG	Rbpjl	ENSMUST00000137427;ENSMUST00000017151
ENSMUSG	Gast	ENSMUST00000017309
ENSMUSG	Gsdma	ENSMUST00000017348
ENSMUSG	Gsdma2	ENSMUST00000093938;ENSMUST00000138686;ENSMUST00000136
ENSMUSG	Pyy	ENSMUST00000177304;ENSMUST00000017455;ENSMUST00000127
ENSMUSG	Mpp2	ENSMUST00000017458;ENSMUST00000100398;ENSMUST00000147
ENSMUSG	Ppy	ENSMUST00000170554;ENSMUST00000017460
ENSMUSG	Vtn	ENSMUST00000128479;ENSMUST00000017488;ENSMUST00000146
ENSMUSG	Stac2	ENSMUST00000017544
ENSMUSG	Pipox	ENSMUST00000017597;ENSMUST00000151383;ENSMUST00000155
ENSMUSG	Zfp334	ENSMUST00000103084
ENSMUSG	Hnf4g	ENSMUST00000108393;ENSMUST00000108394
ENSMUSG	Eppin	ENSMUST00000103100
ENSMUSG	Jph2	ENSMUST00000017961
ENSMUSG	Hspb9	ENSMUST00000169833;ENSMUST00000017976
ENSMUSG	Sgk2	ENSMUST00000018012;ENSMUST00000117123
ENSMUSG	Gdap1l1	ENSMUST00000109420;ENSMUST00000109421;ENSMUST00000018
ENSMUSG	Hnf4a	ENSMUST00000143911;ENSMUST00000109411;ENSMUST00000137
ENSMUSG	Baiap2l	ENSMUST00000165408;ENSMUST00000169462;ENSMUST00000167
ENSMUSG	Wfdc15b	ENSMUST00000164567
ENSMUSG	Gdf9	ENSMUST00000156037;ENSMUST00000145369;ENSMUST00000018
ENSMUSG	Tbx5	ENSMUST00000018407
ENSMUSG	Shroom1	ENSMUST00000109013;ENSMUST00000018531;ENSMUST00000146
ENSMUSG	Slc13a3	ENSMUST00000029208;ENSMUST00000145513;ENSMUST00000143
ENSMUSG	1700125	ENSMUST00000018623
ENSMUSG	Wnt9b	ENSMUST00000018630
ENSMUSG	Adora2b	ENSMUST00000018644
ENSMUSG	1700001	ENSMUST00000107576
ENSMUSG	Slc2a4	ENSMUST00000178809;ENSMUST00000018710;ENSMUST00000141
ENSMUSG	Tbx3	ENSMUST00000202034;ENSMUST00000079719;ENSMUST00000018
ENSMUSG	Mmp20	ENSMUST00000034487
ENSMUSG	Mmp7	ENSMUST00000018767
ENSMUSG	Crhr1	ENSMUST00000093925
ENSMUSG	Dusp14	ENSMUST00000100705;ENSMUST00000018792;ENSMUST00000164
ENSMUSG	Tcaf3	ENSMUST00000069023;ENSMUST00000134707
ENSMUSG	Lhx1	ENSMUST00000018842
ENSMUSG	Abca5	ENSMUST00000043961;ENSMUST00000124714;ENSMUST00000127
ENSMUSG	Wwc1	ENSMUST00000018993;ENSMUST00000131001;ENSMUST00000127
ENSMUSG	Otop3	ENSMUST00000019006
ENSMUSG	Sult4a1	ENSMUST00000082365
ENSMUSG	Pnpla5	ENSMUST00000019012
ENSMUSG	Mb	ENSMUST00000169226
ENSMUSG	P4ha2	ENSMUST00000151218;ENSMUST00000093107;ENSMUST00000125
ENSMUSG	Il3	ENSMUST00000019058
ENSMUSG	Csf2	ENSMUST00000019060
ENSMUSG	Tm4sf5	ENSMUST00000019063;ENSMUST00000179986;ENSMUST00000141
ENSMUSG	Ccl4	ENSMUST00000019074
ENSMUSG	Aldh3a1	ENSMUST00000019246

ENSMUSG	Cacng2	ENSMUST00000019290
ENSMUSG	Lhx9	ENSMUST00000019374;ENSMUST00000046870;ENSMUST00000192
ENSMUSG	Etnppl	ENSMUST00000072271;ENSMUST00000199940;ENSMUST00000170
ENSMUSG	Gdpd2	ENSMUST00000144912;ENSMUST00000156336;ENSMUST00000019
ENSMUSG	Sec14l4	ENSMUST00000131171;ENSMUST00000019512;ENSMUST00000136
ENSMUSG	Ffar3	ENSMUST00000094583;ENSMUST00000185748
ENSMUSG	Cd70	ENSMUST00000019633
ENSMUSG	Cyb561	ENSMUST00000019734;ENSMUST00000184086;ENSMUST00000146
ENSMUSG	Sema6a	ENSMUST00000123228;ENSMUST00000019791;ENSMUST00000115
ENSMUSG	L3hypdh	ENSMUST00000019862;ENSMUST00000124405;ENSMUST00000138
ENSMUSG	Prl8a1	ENSMUST00000095926
ENSMUSG	Iyd	ENSMUST00000019896;ENSMUST00000141745
ENSMUSG	Ccdc170	ENSMUST00000138112;ENSMUST00000019901;ENSMUST00000157
ENSMUSG	Vip	ENSMUST00000019906;ENSMUST00000217331
ENSMUSG	Rgs17	ENSMUST00000131996;ENSMUST00000064225;ENSMUST00000019
ENSMUSG	Frk	ENSMUST00000019913;ENSMUST00000215144;ENSMUST00000170
ENSMUSG	Trdn	ENSMUST00000219982;ENSMUST00000095762;ENSMUST00000219
ENSMUSG	Hey2	ENSMUST00000019924;ENSMUST00000217504
ENSMUSG	Lrp11	ENSMUST00000134346;ENSMUST00000019931;ENSMUST00000130
ENSMUSG	Nr2e1	ENSMUST00000019938
ENSMUSG	Plagl1	ENSMUST00000193426;ENSMUST00000123135;ENSMUST00000135
ENSMUSG	Grm1	ENSMUST00000044306
ENSMUSG	Wasf1	ENSMUST00000019975;ENSMUST00000105509
ENSMUSG	Amd-ps4	ENSMUST00000118392
ENSMUSG	Popdc3	ENSMUST00000175658;ENSMUST00000019994
ENSMUSG	Arfgef3	ENSMUST00000215836
ENSMUSG	Hebp2	ENSMUST00000020000;ENSMUST00000214548
ENSMUSG	Fam184a	ENSMUST00000020003;ENSMUST00000218659;ENSMUST00000171
ENSMUSG	Fabp7	ENSMUST00000020024;ENSMUST00000165013
ENSMUSG	Pkib	ENSMUST00000075992;ENSMUST00000177299;ENSMUST00000176
ENSMUSG	Rspo3	ENSMUST00000215256;ENSMUST00000092623
ENSMUSG	Mgat4c	ENSMUST00000179929;ENSMUST00000219683;ENSMUST00000218
ENSMUSG	Nts	ENSMUST00000020040
ENSMUSG	Dcbld1	ENSMUST00000069004;ENSMUST00000218278;ENSMUST00000218
ENSMUSG	Lrriq1	ENSMUST00000218841;ENSMUST00000166240;ENSMUST00000217
ENSMUSG	Slc6a15	ENSMUST00000218844;ENSMUST00000074204;ENSMUST00000219
ENSMUSG	Rfx6	ENSMUST00000122922
ENSMUSG	Gprc6a	ENSMUST00000020062
ENSMUSG	Lin7a	ENSMUST00000020057;ENSMUST00000105280;ENSMUST00000218
ENSMUSG	Sim1	ENSMUST00000219436;ENSMUST00000020071
ENSMUSG	Kera	ENSMUST00000105286
ENSMUSG	Mrln	ENSMUST00000188568;ENSMUST00000020090;ENSMUST00000190
ENSMUSG	Epyc	ENSMUST00000105285
ENSMUSG	1700040	ENSMUST00000020103;ENSMUST00000166919
ENSMUSG	Cep290	ENSMUST00000220346;ENSMUST00000219765;ENSMUST00000220
ENSMUSG	Enpp3	ENSMUST00000020169;ENSMUST00000219861;ENSMUST00000218
ENSMUSG	Ctgf	ENSMUST00000020171;ENSMUST00000176228;ENSMUST00000129
ENSMUSG	Moxd1	ENSMUST00000095784;ENSMUST00000154916
ENSMUSG	Il20ra	ENSMUST00000020185
ENSMUSG	Vnn3	ENSMUST00000020190
ENSMUSG	Cfap54	ENSMUST00000168110;ENSMUST00000212902;ENSMUST00000163
ENSMUSG	Ntn4	ENSMUST00000147080;ENSMUST00000020204
ENSMUSG	4930463	ENSMUST00000020222
ENSMUSG	Rfx4	ENSMUST00000060397;ENSMUST00000095388
ENSMUSG	Pah	ENSMUST00000020241
ENSMUSG	Mybpc1	ENSMUST00000121629;ENSMUST00000124144;ENSMUST00000119
ENSMUSG	Slc5a8	ENSMUST00000020255

ENSMUSG	Mypn	ENSMUST00000095580
ENSMUSG	Hkdc1	ENSMUST00000020277;ENSMUST00000159454;ENSMUST0000015[illegible]
ENSMUSG	Tacr2	ENSMUST00000020278
ENSMUSG	2010107[illegible]	ENSMUST00000125704;ENSMUST00000142821;ENSMUST00000124
ENSMUSG	Pald1	ENSMUST00000020289;ENSMUST00000219240;ENSMUST0000021[illegible]
ENSMUSG	Slc16a7	ENSMUST00000211781;ENSMUST00000210780;ENSMUST00000105
ENSMUSG	Lrig3	ENSMUST00000218363;ENSMUST00000217813;ENSMUST0000022[illegible]
ENSMUSG	Srgap1	ENSMUST00000020322;ENSMUST00000081688;ENSMUST00000161
ENSMUSG	Pcsk4	ENSMUST00000137177;ENSMUST00000147132;ENSMUST0000013[illegible]
ENSMUSG	Lgr5	ENSMUST00000020350;ENSMUST00000173619;ENSMUST00000105
ENSMUSG	Gamt	ENSMUST00000105363;ENSMUST00000020359;ENSMUST00000148
ENSMUSG	Ptprr	ENSMUST00000063470;ENSMUST00000155606;ENSMUST00000128
ENSMUSG	Gabrp	ENSMUST00000020366;ENSMUST00000135350
ENSMUSG	Olfr299	ENSMUST00000057734
ENSMUSG	Best3	ENSMUST00000020378
ENSMUSG	Rab36	ENSMUST00000136944;ENSMUST00000147802;ENSMUST00000142
ENSMUSG	9530003[illegible]	ENSMUST00000159193;ENSMUST00000020392
ENSMUSG	Nav3	ENSMUST00000032719;ENSMUST00000161582;ENSMUST00000161
ENSMUSG	Ddc	ENSMUST00000178704;ENSMUST00000136810;ENSMUST0000010[illegible]
ENSMUSG	Jsrp1	ENSMUST00000020435;ENSMUST00000181945
ENSMUSG	Wif1	ENSMUST00000020439
ENSMUSG	Slc5a4b	ENSMUST00000120757
ENSMUSG	D10Wsu1	ENSMUST00000020488
ENSMUSG	Aldh1l2	ENSMUST00000020497;ENSMUST00000146640;ENSMUST00000147
ENSMUSG	Glyctk	ENSMUST00000036382;ENSMUST00000112543;ENSMUST00000217
ENSMUSG	Adarb1	ENSMUST00000020496;ENSMUST00000098374;ENSMUST00000105
ENSMUSG	Il9r	ENSMUST00000145401;ENSMUST00000020518;ENSMUST00000128
ENSMUSG	1700093[illegible]	ENSMUST00000020527;ENSMUST00000169264;ENSMUST0000014[illegible]
ENSMUSG	4930524[illegible]	ENSMUST00000102829
ENSMUSG	Stc2	ENSMUST00000020546;ENSMUST00000139991;ENSMUST0000014[illegible]
ENSMUSG	Madcam1	ENSMUST00000020554;ENSMUST00000217748
ENSMUSG	Shc2	ENSMUST00000168116;ENSMUST00000020564;ENSMUST0000016[illegible]
ENSMUSG	Theg	ENSMUST00000159782;ENSMUST00000020566
ENSMUSG	Acsl6	ENSMUST00000108905;ENSMUST00000101211;ENSMUST00000064
ENSMUSG	Slc22a4	ENSMUST00000020586;ENSMUST00000146351;ENSMUST00000154
ENSMUSG	Zfp354b	ENSMUST00000109124;ENSMUST00000152296
ENSMUSG	Flt4	ENSMUST00000152253;ENSMUST00000020617
ENSMUSG	Zfp354a	ENSMUST00000102766;ENSMUST00000156914;ENSMUST0000010[illegible]
ENSMUSG	Rasgef1[illegible]	ENSMUST00000063444
ENSMUSG	Il13	ENSMUST00000020650
ENSMUSG	Nefh	ENSMUST00000093369
ENSMUSG	Havcr2	ENSMUST00000020668
ENSMUSG	Fam71b	ENSMUST00000139280;ENSMUST00000063166
ENSMUSG	Fabp6	ENSMUST00000020672
ENSMUSG	Upp1	ENSMUST00000020677;ENSMUST00000101525;ENSMUST00000164
ENSMUSG	Hormad2	ENSMUST00000109948;ENSMUST00000130174
ENSMUSG	Igfbp1	ENSMUST00000020704
ENSMUSG	Adcy1	ENSMUST00000020706;ENSMUST00000135398
ENSMUSG	4921536[illegible]	ENSMUST00000020712
ENSMUSG	Osbp2	ENSMUST00000070552;ENSMUST00000155197;ENSMUST0000013[illegible]
ENSMUSG	Gabrg2	ENSMUST00000070725;ENSMUST00000146198
ENSMUSG	Npc1l1	ENSMUST00000004505
ENSMUSG	Myl7	ENSMUST00000102921
ENSMUSG	Btnl10	ENSMUST00000069941;ENSMUST00000142499
ENSMUSG	Gria1	ENSMUST00000036315
ENSMUSG	Myocd	ENSMUST00000108695;ENSMUST00000151483
ENSMUSG	Lrrc72	ENSMUST00000221155

ENSMUSG	Pctp	ENSMUST00000020864
ENSMUSG	Efcab10	ENSMUST00000020878
ENSMUSG	Agr2	ENSMUST00000147861;ENSMUST00000020898
ENSMUSG	Ntsr2	ENSMUST00000111064;ENSMUST00000222957
ENSMUSG	Rgs9	ENSMUST00000020920;ENSMUST00000103062;ENSMUST0000010[illegible]
ENSMUSG	Fam84a	ENSMUST00000020926;ENSMUST00000221405
ENSMUSG	Apob	ENSMUST00000171271;ENSMUST00000037811
ENSMUSG	1700012[illegible]	ENSMUST00000106674;ENSMUST00000020941
ENSMUSG	Abca8b	ENSMUST00000020948;ENSMUST00000106669
ENSMUSG	Nt5c1b	ENSMUST00000218026
ENSMUSG	4933434[illegible]	ENSMUST00000149136;ENSMUST00000153429
ENSMUSG	Dcdc2c	ENSMUST00000189735;ENSMUST00000221349
ENSMUSG	Fkbp1b	ENSMUST00000020964;ENSMUST00000219880
ENSMUSG	Allc	ENSMUST00000020965
ENSMUSG	Pfn4	ENSMUST00000219503;ENSMUST00000020967;ENSMUST0000017[illegible]
ENSMUSG	Slc26a4	ENSMUST00000001253
ENSMUSG	Dnajc27	ENSMUST00000020986;ENSMUST00000049584;ENSMUST0000021[illegible]
ENSMUSG	Efr3b	ENSMUST00000111178;ENSMUST00000219331;ENSMUST0000021[illegible]
ENSMUSG	Pomc	ENSMUST00000219543;ENSMUST00000218089;ENSMUST0000002[illegible]
ENSMUSG	Sntg2	ENSMUST00000021004;ENSMUST00000130062;ENSMUST0000015[illegible]
ENSMUSG	Tpo	ENSMUST00000021005;ENSMUST00000140875;ENSMUST0000015[illegible]
ENSMUSG	Hnf1b	ENSMUST00000021016
ENSMUSG	Rasl10b	ENSMUST00000021022;ENSMUST00000164425
ENSMUSG	Gas2l2	ENSMUST00000052521;ENSMUST00000147301
ENSMUSG	Efcab3	ENSMUST00000154543
ENSMUSG	Mrc2	ENSMUST00000126931;ENSMUST00000100335;ENSMUST00000021
ENSMUSG	Tmem132[illegible]	ENSMUST00000054245;ENSMUST00000202598
ENSMUSG	Ccl1	ENSMUST00000108189;ENSMUST00000138333;ENSMUST00000021
ENSMUSG	5530401[illegible]	ENSMUST00000021044
ENSMUSG	Asic2	ENSMUST00000021045;ENSMUST00000066197
ENSMUSG	Tcam1	ENSMUST00000142472;ENSMUST00000044462
ENSMUSG	Gh	ENSMUST00000103071
ENSMUSG	Cacng1	ENSMUST00000021065
ENSMUSG	Cacng4	ENSMUST00000021066;ENSMUST00000134076
ENSMUSG	Cep112	ENSMUST00000150863;ENSMUST00000061287;ENSMUST0000014[illegible]
ENSMUSG	Grin2c	ENSMUST00000106554
ENSMUSG	Aspa	ENSMUST00000021119;ENSMUST00000184572;ENSMUST00000132
ENSMUSG	Tekt1	ENSMUST00000108502;ENSMUST00000108503;ENSMUST00000021
ENSMUSG	Slc13a5	ENSMUST00000021161
ENSMUSG	Pld2	ENSMUST00000157075;ENSMUST00000018429;ENSMUST0000010[illegible]
ENSMUSG	Slc46a1	ENSMUST00000001126;ENSMUST00000146431;ENSMUST0000018[illegible]
ENSMUSG	Vmo1	ENSMUST00000021179
ENSMUSG	Coro6	ENSMUST00000102493;ENSMUST00000052515;ENSMUST0000010[illegible]
ENSMUSG	Tmigd1	ENSMUST00000102495;ENSMUST00000142166
ENSMUSG	Rph3al	ENSMUST00000121287;ENSMUST00000066504;ENSMUST00000021
ENSMUSG	Doc2b	ENSMUST00000021209
ENSMUSG	Cacna1g	ENSMUST00000107786;ENSMUST00000107789;ENSMUST00000021
ENSMUSG	Dlx4	ENSMUST00000021241;ENSMUST00000146838
ENSMUSG	Hoxb9	ENSMUST00000000010
ENSMUSG	Asgr1	ENSMUST00000108585;ENSMUST00000092959
ENSMUSG	A230052[illegible]	ENSMUST00000050921
ENSMUSG	Gucy2e	ENSMUST00000021259;ENSMUST00000108664;ENSMUST0000015[illegible]
ENSMUSG	Alox8	ENSMUST00000021262;ENSMUST00000144787;ENSMUST0000015[illegible]
ENSMUSG	Aloxe3	ENSMUST00000021268;ENSMUST00000155324;ENSMUST0000017[illegible]
ENSMUSG	Cfap52	ENSMUST00000142929;ENSMUST00000021287
ENSMUSG	Usp43	ENSMUST00000021288;ENSMUST00000108677;ENSMUST00000152
ENSMUSG	Myh3	ENSMUST00000108689;ENSMUST00000165221

ENSMUSG	Krt12	ENSMUST00000017741
ENSMUSG	Krt24	ENSMUST00000017255
ENSMUSG	Krt36	ENSMUST00000107416
ENSMUSG	Lyzl6	ENSMUST00000021328
ENSMUSG	Foxg1	ENSMUST00000135006;ENSMUST00000154930;ENSMUST0000017[illegible]
ENSMUSG	Dtd2	ENSMUST00000085404;ENSMUST00000021339
ENSMUSG	Spata7	ENSMUST00000048402;ENSMUST00000155899;ENSMUST00000222
ENSMUSG	Ptpn21	ENSMUST00000170188;ENSMUST00000223321;ENSMUST0000008[illegible]
ENSMUSG	Npas3	ENSMUST00000223358;ENSMUST00000101432;ENSMUST0000022[illegible]
ENSMUSG	Nova1	ENSMUST00000021438;ENSMUST00000219886;ENSMUST0000021[illegible]
ENSMUSG	Esr2	ENSMUST00000101291
ENSMUSG	Bdkrb2	ENSMUST00000001652
ENSMUSG	Trim9	ENSMUST00000110522;ENSMUST00000222316;ENSMUST00000223
ENSMUSG	Rtn1	ENSMUST00000150156;ENSMUST00000078505;ENSMUST00000021
ENSMUSG	Lrrc9	ENSMUST00000161284;ENSMUST00000162159;ENSMUST00000162
ENSMUSG	Gsc	ENSMUST00000021513
ENSMUSG	4930447[illegible]	ENSMUST00000110489;ENSMUST00000044000;ENSMUST00000132
ENSMUSG	Six6	ENSMUST00000021519
ENSMUSG	Rdh12	ENSMUST00000021548;ENSMUST00000122227;ENSMUST00000151
ENSMUSG	Galnt16	ENSMUST00000219993;ENSMUST00000218648;ENSMUST00000217
ENSMUSG	Gm9745	ENSMUST00000187196;ENSMUST00000021573
ENSMUSG	Fbln5	ENSMUST00000021603;ENSMUST00000222587;ENSMUST00000221
ENSMUSG	Tc2n	ENSMUST00000162735;ENSMUST00000110047
ENSMUSG	Unc79	ENSMUST00000101099;ENSMUST00000179002;ENSMUST0000017[illegible]
ENSMUSG	Ifi27l2[illegible]	ENSMUST00000044687
ENSMUSG	Ppp4r4	ENSMUST00000021631;ENSMUST00000187155;ENSMUST0000019[illegible]
ENSMUSG	Akr1c12	ENSMUST00000021632
ENSMUSG	Akr1c18	ENSMUST00000110704;ENSMUST00000021635
ENSMUSG	Tubal3	ENSMUST00000021639
ENSMUSG	Tshz3	ENSMUST00000021641;ENSMUST00000176413
ENSMUSG	Rgs6	ENSMUST00000186848;ENSMUST00000161801;ENSMUST0000018[illegible]
ENSMUSG	Dpf3	ENSMUST00000178756;ENSMUST00000140327;ENSMUST00000144
ENSMUSG	Acot3	ENSMUST00000021653
ENSMUSG	Vsx2	ENSMUST00000021665;ENSMUST00000169934
ENSMUSG	Fos	ENSMUST00000021674;ENSMUST00000136122;ENSMUST0000014[illegible]
ENSMUSG	Esrrb	ENSMUST00000116402
ENSMUSG	Vash1	ENSMUST00000021681
ENSMUSG	Angel1	ENSMUST00000021682;ENSMUST00000167813;ENSMUST0000016[illegible]
ENSMUSG	Cyp46a1	ENSMUST00000021684;ENSMUST00000221708;ENSMUST00000222
ENSMUSG	Hhipl1	ENSMUST00000021685;ENSMUST00000223395
ENSMUSG	Meg3	ENSMUST00000129245;ENSMUST00000143836;ENSMUST00000124
ENSMUSG	Amn	ENSMUST00000021707;ENSMUST00000220903;ENSMUST0000022[illegible]
ENSMUSG	Tnfaip2	ENSMUST00000102745;ENSMUST00000109792;ENSMUST0000015[illegible]
ENSMUSG	Kif26a	ENSMUST00000128402;ENSMUST00000183816
ENSMUSG	Hecw1	ENSMUST00000110516;ENSMUST00000221863;ENSMUST00000222
ENSMUSG	Gng4	ENSMUST00000138783;ENSMUST00000021734;ENSMUST0000022[illegible]
ENSMUSG	Gpr137b	ENSMUST00000021738;ENSMUST00000222958;ENSMUST00000223
ENSMUSG	Ryr2	ENSMUST00000021750;ENSMUST00000222788;ENSMUST00000221
ENSMUSG	Sfrp4	ENSMUST00000222464;ENSMUST00000002883
ENSMUSG	Aoah	ENSMUST00000021757;ENSMUST00000223364;ENSMUST00000221
ENSMUSG	Slc17a1	ENSMUST00000006785
ENSMUSG	Slc17a4	ENSMUST00000225797
ENSMUSG	Scgn	ENSMUST00000021770
ENSMUSG	Tfap2a	ENSMUST00000021787;ENSMUST00000110193;ENSMUST00000224
ENSMUSG	Gcm2	ENSMUST00000021791
ENSMUSG	Mak	ENSMUST00000225084;ENSMUST00000225789
ENSMUSG	Elovl2	ENSMUST00000021793

ENSMUSG	Edn1	ENSMUST00000021796
ENSMUSG	Barx1	ENSMUST00000021813
ENSMUSG	Nxnl2	ENSMUST00000021828;ENSMUST00000221339
ENSMUSG	Serpinb	ENSMUST00000006392
ENSMUSG	Fam217a	ENSMUST00000021851;ENSMUST00000225242
ENSMUSG	Rpp40	ENSMUST00000171686;ENSMUST00000174852;ENSMUST00000174
ENSMUSG	Ctsq	ENSMUST00000021888
ENSMUSG	Fbp2	ENSMUST00000021907
ENSMUSG	Ror2	ENSMUST00000130235;ENSMUST00000021918
ENSMUSG	Drd1	ENSMUST00000221470
ENSMUSG	Slc34a1	ENSMUST00000225259;ENSMUST00000225538
ENSMUSG	F12	ENSMUST00000021948
ENSMUSG	Pitx1	ENSMUST00000021968
ENSMUSG	Cxcl14	ENSMUST00000224801;ENSMUST00000021970
ENSMUSG	Slc25a4	ENSMUST00000021971
ENSMUSG	1700001	ENSMUST00000022007
ENSMUSG	Adcy2	ENSMUST00000022013
ENSMUSG	Il9	ENSMUST00000022019
ENSMUSG	Lect2	ENSMUST00000062806
ENSMUSG	1700067	ENSMUST00000022028
ENSMUSG	Gkap1	ENSMUST00000091579;ENSMUST00000225098;ENSMUST00000224
ENSMUSG	Slc28a3	ENSMUST00000140760;ENSMUST00000022036;ENSMUST00000151
ENSMUSG	Slc6a19	ENSMUST00000022048;ENSMUST00000120322;ENSMUST00000132
ENSMUSG	Slc6a19	ENSMUST00000022049;ENSMUST00000160958
ENSMUSG	Ahrr	ENSMUST00000022059;ENSMUST00000148438
ENSMUSG	Pcsk1	ENSMUST00000022075;ENSMUST00000135349;ENSMUST00000222
ENSMUSG	Rhobtb3	ENSMUST00000022078;ENSMUST00000123353;ENSMUST00000222
ENSMUSG	Spata9	ENSMUST00000225553;ENSMUST00000022081;ENSMUST00000223
ENSMUSG	Irx4	ENSMUST00000176684;ENSMUST00000022095
ENSMUSG	Slc6a3	ENSMUST00000022100
ENSMUSG	Slc6a18	ENSMUST00000109679;ENSMUST00000109680
ENSMUSG	Mrps36-	ENSMUST00000061311
ENSMUSG	Ocln	ENSMUST00000069756;ENSMUST00000022140
ENSMUSG	Serf1	ENSMUST00000022145;ENSMUST00000132053;ENSMUST0000012[illegible]
ENSMUSG	Cartpt	ENSMUST00000022150;ENSMUST00000224142
ENSMUSG	S100z	ENSMUST00000022186
ENSMUSG	Crhbp	ENSMUST00000221025;ENSMUST00000045583
ENSMUSG	Elovl7	ENSMUST00000022207;ENSMUST00000225550
ENSMUSG	Rab3c	ENSMUST00000167824;ENSMUST00000226040
ENSMUSG	Plk2	ENSMUST00000022212;ENSMUST00000224489;ENSMUST00000223
ENSMUSG	Thbs4	ENSMUST00000022213;ENSMUST00000168299
ENSMUSG	Mtx3	ENSMUST00000223719;ENSMUST00000076169;ENSMUST00000224
ENSMUSG	4933425	ENSMUST00000022232
ENSMUSG	Rgs7bp	ENSMUST00000063551
ENSMUSG	Rnf180	ENSMUST00000226044;ENSMUST00000224662;ENSMUST00000224
ENSMUSG	Htr1a	ENSMUST00000022235
ENSMUSG	Hcn1	ENSMUST00000207599;ENSMUST00000006991
ENSMUSG	Fgf10	ENSMUST00000128103;ENSMUST00000146674;ENSMUST00000022
ENSMUSG	Gm5457	ENSMUST00000022260
ENSMUSG	Oit1	ENSMUST00000022269;ENSMUST00000224529
ENSMUSG	Ddx4	ENSMUST00000099166
ENSMUSG	Fst	ENSMUST00000022287;ENSMUST00000223640;ENSMUST00000223
ENSMUSG	Sftpa1	ENSMUST00000170719
ENSMUSG	Dydc1	ENSMUST00000189865
ENSMUSG	Sftpd	ENSMUST00000077136;ENSMUST00000225892
ENSMUSG	Ldb3	ENSMUST00000090040;ENSMUST00000022327;ENSMUST00000022
ENSMUSG	Opn4	ENSMUST00000168444

ENSMUSG	Rgr	ENSMUST00000225070
ENSMUSG	Bmp4	ENSMUST00000074077;ENSMUST00000135408
ENSMUSG	Samd4	ENSMUST00000022386;ENSMUST00000100672;ENSMUST00000141
ENSMUSG	Otx2	ENSMUST00000118578
ENSMUSG	Slc35f4	ENSMUST00000074368;ENSMUST00000135254;ENSMUST0000014(
ENSMUSG	4933413	ENSMUST00000160661;ENSMUST00000059648;ENSMUST0000015(
ENSMUSG	Dnah12	ENSMUST00000022433;ENSMUST00000073309
ENSMUSG	Sema3g	ENSMUST00000090180
ENSMUSG	Ogdhl	ENSMUST00000022480
ENSMUSG	Chat	ENSMUST00000070125
ENSMUSG	Itih4	ENSMUST00000006703;ENSMUST00000078490
ENSMUSG	Gucy1b2	ENSMUST00000022501
ENSMUSG	Ptpn20	ENSMUST00000022508
ENSMUSG	Gdf10	ENSMUST00000168727
ENSMUSG	Tdh	ENSMUST00000022522;ENSMUST00000170772;ENSMUST00000167
ENSMUSG	4930578	ENSMUST00000224600
ENSMUSG	Prss52	ENSMUST00000022537
ENSMUSG	Fgf9	ENSMUST00000165526;ENSMUST00000022545;ENSMUST00000074
ENSMUSG	1700129	ENSMUST00000022548
ENSMUSG	Amer2	ENSMUST00000022561
ENSMUSG	Wnt5a	ENSMUST00000063465;ENSMUST00000134163
ENSMUSG	Erich6b	ENSMUST00000022579
ENSMUSG	Tnfsf11	ENSMUST00000022592
ENSMUSG	Mtrf1	ENSMUST00000022600
ENSMUSG	Nefm	ENSMUST00000111089;ENSMUST00000022638
ENSMUSG	Nefl	ENSMUST00000022639
ENSMUSG	Adam7	ENSMUST00000022640;ENSMUST00000128069
ENSMUSG	Nkx3-1	ENSMUST00000022646
ENSMUSG	Tnfrsf1	ENSMUST00000022663;ENSMUST00000224533;ENSMUST00000224
ENSMUSG	Klhl1	ENSMUST00000022666
ENSMUSG	Pebp4	ENSMUST00000022678
ENSMUSG	Sftpc	ENSMUST00000022692
ENSMUSG	Dmtn	ENSMUST00000022694;ENSMUST00000110984;ENSMUST00000022
ENSMUSG	Fgf17	ENSMUST00000022697
ENSMUSG	Gpc5	ENSMUST00000176993;ENSMUST00000176912;ENSMUST00000175
ENSMUSG	4930449	ENSMUST00000022711;ENSMUST00000193202;ENSMUST00000188
ENSMUSG	Ednrb	ENSMUST00000022718;ENSMUST00000172237
ENSMUSG	Scel	ENSMUST00000095576
ENSMUSG	Acod1	ENSMUST00000022722
ENSMUSG	Dct	ENSMUST00000022725
ENSMUSG	Gdnf	ENSMUST00000022744
ENSMUSG	Mroh2b	ENSMUST00000045736
ENSMUSG	Mcpt8	ENSMUST00000015594;ENSMUST00000225107
ENSMUSG	Rem2	ENSMUST00000164766;ENSMUST00000170337;ENSMUST00000164
ENSMUSG	C6	ENSMUST00000162585;ENSMUST00000161997;ENSMUST00000022
ENSMUSG	Fbxo4	ENSMUST00000022791;ENSMUST00000148817;ENSMUST00000148
ENSMUSG	Gm5546	ENSMUST00000056145
ENSMUSG	Slc22a1	ENSMUST00000050772
ENSMUSG	Npr3	ENSMUST00000066529
ENSMUSG	Jph4	ENSMUST00000022819;ENSMUST00000132992;ENSMUST00000133
ENSMUSG	Carmil3	ENSMUST00000076236
ENSMUSG	Cpne6	ENSMUST00000074225;ENSMUST00000170155;ENSMUST00000165
ENSMUSG	Tgm1	ENSMUST00000002389;ENSMUST00000168729;ENSMUST00000178
ENSMUSG	Mcpt2	ENSMUST00000015576
ENSMUSG	Mcpt1	ENSMUST00000022836
ENSMUSG	Atp12a	ENSMUST00000007340

ENSMUSG	Ropn1l	ENSMUST00000110408
ENSMUSG	Slc45a2	ENSMUST00000117100
ENSMUSG	Amacr	ENSMUST00000070877
ENSMUSG	Skor1	ENSMUST00000119146;ENSMUST00000116613;ENSMUST00000055
ENSMUSG	Ttc23l	ENSMUST00000167842;ENSMUST00000022857
ENSMUSG	Dnah5	ENSMUST00000067048
ENSMUSG	March11	ENSMUST00000126304
ENSMUSG	Dcstamp	ENSMUST00000022913
ENSMUSG	Dpys	ENSMUST00000022915;ENSMUST00000110306;ENSMUST00000150
ENSMUSG	Zfpm2	ENSMUST00000053467
ENSMUSG	Slc30a8	ENSMUST00000037240
ENSMUSG	Cdh10	ENSMUST00000166873;ENSMUST00000176801;ENSMUST00000176
ENSMUSG	Osr2	ENSMUST00000022952
ENSMUSG	Khdrbs3	ENSMUST00000022954
ENSMUSG	Sybu	ENSMUST00000110267;ENSMUST00000090057
ENSMUSG	Kcnv1	ENSMUST00000022967
ENSMUSG	A1bg	ENSMUST00000096418
ENSMUSG	Slc22a2	ENSMUST00000110196
ENSMUSG	Has2	ENSMUST00000050544
ENSMUSG	Col14a1	ENSMUST00000110221;ENSMUST00000023053;ENSMUST00000110
ENSMUSG	Lrrc6	ENSMUST00000023006
ENSMUSG	Adcy8	ENSMUST00000023007
ENSMUSG	Wnt7b	ENSMUST00000109424;ENSMUST00000167968;ENSMUST00000023
ENSMUSG	Ppara	ENSMUST00000134880;ENSMUST00000109423;ENSMUST00000109
ENSMUSG	Ttll8	ENSMUST00000177180
ENSMUSG	Cacna1i	ENSMUST00000160424;ENSMUST00000161863
ENSMUSG	Dscc1	ENSMUST00000023059;ENSMUST00000110231;ENSMUST00000143
ENSMUSG	Dmc1	ENSMUST00000023065
ENSMUSG	Ribc2	ENSMUST00000023067
ENSMUSG	Upk3a	ENSMUST00000023070
ENSMUSG	C1qtnf6	ENSMUST00000023075;ENSMUST00000147093;ENSMUST00000137
ENSMUSG	Efcab6	ENSMUST00000156187;ENSMUST00000149563
ENSMUSG	Adamts2	ENSMUST00000155907;ENSMUST00000129195;ENSMUST00000128
ENSMUSG	Wbp2nl	ENSMUST00000023089
ENSMUSG	Slc38a4	ENSMUST00000023101;ENSMUST00000166223
ENSMUSG	Col2a1	ENSMUST00000088355;ENSMUST00000023123;ENSMUST00000133
ENSMUSG	Hoxc10	ENSMUST00000001699
ENSMUSG	Hoxc5	ENSMUST00000001709
ENSMUSG	Shisa9	ENSMUST00000182889;ENSMUST00000170672;ENSMUST00000023
ENSMUSG	Tnfrsf1	ENSMUST00000023140
ENSMUSG	Prm1	ENSMUST00000023144
ENSMUSG	Fgf12	ENSMUST00000100024
ENSMUSG	Hrasls	ENSMUST00000089824;ENSMUST00000162747
ENSMUSG	Mefv	ENSMUST00000023180;ENSMUST00000100222
ENSMUSG	Tmem44	ENSMUST00000144491;ENSMUST00000089775;ENSMUST00000144
ENSMUSG	Sept12	ENSMUST00000170323
ENSMUSG	Naprt	ENSMUST00000023237
ENSMUSG	Ly6h	ENSMUST00000163116;ENSMUST00000127095;ENSMUST00000065
ENSMUSG	Rhpn1	ENSMUST00000121137;ENSMUST00000124749;ENSMUST00000143
ENSMUSG	Lypd2	ENSMUST00000023260;ENSMUST00000188716
ENSMUSG	Psca	ENSMUST00000023265
ENSMUSG	Mroh4	ENSMUST00000023271;ENSMUST00000177179
ENSMUSG	Miox	ENSMUST00000023282;ENSMUST00000161437
ENSMUSG	Mapk8ip	ENSMUST00000023291;ENSMUST00000137755
ENSMUSG	Rabl2	ENSMUST00000023294;ENSMUST00000094056;ENSMUST00000165
ENSMUSG	Acr	ENSMUST00000023295
ENSMUSG	Retnlb	ENSMUST00000023328

ENSMUSG	Gcsam	ENSMUST00000161347;ENSMUST00000023339
ENSMUSG	Snai2	ENSMUST00000023356
ENSMUSG	B3gnt5	ENSMUST00000121344;ENSMUST00000079780;ENSMUST00000164
ENSMUSG	Drd3	ENSMUST00000023390
ENSMUSG	Tmem114	ENSMUST00000023400
ENSMUSG	Arl6	ENSMUST00000023405;ENSMUST00000099646;ENSMUST00000118
ENSMUSG	Tmem45a	ENSMUST00000023435;ENSMUST00000148452;ENSMUST00000135
ENSMUSG	Adgrg7	ENSMUST00000023437
ENSMUSG	Slc7a4	ENSMUST00000172164;ENSMUST00000116648;ENSMUST00000164
ENSMUSG	P2rx6	ENSMUST00000171002;ENSMUST00000023441;ENSMUST00000168
ENSMUSG	Lrrc74b	ENSMUST00000100123;ENSMUST00000138839;ENSMUST00000065
ENSMUSG	Ncam2	ENSMUST00000037785
ENSMUSG	Aifm3	ENSMUST00000115685;ENSMUST00000023448;ENSMUST00000149
ENSMUSG	Meltf	ENSMUST00000023464
ENSMUSG	Fgd4	ENSMUST00000161861;ENSMUST00000162671;ENSMUST00000069
ENSMUSG	Igsf11	ENSMUST00000023478;ENSMUST00000210471
ENSMUSG	Maats1	ENSMUST00000023501;ENSMUST00000114740;ENSMUST00000141
ENSMUSG	Nr1i2	ENSMUST00000023504
ENSMUSG	Cyp2ab1	ENSMUST00000023513
ENSMUSG	Pdia5	ENSMUST00000023550
ENSMUSG	Thpo	ENSMUST00000076422;ENSMUST00000115437
ENSMUSG	Tmprss1	ENSMUST00000023566
ENSMUSG	Chodl	ENSMUST00000023568
ENSMUSG	Ahsg	ENSMUST00000023583
ENSMUSG	Fetub	ENSMUST00000170805;ENSMUST00000023587;ENSMUST00000116
ENSMUSG	Kng1	ENSMUST00000039492;ENSMUST00000023589
ENSMUSG	Hrg	ENSMUST00000023590
ENSMUSG	Adamts1	ENSMUST00000023610;ENSMUST00000125897;ENSMUST00000138
ENSMUSG	Adamts5	ENSMUST00000023611
ENSMUSG	Ildr1	ENSMUST00000023617;ENSMUST00000089618;ENSMUST00000153
ENSMUSG	Stfa2	ENSMUST00000023619
ENSMUSG	Grik1	ENSMUST00000072256;ENSMUST00000210700;ENSMUST00000211
ENSMUSG	Ripply3	ENSMUST00000023660
ENSMUSG	Clic6	ENSMUST00000162181;ENSMUST00000023670
ENSMUSG	Krt75	ENSMUST00000042957
ENSMUSG	Zfp641	ENSMUST00000023722;ENSMUST00000169721
ENSMUSG	Lalba	ENSMUST00000023726
ENSMUSG	Wnt10b	ENSMUST00000023732;ENSMUST00000166022
ENSMUSG	Wnt1	ENSMUST00000023734
ENSMUSG	Aqp2	ENSMUST00000023752
ENSMUSG	Cela1	ENSMUST00000023775
ENSMUSG	Slc4a8	ENSMUST00000023776;ENSMUST00000161564;ENSMUST00000160
ENSMUSG	Scn8a	ENSMUST00000201518;ENSMUST00000082209;ENSMUST00000108
ENSMUSG	Krt6b	ENSMUST00000023786
ENSMUSG	Soat2	ENSMUST00000023806;ENSMUST00000160465
ENSMUSG	Amhr2	ENSMUST00000023809;ENSMUST00000161376
ENSMUSG	Rgn	ENSMUST00000023832
ENSMUSG	Slc10a2	ENSMUST00000023835
ENSMUSG	Gm7257	ENSMUST00000041537
ENSMUSG	Gm853	ENSMUST00000023884
ENSMUSG	Sult1c2	ENSMUST00000023886
ENSMUSG	Cpn2	ENSMUST00000064856
ENSMUSG	Grm2	ENSMUST00000023959
ENSMUSG	Epb42	ENSMUST00000102490;ENSMUST00000147444;ENSMUST00000145
ENSMUSG	Serinc2	ENSMUST00000122374;ENSMUST00000105996;ENSMUST00000120
ENSMUSG	Guca2a	ENSMUST00000024015
ENSMUSG	Acy1	ENSMUST00000190851;ENSMUST00000024031;ENSMUST00000189

ENSMUSG	9530002	ENSMUST00000024032
ENSMUSG	Bmp15	ENSMUST00000024049
ENSMUSG	Gcm1	ENSMUST00000024104
ENSMUSG	Wfdc1	ENSMUST00000212901;ENSMUST00000024107
ENSMUSG	Kcnk16	ENSMUST00000225596
ENSMUSG	Dlx2	ENSMUST00000024159
ENSMUSG	Stk31	ENSMUST00000024171
ENSMUSG	Nfatc4	ENSMUST00000024179;ENSMUST00000172271
ENSMUSG	Cela3b	ENSMUST00000102522;ENSMUST00000134565
ENSMUSG	Prph	ENSMUST00000024249;ENSMUST00000047104
ENSMUSG	Iqcf3	ENSMUST00000189866;ENSMUST00000062917
ENSMUSG	Rhebl1	ENSMUST00000024518;ENSMUST00000147015;ENSMUST0000015[illegible]
ENSMUSG	Hes7	ENSMUST00000024543
ENSMUSG	Slc22a3	ENSMUST00000024595
ENSMUSG	Slc22a1	ENSMUST00000024596
ENSMUSG	1700010	ENSMUST00000024650;ENSMUST00000151609;ENSMUST0000013[illegible]
ENSMUSG	Zscan10	ENSMUST00000095595;ENSMUST00000125912;ENSMUST0000013[illegible]
ENSMUSG	Mep1a	ENSMUST00000117137;ENSMUST00000024707;ENSMUST00000152
ENSMUSG	Adgrf4	ENSMUST00000024711
ENSMUSG	Rhag	ENSMUST00000024721
ENSMUSG	Efhb	ENSMUST00000024725
ENSMUSG	Sult1c1	ENSMUST00000024738
ENSMUSG	Slc5a7	ENSMUST00000095712
ENSMUSG	Vegfa	ENSMUST00000024747;ENSMUST00000071648;ENSMUST0000015[illegible]
ENSMUSG	Calcr	ENSMUST00000171613;ENSMUST00000075644
ENSMUSG	Prph2	ENSMUST00000024773;ENSMUST00000162469
ENSMUSG	Guca1b	ENSMUST00000024774
ENSMUSG	Guca1a	ENSMUST00000059348
ENSMUSG	Trem2	ENSMUST00000024791;ENSMUST00000132340;ENSMUST0000011[illegible]
ENSMUSG	Treml1	ENSMUST00000024792;ENSMUST00000223968
ENSMUSG	Tspo2	ENSMUST00000159478;ENSMUST00000024794
ENSMUSG	Kif6	ENSMUST00000024798;ENSMUST00000162029;ENSMUST00000162
ENSMUSG	Cpne5	ENSMUST00000024805;ENSMUST00000126993;ENSMUST00000124
ENSMUSG	Pi16	ENSMUST00000135754;ENSMUST00000114701;ENSMUST00000114
ENSMUSG	Tff2	ENSMUST00000024826
ENSMUSG	Tff1	ENSMUST00000024831
ENSMUSG	Tmprss3	ENSMUST00000114549
ENSMUSG	Cbs	ENSMUST00000067801;ENSMUST00000151597
ENSMUSG	Cryaa	ENSMUST00000019192
ENSMUSG	Arhgap2	ENSMUST00000024840;ENSMUST00000164647;ENSMUST0000017[illegible]
ENSMUSG	Galnt14	ENSMUST00000024858;ENSMUST00000112591;ENSMUST0000014[illegible]
ENSMUSG	Vit	ENSMUST00000024880
ENSMUSG	Qpct	ENSMUST00000040789
ENSMUSG	4930583	ENSMUST00000024896
ENSMUSG	Themis3	ENSMUST00000024914
ENSMUSG	Nrxn1	ENSMUST00000160844;ENSMUST00000198899;ENSMUST00000161
ENSMUSG	Cacna1h	ENSMUST00000159610;ENSMUST00000078496;ENSMUST0000015[illegible]
ENSMUSG	Prss41	ENSMUST00000122936;ENSMUST00000024926;ENSMUST00000151
ENSMUSG	Prss21	ENSMUST00000024928
ENSMUSG	Prss30	ENSMUST00000024936;ENSMUST00000135549;ENSMUST0000012[illegible]
ENSMUSG	Sbpl	ENSMUST00000024939
ENSMUSG	Sbp	ENSMUST00000183252
ENSMUSG	Gtf2a1l	ENSMUST00000024970
ENSMUSG	Prss28	ENSMUST00000015267
ENSMUSG	St6gal2	ENSMUST00000133899;ENSMUST00000086878;ENSMUST0000002[illegible]
ENSMUSG	Tpsab1	ENSMUST00000153118;ENSMUST00000146856
ENSMUSG	Tekt4	ENSMUST00000025002

ENSMUSG	Sox8	ENSMUST00000025003;ENSMUST00000173447
ENSMUSG	Acsbg2	ENSMUST00000043062
ENSMUSG	Ip6k3	ENSMUST00000025046
ENSMUSG	Spdef	ENSMUST00000114870;ENSMUST00000127622
ENSMUSG	Armc12	ENSMUST00000025060
ENSMUSG	Clps	ENSMUST00000025062
ENSMUSG	Pdzph1	ENSMUST00000025064
ENSMUSG	Nudt12	ENSMUST00000025065;ENSMUST00000174108;ENSMUST00000174
ENSMUSG	Bambi	ENSMUST00000025075
ENSMUSG	Dync2li	ENSMUST00000025101
ENSMUSG	Adcyap1	ENSMUST00000064775
ENSMUSG	Syt4	ENSMUST00000025110
ENSMUSG	Adad2	ENSMUST00000098361
ENSMUSG	Celf4	ENSMUST00000115816;ENSMUST00000025117;ENSMUST00000223
ENSMUSG	Cyp4f14	ENSMUST00000179434
ENSMUSG	Dtna	ENSMUST00000222515;ENSMUST00000221471;ENSMUST00000115
ENSMUSG	Cdh2	ENSMUST00000025166;ENSMUST00000115850;ENSMUST00000152
ENSMUSG	Ccdc178	ENSMUST00000025160
ENSMUSG	Mep1b	ENSMUST00000082235
ENSMUSG	Btnl2	ENSMUST00000178562;ENSMUST00000025198
ENSMUSG	Psd2	ENSMUST00000115716;ENSMUST00000176873;ENSMUST00000177
ENSMUSG	Gfra3	ENSMUST00000025224
ENSMUSG	Proc	ENSMUST00000171765
ENSMUSG	Myo7b	ENSMUST00000134663
ENSMUSG	Lims2	ENSMUST00000025254;ENSMUST00000225404;ENSMUST00000224
ENSMUSG	Lta	ENSMUST00000025266
ENSMUSG	Pou5f1	ENSMUST00000025271;ENSMUST00000173934;ENSMUST00000172
ENSMUSG	Psors1c	ENSMUST00000025273
ENSMUSG	Aqp4	ENSMUST00000079081
ENSMUSG	Spry4	ENSMUST00000025295
ENSMUSG	Cabyr	ENSMUST00000080415;ENSMUST00000121018
ENSMUSG	H2-M10.	ENSMUST00000025322
ENSMUSG	Arap3	ENSMUST00000042944
ENSMUSG	H2-M5	ENSMUST00000145260;ENSMUST00000169189;ENSMUST00000176
ENSMUSG	Myot	ENSMUST00000025349
ENSMUSG	Mal2	ENSMUST00000025356
ENSMUSG	Lvrn	ENSMUST00000025358
ENSMUSG	Hbegf	ENSMUST00000025363
ENSMUSG	Pou4f3	ENSMUST00000025374
ENSMUSG	Ppp2r2b	ENSMUST00000136118;ENSMUST00000117687;ENSMUST00000025
ENSMUSG	Jakmip2	ENSMUST00000082254
ENSMUSG	Spink1	ENSMUST00000025381
ENSMUSG	Rab27b	ENSMUST00000121693;ENSMUST00000069749;ENSMUST00000117
ENSMUSG	Dynap	ENSMUST00000025390
ENSMUSG	Rax	ENSMUST00000025396
ENSMUSG	Pmaip1	ENSMUST00000025399
ENSMUSG	Prelid3	ENSMUST00000025411
ENSMUSG	Spire1	ENSMUST00000045105;ENSMUST00000115050;ENSMUST00000224
ENSMUSG	Sncaip	ENSMUST00000115410;ENSMUST00000178678;ENSMUST00000025
ENSMUSG	Snx24	ENSMUST00000165032;ENSMUST00000025417
ENSMUSG	4930546	ENSMUST00000025428
ENSMUSG	Slc14a2	ENSMUST00000025434;ENSMUST00000163367
ENSMUSG	Galr1	ENSMUST00000065224
ENSMUSG	Mapk4	ENSMUST00000162863;ENSMUST00000091851;ENSMUST00000159
ENSMUSG	Sall3	ENSMUST00000057950
ENSMUSG	Il17b	ENSMUST00000025471
ENSMUSG	Megf10	ENSMUST00000075770;ENSMUST00000139892

ENSMUSG	Fbn2	ENSMUST00000025497
ENSMUSG	Slc27a6	ENSMUST00000025500
ENSMUSG	Scgb1a1	ENSMUST00000025554
ENSMUSG	Ms4a2	ENSMUST00000186978;ENSMUST00000025583;ENSMUST00000164
ENSMUSG	Ms4a3	ENSMUST00000186023;ENSMUST00000112984;ENSMUST0000019C
ENSMUSG	Gif	ENSMUST00000025585
ENSMUSG	Pcsk5	ENSMUST00000025618;ENSMUST00000050715
ENSMUSG	Trpm6	ENSMUST00000040489
ENSMUSG	Ms4a8a	ENSMUST00000025636;ENSMUST00000187859
ENSMUSG	Ms4a10	ENSMUST00000072748
ENSMUSG	Zp1	ENSMUST00000168708;ENSMUST00000025641
ENSMUSG	Syt7	ENSMUST00000073899;ENSMUST00000169121;ENSMUST00000225
ENSMUSG	Aldh1a7	ENSMUST00000025656
ENSMUSG	Tmc1	ENSMUST00000039500
ENSMUSG	Gm16437	ENSMUST00000025669
ENSMUSG	Lipo3	ENSMUST00000133269;ENSMUST00000112508;ENSMUST00000025
ENSMUSG	Lipf	ENSMUST00000025680
ENSMUSG	Lipn	ENSMUST00000025682;ENSMUST00000148821
ENSMUSG	Lipk	ENSMUST00000225505;ENSMUST00000054260
ENSMUSG	Ankrd22	ENSMUST00000025686
ENSMUSG	Htr7	ENSMUST00000164639;ENSMUST00000164781;ENSMUST00000165
ENSMUSG	Ankrd1	ENSMUST00000025718
ENSMUSG	Mlana	ENSMUST00000025719
ENSMUSG	Trpd52l	ENSMUST00000025727
ENSMUSG	Gldc	ENSMUST00000025778
ENSMUSG	Dmrt1	ENSMUST00000025755
ENSMUSG	Cst6	ENSMUST00000025764
ENSMUSG	Cabp2	ENSMUST00000159148
ENSMUSG	Mbl2	ENSMUST00000025797
ENSMUSG	Acy3	ENSMUST00000054030
ENSMUSG	Dkk1	ENSMUST00000025803
ENSMUSG	Rin1	ENSMUST00000225427;ENSMUST00000224254;ENSMUST00000224
ENSMUSG	Slc29a2	ENSMUST00000025826
ENSMUSG	Lao1	ENSMUST00000058651
ENSMUSG	Tesmin	ENSMUST00000025840;ENSMUST00000151341;ENSMUST00000127
ENSMUSG	Gal	ENSMUST00000025842
ENSMUSG	Vldlr	ENSMUST00000164509;ENSMUST00000164746;ENSMUST00000172
ENSMUSG	Lgals12	ENSMUST00000159983;ENSMUST00000099729;ENSMUST0000007S
ENSMUSG	Hrasls5	ENSMUST00000131326;ENSMUST00000025929;ENSMUST00000148
ENSMUSG	Tectb	ENSMUST00000025936;ENSMUST00000123763
ENSMUSG	Hhex	ENSMUST00000025944;ENSMUST00000128184
ENSMUSG	Cyp26a1	ENSMUST00000025946
ENSMUSG	Pde6c	ENSMUST00000112329
ENSMUSG	Cyp2c55	ENSMUST00000025966
ENSMUSG	Maoa	ENSMUST00000026013
ENSMUSG	Efhc2	ENSMUST00000026014
ENSMUSG	Dusp21	ENSMUST00000026018
ENSMUSG	Nr0b1	ENSMUST00000026036
ENSMUSG	5430427	ENSMUST00000113976
ENSMUSG	Habp2	ENSMUST00000095948
ENSMUSG	Tdrd1	ENSMUST00000111604
ENSMUSG	Gfra1	ENSMUST00000026076;ENSMUST00000129100;ENSMUST0000016S
ENSMUSG	Hspa12a	ENSMUST00000066285
ENSMUSG	Bnc1	ENSMUST00000026096
ENSMUSG	Bhlhe22	ENSMUST00000026120
ENSMUSG	Pycr1	ENSMUST00000170556;ENSMUST00000026133;ENSMUST0000013З
ENSMUSG	Myadml2	ENSMUST00000026134;ENSMUST00000145047

ENSMUSG	Sectm1a	ENSMUST00000026162;ENSMUST00000100126;ENSMUST0000010€
ENSMUSG	Ankrd2	ENSMUST00000026172
ENSMUSG	Hoga1	ENSMUST00000168414;ENSMUST00000081714;ENSMUST00000167
ENSMUSG	Cnnm1	ENSMUST00000223787;ENSMUST00000165311;ENSMUST00000225
ENSMUSG	Abcc2	ENSMUST00000026208;ENSMUST00000143553;ENSMUST00000099
ENSMUSG	Cpn1	ENSMUST00000026210;ENSMUST00000131882
ENSMUSG	Cyp2c44	ENSMUST00000026211
ENSMUSG	Kazald1	ENSMUST00000111948;ENSMUST00000026234
ENSMUSG	Tlx1	ENSMUST00000026236
ENSMUSG	Kcnip2	ENSMUST00000159210;ENSMUST00000079431;ENSMUST00000111
ENSMUSG	Pitx3	ENSMUST00000026259;ENSMUST00000172980
ENSMUSG	Zfhx4	ENSMUST00000175866;ENSMUST00000175641;ENSMUST00000026
ENSMUSG	Itgb8	ENSMUST00000026360;ENSMUST00000151023;ENSMUST00000137
ENSMUSG	Padi3	ENSMUST00000026377;ENSMUST00000172098
ENSMUSG	Padi1	ENSMUST00000026378
ENSMUSG	Mettl7b	ENSMUST00000026398
ENSMUSG	Itga7	ENSMUST00000099112;ENSMUST00000218290;ENSMUST00000218
ENSMUSG	Cdh9	ENSMUST00000026432
ENSMUSG	Il23a	ENSMUST00000026449;ENSMUST00000218109
ENSMUSG	Pde6g	ENSMUST00000141795;ENSMUST00000155421;ENSMUST00000026
ENSMUSG	Hsd17b6	ENSMUST00000219183
ENSMUSG	Tac2	ENSMUST00000179960;ENSMUST00000026466;ENSMUST00000217
ENSMUSG	Bsnd	ENSMUST00000054472
ENSMUSG	Rnf165	ENSMUST00000026494;ENSMUST00000182294;ENSMUST00000182
ENSMUSG	Crisp1	ENSMUST00000026498
ENSMUSG	Crisp3	ENSMUST00000026499
ENSMUSG	Atp23	ENSMUST00000026504;ENSMUST00000168520;ENSMUST00000220
ENSMUSG	Cd163l1	ENSMUST00000211540;ENSMUST00000084460;ENSMUST00000209
ENSMUSG	Fuom	ENSMUST00000120034;ENSMUST00000148716;ENSMUST00000142
ENSMUSG	Prap1	ENSMUST00000026540
ENSMUSG	Caly	ENSMUST00000026541;ENSMUST00000211044;ENSMUST00000166
ENSMUSG	Msx3	ENSMUST00000055353;ENSMUST00000172775
ENSMUSG	Dpysl4	ENSMUST00000026551;ENSMUST00000154273;ENSMUST00000145
ENSMUSG	Syce1	ENSMUST00000026553
ENSMUSG	Cdhr5	ENSMUST00000080654;ENSMUST00000167263;ENSMUST00000210
ENSMUSG	Muc2	ENSMUST00000185406;ENSMUST00000179227;ENSMUST00000167
ENSMUSG	Tktl2	ENSMUST00000002025;ENSMUST00000183187
ENSMUSG	Zfp711	ENSMUST00000151972;ENSMUST00000113409;ENSMUST00000071
ENSMUSG	Slc15a1	ENSMUST00000088386
ENSMUSG	Rbfox3	ENSMUST00000117731;ENSMUST00000103023;ENSMUST00000017
ENSMUSG	Nptx1	ENSMUST00000026670
ENSMUSG	Cpeb1	ENSMUST00000098331;ENSMUST00000130310;ENSMUST00000178
ENSMUSG	Nat1	ENSMUST00000026677
ENSMUSG	Dach2	ENSMUST00000113380;ENSMUST00000113378;ENSMUST00000146
ENSMUSG	Klhl4	ENSMUST00000040504;ENSMUST00000113371
ENSMUSG	Zfp202	ENSMUST00000168691;ENSMUST00000026693
ENSMUSG	Pfkfb4	ENSMUST00000198140;ENSMUST00000198277;ENSMUST00000199
ENSMUSG	Col7a1	ENSMUST00000112070;ENSMUST00000192424;ENSMUST00000127
ENSMUSG	Arhgef9	ENSMUST00000113876;ENSMUST00000113883;ENSMUST00000182
ENSMUSG	Cnksr2	ENSMUST00000026750;ENSMUST00000112513;ENSMUST00000139
ENSMUSG	Rps6ka6	ENSMUST00000065976;ENSMUST00000096348;ENSMUST00000113
ENSMUSG	Alox5	ENSMUST00000026795;ENSMUST00000167447;ENSMUST00000169
ENSMUSG	Slc28a1	ENSMUST00000119083
ENSMUSG	Crispld	ENSMUST00000159958;ENSMUST00000160305;ENSMUST00000095
ENSMUSG	Gdap1	ENSMUST00000026879;ENSMUST00000150457;ENSMUST00000189
ENSMUSG	St8sia2	ENSMUST00000026896
ENSMUSG	Ccr1	ENSMUST00000026911

ENSMUSG	Homer2	ENSMUST00000098326;ENSMUST00000026922;ENSMUST00000207
ENSMUSG	Dhtkd1	ENSMUST00000095147;ENSMUST00000155977;ENSMUST00000127
ENSMUSG	Fam20c	ENSMUST00000026972;ENSMUST00000160645;ENSMUST00000197
ENSMUSG	Cplx2	ENSMUST00000026985
ENSMUSG	Tspan17	ENSMUST00000026993;ENSMUST00000131692;ENSMUST00000163
ENSMUSG	Unc5a	ENSMUST00000026994;ENSMUST00000109994;ENSMUST00000142
ENSMUSG	Hk3	ENSMUST00000052949;ENSMUST00000132309;ENSMUST00000126
ENSMUSG	Myo5b	ENSMUST00000125882;ENSMUST00000135878;ENSMUST00000074
ENSMUSG	Snca	ENSMUST00000163779;ENSMUST00000114268;ENSMUST00000204
ENSMUSG	Rp1	ENSMUST00000208660;ENSMUST00000208793;ENSMUST00000027
ENSMUSG	Sox17	ENSMUST00000027035;ENSMUST00000195555;ENSMUST00000192
ENSMUSG	Oprk1	ENSMUST00000160777
ENSMUSG	Ppp1r42	ENSMUST00000147000;ENSMUST00000124874;ENSMUST00000027
ENSMUSG	Gm5045	ENSMUST00000027054
ENSMUSG	Tfap2b	ENSMUST00000027059;ENSMUST00000064976;ENSMUST00000186
ENSMUSG	Paqr8	ENSMUST00000189400;ENSMUST00000068880;ENSMUST00000167
ENSMUSG	Tmem14a	ENSMUST00000027065;ENSMUST00000027064
ENSMUSG	Gm4956	ENSMUST00000082032
ENSMUSG	Pth2r	ENSMUST00000140190;ENSMUST00000027083
ENSMUSG	Crygc	ENSMUST00000027089
ENSMUSG	Akr1cl	ENSMUST00000069142;ENSMUST00000159896;ENSMUST00000160
ENSMUSG	Mdh1b	ENSMUST00000114094
ENSMUSG	Ccdc150	ENSMUST00000161988;ENSMUST00000027128;ENSMUST00000160
ENSMUSG	Cps1	ENSMUST00000027144
ENSMUSG	Cdk15	ENSMUST00000160662;ENSMUST00000162906
ENSMUSG	Khdrbs2	ENSMUST00000027226;ENSMUST00000189878;ENSMUST00000188
ENSMUSG	Slc9a4	ENSMUST00000027233;ENSMUST00000192454
ENSMUSG	Il1r2	ENSMUST00000191671;ENSMUST00000027243;ENSMUST00000195
ENSMUSG	Chst10	ENSMUST00000027249;ENSMUST00000193441;ENSMUST00000192
ENSMUSG	Mstn	ENSMUST00000027269
ENSMUSG	Tmeff2	ENSMUST00000114565;ENSMUST00000081851
ENSMUSG	Cnga3	ENSMUST00000027288
ENSMUSG	Cfc1	ENSMUST00000027298
ENSMUSG	Prss39	ENSMUST00000027299;ENSMUST00000191604
ENSMUSG	Col19a1	ENSMUST00000144297;ENSMUST00000115244;ENSMUST00000051
ENSMUSG	Col9a1	ENSMUST00000054588;ENSMUST00000088349;ENSMUST00000124
ENSMUSG	Fam135a	ENSMUST00000187619;ENSMUST00000188712;ENSMUST00000187
ENSMUSG	Sdhaf4	ENSMUST00000027338;ENSMUST00000155767
ENSMUSG	Ogfrl1	ENSMUST00000027343;ENSMUST00000186064
ENSMUSG	Nhej1	ENSMUST00000188033
ENSMUSG	Sphkap	ENSMUST00000160953
ENSMUSG	Ccl20	ENSMUST00000027351;ENSMUST00000113437;ENSMUST00000186
ENSMUSG	Wnt10a	ENSMUST00000006718
ENSMUSG	Cxcr2	ENSMUST00000027372;ENSMUST00000106899
ENSMUSG	Tnp1	ENSMUST00000027374
ENSMUSG	Slc23a3	ENSMUST00000027405;ENSMUST00000188080
ENSMUSG	Speg	ENSMUST00000087122;ENSMUST00000148515;ENSMUST00000113
ENSMUSG	Slc16a1	ENSMUST00000027422;ENSMUST00000186980
ENSMUSG	4933407	ENSMUST00000027426;ENSMUST00000188949
ENSMUSG	Spata3	ENSMUST00000149469;ENSMUST00000152501
ENSMUSG	2810459	ENSMUST00000165824
ENSMUSG	Epha4	ENSMUST00000027451;ENSMUST00000188952;ENSMUST00000188
ENSMUSG	Nppc	ENSMUST00000027449
ENSMUSG	Ecel1	ENSMUST00000161002;ENSMUST00000160810;ENSMUST00000162
ENSMUSG	Chrnd	ENSMUST00000186373
ENSMUSG	Efhd1	ENSMUST00000027472;ENSMUST00000150831
ENSMUSG	Ngef	ENSMUST00000027477;ENSMUST00000068681

ENSMUSG	Agxt	ENSMUST00000027491
ENSMUSG	Spp2	ENSMUST00000027518
ENSMUSG	Iqca	ENSMUST00000113094;ENSMUST00000189690
ENSMUSG	Mlph	ENSMUST00000027528;ENSMUST00000125149;ENSMUST00000136
ENSMUSG	Rab17	ENSMUST00000027529
ENSMUSG	Asb1	ENSMUST00000086843;ENSMUST00000027538;ENSMUST00000186
ENSMUSG	Cdh7	ENSMUST00000112701;ENSMUST00000146282
ENSMUSG	Serpinb	ENSMUST00000112706;ENSMUST00000151283;ENSMUST00000123
ENSMUSG	Htr4	ENSMUST00000027560
ENSMUSG	Serpinb	ENSMUST00000191425;ENSMUST00000027566
ENSMUSG	Slco6d1	ENSMUST00000162468
ENSMUSG	Slc35f5	ENSMUST00000190037;ENSMUST00000027580;ENSMUST00000189
ENSMUSG	Rgs18	ENSMUST00000027603
ENSMUSG	Rgs1	ENSMUST00000189061;ENSMUST00000170870;ENSMUST00000167
ENSMUSG	F13b	ENSMUST00000027615
ENSMUSG	Epb41l5	ENSMUST00000052404;ENSMUST00000163147;ENSMUST00000191
ENSMUSG	Sctr	ENSMUST00000072886;ENSMUST00000189037
ENSMUSG	Marco	ENSMUST00000027639
ENSMUSG	Nr5a2	ENSMUST00000027649;ENSMUST00000168126;ENSMUST00000192
ENSMUSG	Cd55	ENSMUST00000027650;ENSMUST00000122830;ENSMUST00000140
ENSMUSG	Cd55b	ENSMUST00000112488;ENSMUST00000136497
ENSMUSG	C4bp	ENSMUST00000027657;ENSMUST00000190624
ENSMUSG	Cacna1s	ENSMUST00000160641;ENSMUST00000112064;ENSMUST00000159
ENSMUSG	Fcamr	ENSMUST00000112477;ENSMUST00000027670
ENSMUSG	Tnni1	ENSMUST00000187830;ENSMUST00000132795;ENSMUST00000152
ENSMUSG	Il24	ENSMUST00000187650;ENSMUST00000121040
ENSMUSG	Gpr37l1	ENSMUST00000027682
ENSMUSG	Avpr1b	ENSMUST00000027690
ENSMUSG	Cdk18	ENSMUST00000027697;ENSMUST00000188387;ENSMUST00000189
ENSMUSG	Nfasc	ENSMUST00000094569;ENSMUST00000043189;ENSMUST00000187
ENSMUSG	Lrrn2	ENSMUST00000027706;ENSMUST00000159088
ENSMUSG	Chit1	ENSMUST00000159963
ENSMUSG	Ppfia4	ENSMUST00000168515;ENSMUST00000186964;ENSMUST00000189
ENSMUSG	Myog	ENSMUST00000027730
ENSMUSG	Lhx4	ENSMUST00000027740
ENSMUSG	Kif26b	ENSMUST00000193931;ENSMUST00000161017;ENSMUST00000160
ENSMUSG	Mixl1	ENSMUST00000027778
ENSMUSG	Cnih3	ENSMUST00000027795
ENSMUSG	Wdr64	ENSMUST00000171939
ENSMUSG	Rgs7	ENSMUST00000194555;ENSMUST00000193798
ENSMUSG	Spta1	ENSMUST00000027817
ENSMUSG	Dusp23	ENSMUST00000027826
ENSMUSG	Slamf9	ENSMUST00000027830
ENSMUSG	Dusp27	ENSMUST00000192369
ENSMUSG	Adcy10	ENSMUST00000148550;ENSMUST00000027852;ENSMUST00000111
ENSMUSG	Tbx19	ENSMUST00000027859;ENSMUST00000161777;ENSMUST00000161
ENSMUSG	Sele	ENSMUST00000027874
ENSMUSG	Astn1	ENSMUST00000193042;ENSMUST00000046110;ENSMUST00000170
ENSMUSG	Sec16b	ENSMUST00000086130;ENSMUST00000111700;ENSMUST00000146
ENSMUSG	Tex35	ENSMUST00000027884;ENSMUST00000118207;ENSMUST00000119
ENSMUSG	Esrrg	ENSMUST00000127489;ENSMUST00000110939;ENSMUST00000152
ENSMUSG	Slc30a1	ENSMUST00000061093
ENSMUSG	Mark1	ENSMUST00000027929;ENSMUST00000192657
ENSMUSG	Atf3	ENSMUST00000027941;ENSMUST00000195117
ENSMUSG	Batf3	ENSMUST00000194833;ENSMUST00000193771;ENSMUST00000027
ENSMUSG	Acbd7	ENSMUST00000115089
ENSMUSG	Olah	ENSMUST00000027955;ENSMUST00000194918

ENSMUSG	Meig1	ENSMUST00000064685;ENSMUST00000115082;ENSMUST00000115
ENSMUSG	Ucma	ENSMUST00000167607;ENSMUST00000127282;ENSMUST00000115
ENSMUSG	Enkur	ENSMUST00000027992;ENSMUST00000147793;ENSMUST00000156
ENSMUSG	Fmo4	ENSMUST00000111525;ENSMUST00000028014;ENSMUST0000014C
ENSMUSG	Myoc	ENSMUST00000028020
ENSMUSG	Tnfsf4	ENSMUST00000028024
ENSMUSG	Pter	ENSMUST00000154269;ENSMUST00000114796;ENSMUST00000134
ENSMUSG	4921504	ENSMUST00000062060
ENSMUSG	Ptf1a	ENSMUST00000028068
ENSMUSG	Nr5a1	ENSMUST00000112883;ENSMUST00000028084
ENSMUSG	Kif5c	ENSMUST00000028102;ENSMUST00000146247;ENSMUST00000138
ENSMUSG	Itga8	ENSMUST00000028106;ENSMUST00000129370;ENSMUST00000172
ENSMUSG	4931423	ENSMUST00000028113;ENSMUST00000114505
ENSMUSG	Gad2	ENSMUST00000028123
ENSMUSG	Slc2a8	ENSMUST00000191777;ENSMUST00000028129;ENSMUST00000193
ENSMUSG	Barhl1	ENSMUST00000050776;ENSMUST00000124059;ENSMUST00000113
ENSMUSG	Gfi1b	ENSMUST00000164290;ENSMUST00000028156;ENSMUST00000155
ENSMUSG	Ak1	ENSMUST00000113278;ENSMUST00000113277;ENSMUST00000156
ENSMUSG	Ptges2	ENSMUST00000028162;ENSMUST00000123714;ENSMUST0000013C
ENSMUSG	Kcnj3	ENSMUST00000067101;ENSMUST00000112633
ENSMUSG	Nr4a2	ENSMUST00000028166;ENSMUST00000112629;ENSMUST00000183
ENSMUSG	Galnt5	ENSMUST00000112616
ENSMUSG	Ermn	ENSMUST00000090940
ENSMUSG	1700007	ENSMUST00000086370
ENSMUSG	Olfm1	ENSMUST00000152415;ENSMUST00000113920;ENSMUST0000010C
ENSMUSG	Acvr1c	ENSMUST00000028178;ENSMUST00000100085;ENSMUST00000154
ENSMUSG	Fcnb	ENSMUST00000028179;ENSMUST00000117486;ENSMUST00000135
ENSMUSG	Col5a1	ENSMUST00000028280
ENSMUSG	Lamc3	ENSMUST00000138325;ENSMUST00000028187;ENSMUST00000135
ENSMUSG	Fibcd1	ENSMUST00000028188
ENSMUSG	Hc	ENSMUST00000028233;ENSMUST00000156049;ENSMUST00000125
ENSMUSG	4930568	ENSMUST00000028243
ENSMUSG	Ttll11	ENSMUST00000028248;ENSMUST00000161970;ENSMUST0000016C
ENSMUSG	Grb14	ENSMUST00000134020;ENSMUST00000149193;ENSMUST0000015C
ENSMUSG	Lhx6	ENSMUST00000112961;ENSMUST00000112966;ENSMUST00000112
ENSMUSG	Gca	ENSMUST00000028257;ENSMUST00000148083
ENSMUSG	Lcn3	ENSMUST00000028304
ENSMUSG	Lcn12	ENSMUST00000028312
ENSMUSG	Sapcd2	ENSMUST00000028329;ENSMUST00000114293;ENSMUST0000010C
ENSMUSG	Uap1l1	ENSMUST00000102925;ENSMUST00000138985;ENSMUST00000151
ENSMUSG	Grin1	ENSMUST00000114310;ENSMUST00000153465;ENSMUST00000153
ENSMUSG	Lrrc26	ENSMUST00000028337
ENSMUSG	Tmem210	ENSMUST00000028340;ENSMUST00000144102
ENSMUSG	Itgb6	ENSMUST00000059888;ENSMUST00000112517
ENSMUSG	Pax8	ENSMUST00000028355;ENSMUST00000149860;ENSMUST00000133
ENSMUSG	Il1f5	ENSMUST00000168941;ENSMUST00000028360;ENSMUST00000123
ENSMUSG	Il1f6	ENSMUST00000028361
ENSMUSG	Hnmt	ENSMUST00000051416;ENSMUST00000114497
ENSMUSG	Dapl1	ENSMUST00000028369;ENSMUST00000144415
ENSMUSG	Galnt3	ENSMUST00000028378;ENSMUST00000155453;ENSMUST0000015C
ENSMUSG	Frzb	ENSMUST00000028389
ENSMUSG	Zfp385b	ENSMUST00000111830;ENSMUST00000171063;ENSMUST0000009C
ENSMUSG	Xirp2	ENSMUST00000112347
ENSMUSG	Abcb11	ENSMUST00000102709
ENSMUSG	Dhrs9	ENSMUST00000063690
ENSMUSG	Lrp2	ENSMUST00000080953;ENSMUST00000100051;ENSMUST00000128
ENSMUSG	Prg3	ENSMUST00000135939;ENSMUST00000028466

ENSMUSG	Smtnl1	ENSMUST00000028471
ENSMUSG	Tfpi	ENSMUST00000111722;ENSMUST00000111718;ENSMUST0000012[illegible]
ENSMUSG	Hoxd8	ENSMUST00000151380;ENSMUST00000019749;ENSMUST0000007[illegible]
ENSMUSG	Chrna1	ENSMUST00000028515
ENSMUSG	4930430[illegible]	ENSMUST00000028577
ENSMUSG	Depdc7	ENSMUST00000028595;ENSMUST00000144133
ENSMUSG	Elf5	ENSMUST00000164172;ENSMUST00000137973;ENSMUST0000002[illegible]
ENSMUSG	Pamr1	ENSMUST00000028612;ENSMUST00000139090;ENSMUST0000014[illegible]
ENSMUSG	Slc12a1	ENSMUST00000110494;ENSMUST00000147095
ENSMUSG	Terb2	ENSMUST00000028661
ENSMUSG	Creb3l1	ENSMUST00000028663
ENSMUSG	F2	ENSMUST00000028681;ENSMUST00000153182
ENSMUSG	Hao1	ENSMUST00000028704
ENSMUSG	Lamp5	ENSMUST00000123436
ENSMUSG	Snap25	ENSMUST00000028727;ENSMUST00000125486
ENSMUSG	Itpka	ENSMUST00000028758
ENSMUSG	Ltk	ENSMUST00000140224;ENSMUST00000082130;ENSMUST0000012[illegible]
ENSMUSG	Tyro3	ENSMUST00000110783;ENSMUST00000148343;ENSMUST0000002[illegible]
ENSMUSG	Oxt	ENSMUST00000028764
ENSMUSG	Chac1	ENSMUST00000028780
ENSMUSG	Gfra4	ENSMUST00000110240;ENSMUST00000136569;ENSMUST0000011[illegible]
ENSMUSG	Adam33	ENSMUST00000128828;ENSMUST00000110232;ENSMUST0000013[illegible]
ENSMUSG	Adra1d	ENSMUST00000103184
ENSMUSG	Fsip1	ENSMUST00000028821
ENSMUSG	Chgb	ENSMUST00000028826
ENSMUSG	Bmp2	ENSMUST00000028836
ENSMUSG	Slc27a2	ENSMUST00000061491;ENSMUST00000126249;ENSMUST0000014[illegible]
ENSMUSG	Fahd2a	ENSMUST00000028848;ENSMUST00000146757;ENSMUST0000012[illegible]
ENSMUSG	Mal	ENSMUST00000028854
ENSMUSG	Nphp1	ENSMUST00000028857;ENSMUST00000144938;ENSMUST0000011[illegible]
ENSMUSG	Fbln7	ENSMUST00000110324;ENSMUST00000028864
ENSMUSG	Pdyn	ENSMUST00000028883;ENSMUST00000130608;ENSMUST0000015[illegible]
ENSMUSG	Tgm6	ENSMUST00000028888
ENSMUSG	1700020[illegible]	ENSMUST00000028898
ENSMUSG	Otor	ENSMUST00000028902
ENSMUSG	Bfsp1	ENSMUST00000028907
ENSMUSG	Nkx2-2	ENSMUST00000067075
ENSMUSG	Cst8	ENSMUST00000109947
ENSMUSG	Cst9	ENSMUST00000028935
ENSMUSG	9230104[illegible]	ENSMUST00000028937;ENSMUST00000149457
ENSMUSG	Snph	ENSMUST00000094456;ENSMUST00000028951;ENSMUST0000010[illegible]
ENSMUSG	Slc52a3	ENSMUST00000109859;ENSMUST00000073228;ENSMUST0000010[illegible]
ENSMUSG	Mylk2	ENSMUST00000028970
ENSMUSG	Ccm2l	ENSMUST00000127898;ENSMUST00000127549;ENSMUST0000012[illegible]
ENSMUSG	Dnmt3b	ENSMUST00000109773;ENSMUST00000081628;ENSMUST0000010[illegible]
ENSMUSG	Sun5	ENSMUST00000028982
ENSMUSG	Bpifb2	ENSMUST00000028983
ENSMUSG	Bpifa3	ENSMUST00000028984
ENSMUSG	Bpifa5	ENSMUST00000028986
ENSMUSG	Bpifb1	ENSMUST00000081816;ENSMUST00000123017
ENSMUSG	Snta1	ENSMUST00000028991;ENSMUST00000109728;ENSMUST0000013[illegible]
ENSMUSG	Stmn2	ENSMUST00000029002;ENSMUST00000191847;ENSMUST0000019[illegible]
ENSMUSG	Fam209	ENSMUST00000029007
ENSMUSG	Zdbf2	ENSMUST00000114132
ENSMUSG	Edn3	ENSMUST00000029030
ENSMUSG	Phactr3	ENSMUST00000108917;ENSMUST00000108915;ENSMUST0000010[illegible]
ENSMUSG	Fabp9	ENSMUST00000191678;ENSMUST00000029038

ENSMUSG	Fabp12	ENSMUST00000029043
ENSMUSG	Car1	ENSMUST00000181860;ENSMUST00000094365
ENSMUSG	Dok5	ENSMUST00000029075
ENSMUSG	Ntsr1	ENSMUST00000029084
ENSMUSG	Col9a3	ENSMUST00000132527;ENSMUST00000103059;ENSMUST00000141
ENSMUSG	Chrna4	ENSMUST00000198922;ENSMUST00000067120;ENSMUST0000013[illegible]
ENSMUSG	Srms	ENSMUST00000016498
ENSMUSG	Oprl1	ENSMUST00000183693;ENSMUST00000151241;ENSMUST0000010[illegible]
ENSMUSG	Procr	ENSMUST00000029140;ENSMUST00000132608;ENSMUST0000015[illegible]
ENSMUSG	Ghrh	ENSMUST00000109536;ENSMUST00000029172
ENSMUSG	Wisp2	ENSMUST00000138730;ENSMUST00000029188
ENSMUSG	Slc2a10	ENSMUST00000029196
ENSMUSG	Ocstamp	ENSMUST00000029213
ENSMUSG	Pex5l	ENSMUST00000194016;ENSMUST00000108221;ENSMUST0000010[illegible]
ENSMUSG	Mecom	ENSMUST00000172694;ENSMUST00000173495;ENSMUST0000010[illegible]
ENSMUSG	Trpc3	ENSMUST00000029271;ENSMUST00000129322;ENSMUST0000013[illegible]
ENSMUSG	Il21	ENSMUST00000029273;ENSMUST00000161015;ENSMUST0000019[illegible]
ENSMUSG	Il2	ENSMUST00000029275
ENSMUSG	Trpc4	ENSMUST00000198444;ENSMUST00000029311
ENSMUSG	Aadac	ENSMUST00000194896;ENSMUST00000029325;ENSMUST00000151
ENSMUSG	Sucnr1	ENSMUST00000029326;ENSMUST00000195544
ENSMUSG	P2ry1	ENSMUST00000029331;ENSMUST00000193201;ENSMUST0000019[illegible]
ENSMUSG	Il12a	ENSMUST00000029345;ENSMUST00000107816;ENSMUST0000019[illegible]
ENSMUSG	Schip1	ENSMUST00000029346;ENSMUST00000182532;ENSMUST00000192
ENSMUSG	Otol1	ENSMUST00000053013
ENSMUSG	Sis	ENSMUST00000167334;ENSMUST00000191870;ENSMUST0000017[illegible]
ENSMUSG	Bche	ENSMUST00000029367;ENSMUST00000133690;ENSMUST0000013[illegible]
ENSMUSG	Ccna1	ENSMUST00000197238
ENSMUSG	Sohlh2	ENSMUST00000029369
ENSMUSG	Smad9	ENSMUST00000029371
ENSMUSG	Dclk1	ENSMUST00000196745;ENSMUST00000167204;ENSMUST00000054
ENSMUSG	Nbea	ENSMUST00000029374;ENSMUST00000199803;ENSMUST0000019[illegible]
ENSMUSG	Tm4sf1	ENSMUST00000171384;ENSMUST00000196979;ENSMUST0000002[illegible]
ENSMUSG	Tm4sf4	ENSMUST00000029377
ENSMUSG	Mme	ENSMUST00000194836;ENSMUST00000194150;ENSMUST00000194
ENSMUSG	Vmn2r1	ENSMUST00000029406
ENSMUSG	Veph1	ENSMUST00000029419;ENSMUST00000107837
ENSMUSG	Ptx3	ENSMUST00000029421;ENSMUST00000182521
ENSMUSG	Syt6	ENSMUST00000090697
ENSMUSG	Ngf	ENSMUST00000035952;ENSMUST00000198168;ENSMUST0000010[illegible]
ENSMUSG	Casq2	ENSMUST00000029454;ENSMUST00000159833
ENSMUSG	Tbx15	ENSMUST00000029462
ENSMUSG	Hsd3b6	ENSMUST00000029463;ENSMUST00000170847
ENSMUSG	Hao2	ENSMUST00000029464
ENSMUSG	Hsd3b1	ENSMUST00000107016
ENSMUSG	Reg4	ENSMUST00000029469
ENSMUSG	Slc25a5	ENSMUST00000029478
ENSMUSG	1700013[illegible]	ENSMUST00000138375;ENSMUST00000029485
ENSMUSG	Sypl2	ENSMUST00000141387;ENSMUST00000156371
ENSMUSG	Gstm4	ENSMUST00000029489;ENSMUST00000106670
ENSMUSG	Slc6a17	ENSMUST00000169449;ENSMUST00000168211;ENSMUST0000017[illegible]
ENSMUSG	Kcnc4	ENSMUST00000009617
ENSMUSG	Chil6	ENSMUST00000029510
ENSMUSG	Tchhl1	ENSMUST00000029516
ENSMUSG	Lce1m	ENSMUST00000029520
ENSMUSG	Crct1	ENSMUST00000029521
ENSMUSG	Lce1b	ENSMUST00000029531

ENSMUSG	Sprr2j-:	ENSMUST00000029533
ENSMUSG	Creb3l4	ENSMUST00000029547;ENSMUST00000107369
ENSMUSG	Fam198b	ENSMUST00000118853;ENSMUST00000135021;ENSMUST0000002[illegible]
ENSMUSG	Tmem144	ENSMUST00000029568;ENSMUST00000168038;ENSMUST00000194
ENSMUSG	Olfm3	ENSMUST00000081752
ENSMUSG	Col11a1	ENSMUST00000092155
ENSMUSG	Neurog2	ENSMUST00000029587
ENSMUSG	Ndst3	ENSMUST00000154668;ENSMUST00000199825
ENSMUSG	Cyp2u1	ENSMUST00000106337;ENSMUST00000200236;ENSMUST00000198
ENSMUSG	Trim2	ENSMUST00000107692;ENSMUST00000107691;ENSMUST00000107
ENSMUSG	Tlr2	ENSMUST00000029623
ENSMUSG	Sfrp2	ENSMUST00000029625;ENSMUST00000140261;ENSMUST00000154
ENSMUSG	Fga	ENSMUST00000029630;ENSMUST00000166581
ENSMUSG	Lrat	ENSMUST00000029632
ENSMUSG	Gucy1b3	ENSMUST00000029635;ENSMUST00000192508;ENSMUST00000193
ENSMUSG	Snx7	ENSMUST00000029639;ENSMUST00000198499;ENSMUST0000019[illegible]
ENSMUSG	Asic5	ENSMUST00000029641;ENSMUST00000107736
ENSMUSG	1700061	ENSMUST00000029642
ENSMUSG	Egf	ENSMUST00000029653;ENSMUST00000197079;ENSMUST0000019[illegible]
ENSMUSG	Glrb	ENSMUST00000135043;ENSMUST00000029654;ENSMUST00000107
ENSMUSG	Pitx2	ENSMUST00000174661;ENSMUST00000187145;ENSMUST00000042
ENSMUSG	Alpk1	ENSMUST00000198955;ENSMUST00000029662;ENSMUST0000016[illegible]
ENSMUSG	Dkk2	ENSMUST00000029665
ENSMUSG	Ptgfr	ENSMUST00000029670;ENSMUST00000106126
ENSMUSG	Efna3	ENSMUST00000029673;ENSMUST00000139439
ENSMUSG	Myoz2	ENSMUST00000029761;ENSMUST00000141588;ENSMUST0000014[illegible]
ENSMUSG	Abca4	ENSMUST00000013995;ENSMUST00000132199;ENSMUST00000141
ENSMUSG	Tmem56	ENSMUST00000135818;ENSMUST00000128909;ENSMUST00000155
ENSMUSG	Rwdd3	ENSMUST00000106466;ENSMUST00000164968;ENSMUST0000003[illegible]
ENSMUSG	Them4	ENSMUST00000049822;ENSMUST00000196824;ENSMUST00000198
ENSMUSG	Them5	ENSMUST00000029794;ENSMUST00000198083
ENSMUSG	Mttp	ENSMUST00000029805;ENSMUST00000098580;ENSMUST00000197
ENSMUSG	Bdh2	ENSMUST00000120397;ENSMUST00000198491
ENSMUSG	Tacr3	ENSMUST00000029822
ENSMUSG	1810013	ENSMUST00000198623
ENSMUSG	Cth	ENSMUST00000118539;ENSMUST00000129805;ENSMUST00000198
ENSMUSG	Adgrl2	ENSMUST00000198139;ENSMUST00000106128;ENSMUST0000020[illegible]
ENSMUSG	Dnase2b	ENSMUST00000029836;ENSMUST00000200633
ENSMUSG	Uox	ENSMUST00000199489;ENSMUST00000029837;ENSMUST00000138
ENSMUSG	Cyr61	ENSMUST00000029846
ENSMUSG	Col24a1	ENSMUST00000029848;ENSMUST00000139001;ENSMUST00000127
ENSMUSG	Cdh17	ENSMUST00000029871;ENSMUST00000108303
ENSMUSG	Mmp16	ENSMUST00000029881;ENSMUST00000133416
ENSMUSG	Sdr16c5	ENSMUST00000040925;ENSMUST00000126274
ENSMUSG	Atp6v0d	ENSMUST00000029900;ENSMUST00000128605
ENSMUSG	Cyp7a1	ENSMUST00000029905
ENSMUSG	Faxc	ENSMUST00000124440;ENSMUST00000029908;ENSMUST00000123
ENSMUSG	Clca1	ENSMUST00000029919
ENSMUSG	Spaca1	ENSMUST00000084734;ENSMUST00000108148;ENSMUST0000002[illegible]
ENSMUSG	Gabrr1	ENSMUST00000029947
ENSMUSG	Epha7	ENSMUST00000080934;ENSMUST00000029964;ENSMUST0000014[illegible]
ENSMUSG	Cfap206	ENSMUST00000029971;ENSMUST00000108136;ENSMUST00000137
ENSMUSG	Cga	ENSMUST00000029975
ENSMUSG	Toporsl	ENSMUST00000107671
ENSMUSG	Stra6l	ENSMUST00000107783;ENSMUST00000107782;ENSMUST00000128
ENSMUSG	Trmo	ENSMUST00000151903;ENSMUST00000086563;ENSMUST0000003[illegible]
ENSMUSG	Hemgn	ENSMUST00000107764

ENSMUSG	Tmeff1	ENSMUST00000030032;ENSMUST00000123476;ENSMUST0000013C
ENSMUSG	Cavin4	ENSMUST00000030033
ENSMUSG	Brinp1	ENSMUST00000030036;ENSMUST00000156773;ENSMUST0000014C
ENSMUSG	Fmn2	ENSMUST00000030039;ENSMUST00000191821
ENSMUSG	Ambp	ENSMUST00000030041
ENSMUSG	Kif12	ENSMUST00000156618;ENSMUST00000131760
ENSMUSG	Zfp618	ENSMUST00000030043;ENSMUST00000107415;ENSMUST00000064
ENSMUSG	Slc44a5	ENSMUST00000089948;ENSMUST00000144677
ENSMUSG	Svep1	ENSMUST00000042850;ENSMUST00000149152;ENSMUST0000012{
ENSMUSG	Pappa	ENSMUST00000084501
ENSMUSG	Zfp37	ENSMUST00000068822;ENSMUST00000222748;ENSMUST00000222
ENSMUSG	Wdr31	ENSMUST00000146108;ENSMUST00000120095;ENSMUST00000132
ENSMUSG	Mpdz	ENSMUST00000107262;ENSMUST00000107258;ENSMUST00000102
ENSMUSG	Tal2	ENSMUST00000030124
ENSMUSG	Aqp7	ENSMUST00000054945;ENSMUST00000030136;ENSMUST00000144
ENSMUSG	1110017	ENSMUST00000123356;ENSMUST00000123865;ENSMUST00000124
ENSMUSG	Cntfr	ENSMUST00000102961;ENSMUST00000102962;ENSMUST00000151
ENSMUSG	Enho	ENSMUST00000127306
ENSMUSG	Unc13b	ENSMUST00000207569;ENSMUST00000171234;ENSMUST00000107
ENSMUSG	Atp8b5	ENSMUST00000107942;ENSMUST00000107937;ENSMUST00000102
ENSMUSG	Npr2	ENSMUST00000030191;ENSMUST00000107874;ENSMUST00000123
ENSMUSG	Hint2	ENSMUST00000151238;ENSMUST00000130593;ENSMUST00000134
ENSMUSG	Reck	ENSMUST00000128463;ENSMUST00000030198;ENSMUST0000013C
ENSMUSG	Sh3gl2	ENSMUST00000107188;ENSMUST00000030212;ENSMUST00000107
ENSMUSG	Saxo1	ENSMUST00000030216
ENSMUSG	Dab1	ENSMUST00000143294;ENSMUST00000106827;ENSMUST00000143
ENSMUSG	4921539	ENSMUST00000030245;ENSMUST00000097944
ENSMUSG	Ak4	ENSMUST00000102780;ENSMUST00000106946;ENSMUST00000131
ENSMUSG	2610528	ENSMUST00000106367;ENSMUST00000030261
ENSMUSG	Artn	ENSMUST00000070816;ENSMUST00000097913
ENSMUSG	Elavl4	ENSMUST00000106598;ENSMUST00000102722;ENSMUST0000010€
ENSMUSG	Calr4	ENSMUST00000106631
ENSMUSG	Fggy	ENSMUST00000079223;ENSMUST00000176162;ENSMUST00000141
ENSMUSG	Eqtn	ENSMUST00000107097;ENSMUST00000030309
ENSMUSG	1700012	ENSMUST00000030323
ENSMUSG	Pramef1	ENSMUST00000133334
ENSMUSG	Pramef1	ENSMUST00000030326;ENSMUST00000123854
ENSMUSG	Gpx7	ENSMUST00000030332
ENSMUSG	Echdc2	ENSMUST00000052999;ENSMUST00000130942;ENSMUST0000011€
ENSMUSG	Dmrtb1	ENSMUST00000131776;ENSMUST00000069271;ENSMUST00000131
ENSMUSG	Col9a2	ENSMUST00000030372
ENSMUSG	Kcnq4	ENSMUST00000030376
ENSMUSG	Edn2	ENSMUST00000030384
ENSMUSG	Tfap2c	ENSMUST00000030391;ENSMUST00000122982;ENSMUST0000009{
ENSMUSG	Ermap	ENSMUST00000138395;ENSMUST00000127574;ENSMUST0000013{
ENSMUSG	Epha8	ENSMUST00000030420
ENSMUSG	Dmbx1	ENSMUST00000064806
ENSMUSG	Cyp4a14	ENSMUST00000030487;ENSMUST00000124412
ENSMUSG	Pdzk1ip	ENSMUST00000171877;ENSMUST00000030488;ENSMUST0000010€
ENSMUSG	Tal1	ENSMUST00000030489;ENSMUST00000161601;ENSMUST00000162
ENSMUSG	Pax7	ENSMUST00000030508;ENSMUST00000174681
ENSMUSG	Pla2g2f	ENSMUST00000030526
ENSMUSG	Pla2g2c	ENSMUST00000105808;ENSMUST00000030530
ENSMUSG	Vwa5b1	ENSMUST00000030533
ENSMUSG	Cda	ENSMUST00000144986
ENSMUSG	Hcrtr1	ENSMUST00000119423
ENSMUSG	Sema3c	ENSMUST00000030568;ENSMUST00000170348;ENSMUST0000016{

ENSMUSG	Spocd1	ENSMUST00000084263
ENSMUSG	Hpca	ENSMUST00000095807;ENSMUST00000116444;ENSMUST0000011[illegible]
ENSMUSG	Tmem54	ENSMUST00000106064;ENSMUST00000030575;ENSMUST0000003[illegible]
ENSMUSG	Azin2	ENSMUST00000119354;ENSMUST00000106068;ENSMUST0000003[illegible]
ENSMUSG	Csmd2	ENSMUST00000129619;ENSMUST00000144298;ENSMUST00000184
ENSMUSG	Trim63	ENSMUST00000105875;ENSMUST00000134164;ENSMUST00000135
ENSMUSG	Slc30a2	ENSMUST00000105873;ENSMUST00000105874;ENSMUST00000105
ENSMUSG	Extl1	ENSMUST00000030643;ENSMUST00000142730
ENSMUSG	Cnksr1	ENSMUST00000030645;ENSMUST00000146283;ENSMUST00000145
ENSMUSG	Hgf	ENSMUST00000195957;ENSMUST00000199581;ENSMUST0000020[illegible]
ENSMUSG	Cd164l2	ENSMUST00000150744;ENSMUST00000218338;ENSMUST00000105
ENSMUSG	Sema3a	ENSMUST00000196514;ENSMUST00000197582;ENSMUST00000195
ENSMUSG	Smpdl3b	ENSMUST00000030709
ENSMUSG	Arhgef1	ENSMUST00000141834;ENSMUST00000140536;ENSMUST00000138
ENSMUSG	Cdk14	ENSMUST00000115451;ENSMUST00000030763;ENSMUST00000115
ENSMUSG	Galntl5	ENSMUST00000030778
ENSMUSG	Hes2	ENSMUST00000030782
ENSMUSG	Hes3	ENSMUST00000094438
ENSMUSG	Car6	ENSMUST00000030817
ENSMUSG	Slc2a5	ENSMUST00000030826;ENSMUST00000136610;ENSMUST00000132
ENSMUSG	Nos3	ENSMUST00000115090;ENSMUST00000030834
ENSMUSG	Rbp7	ENSMUST00000030848
ENSMUSG	Slc26a5	ENSMUST00000030878
ENSMUSG	Trp73	ENSMUST00000105644;ENSMUST00000105643;ENSMUST00000155
ENSMUSG	Gabrd	ENSMUST00000150423;ENSMUST00000030925
ENSMUSG	Plch2	ENSMUST00000105631;ENSMUST00000127661;ENSMUST00000176
ENSMUSG	Mxra8	ENSMUST00000141883;ENSMUST00000030947;ENSMUST00000133
ENSMUSG	Prom1	ENSMUST00000074113;ENSMUST00000165909;ENSMUST00000087
ENSMUSG	D5Ertd6	ENSMUST00000197016;ENSMUST00000198493
ENSMUSG	Ablim2	ENSMUST00000101280;ENSMUST00000054598;ENSMUST00000114
ENSMUSG	Ppp2r2c	ENSMUST00000031003;ENSMUST00000138124
ENSMUSG	Crmp1	ENSMUST00000202434;ENSMUST00000114158;ENSMUST00000201
ENSMUSG	Evc	ENSMUST00000114154;ENSMUST00000031005;ENSMUST00000146
ENSMUSG	Stk32b	ENSMUST00000094836
ENSMUSG	Rnf32	ENSMUST00000160888;ENSMUST00000160383;ENSMUST00000001
ENSMUSG	Plb1	ENSMUST00000201578;ENSMUST00000200878;ENSMUST00000202
ENSMUSG	Slc30a3	ENSMUST00000031037
ENSMUSG	Spata18	ENSMUST00000071077;ENSMUST00000178631
ENSMUSG	Yipf7	ENSMUST00000031045
ENSMUSG	Ppargc1[illegible]	ENSMUST00000132734;ENSMUST00000031059
ENSMUSG	Dpysl5	ENSMUST00000114729;ENSMUST00000101442;ENSMUST00000127
ENSMUSG	1700001[illegible]	ENSMUST00000127815;ENSMUST00000199129
ENSMUSG	Slc34a2	ENSMUST00000094787;ENSMUST00000147243
ENSMUSG	Sel1l3	ENSMUST00000031090;ENSMUST00000196435;ENSMUST00000196
ENSMUSG	Cckar	ENSMUST00000200691
ENSMUSG	Klb	ENSMUST00000031096;ENSMUST00000203333
ENSMUSG	Slc10a4	ENSMUST00000031127
ENSMUSG	Uchl1	ENSMUST00000031131;ENSMUST00000177194;ENSMUST00000176
ENSMUSG	Nmu	ENSMUST00000031146
ENSMUSG	Gnrhr	ENSMUST00000113372;ENSMUST00000094654
ENSMUSG	Ugt2b34	ENSMUST00000031181;ENSMUST00000113333
ENSMUSG	Pigg	ENSMUST00000119014;ENSMUST00000118910
ENSMUSG	Sult1e1	ENSMUST00000031201
ENSMUSG	Sult1d1	ENSMUST00000113314
ENSMUSG	Smr3a	ENSMUST00000031211
ENSMUSG	Smr2	ENSMUST00000199635
ENSMUSG	Amtn	ENSMUST00000073363

ENSMUSG	Enam	ENSMUST00000031222
ENSMUSG	Ambn	ENSMUST00000031226
ENSMUSG	Spp1	ENSMUST00000031243;ENSMUST00000112748;ENSMUST00000112
ENSMUSG	Ibsp	ENSMUST00000031246
ENSMUSG	Dmp1	ENSMUST00000066708
ENSMUSG	Gpat3	ENSMUST00000144623;ENSMUST00000112887;ENSMUST00000031
ENSMUSG	1700016	ENSMUST00000119824
ENSMUSG	Slc10a6	ENSMUST00000031263
ENSMUSG	Prkg2	ENSMUST00000161490;ENSMUST00000160765
ENSMUSG	Bmp3	ENSMUST00000197143;ENSMUST00000031278;ENSMUST00000200
ENSMUSG	Fgf5	ENSMUST00000031280
ENSMUSG	Crybb1	ENSMUST00000031286;ENSMUST00000131673
ENSMUSG	Asphd2	ENSMUST00000031291;ENSMUST00000200227;ENSMUST00000199
ENSMUSG	Nos1	ENSMUST00000142742;ENSMUST00000086451;ENSMUST00000138
ENSMUSG	Alb	ENSMUST00000031314
ENSMUSG	Afm	ENSMUST00000113179;ENSMUST00000128740
ENSMUSG	Rassf6	ENSMUST00000031317;ENSMUST00000202784;ENSMUST00000202
ENSMUSG	Cxcl5	ENSMUST00000202380
ENSMUSG	Ppbp	ENSMUST00000031319
ENSMUSG	Pf4	ENSMUST00000031320;ENSMUST00000202625;ENSMUST00000201
ENSMUSG	Mthfd2l	ENSMUST00000202852;ENSMUST00000202781;ENSMUST00000071
ENSMUSG	Ereg	ENSMUST00000031324
ENSMUSG	Areg	ENSMUST00000031325
ENSMUSG	Cxcl3	ENSMUST00000031326;ENSMUST00000201840
ENSMUSG	Cxcl1	ENSMUST00000031327;ENSMUST00000201245
ENSMUSG	Shroom3	ENSMUST00000113055;ENSMUST00000168878;ENSMUST00000202
ENSMUSG	Rimbp2	ENSMUST00000199537;ENSMUST00000196085;ENSMUST00000111
ENSMUSG	Piwil1	ENSMUST00000086056
ENSMUSG	Mmp17	ENSMUST00000031390;ENSMUST00000177802
ENSMUSG	Gm13103	ENSMUST00000094522
ENSMUSG	Anxa3	ENSMUST00000199330;ENSMUST00000031447;ENSMUST00000200
ENSMUSG	P2rx2	ENSMUST00000112478;ENSMUST00000195985;ENSMUST00000058
ENSMUSG	Ankrd7	ENSMUST00000031489
ENSMUSG	1700123	ENSMUST00000199968
ENSMUSG	Cyp2w1	ENSMUST00000031521
ENSMUSG	Cabp1	ENSMUST00000031519;ENSMUST00000112113;ENSMUST00000151
ENSMUSG	Uncx	ENSMUST00000172997
ENSMUSG	Tfec	ENSMUST00000031533;ENSMUST00000201406;ENSMUST00000201
ENSMUSG	4930519	ENSMUST00000112153
ENSMUSG	Radil	ENSMUST00000063635;ENSMUST00000085758;ENSMUST00000139
ENSMUSG	Spdye4b	ENSMUST00000031574
ENSMUSG	Lhx5	ENSMUST00000031591
ENSMUSG	Sdsl	ENSMUST00000031594
ENSMUSG	Gm5578	ENSMUST00000121490
ENSMUSG	Wasf3	ENSMUST00000016143
ENSMUSG	Rasl11a	ENSMUST00000031646;ENSMUST00000198570
ENSMUSG	Pdx1	ENSMUST00000085591
ENSMUSG	Cdx2	ENSMUST00000031650
ENSMUSG	C8b	ENSMUST00000031663
ENSMUSG	Wdr95	ENSMUST00000110502;ENSMUST00000201525;ENSMUST00000202
ENSMUSG	Tex26	ENSMUST00000031667;ENSMUST00000201454;ENSMUST00000201
ENSMUSG	Tfpi2	ENSMUST00000031674
ENSMUSG	Tspan12	ENSMUST00000120965;ENSMUST00000031678;ENSMUST00000143
ENSMUSG	Wnt16	ENSMUST00000128245;ENSMUST00000031681
ENSMUSG	Eln	ENSMUST00000015138;ENSMUST00000202570;ENSMUST00000201
ENSMUSG	Hyal6	ENSMUST00000031690
ENSMUSG	Hyal4	ENSMUST00000031691

ENSMUSG	Lmod2	ENSMUST00000031694
ENSMUSG	Asb15	ENSMUST00000031696;ENSMUST00000117688
ENSMUSG	Aass	ENSMUST00000031707
ENSMUSG	Fezf1	ENSMUST00000031709
ENSMUSG	Slc13a1	ENSMUST00000031713;ENSMUST00000177412;ENSMUST0000017[illegible]
ENSMUSG	Pax4	ENSMUST00000031718;ENSMUST00000174423
ENSMUSG	Actl6b	ENSMUST00000139395;ENSMUST00000196154
ENSMUSG	Tfr2	ENSMUST00000198866;ENSMUST00000199957;ENSMUST00000197
ENSMUSG	Cyp3a13	ENSMUST00000031741
ENSMUSG	Nobox	ENSMUST00000031749
ENSMUSG	Dlx6	ENSMUST00000160937;ENSMUST00000172943
ENSMUSG	Dlx5	ENSMUST00000142635;ENSMUST00000052609;ENSMUST00000204
ENSMUSG	Dync1i1	ENSMUST00000115556;ENSMUST00000115555;ENSMUST0000011[illegible]
ENSMUSG	Akr1b8	ENSMUST00000038406;ENSMUST00000133370
ENSMUSG	Plxna4	ENSMUST00000115096;ENSMUST00000200732
ENSMUSG	Ccdc136	ENSMUST00000115275;ENSMUST00000145310;ENSMUST0000009[illegible]
ENSMUSG	Adcyap1	ENSMUST00000070736;ENSMUST00000070756;ENSMUST0000016[illegible]
ENSMUSG	Aoc1	ENSMUST00000204856;ENSMUST00000031835;ENSMUST00000162
ENSMUSG	1600015	ENSMUST00000031837
ENSMUSG	Npy	ENSMUST00000031843
ENSMUSG	Ptn	ENSMUST00000101534;ENSMUST00000201321
ENSMUSG	Slc13a4	ENSMUST00000031868;ENSMUST00000155366;ENSMUST00000122
ENSMUSG	Stra8	ENSMUST00000031876;ENSMUST00000114999;ENSMUST00000114
ENSMUSG	Fam131b	ENSMUST00000143278
ENSMUSG	Clcn1	ENSMUST00000031894;ENSMUST00000170028;ENSMUST0000016[illegible]
ENSMUSG	Kel	ENSMUST00000031899;ENSMUST00000141502;ENSMUST0000015[illegible]
ENSMUSG	1700034	ENSMUST00000031900
ENSMUSG	Ccdc184	ENSMUST00000031914
ENSMUSG	Dbpht2	ENSMUST00000072100
ENSMUSG	2210010	ENSMUST00000031931
ENSMUSG	1700074	ENSMUST00000031935
ENSMUSG	Moxd2	ENSMUST00000031937
ENSMUSG	Prss37	ENSMUST00000031967
ENSMUSG	Prdm5	ENSMUST00000031976;ENSMUST00000173538;ENSMUST00000031
ENSMUSG	Clec5a	ENSMUST00000101491;ENSMUST00000031975;ENSMUST0000012[illegible]
ENSMUSG	Tgfa	ENSMUST00000032066;ENSMUST00000193710;ENSMUST00000194
ENSMUSG	Add2	ENSMUST00000204059;ENSMUST00000203366;ENSMUST00000203
ENSMUSG	Nat8	ENSMUST00000032073
ENSMUSG	Tacr1	ENSMUST00000205041;ENSMUST00000032122;ENSMUST00000203
ENSMUSG	Bmp10	ENSMUST00000032125
ENSMUSG	Gkn3	ENSMUST00000032127
ENSMUSG	Gkn2	ENSMUST00000032128
ENSMUSG	Gkn1	ENSMUST00000032129
ENSMUSG	Prok2	ENSMUST00000101120;ENSMUST00000203738
ENSMUSG	Gxylt2	ENSMUST00000032157;ENSMUST00000141333
ENSMUSG	Cntn3	ENSMUST00000203619
ENSMUSG	Cntn6	ENSMUST00000089215
ENSMUSG	Wnt7a	ENSMUST00000032180
ENSMUSG	Grip2	ENSMUST00000162300;ENSMUST00000162293;ENSMUST00000161
ENSMUSG	Slc6a13	ENSMUST00000064580;ENSMUST00000142144
ENSMUSG	Slc6a12	ENSMUST00000032200;ENSMUST00000165456;ENSMUST0000017[illegible]
ENSMUSG	Ret	ENSMUST00000032201
ENSMUSG	A2m	ENSMUST00000204850;ENSMUST00000032203;ENSMUST00000203
ENSMUSG	Klrg1	ENSMUST00000032207
ENSMUSG	Gdf3	ENSMUST00000032211
ENSMUSG	Lag3	ENSMUST00000032217;ENSMUST00000139571
ENSMUSG	Rasgef1	ENSMUST00000164960;ENSMUST00000203804;ENSMUST00000203

ENSMUSG	Clec4e	ENSMUST00000032239;ENSMUST00000176443
ENSMUSG	Gm8882	ENSMUST00000080849
ENSMUSG	Clec4d	ENSMUST00000204826;ENSMUST00000032240;ENSMUST00000203
ENSMUSG	Zfp248	ENSMUST00000069292;ENSMUST00000161519;ENSMUST00000162
ENSMUSG	Clec4b1	ENSMUST00000077228;ENSMUST00000078559
ENSMUSG	Clec4a2	ENSMUST00000041779;ENSMUST00000032248
ENSMUSG	Clec12b	ENSMUST00000032261
ENSMUSG	Clec1b	ENSMUST00000032262;ENSMUST00000112081;ENSMUST00000133
ENSMUSG	Tmem52b	ENSMUST00000032263
ENSMUSG	Olr1	ENSMUST00000032265
ENSMUSG	Klra5	ENSMUST00000167079;ENSMUST00000014683;ENSMUST00000118
ENSMUSG	Klra13-:	ENSMUST00000205252
ENSMUSG	Klra2	ENSMUST00000032306
ENSMUSG	Tas2r11	ENSMUST00000032315
ENSMUSG	Tas2r10	ENSMUST00000032317
ENSMUSG	Bcl2l14	ENSMUST00000032321;ENSMUST00000127865;ENSMUST00000111
ENSMUSG	Gprc5d	ENSMUST00000032327;ENSMUST00000111922
ENSMUSG	Grin2b	ENSMUST00000053880
ENSMUSG	Erp27	ENSMUST00000032343;ENSMUST00000162243;ENSMUST00000162
ENSMUSG	Rerg	ENSMUST00000117919;ENSMUST00000203003;ENSMUST00000149
ENSMUSG	Lmo3	ENSMUST00000162772;ENSMUST00000163024;ENSMUST00000162
ENSMUSG	Pik3c2g	ENSMUST00000187618;ENSMUST00000185968
ENSMUSG	Plcz1	ENSMUST00000032356
ENSMUSG	Slco1c1	ENSMUST00000203755
ENSMUSG	Slco1b2	ENSMUST00000042812
ENSMUSG	Slco1a4	ENSMUST00000165990;ENSMUST00000204676
ENSMUSG	Gys2	ENSMUST00000032371;ENSMUST00000126939
ENSMUSG	Cpne9	ENSMUST00000041203;ENSMUST00000132372;ENSMUST00000124
ENSMUSG	Smco2	ENSMUST00000032433
ENSMUSG	Atp2b2	ENSMUST00000101045;ENSMUST00000152831;ENSMUST00000101
ENSMUSG	Slc6a11	ENSMUST00000032451
ENSMUSG	Slc6a1	ENSMUST00000032454;ENSMUST00000204600;ENSMUST00000203
ENSMUSG	Timp4	ENSMUST00000032462;ENSMUST00000205131
ENSMUSG	Rho	ENSMUST00000032471;ENSMUST00000203531;ENSMUST00000203
ENSMUSG	Akap3	ENSMUST00000095440
ENSMUSG	Dyrk4	ENSMUST00000078521;ENSMUST00000032495
ENSMUSG	Tspan11	ENSMUST00000032501;ENSMUST00000149801
ENSMUSG	Tead4	ENSMUST00000130454;ENSMUST00000006311;ENSMUST00000112
ENSMUSG	Ceacam1.	ENSMUST00000032520;ENSMUST00000108483
ENSMUSG	Ceacam1	ENSMUST00000094799
ENSMUSG	Slc8a2	ENSMUST00000211649;ENSMUST00000168693
ENSMUSG	Sult2a8	ENSMUST00000209972
ENSMUSG	Mzf1	ENSMUST00000069289;ENSMUST00000182515;ENSMUST00000182
ENSMUSG	Zik1	ENSMUST00000032551
ENSMUSG	Ckm	ENSMUST00000208710
ENSMUSG	Rtn2	ENSMUST00000032559;ENSMUST00000209186;ENSMUST00000108
ENSMUSG	Ppm1n	ENSMUST00000032560;ENSMUST00000205792;ENSMUST00000206
ENSMUSG	Nova2	ENSMUST00000032571;ENSMUST00000220302
ENSMUSG	Pop4	ENSMUST00000032585;ENSMUST00000206221;ENSMUST00000205
ENSMUSG	Zfp939	ENSMUST00000186855;ENSMUST00000061201
ENSMUSG	Tmem238	ENSMUST00000168578;ENSMUST00000206315
ENSMUSG	4933421	ENSMUST00000032648;ENSMUST00000206593
ENSMUSG	Siglecg	ENSMUST00000005592;ENSMUST00000154322;ENSMUST00000124
ENSMUSG	Csrp3	ENSMUST00000167786;ENSMUST00000208146
ENSMUSG	Ceacam1	ENSMUST00000032663
ENSMUSG	Siglece	ENSMUST00000032667;ENSMUST00000206421
ENSMUSG	Cyp2b10	ENSMUST00000072438;ENSMUST00000144140

ENSMUSG	Zfp108	ENSMUST00000206777;ENSMUST00000205982;ENSMUST00000072
ENSMUSG	Tdrd12	ENSMUST00000193633
ENSMUSG	Slc7a9	ENSMUST00000147026
ENSMUSG	Slc17a6	ENSMUST00000032710;ENSMUST00000207945
ENSMUSG	Dbx1	ENSMUST00000032717
ENSMUSG	Cers3	ENSMUST00000066475;ENSMUST00000208521
ENSMUSG	Fam189a	ENSMUST00000119118;ENSMUST00000149851;ENSMUST00000144
ENSMUSG	Trpm1	ENSMUST00000085222;ENSMUST00000205348;ENSMUST00000205
ENSMUSG	Chrna7	ENSMUST00000032738;ENSMUST00000208304
ENSMUSG	Mesp2	ENSMUST00000107394
ENSMUSG	Pex11a	ENSMUST00000032761;ENSMUST00000150606;ENSMUST00000205
ENSMUSG	Rhcg	ENSMUST00000032766;ENSMUST00000161084
ENSMUSG	Rab38	ENSMUST00000107256;ENSMUST00000208478
ENSMUSG	Nox4	ENSMUST00000127138;ENSMUST00000032781
ENSMUSG	Cd22	ENSMUST00000019248;ENSMUST00000190646;ENSMUST00000108
ENSMUSG	Dpf1	ENSMUST00000049977;ENSMUST00000154222;ENSMUST00000126
ENSMUSG	Hapln3	ENSMUST00000206092;ENSMUST00000206402
ENSMUSG	Ccdc83	ENSMUST00000107220
ENSMUSG	Prss23o	ENSMUST00000179361;ENSMUST00000209064;ENSMUST00000208
ENSMUSG	1700010	ENSMUST00000179662;ENSMUST00000178848;ENSMUST00000032
ENSMUSG	Sh3gl3	ENSMUST00000032874;ENSMUST00000179800;ENSMUST00000177
ENSMUSG	Calca	ENSMUST00000032906;ENSMUST00000205560;ENSMUST00000032
ENSMUSG	Tbx6	ENSMUST00000172352;ENSMUST00000094037
ENSMUSG	Klk7	ENSMUST00000032955
ENSMUSG	Nupr1	ENSMUST00000032961;ENSMUST00000131174;ENSMUST00000187
ENSMUSG	Cd19	ENSMUST00000205997;ENSMUST00000032968;ENSMUST00000206
ENSMUSG	Lipt2	ENSMUST00000032967
ENSMUSG	Chrdl2	ENSMUST00000207641;ENSMUST00000032977;ENSMUST00000107
ENSMUSG	Myh14	ENSMUST00000107899;ENSMUST00000048102;ENSMUST00000107
ENSMUSG	Zkscan2	ENSMUST00000042470;ENSMUST00000206148;ENSMUST00000128
ENSMUSG	Myo7a	ENSMUST00000107128;ENSMUST00000156992;ENSMUST00000107
ENSMUSG	Aqp8	ENSMUST00000033023
ENSMUSG	Micalcl	ENSMUST00000033033;ENSMUST00000140202
ENSMUSG	Cox6a2	ENSMUST00000033049
ENSMUSG	Itgam	ENSMUST00000064821;ENSMUST00000106242;ENSMUST00000120
ENSMUSG	Lyve1	ENSMUST00000033050;ENSMUST00000209319
ENSMUSG	Itgax	ENSMUST00000033053;ENSMUST00000205460;ENSMUST00000205
ENSMUSG	Dkkl1	ENSMUST00000033057;ENSMUST00000210741;ENSMUST00000209
ENSMUSG	Fgf21	ENSMUST00000033099
ENSMUSG	Abcc6	ENSMUST00000210962;ENSMUST00000002850;ENSMUST00000209
ENSMUSG	Ush1c	ENSMUST00000078680;ENSMUST00000009667;ENSMUST00000129
ENSMUSG	Chp2	ENSMUST00000033152;ENSMUST00000160828
ENSMUSG	Hpx	ENSMUST00000033185
ENSMUSG	Cnga4	ENSMUST00000033187;ENSMUST00000210344
ENSMUSG	Cckbr	ENSMUST00000033189
ENSMUSG	Crym	ENSMUST00000033198;ENSMUST00000134067
ENSMUSG	Anks4b	ENSMUST00000033201
ENSMUSG	Zp2	ENSMUST00000208874;ENSMUST00000207726;ENSMUST00000207
ENSMUSG	Acsm2	ENSMUST00000167935
ENSMUSG	Umod	ENSMUST00000033263
ENSMUSG	Pdilt	ENSMUST00000033267
ENSMUSG	Acsm5	ENSMUST00000207796;ENSMUST00000066465;ENSMUST00000207
ENSMUSG	D7Ertd4	ENSMUST00000094002;ENSMUST00000172947;ENSMUST00000106
ENSMUSG	Trim66	ENSMUST00000106739;ENSMUST00000137704
ENSMUSG	Stk33	ENSMUST00000121748;ENSMUST00000106745;ENSMUST00000090
ENSMUSG	Tub	ENSMUST00000207583;ENSMUST00000208442;ENSMUST00000033
ENSMUSG	Fgf15	ENSMUST00000207040;ENSMUST00000033389

ENSMUSG	Fgf3	ENSMUST00000105898
ENSMUSG	Zfp300	ENSMUST00000040667;ENSMUST00000154299;ENSMUST0000013[illegible]
ENSMUSG	Gm498	ENSMUST00000208761
ENSMUSG	Slc6a14	ENSMUST00000033414
ENSMUSG	Tnni2	ENSMUST00000105971;ENSMUST00000145287;ENSMUST0000014[illegible]
ENSMUSG	Syt8	ENSMUST00000150192;ENSMUST00000128481;ENSMUST0000011[illegible]
ENSMUSG	Slc25a1	ENSMUST00000128773;ENSMUST00000134257;ENSMUST00000134
ENSMUSG	Igsf1	ENSMUST00000033442
ENSMUSG	1700080[illegible]	ENSMUST00000033449
ENSMUSG	3830403[illegible]	ENSMUST00000033458
ENSMUSG	Brs3	ENSMUST00000033464
ENSMUSG	Vgll1	ENSMUST00000141936
ENSMUSG	F9	ENSMUST00000033477
ENSMUSG	Cacna1f	ENSMUST00000144522;ENSMUST00000115726;ENSMUST00000141
ENSMUSG	Magix	ENSMUST00000130287;ENSMUST00000115695
ENSMUSG	Ccdc120	ENSMUST00000033490;ENSMUST00000224502;ENSMUST0000012[illegible]
ENSMUSG	Glod5	ENSMUST00000033503
ENSMUSG	Porcn	ENSMUST00000082320;ENSMUST00000154695;ENSMUST00000077
ENSMUSG	Slc38a5	ENSMUST00000115590
ENSMUSG	4930447[illegible]	ENSMUST00000033525
ENSMUSG	Rab39b	ENSMUST00000033545
ENSMUSG	Asb12	ENSMUST00000033549
ENSMUSG	Gpr165	ENSMUST00000033554;ENSMUST00000137479
ENSMUSG	Pgr15l	ENSMUST00000033556
ENSMUSG	Ophn1	ENSMUST00000033560;ENSMUST00000147529;ENSMUST00000154
ENSMUSG	Magee2	ENSMUST00000033575
ENSMUSG	Fgf16	ENSMUST00000033581
ENSMUSG	Tbx22	ENSMUST00000168174
ENSMUSG	Tnmd	ENSMUST00000033602
ENSMUSG	Srpx2	ENSMUST00000033606;ENSMUST00000113304
ENSMUSG	Sytl4	ENSMUST00000113297;ENSMUST00000033608;ENSMUST00000113
ENSMUSG	Btk	ENSMUST00000033617;ENSMUST00000113213;ENSMUST00000132
ENSMUSG	Serpina	ENSMUST00000033626
ENSMUSG	Col4a6	ENSMUST00000101205;ENSMUST00000137051
ENSMUSG	Col4a5	ENSMUST00000112931;ENSMUST00000128669
ENSMUSG	Chrdl1	ENSMUST00000063029;ENSMUST00000112878;ENSMUST00000074
ENSMUSG	Pak3	ENSMUST00000033640;ENSMUST00000134402;ENSMUST00000112
ENSMUSG	Dcx	ENSMUST00000033642;ENSMUST00000112851;ENSMUST0000013[illegible]
ENSMUSG	Il13ra2	ENSMUST00000033646;ENSMUST00000112827
ENSMUSG	Lrch2	ENSMUST00000152838;ENSMUST00000136789;ENSMUST00000134
ENSMUSG	Slc7a3	ENSMUST00000138162;ENSMUST00000113710;ENSMUST00000144
ENSMUSG	Adgrg2	ENSMUST00000112408;ENSMUST00000112401;ENSMUST00000112
ENSMUSG	Itgb1bp[illegible]	ENSMUST00000033674;ENSMUST00000143946
ENSMUSG	Dmrtc1a	ENSMUST00000119624;ENSMUST00000120808;ENSMUST0000003[illegible]
ENSMUSG	Cdx4	ENSMUST00000033689
ENSMUSG	Zcchc13	ENSMUST00000033692
ENSMUSG	Gabre	ENSMUST00000064780;ENSMUST00000148499;ENSMUST0000015[illegible]
ENSMUSG	Gabra3	ENSMUST00000055966;ENSMUST00000114554
ENSMUSG	Gabrq	ENSMUST00000114553
ENSMUSG	Zfp185	ENSMUST00000164800;ENSMUST00000114546;ENSMUST00000134
ENSMUSG	Grpr	ENSMUST00000033730
ENSMUSG	Zfp92	ENSMUST00000033740;ENSMUST00000086470
ENSMUSG	Atp2b3	ENSMUST00000114479
ENSMUSG	Bmx	ENSMUST00000112265;ENSMUST00000112263
ENSMUSG	Pir	ENSMUST00000033749;ENSMUST00000127685;ENSMUST0000012[illegible]
ENSMUSG	Vegfd	ENSMUST00000033751;ENSMUST00000124851
ENSMUSG	Asb11	ENSMUST00000036858;ENSMUST00000134085;ENSMUST0000003[illegible]

ENSMUSG	Dusp9	ENSMUST00000019701
ENSMUSG	Asb9	ENSMUST00000033756
ENSMUSG	Nxf7	ENSMUST00000113163
ENSMUSG	Zcchc18	ENSMUST00000113067;ENSMUST00000101227;ENSMUST00000033
ENSMUSG	Vsig1	ENSMUST00000033806
ENSMUSG	Morc4	ENSMUST00000033811;ENSMUST00000136415;ENSMUST00000087
ENSMUSG	Rnf128	ENSMUST00000113027;ENSMUST00000113026;ENSMUST00000113
ENSMUSG	F7	ENSMUST00000033820
ENSMUSG	F10	ENSMUST00000063820;ENSMUST00000033821;ENSMUST00000123
ENSMUSG	Adprhl1	ENSMUST00000204916
ENSMUSG	Atp4b	ENSMUST00000033826;ENSMUST00000210491
ENSMUSG	Grk1	ENSMUST00000209909;ENSMUST00000211027
ENSMUSG	1700029	ENSMUST00000187391;ENSMUST00000209207
ENSMUSG	Myom2	ENSMUST00000033842;ENSMUST00000135908;ENSMUST00000131
ENSMUSG	Tpte	ENSMUST00000211747
ENSMUSG	Slc25a1	ENSMUST00000033871;ENSMUST00000210020
ENSMUSG	Adrb3	ENSMUST00000081438;ENSMUST00000121838;ENSMUST00000117
ENSMUSG	Chrna6	ENSMUST00000033882
ENSMUSG	Chrnb3	ENSMUST00000060943
ENSMUSG	Cd209a	ENSMUST00000012847;ENSMUST00000208960;ENSMUST00000207
ENSMUSG	Cd209d	ENSMUST00000011445
ENSMUSG	Vegfc	ENSMUST00000033919
ENSMUSG	Plat	ENSMUST00000210960;ENSMUST00000033941;ENSMUST00000211
ENSMUSG	Ank1	ENSMUST00000110688;ENSMUST00000121802;ENSMUST00000117
ENSMUSG	Ido2	ENSMUST00000140417;ENSMUST00000121992;ENSMUST00000138
ENSMUSG	Ido1	ENSMUST00000110667;ENSMUST00000033956
ENSMUSG	Adam3	ENSMUST00000033958
ENSMUSG	Adam5	ENSMUST00000118419
ENSMUSG	4930555	ENSMUST00000126285
ENSMUSG	Fgl1	ENSMUST00000034003
ENSMUSG	Slc7a2	ENSMUST00000098816;ENSMUST00000057784;ENSMUST00000141
ENSMUSG	Fgf20	ENSMUST00000034014;ENSMUST00000118639
ENSMUSG	Msmo1	ENSMUST00000034015;ENSMUST00000132013;ENSMUST0000014C
ENSMUSG	Scrg1	ENSMUST00000034023
ENSMUSG	Isx	ENSMUST00000034034
ENSMUSG	Sorbs2	ENSMUST00000209534;ENSMUST00000067065;ENSMUST00000124
ENSMUSG	Anxa10	ENSMUST00000034052;ENSMUST00000210799
ENSMUSG	Tlr3	ENSMUST00000034056;ENSMUST00000167106;ENSMUST0000021C
ENSMUSG	Cbr4	ENSMUST00000034058;ENSMUST00000126575;ENSMUST00000156
ENSMUSG	F11	ENSMUST00000034064
ENSMUSG	Mfap3l	ENSMUST00000161421;ENSMUST00000160719;ENSMUST00000161
ENSMUSG	Triml1	ENSMUST00000211446
ENSMUSG	Cbln1	ENSMUST00000034076;ENSMUST00000211279
ENSMUSG	Nkd1	ENSMUST00000034086;ENSMUST00000211113
ENSMUSG	Sall1	ENSMUST00000034090
ENSMUSG	Cdh11	ENSMUST00000075190;ENSMUST00000210578;ENSMUST0000021C
ENSMUSG	1700011	ENSMUST00000034109
ENSMUSG	Pou4f2	ENSMUST00000034115
ENSMUSG	Mylk3	ENSMUST00000034133;ENSMUST00000121972;ENSMUST00000138
ENSMUSG	Pmfbp1	ENSMUST00000212003
ENSMUSG	Irx3	ENSMUST00000093312;ENSMUST00000175795
ENSMUSG	Crnde	ENSMUST00000178064;ENSMUST00000179421;ENSMUST00000034
ENSMUSG	Irx5	ENSMUST00000210246;ENSMUST00000034184
ENSMUSG	Irx6	ENSMUST00000034185
ENSMUSG	Gnao1	ENSMUST00000125695;ENSMUST00000034198;ENSMUST00000125
ENSMUSG	Mt3	ENSMUST00000034211;ENSMUST00000211930;ENSMUST00000211
ENSMUSG	Slc12a3	ENSMUST00000034218;ENSMUST00000212134;ENSMUST00000212

ENSMUSG	Nudt7	ENSMUST00000073521;ENSMUST00000066514;ENSMUST00000109
ENSMUSG	Cntnap4	ENSMUST00000125976;ENSMUST00000118171;ENSMUST00000127
ENSMUSG	Drc7	ENSMUST00000058479;ENSMUST00000212986
ENSMUSG	Cngb1	ENSMUST00000119870;ENSMUST00000121162;ENSMUST0000013З
ENSMUSG	Necab2	ENSMUST00000098363
ENSMUSG	Pde4c	ENSMUST00000123739;ENSMUST00000034307;ENSMUST0000011C
ENSMUSG	Hsd17b2	ENSMUST00000034304;ENSMUST00000212052
ENSMUSG	Bco1	ENSMUST00000034308
ENSMUSG	1700030	ENSMUST00000070577;ENSMUST00000213068
ENSMUSG	Map3k21	ENSMUST00000034316
ENSMUSG	Bean1	ENSMUST00000171018
ENSMUSG	Cdh16	ENSMUST00000212318;ENSMUST00000212882;ENSMUST00000211
ENSMUSG	Ces2d-p	ENSMUST00000043209
ENSMUSG	Ces2e	ENSMUST00000034355;ENSMUST00000109410
ENSMUSG	1700012	ENSMUST00000060330
ENSMUSG	Hephl1	ENSMUST00000159985
ENSMUSG	Tmem231	ENSMUST00000034429;ENSMUST00000213050
ENSMUSG	Chst5	ENSMUST00000034430
ENSMUSG	Ctrb1	ENSMUST00000034435
ENSMUSG	Bmper	ENSMUST00000071982;ENSMUST00000217648
ENSMUSG	Tbx20	ENSMUST00000052946;ENSMUST00000166018;ENSMUST00000217
ENSMUSG	Glb1l3	ENSMUST00000210274
ENSMUSG	Spata19	ENSMUST00000215326
ENSMUSG	Pou2f3	ENSMUST00000176636;ENSMUST00000034513;ENSMUST0000021З
ENSMUSG	Grik4	ENSMUST00000114865
ENSMUSG	Crtam	ENSMUST00000180872;ENSMUST00000188848;ENSMUST0000018C
ENSMUSG	Jhy	ENSMUST00000034521;ENSMUST00000160120
ENSMUSG	Clmp	ENSMUST00000139577;ENSMUST00000034522;ENSMUST00000141
ENSMUSG	Nxpe2	ENSMUST00000034527;ENSMUST00000137976;ENSMUST00000149
ENSMUSG	Barx2	ENSMUST00000116615
ENSMUSG	Kcnj5	ENSMUST00000034533
ENSMUSG	4930510	ENSMUST00000034550
ENSMUSG	Btg4	ENSMUST00000214452;ENSMUST00000216453;ENSMUST00000114
ENSMUSG	4833427	ENSMUST00000170947
ENSMUSG	Cryab	ENSMUST00000217475;ENSMUST00000214609;ENSMUST00000034
ENSMUSG	Tex12	ENSMUST00000217236;ENSMUST00000034568
ENSMUSG	Bco2	ENSMUST00000119103
ENSMUSG	Apoa5	ENSMUST00000121598;ENSMUST00000215187
ENSMUSG	Apoa4	ENSMUST00000034585
ENSMUSG	Apoc3	ENSMUST00000121916
ENSMUSG	Apoa1	ENSMUST00000034588;ENSMUST00000132155
ENSMUSG	Dscaml1	ENSMUST00000217335;ENSMUST00000217538;ENSMUST0000021З
ENSMUSG	D730048	ENSMUST00000034619
ENSMUSG	Acrv1	ENSMUST00000034620;ENSMUST00000184160
ENSMUSG	Slc37a4	ENSMUST00000215420;ENSMUST00000215121;ENSMUST0000021З
ENSMUSG	Fez1	ENSMUST00000034630;ENSMUST00000159137
ENSMUSG	Robo4	ENSMUST00000156972;ENSMUST00000214185;ENSMUST0000011Б
ENSMUSG	Robo3	ENSMUST00000167089;ENSMUST00000171467;ENSMUST0000011Б
ENSMUSG	Pde4a	ENSMUST00000039413;ENSMUST00000131769;ENSMUST0000014C
ENSMUSG	Bmp5	ENSMUST00000012281;ENSMUST00000137213
ENSMUSG	Scg3	ENSMUST00000034699;ENSMUST00000213324
ENSMUSG	Tmod2	ENSMUST00000098552;ENSMUST00000064433;ENSMUST0000021Б
ENSMUSG	Bcl2l10	ENSMUST00000034709
ENSMUSG	Gnb5	ENSMUST00000213990;ENSMUST00000215875;ENSMUST0000021Б
ENSMUSG	Aqp9	ENSMUST00000074465;ENSMUST00000144618;ENSMUST0000006C
ENSMUSG	Lipc	ENSMUST00000034731;ENSMUST00000216798;ENSMUST00000214
ENSMUSG	Fam81a	ENSMUST00000034749;ENSMUST00000141775

ENSMUSG	Gcnt3	ENSMUST00000034751
ENSMUSG	Irak1bp	ENSMUST00000113245;ENSMUST00000034783
ENSMUSG	Ankk1	ENSMUST00000034792;ENSMUST00000216227
ENSMUSG	Drd2	ENSMUST00000075764
ENSMUSG	Elovl4	ENSMUST00000034796
ENSMUSG	Tmprss5	ENSMUST00000165088;ENSMUST00000171217;ENSMUST00000070
ENSMUSG	Htr3a	ENSMUST00000003826
ENSMUSG	Nnmt	ENSMUST00000136813;ENSMUST00000034808;ENSMUST00000119
ENSMUSG	Cyp19a1	ENSMUST00000215736;ENSMUST00000217040
ENSMUSG	Paqr5	ENSMUST00000135050;ENSMUST00000034817
ENSMUSG	Crabp1	ENSMUST00000034830
ENSMUSG	Nr2e3	ENSMUST00000130831;ENSMUST00000128108
ENSMUSG	Celf6	ENSMUST00000143916;ENSMUST00000118549;ENSMUST00000129
ENSMUSG	Chrna3	ENSMUST00000034851
ENSMUSG	Cyp1a2	ENSMUST00000034860;ENSMUST00000215792
ENSMUSG	Tmem266	ENSMUST00000085754;ENSMUST00000034862;ENSMUST00000130
ENSMUSG	Cyp1a1	ENSMUST00000216433;ENSMUST00000034865
ENSMUSG	Isl2	ENSMUST00000175950;ENSMUST00000034869;ENSMUST00000165
ENSMUSG	Loxl1	ENSMUST00000061799
ENSMUSG	Hcn4	ENSMUST00000034889
ENSMUSG	Impg1	ENSMUST00000085289
ENSMUSG	Mlip	ENSMUST00000183955;ENSMUST00000184944
ENSMUSG	Rasgrf1	ENSMUST00000189545;ENSMUST00000034912;ENSMUST00000190
ENSMUSG	Tinag	ENSMUST00000034911
ENSMUSG	Fam83b	ENSMUST00000183437;ENSMUST00000098546
ENSMUSG	Hcrtr2	ENSMUST00000063140
ENSMUSG	Zic1	ENSMUST00000034927
ENSMUSG	Car12	ENSMUST00000071889;ENSMUST00000152011;ENSMUST00000123
ENSMUSG	Aph1b	ENSMUST00000034934;ENSMUST00000168309;ENSMUST00000113
ENSMUSG	Plscr4	ENSMUST00000034941;ENSMUST00000143866
ENSMUSG	Igdcc3	ENSMUST00000034961;ENSMUST00000217371
ENSMUSG	Ube2cbp	ENSMUST00000034986;ENSMUST00000121189
ENSMUSG	Tbx18	ENSMUST00000034991;ENSMUST00000186698
ENSMUSG	Trim42	ENSMUST00000035026
ENSMUSG	Clstn2	ENSMUST00000035027;ENSMUST00000162295
ENSMUSG	Rbp2	ENSMUST00000189446
ENSMUSG	Cldn18	ENSMUST00000035048
ENSMUSG	Cspg5	ENSMUST00000035058;ENSMUST00000196060;ENSMUST00000196
ENSMUSG	Ptgs2	ENSMUST00000035065;ENSMUST00000190784
ENSMUSG	Prss44	ENSMUST00000196597
ENSMUSG	Tdgf1	ENSMUST00000035075;ENSMUST00000197460;ENSMUST00000199
ENSMUSG	Dclk3	ENSMUST00000111879
ENSMUSG	Stac	ENSMUST00000161995;ENSMUST00000035083;ENSMUST00000162
ENSMUSG	Scn5a	ENSMUST00000065196;ENSMUST00000117911;ENSMUST00000120
ENSMUSG	Mobp	ENSMUST00000093773
ENSMUSG	Hhatl	ENSMUST00000035110;ENSMUST00000214768
ENSMUSG	Ss18l2	ENSMUST00000035113;ENSMUST00000215966;ENSMUST00000214
ENSMUSG	Amotl2	ENSMUST00000145913;ENSMUST00000153965;ENSMUST00000153
ENSMUSG	Cck	ENSMUST00000216176;ENSMUST00000035120;ENSMUST00000215
ENSMUSG	Ephb1	ENSMUST00000035129
ENSMUSG	Rab6b	ENSMUST00000189134;ENSMUST00000035155
ENSMUSG	Cpne4	ENSMUST00000098443;ENSMUST00000057742
ENSMUSG	Col6a4	ENSMUST00000149207;ENSMUST00000218471;ENSMUST00000121
ENSMUSG	Hemk1	ENSMUST00000035196;ENSMUST00000148165;ENSMUST00000194
ENSMUSG	1700102	ENSMUST00000192995;ENSMUST00000035234
ENSMUSG	Oas1d	ENSMUST00000044224
ENSMUSG	Thsd7a	ENSMUST00000172356;ENSMUST00000119581;ENSMUST00000137

ENSMUSG	Colgalt	ENSMUST00000127586;ENSMUST00000044311
ENSMUSG	A930018	ENSMUST00000040374
ENSMUSG	Cd59a	ENSMUST00000168176;ENSMUST00000040423
ENSMUSG	6820408	ENSMUST00000039961;ENSMUST00000153713
ENSMUSG	Nlrp3	ENSMUST00000101148;ENSMUST00000149126
ENSMUSG	Trib3	ENSMUST00000040312;ENSMUST00000124995
ENSMUSG	Mdfi	ENSMUST00000113280;ENSMUST00000066368;ENSMUST00000035
ENSMUSG	Bmp8a	ENSMUST00000102641;ENSMUST00000040496
ENSMUSG	Kap	ENSMUST00000048032
ENSMUSG	Gng11	ENSMUST00000031670
ENSMUSG	Trpa1	ENSMUST00000041447
ENSMUSG	Chrm1	ENSMUST00000177197;ENSMUST00000163785
ENSMUSG	Lama1	ENSMUST00000035471
ENSMUSG	Alox12b	ENSMUST00000036424
ENSMUSG	Loxhd1	ENSMUST00000096547;ENSMUST00000148341
ENSMUSG	Ank2	ENSMUST00000044443;ENSMUST00000182588;ENSMUST00000182
ENSMUSG	Ppp1r9a	ENSMUST00000175889;ENSMUST00000168998;ENSMUST00000176
ENSMUSG	Trpc1	ENSMUST00000189137;ENSMUST00000053785;ENSMUST00000190
ENSMUSG	Alpk2	ENSMUST00000035548;ENSMUST00000141250;ENSMUST00000182
ENSMUSG	Rspo4	ENSMUST00000042217
ENSMUSG	P2ry2	ENSMUST00000037540;ENSMUST00000208340;ENSMUST00000207
ENSMUSG	Ccdc85a	ENSMUST00000093253;ENSMUST00000109502;ENSMUST00000042
ENSMUSG	Rims3	ENSMUST00000132895;ENSMUST00000071093;ENSMUST00000106
ENSMUSG	1700031	ENSMUST00000048061
ENSMUSG	Styk1	ENSMUST00000049150;ENSMUST00000121078;ENSMUST00000135
ENSMUSG	Cspg4	ENSMUST00000214057;ENSMUST00000035661;ENSMUST00000215
ENSMUSG	Adgre4	ENSMUST00000025004
ENSMUSG	Rbm11	ENSMUST00000114249;ENSMUST00000114253
ENSMUSG	Lipi	ENSMUST00000186809
ENSMUSG	Guca2b	ENSMUST00000106309;ENSMUST00000044426
ENSMUSG	5730522	ENSMUST00000144503;ENSMUST00000109511;ENSMUST00000144
ENSMUSG	Slc16a8	ENSMUST00000039752
ENSMUSG	Nlrp4f	ENSMUST00000221659
ENSMUSG	Klra9	ENSMUST00000112032
ENSMUSG	Dhrs7c	ENSMUST00000123862;ENSMUST00000168612
ENSMUSG	Resp18	ENSMUST00000039534;ENSMUST00000191283;ENSMUST00000189
ENSMUSG	Vsx1	ENSMUST00000046095
ENSMUSG	Clec1a	ENSMUST00000203162;ENSMUST00000037481;ENSMUST00000204
ENSMUSG	Cst10	ENSMUST00000047008
ENSMUSG	Card10	ENSMUST00000164826;ENSMUST00000170584;ENSMUST00000071
ENSMUSG	Tie1	ENSMUST00000184261;ENSMUST00000047421;ENSMUST00000153
ENSMUSG	Myh2	ENSMUST00000170159
ENSMUSG	Tpsg1	ENSMUST00000160377;ENSMUST00000162021;ENSMUST00000024
ENSMUSG	Mamdc2	ENSMUST00000036069
ENSMUSG	Slc9c1	ENSMUST00000159945;ENSMUST00000162774
ENSMUSG	Slitrk5	ENSMUST00000042767
ENSMUSG	Gm9758	ENSMUST00000035980
ENSMUSG	Wnt6	ENSMUST00000006716;ENSMUST00000189544
ENSMUSG	Trim45	ENSMUST00000037409;ENSMUST00000106980;ENSMUST00000134
ENSMUSG	Rpgrip1	ENSMUST00000047783;ENSMUST00000139113;ENSMUST00000210
ENSMUSG	Galnt9	ENSMUST00000040001;ENSMUST00000200404
ENSMUSG	Plppr5	ENSMUST00000039564;ENSMUST00000106473
ENSMUSG	Chst2	ENSMUST00000036267
ENSMUSG	Palmd	ENSMUST00000040097;ENSMUST00000119557;ENSMUST00000197
ENSMUSG	Rtp1	ENSMUST00000038730
ENSMUSG	Armcx2	ENSMUST00000119010;ENSMUST00000035559;ENSMUST00000113
ENSMUSG	Adamts1	ENSMUST00000065112;ENSMUST00000217070;ENSMUST00000216

ENSMUSG	BC02658	ENSMUST00000193791;ENSMUST00000046743;ENSMUST00000195
ENSMUSG	Crygs	ENSMUST00000040592
ENSMUSG	Idi2	ENSMUST00000038598
ENSMUSG	Acsm1	ENSMUST00000047929
ENSMUSG	Apol6	ENSMUST00000142405;ENSMUST00000127957;ENSMUST0000012[illegible]
ENSMUSG	Myo6	ENSMUST00000035889;ENSMUST00000183377;ENSMUST0000011[illegible]
ENSMUSG	Tmem35a	ENSMUST00000037687
ENSMUSG	Igf2bp2	ENSMUST00000100052;ENSMUST00000129913;ENSMUST00000134
ENSMUSG	Ndn	ENSMUST00000038775
ENSMUSG	Myo5c	ENSMUST00000036555;ENSMUST00000215437;ENSMUST0000021[illegible]
ENSMUSG	Lgi3	ENSMUST00000047331
ENSMUSG	Nat8f2	ENSMUST00000045008
ENSMUSG	Piwil2	ENSMUST00000048129
ENSMUSG	Gabrb3	ENSMUST00000085240;ENSMUST00000196198;ENSMUST0000015[illegible]
ENSMUSG	Akr1c14	ENSMUST00000118717;ENSMUST00000041768
ENSMUSG	Fndc3c1	ENSMUST00000039447
ENSMUSG	St18	ENSMUST00000140079;ENSMUST00000131467
ENSMUSG	Mnd1	ENSMUST00000047368
ENSMUSG	Calm4	ENSMUST00000042219
ENSMUSG	Nrxn2	ENSMUST00000113462;ENSMUST00000128272;ENSMUST0000011[illegible]
ENSMUSG	Npbwr1	ENSMUST00000044180
ENSMUSG	Dysf	ENSMUST00000113821;ENSMUST00000113823;ENSMUST0000020[illegible]
ENSMUSG	Fgb	ENSMUST00000048246
ENSMUSG	Kcnk10	ENSMUST00000221240;ENSMUST00000221305;ENSMUST00000221
ENSMUSG	Fgg	ENSMUST00000048486;ENSMUST00000194175
ENSMUSG	Best4-p	ENSMUST00000129783
ENSMUSG	Rbm46	ENSMUST00000048647;ENSMUST00000182818
ENSMUSG	Cfhr2	ENSMUST00000094489
ENSMUSG	Map9	ENSMUST00000193559;ENSMUST00000091014;ENSMUST00000195
ENSMUSG	Trim36	ENSMUST00000167364;ENSMUST00000037011
ENSMUSG	Rnf225	ENSMUST00000045870
ENSMUSG	Gria2	ENSMUST00000075316;ENSMUST00000107745;ENSMUST00000192
ENSMUSG	Kcnk1	ENSMUST00000046765
ENSMUSG	Neu4	ENSMUST00000050890
ENSMUSG	Rxfp1	ENSMUST00000078527;ENSMUST00000182491;ENSMUST0000018[illegible]
ENSMUSG	Ccdc182	ENSMUST00000037268
ENSMUSG	Prss29	ENSMUST00000024993
ENSMUSG	Phka1	ENSMUST00000052012;ENSMUST00000120270;ENSMUST00000122
ENSMUSG	Myrfl	ENSMUST00000048229
ENSMUSG	Ypel4	ENSMUST00000090729;ENSMUST00000111643;ENSMUST00000151
ENSMUSG	Zfp551	ENSMUST00000080348;ENSMUST00000120220;ENSMUST0000012[illegible]
ENSMUSG	Nlrp4b	ENSMUST00000117413
ENSMUSG	Ano7	ENSMUST00000058682;ENSMUST00000186641;ENSMUST0000019[illegible]
ENSMUSG	Kctd7	ENSMUST00000040616;ENSMUST00000144878;ENSMUST00000144
ENSMUSG	Tspan8	ENSMUST00000080630;ENSMUST00000179196;ENSMUST0000003[illegible]
ENSMUSG	Serpini	ENSMUST00000039047
ENSMUSG	Tmem63c	ENSMUST00000154801;ENSMUST00000146292;ENSMUST0000011[illegible]
ENSMUSG	Zbbx	ENSMUST00000107778;ENSMUST00000107775
ENSMUSG	2310007	ENSMUST00000043718;ENSMUST00000143419
ENSMUSG	Sept14	ENSMUST00000182381;ENSMUST00000182386
ENSMUSG	Slc38a8	ENSMUST00000036748;ENSMUST00000138061;ENSMUST0000013[illegible]
ENSMUSG	Rhov	ENSMUST00000037360
ENSMUSG	Foxj1	ENSMUST00000036215
ENSMUSG	Gm884	ENSMUST00000059279
ENSMUSG	Sdr42e1	ENSMUST00000037955;ENSMUST00000173522;ENSMUST00000174
ENSMUSG	Tmem132	ENSMUST00000044441;ENSMUST00000200136
ENSMUSG	Tmem132	ENSMUST00000119026;ENSMUST00000134780;ENSMUST0000012[illegible]

ENSMUSG	Fam151b	ENSMUST00000225299;ENSMUST00000040106
ENSMUSG	Gm5689	ENSMUST00000041389
ENSMUSG	Csta1	ENSMUST00000096090
ENSMUSG	Ssu2	ENSMUST00000060847;ENSMUST00000143134;ENSMUST0000012[illegible]
ENSMUSG	Lif	ENSMUST00000066283;ENSMUST00000154927;ENSMUST0000004[illegible]
ENSMUSG	Kcnh5	ENSMUST00000042299
ENSMUSG	Pkd1l2	ENSMUST00000098375;ENSMUST00000109093;ENSMUST0000016[illegible]
ENSMUSG	Gulo	ENSMUST00000059970
ENSMUSG	Uroc1	ENSMUST00000164761
ENSMUSG	Eda2r	ENSMUST00000037353;ENSMUST00000113832;ENSMUST0000012[illegible]
ENSMUSG	Dynlrb2	ENSMUST00000213007;ENSMUST00000078170
ENSMUSG	Rasd2	ENSMUST00000139848;ENSMUST00000132133
ENSMUSG	Uaca	ENSMUST00000214354;ENSMUST00000050183;ENSMUST00000214
ENSMUSG	Gbx2	ENSMUST00000187633
ENSMUSG	Kdelc2	ENSMUST00000037853;ENSMUST00000214164
ENSMUSG	Edil3	ENSMUST00000153974;ENSMUST00000081769;ENSMUST0000014[illegible]
ENSMUSG	Gjc1	ENSMUST00000107075;ENSMUST00000068933;ENSMUST00000092
ENSMUSG	Hsd17b1	ENSMUST00000048118;ENSMUST00000112803
ENSMUSG	Fbxo16	ENSMUST00000224629
ENSMUSG	Scn10a	ENSMUST00000213392
ENSMUSG	Hdx	ENSMUST00000113422;ENSMUST00000038472
ENSMUSG	Tex16	ENSMUST00000130247
ENSMUSG	Exph5	ENSMUST00000051014;ENSMUST00000132410;ENSMUST0000013[illegible]
ENSMUSG	Pof1b	ENSMUST00000039887
ENSMUSG	Tmem5	ENSMUST00000140299;ENSMUST00000038772;ENSMUST0000012[illegible]
ENSMUSG	Prss55	ENSMUST00000171503
ENSMUSG	Setmar	ENSMUST00000049246;ENSMUST00000138140
ENSMUSG	Zyg11a	ENSMUST00000223127;ENSMUST00000106690
ENSMUSG	Lrrn1	ENSMUST00000049285
ENSMUSG	Ppp1r1c	ENSMUST00000090760
ENSMUSG	Nlrp4c	ENSMUST00000037728
ENSMUSG	Dnaic2	ENSMUST00000151053;ENSMUST00000069325;ENSMUST00000092
ENSMUSG	Adgrb1	ENSMUST00000042035;ENSMUST00000190524;ENSMUST0000018[illegible]
ENSMUSG	Pabpc5	ENSMUST00000040961
ENSMUSG	Nagk	ENSMUST00000037376;ENSMUST00000113851;ENSMUST0000015[illegible]
ENSMUSG	Pcdh11x	ENSMUST00000192977;ENSMUST00000195088;ENSMUST00000192
ENSMUSG	1700006[illegible]	ENSMUST00000041178;ENSMUST00000160091;ENSMUST0000015[illegible]
ENSMUSG	Asb16	ENSMUST00000036467
ENSMUSG	BC03086	ENSMUST00000100392;ENSMUST00000133930;ENSMUST0000014[illegible]
ENSMUSG	Dsg1c	ENSMUST00000054128
ENSMUSG	Vax2	ENSMUST00000037807
ENSMUSG	B3galt1	ENSMUST00000188614;ENSMUST00000180887;ENSMUST00000112
ENSMUSG	Celf5	ENSMUST00000118763;ENSMUST00000120856;ENSMUST0000012[illegible]
ENSMUSG	Gnat1	ENSMUST00000010205;ENSMUST00000194802;ENSMUST00000194
ENSMUSG	Larp6	ENSMUST00000038407
ENSMUSG	Acot11	ENSMUST00000102762;ENSMUST00000065253;ENSMUST0000014[illegible]
ENSMUSG	Fam151a	ENSMUST00000047620
ENSMUSG	Sncb	ENSMUST00000036825;ENSMUST00000134110;ENSMUST00000153
ENSMUSG	Pygo1	ENSMUST00000038489
ENSMUSG	Mdga2	ENSMUST00000037181;ENSMUST00000113942;ENSMUST00000101
ENSMUSG	Cdhr2	ENSMUST00000037145
ENSMUSG	Ttc22	ENSMUST00000047922;ENSMUST00000136505
ENSMUSG	Hrh2	ENSMUST00000038101;ENSMUST00000209846;ENSMUST00000211
ENSMUSG	Otoa	ENSMUST00000207598;ENSMUST00000171880;ENSMUST00000047
ENSMUSG	Htr2a	ENSMUST00000036653
ENSMUSG	Epgn	ENSMUST00000202724;ENSMUST00000041516
ENSMUSG	C8a	ENSMUST00000064873

ENSMUSG	Creb3l3	ENSMUST00000117422;ENSMUST00000143969
ENSMUSG	Fam167a	ENSMUST00000121288;ENSMUST00000128313
ENSMUSG	Eva1a	ENSMUST00000150976;ENSMUST00000042974;ENSMUST0000015
ENSMUSG	Gm6781	ENSMUST00000038304
ENSMUSG	Gpr33	ENSMUST00000040161
ENSMUSG	Fam124a	ENSMUST00000162987;ENSMUST00000161899;ENSMUST0000003
ENSMUSG	Ano4	ENSMUST00000182462;ENSMUST00000182341;ENSMUST00000182
ENSMUSG	Rfpl4	ENSMUST00000179971;ENSMUST00000045215;ENSMUST0000020
ENSMUSG	Chrnb4	ENSMUST00000034854
ENSMUSG	Rims4	ENSMUST00000044734
ENSMUSG	Pcyt1b	ENSMUST00000113933;ENSMUST00000045898;ENSMUST0000014
ENSMUSG	Amh	ENSMUST00000036016
ENSMUSG	Hpse	ENSMUST00000045617;ENSMUST00000112908
ENSMUSG	Tpbg	ENSMUST00000006559;ENSMUST00000098500;ENSMUST0000018
ENSMUSG	Arx	ENSMUST00000046565
ENSMUSG	Adrb1	ENSMUST00000038949
ENSMUSG	Ccl12	ENSMUST00000000194;ENSMUST00000124916
ENSMUSG	Ccl7	ENSMUST00000021011
ENSMUSG	Ccl2	ENSMUST00000000193;ENSMUST00000124479
ENSMUSG	Kank4	ENSMUST00000102790;ENSMUST00000137270
ENSMUSG	Fam170a	ENSMUST00000039121
ENSMUSG	Ptprh	ENSMUST00000206999;ENSMUST00000166650
ENSMUSG	Sstr1	ENSMUST00000044299
ENSMUSG	Ccr3	ENSMUST00000039171
ENSMUSG	Foxa1	ENSMUST00000044380;ENSMUST00000218398
ENSMUSG	Samt3	ENSMUST00000047945
ENSMUSG	Prdm8	ENSMUST00000112959
ENSMUSG	Tnni3	ENSMUST00000132621;ENSMUST00000098859;ENSMUST0000012
ENSMUSG	Stab2	ENSMUST00000035288;ENSMUST00000219341;ENSMUST0000021
ENSMUSG	Plk5	ENSMUST00000039836;ENSMUST00000146826
ENSMUSG	Fthl17a	ENSMUST00000048382
ENSMUSG	Reep6	ENSMUST00000105355;ENSMUST00000105357;ENSMUST0000010
ENSMUSG	Slc12a8	ENSMUST00000059056;ENSMUST00000119173;ENSMUST0000014
ENSMUSG	Fbxl21	ENSMUST00000121095;ENSMUST00000121871;ENSMUST0000004
ENSMUSG	Npffr2	ENSMUST00000048557
ENSMUSG	Ccdc180	ENSMUST00000178561
ENSMUSG	Gc	ENSMUST00000049209
ENSMUSG	Igfbpl1	ENSMUST00000044297;ENSMUST00000145102
ENSMUSG	Wdr20rt	ENSMUST00000222822
ENSMUSG	Pcdh17	ENSMUST00000071370
ENSMUSG	L3mbtl1	ENSMUST00000153416;ENSMUST00000035751;ENSMUST0000014
ENSMUSG	Kcnh8	ENSMUST00000039366;ENSMUST00000184279
ENSMUSG	Krt33a	ENSMUST00000018399
ENSMUSG	Chrna5	ENSMUST00000093844;ENSMUST00000217408;ENSMUST0000021
ENSMUSG	Ky	ENSMUST00000039390;ENSMUST00000190609
ENSMUSG	Frmpd1	ENSMUST00000134280;ENSMUST00000044773;ENSMUST00000107
ENSMUSG	Olfr150	ENSMUST00000205900;ENSMUST00000215030;ENSMUST0000004
ENSMUSG	Muc20	ENSMUST00000115116;ENSMUST00000041123
ENSMUSG	Lrfn5	ENSMUST00000138980;ENSMUST00000055815
ENSMUSG	Kcnc2	ENSMUST00000219301;ENSMUST00000092175
ENSMUSG	Caps2	ENSMUST00000092176;ENSMUST00000132994
ENSMUSG	Dagla	ENSMUST00000039327;ENSMUST00000156361
ENSMUSG	Bbs10	ENSMUST00000219990;ENSMUST00000040454
ENSMUSG	Krt20	ENSMUST00000017743
ENSMUSG	Cmtm2b	ENSMUST00000041973
ENSMUSG	Twist1	ENSMUST00000049089
ENSMUSG	Ugt2b35	ENSMUST00000031186

ENSMUSG	Plekhs1	ENSMUST00000039666;ENSMUST00000178590;ENSMUST0000022[illegible]
ENSMUSG	Ppp1r26	ENSMUST00000128199;ENSMUST00000150362;ENSMUST00000154
ENSMUSG	Krt25	ENSMUST00000038004
ENSMUSG	Ugt2b1	ENSMUST00000031183
ENSMUSG	Krt222	ENSMUST00000103132;ENSMUST00000038214;ENSMUST00000154
ENSMUSG	Cdhr3	ENSMUST00000095774;ENSMUST00000219453;ENSMUST0000021[illegible]
ENSMUSG	Tmprss1	ENSMUST00000038448
ENSMUSG	Syt1	ENSMUST00000105276;ENSMUST00000064054;ENSMUST00000147
ENSMUSG	Hykk	ENSMUST00000039742;ENSMUST00000125983;ENSMUST00000124
ENSMUSG	Rnase1	ENSMUST00000080126
ENSMUSG	Dcdc2a	ENSMUST00000036932;ENSMUST00000069614
ENSMUSG	Cd276	ENSMUST00000165365;ENSMUST00000039788;ENSMUST0000021[illegible]
ENSMUSG	Ptprq	ENSMUST00000050702;ENSMUST00000218399
ENSMUSG	Myf6	ENSMUST00000044210
ENSMUSG	Chst4	ENSMUST00000211894
ENSMUSG	Olfr750	ENSMUST00000216202;ENSMUST00000048478
ENSMUSG	Gsx2	ENSMUST00000040477
ENSMUSG	Ascl3	ENSMUST00000035372
ENSMUSG	Odf3l2	ENSMUST00000095464
ENSMUSG	Tmem59l	ENSMUST00000045286
ENSMUSG	Nme5	ENSMUST00000134875;ENSMUST00000079287;ENSMUST0000015[illegible]
ENSMUSG	Fam122c	ENSMUST00000124137
ENSMUSG	1810046[illegible]	ENSMUST00000039959;ENSMUST00000181366
ENSMUSG	Prtg	ENSMUST00000055535
ENSMUSG	Zfp57	ENSMUST00000089968;ENSMUST00000069250;ENSMUST00000174
ENSMUSG	Adamtsl[illegible]	ENSMUST00000091233;ENSMUST00000169787;ENSMUST0000013[illegible]
ENSMUSG	Gm12221	ENSMUST00000125096
ENSMUSG	Phf24	ENSMUST00000151824;ENSMUST00000132173;ENSMUST00000107
ENSMUSG	Slc17a3	ENSMUST00000166467;ENSMUST00000225076
ENSMUSG	Dgkb	ENSMUST00000040500;ENSMUST00000221686
ENSMUSG	Slc17a2	ENSMUST00000006786;ENSMUST00000139572
ENSMUSG	Lmo1	ENSMUST00000036992;ENSMUST00000207178
ENSMUSG	Il5	ENSMUST00000048605
ENSMUSG	Frmd7	ENSMUST00000060650
ENSMUSG	Fam110c	ENSMUST00000041133
ENSMUSG	Hoxc9	ENSMUST00000001706
ENSMUSG	Meox2	ENSMUST00000041183
ENSMUSG	Sostdc1	ENSMUST00000041407
ENSMUSG	Cd200r3	ENSMUST00000114613;ENSMUST00000114611;ENSMUST00000164
ENSMUSG	Rorb	ENSMUST00000112832;ENSMUST00000149635
ENSMUSG	Arhgap3	ENSMUST00000114904
ENSMUSG	Pdzrn4	ENSMUST00000169942;ENSMUST00000035399
ENSMUSG	Agr3	ENSMUST00000042101
ENSMUSG	3632451[illegible]	ENSMUST00000036972
ENSMUSG	Tbc1d21	ENSMUST00000040217
ENSMUSG	Trpm8	ENSMUST00000040210
ENSMUSG	Fstl4	ENSMUST00000036796
ENSMUSG	Lrrk2	ENSMUST00000060642;ENSMUST00000140734;ENSMUST0000015[illegible]
ENSMUSG	Slc2a13	ENSMUST00000109283;ENSMUST00000156971;ENSMUST0000010[illegible]
ENSMUSG	Gpr101	ENSMUST00000057645
ENSMUSG	Glb1l2	ENSMUST00000040398;ENSMUST00000066560;ENSMUST00000162
ENSMUSG	Glt6d1	ENSMUST00000038010
ENSMUSG	Pcdh8	ENSMUST00000039568;ENSMUST00000195034
ENSMUSG	Npy1r	ENSMUST00000212588;ENSMUST00000039303
ENSMUSG	Lcn8	ENSMUST00000038482
ENSMUSG	Wtip	ENSMUST00000038537;ENSMUST00000205914;ENSMUST0000012[illegible]
ENSMUSG	Prss56	ENSMUST00000044533

ENSMUSG	Akp3	ENSMUST00000187662;ENSMUST00000044878
ENSMUSG	Cdh8	ENSMUST00000155527;ENSMUST00000142129;ENSMUST00000093
ENSMUSG	Adamts2	ENSMUST00000040523;ENSMUST00000125988;ENSMUST00000127
ENSMUSG	Sh3tc1	ENSMUST00000070203;ENSMUST00000129664;ENSMUST00000151
ENSMUSG	Lgi4	ENSMUST00000039775;ENSMUST00000169785;ENSMUST00000164
ENSMUSG	Ndrg4	ENSMUST00000160964;ENSMUST00000041318;ENSMUST00000166
ENSMUSG	Grifin	ENSMUST00000042993
ENSMUSG	Fut7	ENSMUST00000100320;ENSMUST00000114278;ENSMUST00000041
ENSMUSG	Cpz	ENSMUST00000038676
ENSMUSG	Ccdc113	ENSMUST00000041569
ENSMUSG	Alx1	ENSMUST00000217946;ENSMUST00000167156;ENSMUST00000164
ENSMUSG	Colec11	ENSMUST00000036136;ENSMUST00000220655
ENSMUSG	Tcaf1	ENSMUST00000045054;ENSMUST00000121083;ENSMUST00000045
ENSMUSG	Zcchc12	ENSMUST00000115258;ENSMUST00000048067;ENSMUST00000115
ENSMUSG	Zscan12	ENSMUST00000225545;ENSMUST00000053293;ENSMUST00000099
ENSMUSG	Ttll7	ENSMUST00000037942;ENSMUST00000124743
ENSMUSG	Pramel5	ENSMUST00000035757
ENSMUSG	Kcnk9	ENSMUST00000044624
ENSMUSG	Dner	ENSMUST00000049126
ENSMUSG	Stpg3	ENSMUST00000155706;ENSMUST00000124661;ENSMUST00000043
ENSMUSG	Klhl13	ENSMUST00000115313;ENSMUST00000115319;ENSMUST00000115
ENSMUSG	Slitrk2	ENSMUST00000166241
ENSMUSG	Fam135b	ENSMUST00000022953
ENSMUSG	Noxa1	ENSMUST00000044018
ENSMUSG	Entpd8	ENSMUST00000135004;ENSMUST00000044078;ENSMUST00000114
ENSMUSG	Dpp10	ENSMUST00000112606;ENSMUST00000187202;ENSMUST00000140
ENSMUSG	Atoh7	ENSMUST00000044059
ENSMUSG	Lpar3	ENSMUST00000039164
ENSMUSG	Plch1	ENSMUST00000048134;ENSMUST00000159374;ENSMUST00000159
ENSMUSG	Syde2	ENSMUST00000039517;ENSMUST00000212479;ENSMUST00000200
ENSMUSG	Smad6	ENSMUST00000041029;ENSMUST00000144323;ENSMUST00000179
ENSMUSG	Abcc12	ENSMUST00000129898;ENSMUST00000131423;ENSMUST00000131
ENSMUSG	Prodh2	ENSMUST00000058280;ENSMUST00000142514
ENSMUSG	Trpv5	ENSMUST00000031901
ENSMUSG	C1ql2	ENSMUST00000037286
ENSMUSG	Piwil4	ENSMUST00000136399;ENSMUST00000115644;ENSMUST00000115
ENSMUSG	Kirrel2	ENSMUST00000140565
ENSMUSG	Stox1	ENSMUST00000148720;ENSMUST00000133371;ENSMUST00000126
ENSMUSG	Mucl2	ENSMUST00000037685
ENSMUSG	C130079	ENSMUST00000049476
ENSMUSG	Lrfn3	ENSMUST00000046351
ENSMUSG	Clca2	ENSMUST00000040465;ENSMUST00000198993;ENSMUST00000197
ENSMUSG	Cfap221	ENSMUST00000174370;ENSMUST00000174458
ENSMUSG	Frem2	ENSMUST00000091137;ENSMUST00000197519;ENSMUST00000199
ENSMUSG	Foxa2	ENSMUST00000109964;ENSMUST00000047315;ENSMUST00000146
ENSMUSG	Gm12800	ENSMUST00000075999
ENSMUSG	Tspan15	ENSMUST00000047883;ENSMUST00000218394;ENSMUST00000219
ENSMUSG	Clca3b	ENSMUST00000159989
ENSMUSG	Inhbb	ENSMUST00000038765
ENSMUSG	Azgp1	ENSMUST00000199145
ENSMUSG	Ttc29	ENSMUST00000117845;ENSMUST00000049395;ENSMUST00000156
ENSMUSG	Fer1l6	ENSMUST00000161028;ENSMUST00000159177
ENSMUSG	Trim58	ENSMUST00000075084
ENSMUSG	Tmprss1	ENSMUST00000034597
ENSMUSG	Myom3	ENSMUST00000105854
ENSMUSG	Tas2r10	ENSMUST00000038750
ENSMUSG	2210407	ENSMUST00000108821;ENSMUST00000048801;ENSMUST00000126

ENSMUSG	Il22ra1	ENSMUST00000102546
ENSMUSG	Wee2	ENSMUST00000202464;ENSMUST00000038907
ENSMUSG	Mgarp	ENSMUST00000108046;ENSMUST00000038154;ENSMUST00000157
ENSMUSG	Spaca5	ENSMUST00000040437
ENSMUSG	Mycn	ENSMUST00000043396;ENSMUST00000130990;ENSMUST00000151
ENSMUSG	Nodal	ENSMUST00000049339
ENSMUSG	Krt80	ENSMUST00000077196
ENSMUSG	Grhl3	ENSMUST00000105855
ENSMUSG	H2-M10.	ENSMUST00000041531
ENSMUSG	Pldi	ENSMUST00000036304
ENSMUSG	Itih2	ENSMUST00000042290
ENSMUSG	Dzank1	ENSMUST00000081982;ENSMUST00000124823
ENSMUSG	Ovol2	ENSMUST00000103171;ENSMUST00000037423;ENSMUST00000138
ENSMUSG	Bbs7	ENSMUST00000040148;ENSMUST00000108156;ENSMUST00000108
ENSMUSG	H2-M1	ENSMUST00000041662
ENSMUSG	Hand1	ENSMUST00000036917
ENSMUSG	Hrh4	ENSMUST00000041676
ENSMUSG	4930578	ENSMUST00000147039
ENSMUSG	Nov	ENSMUST00000050027
ENSMUSG	Spon2	ENSMUST00000046186;ENSMUST00000201199;ENSMUST00000201
ENSMUSG	Muc3	ENSMUST00000041226
ENSMUSG	Nmur2	ENSMUST00000037682
ENSMUSG	Adam32	ENSMUST00000121438
ENSMUSG	Vnn1	ENSMUST00000041416
ENSMUSG	Slc18b1	ENSMUST00000119597;ENSMUST00000179321;ENSMUST00000134
ENSMUSG	Acp7	ENSMUST00000040112
ENSMUSG	Erv3	ENSMUST00000040941
ENSMUSG	Zmat4	ENSMUST00000131410;ENSMUST00000042352;ENSMUST00000123
ENSMUSG	Cib2	ENSMUST00000041901
ENSMUSG	1700021	ENSMUST00000187763;ENSMUST00000042671
ENSMUSG	Aldh8a1	ENSMUST00000042699;ENSMUST00000161285
ENSMUSG	Ephx3	ENSMUST00000087721;ENSMUST00000162117
ENSMUSG	BC03049	ENSMUST00000046361;ENSMUST00000147549;ENSMUST00000122
ENSMUSG	Gm5519	ENSMUST00000042061
ENSMUSG	Kcnmb2	ENSMUST00000178668;ENSMUST00000119970
ENSMUSG	Cldn11	ENSMUST00000046174
ENSMUSG	Rgs22	ENSMUST00000172831;ENSMUST00000173018;ENSMUST00000164
ENSMUSG	Slc25a4	ENSMUST00000047655;ENSMUST00000133699
ENSMUSG	Zbtb42	ENSMUST00000174780;ENSMUST00000169593;ENSMUST00000173
ENSMUSG	Kctd8	ENSMUST00000054095;ENSMUST00000087231
ENSMUSG	Cdkn1c	ENSMUST00000167912;ENSMUST00000037287;ENSMUST00000208
ENSMUSG	Armc3	ENSMUST00000114640
ENSMUSG	Aspg	ENSMUST00000223457;ENSMUST00000079400;ENSMUST00000221
ENSMUSG	Tmem247	ENSMUST00000042172
ENSMUSG	Tecta	ENSMUST00000042190;ENSMUST00000160940
ENSMUSG	Spag6	ENSMUST00000137796;ENSMUST00000138017;ENSMUST00000095
ENSMUSG	Avp	ENSMUST00000046001
ENSMUSG	Actrt3	ENSMUST00000047630
ENSMUSG	Phyhipl	ENSMUST00000046513;ENSMUST00000162470;ENSMUST00000163
ENSMUSG	Ptger2	ENSMUST00000046891
ENSMUSG	Slc16a9	ENSMUST00000046807;ENSMUST00000155933
ENSMUSG	Slc32a1	ENSMUST00000045738
ENSMUSG	Dzip1l	ENSMUST00000131095;ENSMUST00000078367;ENSMUST00000112
ENSMUSG	Adh4	ENSMUST00000162260;ENSMUST00000013458
ENSMUSG	Mat1a	ENSMUST00000225720
ENSMUSG	D630003	ENSMUST00000046944
ENSMUSG	Vstm2l	ENSMUST00000109523

ENSMUSG	Zfp365	ENSMUST00000064656;ENSMUST00000132870;ENSMUST00000127
ENSMUSG	1700017	ENSMUST00000035860
ENSMUSG	Dusp8	ENSMUST00000039926;ENSMUST00000143661
ENSMUSG	Pcdh18	ENSMUST00000035931;ENSMUST00000191794;ENSMUST00000193
ENSMUSG	Ankrd13	ENSMUST00000145934;ENSMUST00000037593;ENSMUST00000143
ENSMUSG	Olfr16	ENSMUST00000215254;ENSMUST00000038432
ENSMUSG	Crp	ENSMUST00000038495;ENSMUST00000194251
ENSMUSG	A4gnt	ENSMUST00000042553
ENSMUSG	Ccdc129	ENSMUST00000044729
ENSMUSG	Muc5ac	ENSMUST00000155534;ENSMUST00000041924
ENSMUSG	6430571	ENSMUST00000042581
ENSMUSG	Wnk2	ENSMUST00000162581;ENSMUST00000035538;ENSMUST0000004
ENSMUSG	Slc9b2	ENSMUST00000145195;ENSMUST00000126034;ENSMUST0000014
ENSMUSG	Igsf9	ENSMUST00000127052;ENSMUST00000140814;ENSMUST00000052
ENSMUSG	Slc24a2	ENSMUST00000107157;ENSMUST00000155361
ENSMUSG	Dnajc22	ENSMUST00000146173;ENSMUST00000061295
ENSMUSG	Prm2	ENSMUST00000037996;ENSMUST00000189593
ENSMUSG	Mindy4	ENSMUST00000204842;ENSMUST00000053094;ENSMUST00000203
ENSMUSG	Tigar	ENSMUST00000039913;ENSMUST00000200988
ENSMUSG	Ptpdc1	ENSMUST00000035824;ENSMUST00000222028;ENSMUST00000221
ENSMUSG	Cntnap5	ENSMUST00000076038
ENSMUSG	Dlec1	ENSMUST00000124213;ENSMUST00000165231;ENSMUST0000014
ENSMUSG	Kcna6	ENSMUST00000040751;ENSMUST00000185333;ENSMUST00000112
ENSMUSG	Hspb2	ENSMUST00000042790;ENSMUST00000217159
ENSMUSG	Hsd3b5	ENSMUST00000044094
ENSMUSG	Atp13a4	ENSMUST00000182627;ENSMUST00000182094;ENSMUST00000182
ENSMUSG	AW55198	ENSMUST00000042485;ENSMUST00000147753;ENSMUST0000012
ENSMUSG	Ano2	ENSMUST00000159111;ENSMUST00000160496
ENSMUSG	Tbc1d32	ENSMUST00000099739;ENSMUST00000219385;ENSMUST00000217
ENSMUSG	Rbm24	ENSMUST00000037923;ENSMUST00000225221;ENSMUST0000022
ENSMUSG	Crygn	ENSMUST00000047119
ENSMUSG	Cldn16	ENSMUST00000161053
ENSMUSG	Prdm1	ENSMUST00000039174;ENSMUST00000105490;ENSMUST0000021
ENSMUSG	P3h2	ENSMUST00000039990;ENSMUST00000160067;ENSMUST00000161
ENSMUSG	Enpp6	ENSMUST00000039840
ENSMUSG	Slamf7	ENSMUST00000192195;ENSMUST00000192024;ENSMUST00000111
ENSMUSG	Cer1	ENSMUST00000048430
ENSMUSG	Hand2	ENSMUST00000040104
ENSMUSG	4931409	ENSMUST00000200634;ENSMUST00000198887;ENSMUST0000019
ENSMUSG	Kcna7	ENSMUST00000107774
ENSMUSG	Hoxa13	ENSMUST00000147595;ENSMUST00000047993
ENSMUSG	Asb10	ENSMUST00000048302;ENSMUST00000117900
ENSMUSG	Itln1	ENSMUST00000043094
ENSMUSG	Gm9765	ENSMUST00000043170
ENSMUSG	Serpinf	ENSMUST00000108437;ENSMUST00000043696;ENSMUST0000012
ENSMUSG	Hoxa9	ENSMUST00000048680;ENSMUST00000174115
ENSMUSG	Fam198a	ENSMUST00000043011;ENSMUST00000214536;ENSMUST00000213
ENSMUSG	Hrc	ENSMUST00000085351
ENSMUSG	Mical2	ENSMUST00000050149;ENSMUST00000106647;ENSMUST0000010
ENSMUSG	Fam50b	ENSMUST00000221037
ENSMUSG	Glra3	ENSMUST00000000275
ENSMUSG	Gdf5	ENSMUST00000040162
ENSMUSG	Ccdc155	ENSMUST00000121017;ENSMUST00000211277
ENSMUSG	Atg9b	ENSMUST00000059401;ENSMUST00000128831
ENSMUSG	Pth2	ENSMUST00000042754
ENSMUSG	Spats2l	ENSMUST00000172287;ENSMUST00000168905;ENSMUST00000172
ENSMUSG	Serpinb	ENSMUST00000075515

ENSMUSG	Satb2	ENSMUST00000042857;ENSMUST00000114415;ENSMUST00000177
ENSMUSG	Pcp4l1	ENSMUST00000111332
ENSMUSG	Foxf2	ENSMUST00000042054
ENSMUSG	1700018	ENSMUST00000150418;ENSMUST00000221561
ENSMUSG	Foxq1	ENSMUST00000042118
ENSMUSG	Fcrla	ENSMUST00000046322;ENSMUST00000159149;ENSMUST0000015
ENSMUSG	Srcin1	ENSMUST00000107596;ENSMUST00000107593;ENSMUST00000107
ENSMUSG	Dennd2a	ENSMUST00000036877;ENSMUST00000149162;ENSMUST0000014
ENSMUSG	Olfml2b	ENSMUST00000046792
ENSMUSG	Nos1ap	ENSMUST00000161221;ENSMUST00000160456;ENSMUST00000162
ENSMUSG	Gdf15	ENSMUST00000003808;ENSMUST00000110103
ENSMUSG	Tbkbp1	ENSMUST00000066078;ENSMUST00000107615;ENSMUST0000011
ENSMUSG	Mfsd4b1	ENSMUST00000163705
ENSMUSG	C1rl	ENSMUST00000049124
ENSMUSG	Mfsd4b5	ENSMUST00000073618;ENSMUST00000164566;ENSMUST0000017
ENSMUSG	Mc3r	ENSMUST00000038532
ENSMUSG	Sp6	ENSMUST00000047997;ENSMUST00000107622
ENSMUSG	Bpifb5	ENSMUST00000045959
ENSMUSG	Susd4	ENSMUST00000155229;ENSMUST00000153348;ENSMUST00000194
ENSMUSG	Sct	ENSMUST00000210928;ENSMUST00000167790
ENSMUSG	Colec10	ENSMUST00000036737
ENSMUSG	AI48187	ENSMUST00000107547
ENSMUSG	Capn8	ENSMUST00000193260;ENSMUST00000048941
ENSMUSG	Slc35f1	ENSMUST00000105473
ENSMUSG	Nepn	ENSMUST00000067085
ENSMUSG	Zkscan1	ENSMUST00000107554
ENSMUSG	Akr1d1	ENSMUST00000040987
ENSMUSG	Sycp2l	ENSMUST00000124093;ENSMUST00000145810
ENSMUSG	Fsd2	ENSMUST00000042318
ENSMUSG	Hoxb4	ENSMUST00000049241
ENSMUSG	Hoxb5	ENSMUST00000190470;ENSMUST00000049272
ENSMUSG	Hoxb7	ENSMUST00000049352;ENSMUST00000151596
ENSMUSG	Ptk6	ENSMUST00000016511
ENSMUSG	Elovl3	ENSMUST00000043739
ENSMUSG	Ttll6	ENSMUST00000167258;ENSMUST00000107680
ENSMUSG	Trhr	ENSMUST00000038856
ENSMUSG	Alpk3	ENSMUST00000107348;ENSMUST00000151115
ENSMUSG	Lmx1b	ENSMUST00000041730
ENSMUSG	9130409	ENSMUST00000036819
ENSMUSG	Scgb3a2	ENSMUST00000187157;ENSMUST00000043803;ENSMUST0000018
ENSMUSG	Six3	ENSMUST00000176081;ENSMUST00000162695
ENSMUSG	Garnl3	ENSMUST00000102810;ENSMUST00000049618;ENSMUST00000137
ENSMUSG	Prl3a1	ENSMUST00000049463
ENSMUSG	Ccdc68	ENSMUST00000151773;ENSMUST00000043929;ENSMUST0000013
ENSMUSG	Soga3	ENSMUST00000092629
ENSMUSG	3930402	ENSMUST00000040608;ENSMUST00000208615
ENSMUSG	E330034	ENSMUST00000161740
ENSMUSG	Tcfl5	ENSMUST00000037877;ENSMUST00000131881
ENSMUSG	Sccpdh	ENSMUST00000040538;ENSMUST00000134287;ENSMUST00000131
ENSMUSG	Slco4a1	ENSMUST00000038259;ENSMUST00000139902;ENSMUST0000003
ENSMUSG	Klk1b16	ENSMUST00000005933
ENSMUSG	Rbbp8nl	ENSMUST00000038529
ENSMUSG	Bloc1s5	ENSMUST00000224115;ENSMUST00000035899;ENSMUST00000224
ENSMUSG	Tspyl5	ENSMUST00000042021
ENSMUSG	Hils1	ENSMUST00000038928
ENSMUSG	Asb17	ENSMUST00000044089
ENSMUSG	Siglecf	ENSMUST00000122423

ENSMUSG	Mtg1	ENSMUST00000036977;ENSMUST00000150667;ENSMUST00000141
ENSMUSG	Myo16	ENSMUST00000208309;ENSMUST00000207477;ENSMUST00000214
ENSMUSG	Hrh3	ENSMUST00000056480;ENSMUST00000163215;ENSMUST0000016€
ENSMUSG	Echdc3	ENSMUST00000042658;ENSMUST00000132810;ENSMUST00000114
ENSMUSG	Cpa4	ENSMUST00000049251
ENSMUSG	Sptlc3	ENSMUST00000110083;ENSMUST00000047370
ENSMUSG	En2	ENSMUST00000036177
ENSMUSG	Rsad1	ENSMUST00000040487;ENSMUST00000132274;ENSMUST0000014З
ENSMUSG	Rln1	ENSMUST00000182039;ENSMUST00000044143
ENSMUSG	Gm9767	ENSMUST00000041011
ENSMUSG	Wdr93	ENSMUST00000035622
ENSMUSG	Htr5a	ENSMUST00000036227
ENSMUSG	Mycbpap	ENSMUST00000093945;ENSMUST00000123496
ENSMUSG	Gipc2	ENSMUST00000152283;ENSMUST00000046614;ENSMUST0000012٤
ENSMUSG	Cdh26	ENSMUST00000108912;ENSMUST00000042092;ENSMUST0000017€
ENSMUSG	Akap7	ENSMUST00000100012;ENSMUST00000175979;ENSMUST0000022C
ENSMUSG	Tekt5	ENSMUST00000043415;ENSMUST00000115831
ENSMUSG	Rlbp1	ENSMUST00000179243;ENSMUST00000206624
ENSMUSG	Ptchd3	ENSMUST00000036690
ENSMUSG	Atf7ip2	ENSMUST00000044005;ENSMUST00000117220
ENSMUSG	Svs1	ENSMUST00000037696
ENSMUSG	Il18	ENSMUST00000213916;ENSMUST00000214117;ENSMUST0000005٤
ENSMUSG	Tgfb2	ENSMUST00000045288;ENSMUST00000195201;ENSMUST00000191
ENSMUSG	Lyplal1	ENSMUST00000045388;ENSMUST00000195481
ENSMUSG	Lgi2	ENSMUST00000199942;ENSMUST00000039750
ENSMUSG	Vstm2b	ENSMUST00000044705;ENSMUST00000186352
ENSMUSG	2300002I	ENSMUST00000174246
ENSMUSG	Pcsk1n	ENSMUST00000041096
ENSMUSG	AF52916	ENSMUST00000044491
ENSMUSG	Clnk	ENSMUST00000169819;ENSMUST00000171633
ENSMUSG	Uts2r	ENSMUST00000039044
ENSMUSG	Igfbp2	ENSMUST00000047328;ENSMUST00000120564;ENSMUST0000015Ł
ENSMUSG	Tex19.1	ENSMUST00000155694
ENSMUSG	Mfsd4b2	ENSMUST00000045526
ENSMUSG	Drd5	ENSMUST00000041646
ENSMUSG	Sectm1b	ENSMUST00000039309;ENSMUST00000081499;ENSMUST0000013C
ENSMUSG	March4	ENSMUST00000047786
ENSMUSG	Synpo2l	ENSMUST00000057090;ENSMUST00000119483
ENSMUSG	Cdh6	ENSMUST00000036439
ENSMUSG	Ccdc81	ENSMUST00000131966;ENSMUST00000041195
ENSMUSG	Prss23	ENSMUST00000041761;ENSMUST00000208402;ENSMUST00000207
ENSMUSG	Prdm16	ENSMUST00000030902;ENSMUST00000070313;ENSMUST0000010Ł
ENSMUSG	Wfs1	ENSMUST00000043964;ENSMUST00000166339
ENSMUSG	Micu3	ENSMUST00000068999;ENSMUST00000136835;ENSMUST00000132
ENSMUSG	Cdnf	ENSMUST00000140610;ENSMUST00000036350;ENSMUST00000132
ENSMUSG	Fam26d	ENSMUST00000048052;ENSMUST00000218239
ENSMUSG	Cdsn	ENSMUST00000044804
ENSMUSG	Atp8b1	ENSMUST00000025482
ENSMUSG	Mmd2	ENSMUST00000037048
ENSMUSG	Sgcz	ENSMUST00000135764
ENSMUSG	Ncam1	ENSMUST00000193547;ENSMUST00000194252;ENSMUST0000016€
ENSMUSG	Ajap1	ENSMUST00000105646;ENSMUST00000149177
ENSMUSG	Ccser1	ENSMUST00000126214;ENSMUST00000133478;ENSMUST00000131
ENSMUSG	Rcan2	ENSMUST00000044895;ENSMUST00000044792
ENSMUSG	Olfr303	ENSMUST00000053958
ENSMUSG	Tmem246	ENSMUST00000042750;ENSMUST00000150664;ENSMUST00000151
ENSMUSG	6430573	ENSMUST00000135373;ENSMUST00000147525;ENSMUST00000152

ENSMUSG	Kcne1	ENSMUST00000051705
ENSMUSG	Dusp26	ENSMUST00000036631;ENSMUST00000162551;ENSMUST00000161
ENSMUSG	Kcne2	ENSMUST00000047383
ENSMUSG	Gm5422	ENSMUST00000216161;ENSMUST00000050717
ENSMUSG	Msantd3	ENSMUST00000061135;ENSMUST00000064807;ENSMUST00000107
ENSMUSG	Ldb2	ENSMUST00000199534;ENSMUST00000070748;ENSMUST0000019[illegible]
ENSMUSG	Plekhg5	ENSMUST00000105662;ENSMUST00000118648;ENSMUST00000105
ENSMUSG	Cplx3	ENSMUST00000045068
ENSMUSG	Got1l1	ENSMUST00000038174;ENSMUST00000211002;ENSMUST0000020[illegible]
ENSMUSG	Slc6a5	ENSMUST00000056442
ENSMUSG	Bahcc1	ENSMUST00000122148;ENSMUST00000044985;ENSMUST00000142
ENSMUSG	Fam71f1	ENSMUST00000090487
ENSMUSG	Il22ra2	ENSMUST00000036564
ENSMUSG	Cep131	ENSMUST00000106227;ENSMUST00000106229;ENSMUST00000145
ENSMUSG	Cercam	ENSMUST00000154464;ENSMUST00000047521;ENSMUST00000134
ENSMUSG	Zfand1	ENSMUST00000037839;ENSMUST00000125734;ENSMUST00000108
ENSMUSG	2600006[illegible]	ENSMUST00000047607
ENSMUSG	Gabbr2	ENSMUST00000107749;ENSMUST00000129328
ENSMUSG	Tbc1d2	ENSMUST00000084621;ENSMUST00000107750
ENSMUSG	Xkr5	ENSMUST00000144987;ENSMUST00000055503;ENSMUST00000095
ENSMUSG	Olig2	ENSMUST00000035608
ENSMUSG	Slc45a1	ENSMUST00000037827;ENSMUST00000117997
ENSMUSG	Srrm3	ENSMUST00000146038;ENSMUST00000144211
ENSMUSG	Slc44a3	ENSMUST00000198335;ENSMUST00000039197;ENSMUST0000019[illegible]
ENSMUSG	Txlnb	ENSMUST00000175676;ENSMUST00000037964
ENSMUSG	Gpr37	ENSMUST00000054867
ENSMUSG	Pak7	ENSMUST00000035264;ENSMUST00000143329
ENSMUSG	Stk32a	ENSMUST00000045477
ENSMUSG	Olfr906	ENSMUST00000214344
ENSMUSG	Magi2	ENSMUST00000197354;ENSMUST00000200518;ENSMUST00000197
ENSMUSG	Gnaz	ENSMUST00000037813;ENSMUST00000159991
ENSMUSG	Saa4	ENSMUST00000006952
ENSMUSG	Saa3	ENSMUST00000006956;ENSMUST00000211660;ENSMUST0000021[illegible]
ENSMUSG	Disp2	ENSMUST00000037547;ENSMUST00000110843;ENSMUST00000142
ENSMUSG	Ift88	ENSMUST00000122063;ENSMUST00000150296;ENSMUST00000154
ENSMUSG	Tph1	ENSMUST00000049298;ENSMUST00000107669;ENSMUST0000017[illegible]
ENSMUSG	Gjb6	ENSMUST00000039380;ENSMUST00000224544;ENSMUST0000016[illegible]
ENSMUSG	Pfpl	ENSMUST00000168148
ENSMUSG	Tnni3k	ENSMUST00000064076
ENSMUSG	Mettl11[illegible]	ENSMUST00000159679;ENSMUST00000160495
ENSMUSG	Cacna2d	ENSMUST00000167946;ENSMUST00000101581;ENSMUST0000003[illegible]
ENSMUSG	Sdr9c7	ENSMUST00000047134;ENSMUST00000155067;ENSMUST0000014[illegible]
ENSMUSG	Gpr176	ENSMUST00000039160
ENSMUSG	Abcc8	ENSMUST00000210655;ENSMUST00000209432;ENSMUST0000021[illegible]
ENSMUSG	Ndp	ENSMUST00000040134
ENSMUSG	9430038	ENSMUST00000145881;ENSMUST00000068996;ENSMUST00000117
ENSMUSG	Tdrd6	ENSMUST00000168073
ENSMUSG	Maob	ENSMUST00000040820;ENSMUST00000173143
ENSMUSG	Wfdc5	ENSMUST00000045127
ENSMUSG	Kcns1	ENSMUST00000045196
ENSMUSG	Cd209c	ENSMUST00000044060;ENSMUST00000208622;ENSMUST0000020[illegible]
ENSMUSG	Cd209e	ENSMUST00000033888
ENSMUSG	Kyat3	ENSMUST00000106218;ENSMUST00000044392;ENSMUST0000019[illegible]
ENSMUSG	Syngr4	ENSMUST00000211254;ENSMUST00000039049;ENSMUST0000012[illegible]
ENSMUSG	Sema3d	ENSMUST00000030868;ENSMUST00000197927;ENSMUST00000196
ENSMUSG	Nxph4	ENSMUST00000095266
ENSMUSG	Accs	ENSMUST00000111246;ENSMUST00000068513;ENSMUST00000041

ENSMUSG	Btnl9	ENSMUST00000046522;ENSMUST00000066531;ENSMUST00000131
ENSMUSG	Stac3	ENSMUST00000160019;ENSMUST00000160610;ENSMUST00000035
ENSMUSG	Ctsg	ENSMUST00000015583;ENSMUST00000225312
ENSMUSG	Tex45	ENSMUST00000047265
ENSMUSG	Sec1	ENSMUST00000040636
ENSMUSG	Cbln3	ENSMUST00000063871
ENSMUSG	Havcr1	ENSMUST00000109224;ENSMUST00000081819;ENSMUST00000109
ENSMUSG	5330417	ENSMUST00000106625;ENSMUST00000140357;ENSMUST00000148
ENSMUSG	Timd2	ENSMUST00000169584;ENSMUST00000109225;ENSMUST00000125
ENSMUSG	Ltb4r2	ENSMUST00000044554
ENSMUSG	Hypm	ENSMUST00000044987
ENSMUSG	Gtpbp10	ENSMUST00000115441;ENSMUST00000088842;ENSMUST00000198
ENSMUSG	Ggt6	ENSMUST00000076443;ENSMUST00000108499;ENSMUST00000100
ENSMUSG	Cfap69	ENSMUST00000054865;ENSMUST00000196165;ENSMUST00000199
ENSMUSG	Lrrc52	ENSMUST00000036643
ENSMUSG	Sox30	ENSMUST00000049038
ENSMUSG	Lrfn2	ENSMUST00000046254
ENSMUSG	Chrm4	ENSMUST00000045537
ENSMUSG	March9	ENSMUST00000040307
ENSMUSG	Tlr8	ENSMUST00000112170;ENSMUST00000049023;ENSMUST00000148
ENSMUSG	Cblc	ENSMUST00000125050;ENSMUST00000148416;ENSMUST00000043
ENSMUSG	Tmem225	ENSMUST00000046333
ENSMUSG	Plppr2	ENSMUST00000046371;ENSMUST00000190387;ENSMUST00000188
ENSMUSG	Slc26a7	ENSMUST00000042221
ENSMUSG	Rundc3b	ENSMUST00000047485;ENSMUST00000115378
ENSMUSG	Abcb1a	ENSMUST00000047753;ENSMUST00000126846;ENSMUST00000138
ENSMUSG	Eps8l3	ENSMUST00000037375
ENSMUSG	Nlrp4a	ENSMUST00000068767
ENSMUSG	Bace2	ENSMUST00000047275
ENSMUSG	Ildr2	ENSMUST00000111416;ENSMUST00000192638;ENSMUST00000192
ENSMUSG	Pck2	ENSMUST00000048781
ENSMUSG	Mael	ENSMUST00000194057
ENSMUSG	Nrl	ENSMUST00000111404;ENSMUST00000178694;ENSMUST00000062
ENSMUSG	Erc2	ENSMUST00000210135;ENSMUST00000211684;ENSMUST00000224
ENSMUSG	Ppp1r14	ENSMUST00000043374
ENSMUSG	1700063	ENSMUST00000043553
ENSMUSG	Sh3bgr	ENSMUST00000048770
ENSMUSG	Col16a1	ENSMUST00000044565;ENSMUST00000132251;ENSMUST00000097
ENSMUSG	A930016	ENSMUST00000208685;ENSMUST00000047020
ENSMUSG	Scamp5	ENSMUST00000046587;ENSMUST00000215208;ENSMUST00000214
ENSMUSG	Hesx1	ENSMUST00000035433
ENSMUSG	Cep126	ENSMUST00000037397;ENSMUST00000217241;ENSMUST00000214
ENSMUSG	Erg	ENSMUST00000113846;ENSMUST00000118113;ENSMUST00000113
ENSMUSG	Myh6	ENSMUST00000081857;ENSMUST00000131892
ENSMUSG	Cmtm5	ENSMUST00000037814
ENSMUSG	Il25	ENSMUST00000037863
ENSMUSG	Iqsec3	ENSMUST00000129815;ENSMUST00000151397
ENSMUSG	Chil3	ENSMUST00000063062;ENSMUST00000149836;ENSMUST00000129
ENSMUSG	Agbl2	ENSMUST00000170320;ENSMUST00000051831;ENSMUST00000037
ENSMUSG	Catsper	ENSMUST00000112979
ENSMUSG	Gm11639	ENSMUST00000212287
ENSMUSG	Plekhh2	ENSMUST00000047206
ENSMUSG	Dlk1	ENSMUST00000174539;ENSMUST00000056110;ENSMUST00000109
ENSMUSG	Rsph6a	ENSMUST00000035521
ENSMUSG	Tmem205	ENSMUST00000217263;ENSMUST00000046831;ENSMUST00000178
ENSMUSG	Foxa3	ENSMUST00000036018
ENSMUSG	Kcnk18	ENSMUST00000065204

ENSMUSG	Atp1a3	ENSMUST00000102858;ENSMUST00000080882;ENSMUST0000019[illegible]
ENSMUSG	4930505[illegible]	ENSMUST00000152718
ENSMUSG	Padi6	ENSMUST00000038749
ENSMUSG	Slc16a1	ENSMUST00000126388;ENSMUST00000171032;ENSMUST0000014[illegible]
ENSMUSG	Mgl2	ENSMUST00000041550;ENSMUST00000132645;ENSMUST0000016[illegible]
ENSMUSG	Asgr2	ENSMUST00000102572;ENSMUST00000124721;ENSMUST0000017[illegible]
ENSMUSG	Slc22a2	ENSMUST00000046959
ENSMUSG	Igsf21	ENSMUST00000039331
ENSMUSG	Gm11992	ENSMUST00000043285
ENSMUSG	Sun3	ENSMUST00000043377;ENSMUST00000102909
ENSMUSG	Mill2	ENSMUST00000072386;ENSMUST00000072415;ENSMUST0000020[illegible]
ENSMUSG	Map7d2	ENSMUST00000112471;ENSMUST00000043151
ENSMUSG	Lrit1	ENSMUST00000120052
ENSMUSG	Ramp3	ENSMUST00000045374;ENSMUST00000139540
ENSMUSG	Mslnl	ENSMUST00000047098
ENSMUSG	Grid1	ENSMUST00000043349;ENSMUST00000173307
ENSMUSG	Ccdc8	ENSMUST00000094805
ENSMUSG	Pde9a	ENSMUST00000047168;ENSMUST00000127929;ENSMUST0000012[illegible]
ENSMUSG	Pnmal1	ENSMUST00000038163
ENSMUSG	Dnah7b	ENSMUST00000188209;ENSMUST00000069293;ENSMUST0000019[illegible]
ENSMUSG	Spem1	ENSMUST00000045771
ENSMUSG	Lamp3	ENSMUST00000081880
ENSMUSG	Kcnj1	ENSMUST00000172015
ENSMUSG	Tmco5b	ENSMUST00000040856
ENSMUSG	Car8	ENSMUST00000066674
ENSMUSG	Dmxl2	ENSMUST00000118163;ENSMUST00000154092;ENSMUST0000011[illegible]
ENSMUSG	Adgrf1	ENSMUST00000047399
ENSMUSG	Cftr	ENSMUST00000140407;ENSMUST00000129452
ENSMUSG	Inhba	ENSMUST00000042603;ENSMUST00000164993
ENSMUSG	Atp1b2	ENSMUST00000047889;ENSMUST00000130394;ENSMUST0000013[illegible]
ENSMUSG	Mup4	ENSMUST00000075973
ENSMUSG	Bdkrb1	ENSMUST00000182899;ENSMUST00000041229
ENSMUSG	Rap1gap	ENSMUST00000139831;ENSMUST00000156683;ENSMUST0000015[illegible]
ENSMUSG	Nutm1	ENSMUST00000043970
ENSMUSG	B4galnt	ENSMUST00000125341;ENSMUST00000057283;ENSMUST0000021[illegible]
ENSMUSG	Htr2c	ENSMUST00000096299
ENSMUSG	Paqr6	ENSMUST00000147948;ENSMUST00000147991;ENSMUST0000014[illegible]
ENSMUSG	Gpr12	ENSMUST00000196096
ENSMUSG	Smpx	ENSMUST00000038007
ENSMUSG	Piezo2	ENSMUST00000047480;ENSMUST00000132576;ENSMUST0000012[illegible]
ENSMUSG	Upk2	ENSMUST00000047740
ENSMUSG	Serpina	ENSMUST00000185595
ENSMUSG	Disp3	ENSMUST00000047720
ENSMUSG	Serpina	ENSMUST00000021495
ENSMUSG	Ptchd1	ENSMUST00000038665;ENSMUST00000141356
ENSMUSG	L3mbtl4	ENSMUST00000139383;ENSMUST00000093007
ENSMUSG	Serpina	ENSMUST00000043915
ENSMUSG	Crx	ENSMUST00000044434
ENSMUSG	Sdk2	ENSMUST00000041627;ENSMUST00000156955;ENSMUST0000012[illegible]
ENSMUSG	Nlrp5-p	ENSMUST00000044683;ENSMUST00000173766;ENSMUST0000019[illegible]
ENSMUSG	5730559[illegible]	ENSMUST00000120339;ENSMUST00000150163;ENSMUST0000014[illegible]
ENSMUSG	Entpd3	ENSMUST00000047687;ENSMUST00000143579
ENSMUSG	Kctd12b	ENSMUST00000039424;ENSMUST00000112572
ENSMUSG	Slc5a12	ENSMUST00000045972;ENSMUST00000127822
ENSMUSG	Klf8	ENSMUST00000143880;ENSMUST00000112574;ENSMUST0000003[illegible]
ENSMUSG	Rragb	ENSMUST00000039720;ENSMUST00000144175
ENSMUSG	Bbox1	ENSMUST00000046233;ENSMUST00000133608

ENSMUSG	Rims1	ENSMUST00000097811
ENSMUSG	Iapp	ENSMUST00000041993;ENSMUST00000141583
ENSMUSG	Amot	ENSMUST00000125271;ENSMUST00000112836;ENSMUST00000112
ENSMUSG	Rasl12	ENSMUST00000217172;ENSMUST00000215605;ENSMUST0000008
ENSMUSG	Slco1a1	ENSMUST00000168119
ENSMUSG	1810011	ENSMUST00000039191
ENSMUSG	Mpped1	ENSMUST00000109470
ENSMUSG	Trpc5	ENSMUST00000040184
ENSMUSG	Khdc1c	ENSMUST00000070223
ENSMUSG	Prrxl1	ENSMUST00000187377;ENSMUST00000189022
ENSMUSG	Gm13178	ENSMUST00000036876
ENSMUSG	Trem3	ENSMUST00000048065
ENSMUSG	Plekha6	ENSMUST00000212252;ENSMUST00000187285;ENSMUST00000105
ENSMUSG	Gpr155	ENSMUST00000076463;ENSMUST00000112043;ENSMUST00000131
ENSMUSG	2700046	ENSMUST00000067450
ENSMUSG	Myrip	ENSMUST00000048121;ENSMUST00000214784;ENSMUST0000013
ENSMUSG	Gck	ENSMUST00000109822;ENSMUST00000109823;ENSMUST00000102
ENSMUSG	Fhdc1	ENSMUST00000107689;ENSMUST00000091002;ENSMUST0000012
ENSMUSG	Rhod	ENSMUST00000048197;ENSMUST00000117462;ENSMUST00000151
ENSMUSG	Oosp1	ENSMUST00000048214;ENSMUST00000186557
ENSMUSG	Il17f	ENSMUST00000039046;ENSMUST00000191111;ENSMUST0000018
ENSMUSG	Macc1	ENSMUST00000222784;ENSMUST00000222058;ENSMUST00000221
ENSMUSG	Shisa4	ENSMUST00000041240;ENSMUST00000159918;ENSMUST0000015
ENSMUSG	Gpr45	ENSMUST00000188621;ENSMUST00000179766;ENSMUST00000114
ENSMUSG	Nol4	ENSMUST00000092015;ENSMUST00000081423;ENSMUST00000097
ENSMUSG	Pkp2	ENSMUST00000162150;ENSMUST00000161342;ENSMUST0000003
ENSMUSG	Rptn	ENSMUST00000045912
ENSMUSG	Elmod1	ENSMUST00000215313;ENSMUST00000048409;ENSMUST0000021
ENSMUSG	Hrnr	ENSMUST00000090856;ENSMUST00000093774
ENSMUSG	Foxn4	ENSMUST00000044790
ENSMUSG	Lce3b	ENSMUST00000046234
ENSMUSG	2010003	ENSMUST00000048482
ENSMUSG	Sln	ENSMUST00000048485
ENSMUSG	Wdr60	ENSMUST00000221748;ENSMUST00000039349;ENSMUST00000222
ENSMUSG	Tmcc2	ENSMUST00000045473;ENSMUST00000138717;ENSMUST00000142
ENSMUSG	Lce1c	ENSMUST00000047055
ENSMUSG	Dao	ENSMUST00000112292;ENSMUST00000199175;ENSMUST00000162
ENSMUSG	Zfp239	ENSMUST00000136316;ENSMUST00000134619;ENSMUST00000172
ENSMUSG	Dmgdh	ENSMUST00000048001
ENSMUSG	Klhdc8a	ENSMUST00000046071
ENSMUSG	Lce1f	ENSMUST00000047153
ENSMUSG	Klhl23	ENSMUST00000135759;ENSMUST00000053087;ENSMUST00000152
ENSMUSG	Sprr2i	ENSMUST00000047264
ENSMUSG	Cdrt4	ENSMUST00000035854
ENSMUSG	Sprr2d	ENSMUST00000047477
ENSMUSG	Sgsm1	ENSMUST00000048112;ENSMUST00000154248;ENSMUST00000112
ENSMUSG	Crybb2	ENSMUST00000112336
ENSMUSG	Pglyrp3	ENSMUST00000047660
ENSMUSG	Tmc7	ENSMUST00000044195;ENSMUST00000139693
ENSMUSG	Pglyrp4	ENSMUST00000169698
ENSMUSG	Pm20d1	ENSMUST00000112393;ENSMUST00000152989;ENSMUST00000132
ENSMUSG	Ptchd4	ENSMUST00000048691
ENSMUSG	Trem1	ENSMUST00000048782
ENSMUSG	Slc26a9	ENSMUST00000049027;ENSMUST00000130544;ENSMUST00000147
ENSMUSG	Nxt2	ENSMUST00000042329;ENSMUST00000112914;ENSMUST0000013
ENSMUSG	H1foo	ENSMUST00000037831;ENSMUST00000162084;ENSMUST00000161
ENSMUSG	Gucy2f	ENSMUST00000042530

ENSMUSG	Specc1	ENSMUST00000201866;ENSMUST00000202178;ENSMUST0000013€
ENSMUSG	Gjb5	ENSMUST00000046498
ENSMUSG	4930433	ENSMUST00000188577
ENSMUSG	Gjb3	ENSMUST00000046532
ENSMUSG	Dmrt3	ENSMUST00000048935
ENSMUSG	Esm1	ENSMUST00000038144
ENSMUSG	Gzmk	ENSMUST00000122399;ENSMUST00000038212;ENSMUST0000014C
ENSMUSG	Dlgap3	ENSMUST00000046659;ENSMUST00000106094;ENSMUST0000010€
ENSMUSG	Crtac1	ENSMUST00000048630
ENSMUSG	Prdm14	ENSMUST00000047577
ENSMUSG	Ccno	ENSMUST00000038404;ENSMUST00000225555;ENSMUST00000224
ENSMUSG	Frmpd3	ENSMUST00000208130
ENSMUSG	Adora1	ENSMUST00000086465;ENSMUST00000038191
ENSMUSG	Pih1h3b	ENSMUST00000044806
ENSMUSG	Mybph	ENSMUST00000038445;ENSMUST00000191577
ENSMUSG	Bpifa2	ENSMUST00000048103;ENSMUST00000132933
ENSMUSG	Ago4	ENSMUST00000084289;ENSMUST00000122951
ENSMUSG	Cpa6	ENSMUST00000035577;ENSMUST00000147575;ENSMUST0000014C
ENSMUSG	Klhl14	ENSMUST00000049105;ENSMUST00000122333
ENSMUSG	Mum1l1	ENSMUST00000113043
ENSMUSG	4933428	ENSMUST00000113046
ENSMUSG	Kcnj12	ENSMUST00000089184;ENSMUST00000108717
ENSMUSG	Golga7b	ENSMUST00000122375;ENSMUST00000087123
ENSMUSG	Acot5	ENSMUST00000046422;ENSMUST00000072505
ENSMUSG	Zp3r	ENSMUST00000039862
ENSMUSG	Nek10	ENSMUST00000224491;ENSMUST00000134866;ENSMUST0000013€
ENSMUSG	Cux2	ENSMUST00000111752;ENSMUST00000134326
ENSMUSG	Fam199x	ENSMUST00000047852
ENSMUSG	Kcna4	ENSMUST00000037012
ENSMUSG	Asb4	ENSMUST00000043294;ENSMUST00000183358
ENSMUSG	Maff	ENSMUST00000096350;ENSMUST00000163691
ENSMUSG	Xkr7	ENSMUST00000037235
ENSMUSG	Rgsl1	ENSMUST00000124558;ENSMUST00000145308;ENSMUST0000018€
ENSMUSG	Fam159b	ENSMUST00000043061;ENSMUST00000225969
ENSMUSG	Dusp15	ENSMUST00000123121;ENSMUST00000185197;ENSMUST00000037
ENSMUSG	Rgs8	ENSMUST00000041776;ENSMUST00000111812;ENSMUST00000147
ENSMUSG	Myo15	ENSMUST00000071880;ENSMUST00000122825
ENSMUSG	Garem1	ENSMUST00000049260
ENSMUSG	Npl	ENSMUST00000041874;ENSMUST00000187747
ENSMUSG	Ppp1r3a	ENSMUST00000045096
ENSMUSG	Map3k9	ENSMUST00000035987;ENSMUST00000222322;ENSMUST00000223
ENSMUSG	Ttc9	ENSMUST00000218362;ENSMUST00000036116
ENSMUSG	Bex2	ENSMUST00000049130
ENSMUSG	Nmnat2	ENSMUST00000186368;ENSMUST00000043313;ENSMUST0000018€
ENSMUSG	Mrap2	ENSMUST00000113149
ENSMUSG	Lgr6	ENSMUST00000044828;ENSMUST00000139369
ENSMUSG	Spata46	ENSMUST00000056991
ENSMUSG	Olfr769	ENSMUST00000216906;ENSMUST00000050915
ENSMUSG	Gpr153	ENSMUST00000105650;ENSMUST00000105651;ENSMUST00000055
ENSMUSG	Hecw2	ENSMUST00000120904;ENSMUST00000150677;ENSMUST00000097
ENSMUSG	Flt3	ENSMUST00000049324;ENSMUST00000110549
ENSMUSG	Trim72	ENSMUST00000106248;ENSMUST00000081042
ENSMUSG	Wfdc12	ENSMUST00000051272
ENSMUSG	Lrrtm3	ENSMUST00000105439;ENSMUST00000148712;ENSMUST00000133
ENSMUSG	Olfr141	ENSMUST00000062353
ENSMUSG	Trp53rk	ENSMUST00000065753;ENSMUST00000151826;ENSMUST00000051
ENSMUSG	Olfr137	ENSMUST00000215806;ENSMUST00000058168

ENSMUSG	Lhfpl4	ENSMUST00000162280
ENSMUSG	Abra	ENSMUST00000054742
ENSMUSG	Olfr648	ENSMUST00000216612;ENSMUST00000052659
ENSMUSG	Mamstr	ENSMUST00000210648;ENSMUST00000210213;ENSMUST0000014
ENSMUSG	Gm5436	ENSMUST00000058103
ENSMUSG	9930038	ENSMUST00000151816
ENSMUSG	Notum	ENSMUST00000106177
ENSMUSG	Onecut1	ENSMUST00000056006
ENSMUSG	Ptgir	ENSMUST00000086101;ENSMUST00000144408
ENSMUSG	Wdr63	ENSMUST00000160285
ENSMUSG	Gm16433	ENSMUST00000062429
ENSMUSG	Trpv3	ENSMUST00000049676
ENSMUSG	Ccdc63	ENSMUST00000058960;ENSMUST00000198618;ENSMUST00000152
ENSMUSG	Disc1	ENSMUST00000074562;ENSMUST00000118942;ENSMUST00000122
ENSMUSG	Usp17le	ENSMUST00000211384
ENSMUSG	Qrfp	ENSMUST00000057407
ENSMUSG	Aqp6	ENSMUST00000023754
ENSMUSG	Gm11292	ENSMUST00000146121
ENSMUSG	Ispd	ENSMUST00000221452;ENSMUST00000062041;ENSMUST0000022
ENSMUSG	Hpdl	ENSMUST00000055436
ENSMUSG	Arl11	ENSMUST00000055159;ENSMUST00000224727
ENSMUSG	Tmem212	ENSMUST00000058077
ENSMUSG	Lor	ENSMUST00000058150
ENSMUSG	4930426	ENSMUST00000144487;ENSMUST00000143378;ENSMUST0000013
ENSMUSG	Gm1840	ENSMUST00000051687
ENSMUSG	Gm4835	ENSMUST00000155839
ENSMUSG	Olfr100	ENSMUST00000216443
ENSMUSG	Fam124b	ENSMUST00000058748;ENSMUST00000161427
ENSMUSG	Fam13c	ENSMUST00000218542;ENSMUST00000105436;ENSMUST0000022
ENSMUSG	Pnpla1	ENSMUST00000056866;ENSMUST00000114737
ENSMUSG	B3galnt	ENSMUST00000061826
ENSMUSG	Kcnj6	ENSMUST00000099508;ENSMUST00000165538
ENSMUSG	Olfr30	ENSMUST00000215691;ENSMUST00000055204
ENSMUSG	Cox8c	ENSMUST00000053611
ENSMUSG	Olfr975	ENSMUST00000216647
ENSMUSG	Rhbdl2	ENSMUST00000124290;ENSMUST00000053202;ENSMUST0000015
ENSMUSG	Hoxd9	ENSMUST00000059272
ENSMUSG	Olfr78	ENSMUST00000217123;ENSMUST00000060187
ENSMUSG	Olfr134	ENSMUST00000216226;ENSMUST00000060562
ENSMUSG	Olfr267	ENSMUST00000216719;ENSMUST00000059608
ENSMUSG	Tmem130	ENSMUST00000061446
ENSMUSG	Gpr135	ENSMUST00000050649
ENSMUSG	Hfm1	ENSMUST00000112690;ENSMUST00000134292;ENSMUST0000020
ENSMUSG	Lrit2	ENSMUST00000057176
ENSMUSG	Chd3os	ENSMUST00000129321;ENSMUST00000218008
ENSMUSG	Ccdc185	ENSMUST00000060041
ENSMUSG	Epop	ENSMUST00000052281
ENSMUSG	Gpr149	ENSMUST00000058535;ENSMUST00000149007
ENSMUSG	Zfp536	ENSMUST00000176129;ENSMUST00000175941;ENSMUST0000005
ENSMUSG	Rab9b	ENSMUST00000058814
ENSMUSG	Adam30	ENSMUST00000050342
ENSMUSG	Gm5867	ENSMUST00000063197
ENSMUSG	Krt34	ENSMUST00000056362
ENSMUSG	Tril	ENSMUST00000127748
ENSMUSG	9330132	ENSMUST00000059118
ENSMUSG	Rai2	ENSMUST00000112338;ENSMUST00000061514
ENSMUSG	Olfr266	ENSMUST00000213616;ENSMUST00000216610;ENSMUST0000005

ENSMUSG	Sorcs1	ENSMUST00000211687;ENSMUST00000164039;ENSMUST00000168
ENSMUSG	Fam90a1	ENSMUST00000044989
ENSMUSG	Olfr13	ENSMUST00000216179;ENSMUST00000059512
ENSMUSG	Mmp3	ENSMUST00000034497
ENSMUSG	Vps37d	ENSMUST00000067935;ENSMUST00000076203
ENSMUSG	Eif5al3	ENSMUST00000135410
ENSMUSG	Ubxn10	ENSMUST00000146415;ENSMUST00000105811;ENSMUST00000105
ENSMUSG	Adamts3	ENSMUST00000061427;ENSMUST00000163159;ENSMUST00000198
ENSMUSG	Rbm20	ENSMUST00000164202;ENSMUST00000162910;ENSMUST00000161
ENSMUSG	Npsr1	ENSMUST00000059650;ENSMUST00000154337
ENSMUSG	Tox3	ENSMUST00000109621;ENSMUST00000176616
ENSMUSG	Diras1	ENSMUST00000055125
ENSMUSG	Kcns3	ENSMUST00000055673;ENSMUST00000217974;ENSMUST00000164
ENSMUSG	Fam25c	ENSMUST00000052126
ENSMUSG	Olfr62	ENSMUST00000102666
ENSMUSG	Capn13	ENSMUST00000095208
ENSMUSG	F830045	ENSMUST00000050309
ENSMUSG	B430306	ENSMUST00000049614;ENSMUST00000125174;ENSMUST00000129
ENSMUSG	Dmrta1	ENSMUST00000131576;ENSMUST00000052478
ENSMUSG	1700048	ENSMUST00000214777;ENSMUST00000215356;ENSMUST00000063
ENSMUSG	Rtn4r	ENSMUST00000059589
ENSMUSG	Adamtsl	ENSMUST00000137571;ENSMUST00000124789;ENSMUST00000128
ENSMUSG	Olfr94	ENSMUST00000222190;ENSMUST00000208047;ENSMUST00000055
ENSMUSG	Tmem145	ENSMUST00000108409
ENSMUSG	Clrn1	ENSMUST00000055636;ENSMUST00000051408;ENSMUST00000161
ENSMUSG	Tas2r11	ENSMUST00000062463
ENSMUSG	Gm5809	ENSMUST00000121854
ENSMUSG	Chil5	ENSMUST00000066537;ENSMUST00000054973;ENSMUST00000200
ENSMUSG	Olfr323	ENSMUST00000070804
ENSMUSG	S1pr2	ENSMUST00000054197;ENSMUST00000216499;ENSMUST00000214
ENSMUSG	Olfr922	ENSMUST00000213164;ENSMUST00000051004
ENSMUSG	Ncmap	ENSMUST00000105858;ENSMUST00000105859;ENSMUST00000105
ENSMUSG	Olfr544	ENSMUST00000051201
ENSMUSG	Klri2	ENSMUST00000050385
ENSMUSG	A530064	ENSMUST00000053612;ENSMUST00000027764
ENSMUSG	Adam6a	ENSMUST00000053086
ENSMUSG	Olfr691	ENSMUST00000213290;ENSMUST00000057528
ENSMUSG	Emx2	ENSMUST00000062216
ENSMUSG	Opn5	ENSMUST00000068355
ENSMUSG	Spata31	ENSMUST00000052978
ENSMUSG	Muc19	ENSMUST00000160242
ENSMUSG	Olfr790	ENSMUST00000218901
ENSMUSG	Olfr178	ENSMUST00000058564
ENSMUSG	Npb	ENSMUST00000106194;ENSMUST00000106195;ENSMUST00000061
ENSMUSG	Krt13	ENSMUST00000007275;ENSMUST00000134282
ENSMUSG	Otos	ENSMUST00000053144;ENSMUST00000159803
ENSMUSG	Olfr225	ENSMUST00000213169;ENSMUST00000055276
ENSMUSG	Efcab8	ENSMUST00000175856
ENSMUSG	Lmod3	ENSMUST00000095655
ENSMUSG	Il1f9	ENSMUST00000057567
ENSMUSG	2900011	ENSMUST00000056521
ENSMUSG	Fam109a	ENSMUST00000198271;ENSMUST00000056654;ENSMUST00000198
ENSMUSG	Tnip3	ENSMUST00000212402;ENSMUST00000114236;ENSMUST00000204
ENSMUSG	Rnf182	ENSMUST00000059986;ENSMUST00000161817
ENSMUSG	Ptx4	ENSMUST00000054930
ENSMUSG	Nkx2-6	ENSMUST00000062437
ENSMUSG	Vsig4	ENSMUST00000050707;ENSMUST00000146830

ENSMUSG	Kcnj4	ENSMUST00000057801
ENSMUSG	Aqp5	ENSMUST00000169082
ENSMUSG	Nkx2-3	ENSMUST00000057178
ENSMUSG	Nxpe4	ENSMUST00000216998;ENSMUST00000215780;ENSMUST0000009З
ENSMUSG	Nhlrc1	ENSMUST00000052747
ENSMUSG	Defb29	ENSMUST00000060598
ENSMUSG	Osbpl1a	ENSMUST00000121808;ENSMUST00000074352;ENSMUST00000121
ENSMUSG	Ctla2a	ENSMUST00000021880;ENSMUST00000223750;ENSMUST00000225
ENSMUSG	Olfm5	ENSMUST00000051137
ENSMUSG	Cnr1	ENSMUST00000057188;ENSMUST00000084736
ENSMUSG	Olfr794	ENSMUST00000059957
ENSMUSG	Krt84	ENSMUST00000023720
ENSMUSG	Zfp879	ENSMUST00000109134;ENSMUST00000049625
ENSMUSG	Apol7c	ENSMUST00000062562
ENSMUSG	Neurog3	ENSMUST00000050103;ENSMUST00000218216
ENSMUSG	Mab21l3	ENSMUST00000061831;ENSMUST00000118411
ENSMUSG	1700001	ENSMUST00000139968;ENSMUST00000050003;ENSMUST0000013C
ENSMUSG	Dsc1	ENSMUST00000224432;ENSMUST00000038710
ENSMUSG	Gm9790	ENSMUST00000054148
ENSMUSG	Ackr3	ENSMUST00000065587;ENSMUST00000159654
ENSMUSG	Aplnr	ENSMUST00000057019
ENSMUSG	Slc25a5	ENSMUST00000113069;ENSMUST00000171738;ENSMUST00000113
ENSMUSG	Sowaha	ENSMUST00000104955
ENSMUSG	P2ry4	ENSMUST00000053373
ENSMUSG	Cxxc4	ENSMUST00000197151;ENSMUST00000181904;ENSMUST00000181
ENSMUSG	Slc16a1	ENSMUST00000060010;ENSMUST00000159867;ENSMUST0000019C
ENSMUSG	BC02707	ENSMUST00000057405
ENSMUSG	Gm13080	ENSMUST00000120556
ENSMUSG	Slc15a5	ENSMUST00000111873;ENSMUST00000171804;ENSMUST0000015C
ENSMUSG	2410080	ENSMUST00000142712
ENSMUSG	Sowahd	ENSMUST00000060057
ENSMUSG	Adig	ENSMUST00000059889;ENSMUST00000148187
ENSMUSG	Cryga	ENSMUST00000154012
ENSMUSG	Gm9791	ENSMUST00000060997
ENSMUSG	Ffar1	ENSMUST00000052700
ENSMUSG	Stk-ps2	ENSMUST00000051224;ENSMUST00000187808
ENSMUSG	Klk1b11	ENSMUST00000007156
ENSMUSG	Foxe3	ENSMUST00000050940
ENSMUSG	Zfp488	ENSMUST00000166737
ENSMUSG	A730020	ENSMUST00000196621;ENSMUST00000050571;ENSMUST00000198
ENSMUSG	Ackr2	ENSMUST00000214340;ENSMUST00000216326
ENSMUSG	Dact1	ENSMUST00000061273;ENSMUST00000150639
ENSMUSG	Tceal3	ENSMUST00000125067;ENSMUST00000060904;ENSMUST00000113
ENSMUSG	Rasip1	ENSMUST00000057927
ENSMUSG	5031434	ENSMUST00000188014;ENSMUST00000053559
ENSMUSG	Serpinb	ENSMUST00000027567
ENSMUSG	Mycs	ENSMUST00000058404
ENSMUSG	Gm9294	ENSMUST00000165198
ENSMUSG	Gm4922	ENSMUST00000216654;ENSMUST00000055107
ENSMUSG	Liph	ENSMUST00000060673;ENSMUST00000074230
ENSMUSG	B530045	ENSMUST00000220146;ENSMUST00000060657
ENSMUSG	Pard6b	ENSMUST00000052125
ENSMUSG	Csrnp3	ENSMUST00000122912;ENSMUST00000129133;ENSMUST0000017б
ENSMUSG	Plppr4	ENSMUST00000061071;ENSMUST00000125664
ENSMUSG	Zfp612	ENSMUST00000213084;ENSMUST00000212754;ENSMUST0000005S
ENSMUSG	Ly6k	ENSMUST00000168815;ENSMUST00000060301
ENSMUSG	Cnpy1	ENSMUST00000141601;ENSMUST00000118882;ENSMUST0000012C

ENSMUSG	Gm13749	ENSMUST00000133244
ENSMUSG	Phf11a	ENSMUST00000062307
ENSMUSG	Dok7	ENSMUST00000114270;ENSMUST00000050709;ENSMUST0000015
ENSMUSG	Erich5	ENSMUST00000060894
ENSMUSG	Defb1	ENSMUST00000051017
ENSMUSG	Abca6	ENSMUST00000044003;ENSMUST00000150988
ENSMUSG	Gm12231	ENSMUST00000119447
ENSMUSG	Sntn	ENSMUST00000061045
ENSMUSG	Fads6	ENSMUST00000056153;ENSMUST00000141006
ENSMUSG	Olfr543	ENSMUST00000219647;ENSMUST00000061482
ENSMUSG	D630023	ENSMUST00000148020;ENSMUST00000050047
ENSMUSG	Oxgr1	ENSMUST00000058213
ENSMUSG	Olfr545	ENSMUST00000216116;ENSMUST00000058750
ENSMUSG	Ankrd45	ENSMUST00000111608
ENSMUSG	Defb36	ENSMUST00000058086
ENSMUSG	Olfr649	ENSMUST00000060440
ENSMUSG	Psg22	ENSMUST00000208221
ENSMUSG	Syt16	ENSMUST00000221220
ENSMUSG	Rassf9	ENSMUST00000219445;ENSMUST00000220230
ENSMUSG	Sstr3	ENSMUST00000053239
ENSMUSG	Klhl31	ENSMUST00000057781
ENSMUSG	Mylk4	ENSMUST00000057428;ENSMUST00000132206
ENSMUSG	Kctd21	ENSMUST00000054107
ENSMUSG	Wdr72	ENSMUST00000098564;ENSMUST00000055879;ENSMUST0000021
ENSMUSG	Olfr124	ENSMUST00000053599
ENSMUSG	Ucn3	ENSMUST00000058610
ENSMUSG	Olfr142	ENSMUST00000217952
ENSMUSG	Fzd5	ENSMUST00000063982;ENSMUST00000116133
ENSMUSG	Acer1	ENSMUST00000056113
ENSMUSG	Prss22	ENSMUST00000041649
ENSMUSG	Ankrd34	ENSMUST00000061594
ENSMUSG	Gm9795	ENSMUST00000062156
ENSMUSG	Prlhr	ENSMUST00000051277
ENSMUSG	Kcng3	ENSMUST00000051482
ENSMUSG	9930022	ENSMUST00000055404
ENSMUSG	Gm9796	ENSMUST00000191115
ENSMUSG	Lingo2	ENSMUST00000164772;ENSMUST00000108122;ENSMUST00000124
ENSMUSG	Arhgef3	ENSMUST00000171629
ENSMUSG	Dmd	ENSMUST00000127295;ENSMUST00000114000;ENSMUST00000147
ENSMUSG	Gm5514	ENSMUST00000060477
ENSMUSG	Olfr144	ENSMUST00000049624
ENSMUSG	Amer3	ENSMUST00000052670
ENSMUSG	Sox3	ENSMUST00000135107
ENSMUSG	Shroom2	ENSMUST00000101141;ENSMUST00000137217;ENSMUST00000062
ENSMUSG	Lrrc3b	ENSMUST00000163937;ENSMUST00000055211;ENSMUST0000022
ENSMUSG	Asxl3	ENSMUST00000153836;ENSMUST00000120223;ENSMUST00000097
ENSMUSG	Olfr115	ENSMUST00000213308
ENSMUSG	A730035	ENSMUST00000057889;ENSMUST00000197382
ENSMUSG	Kcng4	ENSMUST00000164382;ENSMUST00000061828
ENSMUSG	Zfp688	ENSMUST00000136123;ENSMUST00000106300;ENSMUST0000014
ENSMUSG	Tas2r11	ENSMUST00000057633
ENSMUSG	Zfp691	ENSMUST00000052715;ENSMUST00000106355;ENSMUST0000017
ENSMUSG	Gpr20	ENSMUST00000064166
ENSMUSG	Dcaf12l	ENSMUST00000115056;ENSMUST00000060481
ENSMUSG	Ush1g	ENSMUST00000103037
ENSMUSG	E330021	ENSMUST00000204830;ENSMUST00000058713
ENSMUSG	Olfr734	ENSMUST00000217152

ENSMUSG	Lhfpl2	ENSMUST00000054274;ENSMUST00000144838;ENSMUST0000015[illegible]
ENSMUSG	Sowahb	ENSMUST00000061328
ENSMUSG	Adra2c	ENSMUST00000049545
ENSMUSG	Proser2	ENSMUST00000114942;ENSMUST00000054254;ENSMUST0000012[illegible]
ENSMUSG	Fndc7	ENSMUST00000180063;ENSMUST00000123018;ENSMUST00000102
ENSMUSG	4933402[illegible]	ENSMUST00000051414
ENSMUSG	Zfp423	ENSMUST00000165770;ENSMUST00000109655;ENSMUST00000052
ENSMUSG	Olfr167	ENSMUST00000214315;ENSMUST00000054606
ENSMUSG	Fam186a	ENSMUST00000100209
ENSMUSG	Pxt1	ENSMUST00000051526
ENSMUSG	Olfr433	ENSMUST00000214690;ENSMUST00000052975
ENSMUSG	Kcnk13	ENSMUST00000160413;ENSMUST00000177549;ENSMUST0000004[illegible]
ENSMUSG	Olfr139	ENSMUST00000215014;ENSMUST00000062719
ENSMUSG	Insm2	ENSMUST00000051857
ENSMUSG	Ttll13	ENSMUST00000058266;ENSMUST00000206240;ENSMUST00000107
ENSMUSG	Lce3c	ENSMUST00000055375
ENSMUSG	Olfr459	ENSMUST00000214976;ENSMUST00000050412
ENSMUSG	Bhlhe23	ENSMUST00000108878
ENSMUSG	Hcar2	ENSMUST00000057145
ENSMUSG	A930002[illegible]	ENSMUST00000050753
ENSMUSG	Gpr150	ENSMUST00000056130
ENSMUSG	Olfr460	ENSMUST00000216942;ENSMUST00000051540
ENSMUSG	Pou3f3	ENSMUST00000054883
ENSMUSG	Onecut3	ENSMUST00000051773
ENSMUSG	Tssk2	ENSMUST00000055374
ENSMUSG	Olfr891	ENSMUST00000062535
ENSMUSG	Kcna5	ENSMUST00000060972
ENSMUSG	Sprr3	ENSMUST00000058142
ENSMUSG	Fpr1	ENSMUST00000061516
ENSMUSG	Mettl24	ENSMUST00000058747
ENSMUSG	Olfr827	ENSMUST00000058123
ENSMUSG	Sprr4	ENSMUST00000062129
ENSMUSG	Frrs1l	ENSMUST00000053681
ENSMUSG	Olig3	ENSMUST00000053225
ENSMUSG	Dbx2	ENSMUST00000054244
ENSMUSG	Gm5455	ENSMUST00000058710
ENSMUSG	Chrm2	ENSMUST00000172278;ENSMUST00000169004
ENSMUSG	Odf3l1	ENSMUST00000055036;ENSMUST00000215694
ENSMUSG	Vwc2l	ENSMUST00000053922;ENSMUST00000159205
ENSMUSG	Fam216b	ENSMUST00000066437
ENSMUSG	Henmt1	ENSMUST00000197884;ENSMUST00000197427;ENSMUST0000005[illegible]
ENSMUSG	Smtnl2	ENSMUST00000108500;ENSMUST00000050226;ENSMUST0000014[illegible]
ENSMUSG	Olfr148	ENSMUST00000217182
ENSMUSG	Tcf21	ENSMUST00000049930;ENSMUST00000218002
ENSMUSG	Lcn6	ENSMUST00000059693
ENSMUSG	Gm4997	ENSMUST00000154056
ENSMUSG	Smkr-ps	ENSMUST00000124522;ENSMUST00000141679
ENSMUSG	Pnoc	ENSMUST00000059339;ENSMUST00000224594
ENSMUSG	Sprn	ENSMUST00000059241
ENSMUSG	Togaram[illegible]	ENSMUST00000156665;ENSMUST00000097284;ENSMUST00000122
ENSMUSG	Olfr624	ENSMUST00000062144
ENSMUSG	Ccdc149	ENSMUST00000198008;ENSMUST00000059428
ENSMUSG	Olfr984	ENSMUST00000213858
ENSMUSG	Gm9801	ENSMUST00000208965
ENSMUSG	1700101	ENSMUST00000062557
ENSMUSG	Tgif2-p	ENSMUST00000061746
ENSMUSG	Slitrk6	ENSMUST00000078386

ENSMUSG	Adra1a	ENSMUST00000159365;ENSMUST00000054661;ENSMUST0000016C
ENSMUSG	Npas4	ENSMUST00000056129
ENSMUSG	C2cd4c	ENSMUST00000059699
ENSMUSG	Ccdc42	ENSMUST00000154294
ENSMUSG	4933440I	ENSMUST00000205794;ENSMUST00000061823
ENSMUSG	Gm20715	ENSMUST00000054781
ENSMUSG	Gpr158	ENSMUST00000055946;ENSMUST00000155760
ENSMUSG	4930451	ENSMUST00000061695
ENSMUSG	Onecut2	ENSMUST00000175965
ENSMUSG	B3gat1	ENSMUST00000160899;ENSMUST00000161431;ENSMUST0000015S
ENSMUSG	D830044	ENSMUST00000143315
ENSMUSG	Gapt	ENSMUST00000058806
ENSMUSG	Pnlip	ENSMUST00000057270
ENSMUSG	Olfr136	ENSMUST00000213912;ENSMUST00000057453
ENSMUSG	Fam26f	ENSMUST00000062784
ENSMUSG	Rp1l1	ENSMUST00000058229
ENSMUSG	Gm15428	ENSMUST00000121352
ENSMUSG	Clec9a	ENSMUST00000058352;ENSMUST00000088075;ENSMUST00000204
ENSMUSG	Gm9805	ENSMUST00000055305
ENSMUSG	Hpcal4	ENSMUST00000059667;ENSMUST00000106246;ENSMUST0000013€
ENSMUSG	Olig1	ENSMUST00000056882
ENSMUSG	Gldn	ENSMUST00000056740
ENSMUSG	Pabpc6	ENSMUST00000057190
ENSMUSG	Nxph1	ENSMUST00000162942;ENSMUST00000160300;ENSMUST0000006C
ENSMUSG	4930550	ENSMUST00000118305
ENSMUSG	Cd109	ENSMUST00000093812;ENSMUST00000163608
ENSMUSG	Iqub	ENSMUST00000052277
ENSMUSG	Sprr2g	ENSMUST00000056590;ENSMUST00000189634
ENSMUSG	Pnma2	ENSMUST00000089236;ENSMUST00000168010
ENSMUSG	Pik3r6	ENSMUST00000060441;ENSMUST00000102613;ENSMUST0000015З
ENSMUSG	Olfr735	ENSMUST00000216634;ENSMUST00000049729
ENSMUSG	Cym	ENSMUST00000029504
ENSMUSG	Rprml	ENSMUST00000057870
ENSMUSG	Hepacam	ENSMUST00000051839
ENSMUSG	Sprr2h	ENSMUST00000059845
ENSMUSG	C87977	ENSMUST00000105753;ENSMUST00000146836
ENSMUSG	Usp27x	ENSMUST00000178293;ENSMUST00000191497;ENSMUST0000011E
ENSMUSG	Olfr144	ENSMUST00000213606;ENSMUST00000059675
ENSMUSG	She	ENSMUST00000050401;ENSMUST00000197454
ENSMUSG	Pnma3	ENSMUST00000060418
ENSMUSG	AI46413	ENSMUST00000054920;ENSMUST00000149596
ENSMUSG	Stxbp6	ENSMUST00000053768;ENSMUST00000120531;ENSMUST0000013C
ENSMUSG	Ccbe1	ENSMUST00000146610;ENSMUST00000061103;ENSMUST0000013C
ENSMUSG	Hs3st2	ENSMUST00000084628
ENSMUSG	Gpat2	ENSMUST00000062211
ENSMUSG	Smco1	ENSMUST00000093183
ENSMUSG	Gjb2	ENSMUST00000055698
ENSMUSG	Mgat4e	ENSMUST00000172898
ENSMUSG	Gm5590	ENSMUST00000059110;ENSMUST00000186976
ENSMUSG	1700067	ENSMUST00000060357;ENSMUST00000174570;ENSMUST0000014C
ENSMUSG	Irx3os	ENSMUST00000133337;ENSMUST00000133528
ENSMUSG	Gm13078	ENSMUST00000078695
ENSMUSG	Cmtr2	ENSMUST00000189685;ENSMUST00000056972
ENSMUSG	C77370	ENSMUST00000118314;ENSMUST00000087879
ENSMUSG	Olfr71	ENSMUST00000217544;ENSMUST00000055401
ENSMUSG	Sh2d7	ENSMUST00000060242;ENSMUST00000118413
ENSMUSG	5930403	ENSMUST00000188813;ENSMUST00000062667;ENSMUST00000191

ENSMUSG	Olfr231	ENSMUST00000063030
ENSMUSG	Rnf222	ENSMUST00000065213
ENSMUSG	C1qtnf2	ENSMUST00000173002;ENSMUST00000057679;ENSMUST00000151
ENSMUSG	Fam19a4	ENSMUST00000089295;ENSMUST00000203930
ENSMUSG	Spin2c	ENSMUST00000148708;ENSMUST00000123264;ENSMUST00000049
ENSMUSG	Arsj	ENSMUST00000093976
ENSMUSG	4930430	ENSMUST00000054471;ENSMUST00000219410;ENSMUST00000219
ENSMUSG	Zfp518b	ENSMUST00000179555;ENSMUST00000057258;ENSMUST00000180
ENSMUSG	Gm7862	ENSMUST00000121461
ENSMUSG	Cfap58	ENSMUST00000066308
ENSMUSG	Tmem215	ENSMUST00000049655
ENSMUSG	Hrk	ENSMUST00000054836
ENSMUSG	Oacyl	ENSMUST00000115097
ENSMUSG	Gjb4	ENSMUST00000060419;ENSMUST00000106090
ENSMUSG	Olfr218	ENSMUST00000057548
ENSMUSG	Olfr144	ENSMUST00000213657
ENSMUSG	Olfr981	ENSMUST00000215956
ENSMUSG	0610025	ENSMUST00000131324
ENSMUSG	Fam46b	ENSMUST00000051676
ENSMUSG	Enpp7	ENSMUST00000092373;ENSMUST00000106273
ENSMUSG	Slitrk4	ENSMUST00000069926;ENSMUST00000114679;ENSMUST00000156
ENSMUSG	Nxph3	ENSMUST00000058866
ENSMUSG	Tmem45a	ENSMUST00000067173
ENSMUSG	Selenov	ENSMUST00000056589
ENSMUSG	A530053	ENSMUST00000205153;ENSMUST00000060147;ENSMUST00000204
ENSMUSG	Rhoj	ENSMUST00000118602;ENSMUST00000118966;ENSMUST00000055
ENSMUSG	8030474	ENSMUST00000163399
ENSMUSG	Olfr134	ENSMUST00000215117
ENSMUSG	Phgr1	ENSMUST00000130293
ENSMUSG	Lrrc75b	ENSMUST00000220172;ENSMUST00000051129
ENSMUSG	Gltpd2	ENSMUST00000057685
ENSMUSG	Ddit4l	ENSMUST00000167564;ENSMUST00000053855;ENSMUST00000165
ENSMUSG	Fam187b	ENSMUST00000206754;ENSMUST00000129773;ENSMUST00000058
ENSMUSG	Mettl21	ENSMUST00000054801;ENSMUST00000150062
ENSMUSG	Krt1	ENSMUST00000023790
ENSMUSG	Vat1l	ENSMUST00000142591;ENSMUST00000049509;ENSMUST00000124
ENSMUSG	Pip5kl1	ENSMUST00000134204;ENSMUST00000055304;ENSMUST00000100
ENSMUSG	Zfp474	ENSMUST00000072666;ENSMUST00000211585;ENSMUST00000209
ENSMUSG	Myct1	ENSMUST00000051809
ENSMUSG	Vmn1r17	ENSMUST00000049819
ENSMUSG	Csl	ENSMUST00000056085
ENSMUSG	Spz1	ENSMUST00000050658
ENSMUSG	4930432	ENSMUST00000063585
ENSMUSG	Gpr156	ENSMUST00000061274
ENSMUSG	BC05339	ENSMUST00000050937
ENSMUSG	Olfr102	ENSMUST00000215347
ENSMUSG	Gm5637	ENSMUST00000153890
ENSMUSG	Spsb4	ENSMUST00000055433;ENSMUST00000162307;ENSMUST00000159
ENSMUSG	1110032	ENSMUST00000054551
ENSMUSG	Msgn1	ENSMUST00000049877
ENSMUSG	Fbxo41	ENSMUST00000159062
ENSMUSG	Catsper	ENSMUST00000055156
ENSMUSG	Cfap65	ENSMUST00000094844;ENSMUST00000139950;ENSMUST00000130
ENSMUSG	Ankrd33	ENSMUST00000070875
ENSMUSG	Olfr432	ENSMUST00000213381;ENSMUST00000062665
ENSMUSG	Olfr914	ENSMUST00000217057
ENSMUSG	Ofcc1	ENSMUST00000054635

ENSMUSG	Pbp2	ENSMUST00000050471
ENSMUSG	Cldn14	ENSMUST00000050962
ENSMUSG	1700113	ENSMUST00000189456;ENSMUST00000189775
ENSMUSG	Dmrta2	ENSMUST00000061187
ENSMUSG	Olfr105	ENSMUST00000054746
ENSMUSG	Chst9	ENSMUST00000130553
ENSMUSG	Helt	ENSMUST00000058636
ENSMUSG	Gm9818	ENSMUST00000050900
ENSMUSG	Dusp18	ENSMUST00000055931
ENSMUSG	Ccdc36	ENSMUST00000076592
ENSMUSG	Rnase2a	ENSMUST00000061936
ENSMUSG	Gm527	ENSMUST00000058135;ENSMUST00000223388;ENSMUST0000022C
ENSMUSG	A2ml1	ENSMUST00000203291;ENSMUST00000060574;ENSMUST00000205
ENSMUSG	Cldn2	ENSMUST00000054889
ENSMUSG	Fbxw21	ENSMUST00000198076
ENSMUSG	Mageh1	ENSMUST00000051484
ENSMUSG	Krtap1-	ENSMUST00000054532
ENSMUSG	Mc4r	ENSMUST00000057942
ENSMUSG	Gap43	ENSMUST00000102817
ENSMUSG	Olfr370	ENSMUST00000209408;ENSMUST00000058609
ENSMUSG	Gpr15	ENSMUST00000089318
ENSMUSG	Kcnv2	ENSMUST00000056708
ENSMUSG	Lancl3	ENSMUST00000069763
ENSMUSG	Tdg-ps	ENSMUST00000051363
ENSMUSG	Olfr976	ENSMUST00000217360
ENSMUSG	Lcn10	ENSMUST00000058912
ENSMUSG	C2cd6b	ENSMUST00000054653
ENSMUSG	A730013	ENSMUST00000057543
ENSMUSG	Fam180a	ENSMUST00000051176
ENSMUSG	A530099	ENSMUST00000151029
ENSMUSG	Zfp30	ENSMUST00000032803;ENSMUST00000126504;ENSMUST00000122
ENSMUSG	Klhl34	ENSMUST00000087157
ENSMUSG	Dlgap2	ENSMUST00000133298;ENSMUST00000141155;ENSMUST00000141
ENSMUSG	Rnf152	ENSMUST00000058688;ENSMUST00000172299
ENSMUSG	Adamts1	ENSMUST00000061318
ENSMUSG	Mroh7	ENSMUST00000106770;ENSMUST00000135000
ENSMUSG	Mbd3l2	ENSMUST00000213777;ENSMUST00000051008
ENSMUSG	Rtp2	ENSMUST00000061030
ENSMUSG	Acot10	ENSMUST00000052910
ENSMUSG	Nxpe5	ENSMUST00000162519;ENSMUST00000110929;ENSMUST00000161
ENSMUSG	Olfr112	ENSMUST00000215371
ENSMUSG	Ankrd34	ENSMUST00000060700
ENSMUSG	Apof	ENSMUST00000050901
ENSMUSG	Cdcp2	ENSMUST00000062495;ENSMUST00000221740
ENSMUSG	Krt87	ENSMUST00000081945
ENSMUSG	D930020	ENSMUST00000140802;ENSMUST00000120642
ENSMUSG	Gm5454	ENSMUST00000052030
ENSMUSG	Gal3st3	ENSMUST00000061169
ENSMUSG	Msl3l2	ENSMUST00000063138
ENSMUSG	Zcchc5	ENSMUST00000062010
ENSMUSG	Olfr138	ENSMUST00000215226
ENSMUSG	4922502	ENSMUST00000051283;ENSMUST00000203159
ENSMUSG	Lrrc49	ENSMUST00000150060;ENSMUST00000053171;ENSMUST00000114
ENSMUSG	Ankfn1	ENSMUST00000208932;ENSMUST00000207815;ENSMUST00000128
ENSMUSG	Lix1	ENSMUST00000115576;ENSMUST00000140134
ENSMUSG	Gjb1	ENSMUST00000119190
ENSMUSG	Trim16	ENSMUST00000055006;ENSMUST00000147942;ENSMUST00000108

ENSMUSG	Bex4	ENSMUST00000116527
ENSMUSG	Foxi1	ENSMUST00000060271
ENSMUSG	Olfr770	ENSMUST00000062314
ENSMUSG	A4galt	ENSMUST00000164614;ENSMUST00000049530
ENSMUSG	Ang2	ENSMUST00000051274
ENSMUSG	Ripply2	ENSMUST00000058846;ENSMUST00000188187
ENSMUSG	Gm5607	ENSMUST00000186174
ENSMUSG	Gp5	ENSMUST00000061190
ENSMUSG	Olfr186	ENSMUST00000062380
ENSMUSG	Stbd1	ENSMUST00000200941;ENSMUST00000050952
ENSMUSG	Kcna1	ENSMUST00000055168;ENSMUST00000203094
ENSMUSG	C2cd4a	ENSMUST00000054500
ENSMUSG	Fam69c	ENSMUST00000052501
ENSMUSG	Hes5	ENSMUST00000049621;ENSMUST00000151480
ENSMUSG	Catsper	ENSMUST00000055892;ENSMUST00000105878
ENSMUSG	Tmem196	ENSMUST00000223374;ENSMUST00000184069
ENSMUSG	Krt35	ENSMUST00000173988
ENSMUSG	Neurod4	ENSMUST00000061571
ENSMUSG	Tmem229	ENSMUST00000127247
ENSMUSG	Fcrl5	ENSMUST00000194102;ENSMUST00000193229;ENSMUST0000004
ENSMUSG	Ccdc187	ENSMUST00000217256;ENSMUST00000155409
ENSMUSG	Pirt	ENSMUST00000123434
ENSMUSG	H1fnt	ENSMUST00000060855
ENSMUSG	Lmod1	ENSMUST00000059352
ENSMUSG	Tmem72	ENSMUST00000056623
ENSMUSG	Nat8l	ENSMUST00000056355;ENSMUST00000201041
ENSMUSG	Olfr49	ENSMUST00000216214;ENSMUST00000059996
ENSMUSG	H2bfm	ENSMUST00000059808
ENSMUSG	Gm7204	ENSMUST00000062591
ENSMUSG	Olfr132	ENSMUST00000215270;ENSMUST00000219631
ENSMUSG	Gm7075	ENSMUST00000054760
ENSMUSG	Bend7	ENSMUST00000184139;ENSMUST00000115022;ENSMUST0000013
ENSMUSG	Gpr85	ENSMUST00000060442;ENSMUST00000115491
ENSMUSG	Nags	ENSMUST00000129799
ENSMUSG	Fbxo43	ENSMUST00000058643
ENSMUSG	H2-M10.	ENSMUST00000057502
ENSMUSG	Gng7	ENSMUST00000117805;ENSMUST00000118233;ENSMUST0000011
ENSMUSG	Gm4879	ENSMUST00000122190
ENSMUSG	Olfr141	ENSMUST00000214887;ENSMUST00000061235
ENSMUSG	Olfr148	ENSMUST00000216801
ENSMUSG	Gm4884	ENSMUST00000164422
ENSMUSG	Mfsd6l	ENSMUST00000053211
ENSMUSG	Ric3	ENSMUST00000147580;ENSMUST00000055993;ENSMUST0000012
ENSMUSG	Gm8258	ENSMUST00000055593
ENSMUSG	Npy4r	ENSMUST00000052164
ENSMUSG	Pou4f1	ENSMUST00000053016;ENSMUST00000139505
ENSMUSG	Fgfbp1	ENSMUST00000199894;ENSMUST00000061299;ENSMUST0000019
ENSMUSG	Foxi2	ENSMUST00000060356
ENSMUSG	Olfr139	ENSMUST00000216273
ENSMUSG	Scrt1	ENSMUST00000096365
ENSMUSG	Osr1	ENSMUST00000217975;ENSMUST00000057021
ENSMUSG	Fam171b	ENSMUST00000051454;ENSMUST00000148184
ENSMUSG	Olfr843	ENSMUST00000214019;ENSMUST00000061365
ENSMUSG	Tprg	ENSMUST00000056087;ENSMUST00000128595
ENSMUSG	Mlf1	ENSMUST00000077916;ENSMUST00000142538;ENSMUST0000012
ENSMUSG	Ranbp3l	ENSMUST00000166524;ENSMUST00000053308
ENSMUSG	Msx1	ENSMUST00000063116;ENSMUST00000183013

ENSMUSG	Sprr1b	ENSMUST00000062160
ENSMUSG	Sult6b2	ENSMUST00000156662
ENSMUSG	Cxcr1	ENSMUST00000190313;ENSMUST00000053389
ENSMUSG	Bdnf	ENSMUST00000053317;ENSMUST00000111051;ENSMUST00000111
ENSMUSG	Gm7461	ENSMUST00000058918
ENSMUSG	Zbtb8b	ENSMUST00000053042;ENSMUST00000106046;ENSMUST00000124
ENSMUSG	Cd300e	ENSMUST00000062787
ENSMUSG	Tmem136	ENSMUST00000061833;ENSMUST00000213648;ENSMUST00000213
ENSMUSG	5930422	ENSMUST00000059351
ENSMUSG	Thnsl1	ENSMUST00000054591;ENSMUST00000102952;ENSMUST00000138
ENSMUSG	E130311	ENSMUST00000061706;ENSMUST00000162448;ENSMUST0000016C
ENSMUSG	Gja3	ENSMUST00000061614
ENSMUSG	Igf2	ENSMUST00000000033;ENSMUST00000121128;ENSMUST00000105
ENSMUSG	Gm5946	ENSMUST00000182652;ENSMUST00000182340
ENSMUSG	Morc2b	ENSMUST00000131954
ENSMUSG	Nog	ENSMUST00000061728
ENSMUSG	Olfr133	ENSMUST00000214301
ENSMUSG	Klf17	ENSMUST00000062747
ENSMUSG	Samd13	ENSMUST00000125965;ENSMUST00000124931;ENSMUST00000147
ENSMUSG	Hmgb4	ENSMUST00000053830
ENSMUSG	Olfr435	ENSMUST00000215565
ENSMUSG	Vmn1r26	ENSMUST00000049694
ENSMUSG	Krt90	ENSMUST00000023714
ENSMUSG	Lurap1l	ENSMUST00000055922
ENSMUSG	Gm8666	ENSMUST00000117517
ENSMUSG	Zfp454	ENSMUST00000050595;ENSMUST00000163301;ENSMUST00000125
ENSMUSG	Prss50	ENSMUST00000051097
ENSMUSG	Rpl29	ENSMUST00000150576;ENSMUST00000059802;ENSMUST00000213
ENSMUSG	Hoxb3	ENSMUST00000093944;ENSMUST00000123091;ENSMUST00000143
ENSMUSG	Tmprss1	ENSMUST00000116553;ENSMUST00000161625
ENSMUSG	Serpinb	ENSMUST00000027564
ENSMUSG	Pthlh	ENSMUST00000052296
ENSMUSG	Ifnb1	ENSMUST00000055671
ENSMUSG	Slc35e4	ENSMUST00000109995;ENSMUST00000051207
ENSMUSG	Lonrf2	ENSMUST00000147695;ENSMUST00000039612;ENSMUST00000191
ENSMUSG	Vstm2a	ENSMUST00000109645;ENSMUST00000109647;ENSMUST00000208
ENSMUSG	Neurog1	ENSMUST00000058475
ENSMUSG	4930539	ENSMUST00000062357
ENSMUSG	Gm5540	ENSMUST00000131852
ENSMUSG	Nr1h5	ENSMUST00000058899
ENSMUSG	Atp13a5	ENSMUST00000075806
ENSMUSG	Prex2	ENSMUST00000027056;ENSMUST00000190935;ENSMUST00000189
ENSMUSG	Nrsn1	ENSMUST00000057866;ENSMUST00000226054
ENSMUSG	Krt31	ENSMUST00000007318
ENSMUSG	Gphb5	ENSMUST00000051079
ENSMUSG	Elfn1	ENSMUST00000050519;ENSMUST00000198608
ENSMUSG	Prss32	ENSMUST00000144925;ENSMUST00000061725
ENSMUSG	Olfr136	ENSMUST00000205976;ENSMUST00000168629;ENSMUST00000218
ENSMUSG	Ndnf	ENSMUST00000054351;ENSMUST00000169795
ENSMUSG	Olfr873	ENSMUST00000212393
ENSMUSG	Olfr398	ENSMUST00000053874
ENSMUSG	Olfr125	ENSMUST00000111519;ENSMUST00000060795
ENSMUSG	Gm9244	ENSMUST00000056767
ENSMUSG	Olfr147	ENSMUST00000214648
ENSMUSG	Pcdh10	ENSMUST00000193252;ENSMUST00000171554;ENSMUST0000017C
ENSMUSG	Ntf3	ENSMUST00000204542;ENSMUST00000050484
ENSMUSG	Oxtr	ENSMUST00000053306

ENSMUSG	Frmd3	ENSMUST00000098006;ENSMUST00000084474;ENSMUST00000154
ENSMUSG	Catsper	ENSMUST00000208607;ENSMUST00000208371;ENSMUST00000207
ENSMUSG	Ivl	ENSMUST00000053107
ENSMUSG	C5ar1	ENSMUST00000050770;ENSMUST00000168818
ENSMUSG	Flg2	ENSMUST00000194707;ENSMUST00000098884;ENSMUST00000194
ENSMUSG	Nrap	ENSMUST00000073536
ENSMUSG	Plcxd3	ENSMUST00000130332;ENSMUST00000061925
ENSMUSG	Olfr125	ENSMUST00000213720;ENSMUST00000111516
ENSMUSG	Ugt3a2	ENSMUST00000072403;ENSMUST00000144755
ENSMUSG	Fam183b	ENSMUST00000094156;ENSMUST00000060581
ENSMUSG	Tex50	ENSMUST00000135643
ENSMUSG	Myoz3	ENSMUST00000056533
ENSMUSG	Rgag4	ENSMUST00000113631;ENSMUST00000119076;ENSMUST00000188
ENSMUSG	Olfr509	ENSMUST00000213756
ENSMUSG	Iba57	ENSMUST00000054523;ENSMUST00000069631
ENSMUSG	Prss38	ENSMUST00000061481
ENSMUSG	Syt12	ENSMUST00000059295;ENSMUST00000128114;ENSMUST00000133
ENSMUSG	Fut4	ENSMUST00000061498
ENSMUSG	Olfr348	ENSMUST00000056865
ENSMUSG	Zfp2	ENSMUST00000109129;ENSMUST00000116378;ENSMUST00000109
ENSMUSG	Tenm2	ENSMUST00000102801;ENSMUST00000057207;ENSMUST00000146
ENSMUSG	Gm5105	ENSMUST00000053318
ENSMUSG	Rd3	ENSMUST00000180463;ENSMUST00000175680;ENSMUST00000192
ENSMUSG	Olfr118	ENSMUST00000219871
ENSMUSG	Prokr1	ENSMUST00000204682
ENSMUSG	Tmem200	ENSMUST00000217910;ENSMUST00000066049
ENSMUSG	Gm9837	ENSMUST00000057722
ENSMUSG	Upk1b	ENSMUST00000057767
ENSMUSG	Cyp20a1	ENSMUST00000114202;ENSMUST00000060608;ENSMUST00000148
ENSMUSG	Gm8396	ENSMUST00000057361
ENSMUSG	Slc36a3	ENSMUST00000020502;ENSMUST00000128244
ENSMUSG	Sppl2c	ENSMUST00000107000
ENSMUSG	Htr1b	ENSMUST00000183482;ENSMUST00000051005
ENSMUSG	Tceal1	ENSMUST00000055104
ENSMUSG	Tecrl	ENSMUST00000053543
ENSMUSG	Adamts1	ENSMUST00000123552
ENSMUSG	Krt82	ENSMUST00000023713
ENSMUSG	Fzd9	ENSMUST00000062572
ENSMUSG	Lingo1	ENSMUST00000053568;ENSMUST00000210032;ENSMUST00000114
ENSMUSG	Olfr95	ENSMUST00000060728
ENSMUSG	Aknad1	ENSMUST00000133931;ENSMUST00000150998
ENSMUSG	Olfr780	ENSMUST00000063168
ENSMUSG	Zfa-ps	ENSMUST00000180673;ENSMUST00000180526
ENSMUSG	Tsku	ENSMUST00000094161;ENSMUST00000164726;ENSMUST00000167
ENSMUSG	Grm5	ENSMUST00000208791;ENSMUST00000125009;ENSMUST00000155
ENSMUSG	Lce1h	ENSMUST00000051521
ENSMUSG	Hoxb13	ENSMUST00000062709
ENSMUSG	Gpr55	ENSMUST00000086975
ENSMUSG	Prss33	ENSMUST00000059906;ENSMUST00000115444;ENSMUST00000164
ENSMUSG	C1ql3	ENSMUST00000061545
ENSMUSG	Vgll2	ENSMUST00000163017;ENSMUST00000058347
ENSMUSG	Gpr3	ENSMUST00000105911
ENSMUSG	Morn4	ENSMUST00000051772
ENSMUSG	Nckap5	ENSMUST00000161954;ENSMUST00000112583;ENSMUST00000160
ENSMUSG	Tmem239	ENSMUST00000055421
ENSMUSG	BC04867	ENSMUST00000054799
ENSMUSG	Ucn2	ENSMUST00000052724

ENSMUSG	Nlrp10	ENSMUST00000055745
ENSMUSG	Gal3st1	ENSMUST00000063004
ENSMUSG	Mmp12	ENSMUST00000150167;ENSMUST00000005950;ENSMUST0000014[illegible]
ENSMUSG	Rxfp4	ENSMUST00000063119
ENSMUSG	Olfr131	ENSMUST00000215341;ENSMUST00000058176
ENSMUSG	Pmis2	ENSMUST00000051495
ENSMUSG	Crh	ENSMUST00000061294
ENSMUSG	Olfr642	ENSMUST00000052660
ENSMUSG	Armcx4	ENSMUST00000124226
ENSMUSG	Olfr466	ENSMUST00000214214
ENSMUSG	Rbx1-ps	ENSMUST00000173786
ENSMUSG	Ceacam1	ENSMUST00000052605
ENSMUSG	Fam26e	ENSMUST00000069125
ENSMUSG	Gm7984	ENSMUST00000060640
ENSMUSG	4921517[illegible]	ENSMUST00000061597
ENSMUSG	Tmem17	ENSMUST00000059319
ENSMUSG	Olfr921	ENSMUST00000217114;ENSMUST00000213958;ENSMUST00000071
ENSMUSG	Lpar4	ENSMUST00000060576
ENSMUSG	Lrrc25	ENSMUST00000210609;ENSMUST00000052437
ENSMUSG	Ppp1r3d	ENSMUST00000058678
ENSMUSG	Apol10b	ENSMUST00000089465
ENSMUSG	Olfr728	ENSMUST00000051563
ENSMUSG	Klk6	ENSMUST00000107968
ENSMUSG	Zfp697	ENSMUST00000196741;ENSMUST00000196194;ENSMUST0000005[illegible]
ENSMUSG	Grem2	ENSMUST00000055294
ENSMUSG	Spink8	ENSMUST00000198988
ENSMUSG	Sprr2b	ENSMUST00000061038
ENSMUSG	Ces2b	ENSMUST00000059449;ENSMUST00000163042
ENSMUSG	Vmn1r23	ENSMUST00000060603
ENSMUSG	Agmo	ENSMUST00000049874;ENSMUST00000160768;ENSMUST0000016[illegible]
ENSMUSG	Grrp1	ENSMUST00000060050;ENSMUST00000138366
ENSMUSG	Bpifc	ENSMUST00000061699;ENSMUST00000105304
ENSMUSG	Prdx6b	ENSMUST00000057072
ENSMUSG	Vwa3b	ENSMUST00000124404;ENSMUST00000067178;ENSMUST00000027
ENSMUSG	Olfr102	ENSMUST00000213441
ENSMUSG	Sarm1	ENSMUST00000108287;ENSMUST00000153534;ENSMUST0000017[illegible]
ENSMUSG	Olfr430	ENSMUST00000213425;ENSMUST00000055873
ENSMUSG	Olfr165	ENSMUST00000206110;ENSMUST00000052516;ENSMUST0000020[illegible]
ENSMUSG	Mchr1	ENSMUST00000166855
ENSMUSG	Olfr768	ENSMUST00000216681;ENSMUST00000063031
ENSMUSG	Otop2	ENSMUST00000055490
ENSMUSG	Pla2g4e	ENSMUST00000136845;ENSMUST00000090071;ENSMUST00000127
ENSMUSG	Olfr70	ENSMUST00000055545
ENSMUSG	Lgsn	ENSMUST00000062560
ENSMUSG	Il17d	ENSMUST00000089494
ENSMUSG	Klre1	ENSMUST00000053708;ENSMUST00000203322;ENSMUST0000020[illegible]
ENSMUSG	Evc2	ENSMUST00000056365;ENSMUST00000101258;ENSMUST0000015[illegible]
ENSMUSG	Olfr809	ENSMUST00000218237
ENSMUSG	Olfr690	ENSMUST00000061920
ENSMUSG	Tmem220	ENSMUST00000079077;ENSMUST00000061786
ENSMUSG	Dscam	ENSMUST00000056102
ENSMUSG	Mrgprg	ENSMUST00000058092;ENSMUST00000208986
ENSMUSG	Olfr630	ENSMUST00000106880
ENSMUSG	Foxc1	ENSMUST00000062292
ENSMUSG	Abca12	ENSMUST00000087268;ENSMUST00000191520;ENSMUST0000018[illegible]
ENSMUSG	Neto1	ENSMUST00000058829
ENSMUSG	Ndufaf6	ENSMUST00000058183;ENSMUST00000144475;ENSMUST0000012[illegible]

ENSMUSG	C130071(	ENSMUST00000131907
ENSMUSG	4930486I	ENSMUST00000091569
ENSMUSG	Olfr524	ENSMUST00000215815;ENSMUST00000051943
ENSMUSG	Hoxd10	ENSMUST00000061745
ENSMUSG	Armcx6	ENSMUST00000113194;ENSMUST00000052431;ENSMUST0000015C
ENSMUSG	Tnfsf15	ENSMUST00000062246
ENSMUSG	Foxl2	ENSMUST00000051312
ENSMUSG	Ppp1r3g	ENSMUST00000225537;ENSMUST00000132661;ENSMUST00000172
ENSMUSG	Pnma5	ENSMUST00000051569
ENSMUSG	Mrgprb2	ENSMUST00000052730
ENSMUSG	Cyp8b1	ENSMUST00000062474
ENSMUSG	Lypd6	ENSMUST00000053208;ENSMUST00000126337
ENSMUSG	Krt78	ENSMUST00000164932
ENSMUSG	Slc35d3	ENSMUST00000059805
ENSMUSG	Gm8394	ENSMUST00000079684
ENSMUSG	Fam167b	ENSMUST00000142699;ENSMUST00000052835
ENSMUSG	Pcdh20	ENSMUST00000061628
ENSMUSG	Oprd1	ENSMUST00000056336
ENSMUSG	Cldn8	ENSMUST00000049697
ENSMUSG	4933406I	ENSMUST00000162187
ENSMUSG	Fam171a	ENSMUST00000072955;ENSMUST00000115099;ENSMUST00000091
ENSMUSG	Htr5b	ENSMUST00000055884
ENSMUSG	B230217(	ENSMUST00000054783;ENSMUST00000155954;ENSMUST00000164
ENSMUSG	Adra1b	ENSMUST00000124306;ENSMUST00000167574;ENSMUST00000067
ENSMUSG	Fam228b	ENSMUST00000218905;ENSMUST00000219898;ENSMUST0000021{
ENSMUSG	Gk2	ENSMUST00000059657
ENSMUSG	Mmp13	ENSMUST00000015394
ENSMUSG	Lrrc4c	ENSMUST00000135431;ENSMUST00000059049
ENSMUSG	Txndc2	ENSMUST00000050236
ENSMUSG	Ccdc121	ENSMUST00000058825
ENSMUSG	Sprr2f	ENSMUST00000050397
ENSMUSG	Defb19	ENSMUST00000053180
ENSMUSG	Mrgpra1	ENSMUST00000098438
ENSMUSG	Trhde	ENSMUST00000061632;ENSMUST00000152702;ENSMUST00000152
ENSMUSG	Vstm4	ENSMUST00000053175
ENSMUSG	Ccdc96	ENSMUST00000060100
ENSMUSG	Ccdc54	ENSMUST00000062439
ENSMUSG	Emilin3	ENSMUST00000109454;ENSMUST00000057169
ENSMUSG	Scg2	ENSMUST00000185234;ENSMUST00000049972
ENSMUSG	Pgbd5	ENSMUST00000172566;ENSMUST00000140012;ENSMUST0000012{
ENSMUSG	Defb6	ENSMUST00000063112
ENSMUSG	Gp1bb	ENSMUST00000167388;ENSMUST00000051160
ENSMUSG	Prss27	ENSMUST00000059482
ENSMUSG	Olfr139	ENSMUST00000059379
ENSMUSG	Olfr112	ENSMUST00000215909;ENSMUST00000062494
ENSMUSG	Olfr131	ENSMUST00000214801;ENSMUST00000060098
ENSMUSG	Muc15	ENSMUST00000111016;ENSMUST00000090332
ENSMUSG	Olfr144	ENSMUST00000216506;ENSMUST00000059033
ENSMUSG	Olfr330	ENSMUST00000134055
ENSMUSG	Slc29a4	ENSMUST00000198728;ENSMUST00000058418
ENSMUSG	Cdh20	ENSMUST00000062528
ENSMUSG	Tmem125	ENSMUST00000060214;ENSMUST00000150044;ENSMUST0000015(
ENSMUSG	Clrn3	ENSMUST00000053716
ENSMUSG	Spata31(	ENSMUST00000224982
ENSMUSG	Gm7327	ENSMUST00000120925
ENSMUSG	Mtnr1b	ENSMUST00000182947
ENSMUSG	Fgf4	ENSMUST00000060336

ENSMUSG	Dcaf12l	ENSMUST00000115057
ENSMUSG	Map10	ENSMUST00000053078
ENSMUSG	Sgms2	ENSMUST00000090246;ENSMUST00000126569;ENSMUST00000197
ENSMUSG	Kcns2	ENSMUST00000072868
ENSMUSG	Lin28a	ENSMUST00000051674;ENSMUST00000176897
ENSMUSG	Creg2	ENSMUST00000053355
ENSMUSG	Gm9847	ENSMUST00000052528
ENSMUSG	Apol10a	ENSMUST00000060551
ENSMUSG	Adgb	ENSMUST00000179956;ENSMUST00000172530;ENSMUST00000208
ENSMUSG	Gm9848	ENSMUST00000053398
ENSMUSG	Olfr161	ENSMUST00000216259
ENSMUSG	Hs3st1	ENSMUST00000117944;ENSMUST00000053116;ENSMUST00000137
ENSMUSG	Serpinb	ENSMUST00000016951
ENSMUSG	Ttc24	ENSMUST00000050258;ENSMUST00000064550
ENSMUSG	Spink14	ENSMUST00000060328
ENSMUSG	Olfr523	ENSMUST00000209314;ENSMUST00000214272;ENSMUST00000215
ENSMUSG	1700014	ENSMUST00000180139;ENSMUST00000055343
ENSMUSG	Gja10	ENSMUST00000056517
ENSMUSG	Mb21d2	ENSMUST00000100023
ENSMUSG	Lingo3	ENSMUST00000053986
ENSMUSG	Vtcn1	ENSMUST00000054791
ENSMUSG	Rgs13	ENSMUST00000052375;ENSMUST00000111941
ENSMUSG	Gm4847	ENSMUST00000046662
ENSMUSG	Olfr986	ENSMUST00000218134;ENSMUST00000062229
ENSMUSG	Olfr77	ENSMUST00000057596
ENSMUSG	Ghsr	ENSMUST00000057186
ENSMUSG	Tas2r10	ENSMUST00000053652
ENSMUSG	Cited1	ENSMUST00000101336
ENSMUSG	Olfr853	ENSMUST00000058296
ENSMUSG	Olfr522	ENSMUST00000050585
ENSMUSG	Olfr655	ENSMUST00000057385
ENSMUSG	Fam174a	ENSMUST00000059975;ENSMUST00000186780
ENSMUSG	4930548	ENSMUST00000190364
ENSMUSG	Mrgprd	ENSMUST00000062163
ENSMUSG	Gpr119	ENSMUST00000053970
ENSMUSG	Fam83a	ENSMUST00000160942;ENSMUST00000050374;ENSMUST00000161
ENSMUSG	Nyx	ENSMUST00000050434
ENSMUSG	Nhlh1	ENSMUST00000059794
ENSMUSG	Olfr136	ENSMUST00000051874
ENSMUSG	Gdf6	ENSMUST00000057613
ENSMUSG	2410124	ENSMUST00000160494
ENSMUSG	Olfr126	ENSMUST00000111508
ENSMUSG	Ffar2	ENSMUST00000053156;ENSMUST00000163504;ENSMUST00000168
ENSMUSG	1500011	ENSMUST00000135091
ENSMUSG	Pcdh19	ENSMUST00000149154;ENSMUST00000167944;ENSMUST0000006C
ENSMUSG	Olfr645	ENSMUST00000057104
ENSMUSG	6030498	ENSMUST00000050744
ENSMUSG	Kctd16	ENSMUST00000091927
ENSMUSG	Olfr829	ENSMUST00000212229;ENSMUST00000058411
ENSMUSG	Fhad1	ENSMUST00000105780;ENSMUST00000137618;ENSMUST00000036
ENSMUSG	Ubqlnl	ENSMUST00000059121
ENSMUSG	Hspb3	ENSMUST00000054650
ENSMUSG	Krtap31	ENSMUST00000058761
ENSMUSG	Foxd4	ENSMUST00000058600
ENSMUSG	Kcnj16	ENSMUST00000180023;ENSMUST00000106636;ENSMUST00000106
ENSMUSG	Tlr6	ENSMUST00000062315;ENSMUST00000201307
ENSMUSG	Zfp786	ENSMUST00000058844;ENSMUST00000204984

ENSMUSG	Siglech	ENSMUST00000172988;ENSMUST00000121492;ENSMUST00000171
ENSMUSG	Fam181b	ENSMUST00000051179
ENSMUSG	Usp29	ENSMUST00000200535;ENSMUST00000208642;ENSMUST00000198
ENSMUSG	Gm5124	ENSMUST00000132683
ENSMUSG	Olfr697	ENSMUST00000217734
ENSMUSG	Ccnb3	ENSMUST00000115752
ENSMUSG	Olfr272	ENSMUST00000107667;ENSMUST00000051600
ENSMUSG	Otop1	ENSMUST00000063136;ENSMUST00000114099;ENSMUST00000185
ENSMUSG	2010001	ENSMUST00000224305;ENSMUST00000224451;ENSMUST0000006C
ENSMUSG	Olfr112	ENSMUST00000213274
ENSMUSG	Ubqln3	ENSMUST00000057254
ENSMUSG	Gm9857	ENSMUST00000050914
ENSMUSG	Kctd19	ENSMUST00000063071
ENSMUSG	Lrrc3	ENSMUST00000057608
ENSMUSG	Treml4	ENSMUST00000059873;ENSMUST00000136272;ENSMUST00000154
ENSMUSG	Kcnf1	ENSMUST00000170580
ENSMUSG	Kctd14	ENSMUST00000206658;ENSMUST00000121987;ENSMUST00000143
ENSMUSG	4930563	ENSMUST00000062638
ENSMUSG	Pabpc2	ENSMUST00000063219
ENSMUSG	Ttn	ENSMUST00000011934;ENSMUST00000099981;ENSMUST00000111
ENSMUSG	Wfdc21	ENSMUST00000070832
ENSMUSG	4930544	ENSMUST00000181553
ENSMUSG	Wfdc15a	ENSMUST00000063251
ENSMUSG	Iqcj	ENSMUST00000063263
ENSMUSG	Olfr284	ENSMUST00000206647
ENSMUSG	Adam6b	ENSMUST00000063317
ENSMUSG	Gypa	ENSMUST00000063359
ENSMUSG	Samd7	ENSMUST00000108262;ENSMUST00000192872
ENSMUSG	Krt71	ENSMUST00000023710
ENSMUSG	Abca16	ENSMUST00000120490
ENSMUSG	Cd209f	ENSMUST00000145007;ENSMUST00000207294;ENSMUST00000156
ENSMUSG	Xkr4	ENSMUST00000162897;ENSMUST00000159265;ENSMUST0000007C
ENSMUSG	Olfr371	ENSMUST00000070849
ENSMUSG	Nanos2	ENSMUST00000063563
ENSMUSG	Tlr11	ENSMUST00000185091;ENSMUST00000063570
ENSMUSG	Prdm9	ENSMUST00000167994;ENSMUST00000128267;ENSMUST0000013C
ENSMUSG	Casr	ENSMUST00000063597;ENSMUST00000172826
ENSMUSG	Igfn1	ENSMUST00000166193
ENSMUSG	Gm9864	ENSMUST00000090043
ENSMUSG	Slc6a7	ENSMUST00000025520
ENSMUSG	1700029	ENSMUST00000091377;ENSMUST00000221277
ENSMUSG	Prss51	ENSMUST00000063785;ENSMUST00000224112
ENSMUSG	D630039	ENSMUST00000063816
ENSMUSG	Akr1b7	ENSMUST00000007449
ENSMUSG	Sema5b	ENSMUST00000133554;ENSMUST00000120756;ENSMUST00000128
ENSMUSG	Foxo6	ENSMUST00000102656
ENSMUSG	Gm9869	ENSMUST00000214999
ENSMUSG	Plpp2	ENSMUST00000163602;ENSMUST00000063879
ENSMUSG	Serpinb	ENSMUST00000110273;ENSMUST00000172184
ENSMUSG	Olfr849	ENSMUST00000217273
ENSMUSG	Hbb-y	ENSMUST00000033229
ENSMUSG	Gm14964	ENSMUST00000149574
ENSMUSG	Gm5963	ENSMUST00000143451
ENSMUSG	Cd177	ENSMUST00000063956;ENSMUST00000206160
ENSMUSG	Hbb-bh1	ENSMUST00000063957
ENSMUSG	Ppp1r36	ENSMUST00000063977;ENSMUST00000218732;ENSMUST0000021S
ENSMUSG	Gpr17	ENSMUST00000064016

ENSMUSG	Fpr2	ENSMUST00000149944;ENSMUST00000064068
ENSMUSG	Ostn	ENSMUST00000066852
ENSMUSG	8030423	ENSMUST00000064097
ENSMUSG	Tbc1d30	ENSMUST00000064107;ENSMUST00000218688;ENSMUST0000021
ENSMUSG	Mrgpra6	ENSMUST00000186456
ENSMUSG	Lrrc15	ENSMUST00000064606
ENSMUSG	Cemip	ENSMUST00000064174;ENSMUST00000145171;ENSMUST0000015
ENSMUSG	Zdhhc19	ENSMUST00000064192;ENSMUST00000160832
ENSMUSG	B630019	ENSMUST00000064196
ENSMUSG	Gm9873	ENSMUST00000180562;ENSMUST00000063891
ENSMUSG	Hoxd3os	ENSMUST00000132326;ENSMUST00000156342;ENSMUST0000013
ENSMUSG	Il1rapl	ENSMUST00000113966
ENSMUSG	Mpp3	ENSMUST00000132094;ENSMUST00000127053;ENSMUST00000107
ENSMUSG	Actn2	ENSMUST00000064204;ENSMUST00000222034;ENSMUST0000011
ENSMUSG	4921508	ENSMUST00000061679
ENSMUSG	Trpm3	ENSMUST00000074770;ENSMUST00000087576;ENSMUST0000009
ENSMUSG	Acot4	ENSMUST00000021652;ENSMUST00000128162;ENSMUST00000221
ENSMUSG	Mogat2	ENSMUST00000064231
ENSMUSG	Tchh	ENSMUST00000064257
ENSMUSG	Gm9877	ENSMUST00000064271
ENSMUSG	Bmpr1b	ENSMUST00000029948;ENSMUST00000106232;ENSMUST0000010
ENSMUSG	Cebpe	ENSMUST00000064290
ENSMUSG	Pmp2	ENSMUST00000029034
ENSMUSG	Gm9881	ENSMUST00000068783
ENSMUSG	A330008	ENSMUST00000064349;ENSMUST00000153366
ENSMUSG	Gm8174	ENSMUST00000064353
ENSMUSG	Tmem171	ENSMUST00000064347
ENSMUSG	Pkdrej	ENSMUST00000064370
ENSMUSG	Epha3	ENSMUST00000064405
ENSMUSG	Robo2	ENSMUST00000117785;ENSMUST00000117200;ENSMUST0000014
ENSMUSG	Serp2	ENSMUST00000064517
ENSMUSG	A1cf	ENSMUST00000075838;ENSMUST00000225165
ENSMUSG	Isoc2b	ENSMUST00000064547;ENSMUST00000207699;ENSMUST0000020
ENSMUSG	Sh2d6	ENSMUST00000159577;ENSMUST00000089687;ENSMUST00000162
ENSMUSG	4930504	ENSMUST00000064614
ENSMUSG	5830454	ENSMUST00000213974;ENSMUST00000064646
ENSMUSG	Zfp112	ENSMUST00000120006;ENSMUST00000005413;ENSMUST0000020
ENSMUSG	Tln2	ENSMUST00000040025;ENSMUST00000217550;ENSMUST00000214
ENSMUSG	Kcnt2	ENSMUST00000120709;ENSMUST00000119786;ENSMUST00000192
ENSMUSG	Map1b	ENSMUST00000064762;ENSMUST00000224702;ENSMUST0000022
ENSMUSG	Trim30b	ENSMUST00000145982;ENSMUST00000171830;ENSMUST0000010
ENSMUSG	Gm12703	ENSMUST00000145411
ENSMUSG	Oas1a	ENSMUST00000080322
ENSMUSG	4833418	ENSMUST00000064826
ENSMUSG	Cysltr1	ENSMUST00000113480;ENSMUST00000064892
ENSMUSG	C130026	ENSMUST00000198458
ENSMUSG	Tas2r13	ENSMUST00000064932
ENSMUSG	Nrk	ENSMUST00000113052
ENSMUSG	Dnah6	ENSMUST00000064948;ENSMUST00000114038
ENSMUSG	Gm9894	ENSMUST00000171257
ENSMUSG	Cyp2j6	ENSMUST00000030303
ENSMUSG	Arhgef1	ENSMUST00000065040;ENSMUST00000108671;ENSMUST00000151
ENSMUSG	Bpi	ENSMUST00000065039
ENSMUSG	Glis3	ENSMUST00000162022;ENSMUST00000159520;ENSMUST0000006
ENSMUSG	C130021	ENSMUST00000152118;ENSMUST00000147294
ENSMUSG	Cpvl	ENSMUST00000166545
ENSMUSG	Hrh1	ENSMUST00000088987;ENSMUST00000160212;ENSMUST00000161

ENSMUSG	Sv2b	ENSMUST00000165175;ENSMUST00000085164;ENSMUST00000206
ENSMUSG	Spink2	ENSMUST00000065216;ENSMUST00000121825
ENSMUSG	Gm6180	ENSMUST00000065243
ENSMUSG	Adh6a	ENSMUST00000013455
ENSMUSG	Clec12a	ENSMUST00000151671;ENSMUST00000147106;ENSMUST00000065
ENSMUSG	9230110	ENSMUST00000065291
ENSMUSG	Lins1	ENSMUST00000153007;ENSMUST00000121777;ENSMUST00000150
ENSMUSG	Myh7	ENSMUST00000102803;ENSMUST00000168485;ENSMUST00000149
ENSMUSG	Gpr141	ENSMUST00000065335;ENSMUST00000222664;ENSMUST00000222
ENSMUSG	E330013	ENSMUST00000065383;ENSMUST00000190409;ENSMUST00000190
ENSMUSG	Ptprt	ENSMUST00000109443;ENSMUST00000109445;ENSMUST00000109
ENSMUSG	Daw1	ENSMUST00000133079;ENSMUST00000113436;ENSMUST00000065
ENSMUSG	Cdh22	ENSMUST00000065438
ENSMUSG	Gm609	ENSMUST00000144346;ENSMUST00000114585
ENSMUSG	Spaca3	ENSMUST00000103223
ENSMUSG	Gm9898	ENSMUST00000065469
ENSMUSG	Cib4	ENSMUST00000065486
ENSMUSG	Prx	ENSMUST00000108355;ENSMUST00000125990;ENSMUST00000065
ENSMUSG	Kat2b-p	ENSMUST00000128468
ENSMUSG	Btn2a2	ENSMUST00000110433;ENSMUST00000110432
ENSMUSG	Tas2r13	ENSMUST00000065532
ENSMUSG	Dspp	ENSMUST00000112771
ENSMUSG	AI85470	ENSMUST00000095938;ENSMUST00000203088;ENSMUST00000154
ENSMUSG	Slamf8	ENSMUST00000065679;ENSMUST00000193843
ENSMUSG	Tarm1	ENSMUST00000203020;ENSMUST00000203821;ENSMUST00000065
ENSMUSG	Epp13	ENSMUST00000121024;ENSMUST00000128903
ENSMUSG	Rxfp2	ENSMUST00000143989;ENSMUST00000065745;ENSMUST00000110
ENSMUSG	Zfp952	ENSMUST00000129057;ENSMUST00000087666;ENSMUST00000141
ENSMUSG	Cacng8	ENSMUST00000182222;ENSMUST00000203617
ENSMUSG	Adamts1	ENSMUST00000093113;ENSMUST00000212665;ENSMUST00000213
ENSMUSG	Adamts1	ENSMUST00000052907
ENSMUSG	Hs6st3	ENSMUST00000065904
ENSMUSG	Tnfaip6	ENSMUST00000065927
ENSMUSG	Lgals7	ENSMUST00000138272;ENSMUST00000081457;ENSMUST00000128
ENSMUSG	A530021	ENSMUST00000208699
ENSMUSG	6430503	ENSMUST00000144411
ENSMUSG	Shisa7	ENSMUST00000066041;ENSMUST00000157068;ENSMUST00000132
ENSMUSG	Ebf4	ENSMUST00000110288;ENSMUST00000110286;ENSMUST00000134
ENSMUSG	Tanc2	ENSMUST00000168598;ENSMUST00000100330;ENSMUST00000124
ENSMUSG	Fate1	ENSMUST00000114569;ENSMUST00000137031
ENSMUSG	Notumos	ENSMUST00000151998
ENSMUSG	Gykl1	ENSMUST00000066193
ENSMUSG	Tll1	ENSMUST00000066166;ENSMUST00000209451
ENSMUSG	Krt42	ENSMUST00000107406;ENSMUST00000017270
ENSMUSG	Tgm5	ENSMUST00000028721;ENSMUST00000142674
ENSMUSG	BC04840	ENSMUST00000065600;ENSMUST00000136432
ENSMUSG	Klk1b26	ENSMUST00000048945
ENSMUSG	Gm5114	ENSMUST00000108017
ENSMUSG	Sox14	ENSMUST00000054819
ENSMUSG	Oas1f	ENSMUST00000057814
ENSMUSG	Rdh8	ENSMUST00000066387
ENSMUSG	1700016	ENSMUST00000066408
ENSMUSG	Lipg	ENSMUST00000066532
ENSMUSG	Adgrg4	ENSMUST00000153784
ENSMUSG	Gm9925	ENSMUST00000066583
ENSMUSG	Slc51b	ENSMUST00000065894
ENSMUSG	Slc39a8	ENSMUST00000081978;ENSMUST00000139953;ENSMUST00000180

ENSMUSG	Ang5	ENSMUST00000066719
ENSMUSG	Stum	ENSMUST00000136521;ENSMUST00000210737
ENSMUSG	Gm9930	ENSMUST00000066742
ENSMUSG	Tusc1	ENSMUST00000066774
ENSMUSG	Tceal5	ENSMUST00000066819
ENSMUSG	Olfr279	ENSMUST00000216822
ENSMUSG	Rdh19	ENSMUST00000077530
ENSMUSG	A930004	ENSMUST00000066163;ENSMUST00000151857
ENSMUSG	Gm9934	ENSMUST00000098303;ENSMUST00000191246
ENSMUSG	Skida1	ENSMUST00000173604;ENSMUST00000091420;ENSMUST0000006
ENSMUSG	Zdhhc25	ENSMUST00000066949
ENSMUSG	Umodl1	ENSMUST00000066981;ENSMUST00000114555
ENSMUSG	Adm2	ENSMUST00000066991
ENSMUSG	Olfr24	ENSMUST00000066997
ENSMUSG	Vmn1r23	ENSMUST00000066998
ENSMUSG	Gm4894	ENSMUST00000216428
ENSMUSG	Nkx2-4	ENSMUST00000067020
ENSMUSG	Spock3	ENSMUST00000117377;ENSMUST00000138398;ENSMUST0000011
ENSMUSG	Ceacam1	ENSMUST00000038069;ENSMUST00000206850;ENSMUST0000020
ENSMUSG	Klf1	ENSMUST00000067060
ENSMUSG	Cthrc1	ENSMUST00000067072;ENSMUST00000144800
ENSMUSG	Ffar4	ENSMUST00000067098
ENSMUSG	Ifi205	ENSMUST00000059226;ENSMUST00000192166
ENSMUSG	Gzmm	ENSMUST00000020549;ENSMUST00000151213
ENSMUSG	Sprr2k	ENSMUST00000067102
ENSMUSG	Fgfr3	ENSMUST00000067150;ENSMUST00000114411;ENSMUST0000016
ENSMUSG	Msi1	ENSMUST00000150779;ENSMUST00000151444;ENSMUST00000067
ENSMUSG	Gm5082	ENSMUST00000221920;ENSMUST00000221546;ENSMUST00000067
ENSMUSG	Serpinb	ENSMUST00000067198
ENSMUSG	Lrrc36	ENSMUST00000139900;ENSMUST00000213547;ENSMUST0000010
ENSMUSG	4930553	ENSMUST00000057837
ENSMUSG	Bsx	ENSMUST00000067375
ENSMUSG	Olfr411	ENSMUST00000214769
ENSMUSG	Tmem74	ENSMUST00000067469
ENSMUSG	Cyp3a44	ENSMUST00000067479
ENSMUSG	2900041	ENSMUST00000155384
ENSMUSG	Fabp1	ENSMUST00000067492
ENSMUSG	Gm9945	ENSMUST00000067523
ENSMUSG	Sytl5	ENSMUST00000067529
ENSMUSG	9430021	ENSMUST00000145985
ENSMUSG	Vsnl1	ENSMUST00000220506;ENSMUST00000072299
ENSMUSG	Thnsl2	ENSMUST00000160918;ENSMUST00000074241;ENSMUST0000017
ENSMUSG	Kcnn2	ENSMUST00000066890;ENSMUST00000184101
ENSMUSG	Gm14461	ENSMUST00000067618;ENSMUST00000166653
ENSMUSG	Olfr150	ENSMUST00000217079
ENSMUSG	Ugt1a6a	ENSMUST00000113134;ENSMUST00000124852;ENSMUST0000014
ENSMUSG	Usp17la	ENSMUST00000067695
ENSMUSG	Pla2r1	ENSMUST00000112525;ENSMUST00000126327;ENSMUST0000013
ENSMUSG	Gm9951	ENSMUST00000139529;ENSMUST00000149618
ENSMUSG	Xlr	ENSMUST00000067782;ENSMUST00000067763;ENSMUST00000114
ENSMUSG	Ugt2b5	ENSMUST00000067790
ENSMUSG	Mmrn1	ENSMUST00000145763;ENSMUST00000129603
ENSMUSG	Pm20d2	ENSMUST00000098181;ENSMUST00000119167
ENSMUSG	Ano9	ENSMUST00000067836;ENSMUST00000210496;ENSMUST00000211
ENSMUSG	Olfr63	ENSMUST00000217023;ENSMUST00000067840
ENSMUSG	Irs4	ENSMUST00000067841
ENSMUSG	5830411	ENSMUST00000210212;ENSMUST00000059882;ENSMUST00000164

ENSMUSG	Srsf12	ENSMUST00000067864;ENSMUST00000108159
ENSMUSG	Emcn	ENSMUST00000122064;ENSMUST00000128985;ENSMUST0000011[illegible]
ENSMUSG	1700013[illegible]	ENSMUST00000067940
ENSMUSG	Phactr1	ENSMUST00000128646;ENSMUST00000132050;ENSMUST0000014[illegible]
ENSMUSG	AU01809	ENSMUST00000205185;ENSMUST00000171749
ENSMUSG	Defb42	ENSMUST00000067990;ENSMUST00000142140;ENSMUST00000111
ENSMUSG	Mtnr1a	ENSMUST00000209488
ENSMUSG	Smim10l[illegible]	ENSMUST00000068106
ENSMUSG	Rnd1	ENSMUST00000109149;ENSMUST00000003451;ENSMUST0000012[illegible]
ENSMUSG	Fam19a5	ENSMUST00000068088
ENSMUSG	Tmem158	ENSMUST00000068140
ENSMUSG	Olfr153	ENSMUST00000217519;ENSMUST00000068163
ENSMUSG	Stfa3	ENSMUST00000068182
ENSMUSG	Zkscan4	ENSMUST00000062609;ENSMUST00000225845
ENSMUSG	Afp	ENSMUST00000042755;ENSMUST00000202209
ENSMUSG	Olfr134	ENSMUST00000215302;ENSMUST00000056144
ENSMUSG	5330416[illegible]	ENSMUST00000144993;ENSMUST00000123535
ENSMUSG	9130008[illegible]	ENSMUST00000068258
ENSMUSG	Nt5c1a	ENSMUST00000068262
ENSMUSG	Lmntd1	ENSMUST00000148739;ENSMUST00000111706;ENSMUST00000111
ENSMUSG	Nyap2	ENSMUST00000068275;ENSMUST00000113494;ENSMUST0000012[illegible]
ENSMUSG	Kbtbd13	ENSMUST00000068307
ENSMUSG	Sec14l3	ENSMUST00000068322
ENSMUSG	Agtr1b	ENSMUST00000068316
ENSMUSG	Cntn1	ENSMUST00000141187;ENSMUST00000169825;ENSMUST0000006[illegible]
ENSMUSG	Sprr2e	ENSMUST00000068399
ENSMUSG	Olfr420	ENSMUST00000213748
ENSMUSG	Gm11190	ENSMUST00000132487
ENSMUSG	Gm9962	ENSMUST00000186595
ENSMUSG	Gabra5	ENSMUST00000068456
ENSMUSG	Olfr354	ENSMUST00000217479
ENSMUSG	Zfp819	ENSMUST00000032661
ENSMUSG	Anxa13	ENSMUST00000068515
ENSMUSG	M5C1000	ENSMUST00000068526;ENSMUST00000196844
ENSMUSG	Cgrrf1	ENSMUST00000068532;ENSMUST00000140114;ENSMUST0000013[illegible]
ENSMUSG	9130017[illegible]	ENSMUST00000135670;ENSMUST00000068548
ENSMUSG	Gm4861	ENSMUST00000198629
ENSMUSG	Zfp78	ENSMUST00000207347;ENSMUST00000208763;ENSMUST0000020[illegible]
ENSMUSG	Ifng	ENSMUST00000068592;ENSMUST00000220309
ENSMUSG	Cstl1	ENSMUST00000109955
ENSMUSG	Klk15	ENSMUST00000068625;ENSMUST00000206955
ENSMUSG	Actbl2	ENSMUST00000054716
ENSMUSG	Fev	ENSMUST00000068631
ENSMUSG	Foxd2	ENSMUST00000068654
ENSMUSG	Pld5	ENSMUST00000065967;ENSMUST00000144340;ENSMUST0000015[illegible]
ENSMUSG	Ntrk2	ENSMUST00000224259;ENSMUST00000079828;ENSMUST00000224
ENSMUSG	Ctsj	ENSMUST00000224224
ENSMUSG	Adh7	ENSMUST00000090171;ENSMUST00000199865
ENSMUSG	Zfp93	ENSMUST00000108438;ENSMUST00000155851;ENSMUST00000032
ENSMUSG	Them7	ENSMUST00000068813;ENSMUST00000131060
ENSMUSG	Prl2c5	ENSMUST00000021778;ENSMUST00000151144
ENSMUSG	Slc6a2	ENSMUST00000072939
ENSMUSG	Fut9	ENSMUST00000084770
ENSMUSG	Rnf212	ENSMUST00000200159
ENSMUSG	4933427[illegible]	ENSMUST00000168837;ENSMUST00000068968
ENSMUSG	Map6	ENSMUST00000122101;ENSMUST00000207883;ENSMUST0000020[illegible]
ENSMUSG	Hottip	ENSMUST00000141300

ENSMUSG	Atp10b	ENSMUST00000148911;ENSMUST00000077659;ENSMUST00000137
ENSMUSG	Fam136b	ENSMUST00000068987
ENSMUSG	Gm9972	ENSMUST00000069051
ENSMUSG	Alk	ENSMUST00000086639
ENSMUSG	Ano5	ENSMUST00000043944
ENSMUSG	Epm2a	ENSMUST00000069106;ENSMUST00000161438
ENSMUSG	Gm14168	ENSMUST00000144818
ENSMUSG	Gucy2g	ENSMUST00000069183
ENSMUSG	Epha6	ENSMUST00000068860
ENSMUSG	4930502	ENSMUST00000069209
ENSMUSG	Spink5	ENSMUST00000069245
ENSMUSG	Unc80	ENSMUST00000061620
ENSMUSG	Hba-x	ENSMUST00000020531
ENSMUSG	Olfr307	ENSMUST00000217494;ENSMUST00000069279
ENSMUSG	B4galnt	ENSMUST00000048002;ENSMUST00000210203;ENSMUST0000020[illegible]
ENSMUSG	Hmcn2	ENSMUST00000113532;ENSMUST00000138821;ENSMUST00000142
ENSMUSG	Dach1	ENSMUST00000069334;ENSMUST00000071533;ENSMUST0000015[illegible]
ENSMUSG	Ubqln5	ENSMUST00000053743
ENSMUSG	Gm9978	ENSMUST00000069431
ENSMUSG	Ces2a	ENSMUST00000034346;ENSMUST00000164182;ENSMUST0000015[illegible]
ENSMUSG	Nap1l3	ENSMUST00000079490
ENSMUSG	Gsdmc4	ENSMUST00000188108;ENSMUST00000063530
ENSMUSG	Rybp-ps	ENSMUST00000069489
ENSMUSG	Gm7936	ENSMUST00000069529
ENSMUSG	Myh8	ENSMUST00000019625;ENSMUST00000108685;ENSMUST0000013[illegible]
ENSMUSG	Abcd2	ENSMUST00000069511
ENSMUSG	Dnaaf3	ENSMUST00000094897
ENSMUSG	Cldn17	ENSMUST00000069549
ENSMUSG	Olfr967	ENSMUST00000213358
ENSMUSG	Gsdmc3	ENSMUST00000185526;ENSMUST00000190682;ENSMUST0000008[illegible]
ENSMUSG	Foxi3	ENSMUST00000163089
ENSMUSG	Abhd16b	ENSMUST00000069649
ENSMUSG	Ubl4b	ENSMUST00000052853
ENSMUSG	Oosp2	ENSMUST00000121793
ENSMUSG	Krt28	ENSMUST00000006963
ENSMUSG	E130018	ENSMUST00000069741
ENSMUSG	BC05107	ENSMUST00000199477
ENSMUSG	Triqk	ENSMUST00000143186;ENSMUST00000183345
ENSMUSG	2810407	ENSMUST00000069809;ENSMUST00000189929
ENSMUSG	Cldn23	ENSMUST00000060128
ENSMUSG	Fut2	ENSMUST00000069800;ENSMUST00000210620;ENSMUST00000211
ENSMUSG	Nod2	ENSMUST00000054324;ENSMUST00000118370;ENSMUST00000124
ENSMUSG	9330182	ENSMUST00000152095;ENSMUST00000131626;ENSMUST0000006[illegible]
ENSMUSG	Gm11541	ENSMUST00000069852
ENSMUSG	Ccdc7b	ENSMUST00000148234;ENSMUST00000026912;ENSMUST0000010[illegible]
ENSMUSG	Gm9989	ENSMUST00000069880
ENSMUSG	9330154	ENSMUST00000185960;ENSMUST00000187891
ENSMUSG	Cyp3a11	ENSMUST00000035918;ENSMUST00000199857
ENSMUSG	Rgs9bp	ENSMUST00000069912
ENSMUSG	Gata5os	ENSMUST00000069943
ENSMUSG	Grik2	ENSMUST00000218823;ENSMUST00000217673;ENSMUST0000021[illegible]
ENSMUSG	Lipm	ENSMUST00000025685
ENSMUSG	Gm5468	ENSMUST00000069992
ENSMUSG	St3gal5	ENSMUST00000069994;ENSMUST00000114112;ENSMUST0000018[illegible]
ENSMUSG	B4galt6	ENSMUST00000070080
ENSMUSG	Gm9991	ENSMUST00000070048
ENSMUSG	Gm9992	ENSMUST00000070059

ENSMUSG	Car10	ENSMUST00000107861;ENSMUST00000092780
ENSMUSG	Cndp1	ENSMUST00000070139;ENSMUST00000130579
ENSMUSG	Olfr283	ENSMUST00000057386
ENSMUSG	Lrguk	ENSMUST00000070189;ENSMUST00000101564
ENSMUSG	Tmem229	ENSMUST00000168554
ENSMUSG	Pla2g4a	ENSMUST00000070200;ENSMUST00000111926;ENSMUST0000019C
ENSMUSG	Spock1	ENSMUST00000189373;ENSMUST00000185502;ENSMUST0000018€
ENSMUSG	Prr9	ENSMUST00000070284
ENSMUSG	Lman1l	ENSMUST00000044937;ENSMUST00000093832
ENSMUSG	Gsdmc2	ENSMUST00000089900;ENSMUST00000188404;ENSMUST0000018ɛ
ENSMUSG	Synpr	ENSMUST00000223583;ENSMUST00000145700;ENSMUST00000147
ENSMUSG	Sertm1	ENSMUST00000070342
ENSMUSG	Myh1	ENSMUST00000124516;ENSMUST00000018637;ENSMUST0000012S
ENSMUSG	D830013	ENSMUST00000221180
ENSMUSG	Actr3b	ENSMUST00000128727;ENSMUST00000088244;ENSMUST0000019ɛ
ENSMUSG	Taar1	ENSMUST00000051532
ENSMUSG	Gpr50	ENSMUST00000070449
ENSMUSG	Prss34	ENSMUST00000037453
ENSMUSG	Adap1	ENSMUST00000151450;ENSMUST00000110865;ENSMUST0000019€
ENSMUSG	Uts2b	ENSMUST00000070531
ENSMUSG	Nat3	ENSMUST00000070514
ENSMUSG	Cyct	ENSMUST00000028430
ENSMUSG	Chn1	ENSMUST00000112024;ENSMUST00000180045;ENSMUST00000102
ENSMUSG	Cngb3	ENSMUST00000102999
ENSMUSG	Ptprn2	ENSMUST00000185505;ENSMUST00000189009;ENSMUST0000019C
ENSMUSG	Trnp1	ENSMUST00000125541
ENSMUSG	Olfr90	ENSMUST00000216488;ENSMUST00000070808
ENSMUSG	Krt72	ENSMUST00000071104
ENSMUSG	Dsg3	ENSMUST00000070892
ENSMUSG	Chst13	ENSMUST00000070890
ENSMUSG	Hoxb8	ENSMUST00000052650
ENSMUSG	Apol8	ENSMUST00000089450;ENSMUST00000070911
ENSMUSG	Gpr173	ENSMUST00000070316;ENSMUST00000137840
ENSMUSG	Olfr135	ENSMUST00000070985
ENSMUSG	Gm5533	ENSMUST00000070987
ENSMUSG	Grm7	ENSMUST00000174018;ENSMUST00000071076;ENSMUST00000172
ENSMUSG	Hmga2	ENSMUST00000072777;ENSMUST00000159699;ENSMUST0000015S
ENSMUSG	Gm10010	ENSMUST00000071101
ENSMUSG	Rps6-ps	ENSMUST00000079841
ENSMUSG	Rpl21-p	ENSMUST00000072532
ENSMUSG	1700028	ENSMUST00000178540
ENSMUSG	Olfr166	ENSMUST00000216465;ENSMUST00000074739
ENSMUSG	Zfp663	ENSMUST00000103085;ENSMUST00000136482
ENSMUSG	Foxb2	ENSMUST00000072915
ENSMUSG	Ttc26	ENSMUST00000162554;ENSMUST00000161751;ENSMUST0000015S
ENSMUSG	Gm16415	ENSMUST00000080097
ENSMUSG	Olfr765	ENSMUST00000216460
ENSMUSG	Jakmip3	ENSMUST00000171843
ENSMUSG	Gulp1	ENSMUST00000074525;ENSMUST00000160854;ENSMUST00000161
ENSMUSG	Olfr533	ENSMUST00000216258
ENSMUSG	Gm4559	ENSMUST00000080669
ENSMUSG	Usp13	ENSMUST00000072312
ENSMUSG	Tas2r10	ENSMUST00000069268
ENSMUSG	Vmn2r10	ENSMUST00000042090
ENSMUSG	Olfr394	ENSMUST00000071478
ENSMUSG	Tas2r11	ENSMUST00000079035
ENSMUSG	Mab21l1	ENSMUST00000075422

ENSMUSG	Olfr315	ENSMUST00000203256;ENSMUST00000081533
ENSMUSG	Gjc3	ENSMUST00000077119;ENSMUST00000199083
ENSMUSG	Magel2	ENSMUST00000080403
ENSMUSG	Fam71d	ENSMUST00000077968;ENSMUST00000218697
ENSMUSG	Nxf3	ENSMUST00000080929
ENSMUSG	Myh4	ENSMUST00000018632
ENSMUSG	Vmn2r-p	ENSMUST00000074107
ENSMUSG	1700010	ENSMUST00000073081
ENSMUSG	Slc35f3	ENSMUST00000108759
ENSMUSG	Fam47e	ENSMUST00000146417;ENSMUST00000176621;ENSMUST00000131
ENSMUSG	Ces1g	ENSMUST00000044602
ENSMUSG	Fxyd3	ENSMUST00000167369;ENSMUST00000169313;ENSMUST00000165
ENSMUSG	AF36626	ENSMUST00000071308
ENSMUSG	Scimp	ENSMUST00000108534
ENSMUSG	Olfr130	ENSMUST00000207590
ENSMUSG	Gm6054	ENSMUST00000078052
ENSMUSG	Gm16372	ENSMUST00000073088
ENSMUSG	Rfx8	ENSMUST00000151913
ENSMUSG	Scn3a	ENSMUST00000066432;ENSMUST00000100069;ENSMUST00000155
ENSMUSG	Ceacam1	ENSMUST00000081703;ENSMUST00000108488
ENSMUSG	BC05114	ENSMUST00000114175;ENSMUST00000149121;ENSMUST00000139
ENSMUSG	Musk	ENSMUST00000081919;ENSMUST00000177951;ENSMUST00000084
ENSMUSG	Gm15732	ENSMUST00000079794
ENSMUSG	Chst3	ENSMUST00000135158;ENSMUST00000068690;ENSMUST00000167
ENSMUSG	Apol9a	ENSMUST00000089452;ENSMUST00000081776
ENSMUSG	Tas2r12	ENSMUST00000071696
ENSMUSG	Ces1c	ENSMUST00000034189
ENSMUSG	Cldn34b	ENSMUST00000076671
ENSMUSG	Olfr910	ENSMUST00000215191
ENSMUSG	Gm6177	ENSMUST00000085589
ENSMUSG	Olfr150	ENSMUST00000206520
ENSMUSG	Vmn1r48	ENSMUST00000073415
ENSMUSG	Trim30d	ENSMUST00000071069;ENSMUST00000033211;ENSMUST00000141
ENSMUSG	Lmcd1	ENSMUST00000032376
ENSMUSG	Lce1a1	ENSMUST00000074142
ENSMUSG	Ldoc1	ENSMUST00000075983
ENSMUSG	Olfr328	ENSMUST00000216725;ENSMUST00000073933
ENSMUSG	Gm11938	ENSMUST00000072306
ENSMUSG	Rpl30-p	ENSMUST00000119767
ENSMUSG	Tas2r13	ENSMUST00000082085
ENSMUSG	9630041	ENSMUST00000112841
ENSMUSG	Tmem178	ENSMUST00000180886;ENSMUST00000061740;ENSMUST00000180
ENSMUSG	Sh3rf2	ENSMUST00000072008;ENSMUST00000074679;ENSMUST00000128
ENSMUSG	Lepr	ENSMUST00000102777;ENSMUST00000106921;ENSMUST00000037
ENSMUSG	Krt33b	ENSMUST00000073890
ENSMUSG	Serpinb	ENSMUST00000081927
ENSMUSG	Tas2r10	ENSMUST00000076756
ENSMUSG	Ankrd29	ENSMUST00000118525
ENSMUSG	Gm10030	ENSMUST00000197321;ENSMUST00000197279
ENSMUSG	1700007	ENSMUST00000080333;ENSMUST00000197724;ENSMUST00000197
ENSMUSG	Olfr144	ENSMUST00000216805;ENSMUST00000081236;ENSMUST00000207
ENSMUSG	Gm5278	ENSMUST00000055957
ENSMUSG	Zfp105	ENSMUST00000051667;ENSMUST00000148851
ENSMUSG	Adgrf2	ENSMUST00000113614
ENSMUSG	Gm10032	ENSMUST00000071522;ENSMUST00000225384
ENSMUSG	Fgf18	ENSMUST00000020507
ENSMUSG	Skint11	ENSMUST00000164297

ENSMUSG	Olfr541	ENSMUST00000210357;ENSMUST00000080681
ENSMUSG	Tdpoz3	ENSMUST00000081780
ENSMUSG	Serpinb	ENSMUST00000023861
ENSMUSG	Ces5a	ENSMUST00000212009;ENSMUST00000080391;ENSMUST00000212
ENSMUSG	Adtrp	ENSMUST00000072012;ENSMUST00000121404;ENSMUST0000022C
ENSMUSG	Zscan5b	ENSMUST00000072662
ENSMUSG	Vmn1r23	ENSMUST00000077301
ENSMUSG	Trim31	ENSMUST00000078438
ENSMUSG	Gm11189	ENSMUST00000117979
ENSMUSG	Olfr127	ENSMUST00000076331
ENSMUSG	H2-M10.	ENSMUST00000073546
ENSMUSG	Chsy3	ENSMUST00000080721
ENSMUSG	Sez6l	ENSMUST00000079491;ENSMUST00000212480;ENSMUST00000075
ENSMUSG	Gm5407	ENSMUST00000080694
ENSMUSG	Olfr66	ENSMUST00000079117
ENSMUSG	Olfr506	ENSMUST00000209296
ENSMUSG	Gm10037	ENSMUST00000221266;ENSMUST00000222626
ENSMUSG	Tas2r13	ENSMUST00000076565
ENSMUSG	Tcp11x2	ENSMUST00000078605
ENSMUSG	Olfr412	ENSMUST00000216362
ENSMUSG	Gm12253	ENSMUST00000108825
ENSMUSG	Tas2r11	ENSMUST00000068302
ENSMUSG	Krt6a	ENSMUST00000023788
ENSMUSG	Qrfpr	ENSMUST00000197447;ENSMUST00000091227;ENSMUST00000197
ENSMUSG	Zfp420	ENSMUST00000074876;ENSMUST00000148489;ENSMUST00000145
ENSMUSG	Cxcl2	ENSMUST00000200681;ENSMUST00000202317;ENSMUST0000020C
ENSMUSG	Btnl4	ENSMUST00000065841
ENSMUSG	Gm5471	ENSMUST00000072732
ENSMUSG	Kl	ENSMUST00000078856;ENSMUST00000202096
ENSMUSG	Olfr869	ENSMUST00000212969
ENSMUSG	Pip	ENSMUST00000203244;ENSMUST00000204575
ENSMUSG	Gm10042	ENSMUST00000079884
ENSMUSG	Mup5	ENSMUST00000082287
ENSMUSG	Gpc6	ENSMUST00000078849;ENSMUST00000123239;ENSMUST00000125
ENSMUSG	Vmn1r21	ENSMUST00000081186
ENSMUSG	Cyp2d41	ENSMUST00000180453
ENSMUSG	AY76118	ENSMUST00000071886
ENSMUSG	Adra2b	ENSMUST00000071902
ENSMUSG	Gm5265	ENSMUST00000076828
ENSMUSG	En1	ENSMUST00000079721
ENSMUSG	Nkx2-9	ENSMUST00000072631
ENSMUSG	Gm4763	ENSMUST00000205376;ENSMUST00000081657
ENSMUSG	Cd300c	ENSMUST00000106580;ENSMUST00000092466;ENSMUST00000061
ENSMUSG	Kcnt1	ENSMUST00000150788;ENSMUST00000131529;ENSMUST00000128
ENSMUSG	Prr19	ENSMUST00000080288
ENSMUSG	Gm9174	ENSMUST00000078149
ENSMUSG	Osm	ENSMUST00000131764;ENSMUST00000075221
ENSMUSG	Nfe2	ENSMUST00000075192;ENSMUST00000149111;ENSMUST00000134
ENSMUSG	Olfr97	ENSMUST00000213328;ENSMUST00000207414
ENSMUSG	Col13a1	ENSMUST00000105451;ENSMUST00000105452;ENSMUST00000105
ENSMUSG	Ppp1r2-	ENSMUST00000078012
ENSMUSG	Olfr952	ENSMUST00000073895
ENSMUSG	Gm4832	ENSMUST00000182679
ENSMUSG	Gm5582	ENSMUST00000075251
ENSMUSG	Olfr102	ENSMUST00000213474
ENSMUSG	Col25a1	ENSMUST00000143830;ENSMUST00000183368;ENSMUST00000106
ENSMUSG	C1qtnf3	ENSMUST00000022853;ENSMUST00000110523

ENSMUSG	Vmn1r6	ENSMUST00000079669
ENSMUSG	Gm2174	ENSMUST00000079663
ENSMUSG	Tex48	ENSMUST00000102861
ENSMUSG	Cfi	ENSMUST00000077918
ENSMUSG	Kcnc1	ENSMUST00000025202;ENSMUST00000160433;ENSMUST0000016C
ENSMUSG	Usp17lc	ENSMUST00000106892
ENSMUSG	Olfr140	ENSMUST00000201132;ENSMUST00000072395
ENSMUSG	Grin2a	ENSMUST00000199708;ENSMUST00000197242
ENSMUSG	Kcp	ENSMUST00000159482;ENSMUST00000159479;ENSMUST00000091
ENSMUSG	Klk1b9	ENSMUST00000081399
ENSMUSG	Olfr15	ENSMUST00000214590
ENSMUSG	Frem1	ENSMUST00000170248;ENSMUST00000107230;ENSMUST0000013C
ENSMUSG	Fcgr4	ENSMUST00000078825
ENSMUSG	Olfr961	ENSMUST00000076548
ENSMUSG	Ifitm6	ENSMUST00000209328;ENSMUST00000081924
ENSMUSG	Lrrc74a	ENSMUST00000223197
ENSMUSG	Olfr539	ENSMUST00000218865
ENSMUSG	Zfp945	ENSMUST00000160457;ENSMUST00000095615;ENSMUST0000008ɛ
ENSMUSG	Krt40	ENSMUST00000074253
ENSMUSG	Pde1a	ENSMUST00000102655;ENSMUST00000102653;ENSMUST0000009C
ENSMUSG	Lep	ENSMUST00000069789
ENSMUSG	Ppp1r2-:	ENSMUST00000112182
ENSMUSG	Gm10062	ENSMUST00000196473;ENSMUST00000072180
ENSMUSG	Tpt1-ps	ENSMUST00000112150
ENSMUSG	Olfr224	ENSMUST00000217009;ENSMUST00000081102
ENSMUSG	Vpreb2	ENSMUST00000075371
ENSMUSG	Olfr295	ENSMUST00000215365
ENSMUSG	Hopx	ENSMUST00000120827;ENSMUST00000113453;ENSMUST00000081
ENSMUSG	Zfp36l3	ENSMUST00000071711
ENSMUSG	Aldoart	ENSMUST00000078617
ENSMUSG	Olfr427	ENSMUST00000080831
ENSMUSG	Gfral	ENSMUST00000074880
ENSMUSG	Nkapl	ENSMUST00000068235
ENSMUSG	Mamld1	ENSMUST00000114629;ENSMUST00000082088
ENSMUSG	Mrgprh	ENSMUST00000075296
ENSMUSG	Tas2r12	ENSMUST00000076119
ENSMUSG	Olfr434	ENSMUST00000076752
ENSMUSG	Olfr365	ENSMUST00000218602
ENSMUSG	Actg2	ENSMUST00000075161;ENSMUST00000205926;ENSMUST00000141
ENSMUSG	Gckr	ENSMUST00000201166;ENSMUST00000202909;ENSMUST00000202
ENSMUSG	Plac8l1	ENSMUST00000081374
ENSMUSG	Olfr286	ENSMUST00000205305
ENSMUSG	Spag11b	ENSMUST00000110767
ENSMUSG	Olfr979	ENSMUST00000080835
ENSMUSG	Plg	ENSMUST00000014578
ENSMUSG	Olfr314	ENSMUST00000205189;ENSMUST00000076393
ENSMUSG	Fbxw26	ENSMUST00000071917
ENSMUSG	Ube2nl	ENSMUST00000085228
ENSMUSG	Rnase2b	ENSMUST00000075648
ENSMUSG	Olfr222	ENSMUST00000214351
ENSMUSG	Olfr39	ENSMUST00000212983
ENSMUSG	Sohlh1	ENSMUST00000076989
ENSMUSG	Krtap8-	ENSMUST00000072280
ENSMUSG	Clec4a4	ENSMUST00000079379
ENSMUSG	Gm7361	ENSMUST00000074148
ENSMUSG	Rnase11	ENSMUST00000076106
ENSMUSG	Reg1	ENSMUST00000204687

ENSMUSG	Stfa2l1	ENSMUST00000079184
ENSMUSG	Krt4	ENSMUST00000023797
ENSMUSG	Olfr138	ENSMUST00000214948
ENSMUSG	Kcnh7	ENSMUST00000075052;ENSMUST00000112454;ENSMUST00000131
ENSMUSG	Olfr767	ENSMUST00000213579
ENSMUSG	Gm16380	ENSMUST00000077997
ENSMUSG	Kprp	ENSMUST00000072363
ENSMUSG	Rpl13-p	ENSMUST00000079960
ENSMUSG	Hydin	ENSMUST00000043141;ENSMUST00000212218;ENSMUST00000212
ENSMUSG	Ntng1	ENSMUST00000156177;ENSMUST00000133268;ENSMUST00000072
ENSMUSG	Olfr139	ENSMUST00000213323;ENSMUST00000217626;ENSMUST00000078
ENSMUSG	Olfr960	ENSMUST00000077060
ENSMUSG	Olfr150	ENSMUST00000206062;ENSMUST00000073571;ENSMUST00000206
ENSMUSG	Tsks	ENSMUST00000120929
ENSMUSG	Tmem40	ENSMUST00000112946;ENSMUST00000127728;ENSMUST00000151
ENSMUSG	Adamts1	ENSMUST00000092486;ENSMUST00000120336
ENSMUSG	Mug1	ENSMUST00000032228
ENSMUSG	Olfr126	ENSMUST00000080311
ENSMUSG	Rpl21-p	ENSMUST00000074828
ENSMUSG	Prh1	ENSMUST00000177523;ENSMUST00000111972
ENSMUSG	Serpinb	ENSMUST00000081277;ENSMUST00000126812
ENSMUSG	Hspa2	ENSMUST00000080449;ENSMUST00000219555
ENSMUSG	Ntm	ENSMUST00000075069;ENSMUST00000155308;ENSMUST00000124
ENSMUSG	Nptx2	ENSMUST00000071782
ENSMUSG	Fcrl1	ENSMUST00000163661;ENSMUST00000072480;ENSMUST00000167
ENSMUSG	Gm10073	ENSMUST00000073722
ENSMUSG	Vmn1r21	ENSMUST00000076897
ENSMUSG	Olfr317	ENSMUST00000075607
ENSMUSG	Tmem26	ENSMUST00000219392;ENSMUST00000080995;ENSMUST00000218
ENSMUSG	Olfr235	ENSMUST00000214638;ENSMUST00000073507
ENSMUSG	Olfr60	ENSMUST00000215023;ENSMUST00000211031;ENSMUST00000216
ENSMUSG	Gm2710	ENSMUST00000073196
ENSMUSG	Klk1b7-:	ENSMUST00000183762
ENSMUSG	Kif27	ENSMUST00000225388;ENSMUST00000225680
ENSMUSG	Klk1b22	ENSMUST00000077528
ENSMUSG	Myh13	ENSMUST00000108684
ENSMUSG	Lrrc10	ENSMUST00000073834
ENSMUSG	Olfr57	ENSMUST00000082244
ENSMUSG	Defa17	ENSMUST00000079528
ENSMUSG	Pyroxd2	ENSMUST00000076505
ENSMUSG	Scrt2	ENSMUST00000064061
ENSMUSG	Nrg2	ENSMUST00000115713;ENSMUST00000115712;ENSMUST00000115
ENSMUSG	Sp7	ENSMUST00000078508
ENSMUSG	Mucl1	ENSMUST00000078842
ENSMUSG	Tmc2	ENSMUST00000077988
ENSMUSG	Caln1	ENSMUST00000111288
ENSMUSG	Chst8	ENSMUST00000078686;ENSMUST00000154629;ENSMUST00000135
ENSMUSG	Olfr136	ENSMUST00000213922
ENSMUSG	Tas2r12	ENSMUST00000076150
ENSMUSG	Gm839	ENSMUST00000079186
ENSMUSG	Pantr1	ENSMUST00000181725;ENSMUST00000181593;ENSMUST00000078
ENSMUSG	Sycp2	ENSMUST00000081134
ENSMUSG	Gm10080	ENSMUST00000075585
ENSMUSG	Samd5	ENSMUST00000100070;ENSMUST00000221079
ENSMUSG	Olfr715	ENSMUST00000214919
ENSMUSG	Nlrp9b	ENSMUST00000073151
ENSMUSG	0610040	ENSMUST00000081747;ENSMUST00000196575;ENSMUST00000196

ENSMUSG	Olfr726	ENSMUST00000213345
ENSMUSG	A930017I	ENSMUST00000081361
ENSMUSG	Dcc	ENSMUST00000114943;ENSMUST00000126030
ENSMUSG	Olfr141	ENSMUST00000214796;ENSMUST00000079875
ENSMUSG	Ces4a	ENSMUST00000161289
ENSMUSG	Gm5591	ENSMUST00000079759
ENSMUSG	Rpl21-p	ENSMUST00000080223
ENSMUSG	Olfr881	ENSMUST00000212489;ENSMUST00000074611
ENSMUSG	Layn	ENSMUST00000217212;ENSMUST00000098782;ENSMUST00000214
ENSMUSG	Cyp2c70	ENSMUST00000051846
ENSMUSG	Ang4	ENSMUST00000073860
ENSMUSG	Gm7099	ENSMUST00000080066
ENSMUSG	Olfr175	ENSMUST00000215069;ENSMUST00000079955
ENSMUSG	1700019	ENSMUST00000190748
ENSMUSG	Vmn1r47	ENSMUST00000074881
ENSMUSG	Rxfp3	ENSMUST00000058007
ENSMUSG	Olfr464	ENSMUST00000217112;ENSMUST00000215062;ENSMUST00000074
ENSMUSG	Intu	ENSMUST00000061590;ENSMUST00000091186;ENSMUST00000204
ENSMUSG	B9d1os	ENSMUST00000137010
ENSMUSG	Gm10088	ENSMUST00000074116
ENSMUSG	Ctnna3	ENSMUST00000105441;ENSMUST00000105440;ENSMUST00000133
ENSMUSG	Tcl1b2	ENSMUST00000146174
ENSMUSG	Olfr142	ENSMUST00000217281;ENSMUST00000072784
ENSMUSG	Kcnd2	ENSMUST00000081542
ENSMUSG	Trim55	ENSMUST00000029139
ENSMUSG	Dmkn	ENSMUST00000165887;ENSMUST00000054427;ENSMUST00000188
ENSMUSG	Irx1	ENSMUST00000077337;ENSMUST00000223379
ENSMUSG	Olfr530	ENSMUST00000216727;ENSMUST00000074177
ENSMUSG	Galnt13	ENSMUST00000112634
ENSMUSG	Mkx	ENSMUST00000176586;ENSMUST00000079788;ENSMUST00000188
ENSMUSG	Cyp4f39	ENSMUST00000003413
ENSMUSG	Spink12	ENSMUST00000081271
ENSMUSG	Olfr160	ENSMUST00000104875
ENSMUSG	Slc38a1	ENSMUST00000127623;ENSMUST00000112420;ENSMUST00000152
ENSMUSG	Tmprss1	ENSMUST00000059424
ENSMUSG	Olfr47	ENSMUST00000078057
ENSMUSG	Gm14173	ENSMUST00000117690
ENSMUSG	Dnaic1	ENSMUST00000102963;ENSMUST00000143198
ENSMUSG	Olfr116	ENSMUST00000216128;ENSMUST00000072265
ENSMUSG	Olfr116	ENSMUST00000215996
ENSMUSG	Nuggc	ENSMUST00000150897
ENSMUSG	Olfr168	ENSMUST00000213480
ENSMUSG	Csn1s2b	ENSMUST00000072539;ENSMUST00000200098
ENSMUSG	Krt79	ENSMUST00000023799
ENSMUSG	Tmem151	ENSMUST00000143677;ENSMUST00000077066;ENSMUST00000136
ENSMUSG	Rpl27a-	ENSMUST00000075382
ENSMUSG	Olfr525	ENSMUST00000214594;ENSMUST00000078103
ENSMUSG	Ube2d4	ENSMUST00000075109
ENSMUSG	Gm10101	ENSMUST00000181311;ENSMUST00000074862
ENSMUSG	Sox21	ENSMUST00000170662
ENSMUSG	Tmem236	ENSMUST00000077517
ENSMUSG	C1qtnf7	ENSMUST00000076939;ENSMUST00000144558;ENSMUST00000121
ENSMUSG	Zfp229	ENSMUST00000182334;ENSMUST00000182923;ENSMUST00000065
ENSMUSG	Ksr2	ENSMUST00000073347;ENSMUST00000180430
ENSMUSG	Lyg2	ENSMUST00000078307
ENSMUSG	Pclo	ENSMUST00000182407;ENSMUST00000030691;ENSMUST00000182
ENSMUSG	Olfr12	ENSMUST00000213247;ENSMUST00000081274

ENSMUSG	Vma21-p	ENSMUST00000114578
ENSMUSG	Rpl34-p	ENSMUST00000081679
ENSMUSG	Btnl7-p	ENSMUST00000023469
ENSMUSG	Slc22a1	ENSMUST00000113451
ENSMUSG	Tac1	ENSMUST00000185187;ENSMUST00000090679;ENSMUST00000184
ENSMUSG	Olfr120	ENSMUST00000213283
ENSMUSG	Armc4	ENSMUST00000081275
ENSMUSG	Rufy4	ENSMUST00000080167;ENSMUST00000127134
ENSMUSG	Myl1	ENSMUST00000186202;ENSMUST00000027151;ENSMUST0000011
ENSMUSG	Ces2c	ENSMUST00000055052;ENSMUST00000162970
ENSMUSG	Defb39	ENSMUST00000075504
ENSMUSG	BC04867	ENSMUST00000073406;ENSMUST00000144156
ENSMUSG	Myt1l	ENSMUST00000049784;ENSMUST00000219899;ENSMUST00000217
ENSMUSG	Dsg1b	ENSMUST00000076737
ENSMUSG	Csn1s2a	ENSMUST00000076379;ENSMUST00000200210;ENSMUST00000197
ENSMUSG	Serpina	ENSMUST00000044231
ENSMUSG	Gm14851	ENSMUST00000077452
ENSMUSG	Ces1e	ENSMUST00000034173;ENSMUST00000175983
ENSMUSG	Cldn24	ENSMUST00000079639
ENSMUSG	Tas2r10	ENSMUST00000072404
ENSMUSG	Rpl10a-	ENSMUST00000071184
ENSMUSG	Zfp13	ENSMUST00000115516;ENSMUST00000057029
ENSMUSG	Abca14	ENSMUST00000143257;ENSMUST00000084640
ENSMUSG	Irgc1	ENSMUST00000080594;ENSMUST00000205776;ENSMUST0000020
ENSMUSG	4932415	ENSMUST00000184645;ENSMUST00000077153
ENSMUSG	Obp2a	ENSMUST00000077667
ENSMUSG	Slc2a7	ENSMUST00000059893
ENSMUSG	Cd200r4	ENSMUST00000176819;ENSMUST00000176321;ENSMUST00000114
ENSMUSG	Rpl13a-	ENSMUST00000071866
ENSMUSG	Zfp119b	ENSMUST00000056147
ENSMUSG	Olfr149	ENSMUST00000082027
ENSMUSG	Olfr20	ENSMUST00000108465
ENSMUSG	Ear6	ENSMUST00000169070;ENSMUST00000074477
ENSMUSG	Rpl21-p	ENSMUST00000080279
ENSMUSG	Tex33	ENSMUST00000074380
ENSMUSG	Ifnlr1	ENSMUST00000074408
ENSMUSG	Mageb1	ENSMUST00000072269
ENSMUSG	Ppef1	ENSMUST00000071204;ENSMUST00000136888
ENSMUSG	Fmr1nb	ENSMUST00000071848
ENSMUSG	Ces3b	ENSMUST00000093221
ENSMUSG	Hs6st2	ENSMUST00000088172;ENSMUST00000114871;ENSMUST00000142
ENSMUSG	Erbb4	ENSMUST00000121473;ENSMUST00000153432
ENSMUSG	Gm5341	ENSMUST00000058881
ENSMUSG	Olfr170	ENSMUST00000218837
ENSMUSG	Opcml	ENSMUST00000134002;ENSMUST00000115243;ENSMUST00000214
ENSMUSG	Fbxw24	ENSMUST00000073962
ENSMUSG	Gm10237	ENSMUST00000078393
ENSMUSG	Gm10113	ENSMUST00000072437
ENSMUSG	Trank1	ENSMUST00000078626;ENSMUST00000200151
ENSMUSG	Rpp25	ENSMUST00000080514
ENSMUSG	Glrp1	ENSMUST00000067625
ENSMUSG	T	ENSMUST00000074667;ENSMUST00000136922
ENSMUSG	Cytl1	ENSMUST00000073554;ENSMUST00000201048
ENSMUSG	Serpinb	ENSMUST00000071873
ENSMUSG	Serpinb	ENSMUST00000009356;ENSMUST00000064916;ENSMUST00000143
ENSMUSG	Gm4968	ENSMUST00000071458
ENSMUSG	Usp17lb	ENSMUST00000106814;ENSMUST00000076501

ENSMUSG	Otof	ENSMUST00000114747;ENSMUST00000074171;ENSMUST00000144
ENSMUSG	4932435	ENSMUST00000148405
ENSMUSG	Dgkk	ENSMUST00000067410;ENSMUST00000145302
ENSMUSG	Hsd3b3	ENSMUST00000107019
ENSMUSG	Olfr304	ENSMUST00000077210
ENSMUSG	Cyp26c1	ENSMUST00000073391
ENSMUSG	Ap3b2	ENSMUST00000147624;ENSMUST00000082090;ENSMUST00000152
ENSMUSG	Cyp4f37	ENSMUST00000077639
ENSMUSG	Gm4945	ENSMUST00000075152
ENSMUSG	4930431	ENSMUST00000199309
ENSMUSG	Ncr1	ENSMUST00000006792;ENSMUST00000126417
ENSMUSG	Tas2r10	ENSMUST00000067539
ENSMUSG	Syt9	ENSMUST00000130414;ENSMUST00000073459;ENSMUST00000144
ENSMUSG	Gsdmcl1	ENSMUST00000071847
ENSMUSG	Olfr709	ENSMUST00000215468;ENSMUST00000072368
ENSMUSG	Gm10118	ENSMUST00000079279
ENSMUSG	Cys1	ENSMUST00000156453;ENSMUST00000074163;ENSMUST00000186
ENSMUSG	Cyp2c67	ENSMUST00000067328
ENSMUSG	Olfr114	ENSMUST00000216249;ENSMUST00000076914
ENSMUSG	Btnl1	ENSMUST00000080254
ENSMUSG	Ncs1	ENSMUST00000000199;ENSMUST00000150670
ENSMUSG	9430002	ENSMUST00000074808;ENSMUST00000185395
ENSMUSG	Olfr531	ENSMUST00000217167
ENSMUSG	Sim2	ENSMUST00000072182
ENSMUSG	Tex101	ENSMUST00000206390
ENSMUSG	Chia1	ENSMUST00000079132
ENSMUSG	Olfr527	ENSMUST00000214858;ENSMUST00000172230
ENSMUSG	Kcnc3	ENSMUST00000209177;ENSMUST00000207493;ENSMUST00000107
ENSMUSG	Vmn1r51	ENSMUST00000072859
ENSMUSG	Zfp28	ENSMUST00000081022;ENSMUST00000207086;ENSMUST00000208
ENSMUSG	Gm4978	ENSMUST00000071565
ENSMUSG	Rpl31-p	ENSMUST00000119898
ENSMUSG	Zfp938	ENSMUST00000041264;ENSMUST00000156218
ENSMUSG	Tas2r11	ENSMUST00000082014
ENSMUSG	Kdr	ENSMUST00000113516;ENSMUST00000202473;ENSMUST00000149
ENSMUSG	Ccdc177	ENSMUST00000073251
ENSMUSG	Gm6378	ENSMUST00000084199
ENSMUSG	Cped1	ENSMUST00000115383;ENSMUST00000141064;ENSMUST00000153
ENSMUSG	Nrg1	ENSMUST00000207470;ENSMUST00000207417;ENSMUST00000209
ENSMUSG	Ica1	ENSMUST00000115519;ENSMUST00000115520;ENSMUST00000038
ENSMUSG	Gm9701	ENSMUST00000077976
ENSMUSG	Msln	ENSMUST00000075884
ENSMUSG	2010204	ENSMUST00000154346;ENSMUST00000147870;ENSMUST00000128
ENSMUSG	Sox7	ENSMUST00000079652
ENSMUSG	Klk1b8	ENSMUST00000072204
ENSMUSG	Olfr151	ENSMUST00000215928;ENSMUST00000079459
ENSMUSG	Olfr410	ENSMUST00000214490
ENSMUSG	Aldoart	ENSMUST00000080123
ENSMUSG	Klk1b1	ENSMUST00000078835
ENSMUSG	Olfr177	ENSMUST00000217377
ENSMUSG	Bbs5	ENSMUST00000134659;ENSMUST00000074963;ENSMUST00000149
ENSMUSG	Olfr811	ENSMUST00000076437
ENSMUSG	Klk1b27	ENSMUST00000079859
ENSMUSG	Olfr107	ENSMUST00000215947;ENSMUST00000216844;ENSMUST00000077
ENSMUSG	Cd300lb	ENSMUST00000149663;ENSMUST00000106581
ENSMUSG	Olfr135	ENSMUST00000216030
ENSMUSG	Olfr917	ENSMUST00000076542

ENSMUSG	Olfr535	ENSMUST00000217235
ENSMUSG	Gpr84	ENSMUST00000079824
ENSMUSG	Grm4	ENSMUST00000118489;ENSMUST00000118161;ENSMUST0000014[illegible]
ENSMUSG	Syt10	ENSMUST00000029441
ENSMUSG	Gm10128	ENSMUST00000171360
ENSMUSG	Tmem117	ENSMUST00000080141
ENSMUSG	Luzp2	ENSMUST00000082373
ENSMUSG	1190007	ENSMUST00000176200;ENSMUST00000079648;ENSMUST0000018[illegible]
ENSMUSG	Olfr874	ENSMUST00000216982
ENSMUSG	Slc39a4	ENSMUST00000073428
ENSMUSG	Ifna13	ENSMUST00000105149
ENSMUSG	BC02310	ENSMUST00000073997
ENSMUSG	Ddo	ENSMUST00000019977;ENSMUST00000214102;ENSMUST0000021[illegible]
ENSMUSG	Wscd2	ENSMUST00000138757;ENSMUST00000094452;ENSMUST0000018[illegible]
ENSMUSG	Sorcs3	ENSMUST00000078880
ENSMUSG	Gm7117	ENSMUST00000071515
ENSMUSG	Plppr1	ENSMUST00000137884;ENSMUST00000076670;ENSMUST00000151
ENSMUSG	Tas2r11	ENSMUST00000080619
ENSMUSG	Gm6756	ENSMUST00000180647
ENSMUSG	Stmnd1	ENSMUST00000076622
ENSMUSG	Sema3e	ENSMUST00000130116;ENSMUST00000073957;ENSMUST0000019[illegible]
ENSMUSG	Zfp773	ENSMUST00000211240;ENSMUST00000032622
ENSMUSG	Gm4825	ENSMUST00000079690
ENSMUSG	Olfr322	ENSMUST00000214392
ENSMUSG	Olfr141	ENSMUST00000217316
ENSMUSG	Gm5513	ENSMUST00000072982
ENSMUSG	Gng8	ENSMUST00000078182;ENSMUST00000205716;ENSMUST0000020[illegible]
ENSMUSG	Egfem1	ENSMUST00000126420;ENSMUST00000119598
ENSMUSG	Gm10134	ENSMUST00000074761
ENSMUSG	Gm7665	ENSMUST00000076710
ENSMUSG	Sox11	ENSMUST00000079063
ENSMUSG	Cnfn	ENSMUST00000167591;ENSMUST00000076276
ENSMUSG	Slc22a2	ENSMUST00000076493;ENSMUST00000129301;ENSMUST0000014[illegible]
ENSMUSG	Krt73	ENSMUST00000063292
ENSMUSG	Nkx6-3	ENSMUST00000071588
ENSMUSG	Crb1	ENSMUST00000059825;ENSMUST00000199479;ENSMUST0000019[illegible]
ENSMUSG	Glyat	ENSMUST00000119960
ENSMUSG	Klk1b24	ENSMUST00000073713
ENSMUSG	Tnfrsf1	ENSMUST00000079772
ENSMUSG	Hsd3b2	ENSMUST00000107022
ENSMUSG	Gm4963	ENSMUST00000072718
ENSMUSG	Rnf217	ENSMUST00000081989
ENSMUSG	Tas2r12	ENSMUST00000070991
ENSMUSG	S100a7a	ENSMUST00000212471;ENSMUST00000079286
ENSMUSG	Chil4	ENSMUST00000082219;ENSMUST00000196128
ENSMUSG	Olfr282	ENSMUST00000075851
ENSMUSG	Slc22a8	ENSMUST00000010251
ENSMUSG	Lin28b	ENSMUST00000079390;ENSMUST00000214555
ENSMUSG	Olfr532	ENSMUST00000073226
ENSMUSG	Olfr138	ENSMUST00000213707;ENSMUST00000217564
ENSMUSG	Gm6065	ENSMUST00000081336
ENSMUSG	Gm21731	ENSMUST00000079524
ENSMUSG	Olfr151	ENSMUST00000078171
ENSMUSG	Srrm4	ENSMUST00000150127;ENSMUST00000076124;ENSMUST00000201
ENSMUSG	Cyp4a32	ENSMUST00000084342
ENSMUSG	Zar1	ENSMUST00000200883;ENSMUST00000073528

ENSMUSG	Slco1a5	ENSMUST00000111825;ENSMUST00000081380;ENSMUST00000111
ENSMUSG	Olfr190	ENSMUST00000216495
ENSMUSG	Dppa1	ENSMUST00000077173
ENSMUSG	Gm648	ENSMUST00000081133
ENSMUSG	Mro	ENSMUST00000120033;ENSMUST00000179472;ENSMUST0000013[illegible]
ENSMUSG	Ccr1l1	ENSMUST00000071404
ENSMUSG	BC04850	ENSMUST00000078471
ENSMUSG	Gm8973	ENSMUST00000079818
ENSMUSG	Cadm2	ENSMUST00000114292;ENSMUST00000120594
ENSMUSG	Olfr96	ENSMUST00000214482;ENSMUST00000078209
ENSMUSG	Trim38	ENSMUST00000226039
ENSMUSG	Prol1	ENSMUST00000170832
ENSMUSG	Izumo1	ENSMUST00000033100;ENSMUST00000209590
ENSMUSG	Tnnt1	ENSMUST00000108587;ENSMUST00000168111;ENSMUST0000008[illegible]
ENSMUSG	Krt2	ENSMUST00000023712
ENSMUSG	Gsdma3	ENSMUST00000073295;ENSMUST00000107508
ENSMUSG	Paqr9	ENSMUST00000079597
ENSMUSG	Chil1	ENSMUST00000156873;ENSMUST00000082060;ENSMUST0000015[illegible]
ENSMUSG	Olfr329	ENSMUST00000219448;ENSMUST00000108823
ENSMUSG	Cntn4	ENSMUST00000113261;ENSMUST00000113264;ENSMUST0000008[illegible]
ENSMUSG	Aox3	ENSMUST00000161549;ENSMUST00000040999;ENSMUST0000015[illegible]
ENSMUSG	2410137	ENSMUST00000039846
ENSMUSG	Zpld1	ENSMUST00000143914
ENSMUSG	Gm23623	ENSMUST00000082461
ENSMUSG	Gm23628	ENSMUST00000082464
ENSMUSG	Gm23927	ENSMUST00000082466
ENSMUSG	Gm22246	ENSMUST00000082478
ENSMUSG	DQ26710	ENSMUST00000082483
ENSMUSG	Gm26227	ENSMUST00000082510
ENSMUSG	Gm24564	ENSMUST00000082518
ENSMUSG	Gm23406	ENSMUST00000082537
ENSMUSG	Gm23296	ENSMUST00000082555
ENSMUSG	Gm26438	ENSMUST00000082593
ENSMUSG	Gm23613	ENSMUST00000082596
ENSMUSG	Gm22982	ENSMUST00000082613
ENSMUSG	Gm24623	ENSMUST00000082616
ENSMUSG	Gm24622	ENSMUST00000082619
ENSMUSG	Gm25592	ENSMUST00000082635
ENSMUSG	Gm25802	ENSMUST00000082639
ENSMUSG	Gm25132	ENSMUST00000082652
ENSMUSG	Gm26922	ENSMUST00000082687
ENSMUSG	Gm24859	ENSMUST00000082729
ENSMUSG	Gm22807	ENSMUST00000082737
ENSMUSG	Gm25357	ENSMUST00000082745
ENSMUSG	Gm25810	ENSMUST00000082752
ENSMUSG	Gm24146	ENSMUST00000082760
ENSMUSG	Gm22507	ENSMUST00000082773
ENSMUSG	Gm24208	ENSMUST00000082783
ENSMUSG	Gm25929	ENSMUST00000082784
ENSMUSG	Gm25856	ENSMUST00000082786
ENSMUSG	Gm25854	ENSMUST00000082792
ENSMUSG	Gm24705	ENSMUST00000082808
ENSMUSG	Gm26328	ENSMUST00000082821
ENSMUSG	Gm23514	ENSMUST00000082827
ENSMUSG	Gm25204	ENSMUST00000082843
ENSMUSG	Gm24036	ENSMUST00000082868
ENSMUSG	Gm23939	ENSMUST00000082880

ENSMUSG	Gm25698	ENSMUST00000082897
ENSMUSG	Gm22505	ENSMUST00000082956
ENSMUSG	Gm25402	ENSMUST00000082966
ENSMUSG	Gm25404	ENSMUST00000082970
ENSMUSG	Gm22229	ENSMUST00000082987
ENSMUSG	n-R5s36	ENSMUST00000082998
ENSMUSG	Rny3	ENSMUST00000083011
ENSMUSG	Gm23241	ENSMUST00000083014
ENSMUSG	Gm25259	ENSMUST00000083048
ENSMUSG	Gm26326	ENSMUST00000083070
ENSMUSG	Gm26325	ENSMUST00000083072
ENSMUSG	Gm23511	ENSMUST00000083084
ENSMUSG	AF35734	ENSMUST00000083088
ENSMUSG	n-R5s16	ENSMUST00000083096
ENSMUSG	Gm22363	ENSMUST00000083100
ENSMUSG	Gm25704	ENSMUST00000083116
ENSMUSG	Gm24533	ENSMUST00000083138
ENSMUSG	Gm24536	ENSMUST00000083144
ENSMUSG	Gm23677	ENSMUST00000083156
ENSMUSG	Gm24458	ENSMUST00000083204
ENSMUSG	Gm25481	ENSMUST00000083221
ENSMUSG	Gm25139	ENSMUST00000083247
ENSMUSG	Gm25138	ENSMUST00000083248
ENSMUSG	Gm25140	ENSMUST00000083254
ENSMUSG	Gm23460	ENSMUST00000083258
ENSMUSG	Gm23459	ENSMUST00000083259
ENSMUSG	Gm23461	ENSMUST00000083265
ENSMUSG	Gm24613	ENSMUST00000083266
ENSMUSG	Gm25792	ENSMUST00000083289
ENSMUSG	Gm25790	ENSMUST00000083293
ENSMUSG	Gm22294	ENSMUST00000083308
ENSMUSG	Gm22296	ENSMUST00000083310
ENSMUSG	Gm22293	ENSMUST00000083314
ENSMUSG	Gm23972	ENSMUST00000083322
ENSMUSG	Gm23453	ENSMUST00000083326
ENSMUSG	Gm25127	ENSMUST00000083336
ENSMUSG	Gm25125	ENSMUST00000083340
ENSMUSG	Gm25124	ENSMUST00000083345
ENSMUSG	Gm24454	ENSMUST00000083349
ENSMUSG	Gm26124	ENSMUST00000083359
ENSMUSG	Gm26066	ENSMUST00000083385
ENSMUSG	Gm22054	ENSMUST00000083395
ENSMUSG	Gm25755	ENSMUST00000083396
ENSMUSG	Gm25579	ENSMUST00000083407
ENSMUSG	Gm25581	ENSMUST00000083410
ENSMUSG	Gm24414	ENSMUST00000083434
ENSMUSG	Gm22739	ENSMUST00000083437
ENSMUSG	Gm22738	ENSMUST00000083440
ENSMUSG	Gm23093	ENSMUST00000083450
ENSMUSG	Mir193a	ENSMUST00000083461
ENSMUSG	Mir31	ENSMUST00000083474
ENSMUSG	Mir221	ENSMUST00000083488
ENSMUSG	Mir325	ENSMUST00000083491
ENSMUSG	Mir134	ENSMUST00000083492
ENSMUSG	Mir382	ENSMUST00000083494
ENSMUSG	Mir208a	ENSMUST00000083498
ENSMUSG	Mir377	ENSMUST00000083504

ENSMUSG	Mir448	ENSMUST00000083516
ENSMUSG	Mir133a	ENSMUST00000083526
ENSMUSG	Mir222	ENSMUST00000083537
ENSMUSG	Mir411	ENSMUST00000083543
ENSMUSG	Mir133b	ENSMUST00000083546
ENSMUSG	Mir346	ENSMUST00000083547
ENSMUSG	Mir130a	ENSMUST00000083550
ENSMUSG	Mir219a	ENSMUST00000083551
ENSMUSG	Mir196a	ENSMUST00000083554
ENSMUSG	Mir365-	ENSMUST00000083555
ENSMUSG	Mir410	ENSMUST00000083563
ENSMUSG	Mir379	ENSMUST00000083564
ENSMUSG	Mir10b	ENSMUST00000083566
ENSMUSG	Mir148a	ENSMUST00000083571
ENSMUSG	Mir204	ENSMUST00000083573
ENSMUSG	Mir10a	ENSMUST00000083585
ENSMUSG	Mir192	ENSMUST00000083589
ENSMUSG	Mir135a	ENSMUST00000083590
ENSMUSG	Mir320	ENSMUST00000083594
ENSMUSG	Mir224	ENSMUST00000083608
ENSMUSG	Mir196a	ENSMUST00000083612
ENSMUSG	Mir210	ENSMUST00000083617
ENSMUSG	Mir206	ENSMUST00000083625
ENSMUSG	Mir30c-	ENSMUST00000083633
ENSMUSG	Mir412	ENSMUST00000083636
ENSMUSG	Mir194-	ENSMUST00000083648
ENSMUSG	Mir211	ENSMUST00000083651
ENSMUSG	Gm24803	ENSMUST00000083693
ENSMUSG	Gm24253	ENSMUST00000083701
ENSMUSG	Gm23875	ENSMUST00000083711
ENSMUSG	n-R5s17	ENSMUST00000083726
ENSMUSG	Rny1	ENSMUST00000083767
ENSMUSG	Gm22341	ENSMUST00000083775
ENSMUSG	Gm26166	ENSMUST00000083788
ENSMUSG	Gm25008	ENSMUST00000083809
ENSMUSG	Gm23347	ENSMUST00000083823
ENSMUSG	n-R5s54	ENSMUST00000083837
ENSMUSG	Gm25869	ENSMUST00000083855
ENSMUSG	Gm26314	ENSMUST00000083891
ENSMUSG	Gm23502	ENSMUST00000083902
ENSMUSG	Gm25189	ENSMUST00000083911
ENSMUSG	Gm22350	ENSMUST00000083924
ENSMUSG	Gm22866	ENSMUST00000083947
ENSMUSG	Gm26110	ENSMUST00000083971
ENSMUSG	Gm22798	ENSMUST00000083992
ENSMUSG	Ifitm7	ENSMUST00000178021
ENSMUSG	Cd209b	ENSMUST00000111014;ENSMUST00000084086
ENSMUSG	Zfp979	ENSMUST00000105720;ENSMUST00000127826
ENSMUSG	Gm10436	ENSMUST00000071580
ENSMUSG	Gm13023	ENSMUST00000085144
ENSMUSG	Gm12866	ENSMUST00000128998
ENSMUSG	Cyp4a12	ENSMUST00000084343
ENSMUSG	Cyp4a10	ENSMUST00000058785
ENSMUSG	Adamtsl	ENSMUST00000147066;ENSMUST00000130474;ENSMUST00000107
ENSMUSG	Gm10154	ENSMUST00000084445
ENSMUSG	Olfr45	ENSMUST00000214637;ENSMUST00000084454
ENSMUSG	Gm11232	ENSMUST00000084489

ENSMUSG	Mup3	ENSMUST00000107488
ENSMUSG	AY51293	ENSMUST00000139751;ENSMUST00000166107
ENSMUSG	Gm12511	ENSMUST00000135987
ENSMUSG	6030445	ENSMUST00000084593
ENSMUSG	Cacng3	ENSMUST00000084615
ENSMUSG	Spag8	ENSMUST00000149575;ENSMUST00000084646;ENSMUST00000107
ENSMUSG	Gpr139	ENSMUST00000084650
ENSMUSG	Pomgnt2	ENSMUST00000217610;ENSMUST00000084743;ENSMUST00000216
ENSMUSG	Olfr639	ENSMUST00000215653
ENSMUSG	Olfr575	ENSMUST00000213477
ENSMUSG	Olfr33	ENSMUST00000216312;ENSMUST00000084817
ENSMUSG	Rtp3	ENSMUST00000199891;ENSMUST00000084922;ENSMUST00000198
ENSMUSG	Tcl1b1	ENSMUST00000085043
ENSMUSG	Serpina	ENSMUST00000085050
ENSMUSG	Iqcf5	ENSMUST00000085113
ENSMUSG	Iqcf1	ENSMUST00000215037
ENSMUSG	Rpl31-p	ENSMUST00000085128
ENSMUSG	Nrxn3	ENSMUST00000167734;ENSMUST00000110138;ENSMUST00000190
ENSMUSG	Plekhd1	ENSMUST00000140770;ENSMUST00000153762
ENSMUSG	Gm5745	ENSMUST00000183748
ENSMUSG	Gm6265	ENSMUST00000149263
ENSMUSG	Klk1b5	ENSMUST00000074359
ENSMUSG	Klk1b4	ENSMUST00000077354
ENSMUSG	Klk1b3	ENSMUST00000085450
ENSMUSG	Klk1b21	ENSMUST00000085455
ENSMUSG	Scgb1b2	ENSMUST00000085618
ENSMUSG	Scgb2b2	ENSMUST00000085620;ENSMUST00000141890
ENSMUSG	Gm7589	ENSMUST00000085631
ENSMUSG	6030419	ENSMUST00000085658;ENSMUST00000217624;ENSMUST00000216
ENSMUSG	Gm5121	ENSMUST00000085711
ENSMUSG	Rpl36-p	ENSMUST00000085720
ENSMUSG	Lefty2	ENSMUST00000085797;ENSMUST00000155546
ENSMUSG	Olfr417	ENSMUST00000220394
ENSMUSG	Pilrb2	ENSMUST00000197411;ENSMUST00000198024;ENSMUST00000196
ENSMUSG	Pilrb1	ENSMUST00000197403;ENSMUST00000197914;ENSMUST00000110
ENSMUSG	Gm13083	ENSMUST00000105773
ENSMUSG	Cyp2b19	ENSMUST00000077855;ENSMUST00000132315;ENSMUST00000138
ENSMUSG	Fxyd6	ENSMUST00000085939;ENSMUST00000217381;ENSMUST00000215
ENSMUSG	Olfr145	ENSMUST00000086062
ENSMUSG	Olfr877	ENSMUST00000213956;ENSMUST00000086063
ENSMUSG	Igfl3	ENSMUST00000152973
ENSMUSG	Psg16	ENSMUST00000071399;ENSMUST00000152671
ENSMUSG	Gm5614	ENSMUST00000091097
ENSMUSG	Zfp648	ENSMUST00000086195
ENSMUSG	Vmn1r84	ENSMUST00000086228
ENSMUSG	Gm10179	ENSMUST00000086233
ENSMUSG	Gm10180	ENSMUST00000086236
ENSMUSG	Zfp772	ENSMUST00000074455
ENSMUSG	Hmcn1	ENSMUST00000074783;ENSMUST00000137197;ENSMUST00000151
ENSMUSG	Oas1g	ENSMUST00000086368;ENSMUST00000162096;ENSMUST00000161
ENSMUSG	Olfr18	ENSMUST00000212943;ENSMUST00000211895;ENSMUST00000211
ENSMUSG	Gm10188	ENSMUST00000086544
ENSMUSG	Gm16391	ENSMUST00000086601
ENSMUSG	Tmem211	ENSMUST00000086615
ENSMUSG	Cryba4	ENSMUST00000112385;ENSMUST00000112383
ENSMUSG	Serpinb	ENSMUST00000154538;ENSMUST00000086690
ENSMUSG	Serpinb	ENSMUST00000086701;ENSMUST00000188745;ENSMUST00000112

ENSMUSG	Cntnap5	ENSMUST00000086738;ENSMUST00000188735;ENSMUST0000018
ENSMUSG	Olfr719	ENSMUST00000137795
ENSMUSG	Gm6990	ENSMUST00000086830
ENSMUSG	Olfr141	ENSMUST00000086837
ENSMUSG	Asb18	ENSMUST00000086882
ENSMUSG	1700010	ENSMUST00000189748;ENSMUST00000187592
ENSMUSG	AY70210	ENSMUST00000180996;ENSMUST00000086914
ENSMUSG	Gm5560	ENSMUST00000086918
ENSMUSG	Mrgpra4	ENSMUST00000087092
ENSMUSG	Olfr132	ENSMUST00000216804
ENSMUSG	H2-M9	ENSMUST00000087165;ENSMUST00000087167
ENSMUSG	Usp51	ENSMUST00000095755
ENSMUSG	Cyp2c65	ENSMUST00000087236
ENSMUSG	Lgi1	ENSMUST00000198518;ENSMUST00000087252;ENSMUST0000019
ENSMUSG	Foxd3	ENSMUST00000087285
ENSMUSG	Capn6	ENSMUST00000087316;ENSMUST00000151154
ENSMUSG	Ppp1r3c	ENSMUST00000087321
ENSMUSG	Crygd	ENSMUST00000045028;ENSMUST00000127762
ENSMUSG	Hmx1	ENSMUST00000172923;ENSMUST00000087674
ENSMUSG	Olfr76	ENSMUST00000087822
ENSMUSG	Olfr142	ENSMUST00000214103
ENSMUSG	Olfr142	ENSMUST00000215374
ENSMUSG	Olfr142	ENSMUST00000214918;ENSMUST00000087828
ENSMUSG	Olfr142	ENSMUST00000207681;ENSMUST00000087830
ENSMUSG	Olfr142	ENSMUST00000214472;ENSMUST00000087831
ENSMUSG	A630073	ENSMUST00000203888
ENSMUSG	Prb1	ENSMUST00000087853
ENSMUSG	Olfr141	ENSMUST00000217617;ENSMUST00000087857
ENSMUSG	Ms4a15	ENSMUST00000087923
ENSMUSG	Cbln4	ENSMUST00000087950
ENSMUSG	Klra3	ENSMUST00000111998;ENSMUST00000088017
ENSMUSG	Krt77	ENSMUST00000087996
ENSMUSG	Krt74	ENSMUST00000088018
ENSMUSG	Dgat2l6	ENSMUST00000140551
ENSMUSG	Klra7	ENSMUST00000112013;ENSMUST00000049304;ENSMUST0000008
ENSMUSG	Nms	ENSMUST00000088029;ENSMUST00000160214
ENSMUSG	Klri1	ENSMUST00000088046
ENSMUSG	Krt83	ENSMUST00000159029;ENSMUST00000023718
ENSMUSG	Krt86	ENSMUST00000088049
ENSMUSG	Krt81	ENSMUST00000061185
ENSMUSG	Mageb18	ENSMUST00000113955
ENSMUSG	Gm10220	ENSMUST00000088236
ENSMUSG	Gm5862	ENSMUST00000072286
ENSMUSG	Wfdc13	ENSMUST00000088260
ENSMUSG	Khdc1a	ENSMUST00000088407
ENSMUSG	Clec4b2	ENSMUST00000088455
ENSMUSG	Pi15	ENSMUST00000088476
ENSMUSG	4930444	ENSMUST00000115367
ENSMUSG	Xkr9	ENSMUST00000088542
ENSMUSG	Tcea1-p	ENSMUST00000117942
ENSMUSG	Map7d3	ENSMUST00000114766
ENSMUSG	Casp16-	ENSMUST00000182769;ENSMUST00000088673
ENSMUSG	Gm7168	ENSMUST00000088809
ENSMUSG	Bpifb9b	ENSMUST00000088921
ENSMUSG	Bpifb9a	ENSMUST00000088924
ENSMUSG	Bpifb3	ENSMUST00000088950
ENSMUSG	Bpifb6	ENSMUST00000088955

ENSMUSG	Gm10229	ENSMUST00000089106
ENSMUSG	Tcf15	ENSMUST00000089112
ENSMUSG	Cyp2d9	ENSMUST00000089129
ENSMUSG	1500009	ENSMUST00000143238
ENSMUSG	Tnfrsf1	ENSMUST00000089161;ENSMUST00000109535
ENSMUSG	Mei1	ENSMUST00000089178;ENSMUST00000189540
ENSMUSG	Agtr2	ENSMUST00000089188
ENSMUSG	Zfp442	ENSMUST00000109916
ENSMUSG	Insm1	ENSMUST00000089257
ENSMUSG	Csnka2i	ENSMUST00000209382
ENSMUSG	Col8a1	ENSMUST00000089332
ENSMUSG	Pick1	ENSMUST00000163571;ENSMUST00000018295;ENSMUST0000005
ENSMUSG	Ssxb9	ENSMUST00000115584
ENSMUSG	Gm6723	ENSMUST00000180449
ENSMUSG	Apol9b	ENSMUST00000109775
ENSMUSG	Apol7b	ENSMUST00000089469
ENSMUSG	Olfr461	ENSMUST00000215009;ENSMUST00000089490
ENSMUSG	Efcc1	ENSMUST00000160743;ENSMUST00000159570;ENSMUST0000003
ENSMUSG	Shroom4	ENSMUST00000103005;ENSMUST00000143641;ENSMUST0000008
ENSMUSG	Cma2	ENSMUST00000089555
ENSMUSG	Nat8f4	ENSMUST00000159755;ENSMUST00000095757
ENSMUSG	Noto	ENSMUST00000089578
ENSMUSG	Spr-ps1	ENSMUST00000187705;ENSMUST00000089584;ENSMUST0000018
ENSMUSG	Slc4a5	ENSMUST00000039212;ENSMUST00000131432
ENSMUSG	Gml	ENSMUST00000096400
ENSMUSG	Rnase13	ENSMUST00000089771
ENSMUSG	Rnase12	ENSMUST00000089798
ENSMUSG	Gmnc	ENSMUST00000089832
ENSMUSG	Olfr742	ENSMUST00000217437
ENSMUSG	Duox2	ENSMUST00000053734;ENSMUST00000155820
ENSMUSG	Aard	ENSMUST00000090025
ENSMUSG	Olfr169	ENSMUST00000215040;ENSMUST00000215476;ENSMUST0000009
ENSMUSG	Doxl2	ENSMUST00000184917
ENSMUSG	Clca4a	ENSMUST00000029923
ENSMUSG	Mgam	ENSMUST00000071535;ENSMUST00000201148;ENSMUST0000020
ENSMUSG	Actc1	ENSMUST00000090269
ENSMUSG	Gjd2	ENSMUST00000090275
ENSMUSG	Efcab1	ENSMUST00000090277
ENSMUSG	Gm6155	ENSMUST00000173735
ENSMUSG	Gm7676	ENSMUST00000084055
ENSMUSG	Gm10247	ENSMUST00000090395
ENSMUSG	Cd59b	ENSMUST00000111131;ENSMUST00000111132;ENSMUST0000012
ENSMUSG	Rps19-p	ENSMUST00000161787
ENSMUSG	Gpr88	ENSMUST00000090473
ENSMUSG	Myoz1	ENSMUST00000090469;ENSMUST00000224472;ENSMUST0000022
ENSMUSG	Flnc	ENSMUST00000148404;ENSMUST00000065090;ENSMUST0000010
ENSMUSG	Gstm6	ENSMUST00000106685;ENSMUST00000106684;ENSMUST0000010
ENSMUSG	Col28a1	ENSMUST00000115537;ENSMUST00000134731
ENSMUSG	Olfr125	ENSMUST00000214846;ENSMUST00000090695
ENSMUSG	Olfr113	ENSMUST00000213302;ENSMUST00000090712
ENSMUSG	Gm5773	ENSMUST00000090853
ENSMUSG	Lce1e	ENSMUST00000090867
ENSMUSG	Sprr2a2	ENSMUST00000090871;ENSMUST00000071805
ENSMUSG	Olfr338	ENSMUST00000072114;ENSMUST00000091006
ENSMUSG	Zfp619	ENSMUST00000108015
ENSMUSG	Zfp114	ENSMUST00000205309;ENSMUST00000206547;ENSMUST0000008
ENSMUSG	Gm6361	ENSMUST00000091031

ENSMUSG	Slc25a3	ENSMUST00000091184
ENSMUSG	Usp9y	ENSMUST00000091188
ENSMUSG	Slc7a14	ENSMUST00000091259
ENSMUSG	Gm10258	ENSMUST00000091270
ENSMUSG	Dytn	ENSMUST00000090313;ENSMUST00000126954
ENSMUSG	Zbtb10	ENSMUST00000155203
ENSMUSG	Nxph2	ENSMUST00000102945
ENSMUSG	Nr2f1	ENSMUST00000091458;ENSMUST00000150498;ENSMUST00000127
ENSMUSG	Zfp72	ENSMUST00000223163;ENSMUST00000091481
ENSMUSG	Zdhhc11	ENSMUST00000222487
ENSMUSG	Gprin1	ENSMUST00000135343;ENSMUST00000099506
ENSMUSG	Gm7251	ENSMUST00000182661
ENSMUSG	Serpinb	ENSMUST00000110275
ENSMUSG	Vmn1r22	ENSMUST00000091719
ENSMUSG	Vmn1r19	ENSMUST00000091732
ENSMUSG	Vmn1r18	ENSMUST00000091739
ENSMUSG	Gm5096	ENSMUST00000091776
ENSMUSG	Prdm6	ENSMUST00000091900;ENSMUST00000115398
ENSMUSG	Gm4950	ENSMUST00000091905
ENSMUSG	Dsg1a	ENSMUST00000077146
ENSMUSG	Rdh16	ENSMUST00000125163;ENSMUST00000071646
ENSMUSG	I830134	ENSMUST00000092265
ENSMUSG	Tspear	ENSMUST00000092366;ENSMUST00000125241
ENSMUSG	Krtap10	ENSMUST00000092369
ENSMUSG	Krtap12	ENSMUST00000092370
ENSMUSG	Gm10272	ENSMUST00000092371
ENSMUSG	Gm5250	ENSMUST00000059065
ENSMUSG	Nkain2	ENSMUST00000191234;ENSMUST00000219125;ENSMUST0000021
ENSMUSG	Taar5	ENSMUST00000092659
ENSMUSG	Taar4	ENSMUST00000092660
ENSMUSG	4930444	ENSMUST00000092672
ENSMUSG	Krtap3-	ENSMUST00000092699
ENSMUSG	Krtap3-	ENSMUST00000092700
ENSMUSG	Ube2u	ENSMUST00000133493
ENSMUSG	Tmem100	ENSMUST00000092788
ENSMUSG	Gm11444	ENSMUST00000146433
ENSMUSG	Gm11426	ENSMUST00000125600
ENSMUSG	Fbp1	ENSMUST00000092888;ENSMUST00000134814
ENSMUSG	Cacng7	ENSMUST00000092891;ENSMUST00000203497
ENSMUSG	Ccdc92b	ENSMUST00000100866
ENSMUSG	Spata22	ENSMUST00000117445
ENSMUSG	Nlrp1a	ENSMUST00000048514
ENSMUSG	Sat2	ENSMUST00000108656;ENSMUST00000151255
ENSMUSG	Slc47a2	ENSMUST00000093029;ENSMUST00000147450
ENSMUSG	4930438	ENSMUST00000108834
ENSMUSG	1700024	ENSMUST00000093146
ENSMUSG	Gm12166	ENSMUST00000093169;ENSMUST00000154156
ENSMUSG	Ces3a	ENSMUST00000093222
ENSMUSG	Gm5356	ENSMUST00000093326
ENSMUSG	Gm8054	ENSMUST00000093411
ENSMUSG	Fam149a	ENSMUST00000135912;ENSMUST00000093526;ENSMUST00000155
ENSMUSG	Mir486	ENSMUST00000093576
ENSMUSG	Mir341	ENSMUST00000093593
ENSMUSG	Mir488	ENSMUST00000093595
ENSMUSG	Gm10286	ENSMUST00000093603
ENSMUSG	Mir485	ENSMUST00000093620
ENSMUSG	Mir496a	ENSMUST00000093627

ENSMUSG	Mir452	ENSMUST00000093629
ENSMUSG	Mir1a-1	ENSMUST00000093635
ENSMUSG	Gm24163	ENSMUST00000093660
ENSMUSG	Gm24817	ENSMUST00000093672
ENSMUSG	Gm22365	ENSMUST00000093750
ENSMUSG	Olfr894	ENSMUST00000093866
ENSMUSG	Mmp27	ENSMUST00000120900;ENSMUST00000151853;ENSMUST00000152
ENSMUSG	Tmem235	ENSMUST00000093905
ENSMUSG	Trim80	ENSMUST00000093914
ENSMUSG	Krtap31	ENSMUST00000093935
ENSMUSG	Fbxo47	ENSMUST00000125403;ENSMUST00000093939
ENSMUSG	Gm10287	ENSMUST00000093950
ENSMUSG	Prok1	ENSMUST00000197758;ENSMUST00000198732;ENSMUST0000004
ENSMUSG	Itgad	ENSMUST00000106237;ENSMUST00000033051;ENSMUST00000177
ENSMUSG	Prss36	ENSMUST00000153110;ENSMUST00000152697;ENSMUST00000094
ENSMUSG	Olfr406	ENSMUST00000214303;ENSMUST00000133561
ENSMUSG	Olfr43	ENSMUST00000205664;ENSMUST00000078936
ENSMUSG	Nlrp1b	ENSMUST00000108515;ENSMUST00000136493;ENSMUST00000108
ENSMUSG	Cyp3a57	ENSMUST00000079268
ENSMUSG	Olfr658	ENSMUST00000210641;ENSMUST00000089296
ENSMUSG	Olfr558	ENSMUST00000094124
ENSMUSG	Olfr313	ENSMUST00000217506;ENSMUST00000082220
ENSMUSG	Olfr290	ENSMUST00000073468
ENSMUSG	Gm9696	ENSMUST00000094227
ENSMUSG	Adamtsl	ENSMUST00000172490;ENSMUST00000173287;ENSMUST00000173
ENSMUSG	Erich6	ENSMUST00000041115
ENSMUSG	Fam217b	ENSMUST00000094251;ENSMUST00000129003
ENSMUSG	Tmem132	ENSMUST00000185104;ENSMUST00000031446
ENSMUSG	Fcrl6	ENSMUST00000094303;ENSMUST00000193566;ENSMUST00000193
ENSMUSG	Nsmce3	ENSMUST00000094331
ENSMUSG	Fcrlb	ENSMUST00000094337
ENSMUSG	Peg12	ENSMUST00000094339
ENSMUSG	Mkrn3	ENSMUST00000094340
ENSMUSG	Wfdc16	ENSMUST00000109336
ENSMUSG	Ccdc190	ENSMUST00000094348;ENSMUST00000175731
ENSMUSG	Mrgprb1	ENSMUST00000094384
ENSMUSG	Mrgprb4	ENSMUST00000094388
ENSMUSG	Mrgprx1	ENSMUST00000094390
ENSMUSG	Ntn5	ENSMUST00000182750;ENSMUST00000107742
ENSMUSG	Slc6a21	ENSMUST00000210861;ENSMUST00000209886
ENSMUSG	Slc17a7	ENSMUST00000085374;ENSMUST00000210498;ENSMUST0000021
ENSMUSG	2310016	ENSMUST00000209111
ENSMUSG	Mn1	ENSMUST00000138784;ENSMUST00000094463
ENSMUSG	Gm10298	ENSMUST00000074801
ENSMUSG	Gm13089	ENSMUST00000073532
ENSMUSG	Sox13	ENSMUST00000144386;ENSMUST00000153799;ENSMUST00000094
ENSMUSG	Etnk2	ENSMUST00000135222;ENSMUST00000147909
ENSMUSG	Ren1	ENSMUST00000094556
ENSMUSG	Rpl31-p	ENSMUST00000116034
ENSMUSG	Lactbl1	ENSMUST00000178843
ENSMUSG	Htr1d	ENSMUST00000088677;ENSMUST00000121571;ENSMUST00000133
ENSMUSG	5830473	ENSMUST00000094615;ENSMUST00000200765
ENSMUSG	Rps12-p	ENSMUST00000117447
ENSMUSG	Cntnap5	ENSMUST00000043725
ENSMUSG	Csn1s1	ENSMUST00000200567;ENSMUST00000196500;ENSMUST0000020
ENSMUSG	Gtsf1l	ENSMUST00000094653
ENSMUSG	Zfp974	ENSMUST00000098639;ENSMUST00000129341;ENSMUST00000145

ENSMUSG	Pla2g4d	ENSMUST00000094665
ENSMUSG	Tmem200	ENSMUST00000094666
ENSMUSG	Rbm44	ENSMUST00000190965;ENSMUST00000094698
ENSMUSG	Ceacam2	ENSMUST00000094753;ENSMUST00000207248
ENSMUSG	Rpl7a-p	ENSMUST00000094754
ENSMUSG	Psg21	ENSMUST00000094793;ENSMUST00000182128;ENSMUST00000182
ENSMUSG	Psg27	ENSMUST00000094794
ENSMUSG	Psg25	ENSMUST00000094795
ENSMUSG	Psg26	ENSMUST00000094798
ENSMUSG	Vmn1r85	ENSMUST00000094829
ENSMUSG	Gm13015	ENSMUST00000118674
ENSMUSG	Aurkc	ENSMUST00000208049
ENSMUSG	Vmn2r30	ENSMUST00000174368
ENSMUSG	Gm1673	ENSMUST00000094869;ENSMUST00000206020;ENSMUST00000114
ENSMUSG	Zfp804a	ENSMUST00000047527
ENSMUSG	Gad1	ENSMUST00000094934;ENSMUST00000155979;ENSMUST0000013C
ENSMUSG	Gm12689	ENSMUST00000094955
ENSMUSG	Gm13288	ENSMUST00000094977
ENSMUSG	Speer4d	ENSMUST00000119100
ENSMUSG	Acnat1	ENSMUST00000107697;ENSMUST00000095086;ENSMUST0000013E
ENSMUSG	Foxe1	ENSMUST00000095097
ENSMUSG	Olfr155	ENSMUST00000095108
ENSMUSG	Hrct1	ENSMUST00000095109
ENSMUSG	Sdr16c6	ENSMUST00000095151;ENSMUST00000108383
ENSMUSG	Gm5499	ENSMUST00000090731
ENSMUSG	Gm9386	ENSMUST00000095195
ENSMUSG	Rasgrp3	ENSMUST00000164192;ENSMUST00000095204
ENSMUSG	Ces1a	ENSMUST00000095211
ENSMUSG	Rpl7a-p	ENSMUST00000095218
ENSMUSG	Trim75	ENSMUST00000095295
ENSMUSG	Gm10313	ENSMUST00000095320
ENSMUSG	Ccdc110	ENSMUST00000095326
ENSMUSG	Spx	ENSMUST00000126521;ENSMUST00000211094;ENSMUST00000141
ENSMUSG	Mboat4	ENSMUST00000095345
ENSMUSG	Tas2r14	ENSMUST00000095391
ENSMUSG	Tas2r11	ENSMUST00000095394
ENSMUSG	Tas2r12	ENSMUST00000095395
ENSMUSG	Gm10318	ENSMUST00000186874
ENSMUSG	Npw	ENSMUST00000095544;ENSMUST00000213213
ENSMUSG	Syndig1	ENSMUST00000095550;ENSMUST00000221793
ENSMUSG	Vrtn	ENSMUST00000221915;ENSMUST00000166772;ENSMUST00000167
ENSMUSG	Zfp213	ENSMUST00000095606
ENSMUSG	Zfp957	ENSMUST00000161649;ENSMUST00000040802;ENSMUST00000162
ENSMUSG	1700086	ENSMUST00000185822
ENSMUSG	Zfp946	ENSMUST00000141831;ENSMUST00000120222;ENSMUST0000008E
ENSMUSG	Bves	ENSMUST00000095715;ENSMUST00000213557
ENSMUSG	Tcp10a	ENSMUST00000128533
ENSMUSG	Armc2	ENSMUST00000160262;ENSMUST00000105503;ENSMUST0000009E
ENSMUSG	Mfsd4b3	ENSMUST00000095749
ENSMUSG	Egr4	ENSMUST00000095759
ENSMUSG	Gm10327	ENSMUST00000105222
ENSMUSG	Reg3b	ENSMUST00000096904;ENSMUST00000204760
ENSMUSG	Mcpt9	ENSMUST00000095798
ENSMUSG	2410004	ENSMUST00000095823;ENSMUST00000189479
ENSMUSG	Rps15-p	ENSMUST00000121788
ENSMUSG	Ccnb1ip	ENSMUST00000095932
ENSMUSG	Rpl21-p	ENSMUST00000095940

ENSMUSG	Olfr437	ENSMUST00000060243
ENSMUSG	Ptgdr	ENSMUST00000095959
ENSMUSG	Vmn1r20	ENSMUST00000095961
ENSMUSG	Olfr455	ENSMUST00000217978;ENSMUST00000171307
ENSMUSG	Olfr263	ENSMUST00000215804;ENSMUST00000096006
ENSMUSG	Gprin2	ENSMUST00000096019
ENSMUSG	3425401	ENSMUST00000096038
ENSMUSG	Akr1c19	ENSMUST00000118663
ENSMUSG	Cpa2	ENSMUST00000096066
ENSMUSG	BC10053	ENSMUST00000096089;ENSMUST00000146691
ENSMUSG	Gm5874	ENSMUST00000096101
ENSMUSG	Rnls	ENSMUST00000096114;ENSMUST00000163093
ENSMUSG	Fam189a	ENSMUST00000096164
ENSMUSG	Gm10340	ENSMUST00000170790
ENSMUSG	Gm4952	ENSMUST00000181868;ENSMUST00000092931
ENSMUSG	Rimbp3	ENSMUST00000169803
ENSMUSG	Foxr2	ENSMUST00000096265
ENSMUSG	Gm10343	ENSMUST00000096279
ENSMUSG	Zcchc16	ENSMUST00000112843;ENSMUST00000096301
ENSMUSG	Tmem28	ENSMUST00000096363
ENSMUSG	Spin4	ENSMUST00000096367
ENSMUSG	Gspt2	ENSMUST00000096368;ENSMUST00000137033
ENSMUSG	Gm5941	ENSMUST00000096371
ENSMUSG	Cldn34b	ENSMUST00000096389
ENSMUSG	C230004	ENSMUST00000137153;ENSMUST00000144853
ENSMUSG	Olfr132	ENSMUST00000096449
ENSMUSG	Rhox12	ENSMUST00000096453
ENSMUSG	Rhox4e	ENSMUST00000089075;ENSMUST00000184330
ENSMUSG	Rhox4b	ENSMUST00000096458;ENSMUST00000122828
ENSMUSG	Ssxb5	ENSMUST00000089370
ENSMUSG	Ccdc112	ENSMUST00000072835
ENSMUSG	Gm94	ENSMUST00000096570
ENSMUSG	2900055	ENSMUST00000096572
ENSMUSG	Fndc1	ENSMUST00000097425;ENSMUST00000097424;ENSMUST00000114
ENSMUSG	Sept5	ENSMUST00000096987
ENSMUSG	Trim6	ENSMUST00000144538;ENSMUST00000098180;ENSMUST00000144
ENSMUSG	Gm5592	ENSMUST00000097044
ENSMUSG	C2cd6	ENSMUST00000097080
ENSMUSG	Gm10355	ENSMUST00000097146
ENSMUSG	Gm10356	ENSMUST00000097152
ENSMUSG	Gm6419	ENSMUST00000097166
ENSMUSG	Nanos1	ENSMUST00000088237
ENSMUSG	Atp5l-p	ENSMUST00000097132
ENSMUSG	1700024	ENSMUST00000100723
ENSMUSG	Gm9008	ENSMUST00000097218
ENSMUSG	Gm10364	ENSMUST00000076633;ENSMUST00000153034
ENSMUSG	Hhla1	ENSMUST00000100584
ENSMUSG	Eddm3b	ENSMUST00000100645
ENSMUSG	Gm16020	ENSMUST00000084281
ENSMUSG	Gm10371	ENSMUST00000127429
ENSMUSG	5930412	ENSMUST00000140028;ENSMUST00000154305;ENSMUST00000137
ENSMUSG	Gm16439	ENSMUST00000100687
ENSMUSG	Ang6	ENSMUST00000160223;ENSMUST00000096869
ENSMUSG	Ear-ps2	ENSMUST00000100689
ENSMUSG	Ear-ps9	ENSMUST00000161963
ENSMUSG	Ear1	ENSMUST00000100691;ENSMUST00000179200
ENSMUSG	Gm10384	ENSMUST00000100713

ENSMUSG	Zfp9	ENSMUST00000161170;ENSMUST00000204066
ENSMUSG	Gm5460	ENSMUST00000100719
ENSMUSG	Gdf2	ENSMUST00000100720
ENSMUSG	Spef2	ENSMUST00000160236;ENSMUST00000208854;ENSMUST00000041
ENSMUSG	Duxbl3	ENSMUST00000174494
ENSMUSG	D6Ertd4	ENSMUST00000159793;ENSMUST00000160188;ENSMUST00000161
ENSMUSG	Rpl21-p	ENSMUST00000080237
ENSMUSG	Myo18b	ENSMUST00000086617;ENSMUST00000182189;ENSMUST00000183
ENSMUSG	Gm6588	ENSMUST00000100882
ENSMUSG	Gm10044	ENSMUST00000081331;ENSMUST00000166813;ENSMUST00000163
ENSMUSG	Gm3715	ENSMUST00000172308
ENSMUSG	Gm16440	ENSMUST00000100900
ENSMUSG	9230020	ENSMUST00000151841
ENSMUSG	Lrcol1	ENSMUST00000185691;ENSMUST00000190060
ENSMUSG	Gm10418	ENSMUST00000100943
ENSMUSG	Gm10419	ENSMUST00000197854;ENSMUST00000196693;ENSMUST00000198
ENSMUSG	Abcb5	ENSMUST00000035515
ENSMUSG	BC06121	ENSMUST00000094587
ENSMUSG	Tmprss1	ENSMUST00000101073;ENSMUST00000198504
ENSMUSG	Gm6116	ENSMUST00000101121
ENSMUSG	Gm10433	ENSMUST00000146364;ENSMUST00000138678
ENSMUSG	Gm2016	ENSMUST00000110147
ENSMUSG	Trpc5os	ENSMUST00000155206
ENSMUSG	Nup62cl	ENSMUST00000101212;ENSMUST00000154385;ENSMUST00000133
ENSMUSG	Ripply1	ENSMUST00000101217
ENSMUSG	Gm10444	ENSMUST00000126708
ENSMUSG	Gm5878	ENSMUST00000203386
ENSMUSG	Tmsb15l	ENSMUST00000127404
ENSMUSG	Gm16401	ENSMUST00000197463
ENSMUSG	Gm10447	ENSMUST00000084653
ENSMUSG	Gm10451	ENSMUST00000219367;ENSMUST00000101281;ENSMUST0000022C
ENSMUSG	Gm5127	ENSMUST00000101297
ENSMUSG	Fnd3c2	ENSMUST00000091282
ENSMUSG	Dmrtc1b	ENSMUST00000101324;ENSMUST00000118842
ENSMUSG	Atoh1	ENSMUST00000101351
ENSMUSG	Gm14812	ENSMUST00000156494
ENSMUSG	D130052	ENSMUST00000101371
ENSMUSG	Gm7173	ENSMUST00000197180;ENSMUST00000196318
ENSMUSG	Smim9	ENSMUST00000101433
ENSMUSG	Drc1	ENSMUST00000101448;ENSMUST00000200183;ENSMUST00000198
ENSMUSG	Gm10466	ENSMUST00000101450
ENSMUSG	Gm10471	ENSMUST00000094946
ENSMUSG	Gm7347	ENSMUST00000095004
ENSMUSG	Speer4a	ENSMUST00000079447
ENSMUSG	Gm1141	ENSMUST00000154780;ENSMUST00000101495
ENSMUSG	4930567	ENSMUST00000092405;ENSMUST00000193512
ENSMUSG	9330158	ENSMUST00000160156;ENSMUST00000159965
ENSMUSG	Gm29254	ENSMUST00000101556;ENSMUST00000189302
ENSMUSG	Zfp449	ENSMUST00000101560
ENSMUSG	Ccdc160	ENSMUST00000101588
ENSMUSG	Speer4c	ENSMUST00000095005;ENSMUST00000179506
ENSMUSG	Klf14	ENSMUST00000101589
ENSMUSG	Gm8773	ENSMUST00000101627
ENSMUSG	Gm9	ENSMUST00000101636
ENSMUSG	Gm14636	ENSMUST00000143129;ENSMUST00000150801
ENSMUSG	Nudt10	ENSMUST00000103006
ENSMUSG	Nudt11	ENSMUST00000103007

ENSMUSG	Gm6594	ENSMUST00000097278
ENSMUSG	Lrrc30	ENSMUST00000097290
ENSMUSG	9830107	ENSMUST00000063481
ENSMUSG	Esp1	ENSMUST00000161110
ENSMUSG	Trim40	ENSMUST00000087158;ENSMUST00000172711;ENSMUST00000172
ENSMUSG	Trim10	ENSMUST00000060524
ENSMUSG	Btnl5-p	ENSMUST00000097346;ENSMUST00000173333
ENSMUSG	Cyp4f15	ENSMUST00000168171
ENSMUSG	Gm10505	ENSMUST00000151398
ENSMUSG	Sh2d1b2	ENSMUST00000162752
ENSMUSG	AA79289	ENSMUST00000202253
ENSMUSG	Dok6	ENSMUST00000097495;ENSMUST00000160328
ENSMUSG	Gm10530	ENSMUST00000097514
ENSMUSG	Gm7276	ENSMUST00000097520
ENSMUSG	Gm5532	ENSMUST00000097531;ENSMUST00000194133
ENSMUSG	Gm6960	ENSMUST00000051629
ENSMUSG	Prr16	ENSMUST00000116639
ENSMUSG	Arl14ep	ENSMUST00000097580
ENSMUSG	Gm10540	ENSMUST00000097581
ENSMUSG	Spink11	ENSMUST00000185266
ENSMUSG	Serpinb	ENSMUST00000027565
ENSMUSG	Serpinb	ENSMUST00000086694
ENSMUSG	Catip	ENSMUST00000097697;ENSMUST00000128445;ENSMUST00000186
ENSMUSG	Crygb	ENSMUST00000027090
ENSMUSG	Pgap1	ENSMUST00000097739;ENSMUST00000185817;ENSMUST00000187
ENSMUSG	Mxra8os	ENSMUST00000097740
ENSMUSG	Gm10564	ENSMUST00000097750
ENSMUSG	4931408	ENSMUST00000097801
ENSMUSG	4933415	ENSMUST00000073179
ENSMUSG	Rsg1	ENSMUST00000097813;ENSMUST00000151475
ENSMUSG	Gm10566	ENSMUST00000086739
ENSMUSG	Gm7534	ENSMUST00000097849
ENSMUSG	4933407	ENSMUST00000133499
ENSMUSG	4933427	ENSMUST00000097896
ENSMUSG	Gm12888	ENSMUST00000097905
ENSMUSG	Gm12863	ENSMUST00000155069
ENSMUSG	Olfr133	ENSMUST00000215186;ENSMUST00000094830
ENSMUSG	Olfr133	ENSMUST00000106360;ENSMUST00000094831
ENSMUSG	Kncn	ENSMUST00000097918;ENSMUST00000128222
ENSMUSG	Gm6471	ENSMUST00000148734
ENSMUSG	Krtap5-	ENSMUST00000097942
ENSMUSG	I0C0044	ENSMUST00000097964;ENSMUST00000168045
ENSMUSG	4930544	ENSMUST00000097982
ENSMUSG	Nps	ENSMUST00000210907
ENSMUSG	Ifna12	ENSMUST00000102806
ENSMUSG	Gm21957	ENSMUST00000073056
ENSMUSG	Olfr472	ENSMUST00000216937
ENSMUSG	Olfr713	ENSMUST00000213623;ENSMUST00000098139
ENSMUSG	Olfr692	ENSMUST00000098152;ENSMUST00000217827
ENSMUSG	Olfr689	ENSMUST00000215413;ENSMUST00000098153
ENSMUSG	Olfr687	ENSMUST00000098154
ENSMUSG	Olfr688	ENSMUST00000098155
ENSMUSG	Olfr676	ENSMUST00000098162
ENSMUSG	Olfr669	ENSMUST00000098164
ENSMUSG	Olfr657	ENSMUST00000215454;ENSMUST00000098171
ENSMUSG	Olfr656	ENSMUST00000098172
ENSMUSG	Olfr654	ENSMUST00000213984;ENSMUST00000210457

ENSMUSG	Olfr653	ENSMUST00000217466;ENSMUST00000098174
ENSMUSG	Olfr652	ENSMUST00000098175
ENSMUSG	Olfr646	ENSMUST00000214099;ENSMUST00000098182
ENSMUSG	Olfr618	ENSMUST00000098197
ENSMUSG	Olfr608	ENSMUST00000213546
ENSMUSG	Olfr561	ENSMUST00000098217
ENSMUSG	Olfr556	ENSMUST00000213485;ENSMUST00000098219
ENSMUSG	Olfr554	ENSMUST00000098221
ENSMUSG	Olfr553	ENSMUST00000098222
ENSMUSG	Olfr552	ENSMUST00000215712
ENSMUSG	Olfr551	ENSMUST00000098224
ENSMUSG	Olfr550	ENSMUST00000216524
ENSMUSG	1700026	ENSMUST00000131357
ENSMUSG	Olfr549	ENSMUST00000098227
ENSMUSG	Olfr548	ENSMUST00000098228
ENSMUSG	Olfr547	ENSMUST00000098229
ENSMUSG	Ttpa	ENSMUST00000121491;ENSMUST00000117632;ENSMUST00000098
ENSMUSG	Klhl40	ENSMUST00000216358;ENSMUST00000098272
ENSMUSG	Gucy2d	ENSMUST00000206435;ENSMUST00000205350;ENSMUST0000020€
ENSMUSG	Gm10608	ENSMUST00000093527
ENSMUSG	Gm5921	ENSMUST00000075898
ENSMUSG	Fbxw18	ENSMUST00000098359
ENSMUSG	Fbxw16	ENSMUST00000196410
ENSMUSG	Osgin1	ENSMUST00000131448;ENSMUST00000152420;ENSMUST00000145
ENSMUSG	Gm10619	ENSMUST00000186073;ENSMUST00000189466
ENSMUSG	Gm6181	ENSMUST00000117187
ENSMUSG	Svip	ENSMUST00000098414;ENSMUST00000207457
ENSMUSG	Rpl10-p	ENSMUST00000098432
ENSMUSG	Mrgpra9	ENSMUST00000179005
ENSMUSG	Saa1	ENSMUST00000210547;ENSMUST00000128088
ENSMUSG	Klk5	ENSMUST00000205919;ENSMUST00000048444
ENSMUSG	Ces1h	ENSMUST00000145041
ENSMUSG	Vmn2r-p	ENSMUST00000150856;ENSMUST00000150147;ENSMUST00000182
ENSMUSG	Zfp791	ENSMUST00000211109;ENSMUST00000098550
ENSMUSG	Clca4b	ENSMUST00000098549
ENSMUSG	Krtdap	ENSMUST00000190480;ENSMUST00000189673;ENSMUST0000019C
ENSMUSG	Adh6b	ENSMUST00000199673;ENSMUST00000199718;ENSMUST0000009C
ENSMUSG	2210011	ENSMUST00000190457;ENSMUST00000185457
ENSMUSG	4932431	ENSMUST00000141713
ENSMUSG	Gm10647	ENSMUST00000098612
ENSMUSG	Gm9387	ENSMUST00000098624
ENSMUSG	Cib3	ENSMUST00000211946;ENSMUST00000098630
ENSMUSG	Gm10651	ENSMUST00000118212
ENSMUSG	Gm10654	ENSMUST00000098653
ENSMUSG	Cyp2a4	ENSMUST00000098657
ENSMUSG	Gramd2	ENSMUST00000128944;ENSMUST00000098661;ENSMUST00000133
ENSMUSG	BC03087	ENSMUST00000098713
ENSMUSG	I830077	ENSMUST00000098758;ENSMUST00000196099;ENSMUST0000012€
ENSMUSG	Tmigd3	ENSMUST00000197907;ENSMUST00000200482;ENSMUST0000010
ENSMUSG	Tnfaip8	ENSMUST00000098760;ENSMUST00000214656
ENSMUSG	Psg23	ENSMUST00000057810
ENSMUSG	C5ar2	ENSMUST00000098792;ENSMUST00000171425
ENSMUSG	Crxos	ENSMUST00000125993;ENSMUST00000098801;ENSMUST0000015C
ENSMUSG	AI42921	ENSMUST00000098825
ENSMUSG	Gm10689	ENSMUST00000098864
ENSMUSG	Gm10696	ENSMUST00000167916;ENSMUST00000161475
ENSMUSG	Smcp	ENSMUST00000098888

ENSMUSG	Defa29	ENSMUST00000098890
ENSMUSG	Defa3	ENSMUST00000098893
ENSMUSG	Defa30	ENSMUST00000098898
ENSMUSG	Sprr2a3	ENSMUST00000193337;ENSMUST00000090872
ENSMUSG	Gm5615	ENSMUST00000098900
ENSMUSG	Defb33	ENSMUST00000098908
ENSMUSG	Zfp872	ENSMUST00000178901
ENSMUSG	A230072	ENSMUST00000098935
ENSMUSG	Bglap	ENSMUST00000076048;ENSMUST00000128732
ENSMUSG	Gm7381	ENSMUST00000098958
ENSMUSG	Bglap2	ENSMUST00000098956
ENSMUSG	Bglap3	ENSMUST00000152477;ENSMUST00000140259;ENSMUST0000007[illegible]
ENSMUSG	Zfp558	ENSMUST00000159596;ENSMUST00000034647;ENSMUST00000034
ENSMUSG	Fat3	ENSMUST00000217187;ENSMUST00000217308;ENSMUST00000082
ENSMUSG	Gm10706	ENSMUST00000128327
ENSMUSG	Gm10709	ENSMUST00000213318
ENSMUSG	Eif1-ps	ENSMUST00000086606
ENSMUSG	Kcng1	ENSMUST00000109191
ENSMUSG	Lekr1	ENSMUST00000099075;ENSMUST00000107848;ENSMUST0000019[illegible]
ENSMUSG	Ankub1	ENSMUST00000200665;ENSMUST00000099091
ENSMUSG	Wfdc6a	ENSMUST00000099096
ENSMUSG	Tox2	ENSMUST00000109428;ENSMUST00000128999;ENSMUST0000009[illegible]
ENSMUSG	Gm826	ENSMUST00000109475;ENSMUST00000138905;ENSMUST0000009[illegible]
ENSMUSG	Arhgap4	ENSMUST00000099133
ENSMUSG	Sox2	ENSMUST00000099151
ENSMUSG	Rdh16f2	ENSMUST00000092058
ENSMUSG	Mcidas	ENSMUST00000092089
ENSMUSG	Lrrc31	ENSMUST00000126658;ENSMUST00000108263;ENSMUST00000194
ENSMUSG	Bpifb4	ENSMUST00000109757;ENSMUST00000099181
ENSMUSG	Foxs1	ENSMUST00000099200
ENSMUSG	Sirpb1c	ENSMUST00000108352;ENSMUST00000050623;ENSMUST0000010[illegible]
ENSMUSG	Defb25	ENSMUST00000099203
ENSMUSG	Defb23	ENSMUST00000099206
ENSMUSG	Zcchc3	ENSMUST00000099207
ENSMUSG	Il22	ENSMUST00000096691
ENSMUSG	Ccl28	ENSMUST00000099241;ENSMUST00000225880
ENSMUSG	Zfp345	ENSMUST00000109914
ENSMUSG	Syndig1	ENSMUST00000109934
ENSMUSG	Sel1l2	ENSMUST00000122367;ENSMUST00000134475
ENSMUSG	Ism1	ENSMUST00000184404
ENSMUSG	Bhmt	ENSMUST00000099309;ENSMUST00000124193
ENSMUSG	Ankef1	ENSMUST00000154394;ENSMUST00000150189;ENSMUST0000014[illegible]
ENSMUSG	Gm10754	ENSMUST00000220233
ENSMUSG	Anapc15	ENSMUST00000218771
ENSMUSG	AU01999	ENSMUST00000143964;ENSMUST00000153028;ENSMUST0000013[illegible]
ENSMUSG	AU01522	ENSMUST00000121103
ENSMUSG	Spdye4c	ENSMUST00000155430;ENSMUST00000144559
ENSMUSG	Papolb	ENSMUST00000099400
ENSMUSG	Cbx3-ps	ENSMUST00000091539
ENSMUSG	Gm10775	ENSMUST00000099425
ENSMUSG	Spata31	ENSMUST00000099427
ENSMUSG	Ctsm	ENSMUST00000223778
ENSMUSG	Ctla2b	ENSMUST00000225439;ENSMUST00000225732;ENSMUST00000021
ENSMUSG	Gm10780	ENSMUST00000099459
ENSMUSG	Eif4e1b	ENSMUST00000110003
ENSMUSG	Sptbn5	ENSMUST00000125737;ENSMUST00000110756;ENSMUST00000134
ENSMUSG	Chst14	ENSMUST00000099546;ENSMUST00000110837

ENSMUSG	Pak6	ENSMUST00000099557;ENSMUST00000152550;ENSMUST00000132
ENSMUSG	Grem1	ENSMUST00000099575
ENSMUSG	Chrm5	ENSMUST00000099589
ENSMUSG	Olfr131	ENSMUST00000207976
ENSMUSG	Olfr131	ENSMUST00000099600
ENSMUSG	Ano3	ENSMUST00000099623;ENSMUST00000140777
ENSMUSG	Fibin	ENSMUST00000099626
ENSMUSG	Wt1os	ENSMUST00000135153;ENSMUST00000172701;ENSMUST0000009
ENSMUSG	Fjx1	ENSMUST00000099678
ENSMUSG	Accsl	ENSMUST00000099690;ENSMUST00000151579
ENSMUSG	Gm10805	ENSMUST00000099710
ENSMUSG	4930431	ENSMUST00000166127;ENSMUST00000166072
ENSMUSG	Gm4981	ENSMUST00000099726
ENSMUSG	Duxf3	ENSMUST00000176875
ENSMUSG	Yae1d1	ENSMUST00000099735;ENSMUST00000222796;ENSMUST00000222
ENSMUSG	Olfr150	ENSMUST00000213994
ENSMUSG	Olfr48	ENSMUST00000216674
ENSMUSG	Olfr125	ENSMUST00000099763
ENSMUSG	Olfr125	ENSMUST00000215730
ENSMUSG	Olfr124	ENSMUST00000111532
ENSMUSG	Olfr120	ENSMUST00000099811
ENSMUSG	Olfr119	ENSMUST00000215529
ENSMUSG	Olfr119	ENSMUST00000099815
ENSMUSG	Olfr119	ENSMUST00000104892
ENSMUSG	Olfr117	ENSMUST00000216887
ENSMUSG	Olfr116	ENSMUST00000099834
ENSMUSG	Olfr113	ENSMUST00000214209
ENSMUSG	Olfr113	ENSMUST00000077471
ENSMUSG	Olfr113	ENSMUST00000099855
ENSMUSG	Olfr110	ENSMUST00000099869
ENSMUSG	F930017	ENSMUST00000215599;ENSMUST00000213342;ENSMUST0000009
ENSMUSG	Olfr106	ENSMUST00000099889
ENSMUSG	Olfr101	ENSMUST00000213515;ENSMUST00000102634
ENSMUSG	Olfr101	ENSMUST00000099915
ENSMUSG	Olfr100	ENSMUST00000099920
ENSMUSG	4833423	ENSMUST00000099923
ENSMUSG	Olfr995	ENSMUST00000099924
ENSMUSG	Olfr994	ENSMUST00000099925
ENSMUSG	Olfr992	ENSMUST00000213749
ENSMUSG	Olfr988	ENSMUST00000215511;ENSMUST00000099928
ENSMUSG	Olfr987	ENSMUST00000216347
ENSMUSG	Lrrc55	ENSMUST00000099930;ENSMUST00000111601
ENSMUSG	Cenpw	ENSMUST00000216853;ENSMUST00000099985
ENSMUSG	Dfnb59	ENSMUST00000099986
ENSMUSG	Bex6	ENSMUST00000099990
ENSMUSG	Ttc30a1	ENSMUST00000099994
ENSMUSG	Ttc30a2	ENSMUST00000099995
ENSMUSG	Ttc30b	ENSMUST00000099996
ENSMUSG	Haglr	ENSMUST00000100000;ENSMUST00000152462
ENSMUSG	Erich2	ENSMUST00000100041;ENSMUST00000134607;ENSMUST00000136
ENSMUSG	Sp5	ENSMUST00000100043
ENSMUSG	Klhl41	ENSMUST00000100050
ENSMUSG	Gm1322	ENSMUST00000134620
ENSMUSG	Fign	ENSMUST00000131615;ENSMUST00000102728;ENSMUST00000126
ENSMUSG	Gm13582	ENSMUST00000146034
ENSMUSG	Rprm	ENSMUST00000100089
ENSMUSG	Igll1	ENSMUST00000124890;ENSMUST00000100136;ENSMUST00000124

ENSMUSG	Olfr361	ENSMUST00000214969
ENSMUSG	2810410	ENSMUST00000155681;ENSMUST00000145437
ENSMUSG	Hoxc4	ENSMUST00000165375;ENSMUST00000100164
ENSMUSG	Krt76	ENSMUST00000100179
ENSMUSG	9430097	ENSMUST00000100190
ENSMUSG	6030408	ENSMUST00000071328;ENSMUST00000191426
ENSMUSG	Gm14488	ENSMUST00000134755;ENSMUST00000131207
ENSMUSG	Olfr288	ENSMUST00000205524;ENSMUST00000205605;ENSMUST00000154
ENSMUSG	Gm11691	ENSMUST00000130963
ENSMUSG	Gm11681	ENSMUST00000127337
ENSMUSG	Slitrk1	ENSMUST00000100322
ENSMUSG	Gm1587	ENSMUST00000100372
ENSMUSG	Cyp2d37	ENSMUST00000100380
ENSMUSG	4930407	ENSMUST00000100396
ENSMUSG	Urad	ENSMUST00000100433
ENSMUSG	Gm10863	ENSMUST00000131710;ENSMUST00000147620;ENSMUST00000148
ENSMUSG	Krtap4-	ENSMUST00000100476
ENSMUSG	Krt26	ENSMUST00000148770
ENSMUSG	Defb30	ENSMUST00000111207
ENSMUSG	Hoxb2	ENSMUST00000182030;ENSMUST00000100523
ENSMUSG	Gm11536	ENSMUST00000130733
ENSMUSG	Nynrin	ENSMUST00000168479
ENSMUSG	Smok3c	ENSMUST00000178402
ENSMUSG	Cyp11b1	ENSMUST00000170259
ENSMUSG	Slurp2	ENSMUST00000057932
ENSMUSG	Tmem92	ENSMUST00000100554;ENSMUST00000143620;ENSMUST00000162
ENSMUSG	Gm11545	ENSMUST00000146507
ENSMUSG	Dio3	ENSMUST00000173014;ENSMUST00000097228
ENSMUSG	Gm24121	ENSMUST00000102029
ENSMUSG	Mir376c	ENSMUST00000102052
ENSMUSG	Gm22751	ENSMUST00000102077
ENSMUSG	Mir450b	ENSMUST00000102095
ENSMUSG	Mir541	ENSMUST00000102097
ENSMUSG	Mir223	ENSMUST00000102112
ENSMUSG	Mir680-	ENSMUST00000102163
ENSMUSG	Gm26384	ENSMUST00000102183
ENSMUSG	Mir542	ENSMUST00000102186
ENSMUSG	Mir711	ENSMUST00000102188
ENSMUSG	Mir679	ENSMUST00000102191
ENSMUSG	Mir694	ENSMUST00000102193
ENSMUSG	Gm24938	ENSMUST00000102225
ENSMUSG	Mir487b	ENSMUST00000102265
ENSMUSG	Gm22758	ENSMUST00000102275
ENSMUSG	Gm22760	ENSMUST00000102277
ENSMUSG	Gm22757	ENSMUST00000102279
ENSMUSG	Gm26300	ENSMUST00000102282
ENSMUSG	Mir543	ENSMUST00000102286
ENSMUSG	Mir680-	ENSMUST00000102298
ENSMUSG	Mir666	ENSMUST00000102317
ENSMUSG	Mir675	ENSMUST00000102320
ENSMUSG	Mir665	ENSMUST00000102358
ENSMUSG	Gm26339	ENSMUST00000102368
ENSMUSG	Gm24715	ENSMUST00000102377
ENSMUSG	Gm22369	ENSMUST00000102415
ENSMUSG	Gm22368	ENSMUST00000102416
ENSMUSG	Mir190a	ENSMUST00000102424
ENSMUSG	Mir667	ENSMUST00000102441

ENSMUSG	Mir450-	ENSMUST00000102444
ENSMUSG	Oxct2a	ENSMUST00000102640
ENSMUSG	Mog	ENSMUST00000102665
ENSMUSG	Mir763	ENSMUST00000103249
ENSMUSG	Mir804	ENSMUST00000103251
ENSMUSG	Mir770	ENSMUST00000103252
ENSMUSG	Gm20730	ENSMUST00000103301
ENSMUSG	Igkv1-1	ENSMUST00000103306
ENSMUSG	Igkv1-9	ENSMUST00000168090
ENSMUSG	Igkv11-	ENSMUST00000103331
ENSMUSG	Igkv4-9	ENSMUST00000103332
ENSMUSG	Igkv4-9	ENSMUST00000103334
ENSMUSG	Igkv12-	ENSMUST00000198880
ENSMUSG	Igkv5-3	ENSMUST00000103372
ENSMUSG	Igkv1-3	ENSMUST00000197820
ENSMUSG	Igkv7-3	ENSMUST00000197429
ENSMUSG	Igkv8-2	ENSMUST00000103382
ENSMUSG	Igkv8-1	ENSMUST00000103390
ENSMUSG	Igkv3-7	ENSMUST00000197560;ENSMUST00000103399
ENSMUSG	Ighv2-6	ENSMUST00000192911
ENSMUSG	Ighv13-	ENSMUST00000183954;ENSMUST00000125484
ENSMUSG	Ighv12-	ENSMUST00000103485
ENSMUSG	Ighv15-	ENSMUST00000103497;ENSMUST00000195337
ENSMUSG	Ighv1-4	ENSMUST00000103518;ENSMUST00000194865
ENSMUSG	Tcrg-V3	ENSMUST00000198663
ENSMUSG	Trav1	ENSMUST00000103567
ENSMUSG	Trav3-1	ENSMUST00000103569
ENSMUSG	Trav6-1	ENSMUST00000103571
ENSMUSG	Trav6-2	ENSMUST00000103573;ENSMUST00000198058
ENSMUSG	Trav18	ENSMUST00000103673
ENSMUSG	Gm22065	ENSMUST00000103753
ENSMUSG	Gm22066	ENSMUST00000103761
ENSMUSG	Gm23251	ENSMUST00000103773
ENSMUSG	Mir544	ENSMUST00000103813
ENSMUSG	Gm22191	ENSMUST00000103831
ENSMUSG	Mir493	ENSMUST00000103837
ENSMUSG	Gm23697	ENSMUST00000103897
ENSMUSG	Gm22117	ENSMUST00000103902
ENSMUSG	Gm23577	ENSMUST00000103950
ENSMUSG	Gm22936	ENSMUST00000103955
ENSMUSG	Gm22934	ENSMUST00000103961
ENSMUSG	n-R5s90	ENSMUST00000103968
ENSMUSG	Gm24578	ENSMUST00000103970
ENSMUSG	Gm24117	ENSMUST00000103973
ENSMUSG	Gm25762	ENSMUST00000103984
ENSMUSG	Gm25757	ENSMUST00000103986
ENSMUSG	Gm25759	ENSMUST00000103988
ENSMUSG	Gm25093	ENSMUST00000103993
ENSMUSG	Gm25092	ENSMUST00000103996
ENSMUSG	Snord64	ENSMUST00000104003
ENSMUSG	Gm22267	ENSMUST00000104008
ENSMUSG	Gm22439	ENSMUST00000104048
ENSMUSG	Gm23270	ENSMUST00000104055
ENSMUSG	Gm23275	ENSMUST00000104060
ENSMUSG	Gm24429	ENSMUST00000104076
ENSMUSG	Gm22784	ENSMUST00000104088
ENSMUSG	Gm24125	ENSMUST00000104105

ENSMUSG	Gm22473	ENSMUST00000104114
ENSMUSG	Gm22469	ENSMUST00000104118
ENSMUSG	n-R5s8	ENSMUST00000104121
ENSMUSG	Gm24171	ENSMUST00000104126
ENSMUSG	n-R5s17	ENSMUST00000104128
ENSMUSG	Gm24173	ENSMUST00000104130
ENSMUSG	Gm25832	ENSMUST00000104140
ENSMUSG	Gm23014	ENSMUST00000104144
ENSMUSG	Gm23011	ENSMUST00000104148
ENSMUSG	Gm23012	ENSMUST00000104149
ENSMUSG	Gm23484	ENSMUST00000104178
ENSMUSG	Gm24334	ENSMUST00000104210
ENSMUSG	Gm25417	ENSMUST00000104212
ENSMUSG	Gm25418	ENSMUST00000104214
ENSMUSG	Gm25421	ENSMUST00000104218
ENSMUSG	Gm25422	ENSMUST00000104219
ENSMUSG	Gm24281	ENSMUST00000104234
ENSMUSG	Gm24279	ENSMUST00000104236
ENSMUSG	Gm22593	ENSMUST00000104245
ENSMUSG	Gm23129	ENSMUST00000104259
ENSMUSG	Gm23127	ENSMUST00000104261
ENSMUSG	Gm25915	ENSMUST00000104265
ENSMUSG	Gm26420	ENSMUST00000104273
ENSMUSG	Gm26418	ENSMUST00000104275
ENSMUSG	Gm24789	ENSMUST00000104282
ENSMUSG	Gm24791	ENSMUST00000104284
ENSMUSG	Gm25274	ENSMUST00000104296
ENSMUSG	Gm25275	ENSMUST00000104297
ENSMUSG	Gm23440	ENSMUST00000104299
ENSMUSG	Gm25276	ENSMUST00000104300
ENSMUSG	Gm23596	ENSMUST00000104304
ENSMUSG	Gm24231	ENSMUST00000104315
ENSMUSG	Gm25875	ENSMUST00000104323
ENSMUSG	n-R5s21	ENSMUST00000104328
ENSMUSG	Gm25879	ENSMUST00000104330
ENSMUSG	Gm22023	ENSMUST00000104354
ENSMUSG	Gm22020	ENSMUST00000104358
ENSMUSG	Gm22019	ENSMUST00000104359
ENSMUSG	Gm23709	ENSMUST00000104364
ENSMUSG	Gm25631	ENSMUST00000104390
ENSMUSG	Gm24891	ENSMUST00000104391
ENSMUSG	Gm26016	ENSMUST00000104403
ENSMUSG	Gm25803	ENSMUST00000104419
ENSMUSG	Gm25498	ENSMUST00000104424
ENSMUSG	Gm25501	ENSMUST00000104429
ENSMUSG	Gm25496	ENSMUST00000104430
ENSMUSG	Gm23769	ENSMUST00000104441
ENSMUSG	Gm22149	ENSMUST00000104442
ENSMUSG	Gm24986	ENSMUST00000104452
ENSMUSG	Gm23320	ENSMUST00000104462
ENSMUSG	Gm23319	ENSMUST00000104467
ENSMUSG	Gm23323	ENSMUST00000104471
ENSMUSG	Gm25344	ENSMUST00000104503
ENSMUSG	Gm24071	ENSMUST00000104520
ENSMUSG	Gm23609	ENSMUST00000104542
ENSMUSG	Gm23529	ENSMUST00000104562
ENSMUSG	Gm22371	ENSMUST00000104588

ENSMUSG	Gm25564	ENSMUST00000104604
ENSMUSG	Gm25565	ENSMUST00000104605
ENSMUSG	Mir105	ENSMUST00000104655
ENSMUSG	Mir453	ENSMUST00000104732
ENSMUSG	Mir874	ENSMUST00000104769
ENSMUSG	Mir190b	ENSMUST00000104803
ENSMUSG	Gm26301	ENSMUST00000104813
ENSMUSG	Gm10912	ENSMUST00000104891
ENSMUSG	Gm10913	ENSMUST00000133732
ENSMUSG	F630028	ENSMUST00000133125;ENSMUST00000147681
ENSMUSG	Krtap1-	ENSMUST00000104930
ENSMUSG	Gm2399	ENSMUST00000104944
ENSMUSG	Erich3	ENSMUST00000098496;ENSMUST00000051862;ENSMUST0000018
ENSMUSG	Rbm8a2	ENSMUST00000104983
ENSMUSG	Gm7290	ENSMUST00000104990
ENSMUSG	Klhdc7a	ENSMUST00000105031
ENSMUSG	Fam43b	ENSMUST00000105032
ENSMUSG	Krtap17	ENSMUST00000105049
ENSMUSG	Krtap16	ENSMUST00000105050
ENSMUSG	Krtap29	ENSMUST00000105051
ENSMUSG	Gm11565	ENSMUST00000105053
ENSMUSG	Foxd1	ENSMUST00000105098
ENSMUSG	AI59344	ENSMUST00000213937
ENSMUSG	Gm7293	ENSMUST00000089534
ENSMUSG	Gm14762	ENSMUST00000105109;ENSMUST00000122953
ENSMUSG	Sf3b5	ENSMUST00000105139
ENSMUSG	Ifna2	ENSMUST00000105147
ENSMUSG	Mos	ENSMUST00000105158
ENSMUSG	Mrpl48-	ENSMUST00000121413
ENSMUSG	Aadacl3	ENSMUST00000105749
ENSMUSG	Gm13128	ENSMUST00000105751
ENSMUSG	Pramef6	ENSMUST00000105767
ENSMUSG	Nkain1	ENSMUST00000180884;ENSMUST00000105993
ENSMUSG	B020031	ENSMUST00000180768;ENSMUST00000080505
ENSMUSG	Tmco2	ENSMUST00000106268
ENSMUSG	Gm11735	ENSMUST00000106377
ENSMUSG	Hs3st4	ENSMUST00000106437
ENSMUSG	Gm4609	ENSMUST00000106443
ENSMUSG	Cyp4a12	ENSMUST00000094887
ENSMUSG	Skint5	ENSMUST00000169631
ENSMUSG	Gm12525	ENSMUST00000125412
ENSMUSG	E030025	ENSMUST00000106710
ENSMUSG	Trim30c	ENSMUST00000106828
ENSMUSG	Olfr613	ENSMUST00000106888
ENSMUSG	Gm12790	ENSMUST00000106916
ENSMUSG	March10	ENSMUST00000049995;ENSMUST00000130685;ENSMUST0000010
ENSMUSG	Lrrc37a	ENSMUST00000153273
ENSMUSG	Gm12695	ENSMUST00000107071
ENSMUSG	Crnn	ENSMUST00000107300;ENSMUST00000195515
ENSMUSG	Sprr2a1	ENSMUST00000074449
ENSMUSG	Fam174b	ENSMUST00000107456;ENSMUST00000206337
ENSMUSG	Mup16	ENSMUST00000107483
ENSMUSG	Mup10	ENSMUST00000098047;ENSMUST00000148302
ENSMUSG	Mup9	ENSMUST00000122177
ENSMUSG	Mup6	ENSMUST00000107517
ENSMUSG	Gm6821	ENSMUST00000107540
ENSMUSG	Gm53	ENSMUST00000129907;ENSMUST00000150490;ENSMUST0000014

ENSMUSG	Gm2128	ENSMUST00000108009
ENSMUSG	Fam205a	ENSMUST00000108026
ENSMUSG	Scgb1b3	ENSMUST00000108083
ENSMUSG	Gm5150	ENSMUST00000194367
ENSMUSG	Gm9733	ENSMUST00000108361
ENSMUSG	Dact3	ENSMUST00000108493
ENSMUSG	Ceacam1	ENSMUST00000108498
ENSMUSG	Zfp541	ENSMUST00000209369
ENSMUSG	Gp6	ENSMUST00000206928
ENSMUSG	Cacng6	ENSMUST00000183200
ENSMUSG	Nlrp12	ENSMUST00000108653
ENSMUSG	Gm17382	ENSMUST00000108741
ENSMUSG	Gm10999	ENSMUST00000108743
ENSMUSG	Gm14308	ENSMUST00000126807;ENSMUST00000108990;ENSMUST0000011[illegible]
ENSMUSG	Gm14434	ENSMUST00000122097;ENSMUST00000120959;ENSMUST0000012[illegible]
ENSMUSG	Gm11009	ENSMUST00000108961
ENSMUSG	Gm14393	ENSMUST00000072895;ENSMUST00000109066
ENSMUSG	Gm14444	ENSMUST00000109069;ENSMUST00000109070
ENSMUSG	Gm11281	ENSMUST00000141391
ENSMUSG	Gm12169	ENSMUST00000109228
ENSMUSG	D030018[illegible]	ENSMUST00000139808;ENSMUST00000140253
ENSMUSG	CN72542	ENSMUST00000190436
ENSMUSG	R3hdml	ENSMUST00000109416
ENSMUSG	Lncenc1	ENSMUST00000109431;ENSMUST00000179324
ENSMUSG	Gm11020	ENSMUST00000109447
ENSMUSG	Gm14222	ENSMUST00000128681
ENSMUSG	Gm14221	ENSMUST00000109473;ENSMUST00000209603
ENSMUSG	1700060[illegible]	ENSMUST00000109506
ENSMUSG	Atp6ap1[illegible]	ENSMUST00000109541;ENSMUST00000182932
ENSMUSG	Ces1b	ENSMUST00000109582
ENSMUSG	Bpifa6	ENSMUST00000109753
ENSMUSG	Dnmt3bo	ENSMUST00000124988
ENSMUSG	Tcl1b4	ENSMUST00000222867
ENSMUSG	Gm14124	ENSMUST00000109922
ENSMUSG	Gm14139	ENSMUST00000109926
ENSMUSG	Col22a1	ENSMUST00000159993
ENSMUSG	Gsdmc	ENSMUST00000110125
ENSMUSG	Gm5662	ENSMUST00000101168
ENSMUSG	BB28746	ENSMUST00000110149
ENSMUSG	Gm8300	ENSMUST00000110152
ENSMUSG	Prox1os	ENSMUST00000110279
ENSMUSG	4933413[illegible]	ENSMUST00000111588;ENSMUST00000110292
ENSMUSG	Serpinb	ENSMUST00000021834
ENSMUSG	Slc8a3	ENSMUST00000085238;ENSMUST00000064594;ENSMUST00000182
ENSMUSG	Gm11042	ENSMUST00000110441
ENSMUSG	Gm3985	ENSMUST00000132101
ENSMUSG	Gm2904	ENSMUST00000170684
ENSMUSG	Tgm7	ENSMUST00000110675
ENSMUSG	Fam90a1[illegible]	ENSMUST00000110741
ENSMUSG	AY76118	ENSMUST00000110758
ENSMUSG	Rpl27-p	ENSMUST00000088784
ENSMUSG	Gm6374	ENSMUST00000159051
ENSMUSG	Fam155a	ENSMUST00000110969;ENSMUST00000208171;ENSMUST00000207
ENSMUSG	Tlr5	ENSMUST00000193687;ENSMUST00000110997;ENSMUST0000019[illegible]
ENSMUSG	Cd209g	ENSMUST00000207281;ENSMUST00000130372
ENSMUSG	Gm13941	ENSMUST00000127918
ENSMUSG	Gm3054	ENSMUST00000111075

ENSMUSG	Gzmc	ENSMUST00000015585
ENSMUSG	Ccr5	ENSMUST00000111442;ENSMUST00000169553;ENSMUST0000016[illegible]
ENSMUSG	Gm11065	ENSMUST00000111462
ENSMUSG	Ccdc13	ENSMUST00000135986
ENSMUSG	Gm13691	ENSMUST00000099947
ENSMUSG	Calhm1	ENSMUST00000111813
ENSMUSG	Gm15217	ENSMUST00000111826
ENSMUSG	Gm6614	ENSMUST00000181791
ENSMUSG	Gm5930	ENSMUST00000111863
ENSMUSG	Hoxd3	ENSMUST00000111983;ENSMUST00000144544;ENSMUST00000111
ENSMUSG	Tmem233	ENSMUST00000111997
ENSMUSG	Lemd1	ENSMUST00000188169;ENSMUST00000188175;ENSMUST0000018[illegible]
ENSMUSG	Ifit1bl	ENSMUST00000168254;ENSMUST00000112467
ENSMUSG	Gm3558	ENSMUST00000163790
ENSMUSG	Gm3476	ENSMUST00000167913
ENSMUSG	Gm8271	ENSMUST00000171053
ENSMUSG	Gm3443	ENSMUST00000112772
ENSMUSG	Gm2974	ENSMUST00000164512;ENSMUST00000180184
ENSMUSG	Gm3411	ENSMUST00000170104
ENSMUSG	Gm3020	ENSMUST00000167430;ENSMUST00000112801
ENSMUSG	1700110	ENSMUST00000112775
ENSMUSG	Gm5795	ENSMUST00000171150
ENSMUSG	Gm2897	ENSMUST00000166494
ENSMUSG	Gm11111	ENSMUST00000112956
ENSMUSG	Gm15023	ENSMUST00000113183
ENSMUSG	Neu2	ENSMUST00000166055;ENSMUST00000172222;ENSMUST00000164
ENSMUSG	Alpi	ENSMUST00000113270
ENSMUSG	B3gnt7	ENSMUST00000189618;ENSMUST00000113306
ENSMUSG	Cldn34c	ENSMUST00000113343
ENSMUSG	Tmprss1	ENSMUST00000134179;ENSMUST00000199703
ENSMUSG	4933403[illegible]	ENSMUST00000152343
ENSMUSG	Prdm12	ENSMUST00000113470
ENSMUSG	Mymx	ENSMUST00000178858;ENSMUST00000113529
ENSMUSG	1700011[illegible]	ENSMUST00000113602
ENSMUSG	Nhsl2	ENSMUST00000125115;ENSMUST00000124279;ENSMUST00000101
ENSMUSG	Nat8f5	ENSMUST00000032074
ENSMUSG	Nat8f6	ENSMUST00000174143
ENSMUSG	Gm13420	ENSMUST00000129574
ENSMUSG	Reg3a	ENSMUST00000101272
ENSMUSG	Clnkos	ENSMUST00000114080
ENSMUSG	Gm5936	ENSMUST00000114116
ENSMUSG	Gm5640	ENSMUST00000114119
ENSMUSG	Obp2b	ENSMUST00000114192
ENSMUSG	Igkv13-	ENSMUST00000114212
ENSMUSG	Gm11149	ENSMUST00000216483;ENSMUST00000068730;ENSMUST0000021[illegible]
ENSMUSG	Tmem182	ENSMUST00000114765
ENSMUSG	Gm5483	ENSMUST00000114858
ENSMUSG	Clec2l	ENSMUST00000114874
ENSMUSG	Gm13218	ENSMUST00000128180
ENSMUSG	Gm13219	ENSMUST00000144870;ENSMUST00000154223
ENSMUSG	Gm595	ENSMUST00000114928
ENSMUSG	Muc4	ENSMUST00000132475;ENSMUST00000096106;ENSMUST0000014[illegible]
ENSMUSG	Tm4sf19	ENSMUST00000115149
ENSMUSG	Rhox4g	ENSMUST00000115166;ENSMUST00000184625
ENSMUSG	Rhox4f	ENSMUST00000115170
ENSMUSG	Rhox4c	ENSMUST00000115183;ENSMUST00000173214
ENSMUSG	Rhox4a	ENSMUST00000115194;ENSMUST00000173298

ENSMUSG	Gm1110	ENSMUST00000217197;ENSMUST00000214461
ENSMUSG	Prrt4	ENSMUST00000159200
ENSMUSG	Vmn1r-p	ENSMUST00000176659
ENSMUSG	Gm14862	ENSMUST00000115553
ENSMUSG	Gm5751	ENSMUST00000115561
ENSMUSG	Gm6592	ENSMUST00000115563
ENSMUSG	Fpr3	ENSMUST00000115565
ENSMUSG	Ttll2	ENSMUST00000115747
ENSMUSG	Gm11172	ENSMUST00000115822
ENSMUSG	Nlrp4g	ENSMUST00000067621;ENSMUST00000214229
ENSMUSG	Klra1	ENSMUST00000032288;ENSMUST00000127570
ENSMUSG	Gm11951	ENSMUST00000116005
ENSMUSG	Gm11970	ENSMUST00000116105
ENSMUSG	Gm11799	ENSMUST00000116110
ENSMUSG	Gm5566	ENSMUST00000116118
ENSMUSG	Rps19-p	ENSMUST00000116122
ENSMUSG	Rpl12-p	ENSMUST00000116168
ENSMUSG	Gm11864	ENSMUST00000116179
ENSMUSG	Gm14448	ENSMUST00000116383
ENSMUSG	Gm13768	ENSMUST00000116469
ENSMUSG	Fam220-	ENSMUST00000116535
ENSMUSG	Gm14806	ENSMUST00000116606
ENSMUSG	Gm22468	ENSMUST00000116680
ENSMUSG	Gm25111	ENSMUST00000116686
ENSMUSG	Gm24587	ENSMUST00000116697
ENSMUSG	Gm26248	ENSMUST00000116700
ENSMUSG	Mir1247	ENSMUST00000116706
ENSMUSG	Gm25778	ENSMUST00000116713
ENSMUSG	Gm25775	ENSMUST00000116716
ENSMUSG	Gm25774	ENSMUST00000116717
ENSMUSG	Gm22102	ENSMUST00000116730
ENSMUSG	Gm22103	ENSMUST00000116733
ENSMUSG	Mir1188	ENSMUST00000116756
ENSMUSG	Mir1193	ENSMUST00000116761
ENSMUSG	Gm24831	ENSMUST00000116778
ENSMUSG	Gm23492	ENSMUST00000116837
ENSMUSG	Gm25338	ENSMUST00000116849
ENSMUSG	Gm25042	ENSMUST00000116856
ENSMUSG	Gm22197	ENSMUST00000116866
ENSMUSG	Gm23109	ENSMUST00000116867
ENSMUSG	Gm23882	ENSMUST00000116879
ENSMUSG	Gm25541	ENSMUST00000116888
ENSMUSG	Gm23844	ENSMUST00000116959
ENSMUSG	Gm25510	ENSMUST00000116986
ENSMUSG	Gm24482	ENSMUST00000117005
ENSMUSG	Gm24484	ENSMUST00000117009
ENSMUSG	Mir1199	ENSMUST00000117015
ENSMUSG	Gm23335	ENSMUST00000117021
ENSMUSG	Gm23336	ENSMUST00000117023
ENSMUSG	Gm23989	ENSMUST00000117030
ENSMUSG	Gm23990	ENSMUST00000117031
ENSMUSG	Gm6031	ENSMUST00000121314
ENSMUSG	Gm8107	ENSMUST00000117477
ENSMUSG	Gm5406	ENSMUST00000120054
ENSMUSG	Gm15366	ENSMUST00000118433
ENSMUSG	Gm13616	ENSMUST00000120600
ENSMUSG	Gm8084	ENSMUST00000120807

ENSMUSG	Gm6982	ENSMUST00000118752
ENSMUSG	Mup-ps2	ENSMUST00000121611
ENSMUSG	Gm7150	ENSMUST00000122032
ENSMUSG	Rpsa-ps	ENSMUST00000120596
ENSMUSG	Gm15661	ENSMUST00000117390
ENSMUSG	Gm12633	ENSMUST00000117742
ENSMUSG	Gm15595	ENSMUST00000120840
ENSMUSG	Gm12219	ENSMUST00000119198
ENSMUSG	Gm12955	ENSMUST00000119689
ENSMUSG	Gm9143	ENSMUST00000120745
ENSMUSG	Gm6368	ENSMUST00000119727
ENSMUSG	Gm15365	ENSMUST00000122392
ENSMUSG	Gm7199	ENSMUST00000118327
ENSMUSG	Gm8731	ENSMUST00000118088
ENSMUSG	Gm7841	ENSMUST00000122108
ENSMUSG	Gm14007	ENSMUST00000118288
ENSMUSG	Gm14367	ENSMUST00000119214
ENSMUSG	Gm15760	ENSMUST00000119553
ENSMUSG	Gm15166	ENSMUST00000120790
ENSMUSG	Gm13310	ENSMUST00000120962
ENSMUSG	Gm13117	ENSMUST00000120894
ENSMUSG	Gm12608	ENSMUST00000122013
ENSMUSG	Gm14513	ENSMUST00000117403
ENSMUSG	Gm12314	ENSMUST00000118104
ENSMUSG	Gm12447	ENSMUST00000119110
ENSMUSG	Gm13751	ENSMUST00000120280
ENSMUSG	Gm9434	ENSMUST00000118975
ENSMUSG	Gm13197	ENSMUST00000117204
ENSMUSG	Gm9001	ENSMUST00000121642
ENSMUSG	M6pr-ps	ENSMUST00000120481
ENSMUSG	Gm11625	ENSMUST00000119535
ENSMUSG	Gm11349	ENSMUST00000122209
ENSMUSG	Gm14880	ENSMUST00000117581
ENSMUSG	Gm15400	ENSMUST00000118944
ENSMUSG	Gm9115	ENSMUST00000120192
ENSMUSG	Gm13379	ENSMUST00000118053
ENSMUSG	Gm12439	ENSMUST00000117162
ENSMUSG	Gm13815	ENSMUST00000119963
ENSMUSG	Gm14523	ENSMUST00000117260
ENSMUSG	Eif1-ps	ENSMUST00000121450
ENSMUSG	Gm5687	ENSMUST00000118252
ENSMUSG	Gm14111	ENSMUST00000119851
ENSMUSG	Gm15010	ENSMUST00000117153
ENSMUSG	Rpl22-p	ENSMUST00000118652
ENSMUSG	Gm15618	ENSMUST00000121610
ENSMUSG	Gm14278	ENSMUST00000117381
ENSMUSG	Gm11645	ENSMUST00000121589
ENSMUSG	Gm14567	ENSMUST00000117155
ENSMUSG	Gm11966	ENSMUST00000119907
ENSMUSG	Gm7129	ENSMUST00000117402
ENSMUSG	Gm12463	ENSMUST00000118271
ENSMUSG	Gm5400	ENSMUST00000118494
ENSMUSG	Gm10224	ENSMUST00000119496
ENSMUSG	Olfr337	ENSMUST00000120380
ENSMUSG	Zfp36l1	ENSMUST00000118349
ENSMUSG	Gm13130	ENSMUST00000119195
ENSMUSG	Gm12967	ENSMUST00000122147

ENSMUSG	Gm5379	ENSMUST00000120973
ENSMUSG	Gm14263	ENSMUST00000117955
ENSMUSG	Gm11453	ENSMUST00000117318
ENSMUSG	Gm12372	ENSMUST00000121654
ENSMUSG	Gm9009	ENSMUST00000117321
ENSMUSG	Gm13772	ENSMUST00000118431
ENSMUSG	Gm12297	ENSMUST00000121380
ENSMUSG	Gm13675	ENSMUST00000120616
ENSMUSG	1700071	ENSMUST00000117522
ENSMUSG	Gm12161	ENSMUST00000121454
ENSMUSG	Gm14469	ENSMUST00000118363
ENSMUSG	Olfr118	ENSMUST00000122036
ENSMUSG	Gm13464	ENSMUST00000119418
ENSMUSG	Gm11885	ENSMUST00000118473
ENSMUSG	Gm14240	ENSMUST00000120353
ENSMUSG	Gm11543	ENSMUST00000120758
ENSMUSG	Gm13880	ENSMUST00000118421
ENSMUSG	Gm13714	ENSMUST00000117313
ENSMUSG	Olfr139	ENSMUST00000121388
ENSMUSG	Gm15936	ENSMUST00000118706
ENSMUSG	Gm15766	ENSMUST00000118025
ENSMUSG	Gm14627	ENSMUST00000118931
ENSMUSG	Gm11855	ENSMUST00000121562
ENSMUSG	Gm12704	ENSMUST00000119789
ENSMUSG	Gm5944	ENSMUST00000118182
ENSMUSG	Psmb7-p	ENSMUST00000118546
ENSMUSG	AU01583	ENSMUST00000121565
ENSMUSG	Gm12025	ENSMUST00000117768
ENSMUSG	Gm12817	ENSMUST00000119622
ENSMUSG	Gm9836	ENSMUST00000117132
ENSMUSG	Rpsa-ps	ENSMUST00000121691
ENSMUSG	Gm6079	ENSMUST00000121356
ENSMUSG	Gm5638	ENSMUST00000118618
ENSMUSG	Gm12565	ENSMUST00000119004
ENSMUSG	Gm12691	ENSMUST00000222516
ENSMUSG	Gm12135	ENSMUST00000119170
ENSMUSG	Gm14630	ENSMUST00000118057
ENSMUSG	Gm12024	ENSMUST00000118819
ENSMUSG	Gm12235	ENSMUST00000118596
ENSMUSG	Gm12745	ENSMUST00000121767
ENSMUSG	Gm5913	ENSMUST00000119397
ENSMUSG	Gm7308	ENSMUST00000121087
ENSMUSG	Gm15390	ENSMUST00000120323
ENSMUSG	Gm12791	ENSMUST00000120788
ENSMUSG	Gm8217	ENSMUST00000119136
ENSMUSG	Gm12990	ENSMUST00000120372
ENSMUSG	Gm12834	ENSMUST00000121855
ENSMUSG	Gm13333	ENSMUST00000119424
ENSMUSG	Gm6024	ENSMUST00000117850
ENSMUSG	Gm12684	ENSMUST00000118675
ENSMUSG	Gm11448	ENSMUST00000120427
ENSMUSG	Gm11401	ENSMUST00000117212
ENSMUSG	Gm12783	ENSMUST00000121425
ENSMUSG	Gm8722	ENSMUST00000121466
ENSMUSG	Gm11783	ENSMUST00000119549
ENSMUSG	Gm8880	ENSMUST00000121800
ENSMUSG	Gm12551	ENSMUST00000122073

ENSMUSG	Gm13500	ENSMUST00000119348
ENSMUSG	H3f3a-p	ENSMUST00000119544
ENSMUSG	Gm4852	ENSMUST00000121202
ENSMUSG	Gm14621	ENSMUST00000117709
ENSMUSG	Hspd1-p	ENSMUST00000121815
ENSMUSG	Gm8424	ENSMUST00000117716
ENSMUSG	Gm13858	ENSMUST00000118862
ENSMUSG	Gm16407	ENSMUST00000122134
ENSMUSG	Gm15615	ENSMUST00000118876
ENSMUSG	Gm13220	ENSMUST00000121535
ENSMUSG	Gm15268	ENSMUST00000118845
ENSMUSG	Gm14542	ENSMUST00000118460
ENSMUSG	Cyp2j12	ENSMUST00000121694;ENSMUST00000097972
ENSMUSG	Gm14373	ENSMUST00000122467
ENSMUSG	Gm11385	ENSMUST00000121513
ENSMUSG	Gm15836	ENSMUST00000119952
ENSMUSG	Gm11836	ENSMUST00000121022
ENSMUSG	Gm13529	ENSMUST00000121118
ENSMUSG	Gm12164	ENSMUST00000121695
ENSMUSG	Gm16032	ENSMUST00000118534
ENSMUSG	Gm12112	ENSMUST00000117772
ENSMUSG	Gm5380	ENSMUST00000119296
ENSMUSG	Gm13607	ENSMUST00000117875
ENSMUSG	Gm6893	ENSMUST00000118385
ENSMUSG	Gm15370	ENSMUST00000117158
ENSMUSG	Gm12261	ENSMUST00000117499
ENSMUSG	Gm11282	ENSMUST00000119018
ENSMUSG	Gm11653	ENSMUST00000119992
ENSMUSG	Gm14883	ENSMUST00000120179
ENSMUSG	Gm15285	ENSMUST00000119271
ENSMUSG	Gm6274	ENSMUST00000118173
ENSMUSG	Gm18859	ENSMUST00000118792
ENSMUSG	Olfr333	ENSMUST00000122311
ENSMUSG	Gm14857	ENSMUST00000117862
ENSMUSG	Gm12520	ENSMUST00000117493
ENSMUSG	Gm6275	ENSMUST00000118971
ENSMUSG	Gm13603	ENSMUST00000119404
ENSMUSG	Gm16011	ENSMUST00000121567
ENSMUSG	Gm11509	ENSMUST00000117358
ENSMUSG	Gm12879	ENSMUST00000120003
ENSMUSG	Gm15266	ENSMUST00000119635
ENSMUSG	Gm14480	ENSMUST00000118521
ENSMUSG	Gm14132	ENSMUST00000117188
ENSMUSG	Gm16205	ENSMUST00000117720
ENSMUSG	Gm12757	ENSMUST00000119465
ENSMUSG	Gm15304	ENSMUST00000122150
ENSMUSG	Rpl7l1-	ENSMUST00000118822
ENSMUSG	Gm15151	ENSMUST00000117106
ENSMUSG	Gm12664	ENSMUST00000118306
ENSMUSG	Gm12307	ENSMUST00000120581
ENSMUSG	Vmn1r-p	ENSMUST00000122016
ENSMUSG	Gm5388	ENSMUST00000120059
ENSMUSG	Gm12389	ENSMUST00000117696
ENSMUSG	Gm14894	ENSMUST00000117170
ENSMUSG	Xlr4e-p	ENSMUST00000122102
ENSMUSG	Gm8595	ENSMUST00000118250
ENSMUSG	Gm9105	ENSMUST00000120160

ENSMUSG	Gm16436	ENSMUST00000121510
ENSMUSG	Gm12752	ENSMUST00000121657
ENSMUSG	Gm11668	ENSMUST00000119655
ENSMUSG	Gm13215	ENSMUST00000117559
ENSMUSG	Gm14879	ENSMUST00000117308
ENSMUSG	Gm14921	ENSMUST00000118643
ENSMUSG	Gm15455	ENSMUST00000117459
ENSMUSG	Gm15047	ENSMUST00000118891
ENSMUSG	Gm3943	ENSMUST00000118606
ENSMUSG	Gm15176	ENSMUST00000117734
ENSMUSG	Gm14830	ENSMUST00000121899
ENSMUSG	Gm13445	ENSMUST00000121664
ENSMUSG	Gm14282	ENSMUST00000119484
ENSMUSG	Gm12882	ENSMUST00000121696
ENSMUSG	Gm14774	ENSMUST00000117857
ENSMUSG	Gm12593	ENSMUST00000120981
ENSMUSG	Serpinb	ENSMUST00000118548
ENSMUSG	Gm5912	ENSMUST00000122282
ENSMUSG	Gm12464	ENSMUST00000121385
ENSMUSG	Gm3880	ENSMUST00000122154
ENSMUSG	Gm11432	ENSMUST00000117334
ENSMUSG	Gm14761	ENSMUST00000121363
ENSMUSG	Gm6767	ENSMUST00000118650
ENSMUSG	Gm7781	ENSMUST00000117258
ENSMUSG	Rpl30-p	ENSMUST00000119630
ENSMUSG	Gm13337	ENSMUST00000119183
ENSMUSG	Gm14131	ENSMUST00000119636
ENSMUSG	Gm14735	ENSMUST00000121772
ENSMUSG	Gm4853	ENSMUST00000119359
ENSMUSG	Gm14558	ENSMUST00000117458
ENSMUSG	AA62394	ENSMUST00000121794
ENSMUSG	Gm15368	ENSMUST00000120438
ENSMUSG	Gm12778	ENSMUST00000122341
ENSMUSG	Gm14130	ENSMUST00000118417
ENSMUSG	Gm12508	ENSMUST00000121282
ENSMUSG	Gm8644	ENSMUST00000118619
ENSMUSG	Gm12654	ENSMUST00000120387
ENSMUSG	Gm16204	ENSMUST00000117864
ENSMUSG	Gm13058	ENSMUST00000117097
ENSMUSG	Gm11658	ENSMUST00000120913
ENSMUSG	Gm13325	ENSMUST00000118746
ENSMUSG	Gm7219	ENSMUST00000119619
ENSMUSG	Gm12646	ENSMUST00000119144
ENSMUSG	Vmn1r-p	ENSMUST00000121318
ENSMUSG	Rps13-p	ENSMUST00000118906
ENSMUSG	Gm11990	ENSMUST00000121505
ENSMUSG	Gm12538	ENSMUST00000120064
ENSMUSG	Gm13077	ENSMUST00000121941
ENSMUSG	Gm11692	ENSMUST00000122121
ENSMUSG	Gm14654	ENSMUST00000118620
ENSMUSG	Gm12328	ENSMUST00000118611
ENSMUSG	Ccdc50-:	ENSMUST00000118044
ENSMUSG	Gm15778	ENSMUST00000118469
ENSMUSG	Gm5697	ENSMUST00000122295
ENSMUSG	Gm12635	ENSMUST00000117285
ENSMUSG	Gm15032	ENSMUST00000118547
ENSMUSG	Gm14429	ENSMUST00000121881

ENSMUSG	Gm14769	ENSMUST00000118590
ENSMUSG	Gm11544	ENSMUST00000120215
ENSMUSG	Gm15968	ENSMUST00000119342
ENSMUSG	Gm14506	ENSMUST00000121165
ENSMUSG	Gm15369	ENSMUST00000119681
ENSMUSG	Gm13651	ENSMUST00000119086
ENSMUSG	Gm12109	ENSMUST00000121140
ENSMUSG	Gm14247	ENSMUST00000120950
ENSMUSG	5033423	ENSMUST00000202802;ENSMUST00000117311
ENSMUSG	Gm14633	ENSMUST00000121152
ENSMUSG	Gm12793	ENSMUST00000119268
ENSMUSG	Gm5937	ENSMUST00000119715
ENSMUSG	Rps12-p	ENSMUST00000117169
ENSMUSG	Eif3s6-	ENSMUST00000117907
ENSMUSG	Gm15071	ENSMUST00000118189
ENSMUSG	Gm15901	ENSMUST00000117663
ENSMUSG	Rplp0-p	ENSMUST00000122125
ENSMUSG	Gm13532	ENSMUST00000120373
ENSMUSG	Gm15060	ENSMUST00000119550
ENSMUSG	Gm13022	ENSMUST00000121181
ENSMUSG	Gm13423	ENSMUST00000117205
ENSMUSG	Gm11787	ENSMUST00000121996
ENSMUSG	Gm11207	ENSMUST00000117824
ENSMUSG	Gm15544	ENSMUST00000118776
ENSMUSG	Gm11677	ENSMUST00000121349
ENSMUSG	Gm12883	ENSMUST00000117867
ENSMUSG	Gm7869	ENSMUST00000121457
ENSMUSG	Gm14794	ENSMUST00000118181
ENSMUSG	Gm6184	ENSMUST00000120403
ENSMUSG	Gm12712	ENSMUST00000120676
ENSMUSG	Gm11874	ENSMUST00000117437
ENSMUSG	Fzd10	ENSMUST00000117102
ENSMUSG	Gm9166	ENSMUST00000120293
ENSMUSG	Gm12317	ENSMUST00000122307
ENSMUSG	Gm13297	ENSMUST00000120205
ENSMUSG	Rpsa-ps	ENSMUST00000121462
ENSMUSG	Gm14714	ENSMUST00000119743
ENSMUSG	Gm11785	ENSMUST00000120165
ENSMUSG	Rps11-p	ENSMUST00000117608
ENSMUSG	Gm8261	ENSMUST00000119275
ENSMUSG	Gm12391	ENSMUST00000117626
ENSMUSG	Olfr129	ENSMUST00000122318
ENSMUSG	Mdm4-ps	ENSMUST00000121299
ENSMUSG	Gm14279	ENSMUST00000117142
ENSMUSG	Gm5721	ENSMUST00000119845
ENSMUSG	Rpl7a-p	ENSMUST00000117118
ENSMUSG	Gm6298	ENSMUST00000122262
ENSMUSG	Gm12067	ENSMUST00000119755
ENSMUSG	Gm15381	ENSMUST00000121501
ENSMUSG	Gm13131	ENSMUST00000122228
ENSMUSG	Gm13866	ENSMUST00000118901
ENSMUSG	Gm14159	ENSMUST00000119832
ENSMUSG	Gm6325	ENSMUST00000122015
ENSMUSG	Gm13991	ENSMUST00000120190
ENSMUSG	Gm11893	ENSMUST00000117829
ENSMUSG	Anp32b-	ENSMUST00000122215
ENSMUSG	5730416	ENSMUST00000120648

ENSMUSG	Gm9005	ENSMUST00000119908
ENSMUSG	Gm15282	ENSMUST00000117707
ENSMUSG	Gm14901	ENSMUST00000119001
ENSMUSG	Gm11878	ENSMUST00000117908
ENSMUSG	Gm15528	ENSMUST00000117821
ENSMUSG	Gm13590	ENSMUST00000120183
ENSMUSG	Gm15626	ENSMUST00000117478
ENSMUSG	Cyp4a28	ENSMUST00000121884
ENSMUSG	Gm6897	ENSMUST00000117714
ENSMUSG	Gm13669	ENSMUST00000122366
ENSMUSG	Gm13770	ENSMUST00000117149
ENSMUSG	Gm11790	ENSMUST00000119569
ENSMUSG	Gm13444	ENSMUST00000119445
ENSMUSG	Gm11241	ENSMUST00000117371
ENSMUSG	Gm14925	ENSMUST00000120611
ENSMUSG	Gm15847	ENSMUST00000121417
ENSMUSG	Gm12281	ENSMUST00000119536
ENSMUSG	Gm14514	ENSMUST00000121680
ENSMUSG	Olfr650	ENSMUST00000121432
ENSMUSG	Gm12413	ENSMUST00000121508
ENSMUSG	Gm12780	ENSMUST00000122458
ENSMUSG	Gm11488	ENSMUST00000117277
ENSMUSG	Gm15223	ENSMUST00000120989
ENSMUSG	Gm12644	ENSMUST00000119935
ENSMUSG	Gm11933	ENSMUST00000121550
ENSMUSG	Gm5385	ENSMUST00000118988
ENSMUSG	Gm15574	ENSMUST00000119937
ENSMUSG	Gm11821	ENSMUST00000117335
ENSMUSG	Spcs2-p	ENSMUST00000122326
ENSMUSG	Gm8864	ENSMUST00000118653
ENSMUSG	Gm5389	ENSMUST00000120893
ENSMUSG	Gm13419	ENSMUST00000121711
ENSMUSG	Gm11370	ENSMUST00000120583
ENSMUSG	Gm11341	ENSMUST00000122316
ENSMUSG	Gm15986	ENSMUST00000122153
ENSMUSG	Gm12858	ENSMUST00000121913
ENSMUSG	Gm15105	ENSMUST00000117777
ENSMUSG	Gm12331	ENSMUST00000119927
ENSMUSG	Gm12193	ENSMUST00000117250
ENSMUSG	Gm14599	ENSMUST00000120469
ENSMUSG	Gm11218	ENSMUST00000121364
ENSMUSG	Gm15149	ENSMUST00000119370
ENSMUSG	Gm9078	ENSMUST00000119126
ENSMUSG	Gm11472	ENSMUST00000121305
ENSMUSG	Olfr119	ENSMUST00000217379
ENSMUSG	Gm15352	ENSMUST00000118696
ENSMUSG	Gm12178	ENSMUST00000120545
ENSMUSG	Gm13626	ENSMUST00000119435
ENSMUSG	Olfr999	ENSMUST00000122319
ENSMUSG	Ak3l2-p	ENSMUST00000120282
ENSMUSG	Gm16480	ENSMUST00000120439
ENSMUSG	Gm16471	ENSMUST00000121940
ENSMUSG	Gm16397	ENSMUST00000122201
ENSMUSG	Gm12482	ENSMUST00000120435
ENSMUSG	Gm14895	ENSMUST00000121858
ENSMUSG	Rps8-ps	ENSMUST00000121011
ENSMUSG	Dnajb3	ENSMUST00000119972

ENSMUSG	Gm13859	ENSMUST00000119936
ENSMUSG	Olfr117	ENSMUST00000118079
ENSMUSG	Gm11371	ENSMUST00000120294
ENSMUSG	Gm13452	ENSMUST00000120896
ENSMUSG	Gm6322	ENSMUST00000118789
ENSMUSG	Gm15231	ENSMUST00000119174
ENSMUSG	Gm11794	ENSMUST00000119446
ENSMUSG	Gm1826	ENSMUST00000118646
ENSMUSG	Gm13081	ENSMUST00000121527
ENSMUSG	Gm8097	ENSMUST00000117903
ENSMUSG	Gm12271	ENSMUST00000121719
ENSMUSG	Gm13724	ENSMUST00000122395
ENSMUSG	Gm12071	ENSMUST00000121968
ENSMUSG	Gm11585	ENSMUST00000120901
ENSMUSG	Gm14464	ENSMUST00000119658
ENSMUSG	Gm12948	ENSMUST00000119406
ENSMUSG	Gm16072	ENSMUST00000119574
ENSMUSG	Psmb6-p	ENSMUST00000117520
ENSMUSG	Rpl5-ps	ENSMUST00000119057
ENSMUSG	Gm13904	ENSMUST00000121901
ENSMUSG	Gm1866	ENSMUST00000120701
ENSMUSG	Dnmt3c	ENSMUST00000119996
ENSMUSG	Gm9246	ENSMUST00000117587
ENSMUSG	Gm13230	ENSMUST00000120870
ENSMUSG	Gm12138	ENSMUST00000122180
ENSMUSG	Gm15928	ENSMUST00000122455
ENSMUSG	Gm14039	ENSMUST00000118705
ENSMUSG	Gm12013	ENSMUST00000121971
ENSMUSG	Glns-ps	ENSMUST00000120291
ENSMUSG	Slfn14	ENSMUST00000163961
ENSMUSG	Rpl19-p	ENSMUST00000121251
ENSMUSG	Gm12479	ENSMUST00000120731
ENSMUSG	Gm14445	ENSMUST00000117051
ENSMUSG	Gm13489	ENSMUST00000121234
ENSMUSG	Gm15490	ENSMUST00000119894
ENSMUSG	Cyp2j14	ENSMUST00000117776
ENSMUSG	Gm11824	ENSMUST00000120191
ENSMUSG	Gm14502	ENSMUST00000118154
ENSMUSG	Gm13094	ENSMUST00000117930
ENSMUSG	Gm13518	ENSMUST00000120018
ENSMUSG	Gm12864	ENSMUST00000122207
ENSMUSG	Gm12788	ENSMUST00000119090
ENSMUSG	Gm13655	ENSMUST00000119695
ENSMUSG	Gm16464	ENSMUST00000117815
ENSMUSG	Gm12008	ENSMUST00000117791
ENSMUSG	Gm12038	ENSMUST00000117322
ENSMUSG	Gm11578	ENSMUST00000118530
ENSMUSG	Gm7091	ENSMUST00000116438
ENSMUSG	Gm8617	ENSMUST00000118796
ENSMUSG	Rpl27-p	ENSMUST00000120378
ENSMUSG	Gm16005	ENSMUST00000120515
ENSMUSG	Gm12428	ENSMUST00000119844
ENSMUSG	Gm13670	ENSMUST00000120947
ENSMUSG	Gm4784	ENSMUST00000117365
ENSMUSG	Gm14719	ENSMUST00000121891
ENSMUSG	Gm12444	ENSMUST00000121512
ENSMUSG	Gm13034	ENSMUST00000121342

ENSMUSG	Gm8577	ENSMUST00000122420
ENSMUSG	Gm12871	ENSMUST00000118758
ENSMUSG	Gm15379	ENSMUST00000121224
ENSMUSG	Gm15826	ENSMUST00000121328
ENSMUSG	Gm14314	ENSMUST00000118225
ENSMUSG	Olfr271	ENSMUST00000121614
ENSMUSG	Gm1401	ENSMUST00000122048
ENSMUSG	Gm13602	ENSMUST00000122405
ENSMUSG	Gm12943	ENSMUST00000119616
ENSMUSG	Gm15079	ENSMUST00000121943
ENSMUSG	Gm6506	ENSMUST00000119775
ENSMUSG	Gm13326	ENSMUST00000118730
ENSMUSG	Gng5-ps	ENSMUST00000120931
ENSMUSG	Gm12443	ENSMUST00000122308
ENSMUSG	Gm6292	ENSMUST00000120712
ENSMUSG	Gm12997	ENSMUST00000120207
ENSMUSG	Gm11204	ENSMUST00000122438
ENSMUSG	Gm14639	ENSMUST00000120850
ENSMUSG	Gm11822	ENSMUST00000119177
ENSMUSG	Gm7429	ENSMUST00000118664
ENSMUSG	Gm14999	ENSMUST00000121039
ENSMUSG	Gm14730	ENSMUST00000122378
ENSMUSG	Gm12799	ENSMUST00000118151
ENSMUSG	Gm11675	ENSMUST00000121873
ENSMUSG	Gm14854	ENSMUST00000117758
ENSMUSG	Gm13885	ENSMUST00000117569
ENSMUSG	Gm13471	ENSMUST00000122057
ENSMUSG	Gm5847	ENSMUST00000178959
ENSMUSG	Gm15596	ENSMUST00000121139
ENSMUSG	Gm12133	ENSMUST00000121775
ENSMUSG	Gm13633	ENSMUST00000120668
ENSMUSG	Gm15770	ENSMUST00000117498
ENSMUSG	Gm12904	ENSMUST00000117451
ENSMUSG	Gm13296	ENSMUST00000117854
ENSMUSG	Vmn1r22	ENSMUST00000122275
ENSMUSG	Gm14162	ENSMUST00000121985
ENSMUSG	Gm11877	ENSMUST00000118921
ENSMUSG	Gm15314	ENSMUST00000120740
ENSMUSG	Gm14615	ENSMUST00000117575
ENSMUSG	Gm14287	ENSMUST00000121060
ENSMUSG	Gm8401	ENSMUST00000120248
ENSMUSG	Gm5395	ENSMUST00000121650
ENSMUSG	Gm15565	ENSMUST00000118890
ENSMUSG	Gm8814	ENSMUST00000119072
ENSMUSG	Btc	ENSMUST00000121044;ENSMUST00000201252;ENSMUST00000201
ENSMUSG	Gm5187	ENSMUST00000117317
ENSMUSG	Gm11955	ENSMUST00000119801
ENSMUSG	Gm15754	ENSMUST00000119191
ENSMUSG	Gm3712	ENSMUST00000118523
ENSMUSG	Gm14415	ENSMUST00000118260
ENSMUSG	Gm13884	ENSMUST00000118103
ENSMUSG	Gm12692	ENSMUST00000121092
ENSMUSG	Mkrn1-p	ENSMUST00000122035
ENSMUSG	Gm12136	ENSMUST00000120460
ENSMUSG	Zfp389	ENSMUST00000224437;ENSMUST00000118425
ENSMUSG	Gm11927	ENSMUST00000118472
ENSMUSG	Gm14799	ENSMUST00000117502

ENSMUSG	Gm16021	ENSMUST00000121176
ENSMUSG	Gm13545	ENSMUST00000119044
ENSMUSG	Gm13303	ENSMUST00000117125
ENSMUSG	Gm12486	ENSMUST00000120887
ENSMUSG	Gm11414	ENSMUST00000121795
ENSMUSG	Gm13740	ENSMUST00000120590
ENSMUSG	Gm13126	ENSMUST00000117079
ENSMUSG	Tdpx-ps	ENSMUST00000117372
ENSMUSG	Gm9025	ENSMUST00000122415
ENSMUSG	Gm9430	ENSMUST00000121232
ENSMUSG	Gm8844	ENSMUST00000119460
ENSMUSG	Rab9b-p	ENSMUST00000119785
ENSMUSG	Gm6743	ENSMUST00000117345
ENSMUSG	Gm12980	ENSMUST00000118912
ENSMUSG	Gm13501	ENSMUST00000118477
ENSMUSG	Kif22-p	ENSMUST00000119302
ENSMUSG	Gm8213	ENSMUST00000118191
ENSMUSG	Gm12242	ENSMUST00000118824
ENSMUSG	Gm4746	ENSMUST00000120395
ENSMUSG	Gm15693	ENSMUST00000120470
ENSMUSG	Gm5909	ENSMUST00000119768
ENSMUSG	Gm14871	ENSMUST00000119056
ENSMUSG	Gm13687	ENSMUST00000119857
ENSMUSG	Gm40	ENSMUST00000122131
ENSMUSG	Gm13937	ENSMUST00000121000
ENSMUSG	Gm15374	ENSMUST00000119313
ENSMUSG	Gm11452	ENSMUST00000122376
ENSMUSG	Gm13342	ENSMUST00000121055
ENSMUSG	Gm5757	ENSMUST00000117494
ENSMUSG	Rpl31-p	ENSMUST00000119959
ENSMUSG	Gm13712	ENSMUST00000117484
ENSMUSG	Gm14704	ENSMUST00000120300
ENSMUSG	Gm15207	ENSMUST00000119259
ENSMUSG	Gm14336	ENSMUST00000117491
ENSMUSG	Gm12196	ENSMUST00000120954
ENSMUSG	Gm14813	ENSMUST00000122291
ENSMUSG	Gm15065	ENSMUST00000121699
ENSMUSG	Gm5387	ENSMUST00000119776
ENSMUSG	Gm15157	ENSMUST00000121852
ENSMUSG	Gm15586	ENSMUST00000121218
ENSMUSG	Gm13050	ENSMUST00000122149
ENSMUSG	Gm11979	ENSMUST00000119629
ENSMUSG	Gm13182	ENSMUST00000122241
ENSMUSG	Gm15443	ENSMUST00000118947
ENSMUSG	Gm11331	ENSMUST00000119764
ENSMUSG	Gm14793	ENSMUST00000118739
ENSMUSG	Gm9673	ENSMUST00000119287
ENSMUSG	Gm15021	ENSMUST00000117832
ENSMUSG	Gm7670	ENSMUST00000121272
ENSMUSG	Smt3h2-:	ENSMUST00000121084
ENSMUSG	Gm14852	ENSMUST00000120065
ENSMUSG	Gm12535	ENSMUST00000121025
ENSMUSG	Gm15204	ENSMUST00000119454
ENSMUSG	Gm12566	ENSMUST00000121479
ENSMUSG	Gm5639	ENSMUST00000122305
ENSMUSG	Rpl30-p	ENSMUST00000117104
ENSMUSG	Gm12754	ENSMUST00000121908

ENSMUSG	Gm14725	ENSMUST00000120324
ENSMUSG	Gm14736	ENSMUST00000121470
ENSMUSG	Gm14612	ENSMUST00000121522
ENSMUSG	Gm11590	ENSMUST00000118999
ENSMUSG	Gm11348	ENSMUST00000118813
ENSMUSG	Gm11857	ENSMUST00000119817
ENSMUSG	Gm11914	ENSMUST00000117094
ENSMUSG	Gm6382	ENSMUST00000118873
ENSMUSG	Gm15371	ENSMUST00000117057
ENSMUSG	Gm12697	ENSMUST00000118008
ENSMUSG	Gm12124	ENSMUST00000120504
ENSMUSG	Gm12515	ENSMUST00000120116
ENSMUSG	Gm14928	ENSMUST00000120198
ENSMUSG	Gm14416	ENSMUST00000118630
ENSMUSG	Gm11424	ENSMUST00000121929
ENSMUSG	Gm14845	ENSMUST00000121259
ENSMUSG	Gm11363	ENSMUST00000121672
ENSMUSG	Gm15153	ENSMUST00000119867
ENSMUSG	Gm5381	ENSMUST00000117975
ENSMUSG	Gm12026	ENSMUST00000120961
ENSMUSG	Gm14127	ENSMUST00000117906
ENSMUSG	Gm12156	ENSMUST00000119188
ENSMUSG	Gm8692	ENSMUST00000118102
ENSMUSG	Gm13024	ENSMUST00000121756
ENSMUSG	Gm14411	ENSMUST00000121798
ENSMUSG	Gm11622	ENSMUST00000120525
ENSMUSG	Gm12727	ENSMUST00000119885
ENSMUSG	Gm381	ENSMUST00000117443
ENSMUSG	Gm15058	ENSMUST00000117689
ENSMUSG	Gm7061	ENSMUST00000118127
ENSMUSG	Gm15191	ENSMUST00000117933
ENSMUSG	Gm14489	ENSMUST00000120868
ENSMUSG	Gm12155	ENSMUST00000121267
ENSMUSG	Gm4875	ENSMUST00000118257
ENSMUSG	Gm6806	ENSMUST00000120092
ENSMUSG	Gm13193	ENSMUST00000117275
ENSMUSG	Gm12015	ENSMUST00000117065
ENSMUSG	Rpl17-p	ENSMUST00000119156
ENSMUSG	Trav4d-	ENSMUST00000120914
ENSMUSG	Gm13803	ENSMUST00000117547
ENSMUSG	Gm15828	ENSMUST00000119911
ENSMUSG	Mif-ps3	ENSMUST00000118636
ENSMUSG	Gm12982	ENSMUST00000117408
ENSMUSG	Gm13612	ENSMUST00000119276
ENSMUSG	Gm15373	ENSMUST00000121495
ENSMUSG	Gm14519	ENSMUST00000118490
ENSMUSG	Gm16421	ENSMUST00000117616
ENSMUSG	Gm11931	ENSMUST00000121630
ENSMUSG	Gm12021	ENSMUST00000121061
ENSMUSG	Gm12293	ENSMUST00000121898
ENSMUSG	Gm11746	ENSMUST00000119843
ENSMUSG	Gm13358	ENSMUST00000121427
ENSMUSG	Vmn1r-p	ENSMUST00000117599
ENSMUSG	Gm12165	ENSMUST00000117047
ENSMUSG	Gm13263	ENSMUST00000121271
ENSMUSG	Gm15773	ENSMUST00000118665
ENSMUSG	Rpl15-p	ENSMUST00000120694

ENSMUSG	Gm11889	ENSMUST00000118402
ENSMUSG	Gm12583	ENSMUST00000120677
ENSMUSG	Gm13525	ENSMUST00000120607
ENSMUSG	Gm12934	ENSMUST00000119221
ENSMUSG	Gm6480	ENSMUST00000121552
ENSMUSG	Gm12041	ENSMUST00000121606
ENSMUSG	Gm11557	ENSMUST00000120200
ENSMUSG	Smt3h1-:	ENSMUST00000117126
ENSMUSG	Gm14758	ENSMUST00000119104
ENSMUSG	Gm14119	ENSMUST00000119511
ENSMUSG	Gm11462	ENSMUST00000117749
ENSMUSG	Gm5863	ENSMUST00000121760
ENSMUSG	Cyp2j8	ENSMUST00000124729
ENSMUSG	Gm14103	ENSMUST00000119075
ENSMUSG	Gm13355	ENSMUST00000117996
ENSMUSG	Gm7834	ENSMUST00000117560
ENSMUSG	Gm13359	ENSMUST00000119000
ENSMUSG	Gm12507	ENSMUST00000119155
ENSMUSG	Gm13217	ENSMUST00000120466
ENSMUSG	Gm12731	ENSMUST00000120932
ENSMUSG	Gm15131	ENSMUST00000119124
ENSMUSG	Gm10601	ENSMUST00000120436
ENSMUSG	Gm14581	ENSMUST00000121726
ENSMUSG	Gm15214	ENSMUST00000122155
ENSMUSG	Gm15056	ENSMUST00000167686;ENSMUST00000122025
ENSMUSG	Rpsa-ps	ENSMUST00000118446
ENSMUSG	Gm12318	ENSMUST00000121235
ENSMUSG	Gm14042	ENSMUST00000120139
ENSMUSG	Rps13-p	ENSMUST00000120038
ENSMUSG	Gm13975	ENSMUST00000119253
ENSMUSG	Gm11221	ENSMUST00000117230
ENSMUSG	Gm13250	ENSMUST00000117209
ENSMUSG	Gm13413	ENSMUST00000120953
ENSMUSG	Gm1335	ENSMUST00000121653
ENSMUSG	Gm11764	ENSMUST00000120221
ENSMUSG	Gm16518	ENSMUST00000117960
ENSMUSG	Gm12618	ENSMUST00000121754
ENSMUSG	Gm12163	ENSMUST00000121597
ENSMUSG	Gm14982	ENSMUST00000121102
ENSMUSG	Olfr334	ENSMUST00000121252
ENSMUSG	Gm14900	ENSMUST00000120304
ENSMUSG	Gm12416	ENSMUST00000120911
ENSMUSG	Gm14699	ENSMUST00000122340
ENSMUSG	Gm14822	ENSMUST00000122247
ENSMUSG	Gm12285	ENSMUST00000117347
ENSMUSG	Gm12986	ENSMUST00000119037
ENSMUSG	Gm13356	ENSMUST00000121678
ENSMUSG	Gm7785	ENSMUST00000117121
ENSMUSG	Gm13195	ENSMUST00000117229
ENSMUSG	Gm13668	ENSMUST00000119237
ENSMUSG	Gm11980	ENSMUST00000121445
ENSMUSG	Gm13223	ENSMUST00000118609
ENSMUSG	Gm15382	ENSMUST00000118011
ENSMUSG	Gm14153	ENSMUST00000119923
ENSMUSG	Gm4760	ENSMUST00000119663
ENSMUSG	Gm11736	ENSMUST00000121468
ENSMUSG	Gm15385	ENSMUST00000118725

ENSMUSG	Gm13430	ENSMUST00000121366
ENSMUSG	Gm6429	ENSMUST00000117144
ENSMUSG	Gm14073	ENSMUST00000117344
ENSMUSG	Rpl10-p	ENSMUST00000117120
ENSMUSG	Gm14790	ENSMUST00000117760
ENSMUSG	Gm14109	ENSMUST00000118814
ENSMUSG	Gm15189	ENSMUST00000121164
ENSMUSG	Cyp4a29	ENSMUST00000118278
ENSMUSG	Gm12288	ENSMUST00000121054
ENSMUSG	Gm11892	ENSMUST00000117711
ENSMUSG	Vmn1r-p	ENSMUST00000118338
ENSMUSG	Gm14201	ENSMUST00000117627
ENSMUSG	Gm6580	ENSMUST00000117504
ENSMUSG	D11Bhm1	ENSMUST00000122252
ENSMUSG	Gm15272	ENSMUST00000120865
ENSMUSG	Gm9432	ENSMUST00000117810
ENSMUSG	Gm12274	ENSMUST00000120062
ENSMUSG	Gm14875	ENSMUST00000120881
ENSMUSG	Gm16425	ENSMUST00000118143
ENSMUSG	Gm12919	ENSMUST00000119855
ENSMUSG	Gm11255	ENSMUST00000120448
ENSMUSG	Gm7831	ENSMUST00000117990
ENSMUSG	Gm11779	ENSMUST00000118541
ENSMUSG	Gm14158	ENSMUST00000118409
ENSMUSG	Gm12262	ENSMUST00000119915
ENSMUSG	Gm13453	ENSMUST00000118708
ENSMUSG	Gm13259	ENSMUST00000120792
ENSMUSG	Gm15180	ENSMUST00000117745
ENSMUSG	Gm11185	ENSMUST00000117732
ENSMUSG	Gm5403	ENSMUST00000117105
ENSMUSG	Gm15568	ENSMUST00000118589
ENSMUSG	Gm5939	ENSMUST00000119613
ENSMUSG	Gm14470	ENSMUST00000119085
ENSMUSG	Gm14874	ENSMUST00000118995
ENSMUSG	Gm11461	ENSMUST00000119909
ENSMUSG	Gm13498	ENSMUST00000119159
ENSMUSG	Gm15376	ENSMUST00000122258
ENSMUSG	Pex13-p	ENSMUST00000118184
ENSMUSG	Gm13332	ENSMUST00000120451
ENSMUSG	Gm15361	ENSMUST00000119320
ENSMUSG	Gm13688	ENSMUST00000121557
ENSMUSG	Gm15815	ENSMUST00000120569
ENSMUSG	Csnk2a1	ENSMUST00000118846
ENSMUSG	Gm12638	ENSMUST00000119531
ENSMUSG	Gm12085	ENSMUST00000119263
ENSMUSG	AA41476	ENSMUST00000121924
ENSMUSG	Gm11871	ENSMUST00000122429
ENSMUSG	Gm15185	ENSMUST00000117397
ENSMUSG	Gm14121	ENSMUST00000121441
ENSMUSG	Gm11826	ENSMUST00000118781
ENSMUSG	Gm12400	ENSMUST00000120915
ENSMUSG	Gm7599	ENSMUST00000121453
ENSMUSG	Gm13397	ENSMUST00000119562
ENSMUSG	Gm11539	ENSMUST00000119837
ENSMUSG	Gm12475	ENSMUST00000119476
ENSMUSG	Gm14238	ENSMUST00000120227
ENSMUSG	Gm7363	ENSMUST00000119883

ENSMUSG	Gm11581	ENSMUST00000122024
ENSMUSG	Olfr100	ENSMUST00000120361
ENSMUSG	Gm11235	ENSMUST00000117763
ENSMUSG	Gm13392	ENSMUST00000117708
ENSMUSG	Gm11449	ENSMUST00000119175
ENSMUSG	Gm15057	ENSMUST00000121516
ENSMUSG	Gm4919	ENSMUST00000117825
ENSMUSG	Gm6335	ENSMUST00000118081
ENSMUSG	Gm11203	ENSMUST00000122169
ENSMUSG	Gm14394	ENSMUST00000120307
ENSMUSG	Gm13176	ENSMUST00000117910
ENSMUSG	Gm14776	ENSMUST00000121029
ENSMUSG	Gm13221	ENSMUST00000121712
ENSMUSG	Gm14591	ENSMUST00000120407
ENSMUSG	Gm13006	ENSMUST00000118683
ENSMUSG	Gm11471	ENSMUST00000121824
ENSMUSG	Gm5402	ENSMUST00000119588
ENSMUSG	Gm15519	ENSMUST00000120088
ENSMUSG	Gm15038	ENSMUST00000122144
ENSMUSG	Gm12826	ENSMUST00000121841
ENSMUSG	Gm13487	ENSMUST00000122240
ENSMUSG	Gm14619	ENSMUST00000120480
ENSMUSG	Gm12182	ENSMUST00000118655
ENSMUSG	Gm12935	ENSMUST00000119352
ENSMUSG	Gm11652	ENSMUST00000117842
ENSMUSG	Gm15840	ENSMUST00000122099
ENSMUSG	Gm9431	ENSMUST00000119765
ENSMUSG	Gm14281	ENSMUST00000121700
ENSMUSG	Nsa2-ps	ENSMUST00000120785
ENSMUSG	Gm5555	ENSMUST00000120352
ENSMUSG	Gm14706	ENSMUST00000117744
ENSMUSG	Gm14044	ENSMUST00000117531
ENSMUSG	Gm14521	ENSMUST00000119545
ENSMUSG	Gm14515	ENSMUST00000121327
ENSMUSG	Gm13877	ENSMUST00000120614
ENSMUSG	Gm6027	ENSMUST00000121467
ENSMUSG	Olfr647	ENSMUST00000121599
ENSMUSG	Gm15196	ENSMUST00000121406
ENSMUSG	Gm9429	ENSMUST00000117804
ENSMUSG	Gm6418	ENSMUST00000118325
ENSMUSG	Gm13867	ENSMUST00000120844
ENSMUSG	H2-M6-p	ENSMUST00000119193
ENSMUSG	Gm14381	ENSMUST00000122105
ENSMUSG	Gm15808	ENSMUST00000119046
ENSMUSG	Oat-rs1	ENSMUST00000122186
ENSMUSG	Gm13320	ENSMUST00000120155
ENSMUSG	Tpt1-ps	ENSMUST00000120257
ENSMUSG	Gm11514	ENSMUST00000117670
ENSMUSG	Gm11989	ENSMUST00000117240
ENSMUSG	Gm9043	ENSMUST00000119677
ENSMUSG	Gm6877	ENSMUST00000119347
ENSMUSG	Gm14939	ENSMUST00000120347
ENSMUSG	Gm11451	ENSMUST00000118946
ENSMUSG	Gm15393	ENSMUST00000120499
ENSMUSG	Gm8688	ENSMUST00000118240
ENSMUSG	Gm13722	ENSMUST00000117964
ENSMUSG	Gm9200	ENSMUST00000118513

ENSMUSG	Gm14810	ENSMUST00000121628
ENSMUSG	Pou2f3-	ENSMUST00000120271
ENSMUSG	H2afb3	ENSMUST00000122050
ENSMUSG	Gm11771	ENSMUST00000118124
ENSMUSG	Gm12469	ENSMUST00000117623
ENSMUSG	Gm15188	ENSMUST00000121264
ENSMUSG	Gm12502	ENSMUST00000120197
ENSMUSG	Gm15175	ENSMUST00000117629
ENSMUSG	Gm12787	ENSMUST00000119234
ENSMUSG	Gm6088	ENSMUST00000117257
ENSMUSG	Rasl2-9	ENSMUST00000147835
ENSMUSG	Gm11524	ENSMUST00000119349
ENSMUSG	Gm11384	ENSMUST00000121376
ENSMUSG	Gm12245	ENSMUST00000117266
ENSMUSG	Gm12152	ENSMUST00000122465
ENSMUSG	Gm12358	ENSMUST00000121842
ENSMUSG	Gm5648	ENSMUST00000122244
ENSMUSG	Zfp133-	ENSMUST00000124788;ENSMUST00000149523;ENSMUST00000137
ENSMUSG	Gm11699	ENSMUST00000119016
ENSMUSG	Gm12989	ENSMUST00000122331
ENSMUSG	Gm12892	ENSMUST00000117367
ENSMUSG	Gm6539	ENSMUST00000119157
ENSMUSG	Gm15154	ENSMUST00000117590
ENSMUSG	Gm14465	ENSMUST00000120603
ENSMUSG	Gm9575	ENSMUST00000122140
ENSMUSG	Gm11971	ENSMUST00000117715
ENSMUSG	Gm4912	ENSMUST00000122212
ENSMUSG	Gm3931	ENSMUST00000119038
ENSMUSG	Gm5883	ENSMUST00000120735
ENSMUSG	Gm14202	ENSMUST00000120121
ENSMUSG	Gm13865	ENSMUST00000120957
ENSMUSG	Gm15161	ENSMUST00000117340
ENSMUSG	Adgrd2-	ENSMUST00000118797
ENSMUSG	Rpl23a-	ENSMUST00000118777
ENSMUSG	Gm11865	ENSMUST00000118093
ENSMUSG	Gm16202	ENSMUST00000117532
ENSMUSG	Gm14197	ENSMUST00000117480
ENSMUSG	Gm14592	ENSMUST00000117201
ENSMUSG	Gm14824	ENSMUST00000119421
ENSMUSG	Gm11662	ENSMUST00000121389
ENSMUSG	Gm14108	ENSMUST00000122234
ENSMUSG	Gm14534	ENSMUST00000120045
ENSMUSG	Rps15a-	ENSMUST00000119415
ENSMUSG	Gm14524	ENSMUST00000121066
ENSMUSG	Gm11405	ENSMUST00000118689
ENSMUSG	Gm15362	ENSMUST00000121459
ENSMUSG	Gm15566	ENSMUST00000121130
ENSMUSG	Gm12728	ENSMUST00000119740
ENSMUSG	Gm11660	ENSMUST00000120955
ENSMUSG	Rps18-p	ENSMUST00000119493
ENSMUSG	Gm16123	ENSMUST00000118647
ENSMUSG	Gm12455	ENSMUST00000121242
ENSMUSG	Gm15502	ENSMUST00000121449
ENSMUSG	Gm5133	ENSMUST00000118940
ENSMUSG	Gm13435	ENSMUST00000120111
ENSMUSG	Gm14400	ENSMUST00000117234
ENSMUSG	Gm14908	ENSMUST00000120930

ENSMUSG	Gm11977	ENSMUST00000118511
ENSMUSG	Gm6415	ENSMUST00000118488
ENSMUSG	Amd-ps1	ENSMUST00000120127
ENSMUSG	Gm2199	ENSMUST00000119065
ENSMUSG	Gm14248	ENSMUST00000117790
ENSMUSG	Gm11898	ENSMUST00000117161
ENSMUSG	Gm14427	ENSMUST00000117183
ENSMUSG	Gm11957	ENSMUST00000118304
ENSMUSG	Gm1947	ENSMUST00000122451
ENSMUSG	Gm13797	ENSMUST00000121792
ENSMUSG	Xlr3d-p	ENSMUST00000121687
ENSMUSG	Gm7309	ENSMUST00000121670
ENSMUSG	Gm12365	ENSMUST00000117931
ENSMUSG	Gm13007	ENSMUST00000121866
ENSMUSG	Dnajc19	ENSMUST00000120890
ENSMUSG	Gm12584	ENSMUST00000117109
ENSMUSG	Gm14979	ENSMUST00000121561
ENSMUSG	Gm13313	ENSMUST00000118938
ENSMUSG	Gm12442	ENSMUST00000118372
ENSMUSG	Vmn1r-p	ENSMUST00000120775
ENSMUSG	Gm12034	ENSMUST00000119032
ENSMUSG	Gm14038	ENSMUST00000121500
ENSMUSG	Gm11369	ENSMUST00000120812
ENSMUSG	E530001	ENSMUST00000120991
ENSMUSG	Gm15703	ENSMUST00000120250
ENSMUSG	Gm1848	ENSMUST00000120711
ENSMUSG	Gm13347	ENSMUST00000120298
ENSMUSG	Gm13294	ENSMUST00000121757
ENSMUSG	Gm5405	ENSMUST00000120478
ENSMUSG	Gm13604	ENSMUST00000119332
ENSMUSG	Rps18-p	ENSMUST00000120838
ENSMUSG	Gm13932	ENSMUST00000120873
ENSMUSG	Gm15476	ENSMUST00000118247
ENSMUSG	Gm14987	ENSMUST00000117820
ENSMUSG	Gm14783	ENSMUST00000118900
ENSMUSG	Gm14749	ENSMUST00000121269
ENSMUSG	Gm11438	ENSMUST00000121582
ENSMUSG	Gm12368	ENSMUST00000122251
ENSMUSG	Cct3-ps	ENSMUST00000118023
ENSMUSG	Gm11298	ENSMUST00000120503
ENSMUSG	Gm12179	ENSMUST00000118344
ENSMUSG	Gm12742	ENSMUST00000117326
ENSMUSG	Gm13725	ENSMUST00000122139
ENSMUSG	Gm15707	ENSMUST00000118804
ENSMUSG	Gm14122	ENSMUST00000122076
ENSMUSG	Gm13482	ENSMUST00000119842
ENSMUSG	Olfr105	ENSMUST00000119802
ENSMUSG	Gm5943	ENSMUST00000119458
ENSMUSG	Gm14802	ENSMUST00000121944
ENSMUSG	Gm13459	ENSMUST00000121558
ENSMUSG	Gm15363	ENSMUST00000118897
ENSMUSG	Gm14650	ENSMUST00000120400
ENSMUSG	Gm12920	ENSMUST00000121818
ENSMUSG	Gm14245	ENSMUST00000118939
ENSMUSG	Gm12969	ENSMUST00000120199
ENSMUSG	Gm11956	ENSMUST00000119525
ENSMUSG	Gm14270	ENSMUST00000118388

ENSMUSG	Gm11895	ENSMUST00000122165
ENSMUSG	Gm16459	ENSMUST00000121443
ENSMUSG	Gm13983	ENSMUST00000118018
ENSMUSG	Gm8855	ENSMUST00000118888
ENSMUSG	Gm12991	ENSMUST00000120561
ENSMUSG	Gm11295	ENSMUST00000121379
ENSMUSG	Gm11833	ENSMUST00000118393
ENSMUSG	Gm14439	ENSMUST00000122320
ENSMUSG	Gm12004	ENSMUST00000117869
ENSMUSG	Gm12079	ENSMUST00000112483
ENSMUSG	Gm15159	ENSMUST00000117062
ENSMUSG	Xlr4d-p	ENSMUST00000118894
ENSMUSG	Gm14269	ENSMUST00000120455
ENSMUSG	Gm12306	ENSMUST00000117256
ENSMUSG	Gm11285	ENSMUST00000118443
ENSMUSG	Gm14600	ENSMUST00000121032
ENSMUSG	Gm11631	ENSMUST00000119220
ENSMUSG	Gm5761	ENSMUST00000119238
ENSMUSG	Gm13961	ENSMUST00000119282
ENSMUSG	Vmn1r-p	ENSMUST00000120909
ENSMUSG	Gm13422	ENSMUST00000118834
ENSMUSG	Gm13578	ENSMUST00000120849
ENSMUSG	Gm15794	ENSMUST00000120182
ENSMUSG	Gm4992	ENSMUST00000120256
ENSMUSG	Olfr106	ENSMUST00000118807
ENSMUSG	Gm12351	ENSMUST00000118727
ENSMUSG	Gm11624	ENSMUST00000118579
ENSMUSG	Gm13588	ENSMUST00000120026
ENSMUSG	Gm8757	ENSMUST00000119660
ENSMUSG	Gm15844	ENSMUST00000118016
ENSMUSG	Gm12856	ENSMUST00000119471
ENSMUSG	Gm14915	ENSMUST00000120747
ENSMUSG	Gm9103	ENSMUST00000119371
ENSMUSG	Gm14395	ENSMUST00000120494
ENSMUSG	Gm11577	ENSMUST00000118030
ENSMUSG	Gm15576	ENSMUST00000117806
ENSMUSG	Gm13345	ENSMUST00000121928
ENSMUSG	Gm13902	ENSMUST00000120168
ENSMUSG	Gm11954	ENSMUST00000121403
ENSMUSG	Gm13508	ENSMUST00000121639
ENSMUSG	Taar7c-]	ENSMUST00000122123
ENSMUSG	Gm13474	ENSMUST00000122466
ENSMUSG	Gm8089	ENSMUST00000122463
ENSMUSG	Gm13282	ENSMUST00000121577
ENSMUSG	Gm6823	ENSMUST00000120791
ENSMUSG	Gm15372	ENSMUST00000120729
ENSMUSG	Gm9089	ENSMUST00000122027
ENSMUSG	Gm5398	ENSMUST00000121933
ENSMUSG	Gm15593	ENSMUST00000122085
ENSMUSG	Gm12062	ENSMUST00000117533
ENSMUSG	Gm12792	ENSMUST00000117669
ENSMUSG	Gm13811	ENSMUST00000117800
ENSMUSG	Gm11949	ENSMUST00000117640
ENSMUSG	Gm11814	ENSMUST00000119515
ENSMUSG	Gm7973	ENSMUST00000122107
ENSMUSG	Gm15488	ENSMUST00000121682
ENSMUSG	Gm14876	ENSMUST00000120795

ENSMUSG	Gm12387	ENSMUST00000118573
ENSMUSG	Gm12337	ENSMUST00000119634
ENSMUSG	Gm8080	ENSMUST00000122046
ENSMUSG	Gm15383	ENSMUST00000121889
ENSMUSG	Gm13416	ENSMUST00000117264
ENSMUSG	Gm12310	ENSMUST00000118013
ENSMUSG	Gm12040	ENSMUST00000118357
ENSMUSG	Gm12844	ENSMUST00000119463
ENSMUSG	Gm8317	ENSMUST00000121261
ENSMUSG	Gm7856	ENSMUST00000117514
ENSMUSG	Gm12194	ENSMUST00000117756
ENSMUSG	Gm8216	ENSMUST00000117232
ENSMUSG	Gm11950	ENSMUST00000121615
ENSMUSG	Gm11257	ENSMUST00000121313
ENSMUSG	Gm13122	ENSMUST00000118380
ENSMUSG	Vmn1r-p	ENSMUST00000121211
ENSMUSG	Gm14125	ENSMUST00000113442;ENSMUST00000120921
ENSMUSG	Gm14046	ENSMUST00000119389
ENSMUSG	Gm11224	ENSMUST00000120465
ENSMUSG	Gm16198	ENSMUST00000118766
ENSMUSG	Rpl17-p	ENSMUST00000120241
ENSMUSG	Gm14608	ENSMUST00000120030
ENSMUSG	Gm11572	ENSMUST00000119164
ENSMUSG	Gm15442	ENSMUST00000117905
ENSMUSG	Gm12957	ENSMUST00000117835
ENSMUSG	Gm12251	ENSMUST00000117893
ENSMUSG	Gm14101	ENSMUST00000121502
ENSMUSG	Gm12017	ENSMUST00000122453
ENSMUSG	Gm12837	ENSMUST00000120131
ENSMUSG	Gm25107	ENSMUST00000122472
ENSMUSG	n-R5s20	ENSMUST00000122497
ENSMUSG	Gm22955	ENSMUST00000122519
ENSMUSG	Gm22956	ENSMUST00000122520
ENSMUSG	Gm25782	ENSMUST00000122526
ENSMUSG	Gm26424	ENSMUST00000122530
ENSMUSG	Gm23789	ENSMUST00000122536
ENSMUSG	Gm23787	ENSMUST00000122538
ENSMUSG	Gm22514	ENSMUST00000122547
ENSMUSG	Gm22482	ENSMUST00000122563
ENSMUSG	Gm22477	ENSMUST00000122569
ENSMUSG	Mir1897	ENSMUST00000122581
ENSMUSG	Mir1906	ENSMUST00000122586
ENSMUSG	Gm25844	ENSMUST00000122587
ENSMUSG	DQ26710	ENSMUST00000122600
ENSMUSG	Gm25270	ENSMUST00000122606
ENSMUSG	Mir1906	ENSMUST00000122610
ENSMUSG	Gm26477	ENSMUST00000122617
ENSMUSG	n-R5s30	ENSMUST00000122624
ENSMUSG	Gm22344	ENSMUST00000122642
ENSMUSG	n-R5s34	ENSMUST00000122653
ENSMUSG	Gm22205	ENSMUST00000122655
ENSMUSG	Gm22208	ENSMUST00000122658
ENSMUSG	Gm25047	ENSMUST00000122667
ENSMUSG	Gm22073	ENSMUST00000122668
ENSMUSG	Gm25112	ENSMUST00000122680
ENSMUSG	Gm23883	ENSMUST00000122686
ENSMUSG	Gm23884	ENSMUST00000122687

ENSMUSG	Gm24380	ENSMUST00000122696
ENSMUSG	Gm26091	ENSMUST00000122704
ENSMUSG	Gm22024	ENSMUST00000122733
ENSMUSG	n-R5s43	ENSMUST00000122739
ENSMUSG	Gm24895	ENSMUST00000122742
ENSMUSG	Gm24893	ENSMUST00000122745
ENSMUSG	Gm24627	ENSMUST00000122764
ENSMUSG	Gm25809	ENSMUST00000122789
ENSMUSG	Gm25295	ENSMUST00000122794
ENSMUSG	Gm25292	ENSMUST00000122797
ENSMUSG	Gm25289	ENSMUST00000122799
ENSMUSG	Gm12532	ENSMUST00000148448
ENSMUSG	Gm13881	ENSMUST00000149025
ENSMUSG	Gm12406	ENSMUST00000155382
ENSMUSG	Platr3	ENSMUST00000146980
ENSMUSG	Gm12714	ENSMUST00000143391
ENSMUSG	Gm13605	ENSMUST00000124489
ENSMUSG	Gm14110	ENSMUST00000127688
ENSMUSG	Gm16741	ENSMUST00000145085
ENSMUSG	Gm11213	ENSMUST00000210529;ENSMUST00000136620
ENSMUSG	Gm12436	ENSMUST00000131099
ENSMUSG	Gm12974	ENSMUST00000129875
ENSMUSG	Gm2568	ENSMUST00000146126
ENSMUSG	Gm15232	ENSMUST00000146929
ENSMUSG	Gm5526	ENSMUST00000153604
ENSMUSG	Myadml2	ENSMUST00000126642
ENSMUSG	2410152	ENSMUST00000137405
ENSMUSG	Gm14539	ENSMUST00000117527
ENSMUSG	Pabpc1l	ENSMUST00000124538
ENSMUSG	B230312	ENSMUST00000145549
ENSMUSG	Hoxb3os	ENSMUST00000147410;ENSMUST00000131275
ENSMUSG	Gm15507	ENSMUST00000140263;ENSMUST00000133523
ENSMUSG	4933408	ENSMUST00000139864
ENSMUSG	Gm16105	ENSMUST00000156926
ENSMUSG	Gm12092	ENSMUST00000125194
ENSMUSG	Gm11791	ENSMUST00000139327
ENSMUSG	Gm12678	ENSMUST00000154177
ENSMUSG	Gm11528	ENSMUST00000156822
ENSMUSG	Gm1980	ENSMUST00000123131
ENSMUSG	1700095	ENSMUST00000128017
ENSMUSG	Gm12906	ENSMUST00000154610
ENSMUSG	3010001	ENSMUST00000130625;ENSMUST00000148651;ENSMUST0000014
ENSMUSG	A830036	ENSMUST00000126470;ENSMUST00000139095;ENSMUST0000012
ENSMUSG	AA67265	ENSMUST00000151550
ENSMUSG	Gm281	ENSMUST00000144914
ENSMUSG	Gm14827	ENSMUST00000146644
ENSMUSG	C630043	ENSMUST00000122923
ENSMUSG	Gm7616	ENSMUST00000143112
ENSMUSG	Gm12708	ENSMUST00000136941
ENSMUSG	Ptprtos	ENSMUST00000151688
ENSMUSG	Gm15230	ENSMUST00000153701
ENSMUSG	Gm13838	ENSMUST00000132130
ENSMUSG	Gm5724	ENSMUST00000148411
ENSMUSG	Gm15156	ENSMUST00000148217
ENSMUSG	Gm14161	ENSMUST00000137020
ENSMUSG	Gm11228	ENSMUST00000143796
ENSMUSG	BB55794	ENSMUST00000126549

ENSMUSG	Gm830	ENSMUST00000142458
ENSMUSG	C030037	ENSMUST00000155211
ENSMUSG	Gm15594	ENSMUST00000137958
ENSMUSG	1700061	ENSMUST00000139488;ENSMUST00000147221
ENSMUSG	Gm16351	ENSMUST00000209660;ENSMUST00000142008
ENSMUSG	Gm14936	ENSMUST00000137765
ENSMUSG	Gm13857	ENSMUST00000150561;ENSMUST00000139645
ENSMUSG	Slc36a1	ENSMUST00000157010
ENSMUSG	Gm13134	ENSMUST00000132106
ENSMUSG	Crocc2	ENSMUST00000138595
ENSMUSG	Gm11419	ENSMUST00000150772
ENSMUSG	Gm12984	ENSMUST00000145711
ENSMUSG	Gm11549	ENSMUST00000124606
ENSMUSG	Dbhos	ENSMUST00000150024
ENSMUSG	Gm13412	ENSMUST00000150621
ENSMUSG	2310081	ENSMUST00000128591
ENSMUSG	Gm5860	ENSMUST00000137810;ENSMUST00000151038
ENSMUSG	C230035	ENSMUST00000141543;ENSMUST00000153753
ENSMUSG	Gm13715	ENSMUST00000136578
ENSMUSG	Srrm3os	ENSMUST00000131896
ENSMUSG	Gm11840	ENSMUST00000156947
ENSMUSG	Gm11217	ENSMUST00000141162
ENSMUSG	1700109	ENSMUST00000150735
ENSMUSG	Gm14809	ENSMUST00000152025
ENSMUSG	Gm13415	ENSMUST00000131886
ENSMUSG	8030453	ENSMUST00000128919;ENSMUST00000135363;ENSMUST0000020C
ENSMUSG	Gm11750	ENSMUST00000146016
ENSMUSG	Gm13111	ENSMUST00000136217
ENSMUSG	Gm14066	ENSMUST00000134801
ENSMUSG	Khdc1b	ENSMUST00000147615
ENSMUSG	Gm4764	ENSMUST00000130207
ENSMUSG	A530072	ENSMUST00000151122
ENSMUSG	B130011	ENSMUST00000151765
ENSMUSG	Rubie	ENSMUST00000151828
ENSMUSG	Gm12766	ENSMUST00000140298
ENSMUSG	Gm16070	ENSMUST00000155588;ENSMUST00000127328;ENSMUST0000014
ENSMUSG	4930444	ENSMUST00000146746
ENSMUSG	5031425	ENSMUST00000127920
ENSMUSG	Gm11417	ENSMUST00000150536
ENSMUSG	Gm13713	ENSMUST00000155472;ENSMUST00000139377
ENSMUSG	A730046	ENSMUST00000135687
ENSMUSG	Gm13429	ENSMUST00000123224;ENSMUST00000138755;ENSMUST0000012
ENSMUSG	Eif2c5	ENSMUST00000125609
ENSMUSG	Gm12609	ENSMUST00000129908
ENSMUSG	Gm14264	ENSMUST00000147141
ENSMUSG	Gm13617	ENSMUST00000155350
ENSMUSG	Gm2869	ENSMUST00000124359
ENSMUSG	AI83859	ENSMUST00000141405;ENSMUST00000136306;ENSMUST0000013
ENSMUSG	Gm13596	ENSMUST00000153354
ENSMUSG	Gm11963	ENSMUST00000149826
ENSMUSG	6030471	ENSMUST00000125884
ENSMUSG	BB21858	ENSMUST00000135180;ENSMUST00000123623;ENSMUST0000013
ENSMUSG	Gm13974	ENSMUST00000144368
ENSMUSG	Gm13425	ENSMUST00000145389
ENSMUSG	Gm7855	ENSMUST00000137737
ENSMUSG	Gm12576	ENSMUST00000135268
ENSMUSG	4930479	ENSMUST00000146754

ENSMUSG	8430437	ENSMUST00000124829
ENSMUSG	Gm13264	ENSMUST00000138416
ENSMUSG	Gm13814	ENSMUST00000141341
ENSMUSG	Gm14235	ENSMUST00000142445
ENSMUSG	E130006	ENSMUST00000137398
ENSMUSG	Sbk3	ENSMUST00000144863;ENSMUST00000133272
ENSMUSG	Gm14487	ENSMUST00000145413
ENSMUSG	Gm15812	ENSMUST00000162331
ENSMUSG	Gm16129	ENSMUST00000152628
ENSMUSG	Gm12853	ENSMUST00000134971
ENSMUSG	Gm13817	ENSMUST00000142249
ENSMUSG	4930430	ENSMUST00000139214;ENSMUST00000145448
ENSMUSG	Gm12410	ENSMUST00000124989
ENSMUSG	D330050	ENSMUST00000126467;ENSMUST00000155323
ENSMUSG	Gm13330	ENSMUST00000136400
ENSMUSG	Gm11548	ENSMUST00000140203
ENSMUSG	Gm17131	ENSMUST00000164420
ENSMUSG	1700030	ENSMUST00000125317
ENSMUSG	Gm11732	ENSMUST00000151381
ENSMUSG	Gm16064	ENSMUST00000154997
ENSMUSG	Myhas	ENSMUST00000145021;ENSMUST00000137662
ENSMUSG	1700092	ENSMUST00000135792;ENSMUST00000169209
ENSMUSG	Gm3160	ENSMUST00000128343
ENSMUSG	Gm12506	ENSMUST00000123620
ENSMUSG	Gm11415	ENSMUST00000128214
ENSMUSG	1700003	ENSMUST00000139884
ENSMUSG	2210414	ENSMUST00000139600
ENSMUSG	Gm13056	ENSMUST00000142429
ENSMUSG	Foxd2os	ENSMUST00000123272;ENSMUST00000144002
ENSMUSG	Slc39a1	ENSMUST00000120862
ENSMUSG	Gm12111	ENSMUST00000150073
ENSMUSG	1700095	ENSMUST00000143715
ENSMUSG	Gm14319	ENSMUST00000142937
ENSMUSG	Halr1	ENSMUST00000125374;ENSMUST00000189071;ENSMUST00000155
ENSMUSG	1700019	ENSMUST00000130004
ENSMUSG	Gm11734	ENSMUST00000132261
ENSMUSG	4732490	ENSMUST00000130373
ENSMUSG	Fhad1os	ENSMUST00000146094
ENSMUSG	6430710	ENSMUST00000136807
ENSMUSG	Gm14344	ENSMUST00000139338
ENSMUSG	Gm13485	ENSMUST00000146690
ENSMUSG	Sorbs2o	ENSMUST00000135116
ENSMUSG	Gm15520	ENSMUST00000132097
ENSMUSG	Gm13686	ENSMUST00000122851
ENSMUSG	Gm15137	ENSMUST00000125161
ENSMUSG	Gm12272	ENSMUST00000138136
ENSMUSG	Hmgn2l6	ENSMUST00000115986
ENSMUSG	1700012	ENSMUST00000132023
ENSMUSG	Gm15691	ENSMUST00000138626
ENSMUSG	Gm16686	ENSMUST00000141816
ENSMUSG	Gm11851	ENSMUST00000132219
ENSMUSG	9430073	ENSMUST00000145346
ENSMUSG	Gm13539	ENSMUST00000211245;ENSMUST00000153913
ENSMUSG	Gm11634	ENSMUST00000154147;ENSMUST00000209862
ENSMUSG	4930557	ENSMUST00000155432
ENSMUSG	Gm15985	ENSMUST00000137731
ENSMUSG	Gm16976	ENSMUST00000099718;ENSMUST00000127408

ENSMUSG	Gm11772	ENSMUST00000136542
ENSMUSG	Gm12320	ENSMUST00000127333
ENSMUSG	Gm2709	ENSMUST00000142979
ENSMUSG	C630028	ENSMUST00000141452
ENSMUSG	Gm12963	ENSMUST00000131846;ENSMUST00000147555;ENSMUST00000156
ENSMUSG	Gm13703	ENSMUST00000150705
ENSMUSG	Gm15945	ENSMUST00000146755
ENSMUSG	Gm16083	ENSMUST00000125681
ENSMUSG	Gm13901	ENSMUST00000137252
ENSMUSG	Slc36a3	ENSMUST00000155883
ENSMUSG	B430319	ENSMUST00000123420
ENSMUSG	Gm16010	ENSMUST00000145410;ENSMUST00000124366
ENSMUSG	Gm13553	ENSMUST00000133025
ENSMUSG	Gm13767	ENSMUST00000124074
ENSMUSG	4930412	ENSMUST00000124122;ENSMUST00000138390;ENSMUST00000146
ENSMUSG	2610028	ENSMUST00000156350
ENSMUSG	Gm12198	ENSMUST00000154068
ENSMUSG	Gm15721	ENSMUST00000138406
ENSMUSG	Gm12602	ENSMUST00000154352;ENSMUST00000138655
ENSMUSG	Gm15485	ENSMUST00000149095
ENSMUSG	Dpy19l2	ENSMUST00000133010
ENSMUSG	3110099	ENSMUST00000134580
ENSMUSG	Rgag1	ENSMUST00000165829
ENSMUSG	Gm12223	ENSMUST00000135468
ENSMUSG	Gm13479	ENSMUST00000130773
ENSMUSG	Gm11551	ENSMUST00000147438
ENSMUSG	Gm13449	ENSMUST00000153559
ENSMUSG	Gm16354	ENSMUST00000155376
ENSMUSG	Gm13850	ENSMUST00000140465
ENSMUSG	G630018	ENSMUST00000221171
ENSMUSG	Gm11222	ENSMUST00000119005
ENSMUSG	Gm11697	ENSMUST00000149226
ENSMUSG	Gm15521	ENSMUST00000141752
ENSMUSG	4930465	ENSMUST00000130564;ENSMUST00000144556
ENSMUSG	A730032	ENSMUST00000143202
ENSMUSG	Gm15704	ENSMUST00000144757
ENSMUSG	Gm12434	ENSMUST00000141580
ENSMUSG	Gm12059	ENSMUST00000128996
ENSMUSG	Gm35202	ENSMUST00000130083
ENSMUSG	Gm12022	ENSMUST00000143311
ENSMUSG	4930594	ENSMUST00000144981
ENSMUSG	Gm16328	ENSMUST00000126985
ENSMUSG	9130409	ENSMUST00000211300;ENSMUST00000150220;ENSMUST00000127
ENSMUSG	Gm16838	ENSMUST00000144594
ENSMUSG	Hbb-bh0	ENSMUST00000124800
ENSMUSG	Gm12968	ENSMUST00000138291
ENSMUSG	Mecomos	ENSMUST00000125611;ENSMUST00000173827
ENSMUSG	Gm12212	ENSMUST00000141032
ENSMUSG	Gm16063	ENSMUST00000156235
ENSMUSG	Gm15163	ENSMUST00000120749
ENSMUSG	Gm15124	ENSMUST00000142053
ENSMUSG	Hmgb4os	ENSMUST00000131581
ENSMUSG	Gm12796	ENSMUST00000131864
ENSMUSG	Gm15915	ENSMUST00000147379
ENSMUSG	Gm15873	ENSMUST00000123390;ENSMUST00000205662
ENSMUSG	Gm11684	ENSMUST00000144975
ENSMUSG	Gm13442	ENSMUST00000135749

ENSMUSG	8430419	ENSMUST00000128654;ENSMUST00000156831;ENSMUST00000133
ENSMUSG	Gm12280	ENSMUST00000155836
ENSMUSG	1700019	ENSMUST00000133350
ENSMUSG	1700061	ENSMUST00000154159
ENSMUSG	Smc2os	ENSMUST00000141473
ENSMUSG	2810430	ENSMUST00000154246
ENSMUSG	D630024	ENSMUST00000129425;ENSMUST00000126265
ENSMUSG	Gm16233	ENSMUST00000136170
ENSMUSG	Gm13055	ENSMUST00000127875
ENSMUSG	Gm12729	ENSMUST00000130088
ENSMUSG	Vax2os	ENSMUST00000123402;ENSMUST00000155159
ENSMUSG	A530010	ENSMUST00000146845
ENSMUSG	Gm12056	ENSMUST00000148364
ENSMUSG	Gm12023	ENSMUST00000145182
ENSMUSG	Gm14718	ENSMUST00000138082
ENSMUSG	Gm16325	ENSMUST00000141199
ENSMUSG	Cep112i	ENSMUST00000143781
ENSMUSG	Gm15843	ENSMUST00000146593
ENSMUSG	Gm13267	ENSMUST00000150099
ENSMUSG	Platr9	ENSMUST00000125820
ENSMUSG	Grin1os	ENSMUST00000129723
ENSMUSG	1700003	ENSMUST00000131342
ENSMUSG	Gm13003	ENSMUST00000151040
ENSMUSG	Gm15622	ENSMUST00000143025
ENSMUSG	Gm13074	ENSMUST00000124848
ENSMUSG	Chn1os1	ENSMUST00000123613
ENSMUSG	Gm11261	ENSMUST00000132470;ENSMUST00000123051;ENSMUST0000012(
ENSMUSG	Kank4os	ENSMUST00000133455
ENSMUSG	Gm11690	ENSMUST00000128183
ENSMUSG	Gm13944	ENSMUST00000146495;ENSMUST00000126572
ENSMUSG	2410003	ENSMUST00000131687;ENSMUST00000145262
ENSMUSG	Gm12801	ENSMUST00000153228
ENSMUSG	C330019	ENSMUST00000149428
ENSMUSG	Gm5834	ENSMUST00000126680
ENSMUSG	Gm12829	ENSMUST00000146346
ENSMUSG	Ube2uos	ENSMUST00000142634
ENSMUSG	Gm12409	ENSMUST00000147231
ENSMUSG	Gm15229	ENSMUST00000146636
ENSMUSG	Gm12224	ENSMUST00000127107
ENSMUSG	5330429	ENSMUST00000154615
ENSMUSG	Gm6689	ENSMUST00000130905
ENSMUSG	Gm15338	ENSMUST00000151884
ENSMUSG	Gm12200	ENSMUST00000144876
ENSMUSG	Trp53co	ENSMUST00000133221
ENSMUSG	Gm8091	ENSMUST00000150506
ENSMUSG	Gm13032	ENSMUST00000151848
ENSMUSG	Fhad1os	ENSMUST00000152351
ENSMUSG	Rtl1	ENSMUST00000149046
ENSMUSG	4933427	ENSMUST00000153921;ENSMUST00000138672
ENSMUSG	Gm13421	ENSMUST00000142511;ENSMUST00000132898
ENSMUSG	Gm12648	ENSMUST00000126270
ENSMUSG	Tmem61	ENSMUST00000148907;ENSMUST00000177702
ENSMUSG	AA38720	ENSMUST00000147463
ENSMUSG	Gm14275	ENSMUST00000152112
ENSMUSG	Gm13589	ENSMUST00000146404
ENSMUSG	Gm15904	ENSMUST00000154515
ENSMUSG	Gm12530	ENSMUST00000152074

ENSMUSG	Gm13027	ENSMUST00000135858
ENSMUSG	Gm15600	ENSMUST00000153522
ENSMUSG	Gm14742	ENSMUST00000148284
ENSMUSG	Gm13816	ENSMUST00000152230
ENSMUSG	Gm12408	ENSMUST00000211577
ENSMUSG	9530051	ENSMUST00000127673
ENSMUSG	Gm11682	ENSMUST00000144689
ENSMUSG	Gm13403	ENSMUST00000127936
ENSMUSG	Gm11479	ENSMUST00000134275
ENSMUSG	Gm11515	ENSMUST00000150818
ENSMUSG	AW82225	ENSMUST00000192662;ENSMUST00000187290;ENSMUST00000148
ENSMUSG	Gm13411	ENSMUST00000149429
ENSMUSG	Gm12493	ENSMUST00000150111
ENSMUSG	B230206	ENSMUST00000132584;ENSMUST00000125406
ENSMUSG	Gm13293	ENSMUST00000131188
ENSMUSG	Gm8817	ENSMUST00000133867;ENSMUST00000126769
ENSMUSG	4930587	ENSMUST00000128588
ENSMUSG	Gm15318	ENSMUST00000146320
ENSMUSG	Gm15902	ENSMUST00000146949;ENSMUST00000219623
ENSMUSG	Gm15706	ENSMUST00000139612
ENSMUSG	Gm12239	ENSMUST00000134285
ENSMUSG	Gm15767	ENSMUST00000153786
ENSMUSG	Rad51ap	ENSMUST00000124065
ENSMUSG	4933406	ENSMUST00000144854
ENSMUSG	Gm12243	ENSMUST00000128521
ENSMUSG	Gm15201	ENSMUST00000136472
ENSMUSG	Gm12610	ENSMUST00000139272
ENSMUSG	Gm12473	ENSMUST00000137535
ENSMUSG	Gm15178	ENSMUST00000132100
ENSMUSG	Gm4846	ENSMUST00000143922
ENSMUSG	Gm12257	ENSMUST00000148244
ENSMUSG	8430436	ENSMUST00000129988
ENSMUSG	Gm12354	ENSMUST00000124319
ENSMUSG	Gm13274	ENSMUST00000123455
ENSMUSG	Gm14396	ENSMUST00000126966
ENSMUSG	Gm15323	ENSMUST00000156109
ENSMUSG	Gm15328	ENSMUST00000209584;ENSMUST00000210201;ENSMUST00000144
ENSMUSG	Gm12688	ENSMUST00000136525
ENSMUSG	Gm11636	ENSMUST00000152172
ENSMUSG	Gm15326	ENSMUST00000128198;ENSMUST00000127817
ENSMUSG	Gm15733	ENSMUST00000132957
ENSMUSG	Gm12065	ENSMUST00000149592
ENSMUSG	A530076	ENSMUST00000138379
ENSMUSG	Gm16331	ENSMUST00000144274
ENSMUSG	Gm12718	ENSMUST00000156144
ENSMUSG	Gm16248	ENSMUST00000124238
ENSMUSG	4930471	ENSMUST00000143070
ENSMUSG	Gm11379	ENSMUST00000154678
ENSMUSG	Gm15729	ENSMUST00000125829
ENSMUSG	Gm15271	ENSMUST00000134627
ENSMUSG	Gm11454	ENSMUST00000144092
ENSMUSG	Gm12923	ENSMUST00000127060
ENSMUSG	Gm15389	ENSMUST00000153774
ENSMUSG	Gm16342	ENSMUST00000124025
ENSMUSG	Gm14206	ENSMUST00000132973
ENSMUSG	Gm13029	ENSMUST00000137283
ENSMUSG	Gm14342	ENSMUST00000137629

ENSMUSG	Gm12144	ENSMUST00000136288
ENSMUSG	2700068	ENSMUST00000137882
ENSMUSG	Gm6425	ENSMUST00000136332
ENSMUSG	Gm15802	ENSMUST00000209595
ENSMUSG	Gm12860	ENSMUST00000148457
ENSMUSG	Gm15169	ENSMUST00000126095
ENSMUSG	Gm15462	ENSMUST00000133653
ENSMUSG	4930579	ENSMUST00000130943
ENSMUSG	Gm12679	ENSMUST00000123899
ENSMUSG	4933405	ENSMUST00000131019
ENSMUSG	Gm12462	ENSMUST00000140826
ENSMUSG	Gm14062	ENSMUST00000134795
ENSMUSG	Srrm4os	ENSMUST00000126959
ENSMUSG	4930412	ENSMUST00000149239
ENSMUSG	2700069	ENSMUST00000177440
ENSMUSG	Gm12660	ENSMUST00000123764
ENSMUSG	Gm4887	ENSMUST00000122230
ENSMUSG	Gm14329	ENSMUST00000136027
ENSMUSG	Gm12121	ENSMUST00000131630
ENSMUSG	Gm12724	ENSMUST00000140196
ENSMUSG	Gm11534	ENSMUST00000125956
ENSMUSG	Gm12052	ENSMUST00000142151
ENSMUSG	Gm3765	ENSMUST00000130985
ENSMUSG	A930031	ENSMUST00000130882;ENSMUST00000153810
ENSMUSG	Marcksl	ENSMUST00000132531
ENSMUSG	Igf2os	ENSMUST00000141681
ENSMUSG	BC03996	ENSMUST00000141582
ENSMUSG	Rbm46os	ENSMUST00000131274
ENSMUSG	1700121	ENSMUST00000137546
ENSMUSG	Olfr75-	ENSMUST00000144832
ENSMUSG	2810433	ENSMUST00000154798;ENSMUST00000147541
ENSMUSG	Frmpd1o	ENSMUST00000134640
ENSMUSG	Gm15138	ENSMUST00000151778
ENSMUSG	D030055	ENSMUST00000143956;ENSMUST00000150953
ENSMUSG	Gm13790	ENSMUST00000156209
ENSMUSG	Gm11754	ENSMUST00000156576
ENSMUSG	Gm11413	ENSMUST00000155822
ENSMUSG	1700120	ENSMUST00000152951
ENSMUSG	Gm12107	ENSMUST00000132857
ENSMUSG	Gm11535	ENSMUST00000145517
ENSMUSG	Gm13481	ENSMUST00000127672
ENSMUSG	Gm13441	ENSMUST00000140104
ENSMUSG	A330102	ENSMUST00000146336;ENSMUST00000130109;ENSMUST0000014C
ENSMUSG	E330017	ENSMUST00000145196;ENSMUST00000141731
ENSMUSG	Gm11978	ENSMUST00000127510;ENSMUST00000122315
ENSMUSG	Gm12127	ENSMUST00000123278
ENSMUSG	4930529	ENSMUST00000131367
ENSMUSG	AV06450	ENSMUST00000124572
ENSMUSG	Chrna1o	ENSMUST00000125413;ENSMUST00000147235
ENSMUSG	Gm12108	ENSMUST00000145338
ENSMUSG	Gm15998	ENSMUST00000135144
ENSMUSG	1700042	ENSMUST00000151032
ENSMUSG	4930555	ENSMUST00000123210
ENSMUSG	Gm15322	ENSMUST00000142621
ENSMUSG	Gm6787	ENSMUST00000123120
ENSMUSG	9330198	ENSMUST00000146702
ENSMUSG	Gm11588	ENSMUST00000141758

ENSMUSG	Gm14002	ENSMUST00000123574
ENSMUSG	Gm12996	ENSMUST00000151670
ENSMUSG	4930421	ENSMUST00000147322
ENSMUSG	F730016	ENSMUST00000156508;ENSMUST00000128888
ENSMUSG	Hoxa11o	ENSMUST00000134512
ENSMUSG	Asb17os	ENSMUST00000200329;ENSMUST00000156292
ENSMUSG	Gm15046	ENSMUST00000130153
ENSMUSG	4933421	ENSMUST00000135313
ENSMUSG	Gm13191	ENSMUST00000136489
ENSMUSG	9030204	ENSMUST00000139455
ENSMUSG	Platr14	ENSMUST00000125854;ENSMUST00000127350;ENSMUST00000127
ENSMUSG	Gm11815	ENSMUST00000139423
ENSMUSG	Gm12236	ENSMUST00000152968
ENSMUSG	4930571	ENSMUST00000132871
ENSMUSG	9130204	ENSMUST00000150312;ENSMUST00000131441;ENSMUST0000015
ENSMUSG	Platr13	ENSMUST00000133945
ENSMUSG	Gm14102	ENSMUST00000147678
ENSMUSG	Gm15287	ENSMUST00000132564
ENSMUSG	Gm11707	ENSMUST00000150366
ENSMUSG	Ift88os	ENSMUST00000149702
ENSMUSG	Gm16362	ENSMUST00000135189
ENSMUSG	2210417	ENSMUST00000150265
ENSMUSG	Gm14204	ENSMUST00000123944;ENSMUST00000123016
ENSMUSG	Gm12681	ENSMUST00000132337
ENSMUSG	4930597	ENSMUST00000128652
ENSMUSG	Nkx2-2o	ENSMUST00000136998
ENSMUSG	Erich2o	ENSMUST00000150702
ENSMUSG	Pabpc1l	ENSMUST00000128361
ENSMUSG	Has2os	ENSMUST00000125401;ENSMUST00000166290;ENSMUST0000016
ENSMUSG	Chn1os3	ENSMUST00000147420;ENSMUST00000149231
ENSMUSG	Gm13709	ENSMUST00000138573
ENSMUSG	Gm12972	ENSMUST00000152986
ENSMUSG	Dlx4os	ENSMUST00000156477
ENSMUSG	Gm14426	ENSMUST00000128562
ENSMUSG	Gm13372	ENSMUST00000123129
ENSMUSG	Gm15540	ENSMUST00000136052
ENSMUSG	Cd101	ENSMUST00000147399;ENSMUST00000167086
ENSMUSG	Gm13133	ENSMUST00000143888
ENSMUSG	Gm2830	ENSMUST00000140330
ENSMUSG	Gm13660	ENSMUST00000142828
ENSMUSG	Gm13583	ENSMUST00000151931;ENSMUST00000168789;ENSMUST0000012
ENSMUSG	Gm15280	ENSMUST00000126961
ENSMUSG	Gm16126	ENSMUST00000126025
ENSMUSG	Gm11729	ENSMUST00000124901
ENSMUSG	Gm12682	ENSMUST00000144637
ENSMUSG	Nudt12o	ENSMUST00000145848;ENSMUST00000152750
ENSMUSG	Gm11635	ENSMUST00000139424
ENSMUSG	Rspo4os	ENSMUST00000152229
ENSMUSG	Gm13348	ENSMUST00000147324
ENSMUSG	Gm14397	ENSMUST00000124728
ENSMUSG	Gm12784	ENSMUST00000127462;ENSMUST00000206001
ENSMUSG	Gm16081	ENSMUST00000155992
ENSMUSG	Gm12949	ENSMUST00000143382
ENSMUSG	Gm14040	ENSMUST00000139058
ENSMUSG	Gm13470	ENSMUST00000123911
ENSMUSG	Gm14260	ENSMUST00000125121
ENSMUSG	Plxna4o	ENSMUST00000152538

ENSMUSG	Tvp23bo	ENSMUST00000148598
ENSMUSG	F630206	ENSMUST00000137002
ENSMUSG	Gm11560	ENSMUST00000117567
ENSMUSG	Gm15432	ENSMUST00000121106
ENSMUSG	Gm16237	ENSMUST00000156666
ENSMUSG	Gm16510	ENSMUST00000153501
ENSMUSG	Gm13595	ENSMUST00000145994
ENSMUSG	4930507	ENSMUST00000137411
ENSMUSG	Gm15848	ENSMUST00000125056;ENSMUST00000136015
ENSMUSG	Gm12088	ENSMUST00000137364
ENSMUSG	Gm16263	ENSMUST00000144680
ENSMUSG	AI42780	ENSMUST00000123127;ENSMUST00000098075;ENSMUST0000018
ENSMUSG	Plxna4o	ENSMUST00000127668
ENSMUSG	Gm13750	ENSMUST00000117621
ENSMUSG	Gm15499	ENSMUST00000141390
ENSMUSG	Gm12833	ENSMUST00000136112
ENSMUSG	Gm9458	ENSMUST00000135273
ENSMUSG	Gm11685	ENSMUST00000141559
ENSMUSG	Gm15816	ENSMUST00000141930
ENSMUSG	Gm15222	ENSMUST00000127359
ENSMUSG	Gm12037	ENSMUST00000123160
ENSMUSG	Gm11770	ENSMUST00000135048
ENSMUSG	Gm11674	ENSMUST00000124855
ENSMUSG	Gm2309	ENSMUST00000149994
ENSMUSG	Cep112o	ENSMUST00000137907
ENSMUSG	Plxna4o	ENSMUST00000150314
ENSMUSG	Gm15587	ENSMUST00000146326;ENSMUST00000156403
ENSMUSG	1700080	ENSMUST00000156401
ENSMUSG	Snhg7os	ENSMUST00000131841
ENSMUSG	Isoc2a	ENSMUST00000125249
ENSMUSG	Lyrm7os	ENSMUST00000154044
ENSMUSG	4930556	ENSMUST00000134422
ENSMUSG	Platr21	ENSMUST00000126339
ENSMUSG	Gm11981	ENSMUST00000139769
ENSMUSG	Gm13657	ENSMUST00000135682
ENSMUSG	5330413	ENSMUST00000143453;ENSMUST00000210606;ENSMUST0000021
ENSMUSG	4930448	ENSMUST00000149958
ENSMUSG	Gm15675	ENSMUST00000130486
ENSMUSG	Gm15579	ENSMUST00000141299
ENSMUSG	Gm12440	ENSMUST00000155224
ENSMUSG	Gm15681	ENSMUST00000137501
ENSMUSG	E030013	ENSMUST00000148089
ENSMUSG	Lce6a	ENSMUST00000138403
ENSMUSG	Gm13257	ENSMUST00000132840
ENSMUSG	Gm1720	ENSMUST00000135086
ENSMUSG	Gm15325	ENSMUST00000149661
ENSMUSG	Gm15963	ENSMUST00000131387
ENSMUSG	Hotair	ENSMUST00000151949
ENSMUSG	Gm13404	ENSMUST00000156633
ENSMUSG	Gm13716	ENSMUST00000126401
ENSMUSG	Gm15298	ENSMUST00000145916
ENSMUSG	Gm15755	ENSMUST00000133698
ENSMUSG	Gm12027	ENSMUST00000146531
ENSMUSG	Gm14019	ENSMUST00000131514
ENSMUSG	Gm11630	ENSMUST00000152825
ENSMUSG	Gm11497	ENSMUST00000138295
ENSMUSG	4930406	ENSMUST00000147951;ENSMUST00000152827

ENSMUSG	Gm15063	ENSMUST00000152648
ENSMUSG	Gm12746	ENSMUST00000151369
ENSMUSG	4930522	ENSMUST00000155178
ENSMUSG	Gm14697	ENSMUST00000147144
ENSMUSG	Gm12596	ENSMUST00000124589
ENSMUSG	4933431	ENSMUST00000131345;ENSMUST00000152663;ENSMUST0000014
ENSMUSG	Gm15208	ENSMUST00000134448
ENSMUSG	Gm12171	ENSMUST00000150895
ENSMUSG	Gm12484	ENSMUST00000156311
ENSMUSG	Gm13544	ENSMUST00000131189
ENSMUSG	Bcas1os	ENSMUST00000131931
ENSMUSG	Gm15475	ENSMUST00000137673
ENSMUSG	Cntrobo	ENSMUST00000132834
ENSMUSG	Gm5530	ENSMUST00000117185
ENSMUSG	9130024	ENSMUST00000185204;ENSMUST00000195462
ENSMUSG	B230119	ENSMUST00000154072
ENSMUSG	Gm14133	ENSMUST00000139471
ENSMUSG	Gm13874	ENSMUST00000151028
ENSMUSG	Gm14155	ENSMUST00000127230
ENSMUSG	Tmem74b	ENSMUST00000149319;ENSMUST00000137422
ENSMUSG	Gm14033	ENSMUST00000128307
ENSMUSG	Gm11611	ENSMUST00000134289
ENSMUSG	1700042	ENSMUST00000152001
ENSMUSG	4930515	ENSMUST00000140588
ENSMUSG	Gm12730	ENSMUST00000125277
ENSMUSG	Gm12405	ENSMUST00000141120
ENSMUSG	Gm11730	ENSMUST00000147186
ENSMUSG	Gm12339	ENSMUST00000127424;ENSMUST00000139361
ENSMUSG	Gm11532	ENSMUST00000128111
ENSMUSG	Gm12505	ENSMUST00000146041
ENSMUSG	Trp73os	ENSMUST00000143429
ENSMUSG	A230065	ENSMUST00000150384
ENSMUSG	Gm12199	ENSMUST00000137567
ENSMUSG	Gm13016	ENSMUST00000126252
ENSMUSG	Emx2os	ENSMUST00000136990
ENSMUSG	Gm11782	ENSMUST00000131675
ENSMUSG	Tmem132	ENSMUST00000145064;ENSMUST00000155106;ENSMUST00000154
ENSMUSG	Gm11264	ENSMUST00000143755
ENSMUSG	Atg4a-p	ENSMUST00000117271
ENSMUSG	Gm7847	ENSMUST00000151645
ENSMUSG	Gm6706	ENSMUST00000145087
ENSMUSG	Gm13839	ENSMUST00000151639
ENSMUSG	A230108	ENSMUST00000151782
ENSMUSG	Gm12756	ENSMUST00000126294
ENSMUSG	A930001	ENSMUST00000154407;ENSMUST00000133871;ENSMUST0000013
ENSMUSG	Gm16096	ENSMUST00000149386
ENSMUSG	Gm15864	ENSMUST00000145950
ENSMUSG	Gm11683	ENSMUST00000147006
ENSMUSG	A830082	ENSMUST00000145613;ENSMUST00000137199;ENSMUST00000144
ENSMUSG	Gm15205	ENSMUST00000129329
ENSMUSG	Gm14228	ENSMUST00000131908
ENSMUSG	Itih5l-	ENSMUST00000145639
ENSMUSG	Gm16336	ENSMUST00000126011
ENSMUSG	Gm14244	ENSMUST00000155688
ENSMUSG	Nr5a1os	ENSMUST00000146954
ENSMUSG	L1td1	ENSMUST00000152889;ENSMUST00000154279;ENSMUST0000017
ENSMUSG	Gm15891	ENSMUST00000127307

ENSMUSG	D230022	ENSMUST00000144735
ENSMUSG	Gm11650	ENSMUST00000141271
ENSMUSG	Gm13872	ENSMUST00000143995;ENSMUST00000147082
ENSMUSG	Gm13431	ENSMUST00000122948;ENSMUST00000129699
ENSMUSG	4930412	ENSMUST00000146501;ENSMUST00000139222
ENSMUSG	Gm13373	ENSMUST00000144634
ENSMUSG	Gm15818	ENSMUST00000134692
ENSMUSG	Gm15813	ENSMUST00000142286
ENSMUSG	Gm13986	ENSMUST00000148587
ENSMUSG	1700073	ENSMUST00000146858;ENSMUST00000136180;ENSMUST00000148
ENSMUSG	Gm5125	ENSMUST00000148614
ENSMUSG	Gm11377	ENSMUST00000131539
ENSMUSG	Gm14014	ENSMUST00000156718
ENSMUSG	Gm14015	ENSMUST00000130634
ENSMUSG	Mroh3	ENSMUST00000146112;ENSMUST00000133030;ENSMUST00000212
ENSMUSG	Gm4750	ENSMUST00000151942
ENSMUSG	Gm12865	ENSMUST00000137353
ENSMUSG	Gm7284	ENSMUST00000131667
ENSMUSG	Gm12126	ENSMUST00000147717
ENSMUSG	Alkal1	ENSMUST00000133144
ENSMUSG	Gm14379	ENSMUST00000128858
ENSMUSG	Gm6985	ENSMUST00000121255
ENSMUSG	Gm12349	ENSMUST00000153030
ENSMUSG	4933427	ENSMUST00000156275
ENSMUSG	Gm14486	ENSMUST00000147959;ENSMUST00000144704
ENSMUSG	Gm12983	ENSMUST00000120974
ENSMUSG	Gm13203	ENSMUST00000139895
ENSMUSG	2010013	ENSMUST00000126922
ENSMUSG	A930006	ENSMUST00000139065
ENSMUSG	Gm13580	ENSMUST00000131457
ENSMUSG	4933424	ENSMUST00000203451;ENSMUST00000132609
ENSMUSG	Gm15866	ENSMUST00000149048;ENSMUST00000152116
ENSMUSG	Lipo2	ENSMUST00000147153
ENSMUSG	A230004	ENSMUST00000125365
ENSMUSG	4930528	ENSMUST00000152815
ENSMUSG	Gm15907	ENSMUST00000134284
ENSMUSG	Gm16075	ENSMUST00000135873
ENSMUSG	Gm6038	ENSMUST00000154310
ENSMUSG	Gm13652	ENSMUST00000143898
ENSMUSG	AW49522	ENSMUST00000149839;ENSMUST00000148316
ENSMUSG	Gm15228	ENSMUST00000142044
ENSMUSG	1700021	ENSMUST00000131110
ENSMUSG	Gm16254	ENSMUST00000131528
ENSMUSG	Gm12498	ENSMUST00000138374
ENSMUSG	4930453	ENSMUST00000125569;ENSMUST00000178516
ENSMUSG	Gm15104	ENSMUST00000165760
ENSMUSG	Gm13398	ENSMUST00000149378
ENSMUSG	C430049	ENSMUST00000155622;ENSMUST00000129764;ENSMUST00000152
ENSMUSG	Gm14696	ENSMUST00000144294
ENSMUSG	Gm15541	ENSMUST00000147449
ENSMUSG	Gm15457	ENSMUST00000129513
ENSMUSG	Gm12414	ENSMUST00000150794
ENSMUSG	Gm12446	ENSMUST00000123933
ENSMUSG	Gm15592	ENSMUST00000143135
ENSMUSG	Gm7598	ENSMUST00000130251
ENSMUSG	Gm15270	ENSMUST00000146208;ENSMUST00000136908;ENSMUST00000141
ENSMUSG	2310065	ENSMUST00000125538;ENSMUST00000153497

ENSMUSG	Gm14529	ENSMUST00000117894
ENSMUSG	Gm15906	ENSMUST00000124746
ENSMUSG	Gm13853	ENSMUST00000155496
ENSMUSG	Gm13994	ENSMUST00000137397
ENSMUSG	Gm7799	ENSMUST00000133080
ENSMUSG	A630031	ENSMUST00000146587
ENSMUSG	Gm13383	ENSMUST00000131190
ENSMUSG	Ppp2r2c	ENSMUST00000152862
ENSMUSG	Gm15241	ENSMUST00000125700
ENSMUSG	Tbx3os1	ENSMUST00000202307;ENSMUST00000200851;ENSMUST00000134
ENSMUSG	Gm371	ENSMUST00000142240
ENSMUSG	Gm15533	ENSMUST00000206216;ENSMUST00000126205
ENSMUSG	Gm15606	ENSMUST00000134006
ENSMUSG	Gm13584	ENSMUST00000137134
ENSMUSG	Gm11412	ENSMUST00000144875
ENSMUSG	Hopxos	ENSMUST00000148568
ENSMUSG	Gm15878	ENSMUST00000127561
ENSMUSG	Gm13923	ENSMUST00000153283
ENSMUSG	Platr27	ENSMUST00000131365
ENSMUSG	Maats1o	ENSMUST00000133496
ENSMUSG	Gm8464	ENSMUST00000140208;ENSMUST00000146697
ENSMUSG	Gm12976	ENSMUST00000153817;ENSMUST00000150171
ENSMUSG	Ighv6-7	ENSMUST00000193397
ENSMUSG	Gm6938	ENSMUST00000123014
ENSMUSG	Gm14635	ENSMUST00000146535
ENSMUSG	Uchl1os	ENSMUST00000177319
ENSMUSG	Cdrt4os	ENSMUST00000153180;ENSMUST00000130177
ENSMUSG	4930458	ENSMUST00000142871
ENSMUSG	Gm13855	ENSMUST00000145644
ENSMUSG	Pnpla1o	ENSMUST00000146779
ENSMUSG	5330434	ENSMUST00000136406
ENSMUSG	Gm12290	ENSMUST00000145095
ENSMUSG	4930419	ENSMUST00000125494
ENSMUSG	Gm13028	ENSMUST00000128563
ENSMUSG	Gm4660	ENSMUST00000131499
ENSMUSG	Gm1667	ENSMUST00000151651
ENSMUSG	E130018	ENSMUST00000129713
ENSMUSG	Gm11186	ENSMUST00000137096
ENSMUSG	4930447	ENSMUST00000141289
ENSMUSG	Gm11762	ENSMUST00000135630
ENSMUSG	Gm14120	ENSMUST00000129067
ENSMUSG	1200007	ENSMUST00000143649
ENSMUSG	1700122	ENSMUST00000147538;ENSMUST00000141347
ENSMUSG	Gm16031	ENSMUST00000153253
ENSMUSG	Gm15674	ENSMUST00000143266
ENSMUSG	Gm16191	ENSMUST00000148464
ENSMUSG	A530058	ENSMUST00000147411
ENSMUSG	Gm16291	ENSMUST00000147378
ENSMUSG	Gm13575	ENSMUST00000142353
ENSMUSG	Gm15650	ENSMUST00000135451
ENSMUSG	Gm25828	ENSMUST00000157087
ENSMUSG	Gm25826	ENSMUST00000157090
ENSMUSG	Gm26274	ENSMUST00000157103
ENSMUSG	Gm24647	ENSMUST00000157112
ENSMUSG	Gm23827	ENSMUST00000157116
ENSMUSG	Gm25149	ENSMUST00000157119
ENSMUSG	Gm23466	ENSMUST00000157133

ENSMUSG	Gm22755	ENSMUST00000157143
ENSMUSG	Gm22320	ENSMUST00000157149
ENSMUSG	Rprl2	ENSMUST00000157150
ENSMUSG	Gm25962	ENSMUST00000157173
ENSMUSG	Gm25961	ENSMUST00000157174
ENSMUSG	Gm25980	ENSMUST00000157176
ENSMUSG	Gm25979	ENSMUST00000157177
ENSMUSG	Gm22492	ENSMUST00000157192
ENSMUSG	Gm22277	ENSMUST00000157204
ENSMUSG	Gm22792	ENSMUST00000157225
ENSMUSG	Gm22794	ENSMUST00000157228
ENSMUSG	Gm22796	ENSMUST00000157234
ENSMUSG	Gm24442	ENSMUST00000157238
ENSMUSG	Gm24444	ENSMUST00000157244
ENSMUSG	Gm26104	ENSMUST00000157250
ENSMUSG	Gm23600	ENSMUST00000157260
ENSMUSG	Gm23599	ENSMUST00000157261
ENSMUSG	Gm25271	ENSMUST00000157273
ENSMUSG	Gm22038	ENSMUST00000157277
ENSMUSG	Gm22037	ENSMUST00000157280
ENSMUSG	Gm24914	ENSMUST00000157285
ENSMUSG	Gm23229	ENSMUST00000157297
ENSMUSG	Gm26048	ENSMUST00000157305
ENSMUSG	Gm26047	ENSMUST00000157308
ENSMUSG	Gm23675	ENSMUST00000157311
ENSMUSG	Gm23676	ENSMUST00000157314
ENSMUSG	Gm24251	ENSMUST00000157324
ENSMUSG	Gm22561	ENSMUST00000157331
ENSMUSG	Gm22227	ENSMUST00000157356
ENSMUSG	Gm22221	ENSMUST00000157362
ENSMUSG	Gm25055	ENSMUST00000157369
ENSMUSG	Gm25054	ENSMUST00000157370
ENSMUSG	Gm23740	ENSMUST00000157385
ENSMUSG	Gm22141	ENSMUST00000157403
ENSMUSG	Gm25261	ENSMUST00000157416
ENSMUSG	Gm23326	ENSMUST00000157419
ENSMUSG	Gm26150	ENSMUST00000157426
ENSMUSG	Gm24475	ENSMUST00000157435
ENSMUSG	Gm24480	ENSMUST00000157442
ENSMUSG	Gm22819	ENSMUST00000157445
ENSMUSG	Gm22816	ENSMUST00000157450
ENSMUSG	Gm22811	ENSMUST00000157468
ENSMUSG	Gm22379	ENSMUST00000157480
ENSMUSG	Gm24065	ENSMUST00000157487
ENSMUSG	Gm26069	ENSMUST00000157497
ENSMUSG	Gm22867	ENSMUST00000157499
ENSMUSG	Gm22586	ENSMUST00000157507
ENSMUSG	Gm27343	ENSMUST00000157514
ENSMUSG	Gm24719	ENSMUST00000157520
ENSMUSG	Mir1983	ENSMUST00000157523
ENSMUSG	Gm24716	ENSMUST00000157524
ENSMUSG	Gm26346	ENSMUST00000157525
ENSMUSG	Gm26350	ENSMUST00000157531
ENSMUSG	Gm23539	ENSMUST00000157537
ENSMUSG	Gm23533	ENSMUST00000157540
ENSMUSG	Gm23098	ENSMUST00000157552
ENSMUSG	Gm22216	ENSMUST00000157557

ENSMUSG	Gm23905	ENSMUST00000157568
ENSMUSG	Gm23755	ENSMUST00000157578
ENSMUSG	Gm23391	ENSMUST00000157579
ENSMUSG	Gm23392	ENSMUST00000157580
ENSMUSG	Gm22082	ENSMUST00000157587
ENSMUSG	Gm22601	ENSMUST00000157595
ENSMUSG	Gm22604	ENSMUST00000157604
ENSMUSG	Gm25429	ENSMUST00000157607
ENSMUSG	Gm25430	ENSMUST00000157614
ENSMUSG	Mir1969	ENSMUST00000157623
ENSMUSG	Gm24286	ENSMUST00000157632
ENSMUSG	Gm24781	ENSMUST00000157636
ENSMUSG	Gm23589	ENSMUST00000157660
ENSMUSG	Gm26412	ENSMUST00000157666
ENSMUSG	AF35742	ENSMUST00000157669
ENSMUSG	Gm26410	ENSMUST00000157670
ENSMUSG	Gm26409	ENSMUST00000157671
ENSMUSG	Gm24505	ENSMUST00000157682
ENSMUSG	Gm26184	ENSMUST00000157686
ENSMUSG	Gm26187	ENSMUST00000157693
ENSMUSG	Gm25661	ENSMUST00000157704
ENSMUSG	Gm22842	ENSMUST00000157709
ENSMUSG	Gm22338	ENSMUST00000157715
ENSMUSG	Gm24013	ENSMUST00000157727
ENSMUSG	Gm24014	ENSMUST00000157728
ENSMUSG	Gm23500	ENSMUST00000157735
ENSMUSG	Gm24690	ENSMUST00000157759
ENSMUSG	Gm26307	ENSMUST00000157767
ENSMUSG	Gm26306	ENSMUST00000157773
ENSMUSG	Gm25887	ENSMUST00000157777
ENSMUSG	Gm25888	ENSMUST00000157778
ENSMUSG	Gm25884	ENSMUST00000157782
ENSMUSG	Gm25882	ENSMUST00000157784
ENSMUSG	Gm24240	ENSMUST00000157785
ENSMUSG	Gm24237	ENSMUST00000157790
ENSMUSG	Gm26162	ENSMUST00000157793
ENSMUSG	Gm24422	ENSMUST00000157800
ENSMUSG	Gm25383	ENSMUST00000157810
ENSMUSG	Gm25382	ENSMUST00000157811
ENSMUSG	Gm26371	ENSMUST00000157829
ENSMUSG	Mir1941	ENSMUST00000157834
ENSMUSG	Gm24751	ENSMUST00000157844
ENSMUSG	Gm23078	ENSMUST00000157848
ENSMUSG	Gm23085	ENSMUST00000157849
ENSMUSG	Gm22651	ENSMUST00000157872
ENSMUSG	Gm26490	ENSMUST00000157879
ENSMUSG	Gm24409	ENSMUST00000157881
ENSMUSG	Gm22789	ENSMUST00000157884
ENSMUSG	Gm26086	ENSMUST00000157902
ENSMUSG	Gm23279	ENSMUST00000157912
ENSMUSG	Gm24962	ENSMUST00000157917
ENSMUSG	Gm24965	ENSMUST00000157922
ENSMUSG	Gm22101	ENSMUST00000157928
ENSMUSG	Gm25455	ENSMUST00000157946
ENSMUSG	Gm24532	ENSMUST00000157951
ENSMUSG	Gm25459	ENSMUST00000157953
ENSMUSG	Gm22965	ENSMUST00000157958

ENSMUSG	Gm24190	ENSMUST00000157976
ENSMUSG	Gm26001	ENSMUST00000157989
ENSMUSG	Gm26000	ENSMUST00000157991
ENSMUSG	Gm22484	ENSMUST00000157992
ENSMUSG	Gm23033	ENSMUST00000157996
ENSMUSG	Gm22313	ENSMUST00000158008
ENSMUSG	Gm22315	ENSMUST00000158010
ENSMUSG	Gm25846	ENSMUST00000158013
ENSMUSG	Gm26484	ENSMUST00000158018
ENSMUSG	Gm26262	ENSMUST00000158024
ENSMUSG	Gm25333	ENSMUST00000158035
ENSMUSG	Gm25335	ENSMUST00000158043
ENSMUSG	Gm23664	ENSMUST00000158047
ENSMUSG	Gm24858	ENSMUST00000158058
ENSMUSG	Gm23994	ENSMUST00000158059
ENSMUSG	Gm24857	ENSMUST00000158061
ENSMUSG	Gm22730	ENSMUST00000158085
ENSMUSG	Gm22727	ENSMUST00000158090
ENSMUSG	Gm23898	ENSMUST00000158111
ENSMUSG	Gm23221	ENSMUST00000158118
ENSMUSG	Gm23224	ENSMUST00000158122
ENSMUSG	Mir1934	ENSMUST00000158127
ENSMUSG	Gm24394	ENSMUST00000158141
ENSMUSG	Gm25494	ENSMUST00000158143
ENSMUSG	Gm26040	ENSMUST00000158147
ENSMUSG	Gm26036	ENSMUST00000158149
ENSMUSG	Gm26034	ENSMUST00000158154
ENSMUSG	Gm25371	ENSMUST00000158162
ENSMUSG	Gm22541	ENSMUST00000158165
ENSMUSG	Gm22542	ENSMUST00000158166
ENSMUSG	Gm22535	ENSMUST00000158174
ENSMUSG	Gm24074	ENSMUST00000158178
ENSMUSG	Gm23543	ENSMUST00000158207
ENSMUSG	Gm26358	ENSMUST00000158215
ENSMUSG	Gm24725	ENSMUST00000158229
ENSMUSG	Gm23068	ENSMUST00000158239
ENSMUSG	Gm25866	ENSMUST00000158245
ENSMUSG	Gm25863	ENSMUST00000158249
ENSMUSG	Gm24211	ENSMUST00000158264
ENSMUSG	Gm22519	ENSMUST00000158270
ENSMUSG	Gm25433	ENSMUST00000158283
ENSMUSG	Gm25898	ENSMUST00000158303
ENSMUSG	Gm25921	ENSMUST00000158305
ENSMUSG	Gm25924	ENSMUST00000158310
ENSMUSG	Gm22090	ENSMUST00000158335
ENSMUSG	Gm23762	ENSMUST00000158348
ENSMUSG	Gm23759	ENSMUST00000158351
ENSMUSG	Gm25603	ENSMUST00000158361
ENSMUSG	Gm25605	ENSMUST00000158364
ENSMUSG	Gm22769	ENSMUST00000158367
ENSMUSG	Gm23859	ENSMUST00000158383
ENSMUSG	Gm25016	ENSMUST00000158399
ENSMUSG	Gm25010	ENSMUST00000158403
ENSMUSG	Gm22156	ENSMUST00000158409
ENSMUSG	Gm22157	ENSMUST00000158410
ENSMUSG	Gm22164	ENSMUST00000158413
ENSMUSG	Gm26173	ENSMUST00000158416

ENSMUSG	Gm26169	ENSMUST00000158420
ENSMUSG	Gm26171	ENSMUST00000158422
ENSMUSG	Gm26167	ENSMUST00000158423
ENSMUSG	Gm23352	ENSMUST00000158428
ENSMUSG	Gm22836	ENSMUST00000158441
ENSMUSG	Gm23380	ENSMUST00000158448
ENSMUSG	Gm23916	ENSMUST00000158484
ENSMUSG	Gm25907	ENSMUST00000158486
ENSMUSG	Gm22902	ENSMUST00000158488
ENSMUSG	Gm25902	ENSMUST00000158493
ENSMUSG	Gm26405	ENSMUST00000158495
ENSMUSG	Gm24769	ENSMUST00000158506
ENSMUSG	Gm24767	ENSMUST00000158509
ENSMUSG	Gm25264	ENSMUST00000158519
ENSMUSG	Gm25266	ENSMUST00000158521
ENSMUSG	Gm25267	ENSMUST00000158522
ENSMUSG	Gm23579	ENSMUST00000158528
ENSMUSG	Gm23583	ENSMUST00000158533
ENSMUSG	Gm22428	ENSMUST00000158554
ENSMUSG	Gm22946	ENSMUST00000158558
ENSMUSG	Gm22947	ENSMUST00000158559
ENSMUSG	Gm23386	ENSMUST00000158585
ENSMUSG	Gm22203	ENSMUST00000158605
ENSMUSG	Gm22202	ENSMUST00000158608
ENSMUSG	Gm25717	ENSMUST00000158631
ENSMUSG	Gm22893	ENSMUST00000158637
ENSMUSG	Mir1961	ENSMUST00000158643
ENSMUSG	Gm24550	ENSMUST00000158648
ENSMUSG	Gm24548	ENSMUST00000158650
ENSMUSG	Gm26025	ENSMUST00000158655
ENSMUSG	Gm26024	ENSMUST00000158657
ENSMUSG	Gm23208	ENSMUST00000158666
ENSMUSG	Mir2139	ENSMUST00000158669
ENSMUSG	Gm24841	ENSMUST00000158676
ENSMUSG	Mir2137	ENSMUST00000158732
ENSMUSG	Gm25491	ENSMUST00000158733
ENSMUSG	Snord10	ENSMUST00000158750
ENSMUSG	Gm24678	ENSMUST00000158756
ENSMUSG	Gm23312	ENSMUST00000158774
ENSMUSG	Gm24877	ENSMUST00000158779
ENSMUSG	Gm26007	ENSMUST00000158798
ENSMUSG	Gm26005	ENSMUST00000158802
ENSMUSG	Gm23194	ENSMUST00000158813
ENSMUSG	Gm23195	ENSMUST00000158814
ENSMUSG	Gm24225	ENSMUST00000158825
ENSMUSG	Gm24221	ENSMUST00000158833
ENSMUSG	Gm25369	ENSMUST00000158848
ENSMUSG	Gm24739	ENSMUST00000158855
ENSMUSG	Gm24737	ENSMUST00000158861
ENSMUSG	Gm24736	ENSMUST00000158862
ENSMUSG	Gm25643	ENSMUST00000158874
ENSMUSG	Gm23474	ENSMUST00000158884
ENSMUSG	Gm24899	ENSMUST00000158892
ENSMUSG	Gm23831	ENSMUST00000158895
ENSMUSG	Gm25160	ENSMUST00000158914
ENSMUSG	Gm25837	ENSMUST00000158919
ENSMUSG	Gm25839	ENSMUST00000158921

ENSMUSG	Gm25831	ENSMUST00000158924
ENSMUSG	Gm24179	ENSMUST00000158925
ENSMUSG	Gm24175	ENSMUST00000158931
ENSMUSG	Gm24668	ENSMUST00000158935
ENSMUSG	Gm23340	ENSMUST00000158955
ENSMUSG	Gm24352	ENSMUST00000158966
ENSMUSG	Gm22500	ENSMUST00000158982
ENSMUSG	Gm22503	ENSMUST00000158983
ENSMUSG	Nat8b-p	ENSMUST00000162660
ENSMUSG	Gm5766	ENSMUST00000160520
ENSMUSG	Gm15796	ENSMUST00000162135
ENSMUSG	Gm15841	ENSMUST00000160540
ENSMUSG	Ear-ps1	ENSMUST00000161720
ENSMUSG	Gm16271	ENSMUST00000162837
ENSMUSG	Gm3470	ENSMUST00000160819
ENSMUSG	Fcor	ENSMUST00000159364
ENSMUSG	Gm15914	ENSMUST00000161922
ENSMUSG	Gm16581	ENSMUST00000160730
ENSMUSG	Ugt1a8	ENSMUST00000113139
ENSMUSG	Gm16299	ENSMUST00000162724
ENSMUSG	Rab42	ENSMUST00000040411
ENSMUSG	Gm15947	ENSMUST00000161395
ENSMUSG	Gm2343	ENSMUST00000161177
ENSMUSG	Gm15833	ENSMUST00000161563
ENSMUSG	Gm15970	ENSMUST00000160623
ENSMUSG	Gm16564	ENSMUST00000162545
ENSMUSG	Matr3-p	ENSMUST00000160839
ENSMUSG	Ear-ps7	ENSMUST00000161287
ENSMUSG	D030046	ENSMUST00000162318
ENSMUSG	Ier5l	ENSMUST00000065134
ENSMUSG	Tmsb15b	ENSMUST00000113071
ENSMUSG	Skint1	ENSMUST00000162885;ENSMUST00000161389;ENSMUST00000162
ENSMUSG	Gm16270	ENSMUST00000160327
ENSMUSG	Gm16306	ENSMUST00000160568
ENSMUSG	Gm454	ENSMUST00000160126
ENSMUSG	Gm3555	ENSMUST00000160287
ENSMUSG	Rps19-p	ENSMUST00000159750
ENSMUSG	Gm7241	ENSMUST00000159853
ENSMUSG	Gm5083	ENSMUST00000163056;ENSMUST00000159595
ENSMUSG	Ear-ps8	ENSMUST00000161935
ENSMUSG	Gm15679	ENSMUST00000161244
ENSMUSG	Gm7097	ENSMUST00000161841
ENSMUSG	Gm2962	ENSMUST00000159266
ENSMUSG	Gm4491	ENSMUST00000159455
ENSMUSG	Gm13769	ENSMUST00000214797
ENSMUSG	Gm16139	ENSMUST00000160015
ENSMUSG	Gm15699	ENSMUST00000159213
ENSMUSG	Gm15562	ENSMUST00000161397
ENSMUSG	Mup13	ENSMUST00000098042;ENSMUST00000072678
ENSMUSG	Gm16102	ENSMUST00000160955
ENSMUSG	4930448	ENSMUST00000159838
ENSMUSG	Gm16280	ENSMUST00000162458
ENSMUSG	4930428	ENSMUST00000225654;ENSMUST00000038250;ENSMUST00000224
ENSMUSG	Gm16563	ENSMUST00000162673
ENSMUSG	Gm43517	ENSMUST00000110746
ENSMUSG	Gm16114	ENSMUST00000161930
ENSMUSG	4930473	ENSMUST00000161975

ENSMUSG	Ugt1a5	ENSMUST00000097659
ENSMUSG	Gm16215	ENSMUST00000161990
ENSMUSG	Gm14435	ENSMUST00000108995;ENSMUST00000127687;ENSMUST00000172
ENSMUSG	4933413	ENSMUST00000162380
ENSMUSG	Rnf224	ENSMUST00000091318;ENSMUST00000205192
ENSMUSG	A830011	ENSMUST00000162868
ENSMUSG	Gm16268	ENSMUST00000159445
ENSMUSG	Ugt1a1	ENSMUST00000073049
ENSMUSG	Gm16550	ENSMUST00000162550
ENSMUSG	Vmn1r19	ENSMUST00000155950
ENSMUSG	Gm7420	ENSMUST00000162836
ENSMUSG	Crb1-ps	ENSMUST00000160441
ENSMUSG	Gm16238	ENSMUST00000159343
ENSMUSG	Gm15852	ENSMUST00000159701
ENSMUSG	Gm16332	ENSMUST00000134229
ENSMUSG	Tmsb15b	ENSMUST00000069803;ENSMUST00000180194
ENSMUSG	Gm16555	ENSMUST00000159971
ENSMUSG	Gm16578	ENSMUST00000160797
ENSMUSG	Gm15900	ENSMUST00000161584
ENSMUSG	Gm16297	ENSMUST00000161466
ENSMUSG	Gm8194	ENSMUST00000159783
ENSMUSG	Olfr106	ENSMUST00000217481;ENSMUST00000105213
ENSMUSG	Nwd2	ENSMUST00000160066;ENSMUST00000159584;ENSMUST00000162
ENSMUSG	Dlx6os1	ENSMUST00000159827;ENSMUST00000159568
ENSMUSG	1110002	ENSMUST00000163080;ENSMUST00000184925
ENSMUSG	Cdk5r2	ENSMUST00000160379
ENSMUSG	Gm15849	ENSMUST00000159257
ENSMUSG	Slmapos	ENSMUST00000160089
ENSMUSG	Ear-ps1	ENSMUST00000161871
ENSMUSG	Gm3076	ENSMUST00000161613
ENSMUSG	Gm15680	ENSMUST00000161597
ENSMUSG	Abhd12b	ENSMUST00000162570;ENSMUST00000169156;ENSMUST00000182
ENSMUSG	Kcne1l	ENSMUST00000134825
ENSMUSG	Ugt1a7c	ENSMUST00000150634;ENSMUST00000058237;ENSMUST00000126
ENSMUSG	Pou3f1	ENSMUST00000053491
ENSMUSG	Olfr287	ENSMUST00000206224;ENSMUST00000170618;ENSMUST00000205
ENSMUSG	Gm16162	ENSMUST00000162296
ENSMUSG	Gm15809	ENSMUST00000159361
ENSMUSG	Ugt1a6b	ENSMUST00000113138;ENSMUST00000113137
ENSMUSG	Gm8580	ENSMUST00000160295
ENSMUSG	4930480	ENSMUST00000162121
ENSMUSG	Gm6808	ENSMUST00000162341
ENSMUSG	Ugt1a10	ENSMUST00000138182;ENSMUST00000113142
ENSMUSG	Ear10	ENSMUST00000163652
ENSMUSG	D630014	ENSMUST00000160562
ENSMUSG	Ugt1a2	ENSMUST00000049289
ENSMUSG	Fbxw10	ENSMUST00000036085;ENSMUST00000150989;ENSMUST00000177
ENSMUSG	Ugt1a9	ENSMUST00000073772
ENSMUSG	Cd200r2	ENSMUST00000102805
ENSMUSG	Gm16307	ENSMUST00000161269
ENSMUSG	Ang-ps1	ENSMUST00000162314
ENSMUSG	9230105	ENSMUST00000161291
ENSMUSG	4930503	ENSMUST00000049703
ENSMUSG	AU01533	ENSMUST00000159040
ENSMUSG	4930516	ENSMUST00000074064
ENSMUSG	Pcp4	ENSMUST00000061739
ENSMUSG	Gm16554	ENSMUST00000159471

ENSMUSG	Rpl17-p	ENSMUST00000161946
ENSMUSG	Gm3287	ENSMUST00000163681
ENSMUSG	Gm1965	ENSMUST00000203378;ENSMUST00000161362;ENSMUST0000016C
ENSMUSG	4921534	ENSMUST00000162370;ENSMUST00000162911
ENSMUSG	Gm4524	ENSMUST00000161614
ENSMUSG	D730045	ENSMUST00000159260
ENSMUSG	Gm9550	ENSMUST00000161130
ENSMUSG	Gm17226	ENSMUST00000166637
ENSMUSG	Lrrc10b	ENSMUST00000171400
ENSMUSG	Gm17152	ENSMUST00000172430
ENSMUSG	Vmn2r-p	ENSMUST00000176077
ENSMUSG	Gm5174	ENSMUST00000171131
ENSMUSG	2310050	ENSMUST00000163439
ENSMUSG	Gm17223	ENSMUST00000171413
ENSMUSG	Gm4462	ENSMUST00000166453
ENSMUSG	Dthd1	ENSMUST00000170704
ENSMUSG	Gm17073	ENSMUST00000168069
ENSMUSG	4932443	ENSMUST00000166277;ENSMUST00000209042;ENSMUST00000214
ENSMUSG	Vmn1r5	ENSMUST00000164307
ENSMUSG	Gm17555	ENSMUST00000164042
ENSMUSG	Teddm3	ENSMUST00000170665
ENSMUSG	Gm2822	ENSMUST00000164299
ENSMUSG	Gm3512	ENSMUST00000172431
ENSMUSG	4933411	ENSMUST00000164518
ENSMUSG	Gm8229	ENSMUST00000168161
ENSMUSG	Gm17402	ENSMUST00000166353
ENSMUSG	Gm17225	ENSMUST00000172051
ENSMUSG	Gm6133	ENSMUST00000164064
ENSMUSG	4930571	ENSMUST00000206013
ENSMUSG	Iltifb	ENSMUST00000163808
ENSMUSG	Gm2840	ENSMUST00000048361
ENSMUSG	Gm4553	ENSMUST00000210925
ENSMUSG	Gm6245	ENSMUST00000164699
ENSMUSG	BC03594	ENSMUST00000170511
ENSMUSG	Gm2888	ENSMUST00000170521
ENSMUSG	Gm3424	ENSMUST00000164585
ENSMUSG	Gm5538	ENSMUST00000168156
ENSMUSG	Gm4675	ENSMUST00000164755
ENSMUSG	Cdr1	ENSMUST00000166381
ENSMUSG	Rpl28-p	ENSMUST00000170447
ENSMUSG	Prlh	ENSMUST00000166281
ENSMUSG	Gm17158	ENSMUST00000168150
ENSMUSG	Olfr753	ENSMUST00000208970;ENSMUST00000219487;ENSMUST00000174
ENSMUSG	Gm8323	ENSMUST00000168436
ENSMUSG	4933406	ENSMUST00000180508;ENSMUST00000181409
ENSMUSG	Gm17180	ENSMUST00000172211
ENSMUSG	Gm8164	ENSMUST00000169120
ENSMUSG	Gm5611	ENSMUST00000164523
ENSMUSG	Gm17052	ENSMUST00000165496
ENSMUSG	Gm3571	ENSMUST00000170703
ENSMUSG	Gm8379	ENSMUST00000165448
ENSMUSG	Gm17060	ENSMUST00000172394
ENSMUSG	Gm8126	ENSMUST00000169587
ENSMUSG	Gm17069	ENSMUST00000172256
ENSMUSG	Gm6189	ENSMUST00000164487
ENSMUSG	Prss47	ENSMUST00000222769
ENSMUSG	Zfp493	ENSMUST00000220570;ENSMUST00000164936;ENSMUST00000223

ENSMUSG	Gad1-ps	ENSMUST00000167243
ENSMUSG	Gm765	ENSMUST00000164491;ENSMUST00000204914
ENSMUSG	Gm5067	ENSMUST00000170449
ENSMUSG	Olfr111	ENSMUST00000214259;ENSMUST00000215424;ENSMUST00000097
ENSMUSG	Gm17221	ENSMUST00000163909
ENSMUSG	Gm9047	ENSMUST00000171317
ENSMUSG	Gm3667	ENSMUST00000171906
ENSMUSG	Apold1	ENSMUST00000167323
ENSMUSG	Cyp4f40	ENSMUST00000165061
ENSMUSG	Gm8237	ENSMUST00000164484
ENSMUSG	Gm17173	ENSMUST00000168000
ENSMUSG	Gm6788	ENSMUST00000166459
ENSMUSG	Gm6871	ENSMUST00000110214
ENSMUSG	Esp8	ENSMUST00000171813
ENSMUSG	Rpl31-p	ENSMUST00000079114
ENSMUSG	Gm17116	ENSMUST00000169175
ENSMUSG	Klhl33	ENSMUST00000164415
ENSMUSG	Gm6729	ENSMUST00000165173
ENSMUSG	Gm4450	ENSMUST00000167753;ENSMUST00000188356
ENSMUSG	Gm8161	ENSMUST00000168739
ENSMUSG	Olfr134	ENSMUST00000168341
ENSMUSG	Gm8297	ENSMUST00000171964
ENSMUSG	Gm17094	ENSMUST00000169881
ENSMUSG	Heatr4	ENSMUST00000164935
ENSMUSG	Gm4340	ENSMUST00000181615;ENSMUST00000163113
ENSMUSG	A530084	ENSMUST00000170573
ENSMUSG	Gm3239	ENSMUST00000163525
ENSMUSG	Olfr733	ENSMUST00000216195
ENSMUSG	Gm6904	ENSMUST00000168702
ENSMUSG	D6Ertd5	ENSMUST00000170124;ENSMUST00000204927;ENSMUST00000203
ENSMUSG	Olfr110	ENSMUST00000168318;ENSMUST00000216472
ENSMUSG	Pabpc4l	ENSMUST00000166505;ENSMUST00000192793;ENSMUST00000195
ENSMUSG	1810064	ENSMUST00000187399;ENSMUST00000188595;ENSMUST00000167
ENSMUSG	Gm17097	ENSMUST00000166414
ENSMUSG	Gm7535	ENSMUST00000164167
ENSMUSG	Vmn2r8	ENSMUST00000172140
ENSMUSG	Olfr718	ENSMUST00000215102;ENSMUST00000218872
ENSMUSG	Gm17197	ENSMUST00000163160
ENSMUSG	Gm6153	ENSMUST00000172004
ENSMUSG	Gm7244	ENSMUST00000164658
ENSMUSG	Gm6872	ENSMUST00000165001
ENSMUSG	D630036	ENSMUST00000171007
ENSMUSG	Gm17107	ENSMUST00000170983
ENSMUSG	Vmn1r1	ENSMUST00000169123
ENSMUSG	Gm5244	ENSMUST00000189517;ENSMUST00000164001
ENSMUSG	Gm5828	ENSMUST00000071842
ENSMUSG	Gm3115	ENSMUST00000166928
ENSMUSG	Gm11107	ENSMUST00000165414
ENSMUSG	Gm17660	ENSMUST00000164471;ENSMUST00000198485
ENSMUSG	Glyatl3	ENSMUST00000166343
ENSMUSG	Gm1979	ENSMUST00000168875
ENSMUSG	Gm17538	ENSMUST00000168236
ENSMUSG	7420461	ENSMUST00000193257;ENSMUST00000193898;ENSMUST00000169
ENSMUSG	Gm4535	ENSMUST00000182274
ENSMUSG	A930018	ENSMUST00000170855
ENSMUSG	Gm4302	ENSMUST00000168769
ENSMUSG	Gm17109	ENSMUST00000163985

ENSMUSG	Lgals1-]	ENSMUST00000170221
ENSMUSG	Gm3278	ENSMUST00000164459
ENSMUSG	Sardhos	ENSMUST00000170435
ENSMUSG	Gm5218	ENSMUST00000172270
ENSMUSG	Gm7271	ENSMUST00000191515
ENSMUSG	Gm8775	ENSMUST00000170943
ENSMUSG	Gm6970	ENSMUST00000169692
ENSMUSG	Vgll3	ENSMUST00000168064
ENSMUSG	Vmn2r19	ENSMUST00000073948
ENSMUSG	Gm6682	ENSMUST00000169666
ENSMUSG	Gm17641	ENSMUST00000166328;ENSMUST00000215570
ENSMUSG	Gm3248	ENSMUST00000163885
ENSMUSG	Gm17234	ENSMUST00000172128
ENSMUSG	Gm17051	ENSMUST00000170787
ENSMUSG	Gm3275	ENSMUST00000169145
ENSMUSG	Gm6047	ENSMUST00000167221
ENSMUSG	Gm8439	ENSMUST00000171482
ENSMUSG	Speer6-]	ENSMUST00000223482
ENSMUSG	Spata31	ENSMUST00000165133
ENSMUSG	Gm5415	ENSMUST00000171322
ENSMUSG	Speer5-]	ENSMUST00000217649;ENSMUST00000169525
ENSMUSG	Gm5277	ENSMUST00000164923
ENSMUSG	Aadacl2	ENSMUST00000169794
ENSMUSG	Vmn2r83	ENSMUST00000167976
ENSMUSG	Gcnt4	ENSMUST00000171324
ENSMUSG	5330438	ENSMUST00000168347
ENSMUSG	Gm6356	ENSMUST00000166618
ENSMUSG	Gm9252	ENSMUST00000172364
ENSMUSG	Gm3140	ENSMUST00000163523
ENSMUSG	Ak9	ENSMUST00000173494
ENSMUSG	Gm6327	ENSMUST00000171185
ENSMUSG	Gm17031	ENSMUST00000016698
ENSMUSG	Otogl	ENSMUST00000165341
ENSMUSG	Gm7808	ENSMUST00000082002
ENSMUSG	Gm3739	ENSMUST00000165744
ENSMUSG	2810468	ENSMUST00000163493
ENSMUSG	Vmn2r11	ENSMUST00000168440
ENSMUSG	Vmn2r87	ENSMUST00000164227
ENSMUSG	Skor2	ENSMUST00000166956
ENSMUSG	BB01943	ENSMUST00000218015;ENSMUST00000167536
ENSMUSG	Vmn1r9	ENSMUST00000171186
ENSMUSG	Gm17572	ENSMUST00000171455
ENSMUSG	Gm6811	ENSMUST00000169389
ENSMUSG	Gm14569	ENSMUST00000169499
ENSMUSG	Gm8108	ENSMUST00000170207;ENSMUST00000167397
ENSMUSG	Gm8206	ENSMUST00000171428;ENSMUST00000164139;ENSMUST0000016[illegible]
ENSMUSG	Gm17033	ENSMUST00000146663
ENSMUSG	Gm17067	ENSMUST00000166837
ENSMUSG	Olfr93	ENSMUST00000219235;ENSMUST00000171679
ENSMUSG	Gm17046	ENSMUST00000168078
ENSMUSG	Gm17657	ENSMUST00000168877
ENSMUSG	Gm3752	ENSMUST00000164366
ENSMUSG	H60c	ENSMUST00000170893
ENSMUSG	Klhdc7b	ENSMUST00000225666;ENSMUST00000166926
ENSMUSG	Tcrg-C3	ENSMUST00000164407;ENSMUST00000198163
ENSMUSG	4930471	ENSMUST00000166038
ENSMUSG	4930433	ENSMUST00000206529

ENSMUSG	Gm17669	ENSMUST00000163749
ENSMUSG	Gm7876	ENSMUST00000171305
ENSMUSG	Gm4222	ENSMUST00000165784
ENSMUSG	Gm17189	ENSMUST00000163707
ENSMUSG	Sec14l5	ENSMUST00000165810
ENSMUSG	Gm17126	ENSMUST00000172276
ENSMUSG	Gimd1	ENSMUST00000212804
ENSMUSG	Siah3	ENSMUST00000164848
ENSMUSG	Gm8220	ENSMUST00000164663;ENSMUST00000177877
ENSMUSG	Vmn1r29	ENSMUST00000168416
ENSMUSG	Gm5093	ENSMUST00000166852
ENSMUSG	Gm5705	ENSMUST00000066241
ENSMUSG	Tspy-ps	ENSMUST00000169014;ENSMUST00000186546
ENSMUSG	Gm3095	ENSMUST00000164696
ENSMUSG	Vmn2r10	ENSMUST00000172203
ENSMUSG	Gm16494	ENSMUST00000165525
ENSMUSG	Gm8340	ENSMUST00000165508
ENSMUSG	Gm21293	ENSMUST00000181634
ENSMUSG	Rpl29-p	ENSMUST00000164794
ENSMUSG	Vmn2r18	ENSMUST00000165928
ENSMUSG	Olfr721	ENSMUST00000215479;ENSMUST00000165291
ENSMUSG	Ear-ps5	ENSMUST00000164700
ENSMUSG	Gm17141	ENSMUST00000167578
ENSMUSG	Speer4f	ENSMUST00000166086
ENSMUSG	Gm17188	ENSMUST00000169417;ENSMUST00000172055
ENSMUSG	Gm17102	ENSMUST00000170929
ENSMUSG	Gm6337	ENSMUST00000168088
ENSMUSG	A830073	ENSMUST00000205693;ENSMUST00000169090
ENSMUSG	Gm17138	ENSMUST00000165702
ENSMUSG	C2cd4b	ENSMUST00000171652
ENSMUSG	Gm17482	ENSMUST00000166681
ENSMUSG	Gm4027	ENSMUST00000164838
ENSMUSG	Gm2108	ENSMUST00000165058
ENSMUSG	Peg10	ENSMUST00000166678;ENSMUST00000176204;ENSMUST0000017
ENSMUSG	Gm5501	ENSMUST00000165917
ENSMUSG	Kif4-ps	ENSMUST00000184686
ENSMUSG	Bend4	ENSMUST00000169190;ENSMUST00000201705;ENSMUST00000201
ENSMUSG	Serpina	ENSMUST00000166601
ENSMUSG	Gm3294	ENSMUST00000164104
ENSMUSG	Gm10320	ENSMUST00000170205
ENSMUSG	Vmn2r90	ENSMUST00000169805
ENSMUSG	BC05097	ENSMUST00000168475;ENSMUST00000169420;ENSMUST0000016
ENSMUSG	Vmn2r86	ENSMUST00000170257
ENSMUSG	Rergl	ENSMUST00000170650
ENSMUSG	Gm3696	ENSMUST00000167923
ENSMUSG	Gm9075	ENSMUST00000168738
ENSMUSG	4833427	ENSMUST00000172874
ENSMUSG	Gm45351	ENSMUST00000210683
ENSMUSG	Cd9-ps	ENSMUST00000174687
ENSMUSG	Gm38505	ENSMUST00000174005
ENSMUSG	A530058	ENSMUST00000131299
ENSMUSG	A930009	ENSMUST00000173620
ENSMUSG	Gm19345	ENSMUST00000172815
ENSMUSG	Vmn2r-p	ENSMUST00000174119
ENSMUSG	Gm20443	ENSMUST00000173840
ENSMUSG	Gm20528	ENSMUST00000173165;ENSMUST00000174821
ENSMUSG	Gm20506	ENSMUST00000172952

ENSMUSG	Gm19807	ENSMUST00000173971
ENSMUSG	Gm18889	ENSMUST00000174284
ENSMUSG	1700031.	ENSMUST00000174380
ENSMUSG	Gm20515	ENSMUST00000174440
ENSMUSG	Esp34	ENSMUST00000178654
ENSMUSG	Gm2308	ENSMUST00000166539
ENSMUSG	Gm7031	ENSMUST00000173709
ENSMUSG	Gm19301	ENSMUST00000174283
ENSMUSG	Gm20465	ENSMUST00000174768
ENSMUSG	Olfr113	ENSMUST00000216181
ENSMUSG	Gm18200	ENSMUST00000173265
ENSMUSG	Gm6887	ENSMUST00000080945
ENSMUSG	Prps1l1	ENSMUST00000134550
ENSMUSG	Gm6633	ENSMUST00000173851
ENSMUSG	Vmn2r-p	ENSMUST00000173861
ENSMUSG	Zfp977	ENSMUST00000173283
ENSMUSG	Gm4831	ENSMUST00000174626
ENSMUSG	Tlx1os	ENSMUST00000173437
ENSMUSG	Vmn1r-p	ENSMUST00000173746
ENSMUSG	Gm20539	ENSMUST00000174811
ENSMUSG	Vmn1r-p	ENSMUST00000174638
ENSMUSG	Gm20391	ENSMUST00000174722
ENSMUSG	Olfr296	ENSMUST00000173904
ENSMUSG	H2-Pb	ENSMUST00000172731
ENSMUSG	Gm20541	ENSMUST00000173035
ENSMUSG	Gm20546	ENSMUST00000173141
ENSMUSG	Gm20445	ENSMUST00000061905
ENSMUSG	Olfr758	ENSMUST00000173901
ENSMUSG	C130080	ENSMUST00000150005
ENSMUSG	Olfr300	ENSMUST00000174262
ENSMUSG	Gm20485	ENSMUST00000174452
ENSMUSG	Gm9921	ENSMUST00000173293
ENSMUSG	Gm20462	ENSMUST00000172623
ENSMUSG	Vhl-ps1	ENSMUST00000174813
ENSMUSG	Gm9613	ENSMUST00000171662
ENSMUSG	Olfr115	ENSMUST00000076936
ENSMUSG	Gm20545	ENSMUST00000174739
ENSMUSG	Gm18958	ENSMUST00000174258
ENSMUSG	Gm7696	ENSMUST00000174727
ENSMUSG	Vmn1r-p	ENSMUST00000174112
ENSMUSG	Gm2991	ENSMUST00000173338
ENSMUSG	Gm18737	ENSMUST00000173594
ENSMUSG	Gm8902	ENSMUST00000174341
ENSMUSG	Gm18808	ENSMUST00000173316
ENSMUSG	Gm20421	ENSMUST00000153344
ENSMUSG	Gm20482	ENSMUST00000172746;ENSMUST00000174729
ENSMUSG	1700092	ENSMUST00000172547
ENSMUSG	Gm20394	ENSMUST00000173304
ENSMUSG	C87198	ENSMUST00000173553
ENSMUSG	Fam71e2	ENSMUST00000163574
ENSMUSG	Actl9	ENSMUST00000174088
ENSMUSG	Gm20389	ENSMUST00000174217
ENSMUSG	Gm18191	ENSMUST00000174624
ENSMUSG	Olfr756	ENSMUST00000173450
ENSMUSG	Gm20461	ENSMUST00000174600
ENSMUSG	Vmn2r-p	ENSMUST00000172586
ENSMUSG	Gm20501	ENSMUST00000211692;ENSMUST00000211303

ENSMUSG	Gm20468	ENSMUST00000172664
ENSMUSG	Olfr757	ENSMUST00000172798
ENSMUSG	Gm20491	ENSMUST00000173765
ENSMUSG	Stk-ps1	ENSMUST00000173340
ENSMUSG	Gm8696	ENSMUST00000174777
ENSMUSG	Olfr759	ENSMUST00000174238
ENSMUSG	Plut	ENSMUST00000174285
ENSMUSG	Gm4134	ENSMUST00000173244
ENSMUSG	Serpinb	ENSMUST00000172762;ENSMUST00000194951
ENSMUSG	2810047	ENSMUST00000210322;ENSMUST00000172930
ENSMUSG	Gm18199	ENSMUST00000173820
ENSMUSG	1700010	ENSMUST00000174502
ENSMUSG	Gm20442	ENSMUST00000174630
ENSMUSG	4931413	ENSMUST00000173637
ENSMUSG	Vmn1r-p	ENSMUST00000173843
ENSMUSG	D130058	ENSMUST00000173856;ENSMUST00000189763
ENSMUSG	Mir3962	ENSMUST00000174892
ENSMUSG	Gm25243	ENSMUST00000174912
ENSMUSG	Gm26241	ENSMUST00000174944
ENSMUSG	Gm24515	ENSMUST00000174960
ENSMUSG	Gm24514	ENSMUST00000174961
ENSMUSG	Gm24510	ENSMUST00000174965
ENSMUSG	Gm22859	ENSMUST00000174978
ENSMUSG	Mir5120	ENSMUST00000175020
ENSMUSG	Gm23366	ENSMUST00000175022
ENSMUSG	Gm22694	ENSMUST00000175051
ENSMUSG	Gm26463	ENSMUST00000175065
ENSMUSG	Mir3086	ENSMUST00000175072
ENSMUSG	Gm24819	ENSMUST00000175094
ENSMUSG	Gm24332	ENSMUST00000175102
ENSMUSG	Gm24328	ENSMUST00000175104
ENSMUSG	Gm24327	ENSMUST00000175108
ENSMUSG	Mir344g	ENSMUST00000175109
ENSMUSG	Mir5103	ENSMUST00000175111
ENSMUSG	Gm25641	ENSMUST00000175135
ENSMUSG	Gm24084	ENSMUST00000175173
ENSMUSG	Mir3472	ENSMUST00000175176
ENSMUSG	Gm22904	ENSMUST00000175190
ENSMUSG	Gm26217	ENSMUST00000175212
ENSMUSG	Gm26220	ENSMUST00000175215
ENSMUSG	Gm22242	ENSMUST00000175224
ENSMUSG	Gm25072	ENSMUST00000175233
ENSMUSG	Gm24159	ENSMUST00000175264
ENSMUSG	Gm22460	ENSMUST00000175269
ENSMUSG	Gm22463	ENSMUST00000175274
ENSMUSG	Gm25303	ENSMUST00000175280
ENSMUSG	Gm27826	ENSMUST00000175282
ENSMUSG	Gm25306	ENSMUST00000175288
ENSMUSG	Gm26455	ENSMUST00000175300
ENSMUSG	Gm24809	ENSMUST00000175318
ENSMUSG	Gm25948	ENSMUST00000175334
ENSMUSG	Gm24323	ENSMUST00000175342
ENSMUSG	Mir3073	ENSMUST00000175349
ENSMUSG	Mir3089	ENSMUST00000175350
ENSMUSG	Gm22701	ENSMUST00000175371
ENSMUSG	Gm22696	ENSMUST00000175373
ENSMUSG	Gm23374	ENSMUST00000175408

ENSMUSG	Gm25037	ENSMUST00000175418
ENSMUSG	Gm22773	ENSMUST00000175419
ENSMUSG	Gm22994	ENSMUST00000175422
ENSMUSG	Gm22993	ENSMUST00000175423
ENSMUSG	Gm22414	ENSMUST00000175466
ENSMUSG	Gm25252	ENSMUST00000175477
ENSMUSG	Gm27903	ENSMUST00000175482
ENSMUSG	Gm25750	ENSMUST00000175483
ENSMUSG	Gm25749	ENSMUST00000175485
ENSMUSG	Mir1251	ENSMUST00000175486
ENSMUSG	Mir9-3	ENSMUST00000175497
ENSMUSG	Gm26235	ENSMUST00000175533
ENSMUSG	Mir5118	ENSMUST00000175551
ENSMUSG	Gm23802	ENSMUST00000175565
ENSMUSG	Gm24973	ENSMUST00000175588
ENSMUSG	Gm22120	ENSMUST00000175590
ENSMUSG	Gm26117	ENSMUST00000175599
ENSMUSG	Gm23291	ENSMUST00000175611
ENSMUSG	Gm23292	ENSMUST00000175618
ENSMUSG	Gm20685	ENSMUST00000176654
ENSMUSG	A330044	ENSMUST00000176108
ENSMUSG	Gm20621	ENSMUST00000176618;ENSMUST00000176338
ENSMUSG	Vmn1r-p	ENSMUST00000175863
ENSMUSG	Gm20694	ENSMUST00000176943
ENSMUSG	Gm5139	ENSMUST00000175943
ENSMUSG	Vmn1r-p	ENSMUST00000176131
ENSMUSG	Six3os1	ENSMUST00000177220;ENSMUST00000176556
ENSMUSG	Gm20659	ENSMUST00000176599
ENSMUSG	Gm20629	ENSMUST00000176157
ENSMUSG	Gm20619	ENSMUST00000176283;ENSMUST00000181343
ENSMUSG	Gm20657	ENSMUST00000177079
ENSMUSG	Gm20625	ENSMUST00000177065
ENSMUSG	Gm20651	ENSMUST00000177219
ENSMUSG	Gm20713	ENSMUST00000176344
ENSMUSG	Gm25442	ENSMUST00000177270
ENSMUSG	Obox3-p	ENSMUST00000177242
ENSMUSG	Gm19553	ENSMUST00000175723
ENSMUSG	Vmn1r-p	ENSMUST00000177047
ENSMUSG	Vmn1r-p	ENSMUST00000176946
ENSMUSG	Smim17	ENSMUST00000161855;ENSMUST00000159318;ENSMUST0000016C
ENSMUSG	Gm7584	ENSMUST00000175829
ENSMUSG	4930505	ENSMUST00000175854
ENSMUSG	Gm6695	ENSMUST00000177264
ENSMUSG	Gm20611	ENSMUST00000177492
ENSMUSG	Gm24756	ENSMUST00000177002
ENSMUSG	Gm20554	ENSMUST00000177421
ENSMUSG	Gm18367	ENSMUST00000176139
ENSMUSG	Gm19459	ENSMUST00000176240
ENSMUSG	Gm20703	ENSMUST00000175668
ENSMUSG	Gm18953	ENSMUST00000175694
ENSMUSG	Vmn1r38	ENSMUST00000176121
ENSMUSG	Gm42419	ENSMUST00000177375
ENSMUSG	Gm19560	ENSMUST00000177510
ENSMUSG	Vmn1r-p	ENSMUST00000176775
ENSMUSG	Gm20614	ENSMUST00000177106
ENSMUSG	Gm20631	ENSMUST00000177239;ENSMUST00000177268
ENSMUSG	Gm5873	ENSMUST00000177157

ENSMUSG	Gm20655	ENSMUST00000181428;ENSMUST00000176132
ENSMUSG	Gm20644	ENSMUST00000176721
ENSMUSG	Gm20618	ENSMUST00000177482
ENSMUSG	Vmn2r-p	ENSMUST00000176738
ENSMUSG	Gm19815	ENSMUST00000175818
ENSMUSG	Gm20667	ENSMUST00000175674
ENSMUSG	Obox3-p	ENSMUST00000176412
ENSMUSG	Vmn1r-p	ENSMUST00000176901
ENSMUSG	AI60647	ENSMUST00000177201
ENSMUSG	Gm18417	ENSMUST00000176564
ENSMUSG	Gm24420	ENSMUST00000176676
ENSMUSG	Gm25591	ENSMUST00000177317
ENSMUSG	Gm20658	ENSMUST00000175871;ENSMUST00000177059
ENSMUSG	Vmn2r-p	ENSMUST00000176920
ENSMUSG	Obox3-p	ENSMUST00000176026
ENSMUSG	AU02307	ENSMUST00000176972
ENSMUSG	Vmn1r-p	ENSMUST00000176956
ENSMUSG	Vmn1r10	ENSMUST00000176073
ENSMUSG	Gm20796	ENSMUST00000180355
ENSMUSG	Asmt	ENSMUST00000178693
ENSMUSG	Gm23620	ENSMUST00000116812
ENSMUSG	Gm5627	ENSMUST00000178539
ENSMUSG	Gm9637	ENSMUST00000179141
ENSMUSG	Gm6900	ENSMUST00000178971
ENSMUSG	Olfr462	ENSMUST00000213928
ENSMUSG	Gm5039	ENSMUST00000218054;ENSMUST00000177747
ENSMUSG	Gm7083	ENSMUST00000178617
ENSMUSG	Gm5117	ENSMUST00000178878
ENSMUSG	Lrit3	ENSMUST00000185462
ENSMUSG	Gsdmcl2	ENSMUST00000185391;ENSMUST00000177709;ENSMUST00000185
ENSMUSG	Gm24540	ENSMUST00000177627
ENSMUSG	Olfr134	ENSMUST00000213549
ENSMUSG	Gm25458	ENSMUST00000178981
ENSMUSG	Olfr239	ENSMUST00000214406;ENSMUST00000112165
ENSMUSG	Rpl35a-	ENSMUST00000137013
ENSMUSG	Gm3033	ENSMUST00000180934
ENSMUSG	Ang-ps2	ENSMUST00000178173
ENSMUSG	Gm2866	ENSMUST00000166053
ENSMUSG	Gm3629	ENSMUST00000171706
ENSMUSG	Igkv9-1	ENSMUST00000200578
ENSMUSG	Zfp853	ENSMUST00000212355;ENSMUST00000212715;ENSMUST0000018C
ENSMUSG	Gm379	ENSMUST00000178160
ENSMUSG	Gm8660	ENSMUST00000105187
ENSMUSG	Olfr463	ENSMUST00000217095
ENSMUSG	Olfr971	ENSMUST00000214242;ENSMUST00000075467
ENSMUSG	Olfr46	ENSMUST00000214180
ENSMUSG	Gm22832	ENSMUST00000178022
ENSMUSG	Ighv1-3	ENSMUST00000103512
ENSMUSG	Gm8165	ENSMUST00000095981
ENSMUSG	Snord11	ENSMUST00000177982
ENSMUSG	Mrgpra2	ENSMUST00000159004
ENSMUSG	Gm14362	ENSMUST00000121679
ENSMUSG	Gm12612	ENSMUST00000140182
ENSMUSG	Gm2237	ENSMUST00000170694;ENSMUST00000112595
ENSMUSG	Gm10406	ENSMUST00000100895;ENSMUST00000170738
ENSMUSG	Rps12-p	ENSMUST00000179020
ENSMUSG	Gm26427	ENSMUST00000093648

ENSMUSG	Gm9866	ENSMUST00000222838
ENSMUSG	Gm5128	ENSMUST00000180198
ENSMUSG	Gm12625	ENSMUST00000179122
ENSMUSG	Gm24169	ENSMUST00000180244
ENSMUSG	Vmn1r94	ENSMUST00000164526
ENSMUSG	Gm10024	ENSMUST00000075421
ENSMUSG	Gm25829	ENSMUST00000178275
ENSMUSG	Gm8879	ENSMUST00000170260;ENSMUST00000178863
ENSMUSG	Gm23002	ENSMUST00000179221
ENSMUSG	Gm21762	ENSMUST00000225881;ENSMUST00000177561
ENSMUSG	Gm21833	ENSMUST00000177665
ENSMUSG	Olfr638	ENSMUST00000209757
ENSMUSG	Gm25156	ENSMUST00000179809
ENSMUSG	Gm20826	ENSMUST00000185348
ENSMUSG	Tdpoz1	ENSMUST00000094413
ENSMUSG	Gm17258	ENSMUST00000168462
ENSMUSG	Gm3233	ENSMUST00000168997
ENSMUSG	Gm13698	ENSMUST00000179192
ENSMUSG	Gm3264	ENSMUST00000166776;ENSMUST00000166410
ENSMUSG	Gm5973	ENSMUST00000178542
ENSMUSG	Olfr151	ENSMUST00000214980
ENSMUSG	Gm21312	ENSMUST00000180889;ENSMUST00000179258
ENSMUSG	Gm10142	ENSMUST00000179767
ENSMUSG	Gm8453	ENSMUST00000167253
ENSMUSG	Gm7233	ENSMUST00000168619
ENSMUSG	Slc6a16	ENSMUST00000213347;ENSMUST00000210163
ENSMUSG	Tdpoz5	ENSMUST00000098879
ENSMUSG	Gm5445	ENSMUST00000180224
ENSMUSG	Rpl31-p	ENSMUST00000143596
ENSMUSG	Gm25450	ENSMUST00000179144
ENSMUSG	Olfr237	ENSMUST00000215911
ENSMUSG	Gm25064	ENSMUST00000179535
ENSMUSG	Vmn1r13	ENSMUST00000177936
ENSMUSG	Gm23450	ENSMUST00000178285
ENSMUSG	Gm23448	ENSMUST00000083151
ENSMUSG	Calhm3	ENSMUST00000178630
ENSMUSG	Gm21847	ENSMUST00000178227;ENSMUST00000198681
ENSMUSG	Rps12-p	ENSMUST00000178427
ENSMUSG	Gm5456	ENSMUST00000170706
ENSMUSG	Gm20091	ENSMUST00000179683
ENSMUSG	Mir3471	ENSMUST00000174896
ENSMUSG	Rpl21-p	ENSMUST00000077420
ENSMUSG	Gm14536	ENSMUST00000118324
ENSMUSG	Gm25123	ENSMUST00000179154
ENSMUSG	Gm15068	ENSMUST00000119796
ENSMUSG	Gm23610	ENSMUST00000178659
ENSMUSG	Gm6164	ENSMUST00000169774
ENSMUSG	Btbd35f	ENSMUST00000105015
ENSMUSG	Gm23243	ENSMUST00000180047
ENSMUSG	Igkv4-5	ENSMUST00000177697;ENSMUST00000199437
ENSMUSG	Gm9125	ENSMUST00000178458;ENSMUST00000180746
ENSMUSG	Gm13693	ENSMUST00000178960
ENSMUSG	Gm11942	ENSMUST00000180255
ENSMUSG	Olfr784	ENSMUST00000214064
ENSMUSG	Gm14307	ENSMUST00000118930
ENSMUSG	Btbd35f	ENSMUST00000179069
ENSMUSG	Gm26058	ENSMUST00000180294

ENSMUSG	Gm26057	ENSMUST00000178099
ENSMUSG	Gm3373	ENSMUST00000177556;ENSMUST00000169087
ENSMUSG	Gm5435	ENSMUST00000179295
ENSMUSG	Gm25065	ENSMUST00000177914
ENSMUSG	Gm23400	ENSMUST00000174994
ENSMUSG	Btbd35f	ENSMUST00000179532
ENSMUSG	Gm23143	ENSMUST00000180137
ENSMUSG	Gm24072	ENSMUST00000178506
ENSMUSG	Gm933	ENSMUST00000179384
ENSMUSG	Gm23616	ENSMUST00000177797
ENSMUSG	1700015	ENSMUST00000180038
ENSMUSG	Olfr972	ENSMUST00000215303
ENSMUSG	Gm22624	ENSMUST00000179913
ENSMUSG	Gm21028	ENSMUST00000179470
ENSMUSG	Gm24307	ENSMUST00000178512
ENSMUSG	Gm21897	ENSMUST00000178312
ENSMUSG	Gm23064	ENSMUST00000179866
ENSMUSG	Igkv3-3	ENSMUST00000103402
ENSMUSG	Olfr393	ENSMUST00000213365
ENSMUSG	Igkv1-1	ENSMUST00000103304
ENSMUSG	Gm5294	ENSMUST00000179205
ENSMUSG	Olfr635	ENSMUST00000209473;ENSMUST00000098185
ENSMUSG	Gm22914	ENSMUST00000175195
ENSMUSG	Gm21399	ENSMUST00000180210
ENSMUSG	Gm20945	ENSMUST00000178122
ENSMUSG	Rpl35a-	ENSMUST00000080603
ENSMUSG	Cyp2d34	ENSMUST00000109515
ENSMUSG	Gm25228	ENSMUST00000180121
ENSMUSG	Gm12620	ENSMUST00000119791
ENSMUSG	Gm15027	ENSMUST00000120922
ENSMUSG	Gm7736	ENSMUST00000096981
ENSMUSG	Ms4a18	ENSMUST00000177684
ENSMUSG	Vmn1r11	ENSMUST00000172322
ENSMUSG	Gm24744	ENSMUST00000116833
ENSMUSG	Tpd52-p	ENSMUST00000178097
ENSMUSG	Gm4810	ENSMUST00000177721
ENSMUSG	Gm24694	ENSMUST00000180054
ENSMUSG	Gm25871	ENSMUST00000177816
ENSMUSG	A630076	ENSMUST00000179399
ENSMUSG	Gm4899	ENSMUST00000178708
ENSMUSG	Gm13271	ENSMUST00000094992
ENSMUSG	Gm22524	ENSMUST00000178357
ENSMUSG	Cecr6	ENSMUST00000178687
ENSMUSG	Gm3252	ENSMUST00000165619
ENSMUSG	Gm3468	ENSMUST00000165193
ENSMUSG	Gm7104	ENSMUST00000179741
ENSMUSG	Snord11	ENSMUST00000179053
ENSMUSG	Gm13287	ENSMUST00000179490
ENSMUSG	Gm7102	ENSMUST00000180168
ENSMUSG	Ighv1-4	ENSMUST00000103516;ENSMUST00000191868
ENSMUSG	Gm25359	ENSMUST00000179315
ENSMUSG	Gm25626	ENSMUST00000177631
ENSMUSG	Olfr135	ENSMUST00000217073
ENSMUSG	Olfr857	ENSMUST00000212013
ENSMUSG	Ap3m1-p	ENSMUST00000177652
ENSMUSG	Ighv1-8	ENSMUST00000103548
ENSMUSG	Olfr916	ENSMUST00000081196

ENSMUSG	Gm24485	ENSMUST00000178283
ENSMUSG	Gm22821	ENSMUST00000179288
ENSMUSG	Gm22825	ENSMUST00000179444
ENSMUSG	Gm5416	ENSMUST00000089628
ENSMUSG	Vmn1r95	ENSMUST00000168984
ENSMUSG	Gm17045	ENSMUST00000178600
ENSMUSG	Olfr130	ENSMUST00000099604
ENSMUSG	Gm4498	ENSMUST00000168580
ENSMUSG	Gm1647	ENSMUST00000195406
ENSMUSG	Rpl29-p	ENSMUST00000086508
ENSMUSG	Gm6625	ENSMUST00000177870
ENSMUSG	Mup12	ENSMUST00000117932
ENSMUSG	Vmn1r2	ENSMUST00000105160
ENSMUSG	Olfr311	ENSMUST00000216473;ENSMUST00000071625
ENSMUSG	1810009	ENSMUST00000076638
ENSMUSG	Olfr907	ENSMUST00000052901
ENSMUSG	Gm3194	ENSMUST00000180867
ENSMUSG	Gm10610	ENSMUST00000207184
ENSMUSG	Gm9034	ENSMUST00000182071
ENSMUSG	Gm21962	ENSMUST00000166899
ENSMUSG	Dlx6os2	ENSMUST00000178206
ENSMUSG	Olfr130	ENSMUST00000087129
ENSMUSG	Gm25121	ENSMUST00000179846
ENSMUSG	Olfr105	ENSMUST00000215518;ENSMUST00000178647
ENSMUSG	Olfr55	ENSMUST00000216633;ENSMUST00000112168
ENSMUSG	Gm4791	ENSMUST00000178065
ENSMUSG	Vmn1r20	ENSMUST00000091730
ENSMUSG	Igkv10-	ENSMUST00000198735;ENSMUST00000103329
ENSMUSG	Vmn1r15	ENSMUST00000177632
ENSMUSG	D430019	ENSMUST00000178224
ENSMUSG	Gm9507	ENSMUST00000180157
ENSMUSG	Gm6605	ENSMUST00000031488
ENSMUSG	Gm24760	ENSMUST00000178530
ENSMUSG	Gm16533	ENSMUST00000140035
ENSMUSG	1700122	ENSMUST00000178823
ENSMUSG	Gm3526	ENSMUST00000164544
ENSMUSG	Gm9726	ENSMUST00000180321
ENSMUSG	Gm23770	ENSMUST00000178009
ENSMUSG	Gm23092	ENSMUST00000178688
ENSMUSG	Ighv1-8	ENSMUST00000103551
ENSMUSG	Rhox4a2	ENSMUST00000179915
ENSMUSG	Mir3070	ENSMUST00000175205
ENSMUSG	Gm25255	ENSMUST00000179579
ENSMUSG	Gm8994	ENSMUST00000077886
ENSMUSG	Gm25256	ENSMUST00000177746
ENSMUSG	Gm8653	ENSMUST00000163096
ENSMUSG	Gm23726	ENSMUST00000178937
ENSMUSG	Topaz1	ENSMUST00000178679
ENSMUSG	Igkv4-5	ENSMUST00000103345
ENSMUSG	Gm22046	ENSMUST00000179027
ENSMUSG	Mir465c	ENSMUST00000104757
ENSMUSG	Rps12-p	ENSMUST00000179567
ENSMUSG	Gm20765	ENSMUST00000181703;ENSMUST00000177792
ENSMUSG	Gm3336	ENSMUST00000179347
ENSMUSG	Sirpb1b	ENSMUST00000193767
ENSMUSG	Olfr151	ENSMUST00000089739
ENSMUSG	Gm17577	ENSMUST00000179916

ENSMUSG	Gm22346	ENSMUST00000116918
ENSMUSG	1700001	ENSMUST00000073721;ENSMUST00000188052
ENSMUSG	AF06706	ENSMUST00000179905
ENSMUSG	Gm5866	ENSMUST00000177881
ENSMUSG	Gm8693	ENSMUST00000178871
ENSMUSG	Olfr385	ENSMUST00000071553
ENSMUSG	Gm22128	ENSMUST00000180346
ENSMUSG	Gm23216	ENSMUST00000179545
ENSMUSG	Gm23217	ENSMUST00000178482
ENSMUSG	Ighv1-3	ENSMUST00000103515
ENSMUSG	Gm12616	ENSMUST00000118594
ENSMUSG	Rps12-p	ENSMUST00000179067
ENSMUSG	Pou3f2	ENSMUST00000178174
ENSMUSG	Hsd3b4	ENSMUST00000196861
ENSMUSG	Gm26032	ENSMUST00000178535
ENSMUSG	Gm25548	ENSMUST00000178681
ENSMUSG	Gm25547	ENSMUST00000178877
ENSMUSG	Gm25546	ENSMUST00000103977
ENSMUSG	Rhox5	ENSMUST00000184866
ENSMUSG	Olfr138	ENSMUST00000167248
ENSMUSG	Gm10668	ENSMUST00000179491
ENSMUSG	Gm3005	ENSMUST00000178060;ENSMUST00000178728
ENSMUSG	Gm8305	ENSMUST00000180022
ENSMUSG	Zfp967	ENSMUST00000178443;ENSMUST00000108997
ENSMUSG	Gm6100	ENSMUST00000180253
ENSMUSG	Gm22398	ENSMUST00000174941
ENSMUSG	Gm9944	ENSMUST00000177649
ENSMUSG	Gm4845	ENSMUST00000179202
ENSMUSG	Gm5478	ENSMUST00000100184
ENSMUSG	Gm24753	ENSMUST00000178183
ENSMUSG	Gm24752	ENSMUST00000178240
ENSMUSG	Olfr681	ENSMUST00000216247
ENSMUSG	Gm10697	ENSMUST00000091692
ENSMUSG	Gm21893	ENSMUST00000179119
ENSMUSG	Gm5225	ENSMUST00000178572
ENSMUSG	Gm24242	ENSMUST00000179440
ENSMUSG	Vmn1r14	ENSMUST00000169697
ENSMUSG	Vmn1r10	ENSMUST00000179079
ENSMUSG	Gfy	ENSMUST00000179443
ENSMUSG	Gm3488	ENSMUST00000181562;ENSMUST00000178220
ENSMUSG	Gm12407	ENSMUST00000180219
ENSMUSG	Gm906	ENSMUST00000099521
ENSMUSG	Olfr957	ENSMUST00000051653
ENSMUSG	Mir3470	ENSMUST00000175223
ENSMUSG	Zfp870	ENSMUST00000178401
ENSMUSG	Gm26095	ENSMUST00000179270
ENSMUSG	Gm3892	ENSMUST00000178043;ENSMUST00000107991
ENSMUSG	Igkv3-2	ENSMUST00000103403
ENSMUSG	Gm3594	ENSMUST00000171784
ENSMUSG	Gm14325	ENSMUST00000188914;ENSMUST00000108939;ENSMUST0000015C
ENSMUSG	Gm4567	ENSMUST00000178591
ENSMUSG	Gm21859	ENSMUST00000178424
ENSMUSG	Olfr91	ENSMUST00000216376;ENSMUST00000087144
ENSMUSG	D630033	ENSMUST00000210802;ENSMUST00000213429
ENSMUSG	Gm17662	ENSMUST00000179793
ENSMUSG	Trav6d-	ENSMUST00000180717
ENSMUSG	Gm10681	ENSMUST00000058728

ENSMUSG	Tmem200	ENSMUST00000178545
ENSMUSG	H2al1e	ENSMUST00000178979
ENSMUSG	Gm23923	ENSMUST00000104108
ENSMUSG	Ighv1-1	ENSMUST00000103500;ENSMUST00000195469
ENSMUSG	Gm23606	ENSMUST00000116938
ENSMUSG	H2al1i	ENSMUST00000164729
ENSMUSG	Snord11	ENSMUST00000179889
ENSMUSG	Gm25076	ENSMUST00000104098
ENSMUSG	Ifna1	ENSMUST00000094972
ENSMUSG	Rps12-p	ENSMUST00000179356
ENSMUSG	Gm3147	ENSMUST00000201071
ENSMUSG	Gm17222	ENSMUST00000180135
ENSMUSG	Gm24136	ENSMUST00000083374
ENSMUSG	D13Ertd	ENSMUST00000195797
ENSMUSG	Gm8298	ENSMUST00000194941
ENSMUSG	Gm25287	ENSMUST00000103846
ENSMUSG	Gm21671	ENSMUST00000162387
ENSMUSG	Ighv2-9	ENSMUST00000180013
ENSMUSG	H2al2b	ENSMUST00000187660
ENSMUSG	Gm12350	ENSMUST00000121208
ENSMUSG	Gm7138	ENSMUST00000167669
ENSMUSG	Olfr106	ENSMUST00000214704;ENSMUST00000178794
ENSMUSG	Gm23424	ENSMUST00000103889
ENSMUSG	Gm12624	ENSMUST00000177615
ENSMUSG	Gm6291	ENSMUST00000179994
ENSMUSG	Gm22950	ENSMUST00000082776
ENSMUSG	Olfr143	ENSMUST00000208197
ENSMUSG	Gm21817	ENSMUST00000178584
ENSMUSG	Gm21818	ENSMUST00000178828
ENSMUSG	Plscr5	ENSMUST00000179751
ENSMUSG	H2al1g	ENSMUST00000179859
ENSMUSG	Gm25100	ENSMUST00000179750
ENSMUSG	Gm8281	ENSMUST00000163719;ENSMUST00000100893
ENSMUSG	Igkv3-1	ENSMUST00000103404
ENSMUSG	Gm10662	ENSMUST00000168053
ENSMUSG	Gm23282	ENSMUST00000175306
ENSMUSG	Gm24828	ENSMUST00000178507
ENSMUSG	Gm8871	ENSMUST00000168945;ENSMUST00000163544
ENSMUSG	Gm7137	ENSMUST00000180161
ENSMUSG	Gm23646	ENSMUST00000178119
ENSMUSG	Gm23649	ENSMUST00000179007
ENSMUSG	Vmn2r29	ENSMUST00000170922;ENSMUST00000209613
ENSMUSG	Gm25311	ENSMUST00000104136
ENSMUSG	Igkv11-	ENSMUST00000197406
ENSMUSG	Gm22476	ENSMUST00000158902
ENSMUSG	Gm24180	ENSMUST00000179961
ENSMUSG	Vmn1r11	ENSMUST00000179511
ENSMUSG	Gm23018	ENSMUST00000177928
ENSMUSG	Gm24674	ENSMUST00000179194
ENSMUSG	Mir465c	ENSMUST00000093560
ENSMUSG	Sirpb1a	ENSMUST00000191958
ENSMUSG	Gm26296	ENSMUST00000179457
ENSMUSG	Gm8332	ENSMUST00000179468
ENSMUSG	Gm5728	ENSMUST00000105209
ENSMUSG	Gm5473	ENSMUST00000179326
ENSMUSG	Gm3238	ENSMUST00000168298
ENSMUSG	Tcstv3	ENSMUST00000178349

ENSMUSG	Gm9117	ENSMUST00000193529;ENSMUST00000177735
ENSMUSG	Gm13697	ENSMUST00000099948
ENSMUSG	Tdgf1-p	ENSMUST00000179882
ENSMUSG	Gm22617	ENSMUST00000175199
ENSMUSG	Gm10256	ENSMUST00000188114
ENSMUSG	Ighv1-4	ENSMUST00000103517;ENSMUST00000195417
ENSMUSG	Lce1k	ENSMUST00000179917
ENSMUSG	Ang-ps3	ENSMUST00000177964
ENSMUSG	Gm9630	ENSMUST00000178304
ENSMUSG	Gm3317	ENSMUST00000179659
ENSMUSG	Vmn1r19	ENSMUST00000072369
ENSMUSG	Gm6686	ENSMUST00000178455
ENSMUSG	Olfr204	ENSMUST00000216261
ENSMUSG	Gm16416	ENSMUST00000179660
ENSMUSG	Gm25195	ENSMUST00000177656
ENSMUSG	Gm21708	ENSMUST00000190283
ENSMUSG	6030469	ENSMUST00000218473
ENSMUSG	Gm22360	ENSMUST00000179775
ENSMUSG	1700036	ENSMUST00000190053
ENSMUSG	Gm13120	ENSMUST00000117343
ENSMUSG	Vmn1r13	ENSMUST00000177884
ENSMUSG	Gm7742	ENSMUST00000167648
ENSMUSG	Gm19402	ENSMUST00000180297
ENSMUSG	Vmn1r11	ENSMUST00000164288
ENSMUSG	Gm5891	ENSMUST00000179493
ENSMUSG	Vmn1r3	ENSMUST00000105159
ENSMUSG	Gm17048	ENSMUST00000179266
ENSMUSG	Gm21060	ENSMUST00000179346
ENSMUSG	2610528	ENSMUST00000191317;ENSMUST00000179488
ENSMUSG	Gm3500	ENSMUST00000177986;ENSMUST00000178058
ENSMUSG	Ifna15	ENSMUST00000102809
ENSMUSG	Sox1	ENSMUST00000180353
ENSMUSG	Gm23189	ENSMUST00000178954
ENSMUSG	D830030	ENSMUST00000223972;ENSMUST00000225885;ENSMUST00000112
ENSMUSG	Gm21698	ENSMUST00000191203
ENSMUSG	Gm23681	ENSMUST00000178821
ENSMUSG	Gm10424	ENSMUST00000201031
ENSMUSG	Gm25349	ENSMUST00000177728
ENSMUSG	Gm4761	ENSMUST00000178086
ENSMUSG	Vmn1r22	ENSMUST00000072385
ENSMUSG	Bhmt-ps	ENSMUST00000178743
ENSMUSG	Gm22307	ENSMUST00000180019
ENSMUSG	Gm25144	ENSMUST00000179561
ENSMUSG	Gm2696	ENSMUST00000172205
ENSMUSG	Gm25145	ENSMUST00000177640
ENSMUSG	Rps12-p	ENSMUST00000179420
ENSMUSG	Ankrd66	ENSMUST00000178772
ENSMUSG	Kcnj11	ENSMUST00000209863;ENSMUST00000211674
ENSMUSG	Ighv1-8	ENSMUST00000103552
ENSMUSG	Olfr477	ENSMUST00000214677
ENSMUSG	Gm5472	ENSMUST00000178604
ENSMUSG	Gm21827	ENSMUST00000178247
ENSMUSG	Gm3436	ENSMUST00000179930
ENSMUSG	Olfr156	ENSMUST00000213751;ENSMUST00000112162
ENSMUSG	Gm8055	ENSMUST00000099956
ENSMUSG	Gm22992	ENSMUST00000179496
ENSMUSG	Gm22991	ENSMUST00000178126

ENSMUSG	Gm21761	ENSMUST00000187530;ENSMUST00000179502
ENSMUSG	Gm5070	ENSMUST00000177599
ENSMUSG	Gm23304	ENSMUST00000180209
ENSMUSG	Btbd35f	ENSMUST00000179991
ENSMUSG	Gm26137	ENSMUST00000116701
ENSMUSG	4930555	ENSMUST00000112770
ENSMUSG	Gm26133	ENSMUST00000157363
ENSMUSG	Gm22069	ENSMUST00000179911
ENSMUSG	Olfr510	ENSMUST00000216331
ENSMUSG	Gm4904	ENSMUST00000178243
ENSMUSG	Gm2916	ENSMUST00000181779
ENSMUSG	Lhx8	ENSMUST00000177846;ENSMUST00000205251;ENSMUST00000203
ENSMUSG	Olfr774	ENSMUST00000097164
ENSMUSG	Gm16429	ENSMUST00000201400;ENSMUST00000179824
ENSMUSG	Gm5123	ENSMUST00000121905
ENSMUSG	Gm24269	ENSMUST00000179216
ENSMUSG	Gm24263	ENSMUST00000104059
ENSMUSG	Gm21093	ENSMUST00000084677
ENSMUSG	Rpl31-p	ENSMUST00000181182
ENSMUSG	Gm23287	ENSMUST00000179427
ENSMUSG	Gm6176	ENSMUST00000178209
ENSMUSG	Tcstv1	ENSMUST00000178510
ENSMUSG	Gm21814	ENSMUST00000203277;ENSMUST00000203729;ENSMUST00000144
ENSMUSG	Gm25208	ENSMUST00000178964
ENSMUSG	Gm3898	ENSMUST00000180221
ENSMUSG	Gm20767	ENSMUST00000225683;ENSMUST00000179071
ENSMUSG	Ighv1-7	ENSMUST00000163670;ENSMUST00000199373
ENSMUSG	Gm13694	ENSMUST00000178533
ENSMUSG	Gm6408	ENSMUST00000179032
ENSMUSG	Ighv8-4	ENSMUST00000178949;ENSMUST00000192298
ENSMUSG	Gm5814	ENSMUST00000177586
ENSMUSG	Gm7094	ENSMUST00000178379
ENSMUSG	Gm19668	ENSMUST00000178581
ENSMUSG	Gm25353	ENSMUST00000179366
ENSMUSG	Fam240a	ENSMUST00000177678
ENSMUSG	Gm23816	ENSMUST00000179617
ENSMUSG	Ighv6-5	ENSMUST00000193936
ENSMUSG	Olfr890	ENSMUST00000213458
ENSMUSG	Gm25484	ENSMUST00000178217
ENSMUSG	Gm10100	ENSMUST00000179726
ENSMUSG	Gm24979	ENSMUST00000178626
ENSMUSG	Btbd35f	ENSMUST00000178080
ENSMUSG	Rps12-p	ENSMUST00000118529
ENSMUSG	Gm5808	ENSMUST00000178090
ENSMUSG	Gm2877	ENSMUST00000178248
ENSMUSG	Ighv1-7	ENSMUST00000170551
ENSMUSG	Igkv14-	ENSMUST00000196006
ENSMUSG	Gm22812	ENSMUST00000178329
ENSMUSG	Gm21750	ENSMUST00000098874
ENSMUSG	Olfr488	ENSMUST00000215173
ENSMUSG	Gm5561	ENSMUST00000178932
ENSMUSG	Olfr92	ENSMUST00000216341;ENSMUST00000168659
ENSMUSG	Gm23976	ENSMUST00000175001
ENSMUSG	Gm3250	ENSMUST00000167358
ENSMUSG	Gm23977	ENSMUST00000177871
ENSMUSG	Gm13696	ENSMUST00000180347
ENSMUSG	Gm10409	ENSMUST00000170123

ENSMUSG	Olfr787	ENSMUST00000213329
ENSMUSG	Gm21772	ENSMUST00000169213
ENSMUSG	Gm7903	ENSMUST00000113172
ENSMUSG	Gm25892	ENSMUST00000179867
ENSMUSG	Gm25891	ENSMUST00000180050
ENSMUSG	Gm25729	ENSMUST00000179050
ENSMUSG	Gm2663	ENSMUST00000166306
ENSMUSG	G430049	ENSMUST00000179907
ENSMUSG	Smlr1	ENSMUST00000179685;ENSMUST00000187490
ENSMUSG	Olfr944	ENSMUST00000079650
ENSMUSG	1700009	ENSMUST00000185309;ENSMUST00000178996;ENSMUST00000185
ENSMUSG	Gm2956	ENSMUST00000177786
ENSMUSG	Oog1	ENSMUST00000222112;ENSMUST00000095509
ENSMUSG	Gm17121	ENSMUST00000170414
ENSMUSG	Gm22917	ENSMUST00000180116
ENSMUSG	Gm22918	ENSMUST00000103855
ENSMUSG	Trav6d-	ENSMUST00000181483;ENSMUST00000184883
ENSMUSG	Tpbgl	ENSMUST00000178124
ENSMUSG	7530416	ENSMUST00000178942
ENSMUSG	Gm2871	ENSMUST00000179984
ENSMUSG	Gm2035	ENSMUST00000180053
ENSMUSG	Gm3383	ENSMUST00000096171;ENSMUST00000112758;ENSMUST00000178
ENSMUSG	Gm6223	ENSMUST00000178436
ENSMUSG	Gm15069	ENSMUST00000117792
ENSMUSG	Gm24517	ENSMUST00000122560
ENSMUSG	Olfr498	ENSMUST00000217616
ENSMUSG	Mup17	ENSMUST00000107484
ENSMUSG	Olfr132	ENSMUST00000071979
ENSMUSG	Gm7352	ENSMUST00000167577
ENSMUSG	Olfr699	ENSMUST00000216307
ENSMUSG	Gm5755	ENSMUST00000152394
ENSMUSG	Mrgpra2	ENSMUST00000119798;ENSMUST00000172559
ENSMUSG	Gm13695	ENSMUST00000099951
ENSMUSG	Vmn1r11	ENSMUST00000166937
ENSMUSG	Gm4307	ENSMUST00000177945
ENSMUSG	Trav7d-	ENSMUST00000179789
ENSMUSG	Ftl1-ps	ENSMUST00000169477
ENSMUSG	Rps12-p	ENSMUST00000178365
ENSMUSG	Gm22115	ENSMUST00000103976
ENSMUSG	Gm5796	ENSMUST00000096184
ENSMUSG	Gm24641	ENSMUST00000178055
ENSMUSG	Gm3002	ENSMUST00000170480;ENSMUST00000178256
ENSMUSG	Olfr905	ENSMUST00000051111
ENSMUSG	Gm22996	ENSMUST00000180236
ENSMUSG	Olfr312	ENSMUST00000216407;ENSMUST00000078217
ENSMUSG	Gm25084	ENSMUST00000175556
ENSMUSG	Gm29231	ENSMUST00000179850
ENSMUSG	Gm21425	ENSMUST00000180318
ENSMUSG	Olfr344	ENSMUST00000215879
ENSMUSG	Rps12-p	ENSMUST00000179239
ENSMUSG	Gm25597	ENSMUST00000179388
ENSMUSG	Cyp2d12	ENSMUST00000068861
ENSMUSG	Gm7732	ENSMUST00000170486
ENSMUSG	Scgb2a2	ENSMUST00000179814
ENSMUSG	Gm23936	ENSMUST00000179354
ENSMUSG	Gm21083	ENSMUST00000188807
ENSMUSG	Gm4858	ENSMUST00000194595;ENSMUST00000195847

ENSMUSG	Gm20797	ENSMUST00000177894
ENSMUSG	Gm3029	ENSMUST00000168306
ENSMUSG	Gm3591	ENSMUST00000164408;ENSMUST00000179312
ENSMUSG	Gm8324	ENSMUST00000178911
ENSMUSG	Gm26506	ENSMUST00000179692
ENSMUSG	Galntl6	ENSMUST00000204128
ENSMUSG	Gm16863	ENSMUST00000181476
ENSMUSG	Gm26721	ENSMUST00000181723
ENSMUSG	Gm9530	ENSMUST00000181160
ENSMUSG	A230028	ENSMUST00000181655
ENSMUSG	Gm6345	ENSMUST00000180378;ENSMUST00000180900
ENSMUSG	3300005	ENSMUST00000180902;ENSMUST00000181484;ENSMUST00000181
ENSMUSG	Gm18336	ENSMUST00000180787
ENSMUSG	Gm16386	ENSMUST00000181397
ENSMUSG	Gm10827	ENSMUST00000181897
ENSMUSG	Redrum	ENSMUST00000181269
ENSMUSG	Gm2670	ENSMUST00000180668
ENSMUSG	D930007	ENSMUST00000181306
ENSMUSG	4930598	ENSMUST00000180742
ENSMUSG	Gm26709	ENSMUST00000223049
ENSMUSG	BC00198	ENSMUST00000191393;ENSMUST00000180822
ENSMUSG	Mir9-3h	ENSMUST00000183043;ENSMUST00000181511;ENSMUST00000182
ENSMUSG	Gm26557	ENSMUST00000180756
ENSMUSG	Gm26559	ENSMUST00000181939
ENSMUSG	Ptgs2os	ENSMUST00000181915;ENSMUST00000181460
ENSMUSG	Gm26820	ENSMUST00000180764
ENSMUSG	1110036	ENSMUST00000181151
ENSMUSG	Gm26821	ENSMUST00000181954
ENSMUSG	2610316	ENSMUST00000181047
ENSMUSG	Pafah1b	ENSMUST00000181263
ENSMUSG	Gm10556	ENSMUST00000180904
ENSMUSG	6530411	ENSMUST00000181618
ENSMUSG	Gm9918	ENSMUST00000181801
ENSMUSG	Pantr2	ENSMUST00000180589;ENSMUST00000192894
ENSMUSG	Gm26743	ENSMUST00000180913
ENSMUSG	Gm26760	ENSMUST00000181901
ENSMUSG	4930544	ENSMUST00000181184;ENSMUST00000220451
ENSMUSG	Foxl2os	ENSMUST00000181706;ENSMUST00000188093;ENSMUST0000018[illegible]
ENSMUSG	4833428	ENSMUST00000181230;ENSMUST00000215652
ENSMUSG	Platr7	ENSMUST00000181466
ENSMUSG	4933440	ENSMUST00000181547
ENSMUSG	D930019	ENSMUST00000181841
ENSMUSG	Gm26634	ENSMUST00000181146
ENSMUSG	C330013	ENSMUST00000181034
ENSMUSG	Gm26547	ENSMUST00000181186
ENSMUSG	2310069	ENSMUST00000181839
ENSMUSG	Gm26579	ENSMUST00000181656
ENSMUSG	Platr6	ENSMUST00000180731
ENSMUSG	Gm26729	ENSMUST00000180744
ENSMUSG	A530020	ENSMUST00000181070;ENSMUST00000199889;ENSMUST0000018[illegible]
ENSMUSG	Gm26886	ENSMUST00000181497;ENSMUST00000181007
ENSMUSG	Gm5262	ENSMUST00000180655
ENSMUSG	1110002	ENSMUST00000181710;ENSMUST00000219021
ENSMUSG	Gm26627	ENSMUST00000181540
ENSMUSG	Gm3764	ENSMUST00000181134;ENSMUST00000181550;ENSMUST00000181
ENSMUSG	Gm26512	ENSMUST00000181874;ENSMUST00000221942;ENSMUST0000022[illegible]
ENSMUSG	BC05107	ENSMUST00000181440

ENSMUSG	2210008	ENSMUST00000180805
ENSMUSG	Gm17644	ENSMUST00000181272
ENSMUSG	Gm26829	ENSMUST00000180581
ENSMUSG	A830009	ENSMUST00000181054
ENSMUSG	Gm17501	ENSMUST00000181247;ENSMUST00000197148
ENSMUSG	4632428	ENSMUST00000181485
ENSMUSG	Gm26595	ENSMUST00000181585
ENSMUSG	Gm19426	ENSMUST00000181711
ENSMUSG	Gm26665	ENSMUST00000181378
ENSMUSG	Gm26700	ENSMUST00000181480
ENSMUSG	AU02275	ENSMUST00000180441
ENSMUSG	Gm26552	ENSMUST00000181033;ENSMUST00000180399;ENSMUST0000018C
ENSMUSG	Gm26599	ENSMUST00000180590
ENSMUSG	1010001	ENSMUST00000180789;ENSMUST00000181316
ENSMUSG	Gm26597	ENSMUST00000181648
ENSMUSG	Gm26907	ENSMUST00000181794
ENSMUSG	1500012	ENSMUST00000205989
ENSMUSG	Gm2694	ENSMUST00000180700;ENSMUST00000180806;ENSMUST00000181
ENSMUSG	Gm26889	ENSMUST00000181523
ENSMUSG	Gm26771	ENSMUST00000180472
ENSMUSG	C430042	ENSMUST00000180515
ENSMUSG	Gm5706	ENSMUST00000181525
ENSMUSG	Gm26803	ENSMUST00000181705
ENSMUSG	Gm26650	ENSMUST00000181157;ENSMUST00000181881;ENSMUST00000181
ENSMUSG	AI84905	ENSMUST00000181021
ENSMUSG	Gm26685	ENSMUST00000181831
ENSMUSG	5031415	ENSMUST00000181546
ENSMUSG	Gm26686	ENSMUST00000181951
ENSMUSG	Gm26684	ENSMUST00000181704
ENSMUSG	D130017	ENSMUST00000180951;ENSMUST00000180460;ENSMUST0000019€
ENSMUSG	Gm2155	ENSMUST00000181581
ENSMUSG	D630002	ENSMUST00000181854
ENSMUSG	Gm26654	ENSMUST00000180455
ENSMUSG	Rpl31-p	ENSMUST00000181857
ENSMUSG	Gm26834	ENSMUST00000181815
ENSMUSG	Gm6410	ENSMUST00000181337
ENSMUSG	A930038	ENSMUST00000180412;ENSMUST00000181044
ENSMUSG	Gm26871	ENSMUST00000181926;ENSMUST00000181060;ENSMUST0000020C
ENSMUSG	Gm26569	ENSMUST00000181968
ENSMUSG	Gm26568	ENSMUST00000180496
ENSMUSG	4930426	ENSMUST00000217797
ENSMUSG	A330048	ENSMUST00000180777
ENSMUSG	Gm26671	ENSMUST00000199759;ENSMUST00000180423;ENSMUST0000019S
ENSMUSG	Gm26774	ENSMUST00000181534
ENSMUSG	4930550	ENSMUST00000181629
ENSMUSG	Gm26544	ENSMUST00000181404
ENSMUSG	Gm26717	ENSMUST00000181713
ENSMUSG	Gm26719	ENSMUST00000180395
ENSMUSG	Gm8177	ENSMUST00000181591
ENSMUSG	Gm26545	ENSMUST00000181082
ENSMUSG	Gm26876	ENSMUST00000180875
ENSMUSG	Gm8705	ENSMUST00000180823
ENSMUSG	B230208	ENSMUST00000181073;ENSMUST00000180529
ENSMUSG	Gm26817	ENSMUST00000180446
ENSMUSG	1500026	ENSMUST00000181598
ENSMUSG	Gm26814	ENSMUST00000181178
ENSMUSG	9130213	ENSMUST00000180487

ENSMUSG	4930563	ENSMUST00000180948
ENSMUSG	Mirg	ENSMUST00000183144;ENSMUST00000182074;ENSMUST00000182
ENSMUSG	D030068	ENSMUST00000180524
ENSMUSG	Gm26556	ENSMUST00000180645
ENSMUSG	Gm26555	ENSMUST00000180882
ENSMUSG	Gm26833	ENSMUST00000180899
ENSMUSG	Gm26831	ENSMUST00000181458
ENSMUSG	Gm26670	ENSMUST00000180429
ENSMUSG	Vmn1r18	ENSMUST00000181796
ENSMUSG	Gm8941	ENSMUST00000181451
ENSMUSG	Gm6763	ENSMUST00000181287
ENSMUSG	AW04773	ENSMUST00000181406
ENSMUSG	Gm26520	ENSMUST00000180719
ENSMUSG	Gm16630	ENSMUST00000180741
ENSMUSG	Gm26783	ENSMUST00000181197
ENSMUSG	Gm26630	ENSMUST00000181759
ENSMUSG	Platr22	ENSMUST00000181274
ENSMUSG	Gm26893	ENSMUST00000180602;ENSMUST00000180965
ENSMUSG	Rian	ENSMUST00000182119;ENSMUST00000183215;ENSMUST00000182
ENSMUSG	Gm26891	ENSMUST00000181644
ENSMUSG	Gm16958	ENSMUST00000180603
ENSMUSG	Gm26736	ENSMUST00000180425
ENSMUSG	D430036	ENSMUST00000181447
ENSMUSG	Gm26584	ENSMUST00000180551
ENSMUSG	Gm9499	ENSMUST00000180888
ENSMUSG	D330020	ENSMUST00000181956
ENSMUSG	Gm17634	ENSMUST00000181383;ENSMUST00000189938;ENSMUST00000186
ENSMUSG	Gm26807	ENSMUST00000181132
ENSMUSG	Gm17733	ENSMUST00000181883
ENSMUSG	Gm26651	ENSMUST00000181851
ENSMUSG	Gm26652	ENSMUST00000181672;ENSMUST00000180592
ENSMUSG	4930528	ENSMUST00000225577;ENSMUST00000181528
ENSMUSG	Gm26696	ENSMUST00000181941
ENSMUSG	Gm17619	ENSMUST00000180625
ENSMUSG	4930488	ENSMUST00000181864;ENSMUST00000212638
ENSMUSG	Gm26846	ENSMUST00000181865
ENSMUSG	Platr31	ENSMUST00000180653
ENSMUSG	Rp31-ps	ENSMUST00000180929
ENSMUSG	Gm4349	ENSMUST00000181809
ENSMUSG	1700037	ENSMUST00000181254
ENSMUSG	Gm26752	ENSMUST00000181086
ENSMUSG	Gm26751	ENSMUST00000181218
ENSMUSG	Mir124a	ENSMUST00000180610
ENSMUSG	B230110	ENSMUST00000181388;ENSMUST00000225651;ENSMUST00000181
ENSMUSG	Gm26748	ENSMUST00000180406
ENSMUSG	Gm21304	ENSMUST00000181059
ENSMUSG	Gm26638	ENSMUST00000181762
ENSMUSG	1700125	ENSMUST00000181124
ENSMUSG	4930578	ENSMUST00000180490
ENSMUSG	Gm2907	ENSMUST00000181256
ENSMUSG	Gm26673	ENSMUST00000181126
ENSMUSG	A430010	ENSMUST00000181304;ENSMUST00000204608;ENSMUST00000203
ENSMUSG	Gm7952	ENSMUST00000180540
ENSMUSG	Gm26659	ENSMUST00000181816
ENSMUSG	Gm26509	ENSMUST00000180947
ENSMUSG	Gm2061	ENSMUST00000180623
ENSMUSG	Gm26507	ENSMUST00000181838

ENSMUSG	Gm26562	ENSMUST00000181113
ENSMUSG	Gm5091	ENSMUST00000181658
ENSMUSG	4921504	ENSMUST00000180594
ENSMUSG	Gm2383	ENSMUST00000181461
ENSMUSG	4930552	ENSMUST00000181198
ENSMUSG	Carlr	ENSMUST00000181515
ENSMUSG	Platr4	ENSMUST00000198593;ENSMUST00000199155
ENSMUSG	Gm26862	ENSMUST00000181886
ENSMUSG	Gm26863	ENSMUST00000181144
ENSMUSG	9330185	ENSMUST00000180837
ENSMUSG	4921507	ENSMUST00000181348
ENSMUSG	Mhrt	ENSMUST00000185798
ENSMUSG	Gm26713	ENSMUST00000181827
ENSMUSG	Gm7389	ENSMUST00000181162
ENSMUSG	Gm2093	ENSMUST00000180518
ENSMUSG	A330094	ENSMUST00000180530
ENSMUSG	Gm26761	ENSMUST00000180650
ENSMUSG	Gm26612	ENSMUST00000180785
ENSMUSG	Rps19-p	ENSMUST00000180745
ENSMUSG	4930471	ENSMUST00000180815
ENSMUSG	4930471	ENSMUST00000206475
ENSMUSG	G730013	ENSMUST00000180509;ENSMUST00000181884
ENSMUSG	Gm26905	ENSMUST00000180798
ENSMUSG	4833412	ENSMUST00000181235;ENSMUST00000208257
ENSMUSG	2810429	ENSMUST00000186183;ENSMUST00000181708;ENSMUST0000018
ENSMUSG	Gm5523	ENSMUST00000180415
ENSMUSG	1700026	ENSMUST00000180937
ENSMUSG	Gm26849	ENSMUST00000180561
ENSMUSG	9530036	ENSMUST00000180878;ENSMUST00000181202
ENSMUSG	F630040	ENSMUST00000180558
ENSMUSG	B930082	ENSMUST00000181046
ENSMUSG	D930032	ENSMUST00000181393
ENSMUSG	4930445	ENSMUST00000181345;ENSMUST00000180631
ENSMUSG	E030044	ENSMUST00000181195
ENSMUSG	Gm26535	ENSMUST00000181602
ENSMUSG	D030040	ENSMUST00000181371
ENSMUSG	Gm26688	ENSMUST00000181176
ENSMUSG	Ptgs2os	ENSMUST00000181308
ENSMUSG	4732463	ENSMUST00000180751
ENSMUSG	Miat	ENSMUST00000182953
ENSMUSG	Gm2115	ENSMUST00000180387;ENSMUST00000208409
ENSMUSG	Gm16399	ENSMUST00000181423
ENSMUSG	Gm26901	ENSMUST00000181554;ENSMUST00000193106;ENSMUST0000019
ENSMUSG	Gm26777	ENSMUST00000181852
ENSMUSG	Rpl31-p	ENSMUST00000181130
ENSMUSG	Gm26813	ENSMUST00000180778
ENSMUSG	Gm26655	ENSMUST00000181089
ENSMUSG	Gm16701	ENSMUST00000181309
ENSMUSG	Gm26912	ENSMUST00000180988
ENSMUSG	Gm26903	ENSMUST00000180749
ENSMUSG	Gm26794	ENSMUST00000180810
ENSMUSG	Gm26756	ENSMUST00000181689
ENSMUSG	4930478	ENSMUST00000181875
ENSMUSG	Gm3532	ENSMUST00000181880
ENSMUSG	Gm26859	ENSMUST00000181743
ENSMUSG	Gm26868	ENSMUST00000181927
ENSMUSG	Gm8764	ENSMUST00000181179

ENSMUSG	Gm26543	ENSMUST00000180729
ENSMUSG	Gm26682	ENSMUST00000180850
ENSMUSG	4930455	ENSMUST00000223613
ENSMUSG	Gm16894	ENSMUST00000181756
ENSMUSG	Gm8652	ENSMUST00000181363
ENSMUSG	Raxos1	ENSMUST00000181100
ENSMUSG	5033428	ENSMUST00000181638;ENSMUST00000209896;ENSMUST0000021C
ENSMUSG	Gm26691	ENSMUST00000180471
ENSMUSG	Ascl5	ENSMUST00000180436
ENSMUSG	Gm7784	ENSMUST00000180769
ENSMUSG	Gm26576	ENSMUST00000181018
ENSMUSG	A730020	ENSMUST00000204848;ENSMUST00000180421
ENSMUSG	Gm26578	ENSMUST00000181702
ENSMUSG	Tunar	ENSMUST00000222718;ENSMUST00000180458
ENSMUSG	C330002	ENSMUST00000181623
ENSMUSG	6720483	ENSMUST00000181654
ENSMUSG	Gm3809	ENSMUST00000180973
ENSMUSG	Gm6967	ENSMUST00000182599
ENSMUSG	Gm8601	ENSMUST00000182096
ENSMUSG	Gm7833	ENSMUST00000183288
ENSMUSG	Gm17799	ENSMUST00000183270
ENSMUSG	Gm8343	ENSMUST00000182705
ENSMUSG	Gm26970	ENSMUST00000182990
ENSMUSG	Gm7611	ENSMUST00000182329
ENSMUSG	Gm4217	ENSMUST00000182243
ENSMUSG	Gm26950	ENSMUST00000182734
ENSMUSG	Gm8492	ENSMUST00000182375
ENSMUSG	Gm6931	ENSMUST00000183137
ENSMUSG	Gm27000	ENSMUST00000182943
ENSMUSG	Gm21045	ENSMUST00000183058
ENSMUSG	Gm9423	ENSMUST00000183173
ENSMUSG	Gm27028	ENSMUST00000182303
ENSMUSG	Gm26917	ENSMUST00000192833
ENSMUSG	Gm4929	ENSMUST00000182021
ENSMUSG	Gm10605	ENSMUST00000183019;ENSMUST00000182342
ENSMUSG	Gm8079	ENSMUST00000182695
ENSMUSG	Gm26918	ENSMUST00000182496
ENSMUSG	Gm5652	ENSMUST00000182336
ENSMUSG	Gm4691	ENSMUST00000182339;ENSMUST00000182989
ENSMUSG	Gm7583	ENSMUST00000182941
ENSMUSG	Gm8167	ENSMUST00000183320
ENSMUSG	Gm26953	ENSMUST00000212309
ENSMUSG	Gm7656	ENSMUST00000182468
ENSMUSG	Gm9061	ENSMUST00000182707
ENSMUSG	Gm27048	ENSMUST00000182891
ENSMUSG	Gm9658	ENSMUST00000182757
ENSMUSG	Gm8565	ENSMUST00000183259
ENSMUSG	Gm8614	ENSMUST00000182528
ENSMUSG	Gm21542	ENSMUST00000182307
ENSMUSG	Gm26967	ENSMUST00000182878
ENSMUSG	Gm2546	ENSMUST00000182630
ENSMUSG	Gm8824	ENSMUST00000182962
ENSMUSG	Gm7745	ENSMUST00000182235
ENSMUSG	Gm6771	ENSMUST00000182716
ENSMUSG	Gm8793	ENSMUST00000183069
ENSMUSG	Gm20757	ENSMUST00000182870
ENSMUSG	Gm5710	ENSMUST00000183020

ENSMUSG	Gm26980	ENSMUST00000183321
ENSMUSG	Gm8254	ENSMUST00000182326
ENSMUSG	Gm18822	ENSMUST00000182857
ENSMUSG	Gm8330	ENSMUST00000182754
ENSMUSG	Gm27032	ENSMUST00000183124
ENSMUSG	Gm5300	ENSMUST00000182201
ENSMUSG	Gm8192	ENSMUST00000182196
ENSMUSG	Gm26947	ENSMUST00000182497
ENSMUSG	Gm8100	ENSMUST00000182906
ENSMUSG	Gm8529	ENSMUST00000183298
ENSMUSG	Gm26945	ENSMUST00000183191
ENSMUSG	Gm9192	ENSMUST00000183221
ENSMUSG	Gm5177	ENSMUST00000183337;ENSMUST00000183092
ENSMUSG	Gm7909	ENSMUST00000182688
ENSMUSG	Gm8609	ENSMUST00000182967
ENSMUSG	Gm26995	ENSMUST00000182216
ENSMUSG	Gm6981	ENSMUST00000182467
ENSMUSG	Gm6773	ENSMUST00000182377
ENSMUSG	Gm20574	ENSMUST00000182867
ENSMUSG	Gm26915	ENSMUST00000183080
ENSMUSG	Gm16470	ENSMUST00000182456
ENSMUSG	Gm2076	ENSMUST00000182524
ENSMUSG	6530403	ENSMUST00000182124;ENSMUST00000183168
ENSMUSG	Gm2467	ENSMUST00000182266
ENSMUSG	Gm8825	ENSMUST00000182565
ENSMUSG	Gm27007	ENSMUST00000183264
ENSMUSG	Gm4654	ENSMUST00000183305
ENSMUSG	Gm2445	ENSMUST00000182798
ENSMUSG	Gm26957	ENSMUST00000182645
ENSMUSG	Gm7191	ENSMUST00000182678
ENSMUSG	Gm4518	ENSMUST00000182638
ENSMUSG	Gm27036	ENSMUST00000182465
ENSMUSG	Gm3693	ENSMUST00000182077
ENSMUSG	Rassf10	ENSMUST00000182858
ENSMUSG	Gm6946	ENSMUST00000182237
ENSMUSG	Gm26988	ENSMUST00000183296
ENSMUSG	Gm6944	ENSMUST00000182449
ENSMUSG	Gm26937	ENSMUST00000182628
ENSMUSG	1700029	ENSMUST00000190628;ENSMUST00000220639
ENSMUSG	Gapdh-p	ENSMUST00000183246
ENSMUSG	Gm10292	ENSMUST00000183142
ENSMUSG	Gm5787	ENSMUST00000183049
ENSMUSG	Gm27023	ENSMUST00000182372
ENSMUSG	Gm9022	ENSMUST00000183285;ENSMUST00000182482
ENSMUSG	Gm21614	ENSMUST00000183260
ENSMUSG	Platr11	ENSMUST00000182885
ENSMUSG	Gm5205	ENSMUST00000182253
ENSMUSG	Gm26973	ENSMUST00000182733;ENSMUST00000183231
ENSMUSG	Gm5430	ENSMUST00000182525
ENSMUSG	Gm7132	ENSMUST00000182302
ENSMUSG	Gm8927	ENSMUST00000181978
ENSMUSG	Gm7286	ENSMUST00000182691
ENSMUSG	Gm9358	ENSMUST00000183067
ENSMUSG	B830012	ENSMUST00000182785
ENSMUSG	Gm26984	ENSMUST00000182345
ENSMUSG	Arl14	ENSMUST00000183126
ENSMUSG	Gm8349	ENSMUST00000182720

ENSMUSG	Gm7481	ENSMUST00000182020
ENSMUSG	Gm8318	ENSMUST00000182954
ENSMUSG	Gm4891	ENSMUST00000182279
ENSMUSG	Gm7719	ENSMUST00000182374
ENSMUSG	1700095	ENSMUST00000182349
ENSMUSG	Gm3671	ENSMUST00000182463
ENSMUSG	Gm26954	ENSMUST00000182753
ENSMUSG	Gm7734	ENSMUST00000182832
ENSMUSG	Gm7577	ENSMUST00000182543
ENSMUSG	Gm8756	ENSMUST00000183250
ENSMUSG	Gm4575	ENSMUST00000182991
ENSMUSG	Gm26929	ENSMUST00000182703
ENSMUSG	Gm7627	ENSMUST00000183205
ENSMUSG	Mir6361	ENSMUST00000185079
ENSMUSG	Gm27169	ENSMUST00000183344
ENSMUSG	Mir6369	ENSMUST00000183772
ENSMUSG	Gm27828	ENSMUST00000184108
ENSMUSG	Mir6358	ENSMUST00000184549
ENSMUSG	A330093	ENSMUST00000184994
ENSMUSG	Mir6980	ENSMUST00000185063
ENSMUSG	Mir7224	ENSMUST00000184202
ENSMUSG	Gm27953	ENSMUST00000185171
ENSMUSG	Mir7683	ENSMUST00000183842
ENSMUSG	Vis1	ENSMUST00000183957
ENSMUSG	Gm27158	ENSMUST00000183813
ENSMUSG	Gm27647	ENSMUST00000184709
ENSMUSG	Gm28001	ENSMUST00000183561
ENSMUSG	Gm27999	ENSMUST00000183539
ENSMUSG	Gm27998	ENSMUST00000184902
ENSMUSG	Mir6377	ENSMUST00000183760
ENSMUSG	Gm27409	ENSMUST00000184727
ENSMUSG	Mir7222	ENSMUST00000183740
ENSMUSG	Gm27410	ENSMUST00000183851
ENSMUSG	Mir6909	ENSMUST00000184212
ENSMUSG	Gm27202	ENSMUST00000183838
ENSMUSG	2010110	ENSMUST00000184161
ENSMUSG	Mir7681	ENSMUST00000184834
ENSMUSG	Gm27308	ENSMUST00000184807
ENSMUSG	Gm27350	ENSMUST00000185109
ENSMUSG	Gm27203	ENSMUST00000183677
ENSMUSG	Gm27853	ENSMUST00000184176
ENSMUSG	Gm27387	ENSMUST00000184583
ENSMUSG	Gm27385	ENSMUST00000184747
ENSMUSG	Phf20-p	ENSMUST00000185066
ENSMUSG	Rgs21	ENSMUST00000184008;ENSMUST00000184189
ENSMUSG	Gm27663	ENSMUST00000184839
ENSMUSG	Ranbp2-	ENSMUST00000184001
ENSMUSG	Gm27366	ENSMUST00000184967
ENSMUSG	Zfp572	ENSMUST00000184208
ENSMUSG	Gm27167	ENSMUST00000183752
ENSMUSG	Gm27535	ENSMUST00000183814
ENSMUSG	Gm5269	ENSMUST00000184038
ENSMUSG	Gm27217	ENSMUST00000183438
ENSMUSG	Mir6402	ENSMUST00000183406
ENSMUSG	Gm27443	ENSMUST00000184660
ENSMUSG	Mir7044	ENSMUST00000184597
ENSMUSG	Gm1113	ENSMUST00000183573

ENSMUSG	Gm27631	ENSMUST00000184163
ENSMUSG	Gm27627	ENSMUST00000183644
ENSMUSG	Mir6981	ENSMUST00000184293
ENSMUSG	Gm27300	ENSMUST00000184085
ENSMUSG	Mir6401	ENSMUST00000184499
ENSMUSG	4930524(	ENSMUST00000183387
ENSMUSG	Gm27976	ENSMUST00000183635
ENSMUSG	Gm27741	ENSMUST00000185060
ENSMUSG	Gm27977	ENSMUST00000185159
ENSMUSG	Mir6239	ENSMUST00000183564
ENSMUSG	Gm27582	ENSMUST00000184084
ENSMUSG	Mir6958	ENSMUST00000184207
ENSMUSG	Gm27588	ENSMUST00000185096
ENSMUSG	Mroh6	ENSMUST00000184858
ENSMUSG	Gm27246	ENSMUST00000183611
ENSMUSG	Mir6973	ENSMUST00000184185
ENSMUSG	Gm27316	ENSMUST00000184884
ENSMUSG	Gm27506	ENSMUST00000184558
ENSMUSG	Gm27617	ENSMUST00000184321
ENSMUSG	Gm27525	ENSMUST00000184465
ENSMUSG	Gm27527	ENSMUST00000183903
ENSMUSG	Gm3428	ENSMUST00000192304
ENSMUSG	Gm27236	ENSMUST00000184147
ENSMUSG	Rps2-ps	ENSMUST00000184266
ENSMUSG	Gm27335	ENSMUST00000184758
ENSMUSG	Mir6417	ENSMUST00000183815
ENSMUSG	Mir6335	ENSMUST00000184081
ENSMUSG	Gm27151	ENSMUST00000185015
ENSMUSG	Obox4-p	ENSMUST00000185088
ENSMUSG	Gm27216	ENSMUST00000184100
ENSMUSG	Gm27313	ENSMUST00000183500
ENSMUSG	Nkx2-2o	ENSMUST00000184407
ENSMUSG	Gm27440	ENSMUST00000184785
ENSMUSG	Gm27342	ENSMUST00000183530
ENSMUSG	Gm27163	ENSMUST00000184767
ENSMUSG	Mir300	ENSMUST00000083485
ENSMUSG	Gm27785	ENSMUST00000184268
ENSMUSG	Gm27783	ENSMUST00000183534
ENSMUSG	Gm27284	ENSMUST00000184357
ENSMUSG	Mir8111	ENSMUST00000184463
ENSMUSG	Kdm4dl	ENSMUST00000184843
ENSMUSG	Mark1-p	ENSMUST00000184922
ENSMUSG	Gm27466	ENSMUST00000184396
ENSMUSG	Gm27753	ENSMUST00000184742
ENSMUSG	Gm21013	ENSMUST00000184952
ENSMUSG	Gm18190	ENSMUST00000174801;ENSMUST00000209485
ENSMUSG	Mir6416	ENSMUST00000183664
ENSMUSG	Mir8120	ENSMUST00000185035
ENSMUSG	Gm27636	ENSMUST00000184528
ENSMUSG	Mir8105	ENSMUST00000184718
ENSMUSG	Gm27699	ENSMUST00000183553
ENSMUSG	Gm27843	ENSMUST00000184273
ENSMUSG	Gm27544	ENSMUST00000185069
ENSMUSG	Gm28003	ENSMUST00000184529
ENSMUSG	Gm27219	ENSMUST00000184633
ENSMUSG	Gm27596	ENSMUST00000185061
ENSMUSG	Mir6974	ENSMUST00000184381

ENSMUSG	Gm27357	ENSMUST00000183754
ENSMUSG	Mir6372	ENSMUST00000185114
ENSMUSG	Gm27227	ENSMUST00000183413
ENSMUSG	Gm27159	ENSMUST00000185132
ENSMUSG	Gm27162	ENSMUST00000185169
ENSMUSG	Tcf24	ENSMUST00000097824
ENSMUSG	2810039	ENSMUST00000183928;ENSMUST00000184602;ENSMUST0000018‌4
ENSMUSG	Mir378c	ENSMUST00000184478
ENSMUSG	Gm27554	ENSMUST00000185074
ENSMUSG	Gm27793	ENSMUST00000183405
ENSMUSG	Gm27242	ENSMUST00000184211
ENSMUSG	Zfp-ps	ENSMUST00000185025
ENSMUSG	Gm19569	ENSMUST00000184658;ENSMUST00000184563
ENSMUSG	Gm11983	ENSMUST00000184257
ENSMUSG	Gm27369	ENSMUST00000185182
ENSMUSG	Gm27193	ENSMUST00000184217
ENSMUSG	Mir7076	ENSMUST00000183519
ENSMUSG	Gm27604	ENSMUST00000182333
ENSMUSG	Gm27608	ENSMUST00000184822
ENSMUSG	Mir6352	ENSMUST00000184495
ENSMUSG	0610031	ENSMUST00000184166;ENSMUST00000185046
ENSMUSG	Gm3331	ENSMUST00000183423
ENSMUSG	Mir6969	ENSMUST00000183777
ENSMUSG	Mir7671	ENSMUST00000184599
ENSMUSG	Gm27804	ENSMUST00000185135
ENSMUSG	Gm27801	ENSMUST00000184174
ENSMUSG	Gm27715	ENSMUST00000184714
ENSMUSG	5730403	ENSMUST00000183878
ENSMUSG	Mir7662	ENSMUST00000184761
ENSMUSG	Gm27248	ENSMUST00000184004
ENSMUSG	Gm27944	ENSMUST00000185155
ENSMUSG	Gm10637	ENSMUST00000184702
ENSMUSG	Gm27684	ENSMUST00000183778
ENSMUSG	Gm27379	ENSMUST00000183730
ENSMUSG	Gm28018	ENSMUST00000103869
ENSMUSG	Gm27882	ENSMUST00000184904
ENSMUSG	Mir7216	ENSMUST00000185107
ENSMUSG	Obox4-p	ENSMUST00000183474
ENSMUSG	Gm27538	ENSMUST00000185065
ENSMUSG	Gm27265	ENSMUST00000183559
ENSMUSG	Gm7863	ENSMUST00000184700
ENSMUSG	Mir7047	ENSMUST00000184595
ENSMUSG	Gm27452	ENSMUST00000184920
ENSMUSG	Mir7235	ENSMUST00000183796
ENSMUSG	Gm11214	ENSMUST00000184810
ENSMUSG	Gm27458	ENSMUST00000185108
ENSMUSG	Gm27419	ENSMUST00000184576
ENSMUSG	Mir7085	ENSMUST00000184353
ENSMUSG	Mir7664	ENSMUST00000184681
ENSMUSG	Gm28017	ENSMUST00000183434
ENSMUSG	Gm28011	ENSMUST00000184097
ENSMUSG	Gm29540	ENSMUST00000188207
ENSMUSG	1700047	ENSMUST00000191278;ENSMUST00000186347;ENSMUST0000019C
ENSMUSG	5730419	ENSMUST00000190504
ENSMUSG	Gm2497	ENSMUST00000189539
ENSMUSG	Platr10	ENSMUST00000190602;ENSMUST00000191479
ENSMUSG	Gm6159	ENSMUST00000191249

ENSMUSG	1700067	ENSMUST00000186180
ENSMUSG	Gm18303	ENSMUST00000190055
ENSMUSG	1700110	ENSMUST00000187397;ENSMUST00000188715
ENSMUSG	Gm29225	ENSMUST00000188647
ENSMUSG	Ms4a14	ENSMUST00000187467
ENSMUSG	Gm28172	ENSMUST00000189880
ENSMUSG	Gm29408	ENSMUST00000187536
ENSMUSG	2310015	ENSMUST00000187920
ENSMUSG	Gm17767	ENSMUST00000190169;ENSMUST00000189410
ENSMUSG	Gm28977	ENSMUST00000188678
ENSMUSG	H2al1h	ENSMUST00000177926
ENSMUSG	Gm18801	ENSMUST00000186616
ENSMUSG	Gm28401	ENSMUST00000190769;ENSMUST00000188856
ENSMUSG	Gm6799	ENSMUST00000186919
ENSMUSG	Gm18248	ENSMUST00000191164
ENSMUSG	Gm29288	ENSMUST00000191582
ENSMUSG	Gm28556	ENSMUST00000187489
ENSMUSG	Gm3830	ENSMUST00000185540
ENSMUSG	Gm28271	ENSMUST00000191369
ENSMUSG	Gm29340	ENSMUST00000188763
ENSMUSG	Gm29539	ENSMUST00000188031
ENSMUSG	4930488	ENSMUST00000188802
ENSMUSG	Gm5525	ENSMUST00000190438
ENSMUSG	Gm28640	ENSMUST00000190918
ENSMUSG	Gm18970	ENSMUST00000186040
ENSMUSG	Gm21819	ENSMUST00000187438
ENSMUSG	Gm28169	ENSMUST00000190342
ENSMUSG	Gm18699	ENSMUST00000190713
ENSMUSG	9830004	ENSMUST00000185548
ENSMUSG	Gm29538	ENSMUST00000190783
ENSMUSG	Mrgprx3	ENSMUST00000187741
ENSMUSG	Gm28729	ENSMUST00000190704
ENSMUSG	Gm29040	ENSMUST00000189899
ENSMUSG	Gm29014	ENSMUST00000191422
ENSMUSG	Gm5264	ENSMUST00000190584
ENSMUSG	Zbed4-p	ENSMUST00000187149
ENSMUSG	Gm29126	ENSMUST00000188686
ENSMUSG	Vmn1r18	ENSMUST00000186062
ENSMUSG	Mrgprb1	ENSMUST00000188191
ENSMUSG	Gm29325	ENSMUST00000190705
ENSMUSG	Gm6757	ENSMUST00000189810
ENSMUSG	Gm28427	ENSMUST00000190144
ENSMUSG	Gm8641	ENSMUST00000186995
ENSMUSG	Gm29462	ENSMUST00000191458
ENSMUSG	1700084	ENSMUST00000191362
ENSMUSG	Gm5776	ENSMUST00000122119
ENSMUSG	Gm5975	ENSMUST00000185256
ENSMUSG	Gm28980	ENSMUST00000187877
ENSMUSG	Gm29012	ENSMUST00000187882
ENSMUSG	Gm28564	ENSMUST00000190864
ENSMUSG	Gm28214	ENSMUST00000185663
ENSMUSG	Gm29460	ENSMUST00000188607
ENSMUSG	2900052	ENSMUST00000191603;ENSMUST00000189828
ENSMUSG	Gm29013	ENSMUST00000187431
ENSMUSG	Gm9451	ENSMUST00000188491
ENSMUSG	Gm17752	ENSMUST00000185787
ENSMUSG	1700122	ENSMUST00000188950

ENSMUSG	Gm10830	ENSMUST00000186379
ENSMUSG	Gm21149	ENSMUST00000197995
ENSMUSG	Gm28884	ENSMUST00000190367
ENSMUSG	Gm19257	ENSMUST00000186121
ENSMUSG	Gm29341	ENSMUST00000190179
ENSMUSG	Gm8228	ENSMUST00000189949
ENSMUSG	Vmn1r19	ENSMUST00000072632
ENSMUSG	Gm7893	ENSMUST00000189666
ENSMUSG	Gm29168	ENSMUST00000189953
ENSMUSG	Gm28493	ENSMUST00000188427
ENSMUSG	Gm29509	ENSMUST00000185716
ENSMUSG	Gm28552	ENSMUST00000190725
ENSMUSG	1810012	ENSMUST00000190830
ENSMUSG	Gm29025	ENSMUST00000186756
ENSMUSG	Gm29668	ENSMUST00000187802
ENSMUSG	Gm29374	ENSMUST00000187198
ENSMUSG	Gm28906	ENSMUST00000191371
ENSMUSG	Gm29328	ENSMUST00000208988;ENSMUST00000207120
ENSMUSG	Gm7200	ENSMUST00000188426
ENSMUSG	1700074	ENSMUST00000189831
ENSMUSG	Gm28178	ENSMUST00000188544
ENSMUSG	Gm2324	ENSMUST00000187464
ENSMUSG	Mrgprb1	ENSMUST00000187816
ENSMUSG	Gm5575	ENSMUST00000190879
ENSMUSG	Gm28153	ENSMUST00000189853
ENSMUSG	Zbed4-p	ENSMUST00000187003
ENSMUSG	Gm12115	ENSMUST00000119969
ENSMUSG	Gm28653	ENSMUST00000185494
ENSMUSG	Gm10421	ENSMUST00000185746
ENSMUSG	Gm28066	ENSMUST00000188657
ENSMUSG	Gm28320	ENSMUST00000187777
ENSMUSG	Gm17983	ENSMUST00000190766
ENSMUSG	1700126	ENSMUST00000185283
ENSMUSG	9130227	ENSMUST00000189293;ENSMUST00000188040
ENSMUSG	Gm9226	ENSMUST00000188352
ENSMUSG	2610027	ENSMUST00000185443
ENSMUSG	Gm28287	ENSMUST00000186612
ENSMUSG	Gm5896	ENSMUST00000189662
ENSMUSG	Gm29065	ENSMUST00000188786
ENSMUSG	Tubb4b-	ENSMUST00000187481
ENSMUSG	Taar8c	ENSMUST00000170267
ENSMUSG	1700010	ENSMUST00000187729
ENSMUSG	Gm29290	ENSMUST00000185385
ENSMUSG	Gm7776	ENSMUST00000191211
ENSMUSG	Gm18766	ENSMUST00000189811
ENSMUSG	Gm19086	ENSMUST00000190772
ENSMUSG	Gm29590	ENSMUST00000190396;ENSMUST00000224398
ENSMUSG	Gm8337	ENSMUST00000187358
ENSMUSG	Gm28671	ENSMUST00000187036
ENSMUSG	Gm5702	ENSMUST00000185629
ENSMUSG	Gm28154	ENSMUST00000186267
ENSMUSG	Gm17944	ENSMUST00000186351
ENSMUSG	Gm18558	ENSMUST00000186253
ENSMUSG	Gm6170	ENSMUST00000187672
ENSMUSG	Gm29649	ENSMUST00000185649
ENSMUSG	1810008	ENSMUST00000187848;ENSMUST00000185464
ENSMUSG	Gm28760	ENSMUST00000185995

ENSMUSG	Gm5644	ENSMUST00000122039
ENSMUSG	Gm28856	ENSMUST00000189150
ENSMUSG	Gm28100	ENSMUST00000186560
ENSMUSG	A330087	ENSMUST00000187265
ENSMUSG	2700089	ENSMUST00000190204
ENSMUSG	Gm553	ENSMUST00000189527
ENSMUSG	Gm29064	ENSMUST00000187014
ENSMUSG	1700065	ENSMUST00000186872
ENSMUSG	Gm6445	ENSMUST00000189515
ENSMUSG	1700020	ENSMUST00000188320
ENSMUSG	Gm28382	ENSMUST00000187902
ENSMUSG	Gm29598	ENSMUST00000188996
ENSMUSG	Gm29052	ENSMUST00000187700
ENSMUSG	Gm2109	ENSMUST00000189326
ENSMUSG	2310034	ENSMUST00000185221;ENSMUST00000188902
ENSMUSG	Gm28527	ENSMUST00000185210
ENSMUSG	Gm29087	ENSMUST00000186462
ENSMUSG	Gm28942	ENSMUST00000186334
ENSMUSG	Gm28719	ENSMUST00000187779
ENSMUSG	1700025	ENSMUST00000191524
ENSMUSG	1810006	ENSMUST00000191060;ENSMUST00000186473
ENSMUSG	Taar8b	ENSMUST00000092654
ENSMUSG	Gm7740	ENSMUST00000186171
ENSMUSG	1700030	ENSMUST00000185546
ENSMUSG	H2al1m	ENSMUST00000073949
ENSMUSG	Gm28793	ENSMUST00000190845
ENSMUSG	1700064	ENSMUST00000185726
ENSMUSG	Gm5522	ENSMUST00000189483
ENSMUSG	Gm28650	ENSMUST00000188413
ENSMUSG	Slc18a3	ENSMUST00000191501
ENSMUSG	A530053	ENSMUST00000187918
ENSMUSG	Btbd35f	ENSMUST00000189190
ENSMUSG	Mir124-	ENSMUST00000188983;ENSMUST00000189467;ENSMUST00000185
ENSMUSG	1700020	ENSMUST00000188956
ENSMUSG	Gm28196	ENSMUST00000187719
ENSMUSG	1810053	ENSMUST00000188056;ENSMUST00000185971;ENSMUST00000185
ENSMUSG	2310069	ENSMUST00000186147
ENSMUSG	Gm18799	ENSMUST00000189104
ENSMUSG	Gm5256	ENSMUST00000191412
ENSMUSG	C230024	ENSMUST00000190663
ENSMUSG	2310008	ENSMUST00000210732;ENSMUST00000210263
ENSMUSG	Gm29601	ENSMUST00000187961
ENSMUSG	Gm5066	ENSMUST00000188479
ENSMUSG	Gm28996	ENSMUST00000190316
ENSMUSG	Gm28188	ENSMUST00000190483
ENSMUSG	Gm28181	ENSMUST00000191460
ENSMUSG	Gm28649	ENSMUST00000189153
ENSMUSG	Gm29336	ENSMUST00000186042
ENSMUSG	Gm29669	ENSMUST00000188605
ENSMUSG	Gm17867	ENSMUST00000188981
ENSMUSG	Gm28094	ENSMUST00000185262
ENSMUSG	Gm28688	ENSMUST00000187301
ENSMUSG	Rbm6-ps	ENSMUST00000186440
ENSMUSG	Gm28935	ENSMUST00000185737
ENSMUSG	Gm29491	ENSMUST00000189481
ENSMUSG	Gm29367	ENSMUST00000190911
ENSMUSG	Gm29326	ENSMUST00000185541

ENSMUSG	Gm28911	ENSMUST00000188837
ENSMUSG	Gm28578	ENSMUST00000189196
ENSMUSG	Gm29152	ENSMUST00000189284
ENSMUSG	C530043	ENSMUST00000189247
ENSMUSG	H2al1k	ENSMUST00000178196
ENSMUSG	Gm9443	ENSMUST00000188406
ENSMUSG	D830032	ENSMUST00000186875
ENSMUSG	Olfr106	ENSMUST00000118335
ENSMUSG	Gm29572	ENSMUST00000190305
ENSMUSG	Gm29479	ENSMUST00000190352
ENSMUSG	Gm7114	ENSMUST00000190159
ENSMUSG	Gm4131	ENSMUST00000186010;ENSMUST00000209021
ENSMUSG	Gm28455	ENSMUST00000189105
ENSMUSG	Gm28639	ENSMUST00000191519
ENSMUSG	Gm28624	ENSMUST00000186430
ENSMUSG	Gm28286	ENSMUST00000188704
ENSMUSG	Nanog-p	ENSMUST00000188967
ENSMUSG	AV02606	ENSMUST00000226075;ENSMUST00000225995;ENSMUST0000018
ENSMUSG	1700111	ENSMUST00000188663
ENSMUSG	Gm29608	ENSMUST00000190444
ENSMUSG	Gm29483	ENSMUST00000188188
ENSMUSG	2310039	ENSMUST00000220058;ENSMUST00000219492
ENSMUSG	Gm19503	ENSMUST00000187109
ENSMUSG	Gm17751	ENSMUST00000190362;ENSMUST00000189132
ENSMUSG	Gm7690	ENSMUST00000190885
ENSMUSG	Gm8173	ENSMUST00000186797
ENSMUSG	1700025	ENSMUST00000185238
ENSMUSG	1700081	ENSMUST00000190550
ENSMUSG	Vmn1r90	ENSMUST00000185220
ENSMUSG	Gm4785	ENSMUST00000191025
ENSMUSG	2810414	ENSMUST00000186444
ENSMUSG	Gm19087	ENSMUST00000191430
ENSMUSG	A230077	ENSMUST00000189574;ENSMUST00000188579
ENSMUSG	1700129	ENSMUST00000189291
ENSMUSG	Gm29243	ENSMUST00000188318
ENSMUSG	1700016	ENSMUST00000186819;ENSMUST00000186843
ENSMUSG	H2al1a	ENSMUST00000188439
ENSMUSG	A830008	ENSMUST00000185465;ENSMUST00000190485
ENSMUSG	Gm3551	ENSMUST00000190750
ENSMUSG	Gm28230	ENSMUST00000053932
ENSMUSG	Gm7634	ENSMUST00000185778
ENSMUSG	F730311	ENSMUST00000189288
ENSMUSG	Gm19029	ENSMUST00000189923
ENSMUSG	6030442	ENSMUST00000186618
ENSMUSG	1700007	ENSMUST00000188418
ENSMUSG	1810044	ENSMUST00000186472
ENSMUSG	Taar7e	ENSMUST00000092656
ENSMUSG	Gm4322	ENSMUST00000191094
ENSMUSG	Gm8419	ENSMUST00000189886
ENSMUSG	Gm19587	ENSMUST00000191395
ENSMUSG	Gm29461	ENSMUST00000189232
ENSMUSG	1700120	ENSMUST00000190881;ENSMUST00000187267
ENSMUSG	Gm9271	ENSMUST00000185988
ENSMUSG	4932411	ENSMUST00000190493
ENSMUSG	Gm5147	ENSMUST00000185999
ENSMUSG	Gm17764	ENSMUST00000185925
ENSMUSG	Gm4035	ENSMUST00000189445;ENSMUST00000190684

ENSMUSG	Gm29107	ENSMUST00000190910
ENSMUSG	4930598	ENSMUST00000187352
ENSMUSG	Gm28231	ENSMUST00000190118
ENSMUSG	Gm21122	ENSMUST00000191548
ENSMUSG	Gm29508	ENSMUST00000189971
ENSMUSG	Gm28199	ENSMUST00000187127
ENSMUSG	Gm29667	ENSMUST00000190817
ENSMUSG	Gm19589	ENSMUST00000189394
ENSMUSG	Gm29521	ENSMUST00000187746
ENSMUSG	Gm29086	ENSMUST00000186774
ENSMUSG	Gm2693	ENSMUST00000189119
ENSMUSG	Itpa-ps	ENSMUST00000188629
ENSMUSG	Snhg14	ENSMUST00000189408;ENSMUST00000185693;ENSMUST0000018
ENSMUSG	Gm29069	ENSMUST00000185857
ENSMUSG	Gm28441	ENSMUST00000190927
ENSMUSG	1700063	ENSMUST00000213914;ENSMUST00000214568
ENSMUSG	Gm8307	ENSMUST00000187682
ENSMUSG	1700041	ENSMUST00000190908
ENSMUSG	1700065	ENSMUST00000190576;ENSMUST00000188648
ENSMUSG	Gm20849	ENSMUST00000189065
ENSMUSG	1700085	ENSMUST00000187123
ENSMUSG	1700027	ENSMUST00000189767
ENSMUSG	Gm8520	ENSMUST00000186517
ENSMUSG	Olfr10	ENSMUST00000187570
ENSMUSG	Gm28064	ENSMUST00000185639
ENSMUSG	Gm29459	ENSMUST00000191359
ENSMUSG	Gm28865	ENSMUST00000187685
ENSMUSG	Gm29229	ENSMUST00000188874
ENSMUSG	Gm28900	ENSMUST00000185232
ENSMUSG	Gm29529	ENSMUST00000187154
ENSMUSG	Gm7654	ENSMUST00000186738
ENSMUSG	Gm29133	ENSMUST00000189155
ENSMUSG	Gm28372	ENSMUST00000188900
ENSMUSG	Gm28449	ENSMUST00000187655
ENSMUSG	Gm29100	ENSMUST00000187564
ENSMUSG	Gm1627	ENSMUST00000188504
ENSMUSG	Gm7891	ENSMUST00000190284
ENSMUSG	Gm28864	ENSMUST00000189251
ENSMUSG	Gm3121	ENSMUST00000187086
ENSMUSG	Gm29541	ENSMUST00000191351
ENSMUSG	1700108	ENSMUST00000189375;ENSMUST00000190818;ENSMUST0000018
ENSMUSG	Gm21152	ENSMUST00000190271
ENSMUSG	D730001	ENSMUST00000186014
ENSMUSG	Gm28723	ENSMUST00000188640
ENSMUSG	Gm29439	ENSMUST00000189996
ENSMUSG	Platr1	ENSMUST00000188474
ENSMUSG	Gm29007	ENSMUST00000190633
ENSMUSG	Gm28651	ENSMUST00000185335
ENSMUSG	Gm10538	ENSMUST00000189499
ENSMUSG	Gm19637	ENSMUST00000185933
ENSMUSG	Gm21139	ENSMUST00000186594
ENSMUSG	Gm7593	ENSMUST00000187246
ENSMUSG	Gm29519	ENSMUST00000186994
ENSMUSG	Hoxd4	ENSMUST00000144040;ENSMUST00000047904;ENSMUST00000111
ENSMUSG	Gm29455	ENSMUST00000186504
ENSMUSG	Gm2914	ENSMUST00000186120
ENSMUSG	1700029	ENSMUST00000187517

ENSMUSG	Gm9239	ENSMUST00000186942
ENSMUSG	Gm6866	ENSMUST00000206653;ENSMUST00000188559
ENSMUSG	Gm8524	ENSMUST00000186232
ENSMUSG	Gm28720	ENSMUST00000190728
ENSMUSG	Gm28818	ENSMUST00000187553
ENSMUSG	Gm6717	ENSMUST00000185549
ENSMUSG	Gm28209	ENSMUST00000187956
ENSMUSG	Gm20954	ENSMUST00000189078
ENSMUSG	1700007	ENSMUST00000186196;ENSMUST00000190494
ENSMUSG	Gm6750	ENSMUST00000189273
ENSMUSG	2310015	ENSMUST00000191452
ENSMUSG	Gm21912	ENSMUST00000186393
ENSMUSG	Gm8449	ENSMUST00000185532
ENSMUSG	Gm28166	ENSMUST00000187637
ENSMUSG	Gm28175	ENSMUST00000209336
ENSMUSG	Gm7358	ENSMUST00000189454
ENSMUSG	Rpl30-p	ENSMUST00000163337
ENSMUSG	Gm10193	ENSMUST00000188288
ENSMUSG	Gm4753	ENSMUST00000188327
ENSMUSG	1700028	ENSMUST00000185938
ENSMUSG	Gm28876	ENSMUST00000188137
ENSMUSG	Gm2495	ENSMUST00000191160
ENSMUSG	Gm7356	ENSMUST00000186636
ENSMUSG	Gm29139	ENSMUST00000185794
ENSMUSG	Gm9114	ENSMUST00000188558
ENSMUSG	Gm5261	ENSMUST00000185860
ENSMUSG	Pced1c-	ENSMUST00000187740
ENSMUSG	Ms4a4a	ENSMUST00000188995
ENSMUSG	Olfr121	ENSMUST00000216592
ENSMUSG	Gm8241	ENSMUST00000189286
ENSMUSG	Mug-ps1	ENSMUST00000186891
ENSMUSG	Gm28673	ENSMUST00000186562
ENSMUSG	Gm28758	ENSMUST00000186123
ENSMUSG	Gm28581	ENSMUST00000186086
ENSMUSG	Gm29101	ENSMUST00000188329
ENSMUSG	Gm29441	ENSMUST00000191584
ENSMUSG	Gm28826	ENSMUST00000190006
ENSMUSG	Gm17791	ENSMUST00000185660
ENSMUSG	Gm1988	ENSMUST00000188190
ENSMUSG	Gm19193	ENSMUST00000190263
ENSMUSG	Gm3052	ENSMUST00000186409;ENSMUST00000188254
ENSMUSG	Gm7761	ENSMUST00000190510
ENSMUSG	Gm21129	ENSMUST00000185932
ENSMUSG	Gm29570	ENSMUST00000185813
ENSMUSG	1700123	ENSMUST00000190515
ENSMUSG	Gm28883	ENSMUST00000189256
ENSMUSG	Gm8885	ENSMUST00000189382
ENSMUSG	Gm2950	ENSMUST00000185530
ENSMUSG	Rps27a-	ENSMUST00000191424
ENSMUSG	Rpl7a-p	ENSMUST00000190579
ENSMUSG	1700109	ENSMUST00000188538;ENSMUST00000186084
ENSMUSG	Gm28385	ENSMUST00000185671
ENSMUSG	Gm5681	ENSMUST00000189217
ENSMUSG	Gm5524	ENSMUST00000185409
ENSMUSG	Ska2l-p	ENSMUST00000186208
ENSMUSG	Gm28465	ENSMUST00000220779
ENSMUSG	Gm18352	ENSMUST00000189113

ENSMUSG	Gm9220	ENSMUST00000190721
ENSMUSG	Gm29630	ENSMUST00000185761
ENSMUSG	Gm7364	ENSMUST00000188760
ENSMUSG	Gm5703	ENSMUST00000186658
ENSMUSG	1700003	ENSMUST00000190074
ENSMUSG	1700040	ENSMUST00000187158
ENSMUSG	Gm21181	ENSMUST00000186249
ENSMUSG	C430002	ENSMUST00000189815;ENSMUST00000188819;ENSMUST0000018
ENSMUSG	Gm28265	ENSMUST00000188201
ENSMUSG	Rbm6-ps	ENSMUST00000186192
ENSMUSG	Gm28555	ENSMUST00000187303
ENSMUSG	Gm28730	ENSMUST00000191072;ENSMUST00000185939
ENSMUSG	Ace3	ENSMUST00000190995
ENSMUSG	Gm7933	ENSMUST00000191215
ENSMUSG	Gm8495	ENSMUST00000188740
ENSMUSG	1700066	ENSMUST00000187091
ENSMUSG	Gm29560	ENSMUST00000191526
ENSMUSG	Gm28307	ENSMUST00000190685
ENSMUSG	Gm29388	ENSMUST00000190718
ENSMUSG	1700028	ENSMUST00000187272
ENSMUSG	Gm19461	ENSMUST00000191207;ENSMUST00000191132;ENSMUST0000018
ENSMUSG	4930521	ENSMUST00000186732
ENSMUSG	1700072	ENSMUST00000189460
ENSMUSG	Gm28453	ENSMUST00000185975
ENSMUSG	Gm29125	ENSMUST00000187121
ENSMUSG	Gm5733	ENSMUST00000190232
ENSMUSG	2310043	ENSMUST00000189507
ENSMUSG	Olfr139	ENSMUST00000214516;ENSMUST00000215861;ENSMUST0000021
ENSMUSG	Gm2987	ENSMUST00000187673
ENSMUSG	Gm29068	ENSMUST00000191607
ENSMUSG	Gm28268	ENSMUST00000188966
ENSMUSG	Gm7255	ENSMUST00000187371
ENSMUSG	Gm7553	ENSMUST00000185805
ENSMUSG	2210011	ENSMUST00000190690
ENSMUSG	Gm5835	ENSMUST00000185806
ENSMUSG	Gm29266	ENSMUST00000190392
ENSMUSG	Rnf170-	ENSMUST00000181188
ENSMUSG	Gm28419	ENSMUST00000189108
ENSMUSG	H2al1b	ENSMUST00000189531
ENSMUSG	Gm29438	ENSMUST00000191298
ENSMUSG	Gm28686	ENSMUST00000187404
ENSMUSG	Gm5829	ENSMUST00000191383
ENSMUSG	Gm28746	ENSMUST00000187453
ENSMUSG	Gm20749	ENSMUST00000186319
ENSMUSG	4933417	ENSMUST00000188213
ENSMUSG	1700026	ENSMUST00000211950;ENSMUST00000212266;ENSMUST0000017
ENSMUSG	1700010	ENSMUST00000188166
ENSMUSG	Olfr139	ENSMUST00000215671;ENSMUST00000187509
ENSMUSG	Gm29282	ENSMUST00000190720
ENSMUSG	1700025	ENSMUST00000187479
ENSMUSG	Gm28981	ENSMUST00000190863
ENSMUSG	Gm6244	ENSMUST00000178732
ENSMUSG	Mrgprb1	ENSMUST00000186150
ENSMUSG	Gm29291	ENSMUST00000188927;ENSMUST00000186055
ENSMUSG	Mrgprc2	ENSMUST00000188039
ENSMUSG	Gm18501	ENSMUST00000185363
ENSMUSG	Gm28825	ENSMUST00000186233

ENSMUSG	Gm29041	ENSMUST00000189199
ENSMUSG	Gm5441	ENSMUST00000190132;ENSMUST00000186454
ENSMUSG	Gm10074	ENSMUST00000174543
ENSMUSG	Gm28164	ENSMUST00000190143
ENSMUSG	Gm29376	ENSMUST00000189357
ENSMUSG	1700001	ENSMUST00000190330
ENSMUSG	Gm5248	ENSMUST00000186108
ENSMUSG	1700027	ENSMUST00000185768;ENSMUST00000188349;ENSMUST0000019C
ENSMUSG	Gm20125	ENSMUST00000185722;ENSMUST00000208005
ENSMUSG	Gm28976	ENSMUST00000188070
ENSMUSG	Gm28929	ENSMUST00000185330
ENSMUSG	Gm18875	ENSMUST00000188563
ENSMUSG	Gm28591	ENSMUST00000185259
ENSMUSG	E330023	ENSMUST00000186387
ENSMUSG	Gm6124	ENSMUST00000188240
ENSMUSG	Gm29530	ENSMUST00000187556
ENSMUSG	Gm29106	ENSMUST00000186264
ENSMUSG	Gm28263	ENSMUST00000185884
ENSMUSG	2010010	ENSMUST00000190465;ENSMUST00000185628;ENSMUST0000018€
ENSMUSG	Gm28387	ENSMUST00000189245
ENSMUSG	1700061	ENSMUST00000185439
ENSMUSG	Gm28706	ENSMUST00000186830
ENSMUSG	Gm28625	ENSMUST00000188309
ENSMUSG	Gm29230	ENSMUST00000189687
ENSMUSG	Mrgprc7	ENSMUST00000187169
ENSMUSG	Gm6818	ENSMUST00000190556
ENSMUSG	Gm5259	ENSMUST00000191231
ENSMUSG	Olfr103	ENSMUST00000213496
ENSMUSG	C730036	ENSMUST00000188305
ENSMUSG	1700101	ENSMUST00000187396
ENSMUSG	2310016	ENSMUST00000190206
ENSMUSG	2310043	ENSMUST00000185323
ENSMUSG	Gm37108	ENSMUST00000194605
ENSMUSG	Rpl28-p	ENSMUST00000194739
ENSMUSG	Gm37109	ENSMUST00000195475
ENSMUSG	Gm38058	ENSMUST00000194323
ENSMUSG	4930429	ENSMUST00000194530;ENSMUST00000194547
ENSMUSG	Gm9723	ENSMUST00000192100
ENSMUSG	Gm37475	ENSMUST00000193107
ENSMUSG	Gm2367	ENSMUST00000192374
ENSMUSG	Gm36945	ENSMUST00000192040
ENSMUSG	Gm36947	ENSMUST00000194709
ENSMUSG	Gm36943	ENSMUST00000194422
ENSMUSG	Gm37871	ENSMUST00000194973
ENSMUSG	Gm37869	ENSMUST00000193385
ENSMUSG	Gm37189	ENSMUST00000192131
ENSMUSG	Gm37190	ENSMUST00000193580
ENSMUSG	Gm37192	ENSMUST00000194479
ENSMUSG	5033404	ENSMUST00000192616
ENSMUSG	Gm37194	ENSMUST00000193092
ENSMUSG	Gm38166	ENSMUST00000191660
ENSMUSG	Gm7299	ENSMUST00000194441
ENSMUSG	Gm38171	ENSMUST00000195716
ENSMUSG	Gm38170	ENSMUST00000195404
ENSMUSG	Gm38173	ENSMUST00000193126
ENSMUSG	Gm36958	ENSMUST00000192388
ENSMUSG	Gm10344	ENSMUST00000193498

ENSMUSG	Gm37489	ENSMUST00000191883
ENSMUSG	Gm6606	ENSMUST00000193613
ENSMUSG	Gm37302	ENSMUST00000192958
ENSMUSG	Gm37299	ENSMUST00000193951
ENSMUSG	Gm37887	ENSMUST00000192752
ENSMUSG	4930423	ENSMUST00000193863
ENSMUSG	Snrpn	ENSMUST00000059305;ENSMUST00000098402
ENSMUSG	Gm38258	ENSMUST00000191792
ENSMUSG	Gm38106	ENSMUST00000194107
ENSMUSG	Gm8826	ENSMUST00000193161
ENSMUSG	Gm7357	ENSMUST00000195361
ENSMUSG	Gm7512	ENSMUST00000195217
ENSMUSG	Gm37145	ENSMUST00000193540
ENSMUSG	Gm36999	ENSMUST00000192756
ENSMUSG	Gm37000	ENSMUST00000193058
ENSMUSG	Gm37004	ENSMUST00000193021
ENSMUSG	Gm37003	ENSMUST00000194702
ENSMUSG	Gm37542	ENSMUST00000192563
ENSMUSG	5730585	ENSMUST00000193457
ENSMUSG	Gm33707	ENSMUST00000195135
ENSMUSG	Ighv8-2	ENSMUST00000195037
ENSMUSG	Gm38194	ENSMUST00000194529
ENSMUSG	2310046	ENSMUST00000193521
ENSMUSG	Gm5543	ENSMUST00000193329
ENSMUSG	Gm37626	ENSMUST00000194332
ENSMUSG	Gm37792	ENSMUST00000195761
ENSMUSG	Gm37234	ENSMUST00000193259
ENSMUSG	Gm19938	ENSMUST00000192692
ENSMUSG	Gm37233	ENSMUST00000192173
ENSMUSG	Gm37232	ENSMUST00000194887
ENSMUSG	Gm38345	ENSMUST00000192635
ENSMUSG	Gm37043	ENSMUST00000194609
ENSMUSG	4930509	ENSMUST00000194514
ENSMUSG	Gm37045	ENSMUST00000192016
ENSMUSG	9530018	ENSMUST00000192253
ENSMUSG	Gm38140	ENSMUST00000193617
ENSMUSG	Gm38143	ENSMUST00000191751
ENSMUSG	Gm38144	ENSMUST00000192790
ENSMUSG	D630023	ENSMUST00000191913
ENSMUSG	Gm37579	ENSMUST00000194291
ENSMUSG	Gm37576	ENSMUST00000194228
ENSMUSG	Gm37580	ENSMUST00000192408
ENSMUSG	Gm5049	ENSMUST00000195878
ENSMUSG	5530400	ENSMUST00000193511
ENSMUSG	Gm37863	ENSMUST00000194911
ENSMUSG	4930568	ENSMUST00000193317;ENSMUST00000195635
ENSMUSG	Gm36935	ENSMUST00000192526
ENSMUSG	Sh2d1b1	ENSMUST00000179976
ENSMUSG	Gm37464	ENSMUST00000192431
ENSMUSG	Gm21949	ENSMUST00000182006
ENSMUSG	Gm38051	ENSMUST00000193740
ENSMUSG	Gm29856	ENSMUST00000191905
ENSMUSG	Gm17953	ENSMUST00000192061
ENSMUSG	Flg	ENSMUST00000216295;ENSMUST00000214837;ENSMUST0000018
ENSMUSG	Gm37102	ENSMUST00000195740
ENSMUSG	Pisrt1	ENSMUST00000195532
ENSMUSG	Gm38197	ENSMUST00000192320

ENSMUSG	Gm37166	ENSMUST00000195473
ENSMUSG	1700003	ENSMUST00000194834
ENSMUSG	Gm37167	ENSMUST00000194946
ENSMUSG	Gm37243	ENSMUST00000193407
ENSMUSG	Gm37926	ENSMUST00000191922
ENSMUSG	Gm37925	ENSMUST00000195352
ENSMUSG	Gm37924	ENSMUST00000195470
ENSMUSG	Catsper	ENSMUST00000194528
ENSMUSG	Gm37920	ENSMUST00000192952
ENSMUSG	Gm36985	ENSMUST00000195595
ENSMUSG	Gm36986	ENSMUST00000195320
ENSMUSG	Gm26524	ENSMUST00000192339;ENSMUST00000195143
ENSMUSG	Gm19445	ENSMUST00000193649
ENSMUSG	Gm37371	ENSMUST00000194837
ENSMUSG	Gm37367	ENSMUST00000191771
ENSMUSG	Gm35323	ENSMUST00000191836
ENSMUSG	Gm38340	ENSMUST00000195039
ENSMUSG	Gm38339	ENSMUST00000195479
ENSMUSG	Gm37781	ENSMUST00000194773
ENSMUSG	1110002	ENSMUST00000194261
ENSMUSG	1700039	ENSMUST00000194370
ENSMUSG	Gm38133	ENSMUST00000195109
ENSMUSG	Gm5103	ENSMUST00000192081;ENSMUST00000195545
ENSMUSG	Gm9722	ENSMUST00000194568
ENSMUSG	Gm16701	ENSMUST00000180995
ENSMUSG	Gm38137	ENSMUST00000193735
ENSMUSG	Gm8276	ENSMUST00000192231
ENSMUSG	Gm37035	ENSMUST00000194809
ENSMUSG	Gm37037	ENSMUST00000194475
ENSMUSG	Gm37966	ENSMUST00000193122
ENSMUSG	Gm33206	ENSMUST00000193544
ENSMUSG	Gm10576	ENSMUST00000192528
ENSMUSG	Gm37965	ENSMUST00000195304
ENSMUSG	Gm37443	ENSMUST00000193458
ENSMUSG	Gm38383	ENSMUST00000194131
ENSMUSG	Gm30097	ENSMUST00000193581
ENSMUSG	Gm20203	ENSMUST00000192352
ENSMUSG	Olfr431	ENSMUST00000192435
ENSMUSG	Rpl21-p	ENSMUST00000193804
ENSMUSG	Gm37267	ENSMUST00000194222
ENSMUSG	Gm37846	ENSMUST00000191840
ENSMUSG	Gm37843	ENSMUST00000195213
ENSMUSG	Gm37848	ENSMUST00000195288
ENSMUSG	Gm37849	ENSMUST00000194722
ENSMUSG	Trbv7	ENSMUST00000193339
ENSMUSG	Gm37673	ENSMUST00000194880
ENSMUSG	Snurf	ENSMUST00000194059
ENSMUSG	Gm37671	ENSMUST00000193198
ENSMUSG	Gm29999	ENSMUST00000192423
ENSMUSG	Gm37786	ENSMUST00000191714
ENSMUSG	Gm38224	ENSMUST00000191648
ENSMUSG	Gm37078	ENSMUST00000193314
ENSMUSG	Gm37899	ENSMUST00000193116
ENSMUSG	Gm19901	ENSMUST00000192191
ENSMUSG	8030442	ENSMUST00000192626
ENSMUSG	Gm37435	ENSMUST00000192078
ENSMUSG	Gm37221	ENSMUST00000194472

ENSMUSG	Gm37223	ENSMUST00000195070;ENSMUST00000193592
ENSMUSG	Gm37374	ENSMUST00000194954
ENSMUSG	Gm37373	ENSMUST00000194005
ENSMUSG	Gm37220	ENSMUST00000195179
ENSMUSG	Gm37219	ENSMUST00000192036
ENSMUSG	Gm37217	ENSMUST00000194017
ENSMUSG	Gm37779	ENSMUST00000194221
ENSMUSG	Gm37496	ENSMUST00000191930
ENSMUSG	Gm37777	ENSMUST00000195546
ENSMUSG	4930433	ENSMUST00000192010
ENSMUSG	Gm37260	ENSMUST00000193748
ENSMUSG	4632432	ENSMUST00000191824
ENSMUSG	Gm7417	ENSMUST00000192950
ENSMUSG	Gm38308	ENSMUST00000194934
ENSMUSG	Gm6209	ENSMUST00000194629;ENSMUST00000193264
ENSMUSG	Gm5099	ENSMUST00000194892
ENSMUSG	Gm37759	ENSMUST00000194155
ENSMUSG	Gm37761	ENSMUST00000194989
ENSMUSG	Gm37936	ENSMUST00000194647
ENSMUSG	Gm37934	ENSMUST00000192902
ENSMUSG	Gm7369	ENSMUST00000192738
ENSMUSG	Gm37599	ENSMUST00000193067
ENSMUSG	Gm2979	ENSMUST00000193442
ENSMUSG	Gm37600	ENSMUST00000193764
ENSMUSG	Gm37601	ENSMUST00000194922
ENSMUSG	Gm7694	ENSMUST00000179801
ENSMUSG	Gm37054	ENSMUST00000193481
ENSMUSG	Gm10463	ENSMUST00000195772
ENSMUSG	Gm37184	ENSMUST00000194720
ENSMUSG	Gm6420	ENSMUST00000193885;ENSMUST00000195365
ENSMUSG	Gm19002	ENSMUST00000192649
ENSMUSG	Gm33320	ENSMUST00000192232
ENSMUSG	Ighv1-1	ENSMUST00000194849
ENSMUSG	Gm38165	ENSMUST00000191654
ENSMUSG	Gm38251	ENSMUST00000192968
ENSMUSG	Gm2447	ENSMUST00000193813;ENSMUST00000191954
ENSMUSG	Gm37708	ENSMUST00000192337
ENSMUSG	Gm37712	ENSMUST00000194295
ENSMUSG	Gm6236	ENSMUST00000193785
ENSMUSG	Gm37711	ENSMUST00000194907
ENSMUSG	Gm37478	ENSMUST00000193624
ENSMUSG	Mgam2-p	ENSMUST00000216691;ENSMUST00000215813;ENSMUST0000019
ENSMUSG	Gm37813	ENSMUST00000195003
ENSMUSG	Gm6185	ENSMUST00000193379;ENSMUST00000212885
ENSMUSG	Gm36990	ENSMUST00000191961
ENSMUSG	1700029	ENSMUST00000194040
ENSMUSG	Gm18956	ENSMUST00000192857
ENSMUSG	Gm37086	ENSMUST00000193082
ENSMUSG	Gm37638	ENSMUST00000194337
ENSMUSG	Gm9839	ENSMUST00000195252
ENSMUSG	Gm37636	ENSMUST00000193583
ENSMUSG	Gm37633	ENSMUST00000192858
ENSMUSG	Gm17868	ENSMUST00000192761
ENSMUSG	4930517	ENSMUST00000193095
ENSMUSG	Gm30667	ENSMUST00000193120
ENSMUSG	Gm10857	ENSMUST00000195343
ENSMUSG	Ighv1-1	ENSMUST00000191801

ENSMUSG	Gm7626	ENSMUST00000195801
ENSMUSG	Gm37851	ENSMUST00000193871
ENSMUSG	5830415	ENSMUST00000192543
ENSMUSG	Gm37811	ENSMUST00000192416
ENSMUSG	Ighv5-1	ENSMUST00000193324
ENSMUSG	Gm38263	ENSMUST00000195879
ENSMUSG	Gm38266	ENSMUST00000194931
ENSMUSG	Gm32460	ENSMUST00000195647
ENSMUSG	Gm34176	ENSMUST00000192621
ENSMUSG	Gm37151	ENSMUST00000194631
ENSMUSG	Gm9116	ENSMUST00000192400
ENSMUSG	Gm38117	ENSMUST00000194653
ENSMUSG	Gm35584	ENSMUST00000191767
ENSMUSG	Gm37551	ENSMUST00000195556
ENSMUSG	Gm37883	ENSMUST00000194970
ENSMUSG	Gm6101	ENSMUST00000195384
ENSMUSG	Gm37016	ENSMUST00000195507
ENSMUSG	Gm37945	ENSMUST00000192536
ENSMUSG	Gm33366	ENSMUST00000195583
ENSMUSG	A730062	ENSMUST00000192843
ENSMUSG	Gm37348	ENSMUST00000193104
ENSMUSG	Gm37347	ENSMUST00000193969
ENSMUSG	Gm38322	ENSMUST00000194074
ENSMUSG	4930456	ENSMUST00000193054
ENSMUSG	A330074	ENSMUST00000194667
ENSMUSG	Gm37762	ENSMUST00000194515
ENSMUSG	Gm38076	ENSMUST00000193889
ENSMUSG	Gm37504	ENSMUST00000192537
ENSMUSG	Gm8860	ENSMUST00000193160
ENSMUSG	Gm37548	ENSMUST00000194519
ENSMUSG	Gm37549	ENSMUST00000194096
ENSMUSG	Gm37686	ENSMUST00000194099
ENSMUSG	Gm37304	ENSMUST00000193833
ENSMUSG	Gm37306	ENSMUST00000193447
ENSMUSG	Gm7871	ENSMUST00000195346
ENSMUSG	Gm38271	ENSMUST00000192296
ENSMUSG	Gm38268	ENSMUST00000195813
ENSMUSG	Gm18186	ENSMUST00000192239
ENSMUSG	Gm36969	ENSMUST00000194551
ENSMUSG	Gm36967	ENSMUST00000193814
ENSMUSG	Gm37903	ENSMUST00000192122
ENSMUSG	Gm38110	ENSMUST00000194594
ENSMUSG	Gm36307	ENSMUST00000195831
ENSMUSG	Gm38123	ENSMUST00000194990
ENSMUSG	Gm38122	ENSMUST00000192679
ENSMUSG	Gm37049	ENSMUST00000194356
ENSMUSG	Gm18752	ENSMUST00000192876;ENSMUST00000193627
ENSMUSG	Gm9867	ENSMUST00000192212
ENSMUSG	Gm37586	ENSMUST00000193654
ENSMUSG	Gm32200	ENSMUST00000192778
ENSMUSG	4930539	ENSMUST00000194792
ENSMUSG	Gm37583	ENSMUST00000195460
ENSMUSG	Gm37389	ENSMUST00000194885
ENSMUSG	Gm37980	ENSMUST00000194420
ENSMUSG	Gm37495	ENSMUST00000194590
ENSMUSG	Gm37743	ENSMUST00000194645
ENSMUSG	Gm37742	ENSMUST00000193263

ENSMUSG	Gm37744	ENSMUST00000194616
ENSMUSG	Gm7341	ENSMUST00000192183
ENSMUSG	AA91442	ENSMUST00000193194
ENSMUSG	Gm8790	ENSMUST00000194108
ENSMUSG	Gm8109	ENSMUST00000194076
ENSMUSG	Gm38294	ENSMUST00000192831
ENSMUSG	Gm31925	ENSMUST00000193561
ENSMUSG	Gm38148	ENSMUST00000195166
ENSMUSG	Gm10537	ENSMUST00000192625
ENSMUSG	Gm37170	ENSMUST00000194488
ENSMUSG	Gm37148	ENSMUST00000195097
ENSMUSG	Gm37168	ENSMUST00000192965
ENSMUSG	Gm37169	ENSMUST00000195599
ENSMUSG	Gm18427	ENSMUST00000195422
ENSMUSG	Gm37091	ENSMUST00000195455
ENSMUSG	Gm37087	ENSMUST00000192966
ENSMUSG	Gm37092	ENSMUST00000193381
ENSMUSG	Gm19066	ENSMUST00000193232
ENSMUSG	Gm37641	ENSMUST00000193837
ENSMUSG	Gm37329	ENSMUST00000192973
ENSMUSG	Gm38139	ENSMUST00000195806
ENSMUSG	Gm8515	ENSMUST00000194852
ENSMUSG	Gm5852	ENSMUST00000194119
ENSMUSG	Gm38388	ENSMUST00000195569
ENSMUSG	Gm37787	ENSMUST00000192524
ENSMUSG	Gm37730	ENSMUST00000192432
ENSMUSG	Gm2077	ENSMUST00000195017
ENSMUSG	Gm37732	ENSMUST00000194377
ENSMUSG	Gm37727	ENSMUST00000191637
ENSMUSG	Gm37158	ENSMUST00000191652
ENSMUSG	Gm18547	ENSMUST00000192439
ENSMUSG	Lce1d	ENSMUST00000029524
ENSMUSG	Gm38088	ENSMUST00000195829
ENSMUSG	Gm37518	ENSMUST00000194930
ENSMUSG	2610105	ENSMUST00000193707
ENSMUSG	Gm8146	ENSMUST00000192106
ENSMUSG	Gm36981	ENSMUST00000192340
ENSMUSG	Gm36979	ENSMUST00000193616
ENSMUSG	Gm37913	ENSMUST00000192925
ENSMUSG	Gm36983	ENSMUST00000192280
ENSMUSG	Gm37916	ENSMUST00000192153
ENSMUSG	Gm5850	ENSMUST00000192923
ENSMUSG	Gm31256	ENSMUST00000194705
ENSMUSG	Gm35048	ENSMUST00000192105
ENSMUSG	Gm38233	ENSMUST00000194259
ENSMUSG	Gm38231	ENSMUST00000192030
ENSMUSG	Gm6677	ENSMUST00000195100
ENSMUSG	4933403	ENSMUST00000193857
ENSMUSG	Gm37404	ENSMUST00000195726
ENSMUSG	Gm10531	ENSMUST00000193527
ENSMUSG	Gm42492	ENSMUST00000195780
ENSMUSG	Gm37995	ENSMUST00000194958
ENSMUSG	Gm37994	ENSMUST00000192124
ENSMUSG	Ighv1-1	ENSMUST00000194912
ENSMUSG	Gm10723	ENSMUST00000195366
ENSMUSG	C230057	ENSMUST00000194944
ENSMUSG	Gm37596	ENSMUST00000193565

ENSMUSG	Gm38083	ENSMUST00000194758
ENSMUSG	Gm2147	ENSMUST00000193588
ENSMUSG	Gm7804	ENSMUST00000192784;ENSMUST00000194187
ENSMUSG	Tdpoz2	ENSMUST00000191960
ENSMUSG	C630004	ENSMUST00000195673
ENSMUSG	Gm36927	ENSMUST00000192504
ENSMUSG	Gm37181	ENSMUST00000192513
ENSMUSG	Gm37180	ENSMUST00000195335
ENSMUSG	Gm37177	ENSMUST00000193768
ENSMUSG	Gm37754	ENSMUST00000194406
ENSMUSG	Gm37752	ENSMUST00000193494
ENSMUSG	1700030	ENSMUST00000192978
ENSMUSG	Gm37757	ENSMUST00000193072
ENSMUSG	Gm10152	ENSMUST00000193475
ENSMUSG	Gm29747	ENSMUST00000194764
ENSMUSG	Gm37929	ENSMUST00000195116
ENSMUSG	Gm37930	ENSMUST00000194308
ENSMUSG	Gm5541	ENSMUST00000193248
ENSMUSG	Gm37339	ENSMUST00000194142
ENSMUSG	Gm29808	ENSMUST00000194419
ENSMUSG	Gm5845	ENSMUST00000195812
ENSMUSG	Gm37535	ENSMUST00000194442
ENSMUSG	Gm2272	ENSMUST00000194903
ENSMUSG	Gm36994	ENSMUST00000192941
ENSMUSG	Gm36991	ENSMUST00000191800
ENSMUSG	Gm37131	ENSMUST00000192502
ENSMUSG	7530428	ENSMUST00000191725
ENSMUSG	Gm37133	ENSMUST00000192705
ENSMUSG	Gm33973	ENSMUST00000195148
ENSMUSG	Gm38098	ENSMUST00000194115
ENSMUSG	Gm37616	ENSMUST00000193974
ENSMUSG	Gm9910	ENSMUST00000195385
ENSMUSG	Gm37698	ENSMUST00000192605
ENSMUSG	Gm37999	ENSMUST00000195178
ENSMUSG	Gm10657	ENSMUST00000193528
ENSMUSG	Gm37412	ENSMUST00000194866
ENSMUSG	Gm37410	ENSMUST00000193623
ENSMUSG	Gm34780	ENSMUST00000193896
ENSMUSG	2210017	ENSMUST00000195278
ENSMUSG	Gm18900	ENSMUST00000193218
ENSMUSG	2610300	ENSMUST00000192930
ENSMUSG	Gm37835	ENSMUST00000193305
ENSMUSG	Gm37834	ENSMUST00000194517
ENSMUSG	1700012	ENSMUST00000193492
ENSMUSG	Gm37665	ENSMUST00000192353
ENSMUSG	Gm37670	ENSMUST00000193622
ENSMUSG	Gm17895	ENSMUST00000194028
ENSMUSG	Gm38221	ENSMUST00000195146
ENSMUSG	Gm35067	ENSMUST00000194355
ENSMUSG	Gm37114	ENSMUST00000195065
ENSMUSG	9330162	ENSMUST00000195397
ENSMUSG	Gm37113	ENSMUST00000195675
ENSMUSG	Gm38325	ENSMUST00000194507
ENSMUSG	Gm18445	ENSMUST00000195502
ENSMUSG	Gm38364	ENSMUST00000191814
ENSMUSG	Gm37355	ENSMUST00000193345
ENSMUSG	Gm19777	ENSMUST00000191940

ENSMUSG	Gm37356	ENSMUST00000193362
ENSMUSG	Gm37020	ENSMUST00000195303
ENSMUSG	Gm412	ENSMUST00000193360
ENSMUSG	Gm37021	ENSMUST00000195835
ENSMUSG	4930442	ENSMUST00000193163
ENSMUSG	Gm37022	ENSMUST00000192517
ENSMUSG	Gm37947	ENSMUST00000192740
ENSMUSG	Gm37948	ENSMUST00000192846
ENSMUSG	1700018.	ENSMUST00000195587
ENSMUSG	Gm37359	ENSMUST00000195243
ENSMUSG	Gm37357	ENSMUST00000192386
ENSMUSG	Gm5694	ENSMUST00000195648
ENSMUSG	Gm37242	ENSMUST00000191873
ENSMUSG	Gm38061	ENSMUST00000192490
ENSMUSG	Gm31406	ENSMUST00000192297
ENSMUSG	Gm19024	ENSMUST00000194681
ENSMUSG	Gm37769	ENSMUST00000193753
ENSMUSG	Gm37770	ENSMUST00000192855
ENSMUSG	Gm37707	ENSMUST00000195309
ENSMUSG	Gm37209	ENSMUST00000194109
ENSMUSG	Gm34294	ENSMUST00000191912
ENSMUSG	Ighv1-8	ENSMUST00000192236
ENSMUSG	Gm38180	ENSMUST00000192381
ENSMUSG	Gm37073	ENSMUST00000192469
ENSMUSG	4930503	ENSMUST00000193395
ENSMUSG	Gm37530	ENSMUST00000191759
ENSMUSG	A830029	ENSMUST00000193672
ENSMUSG	Gm17877	ENSMUST00000192578
ENSMUSG	Nras-ps.	ENSMUST00000195269
ENSMUSG	D030062	ENSMUST00000192660
ENSMUSG	Gm2136	ENSMUST00000193945
ENSMUSG	Gm19430	ENSMUST00000193847
ENSMUSG	Gm37095	ENSMUST00000195421
ENSMUSG	Gm7103	ENSMUST00000195642
ENSMUSG	Gm30074	ENSMUST00000193192
ENSMUSG	Gm37099	ENSMUST00000191902
ENSMUSG	Gm38315	ENSMUST00000194286
ENSMUSG	Gm6162	ENSMUST00000194870
ENSMUSG	Gm38317	ENSMUST00000195564
ENSMUSG	Gm37652	ENSMUST00000195290
ENSMUSG	Gm38238	ENSMUST00000194334
ENSMUSG	Gm38237	ENSMUST00000193366
ENSMUSG	Gm37283	ENSMUST00000193111
ENSMUSG	Gm37284	ENSMUST00000193607
ENSMUSG	Gm37805	ENSMUST00000192612
ENSMUSG	Gm8009	ENSMUST00000191672
ENSMUSG	Gm37859	ENSMUST00000195529
ENSMUSG	Gm37861	ENSMUST00000193534
ENSMUSG	Gm37857	ENSMUST00000191784
ENSMUSG	Gm37858	ENSMUST00000192759
ENSMUSG	Gm7343	ENSMUST00000194510
ENSMUSG	Gm36997	ENSMUST00000195090
ENSMUSG	Gm37523	ENSMUST00000194901
ENSMUSG	Gm35025	ENSMUST00000193114;ENSMUST00000192885
ENSMUSG	Gm37525	ENSMUST00000193499
ENSMUSG	Gm37527	ENSMUST00000192406
ENSMUSG	Ighv1-1	ENSMUST00000194435

ENSMUSG	4930590	ENSMUST00000193999
ENSMUSG	Trbv6	ENSMUST00000193743
ENSMUSG	Gm37061	ENSMUST00000195066
ENSMUSG	Gm38005	ENSMUST00000194845
ENSMUSG	Gm38004	ENSMUST00000192972
ENSMUSG	Gm38007	ENSMUST00000192187
ENSMUSG	Gm19194	ENSMUST00000192015
ENSMUSG	Gm38006	ENSMUST00000192421
ENSMUSG	6030460	ENSMUST00000191764
ENSMUSG	Gm7899	ENSMUST00000192935
ENSMUSG	Gm38177	ENSMUST00000192213
ENSMUSG	Gm37608	ENSMUST00000195696
ENSMUSG	Gm17934	ENSMUST00000192818
ENSMUSG	Gm38362	ENSMUST00000192999
ENSMUSG	Gm21358	ENSMUST00000191645
ENSMUSG	Gm8197	ENSMUST00000193422;ENSMUST00000192906
ENSMUSG	Gm37009	ENSMUST00000193297
ENSMUSG	Gm10748	ENSMUST00000194396;ENSMUST00000194070
ENSMUSG	Gm37452	ENSMUST00000194416
ENSMUSG	Gm37007	ENSMUST00000194234
ENSMUSG	Gm37128	ENSMUST00000192599
ENSMUSG	Gm30238	ENSMUST00000195528
ENSMUSG	Gm38074	ENSMUST00000193467
ENSMUSG	Gm38073	ENSMUST00000195141
ENSMUSG	Gm38072	ENSMUST00000192127
ENSMUSG	Gm5841	ENSMUST00000195307
ENSMUSG	Gm37896	ENSMUST00000195611
ENSMUSG	Gm9932	ENSMUST00000194154
ENSMUSG	Gm29718	ENSMUST00000193396
ENSMUSG	Gm37119	ENSMUST00000195691
ENSMUSG	Gm36388	ENSMUST00000191663
ENSMUSG	Gm8407	ENSMUST00000192719
ENSMUSG	Gm30173	ENSMUST00000195035
ENSMUSG	Gm37120	ENSMUST00000193350
ENSMUSG	Gm38214	ENSMUST00000192581
ENSMUSG	2610509	ENSMUST00000193035
ENSMUSG	Gm37679	ENSMUST00000192868
ENSMUSG	BC05530	ENSMUST00000193380
ENSMUSG	Gm37450	ENSMUST00000195218
ENSMUSG	Gm37449	ENSMUST00000193016
ENSMUSG	Gm38031	ENSMUST00000191819
ENSMUSG	Gm38029	ENSMUST00000192669
ENSMUSG	Gm38030	ENSMUST00000192376
ENSMUSG	5730488	ENSMUST00000192889
ENSMUSG	Gm38025	ENSMUST00000193926
ENSMUSG	Gm38335	ENSMUST00000195727
ENSMUSG	Gm38334	ENSMUST00000195060
ENSMUSG	Gm38337	ENSMUST00000193986
ENSMUSG	Gm10745	ENSMUST00000192022
ENSMUSG	Gm37363	ENSMUST00000192336
ENSMUSG	E330040	ENSMUST00000192299
ENSMUSG	Gm5851	ENSMUST00000194132
ENSMUSG	Gm37210	ENSMUST00000192484
ENSMUSG	Gm17771	ENSMUST00000194477
ENSMUSG	Gm37773	ENSMUST00000194141
ENSMUSG	Gm37776	ENSMUST00000193471
ENSMUSG	Gm37771	ENSMUST00000193121

ENSMUSG	Gm37562	ENSMUST00000194763
ENSMUSG	Gm6525	ENSMUST00000193882
ENSMUSG	Gm37567	ENSMUST00000193241
ENSMUSG	Gm38130	ENSMUST00000194311
ENSMUSG	Etv3l	ENSMUST00000193196
ENSMUSG	Gm9124	ENSMUST00000191789
ENSMUSG	Gm37199	ENSMUST00000193881
ENSMUSG	5430433	ENSMUST00000193978
ENSMUSG	Gm37026	ENSMUST00000191917
ENSMUSG	Gm37024	ENSMUST00000192224
ENSMUSG	Gm38279	ENSMUST00000194067
ENSMUSG	Gm7115	ENSMUST00000193706
ENSMUSG	1700039	ENSMUST00000195377
ENSMUSG	Gm38277	ENSMUST00000195172
ENSMUSG	Gm38275	ENSMUST00000193293
ENSMUSG	Gm37317	ENSMUST00000192198
ENSMUSG	AA61974	ENSMUST00000195095
ENSMUSG	Gm37877	ENSMUST00000191630
ENSMUSG	Gm30292	ENSMUST00000194198
ENSMUSG	Gm37293	ENSMUST00000191658
ENSMUSG	Gm36955	ENSMUST00000194316
ENSMUSG	Gm38103	ENSMUST00000191707
ENSMUSG	Gm38100	ENSMUST00000194391
ENSMUSG	Gm37619	ENSMUST00000193219
ENSMUSG	Gm38249	ENSMUST00000192560
ENSMUSG	Gm38247	ENSMUST00000194675
ENSMUSG	A030012	ENSMUST00000192385
ENSMUSG	Gm37701	ENSMUST00000192947
ENSMUSG	Gm9916	ENSMUST00000192943
ENSMUSG	1700054	ENSMUST00000193604
ENSMUSG	Gm37820	ENSMUST00000194021
ENSMUSG	Gm37821	ENSMUST00000195476
ENSMUSG	Gm37822	ENSMUST00000192618
ENSMUSG	Gm10472	ENSMUST00000193014;ENSMUST00000195796
ENSMUSG	Gm37253	ENSMUST00000195679
ENSMUSG	4930420	ENSMUST00000191831
ENSMUSG	Gm8101	ENSMUST00000192796
ENSMUSG	Gm37396	ENSMUST00000191827
ENSMUSG	Gm37400	ENSMUST00000193148
ENSMUSG	Gm37984	ENSMUST00000191641
ENSMUSG	Gm37985	ENSMUST00000194728
ENSMUSG	4930578	ENSMUST00000193416
ENSMUSG	Gm37693	ENSMUST00000194562
ENSMUSG	Gm37591	ENSMUST00000193283
ENSMUSG	4930533	ENSMUST00000194425
ENSMUSG	Gm37587	ENSMUST00000193450
ENSMUSG	Gm38151	ENSMUST00000195085
ENSMUSG	Gm18036	ENSMUST00000195256
ENSMUSG	9430062	ENSMUST00000192703
ENSMUSG	Gm8869	ENSMUST00000195222
ENSMUSG	Gm37866	ENSMUST00000191950
ENSMUSG	Gm38299	ENSMUST00000193214
ENSMUSG	Gm37461	ENSMUST00000193929
ENSMUSG	Gm37460	ENSMUST00000191796
ENSMUSG	Gm37459	ENSMUST00000192259;ENSMUST00000195007
ENSMUSG	Gm38046	ENSMUST00000192733
ENSMUSG	Gm38040	ENSMUST00000193505

ENSMUSG	Gm9924	ENSMUST00000191780
ENSMUSG	Gm36976	ENSMUST00000192632
ENSMUSG	Gm36974	ENSMUST00000194200
ENSMUSG	Gm36973	ENSMUST00000193942
ENSMUSG	Gm5846	ENSMUST00000193146
ENSMUSG	Gm4081	ENSMUST00000195287
ENSMUSG	Gm37912	ENSMUST00000191895
ENSMUSG	Gm37911	ENSMUST00000193099
ENSMUSG	Gm37907	ENSMUST00000195644
ENSMUSG	Gm37322	ENSMUST00000193409
ENSMUSG	Gm37323	ENSMUST00000195771
ENSMUSG	Gm37324	ENSMUST00000194500
ENSMUSG	Gm38285	ENSMUST00000192928
ENSMUSG	Gm38284	ENSMUST00000194803
ENSMUSG	Gm38282	ENSMUST00000192751
ENSMUSG	Gm34240	ENSMUST00000192500
ENSMUSG	Gm10522	ENSMUST00000194503
ENSMUSG	5730408	ENSMUST00000194955
ENSMUSG	Gm37125	ENSMUST00000195877
ENSMUSG	Gm7003	ENSMUST00000195089
ENSMUSG	Gm38080	ENSMUST00000194035
ENSMUSG	Gm37928	ENSMUST00000193265
ENSMUSG	Gm38081	ENSMUST00000192680
ENSMUSG	4933409	ENSMUST00000193674
ENSMUSG	Gm38079	ENSMUST00000195603
ENSMUSG	Gm37515	ENSMUST00000193321
ENSMUSG	4930402	ENSMUST00000192586
ENSMUSG	Gm37030	ENSMUST00000194254
ENSMUSG	Gm17967	ENSMUST00000195319
ENSMUSG	Gm37957	ENSMUST00000192182
ENSMUSG	Gm37964	ENSMUST00000192459
ENSMUSG	Gm20750	ENSMUST00000194274
ENSMUSG	Gm38011	ENSMUST00000193312
ENSMUSG	Gm37066	ENSMUST00000192713
ENSMUSG	Gm37068	ENSMUST00000195168
ENSMUSG	2900092	ENSMUST00000191770
ENSMUSG	E230020	ENSMUST00000194646
ENSMUSG	A030005	ENSMUST00000193762
ENSMUSG	Gm18299	ENSMUST00000192026
ENSMUSG	Gm37426	ENSMUST00000195241
ENSMUSG	Gm37427	ENSMUST00000192356
ENSMUSG	Gm19030	ENSMUST00000194947
ENSMUSG	Gm37831	ENSMUST00000191685
ENSMUSG	Gm37827	ENSMUST00000194486
ENSMUSG	Gm37824	ENSMUST00000193430
ENSMUSG	Gm37661	ENSMUST00000191863
ENSMUSG	BC00218	ENSMUST00000195674
ENSMUSG	Gm38207	ENSMUST00000193009
ENSMUSG	Gm38208	ENSMUST00000192476
ENSMUSG	Gm38066	ENSMUST00000193223
ENSMUSG	Gm31373	ENSMUST00000194403
ENSMUSG	Gm5837	ENSMUST00000191844
ENSMUSG	Gm37110	ENSMUST00000192993
ENSMUSG	Gm37737	ENSMUST00000195013
ENSMUSG	Gm33994	ENSMUST00000193724
ENSMUSG	Gm18843	ENSMUST00000194982
ENSMUSG	Gm37161	ENSMUST00000191994

ENSMUSG	Gm37336	ENSMUST00000192794;ENSMUST00000193705
ENSMUSG	Gm43314	ENSMUST00000196744
ENSMUSG	Gm43731	ENSMUST00000196150
ENSMUSG	Gm18660	ENSMUST00000199542
ENSMUSG	Gm42686	ENSMUST00000199172
ENSMUSG	E030032	ENSMUST00000198813
ENSMUSG	Gm43022	ENSMUST00000197067
ENSMUSG	Gm43142	ENSMUST00000198670
ENSMUSG	Gm43192	ENSMUST00000198002
ENSMUSG	Gm43041	ENSMUST00000200022
ENSMUSG	Gm43209	ENSMUST00000198949
ENSMUSG	Gm43255	ENSMUST00000198885
ENSMUSG	Gm6641	ENSMUST00000196100
ENSMUSG	4921539	ENSMUST00000197200;ENSMUST00000200072
ENSMUSG	Gm43666	ENSMUST00000197116
ENSMUSG	Gm42721	ENSMUST00000197165
ENSMUSG	Gm42779	ENSMUST00000198807
ENSMUSG	Gm567	ENSMUST00000197127
ENSMUSG	Fbxw27	ENSMUST00000199430;ENSMUST00000212674;ENSMUST00000212
ENSMUSG	Gm6069	ENSMUST00000196501
ENSMUSG	Gm43356	ENSMUST00000196582
ENSMUSG	Gm42541	ENSMUST00000199871
ENSMUSG	Gm6394	ENSMUST00000197077
ENSMUSG	Gm42448	ENSMUST00000197122
ENSMUSG	Gm42675	ENSMUST00000197552
ENSMUSG	Trgj3	ENSMUST00000103562
ENSMUSG	Gm42909	ENSMUST00000196982
ENSMUSG	Gm42461	ENSMUST00000198627
ENSMUSG	Gm9207	ENSMUST00000198773
ENSMUSG	Gm8862	ENSMUST00000198379
ENSMUSG	Gm2314	ENSMUST00000200391
ENSMUSG	Gm21168	ENSMUST00000199646
ENSMUSG	AL92856	ENSMUST00000197891
ENSMUSG	Gm43619	ENSMUST00000197267
ENSMUSG	Gm43601	ENSMUST00000196294
ENSMUSG	Gm3203	ENSMUST00000197670
ENSMUSG	Gm43366	ENSMUST00000199829
ENSMUSG	Gm4961	ENSMUST00000197982
ENSMUSG	Vmn2r-p	ENSMUST00000199364
ENSMUSG	Gm9369	ENSMUST00000196009
ENSMUSG	Gm43665	ENSMUST00000198672
ENSMUSG	Gm43376	ENSMUST00000199747;ENSMUST00000198939
ENSMUSG	Gm9415	ENSMUST00000200462
ENSMUSG	5430427	ENSMUST00000200007
ENSMUSG	Gm19202	ENSMUST00000198096
ENSMUSG	Gm42602	ENSMUST00000200288
ENSMUSG	4930405	ENSMUST00000199950
ENSMUSG	Gm42503	ENSMUST00000199080
ENSMUSG	4933437	ENSMUST00000197910
ENSMUSG	Gm2860	ENSMUST00000199226
ENSMUSG	Gm42500	ENSMUST00000199898
ENSMUSG	Gm43249	ENSMUST00000200464
ENSMUSG	Gm33609	ENSMUST00000198559
ENSMUSG	Gm3283	ENSMUST00000199319
ENSMUSG	Mir292b	ENSMUST00000196199
ENSMUSG	n-TStga	ENSMUST00000199903
ENSMUSG	Gm5565	ENSMUST00000199463

ENSMUSG	Gm43116	ENSMUST00000199789
ENSMUSG	Mir691	ENSMUST00000199719
ENSMUSG	Igkv3-8	ENSMUST00000197635
ENSMUSG	Gm43066	ENSMUST00000199589
ENSMUSG	Igkv8-3	ENSMUST00000196959
ENSMUSG	Gm8920	ENSMUST00000196153
ENSMUSG	Gm43691	ENSMUST00000196625
ENSMUSG	Gm4865	ENSMUST00000196284
ENSMUSG	Gm43559	ENSMUST00000198755
ENSMUSG	Gm19843	ENSMUST00000198144
ENSMUSG	Gm43303	ENSMUST00000200133
ENSMUSG	Gm43326	ENSMUST00000198459
ENSMUSG	Gm43573	ENSMUST00000196445
ENSMUSG	Gm36448	ENSMUST00000199916
ENSMUSG	Gm43756	ENSMUST00000199788
ENSMUSG	Gm44354	ENSMUST00000196017
ENSMUSG	Mir1897	ENSMUST00000198006
ENSMUSG	Gm20121	ENSMUST00000198547
ENSMUSG	Gm36017	ENSMUST00000199005
ENSMUSG	Gm43834	ENSMUST00000197097
ENSMUSG	Gm42619	ENSMUST00000197564
ENSMUSG	Gm5684	ENSMUST00000198419
ENSMUSG	Gm43473	ENSMUST00000200442
ENSMUSG	4930515	ENSMUST00000199440
ENSMUSG	Gm21663	ENSMUST00000196214
ENSMUSG	Gm24826	ENSMUST00000197298
ENSMUSG	Gm43685	ENSMUST00000198876
ENSMUSG	B020017	ENSMUST00000197444
ENSMUSG	Gm42489	ENSMUST00000198964
ENSMUSG	Gm5547	ENSMUST00000200182;ENSMUST00000198306;ENSMUST0000019
ENSMUSG	Gm43710	ENSMUST00000199583
ENSMUSG	Mir7230	ENSMUST00000195977
ENSMUSG	Gm43020	ENSMUST00000197178
ENSMUSG	Gm43177	ENSMUST00000197032
ENSMUSG	E030026	ENSMUST00000200395
ENSMUSG	Gm42510	ENSMUST00000196470
ENSMUSG	3110039	ENSMUST00000127041;ENSMUST00000145291
ENSMUSG	Gm43655	ENSMUST00000197367
ENSMUSG	Gm42708	ENSMUST00000196332
ENSMUSG	Gm43728	ENSMUST00000198817
ENSMUSG	Gm9954	ENSMUST00000198686
ENSMUSG	Gm42544	ENSMUST00000197868
ENSMUSG	Gm43397	ENSMUST00000196422
ENSMUSG	Gm43096	ENSMUST00000196173
ENSMUSG	Gm43000	ENSMUST00000198482
ENSMUSG	Gm43158	ENSMUST00000198484
ENSMUSG	1500005	ENSMUST00000199867
ENSMUSG	Gm43507	ENSMUST00000196411;ENSMUST00000200281
ENSMUSG	Gm43855	ENSMUST00000197462
ENSMUSG	Gm42692	ENSMUST00000199482
ENSMUSG	9330198	ENSMUST00000196846
ENSMUSG	Gm43718	ENSMUST00000197524
ENSMUSG	Gm43023	ENSMUST00000197583
ENSMUSG	Gm42944	ENSMUST00000198383
ENSMUSG	Gm42617	ENSMUST00000197640
ENSMUSG	Gm23927	ENSMUST00000199928
ENSMUSG	Gm5865	ENSMUST00000198713

ENSMUSG	Gm42565	ENSMUST00000197839
ENSMUSG	Gm43243	ENSMUST00000199964
ENSMUSG	Gm43414	ENSMUST00000197710
ENSMUSG	Gm42840	ENSMUST00000200303
ENSMUSG	Gm33651	ENSMUST00000200405
ENSMUSG	Gm8953	ENSMUST00000198497
ENSMUSG	Gm43405	ENSMUST00000198642
ENSMUSG	Gm44376	ENSMUST00000197570
ENSMUSG	Gm42694	ENSMUST00000196951
ENSMUSG	1700016	ENSMUST00000198159
ENSMUSG	4930429	ENSMUST00000198193
ENSMUSG	Gm18131	ENSMUST00000199241
ENSMUSG	Gm43835	ENSMUST00000195932
ENSMUSG	Gm42974	ENSMUST00000199556
ENSMUSG	9430087	ENSMUST00000196548
ENSMUSG	Gm43686	ENSMUST00000197351
ENSMUSG	Gm31274	ENSMUST00000197356
ENSMUSG	Gm43736	ENSMUST00000196377
ENSMUSG	Gm32554	ENSMUST00000196310
ENSMUSG	Gm43622	ENSMUST00000200140
ENSMUSG	Gm43504	ENSMUST00000196056
ENSMUSG	Gm43510	ENSMUST00000197922
ENSMUSG	Gm43822	ENSMUST00000200285
ENSMUSG	Gm43616	ENSMUST00000199347
ENSMUSG	Gm40055	ENSMUST00000196091
ENSMUSG	Gm9484	ENSMUST00000195892
ENSMUSG	Gm43652	ENSMUST00000196219
ENSMUSG	Gm43210	ENSMUST00000196743
ENSMUSG	Gm43546	ENSMUST00000196721
ENSMUSG	Gm7205	ENSMUST00000196032
ENSMUSG	Gm8234	ENSMUST00000198523
ENSMUSG	Gm43537	ENSMUST00000199064
ENSMUSG	Gm18747	ENSMUST00000198129
ENSMUSG	Gm43117	ENSMUST00000198531
ENSMUSG	Gm9682	ENSMUST00000197505
ENSMUSG	Gm42676	ENSMUST00000197609
ENSMUSG	Igkv1-1	ENSMUST00000198926
ENSMUSG	Rpl9-ps	ENSMUST00000198343
ENSMUSG	Gm42533	ENSMUST00000196247
ENSMUSG	Gm42966	ENSMUST00000197883
ENSMUSG	Gm42805	ENSMUST00000196158
ENSMUSG	Gm42498	ENSMUST00000199981
ENSMUSG	Gm43113	ENSMUST00000199713
ENSMUSG	Gm42513	ENSMUST00000197831
ENSMUSG	Gm43818	ENSMUST00000200605
ENSMUSG	Gm4859	ENSMUST00000199625
ENSMUSG	Gm42552	ENSMUST00000196754
ENSMUSG	Gm35507	ENSMUST00000197932
ENSMUSG	Gm43657	ENSMUST00000200336
ENSMUSG	Gm43310	ENSMUST00000198975
ENSMUSG	Amd-ps5	ENSMUST00000198406
ENSMUSG	Gm43765	ENSMUST00000196303
ENSMUSG	Gm19566	ENSMUST00000199576
ENSMUSG	AC06930	ENSMUST00000197243
ENSMUSG	8030487	ENSMUST00000200244
ENSMUSG	Gm19391	ENSMUST00000196653;ENSMUST00000198512
ENSMUSG	Mir7214	ENSMUST00000198396

ENSMUSG	Gm35409	ENSMUST00000196412
ENSMUSG	Gm42910	ENSMUST00000198917
ENSMUSG	Gm42862	ENSMUST00000197494
ENSMUSG	Gm42988	ENSMUST00000198116
ENSMUSG	Gm30735	ENSMUST00000199488
ENSMUSG	Gm19166	ENSMUST00000196041
ENSMUSG	Gm5288	ENSMUST00000198323
ENSMUSG	Gm43353	ENSMUST00000199224
ENSMUSG	Gm43097	ENSMUST00000196867
ENSMUSG	Gm19620	ENSMUST00000195972
ENSMUSG	Gm42564	ENSMUST00000196013
ENSMUSG	Gm43778	ENSMUST00000199438
ENSMUSG	Gm7774	ENSMUST00000200064
ENSMUSG	C230031	ENSMUST00000197856
ENSMUSG	Gm4870	ENSMUST00000198525
ENSMUSG	Gm42927	ENSMUST00000200624
ENSMUSG	Gm43433	ENSMUST00000196573
ENSMUSG	Gm3364	ENSMUST00000196016
ENSMUSG	Gm20568	ENSMUST00000197129
ENSMUSG	Gm43444	ENSMUST00000198123
ENSMUSG	Gm42519	ENSMUST00000199282
ENSMUSG	Gm42753	ENSMUST00000196718
ENSMUSG	Gm43144	ENSMUST00000198256
ENSMUSG	Gm40038	ENSMUST00000197878
ENSMUSG	Gm40040	ENSMUST00000196337;ENSMUST00000196123
ENSMUSG	Gm43333	ENSMUST00000198340
ENSMUSG	Gm42716	ENSMUST00000196003;ENSMUST00000197140;ENSMUST0000019
ENSMUSG	Sox2ot	ENSMUST00000198032;ENSMUST00000196058;ENSMUST0000019
ENSMUSG	Gm42863	ENSMUST00000197875
ENSMUSG	Gm42875	ENSMUST00000197674
ENSMUSG	Gm9504	ENSMUST00000196322
ENSMUSG	Gm42630	ENSMUST00000195908
ENSMUSG	Gm17835	ENSMUST00000196870
ENSMUSG	Gm42981	ENSMUST00000200184
ENSMUSG	Gm33370	ENSMUST00000195935
ENSMUSG	Gm42693	ENSMUST00000196779
ENSMUSG	Gm43577	ENSMUST00000197090
ENSMUSG	Gm42843	ENSMUST00000196328
ENSMUSG	Gm19708	ENSMUST00000199386
ENSMUSG	1700052	ENSMUST00000196142
ENSMUSG	Cldn25-	ENSMUST00000197641
ENSMUSG	Gm30613	ENSMUST00000197277
ENSMUSG	Gm19817	ENSMUST00000199764
ENSMUSG	Gm42953	ENSMUST00000199363
ENSMUSG	Gbp2-ps	ENSMUST00000198870
ENSMUSG	Gm43751	ENSMUST00000200275
ENSMUSG	Gm42705	ENSMUST00000200176
ENSMUSG	Gm9409	ENSMUST00000197017
ENSMUSG	Mir7215	ENSMUST00000200383
ENSMUSG	Gm42680	ENSMUST00000196238
ENSMUSG	Gm42423	ENSMUST00000196396
ENSMUSG	AU02213	ENSMUST00000200601
ENSMUSG	1700094	ENSMUST00000198047;ENSMUST00000197695
ENSMUSG	Gm43244	ENSMUST00000196994
ENSMUSG	Gm43503	ENSMUST00000200033
ENSMUSG	Gm43157	ENSMUST00000198223
ENSMUSG	C030018	ENSMUST00000196829

ENSMUSG	Gm42428	ENSMUST00000198677
ENSMUSG	D130017	ENSMUST00000199836
ENSMUSG	Gm42603	ENSMUST00000199893
ENSMUSG	Gm43719	ENSMUST00000200088
ENSMUSG	Gm42718	ENSMUST00000197319
ENSMUSG	Mir327	ENSMUST00000199328
ENSMUSG	Gm36535	ENSMUST00000198395;ENSMUST00000200641
ENSMUSG	Gm43148	ENSMUST00000198237
ENSMUSG	Gm43396	ENSMUST00000197506
ENSMUSG	Gm29681	ENSMUST00000198696
ENSMUSG	BB18769	ENSMUST00000198619
ENSMUSG	Gm42684	ENSMUST00000197663
ENSMUSG	Gm6057	ENSMUST00000195927
ENSMUSG	Gm43471	ENSMUST00000197550
ENSMUSG	Gm22897	ENSMUST00000197860
ENSMUSG	Gm42949	ENSMUST00000197054
ENSMUSG	Gm43618	ENSMUST00000197392
ENSMUSG	Gm43247	ENSMUST00000198638
ENSMUSG	Gm43205	ENSMUST00000198622
ENSMUSG	Gm43218	ENSMUST00000196275
ENSMUSG	Gm42450	ENSMUST00000199286
ENSMUSG	Gm31693	ENSMUST00000200150
ENSMUSG	Gm8041	ENSMUST00000199755
ENSMUSG	Gm42614	ENSMUST00000198724
ENSMUSG	Gm43837	ENSMUST00000200306
ENSMUSG	Gm10727	ENSMUST00000197107
ENSMUSG	Gm42972	ENSMUST00000196937
ENSMUSG	Gm22813	ENSMUST00000199948
ENSMUSG	Gm5276	ENSMUST00000196683
ENSMUSG	Gm43745	ENSMUST00000198085
ENSMUSG	Gm4742	ENSMUST00000200261
ENSMUSG	A930036	ENSMUST00000196018
ENSMUSG	Gm35439	ENSMUST00000200051
ENSMUSG	Gm43285	ENSMUST00000196563
ENSMUSG	6430500	ENSMUST00000198286
ENSMUSG	Gm42775	ENSMUST00000198407
ENSMUSG	Gm42447	ENSMUST00000200102
ENSMUSG	Gm2505	ENSMUST00000200413
ENSMUSG	Gm44310	ENSMUST00000175424
ENSMUSG	Gm42677	ENSMUST00000196363
ENSMUSG	5330426	ENSMUST00000200008
ENSMUSG	Gm43743	ENSMUST00000197898;ENSMUST00000198353
ENSMUSG	Gm33758	ENSMUST00000199230
ENSMUSG	Gm36823	ENSMUST00000199408;ENSMUST00000196546
ENSMUSG	Gm42837	ENSMUST00000197071
ENSMUSG	Gm43490	ENSMUST00000197593
ENSMUSG	Gm43519	ENSMUST00000197239
ENSMUSG	Gm9480	ENSMUST00000198038
ENSMUSG	Gm5279	ENSMUST00000199575
ENSMUSG	Gm7988	ENSMUST00000196623
ENSMUSG	Gm34248	ENSMUST00000200027
ENSMUSG	Gm43540	ENSMUST00000196320
ENSMUSG	Gm35585	ENSMUST00000195996
ENSMUSG	Mir3966	ENSMUST00000198473
ENSMUSG	Gm42744	ENSMUST00000196517
ENSMUSG	Gm8891	ENSMUST00000198546
ENSMUSG	Gm42971	ENSMUST00000199228

ENSMUSG	Gm43662	ENSMUST00000198310
ENSMUSG	Gm34599	ENSMUST00000198924
ENSMUSG	Gm43663	ENSMUST00000198643
ENSMUSG	Gm43251	ENSMUST00000198339
ENSMUSG	Gm10636	ENSMUST00000197639
ENSMUSG	Ighv1-8	ENSMUST00000200562
ENSMUSG	9530034	ENSMUST00000197919
ENSMUSG	Gm42615	ENSMUST00000197704
ENSMUSG	Gm43548	ENSMUST00000197139
ENSMUSG	Gm32158	ENSMUST00000200135
ENSMUSG	Gm43367	ENSMUST00000199986
ENSMUSG	Gm44469	ENSMUST00000198558
ENSMUSG	Gm42516	ENSMUST00000198691
ENSMUSG	Gm5853	ENSMUST00000197183
ENSMUSG	Gm43134	ENSMUST00000199434
ENSMUSG	Gm19739	ENSMUST00000195941
ENSMUSG	Gm43505	ENSMUST00000200579
ENSMUSG	Gm43494	ENSMUST00000197672
ENSMUSG	1700017	ENSMUST00000198307
ENSMUSG	4930519	ENSMUST00000200254;ENSMUST00000197683
ENSMUSG	Gm42975	ENSMUST00000200612
ENSMUSG	Gm6639	ENSMUST00000198751;ENSMUST00000196841
ENSMUSG	Gm43606	ENSMUST00000195938
ENSMUSG	Gm18341	ENSMUST00000197538
ENSMUSG	Gm43428	ENSMUST00000200108
ENSMUSG	Gm6157	ENSMUST00000199935
ENSMUSG	Gm43120	ENSMUST00000196228
ENSMUSG	Gm43327	ENSMUST00000199285
ENSMUSG	Gm19211	ENSMUST00000200251
ENSMUSG	Gm31881	ENSMUST00000198961
ENSMUSG	Gm43703	ENSMUST00000200523
ENSMUSG	Gm42587	ENSMUST00000198462
ENSMUSG	Gm43305	ENSMUST00000199958;ENSMUST00000225083
ENSMUSG	AC17478	ENSMUST00000199415
ENSMUSG	Gm43827	ENSMUST00000200623
ENSMUSG	Gm18187	ENSMUST00000196476
ENSMUSG	Gm8828	ENSMUST00000196786
ENSMUSG	5330425	ENSMUST00000196714
ENSMUSG	Gm43838	ENSMUST00000196357
ENSMUSG	Gm42973	ENSMUST00000197913
ENSMUSG	4933415	ENSMUST00000195895
ENSMUSG	Gm42685	ENSMUST00000200378
ENSMUSG	Gm43141	ENSMUST00000199695
ENSMUSG	Gm43595	ENSMUST00000196464
ENSMUSG	Gm43130	ENSMUST00000197721
ENSMUSG	Gm43211	ENSMUST00000196901
ENSMUSG	Gm43605	ENSMUST00000199663
ENSMUSG	Gm43607	ENSMUST00000199524
ENSMUSG	Gm42496	ENSMUST00000197976
ENSMUSG	Gm10290	ENSMUST00000200451
ENSMUSG	Igkv8-3	ENSMUST00000200040
ENSMUSG	Gm3970	ENSMUST00000198001
ENSMUSG	Gm5149	ENSMUST00000199980
ENSMUSG	Gm43345	ENSMUST00000199484
ENSMUSG	Gm42707	ENSMUST00000196668;ENSMUST00000196620
ENSMUSG	Mir703	ENSMUST00000197105
ENSMUSG	D030025	ENSMUST00000197413

ENSMUSG	Igkv15-	ENSMUST00000197637
ENSMUSG	Gm42969	ENSMUST00000199858
ENSMUSG	Gm44456	ENSMUST00000196554
ENSMUSG	Mir409	ENSMUST00000198153
ENSMUSG	Gm2861	ENSMUST00000199885
ENSMUSG	Gm8925	ENSMUST00000197212
ENSMUSG	Gm42817	ENSMUST00000198962
ENSMUSG	Gm19185	ENSMUST00000196365
ENSMUSG	Gm43508	ENSMUST00000197251
ENSMUSG	Gm10484	ENSMUST00000196372
ENSMUSG	Gm34144	ENSMUST00000197039
ENSMUSG	Gm42517	ENSMUST00000197216
ENSMUSG	Gm43147	ENSMUST00000196944
ENSMUSG	Gm43572	ENSMUST00000196494
ENSMUSG	Gm22813	ENSMUST00000200346
ENSMUSG	BC03715	ENSMUST00000196993
ENSMUSG	A230001	ENSMUST00000196720;ENSMUST00000198712
ENSMUSG	Gm35013	ENSMUST00000197218
ENSMUSG	Gm7709	ENSMUST00000199391
ENSMUSG	Gm10440	ENSMUST00000197424
ENSMUSG	Gm43612	ENSMUST00000196087
ENSMUSG	Gm5408	ENSMUST00000197998
ENSMUSG	A830019	ENSMUST00000198056
ENSMUSG	Gm43386	ENSMUST00000199654
ENSMUSG	Gm9256	ENSMUST00000198106
ENSMUSG	Gm43575	ENSMUST00000196420
ENSMUSG	Gm43322	ENSMUST00000196232
ENSMUSG	Gm43175	ENSMUST00000198450
ENSMUSG	Gm2965	ENSMUST00000198365
ENSMUSG	Gm5075	ENSMUST00000196916
ENSMUSG	Gm40153	ENSMUST00000197904
ENSMUSG	Gm42793	ENSMUST00000199139
ENSMUSG	Igkv12-	ENSMUST00000199208
ENSMUSG	D530037	ENSMUST00000199255
ENSMUSG	Gm40123	ENSMUST00000198175
ENSMUSG	Gm42609	ENSMUST00000196362;ENSMUST00000196877
ENSMUSG	Gm43555	ENSMUST00000196588
ENSMUSG	Tex13c2	ENSMUST00000197237
ENSMUSG	Gm43427	ENSMUST00000198188
ENSMUSG	Gm44324	ENSMUST00000198517
ENSMUSG	Gm16508	ENSMUST00000197066
ENSMUSG	Gm42920	ENSMUST00000196616
ENSMUSG	Gm42616	ENSMUST00000198989
ENSMUSG	Gm43833	ENSMUST00000198828
ENSMUSG	Gm42980	ENSMUST00000200582
ENSMUSG	Igkv4-5	ENSMUST00000198436
ENSMUSG	Gm44316	ENSMUST00000199274
ENSMUSG	Gm8848	ENSMUST00000197270
ENSMUSG	Gm43608	ENSMUST00000197703
ENSMUSG	Cyp3a63	ENSMUST00000196626
ENSMUSG	Gm42860	ENSMUST00000196492
ENSMUSG	Gm42444	ENSMUST00000199366
ENSMUSG	Ass-ps1	ENSMUST00000197717
ENSMUSG	Gm43623	ENSMUST00000199289
ENSMUSG	Gm9364	ENSMUST00000199220
ENSMUSG	Gm42488	ENSMUST00000197648
ENSMUSG	Igkv2-1	ENSMUST00000196931

ENSMUSG	Gm7902	ENSMUST00000198842
ENSMUSG	Gm9497	ENSMUST00000199908
ENSMUSG	Gm43135	ENSMUST00000196442
ENSMUSG	Gm42892	ENSMUST00000196227
ENSMUSG	Gm43033	ENSMUST00000199682
ENSMUSG	Gm8836	ENSMUST00000198196
ENSMUSG	Gm9419	ENSMUST00000200672
ENSMUSG	Gm43704	ENSMUST00000196164
ENSMUSG	Gm42722	ENSMUST00000198855
ENSMUSG	Gm18916	ENSMUST00000198264
ENSMUSG	Gm43438	ENSMUST00000198133
ENSMUSG	Gm43081	ENSMUST00000199082
ENSMUSG	Gm42660	ENSMUST00000197733
ENSMUSG	Gm43190	ENSMUST00000187850
ENSMUSG	Gm43286	ENSMUST00000197420
ENSMUSG	Gm18830	ENSMUST00000199036
ENSMUSG	Gm5284	ENSMUST00000199053
ENSMUSG	Gm42697	ENSMUST00000198992
ENSMUSG	Gm42679	ENSMUST00000197086
ENSMUSG	Gm42638	ENSMUST00000196065
ENSMUSG	Gm43520	ENSMUST00000197996
ENSMUSG	Gm19210	ENSMUST00000199562
ENSMUSG	Gm36382	ENSMUST00000198892
ENSMUSG	Gm42493	ENSMUST00000197559
ENSMUSG	Gm30648	ENSMUST00000197430;ENSMUST00000200648
ENSMUSG	Gm42208	ENSMUST00000198215
ENSMUSG	Gm18330	ENSMUST00000199246
ENSMUSG	Gm43972	ENSMUST00000181398
ENSMUSG	Gm43045	ENSMUST00000198725
ENSMUSG	Gm43495	ENSMUST00000200060
ENSMUSG	4930555	ENSMUST00000196524;ENSMUST00000196052;ENSMUST00000197
ENSMUSG	Gm26047	ENSMUST00000198840
ENSMUSG	Gm43538	ENSMUST00000200416
ENSMUSG	Gm42764	ENSMUST00000197835
ENSMUSG	Gm43730	ENSMUST00000199863
ENSMUSG	Gm43491	ENSMUST00000197133
ENSMUSG	Gm42987	ENSMUST00000197115
ENSMUSG	Gm18893	ENSMUST00000198416
ENSMUSG	Igkv15-	ENSMUST00000196661
ENSMUSG	Gm9710	ENSMUST00000198053
ENSMUSG	Gm42933	ENSMUST00000196547
ENSMUSG	Gm20768	ENSMUST00000199527
ENSMUSG	Gm42818	ENSMUST00000198869
ENSMUSG	Gm44340	ENSMUST00000197487
ENSMUSG	Gm7815	ENSMUST00000196104
ENSMUSG	Gm43727	ENSMUST00000198021
ENSMUSG	Gm21009	ENSMUST00000197169
ENSMUSG	1700001	ENSMUST00000199380
ENSMUSG	Gm42842	ENSMUST00000197736
ENSMUSG	Gm43112	ENSMUST00000196665
ENSMUSG	Gm43368	ENSMUST00000200638
ENSMUSG	5830416	ENSMUST00000198687
ENSMUSG	2400006	ENSMUST00000197509
ENSMUSG	Gm43564	ENSMUST00000196185
ENSMUSG	Gm18611	ENSMUST00000200334
ENSMUSG	Gm43236	ENSMUST00000197414
ENSMUSG	Gm9260	ENSMUST00000199781

ENSMUSG	Gm18867	ENSMUST00000198022
ENSMUSG	Gm36793	ENSMUST00000199211
ENSMUSG	Igkv12-	ENSMUST00000196961
ENSMUSG	Rpl7a-p	ENSMUST00000198361
ENSMUSG	Gm43752	ENSMUST00000198475
ENSMUSG	Gm22897	ENSMUST00000197782
ENSMUSG	Gm43767	ENSMUST00000198933
ENSMUSG	Gm42495	ENSMUST00000200324
ENSMUSG	Gm43429	ENSMUST00000197889
ENSMUSG	Gm43174	ENSMUST00000195914
ENSMUSG	Gm42932	ENSMUST00000197280
ENSMUSG	4632404	ENSMUST00000197326
ENSMUSG	Gm42765	ENSMUST00000200017
ENSMUSG	Gm42518	ENSMUST00000196277
ENSMUSG	Gm42911	ENSMUST00000199360
ENSMUSG	4933401	ENSMUST00000200529;ENSMUST00000199058
ENSMUSG	Gm40414	ENSMUST00000197608
ENSMUSG	Gm43523	ENSMUST00000197397
ENSMUSG	Gm43755	ENSMUST00000200001
ENSMUSG	Gm9402	ENSMUST00000196849
ENSMUSG	Gm42720	ENSMUST00000196813
ENSMUSG	Gm43467	ENSMUST00000197441
ENSMUSG	Igkv2-9	ENSMUST00000196774
ENSMUSG	Gm42601	ENSMUST00000197025
ENSMUSG	Gm43674	ENSMUST00000196313
ENSMUSG	Mir7228	ENSMUST00000196812
ENSMUSG	Gm43222	ENSMUST00000199013
ENSMUSG	Gm10652	ENSMUST00000199790
ENSMUSG	Gm5309	ENSMUST00000197416
ENSMUSG	Gm43506	ENSMUST00000200675
ENSMUSG	Gm43645	ENSMUST00000200129
ENSMUSG	Gm43489	ENSMUST00000196810
ENSMUSG	Gm18430	ENSMUST00000197534
ENSMUSG	Gm4869	ENSMUST00000197549
ENSMUSG	5033403	ENSMUST00000198248
ENSMUSG	Gm43726	ENSMUST00000199110
ENSMUSG	Gm42607	ENSMUST00000200221
ENSMUSG	n-TSaga	ENSMUST00000197675
ENSMUSG	Gm8013	ENSMUST00000200139
ENSMUSG	Gm35066	ENSMUST00000198898
ENSMUSG	Gm43568	ENSMUST00000199116;ENSMUST00000198040;ENSMUST0000019[illegible]
ENSMUSG	Gm43355	ENSMUST00000196541
ENSMUSG	Gm44300	ENSMUST00000196792
ENSMUSG	Gm3519	ENSMUST00000196113
ENSMUSG	Gm5551	ENSMUST00000199720
ENSMUSG	Gm5870	ENSMUST00000196461
ENSMUSG	Gm43357	ENSMUST00000197132
ENSMUSG	Igkv20-	ENSMUST00000200406
ENSMUSG	Gm29755	ENSMUST00000196976
ENSMUSG	Gm42445	ENSMUST00000196996
ENSMUSG	Gm5857	ENSMUST00000198953
ENSMUSG	Gm5550	ENSMUST00000198658
ENSMUSG	Gm36211	ENSMUST00000198171
ENSMUSG	Gm3103	ENSMUST00000198073
ENSMUSG	Gm42921	ENSMUST00000196670
ENSMUSG	Gm42970	ENSMUST00000196407
ENSMUSG	Gm5711	ENSMUST00000197896

ENSMUSG	Gm9678	ENSMUST00000198979
ENSMUSG	Gm43245	ENSMUST00000197603
ENSMUSG	Gm20755	ENSMUST00000196893
ENSMUSG	Gm43725	ENSMUST00000200122
ENSMUSG	Gm40348	ENSMUST00000199648
ENSMUSG	Gm43111	ENSMUST00000197445
ENSMUSG	Gm36551	ENSMUST00000196787
ENSMUSG	Gm32736	ENSMUST00000200427
ENSMUSG	3830422	ENSMUST00000200199
ENSMUSG	Gm9771	ENSMUST00000198267
ENSMUSG	Gm42446	ENSMUST00000199783
ENSMUSG	Gm29707	ENSMUST00000198744;ENSMUST00000196325
ENSMUSG	Igkv2-1	ENSMUST00000196000
ENSMUSG	Gm42755	ENSMUST00000198533
ENSMUSG	Gm7733	ENSMUST00000199038
ENSMUSG	Gm43032	ENSMUST00000197967
ENSMUSG	Gm42540	ENSMUST00000200319
ENSMUSG	Gm43529	ENSMUST00000199178
ENSMUSG	Gm43056	ENSMUST00000197865
ENSMUSG	Gm43521	ENSMUST00000197838
ENSMUSG	Gm43078	ENSMUST00000197222
ENSMUSG	Gm30382	ENSMUST00000199007;ENSMUST00000199503
ENSMUSG	Gm38509	ENSMUST00000197492
ENSMUSG	Gm8706	ENSMUST00000197372
ENSMUSG	Gm44343	ENSMUST00000196702
ENSMUSG	Gm42706	ENSMUST00000199733
ENSMUSG	Gm42841	ENSMUST00000199806
ENSMUSG	Gm43233	ENSMUST00000200491
ENSMUSG	Gm30301	ENSMUST00000199474
ENSMUSG	B230303	ENSMUST00000199119
ENSMUSG	Gm42653	ENSMUST00000200644
ENSMUSG	Gm1969	ENSMUST00000198125
ENSMUSG	Gm33474	ENSMUST00000199486
ENSMUSG	Gm43680	ENSMUST00000199466
ENSMUSG	Gm43119	ENSMUST00000196957
ENSMUSG	Gm5981	ENSMUST00000199912
ENSMUSG	Gm8969	ENSMUST00000199694
ENSMUSG	Gm43836	ENSMUST00000196532
ENSMUSG	Gm42814	ENSMUST00000199370
ENSMUSG	4933417	ENSMUST00000199732;ENSMUST00000199681
ENSMUSG	Gm42613	ENSMUST00000199975
ENSMUSG	Mir7217	ENSMUST00000200304
ENSMUSG	4930502	ENSMUST00000200282
ENSMUSG	Gm43509	ENSMUST00000196551
ENSMUSG	Gm44365	ENSMUST00000197671
ENSMUSG	Gm43750	ENSMUST00000199027
ENSMUSG	Gm9353	ENSMUST00000200472
ENSMUSG	Gm42752	ENSMUST00000198394
ENSMUSG	Gm8853	ENSMUST00000198366
ENSMUSG	Gm21680	ENSMUST00000200447
ENSMUSG	Gm43558	ENSMUST00000200155
ENSMUSG	Gm43431	ENSMUST00000198577
ENSMUSG	Gm43121	ENSMUST00000199065
ENSMUSG	Gm42523	ENSMUST00000199378
ENSMUSG	Gm42608	ENSMUST00000197563
ENSMUSG	Mir299a	ENSMUST00000198411
ENSMUSG	Gm43587	ENSMUST00000199590

ENSMUSG	Gm43374	ENSMUST00000202498
ENSMUSG	Gm20005	ENSMUST00000202583
ENSMUSG	Gm43798	ENSMUST00000202056
ENSMUSG	Ugt2a1	ENSMUST00000147854
ENSMUSG	Gm43852	ENSMUST00000202098
ENSMUSG	Gm43263	ENSMUST00000201619
ENSMUSG	Gm18345	ENSMUST00000200710
ENSMUSG	Gm42894	ENSMUST00000200788
ENSMUSG	Vmn2r-p	ENSMUST00000202039
ENSMUSG	Gm42799	ENSMUST00000201331
ENSMUSG	Gm30270	ENSMUST00000202675
ENSMUSG	Gm19649	ENSMUST00000201126
ENSMUSG	Gm42546	ENSMUST00000201584
ENSMUSG	Gm43363	ENSMUST00000201590
ENSMUSG	Gm36186	ENSMUST00000200820
ENSMUSG	A330058	ENSMUST00000200757
ENSMUSG	Gm42573	ENSMUST00000202327
ENSMUSG	Gm5	ENSMUST00000202386;ENSMUST00000202066
ENSMUSG	4930513	ENSMUST00000202800;ENSMUST00000202742
ENSMUSG	Gm42847	ENSMUST00000201732
ENSMUSG	Gm43342	ENSMUST00000201027
ENSMUSG	2900064	ENSMUST00000202166
ENSMUSG	Gm43025	ENSMUST00000201764
ENSMUSG	Gm3348	ENSMUST00000177813
ENSMUSG	Gm43230	ENSMUST00000200980
ENSMUSG	Gm43101	ENSMUST00000201733
ENSMUSG	Gm32780	ENSMUST00000201402
ENSMUSG	4930478	ENSMUST00000202026
ENSMUSG	4933424	ENSMUST00000200749
ENSMUSG	C130093	ENSMUST00000202489
ENSMUSG	Gm42781	ENSMUST00000201317
ENSMUSG	4930553	ENSMUST00000201164
ENSMUSG	Gm43050	ENSMUST00000201062;ENSMUST00000201152
ENSMUSG	Gm20756	ENSMUST00000201324;ENSMUST00000201080
ENSMUSG	Gm42885	ENSMUST00000200763
ENSMUSG	Gm42745	ENSMUST00000200904
ENSMUSG	Gm28563	ENSMUST00000201179
ENSMUSG	Gm43498	ENSMUST00000202002
ENSMUSG	Gm42583	ENSMUST00000202678
ENSMUSG	Gm7556	ENSMUST00000201178
ENSMUSG	Gm42632	ENSMUST00000201707
ENSMUSG	Gm42458	ENSMUST00000201914
ENSMUSG	Gm43156	ENSMUST00000202338
ENSMUSG	Gm7492	ENSMUST00000201745
ENSMUSG	5330437	ENSMUST00000201318
ENSMUSG	Gm42811	ENSMUST00000201951
ENSMUSG	Gm42109	ENSMUST00000200893;ENSMUST00000200783
ENSMUSG	Gm3289	ENSMUST00000200819
ENSMUSG	BC04973	ENSMUST00000202626
ENSMUSG	Gm2566	ENSMUST00000201267
ENSMUSG	Gm34091	ENSMUST00000200875
ENSMUSG	Gm7463	ENSMUST00000201541
ENSMUSG	Gm4754	ENSMUST00000201337
ENSMUSG	G630022	ENSMUST00000202923
ENSMUSG	Gm43027	ENSMUST00000202906
ENSMUSG	Gm43125	ENSMUST00000201724
ENSMUSG	Gm42741	ENSMUST00000201340

ENSMUSG	9430007I	ENSMUST00000201947
ENSMUSG	Gm42655	ENSMUST00000202782
ENSMUSG	Gm7832	ENSMUST00000202613
ENSMUSG	Gm42857	ENSMUST00000200823
ENSMUSG	Gm6450	ENSMUST00000200896
ENSMUSG	Gm42506	ENSMUST00000202009
ENSMUSG	Gm43843	ENSMUST00000202396
ENSMUSG	Gm42961	ENSMUST00000202023
ENSMUSG	Gm42785	ENSMUST00000202316
ENSMUSG	Gm43085	ENSMUST00000202859
ENSMUSG	Gm35960	ENSMUST00000202712
ENSMUSG	Gm8675	ENSMUST00000201408
ENSMUSG	Gm43377	ENSMUST00000201640
ENSMUSG	Gm42965	ENSMUST00000201254
ENSMUSG	Gm43690	ENSMUST00000201213
ENSMUSG	Gm42887	ENSMUST00000202469
ENSMUSG	Tsg101-]	ENSMUST00000201023
ENSMUSG	Gm5989	ENSMUST00000096092
ENSMUSG	Gm43399	ENSMUST00000202546
ENSMUSG	Gm10829	ENSMUST00000202084
ENSMUSG	Gm33167	ENSMUST00000201933
ENSMUSG	Gm42734	ENSMUST00000201656
ENSMUSG	Gm43105	ENSMUST00000202987
ENSMUSG	Gm2762	ENSMUST00000201517;ENSMUST00000202502;ENSMUST00000202
ENSMUSG	Gm6051	ENSMUST00000201529
ENSMUSG	Gm42853	ENSMUST00000201273
ENSMUSG	Gm7504	ENSMUST00000201449
ENSMUSG	Gm5043	ENSMUST00000202103
ENSMUSG	Gm43123	ENSMUST00000201893
ENSMUSG	Gm44942	ENSMUST00000096093
ENSMUSG	Gm29793	ENSMUST00000202874
ENSMUSG	4921513I	ENSMUST00000201403
ENSMUSG	Gm43636	ENSMUST00000201501
ENSMUSG	1700021I	ENSMUST00000202665
ENSMUSG	Gm19590	ENSMUST00000201551
ENSMUSG	Gm43155	ENSMUST00000202661
ENSMUSG	Gm35191	ENSMUST00000200925
ENSMUSG	Gm43090	ENSMUST00000202459
ENSMUSG	Gm36840	ENSMUST00000202688
ENSMUSG	Gm43443	ENSMUST00000202337
ENSMUSG	Gm42584	ENSMUST00000201404
ENSMUSG	Gm43760	ENSMUST00000202120
ENSMUSG	Gm9936	ENSMUST00000201635
ENSMUSG	Gm43842	ENSMUST00000202890
ENSMUSG	Vmn2r-p	ENSMUST00000202322
ENSMUSG	Gm3822	ENSMUST00000201941
ENSMUSG	Gm40289	ENSMUST00000201470
ENSMUSG	Gm15997	ENSMUST00000202188
ENSMUSG	Gm42528	ENSMUST00000200697
ENSMUSG	Gm42475	ENSMUST00000201212
ENSMUSG	Gm42888	ENSMUST00000202425
ENSMUSG	Gm38404	ENSMUST00000202005
ENSMUSG	Gm3170	ENSMUST00000168753
ENSMUSG	Gm7919	ENSMUST00000202043
ENSMUSG	Gm42666	ENSMUST00000201954
ENSMUSG	4933425I	ENSMUST00000201035
ENSMUSG	Gm34653	ENSMUST00000201786

ENSMUSG	4933430	ENSMUST00000200924
ENSMUSG	Gm43266	ENSMUST00000201049
ENSMUSG	Gm43845	ENSMUST00000202462
ENSMUSG	Gm43268	ENSMUST00000201536
ENSMUSG	Gm42642	ENSMUST00000200683
ENSMUSG	Gm42689	ENSMUST00000202403
ENSMUSG	Gm19619	ENSMUST00000200707
ENSMUSG	Gm42550	ENSMUST00000201553
ENSMUSG	Gm7542	ENSMUST00000201140
ENSMUSG	D130004	ENSMUST00000201492
ENSMUSG	4930500	ENSMUST00000200855
ENSMUSG	Gm18327	ENSMUST00000202426
ENSMUSG	Gm43371	ENSMUST00000202838
ENSMUSG	Gm43231	ENSMUST00000202960
ENSMUSG	Gm43102	ENSMUST00000201004
ENSMUSG	Gm13821	ENSMUST00000202029
ENSMUSG	Gm43028	ENSMUST00000202561
ENSMUSG	Gm5298	ENSMUST00000201122
ENSMUSG	Trmt112	ENSMUST00000202320
ENSMUSG	Tgif1-p	ENSMUST00000201864
ENSMUSG	Gm34583	ENSMUST00000202537;ENSMUST00000202586
ENSMUSG	Gm9495	ENSMUST00000200846
ENSMUSG	Gm43630	ENSMUST00000201550
ENSMUSG	Gm43458	ENSMUST00000200791
ENSMUSG	Gm43228	ENSMUST00000200735
ENSMUSG	Gm43279	ENSMUST00000202376
ENSMUSG	Gm42935	ENSMUST00000202004
ENSMUSG	Gm43229	ENSMUST00000201812
ENSMUSG	BC08069	ENSMUST00000202858
ENSMUSG	Gm30003	ENSMUST00000200700
ENSMUSG	4933439	ENSMUST00000200866
ENSMUSG	Gm7478	ENSMUST00000201054
ENSMUSG	Gm43008	ENSMUST00000202346
ENSMUSG	Gm43596	ENSMUST00000202818
ENSMUSG	Gm2559	ENSMUST00000201323
ENSMUSG	Gm43225	ENSMUST00000201381
ENSMUSG	4930430	ENSMUST00000202548;ENSMUST00000202251
ENSMUSG	Gm43295	ENSMUST00000201439
ENSMUSG	Gm42558	ENSMUST00000200982
ENSMUSG	Gm43373	ENSMUST00000202552
ENSMUSG	Gstm2-p	ENSMUST00000201478
ENSMUSG	Gm42668	ENSMUST00000200747
ENSMUSG	Gm42557	ENSMUST00000202037
ENSMUSG	C330024	ENSMUST00000201973;ENSMUST00000202634
ENSMUSG	Gm42800	ENSMUST00000202494
ENSMUSG	Gm43124	ENSMUST00000201878
ENSMUSG	4930425	ENSMUST00000205073
ENSMUSG	4930402	ENSMUST00000203115
ENSMUSG	Gm44306	ENSMUST00000203058
ENSMUSG	Gm6312	ENSMUST00000204060
ENSMUSG	Gm44115	ENSMUST00000203917
ENSMUSG	Gm20383	ENSMUST00000204844
ENSMUSG	Gm20553	ENSMUST00000204205
ENSMUSG	Olfr138	ENSMUST00000077143
ENSMUSG	Gm4782	ENSMUST00000168705
ENSMUSG	Gm44129	ENSMUST00000203511
ENSMUSG	Gm31532	ENSMUST00000203555

ENSMUSG	Gm44114	ENSMUST00000203270
ENSMUSG	Gm4640	ENSMUST00000203927
ENSMUSG	Gm40377	ENSMUST00000204604
ENSMUSG	Gm7468	ENSMUST00000203900
ENSMUSG	Gm44054	ENSMUST00000203814
ENSMUSG	Gm19173	ENSMUST00000204494
ENSMUSG	Gm44186	ENSMUST00000204118
ENSMUSG	Gm7384	ENSMUST00000203682
ENSMUSG	Gm44425	ENSMUST00000203739
ENSMUSG	Gm8992	ENSMUST00000204463
ENSMUSG	Gm44165	ENSMUST00000203093
ENSMUSG	Gm44156	ENSMUST00000203885
ENSMUSG	Gm31579	ENSMUST00000203306
ENSMUSG	Gm18325	ENSMUST00000203228
ENSMUSG	4933431	ENSMUST00000203905
ENSMUSG	Gm29781	ENSMUST00000203862
ENSMUSG	Gm44083	ENSMUST00000203114
ENSMUSG	Tgif2-p	ENSMUST00000203590
ENSMUSG	Gm30784	ENSMUST00000204491
ENSMUSG	Gm44046	ENSMUST00000203145
ENSMUSG	Gm43994	ENSMUST00000203809
ENSMUSG	Gm44451	ENSMUST00000203767
ENSMUSG	1700003	ENSMUST00000205198
ENSMUSG	Gm44442	ENSMUST00000204298
ENSMUSG	Gm44307	ENSMUST00000204466
ENSMUSG	Gm20560	ENSMUST00000204577
ENSMUSG	Gm43866	ENSMUST00000203894
ENSMUSG	Gm44096	ENSMUST00000203171
ENSMUSG	2010109	ENSMUST00000204605
ENSMUSG	Gm44193	ENSMUST00000204822
ENSMUSG	4930480	ENSMUST00000203679
ENSMUSG	Olfr138	ENSMUST00000216641;ENSMUST00000203369
ENSMUSG	Gm17834	ENSMUST00000204322
ENSMUSG	Gm32536	ENSMUST00000205209
ENSMUSG	Gm44124	ENSMUST00000204159
ENSMUSG	Gm18716	ENSMUST00000204507
ENSMUSG	3300002	ENSMUST00000204020
ENSMUSG	Gm43897	ENSMUST00000204019
ENSMUSG	Gm6794	ENSMUST00000203829
ENSMUSG	Tas2r14	ENSMUST00000204526
ENSMUSG	Tas2r14	ENSMUST00000204651
ENSMUSG	Gm18913	ENSMUST00000204311
ENSMUSG	Gm44117	ENSMUST00000204442
ENSMUSG	Gm44196	ENSMUST00000203852
ENSMUSG	Gm32479	ENSMUST00000204226
ENSMUSG	Gm44256	ENSMUST00000203304
ENSMUSG	4930512	ENSMUST00000203704;ENSMUST00000205184
ENSMUSG	Gm44070	ENSMUST00000203389
ENSMUSG	1700069	ENSMUST00000204240
ENSMUSG	Tas2r14	ENSMUST00000205072
ENSMUSG	Olfr764	ENSMUST00000204763;ENSMUST00000218589
ENSMUSG	Gm44166	ENSMUST00000204070
ENSMUSG	Gm31520	ENSMUST00000204874;ENSMUST00000203048
ENSMUSG	Gm44446	ENSMUST00000204864
ENSMUSG	Gm6559	ENSMUST00000204678
ENSMUSG	C530044	ENSMUST00000204275
ENSMUSG	Gm44137	ENSMUST00000203670

ENSMUSG	Gm3017	ENSMUST00000203603
ENSMUSG	Gm44277	ENSMUST00000205128
ENSMUSG	Olfr316	ENSMUST00000215071;ENSMUST00000203731
ENSMUSG	Rab5a-p	ENSMUST00000203252
ENSMUSG	Gm44386	ENSMUST00000204288
ENSMUSG	Gm44312	ENSMUST00000203536
ENSMUSG	Gm8921	ENSMUST00000204306
ENSMUSG	Gm44289	ENSMUST00000204144
ENSMUSG	Gm44171	ENSMUST00000203701
ENSMUSG	Gm44399	ENSMUST00000205043
ENSMUSG	Gm43970	ENSMUST00000204346
ENSMUSG	Gm36355	ENSMUST00000203752
ENSMUSG	Gm5307	ENSMUST00000204772
ENSMUSG	Gm6681	ENSMUST00000205215
ENSMUSG	Gm34933	ENSMUST00000203245
ENSMUSG	Gm44135	ENSMUST00000205221
ENSMUSG	Gm44204	ENSMUST00000204722
ENSMUSG	Gm30055	ENSMUST00000203175
ENSMUSG	Gm18765	ENSMUST00000203166
ENSMUSG	Gm43917	ENSMUST00000191984
ENSMUSG	Gm18690	ENSMUST00000204488
ENSMUSG	Gm5881	ENSMUST00000204737
ENSMUSG	Olfr766	ENSMUST00000204515;ENSMUST00000220119
ENSMUSG	Gm6749	ENSMUST00000203838
ENSMUSG	Mageb16	ENSMUST00000204513
ENSMUSG	Gm43898	ENSMUST00000204456
ENSMUSG	Gm44073	ENSMUST00000204173
ENSMUSG	Gm44448	ENSMUST00000205104
ENSMUSG	Gm44275	ENSMUST00000203442
ENSMUSG	Gm43959	ENSMUST00000203740
ENSMUSG	Gm8956	ENSMUST00000203939
ENSMUSG	Gm45083	ENSMUST00000205216
ENSMUSG	Gm6266	ENSMUST00000203844
ENSMUSG	Gm43914	ENSMUST00000203592
ENSMUSG	Gm18689	ENSMUST00000203969
ENSMUSG	Gm44209	ENSMUST00000204556
ENSMUSG	Gm18609	ENSMUST00000203370
ENSMUSG	Gm43935	ENSMUST00000203569
ENSMUSG	Gm44257	ENSMUST00000204469
ENSMUSG	Gm8038	ENSMUST00000164674
ENSMUSG	Olfr135	ENSMUST00000204477
ENSMUSG	Gm6443	ENSMUST00000203599
ENSMUSG	4930434	ENSMUST00000203259
ENSMUSG	Gm44185	ENSMUST00000204539
ENSMUSG	Gm35736	ENSMUST00000203681
ENSMUSG	Gm18584	ENSMUST00000203098
ENSMUSG	Gm44389	ENSMUST00000203795
ENSMUSG	Gm19065	ENSMUST00000203910
ENSMUSG	Gm43936	ENSMUST00000203987
ENSMUSG	Gm44106	ENSMUST00000203153
ENSMUSG	9330102	ENSMUST00000204642
ENSMUSG	Gm44278	ENSMUST00000204082
ENSMUSG	Gm44062	ENSMUST00000203312
ENSMUSG	Gm44138	ENSMUST00000204939
ENSMUSG	Gm5051	ENSMUST00000204085
ENSMUSG	Gm44192	ENSMUST00000204195
ENSMUSG	Gm32592	ENSMUST00000205146

ENSMUSG	Gm43930	ENSMUST00000203868
ENSMUSG	Gm5580	ENSMUST00000122096
ENSMUSG	Gm8574	ENSMUST00000203207
ENSMUSG	Gm43895	ENSMUST00000203219
ENSMUSG	Gm18011	ENSMUST00000203717
ENSMUSG	Gm7783	ENSMUST00000204795
ENSMUSG	Gm44285	ENSMUST00000203557
ENSMUSG	Gm43922	ENSMUST00000205071
ENSMUSG	Gm44090	ENSMUST00000204669
ENSMUSG	Gm44162	ENSMUST00000203543
ENSMUSG	Gm44475	ENSMUST00000204041
ENSMUSG	Gm30498	ENSMUST00000204033
ENSMUSG	4930447	ENSMUST00000204503
ENSMUSG	Gm44259	ENSMUST00000204996
ENSMUSG	Gm44094	ENSMUST00000204703
ENSMUSG	Gm44419	ENSMUST00000203338
ENSMUSG	Gm18198	ENSMUST00000204986
ENSMUSG	Gm44043	ENSMUST00000203426
ENSMUSG	Gm44121	ENSMUST00000205169
ENSMUSG	Gm6064	ENSMUST00000203999
ENSMUSG	Gm19692	ENSMUST00000203856
ENSMUSG	Gm44208	ENSMUST00000205199
ENSMUSG	Gm38708	ENSMUST00000203855
ENSMUSG	Gm44380	ENSMUST00000203125
ENSMUSG	Gm32591	ENSMUST00000208679
ENSMUSG	Gm8039	ENSMUST00000204758
ENSMUSG	6820426	ENSMUST00000204274
ENSMUSG	Gm7298	ENSMUST00000204124
ENSMUSG	Gm44007	ENSMUST00000203845
ENSMUSG	D030044	ENSMUST00000204220
ENSMUSG	Gm43925	ENSMUST00000203498
ENSMUSG	Gm5576	ENSMUST00000075809
ENSMUSG	Gm43924	ENSMUST00000204851
ENSMUSG	Gm43990	ENSMUST00000203790
ENSMUSG	Gm8479	ENSMUST00000203825
ENSMUSG	4921529	ENSMUST00000203047
ENSMUSG	Gm44028	ENSMUST00000205205
ENSMUSG	Hspd1-p	ENSMUST00000203513
ENSMUSG	Gm3455	ENSMUST00000204812
ENSMUSG	Gm44210	ENSMUST00000205253
ENSMUSG	Mug4-ps	ENSMUST00000203118
ENSMUSG	Gm6786	ENSMUST00000203165
ENSMUSG	Gm44229	ENSMUST00000203211
ENSMUSG	Gm3148	ENSMUST00000203594
ENSMUSG	Gm6637	ENSMUST00000204884
ENSMUSG	Gm44189	ENSMUST00000204387
ENSMUSG	Gm31108	ENSMUST00000204101
ENSMUSG	Gm5340	ENSMUST00000203733
ENSMUSG	Gm5886	ENSMUST00000203983
ENSMUSG	Gm5312	ENSMUST00000203728
ENSMUSG	Gm7039	ENSMUST00000205138
ENSMUSG	Gm43891	ENSMUST00000205132
ENSMUSG	Gm12845	ENSMUST00000203629
ENSMUSG	Gm18485	ENSMUST00000203788
ENSMUSG	Gm7498	ENSMUST00000203282
ENSMUSG	Gm38811	ENSMUST00000203781
ENSMUSG	Gm33201	ENSMUST00000204791

ENSMUSG	Gm32914	ENSMUST00000203815
ENSMUSG	Gm43864	ENSMUST00000204452
ENSMUSG	Gm21054	ENSMUST00000203981
ENSMUSG	Gm44231	ENSMUST00000204106
ENSMUSG	Gm44274	ENSMUST00000203417
ENSMUSG	4930511	ENSMUST00000203778
ENSMUSG	Gm29803	ENSMUST00000205069
ENSMUSG	1700097	ENSMUST00000204079
ENSMUSG	Gm44180	ENSMUST00000203671
ENSMUSG	Gm19252	ENSMUST00000204705
ENSMUSG	Gm44427	ENSMUST00000204123
ENSMUSG	Gm32222	ENSMUST00000203296
ENSMUSG	Gm44056	ENSMUST00000204623
ENSMUSG	Gm44203	ENSMUST00000203134
ENSMUSG	Gm43933	ENSMUST00000203893
ENSMUSG	Gm31861	ENSMUST00000204563
ENSMUSG	6430584	ENSMUST00000204412
ENSMUSG	1700102	ENSMUST00000203859
ENSMUSG	Gm4045	ENSMUST00000204076
ENSMUSG	Gm44428	ENSMUST00000204474
ENSMUSG	0610033	ENSMUST00000205236
ENSMUSG	Gm44252	ENSMUST00000204187
ENSMUSG	Gm43896	ENSMUST00000203440
ENSMUSG	Gm43960	ENSMUST00000204249
ENSMUSG	Tas2r11	ENSMUST00000203794
ENSMUSG	Gm44112	ENSMUST00000204787
ENSMUSG	Gm43908	ENSMUST00000205074
ENSMUSG	Gm16499	ENSMUST00000204633;ENSMUST00000104970
ENSMUSG	Gm44251	ENSMUST00000204963
ENSMUSG	Gm43913	ENSMUST00000203924
ENSMUSG	Gm6727	ENSMUST00000204102
ENSMUSG	Tas2r14	ENSMUST00000203065
ENSMUSG	Gm43867	ENSMUST00000203860
ENSMUSG	Gm5305	ENSMUST00000204010
ENSMUSG	Gm36640	ENSMUST00000203686
ENSMUSG	Gm44024	ENSMUST00000203008
ENSMUSG	Gm8703	ENSMUST00000203793
ENSMUSG	Gm44182	ENSMUST00000204052
ENSMUSG	Gm44202	ENSMUST00000203549
ENSMUSG	Gm44120	ENSMUST00000203022
ENSMUSG	Gm44167	ENSMUST00000204957
ENSMUSG	Gm44640	ENSMUST00000206894
ENSMUSG	Ceacam-	ENSMUST00000205380;ENSMUST00000206683
ENSMUSG	Gm45218	ENSMUST00000205533
ENSMUSG	Gm44737	ENSMUST00000206950
ENSMUSG	Gm45052	ENSMUST00000206457
ENSMUSG	Gm44601	ENSMUST00000206678
ENSMUSG	Gm45170	ENSMUST00000206746
ENSMUSG	Gm44945	ENSMUST00000206764
ENSMUSG	Gm44898	ENSMUST00000205934
ENSMUSG	Gm17838	ENSMUST00000205872
ENSMUSG	Gm45196	ENSMUST00000206751
ENSMUSG	Gm44882	ENSMUST00000205702
ENSMUSG	Gm30771	ENSMUST00000205683
ENSMUSG	Gm45215	ENSMUST00000206407
ENSMUSG	Gm4971	ENSMUST00000205991
ENSMUSG	4933435	ENSMUST00000205892

ENSMUSG	Gm18599	ENSMUST00000206729
ENSMUSG	Gm36736	ENSMUST00000206060
ENSMUSG	4931431	ENSMUST00000205522
ENSMUSG	9130221	ENSMUST00000206867
ENSMUSG	Snrp1c-	ENSMUST00000205464
ENSMUSG	Gm36159	ENSMUST00000206737
ENSMUSG	Gm18561	ENSMUST00000206027
ENSMUSG	Gm38832	ENSMUST00000205674
ENSMUSG	Gm45168	ENSMUST00000206461;ENSMUST00000205591
ENSMUSG	Gm45003	ENSMUST00000205875
ENSMUSG	Gm9299	ENSMUST00000206392
ENSMUSG	4930448	ENSMUST00000205988
ENSMUSG	Gm6567	ENSMUST00000205529
ENSMUSG	Gm9463	ENSMUST00000206707
ENSMUSG	Gm34811	ENSMUST00000206580
ENSMUSG	Gm6905	ENSMUST00000205729
ENSMUSG	Gm45171	ENSMUST00000206360
ENSMUSG	Gm30191	ENSMUST00000205501
ENSMUSG	Klk1b14	ENSMUST00000206059
ENSMUSG	Rpl19-p	ENSMUST00000205555
ENSMUSG	Klk1b15	ENSMUST00000206273
ENSMUSG	1700025	ENSMUST00000205586
ENSMUSG	Gm44740	ENSMUST00000205603
ENSMUSG	Gm45005	ENSMUST00000206640
ENSMUSG	BC02676	ENSMUST00000206555
ENSMUSG	Gm30928	ENSMUST00000206881;ENSMUST00000205858
ENSMUSG	Gm44765	ENSMUST00000206927
ENSMUSG	Gm44706	ENSMUST00000206822
ENSMUSG	Gm45058	ENSMUST00000205810
ENSMUSG	Gm42375	ENSMUST00000206226
ENSMUSG	Gm44756	ENSMUST00000206172
ENSMUSG	Gm45768	ENSMUST00000206763
ENSMUSG	Gm19366	ENSMUST00000206996
ENSMUSG	Gm9278	ENSMUST00000205508
ENSMUSG	Klk2-ps	ENSMUST00000206865
ENSMUSG	Gm44997	ENSMUST00000205970;ENSMUST00000205775
ENSMUSG	Gm21269	ENSMUST00000206521
ENSMUSG	Gm44507	ENSMUST00000206249
ENSMUSG	4732496	ENSMUST00000206533
ENSMUSG	Gm44605	ENSMUST00000206892
ENSMUSG	Gm45211	ENSMUST00000206208
ENSMUSG	Gm45054	ENSMUST00000206525
ENSMUSG	Gm4598	ENSMUST00000206756
ENSMUSG	Gm44739	ENSMUST00000205294
ENSMUSG	Gm44895	ENSMUST00000206670
ENSMUSG	2200002	ENSMUST00000206633
ENSMUSG	Gm33248	ENSMUST00000206954
ENSMUSG	Gm45184	ENSMUST00000206354
ENSMUSG	Gm45026	ENSMUST00000205292
ENSMUSG	Gm4265	ENSMUST00000206701
ENSMUSG	Gm44705	ENSMUST00000206719
ENSMUSG	6720469	ENSMUST00000206785
ENSMUSG	Gm44978	ENSMUST00000206635
ENSMUSG	Gm7238	ENSMUST00000206467
ENSMUSG	Gm30075	ENSMUST00000205509
ENSMUSG	Olfr717	ENSMUST00000205589
ENSMUSG	4833421	ENSMUST00000206146

ENSMUSG	Gm7443	ENSMUST00000206355
ENSMUSG	Gm29759	ENSMUST00000205860
ENSMUSG	Gm39091	ENSMUST00000206189
ENSMUSG	Gm32647	ENSMUST00000206579
ENSMUSG	Gm45197	ENSMUST00000205288
ENSMUSG	Klk1b12	ENSMUST00000206650
ENSMUSG	Gm44613	ENSMUST00000205637
ENSMUSG	Gm45006	ENSMUST00000206078
ENSMUSG	Gm38569	ENSMUST00000205476
ENSMUSG	Gm44824	ENSMUST00000207021
ENSMUSG	Gm45219	ENSMUST00000205602
ENSMUSG	Gm45031	ENSMUST00000206800
ENSMUSG	Gm4593	ENSMUST00000205334
ENSMUSG	Gm44883	ENSMUST00000206063
ENSMUSG	Gm35665	ENSMUST00000206742
ENSMUSG	Gm45700	ENSMUST00000205866
ENSMUSG	Gm44896	ENSMUST00000206190
ENSMUSG	Gm18973	ENSMUST00000205814
ENSMUSG	Gm44854	ENSMUST00000205493
ENSMUSG	Gm45225	ENSMUST00000206852
ENSMUSG	Gm38973	ENSMUST00000206810
ENSMUSG	Gm44581	ENSMUST00000206227
ENSMUSG	Itgam	ENSMUST00000098015
ENSMUSG	Gm44708	ENSMUST00000205580
ENSMUSG	Gm32884	ENSMUST00000205528
ENSMUSG	Klk1b10	ENSMUST00000205895
ENSMUSG	Gm44850	ENSMUST00000205621
ENSMUSG	Gm7424	ENSMUST00000206919
ENSMUSG	Gm29725	ENSMUST00000206614
ENSMUSG	Gm4973	ENSMUST00000206925
ENSMUSG	Gm35040	ENSMUST00000206829
ENSMUSG	Gm20670	ENSMUST00000206468
ENSMUSG	Gm45079	ENSMUST00000205627
ENSMUSG	Gm5037	ENSMUST00000205327
ENSMUSG	Gm44720	ENSMUST00000206076
ENSMUSG	Gm4290	ENSMUST00000206897
ENSMUSG	Gm45150	ENSMUST00000206051
ENSMUSG	Gm44920	ENSMUST00000206503
ENSMUSG	Gm44722	ENSMUST00000206473
ENSMUSG	Gm44855	ENSMUST00000205725
ENSMUSG	Klk1b2-	ENSMUST00000206099
ENSMUSG	Gm35611	ENSMUST00000205323
ENSMUSG	Gm45138	ENSMUST00000206141
ENSMUSG	Gm45172	ENSMUST00000206054
ENSMUSG	Gm44897	ENSMUST00000206187
ENSMUSG	Gm44815	ENSMUST00000206812
ENSMUSG	2700080	ENSMUST00000205314
ENSMUSG	Gm44970	ENSMUST00000206318
ENSMUSG	1700123	ENSMUST00000206035
ENSMUSG	Gm29754	ENSMUST00000206269
ENSMUSG	Gm45149	ENSMUST00000206459
ENSMUSG	Gm45080	ENSMUST00000206648
ENSMUSG	Gm45007	ENSMUST00000206237
ENSMUSG	Gm7287	ENSMUST00000205267
ENSMUSG	Gm2511	ENSMUST00000206605
ENSMUSG	Gm5738	ENSMUST00000206016
ENSMUSG	4930486	ENSMUST00000206668

ENSMUSG	Gm44793	ENSMUST00000136923
ENSMUSG	Gm18905	ENSMUST00000205454
ENSMUSG	Gm29977	ENSMUST00000206804
ENSMUSG	4933431	ENSMUST00000205571
ENSMUSG	Gm38991	ENSMUST00000205504
ENSMUSG	Gm44940	ENSMUST00000206225
ENSMUSG	Gm34225	ENSMUST00000205469;ENSMUST00000206921;ENSMUST00000205
ENSMUSG	Gm44837	ENSMUST00000206863
ENSMUSG	Gm44734	ENSMUST00000206025
ENSMUSG	Gm17831	ENSMUST00000205579
ENSMUSG	Gm45135	ENSMUST00000206787
ENSMUSG	Gm6008	ENSMUST00000206542
ENSMUSG	Gm17908	ENSMUST00000205552
ENSMUSG	Gm45198	ENSMUST00000206796
ENSMUSG	Gm44767	ENSMUST00000206634
ENSMUSG	Gm45217	ENSMUST00000206576
ENSMUSG	Gm44748	ENSMUST00000206040
ENSMUSG	Gm44894	ENSMUST00000205280
ENSMUSG	Gm45202	ENSMUST00000205955
ENSMUSG	Gm36028	ENSMUST00000205465;ENSMUST00000205931
ENSMUSG	Gm2926	ENSMUST00000205625
ENSMUSG	Gm29763	ENSMUST00000205306
ENSMUSG	Gm44719	ENSMUST00000206404
ENSMUSG	Gm45169	ENSMUST00000205717
ENSMUSG	Gm20083	ENSMUST00000205849;ENSMUST00000206663
ENSMUSG	Gm45691	ENSMUST00000206905
ENSMUSG	Gm4259	ENSMUST00000206489
ENSMUSG	2200007	ENSMUST00000206367
ENSMUSG	Gm44806	ENSMUST00000205574
ENSMUSG	Gm45195	ENSMUST00000205597
ENSMUSG	Gm9521	ENSMUST00000206656
ENSMUSG	Glud-ps	ENSMUST00000206310
ENSMUSG	Gm44629	ENSMUST00000206951
ENSMUSG	4933432	ENSMUST00000206276
ENSMUSG	Rpl19-p	ENSMUST00000205423
ENSMUSG	Gm30717	ENSMUST00000206549
ENSMUSG	Gm44620	ENSMUST00000209183
ENSMUSG	A230057	ENSMUST00000208961
ENSMUSG	Gm34549	ENSMUST00000207057;ENSMUST00000207356
ENSMUSG	Gm7957	ENSMUST00000207404
ENSMUSG	Gm44832	ENSMUST00000208849
ENSMUSG	Gm44566	ENSMUST00000207638
ENSMUSG	Gm5320	ENSMUST00000207163
ENSMUSG	Frmpd2	ENSMUST00000208853;ENSMUST00000208577;ENSMUST00000208
ENSMUSG	Gm45001	ENSMUST00000208279
ENSMUSG	Gm10623	ENSMUST00000209081
ENSMUSG	B830042	ENSMUST00000208333
ENSMUSG	Gm44911	ENSMUST00000208436
ENSMUSG	D830036	ENSMUST00000208263
ENSMUSG	Gm44776	ENSMUST00000207212
ENSMUSG	Gm6070	ENSMUST00000207275
ENSMUSG	5930435	ENSMUST00000207572
ENSMUSG	Gm44617	ENSMUST00000207408;ENSMUST00000209122
ENSMUSG	Gm35082	ENSMUST00000207308
ENSMUSG	Gm32850	ENSMUST00000209149
ENSMUSG	Gm45157	ENSMUST00000208729
ENSMUSG	Gm45792	ENSMUST00000207795

ENSMUSG	Olfr155	ENSMUST00000219261
ENSMUSG	Gm9449	ENSMUST00000207265
ENSMUSG	Gm44927	ENSMUST00000207573
ENSMUSG	Gm45070	ENSMUST00000208100
ENSMUSG	Gm20136	ENSMUST00000207501
ENSMUSG	Gm44811	ENSMUST00000209120
ENSMUSG	Gm45029	ENSMUST00000208702
ENSMUSG	Gm44618	ENSMUST00000208431
ENSMUSG	Gm8314	ENSMUST00000208964
ENSMUSG	Gm9373	ENSMUST00000208724
ENSMUSG	Gm45113	ENSMUST00000207518
ENSMUSG	Gm2448	ENSMUST00000207080
ENSMUSG	Gm44966	ENSMUST00000208096
ENSMUSG	Gm44802	ENSMUST00000208026
ENSMUSG	4933436	ENSMUST00000208150
ENSMUSG	Vmn2r-p	ENSMUST00000207286
ENSMUSG	Cc2d2b	ENSMUST00000207801;ENSMUST00000224596;ENSMUST00000207
ENSMUSG	Gm10648	ENSMUST00000207552
ENSMUSG	Olfr128	ENSMUST00000062407
ENSMUSG	Gm31463	ENSMUST00000208799
ENSMUSG	Gm44732	ENSMUST00000208055
ENSMUSG	Gm8745	ENSMUST00000207846
ENSMUSG	Gm18908	ENSMUST00000207125
ENSMUSG	Gm44660	ENSMUST00000207134
ENSMUSG	Olfr142	ENSMUST00000208076
ENSMUSG	Gm45069	ENSMUST00000208604
ENSMUSG	Gm45179	ENSMUST00000208452
ENSMUSG	Gm21032	ENSMUST00000208108
ENSMUSG	Gm32540	ENSMUST00000207174
ENSMUSG	Gm45119	ENSMUST00000207612
ENSMUSG	Gm45181	ENSMUST00000207878
ENSMUSG	Gm45011	ENSMUST00000207620
ENSMUSG	Gm45044	ENSMUST00000208078
ENSMUSG	Gm45906	ENSMUST00000208911
ENSMUSG	Gm18210	ENSMUST00000209001
ENSMUSG	Gm7675	ENSMUST00000208828
ENSMUSG	Mlnr-ps	ENSMUST00000208959
ENSMUSG	Gm29797	ENSMUST00000207856
ENSMUSG	Gm44689	ENSMUST00000208786
ENSMUSG	Gm15262	ENSMUST00000207320
ENSMUSG	Gm17988	ENSMUST00000208796
ENSMUSG	Gm44926	ENSMUST00000207898
ENSMUSG	Gm45056	ENSMUST00000208140
ENSMUSG	Gm44846	ENSMUST00000207127
ENSMUSG	Olfr485	ENSMUST00000208296
ENSMUSG	B230209	ENSMUST00000209115
ENSMUSG	Gm44929	ENSMUST00000209134
ENSMUSG	Gm44723	ENSMUST00000207369
ENSMUSG	Gm44809	ENSMUST00000208305
ENSMUSG	Gm44812	ENSMUST00000207627
ENSMUSG	Gm35842	ENSMUST00000207859
ENSMUSG	Olfr142	ENSMUST00000208900
ENSMUSG	Gm45115	ENSMUST00000208546
ENSMUSG	Gm45012	ENSMUST00000208323
ENSMUSG	Gm2676	ENSMUST00000207279
ENSMUSG	A630009	ENSMUST00000208485
ENSMUSG	Gm44741	ENSMUST00000207503

ENSMUSG	Gm9354	ENSMUST00000209075
ENSMUSG	Gm44622	ENSMUST00000209128
ENSMUSG	Gm44840	ENSMUST00000208983
ENSMUSG	Gm45162	ENSMUST00000209013
ENSMUSG	Gm38944	ENSMUST00000208218
ENSMUSG	Gm44586	ENSMUST00000207166
ENSMUSG	Gm44992	ENSMUST00000209615;ENSMUST00000210787;ENSMUST00000208
ENSMUSG	4833411	ENSMUST00000207499;ENSMUST00000207914
ENSMUSG	Gm44827	ENSMUST00000207963
ENSMUSG	Gm19950	ENSMUST00000208167
ENSMUSG	Gm44799	ENSMUST00000207639
ENSMUSG	Gm29683	ENSMUST00000208511;ENSMUST00000209071
ENSMUSG	Gm45132	ENSMUST00000207234
ENSMUSG	4933430	ENSMUST00000209078
ENSMUSG	Gm44781	ENSMUST00000208480
ENSMUSG	6530437	ENSMUST00000207515;ENSMUST00000209089
ENSMUSG	Gm32916	ENSMUST00000207903
ENSMUSG	Gm44753	ENSMUST00000207739
ENSMUSG	Sycp1-p	ENSMUST00000207882
ENSMUSG	Gm18393	ENSMUST00000208349
ENSMUSG	A230103	ENSMUST00000207198
ENSMUSG	Gm44516	ENSMUST00000208794
ENSMUSG	Gm45159	ENSMUST00000208630
ENSMUSG	Gm31135	ENSMUST00000208460
ENSMUSG	Gm45098	ENSMUST00000207179
ENSMUSG	Gm45076	ENSMUST00000207517
ENSMUSG	Gm44798	ENSMUST00000208123
ENSMUSG	Gm44600	ENSMUST00000208540
ENSMUSG	Gm45194	ENSMUST00000207962
ENSMUSG	Gm45166	ENSMUST00000209073
ENSMUSG	2900027	ENSMUST00000207411
ENSMUSG	Gm45085	ENSMUST00000209100
ENSMUSG	Gm44652	ENSMUST00000208648
ENSMUSG	Gm45086	ENSMUST00000209052
ENSMUSG	Gm21035	ENSMUST00000207867
ENSMUSG	Gm21036	ENSMUST00000207544
ENSMUSG	Zfp264	ENSMUST00000208781
ENSMUSG	Gm3257	ENSMUST00000207217
ENSMUSG	Gm5605	ENSMUST00000208940
ENSMUSG	Gm44621	ENSMUST00000207643
ENSMUSG	Gm39027	ENSMUST00000208560
ENSMUSG	Gm44570	ENSMUST00000208858;ENSMUST00000207526
ENSMUSG	Gm45038	ENSMUST00000207537
ENSMUSG	Gm44715	ENSMUST00000209049
ENSMUSG	Gm44969	ENSMUST00000207829
ENSMUSG	Gm21037	ENSMUST00000209086
ENSMUSG	Gm9445	ENSMUST00000207454
ENSMUSG	Gm44954	ENSMUST00000208513
ENSMUSG	Gm45104	ENSMUST00000208748
ENSMUSG	Gm44659	ENSMUST00000207269
ENSMUSG	4930453	ENSMUST00000209053
ENSMUSG	Gm9375	ENSMUST00000208899
ENSMUSG	Gm10297	ENSMUST00000207136
ENSMUSG	Gm45116	ENSMUST00000207392
ENSMUSG	Gm29791	ENSMUST00000207888;ENSMUST00000207144
ENSMUSG	Fam81b	ENSMUST00000208418
ENSMUSG	Gm44577	ENSMUST00000207413

ENSMUSG	9130214	ENSMUST00000208727
ENSMUSG	Gm35060	ENSMUST00000207669;ENSMUST00000207749;ENSMUST00000207
ENSMUSG	Gm44751	ENSMUST00000208302
ENSMUSG	Gm44610	ENSMUST00000209012
ENSMUSG	Gm18453	ENSMUST00000208000
ENSMUSG	Gm44744	ENSMUST00000208234
ENSMUSG	Gm39121	ENSMUST00000208847
ENSMUSG	B230311	ENSMUST00000208859;ENSMUST00000207610
ENSMUSG	Gm44541	ENSMUST00000207837
ENSMUSG	Gm2814	ENSMUST00000207074
ENSMUSG	Gm44808	ENSMUST00000208887
ENSMUSG	Gm45112	ENSMUST00000208576
ENSMUSG	Gm44693	ENSMUST00000208141
ENSMUSG	A330076	ENSMUST00000208822;ENSMUST00000207694;ENSMUST00000207
ENSMUSG	Gm9165	ENSMUST00000208467
ENSMUSG	Gm33926	ENSMUST00000208755
ENSMUSG	Gm44754	ENSMUST00000208155
ENSMUSG	Gm30790	ENSMUST00000208527
ENSMUSG	Gm45030	ENSMUST00000207575
ENSMUSG	Gm30873	ENSMUST00000207512
ENSMUSG	A26c2	ENSMUST00000207359
ENSMUSG	Gm44515	ENSMUST00000209045
ENSMUSG	Gm45175	ENSMUST00000208295
ENSMUSG	Gm44632	ENSMUST00000207245
ENSMUSG	Gm18256	ENSMUST00000209087
ENSMUSG	Olfr706	ENSMUST00000213918
ENSMUSG	Gm4880	ENSMUST00000207645
ENSMUSG	Gm44797	ENSMUST00000208780
ENSMUSG	Gm44946	ENSMUST00000208425
ENSMUSG	Gm44948	ENSMUST00000209050
ENSMUSG	Gm44698	ENSMUST00000207382
ENSMUSG	Gm44626	ENSMUST00000207035
ENSMUSG	Gm19410	ENSMUST00000207505
ENSMUSG	Gm44826	ENSMUST00000207648
ENSMUSG	Gm36584	ENSMUST00000209046
ENSMUSG	Gm17931	ENSMUST00000208132
ENSMUSG	Gm44518	ENSMUST00000208080
ENSMUSG	Gm5737	ENSMUST00000208668
ENSMUSG	A230057	ENSMUST00000207061
ENSMUSG	Gm45199	ENSMUST00000207640
ENSMUSG	Gm3854	ENSMUST00000207173;ENSMUST00000208865
ENSMUSG	Cpeb1os	ENSMUST00000207156
ENSMUSG	Gm45040	ENSMUST00000207742;ENSMUST00000208448
ENSMUSG	Gm19963	ENSMUST00000208413
ENSMUSG	Gm34664	ENSMUST00000207661
ENSMUSG	Gm9343	ENSMUST00000209016
ENSMUSG	Gm45165	ENSMUST00000207763
ENSMUSG	Gm44859	ENSMUST00000207831
ENSMUSG	Gm44800	ENSMUST00000207510
ENSMUSG	Gm45163	ENSMUST00000207606
ENSMUSG	Gm44998	ENSMUST00000207091
ENSMUSG	Gm45174	ENSMUST00000208884
ENSMUSG	Gm44658	ENSMUST00000207965
ENSMUSG	Gm45075	ENSMUST00000208426
ENSMUSG	Gm9195	ENSMUST00000208955
ENSMUSG	Gm3198	ENSMUST00000208725
ENSMUSG	Gm45224	ENSMUST00000208203

ENSMUSG	Gm44530	ENSMUST00000207920
ENSMUSG	Gm44889	ENSMUST00000208346;ENSMUST00000207674
ENSMUSG	Gm45591	ENSMUST00000129058
ENSMUSG	Gm44848	ENSMUST00000207808
ENSMUSG	Gm19032	ENSMUST00000208584
ENSMUSG	Gm30646	ENSMUST00000207662
ENSMUSG	Gm18943	ENSMUST00000207357
ENSMUSG	B930025	ENSMUST00000207718;ENSMUST00000208358
ENSMUSG	Gm21034	ENSMUST00000207390
ENSMUSG	Gm44919	ENSMUST00000208587
ENSMUSG	Gm45042	ENSMUST00000207377
ENSMUSG	Gm6828	ENSMUST00000207902
ENSMUSG	Gm7451	ENSMUST00000208382
ENSMUSG	Gm21033	ENSMUST00000208590
ENSMUSG	AC16289	ENSMUST00000207732
ENSMUSG	Gm18672	ENSMUST00000208035
ENSMUSG	Gm44786	ENSMUST00000208165
ENSMUSG	Gm44956	ENSMUST00000208277
ENSMUSG	Gm45049	ENSMUST00000207586
ENSMUSG	Gm6882	ENSMUST00000177741
ENSMUSG	Olfr131	ENSMUST00000208550
ENSMUSG	Gm44801	ENSMUST00000207644
ENSMUSG	Gm6112	ENSMUST00000208037
ENSMUSG	Gm44788	ENSMUST00000207605
ENSMUSG	Gm45066	ENSMUST00000207528
ENSMUSG	4930435	ENSMUST00000208065
ENSMUSG	Gm44531	ENSMUST00000208364
ENSMUSG	A230057	ENSMUST00000208953
ENSMUSG	Gm38843	ENSMUST00000209189
ENSMUSG	Gm8463	ENSMUST00000208573
ENSMUSG	Ankrd31	ENSMUST00000208758
ENSMUSG	Gm45178	ENSMUST00000207744
ENSMUSG	Muc16	ENSMUST00000034653;ENSMUST00000207857;ENSMUST0000020
ENSMUSG	Gm6579	ENSMUST00000208224
ENSMUSG	Cfap99	ENSMUST00000207754
ENSMUSG	Gm8268	ENSMUST00000209683
ENSMUSG	Gm45417	ENSMUST00000209533
ENSMUSG	Gm36356	ENSMUST00000209224
ENSMUSG	Gm32389	ENSMUST00000209312
ENSMUSG	Gm45346	ENSMUST00000209925
ENSMUSG	Gm4972	ENSMUST00000211108
ENSMUSG	Gm45253	ENSMUST00000210694
ENSMUSG	Gm7669	ENSMUST00000210184
ENSMUSG	Gm45370	ENSMUST00000211274
ENSMUSG	Gm45517	ENSMUST00000209973
ENSMUSG	BC02438	ENSMUST00000210567;ENSMUST00000209406
ENSMUSG	Gm45364	ENSMUST00000210883
ENSMUSG	Gm31479	ENSMUST00000209936
ENSMUSG	C230079	ENSMUST00000209560
ENSMUSG	Gm45474	ENSMUST00000209471
ENSMUSG	Gm31545	ENSMUST00000209358
ENSMUSG	Gm45246	ENSMUST00000210820
ENSMUSG	Gm45523	ENSMUST00000210712
ENSMUSG	Gm3912	ENSMUST00000173575;ENSMUST00000211024
ENSMUSG	Gm29735	ENSMUST00000209599
ENSMUSG	Gm45660	ENSMUST00000210267
ENSMUSG	Gm45308	ENSMUST00000210610

ENSMUSG	Gm9520	ENSMUST00000209460
ENSMUSG	Gm45458	ENSMUST00000209900
ENSMUSG	Gm45470	ENSMUST00000209685
ENSMUSG	Nxpe1-p	ENSMUST00000210160;ENSMUST00000209981;ENSMUST0000021C
ENSMUSG	Gm45562	ENSMUST00000210015
ENSMUSG	Gm34597	ENSMUST00000211189
ENSMUSG	Gm21123	ENSMUST00000210822
ENSMUSG	Gm45674	ENSMUST00000210043
ENSMUSG	Gm45299	ENSMUST00000211011;ENSMUST00000210199
ENSMUSG	Gm38414	ENSMUST00000209819;ENSMUST00000211253
ENSMUSG	Gm19096	ENSMUST00000209705
ENSMUSG	Fth-ps3	ENSMUST00000210405
ENSMUSG	Gm8667	ENSMUST00000211112
ENSMUSG	Gm35021	ENSMUST00000210105
ENSMUSG	Gm42196	ENSMUST00000209618
ENSMUSG	Gm45425	ENSMUST00000211727
ENSMUSG	Gm45272	ENSMUST00000211111
ENSMUSG	Gm39566	ENSMUST00000210714
ENSMUSG	Gm33096	ENSMUST00000210189
ENSMUSG	Gm7561	ENSMUST00000211203
ENSMUSG	Gm45255	ENSMUST00000209943
ENSMUSG	Gm45461	ENSMUST00000211726
ENSMUSG	1700018	ENSMUST00000211116
ENSMUSG	Gm35392	ENSMUST00000209934
ENSMUSG	Hmgb1-r	ENSMUST00000210634
ENSMUSG	Gm5339	ENSMUST00000210551
ENSMUSG	Gm7128	ENSMUST00000211639
ENSMUSG	1700011	ENSMUST00000210613
ENSMUSG	Gm6503	ENSMUST00000210829
ENSMUSG	5430403	ENSMUST00000210717
ENSMUSG	Gm18227	ENSMUST00000211082
ENSMUSG	Gm45415	ENSMUST00000209644
ENSMUSG	Gm45446	ENSMUST00000209348
ENSMUSG	Gm30085	ENSMUST00000209694
ENSMUSG	Gm35315	ENSMUST00000209865;ENSMUST00000210052;ENSMUST00000211
ENSMUSG	Gm39132	ENSMUST00000210446
ENSMUSG	Gm45297	ENSMUST00000209893
ENSMUSG	Gm45384	ENSMUST00000210451
ENSMUSG	Gm45447	ENSMUST00000209874
ENSMUSG	Gm45542	ENSMUST00000210456
ENSMUSG	Gm2033	ENSMUST00000210753
ENSMUSG	Gm45426	ENSMUST00000209393
ENSMUSG	Gm10043	ENSMUST00000210691
ENSMUSG	Gm5130	ENSMUST00000210233
ENSMUSG	Olfr131	ENSMUST00000213696
ENSMUSG	Gm45604	ENSMUST00000209665
ENSMUSG	5430402	ENSMUST00000211799
ENSMUSG	Olfr127	ENSMUST00000216697
ENSMUSG	4930483	ENSMUST00000209850
ENSMUSG	B930018	ENSMUST00000211381
ENSMUSG	Gm6593	ENSMUST00000211165
ENSMUSG	Gm8556	ENSMUST00000210437
ENSMUSG	Olfr643	ENSMUST00000217217
ENSMUSG	Gm6213	ENSMUST00000209966
ENSMUSG	Gm45662	ENSMUST00000210054
ENSMUSG	Gm45605	ENSMUST00000210265
ENSMUSG	Gm45677	ENSMUST00000209975

ENSMUSG	Gm17841	ENSMUST00000210711
ENSMUSG	Gm45627	ENSMUST00000210130
ENSMUSG	Gm33045	ENSMUST00000210049
ENSMUSG	Gm5338	ENSMUST00000209530
ENSMUSG	Gm39158	ENSMUST00000210178
ENSMUSG	Gm35850	ENSMUST00000210956;ENSMUST00000211705
ENSMUSG	Gm45276	ENSMUST00000209549
ENSMUSG	Gm45456	ENSMUST00000210841
ENSMUSG	Gm34096	ENSMUST00000211296
ENSMUSG	Gm18706	ENSMUST00000210053
ENSMUSG	Gm45271	ENSMUST00000211017
ENSMUSG	Gm28756	ENSMUST00000209405
ENSMUSG	AU02376	ENSMUST00000209897
ENSMUSG	Gm5904	ENSMUST00000210286
ENSMUSG	6330420	ENSMUST00000211371
ENSMUSG	Gm6012	ENSMUST00000210821
ENSMUSG	AC15858	ENSMUST00000211738
ENSMUSG	Gm7380	ENSMUST00000211599
ENSMUSG	Gm45663	ENSMUST00000210429
ENSMUSG	Gm30694	ENSMUST00000209278
ENSMUSG	7420700	ENSMUST00000209524
ENSMUSG	Gm33326	ENSMUST00000209741
ENSMUSG	Gm20948	ENSMUST00000211091
ENSMUSG	Gm34730	ENSMUST00000210674
ENSMUSG	Gm39929	ENSMUST00000209672
ENSMUSG	Gm45889	ENSMUST00000209535;ENSMUST00000211255
ENSMUSG	Gm6009	ENSMUST00000210848
ENSMUSG	Gm45601	ENSMUST00000211483
ENSMUSG	D2Bwg14	ENSMUST00000209765
ENSMUSG	Gm18644	ENSMUST00000211458
ENSMUSG	Gm45428	ENSMUST00000209619
ENSMUSG	Gm32786	ENSMUST00000211351
ENSMUSG	Gm45575	ENSMUST00000210283
ENSMUSG	Gm31983	ENSMUST00000210553;ENSMUST00000209999
ENSMUSG	Gm45612	ENSMUST00000211320
ENSMUSG	Zscan4-	ENSMUST00000210559
ENSMUSG	Gm45397	ENSMUST00000209816
ENSMUSG	Gm19140	ENSMUST00000209790
ENSMUSG	Gm45538	ENSMUST00000210771
ENSMUSG	B020031	ENSMUST00000209977
ENSMUSG	Gm31898	ENSMUST00000209232
ENSMUSG	9430099	ENSMUST00000210409
ENSMUSG	Gm45437	ENSMUST00000211418;ENSMUST00000209998
ENSMUSG	Gm35368	ENSMUST00000209554
ENSMUSG	Gm45352	ENSMUST00000210140
ENSMUSG	Olfr644	ENSMUST00000213184;ENSMUST00000077417
ENSMUSG	Gm5361	ENSMUST00000211041
ENSMUSG	Gm33968	ENSMUST00000211015
ENSMUSG	Gm20751	ENSMUST00000210260
ENSMUSG	Gm45237	ENSMUST00000211673;ENSMUST00000211157
ENSMUSG	5430437	ENSMUST00000211750
ENSMUSG	C030029	ENSMUST00000210356
ENSMUSG	Gm45546	ENSMUST00000211053
ENSMUSG	Gm18205	ENSMUST00000209215
ENSMUSG	4930543	ENSMUST00000209503
ENSMUSG	Gm7328	ENSMUST00000211123
ENSMUSG	Gm45322	ENSMUST00000211642

ENSMUSG	1700020(	ENSMUST00000211264
ENSMUSG	Gm5907	ENSMUST00000209784;ENSMUST00000210969
ENSMUSG	Olfr528	ENSMUST00000211655
ENSMUSG	Gm9860	ENSMUST00000211426
ENSMUSG	Gm45337	ENSMUST00000210537
ENSMUSG	Gm31045	ENSMUST00000211266
ENSMUSG	Gm18320	ENSMUST00000211304
ENSMUSG	Gm45588	ENSMUST00000211213
ENSMUSG	Gm45422	ENSMUST00000211476
ENSMUSG	Gm45512	ENSMUST00000210432
ENSMUSG	Gm2875	ENSMUST00000211333
ENSMUSG	Gm45305	ENSMUST00000209799
ENSMUSG	Gm45241	ENSMUST00000211237
ENSMUSG	Gm6145	ENSMUST00000211251
ENSMUSG	Gm19164	ENSMUST00000210667
ENSMUSG	Gm45257	ENSMUST00000210301
ENSMUSG	Gm36243	ENSMUST00000211546
ENSMUSG	Gm39115	ENSMUST00000211350
ENSMUSG	Gm6249	ENSMUST00000211142
ENSMUSG	Gm9132	ENSMUST00000210182
ENSMUSG	Gm45844	ENSMUST00000209833;ENSMUST00000209325;ENSMUST0000021C
ENSMUSG	Gm5599	ENSMUST00000209602
ENSMUSG	Gm45471	ENSMUST00000211270
ENSMUSG	Gm32486	ENSMUST00000209810
ENSMUSG	4933430.	ENSMUST00000210943
ENSMUSG	Gm5609	ENSMUST00000210008
ENSMUSG	Gm18562	ENSMUST00000209949
ENSMUSG	Gm33148	ENSMUST00000210835
ENSMUSG	Gm8168	ENSMUST00000209631
ENSMUSG	Gm45839	ENSMUST00000209884
ENSMUSG	Gm45577	ENSMUST00000210206
ENSMUSG	Gm45783	ENSMUST00000210877
ENSMUSG	5430421	ENSMUST00000209774
ENSMUSG	Gm45684	ENSMUST00000210244
ENSMUSG	Gm45487	ENSMUST00000211039
ENSMUSG	Gm38416	ENSMUST00000209648
ENSMUSG	Gm17766	ENSMUST00000209223
ENSMUSG	Gm9731	ENSMUST00000209480
ENSMUSG	Gm32507	ENSMUST00000209552
ENSMUSG	Gm45690	ENSMUST00000210775
ENSMUSG	Gm35520	ENSMUST00000209235
ENSMUSG	Igip	ENSMUST00000210490
ENSMUSG	Gm45496	ENSMUST00000209918
ENSMUSG	Gm45452	ENSMUST00000210905
ENSMUSG	Gm7192	ENSMUST00000210793
ENSMUSG	Olfr542	ENSMUST00000218525;ENSMUST00000211706
ENSMUSG	Gm45316	ENSMUST00000210128
ENSMUSG	Gm33594	ENSMUST00000209502
ENSMUSG	Gm33586	ENSMUST00000210768
ENSMUSG	Gm33756	ENSMUST00000211358
ENSMUSG	Gm45587	ENSMUST00000211548
ENSMUSG	Gm36325	ENSMUST00000210487
ENSMUSG	Gm36210	ENSMUST00000210663
ENSMUSG	Gm39149	ENSMUST00000210258
ENSMUSG	Gm45528	ENSMUST00000209854
ENSMUSG	Gm45634	ENSMUST00000209736
ENSMUSG	Gm6329	ENSMUST00000209210

ENSMUSG	Gm40493	ENSMUST00000210223
ENSMUSG	Gm45486	ENSMUST00000211767
ENSMUSG	Gm45506	ENSMUST00000211620
ENSMUSG	G630064	ENSMUST00000209814
ENSMUSG	C130073	ENSMUST00000211313
ENSMUSG	Gm45269	ENSMUST00000210359
ENSMUSG	9330121	ENSMUST00000211464
ENSMUSG	Gm2996	ENSMUST00000211398
ENSMUSG	Olfr529	ENSMUST00000211097
ENSMUSG	Olfr65	ENSMUST00000214300;ENSMUST00000209528
ENSMUSG	Gm30504	ENSMUST00000209762
ENSMUSG	Gm45485	ENSMUST00000211229
ENSMUSG	Gm45614	ENSMUST00000209994
ENSMUSG	E330018	ENSMUST00000209455
ENSMUSG	Gm45331	ENSMUST00000209868
ENSMUSG	Gm32742	ENSMUST00000210433
ENSMUSG	Gm45258	ENSMUST00000210042
ENSMUSG	Gm45330	ENSMUST00000209681
ENSMUSG	Gm5608	ENSMUST00000210253
ENSMUSG	Gm45678	ENSMUST00000210509
ENSMUSG	Gm45336	ENSMUST00000210010
ENSMUSG	Gm45557	ENSMUST00000210004
ENSMUSG	Gm36380	ENSMUST00000210339
ENSMUSG	Gm18192	ENSMUST00000210174
ENSMUSG	Gm45516	ENSMUST00000211588
ENSMUSG	Gm7419	ENSMUST00000209962
ENSMUSG	Gm45410	ENSMUST00000211331
ENSMUSG	Gm45420	ENSMUST00000211243
ENSMUSG	Gm45668	ENSMUST00000211271
ENSMUSG	Gm31152	ENSMUST00000210319
ENSMUSG	Gm45304	ENSMUST00000210303;ENSMUST00000210672
ENSMUSG	1700015	ENSMUST00000209585
ENSMUSG	Gm45584	ENSMUST00000211586
ENSMUSG	Vmn2r-p	ENSMUST00000169206
ENSMUSG	Gm45424	ENSMUST00000210575
ENSMUSG	Gm4157	ENSMUST00000211647
ENSMUSG	Gm32050	ENSMUST00000209793
ENSMUSG	Gm45687	ENSMUST00000209454
ENSMUSG	Gm45531	ENSMUST00000211096
ENSMUSG	Gm7514	ENSMUST00000209475
ENSMUSG	A030001	ENSMUST00000211533
ENSMUSG	Gm45566	ENSMUST00000209883
ENSMUSG	Gm45576	ENSMUST00000210981
ENSMUSG	Gm38947	ENSMUST00000209940
ENSMUSG	Gm45885	ENSMUST00000211520
ENSMUSG	Gm34853	ENSMUST00000211662
ENSMUSG	Gm45518	ENSMUST00000210776
ENSMUSG	Gm9619	ENSMUST00000211587
ENSMUSG	Gm45363	ENSMUST00000210196
ENSMUSG	Gm29895	ENSMUST00000209933
ENSMUSG	Gm45580	ENSMUST00000210430
ENSMUSG	Gm45301	ENSMUST00000211359
ENSMUSG	Gm30329	ENSMUST00000211572
ENSMUSG	Gm45579	ENSMUST00000209367
ENSMUSG	Gm45869	ENSMUST00000210335
ENSMUSG	Gm7269	ENSMUST00000210819
ENSMUSG	Gm9908	ENSMUST00000211534

ENSMUSG	Gm45457	ENSMUST00000209931
ENSMUSG	Gm6203	ENSMUST00000209575
ENSMUSG	Gm31727	ENSMUST00000210102
ENSMUSG	Gm45757	ENSMUST00000211835
ENSMUSG	4933406	ENSMUST00000212602
ENSMUSG	Rps7-ps	ENSMUST00000211928
ENSMUSG	Gm3635	ENSMUST00000212314
ENSMUSG	Gm45876	ENSMUST00000212698
ENSMUSG	Gm21320	ENSMUST00000211875
ENSMUSG	Gm45904	ENSMUST00000213082
ENSMUSG	Gm45860	ENSMUST00000212700
ENSMUSG	Gm45815	ENSMUST00000220241
ENSMUSG	Gm10358	ENSMUST00000212864
ENSMUSG	Gm3045	ENSMUST00000212844
ENSMUSG	Gm18000	ENSMUST00000212153
ENSMUSG	Gm45712	ENSMUST00000212417
ENSMUSG	Gm18035	ENSMUST00000211962
ENSMUSG	Vmn2r-p	ENSMUST00000213039
ENSMUSG	Gm45879	ENSMUST00000212521
ENSMUSG	Gm32568	ENSMUST00000212782
ENSMUSG	8030455	ENSMUST00000213014
ENSMUSG	Gm45908	ENSMUST00000212977
ENSMUSG	Gm7688	ENSMUST00000212857
ENSMUSG	Gm9644	ENSMUST00000212828
ENSMUSG	Olfr839	ENSMUST00000212127
ENSMUSG	Gm45776	ENSMUST00000212366
ENSMUSG	Olfr856	ENSMUST00000212177
ENSMUSG	Gm39228	ENSMUST00000212456
ENSMUSG	LOC6654	ENSMUST00000212284
ENSMUSG	Gm45708	ENSMUST00000212785
ENSMUSG	Gm39139	ENSMUST00000212619;ENSMUST00000212621
ENSMUSG	Gm35256	ENSMUST00000212780
ENSMUSG	Gm9204	ENSMUST00000212641
ENSMUSG	Gm45791	ENSMUST00000212908
ENSMUSG	Gm45778	ENSMUST00000211959
ENSMUSG	Gm30052	ENSMUST00000211951
ENSMUSG	4930488	ENSMUST00000212830
ENSMUSG	Gm8798	ENSMUST00000211877;ENSMUST00000213048
ENSMUSG	Gm8838	ENSMUST00000212978
ENSMUSG	Gm7769	ENSMUST00000212900
ENSMUSG	Gm45817	ENSMUST00000212738
ENSMUSG	Gm36879	ENSMUST00000212313
ENSMUSG	Gm5910	ENSMUST00000211819
ENSMUSG	Gm45733	ENSMUST00000212988
ENSMUSG	Gm16486	ENSMUST00000212436
ENSMUSG	Gm45805	ENSMUST00000212695
ENSMUSG	4930567	ENSMUST00000212260;ENSMUST00000213085
ENSMUSG	Gm32122	ENSMUST00000212119
ENSMUSG	Gm45895	ENSMUST00000212248
ENSMUSG	Gm39397	ENSMUST00000212331
ENSMUSG	Gm6831	ENSMUST00000213025;ENSMUST00000159332
ENSMUSG	Gm45903	ENSMUST00000212891
ENSMUSG	Gm45826	ENSMUST00000212226;ENSMUST00000212965
ENSMUSG	Olfr369	ENSMUST00000212589
ENSMUSG	Gm17745	ENSMUST00000212186
ENSMUSG	Gm38574	ENSMUST00000211855
ENSMUSG	Gm45770	ENSMUST00000212105

ENSMUSG	Gm45746	ENSMUST00000212623
ENSMUSG	Gm7684	ENSMUST00000211891
ENSMUSG	Gm45727	ENSMUST00000212886
ENSMUSG	Gm31805	ENSMUST00000211921
ENSMUSG	Gm31786	ENSMUST00000212305
ENSMUSG	Gm30132	ENSMUST00000212816;ENSMUST00000212930
ENSMUSG	Gm45786	ENSMUST00000213021
ENSMUSG	Olfr879	ENSMUST00000212455
ENSMUSG	Gm6796	ENSMUST00000211919
ENSMUSG	Gm5373	ENSMUST00000212931
ENSMUSG	Gm45764	ENSMUST00000213047
ENSMUSG	Gm45899	ENSMUST00000212196
ENSMUSG	Gm45875	ENSMUST00000212178
ENSMUSG	Gm20946	ENSMUST00000212367
ENSMUSG	Gm5357	ENSMUST00000212720
ENSMUSG	Gm6013	ENSMUST00000212264
ENSMUSG	Gnb1-ps	ENSMUST00000212191
ENSMUSG	C78859	ENSMUST00000212800
ENSMUSG	Gm39662	ENSMUST00000212057
ENSMUSG	Gm45910	ENSMUST00000212493
ENSMUSG	AC11847	ENSMUST00000215769
ENSMUSG	CT02561	ENSMUST00000214294
ENSMUSG	AC16409	ENSMUST00000215635
ENSMUSG	AC11847	ENSMUST00000214714
ENSMUSG	AC15863	ENSMUST00000215429
ENSMUSG	AC16635	ENSMUST00000215483
ENSMUSG	AC16012	ENSMUST00000213443
ENSMUSG	AC13319	ENSMUST00000217456
ENSMUSG	AC15197	ENSMUST00000217474
ENSMUSG	AC15930	ENSMUST00000217297
ENSMUSG	AC15372	ENSMUST00000214055
ENSMUSG	AC15930	ENSMUST00000215035
ENSMUSG	AC17120	ENSMUST00000215964
ENSMUSG	AC15429	ENSMUST00000217210
ENSMUSG	AC13485	ENSMUST00000214727
ENSMUSG	AC16033	ENSMUST00000215935
ENSMUSG	AC15862	ENSMUST00000216947
ENSMUSG	AC15564	ENSMUST00000213121
ENSMUSG	AC11672	ENSMUST00000215028
ENSMUSG	AC16488	ENSMUST00000215648
ENSMUSG	AC15683	ENSMUST00000216059
ENSMUSG	AC15184	ENSMUST00000216155;ENSMUST00000214657
ENSMUSG	CAAA012	ENSMUST00000215882
ENSMUSG	AC15335	ENSMUST00000217044
ENSMUSG	AC16293	ENSMUST00000216789
ENSMUSG	AC17120	ENSMUST00000213612
ENSMUSG	AC15989	ENSMUST00000215264
ENSMUSG	CT03072	ENSMUST00000216981
ENSMUSG	AC13166	ENSMUST00000216555
ENSMUSG	AC07924	ENSMUST00000215680
ENSMUSG	AC12233	ENSMUST00000216532
ENSMUSG	AC16797	ENSMUST00000214113;ENSMUST00000216190
ENSMUSG	AC13394	ENSMUST00000213336
ENSMUSG	CT03018	ENSMUST00000213150
ENSMUSG	AC15989	ENSMUST00000213295;ENSMUST00000217220
ENSMUSG	AC15859	ENSMUST00000215560
ENSMUSG	AC15386	ENSMUST00000215700

ENSMUSG	AC15382	ENSMUST00000214355
ENSMUSG	AC12758	ENSMUST00000214084
ENSMUSG	AC10766	ENSMUST00000215790
ENSMUSG	AC16124	ENSMUST00000217207
ENSMUSG	AC15747	ENSMUST00000215555
ENSMUSG	CT02561	ENSMUST00000214447
ENSMUSG	AC16410	ENSMUST00000214913
ENSMUSG	AC11308.	ENSMUST00000217324
ENSMUSG	AC12198	ENSMUST00000213571;ENSMUST00000215085
ENSMUSG	AL77358	ENSMUST00000216831
ENSMUSG	AC15524	ENSMUST00000216345
ENSMUSG	AC16416	ENSMUST00000217446
ENSMUSG	AL60363	ENSMUST00000214564
ENSMUSG	AC12625	ENSMUST00000213195
ENSMUSG	AC12383.	ENSMUST00000214120
ENSMUSG	AC11672	ENSMUST00000217629
ENSMUSG	AC15386.	ENSMUST00000214178;ENSMUST00000216664
ENSMUSG	AC11669	ENSMUST00000217638
ENSMUSG	AC13460	ENSMUST00000216324
ENSMUSG	AC12625	ENSMUST00000216414
ENSMUSG	AC13829.	ENSMUST00000216459
ENSMUSG	AL60363	ENSMUST00000216642
ENSMUSG	AC15663	ENSMUST00000215213
ENSMUSG	AC15862.	ENSMUST00000217047
ENSMUSG	AL66991	ENSMUST00000217640
ENSMUSG	AC16159	ENSMUST00000215261
ENSMUSG	AC10781	ENSMUST00000214053
ENSMUSG	AC15389	ENSMUST00000213761
ENSMUSG	AC15372	ENSMUST00000214434
ENSMUSG	AC13712	ENSMUST00000213466
ENSMUSG	CT02568	ENSMUST00000216399
ENSMUSG	AC12254.	ENSMUST00000213861
ENSMUSG	Olfr156	ENSMUST00000215406;ENSMUST00000079465
ENSMUSG	BX29466	ENSMUST00000215967
ENSMUSG	AC15835	ENSMUST00000215895
ENSMUSG	AC15397	ENSMUST00000216122
ENSMUSG	AC16371	ENSMUST00000215154;ENSMUST00000214500
ENSMUSG	AC10781	ENSMUST00000217455
ENSMUSG	AC14040	ENSMUST00000216204
ENSMUSG	AC16039	ENSMUST00000216012
ENSMUSG	AC16508	ENSMUST00000215257
ENSMUSG	AC15947	ENSMUST00000215786
ENSMUSG	CT01051	ENSMUST00000214088
ENSMUSG	AC12901	ENSMUST00000213973
ENSMUSG	CT01048	ENSMUST00000216866
ENSMUSG	AC12232	ENSMUST00000214199;ENSMUST00000214288
ENSMUSG	AC16012	ENSMUST00000215539
ENSMUSG	AC10393	ENSMUST00000216350
ENSMUSG	AC16488	ENSMUST00000217623
ENSMUSG	CAAA011.	ENSMUST00000215277
ENSMUSG	AC15395	ENSMUST00000217458
ENSMUSG	AC13873	ENSMUST00000216588
ENSMUSG	AC12625	ENSMUST00000215507
ENSMUSG	AC15795	ENSMUST00000215316
ENSMUSG	AC16462	ENSMUST00000214034
ENSMUSG	AC15638	ENSMUST00000215200;ENSMUST00000213206
ENSMUSG	AC13180	ENSMUST00000216477

ENSMUSG	AC15683.	ENSMUST00000217609
ENSMUSG	AC16409.	ENSMUST00000214808
ENSMUSG	AC15395	ENSMUST00000215835
ENSMUSG	AC13211	ENSMUST00000214720
ENSMUSG	AC15382	ENSMUST00000216808
ENSMUSG	AC15197	ENSMUST00000214694
ENSMUSG	AC15924	ENSMUST00000217438
ENSMUSG	AC16005	ENSMUST00000213341
ENSMUSG	AC15356	ENSMUST00000216441
ENSMUSG	AC02770	ENSMUST00000215400
ENSMUSG	AC15616	ENSMUST00000216958
ENSMUSG	AC16013	ENSMUST00000213450
ENSMUSG	AC14573	ENSMUST00000216269
ENSMUSG	AC15426	ENSMUST00000214060
ENSMUSG	CT01048	ENSMUST00000214890
ENSMUSG	AC15355	ENSMUST00000215135
ENSMUSG	AC16544.	ENSMUST00000214172
ENSMUSG	AC15184	ENSMUST00000215367
ENSMUSG	AC15765.	ENSMUST00000214505
ENSMUSG	AC16334.	ENSMUST00000216660;ENSMUST00000215241
ENSMUSG	AC15395	ENSMUST00000216739
ENSMUSG	AC15386	ENSMUST00000213468
ENSMUSG	AC10884	ENSMUST00000214817
ENSMUSG	AC24268.	ENSMUST00000213650
ENSMUSG	AC13861	ENSMUST00000215231
ENSMUSG	AL66991	ENSMUST00000213855
ENSMUSG	AC16124	ENSMUST00000217424
ENSMUSG	CT00972	ENSMUST00000213590
ENSMUSG	AC15683.	ENSMUST00000216217
ENSMUSG	Olfr133	ENSMUST00000077247
ENSMUSG	AC10040	ENSMUST00000217305
ENSMUSG	CT03018	ENSMUST00000216682
ENSMUSG	AC16005	ENSMUST00000217076;ENSMUST00000216597;ENSMUST00000217
ENSMUSG	AL66991	ENSMUST00000215976
ENSMUSG	AC16039	ENSMUST00000215460;ENSMUST00000215586
ENSMUSG	AC15356	ENSMUST00000215943
ENSMUSG	AC15485	ENSMUST00000214849
ENSMUSG	AC15795	ENSMUST00000217561
ENSMUSG	AC15389	ENSMUST00000213102
ENSMUSG	Olfr996	ENSMUST00000077075
ENSMUSG	AC15879	ENSMUST00000214686;ENSMUST00000217441
ENSMUSG	AC13239.	ENSMUST00000215412;ENSMUST00000216652
ENSMUSG	AC14040	ENSMUST00000213907;ENSMUST00000216792
ENSMUSG	CAAA012	ENSMUST00000216416
ENSMUSG	AC05581	ENSMUST00000213924
ENSMUSG	AC16159	ENSMUST00000216389
ENSMUSG	AC15981	ENSMUST00000216151
ENSMUSG	AC14227	ENSMUST00000213723
ENSMUSG	AC09152.	ENSMUST00000213934
ENSMUSG	AC11329	ENSMUST00000215490
ENSMUSG	AC11847	ENSMUST00000215206
ENSMUSG	AC13177	ENSMUST00000214742
ENSMUSG	AC15988	ENSMUST00000214737
ENSMUSG	AC15372	ENSMUST00000213319
ENSMUSG	Olfr132	ENSMUST00000215312;ENSMUST00000081960
ENSMUSG	CU02491	ENSMUST00000214310
ENSMUSG	AC15431	ENSMUST00000216198

ENSMUSG	AC12730	ENSMUST00000215973
ENSMUSG	CT57342	ENSMUST00000216858
ENSMUSG	AC11650	ENSMUST00000215445
ENSMUSG	AC17219	ENSMUST00000214246
ENSMUSG	AC16056	ENSMUST00000215132;ENSMUST00000216854
ENSMUSG	AC15982	ENSMUST00000215204
ENSMUSG	Olfr106	ENSMUST00000216165
ENSMUSG	AC15603	ENSMUST00000213107
ENSMUSG	AC15616	ENSMUST00000217004
ENSMUSG	AC12233	ENSMUST00000214384
ENSMUSG	AC15863	ENSMUST00000214387
ENSMUSG	AC15862	ENSMUST00000217107
ENSMUSG	AC13177	ENSMUST00000217302
ENSMUSG	AC15989	ENSMUST00000215904
ENSMUSG	AC16033	ENSMUST00000217068
ENSMUSG	AC16508	ENSMUST00000217162
ENSMUSG	AC15981	ENSMUST00000216940
ENSMUSG	AL66991	ENSMUST00000214874
ENSMUSG	AC15982	ENSMUST00000217265
ENSMUSG	AC13394	ENSMUST00000216737
ENSMUSG	AC15384	ENSMUST00000213596
ENSMUSG	AC16039	ENSMUST00000214108
ENSMUSG	AC11873	ENSMUST00000213986
ENSMUSG	AC14036	ENSMUST00000214327
ENSMUSG	AC11009	ENSMUST00000215107;ENSMUST00000216718
ENSMUSG	AC15395	ENSMUST00000216787
ENSMUSG	AC11719	ENSMUST00000213359
ENSMUSG	CT02575	ENSMUST00000213455
ENSMUSG	AC10255	ENSMUST00000216236
ENSMUSG	AC15353	ENSMUST00000214129
ENSMUSG	AC15747	ENSMUST00000217574
ENSMUSG	AC12532	ENSMUST00000214169
ENSMUSG	CT02575	ENSMUST00000213870
ENSMUSG	AC16409	ENSMUST00000213810
ENSMUSG	AC16416	ENSMUST00000214802
ENSMUSG	AC15338	ENSMUST00000214959
ENSMUSG	AC10785	ENSMUST00000217400;ENSMUST00000215827
ENSMUSG	AC15708	ENSMUST00000216343
ENSMUSG	AC10774	ENSMUST00000216972
ENSMUSG	AC14143	ENSMUST00000215068
ENSMUSG	AC15898	ENSMUST00000214586
ENSMUSG	AC15353	ENSMUST00000213330;ENSMUST00000217501;ENSMUST00000215
ENSMUSG	CT48560	ENSMUST00000214650
ENSMUSG	AC09871	ENSMUST00000214960;ENSMUST00000217021
ENSMUSG	AC12384	ENSMUST00000217622
ENSMUSG	AC15859	ENSMUST00000214349
ENSMUSG	CT03379	ENSMUST00000214262
ENSMUSG	AC13828	ENSMUST00000213982
ENSMUSG	AC11618	ENSMUST00000217447
ENSMUSG	AC16056	ENSMUST00000216374
ENSMUSG	AC16362	ENSMUST00000214545
ENSMUSG	AC12222	ENSMUST00000214812
ENSMUSG	CT48560	ENSMUST00000213187
ENSMUSG	AC12244	ENSMUST00000214851
ENSMUSG	CT02556	ENSMUST00000213430
ENSMUSG	AC11300	ENSMUST00000217248
ENSMUSG	AC15863	ENSMUST00000215162

ENSMUSG	AC15031	ENSMUST00000214798
ENSMUSG	AC15482	ENSMUST00000217369
ENSMUSG	AL66991	ENSMUST00000214610
ENSMUSG	CT03024	ENSMUST00000216353
ENSMUSG	CT00970	ENSMUST00000213736
ENSMUSG	AC15031	ENSMUST00000214766
ENSMUSG	AC15482	ENSMUST00000216531;ENSMUST00000216103;ENSMUST00000213
ENSMUSG	AC13424	ENSMUST00000216113
ENSMUSG	AC15395	ENSMUST00000213122
ENSMUSG	AC11009	ENSMUST00000213406;ENSMUST00000217104
ENSMUSG	AC16096	ENSMUST00000216860
ENSMUSG	AC11268	ENSMUST00000215435
ENSMUSG	AC16690	ENSMUST00000213191
ENSMUSG	CAAA011	ENSMUST00000217222
ENSMUSG	AC16159	ENSMUST00000214286
ENSMUSG	AC13712	ENSMUST00000215086;ENSMUST00000214671
ENSMUSG	AC15395	ENSMUST00000217008;ENSMUST00000214824
ENSMUSG	AC16461	ENSMUST00000214567
ENSMUSG	AL92922	ENSMUST00000217634
ENSMUSG	AC12218	ENSMUST00000216311
ENSMUSG	AC11724	ENSMUST00000217495
ENSMUSG	AC12303	ENSMUST00000213846
ENSMUSG	AC11503	ENSMUST00000215393
ENSMUSG	AC15947	ENSMUST00000216549
ENSMUSG	CT48560	ENSMUST00000216728
ENSMUSG	AC10775	ENSMUST00000215645;ENSMUST00000215724
ENSMUSG	AC11305	ENSMUST00000217423
ENSMUSG	AC15946	ENSMUST00000214968
ENSMUSG	AL77358	ENSMUST00000121340
ENSMUSG	CT00974	ENSMUST00000216896
ENSMUSG	AC15388	ENSMUST00000214448
ENSMUSG	AC06956	ENSMUST00000217321
ENSMUSG	AC15859	ENSMUST00000213397
ENSMUSG	AC16033	ENSMUST00000217247
ENSMUSG	AL92879	ENSMUST00000214405
ENSMUSG	AC13166	ENSMUST00000215630
ENSMUSG	AC12233	ENSMUST00000216029
ENSMUSG	AC12237	ENSMUST00000216741
ENSMUSG	AC16827	ENSMUST00000214611
ENSMUSG	AC12384	ENSMUST00000216137
ENSMUSG	Olfr157	ENSMUST00000214281;ENSMUST00000215442
ENSMUSG	CT48560	ENSMUST00000213361
ENSMUSG	AC10040	ENSMUST00000215709
ENSMUSG	AC17445	ENSMUST00000215932
ENSMUSG	AC09871	ENSMUST00000213527
ENSMUSG	AC15463	ENSMUST00000215334
ENSMUSG	AC11873	ENSMUST00000217387
ENSMUSG	CT00970	ENSMUST00000216464
ENSMUSG	AC13394	ENSMUST00000214867
ENSMUSG	AC12625	ENSMUST00000213112
ENSMUSG	AC13784	ENSMUST00000215190
ENSMUSG	AC12667	ENSMUST00000217096
ENSMUSG	AC15395	ENSMUST00000217069
ENSMUSG	AC15391	ENSMUST00000215353;ENSMUST00000217378
ENSMUSG	AC12680	ENSMUST00000217349
ENSMUSG	AC15440	ENSMUST00000215220
ENSMUSG	AC12679	ENSMUST00000217529

ENSMUSG	AC13168	ENSMUST00000217206
ENSMUSG	AC18326	ENSMUST00000215015
ENSMUSG	AC15627	ENSMUST00000214912
ENSMUSG	AC15356	ENSMUST00000213841
ENSMUSG	AC15426	ENSMUST00000216064
ENSMUSG	AC12730	ENSMUST00000215480
ENSMUSG	AC15592	ENSMUST00000215676
ENSMUSG	AC06956	ENSMUST00000215291
ENSMUSG	AC12537	ENSMUST00000214983
ENSMUSG	AC11847	ENSMUST00000215662
ENSMUSG	CT00972	ENSMUST00000217258
ENSMUSG	AC13177	ENSMUST00000214749
ENSMUSG	CAAA012	ENSMUST00000217183
ENSMUSG	AC15336	ENSMUST00000213572
ENSMUSG	AC16011	ENSMUST00000213706
ENSMUSG	Olfr113	ENSMUST00000099857
ENSMUSG	AC10038	ENSMUST00000215824;ENSMUST00000214496;ENSMUST00000213
ENSMUSG	AC15751	ENSMUST00000217201
ENSMUSG	AC12401	ENSMUST00000217030
ENSMUSG	AC16607	ENSMUST00000217211
ENSMUSG	AC15184	ENSMUST00000215960
ENSMUSG	AC16690	ENSMUST00000216696
ENSMUSG	AC12380	ENSMUST00000216093
ENSMUSG	AC12680	ENSMUST00000216630
ENSMUSG	AC12039	ENSMUST00000214091
ENSMUSG	AC14040	ENSMUST00000215601
ENSMUSG	AC12038	ENSMUST00000213434
ENSMUSG	AC15426	ENSMUST00000216569
ENSMUSG	AC12179	ENSMUST00000217127
ENSMUSG	AC16039	ENSMUST00000217392
ENSMUSG	AC12230	ENSMUST00000213594
ENSMUSG	Olfr896	ENSMUST00000215219
ENSMUSG	AC10766	ENSMUST00000216090
ENSMUSG	AC12370	ENSMUST00000214719
ENSMUSG	AC11464	ENSMUST00000216282
ENSMUSG	AC15862	ENSMUST00000217363
ENSMUSG	AC16637	ENSMUST00000213227
ENSMUSG	AC16334	ENSMUST00000216533
ENSMUSG	AL92868	ENSMUST00000215677
ENSMUSG	AC14036	ENSMUST00000217562;ENSMUST00000214646;ENSMUST00000213
ENSMUSG	AC16607	ENSMUST00000216894
ENSMUSG	AC15980	ENSMUST00000214276;ENSMUST00000214073
ENSMUSG	AC14046	ENSMUST00000216827
ENSMUSG	AC11352	ENSMUST00000217109
ENSMUSG	AC16159	ENSMUST00000216695
ENSMUSG	AC17334	ENSMUST00000216693
ENSMUSG	AC15372	ENSMUST00000217582
ENSMUSG	AC15627	ENSMUST00000214570
ENSMUSG	AC11330	ENSMUST00000214014
ENSMUSG	AC16409	ENSMUST00000214575
ENSMUSG	AC15679	ENSMUST00000217543
ENSMUSG	AC15823	ENSMUST00000214125
ENSMUSG	AC12901	ENSMUST00000217098
ENSMUSG	AC15395	ENSMUST00000218609
ENSMUSG	AC15190	ENSMUST00000218279
ENSMUSG	AC12240	ENSMUST00000218234
ENSMUSG	AC15929	ENSMUST00000218689

ENSMUSG	AC15184	ENSMUST00000218521
ENSMUSG	CR97459	ENSMUST00000220422
ENSMUSG	AC15388	ENSMUST00000218494
ENSMUSG	AC15880	ENSMUST00000219804
ENSMUSG	AC09346	ENSMUST00000219952
ENSMUSG	AC15571	ENSMUST00000219704
ENSMUSG	AC16290	ENSMUST00000219164
ENSMUSG	AC15294	ENSMUST00000218446
ENSMUSG	AC15349	ENSMUST00000219775
ENSMUSG	Gm3285	ENSMUST00000218443
ENSMUSG	AC15757	ENSMUST00000218052
ENSMUSG	AC15583	ENSMUST00000220050
ENSMUSG	CT01044	ENSMUST00000219471
ENSMUSG	AC15393	ENSMUST00000219466
ENSMUSG	AC12441	ENSMUST00000219800
ENSMUSG	AC10059	ENSMUST00000220318
ENSMUSG	AC16125	ENSMUST00000220310
ENSMUSG	AC10840	ENSMUST00000218323
ENSMUSG	AC15298	ENSMUST00000218320
ENSMUSG	AC15929	ENSMUST00000219567
ENSMUSG	AC14026	ENSMUST00000218322
ENSMUSG	AC15389	ENSMUST00000220021
ENSMUSG	AC15974	ENSMUST00000219903
ENSMUSG	AC13112	ENSMUST00000217728
ENSMUSG	AC15592	ENSMUST00000220217
ENSMUSG	AC15970	ENSMUST00000219576
ENSMUSG	AC15355	ENSMUST00000217665
ENSMUSG	AC15394	ENSMUST00000219384
ENSMUSG	AC12756	ENSMUST00000219270
ENSMUSG	AC12439	ENSMUST00000217847
ENSMUSG	AC15394	ENSMUST00000218173
ENSMUSG	AC16246	ENSMUST00000217825
ENSMUSG	AC15880	ENSMUST00000219495
ENSMUSG	AC15860	ENSMUST00000218203
ENSMUSG	AC13226	ENSMUST00000219233
ENSMUSG	AC10921	ENSMUST00000218168
ENSMUSG	AC16086	ENSMUST00000218412
ENSMUSG	AC15396	ENSMUST00000219391
ENSMUSG	AC15947	ENSMUST00000220010;ENSMUST00000219536;ENSMUST0000021
ENSMUSG	AC15190	ENSMUST00000218683
ENSMUSG	AC13238	ENSMUST00000219908
ENSMUSG	AC15350	ENSMUST00000218156
ENSMUSG	AC16720	ENSMUST00000219637
ENSMUSG	AC15385	ENSMUST00000219681
ENSMUSG	AC15386	ENSMUST00000219972
ENSMUSG	AC15352	ENSMUST00000220047
ENSMUSG	AC14026	ENSMUST00000219668;ENSMUST00000218746
ENSMUSG	AC15281	ENSMUST00000220435
ENSMUSG	AC15393	ENSMUST00000218734
ENSMUSG	AC15860	ENSMUST00000219760
ENSMUSG	AC07968	ENSMUST00000218350
ENSMUSG	AC16002	ENSMUST00000219594
ENSMUSG	AC13317	ENSMUST00000219319
ENSMUSG	AC12201	ENSMUST00000217769
ENSMUSG	AC15386	ENSMUST00000218482
ENSMUSG	CT01044	ENSMUST00000219963
ENSMUSG	Gm5136	ENSMUST00000218023

ENSMUSG	AC10488	ENSMUST00000218277;ENSMUST00000219371;ENSMUST0000021[illegible]
ENSMUSG	AL71398	ENSMUST00000219030
ENSMUSG	AC11081	ENSMUST00000218559
ENSMUSG	AC15295	ENSMUST00000217918
ENSMUSG	AC13453	ENSMUST00000219205
ENSMUSG	AC13394	ENSMUST00000217850
ENSMUSG	AC15353	ENSMUST00000218126
ENSMUSG	CT03003	ENSMUST00000219336;ENSMUST00000219411;ENSMUST0000021[illegible]
ENSMUSG	AC16111	ENSMUST00000218420
ENSMUSG	AC16111	ENSMUST00000218223
ENSMUSG	AC12443	ENSMUST00000217917
ENSMUSG	AC11723	ENSMUST00000217829
ENSMUSG	AC15860	ENSMUST00000220078
ENSMUSG	CT01046	ENSMUST00000218145;ENSMUST00000218836
ENSMUSG	AC13172	ENSMUST00000219113
ENSMUSG	AC11764	ENSMUST00000219777
ENSMUSG	AC16461	ENSMUST00000219348
ENSMUSG	AC15350	ENSMUST00000218819
ENSMUSG	AC10188	ENSMUST00000218838
ENSMUSG	AC15397	ENSMUST00000219416
ENSMUSG	AC16011	ENSMUST00000219139
ENSMUSG	AC13233	ENSMUST00000219429
ENSMUSG	AC12282	ENSMUST00000219396
ENSMUSG	AC13238	ENSMUST00000219985
ENSMUSG	AC12932	ENSMUST00000219787
ENSMUSG	AC15932	ENSMUST00000219989
ENSMUSG	AC15527	ENSMUST00000219719
ENSMUSG	AC15570	ENSMUST00000218794
ENSMUSG	AC12178	ENSMUST00000219700
ENSMUSG	AC15946	ENSMUST00000219578
ENSMUSG	AC15860	ENSMUST00000156803;ENSMUST00000217860
ENSMUSG	AC15430	ENSMUST00000219735
ENSMUSG	AC11529	ENSMUST00000219764
ENSMUSG	RMST_1	ENSMUST00000218060;ENSMUST00000218255;ENSMUST0000022[illegible]
ENSMUSG	AC15337	ENSMUST00000219456
ENSMUSG	AC16390	ENSMUST00000220322;ENSMUST00000219184;ENSMUST0000022[illegible]
ENSMUSG	AC16462	ENSMUST00000220250
ENSMUSG	Pbld1	ENSMUST00000219204;ENSMUST00000219687;ENSMUST0000002[illegible]
ENSMUSG	AC16827	ENSMUST00000218657
ENSMUSG	AC15385	ENSMUST00000219232
ENSMUSG	AC10231	ENSMUST00000218600;ENSMUST00000219530
ENSMUSG	AC11400	ENSMUST00000218097
ENSMUSG	AC11589	ENSMUST00000219161
ENSMUSG	AC15525	ENSMUST00000219812
ENSMUSG	AC14778	ENSMUST00000219747
ENSMUSG	AC15394	ENSMUST00000219080
ENSMUSG	AC10236	ENSMUST00000218415
ENSMUSG	AC16003	ENSMUST00000219914
ENSMUSG	AC16006	ENSMUST00000217700
ENSMUSG	AC15592	ENSMUST00000218541
ENSMUSG	AC15860	ENSMUST00000219737
ENSMUSG	AC11457	ENSMUST00000219468
ENSMUSG	CT00950	ENSMUST00000218252
ENSMUSG	AC15386	ENSMUST00000220084
ENSMUSG	AC15525	ENSMUST00000220213
ENSMUSG	CT00950	ENSMUST00000217951
ENSMUSG	AC16462	ENSMUST00000217916

ENSMUSG	AC15528	ENSMUST00000218632
ENSMUSG	AC15701	ENSMUST00000220148
ENSMUSG	AC13975	ENSMUST00000219418
ENSMUSG	AC15880	ENSMUST00000219452
ENSMUSG	AC15393	ENSMUST00000218485;ENSMUST00000219583
ENSMUSG	AC16702	ENSMUST00000218647
ENSMUSG	AC12933	ENSMUST00000220224
ENSMUSG	AC15314	ENSMUST00000217958
ENSMUSG	AC15354	ENSMUST00000218501
ENSMUSG	AC11457	ENSMUST00000218634
ENSMUSG	AC15353	ENSMUST00000219395
ENSMUSG	AC15385	ENSMUST00000219147
ENSMUSG	AC16456	ENSMUST00000218175
ENSMUSG	AC13975	ENSMUST00000219550
ENSMUSG	Krtap10	ENSMUST00000218843
ENSMUSG	AC15297	ENSMUST00000219702
ENSMUSG	AC15701	ENSMUST00000218833
ENSMUSG	AC15393	ENSMUST00000220354
ENSMUSG	AC13975	ENSMUST00000218037
ENSMUSG	AC15860	ENSMUST00000219191
ENSMUSG	AL58966	ENSMUST00000217799
ENSMUSG	AC12187	ENSMUST00000219123
ENSMUSG	AC16003	ENSMUST00000218242
ENSMUSG	AC15385	ENSMUST00000219565
ENSMUSG	AC12553	ENSMUST00000219849
ENSMUSG	AC15189	ENSMUST00000219025
ENSMUSG	AC12180	ENSMUST00000218509
ENSMUSG	AC15431	ENSMUST00000217666
ENSMUSG	AC16831	ENSMUST00000217977
ENSMUSG	AC16722	ENSMUST00000219379
ENSMUSG	AC10188	ENSMUST00000220432;ENSMUST00000219484
ENSMUSG	AC15354	ENSMUST00000218014
ENSMUSG	AC12727	ENSMUST00000220140
ENSMUSG	AC07968	ENSMUST00000219398
ENSMUSG	AC15396	ENSMUST00000218182;ENSMUST00000164429
ENSMUSG	AC15393	ENSMUST00000218396
ENSMUSG	AC15393	ENSMUST00000218518
ENSMUSG	AC16827	ENSMUST00000218963
ENSMUSG	AC15294	ENSMUST00000219071
ENSMUSG	AC15380	ENSMUST00000219875
ENSMUSG	AC12040	ENSMUST00000220362
ENSMUSG	AC15991	ENSMUST00000219489
ENSMUSG	AC16040	ENSMUST00000218207
ENSMUSG	AC15592	ENSMUST00000217963
ENSMUSG	AC11227	ENSMUST00000218957
ENSMUSG	CT57124	ENSMUST00000218642
ENSMUSG	AC15571	ENSMUST00000220398
ENSMUSG	AC11597	ENSMUST00000218910
ENSMUSG	AC15241	ENSMUST00000218784
ENSMUSG	AC15354	ENSMUST00000219283
ENSMUSG	AC15384	ENSMUST00000219685
ENSMUSG	AC11655	ENSMUST00000219494
ENSMUSG	AC11004	ENSMUST00000220278
ENSMUSG	AC11529	ENSMUST00000219802
ENSMUSG	AC16805	ENSMUST00000219521
ENSMUSG	AC15839	ENSMUST00000218356
ENSMUSG	AC15353	ENSMUST00000219753

ENSMUSG	AC15938	ENSMUST00000218096
ENSMUSG	AC15355	ENSMUST00000218840
ENSMUSG	AC15859	ENSMUST00000217989
ENSMUSG	AC10880	ENSMUST00000219501
ENSMUSG	AC13238	ENSMUST00000220198
ENSMUSG	AC15352	ENSMUST00000220145
ENSMUSG	AC15294	ENSMUST00000219424
ENSMUSG	AC15854	ENSMUST00000219657
ENSMUSG	AC11723	ENSMUST00000217817
ENSMUSG	AC15583	ENSMUST00000218326
ENSMUSG	AC15354	ENSMUST00000219066
ENSMUSG	AC12253	ENSMUST00000220207
ENSMUSG	AC15860	ENSMUST00000219969
ENSMUSG	AC15351	ENSMUST00000220363
ENSMUSG	AC10840	ENSMUST00000219740
ENSMUSG	AC15929	ENSMUST00000218335
ENSMUSG	AC12282	ENSMUST00000219743
ENSMUSG	AC15336	ENSMUST00000220000
ENSMUSG	AC16086	ENSMUST00000218392
ENSMUSG	AC15928	ENSMUST00000220232;ENSMUST00000219472
ENSMUSG	AC15355	ENSMUST00000219474
ENSMUSG	AC15583	ENSMUST00000219077
ENSMUSG	CT00950	ENSMUST00000218349
ENSMUSG	AC10236	ENSMUST00000219165
ENSMUSG	AC15190	ENSMUST00000218144
ENSMUSG	AC15860	ENSMUST00000179851
ENSMUSG	AC15663	ENSMUST00000218639
ENSMUSG	AC12536	ENSMUST00000220065
ENSMUSG	AC12201	ENSMUST00000218775
ENSMUSG	AC15336	ENSMUST00000218604
ENSMUSG	AC15351	ENSMUST00000220426
ENSMUSG	AC10231	ENSMUST00000220039
ENSMUSG	AC12221	ENSMUST00000220216
ENSMUSG	AC15354	ENSMUST00000218300
ENSMUSG	AC16722	ENSMUST00000219947
ENSMUSG	AC16636	ENSMUST00000218567
ENSMUSG	AC11646	ENSMUST00000220324
ENSMUSG	AC13501	ENSMUST00000218113
ENSMUSG	AC12236	ENSMUST00000217737
ENSMUSG	AC15352	ENSMUST00000217713
ENSMUSG	AC16456	ENSMUST00000219363
ENSMUSG	AC15350	ENSMUST00000219287
ENSMUSG	AC15571	ENSMUST00000220248
ENSMUSG	AC15663	ENSMUST00000218845
ENSMUSG	AC16237	ENSMUST00000220120
ENSMUSG	AC15860	ENSMUST00000177744
ENSMUSG	AC09971	ENSMUST00000217919
ENSMUSG	AL69150	ENSMUST00000219965
ENSMUSG	AC15394	ENSMUST00000217798
ENSMUSG	AC15294	ENSMUST00000218988
ENSMUSG	AC24153	ENSMUST00000218537
ENSMUSG	AC15336	ENSMUST00000220081
ENSMUSG	AC15351	ENSMUST00000217783
ENSMUSG	AC12223	ENSMUST00000219622
ENSMUSG	AC15352	ENSMUST00000218389
ENSMUSG	AC11109	ENSMUST00000220181
ENSMUSG	AC10921	ENSMUST00000218403

ENSMUSG AC16462 ENSMUST00000218505
ENSMUSG AC12202 ENSMUST00000220086
ENSMUSG AC13501 ENSMUST00000219949
ENSMUSG AC12441 ENSMUST00000220364;ENSMUST00000218852
ENSMUSG AC15352 ENSMUST00000218919
ENSMUSG AC14763 ENSMUST00000217857;ENSMUST00000218418;ENSMUST0000022C
ENSMUSG AC06907 ENSMUST00000220339
ENSMUSG AC16002 ENSMUST00000219645
ENSMUSG AC12756 ENSMUST00000219006
ENSMUSG AC12441 ENSMUST00000218056
ENSMUSG AC16006 ENSMUST00000217840
ENSMUSG AC14778 ENSMUST00000218390
ENSMUSG AC12198 ENSMUST00000218588
ENSMUSG AC15529 ENSMUST00000217973
ENSMUSG AC14761 ENSMUST00000219876
ENSMUSG AC11578 ENSMUST00000219480
ENSMUSG AC13585 ENSMUST00000217808
ENSMUSG AC12933 ENSMUST00000218053
ENSMUSG AC15395 ENSMUST00000219916
ENSMUSG AC16529 ENSMUST00000219669
ENSMUSG AC15385 ENSMUST00000218318
ENSMUSG AC15352 ENSMUST00000218661
ENSMUSG AC15350 ENSMUST00000219794
ENSMUSG CT57124 ENSMUST00000219694
ENSMUSG CT01046 ENSMUST00000219954
ENSMUSG AC15356 ENSMUST00000218424
ENSMUSG AC16470 ENSMUST00000218711
ENSMUSG AC12180 ENSMUST00000218808
ENSMUSG AC13238 ENSMUST00000217862
ENSMUSG AC16462 ENSMUST00000220387
ENSMUSG AC15350 ENSMUST00000219540
ENSMUSG AC14710 ENSMUST00000219926
ENSMUSG AC13238 ENSMUST00000219485
ENSMUSG AC10059 ENSMUST00000219355
ENSMUSG CT01043 ENSMUST00000218781
ENSMUSG AC14724 ENSMUST00000218767
ENSMUSG AC15349 ENSMUST00000218664
ENSMUSG CT57336 ENSMUST00000218233
ENSMUSG AC16086 ENSMUST00000220129
ENSMUSG AC16124 ENSMUST00000217744
ENSMUSG CR97458 ENSMUST00000220112
ENSMUSG AC12536 ENSMUST00000218548
ENSMUSG AC15385 ENSMUST00000218405
ENSMUSG AC12504 ENSMUST00000219023
ENSMUSG AC16002 ENSMUST00000218937
ENSMUSG AC16456 ENSMUST00000220414
ENSMUSG AC12282 ENSMUST00000218164
ENSMUSG AC12285 ENSMUST00000220380
ENSMUSG AC12178 ENSMUST00000218915
ENSMUSG AC15354 ENSMUST00000218080
ENSMUSG AC16722 ENSMUST00000220057
ENSMUSG CT02562 ENSMUST00000217786
ENSMUSG AC13238 ENSMUST00000219676
ENSMUSG AC11580 ENSMUST00000217680
ENSMUSG AC15206 ENSMUST00000218129
ENSMUSG AC11457 ENSMUST00000220417
ENSMUSG AC15964 ENSMUST00000219860

ENSMUSG	AC15387	ENSMUST00000218749
ENSMUSG	AC16636	ENSMUST00000218578;ENSMUST00000219560;ENSMUST0000021[illegible]
ENSMUSG	AC16041	ENSMUST00000219298
ENSMUSG	AC16636	ENSMUST00000219269
ENSMUSG	AC15448	ENSMUST00000219713
ENSMUSG	AC13238	ENSMUST00000219608
ENSMUSG	AC15353	ENSMUST00000218660
ENSMUSG	AC15593	ENSMUST00000219498
ENSMUSG	AC15571	ENSMUST00000219913
ENSMUSG	AC15564	ENSMUST00000219975
ENSMUSG	AC15314	ENSMUST00000218636
ENSMUSG	AC16457	ENSMUST00000218783
ENSMUSG	AC15394	ENSMUST00000220149
ENSMUSG	AC15388	ENSMUST00000219160
ENSMUSG	AC15354	ENSMUST00000219115
ENSMUSG	AC13238	ENSMUST00000219556
ENSMUSG	AC15592	ENSMUST00000218493
ENSMUSG	AC16328	ENSMUST00000218103
ENSMUSG	AC11104	ENSMUST00000217715
ENSMUSG	AC11989	ENSMUST00000217997
ENSMUSG	AC10231	ENSMUST00000219399
ENSMUSG	AC16524	ENSMUST00000218693
ENSMUSG	AC13501	ENSMUST00000217849
ENSMUSG	AC15294	ENSMUST00000218030
ENSMUSG	AC12722	ENSMUST00000218024
ENSMUSG	AC12548	ENSMUST00000218701
ENSMUSG	AC13452	ENSMUST00000219022
ENSMUSG	AC16827	ENSMUST00000218472
ENSMUSG	AC15626	ENSMUST00000218495
ENSMUSG	AC13176	ENSMUST00000219035
ENSMUSG	AC15937	ENSMUST00000218504
ENSMUSG	AC16722	ENSMUST00000220014
ENSMUSG	AC12369	ENSMUST00000219525
ENSMUSG	AC15583	ENSMUST00000220270
ENSMUSG	AC15594	ENSMUST00000218925;ENSMUST00000218707
ENSMUSG	AC15695	ENSMUST00000219604
ENSMUSG	AC15594	ENSMUST00000218774
ENSMUSG	AC15721	ENSMUST00000218295
ENSMUSG	AC07968	ENSMUST00000217890
ENSMUSG	AC15393	ENSMUST00000218388
ENSMUSG	AC13963	ENSMUST00000219785
ENSMUSG	AC13452	ENSMUST00000219611
ENSMUSG	AC13432	ENSMUST00000217897
ENSMUSG	AC13159	ENSMUST00000219638
ENSMUSG	AC16237	ENSMUST00000218927
ENSMUSG	AC12283	ENSMUST00000218818;ENSMUST00000220293
ENSMUSG	CT03003	ENSMUST00000218809
ENSMUSG	AC16086	ENSMUST00000218099
ENSMUSG	AC15583	ENSMUST00000220033
ENSMUSG	AC16885	ENSMUST00000218409
ENSMUSG	AC16006	ENSMUST00000220074
ENSMUSG	AC15860	ENSMUST00000218329
ENSMUSG	AC15396	ENSMUST00000218195
ENSMUSG	AC12128	ENSMUST00000218069
ENSMUSG	AC16329	ENSMUST00000220177
ENSMUSG	AC11081	ENSMUST00000220360
ENSMUSG	AC14249	ENSMUST00000219818

ENSMUSG	AC15757	ENSMUST00000219221;ENSMUST00000218017
ENSMUSG	AC12369	ENSMUST00000220040
ENSMUSG	AC15529	ENSMUST00000219793
ENSMUSG	AC15396	ENSMUST00000217878
ENSMUSG	AC10231	ENSMUST00000218890
ENSMUSG	AC15932	ENSMUST00000217933
ENSMUSG	AC15350	ENSMUST00000219984;ENSMUST00000217795;ENSMUST00000219
ENSMUSG	CT01049	ENSMUST00000220115
ENSMUSG	AC15860	ENSMUST00000177762
ENSMUSG	AC15880	ENSMUST00000220024
ENSMUSG	AC15383	ENSMUST00000218522
ENSMUSG	AC24153	ENSMUST00000217932;ENSMUST00000220320
ENSMUSG	AC11989	ENSMUST00000220132
ENSMUSG	AC11227	ENSMUST00000218436
ENSMUSG	AC12930	ENSMUST00000218870
ENSMUSG	AC16456	ENSMUST00000217743
ENSMUSG	AC02591	ENSMUST00000220136;ENSMUST00000219248
ENSMUSG	AC15297	ENSMUST00000220221
ENSMUSG	AC15431	ENSMUST00000217755
ENSMUSG	AC15336	ENSMUST00000218188
ENSMUSG	CAAA011	ENSMUST00000220168
ENSMUSG	AC15403	ENSMUST00000219032
ENSMUSG	AC15396	ENSMUST00000220106
ENSMUSG	Gm4305	ENSMUST00000219159
ENSMUSG	AC12455	ENSMUST00000219581
ENSMUSG	AC15860	ENSMUST00000218133
ENSMUSG	AC15525	ENSMUST00000218751
ENSMUSG	AC15396	ENSMUST00000219333
ENSMUSG	AC11580	ENSMUST00000219170
ENSMUSG	AC12219	ENSMUST00000219196
ENSMUSG	AC15529	ENSMUST00000220011
ENSMUSG	AC16625	ENSMUST00000219081
ENSMUSG	AC12303	ENSMUST00000220383
ENSMUSG	AC13238	ENSMUST00000219613
ENSMUSG	AC15701	ENSMUST00000218354
ENSMUSG	AC12253	ENSMUST00000218980
ENSMUSG	AC11719	ENSMUST00000219940
ENSMUSG	AC13112	ENSMUST00000219845
ENSMUSG	AC11823	ENSMUST00000220196
ENSMUSG	AC15295	ENSMUST00000218293
ENSMUSG	AC15860	ENSMUST00000178998
ENSMUSG	AC13230	ENSMUST00000218193
ENSMUSG	CT57125	ENSMUST00000218047
ENSMUSG	Gm4303	ENSMUST00000218922
ENSMUSG	AC16701	ENSMUST00000222879
ENSMUSG	CT01047	ENSMUST00000223429
ENSMUSG	AC16444	ENSMUST00000221369;ENSMUST00000223430
ENSMUSG	AC12655	ENSMUST00000222884
ENSMUSG	AC12473	ENSMUST00000223521
ENSMUSG	CT57298	ENSMUST00000220599
ENSMUSG	AC21187	ENSMUST00000221137
ENSMUSG	AC12255	ENSMUST00000222329
ENSMUSG	AC15455	ENSMUST00000222969
ENSMUSG	AL51302	ENSMUST00000222963;ENSMUST00000221792
ENSMUSG	AC13260	ENSMUST00000220808
ENSMUSG	AC16699	ENSMUST00000222922
ENSMUSG	AC15656	ENSMUST00000221425

ENSMUSG	AC15663.	ENSMUST00000220626
ENSMUSG	CT01045	ENSMUST00000222789
ENSMUSG	AC12231	ENSMUST00000221381
ENSMUSG	CT03378	ENSMUST00000220881
ENSMUSG	AC12284	ENSMUST00000221476
ENSMUSG	AC12451	ENSMUST00000221845
ENSMUSG	AC15435.	ENSMUST00000221477
ENSMUSG	AC15924	ENSMUST00000221663
ENSMUSG	AC16011	ENSMUST00000222369
ENSMUSG	AC11722	ENSMUST00000221635
ENSMUSG	AC11768	ENSMUST00000222104
ENSMUSG	AC12219	ENSMUST00000222187
ENSMUSG	AC15926	ENSMUST00000222236
ENSMUSG	AC21187	ENSMUST00000220838
ENSMUSG	AC13308	ENSMUST00000221736
ENSMUSG	AL51302.	ENSMUST00000220489
ENSMUSG	CT03016	ENSMUST00000222654
ENSMUSG	AC12219	ENSMUST00000221429;ENSMUST00000220837
ENSMUSG	CT00975	ENSMUST00000223489
ENSMUSG	AC15863	ENSMUST00000222959
ENSMUSG	AC13994	ENSMUST00000221517
ENSMUSG	AC14716.	ENSMUST00000221947
ENSMUSG	CT02555	ENSMUST00000222965
ENSMUSG	AC14047	ENSMUST00000222354
ENSMUSG	AC11591	ENSMUST00000221188
ENSMUSG	AC15656	ENSMUST00000220530
ENSMUSG	AC10489	ENSMUST00000222638
ENSMUSG	AC07964	ENSMUST00000220586
ENSMUSG	AC12544	ENSMUST00000222600
ENSMUSG	AC13262	ENSMUST00000221818
ENSMUSG	AC07964	ENSMUST00000222108
ENSMUSG	AC16365	ENSMUST00000222758;ENSMUST00000222985
ENSMUSG	CT03378	ENSMUST00000221347
ENSMUSG	AC12417	ENSMUST00000222678
ENSMUSG	CT02561	ENSMUST00000222331
ENSMUSG	AC15981	ENSMUST00000223227
ENSMUSG	AL80782	ENSMUST00000223151
ENSMUSG	AC13262	ENSMUST00000223323;ENSMUST00000221094
ENSMUSG	AC16099	ENSMUST00000221765
ENSMUSG	AC15657	ENSMUST00000222315
ENSMUSG	AC12475	ENSMUST00000222716
ENSMUSG	CT48561.	ENSMUST00000223359
ENSMUSG	AC13308	ENSMUST00000222616
ENSMUSG	CT03018	ENSMUST00000220846
ENSMUSG	AC15563	ENSMUST00000221687
ENSMUSG	CT02557	ENSMUST00000220862
ENSMUSG	AC13223	ENSMUST00000220950
ENSMUSG	AL92905	ENSMUST00000223545
ENSMUSG	CT02571	ENSMUST00000222949
ENSMUSG	AC11722	ENSMUST00000223449;ENSMUST00000221251;ENSMUST0000022C
ENSMUSG	AC12417	ENSMUST00000222428
ENSMUSG	CT03013	ENSMUST00000222223
ENSMUSG	AC12535	ENSMUST00000221438
ENSMUSG	AC15923	ENSMUST00000220765;ENSMUST00000223513
ENSMUSG	AC13241	ENSMUST00000223085
ENSMUSG	AC13830	ENSMUST00000222541
ENSMUSG	AC09145	ENSMUST00000221773

ENSMUSG	AC19261	ENSMUST00000091545
ENSMUSG	AC15474	ENSMUST00000223477
ENSMUSG	CT00976	ENSMUST00000221353
ENSMUSG	CR97448	ENSMUST00000220925
ENSMUSG	AC15988	ENSMUST00000222636
ENSMUSG	AC15478	ENSMUST00000223522
ENSMUSG	CT02561	ENSMUST00000221428
ENSMUSG	AC11647	ENSMUST00000222381
ENSMUSG	AC16410	ENSMUST00000221671
ENSMUSG	AC12507	ENSMUST00000222073
ENSMUSG	AC13223	ENSMUST00000223062;ENSMUST00000222335
ENSMUSG	AC15919	ENSMUST00000220879
ENSMUSG	AC15854	ENSMUST00000220533
ENSMUSG	AC15924	ENSMUST00000223195
ENSMUSG	AC13132	ENSMUST00000221204
ENSMUSG	AC15920	ENSMUST00000222039
ENSMUSG	CT03065	ENSMUST00000222945
ENSMUSG	CT03378	ENSMUST00000222807
ENSMUSG	AC15524	ENSMUST00000221547
ENSMUSG	AC09883	ENSMUST00000223038
ENSMUSG	AL60678	ENSMUST00000221898
ENSMUSG	AC10256	ENSMUST00000221563
ENSMUSG	AC21187	ENSMUST00000222629
ENSMUSG	AC16416	ENSMUST00000223245
ENSMUSG	AC17321	ENSMUST00000222059
ENSMUSG	AC16416	ENSMUST00000222736
ENSMUSG	AC12237	ENSMUST00000223164
ENSMUSG	AC12235	ENSMUST00000221234
ENSMUSG	AC12459	ENSMUST00000221909
ENSMUSG	CT03016	ENSMUST00000223203
ENSMUSG	AC17321	ENSMUST00000222341
ENSMUSG	AC12201	ENSMUST00000222380
ENSMUSG	CT00948	ENSMUST00000223306
ENSMUSG	AC14958	ENSMUST00000223088;ENSMUST00000222997
ENSMUSG	AC12237	ENSMUST00000220876
ENSMUSG	AC11350	ENSMUST00000222964
ENSMUSG	AC16634	ENSMUST00000220866
ENSMUSG	AC17321	ENSMUST00000221778
ENSMUSG	CT03015	ENSMUST00000222077
ENSMUSG	AC15424	ENSMUST00000220900
ENSMUSG	AC14028	ENSMUST00000222673
ENSMUSG	AC16304	ENSMUST00000221984
ENSMUSG	AL60652	ENSMUST00000222648;ENSMUST00000223473
ENSMUSG	AC12531	ENSMUST00000221002
ENSMUSG	AC15926	ENSMUST00000220746
ENSMUSG	AC15282	ENSMUST00000221143
ENSMUSG	AC12727	ENSMUST00000221441
ENSMUSG	AL45032	ENSMUST00000220600
ENSMUSG	AC15981	ENSMUST00000223362
ENSMUSG	AC12655	ENSMUST00000220966
ENSMUSG	CT48561	ENSMUST00000222555
ENSMUSG	AC15434	ENSMUST00000221223
ENSMUSG	AC15490	ENSMUST00000222121
ENSMUSG	AC19261	ENSMUST00000221482
ENSMUSG	AC12679	ENSMUST00000223112
ENSMUSG	AC13243	ENSMUST00000221553
ENSMUSG	AC16291	ENSMUST00000221219

ENSMUSG AC16013 ENSMUST00000220734
ENSMUSG AC15366 ENSMUST00000222696
ENSMUSG AC16444 ENSMUST00000220818
ENSMUSG AC15657 ENSMUST00000221316
ENSMUSG CT02555 ENSMUST00000223406
ENSMUSG AC10222 ENSMUST00000222882
ENSMUSG AC15523 ENSMUST00000223301;ENSMUST00000222644
ENSMUSG AL59050 ENSMUST00000222053
ENSMUSG AC07964 ENSMUST00000222643
ENSMUSG AC15961 ENSMUST00000221134
ENSMUSG AC12451 ENSMUST00000222659
ENSMUSG AL71402 ENSMUST00000221461
ENSMUSG AC15931 ENSMUST00000221508
ENSMUSG CT03016 ENSMUST00000223027
ENSMUSG AL58973 ENSMUST00000221163
ENSMUSG AC16011 ENSMUST00000220498;ENSMUST00000222732
ENSMUSG AC16335 ENSMUST00000220847
ENSMUSG AC13234 ENSMUST00000221289
ENSMUSG AC15426 ENSMUST00000223190
ENSMUSG Gm16381 ENSMUST00000220528
ENSMUSG AC15926 ENSMUST00000222493
ENSMUSG AC17120 ENSMUST00000220697
ENSMUSG AC14833 ENSMUST00000220853
ENSMUSG CT00975 ENSMUST00000222946
ENSMUSG AC15455 ENSMUST00000220741
ENSMUSG AC12537 ENSMUST00000222518
ENSMUSG AC15448 ENSMUST00000223248
ENSMUSG AC15931 ENSMUST00000220981
ENSMUSG AL59120 ENSMUST00000223299
ENSMUSG AC15423 ENSMUST00000222962
ENSMUSG AC12284 ENSMUST00000220915
ENSMUSG CT00974 ENSMUST00000222877
ENSMUSG AC15448 ENSMUST00000222526
ENSMUSG AC10483 ENSMUST00000222938
ENSMUSG AC12534 ENSMUST00000222565
ENSMUSG AC13482 ENSMUST00000223313
ENSMUSG AC06845 ENSMUST00000223443
ENSMUSG AC14454 ENSMUST00000221840
ENSMUSG CT57299 ENSMUST00000222755
ENSMUSG AC12288 ENSMUST00000223330
ENSMUSG AC12548 ENSMUST00000221672;ENSMUST00000221989
ENSMUSG AC17348 ENSMUST00000223351
ENSMUSG AC15921 ENSMUST00000221315
ENSMUSG AC16514 ENSMUST00000222594
ENSMUSG AC10483 ENSMUST00000222611
ENSMUSG AC12451 ENSMUST00000222149
ENSMUSG CT02560 ENSMUST00000221355
ENSMUSG AC11658 ENSMUST00000099421
ENSMUSG AC11052 ENSMUST00000221823
ENSMUSG CT03026 ENSMUST00000223243
ENSMUSG AL59120 ENSMUST00000220800;ENSMUST00000220793;ENSMUST00000222
ENSMUSG AC15981 ENSMUST00000221762
ENSMUSG CT48561 ENSMUST00000220589
ENSMUSG AC13232 ENSMUST00000221434
ENSMUSG CT02561 ENSMUST00000222537
ENSMUSG CT01047 ENSMUST00000223481
ENSMUSG AC15920 ENSMUST00000220980

ENSMUSG AC09145 ENSMUST00000222054
ENSMUSG AC15749 ENSMUST00000221127
ENSMUSG AC16280 ENSMUST00000220612
ENSMUSG AC12451 ENSMUST00000220507
ENSMUSG AC12468 ENSMUST00000222361
ENSMUSG AC15929 ENSMUST00000221907;ENSMUST00000223144
ENSMUSG CT48561 ENSMUST00000221159
ENSMUSG AC15463 ENSMUST00000221433
ENSMUSG AC09271 ENSMUST00000221878
ENSMUSG AC12237 ENSMUST00000220583
ENSMUSG AC12519 ENSMUST00000222046
ENSMUSG AC09271 ENSMUST00000220479
ENSMUSG AC12451 ENSMUST00000221455
ENSMUSG AC15664 ENSMUST00000223066
ENSMUSG AC13938 ENSMUST00000221402
ENSMUSG CT02553 ENSMUST00000221862
ENSMUSG AC16335 ENSMUST00000220620;ENSMUST00000221460;ENSMUST00000222
ENSMUSG CT03018 ENSMUST00000220991
ENSMUSG AC15315 ENSMUST00000222188
ENSMUSG AC15580 ENSMUST00000222419
ENSMUSG CT00975 ENSMUST00000221298
ENSMUSG AC15923 ENSMUST00000222396;ENSMUST00000222409
ENSMUSG AC12458 ENSMUST00000221673
ENSMUSG AC14958 ENSMUST00000222647
ENSMUSG AC12158 ENSMUST00000220672;ENSMUST00000220728;ENSMUST00000221
ENSMUSG AC15282 ENSMUST00000221819
ENSMUSG AL64570 ENSMUST00000221642
ENSMUSG AC15198 ENSMUST00000223546
ENSMUSG AC13232 ENSMUST00000222619
ENSMUSG AC16293 ENSMUST00000223516
ENSMUSG AC11344 ENSMUST00000221538
ENSMUSG AC10243 ENSMUST00000222199;ENSMUST00000222189;ENSMUST00000221
ENSMUSG AC16076 ENSMUST00000222580;ENSMUST00000223209
ENSMUSG AC16363 ENSMUST00000221012;ENSMUST00000223077
ENSMUSG AC15962 ENSMUST00000221010
ENSMUSG AC12284 ENSMUST00000221474
ENSMUSG AC16335 ENSMUST00000221995
ENSMUSG CT00975 ENSMUST00000222318
ENSMUSG AC15657 ENSMUST00000220840
ENSMUSG CR97457 ENSMUST00000221733
ENSMUSG AC13223 ENSMUST00000223157
ENSMUSG AC13446 ENSMUST00000220693
ENSMUSG CT01045 ENSMUST00000222665
ENSMUSG AC12054 ENSMUST00000221716
ENSMUSG AC13262 ENSMUST00000221314;ENSMUST00000221669;ENSMUST00000223
ENSMUSG AC16010 ENSMUST00000222495
ENSMUSG AC16525 ENSMUST00000220736;ENSMUST00000222925
ENSMUSG AC16455 ENSMUST00000222961
ENSMUSG AC15282 ENSMUST00000222264
ENSMUSG AC13994 ENSMUST00000221565
ENSMUSG AC13697 ENSMUST00000221440
ENSMUSG CT03017 ENSMUST00000220822
ENSMUSG AC15962 ENSMUST00000220994
ENSMUSG AC15927 ENSMUST00000220972
ENSMUSG AC12655 ENSMUST00000222919
ENSMUSG AC16416 ENSMUST00000221809
ENSMUSG AC13175 ENSMUST00000221636

ENSMUSG	AC15920	ENSMUST00000221326
ENSMUSG	CT48561.	ENSMUST00000222722
ENSMUSG	AC16092	ENSMUST00000221685
ENSMUSG	AC12477	ENSMUST00000220656
ENSMUSG	CU02489	ENSMUST00000221361
ENSMUSG	AC12901	ENSMUST00000220725
ENSMUSG	AC12544	ENSMUST00000222182
ENSMUSG	CT57298	ENSMUST00000221057
ENSMUSG	AC12451	ENSMUST00000221302
ENSMUSG	AC15657	ENSMUST00000222752
ENSMUSG	CT95432	ENSMUST00000223310;ENSMUST00000220979
ENSMUSG	CR97442	ENSMUST00000220877
ENSMUSG	AC16524	ENSMUST00000221285;ENSMUST00000221085
ENSMUSG	AC15438	ENSMUST00000223009
ENSMUSG	CT48561.	ENSMUST00000220683
ENSMUSG	CU02488	ENSMUST00000221660
ENSMUSG	AC12475.	ENSMUST00000222131
ENSMUSG	AC13295	ENSMUST00000221529
ENSMUSG	AC16527	ENSMUST00000222780
ENSMUSG	AC12219	ENSMUST00000223408
ENSMUSG	AC15524	ENSMUST00000222266
ENSMUSG	AC12417	ENSMUST00000221151
ENSMUSG	CT02561	ENSMUST00000220475
ENSMUSG	AC15420	ENSMUST00000221924
ENSMUSG	AC11763	ENSMUST00000220568
ENSMUSG	AC11350	ENSMUST00000220833
ENSMUSG	AC12156	ENSMUST00000223136
ENSMUSG	CT03016	ENSMUST00000220640
ENSMUSG	AL60678	ENSMUST00000220450
ENSMUSG	AC15656	ENSMUST00000222978
ENSMUSG	AC12544	ENSMUST00000222426
ENSMUSG	AC13262	ENSMUST00000222670
ENSMUSG	AC13424	ENSMUST00000222069
ENSMUSG	AC15920	ENSMUST00000223378
ENSMUSG	AC16105	ENSMUST00000223158
ENSMUSG	AC15455	ENSMUST00000222278
ENSMUSG	AC13174	ENSMUST00000222096
ENSMUSG	AC07964	ENSMUST00000220924
ENSMUSG	AC15924	ENSMUST00000221505
ENSMUSG	AC15749	ENSMUST00000222392
ENSMUSG	CT48561.	ENSMUST00000222597
ENSMUSG	AC15470	ENSMUST00000222304
ENSMUSG	AC15523	ENSMUST00000223133
ENSMUSG	AC21187	ENSMUST00000223236
ENSMUSG	AC12727	ENSMUST00000221337
ENSMUSG	AC13222	ENSMUST00000220577
ENSMUSG	AC09168	ENSMUST00000220592
ENSMUSG	AC15929	ENSMUST00000222553
ENSMUSG	CT00974.	ENSMUST00000222507
ENSMUSG	AC15926	ENSMUST00000221593
ENSMUSG	AC12544	ENSMUST00000223400
ENSMUSG	AC10243	ENSMUST00000221011
ENSMUSG	AC12392	ENSMUST00000221449
ENSMUSG	AC15451	ENSMUST00000222578
ENSMUSG	AC11441.	ENSMUST00000221491;ENSMUST00000222894
ENSMUSG	CT01046.	ENSMUST00000220554;ENSMUST00000222225
ENSMUSG	AC21187	ENSMUST00000221697

ENSMUSG CT03016 ENSMUST00000222249
ENSMUSG CT57299 ENSMUST00000222440
ENSMUSG AC16514 ENSMUST00000223275
ENSMUSG AC13295 ENSMUST00000222400
ENSMUSG AC12196 ENSMUST00000221458
ENSMUSG AC16410 ENSMUST00000220524
ENSMUSG AC12544 ENSMUST00000223536
ENSMUSG AC16527 ENSMUST00000221478
ENSMUSG AC15735 ENSMUST00000221201
ENSMUSG AC15423 ENSMUST00000220564
ENSMUSG CT48561 ENSMUST00000222179
ENSMUSG AC15486 ENSMUST00000221128;ENSMUST00000222033
ENSMUSG CT03024 ENSMUST00000222704
ENSMUSG AC15445 ENSMUST00000222569
ENSMUSG AC11668 ENSMUST00000222804
ENSMUSG AC16293 ENSMUST00000222366
ENSMUSG AC12237 ENSMUST00000223402
ENSMUSG CT03019 ENSMUST00000222586;ENSMUST00000223183
ENSMUSG AC15530 ENSMUST00000221935
ENSMUSG AC13349 ENSMUST00000222871;ENSMUST00000223254
ENSMUSG AC11650 ENSMUST00000220989
ENSMUSG AC14833 ENSMUST00000221222
ENSMUSG AC14833 ENSMUST00000220897
ENSMUSG AC12655 ENSMUST00000221055
ENSMUSG AC12453 ENSMUST00000220872
ENSMUSG AC15526 ENSMUST00000222787
ENSMUSG CT01043 ENSMUST00000221249
ENSMUSG AC12306 ENSMUST00000221287
ENSMUSG AC12507 ENSMUST00000220773
ENSMUSG CT00975 ENSMUST00000221430
ENSMUSG CT00954 ENSMUST00000221351
ENSMUSG AC16310 ENSMUST00000220789
ENSMUSG AC13223 ENSMUST00000220802
ENSMUSG AC15424 ENSMUST00000222072
ENSMUSG AC16335 ENSMUST00000221741
ENSMUSG AC12446 ENSMUST00000222349
ENSMUSG AC15926 ENSMUST00000223434
ENSMUSG AC16742 ENSMUST00000222999
ENSMUSG CT02555 ENSMUST00000223032
ENSMUSG AC13234 ENSMUST00000221617
ENSMUSG AC12902 ENSMUST00000223284
ENSMUSG AC16010 ENSMUST00000221931
ENSMUSG CT48561 ENSMUST00000223405
ENSMUSG AC16304 ENSMUST00000223462
ENSMUSG CT03018 ENSMUST00000222868
ENSMUSG AC11574 ENSMUST00000222398
ENSMUSG AC17408 ENSMUST00000222094
ENSMUSG AL60649 ENSMUST00000220673
ENSMUSG AC15471 ENSMUST00000221000
ENSMUSG AC14574 ENSMUST00000220590
ENSMUSG CT86873 ENSMUST00000221386
ENSMUSG AC12901 ENSMUST00000222063
ENSMUSG AC12194 ENSMUST00000221755
ENSMUSG AC15981 ENSMUST00000221516
ENSMUSG AC15919 ENSMUST00000221990
ENSMUSG AC12537 ENSMUST00000221928
ENSMUSG AC12201 ENSMUST00000222653

ENSMUSG AL59120 ENSMUST00000222991
ENSMUSG AC13259 ENSMUST00000223043
ENSMUSG AC09178 ENSMUST00000220913
ENSMUSG AC15455 ENSMUST00000221086
ENSMUSG AC15463 ENSMUST00000225225
ENSMUSG AC15529 ENSMUST00000225350
ENSMUSG AC12997 ENSMUST00000224816
ENSMUSG AC15484 ENSMUST00000224873
ENSMUSG CT03064 ENSMUST00000225049
ENSMUSG AC15930 ENSMUST00000225130;ENSMUST00000225860
ENSMUSG AC15435 ENSMUST00000224871
ENSMUSG AC11328 ENSMUST00000225278
ENSMUSG AC15680 ENSMUST00000224407
ENSMUSG AC15919 ENSMUST00000225867
ENSMUSG AC11800 ENSMUST00000224211
ENSMUSG AC16363 ENSMUST00000225041
ENSMUSG AC15422 ENSMUST00000223882
ENSMUSG AC13246 ENSMUST00000224706
ENSMUSG AC12417 ENSMUST00000223701
ENSMUSG AC15435 ENSMUST00000224604
ENSMUSG AC13071 ENSMUST00000224277
ENSMUSG AC15459 ENSMUST00000225533
ENSMUSG AC16514 ENSMUST00000223777
ENSMUSG CU07440 ENSMUST00000224411
ENSMUSG AC15839 ENSMUST00000223720
ENSMUSG AC09065 ENSMUST00000224334
ENSMUSG AC15853 ENSMUST00000224637
ENSMUSG AC15451 ENSMUST00000226085
ENSMUSG AC13486 ENSMUST00000225277
ENSMUSG AC15446 ENSMUST00000224207
ENSMUSG AC16335 ENSMUST00000225539
ENSMUSG CT00972 ENSMUST00000225228
ENSMUSG CU06262 ENSMUST00000225248
ENSMUSG AC17018 ENSMUST00000223710
ENSMUSG AC15446 ENSMUST00000225462
ENSMUSG AC12930 ENSMUST00000223800
ENSMUSG CT02561 ENSMUST00000224552
ENSMUSG AC12921 ENSMUST00000224336
ENSMUSG AC12288 ENSMUST00000225757
ENSMUSG AC16888 ENSMUST00000226018
ENSMUSG AC13866 ENSMUST00000225472
ENSMUSG AC15459 ENSMUST00000225067
ENSMUSG AC13821 ENSMUST00000223838
ENSMUSG AC24159 ENSMUST00000224768
ENSMUSG AC14493 ENSMUST00000223994
ENSMUSG AC15425 ENSMUST00000224811;ENSMUST00000224729
ENSMUSG CT00952 ENSMUST00000224205
ENSMUSG AC15463 ENSMUST00000225983
ENSMUSG AC15429 ENSMUST00000171334
ENSMUSG AC15474 ENSMUST00000225265
ENSMUSG AC16515 ENSMUST00000223825
ENSMUSG AC13915 ENSMUST00000226043
ENSMUSG AC15432 ENSMUST00000224408
ENSMUSG AC09065 ENSMUST00000225584
ENSMUSG CT00960 ENSMUST00000225567
ENSMUSG AC12439 ENSMUST00000224296
ENSMUSG AC13259 ENSMUST00000224374

ENSMUSG	CT00971	ENSMUST00000225532
ENSMUSG	AC16515	ENSMUST00000224080
ENSMUSG	AC16099	ENSMUST00000224924
ENSMUSG	AC12224	ENSMUST00000224650
ENSMUSG	AC15765	ENSMUST00000224130
ENSMUSG	AC12419	ENSMUST00000225970
ENSMUSG	CT02560	ENSMUST00000224040
ENSMUSG	AC15853	ENSMUST00000225870
ENSMUSG	AC15853	ENSMUST00000225455
ENSMUSG	CT03018	ENSMUST00000225935
ENSMUSG	AC12468	ENSMUST00000223923
ENSMUSG	AC15470	ENSMUST00000225482
ENSMUSG	AC23894	ENSMUST00000225106
ENSMUSG	AC09065	ENSMUST00000225378
ENSMUSG	CT00971	ENSMUST00000224987
ENSMUSG	AC16442	ENSMUST00000224210
ENSMUSG	AC15910	ENSMUST00000224421
ENSMUSG	AC13288	ENSMUST00000223780
ENSMUSG	AC12458	ENSMUST00000225416
ENSMUSG	AC16368	ENSMUST00000223766
ENSMUSG	AC15436	ENSMUST00000225694
ENSMUSG	AC16528	ENSMUST00000225713
ENSMUSG	CT00953	ENSMUST00000223774
ENSMUSG	CT03003	ENSMUST00000223964
ENSMUSG	AL67075	ENSMUST00000223668
ENSMUSG	AC16096	ENSMUST00000225802;ENSMUST00000225859
ENSMUSG	AC11800	ENSMUST00000224167
ENSMUSG	AC15910	ENSMUST00000223732
ENSMUSG	AC12930	ENSMUST00000225493
ENSMUSG	AC12501	ENSMUST00000224809
ENSMUSG	AC12386	ENSMUST00000224990
ENSMUSG	AC15919	ENSMUST00000225162
ENSMUSG	AC12361	ENSMUST00000224431;ENSMUST00000226114;ENSMUST00000225
ENSMUSG	AC15460	ENSMUST00000224355
ENSMUSG	AC15919	ENSMUST00000225524
ENSMUSG	CT01048	ENSMUST00000224654
ENSMUSG	AC12439	ENSMUST00000225575
ENSMUSG	CT48578	ENSMUST00000225617
ENSMUSG	AC17348	ENSMUST00000225492
ENSMUSG	AC12921	ENSMUST00000224324
ENSMUSG	AC12627	ENSMUST00000224881
ENSMUSG	AC15436	ENSMUST00000224747
ENSMUSG	CT02568	ENSMUST00000225453
ENSMUSG	AL67219	ENSMUST00000224841
ENSMUSG	AC16125	ENSMUST00000225536
ENSMUSG	CT02555	ENSMUST00000223996
ENSMUSG	AC15462	ENSMUST00000226070
ENSMUSG	AC16125	ENSMUST00000224978
ENSMUSG	CT01048	ENSMUST00000224609
ENSMUSG	AC19040	ENSMUST00000224515
ENSMUSG	AC12234	ENSMUST00000225266;ENSMUST00000225586
ENSMUSG	CT02554	ENSMUST00000225127
ENSMUSG	CT03065	ENSMUST00000224382
ENSMUSG	CT00973	ENSMUST00000225322;ENSMUST00000224949
ENSMUSG	AC11760	ENSMUST00000225500;ENSMUST00000224074;ENSMUST00000225
ENSMUSG	AC15523	ENSMUST00000225610
ENSMUSG	CU06262	ENSMUST00000223953

ENSMUSG	AC16634	ENSMUST00000224139;ENSMUST00000225166
ENSMUSG	AC11800	ENSMUST00000223948
ENSMUSG	AC15464	ENSMUST00000225011
ENSMUSG	CT00971	ENSMUST00000224509
ENSMUSG	AC16514	ENSMUST00000224795
ENSMUSG	AC12361	ENSMUST00000225097
ENSMUSG	AC15457	ENSMUST00000224174
ENSMUSG	AC16098	ENSMUST00000224136
ENSMUSG	AC11328	ENSMUST00000224012
ENSMUSG	AC16010	ENSMUST00000225592
ENSMUSG	AC16998	ENSMUST00000223648
ENSMUSG	AC11328	ENSMUST00000224757
ENSMUSG	CT02560	ENSMUST00000224369
ENSMUSG	CT57125	ENSMUST00000226045
ENSMUSG	AC15920	ENSMUST00000224614
ENSMUSG	CT02560	ENSMUST00000224031
ENSMUSG	AC13290	ENSMUST00000224345
ENSMUSG	AC15457	ENSMUST00000223966
ENSMUSG	AC16125	ENSMUST00000225465
ENSMUSG	AC10072	ENSMUST00000225458
ENSMUSG	AC12042	ENSMUST00000225213
ENSMUSG	AC16609	ENSMUST00000223731;ENSMUST00000224919;ENSMUST00000224
ENSMUSG	AC15468	ENSMUST00000223853
ENSMUSG	AC15529	ENSMUST00000225485
ENSMUSG	CT02553	ENSMUST00000226083
ENSMUSG	AC17479	ENSMUST00000224578;ENSMUST00000225749
ENSMUSG	CT00950	ENSMUST00000223855
ENSMUSG	AC15452	ENSMUST00000224647
ENSMUSG	CT01049	ENSMUST00000225690
ENSMUSG	AC15920	ENSMUST00000225467
ENSMUSG	AC16714	ENSMUST00000224710
ENSMUSG	AC15443	ENSMUST00000223938
ENSMUSG	AC13486	ENSMUST00000223592
ENSMUSG	AC06956	ENSMUST00000224206
ENSMUSG	AC15472	ENSMUST00000224481
ENSMUSG	AC15422	ENSMUST00000225848
ENSMUSG	AC15486	ENSMUST00000225060
ENSMUSG	AC15919	ENSMUST00000224082
ENSMUSG	AC16461	ENSMUST00000224770
ENSMUSG	AC12448	ENSMUST00000225852
ENSMUSG	AC16805	ENSMUST00000223591
ENSMUSG	CT01047	ENSMUST00000225364
ENSMUSG	CT00971	ENSMUST00000224508;ENSMUST00000225065;ENSMUST00000224
ENSMUSG	CT00949	ENSMUST00000224221
ENSMUSG	CU02489	ENSMUST00000224869
ENSMUSG	AC11824	ENSMUST00000224822
ENSMUSG	AC11765	ENSMUST00000225826
ENSMUSG	AC15457	ENSMUST00000225105
ENSMUSG	AC16098	ENSMUST00000225131
ENSMUSG	AC12908	ENSMUST00000224536
ENSMUSG	AC12448	ENSMUST00000223930
ENSMUSG	AC11328	ENSMUST00000225273
ENSMUSG	AC16606	ENSMUST00000225163
ENSMUSG	AC17348	ENSMUST00000225096
ENSMUSG	CT03018	ENSMUST00000225986
ENSMUSG	CT02559	ENSMUST00000225140
ENSMUSG	AC11765	ENSMUST00000224931

ENSMUSG	CT01048	ENSMUST00000224555
ENSMUSG	AC16805	ENSMUST00000225395
ENSMUSG	CT00971	ENSMUST00000223615
ENSMUSG	AC14037	ENSMUST00000225421
ENSMUSG	CT00971	ENSMUST00000224689
ENSMUSG	AC15484	ENSMUST00000225216
ENSMUSG	AC19233	ENSMUST00000224620
ENSMUSG	AC12238	ENSMUST00000225869
ENSMUSG	AC23987	ENSMUST00000224048
ENSMUSG	CT00971	ENSMUST00000225448;ENSMUST00000224732
ENSMUSG	AC13118	ENSMUST00000223936
ENSMUSG	AC12234	ENSMUST00000224326
ENSMUSG	AC15424	ENSMUST00000223952
ENSMUSG	AC15440	ENSMUST00000223601
ENSMUSG	AC16099	ENSMUST00000223784
ENSMUSG	AC12759	ENSMUST00000225697
ENSMUSG	Galr3	ENSMUST00000058004
ENSMUSG	AC15472	ENSMUST00000225782
ENSMUSG	CT02568	ENSMUST00000224316
ENSMUSG	AC13866	ENSMUST00000224880
ENSMUSG	AC15463	ENSMUST00000224589
ENSMUSG	AC16099	ENSMUST00000225675
ENSMUSG	CT00973	ENSMUST00000225957;ENSMUST00000224171
ENSMUSG	AC12693	ENSMUST00000225989
ENSMUSG	AC16127	ENSMUST00000224668
ENSMUSG	CT00971	ENSMUST00000223604
ENSMUSG	AC15529	ENSMUST00000225307;ENSMUST00000225030
ENSMUSG	AC11765	ENSMUST00000225007
ENSMUSG	AC15418	ENSMUST00000224375
ENSMUSG	AC23894	ENSMUST00000224202
ENSMUSG	AC14704	ENSMUST00000223850
ENSMUSG	AC15431	ENSMUST00000223995
ENSMUSG	AL59061	ENSMUST00000225374
ENSMUSG	AC11719	ENSMUST00000224847
ENSMUSG	CT00971	ENSMUST00000225013;ENSMUST00000224151
ENSMUSG	AC15469	ENSMUST00000223821
ENSMUSG	AC06956	ENSMUST00000224644
ENSMUSG	AC15893	ENSMUST00000225372
ENSMUSG	AC09123	ENSMUST00000224911
ENSMUSG	AC12458	ENSMUST00000224270
ENSMUSG	AC15453	ENSMUST00000226101
ENSMUSG	AC12238	ENSMUST00000225128
ENSMUSG	AC16526	ENSMUST00000224190
ENSMUSG	AC15446	ENSMUST00000224878
ENSMUSG	AC09662	ENSMUST00000225443
ENSMUSG	AC12454	ENSMUST00000225541
ENSMUSG	CU06262	ENSMUST00000225522
ENSMUSG	AC11215	ENSMUST00000226016
ENSMUSG	CT00951	ENSMUST00000224691
ENSMUSG	AC15919	ENSMUST00000224307
ENSMUSG	AC14021	ENSMUST00000224961
ENSMUSG	AC12385	ENSMUST00000225756
ENSMUSG	AC16303	ENSMUST00000223945
ENSMUSG	AC12997	ENSMUST00000223705
ENSMUSG	CU02489	ENSMUST00000224608
ENSMUSG	AC12693	ENSMUST00000226061
ENSMUSG	CT00955	ENSMUST00000225750

ENSMUSG	AC13486	ENSMUST00000224724;ENSMUST00000224350
ENSMUSG	AC09178	ENSMUST00000224928
ENSMUSG	AC15451	ENSMUST00000225081
ENSMUSG	AC13486	ENSMUST00000226038
ENSMUSG	AC15446	ENSMUST00000225429
ENSMUSG	AC12539	ENSMUST00000224236
ENSMUSG	AC16509	ENSMUST00000225025
ENSMUSG	AC13871	ENSMUST00000225947
ENSMUSG	AC12759	ENSMUST00000223947
ENSMUSG	AC12458	ENSMUST00000225874
ENSMUSG	CT03025	ENSMUST00000225959
ENSMUSG	AC16303	ENSMUST00000224942
ENSMUSG	CT03013	ENSMUST00000225255
ENSMUSG	AC15459	ENSMUST00000225171
ENSMUSG	AC11081	ENSMUST00000225706
ENSMUSG	AC12383	ENSMUST00000224038
ENSMUSG	AC11328	ENSMUST00000224330
ENSMUSG	AC15463	ENSMUST00000226077
ENSMUSG	AC17479	ENSMUST00000225055
ENSMUSG	AC12522	ENSMUST00000223588
ENSMUSG	AC19243	ENSMUST00000225464
ENSMUSG	AC13359	ENSMUST00000224937
ENSMUSG	AC15431	ENSMUST00000223677
ENSMUSG	AC12693	ENSMUST00000225360
ENSMUSG	AC15418	ENSMUST00000225620
ENSMUSG	CT02562	ENSMUST00000223735
MSTRG.1	Gm6085	ENSMUST00000182774
MSTRG.1	Lypla1	MSTRG.10.1
MSTRG.1	Gm38376	ENSMUST00000195493
MSTRG.1	Gm37447	ENSMUST00000195625
MSTRG.1	Parg	MSTRG.10000.1
MSTRG.1	Gm17210	ENSMUST00000169975
MSTRG.10002		MSTRG.10002.1
MSTRG.10003		MSTRG.10003.1
MSTRG.1	Ercc6	ENSMUST00000066807
MSTRG.1	Arhgap2	ENSMUST00000165792;ENSMUST00000111956;ENSMUST00000131
MSTRG.1	Gm15512	ENSMUST00000137359
MSTRG.1	Arhgap2	MSTRG.10007.1
MSTRG.1	Wdfy4	ENSMUST00000061753;ENSMUST00000130509;MSTRG.10008.2;M
MSTRG.1	Lrrc18	ENSMUST00000038956;ENSMUST00000120866;ENSMUST0000012C
MSTRG.1	Akp-ps1	ENSMUST00000212812
MSTRG.1	Mapk8	ENSMUST00000111945;ENSMUST00000022504;ENSMUST00000111
MSTRG.1	Mapk8	MSTRG.10011.1
MSTRG.1	Mapk8	MSTRG.10012.1
MSTRG.10013		MSTRG.10013.1
MSTRG.1	Rpl23a-]	ENSMUST00000104925
MSTRG.1	A630023	ENSMUST00000178958
MSTRG.1	Antxrl	ENSMUST00000058725
MSTRG.1	Anxa8	ENSMUST00000022519;ENSMUST00000120077
MSTRG.1	Sncg	ENSMUST00000023826
MSTRG.1	Mmrn2	ENSMUST00000111908
MSTRG.1	Gm29371	MSTRG.1002.1
MSTRG.1	Syt15	ENSMUST00000147298;ENSMUST00000119693;ENSMUST0000003E
MSTRG.1	Fam35a	ENSMUST00000111917;MSTRG.10021.2
MSTRG.1	Fam35a	MSTRG.10022.1
MSTRG.1	Glud1	ENSMUST00000022322;ENSMUST00000162912;ENSMUST0000015S
MSTRG.1	Bmpr1a	ENSMUST00000049005;ENSMUST00000165280;ENSMUST00000171

MSTRG.1 Gm17110 ENSMUST00000171248;MSTRG.10025.2
MSTRG.10026 MSTRG.10026.1
MSTRG.10027 MSTRG.10027.1
MSTRG.1 Wapl MSTRG.10028.1;MSTRG.10028.2;ENSMUST00000048263;MSTRG.
MSTRG.1 Wapl MSTRG.10029.1
MSTRG.1 Gm29371 ENSMUST00000186823
MSTRG.1 Wapl MSTRG.10030.1;MSTRG.10030.2;MSTRG.10030.3;MSTRG.1003
MSTRG.1 Cdhr1 ENSMUST00000022337
MSTRG.1 Ccser2 ENSMUST00000067700;ENSMUST00000090024;ENSMUST0000018
MSTRG.1 Ccser2 MSTRG.10033.1
MSTRG.1 Ccser2 MSTRG.10034.1
MSTRG.1 Ghitm ENSMUST00000165649;ENSMUST00000042564;ENSMUST0000022
MSTRG.1 AC16098 ENSMUST00000225855
MSTRG.1 AC16098 ENSMUST00000224927
MSTRG.10038 MSTRG.10038.1
MSTRG.10039 MSTRG.10039.1
MSTRG.1 Alppl2 ENSMUST00000027455
MSTRG.1 Nrg3 ENSMUST00000166968;ENSMUST00000176122
MSTRG.1 Nrg3 MSTRG.10041.1
MSTRG.1 Nrg3 MSTRG.10045.1
MSTRG.1 Sh2d4b MSTRG.10047.1;ENSMUST00000070328;ENSMUST00000096000;
MSTRG.1 Tspan14 MSTRG.10048.1;MSTRG.10048.2;ENSMUST00000047652;ENSMU
MSTRG.1 Dydc2 ENSMUST00000022316
MSTRG.1005 MSTRG.1005.1
MSTRG.1 AC15459 ENSMUST00000224095
MSTRG.1 Fam213a ENSMUST00000022317;ENSMUST00000118466;ENSMUST0000015
MSTRG.10052 MSTRG.10052.1
MSTRG.10053 MSTRG.10053.1
MSTRG.10054 MSTRG.10054.1
MSTRG.10055 MSTRG.10055.1
MSTRG.10056 MSTRG.10056.1
MSTRG.10057 MSTRG.10057.1
MSTRG.1 Cdv3-ps MSTRG.10058.1
MSTRG.1 Cdv3-ps ENSMUST00000169632
MSTRG.1006 MSTRG.1006.1
MSTRG.1 Gm7324 ENSMUST00000094051
MSTRG.1 Gm8113 MSTRG.10062.1;ENSMUST00000160298
MSTRG.1 Gm16545 ENSMUST00000161968
MSTRG.1 Ear2 ENSMUST00000074839
MSTRG.10067 MSTRG.10067.1
MSTRG.1 Psmc6 ENSMUST00000022380;MSTRG.10068.1;MSTRG.10068.2;MSTRG.
MSTRG.1 Txndc16 ENSMUST00000022377;ENSMUST00000139526;ENSMUST0000012
MSTRG.1 Gigyf2 ENSMUST00000174846;ENSMUST00000027475;ENSMUST00000172
MSTRG.1 Gm15765 ENSMUST00000118407
MSTRG.1 Gpr137c ENSMUST00000147957;ENSMUST00000146150;ENSMUST00000151
MSTRG.1 Ero1l ENSMUST00000022378;MSTRG.10072.2
MSTRG.1 Styx MSTRG.10073.1;ENSMUST00000111835;MSTRG.10073.3
MSTRG.1 Styx MSTRG.10074.1
MSTRG.1 Gnpnat1 ENSMUST00000046191
MSTRG.1 Fermt2 ENSMUST00000045905;ENSMUST00000141424;ENSMUST0000015
MSTRG.1 Gm22637 ENSMUST00000158144
MSTRG.1 Gm15516 ENSMUST00000117292
MSTRG.1 Gm7206 ENSMUST00000117730
MSTRG.1 Gm22549 ENSMUST00000178461
MSTRG.1 Gm1821 ENSMUST00000111388
MSTRG.1 Gm1821 MSTRG.10081.1
MSTRG.1 Gm15601 ENSMUST00000122972

MSTRG.1	Ddhd1;M	ENSMUST00000051310;ENSMUST00000152110;ENSMUST00000087
MSTRG.1	Ddhd1	MSTRG.10084.1
MSTRG.1	Ddhd1	MSTRG.10085.1
MSTRG.1	Ddhd1	MSTRG.10086.1
MSTRG.1	Ddhd1	MSTRG.10087.1
MSTRG.1	Cdkn3	ENSMUST00000067426;MSTRG.10088.2
MSTRG.1	Cdkn3	MSTRG.10089.1
MSTRG.1	Gigyf2	MSTRG.1009.1
MSTRG.1	Cdkn3	MSTRG.10090.1
MSTRG.1	Cnih1	ENSMUST00000015903;ENSMUST00000146629
MSTRG.1	Gmfb	MSTRG.10092.1
MSTRG.1	Gmfb	MSTRG.10093.1;ENSMUST00000079314;ENSMUST00000111817;E
MSTRG.1	Gch1	ENSMUST00000089959
MSTRG.1	Gch1	MSTRG.10095.1
MSTRG.1	Gch1	MSTRG.10096.1
MSTRG.1	Gch1	MSTRG.10097.1
MSTRG.1	Wdhd1	ENSMUST00000111792;ENSMUST00000111791;MSTRG.10098.3;E
MSTRG.1	Gm24378	ENSMUST00000122699
MSTRG.1	Gm27881	MSTRG.101.1;ENSMUST00000183805;MSTRG.101.3
MSTRG.1	Gigyf2	MSTRG.1010.1
MSTRG.1	Mapk1ip	MSTRG.10100.1
MSTRG.1	Socs4	ENSMUST00000065562
MSTRG.1	Socs4	MSTRG.10102.1
MSTRG.1	Socs4	MSTRG.10103.1
MSTRG.1	Mapk1ip	ENSMUST00000166743;ENSMUST00000164235;MSTRG.10104.3
MSTRG.1	Mapk1ip	MSTRG.10105.1
MSTRG.1	Lgals3	ENSMUST00000151405;ENSMUST00000142734;ENSMUST00000150
MSTRG.1	Fbxo34	ENSMUST00000043112;ENSMUST00000163324;ENSMUST00000168
MSTRG.1	Fbxo34	MSTRG.10108.1
MSTRG.1	Fbxo34	MSTRG.10109.1
MSTRG.1	Kcnj13	ENSMUST00000113212;MSTRG.1011.2;ENSMUST00000174179
MSTRG.1	Atg14	ENSMUST00000042988
MSTRG.1	Atg14	MSTRG.10111.1
MSTRG.1	Dlgap5	ENSMUST00000043296;ENSMUST00000177822;ENSMUST00000180
MSTRG.1	Dlgap5	MSTRG.10113.1
MSTRG.1	Dlgap5	MSTRG.10114.1
MSTRG.1	Gm37874	ENSMUST00000192676
MSTRG.10116		MSTRG.10116.1
MSTRG.1	Gm6055	ENSMUST00000180532
MSTRG.1	Peli2	ENSMUST00000073150
MSTRG.1	Peli2	MSTRG.10119.1
MSTRG.1	Gm23504	ENSMUST00000158988
MSTRG.1	Peli2	MSTRG.10120.1
MSTRG.1	Gm6498	ENSMUST00000081029;ENSMUST00000181413
MSTRG.1	Peli2	MSTRG.10122.1
MSTRG.1	Gm3534	ENSMUST00000182522
MSTRG.1	Ktn1	ENSMUST00000187839;ENSMUST00000190252;MSTRG.10124.3;E
MSTRG.1	Ktn1	MSTRG.10125.1
MSTRG.1	Ktn1	MSTRG.10126.1
MSTRG.1	Ktn1	MSTRG.10128.1
MSTRG.1	Tmem260	ENSMUST00000124720;ENSMUST00000133744;ENSMUST00000111
MSTRG.1	Inpp5d	ENSMUST00000169754;ENSMUST00000163222;MSTRG.1013.2;EN
MSTRG.1	Tmem260	MSTRG.10130.1
MSTRG.1	Tmem260	MSTRG.10131.1
MSTRG.1	Tmem260	MSTRG.10132.1
MSTRG.1	Gm23470	ENSMUST00000157129
MSTRG.1	Exoc5	ENSMUST00000162175;MSTRG.10134.2;ENSMUST00000161504;M

MSTRG.1	Exoc5	MSTRG.10135.1
MSTRG.1	Ap5m1	ENSMUST00000037473;MSTRG.10136.1;MSTRG.10136.3
MSTRG.1	Naa30	ENSMUST00000153488;MSTRG.10137.2;ENSMUST00000037362;E
MSTRG.1	Naa30	MSTRG.10138.1
MSTRG.1	1700011	ENSMUST00000022398;ENSMUST00000130853
MSTRG.1	Atg16l1	ENSMUST00000144047;ENSMUST00000027512;ENSMUST0000013
MSTRG.1	Ttc5	ENSMUST00000006451;MSTRG.10140.2;MSTRG.10140.3;MSTRG.
MSTRG.1	Gm10916	ENSMUST00000080330
MSTRG.1	Rpph1	MSTRG.10142.1
MSTRG.1	Rpph1	ENSMUST00000175096
MSTRG.1	Parp2	ENSMUST00000036126;MSTRG.10144.1;MSTRG.10144.3
MSTRG.1	Tep1	MSTRG.10145.1;ENSMUST00000006444
MSTRG.1	Gm26782	ENSMUST00000181697
MSTRG.10147		MSTRG.10147.1
MSTRG.1	Ang;Rna	ENSMUST00000171688;ENSMUST00000022428;ENSMUST0000006
MSTRG.1	Rnase6	ENSMUST00000095923
MSTRG.1	Atg16l1	MSTRG.1015.1
MSTRG.1	Osgep	ENSMUST00000160393;ENSMUST00000159292;ENSMUST0000000
MSTRG.1	Osgep	MSTRG.10151.1
MSTRG.1	Osgep	MSTRG.10152.1
MSTRG.1	Gm24689	ENSMUST00000157760
MSTRG.1	Osgep	MSTRG.10154.1
MSTRG.1	Apex1	ENSMUST00000049411;ENSMUST00000136753;ENSMUST00000154
MSTRG.1	Tmem55b	ENSMUST00000160835;ENSMUST00000162957;ENSMUST00000161
MSTRG.1	Pnp;Pnp	ENSMUST00000048615;MSTRG.10157.2;ENSMUST00000178092;E
MSTRG.1	Gm38316	ENSMUST00000194838
MSTRG.1	Ndrg2	ENSMUST00000004673;ENSMUST00000111632;MSTRG.10159.1
MSTRG.1	Atg16l1	MSTRG.1016.1
MSTRG.1	Mettl17	ENSMUST00000047899;ENSMUST00000168413;ENSMUST00000168
MSTRG.1	Mettl17	MSTRG.10161.1
MSTRG.1	Arhgef4	ENSMUST00000182649;MSTRG.10162.1;ENSMUST00000182905;E
MSTRG.1	Gm16617	ENSMUST00000181008
MSTRG.1	Arhgef4	MSTRG.10164.1
MSTRG.1	Hnrnpc	MSTRG.10165.1
MSTRG.1	Zfp219	ENSMUST00000067549;MSTRG.10166.2;MSTRG.10166.3;ENSMUS
MSTRG.1	Tmem253	ENSMUST00000100638;MSTRG.10167.2
MSTRG.1	Olfr221	ENSMUST00000214071;ENSMUST00000214342
MSTRG.1	Gm22354	ENSMUST00000083919
MSTRG.1	Gm25395	ENSMUST00000157343
MSTRG.1	Gm22354	MSTRG.10170.1
MSTRG.1	Hnrnpc	MSTRG.10171.6;MSTRG.10171.5;MSTRG.10171.9;MSTRG.10171
MSTRG.1	Hnrnpc	MSTRG.10172.1
MSTRG.1	Hnrnpc	MSTRG.10173.1
MSTRG.1	Hnrnpc	MSTRG.10174.1
MSTRG.1	Sall2	ENSMUST00000135523
MSTRG.1	Trav2	ENSMUST00000196939
MSTRG.1	Trav5-1	ENSMUST00000103570
MSTRG.1	Trav7-1	ENSMUST00000198019
MSTRG.1	Trav7d-	ENSMUST00000200127
MSTRG.1	Scarna6	ENSMUST00000158656
MSTRG.1	Trav7d-	ENSMUST00000178768
MSTRG.1	Trav8d-	ENSMUST00000103580
MSTRG.1	Rpgrip1	ENSMUST00000111603;ENSMUST00000180646;ENSMUST0000018C
MSTRG.1	Supt16	ENSMUST00000046709;MSTRG.10183.2;MSTRG.10183.3;MSTRG.
MSTRG.1	Gm26590	ENSMUST00000180857
MSTRG.1	Chd8;Sn	ENSMUST00000089752;ENSMUST00000200169;ENSMUST0000014
MSTRG.1	Chd8	MSTRG.10186.1

MSTRG.1	Gm43766	ENSMUST00000200590
MSTRG.1	Rab2b	ENSMUST00000022765;ENSMUST00000167116;ENSMUST00000174
MSTRG.1	Tox4	MSTRG.10189.1
MSTRG.1019		MSTRG.1019.1
MSTRG.1	Rab2b	MSTRG.10190.1
MSTRG.1	Gm23758	ENSMUST00000158354
MSTRG.1	Tox4	ENSMUST00000022766;ENSMUST00000152493;ENSMUST00000172
MSTRG.1	Mettl3	ENSMUST00000153539;ENSMUST00000022767;ENSMUST00000174
MSTRG.1	Dad1	ENSMUST00000022781;ENSMUST00000128231
MSTRG.1	Abhd4	ENSMUST00000180359;ENSMUST00000199338;ENSMUST00000041
MSTRG.1	Oxa1l	ENSMUST00000198107;ENSMUST00000000985;ENSMUST0000019[illegible]
MSTRG.1	Slc7a7	ENSMUST00000000984;MSTRG.10197.1;ENSMUST00000197440;[illegible]
MSTRG.1	Slc7a7	MSTRG.10198.1
MSTRG.1	Mrpl52	ENSMUST00000010550;ENSMUST00000199153;ENSMUST0000020[illegible]
MSTRG.1	Gm38223	ENSMUST00000194484
MSTRG.1	Sag	ENSMUST00000156381;ENSMUST00000130886;ENSMUST0000015[illegible]
MSTRG.1	Mmp14	ENSMUST00000089688;ENSMUST00000197874;ENSMUST0000022[illegible]
MSTRG.1	Lrp10	ENSMUST00000022782;MSTRG.10201.1
MSTRG.1	Trav19;[illegible]	ENSMUST00000103674;MSTRG.10202.1;MSTRG.10202.3;MSTRG.
MSTRG.1	Trav20	ENSMUST00000142754
MSTRG.1	Trav21-[illegible]	ENSMUST00000180938
MSTRG.1	Trdv1	ENSMUST00000103676
MSTRG.1	Gm43647	MSTRG.10206.1;ENSMUST00000198142;MSTRG.10206.3;MSTRG.
MSTRG.1	Trav22	MSTRG.10207.1
MSTRG.1	Trdv2-2	MSTRG.10208.1;MSTRG.10208.2;ENSMUST00000197130;ENSMUS
MSTRG.1	Trav23	ENSMUST00000199137
MSTRG.1	Sag	MSTRG.1021.1
MSTRG.1	Trdv3	ENSMUST00000198551
MSTRG.1	Gm30275	ENSMUST00000198124;ENSMUST00000197620
MSTRG.1	Trdv4	ENSMUST00000103679
MSTRG.1	Trdd1	ENSMUST00000196221;ENSMUST00000179664
MSTRG.1	Trdc;Tr[illegible]	MSTRG.10215.1;ENSMUST00000177564;ENSMUST00000103682;[illegible]
MSTRG.1	Gm43434	MSTRG.10216.1
MSTRG.1	Gm43434	ENSMUST00000196615
MSTRG.1	Trdv5	ENSMUST00000103685;MSTRG.10218.1
MSTRG.1	Trac;Tr[illegible]	MSTRG.10219.1;ENSMUST00000103691;ENSMUST00000103692;[illegible]
MSTRG.1	Dgkd;Gm	MSTRG.1022.1;MSTRG.1022.2;ENSMUST00000027517;MSTRG.1[illegible]
MSTRG.1	Traj48	ENSMUST00000200046;ENSMUST00000103695
MSTRG.1	Traj47	ENSMUST00000197949
MSTRG.1	Traj46	ENSMUST00000103696
MSTRG.1	Traj45	ENSMUST00000103697
MSTRG.1	Traj44	ENSMUST00000103698
MSTRG.1	Traj43	ENSMUST00000103699
MSTRG.1	Traj35;[illegible]	MSTRG.10226.1;ENSMUST00000103700;ENSMUST00000103701;[illegible]
MSTRG.1	Traj36	ENSMUST00000167829
MSTRG.1	Traj32	ENSMUST00000103709
MSTRG.1	Traj17	ENSMUST00000103724
MSTRG.1	Gm19582	MSTRG.1023.1;ENSMUST00000187511;MSTRG.1023.2
MSTRG.1	Traj16	ENSMUST00000103725
MSTRG.1	Traj15	ENSMUST00000103726
MSTRG.1	Traj14	ENSMUST00000103727
MSTRG.1	Traj13	ENSMUST00000103728
MSTRG.1	Traj12	ENSMUST00000103729
MSTRG.1	Traj11	ENSMUST00000103730
MSTRG.1	Traj9	ENSMUST00000103731
MSTRG.1	Traj8	ENSMUST00000103732
MSTRG.1	Traj7	ENSMUST00000103733

MSTRG.1	Traj6	ENSMUST00000103734
MSTRG.1	4833421	ENSMUST00000191704
MSTRG.1	Traj5	ENSMUST00000103735
MSTRG.1	Traj4	ENSMUST00000103736
MSTRG.1	Traj3	ENSMUST00000103737
MSTRG.1	Traj2	ENSMUST00000103738
MSTRG.1	Traj1	ENSMUST00000103739
MSTRG.1	Prmt5	ENSMUST00000023873;ENSMUST00000139964;ENSMUST00000132
MSTRG.10246		MSTRG.10246.1
MSTRG.1	Haus4	ENSMUST00000022784;MSTRG.10247.2
MSTRG.1	Ajuba	ENSMUST00000054487
MSTRG.1	4931414	ENSMUST00000022786
MSTRG.1	Usp40	MSTRG.1025.2;MSTRG.1025.1;MSTRG.1025.3;MSTRG.1025.4;E
MSTRG.1	Psmb5;M	ENSMUST00000022803;MSTRG.10250.1;ENSMUST00000102174
MSTRG.1	Slc7a8	ENSMUST00000022787
MSTRG.1	Ppp1r3e	ENSMUST00000182700;ENSMUST00000194094;ENSMUST00000168
MSTRG.1	Ppp1r3e	MSTRG.10253.1
MSTRG.1	Gm37271	ENSMUST00000194113
MSTRG.1	Gm10332	ENSMUST00000220208;ENSMUST00000219519
MSTRG.1	Gm8865	ENSMUST00000125697
MSTRG.1	Homez	ENSMUST00000081162;ENSMUST00000142283;ENSMUST00000176
MSTRG.1	Trav9d-	ENSMUST00000178426;MSTRG.10258.1;ENSMUST00000180687;M
MSTRG.1	Trav10d	ENSMUST00000103646
MSTRG.1	Usp40	MSTRG.1026.1
MSTRG.1	Trav11d	ENSMUST00000103648
MSTRG.1	Trav7d-	ENSMUST00000197128
MSTRG.1	Trav12d	ENSMUST00000181360
MSTRG.1	Trav13d	ENSMUST00000103588
MSTRG.1	Trav14d	ENSMUST00000181038
MSTRG.1	Gm6740	ENSMUST00000100617
MSTRG.1	Trav15d	MSTRG.10266.1;ENSMUST00000103616
MSTRG.1	Trav3d-	ENSMUST00000199874
MSTRG.1	Trav9d-	ENSMUST00000199746
MSTRG.1	Trav4d-	ENSMUST00000103592
MSTRG.1	Mroh2a	ENSMUST00000061013;ENSMUST00000113130;ENSMUST00000142
MSTRG.1	Rps19-p	ENSMUST00000100614
MSTRG.1	Trav5d-	ENSMUST00000196107
MSTRG.1	Trav12d	ENSMUST00000197007;ENSMUST00000103593
MSTRG.1	Trav9d-	ENSMUST00000178252;MSTRG.10273.1;ENSMUST00000198913
MSTRG.1	Trav5d-	ENSMUST00000170041
MSTRG.1	Trav12d	ENSMUST00000177703
MSTRG.1	Trav14d	MSTRG.10276.1;ENSMUST00000197954;MSTRG.10276.2;ENSMUS
MSTRG.1	Trav14d	ENSMUST00000196802;MSTRG.10277.1;MSTRG.10277.2;ENSMUS
MSTRG.1	Trav15d	ENSMUST00000199800
MSTRG.10279		MSTRG.10279.1
MSTRG.1	Hjurp	MSTRG.1028.1;ENSMUST00000054674;ENSMUST00000065420;EN
MSTRG.1	Gm8704	ENSMUST00000197181
MSTRG.1	Trav3d-	ENSMUST00000196023;ENSMUST00000103599
MSTRG.1	Trav9d-	ENSMUST00000200548;ENSMUST00000167409
MSTRG.1	Trav4d-	ENSMUST00000103600
MSTRG.1	Gm43104	ENSMUST00000197234
MSTRG.1	Trav5d-	ENSMUST00000179701
MSTRG.1	C920008	ENSMUST00000196756
MSTRG.1	Gm30214	ENSMUST00000199271
MSTRG.1	Trav13d	ENSMUST00000179512
MSTRG.1	Trav8d-	ENSMUST00000103605;ENSMUST00000198439;MSTRG.10289.1;E
MSTRG.1	Hjurp	MSTRG.1029.1

MSTRG.1	Trav15d	ENSMUST00000198604
MSTRG.1	Trav7n-	ENSMUST00000103609
MSTRG.1	Trav9n-	ENSMUST00000185224
MSTRG.1	Trav10n	ENSMUST00000103612
MSTRG.1	Trav11n	ENSMUST00000200113
MSTRG.1	Trav7n-	ENSMUST00000199753;MSTRG.10295.1;ENSMUST00000200609
MSTRG.1	Trav12n	ENSMUST00000198682
MSTRG.1	Trav13n	ENSMUST00000198359
MSTRG.1	Trav9n-	MSTRG.10298.1
MSTRG.1	Gm6755	ENSMUST00000100598
MSTRG.1	Gm37702	ENSMUST00000194357
MSTRG.1	Arl4c	ENSMUST00000051236;MSTRG.1030.3;MSTRG.1030.4;ENSMUSTC
MSTRG.1	Trav15n	MSTRG.10300.1;ENSMUST00000103590;ENSMUST00000197433
MSTRG.1	Gm43650	MSTRG.10301.1
MSTRG.1	Trav9n-	MSTRG.10302.1
MSTRG.1	Trav3n-	ENSMUST00000197693
MSTRG.1	Trav4n-	ENSMUST00000103618
MSTRG.1	Rps19-p	ENSMUST00000168472
MSTRG.1	Trav5n-	ENSMUST00000197136
MSTRG.1	Trav12n	ENSMUST00000196674;ENSMUST00000103619
MSTRG.1	Trav9n-	ENSMUST00000177705
MSTRG.1	Trav5n-	ENSMUST00000164511
MSTRG.1	Sh3bp4	ENSMUST00000066279;ENSMUST00000123745
MSTRG.1	Trav12n	ENSMUST00000179583
MSTRG.1	Trav13n	ENSMUST00000196941;ENSMUST00000103622
MSTRG.1	Trav14n	ENSMUST00000197614;MSTRG.10312.1;ENSMUST00000103623;E
MSTRG.1	Trav15n	ENSMUST00000199112
MSTRG.1	Gm43650	MSTRG.10314.1
MSTRG.1	Trav9n-	MSTRG.10315.1
MSTRG.1	Gm43650	MSTRG.10316.1;ENSMUST00000200519;ENSMUST00000197221
MSTRG.1	Trav3n-	ENSMUST00000197557;ENSMUST00000103625
MSTRG.1	Gm43650	MSTRG.10318.1
MSTRG.1	Gm43650	MSTRG.10319.1
MSTRG.1	Gm38312	ENSMUST00000195426
MSTRG.1	Trav9n-	ENSMUST00000103626
MSTRG.1	Trav4n-	ENSMUST00000103627
MSTRG.1	Gm43650	MSTRG.10322.1
MSTRG.1	Trav5n-	ENSMUST00000179997
MSTRG.1	Trav7n-	ENSMUST00000178100
MSTRG.1	Gm43650	ENSMUST00000197337
MSTRG.1	Trav13n	ENSMUST00000179580
MSTRG.1	Trav8n-	ENSMUST00000103632
MSTRG.1	Trav16n	ENSMUST00000199280;ENSMUST00000103633
MSTRG.1	Trav7-4	MSTRG.10329.1
MSTRG.1	Agap1	ENSMUST00000027521;ENSMUST00000190096;ENSMUST00000185
MSTRG.1	Trav15n	ENSMUST00000200498
MSTRG.1	Trav7-2	ENSMUST00000103636
MSTRG.1	Trav4-2	ENSMUST00000103637
MSTRG.1	Trav6-3	ENSMUST00000180549;ENSMUST00000183604
MSTRG.1	Trav7-3	ENSMUST00000177622
MSTRG.1	Trav6-4	ENSMUST00000180711
MSTRG.1	Trav7-4	ENSMUST00000181728
MSTRG.1	Gm18711	ENSMUST00000199341
MSTRG.1	Trav8-1	ENSMUST00000103643
MSTRG.1	Trav9-1	ENSMUST00000103581
MSTRG.1	Agap1	MSTRG.1034.1
MSTRG.10340		MSTRG.10340.1

MSTRG.1	Trav6-5	ENSMUST00000181210;ENSMUST00000183488
MSTRG.1	Trav10	ENSMUST00000103583
MSTRG.1	Trav11	ENSMUST00000103585
MSTRG.1	Trav12-	MSTRG.10344.1;ENSMUST00000200115
MSTRG.1	Trav13-	ENSMUST00000103651
MSTRG.1	Trav14-	MSTRG.10346.1;ENSMUST00000198297
MSTRG.1	Trav15-	MSTRG.10347.1;ENSMUST00000200101
MSTRG.1	Trav3-2	ENSMUST00000198999
MSTRG.1	Trav9-2	ENSMUST00000103654
MSTRG.1	Gm37297	ENSMUST00000192974
MSTRG.1	Trav4-3	ENSMUST00000103655;MSTRG.10350.1;ENSMUST00000103663
MSTRG.1	Trav5-2	ENSMUST00000199855
MSTRG.1	Trav12-	ENSMUST00000180972
MSTRG.1	Trav12-	ENSMUST00000103657
MSTRG.1	Trav13-	ENSMUST00000103658
MSTRG.1	Trav15-	ENSMUST00000103660
MSTRG.1	Trav3-3	MSTRG.10356.1;ENSMUST00000181768;ENSMUST00000183835
MSTRG.1	Trav9-4	ENSMUST00000103662
MSTRG.1	Gm43550	ENSMUST00000200597
MSTRG.1	Trav5-4	ENSMUST00000103664
MSTRG.1	Gm25180	ENSMUST00000157746
MSTRG.1	Trav6-7	ENSMUST00000103638
MSTRG.1	Trav7-6	ENSMUST00000103641;ENSMUST00000184687
MSTRG.1	Trav13-	ENSMUST00000117226
MSTRG.1	Trav8-2	ENSMUST00000130122
MSTRG.1	Trav16	ENSMUST00000103667
MSTRG.1	Trav13-	ENSMUST00000180380;ENSMUST00000184905
MSTRG.1	Trav14-	ENSMUST00000103589
MSTRG.1	Trav15-	ENSMUST00000199403
MSTRG.1	Trav3-4	ENSMUST00000103670
MSTRG.1	Trav3-4	MSTRG.10369.1
MSTRG.1	Gm28499	ENSMUST00000191576
MSTRG.1	Trav12-	ENSMUST00000200663
MSTRG.1	B230359	ENSMUST00000103671
MSTRG.10372		MSTRG.10372.1
MSTRG.1	Trav17	ENSMUST00000103672;ENSMUST00000186545
MSTRG.10374		MSTRG.10374.1
MSTRG.1	Psmb11	ENSMUST00000097177
MSTRG.1	Cdh24;A	MSTRG.10376.1;MSTRG.10376.2;ENSMUST00000067784;MSTRG.
MSTRG.1	Gm20726	ENSMUST00000173083
MSTRG.1	Gm17606	ENSMUST00000169818
MSTRG.1	4930579	ENSMUST00000167763
MSTRG.1	4933400	ENSMUST00000186098;ENSMUST00000187295;ENSMUST00000187
MSTRG.1	Acin1	MSTRG.10380.1
MSTRG.1	Acin1	MSTRG.10381.1
MSTRG.1	Acin1	MSTRG.10382.1
MSTRG.1	1700123	ENSMUST00000038539;MSTRG.10383.1;MSTRG.10383.2
MSTRG.1	Bcl2l2;	ENSMUST00000022806;ENSMUST00000131243;ENSMUST00000134
MSTRG.1	Efs	ENSMUST00000022813;MSTRG.10385.2
MSTRG.1	Ngdn	MSTRG.10386.1;ENSMUST00000022815
MSTRG.1	Dhrs2	ENSMUST00000022820
MSTRG.1	Dhrs2	MSTRG.10388.1
MSTRG.1	Gm8894	ENSMUST00000074642
MSTRG.1	Gm26037	ENSMUST00000158150
MSTRG.1	Dhrs4	ENSMUST00000022821
MSTRG.1	Gm20687	ENSMUST00000176872;ENSMUST00000183822;ENSMUST00000185
MSTRG.1	Zfhx2	ENSMUST00000036328;ENSMUST00000176665;ENSMUST00000185

MSTRG.1	Gm37034	ENSMUST00000191729
MSTRG.1	Gm38129	ENSMUST00000192739
MSTRG.1	Ap1g2	ENSMUST00000151314;ENSMUST00000139536;ENSMUST00000131
MSTRG.1	A730061	ENSMUST00000066106
MSTRG.1	Dcaf11	ENSMUST00000117236;ENSMUST00000147981;ENSMUST00000151
MSTRG.1	Gm15932	ENSMUST00000135068
MSTRG.1	Fitm1	ENSMUST00000022826
MSTRG.1	Gm23169	ENSMUST00000157157
MSTRG.1	Agap1	MSTRG.1040.1
MSTRG.1	Psme1	ENSMUST00000174484;ENSMUST00000174259;ENSMUST00000172
MSTRG.1	Emc9	ENSMUST00000174352;ENSMUST00000173169;ENSMUST00000022
MSTRG.1	Psme2	ENSMUST00000161807;ENSMUST00000111378;ENSMUST00000161
MSTRG.1	Rnf31;I	ENSMUST00000019443;MSTRG.10403.1;ENSMUST00000137296;E
MSTRG.1	Nedd8	ENSMUST00000163750;ENSMUST00000010520
MSTRG.1	Gmpr2	MSTRG.10405.1;ENSMUST00000002397;MSTRG.10405.3
MSTRG.1	Tinf2	MSTRG.10406.1;MSTRG.10406.2;ENSMUST00000007733;MSTRG.
MSTRG.1	Rec8	ENSMUST00000002395;ENSMUST00000140335;ENSMUST00000141
MSTRG.1	Ipo4;Tm	ENSMUST00000047131;ENSMUST00000148351;ENSMUST00000141
MSTRG.1	Tssk4	ENSMUST00000164809;ENSMUST00000007735
MSTRG.1	Gm37521	ENSMUST00000193177
MSTRG.1	Mdp1	ENSMUST00000002400;MSTRG.10410.1
MSTRG.1	Rabggta	ENSMUST00000062861;ENSMUST00000169237;MSTRG.10411.3;E
MSTRG.1	Ltb4r1	ENSMUST00000057569
MSTRG.1	Dhrs1	ENSMUST00000155969;ENSMUST00000128802;ENSMUST00000002
MSTRG.1	Nop9	ENSMUST00000019441
MSTRG.1	Cideb	ENSMUST00000001497;ENSMUST00000136270
MSTRG.10416		MSTRG.10416.1;MSTRG.10416.2
MSTRG.1	Adcy4	ENSMUST00000170223;ENSMUST00000002398
MSTRG.1	Ripk3	ENSMUST00000022830;ENSMUST00000168716;ENSMUST00000178
MSTRG.1	Cma1	ENSMUST00000022834
MSTRG.1	Cops8	ENSMUST00000036153;ENSMUST00000191510;ENSMUST0000018
MSTRG.10420		MSTRG.10420.1
MSTRG.1	Khnyn	MSTRG.10421.2;ENSMUST00000022831;MSTRG.10421.1
MSTRG.1	Sdr39u1	MSTRG.10422.1;MSTRG.10422.2;MSTRG.10422.3;MSTRG.10422
MSTRG.1	Gm5801	ENSMUST00000182247
MSTRG.1	Mcpt4	ENSMUST00000043249
MSTRG.1	Rnf17	ENSMUST00000095793;ENSMUST00000225361
MSTRG.1	Cenpj	ENSMUST00000065302;MSTRG.10427.1;ENSMUST00000225951;M
MSTRG.1	Gm22431	ENSMUST00000158545
MSTRG.1	Parp4	ENSMUST00000161553;MSTRG.10429.2;ENSMUST00000162312
MSTRG.1	Col6a3	ENSMUST00000056925;ENSMUST00000097653;ENSMUST0000015
MSTRG.1	Parp4	MSTRG.10430.1
MSTRG.1	Gm16573	ENSMUST00000160334;ENSMUST00000161382
MSTRG.1	Gm4032	ENSMUST00000211600;ENSMUST00000210886;ENSMUST0000021
MSTRG.1	Mphosph	ENSMUST00000116468;MSTRG.10433.2;ENSMUST00000130246;E
MSTRG.1	Gm16973	ENSMUST00000180752;MSTRG.10434.1;ENSMUST00000180682;E
MSTRG.1	Zmym5	ENSMUST00000039812;ENSMUST00000173954;ENSMUST00000111
MSTRG.1	2410022	ENSMUST00000186474
MSTRG.1	Zmym2	ENSMUST00000223669;MSTRG.10437.1;MSTRG.10437.3;ENSMUS
MSTRG.10438		MSTRG.10438.1
MSTRG.1	Cryl1	ENSMUST00000022517;ENSMUST00000223986;ENSMUST00000224
MSTRG.1	Gm36970	ENSMUST00000193688
MSTRG.1	Gm23593	ENSMUST00000104309
MSTRG.1	Cryl1	MSTRG.10441.1
MSTRG.1	CT01050	ENSMUST00000223673
MSTRG.1	CT01050	ENSMUST00000225457
MSTRG.1	Eef1akm	ENSMUST00000022518;ENSMUST00000225504

MSTRG.1	1700039I	ENSMUST00000180397
MSTRG.1	Xpo4;La	ENSMUST00000174545;MSTRG.10446.2;MSTRG.10446.3;ENSMUS
MSTRG.1	Xpo4	MSTRG.10447.1
MSTRG.1	Gm26440	ENSMUST00000082591
MSTRG.1	Gm20430	ENSMUST00000172474
MSTRG.1045		MSTRG.1045.1
MSTRG.1	Lats2	MSTRG.10450.1
MSTRG.1	Sap18;M	ENSMUST00000128764;ENSMUST00000111269;ENSMUST00000131
MSTRG.1	Ska3	MSTRG.10452.1;ENSMUST00000022536;ENSMUST00000224108;M
MSTRG.1	Ska3	MSTRG.10453.1
MSTRG.1	Mrpl57	ENSMUST00000022538;ENSMUST00000224313
MSTRG.1	Zdhhc20	ENSMUST00000089473;ENSMUST00000226057;ENSMUST0000022[illegible]
MSTRG.1	Zdhhc20	MSTRG.10456.1
MSTRG.1	Micu2	ENSMUST00000225961;ENSMUST00000022543;ENSMUST00000224
MSTRG.1	3110083[illegible]	ENSMUST00000180534;ENSMUST00000224786
MSTRG.1	Gm5142	ENSMUST00000224214
MSTRG.1	Ramp1	MSTRG.1046.1
MSTRG.1	Gm5142	MSTRG.10460.1
MSTRG.1	Rcbtb1	ENSMUST00000173547;MSTRG.10461.1;ENSMUST00000043227;E
MSTRG.1	Phf11b	ENSMUST00000166121;MSTRG.10462.1
MSTRG.1	Shisa2	ENSMUST00000053949;ENSMUST00000224155
MSTRG.1	Phf11d	ENSMUST00000095157;ENSMUST00000161754;ENSMUST0000016[illegible]
MSTRG.1	Phf11c	ENSMUST00000166912
MSTRG.1	Setdb2	ENSMUST00000161459;ENSMUST00000095775;ENSMUST0000015[illegible]
MSTRG.1	Setdb2	MSTRG.10467.1
MSTRG.1	Gm16301	ENSMUST00000161959
MSTRG.1	Cab39l	ENSMUST00000225149;ENSMUST00000224281;ENSMUST00000022
MSTRG.1	Ramp1	ENSMUST00000165855;ENSMUST00000188475;MSTRG.1047.4;MS
MSTRG.1	Cab39l	MSTRG.10470.1
MSTRG.1	Cdadc1	ENSMUST00000225839;ENSMUST00000167100;MSTRG.10471.4;E
MSTRG.1	Cab39l	MSTRG.10472.1
MSTRG.10473		MSTRG.10473.1
MSTRG.10474		MSTRG.10474.1
MSTRG.10475		MSTRG.10475.1
MSTRG.1	Mtmr6	MSTRG.10476.1;ENSMUST00000022563;MSTRG.10476.2;ENSMUS
MSTRG.1	Atp8a2;[illegible]	ENSMUST00000080368;ENSMUST00000145071;ENSMUST00000131
MSTRG.1	Gm9013	ENSMUST00000132939
MSTRG.1	Ramp1	MSTRG.1048.1
MSTRG.1	4930563	ENSMUST00000154835
MSTRG.1	Gm6913	ENSMUST00000100019
MSTRG.1	Gm16261	ENSMUST00000096813
MSTRG.1	AC13168	ENSMUST00000225931
MSTRG.1	Nupl1	MSTRG.10484.1
MSTRG.1	C1qtnf9	ENSMUST00000025940
MSTRG.1	Mipepos	ENSMUST00000155855
MSTRG.1	Mipep	ENSMUST00000063562;ENSMUST00000225506;ENSMUST00000224
MSTRG.1	Mipep	MSTRG.10488.1
MSTRG.1	Spata13	ENSMUST00000160973;ENSMUST00000022566;ENSMUST0000015[illegible]
MSTRG.1	Lrrfip1	ENSMUST00000189505;MSTRG.1049.1;ENSMUST00000185531;EN
MSTRG.1	Tnfrsf1	ENSMUST00000111236;ENSMUST00000111234;ENSMUST0000022[illegible]
MSTRG.1	Sacs	ENSMUST00000089394;ENSMUST00000119509;ENSMUST0000011[illegible]
MSTRG.1	Sgcg	ENSMUST00000077954;ENSMUST00000129886
MSTRG.1	Ebpl	ENSMUST00000022494;ENSMUST00000223560;ENSMUST0000022[illegible]
MSTRG.1	Gm25716	ENSMUST00000158628
MSTRG.1	Ebpl	MSTRG.10495.1
MSTRG.1	Kpna3	MSTRG.10496.1;ENSMUST00000022496;ENSMUST00000225207
MSTRG.1	Spryd7	ENSMUST00000022497;ENSMUST00000100496

MSTRG.1	Trim13;	MSTRG.10498.1;MSTRG.10498.2;ENSMUST00000165015;ENSMUS
MSTRG.1	Dleu2;G	MSTRG.10499.1;ENSMUST00000182768;ENSMUST00000182259;
MSTRG.1	Ncoa2	MSTRG.105.1
MSTRG.1	Lrrfip1	MSTRG.1050.1
MSTRG.1	Gm26916	ENSMUST00000183304
MSTRG.1	Mir15a	ENSMUST00000175266
MSTRG.1	Gm27017	ENSMUST00000183031;ENSMUST00000183747
MSTRG.1	Dleu7	ENSMUST00000063169;ENSMUST00000225120
MSTRG.1	Rnaseh2	ENSMUST00000166879;ENSMUST00000022499;ENSMUST0000016
MSTRG.1	Serpine	ENSMUST00000171692
MSTRG.1	Gm25162	ENSMUST00000158906
MSTRG.1	Ube2f	ENSMUST00000188576;ENSMUST00000059743;ENSMUST0000017
MSTRG.1	Ints6	ENSMUST00000053959;MSTRG.10510.1;MSTRG.10510.4;MSTRG.
MSTRG.1	Ints6	MSTRG.10511.1
MSTRG.1	4931440	ENSMUST00000181344
MSTRG.1	AC15486	ENSMUST00000223767
MSTRG.1	Gm26536	ENSMUST00000181072;MSTRG.10514.2
MSTRG.1	Wdfy2;M	ENSMUST00000014691;ENSMUST00000224948;MSTRG.10515.2;
MSTRG.1	Wdfy2	MSTRG.10516.1
MSTRG.1	Mir6541	ENSMUST00000183649
MSTRG.1	n-R5s47	ENSMUST00000122516
MSTRG.10519		MSTRG.10519.1
MSTRG.1	Gm26683	ENSMUST00000181737
MSTRG.1	Blk	ENSMUST00000014597;ENSMUST00000224965
MSTRG.1	Ctsb	ENSMUST00000006235;ENSMUST00000225639;ENSMUST0000022
MSTRG.1	Fdft1	ENSMUST00000224625;ENSMUST00000054963;ENSMUST0000022
MSTRG.1	Fdft1	MSTRG.10523.1
MSTRG.1	Neil2	ENSMUST00000038229;ENSMUST00000223723
MSTRG.1	Gata4	ENSMUST00000118022;ENSMUST00000067417;ENSMUST00000121
MSTRG.1	Mtmr9	MSTRG.10526.1;ENSMUST00000058679
MSTRG.1	Gm15918	ENSMUST00000137259
MSTRG.1	Xkr6	ENSMUST00000119973;ENSMUST00000176510;ENSMUST00000177
MSTRG.1	Xkr6	MSTRG.10529.1
MSTRG.1	Scly	ENSMUST00000027532;ENSMUST00000123651;ENSMUST00000142
MSTRG.1	Xkr6	MSTRG.10530.1
MSTRG.1	Mir598	ENSMUST00000102094
MSTRG.1	Pinx1	ENSMUST00000022528;MSTRG.10532.2;ENSMUST00000225644;
MSTRG.10533		MSTRG.10533.1
MSTRG.1	AC16699	ENSMUST00000225122
MSTRG.1	Msra	ENSMUST00000067927;ENSMUST00000210428;ENSMUST0000021
MSTRG.1	Msra	MSTRG.10536.2;MSTRG.10536.1
MSTRG.1	Msra	MSTRG.10537.1
MSTRG.1	Kif13b	ENSMUST00000224126;ENSMUST00000100473;ENSMUST00000224
MSTRG.1	Kif13b	MSTRG.10539.1
MSTRG.1	Gm17090	ENSMUST00000171768
MSTRG.1	Gm26225	ENSMUST00000082508
MSTRG.1	Fzd3	ENSMUST00000131309;ENSMUST00000127272
MSTRG.1	Gm27263	ENSMUST00000184760
MSTRG.1	AC15216	ENSMUST00000224558
MSTRG.1	Hmbox1;	MSTRG.10544.3;MSTRG.10544.4;MSTRG.10544.1;MSTRG.10544
MSTRG.1	Gm20111	ENSMUST00000184830
MSTRG.1	AC14142	ENSMUST00000224989
MSTRG.1	Hmbox1	MSTRG.10547.1
MSTRG.1	Hmbox1	MSTRG.10548.1
MSTRG.1	Ints9	ENSMUST00000043914;ENSMUST00000224593;ENSMUST0000022
MSTRG.1	Espnl	ENSMUST00000088904
MSTRG.1	Extl3	ENSMUST00000022550;MSTRG.10550.1;ENSMUST00000225633;

MSTRG.1	Extl3	MSTRG.10551.1
MSTRG.1	AC15898	ENSMUST00000225691
MSTRG.1	Zfp395	ENSMUST00000224623;ENSMUST00000225865;ENSMUST00000066
MSTRG.1	Zfp395	MSTRG.10554.1
MSTRG.1	Elp3	ENSMUST00000225355;ENSMUST00000022609;ENSMUST00000224
MSTRG.1	Scara5	ENSMUST00000022610;ENSMUST00000069226;ENSMUST00000154
MSTRG.1	Pbk	ENSMUST00000022612;ENSMUST00000152130;MSTRG.10557.2;E
MSTRG.1	Esco2	ENSMUST00000022613;ENSMUST00000136920;ENSMUST00000131
MSTRG.1	Ccdc25	ENSMUST00000022614;ENSMUST00000225823;ENSMUST00000226
MSTRG.1	Hes6	ENSMUST00000132188;ENSMUST00000086851;ENSMUST00000126
MSTRG.1	Ccdc25	MSTRG.10560.1
MSTRG.1	Scara3	ENSMUST00000042046
MSTRG.1	Clu	ENSMUST00000022616;ENSMUST00000138665;ENSMUST00000146
MSTRG.1	Gm10233	ENSMUST00000089275
MSTRG.1	Ephx2	ENSMUST00000224698;ENSMUST00000225309;ENSMUST00000070
MSTRG.1	1700001	ENSMUST00000153646;ENSMUST00000145637;ENSMUST00000143
MSTRG.1	Trim35	ENSMUST00000022623;ENSMUST00000223737;MSTRG.10567.2;E
MSTRG.1	Chrna2	ENSMUST00000022620
MSTRG.1	Ptk2b	ENSMUST00000022622;ENSMUST00000178730;MSTRG.10569.2;E
MSTRG.1	Per2	ENSMUST00000069620;ENSMUST00000185298
MSTRG.1	Ptk2b	MSTRG.10570.1
MSTRG.1	Ptk2b	MSTRG.10571.1;MSTRG.10571.2
MSTRG.1	Ptk2b	MSTRG.10572.1
MSTRG.1	Dpysl2	ENSMUST00000022629;MSTRG.10573.2
MSTRG.1	Gm5464	ENSMUST00000100453;ENSMUST00000225817
MSTRG.1	Gm24258	ENSMUST00000083739
MSTRG.10576		MSTRG.10576.1
MSTRG.1	Bnip3l	ENSMUST00000111115;ENSMUST00000022634;ENSMUST00000138
MSTRG.1	Bnip3l	MSTRG.10578.1
MSTRG.1	Ppp2r2a	ENSMUST00000089230;ENSMUST00000225380;ENSMUST00000225
MSTRG.1	Traf3ip	ENSMUST00000189341;ENSMUST00000047242;ENSMUST00000187
MSTRG.1	Gm20675	ENSMUST00000175861
MSTRG.1	Ebf2	ENSMUST00000022637;ENSMUST00000176161;ENSMUST00000177
MSTRG.1	Ebf2	MSTRG.10582.1
MSTRG.1	Gm6878	ENSMUST00000100448
MSTRG.1	1700120	ENSMUST00000177127
MSTRG.1	4930438	ENSMUST00000176841
MSTRG.1	Gm10860	ENSMUST00000176934
MSTRG.1	Cdca2	ENSMUST00000150006;MSTRG.10588.2;ENSMUST00000163100;M
MSTRG.1	Kctd9	ENSMUST00000152243;ENSMUST00000078053;ENSMUST00000145
MSTRG.1	Traf3ip	MSTRG.1059.1
MSTRG.1	Gnrh1	ENSMUST00000223929;ENSMUST00000111095;ENSMUST00000223
MSTRG.1	Dock5	ENSMUST00000039135;ENSMUST00000226033;ENSMUST00000224
MSTRG.1	Mir6539	ENSMUST00000183732
MSTRG.1	Adamdec	ENSMUST00000022641;MSTRG.10593.2;ENSMUST00000111072;E
MSTRG.1	Chmp7	ENSMUST00000224964;ENSMUST00000036381;ENSMUST00000225
MSTRG.1	4930480	ENSMUST00000180465;ENSMUST00000181430
MSTRG.1	AC16293	ENSMUST00000223676
MSTRG.1	Rhobtb2	ENSMUST00000022665;ENSMUST00000224535;ENSMUST00000223
MSTRG.1	Slc25a3	ENSMUST00000037064;ENSMUST00000184497;ENSMUST00000184
MSTRG.1	Gm27222	ENSMUST00000183398
MSTRG.1	Eya1	ENSMUST00000027066;ENSMUST00000168081;MSTRG.106.3;ENS
MSTRG.1	Klhl30	ENSMUST00000027533
MSTRG.1	Gm20236	ENSMUST00000193037
MSTRG.1	Gm31748	ENSMUST00000194938
MSTRG.1	Synb	ENSMUST00000059362
MSTRG.1	Entpd4	ENSMUST00000184973;ENSMUST00000184165;ENSMUST00000184

MSTRG.1	Gm27221	ENSMUST00000183485
MSTRG.1	Gm16867	ENSMUST00000183917;ENSMUST00000183695;ENSMUST0000018
MSTRG.1	Gm16867	MSTRG.10607.1
MSTRG.1	Gm21451	ENSMUST00000111062
MSTRG.1	Gm38378	ENSMUST00000195153
MSTRG.1	Erfe	ENSMUST00000086861;MSTRG.1061.2
MSTRG.1	Gm16867	MSTRG.10610.1
MSTRG.1	Gm16867	MSTRG.10611.1
MSTRG.1	Gm37094	ENSMUST00000193352
MSTRG.1	Gm16867	MSTRG.10613.1
MSTRG.1	Gm37847	ENSMUST00000192163
MSTRG.1	Gm27179	ENSMUST00000184652
MSTRG.1	Entpd4b	ENSMUST00000185072;ENSMUST00000183906;ENSMUST0000006
MSTRG.1	Gm27176	ENSMUST00000183642
MSTRG.1	Gm27177	ENSMUST00000183339;ENSMUST00000184034;ENSMUST0000018
MSTRG.1	Ilkap	ENSMUST00000187049;ENSMUST00000190747;MSTRG.1062.3;EN
MSTRG.1	Gm27177	MSTRG.10620.1
MSTRG.1	Loxl2;M	ENSMUST00000022660;ENSMUST00000184438
MSTRG.1	Gm37513	ENSMUST00000194830
MSTRG.1	Gm27177	ENSMUST00000183514
MSTRG.1	R3hcc1	ENSMUST00000118374;ENSMUST00000121142;ENSMUST0000021
MSTRG.1	Egr3	ENSMUST00000035908;ENSMUST00000225200;ENSMUST0000022
MSTRG.1	Bin3	ENSMUST00000022680;MSTRG.10626.1;MSTRG.10626.3
MSTRG.1	Ccar2;9	ENSMUST00000035612;MSTRG.10627.1;ENSMUST00000058240;E
MSTRG.1	Pdlim2	ENSMUST00000022681;ENSMUST00000153735;ENSMUST0000012
MSTRG.1	Sorbs3	ENSMUST00000022682
MSTRG.1	Ndufa10	ENSMUST00000027478;ENSMUST00000189503;ENSMUST0000015
MSTRG.1	Gm26908	ENSMUST00000181856
MSTRG.1	Ppp3cc	ENSMUST00000078434
MSTRG.1	Ppp3cc	MSTRG.10632.1
MSTRG.1	Ppp3cc	MSTRG.10633.1
MSTRG.1	Slc39a1	ENSMUST00000068044;ENSMUST00000152067;MSTRG.10634.3;E
MSTRG.1	Bmp1	ENSMUST00000022693
MSTRG.1	Nudt18	ENSMUST00000089049
MSTRG.1	Reep4	ENSMUST00000047218;MSTRG.10637.1;MSTRG.10637.3
MSTRG.1	Hr	ENSMUST00000159959;ENSMUST00000161468;ENSMUST0000016
MSTRG.1	Fam160b	ENSMUST00000022690;MSTRG.10639.2;MSTRG.10639.3
MSTRG.1	Mir6900	ENSMUST00000183483
MSTRG.1	Fam160b	MSTRG.10640.1
MSTRG.1	Gfra2	ENSMUST00000022699
MSTRG.1	Npm2	ENSMUST00000062629
MSTRG.1	Xpo7	ENSMUST00000022696;ENSMUST00000167242;MSTRG.10643.3;M
MSTRG.1	Xpo7	MSTRG.10644.1
MSTRG.1	Xpo7	MSTRG.10645.1
MSTRG.1	Xpo7	MSTRG.10646.1
MSTRG.1	Dok2	ENSMUST00000171481;ENSMUST00000169599;ENSMUST0000016
MSTRG.10648		MSTRG.10648.1
MSTRG.10649		MSTRG.10649.1
MSTRG.1	Cops9	ENSMUST00000154129;ENSMUST00000112999;ENSMUST0000014
MSTRG.1	Cysltr2	ENSMUST00000044664;ENSMUST00000169168
MSTRG.1	Fndc3a	ENSMUST00000089017;ENSMUST00000162825;MSTRG.10651.2;E
MSTRG.1	Gm16549	ENSMUST00000160445
MSTRG.1	Fndc3a	MSTRG.10653.1
MSTRG.1	Fndc3a	MSTRG.10654.1
MSTRG.1	Fndc3a	MSTRG.10655.1
MSTRG.1	Fndc3a	MSTRG.10656.1
MSTRG.10657		MSTRG.10657.1

MSTRG.1	Gm16409	ENSMUST00000100510
MSTRG.1	Med4	ENSMUST00000022705
MSTRG.1	Hdac4	MSTRG.1066.1;ENSMUST00000097644;ENSMUST00000008995;EN
MSTRG.1	Med4	MSTRG.10660.1
MSTRG.1	Nudt15	ENSMUST00000043813
MSTRG.1	Sucla2	ENSMUST00000162691;ENSMUST00000162723;ENSMUST0000016C
MSTRG.1	Sucla2	MSTRG.10663.1
MSTRG.1	Cbx3-ps	ENSMUST00000181108
MSTRG.1	Rcbtb2	ENSMUST00000163339;ENSMUST00000163650;ENSMUST00000171
MSTRG.1	Rcbtb2	MSTRG.10666.1
MSTRG.1	Gm17233	ENSMUST00000168470
MSTRG.1	Rb1	ENSMUST00000163932
MSTRG.1	Rb1;Itm	MSTRG.10669.1;MSTRG.10669.2;MSTRG.10669.3;MSTRG.1066S
MSTRG.1	Gm37048	ENSMUST00000194827
MSTRG.1	Mir687	ENSMUST00000102166
MSTRG.1	Lpar6	MSTRG.10671.1
MSTRG.1	Lpar6	ENSMUST00000044405
MSTRG.1	Rb1	ENSMUST00000170967
MSTRG.1	Rubcnl	ENSMUST00000036072
MSTRG.1	Esd	ENSMUST00000022573;ENSMUST00000177445;ENSMUST0000017€
MSTRG.1	Lrch1	MSTRG.10676.2;ENSMUST00000088970;MSTRG.10676.1;MSTRG.
MSTRG.1	Lrrc63	ENSMUST00000022574
MSTRG.1	Lcp1	ENSMUST00000143539;ENSMUST00000124608;ENSMUST00000134
MSTRG.1	Gm26720	ENSMUST00000181260
MSTRG.1	Gm15628	ENSMUST00000162051
MSTRG.1	Gm15629	ENSMUST00000161819
MSTRG.1	Cpb2	ENSMUST00000022576
MSTRG.1	Zc3h13	ENSMUST00000022577;MSTRG.10684.1;MSTRG.10684.2
MSTRG.1	Cog3;Gm	ENSMUST00000049168;MSTRG.10685.1;ENSMUST00000158780
MSTRG.10686		MSTRG.10686.1
MSTRG.1	Slc25a3	MSTRG.10687.1;ENSMUST00000022580
MSTRG.1	Gm4285	ENSMUST00000131222
MSTRG.1	Tpt1	ENSMUST00000110894;ENSMUST00000142061;ENSMUST0000015C
MSTRG.1	Gm37036	ENSMUST00000192836
MSTRG.1	Snora31	ENSMUST00000083213
MSTRG.1	Gtf2f2	ENSMUST00000088922;MSTRG.10691.2
MSTRG.1	Gtf2f2	MSTRG.10692.1
MSTRG.1	Kctd4	MSTRG.10693.1
MSTRG.1	Gm25517	ENSMUST00000083865
MSTRG.1	Gtf2f2	MSTRG.10695.1
MSTRG.1	Gtf2f2	MSTRG.10696.1
MSTRG.1	Gpalpp1	ENSMUST00000022585
MSTRG.1	Nufip1	MSTRG.10698.1;ENSMUST00000022586
MSTRG.1	Nufip1	MSTRG.10699.1
MSTRG.1	Gm22616	ENSMUST00000179783
MSTRG.1	Hdac4	MSTRG.1070.1
MSTRG.1	Rps2-ps	ENSMUST00000095471
MSTRG.1	Tsc22d1	MSTRG.10701.1;ENSMUST00000110888;ENSMUST00000048371;E
MSTRG.1	Tsc22d1	MSTRG.10702.1
MSTRG.1	Tsc22d1	MSTRG.10703.1
MSTRG.1	Lacc1	ENSMUST00000062789;ENSMUST00000139809;ENSMUST00000142
MSTRG.1	Ccdc122	ENSMUST00000048208;ENSMUST00000095625;ENSMUST0000017E
MSTRG.1	Enox1	ENSMUST00000022589
MSTRG.1	Gm10132	ENSMUST00000099431
MSTRG.1	Gm6994	ENSMUST00000088880
MSTRG.1	n-R5s48	ENSMUST00000122482
MSTRG.1	Gm29481	MSTRG.1071.1

MSTRG.1 Enox1 MSTRG.10710.1
MSTRG.1 Gm23823 ENSMUST00000158740
MSTRG.1 Dnajc15 ENSMUST00000022590
MSTRG.1 Epsti1 ENSMUST00000022591;ENSMUST00000169978
MSTRG.1 Akap11 ENSMUST00000123853;MSTRG.10714.1;ENSMUST00000022593
MSTRG.1 Akap11 MSTRG.10715.1
MSTRG.10716 MSTRG.10716.1
MSTRG.10717 MSTRG.10717.1
MSTRG.1 Dgkh ENSMUST00000074729
MSTRG.1 Gm26251 ENSMUST00000122498
MSTRG.1 Gm29481 ENSMUST00000189080
MSTRG.1 Dgkh MSTRG.10720.1
MSTRG.1 Dgkh MSTRG.10721.1
MSTRG.1 Dgkh MSTRG.10722.1
MSTRG.1 Vwa8 ENSMUST00000040990
MSTRG.10724 MSTRG.10724.1
MSTRG.1 Rgcc MSTRG.10725.1;ENSMUST00000022595;MSTRG.10725.2;MSTRG.
MSTRG.1 Kbtbd7 ENSMUST00000061222
MSTRG.1 Kbtbd6 ENSMUST00000100359
MSTRG.1 Naa16 ENSMUST00000171928;MSTRG.10728.2;MSTRG.10728.3;ENSMUS
MSTRG.1 Naa16 MSTRG.10729.1
MSTRG.1 Gm29481 MSTRG.1073.1
MSTRG.1 Naa16 MSTRG.10730.1
MSTRG.1 Wbp4 ENSMUST00000022601
MSTRG.1 Elf1 MSTRG.10732.1;MSTRG.10732.2;ENSMUST00000040131;ENSMUS
MSTRG.1 Elf1 MSTRG.10733.1
MSTRG.1 Elf1 MSTRG.10734.1
MSTRG.1 Elf1 MSTRG.10735.1
MSTRG.1 Gm27857 ENSMUST00000176930
MSTRG.1 Sugt1 MSTRG.10737.1;ENSMUST00000054908
MSTRG.1 Sugt1 MSTRG.10738.1
MSTRG.1 Sugt1 MSTRG.10739.1
MSTRG.1 Gpc1 ENSMUST00000045970;ENSMUST00000191373;ENSMUST0000019C
MSTRG.1 Cnmd ENSMUST00000165835;ENSMUST00000022603;ENSMUST0000016[illegible]
MSTRG.1 Gm6999 ENSMUST00000180427
MSTRG.1 Gm10845 ENSMUST00000179430
MSTRG.1 Gm6999 MSTRG.10743.1
MSTRG.1 Olfm4 ENSMUST00000088735
MSTRG.10745 MSTRG.10745.1
MSTRG.1 Diaph3 ENSMUST00000022599;ENSMUST00000168889
MSTRG.1 Diaph3 MSTRG.10747.1
MSTRG.1 Gm23438 ENSMUST00000104755
MSTRG.1 Tdrd3 ENSMUST00000169504;MSTRG.10749.1;ENSMUST00000168275;E
MSTRG.1 Gpc1 MSTRG.1075.1
MSTRG.1 Tdrd3 MSTRG.10750.1
MSTRG.1 Tdrd3 MSTRG.10751.1
MSTRG.1 Tdrd3 MSTRG.10752.1
MSTRG.1 Tdrd3 MSTRG.10753.1
MSTRG.1 Tdrd3 MSTRG.10754.1
MSTRG.1 Tdrd3 MSTRG.10755.1
MSTRG.1 Tdrd3 MSTRG.10756.1
MSTRG.1 Tdrd3 MSTRG.10757.1
MSTRG.1 Rps3a2 ENSMUST00000074245
MSTRG.1 Gm10110 ENSMUST00000081204
MSTRG.1 Gm29480 ENSMUST00000188589;MSTRG.1076.2
MSTRG.10760 MSTRG.10760.1
MSTRG.10761 MSTRG.10761.1

MSTRG.10762		MSTRG.10762.1
MSTRG.10763		MSTRG.10763.1
MSTRG.10764		MSTRG.10764.1
MSTRG.1	Pcdh9	ENSMUST00000195826
MSTRG.1	Pcdh9	MSTRG.10767.1
MSTRG.10768		MSTRG.10768.1
MSTRG.1	Rpl36a-]	ENSMUST00000081987
MSTRG.1	Mir149	ENSMUST00000083536
MSTRG.1	Mzt1	MSTRG.10770.2;MSTRG.10770.1;MSTRG.10770.3;ENSMUST0000
MSTRG.1	Klf5	ENSMUST00000005279
MSTRG.1	Bora	ENSMUST00000022656;MSTRG.10772.1
MSTRG.1	Bora	MSTRG.10773.1;MSTRG.10773.2
MSTRG.1	Dis3	MSTRG.10774.1;ENSMUST00000042471
MSTRG.1	Pibf1	ENSMUST00000022650
MSTRG.1	Pibf1	MSTRG.10776.1
MSTRG.1	Pibf1	MSTRG.10777.1
MSTRG.1	Pibf1	MSTRG.10778.1
MSTRG.1	Pibf1	MSTRG.10779.1
MSTRG.1	Ankmy1	ENSMUST00000212504;ENSMUST00000112998;ENSMUST0000016C
MSTRG.1	Pibf1	MSTRG.10780.1
MSTRG.1	Pibf1	MSTRG.10781.1
MSTRG.1	Klf12	ENSMUST00000097079
MSTRG.1	Klf12	MSTRG.10783.1
MSTRG.1	4930517(	ENSMUST00000140320;ENSMUST00000145018;ENSMUST00000137
MSTRG.1	Gm16260	ENSMUST00000155141
MSTRG.1	4930517(	MSTRG.10786.1
MSTRG.10787		MSTRG.10787.1
MSTRG.1	Commd6	MSTRG.10788.1;MSTRG.10788.2;ENSMUST00000100339;ENSMUS
MSTRG.1	Tbc1d4	ENSMUST00000161991;ENSMUST00000161304;ENSMUST0000015
MSTRG.1	Dusp28	ENSMUST00000060913
MSTRG.1	Uchl3	ENSMUST00000002289;MSTRG.10790.2
MSTRG.1	Uchl3	MSTRG.10791.1
MSTRG.1	Uchl3	MSTRG.10792.1
MSTRG.10793		MSTRG.10793.1
MSTRG.1	Gm9922	ENSMUST00000066461
MSTRG.1	Lmo7	ENSMUST00000100337;ENSMUST00000159769;ENSMUST0000015
MSTRG.1	Kctd12;I	ENSMUST00000184744;ENSMUST00000183756;MSTRG.10796.3;E
MSTRG.1	Cln5	ENSMUST00000022721
MSTRG.1	Fbxl3	ENSMUST00000022720;ENSMUST00000145693;ENSMUST00000132
MSTRG.1	Eya1	MSTRG.108.1
MSTRG.1	5033417	ENSMUST00000181575;MSTRG.1080.2
MSTRG.1	Mycbp2	ENSMUST00000161008;MSTRG.10800.1;ENSMUST00000160758;E
MSTRG.1	Mycbp2	MSTRG.10801.1
MSTRG.1	Slain1	ENSMUST00000069443;ENSMUST00000160805;ENSMUST00000162
MSTRG.1	Slain1	MSTRG.10805.1
MSTRG.1	Slain1o	MSTRG.10806.1;MSTRG.10806.2
MSTRG.1	Rnf219	ENSMUST00000022716
MSTRG.1	Rbm26;G	MSTRG.10808.1;ENSMUST00000172122;MSTRG.10808.2;ENSMUS
MSTRG.1	Rbm26	MSTRG.10809.1
MSTRG.1	Rnpepl1	ENSMUST00000027487;ENSMUST00000179854;ENSMUST0000018C
MSTRG.1	Ndfip2	ENSMUST00000181969;ENSMUST00000138283;ENSMUST00000155
MSTRG.1	Gm24207	ENSMUST00000082781
MSTRG.1	Ndfip2	MSTRG.10812.1
MSTRG.10814		MSTRG.10814.1
MSTRG.10815		MSTRG.10815.1
MSTRG.1	Gm10076	ENSMUST00000073238
MSTRG.1	Spry2	ENSMUST00000022709

MSTRG.10818		MSTRG.10818.1
MSTRG.10819		MSTRG.10819.1
MSTRG.1	9430060	ENSMUST00000191563
MSTRG.10820		MSTRG.10820.1
MSTRG.10821		MSTRG.10821.1
MSTRG.10822		MSTRG.10822.1
MSTRG.1	Tpm3-rs	ENSMUST00000072359
MSTRG.10824		MSTRG.10824.1
MSTRG.1	Mir17hg	ENSMUST00000134140;ENSMUST00000083574;ENSMUST0000008З
MSTRG.1	Gm26773	ENSMUST00000180772
MSTRG.1	Tgds	ENSMUST00000022727
MSTRG.1	Gpr180	ENSMUST00000022728;MSTRG.10828.2
MSTRG.1	Abcc4	ENSMUST00000036554;ENSMUST00000166646;MSTRG.10829.3
MSTRG.1	Capn10	ENSMUST00000027488;ENSMUST00000117814;ENSMUST00000152
MSTRG.1	Abcc4	MSTRG.10830.1
MSTRG.1	Gm23302	ENSMUST00000082710
MSTRG.1	Abcc4	MSTRG.10832.1
MSTRG.1	Mir6391	ENSMUST00000184391
MSTRG.1	Abcc4	MSTRG.10834.1
MSTRG.1	Abcc4	MSTRG.10835.1
MSTRG.1	Cldn10	ENSMUST00000047761;ENSMUST00000100314
MSTRG.1	n-R5s51	ENSMUST00000104473
MSTRG.1	Dzip1	ENSMUST00000047208;MSTRG.10838.1;MSTRG.10838.3;MSTRG.
MSTRG.10839		MSTRG.10839.1
MSTRG.1	Gpr35	ENSMUST00000185421;ENSMUST00000189697;ENSMUST0000018б
MSTRG.1	Dnajc3	ENSMUST00000022734;MSTRG.10840.2;MSTRG.10840.3
MSTRG.1	Uggt2	ENSMUST00000148022;ENSMUST00000156203;ENSMUST0000012З
MSTRG.1	Gm16835	ENSMUST00000140488
MSTRG.10843		MSTRG.10843.1
MSTRG.1	Rap2a	ENSMUST00000062117;ENSMUST00000156694
MSTRG.1	Rap2a	MSTRG.10845.1
MSTRG.1	Rap2a	MSTRG.10846.1
MSTRG.1	Mbnl2	MSTRG.10847.2;MSTRG.10847.4;ENSMUST00000088419;ENSMUS
MSTRG.1	Mbnl2	MSTRG.10848.1
MSTRG.1	Mbnl2	MSTRG.10849.1
MSTRG.1	Sned1;M	ENSMUST00000062202;ENSMUST00000172289;ENSMUST0000016
MSTRG.1	Mbnl2	MSTRG.10850.1
MSTRG.1	Mbnl2	MSTRG.10851.1
MSTRG.1	Gm26679	ENSMUST00000181685;MSTRG.10852.2
MSTRG.1	Mbnl2	MSTRG.10853.1
MSTRG.10854		MSTRG.10854.1
MSTRG.10855		MSTRG.10855.1
MSTRG.1	Ipo5	MSTRG.10856.1;ENSMUST00000032898
MSTRG.1	Farp1	ENSMUST00000135010;ENSMUST00000026635;ENSMUST0000015З
MSTRG.1	B930095	ENSMUST00000130256
MSTRG.1	Stk24	MSTRG.10859.1;MSTRG.10859.2;ENSMUST00000079817;MSTRG.
MSTRG.1	Sned1	MSTRG.1086.1
MSTRG.1	Stk24	MSTRG.10860.1
MSTRG.1	Stk24	MSTRG.10861.1
MSTRG.10862		MSTRG.10862.1
MSTRG.1	Timm8a2	ENSMUST00000045976
MSTRG.1	1810041	MSTRG.10864.1
MSTRG.1	1810041	ENSMUST00000187218;ENSMUST00000187773;ENSMUST0000018
MSTRG.1	Ubac2	ENSMUST00000039803;MSTRG.10866.2
MSTRG.1	Ubac2	MSTRG.10867.1;MSTRG.10867.2
MSTRG.1	Gpr18	MSTRG.10868.1;ENSMUST00000055475
MSTRG.1	Ubac2	MSTRG.10869.1

MSTRG.1	Mterf4	ENSMUST00000112944;ENSMUST00000027492;ENSMUST00000136
MSTRG.1	Gpr183	MSTRG.10870.1;MSTRG.10870.2;ENSMUST00000049872
MSTRG.1	Dock9	ENSMUST00000040700;ENSMUST00000211803;ENSMUST00000212
MSTRG.1	Dock9	MSTRG.10872.1
MSTRG.1	Dock9	MSTRG.10873.1
MSTRG.1	Dock9	MSTRG.10874.1
MSTRG.1	Dock9	MSTRG.10875.1
MSTRG.1	Dock9	MSTRG.10876.1
MSTRG.1	Dock9	MSTRG.10877.1
MSTRG.1	Dock9	MSTRG.10878.1
MSTRG.1	A330035	ENSMUST00000144926;ENSMUST00000131416
MSTRG.1	Gm28535	ENSMUST00000188069
MSTRG.1	Tm9sf2;	ENSMUST00000026624;ENSMUST00000170007;ENSMUST00000171
MSTRG.1	Gm6254	ENSMUST00000168178
MSTRG.1	Clybl	ENSMUST00000026625
MSTRG.1	1700108	ENSMUST00000187423
MSTRG.1	Gm25464	ENSMUST00000175481
MSTRG.1	Pcca	ENSMUST00000148172;ENSMUST00000154206;ENSMUST00000038
MSTRG.1	Pcca	MSTRG.10886.1
MSTRG.1	Ggact	ENSMUST00000110679;ENSMUST00000038075;ENSMUST00000161
MSTRG.1	4930594	ENSMUST00000100291;ENSMUST00000161599;ENSMUST00000162
MSTRG.1	Tmtc4	ENSMUST00000037726;ENSMUST00000143189;ENSMUST00000126
MSTRG.1	Gm7867	ENSMUST00000189856
MSTRG.1	Gm15735	ENSMUST00000136187
MSTRG.1	Itgbl1	ENSMUST00000049681;ENSMUST00000142161
MSTRG.1	Gm9308	ENSMUST00000119073
MSTRG.1	Itgbl1	MSTRG.10893.1
MSTRG.1	Gm25272	ENSMUST00000104293
MSTRG.1	Fgf14	ENSMUST00000095529;ENSMUST00000026631
MSTRG.1	Gm28932	ENSMUST00000186300
MSTRG.1	Gm15422	ENSMUST00000117453
MSTRG.1	1700024	ENSMUST00000190088
MSTRG.1	Fgf14	MSTRG.10899.1
MSTRG.1	Eya1	MSTRG.109.1
MSTRG.1	Pask	ENSMUST00000128655;ENSMUST00000027493;ENSMUST00000133
MSTRG.1	Gm17615	ENSMUST00000166105
MSTRG.1	Olfr277	ENSMUST00000205749
MSTRG.1	Fgf14	MSTRG.10902.1
MSTRG.1	Selenop	ENSMUST00000160787;ENSMUST00000082424;ENSMUST00000159
MSTRG.1	Ccdc152	ENSMUST00000165386
MSTRG.1	Ccdc152	MSTRG.10905.1
MSTRG.10906		MSTRG.10906.1
MSTRG.1	Ghr	ENSMUST00000069451;ENSMUST00000161561;ENSMUST00000110
MSTRG.1	AW54987	MSTRG.10908.2;ENSMUST00000046633;MSTRG.10908.1
MSTRG.1	BC03703	ENSMUST00000194637;ENSMUST00000155191;ENSMUST00000140
MSTRG.1	Ppp1r7	ENSMUST00000027494;MSTRG.1091.2;ENSMUST00000185498;EN
MSTRG.1	Oxct1	ENSMUST00000110690;ENSMUST00000138927;ENSMUST00000131
MSTRG.1	Gm15632	MSTRG.10911.1
MSTRG.1	C7	ENSMUST00000110689
MSTRG.1	Gm25652	ENSMUST00000083780
MSTRG.1	Gm16077	ENSMUST00000121915
MSTRG.1	Card6	ENSMUST00000118365;MSTRG.10915.2;ENSMUST00000141020
MSTRG.1	Rpl37	ENSMUST00000045356
MSTRG.1	Snord72	ENSMUST00000104549
MSTRG.1	Gm10250	ENSMUST00000090488
MSTRG.1	Prkaa1	ENSMUST00000051186;MSTRG.10919.2
MSTRG.1	Mir6902	ENSMUST00000198980

MSTRG.1	Gm15938	ENSMUST00000150079
MSTRG.1	Ttc33	ENSMUST00000118193;ENSMUST00000022751;ENSMUST00000081
MSTRG.1	Ptger4	MSTRG.10922.1;ENSMUST00000120563;MSTRG.10922.3;ENSMUS
MSTRG.1	Gm15937	ENSMUST00000120727
MSTRG.1	Ptger4	MSTRG.10924.1
MSTRG.10925		MSTRG.10925.1
MSTRG.10926		MSTRG.10926.1
MSTRG.1	Dab2	ENSMUST00000078019;ENSMUST00000161040;ENSMUST0000008C
MSTRG.1	Rictor	MSTRG.10929.2;MSTRG.10929.4;MSTRG.10929.3;MSTRG.1092S
MSTRG.1	Mir6345	ENSMUST00000184286
MSTRG.1	Fyb;Gm7	MSTRG.10930.1;MSTRG.10930.2;ENSMUST00000090461;ENSMUS
MSTRG.1	Fyb	MSTRG.10931.2;MSTRG.10931.1
MSTRG.1	Fyb	MSTRG.10932.1
MSTRG.1	Gm2245	MSTRG.10933.1;MSTRG.10933.2;ENSMUST00000160698
MSTRG.1	Osmr	MSTRG.10934.1;ENSMUST00000022746;ENSMUST00000176826;E
MSTRG.1	Lifr	MSTRG.10935.1;MSTRG.10935.2;MSTRG.10935.3;ENSMUST000C
MSTRG.10936		MSTRG.10936.1
MSTRG.1	Lifr	MSTRG.10937.1
MSTRG.1	Egflam	ENSMUST00000058593;ENSMUST00000096494;ENSMUST0000016C
MSTRG.1	Gm16030	ENSMUST00000159098
MSTRG.1	Gm26446	ENSMUST00000082455
MSTRG.1	Gm16029	ENSMUST00000161643
MSTRG.1	Wdr70	ENSMUST00000045766
MSTRG.1	Gm27529	MSTRG.10942.1;ENSMUST00000184975
MSTRG.1	Wdr70	MSTRG.10943.1
MSTRG.1	Wdr70	MSTRG.10944.1
MSTRG.1	Wdr70	MSTRG.10945.1
MSTRG.1	Nup155	ENSMUST00000163765;MSTRG.10946.2
MSTRG.1	Gm2310	MSTRG.10947.1
MSTRG.1	Gm2310	MSTRG.10948.1
MSTRG.1	Gm2348	ENSMUST00000108782
MSTRG.1	Hdlbp	ENSMUST00000170883;ENSMUST00000042498;ENSMUST0000018б
MSTRG.10950		MSTRG.10950.1
MSTRG.10951		MSTRG.10951.1
MSTRG.1	2410089	ENSMUST00000110617;ENSMUST00000150869;ENSMUST0000013C
MSTRG.1	2410089	MSTRG.10953.1
MSTRG.1	2410089	MSTRG.10954.1
MSTRG.1	2410089	MSTRG.10955.1
MSTRG.1	Gm24144	ENSMUST00000082632
MSTRG.1	2410089	MSTRG.10957.1
MSTRG.10958		MSTRG.10958.1
MSTRG.1	Nipbl	MSTRG.10959.1;MSTRG.10959.2;MSTRG.10959.3;ENSMUST000C
MSTRG.1	Gm17415	ENSMUST00000096427
MSTRG.1	Nipbl	MSTRG.10960.1
MSTRG.1	Nipbl	MSTRG.10961.1
MSTRG.10962		MSTRG.10962.1
MSTRG.10963		MSTRG.10963.1
MSTRG.1	Slc1a3	ENSMUST00000005493;ENSMUST00000125997;ENSMUST0000012Ɛ
MSTRG.1	Gm37310	ENSMUST00000195237
MSTRG.1	Slc1a3	MSTRG.10966.1
MSTRG.1	Slc1a3	MSTRG.10967.1
MSTRG.1	Slc1a3	MSTRG.10968.1
MSTRG.1	Nadk2	ENSMUST00000067760;ENSMUST00000100789;ENSMUST0000010C
MSTRG.1	Sept2	ENSMUST00000027495;ENSMUST00000136182;ENSMUST00000131
MSTRG.1	Nadk2	MSTRG.10970.1
MSTRG.1	Skp2	ENSMUST00000096482;ENSMUST00000110585;ENSMUST00000191
MSTRG.1	Lmbrd2	ENSMUST00000090380

MSTRG.1	Capsl	ENSMUST00000042360
MSTRG.1	Il7r	MSTRG.10974.1;MSTRG.10974.2;ENSMUST00000003981;MSTRG.
MSTRG.1	Il7r	MSTRG.10975.1
MSTRG.1	Dnajc21	ENSMUST00000136591;ENSMUST00000145719;ENSMUST0000014[illegible]
MSTRG.1	Brix1	ENSMUST00000022855;MSTRG.10977.2;ENSMUST00000168690;[illegible]
MSTRG.1	Rad1	ENSMUST00000022856;ENSMUST00000170531;ENSMUST0000010[illegible]
MSTRG.1	Rad1	MSTRG.10979.1
MSTRG.1	Farp2	ENSMUST00000120301;ENSMUST00000122402
MSTRG.1	Rad1	MSTRG.10980.1
MSTRG.1	Rai14	ENSMUST00000090339;MSTRG.10981.1;ENSMUST00000169385;[illegible]
MSTRG.1	4930556[illegible]	ENSMUST00000180604
MSTRG.1	4930556[illegible]	ENSMUST00000181739
MSTRG.1	Tars	ENSMUST00000022849
MSTRG.1	Sub1	ENSMUST00000022816;ENSMUST00000110504;ENSMUST0000012[illegible]
MSTRG.10986		MSTRG.10986.1
MSTRG.1	Zfr	MSTRG.10987.1
MSTRG.10988		MSTRG.10988.1
MSTRG.1	Mtmr12	ENSMUST00000038172;ENSMUST00000071993;ENSMUST00000174
MSTRG.1	Gm37250	ENSMUST00000194919
MSTRG.1	Zfr;Mir	ENSMUST00000122941;MSTRG.10990.6;MSTRG.10990.5;MSTRG.
MSTRG.1	Gm25713	ENSMUST00000158625
MSTRG.1	Gm25713	MSTRG.10992.1
MSTRG.1	1810049[illegible]	ENSMUST00000180521
MSTRG.1	Golph3	MSTRG.10994.2;ENSMUST00000059680;MSTRG.10994.1
MSTRG.1	Golph3	MSTRG.10995.1
MSTRG.1	Pdzd2	ENSMUST00000075317;MSTRG.10996.1;ENSMUST00000188549;[illegible]
MSTRG.1	Pdzd2	MSTRG.10997.1
MSTRG.1	Pdzd2	MSTRG.10998.1
MSTRG.1	Gm24135	ENSMUST00000179085
MSTRG.1	Tcea1	MSTRG.11.1
MSTRG.1	Gm37444	ENSMUST00000193605
MSTRG.1	Stk25	ENSMUST00000027498;ENSMUST00000151068;ENSMUST00000154
MSTRG.1	Gm2606	ENSMUST00000100754
MSTRG.1	F830212[illegible]	ENSMUST00000191701
MSTRG.1	Gm24302	ENSMUST00000178350
MSTRG.11003		MSTRG.11003.1
MSTRG.1	6030458[illegible]	MSTRG.11004.1;ENSMUST00000057256
MSTRG.1	Drosha;[illegible]	ENSMUST00000169061;ENSMUST00000090292;ENSMUST00000141
MSTRG.1	Drosha	MSTRG.11006.1
MSTRG.1	Drosha	MSTRG.11007.1
MSTRG.1	Drosha	MSTRG.11008.1
MSTRG.11009		MSTRG.11009.1
MSTRG.1	Gm28536	ENSMUST00000190971
MSTRG.1	Cdh12	ENSMUST00000075132
MSTRG.1	Gm25101	ENSMUST00000179057
MSTRG.1	Gm38234	ENSMUST00000195204
MSTRG.1	Cdh12	MSTRG.11013.1
MSTRG.11014		MSTRG.11014.1
MSTRG.1	Cdh18	ENSMUST00000170626;ENSMUST00000164787
MSTRG.1	Hnrnpa1	ENSMUST00000022493
MSTRG.1	Cdh18	MSTRG.11019.1
MSTRG.1	Pdcd1	ENSMUST00000027507
MSTRG.1	Basp1	ENSMUST00000058845
MSTRG.1	Myo10	ENSMUST00000110457;ENSMUST00000137601;ENSMUST00000127
MSTRG.1	Myo10	MSTRG.11022.1
MSTRG.1	Gm2862	ENSMUST00000161524
MSTRG.1	Myo10	MSTRG.11024.1

MSTRG.1	Myo10	MSTRG.11025.1
MSTRG.1	Retreg1	ENSMUST00000022881;ENSMUST00000110438
MSTRG.1	Retreg1	MSTRG.11027.1
MSTRG.1	Retreg1	MSTRG.11029.1
MSTRG.1	Gm7895	ENSMUST00000187459
MSTRG.1	Mir7212	ENSMUST00000184098
MSTRG.1	Zfp622	ENSMUST00000061875;MSTRG.11031.2
MSTRG.1	Fbxl7	ENSMUST00000059204
MSTRG.1	Fbxl7	MSTRG.11033.1
MSTRG.1	Gm6576	ENSMUST00000169678
MSTRG.1	Ank;Mir	ENSMUST00000022875;MSTRG.11035.2;ENSMUST00000134004;E
MSTRG.1	Ank	MSTRG.11036.1
MSTRG.1	Ank	MSTRG.11037.1
MSTRG.1	Otulin	MSTRG.11038.1;ENSMUST00000059662
MSTRG.1	Otulin	MSTRG.11039.1
MSTRG.1	D2hgdh	ENSMUST00000187321;ENSMUST00000097633;ENSMUST00000112
MSTRG.1	Otulin	MSTRG.11040.1
MSTRG.1	Fam105a	MSTRG.11041.1;MSTRG.11041.2;MSTRG.11041.3;MSTRG.11041
MSTRG.1	Trio	ENSMUST00000090247;MSTRG.11042.2;MSTRG.11042.3
MSTRG.1	Trio	MSTRG.11043.1
MSTRG.1	Trio	MSTRG.11044.1
MSTRG.1	Trio	MSTRG.11045.1
MSTRG.1	Ctnnd2	ENSMUST00000081728
MSTRG.1	Ctnnd2	MSTRG.11047.1
MSTRG.1	Gm23956	ENSMUST00000157211
MSTRG.1	Gm24487	ENSMUST00000178017
MSTRG.1	D2hgdh	MSTRG.1105.1
MSTRG.1	Dap	ENSMUST00000044524;ENSMUST00000186547;ENSMUST00000186
MSTRG.1	Ankrd33	ENSMUST00000110410;ENSMUST00000123325;ENSMUST00000156
MSTRG.1	Ankrd33	MSTRG.11052.1
MSTRG.1	March6	MSTRG.11053.3;MSTRG.11053.1;MSTRG.11053.2;ENSMUST0000
MSTRG.1	March6	MSTRG.11054.1
MSTRG.11055		MSTRG.11055.1
MSTRG.1	Cmbl	ENSMUST00000162532;ENSMUST00000070918;ENSMUST00000161
MSTRG.1	Cct5	ENSMUST00000022842;ENSMUST00000160174;ENSMUST00000159
MSTRG.1	Fam173b	ENSMUST00000160146;ENSMUST00000042702;ENSMUST00000161
MSTRG.11059		MSTRG.11059.1
MSTRG.1	D2hgdh	MSTRG.1106.1
MSTRG.1	Snhg18	ENSMUST00000181536
MSTRG.1	Snord12	ENSMUST00000158692
MSTRG.1	Sema5a	ENSMUST00000067458
MSTRG.1	Sdc2	ENSMUST00000022871
MSTRG.1	Cpq	ENSMUST00000042167
MSTRG.1	Cpq	MSTRG.11065.1
MSTRG.1	Cpq	MSTRG.11066.1;MSTRG.11066.2
MSTRG.1	1700084	ENSMUST00000187707;ENSMUST00000079057
MSTRG.1	Gm24476	ENSMUST00000157437
MSTRG.1	Laptm4b	ENSMUST00000022867
MSTRG.1	D2hgdh	MSTRG.1107.1
MSTRG.1	Mtdh	ENSMUST00000172082;MSTRG.11070.1;ENSMUST00000022865;E
MSTRG.1	Mtdh	MSTRG.11071.1
MSTRG.1	Mtdh	MSTRG.11072.1
MSTRG.1	Gm24500	ENSMUST00000158447
MSTRG.1	Matn2	ENSMUST00000163455;ENSMUST00000022947
MSTRG.1	9430069	ENSMUST00000179647
MSTRG.1	Rpl30;G	ENSMUST00000142643;ENSMUST00000135417;ENSMUST00000079
MSTRG.1	Rida	ENSMUST00000022946;ENSMUST00000125286;ENSMUST00000141

MSTRG.1	Pop1	ENSMUST00000052290;ENSMUST00000079028;ENSMUST0000012(
MSTRG.1	Pop1	MSTRG.11079.1
MSTRG.1	Gal3st2	ENSMUST00000190710;ENSMUST00000094663
MSTRG.1	Nipal2	ENSMUST00000040791
MSTRG.1	Nipal2	MSTRG.11081.1
MSTRG.1	Stk3	MSTRG.11082.1;ENSMUST00000018476;ENSMUST00000067033;E
MSTRG.1	Stk3	MSTRG.11083.1
MSTRG.1	Stk3	MSTRG.11084.1
MSTRG.1	Stk3	MSTRG.11085.1
MSTRG.1	Stk3	MSTRG.11086.1
MSTRG.1	Stk3	MSTRG.11087.1
MSTRG.1	Vps13b	MSTRG.11088.1;ENSMUST00000048646;MSTRG.11088.3
MSTRG.1	Gm24771	ENSMUST00000158508
MSTRG.1	Gm18666	MSTRG.1109.1
MSTRG.1	Vps13b	MSTRG.11090.1
MSTRG.1	Mir599	ENSMUST00000102378
MSTRG.1	Mir875	ENSMUST00000104698
MSTRG.1	Vps13b	MSTRG.11093.1
MSTRG.1	Vps13b	MSTRG.11094.1
MSTRG.1	Vps13b	MSTRG.11095.1
MSTRG.1	Vps13b	MSTRG.11096.1
MSTRG.1	Vps13b	MSTRG.11097.1
MSTRG.1	Cox6c	ENSMUST00000155638;MSTRG.11098.1;ENSMUST00000014457;E
MSTRG.1	Rpl7a-p	ENSMUST00000090170
MSTRG.1	Gm9947	ENSMUST00000187401;ENSMUST00000067599
MSTRG.1	Gal3st2	ENSMUST00000178757;ENSMUST00000177958
MSTRG.1	Polr2k	ENSMUST00000180159;ENSMUST00000057177
MSTRG.1	Spag1	ENSMUST00000171205
MSTRG.1	Gm23754	ENSMUST00000157576
MSTRG.1	Spag1	MSTRG.11103.1
MSTRG.1	Rnf19a;I	ENSMUST00000022890;ENSMUST00000185150;MSTRG.11104.2;M
MSTRG.1	Ankrd46	MSTRG.11105.1;ENSMUST00000057486
MSTRG.1	Gm44310	MSTRG.11106.1
MSTRG.1	Pabpc1	ENSMUST00000001809;ENSMUST00000155116;MSTRG.11107.2;E
MSTRG.1	Ywhaz	ENSMUST00000022894;ENSMUST00000110361;ENSMUST0000011C
MSTRG.1	Zfp706	ENSMUST00000078976
MSTRG.1	Gm7889	ENSMUST00000185591
MSTRG.1	Gm26766	ENSMUST00000181411
MSTRG.11111		MSTRG.11111.1
MSTRG.11112		MSTRG.11112.1
MSTRG.1	Rrm2b	ENSMUST00000022901;ENSMUST00000137636;ENSMUST0000014(
MSTRG.1	Gm3362	ENSMUST00000148102
MSTRG.1	Ubr5	MSTRG.11115.1
MSTRG.1	Gm16136	ENSMUST00000162520;ENSMUST00000160264
MSTRG.1	Grhl2	ENSMUST00000161405;ENSMUST00000022895;ENSMUST00000161
MSTRG.1	Gm16137	ENSMUST00000161571
MSTRG.1	Grhl2	MSTRG.11119.1
MSTRG.1	Gal3st2	ENSMUST00000097632
MSTRG.1	Gm16138	ENSMUST00000159341
MSTRG.1	Ncald;G	ENSMUST00000168992;ENSMUST00000090150;ENSMUST0000012C
MSTRG.1	Gm23654	ENSMUST00000116758
MSTRG.1	Gm8668	ENSMUST00000120559
MSTRG.1	4930447	ENSMUST00000022897
MSTRG.1	Gm3267	ENSMUST00000118671
MSTRG.1	Gm24833	ENSMUST00000116775
MSTRG.1	Gm15942	ENSMUST00000136305
MSTRG.1	Ncald	MSTRG.11129.1

MSTRG.1	Bok	ENSMUST00000027499;ENSMUST00000201863;ENSMUST0000018[illegible]
MSTRG.1	Ncald	MSTRG.11130.1
MSTRG.1	Gm15940	ENSMUST00000156011
MSTRG.1	Klf10	MSTRG.11132.1;ENSMUST00000074043
MSTRG.1	Ubr5	ENSMUST00000110336;MSTRG.11133.1;MSTRG.11133.3
MSTRG.1	Gm22353	ENSMUST00000083918
MSTRG.1	Ubr5	MSTRG.11135.1
MSTRG.11136		MSTRG.11136.1
MSTRG.1	Azin1	ENSMUST00000065308;ENSMUST00000110329;ENSMUST0000012[illegible]
MSTRG.1	Mir6951	ENSMUST00000183910
MSTRG.1	Atp6v1c	ENSMUST00000022904;MSTRG.11139.2
MSTRG.1	Thap4	ENSMUST00000190116;ENSMUST00000189728;ENSMUST00000112
MSTRG.1	Gm26704	ENSMUST00000181362
MSTRG.1	Fzd6	ENSMUST00000179165;ENSMUST00000022906
MSTRG.1	Slc25a3	MSTRG.11143.1
MSTRG.1	Slc25a3	ENSMUST00000022908
MSTRG.1	Slc25a3	MSTRG.11145.1
MSTRG.1	Slc25a3	MSTRG.11146.1
MSTRG.1	Dcaf13	ENSMUST00000022909;MSTRG.11147.2
MSTRG.1	n-R5s39	ENSMUST00000122613
MSTRG.1	Gm9522	MSTRG.11149.1;MSTRG.11149.2;ENSMUST00000182394
MSTRG.1	Thap4	MSTRG.1115.1
MSTRG.1	Rims2	ENSMUST00000082054;ENSMUST00000042917
MSTRG.1	Rims2	MSTRG.11151.1
MSTRG.1	Rims2	MSTRG.11153.1
MSTRG.1	Lrp12	ENSMUST00000022916;ENSMUST00000110305;ENSMUST0000014[illegible]
MSTRG.1	Lrp12	MSTRG.11155.1
MSTRG.1	Lrp12	MSTRG.11156.1
MSTRG.1	Lrp12	MSTRG.11157.1
MSTRG.1	Lrp12	MSTRG.11158.1
MSTRG.1	Lrp12	MSTRG.11159.1
MSTRG.1	Gm10550	ENSMUST00000188846
MSTRG.1	Oxr1	ENSMUST00000090096;ENSMUST00000110297;ENSMUST0000017[illegible]
MSTRG.1	Oxr1	MSTRG.11161.1
MSTRG.1	Oxr1	MSTRG.11162.1
MSTRG.1	Gm26854	ENSMUST00000180418
MSTRG.1	Oxr1	MSTRG.11164.1
MSTRG.11165		MSTRG.11165.1
MSTRG.1	Angpt1	ENSMUST00000022921;MSTRG.11166.2
MSTRG.1	Gm17473	ENSMUST00000180585
MSTRG.1	Eif3e	ENSMUST00000022960;MSTRG.11168.2
MSTRG.1	Gm10373	ENSMUST00000100683
MSTRG.1	Atg4b	MSTRG.1117.1
MSTRG.1	Emc2	ENSMUST00000022962;MSTRG.11170.1
MSTRG.1	Emc2	MSTRG.11171.1
MSTRG.11172		MSTRG.11172.1
MSTRG.1	Nudcd1	ENSMUST00000038719;MSTRG.11173.1
MSTRG.1	Eny2	ENSMUST00000060652
MSTRG.1	Gm22392	ENSMUST00000177971
MSTRG.1	Ebag9	MSTRG.11176.1;ENSMUST00000022964
MSTRG.1	Pkhd1l1	ENSMUST00000038336;ENSMUST00000166957;ENSMUST0000020[illegible]
MSTRG.11178		MSTRG.11178.1
MSTRG.1	Atg4b	ENSMUST00000149436;ENSMUST00000027502;ENSMUST00000187
MSTRG.1	1700022	MSTRG.11180.1
MSTRG.11181		MSTRG.11181.1
MSTRG.11182		MSTRG.11182.1
MSTRG.1	Csmd3	ENSMUST00000100670

MSTRG.1	Csmd3	MSTRG.11187.1
MSTRG.11188		MSTRG.11188.1
MSTRG.1	Trps1;M	ENSMUST00000165201;ENSMUST00000184458;ENSMUST00000183
MSTRG.1	Dtymk	MSTRG.1119.1;MSTRG.1119.2;ENSMUST00000027503;ENSMUST0
MSTRG.1	Trps1	MSTRG.11190.1
MSTRG.1	Trps1	MSTRG.11191.1
MSTRG.1	Trps1	MSTRG.11192.1
MSTRG.1	Trps1	MSTRG.11193.1
MSTRG.1	Trps1	MSTRG.11194.1
MSTRG.1	Trps1	MSTRG.11195.1
MSTRG.1	Trps1	MSTRG.11196.1
MSTRG.1	Eif3h	ENSMUST00000022925
MSTRG.1	Utp23	ENSMUST00000137116;ENSMUST00000059599;ENSMUST00000136
MSTRG.1	Utp23	MSTRG.11199.1
MSTRG.1	Msc	ENSMUST00000027062;ENSMUST00000190719;ENSMUST00000188
MSTRG.1	Ing5	ENSMUST00000027505;ENSMUST00000188402;ENSMUST0000019C
MSTRG.11200		MSTRG.11200.1
MSTRG.1	Rad21	MSTRG.11201.3;MSTRG.11201.4;MSTRG.11201.2;MSTRG.11201
MSTRG.1	Gm10020	ENSMUST00000081993
MSTRG.1	Med30	MSTRG.11203.1;ENSMUST00000037115
MSTRG.1	Ext1	ENSMUST00000077273;ENSMUST00000133362;ENSMUST0000011C
MSTRG.1	Ext1	MSTRG.11205.1;MSTRG.11205.2;MSTRG.11205.3;MSTRG.11205
MSTRG.1	Ext1	MSTRG.11206.1
MSTRG.1	Samd12	ENSMUST00000154119;ENSMUST00000078673;ENSMUST00000132
MSTRG.1	Samd12	MSTRG.11208.1
MSTRG.1	Gm23200	ENSMUST00000104379
MSTRG.1	Gm22089	ENSMUST00000158336
MSTRG.1	Mir28c	ENSMUST00000200096
MSTRG.1	Gm7489	ENSMUST00000100666
MSTRG.1	Enpp2	ENSMUST00000171545;ENSMUST00000041591;ENSMUST00000173
MSTRG.11213		MSTRG.11213.1
MSTRG.1	Taf2	MSTRG.11214.1;ENSMUST00000041733;MSTRG.11214.3
MSTRG.1	Gm9920	ENSMUST00000110230;ENSMUST00000148544;ENSMUST00000137
MSTRG.1	Deptor	ENSMUST00000096433;ENSMUST00000100660;ENSMUST00000023
MSTRG.1	Mrpl13	ENSMUST00000164542;ENSMUST00000172387;ENSMUST00000165
MSTRG.1	Mtbp	ENSMUST00000022998;ENSMUST00000170046;ENSMUST00000169
MSTRG.1	Mtbp	MSTRG.11219.1
MSTRG.1	Ing5	MSTRG.1122.1
MSTRG.1	Mtbp	MSTRG.11220.1
MSTRG.1	Mtbp	MSTRG.11221.1
MSTRG.1	Mtbp	MSTRG.11222.1
MSTRG.1	Sntb1	ENSMUST00000039769;ENSMUST00000110200;ENSMUST0000014C
MSTRG.1	Sntb1	MSTRG.11224.1
MSTRG.1	Sntb1	MSTRG.11225.1
MSTRG.1	Sntb1	MSTRG.11226.1
MSTRG.1	Zhx2	ENSMUST00000096430;ENSMUST00000160990
MSTRG.1	Zhx2	MSTRG.11228.1
MSTRG.1	Gm16006	ENSMUST00000160938
MSTRG.1	Gm15427	ENSMUST00000117288
MSTRG.1	Zhx2	MSTRG.11230.1
MSTRG.1	9330154	ENSMUST00000176703
MSTRG.1	Zhx2	MSTRG.11232.1
MSTRG.11233		MSTRG.11233.1
MSTRG.1	Derl1	ENSMUST00000022993;MSTRG.11234.1;MSTRG.11234.3
MSTRG.1	Tbc1d31	ENSMUST00000022992;ENSMUST00000110175;ENSMUST00000159
MSTRG.1	Tbc1d31	MSTRG.11236.1
MSTRG.1	9130401	ENSMUST00000100655;ENSMUST00000187062;ENSMUST00000176

MSTRG.1	Gm22299	ENSMUST00000082663
MSTRG.1	Zhx1	MSTRG.11239.1
MSTRG.1	St8sia4	ENSMUST00000043336;ENSMUST00000189556;ENSMUST00000187
MSTRG.1	Atad2	MSTRG.11240.2;ENSMUST00000038194;MSTRG.11240.1;MSTRG.
MSTRG.1	Wdyhv1	MSTRG.11241.1;ENSMUST00000067563
MSTRG.1	Gm22488	ENSMUST00000116811
MSTRG.1	Fbxo32	ENSMUST00000022986;MSTRG.11244.2
MSTRG.1	Gm15943	ENSMUST00000147638
MSTRG.1	Klhl38	ENSMUST00000022985
MSTRG.1	Fam91a1	ENSMUST00000037270;MSTRG.11247.2;MSTRG.11247.3;MSTRG.
MSTRG.1	Fam91a1	MSTRG.11248.1
MSTRG.1	Trmt12	ENSMUST00000036937
MSTRG.1	St8sia4	MSTRG.1125.1
MSTRG.1	Gm20712	ENSMUST00000176387
MSTRG.1	Tmem65	MSTRG.11251.1;ENSMUST00000072113
MSTRG.1	Tmem65	MSTRG.11252.1
MSTRG.1	Tmem65	MSTRG.11253.1
MSTRG.1	Gm24041	ENSMUST00000083115
MSTRG.1	Rnf139	ENSMUST00000036904
MSTRG.1	Tatdn1	ENSMUST00000110155;MSTRG.11256.2
MSTRG.1	Ndufb9	ENSMUST00000022980
MSTRG.1	Mtss1	ENSMUST00000080371;MSTRG.11258.1;MSTRG.11258.3;MSTRG.
MSTRG.1	Mtss1	MSTRG.11259.1;MSTRG.11259.2
MSTRG.1	Gm37642	ENSMUST00000193166
MSTRG.1	Trib1	ENSMUST00000067543;ENSMUST00000118228;MSTRG.11260.2
MSTRG.1	Sqle	ENSMUST00000162142;ENSMUST00000022977;ENSMUST0000010C
MSTRG.1	Washc5	ENSMUST00000022976
MSTRG.1	Nsmce2	ENSMUST00000168722;ENSMUST00000079703;MSTRG.11263.3;M
MSTRG.1	Nsmce2	MSTRG.11264.1;MSTRG.11264.2
MSTRG.1	Nsmce2	MSTRG.11265.1
MSTRG.11266		MSTRG.11266.1
MSTRG.11267		MSTRG.11267.1
MSTRG.1	Fam84b	ENSMUST00000100635;ENSMUST00000186536
MSTRG.1	9930014	ENSMUST00000180730;ENSMUST00000192865;ENSMUST00000181
MSTRG.1127		MSTRG.1127.1
MSTRG.11270		MSTRG.11270.1;MSTRG.11270.2;MSTRG.11270.3;MSTRG.1127C
MSTRG.11271		MSTRG.11271.1
MSTRG.11272		MSTRG.11272.1;MSTRG.11272.2;MSTRG.11272.3
MSTRG.11273		MSTRG.11273.1
MSTRG.1	Myc	ENSMUST00000161976;ENSMUST00000159327;ENSMUST00000167
MSTRG.1	Pvt1;Gm	ENSMUST00000182956;MSTRG.11275.1;ENSMUST00000184473;E
MSTRG.1	Pvt1	MSTRG.11276.1
MSTRG.1	Pvt1	MSTRG.11277.1
MSTRG.1	Pvt1	MSTRG.11278.1
MSTRG.1	Gm27782	ENSMUST00000185059
MSTRG.1	Gm28901	ENSMUST00000188810
MSTRG.1	Pvt1	MSTRG.11280.1
MSTRG.1	Pvt1	MSTRG.11281.1
MSTRG.1	H2afy3	ENSMUST00000151919
MSTRG.1	Gsdmcl-	ENSMUST00000191285;ENSMUST00000185574;ENSMUST0000018E
MSTRG.1	Fam49b	ENSMUST00000063838;MSTRG.11284.2;ENSMUST00000164532;M
MSTRG.1	Gm25628	MSTRG.11285.1;ENSMUST00000084002;MSTRG.11285.3
MSTRG.1	Asap1	ENSMUST00000177371;ENSMUST00000177083;ENSMUST00000177
MSTRG.1	Gm20717	ENSMUST00000176762
MSTRG.1	Asap1	MSTRG.11288.1
MSTRG.1	Asap1	MSTRG.11289.1
MSTRG.1	Gm6430	ENSMUST00000054664

MSTRG.1	Asap1	MSTRG.11290.1
MSTRG.1	Asap1	MSTRG.11291.1
MSTRG.1	Asap1	MSTRG.11292.1
MSTRG.1	Efr3a	ENSMUST00000174135;ENSMUST00000015146;ENSMUST0000009[illegible]
MSTRG.1	Gm20405	ENSMUST00000174002
MSTRG.1	Oc90	ENSMUST00000060522
MSTRG.1	Kcnq3	ENSMUST00000070256;ENSMUST00000183354;ENSMUST0000018[illegible]
MSTRG.1	Gm27153	ENSMUST00000184492
MSTRG.1	Kcnq3	MSTRG.11298.1
MSTRG.1	Kcnq3	MSTRG.11299.1
MSTRG.1	Smt3h2-[illegible]	ENSMUST00000121569
MSTRG.1	Slco4c1	ENSMUST00000071985
MSTRG.1	Kcnq3	MSTRG.11300.1
MSTRG.1	Kcnq3	MSTRG.11301.1
MSTRG.1	Kcnq3	MSTRG.11302.1
MSTRG.1	Kcnq3	MSTRG.11303.1
MSTRG.1	Kcnq3	MSTRG.11304.1
MSTRG.1	Kcnq3	MSTRG.11305.1
MSTRG.1	Kcnq3	MSTRG.11306.1
MSTRG.1	Kcnq3	MSTRG.11307.1
MSTRG.1	Kcnq3	MSTRG.11308.1
MSTRG.1	Kcnq3	MSTRG.11309.1
MSTRG.1	Panct2	ENSMUST00000188631;ENSMUST00000181489
MSTRG.11310		MSTRG.11310.1
MSTRG.1	Tmem71	ENSMUST00000048372
MSTRG.1	Tmem71	MSTRG.11312.1
MSTRG.1	Phf20l1	MSTRG.11313.1
MSTRG.1	Phf20l1	ENSMUST00000048188;MSTRG.11314.2;MSTRG.11314.3;MSTRG.
MSTRG.1	Tg	ENSMUST00000065916;ENSMUST00000167512;ENSMUST00000171
MSTRG.1	Gm17140	ENSMUST00000169163
MSTRG.1	Tg	MSTRG.11317.1
MSTRG.1	Sla	ENSMUST00000164163;ENSMUST00000100572;ENSMUST0000016[illegible]
MSTRG.1	Ndrg1	ENSMUST00000005256;ENSMUST00000165966;ENSMUST0000016[illegible]
MSTRG.1	Gm2895	ENSMUST00000171591
MSTRG.1	St3gal1	ENSMUST00000092640;MSTRG.11322.1;MSTRG.11322.3;MSTRG.
MSTRG.11323		MSTRG.11323.1
MSTRG.1	1700012	MSTRG.11325.1
MSTRG.1	1700012	MSTRG.11326.1
MSTRG.1	Zfat	ENSMUST00000162054;ENSMUST00000162173;ENSMUST0000016[illegible]
MSTRG.1	Zfat	MSTRG.11328.1
MSTRG.1	Zfat	MSTRG.11329.1
MSTRG.1	Slco6b1	MSTRG.1133.1
MSTRG.1	Gm10240	ENSMUST00000089785
MSTRG.1	Gm37320	ENSMUST00000192781
MSTRG.1	Gm26621	MSTRG.11332.1;ENSMUST00000194661;ENSMUST00000180413;[illegible]
MSTRG.1	Gm37296	ENSMUST00000192728;MSTRG.11333.1;ENSMUST00000175699;[illegible]
MSTRG.1	Gm7125	ENSMUST00000093430
MSTRG.11335		MSTRG.11335.1
MSTRG.11336		MSTRG.11336.1
MSTRG.1	Trappc9	ENSMUST00000023276;MSTRG.11337.1;ENSMUST00000089770;[illegible]
MSTRG.1	Gm28020	ENSMUST00000157739
MSTRG.1	Peg13	ENSMUST00000202826
MSTRG.1	Gm7135	ENSMUST00000191257;ENSMUST00000187538
MSTRG.1	Trappc9	MSTRG.11340.1
MSTRG.1	Trappc9	MSTRG.11341.1
MSTRG.1	Gm3150	ENSMUST00000166418
MSTRG.1	Chrac1	ENSMUST00000089765;ENSMUST00000134353;ENSMUST00000137

MSTRG.1	Ago2	ENSMUST00000044113;MSTRG.11344.1;MSTRG.11344.3
MSTRG.1	Ago2	MSTRG.11345.1
MSTRG.1	Ptk2	ENSMUST00000110036;ENSMUST00000170939;MSTRG.11346.3
MSTRG.1	Ptk2	MSTRG.11347.1
MSTRG.1	Mir151	ENSMUST00000083678
MSTRG.1	Ptk2	MSTRG.11349.1
MSTRG.1	Gm7135	MSTRG.1135.1
MSTRG.1	Gm24787	ENSMUST00000157380
MSTRG.1	Dennd3	ENSMUST00000043414;ENSMUST00000173292;ENSMUST00000162
MSTRG.1	Dennd3	MSTRG.11352.1
MSTRG.1	Slc45a4	ENSMUST00000151288;MSTRG.11353.2;ENSMUST00000144936;E
MSTRG.1	Slc45a4	MSTRG.11354.1
MSTRG.1	1700010	ENSMUST00000189435;ENSMUST00000188278
MSTRG.1	Ptp4a3	ENSMUST00000165541;ENSMUST00000167582;ENSMUST00000163
MSTRG.1	Gm22106	ENSMUST00000122544
MSTRG.11358		MSTRG.11358.1
MSTRG.1	Mroh5	ENSMUST00000151999;ENSMUST00000126186;ENSMUST00000071
MSTRG.1	Gm37171	ENSMUST00000192636
MSTRG.1	Gm3244	ENSMUST00000120938
MSTRG.1	Gm6569	ENSMUST00000154520
MSTRG.1	Gm7935	ENSMUST00000100555
MSTRG.1	Gm15387	ENSMUST00000120421
MSTRG.11364		MSTRG.11364.1
MSTRG.1	Arc	ENSMUST00000023268;ENSMUST00000110009
MSTRG.1	Jrk	ENSMUST00000050234
MSTRG.1	4933427	ENSMUST00000185751;ENSMUST00000056711
MSTRG.1	Them6	ENSMUST00000070923
MSTRG.1	Slurp1	ENSMUST00000190433;MSTRG.11369.2
MSTRG.1	D1Ertd6	ENSMUST00000053033;ENSMUST00000153115;ENSMUST00000149
MSTRG.1	Slurp1	MSTRG.11370.1
MSTRG.1	Lynx1	ENSMUST00000023259;ENSMUST00000189128;ENSMUST00000189
MSTRG.1	Ly6d	ENSMUST00000040404;MSTRG.11372.1
MSTRG.1	2010109	ENSMUST00000190826;ENSMUST00000077004
MSTRG.1	2010109	MSTRG.11374.1
MSTRG.11375		MSTRG.11375.1
MSTRG.11376		MSTRG.11376.1
MSTRG.11377		MSTRG.11377.1
MSTRG.11378		MSTRG.11378.1
MSTRG.1	Gm6610	ENSMUST00000186324
MSTRG.1	D1Ertd6	MSTRG.1138.1
MSTRG.11380		MSTRG.11380.1
MSTRG.1	Ly6e	ENSMUST00000188866;ENSMUST00000051698;ENSMUST00000187
MSTRG.1	Gpihbp1	ENSMUST00000189944;ENSMUST00000023243;ENSMUST00000189
MSTRG.1	Zfp41	MSTRG.11383.1
MSTRG.1	Zfp41	ENSMUST00000161785;ENSMUST00000054555;ENSMUST00000161
MSTRG.1	Ly6i	ENSMUST00000023250;ENSMUST00000166694
MSTRG.1	Gm8000	ENSMUST00000188378
MSTRG.1	Gm37042	ENSMUST00000193816
MSTRG.1	Ly6a;Ly	ENSMUST00000189068;ENSMUST00000023248;ENSMUST00000187
MSTRG.1	Gm28068	ENSMUST00000190861
MSTRG.1	D1Ertd6	MSTRG.1139.1
MSTRG.1	Gm28069	ENSMUST00000189926
MSTRG.1	Gm5960	MSTRG.11391.1
MSTRG.1	Gm5960	ENSMUST00000188483
MSTRG.1	Gm28502	ENSMUST00000189781
MSTRG.1	I830127	ENSMUST00000187250;ENSMUST00000190944
MSTRG.1	Ly6g	ENSMUST00000190262;ENSMUST00000023246

MSTRG.1	BC02544	ENSMUST00000185307
MSTRG.1	Gm10238	ENSMUST00000089689
MSTRG.1	Rpl5-ps	ENSMUST00000121367
MSTRG.1	Gm7967	ENSMUST00000191042;ENSMUST00000191409;ENSMUST0000019
MSTRG.1	Ly6f	ENSMUST00000023247
MSTRG.1	Gm29517	ENSMUST00000190744
MSTRG.1	Gm28114	ENSMUST00000187232
MSTRG.1	Gm3142	ENSMUST00000190107
MSTRG.1	Mafa	ENSMUST00000062002
MSTRG.1	Zc3h3	ENSMUST00000100538
MSTRG.1	Gm23747	ENSMUST00000104228
MSTRG.1	Gm7967	MSTRG.1141.1
MSTRG.1	Zc3h3	MSTRG.11410.1
MSTRG.1	Zc3h3	MSTRG.11411.1
MSTRG.1	Gsdmd	MSTRG.11412.1;ENSMUST00000023238;MSTRG.11412.2;MSTRG.
MSTRG.11413		MSTRG.11413.1
MSTRG.1	Eef1d	ENSMUST00000154584;ENSMUST00000109972;ENSMUST0000010
MSTRG.1	Tigd5	ENSMUST00000192937
MSTRG.11416		MSTRG.11416.1
MSTRG.1	Pycrl	ENSMUST00000053918
MSTRG.1	Tsta3	ENSMUST00000023231;MSTRG.11418.1
MSTRG.1	Zfp623	ENSMUST00000037260
MSTRG.1	Gm7967	MSTRG.1142.1
MSTRG.1	Zfp707	ENSMUST00000109966;ENSMUST00000131496;ENSMUST0000010
MSTRG.1	Ccdc166	ENSMUST00000182172;ENSMUST00000183130
MSTRG.1	Puf60	MSTRG.11422.2;ENSMUST00000002599;ENSMUST00000100527;
MSTRG.1	Mapk15	ENSMUST00000161961;ENSMUST00000089669;ENSMUST0000016
MSTRG.1	Fam83h	ENSMUST00000170153;ENSMUST00000060807
MSTRG.1	K230010	ENSMUST00000185810;ENSMUST00000187868;ENSMUST0000018
MSTRG.1	Gm23027	ENSMUST00000158771
MSTRG.1	Scrib	ENSMUST00000136390;ENSMUST00000002603;ENSMUST0000006
MSTRG.1	Mir6952	ENSMUST00000185092
MSTRG.1	Nrbp2	ENSMUST00000019516;MSTRG.11429.1;MSTRG.11429.2;MSTRG.
MSTRG.1	Ppip5k2	ENSMUST00000137758;ENSMUST00000042509;ENSMUST00000112
MSTRG.11430		MSTRG.11430.1;MSTRG.11430.2;MSTRG.11430.3
MSTRG.11431		MSTRG.11431.1;MSTRG.11431.2;MSTRG.11431.3;MSTRG.11431
MSTRG.1	BC02413	ENSMUST00000146157;ENSMUST00000054022;ENSMUST0000012
MSTRG.1	Grina	ENSMUST00000023225;MSTRG.11433.1;MSTRG.11433.2
MSTRG.1	Mir6953	ENSMUST00000184745
MSTRG.1	Plec;Pa	ENSMUST00000054449;ENSMUST00000073418;ENSMUST0000008
MSTRG.1	Gm25720	ENSMUST00000157503
MSTRG.1	Mir1942	ENSMUST00000157274
MSTRG.1	Parp10	MSTRG.11438.1
MSTRG.1	Mirt2	ENSMUST00000175179
MSTRG.1	Gin1	ENSMUST00000112844;ENSMUST00000112842;ENSMUST00000027
MSTRG.1	Spatc1	ENSMUST00000074173
MSTRG.1	Mir3079	ENSMUST00000175428
MSTRG.1	Smpd5	ENSMUST00000163991;ENSMUST00000171192;ENSMUST0000009
MSTRG.1	Oplah	ENSMUST00000171340;ENSMUST00000170261;ENSMUST0000002
MSTRG.1	Oplah	MSTRG.11444.1
MSTRG.1	Exosc4	ENSMUST00000059045;ENSMUST00000167515
MSTRG.1	Gpaa1	ENSMUST00000023221;ENSMUST00000164972;ENSMUST0000016
MSTRG.1	Rps6-ps	ENSMUST00000074205
MSTRG.1	Hgh1	ENSMUST00000023213
MSTRG.1	Hgh1	MSTRG.11449.1
MSTRG.1	Gin1	MSTRG.1145.1
MSTRG.1	Tssk5	ENSMUST00000071119

MSTRG.1	Cyc1	ENSMUST00000023210;MSTRG.11451.1
MSTRG.1	Sharpin	ENSMUST00000123112;ENSMUST00000023211;ENSMUST00000154
MSTRG.1	Maf1	ENSMUST00000161527;ENSMUST00000160853;ENSMUST00000023
MSTRG.1	Gm35339	ENSMUST00000208833
MSTRG.1	Dgat1	ENSMUST00000023214;ENSMUST00000160294;MSTRG.11455.3;E
MSTRG.1	Fbxl6	MSTRG.11456.1;MSTRG.11456.2;ENSMUST00000023219
MSTRG.1	Slc52a2	ENSMUST00000023220
MSTRG.11458		MSTRG.11458.1
MSTRG.11459		MSTRG.11459.1
MSTRG.1	Pam	ENSMUST00000159041;ENSMUST00000058762;ENSMUST00000097
MSTRG.11460		MSTRG.11460.1
MSTRG.1	Mroh1;M	ENSMUST00000161305;ENSMUST00000162204;MSTRG.11461.2;E
MSTRG.1	Mroh1	MSTRG.11462.1
MSTRG.1	Bop1	ENSMUST00000023217;MSTRG.11463.1
MSTRG.1	Scx	MSTRG.11464.1;ENSMUST00000043089
MSTRG.1	Bop1	MSTRG.11465.1
MSTRG.1	Hsf1	ENSMUST00000072838;MSTRG.11466.1
MSTRG.1	Adck5	ENSMUST00000162503;ENSMUST00000161732;ENSMUST00000161
MSTRG.1	Cpsf1	ENSMUST00000071898
MSTRG.1	Tonsl	ENSMUST00000078803;MSTRG.11469.1;MSTRG.11469.2;ENSMUS
MSTRG.1	Pam	MSTRG.1147.1
MSTRG.1	Cyhr1	MSTRG.11470.1;ENSMUST00000081291;MSTRG.11470.3;ENSMUS
MSTRG.1	Kifc2	MSTRG.11471.1;ENSMUST00000004294
MSTRG.1	Foxh1	ENSMUST00000037824
MSTRG.1	Zfp647	ENSMUST00000048854
MSTRG.11474		MSTRG.11474.1
MSTRG.11475		MSTRG.11475.1
MSTRG.1	Ppp1r16	ENSMUST00000037551;ENSMUST00000135388;ENSMUST00000138
MSTRG.1	Gpt	ENSMUST00000023203
MSTRG.1	Mfsd3	ENSMUST00000019224
MSTRG.1	Recql4	ENSMUST00000036852
MSTRG.1	Pam	MSTRG.1148.1
MSTRG.1	Lrrc14	ENSMUST00000136840;ENSMUST00000127208;ENSMUST00000036
MSTRG.1	Lrrc24	ENSMUST00000049956;ENSMUST00000146855;ENSMUST00000146
MSTRG.1	Arhgap3	ENSMUST00000036176;ENSMUST00000077821;ENSMUST00000176
MSTRG.1	Arhgap3	MSTRG.11483.1
MSTRG.1	Arhgap3	MSTRG.11484.1
MSTRG.1	Gm17271	ENSMUST00000165535
MSTRG.1	Arhgap3	MSTRG.11486.1
MSTRG.1	Arhgap3	MSTRG.11487.1
MSTRG.1	Arhgap3	MSTRG.11488.1
MSTRG.1	1110038	MSTRG.11489.1;ENSMUST00000071792
MSTRG.1	Gm37140	ENSMUST00000192914
MSTRG.1	Zfp251	MSTRG.11490.1
MSTRG.1	Zfp251	MSTRG.11491.1;MSTRG.11491.2;ENSMUST00000080406
MSTRG.1	Zfp7	ENSMUST00000023179;MSTRG.11492.1;MSTRG.11492.2;MSTRG.
MSTRG.1	Zfp7	MSTRG.11493.1
MSTRG.1	Zfp7	MSTRG.11494.1
MSTRG.11495		MSTRG.11495.1
MSTRG.11496		MSTRG.11496.1
MSTRG.1	Commd5;	ENSMUST00000068407;MSTRG.11497.1;ENSMUST00000109793;M
MSTRG.1	Rbfox2	ENSMUST00000048145;ENSMUST00000111581;ENSMUST00000166
MSTRG.1	1700109	ENSMUST00000181870
MSTRG.1	Kcnb2	ENSMUST00000175681;ENSMUST00000170146
MSTRG.1	Gm25413	ENSMUST00000158544
MSTRG.1	Rbfox2	MSTRG.11500.1
MSTRG.1	Rbfox2	MSTRG.11501.1

MSTRG.1	Rbfox2	MSTRG.11502.1
MSTRG.1	Gm24056	ENSMUST00000158617
MSTRG.1	Rbfox2	MSTRG.11504.1
MSTRG.1	Apol7e	MSTRG.11505.1;ENSMUST00000096358
MSTRG.1	Gm8221	ENSMUST00000178052
MSTRG.1	Apol11b	ENSMUST00000166623;ENSMUST00000180949;ENSMUST00000181
MSTRG.11508		MSTRG.11508.1
MSTRG.11509		MSTRG.11509.1
MSTRG.1	Gm3531	ENSMUST00000162698
MSTRG.11510		MSTRG.11510.1
MSTRG.1	Myh9	ENSMUST00000016771;ENSMUST00000139729;ENSMUST00000126
MSTRG.1	Gm22107	ENSMUST00000122548
MSTRG.1	Txn2	ENSMUST00000173561;ENSMUST00000173631;MSTRG.11513.1;E
MSTRG.1	Eif3d	MSTRG.11514.1;ENSMUST00000100484
MSTRG.1	Gm17638	ENSMUST00000181255
MSTRG.1	Ift27	ENSMUST00000016781
MSTRG.1	Ncf4	ENSMUST00000096357;ENSMUST00000133618;ENSMUST00000147
MSTRG.1	Csf2rb2	ENSMUST00000096356;MSTRG.11518.1;ENSMUST00000183491
MSTRG.1	Csf2rb;I	MSTRG.11519.1;ENSMUST00000096355;ENSMUST00000183523
MSTRG.1	B230216	ENSMUST00000160588;ENSMUST00000159324;ENSMUST00000161
MSTRG.1	Tst	ENSMUST00000058659
MSTRG.11521		MSTRG.11521.1
MSTRG.1	Mpst	ENSMUST00000167140;ENSMUST00000169133;ENSMUST00000043
MSTRG.1	Kctd17	ENSMUST00000162517;ENSMUST00000166142;ENSMUST00000089
MSTRG.1	Tmprss6	ENSMUST00000017086
MSTRG.1	Il2rb	MSTRG.11525.1;MSTRG.11525.2;MSTRG.11525.3;ENSMUST0000
MSTRG.11526		MSTRG.11526.1
MSTRG.1	Rac2	ENSMUST00000043214;MSTRG.11527.1;MSTRG.11527.3
MSTRG.1	Cyth4	ENSMUST00000043069;MSTRG.11528.2
MSTRG.1	Elfn2	MSTRG.11529.1;ENSMUST00000088592;MSTRG.11529.3
MSTRG.1	Gm24465	ENSMUST00000178398
MSTRG.11530		MSTRG.11530.1
MSTRG.1	Mfng	ENSMUST00000018313;MSTRG.11531.1;ENSMUST00000123518;E
MSTRG.1	Cdc42ep	ENSMUST00000059619
MSTRG.1	Lgals2	ENSMUST00000044584
MSTRG.1	Gga1	ENSMUST00000041587;MSTRG.11534.2;MSTRG.11534.3
MSTRG.1	Mir6955	ENSMUST00000183370
MSTRG.1	Lgals1	MSTRG.11536.1
MSTRG.1	Lgals1	ENSMUST00000089377;MSTRG.11537.1
MSTRG.1	Sh3bp1	ENSMUST00000134703;ENSMUST00000001226;ENSMUST00000061
MSTRG.1	1700027	ENSMUST00000150472
MSTRG.1	Pam	MSTRG.1154.1
MSTRG.1	Nol12	ENSMUST00000138880;ENSMUST00000149580;ENSMUST00000041
MSTRG.1	Triobp	ENSMUST00000140228;ENSMUST00000109690;ENSMUST00000109
MSTRG.1	Mir6956	ENSMUST00000183598
MSTRG.1	H1f0	ENSMUST00000180086
MSTRG.1	Ankrd54	ENSMUST00000040676;ENSMUST00000133541;ENSMUST00000156
MSTRG.1	Eif3l	ENSMUST00000040518
MSTRG.1	Micall1	ENSMUST00000040320;MSTRG.11546.2
MSTRG.1	1700088	MSTRG.11547.1;ENSMUST00000185944;ENSMUST00000187550;E
MSTRG.1	Polr2f	ENSMUST00000040077
MSTRG.1	Sox10	ENSMUST00000040019
MSTRG.1	Pam	MSTRG.1155.1
MSTRG.1	Tmem184	MSTRG.11550.1;ENSMUST00000178522;ENSMUST00000074991;E
MSTRG.1	Tmem184	MSTRG.11551.1
MSTRG.1	Pla2g6	ENSMUST00000174021;ENSMUST00000166977;ENSMUST00000173
MSTRG.1	Pla2g6	MSTRG.11553.1

MSTRG.1	Pla2g6	MSTRG.11554.1
MSTRG.1	Gm20420	ENSMUST00000173178
MSTRG.1	Pla2g6	MSTRG.11556.1
MSTRG.1	Csnk1e	ENSMUST00000117786;ENSMUST00000127155;MSTRG.11557.3;E
MSTRG.1	Gm16059	ENSMUST00000135583
MSTRG.1	Csnk1e	MSTRG.11559.1
MSTRG.1	Pam	MSTRG.1156.1
MSTRG.1	Kdelr3	ENSMUST00000010974;MSTRG.11560.2;MSTRG.11560.3
MSTRG.1	Ddx17	ENSMUST00000054014;MSTRG.11561.1;MSTRG.11561.2;MSTRG.
MSTRG.1	Fam227a	ENSMUST00000109648;ENSMUST00000187519;ENSMUST00000191
MSTRG.1	Cby1	ENSMUST00000023064
MSTRG.1	Tomm22	ENSMUST00000023062;ENSMUST00000127292
MSTRG.1	Josd1	ENSMUST00000023061;MSTRG.11565.1;MSTRG.11565.2
MSTRG.1	Gm26884	ENSMUST00000181207;MSTRG.11566.2
MSTRG.1	Gtpbp1;	ENSMUST00000046463;ENSMUST00000082475;MSTRG.11567.2;M
MSTRG.1	Gtpbp1	MSTRG.11568.1
MSTRG.1	Sun2	ENSMUST00000162392;ENSMUST00000089311;ENSMUST0000010C
MSTRG.1157		MSTRG.1157.1
MSTRG.1	Sun2	MSTRG.11570.1
MSTRG.1	Gm16575	MSTRG.11571.1;ENSMUST00000162186
MSTRG.1	Gm16576	MSTRG.11572.1;ENSMUST00000128342
MSTRG.1	Gm16576	MSTRG.11573.1
MSTRG.1	Gm16576	MSTRG.11574.1
MSTRG.1	Dnal4	ENSMUST00000069877;ENSMUST00000023055;ENSMUST00000162
MSTRG.1	Dnal4	MSTRG.11576.1
MSTRG.1	Dnal4	MSTRG.11577.1
MSTRG.1	Nptxr;N	ENSMUST00000175858;ENSMUST00000023057;ENSMUST00000125
MSTRG.1	Pdgfb	ENSMUST00000000500
MSTRG.1	Gm28187	MSTRG.1158.1;ENSMUST00000186885
MSTRG.1	Apobec3	ENSMUST00000177350;ENSMUST00000177483;ENSMUST0000017€
MSTRG.1	Apobec3	MSTRG.11582.1
MSTRG.1	Cbx7	ENSMUST00000109616;ENSMUST00000089293;MSTRG.11583.3;E
MSTRG.1	Tab1	ENSMUST00000023050;MSTRG.11584.2;MSTRG.11584.3;MSTRG.
MSTRG.1	Mgat3	ENSMUST00000044970
MSTRG.1	Mgat3	MSTRG.11586.1
MSTRG.1	Rpl3;Gm	ENSMUST00000188025;ENSMUST00000187578;ENSMUST00000081
MSTRG.1	Snord83	ENSMUST00000104546
MSTRG.1	Gm24204	ENSMUST00000082950
MSTRG.1	Tnfrsf1	ENSMUST00000027559;ENSMUST00000187812;ENSMUST00000187
MSTRG.1	Syngr1	ENSMUST00000009728;ENSMUST00000009727;ENSMUST00000143
MSTRG.1	Rps19bp	ENSMUST00000052499;MSTRG.11591.2
MSTRG.1	Mief1;A	ENSMUST00000023048;MSTRG.11592.2;ENSMUST00000166030;M
MSTRG.1	Fam83f	ENSMUST00000023044;MSTRG.11593.2
MSTRG.1	Fam83f	MSTRG.11594.1
MSTRG.1	Enthd1	ENSMUST00000096347
MSTRG.1	Grap2;M	MSTRG.11597.3;MSTRG.11597.4;MSTRG.11597.5;MSTRG.11597
MSTRG.11598		MSTRG.11598.1
MSTRG.1	Tnrc6b;	MSTRG.11599.5;MSTRG.11599.8;MSTRG.11599.6;MSTRG.1159
MSTRG.1	Gm38116	ENSMUST00000193873
MSTRG.1	Gm7160	ENSMUST00000185535;ENSMUST00000188519
MSTRG.1	Tnrc6b	MSTRG.11600.1
MSTRG.1	Tnrc6b	MSTRG.11601.1
MSTRG.1	Tnrc6b	MSTRG.11602.1
MSTRG.1	Tnrc6b	MSTRG.11603.1
MSTRG.1	Tnrc6b	MSTRG.11604.1
MSTRG.1	Gm6612	ENSMUST00000170777
MSTRG.1	Slc25a1	MSTRG.11606.3;ENSMUST00000023040;MSTRG.11606.1;MSTRG.

MSTRG.1	Slc25a1	MSTRG.11607.1
MSTRG.1	Rbx1	ENSMUST00000023036;MSTRG.11608.2;MSTRG.11608.3
MSTRG.1	St13	ENSMUST00000172107;MSTRG.11609.1;ENSMUST00000023039;E
MSTRG.1	Gm7160	MSTRG.1161.1
MSTRG.1	Xpnpep3	ENSMUST00000163754;ENSMUST00000163296;ENSMUST00000166
MSTRG.1	Dnajb7	ENSMUST00000057236
MSTRG.1	Gm17025	ENSMUST00000133273
MSTRG.1	1110025	ENSMUST00000192082
MSTRG.1	Sgsm3	ENSMUST00000139517;ENSMUST00000143147;ENSMUST00000124
MSTRG.1	Mkl1	MSTRG.11615.1
MSTRG.1	Mkl1	ENSMUST00000134469;ENSMUST00000149582;MSTRG.11616.3;E
MSTRG.1	Gm25131	MSTRG.11617.1
MSTRG.1	Mkl1	MSTRG.11618.1
MSTRG.1	Gm25131	ENSMUST00000082653
MSTRG.1	Zcchc2	ENSMUST00000118196;ENSMUST00000119166;ENSMUST00000186
MSTRG.1	Mkl1	MSTRG.11620.1;MSTRG.11620.2
MSTRG.1	Mkl1	MSTRG.11621.1
MSTRG.1	Mkl1	MSTRG.11623.1
MSTRG.1	Mkl1	MSTRG.11625.1
MSTRG.1	Mkl1	MSTRG.11626.1
MSTRG.1	Mkl1	MSTRG.11627.1
MSTRG.1	Mkl1	MSTRG.11628.1
MSTRG.1	4930483	ENSMUST00000138715
MSTRG.1	Gm28403	ENSMUST00000188743;ENSMUST00000070699;ENSMUST00000186
MSTRG.1	Ep300;G	ENSMUST00000185375;MSTRG.11630.1;ENSMUST00000068387;E
MSTRG.1	L3mbtl2	ENSMUST00000023029;ENSMUST00000174229;ENSMUST00000172
MSTRG.1	Gm18284	ENSMUST00000172604
MSTRG.1	Chadl	ENSMUST00000173598;ENSMUST00000072910;ENSMUST00000173
MSTRG.1	Rangap1	MSTRG.11634.1;ENSMUST00000052374;ENSMUST00000170134;E
MSTRG.1	Zc3h7b	ENSMUST00000109554;MSTRG.11635.2
MSTRG.1	Zc3h7b	MSTRG.11636.1
MSTRG.1	Gm8444;	ENSMUST00000092020;MSTRG.11637.2;ENSMUST00000050467
MSTRG.1	Gm17597	ENSMUST00000181220
MSTRG.1	Tef	ENSMUST00000109553;ENSMUST00000166302;ENSMUST00000170
MSTRG.1	Gm28403	MSTRG.1164.1
MSTRG.1	Tef	MSTRG.11640.1
MSTRG.1	Phf5a	ENSMUST00000023117;ENSMUST00000160415
MSTRG.1	Aco2	ENSMUST00000023116;ENSMUST00000135198;ENSMUST00000157
MSTRG.1	Aco2	MSTRG.11643.1
MSTRG.1	Polr3h	ENSMUST00000023113
MSTRG.1	Csdc2	ENSMUST00000038757;ENSMUST00000135663;ENSMUST00000140
MSTRG.1	Pmm1	ENSMUST00000071462;ENSMUST00000023112;ENSMUST00000129
MSTRG.11647		MSTRG.11647.1
MSTRG.1	Cenpm	ENSMUST00000089155;ENSMUST00000089157
MSTRG.1	Ccdc134	ENSMUST00000089174;MSTRG.11649.2;MSTRG.11649.3
MSTRG.1	Gm3608	ENSMUST00000086725;MSTRG.1165.1
MSTRG.1	Srebf2;	MSTRG.11650.2;MSTRG.11650.1;ENSMUST00000023100;ENSMUS
MSTRG.1	Shisa8	ENSMUST00000179269
MSTRG.1	Sept3	ENSMUST00000023095;ENSMUST00000116423
MSTRG.1	Xrcc6	MSTRG.11653.1
MSTRG.1	Gm5805	ENSMUST00000072779
MSTRG.1	1700029	ENSMUST00000074608
MSTRG.1	Xrcc6	ENSMUST00000165777;MSTRG.11656.2;ENSMUST00000100399;M
MSTRG.1	Desi1	MSTRG.11657.1;ENSMUST00000146628;ENSMUST00000152227;E
MSTRG.1	Xrcc6	MSTRG.11658.1
MSTRG.1	Snu13	ENSMUST00000080622;ENSMUST00000072904;MSTRG.11659.2;E
MSTRG.1	Pign	MSTRG.1166.1

MSTRG.1	Cyp2d22	ENSMUST00000023083;MSTRG.11660.2
MSTRG.1	Naga	ENSMUST00000023088;MSTRG.11661.1
MSTRG.1	Fam109b	ENSMUST00000159942;ENSMUST00000160994;ENSMUST00000162
MSTRG.1	n-R5s41	ENSMUST00000083025
MSTRG.1	Ndufa6	MSTRG.11664.1
MSTRG.1	Ndufa6	ENSMUST00000023085
MSTRG.11666		MSTRG.11666.1
MSTRG.11667		MSTRG.11667.1
MSTRG.11668		MSTRG.11668.1
MSTRG.11669		MSTRG.11669.1
MSTRG.1	Gm28404	ENSMUST00000190242
MSTRG.1	Nfam1	ENSMUST00000109503;ENSMUST00000023076;ENSMUST0000010C
MSTRG.1	Cyp2d26	ENSMUST00000006094
MSTRG.1	Tcf20;G	MSTRG.11672.3;MSTRG.11672.1;MSTRG.11672.2;MSTRG.11672
MSTRG.1	Gm20324	MSTRG.11673.1;MSTRG.11673.2;MSTRG.11673.3;MSTRG.11673
MSTRG.1	Mir3080	ENSMUST00000175010
MSTRG.1	AW12168	ENSMUST00000181049
MSTRG.1	Rnu12	ENSMUST00000083242
MSTRG.1	Poldip3	ENSMUST00000100375;ENSMUST00000058793;MSTRG.11677.3;M
MSTRG.1	Cyb5r3	ENSMUST00000018186;ENSMUST00000162178;ENSMUST00000162
MSTRG.11679		MSTRG.11679.1
MSTRG.1	Gm20302	ENSMUST00000080671;ENSMUST00000103811
MSTRG.1	Serhl	ENSMUST00000142265;ENSMUST00000167862;ENSMUST00000165
MSTRG.1	Gm17206	ENSMUST00000168123
MSTRG.1	Rrp7a	ENSMUST00000018184;ENSMUST00000166766;ENSMUST00000167
MSTRG.1	Arfgap3	ENSMUST00000067215
MSTRG.1	Arfgap3	MSTRG.11684.1
MSTRG.1	1700001	ENSMUST00000178628;ENSMUST00000179705;ENSMUST0000010C
MSTRG.1	Pacsin2	ENSMUST00000165095;MSTRG.11686.1;MSTRG.11686.2;ENSMUS
MSTRG.1	Gm5417	ENSMUST00000079706
MSTRG.1	Ttll1	ENSMUST00000016897;ENSMUST00000109480;ENSMUST0000010S
MSTRG.1	Mcat	ENSMUST00000061882
MSTRG.1	Pign	MSTRG.1169.1
MSTRG.1	Tspo	ENSMUST00000047419;ENSMUST00000208463
MSTRG.1	Ttll12	ENSMUST00000136066;ENSMUST00000016901;MSTRG.11691.3;E
MSTRG.1	Pnpla3	ENSMUST00000045289
MSTRG.1	Mir6392	ENSMUST00000184924
MSTRG.1	Samm50	ENSMUST00000023071;MSTRG.11694.2
MSTRG.1	Parvb	ENSMUST00000023072;ENSMUST00000122818;ENSMUST0000014€
MSTRG.1	Parvg	MSTRG.11696.1
MSTRG.1	1810041	ENSMUST00000080751;ENSMUST00000189248;ENSMUST0000018S
MSTRG.1	Ldoc1l	ENSMUST00000069476
MSTRG.1	Gm22890	ENSMUST00000158642
MSTRG.1	Kcnb2	MSTRG.117.1
MSTRG.1	Pign	MSTRG.1170.1
MSTRG.1	Parvg	ENSMUST00000127551;MSTRG.11700.1;ENSMUST00000023074;E
MSTRG.1	Prr5	ENSMUST00000171460;ENSMUST00000065499
MSTRG.1	Arhgap8	ENSMUST00000006029;ENSMUST00000172307;ENSMUST00000167
MSTRG.1	Arhgap8	MSTRG.11703.1
MSTRG.1	Arhgap8	MSTRG.11704.1
MSTRG.1	Arhgap8	MSTRG.11705.1
MSTRG.1	Gm29666	ENSMUST00000189185
MSTRG.11707		MSTRG.11707.1
MSTRG.1	Nup50	ENSMUST00000165443;MSTRG.11708.1
MSTRG.1	5031439	ENSMUST00000047144;ENSMUST00000165743;ENSMUST00000124
MSTRG.1	Gm8004	ENSMUST00000187782
MSTRG.1	Fam118a	ENSMUST00000023069

MSTRG.1	Fam118a	MSTRG.11711.1
MSTRG.1	Smc1b	ENSMUST00000023068
MSTRG.1	Gm10923	ENSMUST00000105085
MSTRG.1	Fbln1	ENSMUST00000057410;ENSMUST00000109432;ENSMUST0000016(
MSTRG.1	Atxn10	MSTRG.11715.1;ENSMUST00000163242
MSTRG.1	Gm23517	ENSMUST00000082830
MSTRG.1	Atxn10	MSTRG.11717.1
MSTRG.1	Atxn10	MSTRG.11718.1
MSTRG.1	Atxn10	MSTRG.11719.1
MSTRG.1	Pign	MSTRG.1172.1
MSTRG.1	Atxn10	MSTRG.11720.1
MSTRG.1	D130051	ENSMUST00000189171;MSTRG.11721.1;MSTRG.11721.3;MSTRG.
MSTRG.1	Gm37699	ENSMUST00000194753;MSTRG.11722.1
MSTRG.1	Cdpf1	ENSMUST00000071876;ENSMUST00000134631;ENSMUST00000144
MSTRG.1	Ttc38	ENSMUST00000146088;ENSMUST00000124011;ENSMUST0000013
MSTRG.1	Gtse1	ENSMUST00000170629
MSTRG.1	Trmu	ENSMUST00000162491;ENSMUST00000023019;ENSMUST0000016(
MSTRG.1	Celsr1	ENSMUST00000016172;MSTRG.11727.1;MSTRG.11727.2
MSTRG.1	Celsr1	MSTRG.11728.1
MSTRG.1	Gramd4	ENSMUST00000088931;ENSMUST00000138134;ENSMUST0000012
MSTRG.1	2310035	ENSMUST00000189091;ENSMUST00000186807;ENSMUST0000003
MSTRG.1	Gm15722	ENSMUST00000159271
MSTRG.1	Cerk	ENSMUST00000150767;MSTRG.11731.1;ENSMUST00000044332;
MSTRG.1	Gm24011	ENSMUST00000157732
MSTRG.1	Cerk	MSTRG.11733.1
MSTRG.1	Gm15569	ENSMUST00000133675
MSTRG.1	Tbc1d22	MSTRG.11736.1;ENSMUST00000063414;MSTRG.11736.3;MSTRG.
MSTRG.1	Tbc1d22	MSTRG.11737.1;MSTRG.11737.2;MSTRG.11737.3
MSTRG.1	Tbc1d22	MSTRG.11738.1
MSTRG.1	Zbed4	ENSMUST00000041297;ENSMUST00000160975;MSTRG.11739.2
MSTRG.1	2310035	MSTRG.1174.1
MSTRG.1	Zbed4	MSTRG.11740.1
MSTRG.1	Brd1	MSTRG.11741.3;MSTRG.11741.1;MSTRG.11741.2;ENSMUST000(
MSTRG.1	Brd1	MSTRG.11742.1
MSTRG.1	Pim3	ENSMUST00000042818
MSTRG.1	Alg12	ENSMUST00000160222;ENSMUST00000162183;ENSMUST0000004
MSTRG.1	Creld2	ENSMUST00000024042
MSTRG.1	Mov10l1	ENSMUST00000146993
MSTRG.1	Mov10l1	MSTRG.11749.1
MSTRG.1	Gm37566	ENSMUST00000192363
MSTRG.1	Mov10l1	MSTRG.11750.1
MSTRG.1	Mov10l1	MSTRG.11751.1
MSTRG.1	Panx2	ENSMUST00000161372;ENSMUST00000162424;ENSMUST0000015
MSTRG.1	1810021	ENSMUST00000162579;ENSMUST00000161483;ENSMUST00000161
MSTRG.1	Trabd	ENSMUST00000081702;ENSMUST00000169870;ENSMUST0000016
MSTRG.1	Mapk11	ENSMUST00000088823
MSTRG.1	Mapk11	MSTRG.11756.1
MSTRG.1	Plxnb2	ENSMUST00000143014;MSTRG.11757.2;ENSMUST00000060808;
MSTRG.1	Mir7118	ENSMUST00000197760
MSTRG.1	Selenoo	ENSMUST00000130700;ENSMUST00000082439;MSTRG.11759.1
MSTRG.1176		MSTRG.1176.1
MSTRG.1	Selenoo	MSTRG.11760.1
MSTRG.1	Tubgcp6	ENSMUST00000109353;ENSMUST00000164717;MSTRG.11761.2;
MSTRG.1	Hdac10	ENSMUST00000129398;ENSMUST00000082197;ENSMUST0000014
MSTRG.1	Mapk12	ENSMUST00000088827
MSTRG.1	Mapk12	MSTRG.11764.1
MSTRG.1	Miox	MSTRG.11765.1

MSTRG.1	Gm26798	MSTRG.11766.1;ENSMUST00000181384
MSTRG.11767		MSTRG.11767.1
MSTRG.11768		MSTRG.11768.1
MSTRG.11769		MSTRG.11769.1
MSTRG.1	Gm37372	ENSMUST00000193577
MSTRG.11770		MSTRG.11770.1
MSTRG.1	Ppp6r2	ENSMUST00000088788;MSTRG.11771.2;MSTRG.11771.1;MSTRG.
MSTRG.1	Sbf1	ENSMUST00000123791;ENSMUST00000144585;ENSMUST00000124
MSTRG.1	Mir6959	ENSMUST00000184827
MSTRG.1	Lmf2	MSTRG.11774.1;ENSMUST00000023283
MSTRG.1	Ncaph2	ENSMUST00000036987;ENSMUST00000074552;ENSMUST0000008[illegible]
MSTRG.1	Sco2;Ty[illegible]	ENSMUST00000167643;ENSMUST00000023285
MSTRG.1	Odf3b	ENSMUST00000049968
MSTRG.1	Cpt1b;C[illegible]	ENSMUST00000109313;ENSMUST00000052315;ENSMUST0000016[illegible]
MSTRG.1	C730034[illegible]	ENSMUST00000168646
MSTRG.1	Gm6651	ENSMUST00000189471
MSTRG.11780		MSTRG.11780.1
MSTRG.1	Arsa	ENSMUST00000165199;ENSMUST00000023292;ENSMUST0000016[illegible]
MSTRG.1	C230037[illegible]	ENSMUST00000136218;ENSMUST00000166953;ENSMUST0000016[illegible]
MSTRG.1	Shank3	MSTRG.11783.1;ENSMUST00000135214;ENSMUST00000066545;[illegible]
MSTRG.1	Gm15609	ENSMUST00000155610
MSTRG.11785		MSTRG.11785.1
MSTRG.11786		MSTRG.11786.1
MSTRG.11787		MSTRG.11787.1
MSTRG.1	Alg10b;[illegible]	ENSMUST00000100309;ENSMUST00000183650;MSTRG.11788.2
MSTRG.1	Cpne8	ENSMUST00000088649;ENSMUST00000064391;ENSMUST00000014
MSTRG.1	Gm20753	ENSMUST00000190105
MSTRG.1	Gm6746	ENSMUST00000108774
MSTRG.1	Cpne8	MSTRG.11791.1
MSTRG.1	Rpl31-p	ENSMUST00000181935
MSTRG.1	Kif21a	ENSMUST00000067205;ENSMUST00000088614;ENSMUST0000010[illegible]
MSTRG.1	Gm4335	MSTRG.11794.1
MSTRG.1	Gm4335;[illegible]	MSTRG.11795.1;ENSMUST00000083977
MSTRG.1	Gm4335	ENSMUST00000182581
MSTRG.1	Gxylt1	MSTRG.11797.1;ENSMUST00000049484;ENSMUST00000057896
MSTRG.1	Yaf2	ENSMUST00000080299;ENSMUST00000133736
MSTRG.1	Yaf2	MSTRG.11799.1
MSTRG.1	Gm37138	ENSMUST00000194564
MSTRG.1	Phlpp1	ENSMUST00000061047;ENSMUST00000135810;ENSMUST00000124
MSTRG.1	Yaf2	MSTRG.11800.1
MSTRG.1	Yaf2	MSTRG.11801.1
MSTRG.1	Zcrb1	ENSMUST00000162160;ENSMUST00000076070;ENSMUST0000016[illegible]
MSTRG.1	Pphln1	ENSMUST00000049122;ENSMUST00000068457;ENSMUST0000010[illegible]
MSTRG.1	Prickle	ENSMUST00000109255;ENSMUST00000048982
MSTRG.1	Prickle	MSTRG.11805.1
MSTRG.1	Prickle	MSTRG.11806.1
MSTRG.1	Prickle	MSTRG.11807.1;MSTRG.11807.2;MSTRG.11807.3
MSTRG.1	Pus7l	ENSMUST00000049151;ENSMUST00000147832;ENSMUST00000134
MSTRG.1	Irak4	ENSMUST00000074936;ENSMUST00000138306;ENSMUST0000010[illegible]
MSTRG.1	Phlpp1	MSTRG.1181.1
MSTRG.1	Twf1	MSTRG.11810.3;MSTRG.11810.1;ENSMUST00000023087;MSTRG.
MSTRG.1	Nell2	ENSMUST00000166170;ENSMUST00000075275
MSTRG.1	Nell2	MSTRG.11812.1
MSTRG.11813		MSTRG.11813.1
MSTRG.11814		MSTRG.11814.1
MSTRG.11815		MSTRG.11815.1
MSTRG.1	A130051[illegible]	MSTRG.11816.2;MSTRG.11816.1;MSTRG.11816.3;ENSMUST000[illegible]

MSTRG.1	Gm8849	ENSMUST00000058951
MSTRG.1	Ano6	ENSMUST00000071874;MSTRG.11818.1;MSTRG.11818.2
MSTRG.1	Gm17546	MSTRG.11819.1;ENSMUST00000168960
MSTRG.1	Gm38235	ENSMUST00000194600
MSTRG.1	Ano6	MSTRG.11820.1
MSTRG.1	Gm25070	ENSMUST00000175237
MSTRG.1	Ano6	MSTRG.11822.1
MSTRG.1	Ano6	MSTRG.11823.1;MSTRG.11823.2
MSTRG.1	Scaf11	ENSMUST00000047835;MSTRG.11824.1;MSTRG.11824.3
MSTRG.1	2610037	ENSMUST00000180852;MSTRG.11825.2;ENSMUST00000185067
MSTRG.1	4833422	ENSMUST00000181122;ENSMUST00000181747;ENSMUST00000181
MSTRG.1	2610037	MSTRG.11827.1
MSTRG.1	Arid2;G	MSTRG.11828.1;MSTRG.11828.3;MSTRG.11828.4;MSTRG.1182
MSTRG.1	Slc38a1	MSTRG.11829.1
MSTRG.1	Phlpp1	MSTRG.1183.1
MSTRG.1	Slc38a1	ENSMUST00000100262;MSTRG.11830.1;ENSMUST00000088454;
MSTRG.1	Slc38a1	MSTRG.11831.1
MSTRG.1	Slc38a2	ENSMUST00000023099;MSTRG.11832.1;MSTRG.11832.2;MSTRG.
MSTRG.11833		MSTRG.11833.1
MSTRG.1	Amigo2	ENSMUST00000053106
MSTRG.1	Pced1b	MSTRG.11835.1;ENSMUST00000059433;MSTRG.11835.2;MSTRG.
MSTRG.1	Pced1b	MSTRG.11836.1
MSTRG.1	Pced1b	MSTRG.11837.1
MSTRG.1	Pced1b	MSTRG.11838.1
MSTRG.1	Pced1b	MSTRG.11839.1
MSTRG.1	Kdsr	ENSMUST00000010049;MSTRG.1184.2;ENSMUST00000187319;E
MSTRG.1	Pced1b	MSTRG.11840.1
MSTRG.1	Pced1b	MSTRG.11841.1
MSTRG.1	Pced1b	MSTRG.11842.1
MSTRG.1	Pced1b	MSTRG.11843.1
MSTRG.1	Pced1b	MSTRG.11844.1
MSTRG.1	Pced1b	MSTRG.11845.1
MSTRG.1	Rpap3	ENSMUST00000023104
MSTRG.1	Rpap3	MSTRG.11847.1
MSTRG.1	Endou	ENSMUST00000023105;ENSMUST00000100249;MSTRG.11848.3
MSTRG.1	Rapgef3	MSTRG.11849.1;ENSMUST00000126854;ENSMUST00000129223;
MSTRG.1	Bcl2	ENSMUST00000112751;MSTRG.1185.2;MSTRG.1185.3;MSTRG.11
MSTRG.1	Rapgef3	ENSMUST00000149373;ENSMUST00000124374
MSTRG.1	Vdr	ENSMUST00000023119;ENSMUST00000139656;ENSMUST0000012
MSTRG.1	Tmem106	ENSMUST00000064200;MSTRG.11852.2
MSTRG.11853		MSTRG.11853.1
MSTRG.1	Slc48a1	ENSMUST00000117892;MSTRG.11854.1;MSTRG.11854.3;ENSMUS
MSTRG.1	Hdac7	ENSMUST00000079838;ENSMUST00000116408;ENSMUST0000008
MSTRG.1	Hdac7	MSTRG.11856.1
MSTRG.1	Hdac7	MSTRG.11857.1
MSTRG.1	Senp1	ENSMUST00000044189;ENSMUST00000181855;MSTRG.11858.2;
MSTRG.1	Gm25668	ENSMUST00000157700
MSTRG.1	D630008	ENSMUST00000144260;ENSMUST00000149909;MSTRG.1186.3
MSTRG.1	Pfkm	ENSMUST00000163507;ENSMUST00000051226
MSTRG.1	Gm26513	ENSMUST00000181802
MSTRG.1	Asb8	MSTRG.11862.1;ENSMUST00000143400;ENSMUST00000059112;
MSTRG.1	Asb8	MSTRG.11863.1
MSTRG.1	Ccnt1	MSTRG.11864.1;MSTRG.11864.2;ENSMUST00000169707;ENSMUS
MSTRG.1	Kansl2;	ENSMUST00000023727;ENSMUST00000116400;MSTRG.11865.3;
MSTRG.1	9330020	ENSMUST00000163781
MSTRG.1	4930415	ENSMUST00000023728;MSTRG.11867.2
MSTRG.1	Cacnb3	ENSMUST00000109150;ENSMUST00000003442

MSTRG.1	Mir3473	ENSMUST00000183743
MSTRG.1	Ddx23	ENSMUST00000003450;MSTRG.11870.1;MSTRG.11870.3;ENSMUS
MSTRG.1	Arf3	MSTRG.11871.1;ENSMUST00000053183;ENSMUST00000129354;E
MSTRG.1	Arf3	MSTRG.11872.1
MSTRG.11873		MSTRG.11873.1
MSTRG.1	Lmbr1l	MSTRG.11874.1
MSTRG.1	Dhh	ENSMUST00000023737
MSTRG.1	Lmbr1l	MSTRG.11876.4;ENSMUST00000109127;MSTRG.11876.3;MSTRG.
MSTRG.11877		MSTRG.11877.1
MSTRG.1	Tuba1b;'	ENSMUST00000077577;MSTRG.11878.1;ENSMUST00000140969;E
MSTRG.1	Tuba1b	MSTRG.11879.1
MSTRG.1	D630008	MSTRG.1188.1;MSTRG.1188.3;MSTRG.1188.2
MSTRG.1	Tuba1b	MSTRG.11880.1
MSTRG.1	Ddn	ENSMUST00000075444
MSTRG.1	B130046	ENSMUST00000181510;MSTRG.11882.1
MSTRG.1	Prkag1;	MSTRG.11883.3;MSTRG.11883.2;MSTRG.11883.1;MSTRG.11883
MSTRG.1	Prkag1	MSTRG.11884.1
MSTRG.11885		MSTRG.11885.1
MSTRG.1	Tuba1c	ENSMUST00000058914
MSTRG.1	Troap;M	ENSMUST00000039665;MSTRG.11887.1;ENSMUST00000183449
MSTRG.1	Spats2	ENSMUST00000063517
MSTRG.1	Gm25183	ENSMUST00000157752
MSTRG.1	Gm37053	MSTRG.1189.1;ENSMUST00000192399;MSTRG.1189.3
MSTRG.1	Gm25057	ENSMUST00000157366
MSTRG.1	Kcnh3	ENSMUST00000041415;MSTRG.11891.2
MSTRG.1	Kcnh3	MSTRG.11892.1
MSTRG.1	Mcrs1	ENSMUST00000041190;ENSMUST00000163506
MSTRG.1	1700120	ENSMUST00000187967;ENSMUST00000187706;ENSMUST0000019C
MSTRG.1	Gm25999	ENSMUST00000175398
MSTRG.1	Fam186b	ENSMUST00000109100
MSTRG.1	Prpf40b	ENSMUST00000128352;ENSMUST00000150641;ENSMUST0000013C
MSTRG.1	Fmnl3	ENSMUST00000081224;ENSMUST00000120633;MSTRG.11898.1;E
MSTRG.1	Fmnl3	MSTRG.11899.1
MSTRG.1	Gm25227	ENSMUST00000158204
MSTRG.1	Bcl2	MSTRG.1190.1
MSTRG.1	Fmnl3	MSTRG.11900.1
MSTRG.1	Fmnl3	MSTRG.11901.1
MSTRG.1	Gm22951	ENSMUST00000179471
MSTRG.1	Fmnl3	MSTRG.11903.1
MSTRG.1	Tmbim6	ENSMUST00000023749;MSTRG.11904.2;ENSMUST00000160635;E
MSTRG.11905		MSTRG.11905.1
MSTRG.1	Nckap5l	MSTRG.11906.1;ENSMUST00000023747;ENSMUST00000161004;E
MSTRG.1	Nckap5l	ENSMUST00000159738;MSTRG.11907.2
MSTRG.1	Nckap5l	MSTRG.11908.1;MSTRG.11908.2
MSTRG.11909		MSTRG.11909.1
MSTRG.1	Vps4b	ENSMUST00000112736;ENSMUST00000094646;ENSMUST00000147
MSTRG.11910		MSTRG.11910.1
MSTRG.1	Bcdin3d	ENSMUST00000040313
MSTRG.1	Faim2	ENSMUST00000023750
MSTRG.1	Racgap1	ENSMUST00000171702;MSTRG.11913.2;MSTRG.11913.3;ENSMUS
MSTRG.1	Racgap1	MSTRG.11914.1
MSTRG.1	Asic1	ENSMUST00000023758
MSTRG.1	Gm17349	ENSMUST00000163472
MSTRG.1	Smarcd1	MSTRG.11917.2;ENSMUST00000023759;MSTRG.11917.1;MSTRG.
MSTRG.1	Gm16537	ENSMUST00000161529
MSTRG.1	Gpd1	ENSMUST00000023760;MSTRG.11919.1;ENSMUST00000162194;E
MSTRG.1	Vps4b	MSTRG.1192.1

MSTRG.1	Cox14;G	ENSMUST00000023761;MSTRG.11920.2;MSTRG.11920.1;ENSMUS
MSTRG.1	Cers5	MSTRG.11921.1
MSTRG.1	Cers5	ENSMUST00000109035;ENSMUST00000176248;MSTRG.11922.6;M
MSTRG.1	Cers5	MSTRG.11923.1
MSTRG.1	Lima1	ENSMUST00000109024;ENSMUST00000073691;ENSMUST00000172
MSTRG.1	Lima1	MSTRG.11925.1
MSTRG.1	Gm25897	ENSMUST00000083709
MSTRG.1	Gm4468	ENSMUST00000170206
MSTRG.1	Gm17057	ENSMUST00000169887
MSTRG.1	Larp4	ENSMUST00000057632;ENSMUST00000100206
MSTRG.1193		MSTRG.1193.1
MSTRG.1	2310068	ENSMUST00000188271
MSTRG.1	Larp4	MSTRG.11931.1
MSTRG.1	Mettl7a	ENSMUST00000067752
MSTRG.1	Mettl7a	MSTRG.11933.1
MSTRG.1	Mettl7a	ENSMUST00000075420
MSTRG.1	Dip2b	MSTRG.11935.1;MSTRG.11935.4;ENSMUST00000100203;MSTRG.
MSTRG.1	Dip2b	MSTRG.11936.1
MSTRG.1	Dip2b	MSTRG.11937.1
MSTRG.1	Dip2b	MSTRG.11938.1
MSTRG.1	Atf1	ENSMUST00000023769;ENSMUST00000163855;ENSMUST00000171
MSTRG.1194		MSTRG.1194.1
MSTRG.1	Atf1	MSTRG.11940.1
MSTRG.1	Methig1	ENSMUST00000075675;ENSMUST00000176287
MSTRG.1	Slc11a2	MSTRG.11942.1
MSTRG.1	Slc11a2	ENSMUST00000023774;ENSMUST00000138843;ENSMUST00000124
MSTRG.1	Slc11a2	MSTRG.11944.1
MSTRG.1	Gm5475	ENSMUST00000132119;ENSMUST00000148928
MSTRG.1	5330439	ENSMUST00000180953
MSTRG.1	5330439	MSTRG.11947.1
MSTRG.1	Letmd1	MSTRG.11948.1;ENSMUST00000037001
MSTRG.1	Csrnp2	ENSMUST00000061457
MSTRG.1195		MSTRG.1195.1
MSTRG.1	Tfcp2	ENSMUST00000009877
MSTRG.1	Tfcp2	MSTRG.11951.1
MSTRG.1	Pou6f1	ENSMUST00000176271;ENSMUST00000073837;ENSMUST00000177
MSTRG.1	Gm27209	ENSMUST00000183834
MSTRG.1	Pou6f1	MSTRG.11955.1
MSTRG.1	Dazap2	ENSMUST00000000356;MSTRG.11956.1
MSTRG.1	Smagp	ENSMUST00000066068;ENSMUST00000172334
MSTRG.1	Bin2	ENSMUST00000183211;ENSMUST00000182814;ENSMUST00000182
MSTRG.1	Bin2	MSTRG.11959.2;MSTRG.11959.1
MSTRG.1	Cdh19	ENSMUST00000094626;ENSMUST00000187555
MSTRG.1	Mir6961	ENSMUST00000183874
MSTRG.1	Galnt6	ENSMUST00000159715;ENSMUST00000052069;ENSMUST00000161
MSTRG.1	Galnt6o	MSTRG.11962.1;ENSMUST00000160960
MSTRG.1	Galnt6	MSTRG.11963.1
MSTRG.1	Galnt6	MSTRG.11964.1
MSTRG.1	Fignl2	ENSMUST00000178140;ENSMUST00000213610
MSTRG.1	Fignl2	MSTRG.11966.1
MSTRG.1	Fignl2	MSTRG.11967.1
MSTRG.11968		MSTRG.11968.1
MSTRG.11969		MSTRG.11969.1
MSTRG.1	Cdh19	MSTRG.1197.1
MSTRG.1	Acvr1b	ENSMUST00000000544;MSTRG.11970.2
MSTRG.1	Acvr1b	MSTRG.11971.1
MSTRG.1	Mir6962	ENSMUST00000199512

MSTRG.1	A330009	ENSMUST00000180639
MSTRG.1	Grasp	ENSMUST00000000543
MSTRG.1	Nr4a1	MSTRG.11975.1;ENSMUST00000023779;MSTRG.11975.2
MSTRG.1	Atg101	ENSMUST00000048393;MSTRG.11976.1
MSTRG.1	Krt7	ENSMUST00000141190;ENSMUST00000153021;ENSMUST00000183
MSTRG.1	Krt5	ENSMUST00000023709
MSTRG.1	Gm38255	ENSMUST00000194910
MSTRG.1	Krt8	ENSMUST00000023952
MSTRG.1	Krt18	ENSMUST00000023803
MSTRG.1	Eif4b	ENSMUST00000169681;MSTRG.11982.1
MSTRG.1	Igfbp6	ENSMUST00000023807
MSTRG.1	Tns2	ENSMUST00000046144;ENSMUST00000169627
MSTRG.1	Spryd3	ENSMUST00000139279;ENSMUST00000154032;ENSMUST00000128
MSTRG.1	Spryd3	MSTRG.11986.1
MSTRG.1	Csad	ENSMUST00000023805;MSTRG.11987.2
MSTRG.1	Csad	MSTRG.11988.1
MSTRG.11989		MSTRG.11989.1
MSTRG.1	Gm36944	ENSMUST00000192257
MSTRG.1	Zfp740	ENSMUST00000118729;ENSMUST00000119168;ENSMUST00000141
MSTRG.1	Mfsd5	ENSMUST00000051341
MSTRG.1	Itgb7	MSTRG.11992.1;ENSMUST00000001327;ENSMUST00000127014
MSTRG.1	Rarg	ENSMUST00000043172;ENSMUST00000063339;MSTRG.11993.2;M
MSTRG.1	Espl1	MSTRG.11994.1;ENSMUST00000064924;MSTRG.11994.3;MSTRG.
MSTRG.1	Sp1	ENSMUST00000170884;ENSMUST00000165924;ENSMUST00000163
MSTRG.1	Pfdn5;M	ENSMUST00000166658;ENSMUST00000062492;ENSMUST00000168
MSTRG.1	Aaas	ENSMUST00000041208;MSTRG.11997.1
MSTRG.1	Rpl39-p	ENSMUST00000142604;ENSMUST00000146505
MSTRG.1	Atp5g2;	ENSMUST00000185641;ENSMUST00000075630;ENSMUST00000187
MSTRG.1	Tcea1	MSTRG.12.1
MSTRG.1	Terf1	ENSMUST00000188371;ENSMUST00000186565;ENSMUST00000027
MSTRG.1	Calcoco	MSTRG.12000.1;ENSMUST00000023818;ENSMUST00000168112;E
MSTRG.1	Gm26518	ENSMUST00000181328
MSTRG.1	Prr13	ENSMUST00000164938;ENSMUST00000023810;ENSMUST00000164
MSTRG.1	Pcbp2;G	MSTRG.12004.11;MSTRG.12004.9;MSTRG.12004.10;MSTRG.120
MSTRG.1	Map3k12	MSTRG.12005.1;MSTRG.12005.3;MSTRG.12005.4;ENSMUST0000
MSTRG.1	Gm10337	ENSMUST00000096145
MSTRG.1	Tarbp2	ENSMUST00000131184;ENSMUST00000141266;ENSMUST0000010C
MSTRG.1	Npff;Gm	ENSMUST00000023814;ENSMUST00000184772;ENSMUST00000184
MSTRG.1	Atf7	MSTRG.12009.1
MSTRG.1	Atf7	MSTRG.12010.1
MSTRG.1	Atf7	MSTRG.12011.1
MSTRG.1	Smug1	ENSMUST00000064067
MSTRG.12013		MSTRG.12013.1
MSTRG.12014		MSTRG.12014.1
MSTRG.1	Copz1	ENSMUST00000100162
MSTRG.1	Mir148b	ENSMUST00000083626
MSTRG.1	Cbx5	ENSMUST00000118152;ENSMUST00000108813;ENSMUST00000122
MSTRG.1	Cbx5	MSTRG.12018.1
MSTRG.1	Cbx5	MSTRG.12019.1;MSTRG.12019.2
MSTRG.1	Gm29088	MSTRG.1202.1
MSTRG.1	Hnrnpa1	MSTRG.12020.1;MSTRG.12020.4;MSTRG.12020.2;MSTRG.1202C
MSTRG.1	Zfp385a	ENSMUST00000168828
MSTRG.1	Zfp385a	MSTRG.12022.1
MSTRG.1	Itga5	ENSMUST00000215331;ENSMUST00000023128
MSTRG.1	Mir6963	ENSMUST00000183535
MSTRG.1	Itga5	MSTRG.12025.1
MSTRG.1	Nckap1l	ENSMUST00000047405

MSTRG.1	Nckap1l	MSTRG.12027.1
MSTRG.1	Pde1b	ENSMUST00000023132
MSTRG.1	Ppp1r1a	ENSMUST00000023133
MSTRG.1	Dsel	ENSMUST00000186365;ENSMUST00000035462;ENSMUST00000189
MSTRG.1	Glycam1	ENSMUST00000023134
MSTRG.12031		MSTRG.12031.1
MSTRG.12032		MSTRG.12032.1
MSTRG.12033		MSTRG.12033.1
MSTRG.12034		MSTRG.12034.1
MSTRG.1	Zfp263	ENSMUST00000023176;MSTRG.12035.1;ENSMUST00000159425;E
MSTRG.1	Slx4	ENSMUST00000146569;ENSMUST00000040790;MSTRG.12036.2;E
MSTRG.1	Zfp174	ENSMUST00000041778
MSTRG.1	Zfp597	ENSMUST00000090522;ENSMUST00000138857;ENSMUST00000119
MSTRG.1	Gm26675	ENSMUST00000181068
MSTRG.1204		MSTRG.1204.1
MSTRG.1	Naa60;G	ENSMUST00000151988;ENSMUST00000186375;ENSMUST00000176
MSTRG.1	Zfp597	MSTRG.12041.1
MSTRG.1	1700037	ENSMUST00000177519;ENSMUST00000006139;ENSMUST00000115
MSTRG.1	1700016	ENSMUST00000195807;ENSMUST00000193513
MSTRG.1	Gm23493	ENSMUST00000116835
MSTRG.1	Gm15539	ENSMUST00000118452
MSTRG.1	Gm15537	ENSMUST00000126677
MSTRG.1	Nlrc3	MSTRG.12047.1;MSTRG.12047.2;MSTRG.12047.3;ENSMUST0000
MSTRG.1	Gm15879	ENSMUST00000150906;ENSMUST00000139334
MSTRG.1	Dnase1	MSTRG.12049.1;ENSMUST00000006136;ENSMUST00000157044;E
MSTRG.1205		MSTRG.1205.1
MSTRG.1	Trap1;G	ENSMUST00000006137;MSTRG.12050.1;ENSMUST00000137945;M
MSTRG.1	Adcy9	ENSMUST00000117801;ENSMUST00000120080;ENSMUST00000005
MSTRG.1	Gm6142	ENSMUST00000120290
MSTRG.1	Adcy9	MSTRG.12053.1
MSTRG.1	Adcy9	MSTRG.12054.1
MSTRG.1	Adcy9	MSTRG.12055.1
MSTRG.1	Crebbp;	MSTRG.12056.1;ENSMUST00000023165;ENSMUST00000205765;E
MSTRG.1	Crebbp	MSTRG.12057.1
MSTRG.1	Hmox2	ENSMUST00000154117;MSTRG.12058.1;MSTRG.12058.3;ENSMUS
MSTRG.1	Gm15859	ENSMUST00000132069
MSTRG.1	Gm15859	MSTRG.12060.1
MSTRG.1	Hmox2	MSTRG.12061.1
MSTRG.1	Cdip1	ENSMUST00000147225;MSTRG.12062.3;ENSMUST00000004173;M
MSTRG.1	Gm15835	ENSMUST00000127697
MSTRG.1	Srl	MSTRG.12064.1;ENSMUST00000090500;ENSMUST00000023161;E
MSTRG.1	Gm15885	ENSMUST00000122114
MSTRG.1	Tfap4	ENSMUST00000005862
MSTRG.1	Glis2	ENSMUST00000014447;ENSMUST00000141682;ENSMUST00000122
MSTRG.1	Pam16	MSTRG.12068.1;ENSMUST00000131250;ENSMUST00000156889;E
MSTRG.1	Coro7	ENSMUST00000038552;ENSMUST00000150028;MSTRG.12069.2;M
MSTRG.1	Gm7195	MSTRG.1207.1
MSTRG.1	Vasn;Dn	ENSMUST00000038770;ENSMUST00000138044;ENSMUST00000144
MSTRG.1	Nmral1	ENSMUST00000120056;ENSMUST00000115851;ENSMUST00000079
MSTRG.1	4930562	ENSMUST00000100211;ENSMUST00000175836;ENSMUST00000171
MSTRG.1	Ubald1	MSTRG.12073.1;MSTRG.12073.2;ENSMUST00000037843;ENSMUS
MSTRG.1	Mgrn1	ENSMUST00000023159;ENSMUST00000070658;MSTRG.12074.3;M
MSTRG.1	Gm16861	ENSMUST00000181498
MSTRG.1	Nudt16l	ENSMUST00000050881;MSTRG.12076.1
MSTRG.1	Anks3	MSTRG.12077.1;ENSMUST00000023157;MSTRG.12077.3;MSTRG.
MSTRG.1	4930451	ENSMUST00000090457
MSTRG.1	Smim22	ENSMUST00000184439;ENSMUST00000183821;ENSMUST00000178

MSTRG.1	Gm42477	ENSMUST00000202427
MSTRG.1	Rogdi	ENSMUST00000202281;ENSMUST00000201726;ENSMUST00000202
MSTRG.1	Nagpa	ENSMUST00000147567;ENSMUST00000023911;ENSMUST00000156
MSTRG.1	AU02109	MSTRG.12083.1
MSTRG.1	AU02109	ENSMUST00000050160
MSTRG.1	Mir6364	ENSMUST00000183396
MSTRG.1	Gm23689	ENSMUST00000180354
MSTRG.1	Glyr1	MSTRG.12087.5;MSTRG.12087.6;MSTRG.12087.9;ENSMUST0000
MSTRG.1	Ubn1	ENSMUST00000052449;MSTRG.12089.1;MSTRG.12089.2;MSTRG.
MSTRG.1	Gm28358	MSTRG.1209.1
MSTRG.1	Gm23483	ENSMUST00000104179
MSTRG.1	Ubn1	MSTRG.12091.1
MSTRG.1	Ppl	ENSMUST00000035672
MSTRG.1	Alg1	ENSMUST00000100196;ENSMUST00000153730;ENSMUST00000049
MSTRG.1	Eef2kmt	ENSMUST00000064635;ENSMUST00000132225;ENSMUST00000139
MSTRG.1	Gm15983	ENSMUST00000140000
MSTRG.12096		MSTRG.12096.1
MSTRG.12097		MSTRG.12097.1
MSTRG.1	Mettl22	ENSMUST00000046470;ENSMUST00000150790;ENSMUST00000142
MSTRG.1	Mettl22	MSTRG.12099.1
MSTRG.1	Gm37509	ENSMUST00000194867
MSTRG.1	Gm23706	ENSMUST00000104369
MSTRG.1	Abat	ENSMUST00000065987;ENSMUST00000115838;ENSMUST00000140
MSTRG.1	Gm22137	ENSMUST00000102085;ENSMUST00000118361
MSTRG.12103		MSTRG.12103.1
MSTRG.12104		MSTRG.12104.1
MSTRG.12105		MSTRG.12105.1
MSTRG.12106		MSTRG.12106.1
MSTRG.12107		MSTRG.12107.1
MSTRG.12108		MSTRG.12108.1
MSTRG.12109		MSTRG.12109.1
MSTRG.1	Tsn	ENSMUST00000027623;ENSMUST00000186608;MSTRG.1211.2;EN
MSTRG.12110		MSTRG.12110.1
MSTRG.12111		MSTRG.12111.1
MSTRG.12112		MSTRG.12112.1
MSTRG.12113		MSTRG.12113.1
MSTRG.12114		MSTRG.12114.1
MSTRG.12115		MSTRG.12115.1
MSTRG.12116		MSTRG.12116.1
MSTRG.12117		MSTRG.12117.1
MSTRG.12118		MSTRG.12118.1
MSTRG.12119		MSTRG.12119.1
MSTRG.1	Nifk	ENSMUST00000027626;ENSMUST00000112688;ENSMUST00000161
MSTRG.12120		MSTRG.12120.1
MSTRG.12121		MSTRG.12121.1
MSTRG.12122		MSTRG.12122.1
MSTRG.12123		MSTRG.12123.1
MSTRG.12124		MSTRG.12124.1
MSTRG.12125		MSTRG.12125.1
MSTRG.12126		MSTRG.12126.1
MSTRG.12127		MSTRG.12127.1
MSTRG.12128		MSTRG.12128.1
MSTRG.1	Rbfox1	ENSMUST00000115841;MSTRG.12129.2;ENSMUST00000056416
MSTRG.1	Tfcp2l1	ENSMUST00000190652;ENSMUST00000027629;ENSMUST00000157
MSTRG.1	Rbfox1	MSTRG.12130.1
MSTRG.1	Rbfox1	MSTRG.12131.1
MSTRG.1	Rbfox1	MSTRG.12132.1

MSTRG.1	Rbfox1	MSTRG.12133.1
MSTRG.1	Rbfox1	MSTRG.12134.1
MSTRG.1	Rbfox1	MSTRG.12135.1
MSTRG.1	Rbfox1	MSTRG.12136.1
MSTRG.1	Rbfox1	MSTRG.12137.1
MSTRG.1	Rbfox1	MSTRG.12138.1
MSTRG.1	Rbfox1	MSTRG.12139.1
MSTRG.1	Gm22094	ENSMUST00000158976
MSTRG.1	Rbfox1	MSTRG.12140.1
MSTRG.1	Rbfox1	MSTRG.12141.1
MSTRG.1	Rbfox1	MSTRG.12142.1
MSTRG.1	Rbfox1	MSTRG.12143.1
MSTRG.1	Rbfox1	MSTRG.12144.1
MSTRG.1	Rbfox1	MSTRG.12145.1
MSTRG.1	Rbfox1	MSTRG.12146.1
MSTRG.1	Rbfox1	MSTRG.12147.1
MSTRG.1	Rbfox1	MSTRG.12148.1
MSTRG.1	Rbfox1	MSTRG.12149.1
MSTRG.1	Gm28882	ENSMUST00000185504
MSTRG.1	Rbfox1	MSTRG.12150.1
MSTRG.1	Rbfox1	MSTRG.12151.1
MSTRG.1	Rbfox1	MSTRG.12152.1
MSTRG.1	Rbfox1	MSTRG.12153.1
MSTRG.1	Rbfox1	MSTRG.12154.1
MSTRG.1	Tmem186	ENSMUST00000052505;MSTRG.12155.1
MSTRG.1	Pmm2	ENSMUST00000023396;ENSMUST00000130885;MSTRG.12156.2;E
MSTRG.1	Carhsp1	MSTRG.12157.3;MSTRG.12157.2;ENSMUST00000008537;MSTRG.
MSTRG.1	1810013	ENSMUST00000023150;MSTRG.12158.2;MSTRG.12158.3;MSTRG.
MSTRG.1	Gm22224	ENSMUST00000157357
MSTRG.1	Gli2	ENSMUST00000062483;ENSMUST00000159839;ENSMUST0000016C
MSTRG.12160		MSTRG.12160.1
MSTRG.12161		MSTRG.12161.1
MSTRG.1	Emp2	ENSMUST00000078357
MSTRG.12163		MSTRG.12163.1
MSTRG.1	Gm43786	ENSMUST00000201722;ENSMUST00000202157
MSTRG.1	Usp7	MSTRG.12165.1;MSTRG.12165.2;ENSMUST00000160405;MSTRG.
MSTRG.1	Usp7	MSTRG.12166.1
MSTRG.1	Gm25805	ENSMUST00000122788
MSTRG.1	Nubp1	ENSMUST00000023146;ENSMUST00000140157;ENSMUST0000012S
MSTRG.1	Tvp23a	ENSMUST00000051118;ENSMUST00000128288;ENSMUST0000015З
MSTRG.1	Gli2	MSTRG.1217.1
MSTRG.12170		MSTRG.12170.1
MSTRG.1	Ciita	MSTRG.12171.1;MSTRG.12171.2;ENSMUST00000023147;MSTRG.
MSTRG.1	Ciita	MSTRG.12172.1
MSTRG.1	Dexi	ENSMUST00000184863;MSTRG.12173.3;MSTRG.12173.2;ENSMUS
MSTRG.1	Clec16a	ENSMUST00000066345;ENSMUST00000115824;ENSMUST0000015S
MSTRG.1	Gm15558	MSTRG.12175.1;MSTRG.12175.2;ENSMUST00000126849;MSTRG.
MSTRG.1	Clec16a	MSTRG.12176.1
MSTRG.12177		MSTRG.12177.1
MSTRG.1	Socs1	MSTRG.12178.1;MSTRG.12178.2;ENSMUST00000038099
MSTRG.1	Tnp2	ENSMUST00000051297
MSTRG.1	Mir6346	ENSMUST00000183982
MSTRG.1	Prm3	ENSMUST00000050864
MSTRG.1	Rmi2;Gm	MSTRG.12181.1;MSTRG.12181.2;ENSMUST00000181732;ENSMUS
MSTRG.1	Rmi2	MSTRG.12182.1
MSTRG.1	Litaf	ENSMUST00000023143;ENSMUST00000117360;ENSMUST00000162
MSTRG.1	Gm26268	ENSMUST00000177685

MSTRG.1	Gm4262	ENSMUST00000180624
MSTRG.1	Litaf	MSTRG.12187.1
MSTRG.1	Snn	ENSMUST00000089011;MSTRG.12188.2
MSTRG.1	Txndc11	MSTRG.12189.2;MSTRG.12189.1;ENSMUST00000038424;ENSMUS
MSTRG.1	Gli2	MSTRG.1219.1
MSTRG.1	Snx29	ENSMUST00000180792;MSTRG.12190.1;ENSMUST00000115814;E
MSTRG.1	Snx29	MSTRG.12191.1
MSTRG.1	Snx29	MSTRG.12192.1
MSTRG.1	Gm15897	ENSMUST00000117770
MSTRG.1	Gm4279	MSTRG.12194.1
MSTRG.1	Zc3h7a	MSTRG.12195.1;ENSMUST00000037633;ENSMUST00000138282;E
MSTRG.1	Gm24145	ENSMUST00000082631
MSTRG.1	Zc3h7a	MSTRG.12198.1
MSTRG.1	Zc3h7a	MSTRG.12199.1
MSTRG.1	Sbspon	ENSMUST00000040695
MSTRG.1	Gli2	MSTRG.1220.1
MSTRG.1	4930509	ENSMUST00000181206
MSTRG.1	Gm7638	ENSMUST00000121348
MSTRG.1	Gm6305	ENSMUST00000121027
MSTRG.1	Rsl1d1	ENSMUST00000119953;ENSMUST00000129962;MSTRG.12203.2;E
MSTRG.1	2610020	ENSMUST00000181526;MSTRG.12204.2
MSTRG.1	Gspt1	MSTRG.12205.1;MSTRG.12205.2;ENSMUST00000167571;ENSMUS
MSTRG.1	Mir1945	ENSMUST00000157919
MSTRG.1	Cpped1	ENSMUST00000096272;ENSMUST00000121750;ENSMUST00000145
MSTRG.1	Cpped1	MSTRG.12208.1
MSTRG.1	Gm9961	MSTRG.12209.1;ENSMUST00000068397
MSTRG.1	Gli2	MSTRG.1221.1
MSTRG.12210		MSTRG.12210.1
MSTRG.12211		MSTRG.12211.1
MSTRG.1	Ercc4	ENSMUST00000023206;ENSMUST00000138527;MSTRG.12212.2;E
MSTRG.1	Mkl2	MSTRG.12213.1;ENSMUST00000009713;ENSMUST00000115809;E
MSTRG.1	Mkl2	MSTRG.12214.1
MSTRG.1	Gm15738	MSTRG.12215.1;ENSMUST00000210378;ENSMUST00000140892;E
MSTRG.1	Mkl2	MSTRG.12216.1
MSTRG.1	Mkl2	MSTRG.12217.1
MSTRG.1	Mir193b	ENSMUST00000103784
MSTRG.1	Mir365-	ENSMUST00000083515
MSTRG.1	Gli2	MSTRG.1222.1
MSTRG.1	Gm32817	ENSMUST00000210738;ENSMUST00000210007;ENSMUST0000021C
MSTRG.12221		MSTRG.12221.1
MSTRG.1	Parn	ENSMUST00000058884
MSTRG.1	Parn	MSTRG.12223.1
MSTRG.1	Bfar;31	ENSMUST00000023365;ENSMUST00000225585;ENSMUST00000127
MSTRG.12225		MSTRG.12225.1
MSTRG.1	Pla2g10	ENSMUST00000115807;ENSMUST00000023364
MSTRG.1	Pla2g10	ENSMUST00000141172
MSTRG.1	Rrn3	ENSMUST00000023363;MSTRG.12228.2
MSTRG.1	Gm27734	ENSMUST00000183701;ENSMUST00000023362;ENSMUST00000145
MSTRG.1	Gli2	MSTRG.1223.1
MSTRG.1	Pdxdc1	ENSMUST00000023361;ENSMUST00000127432;ENSMUST00000115
MSTRG.1	Pdxdc1	MSTRG.12231.1
MSTRG.1	Pdxdc1	MSTRG.12232.1
MSTRG.1	Mpv17l	ENSMUST00000128757;MSTRG.12233.2;MSTRG.12233.3;ENSMUS
MSTRG.1	Mpv17l	MSTRG.12234.1
MSTRG.1	Gm15950	ENSMUST00000162902
MSTRG.1	Marf1;G	ENSMUST00000090300;MSTRG.12236.2;MSTRG.12236.3;ENSMUS
MSTRG.1	Mir484	ENSMUST00000093566

MSTRG.12238	MSTRG.12238.1
MSTRG.1 Nde1	ENSMUST00000023359;ENSMUST00000117958;ENSMUST00000132
MSTRG.1 Gli2	MSTRG.1224.1
MSTRG.1 Myh11	ENSMUST00000090287
MSTRG.1 Fopnl	ENSMUST00000023357;ENSMUST00000148278
MSTRG.1 Gm15868	ENSMUST00000144291
MSTRG.1 Gm15869	ENSMUST00000126427
MSTRG.12244	MSTRG.12244.1
MSTRG.12245	MSTRG.12245.1
MSTRG.1 Abcc1	ENSMUST00000130671;ENSMUST00000133454;ENSMUST00000147
MSTRG.1 Abcc1	MSTRG.12247.1
MSTRG.1 Abcc1	MSTRG.12249.1
MSTRG.1 Ralb	ENSMUST00000004565;ENSMUST00000142945;ENSMUST0000015€
MSTRG.1 Abcc1	MSTRG.12250.1
MSTRG.12251	MSTRG.12251.1
MSTRG.1 Ube2v2	ENSMUST00000115777;ENSMUST00000115776;ENSMUST0000011{
MSTRG.1 Ube2v2	MSTRG.12253.1
MSTRG.1 Cebpd	ENSMUST00000096232
MSTRG.1 Spidr	ENSMUST00000040248
MSTRG.1 n-R5s31	ENSMUST00000104162
MSTRG.1 Spidr	MSTRG.12257.1
MSTRG.1 Spidr	MSTRG.12258.1
MSTRG.1 Spidr	MSTRG.12259.1
MSTRG.1 Ralb	MSTRG.1226.1
MSTRG.1 Mcm4	ENSMUST00000023353;MSTRG.12260.2;MSTRG.12260.3
MSTRG.1 Prkdc	ENSMUST00000182134;ENSMUST00000023352
MSTRG.1 Prkdc	MSTRG.12262.1
MSTRG.1 Prkdc	MSTRG.12263.1;MSTRG.12263.2
MSTRG.1 Prkdc	MSTRG.12264.1
MSTRG.1 Mzt2	ENSMUST00000023351;ENSMUST00000137864;ENSMUST00000117
MSTRG.1 Yars2	ENSMUST00000159962;ENSMUST00000161191;ENSMUST0000005€
MSTRG.1 Dnm1l	MSTRG.12267.2;ENSMUST00000023477;ENSMUST00000096229;E
MSTRG.1 Gm7765	ENSMUST00000182585
MSTRG.12269	MSTRG.12269.1
MSTRG.1 Gm29454	ENSMUST00000190805
MSTRG.1 Spag6l	ENSMUST00000023468;ENSMUST00000132033;ENSMUST00000141
MSTRG.1 Rpl31-p	ENSMUST00000119957
MSTRG.1 Mapk1	ENSMUST00000023462;ENSMUST00000069107;ENSMUST0000011£
MSTRG.1 Gm22558	MSTRG.12273.1;ENSMUST00000157326
MSTRG.1 1700056	ENSMUST00000141875
MSTRG.12275	MSTRG.12275.1
MSTRG.12276	MSTRG.12276.1
MSTRG.1 Vpreb1	ENSMUST00000135276;ENSMUST00000075017;ENSMUST00000132
MSTRG.1 Top3b;P	ENSMUST00000139740;ENSMUST00000119787;MSTRG.12278.2;E
MSTRG.1 Sdf2l1	ENSMUST00000023453
MSTRG.1 Gm27184	ENSMUST00000185095
MSTRG.1 2610318	MSTRG.12280.1;ENSMUST00000093336;MSTRG.12280.2;ENSMUS
MSTRG.1 Ypel1	ENSMUST00000035682;ENSMUST00000153489;ENSMUST0000015C
MSTRG.1 Ypel1	MSTRG.12282.1
MSTRG.1 Gm9974	ENSMUST00000120357
MSTRG.1 Ppil2	MSTRG.12284.1;ENSMUST00000164458;ENSMUST00000023455;M
MSTRG.1 Gm15585	MSTRG.12285.1;ENSMUST00000149696
MSTRG.1 Hic2	ENSMUST00000090190
MSTRG.1 Hic2	MSTRG.12287.1
MSTRG.1 Gm26635	ENSMUST00000181077
MSTRG.1 Ccdc116	ENSMUST00000023452;ENSMUST00000115711;ENSMUST0000014£
MSTRG.1 Gm27184	MSTRG.1229.1

MSTRG.1	Ydjc	ENSMUST00000115702;ENSMUST00000137267;ENSMUST0000011[illegible]
MSTRG.1	Ube2l3	ENSMUST00000115699;MSTRG.12292.2;ENSMUST00000090192;[illegible]
MSTRG.1	Gm15648	ENSMUST00000149681
MSTRG.1	Ube2l3	MSTRG.12294.1
MSTRG.1	Crkl	ENSMUST00000006293
MSTRG.1	Gm22770	ENSMUST00000158368
MSTRG.1	Tmem191	ENSMUST00000164950;ENSMUST00000159242;ENSMUST00000161
MSTRG.1	Pi4ka	ENSMUST00000036161;ENSMUST00000139404;ENSMUST00000127
MSTRG.1	Serpind	ENSMUST00000023450;MSTRG.12299.3
MSTRG.1	Sbspon	MSTRG.123.1
MSTRG.1	Tmem185	ENSMUST00000183952;ENSMUST00000183607
MSTRG.1	Gm24927	ENSMUST00000083397
MSTRG.1	Snap29	ENSMUST00000023449;MSTRG.12301.2
MSTRG.1	Lztr1	ENSMUST00000115681;ENSMUST00000023444;ENSMUST0000013[illegible]
MSTRG.1	Thap7	ENSMUST00000100125;ENSMUST00000129297;ENSMUST00000127
MSTRG.1	Smpd4	ENSMUST00000006053;ENSMUST00000171435;ENSMUST00000167
MSTRG.1	Klhl22	ENSMUST00000129199;ENSMUST00000165790;ENSMUST0000014[illegible]
MSTRG.1	Scarf2	ENSMUST00000012161
MSTRG.1	Ccdc74a	ENSMUST00000182822;ENSMUST00000056962;ENSMUST00000183
MSTRG.1	Med15	MSTRG.12308.3;MSTRG.12308.4;MSTRG.12308.6;MSTRG.1230[illegible]
MSTRG.1	Rpl26-p	ENSMUST00000170279
MSTRG.1	3830432	ENSMUST00000188909
MSTRG.1	Med15	MSTRG.12310.1
MSTRG.1	Dgcr14	ENSMUST00000003621;MSTRG.12311.2
MSTRG.1	Car15;G	ENSMUST00000118960;ENSMUST00000150068;ENSMUST0000013[illegible]
MSTRG.1	Tssk1	ENSMUST00000046937
MSTRG.1	Slc25a1	ENSMUST00000003622;ENSMUST00000129270;ENSMUST00000131
MSTRG.1	Dgcr6;G	ENSMUST00000143343;ENSMUST00000148521;MSTRG.12315.3;[illegible]
MSTRG.1	Prodh	ENSMUST00000139861;MSTRG.12316.2;ENSMUST00000003620;[illegible]
MSTRG.12317		MSTRG.12317.1
MSTRG.1	Ccdc188	ENSMUST00000167061
MSTRG.1	Zdhhc8	MSTRG.12319.2;ENSMUST00000076957;MSTRG.12319.1
MSTRG.1	Cldn5	ENSMUST00000043577
MSTRG.1	Cdc45	ENSMUST00000000028;ENSMUST00000096990;ENSMUST0000011[illegible]
MSTRG.1	Ufd1	ENSMUST00000168822;ENSMUST00000172013;MSTRG.12322.4;[illegible]
MSTRG.1	Ranbp1	ENSMUST00000115645;ENSMUST00000052325;MSTRG.12323.2;[illegible]
MSTRG.1	Gm25777	ENSMUST00000116714
MSTRG.1	Trmt2a	ENSMUST00000127239;ENSMUST00000115640;ENSMUST0000014[illegible]
MSTRG.1	Dgcr8;M	ENSMUST00000009321;MSTRG.12326.3;MSTRG.12326.2;ENSMUS
MSTRG.1	Tango2	ENSMUST00000128580;ENSMUST00000115628;ENSMUST0000014[illegible]
MSTRG.1	Tango2	MSTRG.12328.1
MSTRG.1	Mir185	ENSMUST00000083530
MSTRG.1	Tmem177	ENSMUST00000037906
MSTRG.1	Arvcf	ENSMUST00000115614;ENSMUST00000090103;ENSMUST0000011[illegible]
MSTRG.1	Gm26170	ENSMUST00000158419
MSTRG.1	Gm26219	ENSMUST00000175216
MSTRG.1	Comt	ENSMUST00000165430;ENSMUST00000115609;ENSMUST0000000[illegible]
MSTRG.1	Gm15764	ENSMUST00000150389;ENSMUST00000155103
MSTRG.1	Comt	MSTRG.12335.1
MSTRG.1	Txnrd2	ENSMUST00000205679;ENSMUST00000206606;ENSMUST0000011[illegible]
MSTRG.12337		MSTRG.12337.1
MSTRG.1	Gnb1l;R	ENSMUST00000167778;ENSMUST00000139625;ENSMUST0000012[illegible]
MSTRG.1	Gnb1l	MSTRG.12339.1
MSTRG.1	Tmem37	ENSMUST00000056089
MSTRG.1	2510002	ENSMUST00000055413;ENSMUST00000123146;ENSMUST00000191
MSTRG.1	Gm28539	MSTRG.12341.1
MSTRG.1	Mrpl40	ENSMUST00000023391;ENSMUST00000119273

MSTRG.1	Hira	MSTRG.12343.1
MSTRG.1	Hira	MSTRG.12344.1
MSTRG.1	Gm15799	ENSMUST00000120899
MSTRG.1	Gm15798	ENSMUST00000120082
MSTRG.1	Hira	MSTRG.12347.1
MSTRG.1	Hira	MSTRG.12348.1
MSTRG.1	2010309	ENSMUST00000198927
MSTRG.1	Ptpn4	ENSMUST00000064091;ENSMUST00000166624;ENSMUST00000163
MSTRG.1	Iglc1;I	ENSMUST00000200568;MSTRG.12350.3;MSTRG.12350.1;MSTRG.
MSTRG.1	Iglj3p	ENSMUST00000200074
MSTRG.1	Gm18748	ENSMUST00000198507
MSTRG.1	Iglc4	ENSMUST00000197046
MSTRG.1	Iglj4	ENSMUST00000198313
MSTRG.1	Iglc2;I	ENSMUST00000198182;ENSMUST00000103749;MSTRG.12355.2;E
MSTRG.1	Gm43388	ENSMUST00000197818
MSTRG.1	Gm42870	ENSMUST00000199355
MSTRG.1	Iglv3	ENSMUST00000103751
MSTRG.1	A530030	ENSMUST00000199551
MSTRG.1	Ptpn4	MSTRG.1236.1
MSTRG.1	Olfr164	ENSMUST00000216157;ENSMUST00000056727
MSTRG.1	Gm22158	ENSMUST00000158411
MSTRG.1	Gm23612	ENSMUST00000103823
MSTRG.1	A930003	ENSMUST00000100083;MSTRG.12363.1;ENSMUST00000183941
MSTRG.12364		MSTRG.12364.1
MSTRG.12365		MSTRG.12365.1
MSTRG.1	Klhl6	MSTRG.12366.6;MSTRG.12366.4;MSTRG.12366.5;MSTRG.12366
MSTRG.1	Klhl24	ENSMUST00000023509;MSTRG.12367.2;MSTRG.12367.3;MSTRG.
MSTRG.1	Yeats2	ENSMUST00000090052;ENSMUST00000115560;MSTRG.12368.3;M
MSTRG.1	Gm10241	ENSMUST00000117664
MSTRG.1	Gm3851	ENSMUST00000168653
MSTRG.1	Yeats2	MSTRG.12370.1
MSTRG.1	Map6d1	ENSMUST00000040880
MSTRG.1	Parl	ENSMUST00000136252;ENSMUST00000048642;ENSMUST00000155
MSTRG.1	Abcc5	ENSMUST00000079158;ENSMUST00000115547;ENSMUST00000134
MSTRG.1	Eif2b5	ENSMUST00000003320;ENSMUST00000148714
MSTRG.1	Gm16618	ENSMUST00000134992
MSTRG.1	Dvl3	ENSMUST00000003318;ENSMUST00000171572;ENSMUST00000171
MSTRG.1	Dvl3	MSTRG.12377.1
MSTRG.1	Ap2m1	ENSMUST00000090023;ENSMUST00000007216;ENSMUST00000133
MSTRG.1	Abcf3	ENSMUST00000003319;ENSMUST00000152339;ENSMUST00000154
MSTRG.1	Gm37745	ENSMUST00000193304
MSTRG.1	Vwa5b2	ENSMUST00000096197;ENSMUST00000159780
MSTRG.1	Mir1224	ENSMUST00000117019
MSTRG.1	Alg3	ENSMUST00000045918;ENSMUST00000127454;ENSMUST00000123
MSTRG.1	Ece2	ENSMUST00000115522;ENSMUST00000119224;ENSMUST00000120
MSTRG.1	Camk2n2	ENSMUST00000052939
MSTRG.1	Psmd2	ENSMUST00000007212;ENSMUST00000172207;ENSMUST00000169
MSTRG.1	Eif4g1	ENSMUST00000154594;ENSMUST00000128840;ENSMUST00000044
MSTRG.1	Snord66	ENSMUST00000104051
MSTRG.1	Rps10-p	ENSMUST00000127484
MSTRG.1	Fam131a	ENSMUST00000149543;ENSMUST00000118919;ENSMUST00000056
MSTRG.1	Clcn2	ENSMUST00000007207;ENSMUST00000148131;ENSMUST00000153
MSTRG.1	Polr2h	ENSMUST00000021405;ENSMUST00000161038
MSTRG.12392		MSTRG.12392.1
MSTRG.1	Ehhadh	ENSMUST00000023559
MSTRG.1	Gm24898	ENSMUST00000158133
MSTRG.1	Vps8	ENSMUST00000096191;ENSMUST00000096192;ENSMUST00000115

MSTRG.1	Vps8	MSTRG.12396.1
MSTRG.1	Vps8	MSTRG.12397.1
MSTRG.1	2510009	ENSMUST00000053336
MSTRG.1	1300002	ENSMUST00000211443;ENSMUST00000209449;ENSMUST00000181
MSTRG.1	Gm23259	ENSMUST00000158318
MSTRG.1	Gm8338	ENSMUST00000163928
MSTRG.1	1300002	MSTRG.12400.1
MSTRG.12401		MSTRG.12401.1
MSTRG.1	Gm26744	ENSMUST00000181834
MSTRG.1	Map3k13	ENSMUST00000042065
MSTRG.1	Gm6467	ENSMUST00000171547
MSTRG.1	2610020	ENSMUST00000180401
MSTRG.1	Tmem41a	ENSMUST00000023562
MSTRG.1	Tmem41a	MSTRG.12407.1
MSTRG.1	Senp2	ENSMUST00000023561;MSTRG.12408.2;MSTRG.12408.3;MSTRG.
MSTRG.1	Gm4943	ENSMUST00000132879
MSTRG.1	Gm28867	ENSMUST00000190194
MSTRG.1	Tra2b	MSTRG.12410.1;ENSMUST00000161286;ENSMUST00000023564;E
MSTRG.1	Tra2b	MSTRG.12411.1
MSTRG.1	Etv5	ENSMUST00000079601;ENSMUST00000168774;ENSMUST0000017C
MSTRG.1	Tbccd1	ENSMUST00000004576
MSTRG.1	Tbccd1	MSTRG.12414.1
MSTRG.1	Dgkg	ENSMUST00000089925;ENSMUST00000023578;MSTRG.12415.3;E
MSTRG.1	9230117	ENSMUST00000159309
MSTRG.1	Dgkg	MSTRG.12417.1
MSTRG.1	Mir8095	ENSMUST00000185103
MSTRG.1	Gm15651	ENSMUST00000159953
MSTRG.1	Clasp1	MSTRG.1242.1
MSTRG.1	Dgkg	MSTRG.12420.1
MSTRG.1	Gm22481	ENSMUST00000122562
MSTRG.1	Mir6362	ENSMUST00000184329
MSTRG.1	Dnajb11	MSTRG.12423.1;ENSMUST00000004574;ENSMUST00000178320;E
MSTRG.1	Kng2	ENSMUST00000115349;ENSMUST00000100046
MSTRG.1	Gm6493	ENSMUST00000159889
MSTRG.12426		MSTRG.12426.1
MSTRG.1	Adipoq	ENSMUST00000023593;ENSMUST00000171309
MSTRG.1	Eif4a2;	ENSMUST00000023599;ENSMUST00000168891;ENSMUST00000147
MSTRG.1	Rfc4	ENSMUST00000023598;ENSMUST00000115338;ENSMUST00000147
MSTRG.1	Clasp1	ENSMUST00000049404;ENSMUST00000070989;ENSMUST00000178
MSTRG.1	B630019	MSTRG.12430.1;MSTRG.12430.2;MSTRG.12430.4;MSTRG.1243C
MSTRG.1	Gm15746	ENSMUST00000120684
MSTRG.1	BC10617	ENSMUST00000055369
MSTRG.1	St6gal1	ENSMUST00000023601;ENSMUST00000152449;ENSMUST00000178
MSTRG.1	St6gal1	MSTRG.12434.1
MSTRG.1	St6gal1	MSTRG.12435.1
MSTRG.1	Masp1	ENSMUST00000089883
MSTRG.1	Gm45338	ENSMUST00000211460;ENSMUST00000210658;ENSMUST0000020S
MSTRG.1	Bcl6	MSTRG.12438.4;MSTRG.12438.2;MSTRG.12438.3;MSTRG.12438
MSTRG.1	Bcl6	MSTRG.12439.1
MSTRG.1	Clasp1	MSTRG.1244.1
MSTRG.1	Gm37419	ENSMUST00000210705
MSTRG.1	Gm37419	MSTRG.12441.1
MSTRG.1	Lppos	ENSMUST00000181640
MSTRG.1	Lpp	MSTRG.12443.1;ENSMUST00000038053;MSTRG.12443.3;ENSMUS
MSTRG.1	Gm24440	ENSMUST00000157236
MSTRG.1	Lpp	MSTRG.12445.1
MSTRG.1	Lpp	MSTRG.12446.1

MSTRG.1	Lpp	MSTRG.12447.1
MSTRG.1	Lpp	MSTRG.12448.1
MSTRG.1	Lpp	MSTRG.12449.1
MSTRG.1	Gm26080	ENSMUST00000104069
MSTRG.1	Lpp	MSTRG.12450.1
MSTRG.1	Mir28a	ENSMUST00000083560
MSTRG.1	Gm22672	ENSMUST00000104414
MSTRG.1	Lpp	MSTRG.12453.1
MSTRG.1	Lpp	MSTRG.12454.1
MSTRG.1	Trp63	ENSMUST00000115307;ENSMUST00000115308;ENSMUST0000006[illegible]
MSTRG.1	Gm24928	ENSMUST00000083399
MSTRG.1	Gm27005	ENSMUST00000182783
MSTRG.1	Cldn1	ENSMUST00000023154
MSTRG.12459		MSTRG.12459.1
MSTRG.1	2900060[illegible]	ENSMUST00000203191;ENSMUST00000204125;ENSMUST0000011[illegible]
MSTRG.1	Il1rap	ENSMUST00000096129;ENSMUST00000174202;ENSMUST0000002[illegible]
MSTRG.1	Gm20319	ENSMUST00000172522;ENSMUST00000174385
MSTRG.1	Ccdc50	ENSMUST00000100026;ENSMUST00000039443;ENSMUST0000009[illegible]
MSTRG.12463		MSTRG.12463.1
MSTRG.1	Opa1	ENSMUST00000160597;ENSMUST00000159036;ENSMUST0000016[illegible]
MSTRG.1	Opa1	MSTRG.12465.1
MSTRG.1	Opa1	MSTRG.12466.1
MSTRG.1	Hes1	ENSMUST00000160592;ENSMUST00000023171;ENSMUST00000161
MSTRG.1	Atp13a3	MSTRG.12468.3;MSTRG.12468.4;MSTRG.12468.1;ENSMUST000[illegible]
MSTRG.1	Atp13a3	MSTRG.12469.1;MSTRG.12469.2
MSTRG.1	Gm28466	MSTRG.1247.1
MSTRG.1	Atp13a3	MSTRG.12470.1
MSTRG.1	Atp13a3	MSTRG.12471.1
MSTRG.1	Fam43a	ENSMUST00000059078
MSTRG.1	Lsg1	ENSMUST00000117363;ENSMUST00000143170;ENSMUST0000015[illegible]
MSTRG.1	Xxylt1	ENSMUST00000055389;ENSMUST00000140566;ENSMUST0000015[illegible]
MSTRG.1	Gm15742	ENSMUST00000123905
MSTRG.1	Xxylt1	MSTRG.12476.1
MSTRG.1	Xxylt1	MSTRG.12477.1
MSTRG.1	Acap2	ENSMUST00000058033;MSTRG.12478.1;MSTRG.12478.4;MSTRG.
MSTRG.1	Acap2	MSTRG.12479.1
MSTRG.1	Steap3	ENSMUST00000140490;ENSMUST00000112641;ENSMUST00000112
MSTRG.1	Acap2	MSTRG.12480.1
MSTRG.1	Acap2	MSTRG.12481.1
MSTRG.1	Ppp1r2	MSTRG.12482.1;ENSMUST00000060188;ENSMUST00000115233
MSTRG.1	Gm27415	ENSMUST00000183720
MSTRG.12484		MSTRG.12484.1
MSTRG.12485		MSTRG.12485.1
MSTRG.1	Apod	ENSMUST00000130560;ENSMUST00000115230;ENSMUST0000002[illegible]
MSTRG.1	Bdh1	ENSMUST00000089759;ENSMUST00000115227;ENSMUST0000012[illegible]
MSTRG.1	Gm15743	ENSMUST00000182690
MSTRG.1	Bdh1	MSTRG.12489.1
MSTRG.1	Dbi	ENSMUST00000027634;ENSMUST00000132118;ENSMUST00000112
MSTRG.1	Dlg1	ENSMUST00000115205;ENSMUST00000115201;ENSMUST0000010[illegible]
MSTRG.1	Dlg1	MSTRG.12491.1
MSTRG.1	Dlg1	MSTRG.12492.1
MSTRG.1	Gm24879	ENSMUST00000158386
MSTRG.1	Dlg1	MSTRG.12494.1
MSTRG.1	Pigz	ENSMUST00000052174;ENSMUST00000151412;ENSMUST00000134
MSTRG.1	0610012[illegible]	ENSMUST00000202722
MSTRG.1	Ncbp2	ENSMUST00000023460;ENSMUST00000140965;ENSMUST0000012[illegible]
MSTRG.1	Senp5	ENSMUST00000023457;ENSMUST00000155515;ENSMUST0000012[illegible]

MSTRG.1	Gm15696	ENSMUST00000121730
MSTRG.1	Rpl7	ENSMUST00000140597;ENSMUST00000058437;ENSMUST00000141
MSTRG.1	3110009	ENSMUST00000027637;ENSMUST00000112644;ENSMUST0000005€
MSTRG.1	Senp5	MSTRG.12500.1
MSTRG.1	Senp5	MSTRG.12501.1
MSTRG.1	Gm26269	ENSMUST00000180144
MSTRG.1	Gm15694	ENSMUST00000121984
MSTRG.1	Senp5	MSTRG.12504.1
MSTRG.1	Hmgb1-p	ENSMUST00000119503
MSTRG.1	Senp5	MSTRG.12506.1
MSTRG.1	Pak2	MSTRG.12507.1
MSTRG.12508		MSTRG.12508.1
MSTRG.1	Pak2;Pi	MSTRG.12509.1;ENSMUST00000023467;MSTRG.12509.2;ENSMUS
MSTRG.1	3110009	MSTRG.1251.1
MSTRG.1	Gm26419	ENSMUST00000104272
MSTRG.1	Pak2	MSTRG.12511.1
MSTRG.1	Cep19	ENSMUST00000115168;ENSMUST00000042869;ENSMUST0000016§
MSTRG.1	Nrros	ENSMUST00000099991;ENSMUST00000126869;MSTRG.12513.2;E
MSTRG.1	Nrros	MSTRG.12514.1
MSTRG.1	Fbxo45	ENSMUST00000042732
MSTRG.1	Gm25848	ENSMUST00000116790
MSTRG.1	Wdr53	ENSMUST00000141820;ENSMUST00000178573;ENSMUST0000002З
MSTRG.1	Rnf168	ENSMUST00000155649;ENSMUST00000014218;ENSMUST00000171
MSTRG.1	Rnf168	MSTRG.12519.1
MSTRG.1	3110009	MSTRG.1252.1
MSTRG.1	Gm20056	ENSMUST00000194813
MSTRG.1	Gm20056	MSTRG.12521.1
MSTRG.1	Gm20056	MSTRG.12522.1
MSTRG.1	Ubxn7	ENSMUST00000115151;MSTRG.12523.2;MSTRG.12523.3;MSTRG.
MSTRG.1	Ubxn7	MSTRG.12524.1
MSTRG.1	Ubxn7	MSTRG.12525.1
MSTRG.1	Tctex1d	ENSMUST00000014220;ENSMUST00000080316;ENSMUST00000131
MSTRG.1	Pcyt1a	ENSMUST00000115137;ENSMUST00000079791;ENSMUST00000141
MSTRG.1	Slc51a	ENSMUST00000042042
MSTRG.1	Tfrc	ENSMUST00000023486;ENSMUST00000120680;MSTRG.12529.2;E
MSTRG.1	Gm38283	ENSMUST00000193041
MSTRG.1	Tfrc	MSTRG.12530.1
MSTRG.1	Tnk2	ENSMUST00000145627;ENSMUST00000124585;MSTRG.12531.2;E
MSTRG.1	Tnk2	MSTRG.12532.1
MSTRG.1	Tnk2os	MSTRG.12533.1;ENSMUST00000141256
MSTRG.1	Tnk2	MSTRG.12534.1
MSTRG.1	Fyttd1	MSTRG.12535.1
MSTRG.1	Rubcn	ENSMUST00000115105;ENSMUST00000140229;MSTRG.12536.2;E
MSTRG.1	Fyttd1	ENSMUST00000143151;ENSMUST00000023489;ENSMUST00000171
MSTRG.1	Fyttd1	MSTRG.12538.1
MSTRG.1	Fyttd1	MSTRG.12539.1
MSTRG.1	Insig2	ENSMUST00000003818;ENSMUST00000071064;ENSMUST0000016C
MSTRG.1	Gm23916	MSTRG.12540.1
MSTRG.1	Osbpl11	MSTRG.12541.1;ENSMUST00000039733;MSTRG.12541.2
MSTRG.1	Osbpl11	MSTRG.12542.1
MSTRG.1	Osbpl11	MSTRG.12543.1
MSTRG.12544		MSTRG.12544.1
MSTRG.1	Snx4	ENSMUST00000023502
MSTRG.1	Snx4	MSTRG.12546.1
MSTRG.1	Lrch3	ENSMUST00000023491;ENSMUST00000170201;ENSMUST0000017C
MSTRG.1	Gm6611	ENSMUST00000163830
MSTRG.1	Gm17106	ENSMUST00000105610

MSTRG.1	Gm37174	ENSMUST00000194699
MSTRG.1	n-R5s33	ENSMUST00000093713
MSTRG.1	Iqcg	ENSMUST00000115100;ENSMUST00000133219;ENSMUST0000004C
MSTRG.1	Rpl35a	ENSMUST00000078804;ENSMUST00000115079;ENSMUST0000013
MSTRG.1	Lmln	ENSMUST00000023497;ENSMUST00000153549;ENSMUST0000014C
MSTRG.1	Gm22231	ENSMUST00000101027
MSTRG.1	Mir1947	ENSMUST00000158235
MSTRG.1	1700007	ENSMUST00000180923
MSTRG.1	Zfp148	ENSMUST00000165418;MSTRG.12557.2;MSTRG.12557.3;MSTRG.
MSTRG.1	Zfp148	MSTRG.12558.1
MSTRG.1	Heg1	ENSMUST00000154863;MSTRG.12559.1;ENSMUST00000152782;E
MSTRG.1256		MSTRG.1256.1
MSTRG.1	Gm15658	ENSMUST00000148293;ENSMUST00000162677;ENSMUST0000016
MSTRG.1	Muc13	ENSMUST00000115044;ENSMUST00000023520
MSTRG.1	Itgb5	ENSMUST00000115028;ENSMUST00000069345;ENSMUST00000134
MSTRG.1	Umps	ENSMUST00000023510;ENSMUST00000131990
MSTRG.1	Gm15829	ENSMUST00000145415
MSTRG.1	Ccdc14	ENSMUST00000023532
MSTRG.1	Kalrn	ENSMUST00000114973;ENSMUST00000132002;ENSMUST0000007
MSTRG.1	Kalrn	MSTRG.12567.1
MSTRG.1	Kalrn	MSTRG.12568.1
MSTRG.1	Kalrn	MSTRG.12569.1
MSTRG.1	Ccdc93	ENSMUST00000036025;ENSMUST00000156459;ENSMUST0000015
MSTRG.1	Mylk	ENSMUST00000155268;ENSMUST00000023538
MSTRG.1	1700119	ENSMUST00000187558
MSTRG.1	Hacd2	ENSMUST00000061156;MSTRG.12572.2;MSTRG.12572.3
MSTRG.1	Hacd2	MSTRG.12573.1
MSTRG.1	Hacd2	MSTRG.12574.1
MSTRG.1	Hacd2	MSTRG.12575.1
MSTRG.1	Hacd2	MSTRG.12576.1
MSTRG.1	Adcy5	ENSMUST00000114913
MSTRG.1	Sec22a	ENSMUST00000043521;ENSMUST00000154064;ENSMUST00000154
MSTRG.1	Gm15392	ENSMUST00000152863
MSTRG.1	Sec22a	MSTRG.12580.1
MSTRG.1	Sec22a	MSTRG.12581.1
MSTRG.1	Sec22a	MSTRG.12582.1
MSTRG.1	Dirc2;G	MSTRG.12583.1;MSTRG.12583.3;ENSMUST00000023554;MSTRG.
MSTRG.1	Dirc2	MSTRG.12584.1
MSTRG.1	Dirc2	MSTRG.12585.1
MSTRG.1	Hspbap1	ENSMUST00000023555
MSTRG.1	Hspbap1	MSTRG.12587.1
MSTRG.1	Hspbap1	MSTRG.12588.1
MSTRG.1	Gm26838	MSTRG.12589.1
MSTRG.1	Ddx18	ENSMUST00000001724;ENSMUST00000148809;MSTRG.1259.2;EN
MSTRG.1	Parp14	MSTRG.12590.1;ENSMUST00000042665;ENSMUST00000142946;E
MSTRG.1	Wdr5b	ENSMUST00000042203
MSTRG.1	Fam162a	ENSMUST00000004057
MSTRG.1	Fam162a	MSTRG.12593.1
MSTRG.1	Ccdc58	ENSMUST00000163352;ENSMUST00000164916;MSTRG.12594.1;M
MSTRG.1	Ccdc58	MSTRG.12595.1
MSTRG.1	Dtx3l	ENSMUST00000081933;MSTRG.12596.2;MSTRG.12596.3;ENSMUS
MSTRG.1	Parp9	ENSMUST00000114878;ENSMUST00000023622;ENSMUST00000114
MSTRG.1	Gm15564	ENSMUST00000174085;ENSMUST00000173605;ENSMUST00000144
MSTRG.1	Kpna1	ENSMUST00000172991;ENSMUST00000004054;ENSMUST0000017
MSTRG.1	Rdh10	MSTRG.126.1;MSTRG.126.2;ENSMUST00000027053;MSTRG.126.
MSTRG.1260		MSTRG.1260.1
MSTRG.1	Cd86	ENSMUST00000089620;ENSMUST00000135280;ENSMUST0000014

MSTRG.1	Slc15a2	ENSMUST00000023616;ENSMUST00000163471;ENSMUST00000172
MSTRG.1	Slc15a2	MSTRG.12602.1
MSTRG.1	Slc15a2	MSTRG.12603.1
MSTRG.1	Gm8425	ENSMUST00000171395
MSTRG.1	Hcls1	ENSMUST00000023531;ENSMUST00000164050;ENSMUST00000163
MSTRG.1	Fbxo40	ENSMUST00000075869;ENSMUST00000132511;ENSMUST00000127
MSTRG.1	Eaf2	ENSMUST00000075946;ENSMUST00000114829;ENSMUST00000114
MSTRG.1	Iqcb1	ENSMUST00000146272;ENSMUST00000114819;ENSMUST00000023
MSTRG.1	Iqcb1	MSTRG.12609.1
MSTRG.1	Eaf2	MSTRG.12610.1
MSTRG.1	Golgb1	ENSMUST00000135800;ENSMUST00000023534;ENSMUST00000135
MSTRG.1	4930565	ENSMUST00000153572
MSTRG.1	Polq	ENSMUST00000071452;ENSMUST00000183112;ENSMUST00000182
MSTRG.1	Polq	MSTRG.12614.1
MSTRG.1	Polq	MSTRG.12615.1
MSTRG.1	Polq	MSTRG.12616.1
MSTRG.1	Stxbp5l	ENSMUST00000114787;ENSMUST00000149984;ENSMUST00000151
MSTRG.1	Gm15665	ENSMUST00000119116
MSTRG.1	Stxbp5l	MSTRG.12619.1
MSTRG.1	Stxbp5l	MSTRG.12620.1
MSTRG.1	Ndufb4	ENSMUST00000023514;ENSMUST00000135019
MSTRG.1	Gtf2e1	ENSMUST00000023525;ENSMUST00000130350
MSTRG.1	Rabl3	ENSMUST00000023524;ENSMUST00000130028;ENSMUST00000128
MSTRG.1	Hgd	ENSMUST00000160847;ENSMUST00000161193;ENSMUST00000162
MSTRG.1	Fstl1	ENSMUST00000114763;ENSMUST00000152579;ENSMUST00000125
MSTRG.1	Lrrc58;	ENSMUST00000078717;MSTRG.12626.1;ENSMUST00000119947
MSTRG.12627		MSTRG.12627.1
MSTRG.12628		MSTRG.12628.1
MSTRG.1	Gsk3b	MSTRG.12629.1;ENSMUST00000023507;ENSMUST00000114750;E
MSTRG.1	Gm28928	MSTRG.1263.1
MSTRG.1	Gsk3b	MSTRG.12630.1
MSTRG.1	Gsk3b	MSTRG.12631.1
MSTRG.1	Gsk3b	MSTRG.12632.1
MSTRG.1	Gm25617	ENSMUST00000198387;ENSMUST00000179548
MSTRG.1	Gsk3b	MSTRG.12634.1
MSTRG.1	Gm27931	ENSMUST00000184976
MSTRG.1	Pla1a	ENSMUST00000002926
MSTRG.1	Cox17;G	ENSMUST00000050273;ENSMUST00000152868;ENSMUST0000012C
MSTRG.1	Adprh	MSTRG.12638.1
MSTRG.1	Adprh	ENSMUST00000002923
MSTRG.1	Gm37717	ENSMUST00000192647
MSTRG.1	D930030	ENSMUST00000180735
MSTRG.1	Cd80	MSTRG.12641.1;ENSMUST00000099816
MSTRG.1	Cd80	MSTRG.12642.1
MSTRG.1	Timmdc1	ENSMUST00000002925;MSTRG.12643.2;ENSMUST00000147543
MSTRG.1	Gm15953	ENSMUST00000117656
MSTRG.1	Timmdc1	MSTRG.12645.1
MSTRG.1	Poglut1	ENSMUST00000036210;ENSMUST00000153187;ENSMUST00000149
MSTRG.12647		MSTRG.12647.1
MSTRG.1	Tmem39a	ENSMUST00000002924;ENSMUST00000171687;ENSMUST00000163
MSTRG.1	Tmem39a	MSTRG.12649.1
MSTRG.1265		MSTRG.1265.1
MSTRG.1	Gm17103	ENSMUST00000168897
MSTRG.1	Arhgap3	ENSMUST00000023487;ENSMUST00000124866;ENSMUST00000132
MSTRG.1	Gm15530	ENSMUST00000122206
MSTRG.1	Arhgap3	MSTRG.12653.1
MSTRG.1	Arhgap3	MSTRG.12654.1

MSTRG.1	Arhgap3	MSTRG.12655.1
MSTRG.1	Arhgap3	MSTRG.12656.1
MSTRG.1	B4galt4	ENSMUST00000023482;ENSMUST00000114712;ENSMUST00000154
MSTRG.1	B4galt4	MSTRG.12658.1
MSTRG.1	Gm15575	ENSMUST00000119414
MSTRG.1	Actr3	ENSMUST00000027579;ENSMUST00000178474;ENSMUST0000018
MSTRG.12660		MSTRG.12660.1
MSTRG.1	Lsamp	ENSMUST00000189229;ENSMUST00000187695;ENSMUST0000018
MSTRG.1	Lsamp	MSTRG.12662.1
MSTRG.1	Gm22543	ENSMUST00000104347
MSTRG.1	Gm36939	ENSMUST00000194889
MSTRG.1	Gm27887	ENSMUST00000184367
MSTRG.1	Lsamp	MSTRG.12667.1
MSTRG.1	Gm27717	ENSMUST00000183933
MSTRG.1	Gpr39	ENSMUST00000027581;ENSMUST00000159359
MSTRG.1	Gm27587	ENSMUST00000185116
MSTRG.1	Gm28750	ENSMUST00000186327
MSTRG.1	Qtrt2	ENSMUST00000156568;ENSMUST00000023387;MSTRG.12672.3;M
MSTRG.1	Gm25989	ENSMUST00000158761
MSTRG.1	Ccdc191	ENSMUST00000132859;ENSMUST00000122014;ENSMUST0000017
MSTRG.1	Zdhhc23	ENSMUST00000036321;ENSMUST00000165648
MSTRG.1	Gramd1c	ENSMUST00000179565;ENSMUST00000036174;ENSMUST00000214
MSTRG.1	Mir3470	ENSMUST00000175563
MSTRG.1	Gramd1c	MSTRG.12678.1
MSTRG.1	Lypd1	ENSMUST00000159417;ENSMUST00000162899;ENSMUST00000027
MSTRG.1	Zbtb20;	ENSMUST00000126354;ENSMUST00000131689;MSTRG.12680.2;M
MSTRG.1	Zbtb20	MSTRG.12681.1
MSTRG.1	Zbtb20	MSTRG.12682.1;MSTRG.12682.2
MSTRG.1	BC00216	ENSMUST00000121906
MSTRG.1	Zbtb20	MSTRG.12684.1
MSTRG.1	Zbtb20	MSTRG.12685.1
MSTRG.1	Zbtb20	MSTRG.12686.1
MSTRG.1	Gm15712	ENSMUST00000120369
MSTRG.1	Zbtb20	MSTRG.12688.1
MSTRG.1	Gm37946	ENSMUST00000192064
MSTRG.1	Lypd1	MSTRG.1269.1
MSTRG.1	Zbtb20	MSTRG.12690.1
MSTRG.1	Gm15711	ENSMUST00000118889
MSTRG.1	Zbtb20	MSTRG.12692.1
MSTRG.1	Gm9968	MSTRG.12693.1
MSTRG.1	Gm15713	MSTRG.12694.1
MSTRG.1	Zbtb20	MSTRG.12695.1
MSTRG.1	Tigit	ENSMUST00000096065
MSTRG.1	Atp6v1a	ENSMUST00000114666;ENSMUST00000063661;ENSMUST0000013
MSTRG.1	Naa50	ENSMUST00000159514;ENSMUST00000161326;ENSMUST0000006
MSTRG.1	Naa50	MSTRG.12699.1
MSTRG.1	Lypd1	MSTRG.1270.1
MSTRG.1	Usf3	ENSMUST00000119746;ENSMUST00000088356;ENSMUST00000141
MSTRG.1	Usf3	MSTRG.12701.1
MSTRG.1	Sidt1	ENSMUST00000127567;ENSMUST00000136381;ENSMUST00000047
MSTRG.1	Sidt1	MSTRG.12703.1
MSTRG.1	Sidt1	MSTRG.12704.1
MSTRG.1	Sidt1	MSTRG.12705.1
MSTRG.1	Sidt1	MSTRG.12706.1
MSTRG.1	Sidt1	MSTRG.12707.1
MSTRG.1	Sidt1	MSTRG.12708.1
MSTRG.1	Gm26732	ENSMUST00000181807

MSTRG.1	Lypd1	MSTRG.1271.1
MSTRG.1	Spice1	ENSMUST00000050897;ENSMUST00000155661;ENSMUST00000132
MSTRG.1	Spice1	MSTRG.12711.1
MSTRG.1	Spice1	MSTRG.12712.1
MSTRG.12713		MSTRG.12713.1
MSTRG.1	Cfap44	ENSMUST00000147988;ENSMUST00000099742;ENSMUST0000013б
MSTRG.1	Boc	ENSMUST00000114634;MSTRG.12715.1;MSTRG.12715.3
MSTRG.1	Boc	MSTRG.12716.1
MSTRG.1	Mir3081	ENSMUST00000175305
MSTRG.12718		MSTRG.12718.1
MSTRG.1	Nepro	ENSMUST00000048788;ENSMUST00000123673;ENSMUST00000147
MSTRG.1	Lypd1	MSTRG.1272.1
MSTRG.1	Gtpbp8	MSTRG.12720.1;MSTRG.12720.2;ENSMUST00000162512;MSTRG.
MSTRG.12721		MSTRG.12721.1
MSTRG.1	Cd200r1	ENSMUST00000057488;ENSMUST00000134625
MSTRG.1	Cd200r1	MSTRG.12723.1
MSTRG.1	Cd200r1	MSTRG.12724.1
MSTRG.1	Ccdc80	ENSMUST00000139509;ENSMUST00000138048;ENSMUST00000155
MSTRG.1	Gm6030	ENSMUST00000078768
MSTRG.1	Slc35a5	ENSMUST00000023344;ENSMUST00000181177;ENSMUST00000152
MSTRG.1	Atg3	MSTRG.12728.1;ENSMUST00000023343;ENSMUST00000150390
MSTRG.12729		MSTRG.12729.1
MSTRG.1273		MSTRG.1273.1
MSTRG.1	Btla	ENSMUST00000102802;ENSMUST00000063654
MSTRG.1	Btla	MSTRG.12731.1
MSTRG.1	Btla	MSTRG.12732.1
MSTRG.1	Btla	MSTRG.12733.1
MSTRG.1	Cd200	ENSMUST00000163230;ENSMUST00000023341;MSTRG.12734.2;M
MSTRG.1	Cd200	MSTRG.12735.1
MSTRG.1	Gm4737	ENSMUST00000059524
MSTRG.1	Cd96	ENSMUST00000023336;MSTRG.12737.1;MSTRG.12737.3;MSTRG.
MSTRG.1	BC01657	ENSMUST00000036732
MSTRG.1	Tmprss7	ENSMUST00000114562;ENSMUST00000178323
MSTRG.1	Mgat5;G	ENSMUST00000189427;ENSMUST00000038361;MSTRG.1274.4;MS
MSTRG.1	Tagln3	ENSMUST00000096057
MSTRG.1	Abhd10	ENSMUST00000066983;ENSMUST00000128348;ENSMUST0000013‹
MSTRG.1	Abhd10	MSTRG.12742.1
MSTRG.1	Gm15591	ENSMUST00000138539
MSTRG.1	Abhd10	MSTRG.12744.1
MSTRG.1	Phldb2	ENSMUST00000036355;ENSMUST00000076333;ENSMUST00000131
MSTRG.1	Gm15640	ENSMUST00000152070
MSTRG.1	Gm15638	ENSMUST00000154775;ENSMUST00000128159;MSTRG.12747.4
MSTRG.1	Gm15638	MSTRG.12748.1
MSTRG.1	Plcxd2	MSTRG.12749.2;MSTRG.12749.1;ENSMUST00000130481
MSTRG.1	Mgat5	MSTRG.1275.1
MSTRG.1	Nectin3	ENSMUST00000023335;ENSMUST00000121803;ENSMUST0000011
MSTRG.1	Nectin3	MSTRG.12751.1
MSTRG.1	Morc1	ENSMUST00000023330;MSTRG.12752.2
MSTRG.1	Trat1	MSTRG.12753.1;MSTRG.12753.2;ENSMUST00000170861
MSTRG.12754		MSTRG.12754.1
MSTRG.1	Retnla	ENSMUST00000023329
MSTRG.1	Retnlg	ENSMUST00000065666
MSTRG.1	Myh15	ENSMUST00000168680
MSTRG.1	Myh15	MSTRG.12758.1
MSTRG.1	Myh15	MSTRG.12759.1
MSTRG.1	Mgat5	MSTRG.1276.1
MSTRG.1	Dzip3	ENSMUST00000147358;ENSMUST00000121869;ENSMUST0000013

MSTRG.1	Dzip3	MSTRG.12761.1
MSTRG.1	Gm22576	ENSMUST00000083733
MSTRG.1	Dzip3	MSTRG.12763.1
MSTRG.1	Dzip3	MSTRG.12764.1
MSTRG.1	C330027	ENSMUST00000117994;ENSMUST00000048374;MSTRG.12765.3;E
MSTRG.1	Ift57	ENSMUST00000046777;ENSMUST00000142682;ENSMUST00000127
MSTRG.1	Gm15518	ENSMUST00000127115
MSTRG.1	Gm16619	ENSMUST00000181817
MSTRG.12769		MSTRG.12769.1
MSTRG.1	Mgat5	MSTRG.1277.1;MSTRG.1277.3;MSTRG.1277.2
MSTRG.12770		MSTRG.12770.1
MSTRG.12771		MSTRG.12771.1
MSTRG.1	Cd47	MSTRG.12772.1;MSTRG.12772.2;MSTRG.12772.3;MSTRG.12772
MSTRG.12773		MSTRG.12773.1
MSTRG.1	Bbx	MSTRG.12774.1;ENSMUST00000138166;ENSMUST00000066037;E
MSTRG.1	Bbx	MSTRG.12775.1
MSTRG.1	Dubr	ENSMUST00000186944;ENSMUST00000191079;ENSMUST0000018
MSTRG.1	Cblb	MSTRG.12778.1;MSTRG.12778.3;MSTRG.12778.2;ENSMUST000
MSTRG.1	Cblb	MSTRG.12779.1
MSTRG.1	Mgat5	MSTRG.1278.1
MSTRG.1	Cblb	MSTRG.12780.1;MSTRG.12780.2
MSTRG.1	Gm23513	ENSMUST00000158181
MSTRG.1	Alcam	ENSMUST00000023312;ENSMUST00000167115;ENSMUST0000017
MSTRG.12783		MSTRG.12783.1
MSTRG.12784		MSTRG.12784.1
MSTRG.12785		MSTRG.12785.1
MSTRG.12786		MSTRG.12786.1
MSTRG.1	Nfkbiz	ENSMUST00000114458;ENSMUST00000036273;ENSMUST00000114
MSTRG.1	Nxpe3	ENSMUST00000099705;MSTRG.12788.2;MSTRG.12788.3;MSTRG.
MSTRG.1	Nxpe3	MSTRG.12789.1
MSTRG.1	Mgat5	MSTRG.1279.1
MSTRG.1	Nxpe3	MSTRG.12790.1
MSTRG.12791		MSTRG.12791.1
MSTRG.1	Cep97	ENSMUST00000023270;MSTRG.12792.2;ENSMUST00000118500;E
MSTRG.1	Trmt10c	ENSMUST00000059052
MSTRG.1	Gm22422	ENSMUST00000083060
MSTRG.1	Rpl24;Z	ENSMUST00000023269;MSTRG.12795.1;ENSMUST00000122365;E
MSTRG.1	Zbtb11o	ENSMUST00000186480
MSTRG.1	Pcnp	ENSMUST00000119981;ENSMUST00000125040;ENSMUST00000114
MSTRG.1	Senp7	ENSMUST00000201218;ENSMUST00000201476;ENSMUST0000004
MSTRG.1	Gm28095	ENSMUST00000186343
MSTRG.1	Gm8451	ENSMUST00000170338
MSTRG.1	Senp7	MSTRG.12800.1
MSTRG.1	Impg2	ENSMUST00000160116;ENSMUST00000069778;ENSMUST0000006
MSTRG.1	Impg2	MSTRG.12802.1
MSTRG.12803		MSTRG.12803.1
MSTRG.1	Abi3bp	ENSMUST00000048471;ENSMUST00000096012;ENSMUST0000009
MSTRG.1	Tfg	ENSMUST00000065515;ENSMUST00000127994;ENSMUST0000015
MSTRG.1	Tomm70a	ENSMUST00000166897;MSTRG.12806.1
MSTRG.1	Nit2	ENSMUST00000023432
MSTRG.1	Gm16892	MSTRG.12808.1;ENSMUST00000180999
MSTRG.1	Tbc1d23	ENSMUST00000023431;MSTRG.12809.1;MSTRG.12809.3;MSTRG.
MSTRG.1	Mgat5	MSTRG.1281.1
MSTRG.1	Tbc1d23	MSTRG.12810.1
MSTRG.12811		MSTRG.12811.1
MSTRG.12812		MSTRG.12812.1
MSTRG.1	Tmem30c	ENSMUST00000120112

MSTRG.1	Cmss1	ENSMUST00000114371
MSTRG.1	Filip1l	ENSMUST00000159816;ENSMUST00000099667;MSTRG.12816.3;E
MSTRG.1	Cmss1	MSTRG.12817.1
MSTRG.1	Cmss1	MSTRG.12818.1
MSTRG.1	Cmss1	MSTRG.12819.1
MSTRG.1	Gm23370	ENSMUST00000199545;ENSMUST00000197008
MSTRG.1	Cmss1	MSTRG.12820.1
MSTRG.1	Cmss1	MSTRG.12821.1
MSTRG.12822		MSTRG.12822.1
MSTRG.12823		MSTRG.12823.1
MSTRG.1	Cpox	ENSMUST00000060077
MSTRG.1	Dcbld2	ENSMUST00000046663;ENSMUST00000149321;ENSMUST00000134
MSTRG.1	St3gal6	ENSMUST00000137035;ENSMUST00000114358;ENSMUST00000114
MSTRG.1	St3gal6	MSTRG.12827.1
MSTRG.1	St3gal6	MSTRG.12828.1
MSTRG.1	Cldnd1	ENSMUST00000023426;ENSMUST00000162057;ENSMUST0000015[illegible]
MSTRG.1	Gm23734	ENSMUST00000083753
MSTRG.12830		MSTRG.12830.1
MSTRG.12831		MSTRG.12831.1
MSTRG.1	Gm27564	ENSMUST00000185196
MSTRG.1	Gm26019	ENSMUST00000104407
MSTRG.1	Olfr172	ENSMUST00000214139;ENSMUST00000095991
MSTRG.1	Olfr173	ENSMUST00000206205;ENSMUST00000049940
MSTRG.1	Riox2	ENSMUST00000023407;ENSMUST00000120667;ENSMUST0000012[illegible]
MSTRG.1	Crybg3	ENSMUST00000129762;ENSMUST00000044604;ENSMUST0000012[illegible]
MSTRG.1	Crybg3	ENSMUST00000172910
MSTRG.1	Gm9027	ENSMUST00000165914
MSTRG.1	Gm38301	ENSMUST00000195573
MSTRG.12840		MSTRG.12840.1
MSTRG.12841		MSTRG.12841.1
MSTRG.1	Gm9816	ENSMUST00000122292
MSTRG.1	Nsun3	ENSMUST00000063089;ENSMUST00000143314;ENSMUST0000015[illegible]
MSTRG.1	Nsun3	MSTRG.12844.1
MSTRG.1	Nsun3	MSTRG.12845.1
MSTRG.1	Arl13b	ENSMUST00000089289;ENSMUST00000141665;ENSMUST00000132
MSTRG.1	Arl13b	MSTRG.12847.1
MSTRG.1	Gm25986	ENSMUST00000102469
MSTRG.1	Stx19	ENSMUST00000055557
MSTRG.1	Mgat5	MSTRG.1285.1;MSTRG.1285.2;MSTRG.1285.3
MSTRG.1	Arl13b	MSTRG.12850.1
MSTRG.1	Pros1	ENSMUST00000023629;ENSMUST00000155940;ENSMUST00000127
MSTRG.12852		MSTRG.12852.1
MSTRG.1	4930453	ENSMUST00000162732;ENSMUST00000076991
MSTRG.1	Zfp654	ENSMUST00000207826;ENSMUST00000052588;MSTRG.12854.2
MSTRG.1	Zfp654	MSTRG.12855.1
MSTRG.1	Zfp654	MSTRG.12856.1
MSTRG.1	Zfp654	MSTRG.12857.1
MSTRG.1	Cggbp1	ENSMUST00000067744;MSTRG.12858.2;ENSMUST00000136532
MSTRG.1	Htr1f	ENSMUST00000063076
MSTRG.1	Mgat5	MSTRG.1286.1
MSTRG.1	Htr1f	MSTRG.12860.1
MSTRG.1	Chmp2b	ENSMUST00000004965
MSTRG.12862		MSTRG.12862.1
MSTRG.12863		MSTRG.12863.1
MSTRG.12864		MSTRG.12864.1
MSTRG.12865		MSTRG.12865.1
MSTRG.12866		MSTRG.12866.1

MSTRG.1	Gbe1	ENSMUST00000163832;ENSMUST00000099628;ENSMUST00000171
MSTRG.1	Robo1	ENSMUST00000023600
MSTRG.1	Robo1	MSTRG.12869.1
MSTRG.1	Mgat5	MSTRG.1287.1
MSTRG.1	Robo1	MSTRG.12870.1
MSTRG.1	Robo1	MSTRG.12871.1
MSTRG.1	Robo1	MSTRG.12872.1
MSTRG.1	Robo1	MSTRG.12873.1
MSTRG.1	Robo1	MSTRG.12874.1
MSTRG.12875		MSTRG.12875.1
MSTRG.12876		MSTRG.12876.1
MSTRG.12877		MSTRG.12877.1
MSTRG.1	Hspa13	ENSMUST00000046283;ENSMUST00000114244;ENSMUST00000137
MSTRG.1	Gm22923	ENSMUST00000174952
MSTRG.1	Tmem163	ENSMUST00000027585;ENSMUST00000160616;ENSMUST00000185
MSTRG.1	Gm15554	ENSMUST00000120406
MSTRG.1	Samsn1	ENSMUST00000114239;ENSMUST00000114240;ENSMUST0000013S
MSTRG.12883		MSTRG.12883.1
MSTRG.1	Gm9843	ENSMUST00000052867
MSTRG.1	Nrip1	MSTRG.12885.1;MSTRG.12885.2;ENSMUST00000121927;ENSMUS
MSTRG.1	Usp25	MSTRG.12886.1;MSTRG.12886.2;ENSMUST00000023580
MSTRG.1	Mir99ah	ENSMUST00000183333;ENSMUST00000182656;ENSMUST00000183
MSTRG.1	Gm31258	ENSMUST00000194532
MSTRG.1	Gm37466	ENSMUST00000195692
MSTRG.1	Ubxn4	ENSMUST00000190736;MSTRG.1289.1;ENSMUST00000027592;MS
MSTRG.1	Gm21816	ENSMUST00000193546;ENSMUST00000179255
MSTRG.1	9430092	ENSMUST00000194937
MSTRG.1	Gm38071	ENSMUST00000195792
MSTRG.1	9430053	ENSMUST00000192654
MSTRG.1	Gm37063	ENSMUST00000194678
MSTRG.1	Gm37241	ENSMUST00000191736
MSTRG.1	Gm36963	ENSMUST00000193506
MSTRG.1	Gm37606	ENSMUST00000191816
MSTRG.1	Gm11146	ENSMUST00000114231
MSTRG.1	Mir99a	ENSMUST00000083596
MSTRG.1	Stau2	ENSMUST00000162751;ENSMUST00000027052;ENSMUST00000162
MSTRG.1290		MSTRG.1290.1
MSTRG.1	Mirlet7	ENSMUST00000083623
MSTRG.1	Mir99ah	MSTRG.12901.1
MSTRG.1	Mir99ah	MSTRG.12902.1
MSTRG.1	Mir99ah	MSTRG.12903.1
MSTRG.1	Gm37311	ENSMUST00000195541
MSTRG.1	Mir99ah	MSTRG.12905.1
MSTRG.1	Mir99ah	MSTRG.12906.1
MSTRG.1	Mir125b	ENSMUST00000083538;ENSMUST00000193537
MSTRG.1	9530003	ENSMUST00000192663
MSTRG.1	Gm37694	ENSMUST00000191696
MSTRG.1	Cxcr4	ENSMUST00000052172;ENSMUST00000142893
MSTRG.1	E330011	ENSMUST00000211335;ENSMUST00000210839;ENSMUST0000020S
MSTRG.1	Cxadr	ENSMUST00000023572;ENSMUST00000114229
MSTRG.1	Btg3	ENSMUST00000023570;ENSMUST00000148124
MSTRG.1	D16Ertd	MSTRG.12913.1;MSTRG.12913.2;MSTRG.12913.3;ENSMUST0000
MSTRG.1	D16Ertd	MSTRG.12914.1
MSTRG.12915		MSTRG.12915.1
MSTRG.12916		MSTRG.12916.1
MSTRG.12917		MSTRG.12917.2;MSTRG.12917.1
MSTRG.1	Mir155h	ENSMUST00000180991;ENSMUST00000083463

MSTRG.1	Mir155h	MSTRG.12919.1
MSTRG.1	Lct	ENSMUST00000073490
MSTRG.1	Mrpl39	ENSMUST00000116584;MSTRG.12920.2;ENSMUST00000133901
MSTRG.1	App	MSTRG.12921.3;MSTRG.12921.1;MSTRG.12921.2;ENSMUST0000
MSTRG.1	Gm27295	ENSMUST00000184264
MSTRG.1	Jam2	ENSMUST00000114195;ENSMUST00000098407;ENSMUST0000013
MSTRG.1	Gm25908	ENSMUST00000157624
MSTRG.1	Atp5j	ENSMUST00000114193;ENSMUST00000144799;ENSMUST0000011
MSTRG.1	Gabpa	ENSMUST00000114184;MSTRG.12926.1;MSTRG.12926.2;ENSMUS
MSTRG.12927		MSTRG.12927.1
MSTRG.1	Cyyr1;G	ENSMUST00000114174;ENSMUST00000163020;ENSMUST0000016
MSTRG.1	Cyyr1	MSTRG.12929.1
MSTRG.1	Mcm6	ENSMUST00000027601;ENSMUST00000190495;ENSMUST0000019
MSTRG.12930		MSTRG.12930.1
MSTRG.12931		MSTRG.12931.1
MSTRG.1	Rwdd2b	ENSMUST00000039101;ENSMUST00000152430
MSTRG.1	N6amt1	ENSMUST00000054442;ENSMUST00000118310;ENSMUST0000012
MSTRG.1	Ltn1	MSTRG.12934.1;ENSMUST00000039449;MSTRG.12934.3;MSTRG.
MSTRG.1	Ltn1	MSTRG.12935.1
MSTRG.1	Gm25896	ENSMUST00000083713
MSTRG.1	Map3k7c	ENSMUST00000026700
MSTRG.12938		MSTRG.12938.1
MSTRG.1	Usp16	ENSMUST00000026710;ENSMUST00000119504;ENSMUST0000013
MSTRG.1	Mcm6	MSTRG.1294.1
MSTRG.1	Cct8	MSTRG.12940.1;ENSMUST00000026704;MSTRG.12940.3;ENSMUS
MSTRG.1	Cct8	MSTRG.12941.1
MSTRG.1	B130034	ENSMUST00000176296;ENSMUST00000175717
MSTRG.1	Bach1	ENSMUST00000026703;ENSMUST00000151046;ENSMUST0000015
MSTRG.1	Bach1	MSTRG.12944.1
MSTRG.12945		MSTRG.12945.1
MSTRG.1	Tiam1	ENSMUST00000114122;ENSMUST00000002588;ENSMUST0000016
MSTRG.12947		MSTRG.12947.1
MSTRG.12948		MSTRG.12948.1
MSTRG.12949		MSTRG.12949.1
MSTRG.1	Dars	MSTRG.1295.1;ENSMUST00000027602;ENSMUST00000186398;MS
MSTRG.12950		MSTRG.12950.1
MSTRG.1	Hunk	ENSMUST00000065856
MSTRG.1	Sod1	ENSMUST00000023707
MSTRG.1	Scaf4	ENSMUST00000163419;MSTRG.12953.2;MSTRG.12953.3;MSTRG.
MSTRG.1	Scaf4	MSTRG.12954.1
MSTRG.1	Scaf4	MSTRG.12955.1
MSTRG.1	Mis18a	ENSMUST00000099554;ENSMUST00000129171;MSTRG.12956.3;E
MSTRG.1	Gm17518	ENSMUST00000181232
MSTRG.1	Mrap	ENSMUST00000133973;ENSMUST00000138984;ENSMUST0000003
MSTRG.1	Urb1	ENSMUST00000140920;ENSMUST00000140158;ENSMUST0000014
MSTRG.1	Dars	MSTRG.1296.1
MSTRG.1	Eva1c	ENSMUST00000099548;ENSMUST00000130868;ENSMUST0000015
MSTRG.12961		MSTRG.12961.1
MSTRG.1	1110004	ENSMUST00000023694;ENSMUST00000129345;ENSMUST0000012
MSTRG.1	Synj1	ENSMUST00000121759;MSTRG.12963.2;ENSMUST00000170853;E
MSTRG.1	Gm22933	ENSMUST00000175515
MSTRG.1	Gm15965	ENSMUST00000123318
MSTRG.1	4930404	ENSMUST00000154854;ENSMUST00000125902
MSTRG.1	Paxbp1	ENSMUST00000118522;ENSMUST00000023698;ENSMUST0000015
MSTRG.1	4931406	ENSMUST00000147848
MSTRG.1	4932438	ENSMUST00000035689;ENSMUST00000146047
MSTRG.1	Dars	MSTRG.1297.1

MSTRG.1	Ifnar2;(	ENSMUST00000023693;MSTRG.12970.1;ENSMUST00000134491;E
MSTRG.1	Ifnar2	MSTRG.12971.1
MSTRG.1	Gm21970	MSTRG.12972.1
MSTRG.1	Gm7856	MSTRG.12973.1
MSTRG.12974		MSTRG.12974.1
MSTRG.1	A930006	ENSMUST00000149172;MSTRG.12975.1;ENSMUST00000023689;E
MSTRG.1	A930006	MSTRG.12976.1
MSTRG.1	Gm7860	ENSMUST00000121190
MSTRG.1	Ifngr2	MSTRG.12978.1;MSTRG.12978.2;ENSMUST00000023687;ENSMUS
MSTRG.1	Tmem50b	ENSMUST00000023686;ENSMUST00000133180;ENSMUST00000141
MSTRG.1	Acmsd	ENSMUST00000038006;ENSMUST00000186537
MSTRG.1	Gm15964	ENSMUST00000138207
MSTRG.1	Dnajc28	ENSMUST00000169982;ENSMUST00000049244;ENSMUST00000143
MSTRG.1	Gart	ENSMUST00000023684;ENSMUST00000156713;MSTRG.12982.2;M
MSTRG.1	Mrps6;S	ENSMUST00000047429;ENSMUST00000113975;ENSMUST00000131
MSTRG.1	Mrps6	MSTRG.12984.1
MSTRG.1	Gm16310	ENSMUST00000124376
MSTRG.1	Smim11	ENSMUST00000063641;ENSMUST00000118064
MSTRG.1	1700048	ENSMUST00000132861
MSTRG.1	Rcan1	ENSMUST00000023672;ENSMUST00000060005
MSTRG.1	Son	ENSMUST00000117633;ENSMUST00000119368;ENSMUST00000114
MSTRG.1	2900009	ENSMUST00000185310
MSTRG.1	Donson;.	ENSMUST00000133942;ENSMUST00000023682;ENSMUST00000114
MSTRG.1	Gm10785	ENSMUST00000156242
MSTRG.1	Cryzl1	ENSMUST00000073466;ENSMUST00000117644;ENSMUST0000014C
MSTRG.1	Gm10785	MSTRG.12993.1
MSTRG.1	Itsn1	ENSMUST00000114001;ENSMUST00000113999;ENSMUST00000064
MSTRG.1	Itsn1	MSTRG.12995.1
MSTRG.1	Gm25245	ENSMUST00000178139
MSTRG.1	Gm15976	ENSMUST00000129701
MSTRG.1	D430001	ENSMUST00000141344
MSTRG.1	Gm27773	ENSMUST00000184087
MSTRG.1	Gm6104	ENSMUST00000078030
MSTRG.1	Gm7568	ENSMUST00000161626;ENSMUST00000160446
MSTRG.1	2900009	MSTRG.1300.1
MSTRG.1	Runx1	MSTRG.13000.1;ENSMUST00000023673;ENSMUST00000113956;M
MSTRG.1	Gm26626	MSTRG.13001.1;ENSMUST00000180989
MSTRG.1	Runx1	MSTRG.13002.1;MSTRG.13002.2;MSTRG.13002.3;MSTRG.13002
MSTRG.1	Runx1	MSTRG.13003.1
MSTRG.1	Runx1	MSTRG.13004.1
MSTRG.1	Cbr1	ENSMUST00000039659
MSTRG.1	Cbr1	MSTRG.13006.1
MSTRG.1	Setd4	MSTRG.13008.1;ENSMUST00000123756;ENSMUST00000140619;E
MSTRG.13009		MSTRG.13009.1
MSTRG.1	Ccnt2	ENSMUST00000125760;ENSMUST00000126850;ENSMUST0000014S
MSTRG.1	Setd4	MSTRG.13010.1
MSTRG.1	Cbr3	ENSMUST00000039620
MSTRG.13012		MSTRG.13012.1
MSTRG.1	Dopey2	ENSMUST00000045004;MSTRG.13014.2;MSTRG.13014.3;MSTRG.
MSTRG.1	2310043	ENSMUST00000180967
MSTRG.1	Morc3	ENSMUST00000202261;ENSMUST00000044068;ENSMUST00000201
MSTRG.1	Chaf1b	ENSMUST00000023666;ENSMUST00000117099;ENSMUST00000124
MSTRG.1	Hlcs	ENSMUST00000163193;ENSMUST00000099512;MSTRG.13018.2
MSTRG.1	Hlcs	MSTRG.13019.1
MSTRG.1	Map3k19	MSTRG.1302.1
MSTRG.1	Gm23062	ENSMUST00000102349
MSTRG.1	Hlcs	MSTRG.13021.1

MSTRG.1	Hlcs	MSTRG.13022.1
MSTRG.1	Hlcs	MSTRG.13023.1
MSTRG.1	Hlcs	MSTRG.13024.1
MSTRG.1	Hlcs	MSTRG.13025.1
MSTRG.13026		MSTRG.13026.1
MSTRG.1	Dyrk1a;(	MSTRG.13027.1;MSTRG.13027.2;MSTRG.13027.3;MSTRG.13027
MSTRG.1	Pigp;Gm	ENSMUST00000113917;ENSMUST00000117891;ENSMUST00000113
MSTRG.1	Ttc3;Gm	ENSMUST00000117648;ENSMUST00000147352;ENSMUST0000014(
MSTRG.1	Map3k19	ENSMUST00000187653;ENSMUST00000061512;ENSMUST0000020(
MSTRG.1	Dscr3	ENSMUST00000023615;ENSMUST00000125229;ENSMUST00000127
MSTRG.13031		MSTRG.13031.1
MSTRG.1	Kcnj15	ENSMUST00000113861;ENSMUST00000113854;ENSMUST00000037
MSTRG.1	Ets2	ENSMUST00000023612;ENSMUST00000145509;MSTRG.13033.2;E
MSTRG.1	Gm37259	ENSMUST00000193903
MSTRG.13035		MSTRG.13035.1;MSTRG.13035.2
MSTRG.13036		MSTRG.13036.1
MSTRG.1	2810404	ENSMUST00000156615
MSTRG.1	1600002	ENSMUST00000136744;ENSMUST00000127413
MSTRG.1	1600002	MSTRG.13039.1
MSTRG.1	Rab3gap	ENSMUST00000037649;ENSMUST00000186865;ENSMUST00000142
MSTRG.1	Gm15340	ENSMUST00000144984
MSTRG.1	1600002	MSTRG.13041.1
MSTRG.1	1600002	MSTRG.13042.1
MSTRG.1	1600002	MSTRG.13043.1
MSTRG.1	Hmgn1	ENSMUST00000050884;ENSMUST00000145713;ENSMUST00000133
MSTRG.1	Wrb	ENSMUST00000138198;ENSMUST00000140476;ENSMUST00000023
MSTRG.1	Gm15317	ENSMUST00000129569
MSTRG.1	Lca5l	ENSMUST00000113804;ENSMUST00000154445
MSTRG.1	B3galt5	ENSMUST00000113800;ENSMUST00000153586;ENSMUST00000134
MSTRG.1	Psmg1;B	ENSMUST00000023630;MSTRG.13049.1;ENSMUST00000117044;E
MSTRG.1	Rab3gap	MSTRG.1305.1
MSTRG.1	Gm15342	MSTRG.13050.1;ENSMUST00000146222
MSTRG.1	Gm27833	ENSMUST00000184285
MSTRG.1	Brwd1	MSTRG.13052.1
MSTRG.1	1700093	MSTRG.13053.1;ENSMUST00000143671
MSTRG.1	Gm15341	ENSMUST00000137315
MSTRG.1	Fam3b	ENSMUST00000125475;ENSMUST00000049721
MSTRG.1	Gm9242	ENSMUST00000177820
MSTRG.1	Mx1	ENSMUST00000135184;ENSMUST00000155233;ENSMUST00000023
MSTRG.1	Mx2	ENSMUST00000190447;ENSMUST00000188251;ENSMUST00000024
MSTRG.1	Rab3gap	MSTRG.1306.1
MSTRG.1	Mx2	MSTRG.13060.1
MSTRG.1	Tmprss2	ENSMUST00000000395
MSTRG.1	Ripk4	ENSMUST00000113743;ENSMUST00000019386
MSTRG.1	Zbtb21	ENSMUST00000052089;ENSMUST00000063605;ENSMUST00000113
MSTRG.1	Prdm15	ENSMUST00000095849;ENSMUST00000121584;ENSMUST00000142
MSTRG.1	Prdm15	MSTRG.13065.1
MSTRG.1	C2cd2	ENSMUST00000170757
MSTRG.1	C2cd2	MSTRG.13067.1
MSTRG.1	Mir6964	ENSMUST00000200252
MSTRG.13069		MSTRG.13069.1
MSTRG.1	Zranb3	ENSMUST00000086614;ENSMUST00000112538;ENSMUST00000143
MSTRG.1	Gm10232	ENSMUST00000089221
MSTRG.1	C030010	ENSMUST00000181465
MSTRG.1	B230307	ENSMUST00000122450;MSTRG.13072.1;ENSMUST00000183578;E
MSTRG.13073		MSTRG.13073.1
MSTRG.13074		MSTRG.13074.1

MSTRG.13075		MSTRG.13075.1
MSTRG.13076		MSTRG.13076.1
MSTRG.13077		MSTRG.13077.1
MSTRG.1	Gm16569	ENSMUST00000160526
MSTRG.1	Gm5479	ENSMUST00000160578
MSTRG.1	Gm28800	ENSMUST00000186230
MSTRG.1	Gm25909	ENSMUST00000157619
MSTRG.13081		MSTRG.13081.1
MSTRG.1	Pisd-ps	MSTRG.13082.1;ENSMUST00000142526;ENSMUST00000184936;E
MSTRG.1	Tiam2	ENSMUST00000169838
MSTRG.1	Tfb1m	ENSMUST00000041003
MSTRG.1	Cldn20	ENSMUST00000168560
MSTRG.1	Scaf8	MSTRG.13086.2;MSTRG.13086.1
MSTRG.1	Scaf8	ENSMUST00000076734;MSTRG.13087.2;MSTRG.13087.3;MSTRG.
MSTRG.1	Scaf8	MSTRG.13088.1
MSTRG.13089		MSTRG.13089.1
MSTRG.1	Gm38248	ENSMUST00000193744
MSTRG.13090		MSTRG.13090.1
MSTRG.13091		MSTRG.13091.1
MSTRG.13092		MSTRG.13092.1
MSTRG.1	Gm29050	ENSMUST00000190865;ENSMUST00000190732
MSTRG.1	Tmem242	ENSMUST00000005053;MSTRG.13094.1;ENSMUST00000188282;E
MSTRG.1	Gm22475	ENSMUST00000175169
MSTRG.1	Tmem242	MSTRG.13097.1
MSTRG.1	Gm10231	ENSMUST00000181584
MSTRG.1	Gm26622	ENSMUST00000180520
MSTRG.1	Stau2	MSTRG.131.1
MSTRG.1	Zranb3	MSTRG.1310.1
MSTRG.1	Snx9	ENSMUST00000002436
MSTRG.1	Zdhhc14	ENSMUST00000089185;MSTRG.13101.1;MSTRG.13101.2;MSTRG.
MSTRG.1	Zdhhc14	MSTRG.13102.1
MSTRG.1	Zdhhc14	MSTRG.13103.1;MSTRG.13103.2;MSTRG.13103.3
MSTRG.1	Zdhhc14	MSTRG.13104.1;MSTRG.13104.2;MSTRG.13104.3
MSTRG.1	Zdhhc14	MSTRG.13105.1
MSTRG.1	Zdhhc14	MSTRG.13106.1
MSTRG.1	Zdhhc14	MSTRG.13107.1
MSTRG.1	Zdhhc14	MSTRG.13108.1
MSTRG.1	Zdhhc14	MSTRG.13109.1
MSTRG.1	Zranb3	MSTRG.1311.1
MSTRG.1	Zdhhc14	MSTRG.13110.1
MSTRG.1	Zdhhc14	MSTRG.13111.1
MSTRG.1	Arid1b	MSTRG.13112.1;ENSMUST00000124362;ENSMUST00000092723;E
MSTRG.1	Arid1b	MSTRG.13113.1
MSTRG.1	Gm15599	ENSMUST00000122283
MSTRG.1	Arid1b	MSTRG.13115.1
MSTRG.1	Arid1b	MSTRG.13116.1
MSTRG.1	Arid1b	MSTRG.13117.1;MSTRG.13117.2
MSTRG.1	Arid1b	MSTRG.13118.1
MSTRG.1	Synj2	ENSMUST00000146009;ENSMUST00000115791;MSTRG.13119.1;E
MSTRG.1	Rpl28-p	ENSMUST00000121497
MSTRG.1	Synj2	MSTRG.13120.1
MSTRG.1	Serac1	ENSMUST00000097432;ENSMUST00000153325;ENSMUST00000024
MSTRG.1	Serac1	MSTRG.13122.1
MSTRG.1	Serac1	MSTRG.13123.1
MSTRG.1	Gtf2h5	ENSMUST00000039487;ENSMUST00000146035;MSTRG.13124.3;E
MSTRG.1	Gtf2h5	MSTRG.13125.1
MSTRG.13126		MSTRG.13126.1

MSTRG.13127		MSTRG.13127.1
MSTRG.1	Tulp4	ENSMUST00000149838;ENSMUST00000142030;ENSMUST00000151
MSTRG.1	Zranb3	MSTRG.1313.1
MSTRG.1	Gm22774	ENSMUST00000102084;ENSMUST00000078419
MSTRG.1	n-R5s26	ENSMUST00000104601
MSTRG.1	Ppp1r2-	ENSMUST00000179333
MSTRG.1	Gm2885	MSTRG.13133.1;ENSMUST00000181895
MSTRG.1	Gm2885	MSTRG.13134.1
MSTRG.1	Rsph3b	ENSMUST00000024572
MSTRG.1	Tagap1	ENSMUST00000063683;MSTRG.13136.1
MSTRG.1	Rnaset2	ENSMUST00000089119;MSTRG.13137.1;ENSMUST00000179728;M
MSTRG.1	Gm25119	ENSMUST00000158815
MSTRG.1	Rps6ka2	ENSMUST00000024575
MSTRG.1	Gm37625	ENSMUST00000194969
MSTRG.1	Tmem181	ENSMUST00000088940;MSTRG.13140.2;MSTRG.13140.3;MSTRG.
MSTRG.1	Tmem181	MSTRG.13141.1
MSTRG.1	Dynlt1c	MSTRG.13142.1
MSTRG.1	Dynlt1a	ENSMUST00000169415
MSTRG.1	Dynlt1a	MSTRG.13144.1
MSTRG.1	Gm26848	ENSMUST00000181052;MSTRG.13145.2
MSTRG.1	Ppp1r2-	ENSMUST00000178232
MSTRG.1	Dynlt1b	ENSMUST00000179569;MSTRG.13147.2;MSTRG.13147.3;MSTRG.
MSTRG.1	Tmem181	MSTRG.13148.1;ENSMUST00000180035;MSTRG.13148.2;MSTRG.
MSTRG.1	Dynlt1c	ENSMUST00000092966;MSTRG.13149.1;ENSMUST00000149027;E
MSTRG.1	Zranb3	MSTRG.1315.1
MSTRG.1	Dynlt1f	ENSMUST00000179554
MSTRG.1	Ezr	MSTRG.13151.3;MSTRG.13151.1;ENSMUST00000064234;MSTRG.
MSTRG.1	E430024	ENSMUST00000160177;ENSMUST00000160545
MSTRG.1	Tagap	ENSMUST00000036370
MSTRG.1	Rsph3a	ENSMUST00000097423
MSTRG.1	Rnaset2	MSTRG.13155.1;ENSMUST00000097422;MSTRG.13155.3;MSTRG.
MSTRG.1	Gm22416	ENSMUST00000158590
MSTRG.1	Fgfr1op	ENSMUST00000097419;ENSMUST00000162586;MSTRG.13157.3;M
MSTRG.1	Ccr6	MSTRG.13158.1
MSTRG.1	Ccr6	MSTRG.13159.1;ENSMUST00000164411;ENSMUST00000097418;E
MSTRG.1	Gm38026	ENSMUST00000193284
MSTRG.1	Ccr6	MSTRG.13160.1
MSTRG.1	Mpc1;Sf	ENSMUST00000145402;ENSMUST00000155364;ENSMUST00000147
MSTRG.1	4930506	MSTRG.13162.1;ENSMUST00000130639;ENSMUST00000123009;E
MSTRG.1	Sft2d1	MSTRG.13163.1
MSTRG.1	Prr18	ENSMUST00000069742;ENSMUST00000163887
MSTRG.1	Gm16702	ENSMUST00000181180
MSTRG.1	Pde10a	ENSMUST00000115715;ENSMUST00000115722;ENSMUST00000115
MSTRG.1	Gm15425	ENSMUST00000119013
MSTRG.1	Gm17087	ENSMUST00000184255;ENSMUST00000184183
MSTRG.1	R3hdm1	ENSMUST00000191016;MSTRG.1317.1;ENSMUST00000188381;EN
MSTRG.1	Gm17748	ENSMUST00000177906
MSTRG.1	Gm17728	ENSMUST00000076982
MSTRG.1	Qk	MSTRG.13172.1
MSTRG.1	Qk	MSTRG.13173.1;MSTRG.13173.2;ENSMUST00000097414;ENSMUS
MSTRG.1	Qk	MSTRG.13174.1
MSTRG.1	Qk	MSTRG.13175.1
MSTRG.1	Pacrg	ENSMUST00000041463
MSTRG.1	A230009	ENSMUST00000160763;ENSMUST00000159602
MSTRG.1	Gm16168	ENSMUST00000161274;ENSMUST00000161091
MSTRG.1	Gm16169	ENSMUST00000162432
MSTRG.1	Gm29383	ENSMUST00000188284

MSTRG.1	Park2	ENSMUST00000066658;ENSMUST00000186167;ENSMUST00000191
MSTRG.1	Park2	MSTRG.13181.1
MSTRG.1	Gm3145	ENSMUST00000188584
MSTRG.1	Park2	MSTRG.13183.1
MSTRG.1	Gm28505	ENSMUST00000190466
MSTRG.1	Park2	MSTRG.13185.1
MSTRG.1	Park2	MSTRG.13186.1
MSTRG.1	Gm37402	ENSMUST00000194525
MSTRG.1	Gm37332	ENSMUST00000195853
MSTRG.1	Gm28118	ENSMUST00000185957
MSTRG.1	Gm37588	ENSMUST00000195024
MSTRG.1	Gm10513	ENSMUST00000194203
MSTRG.1	Agpat4	ENSMUST00000164143;ENSMUST00000024594;ENSMUST00000172
MSTRG.1	Agpat4	MSTRG.13192.1
MSTRG.1	Agpat4	MSTRG.13193.1
MSTRG.1	Agpat4	MSTRG.13194.1
MSTRG.1	Agpat4	MSTRG.13195.1
MSTRG.1	Tdgf1-p	ENSMUST00000074412
MSTRG.13197		MSTRG.13197.1
MSTRG.1	Map3k4	MSTRG.13198.1;ENSMUST00000089058
MSTRG.1	Map3k4	MSTRG.13199.1
MSTRG.1	Stau2	MSTRG.132.1
MSTRG.1	Gm37868	ENSMUST00000195578
MSTRG.1	Gm24794	ENSMUST00000104288
MSTRG.1	Map3k4	MSTRG.13201.1
MSTRG.1	4732491	ENSMUST00000181774
MSTRG.1	Pnldc1	ENSMUST00000163394
MSTRG.1	Pnldc1	MSTRG.13204.1
MSTRG.1	Igf2r	ENSMUST00000024599;ENSMUST00000161738;MSTRG.13205.3;E
MSTRG.1	Airn;Gm	MSTRG.13206.2;MSTRG.13206.1;ENSMUST00000160932;ENSMUS
MSTRG.1	Airn	MSTRG.13207.1
MSTRG.1	Gm23833	MSTRG.13208.1
MSTRG.1	Gm16574	ENSMUST00000159301
MSTRG.1	Gm37407	ENSMUST00000194793
MSTRG.1	Gm7162	ENSMUST00000161205
MSTRG.1	Airn	MSTRG.13211.1
MSTRG.1	Airn	MSTRG.13212.1
MSTRG.1	Airn	MSTRG.13213.1
MSTRG.1	Mas1	ENSMUST00000089015;ENSMUST00000165020;ENSMUST00000167
MSTRG.1	Gm24085	ENSMUST00000175172
MSTRG.1	Mrpl18	MSTRG.13216.1;ENSMUST00000079121;ENSMUST00000159765;E
MSTRG.1	Tcp1;Gm	ENSMUST00000147724;ENSMUST00000089024;ENSMUST00000151
MSTRG.1	Snora20	ENSMUST00000082703
MSTRG.1	Acat3	ENSMUST00000043923;ENSMUST00000160921
MSTRG.1	Mir128-	ENSMUST00000083586
MSTRG.1	Acat2	ENSMUST00000007005;ENSMUST00000159697;ENSMUST0000015
MSTRG.1	Gm15946	ENSMUST00000160654
MSTRG.1	Wtap	ENSMUST00000159551;ENSMUST00000007007;MSTRG.13222.3;M
MSTRG.13223		MSTRG.13223.1
MSTRG.1	Dact2	ENSMUST00000053218;MSTRG.13224.2
MSTRG.1	Smoc2	ENSMUST00000024660
MSTRG.1	Thbs2	ENSMUST00000170872
MSTRG.1	Gm3222	ENSMUST00000071282
MSTRG.1	Sod2	ENSMUST00000007012
MSTRG.1	Gpr31b	ENSMUST00000091648
MSTRG.1	Thsd7b	ENSMUST00000148051;ENSMUST00000073527;ENSMUST0000004
MSTRG.1	Tcp10b	ENSMUST00000086787;ENSMUST00000116666

MSTRG.1	Unc93a	ENSMUST00000084966
MSTRG.1	Gm23050	ENSMUST00000158775
MSTRG.1	Smok2a	ENSMUST00000080647
MSTRG.1	Smok2b	ENSMUST00000059824
MSTRG.1	Tcp10c	ENSMUST00000097403
MSTRG.1	Gm8597	ENSMUST00000191525
MSTRG.1	Gm25730	ENSMUST00000178753
MSTRG.13239		MSTRG.13239.1
MSTRG.1	Gm38240	ENSMUST00000195110
MSTRG.1	Rnu6	ENSMUST00000178023
MSTRG.1	Tcte2	ENSMUST00000138345;ENSMUST00000137459;ENSMUST0000018[illegible]
MSTRG.1	Gm16050	ENSMUST00000160626
MSTRG.1	Gm8603	ENSMUST00000115649
MSTRG.1	Smok4a	ENSMUST00000144015;ENSMUST00000074512
MSTRG.1	Gm26375	ENSMUST00000101829
MSTRG.1	Tcte2	MSTRG.13246.1
MSTRG.1	Tcte2	MSTRG.13247.1
MSTRG.1	Gm24867	ENSMUST00000104161
MSTRG.1	Gm16046	ENSMUST00000145030;ENSMUST00000150889
MSTRG.1	Gm38330	ENSMUST00000192743
MSTRG.1	Gm16052	ENSMUST00000129681;ENSMUST00000155166
MSTRG.1	Afdn	ENSMUST00000139347;ENSMUST00000156591;ENSMUST0000017[illegible]
MSTRG.1	Afdn	MSTRG.13252.1
MSTRG.1	Afdn	MSTRG.13253.1
MSTRG.1	Gm3355	ENSMUST00000160079
MSTRG.1	Wdr27	ENSMUST00000170386
MSTRG.1	Wdr27	MSTRG.13256.1
MSTRG.1	Wdr27	MSTRG.13257.1
MSTRG.1	Wdr27	MSTRG.13258.1
MSTRG.1	Dll1	ENSMUST00000014917;ENSMUST00000140784;ENSMUST00000152
MSTRG.1	Gm36951	ENSMUST00000193840
MSTRG.1	A930024[illegible]	ENSMUST00000181251
MSTRG.1	Gm7393	ENSMUST00000172650
MSTRG.1	1600012[illegible]	ENSMUST00000052691;ENSMUST00000164837;ENSMUST00000174
MSTRG.1	Phf10	ENSMUST00000024657;ENSMUST00000171526;ENSMUST00000172
MSTRG.1	Gm3448	ENSMUST00000097399;MSTRG.13264.2
MSTRG.1	Ermard	MSTRG.13265.1;ENSMUST00000061688;MSTRG.13265.4;MSTRG.
MSTRG.1	Gm3448;[illegible]	ENSMUST00000179539;ENSMUST00000097400;ENSMUST0000004[illegible]
MSTRG.1	Ermard;[illegible]	MSTRG.13267.1;MSTRG.13267.3;MSTRG.13267.2;MSTRG.13267
MSTRG.1	Fam120b	MSTRG.13268.3;ENSMUST00000055352;MSTRG.13268.1;MSTRG.
MSTRG.1	Fam120b	MSTRG.13269.1
MSTRG.1	Gm15790	ENSMUST00000161109
MSTRG.1	Psmb1	MSTRG.13270.1;ENSMUST00000014913
MSTRG.1	Tbp	ENSMUST00000159197;ENSMUST00000014911;ENSMUST00000162
MSTRG.1	Pdcd2	ENSMUST00000154293;ENSMUST00000054450;ENSMUST0000012[illegible]
MSTRG.1	Rgmb	ENSMUST00000170578
MSTRG.1	Rgmb	MSTRG.13274.1
MSTRG.13275		MSTRG.13275.1
MSTRG.13276		MSTRG.13276.1
MSTRG.1	Chd1	ENSMUST00000024627;ENSMUST00000173311;MSTRG.13277.2;[illegible]
MSTRG.1	Chd1	MSTRG.13278.1
MSTRG.1	Chd1	MSTRG.13279.1
MSTRG.1	Rpl31-p	ENSMUST00000086596
MSTRG.1	Chd1	MSTRG.13280.2;MSTRG.13280.1;MSTRG.13280.3
MSTRG.1	Oaz1-ps	ENSMUST00000052440
MSTRG.1	Chd1	MSTRG.13282.1;ENSMUST00000024620
MSTRG.1	Chd1	MSTRG.13283.1

MSTRG.1	Gm26873	ENSMUST00000181390;MSTRG.13284.2
MSTRG.1	BC00205	ENSMUST00000190415;MSTRG.13285.2;ENSMUST00000071374
MSTRG.1	Zfp97	MSTRG.13286.1;MSTRG.13286.2
MSTRG.1	BC00205	MSTRG.13287.1
MSTRG.1	Zfp97	MSTRG.13288.1;MSTRG.13288.2
MSTRG.1	Zfp97;G	MSTRG.13289.1;MSTRG.13289.2;MSTRG.13289.3;ENSMUST0000
MSTRG.1	Gm37479	ENSMUST00000194169
MSTRG.1	Zfp960;	MSTRG.13290.1;MSTRG.13290.2
MSTRG.13291		MSTRG.13291.1
MSTRG.1	Zfp97	MSTRG.13292.1
MSTRG.1	Zfp97	MSTRG.13293.1
MSTRG.1	Zfp960	ENSMUST00000127027
MSTRG.1	Zfp97	ENSMUST00000147630
MSTRG.1	Zfp97	MSTRG.13296.1
MSTRG.1	Zfp97	MSTRG.13297.1
MSTRG.13298		MSTRG.13298.1
MSTRG.1	Gm6712	MSTRG.13299.1
MSTRG.1	Ube2w	ENSMUST00000182554;ENSMUST00000182080;MSTRG.133.2;ENS
MSTRG.1	Gm37064	ENSMUST00000193851
MSTRG.13300		MSTRG.13300.1
MSTRG.1	Lnpep	MSTRG.13301.1;MSTRG.13301.2;ENSMUST00000041047;MSTRG.
MSTRG.1	Lnpep	MSTRG.13302.1
MSTRG.1	Gm25927	MSTRG.13303.1
MSTRG.1	Mir99b	ENSMUST00000083462
MSTRG.1	Gm25927	ENSMUST00000175058
MSTRG.1	Mirlet7	ENSMUST00000196737
MSTRG.1	Spaca6;	ENSMUST00000133912;ENSMUST00000083545;ENSMUST0000017
MSTRG.1	Has1	ENSMUST00000003762
MSTRG.13309		MSTRG.13309.1
MSTRG.1	Gm37278	ENSMUST00000191619
MSTRG.13311		MSTRG.13311.1
MSTRG.1	Ppp2r1a	ENSMUST00000007708;ENSMUST00000173658;ENSMUST0000017
MSTRG.1	Zfp160	MSTRG.13313.1;ENSMUST00000088811;MSTRG.13313.2;MSTRG.
MSTRG.1	Zfp760	ENSMUST00000073312
MSTRG.1	Zfp760	MSTRG.13315.1
MSTRG.1	Zfp760	MSTRG.13316.1
MSTRG.1	Zfp677	ENSMUST00000056107;ENSMUST00000162659
MSTRG.1	Zfp677	MSTRG.13318.1
MSTRG.1	Zfp54	ENSMUST00000165230;ENSMUST00000007884;ENSMUST0000016
MSTRG.1	Gm29428	ENSMUST00000190086
MSTRG.1	Zfp51	ENSMUST00000039577;MSTRG.13320.2
MSTRG.13321		MSTRG.13321.1
MSTRG.1	Gm26753	ENSMUST00000181938
MSTRG.1	Zfp53	MSTRG.13323.1;ENSMUST00000076664;MSTRG.13323.2
MSTRG.1	Zfp53	MSTRG.13324.1
MSTRG.1	Zfp53	MSTRG.13325.1
MSTRG.1	9330136	ENSMUST00000180842
MSTRG.1	Zfp52	ENSMUST00000079242
MSTRG.1	Zfp948	ENSMUST00000088787
MSTRG.1	Zfp983	MSTRG.13329.1;ENSMUST00000039726
MSTRG.1	Pigr	ENSMUST00000027675;ENSMUST00000133792;ENSMUST0000013
MSTRG.1	Gm10509	MSTRG.13330.1;ENSMUST00000097384;ENSMUST00000072133
MSTRG.13331		MSTRG.13331.1
MSTRG.13332		MSTRG.13332.1
MSTRG.13333		MSTRG.13333.1
MSTRG.1	Gm5224	MSTRG.13334.1
MSTRG.13335		MSTRG.13335.1

MSTRG.1	Gm5493	ENSMUST00000182063
MSTRG.1	Zfp820	ENSMUST00000084141
MSTRG.1	Zfp995	ENSMUST00000106026;ENSMUST00000190066;ENSMUST0000018[illegible]
MSTRG.1	Zfp995	MSTRG.13339.1
MSTRG.1	Fcmr	ENSMUST00000038829;ENSMUST00000149355;ENSMUST0000012[illegible]
MSTRG.1	Zfp942	ENSMUST00000091879;ENSMUST00000074295;ENSMUST0000012[illegible]
MSTRG.1	Zfp942	MSTRG.13341.1
MSTRG.1	Zfp942	MSTRG.13342.1
MSTRG.1	Zfp943	ENSMUST00000055349;ENSMUST00000153985;ENSMUST00000174
MSTRG.1	Gm7805	ENSMUST00000154348
MSTRG.1	Gm7809	ENSMUST00000153199
MSTRG.1	Gm9772	ENSMUST00000075018
MSTRG.1	Gm7052	ENSMUST00000167568
MSTRG.1	Zfp943	MSTRG.13348.1
MSTRG.1	Zfp947	ENSMUST00000080249
MSTRG.1	Gm29427	ENSMUST00000188520;ENSMUST00000189393;ENSMUST00000171
MSTRG.1	Zfp994	ENSMUST00000179996
MSTRG.1	Zfp994	MSTRG.13351.1
MSTRG.13352		MSTRG.13352.1
MSTRG.13353		MSTRG.13353.1
MSTRG.1	Zfp944	ENSMUST00000115535;MSTRG.13354.2
MSTRG.1	Zfp758	ENSMUST00000088765;ENSMUST00000149699;MSTRG.13355.1;[illegible]
MSTRG.1	Zfp758	MSTRG.13356.1
MSTRG.1	Zfp758	MSTRG.13357.1
MSTRG.13358		MSTRG.13358.1
MSTRG.13359		MSTRG.13359.1
MSTRG.1	Gm29427	MSTRG.1336.1
MSTRG.13360		MSTRG.13360.1
MSTRG.1	Mmp25	ENSMUST00000024696
MSTRG.1	Bicdl2	ENSMUST00000062967
MSTRG.1	Thoc6	ENSMUST00000125443;ENSMUST00000047436;ENSMUST0000013[illegible]
MSTRG.1	Hcfc1r1	ENSMUST00000180140;ENSMUST00000024697;ENSMUST0000017[illegible]
MSTRG.1	Tnfrsf1	ENSMUST00000024698;ENSMUST00000167059
MSTRG.1	Cldn6	ENSMUST00000024699
MSTRG.1	Cldn9	ENSMUST00000085989
MSTRG.13368		MSTRG.13368.1
MSTRG.1	1520401	ENSMUST00000201734;ENSMUST00000062827;ENSMUST0000017[illegible]
MSTRG.1	Yod1	ENSMUST00000049813
MSTRG.1	Pkmyt1	ENSMUST00000024701
MSTRG.1	Paqr4	ENSMUST00000024702
MSTRG.1	Kremen2	ENSMUST00000046525
MSTRG.1	9530082	ENSMUST00000181291;ENSMUST00000181605;ENSMUST0000018[illegible]
MSTRG.1	Flywch1	MSTRG.13374.1;MSTRG.13374.2;ENSMUST00000086325;ENSMUS
MSTRG.1	Gm16275	ENSMUST00000147774
MSTRG.1	Flywch2	MSTRG.13376.1
MSTRG.1	Flywch2	ENSMUST00000024704;ENSMUST00000122991;ENSMUST0000012[illegible]
MSTRG.1	D930048	ENSMUST00000180975;ENSMUST00000186961;MSTRG.13378.2;[illegible]
MSTRG.1	Srrm2	MSTRG.13379.1;MSTRG.13379.2;MSTRG.13379.3
MSTRG.1	Gm37163	ENSMUST00000195439
MSTRG.1	Elob	ENSMUST00000069579;MSTRG.13380.1;MSTRG.13380.2
MSTRG.1	Kctd5	ENSMUST00000017090;ENSMUST00000127755
MSTRG.1	Pdpk1	MSTRG.13382.1;MSTRG.13382.2;ENSMUST00000102927;ENSMUS
MSTRG.1	Amdhd2	ENSMUST00000132944;MSTRG.13383.1;ENSMUST00000130520;[illegible]
MSTRG.1	Amdhd2	MSTRG.13384.1
MSTRG.1	Gm43796	ENSMUST00000202853;ENSMUST00000151061;ENSMUST00000024
MSTRG.1	BC02877	ENSMUST00000148704;ENSMUST00000137883
MSTRG.1	1600002	ENSMUST00000024930;ENSMUST00000137648;ENSMUST00000124

MSTRG.1	BC02877	MSTRG.13388.1
MSTRG.1	Ccnf;Mi	MSTRG.13389.3;MSTRG.13389.2;MSTRG.13389.1;ENSMUST0000
MSTRG.1	AA98686	ENSMUST00000039323;ENSMUST00000186185;ENSMUST0000019
MSTRG.1	Abca17	ENSMUST00000121226
MSTRG.1	Gm24427	ENSMUST00000116896
MSTRG.1	Abca3	ENSMUST00000133816;MSTRG.13392.1;ENSMUST00000039013;E
MSTRG.1	Gm25618	ENSMUST00000179484
MSTRG.1	Abca3	MSTRG.13394.1
MSTRG.1	Abca3	MSTRG.13395.1
MSTRG.1	D330041	MSTRG.13396.1;ENSMUST00000181014;ENSMUST00000181259;E
MSTRG.1	Rnps1;E	MSTRG.13397.3;MSTRG.13397.5;MSTRG.13397.4;MSTRG.13397
MSTRG.1	Eci1	MSTRG.13398.1
MSTRG.1	Dnase1l	ENSMUST00000153858;ENSMUST00000088506;ENSMUST0000011
MSTRG.1	Ube2w	MSTRG.134.1
MSTRG.1	Gm28857	ENSMUST00000188068
MSTRG.1	E4f1	ENSMUST00000056032;MSTRG.13400.2;MSTRG.13400.3;MSTRG.
MSTRG.13401		MSTRG.13401.1
MSTRG.1	Pgp	ENSMUST00000053024
MSTRG.1	Mlst8	ENSMUST00000070888;ENSMUST00000179163
MSTRG.1	Bricd5	ENSMUST00000054946
MSTRG.1	Caskin1	ENSMUST00000024958
MSTRG.1	Traf7;R	ENSMUST00000176652;ENSMUST00000177024;ENSMUST0000007
MSTRG.1	Rab26os	ENSMUST00000176921;ENSMUST00000176215;ENSMUST00000082
MSTRG.1	Zfp598	ENSMUST00000047179;MSTRG.13408.2
MSTRG.1	Pkd1	MSTRG.13409.1;MSTRG.13409.2;MSTRG.13409.3;ENSMUST0000
MSTRG.1	Mapkapk	ENSMUST00000016672;ENSMUST00000188459
MSTRG.1	Pkd1	MSTRG.13410.1
MSTRG.1	Gm24589	ENSMUST00000116692
MSTRG.1	Tsc2	ENSMUST00000097373;MSTRG.13412.3;MSTRG.13412.2;MSTRG.
MSTRG.1	Nthl1	ENSMUST00000047611
MSTRG.1	Slc9a3r	ENSMUST00000019684;ENSMUST00000002572
MSTRG.1	Gfer	ENSMUST00000046839
MSTRG.1	Noxo1	ENSMUST00000019464;ENSMUST00000126342;ENSMUST00000141
MSTRG.1	Tbl3	ENSMUST00000126319;ENSMUST00000046534;ENSMUST0000013
MSTRG.1	Rpl3l	ENSMUST00000170239;ENSMUST00000045186
MSTRG.1	Msrb1	ENSMUST00000115262;ENSMUST00000101800
MSTRG.1	Gm25549	ENSMUST00000158080
MSTRG.1	Hs3st6	ENSMUST00000044922
MSTRG.1	Rnf151	ENSMUST00000008626
MSTRG.1	Rps2;Sn	ENSMUST00000152407;MSTRG.13422.2;ENSMUST00000170715;E
MSTRG.1	Snhg9	ENSMUST00000161706
MSTRG.1	Snora78	ENSMUST00000158630
MSTRG.1	Ndufb10	ENSMUST00000141324;ENSMUST00000144285;ENSMUST0000012
MSTRG.1	Ndufb10	MSTRG.13426.1
MSTRG.1	Meiob	ENSMUST00000024972
MSTRG.1	Hagh	ENSMUST00000164251;ENSMUST00000118788;ENSMUST00000154
MSTRG.1	Fahd1	ENSMUST00000049642
MSTRG.1	Gm37084	ENSMUST00000191688
MSTRG.1	Hagh	MSTRG.13430.1
MSTRG.13431		MSTRG.13431.1
MSTRG.1	Hn1l	ENSMUST00000024981
MSTRG.1	Cramp1l	ENSMUST00000073337;MSTRG.13433.1;MSTRG.13433.3
MSTRG.1	Igfals	ENSMUST00000050714
MSTRG.1	Nubp2	ENSMUST00000044252;MSTRG.13435.1
MSTRG.1	Spsb3	ENSMUST00000117890;ENSMUST00000168265;ENSMUST0000006
MSTRG.1	Eme2;Ma	ENSMUST00000119848;MSTRG.13437.2;ENSMUST00000121542;E
MSTRG.1	Mrps34;	ENSMUST00000043907;ENSMUST00000154236;ENSMUST0000013

MSTRG.1	Mapk8ip	MSTRG.13439.1
MSTRG.1	Eif2d	ENSMUST00000139468;MSTRG.1344.1;ENSMUST00000112446;EN
MSTRG.1	Ift140	ENSMUST00000137386;ENSMUST00000024983;ENSMUST00000151
MSTRG.1	Ift140	MSTRG.13441.1
MSTRG.1	Ift140	MSTRG.13442.1
MSTRG.1	Tmem204	ENSMUST00000024984;ENSMUST00000153745
MSTRG.1	Ift140	MSTRG.13444.1
MSTRG.1	Ift140	MSTRG.13445.1
MSTRG.1	Ift140	MSTRG.13446.1
MSTRG.1	Telo2	ENSMUST00000155607;ENSMUST00000115181;ENSMUST00000024
MSTRG.1	Telo2	MSTRG.13448.1
MSTRG.1	BC00396	ENSMUST00000088307;MSTRG.13449.1
MSTRG.1	Rassf5	MSTRG.1345.2;ENSMUST00000068564;MSTRG.1345.1;ENSMUSTC
MSTRG.1	Clcn7	ENSMUST00000040729;ENSMUST00000162722;ENSMUST0000016C
MSTRG.1	Clcn7	MSTRG.13451.1
MSTRG.1	Clcn7	MSTRG.13452.1
MSTRG.1	Ccdc154	ENSMUST00000183178
MSTRG.1	Tpsb2	ENSMUST00000069616
MSTRG.13455		MSTRG.13455.1
MSTRG.1	Unkl	MSTRG.13456.1;ENSMUST00000039734;ENSMUST00000160896;M
MSTRG.1	Unkl	MSTRG.13457.1
MSTRG.1	Unkl	MSTRG.13458.1
MSTRG.1	Gnptg	ENSMUST00000115154;ENSMUST00000038973
MSTRG.1	Rassf5	MSTRG.1346.1
MSTRG.1	Tsr3	ENSMUST00000063574;MSTRG.13460.2
MSTRG.1	Baiap3	ENSMUST00000182126;ENSMUST00000182056;ENSMUST00000182
MSTRG.1	Mir3547	ENSMUST00000175461
MSTRG.1	Ube2i	MSTRG.13463.1;MSTRG.13463.2;ENSMUST00000049911;ENSMUS
MSTRG.1	Lmf1	ENSMUST00000063344;ENSMUST00000116641;ENSMUST0000012€
MSTRG.1	Lmf1	MSTRG.13465.1
MSTRG.1	Lmf1	MSTRG.13466.1
MSTRG.1	Lmf1	MSTRG.13467.1
MSTRG.1	Lmf1	MSTRG.13468.1
MSTRG.1	Lmf1	MSTRG.13469.1
MSTRG.1	Gm17796	ENSMUST00000189015
MSTRG.1	Rpusd1	ENSMUST00000047273;MSTRG.13470.1
MSTRG.1	Gng13	ENSMUST00000172002;ENSMUST00000115108
MSTRG.1	Chtf18	ENSMUST00000170575;ENSMUST00000048054;ENSMUST0000016€
MSTRG.1	Gm8186	ENSMUST00000053575
MSTRG.1	Decr2	ENSMUST00000040907;ENSMUST00000123056;ENSMUST0000015C
MSTRG.1	Nme4	ENSMUST00000025007;ENSMUST00000150280
MSTRG.1	Tmem8	ENSMUST00000025010;ENSMUST00000128597
MSTRG.1	Mrpl28;.	ENSMUST00000025014;ENSMUST00000123132;ENSMUST00000127
MSTRG.1	Axin1	MSTRG.13478.1
MSTRG.1	Axin1	MSTRG.13479.1
MSTRG.1	Ikbke	ENSMUST00000162437;MSTRG.1348.1;ENSMUST00000062108;EN
MSTRG.1	Axin1	MSTRG.13480.1
MSTRG.1	Arhgdig	MSTRG.13481.1
MSTRG.1	Pdia2;A	ENSMUST00000039113;ENSMUST00000148134;ENSMUST0000012C
MSTRG.1	Rgs11	ENSMUST00000152676;ENSMUST00000025020;ENSMUST0000012€
MSTRG.1	Fam234a	ENSMUST00000114988;ENSMUST00000118487;ENSMUST00000153
MSTRG.1	Gm23123	ENSMUST00000157648
MSTRG.1	Luc7l	ENSMUST00000025023;ENSMUST00000114976;MSTRG.13486.3;E
MSTRG.1	Gm16580	ENSMUST00000162696
MSTRG.1	Hba-ps4	MSTRG.13488.1
MSTRG.1	Neurl1b	ENSMUST00000183077;ENSMUST00000053020;MSTRG.13489.4
MSTRG.1	Gm28914	ENSMUST00000188115

MSTRG.1	Neurl1b	MSTRG.13490.1
MSTRG.1	Neurl1b	MSTRG.13491.1
MSTRG.1	Neurl1b	MSTRG.13492.1
MSTRG.1	Dusp1	ENSMUST00000025025;ENSMUST00000126178;ENSMUST0000014б
MSTRG.1	Gm8225	ENSMUST00000090257
MSTRG.1	1700049	ENSMUST00000011196
MSTRG.1	Ergic1	ENSMUST00000167662;ENSMUST00000165021;ENSMUST00000001
MSTRG.1	Gm22420	ENSMUST00000178795
MSTRG.1	Gm17218	ENSMUST00000165911
MSTRG.1	Atp6v0e	ENSMUST00000015719;ENSMUST00000167352
MSTRG.1	Ube2w	MSTRG.135.1
MSTRG.1	Gm28913	ENSMUST00000186240;MSTRG.1350.1
MSTRG.1	Crebrf	ENSMUST00000062519;ENSMUST00000144221;MSTRG.13500.3;E
MSTRG.1	Crebrf	MSTRG.13501.1
MSTRG.1	Bnip1	ENSMUST00000015725;ENSMUST00000128461;ENSMUST0000013
MSTRG.1	Narfl;M	ENSMUST00000134108;ENSMUST00000002350;ENSMUST00000154
MSTRG.1	Haghl	ENSMUST00000140738;ENSMUST00000133071;ENSMUST00000077
MSTRG.1	Ccdc78	ENSMUST00000095500
MSTRG.1	Fam173a	ENSMUST00000072735;MSTRG.13506.3;MSTRG.13506.1;MSTRG.
MSTRG.1	Gm26694	ENSMUST00000180868
MSTRG.1	Fbxl16	ENSMUST00000045692
MSTRG.1	Wdr24	ENSMUST00000026833;ENSMUST00000160349;ENSMUST0000016C
MSTRG.1	Fam72a	ENSMUST00000068613
MSTRG.1	Jmjd8	ENSMUST00000133595;ENSMUST00000026832;ENSMUST0000012
MSTRG.1	Stub1;G	ENSMUST00000044911;MSTRG.13511.1;MSTRG.13511.4;MSTRG.
MSTRG.1	Mcrip2	ENSMUST00000026828
MSTRG.1	Mettl26	ENSMUST00000110456;ENSMUST00000026827;ENSMUST0000016
MSTRG.1	Wfikkn1	ENSMUST00000095487
MSTRG.1	Rab40c	ENSMUST00000166146;ENSMUST00000179998;ENSMUST00000167
MSTRG.1	Gm26694	MSTRG.13516.1
MSTRG.1	Gm10012	ENSMUST00000170379;ENSMUST00000078252
MSTRG.1	Gm16278	ENSMUST00000148064
MSTRG.1	Pigq;Nh	ENSMUST00000026823;ENSMUST00000208043;ENSMUST0000014
MSTRG.1	Fam72a	MSTRG.1352.1
MSTRG.1	A930017	ENSMUST00000162431
MSTRG.1	D630044	MSTRG.13521.1
MSTRG.1	D630044	ENSMUST00000181174
MSTRG.1	Capn15	ENSMUST00000212735;ENSMUST00000212149;ENSMUST00000212
MSTRG.1	Capn15	MSTRG.13524.1
MSTRG.1	Capn15	MSTRG.13525.1
MSTRG.1	1700022	ENSMUST00000180932
MSTRG.1	Rab11fi	ENSMUST00000118828;ENSMUST00000122103;ENSMUST0000012C
MSTRG.1	Rab11fi	MSTRG.13528.1
MSTRG.1	Rab11fi	MSTRG.13529.1
MSTRG.1	Srgap2;	MSTRG.1353.1;ENSMUST00000097588;ENSMUST00000187042;EN
MSTRG.1	Rab11fi	MSTRG.13530.1
MSTRG.1	Rab11fi	MSTRG.13531.1
MSTRG.1	Kifc5b	ENSMUST00000078961;MSTRG.13532.2
MSTRG.1	Gm27775	ENSMUST00000184919
MSTRG.1	Phf1	ENSMUST00000073724;MSTRG.13534.1
MSTRG.1	Mir6968	ENSMUST00000184739
MSTRG.1	Cuta	ENSMUST00000025027;MSTRG.13536.1;MSTRG.13536.2;MSTRG.
MSTRG.1	Syngap1	ENSMUST00000194598;ENSMUST00000201702;ENSMUST00000177
MSTRG.1	Mir3083	ENSMUST00000175377
MSTRG.1	Zbtb9;G	ENSMUST00000120016;MSTRG.13539.2;MSTRG.13539.3;ENSMUS
MSTRG.1	Gm38262	ENSMUST00000194760
MSTRG.1	Ggnbp1	MSTRG.13540.1

MSTRG.1	Ggnbp1	MSTRG.13541.1
MSTRG.1	Ggnbp1	MSTRG.13542.1
MSTRG.1	Bak1	MSTRG.13543.1;ENSMUST00000078691;ENSMUST00000025034;E
MSTRG.1	Itpr3;M	MSTRG.13544.1;ENSMUST00000049308;ENSMUST00000184226
MSTRG.1	Itpr3	MSTRG.13545.1
MSTRG.1	Itpr3os	ENSMUST00000143605
MSTRG.1	Uqcc2	MSTRG.13547.1;ENSMUST00000025045;ENSMUST00000145148;E
MSTRG.1	Lemd2	ENSMUST00000055117;MSTRG.13548.2;MSTRG.13548.3;MSTRG.
MSTRG.1	Gm26724	MSTRG.13549.1
MSTRG.1	Gm37954	ENSMUST00000195400
MSTRG.1	Pacsin1	ENSMUST00000114873;ENSMUST00000045896;ENSMUST00000114
MSTRG.1	Pacsin1	MSTRG.13551.1
MSTRG.1	Pacsin1	MSTRG.13552.1
MSTRG.1	Pacsin1	MSTRG.13553.1
MSTRG.1	Pacsin1	MSTRG.13554.1
MSTRG.1	Gm9703	ENSMUST00000119573
MSTRG.1	Nudt3	MSTRG.13556.1;ENSMUST00000025050;ENSMUST00000114886;E
MSTRG.1	Hmga1	ENSMUST00000117600;ENSMUST00000114888;ENSMUST0000011S
MSTRG.1	AI41358	ENSMUST00000154473;ENSMUST00000080190;MSTRG.13558.4;M
MSTRG.13559		MSTRG.13559.1
MSTRG.1	Gm29488	ENSMUST00000191427
MSTRG.1	Nudt3	MSTRG.13560.1
MSTRG.1	Nudt3	MSTRG.13561.1
MSTRG.1	Nudt3	MSTRG.13562.1
MSTRG.1	Nudt3	MSTRG.13563.1;MSTRG.13563.2
MSTRG.1	Nudt3	MSTRG.13564.1
MSTRG.1	Rps10	ENSMUST00000114882;ENSMUST00000114881;ENSMUST0000002E
MSTRG.1	Gm15420	ENSMUST00000133274
MSTRG.1	Gm15458	ENSMUST00000156950
MSTRG.1	D17Wsu9	MSTRG.13568.1
MSTRG.1	D17Wsu9	ENSMUST00000114863;MSTRG.13569.2;ENSMUST00000114859;E
MSTRG.1	Srgap2	MSTRG.1357.1
MSTRG.1	Rps2-ps	ENSMUST00000121414
MSTRG.1	D17Wsu9	MSTRG.13571.1
MSTRG.1	D17Wsu9	MSTRG.13572.1
MSTRG.1	Snrpc	ENSMUST00000071006;ENSMUST00000137581
MSTRG.1	Uhrf1bp	ENSMUST00000114849;ENSMUST00000130810;ENSMUST00000137
MSTRG.1	Taf11	ENSMUST00000114848;ENSMUST00000025057;ENSMUST0000015E
MSTRG.1	Taf11	MSTRG.13576.1
MSTRG.1	Anks1	MSTRG.13577.1;ENSMUST00000114842;ENSMUST00000025058;E
MSTRG.1	Anks1	MSTRG.13578.1
MSTRG.1	Anks1	MSTRG.13579.1;MSTRG.13579.2
MSTRG.1	Gm29487	ENSMUST00000190146
MSTRG.1	Anks1	MSTRG.13580.1
MSTRG.1	Gm15597	ENSMUST00000145924
MSTRG.1	Anks1	MSTRG.13582.1
MSTRG.1	Gm15598	ENSMUST00000156965
MSTRG.1	Tcp11	ENSMUST00000042692;ENSMUST00000043925;ENSMUST00000114
MSTRG.1	4930526	ENSMUST00000142461;ENSMUST00000134228
MSTRG.1	Scube3	ENSMUST00000043503;ENSMUST00000137147;ENSMUST00000132
MSTRG.1	Scube3	MSTRG.13587.1
MSTRG.1	Zfp523	ENSMUST00000145313;ENSMUST00000073534;ENSMUST00000002
MSTRG.1	Def6	ENSMUST00000002327;MSTRG.13589.1;MSTRG.13589.2;ENSMUS
MSTRG.1	Srgap2	MSTRG.1359.1
MSTRG.1	Def6	MSTRG.13590.1;MSTRG.13590.2
MSTRG.1	Ppard	ENSMUST00000002320;ENSMUST00000123020;ENSMUST0000016E
MSTRG.1	Ppard	MSTRG.13592.1

MSTRG.1	Ppard	MSTRG.13593.1
MSTRG.1	Ppard	MSTRG.13594.1
MSTRG.1	Fance	ENSMUST00000114803;ENSMUST00000124870;ENSMUST0000011[illegible]
MSTRG.13596		MSTRG.13596.1
MSTRG.1	Rpl10a	ENSMUST00000042334;ENSMUST00000128758;ENSMUST0000012[illegible]
MSTRG.1	Tead3	ENSMUST00000154873;ENSMUST00000114799;ENSMUST0000015[illegible]
MSTRG.1	Tead3	MSTRG.13599.1
MSTRG.1	Ube2w	MSTRG.136.1
MSTRG.1	Gm8532	MSTRG.1360.1
MSTRG.1	Tead3	MSTRG.13600.1
MSTRG.1	Tulp1	ENSMUST00000130877;ENSMUST00000041819;ENSMUST0000014[illegible]
MSTRG.1	Gm20109	ENSMUST00000180802
MSTRG.1	E230001[illegible]	ENSMUST00000181029;ENSMUST00000095448
MSTRG.1	Fkbp5	MSTRG.13604.4;MSTRG.13604.3;MSTRG.13604.5;ENSMUST000[illegible]
MSTRG.1	Gm22146	MSTRG.13605.1;MSTRG.13605.2;ENSMUST00000157397
MSTRG.1	Lhfpl5	ENSMUST00000080780
MSTRG.1	Lhfpl5	MSTRG.13607.1
MSTRG.1	Srpk1	MSTRG.13608.1
MSTRG.1	Srpk1	ENSMUST00000130643;ENSMUST00000004987;ENSMUST0000013[illegible]
MSTRG.1	Gm8532	ENSMUST00000179174
MSTRG.1	Srpk1	MSTRG.13610.1
MSTRG.1	Slc26a8	ENSMUST00000114764
MSTRG.1	Mapk14	ENSMUST00000114758;ENSMUST00000004990;ENSMUST00000062
MSTRG.1	Mapk14	MSTRG.13613.1
MSTRG.1	Mapk14	MSTRG.13614.1
MSTRG.1	Brpf3	ENSMUST00000004985;ENSMUST00000136233;ENSMUST0000012[illegible]
MSTRG.1	Brpf3	MSTRG.13616.1
MSTRG.1	Kctd20	ENSMUST00000153462;ENSMUST00000118762;ENSMUST00000057
MSTRG.1	Stk38	ENSMUST00000009138;ENSMUST00000156732;ENSMUST0000011[illegible]
MSTRG.1	Gm23887	ENSMUST00000122683
MSTRG.1	Ctse;Ra[illegible]	ENSMUST00000073350;ENSMUST00000112411;MSTRG.1362.3;MS
MSTRG.1	Srsf3	MSTRG.13620.1;MSTRG.13620.3;ENSMUST00000147265;ENSMUS
MSTRG.1	Gm16196	ENSMUST00000137730
MSTRG.1	Gm16195	ENSMUST00000130607
MSTRG.1	Cdkn1a	ENSMUST00000119901;ENSMUST00000023829;ENSMUST00000122
MSTRG.1	Gm16194	ENSMUST00000141797;ENSMUST00000141239;ENSMUST0000013[illegible]
MSTRG.1	Rab44	ENSMUST00000087942;ENSMUST00000139931
MSTRG.1	Rpl35a-[illegible]	ENSMUST00000147311
MSTRG.1	Ppil1	ENSMUST00000024802;ENSMUST00000136811;MSTRG.13627.2;[illegible]
MSTRG.1	Ppil1	MSTRG.13628.1
MSTRG.1	Ppil1	MSTRG.13629.1
MSTRG.1363		MSTRG.1363.1
MSTRG.1	BC00400	MSTRG.13630.1
MSTRG.1	BC00400	ENSMUST00000064709;ENSMUST00000120346;ENSMUST0000015[illegible]
MSTRG.1	BC00400	MSTRG.13632.1
MSTRG.1	Gm10045	ENSMUST00000178657
MSTRG.1	Mtch1	ENSMUST00000095427;ENSMUST00000118366;ENSMUST00000127
MSTRG.1	Fgd2	ENSMUST00000123989;ENSMUST00000145124;ENSMUST0000014[illegible]
MSTRG.1	Fgd2	MSTRG.13636.1
MSTRG.1	Gm26885	ENSMUST00000181920
MSTRG.1	Pim1	ENSMUST00000024811
MSTRG.1	Slc41a1	ENSMUST00000132585;MSTRG.1364.1;ENSMUST00000086559;E[illegible]
MSTRG.1	Tbc1d22[illegible]	ENSMUST00000048677;ENSMUST00000150388;MSTRG.13640.3;[illegible]
MSTRG.1	Tbc1d22[illegible]	ENSMUST00000163069
MSTRG.1	Mir7667	ENSMUST00000183866
MSTRG.1	Gm28043	MSTRG.13643.1
MSTRG.1	Ccdc167	ENSMUST00000137079;ENSMUST00000123502;ENSMUST0000012[illegible]

MSTRG.1	Mdga1	ENSMUST00000171691;ENSMUST00000073556;MSTRG.13645.2;E
MSTRG.1	Gm16758	ENSMUST00000149552
MSTRG.1	Glp1r	ENSMUST00000114574;MSTRG.13647.2
MSTRG.1	Abcg1	ENSMUST00000024829;ENSMUST00000139013;MSTRG.13648.2;M
MSTRG.1	Gm25447	ENSMUST00000158997
MSTRG.1	Rab29	ENSMUST00000112386;ENSMUST00000027693;ENSMUST00000125
MSTRG.1	Abcg1	MSTRG.13650.1;MSTRG.13650.2
MSTRG.1	Abcg1	MSTRG.13652.1
MSTRG.1	Tff3	ENSMUST00000024827
MSTRG.13654		MSTRG.13654.1
MSTRG.1	Ubash3a	ENSMUST00000048656;ENSMUST00000144772;MSTRG.13655.3;M
MSTRG.1	Rsph1	ENSMUST00000024832
MSTRG.13657		MSTRG.13657.1
MSTRG.1	Slc37a1	ENSMUST00000171233;MSTRG.13658.2;ENSMUST00000165149
MSTRG.1	Slc37a1	MSTRG.13659.1
MSTRG.1	Rab29	MSTRG.1366.1
MSTRG.1	U2af1	MSTRG.13660.1;MSTRG.13660.2;MSTRG.13660.3;ENSMUST0000
MSTRG.1	Wdr4	ENSMUST00000172284;ENSMUST00000171171;ENSMUST0000017
MSTRG.1	Wdr4	MSTRG.13662.1
MSTRG.1	Wdr4	MSTRG.13663.1
MSTRG.1	Ndufv3	ENSMUST00000064798;ENSMUST00000189436;ENSMUST00000046
MSTRG.1	Gm38099	ENSMUST00000193280
MSTRG.1	Gm24970	MSTRG.13666.1
MSTRG.1	4833413	MSTRG.13667.1
MSTRG.1	4833413	ENSMUST00000162059
MSTRG.1	Pknox1	ENSMUST00000175806;ENSMUST00000176895;ENSMUST00000097
MSTRG.1	Nucks1	ENSMUST00000189946;ENSMUST00000062264;ENSMUST00000187
MSTRG.1	Gm24970	ENSMUST00000175582
MSTRG.1	Sik1	ENSMUST00000024839;MSTRG.13671.2
MSTRG.1	Zfand3	MSTRG.13672.1;MSTRG.13672.2;MSTRG.13672.3;ENSMUST0000
MSTRG.1	Zfand3	MSTRG.13673.1;MSTRG.13673.2
MSTRG.1	Zfand3	MSTRG.13674.1
MSTRG.1	Zfand3	MSTRG.13675.1
MSTRG.1	Zfand3	MSTRG.13676.1
MSTRG.1	Zfand3	MSTRG.13677.1
MSTRG.1	Btbd9	ENSMUST00000079924;ENSMUST00000168787;MSTRG.13678.3;M
MSTRG.1	Btbd9	MSTRG.13679.1
MSTRG.1	Gm38067	ENSMUST00000192346
MSTRG.1	Gm25049	ENSMUST00000158597
MSTRG.1	Btbd9	MSTRG.13681.1
MSTRG.1	Btbd9	MSTRG.13682.1
MSTRG.1	Btbd9	MSTRG.13683.1;MSTRG.13683.2
MSTRG.1	Gm9874;	MSTRG.13684.1;MSTRG.13684.2;ENSMUST00000194957;MSTRG.
MSTRG.1	Btbd9	MSTRG.13685.1
MSTRG.1	Btbd9	MSTRG.13686.1
MSTRG.13687		MSTRG.13687.1
MSTRG.1	Glo1;17	ENSMUST00000024823;ENSMUST00000167624;MSTRG.13688.1;M
MSTRG.13689		MSTRG.13689.1
MSTRG.1	Dnah8	MSTRG.13690.8;MSTRG.13690.9;MSTRG.13690.7;MSTRG.1369
MSTRG.1	1700097	MSTRG.13691.1;MSTRG.13691.2
MSTRG.1	Dnah8	MSTRG.13692.1
MSTRG.1	Gm24661	ENSMUST00000083205
MSTRG.1	Dnah8	MSTRG.13694.1;MSTRG.13694.2;MSTRG.13694.3;MSTRG.13694
MSTRG.1	Dnah8	MSTRG.13695.1
MSTRG.13696		MSTRG.13696.1
MSTRG.1	Pdxk-ps	ENSMUST00000159650;ENSMUST00000160550
MSTRG.1	Pdxk-ps	MSTRG.13698.1

MSTRG.1	Rrp1b	ENSMUST00000081339;ENSMUST00000156164;ENSMUST00000151
MSTRG.1	Gm38342	ENSMUST00000195710
MSTRG.1	Nuak2	ENSMUST00000072177;ENSMUST00000082125;ENSMUST00000128
MSTRG.1	Rrp1b	MSTRG.13700.1
MSTRG.1	Gm17276	ENSMUST00000180839
MSTRG.1	Notch3	MSTRG.13702.1;ENSMUST00000087723
MSTRG.1	Brd4	MSTRG.13703.1;ENSMUST00000119123;ENSMUST00000121285;M
MSTRG.1	Brd4	MSTRG.13704.1;MSTRG.13704.2
MSTRG.1	Gm26549	ENSMUST00000181112
MSTRG.1	Gm26549	MSTRG.13706.1
MSTRG.1	Akap8;A	ENSMUST00000002699;MSTRG.13707.3;MSTRG.13707.1;MSTRG.
MSTRG.1	Cyp4f17	ENSMUST00000165999;MSTRG.13708.2;MSTRG.13708.3
MSTRG.1	Cyp4f16	ENSMUST00000169591;ENSMUST00000003416;ENSMUST00000165
MSTRG.1	Gm29103	ENSMUST00000191034;ENSMUST00000177943;ENSMUST00000027
MSTRG.1	Cyp4f16	MSTRG.13710.1
MSTRG.1	Cyp4f16	MSTRG.13711.1
MSTRG.1	Wiz	ENSMUST00000170617;ENSMUST00000087703;MSTRG.13712.5;M
MSTRG.1	Gm26858	ENSMUST00000180608
MSTRG.1	Wiz	MSTRG.13714.1
MSTRG.1	Wiz	MSTRG.13715.1
MSTRG.1	Rasal3	MSTRG.13717.1;MSTRG.13717.2;ENSMUST00000063824;ENSMUS
MSTRG.13718		MSTRG.13718.1
MSTRG.1	Pglyrp2	MSTRG.13719.1;MSTRG.13719.2;MSTRG.13719.3;ENSMUST0000
MSTRG.1	Mfsd4a	MSTRG.1372.1;MSTRG.1372.2;ENSMUST00000126927;ENSMUST0
MSTRG.13720		MSTRG.13720.1
MSTRG.13721		MSTRG.13721.1
MSTRG.1	Zfp871	MSTRG.13722.1;MSTRG.13722.2;ENSMUST00000159086;ENSMUS
MSTRG.1	Zfp871	MSTRG.13723.1
MSTRG.1	Gm17115	ENSMUST00000164075
MSTRG.1	Zfp811	ENSMUST00000200914;ENSMUST00000080905
MSTRG.1	Gm23294	ENSMUST00000104150
MSTRG.1	Gm25555	ENSMUST00000158078
MSTRG.1	Gm26693	ENSMUST00000181318
MSTRG.1	Gm26693	MSTRG.13729.1
MSTRG.1	Gm26892	ENSMUST00000181695
MSTRG.1	Zfp799	ENSMUST00000202988;ENSMUST00000179695;ENSMUST00000201
MSTRG.1	Zfp799	MSTRG.13731.1
MSTRG.1	Zfp472	MSTRG.13732.1
MSTRG.1	Cyp4f13	ENSMUST00000075253;ENSMUST00000139353;ENSMUST00000137
MSTRG.13734		MSTRG.13734.1
MSTRG.1	Cyp4f41	ENSMUST00000126790
MSTRG.1	Gm25667	ENSMUST00000157701
MSTRG.1	Zfp472	ENSMUST00000039132;MSTRG.13737.1;MSTRG.13737.3
MSTRG.1	Zfp763	ENSMUST00000087654;ENSMUST00000124465
MSTRG.1	Zfp763	MSTRG.13739.1
MSTRG.1	Dstyk	ENSMUST00000045110;ENSMUST00000188389;ENSMUST00000188
MSTRG.1	Zfp563	ENSMUST00000131722;ENSMUST00000153072;ENSMUST0000014C
MSTRG.1	Zfp563	MSTRG.13741.1
MSTRG.1	Zfp955a	MSTRG.13742.1;MSTRG.13742.2;ENSMUST00000008830
MSTRG.1	Zfp955b	ENSMUST00000099414
MSTRG.1	Zfp955b	MSTRG.13744.1
MSTRG.1	Zfp955b	MSTRG.13745.1
MSTRG.1	Zfp422-	ENSMUST00000182230
MSTRG.1	Zfp101	MSTRG.13747.1;ENSMUST00000173151;ENSMUST00000167107;E
MSTRG.1	Zfp101	MSTRG.13748.1;MSTRG.13748.2
MSTRG.1	Zfp81	ENSMUST00000054072;MSTRG.13749.2
MSTRG.1	Dstyk	MSTRG.1375.1

MSTRG.1	Adamts1	ENSMUST00000087623;ENSMUST00000173931;ENSMUST00000173
MSTRG.1	Adamts1	MSTRG.13751.1
MSTRG.1	Adamts1	MSTRG.13752.1
MSTRG.1	Myo1f	ENSMUST00000087605;ENSMUST00000173372;ENSMUST00000174
MSTRG.1	Myo1f	MSTRG.13754.1
MSTRG.1	Myo1f	MSTRG.13755.1
MSTRG.1	Rab11b	MSTRG.13756.1
MSTRG.1	Rab11b	ENSMUST00000057373;ENSMUST00000173860;ENSMUST00000172
MSTRG.1	Rab11b	MSTRG.13758.1
MSTRG.1	Gm17251	ENSMUST00000170103
MSTRG.1	6030442	ENSMUST00000180835;ENSMUST00000188203
MSTRG.1	Angptl4	ENSMUST00000002360;ENSMUST00000173869;ENSMUST00000174
MSTRG.1	Zfp414;	ENSMUST00000166627;ENSMUST00000073570;ENSMUST00000172
MSTRG.1	Pram1	MSTRG.13762.1
MSTRG.1	Hnrnpm;	ENSMUST00000148178;ENSMUST00000114385;ENSMUST0000013
MSTRG.1	March2	MSTRG.13764.1
MSTRG.1	Gm7029	ENSMUST00000173226
MSTRG.1	Gm7029	MSTRG.13766.1
MSTRG.1	Kank3	ENSMUST00000048560;ENSMUST00000172649;ENSMUST00000173
MSTRG.1	Rps28	ENSMUST00000173019;ENSMUST00000087342;ENSMUST00000173
MSTRG.1	Ndufa7	ENSMUST00000048249;ENSMUST00000173182;ENSMUST00000173
MSTRG.1	6030442	MSTRG.1377.1
MSTRG.1	Ndufa7	MSTRG.13770.1
MSTRG.1	Ndufa7	MSTRG.13771.1
MSTRG.1	Kifc1	ENSMUST00000173492;ENSMUST00000173982;ENSMUST00000174
MSTRG.1	Kifc1	MSTRG.13773.1
MSTRG.1	Gm27740	ENSMUST00000185129
MSTRG.1	Kifc1	MSTRG.13775.1
MSTRG.1	Platr17	ENSMUST00000172817
MSTRG.1	BC05122	MSTRG.13777.1
MSTRG.1	BC05122	ENSMUST00000174467;ENSMUST00000172526;ENSMUST00000174
MSTRG.1	Daxx	ENSMUST00000173028;ENSMUST00000079421;ENSMUST00000173
MSTRG.1	6030442	MSTRG.1378.1
MSTRG.1	Tapbp;Z	ENSMUST00000172619;ENSMUST00000174463;ENSMUST00000053
MSTRG.1	Gm19412	ENSMUST00000185388;ENSMUST00000189156
MSTRG.1	Rgl2	ENSMUST00000172468;ENSMUST00000047503;ENSMUST00000173
MSTRG.1	Pfdn6	ENSMUST00000025163;ENSMUST00000179418;ENSMUST00000174
MSTRG.1	Wdr46	ENSMUST00000025170;ENSMUST00000173857;ENSMUST00000172
MSTRG.1	B3galt4	ENSMUST00000087543
MSTRG.1	Rps18	ENSMUST00000174758;ENSMUST00000174745;ENSMUST00000174
MSTRG.1	Vps52	ENSMUST00000025178;ENSMUST00000173196;ENSMUST00000173
MSTRG.1	Gm23111	ENSMUST00000122652
MSTRG.1	H2-K2	ENSMUST00000174250;MSTRG.13789.2
MSTRG.1	Rbbp5	ENSMUST00000190825;ENSMUST00000190997;ENSMUST00000187
MSTRG.1	H2-K1	MSTRG.13790.1
MSTRG.1	H2-K2	MSTRG.13791.1
MSTRG.1	Gm4152	ENSMUST00000172675
MSTRG.1	H2-K1	MSTRG.13793.1;ENSMUST00000172912;MSTRG.13793.3;ENSMUS
MSTRG.1	Ring1	ENSMUST00000025183;ENSMUST00000174054;MSTRG.13794.2;E
MSTRG.1	Gm26940	ENSMUST00000174851;ENSMUST00000083621
MSTRG.1	BC05153	ENSMUST00000183290
MSTRG.1	H2-Pa	ENSMUST00000182803
MSTRG.1	H2-Oa	ENSMUST00000025192;ENSMUST00000174670;MSTRG.13799.2
MSTRG.1	Tmem70	ENSMUST00000177501;ENSMUST00000177532;ENSMUST00000065
MSTRG.1	Tmem81	ENSMUST00000058167;ENSMUST00000188789
MSTRG.1	H2-Ke6;	ENSMUST00000173616;ENSMUST00000173425;ENSMUST00000045
MSTRG.1	Rxrb	ENSMUST00000044858;ENSMUST00000174299;ENSMUST00000173

MSTRG.1	Rxrb	MSTRG.13802.1
MSTRG.1	Col11a2	ENSMUST00000131134;ENSMUST00000114252;ENSMUST00000114
MSTRG.1	Col11a2	MSTRG.13804.1
MSTRG.1	H2-DMa	ENSMUST00000042121;MSTRG.13805.2;ENSMUST00000173907
MSTRG.13806		MSTRG.13806.1
MSTRG.1	H2-DMb2	ENSMUST00000173262;ENSMUST00000171231;ENSMUST00000041
MSTRG.1	H2-DMb2	MSTRG.13808.1
MSTRG.1	H2-DMb2	MSTRG.13809.1
MSTRG.1	Cntn2	ENSMUST00000189528;ENSMUST00000188065;ENSMUST0000018€
MSTRG.1	Brd2	ENSMUST00000114242;ENSMUST00000025193;MSTRG.13810.1;M
MSTRG.13811		MSTRG.13811.1
MSTRG.1	Gm20493	MSTRG.13812.1
MSTRG.13813		MSTRG.13813.1
MSTRG.1	Gm20493	ENSMUST00000173706
MSTRG.13815		MSTRG.13815.1
MSTRG.1	H2-Ob	ENSMUST00000095342;ENSMUST00000173681;ENSMUST00000167
MSTRG.1	Psmb9	ENSMUST00000174576;ENSMUST00000178857;ENSMUST00000114
MSTRG.1	Tap1;Psı	ENSMUST00000170086;MSTRG.13818.2;MSTRG.13818.1;ENSMUS
MSTRG.1	Gm20496	ENSMUST00000173770;ENSMUST00000172796
MSTRG.1	Pik3c2b	ENSMUST00000153707;ENSMUST00000145153;ENSMUST00000124
MSTRG.1	Tap2	ENSMUST00000025197;ENSMUST00000138491;MSTRG.13820.3;E
MSTRG.1	Gm15821	MSTRG.13821.2;MSTRG.13821.1;ENSMUST00000121995
MSTRG.1	H2-Ab1	ENSMUST00000040828;ENSMUST00000173103;ENSMUST00000174
MSTRG.1	H2-Aa	ENSMUST00000040655;ENSMUST00000174751;ENSMUST00000165
MSTRG.1	Gm20513	ENSMUST00000174074
MSTRG.1	H2-Eb1	ENSMUST00000074557
MSTRG.1	H2-Eb2	ENSMUST00000050325
MSTRG.1	H2-Ea-p	MSTRG.13827.1;MSTRG.13827.2;MSTRG.13827.3;ENSMUST0000
MSTRG.13828		MSTRG.13828.1
MSTRG.1	Gpsm3;P	ENSMUST00000038244;MSTRG.13829.1;ENSMUST00000150924;E
MSTRG.1	Gm28609	ENSMUST00000186515
MSTRG.1	Ager	ENSMUST00000015596;ENSMUST00000174496;ENSMUST00000184
MSTRG.1	Rnf5	ENSMUST00000015622;ENSMUST00000174475;MSTRG.13831.2;M
MSTRG.1	Gm20463	ENSMUST00000174756
MSTRG.1	Fkbpl	ENSMUST00000036720;ENSMUST00000174796
MSTRG.1	Atf6b	ENSMUST00000015605;ENSMUST00000174614;ENSMUST00000174
MSTRG.1	Agpat1	ENSMUST00000174041;ENSMUST00000173242;ENSMUST0000017З
MSTRG.1	Egfl8;P	ENSMUST00000170850;ENSMUST00000015611;ENSMUST0000016€
MSTRG.1	Prrt1	MSTRG.13837.1;ENSMUST00000015620;MSTRG.13837.2;ENSMUS
MSTRG.1	Prrt1	MSTRG.13838.1
MSTRG.1	Tnxb	ENSMUST00000168533;ENSMUST00000087399
MSTRG.1	Pik3c2b	MSTRG.1384.1
MSTRG.1	Cyp21a2	ENSMUST00000172970
MSTRG.1	C4b;C4a	ENSMUST00000173669;ENSMUST00000069507;MSTRG.13842.1;M
MSTRG.1	Stk19-p	ENSMUST00000174597
MSTRG.1	Skiv2l-	ENSMUST00000173935
MSTRG.1	Tnxa	ENSMUST00000173277
MSTRG.1	Cyp21a1	ENSMUST00000025223
MSTRG.1	Slc44a4	ENSMUST00000173664;ENSMUST00000007249
MSTRG.1	Neu1	ENSMUST00000007253;ENSMUST00000173269;MSTRG.13849.3;E
MSTRG.1	Mdm4	ENSMUST00000186617;ENSMUST00000187244;ENSMUST0000018€
MSTRG.1	Neu1	MSTRG.13850.1
MSTRG.1	Hspa1b	ENSMUST00000172753
MSTRG.1	Hspa1a	ENSMUST00000087328
MSTRG.1	Gm20481	ENSMUST00000173680
MSTRG.1	1110038	ENSMUST00000174464;ENSMUST00000174714;ENSMUST0000017З
MSTRG.1	Stk19	ENSMUST00000077477;ENSMUST00000161885;ENSMUST00000162

MSTRG.1	Dxo	ENSMUST00000173874;ENSMUST00000180043;ENSMUST0000004[illegible]
MSTRG.1	Skiv2l;[illegible]	ENSMUST00000174226;ENSMUST00000046022;ENSMUST00000174
MSTRG.1	Mir6971	ENSMUST00000183497
MSTRG.1	Nelfe	ENSMUST00000097343;ENSMUST00000173357;ENSMUST0000017[illegible]
MSTRG.1	Mdm4	MSTRG.1386.1
MSTRG.1	Cfb;Gm2	ENSMUST00000141295;ENSMUST00000128767;ENSMUST00000154
MSTRG.1	Mir6972	ENSMUST00000184774
MSTRG.1	Zbtb12	ENSMUST00000173093;ENSMUST00000052778
MSTRG.1	Ehmt2	ENSMUST00000114033;ENSMUST00000078061;ENSMUST00000097
MSTRG.1	Tnf	ENSMUST00000025263
MSTRG.1	Nfkbil1	ENSMUST00000048994;ENSMUST00000176748;MSTRG.13865.3
MSTRG.1	Gm16181	ENSMUST00000118793
MSTRG.1	Nfkbil1	MSTRG.13867.1
MSTRG.1	Atp6v1g	ENSMUST00000146711;MSTRG.13868.2;ENSMUST00000130992;[illegible]
MSTRG.1	Ddx39b;[illegible]	ENSMUST00000068056;ENSMUST00000174757;MSTRG.13869.2;[illegible]
MSTRG.1	Gm29257	ENSMUST00000186115
MSTRG.1	Mir6975	ENSMUST00000184879
MSTRG.1	Gm25128	ENSMUST00000083339
MSTRG.1	Ddx39b	MSTRG.13872.1
MSTRG.1	Ddx39b	MSTRG.13873.1
MSTRG.13874		MSTRG.13874.1
MSTRG.13875		MSTRG.13875.1;MSTRG.13875.2
MSTRG.13876		MSTRG.13876.1
MSTRG.1	Tcf19	ENSMUST00000161012;ENSMUST00000160885;MSTRG.13877.3;[illegible]
MSTRG.1	Cchcr1	ENSMUST00000164242;ENSMUST00000174827;ENSMUST0000004[illegible]
MSTRG.1	H2-D1;H	ENSMUST00000172785;MSTRG.13879.2;MSTRG.13879.3;MSTRG.
MSTRG.1	Gm26616	ENSMUST00000195676;ENSMUST00000193049
MSTRG.1	Gm18734	MSTRG.13880.1
MSTRG.1	Gm18734	MSTRG.13881.1
MSTRG.1	Gm18734	MSTRG.13882.1
MSTRG.1	Gm18733	ENSMUST00000174447;ENSMUST00000173950
MSTRG.13884		MSTRG.13884.1
MSTRG.1	Gm18734	MSTRG.13885.1;MSTRG.13885.2
MSTRG.1	H2-Q1	MSTRG.13886.1;MSTRG.13886.2
MSTRG.1	Gm18734	ENSMUST00000172937
MSTRG.1	H2-Q1	MSTRG.13888.1
MSTRG.1	H2-Q1	MSTRG.13889.1
MSTRG.1	Ppp1r15[illegible]	ENSMUST00000052529;ENSMUST00000122956
MSTRG.1	H2-Q1	MSTRG.13890.1
MSTRG.1	H2-Q1	MSTRG.13891.1
MSTRG.1	H2-Q2	MSTRG.13892.1
MSTRG.1	H2-Q1	MSTRG.13893.2;MSTRG.13893.1
MSTRG.1	H2-Q2	MSTRG.13894.1
MSTRG.1	H2-Q1	MSTRG.13895.1
MSTRG.1	Gm8741	ENSMUST00000174736
MSTRG.1	H2-Q2	MSTRG.13897.1
MSTRG.1	H2-Q3	ENSMUST00000172583
MSTRG.1	Gm11131	ENSMUST00000113886;MSTRG.13899.2
MSTRG.1	Eloc	ENSMUST00000188641;ENSMUST00000115352;ENSMUST0000018[illegible]
MSTRG.1	Snrpe	ENSMUST00000166915;ENSMUST00000166291;ENSMUST00000172
MSTRG.1	H2-Q6	MSTRG.13900.1;MSTRG.13900.2;MSTRG.13900.3
MSTRG.1	Gm8750	ENSMUST00000173278
MSTRG.1	Gm8752	ENSMUST00000173427
MSTRG.1	H2-Q10	MSTRG.13903.1
MSTRG.1	Hspa1l	ENSMUST00000007248
MSTRG.1	Gm10501	ENSMUST00000151136
MSTRG.1	Gm10501	MSTRG.13906.1

MSTRG.1	Lsm2	ENSMUST00000172523;ENSMUST00000114011;ENSMUST00000174
MSTRG.1	Msh5;Va	MSTRG.13908.1;MSTRG.13908.2;MSTRG.13908.3;MSTRG.13908
MSTRG.1	Vars	ENSMUST00000087315;ENSMUST00000173584;ENSMUST00000174
MSTRG.1	Gm26706	ENSMUST00000180718
MSTRG.1	Vwa7	ENSMUST00000007245
MSTRG.1	Sapcd1	ENSMUST00000173211;ENSMUST00000174037;ENSMUST0000004C
MSTRG.1	Msh5	ENSMUST00000173124;MSTRG.13912.1;ENSMUST00000007250;E
MSTRG.1	Msh5	MSTRG.13913.1
MSTRG.1	Msh5;Ap	MSTRG.13914.1;MSTRG.13914.2;ENSMUST00000025249
MSTRG.1	Clic1	MSTRG.13915.1;ENSMUST00000007257;MSTRG.13915.3;MSTRG.
MSTRG.1	Gm23421	ENSMUST00000158934
MSTRG.1	Ddah2	ENSMUST00000007255;ENSMUST00000174493;ENSMUST00000173
MSTRG.1	G6b	ENSMUST00000097337;ENSMUST00000174190
MSTRG.1	Ly6g6c	ENSMUST00000173207;ENSMUST00000173478;ENSMUST00000174
MSTRG.1	Lax1	MSTRG.1392.1;MSTRG.1392.2;MSTRG.1392.3;ENSMUST0000016
MSTRG.1	Ly6g6d	ENSMUST00000007259;ENSMUST00000172639;ENSMUST00000172
MSTRG.1	Ly6g6e	ENSMUST00000172494;ENSMUST00000172678;ENSMUST00000013
MSTRG.1	Ly6g6f	ENSMUST00000038507
MSTRG.1	Abhd16a	ENSMUST00000173134;MSTRG.13923.1;ENSMUST00000007251;M
MSTRG.1	Ly6g5c	ENSMUST00000037849
MSTRG.1	Ly6g5b	ENSMUST00000172854;ENSMUST00000062657
MSTRG.1	Csnk2b	ENSMUST00000173114;ENSMUST00000174779;ENSMUST00000025
MSTRG.1	Gpank1	ENSMUST00000173380;ENSMUST00000173043;ENSMUST00000165
MSTRG.1	Gm20522	ENSMUST00000173576
MSTRG.1	D17H6S5	ENSMUST00000174311;ENSMUST00000061859;MSTRG.13929.2
MSTRG.1	Zc3h11a	ENSMUST00000191896;ENSMUST00000027736;MSTRG.1393.3;EN
MSTRG.1	Bag6	ENSMUST00000166426;ENSMUST00000025250;ENSMUST00000172
MSTRG.1	Prrc2a;	ENSMUST00000025253;ENSMUST00000174805;ENSMUST00000173
MSTRG.1	Gm17705	ENSMUST00000172285
MSTRG.1	Aif1	ENSMUST00000173281;ENSMUST00000172693;ENSMUST00000173
MSTRG.1	Ncr3-ps	ENSMUST00000134687
MSTRG.1	Lst1	ENSMUST00000097336
MSTRG.1	Ltb	MSTRG.13936.1;MSTRG.13936.2;ENSMUST00000025262;ENSMUS
MSTRG.1	Mir6973	ENSMUST00000183646
MSTRG.1	Sfta2	ENSMUST00000174521;ENSMUST00000171166
MSTRG.1	Gm9573	ENSMUST00000164502
MSTRG.1	Zc3h11a	MSTRG.1394.1
MSTRG.1	Dpcr1	ENSMUST00000095467
MSTRG.1	Gm20483	ENSMUST00000173162
MSTRG.1	Gm20483	MSTRG.13942.1
MSTRG.1	Gm20483	MSTRG.13943.1
MSTRG.1	Vars2	ENSMUST00000043674;ENSMUST00000165787;MSTRG.13944.2;E
MSTRG.1	Gtf2h4	ENSMUST00000001565;ENSMUST00000162927;ENSMUST0000016C
MSTRG.1	Ddr1;Gm	ENSMUST00000119825;ENSMUST00000117301;ENSMUST00000166
MSTRG.1	Ddr1	MSTRG.13947.1
MSTRG.1	Gm23864	MSTRG.13948.1
MSTRG.1	Gm4577	ENSMUST00000135203
MSTRG.1	Atp2b4;	ENSMUST00000143567;ENSMUST00000167348;ENSMUST00000048
MSTRG.1	Ppp1r18	ENSMUST00000145804
MSTRG.1	Nrm	ENSMUST00000074259;ENSMUST00000174873;MSTRG.13951.2;E
MSTRG.1	Nrm	MSTRG.13952.1
MSTRG.1	Ppp1r18	ENSMUST00000127442;ENSMUST00000144382;ENSMUST00000122
MSTRG.1	Ppp1r18	MSTRG.13955.1
MSTRG.1	Tubb5	MSTRG.13956.1;ENSMUST00000001566;ENSMUST00000173253;E
MSTRG.1	Ier3	ENSMUST00000003635
MSTRG.1	Flot1	ENSMUST00000001569;ENSMUST00000174080;ENSMUST00000173
MSTRG.1	Flot1	MSTRG.13959.1

MSTRG.1	Gm37368	ENSMUST00000193173
MSTRG.1	Tubb5	MSTRG.13960.1
MSTRG.1	Mdc1	ENSMUST00000082337;MSTRG.13961.1;MSTRG.13961.3;ENSMUS
MSTRG.1	Dhx16	ENSMUST00000025292;ENSMUST00000174366;MSTRG.13962.2;E
MSTRG.1	2310061	ENSMUST00000148482;ENSMUST00000148721;ENSMUST00000154
MSTRG.1	Gm16279	ENSMUST00000149452
MSTRG.1	Atat1	ENSMUST00000061052;ENSMUST00000077494;ENSMUST0000014S
MSTRG.1	Mrps18b	ENSMUST00000025305;ENSMUST00000113782;ENSMUST00000174
MSTRG.1	Ppp1r10	ENSMUST00000154856;ENSMUST00000087211;ENSMUST0000013C
MSTRG.1	Rpp21	ENSMUST00000077535;ENSMUST00000173486;ENSMUST0000002S
MSTRG.1	Trim39	ENSMUST00000173665;ENSMUST00000113706;ENSMUST00000042
MSTRG.1	Atp2b4	MSTRG.1397.1
MSTRG.1	H2-M10..	MSTRG.13970.1;ENSMUST00000023845
MSTRG.1	Rnf39	ENSMUST00000040498;ENSMUST00000173072;ENSMUST00000174
MSTRG.1	Ppp1r11	MSTRG.13972.1;ENSMUST00000040402;ENSMUST00000174711
MSTRG.1	Trim26	ENSMUST00000124307;MSTRG.13973.1;ENSMUST00000134325;E
MSTRG.1	Gm25973	ENSMUST00000083717
MSTRG.1	Trim26	MSTRG.13975.1
MSTRG.1	Trim15	ENSMUST00000025329
MSTRG.1	Znrd1	ENSMUST00000113669;ENSMUST00000172518;ENSMUST00000174
MSTRG.1	Znrd1as	ENSMUST00000040177;ENSMUST00000172672;ENSMUST0000017З
MSTRG.1	Optc	ENSMUST00000124245;ENSMUST00000124051;ENSMUST0000015З
MSTRG.1	Gabbr1	ENSMUST00000025338;ENSMUST00000173823;ENSMUST00000174
MSTRG.1	Ubd	ENSMUST00000038844
MSTRG.1	Gm8801	ENSMUST00000173255
MSTRG.1	Gm8801	MSTRG.13983.1
MSTRG.1	Abcf1;H	MSTRG.13984.1;ENSMUST00000043757;ENSMUST00000173111;E
MSTRG.1	Abcf1	MSTRG.13985.1
MSTRG.1	Mir877	ENSMUST00000104738
MSTRG.1	Prr3	ENSMUST00000172429;ENSMUST00000055454;ENSMUST0000017З
MSTRG.1	Gnl1	ENSMUST00000087200;ENSMUST00000174275
MSTRG.1	Gm20508	ENSMUST00000172480
MSTRG.1	Gm15851	ENSMUST00000160564
MSTRG.1	A930015	ENSMUST00000173900
MSTRG.1	H2-T24	MSTRG.13991.1;MSTRG.13991.2;ENSMUST00000113760;ENSMUS
MSTRG.1	A930015	MSTRG.13992.1
MSTRG.1	A930015	MSTRG.13993.1
MSTRG.1	H2-T24	MSTRG.13994.1
MSTRG.1	Gm6034	MSTRG.13995.1
MSTRG.1	Gm6034	MSTRG.13996.1;ENSMUST00000097331
MSTRG.1	Gm11127	ENSMUST00000113742
MSTRG.1	Gm6034	MSTRG.13998.2;MSTRG.13998.1
MSTRG.1	Gm8810	ENSMUST00000172895
MSTRG.14		MSTRG.14.1
MSTRG.1	Eloc	MSTRG.140.1
MSTRG.1	Prelp	ENSMUST00000048432
MSTRG.1	H2-Bl	ENSMUST00000184850;ENSMUST00000173080;ENSMUST00000194
MSTRG.1	H2-T10;	MSTRG.14001.1;MSTRG.14001.2;ENSMUST00000174778;ENSMUS
MSTRG.1	2410017	MSTRG.14002.1
MSTRG.1	Gm8815	ENSMUST00000173809
MSTRG.1	Gm19684	ENSMUST00000173128
MSTRG.1	Gm9574	ENSMUST00000173127
MSTRG.1	2410017	ENSMUST00000056774;ENSMUST00000173025;ENSMUST0000009C
MSTRG.1	Gm17782	ENSMUST00000174345
MSTRG.1	Fmod	ENSMUST00000048183;ENSMUST00000162779
MSTRG.1	Gm6659	ENSMUST00000174752
MSTRG.1	Gm20478	ENSMUST00000173648

MSTRG.1	Gm6623	ENSMUST00000173570
MSTRG.1	Gm20495	ENSMUST00000173902
MSTRG.1	Gm4246	ENSMUST00000172822
MSTRG.1	Gm20447	ENSMUST00000173290
MSTRG.1	Gm8835	ENSMUST00000173096
MSTRG.1	Gm5682	ENSMUST00000173402
MSTRG.1	Gm20392	ENSMUST00000173047
MSTRG.1	Gm20429	ENSMUST00000172477;ENSMUST00000174168
MSTRG.1	Btg2	MSTRG.1402.1;MSTRG.1402.2;MSTRG.1402.3;ENSMUST0000002
MSTRG.1	Olfr754	ENSMUST00000173181
MSTRG.1	Olfr98	ENSMUST00000080759
MSTRG.14022		MSTRG.14022.1
MSTRG.1	Gm25104	ENSMUST00000122476
MSTRG.1	H2-M3	ENSMUST00000038580
MSTRG.1	Olfr755	MSTRG.14025.1
MSTRG.1	Olfr99	ENSMUST00000216328;ENSMUST00000077585
MSTRG.1	Olfr103	MSTRG.14027.1
MSTRG.1	Olfr101	ENSMUST00000215392;ENSMUST00000058046
MSTRG.1	Olfr102	ENSMUST00000169373
MSTRG.1	Tmem183	ENSMUST00000049470;ENSMUST00000191237;ENSMUST0000018
MSTRG.1	Olfr104	ENSMUST00000219092
MSTRG.1	Olfr108	ENSMUST00000218675
MSTRG.1	Olfr760	ENSMUST00000172563
MSTRG.1	Olfr109	ENSMUST00000214668;ENSMUST00000214938;ENSMUST00000031
MSTRG.1	H2-M2	ENSMUST00000016427;MSTRG.14035.2;ENSMUST00000171139
MSTRG.1	Gm7335	MSTRG.14036.1
MSTRG.1	Gm7335	ENSMUST00000172475
MSTRG.14038		MSTRG.14038.1
MSTRG.14039		MSTRG.14039.1
MSTRG.1	Cyb5r1	MSTRG.1404.1;ENSMUST00000154237;ENSMUST00000027726;EN
MSTRG.1	Cenpq	ENSMUST00000087114;ENSMUST00000130890
MSTRG.1	Mut	ENSMUST00000169611
MSTRG.14042		MSTRG.14042.1
MSTRG.14043		MSTRG.14043.1
MSTRG.14044		MSTRG.14044.1
MSTRG.1	Cd2ap	MSTRG.14045.1
MSTRG.1	Cd2ap	ENSMUST00000024709;MSTRG.14046.2;MSTRG.14046.3
MSTRG.1	Cd2ap	MSTRG.14047.1
MSTRG.1	Tnfrsf2	ENSMUST00000024708
MSTRG.14049		MSTRG.14049.1
MSTRG.1	Adipor1	ENSMUST00000027727;ENSMUST00000112237;ENSMUST0000015
MSTRG.1	Adgrf5	ENSMUST00000226087;ENSMUST00000113599;ENSMUST0000022
MSTRG.1	Gm25135	ENSMUST00000082648
MSTRG.1	Pla2g7	ENSMUST00000024706;ENSMUST00000167418;ENSMUST0000016
MSTRG.1	1700071	ENSMUST00000169733;ENSMUST00000172072
MSTRG.1	Slc25a2	ENSMUST00000024705;MSTRG.14054.2
MSTRG.1	Cyp39a1	ENSMUST00000170988
MSTRG.1	Enpp5	ENSMUST00000154166;ENSMUST00000024756;MSTRG.14057.3;E
MSTRG.1	Enpp4	ENSMUST00000024757;ENSMUST00000143137
MSTRG.1	Clic5	ENSMUST00000024755
MSTRG.1	Adipor1	MSTRG.1406.1
MSTRG.1	Clic5	MSTRG.14060.1
MSTRG.1	Runx2	ENSMUST00000162816;ENSMUST00000113572;ENSMUST0000015
MSTRG.1	Runx2os	ENSMUST00000160203
MSTRG.1	n-R5s27	ENSMUST00000093699
MSTRG.1	Runx2os	MSTRG.14064.1
MSTRG.1	Runx2os	ENSMUST00000097320

MSTRG.1	Supt3	ENSMUST00000050630;ENSMUST00000130623;ENSMUST00000127
MSTRG.1	Runx2	MSTRG.14067.1
MSTRG.1	Supt3	MSTRG.14068.1
MSTRG.1	Supt3	MSTRG.14069.1
MSTRG.1	Gm15454	ENSMUST00000179718
MSTRG.1	Supt3	MSTRG.14070.1
MSTRG.1	Supt3	MSTRG.14071.1
MSTRG.14072		MSTRG.14072.1
MSTRG.1	Cdc5l	MSTRG.14073.1;MSTRG.14073.2;ENSMUST00000024727
MSTRG.1	B230354	MSTRG.14074.1;ENSMUST00000181149
MSTRG.1	Spats1	MSTRG.14075.1
MSTRG.1	Spats1	ENSMUST00000024731
MSTRG.1	Gm9104	ENSMUST00000095268
MSTRG.1	Nfkbie	MSTRG.14078.2;ENSMUST00000024742;MSTRG.14078.1
MSTRG.14079		MSTRG.14079.1
MSTRG.1	Gm37935	ENSMUST00000192012
MSTRG.1	Aars2;T	MSTRG.14080.1;MSTRG.14080.2;MSTRG.14080.3;MSTRG.1408C
MSTRG.1	Aars2	ENSMUST00000024733
MSTRG.1	Tmem151	ENSMUST00000180252;MSTRG.14082.2
MSTRG.1	Slc35b2	ENSMUST00000224905;ENSMUST00000225226;MSTRG.14083.2;E
MSTRG.1	Hsp90ab	ENSMUST00000024739;ENSMUST00000145205;ENSMUST00000165
MSTRG.14085		MSTRG.14085.1
MSTRG.1	Capn11	ENSMUST00000120717;ENSMUST00000151350
MSTRG.1	Capn11	MSTRG.14087.1
MSTRG.1	Capn11	MSTRG.14088.1
MSTRG.1	Slc29a1	ENSMUST00000163492;ENSMUST00000171847;ENSMUST00000167
MSTRG.1	Klhl12	ENSMUST00000133721;ENSMUST00000112232;ENSMUST00000027
MSTRG.1	Gm17080	ENSMUST00000087588
MSTRG.14091		MSTRG.14091.1
MSTRG.1	Tmem63b	ENSMUST00000113523;ENSMUST00000151439;ENSMUST00000129
MSTRG.1	Gm16172	ENSMUST00000156224
MSTRG.1	Mrpl14	ENSMUST00000024734;ENSMUST00000149040;ENSMUST00000148
MSTRG.1	Mrpl14	MSTRG.14095.1
MSTRG.1	Mad2l1b	ENSMUST00000171172
MSTRG.1	Mrps18a	ENSMUST00000024763;ENSMUST00000131943;ENSMUST00000123
MSTRG.1	Mrps18a	MSTRG.14098.1
MSTRG.1	Rsph9	ENSMUST00000148581;ENSMUST00000024762;ENSMUST00000152
MSTRG.141		MSTRG.141.1
MSTRG.1	Klhl12	MSTRG.1410.1
MSTRG.1	Gtpbp2	ENSMUST00000169383;ENSMUST00000024748;ENSMUST00000164
MSTRG.1	Dlk2	ENSMUST00000188223;ENSMUST00000061722;ENSMUST00000166
MSTRG.1	Abcc10	ENSMUST00000167360;ENSMUST00000171584;ENSMUST00000047
MSTRG.1	Polh	ENSMUST00000024749;MSTRG.14103.1;MSTRG.14103.3
MSTRG.1	Xpo5	ENSMUST00000087031;MSTRG.14104.1
MSTRG.1	Mir693	ENSMUST00000102187
MSTRG.1	Gm22474	ENSMUST00000179407
MSTRG.1	Polr1c	ENSMUST00000087026;ENSMUST00000124655;ENSMUST00000152
MSTRG.1	Yipf3	ENSMUST00000095263;ENSMUST00000123311;MSTRG.14108.4;E
MSTRG.1	Lrrc73	ENSMUST00000095262
MSTRG.1	Klhl12	MSTRG.1411.1
MSTRG.1	Tjap1	ENSMUST00000180283;ENSMUST00000012440;ENSMUST00000225
MSTRG.1	Gm26785	MSTRG.14111.1;ENSMUST00000181775
MSTRG.1	Zfp318	ENSMUST00000113481;ENSMUST00000138127;ENSMUST00000152
MSTRG.1	Gm23037	ENSMUST00000083874
MSTRG.1	Crip3	ENSMUST00000113465;ENSMUST00000164180;ENSMUST00000024
MSTRG.1	Slc22a7	ENSMUST00000087012
MSTRG.1	Ubr2	ENSMUST00000113337;MSTRG.14116.1;MSTRG.14116.2;ENSMUS

MSTRG.1	Gm23657	ENSMUST00000122623
MSTRG.1	Trerf1	MSTRG.14118.1;MSTRG.14118.2;ENSMUST00000188947;ENSMUS
MSTRG.1	Trerf1	MSTRG.14119.1
MSTRG.1	Rabif	ENSMUST00000047978
MSTRG.1	Trerf1	MSTRG.14120.1
MSTRG.1	Trerf1	MSTRG.14121.1
MSTRG.1	Trerf1	MSTRG.14122.1
MSTRG.1	Trerf1	MSTRG.14123.1
MSTRG.1	Gm26216	ENSMUST00000158583
MSTRG.1	Trerf1	MSTRG.14125.1
MSTRG.1	Trerf1	MSTRG.14126.1
MSTRG.1	Ttbk1	ENSMUST00000047034;ENSMUST00000225808;ENSMUST00000223
MSTRG.1	Gm26904	ENSMUST00000181301
MSTRG.1	AC15127	ENSMUST00000224870
MSTRG.1	Rabif	MSTRG.1413.1
MSTRG.1	Dnph1	ENSMUST00000046497
MSTRG.1	Cul9	ENSMUST00000183312;ENSMUST00000182485;ENSMUST00000183
MSTRG.1	Gm26904	MSTRG.14132.1
MSTRG.1	Srf	ENSMUST00000015749
MSTRG.1	Mir6976	ENSMUST00000199897
MSTRG.1	Ptk7	ENSMUST00000044442
MSTRG.1	Gm26904	MSTRG.14136.1
MSTRG.1	Ptk7	MSTRG.14137.1
MSTRG.1	Klc4	ENSMUST00000003642
MSTRG.1	Gm26904	MSTRG.14139.1
MSTRG.1	Rabif	MSTRG.1414.1
MSTRG.1	Mrpl2	ENSMUST00000002844;ENSMUST00000156464;ENSMUST00000113
MSTRG.1	Cul7	ENSMUST00000043464;ENSMUST00000151002;ENSMUST00000132
MSTRG.1	Rrp36	MSTRG.14142.1;ENSMUST00000024766
MSTRG.1	Rrp36	MSTRG.14143.1
MSTRG.1	Klhdc3	ENSMUST00000165007;ENSMUST00000071841;MSTRG.14144.2
MSTRG.1	Mea1	ENSMUST00000002845
MSTRG.1	Ppp2r5d	ENSMUST00000002839;MSTRG.14146.1;MSTRG.14146.3;MSTRG.
MSTRG.1	Pex6	ENSMUST00000002840;ENSMUST00000144964;ENSMUST00000127
MSTRG.1	Gnmt	ENSMUST00000147112;ENSMUST00000002846
MSTRG.1	Cnpy3	ENSMUST00000059844;ENSMUST00000129200;ENSMUST00000145
MSTRG.1	Gm25612	ENSMUST00000104014
MSTRG.1	Cnpy3	MSTRG.14150.1
MSTRG.1	Ptcra	ENSMUST00000041012;MSTRG.14151.1
MSTRG.1	2310039	ENSMUST00000071430
MSTRG.1	Rpl7l1	ENSMUST00000078286;MSTRG.14153.1
MSTRG.1	Bicral	ENSMUST00000040624;MSTRG.14154.1
MSTRG.1	Gm26904	MSTRG.14155.1
MSTRG.14156		MSTRG.14156.1
MSTRG.1	Tbcc	ENSMUST00000040434
MSTRG.1	Mrps10	ENSMUST00000120737;ENSMUST00000119945;ENSMUST00000135
MSTRG.1	Tfeb	MSTRG.14159.1
MSTRG.1	Kdm5b	MSTRG.1416.1
MSTRG.1	Pgc	ENSMUST00000024782
MSTRG.1	Tfeb	ENSMUST00000086932;ENSMUST00000126258;ENSMUST00000113
MSTRG.1	Tfeb	MSTRG.14162.1
MSTRG.1	Tfeb	MSTRG.14163.1
MSTRG.1	Tfeb	MSTRG.14164.1
MSTRG.14165		MSTRG.14165.1
MSTRG.1	Foxp4	ENSMUST00000113265;ENSMUST00000113263;ENSMUST00000097
MSTRG.1	Foxp4	MSTRG.14167.1
MSTRG.1	Foxp4	MSTRG.14168.1

MSTRG.1	Gm15556	MSTRG.14169.1
MSTRG.1	Kdm5b	ENSMUST00000047714;ENSMUST00000112198;ENSMUST00000112
MSTRG.1	Gm5228	ENSMUST00000117186
MSTRG.14172		MSTRG.14172.1
MSTRG.1	1700001	ENSMUST00000061885;ENSMUST00000125507
MSTRG.1	AI66145	ENSMUST00000037701;ENSMUST00000150819
MSTRG.1	Taf8	MSTRG.14175.2;ENSMUST00000067103;MSTRG.14175.1;MSTRG.
MSTRG.1	Ccnd3	MSTRG.14176.1;MSTRG.14176.2
MSTRG.1	Ccnd3	ENSMUST00000182129;ENSMUST00000171031;ENSMUST00000183
MSTRG.1	Ccnd3	MSTRG.14178.1
MSTRG.1	Ccnd3	MSTRG.14179.2;MSTRG.14179.1;MSTRG.14179.3;MSTRG.14179
MSTRG.1	Kdm5b	MSTRG.1418.1
MSTRG.1	Ccnd3	MSTRG.14180.2;MSTRG.14180.1
MSTRG.1	Ccnd3	MSTRG.14181.1
MSTRG.1	Bysl	MSTRG.14182.1;MSTRG.14182.2;ENSMUST00000024783
MSTRG.1	Bysl	MSTRG.14183.1
MSTRG.1	Gm20517	MSTRG.14184.1;ENSMUST00000024778;ENSMUST00000149893;E
MSTRG.1	Med20	MSTRG.14185.1
MSTRG.1	Gm20517	MSTRG.14187.1
MSTRG.1	Gm20517	MSTRG.14188.1
MSTRG.1	Gm20517	MSTRG.14189.1
MSTRG.1	Tomm6;G	ENSMUST00000113302;ENSMUST00000143789;ENSMUST00000113
MSTRG.1	Tomm6os	ENSMUST00000155812;ENSMUST00000156118;ENSMUST00000113
MSTRG.1	Frs3os	ENSMUST00000136531;ENSMUST00000144979
MSTRG.1	Treml2	ENSMUST00000170941;MSTRG.14193.2
MSTRG.1	Nfya	ENSMUST00000078800;ENSMUST00000162460;ENSMUST00000046
MSTRG.1	Nfya	MSTRG.14195.1
MSTRG.1	Nfya	MSTRG.14196.1
MSTRG.1	Oard1	ENSMUST00000167180;MSTRG.14197.2;MSTRG.14197.3;ENSMUS
MSTRG.1	Apobec2	ENSMUST00000046549
MSTRG.1	Unc5cl	ENSMUST00000074574;ENSMUST00000161587;ENSMUST00000162
MSTRG.1	Ly96	ENSMUST00000191260;MSTRG.142.1;ENSMUST00000026881;ENS
MSTRG.1	Gm4204	ENSMUST00000110798;MSTRG.1420.2
MSTRG.1	Unc5cl	MSTRG.14200.1
MSTRG.1	Unc5cl	MSTRG.14201.1
MSTRG.1	Unc5cl	MSTRG.14202.1
MSTRG.1	Unc5cl	MSTRG.14203.1
MSTRG.1	Gm20540	ENSMUST00000172972
MSTRG.1	Mocs1	ENSMUST00000165390;ENSMUST00000024797;ENSMUST00000173
MSTRG.1	Daam2	ENSMUST00000224954;ENSMUST00000057610;ENSMUST00000224
MSTRG.14207		MSTRG.14207.1
MSTRG.14208		MSTRG.14208.1
MSTRG.1	Rftn1	ENSMUST00000044503;ENSMUST00000113195;ENSMUST00000156
MSTRG.1	Syt2	ENSMUST00000188842;ENSMUST00000121990;ENSMUST00000187
MSTRG.1	Rftn1	MSTRG.14210.1
MSTRG.1	Rftn1	MSTRG.14211.1
MSTRG.1	Rftn1	MSTRG.14212.1
MSTRG.1	Rftn1	MSTRG.14213.1
MSTRG.1	Gm7334	ENSMUST00000061681
MSTRG.1	Plcl2	MSTRG.14215.1;ENSMUST00000043938;MSTRG.14215.3;MSTRG.
MSTRG.1	Plcl2	MSTRG.14216.1;MSTRG.14216.2
MSTRG.1	Plcl2	MSTRG.14217.1
MSTRG.1	Tbc1d5;	ENSMUST00000024717;ENSMUST00000224977;ENSMUST00000224
MSTRG.1	Tbc1d5	MSTRG.14219.1
MSTRG.1	Ppp1r12	MSTRG.1422.1;ENSMUST00000045665;MSTRG.1422.2;MSTRG.14
MSTRG.1	Tbc1d5	MSTRG.14220.1
MSTRG.1	Tbc1d5	MSTRG.14221.1

MSTRG.1	Tbc1d5	MSTRG.14222.1
MSTRG.1	Tbc1d5	MSTRG.14223.1
MSTRG.1	Tbc1d5	MSTRG.14224.1
MSTRG.1	Satb1	MSTRG.14225.1
MSTRG.1	Gm19585	ENSMUST00000186007;ENSMUST00000191539
MSTRG.1	Gm19585	MSTRG.14227.1
MSTRG.14228		MSTRG.14228.1
MSTRG.1	Satb1;E	ENSMUST00000144331;MSTRG.14229.4;MSTRG.14229.2;ENSMUS
MSTRG.1	Gm37949	ENSMUST00000192808
MSTRG.1	Gm37593	MSTRG.14230.1;ENSMUST00000194780;MSTRG.14230.4;MSTRG.
MSTRG.1	Satb1	MSTRG.14231.1
MSTRG.14232		MSTRG.14232.1
MSTRG.14233		MSTRG.14233.1
MSTRG.1	Gm37176	MSTRG.14234.1
MSTRG.14235		MSTRG.14235.2;MSTRG.14235.3;MSTRG.14235.1
MSTRG.14236		MSTRG.14236.1
MSTRG.14237		MSTRG.14237.1
MSTRG.14238		MSTRG.14238.1
MSTRG.14239		MSTRG.14239.1;MSTRG.14239.2
MSTRG.1	Gm22232	ENSMUST00000104654
MSTRG.14240		MSTRG.14240.1;MSTRG.14240.2;MSTRG.14240.3;MSTRG.1424C
MSTRG.14241		MSTRG.14241.1
MSTRG.14242		MSTRG.14242.1
MSTRG.14243		MSTRG.14243.1
MSTRG.14244		MSTRG.14244.1
MSTRG.14245		MSTRG.14245.1
MSTRG.1	Rab5a	ENSMUST00000017975;MSTRG.14246.1
MSTRG.1	Pp2d1	ENSMUST00000056198
MSTRG.1	Pp2d1	MSTRG.14248.1
MSTRG.1	Kat2b	ENSMUST00000163648;MSTRG.14249.1;ENSMUST00000000724;M
MSTRG.1	Gm37677	ENSMUST00000194520
MSTRG.1	Kat2b	MSTRG.14250.1
MSTRG.1	Kat2b	MSTRG.14251.1
MSTRG.1	Kat2b	MSTRG.14252.1
MSTRG.1	Gm6919	ENSMUST00000180049
MSTRG.1	Kat2b	MSTRG.14254.1
MSTRG.1	Kat2b	MSTRG.14255.1
MSTRG.1	Kat2b	MSTRG.14256.1
MSTRG.1	Sgo1	MSTRG.14257.1;ENSMUST00000024736;ENSMUST00000144474;E
MSTRG.1	Sgo1	MSTRG.14258.1
MSTRG.14259		MSTRG.14259.1
MSTRG.1	Gm28892	ENSMUST00000188123;MSTRG.1426.2
MSTRG.1	Pot1b	ENSMUST00000086876;MSTRG.14260.2
MSTRG.1	Pot1b	MSTRG.14261.1
MSTRG.1	Pot1b	MSTRG.14262.1
MSTRG.1	Gm22654	ENSMUST00000157874
MSTRG.1	Pot1b	MSTRG.14264.1
MSTRG.1	Pot1b	MSTRG.14265.1
MSTRG.1	Zfp119a	ENSMUST00000079642
MSTRG.1	Zfp959	ENSMUST00000224379;ENSMUST00000054780;ENSMUST00000224
MSTRG.1	Ebi3	ENSMUST00000003274
MSTRG.1	Gm16712	ENSMUST00000181962
MSTRG.1	Ppp1r12	MSTRG.1427.1
MSTRG.1	Ccdc94	ENSMUST00000086869
MSTRG.1	Ccdc94	MSTRG.14271.1
MSTRG.1	Shd	ENSMUST00000044216;ENSMUST00000225145;ENSMUST00000224
MSTRG.1	Fsd1	ENSMUST00000011733

MSTRG.1	Stap2	ENSMUST00000043785
MSTRG.1	Mpnd	ENSMUST00000149441;ENSMUST00000162883;ENSMUST0000015[illegible]
MSTRG.1	Gm16276	ENSMUST00000131218
MSTRG.1	Sh3gl1	MSTRG.14277.1;MSTRG.14277.2;ENSMUST00000003268
MSTRG.1	Chaf1a	ENSMUST00000002914;MSTRG.14278.2
MSTRG.1	Ubxn6	MSTRG.14279.1;ENSMUST00000019722;ENSMUST00000132804;[illegible]
MSTRG.1	Ppp1r12[illegible]	MSTRG.1428.1
MSTRG.1	Hdgfl2	ENSMUST00000226053;ENSMUST00000002911;MSTRG.14280.4;[illegible]
MSTRG.1	Plin4	ENSMUST00000002908;MSTRG.14281.1;MSTRG.14281.2;ENSMUS[illegible]
MSTRG.1	Plin5	MSTRG.14282.1
MSTRG.1	Plin5	ENSMUST00000019808;ENSMUST00000113072
MSTRG.1	Lrg1	ENSMUST00000041357
MSTRG.1	Fem1a	ENSMUST00000060253
MSTRG.1	Fem1a	MSTRG.14286.1
MSTRG.1	Ticam1	ENSMUST00000058136
MSTRG.1	Tnfaip8	ENSMUST00000077788;MSTRG.14288.1;MSTRG.14288.3
MSTRG.1	Ube2t	ENSMUST00000027687;ENSMUST00000185369;MSTRG.1429.2;E[illegible]
MSTRG.1	Mydgf	ENSMUST00000019723;ENSMUST00000140885;ENSMUST00000151
MSTRG.1	Dpp9	ENSMUST00000223616;MSTRG.14291.1;MSTRG.14291.3;ENSMUS[illegible]
MSTRG.1	Plin3	ENSMUST00000019726;MSTRG.14292.3;MSTRG.14292.2
MSTRG.1	Arrdc5	ENSMUST00000097303
MSTRG.1	Uhrf1	ENSMUST00000001258;MSTRG.14294.5;MSTRG.14294.3;ENSMUS[illegible]
MSTRG.1	Kdm4b	ENSMUST00000139679;MSTRG.14295.1;MSTRG.14295.3;ENSMUS[illegible]
MSTRG.1	Kdm4b	MSTRG.14296.1
MSTRG.1	Ptprs	ENSMUST00000223859;ENSMUST00000086828;MSTRG.14297.3;[illegible]
MSTRG.1	Mir6977	ENSMUST00000184960
MSTRG.1	Ranbp3	MSTRG.14299.1;ENSMUST00000002445;MSTRG.14299.2
MSTRG.143		MSTRG.143.1
MSTRG.1	Timm17a	ENSMUST00000081104;ENSMUST00000152706;ENSMUST00000134
MSTRG.1	Vmac	ENSMUST00000067931;ENSMUST00000164907
MSTRG.1	Ndufa11	ENSMUST00000002452
MSTRG.1	Nrtn	ENSMUST00000044752
MSTRG.1	Dus3l	ENSMUST00000007747;MSTRG.14303.1;MSTRG.14303.2
MSTRG.1	Prr22	ENSMUST00000187612
MSTRG.1	Rfx2	ENSMUST00000002444;ENSMUST00000086801
MSTRG.1	Rfx2	MSTRG.14306.1
MSTRG.1	Rfx2	MSTRG.14307.1
MSTRG.1	Rfx2	MSTRG.14308.1
MSTRG.1	Rfx2	MSTRG.14309.1
MSTRG.1	Gm26642	ENSMUST00000180981
MSTRG.1	Rfx2	MSTRG.14310.1
MSTRG.1	Gm20219	ENSMUST00000209718
MSTRG.1	Safb2	MSTRG.14312.1;ENSMUST00000142752;ENSMUST00000124111;[illegible]
MSTRG.1	Safb	ENSMUST00000182031;MSTRG.14313.1;ENSMUST00000182533;[illegible]
MSTRG.1	2410015[illegible]	ENSMUST00000052832
MSTRG.1	Rpl36	ENSMUST00000080492
MSTRG.1	Lonp1	ENSMUST00000047226;MSTRG.14316.1
MSTRG.1	Clpp	ENSMUST00000002735
MSTRG.1	1700061[illegible]	ENSMUST00000025048;MSTRG.14318.3
MSTRG.1	Mllt1	MSTRG.14319.1;ENSMUST00000025053
MSTRG.1	Elf3	ENSMUST00000003135;ENSMUST00000185752;ENSMUST0000018[illegible]
MSTRG.1	Mllt1	MSTRG.14320.1
MSTRG.1	Gtf2f1	ENSMUST00000002733
MSTRG.1	Gm17949	ENSMUST00000210548
MSTRG.1	Khsrp	ENSMUST00000007814;MSTRG.14323.2;MSTRG.14323.3
MSTRG.1	C3	ENSMUST00000024988;ENSMUST00000177425;MSTRG.14324.2;[illegible]
MSTRG.1	Mir6978	ENSMUST00000184453

MSTRG.1	Gm17168	MSTRG.14326.2
MSTRG.1	Slc25a2	MSTRG.14327.1;ENSMUST00000040280;MSTRG.14327.3;ENSMUS
MSTRG.1	Crb3	ENSMUST00000071826;ENSMUST00000097299;ENSMUST0000016
MSTRG.1	Dennd1c	MSTRG.14329.3;MSTRG.14329.6;ENSMUST00000011623;MSTRG.
MSTRG.1	Rnpep	ENSMUST00000074357;ENSMUST00000077340;ENSMUST00000141
MSTRG.14330		MSTRG.14330.1
MSTRG.1	Gm11110	MSTRG.14331.1
MSTRG.1	Tnfsf9	ENSMUST00000039490
MSTRG.1	Gpr108	ENSMUST00000005975;MSTRG.14334.1;MSTRG.14334.2;MSTRG.
MSTRG.1	Trip10	ENSMUST00000224947;ENSMUST00000019631;ENSMUST00000224
MSTRG.14336		MSTRG.14336.1
MSTRG.1	Adgre1	ENSMUST00000086763;ENSMUST00000004850
MSTRG.1	Vav1	ENSMUST00000169220;ENSMUST00000005889;MSTRG.14338.3;M
MSTRG.1	Gm5815	ENSMUST00000050706
MSTRG.1	Ptprv	ENSMUST00000182120;ENSMUST00000183317;ENSMUST00000182
MSTRG.1	Gm5815	MSTRG.14340.1
MSTRG.14341		MSTRG.14341.1
MSTRG.14342		MSTRG.14342.1
MSTRG.14343		MSTRG.14343.1
MSTRG.14344		MSTRG.14344.1
MSTRG.1	Efna5	MSTRG.14345.1
MSTRG.1	Efna5	ENSMUST00000076840;ENSMUST00000078839
MSTRG.1	Efna5	MSTRG.14347.1
MSTRG.1	Efna5	MSTRG.14348.1
MSTRG.1	Efna5	MSTRG.14349.1
MSTRG.1	Gm10535	ENSMUST00000182275
MSTRG.1	Efna5	MSTRG.14350.1
MSTRG.1	Efna5	MSTRG.14351.1
MSTRG.1	Efna5	MSTRG.14352.1
MSTRG.1	Efna5	MSTRG.14353.1
MSTRG.1	Efna5	MSTRG.14354.1
MSTRG.14355		MSTRG.14355.1
MSTRG.14356		MSTRG.14356.1
MSTRG.1	Fbxl17	ENSMUST00000024761;MSTRG.14357.1;ENSMUST00000112839;E
MSTRG.1	Gm25348	ENSMUST00000158961
MSTRG.1	Fbxl17	MSTRG.14359.1
MSTRG.1	Ptpn7;A	MSTRG.1436.1;MSTRG.1436.2;ENSMUST00000187985;MSTRG.14
MSTRG.1	Fbxl17	MSTRG.14360.1;MSTRG.14360.2;MSTRG.14360.3
MSTRG.1	Fbxl17	MSTRG.14361.1
MSTRG.1	Gm24730	ENSMUST00000158228
MSTRG.1	Fbxl17	MSTRG.14363.1
MSTRG.1	Fbxl17	MSTRG.14364.1
MSTRG.1	Fbxl17	MSTRG.14365.1
MSTRG.1	Fbxl17	MSTRG.14366.1
MSTRG.1	4930405	MSTRG.14367.1;ENSMUST00000164605;ENSMUST00000171379;M
MSTRG.1	Fbxl17	MSTRG.14368.1
MSTRG.1	Gm17228	ENSMUST00000165006
MSTRG.1	Gm26280	ENSMUST00000157096
MSTRG.1	Gm24813	ENSMUST00000175310
MSTRG.1	Fbxl17	MSTRG.14371.1
MSTRG.1	Fbxl17	MSTRG.14372.1
MSTRG.1	Fbxl17	MSTRG.14373.1
MSTRG.1	Pja2	ENSMUST00000172818;ENSMUST00000024888;ENSMUST00000024
MSTRG.1	Pja2	MSTRG.14375.1
MSTRG.1	Gm24194	ENSMUST00000116805
MSTRG.1	Pja2	MSTRG.14377.1
MSTRG.14378		MSTRG.14378.1

MSTRG.1	Man2a1	MSTRG.14379.3;MSTRG.14379.2;MSTRG.14379.1;ENSMUST000C
MSTRG.1	Gm15445	ENSMUST00000146700
MSTRG.1	Gm17133	ENSMUST00000170636
MSTRG.1	Tmem232	ENSMUST00000062161
MSTRG.1	Tmem232	MSTRG.14382.1
MSTRG.1	Gm25731	ENSMUST00000175167
MSTRG.1	Vapa	MSTRG.14384.1
MSTRG.1	Vapa	ENSMUST00000024897;MSTRG.14385.1
MSTRG.1	Rab31	ENSMUST00000070673
MSTRG.1	Twsg1	ENSMUST00000024906
MSTRG.1	Ppp4r1	MSTRG.14388.1;ENSMUST00000161324;MSTRG.14388.3;ENSMUS
MSTRG.1	Ppp4r1	MSTRG.14389.1
MSTRG.1	Ipo9	ENSMUST00000041023;ENSMUST00000161032;ENSMUST00000161
MSTRG.1	Ppp4r1	MSTRG.14390.1
MSTRG.1	Ralbp1	ENSMUST00000024905;ENSMUST00000166543
MSTRG.14392		MSTRG.14392.1
MSTRG.1	Ankrd12	MSTRG.14393.1;ENSMUST00000038116;ENSMUST00000139901;E
MSTRG.1	Ankrd12	MSTRG.14394.1
MSTRG.1	Ndufv2	ENSMUST00000143987;MSTRG.14395.1;ENSMUST00000024909;E
MSTRG.1	Gm23264	ENSMUST00000083372
MSTRG.1	Washc1	ENSMUST00000072383;ENSMUST00000116556
MSTRG.14398		MSTRG.14398.1
MSTRG.1	Ddx11	ENSMUST00000225956;ENSMUST00000163605;ENSMUST00000223
MSTRG.1	Gm28376	ENSMUST00000186604
MSTRG.1	Gm28277	ENSMUST00000187450
MSTRG.1	Ddx11	MSTRG.14400.1
MSTRG.1	Ddx11	MSTRG.14401.1
MSTRG.1	Mtcl1	ENSMUST00000086693;ENSMUST00000097291;ENSMUST00000145
MSTRG.1	Gm4705	ENSMUST00000177415
MSTRG.1	Rab12	ENSMUST00000167962;ENSMUST00000070538;MSTRG.14404.3;M
MSTRG.14405		MSTRG.14405.1
MSTRG.1	Ptprm	ENSMUST00000037974;ENSMUST00000223982;ENSMUST00000225
MSTRG.1	AC13975	ENSMUST00000224424
MSTRG.1	AC13975	ENSMUST00000223962
MSTRG.1	AC16373	ENSMUST00000225696
MSTRG.1	Csrp1	ENSMUST00000097561;ENSMUST00000027677
MSTRG.1	Mir5709	ENSMUST00000176783
MSTRG.1	Ptprm	MSTRG.14411.1
MSTRG.1	Zbtb14	ENSMUST00000112674;ENSMUST00000062369;ENSMUST00000112
MSTRG.1	Zbtb14	MSTRG.14413.1
MSTRG.14414		MSTRG.14414.1
MSTRG.1	A930029	ENSMUST00000181032
MSTRG.1	C030034	ENSMUST00000188481;ENSMUST00000097289
MSTRG.1	Epb41l3	ENSMUST00000223610;ENSMUST00000225062;MSTRG.14417.2;E
MSTRG.1	2410021	ENSMUST00000187090
MSTRG.1	Ndc80	MSTRG.14419.1;ENSMUST00000024851
MSTRG.1	Phlda3	ENSMUST00000038945;ENSMUST00000136445
MSTRG.1	Ndc80	MSTRG.14420.1
MSTRG.1	Gm23115	ENSMUST00000158480
MSTRG.1	Ndc80	MSTRG.14422.1
MSTRG.1	Mettl4-:	ENSMUST00000130218;ENSMUST00000127676;ENSMUST00000124
MSTRG.1	Trmt61b	ENSMUST00000130952;ENSMUST00000137830;ENSMUST00000143
MSTRG.1	Wdr43	ENSMUST00000047086
MSTRG.1	Snord92	ENSMUST00000175548
MSTRG.1	Snord53	ENSMUST00000175146
MSTRG.1	Gm22858	ENSMUST00000174972
MSTRG.1	Tgif1	ENSMUST00000172229;ENSMUST00000059775;ENSMUST00000186

MSTRG.1	Nav1;Gm	ENSMUST00000040599;ENSMUST00000067414;MSTRG.1443.3;EN
MSTRG.1	Gm28727	ENSMUST00000176549;ENSMUST00000184151
MSTRG.1	Mir1195	ENSMUST00000116818
MSTRG.1	Gm26510	ENSMUST00000189974;ENSMUST00000180468;MSTRG.14432.3
MSTRG.1	Gm26561	ENSMUST00000180743
MSTRG.1	Gm26561	MSTRG.14434.1
MSTRG.1	Gm26510	MSTRG.14435.1
MSTRG.1	Gm26510	MSTRG.14436.1
MSTRG.1	Gm26510	MSTRG.14437.1
MSTRG.1	Gm26561	MSTRG.14439.1
MSTRG.1	Mir1231	ENSMUST00000175639
MSTRG.1	Myl12b;l	ENSMUST00000038446;ENSMUST00000137537;MSTRG.14440.4;M
MSTRG.1	Myom1	ENSMUST00000024847;ENSMUST00000073211
MSTRG.1	Lpin2	ENSMUST00000142842;MSTRG.14442.2;ENSMUST00000156570;E
MSTRG.1	Emilin2	ENSMUST00000024849
MSTRG.1	Gm26561	MSTRG.14444.1
MSTRG.1	Smchd1;(	ENSMUST00000138193;MSTRG.14445.1;ENSMUST00000127430;E
MSTRG.1	Gm26561	MSTRG.14446.1
MSTRG.1	Gm37639	ENSMUST00000191623
MSTRG.1	Clip4	ENSMUST00000024854;MSTRG.14448.2
MSTRG.1	Ypel5	MSTRG.14449.1;ENSMUST00000045174
MSTRG.1	2610012(	ENSMUST00000193434
MSTRG.1	Lbh	ENSMUST00000024857;MSTRG.14450.2;ENSMUST00000148556
MSTRG.1	Lclat1	ENSMUST00000130574;ENSMUST00000149064;ENSMUST00000067
MSTRG.1	Lclat1	MSTRG.14452.1
MSTRG.1	Lclat1	MSTRG.14453.1
MSTRG.1	Gm9311	ENSMUST00000118042
MSTRG.1	Gm25406	ENSMUST00000157599
MSTRG.1	Lclat1	MSTRG.14456.1
MSTRG.1	Ehd3	ENSMUST00000024860
MSTRG.1	Xdh	ENSMUST00000024866;MSTRG.14458.2
MSTRG.1	Memo1	MSTRG.14459.1;MSTRG.14459.2;MSTRG.14459.3;ENSMUST0000C
MSTRG.1	Gm37333	ENSMUST00000192904
MSTRG.1	Memo1	MSTRG.14460.1;MSTRG.14460.2
MSTRG.1	Memo1	MSTRG.14461.1
MSTRG.1	Dpy30	ENSMUST00000112571;MSTRG.14462.1;ENSMUST00000164832
MSTRG.1	Spast	MSTRG.14463.1
MSTRG.1	Spast	ENSMUST00000224711;ENSMUST00000024869;ENSMUST00000225
MSTRG.1	Spast	MSTRG.14465.1
MSTRG.1	Spast	MSTRG.14466.1
MSTRG.1	Slc30a6	ENSMUST00000024870;ENSMUST00000179074
MSTRG.1	Nlrc4	ENSMUST00000052124
MSTRG.1	Yipf4	ENSMUST00000024873
MSTRG.1	Gm4793	ENSMUST00000067468
MSTRG.1	Yipf4	MSTRG.14470.1
MSTRG.1	Ltbp1	ENSMUST00000001927;ENSMUST00000146839;ENSMUST00000135
MSTRG.1	Gm6276	ENSMUST00000120159
MSTRG.1	Fam98a	MSTRG.14473.1;MSTRG.14473.2;ENSMUST00000112507;ENSMUS
MSTRG.14474		MSTRG.14474.1
MSTRG.14475		MSTRG.14475.1
MSTRG.1	Crim1	ENSMUST00000112498
MSTRG.1	Fez2	ENSMUST00000070039;ENSMUST00000112487
MSTRG.1	Gm10093	ENSMUST00000079363
MSTRG.1	Pkp1	ENSMUST00000027667;ENSMUST00000163260;ENSMUST00000132
MSTRG.1	Birc6	MSTRG.14480.1
MSTRG.1	Birc6	ENSMUST00000180037;ENSMUST00000182133;ENSMUST00000182
MSTRG.1	Birc6	MSTRG.14482.1

MSTRG.1	Gm26749	ENSMUST00000181387
MSTRG.1	Birc6	MSTRG.14484.1
MSTRG.1	Ttc27	ENSMUST00000024882
MSTRG.1	Ttc27	MSTRG.14486.1
MSTRG.1	Strn	ENSMUST00000145910;ENSMUST00000024881;ENSMUST0000014[illegible]
MSTRG.1	Strn	MSTRG.14488.1
MSTRG.1	Heatr5b	MSTRG.14489.1
MSTRG.1	Gm36938	ENSMUST00000191612
MSTRG.1	Heatr5b	ENSMUST00000097281
MSTRG.1	Gpatch1	ENSMUST00000170759
MSTRG.1	Gm6548	ENSMUST00000167498
MSTRG.1	Eif2ak2	ENSMUST00000024884;ENSMUST00000169940;MSTRG.14493.3;[illegible]
MSTRG.1	n-R5s29	ENSMUST00000082836
MSTRG.1	Eif2ak2	MSTRG.14495.1
MSTRG.1	Eif2ak2	MSTRG.14496.1
MSTRG.1	Gm26637	ENSMUST00000180880
MSTRG.1	Sult6b1	ENSMUST00000169544;ENSMUST00000146474
MSTRG.14499		MSTRG.14499.1
MSTRG.1	4921511[illegible]	ENSMUST00000194790
MSTRG.1	Lad1	ENSMUST00000038760;ENSMUST00000190773;ENSMUST0000018[illegible]
MSTRG.1	Cdc42ep	ENSMUST00000068958;MSTRG.14500.1
MSTRG.1	Gm17315	ENSMUST00000181408
MSTRG.1	Cebpzos	ENSMUST00000180077;ENSMUST00000063817;ENSMUST00000192
MSTRG.1	Cebpz	MSTRG.14503.3;MSTRG.14503.2;ENSMUST00000024885;MSTRG.
MSTRG.1	Ndufaf7	ENSMUST00000024887
MSTRG.1	Prkd3	ENSMUST00000003191;ENSMUST00000168887;MSTRG.14505.3;[illegible]
MSTRG.1	Cyp1b1	ENSMUST00000024894
MSTRG.14507		MSTRG.14507.1
MSTRG.1	Rmdn2	ENSMUST00000223924;ENSMUST00000224014;ENSMUST0000004[illegible]
MSTRG.1	Atl2	MSTRG.14509.1
MSTRG.1	Tnnt2	ENSMUST00000188028;ENSMUST00000178204;ENSMUST00000191
MSTRG.1	Hnrnpll	MSTRG.14510.1
MSTRG.1	Atl2	ENSMUST00000068282;ENSMUST00000223273;MSTRG.14511.2;[illegible]
MSTRG.1	Atl2	MSTRG.14512.1
MSTRG.1	Atl2	MSTRG.14513.1;MSTRG.14513.2
MSTRG.14514		MSTRG.14514.1
MSTRG.14515		MSTRG.14515.1
MSTRG.1	Galm	ENSMUST00000039205;ENSMUST00000133955
MSTRG.1	Hnrnpll	ENSMUST00000184635;ENSMUST00000061331;MSTRG.14517.5;[illegible]
MSTRG.1	Gm22215	ENSMUST00000157558
MSTRG.1	Srsf7;G[illegible]	MSTRG.14519.1;ENSMUST00000157828;MSTRG.14519.10;MSTR[illegible]
MSTRG.1	Tmem9	ENSMUST00000165125;ENSMUST00000063719;ENSMUST00000117
MSTRG.1	Ttc39d	ENSMUST00000134652;ENSMUST00000053168
MSTRG.1	Cdkl4	ENSMUST00000086545
MSTRG.14522		MSTRG.14522.1
MSTRG.14523		MSTRG.14523.1
MSTRG.1	Gemin6	ENSMUST00000156869;ENSMUST00000069486
MSTRG.1	Dhx57	ENSMUST00000038166;ENSMUST00000086555;MSTRG.14525.3
MSTRG.1	Morn2	ENSMUST00000061703
MSTRG.1	Arhgef3	ENSMUST00000068175;ENSMUST00000223878;ENSMUST00000224
MSTRG.1	Gm10190	ENSMUST00000086549
MSTRG.1	Sos1	ENSMUST00000068714;MSTRG.14529.2
MSTRG.1	A430034[illegible]	ENSMUST00000194339
MSTRG.1	Sos1	MSTRG.14530.1
MSTRG.1	Sos1	MSTRG.14531.1
MSTRG.14532		MSTRG.14532.1
MSTRG.1	Map4k3	ENSMUST00000025089;ENSMUST00000112389;MSTRG.14533.1

MSTRG.1	Map4k3	MSTRG.14534.1
MSTRG.1	Gm9959	ENSMUST00000068277
MSTRG.1	Tmem178	ENSMUST00000025092
MSTRG.1	Tmem178	MSTRG.14537.1;MSTRG.14537.2
MSTRG.14538		MSTRG.14538.1
MSTRG.1	Thumpd2	ENSMUST00000025093
MSTRG.1	A430034	MSTRG.1454.1
MSTRG.1	Thumpd2	MSTRG.14540.1
MSTRG.14541		MSTRG.14541.1
MSTRG.1	Slc8a1	ENSMUST00000086538;ENSMUST00000163123;ENSMUST00000163
MSTRG.1	Slc8a1	MSTRG.14544.1
MSTRG.1	Slc8a1	MSTRG.14545.1
MSTRG.1	Slc8a1	MSTRG.14546.1
MSTRG.1	Pkdcc	ENSMUST00000170794;ENSMUST00000166528;ENSMUST0000017C
MSTRG.1	Cox7a2l	MSTRG.14548.1
MSTRG.1	Cox7a2l	ENSMUST00000025095;ENSMUST00000167741
MSTRG.1	Gm37552	ENSMUST00000193939
MSTRG.1	Eml4	ENSMUST00000049503;ENSMUST00000096766;ENSMUST00000112
MSTRG.1	Eml4	MSTRG.14551.1
MSTRG.14552		MSTRG.14552.1
MSTRG.1	Mta3	MSTRG.14553.2;MSTRG.14553.1;ENSMUST00000112350;ENSMUS
MSTRG.14554		MSTRG.14554.1
MSTRG.1	Haao	ENSMUST00000000687
MSTRG.14556		MSTRG.14556.1
MSTRG.14557		MSTRG.14557.1
MSTRG.14558		MSTRG.14558.1
MSTRG.14559		MSTRG.14559.1
MSTRG.1	Gm15850	ENSMUST00000134998;ENSMUST00000146091;ENSMUST00000165
MSTRG.14560		MSTRG.14560.1
MSTRG.1	Zfp36l2	ENSMUST00000060366;MSTRG.14561.2;MSTRG.14561.1;ENSMUS
MSTRG.1	Thada	MSTRG.14562.1;MSTRG.14562.2;MSTRG.14562.3;MSTRG.14562
MSTRG.1	Thada	MSTRG.14563.1
MSTRG.1	Thada	MSTRG.14564.1
MSTRG.1	Gm24492	ENSMUST00000083798
MSTRG.1	Thada	MSTRG.14566.1
MSTRG.1	Gm24722	ENSMUST00000179017
MSTRG.1	Abcg5	ENSMUST00000066175;ENSMUST00000171544
MSTRG.1	Abcg8	ENSMUST00000045714;ENSMUST00000171915
MSTRG.1	Kif21b	ENSMUST00000075164;ENSMUST00000130864;ENSMUST00000122
MSTRG.1	Lrpprc	MSTRG.14570.1;ENSMUST00000112308;ENSMUST00000162799;E
MSTRG.1	Lrpprc	MSTRG.14571.1
MSTRG.1	Six2	ENSMUST00000163568;ENSMUST00000024947
MSTRG.1	1110020	ENSMUST00000181147
MSTRG.1	Ppm1b	ENSMUST00000112305;ENSMUST00000080217;ENSMUST00000112
MSTRG.1	Ppm1b	MSTRG.14575.1
MSTRG.1	Ppm1b	MSTRG.14576.1
MSTRG.1	Slc3a1	ENSMUST00000024944
MSTRG.1	Prepl	ENSMUST00000072406;ENSMUST00000171795;MSTRG.14578.1
MSTRG.1	Camkmt	MSTRG.14579.1;ENSMUST00000095188
MSTRG.1	Gm26781	MSTRG.1458.1;ENSMUST00000181385
MSTRG.1	Camkmt	MSTRG.14580.1
MSTRG.14581		MSTRG.14581.1;MSTRG.14581.2
MSTRG.1	Srbd1	ENSMUST00000095187;MSTRG.14582.2
MSTRG.1	Srbd1	MSTRG.14583.1
MSTRG.1	Srbd1	MSTRG.14584.1
MSTRG.1	Epas1	ENSMUST00000024954;MSTRG.14585.1
MSTRG.1	Prkce	ENSMUST00000097275;ENSMUST00000142003;ENSMUST00000097

MSTRG.1	Prkce	MSTRG.14587.1
MSTRG.1	Prkce	MSTRG.14588.1
MSTRG.1	Prkce	MSTRG.14589.1
MSTRG.1	Gpr25	ENSMUST00000086395
MSTRG.1	Prkce	MSTRG.14590.1
MSTRG.1	Prkce	MSTRG.14591.1
MSTRG.1	Prkce	MSTRG.14592.1
MSTRG.1	Prkce	MSTRG.14593.1
MSTRG.1	2010106	ENSMUST00000191222
MSTRG.1	Prkce	MSTRG.14595.1
MSTRG.1	Prkce	MSTRG.14596.1
MSTRG.1	Prkce	MSTRG.14597.1
MSTRG.1	Prkce	MSTRG.14598.1
MSTRG.1	Gm10309	ENSMUST00000095186
MSTRG.1	Jph1	ENSMUST00000186024;ENSMUST00000038382
MSTRG.1	Gm26781	MSTRG.1460.1
MSTRG.1	Gm5499	MSTRG.14600.1
MSTRG.1	Rhoq	ENSMUST00000024956
MSTRG.1	Pigf	ENSMUST00000024957
MSTRG.1	Pigf	MSTRG.14603.1
MSTRG.1	Cript	ENSMUST00000024959;ENSMUST00000160824;ENSMUST0000016C
MSTRG.14605		MSTRG.14605.1
MSTRG.14606		MSTRG.14606.1
MSTRG.1	Socs5	MSTRG.14607.1;ENSMUST00000041369
MSTRG.1	Mcfd2	ENSMUST00000024963;ENSMUST00000144236;ENSMUST0000012
MSTRG.1	Mcfd2	MSTRG.14609.1
MSTRG.1	Camsap2	ENSMUST00000048309;ENSMUST00000194808;ENSMUST00000192
MSTRG.1	Calm2	MSTRG.14610.1
MSTRG.1	Stpg4	ENSMUST00000040609;ENSMUST00000186211;ENSMUST0000018
MSTRG.1	4833418	ENSMUST00000137539;ENSMUST00000146560;ENSMUST0000016C
MSTRG.1	Ttc7	ENSMUST00000041110;ENSMUST00000126497;MSTRG.14613.3;M
MSTRG.1	Gm15978	MSTRG.14614.1
MSTRG.1	0610012	ENSMUST00000159031
MSTRG.1	Calm2	ENSMUST00000040440;ENSMUST00000143965;ENSMUST0000015C
MSTRG.1	Msh2	MSTRG.14617.1
MSTRG.1	Epcam	ENSMUST00000053577
MSTRG.1	Msh2	ENSMUST00000024967;ENSMUST00000174240;ENSMUST0000017
MSTRG.1	Camsap2	MSTRG.1462.1
MSTRG.1	Msh2	MSTRG.14620.1
MSTRG.1	Kcnk12	ENSMUST00000055221
MSTRG.1	Kcnk12	MSTRG.14622.1
MSTRG.14623		MSTRG.14623.1
MSTRG.1	Msh6	ENSMUST00000005503;MSTRG.14624.3;MSTRG.14624.1;MSTRG.
MSTRG.1	Fbxo11	ENSMUST00000130379;ENSMUST00000005504;MSTRG.14625.2;M
MSTRG.1	Fbxo11	MSTRG.14626.1;MSTRG.14626.2;MSTRG.14626.3
MSTRG.1	Fbxo11	MSTRG.14627.1
MSTRG.1	Fbxo11	MSTRG.14628.1
MSTRG.1	Fbxo11	MSTRG.14629.1
MSTRG.1	9230116	ENSMUST00000181746;ENSMUST00000194227
MSTRG.1	Foxn2	ENSMUST00000112238;ENSMUST00000155640;MSTRG.14630.3;E
MSTRG.1	Foxn2	MSTRG.14631.1
MSTRG.1	Ppp1r21	ENSMUST00000038551;ENSMUST00000189734;MSTRG.14632.2;E
MSTRG.1	Gm38109	ENSMUST00000195386
MSTRG.1	Ston1	ENSMUST00000137138;ENSMUST00000163588;ENSMUST00000064
MSTRG.1	Ston1	MSTRG.14635.1
MSTRG.1	Fshr	MSTRG.14637.1
MSTRG.14638		MSTRG.14638.1

MSTRG.1	Gm10184	ENSMUST00000086423
MSTRG.1	Ddx59	ENSMUST00000129218;ENSMUST00000027655;MSTRG.1464.3;EN
MSTRG.14640		MSTRG.14640.1
MSTRG.1	Gm6741	ENSMUST00000057074
MSTRG.1	Mettl4	MSTRG.14642.1;ENSMUST00000171284
MSTRG.1	2700099	ENSMUST00000182402;ENSMUST00000182313;ENSMUST00000182
MSTRG.1	Gm1976	ENSMUST00000182639;MSTRG.14645.1;ENSMUST00000084298;M
MSTRG.1	Gm23964	ENSMUST00000157966
MSTRG.1	Gm1976	MSTRG.14647.1
MSTRG.1	Gm1976	MSTRG.14648.1
MSTRG.1	Gm26734	ENSMUST00000181769
MSTRG.1	Ddx59	MSTRG.1465.1
MSTRG.1	Gm23102	ENSMUST00000083724
MSTRG.1	Gm20939	ENSMUST00000108007
MSTRG.1	Cul2	ENSMUST00000025073;ENSMUST00000080089;ENSMUST00000162
MSTRG.14653		MSTRG.14653.1
MSTRG.1	Gm10557	MSTRG.14654.1
MSTRG.1	Gm10557	ENSMUST00000097690
MSTRG.1	Crem	ENSMUST00000151311;ENSMUST00000150235;ENSMUST00000122
MSTRG.1	Crem	MSTRG.14657.1
MSTRG.1	Crem	MSTRG.14658.1
MSTRG.1	Crem	MSTRG.14659.1
MSTRG.1	Kif14	ENSMUST00000187963;ENSMUST00000201676;ENSMUST0000018[illegible]
MSTRG.1	Crem	MSTRG.14660.1
MSTRG.1	Crem	MSTRG.14661.1
MSTRG.1	Gm6225	ENSMUST00000181558
MSTRG.1	Map3k8	ENSMUST00000025078;ENSMUST00000173930;ENSMUST00000105
MSTRG.1	Map3k8	MSTRG.14664.1
MSTRG.1	Jcad	ENSMUST00000037029
MSTRG.1	Gm26865	ENSMUST00000180970
MSTRG.1	Mtpap	ENSMUST00000025077;MSTRG.14667.3;MSTRG.14667.2;MSTRG.
MSTRG.1	Svil	ENSMUST00000126977;ENSMUST00000131210;MSTRG.14668.5;M
MSTRG.1	Svil	MSTRG.14669.1
MSTRG.1	Gm29486	ENSMUST00000088648
MSTRG.1	Svil	MSTRG.14670.1
MSTRG.1	Zfp438	MSTRG.14671.1;ENSMUST00000063989
MSTRG.1	Zfp438	MSTRG.14672.1
MSTRG.1	Zfp438	MSTRG.14673.1
MSTRG.1	Gm10125	ENSMUST00000161921;ENSMUST00000177190;ENSMUST0000017[illegible]
MSTRG.1	Gm2238	ENSMUST00000192074
MSTRG.1	Zeb1	ENSMUST00000025081;ENSMUST00000160522;ENSMUST0000015[illegible]
MSTRG.1	Gm16147	MSTRG.14677.1;ENSMUST00000162143
MSTRG.1	Gm22709	ENSMUST00000116893
MSTRG.1	Zeb1	MSTRG.14679.1;MSTRG.14679.2;MSTRG.14679.3
MSTRG.1	Gm29485	ENSMUST00000190220
MSTRG.1	Zeb1	MSTRG.14680.1
MSTRG.1	Zeb1	MSTRG.14681.1
MSTRG.1	Arhgap1	ENSMUST00000182066;ENSMUST00000077128;ENSMUST00000182
MSTRG.1	Arhgap1	MSTRG.14683.1
MSTRG.1	Arhgap1	MSTRG.14684.1
MSTRG.1	Arhgap1	MSTRG.14685.1
MSTRG.1	Arhgap1	MSTRG.14686.1
MSTRG.1	Arhgap1	MSTRG.14687.1
MSTRG.1	Arhgap1	MSTRG.14688.1
MSTRG.1	Arhgap1	MSTRG.14689.1
MSTRG.1	Gm37799	ENSMUST00000194018
MSTRG.1	Arhgap1	MSTRG.14690.1

MSTRG.1	Rpl27-p	ENSMUST00000097682
MSTRG.14692		MSTRG.14692.1
MSTRG.1	Epc1	MSTRG.14693.1
MSTRG.14694		MSTRG.14694.1
MSTRG.1	Kif5b	MSTRG.14695.1;MSTRG.14695.2;MSTRG.14695.3;MSTRG.14695
MSTRG.1	Gm17036	ENSMUST00000168187
MSTRG.1	Kif5b	MSTRG.14697.1
MSTRG.14698		MSTRG.14698.1
MSTRG.14699		MSTRG.14699.1;MSTRG.14699.2;MSTRG.14699.3;MSTRG.14699
MSTRG.1	Jph1	MSTRG.147.1
MSTRG.1	Platr23	ENSMUST00000180797
MSTRG.14700		MSTRG.14700.1
MSTRG.1	Rab18	ENSMUST00000097680
MSTRG.1	Epc1;Mi	ENSMUST00000115870;ENSMUST00000028100;ENSMUST00000124
MSTRG.1	Epc1	MSTRG.14703.1
MSTRG.1	Gm28529	MSTRG.14704.1;ENSMUST00000186159;ENSMUST00000189675
MSTRG.1	Mpp7	ENSMUST00000115869;MSTRG.14705.2
MSTRG.1	Fabp5l2	ENSMUST00000072582
MSTRG.1	Mpp7	MSTRG.14707.1
MSTRG.1	Mpp7	MSTRG.14708.1
MSTRG.1	Mpp7	MSTRG.14709.1
MSTRG.1	Zfp281	ENSMUST00000047734;ENSMUST00000112046
MSTRG.1	Mpp7	MSTRG.14710.1
MSTRG.14711		MSTRG.14711.1
MSTRG.14712		MSTRG.14712.1
MSTRG.14713		MSTRG.14713.1
MSTRG.14714		MSTRG.14714.1
MSTRG.1	Gm5819	MSTRG.14715.1
MSTRG.1	Gm5819	ENSMUST00000171469
MSTRG.1	Gm5819	MSTRG.14717.1
MSTRG.14718		MSTRG.14718.1
MSTRG.1	Fzd8	ENSMUST00000041080
MSTRG.1	Gm19705	ENSMUST00000180495;ENSMUST00000181491;ENSMUST00000181
MSTRG.1	Gjd4	ENSMUST00000041007
MSTRG.1	Ccny	ENSMUST00000053917
MSTRG.1	Ccny	MSTRG.14722.1
MSTRG.14723		MSTRG.14723.1
MSTRG.14724		MSTRG.14724.1
MSTRG.1	Wac	ENSMUST00000171042;ENSMUST00000166378;ENSMUST00000074
MSTRG.1	Wac	MSTRG.14726.1;MSTRG.14726.2
MSTRG.1	Gm7502	ENSMUST00000169235
MSTRG.1	Colec12	ENSMUST00000040069
MSTRG.1	Gm17430	ENSMUST00000171339
MSTRG.1	Gm4258	MSTRG.1473.1
MSTRG.1	Gm7497	ENSMUST00000183064
MSTRG.1	Gm23350	ENSMUST00000158429
MSTRG.1	Thoc1	MSTRG.14733.1;ENSMUST00000025137
MSTRG.1	Thoc1	MSTRG.14734.1
MSTRG.1	Thoc1	MSTRG.14735.1
MSTRG.1	Thoc1	MSTRG.14736.1
MSTRG.1	Usp14	ENSMUST00000092096;ENSMUST00000116669;ENSMUST00000133
MSTRG.1	Rock1	MSTRG.14738.1;MSTRG.14738.2;MSTRG.14738.3;MSTRG.14738
MSTRG.1	Rock1;G	MSTRG.14739.1;ENSMUST00000067947;MSTRG.14739.2;MSTRG.
MSTRG.1	Gm4258;	ENSMUST00000187561;MSTRG.1474.2;MSTRG.1474.1;ENSMUSTC
MSTRG.1	Snrpd1	ENSMUST00000002551
MSTRG.1	Greb1l	ENSMUST00000173356;ENSMUST00000048977;ENSMUST00000172
MSTRG.1	Gm44409	ENSMUST00000198810

MSTRG.1	Nutf2-p	ENSMUST00000179225
MSTRG.1	Gm24894	ENSMUST00000122744
MSTRG.1	Esco1	ENSMUST00000127099;ENSMUST00000097670;ENSMUST0000011[illegible]
MSTRG.1	Esco1	MSTRG.14746.1
MSTRG.1	Esco1	MSTRG.14747.1
MSTRG.1	Esco1	MSTRG.14748.1
MSTRG.1	Mib1	ENSMUST00000052838;ENSMUST00000131073;ENSMUST00000124
MSTRG.1	Gm37298	MSTRG.1475.1
MSTRG.1	Gm25116	ENSMUST00000157191
MSTRG.1	Mir133a	ENSMUST00000179771
MSTRG.1	Mir133a	ENSMUST00000083465
MSTRG.1	Mir1b	ENSMUST00000117012
MSTRG.14755		MSTRG.14755.1
MSTRG.1	Rbbp8	ENSMUST00000115861;ENSMUST00000047322
MSTRG.1	Rbbp8	MSTRG.14757.1
MSTRG.1	Gm6277;[illegible]	ENSMUST00000180860;ENSMUST00000224036;ENSMUST00000122
MSTRG.1	Gm6277	MSTRG.14759.1
MSTRG.1	Gm26979	MSTRG.1476.1;ENSMUST00000182971
MSTRG.1	Cables1	ENSMUST00000171109;ENSMUST00000046948;ENSMUST0000022[illegible]
MSTRG.1	Riok3	MSTRG.14761.1
MSTRG.1	Tmem241	ENSMUST00000209628;ENSMUST00000050228;ENSMUST0000005[illegible]
MSTRG.1	Tmem241	MSTRG.14763.1
MSTRG.1	Tmem241	MSTRG.14764.1
MSTRG.1	Tmem241	MSTRG.14765.1
MSTRG.1	Tmem241	MSTRG.14766.1
MSTRG.1	Tmem241	MSTRG.14767.1
MSTRG.1	Tmem241	MSTRG.14768.1
MSTRG.1	Tmem241	MSTRG.14769.1
MSTRG.1	Gm4258	MSTRG.1477.1
MSTRG.1	Tmem241	MSTRG.14770.1
MSTRG.1	Riok3	MSTRG.14771.2;ENSMUST00000025270;MSTRG.14771.1;MSTRG.
MSTRG.1	3110002[illegible]	ENSMUST00000126523;MSTRG.14772.1;ENSMUST00000025276;[illegible]
MSTRG.1	Npc1	MSTRG.14773.1;ENSMUST00000025279;ENSMUST00000145771;[illegible]
MSTRG.1	Gm15956	ENSMUST00000153352
MSTRG.1	Lama3	ENSMUST00000092070;ENSMUST00000188815;MSTRG.14775.2
MSTRG.1	Gm29201	ENSMUST00000189120
MSTRG.1	Gm29199	ENSMUST00000187766
MSTRG.1	Gm29200	ENSMUST00000188118
MSTRG.1	Gm22340	ENSMUST00000157717
MSTRG.1	Gm26936	ENSMUST00000182424;ENSMUST00000182680
MSTRG.1	Ttc39c	ENSMUST00000169401;ENSMUST00000141060;ENSMUST0000002[illegible]
MSTRG.1	Ttc39c	MSTRG.14781.1
MSTRG.1	Mir1948	ENSMUST00000157325
MSTRG.1	Impact;[illegible]	MSTRG.14783.1;MSTRG.14783.2;ENSMUST00000158504;ENSMUS
MSTRG.1	Impact	MSTRG.14784.1
MSTRG.1	Zfp521	ENSMUST00000025288
MSTRG.1	Zfp521	MSTRG.14786.1
MSTRG.1	Zfp521	MSTRG.14787.1
MSTRG.1	Gm5160	ENSMUST00000069552
MSTRG.1	Gm6457	ENSMUST00000066378
MSTRG.1	Gm4258	MSTRG.1479.1
MSTRG.14790		MSTRG.14790.1;MSTRG.14790.2;MSTRG.14790.3
MSTRG.14791		MSTRG.14791.1
MSTRG.1	Ss18;Ta	MSTRG.14792.1;MSTRG.14792.2;MSTRG.14792.3;MSTRG.14792
MSTRG.1	Ss18;Gm	ENSMUST00000040964;MSTRG.14793.2;MSTRG.14793.3;ENSMUS
MSTRG.1	Ss18	MSTRG.14794.1
MSTRG.1	Psma8	ENSMUST00000040860

MSTRG.1	Taf4b	ENSMUST00000169862
MSTRG.1	Kctd1	ENSMUST00000168989;ENSMUST00000025992;MSTRG.14797.3
MSTRG.1	Kctd1	MSTRG.14798.1
MSTRG.1	Gm6304	ENSMUST00000096579
MSTRG.148		MSTRG.148.1
MSTRG.1	Gm4258	MSTRG.1480.1;MSTRG.1480.2
MSTRG.1	Kctd1	MSTRG.14800.1
MSTRG.1	Kctd1	MSTRG.14801.1
MSTRG.1	Kctd1	MSTRG.14802.1
MSTRG.1	Kctd1	MSTRG.14803.1;MSTRG.14803.2
MSTRG.1	Kctd1	MSTRG.14804.1
MSTRG.1	Kctd1	MSTRG.14805.1
MSTRG.1	Kctd1	MSTRG.14806.1
MSTRG.1	Kctd1	MSTRG.14807.1
MSTRG.1	Kctd1	MSTRG.14808.1
MSTRG.1	Kctd1	MSTRG.14809.1
MSTRG.1	Mir181a	ENSMUST00000083631;ENSMUST00000182158;ENSMUST0000008[illegible]
MSTRG.14810		MSTRG.14810.1
MSTRG.14811		MSTRG.14811.1;MSTRG.14811.2;MSTRG.14811.3;MSTRG.14811
MSTRG.14812		MSTRG.14812.1
MSTRG.1	Gm10036	ENSMUST00000079733
MSTRG.14814		MSTRG.14814.1
MSTRG.14815		MSTRG.14815.1
MSTRG.1	1700001	ENSMUST00000153022;ENSMUST00000125117
MSTRG.1	1700001	MSTRG.14818.1
MSTRG.1	Gm37632	ENSMUST00000191811
MSTRG.1	Dsc3	ENSMUST00000115848;ENSMUST00000225110;ENSMUST0000022[illegible]
MSTRG.1	Dsc2	ENSMUST00000075214;ENSMUST00000039247;ENSMUST0000015[illegible]
MSTRG.1	Gm10269	ENSMUST00000165229
MSTRG.1	Dsg2	ENSMUST00000059787;ENSMUST00000120102;ENSMUST00000121
MSTRG.1	Gm16090	ENSMUST00000130296
MSTRG.1	Ttr	ENSMUST00000075312
MSTRG.1	Trappc8	ENSMUST00000225661;ENSMUST00000025177;MSTRG.14826.4;[illegible]
MSTRG.1	Gm24959	ENSMUST00000157924
MSTRG.1	Trappc8	MSTRG.14828.1
MSTRG.1	Rnf125	ENSMUST00000050004;MSTRG.14829.2;MSTRG.14829.3
MSTRG.1	Gm23782	ENSMUST00000157939
MSTRG.1	Rnf138	ENSMUST00000052396;ENSMUST00000072847;MSTRG.14830.1
MSTRG.1	Rnf138	MSTRG.14831.1
MSTRG.1	Rnf138	MSTRG.14832.1
MSTRG.1	Rnf138	MSTRG.14833.1
MSTRG.1	Gm15972	ENSMUST00000132571
MSTRG.1	Mapre2	ENSMUST00000132286;ENSMUST00000170802;ENSMUST0000015[illegible]
MSTRG.1	Mir6360	ENSMUST00000184278
MSTRG.1	Gm23207	ENSMUST00000158665
MSTRG.1	Zfp397	ENSMUST00000224107;ENSMUST00000225682;ENSMUST0000006[illegible]
MSTRG.1	Zscan30	ENSMUST00000224144;ENSMUST00000115829
MSTRG.1	Gm4258	MSTRG.1484.1
MSTRG.1	Zfp35	ENSMUST00000074941;MSTRG.14840.1
MSTRG.1	Zfp24	ENSMUST00000066497;ENSMUST00000153337;ENSMUST0000014[illegible]
MSTRG.1	Ino80c	ENSMUST00000141489;ENSMUST00000153360;MSTRG.14842.3;[illegible]
MSTRG.14843		MSTRG.14843.1
MSTRG.1	Galnt1	ENSMUST00000000430;ENSMUST00000170243;ENSMUST00000164
MSTRG.1	Galnt1	MSTRG.14845.1
MSTRG.1	Galnt1	MSTRG.14846.1
MSTRG.1	Gm7701	ENSMUST00000163185
MSTRG.1	2700062	ENSMUST00000097646

MSTRG.1	Rprd1a	ENSMUST00000046206;MSTRG.14849.2
MSTRG.1	Nek7	ENSMUST00000186017;ENSMUST00000187407;ENSMUST00000027
MSTRG.1	Mocos	ENSMUST00000068006
MSTRG.1	n-R5s23	ENSMUST00000122523
MSTRG.1	Slc39a6	ENSMUST00000070726;ENSMUST00000154205;ENSMUST0000012[illegible]
MSTRG.1	Elp2	MSTRG.14853.2;ENSMUST00000025120;MSTRG.14853.1
MSTRG.14854		MSTRG.14854.1
MSTRG.14855		MSTRG.14855.1
MSTRG.1	Fhod3	ENSMUST00000037097
MSTRG.1	Gm24294	ENSMUST00000158295
MSTRG.1	Tpgs2	ENSMUST00000148255;ENSMUST00000115817
MSTRG.1	AW55491	ENSMUST00000159605;ENSMUST00000036619;ENSMUST00000161
MSTRG.1	Gm28501	ENSMUST00000188729
MSTRG.1	AW55491	MSTRG.14860.1
MSTRG.1	AW55491	MSTRG.14861.1
MSTRG.1	Gm16558	ENSMUST00000163015
MSTRG.1	AW55491	MSTRG.14863.1
MSTRG.1	AW55491	MSTRG.14864.1
MSTRG.1	Gm7729	ENSMUST00000050143;MSTRG.14865.1
MSTRG.14866		MSTRG.14866.1
MSTRG.14867		MSTRG.14867.1
MSTRG.1	Pik3c3	ENSMUST00000115812;ENSMUST00000115811;ENSMUST00000091
MSTRG.1	Pik3c3	MSTRG.14869.1
MSTRG.1	Nek7	MSTRG.1487.1
MSTRG.1	Slc25a4	ENSMUST00000060396;MSTRG.14870.2
MSTRG.1	B930094	ENSMUST00000164667
MSTRG.1	Sap130	MSTRG.14872.1;MSTRG.14872.2;MSTRG.14872.3;ENSMUST000[illegible]
MSTRG.1	Ammecr1	ENSMUST00000115808;MSTRG.14873.1;MSTRG.14873.3
MSTRG.1	Gm26533	ENSMUST00000180550
MSTRG.1	Gm26533	MSTRG.14875.1
MSTRG.1	Polr2d	ENSMUST00000025106;MSTRG.14876.1
MSTRG.1	Wdr33	ENSMUST00000082319;MSTRG.14877.1;MSTRG.14877.3;ENSMUS[illegible]
MSTRG.1	Gm6665	MSTRG.14878.1
MSTRG.1	Gm6665	ENSMUST00000165131
MSTRG.1	Nek7	MSTRG.1488.1
MSTRG.1	Wdr33	MSTRG.14880.1
MSTRG.1	Gm26823	MSTRG.14881.1;ENSMUST00000181425
MSTRG.1	Wdr33	MSTRG.14882.1
MSTRG.1	Sft2d3	ENSMUST00000054984
MSTRG.1	Iws1	ENSMUST00000025243;ENSMUST00000212675;ENSMUST00000212
MSTRG.1	Iws1	MSTRG.14885.1
MSTRG.1	Map3k2	ENSMUST00000096575;MSTRG.14886.2;MSTRG.14886.3
MSTRG.1	Map3k2	MSTRG.14887.1
MSTRG.1	Ercc3	ENSMUST00000025241;MSTRG.14888.3;ENSMUST00000142213;[illegible]
MSTRG.1	A830052	ENSMUST00000181729
MSTRG.1	Gm37080	ENSMUST00000194804
MSTRG.1	Bin1	MSTRG.14890.1;ENSMUST00000025239;ENSMUST00000091967
MSTRG.1	Gypc	ENSMUST00000174459;ENSMUST00000174000;ENSMUST00000172
MSTRG.1	Gypc	MSTRG.14892.1
MSTRG.1	Gypc	MSTRG.14893.1
MSTRG.1	Tslp	ENSMUST00000025237
MSTRG.1	Gm26378	ENSMUST00000179093
MSTRG.1	Wdr36	ENSMUST00000166214;ENSMUST00000053663;MSTRG.14897.3
MSTRG.1	Stard4	MSTRG.14898.1;MSTRG.14898.2;MSTRG.14898.3;ENSMUST000[illegible]
MSTRG.14899		MSTRG.14899.1
MSTRG.1	Gm37101	ENSMUST00000191656
MSTRG.1	2310026	ENSMUST00000180941

MSTRG.1	Camk4	MSTRG.14901.1;ENSMUST00000042868;MSTRG.14901.4;MSTRG.
MSTRG.1	Gm22814	MSTRG.14902.1;ENSMUST00000157452
MSTRG.1	Camk4	MSTRG.14903.1
MSTRG.1	Camk4	MSTRG.14904.1
MSTRG.1	Camk4	MSTRG.14905.1;MSTRG.14905.2;MSTRG.14905.3
MSTRG.1	Camk4	MSTRG.14907.1
MSTRG.1	Camk4	MSTRG.14908.1
MSTRG.1	Camk4	MSTRG.14909.1
MSTRG.1	Nek7	MSTRG.1491.1
MSTRG.1	Nrep	ENSMUST00000051087;ENSMUST00000171533;ENSMUST0000016[illegible]
MSTRG.1	Epb41l4	ENSMUST00000146010;ENSMUST00000179138
MSTRG.1	Gm23639	ENSMUST00000175078
MSTRG.1	Epb41l4	ENSMUST00000025234
MSTRG.1	Reep5	ENSMUST00000006027;MSTRG.14914.2
MSTRG.1	Gm10548	ENSMUST00000097631;ENSMUST00000165672;MSTRG.14915.3
MSTRG.1	Apc	ENSMUST00000171187;ENSMUST00000079362;ENSMUST0000011[illegible]
MSTRG.1	Apc	MSTRG.14917.1
MSTRG.1	Apc	MSTRG.14918.1
MSTRG.1	Apc	MSTRG.14919.1
MSTRG.1	Nek7	MSTRG.1492.1
MSTRG.1	Srp19	ENSMUST00000126518;ENSMUST00000131713;ENSMUST0000014[illegible]
MSTRG.1	4933408	ENSMUST00000056932;ENSMUST00000129566
MSTRG.1	Brd8	MSTRG.14922.1
MSTRG.1	Pkd2l2	ENSMUST00000166156;ENSMUST00000014647;MSTRG.14923.3
MSTRG.1	Fam13b	MSTRG.14924.3;MSTRG.14924.2;MSTRG.14924.1;MSTRG.14924
MSTRG.1	n-R5s24	ENSMUST00000122518
MSTRG.1	Cdc25c	MSTRG.14926.1;ENSMUST00000060710
MSTRG.1	Gm3550	ENSMUST00000105038
MSTRG.1	Brd8	ENSMUST00000003876;ENSMUST00000115766;ENSMUST00000152
MSTRG.1	Brd8	MSTRG.14929.1
MSTRG.1	Nek7	MSTRG.1493.1
MSTRG.1	Kif20a	MSTRG.14930.1;MSTRG.14930.2;ENSMUST00000167161;ENSMUS
MSTRG.1	Cdc23	MSTRG.14931.1;ENSMUST00000025228;ENSMUST00000133181;E
MSTRG.1	Etf1;Hs	MSTRG.14933.2;ENSMUST00000025218;MSTRG.14933.1;ENSMUS
MSTRG.1	Gm22200	ENSMUST00000158614
MSTRG.1	Gm26109	ENSMUST00000083970
MSTRG.1	Ctnna1	ENSMUST00000042345;MSTRG.14936.2
MSTRG.1	Ctnna1	MSTRG.14937.1
MSTRG.1	Lrrtm2	ENSMUST00000091636
MSTRG.1	2010110	ENSMUST00000181453;ENSMUST00000181641
MSTRG.1	2310009	ENSMUST00000112025;ENSMUST00000195075;ENSMUST00000193
MSTRG.1	Fam53c	ENSMUST00000049281;MSTRG.14940.2;ENSMUST00000097622
MSTRG.1	Kdm3b;R	ENSMUST00000043775;ENSMUST00000225195;MSTRG.14941.1;E
MSTRG.1	Gm26538	ENSMUST00000180512
MSTRG.1	Egr1	ENSMUST00000064795
MSTRG.1	Sil1	ENSMUST00000025215
MSTRG.1	Sil1	MSTRG.14945.1
MSTRG.1	Snhg4;G	ENSMUST00000181664;ENSMUST00000187513;ENSMUST0000018[illegible]
MSTRG.1	Matr3	MSTRG.14947.1
MSTRG.1	Gm36989	ENSMUST00000193225
MSTRG.1	Prob1	ENSMUST00000190196;ENSMUST00000186951;ENSMUST00000097
MSTRG.1	Ptprc	ENSMUST00000112036;MSTRG.1495.1;ENSMUST00000182283;EN
MSTRG.1	Spata24	ENSMUST00000025209;ENSMUST00000096573
MSTRG.1	Paip2	ENSMUST00000041314;ENSMUST00000115737;ENSMUST0000011[illegible]
MSTRG.1	Slc23a1	MSTRG.14952.1;ENSMUST00000025212;ENSMUST00000150877;E
MSTRG.14954		MSTRG.14954.1
MSTRG.1	Mzb1	ENSMUST00000025211

MSTRG.1	Tmem173	ENSMUST00000115728
MSTRG.1	Dnajc18	ENSMUST00000025208;MSTRG.14957.2
MSTRG.1	Dnajc18	MSTRG.14958.1
MSTRG.1	Dnajc18	MSTRG.14959.1
MSTRG.1	Ptprc	MSTRG.1496.1
MSTRG.1	Dnajc18	MSTRG.14960.1
MSTRG.1	1700066	ENSMUST00000143778;ENSMUST00000097617
MSTRG.1	Ecscr	ENSMUST00000097618;ENSMUST00000133064;ENSMUST00000134
MSTRG.1	Cxxc5	MSTRG.14963.1
MSTRG.1	Ube2d2a	MSTRG.14964.1;MSTRG.14964.2;MSTRG.14964.3;MSTRG.14964
MSTRG.1	Gm16490	ENSMUST00000165299
MSTRG.1	Ube2d2a	ENSMUST00000170693;MSTRG.14966.1;ENSMUST00000167406;M
MSTRG.14967		MSTRG.14967.1
MSTRG.1	Gm29417	ENSMUST00000188160;MSTRG.14968.1
MSTRG.1	Pura;Gm	MSTRG.14969.1;ENSMUST00000051301;ENSMUST00000209540
MSTRG.1	Ptprc	MSTRG.1497.1
MSTRG.1	Pura	MSTRG.14970.1
MSTRG.1	Pfdn1	ENSMUST00000025204
MSTRG.14972		MSTRG.14972.1
MSTRG.14973		MSTRG.14973.1
MSTRG.1	Cystm1	ENSMUST00000139727;ENSMUST00000050584;ENSMUST00000145
MSTRG.1	Slc4a9	ENSMUST00000074298;ENSMUST00000115694
MSTRG.1	Ankhd1;	MSTRG.14976.1;ENSMUST00000134146;MSTRG.14976.2;MSTRG.
MSTRG.1	Sra1	ENSMUST00000173875;ENSMUST00000173202;ENSMUST0000006
MSTRG.1	Slc35a4	ENSMUST00000186538;ENSMUST00000050476;ENSMUST0000017C
MSTRG.1	E230025	ENSMUST00000115682
MSTRG.1	A130071	ENSMUST00000194467
MSTRG.1	Cd14	ENSMUST00000061829
MSTRG.1	Tmco6	ENSMUST00000007046
MSTRG.1	Ndufa2	ENSMUST00000014438
MSTRG.1	Ik	ENSMUST00000007042;MSTRG.14983.2;MSTRG.14983.3;ENSMUS
MSTRG.1	Vaultrc	ENSMUST00000083211
MSTRG.1	Wdr55	ENSMUST00000049323;MSTRG.14985.1
MSTRG.1	Dnd1;Ha	ENSMUST00000155827;MSTRG.14986.2;ENSMUST00000061522;E
MSTRG.1	Hars2	ENSMUST00000134122;ENSMUST00000152954;ENSMUST0000001S
MSTRG.1	Zmat2	ENSMUST00000001419;MSTRG.14988.1;MSTRG.14988.2;MSTRG.
MSTRG.1	Diaph1;	ENSMUST00000115634;MSTRG.14989.2;ENSMUST00000025337;E
MSTRG.1	Dennd1b	ENSMUST00000200533;MSTRG.1499.1;ENSMUST00000168527;EN
MSTRG.1	Hdac3	ENSMUST00000143660;ENSMUST00000144471;ENSMUST00000043
MSTRG.1	Rell2	ENSMUST00000163128;ENSMUST00000168056;ENSMUST0000017€
MSTRG.1	Fchsd1	ENSMUST00000165643;ENSMUST00000043437;ENSMUST0000016€
MSTRG.14993		MSTRG.14993.1
MSTRG.14994		MSTRG.14994.1
MSTRG.14995		MSTRG.14995.1
MSTRG.14996		MSTRG.14996.1
MSTRG.1	Pcdh1	MSTRG.14997.1
MSTRG.1	Pcdh1	ENSMUST00000160721;ENSMUST00000161701;ENSMUST00000057
MSTRG.1	2010320	ENSMUST00000160935
MSTRG.1	Gm37277	ENSMUST00000194052
MSTRG.1	Tfap2d	ENSMUST00000037294
MSTRG.1	Gm37423	ENSMUST00000194246;MSTRG.1500.1;ENSMUST00000200494
MSTRG.1	1700086	ENSMUST00000181871;ENSMUST00000181757
MSTRG.1	Gm10268	ENSMUST00000085340
MSTRG.1	0610009	ENSMUST00000025314;MSTRG.15002.2;MSTRG.15002.3
MSTRG.1	Pcdh12	ENSMUST00000195747;ENSMUST00000194012;ENSMUST00000193
MSTRG.1	Rnf14;G	ENSMUST00000072376;ENSMUST00000170811;MSTRG.15004.3;M
MSTRG.15005		MSTRG.15005.1

MSTRG.15006		MSTRG.15006.1
MSTRG.1	Gnpda1	MSTRG.15007.1;ENSMUST00000063814;ENSMUST00000153291;E
MSTRG.1	Gnpda1	MSTRG.15008.1
MSTRG.1	Pcdha1;	ENSMUST00000193839;ENSMUST00000070797;ENSMUST0000011
MSTRG.1	Dennd1b	MSTRG.1501.1
MSTRG.1	Gm37751	ENSMUST00000192440
MSTRG.1	Gm36858	ENSMUST00000194501
MSTRG.1	Gm10545	ENSMUST00000097612
MSTRG.1	Gm19035	ENSMUST00000193299;ENSMUST00000194575
MSTRG.1	Gm10544	ENSMUST00000180516;ENSMUST00000181887;ENSMUST00000192
MSTRG.1	Gm38097	ENSMUST00000192936
MSTRG.1	Pcdhb1	ENSMUST00000052366
MSTRG.1	Pcdhb2	ENSMUST00000056522
MSTRG.1	Gm18553	ENSMUST00000194152
MSTRG.1	Pcdhb3	ENSMUST00000051754
MSTRG.1	Gm18529	ENSMUST00000193025
MSTRG.1	Pcdhb4	ENSMUST00000056712
MSTRG.1	Pcdhb5	ENSMUST00000078271
MSTRG.1	Pcdhb6	ENSMUST00000061717
MSTRG.1	Pcdhb7	ENSMUST00000053037
MSTRG.1	Pcdhb8	ENSMUST00000192867;ENSMUST00000051163
MSTRG.1	Pcdhb9	ENSMUST00000057228
MSTRG.1	Pcdhb10	ENSMUST00000051126
MSTRG.1	Pcdhb11	ENSMUST00000053073
MSTRG.1	Dennd1b	MSTRG.1503.1
MSTRG.1	Gm37446	ENSMUST00000193015
MSTRG.1	Gm20162	ENSMUST00000193629
MSTRG.1	Pcdhb12	ENSMUST00000055495
MSTRG.1	Pcdhb13	ENSMUST00000056915
MSTRG.1	Pcdhb14	ENSMUST00000052387
MSTRG.1	Pcdhb15	ENSMUST00000050034
MSTRG.1	Pcdhb16	ENSMUST00000051442
MSTRG.1	Pcdhb17	ENSMUST00000053856
MSTRG.1	Pcdhb18	ENSMUST00000055949
MSTRG.1	Pcdhb19	ENSMUST00000059571
MSTRG.1	Gm16305	ENSMUST00000140747
MSTRG.1	Pcdhb20	ENSMUST00000052179
MSTRG.1	Pcdhb21	ENSMUST00000061405;ENSMUST00000192409
MSTRG.1	4930517	ENSMUST00000191880
MSTRG.1	Slc25a2	ENSMUST00000058635
MSTRG.1	Taf7	ENSMUST00000066272
MSTRG.1	Gm8242	ENSMUST00000193029
MSTRG.1	3222401	ENSMUST00000192137;ENSMUST00000097608
MSTRG.1	Gm24401	ENSMUST00000157868
MSTRG.1	Gm37165	ENSMUST00000193815
MSTRG.1	Dennd1b	MSTRG.1505.1
MSTRG.1	Pcdhga6	MSTRG.15050.1
MSTRG.1	Gm26672	ENSMUST00000180931;ENSMUST00000192501;ENSMUST00000192
MSTRG.1	Pcdhga1	MSTRG.15052.1
MSTRG.1	BC03703	ENSMUST00000192003
MSTRG.1	Gm29994	ENSMUST00000195495
MSTRG.1	Ndfip1	ENSMUST00000025293
MSTRG.1	Ndfip1	MSTRG.15056.1
MSTRG.15057		MSTRG.15057.1
MSTRG.1	Arhgap2	ENSMUST00000145090;ENSMUST00000148850;ENSMUST0000011
MSTRG.1	Gm19774	ENSMUST00000190667
MSTRG.1	Dennd1b	MSTRG.1506.1

MSTRG.1	Gm15335	ENSMUST00000122442
MSTRG.1	Gm8302	ENSMUST00000119217
MSTRG.1	Fgf1	ENSMUST00000040647;MSTRG.15062.1;ENSMUST00000131348;E
MSTRG.1	Gm15334	MSTRG.15063.1
MSTRG.1	Arhgap2	MSTRG.15064.1
MSTRG.1	Arhgap2	MSTRG.15065.1
MSTRG.1	Gm15336	ENSMUST00000128894
MSTRG.1	Arhgap2	MSTRG.15067.1
MSTRG.1	Arhgap2	MSTRG.15068.1
MSTRG.1	Gm15333	ENSMUST00000118825
MSTRG.1	Gm37470	ENSMUST00000193378
MSTRG.1	Gm15337	ENSMUST00000135378
MSTRG.1	Nr3c1	ENSMUST00000115567;ENSMUST00000115571;MSTRG.15071.4;M
MSTRG.1	Nr3c1	MSTRG.15072.1
MSTRG.1	Yipf5	ENSMUST00000025364;MSTRG.15073.2;MSTRG.15073.3
MSTRG.1	Prelid2	ENSMUST00000070949
MSTRG.1	Prelid2	MSTRG.15075.1
MSTRG.1	Prelid2	MSTRG.15076.1
MSTRG.1	Lars	ENSMUST00000097590;MSTRG.15077.2;MSTRG.15077.3
MSTRG.1	Rps19-p	ENSMUST00000091925
MSTRG.1	Rbm27;G	MSTRG.15079.3;MSTRG.15079.1;ENSMUST00000046972;ENSMUS
MSTRG.1	Gm43544	ENSMUST00000196474
MSTRG.1	Rbm27	MSTRG.15080.1
MSTRG.1	Gpr151	ENSMUST00000054738
MSTRG.1	Tcerg1;	ENSMUST00000025375;ENSMUST00000173642;MSTRG.15082.2;M
MSTRG.1	Gm8181	ENSMUST00000062242
MSTRG.1	Dpysl3	ENSMUST00000121805;ENSMUST00000135547;ENSMUST00000118
MSTRG.1	Gm3650	ENSMUST00000180600;MSTRG.15085.1;MSTRG.15085.2
MSTRG.1	Gm3650	MSTRG.15086.1
MSTRG.1	Eif3j2;	ENSMUST00000057110;ENSMUST00000175467
MSTRG.1	Dcp2	ENSMUST00000202306;MSTRG.15088.1;ENSMUST00000025350;M
MSTRG.1	Dcp2	MSTRG.15089.1
MSTRG.1	Dennd1b	MSTRG.1509.1
MSTRG.1	Mcc	ENSMUST00000089874;ENSMUST00000164666;ENSMUST00000202
MSTRG.1	Mcc	ENSMUST00000202090
MSTRG.1	Mcc	MSTRG.15092.1
MSTRG.1	Mcc	MSTRG.15093.1
MSTRG.1	A930012	ENSMUST00000181094;ENSMUST00000202771
MSTRG.1	A930012	MSTRG.15095.1
MSTRG.1	Mcc	MSTRG.15096.1
MSTRG.1	Mcc	MSTRG.15097.1
MSTRG.1	Mcc	MSTRG.15098.1
MSTRG.1	Gm43425	ENSMUST00000201793
MSTRG.1	Gm10075	ENSMUST00000162030
MSTRG.1	Gm37655	ENSMUST00000193968
MSTRG.1	Ythdc2	ENSMUST00000037763;MSTRG.15100.2;ENSMUST00000201507;E
MSTRG.1	Ythdc2	ENSMUST00000201099
MSTRG.1	Ythdc2	MSTRG.15102.1
MSTRG.1	Ythdc2	MSTRG.15103.1
MSTRG.15104		MSTRG.15104.1
MSTRG.1	Pggt1b	MSTRG.15105.1;ENSMUST00000025354
MSTRG.1	Pggt1b	MSTRG.15106.1
MSTRG.1	Pggt1b	MSTRG.15107.1
MSTRG.1	Gm24617	ENSMUST00000116705
MSTRG.15109		MSTRG.15109.1
MSTRG.1	Mospd4	ENSMUST00000180618
MSTRG.1	Gm24076	ENSMUST00000158179

MSTRG.15112		MSTRG.15112.1
MSTRG.1	Fem1c	ENSMUST00000036226;MSTRG.15113.2;MSTRG.15113.3
MSTRG.1	Fem1c	MSTRG.15114.1
MSTRG.1	Fem1c	MSTRG.15115.1
MSTRG.1	Fem1c	MSTRG.15116.1
MSTRG.1	Ticam2;'	ENSMUST00000070084;ENSMUST00000149929;ENSMUST0000014З
MSTRG.1	Eif1a	ENSMUST00000078079;MSTRG.15118.1;ENSMUST00000168382
MSTRG.1	Cdo1;At	ENSMUST00000035804;MSTRG.15119.1;ENSMUST00000035648;M
MSTRG.1	Dennd1b	MSTRG.1512.1
MSTRG.1	Ap3s1	ENSMUST00000025357;ENSMUST00000225520;MSTRG.15120.2;E
MSTRG.1	Ap3s1	MSTRG.15121.1
MSTRG.1	Ap3s1	MSTRG.15122.1
MSTRG.1	Commd10	ENSMUST00000049388
MSTRG.1	Gm22791	ENSMUST00000157232
MSTRG.1	Commd10	MSTRG.15125.1
MSTRG.1	Commd10	MSTRG.15127.1
MSTRG.15128		MSTRG.15128.1
MSTRG.15129		MSTRG.15129.1
MSTRG.1	Dennd1b	MSTRG.1513.1
MSTRG.15130		MSTRG.15130.1
MSTRG.1	Eno1b	ENSMUST00000076155
MSTRG.15132		MSTRG.15132.1
MSTRG.1	Dtwd2	ENSMUST00000163590;ENSMUST00000025383;ENSMUST0000013
MSTRG.1	Dtwd2	MSTRG.15134.1
MSTRG.1	Dtwd2	MSTRG.15135.1
MSTRG.1	Dtwd2	MSTRG.15136.1
MSTRG.1	Gm16283	ENSMUST00000149935
MSTRG.15138		MSTRG.15138.1
MSTRG.1	Hsd17b4	ENSMUST00000025385;MSTRG.15139.2;ENSMUST00000152388;E
MSTRG.1	Gm37718	ENSMUST00000191655
MSTRG.1	Cd63-ps	ENSMUST00000145417;ENSMUST00000154992
MSTRG.15141		MSTRG.15141.1
MSTRG.1	Dmxl1	MSTRG.15142.2;MSTRG.15142.1;ENSMUST00000180611;ENSMUS
MSTRG.1	Dmxl1	MSTRG.15143.1
MSTRG.1	C030005	MSTRG.15144.1;MSTRG.15144.2;ENSMUST00000151719;ENSMUS
MSTRG.1	Tnfaip8	MSTRG.15145.1
MSTRG.1	Tnfaip8	ENSMUST00000148989;ENSMUST00000087655;ENSMUST0000017
MSTRG.1	Tnfaip8	MSTRG.15147.1
MSTRG.1	Tnfaip8	MSTRG.15148.1
MSTRG.1	Srfbp1	ENSMUST00000025406;MSTRG.15149.2
MSTRG.1	Gm4788	ENSMUST00000111989
MSTRG.1	Gm27026	ENSMUST00000183003
MSTRG.1	Srfbp1	MSTRG.15151.1
MSTRG.1	Lox	ENSMUST00000171470;ENSMUST00000025409
MSTRG.1	Snx2	MSTRG.15153.3;MSTRG.15153.1;MSTRG.15153.2;ENSMUST000C
MSTRG.1	Snx2	MSTRG.15154.1
MSTRG.1	Ppic	ENSMUST00000025419
MSTRG.1	Cep120	ENSMUST00000049811;MSTRG.15156.1;MSTRG.15156.3;MSTRG.
MSTRG.1	Csnk1g3	ENSMUST00000069597;MSTRG.15157.1;MSTRG.15157.2;MSTRG.
MSTRG.1	Csnk1g3	MSTRG.15158.1
MSTRG.1	Csnk1g3	MSTRG.15159.1
MSTRG.1	Gm4788	MSTRG.1516.1
MSTRG.1	Gm5507	ENSMUST00000091897
MSTRG.1	Zfp608	ENSMUST00000064763
MSTRG.1	Gm26742	ENSMUST00000181829
MSTRG.1	Gm4221	ENSMUST00000181538;ENSMUST00000181765
MSTRG.15164		MSTRG.15164.1

MSTRG.1	Gm23555	ENSMUST00000157815
MSTRG.15166		MSTRG.15166.1
MSTRG.1	Gm25476	ENSMUST00000175125
MSTRG.15168		MSTRG.15168.1
MSTRG.15169		MSTRG.15169.1
MSTRG.1	Zbtb41	ENSMUST00000200243;MSTRG.1517.1;ENSMUST00000039867;EN
MSTRG.15170		MSTRG.15170.1
MSTRG.15171		MSTRG.15171.1
MSTRG.15172		MSTRG.15172.1
MSTRG.15173		MSTRG.15173.1
MSTRG.1	Gramd3	ENSMUST00000070166;MSTRG.15174.2;MSTRG.15174.3;MSTRG.
MSTRG.1	Gramd3	MSTRG.15175.1
MSTRG.1	Gramd3	MSTRG.15176.1
MSTRG.1	Aldh7a1	ENSMUST00000172902;ENSMUST00000174518;ENSMUST00000172
MSTRG.1	Aldh7a1	MSTRG.15178.1
MSTRG.1	Mir1258	ENSMUST00000183892
MSTRG.1	Aspm	ENSMUST00000200083;ENSMUST00000199998;ENSMUST0000005З
MSTRG.1	Aldh7a1	MSTRG.15180.1
MSTRG.1	Phax	ENSMUST00000130163;ENSMUST00000132628;ENSMUST0000000
MSTRG.1	Tex43	ENSMUST00000035640;ENSMUST00000153248
MSTRG.1	Lmnb1	ENSMUST00000025486;MSTRG.15183.2;ENSMUST00000128651;M
MSTRG.1	Lmnb1	MSTRG.15184.1
MSTRG.1	Lmnb1	MSTRG.15185.1
MSTRG.1	March3	ENSMUST00000102912;ENSMUST00000035278;ENSMUST0000015
MSTRG.1	Gm15345	ENSMUST00000128240
MSTRG.1	March3	MSTRG.15188.1
MSTRG.1	March3	MSTRG.15189.1
MSTRG.1	Gm38019	ENSMUST00000195133
MSTRG.1	March3	MSTRG.15190.1
MSTRG.1	March3	MSTRG.15191.1
MSTRG.1	March3	MSTRG.15192.1
MSTRG.1	C330018	ENSMUST00000025488;ENSMUST00000139243;ENSMUST00000152
MSTRG.1	Prrc1	ENSMUST00000025490
MSTRG.1	4930511	ENSMUST00000130696;ENSMUST00000150994;ENSMUST0000015
MSTRG.1	Ctxn3	ENSMUST00000091892
MSTRG.1	1700011	ENSMUST00000079738
MSTRG.1	1700011	MSTRG.15198.1
MSTRG.1	Pkhd1	ENSMUST00000088448;ENSMUST00000147480
MSTRG.1	Gm38019	MSTRG.1520.1
MSTRG.1	Slc12a2	ENSMUST00000115366
MSTRG.1	Isoc1	ENSMUST00000025503;MSTRG.15202.2
MSTRG.1	Isoc1	MSTRG.15203.1
MSTRG.1	A730017	ENSMUST00000117064;ENSMUST00000165666;ENSMUST0000005
MSTRG.1	Gm4951	ENSMUST00000031549
MSTRG.1	Gm4951	MSTRG.15206.1
MSTRG.1	Gm5970	ENSMUST00000131638
MSTRG.1	Gm4951	MSTRG.15208.1
MSTRG.1	Gm4841	ENSMUST00000090260
MSTRG.1	Cfh	ENSMUST00000192880;ENSMUST00000111976;ENSMUST00000111
MSTRG.1	F830016	ENSMUST00000171297
MSTRG.1	Iigp1	MSTRG.15211.1;MSTRG.15211.2;ENSMUST00000032473;ENSMUS
MSTRG.1	Smim3	ENSMUST00000042710
MSTRG.1	Dctn4	ENSMUST00000223984;ENSMUST00000025505;ENSMUST0000022
MSTRG.1	Rbm22	MSTRG.15214.1
MSTRG.1	Rbm22	ENSMUST00000025506;ENSMUST00000160020;ENSMUST00000161
MSTRG.1	Synpo	ENSMUST00000097566;ENSMUST00000130044;ENSMUST0000013
MSTRG.1	Ndst1	ENSMUST00000169273;MSTRG.15217.2

MSTRG.15218	MSTRG.15218.1
MSTRG.15219	MSTRG.15219.1
MSTRG.1 Cfh	MSTRG.1522.1
MSTRG.15220	MSTRG.15220.1
MSTRG.15221	MSTRG.15221.1
MSTRG.15222	MSTRG.15222.1
MSTRG.1 Rps14	ENSMUST00000025511;ENSMUST00000142980;ENSMUST0000011[illegible]
MSTRG.15224	MSTRG.15224.1
MSTRG.15225	MSTRG.15225.1
MSTRG.1 Tcof1	MSTRG.15226.1;MSTRG.15226.2;ENSMUST00000175934;ENSMUS
MSTRG.1 Cd74;Mi	ENSMUST00000050487;ENSMUST00000097563;ENSMUST00000167
MSTRG.1 Arsi	ENSMUST00000040359;MSTRG.15228.2;MSTRG.15228.3
MSTRG.1 Camk2a	ENSMUST00000102888;ENSMUST00000134496;ENSMUST0000002[illegible]
MSTRG.1523	MSTRG.1523.1
MSTRG.1 Mir6982	ENSMUST00000184354
MSTRG.1 Cdx1	ENSMUST00000025521
MSTRG.1 Pdgfrb	ENSMUST00000025522;MSTRG.15233.2;ENSMUST00000115274
MSTRG.1 Slc26a2	ENSMUST00000146409;ENSMUST00000037603;ENSMUST0000014[illegible]
MSTRG.1 Pde6a	ENSMUST00000025468
MSTRG.1 Rps2-ps	ENSMUST00000170335;ENSMUST00000076842
MSTRG.1 Csf1r	MSTRG.15237.1;ENSMUST00000025523;ENSMUST00000115268
MSTRG.1 Mir6983	ENSMUST00000183458
MSTRG.1 Hmgxb3	ENSMUST00000091884;MSTRG.15239.2;MSTRG.15239.3;MSTRG.
MSTRG.1524	MSTRG.1524.1
MSTRG.1 Gm26403	ENSMUST00000158500
MSTRG.1 Hmgxb3	MSTRG.15241.1
MSTRG.1 Pde6a	MSTRG.15242.1
MSTRG.1 Ppargc1	ENSMUST00000075299;ENSMUST00000063307;ENSMUST00000142
MSTRG.1 Ppargc1	MSTRG.15244.1
MSTRG.1 Ppargc1	MSTRG.15245.1
MSTRG.1 Ppargc1	MSTRG.15246.1
MSTRG.1 Ppargc1	MSTRG.15247.1
MSTRG.1 Ppargc1	MSTRG.15248.1
MSTRG.1 Ppargc1	MSTRG.15249.1
MSTRG.1525	MSTRG.1525.1
MSTRG.1 Gm25301	ENSMUST00000175285
MSTRG.1 Csnk1a1	ENSMUST00000165123;ENSMUST00000165721;ENSMUST0000011[illegible]
MSTRG.1 Bvht	ENSMUST00000183083;ENSMUST00000183087;MSTRG.15253.3
MSTRG.1 Mir145a	ENSMUST00000083658
MSTRG.1 Mir143	ENSMUST00000083511
MSTRG.1 Carmn	ENSMUST00000182244;MSTRG.15256.1;ENSMUST00000181155
MSTRG.1 Pcyox1l	ENSMUST00000025472;ENSMUST00000195229;MSTRG.15257.3;[illegible]
MSTRG.1 1500015	ENSMUST00000184181;ENSMUST00000184678;ENSMUST00000184
MSTRG.1 Afap1l1	ENSMUST00000120472;ENSMUST00000147278
MSTRG.1526	MSTRG.1526.1
MSTRG.1 Ablim3	ENSMUST00000049378;ENSMUST00000166783
MSTRG.1 Ablim3	MSTRG.15261.1
MSTRG.1 Sh3tc2	ENSMUST00000051720
MSTRG.1 Sh3tc2	MSTRG.15263.1
MSTRG.15264	MSTRG.15264.1
MSTRG.1 Adrb2	ENSMUST00000053640
MSTRG.1 Gm9949	ENSMUST00000067743
MSTRG.15267	MSTRG.15267.1
MSTRG.1 Apcdd1	ENSMUST00000096554
MSTRG.1 Fbxo38	MSTRG.15269.1;ENSMUST00000048688;MSTRG.15269.3;MSTRG.
MSTRG.1 Cdc73	ENSMUST00000018337;ENSMUST00000162638;MSTRG.1527.3;E[illegible]
MSTRG.1 Spink10	ENSMUST00000055725;ENSMUST00000160316

MSTRG.1	Gm45787	ENSMUST00000212026
MSTRG.1	Gm17732	ENSMUST00000163259
MSTRG.1	Napg	ENSMUST00000025474;ENSMUST00000126870;MSTRG.15275.2;E
MSTRG.15276		MSTRG.15276.1
MSTRG.1	Txnl1	ENSMUST00000025476
MSTRG.1	Amd-ps3	ENSMUST00000072796
MSTRG.1	Wdr7	MSTRG.15279.1;ENSMUST00000072726
MSTRG.1	Cdc73	MSTRG.1528.1
MSTRG.1	Gm24384	ENSMUST00000122693
MSTRG.1	Fech	ENSMUST00000025484
MSTRG.1	Nars	MSTRG.15282.2;ENSMUST00000025483;MSTRG.15282.1
MSTRG.1	Malt1	ENSMUST00000224265;MSTRG.15283.1;ENSMUST00000049248;E
MSTRG.1	AC13170	ENSMUST00000224731
MSTRG.1	Nedd4l	ENSMUST00000225057;MSTRG.15285.1;ENSMUST00000163516;E
MSTRG.1	Nedd4l	MSTRG.15286.1
MSTRG.1	Nedd4l	MSTRG.15287.1
MSTRG.1	Nedd4l	MSTRG.15288.1
MSTRG.1	Nedd4l	MSTRG.15289.1
MSTRG.1	Cdc73	MSTRG.1529.1
MSTRG.1	Nedd4l	MSTRG.15290.1
MSTRG.1	Nedd4l	MSTRG.15291.1
MSTRG.1	Nedd4l	MSTRG.15292.1
MSTRG.1	Nedd4l	MSTRG.15293.1
MSTRG.1	Nedd4l	MSTRG.15294.1
MSTRG.1	Zfp532	ENSMUST00000049016;ENSMUST00000183236;ENSMUST00000182
MSTRG.1	Zfp532	MSTRG.15296.1
MSTRG.1	Gm37643	ENSMUST00000195369
MSTRG.1	Zfp532	MSTRG.15298.1
MSTRG.1	Zfp532	MSTRG.15299.1
MSTRG.1	B3galt2	ENSMUST00000038252
MSTRG.1	Sec11c	MSTRG.15300.1
MSTRG.1	Sec11c	ENSMUST00000025394;ENSMUST00000126432;ENSMUST00000153
MSTRG.1	Grp	ENSMUST00000173985;ENSMUST00000173530;ENSMUST00000025
MSTRG.1	Lman1	ENSMUST00000048260;ENSMUST00000120461;ENSMUST00000155
MSTRG.15304		MSTRG.15304.1;MSTRG.15304.2
MSTRG.15305		MSTRG.15305.1
MSTRG.15306		MSTRG.15306.1
MSTRG.1	Gnal	ENSMUST00000025402
MSTRG.1	Gm23512	ENSMUST00000180237
MSTRG.1	Chmp1b	ENSMUST00000209359;ENSMUST00000210564
MSTRG.1	B3galt2	MSTRG.1531.1
MSTRG.1	Mppe1	ENSMUST00000073054;MSTRG.15310.1
MSTRG.1	Tubb6	ENSMUST00000001513
MSTRG.1	Impa2	ENSMUST00000025403
MSTRG.1	Impa2	MSTRG.15313.1
MSTRG.1	Impa2	MSTRG.15314.1
MSTRG.1	B430212	ENSMUST00000144078;ENSMUST00000145081;ENSMUST00000127
MSTRG.1	Cidea	ENSMUST00000025404
MSTRG.1	Afg3l2	ENSMUST00000025408;MSTRG.15317.2;MSTRG.15317.3
MSTRG.1	Afg3l2	MSTRG.15318.1
MSTRG.1	Cep76	ENSMUST00000097542
MSTRG.1	Cdc73	MSTRG.1532.1
MSTRG.1	Psmg2	ENSMUST00000025418;MSTRG.15320.1;MSTRG.15320.3
MSTRG.1	Ptpn2	ENSMUST00000120934;ENSMUST00000025420;ENSMUST00000122
MSTRG.1	Ptpn2	MSTRG.15322.1
MSTRG.1	Ptpn2	MSTRG.15323.1
MSTRG.1	Gm26910	MSTRG.15324.1;ENSMUST00000180450

MSTRG.1	Seh1l	ENSMUST00000025421
MSTRG.1	Cep192	ENSMUST00000025425;ENSMUST00000224387;MSTRG.15326.2;E
MSTRG.1	Cep192	MSTRG.15327.1
MSTRG.1	Mc2r	ENSMUST00000052347
MSTRG.1	Mc2r	MSTRG.15329.1
MSTRG.1	Gm6397	ENSMUST00000161973
MSTRG.1	Mc2r	MSTRG.15330.1
MSTRG.1	Ldlrad4	MSTRG.15331.1;MSTRG.15331.4;MSTRG.15331.3;MSTRG.15331
MSTRG.1	Ldlrad4	MSTRG.15332.1
MSTRG.1	Ldlrad4	MSTRG.15333.1
MSTRG.1	Ldlrad4	MSTRG.15334.1
MSTRG.1	Fam210a	MSTRG.15335.1;ENSMUST00000042852;ENSMUST00000184508;E
MSTRG.1	Fam210a	MSTRG.15336.1
MSTRG.1	Rnmt	ENSMUST00000009679;ENSMUST00000131075;ENSMUST00000151
MSTRG.1	4930503	ENSMUST00000213066;MSTRG.15338.1;ENSMUST00000212683;E
MSTRG.1	4930503	MSTRG.15339.1
MSTRG.1	Rgs2	ENSMUST00000127206;ENSMUST00000027606;ENSMUST00000153
MSTRG.1	Stard6	ENSMUST00000114959;ENSMUST00000168249;ENSMUST00000174
MSTRG.1	Stard6	MSTRG.15341.1
MSTRG.1	Poli	ENSMUST00000161542;ENSMUST00000121674;ENSMUST00000043
MSTRG.1	Mir6357	ENSMUST00000184246
MSTRG.1	Mbd2	ENSMUST00000074058;ENSMUST00000114946;MSTRG.15344.3;M
MSTRG.1	Mex3c	ENSMUST00000091852
MSTRG.1	Mex3c	MSTRG.15346.1
MSTRG.1	Elac1	ENSMUST00000041138
MSTRG.1	Tcf4	ENSMUST00000203002;ENSMUST00000066717;ENSMUST00000114
MSTRG.1	Tcf4	MSTRG.15349.1
MSTRG.1	Glrx2	ENSMUST00000185367;ENSMUST00000145969;ENSMUST00000126
MSTRG.1	Tcf4	MSTRG.15350.1
MSTRG.1	Tcf4	MSTRG.15351.1
MSTRG.1	Tcf4	MSTRG.15352.1
MSTRG.1	Gm24845	ENSMUST00000158034
MSTRG.1	Tcf4	MSTRG.15354.1
MSTRG.1	Smad4	MSTRG.15355.1;ENSMUST00000025393;ENSMUST00000114939;M
MSTRG.1	Smad4	MSTRG.15356.1;MSTRG.15356.2
MSTRG.15357		MSTRG.15357.1
MSTRG.1	Me2	ENSMUST00000025439
MSTRG.15359		MSTRG.15359.1
MSTRG.1	Trove2	ENSMUST00000159879;ENSMUST00000018343
MSTRG.1	Ska1	ENSMUST00000040188;ENSMUST00000177604;MSTRG.15360.3
MSTRG.1	Cxxc1	ENSMUST00000025444;MSTRG.15361.1;MSTRG.15361.2
MSTRG.1	Mbd1	ENSMUST00000224907;MSTRG.15362.1;ENSMUST00000097530;E
MSTRG.1	Cfap53	ENSMUST00000114895;ENSMUST00000176435;ENSMUST00000177
MSTRG.1	Gm14328	ENSMUST00000122178
MSTRG.1	Gm23119	MSTRG.15365.1;ENSMUST00000158610
MSTRG.1	Scarna1	ENSMUST00000158064
MSTRG.15367		MSTRG.15367.1
MSTRG.1	Acaa2	ENSMUST00000041053;MSTRG.15368.2;MSTRG.15368.3
MSTRG.1	Rpl17;S	ENSMUST00000079716;MSTRG.15369.1;ENSMUST00000082937;M
MSTRG.1	Uchl5	ENSMUST00000189936;ENSMUST00000185742;ENSMUST00000018
MSTRG.1	Gm23301	ENSMUST00000082713
MSTRG.1	Gm26202	ENSMUST00000082910
MSTRG.1	Dym	ENSMUST00000039608;MSTRG.15372.2;MSTRG.15372.3
MSTRG.1	Gm27781	ENSMUST00000185185
MSTRG.1	Dym	MSTRG.15374.1
MSTRG.1	Dym	MSTRG.15375.1
MSTRG.1	Gm8807	ENSMUST00000179328

MSTRG.1	Dym	MSTRG.15377.1
MSTRG.1	Dym	MSTRG.15378.1
MSTRG.1	Gm20544	ENSMUST00000174338
MSTRG.1	Gm29170	ENSMUST00000190224;ENSMUST00000157698
MSTRG.1	Smad7	ENSMUST00000026999;MSTRG.15380.1;ENSMUST00000174411;E
MSTRG.1	Ctif	ENSMUST00000165559
MSTRG.1	Ctif	MSTRG.15382.1
MSTRG.1	Gm10532	ENSMUST00000181913
MSTRG.1	Ctif	MSTRG.15384.1
MSTRG.1	Zbtb7c	ENSMUST00000058997;ENSMUST00000167921
MSTRG.1	Zbtb7c	MSTRG.15386.1
MSTRG.15387		MSTRG.15387.1
MSTRG.15388		MSTRG.15388.1
MSTRG.1	Smad2	ENSMUST00000168423;MSTRG.15389.1;ENSMUST00000091831;E
MSTRG.1	Uchl5	MSTRG.1539.1
MSTRG.1	Smad2	MSTRG.15390.1
MSTRG.1	Smad2	MSTRG.15391.1
MSTRG.1	Pias2	ENSMUST00000114777;MSTRG.15392.2;ENSMUST00000114776;E
MSTRG.1	Pias2	MSTRG.15393.1
MSTRG.1	Pias2	MSTRG.15394.1
MSTRG.1	Ier3ip1	ENSMUST00000026487;ENSMUST00000136800;ENSMUST00000128
MSTRG.1	Katnal2	ENSMUST00000126153;ENSMUST00000135029;ENSMUST00000122
MSTRG.1	8030462	MSTRG.15397.3;ENSMUST00000074653;MSTRG.15397.1;MSTRG.
MSTRG.1	8030462	MSTRG.15398.1
MSTRG.1	4930465	MSTRG.15399.1;ENSMUST00000104405
MSTRG.1	Pkhd1	MSTRG.154.1
MSTRG.1	Uchl5	MSTRG.1540.1
MSTRG.1	8030462	MSTRG.15400.1
MSTRG.1	4930465	ENSMUST00000056675
MSTRG.15402		MSTRG.15402.1
MSTRG.1	Haus1	MSTRG.15403.1
MSTRG.1	Haus1	ENSMUST00000048192
MSTRG.1	Atp5a1	ENSMUST00000026495;ENSMUST00000114748;ENSMUST00000135
MSTRG.1	F830208	ENSMUST00000125396;ENSMUST00000143732
MSTRG.1	Pstpip2	MSTRG.15407.1;ENSMUST00000114741
MSTRG.1	Gm23954	ENSMUST00000157213
MSTRG.1	Epg5	MSTRG.15409.1;MSTRG.15409.2;ENSMUST00000044622
MSTRG.1	Uchl5	MSTRG.1541.1
MSTRG.1	Siglec1	MSTRG.15410.1;ENSMUST00000170760
MSTRG.1	Slc14a1	ENSMUST00000091813;ENSMUST00000160292;ENSMUST00000160
MSTRG.1	Slc14a1	MSTRG.15412.1
MSTRG.1	Setbp1	ENSMUST00000025430;MSTRG.15413.2;MSTRG.15413.3
MSTRG.1	Setbp1	MSTRG.15414.1
MSTRG.1	Setbp1	MSTRG.15415.1
MSTRG.1	Setbp1	MSTRG.15416.1
MSTRG.1	Setbp1	MSTRG.15417.1
MSTRG.1	Pard6g	ENSMUST00000070219
MSTRG.1	Gm21886	MSTRG.15419.1
MSTRG.1542		MSTRG.1542.1
MSTRG.1	Pard6g	MSTRG.15421.1
MSTRG.1	Adnp2	ENSMUST00000066743;MSTRG.15422.2
MSTRG.1	Rbfa	MSTRG.15423.1;ENSMUST00000025462
MSTRG.15424		MSTRG.15424.1
MSTRG.1	Hsbp1l1	ENSMUST00000166219
MSTRG.1	Gm16286	ENSMUST00000156400;ENSMUST00000145963;ENSMUST00000125
MSTRG.1	Txnl4a	MSTRG.15427.1
MSTRG.1	Txnl4a	MSTRG.15428.1

MSTRG.1	Txnl4a	MSTRG.15429.1
MSTRG.1	Pqlc1	ENSMUST00000131780;ENSMUST00000157056;ENSMUST0000012
MSTRG.1	Kcng2	ENSMUST00000077962
MSTRG.1	Gm37015	ENSMUST00000192763
MSTRG.1	Gm26676	ENSMUST00000181877
MSTRG.1	D330025	ENSMUST00000191632
MSTRG.1	Ctdp1	ENSMUST00000036229;ENSMUST00000161003;ENSMUST0000016
MSTRG.1	Nfatc1	ENSMUST00000167977;ENSMUST00000078049;ENSMUST0000017
MSTRG.15437		MSTRG.15437.1
MSTRG.15438		MSTRG.15438.1
MSTRG.1	Gm17383	ENSMUST00000078739
MSTRG.1	Gm5263	ENSMUST00000163646
MSTRG.1	Atp9b	ENSMUST00000091790;ENSMUST00000225980;MSTRG.15440.3;
MSTRG.1	Atp9b	MSTRG.15441.1
MSTRG.1	Atp9b	MSTRG.15442.1
MSTRG.1	AC11794	MSTRG.15443.1
MSTRG.1	AC11794	ENSMUST00000225547
MSTRG.1	Atp9b	MSTRG.15445.1
MSTRG.1	Atp9b	MSTRG.15446.1
MSTRG.1	Atp9b	MSTRG.15447.1
MSTRG.1	Gm25071	ENSMUST00000175232
MSTRG.1	Mbp	ENSMUST00000091789;MSTRG.15449.1;ENSMUST00000114676;
MSTRG.1	Gm38187	ENSMUST00000195609
MSTRG.1	Mbp	MSTRG.15450.1
MSTRG.1	Rpl21-p	MSTRG.15451.1;ENSMUST00000130005;MSTRG.15451.3
MSTRG.1	Zfp516	ENSMUST00000171238;ENSMUST00000071233
MSTRG.1	Zfp516	MSTRG.15453.1
MSTRG.1	Zfp516	MSTRG.15454.1
MSTRG.1	Zfp516	MSTRG.15455.1
MSTRG.15456		MSTRG.15456.1
MSTRG.1	Zfp236	ENSMUST00000171071;ENSMUST00000183324;MSTRG.15457.4;
MSTRG.1	Gm10524	MSTRG.15458.1;ENSMUST00000181931
MSTRG.15459		MSTRG.15459.1
MSTRG.1	Brinp3	ENSMUST00000074622;ENSMUST00000132847;ENSMUST0000012
MSTRG.15460		MSTRG.15460.1
MSTRG.1	Tshz1	ENSMUST00000060303;MSTRG.15461.1;ENSMUST00000175783;
MSTRG.1	Tshz1	MSTRG.15462.1
MSTRG.1	Tshz1	MSTRG.15463.1
MSTRG.1	Tshz1	MSTRG.15464.1
MSTRG.1	Zadh2	ENSMUST00000060223;MSTRG.15465.1
MSTRG.1	Zfp407	ENSMUST00000125450;MSTRG.15466.2;MSTRG.15466.3;ENSMUS
MSTRG.1	Zfp407	MSTRG.15467.1
MSTRG.1	Zfp407	MSTRG.15468.1
MSTRG.1	Gm37216	ENSMUST00000193817
MSTRG.1	Gm15583	ENSMUST00000116191
MSTRG.1	Zfp407	MSTRG.15470.1
MSTRG.1	Zfp407	MSTRG.15471.1;MSTRG.15471.2
MSTRG.1	Zfp407	MSTRG.15472.1
MSTRG.1	Cndp2	ENSMUST00000168419;ENSMUST00000025546
MSTRG.1	Cyb5a	MSTRG.15474.1
MSTRG.1	Cyb5a	ENSMUST00000160180;ENSMUST00000025549;ENSMUST0000015
MSTRG.1	Gm16146	MSTRG.15476.1;ENSMUST00000160306
MSTRG.1	Fbxo15	ENSMUST00000224467;ENSMUST00000225445;ENSMUST00000224
MSTRG.1	Timm21	ENSMUST00000025547;MSTRG.15478.1
MSTRG.1	Socs6	ENSMUST00000136173;ENSMUST00000070116;ENSMUST0000012
MSTRG.1	A230059	ENSMUST00000126596;ENSMUST00000191937
MSTRG.1	Socs6	MSTRG.15480.1

MSTRG.1	Rttn;Mi	ENSMUST00000023828;ENSMUST00000183668
MSTRG.1	Cd226	ENSMUST00000037142;MSTRG.15482.2;ENSMUST00000097496;M
MSTRG.1	Cd226	MSTRG.15483.1
MSTRG.1	Cd226	MSTRG.15484.1
MSTRG.15485		MSTRG.15485.1
MSTRG.1	Tmx3	ENSMUST00000025515;MSTRG.15486.2;MSTRG.15486.3
MSTRG.1	Gm45871	ENSMUST00000211710;ENSMUST00000209969;ENSMUST0000020[illegible]
MSTRG.15488		MSTRG.15488.1
MSTRG.1	1700030[illegible]	ENSMUST00000190575;ENSMUST00000191405;ENSMUST0000019[illegible]
MSTRG.1	Gm15584	ENSMUST00000123231
MSTRG.1	1700030[illegible]	MSTRG.15490.1
MSTRG.1	Ighmbp2	ENSMUST00000119292;ENSMUST00000025751;ENSMUST00000154
MSTRG.1	Ighmbp2	MSTRG.15493.1
MSTRG.1	Mrpl21	ENSMUST00000025745;ENSMUST00000139844;ENSMUST00000155
MSTRG.1	Mir6984	ENSMUST00000185105
MSTRG.1	Cpt1a	ENSMUST00000025835;MSTRG.15496.1;MSTRG.15496.3
MSTRG.1	1810055[illegible]	ENSMUST00000039048
MSTRG.1	Lrp5	ENSMUST00000025856;ENSMUST00000177330;ENSMUST00000177
MSTRG.1	Lrp5	MSTRG.15499.1
MSTRG.1	4930486	ENSMUST00000161509
MSTRG.1550		MSTRG.1550.1
MSTRG.1	Lrp5	MSTRG.15500.1
MSTRG.1	Ppp6r3	ENSMUST00000025846;MSTRG.15501.2;MSTRG.15501.3;ENSMUS
MSTRG.1	Ppp6r3	MSTRG.15502.1
MSTRG.1	AC11779	ENSMUST00000224419
MSTRG.1	Gm23940	ENSMUST00000101958
MSTRG.1	AC11779	ENSMUST00000223977
MSTRG.1	Ppp6r3	MSTRG.15506.1;MSTRG.15506.2
MSTRG.1	Aldh3b3	ENSMUST00000179433;ENSMUST00000189124
MSTRG.1	Aldh3b2	ENSMUST00000143380
MSTRG.1	Gstp1	ENSMUST00000169613;MSTRG.15509.2
MSTRG.1551		MSTRG.1551.3;MSTRG.1551.2;MSTRG.1551.1;MSTRG.1551.4;M
MSTRG.1	Gstp2	ENSMUST00000042700
MSTRG.1	Kmt5b	ENSMUST00000052699;ENSMUST00000113972;ENSMUST00000113
MSTRG.1	Gm16066	ENSMUST00000118575
MSTRG.1	Kmt5b	MSTRG.15513.1
MSTRG.1	Chka	ENSMUST00000128694;ENSMUST00000072055;ENSMUST00000125
MSTRG.1	Tcirg1	ENSMUST00000131327;ENSMUST00000145791;ENSMUST00000126
MSTRG.1	Ndufs8;[illegible]	ENSMUST00000075092;MSTRG.15516.1;MSTRG.15516.2;ENSMUS
MSTRG.1	Unc93b1	ENSMUST00000162708;ENSMUST00000162193;MSTRG.15517.4;E
MSTRG.1	Tbx10	ENSMUST00000041871
MSTRG.1	Nudt8;D[illegible]	ENSMUST00000025802;ENSMUST00000155405;ENSMUST00000122
MSTRG.1	Ndufv1	ENSMUST00000134479;ENSMUST00000042497;ENSMUST00000148
MSTRG.1	Gstp3	ENSMUST00000189808;ENSMUST00000076451
MSTRG.15523		MSTRG.15523.3;MSTRG.15523.2;MSTRG.15523.1;MSTRG.15523
MSTRG.1	Cabp4	ENSMUST00000025761
MSTRG.1	Gpr152	ENSMUST00000096338
MSTRG.1	Coro1b	ENSMUST00000123874;ENSMUST00000008893;ENSMUST00000142
MSTRG.1	Cdk2ap2	ENSMUST00000025779;ENSMUST00000174514;ENSMUST00000172
MSTRG.1	Pitpnm1	ENSMUST00000049658;ENSMUST00000151957;ENSMUST00000131
MSTRG.1	Pitpnm1	MSTRG.15529.1
MSTRG.1553		MSTRG.1553.1
MSTRG.1	Aip	ENSMUST00000117831;ENSMUST00000025767;MSTRG.15530.3;M
MSTRG.1	Tmem134	ENSMUST00000169055;ENSMUST00000135257;ENSMUST0000013[illegible]
MSTRG.1	Ptprcap	ENSMUST00000061086
MSTRG.1	Rps6kb2	ENSMUST00000025749;ENSMUST00000127605;ENSMUST00000141
MSTRG.1	Pold4	ENSMUST00000025773;MSTRG.15534.1

MSTRG.1	Carns1;'	ENSMUST00000167055;MSTRG.15535.1;MSTRG.15535.3;MSTRG.
MSTRG.1	Gm17552	ENSMUST00000181211
MSTRG.1	Ppp1ca	ENSMUST00000046094;MSTRG.15537.1
MSTRG.1	Rad9a	ENSMUST00000025740;MSTRG.15538.1;MSTRG.15538.2;MSTRG.
MSTRG.1	Clcf1	ENSMUST00000046506;ENSMUST00000132305;ENSMUST0000013[illegible]
MSTRG.1	Rbm3-ps	ENSMUST00000121422
MSTRG.1	Gm26115	ENSMUST00000175605
MSTRG.1	Ssh3	ENSMUST00000037992;ENSMUST00000113852
MSTRG.1	Mir6985	ENSMUST00000184641
MSTRG.1	Ankrd13	ENSMUST00000169192;ENSMUST00000056888;MSTRG.15543.3;E
MSTRG.1	Grk2	ENSMUST00000025791;ENSMUST00000113837;MSTRG.15544.3;M
MSTRG.1	Kdm2a	ENSMUST00000047898;ENSMUST00000075856;ENSMUST0000017[illegible]
MSTRG.1	Kdm2a	MSTRG.15546.1
MSTRG.1	Kdm2a	MSTRG.15547.1
MSTRG.1	Ctsf	ENSMUST00000119694
MSTRG.1	Actn3	ENSMUST00000138811;ENSMUST00000006626
MSTRG.1555		MSTRG.1555.1
MSTRG.1	Zdhhc24	ENSMUST00000162088;ENSMUST00000006632;ENSMUST00000162
MSTRG.1	Mrpl11	ENSMUST00000025836
MSTRG.1	Bbs1	ENSMUST00000053506
MSTRG.1	Gm25334	ENSMUST00000158036
MSTRG.1	4930533	MSTRG.15554.2;MSTRG.15554.1;ENSMUST00000025851
MSTRG.1	4930533	MSTRG.15555.1
MSTRG.1	Peli3	ENSMUST00000120475;ENSMUST00000025834;ENSMUST0000013[illegible]
MSTRG.1	Pcx	ENSMUST00000068004;ENSMUST00000224726;ENSMUST0000022[illegible]
MSTRG.1	Lrfn4	ENSMUST00000113822;ENSMUST00000053597
MSTRG.1	Pcx	MSTRG.15559.1
MSTRG.1556		MSTRG.1556.1
MSTRG.1	Rce1	ENSMUST00000224707;ENSMUST00000225476;ENSMUST0000002[illegible]
MSTRG.1	Mir6986	ENSMUST00000184483
MSTRG.1	Gm960	ENSMUST00000223788;ENSMUST00000226012;ENSMUST0000022[illegible]
MSTRG.1	Gm960	MSTRG.15563.1
MSTRG.1	Sptbn2;	MSTRG.15564.1;ENSMUST00000182821;ENSMUST00000183008;E
MSTRG.1	Sptbn2	ENSMUST00000008991
MSTRG.1	Gm21992	ENSMUST00000178353;ENSMUST00000180248;ENSMUST0000017[illegible]
MSTRG.1	Gm37206	ENSMUST00000195512
MSTRG.1	Gm21992	MSTRG.15568.1
MSTRG.1	Gm21844	ENSMUST00000178699;MSTRG.15569.2
MSTRG.1	BC00333	ENSMUST00000111913;ENSMUST00000097547;ENSMUST00000094
MSTRG.1	Ccs	ENSMUST00000037246;MSTRG.15570.2;MSTRG.15570.3
MSTRG.1	Ccdc87	ENSMUST00000088653
MSTRG.1	B4gat1	ENSMUST00000053705
MSTRG.1	Brms1	ENSMUST00000116567
MSTRG.1	Cd248	ENSMUST00000070630
MSTRG.1	Yif1a	ENSMUST00000025811
MSTRG.1	Cnih2	ENSMUST00000025805
MSTRG.1	Rab1b	ENSMUST00000025804;MSTRG.15577.1
MSTRG.1	Klc2	ENSMUST00000025798;ENSMUST00000116563;ENSMUST00000142
MSTRG.1	Pacs1	ENSMUST00000025786
MSTRG.1	Tpr	ENSMUST00000124973;ENSMUST00000119161;ENSMUST00000131
MSTRG.1	Gm27468	ENSMUST00000183577
MSTRG.1	Pacs1	MSTRG.15581.1;MSTRG.15581.2;MSTRG.15581.3
MSTRG.1	Pacs1	MSTRG.15582.1
MSTRG.1	Pacs1	MSTRG.15583.1
MSTRG.1	Pacs1	MSTRG.15585.1
MSTRG.1	Pacs1	MSTRG.15586.1
MSTRG.1	Pacs1	MSTRG.15587.1

MSTRG.1	Pacs1	MSTRG.15589.1
MSTRG.1	Tpr	MSTRG.1559.1
MSTRG.1	Pacs1	MSTRG.15590.1
MSTRG.1	Pacs1	MSTRG.15591.1
MSTRG.1	Sf3b2	ENSMUST00000025774
MSTRG.1	Capn1	MSTRG.15593.1;ENSMUST00000025891;ENSMUST00000164843
MSTRG.1	Capn1	MSTRG.15594.1
MSTRG.1	Gm10814	ENSMUST00000180505
MSTRG.1	Znhit2	ENSMUST00000162726
MSTRG.1	Gm8034	MSTRG.15597.1
MSTRG.1	Gm8034	ENSMUST00000178853
MSTRG.1	Syvn1	MSTRG.15599.1;ENSMUST00000138532;ENSMUST00000129081;E
MSTRG.1	Gm15795	ENSMUST00000160969
MSTRG.1	Prg4	ENSMUST00000006171;ENSMUST00000111901;ENSMUST00000111
MSTRG.1	Mir6988	ENSMUST00000184338
MSTRG.1	Mrpl49	ENSMUST00000007482
MSTRG.1	Mrpl49	MSTRG.15602.1
MSTRG.1	Fau	ENSMUST00000043074;ENSMUST00000178310;MSTRG.15603.1;E
MSTRG.1	Arl2	ENSMUST00000134821;ENSMUST00000025893;ENSMUST00000123
MSTRG.1	Batf2	ENSMUST00000134150;ENSMUST00000045042;ENSMUST00000135
MSTRG.1	Gm5510	ENSMUST00000117826
MSTRG.1	Majin	ENSMUST00000025699;ENSMUST00000113528
MSTRG.1	Gpha2	ENSMUST00000025698;ENSMUST00000113526
MSTRG.1	Fam129a	ENSMUST00000134499;ENSMUST00000148810;ENSMUST00000097
MSTRG.1	Ppp2r5b	ENSMUST00000025695;MSTRG.15610.2;ENSMUST00000153155;E
MSTRG.1	Atg2a	ENSMUST00000045351;MSTRG.15611.2;ENSMUST00000145600;E
MSTRG.1	Atg2a	MSTRG.15612.1
MSTRG.1	Gm14963	ENSMUST00000146751
MSTRG.1	Ehd1	ENSMUST00000025684;ENSMUST00000165797;ENSMUST00000171
MSTRG.1	Tm7sf2;'	ENSMUST00000159084;ENSMUST00000025713;ENSMUST00000113
MSTRG.1	Tmem262	ENSMUST00000149347;ENSMUST00000143303
MSTRG.1	Cdca5	ENSMUST00000025704;MSTRG.15617.2;ENSMUST00000156961;E
MSTRG.15618		MSTRG.15618.1
MSTRG.1	Naaladl	ENSMUST00000044451
MSTRG.1	Edem3	ENSMUST00000188145;ENSMUST00000059498;ENSMUST00000187
MSTRG.1	Sac3d1	ENSMUST00000113533
MSTRG.1	Snx15	ENSMUST00000025702;ENSMUST00000154601;ENSMUST00000124
MSTRG.1	Cdc42bp	ENSMUST00000025681;ENSMUST00000141854
MSTRG.1	Banf1	ENSMUST00000170010;ENSMUST00000025762
MSTRG.1	Eif1ad	ENSMUST00000025759
MSTRG.1	Sart1;T	ENSMUST00000044207;MSTRG.15625.1;ENSMUST00000044527
MSTRG.1	Ltbp3;G	MSTRG.15626.1;MSTRG.15626.2;ENSMUST00000192176
MSTRG.1	4930481	ENSMUST00000128542
MSTRG.1	Drap1;E	ENSMUST00000124478;ENSMUST00000148219;ENSMUST00000125
MSTRG.1	Gm10138	ENSMUST00000185848;ENSMUST00000186597;ENSMUST00000206
MSTRG.1	AI83718	ENSMUST00000054477;ENSMUST00000159759
MSTRG.1	Gm25432	ENSMUST00000158284
MSTRG.1	Fosl1	ENSMUST00000025850
MSTRG.1	Ccdc85b	ENSMUST00000179549
MSTRG.1	Fibp	ENSMUST00000225796;ENSMUST00000225917;ENSMUST00000225
MSTRG.1	Ctsw	ENSMUST00000025844
MSTRG.1	Efemp2	ENSMUST00000167304;ENSMUST00000166303;ENSMUST00000165
MSTRG.1	Mus81	ENSMUST00000133436;ENSMUST00000124334;ENSMUST00000140
MSTRG.1	Cfl1	ENSMUST00000209469;ENSMUST00000116560
MSTRG.1	Snx32	ENSMUST00000070172
MSTRG.1	Ivns1ab	ENSMUST00000023918;ENSMUST00000111887;MSTRG.1564.2;EN
MSTRG.1	1700020	ENSMUST00000189704

MSTRG.1	Gm31166	ENSMUST00000210638;ENSMUST00000211026;ENSMUST0000020[illegible]
MSTRG.1	Ovol1	ENSMUST00000025861
MSTRG.1	Ap5b1	MSTRG.15643.1;ENSMUST00000096318
MSTRG.1	Rnaseh2	ENSMUST00000025864;ENSMUST00000127071;ENSMUST0000015[illegible]
MSTRG.1	Kat5	ENSMUST00000113641;MSTRG.15645.1;MSTRG.15645.3
MSTRG.1	Rela	ENSMUST00000025867;MSTRG.15646.2
MSTRG.1	Sipa1;P	ENSMUST00000071857;ENSMUST00000080824;ENSMUST00000164
MSTRG.1	Mir6987	ENSMUST00000184789
MSTRG.1	Map3k11	MSTRG.15649.1;MSTRG.15649.2;ENSMUST00000004156
MSTRG.1	Swt1	ENSMUST00000064771;ENSMUST00000111883;ENSMUST00000124
MSTRG.1	Kcnk7	ENSMUST00000052448
MSTRG.1	Gm16538	ENSMUST00000162407
MSTRG.1	Fam89b;	ENSMUST00000116558;ENSMUST00000099955;ENSMUST00000161
MSTRG.1	Ltbp3	ENSMUST00000081496;MSTRG.15653.2
MSTRG.1	Scyl1	ENSMUST00000025890
MSTRG.1	Gm20417	ENSMUST00000174784
MSTRG.1	Gm27702	ENSMUST00000184512
MSTRG.1	Neat1;G	ENSMUST00000173672;ENSMUST00000174287;ENSMUST00000174
MSTRG.1	Frmd8os	ENSMUST00000187697;ENSMUST00000135347
MSTRG.1	Frmd8	MSTRG.15659.1;ENSMUST00000025728;MSTRG.15659.3;ENSMUS
MSTRG.1	Swt1	MSTRG.1566.1
MSTRG.1	Slc25a4	ENSMUST00000145200;ENSMUST00000025732;ENSMUST0000012[illegible]
MSTRG.1	Tigd3	ENSMUST00000055911
MSTRG.1	Dpf2	ENSMUST00000136983;ENSMUST00000025746;ENSMUST0000011[illegible]
MSTRG.1	Gm42067	ENSMUST00000209642;ENSMUST00000210917
MSTRG.1	Cdc42ep	ENSMUST00000055458
MSTRG.1	Pola2	ENSMUST00000025752;MSTRG.15665.1;MSTRG.15665.2;ENSMUS
MSTRG.1	Hmgb1-p	MSTRG.15666.1
MSTRG.1	Hmgb1-p	ENSMUST00000118281
MSTRG.1	Pygm	ENSMUST00000035269;ENSMUST00000113483;ENSMUST00000142
MSTRG.1	Rasgrp2	ENSMUST00000113476;ENSMUST00000113475;ENSMUST0000011[illegible]
MSTRG.1	Swt1	MSTRG.1567.1
MSTRG.1	Gm14965	ENSMUST00000131314
MSTRG.1	Rps6ka4	ENSMUST00000025903;ENSMUST00000170516;ENSMUST0000019[illegible]
MSTRG.1	Men1	ENSMUST00000113502;ENSMUST00000113504;ENSMUST0000007[illegible]
MSTRG.1	Map4k2;	ENSMUST00000133696;ENSMUST00000152349;MSTRG.15673.2;[illegible]
MSTRG.1	Gm14966	ENSMUST00000129675;ENSMUST00000113496;MSTRG.15674.3
MSTRG.1	Gm14966	MSTRG.15675.1
MSTRG.1	Sf1	ENSMUST00000131252;ENSMUST00000113489;ENSMUST0000011[illegible]
MSTRG.1	Ccdc88b	MSTRG.15677.2;ENSMUST00000113440;MSTRG.15677.3;MSTRG.
MSTRG.15678		MSTRG.15678.1
MSTRG.1	Trmt112	ENSMUST00000088257;ENSMUST00000116551;ENSMUST00000172
MSTRG.1	Swt1	MSTRG.1568.1
MSTRG.1	Esrra;M	ENSMUST00000025906;MSTRG.15680.2;ENSMUST00000173635;[illegible]
MSTRG.1	Fkbp2	MSTRG.15681.1;ENSMUST00000070878;ENSMUST00000177752
MSTRG.1	Vegfb	ENSMUST00000130048;ENSMUST00000025914;ENSMUST00000147
MSTRG.1	Catsper	ENSMUST00000057716
MSTRG.1	Kcnk4	ENSMUST00000025908
MSTRG.1	Gpr137	ENSMUST00000099774;ENSMUST00000099776;ENSMUST00000166
MSTRG.1	Bad	ENSMUST00000025910;ENSMUST00000113426;ENSMUST0000011[illegible]
MSTRG.1	Bad	MSTRG.15687.1
MSTRG.1	Plcb3	ENSMUST00000025912
MSTRG.1	Ppp1r14	ENSMUST00000070850;MSTRG.15689.2
MSTRG.1	Trmt1l	ENSMUST00000188179;ENSMUST00000065625;ENSMUST0000018[illegible]
MSTRG.1	Dnajc4;	ENSMUST00000179118;ENSMUST00000025915;MSTRG.15690.1;[illegible]
MSTRG.1	Trpt1	ENSMUST00000088223
MSTRG.1	Fermt3;	ENSMUST00000040772;MSTRG.15692.1;MSTRG.15692.4;MSTRG.

MSTRG.1	Fermt3	MSTRG.15693.1
MSTRG.1	Atl3	MSTRG.15694.1;ENSMUST00000170373;ENSMUST00000025668;M
MSTRG.1	2700081	ENSMUST00000159348;MSTRG.15695.2;MSTRG.15695.3;ENSMUS
MSTRG.1	Mir6991	ENSMUST00000183837
MSTRG.1	Rtn3	MSTRG.15697.2;MSTRG.15697.3;MSTRG.15697.1;ENSMUST0000
MSTRG.1	Pla2g16	ENSMUST00000136465;ENSMUST00000025925;ENSMUST00000141
MSTRG.1	Mir7682	ENSMUST00000198467
MSTRG.1	Slc22a2	ENSMUST00000075619
MSTRG.15702		MSTRG.15702.1
MSTRG.1	Slc22a2	ENSMUST00000065651
MSTRG.1	Slc22a2	ENSMUST00000113298
MSTRG.1	Macrod1	ENSMUST00000040261;MSTRG.15705.1
MSTRG.1	Macrod1	MSTRG.15706.1
MSTRG.1	Flrt1	ENSMUST00000113383
MSTRG.1	Otub1	ENSMUST00000025679;ENSMUST00000123594;ENSMUST00000127
MSTRG.1	Cox8a	ENSMUST00000039758
MSTRG.1	Gm37893	ENSMUST00000192826
MSTRG.1	Rcor2;G	MSTRG.15710.1;ENSMUST00000170286
MSTRG.1	Naa40	ENSMUST00000025675
MSTRG.1	Gm23997	ENSMUST00000158055
MSTRG.1	Rcor2	ENSMUST00000140442;ENSMUST00000066646;ENSMUST00000113
MSTRG.1	Mark2	ENSMUST00000032557;ENSMUST00000164205;ENSMUST00000025
MSTRG.1	Mark2;1	MSTRG.15716.1;MSTRG.15716.2;MSTRG.15716.3
MSTRG.1	Mark2	MSTRG.15717.1
MSTRG.1	AI84614	MSTRG.15718.1;ENSMUST00000025924;MSTRG.15718.3
MSTRG.1	1700105	ENSMUST00000186160
MSTRG.1	Rnf2;Gm	ENSMUST00000190070;ENSMUST00000187048;MSTRG.1572.3;EN
MSTRG.1	Rab11b-	ENSMUST00000178377
MSTRG.1	Gm8189	ENSMUST00000211035;MSTRG.15721.1;ENSMUST00000209457;M
MSTRG.1	Slc3a2	ENSMUST00000170157;ENSMUST00000206598;ENSMUST0000001C
MSTRG.1	Snhg1;G	ENSMUST00000206155;ENSMUST00000205539;ENSMUST00000206
MSTRG.1	Wdr74;G	ENSMUST00000210512;ENSMUST00000049424;MSTRG.15724.2;E
MSTRG.1	Stx5a;M	ENSMUST00000177322;ENSMUST00000176381;ENSMUST00000175
MSTRG.1	Stx5a	MSTRG.15726.1
MSTRG.1	Polr2g	ENSMUST00000096261
MSTRG.1	Polr2g	MSTRG.15728.1
MSTRG.1	Nxf1	ENSMUST00000010241;MSTRG.15729.1;MSTRG.15729.3;ENSMUS
MSTRG.1	1700025	ENSMUST00000187603;ENSMUST00000044581;ENSMUST00000128
MSTRG.1	Taf6l	ENSMUST00000010249;MSTRG.15730.1;MSTRG.15730.2;MSTRG.
MSTRG.1	Ubxn1	ENSMUST00000166407;ENSMUST00000096255
MSTRG.1	Uqcc3	ENSMUST00000096253
MSTRG.1	Zbtb3	ENSMUST00000172175
MSTRG.1	Ttc9c	MSTRG.15734.1;MSTRG.15734.2;MSTRG.15734.3;MSTRG.15734
MSTRG.1	Hnrnpul	ENSMUST00000096753;MSTRG.15735.1;MSTRG.15735.2;MSTRG.
MSTRG.1	Gng3	ENSMUST00000096259
MSTRG.1	Bscl2	ENSMUST00000171649;ENSMUST00000159770;ENSMUST00000159
MSTRG.1	Lrrn4cl	ENSMUST00000096257
MSTRG.1	Lbhd1;1	ENSMUST00000187504;ENSMUST00000189165;ENSMUST00000177
MSTRG.1	Gm25279	ENSMUST00000122608
MSTRG.1	Snora57	ENSMUST00000093658
MSTRG.1	Mir5136	ENSMUST00000175573
MSTRG.1	B3gat3	MSTRG.15742.1;ENSMUST00000096243
MSTRG.1	Rom1	ENSMUST00000096242
MSTRG.1	Eml3	MSTRG.15744.1;MSTRG.15744.2;ENSMUST00000096241;ENSMUS
MSTRG.1	AC12921	ENSMUST00000225181
MSTRG.1	Mta2	ENSMUST00000096240;ENSMUST00000137956;ENSMUST00000135
MSTRG.1	Ahnak	ENSMUST00000092956;MSTRG.15747.1;ENSMUST00000092955

MSTRG.1	Ahnak	MSTRG.15748.1
MSTRG.1	Ahnak	MSTRG.15749.1
MSTRG.1	Gm28610	ENSMUST00000187716
MSTRG.1	Ahnak	MSTRG.15750.1
MSTRG.1	Ahnak	MSTRG.15751.1
MSTRG.1	Tut1;Ee	MSTRG.15752.2;ENSMUST00000096239;MSTRG.15752.1;MSTRG.
MSTRG.1	Pcna-ps	ENSMUST00000088040
MSTRG.1	Asrgl1	ENSMUST00000049948;MSTRG.15754.1;ENSMUST00000150353
MSTRG.1	Gm9750	ENSMUST00000142025
MSTRG.1	Gm9750	MSTRG.15756.1
MSTRG.1	Stxbp3-	ENSMUST00000090527
MSTRG.1	Incenp	MSTRG.15759.1;MSTRG.15759.2;MSTRG.15759.3;ENSMUST0000
MSTRG.1	1700025	MSTRG.1576.1
MSTRG.1	Fth1	ENSMUST00000025563
MSTRG.1	Best1	ENSMUST00000117346
MSTRG.1	Rab3il1	ENSMUST00000121418;ENSMUST00000149967;ENSMUST0000011
MSTRG.1	Fads3	ENSMUST00000115995
MSTRG.1	Fads2	ENSMUST00000025567
MSTRG.1	Fads1;M	ENSMUST00000010807;MSTRG.15765.2;ENSMUST00000184912
MSTRG.1	Gm10143	ENSMUST00000150038;ENSMUST00000081739
MSTRG.1	Fen1	ENSMUST00000156291;ENSMUST00000116542;ENSMUST0000002
MSTRG.1	Tmem258	ENSMUST00000040372;ENSMUST00000153854;ENSMUST00000127
MSTRG.1	Myrf	ENSMUST00000088013;ENSMUST00000190922;ENSMUST0000018
MSTRG.1	1700025	MSTRG.1577.1
MSTRG.1	Ppp1r32	ENSMUST00000038842
MSTRG.1	Sdhaf2	MSTRG.15771.1
MSTRG.1	Sdhaf2	ENSMUST00000025570;MSTRG.15772.1;MSTRG.15772.3
MSTRG.1	Sdhaf2	MSTRG.15773.1
MSTRG.1	Sdhaf2	MSTRG.15774.1
MSTRG.1	Cpsf7	ENSMUST00000038379;MSTRG.15775.2;MSTRG.15775.3;MSTRG.
MSTRG.1	Tmem216	ENSMUST00000145210;ENSMUST00000123788;ENSMUST0000002
MSTRG.15777		MSTRG.15777.1
MSTRG.1	Tmem138	ENSMUST00000025568
MSTRG.1	Cyb561a	ENSMUST00000168445;MSTRG.15779.1
MSTRG.1	1700025	MSTRG.1578.1
MSTRG.1	Tkfc	ENSMUST00000037678
MSTRG.1	Ddb1	MSTRG.15781.2;MSTRG.15781.1;ENSMUST00000025649
MSTRG.1	Vwce	ENSMUST00000055115
MSTRG.1	Cd5	MSTRG.15783.1;MSTRG.15783.2;ENSMUST00000025571;MSTRG.
MSTRG.1	Pga5	ENSMUST00000025647;ENSMUST00000225135
MSTRG.1	Vps37c	ENSMUST00000087951;MSTRG.15786.2
MSTRG.1	A430093	MSTRG.15787.1;ENSMUST00000186596;ENSMUST00000087947;E
MSTRG.1	A430093	MSTRG.15788.1
MSTRG.1	A430093	MSTRG.15789.1;MSTRG.15789.2;MSTRG.15789.3
MSTRG.1	Tsen15	ENSMUST00000015124;ENSMUST00000161717;ENSMUST00000162
MSTRG.1	Cd6	ENSMUST00000080292;ENSMUST00000025572;MSTRG.15790.2;E
MSTRG.1	Slc15a3	ENSMUST00000138380;ENSMUST00000025646;ENSMUST00000131
MSTRG.1	Tmem132	ENSMUST00000025645;ENSMUST00000120524
MSTRG.1	Tmem109	ENSMUST00000038128;ENSMUST00000133303;ENSMUST00000138
MSTRG.1	Ptgdr2	ENSMUST00000037261
MSTRG.1	Ccdc86	ENSMUST00000133836;ENSMUST00000025639;ENSMUST0000018
MSTRG.1	Prpf19	ENSMUST00000025642;ENSMUST00000186416;ENSMUST00000187
MSTRG.1	AW11201	ENSMUST00000099676
MSTRG.1	Gm336	ENSMUST00000186376
MSTRG.1	1700017	ENSMUST00000025635;ENSMUST00000188756
MSTRG.1	Pkhd1	MSTRG.158.1
MSTRG.1	Tsen15	MSTRG.1580.1

MSTRG.1	Gm6501	ENSMUST00000187178
MSTRG.1	4930526	ENSMUST00000181137
MSTRG.1	Ms4a1	ENSMUST00000169159;ENSMUST00000185851;ENSMUST00000187
MSTRG.1	Ms4a5	ENSMUST00000067673
MSTRG.1	Ms4a7	ENSMUST00000025574;ENSMUST00000067532;ENSMUST0000056
MSTRG.1	Ms4a6d	ENSMUST00000025582;ENSMUST00000155669;ENSMUST00000125
MSTRG.1	Ms4a4c	ENSMUST00000153546;ENSMUST00000072729;ENSMUST0000011
MSTRG.1	Ms4a4b	MSTRG.15809.1;ENSMUST00000136366;ENSMUST00000145160;E
MSTRG.1	Rgl1	ENSMUST00000111859;ENSMUST00000027760;ENSMUST00000111
MSTRG.1	Gm5511	ENSMUST00000189380
MSTRG.1	Gm37387	ENSMUST00000192201
MSTRG.1	Ms4a6c;	ENSMUST00000165310;ENSMUST00000187462;MSTRG.15812.2;M
MSTRG.1	BE69200	MSTRG.15813.1;ENSMUST00000189306;ENSMUST00000186500
MSTRG.1	Gm8369	ENSMUST00000189635;ENSMUST00000190498;ENSMUST00000185
MSTRG.1	Gm19261	ENSMUST00000189628
MSTRG.1	Ms4a6b;	ENSMUST00000163078;ENSMUST00000025580;MSTRG.15816.2;E
MSTRG.1	Gm10212	ENSMUST00000087884
MSTRG.1	Gm6365	MSTRG.15818.1
MSTRG.1	Gm6365	ENSMUST00000054711
MSTRG.1	Rgl1	MSTRG.1582.1
MSTRG.1	Mrpl16	ENSMUST00000167199
MSTRG.1	Stx3	ENSMUST00000047698;ENSMUST00000069285;ENSMUST00000211
MSTRG.1	Patl1	MSTRG.15822.1;ENSMUST00000061618
MSTRG.1	Mir6994	ENSMUST00000198985
MSTRG.1	Patl1	MSTRG.15824.1
MSTRG.1	Gm22272	ENSMUST00000104039
MSTRG.15826		MSTRG.15826.1
MSTRG.1	Osbp	ENSMUST00000025590;MSTRG.15827.2;MSTRG.15827.3
MSTRG.1	Mpeg1	MSTRG.15828.2;MSTRG.15828.1;ENSMUST00000081035;MSTRG.
MSTRG.1	Dtx4	ENSMUST00000045521
MSTRG.1	Gm15479	ENSMUST00000139309
MSTRG.1	A330040	ENSMUST00000224046;ENSMUST00000136697
MSTRG.1	AC15173	ENSMUST00000226106
MSTRG.1	A330040	MSTRG.15832.1
MSTRG.1	AC15173	ENSMUST00000217186
MSTRG.1	A330040	MSTRG.15834.1
MSTRG.1	AC15173	ENSMUST00000224027
MSTRG.1	Fam111a	ENSMUST00000139270;MSTRG.15836.2;ENSMUST00000151307;E
MSTRG.1	Gm16274	ENSMUST00000139251
MSTRG.1	A330040	MSTRG.15838.1
MSTRG.1	Cntf;Zf	ENSMUST00000112933;ENSMUST00000142247;ENSMUST0000003
MSTRG.1	Gm7278	ENSMUST00000119836
MSTRG.1	Lpxn	ENSMUST00000025601
MSTRG.1	AC13563	ENSMUST00000220222;ENSMUST00000218013
MSTRG.1	Olfr145	ENSMUST00000213493
MSTRG.15843		MSTRG.15843.1
MSTRG.1	Tle4	ENSMUST00000052011;ENSMUST00000167776
MSTRG.1	Psat1	ENSMUST00000025542;ENSMUST00000162053;ENSMUST00000161
MSTRG.1	Cep78	ENSMUST00000047704
MSTRG.1	C130060	ENSMUST00000181731
MSTRG.1	Gnaq	ENSMUST00000025541;ENSMUST00000170229;ENSMUST00000167
MSTRG.1	Gm28792	ENSMUST00000191482
MSTRG.1	Gnaq	MSTRG.15850.1
MSTRG.1	E030024	ENSMUST00000075293
MSTRG.1	Gnaq	MSTRG.15852.1
MSTRG.1	Gnaq	MSTRG.15853.1
MSTRG.1	Gnaq	MSTRG.15854.1

MSTRG.1	Gm10819	ENSMUST00000099988
MSTRG.1	Gnaq	MSTRG.15856.1
MSTRG.1	Gm17068	ENSMUST00000163135
MSTRG.1	Gnaq	MSTRG.15858.1
MSTRG.1	Gnaq	MSTRG.15859.1
MSTRG.1	Apobec4	ENSMUST00000068875
MSTRG.1	Mir496b	ENSMUST00000184935
MSTRG.1	Gnaq	MSTRG.15861.1
MSTRG.1	Gnaq	MSTRG.15862.1
MSTRG.1	Gnaq	MSTRG.15863.1
MSTRG.1	Gnaq	MSTRG.15864.1
MSTRG.1	Gna14	ENSMUST00000025602
MSTRG.1	Gna14	MSTRG.15866.1
MSTRG.1	Gna14	MSTRG.15867.1
MSTRG.1	Prune2	ENSMUST00000224145;ENSMUST00000087689;ENSMUST0000022[illegible]
MSTRG.1	Prune2	MSTRG.15869.1
MSTRG.1	Arpc5	ENSMUST00000077755;ENSMUST00000097536;MSTRG.1587.3;MS
MSTRG.1	Gcnt1	ENSMUST00000174236;ENSMUST00000169897;MSTRG.15872.3;E
MSTRG.1	Gcnt1	MSTRG.15873.1
MSTRG.1	Vps13a	ENSMUST00000223667;MSTRG.15874.2;MSTRG.15874.3;ENSMUS
MSTRG.1	Vps13a	MSTRG.15875.1
MSTRG.1	Vps13a	MSTRG.15876.1
MSTRG.1	AC13537	ENSMUST00000223692
MSTRG.1	Vps13a	MSTRG.15878.1
MSTRG.1	Vps13a	MSTRG.15879.1
MSTRG.1	Ncf2	ENSMUST00000186568;ENSMUST00000190323;ENSMUST00000027
MSTRG.1	Vps13a	MSTRG.15880.1
MSTRG.1	Vps13a	MSTRG.15881.1
MSTRG.1	Vps13a	MSTRG.15882.1
MSTRG.1	Vps13a	MSTRG.15883.1
MSTRG.1	Gm9806	ENSMUST00000051080
MSTRG.1	Rfk	ENSMUST00000025617;MSTRG.15885.1;ENSMUST00000146171
MSTRG.15886		MSTRG.15886.1
MSTRG.15887		MSTRG.15887.1
MSTRG.15888		MSTRG.15888.1
MSTRG.1	Ostf1	ENSMUST00000025631;MSTRG.15889.2;ENSMUST00000156908;E
MSTRG.1	Smg7	ENSMUST00000073441;MSTRG.1589.2;MSTRG.1589.3;MSTRG.15
MSTRG.1	Gm8250	ENSMUST00000117398
MSTRG.1	Ostf1	MSTRG.15891.1
MSTRG.1	Ostf1	MSTRG.15892.1
MSTRG.1	Nmrk1	MSTRG.15893.1
MSTRG.1	Nmrk1	ENSMUST00000159061;ENSMUST00000042392;ENSMUST0000015[illegible]
MSTRG.1	Carnmt1	ENSMUST00000025632
MSTRG.1	Carnmt1	MSTRG.15896.1
MSTRG.1	Carnmt1	MSTRG.15897.1
MSTRG.1	Carnmt1	MSTRG.15898.1
MSTRG.1	Carnmt1	MSTRG.15899.1
MSTRG.1	Mcm3	ENSMUST00000053266;ENSMUST00000185296;ENSMUST0000018[illegible]
MSTRG.1	Gm28791	ENSMUST00000185888
MSTRG.1	D030056	ENSMUST00000062753;ENSMUST00000055792
MSTRG.1	Gm23238	ENSMUST00000083007
MSTRG.1	Anxa1	ENSMUST00000025561;ENSMUST00000163902;ENSMUST00000167
MSTRG.1	Aldh1a1	ENSMUST00000224807;ENSMUST00000225337;ENSMUST00000224
MSTRG.1	AC15216	ENSMUST00000225910
MSTRG.1	Gm6684	ENSMUST00000053717
MSTRG.1	Zfand5	ENSMUST00000025659;ENSMUST00000149144;ENSMUST00000152
MSTRG.15907		MSTRG.15907.1

MSTRG.1	Gda	ENSMUST00000087600;ENSMUST00000121725;ENSMUST0000013€
MSTRG.15909		MSTRG.15909.1
MSTRG.1	Smg7	MSTRG.1591.1
MSTRG.1	1110059	ENSMUST00000177577;ENSMUST00000038830;MSTRG.15910.2;M
MSTRG.1	1110059	MSTRG.15911.1
MSTRG.1	1110059	MSTRG.15912.1
MSTRG.1	Abhd17b	MSTRG.15913.1;ENSMUST00000052556
MSTRG.1	Tmem2	MSTRG.15914.2;MSTRG.15914.1;ENSMUST00000025663;ENSMUS
MSTRG.1	Klf9	ENSMUST00000036884
MSTRG.1	Mir1192	ENSMUST00000116976
MSTRG.1	1700028	ENSMUST00000035849
MSTRG.1	Gm9493	ENSMUST00000104916
MSTRG.1	Gm22144	ENSMUST00000157402
MSTRG.1	Smg7	MSTRG.1592.1
MSTRG.1	Gm6563	ENSMUST00000056396
MSTRG.1	Smc5	MSTRG.15921.1;MSTRG.15921.2;ENSMUST00000087556;ENSMUS
MSTRG.1	Ptar1;G	ENSMUST00000099560;MSTRG.15922.1;ENSMUST00000067077
MSTRG.1	Apba1	ENSMUST00000025830;MSTRG.15923.2
MSTRG.1	Tjp2	ENSMUST00000099558;MSTRG.15924.2
MSTRG.1	Fxn	ENSMUST00000081333;ENSMUST00000123684;ENSMUST00000132
MSTRG.1	Fxn	MSTRG.15926.1
MSTRG.1	Gm16216	ENSMUST00000118970
MSTRG.1	Pip5k1b	ENSMUST00000112673;ENSMUST00000025800;ENSMUST00000134
MSTRG.1	Pip5k1b	MSTRG.15929.1
MSTRG.1	Smg7	MSTRG.1593.1
MSTRG.1	Pip5k1b	MSTRG.15930.1
MSTRG.1	Pip5k1b	ENSMUST00000126936
MSTRG.1	Pip5k1b	MSTRG.15932.1
MSTRG.1	Pip5k1b	MSTRG.15933.1
MSTRG.1	Fam122a	ENSMUST00000099556
MSTRG.1	Pip5k1b	MSTRG.15935.1
MSTRG.1	Pip5k1b	MSTRG.15936.1
MSTRG.1	Pip5k1b	MSTRG.15937.1
MSTRG.1	Pip5k1b	MSTRG.15938.1
MSTRG.1	Pip5k1b	MSTRG.15939.1
MSTRG.1594		MSTRG.1594.1
MSTRG.1	Pip5k1b	MSTRG.15940.1
MSTRG.1	Pip5k1b	MSTRG.15941.1
MSTRG.1	Gm10053	ENSMUST00000073080
MSTRG.1	Tmem252	ENSMUST00000057243
MSTRG.1	Pgm5	ENSMUST00000047666;ENSMUST00000133692;ENSMUST0000015C
MSTRG.1	Gm17800	ENSMUST00000182466
MSTRG.1	Pgm5	MSTRG.15946.1
MSTRG.1	Cbwd1	ENSMUST00000025815
MSTRG.1	Mir3084	ENSMUST00000198203;ENSMUST00000083233
MSTRG.1	Kank1	ENSMUST00000049400;ENSMUST00000137260;MSTRG.15949.3;E
MSTRG.1	Lamc2	ENSMUST00000185356;ENSMUST00000189005;ENSMUST0000018€
MSTRG.1	Kank1	MSTRG.15950.1
MSTRG.1	2610016	ENSMUST00000185211;ENSMUST00000188711;ENSMUST0000018€
MSTRG.1	Dmrt2	ENSMUST00000053068
MSTRG.1	Dmrt2	MSTRG.15953.1
MSTRG.15954		MSTRG.15954.1
MSTRG.15955		MSTRG.15955.1
MSTRG.1	Dock8	ENSMUST00000025831;MSTRG.15956.1;MSTRG.15956.2;MSTRG.
MSTRG.1	Dock8	MSTRG.15957.1
MSTRG.1	Dock8	MSTRG.15958.1
MSTRG.1	Dock8	MSTRG.15959.1

MSTRG.1596		MSTRG.1596.1
MSTRG.1	Dock8	MSTRG.15960.1
MSTRG.1	Gm24252	ENSMUST00000083700
MSTRG.15962		MSTRG.15962.1
MSTRG.1	Pum3	ENSMUST00000076219
MSTRG.1	Rfx3	ENSMUST00000165566;ENSMUST00000046898;ENSMUST00000174
MSTRG.1	Gm8412	ENSMUST00000077493
MSTRG.1	Rfx3	MSTRG.15966.1
MSTRG.1	Rfx3	MSTRG.15967.1
MSTRG.1	Rfx3	MSTRG.15968.1
MSTRG.1	Rfx3	MSTRG.15969.1
MSTRG.1	Lamc1	ENSMUST00000161744;ENSMUST00000027752;ENSMUST0000016
MSTRG.1	Rfx3	MSTRG.15970.1
MSTRG.1	Gm20407	MSTRG.15971.1;ENSMUST00000172891
MSTRG.1	Rfx3	MSTRG.15973.1
MSTRG.1	Smarca2	ENSMUST00000176769;ENSMUST00000208163;ENSMUST0000002
MSTRG.1	Smarca2	MSTRG.15975.1
MSTRG.1	Smarca2	MSTRG.15976.1
MSTRG.1	Gm20616	ENSMUST00000177262;ENSMUST00000177453
MSTRG.1	Smarca2	MSTRG.15978.1
MSTRG.1	Smarca2	MSTRG.15979.1
MSTRG.1	Gm38009	ENSMUST00000194884
MSTRG.1	Smarca2	MSTRG.15980.1
MSTRG.1	4931403	ENSMUST00000177048
MSTRG.1	Slc1a1	ENSMUST00000161340;ENSMUST00000025875;ENSMUST00000161
MSTRG.1	Gm5822	ENSMUST00000161562
MSTRG.1	4430402	ENSMUST00000175647;ENSMUST00000025872;ENSMUST00000162
MSTRG.1	Plpp6	ENSMUST00000045674
MSTRG.1	Cdc37l1	ENSMUST00000225310;ENSMUST00000225210;ENSMUST00000224
MSTRG.1	Gm10136	ENSMUST00000183196
MSTRG.1	Cdc37l1	MSTRG.15988.1
MSTRG.1	Ak3	MSTRG.15989.1;ENSMUST00000025696;ENSMUST00000125587;
MSTRG.1	Gm8818	ENSMUST00000110797
MSTRG.1	1700018	ENSMUST00000187897
MSTRG.1	Rcl1	ENSMUST00000064393
MSTRG.1	Mir101b	ENSMUST00000083622
MSTRG.1	Gm5518	ENSMUST00000084171
MSTRG.1	Insl6	ENSMUST00000052380
MSTRG.1	Jak2	ENSMUST00000025705;ENSMUST00000065796;MSTRG.15995.1;
MSTRG.1	Pdcd1lg	ENSMUST00000112576
MSTRG.1	Plgrkt	ENSMUST00000143467;MSTRG.15997.1;ENSMUST00000152936;
MSTRG.1	Cd274	MSTRG.15998.1;ENSMUST00000016640;MSTRG.15998.3;MSTRG.
MSTRG.1	9930021	ENSMUST00000175726;ENSMUST00000175764;ENSMUST00000177
MSTRG.1	Rgs20	ENSMUST00000002533;ENSMUST00000118000;ENSMUST0000011
MSTRG.1	Efhc1	ENSMUST00000160782;ENSMUST00000038447
MSTRG.1	Lamc1	MSTRG.1600.1
MSTRG.1	Ranbp6	ENSMUST00000099525
MSTRG.1	Il33	ENSMUST00000120388;ENSMUST00000144528;ENSMUST0000002
MSTRG.16002		MSTRG.16002.1
MSTRG.1	A930007	ENSMUST00000181726;ENSMUST00000180986;ENSMUST0000018
MSTRG.1	Ric1	ENSMUST00000043610;ENSMUST00000162184;MSTRG.16004.2;
MSTRG.1	Ermp1	MSTRG.16005.1;ENSMUST00000159692;ENSMUST00000161749;
MSTRG.1	Uhrf2	ENSMUST00000134518;ENSMUST00000137368;MSTRG.16006.2;
MSTRG.1	Prkg1	ENSMUST00000065067;ENSMUST00000073581;ENSMUST0000018
MSTRG.1	Cstf2t	ENSMUST00000066039
MSTRG.1	E330020	ENSMUST00000186785;ENSMUST00000097535;ENSMUST0000018
MSTRG.1	Asah2	ENSMUST00000096119;MSTRG.16010.1

MSTRG.1	Rpl9-ps	ENSMUST00000078034
MSTRG.1	Minpp1	ENSMUST00000025827;MSTRG.16012.2;MSTRG.16012.3
MSTRG.1	Minpp1	MSTRG.16014.1
MSTRG.1	Sgms1	ENSMUST00000099514;ENSMUST00000142618;ENSMUST00000151
MSTRG.1	Sgms1	MSTRG.16016.1
MSTRG.1	Sgms1	MSTRG.16017.1
MSTRG.1	Sgms1	MSTRG.16018.1
MSTRG.1	Sgms1	MSTRG.16019.1
MSTRG.1	E330020	MSTRG.1602.1
MSTRG.1	Sgms1	MSTRG.16020.1
MSTRG.1	2700046	ENSMUST00000181612
MSTRG.1	Papss2	MSTRG.16022.1;ENSMUST00000025833;MSTRG.16022.3
MSTRG.1	Atad1	ENSMUST00000070210;MSTRG.16023.1;MSTRG.16023.3;MSTRG.
MSTRG.1	Pten	ENSMUST00000013807;MSTRG.16024.3;ENSMUST00000140014;M
MSTRG.1	Pten	MSTRG.16025.1
MSTRG.1	Pten	MSTRG.16026.1
MSTRG.16027		MSTRG.16027.1
MSTRG.1	Acta2	ENSMUST00000039631
MSTRG.1	Stambpl	MSTRG.16029.1;MSTRG.16029.2;MSTRG.16029.3;ENSMUST0000
MSTRG.1	Gm6652	ENSMUST00000186414
MSTRG.1	Stambpl	MSTRG.16030.1
MSTRG.1	Stambpl	MSTRG.16031.1
MSTRG.1	Stambpl	MSTRG.16032.1
MSTRG.1	Fas;Gm2	ENSMUST00000112472;MSTRG.16033.3;MSTRG.16033.1;MSTRG.
MSTRG.1	Ch25h	ENSMUST00000050562
MSTRG.1	Gm26902	ENSMUST00000181777;ENSMUST00000181307
MSTRG.1	Lipa	ENSMUST00000049572;ENSMUST00000178114;MSTRG.16037.3;M
MSTRG.1	Ifit2	ENSMUST00000149829;MSTRG.16038.2;ENSMUST00000102826
MSTRG.1	Ifit2	MSTRG.16039.1
MSTRG.1	Shcbp1l	ENSMUST00000042373;ENSMUST00000136614;ENSMUST00000137
MSTRG.1	Ifit3	ENSMUST00000102825
MSTRG.1	Ifit3b	ENSMUST00000076249
MSTRG.1	Ifit1bl	ENSMUST00000112463;ENSMUST00000087357
MSTRG.1	Ifit1	ENSMUST00000102824
MSTRG.1	A830019	ENSMUST00000178904
MSTRG.1	A830019	MSTRG.16045.1
MSTRG.1	Pank1	ENSMUST00000036584;ENSMUST00000112460
MSTRG.1	Kif20b	ENSMUST00000087341;ENSMUST00000223907;ENSMUST00000223
MSTRG.1	Kif20b	MSTRG.16049.1
MSTRG.1	Gm25588	ENSMUST00000082501
MSTRG.1	Kif20b	MSTRG.16050.1
MSTRG.1	Mir1950	ENSMUST00000158526
MSTRG.16052		MSTRG.16052.1
MSTRG.16053		MSTRG.16053.1
MSTRG.1	Rpp30	ENSMUST00000025714
MSTRG.1	Rpp30	MSTRG.16055.1
MSTRG.1	Rpp30	MSTRG.16056.1
MSTRG.1	AC11776	ENSMUST00000225679
MSTRG.1	Pcgf5;A	ENSMUST00000225920;ENSMUST00000224805;MSTRG.16058.3;M
MSTRG.1	Gm23170	ENSMUST00000157158
MSTRG.1	Gm37669	ENSMUST00000192184
MSTRG.1	Pcgf5	MSTRG.16060.1
MSTRG.1	AC11776	ENSMUST00000225814
MSTRG.1	AC11776	MSTRG.16062.1
MSTRG.1	Hectd2	ENSMUST00000139215;ENSMUST00000169036;ENSMUST0000014
MSTRG.1	Hectd2	MSTRG.16064.1
MSTRG.1	Hectd2o	ENSMUST00000149601;ENSMUST00000142067;ENSMUST00000147

MSTRG.1	Tnks2	ENSMUST00000025729;ENSMUST00000168656;MSTRG.16066.2;E
MSTRG.1	Tnks2	MSTRG.16067.1
MSTRG.1	Fgfbp3	ENSMUST00000057337
MSTRG.1	Btaf1	ENSMUST00000099494;MSTRG.16069.2;MSTRG.16069.3;MSTRG.
MSTRG.1	Shcbp1l	MSTRG.1607.1
MSTRG.1	Cpeb3	ENSMUST00000079754;ENSMUST00000124158;ENSMUST00000154
MSTRG.1	Gm22714	ENSMUST00000116894
MSTRG.1	A330032	ENSMUST00000180609
MSTRG.1	Gm23918	ENSMUST00000175253
MSTRG.1	March5	ENSMUST00000024078;ENSMUST00000112391;ENSMUST00000148
MSTRG.1	Ide	ENSMUST00000131070;MSTRG.16075.2;MSTRG.16075.1;ENSMUS
MSTRG.1	Rpl10-p	ENSMUST00000121321
MSTRG.16077		MSTRG.16077.1
MSTRG.1	Kif11	MSTRG.16078.1;ENSMUST00000012587;MSTRG.16078.3
MSTRG.1	Exoc6	ENSMUST00000066439
MSTRG.1	Shcbp1l	MSTRG.1608.1
MSTRG.1	Exoc6	MSTRG.16080.1
MSTRG.1	Exoc6	MSTRG.16081.1
MSTRG.1	Cep55	ENSMUST00000096096;ENSMUST00000116506;ENSMUST0000016(
MSTRG.1	Gm23300	ENSMUST00000082712
MSTRG.1	Myof	ENSMUST00000172095;ENSMUST00000041475;ENSMUST0000022(
MSTRG.1	Rbp4	ENSMUST00000112335;ENSMUST00000025951
MSTRG.1	Fra10ac	ENSMUST00000067167;MSTRG.16086.2
MSTRG.1	Fra10ac	MSTRG.16087.1
MSTRG.16088		MSTRG.16088.1;MSTRG.16088.2
MSTRG.16089		MSTRG.16089.1
MSTRG.1	Dhx9	ENSMUST00000186380;ENSMUST00000042141;MSTRG.1609.3;EN
MSTRG.16090		MSTRG.16090.1
MSTRG.1	Slc35g1	ENSMUST00000054098
MSTRG.1	Slc35g1	MSTRG.16092.1
MSTRG.1	Plce1	ENSMUST00000181994;ENSMUST00000182481;ENSMUST0000016(
MSTRG.1	Gm28991	ENSMUST00000186584
MSTRG.1	Noc3l	ENSMUST00000025963
MSTRG.1	Tbc1d12	ENSMUST00000037302
MSTRG.1	Tbc1d12	MSTRG.16097.1
MSTRG.1	Tbc1d12	MSTRG.16098.1
MSTRG.1	Tbc1d12	MSTRG.16099.1
MSTRG.1	Tram2	ENSMUST00000037998;ENSMUST00000187210
MSTRG.1	Rgs16	ENSMUST00000027748
MSTRG.1	Gm25838	ENSMUST00000158920
MSTRG.1	Tbc1d12	MSTRG.16101.1
MSTRG.1	Hells	ENSMUST00000025965;ENSMUST00000138292;ENSMUST00000145
MSTRG.16103		MSTRG.16103.1
MSTRG.16105		MSTRG.16105.1
MSTRG.1	Cyp2c70	MSTRG.16106.1
MSTRG.1	Cyp2c54	MSTRG.16108.1
MSTRG.1	Pdlim1	ENSMUST00000068439;MSTRG.16109.1;ENSMUST00000182540;E
MSTRG.1	Gm28513	ENSMUST00000191474
MSTRG.1	Sorbs1	MSTRG.16110.1;ENSMUST00000226047;ENSMUST00000224247;E
MSTRG.1	AC14494	ENSMUST00000225358
MSTRG.1	Sorbs1	MSTRG.16112.1
MSTRG.1	Sorbs1	MSTRG.16113.1
MSTRG.1	Ccnj	ENSMUST00000025983;ENSMUST00000120057;ENSMUST0000011(
MSTRG.1	Ccnj	MSTRG.16115.1
MSTRG.16116		MSTRG.16116.1
MSTRG.1	Aldh18a	ENSMUST00000176939;MSTRG.16117.1;ENSMUST00000025979;E
MSTRG.1	Gm15801	ENSMUST00000120920

MSTRG.1	Gm27042	ENSMUST00000181109
MSTRG.1	Rnasel;	ENSMUST00000086209;MSTRG.1612.2;ENSMUST00000182722;EN
MSTRG.1	Tctn3	ENSMUST00000025981;ENSMUST00000135795;ENSMUST00000132
MSTRG.1	Entpd1	ENSMUST00000163023;ENSMUST00000134063;ENSMUST00000112
MSTRG.1	Gm8783	ENSMUST00000159764
MSTRG.1	Mir8092	ENSMUST00000183735;MSTRG.16124.1;ENSMUST00000050092
MSTRG.1	Blnk	ENSMUST00000117695;ENSMUST00000054769;ENSMUST00000134
MSTRG.16126		MSTRG.16126.1;MSTRG.16126.2;MSTRG.16126.4;MSTRG.1612
MSTRG.1	Dntt	MSTRG.16127.1;MSTRG.16127.2
MSTRG.16128		MSTRG.16128.1
MSTRG.1	Dntt	MSTRG.16129.1;ENSMUST00000051806;ENSMUST00000112200;M
MSTRG.1	Teddm2	ENSMUST00000059607;ENSMUST00000123490;ENSMUST0000019C
MSTRG.16130		MSTRG.16130.1
MSTRG.1	Dntt	MSTRG.16131.1
MSTRG.1	Dntt	MSTRG.16132.1;MSTRG.16132.2
MSTRG.1	Opalin	ENSMUST00000087176
MSTRG.1	Tll2	ENSMUST00000025986;ENSMUST00000169941
MSTRG.1	Tll2	MSTRG.16135.1
MSTRG.1	Tm9sf3	ENSMUST00000025989;MSTRG.16136.1;MSTRG.16136.3
MSTRG.1	Tm9sf3	MSTRG.16137.1;MSTRG.16137.2
MSTRG.1	Pik3ap1	ENSMUST00000059672
MSTRG.1	AI60618	MSTRG.16139.1
MSTRG.1	Teddm1b	ENSMUST00000055314
MSTRG.1	Slit1	ENSMUST00000169141;ENSMUST00000169416
MSTRG.1	Lcor;Gm	MSTRG.16141.1;ENSMUST00000067795;MSTRG.16141.3;MSTRG.
MSTRG.1	Gm9788	ENSMUST00000057038
MSTRG.1	Lcor	MSTRG.16143.1
MSTRG.1	Lcor	MSTRG.16144.1
MSTRG.1	Frat1	ENSMUST00000087155
MSTRG.1	Arhgap1	ENSMUST00000026150;ENSMUST00000163265;ENSMUST0000017
MSTRG.1	Arhgap1	MSTRG.16147.1
MSTRG.1	Arhgap1	MSTRG.16148.1
MSTRG.1	Arhgap1	MSTRG.16149.1
MSTRG.1	Gm38352	ENSMUST00000194459
MSTRG.1	Frat2	ENSMUST00000059231
MSTRG.1	Rrp12	ENSMUST00000038677
MSTRG.1	Pi4k2a	ENSMUST00000066778
MSTRG.1	Avpi1	ENSMUST00000018965;ENSMUST00000161873
MSTRG.1	Marveld	ENSMUST00000061111;ENSMUST00000184186
MSTRG.1	Zfyve27	ENSMUST00000164455;ENSMUST00000169536;ENSMUST0000009
MSTRG.1	Sfrp5	ENSMUST00000018966
MSTRG.1	Pgam1	ENSMUST00000011896;MSTRG.16157.2;MSTRG.16157.3
MSTRG.1	Exosc1	ENSMUST00000075280;ENSMUST00000112123
MSTRG.1	Zdhhc16	ENSMUST00000224258;MSTRG.16159.1;ENSMUST00000026154;E
MSTRG.1	Teddm1a	ENSMUST00000050660
MSTRG.1	Mms19	ENSMUST00000167927;ENSMUST00000171561;ENSMUST0000017C
MSTRG.1	Gm23113	ENSMUST00000158477
MSTRG.1	Ubtd1	ENSMUST00000026170
MSTRG.1	Ubtd1	MSTRG.16163.1
MSTRG.1	Ubtd1	MSTRG.16164.1
MSTRG.1	Ubtd1	MSTRG.16165.1
MSTRG.1	R3hcc1l	ENSMUST00000026188;ENSMUST00000160107;ENSMUST00000161
MSTRG.1	R3hcc1l	MSTRG.16167.1
MSTRG.1	Gm16541	ENSMUST00000160992;ENSMUST00000162651;ENSMUST00000162
MSTRG.1	Loxl4	ENSMUST00000026190;ENSMUST00000164786;ENSMUST00000164
MSTRG.1	Mir8114	ENSMUST00000183582;ENSMUST00000086199;ENSMUST0000013
MSTRG.1	Hps1	ENSMUST00000162004;ENSMUST00000026194;ENSMUST0000015

MSTRG.1	Gm16244	ENSMUST00000159313;ENSMUST00000160001
MSTRG.1	Hpse2	ENSMUST00000099428
MSTRG.1	Gm22135	ENSMUST00000158752
MSTRG.1	Gm6776	ENSMUST00000057752
MSTRG.1	Got1	ENSMUST00000026196;ENSMUST00000133325;ENSMUST00000132
MSTRG.1	Slc25a2	ENSMUST00000046038;MSTRG.16176.1
MSTRG.1	Entpd7	ENSMUST00000081079;ENSMUST00000152786;ENSMUST00000144
MSTRG.1	Cox15	ENSMUST00000045562;MSTRG.16178.2;MSTRG.16178.3;ENSMUS
MSTRG.1	Cutc	ENSMUST00000026199;ENSMUST00000112047;ENSMUST00000153
MSTRG.1	Cacna1e	ENSMUST00000187541;ENSMUST00000004214;ENSMUST00000211
MSTRG.1	Dnmbp	MSTRG.16180.1;ENSMUST00000212396;ENSMUST00000212592;E
MSTRG.1	Gm24400	ENSMUST00000157865
MSTRG.1	n-R5s21	ENSMUST00000122692
MSTRG.1	Dnmbp	MSTRG.16183.1
MSTRG.1	Cwf19l1	ENSMUST00000026218;MSTRG.16184.2
MSTRG.1	Gm24336	ENSMUST00000104203
MSTRG.1	Bloc1s2	MSTRG.16186.1;ENSMUST00000079033
MSTRG.1	Pkd2l1	ENSMUST00000042026;ENSMUST00000161357
MSTRG.1	Pkd2l1	MSTRG.16188.1
MSTRG.1	Erlin1	ENSMUST00000170801;ENSMUST00000071698;ENSMUST00000112
MSTRG.1	Cacna1e	MSTRG.1619.1
MSTRG.1	Erlin1	MSTRG.16190.1
MSTRG.1	Erlin1	MSTRG.16191.1
MSTRG.1	Chuk	ENSMUST00000026217;MSTRG.16192.2;ENSMUST00000119591;E
MSTRG.1	Scd3	ENSMUST00000026220
MSTRG.1	Scd3	MSTRG.16194.1
MSTRG.1	Scd2;Mi	MSTRG.16195.1;ENSMUST00000026221;ENSMUST00000175574
MSTRG.16196		MSTRG.16196.1
MSTRG.1	Scd4	MSTRG.16197.1
MSTRG.16198		MSTRG.16198.1
MSTRG.16199		MSTRG.16199.1
MSTRG.1	Tram2	MSTRG.162.1
MSTRG.1	Cacna1e	MSTRG.1620.1
MSTRG.1	Scd4	ENSMUST00000133314;ENSMUST00000058856
MSTRG.1	Scd1	ENSMUST00000041331;MSTRG.16201.1;MSTRG.16201.4;MSTRG.
MSTRG.1	Hif1an	ENSMUST00000131032;ENSMUST00000040455
MSTRG.1	Hif1an	MSTRG.16203.1
MSTRG.1	Wnt8b	ENSMUST00000041163
MSTRG.1	Sec31b;	ENSMUST00000063632;ENSMUST00000111985;ENSMUST00000165
MSTRG.1	Gm20538	MSTRG.16206.1
MSTRG.1	Slf2;Gm	MSTRG.16207.1;ENSMUST00000096053;MSTRG.16207.3;ENSMUS
MSTRG.1	Sema4g	ENSMUST00000026225;ENSMUST00000130549;ENSMUST0000017
MSTRG.1	Mrpl43	ENSMUST00000097715
MSTRG.1	Cacna1e	MSTRG.1621.1
MSTRG.1	Twnk	ENSMUST00000026227
MSTRG.1	Lzts2	ENSMUST00000039016;ENSMUST00000179108;ENSMUST0000017
MSTRG.1	Lzts2	MSTRG.16212.1
MSTRG.1	Lzts2	MSTRG.16213.1
MSTRG.1	Pdzd7	ENSMUST00000169459;ENSMUST00000145391
MSTRG.1	Sfxn3	MSTRG.16215.1;ENSMUST00000062213;ENSMUST00000084493;E
MSTRG.1	Btrc	ENSMUST00000223764;ENSMUST00000065601;ENSMUST0000022
MSTRG.1	Btrc	MSTRG.16217.1
MSTRG.1	Btrc	MSTRG.16218.1
MSTRG.1	Btrc	MSTRG.16219.1
MSTRG.1	Ier5	ENSMUST00000055322
MSTRG.1	Gm6807	ENSMUST00000077435
MSTRG.1	Btrc	MSTRG.16221.1

MSTRG.1	Poll	ENSMUST00000026239
MSTRG.1	Gm17018	ENSMUST00000047057;ENSMUST00000161373
MSTRG.1	Fbxw4	ENSMUST00000046869;ENSMUST00000160018;ENSMUST00000162
MSTRG.1	Fbxw4	MSTRG.16225.1
MSTRG.1	Npm3	ENSMUST00000070215;ENSMUST00000144165
MSTRG.1	Gm15491	ENSMUST00000133963
MSTRG.1	Mgea5	ENSMUST00000026243;MSTRG.16228.1;MSTRG.16228.10;MSTRG
MSTRG.1	9130011	ENSMUST00000045396
MSTRG.1	Mr1	ENSMUST00000194612;ENSMUST00000191773;MSTRG.1623.2;EN
MSTRG.1	9130011	MSTRG.16230.1
MSTRG.1	4930505	ENSMUST00000181820
MSTRG.1	Hps6	ENSMUST00000099393
MSTRG.1	Nolc1	ENSMUST00000165017;ENSMUST00000223741;ENSMUST00000225
MSTRG.1	Ldb1	MSTRG.16234.1;ENSMUST00000152946;ENSMUST00000056931;E
MSTRG.1	Pprc1	ENSMUST00000111899;ENSMUST00000099392;MSTRG.16235.4;M
MSTRG.1	Mfsd13a	ENSMUST00000128041;ENSMUST00000086969;ENSMUST00000142
MSTRG.1	2310034	ENSMUST00000189008
MSTRG.1	Gm24610	ENSMUST00000083270
MSTRG.1	Xpr1	ENSMUST00000027741;MSTRG.1624.2;MSTRG.1624.3;MSTRG.16
MSTRG.1	Actr1a	MSTRG.16240.1;MSTRG.16240.2;ENSMUST00000040270
MSTRG.16241		MSTRG.16241.1
MSTRG.1	Sufu	ENSMUST00000039922;ENSMUST00000111867;ENSMUST0000012C
MSTRG.1	Trim8	ENSMUST00000026008;MSTRG.16243.1;MSTRG.16243.2;MSTRG.
MSTRG.1	Borcs7	ENSMUST00000074912;MSTRG.16244.2
MSTRG.1	Borcs7	MSTRG.16245.1
MSTRG.1	Borcs7	MSTRG.16246.1
MSTRG.1	Borcs7	MSTRG.16247.1
MSTRG.1	As3mt	ENSMUST00000003655
MSTRG.1	Gbf1	ENSMUST00000026254;ENSMUST00000175868;MSTRG.16249.2;E
MSTRG.1	Gm22569	ENSMUST00000175545
MSTRG.1	Gm8974	ENSMUST00000175770
MSTRG.1	Gbf1	MSTRG.16251.1
MSTRG.1	Gbf1	MSTRG.16252.1
MSTRG.1	Gm16726	ENSMUST00000130184
MSTRG.1	Nfkb2	MSTRG.16254.1;ENSMUST00000073116;ENSMUST00000111881
MSTRG.1	Psd	MSTRG.16255.1;ENSMUST00000225748;ENSMUST00000224556;E
MSTRG.1	Fbxl15	ENSMUST00000026256
MSTRG.1	Cuedc2	ENSMUST00000051234;ENSMUST00000167861;MSTRG.16257.3;M
MSTRG.1	Gm26792	MSTRG.16258.1;ENSMUST00000180538
MSTRG.1	Arl3	ENSMUST00000026009
MSTRG.1	Xpr1	MSTRG.1626.1
MSTRG.1	Arl3	MSTRG.16260.1
MSTRG.1	Sfxn2	ENSMUST00000026011
MSTRG.1	Wbp1l	ENSMUST00000138302;ENSMUST00000132202;ENSMUST0000009
MSTRG.1	Wbp1l	MSTRG.16263.1
MSTRG.1	Wbp1l	MSTRG.16264.1
MSTRG.1	Wbp1l	MSTRG.16265.1
MSTRG.1	Cyp17a1	ENSMUST00000026012;ENSMUST00000131142;ENSMUST0000015
MSTRG.1	Pcgf6	ENSMUST00000026032
MSTRG.1	Pcgf6	MSTRG.16268.1
MSTRG.1	Cnnm2	ENSMUST00000077666;ENSMUST00000099373
MSTRG.1	Stx6;Gm	ENSMUST00000027743;ENSMUST00000193059;ENSMUST00000195
MSTRG.1	Cnnm2	MSTRG.16270.1
MSTRG.1	Cnnm2	MSTRG.16271.1
MSTRG.1	Nt5c2	MSTRG.16272.1;ENSMUST00000168536;ENSMUST00000086961;E
MSTRG.1	Ina	ENSMUST00000037636
MSTRG.16274		MSTRG.16274.1

MSTRG.1	Taf5	ENSMUST00000026027
MSTRG.1	Usmg5	ENSMUST00000096014
MSTRG.1	Pdcd11	ENSMUST00000072141;MSTRG.16277.2
MSTRG.1	Calhm2	ENSMUST00000140512;ENSMUST00000035822
MSTRG.1	Neurl1a	ENSMUST00000111808;ENSMUST00000111807
MSTRG.1	Stx6	MSTRG.1628.1
MSTRG.1	Sh3pxd2.	ENSMUST00000081619;ENSMUST00000111800;MSTRG.16280.3
MSTRG.1	Sh3pxd2.	MSTRG.16281.1
MSTRG.1	Mir6995	ENSMUST00000183658
MSTRG.1	Sh3pxd2.	MSTRG.16283.1
MSTRG.1	Stn1	ENSMUST00000049369;ENSMUST00000182873;MSTRG.16284.3;E
MSTRG.1	Gm19557	ENSMUST00000182109
MSTRG.1	Sfr1	ENSMUST00000099353;MSTRG.16286.2
MSTRG.1	Sfr1	MSTRG.16287.2;MSTRG.16287.3;MSTRG.16287.1;MSTRG.16287
MSTRG.1	Slk	ENSMUST00000026043;MSTRG.16288.2;ENSMUST00000051691;M
MSTRG.1	Slk	MSTRG.16289.1
MSTRG.1	Stx6	MSTRG.1629.1
MSTRG.1	Slk	MSTRG.16291.1
MSTRG.1	Gm16068	ENSMUST00000136557
MSTRG.1	Col17a1	ENSMUST00000026045;ENSMUST00000086923;ENSMUST00000151
MSTRG.1	Cfap43;(	ENSMUST00000160247;ENSMUST00000162657;ENSMUST0000015S
MSTRG.1	Mir8090	ENSMUST00000185078
MSTRG.1	Gsto1	ENSMUST00000026050;ENSMUST00000130157;ENSMUST00000143
MSTRG.1	Gsto2	ENSMUST00000120645;ENSMUST00000056159;ENSMUST00000135
MSTRG.1	Cfap43	MSTRG.16298.1
MSTRG.1	Tram2	MSTRG.163.1
MSTRG.1	BC03409	ENSMUST00000185937;ENSMUST00000186156;ENSMUST00000035
MSTRG.1	Xpnpep1	ENSMUST00000182500;ENSMUST00000182877;ENSMUST00000183
MSTRG.1	Xpnpep1	MSTRG.16301.1
MSTRG.1	Mir6407	ENSMUST00000184510
MSTRG.1	Add3	ENSMUST00000111741;ENSMUST00000050096;ENSMUST00000025
MSTRG.1	Add3	MSTRG.16304.1
MSTRG.1	Gm24724	ENSMUST00000158230
MSTRG.1	Mxi1	ENSMUST00000003870;ENSMUST00000025998;ENSMUST00000111
MSTRG.1	Mxi1	MSTRG.16307.1
MSTRG.1	Gm10197	ENSMUST00000086887
MSTRG.1	Smndc1	ENSMUST00000025997;MSTRG.16309.1;MSTRG.16309.3
MSTRG.1	Gm37622	ENSMUST00000192162
MSTRG.16310		MSTRG.16310.1
MSTRG.1	Mirt1	MSTRG.16311.1;ENSMUST00000180489;ENSMUST00000180667;E
MSTRG.1	Mirt1;4	MSTRG.16313.1;MSTRG.16313.2
MSTRG.1	4833407	ENSMUST00000180442;MSTRG.16314.1
MSTRG.1	Dusp5	ENSMUST00000038287;MSTRG.16315.1;MSTRG.16315.3;MSTRG.
MSTRG.1	Nutf2-p	ENSMUST00000095978
MSTRG.1	Smc3	MSTRG.16317.1
MSTRG.1	Smc3	MSTRG.16318.1;MSTRG.16318.2;ENSMUST00000025930;MSTRG.
MSTRG.1	Shoc2	MSTRG.16319.1
MSTRG.1	BC03409	MSTRG.1632.1
MSTRG.1	Adra2a	ENSMUST00000036700
MSTRG.1	Gpam	MSTRG.16321.1;ENSMUST00000061856
MSTRG.1	Pdcd4;S	MSTRG.16322.1;ENSMUST00000074371;ENSMUST00000025931;E
MSTRG.1	Bbip1	MSTRG.16323.1;ENSMUST00000135402;MSTRG.16323.2;MSTRG.
MSTRG.1	Shoc2	MSTRG.16324.1
MSTRG.1	Acsl5	ENSMUST00000225551;ENSMUST00000224337;ENSMUST00000043
MSTRG.1	Zdhhc6	ENSMUST00000225963;ENSMUST00000226103;ENSMUST00000225
MSTRG.1	Zdhhc6	MSTRG.16327.1
MSTRG.1	Vti1a	ENSMUST00000225529;ENSMUST00000226069;ENSMUST00000225

MSTRG.1	Vti1a	MSTRG.16329.1
MSTRG.1	Acbd6	ENSMUST00000080138;ENSMUST00000035560;ENSMUST00000097
MSTRG.1	Vti1a	MSTRG.16330.1
MSTRG.1	Vti1a	MSTRG.16331.1;MSTRG.16331.2
MSTRG.1	Vti1a	MSTRG.16332.1
MSTRG.1	Vti1a	MSTRG.16333.1
MSTRG.1	Vti1a	MSTRG.16334.1
MSTRG.1	Vti1a	MSTRG.16335.1
MSTRG.1	Vti1a	MSTRG.16336.1
MSTRG.1	Vti1a	MSTRG.16337.1
MSTRG.1	Vti1a	MSTRG.16338.1
MSTRG.1	Casp7	ENSMUST00000026062
MSTRG.1	Acbd6	MSTRG.1634.1
MSTRG.1	Tcf7l2	ENSMUST00000061496;ENSMUST00000111657;ENSMUST00000111
MSTRG.1	Tcf7l2	MSTRG.16341.1
MSTRG.1	Tcf7l2	MSTRG.16342.1
MSTRG.1	Tcf7l2	MSTRG.16343.1
MSTRG.1	Tcf7l2	MSTRG.16344.1
MSTRG.1	Tcf7l2	MSTRG.16345.1
MSTRG.1	Ppnr	ENSMUST00000190974;MSTRG.16346.1
MSTRG.1	Tcf7l2	MSTRG.16347.1
MSTRG.1	Tcf7l2	MSTRG.16348.1
MSTRG.16349		MSTRG.16349.1
MSTRG.1	Acbd6	MSTRG.1635.1
MSTRG.1	Dclre1a	ENSMUST00000183143;ENSMUST00000182058;ENSMUST00000182
MSTRG.1	Nhlrc2	ENSMUST00000071423;MSTRG.16351.2
MSTRG.1	Nhlrc2	MSTRG.16352.1
MSTRG.1	Nhlrc2	MSTRG.16353.1
MSTRG.1	Ccdc186	ENSMUST00000118592;ENSMUST00000135666;ENSMUST0000007€
MSTRG.1	Vwa2	ENSMUST00000026068
MSTRG.1	Afap1l2	ENSMUST00000118800;ENSMUST00000111584;ENSMUST00000122
MSTRG.1	Afap1l2	MSTRG.16357.1
MSTRG.1	B230217(	ENSMUST00000181921
MSTRG.1	B230217(	MSTRG.16359.1
MSTRG.1	Acbd6	MSTRG.1636.1
MSTRG.1	B230217(	MSTRG.16360.1
MSTRG.1	Fam160b	ENSMUST00000036407;MSTRG.16361.3;MSTRG.16361.2;MSTRG.
MSTRG.1	Trub1	ENSMUST00000026072;ENSMUST00000026073
MSTRG.1	Trub1	MSTRG.16363.1
MSTRG.1	Gm26874	ENSMUST00000181959
MSTRG.1	Ablim1	ENSMUST00000079360;ENSMUST00000111529;ENSMUST00000104
MSTRG.1	Ablim1	MSTRG.16366.1
MSTRG.1	Ablim1	MSTRG.16367.1;MSTRG.16367.2
MSTRG.1	Ablim1	MSTRG.16368.1
MSTRG.1	Ablim1	MSTRG.16369.1;MSTRG.16369.2
MSTRG.1	Acbd6	MSTRG.1637.1
MSTRG.1	Ablim1	MSTRG.16370.1
MSTRG.1	Atrnl1	MSTRG.16371.1;ENSMUST00000077282
MSTRG.1	Atrnl1	MSTRG.16372.1
MSTRG.1	Atrnl1	MSTRG.16373.1
MSTRG.1	Atrnl1	MSTRG.16374.1
MSTRG.1	Atrnl1	MSTRG.16375.1
MSTRG.1	Atrnl1	MSTRG.16376.1
MSTRG.1	Mir5623	ENSMUST00000176631
MSTRG.1	Atrnl1	MSTRG.16378.1
MSTRG.1	Atrnl1	MSTRG.16379.1
MSTRG.1	Acbd6	MSTRG.1638.1

MSTRG.1	Eno4	ENSMUST00000054280;ENSMUST00000200910
MSTRG.1	Shtn1	ENSMUST00000047511
MSTRG.1	Gm29261	ENSMUST00000188535
MSTRG.1	Slc18a2	ENSMUST00000026084
MSTRG.1	Pdzd8	MSTRG.16384.1;ENSMUST00000099274
MSTRG.1	Rps12-p	ENSMUST00000086764
MSTRG.1	Pdzd8	MSTRG.16386.1
MSTRG.16387		MSTRG.16387.1
MSTRG.1	Rab11fi	MSTRG.16388.1;ENSMUST00000164379;MSTRG.16388.3;ENSMUS
MSTRG.1	Gm17203	MSTRG.16389.1
MSTRG.1	Acbd6	MSTRG.1639.1
MSTRG.1	Gm4219	ENSMUST00000168140
MSTRG.16391		MSTRG.16391.1
MSTRG.1	Fam204a	MSTRG.16392.1;ENSMUST00000065286;MSTRG.16392.3
MSTRG.1	Fam204a	MSTRG.16393.1
MSTRG.1	4933412	ENSMUST00000061046
MSTRG.1	Cacul1	ENSMUST00000081790;ENSMUST00000111460;ENSMUST0000016
MSTRG.1	Cacul1	MSTRG.16396.1
MSTRG.16398		MSTRG.16398.1
MSTRG.1	Eif3a	ENSMUST00000025955;MSTRG.16399.1
MSTRG.1	Gsta3	ENSMUST00000027067;ENSMUST00000121676;ENSMUST00000124
MSTRG.1	Acbd6	MSTRG.1640.1
MSTRG.1	Mir3084	ENSMUST00000199618;ENSMUST00000082962
MSTRG.1	Gm26158	ENSMUST00000158973
MSTRG.1	Fam45a	ENSMUST00000128357;ENSMUST00000119633;ENSMUST00000147
MSTRG.1	Fam45a	MSTRG.16403.1
MSTRG.1	Fam45a	MSTRG.16405.1;MSTRG.16405.2
MSTRG.1	Sfxn4	ENSMUST00000080806;ENSMUST00000135808;ENSMUST00000124
MSTRG.1	Prdx3	ENSMUST00000025961
MSTRG.1	Prdx3	MSTRG.16408.1
MSTRG.1	Csf2ra	ENSMUST00000076046
MSTRG.1	Acbd6	MSTRG.1641.1
MSTRG.16410		MSTRG.16410.1
MSTRG.1	Grk5	ENSMUST00000003313
MSTRG.1	Grk5	MSTRG.16412.1
MSTRG.1	Grk5	MSTRG.16413.1
MSTRG.1	Grk5	MSTRG.16414.1
MSTRG.1	Grk5	MSTRG.16415.1
MSTRG.1	Grk5	MSTRG.16416.1
MSTRG.1	Grk5	MSTRG.16417.1
MSTRG.1	Gm9529	ENSMUST00000177598
MSTRG.1	Grk5	MSTRG.16419.1
MSTRG.1	Qsox1	ENSMUST00000195419;ENSMUST00000035325;ENSMUST00000111
MSTRG.1	Gm22520	ENSMUST00000158269
MSTRG.1	Grk5	MSTRG.16421.1
MSTRG.1	Grk5	MSTRG.16422.1
MSTRG.1	Zfp950	ENSMUST00000122927;MSTRG.16423.2;ENSMUST00000127290;E
MSTRG.1	Zfp950	MSTRG.16424.1
MSTRG.1	Nmt2	ENSMUST00000081932;ENSMUST00000102989;ENSMUST00000091
MSTRG.1	Nmt2	MSTRG.16426.1
MSTRG.1	Gm22005	ENSMUST00000082795
MSTRG.1	Nmt2	MSTRG.16428.1
MSTRG.1	Rpp38	ENSMUST00000062672
MSTRG.1	Gm37539	ENSMUST00000192644
MSTRG.1	Hspa14;	ENSMUST00000027961;ENSMUST00000129505;ENSMUST0000015
MSTRG.1	Dclre1c	ENSMUST00000100463;ENSMUST00000061852;ENSMUST00000132
MSTRG.1	Suv39h2	ENSMUST00000027956;ENSMUST00000100458;ENSMUST00000127

MSTRG.1	Gm13184	ENSMUST00000149932
MSTRG.1	Fam107b	ENSMUST00000115052;ENSMUST00000115055;ENSMUST0000011[illegible]
MSTRG.1	Gm13183	ENSMUST00000118615
MSTRG.1	Gm13181	ENSMUST00000120117
MSTRG.1	Fam107b	MSTRG.16437.1
MSTRG.1	Fam107b	MSTRG.16438.1
MSTRG.1	Fam107b	MSTRG.16439.1
MSTRG.1	Gm37571	ENSMUST00000195048
MSTRG.1	Gm13185	MSTRG.16440.1
MSTRG.1	Gm13185	MSTRG.16441.1
MSTRG.1	Gm13185	ENSMUST00000131044
MSTRG.1	Prpf18	ENSMUST00000035721;ENSMUST00000153881;MSTRG.16443.3;[illegible]
MSTRG.1	Frmd4a	ENSMUST00000138735;ENSMUST00000137809;ENSMUST00000177
MSTRG.1	Gm2639	ENSMUST00000156881
MSTRG.1	Gm10862	ENSMUST00000193956
MSTRG.1	Gm38085	ENSMUST00000195785
MSTRG.1	1700080[illegible]	ENSMUST00000141488;ENSMUST00000155091;ENSMUST00000154
MSTRG.1	Cep350	ENSMUST00000138762;ENSMUST00000132189;ENSMUST0000012[illegible]
MSTRG.1	Gm13189	ENSMUST00000138462
MSTRG.1	Gm37814	ENSMUST00000195820
MSTRG.1	Gm37107	ENSMUST00000195326
MSTRG.1	Frmd4a	MSTRG.16453.1
MSTRG.1	Gm38348	ENSMUST00000191718
MSTRG.1	Gm13187	ENSMUST00000132211;ENSMUST00000133873
MSTRG.1	Gm36988	ENSMUST00000194090
MSTRG.1	Gm23691	ENSMUST00000101878
MSTRG.1	Gm13175	ENSMUST00000139479
MSTRG.1	Frmd4a	MSTRG.16459.1
MSTRG.1	Gm38038	ENSMUST00000193803
MSTRG.1	Gm13179	ENSMUST00000151994
MSTRG.1	Gm13176	MSTRG.16461.1
MSTRG.1	Sephs1	MSTRG.16462.1;ENSMUST00000027973;ENSMUST00000115019;[illegible]
MSTRG.1	Sephs1	MSTRG.16463.1
MSTRG.1	Sephs1	MSTRG.16464.1
MSTRG.1	Gm13192	ENSMUST00000101199
MSTRG.1	Phyh	ENSMUST00000027975;ENSMUST00000146933;ENSMUST0000012[illegible]
MSTRG.1	Gm13194	ENSMUST00000142299
MSTRG.1	Mcm10	ENSMUST00000102985;ENSMUST00000125349;ENSMUST0000015[illegible]
MSTRG.16469		MSTRG.16469.1
MSTRG.1	Gm36934	ENSMUST00000192007
MSTRG.1	Optn	ENSMUST00000125203;ENSMUST00000114996;ENSMUST00000027
MSTRG.1	4930551[illegible]	ENSMUST00000145585
MSTRG.1	Ccdc3	ENSMUST00000027988
MSTRG.1	Gm13198	ENSMUST00000119322
MSTRG.16475		MSTRG.16475.1
MSTRG.1	Cdc123	ENSMUST00000043864;ENSMUST00000130142;ENSMUST00000124
MSTRG.1	Nudt5	ENSMUST00000026927;ENSMUST00000179748;ENSMUST0000013[illegible]
MSTRG.1	Gm13199	ENSMUST00000142918;ENSMUST00000071016
MSTRG.1	Sec61a2	ENSMUST00000102981;ENSMUST00000193792;ENSMUST00000132
MSTRG.1	Gm37383	ENSMUST00000191750
MSTRG.1	Camk1d	MSTRG.16480.1;ENSMUST00000044009;MSTRG.16480.3;ENSMUS[illegible]
MSTRG.1	Camk1d	MSTRG.16481.1
MSTRG.1	Camk1d	MSTRG.16482.1
MSTRG.1	Camk1d	MSTRG.16483.1
MSTRG.1	Camk1d	MSTRG.16484.1
MSTRG.1	Camk1d	MSTRG.16485.1
MSTRG.1	Camk1d	MSTRG.16486.1

MSTRG.1	Camk1d	MSTRG.16487.1
MSTRG.1	Camk1d	MSTRG.16488.1
MSTRG.1	Camk1d	MSTRG.16489.1
MSTRG.1	Gm23744	ENSMUST00000104231
MSTRG.1	Camk1d	MSTRG.16490.1
MSTRG.1	Camk1d	MSTRG.16491.1
MSTRG.1	Camk1d	MSTRG.16492.1
MSTRG.1	Camk1d	MSTRG.16493.1
MSTRG.1	Camk1d	MSTRG.16494.1
MSTRG.1	Camk1d	MSTRG.16495.1
MSTRG.1	Gm13216	ENSMUST00000117145
MSTRG.1	Camk1d	MSTRG.16497.1;MSTRG.16497.2;MSTRG.16497.3;MSTRG.16497
MSTRG.1	Camk1d	MSTRG.16498.1;MSTRG.16498.2
MSTRG.1	Upf2	ENSMUST00000060092;MSTRG.16499.1;MSTRG.16499.3;MSTRG.
MSTRG.1	Gm28836	ENSMUST00000185842
MSTRG.1	Gm9694	ENSMUST00000193347
MSTRG.1	Upf2	MSTRG.16500.1
MSTRG.1	Upf2	MSTRG.16501.1
MSTRG.1	Upf2	MSTRG.16502.1
MSTRG.1	Usp6nl	ENSMUST00000114937;ENSMUST00000132391;ENSMUST00000042
MSTRG.1	Usp6nl	MSTRG.16504.1
MSTRG.1	Usp6nl	MSTRG.16505.1
MSTRG.1	Usp6nl	MSTRG.16506.1
MSTRG.1	Usp6nl	MSTRG.16507.1
MSTRG.1	Usp6nl	MSTRG.16508.1
MSTRG.1	Gm13388	ENSMUST00000126659
MSTRG.1	Tor1aip	ENSMUST00000169241;MSTRG.1651.2;ENSMUST00000097527;EN
MSTRG.1	Gm13391	ENSMUST00000147685;ENSMUST00000128589
MSTRG.1	Gm38386	ENSMUST00000194293
MSTRG.16512		MSTRG.16512.1
MSTRG.1	Celf2	ENSMUST00000002176;ENSMUST00000100429;ENSMUST00000114
MSTRG.1	Celf2	MSTRG.16514.1
MSTRG.1	Gm13389	ENSMUST00000142774
MSTRG.1	Gm10855	ENSMUST00000100426
MSTRG.1	Gm37340	ENSMUST00000193124
MSTRG.1	Gm10115	ENSMUST00000076071
MSTRG.1	Gm26315	ENSMUST00000083890
MSTRG.1	Tor1aip	MSTRG.1652.1
MSTRG.1	Gm26565	ENSMUST00000181642
MSTRG.1	Celf2	MSTRG.16522.1
MSTRG.16523		MSTRG.16523.1
MSTRG.16524		MSTRG.16524.1
MSTRG.1	Gata3;9	ENSMUST00000102976;MSTRG.16525.2;MSTRG.16525.1;ENSMUS
MSTRG.1	Gata3	MSTRG.16526.1
MSTRG.1	Gm13256	MSTRG.16527.1;ENSMUST00000131297
MSTRG.1	4930412	ENSMUST00000026889;ENSMUST00000114919
MSTRG.1	Gm13262	ENSMUST00000149014
MSTRG.1	Tor1aip	ENSMUST00000111757;ENSMUST00000111754;MSTRG.1653.2;EN
MSTRG.1	Taf3	ENSMUST00000026888;ENSMUST00000114909;MSTRG.16530.3;E
MSTRG.1	Taf3	MSTRG.16531.1
MSTRG.1	Taf3	MSTRG.16532.1
MSTRG.1	Taf3	MSTRG.16533.1
MSTRG.1	C630004	ENSMUST00000194765
MSTRG.1	Itih5	ENSMUST00000026886
MSTRG.1	Itih5	MSTRG.16536.1
MSTRG.1	Atp5c1	ENSMUST00000026887;ENSMUST00000114896;ENSMUST00000114
MSTRG.1	Gm23608	ENSMUST00000104545

MSTRG.1	Kin	ENSMUST00000142599;ENSMUST00000042512;MSTRG.16539.2;M
MSTRG.1	Tor1aip	MSTRG.1654.1
MSTRG.16540		MSTRG.16540.1
MSTRG.1	Gm13292	ENSMUST00000120014
MSTRG.1	Prkcq;G	ENSMUST00000028118;ENSMUST00000114853;MSTRG.16542.3;E
MSTRG.1	Gm36932	ENSMUST00000195226
MSTRG.1	Gm37520	MSTRG.16544.1;ENSMUST00000194883;MSTRG.16544.3
MSTRG.1	Pfkfb3	ENSMUST00000192949;ENSMUST00000179584;ENSMUST0000002[illegible]
MSTRG.1	Pfkfb3	MSTRG.16546.1
MSTRG.1	Gm10851	MSTRG.16547.1;ENSMUST00000192087
MSTRG.1	Pfkfb3	MSTRG.16548.1
MSTRG.1	Pfkfb3	MSTRG.16549.1
MSTRG.1	Gm10031	ENSMUST00000190422
MSTRG.1	Gm37975	ENSMUST00000195494;MSTRG.16550.1;MSTRG.16550.3;MSTRG.
MSTRG.1	Rbm17	ENSMUST00000040314;ENSMUST00000125808;MSTRG.16552.2;E
MSTRG.1	Gm13260	ENSMUST00000117797
MSTRG.1	Gm17490	ENSMUST00000166779
MSTRG.1	Il2ra	ENSMUST00000028111;ENSMUST00000150890
MSTRG.1	Il2ra	MSTRG.16556.1
MSTRG.1	Il2ra	ENSMUST00000195427
MSTRG.1	Gm37881	ENSMUST00000193001
MSTRG.1	Il15ra	ENSMUST00000138349;ENSMUST00000091456;ENSMUST0000013[illegible]
MSTRG.1	Tdrd5	ENSMUST00000121146;ENSMUST00000167528;ENSMUST0000014[illegible]
MSTRG.1	Il15ra	MSTRG.16560.1
MSTRG.1	Fbxo18	ENSMUST00000139292;ENSMUST00000071564;ENSMUST0000015[illegible]
MSTRG.1	Ankrd16	MSTRG.16562.1
MSTRG.1	Ankrd16	ENSMUST00000130186;MSTRG.16563.1;ENSMUST00000056108;E
MSTRG.16564		MSTRG.16564.1
MSTRG.1	Mindy3;	ENSMUST00000155530;ENSMUST00000129489;ENSMUST00000154
MSTRG.1	Gm37255	ENSMUST00000193302
MSTRG.1	Mindy3	MSTRG.16567.1
MSTRG.1	Gm13261	ENSMUST00000137351
MSTRG.1	Sfmbt2	ENSMUST00000114862;ENSMUST00000114861;ENSMUST00000114
MSTRG.1	A430050	ENSMUST00000195348
MSTRG.1	Sfmbt2	MSTRG.16570.1
MSTRG.1	Sfmbt2	MSTRG.16571.1
MSTRG.1	Sfmbt2	MSTRG.16572.1
MSTRG.1	Gm22072	ENSMUST00000116850
MSTRG.1	Gm22677	ENSMUST00000116977
MSTRG.1	Mir466m	ENSMUST00000175052
MSTRG.1	Mir466f	ENSMUST00000104779
MSTRG.1	Mir669f	ENSMUST00000103850
MSTRG.1	Mir669e	ENSMUST00000117003
MSTRG.1	Mir669b	ENSMUST00000102172
MSTRG.1	Nphs2	MSTRG.1658.1
MSTRG.1	Gm23578	ENSMUST00000103943
MSTRG.1	Mir669d	ENSMUST00000104644
MSTRG.1	Mir466f	ENSMUST00000104805
MSTRG.1	Mir669l	ENSMUST00000103771
MSTRG.1	Mir669d	ENSMUST00000116854
MSTRG.1	Mir466f	ENSMUST00000104788
MSTRG.1	Mir297a	ENSMUST00000103795
MSTRG.1	Mir466o	ENSMUST00000104832
MSTRG.1	Gm26073	ENSMUST00000179906
MSTRG.1	Mir467c	ENSMUST00000103861
MSTRG.1	4930518	ENSMUST00000195064
MSTRG.1	Mir466b	ENSMUST00000177988

MSTRG.1	Mir669a	ENSMUST00000102074
MSTRG.1	Mir669k	ENSMUST00000177742
MSTRG.1	Gm26491	ENSMUST00000179268
MSTRG.1	Mir467a	ENSMUST00000102207
MSTRG.1	Mir466b	ENSMUST00000103883
MSTRG.1	Mir669a	ENSMUST00000180238
MSTRG.1	Mir669g	ENSMUST00000116809
MSTRG.1	Gm25701	ENSMUST00000179988
MSTRG.1	Mir669j	ENSMUST00000179023
MSTRG.166		MSTRG.166.1
MSTRG.1	Nphs2	ENSMUST00000027896;MSTRG.1660.1;MSTRG.1660.2;ENSMUST0
MSTRG.1	Mir467a	ENSMUST00000179447
MSTRG.1	Mir466e	ENSMUST00000198862
MSTRG.1	Mir669a	ENSMUST00000198665
MSTRG.1	Gm24315	ENSMUST00000177835
MSTRG.1	Gm25451	ENSMUST00000177832
MSTRG.1	Mir467b	ENSMUST00000102089
MSTRG.1	Mir466c	ENSMUST00000200654
MSTRG.1	Mir669a	ENSMUST00000197439
MSTRG.1	Gm24761	ENSMUST00000179589
MSTRG.1	Gm23651	ENSMUST00000116755
MSTRG.1	Axdnd1;	ENSMUST00000177824;ENSMUST00000178036;ENSMUST0000021З
MSTRG.1	Mir467a	ENSMUST00000178082
MSTRG.1	Mir466c	ENSMUST00000180211
MSTRG.1	Mir669a	ENSMUST00000196127
MSTRG.1	Gm23108	ENSMUST00000178295
MSTRG.1	Gm25680	ENSMUST00000116917
MSTRG.1	Gm22775	ENSMUST00000177630
MSTRG.1	Mir467a	ENSMUST00000179923
MSTRG.1	Mir466b	ENSMUST00000178801
MSTRG.1	Mir669a	ENSMUST00000180364
MSTRG.1	Gm26060	ENSMUST00000180094
MSTRG.1	Axdnd1	MSTRG.1662.1
MSTRG.1	Gm26431	ENSMUST00000180199
MSTRG.1	Gm26229	ENSMUST00000179699
MSTRG.1	Mir467a	ENSMUST00000179352
MSTRG.1	Mir466b	ENSMUST00000103900
MSTRG.1	Mir669p	ENSMUST00000103884
MSTRG.1	Gm24167	ENSMUST00000177733
MSTRG.1	Gm24640	ENSMUST00000116949
MSTRG.1	Gm23288	ENSMUST00000179213
MSTRG.1	Mir467a	ENSMUST00000177790
MSTRG.1	Gm25285	ENSMUST00000178951
MSTRG.1	Axdnd1	MSTRG.1663.1
MSTRG.1	Mir669a	ENSMUST00000200268
MSTRG.1	Gm24673	ENSMUST00000116948
MSTRG.1	Gm22635	ENSMUST00000116962
MSTRG.1	Gm23569	ENSMUST00000180120
MSTRG.1	Mir467a	ENSMUST00000178635
MSTRG.1	Mir466b	ENSMUST00000180061
MSTRG.1	Mir669a	ENSMUST00000200087
MSTRG.1	Gm26426	ENSMUST00000177866
MSTRG.1	Gm24488	ENSMUST00000179934
MSTRG.1	Gm25158	ENSMUST00000177681
MSTRG.1	Axdnd1	MSTRG.1664.1
MSTRG.1	Mir467a	ENSMUST00000180070
MSTRG.1	Mir466b	ENSMUST00000103804

MSTRG.1	Mir669p	ENSMUST00000103868
MSTRG.1	Gm24028	ENSMUST00000178991
MSTRG.1	Gm22626	ENSMUST00000116990
MSTRG.1	Gm22166	ENSMUST00000178419
MSTRG.1	Mir467a	ENSMUST00000179752
MSTRG.1	Mir466b	ENSMUST00000103778
MSTRG.1	Mir669a	ENSMUST00000197745
MSTRG.1	Gm26156	ENSMUST00000116999
MSTRG.1	Gm2000	ENSMUST00000102782
MSTRG.1	Gm23571	ENSMUST00000180073
MSTRG.1	Gm25316	ENSMUST00000116826
MSTRG.1	Gm25022	ENSMUST00000102205
MSTRG.1	Gm25943	ENSMUST00000103791
MSTRG.1	Mir669a	ENSMUST00000197952
MSTRG.1	Gm23988	ENSMUST00000117034
MSTRG.1	Gm25673	ENSMUST00000179957
MSTRG.1	Gm25753	ENSMUST00000116792
MSTRG.1	Mir467a	ENSMUST00000179320
MSTRG.1	Mir466b	ENSMUST00000103806
MSTRG.1	Soat1	MSTRG.1666.1;MSTRG.1666.2
MSTRG.1	Mir669a	ENSMUST00000197274
MSTRG.1	Gm26398	ENSMUST00000179015
MSTRG.1	Mir467e	ENSMUST00000103760
MSTRG.1	Mir466p	ENSMUST00000103925
MSTRG.1	Gm22051	ENSMUST00000117002
MSTRG.1	Gm22678	ENSMUST00000116974
MSTRG.1	Mir467d	ENSMUST00000103833
MSTRG.1	Mir466	ENSMUST00000093591
MSTRG.1	Gm22091	ENSMUST00000116908
MSTRG.1	Mir297c	ENSMUST00000103788
MSTRG.1	Gm37648	ENSMUST00000192072
MSTRG.1	Mir669c	ENSMUST00000084675
MSTRG.1	Gm24342	ENSMUST00000103907
MSTRG.1	Gm25263	ENSMUST00000103916
MSTRG.1	Mir669a	ENSMUST00000102060
MSTRG.1	Gm22239	ENSMUST00000175220
MSTRG.1	Gm22925	ENSMUST00000174956
MSTRG.1	Gm23542	ENSMUST00000158208
MSTRG.1	Mir297b	ENSMUST00000102268
MSTRG.1	Mir466d	ENSMUST00000104837
MSTRG.1	Gm26092	ENSMUST00000179798
MSTRG.1	Soat1	MSTRG.1668.1
MSTRG.1	Mir669m	ENSMUST00000158945
MSTRG.1	Mir669m	ENSMUST00000158355
MSTRG.1	Mir466n	ENSMUST00000104798
MSTRG.1	Mir669o	ENSMUST00000103898
MSTRG.1	Mir466g	ENSMUST00000104831
MSTRG.1	Mir466h	ENSMUST00000104748
MSTRG.1	Mir297a	ENSMUST00000103768
MSTRG.1	Mir466l	ENSMUST00000104866
MSTRG.1	Mir297a	ENSMUST00000104761
MSTRG.1	Gm24806	ENSMUST00000117036
MSTRG.1	Abl2	ENSMUST00000173929;ENSMUST00000166172;ENSMUST00000027
MSTRG.1	Mir669i	ENSMUST00000117007
MSTRG.1	Mir669h	ENSMUST00000116944
MSTRG.1	Trdmt1	ENSMUST00000124488;ENSMUST00000028055;ENSMUST00000144
MSTRG.1	Trdmt1	MSTRG.16693.1

MSTRG.16694		MSTRG.16694.1
MSTRG.1	Vim	ENSMUST00000141365;ENSMUST00000028062;ENSMUST0000019[illegible]
MSTRG.1	Rsu1	ENSMUST00000114791;ENSMUST00000028059;ENSMUST0000016[illegible]
MSTRG.1	Gm38156	ENSMUST00000194601
MSTRG.1	Rsu1	MSTRG.16698.1
MSTRG.1	Gm37780	ENSMUST00000194117
MSTRG.1	Mir30a	ENSMUST00000083471
MSTRG.1	Abl2	MSTRG.1670.1
MSTRG.1	Gm27540	ENSMUST00000184950
MSTRG.1	Gm37160	ENSMUST00000193081
MSTRG.1	Gm13270	ENSMUST00000149137;ENSMUST00000193685
MSTRG.1	Cubn	ENSMUST00000091436;ENSMUST00000195447
MSTRG.1	St8sia6	ENSMUST00000003509;ENSMUST00000150781
MSTRG.1	Gm37126	ENSMUST00000195289
MSTRG.1	St8sia6	MSTRG.16706.1
MSTRG.1	Hacd1	ENSMUST00000131730;ENSMUST00000091429;ENSMUST0000011[illegible]
MSTRG.1	D930036[illegible]	ENSMUST00000191894
MSTRG.1	Gm37894	ENSMUST00000195416
MSTRG.1	Abl2	MSTRG.1671.1
MSTRG.1	Stamos	ENSMUST00000152524;ENSMUST00000194771
MSTRG.1	Stam	ENSMUST00000102960;ENSMUST00000028050;ENSMUST0000019[illegible]
MSTRG.1	Stam	MSTRG.16712.1
MSTRG.1	Stam	MSTRG.16713.1
MSTRG.1	Gm37499	ENSMUST00000194683
MSTRG.1	Mrc1	ENSMUST00000028045;ENSMUST00000146168
MSTRG.1	Mir511	ENSMUST00000104704
MSTRG.1	Gm37697	ENSMUST00000195680
MSTRG.1	Slc39a1[illegible]	ENSMUST00000114731;ENSMUST00000082290
MSTRG.16719		MSTRG.16719.1
MSTRG.1	Abl2	MSTRG.1672.1
MSTRG.1	Malrd1	ENSMUST00000146205
MSTRG.1	Gm37416	MSTRG.16723.1;ENSMUST00000193596
MSTRG.1	Cacnb2	ENSMUST00000193730;ENSMUST00000114723;ENSMUST0000015[illegible]
MSTRG.1	Gm10849	ENSMUST00000100397
MSTRG.1	Gm38105	ENSMUST00000192112
MSTRG.1	Gm13268	ENSMUST00000117485
MSTRG.1	Gm13312	ENSMUST00000119631
MSTRG.1	2900092[illegible]	ENSMUST00000193601
MSTRG.1	Gm38043	MSTRG.1673.1;ENSMUST00000195501
MSTRG.1	Gm13314	ENSMUST00000118742
MSTRG.1	Gm13311	ENSMUST00000117863
MSTRG.1	Gm10848	ENSMUST00000192361
MSTRG.1	Gm37006	ENSMUST00000195253
MSTRG.1	Gm13315	ENSMUST00000120376
MSTRG.1	Gm38273	ENSMUST00000194987
MSTRG.1	Gm13269	ENSMUST00000143458;ENSMUST00000147019
MSTRG.1	Gm13316	ENSMUST00000140537
MSTRG.1	Gm37437	ENSMUST00000194451
MSTRG.1	Gm37891	ENSMUST00000195585
MSTRG.1	Abl2	MSTRG.1674.1;MSTRG.1674.2
MSTRG.1	Nsun6	ENSMUST00000028034;ENSMUST00000076435;ENSMUST0000019[illegible]
MSTRG.1	Nsun6	MSTRG.16741.1
MSTRG.1	Nsun6	MSTRG.16742.1
MSTRG.1	Nsun6	MSTRG.16743.1
MSTRG.1	Gm36930	ENSMUST00000195431
MSTRG.1	Arl5b	ENSMUST00000017562;ENSMUST00000069870;ENSMUST0000012[illegible]
MSTRG.1	Gm38257	ENSMUST00000195881

MSTRG.1	Plxdc2	MSTRG.16747.2;MSTRG.16747.1;MSTRG.16747.3;MSTRG.16747
MSTRG.1	Plxdc2	MSTRG.16748.1
MSTRG.1	Plxdc2	MSTRG.16749.1;MSTRG.16749.2
MSTRG.1	Abl2	MSTRG.1675.1
MSTRG.1	Plxdc2	MSTRG.16750.1
MSTRG.1	Plxdc2	MSTRG.16751.1
MSTRG.16752		MSTRG.16752.1
MSTRG.1	Nebl	MSTRG.16753.1;MSTRG.16753.3;MSTRG.16753.2;ENSMUST0000
MSTRG.1	Gm13324	ENSMUST00000148607
MSTRG.1	Commd3	ENSMUST00000171845;ENSMUST00000061158;ENSMUST00000151
MSTRG.1	Bmi1	ENSMUST00000028071;ENSMUST00000132014;ENSMUST0000015C
MSTRG.1	Pip4k2a	MSTRG.16758.2;MSTRG.16758.1;ENSMUST00000006912;ENSMUS
MSTRG.1	Pip4k2a	MSTRG.16759.1
MSTRG.1	Tor3a	ENSMUST00000156861;ENSMUST00000079625;ENSMUST0000015C
MSTRG.1	Pip4k2a	MSTRG.16760.1
MSTRG.1	Pip4k2a	MSTRG.16761.1
MSTRG.1	4930426	ENSMUST00000028069
MSTRG.16763		MSTRG.16763.1
MSTRG.1	1810059	ENSMUST00000125173
MSTRG.1	Mllt10;	ENSMUST00000028076;ENSMUST00000150422;ENSMUST00000114
MSTRG.1	Mir7655	ENSMUST00000183376
MSTRG.1	Mllt10	MSTRG.16767.1
MSTRG.1	Dnajc1	MSTRG.16768.1;MSTRG.16768.2;ENSMUST00000148401;ENSMUS
MSTRG.1	Gm23638	ENSMUST00000175073
MSTRG.1	Tor3a	MSTRG.1677.1
MSTRG.1	Mllt10	MSTRG.16770.1
MSTRG.1	Gm13351	ENSMUST00000118087
MSTRG.1	Mllt10	MSTRG.16772.1
MSTRG.1	Gm13350	ENSMUST00000120519
MSTRG.1	Dnajc1	MSTRG.16774.1
MSTRG.1	Dnajc1	MSTRG.16775.1
MSTRG.1	Dnajc1	MSTRG.16776.1
MSTRG.1	Gm13349	ENSMUST00000118436
MSTRG.1	Dnajc1	MSTRG.16778.1
MSTRG.1	Dnajc1	MSTRG.16779.1;MSTRG.16779.2
MSTRG.1	Fam20b	ENSMUST00000122424;MSTRG.1678.2;ENSMUST00000086153;EN
MSTRG.1	Dnajc1	MSTRG.16780.1
MSTRG.1	Msrb2	ENSMUST00000137812;ENSMUST00000023856;ENSMUST0000012€
MSTRG.1	Msrb2	MSTRG.16782.1
MSTRG.1	Msrb2	MSTRG.16783.1
MSTRG.16784		MSTRG.16784.1
MSTRG.1	Gm13343	ENSMUST00000116474
MSTRG.1	Otud1	ENSMUST00000052168
MSTRG.1	Etl4	ENSMUST00000114610;ENSMUST00000114627;ENSMUST00000131
MSTRG.1	Gm13363	ENSMUST00000122008
MSTRG.1	Gm13328	ENSMUST00000120816
MSTRG.1	Gm17171	ENSMUST00000168048
MSTRG.1	Gm13335	ENSMUST00000129994;ENSMUST00000149398
MSTRG.1	Etl4	MSTRG.16796.1
MSTRG.1	Gm27446	ENSMUST00000183963
MSTRG.1	Gm13362	ENSMUST00000137186
MSTRG.1	Gm13378	ENSMUST00000117483
MSTRG.168		MSTRG.168.1
MSTRG.1	4930439	MSTRG.1680.1
MSTRG.16800		MSTRG.16800.1
MSTRG.16801		MSTRG.16801.1
MSTRG.1	Arhgap2	ENSMUST00000154230;ENSMUST00000114594;ENSMUST00000173

MSTRG.1	Arhgap2	MSTRG.16803.1
MSTRG.1	Gm13380	ENSMUST00000124322
MSTRG.1	Gm13375	ENSMUST00000126967
MSTRG.16806		MSTRG.16806.1
MSTRG.1	Apbb1ip	ENSMUST00000131195;ENSMUST00000014290;ENSMUST0000014ε
MSTRG.1	Apbb1ip	MSTRG.16808.1
MSTRG.1	Acbd5	ENSMUST00000028121;ENSMUST00000114523;MSTRG.16809.3;E
MSTRG.1	Rasal2	MSTRG.1681.1;ENSMUST00000078308;ENSMUST00000132699;EN
MSTRG.1	Acbd5	MSTRG.16810.1
MSTRG.1	Mastl	ENSMUST00000028119;ENSMUST00000136207
MSTRG.1	Mastl	MSTRG.16812.1
MSTRG.1	Mastl	MSTRG.16813.1
MSTRG.1	Yme1l1	ENSMUST00000028117;ENSMUST00000125004;ENSMUST0000014S
MSTRG.1	Gm38309	ENSMUST00000192063
MSTRG.1	A130006	ENSMUST00000091396
MSTRG.1	Pdss1	ENSMUST00000148072;MSTRG.16817.1;ENSMUST00000053729;E
MSTRG.1	Gm25544	ENSMUST00000122676
MSTRG.1	Gm14441	ENSMUST00000121960
MSTRG.1	Rasal2	MSTRG.1682.1
MSTRG.1	Abi1;Gm	ENSMUST00000126112;ENSMUST00000140164;ENSMUST0000014S
MSTRG.1	Abi1	MSTRG.16821.1
MSTRG.1	Abi1	MSTRG.16822.1;MSTRG.16822.2
MSTRG.1	Abi1	MSTRG.16823.1
MSTRG.16824		MSTRG.16824.1
MSTRG.16825		MSTRG.16825.1
MSTRG.1	Gm13410	ENSMUST00000117058
MSTRG.1	Spopl	MSTRG.16827.1;MSTRG.16827.2;MSTRG.16827.3;ENSMUST0000C
MSTRG.1	Il1rn	ENSMUST00000114487;ENSMUST00000114486;ENSMUST00000142
MSTRG.1	Gm37788	ENSMUST00000193958
MSTRG.1	Psd4	ENSMUST00000140547;ENSMUST00000102942;ENSMUST0000005ϵ
MSTRG.1	Zmynd19	ENSMUST00000028350;ENSMUST00000148042
MSTRG.1	Zmynd19	MSTRG.16832.1
MSTRG.1	Arrdc1	MSTRG.16833.1;ENSMUST00000102935;ENSMUST00000028349;E
MSTRG.1	Arrdc1	MSTRG.16834.1
MSTRG.1	Arrdc1	MSTRG.16835.1
MSTRG.1	Gm22875	ENSMUST00000175449
MSTRG.1	Ehmt1;G	ENSMUST00000147147;ENSMUST00000114418;ENSMUST00000134
MSTRG.1	Ehmt1	MSTRG.16838.1
MSTRG.1	Ehmt1	MSTRG.16839.1
MSTRG.1	Gm37578	ENSMUST00000192256
MSTRG.1	Ehmt1	MSTRG.16840.1
MSTRG.1	Ehmt1	MSTRG.16841.1;MSTRG.16841.2
MSTRG.1	Dph7	ENSMUST00000028351;ENSMUST00000126909;ENSMUST0000013E
MSTRG.1	Mrpl41	ENSMUST00000045604;ENSMUST00000194392
MSTRG.1	Pnpla7	ENSMUST00000045295;ENSMUST00000137913;ENSMUST0000015З
MSTRG.1	Gm13370	ENSMUST00000121309
MSTRG.1	Gm13369	ENSMUST00000119442
MSTRG.1	Gm25518	ENSMUST00000083864
MSTRG.1	Pnpla7	MSTRG.16848.1
MSTRG.1	Gm24875	ENSMUST00000158888
MSTRG.1	Gm6058	ENSMUST00000154261
MSTRG.1	Pnpla7	MSTRG.16850.1
MSTRG.1	Fam166a	ENSMUST00000028346
MSTRG.1	Tubb4b	ENSMUST00000043584;ENSMUST00000145607
MSTRG.1	Gm13387	ENSMUST00000123483;ENSMUST00000131147;ENSMUST0000014S
MSTRG.1	Nrarp	ENSMUST00000104999
MSTRG.1	Gm38287	ENSMUST00000195193

MSTRG.1	Tor4a	ENSMUST00000081869;ENSMUST00000142857;ENSMUST00000137
MSTRG.1	Nelfb	ENSMUST00000059849;ENSMUST00000128876;ENSMUST0000012[illegible]
MSTRG.1	Gm22572	ENSMUST00000082985
MSTRG.1	Tmem203	MSTRG.16859.1
MSTRG.1	Gm37060	ENSMUST00000194997
MSTRG.1	Cysrt1	ENSMUST00000186719
MSTRG.1	Rnf208	ENSMUST00000154498;ENSMUST00000114355;ENSMUST0000006[illegible]
MSTRG.1	Ndor1	ENSMUST00000100329;MSTRG.16862.2;ENSMUST00000114349;[illegible]
MSTRG.1	Tmem203	ENSMUST00000104998
MSTRG.1	Tprn	ENSMUST00000114336;ENSMUST00000141509;ENSMUST00000155
MSTRG.1	Ssna1	ENSMUST00000133697;ENSMUST00000028342;MSTRG.16865.3;[illegible]
MSTRG.1	Anapc2	ENSMUST00000028341;ENSMUST00000129265;MSTRG.16866.2;[illegible]
MSTRG.1	AA54318	ENSMUST00000180841
MSTRG.1	Man1b1	ENSMUST00000042390;ENSMUST00000149464;ENSMUST00000152
MSTRG.1	Gm25220	ENSMUST00000157546
MSTRG.1	2810025[illegible]	ENSMUST00000061537;ENSMUST00000187761;ENSMUST00000194
MSTRG.1	Dpp7	ENSMUST00000139108;ENSMUST00000139971;ENSMUST0000014[illegible]
MSTRG.1	Entpd2;[illegible]	ENSMUST00000028328;ENSMUST00000148859;ENSMUST0000014[illegible]
MSTRG.1	Abca2	MSTRG.16872.1;ENSMUST00000102919;MSTRG.16872.3;ENSMUS
MSTRG.1	Mir3087	ENSMUST00000199405
MSTRG.1	Paxx	ENSMUST00000132596;MSTRG.16874.1;ENSMUST00000133919;[illegible]
MSTRG.1	Clic3	MSTRG.16875.1;ENSMUST00000140022;ENSMUST00000114265;[illegible]
MSTRG.1	C8g	ENSMUST00000040042;ENSMUST00000015227;ENSMUST0000015[illegible]
MSTRG.1	Fbxw5	ENSMUST00000015239;ENSMUST00000124258;ENSMUST0000012[illegible]
MSTRG.1	Traf2	MSTRG.16878.1;ENSMUST00000028311;MSTRG.16878.3;ENSMUS
MSTRG.1	Gm13433	ENSMUST00000121701
MSTRG.1	Ralgps2	ENSMUST00000172057;ENSMUST00000027886;ENSMUST00000192
MSTRG.1	Traf2	MSTRG.16880.1
MSTRG.1	Gm13432	ENSMUST00000118861
MSTRG.1	Edf1	ENSMUST00000015236;ENSMUST00000153008;ENSMUST00000134
MSTRG.1	Mamdc4;[illegible]	ENSMUST00000095117;ENSMUST00000114223;ENSMUST00000152
MSTRG.1	Gm996	ENSMUST00000114217;ENSMUST00000191602;ENSMUST0000018[illegible]
MSTRG.1	A230005[illegible]	ENSMUST00000137661;ENSMUST00000151069
MSTRG.1	Bmyc	ENSMUST00000061483
MSTRG.1	Rabl6	MSTRG.16888.1;ENSMUST00000058137;ENSMUST00000150206;[illegible]
MSTRG.1	Rabl6	MSTRG.16889.1
MSTRG.1	Angptl1	ENSMUST00000027885;ENSMUST00000111720
MSTRG.1	Fcnaos	ENSMUST00000155684;ENSMUST00000127642
MSTRG.1	Ccdc183	ENSMUST00000028309;ENSMUST00000129520
MSTRG.1	Tmem141	ENSMUST00000028308;ENSMUST00000142087;ENSMUST0000015[illegible]
MSTRG.1	Fcna	ENSMUST00000028307;ENSMUST00000142371
MSTRG.1	Camsap1	MSTRG.16894.2;ENSMUST00000091268;ENSMUST00000114167;[illegible]
MSTRG.1	Gm13542	ENSMUST00000118484
MSTRG.1	Ubac1	ENSMUST00000123275;ENSMUST00000036509;ENSMUST00000154
MSTRG.1	Nacc2	ENSMUST00000114159;ENSMUST00000028300;ENSMUST0000012[illegible]
MSTRG.1	Tmem250[illegible]	ENSMUST00000133808;ENSMUST00000163836
MSTRG.169		MSTRG.169.1
MSTRG.1	Angptl1	MSTRG.1690.1
MSTRG.1	1810012[illegible]	MSTRG.16900.2
MSTRG.1	Lhx3	ENSMUST00000028302
MSTRG.1	Gm27196	ENSMUST00000184787
MSTRG.1	Qsox2	ENSMUST00000091263;MSTRG.16903.1;ENSMUST00000036187
MSTRG.1	Gpsm1	ENSMUST00000066936;MSTRG.16904.3;MSTRG.16904.5;ENSMUS
MSTRG.1	Dnlz	ENSMUST00000125848;ENSMUST00000028295
MSTRG.1	Card9	ENSMUST00000100303;ENSMUST00000153370;ENSMUST0000002[illegible]
MSTRG.1	Gm13562	ENSMUST00000125198;ENSMUST00000137869
MSTRG.1	Snapc4	ENSMUST00000114115;ENSMUST00000135171;ENSMUST0000003[illegible]

MSTRG.1	0610009	ENSMUST00000133463;ENSMUST00000151521;ENSMUST0000013
MSTRG.1	Ralgps2	MSTRG.1691.1
MSTRG.1	Notch1	ENSMUST00000028288;MSTRG.16910.1;MSTRG.16910.3;ENSMUS
MSTRG.1	Mir6996	ENSMUST00000183922
MSTRG.1	Gm13563	ENSMUST00000125518;MSTRG.16912.2;ENSMUST00000152375;E
MSTRG.1	Sdccag3	ENSMUST00000114100;ENSMUST00000077983;ENSMUST0000002
MSTRG.1	Pmpca	ENSMUST00000076431;ENSMUST00000114093;ENSMUST00000137
MSTRG.1	Inpp5e	ENSMUST00000145701;ENSMUST00000028291;MSTRG.16915.4;E
MSTRG.1	Sec16a	ENSMUST00000156442;ENSMUST00000114082;MSTRG.16916.3;M
MSTRG.1	Lcn4	ENSMUST00000028283
MSTRG.1	Abo	MSTRG.16918.1
MSTRG.1	Egfl7;G	ENSMUST00000166920;ENSMUST00000100290;ENSMUST00000102
MSTRG.1	Ralgps2	MSTRG.1692.1
MSTRG.1	Agpat2	ENSMUST00000131940;ENSMUST00000028286;ENSMUST0000014C
MSTRG.1	Snhg7	MSTRG.16922.1
MSTRG.1	Snhg7;S	ENSMUST00000181621;ENSMUST00000082924
MSTRG.1	Snora17	ENSMUST00000104004
MSTRG.1	Adamts1	ENSMUST00000014996;ENSMUST00000102891;MSTRG.16925.3
MSTRG.1	Adamts1	MSTRG.16926.1
MSTRG.1	Cacfd1	ENSMUST00000114005;ENSMUST00000114004;MSTRG.16927.1;E
MSTRG.1	Slc2a6	ENSMUST00000102890;ENSMUST00000145742;ENSMUST0000012
MSTRG.1	Surf6	ENSMUST00000140392;MSTRG.16929.1;ENSMUST00000129554;E
MSTRG.1	Ralgps2	MSTRG.1693.1
MSTRG.1	Med22	ENSMUST00000102899;ENSMUST00000015920;MSTRG.16930.2;E
MSTRG.1	Rpl7a;G	ENSMUST00000102898;ENSMUST00000136269;ENSMUST00000137
MSTRG.1	Surf1	ENSMUST00000142967;ENSMUST00000183520;MSTRG.16932.2;E
MSTRG.1	Surf2	ENSMUST00000015017;ENSMUST00000126947;MSTRG.16933.3;E
MSTRG.1	Surf4	ENSMUST00000015011;ENSMUST00000153641;ENSMUST00000154
MSTRG.1	Stkld1	ENSMUST00000153771;ENSMUST00000055406
MSTRG.1	Rexo4	ENSMUST00000064244;ENSMUST00000114020;ENSMUST00000123
MSTRG.1	Vav2	MSTRG.16937.1;ENSMUST00000185188;ENSMUST00000056176;E
MSTRG.1	AA64544	ENSMUST00000156020
MSTRG.1	Gm24049	ENSMUST00000102409
MSTRG.1	4930439	ENSMUST00000186765
MSTRG.1	Vav2	MSTRG.16940.1
MSTRG.1	Rxra	ENSMUST00000129514;ENSMUST00000077257;ENSMUST0000010C
MSTRG.1	Rxra	MSTRG.16942.1
MSTRG.1	Brd3;Mi	ENSMUST00000028282;ENSMUST00000077737;ENSMUST00000113
MSTRG.1	Wdr5	MSTRG.16944.1;ENSMUST00000144315;ENSMUST00000113952;E
MSTRG.1	Rpsa-ps	ENSMUST00000120702
MSTRG.1	Mrps2	ENSMUST00000038600;ENSMUST00000123533;ENSMUST00000146
MSTRG.1	Gbgt1	ENSMUST00000127958;ENSMUST00000163121;ENSMUST0000002
MSTRG.1	Ralgds	ENSMUST00000135803;ENSMUST00000028170;ENSMUST00000113
MSTRG.1	Cel	ENSMUST00000028161
MSTRG.1	Pappa2;	ENSMUST00000159861;ENSMUST00000159892;ENSMUST00000062
MSTRG.1	Gm13381	ENSMUST00000177012
MSTRG.1	Gtf3c5	ENSMUST00000113889;ENSMUST00000028157;ENSMUST0000013
MSTRG.1	Tsc1	ENSMUST00000156857;ENSMUST00000028155;ENSMUST00000133
MSTRG.1	Tsc1	MSTRG.16953.1
MSTRG.1	Spaca9	ENSMUST00000102877;ENSMUST00000124840;ENSMUST00000144
MSTRG.1	Ak8	ENSMUST00000074156;ENSMUST00000137588
MSTRG.1	Mir3088	ENSMUST00000175091
MSTRG.1	Gm13385	ENSMUST00000121089
MSTRG.1	Gm13386	ENSMUST00000117677
MSTRG.1	Gtf3c4	ENSMUST00000171404;ENSMUST00000037117;ENSMUST00000156
MSTRG.1	Pappa2	MSTRG.1696.1
MSTRG.1	Ddx31	ENSMUST00000147779;ENSMUST00000113853;ENSMUST00000147

MSTRG.1	Ddx31	MSTRG.16961.1
MSTRG.1	Ddx31	MSTRG.16962.1
MSTRG.1	Cfap77	ENSMUST00000189027;ENSMUST00000189711;ENSMUST00000190
MSTRG.1	Gm13394	ENSMUST00000121658
MSTRG.1	Ttf1	ENSMUST00000100237;ENSMUST00000142786;MSTRG.16965.3;E
MSTRG.1	Gm13393	ENSMUST00000141935
MSTRG.1	Setx	ENSMUST00000129544;MSTRG.16968.1;ENSMUST00000061578;E
MSTRG.1	Ntng2;6	ENSMUST00000183583;ENSMUST00000177689;ENSMUST00000048
MSTRG.1	Pappa2	MSTRG.1697.1
MSTRG.1	Med27	ENSMUST00000028139;ENSMUST00000113830;ENSMUST00000162
MSTRG.1	Gm13402	MSTRG.16971.1;MSTRG.16971.2;ENSMUST00000153600
MSTRG.1	Gm13402	MSTRG.16972.1
MSTRG.1	Med27	MSTRG.16973.1
MSTRG.1	Gm24976	ENSMUST00000158972
MSTRG.1	Mir133c	ENSMUST00000184068
MSTRG.1	Gm13401	ENSMUST00000117074
MSTRG.1	Rapgef1	MSTRG.16977.1
MSTRG.1	Rapgef1	ENSMUST00000095087;ENSMUST00000091146;MSTRG.16978.4;M
MSTRG.1	Rfwd2	ENSMUST00000195800;ENSMUST00000076894;ENSMUST00000192
MSTRG.1	Gm13547	ENSMUST00000177379;ENSMUST00000113810
MSTRG.1	Trub2	ENSMUST00000113807;ENSMUST00000156846;ENSMUST00000048
MSTRG.1	Coq4	ENSMUST00000143616;ENSMUST00000176348;ENSMUST00000028
MSTRG.1	Slc27a4	ENSMUST00000080065;ENSMUST00000136444
MSTRG.1	Urm1	ENSMUST00000149379;ENSMUST00000156944;ENSMUST00000091
MSTRG.1	Gle1	ENSMUST00000019859;ENSMUST00000154490;ENSMUST00000148
MSTRG.1	Odf2	ENSMUST00000123335;ENSMUST00000148883;ENSMUST00000113
MSTRG.1	Sptan1	ENSMUST00000113719;ENSMUST00000113717;ENSMUST00000143
MSTRG.1	Sptan1	MSTRG.16989.1
MSTRG.1	Gm37115	ENSMUST00000193750
MSTRG.1	Mir6997	ENSMUST00000184313
MSTRG.1	Wdr34	ENSMUST00000113711;ENSMUST00000139145;ENSMUST00000143
MSTRG.1	Tbc1d13	ENSMUST00000136183;ENSMUST00000044556;ENSMUST00000150
MSTRG.1	Set	ENSMUST00000134364;MSTRG.16993.2;ENSMUST00000149578;E
MSTRG.1	Pkn3	ENSMUST00000125346;ENSMUST00000148650;ENSMUST00000150
MSTRG.1	Pkn3	MSTRG.16995.1
MSTRG.1	Zdhhc12	ENSMUST00000148717;ENSMUST00000102865;MSTRG.16996.3;E
MSTRG.1	Zer1	MSTRG.16997.1;ENSMUST00000044751;ENSMUST00000113677;M
MSTRG.1	Endog	ENSMUST00000015481
MSTRG.1	Spout1	ENSMUST00000142669;ENSMUST00000127309;ENSMUST00000136
MSTRG.1	Gm17100	ENSMUST00000168963
MSTRG.1	Kcnq5	ENSMUST00000029667;ENSMUST00000115300;ENSMUST00000173
MSTRG.1	Rfwd2	MSTRG.1700.1
MSTRG.1	Kyat1	ENSMUST00000044038;ENSMUST00000113662;ENSMUST00000148
MSTRG.1	Kyat1	MSTRG.17001.1
MSTRG.1	1700084	ENSMUST00000187343;ENSMUST00000205624
MSTRG.1	Lrrc8a;	ENSMUST00000113654;ENSMUST00000095078;ENSMUST00000139
MSTRG.1	Gm28035	MSTRG.17004.1
MSTRG.1	Dolk	ENSMUST00000100219
MSTRG.1	Gm14471	ENSMUST00000141035
MSTRG.1	Sh3glb2	ENSMUST00000100215;ENSMUST00000028214;ENSMUST00000113
MSTRG.1	Ptpa	MSTRG.17008.1
MSTRG.17009		MSTRG.17009.1
MSTRG.1	Rfwd2	MSTRG.1701.1;MSTRG.1701.2
MSTRG.17010		MSTRG.17010.1
MSTRG.1	Miga2;A	ENSMUST00000077977;ENSMUST00000146308;ENSMUST00000140
MSTRG.1	Dolpp1	ENSMUST00000028209;ENSMUST00000113612;ENSMUST00000123
MSTRG.1	Crat	ENSMUST00000102855;ENSMUST00000146603;ENSMUST00000028

MSTRG.1	Gm16323	ENSMUST00000134920
MSTRG.1	Ptpa	ENSMUST00000042055;ENSMUST00000113601;ENSMUST00000152
MSTRG.17016		MSTRG.17016.1
MSTRG.1	Cstad	ENSMUST00000102853;ENSMUST00000129719
MSTRG.1	Cstad	MSTRG.17018.1
MSTRG.1	Ntmt1	ENSMUST00000041830;MSTRG.17019.2;ENSMUST00000138889;E
MSTRG.1	Rfwd2	MSTRG.1702.1
MSTRG.1	Ntmt1	MSTRG.17020.1
MSTRG.1	Asb6	ENSMUST00000148036;ENSMUST00000041726;ENSMUST0000012[illegible]
MSTRG.1	Tor1a	ENSMUST00000028200;ENSMUST00000137694;ENSMUST00000144
MSTRG.1	Prrx2	ENSMUST00000113592;ENSMUST00000041659;ENSMUST0000015[illegible]
MSTRG.1	Ptges	ENSMUST00000102852;MSTRG.17024.1;ENSMUST00000056046;E
MSTRG.1	Gm13400	ENSMUST00000117436
MSTRG.1	Tor1b	ENSMUST00000028199;ENSMUST00000133544;ENSMUST0000013[illegible]
MSTRG.1	Gpr107	MSTRG.17027.1
MSTRG.1	Ass1	ENSMUST00000102840;ENSMUST00000126474;ENSMUST00000192
MSTRG.1	Fubp3	ENSMUST00000113482;ENSMUST00000137889;ENSMUST00000137
MSTRG.1	Rfwd2	MSTRG.1703.1
MSTRG.1	Fubp3	MSTRG.17030.1
MSTRG.1	Mir6998	ENSMUST00000183343
MSTRG.1	BC00562	ENSMUST00000028205;MSTRG.17032.2;ENSMUST00000152007;E
MSTRG.1	Usp20	ENSMUST00000102849;ENSMUST00000192882;ENSMUST00000061
MSTRG.1	Fnbp1	MSTRG.17034.1;ENSMUST00000073879;ENSMUST00000113559;E
MSTRG.1	Fnbp1	MSTRG.17035.1
MSTRG.1	D330023	MSTRG.17036.1
MSTRG.1	D330023	ENSMUST00000148230;ENSMUST00000133550
MSTRG.1	Gpr107	ENSMUST00000056433;ENSMUST00000153032;MSTRG.17038.2;E
MSTRG.1	Gm22516	ENSMUST00000158266
MSTRG.1	Rfwd2	MSTRG.1704.1
MSTRG.1	Gm13405	ENSMUST00000146769
MSTRG.1	Exosc2	ENSMUST00000038474;ENSMUST00000149094;ENSMUST00000137
MSTRG.1	Gm13427	ENSMUST00000154296
MSTRG.1	Abl1	ENSMUST00000146537;ENSMUST00000156736;ENSMUST0000007[illegible]
MSTRG.1	Abl1	MSTRG.17044.1
MSTRG.1	Abl1	MSTRG.17045.1
MSTRG.1	Plpp7	ENSMUST00000057423;ENSMUST00000146502
MSTRG.1	Nup214;	ENSMUST00000065398;ENSMUST00000130975;ENSMUST0000012[illegible]
MSTRG.1	Gm13608	ENSMUST00000121807
MSTRG.1	Nup214	MSTRG.17049.1
MSTRG.1	Rfwd2	MSTRG.1705.1
MSTRG.1	Gm13609	ENSMUST00000141023
MSTRG.1	Fam78a;	MSTRG.17051.1;MSTRG.17051.2;MSTRG.17051.3;ENSMUST000[illegible]
MSTRG.1	Fam78a	MSTRG.17052.1
MSTRG.1	Gm13611	ENSMUST00000118269
MSTRG.1	Lcn2	ENSMUST00000147219;ENSMUST00000050785;ENSMUST00000144
MSTRG.1	Prrc2b;	ENSMUST00000036691;MSTRG.17055.8;ENSMUST00000069817;M
MSTRG.1	Prrc2b	MSTRG.17056.1;MSTRG.17056.2;MSTRG.17056.3
MSTRG.1	Gm27805	ENSMUST00000184513
MSTRG.1	Gm13610	ENSMUST00000128298
MSTRG.1	Pomt1	ENSMUST00000036473;ENSMUST00000126784;ENSMUST00000154
MSTRG.1	Gm37088	ENSMUST00000193887
MSTRG.1	Uck1	ENSMUST00000002625;ENSMUST00000133804;MSTRG.17060.2;E
MSTRG.1	Slc25a2	ENSMUST00000052119;ENSMUST00000028160;ENSMUST00000113
MSTRG.1	Naif1	ENSMUST00000048431
MSTRG.1	Fam102a	ENSMUST00000136816;ENSMUST00000133512;MSTRG.17063.2;M
MSTRG.1	Dpm2	ENSMUST00000140592;ENSMUST00000028151;ENSMUST0000012[illegible]
MSTRG.1	Swi5	ENSMUST00000113395;ENSMUST00000140406;ENSMUST00000113

MSTRG.1	Golga2	ENSMUST00000139494;ENSMUST00000113377;ENSMUST0000010C
MSTRG.1	Golga2	MSTRG.17067.1
MSTRG.1	Dnm1	ENSMUST00000113352;ENSMUST00000091089;ENSMUST0000007E
MSTRG.1	Scarna3	ENSMUST00000158911;ENSMUST00000198115
MSTRG.1	Mir199b	ENSMUST00000175066
MSTRG.1	Ciz1	ENSMUST00000125769;ENSMUST00000132028;ENSMUST0000013E
MSTRG.1	1110008	ENSMUST00000146423;ENSMUST00000048792
MSTRG.1	St6galn	ENSMUST00000102818;ENSMUST00000123080;ENSMUST00000192
MSTRG.1	Gm23369	ENSMUST00000175406
MSTRG.1	St6galn	ENSMUST00000131229;ENSMUST00000140983;ENSMUST00000113
MSTRG.1	Sh2d3c	ENSMUST00000074248;MSTRG.17076.1;ENSMUST00000124133;E
MSTRG.1	6330409	ENSMUST00000151536;ENSMUST00000009695
MSTRG.1	Eng	ENSMUST00000113272;ENSMUST00000009705;ENSMUST00000167
MSTRG.1	Mir1954	ENSMUST00000175595
MSTRG.1	Rfwd2	MSTRG.1708.1
MSTRG.1	AL77227	ENSMUST00000196848;ENSMUST00000175536
MSTRG.1	Eng	MSTRG.17081.1
MSTRG.1	Fpgs;Mi	ENSMUST00000028148;ENSMUST00000130164;ENSMUST00000132
MSTRG.1	Cdk9;Mi	ENSMUST00000125058;MSTRG.17083.1;MSTRG.17083.3;ENSMUS
MSTRG.1	Ttc16	ENSMUST00000091059;ENSMUST00000066478;ENSMUST00000161
MSTRG.1	Tor2a	ENSMUST00000009707;ENSMUST00000177382;ENSMUST0000015E
MSTRG.1	Ptrh1	ENSMUST00000066352;ENSMUST00000192262;ENSMUST0000013E
MSTRG.1	Cfap157	ENSMUST00000102813;ENSMUST00000150918
MSTRG.1	Stxbp1	ENSMUST00000050000;ENSMUST00000113222;ENSMUST00000077
MSTRG.1	Gm24165	ENSMUST00000082718
MSTRG.1	Mrps14	ENSMUST00000078878;ENSMUST00000135680;ENSMUST00000097
MSTRG.1	Stxbp1	MSTRG.17090.1
MSTRG.1	Gm13524	ENSMUST00000125869
MSTRG.1	Gm13523	ENSMUST00000151820
MSTRG.1	Fam129b	ENSMUST00000028135;ENSMUST00000138781;ENSMUST00000125
MSTRG.1	Lrsam1	ENSMUST00000028132;ENSMUST00000113200;ENSMUST00000195
MSTRG.1	Rpl12;S	ENSMUST00000126610;ENSMUST00000134912;ENSMUST00000154
MSTRG.1	Zbtb34	ENSMUST00000091037;ENSMUST00000113158;ENSMUST00000134
MSTRG.1	Zbtb34	MSTRG.17097.1
MSTRG.1	Zbtb34	MSTRG.17098.1
MSTRG.1	Zbtb34	MSTRG.17099.1
MSTRG.1	Gm36949	ENSMUST00000192487
MSTRG.1	Cacybp	ENSMUST00000014370;MSTRG.1710.2;ENSMUST00000195654;EN
MSTRG.1	Zbtb34	MSTRG.17100.1
MSTRG.1	Zbtb34	MSTRG.17101.1
MSTRG.1	Ralgps1	ENSMUST00000131298;MSTRG.17102.2;ENSMUST00000042615;E
MSTRG.1	Gm13528	ENSMUST00000139288
MSTRG.1	Gm23546	ENSMUST00000158211
MSTRG.1	Angptl2	ENSMUST00000004208;ENSMUST00000143252;ENSMUST0000013S
MSTRG.1	Gm13537	ENSMUST00000120467
MSTRG.1	Gm25998	ENSMUST00000175397
MSTRG.1	Ralgps1	MSTRG.17108.1
MSTRG.1	Gm13536	ENSMUST00000132913
MSTRG.1	4933417	ENSMUST00000193962
MSTRG.1	Zbtb43	ENSMUST00000095035;ENSMUST00000028125;ENSMUST00000113
MSTRG.1	Mvb12b	ENSMUST00000041555;MSTRG.17111.2;MSTRG.17111.3;MSTRG.
MSTRG.1	Nron	ENSMUST00000156297;ENSMUST00000151761;ENSMUST0000014C
MSTRG.1	Mvb12b	MSTRG.17113.1
MSTRG.1	Pbx3	ENSMUST00000113132;ENSMUST00000040638;ENSMUST0000017E
MSTRG.1	Pbx3	MSTRG.17115.1
MSTRG.1	Pbx3	MSTRG.17116.1
MSTRG.1	C79798	ENSMUST00000131588

MSTRG.1	Pbx3	MSTRG.17118.1
MSTRG.1	Pbx3	MSTRG.17119.1
MSTRG.1	Gm37294	ENSMUST00000193461
MSTRG.1	Gm13414	ENSMUST00000117516
MSTRG.1	Mapkap1	ENSMUST00000149383;ENSMUST00000124443;ENSMUST00000141
MSTRG.1	Gm13407	ENSMUST00000118256
MSTRG.1	Mapkap1	MSTRG.17123.1
MSTRG.1	Gm13406	ENSMUST00000121991
MSTRG.1	Gm13408	ENSMUST00000119822
MSTRG.1	Gapvd1	ENSMUST00000102800;ENSMUST00000113111;ENSMUST0000002[illegible]
MSTRG.1	Psmd5	ENSMUST00000028225;ENSMUST00000143456;ENSMUST0000013[illegible]
MSTRG.1	Psmd5	ENSMUST00000202917
MSTRG.1	Cutal	ENSMUST00000113068;ENSMUST00000149250;ENSMUST0000002[illegible]
MSTRG.1	4930523[illegible]	ENSMUST00000191933;ENSMUST00000168250;ENSMUST0000016[illegible]
MSTRG.1	Phf19	ENSMUST00000028232;ENSMUST00000128663;ENSMUST0000012[illegible]
MSTRG.1	Phf19	MSTRG.17131.1
MSTRG.1	Gm13448	ENSMUST00000143469
MSTRG.1	Gm24905	ENSMUST00000158129
MSTRG.1	Hspa5	ENSMUST00000028222;MSTRG.17134.2;ENSMUST00000100171;[illegible]
MSTRG.1	Rabepk	ENSMUST00000145903;ENSMUST00000113086;ENSMUST0000011[illegible]
MSTRG.1	Fbxw2	MSTRG.17136.1;ENSMUST0000156130;ENSMUST00000028220;[illegible]
MSTRG.1	Traf1	ENSMUST00000172159;MSTRG.17137.1;ENSMUST00000113064;[illegible]
MSTRG.1	Gm13450	ENSMUST00000121107
MSTRG.1	Rab14	MSTRG.17139.1;MSTRG.17139.2;ENSMUST00000028238;ENSMUS
MSTRG.1	4930523[illegible]	MSTRG.1714.1
MSTRG.1	AI18237	ENSMUST00000045776;ENSMUST00000151161;ENSMUST00000154
MSTRG.1	Cntrl	MSTRG.17141.1;MSTRG.17141.2;ENSMUST00000028235;MSTRG.
MSTRG.1	Stom	ENSMUST00000028241;ENSMUST00000202568
MSTRG.1	Gm13443	ENSMUST00000120770
MSTRG.1	Gsn	ENSMUST00000201185;ENSMUST00000202990;ENSMUST00000202
MSTRG.1	Gsn	MSTRG.17145.1
MSTRG.1	Gm43128	ENSMUST00000202212
MSTRG.1	4930402[illegible]	ENSMUST00000113010
MSTRG.1	Ggta1	ENSMUST00000079424;ENSMUST00000113002;MSTRG.17148.3;[illegible]
MSTRG.1	Ggta1	MSTRG.17149.1
MSTRG.1	4930562[illegible]	ENSMUST00000028061;ENSMUST00000164249;ENSMUST0000016[illegible]
MSTRG.1	Ggta1	MSTRG.17150.1
MSTRG.1	Ggta1	MSTRG.17151.1
MSTRG.1	Gm43761	ENSMUST00000202132
MSTRG.1	Ggta1	MSTRG.17153.1
MSTRG.1	Ggta1	MSTRG.17154.1
MSTRG.1	Ndufa8	ENSMUST00000070112
MSTRG.1	Morn5	ENSMUST00000028256
MSTRG.1	Gm43764	ENSMUST00000202553
MSTRG.1	Dab2ip	ENSMUST00000112986;ENSMUST00000112987;MSTRG.17159.3;[illegible]
MSTRG.1	Tnn	ENSMUST00000039178
MSTRG.1	Rbm18	ENSMUST00000028251;ENSMUST00000200733;ENSMUST00000122
MSTRG.1	Mrrf	ENSMUST00000028250;ENSMUST00000200701;ENSMUST00000132
MSTRG.1	Gm13428	ENSMUST00000117540
MSTRG.1	Gm13436	ENSMUST00000120418
MSTRG.17164		MSTRG.17164.1
MSTRG.1	Ptgs1	ENSMUST00000062069;ENSMUST00000144319;ENSMUST0000014[illegible]
MSTRG.1	Gm43621	ENSMUST00000202733
MSTRG.1	Pdcl	ENSMUST00000009174;ENSMUST00000112940;ENSMUST0000013[illegible]
MSTRG.1	Rc3h2	ENSMUST00000112934;ENSMUST00000100143;MSTRG.17168.2;[illegible]
MSTRG.1	Snord90	ENSMUST00000104568
MSTRG.1717		MSTRG.1717.1

MSTRG.1	Zbtb6;Z	ENSMUST00000053098;ENSMUST00000112932;ENSMUST0000067
MSTRG.1	Rabgap1	ENSMUST00000133434;MSTRG.17171.1;ENSMUST00000061179;E
MSTRG.1	Strbp	ENSMUST00000183690;ENSMUST00000028279;MSTRG.17172.2;E
MSTRG.1	Gm17893	ENSMUST00000203017
MSTRG.1	Rabgap1	MSTRG.17174.1
MSTRG.1	Strbp	MSTRG.17175.1
MSTRG.1	Gm44063	ENSMUST00000203307
MSTRG.1	Strbp	MSTRG.17177.1
MSTRG.1	Strbp	MSTRG.17178.1
MSTRG.1	Gm13543	ENSMUST00000121230
MSTRG.1	Rabgap1	MSTRG.1718.1;ENSMUST00000028052;ENSMUST00000192019;EN
MSTRG.1	Strbp	MSTRG.17180.1
MSTRG.1	Mir5128	MSTRG.17181.1;ENSMUST00000175217;MSTRG.17181.3
MSTRG.1	Strbp	MSTRG.17182.1
MSTRG.1	Crb2	ENSMUST00000050372;ENSMUST00000137693
MSTRG.1	Dennd1a	ENSMUST00000102787;ENSMUST00000136460;ENSMUST0000014C
MSTRG.1	Dennd1a	MSTRG.17185.1
MSTRG.1	Dennd1a	MSTRG.17186.1
MSTRG.1	Dennd1a	MSTRG.17187.1
MSTRG.1	Gm44283	ENSMUST00000204461
MSTRG.1	Gm44443	ENSMUST00000204396
MSTRG.1	Gm36975	ENSMUST00000194105;MSTRG.1719.2
MSTRG.1	Dennd1a	MSTRG.17190.1
MSTRG.1	Dennd1a	MSTRG.17191.1
MSTRG.1	Dennd1a	MSTRG.17192.1
MSTRG.1	Gm44291	ENSMUST00000203102
MSTRG.1	Dennd1a	MSTRG.17194.1
MSTRG.1	Dennd1a	MSTRG.17195.1
MSTRG.1	Dennd1a	MSTRG.17196.1
MSTRG.1	Gm44455	ENSMUST00000200061
MSTRG.1	Dennd1a	MSTRG.17198.1
MSTRG.1	Gm13556	ENSMUST00000132692
MSTRG.1	Gm7658	ENSMUST00000119064
MSTRG.1	Mir1927	ENSMUST00000158579
MSTRG.1	Nek6	ENSMUST00000054234;MSTRG.17200.4;ENSMUST00000151683
MSTRG.1	Gm35808	ENSMUST00000204769
MSTRG.1	Gm44183	ENSMUST00000203964
MSTRG.1	Psmb7	ENSMUST00000028083;ENSMUST00000151981;MSTRG.17203.3
MSTRG.1	Gm13586	ENSMUST00000121784
MSTRG.1	Gm27634	ENSMUST00000175060
MSTRG.1	Olfml2a	ENSMUST00000057279
MSTRG.1	Gm13473	ENSMUST00000119289
MSTRG.1	Nr6a1	ENSMUST00000112877;ENSMUST00000076275;ENSMUST00000123
MSTRG.1	Gm44146	ENSMUST00000203508
MSTRG.1	Gm37529	ENSMUST00000195618
MSTRG.1	Rpl15-p	ENSMUST00000122372
MSTRG.1	Nr6a1	MSTRG.17211.1
MSTRG.1	Nr6a1	MSTRG.17212.1
MSTRG.1	Nr6a1os	ENSMUST00000129089;ENSMUST00000151229
MSTRG.1	Nr6a1	MSTRG.17214.1
MSTRG.1	Nr6a1	MSTRG.17215.1
MSTRG.1	Nr6a1	MSTRG.17216.1
MSTRG.1	Mir181a	ENSMUST00000083489
MSTRG.1	Gm44144	ENSMUST00000203683
MSTRG.1	Gm36975	MSTRG.1722.1
MSTRG.1	Nr6a1	MSTRG.17220.1
MSTRG.1	5730507	ENSMUST00000204297

MSTRG.1	Nr6a1	MSTRG.17222.1
MSTRG.1	Nr6a1	MSTRG.17223.1
MSTRG.1	Nr6a1	MSTRG.17224.1
MSTRG.1	Nr6a1	MSTRG.17225.1
MSTRG.1	Nr6a1	MSTRG.17226.1
MSTRG.1	Gm13496	ENSMUST00000141133
MSTRG.1	Wdr38;A	ENSMUST00000039535;ENSMUST00000112872;ENSMUST00000141
MSTRG.1	Rpl35	ENSMUST00000080861;ENSMUST00000152441;ENSMUST0000012€
MSTRG.1	Rabgap1	MSTRG.1723.1
MSTRG.1	Golga1	ENSMUST00000039165;ENSMUST00000112850;ENSMUST0000015З
MSTRG.1	Gm26127	ENSMUST00000083193
MSTRG.17232		MSTRG.17232.1
MSTRG.17233		MSTRG.17233.1
MSTRG.1	Gm13456	ENSMUST00000118928
MSTRG.17235		MSTRG.17235.1
MSTRG.1	Scai	ENSMUST00000038874;ENSMUST00000204404;ENSMUST00000204
MSTRG.1	Scai	MSTRG.17237.1
MSTRG.1	Scai	MSTRG.17238.1
MSTRG.1	Scai	MSTRG.17239.1
MSTRG.1	Rabgap1	MSTRG.1724.1
MSTRG.1	Scai	MSTRG.17240.1
MSTRG.1	Gm18791	ENSMUST00000204611
MSTRG.1	Scai	MSTRG.17242.1
MSTRG.1	Gm16523	ENSMUST00000203640
MSTRG.1	Scai	MSTRG.17244.1
MSTRG.1	Gm44027	ENSMUST00000203419
MSTRG.1	Scai	MSTRG.17246.1
MSTRG.1	Ppp6c	MSTRG.17247.1
MSTRG.1	Ppp6c	ENSMUST00000028087;ENSMUST00000204257;MSTRG.17248.3;E
MSTRG.1	Gm13511	ENSMUST00000121167
MSTRG.1	Rabgap1	MSTRG.1725.1
MSTRG.1	Kynu	ENSMUST00000138540;ENSMUST00000028223;ENSMUST00000112
MSTRG.1	Kynu	MSTRG.17251.1
MSTRG.1	Lrp1b;G	ENSMUST00000129974;ENSMUST00000052550;ENSMUST00000184
MSTRG.1	Gm13462	ENSMUST00000121472
MSTRG.1	Gm13460	ENSMUST00000121274
MSTRG.1	Gm13461	ENSMUST00000120980
MSTRG.1	Gm13463	ENSMUST00000119020
MSTRG.1	Arhgap1	ENSMUST00000112824;ENSMUST00000112822;ENSMUST0000014C
MSTRG.1	Arhgap1	MSTRG.17259.1
MSTRG.1	Gm37212	ENSMUST00000192582
MSTRG.1	Arhgap1	MSTRG.17260.1
MSTRG.1	Arhgap1	MSTRG.17261.1
MSTRG.1	Arhgap1	MSTRG.17262.1
MSTRG.1	Arhgap1	ENSMUST00000139948
MSTRG.1	Arhgap1	MSTRG.17264.1
MSTRG.1	Arhgap1	MSTRG.17265.1
MSTRG.1	Gtdc1	ENSMUST00000112810;ENSMUST00000049051;ENSMUST00000134
MSTRG.1	Gtdc1	MSTRG.17267.1
MSTRG.1	Gtdc1	MSTRG.17268.1
MSTRG.1	Gm13477	ENSMUST00000121620
MSTRG.1	Gm37215	MSTRG.1727.1;ENSMUST00000194927
MSTRG.1	Gtdc1	MSTRG.17270.1
MSTRG.1	Gtdc1	MSTRG.17271.1
MSTRG.17272		MSTRG.17272.1
MSTRG.17273		MSTRG.17273.1
MSTRG.17274		MSTRG.17274.1

MSTRG.17275		MSTRG.17275.1
MSTRG.1	Zeb2;Mi	ENSMUST00000176438;ENSMUST00000176732;ENSMUST00000028
MSTRG.1	Zeb2	MSTRG.17277.1
MSTRG.1	Zeb2os;	ENSMUST00000127150;ENSMUST00000184091;ENSMUST00000130
MSTRG.1	Gm27676	ENSMUST00000183669
MSTRG.1	Gm38302	ENSMUST00000191676
MSTRG.1	Epc2	ENSMUST00000092123;MSTRG.17280.2;MSTRG.17280.3;ENSMUS
MSTRG.1	Epc2	MSTRG.17281.1
MSTRG.1	Epc2	MSTRG.17282.1
MSTRG.1	Acvr2a	ENSMUST00000063886;ENSMUST00000156681
MSTRG.1	Orc4	ENSMUST00000028098;ENSMUST00000156956;ENSMUST00000154
MSTRG.1	Mbd5	ENSMUST00000112745;ENSMUST00000154653;ENSMUST00000112
MSTRG.1	Gm13510	ENSMUST00000118659
MSTRG.1	Mbd5	MSTRG.17287.1
MSTRG.1	Gm13509	ENSMUST00000119733
MSTRG.1	Lypd6b	ENSMUST00000028103;ENSMUST00000153422;ENSMUST00000129
MSTRG.1	6720464	ENSMUST00000193946
MSTRG.1	Gm13480	ENSMUST00000125095
MSTRG.1	Gm25223	ENSMUST00000158200
MSTRG.1	Lypd6b	MSTRG.17292.1
MSTRG.1	Lypd6b	MSTRG.17293.1
MSTRG.1	Mmadhc	ENSMUST00000147345;ENSMUST00000102769;ENSMUST00000134
MSTRG.1	Mmadhc	MSTRG.17295.1
MSTRG.1	Gm13483	ENSMUST00000138528;ENSMUST00000123699
MSTRG.1	Gm13486	ENSMUST00000119910
MSTRG.1	Rnd3	ENSMUST00000017288;ENSMUST00000154545;ENSMUST00000140
MSTRG.1	Gm13493	ENSMUST00000120318
MSTRG.1	Gm38243	ENSMUST00000194737
MSTRG.1	Gm23528	ENSMUST00000104571
MSTRG.1	Rbm43	ENSMUST00000102767;ENSMUST00000102768;ENSMUST00000165
MSTRG.1	Gm13495	ENSMUST00000121156
MSTRG.17302		MSTRG.17302.1
MSTRG.1	Nmi	ENSMUST00000028314;ENSMUST00000145481;ENSMUST00000112
MSTRG.1	Nmi	MSTRG.17304.1
MSTRG.1	Nmi	MSTRG.17305.1
MSTRG.1	Arl5a	ENSMUST00000036541;ENSMUST00000130357
MSTRG.1	A430018	ENSMUST00000124210;ENSMUST00000143821
MSTRG.1	Cacnb4	ENSMUST00000178799;ENSMUST00000102761;ENSMUST00000078
MSTRG.1	Gm13541	ENSMUST00000119519
MSTRG.1	Gm37873	ENSMUST00000194571
MSTRG.1	Bloc1s2	ENSMUST00000183232
MSTRG.1	Stam2;G	ENSMUST00000102759;ENSMUST00000127316;ENSMUST00000150
MSTRG.1	Fmnl2	ENSMUST00000090952;ENSMUST00000050719;ENSMUST00000049
MSTRG.1	Gm26603	ENSMUST00000181380
MSTRG.1	Fmnl2	MSTRG.17314.1
MSTRG.1	Fmnl2	MSTRG.17315.1
MSTRG.1	Fmnl2	MSTRG.17316.1
MSTRG.1	Fmnl2	MSTRG.17317.1
MSTRG.1	Fmnl2	MSTRG.17318.1
MSTRG.1	Arl6ip6	ENSMUST00000155517;ENSMUST00000028336
MSTRG.1	Gm37328	ENSMUST00000193191
MSTRG.1	Rif1	ENSMUST00000112693;ENSMUST00000069794;ENSMUST00000126
MSTRG.1	Neb	MSTRG.17321.1;ENSMUST00000036934;ENSMUST00000028320;E
MSTRG.1	Gm13521	ENSMUST00000118922
MSTRG.1	Gm13522	ENSMUST00000209682;ENSMUST00000124622
MSTRG.1	Prpf40a	ENSMUST00000076313;ENSMUST00000210789;ENSMUST00000133
MSTRG.1	Gm13502	ENSMUST00000121574

MSTRG.17327		MSTRG.17327.1
MSTRG.1	Gm13526	ENSMUST00000117970
MSTRG.17329		MSTRG.17329.1
MSTRG.1	Gm38329	ENSMUST00000193473
MSTRG.17330		MSTRG.17330.1
MSTRG.1	A930012	ENSMUST00000146529;ENSMUST00000156418
MSTRG.1	Gpd2	ENSMUST00000141536;ENSMUST00000169687;ENSMUST00000112
MSTRG.1	Gpd2	MSTRG.17333.1
MSTRG.1	Gm13535	ENSMUST00000118714
MSTRG.1	Cytip	MSTRG.17335.1;ENSMUST00000028175;ENSMUST00000144117;E
MSTRG.1	Gm13534	ENSMUST00000121169
MSTRG.1	Gm13546	ENSMUST00000138444;ENSMUST00000135735
MSTRG.1	Acvr1	ENSMUST00000145495;ENSMUST00000112599;ENSMUST0000009C
MSTRG.1	Gm17409	ENSMUST00000167423
MSTRG.1	Gm38126	ENSMUST00000195297
MSTRG.1	Acvr1	MSTRG.17340.1
MSTRG.1	Upp2	ENSMUST00000135737;ENSMUST00000137007;ENSMUST00000071
MSTRG.1	Gm13558	ENSMUST00000140464
MSTRG.1	Gm13559	ENSMUST00000120476
MSTRG.1	Gm13552	ENSMUST00000118837
MSTRG.1	Ccdc148	ENSMUST00000077687;ENSMUST00000134708;ENSMUST00000131
MSTRG.1	Gm11099	ENSMUST00000112589
MSTRG.1	Gm13560	ENSMUST00000121865
MSTRG.1	Gm13557	ENSMUST00000120771
MSTRG.1	Gm13550	ENSMUST00000118185
MSTRG.1	Rabgap1	MSTRG.1735.1
MSTRG.1	Pkp4	ENSMUST00000168631;ENSMUST00000102754;ENSMUST00000183
MSTRG.1	Tanc1	ENSMUST00000112568;ENSMUST00000037526;ENSMUST00000142
MSTRG.1	Tanc1	MSTRG.17352.1
MSTRG.1	Wdsub1	ENSMUST00000028368;ENSMUST00000102751;ENSMUST00000144
MSTRG.1	Wdsub1	MSTRG.17354.1
MSTRG.1	Ly75	ENSMUST00000112533;ENSMUST00000028362;MSTRG.17355.3;M
MSTRG.1	Gm13571	ENSMUST00000151984;ENSMUST00000124654;MSTRG.17356.3
MSTRG.1	Rbms1	MSTRG.17357.1;ENSMUST00000028347;ENSMUST00000164147;E
MSTRG.1	Rbms1	MSTRG.17358.1
MSTRG.17359		MSTRG.17359.1
MSTRG.1	Rabgap1	MSTRG.1736.1
MSTRG.1	Baz2b	MSTRG.17360.1
MSTRG.1	Baz2b;G	ENSMUST00000112550;ENSMUST00000090925;MSTRG.17361.2;E
MSTRG.1	Gm13572	MSTRG.17362.1
MSTRG.1	Baz2b	MSTRG.17363.1
MSTRG.1	Baz2b	MSTRG.17364.1
MSTRG.1	Gm13620	ENSMUST00000131198
MSTRG.1	Ldha-ps	ENSMUST00000119390
MSTRG.1	Baz2b	MSTRG.17367.1
MSTRG.1	Baz2b	MSTRG.17368.1
MSTRG.1	March7	ENSMUST00000102748;ENSMUST00000143806;ENSMUST00000102
MSTRG.1	Gm37083	ENSMUST00000195784
MSTRG.1	March7	MSTRG.17370.1
MSTRG.1	Cd302	ENSMUST00000028356;ENSMUST00000074606
MSTRG.1	Gm13573	ENSMUST00000116410
MSTRG.1	Tank	ENSMUST00000112502;ENSMUST00000078074;ENSMUST00000145
MSTRG.17374		MSTRG.17374.1
MSTRG.1	Psmd14	ENSMUST00000028278;ENSMUST00000135265;ENSMUST00000146
MSTRG.1	Psmd14	MSTRG.17376.1
MSTRG.1	Gm22403	ENSMUST00000174919
MSTRG.1	Psmd14	MSTRG.17378.1

MSTRG.1	Gm25966	ENSMUST00000157169
MSTRG.1	Gas5	MSTRG.1738.1
MSTRG.1	Tbr1	ENSMUST00000136867;ENSMUST00000048934;ENSMUST00000102
MSTRG.1	Ifih1	ENSMUST00000028259;ENSMUST00000112459;ENSMUST00000176
MSTRG.1	Ifih1	MSTRG.17382.1
MSTRG.1	Slc4a10	ENSMUST00000102735;ENSMUST00000147917
MSTRG.1	Dpp4	ENSMUST00000047812;MSTRG.17384.2;ENSMUST00000156871;M
MSTRG.1	Gm13561	ENSMUST00000136879
MSTRG.1	Gm13565	ENSMUST00000133596
MSTRG.1	Gm13577	ENSMUST00000121365
MSTRG.1	Cobll1	ENSMUST00000112429;ENSMUST00000090896;ENSMUST00000102
MSTRG.1	Dars2	ENSMUST00000035430;ENSMUST00000162654;ENSMUST00000161
MSTRG.1	Cobll1	MSTRG.17390.1
MSTRG.1	Cobll1	MSTRG.17391.1
MSTRG.1	Cobll1	MSTRG.17392.1
MSTRG.1	Gm13594	ENSMUST00000137582
MSTRG.1	Scn2a	ENSMUST00000144254;ENSMUST00000028377;ENSMUST00000202
MSTRG.1	Scn2a	MSTRG.17395.1;MSTRG.17395.2
MSTRG.1	Ttc21b	ENSMUST00000102718;ENSMUST00000125446;ENSMUST00000169
MSTRG.1	Gm13618	ENSMUST00000128859
MSTRG.1	Ttc21b	MSTRG.17398.1
MSTRG.1	Scn7a	ENSMUST00000042792
MSTRG.1	Kcnq5	MSTRG.174.1
MSTRG.1	Gm37634	ENSMUST00000195700
MSTRG.1	Scn1a	ENSMUST00000200839;ENSMUST00000112366;ENSMUST00000112
MSTRG.1	Gm13630	ENSMUST00000127849
MSTRG.1	Gm13629	ENSMUST00000141149;ENSMUST00000152740
MSTRG.1	Rpl35-p	ENSMUST00000118070
MSTRG.1	Scn9a	ENSMUST00000100063;ENSMUST00000100064;ENSMUST00000169
MSTRG.1	Gm13627	ENSMUST00000119700
MSTRG.1	Gm23046	ENSMUST00000158369
MSTRG.1	Gm13597	ENSMUST00000121239
MSTRG.1	Stk39	ENSMUST00000102715;ENSMUST00000126663;ENSMUST00000130
MSTRG.1	Stk39	MSTRG.17409.1
MSTRG.1	Cenpl	ENSMUST00000028035;ENSMUST00000111618;ENSMUST00000111
MSTRG.1	Stk39	MSTRG.17410.1
MSTRG.1	Stk39	MSTRG.17411.1
MSTRG.1	Stk39	MSTRG.17412.1
MSTRG.1	Stk39	MSTRG.17413.1
MSTRG.1	Stk39	MSTRG.17414.1
MSTRG.1	Stk39	MSTRG.17415.1
MSTRG.1	Cers6	ENSMUST00000028426;ENSMUST00000183755;ENSMUST00000175
MSTRG.1	Cers6	MSTRG.17417.1
MSTRG.1	Gm37159	ENSMUST00000192720
MSTRG.1	Gm38236	ENSMUST00000192661
MSTRG.1	Cenpl	MSTRG.1742.1
MSTRG.1	Gm37651	ENSMUST00000194679
MSTRG.1	Cers6	MSTRG.17421.1;MSTRG.17421.2
MSTRG.1	Gm37747	ENSMUST00000193717
MSTRG.1	Gm37666	ENSMUST00000194110
MSTRG.1	Gm38377	ENSMUST00000193253
MSTRG.1	Nostrin	ENSMUST00000041865;ENSMUST00000141276
MSTRG.1	Spc25	ENSMUST00000112320;ENSMUST00000127243;ENSMUST00000005
MSTRG.1	Fastkd1	ENSMUST00000073152;ENSMUST00000102706;ENSMUST00000140
MSTRG.1	Ppig	ENSMUST00000153772;ENSMUST00000090858;ENSMUST00000124
MSTRG.1	Ppig	MSTRG.17429.1
MSTRG.1	Klhl20	ENSMUST00000111611;ENSMUST00000140905;ENSMUST00000117

MSTRG.1	Ppig	MSTRG.17430.1
MSTRG.1	Gm13622	ENSMUST00000120658
MSTRG.1	Ppig	MSTRG.17432.1
MSTRG.1	Ccdc173	ENSMUST00000094942;ENSMUST00000148190
MSTRG.1	Gm13623	ENSMUST00000118359
MSTRG.1	Phospho	ENSMUST00000151298;ENSMUST00000028494;ENSMUST00000112
MSTRG.1	Gm13624	ENSMUST00000120263
MSTRG.1	Mettl5;	MSTRG.17437.1;ENSMUST00000060447;ENSMUST00000142127;E
MSTRG.1	Ssb	ENSMUST00000090852;ENSMUST00000166411;MSTRG.17438.3;M
MSTRG.1	Mettl5	MSTRG.17439.1
MSTRG.1	Prdx6	ENSMUST00000071718;MSTRG.1744.2;ENSMUST00000192639;EN
MSTRG.1	Mettl5o	ENSMUST00000129114
MSTRG.1	Ubr3	ENSMUST00000055758;ENSMUST00000112251;ENSMUST00000131
MSTRG.1	Ubr3	MSTRG.17442.1
MSTRG.1	Ubr3	MSTRG.17443.1
MSTRG.1	Ubr3	MSTRG.17444.1
MSTRG.1	Ubr3	MSTRG.17445.1
MSTRG.1	Rpl9-ps	ENSMUST00000140507
MSTRG.1	Gm13631	ENSMUST00000140978;ENSMUST00000060208;ENSMUST00000112
MSTRG.1	Myo3b	MSTRG.17448.1
MSTRG.1	Gm26558	ENSMUST00000180559
MSTRG.1	Rc3h1	ENSMUST00000161609;ENSMUST00000035911;MSTRG.1745.2;EN
MSTRG.1	Gorasp2	ENSMUST00000112201;ENSMUST00000028509;ENSMUST00000133
MSTRG.1	Gorasp2	MSTRG.17451.1
MSTRG.1	Tlk1	ENSMUST00000135128;ENSMUST00000038584;MSTRG.17452.3;M
MSTRG.1	Tlk1	MSTRG.17453.1;MSTRG.17453.2
MSTRG.1	Dync1i2	MSTRG.17454.1
MSTRG.1	Mettl8	ENSMUST00000100037;ENSMUST00000112179;ENSMUST00000112
MSTRG.1	Mettl8	MSTRG.17456.1
MSTRG.1	Mettl8	MSTRG.17457.1
MSTRG.1	Mettl8	MSTRG.17458.1
MSTRG.1	Dcaf17	MSTRG.17459.1;ENSMUST00000130292;ENSMUST00000154704;E
MSTRG.1	Gm37052	MSTRG.1746.1;ENSMUST00000194929
MSTRG.1	Dcaf17	MSTRG.17460.1
MSTRG.1	Cybrd1	ENSMUST00000028403
MSTRG.1	Dync1i2	ENSMUST00000112142;ENSMUST00000112139;ENSMUST00000112
MSTRG.1	Slc25a1	ENSMUST00000151937;MSTRG.17463.2;ENSMUST00000184169;E
MSTRG.1	Gm13641	ENSMUST00000117993
MSTRG.1	Slc25a1	MSTRG.17465.1
MSTRG.1	Hat1	MSTRG.17466.1;ENSMUST00000112122;ENSMUST00000028408;E
MSTRG.1	Gm13642	ENSMUST00000121697
MSTRG.1	Metap1d	ENSMUST00000037210;ENSMUST00000123858;ENSMUST0000013
MSTRG.1	Gm23253	ENSMUST00000157441
MSTRG.1	Gm37809	ENSMUST00000195050
MSTRG.1	Dlx1as	ENSMUST00000140271;ENSMUST00000122990
MSTRG.1	Dlx1	ENSMUST00000037119;ENSMUST00000172629
MSTRG.1	Pdk1	ENSMUST00000006669;ENSMUST00000152696;ENSMUST00000134
MSTRG.17473		MSTRG.17473.1
MSTRG.17474		MSTRG.17474.1
MSTRG.1	Gm13647	ENSMUST00000154654
MSTRG.1	Itga6	ENSMUST00000133124;ENSMUST00000152009;MSTRG.17476.2;E
MSTRG.1	Gm13663	ENSMUST00000145563;ENSMUST00000127600;ENSMUST00000157
MSTRG.1	Gm17250	ENSMUST00000180494
MSTRG.1	Gm37653	ENSMUST00000195401
MSTRG.1	Itga6	MSTRG.17480.1
MSTRG.1	Itga6	MSTRG.17481.1
MSTRG.1	Itga6	MSTRG.17482.1

MSTRG.1	Map3k20	ENSMUST00000090824;ENSMUST00000135469;ENSMUST00000112
MSTRG.1	Zakit	ENSMUST00000157677
MSTRG.1	Rpl36a-]	ENSMUST00000121019
MSTRG.1	Map3k20	MSTRG.17486.1
MSTRG.1	Map3k20	MSTRG.17487.1
MSTRG.1	Rapgef4	ENSMUST00000090826;ENSMUST00000149421;MSTRG.17488.3;E
MSTRG.1	Rc3h1	MSTRG.1749.1
MSTRG.1	Rapgef4	ENSMUST00000126462
MSTRG.1	Rapgef4	MSTRG.17491.1
MSTRG.1	Rapgef4	MSTRG.17492.1
MSTRG.1	Rapgef4	MSTRG.17493.1
MSTRG.1	Rapgef4	MSTRG.17494.1
MSTRG.1	Rapgef4	MSTRG.17495.1
MSTRG.1	Rapgef4	MSTRG.17496.1
MSTRG.1	Rapgef4	ENSMUST00000128165
MSTRG.1	Rapgef4	MSTRG.17498.1
MSTRG.1	Rapgef4	ENSMUST00000132792
MSTRG.1	Gm26580	MSTRG.175.1;ENSMUST00000181002
MSTRG.1	Serpinc	ENSMUST00000193477;ENSMUST00000195438;ENSMUST00000064
MSTRG.1	Rapgef4	MSTRG.17500.1
MSTRG.1	Cdca7	MSTRG.17501.1;MSTRG.17501.2;ENSMUST00000102691;ENSMUS
MSTRG.1	Sp3;Gm2	ENSMUST00000102689;ENSMUST00000066003;MSTRG.17502.4;E
MSTRG.1	Hspe1-p	ENSMUST00000118080
MSTRG.1	Sp3	MSTRG.17504.1
MSTRG.1	Sp3os	ENSMUST00000135297;ENSMUST00000123087;ENSMUST00000146
MSTRG.1	Ola1	ENSMUST00000028517;ENSMUST00000152608;ENSMUST00000112
MSTRG.1	Gm13665	ENSMUST00000120830
MSTRG.1	Gm13666	MSTRG.17508.1
MSTRG.1	Gm13666	ENSMUST00000120032
MSTRG.1	Gm37729	ENSMUST00000194761
MSTRG.1	Gm13664	ENSMUST00000117304
MSTRG.1	Ola1	MSTRG.17511.1
MSTRG.1	Sp9	ENSMUST00000090813;ENSMUST00000147133
MSTRG.1	Cir1	MSTRG.17513.1;MSTRG.17513.2;MSTRG.17513.3;ENSMUST0000
MSTRG.1	Scrn3	ENSMUST00000090811;ENSMUST00000112050;ENSMUST00000155
MSTRG.1	Gm13707	ENSMUST00000123663
MSTRG.1	Wipf1	ENSMUST00000094681;ENSMUST00000102680;MSTRG.17516.2;M
MSTRG.1	Gm13708	ENSMUST00000145170;MSTRG.17517.2;MSTRG.17517.3
MSTRG.1	Atp5g3	MSTRG.17518.1;ENSMUST00000111996;ENSMUST00000018914;E
MSTRG.1	Gm13640	ENSMUST00000118445
MSTRG.1	Gm37072	ENSMUST00000194570
MSTRG.1	Atf2;Gm	ENSMUST00000124737;ENSMUST00000112010;ENSMUST0000009C
MSTRG.1	Gm10822	ENSMUST00000181596
MSTRG.1	Gm10822	MSTRG.17522.1
MSTRG.1	Mrpl23-]	ENSMUST00000173086
MSTRG.1	Lnpk	MSTRG.17524.1
MSTRG.17525		MSTRG.17525.1
MSTRG.1	Lnpk	ENSMUST00000111993;ENSMUST00000064503;ENSMUST00000127
MSTRG.1	Lnpk	MSTRG.17527.1
MSTRG.1	Lnpk	MSTRG.17528.1
MSTRG.1	Lnpk	MSTRG.17529.1
MSTRG.1	Zbtb37	ENSMUST00000162226;ENSMUST00000163079;MSTRG.1753.3;EN
MSTRG.1	Mtx2	ENSMUST00000028511;ENSMUST00000155844;ENSMUST0000012S
MSTRG.1	Gm14425	ENSMUST00000121240
MSTRG.1	2600014	ENSMUST00000186411
MSTRG.1	A330043	ENSMUST00000139823
MSTRG.1	Rps6-ps	ENSMUST00000118499

MSTRG.1	Gm13653	ENSMUST00000122260
MSTRG.17537		MSTRG.17537.1
MSTRG.17538		MSTRG.17538.1
MSTRG.1	Nfe2l2	ENSMUST00000102672;MSTRG.17539.1;ENSMUST00000152371
MSTRG.1	Gm37362	ENSMUST00000194567
MSTRG.1	Gm24996	ENSMUST00000158390
MSTRG.1	Nfe2l2	MSTRG.17541.1
MSTRG.1	E030042	ENSMUST00000135244
MSTRG.1	Gm24574	ENSMUST00000175501;ENSMUST00000111964;ENSMUST00000111
MSTRG.1	Agps	ENSMUST00000047232;ENSMUST00000111952;MSTRG.17544.3;E
MSTRG.1	Pde11a	ENSMUST00000124825;ENSMUST00000099992;ENSMUST00000144
MSTRG.1	Pde11a	MSTRG.17547.1
MSTRG.1	Pde11a	MSTRG.17548.1
MSTRG.1	Pde11a	MSTRG.17549.1
MSTRG.1	Gas5;Gm	ENSMUST00000160152;ENSMUST00000161005;ENSMUST00000159
MSTRG.1	Rbm45	ENSMUST00000046389;MSTRG.17551.2;MSTRG.17551.3
MSTRG.1	Rbm45	MSTRG.17552.1
MSTRG.17553		MSTRG.17553.1
MSTRG.1	Osbpl6	ENSMUST00000133275;ENSMUST00000077972;ENSMUST00000111
MSTRG.1	Osbpl6	MSTRG.17555.1
MSTRG.1	Gm23164	ENSMUST00000175084
MSTRG.1	Prkra	ENSMUST00000002808;ENSMUST00000140493
MSTRG.1	Plekha3	MSTRG.17558.1;ENSMUST00000111920;ENSMUST00000125119
MSTRG.1	Ccdc141	ENSMUST00000049544;MSTRG.17559.1;ENSMUST00000164114;E
MSTRG.1	Tnfsf18	ENSMUST00000086084
MSTRG.1	Gm44360	ENSMUST00000200081
MSTRG.1	Sestd1	ENSMUST00000102660;MSTRG.17561.1;ENSMUST00000102659;E
MSTRG.1	Gm13650	ENSMUST00000117729
MSTRG.1	Sestd1	MSTRG.17563.1
MSTRG.1	Gm13727	ENSMUST00000154480;ENSMUST00000111821;ENSMUST00000065
MSTRG.1	Cwc22	MSTRG.17565.1
MSTRG.1	Cwc22	MSTRG.17566.1
MSTRG.17567		MSTRG.17567.1
MSTRG.1	Ube2e3	ENSMUST00000124120;ENSMUST00000130914;ENSMUST00000028
MSTRG.17569		MSTRG.17569.1
MSTRG.1	Gm25488	ENSMUST00000158728
MSTRG.1	Ssfa2	MSTRG.17570.1
MSTRG.1	Ssfa2	ENSMUST00000111785;ENSMUST00000111784;ENSMUST00000124
MSTRG.1	Ssfa2	MSTRG.17572.1
MSTRG.17573		MSTRG.17573.1
MSTRG.17574		MSTRG.17574.1
MSTRG.17575		MSTRG.17575.1
MSTRG.17576		MSTRG.17576.1
MSTRG.17577		MSTRG.17577.1
MSTRG.17578		MSTRG.17578.1
MSTRG.1	Itga4	ENSMUST00000155105;MSTRG.17579.1;ENSMUST00000099972;E
MSTRG.1758		MSTRG.1758.1
MSTRG.1	Itga4	MSTRG.17580.1
MSTRG.1	Itga4	MSTRG.17581.1
MSTRG.1	Cerkl;N	ENSMUST00000156731;ENSMUST00000099974;ENSMUST00000147
MSTRG.1	Gm22606	ENSMUST00000158290
MSTRG.1	Gm13673	ENSMUST00000122056
MSTRG.1	Dnajc10	ENSMUST00000125000;ENSMUST00000028392;ENSMUST00000133
MSTRG.1	Nckap1	ENSMUST00000028386;ENSMUST00000111760;ENSMUST00000154
MSTRG.1	Gm13689	ENSMUST00000119360
MSTRG.1	Gm24461	ENSMUST00000082735
MSTRG.1	Gm15429	ENSMUST00000151268

MSTRG.1	Nckap1	MSTRG.17590.1
MSTRG.1	Dusp19;	ENSMUST00000028384;ENSMUST00000147290;ENSMUST00000148
MSTRG.1	Mir684-	ENSMUST00000196622
MSTRG.1	Gm13690	ENSMUST00000118989
MSTRG.1	Nup35	MSTRG.17594.1
MSTRG.1	Nup35	MSTRG.17595.1
MSTRG.1	Gm4735	MSTRG.17596.1;ENSMUST00000138679
MSTRG.17597		MSTRG.17597.1
MSTRG.17598		MSTRG.17598.1
MSTRG.1	Gm13680	ENSMUST00000119790
MSTRG.1	B3gat2	ENSMUST00000144602;ENSMUST00000063663;ENSMUST0000014C
MSTRG.1	Suco	ENSMUST00000048377;ENSMUST00000192422;MSTRG.1760.4;MS
MSTRG.17600		MSTRG.17600.1
MSTRG.1	Zc3h15	ENSMUST00000081591;ENSMUST00000130948;MSTRG.17601.2;E
MSTRG.1	Zc3h15	MSTRG.17602.1
MSTRG.17603		MSTRG.17603.1
MSTRG.17604		MSTRG.17604.1
MSTRG.1	Itgav	ENSMUST00000125360;ENSMUST00000028499;ENSMUST00000141
MSTRG.1	Gm13684	ENSMUST00000152569
MSTRG.1	Dnmt3l-	ENSMUST00000118734
MSTRG.1	Gm13711	ENSMUST00000119708
MSTRG.1	Calcrl	ENSMUST00000074262;ENSMUST00000099944;ENSMUST00000151
MSTRG.1	Gm7496	ENSMUST00000192573
MSTRG.1	Calcrl	MSTRG.17610.1
MSTRG.1	Calcrl	MSTRG.17611.1
MSTRG.1	Calcrl	MSTRG.17612.1
MSTRG.17613		MSTRG.17613.1
MSTRG.1	Gm13710	ENSMUST00000126679;ENSMUST00000139492
MSTRG.1	Gm13710	MSTRG.17615.1
MSTRG.17616		MSTRG.17616.1
MSTRG.17617		MSTRG.17617.1
MSTRG.1	Clp1	MSTRG.17618.1
MSTRG.1	Clp1	ENSMUST00000165219;ENSMUST00000028475
MSTRG.1	Mettl13	ENSMUST00000028017;ENSMUST00000159316;ENSMUST00000159
MSTRG.1	Serping	ENSMUST00000023994;MSTRG.17620.2;ENSMUST00000111641;E
MSTRG.1	Ube2l6	ENSMUST00000102642;ENSMUST00000150325;ENSMUST00000154
MSTRG.1	Timm10	ENSMUST00000028470;ENSMUST00000111631
MSTRG.1	Slc43a1	ENSMUST00000121114;ENSMUST00000146816;MSTRG.17623.2;E
MSTRG.1	Rtn4rl2	ENSMUST00000151799;ENSMUST00000054514;ENSMUST00000151
MSTRG.1	Ctnnd1;	ENSMUST00000099941;ENSMUST00000111675;ENSMUST00000111
MSTRG.1	Btbd18	ENSMUST00000133437
MSTRG.1	Selenoh	ENSMUST00000117299;ENSMUST00000102647;ENSMUST00000189
MSTRG.1	Med19	ENSMUST00000102645;ENSMUST00000141595
MSTRG.1	Zdhhc5	MSTRG.17629.1;ENSMUST00000035840;MSTRG.17629.3;MSTRG.
MSTRG.1	4930558	ENSMUST00000162676;ENSMUST00000141397
MSTRG.1	n-R5s20	ENSMUST00000104194
MSTRG.1	Gm27942	ENSMUST00000184669
MSTRG.1	Prg2	ENSMUST00000028467
MSTRG.1	Slc43a3	ENSMUST00000130278;MSTRG.17633.1;ENSMUST00000138719;E
MSTRG.1	P2rx3	ENSMUST00000028465;ENSMUST00000111613;ENSMUST00000127
MSTRG.1	Ssrp1	ENSMUST00000077798;ENSMUST00000168266;ENSMUST00000123
MSTRG.1	Tnks1bp	ENSMUST00000139915;ENSMUST00000151092;ENSMUST0000015C
MSTRG.1	Tnks1bp	MSTRG.17637.1
MSTRG.1	4930443	ENSMUST00000135659
MSTRG.17639		MSTRG.17639.1
MSTRG.1	Pigc;Gm	ENSMUST00000111594;ENSMUST00000028021;ENSMUST00000161
MSTRG.17640		MSTRG.17640.1

MSTRG.1	Olfr103	MSTRG.17641.1
MSTRG.17642		MSTRG.17642.1
MSTRG.1	Olfr103	ENSMUST00000213774
MSTRG.1	Gm13736	ENSMUST00000119272
MSTRG.17645		MSTRG.17645.1
MSTRG.17646		MSTRG.17646.1
MSTRG.17647		MSTRG.17647.1
MSTRG.17648		MSTRG.17648.1
MSTRG.17649		MSTRG.17649.1
MSTRG.1	Dnm3	ENSMUST00000159763;ENSMUST00000195403;ENSMUST0000008
MSTRG.17650		MSTRG.17650.1
MSTRG.17651		MSTRG.17651.1
MSTRG.1	Ptprj	ENSMUST00000129323;ENSMUST00000111495;ENSMUST0000011
MSTRG.1	4933423	ENSMUST00000126219;ENSMUST00000126953
MSTRG.1	Ptprj	MSTRG.17654.1
MSTRG.1	Nup160	ENSMUST00000057481;ENSMUST00000154036;ENSMUST0000014
MSTRG.1	Fnbp4	ENSMUST00000013759;MSTRG.17656.1;MSTRG.17656.3;ENSMUS
MSTRG.1	Rpl30-p	ENSMUST00000117928
MSTRG.1	Mtch2	ENSMUST00000136872;ENSMUST00000111468;ENSMUST0000014
MSTRG.1	C1qtnf4	ENSMUST00000111466
MSTRG.1	2810442	ENSMUST00000161583
MSTRG.1	Ndufs3	ENSMUST00000140248;ENSMUST00000005647;ENSMUST0000012
MSTRG.1	Kbtbd4	ENSMUST00000111464;ENSMUST00000090682;ENSMUST0000012
MSTRG.1	Ptpmt1	MSTRG.17662.1;ENSMUST00000125001;ENSMUST00000077874;E
MSTRG.1	Gm17661	ENSMUST00000170317
MSTRG.1	Ptpmt1	MSTRG.17664.1
MSTRG.1	Celf1	ENSMUST00000150546;MSTRG.17665.1;ENSMUST00000005643;E
MSTRG.1	Celf1	MSTRG.17666.1
MSTRG.1	Celf1	MSTRG.17667.1
MSTRG.1	Celf1	MSTRG.17668.1
MSTRG.1	Gm13777	ENSMUST00000118208
MSTRG.1	Gm37767	ENSMUST00000193239
MSTRG.1	Spi1	ENSMUST00000169852;ENSMUST00000131400;ENSMUST0000000
MSTRG.1	Rapsn	ENSMUST00000111445;ENSMUST00000050323;ENSMUST0000012
MSTRG.1	Gm13778	ENSMUST00000146633;ENSMUST00000144822
MSTRG.1	Gm13778	MSTRG.17673.1
MSTRG.1	Gm13778	MSTRG.17674.1
MSTRG.1	Psmc3	ENSMUST00000067663;ENSMUST00000002171;ENSMUST0000015
MSTRG.1	Slc39a1	ENSMUST00000079976;MSTRG.17676.1;ENSMUST00000073575;E
MSTRG.1	Lrp4	ENSMUST00000143874;ENSMUST00000028689;ENSMUST0000012
MSTRG.1	Arfgap2	ENSMUST00000080008;ENSMUST00000028691;ENSMUST0000015
MSTRG.1	1110051	MSTRG.17679.1;ENSMUST00000102594;ENSMUST00000094835;E
MSTRG.1	Dnm3os;	ENSMUST00000178978;ENSMUST00000139662;ENSMUST0000009
MSTRG.1	Gm13787	ENSMUST00000120422
MSTRG.1	Ckap5;S	ENSMUST00000111338;ENSMUST00000123253;MSTRG.17682.2;E
MSTRG.1	Mybpc3	ENSMUST00000169776
MSTRG.1	Madd	ENSMUST00000111373;ENSMUST00000111375;ENSMUST0000011
MSTRG.1	Nr1h3;G	ENSMUST00000150654;ENSMUST00000111356;ENSMUST0000000
MSTRG.1	Gm23390	ENSMUST00000157393
MSTRG.1	Acp2	ENSMUST00000150403;ENSMUST00000002172;ENSMUST0000015
MSTRG.1	Ddb2	ENSMUST00000152277;ENSMUST00000150427;ENSMUST0000011
MSTRG.1	A330069	ENSMUST00000181191
MSTRG.1	Dnm3	MSTRG.1769.1
MSTRG.1	Gm17281	ENSMUST00000180732
MSTRG.1	Pacsin3	ENSMUST00000134699;ENSMUST00000143883;ENSMUST0000002
MSTRG.1	Atg13	ENSMUST00000076803;ENSMUST00000028678;ENSMUST0000013
MSTRG.1	Harbi1	ENSMUST00000128140;ENSMUST00000140183;ENSMUST0000014

MSTRG.1	Gm13776	ENSMUST00000122236
MSTRG.1	Gm13771	ENSMUST00000117988
MSTRG.1	Zfp408	ENSMUST00000099714;MSTRG.17696.2;ENSMUST00000111333;E
MSTRG.1	Arhgap1	ENSMUST00000111329;MSTRG.17697.2;ENSMUST00000111331;M
MSTRG.1	Ambra1	MSTRG.17698.1
MSTRG.1	Ambra1	ENSMUST00000099712;ENSMUST00000111317;ENSMUST00000124
MSTRG.1	Smap1	ENSMUST00000149737;MSTRG.177.2;ENSMUST00000027339;ENS
MSTRG.1	Gm16101	ENSMUST00000160695
MSTRG.1	Ambra1	MSTRG.17700.1
MSTRG.1	Ambra1	MSTRG.17701.1
MSTRG.1	Ambra1	MSTRG.17702.1
MSTRG.1	Ambra1	MSTRG.17703.1;MSTRG.17703.2
MSTRG.1	Ambra1	MSTRG.17704.1
MSTRG.1	Ambra1	MSTRG.17705.1
MSTRG.1	Mdk	ENSMUST00000111309;ENSMUST00000069423;ENSMUST00000028
MSTRG.1	Dgkz;Mi	ENSMUST00000128152;ENSMUST00000099709;ENSMUST00000028
MSTRG.1	Gm9821	ENSMUST00000178895
MSTRG.1	Dgkz	MSTRG.17709.1
MSTRG.1	Slc35c1	ENSMUST00000067631;ENSMUST00000125276;ENSMUST00000136
MSTRG.1	Chst1	ENSMUST00000065797
MSTRG.1	Gm13791	ENSMUST00000123263
MSTRG.1	Gm13792	ENSMUST00000121350
MSTRG.1	Syt13	MSTRG.17714.1;ENSMUST00000028648;MSTRG.17714.2
MSTRG.1	Prdm11	ENSMUST00000111274;MSTRG.17715.2;ENSMUST00000178666;E
MSTRG.1	Prdm11	MSTRG.17716.1
MSTRG.1	4631405	MSTRG.17717.1;ENSMUST00000111270;ENSMUST00000136401;E
MSTRG.1	Gm13780	ENSMUST00000150482
MSTRG.1	Vamp4	ENSMUST00000132158;ENSMUST00000135241;ENSMUST00000128
MSTRG.1	Phf21a;I	ENSMUST00000044036;ENSMUST00000068702;ENSMUST00000111
MSTRG.1	Phf21a	MSTRG.17721.1
MSTRG.1	Gm13786	ENSMUST00000122371
MSTRG.1	Phf21a	MSTRG.17724.1
MSTRG.1	Gm13785	ENSMUST00000119099
MSTRG.1	Large2	ENSMUST00000176774;ENSMUST00000068586;ENSMUST00000130
MSTRG.1	Pex16;1	ENSMUST00000155891;ENSMUST00000028650;ENSMUST00000154
MSTRG.1	Mapk8ip	ENSMUST00000111279;ENSMUST00000050312;ENSMUST00000149
MSTRG.1	Mir7000	ENSMUST00000183506
MSTRG.1	Vamp4	MSTRG.1773.1
MSTRG.1	Cry2	ENSMUST00000090559;ENSMUST00000111278;ENSMUST00000125
MSTRG.1	D930015I	ENSMUST00000126002
MSTRG.1	D930015I	MSTRG.17732.1
MSTRG.1	Cry2	MSTRG.17733.1
MSTRG.1	Gm13807	ENSMUST00000137073
MSTRG.1	Gm13807	MSTRG.17735.1
MSTRG.1	Trp53i1	ENSMUST00000150462;ENSMUST00000111266;ENSMUST00000147
MSTRG.1	Tspan18	ENSMUST00000111265;ENSMUST00000028646;ENSMUST00000154
MSTRG.1	Cd82	ENSMUST00000099696;ENSMUST00000028644;ENSMUST00000116
MSTRG.1	Mir7001	ENSMUST00000184769
MSTRG.1	Fmo1	ENSMUST00000046049;ENSMUST00000136120;ENSMUST00000143
MSTRG.1	Gm10804	MSTRG.17740.1;ENSMUST00000099693;ENSMUST00000163052;E
MSTRG.17741		MSTRG.17741.1
MSTRG.1	Alx4	ENSMUST00000111254
MSTRG.1	Ext2	ENSMUST00000184931;MSTRG.17743.3;MSTRG.17743.2;ENSMUS
MSTRG.1	Gm13818	ENSMUST00000119441
MSTRG.1	Hsd17b1	ENSMUST00000145967;ENSMUST00000028619;ENSMUST00000127
MSTRG.1	Hsd17b1	MSTRG.17746.1
MSTRG.1	Hsd17b1	MSTRG.17747.1

MSTRG.1	Gm13889	ENSMUST00000148314;ENSMUST00000099689;ENSMUST00000183
MSTRG.1	Alkbh3o	ENSMUST00000136886;MSTRG.17749.2
MSTRG.1	Fmo1	MSTRG.1775.1
MSTRG.1	Alkbh3	MSTRG.17750.1
MSTRG.1	Mir670h	ENSMUST00000210851;ENSMUST00000209947;ENSMUST00000211
MSTRG.17755		MSTRG.17755.1
MSTRG.17756		MSTRG.17756.1
MSTRG.17757		MSTRG.17757.1
MSTRG.17758		MSTRG.17758.1
MSTRG.1	Ttc17;I	ENSMUST00000055081;MSTRG.17759.1;MSTRG.17759.2;ENSMUS
MSTRG.1	Fmo2	ENSMUST00000045902;ENSMUST00000194061;ENSMUST00000111
MSTRG.1	Ttc17	MSTRG.17760.1
MSTRG.1	Ttc17	MSTRG.17761.1
MSTRG.1	Ttc17	MSTRG.17762.1
MSTRG.1	Ttc17	MSTRG.17763.1
MSTRG.1	2810002	ENSMUST00000152313
MSTRG.1	Api5	ENSMUST00000028617;ENSMUST00000144390;MSTRG.17765.3;E
MSTRG.17766		MSTRG.17766.1
MSTRG.17767		MSTRG.17767.1
MSTRG.1	Gm10801	ENSMUST00000099684
MSTRG.1	Gm10800	MSTRG.17769.1;MSTRG.17769.2;ENSMUST00000099683
MSTRG.1	Fmo6	ENSMUST00000195576
MSTRG.17770		MSTRG.17770.1
MSTRG.17771		MSTRG.17771.1
MSTRG.1	Traf6	ENSMUST00000143341;MSTRG.17772.1;MSTRG.17772.3;ENSMUS
MSTRG.1	B230118	ENSMUST00000111231;ENSMUST00000136601;ENSMUST0000009C
MSTRG.1	Gm20693	ENSMUST00000177431
MSTRG.1	B230118	MSTRG.17775.1
MSTRG.1	B230118	MSTRG.17776.1
MSTRG.1	Rag2	ENSMUST00000044031;MSTRG.17777.1;ENSMUST00000111227;M
MSTRG.17778		MSTRG.17778.1
MSTRG.1	Prr5l	ENSMUST00000171088;ENSMUST00000163762;ENSMUST00000043
MSTRG.1	Fmo3	ENSMUST00000028010;ENSMUST00000142759
MSTRG.1	Commd9	ENSMUST00000131287;ENSMUST00000028584;ENSMUST00000133
MSTRG.1	Commd9	MSTRG.17782.1
MSTRG.1	Trim44	ENSMUST00000102573;ENSMUST00000152929
MSTRG.1	Gm13868	ENSMUST00000122411
MSTRG.1	Ldlrad3	ENSMUST00000111222;ENSMUST00000058790;ENSMUST00000127
MSTRG.1	Ldlrad3	MSTRG.17786.1;MSTRG.17786.2;MSTRG.17786.3;MSTRG.1778€
MSTRG.1	Ldlrad3	MSTRG.17787.1
MSTRG.1	Ldlrad3	MSTRG.17788.1
MSTRG.1	Ldlrad3	MSTRG.17789.1
MSTRG.1	Ldlrad3	MSTRG.17790.1
MSTRG.1	Gm13921	ENSMUST00000119411
MSTRG.1	Ldlrad3	MSTRG.17792.1
MSTRG.1	Cd44	ENSMUST00000005218;ENSMUST00000111194;ENSMUST0000006C
MSTRG.1	Gm23731	ENSMUST00000083749
MSTRG.1	Pdhx	ENSMUST00000011058;ENSMUST00000132449;ENSMUST00000111
MSTRG.1	Apip	ENSMUST00000011055;MSTRG.17796.1;ENSMUST00000124540;E
MSTRG.1	Gm10038	ENSMUST00000077233
MSTRG.1	Ehf	ENSMUST00000090475;ENSMUST00000111174;ENSMUST00000137
MSTRG.1	Cat	ENSMUST00000028610;ENSMUST00000111168;MSTRG.17799.3
MSTRG.1	Smap1	MSTRG.178.1
MSTRG.1	Mroh9	ENSMUST00000096608
MSTRG.1	Abtb2	ENSMUST00000076212;MSTRG.17800.2;MSTRG.17800.3;MSTRG.
MSTRG.1	Abtb2	MSTRG.17801.2;MSTRG.17801.1
MSTRG.1	Abtb2	MSTRG.17802.1

MSTRG.1	Abtb2	MSTRG.17803.1
MSTRG.1	Abtb2	MSTRG.17804.1;MSTRG.17804.2
MSTRG.17805		MSTRG.17805.1
MSTRG.1	4930547	ENSMUST00000143559
MSTRG.1	Lmo2	ENSMUST00000170926;ENSMUST00000123437;ENSMUST00000111
MSTRG.1	4931422	ENSMUST00000123966;ENSMUST00000138867
MSTRG.1	Fbxo3;G	ENSMUST00000028603;ENSMUST00000111135;ENSMUST00000111
MSTRG.1	Nat10	ENSMUST00000028608;ENSMUST00000143017;ENSMUST00000133
MSTRG.1	Caprin1	ENSMUST00000028607;MSTRG.17811.2;ENSMUST00000111147;E
MSTRG.1	Caprin1	MSTRG.17812.1
MSTRG.1	D430041	ENSMUST00000141159;ENSMUST00000089726;ENSMUST00000136
MSTRG.1	Gm22501	ENSMUST00000158981
MSTRG.1	Gm13883	ENSMUST00000151310;ENSMUST00000150450
MSTRG.1	D430041	MSTRG.17816.1
MSTRG.1	D430041	MSTRG.17817.1
MSTRG.1	Gm25554	ENSMUST00000158075
MSTRG.1	Gm13882	ENSMUST00000120886
MSTRG.1	Prrx1	ENSMUST00000075805;ENSMUST00000027878;ENSMUST00000183
MSTRG.1	D430041	MSTRG.17820.1
MSTRG.1	D430041	MSTRG.17821.1
MSTRG.1	D430041	MSTRG.17822.1
MSTRG.1	D430041	MSTRG.17823.1
MSTRG.1	D430041	MSTRG.17824.1
MSTRG.1	D430041	MSTRG.17825.1
MSTRG.1	D430041	MSTRG.17826.1
MSTRG.1	D430041	MSTRG.17827.1
MSTRG.1	Hipk3;M	ENSMUST00000028600;ENSMUST00000122567;MSTRG.17828.3;E
MSTRG.1	Qser1	ENSMUST00000117237
MSTRG.1	Gm20471	ENSMUST00000172793
MSTRG.1	Gm13886	ENSMUST00000117876
MSTRG.1	Prrg4	ENSMUST00000028593;ENSMUST00000126824
MSTRG.17832		MSTRG.17832.1
MSTRG.1	Cstf3	ENSMUST00000028599;ENSMUST00000122900;MSTRG.17833.2;E
MSTRG.1	Cstf3	MSTRG.17834.1
MSTRG.1	Cstf3	MSTRG.17835.1
MSTRG.1	Cstf3	MSTRG.17836.1
MSTRG.1	Tcp11l1	ENSMUST00000129571;ENSMUST00000111118;ENSMUST00000028
MSTRG.1	Tcp11l1	MSTRG.17838.1
MSTRG.1	Ccdc73	ENSMUST00000111114;MSTRG.17839.4;ENSMUST00000130611
MSTRG.1	Prrx1	MSTRG.1784.1
MSTRG.1	Gm13887	ENSMUST00000119009
MSTRG.1	Eif3m	ENSMUST00000131266;ENSMUST00000028592;ENSMUST00000148
MSTRG.1	Rcn1	ENSMUST00000006128;ENSMUST00000127019
MSTRG.1	Arl14ep	ENSMUST00000028536;ENSMUST00000093883;ENSMUST00000146
MSTRG.1	Rpl35a-	ENSMUST00000121779
MSTRG.1	Gm13910	ENSMUST00000121752
MSTRG.17846		MSTRG.17846.1
MSTRG.1	Rpl10-p	ENSMUST00000119438
MSTRG.1	Pax6os1	MSTRG.17849.1
MSTRG.1	Gm26523	ENSMUST00000180713
MSTRG.1	Pax6	ENSMUST00000111086;ENSMUST00000111088;ENSMUST00000111
MSTRG.1	Elp4	ENSMUST00000122965;ENSMUST00000028588;ENSMUST00000152
MSTRG.1	Gm13954	ENSMUST00000123441
MSTRG.1	Elp4	MSTRG.17854.1
MSTRG.1	Elp4	MSTRG.17855.1
MSTRG.1	Elp4	MSTRG.17856.1
MSTRG.1	Elp4	MSTRG.17857.1

MSTRG.1	Elp4	MSTRG.17858.1
MSTRG.1	Elp4	MSTRG.17859.1
MSTRG.1	Gm37644	ENSMUST00000192190
MSTRG.1	Immp1l	ENSMUST00000037499;MSTRG.17860.1
MSTRG.1	Dnajc24	ENSMUST00000155811;ENSMUST00000102555;MSTRG.17861.3;E
MSTRG.1	Dnajc24	MSTRG.17862.1
MSTRG.1	Gm22002	ENSMUST00000175622
MSTRG.1	Dnajc24	MSTRG.17864.1
MSTRG.1	n-R5s20	ENSMUST00000122622
MSTRG.1	Dnajc24	MSTRG.17866.1
MSTRG.1	Dnajc24	MSTRG.17867.1
MSTRG.1	Dcdc5	ENSMUST00000126340;ENSMUST00000122388;ENSMUST00000152
MSTRG.1	Gm13992	ENSMUST00000120558
MSTRG.1	Rpsa-ps	ENSMUST00000191040
MSTRG.1	Gm29053	ENSMUST00000187484
MSTRG.1	Gm14013	ENSMUST00000119006
MSTRG.1	Mettl15	ENSMUST00000081631;ENSMUST00000147376;ENSMUST00000147
MSTRG.1	Gm24115	ENSMUST00000103799
MSTRG.1	Mettl15	MSTRG.17874.1
MSTRG.1	Mettl15	MSTRG.17875.1
MSTRG.1	Mettl15	MSTRG.17876.1
MSTRG.1	Kif18a	ENSMUST00000028527;ENSMUST00000130137;ENSMUST00000151
MSTRG.1	Kif18a	MSTRG.17878.1
MSTRG.1	Lin7c	ENSMUST00000028583;ENSMUST00000151163
MSTRG.1	Gorab	ENSMUST00000045138;ENSMUST00000186402;ENSMUST00000185
MSTRG.1	Platr8	ENSMUST00000146110
MSTRG.1	Lgr4	ENSMUST00000046548;ENSMUST00000152584;ENSMUST00000142
MSTRG.1	Ccdc34	ENSMUST00000028580;ENSMUST00000150183;ENSMUST00000127
MSTRG.1	Ccdc34	MSTRG.17883.1
MSTRG.1	Ccdc34o	ENSMUST00000153332;ENSMUST00000123633
MSTRG.17885		MSTRG.17885.1
MSTRG.1	Nop10	ENSMUST00000028553
MSTRG.1	Lpcat4	ENSMUST00000028554;ENSMUST00000136219;ENSMUST00000132
MSTRG.1	Emc7	ENSMUST00000069747
MSTRG.1	Gm24940	ENSMUST00000158326
MSTRG.1	Slc12a6	ENSMUST00000110991;ENSMUST00000141047;ENSMUST00000110
MSTRG.1	Gm21985	MSTRG.17891.1
MSTRG.1	Gm21985	MSTRG.17892.1
MSTRG.1	Emc4	ENSMUST00000028551;MSTRG.17893.2;MSTRG.17893.3;ENSMUS
MSTRG.1	Gm21985	MSTRG.17894.1
MSTRG.1	Gm21985	MSTRG.17895.1
MSTRG.1	Gm13940	ENSMUST00000121023;MSTRG.17896.1
MSTRG.1	Gm21985	MSTRG.17897.1
MSTRG.1	Gm21985	MSTRG.17898.1
MSTRG.1	Fmn1	ENSMUST00000099576;ENSMUST00000110954;ENSMUST00000102
MSTRG.1	Smap1	MSTRG.179.1
MSTRG.1790		MSTRG.1790.1
MSTRG.1	Gm13967	ENSMUST00000120404
MSTRG.1	Gm13963	ENSMUST00000143525;ENSMUST00000148420
MSTRG.1	Gm13965	ENSMUST00000154834
MSTRG.1	Gm44465	ENSMUST00000199536
MSTRG.1	Gm13964	ENSMUST00000133815
MSTRG.1	Fmn1	MSTRG.17905.1
MSTRG.1	4930533	ENSMUST00000123377
MSTRG.1	Aven	ENSMUST00000003705;ENSMUST00000127894;ENSMUST00000099
MSTRG.1	Aven	MSTRG.17908.1
MSTRG.1	Gm22676	ENSMUST00000116972

MSTRG.1	Prrc2c;	ENSMUST00000182149;ENSMUST00000182660;ENSMUST00000183
MSTRG.1	Aven	MSTRG.17910.1
MSTRG.1	Aven	MSTRG.17911.1
MSTRG.1	Aven	MSTRG.17912.1
MSTRG.1	Aven	MSTRG.17913.1
MSTRG.1	Aven	MSTRG.17914.1
MSTRG.1	Aven	MSTRG.17915.1
MSTRG.1	Gm44374	ENSMUST00000199821
MSTRG.1	Ryr3	ENSMUST00000208151;ENSMUST00000208290;ENSMUST00000091
MSTRG.1	Scg5	ENSMUST00000024005;ENSMUST00000139975
MSTRG.1	Scg5	MSTRG.17919.1
MSTRG.1	Kifap3	ENSMUST00000027877;ENSMUST00000077642
MSTRG.1	Arhgap1	ENSMUST00000110948;ENSMUST00000110949;MSTRG.17920.3;E
MSTRG.1	Gm21596	ENSMUST00000178049
MSTRG.1	Zfp770	ENSMUST00000050668;ENSMUST00000123562
MSTRG.1	Aqr	MSTRG.17923.1;MSTRG.17923.2;ENSMUST00000043160;ENSMUS
MSTRG.1	Mir7002	ENSMUST00000184524
MSTRG.1	Dph6	ENSMUST00000028640;ENSMUST00000102542;ENSMUST00000055
MSTRG.1	Dph6	MSTRG.17926.1
MSTRG.1	Gm13972	ENSMUST00000153726
MSTRG.1	Gm13977	ENSMUST00000133648
MSTRG.1	BC05204	ENSMUST00000166472;ENSMUST00000110918;ENSMUST00000147
MSTRG.1	Scyl3	ENSMUST00000161908;ENSMUST00000027876;ENSMUST0000017C
MSTRG.1	BC05204	MSTRG.17930.1
MSTRG.1	Mir1951	ENSMUST00000157981
MSTRG.1	BC05204	MSTRG.17932.1
MSTRG.1	BC05204	MSTRG.17933.1
MSTRG.1	BC05204	MSTRG.17934.1
MSTRG.1	BC05204	MSTRG.17935.1
MSTRG.1	2700033	ENSMUST00000140185
MSTRG.1	G630016	ENSMUST00000152412;ENSMUST00000156095
MSTRG.1	G630016	MSTRG.17938.1
MSTRG.1	2810405	ENSMUST00000195368
MSTRG.1	Scyl3	MSTRG.1794.1
MSTRG.1	Spred1	ENSMUST00000028829;ENSMUST00000110901
MSTRG.1	Spred1	MSTRG.17941.1
MSTRG.1	Spred1	MSTRG.17942.1
MSTRG.1	Mir674	ENSMUST00000102421
MSTRG.1	Fam98b	ENSMUST00000140936;ENSMUST00000028825;ENSMUST00000143
MSTRG.1	Meis2	ENSMUST00000028639;ENSMUST00000102538;ENSMUST0000011C
MSTRG.1	Meis2	MSTRG.17946.1
MSTRG.1	Meis2	MSTRG.17947.1
MSTRG.1	Gm13982	MSTRG.17948.1
MSTRG.1	Gm13982	ENSMUST00000138372
MSTRG.1	BC05532	ENSMUST00000097493;ENSMUST00000045876;ENSMUST0000015S
MSTRG.17950		MSTRG.17950.1
MSTRG.17951		MSTRG.17951.1;MSTRG.17951.2;MSTRG.17951.3
MSTRG.17952		MSTRG.17952.1
MSTRG.1	Rasgrp1	ENSMUST00000102534;ENSMUST00000178884;MSTRG.17953.2;M
MSTRG.1	Thbs1	ENSMUST00000039559;ENSMUST00000142588
MSTRG.17956		MSTRG.17956.1
MSTRG.17957		MSTRG.17957.1
MSTRG.1	Eif2ak4	ENSMUST00000110877;ENSMUST00000005233;ENSMUST0000011C
MSTRG.1	Eif2ak4	MSTRG.17959.1
MSTRG.1	BC05532	MSTRG.1796.1
MSTRG.1	Eif2ak4	MSTRG.17960.1
MSTRG.1	Eif2ak4	MSTRG.17961.1

MSTRG.1	Eif2ak4	MSTRG.17962.1
MSTRG.1	Eif2ak4	MSTRG.17963.1
MSTRG.1	Eif2ak4	MSTRG.17964.1
MSTRG.1	Srp14	ENSMUST00000009693;ENSMUST00000149615;ENSMUST0000013[illegible]
MSTRG.1	Bmf	MSTRG.17966.1;ENSMUST00000090219;ENSMUST00000110859;[illegible]
MSTRG.1	Bmf	MSTRG.17967.1
MSTRG.1	Bub1b	ENSMUST00000038341;ENSMUST00000127356;ENSMUST0000013[illegible]
MSTRG.1	Bub1b	MSTRG.17969.1
MSTRG.1	BC05532	MSTRG.1797.1
MSTRG.1	Inafm2	ENSMUST00000149978;MSTRG.17970.1
MSTRG.1	A430105	ENSMUST00000059997;ENSMUST00000151270
MSTRG.1	Knstrn	ENSMUST00000134661;ENSMUST00000177103;ENSMUST00000134
MSTRG.1	Knstrn	MSTRG.17973.1
MSTRG.1	Gm14091	ENSMUST00000133534
MSTRG.1	Ivd	ENSMUST00000028807;ENSMUST00000126082;ENSMUST00000141
MSTRG.1	Bahd1	ENSMUST00000151162;MSTRG.17976.1;ENSMUST00000036578
MSTRG.1	Ankrd63	ENSMUST00000104937;MSTRG.17977.2;MSTRG.17977.3;ENSMUS[illegible]
MSTRG.17978		MSTRG.17978.1
MSTRG.17979		MSTRG.17979.1
MSTRG.1	Mettl18	ENSMUST00000045694;ENSMUST00000111490
MSTRG.1	Ccdc32	ENSMUST00000036470;MSTRG.17980.2;MSTRG.17980.3;ENSMUS[illegible]
MSTRG.1	Rpusd2	ENSMUST00000028796
MSTRG.1	Knl1	ENSMUST00000152380;ENSMUST00000099542;ENSMUST0000015[illegible]
MSTRG.1	Knl1	MSTRG.17983.1
MSTRG.1	Knl1	MSTRG.17984.1
MSTRG.1	Rad51	ENSMUST00000140939;ENSMUST00000028795;ENSMUST0000011[illegible]
MSTRG.1	Rad51	MSTRG.17986.1
MSTRG.1	Rmdn3	ENSMUST00000094695;ENSMUST00000127965;MSTRG.17987.2;[illegible]
MSTRG.1	Ppp1r14[illegible]	ENSMUST00000076084;ENSMUST00000110820
MSTRG.1	Spint1	ENSMUST00000028783;ENSMUST00000110816;ENSMUST0000011[illegible]
MSTRG.1	Sell	ENSMUST00000192047;ENSMUST00000027871;MSTRG.1799.2;M[illegible]
MSTRG.1	Vps18	ENSMUST00000139367;ENSMUST00000037280;ENSMUST00000151
MSTRG.1	Gm14207	ENSMUST00000147929;ENSMUST00000156538;ENSMUST00000124
MSTRG.1	Dll4	ENSMUST00000102517;ENSMUST00000127102
MSTRG.1	Gchfr	ENSMUST00000057454
MSTRG.1	Dnajc17	ENSMUST00000038439;ENSMUST00000141009;ENSMUST0000012[illegible]
MSTRG.1	Gm14137	ENSMUST00000069711
MSTRG.1	Gm14138	ENSMUST00000122061
MSTRG.1	Dnajc17	MSTRG.17997.1
MSTRG.1	Zfyve19	ENSMUST00000149036;ENSMUST00000090174;ENSMUST00000142
MSTRG.1	Ino80	MSTRG.17999.1;MSTRG.17999.2;ENSMUST00000049920;MSTRG.
MSTRG.1	Gm37079	ENSMUST00000194114
MSTRG.180		MSTRG.180.1
MSTRG.1	Slc19a2	ENSMUST00000044021;ENSMUST00000159230;ENSMUST0000016[illegible]
MSTRG.1	Ino80	MSTRG.18000.1
MSTRG.1	Ino80	MSTRG.18001.1
MSTRG.1	Ino80	MSTRG.18002.1
MSTRG.1	Ino80	MSTRG.18003.1
MSTRG.1	H3f3c	ENSMUST00000119459
MSTRG.1	Ndufaf1	ENSMUST00000110801;ENSMUST00000110802;ENSMUST0000002[illegible]
MSTRG.1	Gm14208	ENSMUST00000119645
MSTRG.1	Exd1	ENSMUST00000060009;ENSMUST00000133668
MSTRG.1	Chp1	ENSMUST00000014221;ENSMUST00000119172;ENSMUST00000154
MSTRG.1	Chp1	MSTRG.18009.1
MSTRG.1	Ccdc181	ENSMUST00000027867
MSTRG.1	Chp1	MSTRG.18010.1
MSTRG.1	Chp1	MSTRG.18011.1

MSTRG.1	1700020	ENSMUST00000147425;ENSMUST00000153581
MSTRG.1	Oip5	ENSMUST00000110805;ENSMUST00000123818;MSTRG.18013.2
MSTRG.1	Nusap1	ENSMUST00000068225;ENSMUST00000153036;MSTRG.18014.4;M
MSTRG.1	Nusap1	MSTRG.18015.1
MSTRG.1	n-R5s20	ENSMUST00000082972
MSTRG.1	Rtf1	ENSMUST00000028767;ENSMUST00000136321;ENSMUST00000149
MSTRG.1	Rpap1	ENSMUST00000110793;ENSMUST00000099529;ENSMUST00000048
MSTRG.1	Mga	MSTRG.18019.1;ENSMUST00000046717;ENSMUST00000079934;E
MSTRG.1	Selp	ENSMUST00000162746;ENSMUST00000160000;ENSMUST00000161
MSTRG.1	Gm13998	ENSMUST00000120952
MSTRG.1	Mga	MSTRG.18021.1;MSTRG.18021.2
MSTRG.1	Mga	MSTRG.18022.1
MSTRG.1	Ehd4	ENSMUST00000028755;ENSMUST00000150240;ENSMUST00000152
MSTRG.1	Mapkbp1	ENSMUST00000066058;ENSMUST00000137760;ENSMUST00000128
MSTRG.1	Mapkbp1	MSTRG.18025.1
MSTRG.1	Mapkbp1	MSTRG.18026.1
MSTRG.1	Mir7665	ENSMUST00000184907
MSTRG.1	Gm13999	ENSMUST00000141511
MSTRG.1	Jmjd7;G	ENSMUST00000044675;ENSMUST00000162393;ENSMUST00000135
MSTRG.1	Gm16587	ENSMUST00000161943
MSTRG.1	Sptbn5	ENSMUST00000156159
MSTRG.1	Pla2g4f	ENSMUST00000142183;ENSMUST00000054651
MSTRG.1	Vps39	ENSMUST00000028752;ENSMUST00000102501;ENSMUST00000129
MSTRG.1	Snap23	ENSMUST00000110711;ENSMUST00000028743;ENSMUST00000116
MSTRG.1	Snap23	MSTRG.18034.1
MSTRG.1	Snap23	MSTRG.18035.1
MSTRG.1	Lrrc57	MSTRG.18036.1;ENSMUST00000102496;ENSMUST00000102497;E
MSTRG.1	Haus2	ENSMUST00000028741;ENSMUST00000124187;MSTRG.18037.3
MSTRG.1	Haus2	MSTRG.18038.1
MSTRG.1	Tmem62	ENSMUST00000067582;ENSMUST00000139428;ENSMUST00000110
MSTRG.1	Gm16548	ENSMUST00000133341
MSTRG.1	Tmem62	MSTRG.18041.1
MSTRG.1	Gm22865	ENSMUST00000083949
MSTRG.1	Tmem87a	ENSMUST00000090042;ENSMUST00000090046;ENSMUST00000136
MSTRG.1	Tmem87a	MSTRG.18044.1
MSTRG.1	Ganc;Ca	ENSMUST00000154574;ENSMUST00000154963;ENSMUST00000131
MSTRG.1	Ganc	MSTRG.18046.1
MSTRG.1	Ganc	MSTRG.18047.1
MSTRG.1	Capn3	MSTRG.18048.1
MSTRG.1	Zfp106	ENSMUST00000055241;ENSMUST00000171215;ENSMUST00000130
MSTRG.1	F5	ENSMUST00000086040
MSTRG.1	Gm26899	MSTRG.18050.2
MSTRG.1	Zfp106	MSTRG.18051.1
MSTRG.1	Zfp106	MSTRG.18052.1
MSTRG.1	Ccndbp1	ENSMUST00000060455;ENSMUST00000099488;ENSMUST00000099
MSTRG.1	Stard9	ENSMUST00000150912;ENSMUST00000180041;ENSMUST00000140
MSTRG.1	n-R5s20	ENSMUST00000083149
MSTRG.1	Cdan1;T	MSTRG.18056.1;MSTRG.18056.2;ENSMUST00000110701;ENSMUS
MSTRG.1	Cdan1	MSTRG.18057.1
MSTRG.1	Cdan1	MSTRG.18058.1
MSTRG.1	Cdan1	MSTRG.18059.1
MSTRG.1	AV03930	ENSMUST00000133643;ENSMUST00000124795;ENSMUST00000171
MSTRG.1	Ubr1	MSTRG.18061.3;MSTRG.18061.1;MSTRG.18061.2;ENSMUST0000
MSTRG.1	Ubr1	MSTRG.18062.1
MSTRG.1	Ubr1	MSTRG.18063.1
MSTRG.1	Ubr1	MSTRG.18064.1
MSTRG.1	Ubr1	MSTRG.18065.1

MSTRG.1	Lcmt2	ENSMUST00000099486;ENSMUST00000110674
MSTRG.1	Gm23858	ENSMUST00000158384
MSTRG.1	Adal	ENSMUST00000130221;ENSMUST00000028702;ENSMUST00000110
MSTRG.1	Adal	MSTRG.18069.1
MSTRG.1807		MSTRG.1807.1;MSTRG.1807.2
MSTRG.1	Adal	MSTRG.18070.1
MSTRG.1	Zscan29	ENSMUST00000110661;MSTRG.18071.2;MSTRG.18071.3;MSTRG.
MSTRG.1	Tubgcp4	ENSMUST00000144123;ENSMUST00000110658;ENSMUST00000186
MSTRG.1	Trp53bp	ENSMUST00000135890;MSTRG.18073.3;MSTRG.18073.2;MSTRG.
MSTRG.1	Map1a	ENSMUST00000094639;ENSMUST00000131901;ENSMUST00000110
MSTRG.1	Gm14978	ENSMUST00000129000
MSTRG.1	Ppip5k1	ENSMUST00000052029;ENSMUST00000110627;ENSMUST00000110
MSTRG.1	Ppip5k1	MSTRG.18077.1
MSTRG.1	Ckmt1	ENSMUST00000125812;ENSMUST00000078222;ENSMUST00000125
MSTRG.1	Strc	ENSMUST00000038389
MSTRG.1808		MSTRG.1808.1
MSTRG.1	Catsper	ENSMUST00000154604;ENSMUST00000038073;ENSMUST00000146
MSTRG.1	Gm14001	ENSMUST00000118847
MSTRG.1	Gm14000	ENSMUST00000118670
MSTRG.1	Pdia3	ENSMUST00000028683;ENSMUST00000153378;ENSMUST00000135
MSTRG.1	Wdr76;G	MSTRG.18084.1;MSTRG.18084.2;ENSMUST00000028676;ENSMUS
MSTRG.1	Ell3;Se	ENSMUST00000028679;ENSMUST00000116432;ENSMUST00000140
MSTRG.1	Serf2;H	ENSMUST00000139253;ENSMUST00000134412;ENSMUST00000110
MSTRG.1	Mfap1b;	ENSMUST00000056732;MSTRG.18088.1;MSTRG.18088.3;MSTRG.
MSTRG.1	Mfap1b	MSTRG.18089.1
MSTRG.1	Blzf1	ENSMUST00000027866;ENSMUST00000120447;ENSMUST00000124
MSTRG.1	Gm14017	ENSMUST00000121934
MSTRG.1	Frmd5	ENSMUST00000110593;ENSMUST00000138157;ENSMUST00000121
MSTRG.1	Gm14021	ENSMUST00000117683
MSTRG.1	Gm14020	ENSMUST00000118165
MSTRG.1	Patl2	ENSMUST00000028665
MSTRG.1	B2m	ENSMUST00000102476
MSTRG.1	Patl2	MSTRG.18096.1
MSTRG.1	Trim69	ENSMUST00000036089;ENSMUST00000143088
MSTRG.1	Sord	ENSMUST00000110551
MSTRG.1	Casc4	ENSMUST00000110586;ENSMUST00000078752;ENSMUST00000127
MSTRG.181		MSTRG.181.1
MSTRG.1	Nme7	ENSMUST00000193808;ENSMUST00000193683;ENSMUST00000195
MSTRG.1	Casc4	MSTRG.18100.1
MSTRG.1	Casc4	MSTRG.18101.1
MSTRG.1	Casc4	MSTRG.18102.1
MSTRG.1	Mageb3	MSTRG.18103.1
MSTRG.1	Gm23983	ENSMUST00000157142
MSTRG.1	Casc4	MSTRG.18105.1
MSTRG.1	Casc4	MSTRG.18106.1
MSTRG.1	Mageb3;	MSTRG.18107.1;MSTRG.18107.2
MSTRG.1	Casc4	MSTRG.18108.1
MSTRG.1	Mageb3	ENSMUST00000104936
MSTRG.1	Nme7	MSTRG.1811.1
MSTRG.1	Ctdspl2	ENSMUST00000036647;ENSMUST00000110578;ENSMUST00000138
MSTRG.1	Ctdspl2	MSTRG.18111.1
MSTRG.1	Ctdspl2	MSTRG.18112.1
MSTRG.1	Ctdspl2	MSTRG.18113.1
MSTRG.18114		MSTRG.18114.1
MSTRG.1	Eif3j1;	MSTRG.18115.1;ENSMUST00000028668;ENSMUST00000197244;E
MSTRG.1	Eif3j1	MSTRG.18116.1
MSTRG.1	Spg11	ENSMUST00000036450;MSTRG.18117.2;MSTRG.18117.3;MSTRG.

MSTRG.1	Spg11	MSTRG.18118.1
MSTRG.18119		MSTRG.18119.1
MSTRG.1	Nme7	MSTRG.1812.1
MSTRG.1	Duoxa2	ENSMUST00000028656
MSTRG.1	Duoxa1	ENSMUST00000110538;ENSMUST00000110537;ENSMUST00000155
MSTRG.1	Duox1	ENSMUST00000099461
MSTRG.1	Shf	ENSMUST00000048635;ENSMUST00000110531;ENSMUST00000139
MSTRG.1	Gatm	ENSMUST00000028624;ENSMUST00000154598;ENSMUST0000014C
MSTRG.1	Spata5l	ENSMUST00000149090;ENSMUST00000152813
MSTRG.1	AA46719	ENSMUST00000142767;ENSMUST00000047498;ENSMUST00000175
MSTRG.1	Slc28a2	ENSMUST00000110525;MSTRG.18127.3;ENSMUST00000028652;E
MSTRG.1	Bambi-p	MSTRG.18129.1
MSTRG.1	Nme7	MSTRG.1813.1
MSTRG.1	Bambi-p	MSTRG.18130.1
MSTRG.1	Bambi-p	ENSMUST00000121525
MSTRG.1	Slc30a4	ENSMUST00000005952;ENSMUST00000099457
MSTRG.1	4930417	ENSMUST00000134411;ENSMUST00000123403
MSTRG.1	Bloc1s6	ENSMUST00000150154
MSTRG.1	Bloc1s6	ENSMUST00000005954;ENSMUST00000127595;ENSMUST00000148
MSTRG.1	Sema6d	ENSMUST00000103241;ENSMUST00000132088;ENSMUST00000051
MSTRG.1	Sema6d	MSTRG.18139.1
MSTRG.1	Gm37411	ENSMUST00000194180
MSTRG.1	Dut	ENSMUST00000082122;MSTRG.18140.2;ENSMUST00000051605;E
MSTRG.1	Slc24a5	ENSMUST00000070353;ENSMUST00000139615;MSTRG.18141.3
MSTRG.1	Myef2	MSTRG.18142.1;MSTRG.18142.2;MSTRG.18142.3;ENSMUST000C
MSTRG.1	Gm22352	ENSMUST00000083917
MSTRG.1	Fbn1	ENSMUST00000028633;ENSMUST00000103234;ENSMUST00000148
MSTRG.1	Gm9913	ENSMUST00000066157
MSTRG.1	Cep152	ENSMUST00000089776;MSTRG.18146.2
MSTRG.1	Cep152	MSTRG.18147.1
MSTRG.1	Shc4	ENSMUST00000110480;ENSMUST00000042246;ENSMUST0000011C
MSTRG.1	Eid1	ENSMUST00000164756
MSTRG.1	Atp1b1	ENSMUST00000027863;MSTRG.1815.2;ENSMUST00000193980;EN
MSTRG.1	Secisbp	ENSMUST00000053699;MSTRG.18150.2;ENSMUST00000139944;M
MSTRG.1	Cops2	MSTRG.18151.1
MSTRG.1	Cops2	ENSMUST00000110463;MSTRG.18152.3;ENSMUST00000028635;E
MSTRG.1	Cops2	MSTRG.18153.1
MSTRG.1	Galk2	ENSMUST00000135782;ENSMUST00000028636;ENSMUST00000125
MSTRG.1	Fam227b	ENSMUST00000110446
MSTRG.1	Fam227b	MSTRG.18156.1
MSTRG.1	Fgf7	ENSMUST00000138576;ENSMUST00000064794;ENSMUST0000011C
MSTRG.1	Gm26496	ENSMUST00000083150
MSTRG.1	Dtwd1	ENSMUST00000170908;ENSMUST00000110437
MSTRG.1	Gm32391	ENSMUST00000191839
MSTRG.1	Atp8b4	ENSMUST00000040128;ENSMUST00000040149;ENSMUST00000134
MSTRG.1	Atp8b4	MSTRG.18161.1
MSTRG.1	Atp8b4	MSTRG.18162.1
MSTRG.1	Hdc	ENSMUST00000028838;ENSMUST00000124396;ENSMUST00000138
MSTRG.1	Gabpb1	ENSMUST00000110425;ENSMUST00000110424;MSTRG.18164.3;M
MSTRG.1	Gm23753	ENSMUST00000157575
MSTRG.1	Gm27003	MSTRG.18166.1;ENSMUST00000182590
MSTRG.1	Gabpb1	MSTRG.18167.1
MSTRG.1	Usp8	ENSMUST00000138859;ENSMUST00000028841;MSTRG.18168.2
MSTRG.1	Usp50;G	ENSMUST00000136319;ENSMUST00000188689;ENSMUST00000145
MSTRG.1	Dpt	ENSMUST00000027861
MSTRG.1	Usp50	MSTRG.18170.1
MSTRG.1	Sppl2a	ENSMUST00000028844;ENSMUST00000143700;MSTRG.18171.2;E

MSTRG.1	Mir3473	ENSMUST00000183494
MSTRG.1	Trpm7	ENSMUST00000103224;ENSMUST00000028843;ENSMUST00000127
MSTRG.1	Gm14212	ENSMUST00000122023
MSTRG.1	Trpm7	MSTRG.18175.1
MSTRG.1	Ap4e1	ENSMUST00000149254;ENSMUST00000129859;ENSMUST0000011C
MSTRG.1	Ap4e1	MSTRG.18177.1
MSTRG.1	Gm24081	ENSMUST00000175177
MSTRG.1	Ap4e1	MSTRG.18179.1
MSTRG.1	Gm32569	ENSMUST00000194659;ENSMUST00000193955
MSTRG.1	Blvra	ENSMUST00000142861;ENSMUST00000002064;ENSMUST00000135
MSTRG.1	Itpripl	ENSMUST00000132773;ENSMUST00000110386;ENSMUST00000154
MSTRG.1	Ncaph	ENSMUST00000110387;ENSMUST00000152806;MSTRG.18182.3;M
MSTRG.1	1810024	ENSMUST00000056146;ENSMUST00000150956
MSTRG.1	Snrnp20	ENSMUST00000142327;MSTRG.18184.2;ENSMUST00000103220;E
MSTRG.1	Ciao1	ENSMUST00000003759;ENSMUST00000174030;ENSMUST00000143
MSTRG.1	Tmem127	ENSMUST00000035871;ENSMUST00000174503;MSTRG.18186.2;E
MSTRG.1	Gm10766	ENSMUST00000194123;ENSMUST00000192976
MSTRG.1	Stard7	ENSMUST00000131196;ENSMUST00000110375;ENSMUST00000125
MSTRG.18189		MSTRG.18189.1
MSTRG.1	Gm20743	ENSMUST00000191735;ENSMUST00000194322
MSTRG.18190		MSTRG.18190.1
MSTRG.1	Dusp2;A	ENSMUST00000028846;MSTRG.18191.2;MSTRG.18191.3;MSTRG.
MSTRG.1	Kcnip3	ENSMUST00000028850;ENSMUST00000103215;ENSMUST00000088
MSTRG.1	Gm22613	ENSMUST00000122679
MSTRG.1	Kcnip3	MSTRG.18194.1
MSTRG.1	Kcnip3	MSTRG.18195.1
MSTRG.1	Prom2	ENSMUST00000028855;ENSMUST00000103214;MSTRG.18196.2;M
MSTRG.1	Mall	ENSMUST00000028856
MSTRG.1	Zfp661	ENSMUST00000110366;ENSMUST00000110368;ENSMUST00000077
MSTRG.1	Mrps5	ENSMUST00000028852;ENSMUST00000134101;ENSMUST00000128
MSTRG.1	Lmbrd1	ENSMUST00000191471;ENSMUST00000186096;MSTRG.182.4;ENS
MSTRG.1	Gm37403	ENSMUST00000193775
MSTRG.1	Bub1	ENSMUST00000028858;ENSMUST00000138624;MSTRG.18200.2;M
MSTRG.1	Acoxl	ENSMUST00000126410;ENSMUST00000028859;ENSMUST0000011C
MSTRG.1	Acoxl	MSTRG.18203.1
MSTRG.1	Gm23101	ENSMUST00000157550
MSTRG.1	Bcl2l11	ENSMUST00000146023;ENSMUST00000089634;ENSMUST0000001S
MSTRG.1	Gm14005	ENSMUST00000225087;ENSMUST00000224993;ENSMUST00000143
MSTRG.1	Gm14009	ENSMUST00000152439
MSTRG.1	Gm14005	MSTRG.18208.1
MSTRG.1	Gm14010	ENSMUST00000131961
MSTRG.1	Xcl1	ENSMUST00000027860
MSTRG.1	AL84458	ENSMUST00000224297
MSTRG.1	Mertk	ENSMUST00000014505;ENSMUST00000140221
MSTRG.1	Gm14011	ENSMUST00000145178
MSTRG.1	Gm23172	ENSMUST00000157160
MSTRG.1	Anapc1	ENSMUST00000014499;ENSMUST00000124418;ENSMUST00000151
MSTRG.1	Anapc1	MSTRG.18215.1
MSTRG.1	Anapc1	MSTRG.18216.1
MSTRG.1	Tmem87b	ENSMUST00000110325;MSTRG.18217.2;MSTRG.18217.3;ENSMUS
MSTRG.1	Zc3h8	ENSMUST00000028866
MSTRG.1	Zc3h8	MSTRG.18219.1
MSTRG.1	Sft2d2	ENSMUST00000043338;ENSMUST00000192185;ENSMUST00000192
MSTRG.1	Gm10762	ENSMUST00000099385
MSTRG.1	Zc3h6;G	ENSMUST00000110320;ENSMUST00000110319;ENSMUST00000135
MSTRG.1	Zc3h6	MSTRG.18222.1
MSTRG.1	Zc3h6	MSTRG.18223.1

MSTRG.1	Gm14025	ENSMUST00000145798
MSTRG.1	Gm14022	ENSMUST00000143857
MSTRG.1	Ttl	ENSMUST00000035812;ENSMUST00000144730;ENSMUST00000144
MSTRG.1	Polr1b;	ENSMUST00000103205;MSTRG.18227.1;ENSMUST00000028874;E
MSTRG.1	AI84715	ENSMUST00000148997;ENSMUST00000141786;ENSMUST00000137
MSTRG.1	Gm14026	ENSMUST00000120625
MSTRG.1	Tiprl	ENSMUST00000043235;ENSMUST00000192726;ENSMUST00000192
MSTRG.1	AI84715	MSTRG.18230.1
MSTRG.1	Gm14029	ENSMUST00000151051;MSTRG.18231.1
MSTRG.18232		MSTRG.18232.1
MSTRG.1	Ckap2l	ENSMUST00000052708;MSTRG.18234.1
MSTRG.1	Gm14023	ENSMUST00000144178
MSTRG.1	Il1a	ENSMUST00000028882
MSTRG.1	Gm4430	ENSMUST00000193902
MSTRG.1	Gm4430	ENSMUST00000192413
MSTRG.1	Slc20a1	ENSMUST00000028880;ENSMUST00000140907;MSTRG.18239.2;E
MSTRG.1	Gm38190	ENSMUST00000192350
MSTRG.1	9830144	ENSMUST00000127525;ENSMUST00000126080
MSTRG.1	A730036	ENSMUST00000143346;ENSMUST00000148548
MSTRG.1	Il1b	ENSMUST00000028881;ENSMUST00000141979;ENSMUST00000155
MSTRG.1	Il1bos	ENSMUST00000156601;ENSMUST00000168775
MSTRG.1	Stk35	ENSMUST00000165413;ENSMUST00000166282;MSTRG.18244.1
MSTRG.1	Sirpa	ENSMUST00000127751;ENSMUST00000136153;ENSMUST00000153
MSTRG.1	Tgm3	ENSMUST00000110299
MSTRG.1	Mir6339	ENSMUST00000185120
MSTRG.1	Snrpb	MSTRG.18248.1;ENSMUST00000139225;ENSMUST00000103199;E
MSTRG.1	Cpxm1	ENSMUST00000028897;ENSMUST00000130533
MSTRG.1	Gpr161	ENSMUST00000178700;ENSMUST00000111450
MSTRG.1	Nop56;G	ENSMUST00000103198;ENSMUST00000150745;ENSMUST00000161
MSTRG.1	Snord11	ENSMUST00000116960
MSTRG.1	Idh3b	ENSMUST00000028892;ENSMUST00000184538;MSTRG.18252.2;E
MSTRG.1	Pced1a	ENSMUST00000131561;ENSMUST00000089581;ENSMUST00000123
MSTRG.1	Vps16	ENSMUST00000125973;MSTRG.18254.1;ENSMUST00000128994;E
MSTRG.1	Vps16	MSTRG.18255.1
MSTRG.1	Vps16	MSTRG.18256.1
MSTRG.1	Ptpra	ENSMUST00000028769;ENSMUST00000077303;MSTRG.18257.3
MSTRG.1	Ptpra	MSTRG.18258.1
MSTRG.1	Ptpra	MSTRG.18259.1;MSTRG.18259.2
MSTRG.1	Dcaf6	ENSMUST00000027856;ENSMUST00000193353;MSTRG.1826.2;EN
MSTRG.1	Ptpra	MSTRG.18260.1
MSTRG.1	Gm23876	ENSMUST00000103816
MSTRG.1	4930473	MSTRG.18262.1;ENSMUST00000136793
MSTRG.1	4930473	MSTRG.18263.1
MSTRG.1	4930473	MSTRG.18264.1
MSTRG.1	4930473	MSTRG.18265.1
MSTRG.1	Mrps26	ENSMUST00000046149;ENSMUST00000145614;MSTRG.18266.3
MSTRG.1	Ubox5;F	MSTRG.18267.1;ENSMUST00000028761;MSTRG.18267.3;ENSMUS
MSTRG.1	Ubox5	MSTRG.18268.1
MSTRG.1	Ubox5	MSTRG.18269.1
MSTRG.1	Dcaf6	MSTRG.1827.1
MSTRG.1	Ubox5	MSTRG.18270.1
MSTRG.1	Ubox5	MSTRG.18271.1
MSTRG.1	Lzts3	ENSMUST00000089561;ENSMUST00000110260;ENSMUST00000045
MSTRG.1	Ddrgk1	ENSMUST00000089559;ENSMUST00000153271;MSTRG.18273.2;E
MSTRG.1	Itpa	ENSMUST00000103193;ENSMUST00000156382;ENSMUST00000133
MSTRG.1	Itpa	MSTRG.18275.1
MSTRG.1	Slc4a11	ENSMUST00000144945;ENSMUST00000099362;ENSMUST00000134

MSTRG.1	Siglec1	ENSMUST00000028794;ENSMUST00000110227;ENSMUST0000011C
MSTRG.1	4930402	ENSMUST00000044766;ENSMUST00000119422;ENSMUST0000013S
MSTRG.1	4930402	MSTRG.18279.1
MSTRG.1	Gm38368	ENSMUST00000193181
MSTRG.1	4930402	MSTRG.18280.1
MSTRG.1	4930402	MSTRG.18281.1
MSTRG.1	4930402	MSTRG.18282.1
MSTRG.1	4930402	MSTRG.18283.1
MSTRG.1	Gm14048	ENSMUST00000121864
MSTRG.1	Atrn	ENSMUST00000028781;ENSMUST00000134964;ENSMUST0000015€
MSTRG.1	Atrn	MSTRG.18286.1
MSTRG.1	Gm14049	ENSMUST00000121009
MSTRG.1	Atrn	MSTRG.18288.1
MSTRG.1	Ap5s1	ENSMUST00000143546;ENSMUST00000134086;ENSMUST0000015З
MSTRG.1	Gm24637	ENSMUST00000158674
MSTRG.1	Hspa12b	ENSMUST00000099349;ENSMUST00000100763;ENSMUST00000127
MSTRG.1	Gm11037	ENSMUST00000110225
MSTRG.1	1700037	ENSMUST00000184535;ENSMUST00000136838;ENSMUST0000010З
MSTRG.1	Gm14232	ENSMUST00000137176
MSTRG.1	Spef1;C	ENSMUST00000028801;ENSMUST00000110218;MSTRG.18294.3;E
MSTRG.1	Cdc25b	ENSMUST00000028804;ENSMUST00000079857;ENSMUST00000125
MSTRG.1	Mavs	ENSMUST00000130597;ENSMUST00000041362;ENSMUST0000011C
MSTRG.1	Pank2;M	ENSMUST00000131779;ENSMUST00000150843;ENSMUST00000184
MSTRG.1	Pank2	MSTRG.18298.1
MSTRG.1	Pank2	MSTRG.18299.1
MSTRG.1	Lmbrd1	MSTRG.183.1
MSTRG.1	Dcaf6	MSTRG.1830.1
MSTRG.1	Rnf24	ENSMUST00000059372;ENSMUST00000165420;ENSMUST0000011C
MSTRG.1	Snrpert	ENSMUST00000121967
MSTRG.1	Smox	ENSMUST00000129143;ENSMUST00000028806;ENSMUST0000018З
MSTRG.1	Prnp;Pr	ENSMUST00000091288;ENSMUST00000142070;ENSMUST00000124
MSTRG.1	Rassf2	ENSMUST00000028814;MSTRG.18304.1;MSTRG.18304.5;MSTRG.
MSTRG.1	Slc23a2	ENSMUST00000028815;ENSMUST00000148749;ENSMUST00000137
MSTRG.1	Gm14052	ENSMUST00000121434
MSTRG.1	Slc23a2	MSTRG.18307.1
MSTRG.1	Gm14051	ENSMUST00000131603
MSTRG.1	Tmem230	ENSMUST00000110164;ENSMUST00000110163;ENSMUST0000002Е
MSTRG.1	Gm37864	ENSMUST00000195308
MSTRG.1	Prokr2	ENSMUST00000110157;ENSMUST00000049997;ENSMUST00000142
MSTRG.1	Pcna	ENSMUST00000140338;ENSMUST00000028817;ENSMUST0000013€
MSTRG.1	AV09932	ENSMUST00000124336
MSTRG.1	AV09932	MSTRG.18313.1
MSTRG.1	Cds2	ENSMUST00000110158;ENSMUST00000103181;MSTRG.18314.1;M
MSTRG.1	Gm14095	ENSMUST00000134264
MSTRG.1	1700026	ENSMUST00000151417;ENSMUST00000130884;ENSMUST0000012€
MSTRG.1	Gpcpd1	MSTRG.18317.1
MSTRG.1	Gpcpd1	ENSMUST00000127712;ENSMUST00000148833;ENSMUST0000011C
MSTRG.1	Gpcpd1	MSTRG.18319.1
MSTRG.1	Dcaf6	MSTRG.1832.1
MSTRG.1	1110034	ENSMUST00000061891;ENSMUST00000148271;ENSMUST0000011C
MSTRG.1	1110034	MSTRG.18321.1
MSTRG.1	Gm14097	ENSMUST00000147868
MSTRG.1	Gm22245	ENSMUST00000082479
MSTRG.1	1110034	MSTRG.18324.1
MSTRG.1	Trmt6	ENSMUST00000152836;ENSMUST00000039554;ENSMUST00000147
MSTRG.1	Mcm8	ENSMUST00000028831;ENSMUST00000066559;ENSMUST0000013S
MSTRG.18327		MSTRG.18327.1

MSTRG.18328		MSTRG.18328.1
MSTRG.1	Gm14104	ENSMUST00000135990
MSTRG.1	Mpc2	ENSMUST00000195026;ENSMUST00000027853;ENSMUST00000128
MSTRG.1	Crls1	ENSMUST00000028835;ENSMUST00000110122;ENSMUST00000124
MSTRG.1	Crls1	MSTRG.18331.1
MSTRG.1	Lrrn4	ENSMUST00000049787
MSTRG.1	Fermt1	ENSMUST00000134937;ENSMUST00000038280;ENSMUST00000144
MSTRG.1	Fermt1	MSTRG.18334.1
MSTRG.18335		MSTRG.18335.1
MSTRG.18336		MSTRG.18336.1
MSTRG.18337		MSTRG.18337.1
MSTRG.1	Tmx4	ENSMUST00000038228;ENSMUST00000137377;ENSMUST00000110
MSTRG.1	Plcb1	ENSMUST00000131552;ENSMUST00000110116;ENSMUST00000130
MSTRG.1	Gm37860	ENSMUST00000195380
MSTRG.1	Gm14036	ENSMUST00000118248
MSTRG.1	4930545	ENSMUST00000138303
MSTRG.1	9630028	ENSMUST00000153402;ENSMUST00000129868
MSTRG.1	Plcb4	ENSMUST00000144931;ENSMUST00000139480;ENSMUST00000146
MSTRG.1	Plcb4	MSTRG.18344.1
MSTRG.1	Plcb4	MSTRG.18345.1
MSTRG.1	Gm14209	ENSMUST00000154946
MSTRG.1	Gm14211	ENSMUST00000152250
MSTRG.1	Plcb4	MSTRG.18348.1
MSTRG.1	Plcb4	MSTRG.18349.1
MSTRG.1	Mpzl1	ENSMUST00000068705;ENSMUST00000111435;ENSMUST00000191
MSTRG.1	Plcb4	MSTRG.18350.1
MSTRG.1	Gm14210	ENSMUST00000140949
MSTRG.1	Plcb4	MSTRG.18352.1
MSTRG.1	Jag1	ENSMUST00000028735;ENSMUST00000133640
MSTRG.18354		MSTRG.18354.1
MSTRG.18355		MSTRG.18355.1
MSTRG.1	Mkks	ENSMUST00000028730;ENSMUST00000110089;ENSMUST00000144
MSTRG.1	Slx4ip	ENSMUST00000028737;ENSMUST00000141463;ENSMUST00000135
MSTRG.1	Slx4ip	MSTRG.18358.1
MSTRG.1	Slx4ip	MSTRG.18359.1
MSTRG.1	Gm37559	ENSMUST00000193556
MSTRG.1	Slx4ip	MSTRG.18360.1
MSTRG.1	Slx4ip	MSTRG.18361.1
MSTRG.1	Slx4ip	MSTRG.18362.1
MSTRG.1	Slx4ip	MSTRG.18363.1
MSTRG.1	Slx4ip	MSTRG.18364.1
MSTRG.18365		MSTRG.18365.1
MSTRG.18366		MSTRG.18366.1
MSTRG.1	Btbd3	ENSMUST00000091556;ENSMUST00000128656;ENSMUST00000075
MSTRG.1	Btbd3	MSTRG.18368.1
MSTRG.1	Gm44458	MSTRG.1837.1;MSTRG.1837.2;MSTRG.1837.3;MSTRG.1837.4;M
MSTRG.1	Tasp1	ENSMUST00000046656;ENSMUST00000099304;ENSMUST00000110
MSTRG.1	Gm14070	ENSMUST00000120773
MSTRG.1	Tasp1	MSTRG.18372.1
MSTRG.1	Tasp1	MSTRG.18373.1
MSTRG.1	Tasp1	MSTRG.18374.1
MSTRG.1	Tasp1	MSTRG.18375.1
MSTRG.1	Tasp1	MSTRG.18376.1
MSTRG.1	Tasp1	MSTRG.18377.1
MSTRG.1	Esf1	ENSMUST00000046030;ENSMUST00000153769;MSTRG.18378.3;E
MSTRG.1	Gm17374	ENSMUST00000104994
MSTRG.1	Gm18407	ENSMUST00000192126

MSTRG.1	Esf1	MSTRG.18380.1
MSTRG.1	Ndufaf5	ENSMUST00000044825;ENSMUST00000123078;ENSMUST00000125
MSTRG.1	Ndufaf5	MSTRG.18382.1
MSTRG.1	Ndufaf5	MSTRG.18383.1
MSTRG.1	Macrod2	ENSMUST00000110067;ENSMUST00000110063;ENSMUST0000011
MSTRG.1	Gm23846	ENSMUST00000083835
MSTRG.1	Macrod2	MSTRG.18386.1
MSTRG.1	Flrt3	ENSMUST00000172544;ENSMUST00000110057;ENSMUST0000005
MSTRG.1	Calr-ps	ENSMUST00000121776
MSTRG.1	Zcchc9-	ENSMUST00000117884
MSTRG.1	Rcsd1	MSTRG.1839.1;ENSMUST00000097474;ENSMUST00000040357;MS
MSTRG.1	Macrod2	ENSMUST00000143227;ENSMUST00000151538;ENSMUST0000017
MSTRG.1	Gm25583	ENSMUST00000083413
MSTRG.1	Fau-ps1	ENSMUST00000119675
MSTRG.1	Macrod2	MSTRG.18393.1
MSTRG.1	Gm23179	ENSMUST00000158693
MSTRG.1	Snrpb2	ENSMUST00000008477;ENSMUST00000139114;MSTRG.18395.2;E
MSTRG.1	Rbmx2-p	ENSMUST00000119377
MSTRG.1	Kif16b	ENSMUST00000043589;ENSMUST00000142078;ENSMUST00000211
MSTRG.1	Gm22310	ENSMUST00000157509
MSTRG.1	Lmbrd1	MSTRG.184.1
MSTRG.1840		MSTRG.1840.1
MSTRG.1	Kif16b	MSTRG.18400.1
MSTRG.1	Kif16b	MSTRG.18401.1
MSTRG.1	Kif16b	MSTRG.18402.1
MSTRG.1	Kif16bo	ENSMUST00000124774
MSTRG.1	Pcsk2os	ENSMUST00000126735
MSTRG.1	Pcsk2	ENSMUST00000028905;ENSMUST00000124751
MSTRG.1	Pcsk2os	ENSMUST00000153878
MSTRG.1	Pcsk2	MSTRG.18407.1
MSTRG.1	Dstn	ENSMUST00000103172
MSTRG.1	Rrbp1	ENSMUST00000154649;MSTRG.18409.1;MSTRG.18409.2;ENSMUS
MSTRG.1841		MSTRG.1841.1
MSTRG.1	Gm5535	ENSMUST00000210200;ENSMUST00000099292;ENSMUST00000211
MSTRG.1	Gm11687	ENSMUST00000118911
MSTRG.1	Snx5;Gm	ENSMUST00000028909;ENSMUST00000110030;ENSMUST0000013
MSTRG.1	Gm26165	ENSMUST00000083791
MSTRG.1	Snord17	ENSMUST00000104526
MSTRG.1	Mgme1	ENSMUST00000110028;ENSMUST00000028910;ENSMUST0000011
MSTRG.1	Mgme1	MSTRG.18416.1
MSTRG.18417		MSTRG.18417.1
MSTRG.18418		MSTRG.18418.1
MSTRG.1	Polr3f	ENSMUST00000028914;ENSMUST00000136999;ENSMUST0000011
MSTRG.1	Gm6286	ENSMUST00000145247
MSTRG.1	Rbbp9	ENSMUST00000028915;ENSMUST00000140340
MSTRG.1	Gm11701	ENSMUST00000121277
MSTRG.1	Kat14;P	ENSMUST00000028911;ENSMUST00000171261;ENSMUST0000018
MSTRG.1	Smim26	ENSMUST00000136628
MSTRG.1	Sec23b	ENSMUST00000143573;MSTRG.18424.1;ENSMUST00000028916;E
MSTRG.1	Dtd1	ENSMUST00000028917;ENSMUST00000145814;ENSMUST00000137
MSTRG.1	Gm24823	ENSMUST00000175092
MSTRG.1	Dtd1	MSTRG.18427.1
MSTRG.1	Dtd1	MSTRG.18428.1
MSTRG.1	Dtd1	MSTRG.18429.1
MSTRG.1	Gm15853	ENSMUST00000195518;ENSMUST00000160268
MSTRG.1	Gm11743	ENSMUST00000117385
MSTRG.18431		MSTRG.18431.1

MSTRG.1	Slc24a3	ENSMUST00000110007;ENSMUST00000137908;ENSMUST00000081
MSTRG.1	Gm14093	ENSMUST00000145579
MSTRG.1	Gm22731	ENSMUST00000158086
MSTRG.1	Gm22390	ENSMUST00000157588
MSTRG.1	Gm11759	ENSMUST00000121742
MSTRG.1	Gm14092	ENSMUST00000136794
MSTRG.1	Slc24a3	MSTRG.18438.1
MSTRG.1	BC03977	ENSMUST00000144661;MSTRG.18439.1;MSTRG.18439.2;ENSMUS
MSTRG.1	Tada1	ENSMUST00000027846;ENSMUST00000138148;ENSMUST0000013
MSTRG.1	Gm11763	ENSMUST00000119964
MSTRG.1	Gm25026	ENSMUST00000175017
MSTRG.1	Naa20	ENSMUST00000150316;ENSMUST00000002805;ENSMUST0000011
MSTRG.1	Crnkl1	ENSMUST00000001818;ENSMUST00000145425;ENSMUST00000147
MSTRG.1	Cfap61	ENSMUST00000138774;ENSMUST00000133433;ENSMUST0000011
MSTRG.1	Kiz	ENSMUST00000099278;ENSMUST00000156232;ENSMUST0000013
MSTRG.1	Kiz	MSTRG.18446.1
MSTRG.1	Kiz	MSTRG.18447.1
MSTRG.1	Kiz	MSTRG.18448.1
MSTRG.1	Kiz	MSTRG.18449.1
MSTRG.1	Pogk	ENSMUST00000135673;ENSMUST00000131487;ENSMUST0000016
MSTRG.1	Kiz	MSTRG.18450.1
MSTRG.1	Kiz	MSTRG.18451.1
MSTRG.1	Ralgapa	ENSMUST00000109986;ENSMUST00000149499;ENSMUST00000124
MSTRG.1	Ralgapa	MSTRG.18453.1
MSTRG.1	A930019	ENSMUST00000135360
MSTRG.1	Ralgapa	MSTRG.18455.1
MSTRG.1	Ralgapa	MSTRG.18456.1
MSTRG.1	Ralgapa	MSTRG.18457.1
MSTRG.1	Ralgapa	MSTRG.18458.1
MSTRG.1	Ralgapa	MSTRG.18459.1
MSTRG.1	Fam78b	ENSMUST00000165874;ENSMUST00000190081;ENSMUST0000015
MSTRG.1	Gm14117	ENSMUST00000122417
MSTRG.1	Ralgapa	MSTRG.18461.1
MSTRG.1	Ralgapa	MSTRG.18462.1
MSTRG.1	Xrn2	MSTRG.18463.1;ENSMUST00000028921;ENSMUST00000147057;
MSTRG.18464		MSTRG.18464.1
MSTRG.1	AI64651	MSTRG.18465.1
MSTRG.1	AI64651	ENSMUST00000140987
MSTRG.1	Pax1	MSTRG.18467.1;ENSMUST00000126068;MSTRG.18467.2;MSTRG.
MSTRG.18468		MSTRG.18468.1
MSTRG.1	Cd93	ENSMUST00000099269
MSTRG.1	Fam78b	MSTRG.1847.1
MSTRG.1	Sstr4	ENSMUST00000109962
MSTRG.1	Thbd	ENSMUST00000099270
MSTRG.18472		MSTRG.18472.1;MSTRG.18472.3;MSTRG.18472.2
MSTRG.1	Nxt1	ENSMUST00000109961;ENSMUST00000047177
MSTRG.1	Gzf1	ENSMUST00000028928;ENSMUST00000131292;ENSMUST00000151
MSTRG.1	Napb	ENSMUST00000136513;ENSMUST00000028926;ENSMUST0000014
MSTRG.1	Cst3	MSTRG.18476.1
MSTRG.1	Cst3	ENSMUST00000028938;ENSMUST00000149691;ENSMUST00000144
MSTRG.1	Zfp120	MSTRG.18478.1
MSTRG.1	Gm14140	ENSMUST00000119999
MSTRG.1	Fam78b	MSTRG.1848.1
MSTRG.1	Zfp120	ENSMUST00000109931;ENSMUST00000122859;ENSMUST0000008
MSTRG.1	Zfp120	MSTRG.18481.1
MSTRG.1	Zfp120	MSTRG.18482.1
MSTRG.1	Zfp937	ENSMUST00000073782

MSTRG.1	Gm21994	ENSMUST00000063463
MSTRG.18485		MSTRG.18485.1
MSTRG.1	3300002	ENSMUST00000051153;ENSMUST00000124945;MSTRG.18486.3;E
MSTRG.1	Gm10130	ENSMUST00000133235
MSTRG.1	3300002	MSTRG.18488.1
MSTRG.1	Cst7	ENSMUST00000089200
MSTRG.1	Fam78b	MSTRG.1849.1
MSTRG.1	Apmap	ENSMUST00000046399;ENSMUST00000126704;ENSMUST00000145
MSTRG.1	Gm14123	ENSMUST00000156345
MSTRG.1	Acss1	MSTRG.18492.1
MSTRG.1	Gm26681	ENSMUST00000180851
MSTRG.1	Entpd6	ENSMUST00000094467;ENSMUST00000147223;ENSMUST00000146
MSTRG.1	Acss1	MSTRG.18495.5;MSTRG.18495.2;MSTRG.18495.3;MSTRG.18495
MSTRG.1	E130215	ENSMUST00000145525
MSTRG.1	Pygb	MSTRG.18497.1
MSTRG.1	Pygb	MSTRG.18498.1;MSTRG.18498.2;ENSMUST00000045441;ENSMUS
MSTRG.1	Abhd12	ENSMUST00000156641;ENSMUST00000056149;ENSMUST00000141
MSTRG.1	Lmbrd1	MSTRG.185.1
MSTRG.1	Gm36972	MSTRG.1850.1;MSTRG.1850.4;MSTRG.1850.2;MSTRG.1850.3;M
MSTRG.1	Abhd12	MSTRG.18500.1
MSTRG.1	Gm23598	ENSMUST00000157262
MSTRG.1	Abhd12	MSTRG.18502.1
MSTRG.1	Gins1	ENSMUST00000152991;ENSMUST00000028948;ENSMUST0000014C
MSTRG.1	Gm14149	ENSMUST00000123048
MSTRG.1	Gm14149	MSTRG.18505.1
MSTRG.18506		MSTRG.18506.1
MSTRG.1	Gm14150	ENSMUST00000122404
MSTRG.1	Gm14143	ENSMUST00000121686
MSTRG.1	Ninl;Na	ENSMUST00000109896;ENSMUST00000150036;ENSMUST00000124
MSTRG.1	Gm34767	ENSMUST00000191813
MSTRG.1	Gm14142	ENSMUST00000117833
MSTRG.1	Gm14141	ENSMUST00000120236
MSTRG.1	Nsfl1c	ENSMUST00000089140;ENSMUST00000153333;MSTRG.18512.2;E
MSTRG.1	Tmem74b	ENSMUST00000060196;ENSMUST00000109872
MSTRG.1	Psmf1	ENSMUST00000042452;ENSMUST00000125977;MSTRG.18514.2;E
MSTRG.1	Gm14167	ENSMUST00000148705
MSTRG.1	Fkbp1a	ENSMUST00000044011;ENSMUST00000142985;ENSMUST00000126
MSTRG.1	Fkbp1a	MSTRG.18517.1
MSTRG.1	Sdcbp2	ENSMUST00000028950
MSTRG.1	Angpt4	ENSMUST00000028955
MSTRG.1	Creg1	ENSMUST00000111432;ENSMUST00000140769;ENSMUST0000004C
MSTRG.1	Gm14154	ENSMUST00000142642
MSTRG.18521		MSTRG.18521.1
MSTRG.1	Mir1953	ENSMUST00000157866
MSTRG.1	Fam110a	ENSMUST00000109865;ENSMUST00000109864;ENSMUST00000062
MSTRG.1	Srxn1	ENSMUST00000137751;ENSMUST00000128882;ENSMUST00000041
MSTRG.1	Rps15a-	ENSMUST00000117430
MSTRG.1	Csnk2a1	MSTRG.18526.1
MSTRG.1	Csnk2a1	ENSMUST00000099224;ENSMUST00000136063;MSTRG.18527.2;E
MSTRG.1	Gm14164	ENSMUST00000128577;ENSMUST00000089083
MSTRG.1	Gm14165	ENSMUST00000121456
MSTRG.1	Gm36972	ENSMUST00000194000
MSTRG.1	Nrsn2	ENSMUST00000079278
MSTRG.1	Sox12	ENSMUST00000182625
MSTRG.1	Tbc1d20	ENSMUST00000028963;ENSMUST00000144252;ENSMUST0000014C
MSTRG.1	Tbc1d20	MSTRG.18533.1
MSTRG.1	Rbck1	ENSMUST00000130165;MSTRG.18534.2;MSTRG.18534.1;ENSMUS

MSTRG.1	Cox4i2	ENSMUST00000010020;ENSMUST00000109821
MSTRG.1	Cox4i2	MSTRG.18536.1
MSTRG.1	Gm23802	MSTRG.18537.1
MSTRG.1	Bcl2l1	ENSMUST00000007803;ENSMUST00000109820;MSTRG.18538.4;E
MSTRG.1	H13;Mct	ENSMUST00000125366;ENSMUST00000148156;MSTRG.18539.2;E
MSTRG.1	Cd247;G	MSTRG.1854.1;ENSMUST00000159138;ENSMUST00000193086;EN
MSTRG.1	Gm26841	MSTRG.18540.1
MSTRG.1	H13	MSTRG.18541.1
MSTRG.1	Tpx2;Mi	ENSMUST00000164120;ENSMUST00000178997;ENSMUST0000010
MSTRG.1	Tpx2	MSTRG.18543.1
MSTRG.1	Ttll9	ENSMUST00000099197;ENSMUST00000152158;ENSMUST00000165
MSTRG.1	Gm14199	ENSMUST00000130021
MSTRG.1	Pdrg1	ENSMUST00000028972;ENSMUST00000150545;ENSMUST00000151
MSTRG.1	Hck	ENSMUST00000189688;ENSMUST00000109799;ENSMUST00000191
MSTRG.1	Tspyl3	ENSMUST00000099194
MSTRG.1	Tm9sf4	ENSMUST00000137446;ENSMUST00000089027;ENSMUST00000141
MSTRG.1	Gm24808	ENSMUST00000157993
MSTRG.1	Tm9sf4	MSTRG.18550.1
MSTRG.1	Tm9sf4	MSTRG.18551.1
MSTRG.1	Plagl2	ENSMUST00000056924;ENSMUST00000109795;MSTRG.18552.3
MSTRG.1	Plagl2	MSTRG.18553.1
MSTRG.1	Pofut1	ENSMUST00000049863;ENSMUST00000099191;ENSMUST0000009
MSTRG.1	Kif3b	ENSMUST00000028977
MSTRG.1	Kif3b	MSTRG.18556.1
MSTRG.1	Kif3b	MSTRG.18557.1
MSTRG.1	2500004	ENSMUST00000128026;ENSMUST00000125345;ENSMUST0000013
MSTRG.1	Asxl1	MSTRG.18559.2;MSTRG.18559.1;ENSMUST00000109790;MSTRG.
MSTRG.1	Pou2f1	ENSMUST00000111427;ENSMUST00000160260;ENSMUST0000015
MSTRG.1	Nol4l	ENSMUST00000035346;ENSMUST00000109784;ENSMUST00000132
MSTRG.1	Gm14472	ENSMUST00000119815
MSTRG.1	4930404	ENSMUST00000109783
MSTRG.1	Nol4l	MSTRG.18563.1
MSTRG.1	Commd7	ENSMUST00000071852;ENSMUST00000147822;ENSMUST0000010
MSTRG.1	Commd7	MSTRG.18565.1
MSTRG.1	Mapre1;	ENSMUST00000028981;MSTRG.18566.1;ENSMUST00000123795;E
MSTRG.1	Efcab8	ENSMUST00000126656
MSTRG.1	Gm14494	ENSMUST00000118786
MSTRG.1	Gm14497	MSTRG.18569.1
MSTRG.1	Gm17976	ENSMUST00000184037
MSTRG.1	Gm14497	ENSMUST00000119537
MSTRG.1	Bpifa1	ENSMUST00000028985;ENSMUST00000144665;ENSMUST0000014
MSTRG.1	Cdk5rap	ENSMUST00000109731;ENSMUST00000028990;ENSMUST0000014
MSTRG.1	Cdk5rap	MSTRG.18573.1
MSTRG.1	Cdk5rap	MSTRG.18574.1
MSTRG.1	Cdk5rap	MSTRG.18575.1
MSTRG.1	Cbfa2t2	MSTRG.18576.1
MSTRG.1	Cbfa2t2	ENSMUST00000109725;ENSMUST00000099178;ENSMUST00000045
MSTRG.1	Cbfa2t2	MSTRG.18578.1
MSTRG.1	Cbfa2t2	MSTRG.18579.1
MSTRG.1	Pou2f1	MSTRG.1858.1
MSTRG.1	Necab3;	ENSMUST00000109716;ENSMUST00000000895;ENSMUST0000013
MSTRG.1	1700003	ENSMUST00000135641;ENSMUST00000045116
MSTRG.1	Actl10	ENSMUST00000104928
MSTRG.1	Chmp4b	ENSMUST00000151668;ENSMUST00000044277;ENSMUST0000013
MSTRG.1	Chmp4b	MSTRG.18584.1
MSTRG.1	Chmp4b	MSTRG.18585.1
MSTRG.1	Pxmp4	MSTRG.18586.1;MSTRG.18586.2;MSTRG.18586.3;MSTRG.1858

MSTRG.1	4930519	ENSMUST00000181369
MSTRG.1	Zfp341	ENSMUST00000081926;ENSMUST00000126421
MSTRG.1	Gm14198	ENSMUST00000153208
MSTRG.1	Pou2f1	MSTRG.1859.1
MSTRG.1	Ahcy	ENSMUST00000054607;MSTRG.18590.2;ENSMUST00000137242;E
MSTRG.1	Gm45609	ENSMUST00000209837;ENSMUST00000209399;ENSMUST0000021C
MSTRG.18593		MSTRG.18593.1
MSTRG.1	Raly	MSTRG.18595.1;MSTRG.18595.2
MSTRG.1	Raly;a	MSTRG.18597.1;MSTRG.18597.2
MSTRG.1	Raly;a	ENSMUST00000109701;MSTRG.18598.1;ENSMUST00000129137;M
MSTRG.1	Raly	MSTRG.18599.1
MSTRG.1	Lmbrd1	MSTRG.186.1
MSTRG.1	Pou2f1	MSTRG.1860.1
MSTRG.1	Raly	MSTRG.18600.1
MSTRG.1	Eif2s2	ENSMUST00000099173;MSTRG.18601.3;ENSMUST00000147136;E
MSTRG.1	Eif2s2	MSTRG.18602.1
MSTRG.1	Eif2s2	MSTRG.18603.1
MSTRG.1	a	MSTRG.18604.1
MSTRG.1	Gm14226	ENSMUST00000130870
MSTRG.18606		MSTRG.18606.1
MSTRG.1	Map1lc3	ENSMUST00000029128;ENSMUST00000145086;ENSMUST00000154
MSTRG.1	Pigu	MSTRG.18608.1;ENSMUST00000139854;ENSMUST00000077626;E
MSTRG.1	Pigu	MSTRG.18609.1
MSTRG.1	Pou2f1	MSTRG.1861.1
MSTRG.1	Mir695	ENSMUST00000102261
MSTRG.1	Itch	ENSMUST00000126648;ENSMUST00000155360;ENSMUST0000002S
MSTRG.1	Itch	MSTRG.18612.1
MSTRG.1	Itch	MSTRG.18613.1
MSTRG.1	Gm14227	ENSMUST00000119431
MSTRG.1	Dynlrb1	ENSMUST00000054912;MSTRG.18615.2;ENSMUST00000109682;E
MSTRG.1	Trp53in	ENSMUST00000151466;ENSMUST00000043237;ENSMUST00000174
MSTRG.1	Ncoa6	ENSMUST00000043126;ENSMUST00000109670;ENSMUST00000143
MSTRG.1	Ncoa6	MSTRG.18618.1
MSTRG.1	Ncoa6	MSTRG.18619.1
MSTRG.1	Pou2f1	MSTRG.1862.1
MSTRG.1	Ncoa6	MSTRG.18620.1;MSTRG.18620.2
MSTRG.1	Gm14239	MSTRG.18621.1
MSTRG.1	Ncoa6	MSTRG.18622.1
MSTRG.1	Ncoa6	MSTRG.18623.1
MSTRG.1	Ggt7	ENSMUST00000029131;ENSMUST00000138033;ENSMUST00000148
MSTRG.1	Acss2	ENSMUST00000029135;ENSMUST00000149788;ENSMUST00000065
MSTRG.1	Mipep-p	ENSMUST00000177439
MSTRG.1	Gm20637	ENSMUST00000175715
MSTRG.1	Acss2os	ENSMUST00000148685
MSTRG.1	Gss	ENSMUST00000079691;ENSMUST00000175993;ENSMUST0000014C
MSTRG.1863		MSTRG.1863.1
MSTRG.1	Gssos2;	ENSMUST00000130859;ENSMUST00000155884;ENSMUST00000142
MSTRG.1	Gm14257	ENSMUST00000144649
MSTRG.1	Edem2	ENSMUST00000040833;MSTRG.18632.2;ENSMUST00000148571;E
MSTRG.1	Edem2	MSTRG.18633.1
MSTRG.1	Mmp24	ENSMUST00000029141;ENSMUST00000135945
MSTRG.1	BC02972	ENSMUST00000124586;MSTRG.18635.2;ENSMUST00000029142;E
MSTRG.1	Gm16098	ENSMUST00000141926
MSTRG.1	Fam83c	ENSMUST00000029143
MSTRG.1	Myh7b	ENSMUST00000092995;MSTRG.18638.3;ENSMUST00000154656
MSTRG.1	Tmco1	ENSMUST00000195015;ENSMUST00000193446;ENSMUST00000097
MSTRG.1	Trpc4ap	ENSMUST00000041059;MSTRG.18640.3;MSTRG.18640.2;ENSMUS

MSTRG.1	Trpc4ap	MSTRG.18641.1;MSTRG.18641.2;MSTRG.18641.3;MSTRG.18641
MSTRG.1	Trpc4ap	MSTRG.18642.1;MSTRG.18642.2
MSTRG.1	Trpc4ap	MSTRG.18643.1
MSTRG.1	Uqcc1	ENSMUST00000136933;ENSMUST00000152818;ENSMUST00000151
MSTRG.1	Uqcc1	MSTRG.18645.1
MSTRG.1	Uqcc1	MSTRG.18646.1
MSTRG.1	Uqcc1	MSTRG.18647.1
MSTRG.1	Uqcc1	MSTRG.18648.1
MSTRG.1	Cep250	ENSMUST00000149905;MSTRG.18649.1;ENSMUST00000039994;E
MSTRG.1	Uck2	ENSMUST00000053686;ENSMUST00000192702;ENSMUST00000195
MSTRG.1	6430550	ENSMUST00000124812;ENSMUST00000109617;ENSMUST00000086
MSTRG.1	6430550	MSTRG.18651.1
MSTRG.1	Ergic3	ENSMUST00000006035;MSTRG.18652.2;ENSMUST00000088650;E
MSTRG.1	Fer1l4	ENSMUST00000109611;ENSMUST00000138297
MSTRG.1	2900097	MSTRG.18654.1;MSTRG.18654.2;ENSMUST00000192863;ENSMUS
MSTRG.1	Gm22316	ENSMUST00000158012
MSTRG.1	Rpl21-p	ENSMUST00000119897
MSTRG.1	Phf20	MSTRG.18657.1
MSTRG.1	Phf20	ENSMUST00000145731;ENSMUST00000037401;ENSMUST00000152
MSTRG.1	Phf20	MSTRG.18659.1
MSTRG.1	Gm37982	MSTRG.1866.1;ENSMUST00000193076;MSTRG.1866.3
MSTRG.1	Phf20	MSTRG.18660.1
MSTRG.1	Phf20	MSTRG.18661.1
MSTRG.1	Phf20	MSTRG.18662.1
MSTRG.1	Scand1	ENSMUST00000079125
MSTRG.1	Cnbd2	ENSMUST00000037096;ENSMUST00000125395;ENSMUST00000073
MSTRG.1	Gm14251	ENSMUST00000122133;ENSMUST00000102276
MSTRG.1	Gm14252	ENSMUST00000131801
MSTRG.1	Gm14253	ENSMUST00000122112
MSTRG.1	Cnbd2	MSTRG.18668.1
MSTRG.1	Gm14170	ENSMUST00000133751
MSTRG.1	Uck2	MSTRG.1867.1
MSTRG.1	Aar2	ENSMUST00000109570;ENSMUST00000029158;ENSMUST00000137
MSTRG.1	Epb41l1	ENSMUST00000103137;ENSMUST00000109574;ENSMUST00000142
MSTRG.1	Gm14225	ENSMUST00000142310
MSTRG.1	Gm14224	ENSMUST00000147187
MSTRG.1	Epb41l1	MSTRG.18674.1
MSTRG.1	Myl9	ENSMUST00000088552;ENSMUST00000145299;ENSMUST00000131
MSTRG.1	Spag4	ENSMUST00000133511;ENSMUST00000125996;ENSMUST00000136
MSTRG.1	Spag4	MSTRG.18677.1
MSTRG.1	Cpne1;G	ENSMUST00000184265;ENSMUST00000127956;MSTRG.18678.2;E
MSTRG.1	Cpne1	MSTRG.18679.1
MSTRG.1	Uck2	MSTRG.1868.1
MSTRG.1	Romo1	ENSMUST00000088610;ENSMUST00000109597;ENSMUST00000109
MSTRG.1	Rbm39	ENSMUST00000109587;ENSMUST00000146297;MSTRG.18681.3;E
MSTRG.1	Dlgap4	ENSMUST00000109567;ENSMUST00000169464;ENSMUST00000137
MSTRG.1	Gm14172	ENSMUST00000118255
MSTRG.1	4930405	ENSMUST00000139042;ENSMUST00000130089
MSTRG.1	Gm14230	ENSMUST00000137463;ENSMUST00000149275;ENSMUST00000134
MSTRG.1	Tgif2	ENSMUST00000081335;ENSMUST00000073352;ENSMUST00000150
MSTRG.1	5430405	ENSMUST00000185402;ENSMUST00000153616;ENSMUST00000176
MSTRG.1	Gm37790	ENSMUST00000193136
MSTRG.1	Gm2453	ENSMUST00000195508
MSTRG.1	4930518	ENSMUST00000099141
MSTRG.1	5430405	MSTRG.18691.1
MSTRG.1	5430405	MSTRG.18692.1
MSTRG.1	Gm27651	ENSMUST00000183465

MSTRG.1	5430405	MSTRG.18694.1
MSTRG.1	1110008	ENSMUST00000029165;ENSMUST00000152551;ENSMUST00000152
MSTRG.1	Sla2	ENSMUST00000029164;MSTRG.18696.3;MSTRG.18696.2;ENSMUS
MSTRG.1	Ndrg3	ENSMUST00000069600;ENSMUST00000072298;ENSMUST0000014
MSTRG.1	Gm27653	ENSMUST00000185037
MSTRG.1	Soga1	ENSMUST00000069098;ENSMUST00000133571
MSTRG.1	Lmbrd1	MSTRG.187.1
MSTRG.1	Mgst3	ENSMUST00000028005
MSTRG.1	Gm14276	ENSMUST00000117941
MSTRG.1	Dsn1	ENSMUST00000103129;ENSMUST00000103130;ENSMUST0000014
MSTRG.1	Tldc2	ENSMUST00000166140;ENSMUST00000099140
MSTRG.1	Samhd1	ENSMUST00000057725;ENSMUST00000123932;ENSMUST0000013
MSTRG.1	Manbal	ENSMUST00000081202
MSTRG.1	Manbal	MSTRG.18705.1
MSTRG.1	Gm14286	ENSMUST00000155674
MSTRG.1	Blcap	MSTRG.18707.1
MSTRG.1	Rbl1	ENSMUST00000029170;MSTRG.18708.2;ENSMUST00000154721;E
MSTRG.1	Rbl1	MSTRG.18709.1
MSTRG.1	Aldh9a1	ENSMUST00000028004;ENSMUST00000194843;ENSMUST00000193
MSTRG.1	Gm14277	ENSMUST00000121195
MSTRG.1	Rbl1	MSTRG.18711.1
MSTRG.1	Rbl1	MSTRG.18712.1
MSTRG.1	Mroh8	ENSMUST00000143663;ENSMUST00000150035
MSTRG.1	Rpn2	ENSMUST00000142463;ENSMUST00000116380;ENSMUST0000002
MSTRG.1	Src	ENSMUST00000109533;ENSMUST00000145715;ENSMUST0000002
MSTRG.1	Blcap	ENSMUST00000109528;ENSMUST00000088494;ENSMUST00000134
MSTRG.1	Nnat	ENSMUST00000153739;ENSMUST00000173595;ENSMUST0000010
MSTRG.1872		MSTRG.1872.1
MSTRG.1	Gm14176	ENSMUST00000121302
MSTRG.1	Ctnnbl1	ENSMUST00000029178;MSTRG.18721.2;ENSMUST00000155479;E
MSTRG.1	Ctnnbl1	MSTRG.18722.1
MSTRG.1	Snhg17;	ENSMUST00000186993;MSTRG.18723.1;ENSMUST00000141809;E
MSTRG.1	Tti1	ENSMUST00000029179;ENSMUST00000109522;MSTRG.18724.3;E
MSTRG.1	Rprd1b	ENSMUST00000103123;ENSMUST00000109519;ENSMUST0000015
MSTRG.1	2010009	ENSMUST00000186977
MSTRG.1	Tgm2	ENSMUST00000103122;ENSMUST00000174718;ENSMUST0000014
MSTRG.1	Gm20412	ENSMUST00000173249
MSTRG.1	Snhg11;	ENSMUST00000109488;ENSMUST00000148965;MSTRG.18729.2;E
MSTRG.1	Lmx1a	ENSMUST00000028003
MSTRG.1	Gm25129	ENSMUST00000082651
MSTRG.1	Ralgapb	ENSMUST00000109486;ENSMUST00000046274;ENSMUST0000015
MSTRG.1	Gm14200	ENSMUST00000122256
MSTRG.1	Ralgapb	MSTRG.18733.1
MSTRG.1	Actr5;M	ENSMUST00000125390;ENSMUST00000142531;ENSMUST00000183
MSTRG.1	Fam83d	ENSMUST00000029183;ENSMUST00000151801
MSTRG.1	Ppp1r16	ENSMUST00000129902;ENSMUST00000052927;ENSMUST00000103
MSTRG.1	Ppp1r16	MSTRG.18737.1
MSTRG.1	Gm26141	ENSMUST00000175299
MSTRG.18739		MSTRG.18739.1
MSTRG.1	Lmx1a	MSTRG.1874.1
MSTRG.1	Dhx35	ENSMUST00000029186;ENSMUST00000109478;ENSMUST0000014
MSTRG.1	Dhx35	MSTRG.18742.1
MSTRG.1	Mafb	MSTRG.18743.1;ENSMUST00000099126
MSTRG.18744		MSTRG.18744.1
MSTRG.18745		MSTRG.18745.1
MSTRG.1	Gm29229	MSTRG.18746.1
MSTRG.1	Chd6	MSTRG.18747.1;ENSMUST00000039782;MSTRG.18747.3;ENSMUS

MSTRG.1	Gm25561	ENSMUST00000104607
MSTRG.1	Chd6	MSTRG.18749.1
MSTRG.1	Lmx1a	MSTRG.1875.1
MSTRG.1	Chd6	MSTRG.18750.1
MSTRG.1	Chd6	MSTRG.18751.1
MSTRG.1	Top1	ENSMUST00000109468;ENSMUST00000133262;ENSMUST0000012[illegible]
MSTRG.1	Plcg1	ENSMUST00000151590;ENSMUST00000103115;ENSMUST00000174
MSTRG.1	Zhx3;Gm	MSTRG.18754.1;MSTRG.18754.2;ENSMUST00000133937;ENSMUS
MSTRG.1	Zhx3	MSTRG.18755.1
MSTRG.1	Zhx3	MSTRG.18756.1
MSTRG.1	Gm16751	ENSMUST00000142212
MSTRG.1	Gm11447	ENSMUST00000118983
MSTRG.1	Zhx3	MSTRG.18759.1
MSTRG.1	Gm20711	ENSMUST00000177351
MSTRG.1	Lpin3	ENSMUST00000109456;ENSMUST00000040872;ENSMUST0000010[illegible]
MSTRG.1	Srsf6	ENSMUST00000193611;ENSMUST00000130411;ENSMUST00000017
MSTRG.1	Ift52;M	ENSMUST00000018002;MSTRG.18762.1;ENSMUST00000150396;E
MSTRG.1	Mir7003	ENSMUST00000184607
MSTRG.1	Fitm2	ENSMUST00000109418
MSTRG.1	2310001	ENSMUST00000186760;ENSMUST00000065731
MSTRG.1	Oser1	ENSMUST00000046908;ENSMUST00000104954;ENSMUST0000014[illegible]
MSTRG.1	2900093	ENSMUST00000190930
MSTRG.1	Oser1	MSTRG.18768.1
MSTRG.1	Ttpal	ENSMUST00000171696;ENSMUST00000109408;ENSMUST00000152
MSTRG.1	Pbx1	ENSMUST00000188912;ENSMUST00000176540;ENSMUST0000017[illegible]
MSTRG.1	Serinc3	MSTRG.18770.1;ENSMUST00000017851;ENSMUST00000129854
MSTRG.1	0610039	ENSMUST00000076667;ENSMUST00000184724
MSTRG.1	Pkig	ENSMUST00000147385;ENSMUST00000164399;ENSMUST00000064
MSTRG.1	Pkig	MSTRG.18773.1
MSTRG.1	Gm16316	ENSMUST00000154065
MSTRG.1	Pkig	MSTRG.18775.1
MSTRG.1	Pkig	MSTRG.18776.1
MSTRG.1	Ada	ENSMUST00000017841;MSTRG.18777.1;ENSMUST00000156939
MSTRG.1	Ywhab	ENSMUST00000018470;ENSMUST00000131288
MSTRG.1	Slpi;Mi	ENSMUST00000109367;MSTRG.18779.1;ENSMUST00000165980;M
MSTRG.1	Pbx1	MSTRG.1878.1
MSTRG.1	Sdc4	ENSMUST00000017153;MSTRG.18780.2;ENSMUST00000142909
MSTRG.1	Pabpc1l	ENSMUST00000067715;ENSMUST00000141671;ENSMUST0000015[illegible]
MSTRG.1	Tomm34	ENSMUST00000132602;ENSMUST00000018466;MSTRG.18782.1;M
MSTRG.1	Stk4;Gm	ENSMUST00000134078;MSTRG.18783.1;ENSMUST00000018353;M
MSTRG.1	Stk4	MSTRG.18784.2;MSTRG.18784.1;MSTRG.18784.4;MSTRG.18784
MSTRG.1	Sys1;Gm	ENSMUST00000142347;ENSMUST00000109352;ENSMUST0000015[illegible]
MSTRG.1	Gm14302	ENSMUST00000122806
MSTRG.1	Trp53tg	ENSMUST00000017864
MSTRG.1	Wfdc2	ENSMUST00000146270;ENSMUST00000017867;ENSMUST0000012[illegible]
MSTRG.1	Pigt	ENSMUST00000103101;ENSMUST00000117066;MSTRG.18789.2;E
MSTRG.1	Pbx1	MSTRG.1879.1
MSTRG.1	Wfdc3	ENSMUST00000103096;ENSMUST00000130227
MSTRG.1	Dnttip1	ENSMUST00000017443;ENSMUST00000109326;MSTRG.18791.2;M
MSTRG.1	Gm11457	ENSMUST00000124435;ENSMUST00000141843;ENSMUST0000014[illegible]
MSTRG.1	Ube2c	ENSMUST00000088248;MSTRG.18793.1;ENSMUST00000001439
MSTRG.1	Tnnc2	ENSMUST00000103095
MSTRG.1	Snx21	ENSMUST00000174070;ENSMUST00000172577;ENSMUST0000005[illegible]
MSTRG.1	Acot8	ENSMUST00000133053;MSTRG.18796.2;MSTRG.18796.1;ENSMUS
MSTRG.1	Zswim3	ENSMUST00000052107
MSTRG.1	Zswim1	ENSMUST00000017908;ENSMUST00000132282
MSTRG.1	Spata25	ENSMUST00000141411;ENSMUST00000017911

MSTRG.1 Lmbrd1 MSTRG.188.1
MSTRG.1 Pbx1 MSTRG.1880.1
MSTRG.1 Neurl2 ENSMUST00000042775
MSTRG.1 Neurl2 MSTRG.18801.1
MSTRG.1 Ctsa ENSMUST00000103093;ENSMUST00000017904;ENSMUST00000133
MSTRG.1 Pltp ENSMUST00000109316;ENSMUST00000059954;ENSMUST00000142
MSTRG.1 Pltp MSTRG.18804.1
MSTRG.18805 MSTRG.18805.1
MSTRG.1 Mmp9 ENSMUST00000137626;ENSMUST00000017881;ENSMUST00000134
MSTRG.1 Cd40 ENSMUST00000154443;ENSMUST00000017799;ENSMUST00000073
MSTRG.1 Cd40 MSTRG.18808.1
MSTRG.18809 MSTRG.18809.1
MSTRG.1 Pbx1 MSTRG.1881.1
MSTRG.1 Pcif1 ENSMUST00000145327;MSTRG.18810.1;ENSMUST00000041643;M
MSTRG.1 Zfp335 MSTRG.18811.1;ENSMUST00000041361;ENSMUST00000183830;E
MSTRG.1 Zfp335o MSTRG.18812.1;ENSMUST00000129364;ENSMUST00000142338
MSTRG.1 Slc12a5 ENSMUST00000125867;ENSMUST00000124372;ENSMUST00000202
MSTRG.1 Slc12a5 MSTRG.18814.1
MSTRG.1 Ncoa5 ENSMUST00000040381;ENSMUST00000121377;ENSMUST00000122
MSTRG.1 Trp53rk MSTRG.18816.1;ENSMUST00000039007;ENSMUST00000125497
MSTRG.1 Slc35c2 ENSMUST00000125550;ENSMUST00000109298;ENSMUST00000132
MSTRG.1 Elmo2 ENSMUST00000103091;ENSMUST00000148643;MSTRG.18818.2;E
MSTRG.1 Elmo2 MSTRG.18819.1
MSTRG.1 Pbx1 MSTRG.1882.1
MSTRG.1 Elmo2 MSTRG.18820.1
MSTRG.18821 MSTRG.18821.1
MSTRG.18822 MSTRG.18822.1
MSTRG.1 Eya2 ENSMUST00000088132;MSTRG.18823.2;ENSMUST00000063433;E
MSTRG.1 Eya2 MSTRG.18824.1
MSTRG.1 Gm28163 ENSMUST00000187099
MSTRG.1 Eya2 MSTRG.18826.1
MSTRG.1 Gm11460 ENSMUST00000118200
MSTRG.1 Eya2 MSTRG.18828.1
MSTRG.1 Eya2 MSTRG.18829.1
MSTRG.1 Gm37524 ENSMUST00000192272
MSTRG.1 Eya2 MSTRG.18830.1
MSTRG.1 Eya2 MSTRG.18831.1
MSTRG.1 Eya2 MSTRG.18832.1
MSTRG.1 Eya2 MSTRG.18833.1
MSTRG.1 Eya2 MSTRG.18834.1
MSTRG.1 Zmynd8 ENSMUST00000109266;ENSMUST00000177633;ENSMUST00000099
MSTRG.1 Zmynd8 MSTRG.18836.1
MSTRG.1 BC04640 MSTRG.18837.1
MSTRG.1 Gm11465 MSTRG.18838.1
MSTRG.1 Platr29 ENSMUST00000130022
MSTRG.1 Pbx1 MSTRG.1884.1
MSTRG.1 Gm11464 ENSMUST00000146963
MSTRG.1 Ncoa3 ENSMUST00000109252;ENSMUST00000088095;MSTRG.18841.2;M
MSTRG.1 Ncoa3 MSTRG.18842.1;MSTRG.18842.2
MSTRG.1 Ncoa3 MSTRG.18843.1
MSTRG.1 Sulf2 ENSMUST00000109249;ENSMUST00000088086;ENSMUST00000146
MSTRG.1 Arfgef2 ENSMUST00000099078;MSTRG.18845.2;ENSMUST00000144847
MSTRG.1 Arfgef2 MSTRG.18846.1
MSTRG.1 Arfgef2 MSTRG.18847.1
MSTRG.1 Prex1 ENSMUST00000036719;ENSMUST00000099080;MSTRG.18848.4;E
MSTRG.1 Gm23152 ENSMUST00000175322
MSTRG.1 Pbx1 MSTRG.1885.1

MSTRG.1	Prex1	MSTRG.18850.1
MSTRG.1	Gm25265	MSTRG.18851.1;ENSMUST00000158520
MSTRG.1	Stau1	ENSMUST00000109235;ENSMUST00000142481;MSTRG.18852.2;E
MSTRG.1	Stau1	MSTRG.18853.1
MSTRG.1	Stau1	MSTRG.18854.1
MSTRG.1	Stau1	MSTRG.18855.1
MSTRG.1	Ddx27	ENSMUST00000018143;ENSMUST00000176066;ENSMUST00000150
MSTRG.1	Cse1l;G	ENSMUST00000171410;ENSMUST00000002790;ENSMUST00000169
MSTRG.1	Znfx1	ENSMUST00000048988;ENSMUST00000067584;MSTRG.18858.2;E
MSTRG.1	Zfas1;G	ENSMUST00000189909;ENSMUST00000145859;ENSMUST00000184
MSTRG.1	Gm38381	ENSMUST00000193594
MSTRG.1	Kcnb1	ENSMUST00000207917;ENSMUST00000144351;ENSMUST00000148
MSTRG.1	1110018	ENSMUST00000138928;ENSMUST00000147446
MSTRG.1	Ptgis	ENSMUST00000018113;ENSMUST00000136271
MSTRG.1	Gm14291	ENSMUST00000143645
MSTRG.18864		MSTRG.18864.1
MSTRG.1	B4galt5	ENSMUST00000109221;ENSMUST00000149698;ENSMUST00000155
MSTRG.1	Slc9a8	ENSMUST00000047815;ENSMUST00000109218;ENSMUST00000073
MSTRG.1	Slc9a8	MSTRG.18867.1
MSTRG.1	Spata2	ENSMUST00000057627;MSTRG.18868.2;ENSMUST00000109211;E
MSTRG.1	Rnf114	ENSMUST00000109214;ENSMUST00000078050;MSTRG.18869.4;E
MSTRG.1	Pbx1	MSTRG.1887.1
MSTRG.1	Gm14321	MSTRG.18870.1;MSTRG.18870.2;ENSMUST00000139927;ENSMUS
MSTRG.1	Gm11476	MSTRG.18871.1;MSTRG.18871.2
MSTRG.1	Gm11474	ENSMUST00000120814
MSTRG.1	Snai1	ENSMUST00000052631
MSTRG.1	Gm11476	ENSMUST00000127006
MSTRG.1	Gm11476	MSTRG.18875.1
MSTRG.1	Gm11476	MSTRG.18876.1
MSTRG.1	Gm11475	ENSMUST00000118482
MSTRG.1	Ube2v1;	ENSMUST00000109207;ENSMUST00000060645;ENSMUST00000125
MSTRG.1	Gm14320	ENSMUST00000137275
MSTRG.1	Pbx1	MSTRG.1888.1
MSTRG.1	Cebpb	ENSMUST00000070642
MSTRG.1	A530013	ENSMUST00000141923;ENSMUST00000130679
MSTRG.1	Ptpn1	ENSMUST00000147210;ENSMUST00000029053;ENSMUST00000151
MSTRG.1	Ripor3	ENSMUST00000123782;ENSMUST00000099073;ENSMUST00000131
MSTRG.1	Gm14236	ENSMUST00000142374
MSTRG.18885		MSTRG.18885.1
MSTRG.18886		MSTRG.18886.1
MSTRG.1	Gm20716	MSTRG.18887.1;ENSMUST00000175182;ENSMUST00000057793;M
MSTRG.1	Mocs3	ENSMUST00000099071
MSTRG.1	Nfatc2	ENSMUST00000074618;ENSMUST00000109184;ENSMUST00000151
MSTRG.1	Pbx1	MSTRG.1889.1
MSTRG.1	Nfatc2	MSTRG.18890.1
MSTRG.1	Atp9a	ENSMUST00000109175;ENSMUST00000029060;ENSMUST00000109
MSTRG.1	Mir7004	ENSMUST00000200215
MSTRG.1	Atp9a	MSTRG.18893.1
MSTRG.1	Atp9a	MSTRG.18894.1
MSTRG.1	Zfp64	MSTRG.18895.1
MSTRG.1	Gm25214	ENSMUST00000104573
MSTRG.1	Zfp64	ENSMUST00000109162;ENSMUST00000124599;ENSMUST00000087
MSTRG.1	Zfp64	MSTRG.18898.1
MSTRG.1	Zfp64	MSTRG.18899.1
MSTRG.189		MSTRG.189.1
MSTRG.1	Rgs5	ENSMUST00000027997;ENSMUST00000152809
MSTRG.1	Tshz2	ENSMUST00000109159;ENSMUST00000109157;ENSMUST00000123

MSTRG.1	Gm26883	ENSMUST00000181446
MSTRG.1	Gm26883	MSTRG.18902.1
MSTRG.1	Gm26883	MSTRG.18903.1
MSTRG.1	Gm26883	MSTRG.18904.1
MSTRG.1	Gm26883	MSTRG.18905.1
MSTRG.1	Gm26883	MSTRG.18906.1
MSTRG.1	Gm26883	MSTRG.18907.1
MSTRG.1	Gm26883	MSTRG.18908.1
MSTRG.1	Gm26883	MSTRG.18909.1
MSTRG.1	Nuf2	ENSMUST00000111368;ENSMUST00000028000;ENSMUST00000192
MSTRG.1	AY70210	ENSMUST00000194651;ENSMUST00000194482
MSTRG.1	A630075	ENSMUST00000180895
MSTRG.1	Zfp217	ENSMUST00000063710;MSTRG.18912.2;MSTRG.18912.1;ENSMUS
MSTRG.1	Cyp24a1	ENSMUST00000038824
MSTRG.1	Pfdn4	ENSMUST00000075087;ENSMUST00000063682;ENSMUST00000136
MSTRG.1	Gm16796	ENSMUST00000126086
MSTRG.1	Fam210b	ENSMUST00000028995
MSTRG.1	Aurka	ENSMUST00000109140;ENSMUST00000109139;ENSMUST00000128
MSTRG.1	Cstf1	ENSMUST00000151511;MSTRG.18918.1;ENSMUST00000116375
MSTRG.1	Gm14303	ENSMUST00000121320
MSTRG.1	Nuf2	MSTRG.1892.1
MSTRG.18920		MSTRG.18920.1
MSTRG.1	Cass4	ENSMUST00000099061;ENSMUST00000103073;ENSMUST00000109
MSTRG.1	Gm14455	ENSMUST00000138288
MSTRG.1	Rtfdc1	ENSMUST00000029005;ENSMUST00000109132;MSTRG.18923.2;E
MSTRG.1	Gcnt7	ENSMUST00000099060;ENSMUST00000161334
MSTRG.1	Bmp7	ENSMUST00000009143;ENSMUST00000137247
MSTRG.18926		MSTRG.18926.1
MSTRG.1	Rae1	ENSMUST00000029013;MSTRG.18927.2;MSTRG.18927.3;MSTRG.
MSTRG.18928		MSTRG.18928.1;MSTRG.18928.2
MSTRG.1	Spo11	MSTRG.18929.1;MSTRG.18929.2;ENSMUST00000144044;ENSMUS
MSTRG.1	Rgs4	ENSMUST00000027991;ENSMUST00000132567
MSTRG.1	Rbm38	ENSMUST00000173393;ENSMUST00000173878;ENSMUST00000029
MSTRG.1	Gm20490	ENSMUST00000173622
MSTRG.1	Gm14453	ENSMUST00000139677
MSTRG.1	Gm14453	MSTRG.18933.1
MSTRG.18934		MSTRG.18934.1
MSTRG.1	Ctcfl	ENSMUST00000094287
MSTRG.1	Ctcflos	ENSMUST00000144256;ENSMUST00000142207;ENSMUST00000142
MSTRG.1	Pck1	ENSMUST00000029017;ENSMUST00000151269;ENSMUST00000150
MSTRG.18938		MSTRG.18938.1
MSTRG.1	Zbp1	MSTRG.18939.1
MSTRG.1	Hsd17b7	ENSMUST00000027989;ENSMUST00000133563;ENSMUST00000111
MSTRG.1	Zbp1	ENSMUST00000029018;ENSMUST00000146802;MSTRG.18940.3;E
MSTRG.1	Pmepa1	ENSMUST00000036248;ENSMUST00000139306;ENSMUST00000124
MSTRG.1	Pmepa1	MSTRG.18942.1
MSTRG.1	Pmepa1	MSTRG.18943.1
MSTRG.1	Pmepa1o	ENSMUST00000150185
MSTRG.1	Vapb	ENSMUST00000067530;MSTRG.18945.1;ENSMUST00000154306
MSTRG.1	Gm14371	ENSMUST00000138925
MSTRG.1	Vamp7-p	MSTRG.18947.1
MSTRG.1	Vamp7-p	ENSMUST00000120334
MSTRG.1	Ankrd60	ENSMUST00000119453;ENSMUST00000147693
MSTRG.1	3110045	ENSMUST00000180638
MSTRG.1	Ppp4r1l	ENSMUST00000141051;ENSMUST00000151763;ENSMUST00000132
MSTRG.1	Ppp4r1l	MSTRG.18951.1
MSTRG.1	Ppp4r1l	MSTRG.18952.1

MSTRG.1	Ppp4r1l	MSTRG.18953.1
MSTRG.1	Ppp4r1l	MSTRG.18954.1
MSTRG.1	Ppp4r1l	MSTRG.18955.1
MSTRG.1	Rab22a	ENSMUST00000142820;ENSMUST00000029024;ENSMUST0000010[illegible]
MSTRG.1	Rab22a	MSTRG.18957.1
MSTRG.1	Stx16	ENSMUST00000155000;ENSMUST00000134876;MSTRG.18959.2;[illegible]
MSTRG.1	Ddr2	ENSMUST00000194690;ENSMUST00000170800;MSTRG.1896.3;EN[illegible]
MSTRG.1	Npepl1	ENSMUST00000044415;ENSMUST00000125502;ENSMUST0000015[illegible]
MSTRG.1	Gm10714	ENSMUST00000131346
MSTRG.1	Atp5e	ENSMUST00000016396;ENSMUST00000149191;ENSMUST0000015[illegible]
MSTRG.1	Nelfcd	ENSMUST00000109075;ENSMUST00000016397;ENSMUST0000012[illegible]
MSTRG.1	Ctsz	MSTRG.18964.1;ENSMUST00000016400
MSTRG.1	Mir296	MSTRG.18965.1
MSTRG.1	Mir296	ENSMUST00000083587
MSTRG.1	Mir298	ENSMUST00000083476
MSTRG.18968		MSTRG.18968.1
MSTRG.1	Nespas	MSTRG.18969.1;ENSMUST00000151472;ENSMUST00000150276;[illegible]
MSTRG.1	Uap1	ENSMUST00000111350;ENSMUST00000111351;ENSMUST00000027
MSTRG.1	Gnas	ENSMUST00000109095;ENSMUST00000180362;ENSMUST0000010[illegible]
MSTRG.1	Gm27899	ENSMUST00000184715
MSTRG.1	Gnas	MSTRG.18972.1
MSTRG.1	Nespas	MSTRG.18973.1
MSTRG.1	Gm27849	ENSMUST00000183673
MSTRG.1	Gm20721	ENSMUST00000168292
MSTRG.1	Prelid3[illegible]	MSTRG.18977.1;MSTRG.18977.2;ENSMUST00000016401;ENSMUS[illegible]
MSTRG.18978		MSTRG.18978.1
MSTRG.1	Vmn1r-p	ENSMUST00000118738
MSTRG.1	Gm37748	ENSMUST00000194379
MSTRG.18980		MSTRG.18980.1
MSTRG.18981		MSTRG.18981.1
MSTRG.18982		MSTRG.18982.1
MSTRG.1	Zfp831	MSTRG.18983.1;ENSMUST00000059452
MSTRG.1	Zfp831	MSTRG.18984.1
MSTRG.1	Zfp831	MSTRG.18985.1
MSTRG.1	Zfp831	MSTRG.18986.1
MSTRG.1	Gm14401	ENSMUST00000134614;ENSMUST00000108969;ENSMUST0000010[illegible]
MSTRG.1	Gm14401	MSTRG.18988.1
MSTRG.1	Gm14407	ENSMUST00000121260
MSTRG.1	Gm37502	ENSMUST00000192981
MSTRG.1	Gm14387	ENSMUST00000121827
MSTRG.1	Gm14392	ENSMUST00000120332
MSTRG.1	Gm14399	ENSMUST00000109062;ENSMUST00000108929;ENSMUST0000010[illegible]
MSTRG.1	Gm16355	ENSMUST00000128606
MSTRG.1	Gm14443	ENSMUST00000127251;ENSMUST00000109054
MSTRG.1	Gm14391	ENSMUST00000165892;ENSMUST00000109056;ENSMUST0000010[illegible]
MSTRG.1	Gm14438	ENSMUST00000121572
MSTRG.1	Gm14440	ENSMUST00000109051;ENSMUST00000098998
MSTRG.1	Gm14298	ENSMUST00000139032
MSTRG.1	Gm16041	ENSMUST00000144339
MSTRG.1	Gm7846	ENSMUST00000145844
MSTRG.1	Uhmk1	ENSMUST00000150821;ENSMUST00000027979;ENSMUST0000012[illegible]
MSTRG.1	Gm4631	ENSMUST00000121393;ENSMUST00000109050
MSTRG.1	Zfp968	ENSMUST00000131676;ENSMUST00000109048;ENSMUST0000010[illegible]
MSTRG.1	Gm14297	ENSMUST00000129110
MSTRG.1	Gm4724	ENSMUST00000109046;ENSMUST00000109045;ENSMUST00000152
MSTRG.1	Zfp965	ENSMUST00000109042;ENSMUST00000109002;ENSMUST0000010[illegible]
MSTRG.1	Gm11007	ENSMUST00000177700;ENSMUST00000178120;ENSMUST0000018[illegible]

MSTRG.1	Gm16359	ENSMUST00000124511
MSTRG.1	Zfp969	ENSMUST00000109027;ENSMUST00000179061;ENSMUST00000131
MSTRG.1	Gm2007	ENSMUST00000177981;ENSMUST00000178133;ENSMUST0000018[illegible]
MSTRG.1	Gm16358	ENSMUST00000146046
MSTRG.1	4930500[illegible]	ENSMUST00000159201;ENSMUST00000194988
MSTRG.1	Gm14288	ENSMUST00000109020;ENSMUST00000123863
MSTRG.1	Rps8-ps[illegible]	ENSMUST00000118709
MSTRG.1	Gm6710	ENSMUST00000109011;ENSMUST00000147820;ENSMUST0000014[illegible]
MSTRG.1	Gm6710	MSTRG.19014.1
MSTRG.1	Gm16356	ENSMUST00000143300
MSTRG.1	Zfp966	ENSMUST00000108949;ENSMUST00000109018
MSTRG.1	Gm2026	ENSMUST00000109007;ENSMUST00000174416;ENSMUST0000010[illegible]
MSTRG.1	Gm11008	ENSMUST00000178432;ENSMUST00000109032
MSTRG.1	Gm16357	ENSMUST00000144091
MSTRG.1	Atf6	ENSMUST00000027974;ENSMUST00000182787
MSTRG.1	Gm2004	ENSMUST00000109049;ENSMUST00000178872
MSTRG.1	2210418[illegible]	ENSMUST00000099009;ENSMUST00000136719;ENSMUST0000013[illegible]
MSTRG.19023		MSTRG.19023.1
MSTRG.19024		MSTRG.19024.1
MSTRG.1	Gm14413	MSTRG.19025.1
MSTRG.1	Gm14413	ENSMUST00000122158
MSTRG.19027		MSTRG.19027.1
MSTRG.1	Gm14410	ENSMUST00000156269;ENSMUST00000133301;ENSMUST0000010[illegible]
MSTRG.1	Zfp973;[illegible]	ENSMUST00000121956;ENSMUST00000108985;ENSMUST00000141
MSTRG.1	Gm23523	ENSMUST00000180190
MSTRG.19030		MSTRG.19030.1
MSTRG.1	Gm14301	ENSMUST00000120181
MSTRG.1	Gm14390	ENSMUST00000117505
MSTRG.1	Gm14295	ENSMUST00000118012;ENSMUST00000132883;ENSMUST0000017[illegible]
MSTRG.1	Gm14405	ENSMUST00000117808
MSTRG.1	Gm14296	ENSMUST00000134982;ENSMUST00000099007;ENSMUST0000012[illegible]
MSTRG.1	Gm14419	ENSMUST00000108975;ENSMUST00000156844;ENSMUST0000012[illegible]
MSTRG.1	Gm25235	ENSMUST00000179933
MSTRG.1	Gm14421	ENSMUST00000121755
MSTRG.1	Gm14323	ENSMUST00000122418
MSTRG.19042		MSTRG.19042.1
MSTRG.19043		MSTRG.19043.1
MSTRG.19044		MSTRG.19044.1
MSTRG.1	Gm14409	ENSMUST00000108963
MSTRG.1	Gm14412	ENSMUST00000108959
MSTRG.1	Gm14418	ENSMUST00000128657;ENSMUST00000149689;ENSMUST0000010[illegible]
MSTRG.1	Gm14414	ENSMUST00000120833
MSTRG.1	Gm9929	ENSMUST00000171747
MSTRG.1	Zfp970	ENSMUST00000099002;ENSMUST00000108952
MSTRG.1	Gm14403	ENSMUST00000142549;ENSMUST00000108947;ENSMUST0000010[illegible]
MSTRG.1	Gm16363	ENSMUST00000132460
MSTRG.19053		MSTRG.19053.1
MSTRG.1	Gm14406	ENSMUST00000108943
MSTRG.1	Gm14324	ENSMUST00000121358
MSTRG.1	Gm14322	ENSMUST00000148388;ENSMUST00000119838;ENSMUST0000010[illegible]
MSTRG.1	Gm14327	ENSMUST00000108935;ENSMUST00000099001
MSTRG.1	Gm14326	MSTRG.19058.1;ENSMUST00000181241
MSTRG.1	Zfp972	ENSMUST00000108934;ENSMUST00000081529;ENSMUST0000010[illegible]
MSTRG.1	Dusp12	ENSMUST00000170420;ENSMUST00000171447;ENSMUST0000016[illegible]
MSTRG.1	Gm14326	ENSMUST00000108932;ENSMUST00000178829;ENSMUST0000010[illegible]
MSTRG.1	Zfp971	ENSMUST00000108925;ENSMUST00000108926
MSTRG.19063		MSTRG.19063.1

MSTRG.1	Zfp931	ENSMUST00000108924;ENSMUST00000131702;ENSMUST00000123
MSTRG.1	Gm14292	ENSMUST00000118761
MSTRG.1	Cdh4	ENSMUST00000000314;MSTRG.19066.2;ENSMUST00000108911;E
MSTRG.1	Gm14300	ENSMUST00000120800
MSTRG.1	Cdh4	MSTRG.19068.1
MSTRG.1	Taf4	ENSMUST00000041618;MSTRG.19069.2;MSTRG.19069.3;MSTRG.
MSTRG.1	Gm9929	MSTRG.1907.1
MSTRG.1	Taf4	MSTRG.19070.1
MSTRG.1	4921531	ENSMUST00000153188;ENSMUST00000150145
MSTRG.1	Lsm14b	MSTRG.19072.1;ENSMUST00000055485;MSTRG.19072.2;ENSMUS
MSTRG.1	Osbpl2	ENSMUST00000040668;ENSMUST00000140210;ENSMUST00000142
MSTRG.1	Osbpl2	MSTRG.19074.1
MSTRG.1	Psma7	ENSMUST00000129529;ENSMUST00000126021;ENSMUST0000002
MSTRG.1	Ss18l1	ENSMUST00000041126;MSTRG.19076.2
MSTRG.1	Ss18l1	MSTRG.19077.1
MSTRG.1	Mtg2	ENSMUST00000147264;MSTRG.19078.2;ENSMUST00000142608;E
MSTRG.1	Rps21	ENSMUST00000059080;ENSMUST00000148629;ENSMUST00000125
MSTRG.1	Gm26620	ENSMUST00000180542
MSTRG.1	Mir3091	ENSMUST00000175086
MSTRG.1	Cables2	ENSMUST00000108891;ENSMUST00000136706;ENSMUST00000141
MSTRG.1	Adrm1	ENSMUST00000061437;ENSMUST00000155357;ENSMUST00000147
MSTRG.1	Lama5	ENSMUST00000130796;ENSMUST00000015791;ENSMUST00000123
MSTRG.1	Mir7005	ENSMUST00000185089
MSTRG.1	Mrgbp	ENSMUST00000169630;ENSMUST00000029085;ENSMUST0000015
MSTRG.1	Ogfr	ENSMUST00000029087;ENSMUST00000147070;MSTRG.19086.2
MSTRG.1	Dido1	ENSMUST00000087517;MSTRG.19087.1;MSTRG.19087.3;ENSMUS
MSTRG.1	Gm22502	ENSMUST00000082488
MSTRG.1	Gid8	MSTRG.19089.1;ENSMUST00000029090;MSTRG.19089.3;ENSMUS
MSTRG.1	Fcgr2b	ENSMUST00000027966;ENSMUST00000081103;MSTRG.1909.2;EN
MSTRG.1	Slc17a9	MSTRG.19090.1;ENSMUST00000094218;ENSMUST00000151882;E
MSTRG.1	Ythdf1	MSTRG.19091.1;MSTRG.19091.2;ENSMUST00000037299;ENSMUS
MSTRG.1	Ppdpf	ENSMUST00000016488;ENSMUST00000127971;ENSMUST00000108
MSTRG.1	Ptk6	MSTRG.19093.1
MSTRG.1	Helz2	MSTRG.19094.1
MSTRG.1	Gm14398	ENSMUST00000117287
MSTRG.1	Fndc11	ENSMUST00000050026;ENSMUST00000108835
MSTRG.1	Helz2	ENSMUST00000108831;ENSMUST00000094203;ENSMUST00000121
MSTRG.1	Gmeb2	MSTRG.19098.1;MSTRG.19098.2;ENSMUST00000049032;ENSMUS
MSTRG.1	Gmeb2	MSTRG.19099.1
MSTRG.191		MSTRG.191.1
MSTRG.1	Gm38204	ENSMUST00000194835
MSTRG.1	Gm7645	MSTRG.19100.1;ENSMUST00000119008
MSTRG.1	Stmn3	ENSMUST00000103045
MSTRG.1	Birc7	ENSMUST00000137859
MSTRG.1	Nkain4	ENSMUST00000151494;ENSMUST00000103053;ENSMUST0000013
MSTRG.1	Gm14341	ENSMUST00000154616
MSTRG.1	Arfgap1	ENSMUST00000108862;ENSMUST00000029092;ENSMUST00000108
MSTRG.1	Slc2a4r	ENSMUST00000127559;MSTRG.19106.1;ENSMUST00000176590
MSTRG.1	Zbtb46	ENSMUST00000029106;ENSMUST00000180222;ENSMUST00000087
MSTRG.1	Gm22708	ENSMUST00000158987
MSTRG.1	Tpd52l2	ENSMUST00000184588;ENSMUST00000183956;ENSMUST0000014
MSTRG.1	Fcgr3	ENSMUST00000168817;ENSMUST00000164044;MSTRG.1911.2;EN
MSTRG.1	Tpd52l2	MSTRG.19110.1
MSTRG.1	Dnajc5	MSTRG.19111.1
MSTRG.1	Dnajc5	ENSMUST00000072334;ENSMUST00000116365;MSTRG.19112.6;E
MSTRG.1	Rtel1	ENSMUST00000098971;ENSMUST00000146273;MSTRG.19113.2;E
MSTRG.1	Rtel1	MSTRG.19114.1

MSTRG.1	Rtel1	MSTRG.19115.1
MSTRG.1	Arfrp1	ENSMUST00000170190;ENSMUST00000185118;ENSMUST0000012[illegible]
MSTRG.1	Zgpat;L	ENSMUST00000029105;MSTRG.19117.1;ENSMUST00000108807;[illegible]
MSTRG.1	Sox18	ENSMUST00000054491
MSTRG.1	Tcea2	ENSMUST00000129686;ENSMUST00000103042;ENSMUST0000012[illegible]
MSTRG.1	Mir6546	ENSMUST00000184460
MSTRG.1	Rgs19	ENSMUST00000144476;MSTRG.19120.1;ENSMUST00000108779;[illegible]
MSTRG.1	Lkaaear	ENSMUST00000052416;ENSMUST00000132409
MSTRG.1	Uckl1;Z	ENSMUST00000134340;ENSMUST00000057816;ENSMUST0000013[illegible]
MSTRG.1	Uckl1os	ENSMUST00000152365;ENSMUST00000123110;MSTRG.19123.2;[illegible]
MSTRG.1	Mir7006	ENSMUST00000183624
MSTRG.1	Prpf6	ENSMUST00000132714;ENSMUST00000136481;ENSMUST0000000[illegible]
MSTRG.1	Samd10	ENSMUST00000060173;ENSMUST00000183836;ENSMUST0000013[illegible]
MSTRG.1	Pcmtd2	ENSMUST00000029116;ENSMUST00000108754;ENSMUST0000013[illegible]
MSTRG.19128		MSTRG.19128.1
MSTRG.1	Polr3k	ENSMUST00000039551;MSTRG.19129.1;ENSMUST00000153214
MSTRG.1	Gm27552	MSTRG.1913.1;MSTRG.1913.3;MSTRG.1913.2
MSTRG.19130		MSTRG.19130.1
MSTRG.1	Gm5466	ENSMUST00000119395
MSTRG.19132		MSTRG.19132.1
MSTRG.19133		MSTRG.19133.1
MSTRG.19134		MSTRG.19134.1
MSTRG.19135		MSTRG.19135.1
MSTRG.19136		MSTRG.19136.1
MSTRG.19137		MSTRG.19137.1
MSTRG.19138		MSTRG.19138.1
MSTRG.1	Gm8797	ENSMUST00000192045
MSTRG.1	Gm27552	ENSMUST00000183351
MSTRG.19140		MSTRG.19140.1
MSTRG.1	Pex2	ENSMUST00000164828;ENSMUST00000165309;ENSMUST00000195
MSTRG.1	Pkia	ENSMUST00000192468;ENSMUST00000028999;ENSMUST00000193
MSTRG.1	Pkia	MSTRG.19143.1
MSTRG.1	Gm37918	ENSMUST00000192071
MSTRG.1	Zc2hc1a	ENSMUST00000051064;ENSMUST00000192835
MSTRG.19146		MSTRG.19146.1
MSTRG.1	Il7	ENSMUST00000194279;ENSMUST00000168269;ENSMUST00000194
MSTRG.1	Il7	MSTRG.19148.1
MSTRG.1	Gm16685	ENSMUST00000181286
MSTRG.1	Gm27844	ENSMUST00000183613
MSTRG.1	Gm16685	MSTRG.19150.1
MSTRG.1	Gm16685	MSTRG.19151.1
MSTRG.19152		MSTRG.19152.1
MSTRG.19153		MSTRG.19153.1
MSTRG.19154		MSTRG.19154.1
MSTRG.1	Hey1	ENSMUST00000042412;ENSMUST00000192550;ENSMUST00000192
MSTRG.1	C030034	ENSMUST00000180874;ENSMUST00000181331;ENSMUST00000181
MSTRG.1	C030034	MSTRG.19157.1
MSTRG.1	C030034	MSTRG.19158.1
MSTRG.1	Zfp704	MSTRG.19159.1;ENSMUST00000041124;ENSMUST00000193947;[illegible]
MSTRG.1	Cfap126	ENSMUST00000129651;ENSMUST00000151340;ENSMUST00000027
MSTRG.1	Gm38001	MSTRG.19160.1
MSTRG.1	Gm38001	ENSMUST00000192900
MSTRG.1	Zfp704	MSTRG.19162.1
MSTRG.1	Zfp704	MSTRG.19163.1
MSTRG.1	Mrps28;[illegible]	ENSMUST00000042148;MSTRG.19164.1;ENSMUST00000120143;[illegible]
MSTRG.1	Mrps28	MSTRG.19165.1
MSTRG.1	Mrps28	MSTRG.19166.1

MSTRG.1	Gm15467	ENSMUST00000120582
MSTRG.1	Gm15466	ENSMUST00000122299
MSTRG.1	Tpd52	MSTRG.19169.1
MSTRG.1	Sdhc	ENSMUST00000111336;ENSMUST00000081560;ENSMUST0000015
MSTRG.1	Tpd52	MSTRG.19170.1
MSTRG.1	Tpd52	MSTRG.19171.1
MSTRG.1	Pag1;Gm	MSTRG.19172.2;MSTRG.19172.1;ENSMUST00000161949;MSTRG.
MSTRG.1	Gm17806	ENSMUST00000195844
MSTRG.1	Gm16337	ENSMUST00000160368
MSTRG.1	Pag1	MSTRG.19175.1
MSTRG.1	Pag1	MSTRG.19176.1
MSTRG.1	Fabp5	ENSMUST00000029046;ENSMUST00000123744
MSTRG.1	Gm9833	ENSMUST00000061419;MSTRG.19178.2
MSTRG.1	Gm37308	ENSMUST00000194684
MSTRG.1	Mpz	ENSMUST00000070758;ENSMUST00000125565
MSTRG.1	Fabp4	ENSMUST00000029041;ENSMUST00000191757;MSTRG.19180.2
MSTRG.1	A930014	ENSMUST00000194572
MSTRG.1	A930014	MSTRG.19182.1
MSTRG.1	Gm38364	MSTRG.19183.1
MSTRG.1	Impa1;G	ENSMUST00000065938;ENSMUST00000118410;ENSMUST0000019
MSTRG.1	Chmp4c	ENSMUST00000029049;ENSMUST00000191826
MSTRG.1	Snx16	ENSMUST00000099223;ENSMUST00000029047;ENSMUST0000019
MSTRG.1	Snx16	MSTRG.19187.1
MSTRG.1	Snx16	MSTRG.19188.1
MSTRG.19189		MSTRG.19189.1
MSTRG.1	Fcer1g	ENSMUST00000079957;ENSMUST00000193973;ENSMUST0000015
MSTRG.1	Slc7a12	ENSMUST00000037321
MSTRG.1	Slc7a12	MSTRG.19191.1
MSTRG.1	Ralyl	ENSMUST00000193117;ENSMUST00000108373
MSTRG.1	Gm2464	ENSMUST00000180838
MSTRG.1	Gm37164	ENSMUST00000192809
MSTRG.1	Gm2474	ENSMUST00000195162
MSTRG.1	Gm19114	ENSMUST00000193972
MSTRG.1	Gm25981	ENSMUST00000157921
MSTRG.1	Gm6473	ENSMUST00000188572;MSTRG.192.2
MSTRG.1	Ndufs2	ENSMUST00000138069;ENSMUST00000150108;ENSMUST0000001
MSTRG.1	Car13	ENSMUST00000029071
MSTRG.1	E2f5	ENSMUST00000029069;ENSMUST00000165922
MSTRG.1	1810022	ENSMUST00000185384;ENSMUST00000108365;ENSMUST0000018
MSTRG.1	Car3	ENSMUST00000195575;ENSMUST00000029076;ENSMUST0000019
MSTRG.1	Lrrcc1;	ENSMUST00000108370;ENSMUST00000169079;ENSMUST0000009
MSTRG.1	Car2	ENSMUST00000192609;ENSMUST00000029078;ENSMUST0000019
MSTRG.19207		MSTRG.19207.1
MSTRG.1	Gm33819	ENSMUST00000194008
MSTRG.19209		MSTRG.19209.1
MSTRG.1	Adamts4	ENSMUST00000111315;ENSMUST00000111314
MSTRG.1	Ythdf3	ENSMUST00000108345;MSTRG.19210.1;ENSMUST00000193598;E
MSTRG.1	Gm5844	ENSMUST00000119417
MSTRG.19212		MSTRG.19212.1
MSTRG.1	Cyp7b1	ENSMUST00000035625
MSTRG.1	Gm23686	ENSMUST00000083241
MSTRG.1	Cyp7b1	MSTRG.19215.1
MSTRG.1	Armc1	MSTRG.19216.1;ENSMUST00000029125;MSTRG.19216.3
MSTRG.1	Gm37979	ENSMUST00000193065
MSTRG.1	Dnajc5b	ENSMUST00000029132;ENSMUST00000118735
MSTRG.1	B4galt3	ENSMUST00000064272;ENSMUST00000151863;ENSMUST0000014
MSTRG.1	Gm6141	ENSMUST00000117181

MSTRG.1	1700064	ENSMUST00000119133
MSTRG.1	4632415	ENSMUST00000059704;ENSMUST00000198527
MSTRG.1	Mtfr1	ENSMUST00000130806;ENSMUST00000117529;ENSMUST0000011
MSTRG.1	Pde7a	ENSMUST00000099195;MSTRG.19224.6;ENSMUST00000091314;M
MSTRG.1	Gm16093	ENSMUST00000141455
MSTRG.1	Pde7a	MSTRG.19226.1
MSTRG.1	Pde7a	MSTRG.19227.1
MSTRG.1	Gm22282	ENSMUST00000158524
MSTRG.1	Cp	ENSMUST00000091309;ENSMUST00000108329;ENSMUST0000012
MSTRG.1	Ppox	ENSMUST00000155083;MSTRG.1923.2;ENSMUST00000073120;EN
MSTRG.1	Hps3	ENSMUST00000012580;ENSMUST00000130171;ENSMUST00000141
MSTRG.1	Cp	MSTRG.19231.1
MSTRG.1	Hps3	MSTRG.19232.1
MSTRG.1	Hps3	MSTRG.19233.1
MSTRG.1	Gyg	ENSMUST00000118015;ENSMUST00000178328;ENSMUST00000184
MSTRG.1	Hltf	ENSMUST00000143005;ENSMUST00000002502;ENSMUST0000012
MSTRG.19236		MSTRG.19236.1
MSTRG.1	Cpa3	ENSMUST00000001921;ENSMUST00000191659
MSTRG.1	Mir7007	ENSMUST00000184606
MSTRG.19239		MSTRG.19239.1
MSTRG.1	Gm20045	ENSMUST00000192658
MSTRG.1	Gm7488	ENSMUST00000117148
MSTRG.1	Gm43672	MSTRG.19241.1
MSTRG.1	Gm43672	ENSMUST00000199072
MSTRG.19243		MSTRG.19243.1
MSTRG.1	Tbl1xr1	ENSMUST00000193734;ENSMUST00000202747;ENSMUST00000202
MSTRG.1	Gm5842	ENSMUST00000195073
MSTRG.1	Tbl1xr1	MSTRG.19246.1
MSTRG.1	Tbl1xr1	MSTRG.19247.1
MSTRG.1	Gm10259	ENSMUST00000091289
MSTRG.1	Naaladl	ENSMUST00000203414;ENSMUST00000192246;ENSMUST00000203
MSTRG.1	Ufc1	ENSMUST00000080001;ENSMUST00000138974;ENSMUST00000144
MSTRG.1	Gm7536	ENSMUST00000077389
MSTRG.1	Gm24704	ENSMUST00000093715
MSTRG.1	Gm24062	ENSMUST00000157490
MSTRG.1	Gm42774	ENSMUST00000198854
MSTRG.1	Gm37136	ENSMUST00000194368
MSTRG.19255		MSTRG.19255.1
MSTRG.1	Spata16	MSTRG.19257.1
MSTRG.1	Nlgn1	ENSMUST00000193603;ENSMUST00000075054;ENSMUST0000010
MSTRG.1	Gm24250	ENSMUST00000157320
MSTRG.1	Ufc1	MSTRG.1926.1
MSTRG.1	Gm37573	ENSMUST00000193993
MSTRG.1	Nlgn1	MSTRG.19261.1
MSTRG.1	Gm6197	ENSMUST00000192273
MSTRG.1	Nlgn1	MSTRG.19263.1
MSTRG.1	Nlgn1	MSTRG.19264.1
MSTRG.1	Nlgn1	MSTRG.19265.1
MSTRG.1	Nlgn1	MSTRG.19266.1
MSTRG.1	Nlgn1	MSTRG.19267.1
MSTRG.1	Nlgn1	MSTRG.19268.1
MSTRG.1	Nlgn1	MSTRG.19269.1
MSTRG.1	Ufc1	MSTRG.1927.1
MSTRG.1	Nlgn1	MSTRG.19270.1
MSTRG.1	A830092	ENSMUST00000195446
MSTRG.1	Nceh1	ENSMUST00000046515;ENSMUST00000138947;ENSMUST0000012
MSTRG.1	Gm37191	ENSMUST00000193346

MSTRG.1	Tnfsf10	ENSMUST00000046383;ENSMUST00000174840
MSTRG.1	Tnfsf10	MSTRG.19275.1
MSTRG.1	Tnfsf10	MSTRG.19276.1
MSTRG.1	Gm25152	MSTRG.19277.1;ENSMUST00000157117
MSTRG.1	Gm7558	ENSMUST00000193221
MSTRG.1	Ect2	ENSMUST00000108300;ENSMUST00000108298;ENSMUST0000017€
MSTRG.1	Usp21	ENSMUST00000065941;ENSMUST00000111305;ENSMUST00000144
MSTRG.1	Fndc3b	ENSMUST00000195008;ENSMUST00000046157;MSTRG.19280.1;E
MSTRG.1	Gm43344	ENSMUST00000198723
MSTRG.1	Mir3092	ENSMUST00000174954
MSTRG.1	Pld1	ENSMUST00000120834;ENSMUST00000127139;ENSMUST00000067
MSTRG.1	Slc2a2	ENSMUST00000029240;ENSMUST00000169047
MSTRG.1	1700112	ENSMUST00000167704;ENSMUST00000193387
MSTRG.1	Gm6505	ENSMUST00000094335
MSTRG.1	Eif5a2	ENSMUST00000060500
MSTRG.1	Gm37867	MSTRG.19288.1
MSTRG.1	Gm32950	ENSMUST00000193336
MSTRG.1	B930036	ENSMUST00000181170;ENSMUST00000165827;ENSMUST0000008З
MSTRG.1	Rpl22l1	ENSMUST00000043867;ENSMUST00000194649;ENSMUST00000195
MSTRG.1	Gm33051	ENSMUST00000192940
MSTRG.19292		MSTRG.19292.1
MSTRG.1	Tnik;Mi	ENSMUST00000160934;ENSMUST00000159236;ENSMUST00000161
MSTRG.1	Tnik	MSTRG.19294.1
MSTRG.1	Tnik	MSTRG.19295.1;MSTRG.19295.2
MSTRG.1	Tnik	MSTRG.19296.1
MSTRG.1	Gm37537	ENSMUST00000192154
MSTRG.1	Tnik	MSTRG.19298.1;MSTRG.19298.2;MSTRG.19298.3
MSTRG.1	Tnik	MSTRG.19299.1
MSTRG.1	Adgrb3	ENSMUST00000151309;ENSMUST00000153568;ENSMUST0000019C
MSTRG.1	Alyref2	ENSMUST00000081527
MSTRG.1	Tnik	MSTRG.19300.1
MSTRG.1	Tnik	MSTRG.19301.1
MSTRG.1	Tnik	MSTRG.19302.1
MSTRG.1	Tnik	MSTRG.19303.1
MSTRG.1	Tnik	MSTRG.19304.1
MSTRG.1	Tnik	MSTRG.19305.1
MSTRG.1	4933429	ENSMUST00000187361;ENSMUST00000180833
MSTRG.1	Sec62	ENSMUST00000029256;ENSMUST00000191730;MSTRG.19307.2;E
MSTRG.1	Mynn	ENSMUST00000192715;ENSMUST00000195396;ENSMUST00000047
MSTRG.1	Lrrc34	ENSMUST00000029252
MSTRG.1	Cd244	ENSMUST00000004829;ENSMUST00000194797;ENSMUST00000194
MSTRG.1	Lrriq4	ENSMUST00000108267
MSTRG.19311		MSTRG.19311.1
MSTRG.1	Gpr160	ENSMUST00000130294;ENSMUST00000108259;ENSMUST00000108
MSTRG.1	Phc3;95	MSTRG.19313.1;MSTRG.19313.2;ENSMUST00000168645;ENSMUS
MSTRG.1	Prkci	ENSMUST00000108249;ENSMUST00000130238;ENSMUST00000123
MSTRG.1	Mir7008	ENSMUST00000184398
MSTRG.1	Skil	ENSMUST00000118470;ENSMUST00000144756;ENSMUST0000002S
MSTRG.1	Zmat3	ENSMUST00000029199;ENSMUST00000139660;ENSMUST00000195
MSTRG.1	Zfp639	ENSMUST00000193287;ENSMUST00000191783;ENSMUST00000193
MSTRG.1	Gm37592	ENSMUST00000192741
MSTRG.1	Ly9	ENSMUST00000068878;ENSMUST00000004827;ENSMUST00000143
MSTRG.1	Pik3ca	ENSMUST00000108243;MSTRG.19320.2;MSTRG.19320.3;MSTRG.
MSTRG.1	Gm37123	ENSMUST00000192994
MSTRG.1	Kcnmb3	ENSMUST00000164954
MSTRG.1	Mfn1	ENSMUST00000091257;ENSMUST00000125182;ENSMUST00000137
MSTRG.1	Mfn1	MSTRG.19324.1

MSTRG.1	Gnb4	ENSMUST00000155737;ENSMUST00000108234;ENSMUST00000152
MSTRG.1	Gm37640	ENSMUST00000195407
MSTRG.1	Actl6a	ENSMUST00000194781;ENSMUST00000029214;ENSMUST00000194
MSTRG.1	Gm37353	ENSMUST00000194497
MSTRG.1	Mrpl47	ENSMUST00000043966;ENSMUST00000153564;ENSMUST00000192
MSTRG.1	Dedd	ENSMUST00000111300;ENSMUST00000111299;ENSMUST00000064
MSTRG.1	Ndufb5	ENSMUST00000127477;ENSMUST00000121778;ENSMUST00000195
MSTRG.19331		MSTRG.19331.1
MSTRG.19332		MSTRG.19332.1
MSTRG.1	Ttc14	ENSMUST00000099153;ENSMUST00000199523;ENSMUST0000020
MSTRG.1	Ccdc39	ENSMUST00000029222;ENSMUST00000200536;MSTRG.19334.3;
MSTRG.1	Gm43140	ENSMUST00000197317
MSTRG.19336		MSTRG.19336.1
MSTRG.1	Gm43077	ENSMUST00000200602
MSTRG.1	Fxr1	ENSMUST00000001620;ENSMUST00000200086;ENSMUST00000198
MSTRG.1	Fxr1	MSTRG.19339.1
MSTRG.1	Dedd	MSTRG.1934.1
MSTRG.1	Gm43667	ENSMUST00000198616
MSTRG.1	Dnajc19	ENSMUST00000011029;ENSMUST00000108195;ENSMUST0000012
MSTRG.1	Gm43668	ENSMUST00000197014
MSTRG.1	Gm7741	ENSMUST00000181445
MSTRG.1	Atp11b	ENSMUST00000200351;ENSMUST00000200445;ENSMUST00000196
MSTRG.1	Gm43203	ENSMUST00000200130
MSTRG.1	Atp11b	MSTRG.19346.1
MSTRG.1	D3Ertd2	ENSMUST00000197653;ENSMUST00000205077;ENSMUST00000165
MSTRG.1	Acad9;G	ENSMUST00000011492;ENSMUST00000196648;ENSMUST00000198
MSTRG.1	Acad9	MSTRG.19349.1
MSTRG.1	Nit1	ENSMUST00000146428;ENSMUST00000111296;ENSMUST00000111
MSTRG.1	Acad9	MSTRG.19350.1
MSTRG.1	Dcun1d1	MSTRG.19351.1;ENSMUST00000178098;ENSMUST00000108182;
MSTRG.1	Dcun1d1	MSTRG.19352.1
MSTRG.1	Gm15952	ENSMUST00000122858;ENSMUST00000196662;ENSMUST00000144
MSTRG.1	8430422	ENSMUST00000199608;MSTRG.19354.1;ENSMUST00000199113;
MSTRG.1	Gm43080	ENSMUST00000199006
MSTRG.1	Mccc1os	ENSMUST00000137198;ENSMUST00000144118;ENSMUST00000124
MSTRG.1	Ccdc144	ENSMUST00000200469;ENSMUST00000196964
MSTRG.1	Anxa5	ENSMUST00000029266;ENSMUST00000196097;ENSMUST00000199
MSTRG.1	Pfdn2	ENSMUST00000135941;ENSMUST00000006579;ENSMUST00000138
MSTRG.1	Mir7009	ENSMUST00000184519
MSTRG.1	1810062	ENSMUST00000029268
MSTRG.1	Exosc9	ENSMUST00000155866;ENSMUST00000029269;ENSMUST00000138
MSTRG.1	Ccna2	MSTRG.19363.1;ENSMUST00000029270;ENSMUST00000156575;
MSTRG.1	Gm42922	ENSMUST00000195887
MSTRG.1	Spry1	ENSMUST00000108109;ENSMUST00000038569;ENSMUST00000108
MSTRG.1	Gm43820	ENSMUST00000199414
MSTRG.1	Gm5148	ENSMUST00000079755;ENSMUST00000108105;ENSMUST00000099
MSTRG.1	Rps23-p	ENSMUST00000116115
MSTRG.1	Gm43821	ENSMUST00000199198
MSTRG.1	Pfdn2	MSTRG.1937.1
MSTRG.1	Rpl21-p	ENSMUST00000071237
MSTRG.1	Cetn4	ENSMUST00000071400;ENSMUST00000140956;ENSMUST00000075
MSTRG.1	Gm43439	ENSMUST00000138710;ENSMUST00000057975;ENSMUST00000108
MSTRG.1	Bbs12	MSTRG.19373.1
MSTRG.1	Gm12540	ENSMUST00000121938
MSTRG.1	Fgf2os	ENSMUST00000153785
MSTRG.1	Gm43488	ENSMUST00000198874
MSTRG.1	Gm42923	ENSMUST00000198438

MSTRG.1	Nudt6	ENSMUST00000108117;ENSMUST00000108118;ENSMUST00000052
MSTRG.1	Spata5;	ENSMUST00000108112;ENSMUST00000029277;ENSMUST00000198
MSTRG.1	Pfdn2	MSTRG.1938.1
MSTRG.1	Spata5	MSTRG.19380.1
MSTRG.1	Spata5	MSTRG.19381.1
MSTRG.1	Spata5	MSTRG.19382.1
MSTRG.1	Spata5	MSTRG.19383.1
MSTRG.1	Gm12564	ENSMUST00000119860
MSTRG.1	Spata5	MSTRG.19385.1
MSTRG.1	Ankrd50	ENSMUST00000120875;ENSMUST00000156038;MSTRG.19386.2;M
MSTRG.1	Gm9845	ENSMUST00000056409
MSTRG.1	C230034	ENSMUST00000153659
MSTRG.1	Fat4	MSTRG.19389.1;ENSMUST00000061260;ENSMUST00000129182
MSTRG.1	Gm37154	ENSMUST00000192266
MSTRG.1	Gm43539	ENSMUST00000196190
MSTRG.1	4932438	MSTRG.19391.1;ENSMUST00000198742;ENSMUST00000057272;E
MSTRG.1	Gm12531	ENSMUST00000126099;ENSMUST00000139667
MSTRG.1	Hspa4l	MSTRG.19393.1;MSTRG.19393.3;ENSMUST00000204702;ENSMUS
MSTRG.1	Pgrmc2	ENSMUST00000058578;ENSMUST00000124636
MSTRG.1	Pgrmc2	MSTRG.19395.1
MSTRG.1	Pgrmc2	MSTRG.19396.1
MSTRG.1	Larp1b	ENSMUST00000048490;ENSMUST00000191872;ENSMUST00000200
MSTRG.1	Larp1b	MSTRG.19398.1
MSTRG.1	Larp1b	MSTRG.19399.1
MSTRG.1	Gm15702	ENSMUST00000119680
MSTRG.1	Klhdc9	ENSMUST00000061878
MSTRG.1	Larp1b	MSTRG.19400.1
MSTRG.1	Gm22043	ENSMUST00000082993
MSTRG.1	Gm42436	ENSMUST00000198804
MSTRG.1	Gm42437	ENSMUST00000197263
MSTRG.1	Larp1b	MSTRG.19404.1
MSTRG.1	Larp1b	MSTRG.19405.1
MSTRG.1	Larp1b	ENSMUST00000191882;ENSMUST00000065411;ENSMUST00000197
MSTRG.1	Larp1b	MSTRG.19407.1
MSTRG.1	Plk4	ENSMUST00000026858;MSTRG.19408.1;ENSMUST00000204594;M
MSTRG.1	Mfsd8	ENSMUST00000026859;ENSMUST00000204054;MSTRG.19409.3;E
MSTRG.1	Gm37097	ENSMUST00000193676
MSTRG.1	Mfsd8	MSTRG.19410.1
MSTRG.1	Gm2011;	ENSMUST00000203124;ENSMUST00000108078;ENSMUST00000203
MSTRG.1	Gm10731	ENSMUST00000181171
MSTRG.1	Gm44136	ENSMUST00000203819
MSTRG.1	Gm44524	ENSMUST00000203899
MSTRG.1	Abhd18	MSTRG.19415.1
MSTRG.1	6430590	ENSMUST00000181813;ENSMUST00000203407
MSTRG.1	1700034	ENSMUST00000099123
MSTRG.1	Abhd18	MSTRG.19418.1
MSTRG.1	Abhd18	MSTRG.19419.1
MSTRG.1	Nectin4	ENSMUST00000111286;ENSMUST00000006578;ENSMUST00000094
MSTRG.1	Abhd18	MSTRG.19420.1
MSTRG.19421		MSTRG.19421.1
MSTRG.19422		MSTRG.19422.1
MSTRG.1	Jade1	ENSMUST00000026865;ENSMUST00000194181;ENSMUST00000195
MSTRG.1	Gm31266	MSTRG.19424.1
MSTRG.1	Gm31266	ENSMUST00000192060
MSTRG.1	Sclt1	ENSMUST00000026866;ENSMUST00000146125;MSTRG.19426.2;E
MSTRG.1	Sclt1	MSTRG.19427.1
MSTRG.1	Sclt1	MSTRG.19428.1

MSTRG.1	Gm38047	ENSMUST00000193690
MSTRG.1	Arhgap3	ENSMUST00000056449;ENSMUST00000131607;ENSMUST0000013
MSTRG.1	Sclt1	MSTRG.19430.1
MSTRG.1	D3Ertd7	ENSMUST00000120167;ENSMUST00000108065;ENSMUST0000014
MSTRG.1	D3Ertd7	MSTRG.19432.1
MSTRG.1	Gm37268	ENSMUST00000192254
MSTRG.19434		MSTRG.19434.1
MSTRG.19435		MSTRG.19435.1
MSTRG.19436		MSTRG.19436.1
MSTRG.19437		MSTRG.19437.1
MSTRG.1	Slc7a11	ENSMUST00000194462;ENSMUST00000029297;MSTRG.19438.2;M
MSTRG.1	Usf1;Ts	ENSMUST00000159207;ENSMUST00000001284;MSTRG.1944.1;EN
MSTRG.1	Gm37826	ENSMUST00000195329
MSTRG.1	Gm2345	ENSMUST00000193451
MSTRG.1	Slc7a11	MSTRG.19443.1
MSTRG.1	Gm37498	ENSMUST00000193524
MSTRG.1	Slc7a11	MSTRG.19445.1
MSTRG.1	Slc7a11	MSTRG.19446.1
MSTRG.19447		MSTRG.19447.1
MSTRG.1	Gm38246	ENSMUST00000194318
MSTRG.1	Noct	ENSMUST00000023849;ENSMUST00000144826;ENSMUST0000019
MSTRG.1	Gm26641	ENSMUST00000181499
MSTRG.1	Gm38357	ENSMUST00000195382
MSTRG.1	Gm9442	ENSMUST00000194242
MSTRG.1	Ndufc1	ENSMUST00000038108;ENSMUST00000145572;ENSMUST0000013
MSTRG.1	Elf2	ENSMUST00000194641;ENSMUST00000183463;ENSMUST0000006
MSTRG.1	4930577	ENSMUST00000139771;MSTRG.19454.1
MSTRG.1	Elf2	MSTRG.19455.1
MSTRG.1	Elf2	MSTRG.19456.1
MSTRG.1	Gm24282	ENSMUST00000104232
MSTRG.1	Gm37399	ENSMUST00000195224
MSTRG.1	Gm38160	ENSMUST00000193090
MSTRG.1	F11r	ENSMUST00000043839;ENSMUST00000144497;ENSMUST0000015
MSTRG.1	Rab33b	ENSMUST00000195715;ENSMUST00000192172;MSTRG.19460.2;E
MSTRG.1	Gm37968	ENSMUST00000195131
MSTRG.1	Naa15	ENSMUST00000193266;ENSMUST00000029303;MSTRG.19462.2;E
MSTRG.1	Gm37646	ENSMUST00000191756
MSTRG.1	Naa15	MSTRG.19464.1
MSTRG.1	Naa15	MSTRG.19465.1
MSTRG.1	Setd7	ENSMUST00000037141;MSTRG.19466.2;MSTRG.19466.3;MSTRG.
MSTRG.1	Setd7	MSTRG.19467.1
MSTRG.1	Gm38200	ENSMUST00000192083
MSTRG.1	5031434	ENSMUST00000180404;ENSMUST00000181303;ENSMUST0000018
MSTRG.1	Tma7-ps	ENSMUST00000118448
MSTRG.19470		MSTRG.19470.1
MSTRG.1	Foxo1;G	ENSMUST00000053764;MSTRG.19471.2;MSTRG.19471.3;MSTRG.
MSTRG.1	Gm20402	ENSMUST00000174738
MSTRG.1	Foxo1	MSTRG.19473.1;MSTRG.19473.2
MSTRG.1	Mgst2;G	ENSMUST00000160753;ENSMUST00000161627;ENSMUST0000009
MSTRG.1	Maml3	ENSMUST00000118075;ENSMUST00000121440;ENSMUST0000019
MSTRG.1	Gm10729	ENSMUST00000099104
MSTRG.1	Gm37342	ENSMUST00000194588
MSTRG.1	Maml3	MSTRG.19478.1
MSTRG.1	Maml3	MSTRG.19479.1
MSTRG.1	A630035	ENSMUST00000194468;ENSMUST00000194143
MSTRG.1	Maml3	MSTRG.19480.1
MSTRG.1	Maml3	MSTRG.19481.1

MSTRG.1	Maml3	MSTRG.19482.1
MSTRG.1	Maml3	MSTRG.19483.1
MSTRG.1	Gm37229	MSTRG.19484.1
MSTRG.1	n-R5s19	ENSMUST00000083136
MSTRG.1	3110080	ENSMUST00000193469
MSTRG.1	Maml3	MSTRG.19487.1
MSTRG.1	Maml3	MSTRG.19488.1
MSTRG.1	Gm38000	ENSMUST00000192390
MSTRG.1	A630035	MSTRG.1949.1
MSTRG.1	Gm37675	ENSMUST00000192303
MSTRG.1	Gm38252	ENSMUST00000192701
MSTRG.1	Maml3	MSTRG.19492.1
MSTRG.1	Gm10728	ENSMUST00000194665;ENSMUST00000194292
MSTRG.1	Maml3	MSTRG.19494.1
MSTRG.1	Maml3	MSTRG.19495.1
MSTRG.1	Maml3	MSTRG.19496.1
MSTRG.1	Gm20089	ENSMUST00000192391
MSTRG.1	Gm38055	ENSMUST00000195353
MSTRG.1	Gm37526	ENSMUST00000194640
MSTRG.1	A630035	MSTRG.1950.1
MSTRG.1	Maml3	MSTRG.19500.1
MSTRG.1	Maml3	MSTRG.19501.1
MSTRG.1	Gm37465	ENSMUST00000192845
MSTRG.1	Maml3	MSTRG.19503.1
MSTRG.1	Maml3	MSTRG.19504.1
MSTRG.1	Maml3	MSTRG.19505.1
MSTRG.1	C130089	MSTRG.19506.1;ENSMUST00000194534
MSTRG.1	Maml3	MSTRG.19507.1
MSTRG.1	5430420	ENSMUST00000194956
MSTRG.1	Maml3	MSTRG.19509.1
MSTRG.1	Gm37981	ENSMUST00000194280
MSTRG.1	Gm10293	ENSMUST00000076317
MSTRG.1	Cog6	ENSMUST00000036665;ENSMUST00000194779;ENSMUST00000193
MSTRG.19513		MSTRG.19513.1
MSTRG.1	Gm42901	ENSMUST00000197735
MSTRG.1	Lhfp	ENSMUST00000059562;ENSMUST00000196913
MSTRG.1	Gm42898	ENSMUST00000200370
MSTRG.1	Gm42899	ENSMUST00000197034
MSTRG.1	C130075	ENSMUST00000198178
MSTRG.1	Gm43804	ENSMUST00000198280
MSTRG.1	Cd48	ENSMUST00000015499;ENSMUST00000068584
MSTRG.1	Gm43803	ENSMUST00000197765;ENSMUST00000196732
MSTRG.1	Gm43805	ENSMUST00000198488
MSTRG.1	Lhfp	MSTRG.19522.1
MSTRG.1	Proser1	MSTRG.19523.1
MSTRG.1	Stoml3	ENSMUST00000124702;ENSMUST00000029307
MSTRG.1	Nhlrc3	ENSMUST00000056749;ENSMUST00000130348;ENSMUST0000012
MSTRG.1	Gm16206	ENSMUST00000132485
MSTRG.1	Proser1	ENSMUST00000058577;ENSMUST00000198186;ENSMUST00000141
MSTRG.1	Gm6204	ENSMUST00000198872
MSTRG.19529		MSTRG.19529.1
MSTRG.1	Gm37010	ENSMUST00000194539
MSTRG.1	Ufm1	ENSMUST00000146598;ENSMUST00000148338;ENSMUST0000002
MSTRG.1	Gm5641	ENSMUST00000091130;ENSMUST00000198699
MSTRG.1	Postn	ENSMUST00000117373;ENSMUST00000107985;MSTRG.19532.3;E
MSTRG.1	Rfxap	ENSMUST00000044373;ENSMUST00000182246;ENSMUST00000217
MSTRG.1	Gm10254	ENSMUST00000052888

MSTRG.19535		MSTRG.19535.1
MSTRG.1	Supt20	ENSMUST00000197502;ENSMUST00000029315;ENSMUST0000020C
MSTRG.1	Exosc8	ENSMUST00000029316;ENSMUST00000125632;MSTRG.19538.2;M
MSTRG.1	Alg5	ENSMUST00000044567;ENSMUST00000141191;ENSMUST0000015E
MSTRG.1	Vangl2	ENSMUST00000027837;ENSMUST00000111263;ENSMUST00000111
MSTRG.1	Alg5	MSTRG.19540.1
MSTRG.1	Spg20	ENSMUST00000044116;ENSMUST00000199416;ENSMUST00000107
MSTRG.1	Ccdc169	ENSMUST00000052904;ENSMUST00000118963;ENSMUST0000015З
MSTRG.1	Gm22029	ENSMUST00000116940
MSTRG.1	Gm43549	ENSMUST00000198856;MSTRG.19544.2
MSTRG.1	Wwtr1	ENSMUST00000029380;ENSMUST00000120977
MSTRG.1	Gm16016	ENSMUST00000120831
MSTRG.1	Gm43437	ENSMUST00000198535
MSTRG.1	Commd2	ENSMUST00000160959;ENSMUST00000041954;MSTRG.19548.2;E
MSTRG.1	Gm5537	ENSMUST00000091117
MSTRG.1	Vangl2	MSTRG.1955.1
MSTRG.1	Rnf13	ENSMUST00000041826;ENSMUST00000198510;ENSMUST0000019E
MSTRG.1	Gm9645	ENSMUST00000197131
MSTRG.1	Gm17951	ENSMUST00000198536
MSTRG.1	Rnf13	MSTRG.19553.1
MSTRG.1	Pfn2	ENSMUST00000066882;ENSMUST00000119344;ENSMUST00000122
MSTRG.1	Tsc22d2	ENSMUST00000099090;ENSMUST00000199164;ENSMUST0000019S
MSTRG.1	Tsc22d2	MSTRG.19556.1
MSTRG.1	Serp1	ENSMUST00000029385;MSTRG.19557.2;MSTRG.19557.3
MSTRG.1	Eif2a	ENSMUST00000029387;ENSMUST00000137469;ENSMUST0000013E
MSTRG.1	6720482	ENSMUST00000199552
MSTRG.1	Vangl2	MSTRG.1956.1
MSTRG.1	Selenot	ENSMUST00000107924;MSTRG.19560.5;ENSMUST00000125815;M
MSTRG.1	Selenot	MSTRG.19561.1
MSTRG.1	Rpl13-p	ENSMUST00000079761
MSTRG.1	Siah2	ENSMUST00000070368;ENSMUST00000145032
MSTRG.1	Siah2	MSTRG.19564.1
MSTRG.1	Siah2	MSTRG.19565.1
MSTRG.1	4930593	ENSMUST00000203879;ENSMUST00000181694
MSTRG.1	A930028	ENSMUST00000197219
MSTRG.1	Mindy4b	ENSMUST00000191174;ENSMUST00000191233
MSTRG.1	Gm5539	ENSMUST00000192177
MSTRG.1	Vangl2	MSTRG.1957.1
MSTRG.1	4930449	MSTRG.19572.1
MSTRG.1	Gm43570	ENSMUST00000199951
MSTRG.1	Med12l	ENSMUST00000040846;ENSMUST00000199659;ENSMUST0000002S
MSTRG.1	Gpr171	ENSMUST00000085040;MSTRG.19575.2;MSTRG.19575.1
MSTRG.1	P2ry14	ENSMUST00000197841;ENSMUST00000065220;ENSMUST0000019E
MSTRG.1	Med12l	MSTRG.19577.1
MSTRG.1	Gpr87	ENSMUST00000199833;ENSMUST00000200095;ENSMUST0000005E
MSTRG.1	P2ry13	ENSMUST00000040622
MSTRG.1	Slamf1	ENSMUST00000015460;ENSMUST00000163992;MSTRG.1958.2
MSTRG.1	P2ry12	ENSMUST00000196583;ENSMUST00000050360;ENSMUST0000017C
MSTRG.1	Gm43589	ENSMUST00000199400
MSTRG.1	Igsf10	ENSMUST00000039419;ENSMUST00000193455;ENSMUST00000194
MSTRG.1	Gm38186	ENSMUST00000192574
MSTRG.1	Mbnl1	MSTRG.19584.1
MSTRG.19585		MSTRG.19585.1
MSTRG.1	Gm8325	ENSMUST00000193411;ENSMUST00000099086
MSTRG.19587		MSTRG.19587.1
MSTRG.19588		MSTRG.19588.1
MSTRG.1	Gm37696	ENSMUST00000194223

MSTRG.1	Slamf1	MSTRG.1959.1
MSTRG.1	Rap2b	ENSMUST00000049064
MSTRG.1	Mbnl1	ENSMUST00000195724;ENSMUST00000195077;MSTRG.19592.2;E
MSTRG.1	Mbnl1	MSTRG.19593.1
MSTRG.1	Gm37589	ENSMUST00000193609
MSTRG.1	Gm37488	ENSMUST00000195434
MSTRG.1	9330121	ENSMUST00000192267
MSTRG.1	Arhgef2	ENSMUST00000079300;ENSMUST00000163008;ENSMUST00000161
MSTRG.1	Arhgef2	MSTRG.19598.1
MSTRG.1	Dhx36	ENSMUST00000029336;ENSMUST00000160858;MSTRG.19599.3;E
MSTRG.1	Slamf1	MSTRG.1960.1
MSTRG.1	Slc33a1	ENSMUST00000161659;ENSMUST00000029402;ENSMUST00000160
MSTRG.1	Gm26850	ENSMUST00000181653;ENSMUST00000192898
MSTRG.1	Gmps	ENSMUST00000029405;MSTRG.19602.2
MSTRG.1	Gm26939	MSTRG.19604.1
MSTRG.1	Gm38048	ENSMUST00000193778
MSTRG.1	Kcnab1	ENSMUST00000159525;ENSMUST00000049230;ENSMUST00000161
MSTRG.1	A330015	ENSMUST00000195685;ENSMUST00000195489
MSTRG.1	A730090	ENSMUST00000160137
MSTRG.1961		MSTRG.1961.1
MSTRG.1	Kcnab1	MSTRG.19610.1
MSTRG.1	Ssr3	ENSMUST00000029414;ENSMUST00000139785;ENSMUST00000119
MSTRG.1	4931440	ENSMUST00000147424;ENSMUST00000194448;ENSMUST00000184
MSTRG.1	Gm22279	ENSMUST00000157200
MSTRG.1	Tiparp	ENSMUST00000047906;ENSMUST00000130705;ENSMUST00000154
MSTRG.1	Tiparp	MSTRG.19615.1
MSTRG.1	Ccnl1	MSTRG.19616.1
MSTRG.1	Ccnl1	ENSMUST00000129002;ENSMUST00000154585;ENSMUST00000122
MSTRG.1	Gm37305	ENSMUST00000195714
MSTRG.1	Gm35106	ENSMUST00000192731
MSTRG.1	Cd84	ENSMUST00000155802;ENSMUST00000128189;MSTRG.1962.2;EN
MSTRG.1	Shox2	ENSMUST00000029422
MSTRG.1	Rsrc1	ENSMUST00000162362;ENSMUST00000065074;ENSMUST00000162
MSTRG.1	Gm22295	ENSMUST00000083311
MSTRG.1	Rsrc1	MSTRG.19623.1
MSTRG.1	Rsrc1	MSTRG.19624.1
MSTRG.1	Rsrc1	MSTRG.19625.1
MSTRG.1	Rsrc1	MSTRG.19626.1
MSTRG.1	Rsrc1	MSTRG.19627.1
MSTRG.1	Gfm1	ENSMUST00000077271;ENSMUST00000161009;ENSMUST00000160
MSTRG.1	Lxn	ENSMUST00000058981;ENSMUST00000160827;ENSMUST00000160
MSTRG.1	Cd84;Gm	MSTRG.1963.1;MSTRG.1963.3;MSTRG.1963.2;MSTRG.1963.5;M
MSTRG.1	Gm42568	ENSMUST00000195639
MSTRG.1	Rarres1	ENSMUST00000054825
MSTRG.1	Mfsd1	ENSMUST00000029344;ENSMUST00000192776
MSTRG.19633		MSTRG.19633.1
MSTRG.1	Gm10040	ENSMUST00000080726
MSTRG.1	Gm10040	MSTRG.19635.1
MSTRG.19636		MSTRG.19636.1
MSTRG.1	Kpna4	ENSMUST00000029353;ENSMUST00000194558;ENSMUST00000127
MSTRG.1	Gm22009	ENSMUST00000158792
MSTRG.1	Kpna4	MSTRG.19639.1
MSTRG.1	Gm10521	ENSMUST00000097466
MSTRG.1	Gm37558	ENSMUST00000193684
MSTRG.1	Ppm1l	ENSMUST00000029355;ENSMUST00000193662;ENSMUST00000134
MSTRG.1	Gm17214	ENSMUST00000164501
MSTRG.1	Ppm1l	MSTRG.19643.1

MSTRG.1	Gm17212	ENSMUST00000166666
MSTRG.1	Ppm1l	MSTRG.19645.1
MSTRG.1	Ppm1l	MSTRG.19646.1
MSTRG.1	Ppm1l	MSTRG.19647.1
MSTRG.1	Ppm1l	MSTRG.19648.1
MSTRG.1	Ppm1l	MSTRG.19649.1
MSTRG.1	Gm10521	MSTRG.1965.1
MSTRG.1	Ppm1l	MSTRG.19650.1
MSTRG.1	Ppm1l	MSTRG.19651.1
MSTRG.1	Gm17213	ENSMUST00000165161
MSTRG.1	Ppm1l	MSTRG.19653.1
MSTRG.1	Nmd3	MSTRG.19654.1;ENSMUST00000149680;MSTRG.19654.2;ENSMUS
MSTRG.1	Rpl32-p	ENSMUST00000091052
MSTRG.1	Sptssb	ENSMUST00000051239;ENSMUST00000171529
MSTRG.1	Sptssb	MSTRG.19657.1
MSTRG.1	Ift80;A	ENSMUST00000107812;ENSMUST00000136448;MSTRG.19658.3;E
MSTRG.1	Gm20689	ENSMUST00000176033
MSTRG.1966		MSTRG.1966.1
MSTRG.1	Gm20690	ENSMUST00000195126;ENSMUST00000177341
MSTRG.1	Gm18588	ENSMUST00000177308
MSTRG.1	Gm20691	ENSMUST00000176889
MSTRG.1	Ift80	MSTRG.19663.1
MSTRG.1	Smc4	ENSMUST00000042901;ENSMUST00000136502;MSTRG.19664.2;E
MSTRG.1	Mir15b	ENSMUST00000083646
MSTRG.1	Mir16-2	ENSMUST00000083672
MSTRG.1	Trim59	MSTRG.19667.1;MSTRG.19667.2;ENSMUST00000107802;ENSMUS
MSTRG.1	Gm37784	ENSMUST00000193233
MSTRG.1	Gm37784	MSTRG.19669.1
MSTRG.1	Gm37065	MSTRG.1967.1
MSTRG.1	Gm37784	MSTRG.19670.1
MSTRG.19671		MSTRG.19671.1
MSTRG.19672		MSTRG.19672.1
MSTRG.1	Gm6098	MSTRG.19673.1
MSTRG.1	Wdr49	ENSMUST00000193989;ENSMUST00000203169;ENSMUST00000204
MSTRG.1	Wdr49	MSTRG.19676.1
MSTRG.1	Gm17836	ENSMUST00000191976
MSTRG.1	Pdcd10	MSTRG.19678.1
MSTRG.1	Pdcd10	ENSMUST00000161137;ENSMUST00000159519;ENSMUST00000029
MSTRG.1	Slamf6	ENSMUST00000195656;ENSMUST00000171330;ENSMUST00000194
MSTRG.1	Serpini	ENSMUST00000161776;ENSMUST00000029423;ENSMUST00000195
MSTRG.1	Gm37672	ENSMUST00000192682
MSTRG.1	Fstl5	ENSMUST00000038364
MSTRG.1	Gm37897	ENSMUST00000192613
MSTRG.1	Fstl5	MSTRG.19684.1
MSTRG.1	Fstl5	MSTRG.19685.1
MSTRG.1	Golim4	ENSMUST00000038563;ENSMUST00000117242;ENSMUST00000134
MSTRG.1	Golim4	MSTRG.19687.1
MSTRG.1	Golim4	MSTRG.19688.1
MSTRG.1	Gm37685	ENSMUST00000193507
MSTRG.1	Slamf6	MSTRG.1969.1
MSTRG.1	Gm37256	ENSMUST00000193034
MSTRG.1	Gm10291	ENSMUST00000094175
MSTRG.1	Gm9762	ENSMUST00000036976;MSTRG.19692.1
MSTRG.1	Gm17359	ENSMUST00000164216;ENSMUST00000199658
MSTRG.1	Fnip2	ENSMUST00000133154;ENSMUST00000149475;ENSMUST00000076
MSTRG.1	Gm3513	ENSMUST00000161319
MSTRG.1	Fnip2	MSTRG.19696.1

MSTRG.1	Rapgef2	MSTRG.19697.1
MSTRG.1	Rapgef2	ENSMUST00000118100;ENSMUST00000118340;ENSMUST00000195
MSTRG.1	Rapgef2	MSTRG.19699.1
MSTRG.1	Pex19	ENSMUST00000075895;ENSMUST00000147799;MSTRG.1970.2;EN
MSTRG.1	Gm24150	ENSMUST00000082759
MSTRG.1	4921511	ENSMUST00000200412;MSTRG.19701.2;MSTRG.19701.3
MSTRG.1	Rapgef2	MSTRG.19702.1
MSTRG.1	6430573	ENSMUST00000192648;ENSMUST00000195547
MSTRG.1	Ppid	ENSMUST00000161460;ENSMUST00000029382;ENSMUST00000162
MSTRG.1	Etfdh	ENSMUST00000029386;ENSMUST00000120992;ENSMUST00000135
MSTRG.1	Gm37973	ENSMUST00000195767
MSTRG.1	4930579	ENSMUST00000029388
MSTRG.1	Pdgfc	ENSMUST00000029652;ENSMUST00000129285;ENSMUST00000143
MSTRG.1	Gm16000	ENSMUST00000146657
MSTRG.1	Ncstn	MSTRG.1971.1;ENSMUST00000003550;ENSMUST00000140643;MS
MSTRG.1	Pdgfc	MSTRG.19710.1
MSTRG.1	Ctso	ENSMUST00000029649;ENSMUST00000192067;ENSMUST00000143
MSTRG.1	Tdo2	ENSMUST00000029645;ENSMUST00000151089;ENSMUST00000137
MSTRG.1	Gucy1a3	ENSMUST00000193924;ENSMUST00000048976;ENSMUST00000192
MSTRG.19714		MSTRG.19714.1
MSTRG.19715		MSTRG.19715.1
MSTRG.19716		MSTRG.19716.1
MSTRG.1	Plrg1	ENSMUST00000150268;ENSMUST00000029628;ENSMUST00000122
MSTRG.1	Gm10710	ENSMUST00000047876
MSTRG.1	Dchs2	ENSMUST00000191829
MSTRG.1	Copa	ENSMUST00000135192;ENSMUST00000138407;MSTRG.1972.2;EN
MSTRG.1	Dchs2	MSTRG.19720.1
MSTRG.1	Dchs2	MSTRG.19721.1
MSTRG.1	Tmem131	ENSMUST00000191758;ENSMUST00000192095;ENSMUST00000052
MSTRG.1	Tmem131	MSTRG.19723.1;MSTRG.19723.2;MSTRG.19723.3;MSTRG.19723
MSTRG.1	Sh3d19	ENSMUST00000182969;ENSMUST00000182723;ENSMUST00000183
MSTRG.1	Prss48	ENSMUST00000061343
MSTRG.1	Sh3d19	MSTRG.19726.1
MSTRG.1	Sh3d19	MSTRG.19727.1
MSTRG.1	Gm25188	ENSMUST00000083913
MSTRG.1	Rps3a1;	MSTRG.19729.1;ENSMUST00000029722;ENSMUST00000082456
MSTRG.1	Gm38164	ENSMUST00000192810
MSTRG.1	Snord73	ENSMUST00000083050
MSTRG.1	Gm37933	ENSMUST00000193964
MSTRG.1	Gm37493	ENSMUST00000193167
MSTRG.1	Arfip1;	ENSMUST00000098990;ENSMUST00000154148;ENSMUST00000107
MSTRG.1	Gm37240	MSTRG.19734.1
MSTRG.1	Gm37240	MSTRG.19735.1
MSTRG.1	Gm8813	ENSMUST00000155930
MSTRG.1	Gm38241	ENSMUST00000192641
MSTRG.1	Gm37240	MSTRG.19738.1
MSTRG.1	Gm37240	MSTRG.19739.1
MSTRG.1	Gm37756	ENSMUST00000191834
MSTRG.1	Tigd4	ENSMUST00000062623
MSTRG.1	Tmem154	ENSMUST00000107682;ENSMUST00000134890;ENSMUST00000129
MSTRG.1	Tmem154	MSTRG.19742.1
MSTRG.1	Fbxw7	MSTRG.19743.1;MSTRG.19743.2;ENSMUST00000107679;MSTRG.
MSTRG.1	Gm37240	MSTRG.19744.1
MSTRG.1	Fbxw7	MSTRG.19745.1
MSTRG.1	Gm37240	MSTRG.19746.1
MSTRG.1	Gm37726	ENSMUST00000193246
MSTRG.1	Gm37240	MSTRG.19748.1

MSTRG.1	Gm38041	ENSMUST00000194445
MSTRG.1	Copa	MSTRG.1975.1
MSTRG.1	Gm38313	ENSMUST00000191953
MSTRG.1	Gm37240	MSTRG.19751.1
MSTRG.1	Gm45790	ENSMUST00000211986
MSTRG.1	Gm42812	ENSMUST00000199844
MSTRG.1	Gm15535	ENSMUST00000148478;ENSMUST00000128872
MSTRG.1	Gatb	ENSMUST00000107672;ENSMUST00000127348;ENSMUST00000107
MSTRG.1	Gm37240	MSTRG.19759.1
MSTRG.1	Kcnj10	ENSMUST00000056136
MSTRG.1	4933425	ENSMUST00000195991
MSTRG.1	Lrba	MSTRG.19761.1;MSTRG.19761.2;ENSMUST00000192145;ENSMUS
MSTRG.1	Gm37288	ENSMUST00000193963
MSTRG.1	Gm26343	ENSMUST00000104556
MSTRG.1	Gm23850	ENSMUST00000083830
MSTRG.1	Gm25039	ENSMUST00000082933
MSTRG.1	Lrba	MSTRG.19766.1
MSTRG.1	Mab21l2	ENSMUST00000077524;ENSMUST00000191695
MSTRG.1	Lrba	MSTRG.19768.1
MSTRG.1	Gm3788	ENSMUST00000119949
MSTRG.1	Pigm	ENSMUST00000052455
MSTRG.1	Lrba	MSTRG.19770.1
MSTRG.1	Gm37876	ENSMUST00000194490
MSTRG.1	Cd1d2	ENSMUST00000194208;ENSMUST00000041920;ENSMUST00000192
MSTRG.1	Cd1d1	ENSMUST00000107620;ENSMUST00000063869;ENSMUST0000002
MSTRG.1	Dclk2	ENSMUST00000191752;ENSMUST00000192773;ENSMUST0000002
MSTRG.1	Gm37025	MSTRG.19775.1;ENSMUST00000195159
MSTRG.1	Dclk2	MSTRG.19776.1
MSTRG.1	Dclk2	MSTRG.19777.1
MSTRG.1	Dclk2	MSTRG.19778.1
MSTRG.1	Gm10705	ENSMUST00000098980
MSTRG.1	Gm10171	ENSMUST00000126220
MSTRG.1	Kirrel	ENSMUST00000159976;ENSMUST00000107618;ENSMUST00000041
MSTRG.1	Gm37855	ENSMUST00000192571
MSTRG.1	Cd5l	ENSMUST00000015998
MSTRG.1	Etv3	ENSMUST00000119109;ENSMUST00000170036;ENSMUST00000136
MSTRG.1	Sh2d2a	ENSMUST00000107581;ENSMUST00000029709;ENSMUST00000194
MSTRG.1	Prcc	ENSMUST00000005015;MSTRG.19785.2
MSTRG.1	Hdgf	ENSMUST00000005017;ENSMUST00000159492;ENSMUST00000162
MSTRG.1	Arhgef1	ENSMUST00000136072;ENSMUST00000039476;ENSMUST0000012
MSTRG.1	Lrrc71;	ENSMUST00000023846;ENSMUST00000174381;ENSMUST00000172
MSTRG.1	Ntrk1	ENSMUST00000029712
MSTRG.1	Dcaf8	ENSMUST00000193638;ENSMUST00000195394;MSTRG.1979.2;EN
MSTRG.1	Insrr	ENSMUST00000029711;ENSMUST00000166771
MSTRG.1	Mrpl24	ENSMUST00000119968;ENSMUST00000019854;ENSMUST00000137
MSTRG.1	Rrnad1	ENSMUST00000159091;ENSMUST00000160648;ENSMUST0000000
MSTRG.1	Isg20l2	ENSMUST00000055984;MSTRG.19793.1;MSTRG.19793.2
MSTRG.1	Gm19439	ENSMUST00000198195
MSTRG.1	Gpatch4	ENSMUST00000029707;ENSMUST00000166021;ENSMUST00000194
MSTRG.1	Naxe	ENSMUST00000029708;MSTRG.19796.2;MSTRG.19796.3
MSTRG.1	Iqgap3	ENSMUST00000071812;ENSMUST00000193940;ENSMUST00000195
MSTRG.1	Iqgap3	MSTRG.19798.1
MSTRG.1	Iqgap3	MSTRG.19799.1
MSTRG.1	Gm37331	ENSMUST00000192367
MSTRG.1	Dcaf8	MSTRG.1980.1
MSTRG.19800		MSTRG.19800.1
MSTRG.1	Mef2d	ENSMUST00000001455;ENSMUST00000119251;ENSMUST00000107

MSTRG.1	1700113	ENSMUST00000197172;ENSMUST00000181837;MSTRG.19802.2;M
MSTRG.1	1700113	MSTRG.19803.1
MSTRG.1	Rhbg;Gm	ENSMUST00000171887;ENSMUST00000163277;ENSMUST00000107
MSTRG.1	Gm38392	MSTRG.19805.1
MSTRG.1	Gm37116	ENSMUST00000195161
MSTRG.1	Tsacc	MSTRG.19807.1
MSTRG.1	Cct3	ENSMUST00000001452;ENSMUST00000193666;ENSMUST00000167
MSTRG.1	Pmf1	ENSMUST00000193338;ENSMUST00000056370;ENSMUST00000192
MSTRG.1	Dcaf8	MSTRG.1981.1
MSTRG.1	Slc25a4	ENSMUST00000168755;ENSMUST00000193433;MSTRG.19810.3;E
MSTRG.1	Glmp	ENSMUST00000177005;ENSMUST00000193646;ENSMUST00000153
MSTRG.1	Tmem79	ENSMUST00000001456;ENSMUST00000107553;ENSMUST00000107
MSTRG.1	Smg5	ENSMUST00000001451;ENSMUST00000192057;MSTRG.19813.2;E
MSTRG.1	Sema4a;I	ENSMUST00000029700;ENSMUST00000156108;ENSMUST00000135
MSTRG.1	Lmna	ENSMUST00000147537;ENSMUST00000029699;ENSMUST00000135
MSTRG.1	Gm37584	MSTRG.19816.1
MSTRG.1	Gm37584	ENSMUST00000193595
MSTRG.19818		MSTRG.19818.1;MSTRG.19818.2
MSTRG.1	Mex3a	ENSMUST00000172699
MSTRG.1	Pea15a	MSTRG.1982.1;ENSMUST00000013842;ENSMUST00000111247;EN
MSTRG.1	Rab25	ENSMUST00000008745;ENSMUST00000131775;ENSMUST00000141
MSTRG.1	Lamtor2	ENSMUST00000145548;ENSMUST00000029698;ENSMUST00000119
MSTRG.1	Ubqln4	ENSMUST00000008748;ENSMUST00000194303;ENSMUST00000192
MSTRG.1	Gm43714	ENSMUST00000198351
MSTRG.1	2810403	ENSMUST00000029696;ENSMUST00000200588;ENSMUST00000198
MSTRG.1	Ssr2	ENSMUST00000193069;ENSMUST00000194604;MSTRG.19826.4;E
MSTRG.1	Gm10704	ENSMUST00000098952
MSTRG.1	Arhgef2	ENSMUST00000212392;ENSMUST00000176226;ENSMUST00000175
MSTRG.1	Gm20652	ENSMUST00000177314
MSTRG.1	Casq1	ENSMUST00000003554;ENSMUST00000170638
MSTRG.1	Rusc1	MSTRG.19830.1
MSTRG.1	Ash1l	MSTRG.19831.1
MSTRG.1	Gm43738	ENSMUST00000200659;ENSMUST00000166687;ENSMUST0000009C
MSTRG.1	Gm22935	ENSMUST00000103960
MSTRG.1	Dpm3	ENSMUST00000107462;ENSMUST00000040824;ENSMUST00000137
MSTRG.1	Slc50a1	ENSMUST00000185119;ENSMUST00000107460;ENSMUST00000132
MSTRG.1	Efna1	ENSMUST00000029566;ENSMUST00000118587;ENSMUST00000118
MSTRG.1	Rit1	ENSMUST00000029692;ENSMUST00000171645;ENSMUST00000165
MSTRG.1	Gm17146	ENSMUST00000108464
MSTRG.1	Rit1	MSTRG.19839.1
MSTRG.1	Gm17224	ENSMUST00000171429
MSTRG.1	Rit1	MSTRG.19840.1
MSTRG.1	Gm10253	ENSMUST00000194270
MSTRG.1	Syt11	ENSMUST00000107503;ENSMUST00000107505;ENSMUST0000009C
MSTRG.1	5830417	MSTRG.19843.1;ENSMUST00000135913;MSTRG.19843.3;MSTRG.
MSTRG.1	5830417	MSTRG.19844.1
MSTRG.1	1500004	ENSMUST00000184014;MSTRG.19845.2;ENSMUST00000183374;E
MSTRG.1	Gon4l	MSTRG.19846.1
MSTRG.1	Gm43713	ENSMUST00000198251
MSTRG.1	Gm23054	ENSMUST00000082804
MSTRG.1	Msto1	ENSMUST00000107494;ENSMUST00000137243;ENSMUST00000126
MSTRG.1	Atp1a4	ENSMUST00000111243;ENSMUST00000193316
MSTRG.1	Dap3	MSTRG.19850.1;ENSMUST00000090938;ENSMUST00000173711;E
MSTRG.1	Ash1l	MSTRG.19851.1;MSTRG.19851.2;MSTRG.19851.4;MSTRG.19851
MSTRG.1	Ash1l	MSTRG.19852.1
MSTRG.1	Gm26465	MSTRG.19853.1
MSTRG.1	Ash1l	MSTRG.19854.1

MSTRG.1	Gm26465	ENSMUST00000178517
MSTRG.1	Ash1l	MSTRG.19856.1
MSTRG.1	Gm24403	ENSMUST00000104616
MSTRG.1	Pklr	ENSMUST00000151591;ENSMUST00000107482;ENSMUST0000014[illegible]
MSTRG.1	Hcn3	ENSMUST00000029686;ENSMUST00000132156;ENSMUST0000012[illegible]
MSTRG.1	Gm37950	ENSMUST00000191616
MSTRG.1	Clk2;Sc[illegible]	ENSMUST00000121212;ENSMUST00000152205;ENSMUST0000009[illegible]
MSTRG.1	Gm16069	ENSMUST00000131146
MSTRG.1	Fam189b	ENSMUST00000140473;ENSMUST00000041913;MSTRG.19862.2;[illegible]
MSTRG.1	Gba	ENSMUST00000077367;ENSMUST00000197738;ENSMUST0000016[illegible]
MSTRG.1	Mtx1	ENSMUST00000073572;ENSMUST00000174218;ENSMUST0000013[illegible]
MSTRG.1	Gm43737	ENSMUST00000199915
MSTRG.1	Thbs3	ENSMUST00000029682;ENSMUST00000142051;ENSMUST0000012[illegible]
MSTRG.1	Mir92b	ENSMUST00000102300
MSTRG.1	Muc1	ENSMUST00000041142;ENSMUST00000146844;ENSMUST0000013[illegible]
MSTRG.1	Igsf8	ENSMUST00000155363;ENSMUST00000139528;ENSMUST0000012[illegible]
MSTRG.1	Trim46	ENSMUST00000107464;ENSMUST00000090924;ENSMUST0000013[illegible]
MSTRG.1	Krtcap2	ENSMUST00000126691;ENSMUST00000040888;ENSMUST0000016[illegible]
MSTRG.1	Efna4	ENSMUST00000029674
MSTRG.1	4731419	ENSMUST00000180791
MSTRG.1	Adam15	ENSMUST00000184651;ENSMUST00000107448;ENSMUST0000002[illegible]
MSTRG.1	Dcst1	ENSMUST00000070820;ENSMUST00000139467;ENSMUST0000020[illegible]
MSTRG.1	Dcst2	ENSMUST00000208216
MSTRG.1	Zbtb7b	ENSMUST00000107433;ENSMUST00000107435;ENSMUST00000107
MSTRG.1	Gm15417	ENSMUST00000145400;ENSMUST00000126926;ENSMUST0000015[illegible]
MSTRG.1	Atp1a2	ENSMUST00000085913;ENSMUST00000131751;ENSMUST00000191
MSTRG.1	Lenep;F[illegible]	ENSMUST00000057431;ENSMUST00000050398;ENSMUST0000012[illegible]
MSTRG.1	Pmvk	ENSMUST00000029564;ENSMUST00000107410;ENSMUST00000184
MSTRG.1	Kcnn3	ENSMUST00000000811;ENSMUST00000124584
MSTRG.1	Kcnn3	MSTRG.19883.1
MSTRG.1	Cks1b	ENSMUST00000029679;ENSMUST00000107422;ENSMUST0000018[illegible]
MSTRG.1	Shc1	ENSMUST00000039110;ENSMUST00000125036;ENSMUST00000191
MSTRG.1	Pygo2	ENSMUST00000136573;ENSMUST00000060061;ENSMUST0000014[illegible]
MSTRG.1	Pbxip1	ENSMUST00000038942;ENSMUST00000141340;ENSMUST0000013[illegible]
MSTRG.1	Ube2q1	ENSMUST00000038356;ENSMUST00000199427;MSTRG.19888.2;[illegible]
MSTRG.1	Adar	ENSMUST00000197253;MSTRG.19889.1;ENSMUST00000098924;[illegible]
MSTRG.1	Kcnj9	ENSMUST00000062387;ENSMUST00000194204;ENSMUST00000194
MSTRG.1	Chrnb2	ENSMUST00000200558;ENSMUST00000199372;ENSMUST0000002[illegible]
MSTRG.1	4632404[illegible]	MSTRG.19891.1;ENSMUST00000038450;ENSMUST00000195940
MSTRG.1	Il6ra	ENSMUST00000197679;ENSMUST00000029559
MSTRG.1	Il6ra	MSTRG.19893.1
MSTRG.1	Il6ra	MSTRG.19894.1
MSTRG.1	Il6ra	MSTRG.19895.1
MSTRG.1	Il6ra	MSTRG.19896.1
MSTRG.1	Il6ra	MSTRG.19897.1
MSTRG.1	Gm42809	ENSMUST00000198429
MSTRG.1	Tpm3	ENSMUST00000121503;ENSMUST00000119570;ENSMUST0000002[illegible]
MSTRG.1	Gm37211	ENSMUST00000193392
MSTRG.1	Gm36937	ENSMUST00000192957
MSTRG.1	Jtb	MSTRG.19900.2;ENSMUST00000029546;MSTRG.19900.1;ENSMUS[illegible]
MSTRG.1	Tpm3	MSTRG.19901.1
MSTRG.1	Nup210l	ENSMUST00000029548;ENSMUST00000200410;MSTRG.19902.3
MSTRG.1	Gm23723	ENSMUST00000082996
MSTRG.1	Gm43712	ENSMUST00000198020
MSTRG.1	Gm24185	ENSMUST00000122578
MSTRG.1	Rps27	ENSMUST00000196205;ENSMUST00000199031;ENSMUST0000020[illegible]
MSTRG.1	Rab13	ENSMUST00000107373;ENSMUST00000198809;ENSMUST0000006[illegible]

MSTRG.1	Atp8b2	ENSMUST00000069805;ENSMUST00000107396;ENSMUST00000163
MSTRG.1	Aqp10-p	ENSMUST00000199555
MSTRG.1	Tagln2	ENSMUST00000138296;ENSMUST00000111230;ENSMUST00000192
MSTRG.1	Gm24046	ENSMUST00000083111
MSTRG.1	Hax1	ENSMUST00000079724;ENSMUST00000197767;ENSMUST00000197
MSTRG.1	Gm19710	ENSMUST00000200257;ENSMUST00000200320
MSTRG.1	Ubap2l	ENSMUST00000198322;ENSMUST00000029553;ENSMUST00000064
MSTRG.1	Gm24608	ENSMUST00000104381
MSTRG.1	Mir7669	ENSMUST00000183442
MSTRG.1	Ubap2l	ENSMUST00000196952
MSTRG.1	4933434	MSTRG.19917.1;ENSMUST00000160640;ENSMUST00000159202;E
MSTRG.1	Gm16540	ENSMUST00000160263
MSTRG.1	4930537	ENSMUST00000200437
MSTRG.1	Cfap45	ENSMUST00000194051;ENSMUST00000085894;ENSMUST0000016C
MSTRG.1	Gatad2b	ENSMUST00000199754;ENSMUST00000200373;MSTRG.19920.2;M
MSTRG.1	Gm45477	ENSMUST00000200213
MSTRG.1	Gm45354	ENSMUST00000199820
MSTRG.1	Slc39a1	ENSMUST00000015467;MSTRG.19923.2
MSTRG.1	Crtc2	ENSMUST00000029545;ENSMUST00000130002;ENSMUST0000014S
MSTRG.1	Dennd4b	ENSMUST00000129564;ENSMUST00000098914;MSTRG.19925.6;M
MSTRG.1	Mir7012	ENSMUST00000183900
MSTRG.1	Dennd4b	MSTRG.19927.1
MSTRG.19928		MSTRG.19928.1
MSTRG.1	Slc27a3	ENSMUST00000029541;ENSMUST00000144572;ENSMUST00000132
MSTRG.1	4933439	ENSMUST00000132370;MSTRG.1993.2;ENSMUST00000140307;EN
MSTRG.1	Ints3	ENSMUST00000071488;MSTRG.19930.2;ENSMUST00000029542;E
MSTRG.1	Npr1	ENSMUST00000029540;ENSMUST00000152510;ENSMUST00000142
MSTRG.1	Gm16048	ENSMUST00000134250
MSTRG.1	Chtop	MSTRG.19933.1
MSTRG.1	Ilf2	ENSMUST00000196769;ENSMUST00000197644;ENSMUST0000019E
MSTRG.1	Snapin	ENSMUST00000107352;MSTRG.19935.1;ENSMUST00000149884;E
MSTRG.1	S100a1	ENSMUST00000060738;ENSMUST00000107340
MSTRG.1	S100a13	MSTRG.19937.1
MSTRG.1	S100a14	ENSMUST00000199538;ENSMUST00000164481;ENSMUST00000167
MSTRG.1	Chtop	ENSMUST00000107343;ENSMUST00000001043;ENSMUST00000107
MSTRG.1	Vsig8	ENSMUST00000061835;ENSMUST00000177086
MSTRG.1	S100a16	ENSMUST00000098911;ENSMUST00000107335;ENSMUST00000107
MSTRG.1	Gm23464	ENSMUST00000083145
MSTRG.1	S100a3;	ENSMUST00000199225;ENSMUST00000200508;ENSMUST0000020C
MSTRG.1	S100a2	ENSMUST00000179550
MSTRG.1	S100a6	ENSMUST00000001051;ENSMUST00000197189;ENSMUST0000019E
MSTRG.1	S100a8	ENSMUST00000069927;ENSMUST00000198156
MSTRG.1	S100a9	ENSMUST00000069960;ENSMUST00000117167
MSTRG.19947		MSTRG.19947.1
MSTRG.19948		MSTRG.19948.1
MSTRG.19949		MSTRG.19949.1
MSTRG.1	Ackr1;C	ENSMUST00000194298;ENSMUST00000038227;ENSMUST00000194
MSTRG.19950		MSTRG.19950.1
MSTRG.1	Gm9774	ENSMUST00000192538;ENSMUST00000047300
MSTRG.1	Sprr1a	ENSMUST00000054599
MSTRG.1	Gm4202	ENSMUST00000169025
MSTRG.19954		MSTRG.19954.1
MSTRG.1	Gm37361	ENSMUST00000194345
MSTRG.1	S100a11	ENSMUST00000029515
MSTRG.1	S100a10	ENSMUST00000045756;ENSMUST00000148400;ENSMUST0000017C
MSTRG.1	Gm36070	MSTRG.19958.1;ENSMUST00000196531
MSTRG.1	C2cd4d	MSTRG.19959.1;ENSMUST00000169433;ENSMUST00000197526;M

MSTRG.1	Aim2	ENSMUST00000147604;ENSMUST00000174263;MSTRG.1996.2;EN
MSTRG.1	Gm38411	MSTRG.19960.1;MSTRG.19960.2;MSTRG.19960.3;MSTRG.1996C
MSTRG.1	Rorc	MSTRG.19961.1;MSTRG.19961.2;ENSMUST00000029795;ENSMUS
MSTRG.1	Gm38411	MSTRG.19962.1
MSTRG.1	Rorc	MSTRG.19963.1
MSTRG.1	Gm43401	ENSMUST00000198829
MSTRG.1	Lingo4	ENSMUST00000050975
MSTRG.1	Lingo4	MSTRG.19966.1
MSTRG.1	1700040	ENSMUST00000181305
MSTRG.1	Tdrkh	ENSMUST00000166032;ENSMUST00000197876;ENSMUST0000020C
MSTRG.1	Gm42463	ENSMUST00000198041
MSTRG.1	Aim2	MSTRG.1997.1
MSTRG.1	Gm42464	ENSMUST00000198111;ENSMUST00000196717
MSTRG.1	Oaz3	ENSMUST00000199318;ENSMUST00000199678;ENSMUST00000204
MSTRG.1	Mrpl9	ENSMUST00000199009;ENSMUST00000029786;ENSMUST0000019
MSTRG.1	Gm30023	ENSMUST00000197970
MSTRG.1	Riiad1	ENSMUST00000029785;ENSMUST00000183915;ENSMUST00000184
MSTRG.1	Snx27	MSTRG.19975.1
MSTRG.1	Celf3	ENSMUST00000198316;ENSMUST00000197558;ENSMUST0000020C
MSTRG.1	Celf3	MSTRG.19977.1
MSTRG.1	Snx27	MSTRG.19978.2;MSTRG.19978.1;ENSMUST00000029783;ENSMUS
MSTRG.1	Gm43773	ENSMUST00000198548
MSTRG.1	Aim2	MSTRG.1998.1
MSTRG.1	Gm43774	ENSMUST00000196327
MSTRG.1	Snx27	MSTRG.19981.1
MSTRG.1	Gm43768	ENSMUST00000198380
MSTRG.1	Psmd4	ENSMUST00000107237;ENSMUST00000071664;ENSMUST0000014C
MSTRG.1	Psmd4	MSTRG.19984.1
MSTRG.1	Vps72	ENSMUST00000009102;ENSMUST00000136076;MSTRG.19985.3;E
MSTRG.1	Vps72	MSTRG.19986.1
MSTRG.1	Pip5k1a	ENSMUST00000107236;ENSMUST00000107233;ENSMUST00000107
MSTRG.1	Gm24920	ENSMUST00000083018
MSTRG.1	Gm22301	ENSMUST00000082656
MSTRG.1	Ifi206	ENSMUST00000160565;ENSMUST00000169797
MSTRG.1	Pip5k1a	MSTRG.19990.1
MSTRG.1	Tuft1	ENSMUST00000006123;ENSMUST00000196733;ENSMUST0000019
MSTRG.1	Tuft1	MSTRG.19993.1
MSTRG.1	Tuft1	MSTRG.19994.1
MSTRG.1	Gm15234	ENSMUST00000141485
MSTRG.1	BC02176	ENSMUST00000136988;ENSMUST00000126387
MSTRG.1	Selenbp	ENSMUST00000090848;ENSMUST00000132162;ENSMUST00000173
MSTRG.1	Gm15264	ENSMUST00000118050
MSTRG.1	Gm43711	ENSMUST00000195994
MSTRG.2	Gm6119	ENSMUST00000193443
MSTRG.2	Gm17101	ENSMUST00000169520
MSTRG.2	Gm37862	ENSMUST00000193079
MSTRG.2	Ifi214	ENSMUST00000139092;ENSMUST00000090406;ENSMUST00000097
MSTRG.2	Gm15264	MSTRG.20000.1
MSTRG.2	Cgn	ENSMUST00000107272;ENSMUST00000107273;ENSMUST00000153
MSTRG.2	Pogz	MSTRG.20002.1;MSTRG.20002.2
MSTRG.2	Gm15263	ENSMUST00000121556
MSTRG.2	Pogz	ENSMUST00000107270;ENSMUST00000107265;ENSMUST0000012
MSTRG.2	Psmb4	ENSMUST00000005923;ENSMUST00000134133;MSTRG.20005.2;E
MSTRG.2	Selenbp	ENSMUST00000140757;ENSMUST00000090839;ENSMUST0000013
MSTRG.20008		MSTRG.20008.1
MSTRG.2	Rfx5	ENSMUST00000107260;ENSMUST00000142311;MSTRG.20009.2;E
MSTRG.2	Gm44481	ENSMUST00000197288

MSTRG.2	B230398	ENSMUST00000125186
MSTRG.20011		MSTRG.20011.1
MSTRG.2	Pi4kb;M	ENSMUST00000072287;ENSMUST00000167008;ENSMUST00000107
MSTRG.2	A730011	ENSMUST00000141787;MSTRG.20013.1;ENSMUST00000135483;M
MSTRG.2	Zfp687	ENSMUST00000146169;ENSMUST00000019482;ENSMUST00000126
MSTRG.2	4930481	MSTRG.20015.1;ENSMUST00000128160;ENSMUST00000151207
MSTRG.2	Tmod4	ENSMUST00000131597;ENSMUST00000005769;ENSMUST00000107
MSTRG.2	Scnm1	ENSMUST00000172572;ENSMUST00000174508;MSTRG.20017.2;E
MSTRG.2	Lysmd1	ENSMUST00000066386
MSTRG.2	Ifi213	ENSMUST00000150649;ENSMUST00000178186;ENSMUST00000097
MSTRG.2	Tnfaip8	ENSMUST00000013851;MSTRG.20020.2;MSTRG.20020.1
MSTRG.2	Gm9169	ENSMUST00000182930
MSTRG.2	Sema6c	ENSMUST00000142306;ENSMUST00000130814;ENSMUST00000131
MSTRG.2	Gabpb2	ENSMUST00000107209;ENSMUST00000136139;ENSMUST00000098
MSTRG.2	Gabpb2	MSTRG.20024.1
MSTRG.2	Gm16740	ENSMUST00000181819;MSTRG.20025.2
MSTRG.2	Mllt11	ENSMUST00000065482;ENSMUST00000196025;ENSMUST00000198
MSTRG.2	Cdc42se	ENSMUST00000053872;ENSMUST00000149804;MSTRG.20027.2;E
MSTRG.2	Gm128;B	ENSMUST00000090815;ENSMUST00000107195;ENSMUST00000137
MSTRG.2	Ctss	ENSMUST00000015667;ENSMUST00000116304
MSTRG.2	Ifi209	ENSMUST00000056071;ENSMUST00000193727;MSTRG.2003.2;MS
MSTRG.2	Hmgb1-p	ENSMUST00000121058
MSTRG.2	Ctss	MSTRG.20031.1
MSTRG.2	Prune1	ENSMUST00000015855;ENSMUST00000148959
MSTRG.2	Prune1	MSTRG.20033.1
MSTRG.2	Mindy1	ENSMUST00000152640;ENSMUST00000135249;ENSMUST00000147
MSTRG.2	Anxa9;6	ENSMUST00000107183;ENSMUST00000015846;ENSMUST00000164
MSTRG.2	Cers2	ENSMUST00000129267;ENSMUST00000015858;ENSMUST00000133
MSTRG.2	Setdb1	ENSMUST00000124638;MSTRG.20037.1;MSTRG.20037.2;ENSMUS
MSTRG.2	Gm42578	ENSMUST00000199960
MSTRG.2	Gm37500	ENSMUST00000192059
MSTRG.2	Gm44339	ENSMUST00000200598
MSTRG.2	Arnt	ENSMUST00000147765;ENSMUST00000102749;ENSMUST00000090
MSTRG.2	Gm42672	ENSMUST00000197821
MSTRG.2	Ctsk	ENSMUST00000015664
MSTRG.2	Golph3l	ENSMUST00000177390;ENSMUST00000060323;ENSMUST00000098
MSTRG.2	Rps10-p	ENSMUST00000074353
MSTRG.20045		MSTRG.20045.1
MSTRG.2	Aph1a	ENSMUST00000015894;ENSMUST00000197081;ENSMUST00000056
MSTRG.2	Car14	ENSMUST00000036181;ENSMUST00000147962;ENSMUST00000149
MSTRG.2	Anp32e	ENSMUST00000165307;ENSMUST00000015893;ENSMUST00000169
MSTRG.2	Gm43375	ENSMUST00000196705
MSTRG.2	Ifi208	ENSMUST00000182880;MSTRG.2005.2;ENSMUST00000085876;MS
MSTRG.2	Plekho1	ENSMUST00000015889;ENSMUST00000123006;ENSMUST00000130
MSTRG.2	Vps45	ENSMUST00000015891;ENSMUST00000131281;ENSMUST00000140
MSTRG.2	Vps45	MSTRG.20052.1
MSTRG.2	Vps45	MSTRG.20053.1
MSTRG.2	Otud7b	ENSMUST00000132980;ENSMUST00000138206;ENSMUST00000035
MSTRG.2	Otud7b	MSTRG.20055.1
MSTRG.2	Gm24830	ENSMUST00000102024
MSTRG.2	Rnu1b1	ENSMUST00000178250
MSTRG.2	Rnu1b2	ENSMUST00000178073
MSTRG.2	Gm21960	ENSMUST00000181924
MSTRG.2	Fh1	ENSMUST00000027810;ENSMUST00000130996;ENSMUST00000133
MSTRG.2	Terc	ENSMUST00000082862
MSTRG.2	BC10736	ENSMUST00000093126
MSTRG.2	Gm26232	ENSMUST00000083360

MSTRG.2	BC10736	MSTRG.20063.1
MSTRG.2	Gm10685	ENSMUST00000196120
MSTRG.2	Rnu1b6	ENSMUST00000083839
MSTRG.2	Gm43534	ENSMUST00000197685
MSTRG.2	Gm25890	ENSMUST00000177643
MSTRG.2	Mtmr11	MSTRG.20068.1;ENSMUST00000147245;ENSMUST00000054356;E
MSTRG.2	Gm17690	ENSMUST00000180958;ENSMUST00000199721
MSTRG.2	Kmo	ENSMUST00000140474;ENSMUST00000040250;ENSMUST00000142
MSTRG.2	Sf3b4;S	ENSMUST00000076372;MSTRG.20070.1;ENSMUST00000153977;E
MSTRG.2	Gm22027	ENSMUST00000122737
MSTRG.2	Bola1	ENSMUST00000016087;ENSMUST00000107099;ENSMUST00000177
MSTRG.2	Bola1;G	MSTRG.20073.1;ENSMUST00000177363
MSTRG.2	Gm15444	ENSMUST00000148335
MSTRG.2	Hist2h2	ENSMUST00000073115
MSTRG.2	Hist2h2	ENSMUST00000090782
MSTRG.2	Gm44492	ENSMUST00000197012
MSTRG.2	Hist2h2	ENSMUST00000090781
MSTRG.2	Hist2h3	ENSMUST00000167403;MSTRG.20079.1
MSTRG.2	Opn3;Ch	ENSMUST00000027809;ENSMUST00000209720;ENSMUST0000021C
MSTRG.2	Hist2h3	MSTRG.20080.1;ENSMUST00000078756;ENSMUST00000090779;E
MSTRG.2	Gm20632	ENSMUST00000176760;ENSMUST00000198228
MSTRG.2	Hist2h2	ENSMUST00000074976
MSTRG.2	Gm20633	ENSMUST00000176742;ENSMUST00000197780
MSTRG.2	Gm20627	ENSMUST00000176275;ENSMUST00000176143
MSTRG.2	Hist2h4	ENSMUST00000171473
MSTRG.2	Gm38227	ENSMUST00000193036
MSTRG.2	Hist2h3	ENSMUST00000098843
MSTRG.2	Gm42743	ENSMUST00000051089;ENSMUST00000177113
MSTRG.2	Ensa	ENSMUST00000199421;ENSMUST00000058230;ENSMUST00000037
MSTRG.2	Opn3	MSTRG.2009.1
MSTRG.2	E330034	ENSMUST00000200640
MSTRG.2	E330034	MSTRG.20091.1
MSTRG.2	Mcl1	MSTRG.20092.1;MSTRG.20092.2;MSTRG.20092.3;ENSMUST000C
MSTRG.20093		MSTRG.20093.1;MSTRG.20093.2
MSTRG.20094		MSTRG.20094.1;MSTRG.20094.2
MSTRG.20095		MSTRG.20095.1
MSTRG.2	Adamtsl	MSTRG.20096.1;MSTRG.20096.2;MSTRG.20096.3;MSTRG.2009€
MSTRG.20097		MSTRG.20097.1
MSTRG.20098		MSTRG.20098.1
MSTRG.2	Ecm1;Mi	ENSMUST00000117507;ENSMUST00000029753;ENSMUST00000144
MSTRG.2	Gm38286	ENSMUST00000195128
MSTRG.2	Ifi207;	ENSMUST00000042610;MSTRG.2010.2;ENSMUST00000127730;EN
MSTRG.2	Tars2;R	ENSMUST00000199869;ENSMUST00000196077;ENSMUST0000019S
MSTRG.2	Gm43436	ENSMUST00000199308
MSTRG.2	Gm26361	ENSMUST00000158868
MSTRG.2	Gm17815	ENSMUST00000198305
MSTRG.2	Rprd2	MSTRG.20104.1
MSTRG.2	Prpf3	MSTRG.20105.1
MSTRG.2	Prpf3	ENSMUST00000015892;ENSMUST00000159901;ENSMUST0000016C
MSTRG.2	Gm26594	ENSMUST00000180383
MSTRG.2	Mrps21	ENSMUST00000067298;ENSMUST00000072587;ENSMUST00000132
MSTRG.2	Mrps21	MSTRG.20109.1
MSTRG.2	Ifi203	MSTRG.2011.1;MSTRG.2011.2
MSTRG.2	C920021	ENSMUST00000142433;ENSMUST00000120398;ENSMUST0000014З
MSTRG.2	C920021	MSTRG.20111.1
MSTRG.2	Ciart	ENSMUST00000159739;ENSMUST00000036418;ENSMUST00000161
MSTRG.2	BC02852	ENSMUST00000090476;ENSMUST00000036360;ENSMUST00000171

MSTRG.2	Acp6	ENSMUST00000090759;ENSMUST00000199346;ENSMUST00000133
MSTRG.2	Bcl9	ENSMUST00000046521;ENSMUST00000166341;MSTRG.20115.2;E
MSTRG.2	Gm42508	ENSMUST00000199972
MSTRG.2	Gm43073	ENSMUST00000198200
MSTRG.2	Bcl9	MSTRG.20118.1
MSTRG.2	Chd1l	ENSMUST00000029730;ENSMUST00000195962;ENSMUST00000197
MSTRG.2	Mndal	MSTRG.2012.1
MSTRG.2	Chd1l	MSTRG.20120.1
MSTRG.2	Chd1l	MSTRG.20121.1
MSTRG.2	Gm43075	ENSMUST00000198613
MSTRG.2	Fmo5	ENSMUST00000107050;ENSMUST00000029729;ENSMUST00000133
MSTRG.2	Prkab2	ENSMUST00000045743;ENSMUST00000130924;ENSMUST00000132
MSTRG.2	Gm17651	ENSMUST00000166227
MSTRG.2	Sec22b	ENSMUST00000029476;ENSMUST00000130778;ENSMUST00000130
MSTRG.2	Gm5544	ENSMUST00000098828
MSTRG.2	Pde4dip	ENSMUST00000168438;ENSMUST00000090750;ENSMUST00000184
MSTRG.2	Gm31305	ENSMUST00000198557;ENSMUST00000200435
MSTRG.2	Ifi203	MSTRG.2013.1
MSTRG.2	Gm22614	ENSMUST00000178300
MSTRG.2	Polr3c	MSTRG.20131.1
MSTRG.2	Hfe2	ENSMUST00000049208
MSTRG.2	Gm15441	ENSMUST00000098839;ENSMUST00000107095
MSTRG.2	Txnip	ENSMUST00000074519;ENSMUST00000049093;ENSMUST00000151
MSTRG.2	Gm16253	ENSMUST00000148290
MSTRG.2	Polr3gl	MSTRG.20136.1;ENSMUST00000091924;ENSMUST00000131641;E
MSTRG.2	Polr3gl	MSTRG.20137.1
MSTRG.2	Ankrd34	ENSMUST00000058943
MSTRG.2	Lix1l	ENSMUST00000062058;ENSMUST00000199166
MSTRG.2	Ifi203	MSTRG.2014.2;MSTRG.2014.3;MSTRG.2014.1
MSTRG.2	6330549	ENSMUST00000160818;ENSMUST00000059190;ENSMUST00000159
MSTRG.2	Rbm8a	ENSMUST00000048915;ENSMUST00000196456;ENSMUST00000198
MSTRG.2	Pex11b;	ENSMUST00000144962;ENSMUST00000118557;MSTRG.20142.2;E
MSTRG.2	Rpl21-p	ENSMUST00000077278
MSTRG.2	Ankrd35	ENSMUST00000048427;ENSMUST00000122960;ENSMUST00000130
MSTRG.2	Pias3	ENSMUST00000107076;ENSMUST00000162934;ENSMUST00000162
MSTRG.2	Nudt17	ENSMUST00000200387;ENSMUST00000196773;ENSMUST00000171
MSTRG.2	Polr3c;	ENSMUST00000029741;ENSMUST00000154679;MSTRG.20147.3;M
MSTRG.2	Polr3c	MSTRG.20148.1
MSTRG.2	Gm42783	ENSMUST00000197075
MSTRG.2	Gm7897	ENSMUST00000193981
MSTRG.2	Rnf115	ENSMUST00000029740;MSTRG.20150.2;ENSMUST00000199051;M
MSTRG.2	Gm42782	ENSMUST00000197286
MSTRG.20152		MSTRG.20152.1
MSTRG.2	Cd160	ENSMUST00000107074;ENSMUST00000165991;ENSMUST00000047
MSTRG.2	Pdzk1	ENSMUST00000058865;ENSMUST00000107070;ENSMUST00000128
MSTRG.2	4930442	ENSMUST00000145865
MSTRG.2	Gpr89	ENSMUST00000029738;ENSMUST00000146645;ENSMUST00000135
MSTRG.2	Gja5	ENSMUST00000199597;ENSMUST00000072600
MSTRG.2	Gja8	ENSMUST00000062944
MSTRG.2	Ifi203	MSTRG.2016.1
MSTRG.2	Phgdh	ENSMUST00000065793;ENSMUST00000153694;ENSMUST00000152
MSTRG.2	Phgdh	MSTRG.20162.1
MSTRG.2	Hmgcs2	ENSMUST00000090746
MSTRG.2	Gm43189	ENSMUST00000196040
MSTRG.2	Notch2;	ENSMUST00000198324;ENSMUST00000079812;ENSMUST00000200
MSTRG.2	Gm42819	ENSMUST00000199630
MSTRG.2	Wars2	ENSMUST00000004343;ENSMUST00000135960;ENSMUST00000145

MSTRG.2 Gm43024 ENSMUST00000196770
MSTRG.2 Wars2 MSTRG.20169.1
MSTRG.2 Mndal MSTRG.2017.1
MSTRG.2 Wars2 MSTRG.20170.1
MSTRG.2 Gm18982 ENSMUST00000198118
MSTRG.2 Wars2 MSTRG.20172.1
MSTRG.2 Mir6481 ENSMUST00000184617
MSTRG.2 Wars2 MSTRG.20174.1
MSTRG.2 Wars2 MSTRG.20175.1
MSTRG.2 Gm42717 ENSMUST00000197309
MSTRG.2 Fam46c ENSMUST00000128107;ENSMUST00000139833;ENSMUST00000061
MSTRG.2 Gm12474 ENSMUST00000122898
MSTRG.2 Gm42868 ENSMUST00000199168
MSTRG.2 Ifi202b ENSMUST00000000266
MSTRG.2 Spag17 ENSMUST00000127518;ENSMUST00000164539;ENSMUST00000152
MSTRG.2 Spag17o ENSMUST00000145279;ENSMUST00000137776
MSTRG.2 Wdr3 ENSMUST00000052120;ENSMUST00000135812;ENSMUST0000014[illegible]
MSTRG.2 Gdap2 ENSMUST00000029459;ENSMUST00000106997;MSTRG.20185.2;[illegible]
MSTRG.2 Gdap2 MSTRG.20186.1
MSTRG.2 Gdap2 MSTRG.20187.1
MSTRG.20188 MSTRG.20188.1
MSTRG.2 Man1a2 ENSMUST00000008907;MSTRG.20189.2;ENSMUST00000133642;[illegible]
MSTRG.2 Exo1 ENSMUST00000039725;ENSMUST00000195196;ENSMUST00000194
MSTRG.2 Gm43466 ENSMUST00000197836
MSTRG.2 Gm42869 ENSMUST00000199953
MSTRG.2 Gm43462 ENSMUST00000200265
MSTRG.2 Gm43463 ENSMUST00000195967
MSTRG.2 Gm12485 ENSMUST00000119641
MSTRG.2 Gm43464 ENSMUST00000198003
MSTRG.2 Ttf2 ENSMUST00000076941;ENSMUST00000145811;ENSMUST00000127
MSTRG.2 Gm12488 ENSMUST00000122449
MSTRG.2 Ptgfrn ENSMUST00000102694;ENSMUST00000198037;ENSMUST0000020[illegible]
MSTRG.2 Gm43465 ENSMUST00000200367
MSTRG.2 Gm37880 ENSMUST00000192216
MSTRG.2 Exo1 MSTRG.2020.1
MSTRG.20200 MSTRG.20200.1;MSTRG.20200.2
MSTRG.2 Gm12490 ENSMUST00000120255
MSTRG.2 Cd2 MSTRG.20203.1;MSTRG.20203.2;ENSMUST00000029456;ENSMUS[illegible]
MSTRG.20204 MSTRG.20204.1
MSTRG.2 Igsf3 ENSMUST00000198995;ENSMUST00000043983;MSTRG.20205.1;[illegible]
MSTRG.2 Igsf3 MSTRG.20206.1
MSTRG.2 Gm42939 ENSMUST00000197514
MSTRG.2 Gm42938 ENSMUST00000198414
MSTRG.2 Gm42538 ENSMUST00000199431;ENSMUST00000196233
MSTRG.2 Exo1 MSTRG.2021.1
MSTRG.2 Atp1a1 MSTRG.20210.1;ENSMUST00000036493;ENSMUST00000130649;[illegible]
MSTRG.2 Gm42937 ENSMUST00000197360
MSTRG.2 Gm42941 ENSMUST00000200347
MSTRG.2 Slc22a1 ENSMUST00000106928;ENSMUST00000190824;ENSMUST00000182
MSTRG.2 Slc22a1 MSTRG.20214.1
MSTRG.2 Vangl1 ENSMUST00000029453;ENSMUST00000159388;ENSMUST00000161
MSTRG.2 Vangl1 MSTRG.20216.1
MSTRG.2 Gm16160 ENSMUST00000160105
MSTRG.2 Gm42682 ENSMUST00000198349
MSTRG.2 Tspan2o ENSMUST00000146624
MSTRG.2 Gm23805 ENSMUST00000180129
MSTRG.2 Tspan2 ENSMUST00000029451;ENSMUST00000196611;ENSMUST0000011[illegible]

MSTRG.2	Tspan2	MSTRG.20221.1
MSTRG.2	Tshb	ENSMUST00000196958;ENSMUST00000029450
MSTRG.2	Gm42681	ENSMUST00000197999
MSTRG.2	Sycp1	ENSMUST00000029448;ENSMUST00000198651;ENSMUST0000019€
MSTRG.2	Sike1	ENSMUST00000029447;ENSMUST00000151065;MSTRG.20228.3;E
MSTRG.2	Gm43062	ENSMUST00000196302
MSTRG.2	Cep170	ENSMUST00000057037;ENSMUST00000192664;ENSMUST0000019£
MSTRG.2	Ampd1	ENSMUST00000090715;ENSMUST00000155034;ENSMUST0000019¢
MSTRG.2	Gm23820	ENSMUST00000158450
MSTRG.2	Gm43063	ENSMUST00000198198
MSTRG.2	Dennd2c	ENSMUST00000173206;ENSMUST00000166143;MSTRG.20233.3;E
MSTRG.2	Bcas2	ENSMUST00000005830;ENSMUST00000136937;ENSMUST0000013¢
MSTRG.2	Gm27024	ENSMUST00000182561
MSTRG.2	Csde1	ENSMUST00000029446;ENSMUST00000198180;ENSMUST00000197
MSTRG.2	Nras	ENSMUST00000029445;ENSMUST00000200457;ENSMUST0000020C
MSTRG.20238		MSTRG.20238.1
MSTRG.2	Gm25009	ENSMUST00000158404
MSTRG.2	Cep170	MSTRG.2024.1
MSTRG.2	Trim33	ENSMUST00000029444;ENSMUST00000106860;MSTRG.20240.3;E
MSTRG.2	Trim33	MSTRG.20241.1
MSTRG.2	Trim33	MSTRG.20242.1
MSTRG.2	Trim33	MSTRG.20243.1
MSTRG.2	Trim33	MSTRG.20244.1
MSTRG.2	Trim33	MSTRG.20245.1
MSTRG.2	Gm43061	ENSMUST00000198850
MSTRG.2	Trim33	MSTRG.20247.1
MSTRG.2	Olfml3	ENSMUST00000157042;ENSMUST00000029440;ENSMUST0000016¢
MSTRG.2	Gm10964	ENSMUST00000106852
MSTRG.2	3110062(	ENSMUST00000192113
MSTRG.2	Hipk1;G	MSTRG.20250.1;MSTRG.20250.2;ENSMUST00000118317;ENSMUS
MSTRG.2	Gm15886	MSTRG.20251.1;MSTRG.20251.2;ENSMUST00000154868;ENSMUS
MSTRG.2	Dclre1b	ENSMUST00000063502;ENSMUST00000029435;ENSMUST00000147
MSTRG.2	Gm15471	ENSMUST00000149374;ENSMUST00000145893
MSTRG.2	Ap4b1;G	ENSMUST00000076599;ENSMUST00000199710;ENSMUST0000010€
MSTRG.2	Ptpn22;(	MSTRG.20255.2;MSTRG.20255.1;ENSMUST00000146071;ENSMUS
MSTRG.2	Gm15602	ENSMUST00000116162
MSTRG.2	Gm43582	MSTRG.20258.1
MSTRG.2	Gm43582	ENSMUST00000196149
MSTRG.2	Gm38104	ENSMUST00000195121
MSTRG.20260		MSTRG.20260.1
MSTRG.2	Slc16a1	ENSMUST00000046212;ENSMUST00000199375;MSTRG.20261.2
MSTRG.2	Gm43581	ENSMUST00000196766
MSTRG.2	Gm29561	ENSMUST00000187838
MSTRG.2	Lrig2;G	ENSMUST00000199180;MSTRG.20264.2;ENSMUST00000046316;E
MSTRG.2	Gm38412	ENSMUST00000199205
MSTRG.2	Gm9273	ENSMUST00000200606
MSTRG.2	Lrig2	MSTRG.20267.1
MSTRG.2	A530041I	ENSMUST00000199690
MSTRG.2	4930509	ENSMUST00000196088
MSTRG.2	Gm36992	ENSMUST00000195463
MSTRG.2	Rhoc	ENSMUST00000002303;ENSMUST00000106787;ENSMUST0000017€
MSTRG.2	Gm6485;(	ENSMUST00000160676;ENSMUST00000199762;ENSMUST0000013¢
MSTRG.2	Ppm1j	ENSMUST00000002298;ENSMUST00000197264
MSTRG.2	Rsbn1	ENSMUST00000051139;ENSMUST00000068879;ENSMUST00000151
MSTRG.2	Rsbn1	MSTRG.20274.1
MSTRG.2	Rsbn1	MSTRG.20275.1
MSTRG.2	Phtf1os	ENSMUST00000160110

MSTRG.2	Phtf1	ENSMUST00000117150;ENSMUST00000063717;ENSMUST0000014[illegible]
MSTRG.2	Phtf1	MSTRG.20278.1
MSTRG.2	Magi3	ENSMUST00000064371;MSTRG.20279.2;ENSMUST00000121198;[illegible]
MSTRG.2	Mir350	ENSMUST00000083639
MSTRG.2	Gm42696	ENSMUST00000199859
MSTRG.2	Magi3	MSTRG.20281.1
MSTRG.2	9530097[illegible]	ENSMUST00000197819
MSTRG.2	Gm23830	ENSMUST00000157391
MSTRG.2	Gm42700	ENSMUST00000199071
MSTRG.2	Gm42699	ENSMUST00000198104
MSTRG.2	Magi3	MSTRG.20286.1
MSTRG.2	Gm17956	ENSMUST00000199931
MSTRG.2	Magi3	MSTRG.20288.1
MSTRG.2	Gm42702	ENSMUST00000199515
MSTRG.2	Gm25288	ENSMUST00000122800
MSTRG.2	Gm42701	ENSMUST00000196117
MSTRG.2	Gm43258	ENSMUST00000200312
MSTRG.2	C030032[illegible]	ENSMUST00000196427
MSTRG.2	Magi3	MSTRG.20293.1
MSTRG.2	Gm43259	ENSMUST00000198446
MSTRG.2	Gm43260	ENSMUST00000197378
MSTRG.2	Gm43256	ENSMUST00000199995
MSTRG.2	Magi3	MSTRG.20297.1
MSTRG.2	Magi3	MSTRG.20298.1
MSTRG.2	Gm23077	ENSMUST00000157854
MSTRG.2	Adgrb3	MSTRG.203.1
MSTRG.2	Cep170	MSTRG.2030.1
MSTRG.2	Mov10	ENSMUST00000002297;ENSMUST00000168015;ENSMUST0000010[illegible]
MSTRG.2	Mov10	MSTRG.20302.1
MSTRG.2	Mov10	MSTRG.20303.1
MSTRG.2	Gm40117	ENSMUST00000198827
MSTRG.2	Cttnbp2[illegible]	ENSMUST00000077548;ENSMUST00000098763;ENSMUST00000197[illegible]
MSTRG.2	Cttnbp2[illegible]	MSTRG.20306.1
MSTRG.2	Cttnbp2[illegible]	MSTRG.20307.1
MSTRG.2	Fam212b	ENSMUST00000066610;ENSMUST00000098273
MSTRG.2	Capza1	MSTRG.20309.1;ENSMUST00000094028;ENSMUST00000195912;[illegible]
MSTRG.2	Cep170	MSTRG.2031.1
MSTRG.2	Gm42658	ENSMUST00000197199
MSTRG.2	Gm42659	ENSMUST00000196955
MSTRG.2	St7l	MSTRG.20312.1;MSTRG.20312.2;ENSMUST00000199186;MSTRG.[illegible]
MSTRG.2	St7l	MSTRG.20313.1
MSTRG.2	Gm4525	ENSMUST00000198831
MSTRG.20315		MSTRG.20315.1
MSTRG.2	Wnt2b	ENSMUST00000029429;ENSMUST00000198108
MSTRG.2	Gm43846	ENSMUST00000200502
MSTRG.2	Kcnd3os	ENSMUST00000143893;ENSMUST00000130683
MSTRG.2	Kcnd3	ENSMUST00000098761;ENSMUST00000141694;ENSMUST0000011[illegible]
MSTRG.2	Cep170	MSTRG.2032.1
MSTRG.2	Gm43847	ENSMUST00000200070
MSTRG.2	Ddx20	ENSMUST00000090680;ENSMUST00000132008
MSTRG.2	Gm43848	ENSMUST00000198060
MSTRG.2	Rap1a;G[illegible]	MSTRG.20323.1;ENSMUST00000090678;ENSMUST00000199969;[illegible]
MSTRG.2	Gm4540	ENSMUST00000170669
MSTRG.2	Atp5f1	ENSMUST00000118209;ENSMUST00000133320;ENSMUST0000019[illegible]
MSTRG.2	Wdr77;G[illegible]	ENSMUST00000010278;ENSMUST00000167642;ENSMUST00000127[illegible]
MSTRG.20327		MSTRG.20327.1
MSTRG.2	Cd53	ENSMUST00000038845;MSTRG.20328.1;MSTRG.20328.2

MSTRG.2	Lrif1;G	ENSMUST00000098751;ENSMUST00000156544;ENSMUST0000013C
MSTRG.2	Sdccag8	ENSMUST00000150429;ENSMUST00000056773;ENSMUST0000013
MSTRG.2	Gm38244	ENSMUST00000191737
MSTRG.2	2010016	MSTRG.20331.1;ENSMUST00000190356;ENSMUST00000189683;E
MSTRG.2	Dennd2d	ENSMUST00000139062;MSTRG.20332.1;ENSMUST00000029508;E
MSTRG.2	Cept1	ENSMUST00000068301;MSTRG.20333.4;ENSMUST00000121231;M
MSTRG.2	Cept1	MSTRG.20334.1
MSTRG.2	Dram2	ENSMUST00000067630;ENSMUST00000129053;ENSMUST00000134
MSTRG.2	Gm43070	ENSMUST00000196938
MSTRG.2	Dram2	MSTRG.20337.1
MSTRG.2	Gm43071	ENSMUST00000197233
MSTRG.2	Gm27008	ENSMUST00000182400;ENSMUST00000182414;MSTRG.20339.2;M
MSTRG.2	Gm38158	ENSMUST00000194617
MSTRG.2	Kcna3	ENSMUST00000052718
MSTRG.2	AI50443	ENSMUST00000070085
MSTRG.2	Gm27008	MSTRG.20342.1
MSTRG.2	Kcna2;A	MSTRG.20344.1;MSTRG.20344.2;ENSMUST00000060946;ENSMUS
MSTRG.2	Gm27008	MSTRG.20345.1
MSTRG.2	Lamtor5	ENSMUST00000145735;ENSMUST00000199317
MSTRG.2	Slc16a4	ENSMUST00000029502;ENSMUST00000153322;ENSMUST0000010E
MSTRG.2	Slc16a4	MSTRG.20348.1
MSTRG.2	Rbm15	MSTRG.20349.1;ENSMUST00000061772;ENSMUST00000197769
MSTRG.2	Gm38053	ENSMUST00000193938
MSTRG.20350		MSTRG.20350.1
MSTRG.2	Strip1	ENSMUST00000200049;ENSMUST00000064759;MSTRG.20351.1;E
MSTRG.2	Gm10961	ENSMUST00000106703
MSTRG.2	Ahcyl1	ENSMUST00000138091;MSTRG.20353.1;ENSMUST00000029490;E
MSTRG.2	Ahcyl1	MSTRG.20354.1
MSTRG.2	Csf1	ENSMUST00000118593;ENSMUST00000014743;ENSMUST0000012C
MSTRG.2	Gstm2	ENSMUST00000012348;ENSMUST00000066530;ENSMUST0000014C
MSTRG.2	Gstm1	ENSMUST00000004140;ENSMUST00000198532;ENSMUST0000015
MSTRG.2	Ampd2	ENSMUST00000136712;ENSMUST00000102638;ENSMUST00000102
MSTRG.2	Gm12500	ENSMUST00000186471;ENSMUST00000070502
MSTRG.2	Gm25993	ENSMUST00000175391
MSTRG.2	Gnai3	MSTRG.20360.1;ENSMUST00000000001
MSTRG.2	Gnai3	MSTRG.20361.1
MSTRG.2	Gpr61	ENSMUST00000062028
MSTRG.2	Gm12524	ENSMUST00000143109
MSTRG.2	Amigo1	ENSMUST00000050909;ENSMUST00000106659;ENSMUST0000010E
MSTRG.2	Cyb561d	ENSMUST00000106655;ENSMUST00000065664;ENSMUST0000015
MSTRG.2	Atxn7l2	ENSMUST00000117409;ENSMUST00000102633;ENSMUST00000141
MSTRG.2	1700010	ENSMUST00000125852
MSTRG.2	Psma5	ENSMUST00000090569;ENSMUST00000129708
MSTRG.2	Gm12523	ENSMUST00000148844
MSTRG.2	Gm37246	ENSMUST00000192665
MSTRG.2	Sort1	ENSMUST00000135636;ENSMUST00000102632;ENSMUST0000012S
MSTRG.2	Gm43099	ENSMUST00000198943
MSTRG.2	Mybphl	ENSMUST00000090563
MSTRG.2	Gm12522	ENSMUST00000142833
MSTRG.2	Psrc1	ENSMUST00000090561;ENSMUST00000133405;ENSMUST0000013C
MSTRG.2	Celsr2	ENSMUST00000090558;ENSMUST00000147251;ENSMUST0000013
MSTRG.2	Sars	MSTRG.20376.1;ENSMUST00000090553;MSTRG.20376.3;ENSMUS
MSTRG.2	Gm22942	ENSMUST00000158563
MSTRG.2	Scarna2	ENSMUST00000157560
MSTRG.2	Gm43435	ENSMUST00000199996
MSTRG.2	Sdccag8	MSTRG.2038.1
MSTRG.2	Tmem167	ENSMUST00000106622;ENSMUST00000090546

MSTRG.2	Taf13	ENSMUST00000056292;ENSMUST00000143054;ENSMUST00000139
MSTRG.2	Taf13	MSTRG.20382.1
MSTRG.2	Wdr47	ENSMUST00000051145;ENSMUST00000124731;ENSMUST00000139
MSTRG.2	Gm12517	ENSMUST00000122082
MSTRG.2	Wdr47	MSTRG.20385.1
MSTRG.2	Clcc1	ENSMUST00000106609;ENSMUST00000125274;ENSMUST00000130
MSTRG.2	Gpsm2	ENSMUST00000029482;ENSMUST00000154263;ENSMUST00000145
MSTRG.2	Gm27244	ENSMUST00000184260
MSTRG.2	Stxbp3	ENSMUST00000102621;ENSMUST00000124782;MSTRG.20389.2;E
MSTRG.2	Gm37490	ENSMUST00000195566
MSTRG.2	Stxbp3	MSTRG.20390.1
MSTRG.2	Prpf38b	ENSMUST00000129273;ENSMUST00000029480;ENSMUST00000199
MSTRG.2	Fam102b	ENSMUST00000046924;ENSMUST00000171143
MSTRG.2	Gm43221	ENSMUST00000197612
MSTRG.2	Slc25a2	ENSMUST00000029477;ENSMUST00000140786
MSTRG.2	Vav3	ENSMUST00000046864;ENSMUST00000126179;ENSMUST00000137
MSTRG.2	Vav3	MSTRG.20396.1
MSTRG.2	Vav3	MSTRG.20397.1
MSTRG.2	Vav3	MSTRG.20398.1
MSTRG.2	Vav3	MSTRG.20399.1
MSTRG.2	Sdccag8	MSTRG.2040.1
MSTRG.2	Vav3	MSTRG.20400.1
MSTRG.2	Vav3	MSTRG.20401.1
MSTRG.2	Vav3	MSTRG.20402.1
MSTRG.2	Vav3	MSTRG.20403.1
MSTRG.2	Prmt6	ENSMUST00000168412;ENSMUST00000190378;ENSMUST00000106
MSTRG.2	C130013	ENSMUST00000197569
MSTRG.2	Amy2-ps	MSTRG.20407.1
MSTRG.20408		MSTRG.20408.1
MSTRG.2	Amy1;Rn	ENSMUST00000067980;ENSMUST00000106540;ENSMUST00000172
MSTRG.2	Gm15423	ENSMUST00000150600
MSTRG.2	Amy1	MSTRG.20410.1
MSTRG.2	Gm17954	ENSMUST00000195893
MSTRG.20412		MSTRG.20412.1
MSTRG.20413		MSTRG.20413.1
MSTRG.2	S1pr1	ENSMUST00000055676
MSTRG.2	Gm9889	ENSMUST00000064809
MSTRG.2	Vcam1	ENSMUST00000029574;ENSMUST00000197031;ENSMUST00000196
MSTRG.2	Gm43109	ENSMUST00000198269
MSTRG.2	A930005	ENSMUST00000188260;ENSMUST00000185681;ENSMUST00000185
MSTRG.2	Dph5	ENSMUST00000189799;ENSMUST00000200258;ENSMUST00000196
MSTRG.2	Sdccag8	MSTRG.2042.1
MSTRG.2	Dph5	MSTRG.20420.1
MSTRG.2	Gm42687	ENSMUST00000199075
MSTRG.2	Dph5	MSTRG.20422.1
MSTRG.2	Gm43108	ENSMUST00000200525
MSTRG.2	Slc30a7	ENSMUST00000198571;MSTRG.20425.1;ENSMUST00000067485;M
MSTRG.2	Mir669n	ENSMUST00000158636
MSTRG.2	Slc30a7	MSTRG.20428.1
MSTRG.2	Extl2	ENSMUST00000029575;ENSMUST00000106501;ENSMUST00000128
MSTRG.2	Gm37519	ENSMUST00000191687
MSTRG.2	Cdc14a	ENSMUST00000106491;ENSMUST00000090464;MSTRG.20430.3;M
MSTRG.2	Cdc14a	MSTRG.20431.1
MSTRG.2	Cdc14a	MSTRG.20432.1
MSTRG.2	Gm29151	ENSMUST00000196669
MSTRG.2	Cdc14a	MSTRG.20434.1
MSTRG.2	Cdc14a	MSTRG.20435.1

MSTRG.2	Cdc14a	MSTRG.20436.1
MSTRG.2	Cdc14a	MSTRG.20437.1
MSTRG.2	Cdc14a	MSTRG.20438.1
MSTRG.2	Rtca	MSTRG.20439.1;ENSMUST00000000348;ENSMUST00000124486;E
MSTRG.2	Sdccag8	MSTRG.2044.1
MSTRG.2	Gm26530	ENSMUST00000180969
MSTRG.2	Gm9761	ENSMUST00000075650
MSTRG.2	Dbt	ENSMUST00000000349;ENSMUST00000197201;ENSMUST0000019
MSTRG.2	Gm4596	ENSMUST00000117197
MSTRG.2	Dbt	MSTRG.20444.1
MSTRG.2	Gm43651	ENSMUST00000199796
MSTRG.2	Lrrc39	ENSMUST00000203327;ENSMUST00000029573
MSTRG.2	Gm42940	ENSMUST00000200361
MSTRG.2	Lrrc39	MSTRG.20448.1
MSTRG.2	Lrrc39	MSTRG.20449.1
MSTRG.2	Sdccag8	MSTRG.2045.1
MSTRG.2	Trmt13	ENSMUST00000041524;ENSMUST00000184963;ENSMUST0000018
MSTRG.2	Sass6	ENSMUST00000198386;MSTRG.20451.1;MSTRG.20451.3;ENSMUS
MSTRG.2	Mfsd14a	ENSMUST00000029570;ENSMUST00000196274;MSTRG.20453.2;E
MSTRG.2	Gm42457	ENSMUST00000197572
MSTRG.2	Mfsd14a	MSTRG.20455.1
MSTRG.2	4930512	ENSMUST00000197000
MSTRG.2	Agl	ENSMUST00000040603;ENSMUST00000162792;ENSMUST0000016
MSTRG.2	Agl	MSTRG.20458.1
MSTRG.2	Frrs1	ENSMUST00000040260;ENSMUST00000195905;ENSMUST0000019
MSTRG.2	Akt3	ENSMUST00000019843;ENSMUST00000111160;ENSMUST00000111
MSTRG.2	Frrs1	MSTRG.20460.1
MSTRG.2	Frrs1	MSTRG.20461.1
MSTRG.2	Frrs1	MSTRG.20462.1
MSTRG.2	Dpyd	ENSMUST00000039177;ENSMUST00000126254;ENSMUST0000015
MSTRG.2	Gm43410	ENSMUST00000197510
MSTRG.2	Dpyd	MSTRG.20465.1
MSTRG.2	Dpyd	MSTRG.20466.1
MSTRG.2	Gm4332	ENSMUST00000196114
MSTRG.2	Ptbp2;G	ENSMUST00000029780;ENSMUST00000200097;ENSMUST00000197
MSTRG.2	Gm42664	ENSMUST00000199417
MSTRG.2	Gm37960	ENSMUST00000192402
MSTRG.2	Gm42804	ENSMUST00000199701
MSTRG.20471		MSTRG.20471.1
MSTRG.2	Alg14	ENSMUST00000039442;ENSMUST00000198235;ENSMUST0000019
MSTRG.2	Alg14	MSTRG.20473.1
MSTRG.2	Alg14	MSTRG.20474.1
MSTRG.2	Alg14	MSTRG.20475.1
MSTRG.2	Alg14	MSTRG.20476.1
MSTRG.2	Alg14	MSTRG.20477.1
MSTRG.2	Alg14	MSTRG.20478.1
MSTRG.2	Gm26027	ENSMUST00000122725
MSTRG.2	Gm37106	ENSMUST00000193760
MSTRG.2	Gm42928	ENSMUST00000197338
MSTRG.2	Cnn3	ENSMUST00000029773;ENSMUST00000199188;ENSMUST00000197
MSTRG.2	Gm42929	ENSMUST00000198374
MSTRG.2	Cnn3	MSTRG.20483.1
MSTRG.2	Gm43823	ENSMUST00000197391
MSTRG.2	F3	ENSMUST00000029771;ENSMUST00000196746;ENSMUST00000197
MSTRG.2	Gm42777	ENSMUST00000196026
MSTRG.2	Abcd3	ENSMUST00000197383;ENSMUST00000029770;ENSMUST00000197
MSTRG.2	Abcd3	MSTRG.20488.1

MSTRG.2	Gm43609	ENSMUST00000199084
MSTRG.2	Gm37598	ENSMUST00000194506
MSTRG.2	4633401	ENSMUST00000198169
MSTRG.2	Arhgap2	ENSMUST00000196984;ENSMUST00000037958;ENSMUST0000019€
MSTRG.2	Fnbp1l;	ENSMUST00000197259;ENSMUST00000162409;ENSMUST0000019€
MSTRG.2	Gm43569	ENSMUST00000196200
MSTRG.2	Gm42994	ENSMUST00000195964
MSTRG.2	Gclm;Gm	ENSMUST00000029769;ENSMUST00000137540;ENSMUST0000019[illegible]
MSTRG.2	Gm17494	ENSMUST00000072769;ENSMUST00000199060;ENSMUST0000010[illegible]
MSTRG.2	Dnttip2	ENSMUST00000035776;ENSMUST00000199627;ENSMUST0000019€
MSTRG.2	Bcar3	ENSMUST00000196134;ENSMUST00000200544;ENSMUST0000002[illegible]
MSTRG.2	Adgrb3	MSTRG.205.1
MSTRG.2	Gm38146	ENSMUST00000194121
MSTRG.2	Bcar3	MSTRG.20500.1
MSTRG.2	Gm4610	ENSMUST00000198222
MSTRG.2	Gm42836	ENSMUST00000197198
MSTRG.2	Bcar3	MSTRG.20503.1
MSTRG.2	Bcar3	MSTRG.20504.1
MSTRG.2	Gm25153	ENSMUST00000157118
MSTRG.2	Pde5a	ENSMUST00000066728;ENSMUST00000200389;ENSMUST0000019[illegible]
MSTRG.2	4930447	ENSMUST00000196290;ENSMUST00000198715;ENSMUST0000019[illegible]
MSTRG.2	Gm34139	ENSMUST00000196166
MSTRG.2	Pde5a	MSTRG.20509.1
MSTRG.2	Akt3	MSTRG.2051.1
MSTRG.2	4933405	ENSMUST00000180557
MSTRG.2	Fabp2	ENSMUST00000023820
MSTRG.2	Gm10959	ENSMUST00000106431
MSTRG.2	1810037	ENSMUST00000106429
MSTRG.2	Usp53	ENSMUST00000090379;ENSMUST00000198814;ENSMUST0000019[illegible]
MSTRG.2	Gm35065	ENSMUST00000199206
MSTRG.2	Prss12	ENSMUST00000029603;MSTRG.20516.2
MSTRG.2	Gm43590	ENSMUST00000195987
MSTRG.2	Prss12	MSTRG.20518.1
MSTRG.2	Snhg8;S	ENSMUST00000196466;ENSMUST00000198509;ENSMUST0000019[illegible]
MSTRG.2	Gm37706	ENSMUST00000195379
MSTRG.2	Synpo2	ENSMUST00000139160;ENSMUST00000106426;ENSMUST0000010€
MSTRG.2	Gm43744	ENSMUST00000196387
MSTRG.2	Gm43287	ENSMUST00000197747
MSTRG.2	Gm43288	MSTRG.20523.1;ENSMUST00000197594
MSTRG.2	Gm43283	ENSMUST00000199138;MSTRG.20524.1
MSTRG.2	Synpo2	MSTRG.20525.1
MSTRG.2	Sec24d	ENSMUST00000047923;ENSMUST00000198210;ENSMUST0000020[illegible]
MSTRG.2	Mettl14	ENSMUST00000090371;ENSMUST00000174323;ENSMUST0000002[illegible]
MSTRG.2	Mettl14	MSTRG.20528.1
MSTRG.2	Gm4617	MSTRG.20529.1
MSTRG.2	Akt3	MSTRG.2053.1
MSTRG.2	Gm4617	ENSMUST00000178993;MSTRG.20530.1
MSTRG.2	Ugt8a	ENSMUST00000057944;ENSMUST00000198610;ENSMUST0000019€
MSTRG.2	Ugt8a	MSTRG.20532.1
MSTRG.2	Ugt8a	MSTRG.20533.1
MSTRG.2	Ugt8a	MSTRG.20534.1
MSTRG.2	Ugt8a	MSTRG.20535.1
MSTRG.2	Ugt8a	MSTRG.20536.1
MSTRG.2	Ugt8a	MSTRG.20537.1
MSTRG.2	Camk2d	ENSMUST00000143383;ENSMUST00000106402;ENSMUST0000010€
MSTRG.2	Gm43011	ENSMUST00000197897
MSTRG.2	Akt3	MSTRG.2054.1

MSTRG.2	Camk2d	MSTRG.20540.1
MSTRG.2	Gm43010	ENSMUST00000195993
MSTRG.2	Camk2d	MSTRG.20542.1
MSTRG.2	Camk2d	MSTRG.20543.1
MSTRG.2	Gm43009	ENSMUST00000199979
MSTRG.2	Camk2d	MSTRG.20545.1
MSTRG.2	Gm43006	ENSMUST00000197702
MSTRG.2	Gm43005	ENSMUST00000199015
MSTRG.2	Camk2d	MSTRG.20548.1
MSTRG.2	Gm15551	ENSMUST00000132762
MSTRG.2	Akt3	MSTRG.2055.1
MSTRG.2	Camk2d	MSTRG.20550.1
MSTRG.2	Camk2d	MSTRG.20551.1
MSTRG.2	Camk2d	MSTRG.20552.1
MSTRG.2	Camk2d	MSTRG.20553.1
MSTRG.2	Gm42515	ENSMUST00000199936
MSTRG.2	Gm42514	ENSMUST00000196636
MSTRG.2	Camk2d	MSTRG.20556.1
MSTRG.2	Camk2d	MSTRG.20557.1
MSTRG.2	Larp7	MSTRG.20558.1;ENSMUST00000029588;ENSMUST00000195976;E
MSTRG.2	Mir302b	ENSMUST00000102297
MSTRG.2	Akt3	MSTRG.2056.1
MSTRG.2	Mir302c	ENSMUST00000083548
MSTRG.2	Mir302a	ENSMUST00000083618
MSTRG.2	Mir302d	ENSMUST00000083513
MSTRG.2	Mir367	ENSMUST00000083479
MSTRG.2	Zgrf1	MSTRG.20564.1;MSTRG.20564.3;ENSMUST00000200490;ENSMUS
MSTRG.2	Gm43729	ENSMUST00000196949
MSTRG.2	Zgrf1	MSTRG.20566.1
MSTRG.2	5730508	ENSMUST00000199273;ENSMUST00000057198
MSTRG.2	5730508	MSTRG.20568.1
MSTRG.2	5730508	MSTRG.20569.1
MSTRG.2	Akt3	MSTRG.2057.1
MSTRG.2	5730508	MSTRG.20570.1
MSTRG.2	Tifa	ENSMUST00000054483;ENSMUST00000166778;ENSMUST0000016З
MSTRG.2	Ap1ar	ENSMUST00000200409;ENSMUST00000051737;ENSMUST00000198
MSTRG.2	Tifa	MSTRG.20573.1
MSTRG.2	Gm43654	ENSMUST00000200664
MSTRG.2	Gm43653	MSTRG.20575.1;ENSMUST00000198136
MSTRG.2	Enpep	ENSMUST00000029658;ENSMUST00000170918;ENSMUST00000164
MSTRG.2	Gm42628	ENSMUST00000198498
MSTRG.20578		MSTRG.20578.1
MSTRG.2	Gm35986	ENSMUST00000197370
MSTRG.2	Gm37463	ENSMUST00000193819
MSTRG.2	Elovl6	ENSMUST00000199910;ENSMUST00000197070;ENSMUST00000071
MSTRG.2	Gm43072	ENSMUST00000200283
MSTRG.2	Elovl6	MSTRG.20582.1
MSTRG.2	Elovl6	MSTRG.20583.1
MSTRG.2	Elovl6	MSTRG.20584.1
MSTRG.2	Rrh	ENSMUST00000196317;ENSMUST00000029648;ENSMUST00000197
MSTRG.2	Gar1	ENSMUST00000029643;ENSMUST00000134174;ENSMUST00000143
MSTRG.2	Pla2g12	ENSMUST00000029629;ENSMUST00000196838;ENSMUST00000061
MSTRG.2	Pla2g12	MSTRG.20588.1
MSTRG.2	Casp6	ENSMUST00000029626;ENSMUST00000137314;ENSMUST00000197
MSTRG.2	Akt3	MSTRG.2059.1
MSTRG.2	Mcub	ENSMUST00000029624;ENSMUST00000153506;ENSMUST00000122
MSTRG.2	Gm9396	ENSMUST00000076703

MSTRG.2	Sec24b	ENSMUST00000001079;ENSMUST00000170163;MSTRG.20592.2;E
MSTRG.2	Sec24b	MSTRG.20593.1
MSTRG.2	Sec24b	MSTRG.20594.1
MSTRG.2	Ostc	ENSMUST00000043937;ENSMUST00000197152;ENSMUST00000197
MSTRG.2	Gm42998	ENSMUST00000197053
MSTRG.2	Rpl34	ENSMUST00000062601;ENSMUST00000199001;ENSMUST0000007
MSTRG.2	Gm42997	ENSMUST00000200321
MSTRG.2	Adgrb3	MSTRG.206.1
MSTRG.2	Gm38328	ENSMUST00000195582
MSTRG.2	Gm43522	ENSMUST00000196697
MSTRG.2	2010110	ENSMUST00000196438
MSTRG.2	Gm5982	ENSMUST00000196234
MSTRG.2	Gm36520	ENSMUST00000200449
MSTRG.2	Gm26090	ENSMUST00000157897
MSTRG.2	Gm43351	ENSMUST00000198737
MSTRG.2	Gm43350	ENSMUST00000196382
MSTRG.2	2010110	MSTRG.20607.1
MSTRG.2	5830437	ENSMUST00000199219
MSTRG.2	Gm43349	ENSMUST00000200047
MSTRG.2	Gm26801	ENSMUST00000193187;ENSMUST00000180591
MSTRG.2	Gm5855	ENSMUST00000200240
MSTRG.20611		MSTRG.20611.1
MSTRG.2	A430072	MSTRG.20612.1
MSTRG.2	A430072	ENSMUST00000197848
MSTRG.2	Hadh	ENSMUST00000029610;MSTRG.20614.1;ENSMUST00000152427
MSTRG.2	Gm43352	ENSMUST00000196419
MSTRG.2	Lef1;Gm	ENSMUST00000029611;ENSMUST00000066849;ENSMUST0000010
MSTRG.2	Lef1	MSTRG.20617.1
MSTRG.2	Lef1	MSTRG.20618.1
MSTRG.2	Lef1	MSTRG.20619.1;MSTRG.20619.2;MSTRG.20619.3
MSTRG.2	Zbtb18	ENSMUST00000195002;MSTRG.2062.1;ENSMUST00000195549;EN
MSTRG.2	Lef1	MSTRG.20620.1
MSTRG.2	Lef1	MSTRG.20621.1
MSTRG.2	Papss1	ENSMUST00000029666;ENSMUST00000197402;ENSMUST0000019
MSTRG.2	Gm42881	ENSMUST00000198636
MSTRG.2	Papss1	MSTRG.20624.1
MSTRG.2	Npnt	ENSMUST00000042744;ENSMUST00000042729;ENSMUST00000117
MSTRG.2	Gm23196	ENSMUST00000158806
MSTRG.2	Aimp1	ENSMUST00000029663;ENSMUST00000197646;ENSMUST0000020
MSTRG.2	Tbck	ENSMUST00000169172;ENSMUST00000198147;ENSMUST0000002
MSTRG.2	Gm42872	ENSMUST00000200026
MSTRG.2	1700016	ENSMUST00000016106
MSTRG.2	Gm22322	ENSMUST00000157151
MSTRG.2	Gm42876	ENSMUST00000196989
MSTRG.2	Gm29811	ENSMUST00000197386
MSTRG.2	Gm9403	ENSMUST00000196299
MSTRG.20634		MSTRG.20634.1
MSTRG.2	Gstcd	ENSMUST00000029651;ENSMUST00000080583;ENSMUST0000016
MSTRG.2	Gm19148	ENSMUST00000197045
MSTRG.2	Gstcd	MSTRG.20637.1
MSTRG.2	Gm42874	ENSMUST00000198888
MSTRG.2	Gm42873	ENSMUST00000196110
MSTRG.2	1700016	MSTRG.2064.1
MSTRG.2	Ints12	ENSMUST00000029650;ENSMUST00000160036;ENSMUST0000016
MSTRG.2	Arhgef3	ENSMUST00000147041;ENSMUST00000161022;ENSMUST00000161
MSTRG.2	Arhgef3	MSTRG.20642.1
MSTRG.2	Gm43254	ENSMUST00000199187

MSTRG.2	Ppa2	ENSMUST00000029644;MSTRG.20644.2;ENSMUST00000122334;E
MSTRG.2	Gm37228	ENSMUST00000193574
MSTRG.2	Gm37228	MSTRG.20646.1
MSTRG.2	Tet2	ENSMUST00000098603;ENSMUST00000199381;ENSMUST0000019[illegible]
MSTRG.2	Tet2	MSTRG.20648.1
MSTRG.2	Tet2	MSTRG.20649.1
MSTRG.2	Adss	ENSMUST00000016105;ENSMUST00000159184;MSTRG.2065.3;MS
MSTRG.2	Tet2	MSTRG.20650.1
MSTRG.2	Gm42567	ENSMUST00000199268
MSTRG.2	Gm23011	ENSMUST00000197855
MSTRG.2	Cenpe	ENSMUST00000062893;MSTRG.20653.2;ENSMUST00000197369;E
MSTRG.2	Cenpe	MSTRG.20654.1
MSTRG.2	Slc9b1	ENSMUST00000078568;ENSMUST00000160460;ENSMUST00000162
MSTRG.2	Slc9b1	MSTRG.20656.1
MSTRG.20657		MSTRG.20657.1
MSTRG.2	Cisd2;2	MSTRG.20658.1;ENSMUST00000029815;MSTRG.20658.2;ENSMUS
MSTRG.2	Ube2d3	ENSMUST00000196446;ENSMUST00000106291;ENSMUST0000019[illegible]
MSTRG.2	Gm16585	ENSMUST00000161442
MSTRG.2	Gm42807	ENSMUST00000200141
MSTRG.2	Manba	ENSMUST00000131610;ENSMUST00000029814;ENSMUST0000014[illegible]
MSTRG.2	Gm16100	ENSMUST00000120489
MSTRG.2	Oaz2-ps	ENSMUST00000121735
MSTRG.2	Manba	MSTRG.20664.1
MSTRG.2	Manba	MSTRG.20665.1
MSTRG.2	Manba	MSTRG.20666.1
MSTRG.20667		MSTRG.20667.1
MSTRG.2	Nfkb1	ENSMUST00000029812;ENSMUST00000132668;MSTRG.20668.2;E
MSTRG.2	Gm43430	ENSMUST00000196513
MSTRG.2	Adss	MSTRG.2067.1
MSTRG.2	Nfkb1	MSTRG.20670.1
MSTRG.2	Gm31243	ENSMUST00000197498;ENSMUST00000198354
MSTRG.2	Gm5281	ENSMUST00000197542
MSTRG.2	Bank1	ENSMUST00000196159;ENSMUST00000041577;ENSMUST0000019[illegible]
MSTRG.2	Bank1	MSTRG.20675.1
MSTRG.20676		MSTRG.20676.1
MSTRG.2	Ppp3ca;[illegible]	ENSMUST00000124206;ENSMUST00000070198;MSTRG.20677.3;E
MSTRG.2	Ppp3ca	MSTRG.20678.1
MSTRG.2	Ppp3ca	MSTRG.20679.1
MSTRG.2	Gm16432	ENSMUST00000191719;ENSMUST00000094273;ENSMUST00000192
MSTRG.2	4930599[illegible]	MSTRG.20680.1
MSTRG.2	4930599[illegible]	ENSMUST00000198822
MSTRG.2	Ppp3ca	MSTRG.20682.1
MSTRG.2	Ppp3ca	MSTRG.20683.1
MSTRG.2	Gm44327	ENSMUST00000200068
MSTRG.2	Ppp3ca	MSTRG.20685.1
MSTRG.20686		MSTRG.20686.1
MSTRG.2	Gm43403	ENSMUST00000198011
MSTRG.2	H2afz	ENSMUST00000041045;ENSMUST00000174561;ENSMUST0000017[illegible]
MSTRG.2	Dnajb14	ENSMUST00000090178;ENSMUST00000125226;ENSMUST00000197
MSTRG.2	Gm16432	MSTRG.2069.1
MSTRG.2	Gm40155	ENSMUST00000198291
MSTRG.2	Gm43088	ENSMUST00000197530
MSTRG.2	Gm43689	ENSMUST00000195951
MSTRG.2	Lamtor3	ENSMUST00000197064;ENSMUST00000197798;ENSMUST0000016[illegible]
MSTRG.2	Dapp1	MSTRG.20695.1;ENSMUST00000029806;ENSMUST00000184222;E
MSTRG.2	Dapp1	MSTRG.20696.1
MSTRG.2	Dapp1	MSTRG.20697.1

MSTRG.2	Dapp1	MSTRG.20698.1
MSTRG.2	Adh1	ENSMUST00000004232;ENSMUST00000161799
MSTRG.2	Gm37313	ENSMUST00000191673
MSTRG.2	Gm16559	ENSMUST00000160080
MSTRG.2	Adh5	ENSMUST00000005964;ENSMUST00000162024;MSTRG.20701.2;E
MSTRG.2	Metap1	MSTRG.20702.1;ENSMUST00000029804;ENSMUST00000198700;E
MSTRG.2	Metap1	MSTRG.20703.1
MSTRG.2	Eif4e;M	ENSMUST00000029803;ENSMUST00000157748;ENSMUST0000020C
MSTRG.2	Eif4e	MSTRG.20705.1
MSTRG.2	Eif4e	MSTRG.20706.1
MSTRG.20707		MSTRG.20707.1
MSTRG.2	Tspan5	ENSMUST00000029800;ENSMUST00000142890;ENSMUST00000127
MSTRG.2	Tspan5	MSTRG.20709.1
MSTRG.2	Tspan5	MSTRG.20710.1
MSTRG.2	Gm16060	ENSMUST00000153140
MSTRG.2	Rap1gds	ENSMUST00000098574;ENSMUST00000196280;MSTRG.20712.3;E
MSTRG.2	Gm43162	ENSMUST00000198292
MSTRG.2	Rap1gds	MSTRG.20714.1
MSTRG.2	Rap1gds	MSTRG.20715.1
MSTRG.2	Stpg2	ENSMUST00000106239;ENSMUST00000062306
MSTRG.2	Stpg2	MSTRG.20717.1
MSTRG.2	Stpg2	MSTRG.20718.1
MSTRG.2	Gm16432	MSTRG.2072.1
MSTRG.2	Gm24260	ENSMUST00000083741
MSTRG.2	Gm2451	ENSMUST00000199447
MSTRG.20722		MSTRG.20722.1
MSTRG.20723		MSTRG.20723.1
MSTRG.20724		MSTRG.20724.1
MSTRG.20726		MSTRG.20726.1
MSTRG.20727		MSTRG.20727.1
MSTRG.2	Gm15688	ENSMUST00000161394
MSTRG.2	Unc5c	ENSMUST00000106236;ENSMUST00000075282;ENSMUST0000020C
MSTRG.2	Gm16432	MSTRG.2073.1
MSTRG.2	Unc5c	MSTRG.20730.1
MSTRG.2	Unc5c	MSTRG.20731.1
MSTRG.2	Unc5c	MSTRG.20732.1
MSTRG.2	Gm15689	ENSMUST00000160604
MSTRG.2	Gm43826	ENSMUST00000199604
MSTRG.2	Pdlim5	ENSMUST00000029941;ENSMUST00000195975;ENSMUST0000019€
MSTRG.2	Pdlim5	MSTRG.20736.1
MSTRG.2	Gbp5	ENSMUST00000198803;ENSMUST00000090127;ENSMUST0000019€
MSTRG.2	Gbp7;Gm	MSTRG.20738.1;MSTRG.20738.2;ENSMUST00000171263;ENSMUS
MSTRG.2	Gbp3	ENSMUST00000199325;ENSMUST00000106222;ENSMUST0000012€
MSTRG.2	Gm8010	ENSMUST00000194128
MSTRG.2	Gbp2b	ENSMUST00000029936
MSTRG.2	Gbp2	ENSMUST00000165774;ENSMUST00000169572
MSTRG.2	Mir6381	ENSMUST00000183426
MSTRG.2	Gm2574	ENSMUST00000181051
MSTRG.2	Gtf2b	ENSMUST00000029938;ENSMUST00000196311;MSTRG.20744.3
MSTRG.2	Gm24457	ENSMUST00000083196
MSTRG.2	Pkn2	MSTRG.20746.1;ENSMUST00000043812;ENSMUST00000173913;E
MSTRG.2	Gm25965	ENSMUST00000157170
MSTRG.2	Pkn2	MSTRG.20748.1
MSTRG.2	Pkn2	MSTRG.20749.1
MSTRG.2075		MSTRG.2075.1
MSTRG.2	Gm43646	ENSMUST00000199962;ENSMUST00000197517
MSTRG.2	9530052	ENSMUST00000181882

MSTRG.2	Lmo4	ENSMUST00000121796;ENSMUST00000120539;ENSMUST00000121
MSTRG.2	Gm43445	ENSMUST00000198564
MSTRG.20754		MSTRG.20754.1
MSTRG.2	Hs2st1	ENSMUST00000043325;ENSMUST00000160690;ENSMUST0000019
MSTRG.2	Gm43707	ENSMUST00000196451
MSTRG.2	Hs2st1	MSTRG.20757.1
MSTRG.2	Gm43560	ENSMUST00000198100
MSTRG.2	Selenof	ENSMUST00000144859;ENSMUST00000082437;ENSMUST0000010€
MSTRG.2	Gm38300	ENSMUST00000194245
MSTRG.2	Gm24406	ENSMUST00000083026
MSTRG.2	Sh3glb1	MSTRG.20761.1;ENSMUST00000199531;MSTRG.20761.2;ENSMUS
MSTRG.20762		MSTRG.20762.1
MSTRG.2	Clca3a1	ENSMUST00000029932;MSTRG.20763.1;ENSMUST00000059091;E
MSTRG.2	Gm34866	ENSMUST00000196146
MSTRG.2	Gm17743	ENSMUST00000198545
MSTRG.20766		MSTRG.20766.1
MSTRG.2	Gm43240	ENSMUST00000198543
MSTRG.2	Odf2l	ENSMUST00000199435;MSTRG.20768.1;ENSMUST00000098538;E
MSTRG.2	Odf2l	MSTRG.20769.1
MSTRG.2	Desi2	ENSMUST00000161075;ENSMUST00000027783;MSTRG.2077.3;MS
MSTRG.2	Znhit6	ENSMUST00000199033;ENSMUST00000098534;ENSMUST0000020C
MSTRG.2	Znhit6	MSTRG.20771.1
MSTRG.2	Ddah1	ENSMUST00000029845;ENSMUST00000120310;ENSMUST00000127
MSTRG.2	Rpl36a-[illegible]	ENSMUST00000197973
MSTRG.2	Bcl10	ENSMUST00000197842;ENSMUST00000029842;ENSMUST00000198
MSTRG.2	2410004[illegible]	ENSMUST00000039571;ENSMUST00000134575;ENSMUST00000152
MSTRG.2	Mcoln3	ENSMUST00000140214;ENSMUST00000039450;ENSMUST0000014€
MSTRG.2	Ssx2ip	ENSMUST00000106151;ENSMUST00000106153;ENSMUST0000003S
MSTRG.2	Ssx2ip	MSTRG.20778.1
MSTRG.2	Ctbs	ENSMUST00000197166;ENSMUST00000061937;ENSMUST00000196
MSTRG.2	Hnrnpu	MSTRG.2078.1;ENSMUST00000159117;MSTRG.2078.2;ENSMUSTC
MSTRG.2	Spata1	ENSMUST00000200488;ENSMUST00000197980;ENSMUST0000002S
MSTRG.2	Ctbs	MSTRG.20781.1
MSTRG.2	Spata1	MSTRG.20782.1
MSTRG.2	Gng5	ENSMUST00000090031;ENSMUST00000118280;ENSMUST0000011S
MSTRG.2	Rpf1	MSTRG.20784.1;ENSMUST00000029838;ENSMUST00000199174;E
MSTRG.2	Gm10288	ENSMUST00000084614
MSTRG.2	Prkacb	ENSMUST00000102515;ENSMUST00000106137;ENSMUST0000010€
MSTRG.2	Prkacb	MSTRG.20787.1
MSTRG.2	Gm19104	ENSMUST00000198392
MSTRG.20789		MSTRG.20789.1
MSTRG.2	Gm8023	ENSMUST00000195298
MSTRG.20790		MSTRG.20790.1
MSTRG.20791		MSTRG.20791.1
MSTRG.2	Rpsa-ps	ENSMUST00000057740
MSTRG.2	Adgrl4	ENSMUST00000046977;ENSMUST00000196970;ENSMUST0000015S
MSTRG.20794		MSTRG.20794.1
MSTRG.2	Ifi44	ENSMUST00000029671;ENSMUST00000149919;ENSMUST0000013S
MSTRG.2	Ifi44l	ENSMUST00000046739;MSTRG.20796.1
MSTRG.2	Miga1	ENSMUST00000073089;ENSMUST00000068243;ENSMUST0000019S
MSTRG.2	Miga1	MSTRG.20798.1
MSTRG.2	Miga1	MSTRG.20799.1
MSTRG.2	Dnajb4	ENSMUST00000134701;ENSMUST00000050073;ENSMUST00000144
MSTRG.2	Fubp1	ENSMUST00000166984;ENSMUST00000199202;ENSMUST0000020C
MSTRG.2	Nexn;Gm	ENSMUST00000199470;ENSMUST00000046045;ENSMUST00000198
MSTRG.2	Pigk	ENSMUST00000159899;ENSMUST00000162835;ENSMUST0000004S
MSTRG.2	Rpl29-p	ENSMUST00000161849

MSTRG.2	St6galn	ENSMUST00000044278;ENSMUST00000161757
MSTRG.2	Gm16232	ENSMUST00000162856
MSTRG.2	Gm16213	ENSMUST00000160076
MSTRG.2	Gm16231	ENSMUST00000160432
MSTRG.2	Gm42710	ENSMUST00000200608
MSTRG.2	Gm37486	ENSMUST00000195663
MSTRG.2	Usp33	ENSMUST00000117492;ENSMUST00000026507;ENSMUST00000197
MSTRG.2	Usp33	MSTRG.20811.1
MSTRG.2	Gm42967	ENSMUST00000198647
MSTRG.2	Gm43859	ENSMUST00000200075
MSTRG.2	Gm43858	ENSMUST00000199418
MSTRG.2	Gm43857	ENSMUST00000200343
MSTRG.2	Zzz3	MSTRG.20816.1
MSTRG.2	Zzz3	ENSMUST00000106103;ENSMUST00000138115;ENSMUST0000010(
MSTRG.2	Zzz3	MSTRG.20818.1
MSTRG.2	Zzz3	MSTRG.20819.1
MSTRG.2	B230369	ENSMUST00000141846
MSTRG.2	Zzz3	MSTRG.20820.1
MSTRG.2	Zzz3	MSTRG.20821.1
MSTRG.2	Zzz3	MSTRG.20822.1
MSTRG.2	Ak5	ENSMUST00000045262;ENSMUST00000200062;ENSMUST0000019
MSTRG.2	Ak5	MSTRG.20824.1
MSTRG.2	St6galn	ENSMUST00000200397;ENSMUST00000199707;ENSMUST00000064
MSTRG.2	St6galn	MSTRG.20827.1
MSTRG.2	St6galn	MSTRG.20828.1
MSTRG.2	1700012	ENSMUST00000197231
MSTRG.2	Cox20	ENSMUST00000154437;ENSMUST00000027781
MSTRG.2	A530083	ENSMUST00000199877
MSTRG.2	St6galn	MSTRG.20831.1
MSTRG.2	Gm27405	ENSMUST00000184201
MSTRG.2	St6galn	MSTRG.20833.1
MSTRG.2	St6galn	MSTRG.20834.1
MSTRG.2	St6galn	MSTRG.20835.1
MSTRG.2	St6galn	MSTRG.20837.1
MSTRG.2	St6galn	MSTRG.20838.1
MSTRG.2	St6galn	MSTRG.20839.1
MSTRG.2	Efcab2	ENSMUST00000194861;ENSMUST00000027775;ENSMUST00000192
MSTRG.2	1700015	ENSMUST00000197297
MSTRG.2	4930597	ENSMUST00000196286
MSTRG.2	St6galn	MSTRG.20842.1
MSTRG.2	St6galn	MSTRG.20843.1
MSTRG.2	Gm22136	ENSMUST00000158751
MSTRG.2	St6galn	MSTRG.20845.1
MSTRG.2	St6galn	MSTRG.20846.1
MSTRG.2	St6galn	MSTRG.20847.1
MSTRG.2	Gm22206	ENSMUST00000158612
MSTRG.2	St6galn	MSTRG.20849.1
MSTRG.2	Efcab2	MSTRG.2085.1
MSTRG.2	St6galn	MSTRG.20850.1
MSTRG.2	Rabggtb	ENSMUST00000197438;ENSMUST00000167111;ENSMUST0000008
MSTRG.2	Gm24494	ENSMUST00000083804
MSTRG.2	Gm43400	ENSMUST00000197294
MSTRG.2	Acadm	ENSMUST00000072697;ENSMUST00000196688;MSTRG.20854.2;E
MSTRG.2	Tyw3	ENSMUST00000052774;ENSMUST00000170461;ENSMUST00000122
MSTRG.2	Tyw3	MSTRG.20856.1
MSTRG.2	Cryz	ENSMUST00000192462;ENSMUST00000029850;ENSMUST00000184
MSTRG.2	Fpgt	ENSMUST00000066568;ENSMUST00000194095;ENSMUST00000193

MSTRG.2	Gm26456	ENSMUST00000082831
MSTRG.2	Smyd3	ENSMUST00000128302;ENSMUST00000068437;ENSMUST00000131
MSTRG.2	Lrriq3	ENSMUST00000195418;ENSMUST00000029833;ENSMUST00000192
MSTRG.2	Gm37482	ENSMUST00000194899
MSTRG.2	4930570	ENSMUST00000139363;ENSMUST00000140941
MSTRG.2	Negr1	ENSMUST00000074015;ENSMUST00000041425;ENSMUST00000106
MSTRG.2	Gm42946	ENSMUST00000196108
MSTRG.2	Gm42942	ENSMUST00000196086
MSTRG.2	Gm42943	ENSMUST00000199676
MSTRG.2	Gm43528	ENSMUST00000199492
MSTRG.2	Gm15578	ENSMUST00000117320
MSTRG.2	Gm15577	ENSMUST00000144350
MSTRG.2	Smyd3	MSTRG.2087.1
MSTRG.2	Gm43527	ENSMUST00000198749
MSTRG.2	Negr1	MSTRG.20871.1
MSTRG.2	Gm43526	ENSMUST00000196666
MSTRG.2	Negr1	MSTRG.20873.1
MSTRG.2	4930566	ENSMUST00000195173
MSTRG.2	Gm23725	ENSMUST00000177876
MSTRG.2	Ptger3	ENSMUST00000041175;ENSMUST00000196682;ENSMUST00000173
MSTRG.2	4930592	ENSMUST00000196791
MSTRG.2	Zranb2;	ENSMUST00000106058;ENSMUST00000184802;ENSMUST00000198
MSTRG.2	Smyd3	MSTRG.2088.1
MSTRG.2	Ankrd13	ENSMUST00000199727;ENSMUST00000040787;ENSMUST00000197
MSTRG.2	Ankrd13	MSTRG.20881.1
MSTRG.2	Ankrd13	MSTRG.20882.1
MSTRG.2	Ankrd13	MSTRG.20883.1
MSTRG.2	Ankrd13	MSTRG.20884.1
MSTRG.2	Lrrc7	ENSMUST00000200196;ENSMUST00000200137
MSTRG.2	Gm43485	ENSMUST00000199292
MSTRG.2	Lrrc7	MSTRG.20889.1
MSTRG.2	Smyd3	MSTRG.2089.1
MSTRG.2	Depdc1a	ENSMUST00000120272;MSTRG.20891.1;ENSMUST00000029825;E
MSTRG.2	Depdc1a	MSTRG.20892.1
MSTRG.2	Depdc1a	MSTRG.20893.1
MSTRG.2	Srsf11;	ENSMUST00000121326;ENSMUST00000152274;MSTRG.20894.3;E
MSTRG.2	Lrrc40	ENSMUST00000072080;ENSMUST00000156597;ENSMUST0000015C
MSTRG.2	Lrrc40	MSTRG.20896.1
MSTRG.2	B230334	ENSMUST00000200255
MSTRG.2	Wls	ENSMUST00000068952;ENSMUST00000198878;ENSMUST0000020C
MSTRG.2	Gm43307	ENSMUST00000199465
MSTRG.2	Gm37051	ENSMUST00000194535
MSTRG.2	Smyd3	MSTRG.2090.1
MSTRG.2	Gm23941	ENSMUST00000101953
MSTRG.20901		MSTRG.20901.1
MSTRG.20902		MSTRG.20902.1
MSTRG.2	Tmem68	ENSMUST00000154922;ENSMUST00000029891;ENSMUST00000134
MSTRG.2	Tgs1	ENSMUST00000052712;ENSMUST00000132518;MSTRG.20904.2;E
MSTRG.2	Lyn	ENSMUST00000041377;ENSMUST00000103010;ENSMUST00000145
MSTRG.2	Gm26857	ENSMUST00000181580;MSTRG.20906.2;MSTRG.20906.3
MSTRG.2	Gm11805	ENSMUST00000121238
MSTRG.2	Lyn	MSTRG.20908.1
MSTRG.2	Gm22781	ENSMUST00000104083
MSTRG.2	Smyd3	MSTRG.2091.1
MSTRG.2	9430025	ENSMUST00000068663
MSTRG.2	Rps20;G	MSTRG.20911.1;ENSMUST00000138502;ENSMUST00000130128;E
MSTRG.2	Plag1	ENSMUST00000003369;ENSMUST00000137439;ENSMUST00000151

MSTRG.2	Plag1	MSTRG.20913.1
MSTRG.2	Plag1	MSTRG.20914.1
MSTRG.2	Gm11808	ENSMUST00000089430
MSTRG.2	Chchd7	ENSMUST00000108386;ENSMUST00000121110;ENSMUST0000014[illegible]
MSTRG.2	Penk	ENSMUST00000070375;MSTRG.20917.1;ENSMUST00000133567
MSTRG.2	A830012[illegible]	ENSMUST00000131498
MSTRG.2	Impad1	ENSMUST00000084949;MSTRG.20919.2;MSTRG.20919.3
MSTRG.2	Smyd3	MSTRG.2092.1
MSTRG.20920		MSTRG.20920.1
MSTRG.20921		MSTRG.20921.1
MSTRG.20922		MSTRG.20922.1
MSTRG.2	Ubxn2b	ENSMUST00000029907
MSTRG.2	Fam110b	ENSMUST00000124256;ENSMUST00000054857;ENSMUST00000144
MSTRG.2	Gm24337	ENSMUST00000104204
MSTRG.2	Gm11796	ENSMUST00000120208
MSTRG.2	Fam110b	MSTRG.20927.1
MSTRG.2	Gm11797	ENSMUST00000121336
MSTRG.2	Tox	MSTRG.20929.1
MSTRG.2	Smyd3	MSTRG.2093.1
MSTRG.2	Gm11804	ENSMUST00000121663
MSTRG.20931		MSTRG.20931.1
MSTRG.20932		MSTRG.20932.1
MSTRG.2	Sdcbp	ENSMUST00000029912;ENSMUST00000103008;ENSMUST0000015[illegible]
MSTRG.2	Gm37689	ENSMUST00000193139
MSTRG.2	Nsmaf	ENSMUST00000029910;ENSMUST00000143704;ENSMUST0000014[illegible]
MSTRG.2	Tox	MSTRG.20936.3;MSTRG.20936.1;ENSMUST00000039987;MSTRG.
MSTRG.2	Gm11802	MSTRG.20937.2
MSTRG.2	Tox	MSTRG.20938.1
MSTRG.2	Smyd3	MSTRG.2094.1
MSTRG.2	Tox	MSTRG.20941.1;MSTRG.20941.2;MSTRG.20941.3;MSTRG.20941
MSTRG.20942		MSTRG.20942.1;MSTRG.20942.2
MSTRG.2	Gm11804	MSTRG.20943.1
MSTRG.20944		MSTRG.20944.1
MSTRG.20945		MSTRG.20945.1;MSTRG.20945.2
MSTRG.20946		MSTRG.20946.1
MSTRG.20947		MSTRG.20947.1;MSTRG.20947.2;MSTRG.20947.3
MSTRG.20948		MSTRG.20948.1
MSTRG.2	Rab2a	ENSMUST00000060232;MSTRG.20949.2
MSTRG.2	Smyd3	MSTRG.2095.1;MSTRG.2095.2;MSTRG.2095.3
MSTRG.2	Rab2a	MSTRG.20950.1
MSTRG.2	Rab2a	MSTRG.20951.1
MSTRG.2	Rab2a	MSTRG.20952.1
MSTRG.2	Rab2a	MSTRG.20953.1;MSTRG.20953.2
MSTRG.2	Rab2a	MSTRG.20954.1
MSTRG.2	Chd7	ENSMUST00000170457;ENSMUST00000127476;MSTRG.20955.2;[illegible]
MSTRG.2	Gm12918	ENSMUST00000120234
MSTRG.2	2610301[illegible]	ENSMUST00000080517;ENSMUST00000131437;ENSMUST00000101
MSTRG.2	Clvs1	ENSMUST00000128117;ENSMUST00000141085
MSTRG.2	Gm11816	ENSMUST00000149848;ENSMUST00000142744
MSTRG.2	Gm11817	ENSMUST00000155578
MSTRG.2	Asph	ENSMUST00000151232;ENSMUST00000078139;ENSMUST0000010[illegible]
MSTRG.2	Plekhf2	MSTRG.20964.1;ENSMUST00000054776;ENSMUST00000137891
MSTRG.2	Plekhf2	MSTRG.20965.1
MSTRG.2	Plekhf2	MSTRG.20966.1
MSTRG.2	Gm11827	ENSMUST00000151138;ENSMUST00000142297
MSTRG.2	Gm11829	ENSMUST00000121003
MSTRG.2	Smyd3	MSTRG.2097.1

MSTRG.2	Gm11830	ENSMUST00000120175
MSTRG.20971		MSTRG.20971.1
MSTRG.2	Trp53in	ENSMUST00000029865;ENSMUST00000132010;ENSMUST00000132
MSTRG.2	Ccne2	MSTRG.20973.2;MSTRG.20973.1;ENSMUST00000145430;ENSMUS
MSTRG.2	Ints8	ENSMUST00000044616;ENSMUST00000136545;MSTRG.20974.2;E
MSTRG.2	Dpy19l4	ENSMUST00000139385;ENSMUST00000084892;ENSMUST00000128
MSTRG.2	Dpy19l4	MSTRG.20976.1
MSTRG.2	Gm22940	ENSMUST00000158560
MSTRG.2	Dpy19l4	MSTRG.20978.1
MSTRG.2	Dpy19l4	MSTRG.20979.1
MSTRG.2	Smyd3	MSTRG.2098.1
MSTRG.2	Gm26663	ENSMUST00000181105
MSTRG.2	Esrp1	ENSMUST00000043781;ENSMUST00000108313;ENSMUST00000108
MSTRG.2	Virma	ENSMUST00000055372;ENSMUST00000059914;MSTRG.20982.1;E
MSTRG.2	Gm11831	ENSMUST00000141538
MSTRG.2	Gem	ENSMUST00000108304;ENSMUST00000029868
MSTRG.2	Rad54b;	ENSMUST00000179498;ENSMUST00000095145;ENSMUST00000108
MSTRG.2	Rad54b	MSTRG.20986.1
MSTRG.2	Rad54b	MSTRG.20987.1
MSTRG.2	Rad54b	MSTRG.20988.1
MSTRG.2	Gm11832	MSTRG.20989.1
MSTRG.2	Tfb2m	MSTRG.2099.1;ENSMUST00000027769;ENSMUST00000150778;EN
MSTRG.20990		MSTRG.20990.1
MSTRG.2	Pdp1	ENSMUST00000108297;ENSMUST00000108301;ENSMUST00000095
MSTRG.2	1700123	ENSMUST00000154194;ENSMUST00000146154;ENSMUST00000128
MSTRG.2	Gm10604	ENSMUST00000098260
MSTRG.2	Gm26895	ENSMUST00000181295
MSTRG.2	Gm11847	ENSMUST00000125165;MSTRG.20995.1
MSTRG.2	Gm11842	ENSMUST00000130117
MSTRG.2	Tmem67	ENSMUST00000050686;ENSMUST00000131145;ENSMUST00000108
MSTRG.2	Rbm12b2	ENSMUST00000095143;MSTRG.20998.3;MSTRG.20998.5;ENSMUS
MSTRG.2	Gm11841	ENSMUST00000117060
MSTRG.2	Atp6v1h	ENSMUST00000192847;ENSMUST00000044369;ENSMUST00000194
MSTRG.2	Gm38260	ENSMUST00000193573
MSTRG.2	Gm15480	ENSMUST00000120442
MSTRG.2	Gm11843	ENSMUST00000118984
MSTRG.21001		MSTRG.21001.1
MSTRG.2	Fam92a	ENSMUST00000108285;ENSMUST00000177837;ENSMUST00000087
MSTRG.2	Lrrc69	ENSMUST00000023917
MSTRG.2	Gm11838	ENSMUST00000121999
MSTRG.21005		MSTRG.21005.1
MSTRG.2	Otud6b	ENSMUST00000117268;ENSMUST00000151012;MSTRG.21006.3
MSTRG.2	Tmem55a	ENSMUST00000029875
MSTRG.2	Tmem55a	MSTRG.21008.1
MSTRG.2	Gm11837	ENSMUST00000136992
MSTRG.2	Cnst	ENSMUST00000040706;ENSMUST00000144370;ENSMUST00000153
MSTRG.2	Necab1	ENSMUST00000041606;ENSMUST00000141105
MSTRG.2	Necab1	MSTRG.21011.1
MSTRG.2	Gm11844	ENSMUST00000129207
MSTRG.21013		MSTRG.21013.1;MSTRG.21013.2;MSTRG.21013.3
MSTRG.21014		MSTRG.21014.1;MSTRG.21014.2;MSTRG.21014.3
MSTRG.21015		MSTRG.21015.1
MSTRG.2	Tmem64	ENSMUST00000062684
MSTRG.2	Calb1	ENSMUST00000029876;ENSMUST00000136266;ENSMUST00000141
MSTRG.2	Decr1	ENSMUST00000029877;ENSMUST00000150464;ENSMUST00000154
MSTRG.2	Gm37070	ENSMUST00000193272
MSTRG.2	Mir6400	ENSMUST00000184870

MSTRG.2	Nbn	ENSMUST00000029879;ENSMUST00000149069
MSTRG.2	Gm11869	ENSMUST00000118701
MSTRG.2	Osgin2	ENSMUST00000037198;ENSMUST00000134048;ENSMUST00000149
MSTRG.2	Gm11870	ENSMUST00000119255
MSTRG.2	Ripk2	ENSMUST00000037035;ENSMUST00000183871
MSTRG.2	Gm22690	ENSMUST00000175054
MSTRG.2	Kif28;A	ENSMUST00000131716;ENSMUST00000027768;ENSMUST00000140
MSTRG.2	Gm11867	MSTRG.21030.1
MSTRG.2	Gm11867	MSTRG.21031.1
MSTRG.2	Gm11868	ENSMUST00000121594
MSTRG.2	Cpne3	ENSMUST00000029885;MSTRG.21037.2;ENSMUST00000134025;E
MSTRG.2	Ggh	ENSMUST00000098242
MSTRG.2	Rmdn1	ENSMUST00000108253;ENSMUST00000029888;MSTRG.21039.3;M
MSTRG.2	Gm10518	ENSMUST00000097454
MSTRG.2	Wwp1	ENSMUST00000108246;ENSMUST00000035982;MSTRG.21040.3
MSTRG.21041		MSTRG.21041.1
MSTRG.2	Nkain3	ENSMUST00000119374;ENSMUST00000102998
MSTRG.2	Gm11873	ENSMUST00000117877
MSTRG.2	Nkain3	MSTRG.21045.1
MSTRG.2	Pnisr	MSTRG.21046.1
MSTRG.2	Coq3	ENSMUST00000029909;ENSMUST00000142244
MSTRG.2	Coq3	MSTRG.21048.1
MSTRG.2	Fbxl4	ENSMUST00000131972;ENSMUST00000039234;ENSMUST00000185
MSTRG.2	Cdc42bp	ENSMUST00000097453;ENSMUST00000111117;ENSMUST00000134
MSTRG.2	Fbxl4	MSTRG.21050.1
MSTRG.2	Gm24607	ENSMUST00000104373
MSTRG.2	Fbxl4	MSTRG.21052.1
MSTRG.2	Gm37900	ENSMUST00000195510
MSTRG.2	Ccnc	ENSMUST00000065928;ENSMUST00000102997;ENSMUST00000120
MSTRG.2	Tstd3	ENSMUST00000029915
MSTRG.2	Usp45	ENSMUST00000156589;ENSMUST00000125262;MSTRG.21056.2;E
MSTRG.2	Usp45	MSTRG.21057.1;MSTRG.21057.2
MSTRG.2	Usp45	MSTRG.21058.1
MSTRG.2	Pnisr	ENSMUST00000029911;ENSMUST00000098238;ENSMUST00000152
MSTRG.2	Gm38169	ENSMUST00000191822
MSTRG.2	Pnisr	MSTRG.21060.1
MSTRG.2	Ndufaf4	ENSMUST00000029925;ENSMUST00000146721;ENSMUST00000151
MSTRG.2	Mms22l	ENSMUST00000173079;ENSMUST00000108222;ENSMUST00000138
MSTRG.2	Mms22l	MSTRG.21063.1
MSTRG.21064		MSTRG.21064.1
MSTRG.2	Klhl32	ENSMUST00000108218;ENSMUST00000150920;ENSMUST00000108
MSTRG.2	Klhl32	MSTRG.21066.1
MSTRG.2	Gm11899	ENSMUST00000142373
MSTRG.2	C230012	ENSMUST00000133431;ENSMUST00000130085;ENSMUST00000139
MSTRG.2	Gpr63;G	ENSMUST00000151006;ENSMUST00000124792;ENSMUST00000038
MSTRG.2	Gm38331	ENSMUST00000195719
MSTRG.2	Fhl5	ENSMUST00000029922;ENSMUST00000108204
MSTRG.2	Ufl1	ENSMUST00000142949;MSTRG.21071.2;ENSMUST00000102994;M
MSTRG.2	Manea	ENSMUST00000041374;ENSMUST00000133105;ENSMUST00000140
MSTRG.2	Manea	MSTRG.21073.1
MSTRG.21074		MSTRG.21074.1
MSTRG.2	Map3k7	ENSMUST00000037607;MSTRG.21075.2;ENSMUST00000080933;M
MSTRG.2	Gm11926	ENSMUST00000118520
MSTRG.2	Gm26254	ENSMUST00000122491
MSTRG.2	Map3k7	MSTRG.21078.1
MSTRG.2	Gm11929	ENSMUST00000118175
MSTRG.2	Coq8a	ENSMUST00000170472;ENSMUST00000027766;ENSMUST00000160

MSTRG.2	Bach2;B	MSTRG.21080.1;ENSMUST00000108180;ENSMUST00000149201;M
MSTRG.2	Bach2it	MSTRG.21081.1
MSTRG.2	Bach2	MSTRG.21082.1
MSTRG.2	Bach2	MSTRG.21083.1
MSTRG.2	Bach2	MSTRG.21084.1;MSTRG.21084.2
MSTRG.2	Gm24371	ENSMUST00000122662
MSTRG.2	Bach2os	ENSMUST00000126225;ENSMUST00000136149;ENSMUST0000014S
MSTRG.2	Rragd	ENSMUST00000098190;ENSMUST00000029946;ENSMUST00000084
MSTRG.2	Gm11943	ENSMUST00000119473
MSTRG.2	Lyrm2	ENSMUST00000062802;ENSMUST00000135833;ENSMUST0000013C
MSTRG.2	Coq8a	MSTRG.2109.1
MSTRG.2	Ankrd6	ENSMUST00000035719;ENSMUST00000084749;ENSMUST00000084
MSTRG.2	Gm11941	ENSMUST00000119904
MSTRG.2	Ankrd6	MSTRG.21092.1
MSTRG.2	Ankrd6	MSTRG.21093.1
MSTRG.2	Ankrd6	MSTRG.21094.1
MSTRG.2	Ankrd6	MSTRG.21095.1
MSTRG.2	Ankrd6	MSTRG.21096.1
MSTRG.2	Ankrd6	MSTRG.21097.1
MSTRG.2	4933421	ENSMUST00000155691
MSTRG.2	Ube2j1	ENSMUST00000124992;MSTRG.21099.1;MSTRG.21099.2;ENSMUS
MSTRG.2	Gm37628	ENSMUST00000194644
MSTRG.2	Psen2	ENSMUST00000130711;ENSMUST00000111106;ENSMUST00000111
MSTRG.2	Casp8ap	ENSMUST00000124278;MSTRG.21100.1;ENSMUST00000127619;E
MSTRG.2	Casp8ap	MSTRG.21101.1
MSTRG.2	Casp8ap	MSTRG.21102.1
MSTRG.2	Gm11936	ENSMUST00000118323
MSTRG.2	Mdn1	ENSMUST00000071642;ENSMUST00000178134;ENSMUST00000151
MSTRG.2	Mdn1	MSTRG.21105.1
MSTRG.21106		MSTRG.21106.1
MSTRG.21107		MSTRG.21107.1;MSTRG.21107.2
MSTRG.21108		MSTRG.21108.1
MSTRG.21109		MSTRG.21109.1
MSTRG.2	Psen2	MSTRG.2111.1
MSTRG.2	Gabrr2	ENSMUST00000108162;ENSMUST00000131920;ENSMUST00000147
MSTRG.2	Gabrr2	MSTRG.21111.1
MSTRG.2	Pnrc1	ENSMUST00000049357;ENSMUST00000155616;ENSMUST00000137
MSTRG.21113		MSTRG.21113.1
MSTRG.2	Gm11934	ENSMUST00000132553
MSTRG.2	Rngtt	ENSMUST00000108153;ENSMUST00000029942;ENSMUST0000013C
MSTRG.2	Mir8118	ENSMUST00000183962
MSTRG.2	Gm11935	ENSMUST00000118608
MSTRG.2	Rngtt	MSTRG.21118.1
MSTRG.2	Rngtt	MSTRG.21119.1
MSTRG.2	Gm31728	ENSMUST00000192164
MSTRG.2	Rngtt	MSTRG.21120.1
MSTRG.21121		MSTRG.21121.1
MSTRG.2	Gm12751	ENSMUST00000122336
MSTRG.2	Smim8	ENSMUST00000108132;ENSMUST00000108133;ENSMUST0000010E
MSTRG.2	Akirin2	ENSMUST00000084299;ENSMUST00000132230;MSTRG.21124.2;M
MSTRG.2	Orc3	ENSMUST00000108142;ENSMUST00000048706;ENSMUST00000132
MSTRG.2	Rars2	ENSMUST00000029968;ENSMUST00000149620;ENSMUST0000013E
MSTRG.2	Rars2	MSTRG.21127.1
MSTRG.2	Slc35a1	ENSMUST00000029970;MSTRG.21128.2;ENSMUST00000151549;E
MSTRG.2	Gm22849	ENSMUST00000157713
MSTRG.2	Gm5069	ENSMUST00000050581;ENSMUST00000192714
MSTRG.21130		MSTRG.21130.1

MSTRG.2	Zfp292	MSTRG.21131.1;MSTRG.21131.2;ENSMUST00000098163;ENSMUS
MSTRG.2	Gm12363	ENSMUST00000122250
MSTRG.2	Zfp292	MSTRG.21133.1
MSTRG.2	Mob3b	ENSMUST00000102975;ENSMUST00000155725
MSTRG.2	Gm12364	ENSMUST00000144419
MSTRG.2	Mob3b	MSTRG.21136.1
MSTRG.2	Mob3b	MSTRG.21137.1
MSTRG.2	Mob3b	MSTRG.21138.1
MSTRG.2	Ifnk	ENSMUST00000058595
MSTRG.2	Itpkb;G	ENSMUST00000070181;MSTRG.2114.2;MSTRG.2114.3;MSTRG.21
MSTRG.2	3110043	ENSMUST00000084724;ENSMUST00000108126;ENSMUST0000010
MSTRG.2	Gm12366	ENSMUST00000121722
MSTRG.2	Gm12367	ENSMUST00000127015;ENSMUST00000149677;ENSMUST0000014
MSTRG.21143		MSTRG.21143.1
MSTRG.2	Gm12381	MSTRG.21145.1
MSTRG.21147		MSTRG.21147.1
MSTRG.21148		MSTRG.21148.1
MSTRG.2	Aco1	ENSMUST00000102973;ENSMUST00000152911;ENSMUST00000142
MSTRG.2	Gm37033	ENSMUST00000193199
MSTRG.2	Ddx58	ENSMUST00000136435;ENSMUST00000137903;MSTRG.21150.2;E
MSTRG.2	Topors	ENSMUST00000042575;MSTRG.21151.1
MSTRG.2	Toporso	ENSMUST00000129758;ENSMUST00000207780
MSTRG.2	Ndufb6	ENSMUST00000095128;ENSMUST00000108108
MSTRG.2	Aptx	ENSMUST00000068125;ENSMUST00000030119;ENSMUST0000010
MSTRG.2	Aptx	MSTRG.21155.1
MSTRG.2	Gm12396	ENSMUST00000121600
MSTRG.2	Gm12397	ENSMUST00000117674
MSTRG.2	Gm12390	ENSMUST00000117897
MSTRG.2	Gm6297	ENSMUST00000181475
MSTRG.2	Itpkb	MSTRG.2116.1
MSTRG.2	Dnaja1;	ENSMUST00000139406;ENSMUST00000030118;MSTRG.21160.3;M
MSTRG.2	Smu1	MSTRG.21161.1;ENSMUST00000030117;MSTRG.21161.3;ENSMUS
MSTRG.2	B4galt1	MSTRG.21162.3;MSTRG.21162.2;ENSMUST00000030121;MSTRG.
MSTRG.2	B4galt1	MSTRG.21163.1
MSTRG.2	B4galt1	MSTRG.21164.1
MSTRG.2	Mir5123	ENSMUST00000177794
MSTRG.2	Gm24112	ENSMUST00000179159
MSTRG.2	Gm25931	ENSMUST00000175230
MSTRG.2	Spink4	ENSMUST00000030122;ENSMUST00000132943
MSTRG.2	Bag1	ENSMUST00000108089;ENSMUST00000191273;MSTRG.21169.3;E
MSTRG.2	Parp1;M	ENSMUST00000194608;ENSMUST00000027777;ENSMUST00000194
MSTRG.2	Chmp5	ENSMUST00000030128;MSTRG.21170.2
MSTRG.2	Aqp3	ENSMUST00000055327;ENSMUST00000154722
MSTRG.2	Nfx1;Mi	ENSMUST00000091614;MSTRG.21172.1;ENSMUST00000030133;M
MSTRG.2	Ube2r2	MSTRG.21173.1
MSTRG.2	Nol6	ENSMUST00000030138;ENSMUST00000155315;ENSMUST0000012
MSTRG.2	Gm12402	ENSMUST00000120536
MSTRG.2	Ube2r2	MSTRG.21176.2;MSTRG.21176.1;ENSMUST00000040008;MSTRG.
MSTRG.2	Ube2r2	MSTRG.21177.1
MSTRG.2	Gm12403	ENSMUST00000121384
MSTRG.2	Gm24650	ENSMUST00000157105
MSTRG.2	Lin9	ENSMUST00000192561;ENSMUST00000192725;ENSMUST0000008
MSTRG.2	Ubap2;G	ENSMUST00000108068;MSTRG.21180.3;MSTRG.21180.2;ENSMUS
MSTRG.2	Gm22888	ENSMUST00000158640
MSTRG.2	Ubap2	MSTRG.21182.1
MSTRG.2	Gm12401	ENSMUST00000118435
MSTRG.2	Ubap2	MSTRG.21184.1

MSTRG.2	Ubap2	MSTRG.21185.1
MSTRG.2	Ubap2	MSTRG.21186.1
MSTRG.2	Ubap2	MSTRG.21187.1
MSTRG.2	Dcaf12	ENSMUST00000030145;MSTRG.21188.2
MSTRG.2	n-R5s18	ENSMUST00000083939
MSTRG.2	Lin9	MSTRG.2119.1
MSTRG.2	Gm26049	ENSMUST00000157313
MSTRG.2	Nudt2	ENSMUST00000030154
MSTRG.2	Gm26084	ENSMUST00000157903
MSTRG.2	Ubap1	ENSMUST00000108060;ENSMUST00000072866;MSTRG.21193.3;M
MSTRG.2	Ubap1	MSTRG.21194.1
MSTRG.2	Kif24	MSTRG.21195.1;ENSMUST00000108055;MSTRG.21195.3;ENSMUS
MSTRG.2	Kif24	MSTRG.21196.1
MSTRG.2	Kif24	MSTRG.21197.1
MSTRG.2	Kif24	MSTRG.21198.1
MSTRG.2	Fam219a	ENSMUST00000151164
MSTRG.2	8030445	ENSMUST00000192614
MSTRG.2	Acbd3	MSTRG.2120.2;ENSMUST00000027780;MSTRG.2120.1;MSTRG.21
MSTRG.2	Fam219a	ENSMUST00000108049;ENSMUST00000108050;ENSMUST00000108
MSTRG.2	Gm12404	ENSMUST00000122839
MSTRG.2	Gm13307	ENSMUST00000141375;ENSMUST00000142990
MSTRG.2	Gm13302	ENSMUST00000119405
MSTRG.2	Gm21541	ENSMUST00000098128
MSTRG.2	Ccl19-p	ENSMUST00000119621
MSTRG.2	Gm20878	ENSMUST00000151103;ENSMUST00000153997;ENSMUST00000108
MSTRG.2	Gm13304	ENSMUST00000098123
MSTRG.2	Galt;Il	ENSMUST00000084695;ENSMUST00000108038;ENSMUST00000135
MSTRG.2	Rpp25l	MSTRG.21209.1;ENSMUST00000038434
MSTRG.2	Gm37768	ENSMUST00000192748
MSTRG.2	Dctn3	ENSMUST00000171641;ENSMUST00000030158;MSTRG.21210.3;E
MSTRG.2	Arid3c	ENSMUST00000171251;ENSMUST00000084698;ENSMUST0000015
MSTRG.2	Sigmar1	ENSMUST00000059354;ENSMUST00000071561;ENSMUST0000014
MSTRG.2	Ccl27a	ENSMUST00000108033;ENSMUST00000151909;ENSMUST0000010
MSTRG.2	Dnajb5	ENSMUST00000037872;ENSMUST00000098112;ENSMUST0000012
MSTRG.2	Gm13299	ENSMUST00000133644
MSTRG.2	Ccl19-p	ENSMUST00000179634
MSTRG.2	Gm13306	ENSMUST00000131704;ENSMUST00000154600;ENSMUST0000010
MSTRG.2	Gm13305	ENSMUST00000098121;ENSMUST00000152686;ENSMUST0000014
MSTRG.2	1700045	ENSMUST00000194752
MSTRG.2122		MSTRG.2122.1
MSTRG.2	Gm17167	ENSMUST00000169242;ENSMUST00000121728
MSTRG.2	Gm5859	ENSMUST00000165401
MSTRG.2	Gm17081	ENSMUST00000167363
MSTRG.2	Gm10600	ENSMUST00000117202
MSTRG.2	Ccl21b	ENSMUST00000178864
MSTRG.2	Gm21953	ENSMUST00000108002
MSTRG.2	Gm21586	ENSMUST00000107988
MSTRG.2	Gm3883	ENSMUST00000098119
MSTRG.2	Gm38293	ENSMUST00000193209
MSTRG.2	Gm2163	ENSMUST00000179734
MSTRG.2	Fam205a	ENSMUST00000180201
MSTRG.2	Gm2163	MSTRG.21232.1
MSTRG.2	Gm10593	ENSMUST00000178906
MSTRG.2	Gm10591	ENSMUST00000178168
MSTRG.2	Gm2564	ENSMUST00000166898
MSTRG.2	Ccl27b	ENSMUST00000177785
MSTRG.2	Il11ra2	ENSMUST00000108006

MSTRG.21238		MSTRG.21238.1
MSTRG.2	Gm21955	ENSMUST00000194137
MSTRG.2	Gm38293	MSTRG.2124.1
MSTRG.2	4930578	ENSMUST00000145453;ENSMUST00000068158;ENSMUST00000107
MSTRG.2	Gm12395	ENSMUST00000144765
MSTRG.2	Ccl19	ENSMUST00000102957;ENSMUST00000134240;ENSMUST0000014
MSTRG.2	Ccl21a	ENSMUST00000095114
MSTRG.2	Gm12394	ENSMUST00000107981
MSTRG.2	4930578	MSTRG.21245.1
MSTRG.2	Fam205a	ENSMUST00000107979
MSTRG.2	Fam205c	ENSMUST00000055944;ENSMUST00000107978;ENSMUST00000144
MSTRG.2	1700022	ENSMUST00000030163;ENSMUST00000139127
MSTRG.2	Vcp	ENSMUST00000030164;ENSMUST00000154541;ENSMUST00000154
MSTRG.2	Gm38293	MSTRG.2125.1
MSTRG.2	Fancg	ENSMUST00000030165;ENSMUST00000123332;ENSMUST0000014
MSTRG.2	Fancg	MSTRG.21251.1
MSTRG.2	Pigo	ENSMUST00000149333;ENSMUST00000098109;ENSMUST00000067
MSTRG.2	Stoml2	ENSMUST00000030169;ENSMUST00000125180;ENSMUST0000015
MSTRG.2	Gm26643	ENSMUST00000180854
MSTRG.2	Fam214b	ENSMUST00000107958;ENSMUST00000107959;ENSMUST00000107
MSTRG.2	Gm26881	ENSMUST00000209785;ENSMUST00000180426;ENSMUST00000211
MSTRG.2	Rusc2	ENSMUST00000035645;ENSMUST00000131037;ENSMUST0000013
MSTRG.2	Fam166b	ENSMUST00000052829;ENSMUST00000171134;ENSMUST00000107
MSTRG.2	Tesk1	ENSMUST00000060864;ENSMUST00000138981;ENSMUST0000012
MSTRG.2	H3f3a	ENSMUST00000161308;MSTRG.2126.1;ENSMUST00000081026;EN
MSTRG.2	Cd72	ENSMUST00000098104;ENSMUST00000107926;ENSMUST00000107
MSTRG.2	Sit1	MSTRG.21261.1;ENSMUST00000030180;ENSMUST00000123526;
MSTRG.2	Rmrp	ENSMUST00000157463
MSTRG.2	Ccdc107	MSTRG.21263.1;ENSMUST00000143073;MSTRG.21263.3;ENSMUS
MSTRG.2	Arhgef3	ENSMUST00000152134;ENSMUST00000054538;ENSMUST0000012
MSTRG.2	Gm12454	ENSMUST00000149817;MSTRG.21265.2;ENSMUST00000139119
MSTRG.2	Car9	ENSMUST00000030183;ENSMUST00000128232;ENSMUST0000012
MSTRG.2	Tpm2	ENSMUST00000150262;ENSMUST00000030184;ENSMUST00000107
MSTRG.2	Fam221b	ENSMUST00000056474;ENSMUST00000134487
MSTRG.2	Gm17275	MSTRG.2127.1;ENSMUST00000181811
MSTRG.2	Tmem8b	ENSMUST00000107865;ENSMUST00000167153;ENSMUST00000107
MSTRG.2	Gm23257	ENSMUST00000158322
MSTRG.2	Spaar	ENSMUST00000131248;ENSMUST00000142309
MSTRG.2	Spaar	MSTRG.21273.1
MSTRG.2	Tln1	ENSMUST00000030187;MSTRG.21274.1;ENSMUST00000134623;
MSTRG.2	Creb3	ENSMUST00000102944;MSTRG.21275.2;ENSMUST00000167751;
MSTRG.2	Gba2	ENSMUST00000128403;ENSMUST00000152251;ENSMUST0000003
MSTRG.2	Rgp1	ENSMUST00000107886;ENSMUST00000117140;ENSMUST00000131
MSTRG.2	Msmp	ENSMUST00000107884
MSTRG.2	Glipr2	ENSMUST00000030202;ENSMUST00000107855
MSTRG.2	H3f3aos	ENSMUST00000159740;ENSMUST00000097448
MSTRG.2	Glipr2	MSTRG.21280.1
MSTRG.2	Glipr2	MSTRG.21281.1
MSTRG.2	Ccin	ENSMUST00000095107
MSTRG.2	Gm12503	ENSMUST00000146576
MSTRG.21284		MSTRG.21284.1
MSTRG.2	Gm12504	ENSMUST00000120860;ENSMUST00000148026
MSTRG.2	Clta	ENSMUST00000107846;ENSMUST00000107847;ENSMUST0000017
MSTRG.2	Gne	ENSMUST00000030201;ENSMUST00000174522;ENSMUST00000102
MSTRG.2	Gne	MSTRG.21288.1
MSTRG.2	Gm22706	ENSMUST00000157907
MSTRG.2129		MSTRG.2129.1

MSTRG.2	Melk	ENSMUST00000045607;ENSMUST00000137703;ENSMUST00000125
MSTRG.2	Gm12677	ENSMUST00000118668
MSTRG.2	Rnf38	ENSMUST00000098098;ENSMUST00000102934;ENSMUST00000045
MSTRG.2	Mir5106	ENSMUST00000174936
MSTRG.2	Zcchc7	ENSMUST00000154121;ENSMUST00000107824;ENSMUST00000144
MSTRG.2	Zcchc7	MSTRG.21295.1;MSTRG.21295.2
MSTRG.2	Gm22639	ENSMUST00000175591;MSTRG.21296.1
MSTRG.2	Zcchc7	MSTRG.21297.1
MSTRG.2	Grhpr	ENSMUST00000045078;ENSMUST00000128973;ENSMUST00000151
MSTRG.2	Zbtb5	ENSMUST00000055028;ENSMUST00000180217;ENSMUST00000107
MSTRG.2	Gm38198	ENSMUST00000191772
MSTRG.2	Gm24836	ENSMUST00000122609
MSTRG.2	Zbtb5	MSTRG.21300.1
MSTRG.2	1700055	ENSMUST00000186030;ENSMUST00000095105
MSTRG.2	Tomm5	ENSMUST00000107810;ENSMUST00000107809;ENSMUST00000107
MSTRG.2	Polr1e	ENSMUST00000133157;ENSMUST00000029999;ENSMUST00000107
MSTRG.2	Fbxo10	ENSMUST00000052236;ENSMUST00000155583
MSTRG.2	Trmt10b	ENSMUST00000044673;ENSMUST00000144781;ENSMUST00000141
MSTRG.2	Trmt10b	MSTRG.21306.1
MSTRG.2	Trmt10b	MSTRG.21307.1
MSTRG.2	Exosc3	ENSMUST00000030003;ENSMUST00000143111;ENSMUST00000152
MSTRG.2	Sde2	ENSMUST00000038091;MSTRG.2131.1;ENSMUST00000194948;MS
MSTRG.2	Dcaf10	ENSMUST00000155551;ENSMUST00000130821;MSTRG.21310.2;E
MSTRG.2	Slc25a5	MSTRG.21311.1;ENSMUST00000107796;ENSMUST00000116341;E
MSTRG.2	Shb	ENSMUST00000147926;ENSMUST00000061986;MSTRG.21312.3;E
MSTRG.2	Gm22518	ENSMUST00000158267
MSTRG.2	Gm12411	ENSMUST00000119140
MSTRG.2	Aldh1b1	ENSMUST00000044384;ENSMUST00000172750
MSTRG.2	Tdrd7	ENSMUST00000107777;ENSMUST00000102929;ENSMUST00000151
MSTRG.2	Tmod1	ENSMUST00000107773;ENSMUST00000128689;ENSMUST00000155
MSTRG.2	Tstd2	ENSMUST00000107772;ENSMUST00000107770;ENSMUST0000016
MSTRG.2	Gm23250	ENSMUST00000157444
MSTRG.2	Gm20305	ENSMUST00000193226
MSTRG.2	Tstd2	MSTRG.21320.1
MSTRG.2	Tstd2	MSTRG.21321.1
MSTRG.2	Ncbp1	ENSMUST00000030014;ENSMUST00000131187;ENSMUST00000133
MSTRG.2	Ncbp1	MSTRG.21323.1
MSTRG.2	Xpa	ENSMUST00000058232;ENSMUST00000132358;ENSMUST00000141
MSTRG.2	Anp32b	ENSMUST00000102926;MSTRG.21325.1
MSTRG.2	Anp32b	MSTRG.21326.1
MSTRG.2	Nans	ENSMUST00000030018;ENSMUST00000132345;ENSMUST0000015
MSTRG.2	Trim14	ENSMUST00000184112;MSTRG.21328.1;ENSMUST00000136978;E
MSTRG.2	Gm22247	ENSMUST00000082476
MSTRG.2	Pycr2	ENSMUST00000027802;ENSMUST00000194026;ENSMUST00000194
MSTRG.2	Trim14	MSTRG.21330.1
MSTRG.2	Gm16731	ENSMUST00000132432
MSTRG.2	Coro2a	ENSMUST00000030021;ENSMUST00000107757;ENSMUST00000107
MSTRG.2	Gm568	ENSMUST00000135777
MSTRG.2	Anks6	ENSMUST00000107747;ENSMUST00000084616;ENSMUST00000152
MSTRG.2	Gm12425	ENSMUST00000119580
MSTRG.2	Galnt12	ENSMUST00000045041;ENSMUST00000107744;MSTRG.21336.3;M
MSTRG.2	Galnt12	MSTRG.21338.1
MSTRG.2	Lefty1	ENSMUST00000037361;ENSMUST00000132087
MSTRG.2	Col15a1	ENSMUST00000082303;ENSMUST00000102917;ENSMUST00000124
MSTRG.21341		MSTRG.21341.1
MSTRG.2	Gm12430	ENSMUST00000121539
MSTRG.2	Tgfbr1	ENSMUST00000007757;ENSMUST00000044234;ENSMUST00000123

MSTRG.2	Tgfbr1	MSTRG.21344.1
MSTRG.2	Gm12430	MSTRG.21345.1
MSTRG.2	Alg2	ENSMUST00000136912;ENSMUST00000044148;ENSMUST00000143
MSTRG.2	Sec61b	ENSMUST00000065678;ENSMUST00000137461;ENSMUST00000125
MSTRG.21348		MSTRG.21348.1
MSTRG.21349		MSTRG.21349.1
MSTRG.2	Tmem63a	ENSMUST00000161847;MSTRG.2135.1;ENSMUST00000162283;EN
MSTRG.21350		MSTRG.21350.1
MSTRG.2	Nr4a3	ENSMUST00000030025;ENSMUST00000154186;ENSMUST00000153
MSTRG.2	Stx17	ENSMUST00000107721;ENSMUST00000153502;MSTRG.21352.2;E
MSTRG.2	Erp44	ENSMUST00000030028;ENSMUST00000138743;ENSMUST00000132
MSTRG.2	Mir1958	ENSMUST00000157219
MSTRG.2	Invs	ENSMUST00000156602;MSTRG.21355.1;ENSMUST00000151762;E
MSTRG.2	Invs	MSTRG.21356.1
MSTRG.2	Gm12435	ENSMUST00000118949
MSTRG.2	Invs	MSTRG.21358.1
MSTRG.2	Tex10	ENSMUST00000155905;ENSMUST00000030030;ENSMUST00000164
MSTRG.2	2210411	ENSMUST00000128545
MSTRG.2	Tex10	MSTRG.21360.1
MSTRG.2	Acnat2	ENSMUST00000139564
MSTRG.2	Acnat2	MSTRG.21363.1
MSTRG.2	Gm12452	ENSMUST00000117128
MSTRG.2	Baat	ENSMUST00000043056;ENSMUST00000166036
MSTRG.2	Mrpl50	ENSMUST00000057829
MSTRG.2	Zfp189	ENSMUST00000042964;ENSMUST00000107696
MSTRG.2	Aldob	ENSMUST00000029987;ENSMUST00000148415;ENSMUST00000144
MSTRG.2	Rnf20	ENSMUST00000140341;ENSMUST00000156314;ENSMUST00000167
MSTRG.2	Gm36961	ENSMUST00000191980;ENSMUST00000154133;ENSMUST00000036
MSTRG.2	Rnf20	MSTRG.21370.1
MSTRG.21371		MSTRG.21371.1
MSTRG.2	Grin3a	ENSMUST00000076674;ENSMUST00000093859;ENSMUST00000149
MSTRG.2	Ppp3r2	ENSMUST00000029991
MSTRG.2	Gm22042	ENSMUST00000082989
MSTRG.21376		MSTRG.21376.1
MSTRG.2	Smc2	ENSMUST00000102915;ENSMUST00000117280;ENSMUST00000131
MSTRG.2	Tmc4-ps	ENSMUST00000117819
MSTRG.2	Nipsnap	ENSMUST00000015391;ENSMUST00000107665;ENSMUST00000132
MSTRG.2	Nvl	ENSMUST00000027797;ENSMUST00000191721;ENSMUST00000195
MSTRG.2	Abca1	ENSMUST00000030010;ENSMUST00000149127;MSTRG.21380.2;M
MSTRG.2	Slc44a1	ENSMUST00000107646;ENSMUST00000102911;ENSMUST00000136
MSTRG.2	1700060	ENSMUST00000138377
MSTRG.2	Fsd1l	ENSMUST00000132151;ENSMUST00000107643;ENSMUST00000162
MSTRG.2	Gm12470	ENSMUST00000117239
MSTRG.2	Fsd1l	MSTRG.21385.1
MSTRG.2	Fktn	ENSMUST00000128667;ENSMUST00000107638;ENSMUST00000222
MSTRG.2	Gm25445	ENSMUST00000158996
MSTRG.2	Tmem38b	ENSMUST00000030127;ENSMUST00000150803;ENSMUST00000151
MSTRG.2	Tmem38b	MSTRG.21389.1
MSTRG.2	Tmem38b	MSTRG.21390.1
MSTRG.2	Zfp462	ENSMUST00000133895;ENSMUST00000098070;ENSMUST00000030
MSTRG.2	Zfp462	MSTRG.21392.1
MSTRG.2	Zfp462	MSTRG.21393.1
MSTRG.21394		MSTRG.21394.1
MSTRG.2	Rad23b	MSTRG.21395.1
MSTRG.2	Rad23b	ENSMUST00000030134;ENSMUST00000127266;ENSMUST00000148
MSTRG.2	Rad23b	MSTRG.21397.1
MSTRG.2	Gm12516	ENSMUST00000120819

MSTRG.2	Klf4	ENSMUST00000107619;ENSMUST00000129250;ENSMUST00000132
MSTRG.2	Gm37077	ENSMUST00000195723
MSTRG.2	Cnih4	ENSMUST00000193907;ENSMUST00000111059;ENSMUST00000134
MSTRG.2	Epb41l4	ENSMUST00000095076;ENSMUST00000044022;ENSMUST0000003C
MSTRG.21401		MSTRG.21401.1
MSTRG.2	Gm26144	ENSMUST00000104475
MSTRG.2	Actl7b	ENSMUST00000095080
MSTRG.2	Gm26657	ENSMUST00000181745
MSTRG.2	Actl7a	ENSMUST00000095079
MSTRG.2	Ikbkap	ENSMUST00000030140;ENSMUST00000171411;ENSMUST0000015C
MSTRG.2	Gm22952	ENSMUST00000116720
MSTRG.2	Fam206a	ENSMUST00000152638;ENSMUST00000045368;ENSMUST00000131
MSTRG.2	Ctnnal1	ENSMUST00000045142;MSTRG.21409.2;ENSMUST00000131231;E
MSTRG.2	Cnih4	MSTRG.2141.1
MSTRG.2	Tmem245	ENSMUST00000068792;MSTRG.21410.2;MSTRG.21410.3;ENSMUS
MSTRG.2	Mir32	ENSMUST00000083610
MSTRG.2	Gm25053	ENSMUST00000103982;ENSMUST00000199731
MSTRG.2	Gm22300	ENSMUST00000082661
MSTRG.2	Tmem245	MSTRG.21414.1;MSTRG.21414.2
MSTRG.2	Tmem245	MSTRG.21415.1
MSTRG.2	Tmem245	MSTRG.21416.1
MSTRG.2	Gm12537	ENSMUST00000119266
MSTRG.2	Ptpn3	MSTRG.21418.1
MSTRG.2	Ptpn3	ENSMUST00000075637;ENSMUST00000123664;ENSMUST00000153
MSTRG.2	Cnih4	MSTRG.2142.1
MSTRG.2	Ptpn3	MSTRG.21420.1
MSTRG.2	Ptpn3	MSTRG.21421.1
MSTRG.2	Ptpn3	MSTRG.21422.1
MSTRG.2	Ptpn3	MSTRG.21423.1
MSTRG.2	Gm12536	ENSMUST00000129102
MSTRG.21425		MSTRG.21425.1
MSTRG.2	Txn1	ENSMUST00000030051
MSTRG.2	Palm2;P	ENSMUST00000102905;ENSMUST00000102904;ENSMUST00000098
MSTRG.2	Gm12539	ENSMUST00000121549
MSTRG.2	Gm12526	ENSMUST00000154462
MSTRG.2143		MSTRG.2143.1
MSTRG.2	Gm24277	ENSMUST00000158661
MSTRG.2	Pakap	MSTRG.21431.1
MSTRG.2	Pakap	MSTRG.21432.1
MSTRG.2	Pakap	MSTRG.21433.1
MSTRG.2	Gm26138	ENSMUST00000179236
MSTRG.2	Lpar1	ENSMUST00000107570;ENSMUST00000107571;ENSMUST00000055
MSTRG.2	Mir3095	ENSMUST00000175552
MSTRG.2	Gm12577	ENSMUST00000119701
MSTRG.2	Ptgr1	ENSMUST00000030069
MSTRG.2	AI31418	MSTRG.21439.1;MSTRG.21439.2;ENSMUST00000102889;ENSMUS
MSTRG.2144		MSTRG.2144.1
MSTRG.2	AI31418	MSTRG.21440.1
MSTRG.2	Dnajc25	ENSMUST00000148366;ENSMUST00000152199;ENSMUST00000095
MSTRG.2	Dnajc25	MSTRG.21442.1
MSTRG.2	Dnajc25	MSTRG.21443.1
MSTRG.2	Dnajc25	MSTRG.21444.1
MSTRG.2	Gm20503	MSTRG.21445.1
MSTRG.2	Gm20503	MSTRG.21446.1
MSTRG.21447		MSTRG.21447.1
MSTRG.2	Ugcg	ENSMUST00000030074;ENSMUST00000133996;ENSMUST00000128
MSTRG.2	Ugcg	MSTRG.21449.1

MSTRG.2	A430110	ENSMUST00000181680;ENSMUST00000066988
MSTRG.21450		MSTRG.21450.1
MSTRG.21451		MSTRG.21451.1
MSTRG.2	Susd1	MSTRG.21452.1
MSTRG.2	Susd1	ENSMUST00000136942;MSTRG.21453.1;ENSMUST00000040166;E
MSTRG.2	Gm12527	ENSMUST00000117967
MSTRG.2	Susd1	MSTRG.21455.1
MSTRG.2	Susd1	MSTRG.21456.1
MSTRG.2	Susd1	MSTRG.21457.1
MSTRG.2	Ptbp3	ENSMUST00000030076;MSTRG.21458.2;ENSMUST00000174586;E
MSTRG.2	Gm12529	ENSMUST00000119529
MSTRG.2	Wdr26	ENSMUST00000162819;MSTRG.2146.2;MSTRG.2146.3;MSTRG.21
MSTRG.2	Ptbp3	MSTRG.21460.1
MSTRG.2	1700018	ENSMUST00000052502
MSTRG.2	Slc46a2	ENSMUST00000030081;MSTRG.21462.2
MSTRG.2	Hsdl2	ENSMUST00000030078;ENSMUST00000107528;ENSMUST00000128
MSTRG.2	E130308	ENSMUST00000070150;ENSMUST00000052420;ENSMUST00000152
MSTRG.2	E130308	MSTRG.21465.1
MSTRG.2	E130308	MSTRG.21466.1
MSTRG.2	E130308	MSTRG.21467.1
MSTRG.2	E130308	MSTRG.21468.1
MSTRG.2	E130308	MSTRG.21469.1
MSTRG.2	Rpl35a-	ENSMUST00000117444
MSTRG.2	E130308	MSTRG.21470.1
MSTRG.2	E130308	MSTRG.21471.1
MSTRG.2	E130308	MSTRG.21472.1
MSTRG.2	E130308	MSTRG.21473.1
MSTRG.2	Inip	MSTRG.21474.1;MSTRG.21474.2;ENSMUST00000095063;ENSMUS
MSTRG.2	Snx30;G	ENSMUST00000030080;ENSMUST00000123665;ENSMUST0000012C
MSTRG.2	Snx30	MSTRG.21476.1
MSTRG.2	Snx30	MSTRG.21477.1
MSTRG.2	Gm12542	ENSMUST00000133015
MSTRG.21479		MSTRG.21479.1
MSTRG.2148		MSTRG.2148.1
MSTRG.2	Bspry	ENSMUST00000030088;ENSMUST00000107449;ENSMUST00000132
MSTRG.2	Bspry	MSTRG.21481.1
MSTRG.2	Hdhd3	ENSMUST00000037820;ENSMUST00000140038
MSTRG.2	Slc31a2	ENSMUST00000107468;ENSMUST00000084530;MSTRG.21483.2;M
MSTRG.2	Gm23148	ENSMUST00000180367
MSTRG.2	Fkbp15	ENSMUST00000139308;ENSMUST00000084527;MSTRG.21485.3;M
MSTRG.2	Slc31a1	ENSMUST00000084526;ENSMUST00000122092
MSTRG.2	Cdc26	ENSMUST00000134727;ENSMUST00000084525;ENSMUST00000107
MSTRG.2	Cdc26	MSTRG.21488.1
MSTRG.2	Prpf4	MSTRG.21489.1;ENSMUST00000084524;ENSMUST00000148774
MSTRG.2	Dnah14	ENSMUST00000208001;ENSMUST00000195458;ENSMUST00000195
MSTRG.2	Rnf183	ENSMUST00000107455;ENSMUST00000107454;ENSMUST0000007S
MSTRG.2	Alad	ENSMUST00000137448;ENSMUST00000107444;ENSMUST0000003C
MSTRG.2	Pole3	ENSMUST00000030091;ENSMUST00000128100;ENSMUST0000012S
MSTRG.2	4933430	ENSMUST00000062145
MSTRG.2	Rgs3	ENSMUST00000065870;ENSMUST00000098031;MSTRG.21494.2;M
MSTRG.2	Gm11209	ENSMUST00000130206
MSTRG.2	Gm11210	ENSMUST00000150203
MSTRG.2	Gm11211	ENSMUST00000143618
MSTRG.21498		MSTRG.21498.1
MSTRG.2	Col27a1	ENSMUST00000125504;ENSMUST00000148751;ENSMUST0000003G
MSTRG.2	Gm37895	ENSMUST00000192448
MSTRG.2	Gm37664	ENSMUST00000194607

MSTRG.2	Mir455	ENSMUST00000093594
MSTRG.2	Orm1	ENSMUST00000030044
MSTRG.2	Gm11212	ENSMUST00000118337
MSTRG.2	Orm2;Or	MSTRG.21503.1;ENSMUST00000006687;ENSMUST00000075341
MSTRG.2	Akna	ENSMUST00000035724;MSTRG.21504.1;MSTRG.21504.2;MSTRG.
MSTRG.2	Aknaos	ENSMUST00000144095
MSTRG.2	Whrn	ENSMUST00000145133;ENSMUST00000155058;MSTRG.21506.3;E
MSTRG.2	Atp6v1g	ENSMUST00000035301;MSTRG.21507.2;MSTRG.21507.3;MSTRG.
MSTRG.21508		MSTRG.21508.1
MSTRG.2	Tmem268	ENSMUST00000077709;ENSMUST00000126738;ENSMUST00000124
MSTRG.2	Gm11483	ENSMUST00000119839
MSTRG.2	Gm24448	ENSMUST00000083979
MSTRG.2	Tnfsf8	ENSMUST00000030047
MSTRG.2	Tnc	ENSMUST00000030056;ENSMUST00000107372;ENSMUST00000107
MSTRG.2	8030451	ENSMUST00000141428;ENSMUST00000135042;ENSMUST0000013
MSTRG.2	Tnc	MSTRG.21515.1
MSTRG.21517		MSTRG.21517.1
MSTRG.2	Astn2	ENSMUST00000084496;ENSMUST00000068214
MSTRG.2	Trim32	ENSMUST00000050850;ENSMUST00000107366;ENSMUST00000155
MSTRG.2	Dnah14	MSTRG.2152.1
MSTRG.2	Gm11484	ENSMUST00000119808
MSTRG.2	Tlr4	ENSMUST00000048096;ENSMUST00000107365;ENSMUST0000014
MSTRG.2	Gm11249	ENSMUST00000117071
MSTRG.2	Gm11751	MSTRG.21524.1
MSTRG.2	Gm11223	MSTRG.21525.1;MSTRG.21525.2;ENSMUST00000120560
MSTRG.2	Megf9	ENSMUST00000107359;ENSMUST00000037881
MSTRG.2	Megf9	MSTRG.21527.1
MSTRG.2	Cdk5rap	MSTRG.21528.1;ENSMUST00000144099;ENSMUST00000076541;E
MSTRG.2	Cdk5rap	MSTRG.21529.1
MSTRG.2	Lbr;Gm2	ENSMUST00000005003;ENSMUST00000192075;MSTRG.2153.2;EN
MSTRG.2	Cdk5rap	MSTRG.21530.1
MSTRG.2	Cdk5rap	MSTRG.21531.1
MSTRG.2	Cdk5rap	MSTRG.21532.1
MSTRG.2	Cdk5rap	MSTRG.21533.1
MSTRG.21534		MSTRG.21534.1
MSTRG.21535		MSTRG.21535.1
MSTRG.21536		MSTRG.21536.1
MSTRG.2	Tle1	ENSMUST00000074216;ENSMUST00000072695;ENSMUST00000102
MSTRG.2	Gm11250	ENSMUST00000142137
MSTRG.2	Rasef	ENSMUST00000058292;ENSMUST00000102837;ENSMUST00000222
MSTRG.2	Enah	ENSMUST00000193703;ENSMUST00000111024;ENSMUST00000111
MSTRG.2	Gm11240	ENSMUST00000142047
MSTRG.2	Tmem261	ENSMUST00000030103
MSTRG.2	Kdm4c	ENSMUST00000030102;ENSMUST00000156065;ENSMUST0000014
MSTRG.2	A230083	ENSMUST00000126646
MSTRG.2	Kdm4c	MSTRG.21544.1
MSTRG.21545		MSTRG.21545.1
MSTRG.2	Gm11263	ENSMUST00000121853
MSTRG.21547		MSTRG.21547.1
MSTRG.21548		MSTRG.21548.1
MSTRG.2	Ptprd	ENSMUST00000107289;ENSMUST00000102834;ENSMUST0000017
MSTRG.2	2700078	ENSMUST00000194331
MSTRG.2	Ptprd	MSTRG.21550.1
MSTRG.2	Gm11252	ENSMUST00000120572
MSTRG.2	Ptprd	MSTRG.21552.1
MSTRG.2	Gm42303	ENSMUST00000209960
MSTRG.2	Gm11242	ENSMUST00000120103

MSTRG.2 Gm11243 ENSMUST00000122335
MSTRG.2 Gm11244 ENSMUST00000121243
MSTRG.2 Gm11245 ENSMUST00000119419
MSTRG.2 Gm11246 ENSMUST00000120880
MSTRG.2 Gm38359 ENSMUST00000191884
MSTRG.2 Nfib ENSMUST00000050872;ENSMUST00000107245;ENSMUST00000064
MSTRG.2 n-R5s18 ENSMUST00000122646
MSTRG.2 Nfib MSTRG.21563.1
MSTRG.2 Gm11266 ENSMUST00000139835
MSTRG.2 Gm11269 ENSMUST00000157011
MSTRG.2 Zdhhc21 MSTRG.21568.1;ENSMUST00000030110;ENSMUST00000173741;E
MSTRG.2 Zdhhc21 MSTRG.21569.1
MSTRG.2 Gm37018 ENSMUST00000192441
MSTRG.2 Ttc39b MSTRG.21570.1;ENSMUST00000102823;ENSMUST00000143362;E
MSTRG.2 Gm23412 ENSMUST00000083017
MSTRG.2 Snapc3 ENSMUST00000030206;ENSMUST00000123262;MSTRG.21572.1;E
MSTRG.2 Psip1 MSTRG.21573.1;ENSMUST00000030207;ENSMUST00000152382;E
MSTRG.2 Ccdc171 ENSMUST00000053414;ENSMUST00000126429;ENSMUST00000125
MSTRG.2 Gm25899 ENSMUST00000082672
MSTRG.2 Ccdc171 MSTRG.21576.1
MSTRG.2 Gm27046 ENSMUST00000182160
MSTRG.2 Cbx3-ps ENSMUST00000118595
MSTRG.2 Gm26968 ENSMUST00000182894
MSTRG.2 Gm37258 ENSMUST00000191734
MSTRG.2 Bnc2 ENSMUST00000102820;ENSMUST00000107198;ENSMUST00000176
MSTRG.2 Gm12421 ENSMUST00000119071
MSTRG.2 Bnc2 MSTRG.21582.1
MSTRG.2 Cntln ENSMUST00000047023;ENSMUST00000102819;ENSMUST00000169
MSTRG.2 Gm12418 ENSMUST00000118116
MSTRG.2 Cntln MSTRG.21585.1
MSTRG.2 Gm12419 ENSMUST00000116649
MSTRG.2 Rraga ENSMUST00000091064
MSTRG.2 Haus6 MSTRG.21588.1;ENSMUST00000070607;MSTRG.21588.3;MSTRG.
MSTRG.2 Scarna8 ENSMUST00000158333
MSTRG.2 Srp9 ENSMUST00000111018;ENSMUST00000027792;ENSMUST00000191
MSTRG.2 Plin2 ENSMUST00000154999;MSTRG.21590.1;ENSMUST00000000466;E
MSTRG.2 Rps6 ENSMUST00000102814;MSTRG.21591.2;ENSMUST00000136174;E
MSTRG.2 Dennd4c ENSMUST00000045512;ENSMUST00000082026;MSTRG.21592.3;M
MSTRG.2 Dennd4c MSTRG.21593.1
MSTRG.2 Gm12630 ENSMUST00000121575
MSTRG.2 Dennd4c MSTRG.21595.1
MSTRG.2 Acer2 ENSMUST00000045224;ENSMUST00000084433;ENSMUST00000128
MSTRG.21597 MSTRG.21597.1
MSTRG.2 Mllt3 ENSMUST00000078090;ENSMUST00000126353;ENSMUST00000149
MSTRG.2 Mllt3 MSTRG.21599.1
MSTRG.2 Gm36952 ENSMUST00000194923
MSTRG.2 Mllt3 MSTRG.21600.1;MSTRG.21600.2
MSTRG.2 Mllt3 MSTRG.21601.1
MSTRG.2 Mllt3 MSTRG.21602.1
MSTRG.2 Mllt3 MSTRG.21603.1;MSTRG.21603.2;MSTRG.21603.3
MSTRG.2 Mllt3 MSTRG.21604.1
MSTRG.2 Gm12631 ENSMUST00000130576
MSTRG.2 Klhl9 ENSMUST00000094993
MSTRG.2 Gm26566 ENSMUST00000181601
MSTRG.21608 MSTRG.21608.1
MSTRG.2 Focad ENSMUST00000129967;ENSMUST00000097992;ENSMUST00000161
MSTRG.2161 MSTRG.2161.1;MSTRG.2161.2;MSTRG.2161.3

MSTRG.2	Mir491	ENSMUST00000093569
MSTRG.2	Gm12645	ENSMUST00000117297
MSTRG.2	Focad	MSTRG.21612.1
MSTRG.2	Focad	MSTRG.21613.1
MSTRG.2	Hacd4	ENSMUST00000132493;ENSMUST00000151280;ENSMUST0000003C
MSTRG.2	Gm10583	MSTRG.21615.1
MSTRG.2	Gm13284	MSTRG.21616.1;ENSMUST00000191112;ENSMUST00000179425
MSTRG.2	Gm13286	ENSMUST00000117750
MSTRG.2	Gm10583	ENSMUST00000097988
MSTRG.2	Gm10582	ENSMUST00000097987
MSTRG.2	Degs1	ENSMUST00000035295;MSTRG.2162.2;MSTRG.2162.3;ENSMUSTC
MSTRG.2	Gm13284	ENSMUST00000121020
MSTRG.2	Gm10581	ENSMUST00000097986
MSTRG.2	Gm13290	ENSMUST00000177806
MSTRG.2	Gm10580	ENSMUST00000097985
MSTRG.2	Gm13289	ENSMUST00000179158
MSTRG.21625		MSTRG.21625.1
MSTRG.2	Gm13272	MSTRG.21626.1
MSTRG.2	Ifnz	ENSMUST00000105144
MSTRG.21628		MSTRG.21628.1
MSTRG.2	Gm26525	ENSMUST00000180666;MSTRG.21629.2
MSTRG.2	1700047I	MSTRG.2163.1
MSTRG.2	Gm13276	ENSMUST00000179372
MSTRG.2	Gm13277	ENSMUST00000178480
MSTRG.2	Gm13278	ENSMUST00000177771
MSTRG.2	Gm13275	ENSMUST00000178347
MSTRG.2	Gm26867	ENSMUST00000180963
MSTRG.2	Gm13279	ENSMUST00000178602
MSTRG.2	Gm13285	ENSMUST00000179725
MSTRG.2	Mtap	ENSMUST00000058030;ENSMUST00000204571
MSTRG.2	Gm12607	ENSMUST00000120724
MSTRG.2	Cdkn2a	ENSMUST00000060501;ENSMUST00000107131
MSTRG.2	1700047I	ENSMUST00000189594
MSTRG.2	Cdkn2b	ENSMUST00000097981
MSTRG.2	Gm12669	ENSMUST00000122162
MSTRG.2	Gm12671	ENSMUST00000119978
MSTRG.2	Gm12666	ENSMUST00000123179
MSTRG.21644		MSTRG.21644.1
MSTRG.21645		MSTRG.21645.1
MSTRG.2	Fbxo28	MSTRG.2165.1;MSTRG.2165.2;ENSMUST00000051431;ENSMUSTC
MSTRG.2	Gm12649	MSTRG.21650.1
MSTRG.2	Gm12655	ENSMUST00000133884;ENSMUST00000127678
MSTRG.2	Gm12655	MSTRG.21652.1
MSTRG.21653		MSTRG.21653.1
MSTRG.2	Gm22804	ENSMUST00000158052
MSTRG.21655		MSTRG.21655.1
MSTRG.2	Gm12656	ENSMUST00000118149
MSTRG.2	Caap1	ENSMUST00000030313;MSTRG.21657.2;ENSMUST00000153294
MSTRG.2	Caap1	MSTRG.21658.1
MSTRG.2	Caap1	MSTRG.21659.1
MSTRG.2	Gm10517	ENSMUST00000097444
MSTRG.2	Caap1	MSTRG.21660.1
MSTRG.2	Gm10306	ENSMUST00000094969
MSTRG.21662		MSTRG.21662.1
MSTRG.2	Plaa	ENSMUST00000129748;ENSMUST00000107107;ENSMUST00000127
MSTRG.2	Gm12657	ENSMUST00000119560
MSTRG.2	Plaa	MSTRG.21665.1

MSTRG.2	Ift74	ENSMUST00000107104;ENSMUST00000030311;ENSMUST00000152
MSTRG.2	Lrrc19	ENSMUST00000107101
MSTRG.2	Ift74	MSTRG.21668.1
MSTRG.2	Mir872	ENSMUST00000104728
MSTRG.2	Trp53bp	ENSMUST00000117245;ENSMUST00000191804;ENSMUST00000191
MSTRG.2	Gm12693	ENSMUST00000121965
MSTRG.2	Mysm1	MSTRG.21671.1;ENSMUST00000075872;ENSMUST00000124820;E
MSTRG.2	Gm12694	ENSMUST00000149367
MSTRG.2	Jun	ENSMUST00000107094
MSTRG.2	Junos;n	ENSMUST00000142292;ENSMUST00000125917;ENSMUST00000124
MSTRG.2	4930551	ENSMUST00000156086
MSTRG.2	Gm28096	ENSMUST00000191391
MSTRG.2	Cyp2j13	ENSMUST00000132513;ENSMUST00000030305;ENSMUST00000107
MSTRG.2	Cyp2j13	MSTRG.21678.1
MSTRG.2	Cyp2j13	MSTRG.21679.1
MSTRG.2	Trp53bp	MSTRG.2168.1
MSTRG.2	9530080	ENSMUST00000137239
MSTRG.2	Hook1	ENSMUST00000030306;ENSMUST00000125627;ENSMUST00000107
MSTRG.2	Hook1	MSTRG.21683.1
MSTRG.2	Hook1	MSTRG.21684.1
MSTRG.2	Hook1	MSTRG.21685.1
MSTRG.21686		MSTRG.21686.1
MSTRG.2	Gm12696	ENSMUST00000122361
MSTRG.21688		MSTRG.21688.1
MSTRG.2	E130114	ENSMUST00000148653;ENSMUST00000107067;ENSMUST00000126
MSTRG.2	Capn2	ENSMUST00000068505;ENSMUST00000194940;ENSMUST00000194
MSTRG.2	E130114	MSTRG.21690.1
MSTRG.2	Gm12676	ENSMUST00000149078
MSTRG.2	Nfia	MSTRG.21692.1;ENSMUST00000152023;ENSMUST00000075448;E
MSTRG.2	Nfia	MSTRG.21693.1
MSTRG.2	Nfia	MSTRG.21694.1;MSTRG.21694.2
MSTRG.2	Tm2d1	ENSMUST00000125034;ENSMUST00000107051;ENSMUST0000030
MSTRG.2	Tm2d1	MSTRG.21696.1
MSTRG.2	Patj	ENSMUST00000141796;ENSMUST00000041284;ENSMUST00000107
MSTRG.2	Gm26154	ENSMUST00000102339
MSTRG.2	Gm37602	ENSMUST00000195187
MSTRG.2	Gm37885	ENSMUST00000192568
MSTRG.2	Gm12846	ENSMUST00000120431
MSTRG.2	Usp1	ENSMUST00000030289;ENSMUST00000091358;ENSMUST0000016
MSTRG.2	Atg4c	ENSMUST00000030279;ENSMUST00000180278;ENSMUST00000152
MSTRG.2	Atg4c	MSTRG.21703.1
MSTRG.2	Gm10305	ENSMUST00000195331
MSTRG.2	Dock7	ENSMUST00000030286;ENSMUST00000206128;ENSMUST00000127
MSTRG.2	Angptl3	ENSMUST00000030280;ENSMUST00000125546
MSTRG.2	Gm12852	ENSMUST00000119478
MSTRG.2	Gm12705	ENSMUST00000129460
MSTRG.2	Alg6	ENSMUST00000137473;ENSMUST00000144805;ENSMUST00000097
MSTRG.2	Gm37069	ENSMUST00000192742
MSTRG.2	Itgb3bp	ENSMUST00000146258;ENSMUST00000123830;ENSMUST00000102
MSTRG.2	Itgb3bp	MSTRG.21711.1
MSTRG.2	Itgb3bp	MSTRG.21712.1
MSTRG.2	Itgb3bp	MSTRG.21713.1
MSTRG.2	Itgb3bp	MSTRG.21714.1
MSTRG.2	Itgb3bp	MSTRG.21715.1
MSTRG.2	Itgb3bp	MSTRG.21716.1
MSTRG.2	Itgb3bp	MSTRG.21717.1
MSTRG.2	Efcab7	ENSMUST00000143994;ENSMUST00000124547;ENSMUST0000010

MSTRG.2	Efcab7	MSTRG.21719.1
MSTRG.2	Gm24975	ENSMUST00000178492
MSTRG.2	Efcab7	MSTRG.21720.1
MSTRG.2	Gm26425	ENSMUST00000122529
MSTRG.2	Pgm2	ENSMUST00000058351;ENSMUST00000102783
MSTRG.2	Ror1	ENSMUST00000039630
MSTRG.2	Ror1	MSTRG.21724.1
MSTRG.2	Gm12701	MSTRG.21725.1
MSTRG.2	Gm12701	ENSMUST00000134206
MSTRG.2	Ror1	MSTRG.21727.1
MSTRG.2	Ror1	MSTRG.21728.1
MSTRG.2	Ror1	MSTRG.21729.1
MSTRG.2	Disp1	ENSMUST00000171366;MSTRG.2173.1;ENSMUST00000003035;EN
MSTRG.2	Ror1	MSTRG.21730.1
MSTRG.2	Gm12706	ENSMUST00000121588
MSTRG.2	Cachd1	ENSMUST00000030257;ENSMUST00000123475;ENSMUST0000013
MSTRG.2	Cachd1	MSTRG.21733.1
MSTRG.2	Gm10577	ENSMUST00000097954
MSTRG.2	Raver2	ENSMUST00000106955;ENSMUST00000038463;ENSMUST0000014
MSTRG.2	Jak1;Gm	MSTRG.21737.1;ENSMUST00000102781;ENSMUST00000151235;E
MSTRG.2	Gm24468	ENSMUST00000104489
MSTRG.2	Disp1	MSTRG.2174.1
MSTRG.2	Jak1	MSTRG.21740.1
MSTRG.2	E130102	ENSMUST00000144604;ENSMUST00000083517;ENSMUST0000018
MSTRG.2	0610043	ENSMUST00000123107;ENSMUST00000142581
MSTRG.2	Gm12795	ENSMUST00000117594
MSTRG.2	Gm12798	ENSMUST00000124786
MSTRG.2	Leprot	ENSMUST00000030254;ENSMUST00000106927
MSTRG.2	Leprot	MSTRG.21746.1
MSTRG.2	Dnajc6	ENSMUST00000106930;ENSMUST00000154120;ENSMUST0000003
MSTRG.2	Gm22533	ENSMUST00000158821;MSTRG.21748.1
MSTRG.2	Dnajc6	MSTRG.21749.1
MSTRG.2	Disp1	MSTRG.2175.1
MSTRG.21750		MSTRG.21750.1
MSTRG.21751		MSTRG.21751.1
MSTRG.2	Pde4b	ENSMUST00000106911;ENSMUST00000173119;ENSMUST0000010
MSTRG.2	Sgip1	ENSMUST00000097948;ENSMUST00000142430;ENSMUST0000006
MSTRG.2	Sgip1	MSTRG.21754.1
MSTRG.2	Gm12709	ENSMUST00000149348
MSTRG.2	Tctex1d	ENSMUST00000220547;ENSMUST00000223169;ENSMUST0000012
MSTRG.2	Tctex1d	MSTRG.21757.1
MSTRG.2	BB03177	MSTRG.21758.1;ENSMUST00000148892
MSTRG.2	Slc35d1	ENSMUST00000150285;MSTRG.21759.2;MSTRG.21759.3;MSTRG.
MSTRG.2	Disp1	MSTRG.2176.1
MSTRG.21760		MSTRG.21760.1
MSTRG.2	Gm44037	ENSMUST00000204816
MSTRG.2	4930456	ENSMUST00000145577
MSTRG.2	Oma1	ENSMUST00000035780;ENSMUST00000143399;ENSMUST0000013
MSTRG.2	Oma1	MSTRG.21764.1
MSTRG.2	Oma1	MSTRG.21765.1
MSTRG.2	Insl5	ENSMUST00000084382;ENSMUST00000106869
MSTRG.21767		MSTRG.21767.1
MSTRG.2	Wdr78	ENSMUST00000138960;ENSMUST00000036451;ENSMUST0000003
MSTRG.2	Wdr78	MSTRG.21769.1
MSTRG.2	Disp1	MSTRG.2177.1
MSTRG.2	Gm12710	ENSMUST00000119011
MSTRG.2	Wdr78	MSTRG.21771.1

MSTRG.2	Wdr78	MSTRG.21772.1
MSTRG.2	Mier1	ENSMUST00000136092;MSTRG.21773.1;ENSMUST00000106858;E
MSTRG.2	Gm12715	ENSMUST00000121430;ENSMUST00000175624
MSTRG.2	Prkaa2	ENSMUST00000030243
MSTRG.2	Fyb2	ENSMUST00000106803;ENSMUST00000106804;MSTRG.21776.2
MSTRG.2	Gm12721	ENSMUST00000120621
MSTRG.2	Fyb2	MSTRG.21778.1
MSTRG.2	Fyb2	MSTRG.21779.1
MSTRG.2	Disp1	MSTRG.2178.1
MSTRG.2	Prkaa2	MSTRG.21780.1
MSTRG.2	Plpp3	ENSMUST00000064139
MSTRG.2	Plpp3	MSTRG.21782.1
MSTRG.2	Plpp3	MSTRG.21783.1
MSTRG.2	Dhcr24	ENSMUST00000047973
MSTRG.2	Gm12744	ENSMUST00000146976
MSTRG.2	Lexm	ENSMUST00000067387
MSTRG.2	Pars2	ENSMUST00000106782;ENSMUST00000146966;ENSMUST0000005
MSTRG.2	Usp24	ENSMUST00000165709;ENSMUST00000106798;MSTRG.21788.4;M
MSTRG.2	Usp24	MSTRG.21789.1
MSTRG.2	Disp1	MSTRG.2179.1
MSTRG.2	Pcsk9	ENSMUST00000049507
MSTRG.2	Ttc4	ENSMUST00000026480;ENSMUST00000142342;ENSMUST0000013
MSTRG.2	Ssbp3	ENSMUST00000150915;MSTRG.21792.1;ENSMUST00000030367;E
MSTRG.2	Ssbp3	MSTRG.21793.1
MSTRG.2	Gm12786	MSTRG.21795.1;ENSMUST00000129617
MSTRG.2	Tmem59	MSTRG.21796.1
MSTRG.21797		MSTRG.21797.1
MSTRG.2	Ldlrad1	ENSMUST00000094916
MSTRG.2	Gm12802	ENSMUST00000145510
MSTRG.2	BC08527	ENSMUST00000191852;ENSMUST00000195236
MSTRG.2	Tceanc2	ENSMUST00000057043;ENSMUST00000127720;ENSMUST0000012
MSTRG.2	Tceanc2	MSTRG.21801.1
MSTRG.2	Tmem59	ENSMUST00000030361;ENSMUST00000128123;ENSMUST0000010
MSTRG.2	Mrpl37	ENSMUST00000030365;ENSMUST00000141150;MSTRG.21803.2;E
MSTRG.2	Cyb5rl	ENSMUST00000156406;ENSMUST00000106758;ENSMUST0000014
MSTRG.2	Dio1	ENSMUST00000082426;ENSMUST00000134366;ENSMUST0000010
MSTRG.2	Lrrc42	ENSMUST00000030360;ENSMUST00000137837;ENSMUST0000014
MSTRG.2	Hspb11	MSTRG.21807.1;ENSMUST00000152717;ENSMUST00000156934;E
MSTRG.2	Hspb11	MSTRG.21808.1
MSTRG.2	Hspb11	MSTRG.21809.1
MSTRG.2	Brox	ENSMUST00000057062;ENSMUST00000163528;MSTRG.2181.3;EN
MSTRG.2	Gm12850	ENSMUST00000119210
MSTRG.2	Yipf1	ENSMUST00000075693;ENSMUST00000140162;ENSMUST0000013
MSTRG.2	Gm12870	ENSMUST00000121383
MSTRG.2	Yipf1	MSTRG.21813.1
MSTRG.2	Yipf1	MSTRG.21814.1
MSTRG.2	Gm12869	ENSMUST00000156265
MSTRG.2	Ndc1	ENSMUST00000139560;ENSMUST00000030357;ENSMUST0000014
MSTRG.2	Ndc1	MSTRG.21817.1
MSTRG.2	Ndc1	MSTRG.21818.1
MSTRG.2	Glis1	ENSMUST00000046005;ENSMUST00000106738;ENSMUST0000013
MSTRG.2	Aida	ENSMUST00000192252;ENSMUST00000109166;ENSMUST00000191
MSTRG.2	Lrp8os3	ENSMUST00000125464
MSTRG.2	Lrp8	ENSMUST00000143601;ENSMUST00000106733;MSTRG.21821.3;E
MSTRG.2	Lrp8os2	ENSMUST00000188737;ENSMUST00000150323
MSTRG.2	Lrp8os1	ENSMUST00000142425;ENSMUST00000030352
MSTRG.2	Lrp8	MSTRG.21824.1

MSTRG.2	Lrp8	MSTRG.21825.1
MSTRG.2	Lrp8	MSTRG.21826.1
MSTRG.2	Magoh	ENSMUST00000030348;ENSMUST00000141376
MSTRG.2	0610037	ENSMUST00000135454;ENSMUST00000106726;ENSMUST0000003C
MSTRG.2	Cpt2	ENSMUST00000030345;ENSMUST00000106719;ENSMUST0000017
MSTRG.2	Aida	MSTRG.2183.1
MSTRG.21830		MSTRG.21830.1
MSTRG.2	Zyg11b	MSTRG.21831.1
MSTRG.2	Podn	ENSMUST00000106709;ENSMUST00000146851;ENSMUST00000044
MSTRG.2	Gm23354	ENSMUST00000158426
MSTRG.2	Scp2	ENSMUST00000030340;ENSMUST00000145721;ENSMUST00000122
MSTRG.2	Mir6397	ENSMUST00000183919
MSTRG.2	Zyg11b	ENSMUST00000043616;MSTRG.21836.2;ENSMUST00000130508;M
MSTRG.2	Coa7	ENSMUST00000131656;ENSMUST00000141423;ENSMUST00000147
MSTRG.2	Btf3l4	ENSMUST00000102742;ENSMUST00000102741;ENSMUST00000102
MSTRG.2	Btf3l4	MSTRG.21839.1
MSTRG.2	Mia3	ENSMUST00000109158;ENSMUST00000069922;MSTRG.2184.2;MS
MSTRG.2	Btf3l4	MSTRG.21840.1
MSTRG.2	Txndc12	ENSMUST00000030296;ENSMUST00000147581;ENSMUST00000154
MSTRG.2	A730015	ENSMUST00000164855
MSTRG.2	Kti12	ENSMUST00000102738
MSTRG.2	Zcchc11	ENSMUST00000106673;MSTRG.21844.1;ENSMUST00000043368;M
MSTRG.2	Gm24967	ENSMUST00000179791
MSTRG.2	Gm12739	MSTRG.21846.1
MSTRG.2	Gm12739	ENSMUST00000119769
MSTRG.2	Gm12737	ENSMUST00000142835
MSTRG.2	Gm25827	ENSMUST00000157089
MSTRG.2	Mia3	ENSMUST00000194543
MSTRG.2	Gm22345	ENSMUST00000122644
MSTRG.2	Prpf38a	MSTRG.21851.1;ENSMUST00000132951;ENSMUST00000079213;E
MSTRG.2	Orc1	ENSMUST00000139772;ENSMUST00000130162;ENSMUST00000102
MSTRG.2	Orc1	MSTRG.21853.1
MSTRG.2	Gm12743	ENSMUST00000126668
MSTRG.2	Cc2d1b	MSTRG.21855.1;ENSMUST00000030320;MSTRG.21855.3;ENSMUS
MSTRG.2	Gm20731	ENSMUST00000193462
MSTRG.2	Gm17354	ENSMUST00000170933
MSTRG.2	Zfyve9	ENSMUST00000106658;ENSMUST00000106657;ENSMUST00000042
MSTRG.2	3110021	ENSMUST00000178992
MSTRG.2	Mia3	MSTRG.2186.1
MSTRG.2	Gm12741	ENSMUST00000118836
MSTRG.2	Eps15	ENSMUST00000102729;ENSMUST00000176251;ENSMUST00000177
MSTRG.2	Eps15	MSTRG.21862.1
MSTRG.2	Gm12749	ENSMUST00000122138
MSTRG.2	Nrd1;Mi	ENSMUST00000065977;ENSMUST00000102736;ENSMUST0000010
MSTRG.2	Nrd1	MSTRG.21865.1
MSTRG.2	Osbpl9	ENSMUST00000084366;ENSMUST00000194478;ENSMUST0000016C
MSTRG.2	Osbpl9	MSTRG.21867.1
MSTRG.2	Gm23743	ENSMUST00000157389
MSTRG.2	Ttc39ao	ENSMUST00000154561;ENSMUST00000150239
MSTRG.2	Taf1a	MSTRG.2187.1;ENSMUST00000097043;ENSMUST00000192076;MS
MSTRG.2	Ttc39a	ENSMUST00000124209;ENSMUST00000064129;ENSMUST0000010
MSTRG.2	Rnf11	ENSMUST00000030284;ENSMUST00000064167;ENSMUST0000014
MSTRG.2	Gm12811	MSTRG.21873.1
MSTRG.2	Gm12803	ENSMUST00000118907
MSTRG.2	9630013	ENSMUST00000156790;ENSMUST00000131216;ENSMUST00000127
MSTRG.2	Cdkn2c	ENSMUST00000063531;ENSMUST00000097921;MSTRG.21876.3
MSTRG.2	Faf1	ENSMUST00000102724;MSTRG.21877.2;ENSMUST00000143217;E

MSTRG.2	Gm12808	MSTRG.21878.1
MSTRG.2	Gm12808	ENSMUST00000120597
MSTRG.2	Gm37986	ENSMUST00000193285
MSTRG.2	Faf1	MSTRG.21880.1
MSTRG.2	Dmrta2o	ENSMUST00000141893;ENSMUST00000124754
MSTRG.2	Agbl4	ENSMUST00000106592;ENSMUST00000080744;ENSMUST00000136
MSTRG.2	Gm12806	ENSMUST00000119384
MSTRG.2	Gm12807	ENSMUST00000121546
MSTRG.2	Gm12804	ENSMUST00000121030
MSTRG.2	Gm12805	ENSMUST00000117305
MSTRG.2	Bend5	ENSMUST00000030274;ENSMUST00000130523;ENSMUST00000129
MSTRG.2	Agbl4	MSTRG.21888.1
MSTRG.2	n-R5s19	ENSMUST00000122724
MSTRG.2	Hhipl2	ENSMUST00000195809;ENSMUST00000065900;ENSMUST00000193
MSTRG.2	Agbl4	MSTRG.21890.1
MSTRG.2	Agbl4	MSTRG.21891.1
MSTRG.2	Agbl4	MSTRG.21892.1
MSTRG.2	Spata6	MSTRG.21893.1
MSTRG.21894		MSTRG.21894.1
MSTRG.21895		MSTRG.21895.1
MSTRG.2	Spata6	ENSMUST00000038868;ENSMUST00000070513;ENSMUST00000122
MSTRG.2	Spata6	MSTRG.21897.1
MSTRG.2	Gm12961	ENSMUST00000120507
MSTRG.2	Spata6	MSTRG.21899.1
MSTRG.2	Gm37214	ENSMUST00000192860
MSTRG.2	Gm12960	ENSMUST00000120017
MSTRG.2	Slc5a9	ENSMUST00000102721;ENSMUST00000128224;ENSMUST00000102
MSTRG.2	Skint8	ENSMUST00000147395;ENSMUST00000106572;ENSMUST00000124
MSTRG.2	Skint7	ENSMUST00000106568;ENSMUST00000055014;ENSMUST00000163
MSTRG.2	Skint2	ENSMUST00000106560;ENSMUST00000058791;ENSMUST00000186
MSTRG.2	Skint2	MSTRG.21906.1
MSTRG.2	Skint2	MSTRG.21908.1
MSTRG.2	Skint10	ENSMUST00000060327;ENSMUST00000068851;ENSMUST00000137
MSTRG.2	Dusp10	MSTRG.2191.1;ENSMUST00000048655;MSTRG.2191.3;MSTRG.21
MSTRG.2	Skint10	MSTRG.21910.1
MSTRG.2	Skint6	ENSMUST00000138966
MSTRG.2	Gm12823	ENSMUST00000134494
MSTRG.2	Gm12816	ENSMUST00000118069
MSTRG.2	Gm12818	ENSMUST00000117562
MSTRG.2	Trabd2b	ENSMUST00000094894
MSTRG.2	Trabd2b	MSTRG.21916.1
MSTRG.2	Trabd2b	MSTRG.21917.1
MSTRG.2	Trabd2b	MSTRG.21918.1
MSTRG.2	Trabd2b	MSTRG.21919.1
MSTRG.2	1700056	ENSMUST00000193919;ENSMUST00000050306
MSTRG.2	Cmpk1	ENSMUST00000030491;MSTRG.21920.2;ENSMUST00000133066;E
MSTRG.2	Cmpk1	MSTRG.21921.1
MSTRG.2	Cmpk1	MSTRG.21922.1
MSTRG.2	Cmpk1	MSTRG.21923.1
MSTRG.2	Gm24045	ENSMUST00000083113
MSTRG.2	Stil	ENSMUST00000030490;ENSMUST00000141933;MSTRG.21925.4;E
MSTRG.2	Cyp4b1	ENSMUST00000135569;ENSMUST00000102707;ENSMUST00000145
MSTRG.2	Faah	ENSMUST00000125761;ENSMUST00000154249;ENSMUST00000049
MSTRG.2	Faah	MSTRG.21928.1
MSTRG.2	Efcab14	ENSMUST00000106525;ENSMUST00000136593;ENSMUST00000106
MSTRG.2	Hlx	ENSMUST00000048572;ENSMUST00000174257
MSTRG.2	Tex38	ENSMUST00000106521

MSTRG.2	Atpaf1	ENSMUST00000177280;ENSMUST00000176047;MSTRG.21931.3;E
MSTRG.2	Atpaf1	MSTRG.21932.1
MSTRG.2	Gm12847	ENSMUST00000156219
MSTRG.2	Mob3c	ENSMUST00000030477;ENSMUST00000144839;ENSMUST0000014‌8
MSTRG.2	Mob3c	MSTRG.21935.1
MSTRG.2	Mknk1	ENSMUST00000019677;ENSMUST00000144235;ENSMUST00000144
MSTRG.2	Nsun4	ENSMUST00000030475;ENSMUST00000030474;MSTRG.21938.3;M
MSTRG.2	Nsun4	MSTRG.21939.1
MSTRG.2	C130074	ENSMUST00000048308
MSTRG.2	AL67060	ENSMUST00000216692;ENSMUST00000147292;ENSMUST0000003C
MSTRG.2	Llph-ps	ENSMUST00000121281
MSTRG.2	AL67060	MSTRG.21943.1
MSTRG.2	Pik3r3	MSTRG.21944.1
MSTRG.2	Pik3r3	MSTRG.21945.1
MSTRG.2	Mast2	ENSMUST00000106484;ENSMUST00000003908;MSTRG.21946.3;E
MSTRG.2	Mast2	MSTRG.21947.1
MSTRG.2	Gm12950	ENSMUST00000121683
MSTRG.2	Mast2	MSTRG.21949.1
MSTRG.2	Marc1	ENSMUST00000048462;ENSMUST00000110992;ENSMUST0000018‌9
MSTRG.2	Uqcrh	ENSMUST00000050580;ENSMUST00000134760;ENSMUST0000007‌8
MSTRG.2	Gm12854	ENSMUST00000128518
MSTRG.2	Lrrc41	ENSMUST00000030471;ENSMUST00000154274;ENSMUST0000013‌3
MSTRG.2	Lurap1	MSTRG.21953.1;ENSMUST00000030469;ENSMUST00000135982
MSTRG.2	Rad54l	ENSMUST00000139147;MSTRG.21954.2;ENSMUST00000102705;E
MSTRG.2	Pomgnt1	MSTRG.21955.1;ENSMUST00000144311;ENSMUST00000120083;E
MSTRG.2	Tspan1	ENSMUST00000030465;ENSMUST00000143426;ENSMUST00000154
MSTRG.2	Akr1a1	ENSMUST00000030455;ENSMUST00000126146;ENSMUST0000012‌8
MSTRG.2	Ipp	ENSMUST00000106479;ENSMUST00000030461
MSTRG.2	Tmem69	ENSMUST00000106478;MSTRG.21959.2;ENSMUST00000069674
MSTRG.2	Marc2;M	ENSMUST00000068725;ENSMUST00000161359;ENSMUST00000161
MSTRG.2	Gm12953	ENSMUST00000138426
MSTRG.2	Gpbp1l1	MSTRG.21961.4;MSTRG.21961.1;MSTRG.21961.2;MSTRG.21961
MSTRG.2	C530005	ENSMUST00000142815
MSTRG.2	Ccdc17	ENSMUST00000121907;ENSMUST00000051869;ENSMUST0000014‌6
MSTRG.2	Nasp	ENSMUST00000030457;ENSMUST00000081182;ENSMUST0000003C
MSTRG.2	Prdx1	ENSMUST00000135573;ENSMUST00000156145;ENSMUST00000151
MSTRG.2	Zswim5	ENSMUST00000044823
MSTRG.2	Gm26355	ENSMUST00000158220
MSTRG.2	Gm25435	ENSMUST00000158277
MSTRG.2	Gm22092	ENSMUST00000178290
MSTRG.2	Rab3gap	ENSMUST00000194740;ENSMUST00000069652;ENSMUST00000191
MSTRG.2	Urod	ENSMUST00000030446;ENSMUST00000134598;ENSMUST0000015C
MSTRG.2	Hectd3	MSTRG.21972.1;ENSMUST00000050067;ENSMUST00000155267;E
MSTRG.2	1700021	ENSMUST00000138729
MSTRG.2	Mmachc	ENSMUST00000030453;ENSMUST00000143330
MSTRG.2	Ccdc163	ENSMUST00000030452;ENSMUST00000106462;ENSMUST0000012‌5
MSTRG.2	Tesk2	MSTRG.21976.1
MSTRG.2	Tesk2	MSTRG.21977.1
MSTRG.2	Gm27688	ENSMUST00000183634
MSTRG.2	Toe1	ENSMUST00000106455;ENSMUST00000030451;ENSMUST00000154
MSTRG.2	Snora36	ENSMUST00000104383;ENSMUST00000196796
MSTRG.2	Mutyh	ENSMUST00000102699;ENSMUST00000130359;ENSMUST00000124
MSTRG.2	Mutyh	MSTRG.21981.1
MSTRG.2	Eif2b3	ENSMUST00000106448;ENSMUST00000106447;ENSMUST0000007C
MSTRG.2	Gm22335	ENSMUST00000157724
MSTRG.2	Rps8;Sn	ENSMUST00000102696;ENSMUST00000137423;ENSMUST00000127
MSTRG.2	Gm22980	ENSMUST00000082608

MSTRG.2	Snord55	ENSMUST00000174939
MSTRG.2	Ptch2	ENSMUST00000030443;ENSMUST00000144620;ENSMUST00000156
MSTRG.2	Ptch2	MSTRG.21988.1
MSTRG.21989		MSTRG.21989.1
MSTRG.2	Gm34882	ENSMUST00000193245
MSTRG.2	Btbd19	ENSMUST00000153924;ENSMUST00000183310;ENSMUST00000153
MSTRG.2	Tctex1d	ENSMUST00000134074;ENSMUST00000062206;ENSMUST00000144
MSTRG.2	Plk3	ENSMUST00000126135;ENSMUST00000076859;ENSMUST00000147
MSTRG.2	Gm25099	ENSMUST00000093716
MSTRG.2	Kif2c	ENSMUST00000065896;ENSMUST00000106436;MSTRG.21994.2;E
MSTRG.21995		MSTRG.21995.1
MSTRG.2	Gm1661	ENSMUST00000165128
MSTRG.2	Tmem53	ENSMUST00000062824;ENSMUST00000125943;ENSMUST00000154
MSTRG.2	Rnf220	ENSMUST00000030439;ENSMUST00000102690;MSTRG.21998.3;M
MSTRG.2	Gm12843	ENSMUST00000137607;ENSMUST00000139618
MSTRG.2	Atp6v1h	MSTRG.22.1
MSTRG.2	Ptp4a1	ENSMUST00000027232;ENSMUST00000076587;ENSMUST00000153
MSTRG.2	Rab3gap	MSTRG.2200.1
MSTRG.2	Gm12828	ENSMUST00000136115
MSTRG.2	Gm12827	ENSMUST00000132690
MSTRG.22002		MSTRG.22002.1
MSTRG.22003		MSTRG.22003.1
MSTRG.2	Eri3	ENSMUST00000037127;ENSMUST00000144373;MSTRG.22004.2;E
MSTRG.2	Eri3	MSTRG.22005.1
MSTRG.2	Eri3	MSTRG.22006.1
MSTRG.2	Gm25559	ENSMUST00000157182
MSTRG.2	Dmap1	ENSMUST00000135582;ENSMUST00000102687;ENSMUST00000129
MSTRG.2	Gm12840	ENSMUST00000156081;MSTRG.22009.2
MSTRG.2	Iars2	ENSMUST00000027921;ENSMUST00000131770;ENSMUST00000110
MSTRG.2	Ipo13	ENSMUST00000134752;ENSMUST00000036156;MSTRG.22010.1;E
MSTRG.2	Slc6a9	ENSMUST00000063857;ENSMUST00000030269;ENSMUST00000149
MSTRG.2	Gm17114	ENSMUST00000164716;ENSMUST00000164853;ENSMUST00000163
MSTRG.2	Gm12841	ENSMUST00000128263
MSTRG.2	Atp6v0b	ENSMUST00000036380;ENSMUST00000133530;ENSMUST00000150
MSTRG.2	Dph2	ENSMUST00000030265;ENSMUST00000139332;ENSMUST00000183
MSTRG.22016		MSTRG.22016.1
MSTRG.2	St3gal3	ENSMUST00000030263;ENSMUST00000106410;ENSMUST00000097
MSTRG.2	St3gal3	MSTRG.22018.1
MSTRG.2	9530034	ENSMUST00000146982;MSTRG.22019.2
MSTRG.2	2010103	ENSMUST00000192375;ENSMUST00000083628
MSTRG.2	St3gal3	MSTRG.22020.1
MSTRG.2	St3gal3	MSTRG.22021.1
MSTRG.2	Kdm4a	ENSMUST00000106406;ENSMUST00000097911;ENSMUST00000106
MSTRG.2	Gm23382	ENSMUST00000157678
MSTRG.22024		MSTRG.22024.1
MSTRG.22025		MSTRG.22025.1
MSTRG.2	Ptprf	MSTRG.22026.1;MSTRG.22026.3;MSTRG.22026.2;ENSMUST0000
MSTRG.2	Mir7226	ENSMUST00000184953
MSTRG.2	Ptprf	MSTRG.22028.1
MSTRG.2	Elovl1	ENSMUST00000006557;MSTRG.22029.2;ENSMUST00000167636;E
MSTRG.2	Mir194-	ENSMUST00000083647
MSTRG.2	Cdc20	ENSMUST00000006565;MSTRG.22030.1;ENSMUST00000183942;E
MSTRG.2	Hyi	ENSMUST00000194248;ENSMUST00000179719;ENSMUST00000106
MSTRG.2	Szt2	MSTRG.22032.1;MSTRG.22032.2;ENSMUST00000075406;ENSMUS
MSTRG.2	Med8	ENSMUST00000084319;ENSMUST00000106384;ENSMUST00000126
MSTRG.2	Cfap57	ENSMUST00000071972
MSTRG.2	Ebna1bp	ENSMUST00000030501;ENSMUST00000130219;MSTRG.22035.2;E

MSTRG.2 Olfr134	ENSMUST00000216242
MSTRG.2 Olfr134	MSTRG.22037.1;MSTRG.22037.2
MSTRG.2 Slc2a1	ENSMUST00000030398;ENSMUST00000134105;ENSMUST00000144
MSTRG.2 Slc2a1	MSTRG.22039.1
MSTRG.2 Gm37785	ENSMUST00000195783
MSTRG.2 Mir1957	ENSMUST00000157927
MSTRG.2 Gm12867	ENSMUST00000151216
MSTRG.2 Gm12868	ENSMUST00000143801
MSTRG.2 Svbp	ENSMUST00000030395;ENSMUST00000106345;ENSMUST00000097
MSTRG.2 Svbp	MSTRG.22044.1
MSTRG.2 AU02225	ENSMUST00000132778;ENSMUST00000212054;MSTRG.22045.4;E
MSTRG.2 Gm12898	ENSMUST00000144407
MSTRG.2 P3h1	ENSMUST00000121111;ENSMUST00000030393;ENSMUST00000153
MSTRG.2 P3h1	MSTRG.22048.1
MSTRG.2 Bpnt1	ENSMUST00000027916;ENSMUST00000210277;ENSMUST00000151
MSTRG.2 Cldn19	ENSMUST00000084309;ENSMUST00000094823
MSTRG.2 Ybx1	MSTRG.22051.1
MSTRG.2 Ybx1	ENSMUST00000079644;ENSMUST00000127737;ENSMUST00000145
MSTRG.2 Gm37817	ENSMUST00000195276
MSTRG.2 Ppih	MSTRG.22054.2;ENSMUST00000056458;MSTRG.22054.1;ENSMUS
MSTRG.2 Ccdc30	ENSMUST00000154606;ENSMUST00000063642;ENSMUST00000146
MSTRG.2 Ldha-ps	ENSMUST00000118722
MSTRG.2 Ppcs	ENSMUST00000140641;ENSMUST00000106316;ENSMUST0000003C
MSTRG.2 Zmynd12	ENSMUST00000094819;ENSMUST00000129150
MSTRG.2 4930532	ENSMUST00000180632;ENSMUST00000195314
MSTRG.2 Rimkla	ENSMUST00000049994;ENSMUST00000126728
MSTRG.22061	MSTRG.22061.1
MSTRG.2 Rnu6-ps	ENSMUST00000178059
MSTRG.2 Frg2f1	ENSMUST00000079611;ENSMUST00000149305;ENSMUST00000177
MSTRG.2 Foxj3	ENSMUST00000176012;ENSMUST00000044564;ENSMUST00000162
MSTRG.2 Gm12959	ENSMUST00000119396
MSTRG.2 Hivep3	ENSMUST00000144907;ENSMUST00000141605;ENSMUST00000084
MSTRG.2 Hivep3	MSTRG.22067.1
MSTRG.2 Hivep3	MSTRG.22068.1
MSTRG.2 Hivep3	MSTRG.22069.1
MSTRG.2 Eprs	ENSMUST00000192284;MSTRG.2207.1;ENSMUST00000046514;EN
MSTRG.2 Hivep3	MSTRG.22070.1
MSTRG.2 Hivep3	MSTRG.22071.1
MSTRG.2 Hivep3	MSTRG.22072.1
MSTRG.2 Hivep3	MSTRG.22073.1
MSTRG.2 Hivep3	MSTRG.22074.1
MSTRG.2 Hivep3	MSTRG.22075.1
MSTRG.2 Slfnl1	ENSMUST00000062990
MSTRG.2 Ctps	ENSMUST00000030381;ENSMUST00000147980;ENSMUST0000014C
MSTRG.2 Scmh1	ENSMUST00000134375;ENSMUST00000000087;ENSMUST00000151
MSTRG.2 Scmh1	MSTRG.22079.1;MSTRG.22079.2
MSTRG.2 Eprs	MSTRG.2208.1
MSTRG.2 Cited4	ENSMUST00000094814
MSTRG.2 Exo5	ENSMUST00000144114;ENSMUST00000030375;ENSMUST00000177
MSTRG.2 Nfyc	ENSMUST00000120779;ENSMUST00000043429;ENSMUST00000127
MSTRG.2 Mir30c-	ENSMUST00000083556
MSTRG.2 Mir30f	ENSMUST00000196073
MSTRG.2 Mir30e	ENSMUST00000083475
MSTRG.2 Zfp69	ENSMUST00000106280;ENSMUST00000106281;ENSMUST0000013C
MSTRG.2 Zfp69	MSTRG.22087.1
MSTRG.2 Zfp69	MSTRG.22088.1
MSTRG.2 Zfp69	MSTRG.22089.1

MSTRG.2	9630028	ENSMUST00000143787
MSTRG.2	Zfp69	MSTRG.22090.1
MSTRG.22091		MSTRG.22091.1
MSTRG.2	Smap2	MSTRG.22092.1;ENSMUST00000043200;ENSMUST00000151615
MSTRG.2	Mir7015	ENSMUST00000199525
MSTRG.2	Zmpste2	ENSMUST00000058754;MSTRG.22094.2;ENSMUST00000143768;E
MSTRG.2	Gm23540	ENSMUST00000157543
MSTRG.2	Gm12877	ENSMUST00000120383
MSTRG.2	Gm12877	MSTRG.22098.1
MSTRG.2	Rlf	ENSMUST00000168615;MSTRG.22099.1;ENSMUST00000056635;M
MSTRG.2	Gm19680	ENSMUST00000095050
MSTRG.2	Rrp15	ENSMUST00000001339;ENSMUST00000162336
MSTRG.2	Ppt1	ENSMUST00000030412;ENSMUST00000121870;ENSMUST0000012C
MSTRG.2	Ppt1	MSTRG.22101.1
MSTRG.2	Cap1	ENSMUST00000069533;ENSMUST00000106255;ENSMUST00000106
MSTRG.2	Cap1	MSTRG.22103.1
MSTRG.2	Mycl	ENSMUST00000144998;ENSMUST00000106252;ENSMUST0000003C
MSTRG.2	Gm12891	MSTRG.22105.1;MSTRG.22105.2
MSTRG.2	Gm12891	ENSMUST00000119358
MSTRG.2	Gm12891	MSTRG.22107.1
MSTRG.2	Mfsd2a	ENSMUST00000152308;ENSMUST00000030408;ENSMUST0000013E
MSTRG.2	Trit1	ENSMUST00000102649;ENSMUST00000147165;MSTRG.22109.3;E
MSTRG.2	A430105	ENSMUST00000180869
MSTRG.2	Trit1	MSTRG.22110.1
MSTRG.2	Trit1	MSTRG.22111.1
MSTRG.2	Trit1	MSTRG.22112.1
MSTRG.2	Trit1	MSTRG.22113.1
MSTRG.2	Heyl	ENSMUST00000040821;ENSMUST00000151870;ENSMUST00000127
MSTRG.2	Bmp8b	ENSMUST00000002457;ENSMUST00000151850
MSTRG.2	Gm17244	ENSMUST00000192482;ENSMUST00000194481
MSTRG.2	Oxct2b	ENSMUST00000102648
MSTRG.2	Ppie	ENSMUST00000030404;ENSMUST00000136466;ENSMUST0000012€
MSTRG.2	Pabpc4	ENSMUST00000156807;ENSMUST00000106243;ENSMUST0000010€
MSTRG.2	Spata17	ENSMUST00000183819;ENSMUST00000110945;ENSMUST00000183
MSTRG.2	Gm12900	ENSMUST00000120919
MSTRG.2	Gm25788	ENSMUST00000082721
MSTRG.2	Gm22154	ENSMUST00000083844
MSTRG.2	Gm12922	ENSMUST00000120626
MSTRG.2	Ndufs5	ENSMUST00000106207;ENSMUST00000106206;ENSMUST0000003C
MSTRG.2	Akirin1	MSTRG.22125.1;ENSMUST00000102636;ENSMUST00000142778;E
MSTRG.2	Gm26606	ENSMUST00000181292
MSTRG.2	D130007	ENSMUST00000067277
MSTRG.2	Mycbp	ENSMUST00000030400;ENSMUST00000106202;ENSMUST00000147
MSTRG.2	Gm15508	ENSMUST00000160330
MSTRG.2	Gm12905	ENSMUST00000153814;ENSMUST00000163208
MSTRG.2	Rragc	ENSMUST00000030399;MSTRG.22131.2;ENSMUST00000155757;M
MSTRG.2	4930535	ENSMUST00000181410
MSTRG.2	Gm12902	ENSMUST00000118389
MSTRG.2	Utp11	ENSMUST00000030738;ENSMUST00000153088
MSTRG.2	Fhl3	ENSMUST00000106199;ENSMUST00000145942;ENSMUST0000003E
MSTRG.2	Fhl3	MSTRG.22136.1
MSTRG.2	Sf3a3	ENSMUST00000030734;ENSMUST00000154712;ENSMUST00000151
MSTRG.2	Gm12917	ENSMUST00000119850
MSTRG.2	Mir697	ENSMUST00000102316
MSTRG.2	Gm43497	ENSMUST00000201137
MSTRG.2	Macf1	ENSMUST00000123765;ENSMUST00000097897;ENSMUST0000014S
MSTRG.2	Gm12924	ENSMUST00000121747

MSTRG.2 Gm23548	ENSMUST00000158963
MSTRG.2 Macf1	MSTRG.22143.1
MSTRG.2 Macf1	MSTRG.22144.1
MSTRG.2 Mir6398	ENSMUST00000184541
MSTRG.2 Macf1	MSTRG.22146.1
MSTRG.2 Gm12926	ENSMUST00000121158
MSTRG.2 Gm12925	ENSMUST00000140510;ENSMUST00000138861
MSTRG.2 Macf1	MSTRG.22149.1
MSTRG.2 Gm43496	ENSMUST00000201682
MSTRG.2 Gm12915	ENSMUST00000131381
MSTRG.2 Inpp5b;I	ENSMUST00000094782;ENSMUST00000153837;ENSMUST00000184
MSTRG.2 Inpp5b	MSTRG.22153.1
MSTRG.2 Inpp5b	MSTRG.22154.1
MSTRG.2 Inpp5b	MSTRG.22155.1
MSTRG.2 Mtf1	ENSMUST00000106193;ENSMUST00000138807;ENSMUST0000003C
MSTRG.2 Mtf1	MSTRG.22157.1
MSTRG.2 n-R5s19	ENSMUST00000122577
MSTRG.2 Mtf1	MSTRG.22159.1
MSTRG.2 Gm15509	ENSMUST00000162615
MSTRG.2 Mtf1	MSTRG.22160.1
MSTRG.2 1110065	ENSMUST00000184704;ENSMUST00000163946;ENSMUST00000106
MSTRG.2 Yrdc	ENSMUST00000102628;MSTRG.22162.2;ENSMUST00000144851;E
MSTRG.2 Maneal	ENSMUST00000064444
MSTRG.2 Epha10	ENSMUST00000059343;ENSMUST00000149146
MSTRG.2 Cdca8	ENSMUST00000084296;ENSMUST00000030690;ENSMUST00000135
MSTRG.2 9930104	ENSMUST00000136276;ENSMUST00000145046;ENSMUST00000127
MSTRG.2 Gm12930	ENSMUST00000146679
MSTRG.2 Rspo1	ENSMUST00000030687;ENSMUST00000140698
MSTRG.2 1700007	ENSMUST00000160380
MSTRG.2 Gnl2	ENSMUST00000106176;ENSMUST00000030684;ENSMUST00000136
MSTRG.2 Dnali1	ENSMUST00000036383
MSTRG.2 Zc3h12a	MSTRG.22173.1;ENSMUST00000036188;ENSMUST00000131685
MSTRG.2 Snip1	ENSMUST00000052183;MSTRG.22174.2;ENSMUST00000145733
MSTRG.22175	MSTRG.22175.1
MSTRG.2 Gm12932	MSTRG.22176.1
MSTRG.2 Gm12932	ENSMUST00000143705
MSTRG.2 Meaf6	ENSMUST00000154689;ENSMUST00000055213;ENSMUST00000184
MSTRG.2 Grik3	ENSMUST00000030676
MSTRG.2 Gpatch2	ENSMUST00000097443;ENSMUST00000160481;ENSMUST00000065
MSTRG.2 Ftl2-ps	ENSMUST00000118517;ENSMUST00000102081
MSTRG.2 Csf3r	MSTRG.22181.1;ENSMUST00000106162;ENSMUST00000153968;E
MSTRG.2 Lsm10	ENSMUST00000055575;ENSMUST00000179323;ENSMUST00000151
MSTRG.2 Mrps15	ENSMUST00000030675;ENSMUST00000138739;ENSMUST00000124
MSTRG.2 Oscp1	ENSMUST00000035497;ENSMUST00000143712;ENSMUST00000153
MSTRG.2 Rpl28-p	ENSMUST00000116058
MSTRG.2 Stk40	ENSMUST00000116286;ENSMUST00000094761;MSTRG.22186.3;E
MSTRG.2 Trappc3	ENSMUST00000030660;ENSMUST00000126786;MSTRG.22187.2
MSTRG.2 Map7d1	MSTRG.22188.1;ENSMUST00000125981;MSTRG.22188.2;ENSMUS
MSTRG.2 Map7d1	MSTRG.22189.1
MSTRG.2 Gm37254	ENSMUST00000193542
MSTRG.2 Gm12946	ENSMUST00000122969
MSTRG.2 Eva1b	ENSMUST00000052876;ENSMUST00000106152;ENSMUST00000106
MSTRG.2 Sh3d21	ENSMUST00000094760;ENSMUST00000142891;ENSMUST00000097
MSTRG.2 Thrap3;	ENSMUST00000080919;ENSMUST00000140390;MSTRG.22193.2;E
MSTRG.2 Thrap3	MSTRG.22194.1
MSTRG.2 Col8a2	ENSMUST00000070132;ENSMUST00000128435
MSTRG.2 Adprhl2	ENSMUST00000151191;ENSMUST00000102617;ENSMUST00000128

MSTRG.2 Tekt2	ENSMUST00000102616;ENSMUST00000156139;ENSMUST00000131
MSTRG.2 Gm12945	ENSMUST00000140080
MSTRG.2 Ago3	ENSMUST00000132123;MSTRG.22199.2;ENSMUST00000069097;E
MSTRG.2 Phf3	ENSMUST00000186733;ENSMUST00000088310;ENSMUST00000191
MSTRG.2 Gm38155	ENSMUST00000192285
MSTRG.2 Ago3	MSTRG.22200.1
MSTRG.2 Ago3	MSTRG.22201.1
MSTRG.2 Ago3	MSTRG.22202.1
MSTRG.2 Gm12929	ENSMUST00000122406
MSTRG.2 Gm12928	ENSMUST00000117115
MSTRG.2 Ago1	MSTRG.22205.1;ENSMUST00000097888;ENSMUST00000127800;E
MSTRG.2 Zmym6;Tı	ENSMUST00000046751;ENSMUST00000151461;MSTRG.22206.3;E
MSTRG.2 Gm25600	ENSMUST00000158357
MSTRG.2 Smim12	ENSMUST00000142029
MSTRG.2 Gja4	ENSMUST00000053753
MSTRG.2 Ush2a	ENSMUST00000060479;ENSMUST00000127077
MSTRG.2 Tlr12	ENSMUST00000074829;ENSMUST00000133382
MSTRG.2 Zscan20	ENSMUST00000084276;ENSMUST00000097877;ENSMUST00000135
MSTRG.2 Clspn	ENSMUST00000048391;ENSMUST00000147675;ENSMUST00000123
MSTRG.2 Mir7119	ENSMUST00000185054
MSTRG.2 Gm12936	ENSMUST00000121439
MSTRG.2 573040	ENSMUST00000097886;ENSMUST00000164362
MSTRG.2 Gm12933	ENSMUST00000120203
MSTRG.2 Psmb2	ENSMUST00000030642;ENSMUST00000130737;ENSMUST0000015C
MSTRG.2 Tfap2e	ENSMUST00000048194
MSTRG.2 Ncdn	ENSMUST00000030637;ENSMUST00000106116;ENSMUST00000127
MSTRG.2 Ush2a	MSTRG.2222.1
MSTRG.2 AU04032	ENSMUST00000148935;ENSMUST00000154640;ENSMUST00000102
MSTRG.2 Gm12937	MSTRG.22221.1;ENSMUST00000118333
MSTRG.2 Gm24161	ENSMUST00000104592
MSTRG.2 AU04032	MSTRG.22223.1
MSTRG.2 Gm25604	ENSMUST00000158359
MSTRG.2 Zmym4	ENSMUST00000135003;MSTRG.22225.1;ENSMUST00000106108;E
MSTRG.2 Zmym4	MSTRG.22226.1
MSTRG.2 Zmym4	MSTRG.22227.1
MSTRG.2 Zmym4	MSTRG.22228.1
MSTRG.2 Zmym4	MSTRG.22229.1
MSTRG.22231	MSTRG.22231.1
MSTRG.22232	MSTRG.22232.1
MSTRG.2 Sfpq;Gm	ENSMUST00000030623;MSTRG.22233.1;ENSMUST00000140682;M
MSTRG.2 Zmym1	ENSMUST00000106102;ENSMUST00000055013;MSTRG.22234.2;E
MSTRG.2 Zmym1	MSTRG.22235.1
MSTRG.2 Gm12944	ENSMUST00000119262
MSTRG.2 Phc2	ENSMUST00000030588;ENSMUST00000136377;ENSMUST00000148
MSTRG.2 A3galt2	ENSMUST00000106077;ENSMUST00000030585
MSTRG.2 Zfp362	ENSMUST00000106072;ENSMUST00000071108;MSTRG.22239.4;M
MSTRG.2 Trim62	ENSMUST00000147852;ENSMUST00000035667
MSTRG.2 Ak2	ENSMUST00000030583;ENSMUST00000102604;MSTRG.22241.2;E
MSTRG.2 170008	ENSMUST00000149472
MSTRG.2 Rnf19b	ENSMUST00000168461;ENSMUST00000030584;ENSMUST00000152
MSTRG.2 Rnf19b	MSTRG.22244.1
MSTRG.2 S100pbp	ENSMUST00000106061;ENSMUST00000072431;ENSMUST0000010€
MSTRG.2 S100pbp	MSTRG.22246.1
MSTRG.2 Yars	ENSMUST00000106054;ENSMUST00000128287;ENSMUST00000133
MSTRG.2 Yars	MSTRG.22248.1
MSTRG.2 C77080	ENSMUST00000106051;ENSMUST00000097873;ENSMUST00000052
MSTRG.2 Kctd3	ENSMUST00000193143;ENSMUST00000085678;ENSMUST00000192

MSTRG.2 C77080	MSTRG.22250.1
MSTRG.2 Sync	ENSMUST00000102599;ENSMUST00000146448
MSTRG.2 Rbbp4	MSTRG.22252.1;ENSMUST00000102598;ENSMUST00000140291;E
MSTRG.2 Sync	MSTRG.22253.1
MSTRG.2 Gm26722	ENSMUST00000181237
MSTRG.2 Zbtb8os	ENSMUST00000141235;ENSMUST00000146767;ENSMUST0000011
MSTRG.2 Zbtb8a	ENSMUST00000030610
MSTRG.2 Zbtb8a	MSTRG.22257.1
MSTRG.2 Bsdc1	ENSMUST00000048162;ENSMUST00000149587;ENSMUST00000131
MSTRG.2 Bsdc1	MSTRG.22259.1
MSTRG.2 Kctd3	MSTRG.2226.1
MSTRG.2 Tssk3	ENSMUST00000000421
MSTRG.2 Fam229a	ENSMUST00000135698
MSTRG.2 Gm12979	ENSMUST00000145488
MSTRG.2 Marcksl	MSTRG.22263.1
MSTRG.2 Marcksl	ENSMUST00000062356
MSTRG.2 Hdac1	ENSMUST00000102597;ENSMUST00000145628;MSTRG.22265.4;M
MSTRG.2 Hdac1	MSTRG.22266.1
MSTRG.2 Hdac1	MSTRG.22267.1
MSTRG.2 Hdac1	MSTRG.22268.1
MSTRG.2 Hdac1	MSTRG.22269.1
MSTRG.2 Kctd3	MSTRG.2227.1
MSTRG.2 Hdac1	MSTRG.22270.1
MSTRG.2 Lck;Mir	ENSMUST00000167288;ENSMUST00000067240;ENSMUST00000102
MSTRG.2 Lck	MSTRG.22272.1
MSTRG.2 Lck	MSTRG.22273.1
MSTRG.22274	MSTRG.22274.1;MSTRG.22274.2;MSTRG.22274.3
MSTRG.22275	MSTRG.22275.1
MSTRG.2 Tmem39b	ENSMUST00000125445;ENSMUST00000102588;ENSMUST00000154
MSTRG.2 Tmem39b	MSTRG.22277.1;MSTRG.22277.2
MSTRG.2 Mir7016	ENSMUST00000184308
MSTRG.2 Khdrbs1	MSTRG.22279.1;MSTRG.22279.2;ENSMUST00000129342;ENSMUS
MSTRG.2 Kcnk2	ENSMUST00000079451;ENSMUST00000110920;ENSMUST0000018
MSTRG.2 Khdrbs1	MSTRG.22280.1
MSTRG.2 Khdrbs1	MSTRG.22281.1
MSTRG.2 Gm10571	MSTRG.22282.1
MSTRG.2 Ptp4a2	ENSMUST00000132386;ENSMUST00000132411;ENSMUST0000003
MSTRG.2 Ptp4a2	MSTRG.22284.1
MSTRG.2 Gm10571	ENSMUST00000097869
MSTRG.2 Eif3i	ENSMUST00000102593;ENSMUST00000136843;ENSMUST0000013
MSTRG.2 Tmem234	ENSMUST00000172774;ENSMUST00000129515;ENSMUST00000137
MSTRG.2 Dcdc2b	ENSMUST00000179209;MSTRG.22288.2;ENSMUST00000143374;E
MSTRG.2 Iqcc	ENSMUST00000046675;ENSMUST00000127029;MSTRG.22289.3;E
MSTRG.2 2900042	ENSMUST00000194444
MSTRG.2 Ccdc28b	ENSMUST00000126777;ENSMUST00000030586;ENSMUST00000141
MSTRG.2 Txlna	ENSMUST00000046425;ENSMUST00000142577;ENSMUST00000084
MSTRG.2 Kpna6	ENSMUST00000102590;ENSMUST00000003828;ENSMUST00000138
MSTRG.2 Kpna6	MSTRG.22293.1
MSTRG.2 Gm12966	ENSMUST00000118024
MSTRG.2 Pef1	ENSMUST00000118199;ENSMUST00000030563;MSTRG.22295.2
MSTRG.2 Pef1	MSTRG.22296.1
MSTRG.2 Adgrb2	ENSMUST00000120204;ENSMUST00000106017;ENSMUST00000121
MSTRG.2 Tinagl1	ENSMUST00000030560;ENSMUST00000105999;ENSMUST0000014
MSTRG.2 Gm10570	ENSMUST00000097865
MSTRG.2 Gm37522	ENSMUST00000195437
MSTRG.2 Pdcd5-p	ENSMUST00000191699
MSTRG.2 Fabp3	ENSMUST00000070532

MSTRG.2 Fabp3	MSTRG.22301.1
MSTRG.2 Zcchc17	ENSMUST00000134159;ENSMUST00000141615;ENSMUST0000014[illegible]
MSTRG.2 Zcchc17	MSTRG.22303.1
MSTRG.2 Zcchc17	MSTRG.22304.1
MSTRG.2 Snrnp40	MSTRG.22305.1;ENSMUST00000105994;ENSMUST00000180577;E
MSTRG.2 Sdc3	ENSMUST00000070478;ENSMUST00000146093;ENSMUST00000152
MSTRG.2 Gm12970	ENSMUST00000134911
MSTRG.2 Matn1	ENSMUST00000102576
MSTRG.2 A430027	ENSMUST00000191809;ENSMUST00000192797
MSTRG.2 Matn1	MSTRG.22310.1
MSTRG.2 Laptm5	ENSMUST00000151698;ENSMUST00000156225;ENSMUST0000013[illegible]
MSTRG.2 Laptm5	MSTRG.22312.1
MSTRG.2 Pum1;Sn	ENSMUST00000030315;ENSMUST00000105991;ENSMUST00000105
MSTRG.2 Pum1	MSTRG.22314.1
MSTRG.2 Gm12971	ENSMUST00000154720
MSTRG.2 Gm26516	MSTRG.22316.1;ENSMUST00000180943
MSTRG.2 Ptpru	ENSMUST00000105987;ENSMUST00000030741;ENSMUST00000127
MSTRG.2 Srsf4;A	MSTRG.22319.1;ENSMUST00000053819;ENSMUST00000129966;E
MSTRG.2 Gm24092	ENSMUST00000180257
MSTRG.2 Mecr	ENSMUST00000030742;ENSMUST00000137321;ENSMUST0000013[illegible]
MSTRG.2 Srsf4	MSTRG.22321.1
MSTRG.2 Gm12992	MSTRG.22322.1;ENSMUST00000124738;ENSMUST00000121250;E
MSTRG.2 Ythdf2	MSTRG.22323.1
MSTRG.2 Ythdf2	ENSMUST00000152796;ENSMUST00000085181;ENSMUST00000165
MSTRG.2 Ythdf2	MSTRG.22325.1
MSTRG.2 Epb41	ENSMUST00000155990;ENSMUST00000151746;ENSMUST00000146
MSTRG.2 Gm27445	ENSMUST00000184484
MSTRG.2 Gm13063	MSTRG.22328.1;ENSMUST00000132247
MSTRG.2 Gm10300	ENSMUST00000094662
MSTRG.2 Cenpf	MSTRG.2233.1;ENSMUST00000171929;ENSMUST00000165798;EN
MSTRG.2 Gm28872	ENSMUST00000191518
MSTRG.2 Taf12	ENSMUST00000030731;ENSMUST00000124365;ENSMUST00000105
MSTRG.2 Taf12	MSTRG.22333.1
MSTRG.2 Gmeb1;G	ENSMUST00000168553;ENSMUST00000105965;ENSMUST0000003[illegible]
MSTRG.22335	MSTRG.22335.1
MSTRG.22336	MSTRG.22336.1
MSTRG.2 Gm28874	ENSMUST00000190917
MSTRG.2 Snhg12;	ENSMUST00000137343;ENSMUST00000144705;ENSMUST0000015[illegible]
MSTRG.2 Snora16	ENSMUST00000196061;ENSMUST00000083163
MSTRG.2 Gm37239	ENSMUST00000193510
MSTRG.2 Trnau1a	ENSMUST00000105962;ENSMUST00000030730;ENSMUST00000146
MSTRG.2 Trnau1a	MSTRG.22341.1
MSTRG.2 Med18	ENSMUST00000102567;ENSMUST00000123604
MSTRG.2 Rcc1;Sn	ENSMUST00000084250;MSTRG.22343.3;MSTRG.22343.2;ENSMUS
MSTRG.2 Gm17300	MSTRG.22344.1;ENSMUST00000172202
MSTRG.2 Snora73	ENSMUST00000083419
MSTRG.2 Phactr4	ENSMUST00000102568;ENSMUST00000084170;ENSMUST00000084
MSTRG.2 Phactr4	MSTRG.22347.1
MSTRG.2 Phactr4	MSTRG.22348.1
MSTRG.2 Gm25707	ENSMUST00000175120
MSTRG.2 Ptpn14	ENSMUST00000097442;ENSMUST00000194127;MSTRG.2235.2;EN
MSTRG.2 Sesn2	ENSMUST00000030724
MSTRG.2 Gm12981	ENSMUST00000131733
MSTRG.2 Sesn2	MSTRG.22352.1
MSTRG.2 Ptafr	ENSMUST00000070690
MSTRG.2 Atpif1	ENSMUST00000152993;ENSMUST00000067496;ENSMUST00000145
MSTRG.2 Gm12999	ENSMUST00000124471

MSTRG.2	Dnajc8	ENSMUST00000139074;ENSMUST00000094657;ENSMUST00000142
MSTRG.2	Gm24762	ENSMUST00000083689
MSTRG.22358		MSTRG.22358.1
MSTRG.2	Eya3	ENSMUST00000135299;ENSMUST00000020197;ENSMUST00000180
MSTRG.2	Ptpn14	MSTRG.2236.1
MSTRG.2	Eya3	MSTRG.22360.1
MSTRG.2	Eya3	MSTRG.22361.1
MSTRG.2	Eya3	MSTRG.22362.1
MSTRG.2	Eya3	MSTRG.22363.1
MSTRG.2	Xkr8	ENSMUST00000045550;ENSMUST00000150104;ENSMUST00000143
MSTRG.2	Rpa2	ENSMUST00000153046;ENSMUST00000102561;ENSMUST00000130
MSTRG.2	Themis2	ENSMUST00000045154
MSTRG.2	Fam76a	ENSMUST00000030696;ENSMUST00000097856;ENSMUST00000148
MSTRG.2	Ppp1r8	MSTRG.22368.1;ENSMUST00000030702;ENSMUST00000105919;E
MSTRG.2	Gm22767	ENSMUST00000158365
MSTRG.2	Ptpn14	MSTRG.2237.1
MSTRG.2	Stx12	MSTRG.22370.1
MSTRG.2	Stx12	ENSMUST00000030698;ENSMUST00000137109
MSTRG.2	Stx12	MSTRG.22372.1
MSTRG.2	Gm13033	ENSMUST00000117591
MSTRG.2	Fgr	ENSMUST00000124451;ENSMUST00000030693;ENSMUST00000171
MSTRG.22375		MSTRG.22375.1
MSTRG.22376		MSTRG.22376.1
MSTRG.22377		MSTRG.22377.1
MSTRG.2	Ahdc1	MSTRG.22378.1;MSTRG.22378.2
MSTRG.2	Ahdc1	ENSMUST00000154646;ENSMUST00000105915;ENSMUST00000105
MSTRG.2	Gm30280	ENSMUST00000192214
MSTRG.2	Ahdc1	MSTRG.22380.1
MSTRG.2	Ahdc1	MSTRG.22381.1
MSTRG.2	Wasf2	MSTRG.22382.1
MSTRG.2	Ahdc1	MSTRG.22383.1
MSTRG.2	Ahdc1	MSTRG.22384.1
MSTRG.2	Gm26615	ENSMUST00000181522
MSTRG.22386		MSTRG.22386.1
MSTRG.2	Wasf2	MSTRG.22387.1
MSTRG.2	Wasf2;M	ENSMUST00000084241;ENSMUST00000138831;ENSMUST00000136
MSTRG.2	Wasf2	MSTRG.22389.1
MSTRG.2	Gm26574	ENSMUST00000181876
MSTRG.2	Wasf2	MSTRG.22390.1
MSTRG.2	Wasf2	MSTRG.22391.1
MSTRG.2	Wasf2	MSTRG.22392.1
MSTRG.2	Wasf2	MSTRG.22393.1
MSTRG.2	Wasf2	MSTRG.22394.1
MSTRG.2	Gm24636	ENSMUST00000158673
MSTRG.2	Map3k6	ENSMUST00000030677;ENSMUST00000123612;ENSMUST00000127
MSTRG.2	Sytl1	ENSMUST00000105908;ENSMUST00000030674;ENSMUST00000142
MSTRG.2	Tmem222	ENSMUST00000105907;ENSMUST00000137622;ENSMUST00000139
MSTRG.2	Wdtc1	MSTRG.22399.1
MSTRG.2	Gm37906	ENSMUST00000192050
MSTRG.2	Gm37422	ENSMUST00000193794
MSTRG.2	Wdtc1	ENSMUST00000043305;ENSMUST00000105906;MSTRG.22400.2;M
MSTRG.2	Wdtc1	MSTRG.22401.1
MSTRG.2	Wdtc1	MSTRG.22402.1
MSTRG.2	Slc9a1;l	ENSMUST00000030669;ENSMUST00000175004;MSTRG.22403.4;M
MSTRG.2	Slc9a1	MSTRG.22404.1
MSTRG.2	Slc9a1	MSTRG.22405.1
MSTRG.2	Kdf1	ENSMUST00000149807;ENSMUST00000042919;ENSMUST00000153

MSTRG.2 Nudc	ENSMUST00000030665;ENSMUST00000141860;ENSMUST0000014[illegible]
MSTRG.2 Gpatch3	MSTRG.22408.1
MSTRG.2 Nr0b2	ENSMUST00000042706
MSTRG.2 Smyd2	ENSMUST00000027897;ENSMUST00000130804;ENSMUST0000013[illegible]
MSTRG.2 Gm23158	ENSMUST00000175076
MSTRG.2 Gpatch3	ENSMUST00000030662;ENSMUST00000154648
MSTRG.2 Gpn2	ENSMUST00000030661;ENSMUST00000105899;ENSMUST0000014[illegible]
MSTRG.2 Sfn;Zdh[illegible]	ENSMUST00000057311;MSTRG.22413.1;ENSMUST00000084238;[illegible]
MSTRG.2 Zdhhc18	MSTRG.22414.1
MSTRG.2 Gm13213	ENSMUST00000119668
MSTRG.22416	MSTRG.22416.1
MSTRG.2 Hmgn2	MSTRG.22417.1
MSTRG.2 Gm22662	ENSMUST00000175290;MSTRG.22418.1;MSTRG.22418.3;MSTRG.
MSTRG.2 Pigv	MSTRG.22419.1;ENSMUST00000062118;ENSMUST00000067902;[illegible]
MSTRG.2 Smyd2	MSTRG.2242.1
MSTRG.2 Arid1a	ENSMUST00000105897;ENSMUST00000145664;ENSMUST0000000[illegible]
MSTRG.2 Arid1a	MSTRG.22421.1
MSTRG.2 Arid1a	MSTRG.22422.1
MSTRG.2 Mir7227	ENSMUST00000200408
MSTRG.2 Arid1a	MSTRG.22424.1
MSTRG.2 Rps6ka1	ENSMUST00000105894;ENSMUST00000168974;ENSMUST0000000[illegible]
MSTRG.2 Gm12977	ENSMUST00000133742;ENSMUST00000134799
MSTRG.2 Gm12985	ENSMUST00000118376
MSTRG.2 Gm22523	ENSMUST00000158696
MSTRG.2 Hmgn2	ENSMUST00000102553;ENSMUST00000105893;ENSMUST0000014[illegible]
MSTRG.2 Gm38245	ENSMUST00000191702
MSTRG.2 Dhdds	ENSMUST00000144668;ENSMUST00000012262;ENSMUST0000013[illegible]
MSTRG.2 Dhdds	MSTRG.22431.1
MSTRG.2 Dhdds	MSTRG.22432.1
MSTRG.2 Zfp683	ENSMUST00000105884
MSTRG.2 Crybg2	ENSMUST00000219402;ENSMUST00000137053;ENSMUST0000012[illegible]
MSTRG.2 Cd52	ENSMUST00000145122;MSTRG.22435.2;ENSMUST00000000696;[illegible]
MSTRG.2 Zfp593	ENSMUST00000030644;ENSMUST00000129183
MSTRG.2 E130218	ENSMUST00000135228;ENSMUST00000144222;ENSMUST0000012[illegible]
MSTRG.2 Pdik1l	ENSMUST00000105877;ENSMUST00000061234;ENSMUST0000010[illegible]
MSTRG.2 Ubxn11	MSTRG.22439.1;ENSMUST00000074690;ENSMUST00000142442;[illegible]
MSTRG.2 Rps6kc1	ENSMUST00000159624;ENSMUST00000160889;ENSMUST0000015[illegible]
MSTRG.2 Sh3bgrl	ENSMUST00000030651;MSTRG.22440.1;MSTRG.22440.3;ENSMUS[illegible]
MSTRG.2 Cep85	MSTRG.22441.1
MSTRG.2 Cep85	MSTRG.22442.1
MSTRG.2 Cep85	MSTRG.22443.1
MSTRG.2 Gm27619	ENSMUST00000184419
MSTRG.2 Pafah2	ENSMUST00000105869;ENSMUST00000140888;ENSMUST0000013[illegible]
MSTRG.2 Stmn1;G[illegible]	ENSMUST00000030636;ENSMUST00000175071;MSTRG.22446.2;[illegible]
MSTRG.2 Paqr7	ENSMUST00000081525;ENSMUST00000134506;ENSMUST0000013[illegible]
MSTRG.2 Selenon	ENSMUST00000060435;ENSMUST00000127585
MSTRG.2 Aunip	ENSMUST00000105866
MSTRG.2 Rps6kc1	MSTRG.2245.1
MSTRG.2 Mtfr1l	ENSMUST00000102550;ENSMUST00000116279;ENSMUST0000013[illegible]
MSTRG.2 Man1c1	ENSMUST00000054096;ENSMUST00000176606
MSTRG.2 Mir6403	ENSMUST00000185007
MSTRG.2 Ldlrap1	ENSMUST00000037828;ENSMUST00000148479;ENSMUST0000014[illegible]
MSTRG.2 Ldlrap1	MSTRG.22454.1
MSTRG.2 Tmem57	ENSMUST00000150031;ENSMUST00000030628;MSTRG.22455.3;[illegible]
MSTRG.2 Rhd	ENSMUST00000030627;ENSMUST00000136163
MSTRG.2 Tmem50a	MSTRG.22457.1;ENSMUST00000030626;MSTRG.22457.2;MSTRG.
MSTRG.2 Rsrp1;S[illegible]	ENSMUST00000078084;ENSMUST00000148900;MSTRG.22458.1;[illegible]

MSTRG.2 Clic4	ENSMUST00000037099;ENSMUST00000143370
MSTRG.2 Angel2	ENSMUST00000027947;MSTRG.2246.2;ENSMUST00000137608;EN
MSTRG.2 Gm16225	ENSMUST00000146152
MSTRG.2 Runx3	ENSMUST00000056977;ENSMUST00000119564;ENSMUST0000015€
MSTRG.2 Gm16224	MSTRG.22462.1;ENSMUST00000127711
MSTRG.2 Rcan3;M	ENSMUST00000132654;ENSMUST00000030606;ENSMUST00000102
MSTRG.2 Gm15979	ENSMUST00000149741
MSTRG.2 Srrm1	ENSMUST00000105861;MSTRG.22465.2;MSTRG.22465.4;MSTRG.
MSTRG.2 Srrm1	MSTRG.22466.1
MSTRG.2 Nipal3	ENSMUST00000105856;ENSMUST00000102549;ENSMUST0000014C
MSTRG.2 Stpg1	ENSMUST00000153891;ENSMUST00000123221;ENSMUST0000006З
MSTRG.2 Vash2	ENSMUST00000166139;ENSMUST00000047409;ENSMUST00000192
MSTRG.2 Gm25317	ENSMUST00000116824
MSTRG.2 Gm12988	ENSMUST00000119054
MSTRG.2 Pnrc2	ENSMUST00000030436;ENSMUST00000142585;ENSMUST0000018C
MSTRG.2 Cnr2	ENSMUST00000097843;ENSMUST00000068830
MSTRG.2 Gm13000	ENSMUST00000120937
MSTRG.2 Srsf10	ENSMUST00000097844;ENSMUST00000105853;ENSMUST00000102
MSTRG.2 Fuca1;H	ENSMUST00000030434;MSTRG.22476.1;ENSMUST00000153592;E
MSTRG.2 Hmgcl	MSTRG.22477.1
MSTRG.2 Pithd1	ENSMUST00000105851;ENSMUST00000150885;ENSMUST0000012З
MSTRG.2 Hmgcl	MSTRG.22479.1
MSTRG.2 Vash2	MSTRG.2248.1
MSTRG.2 Gale	ENSMUST00000102541;ENSMUST00000128929;ENSMUST0000014З
MSTRG.2 Lypla2	ENSMUST00000105852;ENSMUST00000067567;ENSMUST0000015€
MSTRG.2 Eloa	ENSMUST00000030427;MSTRG.22482.3;MSTRG.22482.2
MSTRG.2 Gm13008	ENSMUST00000142289
MSTRG.2 Gm24362	ENSMUST00000183220
MSTRG.2 Rpl11	ENSMUST00000153223;ENSMUST00000137580;ENSMUST00000102
MSTRG.2 Id3	ENSMUST00000008016;ENSMUST00000151001;MSTRG.22486.2;M
MSTRG.2 E2f2	MSTRG.22487.4;MSTRG.22487.6;MSTRG.22487.5;MSTRG.22487
MSTRG.2 Asap3	ENSMUST00000047526
MSTRG.2 Tcea3	ENSMUST00000136812;ENSMUST00000102533;ENSMUST0000014З
MSTRG.2249	MSTRG.2249.1
MSTRG.2 Hnrnpr	MSTRG.22490.1;MSTRG.22490.2;ENSMUST00000131671;ENSMUS
MSTRG.2 Zfp46	ENSMUST00000144217;ENSMUST00000130223;ENSMUST00000132
MSTRG.2 Gm13013	ENSMUST00000119087
MSTRG.2 Luzp1	ENSMUST00000170102;ENSMUST00000105849;ENSMUST00000142
MSTRG.2 Kdm1a;G	ENSMUST00000116273;ENSMUST00000105847;ENSMUST00000171
MSTRG.2 C1qb	ENSMUST00000046384
MSTRG.2 C1qc	MSTRG.22497.1;ENSMUST00000046332;ENSMUST00000153104
MSTRG.2 C1qa	ENSMUST00000046285
MSTRG.2 Ephb2	ENSMUST00000105845;ENSMUST00000059287;ENSMUST0000010Ѣ
MSTRG.2 Gm37724	ENSMUST00000195240
MSTRG.2 Mfsd7b	ENSMUST00000194917;ENSMUST00000085635;ENSMUST00000192
MSTRG.2 Ephb2	MSTRG.22500.1
MSTRG.2 Ephb2	MSTRG.22501.1
MSTRG.2 Ephb2	MSTRG.22502.1
MSTRG.2 Ephb2	MSTRG.22503.1
MSTRG.2 Ephb2	MSTRG.22504.1
MSTRG.2 Ephb2	MSTRG.22505.1
MSTRG.2 Ephb2	MSTRG.22506.1
MSTRG.2 Ephb2	MSTRG.22507.1
MSTRG.2 Ephb2	MSTRG.22508.1
MSTRG.2 Ephb2	MSTRG.22509.1
MSTRG.2 Mfsd7b	MSTRG.2251.1
MSTRG.2 Ephb2	MSTRG.22510.1

MSTRG.2	Zbtb40	ENSMUST00000049583;MSTRG.22511.2;ENSMUST00000218160;E
MSTRG.2	Zbtb40	MSTRG.22512.1
MSTRG.2	Zbtb40	MSTRG.22513.1
MSTRG.2	Gm23834	ENSMUST00000104436
MSTRG.2	Zbtb40	MSTRG.22515.1
MSTRG.22516		MSTRG.22516.1
MSTRG.22517		MSTRG.22517.1
MSTRG.2	2810405	ENSMUST00000189334
MSTRG.2	Gm13005	MSTRG.22519.1
MSTRG.2	A230020	ENSMUST00000181050;ENSMUST00000192407
MSTRG.2	Gm13005	ENSMUST00000119518
MSTRG.2	Gm13002	ENSMUST00000119286
MSTRG.2	Wnt4	ENSMUST00000045747;MSTRG.22522.1
MSTRG.2	Cdc42	ENSMUST00000051477;ENSMUST00000030417
MSTRG.2	Hspg2	ENSMUST00000030547;MSTRG.22525.2;ENSMUST00000171332;E
MSTRG.2	Mir7018	ENSMUST00000184284
MSTRG.2	Ldlrad2	ENSMUST00000178923
MSTRG.2	Usp48	MSTRG.22528.1
MSTRG.2	Usp48	ENSMUST00000153100;MSTRG.22529.2;MSTRG.22529.1;ENSMUS
MSTRG.2	Spata45	ENSMUST00000192151
MSTRG.2	Usp48	MSTRG.22530.1
MSTRG.2	Usp48	MSTRG.22531.1
MSTRG.2	Usp48	MSTRG.22532.1
MSTRG.2	Ddost	ENSMUST00000030538;MSTRG.22533.1
MSTRG.2	Pink1;M	ENSMUST00000105816;ENSMUST00000030536;ENSMUST0000018З
MSTRG.2	Rpl38-p	ENSMUST00000117723
MSTRG.2	Mul1	ENSMUST00000044058;ENSMUST00000105813;ENSMUST00000105
MSTRG.2	Camk2n1	ENSMUST00000050918
MSTRG.2	Pla2g2d	ENSMUST00000030528;ENSMUST00000105806
MSTRG.2	Pla2g5	ENSMUST00000102513;ENSMUST00000102511;ENSMUST00000127
MSTRG.2	Tatdn3	ENSMUST00000110893;ENSMUST00000085633;ENSMUST0000011C
MSTRG.2	Pla2g2e	MSTRG.22542.1
MSTRG.2	Pla2g2e	ENSMUST00000030531;ENSMUST00000105804
MSTRG.2	Otud3	ENSMUST00000097830
MSTRG.2	Otud3	MSTRG.22546.1
MSTRG.2	Otud3	MSTRG.22547.1
MSTRG.2	Otud3	MSTRG.22548.1
MSTRG.2	Rnf186	ENSMUST00000116094
MSTRG.2	Nsl1	ENSMUST00000076952;ENSMUST00000139340;ENSMUST00000078
MSTRG.2	Tmco4	ENSMUST00000123636;MSTRG.22550.1;ENSMUST00000043042;E
MSTRG.2	Htr6	ENSMUST00000105802
MSTRG.2	Gm45533	MSTRG.22552.1
MSTRG.2	Nbl1	ENSMUST00000042844;ENSMUST00000132940;ENSMUST00000132
MSTRG.2	Minos1	ENSMUST00000143971;MSTRG.22555.2;ENSMUST00000063040;E
MSTRG.2	Minos1	MSTRG.22556.1
MSTRG.2	Gm16287	ENSMUST00000097827
MSTRG.22558		MSTRG.22558.1
MSTRG.2	Capzb	ENSMUST00000102508;ENSMUST00000131912;ENSMUST00000102
MSTRG.2	Gm38188	ENSMUST00000192879
MSTRG.2	Capzb	MSTRG.22560.1
MSTRG.2	Capzb	MSTRG.22561.1
MSTRG.2	Pqlc2	ENSMUST00000139840;ENSMUST00000053862;ENSMUST00000172
MSTRG.2	Pqlc2	MSTRG.22563.1
MSTRG.2	Pqlc2	MSTRG.22564.1
MSTRG.2	Akr7a5	ENSMUST00000073787;ENSMUST00000153645
MSTRG.2	Alpl	ENSMUST00000030551;ENSMUST00000141451;ENSMUST0000013S
MSTRG.2	Alpl;Ec	MSTRG.22567.1;MSTRG.22567.2;MSTRG.22567.3;MSTRG.22567

MSTRG.22568	MSTRG.22568.1
MSTRG.2 Ece1	ENSMUST00000102518;ENSMUST00000130407;MSTRG.22569.2;M
MSTRG.2 D730003	ENSMUST00000139056;ENSMUST00000194375
MSTRG.2 Gm13012	MSTRG.22570.1
MSTRG.2 Ece1	MSTRG.22571.1
MSTRG.2 Eif4g3	MSTRG.22572.1
MSTRG.2 Eif4g3;I	MSTRG.22573.2;MSTRG.22573.1;ENSMUST00000139759;ENSMUS
MSTRG.2 Eif4g3	MSTRG.22574.1
MSTRG.2 Eif4g3	MSTRG.22575.1
MSTRG.2 Eif4g3	MSTRG.22576.1;MSTRG.22576.2
MSTRG.2 Eif4g3	MSTRG.22577.1
MSTRG.2 Eif4g3	MSTRG.22578.1
MSTRG.2 Eif4g3	MSTRG.22579.1
MSTRG.2258	MSTRG.2258.1
MSTRG.2 Eif4g3	MSTRG.22580.1
MSTRG.2 Eif4g3	MSTRG.22581.1
MSTRG.2 Eif4g3	MSTRG.22582.1;MSTRG.22582.2;MSTRG.22582.3;MSTRG.22582
MSTRG.2 Eif4g3	MSTRG.22583.1
MSTRG.2 Eif4g3	MSTRG.22584.1
MSTRG.2 1700095	MSTRG.22585.1
MSTRG.2 Eif4g3	MSTRG.22586.1
MSTRG.2 Eif4g3	MSTRG.22587.1
MSTRG.2 2310026	ENSMUST00000146658
MSTRG.2 Hp1bp3	ENSMUST00000105827;ENSMUST00000142420;ENSMUST00000137
MSTRG.2259	MSTRG.2259.1
MSTRG.2 Sh2d5;K	ENSMUST00000105824;ENSMUST00000124239;ENSMUST0000013
MSTRG.2 Iffo2;G	ENSMUST00000123827;ENSMUST00000040023;ENSMUST00000174
MSTRG.2 Iffo2	MSTRG.22592.1
MSTRG.2 Gm25571	ENSMUST00000178322
MSTRG.2 Gm21969	MSTRG.22594.1
MSTRG.2 Mir7020	ENSMUST00000183787
MSTRG.2 Ccnd3-p	MSTRG.22596.1
MSTRG.2 Ccnd3-p	ENSMUST00000117963
MSTRG.2 Mrto4	ENSMUST00000131052;ENSMUST00000102503;ENSMUST0000003C
MSTRG.2 Emc1;Ub	ENSMUST00000179784;ENSMUST00000082262;ENSMUST00000144
MSTRG.226	MSTRG.226.1
MSTRG.2 Gm37074	ENSMUST00000193444
MSTRG.2 2700016	ENSMUST00000181556
MSTRG.2 Arhgef1	ENSMUST00000138493;ENSMUST00000105797;ENSMUST00000105
MSTRG.2 Arhgef1	MSTRG.22602.1
MSTRG.2 Padi4	MSTRG.22603.1;MSTRG.22603.2;ENSMUST00000026381;ENSMUS
MSTRG.2 Rcc2;Gm	MSTRG.22604.1;ENSMUST00000071169;ENSMUST00000138808;E
MSTRG.2 Rcc2	MSTRG.22605.1
MSTRG.2 Padi2	ENSMUST00000030765;ENSMUST00000148160;ENSMUST0000013C
MSTRG.2 Gm26226	ENSMUST00000082509
MSTRG.2 Gm13031	ENSMUST00000140501
MSTRG.2 Sdhb	ENSMUST00000010007;ENSMUST00000129181;ENSMUST00000125
MSTRG.2 Nenf	ENSMUST00000046770
MSTRG.2 Atp13a2	ENSMUST00000168047;MSTRG.22610.1;ENSMUST00000037055;E
MSTRG.2 Fbxo42	ENSMUST00000030757;ENSMUST00000146768;MSTRG.22611.3
MSTRG.2 Mfap2	ENSMUST00000166376;ENSMUST00000124506;ENSMUST00000071
MSTRG.2 Crocc;S	ENSMUST00000122846;ENSMUST00000151455;ENSMUST00000102
MSTRG.2 Necap2	ENSMUST00000132906;ENSMUST00000030760;ENSMUST0000014
MSTRG.2 Gm13047	ENSMUST00000117116
MSTRG.2 Spata21	ENSMUST00000051907;ENSMUST00000181353
MSTRG.2 Spata21	MSTRG.22617.1
MSTRG.2 4921514	MSTRG.22618.1;MSTRG.22618.2

MSTRG.2 Szrd1	MSTRG.22619.1
MSTRG.2 Tmem206	ENSMUST00000027940;MSTRG.2262.2
MSTRG.2 Tmem82	ENSMUST00000143154;ENSMUST00000053263;ENSMUST0000015[illegible]
MSTRG.2 Slc25a3	ENSMUST00000038661
MSTRG.2 Plekhm2	ENSMUST00000084203;ENSMUST00000030751;ENSMUST0000013[illegible]
MSTRG.2 Plekhm2	MSTRG.22624.1
MSTRG.2 Plekhm2	MSTRG.22625.1
MSTRG.2 Clcnka	ENSMUST00000105790
MSTRG.2 Clcnka	MSTRG.22627.1
MSTRG.2 Gm13075	ENSMUST00000149247
MSTRG.2 Clcnkb	ENSMUST00000105788;ENSMUST00000145725
MSTRG.2 Tmem206	MSTRG.2263.1
MSTRG.2 Hspb7	ENSMUST00000102486
MSTRG.2 Gm694	ENSMUST00000094544
MSTRG.2 Zbtb17	ENSMUST00000006377;ENSMUST00000142438;MSTRG.22632.3;[illegible]
MSTRG.2 Spen	MSTRG.22633.1;MSTRG.22633.2;ENSMUST00000078886;ENSMUS[illegible]
MSTRG.2 Spen	MSTRG.22634.1
MSTRG.2 Spen	MSTRG.22635.1
MSTRG.2 Spen	MSTRG.22636.1
MSTRG.2 Spen	MSTRG.22637.1
MSTRG.2 B330016[illegible]	ENSMUST00000049927
MSTRG.22639	MSTRG.22639.1
MSTRG.2 Ppp2r5a	MSTRG.2264.5;ENSMUST00000067976;MSTRG.2264.1;MSTRG.22
MSTRG.2 Ddi2;Rs[illegible]	ENSMUST00000102484;ENSMUST00000177592;ENSMUST0000010[illegible]
MSTRG.2 Ddi2	MSTRG.22641.1
MSTRG.2 Ddi2	MSTRG.22642.1
MSTRG.2 Agmat	ENSMUST00000038161
MSTRG.2 Dnajc16	ENSMUST00000038014;ENSMUST00000138654;ENSMUST0000015[illegible]
MSTRG.2 Efhd2	ENSMUST00000036854
MSTRG.2 Efhd2	MSTRG.22646.1
MSTRG.2 Casp9	ENSMUST00000128660;ENSMUST00000097805;ENSMUST000000[illegible]
MSTRG.2 Cela2a;[illegible]	ENSMUST00000127962;ENSMUST00000102481;ENSMUST0000015[illegible]
MSTRG.2 Gm37688	ENSMUST00000195195
MSTRG.2 4930455[illegible]	ENSMUST00000126231
MSTRG.2 Tmem51	ENSMUST00000036572
MSTRG.2 Gm13053	ENSMUST00000128385
MSTRG.2 Tmem51o	ENSMUST00000153023;ENSMUST00000141769;ENSMUST000001[illegible]
MSTRG.2 Kazn	ENSMUST00000155023;ENSMUST00000135922;ENSMUST0000017[illegible]
MSTRG.2 Gm13062	ENSMUST00000151337
MSTRG.2 Kazn	MSTRG.22656.1
MSTRG.2 Kazn	MSTRG.22657.1
MSTRG.22658	MSTRG.22658.1
MSTRG.22659	MSTRG.22659.1
MSTRG.2 Gm37432	MSTRG.2266.1;MSTRG.2266.2
MSTRG.22660	MSTRG.22660.1
MSTRG.2 Gm13052	ENSMUST00000125582
MSTRG.22662	MSTRG.22662.1
MSTRG.22663	MSTRG.22663.1
MSTRG.22664	MSTRG.22664.1
MSTRG.22665	MSTRG.22665.1
MSTRG.22666	MSTRG.22666.1
MSTRG.2 Pdpn	ENSMUST00000030317;ENSMUST00000119654
MSTRG.2 Prdm2;M	MSTRG.22668.1;ENSMUST00000105778;MSTRG.22668.3;MSTRG.
MSTRG.2 Gm13038	ENSMUST00000119886
MSTRG.2 Gm37432	ENSMUST00000195294
MSTRG.2 Prdm2	MSTRG.22670.1
MSTRG.2 Gm13039	ENSMUST00000118638

MSTRG.2	Gm22039	ENSMUST00000157278
MSTRG.2	Prdm2	MSTRG.22673.1
MSTRG.2	Pramef8	ENSMUST00000059790;ENSMUST00000132915;ENSMUST00000037
MSTRG.22675		MSTRG.22675.1
MSTRG.22676		MSTRG.22676.1
MSTRG.22677		MSTRG.22677.1
MSTRG.22678		MSTRG.22678.1
MSTRG.2	Gm13123	ENSMUST00000122173
MSTRG.2268		MSTRG.2268.1
MSTRG.2	Gm13127	ENSMUST00000118978
MSTRG.2	Dhrs3	ENSMUST00000105744;ENSMUST00000171001;ENSMUST00000154
MSTRG.2	Dhrs3	MSTRG.22682.1
MSTRG.2	Tnfrsf1	ENSMUST00000030336;ENSMUST00000143055
MSTRG.2	Tnfrsf8	ENSMUST00000030339;ENSMUST00000123027
MSTRG.2	Smarca5	ENSMUST00000171805;ENSMUST00000121843;MSTRG.22685.2
MSTRG.2	Gm13229	ENSMUST00000118238
MSTRG.2	Vps13d	ENSMUST00000036579;ENSMUST00000130704;MSTRG.22687.3;E
MSTRG.2	Vps13d	MSTRG.22688.1
MSTRG.2	Zfp987	ENSMUST00000091878
MSTRG.2269		MSTRG.2269.1
MSTRG.2	Zfp987	MSTRG.22690.1
MSTRG.2	Zfp990	ENSMUST00000105742
MSTRG.2	Gm21978	ENSMUST00000179696
MSTRG.22693		MSTRG.22693.1
MSTRG.2	Gm13226	ENSMUST00000117838
MSTRG.2	Gm13212	ENSMUST00000105739;ENSMUST00000119718;ENSMUST00000097
MSTRG.2	Gm13212	MSTRG.22698.1
MSTRG.2	Gm13212	MSTRG.22699.1
MSTRG.2	Prim2	ENSMUST00000027312;ENSMUST00000189552;ENSMUST00000187
MSTRG.2	9430037	ENSMUST00000192556
MSTRG.2	Gm13212	MSTRG.22700.1
MSTRG.2	Gm26763	ENSMUST00000180968
MSTRG.2	Gm13234	ENSMUST00000120010
MSTRG.2	Gm13231	ENSMUST00000119218
MSTRG.2	Zfp980	ENSMUST00000105738;ENSMUST00000180014
MSTRG.2	Gm13239	ENSMUST00000119920
MSTRG.2	Gm13232	ENSMUST00000121816
MSTRG.2	Gm13244	ENSMUST00000118855
MSTRG.2	Dtl	ENSMUST00000195650;ENSMUST00000027933;ENSMUST00000195
MSTRG.2	Gm13243	ENSMUST00000117481
MSTRG.2	Vmn2r-p	ENSMUST00000180112
MSTRG.2	Znf41-p	ENSMUST00000122051
MSTRG.2	Gm13238	ENSMUST00000117655
MSTRG.2	Gm13246	MSTRG.22714.1
MSTRG.2	Gm13246	ENSMUST00000120994
MSTRG.2	Zfp986	ENSMUST00000123460
MSTRG.2	Gm13137	MSTRG.22717.1
MSTRG.2	Gm16211	ENSMUST00000147722
MSTRG.2	Ints7;G	ENSMUST00000045450;ENSMUST00000193961;ENSMUST00000194
MSTRG.2	Gm13136	ENSMUST00000121031
MSTRG.2	Zfp984	MSTRG.22722.1
MSTRG.2	Gm13148	ENSMUST00000118892
MSTRG.2	Zfp984;	ENSMUST00000105734;ENSMUST00000122309;ENSMUST00000176
MSTRG.2	Rps19-p	ENSMUST00000116174
MSTRG.2	Zfp933	ENSMUST00000105718;ENSMUST00000135798
MSTRG.2	Zfp933	MSTRG.22728.1
MSTRG.2	Plod1	ENSMUST00000019199;ENSMUST00000149129;ENSMUST00000124

MSTRG.2	Lpgat1	ENSMUST00000133076;ENSMUST00000110855;ENSMUST0000013[illegible]
MSTRG.2	2510039[illegible]	ENSMUST00000103232;MSTRG.22730.2
MSTRG.2	Miip	ENSMUST00000030886;ENSMUST00000172710;ENSMUST0000017[illegible]
MSTRG.2	Fv1	ENSMUST00000094481
MSTRG.2	Fv1	MSTRG.22733.1
MSTRG.2	Mfn2	ENSMUST00000105716;ENSMUST00000030884;MSTRG.22734.3;[illegible]
MSTRG.2	Gm13156	ENSMUST00000118348
MSTRG.2	Gm26573	ENSMUST00000181199
MSTRG.2	Zfp600	ENSMUST00000168483;ENSMUST00000092750
MSTRG.2	Gm13165	ENSMUST00000152923
MSTRG.2	Gm22446	ENSMUST00000180108
MSTRG.2	Gm13167	ENSMUST00000118554
MSTRG.2	Gm13169	ENSMUST00000122090
MSTRG.2	Gm13166	ENSMUST00000154592;ENSMUST00000146671;ENSMUST0000014[illegible]
MSTRG.2	Gm13168	ENSMUST00000121771
MSTRG.2	Vmn2r-p	ENSMUST00000178733
MSTRG.2	Gm26573	MSTRG.22745.1
MSTRG.2	Gm26573	MSTRG.22746.1
MSTRG.2	Zfp992	ENSMUST00000105733
MSTRG.2	Gm13241	ENSMUST00000094493
MSTRG.2	Gm13245	ENSMUST00000119646
MSTRG.2	Gm26203	ENSMUST00000082911
MSTRG.2	Zfp981	ENSMUST00000105735
MSTRG.2	Zfp981	MSTRG.22751.1
MSTRG.2	Gm26573	MSTRG.22752.1
MSTRG.2	Gm26880	ENSMUST00000181610
MSTRG.2	1700095[illegible]	ENSMUST00000145269
MSTRG.2	Gm13237	ENSMUST00000122151
MSTRG.2	Gm13233	ENSMUST00000118760
MSTRG.2	Gm13240	ENSMUST00000120042
MSTRG.2	Vmn2r-p	ENSMUST00000178737
MSTRG.2	Zfp993	ENSMUST00000130825;MSTRG.22759.2;ENSMUST00000070932
MSTRG.2	Gm38037	ENSMUST00000192600
MSTRG.2	Gm26573	MSTRG.22760.1
MSTRG.2	Gm21411	ENSMUST00000049821
MSTRG.2	Gm20760	ENSMUST00000179197
MSTRG.2	Gm26573	MSTRG.22763.1
MSTRG.2	Gm26573	MSTRG.22764.1
MSTRG.2	Gm13140	ENSMUST00000119840
MSTRG.2	Zfp989	ENSMUST00000146688;ENSMUST00000132166
MSTRG.2	C230088[illegible]	ENSMUST00000181084;ENSMUST00000181752
MSTRG.2	Rex2	ENSMUST00000075775
MSTRG.2	Nek2	ENSMUST00000027931;ENSMUST00000126446;ENSMUST0000015[illegible]
MSTRG.2	C230088[illegible]	MSTRG.22771.1
MSTRG.2	Gm13160	ENSMUST00000118441
MSTRG.2	Gm13142	ENSMUST00000117213
MSTRG.2	Gm13162	ENSMUST00000121481
MSTRG.2	Vmn2r-p	ENSMUST00000177972
MSTRG.2	Zfp991	ENSMUST00000084149;ENSMUST00000151022
MSTRG.2	Gm26624	ENSMUST00000181486
MSTRG.2	Gm13146	ENSMUST00000118508
MSTRG.2	1700034[illegible]	ENSMUST00000190327;ENSMUST00000154116;ENSMUST0000014[illegible]
MSTRG.2	Gm13163	ENSMUST00000119875
MSTRG.2	C230088[illegible]	MSTRG.22781.1
MSTRG.2	Gm13147	ENSMUST00000125785;ENSMUST00000177526
MSTRG.2	Gm13158	ENSMUST00000122007
MSTRG.2	Gm13135	ENSMUST00000117628

MSTRG.2 Zfp988	ENSMUST00000148762;ENSMUST00000166764
MSTRG.2 Zfp978	ENSMUST00000133078;ENSMUST00000154154
MSTRG.2 Gm13161	ENSMUST00000120609
MSTRG.2 Slc30a1	ENSMUST00000044954;ENSMUST00000161756
MSTRG.2 Vmn2r-p	ENSMUST00000179720
MSTRG.2 Zfp982	ENSMUST00000105721;ENSMUST00000063704
MSTRG.2 Gm15951	ENSMUST00000118377
MSTRG.2 Vmn2r-p	ENSMUST00000179853
MSTRG.2 Gm13141	ENSMUST00000117417
MSTRG.2 Gm16503	ENSMUST00000104964
MSTRG.2 Zfp985	ENSMUST00000081742
MSTRG.2 Nppa	ENSMUST00000103230
MSTRG.2 Clcn6	MSTRG.22798.1;ENSMUST00000137724;MSTRG.22798.4;ENSMUS
MSTRG.2 Gm13207	ENSMUST00000119862
MSTRG.2 Gm29228	ENSMUST00000185489
MSTRG.2280	MSTRG.2280.1
MSTRG.2 Clcn6	MSTRG.22800.1
MSTRG.2 Mthfr	ENSMUST00000097788;ENSMUST00000141283;ENSMUST0000014[illegible]
MSTRG.2 Chchd2-[illegible]	ENSMUST00000117487
MSTRG.2 Gm13201	ENSMUST00000177629;ENSMUST00000144031
MSTRG.2 Agtrap	ENSMUST00000030865;MSTRG.22804.1;ENSMUST00000152323;E
MSTRG.2 Draxin	ENSMUST00000030862
MSTRG.2 Mad2l2	ENSMUST00000084129;ENSMUST00000132698;ENSMUST0000003[illegible]
MSTRG.2 Fbxo6	ENSMUST00000105706;ENSMUST00000168503;ENSMUST0000003[illegible]
MSTRG.2 Mir7022	ENSMUST00000183749
MSTRG.2 Fbxo44	ENSMUST00000167160;ENSMUST00000057907;ENSMUST0000012[illegible]
MSTRG.2281	MSTRG.2281.1
MSTRG.2 Fbxo2	ENSMUST00000047951
MSTRG.2 Ubiad1	ENSMUST00000051633;ENSMUST00000156686
MSTRG.2 Gm23303	ENSMUST00000082714
MSTRG.2 Mtor	ENSMUST00000123566;ENSMUST00000103221;ENSMUST00000057
MSTRG.2 Angptl7	MSTRG.22814.1;ENSMUST00000030840
MSTRG.2 Gm24002	ENSMUST00000158456
MSTRG.2 Gm23318	ENSMUST00000158000
MSTRG.2 Exosc10	ENSMUST00000017408;ENSMUST00000173154;MSTRG.22817.4;M
MSTRG.2 Exosc10	MSTRG.22818.1
MSTRG.2 Gm13204	ENSMUST00000120110
MSTRG.2 Traf5;M	ENSMUST00000194408;ENSMUST00000192628;ENSMUST0000008[illegible]
MSTRG.2 Exosc10	MSTRG.22820.1
MSTRG.2 Gm13209	ENSMUST00000138492
MSTRG.2 Gm572	ENSMUST00000105698
MSTRG.2 Gm572	MSTRG.22823.1
MSTRG.2 Srm	ENSMUST00000006611;ENSMUST00000152701;ENSMUST0000014[illegible]
MSTRG.22825	MSTRG.22825.1
MSTRG.2 Masp2	ENSMUST00000105701;ENSMUST00000052060;ENSMUST00000154
MSTRG.2 Tardbp	ENSMUST00000105702;ENSMUST00000165113;ENSMUST00000172
MSTRG.2 Pgd	ENSMUST00000084124;ENSMUST00000124409;ENSMUST0000015[illegible]
MSTRG.2 Casz1	ENSMUST00000094464;ENSMUST00000122222;ENSMUST0000012[illegible]
MSTRG.2 Traf5	MSTRG.2283.1
MSTRG.2 Gm15969	ENSMUST00000151967
MSTRG.2 Gm13205	ENSMUST00000123548;MSTRG.22831.1;ENSMUST00000103217;E
MSTRG.2 Gm17029	ENSMUST00000145143;ENSMUST00000146124;ENSMUST0000015[illegible]
MSTRG.2 Pex14	MSTRG.22833.1
MSTRG.2 Pex14	MSTRG.22834.1
MSTRG.2 Pex14	MSTRG.22835.1
MSTRG.2 Pex14	MSTRG.22836.1
MSTRG.2 Pex14	MSTRG.22837.1

MSTRG.2	Pex14	MSTRG.22838.1
MSTRG.2	Gm9506	ENSMUST00000118516
MSTRG.2	Gstp-ps	ENSMUST00000195526
MSTRG.2	Dffa	ENSMUST00000103216;ENSMUST00000030816;ENSMUST00000153
MSTRG.2	Cort	ENSMUST00000030815
MSTRG.2	Apitd1	ENSMUST00000105696;ENSMUST00000030813;ENSMUST00000177
MSTRG.2	Kif1b	ENSMUST00000060537;ENSMUST00000150853;ENSMUST00000139
MSTRG.2	Kif1b	MSTRG.22844.1
MSTRG.2	Kif1b	MSTRG.22845.1
MSTRG.2	Trmt112	ENSMUST00000117987
MSTRG.2	Ube4b	MSTRG.22847.2;MSTRG.22847.1;MSTRG.22847.3;ENSMUST0000
MSTRG.2	Ube4bos	ENSMUST00000155845
MSTRG.2	Ube4bos	MSTRG.22849.1
MSTRG.2	Gm26879	ENSMUST00000181127
MSTRG.2	Ube4bos	ENSMUST00000151896;ENSMUST00000141259
MSTRG.2	Ube4b	MSTRG.22851.1
MSTRG.2	Ube4b	MSTRG.22852.1
MSTRG.22853		MSTRG.22853.1
MSTRG.22854		MSTRG.22854.1
MSTRG.2	Nmnat1	ENSMUST00000119921;ENSMUST00000030845;ENSMUST00000105
MSTRG.2	Gm13097	ENSMUST00000210722
MSTRG.2	Lzic;Gm	ENSMUST00000124413;ENSMUST00000141293;ENSMUST00000138
MSTRG.2	Ctnnbip	ENSMUST00000030839;ENSMUST00000105692
MSTRG.2	Rcor3	ENSMUST00000110849;ENSMUST00000073279;ENSMUST00000192
MSTRG.2	Mir5616	ENSMUST00000177217
MSTRG.2	Ctnnbip	MSTRG.22861.1
MSTRG.2	Gm13066	ENSMUST00000145263
MSTRG.2	Clstn1	ENSMUST00000105691;ENSMUST00000039144;MSTRG.22863.2;M
MSTRG.2	Tmem201	ENSMUST00000105687;ENSMUST00000054459;ENSMUST00000139
MSTRG.2	Pik3cd	ENSMUST00000038859;ENSMUST00000105690;ENSMUST00000177
MSTRG.2	Mir7023	ENSMUST00000185093
MSTRG.2	Pik3cd	MSTRG.22867.1
MSTRG.2	Slc25a3	ENSMUST00000105686
MSTRG.2	Gm13068	ENSMUST00000146250
MSTRG.2	Rcor3	MSTRG.2287.1
MSTRG.2	Slc25a3	MSTRG.22870.1
MSTRG.2	Gm16188	ENSMUST00000142019
MSTRG.2	Gm13073	ENSMUST00000134059
MSTRG.2	Gm13065	ENSMUST00000119178
MSTRG.2	Gm13064	ENSMUST00000119125
MSTRG.2	AL62680	ENSMUST00000199807
MSTRG.2	Spsb1	ENSMUST00000038562;ENSMUST00000105685;ENSMUST00000105
MSTRG.2	Gm13070	ENSMUST00000146532
MSTRG.2	Spsb1	MSTRG.22878.1
MSTRG.2	Spsb1	MSTRG.22879.1
MSTRG.2	Rcor3	MSTRG.2288.1
MSTRG.2	Spsb1	MSTRG.22880.1
MSTRG.2	Spsb1	MSTRG.22881.1
MSTRG.22882		MSTRG.22882.1
MSTRG.2	Gm13067	ENSMUST00000134236
MSTRG.2	Gm13067	MSTRG.22884.1
MSTRG.2	Mir34a;	ENSMUST00000083559;ENSMUST00000194216
MSTRG.2	Gpr157	ENSMUST00000094451
MSTRG.22887		MSTRG.22887.1
MSTRG.2	H6pd	MSTRG.22888.1;ENSMUST00000084117;MSTRG.22888.3;ENSMUS
MSTRG.2	H6pd	MSTRG.22889.1
MSTRG.2	Rcor3	MSTRG.2289.1

MSTRG.2	Eno1	ENSMUST00000150175;ENSMUST00000080926;ENSMUST0000013C
MSTRG.2	Rps15a-:	ENSMUST00000121072
MSTRG.2	Errfi1	ENSMUST00000073600;ENSMUST00000128640;ENSMUST0000003C
MSTRG.2	Rere	MSTRG.22895.1;MSTRG.22895.2;MSTRG.22895.3
MSTRG.2	Rere	MSTRG.22896.2;MSTRG.22896.1;ENSMUST00000105682;ENSMUS
MSTRG.2	Rere	MSTRG.22897.1
MSTRG.2	Gm13092	MSTRG.22898.1
MSTRG.2	Gm13092	ENSMUST00000136346
MSTRG.2	Prim2	MSTRG.229.1
MSTRG.2	Gm23209	ENSMUST00000158667
MSTRG.2	Rere	MSTRG.22901.1;MSTRG.22901.2
MSTRG.2	Rere	MSTRG.22902.1
MSTRG.2	Rere	MSTRG.22903.1
MSTRG.2	Gm13091	MSTRG.22904.1
MSTRG.2	Gm13091	MSTRG.22905.1;MSTRG.22905.2;ENSMUST00000128800
MSTRG.2	Rere	MSTRG.22906.1
MSTRG.2	Thap3	MSTRG.22907.1;ENSMUST00000036680;ENSMUST00000105665;M
MSTRG.2	Phf13	ENSMUST00000055688
MSTRG.2	Dnajc11	ENSMUST00000062904;ENSMUST00000139555;ENSMUST0000013З
MSTRG.2	Gm10516	ENSMUST00000180598;ENSMUST00000195083
MSTRG.2	Gm9768	ENSMUST00000206007
MSTRG.2	Klhl21	ENSMUST00000097773
MSTRG.2	Zbtb48	ENSMUST00000066715;ENSMUST00000155389;ENSMUST00000123
MSTRG.2	Tas1r1	ENSMUST00000030792
MSTRG.2	Park7	ENSMUST00000030805;ENSMUST00000105675;ENSMUST00000105
MSTRG.2	Tnfrsf9	ENSMUST00000135169;ENSMUST00000105672;ENSMUST0000003C
MSTRG.2	Camta1	ENSMUST00000169423;ENSMUST00000097774;ENSMUST0000014C
MSTRG.2	Gm23405	ENSMUST00000082542
MSTRG.2	Camta1	MSTRG.22918.1
MSTRG.2	Uts2	ENSMUST00000030803
MSTRG.2	Kcnh1	ENSMUST00000078470;ENSMUST00000110844;ENSMUST00000151
MSTRG.2	Per3	ENSMUST00000103204;ENSMUST00000138052;ENSMUST00000138
MSTRG.2	Vamp3	ENSMUST00000030797;ENSMUST00000155446;ENSMUST00000125
MSTRG.2	Gm13090	ENSMUST00000105667
MSTRG.2	4930589	ENSMUST00000129097
MSTRG.2	Camta1	MSTRG.22924.1
MSTRG.2	Camta1	MSTRG.22925.1
MSTRG.2	Nol9	ENSMUST00000147797;MSTRG.22926.1;ENSMUST00000084116;E
MSTRG.2	Acot7	ENSMUST00000075363;ENSMUST00000124548;ENSMUST00000167
MSTRG.2	Acot7	MSTRG.22928.1
MSTRG.2	Gm27500	ENSMUST00000184331
MSTRG.2	Hhat	ENSMUST00000128619;ENSMUST00000044190;ENSMUST00000192
MSTRG.2	Gm13096	ENSMUST00000127752
MSTRG.2	Acot7	MSTRG.22931.1
MSTRG.2	Tnfrsf2	ENSMUST00000025706;ENSMUST00000035275;ENSMUST00000127
MSTRG.2	Espn	ENSMUST00000030785;ENSMUST00000105659;ENSMUST0000004S
MSTRG.2	Icmt	ENSMUST00000048892;MSTRG.22934.2;ENSMUST00000151372;E
MSTRG.2	Rnf207	ENSMUST00000076183;ENSMUST00000108688;ENSMUST00000135
MSTRG.2	Rpl22	ENSMUST00000142735;ENSMUST00000103191;ENSMUST0000013S
MSTRG.2	A430005	ENSMUST00000105645;ENSMUST00000058393;ENSMUST0000013S
MSTRG.2	Chd5	ENSMUST00000030775;ENSMUST00000164662
MSTRG.2	Gm13645	ENSMUST00000145163
MSTRG.2	Gm22265	ENSMUST00000082947
MSTRG.2	Kcnab2;(	ENSMUST00000159840;ENSMUST00000160884;ENSMUST0000003C
MSTRG.2	Gm16333	ENSMUST00000161416
MSTRG.2	Kcnab2	MSTRG.22942.1
MSTRG.2	Nphp4	ENSMUST00000142027;ENSMUST00000081393;ENSMUST00000153

MSTRG.2	Dffb	ENSMUST00000030893;ENSMUST00000133607;ENSMUST0000021[illegible]
MSTRG.2	Dffb	MSTRG.22945.1
MSTRG.2	Cep104	MSTRG.22946.1;ENSMUST00000047497;ENSMUST00000183790;[illegible]
MSTRG.2	Lrrc47	ENSMUST00000169622;ENSMUST00000030894;MSTRG.22947.3;[illegible]
MSTRG.2	Smim1	ENSMUST00000182191;ENSMUST00000182151;ENSMUST0000012[illegible]
MSTRG.2	Ccdc27	ENSMUST00000047207
MSTRG.2	Sertad4	ENSMUST00000155579;ENSMUST00000125391
MSTRG.2	Wrap73	ENSMUST00000030895;ENSMUST00000142665;MSTRG.22950.2;[illegible]
MSTRG.2	Tprgl	MSTRG.22951.1;ENSMUST00000030896;ENSMUST00000134492;[illegible]
MSTRG.2	Wrap73	MSTRG.22952.1
MSTRG.2	Megf6	ENSMUST00000030897;ENSMUST00000152159;MSTRG.22953.3;[illegible]
MSTRG.2	Gm13132	ENSMUST00000121194
MSTRG.2	Megf6	MSTRG.22955.1
MSTRG.2	Arhgef1	ENSMUST00000143141;ENSMUST00000144145;ENSMUST0000003[illegible]
MSTRG.2	Tnfrsf1	MSTRG.22957.1
MSTRG.2	Ttc34	ENSMUST00000207854;ENSMUST00000137684
MSTRG.2	Gm13112	ENSMUST00000138795
MSTRG.2	Gm15867	ENSMUST00000159407
MSTRG.2	Mmel1	ENSMUST00000079269;ENSMUST00000163732;ENSMUST0000008[illegible]
MSTRG.2	Fam213b	ENSMUST00000123513;ENSMUST00000030935;ENSMUST00000132
MSTRG.2	Tnfrsf1	ENSMUST00000219534;ENSMUST00000123514;MSTRG.22962.4;[illegible]
MSTRG.2	Pank4	ENSMUST00000030931;ENSMUST00000070953;MSTRG.22963.3;[illegible]
MSTRG.2	Pex10	ENSMUST00000103180;ENSMUST00000123395;ENSMUST0000012[illegible]
MSTRG.2	Faap20	ENSMUST00000126803;ENSMUST00000126713;ENSMUST00000122
MSTRG.2	Faap20	MSTRG.22966.1
MSTRG.2	Rer1	MSTRG.22967.1;ENSMUST00000149796;ENSMUST00000030914;[illegible]
MSTRG.2	Morn1	ENSMUST00000132367;ENSMUST00000030915;ENSMUST0000015[illegible]
MSTRG.2	Ski	MSTRG.22969.1;ENSMUST00000129314;ENSMUST00000084103;[illegible]
MSTRG.2	Gm37103	ENSMUST00000191830
MSTRG.2	Ski	MSTRG.22970.1
MSTRG.2	Ski	MSTRG.22971.1
MSTRG.2	Ski	MSTRG.22972.1
MSTRG.2	Prkcz	ENSMUST00000103178;ENSMUST00000030922;ENSMUST0000014[illegible]
MSTRG.2	Prkcz	MSTRG.22974.1
MSTRG.2	Gm27200	ENSMUST00000184626
MSTRG.22976		MSTRG.22976.1
MSTRG.2	Slc35e2	ENSMUST00000105608;ENSMUST00000043829;ENSMUST00000118
MSTRG.2	Gm16023	MSTRG.22978.1;ENSMUST00000137752;ENSMUST00000154931
MSTRG.2	Gm10563	ENSMUST00000115821
MSTRG.2	Gm37103	MSTRG.2298.1
MSTRG.2	Cdk11b	ENSMUST00000135550;ENSMUST00000067081;ENSMUST0000014[illegible]
MSTRG.2	Fndc10	ENSMUST00000099265
MSTRG.2	Mmp23	ENSMUST00000030937;ENSMUST00000156906;ENSMUST0000013[illegible]
MSTRG.2	Mib2	ENSMUST00000128204;MSTRG.22983.1;ENSMUST00000151843;[illegible]
MSTRG.2	5830444[illegible]	ENSMUST00000166921;ENSMUST00000152780;ENSMUST0000014[illegible]
MSTRG.2	Cfap74	ENSMUST00000129481;ENSMUST00000123952;MSTRG.22985.3;[illegible]
MSTRG.2	Cfap74	MSTRG.22986.1
MSTRG.2	Cfap74	MSTRG.22987.1
MSTRG.2	Cfap74	MSTRG.22988.1
MSTRG.2	Tmem52	ENSMUST00000131599;ENSMUST00000178238;ENSMUST0000002[illegible]
MSTRG.2	Diexf	ENSMUST00000194412;ENSMUST00000193460;ENSMUST0000008[illegible]
MSTRG.2	Gnb1	ENSMUST00000165335;ENSMUST00000105616;ENSMUST0000003[illegible]
MSTRG.2	Gnb1	MSTRG.22991.1
MSTRG.2	Gm13171	ENSMUST00000117233
MSTRG.2	Gnb1	MSTRG.22993.1
MSTRG.2	Gnb1	MSTRG.22994.1
MSTRG.2	Gnb1	MSTRG.22995.1

MSTRG.2 Nadk	MSTRG.22996.1
MSTRG.2 Nadk	ENSMUST00000030939;ENSMUST00000105613;ENSMUST00000147
MSTRG.22998	MSTRG.22998.1
MSTRG.2 Aurkaip	ENSMUST00000139651;ENSMUST00000084097;ENSMUST00000151
MSTRG.2 Atp6v1h	MSTRG.23.1
MSTRG.2 Prim2	MSTRG.230.1
MSTRG.2 Diexf	MSTRG.2300.1
MSTRG.2 Ssu72	ENSMUST00000030905;ENSMUST00000140322;MSTRG.23000.2;E
MSTRG.2 Atad3a;	MSTRG.23001.1;ENSMUST00000082982
MSTRG.2 1500002	ENSMUST00000178987;ENSMUST00000197451
MSTRG.2 Tmem240	ENSMUST00000147721;ENSMUST00000176526;ENSMUST00000127
MSTRG.2 Atad3a	ENSMUST00000030903;ENSMUST00000126725;MSTRG.23004.2;M
MSTRG.2 Atad3ao	ENSMUST00000174185;ENSMUST00000067628;ENSMUST00000155
MSTRG.2 Atad3ao	MSTRG.23006.1
MSTRG.2 Vwa1	ENSMUST00000042196
MSTRG.2 Gm26840	ENSMUST00000180784
MSTRG.2 Tmem88b	ENSMUST00000097742
MSTRG.2 Irf6;A1	ENSMUST00000076521;ENSMUST00000138826;ENSMUST0000014C
MSTRG.2 Ankrd65	ENSMUST00000165000;ENSMUST00000105593
MSTRG.2 Mrpl20	ENSMUST00000184339;MSTRG.23012.1;ENSMUST00000030942;E
MSTRG.2 Mrpl20	MSTRG.23013.1
MSTRG.2 Ccnl2	ENSMUST00000030944;ENSMUST00000139066;ENSMUST0000012
MSTRG.2 Dvl1	ENSMUST00000030948;ENSMUST00000168552;ENSMUST0000015
MSTRG.2 Tas1r3	ENSMUST00000030949
MSTRG.2 Cptp	ENSMUST00000030950;ENSMUST00000151961
MSTRG.2 Ints11	ENSMUST00000120794;ENSMUST00000134678;ENSMUST0000003C
MSTRG.2 Ints11	MSTRG.23019.1
MSTRG.2 Traf3ip	ENSMUST00000192020;ENSMUST00000194278;MSTRG.2302.5;MS
MSTRG.2 Ints11	MSTRG.23020.1
MSTRG.2 Ints11	MSTRG.23021.1
MSTRG.2 Pusl1	ENSMUST00000123504;ENSMUST00000097737;MSTRG.23022.3;E
MSTRG.2 Acap3	ENSMUST00000105584;ENSMUST00000079031;ENSMUST0000012
MSTRG.2 Ube2j2;	ENSMUST00000103175;ENSMUST00000166489;ENSMUST00000024
MSTRG.2 Ube2j2	MSTRG.23025.1
MSTRG.2 Ube2j2	MSTRG.23026.1
MSTRG.23027	MSTRG.23027.1
MSTRG.2 Rnf223	ENSMUST00000209248
MSTRG.2 9430015	ENSMUST00000072554;ENSMUST00000125786;ENSMUST0000016
MSTRG.2 Gm15872	MSTRG.2303.1;ENSMUST00000162454
MSTRG.2 B3galt6	ENSMUST00000052185
MSTRG.2 Sdf4	ENSMUST00000105579;ENSMUST00000050078;ENSMUST00000097
MSTRG.2 Sdf4	MSTRG.23032.1;MSTRG.23032.2
MSTRG.2 Tnfrsf4	ENSMUST00000030952;ENSMUST00000143576;MSTRG.23033.2;E
MSTRG.2 Gm37090	ENSMUST00000195694;ENSMUST00000181389;ENSMUST00000151
MSTRG.2 Gm25982	ENSMUST00000157920
MSTRG.2 Tnfrsf1	ENSMUST00000103173;ENSMUST00000141260;ENSMUST0000004C
MSTRG.2 Gm16008	MSTRG.23037.1
MSTRG.2 Ttll10	ENSMUST00000051509;ENSMUST00000184348;ENSMUST00000184
MSTRG.2 Gm13648	ENSMUST00000138361;ENSMUST00000083493
MSTRG.2304	MSTRG.2304.1
MSTRG.2 Mir200a	ENSMUST00000083466
MSTRG.2 Mir200b	ENSMUST00000083615
MSTRG.2 Isg15	ENSMUST00000085425
MSTRG.2 Agrn	ENSMUST00000154494;ENSMUST00000071248;ENSMUST00000105
MSTRG.2 AW01173	ENSMUST00000105140;MSTRG.23044.2;MSTRG.23044.1;MSTRG.
MSTRG.2 Perm1	ENSMUST00000105572
MSTRG.2 Plekhn1	ENSMUST00000219227;ENSMUST00000218699;MSTRG.23046.2;E

MSTRG.2	Gm15772	ENSMUST00000118533
MSTRG.2	Noc2l	MSTRG.23048.1;ENSMUST00000179543;ENSMUST00000179886
MSTRG.2	Samd11	ENSMUST00000179919;ENSMUST00000219866;ENSMUST0000021[illegible]
MSTRG.2	4631405[illegible]	ENSMUST00000180420;ENSMUST00000083599
MSTRG.2	CAAA010	ENSMUST00000217800
MSTRG.2	Gm36548	ENSMUST00000200222;ENSMUST00000197473
MSTRG.23052		MSTRG.23052.1
MSTRG.23053		MSTRG.23053.1
MSTRG.2	Cdk6	MSTRG.23054.1;ENSMUST00000199156;ENSMUST00000197607;E
MSTRG.2	Cdk6	MSTRG.23055.1
MSTRG.2	Cdk6	MSTRG.23056.1
MSTRG.2	Cdk6	MSTRG.23057.1
MSTRG.2	Gm17590	ENSMUST00000197881;ENSMUST00000168422
MSTRG.2	Cdk6	MSTRG.23059.1
MSTRG.2	4930570[illegible]	ENSMUST00000159273
MSTRG.2	Gm42435	ENSMUST00000196630
MSTRG.2	Mterf1b	ENSMUST00000177258
MSTRG.2	Mterf1b	MSTRG.23062.1
MSTRG.2	Fzd1	ENSMUST00000054294
MSTRG.2	Akap9	ENSMUST00000132058;ENSMUST00000143365;MSTRG.23064.2;E
MSTRG.2	Akap9	MSTRG.23065.1
MSTRG.2	Wdr46-p	ENSMUST00000200591
MSTRG.2	Gm43031	ENSMUST00000198500
MSTRG.2	Cyp51	ENSMUST00000001507;ENSMUST00000129448;ENSMUST00000151
MSTRG.2	Fam133b	ENSMUST00000197082;ENSMUST00000198598;ENSMUST0000011[illegible]
MSTRG.2	Lamb3	ENSMUST00000194407;ENSMUST00000194677;ENSMUST00000192
MSTRG.2	Gm43017	ENSMUST00000198296
MSTRG.2	n-R5s16	ENSMUST00000083110
MSTRG.2	Rbm48	MSTRG.23072.1;ENSMUST00000042753;ENSMUST00000176000;E
MSTRG.2	2510017[illegible]	ENSMUST00000198718
MSTRG.2	Pex1	ENSMUST00000006061;ENSMUST00000121291;ENSMUST00000142
MSTRG.2	Gm43825	ENSMUST00000199650
MSTRG.2	Pex1	MSTRG.23076.1
MSTRG.2	4833413[illegible]	ENSMUST00000199632
MSTRG.2	Gatad1	ENSMUST00000140871;MSTRG.23078.1;ENSMUST00000007559;E
MSTRG.2	Tmbim7	ENSMUST00000199959;ENSMUST00000014673;ENSMUST0000015[illegible]
MSTRG.2	Hsd11b1	ENSMUST00000016338;ENSMUST00000160929;ENSMUST00000161
MSTRG.2	Ankib1;[illegible]	MSTRG.23081.2;ENSMUST00000043551;MSTRG.23081.1;ENSMUS
MSTRG.2	Gm22926	ENSMUST00000174957
MSTRG.2	Ankib1	MSTRG.23083.1
MSTRG.2	Ankib1	MSTRG.23084.1
MSTRG.2	Krit1	ENSMUST00000199845;ENSMUST00000080085;ENSMUST0000019[illegible]
MSTRG.2	Lrrd1	ENSMUST00000044039
MSTRG.2	Mterf1a	MSTRG.23087.1;ENSMUST00000044746;ENSMUST00000117463;E
MSTRG.2	Rps4x-p	ENSMUST00000199852
MSTRG.2	Cldn12	ENSMUST00000179804;ENSMUST00000060947;ENSMUST0000011[illegible]
MSTRG.2	Gm38118	ENSMUST00000192052
MSTRG.2	Gm26825	ENSMUST00000181751
MSTRG.2	Gm15459	ENSMUST00000117405
MSTRG.23092		MSTRG.23092.1
MSTRG.2	Zfp804b	ENSMUST00000200317
MSTRG.2	Gm24525	ENSMUST00000082917
MSTRG.2	Tubb4b-[illegible]	ENSMUST00000179460
MSTRG.2	Gm42598	ENSMUST00000200110
MSTRG.2	Gm42597	ENSMUST00000196663
MSTRG.2	Gm43841	ENSMUST00000199491
MSTRG.2	Tex47	ENSMUST00000196680;ENSMUST00000088796;ENSMUST0000016[illegible]

MSTRG.2	Prim2	MSTRG.231.2;MSTRG.231.1
MSTRG.2	G0s2	ENSMUST00000009777
MSTRG.2	Gm30835	ENSMUST00000198825
MSTRG.2	Gm8802	ENSMUST00000118633
MSTRG.2	Gm22196	ENSMUST00000116865
MSTRG.2	Steap4	ENSMUST00000115421
MSTRG.23105		MSTRG.23105.1
MSTRG.2	Gm15731	ENSMUST00000146887
MSTRG.2	Sri	ENSMUST00000088786;ENSMUST00000148633;ENSMUST0000014[illegible]
MSTRG.2	Gm15730	ENSMUST00000121828
MSTRG.2	Adam22	ENSMUST00000050166;ENSMUST00000088761;ENSMUST0000011[illegible]
MSTRG.2	Gm37691	ENSMUST00000192147
MSTRG.2	Gm21759	ENSMUST00000178345
MSTRG.2	Gm24196	ENSMUST00000179161
MSTRG.2	Gm23993	ENSMUST00000157478
MSTRG.2	Gm42490	ENSMUST00000198595
MSTRG.2	Dbf4	ENSMUST00000002368;MSTRG.23114.2;ENSMUST00000171808;E
MSTRG.2	Slc25a4	ENSMUST00000066921;ENSMUST00000199817;ENSMUST0000019[illegible]
MSTRG.2	Slc25a4	MSTRG.23116.1
MSTRG.2	Gm5106	ENSMUST00000181058
MSTRG.2	Abcb1b	ENSMUST00000199955;ENSMUST00000196580;ENSMUST0000000[illegible]
MSTRG.2	Abcb1b	MSTRG.23119.1
MSTRG.2	Gm37650	ENSMUST00000193570
MSTRG.2	7330423	ENSMUST00000197499
MSTRG.2	Abcb4	ENSMUST00000003717;ENSMUST00000197458;ENSMUST0000019[illegible]
MSTRG.2	Gm17936	ENSMUST00000199413
MSTRG.2	Crot;Gm	ENSMUST00000003720;ENSMUST00000146115;MSTRG.23123.3;E
MSTRG.2	Gm15610	ENSMUST00000120584
MSTRG.2	Crot	MSTRG.23125.1
MSTRG.2	Crot	MSTRG.23126.1
MSTRG.2	Gm7332	ENSMUST00000118351
MSTRG.23129		MSTRG.23129.1
MSTRG.2	Camk1g	ENSMUST00000169907;ENSMUST00000016323;ENSMUST0000016[illegible]
MSTRG.23130		MSTRG.23130.1
MSTRG.2	1700003	ENSMUST00000188236
MSTRG.2	Gm8924	ENSMUST00000197405
MSTRG.2	1700003	MSTRG.23133.1
MSTRG.2	Tmem243	ENSMUST00000196029;ENSMUST00000115365;MSTRG.23134.3;E
MSTRG.2	Dmtf1	ENSMUST00000071921;ENSMUST00000184159;ENSMUST0000009[illegible]
MSTRG.2	Gm24666	ENSMUST00000158937
MSTRG.2	Gm10481	ENSMUST00000179228
MSTRG.2	Gm10108	ENSMUST00000078246
MSTRG.23139		MSTRG.23139.1
MSTRG.2	Gm37942	ENSMUST00000191875
MSTRG.2	Gm8991	ENSMUST00000073014
MSTRG.2	Gnat3	ENSMUST00000030561
MSTRG.2	Cd36	ENSMUST00000170051;ENSMUST00000082367;MSTRG.23142.2;E
MSTRG.2	Gnai1	ENSMUST00000074694
MSTRG.2	Gm3527	ENSMUST00000198905
MSTRG.23145		MSTRG.23145.1
MSTRG.2	Phtf2	MSTRG.23146.1
MSTRG.2	Phtf2	ENSMUST00000118174;ENSMUST00000153194;MSTRG.23147.3;E
MSTRG.2	Rpl31-p	ENSMUST00000118086
MSTRG.2	Tmem60	ENSMUST00000115259;ENSMUST00000196723
MSTRG.2	Plxna2	ENSMUST00000194398;ENSMUST00000027952;ENSMUST0000012[illegible]
MSTRG.2	Rsbn1l	MSTRG.23150.2;MSTRG.23150.4;MSTRG.23150.3;ENSMUST000[illegible]
MSTRG.2	A630072	ENSMUST00000190567

MSTRG.2	A630072	MSTRG.23152.1
MSTRG.23153		MSTRG.23153.1
MSTRG.2	Ptpn12	ENSMUST00000030556;ENSMUST00000151366;ENSMUST0000014{8}
MSTRG.2	Ptpn12	MSTRG.23155.1
MSTRG.2	Gm43216	ENSMUST00000196295
MSTRG.2	Ptpn12	MSTRG.23157.1
MSTRG.2	Gsap	ENSMUST00000197522;ENSMUST00000198014;ENSMUST0000003{6}
MSTRG.2	Gm43215	ENSMUST00000199108
MSTRG.2	Gm37783	ENSMUST00000192025
MSTRG.2	Gsap	MSTRG.23160.1
MSTRG.2	Gsap	MSTRG.23161.1
MSTRG.2	Gsap	MSTRG.23162.1
MSTRG.2	Gm43214	ENSMUST00000199242
MSTRG.2	Ccdc146	ENSMUST00000115245;ENSMUST00000129172
MSTRG.2	Fgl2	ENSMUST00000035799
MSTRG.2	Fam185a	ENSMUST00000056045;ENSMUST00000153301;ENSMUST0000014{6}
MSTRG.2	Fam185a	MSTRG.23167.1
MSTRG.2	Fbxl13	ENSMUST00000051358;ENSMUST00000137788;ENSMUST0000015{3}
MSTRG.2	Lrrc17	ENSMUST00000199277;ENSMUST00000035651
MSTRG.2	Gm37674	ENSMUST00000194177
MSTRG.2	Gm6543	ENSMUST00000197353
MSTRG.2	Gm8539	ENSMUST00000199822
MSTRG.2	Armc10	ENSMUST00000148873;ENSMUST00000072896;ENSMUST0000014{6}
MSTRG.2	Napepld	ENSMUST00000115217;ENSMUST00000060899
MSTRG.2	Gm15719	ENSMUST00000120619
MSTRG.2	Psmc2	ENSMUST00000030769;MSTRG.23175.2;MSTRG.23175.3;ENSMUS
MSTRG.2	Pmpcb	ENSMUST00000030882;ENSMUST00000125693;ENSMUST0000014{9}
MSTRG.2	Gm15715	ENSMUST00000118032
MSTRG.2	Dnajc2	ENSMUST00000030771;ENSMUST00000115193;ENSMUST0000013{2}
MSTRG.2	Gm43110	ENSMUST00000198877
MSTRG.2	Gm38036	ENSMUST00000194586
MSTRG.2	Reln	ENSMUST00000161356;ENSMUST00000200318;ENSMUST0000020{0}
MSTRG.2	Gm16111	ENSMUST00000162128
MSTRG.2	Reln	ENSMUST00000199034
MSTRG.2	Gm10475	ENSMUST00000084713
MSTRG.2	Gm19666	ENSMUST00000198176
MSTRG.2	Reln	MSTRG.23186.1
MSTRG.2	Reln	MSTRG.23187.1
MSTRG.2	Gm16113	ENSMUST00000159629
MSTRG.2	Gm16112	ENSMUST00000159075
MSTRG.2	2900035	ENSMUST00000194966
MSTRG.2	Gm16110	ENSMUST00000161473
MSTRG.2	Orc5;Gm	ENSMUST00000135906;ENSMUST00000030872;ENSMUST0000019{9}
MSTRG.2	Gm22887	ENSMUST00000178586
MSTRG.2	6030443	ENSMUST00000195898;ENSMUST00000181764;ENSMUST00000181
MSTRG.2	Gm9057	ENSMUST00000198791
MSTRG.2	Rpl17-p	ENSMUST00000121001
MSTRG.2	Gm24512	ENSMUST00000174963
MSTRG.2	Gm9523	ENSMUST00000199146
MSTRG.2	6030443	MSTRG.23198.1
MSTRG.2	Gm6560	ENSMUST00000198588
MSTRG.2	Gm37618	ENSMUST00000192891
MSTRG.2	Plekhg1	ENSMUST00000120274;ENSMUST00000144543;ENSMUST00000137
MSTRG.2	A930003	ENSMUST00000199056;ENSMUST00000196218
MSTRG.2	A930003	ENSMUST00000197486
MSTRG.2	Lhfpl3	ENSMUST00000197992;ENSMUST00000196406
MSTRG.2	Gm21846	ENSMUST00000179257

MSTRG.2	Gm43169	ENSMUST00000199544
MSTRG.2	Gm6564	ENSMUST00000075377
MSTRG.2	Lhfpl3	MSTRG.23207.1
MSTRG.2	Lhfpl3	MSTRG.23208.1
MSTRG.2	Lhfpl3	MSTRG.23209.1
MSTRG.2	Plekhg1	MSTRG.2321.1
MSTRG.2	Gm42948	ENSMUST00000199085
MSTRG.2	AI50681	MSTRG.23211.1;MSTRG.23211.2;ENSMUST00000198331;ENSMUS
MSTRG.2	Mir3096	ENSMUST00000116685
MSTRG.2	Tomm7	ENSMUST00000030851
MSTRG.2	2700038	ENSMUST00000181574;ENSMUST00000197551
MSTRG.2	Snord93	ENSMUST00000175580
MSTRG.2	2700038	MSTRG.23216.1
MSTRG.2	Pus7	ENSMUST00000119946;ENSMUST00000148618;ENSMUST00000131
MSTRG.2	Pus7	ENSMUST00000195909
MSTRG.2	Rint1	ENSMUST00000120869;ENSMUST00000030852;ENSMUST00000117
MSTRG.2	Plekhg1	MSTRG.2322.1
MSTRG.2	Gm43054	ENSMUST00000199495
MSTRG.2	Rint1	MSTRG.23221.1
MSTRG.2	4933427	ENSMUST00000122963
MSTRG.2	4933427	MSTRG.23223.1
MSTRG.2	4933427	MSTRG.23224.1
MSTRG.2	4933427	MSTRG.23225.1
MSTRG.2	Fam126a	ENSMUST00000101513;ENSMUST00000030849;ENSMUST00000197
MSTRG.2	Fam126a	MSTRG.23227.1
MSTRG.2	Fam126a	MSTRG.23228.1
MSTRG.2	Fam126a	MSTRG.23229.1
MSTRG.2	Gm16149	ENSMUST00000117631
MSTRG.2	Gm6745	ENSMUST00000199216
MSTRG.2	Fam126a	MSTRG.23231.1
MSTRG.2	Fam126a	MSTRG.23232.1
MSTRG.23233		MSTRG.23233.1
MSTRG.2	Gm42951	ENSMUST00000199688
MSTRG.2	5031425	MSTRG.23235.1;ENSMUST00000185943;ENSMUST00000188328;M
MSTRG.2	Gm43722	ENSMUST00000196054
MSTRG.2	4930580	ENSMUST00000197377
MSTRG.2	Gm43058	ENSMUST00000197645
MSTRG.2	Gm42509	ENSMUST00000197390
MSTRG.2	Gm23023	ENSMUST00000158767
MSTRG.2	Kmt2e	ENSMUST00000196889;ENSMUST00000094962;MSTRG.23240.4;M
MSTRG.2	Gm25219	ENSMUST00000157545
MSTRG.2	Kmt2e	MSTRG.23242.1
MSTRG.2	Srpk2	MSTRG.23243.4;ENSMUST00000088392;MSTRG.23243.3;MSTRG.
MSTRG.2	Srpk2	MSTRG.23244.1
MSTRG.2	Srpk2	MSTRG.23245.1
MSTRG.2	Srpk2	MSTRG.23246.1
MSTRG.2	Srpk2	MSTRG.23247.1
MSTRG.2	Srpk2	MSTRG.23248.1
MSTRG.2	Srpk2	MSTRG.23249.1
MSTRG.2	Gm16148	ENSMUST00000119653
MSTRG.2	Srpk2	MSTRG.23250.1
MSTRG.2	Gm24009	ENSMUST00000157734
MSTRG.2	Gm43566	ENSMUST00000196851
MSTRG.2	Klhl7	ENSMUST00000030841;ENSMUST00000200116;ENSMUST00000163
MSTRG.2	Klhl7	MSTRG.23254.1
MSTRG.2	Gm3724	ENSMUST00000197957
MSTRG.2	Gm43389	ENSMUST00000196080

MSTRG.2	Nupl2	ENSMUST00000049887;ENSMUST00000115101;ENSMUST00000147
MSTRG.2	Nupl2	MSTRG.23258.1
MSTRG.23259		MSTRG.23259.1
MSTRG.2	Cd34	ENSMUST00000016638;ENSMUST00000110815;ENSMUST00000194
MSTRG.2	Kcnh2	MSTRG.23260.1;ENSMUST00000115098;ENSMUST00000036092;E
MSTRG.2	Gm15589	ENSMUST00000144193
MSTRG.2	Abcb8	ENSMUST00000138168;ENSMUST00000115077;ENSMUST00000073
MSTRG.2	Asic3	ENSMUST00000049346;ENSMUST00000198241;ENSMUST00000197
MSTRG.2	Cdk5	ENSMUST00000030814;ENSMUST00000198990;ENSMUST00000197
MSTRG.2	Agap3	ENSMUST00000024123;ENSMUST00000212381;ENSMUST0000019
MSTRG.2	Agap3	MSTRG.23266.1
MSTRG.2	Gbx1	ENSMUST00000088311
MSTRG.2	Slc4a2	ENSMUST00000153274;ENSMUST00000127315;ENSMUST00000141
MSTRG.2	Fastk;G	ENSMUST00000115043;ENSMUST00000149537;ENSMUST00000158
MSTRG.2	Gm16897	ENSMUST00000180780
MSTRG.2	Tmub1	ENSMUST00000030799;ENSMUST00000115036;ENSMUST00000115
MSTRG.2	2310074	ENSMUST00000198276
MSTRG.2	Abcf2	MSTRG.23272.2;MSTRG.23272.1;ENSMUST00000030795
MSTRG.2	Gm10221	ENSMUST00000088299
MSTRG.2	Chpf2;M	ENSMUST00000088295;ENSMUST00000198521;ENSMUST00000121
MSTRG.2	Smarcd3	ENSMUST00000144995;ENSMUST00000030791;ENSMUST00000195
MSTRG.2	Smarcd3	MSTRG.23276.1
MSTRG.2	Nub1	ENSMUST00000197407;ENSMUST00000068825;MSTRG.23277.3;E
MSTRG.2	Gm26648	MSTRG.23278.1;ENSMUST00000181925
MSTRG.2	Wdr86	ENSMUST00000068693;ENSMUST00000142607;ENSMUST00000196
MSTRG.2	A330023	ENSMUST00000181226;ENSMUST00000181947;ENSMUST00000181
MSTRG.2	Rheb;Gm	MSTRG.23280.1;ENSMUST00000030787;ENSMUST00000146310;E
MSTRG.2	Prkag2	ENSMUST00000030784;ENSMUST00000114975;ENSMUST00000076
MSTRG.2	Prkag2o	ENSMUST00000123610
MSTRG.2	Prkag2	MSTRG.23283.1
MSTRG.2	Prkag2o	ENSMUST00000134633;MSTRG.23284.2
MSTRG.2	1500035	ENSMUST00000075081
MSTRG.2	2900005	ENSMUST00000171190;ENSMUST00000128569
MSTRG.2	Galnt11	ENSMUST00000196213;ENSMUST00000045737;ENSMUST00000114
MSTRG.2	Galnt11	MSTRG.23289.1
MSTRG.2	A330023	MSTRG.2329.1
MSTRG.2	Kmt2c	MSTRG.23290.1
MSTRG.2	Cct8l1	ENSMUST00000045016
MSTRG.23292		MSTRG.23292.1
MSTRG.2	1700096	ENSMUST00000188465
MSTRG.23294		MSTRG.23294.1
MSTRG.23295		MSTRG.23295.1
MSTRG.23296		MSTRG.23296.1
MSTRG.2	Gm43143	ENSMUST00000196083
MSTRG.2	Xrcc2	ENSMUST00000030773;ENSMUST00000114922;ENSMUST00000134
MSTRG.2	Xrcc2	MSTRG.23299.1
MSTRG.2	Gm17813	ENSMUST00000185377
MSTRG.2	Gm32250	ENSMUST00000191885
MSTRG.2	Kmt2c	ENSMUST00000174734;MSTRG.23300.3;MSTRG.23300.4;MSTRG.
MSTRG.2	Gm42585	ENSMUST00000198731
MSTRG.2	Hspe1-p	ENSMUST00000172726
MSTRG.2	Gm20432	ENSMUST00000173716
MSTRG.2	Gm18343	ENSMUST00000172802
MSTRG.2	Kmt2c	MSTRG.23305.1
MSTRG.2	Kmt2c	MSTRG.23306.1
MSTRG.2	Kmt2c	MSTRG.23307.1
MSTRG.2	4831440	ENSMUST00000181942

MSTRG.2	Gm22656	ENSMUST00000104199
MSTRG.2	Gm37334	ENSMUST00000192325
MSTRG.2	Paxip1	ENSMUST00000002291;ENSMUST00000197625;ENSMUST0000019€
MSTRG.2	Paxip1	MSTRG.23311.1
MSTRG.2	Gm26608	ENSMUST00000180740;ENSMUST00000181503
MSTRG.2	Gm43611	ENSMUST00000196656
MSTRG.2	Insig1	ENSMUST00000059155;ENSMUST00000199728
MSTRG.2	Dpp6	ENSMUST00000149678;ENSMUST00000071500;ENSMUST0000013€
MSTRG.2	Gm29784	ENSMUST00000196217
MSTRG.2	Gm16057	ENSMUST00000120811
MSTRG.2	Gm43776	ENSMUST00000199729
MSTRG.2	9630010	ENSMUST00000192348
MSTRG.2	Gm43502	ENSMUST00000200263
MSTRG.2	Speer4b	ENSMUST00000053257;ENSMUST00000155721;ENSMUST00000124
MSTRG.2	Gm16058	ENSMUST00000124392
MSTRG.2	Rbm33	ENSMUST00000090355;MSTRG.23323.1;ENSMUST00000114884;E
MSTRG.2	Rbm33	MSTRG.23324.1
MSTRG.2	Nom1	ENSMUST00000001611;ENSMUST00000146347
MSTRG.2	Lmbr1	ENSMUST00000055195;ENSMUST00000179191;ENSMUST0000019€
MSTRG.2	C79130	ENSMUST00000199610
MSTRG.2	Lmbr1	MSTRG.23328.1
MSTRG.2	Lmbr1	MSTRG.23329.1
MSTRG.2	Gm37027	MSTRG.2333.1;ENSMUST00000194642
MSTRG.2	Gm23801	ENSMUST00000175561
MSTRG.2	Lmbr1	MSTRG.23331.1
MSTRG.2	4632411	ENSMUST00000181005
MSTRG.2	Ube3c	ENSMUST00000049453;ENSMUST00000199032;MSTRG.23333.2;E
MSTRG.2	Ube3c	MSTRG.23334.1
MSTRG.2	Ube3c	MSTRG.23335.1
MSTRG.23336		MSTRG.23336.1
MSTRG.2	Gm42570	ENSMUST00000195937
MSTRG.2	Gm5129	ENSMUST00000065372
MSTRG.2	Dnajb6	ENSMUST00000008733;MSTRG.23339.2;ENSMUST00000114839;M
MSTRG.2	Cd46;Cr	ENSMUST00000162650;MSTRG.2334.1;ENSMUST00000161772;EN
MSTRG.2	Dnajb6	MSTRG.23340.1
MSTRG.2	Dnajb6	MSTRG.23341.1
MSTRG.2	Gm5552	ENSMUST00000197149
MSTRG.2	Dnajb6	MSTRG.23343.1
MSTRG.2	Gm2213	ENSMUST00000197485
MSTRG.2	A230098	ENSMUST00000200055
MSTRG.2	Il6	ENSMUST00000026845;ENSMUST00000199183;ENSMUST0000019€
MSTRG.2	Tyms	MSTRG.23347.1
MSTRG.2	Tyms	ENSMUST00000141630;MSTRG.23348.2;ENSMUST00000026846;E
MSTRG.23349		MSTRG.23349.1
MSTRG.2	Gm37366	ENSMUST00000194487
MSTRG.23350		MSTRG.23350.1
MSTRG.2	Garem2	ENSMUST00000058045
MSTRG.2	Hadha	ENSMUST00000156859;MSTRG.23353.3;ENSMUST00000026843;E
MSTRG.2	Hadhb	ENSMUST00000026841;ENSMUST00000123980;ENSMUST00000114
MSTRG.2	Hadhb	MSTRG.23355.1
MSTRG.2	Mir1960	ENSMUST00000157488
MSTRG.2	Hadhb	MSTRG.23357.1
MSTRG.2	Adgrf3	ENSMUST00000088117;ENSMUST00000135322
MSTRG.23359		MSTRG.23359.1
MSTRG.2	Gm37132	ENSMUST00000195553
MSTRG.2	Selenoi	ENSMUST00000145167;ENSMUST00000198788;ENSMUST00000151
MSTRG.2	Gm16288	ENSMUST00000137940

MSTRG.2	Gm9899	ENSMUST00000199326;ENSMUST00000065519;ENSMUST0000020C
MSTRG.2	Kcnk3	ENSMUST00000066295;ENSMUST00000197428
MSTRG.2	Slc35f6	ENSMUST00000062962;ENSMUST00000176598;ENSMUST0000019б
MSTRG.2	Cenpa	ENSMUST00000144742;ENSMUST00000133316;ENSMUST00000134
MSTRG.2	Mapre3	ENSMUST00000031058;ENSMUST00000201347;ENSMUST0000020C
MSTRG.2	Gm19409	ENSMUST00000198244
MSTRG.2	Gm15461	ENSMUST00000143488
MSTRG.2	Khk	ENSMUST00000201621;ENSMUST00000201571;ENSMUST00000031
MSTRG.2	Cr2	ENSMUST00000082321;ENSMUST00000195120;ENSMUST0000019З
MSTRG.2	Cgref1	ENSMUST00000031051;ENSMUST00000202627
MSTRG.2	Tmem214	ENSMUST00000201203;ENSMUST00000114716;ENSMUST00000202
MSTRG.2	Gm43808	ENSMUST00000202534
MSTRG.2	Ost4	MSTRG.23373.1;MSTRG.23373.2;ENSMUST00000132253;ENSMUS
MSTRG.2	Agbl5;5	ENSMUST00000200695;ENSMUST00000201167;ENSMUST00000201
MSTRG.2	Emilin1	ENSMUST00000031055;ENSMUST00000201169;ENSMUST00000202
MSTRG.2	Abhd1	ENSMUST00000202401;ENSMUST00000201125;ENSMUST00000201
MSTRG.2	Preb	ENSMUST00000201821;ENSMUST00000074840;ENSMUST00000201
MSTRG.2	Slc5a6	ENSMUST00000202520;ENSMUST00000114668;ENSMUST0000008C
MSTRG.2	Atraid;	ENSMUST00000201174;ENSMUST00000201887;ENSMUST00000013
MSTRG.2	Akap12	MSTRG.2338.1
MSTRG.2	Atraid	MSTRG.23380.1
MSTRG.2	Mpv17;G	ENSMUST00000154241;ENSMUST00000200744;ENSMUST0000020C
MSTRG.2	Mpv17	MSTRG.23382.1
MSTRG.2	Ppm1g	ENSMUST00000202294;ENSMUST00000031032;ENSMUST00000202
MSTRG.2	Gm42798	ENSMUST00000200853
MSTRG.2	Eif2b4	ENSMUST00000077693;ENSMUST00000166769;ENSMUST00000114
MSTRG.2	Snx17	ENSMUST00000031029;ENSMUST00000201679;ENSMUST0000020C
MSTRG.2	Zfp513	ENSMUST00000114590;ENSMUST00000031562;ENSMUST00000201
MSTRG.2	Gm9970	ENSMUST00000068997
MSTRG.2	Nrbp1	ENSMUST00000202576;ENSMUST00000031034;ENSMUST00000202
MSTRG.2	Akap12	ENSMUST00000045730;ENSMUST00000216139;ENSMUST00000215
MSTRG.2	Krtcap3	ENSMUST00000201333;ENSMUST00000054829;ENSMUST00000201
MSTRG.2	Ift172	ENSMUST00000041565;ENSMUST00000201426;ENSMUST00000202
MSTRG.2	Fndc4	ENSMUST00000172435;ENSMUST00000041266;ENSMUST0000020C
MSTRG.2	Gm43659	ENSMUST00000200894
MSTRG.2	4930478	ENSMUST00000201366
MSTRG.2	Zfp512;	ENSMUST00000202061;ENSMUST00000076264;ENSMUST00000201
MSTRG.2	Gm43660	ENSMUST00000200956
MSTRG.2	AI83997	ENSMUST00000201790;ENSMUST00000200738
MSTRG.2	2900076	ENSMUST00000202297
MSTRG.2	4930548	ENSMUST00000202441
MSTRG.2	1700001	ENSMUST00000191779;ENSMUST00000191965
MSTRG.2	Akap12	MSTRG.2340.1
MSTRG.2	Gpn1	ENSMUST00000076949;ENSMUST00000201881;ENSMUST00000202
MSTRG.2	Supt7l	ENSMUST00000065388;ENSMUST00000201769;ENSMUST0000020C
MSTRG.2	Slc4a1a	ENSMUST00000201294;ENSMUST00000114533;ENSMUST00000202
MSTRG.2	Slc4a1a	MSTRG.23404.1
MSTRG.2	Gm43660	MSTRG.23405.1
MSTRG.2	Fosl2	ENSMUST00000031017;ENSMUST00000201110;ENSMUST00000202
MSTRG.2	Gm5864	ENSMUST00000202263
MSTRG.2	Mrpl33;	ENSMUST00000201999;ENSMUST00000200694;ENSMUST00000202
MSTRG.2	Mrpl33	MSTRG.23409.1
MSTRG.2	Akap12	MSTRG.2341.1
MSTRG.2	Rbks;Mr	ENSMUST00000201744;MSTRG.23410.1;ENSMUST00000031018;M
MSTRG.2	Mir3473	ENSMUST00000183932
MSTRG.2	Gm43809	MSTRG.23412.1
MSTRG.2	Gm43809	MSTRG.23413.1

MSTRG.2	Gm43810	ENSMUST00000201018
MSTRG.2	Gm24436	ENSMUST00000157889
MSTRG.2	Babam2	MSTRG.23416.1
MSTRG.2	Gm43811	ENSMUST00000202862
MSTRG.2	Gm17130	MSTRG.23418.1
MSTRG.2	Ppp1cb;	ENSMUST00000015100;ENSMUST00000201360;ENSMUST00000201
MSTRG.2	Akap12	MSTRG.2342.1
MSTRG.2	Gm43313	ENSMUST00000201398
MSTRG.2	Gm15613	ENSMUST00000118396
MSTRG.2	Gm15614	ENSMUST00000155976
MSTRG.2	Gm42964	ENSMUST00000202965
MSTRG.2	Gm43812	ENSMUST00000202701
MSTRG.2	Gm10461	ENSMUST00000202073
MSTRG.2	Yes1	ENSMUST00000202543;ENSMUST00000072311;ENSMUST0000020C
MSTRG.2	Yes1	MSTRG.23427.1
MSTRG.2	Gm43694	ENSMUST00000200773
MSTRG.2	Ywhah	ENSMUST00000019109;ENSMUST00000199651;ENSMUST0000013E
MSTRG.2	Zbtb2	ENSMUST00000100077;ENSMUST00000100078;ENSMUST0000021E
MSTRG.2	Gm2420	ENSMUST00000202748;ENSMUST00000119864;ENSMUST00000202
MSTRG.2	Pisd;Gm	ENSMUST00000142779;ENSMUST00000061895;ENSMUST00000202
MSTRG.2	Gm20671	MSTRG.23432.1
MSTRG.2	Gm43692	ENSMUST00000198236
MSTRG.2	Gm2623	ENSMUST00000199229
MSTRG.2	Ctbp1	MSTRG.23435.1
MSTRG.2	Depdc5;	ENSMUST00000149350;ENSMUST00000118698;ENSMUST0000015C
MSTRG.2	Gm24003	ENSMUST00000158455
MSTRG.2	Depdc5	MSTRG.23438.1
MSTRG.2	Ctbp1	ENSMUST00000079746;ENSMUST00000201575;ENSMUST00000202
MSTRG.2	Zbtb2	MSTRG.2344.1
MSTRG.2	Gm43850	MSTRG.23440.1;MSTRG.23440.2
MSTRG.2	Maea	ENSMUST00000114449;ENSMUST00000200716;ENSMUST0000020C
MSTRG.2	Uvssa	ENSMUST00000200682;ENSMUST00000087864;ENSMUST00000202
MSTRG.2	Uvssa	MSTRG.23443.1
MSTRG.2	Letm1	ENSMUST00000005431;ENSMUST00000149886;MSTRG.23444.3;E
MSTRG.2	Gm10059	ENSMUST00000131139
MSTRG.2	Gm30708	ENSMUST00000202183
MSTRG.2	Fam53a	ENSMUST00000065119;ENSMUST00000065162;MSTRG.23447.2;E
MSTRG.2	Gm9903	ENSMUST00000181102
MSTRG.2	Slbp	ENSMUST00000075670;ENSMUST00000101354;ENSMUST00000057
MSTRG.2	Mthfd1l	ENSMUST00000117291;ENSMUST00000120585;ENSMUST0000004E
MSTRG.2	Gm10459	ENSMUST00000202708
MSTRG.2	Tmem129	ENSMUST00000019439;ENSMUST00000140931;MSTRG.23451.3;E
MSTRG.2	Tacc3	ENSMUST00000074849;ENSMUST00000079534;ENSMUST00000201
MSTRG.2	Nsd2	ENSMUST00000075812;ENSMUST00000114397;MSTRG.23453.2;E
MSTRG.2	Nsd2	MSTRG.23454.1
MSTRG.2	Nelfa	MSTRG.23455.1;ENSMUST00000030993;ENSMUST00000132942
MSTRG.2	Mir7024	ENSMUST00000183676
MSTRG.2	Gm42849	ENSMUST00000202834
MSTRG.2	Gm42851	ENSMUST00000201325
MSTRG.23459		MSTRG.23459.1
MSTRG.2	Gm16074	ENSMUST00000119524
MSTRG.2	Zfyve28	ENSMUST00000094868;MSTRG.23460.2;ENSMUST00000140646;M
MSTRG.2	Gm42848	ENSMUST00000201477
MSTRG.2	Poln;Mx	ENSMUST00000201251;ENSMUST00000042954;ENSMUST00000202
MSTRG.2	Gm19798	ENSMUST00000201502
MSTRG.2	Gm43459	ENSMUST00000202093
MSTRG.2	4930557	ENSMUST00000202325

MSTRG.2	Poln	MSTRG.23466.1
MSTRG.2	Poln	MSTRG.23467.1
MSTRG.2	Gm25918	ENSMUST00000104266
MSTRG.2	Gm42846	ENSMUST00000201771
MSTRG.2	Mthfd1l	MSTRG.2347.1
MSTRG.2	Gm43457	ENSMUST00000200867
MSTRG.2	Tnip2	ENSMUST00000030991;ENSMUST00000087737;ENSMUST00000143
MSTRG.2	Rnf4	ENSMUST00000182709;ENSMUST00000182970;MSTRG.23472.2;E
MSTRG.2	Fam193a	MSTRG.23473.1;MSTRG.23473.2;MSTRG.23473.3;MSTRG.23473
MSTRG.23474		MSTRG.23474.1
MSTRG.2	Fam193a	ENSMUST00000180376;MSTRG.23475.2;ENSMUST00000094867;E
MSTRG.2	Fam193a	MSTRG.23476.1;MSTRG.23476.2
MSTRG.2	Fam193a	MSTRG.23477.1
MSTRG.2	Fam193a	MSTRG.23478.1
MSTRG.2	Gm42559	ENSMUST00000201811
MSTRG.2	Gm25515	ENSMUST00000083858
MSTRG.2	Gm26931	ENSMUST00000182273
MSTRG.2	Sh3bp2	ENSMUST00000125817;MSTRG.23481.2;ENSMUST00000067638;E
MSTRG.2	Gm22847	ENSMUST00000157707
MSTRG.2	Add1;Mi	ENSMUST00000114338;ENSMUST00000147574;ENSMUST00000146
MSTRG.2	Gm42867	ENSMUST00000202995
MSTRG.2	Add1	MSTRG.23485.1
MSTRG.2	Mfsd10	ENSMUST00000001109;ENSMUST00000114331;ENSMUST00000130
MSTRG.2	Nop14	MSTRG.23487.1;ENSMUST00000041364;ENSMUST00000201897;M
MSTRG.2	Gm15522	ENSMUST00000134467
MSTRG.2	Grk4	ENSMUST00000074651;ENSMUST00000001112;ENSMUST00000148
MSTRG.2	Armt1	MSTRG.2349.1
MSTRG.2	Gm42560	ENSMUST00000201216
MSTRG.2	Mir467g	ENSMUST00000116906
MSTRG.2	Htt	ENSMUST00000148953;MSTRG.23492.1;ENSMUST00000080036;E
MSTRG.2	Htt	MSTRG.23493.1
MSTRG.2	Msantd1	ENSMUST00000201636;ENSMUST00000050535
MSTRG.2	Lrpap1	ENSMUST00000030986;ENSMUST00000153664;ENSMUST00000147
MSTRG.2	Gm43860	ENSMUST00000201742
MSTRG.2	Plk-ps1	ENSMUST00000119494
MSTRG.2	Trmt44	ENSMUST00000030980;ENSMUST00000145930;ENSMUST00000128
MSTRG.2	Rgs12	ENSMUST00000030984;ENSMUST00000128161;MSTRG.23499.2;E
MSTRG.2	Bag2	ENSMUST00000044691;ENSMUST00000155484;ENSMUST00000187
MSTRG.2	Gm21781	ENSMUST00000178920
MSTRG.2	Mir3097	ENSMUST00000175356
MSTRG.2	Gm43791	ENSMUST00000202914
MSTRG.2	Hgfac	ENSMUST00000030985;ENSMUST00000201038;ENSMUST00000202
MSTRG.2	Htra3	ENSMUST00000087629;ENSMUST00000114233;ENSMUST00000129
MSTRG.2	4931431	ENSMUST00000124643
MSTRG.2	Acox3	ENSMUST00000068947;ENSMUST00000114237;ENSMUST00000156
MSTRG.2	Afap1	ENSMUST00000064571;ENSMUST00000146300;ENSMUST00000141
MSTRG.2	H2af-ps	ENSMUST00000119066
MSTRG.2	Afap1	MSTRG.23508.1
MSTRG.2	Sorcs2	ENSMUST00000037370;ENSMUST00000137040;ENSMUST00000135
MSTRG.2	Rmnd1	MSTRG.2351.1
MSTRG.2	Psapl1	ENSMUST00000052224
MSTRG.2	Mir7689	ENSMUST00000184943
MSTRG.2	2210406	ENSMUST00000149687;ENSMUST00000122882;ENSMUST00000044
MSTRG.2	Grpel1	ENSMUST00000031099
MSTRG.2	Tada2b	ENSMUST00000031097;ENSMUST00000119916
MSTRG.2	Bloc1s4	ENSMUST00000071949
MSTRG.2	Mrfap1	ENSMUST00000068795

MSTRG.2	Man2b2	ENSMUST00000031002;ENSMUST00000147893;ENSMUST00000124
MSTRG.2	Tbc1d14	ENSMUST00000146430;ENSMUST00000136189;ENSMUST00000031
MSTRG.2	Rmnd1	ENSMUST00000042251;MSTRG.2352.2;MSTRG.2352.3;ENSMUST0
MSTRG.2	Tbc1d14	MSTRG.23520.1
MSTRG.2	D5Ertd5	MSTRG.23521.1
MSTRG.2	Gm42507	ENSMUST00000201187
MSTRG.2	D5Ertd5	MSTRG.23523.1
MSTRG.2	Gm24878	ENSMUST00000158782
MSTRG.2	D5Ertd5	MSTRG.23525.1
MSTRG.2	Jakmip1	ENSMUST00000173836;ENSMUST00000172940;ENSMUST00000172
MSTRG.2	Gm26647	ENSMUST00000180816
MSTRG.2	Jakmip1	MSTRG.23529.1
MSTRG.2	Gm16153	ENSMUST00000132184
MSTRG.2	Jakmip1	MSTRG.23530.1
MSTRG.2	Gm25551	ENSMUST00000158082
MSTRG.2	Jakmip1	MSTRG.23532.1
MSTRG.2	Jakmip1	MSTRG.23533.1
MSTRG.2	Jakmip1	MSTRG.23534.1
MSTRG.2	Gm1043	ENSMUST00000207866;ENSMUST00000213782;ENSMUST00000208
MSTRG.2	Stx18	ENSMUST00000114126;ENSMUST00000031008;ENSMUST00000042
MSTRG.2	Stx18	MSTRG.23537.1
MSTRG.2	Stx18	MSTRG.23538.1
MSTRG.2	Stx18	MSTRG.23539.1
MSTRG.2	Armt1	ENSMUST00000095893;ENSMUST00000118544;ENSMUST00000117
MSTRG.2	Stx18	MSTRG.23540.1
MSTRG.2	Gm42858	ENSMUST00000202512
MSTRG.2	Nsg1	ENSMUST00000031009;ENSMUST00000202129;ENSMUST00000201
MSTRG.2	Tmem128	ENSMUST00000087511;ENSMUST00000201661;ENSMUST0000011[illegible]
MSTRG.2	Zbtb49	ENSMUST00000124939;ENSMUST00000094833;ENSMUST0000012[illegible]
MSTRG.2	Zbtb49	MSTRG.23545.1
MSTRG.2	Lyar	ENSMUST00000087514;ENSMUST00000130721;ENSMUST00000123
MSTRG.2	Lyar	MSTRG.23547.1
MSTRG.2	4930487	ENSMUST00000201983
MSTRG.2	Slc2a9	ENSMUST00000067886;ENSMUST00000067872;ENSMUST00000122
MSTRG.2	Esr1	ENSMUST00000149195;ENSMUST00000105590;ENSMUST00000133
MSTRG.2	Slc2a9	MSTRG.23550.1
MSTRG.2	Wdr1	ENSMUST00000005234;ENSMUST00000200909;MSTRG.23551.2;E
MSTRG.2	Gm7816	ENSMUST00000119884
MSTRG.2	Gm2810	ENSMUST00000118681
MSTRG.23555		MSTRG.23555.1
MSTRG.2	Rab28	ENSMUST00000201422;ENSMUST00000031011;ENSMUST00000202
MSTRG.2	Rab28	MSTRG.23557.1
MSTRG.2	Rab28	MSTRG.23558.1
MSTRG.2	Esr1	MSTRG.2356.1
MSTRG.2	Gm42553	ENSMUST00000196390
MSTRG.23562		MSTRG.23562.1
MSTRG.2	Bod1l	ENSMUST00000050556;ENSMUST00000202908;MSTRG.23563.3;M
MSTRG.2	Gm7854	ENSMUST00000187409;ENSMUST00000140650
MSTRG.2	Gm7854	MSTRG.23565.1
MSTRG.2	Cpeb2	ENSMUST00000169035;ENSMUST00000166713;ENSMUST00000198
MSTRG.2	Gm42551	ENSMUST00000197701
MSTRG.2	6030400	ENSMUST00000199734
MSTRG.2	Cpeb2	MSTRG.23569.1
MSTRG.2357		MSTRG.2357.1
MSTRG.23570		MSTRG.23570.1
MSTRG.2	Cd38	ENSMUST00000030964;ENSMUST00000196600
MSTRG.2	Gm16014	ENSMUST00000123363

MSTRG.2	Gm16015	ENSMUST00000154897
MSTRG.23574		MSTRG.23574.1
MSTRG.2	Gm7879	ENSMUST00000122204;MSTRG.23575.2;ENSMUST00000200338
MSTRG.2	Gm7882	ENSMUST00000177915
MSTRG.2	Gm5292	ENSMUST00000073317
MSTRG.2	Gm43183	ENSMUST00000200632
MSTRG.2	Gm43184	ENSMUST00000197247
MSTRG.2	Fbxo5	ENSMUST00000019907;MSTRG.2358.1;MSTRG.2358.2;ENSMUST0
MSTRG.2	Cc2d2a	ENSMUST00000048150;ENSMUST00000142303;ENSMUST0000012
MSTRG.2	Fbxl5	ENSMUST00000119523;ENSMUST00000141902;ENSMUST0000019
MSTRG.2	Gm42462	ENSMUST00000196543
MSTRG.2	Fbxl5	MSTRG.23583.1
MSTRG.2	Gm43182	ENSMUST00000199476
MSTRG.2	Gm43181	ENSMUST00000197001
MSTRG.2	Gm42555	ENSMUST00000199304
MSTRG.2	Bst1	ENSMUST00000126976;ENSMUST00000101237;ENSMUST0000011
MSTRG.2	Gm42429	ENSMUST00000198579
MSTRG.2	Gm42427	ENSMUST00000200473
MSTRG.2	Mtrf1l	ENSMUST00000019908;ENSMUST00000145718;ENSMUST0000014
MSTRG.2	Tapt1;E	ENSMUST00000055128;MSTRG.23590.2;ENSMUST00000199374;E
MSTRG.2	Gm42982	MSTRG.23591.1;ENSMUST00000199879
MSTRG.2	Gm42984	ENSMUST00000195990
MSTRG.2	Gm42984	MSTRG.23593.1
MSTRG.2	Qdpr	ENSMUST00000198258;ENSMUST00000197946;ENSMUST0000011
MSTRG.2	Gm42413	ENSMUST00000196216
MSTRG.2	Lap3	ENSMUST00000046122;ENSMUST00000137699;ENSMUST0000013
MSTRG.23597		MSTRG.23597.1
MSTRG.2	Med28	ENSMUST00000156481;ENSMUST00000119579;ENSMUST0000011
MSTRG.2	Fam184b	ENSMUST00000198680;ENSMUST00000016023
MSTRG.2	Gm37905	ENSMUST00000195310
MSTRG.2	Mtrf1l	MSTRG.2360.1
MSTRG.2	Gm42521	ENSMUST00000199190
MSTRG.2	Gm42520	ENSMUST00000199642
MSTRG.2	9630001	ENSMUST00000181335
MSTRG.2	Gm7931	ENSMUST00000121537
MSTRG.23604		MSTRG.23604.1
MSTRG.2	1600023	ENSMUST00000196242
MSTRG.2	Ncapg	MSTRG.23606.1;ENSMUST00000117396;MSTRG.23606.3;ENSMUS
MSTRG.2	Lcorl	ENSMUST00000045586;ENSMUST00000190036;ENSMUST0000012
MSTRG.2	Gm36401	ENSMUST00000194255
MSTRG.2	Lcorl	MSTRG.23609.1
MSTRG.2	Syne1	ENSMUST00000215295;MSTRG.2361.2;ENSMUST00000095899;MS
MSTRG.2	Lcorl	MSTRG.23610.1
MSTRG.2	Gm43201	ENSMUST00000195925
MSTRG.2	Lcorl	MSTRG.23612.1
MSTRG.2	Gm29036	ENSMUST00000189967
MSTRG.2	Gm43200	ENSMUST00000200385
MSTRG.2	Lcorl	MSTRG.23615.1
MSTRG.2	Lcorl	MSTRG.23616.1
MSTRG.2	Lcorl	MSTRG.23617.1
MSTRG.2	Lcorl	MSTRG.23618.1;MSTRG.23618.2
MSTRG.2	Lcorl	MSTRG.23619.1
MSTRG.2	Gm25694	ENSMUST00000175448
MSTRG.2	Lcorl	MSTRG.23620.1
MSTRG.2	4930449	ENSMUST00000198191
MSTRG.2	Adgra3	ENSMUST00000030971;ENSMUST00000196177;ENSMUST0000019
MSTRG.2	Gm42460	ENSMUST00000198863

MSTRG.2	Slit2	ENSMUST00000173107;ENSMUST00000174061;ENSMUST00000172
MSTRG.2	4930518	ENSMUST00000172700
MSTRG.2	Gm20475	ENSMUST00000174884
MSTRG.2	Gm43426	ENSMUST00000197287
MSTRG.2	Gm42536	ENSMUST00000197562
MSTRG.2	Gm42537	ENSMUST00000198260
MSTRG.2	Gm10097	ENSMUST00000180100
MSTRG.2	Gm42534	ENSMUST00000196063
MSTRG.2	5730480	ENSMUST00000196604;ENSMUST00000030968;ENSMUST0000019€
MSTRG.2	5730480	MSTRG.23634.1
MSTRG.2	5730480	MSTRG.23635.1
MSTRG.2	5730480	MSTRG.23636.1
MSTRG.2	Kcnip4	ENSMUST00000176978;ENSMUST00000087395;ENSMUST00000175
MSTRG.2	5730480	MSTRG.23638.1
MSTRG.2	Gm20700	ENSMUST00000176749
MSTRG.2	Gm43648	ENSMUST00000197775
MSTRG.2	Gm19031	ENSMUST00000176280
MSTRG.2	Gm43829	ENSMUST00000196191
MSTRG.2	Gm43830	ENSMUST00000198760
MSTRG.2	Gm43319	ENSMUST00000198036
MSTRG.2	Gm42919	ENSMUST00000197753
MSTRG.2	Gm43321	ENSMUST00000198897
MSTRG.2	Gm43320	ENSMUST00000196047
MSTRG.2	Gm10025	ENSMUST00000079568
MSTRG.2	Gm23573	ENSMUST00000104408
MSTRG.2	Gm10048	ENSMUST00000200465
MSTRG.2	Gm43318	ENSMUST00000199939
MSTRG.2	Gm43341	ENSMUST00000200104
MSTRG.2	C030015	ENSMUST00000198148
MSTRG.2	Gm43317	ENSMUST00000197924
MSTRG.2	Gm43316	ENSMUST00000197252
MSTRG.2	Gm43046	ENSMUST00000198771
MSTRG.2	Gm43047	ENSMUST00000200089
MSTRG.2	Gm42767	ENSMUST00000198583
MSTRG.2	Cnksr3	ENSMUST00000150282;ENSMUST00000015346;MSTRG.2366.3
MSTRG.2	Gm42768	ENSMUST00000197734
MSTRG.2	Gm42769	ENSMUST00000198576
MSTRG.2	Gm42770	ENSMUST00000199657
MSTRG.2	Gm43048	ENSMUST00000196101
MSTRG.2	Gm42771	ENSMUST00000198342
MSTRG.2	Gm42772	ENSMUST00000199945
MSTRG.2	Gm19932	ENSMUST00000177033
MSTRG.2	Gm43670	ENSMUST00000199047
MSTRG.2	2900064	ENSMUST00000196567
MSTRG.2	Sod3	ENSMUST00000101208
MSTRG.2	Cnksr3	MSTRG.2367.1
MSTRG.2	Gm43176	ENSMUST00000199653;MSTRG.23670.1;ENSMUST00000031061;E
MSTRG.2	Gm43178	ENSMUST00000197257
MSTRG.2	9230114	ENSMUST00000199547;ENSMUST00000180601;ENSMUST0000019
MSTRG.2	C130083	ENSMUST00000197171
MSTRG.2	Gm43180	ENSMUST00000197541
MSTRG.2	Sepsecs	ENSMUST00000031069;ENSMUST00000126574;MSTRG.23675.3;E
MSTRG.2	Pi4k2b	ENSMUST00000031081;ENSMUST00000131526;ENSMUST00000031
MSTRG.2	Zcchc4	ENSMUST00000113901;ENSMUST00000031077;ENSMUST00000113
MSTRG.2	Anapc4	MSTRG.23678.1;ENSMUST00000031072;MSTRG.23678.3;ENSMUS
MSTRG.2	Anapc4	MSTRG.23679.1
MSTRG.2	Cnksr3	MSTRG.2368.1

MSTRG.2	Smim20	ENSMUST00000203623;ENSMUST00000147148;ENSMUST00000204
MSTRG.2	Smim20	MSTRG.23681.1
MSTRG.2	Gm45495	ENSMUST00000200940
MSTRG.2	Rbpj	ENSMUST00000201991;MSTRG.23683.1;ENSMUST00000201721;E
MSTRG.2	Gm10441	ENSMUST00000200950
MSTRG.2	Gm40304	ENSMUST00000202154
MSTRG.2	Rbpj	MSTRG.23686.1
MSTRG.2	Rbpj	MSTRG.23687.1
MSTRG.2	Tbc1d19	ENSMUST00000202528;ENSMUST00000037337;ENSMUST00000201
MSTRG.2	Tbc1d19	MSTRG.23689.1
MSTRG.2	AC14751	ENSMUST00000220179
MSTRG.2	Stim2	ENSMUST00000117661;ENSMUST00000201469;MSTRG.23690.3;E
MSTRG.2	Stim2	MSTRG.23691.1
MSTRG.2	Stim2	MSTRG.23692.1
MSTRG.2	Gm15820	MSTRG.23693.1
MSTRG.2	Gm15975	ENSMUST00000122385
MSTRG.23695		MSTRG.23695.1
MSTRG.2	Gm43042	ENSMUST00000198676
MSTRG.2	Cbfa2t2	ENSMUST00000117165
MSTRG.2	4932441	ENSMUST00000180708;ENSMUST00000193156
MSTRG.2	Gm8121	ENSMUST00000153583
MSTRG.2	Zfp451	MSTRG.237.1;ENSMUST00000139143;ENSMUST00000019861;ENS
MSTRG.2	AC14751	MSTRG.2370.1
MSTRG.2	4932441	MSTRG.23701.1
MSTRG.2	Pcdh7	ENSMUST00000191837;ENSMUST00000068110;ENSMUST00000094
MSTRG.2	Gm42635	ENSMUST00000200266
MSTRG.2	Gm42481	ENSMUST00000196822
MSTRG.2	Gm42480	ENSMUST00000198434
MSTRG.2	Gm42482	ENSMUST00000198904
MSTRG.2	Gm17977	ENSMUST00000198743
MSTRG.2	Gm43715	ENSMUST00000196307
MSTRG.2371		MSTRG.2371.1
MSTRG.2	Gm42479	ENSMUST00000198654
MSTRG.2	Gm42478	ENSMUST00000199252
MSTRG.2	Gm42639	ENSMUST00000197061
MSTRG.2	Gm42640	ENSMUST00000199792
MSTRG.2	Gm42484	ENSMUST00000196075
MSTRG.2	Gm37720	ENSMUST00000195497
MSTRG.2	Gm42483	ENSMUST00000197556
MSTRG.2	Gm42486	ENSMUST00000200369
MSTRG.23718		MSTRG.23718.1
MSTRG.23719		MSTRG.23719.1
MSTRG.2	Ulbp1	ENSMUST00000218087;ENSMUST00000177585;ENSMUST0000016S
MSTRG.23720		MSTRG.23720.1
MSTRG.23721		MSTRG.23721.1
MSTRG.2	Arap2;Gı	ENSMUST00000160620;MSTRG.23722.1;MSTRG.23722.2;ENSMUS
MSTRG.2	Gm43237	ENSMUST00000196835
MSTRG.2	Arap2	MSTRG.23725.1
MSTRG.2	Rell1	ENSMUST00000154169;ENSMUST00000087327;ENSMUST00000154
MSTRG.2	Rell1	MSTRG.23727.1
MSTRG.2	Gm15819	ENSMUST00000153089
MSTRG.23729		MSTRG.23729.1
MSTRG.2	AC14751	ENSMUST00000217912
MSTRG.2	Gm42566	ENSMUST00000197120
MSTRG.2	Pgm1	ENSMUST00000087324;ENSMUST00000199093;ENSMUST0000012S
MSTRG.2	Gm23967	ENSMUST00000175062
MSTRG.2	Gm22395	ENSMUST00000177427

MSTRG.2	Tbc1d1	ENSMUST00000199270;ENSMUST00000043893;ENSMUST00000121
MSTRG.2	Tbc1d1	MSTRG.23736.1
MSTRG.2	Gm15824	ENSMUST00000119516
MSTRG.2	Tbc1d1	MSTRG.23738.1
MSTRG.2	Tbc1d1	MSTRG.23739.1
MSTRG.2	Ulbp1	MSTRG.2374.1
MSTRG.2	Gm43721	ENSMUST00000199677
MSTRG.2	Tbc1d1	MSTRG.23741.1
MSTRG.2	Gm15823	ENSMUST00000064305
MSTRG.2	Tbc1d1	MSTRG.23743.1
MSTRG.2	Tbc1d1	MSTRG.23744.1
MSTRG.2	Tbc1d1	MSTRG.23745.1
MSTRG.2	Gm6044	ENSMUST00000121819
MSTRG.2	Tbc1d1	MSTRG.23747.1
MSTRG.2	Tbc1d1	MSTRG.23748.1
MSTRG.2	Tbc1d1	MSTRG.23749.1
MSTRG.2375		MSTRG.2375.1
MSTRG.2	Tbc1d1	MSTRG.23750.1
MSTRG.2	Gm3716	ENSMUST00000198582;ENSMUST00000196283
MSTRG.2	Gm3716	MSTRG.23752.1
MSTRG.2	Gm20033	ENSMUST00000180912;ENSMUST00000192228;ENSMUST00000181
MSTRG.2	Klf3	ENSMUST00000165536;ENSMUST00000200587;MSTRG.23754.2;E
MSTRG.2	C230096	ENSMUST00000198074
MSTRG.2	Tlr1	ENSMUST00000197315;ENSMUST00000059349;ENSMUST00000197
MSTRG.2	Fam114a	ENSMUST00000031080;ENSMUST00000139366
MSTRG.2	Mir574	ENSMUST00000103854
MSTRG.2	Gm43627	ENSMUST00000197119
MSTRG.2	Fam114a	MSTRG.23760.1
MSTRG.2	Tmem156	ENSMUST00000203664;ENSMUST00000043352;MSTRG.23761.3;E
MSTRG.2	Tmem156	MSTRG.23762.1
MSTRG.2	Klhl5	ENSMUST00000204097;ENSMUST00000101191;ENSMUST00000204
MSTRG.2	Gm25154	ENSMUST00000157123
MSTRG.2	Wdr19	ENSMUST00000203653;ENSMUST00000041892;ENSMUST00000204
MSTRG.2	Wdr19	MSTRG.23766.1
MSTRG.2	Rfc1	ENSMUST00000203581;ENSMUST00000172732;ENSMUST00000204
MSTRG.2	Rfc1	MSTRG.23768.1
MSTRG.2	Rfc1	MSTRG.23769.1
MSTRG.2	Oprm1;I	ENSMUST00000105607;ENSMUST00000000783;ENSMUST00000154
MSTRG.2	Gm43628	ENSMUST00000197871
MSTRG.2	Rfc1	MSTRG.23771.1
MSTRG.2	Rhoh;Gm	ENSMUST00000031106;ENSMUST00000201533;MSTRG.23772.2;E
MSTRG.23774		MSTRG.23774.1
MSTRG.2	N4bp2os	ENSMUST00000202673;ENSMUST00000202069
MSTRG.2	N4bp2;G	ENSMUST00000201489;ENSMUST00000201503;ENSMUST00000202
MSTRG.2	N4bp2	MSTRG.23777.1
MSTRG.23778		MSTRG.23778.1
MSTRG.23779		MSTRG.23779.1
MSTRG.2	Ipcef1	MSTRG.2378.2;ENSMUST00000105617;MSTRG.2378.1;MSTRG.23
MSTRG.2	Gm42645	ENSMUST00000201573
MSTRG.2	Gm42645	MSTRG.23781.1
MSTRG.2	Gm42646	ENSMUST00000202233
MSTRG.2	Gm43004	ENSMUST00000201872
MSTRG.2	Gm43004	MSTRG.23784.1
MSTRG.2	Rpl9	ENSMUST00000151917;ENSMUST00000150815;ENSMUST00000118
MSTRG.2	Lias;11	ENSMUST00000122026;ENSMUST00000031101;ENSMUST00000139
MSTRG.2	Gm43311	ENSMUST00000200016
MSTRG.2	Ugdh;Gm	ENSMUST00000031103;ENSMUST00000125375;MSTRG.23788.2;E

MSTRG.2	Gm43289	ENSMUST00000200537
MSTRG.2	Ipcef1	MSTRG.2379.1
MSTRG.2	Smim14	MSTRG.23790.1
MSTRG.2	Gm43290	ENSMUST00000200359
MSTRG.2	4930589	ENSMUST00000200768
MSTRG.2	Gm42725	ENSMUST00000199296
MSTRG.2	Ube2k	ENSMUST00000142407;ENSMUST00000201266;ENSMUST00000201
MSTRG.2	Gm40309	MSTRG.23795.1;ENSMUST00000202699
MSTRG.2	Ube2k	MSTRG.23796.2;MSTRG.23796.1
MSTRG.2	Ube2k	MSTRG.23797.1
MSTRG.2	Ube2k	MSTRG.23798.1
MSTRG.2	Ube2k	MSTRG.23799.1
MSTRG.238		MSTRG.238.1
MSTRG.2	Ipcef1	MSTRG.2380.1
MSTRG.2	Gm42726	ENSMUST00000202274;MSTRG.23800.2
MSTRG.2	Pds5a;G	ENSMUST00000201948;ENSMUST00000201673;MSTRG.23801.2;E
MSTRG.2	Gm42727	ENSMUST00000202446
MSTRG.2	Gm42729	ENSMUST00000201770
MSTRG.2	Gm24399	ENSMUST00000157869
MSTRG.2	C030017	ENSMUST00000202959
MSTRG.2	Chrna9;	ENSMUST00000202957;ENSMUST00000201814;MSTRG.23806.2;E
MSTRG.2	Gm43771	ENSMUST00000202931
MSTRG.2	9130230	ENSMUST00000201562;ENSMUST00000201351;ENSMUST00000202
MSTRG.2	Gm25952	ENSMUST00000175329
MSTRG.2	Gm15536	ENSMUST00000121411
MSTRG.2	9130230	MSTRG.23810.1
MSTRG.2	Rbm47;G	ENSMUST00000113726;ENSMUST00000201544;ENSMUST00000094
MSTRG.2	Rbm47	MSTRG.23812.1
MSTRG.2	Gm43775	ENSMUST00000201019
MSTRG.2	Rbm47	MSTRG.23814.1
MSTRG.2	Rbm47	MSTRG.23815.1
MSTRG.2	Gm26725	ENSMUST00000180652
MSTRG.2	Gm22929	ENSMUST00000175517
MSTRG.2	1700126	ENSMUST00000201903
MSTRG.2	Gm43772	ENSMUST00000202052
MSTRG.2	Pcmt1	ENSMUST00000161123;ENSMUST00000162606;ENSMUST0000015
MSTRG.2	Nsun7	ENSMUST00000202994;ENSMUST00000201100;ENSMUST0000020
MSTRG.2	Apbb2	ENSMUST00000162955;ENSMUST00000162366;ENSMUST0000016
MSTRG.2	Gm43790	ENSMUST00000202694
MSTRG.2	Gm23183	ENSMUST00000122595
MSTRG.2	Gm43793	ENSMUST00000201645
MSTRG.2	Gm16273	ENSMUST00000202238
MSTRG.2	Gm43792	ENSMUST00000202492
MSTRG.2	Gm43794	ENSMUST00000200971
MSTRG.2	Apbb2	MSTRG.23828.1
MSTRG.2	Gm43343	ENSMUST00000202454
MSTRG.2	Pcmt1	MSTRG.2383.1
MSTRG.2	Gm43795	ENSMUST00000202051
MSTRG.2	Apbb2	MSTRG.23831.1
MSTRG.2	C530043	ENSMUST00000201865
MSTRG.2	Apbb2	MSTRG.23833.1
MSTRG.2	Gm42670	ENSMUST00000201816
MSTRG.2	Tmem33	ENSMUST00000037918;ENSMUST00000162543;ENSMUST00000161
MSTRG.2	Tmem33	MSTRG.23836.1
MSTRG.2	Limch1	ENSMUST00000201852;ENSMUST00000101164;ENSMUST00000202
MSTRG.2	Limch1	MSTRG.23838.1
MSTRG.2	Gm6517	ENSMUST00000121056

MSTRG.2	Nup43	ENSMUST00000040135;ENSMUST00000162346;ENSMUST00000162
MSTRG.2	Gm43281	ENSMUST00000202522
MSTRG.2	Gm43282	ENSMUST00000201848
MSTRG.2	Gm15948	MSTRG.23842.1;ENSMUST00000150616
MSTRG.2	Limch1	MSTRG.23843.1
MSTRG.2	Gm23841	ENSMUST00000116951
MSTRG.23846		MSTRG.23846.1
MSTRG.2	Slc30a9	ENSMUST00000113676;ENSMUST00000162372;ENSMUST00000201
MSTRG.2	Gm43698	ENSMUST00000202390
MSTRG.2	Gm21905	ENSMUST00000177616;MSTRG.23849.1;MSTRG.23849.3
MSTRG.2	Lats1	ENSMUST00000165952;MSTRG.2385.1;MSTRG.2385.2;ENSMUST0
MSTRG.2	4930425	ENSMUST00000201190
MSTRG.2	Gm5108	ENSMUST00000202730
MSTRG.23852		MSTRG.23852.1
MSTRG.2	1700025	ENSMUST00000200753
MSTRG.2	Shisa3	ENSMUST00000087241;ENSMUST00000142843
MSTRG.2	Atp8a1;	ENSMUST00000135930;ENSMUST00000037380;MSTRG.23855.3;E
MSTRG.2	Gm23617	ENSMUST00000175410
MSTRG.2	Gm42735	ENSMUST00000201718
MSTRG.2	Atp8a1	MSTRG.23858.1
MSTRG.2	Gm15478	MSTRG.23859.1;MSTRG.23859.2;ENSMUST00000145914
MSTRG.2	Katna1	ENSMUST00000019929;ENSMUST00000165806;ENSMUST00000173
MSTRG.2	Atp8a1	MSTRG.23860.1
MSTRG.2	Gm42737	ENSMUST00000201074
MSTRG.2	Atp8a1	MSTRG.23862.1
MSTRG.2	D630030	ENSMUST00000202983
MSTRG.2	Gm15477	ENSMUST00000143945;ENSMUST00000140578
MSTRG.2	Gm42736	ENSMUST00000200723
MSTRG.23866		MSTRG.23866.1
MSTRG.23867		MSTRG.23867.1
MSTRG.23868		MSTRG.23868.1
MSTRG.2	3110031	ENSMUST00000201115
MSTRG.2	Gm20470	ENSMUST00000174654
MSTRG.2	Guf1	ENSMUST00000154728;ENSMUST00000125543;ENSMUST00000132
MSTRG.2	Gnpda2	MSTRG.23871.2;ENSMUST00000031117;ENSMUST00000166298;E
MSTRG.2	Gabra4	ENSMUST00000031121;ENSMUST00000199150
MSTRG.2	Gabrb1	ENSMUST00000031122
MSTRG.2	Gm15616	ENSMUST00000118662
MSTRG.2	Commd8	ENSMUST00000013693;MSTRG.23877.3;ENSMUST00000199767;M
MSTRG.2	Atp10d	ENSMUST00000169617;ENSMUST00000126664;ENSMUST00000156
MSTRG.2	Atp10d	MSTRG.23879.1
MSTRG.2	Zc3h12d	ENSMUST00000039484
MSTRG.2	Atp10d	MSTRG.23880.1
MSTRG.2	Atp10d	MSTRG.23881.1
MSTRG.2	Nfxl1	ENSMUST00000087216;ENSMUST00000135318;ENSMUST00000149
MSTRG.2	Nfxl1	MSTRG.23883.1
MSTRG.2	Gm43815	ENSMUST00000197676
MSTRG.2	Cnga1	ENSMUST00000201463;ENSMUST00000087213;ENSMUST00000126
MSTRG.2	Gm5297	ENSMUST00000198555
MSTRG.2	4933408	ENSMUST00000199037
MSTRG.2	Nipal1	ENSMUST00000087212;ENSMUST00000197837;ENSMUST00000197
MSTRG.2	Txk	MSTRG.23889.1
MSTRG.2	Mir5104	ENSMUST00000175319
MSTRG.2	Gm9870	ENSMUST00000201161;ENSMUST00000063874
MSTRG.2	Slain2	ENSMUST00000144843;ENSMUST00000143829;ENSMUST00000183
MSTRG.2	Slain2	MSTRG.23892.1
MSTRG.2	Gm10135	ENSMUST00000043711

MSTRG.2	Gm24776	ENSMUST00000157643
MSTRG.2	Txk	ENSMUST00000169534;MSTRG.23895.2;MSTRG.23895.1;ENSMUS
MSTRG.2	Gm43717	ENSMUST00000198684
MSTRG.2	Tec	ENSMUST00000071944;ENSMUST00000113594;ENSMUST0000012€
MSTRG.2	Tec	MSTRG.23898.1
MSTRG.2	Tec	MSTRG.23899.1
MSTRG.2	Gm37233	MSTRG.239.1
MSTRG.2	Gm24726	ENSMUST00000158232
MSTRG.2	Gm43499	ENSMUST00000202091
MSTRG.2	Tec	MSTRG.23901.1
MSTRG.2	Tec	MSTRG.23902.1
MSTRG.2	Cwh43	ENSMUST00000031040
MSTRG.2	Cwh43	MSTRG.23904.1
MSTRG.2	Ociad1	ENSMUST00000071081;ENSMUST00000031038;ENSMUST0000016€
MSTRG.2	Gm42732	ENSMUST00000202887
MSTRG.2	Gm42732	MSTRG.23908.1
MSTRG.23909		MSTRG.23909.1
MSTRG.2391		MSTRG.2391.1
MSTRG.2	Ociad2	MSTRG.23910.1;ENSMUST00000200830;ENSMUST00000087195;E
MSTRG.2	Lrrc66	ENSMUST00000087177;ENSMUST00000152215
MSTRG.2	Sgcb	ENSMUST00000081170;ENSMUST00000201416
MSTRG.2	Gm43799	ENSMUST00000201845
MSTRG.2	Dcun1d4	ENSMUST00000087181;ENSMUST00000130087;ENSMUST0000011З
MSTRG.2	Dcun1d4	MSTRG.23916.1
MSTRG.2	Dcun1d4	MSTRG.23917.1
MSTRG.2	Dcun1d4	MSTRG.23918.1
MSTRG.2	Dcun1d4	MSTRG.23919.1
MSTRG.2	Ginm1	ENSMUST00000039763;ENSMUST00000133796;ENSMUST00000124
MSTRG.2	Usp46	ENSMUST00000119154;ENSMUST00000068058;ENSMUST0000012{
MSTRG.2	Usp46	MSTRG.23921.1
MSTRG.2	Usp46os	ENSMUST00000129275
MSTRG.2	Usp46os	ENSMUST00000202793;ENSMUST00000180935;ENSMUST00000152
MSTRG.2	Gm43417	ENSMUST00000201586
MSTRG.2	Gm43415	ENSMUST00000201934
MSTRG.2	Gm43416	ENSMUST00000201916
MSTRG.2	Dancr;S	ENSMUST00000066220;ENSMUST00000132389;ENSMUST0000012C
MSTRG.2	Rasl11b	ENSMUST00000051937;ENSMUST00000183995;ENSMUST00000201
MSTRG.2	Fryl	ENSMUST00000202697;ENSMUST00000101127;ENSMUST00000202
MSTRG.2	B430219	ENSMUST00000180853;ENSMUST00000147230
MSTRG.2	Gm42572	ENSMUST00000202292
MSTRG.2	Fryl	MSTRG.23931.1
MSTRG.2	1700071	MSTRG.23932.1;ENSMUST00000146728
MSTRG.2	Gm42571	ENSMUST00000201197
MSTRG.2	Gm17207	MSTRG.23934.1;MSTRG.23934.2;ENSMUST00000168686
MSTRG.2	Fryl	MSTRG.23935.1
MSTRG.2	Scfd2	ENSMUST00000072857;ENSMUST00000202465;ENSMUST0000014€
MSTRG.2	Gm42599	ENSMUST00000201308
MSTRG.2	Scfd2	MSTRG.23938.1
MSTRG.2	Gm15981	ENSMUST00000119196
MSTRG.2	Ppil4	ENSMUST00000015901;ENSMUST00000138151;MSTRG.2394.2;EN
MSTRG.2	Scfd2	MSTRG.23940.1
MSTRG.2	Scfd2	MSTRG.23941.1
MSTRG.2	Gm15982	ENSMUST00000121681
MSTRG.2	6720475	ENSMUST00000201755
MSTRG.2	Scfd2	MSTRG.23944.1
MSTRG.2	Scfd2	MSTRG.23945.1
MSTRG.2	Scfd2	MSTRG.23946.1

MSTRG.2	Gm43167	ENSMUST00000202001
MSTRG.2	Scfd2	MSTRG.23948.1
MSTRG.2	Scfd2	MSTRG.23949.1
MSTRG.2	Gm15560	ENSMUST00000117107
MSTRG.2	Gm42577	ENSMUST00000201367
MSTRG.2	Chic2	ENSMUST00000075452;ENSMUST00000162397
MSTRG.2	Gm42576	ENSMUST00000202336
MSTRG.2	Chic2	MSTRG.23953.1
MSTRG.2	Chic2	MSTRG.23954.1
MSTRG.2	Fip1l1;	ENSMUST00000080164;ENSMUST00000113536;ENSMUST00000113
MSTRG.2	Lnx1	ENSMUST00000113531;ENSMUST00000039744;ENSMUST00000087
MSTRG.2	Gm15984	ENSMUST00000156466;ENSMUST00000155864
MSTRG.2	Pdgfra;	ENSMUST00000202681;ENSMUST00000000476;ENSMUST00000202
MSTRG.2	Tab2	ENSMUST00000146444;ENSMUST00000143848;MSTRG.2396.2;EN
MSTRG.2	Gm42803	ENSMUST00000201051
MSTRG.2	Kit	ENSMUST00000144270;ENSMUST00000005815;ENSMUST00000143
MSTRG.2	Srd5a3	ENSMUST00000138699;ENSMUST00000031143;ENSMUST00000149
MSTRG.2	Tmem165	ENSMUST00000031144;ENSMUST00000136424;ENSMUST00000119
MSTRG.2	Clock	ENSMUST00000202651;ENSMUST00000075159;ENSMUST00000202
MSTRG.2	Ube2n-p	ENSMUST00000201768
MSTRG.2	Gm43658	ENSMUST00000202348
MSTRG.2	Gm7467	ENSMUST00000185173
MSTRG.2	Exoc1	ENSMUST00000087133;ENSMUST00000113493;ENSMUST00000150
MSTRG.2	Cep135	ENSMUST00000130651;ENSMUST00000121979;ENSMUST00000049
MSTRG.2	Ust	ENSMUST00000219116;ENSMUST00000061601
MSTRG.2	Cep135	MSTRG.23970.1
MSTRG.2	Cep135	MSTRG.23971.1
MSTRG.2	Cep135	MSTRG.23972.1
MSTRG.2	A730089	ENSMUST00000194526
MSTRG.2	C530008	ENSMUST00000086909;ENSMUST00000128112;ENSMUST00000187
MSTRG.2	Gm7494	ENSMUST00000121248
MSTRG.2	Aasdh	ENSMUST00000123682;ENSMUST00000145022;ENSMUST00000120
MSTRG.2	Ppat	ENSMUST00000155272;ENSMUST00000140076;ENSMUST00000031
MSTRG.2	2310040	ENSMUST00000101090;ENSMUST00000145897
MSTRG.2	Paics	ENSMUST00000031160;ENSMUST00000120912;ENSMUST00000117
MSTRG.2	Ust	MSTRG.2398.1
MSTRG.23980		MSTRG.23980.1
MSTRG.2	Srp72	ENSMUST00000101087;ENSMUST00000120550;ENSMUST00000150
MSTRG.2	Arl9	ENSMUST00000071199;ENSMUST00000134197
MSTRG.2	1700112	ENSMUST00000129353
MSTRG.2	Thegl	ENSMUST00000031161
MSTRG.23985		MSTRG.23985.1
MSTRG.2	Rest;Mi	ENSMUST00000080359;ENSMUST00000113449;MSTRG.23986.3;M
MSTRG.2	Noa1	MSTRG.23987.1;ENSMUST00000047860;ENSMUST00000150451;E
MSTRG.2	Polr2b	ENSMUST00000031167;ENSMUST00000138121;ENSMUST00000132
MSTRG.2	Gm42758	ENSMUST00000202146
MSTRG.2	Ust	MSTRG.2399.1
MSTRG.2	Igfbp7	ENSMUST00000046746;ENSMUST00000163898
MSTRG.2	Gm34648	ENSMUST00000202460
MSTRG.2	Rpl7-ps	ENSMUST00000200912
MSTRG.23993		MSTRG.23993.1
MSTRG.23994		MSTRG.23994.1
MSTRG.23995		MSTRG.23995.1
MSTRG.23996		MSTRG.23996.1
MSTRG.2	Epha5	ENSMUST00000053733;ENSMUST00000113399;ENSMUST00000113
MSTRG.2	Hmgn2-p	ENSMUST00000120716
MSTRG.2	Epha5	MSTRG.23999.1

MSTRG.2	Gm19026	ENSMUST00000194240
MSTRG.2	Ccdc115	ENSMUST00000042493;MSTRG.240.1;ENSMUST00000188583;ENS
MSTRG.2400		MSTRG.2400.1;MSTRG.2400.2
MSTRG.2	Epha5	ENSMUST00000200076
MSTRG.2	Adgrl3	ENSMUST00000147226;ENSMUST00000132375;ENSMUST0000014
MSTRG.2	Gm43594	ENSMUST00000201153
MSTRG.2	Adgrl3	MSTRG.24003.1
MSTRG.2	Gm43084	ENSMUST00000200696
MSTRG.2	Adgrl3	MSTRG.24005.1
MSTRG.2	Cenpc1	MSTRG.24008.1;MSTRG.24008.2;ENSMUST00000031170;ENSMUS
MSTRG.2	Cenpc1	MSTRG.24009.1
MSTRG.2401		MSTRG.2401.1;MSTRG.2401.2
MSTRG.2	Cenpc1	MSTRG.24010.1
MSTRG.2	Gm2287	ENSMUST00000197417
MSTRG.2	Tmprss1	ENSMUST00000122377;ENSMUST00000031175
MSTRG.2	Tmprss1	MSTRG.24013.1
MSTRG.2	Stap1;G	ENSMUST00000198435;ENSMUST00000123393;ENSMUST00000151
MSTRG.2	Uba6	MSTRG.24015.1;ENSMUST00000039373;ENSMUST00000113373;E
MSTRG.2	Uba6	MSTRG.24016.1
MSTRG.2	Uba6	MSTRG.24017.1
MSTRG.2	Gm5869	ENSMUST00000200248
MSTRG.24019		MSTRG.24019.1
MSTRG.2402		MSTRG.2402.1
MSTRG.2	Tmprss1	ENSMUST00000161306
MSTRG.2	Gm16579	ENSMUST00000161985;MSTRG.24021.2;ENSMUST00000119339;E
MSTRG.2	Csn2	ENSMUST00000197422;ENSMUST00000197281;ENSMUST0000020C
MSTRG.24024		MSTRG.24024.1
MSTRG.24025		MSTRG.24025.1
MSTRG.2	Jchain	ENSMUST00000164073;ENSMUST00000087033
MSTRG.2	Mob1b;D	ENSMUST00000155298;ENSMUST00000113229;ENSMUST0000000
MSTRG.2	Gm15682	ENSMUST00000121563
MSTRG.2	Gm43432	ENSMUST00000196352
MSTRG.2	Sash1	ENSMUST00000015449;ENSMUST00000212869;ENSMUST00000212
MSTRG.2	Utp3	ENSMUST00000090413
MSTRG.2	Rufy3	ENSMUST00000196894;ENSMUST00000198965;ENSMUST0000019
MSTRG.2	Rufy3	MSTRG.24032.1
MSTRG.2	Grsf1	ENSMUST00000078945;ENSMUST00000113234;ENSMUST0000013C
MSTRG.2	Slc4a4	ENSMUST00000148750;ENSMUST00000135283;ENSMUST0000011
MSTRG.2	Slc4a4	MSTRG.24035.1
MSTRG.2	Slc4a4	MSTRG.24036.1
MSTRG.2	Cox18	ENSMUST00000118816;ENSMUST00000048363;ENSMUST0000013
MSTRG.2	Gm9958	ENSMUST00000068250
MSTRG.2	Gm42530	MSTRG.24039.1
MSTRG.2	Gm25410	ENSMUST00000083758
MSTRG.24040		MSTRG.24040.1
MSTRG.2	Ankrd17	ENSMUST00000014421;ENSMUST00000196919;ENSMUST0000016
MSTRG.2	Ankrd17	MSTRG.24042.1
MSTRG.2	Ankrd17	MSTRG.24043.1
MSTRG.2	Ankrd17	MSTRG.24044.1
MSTRG.2	Ankrd17	MSTRG.24045.1
MSTRG.2	Parm1	ENSMUST00000040576
MSTRG.2	Parm1	MSTRG.24047.1
MSTRG.2	Gm5558	ENSMUST00000178933
MSTRG.2	Gm43688	ENSMUST00000202249
MSTRG.2	AC15356	ENSMUST00000214016
MSTRG.2	Rchy1	MSTRG.24050.1;ENSMUST00000140670;ENSMUST00000031345;M
MSTRG.24051		MSTRG.24051.1

MSTRG.2	Rchy1	MSTRG.24052.1
MSTRG.2	Gm43039	ENSMUST00000202883
MSTRG.2	Thap6	ENSMUST00000193925;ENSMUST00000192939;ENSMUST00000192
MSTRG.2	Gm24113	ENSMUST00000157477
MSTRG.2	Gm1045	ENSMUST00000201568;ENSMUST00000178614
MSTRG.24057		MSTRG.24057.1
MSTRG.2	Cdkl2	ENSMUST00000069937;ENSMUST00000086978;ENSMUST00000113
MSTRG.2	Gm26582	ENSMUST00000181481
MSTRG.2	Gm26674	ENSMUST00000180770
MSTRG.2	G3bp2	ENSMUST00000167918;ENSMUST00000113127;ENSMUST00000164
MSTRG.2	Gm22728	ENSMUST00000158087
MSTRG.2	G3bp2	MSTRG.24062.1
MSTRG.2	Uso1	ENSMUST00000031355;ENSMUST00000202155;ENSMUST00000201
MSTRG.2	Gm15710	ENSMUST00000121821
MSTRG.2	Uso1	MSTRG.24065.1
MSTRG.2	Gm43040	ENSMUST00000202399
MSTRG.2	Gm25290	ENSMUST00000122796
MSTRG.2	U90926	ENSMUST00000201332;ENSMUST00000031356;ENSMUST00000202
MSTRG.2	Ppef2	ENSMUST00000201130
MSTRG.2	AC15356	ENSMUST00000215873
MSTRG.2	Naaa	ENSMUST00000113102;ENSMUST00000159732;ENSMUST0000015[illegible]
MSTRG.2	Cxcl9	ENSMUST00000113093;ENSMUST00000202404
MSTRG.2	Sdad1	ENSMUST00000031364;ENSMUST00000202903;ENSMUST00000201
MSTRG.2	Gm43599	ENSMUST00000202680
MSTRG.2	Gm23031	ENSMUST00000157998
MSTRG.2	Nup54	MSTRG.24075.1;ENSMUST00000038514;ENSMUST00000123694;E
MSTRG.2	Gm26039	ENSMUST00000158152
MSTRG.24077		MSTRG.24077.1
MSTRG.2	Art3	ENSMUST00000154245;ENSMUST00000125462;ENSMUST0000013[illegible]
MSTRG.2	Cxcl10	ENSMUST00000201176;ENSMUST00000038816;ENSMUST0000011[illegible]
MSTRG.2	AC15356	MSTRG.2408.1
MSTRG.2	Cxcl11	ENSMUST00000122808;ENSMUST00000077820
MSTRG.2	Gm2673	ENSMUST00000120958
MSTRG.2	Scarb2	MSTRG.24082.3;MSTRG.24082.2;ENSMUST00000031377;MSTRG.
MSTRG.2	Mir6415	ENSMUST00000184064
MSTRG.2	Scarb2	MSTRG.24084.1
MSTRG.2	Ccdc158	ENSMUST00000060930;ENSMUST00000150359
MSTRG.2	Ccdc158	MSTRG.24086.1
MSTRG.24087		MSTRG.24087.1
MSTRG.2	Gm43172	ENSMUST00000202139
MSTRG.2	Stxbp5	ENSMUST00000038213;ENSMUST00000141722;ENSMUST00000151
MSTRG.2	Gm43173	ENSMUST00000202246
MSTRG.2	Sept11	ENSMUST00000074733;ENSMUST00000201377;ENSMUST00000201
MSTRG.2	Sept11	MSTRG.24092.1
MSTRG.2	Sept11	MSTRG.24093.1
MSTRG.2	Gm43682	ENSMUST00000202150
MSTRG.2	Ccni	MSTRG.24095.2;ENSMUST00000058550;MSTRG.24095.1;ENSMUS
MSTRG.2	Gm43681	ENSMUST00000201581
MSTRG.2	Gm2986	ENSMUST00000121603
MSTRG.2	2010109	ENSMUST00000031330;ENSMUST00000201993
MSTRG.2	Imp4	ENSMUST00000027303;ENSMUST00000187077;ENSMUST00000137
MSTRG.2	Stxbp5	MSTRG.2410.1
MSTRG.2	Ccng2	ENSMUST00000031331;ENSMUST00000152097;MSTRG.24100.2;E
MSTRG.24101		MSTRG.24101.1
MSTRG.24102		MSTRG.24102.1;MSTRG.24102.2
MSTRG.24103		MSTRG.24103.1
MSTRG.2	Gm5559	ENSMUST00000180755

MSTRG.2	Cxcl13	ENSMUST00000023840
MSTRG.2	Gm7993	ENSMUST00000200970
MSTRG.2	Mrpl1	ENSMUST00000117766;ENSMUST00000036437;ENSMUST00000121
MSTRG.2	Mrpl1	MSTRG.24108.1
MSTRG.2	Mrpl1	MSTRG.24109.1
MSTRG.2	Gm25635	ENSMUST00000082675
MSTRG.2	Cnot6l	ENSMUST00000155901;ENSMUST00000152490;MSTRG.24110.2;E
MSTRG.2	Cnot6l	MSTRG.24111.1
MSTRG.2	Fras1	ENSMUST00000036019;ENSMUST00000199841;ENSMUST0000019€
MSTRG.2	Gm33050	ENSMUST00000198132
MSTRG.2	Gm42605	ENSMUST00000199565
MSTRG.2	Fras1	MSTRG.24115.1
MSTRG.2	Gm42604	ENSMUST00000200077
MSTRG.2	Bmp2k	ENSMUST00000112974;ENSMUST00000035635;MSTRG.24117.3
MSTRG.2	Bmp2k	MSTRG.24118.1
MSTRG.2	Gm43145	ENSMUST00000198963
MSTRG.2	Stxbp5	MSTRG.2412.1
MSTRG.2	Paqr3	ENSMUST00000069453;ENSMUST00000126585;ENSMUST0000019€
MSTRG.2	Antxr2	ENSMUST00000031281;ENSMUST00000199088;MSTRG.24121.3;E
MSTRG.2	Antxr2	MSTRG.24122.1
MSTRG.2	C430019	ENSMUST00000199517
MSTRG.2	Gm42759	ENSMUST00000195915
MSTRG.2	Rasgef1	ENSMUST00000031276;ENSMUST00000168092;ENSMUST0000020€
MSTRG.2	Rasgef1	MSTRG.24126.1
MSTRG.2	Gm35394	ENSMUST00000196434
MSTRG.2	Gm35172	ENSMUST00000196037
MSTRG.2	Stxbp5	MSTRG.2413.1
MSTRG.2	Gm16228	ENSMUST00000162286
MSTRG.2	4930405	ENSMUST00000159244
MSTRG.2	Gm25969	ENSMUST00000157166
MSTRG.2	Gm16226	ENSMUST00000159950;ENSMUST00000162639
MSTRG.2	4930522	ENSMUST00000031274
MSTRG.2	Hnrnpdl	MSTRG.24135.1
MSTRG.2	Gm35911	ENSMUST00000212164;ENSMUST00000198837
MSTRG.2	Gm35911	MSTRG.24138.1
MSTRG.2	Hnrnpd	ENSMUST00000172361;ENSMUST00000019128;ENSMUST00000112
MSTRG.2	AC12239	ENSMUST00000218800
MSTRG.2	Gm17092	MSTRG.24140.1;ENSMUST00000166606
MSTRG.2	4930524	ENSMUST00000046721
MSTRG.2	Enoph1	ENSMUST00000169390;ENSMUST00000031268;ENSMUST0000019€
MSTRG.2	Gm26363	ENSMUST00000158866
MSTRG.2	Hnrnpdl	ENSMUST00000135424;ENSMUST00000128187;ENSMUST0000015€
MSTRG.2	Tmem150	ENSMUST00000063192;MSTRG.24145.2;ENSMUST00000146731;E
MSTRG.2	Tmem150	ENSMUST00000143245;ENSMUST00000154539
MSTRG.2	Sec31a	ENSMUST00000182569;ENSMUST00000094578;ENSMUST00000182
MSTRG.2	Sec31a	MSTRG.24148.1
MSTRG.2	5430416	MSTRG.24149.1;ENSMUST00000181873;ENSMUST00000180779;E
MSTRG.2	Gm28905	ENSMUST00000219328
MSTRG.2	Gm42690	ENSMUST00000197921
MSTRG.2	Plac8	ENSMUST00000031264;ENSMUST00000097437;ENSMUST00000112
MSTRG.2	Lin54	ENSMUST00000149714;ENSMUST00000137750;ENSMUST00000154
MSTRG.2	Lin54	MSTRG.24157.1
MSTRG.2	Lin54	MSTRG.24158.1
MSTRG.2	Lin54	MSTRG.24159.1
MSTRG.2	Gm28905	MSTRG.2416.1
MSTRG.2	Lin54	MSTRG.24160.1
MSTRG.2	Lin54	MSTRG.24161.1

MSTRG.2	Gm23222	ENSMUST00000158117
MSTRG.2	Cops4	ENSMUST00000045993;ENSMUST00000147729;MSTRG.24163.2;E
MSTRG.2	Cops4	MSTRG.24164.1
MSTRG.2	Coq2	MSTRG.24165.2;MSTRG.24165.1;ENSMUST00000031262;ENSMUS
MSTRG.2	Gm15777	MSTRG.24166.1
MSTRG.2	Helq	ENSMUST00000044684;ENSMUST00000139203;ENSMUST00000138
MSTRG.2	Helq	MSTRG.24168.1
MSTRG.2	Mrps18c	ENSMUST00000016977;ENSMUST00000112898;ENSMUST0000014C
MSTRG.2	AC12230	ENSMUST00000219625
MSTRG.2	Abraxas	ENSMUST00000153302;ENSMUST00000200657;ENSMUST00000044
MSTRG.2	Gm43514	ENSMUST00000198852
MSTRG.24172		MSTRG.24172.1
MSTRG.2	Cds1	ENSMUST00000031273;ENSMUST00000132213
MSTRG.2	Gm43273	ENSMUST00000199649
MSTRG.2	Gm43272	ENSMUST00000200599
MSTRG.24176		MSTRG.24176.1
MSTRG.2	Wdfy3	MSTRG.24177.2;ENSMUST00000053177;MSTRG.24177.1;ENSMUS
MSTRG.2	Gm20548	ENSMUST00000173528;ENSMUST00000173965;ENSMUST00000174
MSTRG.2	Gm42934	ENSMUST00000196920
MSTRG.2	Gm28905	MSTRG.2418.1
MSTRG.2	Gm43787	ENSMUST00000197792
MSTRG.2	1700013I	ENSMUST00000197709
MSTRG.2	Arhgap2	ENSMUST00000073302;ENSMUST00000094559;ENSMUST00000126
MSTRG.2	Arhgap2	MSTRG.24184.1
MSTRG.2	Arhgap2	MSTRG.24185.1
MSTRG.2	Arhgap2	MSTRG.24186.1
MSTRG.2	Arhgap2	MSTRG.24187.1
MSTRG.2	Arhgap2	MSTRG.24188.1
MSTRG.2	Arhgap2	MSTRG.24189.1
MSTRG.2	AC12230	ENSMUST00000219020
MSTRG.2	Arhgap2	MSTRG.24190.1
MSTRG.2	Mapk10	ENSMUST00000170792;ENSMUST00000086854;ENSMUST00000112
MSTRG.2	Gm42522	ENSMUST00000196071
MSTRG.2	Mapk10	MSTRG.24193.1
MSTRG.2	Gm43789	ENSMUST00000199275
MSTRG.2	Ptpn13	ENSMUST00000199412;ENSMUST00000196014;ENSMUST00000196
MSTRG.2	Gm42620	ENSMUST00000197715
MSTRG.2	Klhl8	ENSMUST00000112811;ENSMUST00000112815;ENSMUST00000031
MSTRG.2	Gm43547	ENSMUST00000197729
MSTRG.2	Gm24897	ENSMUST00000179033
MSTRG.2	Gm28418	MSTRG.242.1
MSTRG.2	Gm8200	ENSMUST00000122390
MSTRG.2	Hsd17b1	ENSMUST00000031251;ENSMUST00000119025
MSTRG.2	Aff1	ENSMUST00000126335;MSTRG.24202.2;MSTRG.24202.1;MSTRG.
MSTRG.2	Aff1	MSTRG.24203.1;MSTRG.24203.2
MSTRG.2	Aff1	MSTRG.24204.1;MSTRG.24204.2
MSTRG.2	Gm43545	ENSMUST00000196765
MSTRG.2	Nudt9	ENSMUST00000150226;MSTRG.24206.2;ENSMUST00000031250;E
MSTRG.2	Mir5619	ENSMUST00000176065
MSTRG.24208		MSTRG.24208.1
MSTRG.2	Sparcl1	ENSMUST00000031249;ENSMUST00000199947
MSTRG.2	Gm26703	ENSMUST00000181370;ENSMUST00000181506
MSTRG.2	Pkd2	ENSMUST00000086831;ENSMUST00000130931;MSTRG.24211.3;E
MSTRG.2	BC00556	ENSMUST00000096452
MSTRG.24213		MSTRG.24213.1
MSTRG.2	D930016	ENSMUST00000181620;ENSMUST00000200034;ENSMUST00000181
MSTRG.24215		MSTRG.24215.1

MSTRG.24216		MSTRG.24216.1
MSTRG.24217		MSTRG.24217.1
MSTRG.24218		MSTRG.24218.1
MSTRG.24219		MSTRG.24219.1
MSTRG.2	AC13829	ENSMUST00000219242
MSTRG.24220		MSTRG.24220.1
MSTRG.24221		MSTRG.24221.1
MSTRG.24222		MSTRG.24222.1
MSTRG.24223		MSTRG.24223.1
MSTRG.24224		MSTRG.24224.1
MSTRG.24225		MSTRG.24225.1
MSTRG.24226		MSTRG.24226.1;MSTRG.24226.2
MSTRG.24227		MSTRG.24227.1
MSTRG.2	Zfp951	MSTRG.24228.1
MSTRG.2	Zfp951	ENSMUST00000186219;ENSMUST00000089906;MSTRG.24229.3;M
MSTRG.2	Zfp951	MSTRG.24230.1
MSTRG.2	Zfp951	MSTRG.24231.1;MSTRG.24231.2
MSTRG.2	Zfp951	MSTRG.24232.1
MSTRG.2	Zfp951	MSTRG.24233.1
MSTRG.2	Zfp951	MSTRG.24234.1
MSTRG.2	Zfp951	MSTRG.24235.1
MSTRG.2	Zfp951	MSTRG.24236.1
MSTRG.2	Zfp951	MSTRG.24237.1
MSTRG.2	Zfp951	MSTRG.24238.1
MSTRG.2	Zfp951	MSTRG.24239.1
MSTRG.2	4930598	ENSMUST00000208997;ENSMUST00000208693;ENSMUST0000020‸
MSTRG.2	Zfp951	MSTRG.24240.1
MSTRG.2	Zfp951	MSTRG.24241.1
MSTRG.2	Abcg3	MSTRG.24242.1;ENSMUST00000031239;MSTRG.24242.3;ENSMUS
MSTRG.2	Gm24097	ENSMUST00000178720
MSTRG.2	Abcg3	MSTRG.24244.1
MSTRG.2	Gm43856	ENSMUST00000197004
MSTRG.2	Gm32051	ENSMUST00000197905
MSTRG.2	Lrrc8b	ENSMUST00000112707;MSTRG.24247.2
MSTRG.2	Lrrc8b	MSTRG.24248.1
MSTRG.2	Gm42141	ENSMUST00000196534;MSTRG.24249.1
MSTRG.2	Gm44555	ENSMUST00000209008
MSTRG.2	Lrrc8c	ENSMUST00000067924;ENSMUST00000150981;ENSMUST0000015
MSTRG.2	Lrrc8c	MSTRG.24251.1
MSTRG.2	Lrrc8c	MSTRG.24252.1
MSTRG.2	Lrrc8c	MSTRG.24253.1
MSTRG.2	Gbp8;Gb	ENSMUST00000199483;ENSMUST00000198326;MSTRG.24255.6;M
MSTRG.2	Gbp8	MSTRG.24256.1
MSTRG.2	Gbp8	MSTRG.24257.1
MSTRG.2	Gbp8	MSTRG.24258.1
MSTRG.2	Gbp8	MSTRG.24259.1
MSTRG.2	Gbp9	MSTRG.24260.1
MSTRG.2	Gm43302	ENSMUST00000050011;ENSMUST00000065588;ENSMUST0000019
MSTRG.2	Gm43302	MSTRG.24262.1
MSTRG.2	Gm43302	MSTRG.24263.1
MSTRG.2	Gbp11	ENSMUST00000100960;ENSMUST00000171587
MSTRG.2	Gm10359	ENSMUST00000180167
MSTRG.2	Lrrc8d	ENSMUST00000060531;ENSMUST00000120847;ENSMUST0000014
MSTRG.2	Lrrc8d	MSTRG.24267.1
MSTRG.2	Lrrc8d	MSTRG.24268.1
MSTRG.2	Lrrc8d	MSTRG.24269.1
MSTRG.2	Rab32	ENSMUST00000019974;ENSMUST00000220018

MSTRG.2	Lrrc8d	MSTRG.24270.1
MSTRG.2	Lrrc8d	MSTRG.24271.1
MSTRG.2	Rps15a-	ENSMUST00000132586
MSTRG.2	Lrrc8do	ENSMUST00000135958
MSTRG.2	Zfp326	ENSMUST00000150440;ENSMUST00000150109;ENSMUST00000031
MSTRG.2	Gm43817	ENSMUST00000196459
MSTRG.2	Zfp326	MSTRG.24276.1
MSTRG.24277		MSTRG.24277.1
MSTRG.24278		MSTRG.24278.1
MSTRG.2	Cdc7	ENSMUST00000117196;ENSMUST00000031221;ENSMUST0000007(
MSTRG.2	Shprh	ENSMUST00000159810;ENSMUST00000044053;ENSMUST00000161
MSTRG.2	Gm28050	ENSMUST00000131029;MSTRG.24280.2;ENSMUST00000124394;E
MSTRG.2	Gm28050	MSTRG.24281.1
MSTRG.2	Gm29464	ENSMUST00000187692
MSTRG.2	Gm17304	MSTRG.24283.1
MSTRG.2	Gm17937	ENSMUST00000197908
MSTRG.2	Zfp644	MSTRG.24285.1
MSTRG.2	Gm24191	MSTRG.24286.1;MSTRG.24286.2;ENSMUST00000157977
MSTRG.2	Zfp644	MSTRG.24287.1
MSTRG.2	Tgfbr3;	ENSMUST00000031224;ENSMUST00000138469;ENSMUST0000013(
MSTRG.2	Gm43423	ENSMUST00000198390
MSTRG.2	Shprh	MSTRG.2429.1
MSTRG.2	Tgfbr3	MSTRG.24290.1
MSTRG.2	Tgfbr3	MSTRG.24291.1
MSTRG.2	Gm8145	ENSMUST00000118220
MSTRG.2	Tgfbr3	MSTRG.24293.1
MSTRG.2	Tgfbr3	MSTRG.24294.1
MSTRG.2	Gm42900	ENSMUST00000197184
MSTRG.2	Brdt	ENSMUST00000031215;MSTRG.24296.4;ENSMUST00000162804
MSTRG.2	Gm8152	ENSMUST00000120842
MSTRG.2	Brdt	MSTRG.24298.1
MSTRG.2	Gm42902	ENSMUST00000199508
MSTRG.2	Ptpn18	ENSMUST00000185610;ENSMUST00000027302;MSTRG.243.2;ENS
MSTRG.2	Shprh	MSTRG.2430.1
MSTRG.2	Gm42902	MSTRG.24300.1
MSTRG.2	n-R5s17	ENSMUST00000082620
MSTRG.2	Ephx4	ENSMUST00000159968;ENSMUST00000049146;ENSMUST00000161
MSTRG.2	Gm17202	ENSMUST00000171723
MSTRG.2	Lpcat2b	ENSMUST00000166599
MSTRG.2	Gfi1	ENSMUST00000031205;MSTRG.24305.3;ENSMUST00000159164;M
MSTRG.2	A430072	ENSMUST00000161480;ENSMUST00000162148
MSTRG.2	A430072	MSTRG.24307.1
MSTRG.2	Evi5	ENSMUST00000112642;ENSMUST00000155897;ENSMUST0000013(
MSTRG.2	Gm43593	ENSMUST00000198605
MSTRG.2	Gm16577	ENSMUST00000161579
MSTRG.2	Ube2d2b	ENSMUST00000072578
MSTRG.2	Gm16007	ENSMUST00000117837
MSTRG.2	Gm43592	ENSMUST00000196922
MSTRG.2	Btbd8;G	ENSMUST00000162298;ENSMUST00000094541;ENSMUST00000212
MSTRG.2	Gm9727	ENSMUST00000161387
MSTRG.2	Btbd8	MSTRG.24315.1
MSTRG.2	Glmn	ENSMUST00000078021;ENSMUST00000100949;ENSMUST00000137
MSTRG.2	Glmn	MSTRG.24317.1
MSTRG.2	Gm42669	MSTRG.24318.1
MSTRG.2	Glmn	MSTRG.24319.1
MSTRG.2	AC16723	ENSMUST00000181330;ENSMUST00000181688
MSTRG.2	Rpap2	ENSMUST00000112650;ENSMUST00000112651;ENSMUST00000112

MSTRG.2	Rpap2	MSTRG.24321.1
MSTRG.2	A930041	ENSMUST00000149156
MSTRG.2	Rpap2	MSTRG.24323.1
MSTRG.2	Gm26692	MSTRG.24324.1;ENSMUST00000180428;MSTRG.24324.2
MSTRG.2	Gm26692	MSTRG.24325.1
MSTRG.2	5830411	ENSMUST00000200000
MSTRG.2	4930428	ENSMUST00000198537
MSTRG.2	Rpl5;Sn	ENSMUST00000082223;ENSMUST00000123183;MSTRG.24328.2;M
MSTRG.2	Fam69a	ENSMUST00000031198;ENSMUST00000153172
MSTRG.2	Fbxo30	ENSMUST00000129456;ENSMUST00000070300
MSTRG.2	Fam69a	MSTRG.24330.1
MSTRG.2	Fam69a	MSTRG.24331.1
MSTRG.2	Fam69a	MSTRG.24332.1
MSTRG.2	Fam69a	MSTRG.24333.1
MSTRG.2	Gm9850	ENSMUST00000117916
MSTRG.2	Dr1	ENSMUST00000031190;ENSMUST00000154862
MSTRG.2	Ccdc18	ENSMUST00000197718;ENSMUST00000047677;ENSMUST0000020C
MSTRG.2	Gm7118	ENSMUST00000200167
MSTRG.2	Ccdc18	MSTRG.24338.1
MSTRG.2	Gm8190	ENSMUST00000199603
MSTRG.2	Gm9797	ENSMUST00000052902;ENSMUST00000218003
MSTRG.2	Gm43083	ENSMUST00000199594
MSTRG.2	Mtf2	ENSMUST00000131291;ENSMUST00000124195;ENSMUST00000141
MSTRG.2	Mtf2	MSTRG.24342.1
MSTRG.2	Mtf2	MSTRG.24343.1
MSTRG.2	Tmed5	ENSMUST00000118036;ENSMUST00000061203;MSTRG.24344.3;M
MSTRG.2	Tmed5	MSTRG.24345.1
MSTRG.2	Pde6b	ENSMUST00000031456;ENSMUST00000134865;MSTRG.24346.3
MSTRG.2	Atp5k	ENSMUST00000049628;ENSMUST00000123184
MSTRG.24348		MSTRG.24348.1
MSTRG.24349		MSTRG.24349.1
MSTRG.2	Stx11	ENSMUST00000042861;ENSMUST00000163425;ENSMUST0000021{8}
MSTRG.2	Gm8473	ENSMUST00000176697
MSTRG.2	Gm8493	ENSMUST00000196881
MSTRG.2	Crlf2	ENSMUST00000200284;ENSMUST00000044579;ENSMUST0000019{8}
MSTRG.24355		MSTRG.24355.1
MSTRG.2	Mfsd7a	ENSMUST00000031455
MSTRG.2	Pcgf3	ENSMUST00000112597;ENSMUST00000046975;MSTRG.24357.1;M
MSTRG.2	Gak;Gm4	MSTRG.24358.1;MSTRG.24358.2;ENSMUST00000156110;ENSMUS
MSTRG.2	Gm10086	ENSMUST00000136334
MSTRG.2436		MSTRG.2436.1
MSTRG.2	Cplx1	ENSMUST00000046892
MSTRG.2	Gm43364	ENSMUST00000196046
MSTRG.2	Tmem175	ENSMUST00000146207;ENSMUST00000063272;MSTRG.24362.3;E
MSTRG.2	Dgkq	ENSMUST00000053913;ENSMUST00000132179;ENSMUST00000144
MSTRG.2	Idua	ENSMUST00000071650;ENSMUST00000140620;ENSMUST00000112
MSTRG.2	Slc26a1	ENSMUST00000051757;ENSMUST00000119270
MSTRG.2	Idua	MSTRG.24366.1
MSTRG.2	Fgfrl1	ENSMUST00000197255;MSTRG.24367.2;ENSMUST00000112560;E
MSTRG.2	5430403	ENSMUST00000092720
MSTRG.2	5430403	MSTRG.24369.1
MSTRG.2	Utrn;AC	ENSMUST00000076817;ENSMUST00000219003;MSTRG.2437.2;MS
MSTRG.2	Zfp932	MSTRG.24370.1
MSTRG.2	4930522	ENSMUST00000112547;ENSMUST00000100937
MSTRG.2	4930522	MSTRG.24372.1
MSTRG.2	Gm8508	ENSMUST00000061226
MSTRG.2	A430073	ENSMUST00000196262

MSTRG.2	A430073	MSTRG.24375.1
MSTRG.2	Gm10416	ENSMUST00000197379
MSTRG.24377		MSTRG.24377.1
MSTRG.2	Gm15446	MSTRG.24378.3;MSTRG.24378.1;MSTRG.24378.2;ENSMUST0000
MSTRG.2	Gm26808	MSTRG.24379.1
MSTRG.2	Utrn	MSTRG.2438.1;MSTRG.2438.2
MSTRG.24380		MSTRG.24380.4;MSTRG.24380.1;MSTRG.24380.2;MSTRG.2438
MSTRG.24381		MSTRG.24381.1
MSTRG.24382		MSTRG.24382.1
MSTRG.24383		MSTRG.24383.1
MSTRG.2	Gm26808	ENSMUST00000181569
MSTRG.2	Gm15446	ENSMUST00000112544;ENSMUST00000146564;ENSMUST0000017
MSTRG.2	Zfp932	MSTRG.24386.1
MSTRG.2	1010001	ENSMUST00000181488
MSTRG.2	Zfp932	ENSMUST00000112540;ENSMUST00000099484;ENSMUST0000012
MSTRG.2	Zfp932	MSTRG.24389.1
MSTRG.2	Utrn	MSTRG.2439.1
MSTRG.24390		MSTRG.24390.1
MSTRG.2	Gm17655	ENSMUST00000210275;ENSMUST00000167969
MSTRG.2	Plcxd1	ENSMUST00000086687;ENSMUST00000141066;ENSMUST0000112
MSTRG.2	Gtpbp6	ENSMUST00000077220;ENSMUST00000138507;MSTRG.24393.2;E
MSTRG.2	Zfp605	MSTRG.24394.1;ENSMUST00000112528;ENSMUST00000144089;E
MSTRG.2	Gm15484	ENSMUST00000118871
MSTRG.2	Chfr	ENSMUST00000200038;MSTRG.24396.1;ENSMUST00000199672;E
MSTRG.2	Gm43136	ENSMUST00000200403
MSTRG.2	Gm15787	ENSMUST00000152014;ENSMUST00000135366;MSTRG.24398.3;E
MSTRG.2	Golga3;	ENSMUST00000112512;ENSMUST00000136900;ENSMUST0000013
MSTRG.2	Gm28417	ENSMUST00000191357;ENSMUST00000185218;ENSMUST0000018
MSTRG.2	Utrn	MSTRG.2440.1
MSTRG.2	Golga3	MSTRG.24400.1
MSTRG.2	Gm43137	ENSMUST00000198052
MSTRG.2	Ankle2	ENSMUST00000197188;ENSMUST00000086674;ENSMUST0000031
MSTRG.2	Pgam5	ENSMUST00000112505;MSTRG.24403.2;ENSMUST00000059229;E
MSTRG.2	Gm26718	ENSMUST00000181048
MSTRG.2	Gm15788	ENSMUST00000140087;ENSMUST00000130123
MSTRG.2	Pxmp2	ENSMUST00000031472;ENSMUST00000141550;ENSMUST0000012
MSTRG.2	Pole	ENSMUST00000007296;ENSMUST00000112482;ENSMUST0000131
MSTRG.2	Gm26277	ENSMUST00000157097
MSTRG.2	Gm15791	ENSMUST00000120365
MSTRG.2	Gm15792	ENSMUST00000137099
MSTRG.2	A630023	ENSMUST00000141532;ENSMUST00000196681;MSTRG.24411.2;E
MSTRG.2	Fbrsl1	ENSMUST00000069483;ENSMUST00000056124;ENSMUST0000019
MSTRG.2	Gm26711	MSTRG.24413.1;ENSMUST00000180866
MSTRG.2	Fbrsl1	MSTRG.24414.1;MSTRG.24414.2
MSTRG.2	Fbrsl1	MSTRG.24415.1
MSTRG.2	Fbrsl1	ENSMUST00000198415
MSTRG.2	Fbrsl1	MSTRG.24417.1
MSTRG.2	Gm42596	ENSMUST00000196305
MSTRG.2	Hscb	ENSMUST00000056937;ENSMUST00000144850;ENSMUST0000014
MSTRG.2	Utrn	MSTRG.2442.1
MSTRG.2	Chek2	MSTRG.24420.1
MSTRG.2	Chek2	ENSMUST00000200172;ENSMUST00000199937;ENSMUST0000006
MSTRG.2	Chek2	MSTRG.24422.1
MSTRG.2	Noc4l	ENSMUST00000042147;ENSMUST00000123380;ENSMUST0000015
MSTRG.2	Ddx51	ENSMUST00000031478;ENSMUST00000136629;MSTRG.24424.2;E
MSTRG.2	Gm42595	ENSMUST00000196771
MSTRG.2	Ep400;M	ENSMUST00000041558;ENSMUST00000112435;MSTRG.24426.3;E

MSTRG.2	Gm22716	ENSMUST00000177596
MSTRG.2	Gm22583	ENSMUST00000178031
MSTRG.2	Gm15559	ENSMUST00000132267
MSTRG.2	Gm23044	ENSMUST00000157891
MSTRG.2	Pus1	ENSMUST00000112426;ENSMUST00000031483;ENSMUST0000008[illegible]
MSTRG.2	Gm26515	ENSMUST00000181141
MSTRG.2	Ulk1	ENSMUST00000031490;ENSMUST00000196094;ENSMUST0000019[illegible]
MSTRG.2	Pitpnb	MSTRG.24433.1
MSTRG.2	Gm27680	ENSMUST00000199221;ENSMUST00000184192
MSTRG.2	Pitpnb	ENSMUST00000086635;MSTRG.24435.3;ENSMUST00000200298;[illegible]
MSTRG.2	Ttc28	ENSMUST00000040111;ENSMUST00000156290;MSTRG.24437.3;[illegible]
MSTRG.2	Gm15989	ENSMUST00000121895
MSTRG.2	Ttc28	MSTRG.24439.1
MSTRG.2	AC12273	ENSMUST00000219425
MSTRG.2	Gm42778	ENSMUST00000199644
MSTRG.2	Ttc28	MSTRG.24441.1
MSTRG.2	Mir701	ENSMUST00000102208
MSTRG.2	Gm15988	ENSMUST00000118677
MSTRG.2	Ttc28	MSTRG.24444.1
MSTRG.2	Gm42780	ENSMUST00000197083
MSTRG.2	Ttc28	MSTRG.24446.1
MSTRG.2	Gm43677	ENSMUST00000198098
MSTRG.2	Gm43676	ENSMUST00000197532
MSTRG.2	Gm27602	ENSMUST00000183861
MSTRG.2	Zc2hc1b	ENSMUST00000019954
MSTRG.24450		MSTRG.24450.1
MSTRG.2	Tpst2	ENSMUST00000031287;ENSMUST00000134071;ENSMUST00000071
MSTRG.2	Tpst2	MSTRG.24452.1
MSTRG.2	Tpst2	MSTRG.24453.1
MSTRG.24454		MSTRG.24454.1
MSTRG.2	Tfip11	ENSMUST00000031288;ENSMUST00000198238;ENSMUST0000012[illegible]
MSTRG.2	Srrd	ENSMUST00000155756;ENSMUST00000146510;ENSMUST00000031
MSTRG.2	Hps4	ENSMUST00000112359;ENSMUST00000176737;MSTRG.24457.2;[illegible]
MSTRG.2	Gm6583	ENSMUST00000051117
MSTRG.2	Gm43759	ENSMUST00000196925
MSTRG.2	Ltv1	MSTRG.2446.1;ENSMUST00000019950;ENSMUST00000219824
MSTRG.2	Gm43758	ENSMUST00000199500
MSTRG.2	Gm20636	ENSMUST00000176660
MSTRG.2	Grk3	ENSMUST00000065167;ENSMUST00000200332;ENSMUST0000019[illegible]
MSTRG.2	Grk3	MSTRG.24463.1
MSTRG.2	Grk3	MSTRG.24464.1
MSTRG.2	Crybb3	ENSMUST00000076069;ENSMUST00000118226;ENSMUST0000015[illegible]
MSTRG.2	2900026	ENSMUST00000212276;ENSMUST00000050125;MSTRG.24466.1;[illegible]
MSTRG.2	Gm43246	ENSMUST00000197597
MSTRG.2	F830115	ENSMUST00000197638
MSTRG.2	4930557	ENSMUST00000200193
MSTRG.2	Phactr2	ENSMUST00000105545;ENSMUST00000105543;ENSMUST0000010[illegible]
MSTRG.2	Gm42161	ENSMUST00000196278
MSTRG.2	2900026	MSTRG.24471.1
MSTRG.2	Gm43094	ENSMUST00000196549
MSTRG.2	Cmklr1	MSTRG.24473.1
MSTRG.2	Cmklr1	ENSMUST00000047936;ENSMUST00000132065;ENSMUST00000142
MSTRG.2	Gm15736	ENSMUST00000141710
MSTRG.2	Tmem119	ENSMUST00000067853;ENSMUST00000160374
MSTRG.24477		MSTRG.24477.1
MSTRG.2	Gm19931	ENSMUST00000198920
MSTRG.2	Selplg	ENSMUST00000201931;ENSMUST00000100874;ENSMUST00000201

MSTRG.2	Phactr2	MSTRG.2448.1
MSTRG.2	Selplg	MSTRG.24480.2;MSTRG.24480.1
MSTRG.2	1700069	ENSMUST00000162388;ENSMUST00000159233
MSTRG.2	Ficd	ENSMUST00000065698
MSTRG.2	Sart3	MSTRG.24483.1;ENSMUST00000019118;ENSMUST00000197041;M
MSTRG.2	Sart3	MSTRG.24484.1
MSTRG.2	Gm3511	ENSMUST00000199823
MSTRG.2	Sart3	MSTRG.24486.1
MSTRG.2	Iscu	ENSMUST00000026937;ENSMUST00000112311;ENSMUST00000112
MSTRG.2	Coro1c	ENSMUST00000164980;ENSMUST00000004646;MSTRG.24488.3;E
MSTRG.2	Gm17122	ENSMUST00000166047
MSTRG.2	Phactr2	MSTRG.2449.1
MSTRG.2	Gm43335	ENSMUST00000198299
MSTRG.2	Gm43336	ENSMUST00000200487
MSTRG.2	Ssh1	MSTRG.24492.1;ENSMUST00000159592;ENSMUST00000112298;E
MSTRG.2	Svop	ENSMUST00000058472;ENSMUST00000140151
MSTRG.2	Usp30	ENSMUST00000162506;ENSMUST00000202603;ENSMUST00000031
MSTRG.2	Gm17833	ENSMUST00000197629
MSTRG.2	Gm42913	ENSMUST00000197936
MSTRG.2	Alkbh2	ENSMUST00000053657;ENSMUST00000112279;ENSMUST00000149
MSTRG.2	Ung	ENSMUST00000031587;ENSMUST00000102584;ENSMUST00000112
MSTRG.2	Acacb	ENSMUST00000102582;ENSMUST00000031583;ENSMUST00000146
MSTRG.2	4930568	ENSMUST00000188292;ENSMUST00000186117
MSTRG.2	Phactr2	MSTRG.2450.1
MSTRG.2	Gm13784	ENSMUST00000119639
MSTRG.2	Myo1h	ENSMUST00000169347;ENSMUST00000202006;ENSMUST00000152
MSTRG.2	Kctd10	ENSMUST00000102581;ENSMUST00000134532;ENSMUST00000001
MSTRG.2	Ube3b;M	ENSMUST00000074002;ENSMUST00000130169;ENSMUST00000151
MSTRG.2	Mmab	ENSMUST00000031560;ENSMUST00000112245;ENSMUST00000123
MSTRG.2	Mvk	ENSMUST00000137167;MSTRG.24505.1;ENSMUST00000112239;E
MSTRG.2	Fam222a	ENSMUST00000043650;ENSMUST00000130983
MSTRG.2	Fam222a	MSTRG.24507.1
MSTRG.2	Gltp	MSTRG.24508.1;ENSMUST00000012028;ENSMUST00000112214;M
MSTRG.24509		MSTRG.24509.1
MSTRG.2	Phactr2	MSTRG.2451.1
MSTRG.2	Tchp	ENSMUST00000094441;ENSMUST00000152999;ENSMUST00000124
MSTRG.2	Gm42789	ENSMUST00000202276
MSTRG.2	Tchp	MSTRG.24512.1
MSTRG.2	Mir7028	ENSMUST00000185016
MSTRG.2	Git2	ENSMUST00000112185;ENSMUST00000178440;ENSMUST00000146
MSTRG.2	4930515	ENSMUST00000173208
MSTRG.2	Ankrd13	ENSMUST00000102578;MSTRG.24516.2;ENSMUST00000140604;M
MSTRG.2	Ankrd13	MSTRG.24517.1
MSTRG.2	1500011	ENSMUST00000112160;ENSMUST00000140374;ENSMUST00000061
MSTRG.2	Ywhaq-p	ENSMUST00000117738
MSTRG.2	Fuca2	ENSMUST00000130865;ENSMUST00000120549;ENSMUST00000060
MSTRG.2	Unc119b	ENSMUST00000060798;ENSMUST00000137140;ENSMUST00000153
MSTRG.2	Rpl37rt	ENSMUST00000100847;ENSMUST00000201888;ENSMUST00000124
MSTRG.2	Rpl37rt	MSTRG.24522.1
MSTRG.2	Rpl37rt	MSTRG.24523.1
MSTRG.2	Acads	ENSMUST00000131726;ENSMUST00000031524;ENSMUST00000153
MSTRG.2	Mlec	ENSMUST00000112121;ENSMUST00000124678
MSTRG.2	Oasl2	ENSMUST00000031542;ENSMUST00000146072;ENSMUST00000150
MSTRG.2	Gm13822	ENSMUST00000127563
MSTRG.2	Oasl1	ENSMUST00000031540;ENSMUST00000112143;ENSMUST00000155
MSTRG.2	2210016	ENSMUST00000031538;ENSMUST00000147743;ENSMUST00000131
MSTRG.2	Pex3	ENSMUST00000170376;ENSMUST00000019945;ENSMUST00000105

MSTRG.2	2210016	MSTRG.24530.1
MSTRG.2	Hnf1a	ENSMUST00000031535;ENSMUST00000184027
MSTRG.2	Sppl3	MSTRG.24532.1;MSTRG.24532.2
MSTRG.2	Sppl3	MSTRG.24533.1
MSTRG.2	Hnf1aos	ENSMUST00000129609;ENSMUST00000138108;ENSMUST00000131
MSTRG.2	1810017	ENSMUST00000202592
MSTRG.2	Hnf1aos	MSTRG.24536.1
MSTRG.2	Sppl3	ENSMUST00000031530;ENSMUST00000150708;ENSMUST00000128
MSTRG.2	Sppl3	MSTRG.24538.1
MSTRG.2	C730045	ENSMUST00000201944
MSTRG.2	Pex3	MSTRG.2454.1
MSTRG.2	Sppl3	MSTRG.24540.1
MSTRG.2	Gm13827	ENSMUST00000148801
MSTRG.2	Sppl3	MSTRG.24542.1
MSTRG.2	Coq5	ENSMUST00000040421;ENSMUST00000201429;ENSMUST00000129
MSTRG.2	Gm13829	ENSMUST00000119600
MSTRG.2	Dynll1	ENSMUST00000009157;ENSMUST00000202980;ENSMUST00000123
MSTRG.2	Gm13830	ENSMUST00000149609;ENSMUST00000201195;ENSMUST00000144
MSTRG.2	Pop5	ENSMUST00000081497;ENSMUST00000154800;ENSMUST00000135
MSTRG.2	Rnf10	ENSMUST00000112097;ENSMUST00000040555;ENSMUST00000112
MSTRG.2	Adat2	ENSMUST00000019944
MSTRG.2	Gm13831	ENSMUST00000118764
MSTRG.2	Rnf10	MSTRG.24551.1
MSTRG.2	Cox6a1	ENSMUST00000040154;ENSMUST00000137766
MSTRG.2	Gm42903	ENSMUST00000202607
MSTRG.2	Srsf9	ENSMUST00000031513;ENSMUST00000209604;ENSMUST00000144
MSTRG.2	Gatc	ENSMUST00000139167;MSTRG.24555.1
MSTRG.2	Triap1	ENSMUST00000031508
MSTRG.2	4930401	ENSMUST00000139500
MSTRG.2	Gm13832	ENSMUST00000146546;ENSMUST00000151159
MSTRG.2	Gm13832	MSTRG.24559.1
MSTRG.2456		MSTRG.2456.1
MSTRG.2	Gm13832	MSTRG.24560.1
MSTRG.2	A930005	ENSMUST00000201293
MSTRG.2	Rplp0	ENSMUST00000086519;MSTRG.24562.2;ENSMUST00000156359;E
MSTRG.2	1110006	ENSMUST00000201728
MSTRG.2	Rab35;M	ENSMUST00000031492;ENSMUST00000138885;MSTRG.24564.3;E
MSTRG.2	Pla2g1b	ENSMUST00000145785;ENSMUST00000031495;ENSMUST00000125
MSTRG.2	Sirt4	ENSMUST00000112067;ENSMUST00000112066;ENSMUST00000154
MSTRG.2	Gm24265	ENSMUST00000101833
MSTRG.2	Gm24407	ENSMUST00000177864
MSTRG.2	Gm13836	ENSMUST00000121159
MSTRG.2	Aig1	ENSMUST00000162610;ENSMUST00000105534;ENSMUST00000019
MSTRG.2	Pxn	ENSMUST00000067268;ENSMUST00000152156;ENSMUST00000086
MSTRG.2	Gm13840	ENSMUST00000149608
MSTRG.2	Pxn	MSTRG.24572.1
MSTRG.2	Gcn1l1;I	ENSMUST00000064454;ENSMUST00000141472;ENSMUST00000136
MSTRG.2	Bicdl1	ENSMUST00000201869;MSTRG.24574.1;ENSMUST00000055408;E
MSTRG.2	Gm13841	ENSMUST00000118829
MSTRG.2	C330018	ENSMUST00000127792
MSTRG.2	Ccdc60	ENSMUST00000050178;ENSMUST00000086483
MSTRG.2	Ccdc60	MSTRG.24578.1
MSTRG.2	Gm14507	ENSMUST00000156169
MSTRG.2	Aig1	MSTRG.2458.1
MSTRG.2	Gm13837	ENSMUST00000126326
MSTRG.2	Hspb8	ENSMUST00000036991;ENSMUST00000133335
MSTRG.2	Cit	ENSMUST00000147330;MSTRG.24582.1;ENSMUST00000137952;E

MSTRG.2	Gm42473	ENSMUST00000201056
MSTRG.2	Gm42471	ENSMUST00000201457
MSTRG.2	Gm14508	ENSMUST00000127049
MSTRG.2	Prkab1	ENSMUST00000031486;ENSMUST00000111999;ENSMUST00000143
MSTRG.2	Prkab1	MSTRG.24587.1
MSTRG.2	Gm13842	ENSMUST00000156331;ENSMUST00000144932
MSTRG.2	Pebp1	ENSMUST00000036951;ENSMUST00000111973;ENSMUST00000142
MSTRG.2	Aig1	MSTRG.2459.1
MSTRG.2	Gm10399	ENSMUST00000100834
MSTRG.2	Vsig10	ENSMUST00000111967;ENSMUST00000145052;ENSMUST00000128
MSTRG.2	Gm15727	ENSMUST00000122416
MSTRG.24593		MSTRG.24593.1
MSTRG.2	Suds3	ENSMUST00000086471;ENSMUST00000202713;MSTRG.24594.3;E
MSTRG.2	9530046	ENSMUST00000149413
MSTRG.2	Taok3	ENSMUST00000111978;ENSMUST00000125738;ENSMUST00000128
MSTRG.2	Taok3	MSTRG.24597.1
MSTRG.2	Taok3	MSTRG.24598.1
MSTRG.2	Gm43637	ENSMUST00000202206
MSTRG.246		MSTRG.246.1
MSTRG.2	Aig1	MSTRG.2460.1
MSTRG.2	Taok3	MSTRG.24600.1;MSTRG.24600.2;MSTRG.24600.3
MSTRG.2	Taok3	MSTRG.24601.1;MSTRG.24601.2
MSTRG.2	n-R5s17	ENSMUST00000122638
MSTRG.2	Wsb2	ENSMUST00000031309;ENSMUST00000125522;MSTRG.24603.2;E
MSTRG.2	Rfc5	ENSMUST00000086461;ENSMUST00000150962;ENSMUST00000129
MSTRG.2	Gm15728	ENSMUST00000140833;ENSMUST00000156391
MSTRG.2	Fbxo21	ENSMUST00000202447;ENSMUST00000201427;ENSMUST00000035
MSTRG.2	Tesc	ENSMUST00000031304;ENSMUST00000124648;ENSMUST00000135
MSTRG.2	Fbxw8	ENSMUST00000049474;ENSMUST00000201545
MSTRG.2	Gm26835	MSTRG.2461.1;MSTRG.2461.3;ENSMUST00000181888;MSTRG.24
MSTRG.2	Gm9754	ENSMUST00000031305
MSTRG.2	Gm26411	ENSMUST00000157668
MSTRG.2	Rnft2	ENSMUST00000117177;ENSMUST00000121369;ENSMUST00000134
MSTRG.2	Gm24050	ENSMUST00000158623
MSTRG.2	2410131	ENSMUST00000049138
MSTRG.2	2410131	MSTRG.24615.1
MSTRG.24616		MSTRG.24616.1
MSTRG.24617		MSTRG.24617.1
MSTRG.2	Med13l;	ENSMUST00000201614;MSTRG.24618.1;ENSMUST00000100816;M
MSTRG.2	Gm43785	ENSMUST00000201036
MSTRG.2	Med13l	MSTRG.24620.1
MSTRG.2	Gm43788	ENSMUST00000201539
MSTRG.2	Gm43275	MSTRG.24622.1;ENSMUST00000202053
MSTRG.2	Rbm19	ENSMUST00000031590;ENSMUST00000202388;MSTRG.24623.2;E
MSTRG.2	Gm10390	ENSMUST00000180812;ENSMUST00000181905
MSTRG.2	Rbm19	MSTRG.24625.1
MSTRG.2	Rbm19	MSTRG.24626.1
MSTRG.2	Sds	ENSMUST00000201684;ENSMUST00000111898;ENSMUST00000124
MSTRG.2	Gm15690	ENSMUST00000128728
MSTRG.2	Plbd2	ENSMUST00000031597;ENSMUST00000131831;ENSMUST00000151
MSTRG.2	Hivep2	MSTRG.2463.1;MSTRG.2463.3;MSTRG.2463.2;ENSMUST0000018
MSTRG.2	Gm15749	ENSMUST00000161848
MSTRG.2	Rasal1	ENSMUST00000125804;ENSMUST00000031606;ENSMUST00000156
MSTRG.2	Rasal1	MSTRG.24632.1
MSTRG.2	Rasal1	MSTRG.24633.1
MSTRG.2	Dtx1	ENSMUST00000031607;ENSMUST00000145174;ENSMUST00000201
MSTRG.2	Dtx1	MSTRG.24635.1

MSTRG.2	Slc8b1	ENSMUST00000068326;ENSMUST00000111890;ENSMUST00000076
MSTRG.2	Tpcn1;2	ENSMUST00000046426;MSTRG.24637.3;MSTRG.24637.2;MSTRG.
MSTRG.2	Tpcn1	MSTRG.24638.1
MSTRG.2	Tpcn1	MSTRG.24639.1
MSTRG.2	Hivep2	MSTRG.2464.1
MSTRG.2	Tpcn1	MSTRG.24640.1
MSTRG.2	Tpcn1	MSTRG.24641.1
MSTRG.2	Iqcd	ENSMUST00000069259;ENSMUST00000094391
MSTRG.2	Rita1	ENSMUST00000111884;ENSMUST00000156356;ENSMUST00000147
MSTRG.2	Ddx54	ENSMUST00000031598;ENSMUST00000202672;MSTRG.24644.2;E
MSTRG.2	Gm43579	ENSMUST00000202387
MSTRG.2	Cfap73	ENSMUST00000177908
MSTRG.2	Oas2	ENSMUST00000053909;ENSMUST00000081491;ENSMUST00000147
MSTRG.2	Gm43068	ENSMUST00000201095
MSTRG.2	Oas3	ENSMUST00000044833;ENSMUST00000165820;ENSMUST0000020C
MSTRG.2	Hivep2	MSTRG.2465.1
MSTRG.2	Oas3	MSTRG.24650.1
MSTRG.2	Gm43069	ENSMUST00000201006
MSTRG.2	Oas1e	ENSMUST00000100785
MSTRG.2	Oas1c	ENSMUST00000117193;ENSMUST00000125547;ENSMUST0000013C
MSTRG.2	Oas1c	MSTRG.24654.1
MSTRG.2	Oas1b	MSTRG.24655.1;ENSMUST00000182725;ENSMUST00000086377;M
MSTRG.2	Rph3a	ENSMUST00000079204;ENSMUST00000202326
MSTRG.2	Gm42656	ENSMUST00000201506
MSTRG.2	Rph3a	MSTRG.24658.1
MSTRG.2	Ptpn11	ENSMUST00000100770;ENSMUST00000148407;MSTRG.24659.3;E
MSTRG.2	Hivep2	MSTRG.2466.1
MSTRG.2	Gm42930	ENSMUST00000200872
MSTRG.2	Rpl6;Gm	ENSMUST00000031617;ENSMUST00000146579;ENSMUST00000197
MSTRG.2	Trafd1	ENSMUST00000133138;ENSMUST00000156158;MSTRG.24662.1;E
MSTRG.2	Gm42917	ENSMUST00000200668
MSTRG.2	Gm42918	ENSMUST00000199249
MSTRG.2	Hectd4	ENSMUST00000042614;MSTRG.24665.2;MSTRG.24665.3;MSTRG.
MSTRG.2	Hectd4	MSTRG.24666.1
MSTRG.2	Gm24671	ENSMUST00000104159
MSTRG.2	Aldh2	ENSMUST00000031411;ENSMUST00000196694;ENSMUST0000019S
MSTRG.2	Gm42879	ENSMUST00000198656
MSTRG.2	Hivep2	MSTRG.2467.1
MSTRG.2	Brap	MSTRG.24670.1
MSTRG.2	Naa25	ENSMUST00000042163;ENSMUST00000173895;ENSMUST00000131
MSTRG.2	Gm43420	ENSMUST00000198012
MSTRG.2	Erp29	MSTRG.24673.1;ENSMUST00000153758;ENSMUST00000130451;M
MSTRG.2	Erp29	MSTRG.24674.1
MSTRG.2	Tmem116	ENSMUST00000111795;ENSMUST00000124247;ENSMUST0000015C
MSTRG.2	Gm15546	ENSMUST00000122095
MSTRG.2	Adam1b	ENSMUST00000079368
MSTRG.2	4930477	ENSMUST00000196484
MSTRG.2	Adam1a;	ENSMUST00000100757;MSTRG.24679.1;ENSMUST00000125946;E
MSTRG.2	Hivep2	MSTRG.2468.1
MSTRG.2	Gm15547	ENSMUST00000122152
MSTRG.2	Acad12	ENSMUST00000041252;ENSMUST00000131338;ENSMUST00000111
MSTRG.2	Acad12	MSTRG.24682.1
MSTRG.2	Acad10	MSTRG.24683.1
MSTRG.2	Acad10	ENSMUST00000031412;ENSMUST00000111770;ENSMUST00000137
MSTRG.2	Acad10	MSTRG.24685.1
MSTRG.2	Brap	ENSMUST00000031414;ENSMUST00000140996;ENSMUST00000132
MSTRG.2	Ppp1cc;	ENSMUST00000102528;MSTRG.24687.2;MSTRG.24687.3;ENSMUS

MSTRG.2	Atxn2	ENSMUST00000162611;ENSMUST00000051950;ENSMUST00000225
MSTRG.2	Gm43571	ENSMUST00000200067
MSTRG.2	Gm28289	ENSMUST00000189331
MSTRG.2	Gm16552	ENSMUST00000162348
MSTRG.2	Atxn2	MSTRG.24691.1
MSTRG.2	Gm42866	ENSMUST00000195919
MSTRG.2	Atxn2	MSTRG.24693.1
MSTRG.2	Atxn2	MSTRG.24694.1
MSTRG.2	Sh2b3;M	ENSMUST00000136960;MSTRG.24695.3;MSTRG.24695.2;ENSMUS
MSTRG.2	1700008	ENSMUST00000198145
MSTRG.24697		MSTRG.24697.1
MSTRG.24698		MSTRG.24698.1
MSTRG.24699		MSTRG.24699.1
MSTRG.2	Plekhb2	ENSMUST00000156687;ENSMUST00000027297;ENSMUST00000152
MSTRG.2	Adgrg6	ENSMUST00000041168;ENSMUST00000208429;ENSMUST00000208
MSTRG.2	Hvcn1	ENSMUST00000145854;ENSMUST00000072602;ENSMUST0000014З
MSTRG.2	Tctn1	MSTRG.24701.1;MSTRG.24701.2;ENSMUST00000111738;MSTRG.
MSTRG.2	Tctn1	MSTRG.24703.1
MSTRG.2	Pptc7;G	ENSMUST00000053426;MSTRG.24704.2;MSTRG.24704.3;MSTRG.
MSTRG.2	Rad9b	ENSMUST00000117263;ENSMUST00000149600;ENSMUST0000004
MSTRG.2	Vps29	ENSMUST00000155671;ENSMUST00000118830;ENSMUST00000117
MSTRG.2	Fam216a	ENSMUST00000031419
MSTRG.2	Gpn3	ENSMUST00000127608;ENSMUST00000031420;ENSMUST0000015
MSTRG.2	Gm42830	ENSMUST00000196642
MSTRG.2	Gm10335	ENSMUST00000096020
MSTRG.24710		MSTRG.24710.1
MSTRG.2	Arpc3	ENSMUST00000102525;ENSMUST00000148913;ENSMUST00000141
MSTRG.2	Gm42829	ENSMUST00000198417
MSTRG.2	Anapc7	ENSMUST00000031422;ENSMUST00000119792;ENSMUST0000014
MSTRG.2	Gm15846	ENSMUST00000116022
MSTRG.24715		MSTRG.24715.1
MSTRG.2	Atp2a2	ENSMUST00000179939;ENSMUST00000177974;ENSMUST00000031
MSTRG.2	Gm43359	MSTRG.24717.1;ENSMUST00000197944
MSTRG.2	Gm43360	ENSMUST00000197656
MSTRG.2	Gm43361	ENSMUST00000196562
MSTRG.2	AC11820	ENSMUST00000220256
MSTRG.2	Ift81	ENSMUST00000031426;ENSMUST00000135956;ENSMUST0000013
MSTRG.2	Gm43541	ENSMUST00000197208
MSTRG.2	Gm43301	ENSMUST00000198918
MSTRG.2	P2rx7;M	ENSMUST00000100737;ENSMUST00000121489;ENSMUST00000031
MSTRG.2	Gm10064	ENSMUST00000071235
MSTRG.2	P2rx4	ENSMUST00000031429;ENSMUST00000195963;MSTRG.24725.2;E
MSTRG.2	Camkk2	MSTRG.24726.1;ENSMUST00000111668;ENSMUST00000200109;E
MSTRG.2	Camkk2	MSTRG.24727.1
MSTRG.2	Camkk2	MSTRG.24728.1
MSTRG.2	Gm43684	ENSMUST00000199279
MSTRG.2	Vta1	ENSMUST00000154132;ENSMUST00000020018;ENSMUST0000015
MSTRG.2	Anapc5	ENSMUST00000086216;ENSMUST00000196423;ENSMUST0000019
MSTRG.2	Anapc5	MSTRG.24731.1
MSTRG.2	Wdr66	ENSMUST00000121964;ENSMUST00000170536;ENSMUST0000014
MSTRG.2	Rpl35a-	ENSMUST00000148282
MSTRG.2	Gm15857	ENSMUST00000133476
MSTRG.2	Gm15860	ENSMUST00000197262;ENSMUST00000137257
MSTRG.2	Bcl7a;G	MSTRG.24736.1;ENSMUST00000120593;ENSMUST00000031391;E
MSTRG.2	5830487	ENSMUST00000198600;ENSMUST00000200118
MSTRG.2	Gm15747	ENSMUST00000141216;ENSMUST00000151096
MSTRG.2	Mlxip	ENSMUST00000111596;MSTRG.24739.1;ENSMUST00000068237;M

MSTRG.2	Gm16234	ENSMUST00000124976
MSTRG.2	Gm42907	ENSMUST00000199793
MSTRG.2	Gm42908	ENSMUST00000197508
MSTRG.2	Gm43813	MSTRG.24742.1;ENSMUST00000200134
MSTRG.2	Mlxip	MSTRG.24743.1
MSTRG.2	Rnf34	ENSMUST00000031434;ENSMUST00000198602
MSTRG.2	Kdm2b;G	ENSMUST00000046073;ENSMUST00000031435;ENSMUST0000011
MSTRG.2	Kdm2b	MSTRG.24746.1
MSTRG.2	Gm43411	ENSMUST00000196235
MSTRG.2	Kdm2b	MSTRG.24748.1
MSTRG.2	Kdm2b	MSTRG.24749.1
MSTRG.2	Vta1	MSTRG.2475.1
MSTRG.2	Kdm2b	MSTRG.24750.1
MSTRG.2	Kdm2b	MSTRG.24751.1
MSTRG.2	A930024	ENSMUST00000148466;MSTRG.24752.2
MSTRG.2	Gm44574	MSTRG.24753.1
MSTRG.2	Orai1	ENSMUST00000051016;ENSMUST00000121652;ENSMUST00000152
MSTRG.24755		MSTRG.24755.1
MSTRG.2	Morn3	ENSMUST00000132775;ENSMUST00000137490;ENSMUST00000031
MSTRG.2	Gm43412	ENSMUST00000197455
MSTRG.2	Gm6444	ENSMUST00000119611
MSTRG.2	Vta1	MSTRG.2476.1
MSTRG.2	Tmem120	ENSMUST00000161660;ENSMUST00000067505;ENSMUST00000161
MSTRG.2	Tmem120	MSTRG.24761.1
MSTRG.2	Rhof;AI	MSTRG.24762.1;ENSMUST00000186469;ENSMUST00000160479;
MSTRG.2	Gm38102	ENSMUST00000192791
MSTRG.2	4932422	ENSMUST00000195992
MSTRG.2	Setd1b	ENSMUST00000100731;ENSMUST00000174836;ENSMUST0000016
MSTRG.2	Psmd9	ENSMUST00000199260;ENSMUST00000133386;ENSMUST0000010
MSTRG.2	Hpd	ENSMUST00000031398;ENSMUST00000155092;ENSMUST00000144
MSTRG.2	Gm43409	ENSMUST00000195921
MSTRG.2	Nmbr	ENSMUST00000191238;ENSMUST00000188021;ENSMUST0000018
MSTRG.2	Lrrc43	ENSMUST00000196809;ENSMUST00000094327
MSTRG.2	Gm15751	ENSMUST00000134552
MSTRG.2	Diablo;	ENSMUST00000125652;MSTRG.24772.2;MSTRG.24772.3;ENSMUS
MSTRG.2	B3gnt4	ENSMUST00000031384
MSTRG.2	Gm24682	ENSMUST00000158764
MSTRG.2	Mir7647	ENSMUST00000183364
MSTRG.2	Vps33a	MSTRG.24776.1;ENSMUST00000031388;MSTRG.24776.3;ENSMUS
MSTRG.2	Vps33a	MSTRG.24777.1
MSTRG.2	Vps33a	MSTRG.24778.1
MSTRG.2	Vps33a	MSTRG.24779.1
MSTRG.2	Gje1	ENSMUST00000020016
MSTRG.2	Gm43133	ENSMUST00000197472
MSTRG.2	Hcar1	ENSMUST00000164267
MSTRG.2	Kntc1	ENSMUST00000199838;MSTRG.24782.2;ENSMUST00000031366;
MSTRG.2	Denr;Gm	ENSMUST00000023869;ENSMUST00000168899;ENSMUST0000019
MSTRG.2	Denr	MSTRG.24784.1
MSTRG.2	Ccdc62	MSTRG.24785.1
MSTRG.2	Clip1	ENSMUST00000111564;ENSMUST00000111566;ENSMUST00000137
MSTRG.2	Gm24840	ENSMUST00000122603
MSTRG.2	Gm42651	ENSMUST00000199851
MSTRG.2	Gm43797	ENSMUST00000198221
MSTRG.2	Gm8355	ENSMUST00000177767
MSTRG.2	Zcchc8	ENSMUST00000196282;ENSMUST00000031376;ENSMUST0000019
MSTRG.2	Rsrc2	ENSMUST00000050827;ENSMUST00000057795;ENSMUST00000182
MSTRG.2	Hip1r;G	ENSMUST00000000939;ENSMUST00000167879;ENSMUST0000016

MSTRG.2	Hip1r	MSTRG.24793.1
MSTRG.2	Mir7032	ENSMUST00000185154
MSTRG.2	Vps37b	MSTRG.24795.1;ENSMUST00000040967;MSTRG.24795.3;ENSMUS
MSTRG.2	Vps37b	MSTRG.24796.1
MSTRG.2	Gm34086	ENSMUST00000196486
MSTRG.2	Gm43661	ENSMUST00000199099
MSTRG.2	Abcb9	MSTRG.24799.1;ENSMUST00000031354;ENSMUST00000153433;E
MSTRG.248		MSTRG.248.1
MSTRG.2	B230364	ENSMUST00000181479
MSTRG.24800		MSTRG.24800.1
MSTRG.2	Gm16001	MSTRG.24801.1;ENSMUST00000127989;ENSMUST00000143062
MSTRG.2	Ogfod2	ENSMUST00000024470;ENSMUST00000130140;ENSMUST0000011
MSTRG.2	Cdk2ap1	ENSMUST00000031341;ENSMUST00000111474;ENSMUST00000111
MSTRG.24804		MSTRG.24804.1
MSTRG.2	Arl6ip4	ENSMUST00000149835;ENSMUST00000031351;MSTRG.24805.2;E
MSTRG.2	Pitpnm2	ENSMUST00000086123;ENSMUST00000162812;MSTRG.24806.1;M
MSTRG.2	Pitpnm2	ENSMUST00000159053;ENSMUST00000162577;ENSMUST0000019
MSTRG.2	Pitpnm2	MSTRG.24809.1
MSTRG.2	AC15337	ENSMUST00000220296
MSTRG.2	Pitpnm2	MSTRG.24810.1;MSTRG.24810.2
MSTRG.2	Gm23171	ENSMUST00000157159
MSTRG.2	Gm43340	ENSMUST00000200448
MSTRG.2	2810006	ENSMUST00000111477;ENSMUST00000077376
MSTRG.2	Kmt5a	ENSMUST00000059580;ENSMUST00000199798;ENSMUST0000019
MSTRG.2	Rilpl2	ENSMUST00000031347;ENSMUST00000198413
MSTRG.2	Gm15621	ENSMUST00000119162
MSTRG.2	Sbno1	ENSMUST00000065263;ENSMUST00000199808;ENSMUST0000016
MSTRG.2	Gm37415	ENSMUST00000192265;ENSMUST00000192373
MSTRG.2	Snrnp35	ENSMUST00000031349;ENSMUST00000136567;ENSMUST00000111
MSTRG.2	AC15337	MSTRG.2482.1;MSTRG.2482.2
MSTRG.2	Rilpl1	ENSMUST00000062153;ENSMUST00000200202;ENSMUST0000019
MSTRG.2	Gm43034	ENSMUST00000198678
MSTRG.2	Tmed2	ENSMUST00000060226;ENSMUST00000200031;ENSMUST00000124
MSTRG.2	Ddx55	ENSMUST00000071057;ENSMUST00000131631;ENSMUST0000013
MSTRG.2	Eif2b1	ENSMUST00000031334;ENSMUST00000152716;ENSMUST0000019
MSTRG.2	Gtf2h3	ENSMUST00000031333;ENSMUST00000200670;ENSMUST0000012
MSTRG.2	Zfp664	ENSMUST00000111417;MSTRG.24826.1;MSTRG.24826.2;MSTRG.
MSTRG.2	Zfp664	MSTRG.24827.1
MSTRG.2	Zfp664	MSTRG.24828.1
MSTRG.2	Rflna	ENSMUST00000036109;ENSMUST00000145946
MSTRG.2	AC15337	ENSMUST00000217920;ENSMUST00000219259;MSTRG.2483.3
MSTRG.2	Rflna	MSTRG.24830.1
MSTRG.2	Tctn2	ENSMUST00000140953;ENSMUST00000185820;ENSMUST00000132
MSTRG.2	Atp6v0a	ENSMUST00000200292;MSTRG.24832.1;ENSMUST00000037865;E
MSTRG.2	Gm24854	ENSMUST00000158025
MSTRG.2	4930404	ENSMUST00000199971;ENSMUST00000199356
MSTRG.2	Dnah10	ENSMUST00000058440;ENSMUST00000126998;ENSMUST00000197
MSTRG.2	Ccdc92	ENSMUST00000036206;ENSMUST00000135495;ENSMUST0000014
MSTRG.2	Ccdc92	MSTRG.24838.1
MSTRG.2	Scarb1	ENSMUST00000086075;ENSMUST00000111390;ENSMUST00000127
MSTRG.2	AC15337	MSTRG.2484.1
MSTRG.2	Gm42633	ENSMUST00000198853
MSTRG.24841		MSTRG.24841.1
MSTRG.2	Ubc	ENSMUST00000136312;MSTRG.24843.6;MSTRG.24843.4;MSTRG.
MSTRG.2	Gm10382	ENSMUST00000100700
MSTRG.24845		MSTRG.24845.1
MSTRG.2	Bri3bp	ENSMUST00000049040;ENSMUST00000198811

MSTRG.2	Dhx37	ENSMUST00000169485;ENSMUST00000196444;ENSMUST0000019[illegible]
MSTRG.2	Dhx37	MSTRG.24848.1
MSTRG.2	Ncor2	ENSMUST00000125053;ENSMUST00000111402;ENSMUST00000111
MSTRG.2	Cited2	ENSMUST00000038107;ENSMUST00000219558;ENSMUST0000021[illegible]
MSTRG.2	Gm42838	ENSMUST00000196503
MSTRG.2	Gm42839	ENSMUST00000198978
MSTRG.2	Ncor2	MSTRG.24852.1
MSTRG.2	Ncor2	MSTRG.24853.1
MSTRG.2	Ncor2	MSTRG.24854.1;MSTRG.24854.2
MSTRG.2	Aacs	ENSMUST00000031445;ENSMUST00000144361;ENSMUST00000131
MSTRG.2	Gm27551	ENSMUST00000184258
MSTRG.24857		MSTRG.24857.1
MSTRG.24858		MSTRG.24858.1
MSTRG.24859		MSTRG.24859.1
MSTRG.2	Heca	ENSMUST00000217949;ENSMUST00000037879;ENSMUST0000021[illegible]
MSTRG.2	Slc15a4	ENSMUST00000031367;MSTRG.24860.2;ENSMUST00000124569;[illegible]
MSTRG.2	Gm43474	ENSMUST00000196415
MSTRG.2	Glt1d1	ENSMUST00000137157;ENSMUST00000118139;ENSMUST00000134
MSTRG.2	Stx2	ENSMUST00000031378;ENSMUST00000100680;ENSMUST0000012[illegible]
MSTRG.2	Stx2	MSTRG.24864.1
MSTRG.2	Ran	ENSMUST00000031383;ENSMUST00000134632;ENSMUST00000111
MSTRG.2	Adgrd1	ENSMUST00000056617;ENSMUST00000155590;ENSMUST0000015[illegible]
MSTRG.2	Rps16-p	ENSMUST00000140155
MSTRG.2	Adgrd1	MSTRG.24868.1
MSTRG.2	Gm6139	ENSMUST00000079261
MSTRG.2	Abracl	ENSMUST00000020002;ENSMUST00000220110;ENSMUST0000021[illegible]
MSTRG.2	Zfp11	ENSMUST00000049778
MSTRG.24871		MSTRG.24871.1
MSTRG.2	Gm40332	ENSMUST00000199847
MSTRG.2	Sfswap;[illegible]	ENSMUST00000053737;ENSMUST00000196698;MSTRG.24873.2;[illegible]
MSTRG.2	Mrps17	ENSMUST00000119576;ENSMUST00000042191;ENSMUST0000011[illegible]
MSTRG.2	Nipsnap[illegible]	MSTRG.24875.1
MSTRG.2	Nipsnap[illegible]	ENSMUST00000086046;ENSMUST00000124342;ENSMUST0000019[illegible]
MSTRG.2	Gm15903	ENSMUST00000155796;ENSMUST00000199631
MSTRG.2	Gusb	ENSMUST00000026613;ENSMUST00000201801;ENSMUST0000012[illegible]
MSTRG.2	Asl	ENSMUST00000161094;ENSMUST00000159096;ENSMUST0000015[illegible]
MSTRG.2	Crcp	ENSMUST00000026608;ENSMUST00000202163;ENSMUST00000202
MSTRG.2	Crcp	MSTRG.24881.1
MSTRG.2	Tpst1	ENSMUST00000118993;ENSMUST00000040721;ENSMUST00000142
MSTRG.2	2210412[illegible]	ENSMUST00000201281
MSTRG.2	Psph	ENSMUST00000118268;ENSMUST00000031399;ENSMUST0000013[illegible]
MSTRG.2	Psph	MSTRG.24885.1
MSTRG.2	Cct6a;S[illegible]	ENSMUST00000201414;ENSMUST00000031402;ENSMUST00000202
MSTRG.2	Gm23245	ENSMUST00000083370
MSTRG.2	Phkg1	ENSMUST00000026617;ENSMUST00000140667;ENSMUST00000154
MSTRG.2	Gm42790	ENSMUST00000201268
MSTRG.2	Abracl	MSTRG.2489.1
MSTRG.2	Chchd2	ENSMUST00000094280;ENSMUST00000131645
MSTRG.2	Phkg1	MSTRG.24891.1
MSTRG.2	Phkg1	MSTRG.24892.1
MSTRG.2	Zbed5	ENSMUST00000041466;ENSMUST00000202430;ENSMUST00000077
MSTRG.2	Nupr1l	ENSMUST00000195766;ENSMUST00000194521;ENSMUST0000017[illegible]
MSTRG.2	Vkorc1l	ENSMUST00000051758;ENSMUST00000201855;ENSMUST0000007[illegible]
MSTRG.2	Gm42986	ENSMUST00000201618
MSTRG.2	Gm43482	ENSMUST00000202797
MSTRG.2	Rabgef1	ENSMUST00000119797;ENSMUST00000148264;ENSMUST0000011[illegible]
MSTRG.2	Rabgef1	MSTRG.24899.1

MSTRG.2	Arhgef4	ENSMUST00000159747;ENSMUST00000047664;ENSMUST00000162
MSTRG.2	Abracl	MSTRG.2490.1
MSTRG.2	Gm15920	ENSMUST00000118998
MSTRG.2	Rabgef1	MSTRG.24901.1
MSTRG.2	Rabgef1	MSTRG.24902.1
MSTRG.2	Rabgef1	MSTRG.24903.1
MSTRG.2	Gm15921	ENSMUST00000119542
MSTRG.2	Tmem248	ENSMUST00000202612;ENSMUST00000202305;MSTRG.24905.2;E
MSTRG.2	Gm6598	ENSMUST00000202719
MSTRG.2	Tmem248	MSTRG.24907.1
MSTRG.2	Sbds	ENSMUST00000026387;MSTRG.24908.1;ENSMUST00000139211;E
MSTRG.2	Tyw1	ENSMUST00000044204;ENSMUST00000100662;ENSMUST0000004
MSTRG.2	Ccdc28a	ENSMUST00000080860;ENSMUST00000174592;ENSMUST00000174
MSTRG.2	Tyw1	MSTRG.24910.1
MSTRG.2	Tyw1	MSTRG.24911.1
MSTRG.2	Wbscr17	ENSMUST00000086023;ENSMUST00000160609;ENSMUST0000016
MSTRG.2	Gm42897	ENSMUST00000201486
MSTRG.2	Gm42896	ENSMUST00000201249
MSTRG.2	Wbscr17	MSTRG.24915.1
MSTRG.2	Gm43165	ENSMUST00000200842
MSTRG.2	Wbscr17	MSTRG.24917.1
MSTRG.2	Gm43166	ENSMUST00000202871
MSTRG.2	Wbscr17	MSTRG.24919.1
MSTRG.2	Ccdc28a	MSTRG.2492.1
MSTRG.24920		MSTRG.24920.1
MSTRG.2	Gm10051	ENSMUST00000145209
MSTRG.2	Gatsl2	ENSMUST00000016088;ENSMUST00000140240
MSTRG.2	Gatsl2	MSTRG.24923.1
MSTRG.2	Rcc1l	ENSMUST00000076228;ENSMUST00000126113;ENSMUST0000015
MSTRG.2	Rcc1l	MSTRG.24925.1
MSTRG.2	B230377	ENSMUST00000192921;ENSMUST00000192229
MSTRG.2	Auts2	ENSMUST00000161226;ENSMUST00000161804;ENSMUST00000182
MSTRG.2	Gm27266	ENSMUST00000184521
MSTRG.2	Gm42589	ENSMUST00000199097
MSTRG.2	Reps1	ENSMUST00000154718;ENSMUST00000155284;ENSMUST0000012
MSTRG.2	Gm42588	ENSMUST00000200538
MSTRG.2	2810432	ENSMUST00000198632
MSTRG.2	Gm42591	ENSMUST00000197249
MSTRG.2	Gm43483	ENSMUST00000196724
MSTRG.2	Auts2	MSTRG.24934.1
MSTRG.2	Gm42443	ENSMUST00000198084
MSTRG.2	Gm42442	ENSMUST00000197951
MSTRG.2	4930563	ENSMUST00000198640
MSTRG.2	C230071	ENSMUST00000196044
MSTRG.2	Gm42441	ENSMUST00000199778
MSTRG.2	Reps1	MSTRG.2494.1
MSTRG.2	Auts2	MSTRG.24940.1
MSTRG.2	Auts2	MSTRG.24941.1
MSTRG.2	Gm42440	ENSMUST00000197906
MSTRG.2	Gm42439	ENSMUST00000195931
MSTRG.2	Gm42438	ENSMUST00000197832
MSTRG.2	Gm42991	ENSMUST00000196468
MSTRG.2	Gm42992	ENSMUST00000197043
MSTRG.2	2610011	ENSMUST00000200128
MSTRG.2	Gm42993	ENSMUST00000196856
MSTRG.2	Gm42995	ENSMUST00000197047
MSTRG.2	Reps1	MSTRG.2495.1

MSTRG.2	Gm42996	ENSMUST00000196116
MSTRG.2	Auts2	MSTRG.24951.1
MSTRG.2	Gm42989	ENSMUST00000200646
MSTRG.2	Gm43019	ENSMUST00000196948
MSTRG.2	Gm43018	ENSMUST00000198075
MSTRG.2	Gm43187	ENSMUST00000200652
MSTRG.2	Auts2	MSTRG.24956.1
MSTRG.2	Gm43188	ENSMUST00000200180
MSTRG.2	Auts2	MSTRG.24958.1
MSTRG.2	Gm43186	ENSMUST00000198130
MSTRG.2	Ect2l	ENSMUST00000215232;ENSMUST00000208948;ENSMUST00000095
MSTRG.2	Auts2	MSTRG.24960.1
MSTRG.2	Gtf2ird	ENSMUST00000123941;ENSMUST00000016086;ENSMUST00000128
MSTRG.2	Gtf2ird	ENSMUST00000201098
MSTRG.2	Gm24139	ENSMUST00000122780
MSTRG.2	Ncf1	ENSMUST00000146354;ENSMUST00000016094;ENSMUST00000111
MSTRG.2	Gtf2i;G	ENSMUST00000059042;ENSMUST00000082057;ENSMUST00000111
MSTRG.2	Gm43481	ENSMUST00000201715
MSTRG.2	Clip2	ENSMUST00000036999;ENSMUST00000100647;ENSMUST00000202
MSTRG.2	2700029	ENSMUST00000202895
MSTRG.2	Gm42884	ENSMUST00000200729
MSTRG.2	Nhsl1	ENSMUST00000207038;ENSMUST00000159299;ENSMUST00000161
MSTRG.2	Syna	ENSMUST00000149604
MSTRG.2	Eif4h	MSTRG.24971.1;ENSMUST00000202622;MSTRG.24971.4;MSTRG.
MSTRG.2	Gtf2ird	ENSMUST00000111244;ENSMUST00000171794;ENSMUST00000073
MSTRG.2	Gtf2ird	MSTRG.24973.1
MSTRG.2	n-R5s17	ENSMUST00000104249
MSTRG.2	Gtf2ird	MSTRG.24975.1
MSTRG.2	Gtf2ird	MSTRG.24977.1;MSTRG.24977.2
MSTRG.2	Gm26340	ENSMUST00000102365
MSTRG.2	Nhsl1	MSTRG.2498.1
MSTRG.2	Gtf2ird	MSTRG.24980.1
MSTRG.2	Gm42882	ENSMUST00000201788
MSTRG.2	Limk1	ENSMUST00000015137;ENSMUST00000111233;ENSMUST00000134
MSTRG.2	Gm10369	ENSMUST00000100641
MSTRG.2	Rfc2	ENSMUST00000202761;ENSMUST00000023867;ENSMUST00000201
MSTRG.2	Lat2	ENSMUST00000077636;ENSMUST00000036362;ENSMUST00000200
MSTRG.24986		MSTRG.24986.1
MSTRG.2	Cldn4	ENSMUST00000051401
MSTRG.2	9030607	ENSMUST00000202228
MSTRG.2	Mettl27	ENSMUST00000047196;ENSMUST00000111219;ENSMUST00000111
MSTRG.2	AC15356	ENSMUST00000213578
MSTRG.2	Mettl27	MSTRG.24990.1
MSTRG.2	Cldn3	ENSMUST00000094245
MSTRG.2	Wbscr25	ENSMUST00000148729
MSTRG.2	Rps29-p	ENSMUST00000122083
MSTRG.2	Gm43372	ENSMUST00000202662
MSTRG.2	Abhd11	ENSMUST00000111216;ENSMUST00000046999;ENSMUST00000201
MSTRG.2	Abhd11o	ENSMUST00000136022;ENSMUST00000154060;ENSMUST00000222
MSTRG.2	Stx1a	ENSMUST00000005509;ENSMUST00000201008;ENSMUST00000150
MSTRG.2	Stx1a	MSTRG.24998.1
MSTRG.2	Stx1a	MSTRG.24999.1
MSTRG.2	Gm19026	MSTRG.25.1
MSTRG.2	Gm37224	ENSMUST00000192878
MSTRG.2	Nhsl1	MSTRG.2500.1
MSTRG.2	Stx1a	MSTRG.25000.1
MSTRG.2	Wbscr22	ENSMUST00000129691;MSTRG.25001.2;ENSMUST00000085984;E

MSTRG.2	Dnajc30	ENSMUST00000071263;MSTRG.25002.1
MSTRG.2	Tbl2	ENSMUST00000153183;ENSMUST00000139565;ENSMUST00000005
MSTRG.2	Tbl2	MSTRG.25004.1
MSTRG.2	Bcl7b	ENSMUST00000031692;ENSMUST00000111187;ENSMUST00000111
MSTRG.2	Baz1b;G	MSTRG.25006.2;MSTRG.25006.3;ENSMUST00000002825;MSTRG.
MSTRG.2	Trim50	ENSMUST00000065785
MSTRG.2	Nsun5	ENSMUST00000000940;ENSMUST00000127746;ENSMUST0000017C
MSTRG.2	Pom121	MSTRG.25009.1;MSTRG.25009.2;ENSMUST00000111171
MSTRG.2	Nhsl1	MSTRG.2501.1
MSTRG.2	Mir7033	ENSMUST00000184259
MSTRG.2	Rhbdd2	MSTRG.25011.1
MSTRG.2	Hip1	ENSMUST00000060311;ENSMUST00000202643;ENSMUST00000201
MSTRG.2	Gm42731	ENSMUST00000201839
MSTRG.2	Hip1	MSTRG.25014.1
MSTRG.25015		MSTRG.25015.1
MSTRG.25016		MSTRG.25016.1
MSTRG.2	Rhbdd2	ENSMUST00000043707;ENSMUST00000136748;ENSMUST0000013C
MSTRG.2	Perp	ENSMUST00000019998
MSTRG.2	Mdh2	MSTRG.25020.1
MSTRG.2	Gm43091	MSTRG.25021.1
MSTRG.2	Gm43091	ENSMUST00000198157
MSTRG.2	Gm43091	MSTRG.25023.1
MSTRG.2	Por	ENSMUST00000153500;ENSMUST00000150058;ENSMUST00000153
MSTRG.2	Gm16061	ENSMUST00000122167
MSTRG.2	Por	MSTRG.25026.1
MSTRG.2	Tmem120	ENSMUST00000043378;ENSMUST00000141779;ENSMUST00000199
MSTRG.2	Mir7034	ENSMUST00000184682
MSTRG.2	Tmem120	MSTRG.25029.1
MSTRG.2	Tnfaip3	ENSMUST00000019997;ENSMUST00000105527;ENSMUST00000154
MSTRG.2	Hspb1	ENSMUST00000005077;ENSMUST00000111155
MSTRG.2	Styxl1	ENSMUST00000053906;ENSMUST00000178515;ENSMUST00000111
MSTRG.2	Mdh2	ENSMUST00000019323;ENSMUST00000197909;MSTRG.25032.2;E
MSTRG.2	Upk3b	ENSMUST00000062606
MSTRG.2	Ywhag	ENSMUST00000198270;ENSMUST00000055808;MSTRG.25034.3;M
MSTRG.2	Dtx2	MSTRG.25035.1;ENSMUST00000111142;ENSMUST00000111145;E
MSTRG.2	Ssc4d	ENSMUST00000111153;ENSMUST00000111152;ENSMUST00000054
MSTRG.2	Zp3	ENSMUST00000005073
MSTRG.2	Dtx2	MSTRG.25038.1
MSTRG.2	Rasa4;M	ENSMUST00000042135;ENSMUST00000134509;ENSMUST00000125
MSTRG.2	AC15862	ENSMUST00000216041;ENSMUST00000215762;ENSMUST00000216
MSTRG.2	Gm22214	ENSMUST00000157563
MSTRG.2	Gm15701	ENSMUST00000129559
MSTRG.2	Rasa4	MSTRG.25042.1
MSTRG.2	Orai2	ENSMUST00000041048;ENSMUST00000196454;MSTRG.25043.3;M
MSTRG.2	Orai2	MSTRG.25044.1
MSTRG.2	Polr2j	ENSMUST00000111127;ENSMUST00000041366;MSTRG.25045.1;E
MSTRG.2	Gm43604	ENSMUST00000199799
MSTRG.2	Lrwd1	MSTRG.25047.2;MSTRG.25047.1;ENSMUST00000006301;ENSMUS
MSTRG.2	Alkbh4	ENSMUST00000136634;ENSMUST00000041100;MSTRG.25048.3;E
MSTRG.25049		MSTRG.25049.1
MSTRG.2	AC15862	MSTRG.2505.1
MSTRG.2	Gm5050	ENSMUST00000197164
MSTRG.2	Prkrip1	ENSMUST00000151786;ENSMUST00000123921;ENSMUST00000149
MSTRG.2	Prkrip1	MSTRG.25052.1
MSTRG.25053		MSTRG.25053.1
MSTRG.25054		MSTRG.25054.1
MSTRG.25055		MSTRG.25055.1

MSTRG.25056		MSTRG.25056.1
MSTRG.2	Ift22	ENSMUST00000200157;ENSMUST00000199101;ENSMUST0000020[illegible]
MSTRG.2	Ift22	MSTRG.25059.1
MSTRG.2	AC15862	MSTRG.2506.1
MSTRG.2	4933404[illegible]	ENSMUST00000181045;ENSMUST00000199264;ENSMUST00000197
MSTRG.2	Fis1	ENSMUST00000111094;ENSMUST00000111097;ENSMUST0000001[illegible]
MSTRG.2	Fis1	MSTRG.25062.1
MSTRG.2	Cldn15	ENSMUST00000111093;ENSMUST00000001790;ENSMUST00000128
MSTRG.2	Sh2b2	ENSMUST00000196397;ENSMUST00000005188;ENSMUST00000197
MSTRG.2	Cux1;A4	ENSMUST00000175998;MSTRG.25065.1;MSTRG.25065.2;ENSMUS
MSTRG.2	Gm16599	ENSMUST00000148082;MSTRG.25066.3;MSTRG.25066.2;MSTRG.
MSTRG.2	Cux1	MSTRG.25067.1
MSTRG.2	Cux1	MSTRG.25068.1
MSTRG.2	Cux1	MSTRG.25069.1
MSTRG.2	AC16182	ENSMUST00000215977
MSTRG.2	Cux1	MSTRG.25070.1;MSTRG.25070.2
MSTRG.2	Gm38082	ENSMUST00000192800
MSTRG.2	Cux1	MSTRG.25072.1
MSTRG.2	Cux1	MSTRG.25073.1;MSTRG.25073.2
MSTRG.2	Znhit1	ENSMUST00000156963;ENSMUST00000085941;ENSMUST00000034
MSTRG.2	Plod3;M	ENSMUST00000004968;ENSMUST00000129896;ENSMUST00000144
MSTRG.2	Vgf	ENSMUST00000190827;ENSMUST00000041543;ENSMUST00000186
MSTRG.2	Ap1s1	ENSMUST00000111080;ENSMUST00000154183;ENSMUST0000015[illegible]
MSTRG.2	Serpine	ENSMUST00000041388;ENSMUST00000199832
MSTRG.2	Trim56	ENSMUST00000054384;MSTRG.25079.1;MSTRG.25079.3;ENSMUS
MSTRG.2	AC16182	ENSMUST00000213234
MSTRG.2	Muc3a	ENSMUST00000196391;ENSMUST00000179412
MSTRG.2	Ephb4	ENSMUST00000061244;ENSMUST00000144296;ENSMUST00000111
MSTRG.2	Zan	ENSMUST00000117564;ENSMUST00000197503;ENSMUST0000015[illegible]
MSTRG.2	Gm42456	MSTRG.25084.1
MSTRG.2	Gm42456	ENSMUST00000196840
MSTRG.2	Ache;Mi	MSTRG.25086.1;MSTRG.25086.3;MSTRG.25086.4;ENSMUST000[illegible]
MSTRG.2	Srrt;Mi	ENSMUST00000040873;ENSMUST00000199243;ENSMUST00000197
MSTRG.2	Srrt	MSTRG.25088.1
MSTRG.2	Gm43013	ENSMUST00000198216
MSTRG.2	Ifngr1	ENSMUST00000020188;ENSMUST00000110989;MSTRG.2509.2;E[illegible]
MSTRG.2	Trip6	ENSMUST00000024119;ENSMUST00000199121;ENSMUST00000197
MSTRG.2	Slc12a9	ENSMUST00000039991;ENSMUST00000141517;ENSMUST0000014[illegible]
MSTRG.2	Slc12a9	MSTRG.25092.1
MSTRG.2	Gm43012	ENSMUST00000198569
MSTRG.2	Gnb2;Mi	ENSMUST00000031726;ENSMUST00000167225;ENSMUST0000014[illegible]
MSTRG.2	Pop7	ENSMUST00000111035;ENSMUST00000031728
MSTRG.2	9130604[illegible]	ENSMUST00000197965
MSTRG.2	Gm8066;[illegible]	ENSMUST00000197762;MSTRG.25097.1;ENSMUST00000197624;[illegible]
MSTRG.2	Gm17112	ENSMUST00000163194
MSTRG.2	Gm15498	ENSMUST00000126693
MSTRG.2	Gm37845	ENSMUST00000194977
MSTRG.2	Pex7	ENSMUST00000020182;ENSMUST00000125942;ENSMUST0000016[illegible]
MSTRG.2	Gm15498	MSTRG.25100.1
MSTRG.25101		MSTRG.25101.1
MSTRG.2	Gm15753	ENSMUST00000119241
MSTRG.2	Nyap1	ENSMUST00000118326;MSTRG.25103.2;ENSMUST00000061789;[illegible]
MSTRG.2	Tsc22d4	ENSMUST00000100540;ENSMUST00000129349;MSTRG.25104.2;[illegible]
MSTRG.2	Mospd3	ENSMUST00000111007;ENSMUST00000037620;MSTRG.25105.1;[illegible]
MSTRG.2	Mospd3	MSTRG.25106.1
MSTRG.2	Gm7285	MSTRG.25107.1;MSTRG.25107.2;ENSMUST00000198801
MSTRG.2	Pcolce;[illegible]	ENSMUST00000128755;ENSMUST00000031731;ENSMUST0000014[illegible]

MSTRG.2	Gm36266	MSTRG.25109.1;MSTRG.25109.2;ENSMUST00000196660
MSTRG.2	Pex7	MSTRG.2511.1
MSTRG.2	Gm16089	ENSMUST00000117679
MSTRG.2	Lrch4;G	ENSMUST00000031734;ENSMUST00000176988;ENSMUST00000177
MSTRG.2	Irs3	ENSMUST00000196511;ENSMUST00000057314
MSTRG.2	Agfg2	MSTRG.25113.1
MSTRG.2	Agfg2	ENSMUST00000031736;ENSMUST00000100544;ENSMUST00000147
MSTRG.2	Agfg2	MSTRG.25115.1
MSTRG.2	Agfg2	MSTRG.25116.1
MSTRG.2	Agfg2	MSTRG.25117.1
MSTRG.25118		MSTRG.25118.1
MSTRG.2	Ppp1r35	ENSMUST00000126126;ENSMUST00000031739;MSTRG.25119.2;E
MSTRG.2	AC17127	ENSMUST00000216902
MSTRG.2	Mepce	MSTRG.25120.1;ENSMUST00000031740;MSTRG.25120.2;ENSMUS
MSTRG.2	Zcwpw1	ENSMUST00000035852;ENSMUST00000200505;ENSMUST00000127
MSTRG.2	Pilra	ENSMUST00000058897;ENSMUST00000130460;ENSMUST0000011C
MSTRG.25123		MSTRG.25123.1
MSTRG.2	Zkscan1	ENSMUST00000019660;ENSMUST00000066617;MSTRG.25124.3;E
MSTRG.2	Zkscan1	MSTRG.25125.1
MSTRG.2	Zkscan1	MSTRG.25126.1
MSTRG.2	Zkscan1	MSTRG.25127.1
MSTRG.2	Zscan21	ENSMUST00000062350;ENSMUST00000110961;ENSMUST0000008C
MSTRG.2	Zscan21	MSTRG.25129.1
MSTRG.2	Pex7	MSTRG.2513.1
MSTRG.2	Zfp113	ENSMUST00000049393;ENSMUST00000165640;ENSMUST00000144
MSTRG.2	Lamtor4	MSTRG.25131.1;ENSMUST00000062067;ENSMUST00000160157
MSTRG.2	Cops6	ENSMUST00000019638;ENSMUST00000110951;ENSMUST00000127
MSTRG.2	Mcm7;Mi	ENSMUST00000000505;ENSMUST00000125316;MSTRG.25133.2;M
MSTRG.2	Ap4m1	ENSMUST00000019662;ENSMUST00000151318;ENSMUST00000157
MSTRG.2	Taf6	ENSMUST00000048698;ENSMUST00000110936;ENSMUST0000011C
MSTRG.2	Cnpy4	ENSMUST00000110934;MSTRG.25136.2;MSTRG.25136.3;ENSMUS
MSTRG.2	Mblac1	ENSMUST00000057773
MSTRG.2	Mblac1	MSTRG.25138.1
MSTRG.2	Pvrig	ENSMUST00000211088
MSTRG.2	Map3k5	ENSMUST00000095806;ENSMUST00000120259;ENSMUST0000012S
MSTRG.25140		MSTRG.25140.1
MSTRG.2	Pvrig-p	ENSMUST00000199472
MSTRG.2	Gm10874	ENSMUST00000100526
MSTRG.2	Got2-ps	ENSMUST00000121655
MSTRG.2	Gm25525	ENSMUST00000177701
MSTRG.2	AC15758	ENSMUST00000218470
MSTRG.2	Gm5564	ENSMUST00000034916
MSTRG.2	Zfp157	ENSMUST00000085856;ENSMUST00000137711;ENSMUST00000134
MSTRG.2	Zfp157	MSTRG.25148.1
MSTRG.2	BC03703	ENSMUST00000048421;ENSMUST00000159649;ENSMUST0000015S
MSTRG.2	Map3k5	MSTRG.2515.1
MSTRG.2	6330418	ENSMUST00000124298;ENSMUST00000141454;ENSMUST00000125
MSTRG.2	Gal3st4	ENSMUST00000161647;ENSMUST00000100530;ENSMUST00000161
MSTRG.2	Stag3	ENSMUST00000160729;ENSMUST00000161113;ENSMUST0000015S
MSTRG.2	A430033	ENSMUST00000200521;ENSMUST00000198958;ENSMUST0000006S
MSTRG.2	Zfp68	ENSMUST00000110905;ENSMUST00000085852;ENSMUST00000197
MSTRG.2	Gm15497	ENSMUST00000119137
MSTRG.2	Gm42815	ENSMUST00000198787
MSTRG.2	Pdgfa	ENSMUST00000076095;ENSMUST00000046901;ENSMUST0000011C
MSTRG.2	Pdgfa	MSTRG.25159.1
MSTRG.2	Map3k5	MSTRG.2516.1
MSTRG.2	6330403	ENSMUST00000187923

MSTRG.2	Prkar1b	ENSMUST00000026973;ENSMUST00000110890;ENSMUST0000013[illegible]
MSTRG.2	Dnaaf5	ENSMUST00000026975;ENSMUST00000127542
MSTRG.2	Cox19	ENSMUST00000049630;ENSMUST00000197520;ENSMUST0000014[illegible]
MSTRG.2	Sun1	ENSMUST00000058716;ENSMUST00000110883;ENSMUST0000011[illegible]
MSTRG.2	4930520[illegible]	MSTRG.25165.1
MSTRG.2	Get4	ENSMUST00000026976;ENSMUST00000130326;ENSMUST0000013[illegible]
MSTRG.2	Zfand2a	ENSMUST00000079996;ENSMUST00000110851;ENSMUST00000132
MSTRG.2	3110082	ENSMUST00000066052;ENSMUST00000198474;ENSMUST0000019[illegible]
MSTRG.2	Mir339	ENSMUST00000083659
MSTRG.2	Map3k5	MSTRG.2517.1
MSTRG.2	3110082	MSTRG.25170.1
MSTRG.2	Gpr146	ENSMUST00000188655;ENSMUST00000137254;ENSMUST0000013[illegible]
MSTRG.2	Gpr146	MSTRG.25172.1
MSTRG.2	C130050[illegible]	ENSMUST00000163940;ENSMUST00000052176;ENSMUST00000164
MSTRG.2	Gper1	ENSMUST00000066211;ENSMUST00000173364
MSTRG.2	Gm26285	ENSMUST00000177923
MSTRG.2	Gm42424	ENSMUST00000198319
MSTRG.2	Micall2	ENSMUST00000170773;ENSMUST00000044642;ENSMUST00000164
MSTRG.2	Ints1;M	ENSMUST00000072607;ENSMUST00000198615;ENSMUST0000020[illegible]
MSTRG.2	Mafk	ENSMUST00000018287;ENSMUST00000151577;ENSMUST0000011[illegible]
MSTRG.2	Map3k5	MSTRG.2518.1
MSTRG.2	Tmem184[illegible]	ENSMUST00000110832;ENSMUST00000044002;ENSMUST00000132
MSTRG.25181		MSTRG.25181.1
MSTRG.2	Snx8	ENSMUST00000197880;ENSMUST00000196130;ENSMUST00000031
MSTRG.2	Snx8	MSTRG.25183.1
MSTRG.2	Snx8	MSTRG.25184.1
MSTRG.2	Snx8	MSTRG.25185.1
MSTRG.2	Eif3b	ENSMUST00000100507;MSTRG.25186.1;ENSMUST00000197632;[illegible]
MSTRG.2	Mir7038	ENSMUST00000184833
MSTRG.2	Chst12	ENSMUST00000043050;ENSMUST00000124142
MSTRG.25189		MSTRG.25189.1
MSTRG.2	Map3k5	MSTRG.2519.1
MSTRG.2	Mad1l1;[illegible]	MSTRG.25190.1;ENSMUST00000110829;ENSMUST00000031534;[illegible]
MSTRG.25191		MSTRG.25191.1
MSTRG.2	Mad1l1	MSTRG.25192.1
MSTRG.2	Gm43702	MSTRG.25193.1
MSTRG.2	Mad1l1	MSTRG.25194.2;MSTRG.25194.3;MSTRG.25194.1;MSTRG.25194
MSTRG.2	Gm16122	ENSMUST00000142625
MSTRG.2	Gm16121	MSTRG.25196.1;ENSMUST00000128520
MSTRG.2	Gm16120	MSTRG.25197.1;MSTRG.25197.2;MSTRG.25197.3;MSTRG.25197
MSTRG.2	Mad1l1	MSTRG.25198.1
MSTRG.2	Gm43338	ENSMUST00000198617
MSTRG.2	Gm38336	ENSMUST00000193040
MSTRG.2	Map7	ENSMUST00000020173;ENSMUST00000116259;ENSMUST00000214
MSTRG.2	Nudt1	ENSMUST00000198660;ENSMUST00000071881;ENSMUST0000005[illegible]
MSTRG.2	Nudt1	MSTRG.25201.1
MSTRG.2	Mrm2	ENSMUST00000031536
MSTRG.2	Lfng	ENSMUST00000031555;MSTRG.25203.1;ENSMUST00000200626;[illegible]
MSTRG.2	Lfng	MSTRG.25204.1
MSTRG.2	Lfng	MSTRG.25205.1
MSTRG.2	Ttyh3	MSTRG.25206.2;MSTRG.25206.1;ENSMUST00000197452;ENSMUS
MSTRG.25207		MSTRG.25207.1
MSTRG.2	Iqce	ENSMUST00000041783;ENSMUST00000147839;MSTRG.25208.2;[illegible]
MSTRG.2	Brat1	ENSMUST00000041588;ENSMUST00000153440;ENSMUST0000011[illegible]
MSTRG.2	Map7	MSTRG.2521.1
MSTRG.2	Amz1	ENSMUST00000126975;ENSMUST00000060918;ENSMUST00000177
MSTRG.2	Amz1	MSTRG.25211.1

MSTRG.2	Amz1	MSTRG.25212.1
MSTRG.2	Gna12	ENSMUST00000000153;ENSMUST00000198447
MSTRG.2	Gna12	MSTRG.25214.1
MSTRG.2	Card11	ENSMUST00000085786;ENSMUST00000196169;MSTRG.25215.2;M
MSTRG.2	Card11	MSTRG.25216.1
MSTRG.2	Card11	MSTRG.25217.1
MSTRG.2	Card11	MSTRG.25218.1
MSTRG.2	Card11	MSTRG.25219.1;MSTRG.25219.2;MSTRG.25219.3
MSTRG.2	Map7	MSTRG.2522.1
MSTRG.2	Card11	MSTRG.25220.1;MSTRG.25220.2;MSTRG.25220.3
MSTRG.2	Sdk1	ENSMUST00000085774;ENSMUST00000129831;ENSMUST00000145
MSTRG.2	Gm6433	ENSMUST00000118634
MSTRG.2	Gm43708	ENSMUST00000198857
MSTRG.2	Gm43556	ENSMUST00000196186
MSTRG.2	Gm43557	ENSMUST00000197100
MSTRG.2	Sdk1	MSTRG.25226.1
MSTRG.2	Gm16035	ENSMUST00000153879
MSTRG.2	Gm24131	ENSMUST00000158539
MSTRG.2	Gm16036	ENSMUST00000150385
MSTRG.2	AC15862	ENSMUST00000214323
MSTRG.2	Foxk1	ENSMUST00000198422;ENSMUST00000072837;MSTRG.25230.2
MSTRG.2	Ap5z1	ENSMUST00000198967;ENSMUST00000198135;ENSMUST00000197
MSTRG.2	Wipi2	ENSMUST00000197864;ENSMUST00000036872;ENSMUST00000153
MSTRG.2	Wipi2	MSTRG.25233.1
MSTRG.2	Actb	MSTRG.25234.1
MSTRG.2	Fbxl18	ENSMUST00000110766;MSTRG.25235.2;ENSMUST00000199067;E
MSTRG.2	D430018	ENSMUST00000181842
MSTRG.2	Fbxl18	MSTRG.25237.1
MSTRG.2	Tnrc18	ENSMUST00000152247;MSTRG.25238.2;MSTRG.25238.3;MSTRG.
MSTRG.2	Actb	ENSMUST00000100497;ENSMUST00000196997;ENSMUST00000167
MSTRG.2	AC15862	ENSMUST00000216127
MSTRG.2	Actb	MSTRG.25240.1
MSTRG.2	Fscn1	ENSMUST00000031565;ENSMUST00000198017;ENSMUST00000129
MSTRG.2	Rbak	ENSMUST00000049861;ENSMUST00000165318;ENSMUST00000166
MSTRG.2	Rnf216	ENSMUST00000053498;ENSMUST00000200607;ENSMUST0000020C
MSTRG.2	Rnf216	MSTRG.25244.1
MSTRG.2	Rnf216	MSTRG.25247.1
MSTRG.2	Gm43378	ENSMUST00000199336
MSTRG.2	Rnf216	MSTRG.25249.1
MSTRG.2	AC15384	ENSMUST00000217535;ENSMUST00000213372;ENSMUST00000215
MSTRG.2	Rnf216	MSTRG.25250.1
MSTRG.2	Rnf216	MSTRG.25252.1
MSTRG.2	Rnf216	MSTRG.25253.1
MSTRG.2	Zfp12	ENSMUST00000077485;ENSMUST00000032591;ENSMUST00000075
MSTRG.2	Zfp316	ENSMUST00000161448;ENSMUST00000197349;ENSMUST00000051
MSTRG.2	BC03034	ENSMUST00000199606
MSTRG.2	Grid2ip	ENSMUST00000120825;ENSMUST00000196148;ENSMUST00000138
MSTRG.2	Gm20635	ENSMUST00000176473
MSTRG.2	Gm42504	ENSMUST00000198312;ENSMUST00000197918
MSTRG.2	Mtfr2	ENSMUST00000169712;ENSMUST00000217608;ENSMUST00000213
MSTRG.2	Gm43398	ENSMUST00000196584
MSTRG.2	Kdelr2	ENSMUST00000110731;MSTRG.25261.2
MSTRG.2	E130309	ENSMUST00000159406;ENSMUST00000046418;ENSMUST0000016C
MSTRG.2	Gm42501	ENSMUST00000200220
MSTRG.2	Zdhhc4	ENSMUST00000001900;ENSMUST00000159813;ENSMUST00000161
MSTRG.2	0610040	ENSMUST00000160231;ENSMUST00000198455;ENSMUST00000162
MSTRG.2	Daglb	ENSMUST00000045593;ENSMUST00000174273;ENSMUST00000199

MSTRG.2	Rac1	ENSMUST00000080537;ENSMUST00000100489;MSTRG.25267.2;M
MSTRG.2	Gm42502	ENSMUST00000198550
MSTRG.2	Fam220a	ENSMUST00000067145;ENSMUST00000169329;ENSMUST0000020C
MSTRG.2	4933406	ENSMUST00000092680
MSTRG.2	Cyth3	ENSMUST00000116456;ENSMUST00000198355;ENSMUST00000177
MSTRG.2	Usp42	ENSMUST00000053287;ENSMUST00000141225;ENSMUST00000141
MSTRG.2	Usp42	MSTRG.25272.1
MSTRG.2	Ccz1	ENSMUST00000031621;ENSMUST00000200619;ENSMUST0000020C
MSTRG.2	Ocm	ENSMUST00000031622;ENSMUST00000085704;ENSMUST0000011C
MSTRG.2	Eif2ak1	ENSMUST00000100487;ENSMUST00000140013;MSTRG.25276.2;E
MSTRG.2	Ankrd61	ENSMUST00000079624;ENSMUST00000110718
MSTRG.2	Aimp2	ENSMUST00000031613;ENSMUST00000100483;ENSMUST00000128
MSTRG.2	Pms2;Gm	ENSMUST00000141942;ENSMUST00000148011;ENSMUST0000017C
MSTRG.2	Bclaf1	ENSMUST00000092678;ENSMUST00000186100;ENSMUST00000043
MSTRG.2	Gm17135	ENSMUST00000167009
MSTRG.2	Pms2	MSTRG.25281.1
MSTRG.2	Lmtk2	ENSMUST00000041804;MSTRG.25282.2
MSTRG.2	Gm26925	ENSMUST00000182470
MSTRG.2	Lmtk2	MSTRG.25284.1
MSTRG.2	Bhlha15	ENSMUST00000060747
MSTRG.2	Tecpr1	ENSMUST00000085701;MSTRG.25286.2;ENSMUST00000153103;M
MSTRG.2	Gm43267	ENSMUST00000199664
MSTRG.25288		MSTRG.25288.1
MSTRG.2	2900089	ENSMUST00000124015;ENSMUST00000153130
MSTRG.2	Pde7b	ENSMUST00000020165;ENSMUST00000169016;ENSMUST00000169
MSTRG.2	Bri3	ENSMUST00000110695;ENSMUST00000155491;ENSMUST00000056
MSTRG.2	Bri3	MSTRG.25291.1
MSTRG.2	Baiap2l	MSTRG.25292.1;ENSMUST00000055190;MSTRG.25292.3;ENSMUS
MSTRG.2	Gm15708	ENSMUST00000156467;ENSMUST00000198873
MSTRG.2	Smurf1	ENSMUST00000126822;ENSMUST00000110677;ENSMUST00000085
MSTRG.2	Mir7039	ENSMUST00000183852
MSTRG.2	Smurf1	MSTRG.25298.1
MSTRG.2	Smurf1	MSTRG.25299.1
MSTRG.2	Fam168b	ENSMUST00000167518;MSTRG.253.2;MSTRG.253.3;MSTRG.253.
MSTRG.2	Gm17229	ENSMUST00000097355;ENSMUST00000166792
MSTRG.2	Smurf1	MSTRG.25300.1
MSTRG.2	Trrap	ENSMUST00000197741;ENSMUST00000136379;MSTRG.25301.2;E
MSTRG.2	Gm23995	ENSMUST00000158060
MSTRG.2	Kpna7	ENSMUST00000110673;ENSMUST00000151196
MSTRG.2	Gm43563	ENSMUST00000196761
MSTRG.2	Gm43562	ENSMUST00000196439
MSTRG.2	Arpc1a	MSTRG.25308.1;ENSMUST00000031625;ENSMUST00000134835;M
MSTRG.2	1700018	ENSMUST00000031624
MSTRG.2	Gm17230	ENSMUST00000171194
MSTRG.2	Arpc1a	MSTRG.25310.1;MSTRG.25310.2
MSTRG.2	Zkscan1	ENSMUST00000031632;ENSMUST00000162220;ENSMUST00000198
MSTRG.2	Zkscan5	ENSMUST00000085671;ENSMUST00000031601;ENSMUST00000161
MSTRG.2	Zkscan5	MSTRG.25313.1
MSTRG.2	Zfp655	MSTRG.25314.1
MSTRG.25315		MSTRG.25315.1
MSTRG.2	Zfp655	ENSMUST00000200039;ENSMUST00000167316;ENSMUST00000196
MSTRG.2	Arpc1b	ENSMUST00000085679;ENSMUST00000129033;MSTRG.25317.2;E
MSTRG.2	Pdap1	ENSMUST00000031627;ENSMUST00000123111;ENSMUST00000143
MSTRG.2	Bud31	ENSMUST00000162594;ENSMUST00000160004;MSTRG.25319.2;E
MSTRG.2	Pde7b	MSTRG.2532.1
MSTRG.2	Ptcd1	MSTRG.25320.1;ENSMUST00000031628;ENSMUST00000161390
MSTRG.2	Cpsf4	ENSMUST00000160629;ENSMUST00000070487;ENSMUST0000016C

MSTRG.2	Atp5j2	ENSMUST00000161741;ENSMUST00000162360;MSTRG.25322.2;E
MSTRG.25323		MSTRG.25323.1
MSTRG.2	Cyp3a16	ENSMUST00000031633
MSTRG.2	Cyp3a16	MSTRG.25325.1
MSTRG.2	Zscan25	MSTRG.25326.1;ENSMUST00000200246;ENSMUST00000199563;E
MSTRG.2	Gm43625	ENSMUST00000199177
MSTRG.2	Rnf6	ENSMUST00000161859;ENSMUST00000169407;ENSMUST00000067
MSTRG.2	Cdk8;Gm	ENSMUST00000162494;ENSMUST00000161181;ENSMUST00000161
MSTRG.2	Pde7b	MSTRG.2533.1
MSTRG.2	Cdk8	MSTRG.25330.1
MSTRG.2	Gm8494	ENSMUST00000178171
MSTRG.2	Usp12	MSTRG.25332.1;ENSMUST00000085614;MSTRG.25332.4;ENSMUS
MSTRG.2	Rpl21	MSTRG.25333.1
MSTRG.2	Usp12	MSTRG.25334.1
MSTRG.2	1700041	ENSMUST00000197536
MSTRG.2	Rpl21;G	ENSMUST00000035983;MSTRG.25336.2;ENSMUST00000154768;E
MSTRG.2	Gtf3a	ENSMUST00000146511;ENSMUST00000133682;ENSMUST00000132
MSTRG.2	Mtif3	ENSMUST00000066675;ENSMUST00000016654;ENSMUST00000110
MSTRG.2	Lnx2	ENSMUST00000016664;ENSMUST00000123092;MSTRG.25339.3
MSTRG.2	Ahi1	ENSMUST00000105525;ENSMUST00000214745;ENSMUST00000214
MSTRG.2	Lnx2	MSTRG.25340.1
MSTRG.2	Lnx2	MSTRG.25341.1
MSTRG.2	Lnx2	MSTRG.25342.1
MSTRG.2	Polr1d	ENSMUST00000050970;ENSMUST00000110557;ENSMUST00000200
MSTRG.2	Rps10-p	ENSMUST00000120308
MSTRG.2	D5Ertd6	ENSMUST00000201128
MSTRG.2	Gm43461	ENSMUST00000202351
MSTRG.2	Gm43460	ENSMUST00000202491
MSTRG.25348		MSTRG.25348.1
MSTRG.2	Flt1	ENSMUST00000031653;ENSMUST00000125272;ENSMUST00000142
MSTRG.2	AC11763	ENSMUST00000215366
MSTRG.2	Flt1	MSTRG.25350.1
MSTRG.2	Pomp	ENSMUST00000031654;ENSMUST00000201376;ENSMUST00000202
MSTRG.2	Gm35648	ENSMUST00000202304
MSTRG.2	Slc46a3	ENSMUST00000118527;ENSMUST00000031655;ENSMUST00000152
MSTRG.2	Pan3	MSTRG.25354.1;ENSMUST00000031651;ENSMUST00000176600;E
MSTRG.2	Pan3	MSTRG.25355.1
MSTRG.2	Gm42883	ENSMUST00000202594
MSTRG.2	Pan3	MSTRG.25357.1
MSTRG.2	Mtus2	ENSMUST00000085558;ENSMUST00000149336;ENSMUST00000201
MSTRG.2	Gm10167	ENSMUST00000150411
MSTRG.2	AC11763	ENSMUST00000214691
MSTRG.2	Gm43553	ENSMUST00000200900
MSTRG.2	Mtus2	MSTRG.25361.1
MSTRG.2	Slc7a1	ENSMUST00000048116;MSTRG.25362.2;ENSMUST00000138257;E
MSTRG.2	Slc7a1	MSTRG.25363.1
MSTRG.2	Slc7a1	MSTRG.25364.1
MSTRG.2	Ubl3	ENSMUST00000079324;ENSMUST00000201595;ENSMUST00000164
MSTRG.2	2310047	ENSMUST00000189493;ENSMUST00000189639
MSTRG.2	Gm29264	ENSMUST00000191240
MSTRG.2	Gm29264	MSTRG.25368.1
MSTRG.2	Gm24105	ENSMUST00000174933
MSTRG.2	Ahi1	MSTRG.2537.1
MSTRG.2	Alox5ap	ENSMUST00000071130;ENSMUST00000200928;ENSMUST00000202
MSTRG.2	n-R5s18	ENSMUST00000082770
MSTRG.2	Gm19719	ENSMUST00000200934
MSTRG.2	Gm20488	MSTRG.25373.1;MSTRG.25373.2;MSTRG.25373.3;ENSMUST0000

MSTRG.2	BC02847	MSTRG.25374.1;MSTRG.25374.2;MSTRG.25374.3;MSTRG.25374
MSTRG.2	Gm43152	ENSMUST00000202495
MSTRG.2	Gm43151	ENSMUST00000201093
MSTRG.2	Gm43150	ENSMUST00000200931
MSTRG.2	B3glct	ENSMUST00000100404;ENSMUST00000202302;ENSMUST00000201
MSTRG.2	B3glct	MSTRG.25379.1
MSTRG.2	Myb	MSTRG.2538.1
MSTRG.2	B3glct	MSTRG.25380.1
MSTRG.2	Hsph1	ENSMUST00000201452;ENSMUST00000202361;ENSMUST00000074
MSTRG.2	Katnal1	ENSMUST00000047257;ENSMUST00000139269;ENSMUST0000011C
MSTRG.2	Gm15410	ENSMUST00000127763
MSTRG.2	8430423	ENSMUST00000110507
MSTRG.2	Gm15409	ENSMUST00000135240;MSTRG.25386.2
MSTRG.2	Gm42791	MSTRG.25387.1
MSTRG.2	Gm42791	MSTRG.25388.1
MSTRG.2	Hmgb1;5	MSTRG.25389.1;ENSMUST00000085546;ENSMUST00000110505;M
MSTRG.2	Myb	ENSMUST00000188495;MSTRG.2539.2;MSTRG.2539.3;ENSMUSTC
MSTRG.2	Gm15408	MSTRG.25390.1;ENSMUST00000124198
MSTRG.2	Hmgb1	MSTRG.25391.1
MSTRG.2	Hmgb1	MSTRG.25392.1
MSTRG.2	Hmgb1	MSTRG.25393.1
MSTRG.2	Gm42787	ENSMUST00000200879;MSTRG.25394.2
MSTRG.2	Gm42788	ENSMUST00000201604
MSTRG.2	Gm23362	ENSMUST00000175023
MSTRG.2	5730422	MSTRG.25397.1
MSTRG.2	5730422	MSTRG.25398.1
MSTRG.2	5730422	MSTRG.25399.1
MSTRG.2	Gm37127	ENSMUST00000192924
MSTRG.2	Gm26577	ENSMUST00000180680
MSTRG.2	Gm8615	ENSMUST00000201946;ENSMUST00000025307
MSTRG.2	5730422	MSTRG.25401.1
MSTRG.2	Hmgb1	MSTRG.25402.1
MSTRG.2	Gm43332	ENSMUST00000202511
MSTRG.2	Uspl1	ENSMUST00000121416;ENSMUST00000050472;ENSMUST00000122
MSTRG.2	Fry	ENSMUST00000200960;ENSMUST00000202530;ENSMUST00000203
MSTRG.2	Fry	MSTRG.25406.1
MSTRG.2	Fry	MSTRG.25407.1
MSTRG.2	Fry	MSTRG.25408.1
MSTRG.2	Fry	MSTRG.25409.1
MSTRG.2	AC15355	ENSMUST00000213692
MSTRG.2	Gm36378	ENSMUST00000201709
MSTRG.2	Gm42556	ENSMUST00000201397
MSTRG.2	Gm18753	ENSMUST00000201000
MSTRG.2	Brca2	ENSMUST00000200686;ENSMUST00000044620;ENSMUST00000202
MSTRG.2	Gm25284	ENSMUST00000180345
MSTRG.2	Gm23641	ENSMUST00000157857
MSTRG.2	Gm42529	ENSMUST00000201843
MSTRG.2	Gm42531	ENSMUST00000201648
MSTRG.2	N4bp2l1	ENSMUST00000016279;ENSMUST00000202031;MSTRG.25418.3;M
MSTRG.2	Gm43597	ENSMUST00000201992;ENSMUST00000201650;MSTRG.25419.2;E
MSTRG.2	AC15355	ENSMUST00000215633;ENSMUST00000216598
MSTRG.2	Pds5b	MSTRG.25420.1
MSTRG.2	Gm36447	ENSMUST00000201101
MSTRG.2	Gm43298	ENSMUST00000200887
MSTRG.2	Pds5b;G	ENSMUST00000202170;ENSMUST00000016569;ENSMUST00000038
MSTRG.2	Gm24630	ENSMUST00000122768
MSTRG.2	Gm26013	ENSMUST00000104404

MSTRG.2	Pds5b	MSTRG.25426.1
MSTRG.2	Stard13	ENSMUST00000110483;ENSMUST00000062015;ENSMUST00000202
MSTRG.2543		MSTRG.2543.1
MSTRG.2	1700028	ENSMUST00000181555;ENSMUST00000181114;ENSMUST00000202
MSTRG.2	Gm42906	MSTRG.25431.1
MSTRG.2	D730045	MSTRG.25432.1
MSTRG.2	AC12001	ENSMUST00000202147
MSTRG.2	Rfc3	ENSMUST00000038131;ENSMUST00000127366;ENSMUST00000156
MSTRG.2	Gm18222	MSTRG.25435.1
MSTRG.2	Gm8579	ENSMUST00000201943
MSTRG.2	Gm8579	MSTRG.25437.1
MSTRG.2	Gm18222	ENSMUST00000202416
MSTRG.2	Gm43196	ENSMUST00000201762
MSTRG.2	AC15355	ENSMUST00000217285
MSTRG.25440		MSTRG.25440.1
MSTRG.25441		MSTRG.25441.1
MSTRG.2	Gm20559	MSTRG.25442.1;ENSMUST00000201831;ENSMUST00000201493;E
MSTRG.2	Gm20559	MSTRG.25444.1
MSTRG.2	Samd9l	ENSMUST00000120087;ENSMUST00000201638
MSTRG.2	Samd9l	MSTRG.25446.1
MSTRG.2	Samd9l	MSTRG.25447.1;MSTRG.25447.2;MSTRG.25447.3
MSTRG.2	Samd9l	MSTRG.25448.1
MSTRG.2	Samd9l	MSTRG.25449.1
MSTRG.2545		MSTRG.2545.1
MSTRG.2	Hepacam	ENSMUST00000049985;ENSMUST00000201276;ENSMUST00000201
MSTRG.2	Vps50	ENSMUST00000001412;ENSMUST00000170873;ENSMUST00000164
MSTRG.2	Vps50	MSTRG.25452.1
MSTRG.2	Vps50	MSTRG.25453.1
MSTRG.2	Vps50	MSTRG.25454.1
MSTRG.2	Gm43294	ENSMUST00000202850
MSTRG.2	Bet1	ENSMUST00000049166;ENSMUST00000204806;ENSMUST0000015C
MSTRG.25457		MSTRG.25457.1
MSTRG.2	Col1a2	ENSMUST00000141483;ENSMUST00000031668;ENSMUST0000013E
MSTRG.2	Gm43921	ENSMUST00000204296
MSTRG.2	Hbs1l	ENSMUST00000020153;ENSMUST00000218032;ENSMUST00000061
MSTRG.2	Casd1	ENSMUST00000015333;ENSMUST00000181734;ENSMUST0000015З
MSTRG.2	Gm44432	ENSMUST00000203342
MSTRG.2	Gmfg-ps	ENSMUST00000121071
MSTRG.2	Gm44250	ENSMUST00000203092
MSTRG.2	Pon1	ENSMUST00000002663;ENSMUST00000176933
MSTRG.2	Pon3	ENSMUST00000129344;ENSMUST00000031773;ENSMUST0000012E
MSTRG.2	Gm44424	MSTRG.25466.1
MSTRG.2	Pon2	MSTRG.25467.1
MSTRG.2	Pon2	ENSMUST00000057792;MSTRG.25468.2;MSTRG.25468.3;MSTRG.
MSTRG.2	Gm44424	ENSMUST00000203438
MSTRG.2	AC15355	MSTRG.2547.1;MSTRG.2547.2;ENSMUST00000218108
MSTRG.2	Pon2	MSTRG.25470.1
MSTRG.2	Pdk4	ENSMUST00000019721;ENSMUST00000203347;ENSMUST00000134
MSTRG.2	Slc25a1	ENSMUST00000015256;MSTRG.25472.2;ENSMUST00000188414;E
MSTRG.2	Sem1	ENSMUST00000041111;MSTRG.25473.2;ENSMUST00000205180
MSTRG.2	Sem1	MSTRG.25474.1
MSTRG.25475		MSTRG.25475.1
MSTRG.2	Sdhaf3	ENSMUST00000040826;ENSMUST00000123848
MSTRG.2	Sdhaf3	MSTRG.25477.1
MSTRG.2	Sdhaf3	MSTRG.25478.1
MSTRG.2	Sdhaf3	MSTRG.25479.1
MSTRG.2	Hbs1l	MSTRG.2548.1

MSTRG.2	Sdhaf3	MSTRG.25480.1
MSTRG.2	Sdhaf3	MSTRG.25481.1
MSTRG.2	Asns	ENSMUST00000115542;ENSMUST00000031766;ENSMUST0000014C
MSTRG.2	Gm22276	ENSMUST00000122469
MSTRG.2	C1galt1	MSTRG.25484.2;ENSMUST00000040159;MSTRG.25484.1;ENSMUS
MSTRG.2	Gm43962	ENSMUST00000203898
MSTRG.2	Gm9825	ENSMUST00000178598
MSTRG.2	Mios	ENSMUST00000040017;ENSMUST00000160265;ENSMUST00000161
MSTRG.2	Mios	MSTRG.25488.1
MSTRG.25489		MSTRG.25489.1
MSTRG.2	Sgk1	ENSMUST00000120509;ENSMUST00000143094;ENSMUST00000124
MSTRG.2	Rpa3	ENSMUST00000012627;MSTRG.25490.2;ENSMUST00000159594
MSTRG.2	Umad1;Gı	ENSMUST00000159335;ENSMUST00000162034;ENSMUST0000016C
MSTRG.2	Umad1	MSTRG.25492.1
MSTRG.2	Umad1	MSTRG.25493.1
MSTRG.2	Gm16055	ENSMUST00000161366
MSTRG.2	Gm8428	ENSMUST00000159919
MSTRG.2	Gm16043	ENSMUST00000160030
MSTRG.2	Gm24059	ENSMUST00000157493
MSTRG.2	A430035	ENSMUST00000148063;ENSMUST00000124104;ENSMUST00000161
MSTRG.2	Glcci1	MSTRG.25499.1
MSTRG.2	Bend6	ENSMUST00000062289;ENSMUST00000195350;ENSMUST0000012€
MSTRG.2	Gm2539;	ENSMUST00000180720;MSTRG.2550.1;ENSMUST00000180781;MS
MSTRG.25501		MSTRG.25501.1
MSTRG.25502		MSTRG.25502.1
MSTRG.25503		MSTRG.25503.1
MSTRG.25504		MSTRG.25504.1
MSTRG.2	Ndufa4	ENSMUST00000204978;ENSMUST00000204084;ENSMUST0000020З
MSTRG.2	Phf14	ENSMUST00000155037;ENSMUST00000203459;ENSMUST0000011€
MSTRG.2	Phf14	MSTRG.25507.1
MSTRG.2	Phf14	MSTRG.25508.1
MSTRG.2	Phf14	MSTRG.25509.1;MSTRG.25509.2
MSTRG.2	Gm2539	MSTRG.2551.1
MSTRG.2	Phf14	MSTRG.25510.1
MSTRG.2	Phf14	MSTRG.25511.1
MSTRG.2	Phf14	MSTRG.25512.1
MSTRG.2	Gm6578	ENSMUST00000080891;ENSMUST00000203684
MSTRG.25514		MSTRG.25514.1
MSTRG.2	Tmem106	ENSMUST00000124234;ENSMUST00000142211;ENSMUST00000031
MSTRG.2	Tmem168	MSTRG.25516.1;ENSMUST00000031554;ENSMUST00000149123;E
MSTRG.2	Bmt2	ENSMUST00000045235;ENSMUST00000203078;MSTRG.25517.3
MSTRG.2	Bmt2	MSTRG.25518.1
MSTRG.2	Bmt2	MSTRG.25519.1
MSTRG.2	E030030	MSTRG.2552.1
MSTRG.2	Bmt2	MSTRG.25520.1
MSTRG.25521		MSTRG.25521.1
MSTRG.2	2610001	ENSMUST00000139231;ENSMUST00000155856;ENSMUST0000018€
MSTRG.2	1110019	ENSMUST00000181090;ENSMUST00000181225
MSTRG.2	Gm44412	ENSMUST00000203018
MSTRG.2	Gm44411	ENSMUST00000204172
MSTRG.2	Foxp2	ENSMUST00000115477;ENSMUST00000204869;ENSMUST0000013€
MSTRG.2	Foxp2	MSTRG.25527.1
MSTRG.2	Gm25470	ENSMUST00000175576
MSTRG.2	Mdfic	ENSMUST00000190255;ENSMUST00000189359;ENSMUST0000014C
MSTRG.2	Gm19791	MSTRG.2553.1
MSTRG.2	Mdfic	MSTRG.25530.1
MSTRG.2	Mdfic	MSTRG.25531.1

MSTRG.2	Mdfic	MSTRG.25532.1
MSTRG.2	Mdfic	MSTRG.25533.1
MSTRG.2	Mdfic	MSTRG.25534.1
MSTRG.2	Gm15473	ENSMUST00000140746
MSTRG.2	Gm4876	ENSMUST00000140709;ENSMUST00000139701;ENSMUST0000013б
MSTRG.2	Tes	ENSMUST00000115467;ENSMUST00000200690;ENSMUST00000154
MSTRG.2	E030030	ENSMUST00000179054;ENSMUST00000181677;ENSMUST00000181
MSTRG.2	Cav2	ENSMUST00000000058;ENSMUST00000115459;ENSMUST00000115
MSTRG.2	Cav1	ENSMUST00000007799;ENSMUST00000115456;ENSMUST00000115
MSTRG.2	Gm29591	ENSMUST00000188215
MSTRG.2	Met	ENSMUST00000115443;ENSMUST00000140070;ENSMUST00000115
MSTRG.2	Capza2	ENSMUST00000148694;ENSMUST00000015877;ENSMUST0000015б
MSTRG.2	St7;Gm2	ENSMUST00000129105;ENSMUST00000184623;ENSMUST0000018З
MSTRG.2	Gm27960	ENSMUST00000183445
MSTRG.2	Gm27365	ENSMUST00000184041
MSTRG.2	Gm27542	ENSMUST00000184184
MSTRG.2	Gm28008	ENSMUST00000184021
MSTRG.2	E030030	MSTRG.2555.1
MSTRG.2	St7	MSTRG.25550.1
MSTRG.2	Gm13898	ENSMUST00000141865
MSTRG.2	Gm13899	ENSMUST00000121519
MSTRG.2	St7	MSTRG.25553.1
MSTRG.2	Gm43533	ENSMUST00000202933
MSTRG.2	St7	MSTRG.25555.1
MSTRG.2	St7	MSTRG.25556.1
MSTRG.2	St7	MSTRG.25557.1
MSTRG.2	Gm42852	ENSMUST00000202649
MSTRG.2	St7	MSTRG.25559.1
MSTRG.2	Gm7678	ENSMUST00000181879
MSTRG.2	St7	MSTRG.25560.1
MSTRG.2	Gm26738	ENSMUST00000181312
MSTRG.2	St7	MSTRG.25562.1
MSTRG.2	Gm27402	ENSMUST00000183890
MSTRG.2	Gm43844	ENSMUST00000202926
MSTRG.25565		MSTRG.25565.1
MSTRG.2	Lsm8;Gm	MSTRG.25566.1;MSTRG.25566.2;ENSMUST00000180863;ENSMUS
MSTRG.2	Gm20186	MSTRG.25567.1;MSTRG.25567.2;ENSMUST00000201775;ENSMUS
MSTRG.25568		MSTRG.25568.1
MSTRG.2	Ing3	ENSMUST00000031680;ENSMUST00000144534;ENSMUST0000013б
MSTRG.2	E030030	MSTRG.2557.1
MSTRG.2	Ing3	MSTRG.25570.1;MSTRG.25570.2
MSTRG.2	Fam3c	ENSMUST00000165576;MSTRG.25571.2;ENSMUST00000163963;E
MSTRG.2	Fam3c	MSTRG.25572.1
MSTRG.2	Ptprz1	ENSMUST00000090568;ENSMUST00000202579;ENSMUST00000202
MSTRG.2	Ndufa5	ENSMUST00000132588;ENSMUST00000118558;ENSMUST0000002З
MSTRG.2	Cadps2	ENSMUST00000166458;ENSMUST00000115358;ENSMUST0000016З
MSTRG.2	Gm27959	ENSMUST00000185172
MSTRG.2	Gm24217	ENSMUST00000158260
MSTRG.2	Rnf133	ENSMUST00000063548;ENSMUST00000115354
MSTRG.2	Rnf148	ENSMUST00000104979
MSTRG.2	E030030	MSTRG.2558.1
MSTRG.2	Cadps2	MSTRG.25580.1
MSTRG.2	Wasl	MSTRG.25581.1;ENSMUST00000031695;ENSMUST00000201867;E
MSTRG.2	Gm42856	ENSMUST00000200848
MSTRG.2	Wasl	MSTRG.25583.1
MSTRG.25584		MSTRG.25584.1
MSTRG.25585		MSTRG.25585.1

MSTRG.2	Pot1a	ENSMUST00000115330;ENSMUST00000134339;ENSMUST0000016
MSTRG.2	Pot1a	MSTRG.25587.1
MSTRG.2	Grm8;Mi	ENSMUST00000115323;ENSMUST00000115324;ENSMUST0000009
MSTRG.2	Gm42801	ENSMUST00000202562
MSTRG.2559		MSTRG.2559.1
MSTRG.2	Gm26310	ENSMUST00000157766
MSTRG.2	Grm8	MSTRG.25591.1
MSTRG.2	Gm42547	ENSMUST00000201159
MSTRG.2	6530409	ENSMUST00000143099;ENSMUST00000143551
MSTRG.2	Gm42549	ENSMUST00000202392
MSTRG.2	Zfp800;	MSTRG.25595.1;ENSMUST00000202669;ENSMUST00000115320;E
MSTRG.2	6530409	MSTRG.25596.1
MSTRG.2	Gm5303	ENSMUST00000117943
MSTRG.2	Gcc1	ENSMUST00000090511;ENSMUST00000064377;ENSMUST0000016
MSTRG.2	Gcc1	MSTRG.25599.1
MSTRG.2	Gm37067	ENSMUST00000195730
MSTRG.2	Slc2a12	ENSMUST00000042261;ENSMUST00000159174
MSTRG.2	Arf5	ENSMUST00000020717;ENSMUST00000169841;ENSMUST00000202
MSTRG.2	Fscn3	ENSMUST00000031719
MSTRG.25602		MSTRG.25602.1
MSTRG.2	Snd1	ENSMUST00000168362;ENSMUST00000164915;ENSMUST00000001
MSTRG.2	Gm37321	ENSMUST00000195742
MSTRG.2	Gm37978	ENSMUST00000194736
MSTRG.2	Snd1	MSTRG.25606.1
MSTRG.2	Lrrc4	ENSMUST00000171353;ENSMUST00000062304
MSTRG.2	Gm37940	ENSMUST00000194243
MSTRG.2	Snd1	MSTRG.25609.1
MSTRG.2	Slc2a12	MSTRG.2561.1
MSTRG.2	Snd1	MSTRG.25610.1
MSTRG.2	Snd1	MSTRG.25611.1
MSTRG.2	Snd1	MSTRG.25612.1
MSTRG.2	Snd1	MSTRG.25613.1;MSTRG.25613.2
MSTRG.2	Rbm28	ENSMUST00000007993;ENSMUST00000169214;MSTRG.25614.2;E
MSTRG.2	Rbm28	MSTRG.25615.1
MSTRG.2	Gm24303	ENSMUST00000178237
MSTRG.2	Hilpda	ENSMUST00000115289;ENSMUST00000054445
MSTRG.2	Fam71f2	ENSMUST00000167131;ENSMUST00000115286
MSTRG.2	Calu	ENSMUST00000173216;ENSMUST00000172974;ENSMUST00000031
MSTRG.2	Tbpl1	ENSMUST00000127698;ENSMUST00000095794;ENSMUST00000141
MSTRG.2	Opn1sw	ENSMUST00000080428;ENSMUST00000147483;ENSMUST00000131
MSTRG.2	Impdh1	ENSMUST00000162739;ENSMUST00000160878;ENSMUST0000007
MSTRG.25622		MSTRG.25622.1
MSTRG.2	Atp6v1f	ENSMUST00000004396;ENSMUST00000143101;ENSMUST0000014
MSTRG.2	Irf5	ENSMUST00000004392;ENSMUST00000167252;ENSMUST00000164
MSTRG.2	Irf5	MSTRG.25625.1
MSTRG.2	Tnpo3	ENSMUST00000115251;ENSMUST00000012679;ENSMUST0000017
MSTRG.2	Gm43029	ENSMUST00000201829
MSTRG.2	Smo	ENSMUST00000201515;MSTRG.25628.2;ENSMUST00000001812
MSTRG.2	Gm13717	ENSMUST00000119868
MSTRG.2	Mir7663	ENSMUST00000183950
MSTRG.2	Ahcyl2	ENSMUST00000136031;MSTRG.25630.1;ENSMUST00000143707;M
MSTRG.2	Ahcyl2	MSTRG.25631.1
MSTRG.2	Ahcyl2	MSTRG.25632.1
MSTRG.2	Ahcyl2	MSTRG.25633.1
MSTRG.2	Ahcyl2	MSTRG.25634.1
MSTRG.2	4930528	ENSMUST00000201689
MSTRG.2	Strip2	ENSMUST00000151738;ENSMUST00000137068;ENSMUST0000011

MSTRG.2	Ube2h	MSTRG.25637.1;ENSMUST00000102993;ENSMUST00000128141;E
MSTRG.2	Ube2h	MSTRG.25638.1
MSTRG.2	Ube2h	MSTRG.25639.1
MSTRG.2	Gm4895	MSTRG.2564.1
MSTRG.2	Ube2h	MSTRG.25640.1
MSTRG.2	Nrf1	ENSMUST00000115212;ENSMUST00000123194;ENSMUST00000115
MSTRG.2	Nrf1	MSTRG.25642.1
MSTRG.2	Nrf1	MSTRG.25643.2;MSTRG.25643.1
MSTRG.2	Nrf1	MSTRG.25645.1
MSTRG.2	Gm25580	ENSMUST00000083409
MSTRG.2	Mir96	ENSMUST00000083652
MSTRG.2	Mir183	ENSMUST00000083685
MSTRG.2	Gm4895	ENSMUST00000181646
MSTRG.2	Rncr4	ENSMUST00000194541
MSTRG.2	Zc3hc1	ENSMUST00000080812;ENSMUST00000152391;ENSMUST00000202
MSTRG.2	n-R5s16	ENSMUST00000083728
MSTRG.2	Klhdc10	ENSMUST00000068240;ENSMUST00000068259;MSTRG.25653.3;M
MSTRG.2	Tmem209	ENSMUST00000151187;ENSMUST00000064330;ENSMUST00000138
MSTRG.2	Ssmem1	ENSMUST00000031797
MSTRG.2	Cpa1	ENSMUST00000031806;ENSMUST00000139004
MSTRG.2	Cep41	ENSMUST00000031810;ENSMUST00000140211;ENSMUST00000115
MSTRG.2	Cep41	MSTRG.25658.1
MSTRG.2	Cep41	MSTRG.25659.1
MSTRG.2	Raet1e;	MSTRG.2566.1;ENSMUST00000181645;ENSMUST00000105522;EN
MSTRG.2	Gm13782	ENSMUST00000149515
MSTRG.2	Mest	ENSMUST00000163949;ENSMUST00000124665;ENSMUST00000131
MSTRG.2	Gm44296	ENSMUST00000204689
MSTRG.2	Mir335	ENSMUST00000083567
MSTRG.2	Copg2	ENSMUST00000048774;ENSMUST00000166192;MSTRG.25666.3;E
MSTRG.2	Copg2	MSTRG.25667.1
MSTRG.2	Copg2	MSTRG.25668.1
MSTRG.2	4930412	ENSMUST00000139006
MSTRG.2	Gm26740	MSTRG.2567.1;MSTRG.2567.2;MSTRG.2567.3
MSTRG.2	Tsga13	ENSMUST00000048580
MSTRG.2	Lncpint	MSTRG.25671.1;MSTRG.25671.2;MSTRG.25671.3;MSTRG.25671
MSTRG.2	Mir29a	ENSMUST00000083676
MSTRG.2	Mir29b-	ENSMUST00000083670;ENSMUST00000183056
MSTRG.2	Gm13833	ENSMUST00000146766
MSTRG.2	5330406	ENSMUST00000195672
MSTRG.2	Lncpint	MSTRG.25676.1
MSTRG.2	Gm37728	ENSMUST00000195025
MSTRG.2	Lncpint	MSTRG.25678.1
MSTRG.2	2210408	ENSMUST00000144612;ENSMUST00000123149;ENSMUST00000155
MSTRG.2	Raet1e	MSTRG.2568.1;MSTRG.2568.2
MSTRG.2	2210408	MSTRG.25680.1
MSTRG.2	2210408	MSTRG.25681.1
MSTRG.2	Gm43800	ENSMUST00000201868
MSTRG.2	Podxl	ENSMUST00000026698;ENSMUST00000136877
MSTRG.2	Gm14532	ENSMUST00000156115
MSTRG.2	Mkln1os	ENSMUST00000144232;ENSMUST00000154820;ENSMUST00000139
MSTRG.2	Gm13844	ENSMUST00000134541
MSTRG.2	Mkln1	ENSMUST00000026699;ENSMUST00000134315;ENSMUST00000137
MSTRG.2	Mkln1	MSTRG.25688.1
MSTRG.2	Gm43154	ENSMUST00000209658
MSTRG.2	Gm26740	ENSMUST00000180456;MSTRG.2569.2;MSTRG.2569.3;ENSMUST0
MSTRG.2	Gm43293	ENSMUST00000202780
MSTRG.2	Gm13849	ENSMUST00000153328

MSTRG.2	Gm13849	MSTRG.25693.1
MSTRG.2	Gm13849	MSTRG.25694.1
MSTRG.2	Gm13849	MSTRG.25695.1
MSTRG.25696		MSTRG.25696.1
MSTRG.2	Chchd3;	ENSMUST00000129069;ENSMUST00000066379;ENSMUST00000124
MSTRG.2	Chchd3	MSTRG.25698.1
MSTRG.2	Chchd3	MSTRG.25699.1
MSTRG.2	Dst	MSTRG.257.1;ENSMUST00000183034;ENSMUST00000187486;ENS
MSTRG.2	Gm26581	ENSMUST00000181953
MSTRG.2	Chchd3	MSTRG.25700.1
MSTRG.2	Chchd3	MSTRG.25701.1
MSTRG.2	Chchd3	MSTRG.25702.1
MSTRG.2	Chchd3	MSTRG.25703.1
MSTRG.2	Chchd3	MSTRG.25704.1
MSTRG.2	Gm14540	ENSMUST00000138298
MSTRG.2	Exoc4	ENSMUST00000052266;ENSMUST00000137043;ENSMUST0000009C
MSTRG.2	Gm13854	ENSMUST00000121183
MSTRG.2	Gm43168	ENSMUST00000201039
MSTRG.2	Exoc4	MSTRG.25709.1
MSTRG.2	Gm1972	ENSMUST00000181606
MSTRG.2	Exoc4	MSTRG.25710.1
MSTRG.2	Exoc4	MSTRG.25711.1
MSTRG.2	Exoc4	MSTRG.25712.1;MSTRG.25712.2
MSTRG.2	Exoc4	MSTRG.25715.1
MSTRG.2	Gm43164	ENSMUST00000202529
MSTRG.2	Exoc4	MSTRG.25717.1
MSTRG.2	Exoc4	MSTRG.25718.1
MSTRG.2	Gm13856	ENSMUST00000126498
MSTRG.2	Hmgb1-p	ENSMUST00000181542
MSTRG.2	Slc35b4	ENSMUST00000019143;ENSMUST00000146514;MSTRG.25720.2;M
MSTRG.2	Gm14546	ENSMUST00000138275
MSTRG.2	Akr1b3	ENSMUST00000201392;ENSMUST00000142761;ENSMUST00000102
MSTRG.2	Akr1b10	ENSMUST00000038383;ENSMUST00000144384;ENSMUST00000115
MSTRG.25724		MSTRG.25724.1
MSTRG.2	Bpgm	ENSMUST00000045372;ENSMUST00000135743;MSTRG.25725.2;E
MSTRG.2	Cald1	ENSMUST00000079391;ENSMUST00000142512;ENSMUST00000155
MSTRG.2	Cald1	MSTRG.25727.1
MSTRG.2	Cald1	MSTRG.25728.1
MSTRG.2	Gm13861	ENSMUST00000137136
MSTRG.2	Gm26740	MSTRG.2573.1
MSTRG.2	Npn2	ENSMUST00000189275;ENSMUST00000075773
MSTRG.2	Gm14547	ENSMUST00000138341
MSTRG.2	Agbl3	ENSMUST00000202017;ENSMUST00000115016;ENSMUST00000135
MSTRG.2	Gm13864	ENSMUST00000119745
MSTRG.2	Gm13862	ENSMUST00000121220
MSTRG.2	Gm13863	ENSMUST00000118782
MSTRG.2	Gm43748	ENSMUST00000202329
MSTRG.2	Tmem140	ENSMUST00000123019;ENSMUST00000074949;ENSMUST00000202
MSTRG.2	Tmem140	MSTRG.25738.1
MSTRG.2	3110062I	ENSMUST00000055097;ENSMUST00000115007;ENSMUST00000115
MSTRG.2	Gm26528	ENSMUST00000181965
MSTRG.2	Wdr91	MSTRG.25740.1
MSTRG.2	Slc23a4	ENSMUST00000044387;ENSMUST00000201355;ENSMUST00000134
MSTRG.2	Slc23a4	MSTRG.25742.1
MSTRG.2	Cnot4	ENSMUST00000200954;MSTRG.25743.1;ENSMUST00000202143;E
MSTRG.2	Cnot4	MSTRG.25744.1
MSTRG.2	Cnot4	MSTRG.25745.1

MSTRG.2	Cnot4	MSTRG.25746.1
MSTRG.2	Cnot4	MSTRG.25747.1
MSTRG.2	Cnot4	MSTRG.25748.1
MSTRG.2	Cnot4	MSTRG.25749.1
MSTRG.2	H60b	MSTRG.2575.1
MSTRG.2	Cnot4	MSTRG.25750.1
MSTRG.2	1810058	ENSMUST00000152147;ENSMUST00000130875;ENSMUST0000013[illegible]
MSTRG.2	Nup205	ENSMUST00000201374;ENSMUST00000043815;ENSMUST00000202
MSTRG.2	Gm23435	ENSMUST00000157183
MSTRG.25754		MSTRG.25754.1
MSTRG.2	Mtpn	ENSMUST00000031866;ENSMUST00000201026;MSTRG.25755.2
MSTRG.2	Gm23053	ENSMUST00000175136
MSTRG.2	Gm43442	ENSMUST00000202865
MSTRG.2	Creb3l2	ENSMUST00000041093;ENSMUST00000128534;MSTRG.25758.3
MSTRG.2	Dgki	ENSMUST00000101532;ENSMUST00000090314;ENSMUST0000015[illegible]
MSTRG.2	Gm26740	MSTRG.2576.1
MSTRG.2	1700111	ENSMUST00000031863
MSTRG.2	Dgki	MSTRG.25761.1
MSTRG.2	Gm7452	ENSMUST00000200764;ENSMUST00000202891
MSTRG.2	Gm15487	ENSMUST00000122373
MSTRG.2	Ybx1-ps	MSTRG.25764.1
MSTRG.2	Ybx1-ps	ENSMUST00000201305
MSTRG.2	Trim24	ENSMUST00000153004;ENSMUST00000149828;MSTRG.25766.1;[illegible]
MSTRG.2	Trim24	MSTRG.25767.1
MSTRG.25768		MSTRG.25768.1
MSTRG.2	Svopl	MSTRG.25769.1
MSTRG.2	Gm5421	ENSMUST00000181698
MSTRG.2	Svopl	ENSMUST00000096040;ENSMUST00000200796
MSTRG.2	Zc3hav1	ENSMUST00000058524;MSTRG.25771.2
MSTRG.2	Atp6v0a	ENSMUST00000114908;ENSMUST00000135594;ENSMUST00000144
MSTRG.2	Tmem213	ENSMUST00000031851
MSTRG.2	Gm23231	ENSMUST00000157299
MSTRG.2	Zc3hav1	ENSMUST00000114900;MSTRG.25775.2;MSTRG.25775.3;ENSMUS
MSTRG.2	Ubn2	MSTRG.25776.1
MSTRG.2	Ubn2;Mi	ENSMUST00000160583;ENSMUST00000039127;ENSMUST00000162
MSTRG.2	Ubn2	MSTRG.25778.1
MSTRG.2	Ubn2	MSTRG.25779.1
MSTRG.2	Gm10825	ENSMUST00000180537
MSTRG.2	Ubn2	MSTRG.25780.1
MSTRG.2	Ubn2	MSTRG.25781.1
MSTRG.2	Fmc1	ENSMUST00000147651;ENSMUST00000019833
MSTRG.2	Gm15697	ENSMUST00000121946
MSTRG.2	Luc7l2	ENSMUST00000163047;ENSMUST00000161538;MSTRG.25784.2;[illegible]
MSTRG.2	Gm26699	ENSMUST00000180825
MSTRG.2	Luc7l2	MSTRG.25786.1
MSTRG.2	Klrg2	MSTRG.25787.1;ENSMUST00000202245
MSTRG.2	Rab19	ENSMUST00000031986
MSTRG.2	Rab19	MSTRG.25789.1
MSTRG.2	Gm16531	ENSMUST00000180658
MSTRG.2	Rab19	MSTRG.25790.1
MSTRG.2	Mkrn1	ENSMUST00000114823;ENSMUST00000031985;ENSMUST0000015[illegible]
MSTRG.2	Gm10244	ENSMUST00000156905;ENSMUST00000090237
MSTRG.25793		MSTRG.25793.1
MSTRG.25794		MSTRG.25794.1
MSTRG.2	Hipk2	ENSMUST00000161779;ENSMUST00000160962;ENSMUST00000162
MSTRG.2	Tbxas1	ENSMUST00000162521;ENSMUST00000003017;ENSMUST0000015[illegible]
MSTRG.2	Gm42962	ENSMUST00000201966

MSTRG.2	Gm23010	ENSMUST00000104151
MSTRG.2	1700025	ENSMUST00000186189
MSTRG.2	Gm37354	ENSMUST00000193333
MSTRG.2	Gm26585	ENSMUST00000180890
MSTRG.2	Parp12	ENSMUST00000038398;ENSMUST00000129916
MSTRG.2	4930599	ENSMUST00000146981
MSTRG.2	Gm42963	ENSMUST00000202943
MSTRG.2	Kdm7a	ENSMUST00000002305;ENSMUST00000127036;MSTRG.25803.3;M
MSTRG.2	Gm43479	ENSMUST00000200801
MSTRG.2	Gm26008	ENSMUST00000158797
MSTRG.2	Slc37a3	MSTRG.25806.1;MSTRG.25806.2;ENSMUST00000090243;ENSMUS
MSTRG.25807		MSTRG.25807.1
MSTRG.2	Slc37a3	MSTRG.25809.1
MSTRG.2581		MSTRG.2581.1
MSTRG.2	Mrps33	ENSMUST00000031978;ENSMUST00000114797;ENSMUST00000201
MSTRG.2	Adck2;G	ENSMUST00000140364;ENSMUST00000145788;MSTRG.25811.3;M
MSTRG.2	Gm16272	ENSMUST00000128218
MSTRG.2	Braf	ENSMUST00000002487;MSTRG.25813.2;ENSMUST00000101497;E
MSTRG.2	Fth-ps2	ENSMUST00000168364
MSTRG.2	Agk	ENSMUST00000031977;ENSMUST00000201342;ENSMUST00000201
MSTRG.2	Agk	MSTRG.25816.1
MSTRG.2	Agk	MSTRG.25817.1
MSTRG.2	Agk	MSTRG.25818.1
MSTRG.2	E330009	ENSMUST00000101492;ENSMUST00000039008;ENSMUST00000201
MSTRG.2582		MSTRG.2582.1
MSTRG.2	Ssbp1	ENSMUST00000114779;ENSMUST00000031971;ENSMUST00000201
MSTRG.2	Ssbp1	MSTRG.25821.1
MSTRG.2	Gm38276	ENSMUST00000192446
MSTRG.2	Trbv1	MSTRG.25823.1;ENSMUST00000103262
MSTRG.25824		MSTRG.25824.1
MSTRG.2	Trbv2;T	MSTRG.25825.1;MSTRG.25825.2;ENSMUST00000103263;ENSMUS
MSTRG.2	5830405	ENSMUST00000193357;MSTRG.25826.2;MSTRG.25826.3;ENSMUS
MSTRG.2	Trbv31	MSTRG.25827.1
MSTRG.2	Trbv31	ENSMUST00000103300;ENSMUST00000193003;MSTRG.25828.3;M
MSTRG.2	Trbv31;	MSTRG.25829.3;MSTRG.25829.2;MSTRG.25829.1;MSTRG.2582
MSTRG.2	Eya4	ENSMUST00000092665;ENSMUST00000074366;ENSMUST0000022C
MSTRG.2	Gm27549	ENSMUST00000183508
MSTRG.2	Trpv6	MSTRG.25832.1;ENSMUST00000031902;ENSMUST00000194405
MSTRG.25833		MSTRG.25833.1
MSTRG.25834		MSTRG.25834.1
MSTRG.25835		MSTRG.25835.1
MSTRG.2	Gm37610	ENSMUST00000191867;ENSMUST00000194020;ENSMUST0000016
MSTRG.2	Trbv9	ENSMUST00000194494
MSTRG.2	Trbv13-	MSTRG.25838.1;ENSMUST00000103267;MSTRG.25838.2;ENSMUS
MSTRG.2	Trbv11	ENSMUST00000194354
MSTRG.2	Eya4	MSTRG.2584.1
MSTRG.2	Trbv12-	MSTRG.25840.2;MSTRG.25840.3;MSTRG.25840.1;ENSMUST000C
MSTRG.2	Trbv12-	ENSMUST00000192675
MSTRG.2	Trbv13-	ENSMUST00000103271
MSTRG.2	Trbv14	ENSMUST00000103272
MSTRG.2	Trbv15	ENSMUST00000103273
MSTRG.2	Trbv16	ENSMUST00000103274
MSTRG.2	Trbv17	MSTRG.25846.1;ENSMUST00000103275;MSTRG.25846.2
MSTRG.2	Trbv18	ENSMUST00000194256
MSTRG.2	Trbv19	ENSMUST00000103276
MSTRG.2	Trbv20	MSTRG.25849.1;ENSMUST00000103277
MSTRG.2	Eya4	MSTRG.2585.1

MSTRG.25850		MSTRG.25850.1;MSTRG.25850.2
MSTRG.25851		MSTRG.25851.1
MSTRG.2	Trbv21	ENSMUST00000195087;ENSMUST00000103278
MSTRG.2	Trbv22	ENSMUST00000191697
MSTRG.2	Trbv23	ENSMUST00000193997
MSTRG.2	Trbv24	ENSMUST00000194614
MSTRG.2	Trbv25	ENSMUST00000194700
MSTRG.2	Gm2719	ENSMUST00000191929
MSTRG.2	Trbv28	ENSMUST00000192804
MSTRG.2	Gm24655	ENSMUST00000178790
MSTRG.2	Trbv29	ENSMUST00000103281
MSTRG.2	Trbv30	ENSMUST00000103282;ENSMUST00000191646
MSTRG.2	Try4	ENSMUST00000031913
MSTRG.2	Try5	ENSMUST00000064324
MSTRG.2	Prss3	ENSMUST00000096003
MSTRG.2	Gm5771	ENSMUST00000049079
MSTRG.2	Eya4	MSTRG.2587.1
MSTRG.2	Prss1	ENSMUST00000031910
MSTRG.2	Gm37301	ENSMUST00000195412
MSTRG.2	Gm29813	ENSMUST00000195856
MSTRG.2	Gm38018	ENSMUST00000195651
MSTRG.2	Prss2	ENSMUST00000070380
MSTRG.2	Trbj1-5	ENSMUST00000103288
MSTRG.2	Trbj1-6	ENSMUST00000103289
MSTRG.2	Trbj1-7	ENSMUST00000103290;ENSMUST00000193061
MSTRG.2	Eya4	MSTRG.2588.1
MSTRG.2	Trbc2	MSTRG.25880.1
MSTRG.2	Trbc2;T	MSTRG.25881.1;MSTRG.25881.2;MSTRG.25881.3;MSTRG.25881
MSTRG.2	Gstk1	ENSMUST00000031897;ENSMUST00000204088;ENSMUST00000203
MSTRG.2	Tmem139	ENSMUST00000095987
MSTRG.2	Casp2;Gı	ENSMUST00000031895;ENSMUST00000139930;MSTRG.25884.3;M
MSTRG.2	Tcaf2	ENSMUST00000031879;ENSMUST00000131341
MSTRG.2	Gm44284	ENSMUST00000203836
MSTRG.2	Zyx	ENSMUST00000203329;ENSMUST00000203846;ENSMUST00000203
MSTRG.2	Epha1	ENSMUST00000073387;ENSMUST00000204357;ENSMUST00000204
MSTRG.2	Eya4	MSTRG.2589.1
MSTRG.2	2010310	ENSMUST00000190889;ENSMUST00000187172
MSTRG.2	Tas2r14	ENSMUST00000057398
MSTRG.2	Tas2r13	ENSMUST00000070178
MSTRG.2	Tas2r12	ENSMUST00000059534
MSTRG.2	Olfr458	ENSMUST00000216650;ENSMUST00000090156
MSTRG.2	Olfr457	ENSMUST00000203396;ENSMUST00000170504
MSTRG.2	Olfr456	ENSMUST00000057251
MSTRG.2	Gm44731	MSTRG.25898.1
MSTRG.2	Gm44731	ENSMUST00000203086
MSTRG.2	Gm28631	ENSMUST00000190740
MSTRG.2	Eya4	MSTRG.2590.1
MSTRG.2	Arhgef5	ENSMUST00000031750;ENSMUST00000183227;ENSMUST00000183
MSTRG.2	Gm44253	ENSMUST00000203387
MSTRG.25902		MSTRG.25902.1
MSTRG.2	Tpk1	ENSMUST00000067888;ENSMUST00000203649;ENSMUST00000114
MSTRG.2	Gm15550	ENSMUST00000120824
MSTRG.2	Tpk1	MSTRG.25905.1
MSTRG.2	Tpk1	MSTRG.25906.1
MSTRG.2	Tpk1	MSTRG.25907.1
MSTRG.2	9430018	ENSMUST00000203260
MSTRG.2	Tpk1	MSTRG.25909.1

MSTRG.2	Eya4	MSTRG.2591.1
MSTRG.2	Tpk1	MSTRG.25910.1
MSTRG.2	Tpk1	MSTRG.25911.1
MSTRG.2	Cntnap2	ENSMUST00000114641;ENSMUST00000196561;ENSMUST0000019
MSTRG.2	B230112	ENSMUST00000203953
MSTRG.2	Gm43879	ENSMUST00000203811
MSTRG.2	Gm43872	ENSMUST00000203218
MSTRG.2	Gm43885	ENSMUST00000204928
MSTRG.2	Gm43889	ENSMUST00000203950
MSTRG.2	Gm43874	ENSMUST00000204853
MSTRG.2	Gm43869	ENSMUST00000204742
MSTRG.2	Rps12;S	ENSMUST00000218142;ENSMUST00000218107;ENSMUST0000022
MSTRG.2	6330419	ENSMUST00000203087
MSTRG.2	Gm43882	ENSMUST00000204871
MSTRG.2	Gm43884	ENSMUST00000204251
MSTRG.2	Gm43880	ENSMUST00000203635
MSTRG.2	Gm43876	ENSMUST00000203831
MSTRG.2	Gm43886	ENSMUST00000205039
MSTRG.2	Gm43871	ENSMUST00000204931
MSTRG.2	Gm43873	ENSMUST00000204505
MSTRG.2	Gm43877	ENSMUST00000205029
MSTRG.2	Stx7	ENSMUST00000218050;ENSMUST00000020174;ENSMUST0000022
MSTRG.2	Gm43888	ENSMUST00000204405
MSTRG.2	Gm43887	ENSMUST00000204727
MSTRG.2	Gm43870	ENSMUST00000205102
MSTRG.2	Gm43881	ENSMUST00000203051
MSTRG.2	Gm43875	ENSMUST00000203319
MSTRG.2	Cntnap2	MSTRG.25936.1
MSTRG.2	Rbpsuh-	ENSMUST00000182751
MSTRG.2	Stx7	MSTRG.2594.1
MSTRG.2	Gm43883	ENSMUST00000204002
MSTRG.2	Gm6352	ENSMUST00000203583
MSTRG.25942		MSTRG.25942.1
MSTRG.25943		MSTRG.25943.1
MSTRG.2	Gm44141	MSTRG.25944.1
MSTRG.2	Gm44141	ENSMUST00000204231
MSTRG.2	Gm25283	ENSMUST00000157272
MSTRG.25947		MSTRG.25947.1
MSTRG.25948		MSTRG.25948.1
MSTRG.25949		MSTRG.25949.1
MSTRG.2595		MSTRG.2595.1
MSTRG.25950		MSTRG.25950.1
MSTRG.25951		MSTRG.25951.1
MSTRG.25952		MSTRG.25952.1;MSTRG.25952.2
MSTRG.25953		MSTRG.25953.1
MSTRG.2	Pdia4;M	ENSMUST00000077290;MSTRG.25954.2;ENSMUST00000102106;E
MSTRG.2	Zfp398	ENSMUST00000146202;ENSMUST00000079881;ENSMUST0000114
MSTRG.2	Gm44041	ENSMUST00000203678
MSTRG.2	Zfp282	ENSMUST00000061890;ENSMUST00000125957
MSTRG.2	Cul1	ENSMUST00000146828;ENSMUST00000152547;ENSMUST0000146
MSTRG.2	A930035	ENSMUST00000064013
MSTRG.2	Enpp1	ENSMUST00000105520;ENSMUST00000039882;ENSMUST0000135
MSTRG.2	Cul1	MSTRG.25960.1
MSTRG.2	Ezh2	ENSMUST00000204243;ENSMUST00000081721;ENSMUST0000114
MSTRG.2	Gm26625	MSTRG.25962.1;ENSMUST00000181519
MSTRG.2	Gm44282	ENSMUST00000203199
MSTRG.2	Ezh2	MSTRG.25965.1

MSTRG.2	Ezh2	MSTRG.25966.1
MSTRG.2	Zfp212	ENSMUST00000009411;MSTRG.25967.2;MSTRG.25967.3;ENSMUS
MSTRG.25968		MSTRG.25968.1
MSTRG.25969		MSTRG.25969.1;MSTRG.25969.2;MSTRG.25969.3
MSTRG.2	Enpp1	MSTRG.2597.1
MSTRG.2	Zfp783;	ENSMUST00000130490;ENSMUST00000114588;ENSMUST00000144
MSTRG.2	Zfp777	ENSMUST00000114583;MSTRG.25971.2;ENSMUST00000095944;E
MSTRG.2	Gm45738	ENSMUST00000203645
MSTRG.2	Zfp746	MSTRG.25974.2;MSTRG.25974.1;ENSMUST00000073124;ENSMUS
MSTRG.2	Gm44696	ENSMUST00000181943
MSTRG.2	Gm7880	ENSMUST00000203748
MSTRG.2	Krba1	ENSMUST00000114571;ENSMUST00000031815;ENSMUST00000204
MSTRG.2	Gm24325	ENSMUST00000175344
MSTRG.2	Atp6v0e	ENSMUST00000040361;ENSMUST00000204277
MSTRG.2	Med23;G	ENSMUST00000092646;ENSMUST00000177522;ENSMUST00000177
MSTRG.2	Zfp862-	ENSMUST00000203848;ENSMUST00000204484;ENSMUST00000203
MSTRG.2	Gm10243	ENSMUST00000117571
MSTRG.2	Gm7887	ENSMUST00000056948
MSTRG.2	Lrrc61	ENSMUST00000114545;ENSMUST00000153222;ENSMUST00000204
MSTRG.2	Rarres2	ENSMUST00000203541;ENSMUST00000009425;ENSMUST00000204
MSTRG.2	Zfp467	ENSMUST00000114566;ENSMUST00000101443;ENSMUST00000114
MSTRG.2	Sspo	ENSMUST00000212740;ENSMUST00000169350;ENSMUST00000043
MSTRG.2	Gm5111;	ENSMUST00000203141;ENSMUST00000181661;ENSMUST00000154
MSTRG.2	Gm38804	ENSMUST00000203998
MSTRG.2	Gm38804	MSTRG.25989.1
MSTRG.2	Med23	MSTRG.2599.1
MSTRG.2	Gimap8	MSTRG.25990.1;ENSMUST00000203083;MSTRG.25990.2;ENSMUS
MSTRG.2	Gm44262	ENSMUST00000204969
MSTRG.2	Gimap4	MSTRG.25992.1
MSTRG.2	Gimap9	ENSMUST00000054050;MSTRG.25993.2;MSTRG.25993.1;ENSMUS
MSTRG.2	Gimap4	ENSMUST00000118802;ENSMUST00000067506;ENSMUST00000119
MSTRG.2	Gimap6	ENSMUST00000053661;ENSMUST00000119315;ENSMUST00000126
MSTRG.2	Gimap3	ENSMUST00000204036;ENSMUST00000038811;MSTRG.25996.2;M
MSTRG.2	Gm28053	ENSMUST00000127537;ENSMUST00000052503;ENSMUST00000204
MSTRG.2	Gm28053	MSTRG.25998.1
MSTRG.2	Gimap1o	MSTRG.25999.1;ENSMUST00000155906;ENSMUST00000205172
MSTRG.2	Rb1cc1	ENSMUST00000159906;ENSMUST00000160871;MSTRG.26.2;ENSM
MSTRG.2	Med23	MSTRG.2600.1
MSTRG.2	Gm44226	MSTRG.26000.1;ENSMUST00000205203
MSTRG.2	4833403	ENSMUST00000204490
MSTRG.2	Gpnmb	ENSMUST00000205103;ENSMUST00000031840;ENSMUST00000203
MSTRG.2	Tmem176	ENSMUST00000166247;ENSMUST00000164733;ENSMUST00000101
MSTRG.2	Tmem176	ENSMUST00000204400;ENSMUST00000101426;ENSMUST00000204
MSTRG.2	Gm44764	ENSMUST00000204912;ENSMUST00000204021
MSTRG.2	Gm7932	ENSMUST00000205047
MSTRG.2	Malsu1	ENSMUST00000128616;ENSMUST00000114500;ENSMUST00000031
MSTRG.2	Gm7890	ENSMUST00000204877
MSTRG.2	Igf2bp3	MSTRG.26009.1;MSTRG.26009.2;MSTRG.26009.3;ENSMUST0000
MSTRG.2	Med23	MSTRG.2601.1
MSTRG.2	Igf2bp3	MSTRG.26010.1
MSTRG.2	Igf2bp3	MSTRG.26011.1
MSTRG.2	Gm18010	ENSMUST00000203553
MSTRG.2	Igf2bp3	MSTRG.26013.1
MSTRG.2	Igf2bp3	MSTRG.26014.1
MSTRG.2	Ccdc126	ENSMUST00000055559;ENSMUST00000114491
MSTRG.2	Ccdc126	MSTRG.26016.1
MSTRG.2	Tra2a	ENSMUST00000204189;ENSMUST00000203820;MSTRG.26017.4;E

MSTRG.2	Gm43980	ENSMUST00000204899
MSTRG.2	Fam221a	ENSMUST00000060561;ENSMUST00000121903;ENSMUST00000134
MSTRG.2	Mir6905	ENSMUST00000184228
MSTRG.2	Fam221a	MSTRG.26020.1
MSTRG.26021		MSTRG.26021.1
MSTRG.2	Mpp6	ENSMUST00000166318;ENSMUST00000036236;ENSMUST00000204
MSTRG.2	Mpp6	MSTRG.26023.1
MSTRG.2	Mpp6	MSTRG.26024.1
MSTRG.2	Dfna5	ENSMUST00000204417;ENSMUST00000165099;ENSMUST00000101
MSTRG.2	Dfna5	MSTRG.26026.1
MSTRG.2	Osbpl3	ENSMUST00000114468;ENSMUST00000141466;ENSMUST0000009C
MSTRG.2	Osbpl3	MSTRG.26028.1
MSTRG.2	Osbpl3	MSTRG.26029.1
MSTRG.2	Arg1	ENSMUST00000020161;ENSMUST00000218260;MSTRG.2603.3;EN
MSTRG.2	Cycs	ENSMUST00000161401;ENSMUST00000077766
MSTRG.2	5430402	ENSMUST00000147260;ENSMUST00000126537
MSTRG.2	Mir6371	ENSMUST00000184470
MSTRG.2	4921507	ENSMUST00000031852
MSTRG.2	5430402	MSTRG.26034.1
MSTRG.2	Cbx3	ENSMUST00000094623;ENSMUST00000031862;ENSMUST0000015€
MSTRG.2	Nfe2l3	ENSMUST00000005103;ENSMUST00000061507;ENSMUST0000016C
MSTRG.2	Nfe2l3	MSTRG.26037.1
MSTRG.2	Hnrnpa2	ENSMUST00000069949;ENSMUST00000203954;ENSMUST0000009C
MSTRG.2	Hnrnpa2	MSTRG.26039.1
MSTRG.2	AC15950	ENSMUST00000219852
MSTRG.26040		MSTRG.26040.1
MSTRG.2	Snx10	ENSMUST00000049152;ENSMUST00000155724;ENSMUST00000137
MSTRG.26042		MSTRG.26042.1
MSTRG.2	Skap2	ENSMUST00000204778;ENSMUST00000078214;MSTRG.26043.3;E
MSTRG.2	Skap2	MSTRG.26044.1
MSTRG.2	Skap2	MSTRG.26045.1
MSTRG.2	Gm44077	ENSMUST00000203453
MSTRG.2	Gm8129	ENSMUST00000118230
MSTRG.2	Hoxa1	ENSMUST00000000964;ENSMUST00000120363
MSTRG.2	Hotairm	ENSMUST00000129546;ENSMUST00000184778;ENSMUST00000132
MSTRG.2605		MSTRG.2605.1
MSTRG.2	Gm27477	ENSMUST00000184649
MSTRG.2	Hoxa2;H	ENSMUST00000014848;ENSMUST00000114434;ENSMUST00000134
MSTRG.2	Hoxaas2	ENSMUST00000155922;ENSMUST00000189230;ENSMUST00000114
MSTRG.2	Gm15050	ENSMUST00000133723
MSTRG.2	Hoxaas3	ENSMUST00000139055;ENSMUST00000136806;ENSMUST00000131
MSTRG.2	5730596	ENSMUST00000070587
MSTRG.2	Gm29430	ENSMUST00000191453
MSTRG.2	Hoxa4	ENSMUST00000101395
MSTRG.2	Hoxa5	ENSMUST00000048794
MSTRG.2	Hoxa6	ENSMUST00000062829
MSTRG.2	AC15354	ENSMUST00000218296
MSTRG.2	Hoxa7	ENSMUST00000153280;ENSMUST00000048715;ENSMUST00000134
MSTRG.2	Hibadh;	MSTRG.26061.1;MSTRG.26061.2;ENSMUST00000031788;ENSMUS
MSTRG.2	Hibadh	MSTRG.26062.1
MSTRG.2	Gm44445	ENSMUST00000204075
MSTRG.2	Hibadh	MSTRG.26064.1
MSTRG.2	Gm44434	MSTRG.26065.1
MSTRG.2	Gm44434	ENSMUST00000203918
MSTRG.26067		MSTRG.26067.1
MSTRG.2	Gm43966	ENSMUST00000203301
MSTRG.2	Tax1bp1	ENSMUST00000080723;ENSMUST00000149588;ENSMUST0000012E

MSTRG.2	Epb41l2	MSTRG.2607.1;ENSMUST00000053748;MSTRG.2607.3;ENSMUSTC
MSTRG.2	Gm43967	ENSMUST00000203787
MSTRG.2	Jazf1	ENSMUST00000128282;ENSMUST00000136250;ENSMUST00000074
MSTRG.2	Gm15572	ENSMUST00000119248
MSTRG.2	Jazf1	MSTRG.26073.1
MSTRG.2	Jazf1	MSTRG.26074.1
MSTRG.2	Jazf1	MSTRG.26075.1
MSTRG.2	Jazf1	MSTRG.26076.1
MSTRG.2	Jazf1	MSTRG.26077.1
MSTRG.2	Gm26215	ENSMUST00000158584
MSTRG.2	Jazf1	MSTRG.26079.1
MSTRG.2	Epb41l2	MSTRG.2608.1
MSTRG.2	Jazf1	MSTRG.26080.1
MSTRG.2	Jazf1	MSTRG.26081.1
MSTRG.2	Gm15573	ENSMUST00000119487
MSTRG.2	Jazf1	MSTRG.26083.1
MSTRG.2	Creb5	ENSMUST00000205222;ENSMUST00000203528;ENSMUST00000205
MSTRG.2	Creb5	MSTRG.26086.1
MSTRG.2	Gm44080	ENSMUST00000204451
MSTRG.2	Fkbp14	ENSMUST00000046520;ENSMUST00000155047;ENSMUST00000117
MSTRG.2	Fkbp14	MSTRG.26089.1
MSTRG.2	Epb41l2	MSTRG.2609.1
MSTRG.2	Plekha8	ENSMUST00000119706;ENSMUST00000101385
MSTRG.2	Gm28402	ENSMUST00000188574
MSTRG.2	Mturn	ENSMUST00000190641;ENSMUST00000187701;ENSMUST00000185
MSTRG.2	Gm44008	ENSMUST00000204345
MSTRG.2	Chn2	ENSMUST00000138930;ENSMUST00000046856;MSTRG.26094.4;E
MSTRG.2	Chn2	MSTRG.26095.1
MSTRG.2	Chn2	MSTRG.26096.1;MSTRG.26096.2
MSTRG.2	Chn2	MSTRG.26097.1
MSTRG.2	Chn2	MSTRG.26098.1
MSTRG.2	Chn2	MSTRG.26099.1
MSTRG.2	Gm38157	ENSMUST00000194178
MSTRG.2	AC15627	MSTRG.2610.1
MSTRG.2	9530036	ENSMUST00000138767
MSTRG.2	Gm15526	ENSMUST00000112504
MSTRG.2	Gm15527	ENSMUST00000146902;ENSMUST00000138259
MSTRG.2	9130019	ENSMUST00000145111;ENSMUST00000135612
MSTRG.2	Prr15	ENSMUST00000204057;ENSMUST00000059138
MSTRG.2	Wipf3	ENSMUST00000126637;ENSMUST00000132855;ENSMUST00000163
MSTRG.2	Gm44026	ENSMUST00000203985
MSTRG.2	Scrn1	ENSMUST00000019268;ENSMUST00000124325;ENSMUST0000014C
MSTRG.2	Znrf2	MSTRG.26108.1
MSTRG.2	Znrf2	MSTRG.26109.1;ENSMUST00000079869;MSTRG.26109.2;ENSMUS
MSTRG.2	Znrf2	MSTRG.26110.1
MSTRG.2	Nod1	ENSMUST00000168172;ENSMUST00000060655;ENSMUST00000203
MSTRG.2	Nod1	MSTRG.26112.1
MSTRG.2	Nod1	MSTRG.26113.1
MSTRG.2	Gars	MSTRG.26114.1;ENSMUST00000003572;ENSMUST00000205258;E
MSTRG.2	Inmt	ENSMUST00000003569;ENSMUST00000152720
MSTRG.2	Aqp1	ENSMUST00000004774
MSTRG.2	Lsm5	ENSMUST00000170382;ENSMUST00000203958;ENSMUST00000175
MSTRG.2	Pde1c	ENSMUST00000044505;ENSMUST00000203372;ENSMUST00000203
MSTRG.2	Gm44413	ENSMUST00000198921
MSTRG.2612		MSTRG.2612.1
MSTRG.2	Pde1c	MSTRG.26120.1
MSTRG.2	Avl9	ENSMUST00000205188;ENSMUST00000031805;ENSMUST00000177

MSTRG.2	Kbtbd2	ENSMUST00000114323;MSTRG.26122.2;MSTRG.26122.3;MSTRG.
MSTRG.2	Gm10209	ENSMUST00000203397
MSTRG.2	Fkbp9	ENSMUST00000031795;ENSMUST00000147159
MSTRG.2	Nt5c3	ENSMUST00000101367;ENSMUST00000031793;ENSMUST00000152
MSTRG.2	Nt5c3	MSTRG.26126.1
MSTRG.2	Nt5c3	MSTRG.26127.1
MSTRG.2	Gm3793	ENSMUST00000204973
MSTRG.2	Vmn1r4	ENSMUST00000204739;ENSMUST00000176838;ENSMUST00000096
MSTRG.2613		MSTRG.2613.1
MSTRG.2	Gm44049	ENSMUST00000204905
MSTRG.2	Gm37397	ENSMUST00000194579;ENSMUST00000053386;ENSMUST00000180
MSTRG.2	4930533	ENSMUST00000176290;ENSMUST00000204686;MSTRG.26132.5;M
MSTRG.2	Ppm1k	ENSMUST00000042766;ENSMUST00000204607;ENSMUST00000203
MSTRG.2	Gm45193	MSTRG.26134.1
MSTRG.2	Gm27896	ENSMUST00000175162
MSTRG.2	Gm19244	ENSMUST00000204340
MSTRG.2	Herc6	MSTRG.26137.1
MSTRG.2	Herc6	MSTRG.26138.1
MSTRG.2	Herc6	MSTRG.26139.1
MSTRG.2	Samd3	ENSMUST00000218301;ENSMUST00000060716;ENSMUST00000220
MSTRG.2	Herc6	MSTRG.26140.1
MSTRG.2	Herc6	MSTRG.26141.1
MSTRG.2	Herc6	MSTRG.26142.1
MSTRG.2	Lancl2	ENSMUST00000072954;ENSMUST00000050077;ENSMUST00000153
MSTRG.2	Vopp1	ENSMUST00000127485;ENSMUST00000114297;ENSMUST00000204
MSTRG.26145		MSTRG.26145.1
MSTRG.26146		MSTRG.26146.1
MSTRG.2	Abcg2	ENSMUST00000148008;ENSMUST00000031822;ENSMUST00000124
MSTRG.2	Fam13a	ENSMUST00000089860;ENSMUST00000173712;ENSMUST00000174
MSTRG.2	Gm45060	ENSMUST00000203293
MSTRG.2	Samd3	MSTRG.2615.1
MSTRG.2	BB36589	ENSMUST00000173978
MSTRG.2	Gm45061	ENSMUST00000204395
MSTRG.2	Fam13a	MSTRG.26152.1
MSTRG.2	Herc3	ENSMUST00000141600;MSTRG.26153.1;ENSMUST00000122981;M
MSTRG.2	Herc3	MSTRG.26154.1
MSTRG.2	Nap1l5	ENSMUST00000059539;ENSMUST00000205101
MSTRG.2	Tigd2	ENSMUST00000062626
MSTRG.26157		MSTRG.26157.1
MSTRG.2	Gprin3	ENSMUST00000051065
MSTRG.26159		MSTRG.26159.1
MSTRG.2	Samd3	MSTRG.2616.1
MSTRG.26160		MSTRG.26160.1
MSTRG.2	9330118	ENSMUST00000203667
MSTRG.2	Grid2	ENSMUST00000159319;ENSMUST00000159561;ENSMUST00000095
MSTRG.2	Gm44075	ENSMUST00000204208
MSTRG.2	Gm44072	ENSMUST00000203666
MSTRG.2	Grid2	MSTRG.26168.1
MSTRG.2	Grid2	MSTRG.26169.1
MSTRG.2	L3mbtl3	ENSMUST00000105519;ENSMUST00000218585;ENSMUST00000040
MSTRG.2	Hpgds	ENSMUST00000031982;ENSMUST00000205250;ENSMUST00000170
MSTRG.2	Hpgds	MSTRG.26171.1
MSTRG.2	Gm17147	ENSMUST00000164176
MSTRG.2	Hpgds	MSTRG.26173.1
MSTRG.2	Smarcad	ENSMUST00000204801;MSTRG.26174.1;ENSMUST00000031984;E
MSTRG.2	Smarcad	MSTRG.26175.1
MSTRG.26176		MSTRG.26176.1

MSTRG.2	Mad2l1	ENSMUST00000116605;ENSMUST00000125430;ENSMUST00000101
MSTRG.2	Vmn1r32	ENSMUST00000079584
MSTRG.2	Gm9794	ENSMUST00000202370
MSTRG.2	Gm27754	ENSMUST00000183340
MSTRG.2	4930597	ENSMUST00000203383;ENSMUST00000065878
MSTRG.2	Gng12	ENSMUST00000043148;ENSMUST00000114228;ENSMUST00000114
MSTRG.2	Gng12	MSTRG.26182.1
MSTRG.2	Gng12	MSTRG.26183.1
MSTRG.2	Gng12	MSTRG.26184.1
MSTRG.2	Gm36816	ENSMUST00000205197;ENSMUST00000204824
MSTRG.2	Gm15644	ENSMUST00000121764
MSTRG.2	Gm29848	ENSMUST00000203107
MSTRG.2	Gm25260	ENSMUST00000083053
MSTRG.2	Gadd45a	ENSMUST00000043098;ENSMUST00000151958;ENSMUST00000204
MSTRG.2	Gm23757	ENSMUST00000158353
MSTRG.2	E230016	ENSMUST00000130657;ENSMUST00000147548;ENSMUST0000014
MSTRG.2	Il23r	ENSMUST00000118364
MSTRG.2	Gm42889	ENSMUST00000199480;ENSMUST00000103302
MSTRG.2	Tacstd2	ENSMUST00000058178
MSTRG.2	Igkv1-1	ENSMUST00000103303
MSTRG.2	Igkv14-	MSTRG.26195.1
MSTRG.2	Igkv17-	ENSMUST00000200586;ENSMUST00000103309
MSTRG.2	Gm38825	ENSMUST00000204022
MSTRG.2	Gm8566	ENSMUST00000120174
MSTRG.2	Mir6896	ENSMUST00000198033
MSTRG.2	L3mbtl3	MSTRG.2620.1
MSTRG.2	Serbp1	ENSMUST00000204810;MSTRG.26200.1;ENSMUST00000042990;E
MSTRG.2	Il12rb2	ENSMUST00000117441;ENSMUST00000018485
MSTRG.2	Igkv9-1	ENSMUST00000103313
MSTRG.2	Igkv9-1	ENSMUST00000103312;ENSMUST00000196768
MSTRG.2	Igkv14-	ENSMUST00000103310
MSTRG.2	Igkv1-1	ENSMUST00000103314
MSTRG.2	Igkv17-	ENSMUST00000197515;ENSMUST00000103315
MSTRG.2	Igkv9-1	ENSMUST00000103316
MSTRG.2	Igkv2-1	ENSMUST00000103322
MSTRG.2	L3mbtl3	MSTRG.2621.1;MSTRG.2621.2
MSTRG.2	Igkv1-1	ENSMUST00000103317;MSTRG.26210.1;ENSMUST00000103321
MSTRG.2	Igkv2-1	ENSMUST00000199327
MSTRG.2	Gm4873	ENSMUST00000199402
MSTRG.2	Igkv2-1	ENSMUST00000103318
MSTRG.2	Igkv14-	MSTRG.26219.1;ENSMUST00000103320
MSTRG.2	AC10167	ENSMUST00000218733
MSTRG.2	Gm25436	ENSMUST00000158275
MSTRG.2	Igkv16-	ENSMUST00000103323
MSTRG.2	Igkv15-	ENSMUST00000103324
MSTRG.2	Igkv14-	ENSMUST00000199510
MSTRG.2	Igkv12-	ENSMUST00000103327;ENSMUST00000196839
MSTRG.2	Igkv10-	ENSMUST00000103328
MSTRG.2	Igkv10-	ENSMUST00000103330
MSTRG.2	Igkv19-	ENSMUST00000183936;ENSMUST00000196863
MSTRG.2	Igkv4-9	ENSMUST00000103333
MSTRG.2	Igkv12-	ENSMUST00000199143
MSTRG.2	AC15863	ENSMUST00000219202
MSTRG.2	Igkv1-8	ENSMUST00000103336
MSTRG.2	Igkv4-8	ENSMUST00000103337;ENSMUST00000200454
MSTRG.2	Igkv13-	ENSMUST00000103339
MSTRG.2	Igkv4-5	ENSMUST00000103354

MSTRG.2	Igkv4-5	ENSMUST00000103356
MSTRG.2	Igkv4-5	ENSMUST00000103357
MSTRG.2	Rn7s6;A	MSTRG.26236.1;MSTRG.26236.2;MSTRG.26236.3;MSTRG.2623
MSTRG.2	Igkv4-6	ENSMUST00000200639
MSTRG.2	Igkv4-5	ENSMUST00000103355;ENSMUST00000197448
MSTRG.2	Igkv4-5	ENSMUST00000103359
MSTRG.2	AC15863	ENSMUST00000219999
MSTRG.2	Igkv4-5	ENSMUST00000166380;ENSMUST00000198328
MSTRG.2	Igkv4-5	ENSMUST00000103363
MSTRG.2	Igkv5-4	ENSMUST00000103364
MSTRG.2	Igkv12-	ENSMUST00000103365
MSTRG.2	Igkv5-4	ENSMUST00000103366
MSTRG.2	Igkv4-8	ENSMUST00000197966;ENSMUST00000103340
MSTRG.2	Igkv13-	ENSMUST00000197926
MSTRG.2	Igkv4-8	ENSMUST00000103341
MSTRG.2	Arhgap1	ENSMUST00000176060;ENSMUST00000039557;ENSMUST0000022
MSTRG.2	Igkv4-7	ENSMUST00000103342
MSTRG.2	Igkv4-7	ENSMUST00000103343
MSTRG.2	Igkv4-7	ENSMUST00000199410
MSTRG.2	Igkv4-7	ENSMUST00000103344
MSTRG.2	Arhgap1	MSTRG.2626.1
MSTRG.2	Igkv4-7	ENSMUST00000199760
MSTRG.2	Igkv4-7	ENSMUST00000103346
MSTRG.2	Igkv4-7	ENSMUST00000101325;ENSMUST00000196201
MSTRG.26264		MSTRG.26264.1
MSTRG.2	Igkv4-7	ENSMUST00000103348
MSTRG.2	Igkv4-6	ENSMUST00000103349
MSTRG.2	Igkv4-6	ENSMUST00000103350
MSTRG.2	Gm10145	ENSMUST00000077776
MSTRG.2	Igkv12-	ENSMUST00000199305
MSTRG.2	Igkv4-6	ENSMUST00000103351;ENSMUST00000196595
MSTRG.2	Igkv4-6	ENSMUST00000177795;ENSMUST00000198345
MSTRG.2	Igkv4-6	ENSMUST00000199160;ENSMUST00000103353
MSTRG.2	Igkv12-	ENSMUST00000103367
MSTRG.2	Igkv5-4	ENSMUST00000103368
MSTRG.2	Igkv12-	ENSMUST00000103369
MSTRG.2	Igkv5-3	ENSMUST00000103370;ENSMUST00000197290
MSTRG.2	Igkv18-	MSTRG.26282.3
MSTRG.2	Igkv6-3	ENSMUST00000103377
MSTRG.2	Igkv8-3	ENSMUST00000103378
MSTRG.2	Igkv6-2	ENSMUST00000197371
MSTRG.2	Igkv8-2	ENSMUST00000103380;ENSMUST00000197525
MSTRG.2	Igkv8-2	ENSMUST00000103381;ENSMUST00000197272
MSTRG.2	Igkv6-2	ENSMUST00000103383
MSTRG.2	Igkv8-2	ENSMUST00000103384
MSTRG.2	Igkv6-2	ENSMUST00000103386
MSTRG.2	Igkv8-2	ENSMUST00000103387
MSTRG.2	Igkv6-2	ENSMUST00000103388
MSTRG.2	Igkv8-1	ENSMUST00000103389;ENSMUST00000196599
MSTRG.2	Igkv6-1	ENSMUST00000103391
MSTRG.2	Igkv8-1	ENSMUST00000103392;ENSMUST00000195945
MSTRG.2	Igkv6-1	ENSMUST00000103393
MSTRG.2	Igkv6-1	ENSMUST00000103394
MSTRG.2	Igkv6-1	ENSMUST00000103395;ENSMUST00000198184
MSTRG.2	Igkv3-1	ENSMUST00000103396
MSTRG.2	Gm26788	ENSMUST00000181692;ENSMUST00000187268
MSTRG.2	Lama2	ENSMUST00000186279;ENSMUST00000092639;ENSMUST0000021

MSTRG.2	Igkv3-1	ENSMUST00000197500
MSTRG.2	Igkv3-1	ENSMUST00000103397;ENSMUST00000196940
MSTRG.2	Igkv3-5	ENSMUST00000103400
MSTRG.2	Igkv3-4	ENSMUST00000103401
MSTRG.2	Gm30211	ENSMUST00000197227
MSTRG.2	Igkj1	ENSMUST00000103405
MSTRG.2	Igkj2	ENSMUST00000198234;ENSMUST00000103406
MSTRG.2	Igkj3	ENSMUST00000103407;ENSMUST00000199487
MSTRG.2	Igkj4	ENSMUST00000103408
MSTRG.2	Igkj5	ENSMUST00000199459;ENSMUST00000103409
MSTRG.2	AC15380	ENSMUST00000218561
MSTRG.2	Igkc	ENSMUST00000103410
MSTRG.2	Rpia	ENSMUST00000066134;ENSMUST00000124633;ENSMUST00000129
MSTRG.2	Rpl34-p	ENSMUST00000121998
MSTRG.2	Eif2ak3	ENSMUST00000034093;ENSMUST00000162950;MSTRG.26313.2;E
MSTRG.2	Gm23346	ENSMUST00000083816
MSTRG.2	Eif2ak3	MSTRG.26315.1
MSTRG.2	Gm43292	ENSMUST00000199580
MSTRG.2	Gm44111	ENSMUST00000204649
MSTRG.2	Gm44110	MSTRG.26318.1;ENSMUST00000203871
MSTRG.2	Smyd1	ENSMUST00000074301;ENSMUST00000114186;ENSMUST00000114
MSTRG.2	AC15380	ENSMUST00000219212
MSTRG.2	Mir8112	ENSMUST00000184382;ENSMUST00000204436;ENSMUST00000205
MSTRG.2	Gm44321	ENSMUST00000204262
MSTRG.2	Gm1070	ENSMUST00000114185
MSTRG.2	Smyd1	MSTRG.26323.1
MSTRG.2	Smyd1	MSTRG.26324.1
MSTRG.2	Smyd1	MSTRG.26325.1
MSTRG.2	Smyd1	MSTRG.26326.1
MSTRG.2	Cd8b1	MSTRG.26327.2;MSTRG.26327.1;ENSMUST00000065248
MSTRG.2	Rmnd5a	MSTRG.26328.1
MSTRG.2	Gm44111	MSTRG.26329.1
MSTRG.2	4930579	ENSMUST00000187815
MSTRG.2	Gm44175	MSTRG.26330.1;MSTRG.26330.2;MSTRG.26330.3;ENSMUST0000
MSTRG.2	Gm44174	ENSMUST00000205022
MSTRG.2	Gm44174	MSTRG.26332.1
MSTRG.2	Cd8a	ENSMUST00000066747;ENSMUST00000205054;ENSMUST00000172
MSTRG.2	Rmnd5a;	ENSMUST00000002292;ENSMUST00000149415;MSTRG.26334.2;E
MSTRG.2	Rnf103	MSTRG.26335.1;ENSMUST00000064637;MSTRG.26335.2;ENSMUS
MSTRG.2	Gm6063	ENSMUST00000206958
MSTRG.2	Reep1	ENSMUST00000121469;ENSMUST00000212792
MSTRG.2	Gm44769	ENSMUST00000206120
MSTRG.2	Gm44770	ENSMUST00000205395
MSTRG.2	Gm23353	ENSMUST00000158425
MSTRG.2	Mrpl35	ENSMUST00000065103;ENSMUST00000205435
MSTRG.2	Gm44194	ENSMUST00000204222
MSTRG.2	1700040	ENSMUST00000205011
MSTRG.2	Chmp3	ENSMUST00000059462;ENSMUST00000127774;ENSMUST00000204
MSTRG.2	Kdm3a	ENSMUST00000065509;ENSMUST00000167220;MSTRG.26344.4;M
MSTRG.2	Gm44771	ENSMUST00000206975
MSTRG.2	Immt	ENSMUST00000064062;ENSMUST00000166975;ENSMUST00000167
MSTRG.2	Immt	MSTRG.26347.1
MSTRG.2	Gm17825	ENSMUST00000206836
MSTRG.2	Ptcd3;G	ENSMUST00000205269;ENSMUST00000082094;ENSMUST00000206
MSTRG.2	Gm36962	ENSMUST00000192114
MSTRG.2	Polr1a	ENSMUST00000206556;ENSMUST00000055296;ENSMUST00000205
MSTRG.2	Gm19078	ENSMUST00000206311

MSTRG.2	Polr1a	MSTRG.26352.1
MSTRG.2	Gm26628	ENSMUST00000181028
MSTRG.2	Atoh8	ENSMUST00000206425;ENSMUST00000042646;ENSMUST00000206
MSTRG.2	Sftpb	ENSMUST00000182014;MSTRG.26355.5
MSTRG.2	Usp39	ENSMUST00000070345;ENSMUST00000154799;ENSMUST00000206
MSTRG.2	Gm45051	ENSMUST00000206838
MSTRG.2	0610030	ENSMUST00000205578;ENSMUST00000205472;ENSMUST00000077
MSTRG.2	Vamp8	ENSMUST00000059983;ENSMUST00000142613
MSTRG.2	Ptprk	ENSMUST00000166468;ENSMUST00000218359;ENSMUST00000218
MSTRG.2	Tmem150	ENSMUST00000069695;ENSMUST00000206064;ENSMUST00000206
MSTRG.2	Rnf181	ENSMUST00000206319;ENSMUST00000069580;ENSMUST00000125
MSTRG.2	Vamp5	ENSMUST00000206154;ENSMUST00000101285;ENSMUST00000074
MSTRG.2	Vamp5	MSTRG.26363.1
MSTRG.2	Ggcx	ENSMUST00000065906;ENSMUST00000207012;ENSMUST00000132
MSTRG.2	Mat2a	ENSMUST00000205335;ENSMUST00000059472;ENSMUST00000205
MSTRG.2	Particl	ENSMUST00000205370
MSTRG.2	Capg	ENSMUST00000071044;ENSMUST00000114072;ENSMUST00000155
MSTRG.2	Elmod3	ENSMUST00000114069;ENSMUST00000070990;MSTRG.26368.3;E
MSTRG.2	Retsat	ENSMUST00000070597;MSTRG.26369.2;ENSMUST00000175824;E
MSTRG.2	Gm22370	ENSMUST00000102422
MSTRG.2	Gm45053	ENSMUST00000206112
MSTRG.2	Tgoln1	ENSMUST00000070524;MSTRG.26371.1
MSTRG.2	Gm20536	ENSMUST00000172588
MSTRG.2	Tcf7l1	ENSMUST00000069536;ENSMUST00000114053;ENSMUST00000141
MSTRG.2	Gm15401	ENSMUST00000156497;ENSMUST00000081929;ENSMUST00000101
MSTRG.2	Gm37969	ENSMUST00000195777
MSTRG.2	Gm38114	ENSMUST00000191877
MSTRG.2	Gm38320	ENSMUST00000192767
MSTRG.2	Gm15402	ENSMUST00000114051
MSTRG.2	Gm37736	ENSMUST00000193564
MSTRG.2638		MSTRG.2638.1
MSTRG.2	Kcmf1	MSTRG.26380.1
MSTRG.2	Kcmf1	MSTRG.26381.1
MSTRG.2	Kcmf1	ENSMUST00000068697;ENSMUST00000204598;ENSMUST00000206
MSTRG.2	Kcmf1	MSTRG.26383.1
MSTRG.2	D530018	ENSMUST00000205107
MSTRG.2	Gm26640	ENSMUST00000180809
MSTRG.2	Tmsb10	ENSMUST00000114050;ENSMUST00000114049;ENSMUST00000114
MSTRG.2	Gm18402	MSTRG.26387.1
MSTRG.2	Suclg1	ENSMUST00000064740;ENSMUST00000123159;ENSMUST00000203
MSTRG.2	AC10766	ENSMUST00000213962
MSTRG.26390		MSTRG.26390.1
MSTRG.26391		MSTRG.26391.1;MSTRG.26391.2;MSTRG.26391.3;MSTRG.26391
MSTRG.26392		MSTRG.26392.1;MSTRG.26392.2
MSTRG.2	Gm6210	ENSMUST00000204734
MSTRG.26394		MSTRG.26394.1
MSTRG.2	Gm44126	ENSMUST00000204831
MSTRG.2	Reg3g	ENSMUST00000176650;ENSMUST00000032089
MSTRG.26397		MSTRG.26397.1
MSTRG.2	Ctnna2	ENSMUST00000160894;ENSMUST00000162991;ENSMUST00000162
MSTRG.2	Lrrtm1	ENSMUST00000020400;ENSMUST00000159616
MSTRG.2	A930005	ENSMUST00000194920
MSTRG.2	Gm9824	ENSMUST00000178707
MSTRG.2	Gm44234	ENSMUST00000204246
MSTRG.2	6330415	ENSMUST00000204215
MSTRG.2	Gm44435	ENSMUST00000204379
MSTRG.2	Gm44433	ENSMUST00000203540

MSTRG.2	Gm44235	ENSMUST00000204312
MSTRG.2	Gm44438	ENSMUST00000204388
MSTRG.2	Gm44286	ENSMUST00000204098
MSTRG.2	Gm44288	ENSMUST00000205061
MSTRG.2	Ctnna2	MSTRG.26408.1
MSTRG.2	Gm44437	ENSMUST00000203832
MSTRG.2	Themis	ENSMUST00000060409;ENSMUST00000056097;ENSMUST00000162
MSTRG.2	Gcfc2	ENSMUST00000043195;ENSMUST00000152996;ENSMUST00000132
MSTRG.2	Gcfc2	MSTRG.26411.1
MSTRG.2	Gcfc2	MSTRG.26412.1
MSTRG.2	Mrpl19	ENSMUST00000032124;ENSMUST00000128374;ENSMUST00000148
MSTRG.2	Pole4	ENSMUST00000161309;ENSMUST00000160281;ENSMUST00000095
MSTRG.2	Hk2	ENSMUST00000000642;ENSMUST00000168725;ENSMUST0000017C
MSTRG.2	Gm17034	ENSMUST00000168990
MSTRG.2	Pcgf1	ENSMUST00000176100;ENSMUST00000165164;ENSMUST00000092
MSTRG.2	Pcgf1	MSTRG.26419.1
MSTRG.2	Themis	MSTRG.2642.1
MSTRG.2	Lbx2	ENSMUST00000041265;ENSMUST00000151508
MSTRG.2	M1ap	ENSMUST00000113980;ENSMUST00000204891;ENSMUST00000144
MSTRG.2	Gm43985	ENSMUST00000203283
MSTRG.2	Gm43981	ENSMUST00000203285
MSTRG.2	Dok1	ENSMUST00000089651;ENSMUST00000149918;ENSMUST00000204
MSTRG.2	Loxl3	ENSMUST00000152679;ENSMUST00000000707;ENSMUST00000204
MSTRG.2	Mir7040	ENSMUST00000184661
MSTRG.2	Htra2	ENSMUST00000113962;ENSMUST00000089645;ENSMUST00000113
MSTRG.2	Aup1	ENSMUST00000092618;ENSMUST00000204895;ENSMUST00000205
MSTRG.2	AC15947	MSTRG.2643.2;MSTRG.2643.1;MSTRG.2643.3;ENSMUST0000021
MSTRG.2	Dqx1	ENSMUST00000077502;ENSMUST00000204803
MSTRG.2	Tlx2	ENSMUST00000174674
MSTRG.2	Ccdc142	ENSMUST00000101254;ENSMUST00000101253;ENSMUST00000203
MSTRG.2	Ccdc142	ENSMUST00000125657;ENSMUST00000141639;ENSMUST00000138
MSTRG.2	Mogs	ENSMUST00000032114;MSTRG.26434.2;MSTRG.26434.3;ENSMUS
MSTRG.2	Wbp1	ENSMUST00000032111;ENSMUST00000113936;ENSMUST00000131
MSTRG.2	Ino80b	ENSMUST00000113935;ENSMUST00000143814;ENSMUST00000032
MSTRG.2	Gm44287	ENSMUST00000203069
MSTRG.2	Rtkn	ENSMUST00000135490;ENSMUST00000065512;ENSMUST00000121
MSTRG.2	Rtkn	MSTRG.26439.1
MSTRG.2	Themis	MSTRG.2644.1;MSTRG.2644.2
MSTRG.2	Wdr54	ENSMUST00000153148;ENSMUST00000144723;ENSMUST00000154
MSTRG.2	1700003	ENSMUST00000032106;ENSMUST00000203203
MSTRG.2	Dctn1	ENSMUST00000077407;MSTRG.26442.2;ENSMUST00000113913;E
MSTRG.2	Gm15624	ENSMUST00000154953;ENSMUST00000204811;ENSMUST00000145
MSTRG.2	Dctn1	MSTRG.26444.1
MSTRG.2	Mthfd2	ENSMUST00000005810;ENSMUST00000203847;ENSMUST00000141
MSTRG.2	Gm43890	ENSMUST00000203437
MSTRG.2	Mob1a	ENSMUST00000055261;ENSMUST00000146420;MSTRG.26447.2;E
MSTRG.2	Bola3	ENSMUST00000122216;ENSMUST00000120040;ENSMUST00000136
MSTRG.2	Tet3;Mi	ENSMUST00000089622;MSTRG.26449.1;MSTRG.26449.4;MSTRG.
MSTRG.2	4930519	ENSMUST00000159347;ENSMUST00000163084
MSTRG.2	B230319	MSTRG.26450.1;ENSMUST00000170986;ENSMUST00000167886
MSTRG.2	Dguok	ENSMUST00000014698;ENSMUST00000113888;ENSMUST00000145
MSTRG.2	Dguok	MSTRG.26452.1
MSTRG.2	Cd207	ENSMUST00000037882
MSTRG.2	Stambp	ENSMUST00000205648;ENSMUST00000206400;ENSMUST00000068
MSTRG.2	Stambp	MSTRG.26455.1
MSTRG.2	Gm18537	MSTRG.26456.1
MSTRG.2	Gm18537	ENSMUST00000206215;ENSMUST00000205709;ENSMUST0000020€

MSTRG.2	Stambp	MSTRG.26458.1
MSTRG.2	Stambp	MSTRG.26459.1
MSTRG.2	4930519	MSTRG.2646.1
MSTRG.2	Gm21284	ENSMUST00000205532
MSTRG.2	1700124	ENSMUST00000189129;ENSMUST00000189308;ENSMUST0000019C
MSTRG.2	Ankrd53	ENSMUST00000014891
MSTRG.2	Tex261	MSTRG.26463.1;MSTRG.26463.2;ENSMUST00000014892;ENSMUS
MSTRG.2	Paip2b	ENSMUST00000058383;ENSMUST00000125493;ENSMUST00000145
MSTRG.2	Zfp638	ENSMUST00000204415;ENSMUST00000203273;ENSMUST00000204
MSTRG.2	Gm44750	ENSMUST00000206185
MSTRG.2	Gm5138	ENSMUST00000113842
MSTRG.2	Gm43919	ENSMUST00000204303
MSTRG.2	Gm43920	ENSMUST00000204953
MSTRG.2	AC15379	ENSMUST00000219942
MSTRG.2	Zfp638	MSTRG.26470.1
MSTRG.2	Cyp26b1	ENSMUST00000205228;ENSMUST00000204146;ENSMUST00000077
MSTRG.2	Npm3-ps	ENSMUST00000119728
MSTRG.2	Exoc6b	ENSMUST00000160197;ENSMUST00000051531;ENSMUST0000004€
MSTRG.2	Exoc6b	MSTRG.26474.1
MSTRG.2	Gm7507	ENSMUST00000183053
MSTRG.2	Gm44012	ENSMUST00000204154
MSTRG.2	Gm44011	ENSMUST00000203336
MSTRG.2	Gm26353	ENSMUST00000158224
MSTRG.2	Exoc6b	MSTRG.26479.1
MSTRG.2	AC15391	ENSMUST00000215448;ENSMUST00000215520
MSTRG.2	Spr	ENSMUST00000204757;ENSMUST00000045986;ENSMUST00000174
MSTRG.2	Emx1	ENSMUST00000045942;ENSMUST00000173919
MSTRG.2	Sfxn5	ENSMUST00000045846;ENSMUST00000134392;ENSMUST0000005S
MSTRG.2	Sfxn5	MSTRG.26483.1
MSTRG.2	Rab11fi	ENSMUST00000060837;ENSMUST00000204087;ENSMUST00000102
MSTRG.2	Smyd5	ENSMUST00000045693;ENSMUST00000152218;ENSMUST00000204
MSTRG.2	Pradc1	ENSMUST00000032080;ENSMUST00000126805;ENSMUST0000014€
MSTRG.2	Cct7	ENSMUST00000032078;ENSMUST00000205094;ENSMUST00000204
MSTRG.2	Alms1	ENSMUST00000072018;ENSMUST00000213058;ENSMUST00000201
MSTRG.2	Gm43588	ENSMUST00000201862
MSTRG.2	AC15391	MSTRG.2649.1
MSTRG.2	Gm42600	ENSMUST00000202081
MSTRG.2	Alms1	MSTRG.26491.1
MSTRG.2	Nat8f7	ENSMUST00000160534;ENSMUST00000179613
MSTRG.2	Alms1-p	ENSMUST00000159498
MSTRG.2	Nat8f3	ENSMUST00000201219;ENSMUST00000050780;ENSMUST0000016E
MSTRG.2	Gm27317	ENSMUST00000183533
MSTRG.2	Alms1-p	ENSMUST00000161983;ENSMUST00000160606
MSTRG.2	Alms1-p	MSTRG.26499.1
MSTRG.2	Hs6st1	ENSMUST00000088174;MSTRG.265.2;MSTRG.265.3;ENSMUST000
MSTRG.2	Echdc1	ENSMUST00000160399;ENSMUST00000161605;ENSMUST0000002C
MSTRG.26500		MSTRG.26500.1
MSTRG.2	Ptges3-	ENSMUST00000160012
MSTRG.2	Nat8f1	ENSMUST00000161198;ENSMUST00000172982;ENSMUST00000174
MSTRG.2	Tprkb	ENSMUST00000202142;ENSMUST00000200680;ENSMUST00000202
MSTRG.2	Gm18290	ENSMUST00000202900
MSTRG.2	Dusp11	MSTRG.26505.1;ENSMUST00000032071;MSTRG.26505.3;ENSMUS
MSTRG.2	Gm19265	ENSMUST00000201818
MSTRG.2	Gm10443	ENSMUST00000119024
MSTRG.2	Fam136a	ENSMUST00000071492;ENSMUST00000203237;ENSMUST00000204
MSTRG.2	Snrpg	ENSMUST00000089558;ENSMUST00000203782;ENSMUST00000204
MSTRG.2	Echdc1	MSTRG.2651.1

MSTRG.2	Gm44941	MSTRG.26510.1;ENSMUST00000204216
MSTRG.2	Pcyox1	ENSMUST00000032065;ENSMUST00000204116;ENSMUST00000131
MSTRG.2	Pcbp1	MSTRG.26512.1
MSTRG.2	Tia1;Gm	ENSMUST00000095754;ENSMUST00000095753;ENSMUST00000203
MSTRG.2	A430078	ENSMUST00000129343;ENSMUST00000154066
MSTRG.2	2310040	ENSMUST00000188135;ENSMUST00000189386
MSTRG.2	Pcbp1	ENSMUST00000053015;MSTRG.26516.1;MSTRG.26516.3
MSTRG.2	1600020	ENSMUST00000204068;ENSMUST00000204008;ENSMUST0000018C
MSTRG.2	1600020	MSTRG.26518.1
MSTRG.2	1600020	MSTRG.26519.1
MSTRG.2	Rnf146	ENSMUST00000160144;ENSMUST00000037548;MSTRG.2652.3;EN
MSTRG.2	1600020	MSTRG.26520.1
MSTRG.2	Gm22184	ENSMUST00000175421
MSTRG.2	Gm44369	ENSMUST00000204543
MSTRG.2	Asprv1	ENSMUST00000043400
MSTRG.2	Mxd1	ENSMUST00000001184;ENSMUST00000203946;ENSMUST00000204
MSTRG.2	Gm44292	ENSMUST00000205194
MSTRG.2	Snrnp27	MSTRG.26527.1
MSTRG.2	Snrnp27	ENSMUST00000001186;ENSMUST00000131674;ENSMUST00000113
MSTRG.2	Gm44214	ENSMUST00000204153
MSTRG.2	Gm25596	ENSMUST00000178368
MSTRG.2	Gmcl1	MSTRG.26530.1;ENSMUST00000001185;ENSMUST00000203741;E
MSTRG.2	Gm44036	ENSMUST00000204551
MSTRG.2	Anxa4	ENSMUST00000113675;ENSMUST00000001187;ENSMUST00000127
MSTRG.26533		MSTRG.26533.1
MSTRG.2	Gfpt1	ENSMUST00000113657;ENSMUST00000113658;ENSMUST00000204
MSTRG.2	2610306	ENSMUST00000185414
MSTRG.2	Aak1;Gm	MSTRG.26536.1;ENSMUST00000089519;ENSMUST00000204414;M
MSTRG.2	Gm44153	MSTRG.26537.1;MSTRG.26537.2;ENSMUST00000204162
MSTRG.2	Gm44152	ENSMUST00000205007
MSTRG.2	Aak1	MSTRG.26539.1;MSTRG.26539.2
MSTRG.2	AC15391	ENSMUST00000215840
MSTRG.2	Aak1	MSTRG.26540.1
MSTRG.2	Gm44091	ENSMUST00000205212
MSTRG.2	Nfu1	ENSMUST00000120240;ENSMUST00000203512;ENSMUST00000032
MSTRG.2	Nfu1	MSTRG.26543.1
MSTRG.26544		MSTRG.26544.1
MSTRG.26545		MSTRG.26545.1
MSTRG.2	Antxr1	ENSMUST00000042025;ENSMUST00000203131;ENSMUST00000204
MSTRG.2	Gm44415	ENSMUST00000204151
MSTRG.2	Gm44416	ENSMUST00000204055
MSTRG.2	Antxr1	MSTRG.26549.1
MSTRG.2	AC15391	ENSMUST00000214789
MSTRG.2	Gm44417	ENSMUST00000203298
MSTRG.2	Gm44418	ENSMUST00000204219
MSTRG.2	9530013	ENSMUST00000203393
MSTRG.2	Gm44414	ENSMUST00000203660
MSTRG.2	Arhgap2	MSTRG.26555.1
MSTRG.2	Arhgap2	ENSMUST00000145128;ENSMUST00000113637;ENSMUST00000101
MSTRG.2	Arhgap2	MSTRG.26557.1;MSTRG.26557.2
MSTRG.2	Gm44198	ENSMUST00000203063
MSTRG.2	Aplf	ENSMUST00000065997;MSTRG.26559.1;ENSMUST00000203209;E
MSTRG.2	Gm10275	ENSMUST00000092620;ENSMUST00000217340;ENSMUST00000168
MSTRG.2	Gm44097	ENSMUST00000204112
MSTRG.2	E230015	ENSMUST00000160577;ENSMUST00000159884
MSTRG.2	1810020	ENSMUST00000162547
MSTRG.2	Gm5879	ENSMUST00000089501

MSTRG.2	Gm43904	ENSMUST00000204945
MSTRG.2	Gp9	ENSMUST00000032133
MSTRG.2	Rab43;G	ENSMUST00000032134;ENSMUST00000078647;ENSMUST00000204
MSTRG.2	Gm44423	ENSMUST00000203746
MSTRG.2	9930120	ENSMUST00000203027
MSTRG.2	B130021	ENSMUST00000205226
MSTRG.2657		MSTRG.2657.1
MSTRG.2	Cnbp	ENSMUST00000032138;ENSMUST00000204653;MSTRG.26570.3;E
MSTRG.2	Gm44064	ENSMUST00000204964
MSTRG.26572		MSTRG.26572.1
MSTRG.26573		MSTRG.26573.1
MSTRG.2	Copg1	ENSMUST00000113607;ENSMUST00000049966;ENSMUST00000203
MSTRG.2	Gm26636	ENSMUST00000181696
MSTRG.2	Hmces	ENSMUST00000032141;ENSMUST00000204614;ENSMUST00000132
MSTRG.2	Hmces	MSTRG.26577.1
MSTRG.2	H1fx;AC	ENSMUST00000056403;ENSMUST00000199539
MSTRG.2	Gm5577	ENSMUST00000134600;MSTRG.26579.1;ENSMUST00000143871;E
MSTRG.2	AC15389	ENSMUST00000214092
MSTRG.2	Gm44168	ENSMUST00000204835
MSTRG.2	4933412	ENSMUST00000203702
MSTRG.2	Rab7	ENSMUST00000113600;ENSMUST00000113598;ENSMUST00000113
MSTRG.2	Rab7	MSTRG.26583.1
MSTRG.2	Rab7	MSTRG.26584.1;MSTRG.26584.2
MSTRG.2	Gm44187	ENSMUST00000204735
MSTRG.2	Rpn1	ENSMUST00000032143;ENSMUST00000204838;ENSMUST00000203
MSTRG.2	Ruvbl1	ENSMUST00000032165;ENSMUST00000129035
MSTRG.2	Dnajb8	ENSMUST00000061866;ENSMUST00000203827
MSTRG.2	Trmt11	ENSMUST00000019927;ENSMUST00000215595;MSTRG.2659.3;EN
MSTRG.2	1700031	ENSMUST00000151901
MSTRG.2	Dnajb8	MSTRG.26591.1
MSTRG.26592		MSTRG.26592.1
MSTRG.2	Eefsec	MSTRG.26593.1;ENSMUST00000165242;ENSMUST00000205179;E
MSTRG.2	Gm44265	ENSMUST00000203631
MSTRG.2	Gm25178	ENSMUST00000122633
MSTRG.2	Eefsec	MSTRG.26596.1
MSTRG.2	Eefsec	MSTRG.26597.1
MSTRG.2	Gm44264	MSTRG.26598.1;ENSMUST00000203049
MSTRG.2	Sec61a1	ENSMUST00000032168;ENSMUST00000136103;ENSMUST00000203
MSTRG.2	AC15389	ENSMUST00000214634
MSTRG.2	Gm44170	ENSMUST00000203181
MSTRG.2	Gm44430	ENSMUST00000203376
MSTRG.2	Kbtbd12	ENSMUST00000120933;ENSMUST00000184743;ENSMUST00000184
MSTRG.2	Kbtbd12	MSTRG.26603.1
MSTRG.2	Gm26588	ENSMUST00000181355;ENSMUST00000181064
MSTRG.2	Mgll	ENSMUST00000113585;ENSMUST00000151699;ENSMUST00000113
MSTRG.2	Gm44005	ENSMUST00000203186
MSTRG.2	Gm43999	ENSMUST00000204286
MSTRG.2	Gm43937	ENSMUST00000204640
MSTRG.2	Gm44001	ENSMUST00000203158
MSTRG.2	Hint3;N	ENSMUST00000161074;ENSMUST00000216617;ENSMUST0000016C
MSTRG.2	Gm36189	ENSMUST00000204113
MSTRG.2	Mcm2	MSTRG.26611.1;ENSMUST00000058011;ENSMUST00000204365;E
MSTRG.2	Abtb1	ENSMUST00000032169;MSTRG.26612.5;MSTRG.26612.3;MSTRG.
MSTRG.2	Podxl2	ENSMUST00000141401;ENSMUST00000038409;ENSMUST00000061
MSTRG.2	Gm15612	ENSMUST00000147542
MSTRG.26615		MSTRG.26615.1
MSTRG.2	Gm44178	ENSMUST00000205229

MSTRG.2	Tpra1	ENSMUST00000055022;ENSMUST00000138415;ENSMUST00000204
MSTRG.2	Gm26811	ENSMUST00000181258
MSTRG.2	Plxna1	MSTRG.26619.1;ENSMUST00000163139;ENSMUST00000049845;E
MSTRG.2	Hint3	MSTRG.2662.1
MSTRG.2	Gm44207	ENSMUST00000204568
MSTRG.2	Chchd6	ENSMUST00000113550;ENSMUST00000032172;ENSMUST00000204
MSTRG.2	Gm43903	ENSMUST00000205126
MSTRG.2	Txnrd3	ENSMUST00000000828;ENSMUST00000101171
MSTRG.2	Gm20426	ENSMUST00000173657
MSTRG.2	Gm20426	MSTRG.26628.1
MSTRG.2	Vmn1r53	ENSMUST00000076086
MSTRG.2	Gm24189	ENSMUST00000157975
MSTRG.2	Zxdc;C0	ENSMUST00000113539;MSTRG.26630.1;ENSMUST00000075117;E
MSTRG.2	Cfap100	ENSMUST00000153843;ENSMUST00000062750;ENSMUST00000165
MSTRG.2	Klf15	ENSMUST00000032174;ENSMUST00000203039;ENSMUST00000113
MSTRG.2	Gm44421	ENSMUST00000205064
MSTRG.2	Gm45901	ENSMUST00000203807;ENSMUST00000127199;ENSMUST00000130
MSTRG.2	Gm44236	ENSMUST00000204492
MSTRG.2	Gm15756	ENSMUST00000161447
MSTRG.2	Slc41a3	ENSMUST00000044019;ENSMUST00000032177
MSTRG.2	Slc41a3	MSTRG.26638.1
MSTRG.2	Iqsec1	ENSMUST00000101153;ENSMUST00000204983;ENSMUST00000101
MSTRG.2	AC15397	ENSMUST00000215097
MSTRG.2	Gm24816	ENSMUST00000175311
MSTRG.2	Gm44123	ENSMUST00000204662
MSTRG.2	Gm44105	ENSMUST00000204401
MSTRG.2	Rpl21-p	ENSMUST00000080061
MSTRG.2	Gm25185	ENSMUST00000157754
MSTRG.2	Ppp1r2-	ENSMUST00000117103
MSTRG.2	Nup210	ENSMUST00000032179;ENSMUST00000142951;ENSMUST00000113
MSTRG.2	Hdac11	ENSMUST00000041736;ENSMUST00000154389;ENSMUST00000143
MSTRG.2	Gm14573	ENSMUST00000150544
MSTRG.2	Ncoa7	MSTRG.2665.1
MSTRG.2	Fbln2	ENSMUST00000113498;ENSMUST00000041544;ENSMUST00000153
MSTRG.2	Chchd4	ENSMUST00000040835;ENSMUST00000205802
MSTRG.2	Tmem43	ENSMUST00000032183;ENSMUST00000153179;ENSMUST00000205
MSTRG.2	Tmem43	MSTRG.26653.1
MSTRG.2	Xpc	ENSMUST00000032182;ENSMUST00000150279
MSTRG.2	Xpc	MSTRG.26655.1
MSTRG.2	Lsm3;Gm	ENSMUST00000040607;ENSMUST00000126253;ENSMUST00000205
MSTRG.2	Slc6a6	ENSMUST00000032185;ENSMUST00000205443;ENSMUST00000206
MSTRG.2	4930517	ENSMUST00000206152
MSTRG.2	Slc6a6	MSTRG.26659.1
MSTRG.2	Ncoa7	MSTRG.2666.1
MSTRG.2	Gm45828	ENSMUST00000206938
MSTRG.2	Gm44981	ENSMUST00000205549
MSTRG.2	Slc6a6	MSTRG.26662.1
MSTRG.2	Gm44712	ENSMUST00000206250
MSTRG.2	Ccdc174	ENSMUST00000138868;ENSMUST00000037783;ENSMUST00000136
MSTRG.2	Ccdc174	MSTRG.26665.1
MSTRG.2	4930590	ENSMUST00000205974;ENSMUST00000205686;ENSMUST00000205
MSTRG.2	Gm44711	ENSMUST00000205414
MSTRG.2	Fgd5	ENSMUST00000089334;ENSMUST00000113466;ENSMUST00000146
MSTRG.2	Gm44095	ENSMUST00000204631
MSTRG.2	Ncoa7	MSTRG.2667.1
MSTRG.2	4930466	ENSMUST00000144105
MSTRG.2	Rbsn	ENSMUST00000014694;ENSMUST00000124635;ENSMUST00000141

MSTRG.2	Nr2c2	MSTRG.26672.1;MSTRG.26672.2;ENSMUST00000113460;ENSMUS
MSTRG.2	Gm44249	ENSMUST00000204538
MSTRG.2	Mrps25	MSTRG.26674.1;ENSMUST00000140438;ENSMUST00000014695;E
MSTRG.2	Prickle	ENSMUST00000113447;ENSMUST00000032093;ENSMUST0000011З
MSTRG.2	Gm17797	ENSMUST00000182175
MSTRG.26677		MSTRG.26677.1
MSTRG.2	Adamts9	ENSMUST00000113438;ENSMUST00000167391;ENSMUST00000125
MSTRG.2	A730049	ENSMUST00000057977;ENSMUST00000133858
MSTRG.2	AC15397	ENSMUST00000214553
MSTRG.2	Adamts9	MSTRG.26680.1
MSTRG.2	Adamts9	MSTRG.26681.1
MSTRG.2	Adamts9	MSTRG.26682.1
MSTRG.2	Gm15737	ENSMUST00000113434
MSTRG.2	Adamts9	MSTRG.26684.1
MSTRG.2	9530026	ENSMUST00000204449;ENSMUST00000181121;ENSMUST00000205
MSTRG.2	9530026	MSTRG.26686.1
MSTRG.2	9530026	MSTRG.26687.1
MSTRG.2	9530026	MSTRG.26688.1
MSTRG.2	Gm44224	ENSMUST00000204131
MSTRG.2	AC12553	ENSMUST00000214944
MSTRG.2	9530026	MSTRG.26690.1
MSTRG.2	Gm5313	ENSMUST00000182893
MSTRG.2	9530026	MSTRG.26692.1
MSTRG.2	Gm43923	ENSMUST00000203992
MSTRG.2	9530026	MSTRG.26694.1
MSTRG.2	9530026	MSTRG.26695.1
MSTRG.2	Gm44220	ENSMUST00000203784
MSTRG.2	Magi1	ENSMUST00000055224;ENSMUST00000089317;ENSMUST00000204
MSTRG.2	Gm23035	ENSMUST00000158002
MSTRG.2	Gm22840	ENSMUST00000158444
MSTRG.2	Neurl3	ENSMUST00000056946;ENSMUST00000188666
MSTRG.2	Ncoa7	MSTRG.2670.1
MSTRG.2	Magi1	MSTRG.26700.1
MSTRG.26701		MSTRG.26701.1
MSTRG.2	Kbtbd8	ENSMUST00000122938;ENSMUST00000032107;ENSMUST0000011
MSTRG.2	Slc25a2	ENSMUST00000061118;ENSMUST00000204764;ENSMUST00000204
MSTRG.2	Slc25a2	MSTRG.26705.1
MSTRG.2	Slc25a2	MSTRG.26706.1
MSTRG.2	Lrig1;M	ENSMUST00000204645;ENSMUST00000032105;ENSMUST00000101
MSTRG.2	Lrig1	MSTRG.26708.1
MSTRG.2	Lrig1	MSTRG.26709.1
MSTRG.2	Hddc2	ENSMUST00000000304;ENSMUST00000162076;ENSMUST0000016C
MSTRG.2	Suclg2	ENSMUST00000204224;ENSMUST00000079847;ENSMUST00000203
MSTRG.2	Fam19a1	ENSMUST00000122120;ENSMUST00000125951;ENSMUST0000012
MSTRG.2	1700123	ENSMUST00000090061
MSTRG.2	Fam19a1	MSTRG.26713.1
MSTRG.2	Eogt	ENSMUST00000054344;ENSMUST00000113387;ENSMUST0000013C
MSTRG.2	Gm44065	ENSMUST00000203184
MSTRG.2	Tmf1;Gm	ENSMUST00000095664;ENSMUST00000124173;ENSMUST00000204
MSTRG.2	Uba3	ENSMUST00000164744;ENSMUST00000204056;ENSMUST0000008
MSTRG.2	Arl6ip5	ENSMUST00000044681;MSTRG.26720.2
MSTRG.2	Arl6ip5	MSTRG.26721.1
MSTRG.2	Frmd4b	ENSMUST00000113355;ENSMUST00000203486;MSTRG.26722.2;E
MSTRG.2	Frmd4b	MSTRG.26723.1
MSTRG.2	Frmd4b	MSTRG.26724.1
MSTRG.2	Frmd4b	MSTRG.26725.1
MSTRG.2	Frmd4b	MSTRG.26726.1

MSTRG.2	Frmd4b	MSTRG.26727.1
MSTRG.2	Gm19653	ENSMUST00000204326
MSTRG.2	Frmd4b	MSTRG.26729.1
MSTRG.2	AC15396	ENSMUST00000214047
MSTRG.2	Frmd4b	MSTRG.26730.1
MSTRG.2	Frmd4b	MSTRG.26731.1
MSTRG.2	Frmd4b	MSTRG.26732.1
MSTRG.2	Gm25852	ENSMUST00000082790
MSTRG.2	Frmd4b	MSTRG.26734.1
MSTRG.2	Gm23510	ENSMUST00000083083
MSTRG.2	Gm44102	ENSMUST00000204031
MSTRG.2	Mitf	ENSMUST00000203884;ENSMUST00000148233;ENSMUST0000004З
MSTRG.2	Mitf	MSTRG.26738.1
MSTRG.2	Mitf	MSTRG.26739.1
MSTRG.2	Mitf	MSTRG.26740.1
MSTRG.2	Mitf	MSTRG.26741.1
MSTRG.2	Mitf	MSTRG.26742.1
MSTRG.2	Mitf	MSTRG.26743.1
MSTRG.2	Gm15531	ENSMUST00000118062
MSTRG.2	Mitf	MSTRG.26745.1
MSTRG.2	Tpt1-ps	ENSMUST00000121046
MSTRG.2	Rybp	ENSMUST00000101118;ENSMUST00000203410;ENSMUST00000205
MSTRG.2	Gm18422	ENSMUST00000203663;ENSMUST00000203798
MSTRG.2	Gm44045	ENSMUST00000204906
MSTRG.2	Gm44044	ENSMUST00000205178
MSTRG.2	Shq1	ENSMUST00000113312;ENSMUST00000170667;ENSMUST0000008[illegible]
MSTRG.2	Shq1	MSTRG.26752.1
MSTRG.2	Gm6565	ENSMUST00000121217
MSTRG.2	Gm44108	ENSMUST00000203525
MSTRG.2	Gm44116	ENSMUST00000204269
MSTRG.2	Ppp4r2	ENSMUST00000063854;MSTRG.26756.2;ENSMUST00000204001;E
MSTRG.2	Gm43948	ENSMUST00000203474
MSTRG.2	Gm43948	MSTRG.26759.1;MSTRG.26759.2
MSTRG.2676		MSTRG.2676.1
MSTRG.2	Gm43942	ENSMUST00000204522
MSTRG.2	Pdzrn3	ENSMUST00000075994;ENSMUST00000124884;ENSMUST00000144
MSTRG.2	Gm26911	ENSMUST00000181770;ENSMUST00000204244;ENSMUST00000204
MSTRG.2	9530086	ENSMUST00000203503
MSTRG.2	Gm43953	ENSMUST00000203709
MSTRG.2	Gm43947	ENSMUST00000203019
MSTRG.2	Gm43946	ENSMUST00000204058
MSTRG.2	Foxp1;A	MSTRG.26768.1;ENSMUST00000113326;ENSMUST00000113322;E
MSTRG.2	Foxp1	MSTRG.26769.1
MSTRG.2	Foxp1	MSTRG.26770.1
MSTRG.2	Foxp1	MSTRG.26771.1;MSTRG.26771.2
MSTRG.2	Foxp1	MSTRG.26772.1
MSTRG.2	Foxp1	MSTRG.26774.1;MSTRG.26774.2
MSTRG.2	Foxp1	MSTRG.26775.1
MSTRG.2	Gm22328	ENSMUST00000158074
MSTRG.2	Gm20696	MSTRG.26777.1
MSTRG.2	Gm20696	MSTRG.26778.1
MSTRG.2	Gm24936	ENSMUST00000122598
MSTRG.2	Rsph4a	ENSMUST00000169670
MSTRG.2	Gm20696	MSTRG.26780.1
MSTRG.26781		MSTRG.26781.1
MSTRG.26782		MSTRG.26782.1
MSTRG.2	Gpr27	ENSMUST00000101122

MSTRG.2 Gm44429 ENSMUST00000203542
MSTRG.26785 MSTRG.26785.1
MSTRG.2 Chl1 ENSMUST00000203912;ENSMUST00000203489;ENSMUST0000006[illegible]
MSTRG.2 Chl1 MSTRG.26787.1
MSTRG.2 Gm44439 ENSMUST00000203379
MSTRG.2 Gm7946 ENSMUST00000203326
MSTRG.2 Zufsp ENSMUST00000218055;ENSMUST00000218275;MSTRG.2679.2;EN[illegible]
MSTRG.2 Gm44441 ENSMUST00000203642
MSTRG.2 Gm24784 ENSMUST00000157640
MSTRG.2 Chl1 MSTRG.26792.1
MSTRG.2 Gm44440 ENSMUST00000203139
MSTRG.2 Gm44436 ENSMUST00000204200
MSTRG.26795 MSTRG.26795.1
MSTRG.2 Il5ra ENSMUST00000204659;ENSMUST00000205004;ENSMUST00000167
MSTRG.2 Trnt1 ENSMUST00000113247;ENSMUST00000113249;ENSMUST0000011[illegible]
MSTRG.2 Crbn MSTRG.26798.2;ENSMUST00000013882;ENSMUST00000113239;[illegible]
MSTRG.26799 MSTRG.26799.1
MSTRG.2 Neurl3 MSTRG.268.1
MSTRG.2 Zufsp MSTRG.2680.1
MSTRG.26800 MSTRG.26800.1
MSTRG.26801 MSTRG.26801.1
MSTRG.2 Sumf1 ENSMUST00000032191;ENSMUST00000166197;ENSMUST00000167
MSTRG.2 Gm4604 ENSMUST00000025040
MSTRG.2 0610040[illegible] ENSMUST00000137478;ENSMUST00000204759
MSTRG.2 Gm17055 ENSMUST00000204397
MSTRG.2 Bhlhe40 ENSMUST00000032194;ENSMUST00000166346;MSTRG.26807.2;[illegible]
MSTRG.2 Itpr1 ENSMUST00000032192;MSTRG.26808.4;MSTRG.26808.5;MSTRG.
MSTRG.2 Itpr1 MSTRG.26809.1
MSTRG.2 Rwdd1 ENSMUST00000019917;ENSMUST00000128699;ENSMUST0000021[illegible]
MSTRG.2 Gm27813 ENSMUST00000184343
MSTRG.2 Gm44101 ENSMUST00000203951
MSTRG.2 Itpr1 MSTRG.26812.1
MSTRG.2 Mir7661 ENSMUST00000197023
MSTRG.2 Itpr1 MSTRG.26814.1
MSTRG.2 Gm44040 ENSMUST00000203360
MSTRG.2 Arl8b;E[illegible] ENSMUST00000032196;MSTRG.26816.1;ENSMUST00000204483;[illegible]
MSTRG.2 Edem1 MSTRG.26817.1
MSTRG.2 Rad18 ENSMUST00000077088;ENSMUST00000113182;ENSMUST0000011[illegible]
MSTRG.2 Gm26799 ENSMUST00000180959
MSTRG.2 AC15396[illegible] ENSMUST00000218530
MSTRG.2 Gm26799 MSTRG.26820.1
MSTRG.2 Srgap3 ENSMUST00000088373;ENSMUST00000113169;MSTRG.26821.3;[illegible]
MSTRG.2 Gm44081 ENSMUST00000204406
MSTRG.2 Mtmr14 ENSMUST00000113146;ENSMUST00000142938;MSTRG.26823.2;[illegible]
MSTRG.2 Gm16161 MSTRG.26824.1;ENSMUST00000162239
MSTRG.2 Gm43868 ENSMUST00000205187
MSTRG.2 Thumpd3 ENSMUST00000032398;ENSMUST00000155378;ENSMUST00000137
MSTRG.2 Gt(ROSA ENSMUST00000167415;ENSMUST00000133467;MSTRG.26827.3;[illegible]
MSTRG.2 Setd5 ENSMUST00000143998;MSTRG.26828.1;ENSMUST00000042889;[illegible]
MSTRG.2 Brpf1 ENSMUST00000113122;ENSMUST00000204198;ENSMUST0000011[illegible]
MSTRG.2 Dse ENSMUST00000048010;ENSMUST00000217051;ENSMUST0000021[illegible]
MSTRG.2 Gm15492 ENSMUST00000156734
MSTRG.2 Ogg1 ENSMUST00000032406;ENSMUST00000129871;MSTRG.26831.2;[illegible]
MSTRG.2 Camk1 ENSMUST00000032409;ENSMUST00000153067;ENSMUST0000014[illegible]
MSTRG.2 Cidec ENSMUST00000113089;ENSMUST00000032416;ENSMUST0000011[illegible]
MSTRG.2 Jagn1 ENSMUST00000101070;ENSMUST00000204287;ENSMUST00000204
MSTRG.2 Gm20589 ENSMUST00000204099

MSTRG.2	Tada3	MSTRG.26836.1;ENSMUST00000032410;ENSMUST00000043333;E
MSTRG.2	Arpc4;T	ENSMUST00000156898;MSTRG.26837.1;ENSMUST00000203578;M
MSTRG.2	Rpusd3	ENSMUST00000113092;ENSMUST00000060634;ENSMUST00000203
MSTRG.2	Gm44280	ENSMUST00000204498
MSTRG.2	AC15395	ENSMUST00000213617
MSTRG.2	Il17re	ENSMUST00000058548;ENSMUST00000204523;ENSMUST00000204
MSTRG.2	Il17rc	ENSMUST00000058300;ENSMUST00000204447;ENSMUST00000205
MSTRG.2	Creld1	ENSMUST00000032422;ENSMUST00000147932;ENSMUST00000156
MSTRG.2	Prrt3	ENSMUST00000204268;ENSMUST00000101059;ENSMUST00000205
MSTRG.2	Brk1	ENSMUST00000035725;MSTRG.26844.1
MSTRG.2	Gm44200	ENSMUST00000204883
MSTRG.2	Gm44199	MSTRG.26846.1;ENSMUST00000205219
MSTRG.2	Vhl	ENSMUST00000035673;ENSMUST00000128770;ENSMUST00000131
MSTRG.2	1700015	ENSMUST00000180964
MSTRG.2	1700015	MSTRG.26849.1
MSTRG.2	Tspyl1	ENSMUST00000061372
MSTRG.2	Emc3	MSTRG.26850.1;ENSMUST00000032425;ENSMUST00000154605;E
MSTRG.2	Gm23365	ENSMUST00000175019
MSTRG.2	Fancd2	ENSMUST00000036340;ENSMUST00000143535;ENSMUST00000101
MSTRG.2	Gm44232	ENSMUST00000204537
MSTRG.2	Fancd2	MSTRG.26854.1
MSTRG.2	Fancd2o	ENSMUST00000125139;ENSMUST00000035870
MSTRG.2	Gm44053	ENSMUST00000203364
MSTRG.2	Sec13	MSTRG.26857.1;ENSMUST00000032440;ENSMUST00000138204;E
MSTRG.2	Irak2	ENSMUST00000059286;ENSMUST00000089023;ENSMUST00000204
MSTRG.2	Gm43964	ENSMUST00000203570
MSTRG.2	Nt5dc1	ENSMUST00000129363;ENSMUST00000105512;ENSMUST00000047
MSTRG.2	Gm43963	ENSMUST00000203466
MSTRG.2	Gm43912	ENSMUST00000204352
MSTRG.2	Irak2	MSTRG.26862.1
MSTRG.2	Tatdn2;l	ENSMUST00000089018;MSTRG.26863.2;MSTRG.26863.3;ENSMUS
MSTRG.2	Ghrl	ENSMUST00000204533;ENSMUST00000064993;ENSMUST00000203
MSTRG.2	Gm44033	ENSMUST00000203699
MSTRG.2	Tamm41	ENSMUST00000032461;ENSMUST00000137654;ENSMUST00000144
MSTRG.2	Tamm41	MSTRG.26867.1
MSTRG.2	Tamm41	MSTRG.26868.1
MSTRG.2	Atg7	ENSMUST00000032457;ENSMUST00000169310;ENSMUST00000183
MSTRG.2	AC11305	ENSMUST00000213462
MSTRG.2	Gm19040	ENSMUST00000203280
MSTRG.2	Gm44332	ENSMUST00000203712
MSTRG.2	Gm44331	ENSMUST00000203217
MSTRG.2	Vgll4	ENSMUST00000152710;ENSMUST00000032459;MSTRG.26873.2;E
MSTRG.2	Vgll4	MSTRG.26874.1
MSTRG.26876		MSTRG.26876.1
MSTRG.2	Pparg	ENSMUST00000171644;ENSMUST00000203732;ENSMUST00000204
MSTRG.2	Pparg	MSTRG.26878.1
MSTRG.2	Gm44079	ENSMUST00000204889
MSTRG.2	Gm26564	ENSMUST00000181185;ENSMUST00000214449
MSTRG.2	Tsen2	ENSMUST00000130425;ENSMUST00000040234;ENSMUST00000123
MSTRG.2	Mkrn2os	ENSMUST00000088962;ENSMUST00000112957
MSTRG.2	Raf1	MSTRG.26882.1
MSTRG.2	Mkrn2	MSTRG.26883.1
MSTRG.2	Mkrn2	ENSMUST00000000449;ENSMUST00000204409;MSTRG.26884.2;E
MSTRG.2	Gm44157	ENSMUST00000204720
MSTRG.2	Raf1;Gm	ENSMUST00000130528;MSTRG.26886.2;ENSMUST00000112949;E
MSTRG.2	Raf1	MSTRG.26887.1
MSTRG.2	D830050	ENSMUST00000203858;ENSMUST00000203121

MSTRG.2	Cand2	ENSMUST00000075995
MSTRG.2	Tspyl4	ENSMUST00000047935;ENSMUST00000213791
MSTRG.2	Mir7660	ENSMUST00000183825
MSTRG.2	Rpl32;S	ENSMUST00000081840;ENSMUST00000203816;ENSMUST00000082
MSTRG.2	Efcab12	ENSMUST00000032468;ENSMUST00000124531
MSTRG.2	Gm44206	ENSMUST00000203044;ENSMUST00000203620
MSTRG.2	Efcab12	MSTRG.26894.1
MSTRG.2	Mbd4	MSTRG.26895.1;ENSMUST00000032469;ENSMUST00000133830;
MSTRG.2	Ift122	ENSMUST00000112925;ENSMUST00000038234;ENSMUST00000141
MSTRG.2	Plxnd1	ENSMUST00000015511;ENSMUST00000205003;ENSMUST0000012
MSTRG.2	Tmcc1	ENSMUST00000088896;ENSMUST00000204353;ENSMUST00000032
MSTRG.2	Tmcc1	MSTRG.26899.1
MSTRG.2	Gm33280	ENSMUST00000193983
MSTRG.2	Nt5dc1	MSTRG.2690.1
MSTRG.2	Tmcc1	MSTRG.26900.1
MSTRG.2	Gm20404	ENSMUST00000174032
MSTRG.2	Tmcc1	MSTRG.26902.1
MSTRG.2	Tmcc1	MSTRG.26904.1
MSTRG.2	Zfp422	ENSMUST00000079749;ENSMUST00000112880;ENSMUST00000057
MSTRG.2	Gm44154	ENSMUST00000204559
MSTRG.2	Rassf4	ENSMUST00000035842;ENSMUST00000203029;ENSMUST0000020
MSTRG.2	Mir7043	ENSMUST00000184219
MSTRG.2	8430408	ENSMUST00000067354;ENSMUST00000178241;ENSMUST00000204
MSTRG.2	Nt5dc1	MSTRG.2691.1
MSTRG.2	Rassf4	MSTRG.26910.1
MSTRG.2	Cxcl12	ENSMUST00000112866;ENSMUST00000112871;ENSMUST00000134
MSTRG.2	9530062	ENSMUST00000203332
MSTRG.2	Washc2	ENSMUST00000036759;ENSMUST00000204476;ENSMUST0000020
MSTRG.2	Gm44103	ENSMUST00000204364
MSTRG.2	Zfand4	ENSMUST00000112900;ENSMUST00000222819;ENSMUST0000022
MSTRG.2	Zfand4	MSTRG.26916.1
MSTRG.2	Zfand4	MSTRG.26917.1
MSTRG.2	Zfand4	MSTRG.26918.1
MSTRG.2	Gm44957	ENSMUST00000203361;ENSMUST00000205137
MSTRG.2	Nt5dc1	MSTRG.2692.1
MSTRG.2	March8	ENSMUST00000079012;ENSMUST00000101032;ENSMUST0000012
MSTRG.2	Gm23527	ENSMUST00000104570
MSTRG.2	Gm44237	ENSMUST00000205183
MSTRG.2	Gm7292	ENSMUST00000194706;ENSMUST00000195277
MSTRG.2	Zfp637	ENSMUST00000127593;ENSMUST00000112859;ENSMUST00000137
MSTRG.2	Hnrnpf	ENSMUST00000177743;ENSMUST00000167182;ENSMUST0000017
MSTRG.2	Hnrnpf	MSTRG.26927.1
MSTRG.2	4933440	MSTRG.26928.1;ENSMUST00000204444;ENSMUST00000035534;
MSTRG.2	Hnrnpf	MSTRG.26929.1
MSTRG.2	Nt5dc1	MSTRG.2693.1
MSTRG.2	Fxyd4	ENSMUST00000203082;ENSMUST00000203433;ENSMUST0000000
MSTRG.2	Gm25535	ENSMUST00000175364
MSTRG.2	Csgalna	ENSMUST00000125531;ENSMUST00000049344;ENSMUST0000013
MSTRG.2	Gm23908	ENSMUST00000104599
MSTRG.2	Csgalna	MSTRG.26934.1
MSTRG.2	Bms1	MSTRG.26935.1;ENSMUST00000032237;ENSMUST00000205207;
MSTRG.2	Ankrd26	ENSMUST00000112830;ENSMUST00000188172;ENSMUST0000018
MSTRG.2	Cacna2d	ENSMUST00000037434;ENSMUST00000190015;ENSMUST0000018
MSTRG.2	Lrtm2	ENSMUST00000112756;ENSMUST00000068351
MSTRG.2	Adipor2	MSTRG.26939.1;ENSMUST00000032272;MSTRG.26939.2;ENSMUS
MSTRG.2	Col10a1	ENSMUST00000105511
MSTRG.2	Wnt5b	ENSMUST00000117171;ENSMUST00000178696;ENSMUST0000011

MSTRG.2	Wnt5b	MSTRG.26941.1
MSTRG.2	Wnt5b	MSTRG.26942.1
MSTRG.2	Wnt5b	MSTRG.26943.1
MSTRG.2	Wnt5b	MSTRG.26944.1
MSTRG.2	Fbxl14	MSTRG.26945.1;ENSMUST00000032094
MSTRG.2	Wnt5b	MSTRG.26946.1
MSTRG.2	Wnt5b	MSTRG.26947.1
MSTRG.2	Wnt5b	MSTRG.26948.1
MSTRG.2	Wnt5b	MSTRG.26949.1
MSTRG.2695		MSTRG.2695.1
MSTRG.2	Wnt5b	MSTRG.26950.1
MSTRG.2	Wnt5b	MSTRG.26951.1
MSTRG.2	Wnt5b	MSTRG.26952.1
MSTRG.2	Cacna1c	ENSMUST00000186889;ENSMUST00000191196;ENSMUST0000018[illegible]
MSTRG.2	Gm18981	ENSMUST00000185373
MSTRG.2	Gm25905	ENSMUST00000158490
MSTRG.2	Dcp1b	ENSMUST00000112777;MSTRG.26956.4;ENSMUST00000073909;[illegible]
MSTRG.2	Cacna1c	MSTRG.26957.1
MSTRG.2	Erc1	MSTRG.26958.1;ENSMUST00000183703;ENSMUST00000032279;[illegible]
MSTRG.2	Gm44317	ENSMUST00000204314
MSTRG.2	Amd2	ENSMUST00000080898
MSTRG.2	Erc1	MSTRG.26960.1
MSTRG.2	4930540[illegible]	ENSMUST00000180527
MSTRG.2	Rad52	MSTRG.26962.1;ENSMUST00000162461;ENSMUST00000161045;[illegible]
MSTRG.2	Rad52	MSTRG.26963.1
MSTRG.2	Ninj2	ENSMUST00000112711;ENSMUST00000035244
MSTRG.2	Ninj2	MSTRG.26965.1
MSTRG.2	Wnk1	ENSMUST00000159903;MSTRG.26967.1;ENSMUST00000060043;[illegible]
MSTRG.2	Gm16199	ENSMUST00000160032
MSTRG.2	Wnk1	MSTRG.26969.1
MSTRG.2	Hs3st5	ENSMUST00000167191;ENSMUST00000058738;ENSMUST00000172
MSTRG.2	Mir706	ENSMUST00000102262
MSTRG.2	Il17ra	ENSMUST00000204239;MSTRG.26971.1;MSTRG.26971.2;MSTRG.
MSTRG.2	Ccdc77	ENSMUST00000032283;ENSMUST00000127146;ENSMUST00000112
MSTRG.2	Ccdc77	MSTRG.26973.1
MSTRG.2	Ccdc77	MSTRG.26974.1
MSTRG.2	Kdm5a	ENSMUST00000135802;ENSMUST00000005108;ENSMUST00000152
MSTRG.2	Kdm5a	MSTRG.26976.1
MSTRG.2	Gm24592	ENSMUST00000122505
MSTRG.26978		MSTRG.26978.1
MSTRG.2	Hdhd5	MSTRG.26979.2;MSTRG.26979.3;MSTRG.26979.1;ENSMUST000[illegible]
MSTRG.2	AC13166	ENSMUST00000214665
MSTRG.2	Gm20692	MSTRG.26980.1;ENSMUST00000177251
MSTRG.2	Gm24391	ENSMUST00000158138
MSTRG.2	Hdhd5	MSTRG.26982.1
MSTRG.2	Cecr2	ENSMUST00000148346;ENSMUST00000112686;ENSMUST0000010[illegible]
MSTRG.2	Slc25a1	ENSMUST00000112682;MSTRG.26984.2;MSTRG.26984.3
MSTRG.2	Atp6v1e	ENSMUST00000203527;ENSMUST00000019354;ENSMUST0000020[illegible]
MSTRG.2	Atp6v1e	MSTRG.26986.1
MSTRG.2	Atp6v1e	MSTRG.26987.1
MSTRG.2	Bcl2l13	ENSMUST00000203584;ENSMUST00000204004;ENSMUST0000020[illegible]
MSTRG.2	Bcl2l13	MSTRG.26989.1
MSTRG.2	Hs3st5	MSTRG.2699.1
MSTRG.2	Bid	ENSMUST00000160684;ENSMUST00000004560;ENSMUST00000161
MSTRG.2	Bid	MSTRG.26991.1
MSTRG.2	Gm17812	ENSMUST00000204935
MSTRG.2	Gm44260	ENSMUST00000204946

MSTRG.2	Pex26	ENSMUST00000118234;ENSMUST00000088561;ENSMUST0000012
MSTRG.2	Gm44014	ENSMUST00000205150
MSTRG.2	Tuba8	ENSMUST00000032233
MSTRG.2	Gm15856	ENSMUST00000124853
MSTRG.26998		MSTRG.26998.1
MSTRG.2	Usp18	ENSMUST00000032198;ENSMUST00000203250;MSTRG.26999.2;
MSTRG.2	4732440	ENSMUST00000191825;ENSMUST00000159618;ENSMUST00000097
MSTRG.2	Uggt1	ENSMUST00000046875;ENSMUST00000173999;ENSMUST00000174
MSTRG.2	Gm44148	ENSMUST00000203718
MSTRG.2	Usp18	MSTRG.27001.1
MSTRG.2	Mical3	ENSMUST00000098457;ENSMUST00000207889;ENSMUST00000212
MSTRG.2	Gm43916	ENSMUST00000205062
MSTRG.2	Gm43915	ENSMUST00000203013
MSTRG.2	Mical3	MSTRG.27005.1
MSTRG.2	Mical3	MSTRG.27006.1
MSTRG.2	Gm4651	ENSMUST00000181738;ENSMUST00000203697
MSTRG.2	Mical3	MSTRG.27008.1
MSTRG.2	Minpp1-	ENSMUST00000203344
MSTRG.2	Hdac2	ENSMUST00000019911;ENSMUST00000105510;MSTRG.2701.2;EN
MSTRG.2	Mug2	ENSMUST00000081777
MSTRG.2	Mug2	MSTRG.27012.1
MSTRG.2	Rimklb	ENSMUST00000146274;ENSMUST00000068242;ENSMUST00000143
MSTRG.2	Gm8430	ENSMUST00000119780
MSTRG.2	Mfap5	ENSMUST00000032210;ENSMUST00000148517;ENSMUST00000121
MSTRG.2	M6pr	ENSMUST00000007602;ENSMUST00000112610;ENSMUST00000123
MSTRG.2	Phc1	ENSMUST00000079560;ENSMUST00000081849;ENSMUST00000161
MSTRG.2	Gm10420	ENSMUST00000121713
MSTRG.2	Aicda	ENSMUST00000043301;ENSMUST00000160685
MSTRG.2	Marcks	ENSMUST00000092584
MSTRG.2	Gm16556	ENSMUST00000162608
MSTRG.2	Gm8459	ENSMUST00000162504
MSTRG.2	Apobec1	ENSMUST00000112586;ENSMUST00000112587;MSTRG.27023.2;E
MSTRG.2	C3ar1	ENSMUST00000042081
MSTRG.2	Slc2a3	ENSMUST00000032476;ENSMUST00000168704;MSTRG.27025.2;E
MSTRG.2	Slc2a3	MSTRG.27026.1
MSTRG.2	Gm4482	ENSMUST00000200876
MSTRG.2	Gm5112	ENSMUST00000204407
MSTRG.2	Gm26826	ENSMUST00000181317
MSTRG.2	Lama4	ENSMUST00000159506;ENSMUST00000019992;ENSMUST00000162
MSTRG.2	Gm26826	MSTRG.27030.1
MSTRG.2	Foxj2;M	ENSMUST00000003238;ENSMUST00000203075;ENSMUST00000196
MSTRG.2	Necap1	ENSMUST00000032477;ENSMUST00000203715;ENSMUST00000203
MSTRG.2	Gm5316	ENSMUST00000185734;ENSMUST00000050391
MSTRG.2	Clec4a1	ENSMUST00000060484
MSTRG.2	Clec4a3	ENSMUST00000117173;ENSMUST00000088468
MSTRG.2	Clec4n	ENSMUST00000112554;ENSMUST00000024118;ENSMUST00000144
MSTRG.27037		MSTRG.27037.1
MSTRG.27038		MSTRG.27038.1
MSTRG.2	Vmn2r27	ENSMUST00000100968
MSTRG.2	Fam229b	ENSMUST00000063204;ENSMUST00000139743;ENSMUST00000137
MSTRG.2	Vmn2r27	MSTRG.27040.1
MSTRG.2	Gm6308	ENSMUST00000203226
MSTRG.27042		MSTRG.27042.1
MSTRG.2	Pex5	ENSMUST00000112532;ENSMUST00000112530;ENSMUST00000112
MSTRG.2	Pex5	MSTRG.27044.1
MSTRG.2	Clstn3	ENSMUST00000112523;ENSMUST00000008297;ENSMUST00000124
MSTRG.2	C1ra	ENSMUST00000068593

MSTRG.2	C1s1;C1	ENSMUST00000160505;ENSMUST00000162443;MSTRG.27047.2;E
MSTRG.2	Gm18840	ENSMUST00000204553
MSTRG.2	Tube1	ENSMUST00000213237;ENSMUST00000019991;MSTRG.2705.2;EN
MSTRG.2	C1rb	ENSMUST00000184647
MSTRG.2	Ptpn6	ENSMUST00000112484;ENSMUST00000004377;ENSMUST00000171
MSTRG.2	Gm20531	ENSMUST00000174861
MSTRG.27053		MSTRG.27053.1
MSTRG.27054		MSTRG.27054.1
MSTRG.2	Spsb2	ENSMUST00000047760;ENSMUST00000143040;ENSMUST00000052
MSTRG.2	Tpi1	ENSMUST00000172132;ENSMUST00000149610;ENSMUST00000133
MSTRG.2	Grcc10;(	ENSMUST00000129823;ENSMUST00000004389;ENSMUST0000014C
MSTRG.2	Eno2	ENSMUST00000147307;ENSMUST00000112476;ENSMUST00000004
MSTRG.2	Lrrc23	ENSMUST00000032218;ENSMUST00000112475;ENSMUST00000132
MSTRG.2	Wisp3	ENSMUST00000076713;MSTRG.2706.2
MSTRG.2	Lpcat3	ENSMUST00000135516;ENSMUST00000004381;ENSMUST00000128
MSTRG.2	Emg1	ENSMUST00000204334;ENSMUST00000004379;ENSMUST00000125
MSTRG.2	Phb2;Gm	ENSMUST00000150847;ENSMUST00000124779;ENSMUST00000004
MSTRG.2	Gm15884	ENSMUST00000143765
MSTRG.2	Gm29008	ENSMUST00000189387;ENSMUST00000083540
MSTRG.2	Mir200c	ENSMUST00000083528
MSTRG.2	Usp5	ENSMUST00000047510;MSTRG.27066.3;MSTRG.27066.2;ENSMUS
MSTRG.2	Cdca3	ENSMUST00000150120;ENSMUST00000024270;ENSMUST00000131
MSTRG.2	Gnb3	MSTRG.27068.1
MSTRG.2	Gnb3	ENSMUST00000024206;ENSMUST00000129251;ENSMUST0000014C
MSTRG.2	Rev3l	MSTRG.2707.1
MSTRG.2	P3h3	ENSMUST00000023958;ENSMUST00000135127;ENSMUST00000126
MSTRG.2	Gpr162	ENSMUST00000046893;ENSMUST00000204161;ENSMUST00000204
MSTRG.2	Cd4	ENSMUST00000024044;MSTRG.27072.1;MSTRG.27072.2;ENSMUS
MSTRG.2	Ptms	ENSMUST00000032216;ENSMUST00000203422;ENSMUST00000204
MSTRG.2	A230083(	ENSMUST00000069553
MSTRG.2	A230083(	MSTRG.27075.1
MSTRG.2	Mlf2	ENSMUST00000204177;ENSMUST00000204815;ENSMUST00000204
MSTRG.2	Lpar5	ENSMUST00000088292;ENSMUST00000140346;ENSMUST00000171
MSTRG.2	2010008(	ENSMUST00000203956
MSTRG.2	Cops7a	ENSMUST00000112439;ENSMUST00000032220;ENSMUST00000135
MSTRG.2	Mfsd4b4	ENSMUST00000178045;ENSMUST00000178563
MSTRG.2	Gm23851	ENSMUST00000083831
MSTRG.2	Pianp	ENSMUST00000032479;ENSMUST00000162000;MSTRG.27081.2;M
MSTRG.2	Pianp	MSTRG.27082.1
MSTRG.2	Zfp384	ENSMUST00000132863;ENSMUST00000054553;ENSMUST00000145
MSTRG.2	4930557I	ENSMUST00000205110
MSTRG.2	4930557I	MSTRG.27085.1
MSTRG.2	4930557I	MSTRG.27086.1
MSTRG.2	Ing4;Gm	ENSMUST00000140131;ENSMUST00000203287;ENSMUST00000128
MSTRG.2	Chd4;Mi	ENSMUST00000112392;ENSMUST00000056889;ENSMUST00000112
MSTRG.2	Gm23751	ENSMUST00000157583
MSTRG.2	4930547I	ENSMUST00000181590;ENSMUST00000213497
MSTRG.2	Nop2	ENSMUST00000044200;ENSMUST00000204185
MSTRG.2	Nop2	MSTRG.27091.1
MSTRG.2	Iffo1	ENSMUST00000119527;ENSMUST00000139071;ENSMUST00000144
MSTRG.2	Iffo1	MSTRG.27093.1
MSTRG.2	Gapdh	ENSMUST00000192506;ENSMUST00000147954;MSTRG.27094.2;E
MSTRG.2	Ncapd2;	ENSMUST00000188119;ENSMUST00000043848;MSTRG.27095.1;E
MSTRG.2	Mrpl51;I	ENSMUST00000136804;ENSMUST00000032485;ENSMUST00000144
MSTRG.2	Vamp1	ENSMUST00000032487;ENSMUST00000100942;ENSMUST00000063
MSTRG.2	Tapbpl;(	MSTRG.27098.9;MSTRG.27098.1;MSTRG.27098.10;MSTRG.2709
MSTRG.2	Vamp1	MSTRG.27099.1

MSTRG.2	Mir6897	ENSMUST00000184790
MSTRG.2	Slc16a1	ENSMUST00000092566;ENSMUST00000213827;ENSMUST0000021З
MSTRG.2	4930417	ENSMUST00000204911;ENSMUST00000204597;MSTRG.27100.2;E
MSTRG.2	Tuba3a	MSTRG.27101.1;MSTRG.27101.2;ENSMUST00000088246
MSTRG.2	Ltbr	ENSMUST00000032489;ENSMUST00000161891;ENSMUST0000016C
MSTRG.2	Scnn1a	ENSMUST00000176655;ENSMUST00000176110;ENSMUST0000017Є
MSTRG.2	Tnfrsf1	ENSMUST00000131434;ENSMUST00000130257;ENSMUST0000003Z
MSTRG.2	Tnfrsf1	MSTRG.27105.1
MSTRG.2	Plekhg6	MSTRG.27106.1;ENSMUST00000042647
MSTRG.2	Cd9	ENSMUST00000032492;ENSMUST00000130132;ENSMUST0000014C
MSTRG.2	Gm28809	ENSMUST00000187542;ENSMUST00000187724
MSTRG.2	Gm26728	ENSMUST00000203423;ENSMUST00000204130;ENSMUST0000018Ƽ
MSTRG.2	Slc16a1	MSTRG.2711.1
MSTRG.2	Vwf	ENSMUST00000100941;ENSMUST00000112254;ENSMUST00000112
MSTRG.2	Ndufa9	ENSMUST00000205002;MSTRG.27111.1;ENSMUST00000088194;E
MSTRG.2	1700018	ENSMUST00000201350
MSTRG.2	Rad51ap	ENSMUST00000112220;ENSMUST00000201102;ENSMUST00000112
MSTRG.2	Gm17808	ENSMUST00000200859
MSTRG.2	D6Wsu16	ENSMUST00000202086;ENSMUST00000032497;ENSMUST00000201
MSTRG.2	D6Wsu16	MSTRG.27116.1
MSTRG.27117		MSTRG.27117.1
MSTRG.2	Gm43635	ENSMUST00000202533
MSTRG.2	Gm43635	MSTRG.27119.1
MSTRG.2	Gm25613	ENSMUST00000104012
MSTRG.2	Gm43126	ENSMUST00000201546
MSTRG.2	Ccnd2	ENSMUST00000000188;MSTRG.27121.2;MSTRG.27121.3;ENSMUS
MSTRG.2	9330179	ENSMUST00000180811;ENSMUST00000180569
MSTRG.2	Gm18995	ENSMUST00000202981
MSTRG.2	Tpi-rs1	ENSMUST00000202145
MSTRG.2	Parp11	MSTRG.27125.1
MSTRG.27126		MSTRG.27126.1
MSTRG.27127		MSTRG.27127.1
MSTRG.2	Parp11	MSTRG.27128.3;MSTRG.27128.1;MSTRG.27128.2;ENSMUST000C
MSTRG.2	Gm43633	ENSMUST00000202455
MSTRG.2	Slc16a1	MSTRG.2713.1
MSTRG.2	Cracr2a	ENSMUST00000212051;ENSMUST00000201303;ENSMUST00000071
MSTRG.2	Cracr2a	MSTRG.27131.1
MSTRG.2	Cracr2a	MSTRG.27132.1
MSTRG.2	Cracr2a	MSTRG.27133.1
MSTRG.2	Tspan9	MSTRG.27134.1;ENSMUST00000112173;ENSMUST00000112171;E
MSTRG.2	Gm26338	ENSMUST00000102372
MSTRG.2	Tspan9	MSTRG.27136.1
MSTRG.2	Rpl18-p	ENSMUST00000118863
MSTRG.2	Gm8724	ENSMUST00000081668
MSTRG.27139		MSTRG.27139.1
MSTRG.2	Slc16a1	MSTRG.2714.1
MSTRG.2	Klrb1a	ENSMUST00000032512;ENSMUST00000203150
MSTRG.2	Klrb1a	MSTRG.27141.1
MSTRG.2	Klrb1c	MSTRG.27142.1
MSTRG.2	Gm5884	ENSMUST00000036712
MSTRG.2	Clec2j	ENSMUST00000203658
MSTRG.2	Gm44216	ENSMUST00000203942
MSTRG.2	Clec2h	ENSMUST00000032518;ENSMUST00000205225
MSTRG.27149		MSTRG.27149.1
MSTRG.2	Gm44215	ENSMUST00000204910
MSTRG.2	Klrb1	ENSMUST00000203869;ENSMUST00000112110
MSTRG.2	Klrb1c	MSTRG.27153.1;MSTRG.27153.2

MSTRG.2	Klrb1c;(	ENSMUST00000167691;ENSMUST00000174404;ENSMUST00000172
MSTRG.2	Gm44511	MSTRG.27155.1
MSTRG.2	Tulp3	MSTRG.27156.1;ENSMUST00000001562;ENSMUST00000128708;E
MSTRG.2	Gm44190	ENSMUST00000205083
MSTRG.2	Tulp3	MSTRG.27158.1
MSTRG.2	Gm26770	ENSMUST00000180586
MSTRG.2	Rpf2	ENSMUST00000045114;MSTRG.2716.2;ENSMUST00000183309;EN
MSTRG.2	Rhno1	ENSMUST00000112152;ENSMUST00000057421;ENSMUST00000223
MSTRG.2	Foxm1;4	ENSMUST00000123988;ENSMUST00000073316;ENSMUST00000112
MSTRG.2	Gm15862	ENSMUST00000146349
MSTRG.2	Itfg2	ENSMUST00000001559;ENSMUST00000203984;ENSMUST00000142
MSTRG.2	Fkbp4	ENSMUST00000139448;ENSMUST00000032508;MSTRG.27165.4;M
MSTRG.2	Gm10069	ENSMUST00000144745;ENSMUST00000204081;ENSMUST00000204
MSTRG.2	Gm10069	MSTRG.27167.1
MSTRG.2	Gm18009	ENSMUST00000203239
MSTRG.2	Rpf2	MSTRG.2717.1
MSTRG.2	Gm10069	MSTRG.27170.1
MSTRG.2	Pzp	ENSMUST00000112132;ENSMUST00000203753
MSTRG.2	Gm27514	ENSMUST00000183692
MSTRG.2	2310001	ENSMUST00000181238;ENSMUST00000181517;MSTRG.27174.3;E
MSTRG.2	2310001	MSTRG.27175.1
MSTRG.2	2310001	MSTRG.27176.1
MSTRG.2	2310001	MSTRG.27177.1
MSTRG.2	AC14219	ENSMUST00000218416;ENSMUST00000219217
MSTRG.2	Cd69	MSTRG.27179.2;MSTRG.27179.4;MSTRG.27179.1;MSTRG.2717
MSTRG.2	Rnu3a	ENSMUST00000196710;ENSMUST00000083936
MSTRG.2	Gm44066	ENSMUST00000203263
MSTRG.2	Gm44067	ENSMUST00000203350
MSTRG.2	Gm26656	ENSMUST00000181825
MSTRG.2	BC03504	MSTRG.27183.1;ENSMUST00000160290;MSTRG.27183.3;ENSMUS
MSTRG.2	BC03504	MSTRG.27184.1
MSTRG.2	Clec2g	MSTRG.27185.1;MSTRG.27185.2
MSTRG.2	Gm8774	ENSMUST00000203621
MSTRG.2	Clec2i;(	ENSMUST00000032519;ENSMUST00000159866;ENSMUST0000015
MSTRG.2	BC03504	MSTRG.27188.1
MSTRG.2	Gm15987	MSTRG.27189.1
MSTRG.2	Fyn	ENSMUST00000148152;MSTRG.2719.1;ENSMUST00000063091;EN
MSTRG.2	Gm44223	ENSMUST00000204854
MSTRG.2	Gm27336	ENSMUST00000185190
MSTRG.2	Clec2g	ENSMUST00000000254;ENSMUST00000146925;ENSMUST00000204
MSTRG.2	Clec2g	MSTRG.27193.1
MSTRG.2	Gm15987	ENSMUST00000141700;ENSMUST00000184433
MSTRG.2	Clec2g	MSTRG.27195.1
MSTRG.2	Gm8778	ENSMUST00000127246
MSTRG.2	BC06407	ENSMUST00000131176;ENSMUST00000122939
MSTRG.2	Clec2f	ENSMUST00000204981
MSTRG.2	Gm16186	ENSMUST00000120132
MSTRG.2	4930403	ENSMUST00000192234
MSTRG.2	Fyn	MSTRG.2720.1;MSTRG.2720.2
MSTRG.2	Klrb1f	ENSMUST00000032257;ENSMUST00000203059;ENSMUST00000204
MSTRG.2	Gm43992	ENSMUST00000204358
MSTRG.2	Gm44000	ENSMUST00000205038
MSTRG.2	Clec2e	ENSMUST00000032258
MSTRG.2	Klrb1-p	ENSMUST00000184388
MSTRG.2	Gm26160	ENSMUST00000158965
MSTRG.2	Clec7a	ENSMUST00000195589;ENSMUST00000112076;ENSMUST00000184
MSTRG.2	1700101	ENSMUST00000203703

MSTRG.2	Gabarap	ENSMUST00000032264;ENSMUST00000204487;ENSMUST00000204
MSTRG.2	Gm44243	ENSMUST00000204323
MSTRG.2	Gm6963;	MSTRG.2721.1;ENSMUST00000147879;MSTRG.2721.3
MSTRG.2	Klrd1	ENSMUST00000032268;ENSMUST00000112063;ENSMUST0000011
MSTRG.2	Klrk1	MSTRG.27211.1;ENSMUST00000168919;ENSMUST00000032252;E
MSTRG.27212		MSTRG.27212.1
MSTRG.2	Klrc3	MSTRG.27213.1
MSTRG.2	Klrc3	ENSMUST00000170148;ENSMUST00000071149
MSTRG.2	Klrc2	MSTRG.27215.1;ENSMUST00000145984;ENSMUST00000071920;E
MSTRG.2	Klrc2	MSTRG.27216.1
MSTRG.2	Klrc1	ENSMUST00000032270;ENSMUST00000169545;ENSMUST00000032
MSTRG.2	Gm156	ENSMUST00000118532;ENSMUST00000095409
MSTRG.2	Gm16242	ENSMUST00000117741
MSTRG.2	Gm16364	ENSMUST00000153722
MSTRG.2	Gm16242	MSTRG.27220.1
MSTRG.2	Klra17	ENSMUST00000014687
MSTRG.2	Klra17	MSTRG.27223.1
MSTRG.2	Gm17631	ENSMUST00000171980
MSTRG.2	Gm33962	MSTRG.27226.1
MSTRG.2	Magohb;	ENSMUST00000032307;ENSMUST00000174488;ENSMUST00000172
MSTRG.2	Ybx3	ENSMUST00000032309;ENSMUST00000087865;ENSMUST00000205
MSTRG.2	Gm22362	ENSMUST00000083102
MSTRG.2	Gm16365	ENSMUST00000122987;ENSMUST00000142723;ENSMUST00000143
MSTRG.2	Gm6619;	ENSMUST00000159229;ENSMUST00000075020;ENSMUST00000161
MSTRG.2	5430401	MSTRG.27232.1
MSTRG.2	Gm16571	MSTRG.27233.1
MSTRG.2	Tas2r13	ENSMUST00000067597
MSTRG.2	Tas2r10	ENSMUST00000065781
MSTRG.27239		MSTRG.27239.1
MSTRG.2	Traf3ip	MSTRG.2724.1;MSTRG.2724.3;MSTRG.2724.2;ENSMUST0000001
MSTRG.2	Smim10l	ENSMUST00000100864;ENSMUST00000191462;ENSMUST00000187
MSTRG.27241		MSTRG.27241.1
MSTRG.2	Gm38910	ENSMUST00000204630
MSTRG.2	Mansc1	ENSMUST00000047443
MSTRG.2	Gm43952	ENSMUST00000203765
MSTRG.2	Lrp6	ENSMUST00000032322;MSTRG.27245.2;ENSMUST00000111950;E
MSTRG.2	Lrp6	MSTRG.27246.1
MSTRG.2	Lrp6	MSTRG.27247.1
MSTRG.2	Borcs5	ENSMUST00000166591;ENSMUST00000062755;ENSMUST00000203
MSTRG.2	Gm44017	ENSMUST00000203231
MSTRG.2	Traf3ip	MSTRG.2725.1
MSTRG.2	Etv6;Gm	ENSMUST00000081028;ENSMUST00000111963;ENSMUST00000164
MSTRG.2	Etv6	MSTRG.27251.1
MSTRG.2	Gm43984	ENSMUST00000204572
MSTRG.2	Etv6	MSTRG.27253.1
MSTRG.2	Etv6	MSTRG.27254.1
MSTRG.2	Etv6	MSTRG.27256.1
MSTRG.2	Gm17088	MSTRG.27257.1
MSTRG.2	Etv6	MSTRG.27258.1
MSTRG.2	Gm17089	MSTRG.27259.1
MSTRG.2	E130307	ENSMUST00000129191;MSTRG.2726.2;ENSMUST00000151921;EN
MSTRG.2	1700051	ENSMUST00000204426
MSTRG.2	Dusp16	ENSMUST00000129433;ENSMUST00000100857;ENSMUST00000204
MSTRG.2	Dusp16	MSTRG.27262.1
MSTRG.2	Gm36328	ENSMUST00000204237
MSTRG.2	Dusp16	MSTRG.27264.1
MSTRG.2	Crebl2	ENSMUST00000046303;ENSMUST00000145643;ENSMUST00000111

MSTRG.2	Gm44084	ENSMUST00000203947
MSTRG.2	Crebl2	MSTRG.27267.1
MSTRG.2	Gpr19	ENSMUST00000165392;ENSMUST00000116515;ENSMUST0000004€
MSTRG.2	Gm44366	ENSMUST00000204933
MSTRG.2	AC16040	ENSMUST00000214635
MSTRG.2	2810454	ENSMUST00000189942
MSTRG.2	Cdkn1b	MSTRG.27271.1;ENSMUST00000003115;ENSMUST00000067327;E
MSTRG.2	Cdkn1b	MSTRG.27272.1
MSTRG.2	Gm44238	ENSMUST00000203300
MSTRG.2	Lockd	ENSMUST00000183867;ENSMUST00000184991;ENSMUST0000018З
MSTRG.2	Gm44238	MSTRG.27275.1;MSTRG.27275.3;MSTRG.27275.2;MSTRG.27275
MSTRG.2	Lockd	MSTRG.27276.1
MSTRG.2	Gprc5a	ENSMUST00000050104
MSTRG.2	Ddx47	ENSMUST00000032326;ENSMUST00000130851;ENSMUST00000205
MSTRG.2	Dynlt1-	ENSMUST00000117045
MSTRG.2	E130307	MSTRG.2728.1
MSTRG.2	Emp1	ENSMUST00000205156;ENSMUST00000032330;ENSMUST00000154
MSTRG.2	Hebp1	ENSMUST00000045855;ENSMUST00000204000;ENSMUST00000204
MSTRG.2	Gm22881	ENSMUST00000157747
MSTRG.2	Hebp1	MSTRG.27283.1
MSTRG.2	Gm34391	ENSMUST00000203706
MSTRG.2	Gm19434	ENSMUST00000204330
MSTRG.2	Fam234b	ENSMUST00000111915;ENSMUST00000111916;ENSMUST0000013C
MSTRG.2	Gsg1	ENSMUST00000087729;ENSMUST00000111911
MSTRG.2	Hist4h4	ENSMUST00000179285
MSTRG.2	Gm43969	ENSMUST00000204833
MSTRG.2	E130307	MSTRG.2729.1
MSTRG.2	Gm43969	MSTRG.27290.1
MSTRG.2	Gucy2c	ENSMUST00000032338
MSTRG.2	Gucy2c	MSTRG.27292.1
MSTRG.2	H2afj	ENSMUST00000074556;ENSMUST00000203982
MSTRG.2	Gm44140	MSTRG.27295.1
MSTRG.2	Gm6728	MSTRG.27296.1
MSTRG.2	Gm6728	ENSMUST00000178117
MSTRG.2	Gm44131	ENSMUST00000203036
MSTRG.2	Rpl36a-	ENSMUST00000120093
MSTRG.2	Arid5a	ENSMUST00000126413;ENSMUST00000142319;ENSMUST0000015C
MSTRG.2	Rev3l	ENSMUST00000019986;MSTRG.2730.2;MSTRG.2730.3;ENSMUSTC
MSTRG.2	Atf7ip	ENSMUST00000203988;ENSMUST00000032335;ENSMUST00000185
MSTRG.2	Gm44140	ENSMUST00000204157
MSTRG.2	Atf7ip	MSTRG.27302.1
MSTRG.2	Atf7ip	MSTRG.27303.1
MSTRG.2	Plbd1	MSTRG.27304.1;ENSMUST00000032336;ENSMUST00000137139;E
MSTRG.2	Gm44401	ENSMUST00000204462
MSTRG.2	Gm44069	ENSMUST00000204898
MSTRG.2	Art4	ENSMUST00000032341
MSTRG.2	Mgp	ENSMUST00000032342;ENSMUST00000203103
MSTRG.2	Wbp11	MSTRG.27309.1;MSTRG.27309.2;ENSMUST00000116514;ENSMUS
MSTRG.2	Gm16210	ENSMUST00000122161
MSTRG.2	BC04971	ENSMUST00000205058;ENSMUST00000052702;ENSMUST0000020З
MSTRG.2	Smco3	ENSMUST00000068293;ENSMUST00000203468;ENSMUST00000111
MSTRG.2	Arhgdib	ENSMUST00000111892;ENSMUST00000032344;MSTRG.27312.1;E
MSTRG.2	Pde6h	ENSMUST00000204627;ENSMUST00000057650
MSTRG.2	Ptpro	ENSMUST00000167679;ENSMUST00000077115;ENSMUST00000167
MSTRG.2	Ptpro	MSTRG.27315.1
MSTRG.2	Strap	ENSMUST00000064910;ENSMUST00000154698;ENSMUST00000137
MSTRG.2	Dera	ENSMUST00000204100;ENSMUST00000203136;ENSMUST00000087

MSTRG.2	Dera	MSTRG.27319.1
MSTRG.2	Rev3l	MSTRG.2732.1
MSTRG.2	Dera	MSTRG.27320.1
MSTRG.2	Mgst1	ENSMUST00000008684;ENSMUST00000120939;ENSMUST00000204
MSTRG.2	Pik3c2g	ENSMUST00000111868;ENSMUST00000187223;ENSMUST00000187
MSTRG.2	Gm43940	ENSMUST00000204549
MSTRG.2	Gm44013	ENSMUST00000204392
MSTRG.2	Aebp2	MSTRG.27326.1;MSTRG.27326.2;ENSMUST00000032359;ENSMUS
MSTRG.2	Aebp2	MSTRG.27327.1
MSTRG.2	Plekha5	ENSMUST00000087622;MSTRG.27328.3;MSTRG.27328.2;ENSMUS
MSTRG.2	Plekha5	MSTRG.27329.1
MSTRG.2	Gm26824	MSTRG.2733.1;ENSMUST00000180506
MSTRG.2	Gm25780	ENSMUST00000122528
MSTRG.2	Gm11077	ENSMUST00000111844
MSTRG.2	Rpl38-p	ENSMUST00000117356
MSTRG.2	Gm30524	ENSMUST00000204271
MSTRG.2	Gm30524	MSTRG.27334.1
MSTRG.2	Gm28523	ENSMUST00000190040
MSTRG.2	Pde3a	ENSMUST00000043259;ENSMUST00000189060
MSTRG.2	Pde3a	MSTRG.27338.1
MSTRG.2	Gm10400	ENSMUST00000203920
MSTRG.2	Gtf3c6	ENSMUST00000217537;ENSMUST00000019982;ENSMUST00000217
MSTRG.2	Gm43958	ENSMUST00000204360
MSTRG.2	Slco1a6	ENSMUST00000111827;ENSMUST00000172984;ENSMUST00000173
MSTRG.2	Gm18159	ENSMUST00000174516
MSTRG.2	Gm20400	ENSMUST00000173695
MSTRG.2	Gm20400	MSTRG.27344.1
MSTRG.2	Pyroxd1	ENSMUST00000041852;ENSMUST00000204971;ENSMUST00000137
MSTRG.2	Recql	ENSMUST00000032370;ENSMUST00000100832;ENSMUST00000141
MSTRG.2	Recql	MSTRG.27347.1
MSTRG.2	Golt1b	ENSMUST00000032372;ENSMUST00000146508
MSTRG.2	Ldhb	ENSMUST00000130817;ENSMUST00000032373;ENSMUST00000204
MSTRG.2	Gtf3c6	MSTRG.2735.1
MSTRG.2	Kcnj8	ENSMUST00000203945
MSTRG.2	Abcc9	ENSMUST00000100827;ENSMUST00000111771;ENSMUST00000204
MSTRG.2	5330439	ENSMUST00000194811
MSTRG.2	Cmas	ENSMUST00000032419;ENSMUST00000133248;MSTRG.27353.3;E
MSTRG.2	Gm7457	ENSMUST00000100821;ENSMUST00000159599;MSTRG.27354.3
MSTRG.2	St8sia1	ENSMUST00000032421;MSTRG.27355.2;ENSMUST00000205149;E
MSTRG.2	St8sia1	MSTRG.27356.1
MSTRG.2	St8sia1	MSTRG.27357.1
MSTRG.2	St8sia1	MSTRG.27358.1
MSTRG.2	St8sia1	MSTRG.27359.1
MSTRG.2	Amd1	MSTRG.2736.1;ENSMUST00000099945;ENSMUST00000213620;EN
MSTRG.2	Etnk1;1	ENSMUST00000204947;ENSMUST00000032413;ENSMUST00000203
MSTRG.2	Etnk1	MSTRG.27361.1
MSTRG.2	1700126	MSTRG.27362.1
MSTRG.2	1700126	MSTRG.27363.1
MSTRG.2	C2cd5	ENSMUST00000087485;ENSMUST00000171349;ENSMUST00000205
MSTRG.2	Sox5	ENSMUST00000038815;ENSMUST00000170367;ENSMUST00000111
MSTRG.2	Gm44068	ENSMUST00000204049
MSTRG.2	Sox5os1	ENSMUST00000156994
MSTRG.2	Sox5	MSTRG.27368.1
MSTRG.2	Sox5	MSTRG.27369.1
MSTRG.2	Cdk19	ENSMUST00000216736;ENSMUST00000044672;ENSMUST00000095
MSTRG.2	Sox5os2	ENSMUST00000156443
MSTRG.2	Sox5it	ENSMUST00000144026

MSTRG.2	Sox5os3	ENSMUST00000139351;ENSMUST00000154167
MSTRG.2	Sox5os4	ENSMUST00000155562
MSTRG.2	Sox5os5	ENSMUST00000152192
MSTRG.2	Sox5	MSTRG.27375.1
MSTRG.2	Bcat1	ENSMUST00000032402;ENSMUST00000111742;ENSMUST0000004٤
MSTRG.2	Gm23071	ENSMUST00000158242
MSTRG.2	Gm26666	ENSMUST00000180763
MSTRG.2	Gm15687	ENSMUST00000141440
MSTRG.2	Cdk19os	ENSMUST00000143103;MSTRG.2738.3
MSTRG.2	Rps25-p	ENSMUST00000119876
MSTRG.2	Tuba3b	ENSMUST00000087445
MSTRG.2	Rassf8	ENSMUST00000111704;ENSMUST00000140966;ENSMUST00000032
MSTRG.2	Rassf8	MSTRG.27383.1
MSTRG.2	Bhlhe41	MSTRG.27384.1;ENSMUST00000032386;ENSMUST00000111703;E
MSTRG.2	Gm43909	ENSMUST00000203443
MSTRG.27386		MSTRG.27386.1
MSTRG.2	Sspn	ENSMUST00000111702;ENSMUST00000032383;ENSMUST00000111
MSTRG.2	Gm43931	ENSMUST00000204817
MSTRG.2	Sspnos	ENSMUST00000139089
MSTRG.2	Cdk19	MSTRG.2739.1
MSTRG.2	Lrmp	MSTRG.27390.2;MSTRG.27390.3;MSTRG.27390.1;ENSMUST0000
MSTRG.2	Lrmp	MSTRG.27391.1
MSTRG.2	Gm43910	ENSMUST00000204561
MSTRG.2	Casc1	MSTRG.27393.1;ENSMUST00000111727;ENSMUST00000111728;E
MSTRG.2	Gm15543	ENSMUST00000141307
MSTRG.2	Etfrf1	ENSMUST00000111725;ENSMUST00000111726;MSTRG.27395.2;E
MSTRG.2	Kras;Gm	ENSMUST00000032399;ENSMUST00000123972;MSTRG.27396.2;E
MSTRG.2	Ints13	MSTRG.27397.1
MSTRG.2	Itpr2;G	ENSMUST00000079573;ENSMUST00000053273;ENSMUST00000137
MSTRG.2	Itpr2	MSTRG.27399.1
MSTRG.2	4930403	MSTRG.274.1
MSTRG.2	Cdk19	MSTRG.2740.1
MSTRG.2	Itpr2	MSTRG.27400.1
MSTRG.2	Itpr2	MSTRG.27401.1;MSTRG.27401.2
MSTRG.2	Gm44086	ENSMUST00000203251
MSTRG.2	Itpr2	MSTRG.27403.1
MSTRG.2	Itpr2	MSTRG.27404.1
MSTRG.2	Gm44087	ENSMUST00000204247
MSTRG.2	Itpr2	MSTRG.27406.1
MSTRG.2	Itpr2	MSTRG.27407.1
MSTRG.2	Itpr2	MSTRG.27408.1
MSTRG.2	Itpr2	MSTRG.27409.1
MSTRG.2	Cdc40	MSTRG.2741.1;ENSMUST00000044166;ENSMUST00000216025;EN
MSTRG.2	Itpr2	MSTRG.27410.1
MSTRG.2	Itpr2	MSTRG.27411.1
MSTRG.2	Itpr2	MSTRG.27412.1
MSTRG.2	Itpr2	MSTRG.27413.1
MSTRG.2	Itpr2	MSTRG.27414.1
MSTRG.27415		MSTRG.27415.1;MSTRG.27415.2
MSTRG.27416		MSTRG.27416.1
MSTRG.27417		MSTRG.27417.1
MSTRG.2	Stk38l	ENSMUST00000001675;ENSMUST00000111644;ENSMUST0000020
MSTRG.2	Gm44270	ENSMUST00000203084
MSTRG.2	Gm22948	ENSMUST00000158561
MSTRG.2	Ints13	ENSMUST00000139979;MSTRG.27420.1;ENSMUST00000203545;E
MSTRG.2	Fgfr1op	ENSMUST00000037836;ENSMUST00000067404;ENSMUST00000111
MSTRG.2	Tm7sf3	ENSMUST00000037709;ENSMUST00000127529;ENSMUST00000147

MSTRG.2	Med21	ENSMUST00000032429;ENSMUST00000125539;ENSMUST0000020З
MSTRG.2	Arntl2	ENSMUST00000123444;ENSMUST00000111639;ENSMUST0000008C
MSTRG.2	Arntl2	MSTRG.27426.1
MSTRG.2	Ppfibp1	ENSMUST00000016631;ENSMUST00000203730;ENSMUST00000111
MSTRG.2	Ppfibp1	MSTRG.27428.1
MSTRG.2	1700034	ENSMUST00000036592
MSTRG.2743		MSTRG.2743.1
MSTRG.2	Gm44085	ENSMUST00000205056
MSTRG.2	Gm35876	ENSMUST00000205044
MSTRG.2	Rep15	ENSMUST00000036194
MSTRG.2	Mrps35	ENSMUST00000149660;ENSMUST00000036111;ENSMUST00000137
MSTRG.2	Mrps35	MSTRG.27434.1
MSTRG.2	Mrps35	MSTRG.27435.1
MSTRG.2	Mansc4	ENSMUST00000100780;ENSMUST00000123367
MSTRG.2	Mansc4	MSTRG.27437.1
MSTRG.2	Klhl42	MSTRG.27438.1;ENSMUST00000036003;MSTRG.27438.3;ENSMUS
MSTRG.2	Klhl42	MSTRG.27439.1;MSTRG.27439.2
MSTRG.2	Fig4	ENSMUST00000043814;MSTRG.2744.2
MSTRG.2	Klhl42	MSTRG.27440.1
MSTRG.2	Gm15762	ENSMUST00000126447
MSTRG.2	Gm15762	MSTRG.27442.1
MSTRG.2	Gm15762	MSTRG.27443.1
MSTRG.2	Gm6288	ENSMUST00000100778;ENSMUST00000128634
MSTRG.2	Gm6288	MSTRG.27445.1
MSTRG.2	Ccdc91	ENSMUST00000203659;ENSMUST00000032441;ENSMUST0000015б
MSTRG.2	Ccdc91	MSTRG.27447.1
MSTRG.2	Ccdc91	MSTRG.27448.1
MSTRG.2	4933406	ENSMUST00000203567
MSTRG.2	Fig4	MSTRG.2745.1
MSTRG.27450		MSTRG.27450.1
MSTRG.27451		MSTRG.27451.1
MSTRG.2	Far2os2	ENSMUST00000149815
MSTRG.2	Far2	ENSMUST00000032443;ENSMUST00000203278;ENSMUST00000111
MSTRG.2	Far2os1	ENSMUST00000162391
MSTRG.2	Ergic2	ENSMUST00000136008;ENSMUST00000032446;ENSMUST0000014З
MSTRG.2	Ergic2	MSTRG.27456.1
MSTRG.2	4732416	ENSMUST00000203932
MSTRG.2	4732416	MSTRG.27459.1
MSTRG.2	Gm25526	ENSMUST00000104332
MSTRG.2	Tmtc1	ENSMUST00000060095;ENSMUST00000100772;ENSMUST0000014C
MSTRG.2	Gm15763	ENSMUST00000120136
MSTRG.2	Rps4l	ENSMUST00000071745;ENSMUST00000204242
MSTRG.2	Gm44020	ENSMUST00000204618
MSTRG.2	Gm44021	ENSMUST00000203055
MSTRG.2	Gm44022	ENSMUST00000203708
MSTRG.2	Tmtc1	MSTRG.27466.1
MSTRG.2	Ipo8	ENSMUST00000136896;ENSMUST00000048418;ENSMUST00000204
MSTRG.2	2610017	ENSMUST00000204670
MSTRG.2	Caprin2	ENSMUST00000203014;ENSMUST00000111569;ENSMUST00000127
MSTRG.2	Fig4	MSTRG.2747.1
MSTRG.2	Caprin2	MSTRG.27470.1
MSTRG.2	Fam60a	ENSMUST00000054080;ENSMUST00000203164;ENSMUST00000204
MSTRG.2	3010003	ENSMUST00000047953;ENSMUST00000154673
MSTRG.2	Dennd5b	ENSMUST00000111557;MSTRG.27473.2;ENSMUST00000129539;E
MSTRG.2	Gm15779	ENSMUST00000117470
MSTRG.2	AC13241	ENSMUST00000204155;ENSMUST00000083730
MSTRG.2	Gm15780	ENSMUST00000121283

MSTRG.2	Gm23462	ENSMUST00000083264
MSTRG.2	Etfbkmt	ENSMUST00000126406;ENSMUST00000179873;ENSMUST00000047
MSTRG.2	Etfbkmt	MSTRG.27481.1
MSTRG.2	Etfbkmt	MSTRG.27482.1
MSTRG.2	Amn1	ENSMUST00000111535;ENSMUST00000127743;MSTRG.27483.2;E
MSTRG.2	Gm10388	ENSMUST00000205001
MSTRG.27485		MSTRG.27485.1
MSTRG.27486		MSTRG.27486.1
MSTRG.2	2810474	ENSMUST00000130664;ENSMUST00000046689;ENSMUST0000019C
MSTRG.2	Gm15782	ENSMUST00000121073
MSTRG.2	Gm15784	ENSMUST00000120260
MSTRG.2749		MSTRG.2749.1
MSTRG.2	Gm15784	MSTRG.27490.1
MSTRG.27491		MSTRG.27491.1
MSTRG.2	Bicd1	ENSMUST00000203502;ENSMUST00000086829;ENSMUST00000111
MSTRG.2	Gm15785	ENSMUST00000119145
MSTRG.2	Bicd1	MSTRG.27494.1
MSTRG.2	Gm7353	MSTRG.27495.1;ENSMUST00000069044
MSTRG.2	Gm3375	ENSMUST00000203929
MSTRG.2	Myadm;P	ENSMUST00000203328;ENSMUST00000203600;ENSMUST0000009€
MSTRG.2	Rps9	ENSMUST00000006496;ENSMUST00000108623;ENSMUST0000012ε
MSTRG.2	Pirb	ENSMUST00000078451;ENSMUST00000136238;ENSMUST0000015Ε
MSTRG.2	Kansl3;I	MSTRG.275.2;ENSMUST00000185912;MSTRG.275.1;ENSMUST000
MSTRG.2	Zbtb24	ENSMUST00000080771;ENSMUST00000213797;ENSMUST0000021€
MSTRG.2	Gm15922	ENSMUST00000118068
MSTRG.2	Gm15922	MSTRG.27501.1
MSTRG.2	Gm15930	ENSMUST00000156603
MSTRG.2	Gm15925	ENSMUST00000120641
MSTRG.2	Gm15448	ENSMUST00000153846
MSTRG.2	Pira2	ENSMUST00000108615;ENSMUST00000119469
MSTRG.2	Gm10693	ENSMUST00000205904
MSTRG.2	Gm14548	ENSMUST00000070639
MSTRG.2	Lilra6	ENSMUST00000206077;ENSMUST00000131255;ENSMUST0000003ε
MSTRG.2	Oscar	ENSMUST00000108645
MSTRG.2	Zbtb24	MSTRG.2751.1
MSTRG.2	Ndufa3	ENSMUST00000076657;ENSMUST00000150497;ENSMUST0000015€
MSTRG.2	Tfpt	ENSMUST00000155592;ENSMUST00000108641;MSTRG.27511.3;E
MSTRG.2	Prpf31	ENSMUST00000179769;ENSMUST00000008517;ENSMUST0000014Ξ
MSTRG.2	Gm15927	ENSMUST00000148641;ENSMUST00000206075;ENSMUST0000020€
MSTRG.2	Cnot3;M	ENSMUST00000206331;MSTRG.27514.1;ENSMUST00000143085;E
MSTRG.2	Leng1;T	ENSMUST00000019878;MSTRG.27515.1;ENSMUST00000038743;E
MSTRG.2	Gm15929	ENSMUST00000138926
MSTRG.2	Mboat7	MSTRG.27517.1;MSTRG.27517.2;ENSMUST00000038608;ENSMUS
MSTRG.2	Tsen34	ENSMUST00000108630;ENSMUST00000123088;ENSMUST0000020Ε
MSTRG.2	Lair1	ENSMUST00000068865;ENSMUST00000086400;ENSMUST0000008€
MSTRG.2752		MSTRG.2752.1
MSTRG.2	Lair1	MSTRG.27520.1
MSTRG.2	Lair1	MSTRG.27521.1
MSTRG.2	Lilra5	ENSMUST00000117550
MSTRG.2	Gm15931	ENSMUST00000118066
MSTRG.2	Gm15931	MSTRG.27524.1
MSTRG.2	9430041	ENSMUST00000154825
MSTRG.2	Gm23741	ENSMUST00000097237
MSTRG.2	Ttyh1	ENSMUST00000126971;ENSMUST00000129423;ENSMUST00000032
MSTRG.2	D030047	ENSMUST00000148318
MSTRG.2	Leng8	ENSMUST00000128756;ENSMUST00000132086;ENSMUST00000037
MSTRG.2	Cd164	ENSMUST00000019962;ENSMUST00000187279;ENSMUST0000018Ω

MSTRG.2	Leng9	ENSMUST00000058358
MSTRG.2	Cdc42ep	ENSMUST00000076831;ENSMUST00000143825;ENSMUST0000014C
MSTRG.2	Rdh13;G	ENSMUST00000119485;ENSMUST00000008579;ENSMUST0000013ε
MSTRG.2	Gm15494	ENSMUST00000146199;ENSMUST00000171665
MSTRG.2	Gm15494	MSTRG.27534.1
MSTRG.2	Eps8l1	ENSMUST00000163804;ENSMUST00000171445;ENSMUST00000167
MSTRG.2	Ppp1r12	ENSMUST00000124248;ENSMUST00000013886;MSTRG.27536.3;E
MSTRG.2	Gm44877	ENSMUST00000206485
MSTRG.2	Tmem86b	ENSMUST00000055085;ENSMUST00000205360;MSTRG.27538.2;M
MSTRG.2	Gm6855	ENSMUST00000206307
MSTRG.2	Mical1	ENSMUST00000019967;ENSMUST00000119962;ENSMUST00000134
MSTRG.2	Gm44878	ENSMUST00000205812
MSTRG.2	Rpl28	ENSMUST00000032597;ENSMUST00000078432;ENSMUST0000020€
MSTRG.2	Ube2s	ENSMUST00000205815;ENSMUST00000206139;ENSMUST0000007S
MSTRG.2	Hspbp1	MSTRG.27543.4;MSTRG.27543.1;MSTRG.27543.2;MSTRG.27543
MSTRG.2	Brsk1	ENSMUST00000048248;ENSMUST00000205666;ENSMUST00000123
MSTRG.2	Tmem150	ENSMUST00000086364;ENSMUST00000086363
MSTRG.2	Kmt5c	ENSMUST00000098853;ENSMUST00000135541;ENSMUST0000013C
MSTRG.2	Cox6b2	ENSMUST00000183971;ENSMUST00000182111;ENSMUST00000184
MSTRG.2	Zfp628	ENSMUST00000116354;MSTRG.27549.1;MSTRG.27549.2
MSTRG.2	Smpd2	MSTRG.2755.1;MSTRG.2755.2;ENSMUST00000126833;MSTRG.27
MSTRG.2	Nat14	ENSMUST00000207506;ENSMUST00000047309;ENSMUST00000207
MSTRG.2	Ssc5d	ENSMUST00000057612;ENSMUST00000207310
MSTRG.2	Sbk2	ENSMUST00000208109;ENSMUST00000182214
MSTRG.2	Zfp579	ENSMUST00000159754;ENSMUST00000162731;ENSMUST0000016C
MSTRG.2	Epn1	ENSMUST00000098845;ENSMUST00000146317;MSTRG.27554.2;E
MSTRG.2	Fiz1	ENSMUST00000167804;ENSMUST00000077385;ENSMUST00000165
MSTRG.2	Zfp524;	ENSMUST00000207901;ENSMUST00000086349;MSTRG.27556.3;E
MSTRG.2	Zfp784	ENSMUST00000062428
MSTRG.2	Gm15510	ENSMUST00000123644
MSTRG.2	Zfp580	ENSMUST00000069324;MSTRG.27559.1
MSTRG.2	Ppil6	ENSMUST00000105507;ENSMUST00000215608
MSTRG.2	Gm38941	ENSMUST00000208606
MSTRG.2	Gm45133	ENSMUST00000207548
MSTRG.2	U2af2	ENSMUST00000005041;ENSMUST00000209099;ENSMUST00000207
MSTRG.2	Zfp787;	ENSMUST00000094870;ENSMUST00000207315;ENSMUST0000020ε
MSTRG.2	4933431	ENSMUST00000208973
MSTRG.2	Zfp787	MSTRG.27565.1
MSTRG.2	Gm36371	ENSMUST00000209043
MSTRG.2	Zfp444;	ENSMUST00000128495;ENSMUST00000108567;ENSMUST00000207
MSTRG.2	Zfp444	MSTRG.27568.1
MSTRG.2	Galp	MSTRG.27569.1
MSTRG.2	Gm9855	ENSMUST00000121229
MSTRG.2	Galp	ENSMUST00000037553
MSTRG.2	Zfp667	ENSMUST00000086327;ENSMUST00000153840;ENSMUST0000017C
MSTRG.2	Zfp667	MSTRG.27572.1
MSTRG.2	Zfp583	ENSMUST00000062765;ENSMUST00000108560;ENSMUST00000165
MSTRG.27574		MSTRG.27574.1
MSTRG.27575		MSTRG.27575.1
MSTRG.2	Zfp954	ENSMUST00000056246;ENSMUST00000210142
MSTRG.2	Gm44697	ENSMUST00000207756
MSTRG.27578		MSTRG.27578.1
MSTRG.2	Zfp418	ENSMUST00000051435;ENSMUST00000209387
MSTRG.2	Ccdc162	MSTRG.2758.1
MSTRG.2	Clcn4	MSTRG.27580.2;ENSMUST00000000619;ENSMUST00000210061;E
MSTRG.2	Gm45631	ENSMUST00000210318
MSTRG.2	Mir5620	ENSMUST00000176903

MSTRG.2	Vmn2r38	MSTRG.27584.1
MSTRG.2	Gm45403	ENSMUST00000209453
MSTRG.27586		MSTRG.27586.1
MSTRG.27587		MSTRG.27587.1
MSTRG.27588		MSTRG.27588.1
MSTRG.2	Zfp606	ENSMUST00000098822;ENSMUST00000123589;MSTRG.27589.2;E
MSTRG.2	Ccdc162	ENSMUST00000159978;ENSMUST00000219054;ENSMUST0000009
MSTRG.2	Gm45593	ENSMUST00000211415
MSTRG.2	Zfp110	ENSMUST00000004614;ENSMUST00000154011;ENSMUST0000014
MSTRG.2	Zscan18	ENSMUST00000211665;ENSMUST00000046245;ENSMUST00000211
MSTRG.2	1700047	ENSMUST00000209738
MSTRG.2	Gm4247	ENSMUST00000211441
MSTRG.2	Gm18194	ENSMUST00000210952;MSTRG.27595.2;ENSMUST00000210455
MSTRG.2	Zfp128	ENSMUST00000081891;ENSMUST00000144578
MSTRG.2	Zscan22	ENSMUST00000055528;ENSMUST00000117189;ENSMUST0000012C
MSTRG.2	Zscan22	MSTRG.27599.1
MSTRG.276		MSTRG.276.1
MSTRG.2	Gm17196	ENSMUST00000164074
MSTRG.27600		MSTRG.27600.1
MSTRG.2	Rps5	ENSMUST00000108539;ENSMUST00000004554;ENSMUST00000147
MSTRG.2	1810019	ENSMUST00000181128;ENSMUST00000191346
MSTRG.2	Gm29638	ENSMUST00000186106
MSTRG.2	Zfp324	ENSMUST00000038701;ENSMUST00000124387;ENSMUST0000012
MSTRG.2	Zfp446	ENSMUST00000108535;ENSMUST00000108537;ENSMUST0000004
MSTRG.2	Slc27a5	ENSMUST00000032539;ENSMUST00000155192
MSTRG.2	Slc27a5	MSTRG.27607.1
MSTRG.2	Trim28	ENSMUST00000209577;ENSMUST00000005705;ENSMUST00000142
MSTRG.2	Zbtb45	ENSMUST00000210282;ENSMUST00000051390;MSTRG.27609.3;E
MSTRG.2	Ccdc162	MSTRG.2761.1
MSTRG.2	Chmp2a;	ENSMUST00000210587;ENSMUST00000211344;ENSMUST0000020
MSTRG.27611		MSTRG.27611.1
MSTRG.27612		MSTRG.27612.1
MSTRG.2	Obox3-p	MSTRG.27613.1
MSTRG.2	Mrip-ps	ENSMUST00000184613
MSTRG.27615		MSTRG.27615.1
MSTRG.27616		MSTRG.27616.1
MSTRG.2	6330408	ENSMUST00000119139;ENSMUST00000108532;ENSMUST0000013
MSTRG.2	6330408	MSTRG.27618.1
MSTRG.2	Lig1	ENSMUST00000148471;ENSMUST00000177588;ENSMUST0000009
MSTRG.2	5730435	ENSMUST00000170047;ENSMUST00000165647
MSTRG.27620		MSTRG.27620.1
MSTRG.2	Pla2g4c	ENSMUST00000167232;ENSMUST00000108528;ENSMUST0000010
MSTRG.2	Rps8-ps	ENSMUST00000092523
MSTRG.2	Gm24909	ENSMUST00000157290
MSTRG.2	Pla2g4c	ENSMUST00000204668
MSTRG.2	Gm45268	ENSMUST00000210046
MSTRG.2	Gm23499	ENSMUST00000157736
MSTRG.2	Obox1	ENSMUST00000067288
MSTRG.2	Ccdc162	MSTRG.2763.1
MSTRG.2	Obox3-p	ENSMUST00000176273
MSTRG.2	Obox3	MSTRG.27639.1
MSTRG.2	Selenow	ENSMUST00000044355
MSTRG.2	Ehd2	ENSMUST00000098799;MSTRG.27641.2;ENSMUST00000144956
MSTRG.2	Nop53	ENSMUST00000044158;ENSMUST00000197477;ENSMUST0000019
MSTRG.2	Snord23	ENSMUST00000116828
MSTRG.2	Gm24576	ENSMUST00000175508
MSTRG.2	Bicra	ENSMUST00000210713;MSTRG.27645.1;ENSMUST00000210781;E

MSTRG.2	Gm38948	ENSMUST00000210035;MSTRG.27646.2
MSTRG.2	Bicra	MSTRG.27647.1
MSTRG.2	Bicra	MSTRG.27648.1;MSTRG.27648.2
MSTRG.2	Napa	ENSMUST00000006181;ENSMUST00000127637;ENSMUST00000124
MSTRG.2	AC16142	ENSMUST00000214137
MSTRG.2	Meis3	ENSMUST00000176342;ENSMUST00000177540;ENSMUST0000017€
MSTRG.2	Inafm1	ENSMUST00000169612
MSTRG.2	Dhx34	ENSMUST00000128452;ENSMUST00000121123;MSTRG.27652.3;E
MSTRG.2	Dhx34	MSTRG.27653.1
MSTRG.2	Ccdc9	ENSMUST00000172670;ENSMUST00000118976;ENSMUST00000041
MSTRG.2	Bbc3	ENSMUST00000209688;ENSMUST00000002152;ENSMUST0000013€
MSTRG.2	Bbc3	MSTRG.27656.1
MSTRG.2	Sae1	ENSMUST00000211741;ENSMUST00000210999;MSTRG.27657.4;E
MSTRG.2	Sae1	MSTRG.27658.1
MSTRG.2	Sae1	MSTRG.27659.1
MSTRG.2	Cep57l1	ENSMUST00000105505;MSTRG.2766.1;ENSMUST00000186239;EN
MSTRG.2	Zc3h4	MSTRG.27660.1;ENSMUST00000214735;MSTRG.27660.2;ENSMUS
MSTRG.2	Gm45508	ENSMUST00000209298
MSTRG.2	Gm45509	ENSMUST00000209624
MSTRG.2	Gm45510	ENSMUST00000210059
MSTRG.2	Tmem160	ENSMUST00000019302;MSTRG.27664.2;ENSMUST00000209536
MSTRG.2	Npas1	MSTRG.27665.1;ENSMUST00000210748;ENSMUST00000002053;E
MSTRG.2	Npas1	MSTRG.27666.1
MSTRG.2	Gm5586	ENSMUST00000206264
MSTRG.2	Arhgap3	ENSMUST00000075845;MSTRG.27669.2;ENSMUST00000171937;M
MSTRG.2	BC04855	ENSMUST00000215667;ENSMUST00000190793;MSTRG.2767.3
MSTRG.2	Gm17826	ENSMUST00000189720
MSTRG.2	Gm29443	MSTRG.27671.1
MSTRG.2	Gm17981	ENSMUST00000191109
MSTRG.2	Arhgap3	MSTRG.27673.1
MSTRG.2	Gm44589	ENSMUST00000207025
MSTRG.2	Gm29442	ENSMUST00000188725
MSTRG.2	Ap2s1	ENSMUST00000086112;ENSMUST00000205607;MSTRG.27677.2;E
MSTRG.2	9330104	MSTRG.27678.1
MSTRG.2	9330104	ENSMUST00000181501;ENSMUST00000205647
MSTRG.2	BC04855	MSTRG.2768.1
MSTRG.2	Pnmal2	ENSMUST00000094807
MSTRG.2	Gm42372	ENSMUST00000206129
MSTRG.2	Calm3	ENSMUST00000019514;ENSMUST00000172594;MSTRG.27682.2;M
MSTRG.2	Gm32772	MSTRG.27684.1
MSTRG.27685		MSTRG.27685.1
MSTRG.27686		MSTRG.27686.1
MSTRG.27687		MSTRG.27687.1
MSTRG.2	Slc1a5	ENSMUST00000108496;MSTRG.27688.2;ENSMUST00000127401;M
MSTRG.2	Slc1a5	MSTRG.27689.1
MSTRG.2	Cep57l1	MSTRG.2769.1
MSTRG.2	Fkrp	MSTRG.27690.1;ENSMUST00000061390;ENSMUST00000206259;E
MSTRG.2	Prkd2;S	MSTRG.27691.1;ENSMUST00000108495;MSTRG.27691.3;ENSMUS
MSTRG.2	Ppp5c	ENSMUST00000003183;MSTRG.27692.2;ENSMUST00000142597;E
MSTRG.2	Hif3a	ENSMUST00000108492;ENSMUST00000037762;ENSMUST0000013S
MSTRG.2	4833404	ENSMUST00000206290
MSTRG.2	Mill1	ENSMUST00000066780
MSTRG.27696		MSTRG.27696.1
MSTRG.2	Pglyrp1	ENSMUST00000206569;ENSMUST00000032573;ENSMUST0000020€
MSTRG.2	Ccdc61	ENSMUST00000206037;MSTRG.27698.1;ENSMUST00000098780;E
MSTRG.2	Irf2bp1	ENSMUST00000053713
MSTRG.2	Cnnm4;A	ENSMUST00000153128;ENSMUST00000192530;ENSMUST0000020C

MSTRG.2	Sesn1	ENSMUST00000099931;MSTRG.2770.2;ENSMUST00000041438;MS
MSTRG.2	Irf2bp1	MSTRG.27700.1
MSTRG.2	Nanos2	MSTRG.27701.1
MSTRG.2	Gm10676	ENSMUST00000098778
MSTRG.2	Mypop	ENSMUST00000059331;ENSMUST00000131087;ENSMUST0000013C
MSTRG.2	Mypopos	ENSMUST00000206304;ENSMUST00000124897;ENSMUST0000020€
MSTRG.2	Mypopos	ENSMUST00000205975
MSTRG.2	Mypop	MSTRG.27706.1
MSTRG.2	Dmwd	ENSMUST00000032570;MSTRG.27707.2;ENSMUST00000108479;E
MSTRG.2	Sympk	ENSMUST00000023882;ENSMUST00000153976;ENSMUST00000137
MSTRG.2	Sympk	MSTRG.27709.1
MSTRG.2	Sesn1	MSTRG.2771.1
MSTRG.2	Fbxo46	ENSMUST00000053109;MSTRG.27710.2;ENSMUST00000165913
MSTRG.2	Qpctl	ENSMUST00000032566;ENSMUST00000205979
MSTRG.2	Snrpd2	ENSMUST00000049294;ENSMUST00000206766
MSTRG.2	Gipr	ENSMUST00000094790
MSTRG.2	Dmpk	ENSMUST00000032568;ENSMUST00000122999;ENSMUST00000154
MSTRG.2	Mir3100	ENSMUST00000174918
MSTRG.2	Six5	ENSMUST00000049454
MSTRG.2	Gm4969	ENSMUST00000141380;ENSMUST00000127433
MSTRG.2	Eml2;Mi	ENSMUST00000117338;ENSMUST00000120595;ENSMUST0000014€
MSTRG.2	Gpr4	ENSMUST00000060225
MSTRG.2	AC11529	ENSMUST00000214902
MSTRG.2	Gpr4	MSTRG.27720.1
MSTRG.2	Opa3	ENSMUST00000063976;ENSMUST00000206335;ENSMUST00000161
MSTRG.2	Gm25134	ENSMUST00000082646
MSTRG.2	Vasp	ENSMUST00000032561;ENSMUST00000161488;MSTRG.27723.2;M
MSTRG.2	1700058	ENSMUST00000159981
MSTRG.27725		MSTRG.27725.1
MSTRG.2	Ercc1	ENSMUST00000003645;ENSMUST00000160192;ENSMUST0000016C
MSTRG.2	Cd3eap	ENSMUST00000047036;ENSMUST00000176333
MSTRG.2	Ppp1r13	ENSMUST00000150448;ENSMUST00000140836;ENSMUST00000127
MSTRG.2	Ercc2;M	ENSMUST00000062831;ENSMUST00000108460;ENSMUST00000127
MSTRG.2	Sesn1	MSTRG.2773.1
MSTRG.2	Gm26852	ENSMUST00000180691
MSTRG.2	Klc3	ENSMUST00000108458;ENSMUST00000047170;ENSMUST0000010€
MSTRG.2	Klc3	MSTRG.27732.1
MSTRG.2	Apoc1	ENSMUST00000108451;ENSMUST00000045035;ENSMUST00000207
MSTRG.2	Mark4	ENSMUST00000085715;ENSMUST00000207767;ENSMUST0000020€
MSTRG.2	Mark4	MSTRG.27735.1
MSTRG.2	Exoc3l2	MSTRG.27736.1
MSTRG.2	Gm23099	ENSMUST00000083454
MSTRG.2	Exoc3l2	ENSMUST00000137613;ENSMUST00000011407
MSTRG.2	Sesn1	MSTRG.2774.1
MSTRG.2	Bloc1s3	ENSMUST00000077408;MSTRG.27740.2
MSTRG.2	Trappc6	ENSMUST00000207730;ENSMUST00000136873;ENSMUST00000147
MSTRG.2	Nkpd1	ENSMUST00000207576
MSTRG.2	Ppp1r37	ENSMUST00000209190;ENSMUST00000058444;MSTRG.27743.1;E
MSTRG.2	Zfp296	ENSMUST00000108453;ENSMUST00000208314
MSTRG.2	Clasrp	ENSMUST00000086041;ENSMUST00000208427;ENSMUST0000020€
MSTRG.2	Gm26890	ENSMUST00000180681
MSTRG.2	Apoe	ENSMUST00000174064;ENSMUST00000167646;ENSMUST00000173
MSTRG.2	Relb	ENSMUST00000049912;ENSMUST00000094762;ENSMUST00000131
MSTRG.2	Clptm1	MSTRG.27749.1
MSTRG.2	Sesn1	MSTRG.2775.1
MSTRG.2	Clptm1	ENSMUST00000055242;ENSMUST00000208554;MSTRG.27750.3;E
MSTRG.2	Clptm1	MSTRG.27751.1

MSTRG.2	Apoc2;G	ENSMUST00000003074;ENSMUST00000134116;ENSMUST00000127
MSTRG.2	Tomm40	ENSMUST00000093552;ENSMUST00000032555;MSTRG.27753.2;E
MSTRG.2	Gm20512	ENSMUST00000172483
MSTRG.2	Nectin2	ENSMUST00000075447;ENSMUST00000108450
MSTRG.2	Bcam	ENSMUST00000003061;ENSMUST00000135632;ENSMUST00000133
MSTRG.2	Gm45114	ENSMUST00000208280
MSTRG.2	Gm26600	ENSMUST00000181637
MSTRG.2	Bcl3	ENSMUST00000135609;MSTRG.27759.1;ENSMUST00000120537;E
MSTRG.2	Foxo3	ENSMUST00000105502;ENSMUST00000056974;MSTRG.2776.4;MS
MSTRG.2	Gm16175	ENSMUST00000128181
MSTRG.2	Gm16174	MSTRG.27761.1;ENSMUST00000137373
MSTRG.27762		MSTRG.27762.1
MSTRG.27763		MSTRG.27763.1
MSTRG.27764		MSTRG.27764.1
MSTRG.2	Pvr	MSTRG.27765.1;ENSMUST00000043517;ENSMUST00000159413;E
MSTRG.2	Gm16251	ENSMUST00000162649
MSTRG.2	Gm16251	MSTRG.27768.1
MSTRG.2	Igsf23	ENSMUST00000208974;ENSMUST00000043440;ENSMUST00000208
MSTRG.2	Foxo3	MSTRG.2777.1
MSTRG.2	Gm44651	ENSMUST00000207343
MSTRG.2	Krt8-ps	ENSMUST00000208495
MSTRG.2	Gm10175	ENSMUST00000086013;ENSMUST00000208752
MSTRG.27774		MSTRG.27774.1
MSTRG.2	Zfp180	ENSMUST00000207002;ENSMUST00000068975;ENSMUST00000206
MSTRG.2	Zfp180	MSTRG.27776.1
MSTRG.2	Zfp180	MSTRG.27777.1
MSTRG.2	Zfp235	ENSMUST00000205834;ENSMUST00000205736;ENSMUST00000205
MSTRG.2	Zfp111	ENSMUST00000086006;ENSMUST00000056683;ENSMUST00000073
MSTRG.2	Afg1l	ENSMUST00000151747;ENSMUST00000041024;ENSMUST00000133
MSTRG.2	Gm44796	ENSMUST00000205272
MSTRG.2	Zfp109	MSTRG.27781.1
MSTRG.2	Zfp109	ENSMUST00000206362;ENSMUST00000206960;ENSMUST00000037
MSTRG.2	Lypd5	ENSMUST00000032683
MSTRG.2	Kcnn4	MSTRG.27784.1;MSTRG.27784.2;MSTRG.27784.3
MSTRG.2	Kcnn4	ENSMUST00000205428;ENSMUST00000171904;ENSMUST00000205
MSTRG.2	Kcnn4	MSTRG.27786.1
MSTRG.2	Zfp61	ENSMUST00000077780;ENSMUST00000165241;ENSMUST00000138
MSTRG.2	Zfp94	ENSMUST00000108436;ENSMUST00000032673;ENSMUST00000146
MSTRG.2	Smg9	ENSMUST00000146381;ENSMUST00000002280;ENSMUST00000139
MSTRG.2	Gm15197	ENSMUST00000120526
MSTRG.2	Plaur	ENSMUST00000002284;ENSMUST00000206693;ENSMUST00000205
MSTRG.2	Irgq	ENSMUST00000049020;MSTRG.27791.1
MSTRG.2	Cadm4	ENSMUST00000068023
MSTRG.2	Cadm4	MSTRG.27793.1
MSTRG.2	Zfp428	ENSMUST00000176880;ENSMUST00000071361;ENSMUST00000177
MSTRG.2	Xrcc1	ENSMUST00000205573;ENSMUST00000063249;ENSMUST00000205
MSTRG.2	Gm9844	ENSMUST00000170837
MSTRG.2	Zfp575	ENSMUST00000094705
MSTRG.2	Ethe1	ENSMUST00000077191
MSTRG.2	Phldb3	MSTRG.27799.1;ENSMUST00000206422;ENSMUST00000073325;E
MSTRG.2	Gm37909	ENSMUST00000193157
MSTRG.2	Afg1l	MSTRG.2780.1
MSTRG.2	Phldb3	MSTRG.27800.1
MSTRG.2	Gm26550	ENSMUST00000180699
MSTRG.2	Lypd3	ENSMUST00000080718
MSTRG.2	Rabac1	ENSMUST00000076961;ENSMUST00000206222;ENSMUST00000206
MSTRG.2	Mir7046	ENSMUST00000183435

MSTRG.2	Grik5	ENSMUST00000205328;ENSMUST00000003468;ENSMUST0000020€
MSTRG.2	Zfp574	MSTRG.27806.1;ENSMUST00000206733;ENSMUST00000206530;E
MSTRG.2	Pou2f2;I	ENSMUST00000108415;ENSMUST00000175774;ENSMUST0000009€
MSTRG.2	D930028I	ENSMUST00000098678;ENSMUST00000186424
MSTRG.2	Erf	MSTRG.27809.1;ENSMUST00000045847;ENSMUST00000116343
MSTRG.2	Afg1l	MSTRG.2781.1
MSTRG.2	Dedd2;M	MSTRG.27810.1;ENSMUST00000206750;ENSMUST00000058702;E
MSTRG.2	Dedd2	MSTRG.27811.1
MSTRG.2	4933430I	ENSMUST00000206788
MSTRG.2	Zfp526	ENSMUST00000055604;MSTRG.27813.2;MSTRG.27813.3;MSTRG.
MSTRG.2	Gsk3a	ENSMUST00000071739;ENSMUST00000108411;ENSMUST0000020€
MSTRG.2	9130221I	ENSMUST00000169914;ENSMUST00000190693
MSTRG.2	Rps19	ENSMUST00000108430;ENSMUST00000129847;ENSMUST0000013€
MSTRG.2	Cd79a	MSTRG.27817.1;ENSMUST00000003469
MSTRG.2	Arhgef1	ENSMUST00000117796;ENSMUST00000047873;ENSMUST0000009€
MSTRG.2	Arhgef1	MSTRG.27819.1
MSTRG.2	Afg1l	MSTRG.2782.1
MSTRG.2	Gm4881	ENSMUST00000206705;MSTRG.27820.2
MSTRG.2	Pafah1b	ENSMUST00000140093;ENSMUST00000108410;ENSMUST0000000€
MSTRG.2	Cic	MSTRG.27823.1;ENSMUST00000169266;ENSMUST00000169392;M
MSTRG.2	Megf8	ENSMUST00000128119;ENSMUST00000153077
MSTRG.2	4732471	ENSMUST00000206436;ENSMUST00000205787;ENSMUST0000006€
MSTRG.2	Lipe	ENSMUST00000003207;ENSMUST00000054301;MSTRG.27826.3;E
MSTRG.2	Cxcl17	ENSMUST00000074040;ENSMUST00000200880
MSTRG.2	Gm5587	ENSMUST00000206270
MSTRG.2	Gm20949	ENSMUST00000206478
MSTRG.2	Afg1l	MSTRG.2783.1
MSTRG.2	Gm15495	ENSMUST00000117570
MSTRG.2	Ceacam1	ENSMUST00000098666;ENSMUST00000098669;ENSMUST0000009€
MSTRG.2	Ceacam2	ENSMUST00000064862;ENSMUST00000066503;ENSMUST0000004€
MSTRG.2	B9d2	ENSMUST00000205658;ENSMUST00000205325;ENSMUST0000020€
MSTRG.2	Atp5sl	ENSMUST00000077338;ENSMUST00000205593;ENSMUST0000008€
MSTRG.2	Bckdha	MSTRG.27835.1;MSTRG.27835.2;ENSMUST00000205808;ENSMUS
MSTRG.2	B3gnt8	ENSMUST00000205281;ENSMUST00000206940;ENSMUST0000007€
MSTRG.2	Exosc5	ENSMUST00000079634;ENSMUST00000206561;ENSMUST0000020€
MSTRG.2	Tmem91	MSTRG.27838.1;ENSMUST00000108405;ENSMUST00000150306;E
MSTRG.2	Tgfb1	ENSMUST00000002678;ENSMUST00000206584;ENSMUST0000020€
MSTRG.2	Afg1l	MSTRG.2784.1
MSTRG.2	Ccdc97	MSTRG.27840.1;MSTRG.27840.2;ENSMUST00000002683;ENSMUS
MSTRG.2	Axl	ENSMUST00000002677;MSTRG.27842.2;ENSMUST00000085948;E
MSTRG.2	Cyp2s1	ENSMUST00000043314;ENSMUST00000108395;ENSMUST00000152
MSTRG.2	Gm45226	ENSMUST00000206602
MSTRG.2	Gm26707	ENSMUST00000181552
MSTRG.2	Gm4613;	ENSMUST00000165488;ENSMUST00000103246
MSTRG.2	Hnrnpul	ENSMUST00000206832;ENSMUST00000043765;MSTRG.27847.4;M
MSTRG.2	Cyp2g1	ENSMUST00000040944;ENSMUST00000205273
MSTRG.27849		MSTRG.27849.1
MSTRG.2	Gm15198	ENSMUST00000117283
MSTRG.27850		MSTRG.27850.1
MSTRG.2	Cyp2a5	ENSMUST00000005685;ENSMUST00000168869;ENSMUST0000016€
MSTRG.27852		MSTRG.27852.1
MSTRG.2	Cyp2f2	ENSMUST00000003100;ENSMUST00000133841;ENSMUST0000020€
MSTRG.2	Gm15883	ENSMUST00000153562
MSTRG.2	Cyp2t4	ENSMUST00000164093;ENSMUST00000108385;MSTRG.27855.3
MSTRG.2	Egln2;G	ENSMUST00000080058;MSTRG.27856.1;ENSMUST00000108382;M
MSTRG.2	Snrpa	MSTRG.27857.1
MSTRG.2	Snrpa	ENSMUST00000148491;ENSMUST00000163311;ENSMUST00000122

MSTRG.2	BC02497	ENSMUST00000179391;ENSMUST00000108379;ENSMUST00000155
MSTRG.2	Snx3	ENSMUST00000105500;ENSMUST00000019939;ENSMUST00000105
MSTRG.2	Itpkc	ENSMUST00000003850;ENSMUST00000147170;ENSMUST00000149
MSTRG.2	Coq8b;G	ENSMUST00000003860;ENSMUST00000108378;ENSMUST00000128
MSTRG.2	Gm15567	ENSMUST00000148699
MSTRG.2	Gm15570	ENSMUST00000122093
MSTRG.2	Ltbp4	ENSMUST00000038618;ENSMUST00000121175;ENSMUST00000118
MSTRG.2	Blvrb	ENSMUST00000037399;ENSMUST00000108358;ENSMUST00000133
MSTRG.2	Pgam1-p	ENSMUST00000117129
MSTRG.2	Sertad3	ENSMUST00000068641
MSTRG.2	Sertad1	ENSMUST00000008528;ENSMUST00000135881
MSTRG.2	Shkbp1;	ENSMUST00000003857;ENSMUST00000132684;ENSMUST00000123
MSTRG.2	Ostm1	ENSMUST00000105497;ENSMUST00000040718;ENSMUST00000154
MSTRG.2	Gm44924	ENSMUST00000138087
MSTRG.2	Hipk4	ENSMUST00000108353;ENSMUST00000037134
MSTRG.2	Hipk4	MSTRG.27872.1
MSTRG.2	Pld3	ENSMUST00000117095;ENSMUST00000117611;ENSMUST00000155
MSTRG.2	2310022	ENSMUST00000067386;ENSMUST00000191126;ENSMUST00000190
MSTRG.2	2310022	MSTRG.27875.1
MSTRG.2	2310022	MSTRG.27876.1
MSTRG.2	Akt2	ENSMUST00000108344;ENSMUST00000167435;ENSMUST00000128
MSTRG.2	Prkcz2	ENSMUST00000206136
MSTRG.27879		MSTRG.27879.1
MSTRG.2	Ostm1	MSTRG.2788.1
MSTRG.27880		MSTRG.27880.1
MSTRG.27881		MSTRG.27881.1
MSTRG.2	Ttc9b	ENSMUST00000008088
MSTRG.2	Map3k10	ENSMUST00000036453;ENSMUST00000108341;ENSMUST00000152
MSTRG.2	Map3k10	MSTRG.27884.1
MSTRG.2	Gm44684	ENSMUST00000205388
MSTRG.2	Zfp607b	ENSMUST00000120004;ENSMUST00000127200;ENSMUST00000124
MSTRG.2	Zfp60	ENSMUST00000108336;ENSMUST00000126884;ENSMUST00000130
MSTRG.2	Gm10046	ENSMUST00000208208;ENSMUST00000208942
MSTRG.2	Zfp626	ENSMUST00000205671
MSTRG.2	Gm15200	ENSMUST00000149950
MSTRG.2	Zfp59	ENSMUST00000205701;ENSMUST00000108331
MSTRG.2	Zfp607a	ENSMUST00000205715;ENSMUST00000205534;ENSMUST00000053
MSTRG.2	Zfp780b	ENSMUST00000205874;ENSMUST00000206685;ENSMUST00000081
MSTRG.2	Zfp850	ENSMUST00000180502;ENSMUST00000099111;ENSMUST00000180
MSTRG.2	Zfp850	MSTRG.27894.1
MSTRG.2	Zfp850	MSTRG.27895.1
MSTRG.2	Psmc4	ENSMUST00000032824;ENSMUST00000138512;MSTRG.27896.2;E
MSTRG.2	Fcgbp	ENSMUST00000076648;ENSMUST00000140004;ENSMUST00000148
MSTRG.27898		MSTRG.27898.1
MSTRG.27899		MSTRG.27899.1
MSTRG.2	Fer1l5	ENSMUST00000179162;ENSMUST00000202030;MSTRG.279.3
MSTRG.2	Sec63	ENSMUST00000019937;ENSMUST00000155410;ENSMUST00000144
MSTRG.2	Eid2	ENSMUST00000059596
MSTRG.2	Eid2b	ENSMUST00000094651
MSTRG.2	9530053	ENSMUST00000059886;ENSMUST00000130693
MSTRG.2	Gm31024	ENSMUST00000207600
MSTRG.2	Fbl	ENSMUST00000042405;ENSMUST00000207956;ENSMUST00000208
MSTRG.2	Dyrk1b	ENSMUST00000085901;ENSMUST00000172761;MSTRG.27905.3;M
MSTRG.2	Dyrk1b	MSTRG.27906.1;MSTRG.27906.2
MSTRG.2	Timm50	ENSMUST00000081946;ENSMUST00000151990;ENSMUST00000134
MSTRG.2	Rps16	ENSMUST00000082134;ENSMUST00000152918;ENSMUST00000155
MSTRG.2	AF35739	ENSMUST00000104523

MSTRG.2	Gm26860	ENSMUST00000181674
MSTRG.2	Supt5	ENSMUST00000207563;ENSMUST00000207879;MSTRG.27910.2;E
MSTRG.2	Gm44709	ENSMUST00000207649
MSTRG.2	Supt5	MSTRG.27912.1
MSTRG.2	Fbxo27	ENSMUST00000039998;ENSMUST00000151227;ENSMUST00000108
MSTRG.27914		MSTRG.27914.1
MSTRG.2	Plekhg2	ENSMUST00000094644;ENSMUST00000129145;MSTRG.27915.6;M
MSTRG.2	Gm44710	ENSMUST00000208885
MSTRG.2	Med29	ENSMUST00000003536
MSTRG.2	Paf1	ENSMUST00000003529;ENSMUST00000146604;ENSMUST00000122
MSTRG.2	Samd4b;	MSTRG.27919.1;ENSMUST00000208199;ENSMUST00000040531;E
MSTRG.2	Sec63	MSTRG.2792.1
MSTRG.2	Gmfg	ENSMUST00000108292;ENSMUST00000108289;ENSMUST0000078
MSTRG.2	Lrfn1	ENSMUST00000190954;ENSMUST00000189877;ENSMUST00000191
MSTRG.2	Ifnl2	ENSMUST00000081684
MSTRG.2	Ifnl3	ENSMUST00000078364
MSTRG.2	Sycn	ENSMUST00000119180
MSTRG.2	Nccrp1	ENSMUST00000057974
MSTRG.2	Mir7049	ENSMUST00000183983
MSTRG.2	Sec63	MSTRG.2793.1
MSTRG.2	Fbxo17	ENSMUST00000032818;ENSMUST00000108278;ENSMUST0000013
MSTRG.2	Fbxo17	MSTRG.27931.1
MSTRG.2	Fbxo17	MSTRG.27932.1
MSTRG.2	Fbxo17	MSTRG.27933.1
MSTRG.2	Mrps12	ENSMUST00000165246;ENSMUST00000171183;ENSMUST0000056
MSTRG.2	Sars2	ENSMUST00000207877;ENSMUST00000094632;ENSMUST00000207
MSTRG.2	Sars2	MSTRG.27936.1
MSTRG.2	Ccer2	ENSMUST00000178767
MSTRG.2	Nfkbib	ENSMUST00000032815;ENSMUST00000085851;ENSMUST00000137
MSTRG.2	Sirt2	ENSMUST00000122915;ENSMUST00000072965;ENSMUST0000017
MSTRG.2	Sec63	MSTRG.2794.1
MSTRG.2	Rinl	ENSMUST00000209035;ENSMUST00000059857;ENSMUST00000207
MSTRG.2	Rinl	MSTRG.27941.1
MSTRG.2	Hnrnpl	ENSMUST00000174548;ENSMUST00000173750;MSTRG.27942.2;E
MSTRG.2	Gm44702	ENSMUST00000209194
MSTRG.2	Ech1;Lg	ENSMUST00000154839;ENSMUST00000066264;MSTRG.27944.1;E
MSTRG.2	Capn12	ENSMUST00000066880;ENSMUST00000208299
MSTRG.2	Gm44700	ENSMUST00000208228
MSTRG.2	Actn4	ENSMUST00000068045;ENSMUST00000144909;ENSMUST00000217
MSTRG.2	Gm44699	ENSMUST00000208229
MSTRG.2	Actn4	MSTRG.27949.1
MSTRG.2	Gm26464	ENSMUST00000157094
MSTRG.2	Actn4	MSTRG.27950.1
MSTRG.2	Eif3k	ENSMUST00000066070;ENSMUST00000207683;ENSMUST0000020
MSTRG.2	Map4k1	ENSMUST00000208227;ENSMUST00000085835;MSTRG.27952.3;E
MSTRG.2	Ryr1	ENSMUST00000214374;ENSMUST00000179893;ENSMUST0000020
MSTRG.2	Gm38979	ENSMUST00000207099
MSTRG.2	Ryr1	MSTRG.27955.1
MSTRG.2	Gm44408	ENSMUST00000200205
MSTRG.2	Gm26604	ENSMUST00000180926;ENSMUST00000209109
MSTRG.2	Yif1b	ENSMUST00000108238;ENSMUST00000032809;MSTRG.27959.3;E
MSTRG.2	Sec63	MSTRG.2796.1
MSTRG.2	2200002	ENSMUST00000032808;ENSMUST00000151339
MSTRG.27961		MSTRG.27961.1
MSTRG.2	Catsper	ENSMUST00000166419;ENSMUST00000085819;ENSMUST0000016
MSTRG.2	Catsper	MSTRG.27964.1
MSTRG.2	Kcnk6	ENSMUST00000085818;ENSMUST00000208807

MSTRG.2	Spint2	ENSMUST00000207601;ENSMUST00000108236;ENSMUST0000009E
MSTRG.2	Spint2	MSTRG.27967.1
MSTRG.2	Ppp1r14	ENSMUST00000207714;ENSMUST00000048187
MSTRG.2	Rasgrp4	ENSMUST00000203070;ENSMUST00000203380;ENSMUST0000020E
MSTRG.2	Scml4	ENSMUST00000063063;ENSMUST00000157071;ENSMUST0000010E
MSTRG.2	Gm16282	MSTRG.27970.1;ENSMUST00000162641
MSTRG.2	Rasgrp4	MSTRG.27971.1
MSTRG.2	Fam98c	ENSMUST00000164589;ENSMUST00000134176;ENSMUST00000147
MSTRG.2	1110035	ENSMUST00000184073
MSTRG.2	Spred3	ENSMUST00000048923;ENSMUST00000207778;ENSMUST0000013C
MSTRG.2	Ggn	ENSMUST00000098609;MSTRG.27975.2;ENSMUST00000033886;E
MSTRG.2	Psmd8	MSTRG.27976.1;ENSMUST00000209034;ENSMUST00000182328;E
MSTRG.27977		MSTRG.27977.1
MSTRG.27978		MSTRG.27978.1
MSTRG.27979		MSTRG.27979.1
MSTRG.2	AC12520	ENSMUST00000217505;ENSMUST00000216467;MSTRG.2798.2;EN
MSTRG.2	Sipa1l3	ENSMUST00000183330;MSTRG.27980.1;ENSMUST00000182223;E
MSTRG.2	Sipa1l3	MSTRG.27981.1
MSTRG.2	Zfp84;M	ENSMUST00000032802;MSTRG.27983.2;MSTRG.27983.3;ENSMUS
MSTRG.2	Zfp790	ENSMUST00000178162;MSTRG.27984.2;ENSMUST00000032796;E
MSTRG.2	Zfp940	ENSMUST00000108223;ENSMUST00000145098;ENSMUST0000008E
MSTRG.2	Gm26554	ENSMUST00000180801
MSTRG.2	Gm17199	ENSMUST00000097296
MSTRG.2	Zfp260	ENSMUST00000123095;ENSMUST00000050735;ENSMUST0000014S
MSTRG.2	Gm23368	ENSMUST00000116939
MSTRG.2	AC12520	MSTRG.2799.1;ENSMUST00000214070
MSTRG.2	Zfp260	MSTRG.27990.1
MSTRG.2	Zfp260	MSTRG.27991.1
MSTRG.2	Zfp27	MSTRG.27992.1
MSTRG.2	Zfp27	ENSMUST00000162592;ENSMUST00000161904;ENSMUST0000005E
MSTRG.2	Zfp27	MSTRG.27994.1
MSTRG.2	Zfp383	ENSMUST00000187028;ENSMUST00000186475
MSTRG.2	Gm44873	ENSMUST00000208490
MSTRG.2	Zfp74	ENSMUST00000108212;ENSMUST00000108211;MSTRG.27998.3;E
MSTRG.2	Zfp74	MSTRG.27999.1
MSTRG.2	Rb1cc1	MSTRG.28.1
MSTRG.2	Fer1l5	MSTRG.280.1
MSTRG.2	AC12520	ENSMUST00000217454
MSTRG.2	C230062	MSTRG.28000.1;ENSMUST00000077142;ENSMUST00000207746;E
MSTRG.2	C230062	MSTRG.28001.1
MSTRG.2	C230062	MSTRG.28002.1
MSTRG.2	C230062	MSTRG.28003.1
MSTRG.2	Zfp568	MSTRG.28005.1;MSTRG.28005.2;ENSMUST00000148442;ENSMUS
MSTRG.2	Zfp568	MSTRG.28006.1
MSTRG.2	Zfp568	MSTRG.28007.1
MSTRG.2	Zfp14	ENSMUST00000207873;ENSMUST00000207072;ENSMUST00000077
MSTRG.2	Zfp14	MSTRG.28009.1
MSTRG.2	Scml4	MSTRG.2801.1
MSTRG.2	Zfp82;G	ENSMUST00000183025;ENSMUST00000182746;ENSMUST00000182
MSTRG.2	Gm26920	MSTRG.28011.1
MSTRG.2	Gm44872	ENSMUST00000207864
MSTRG.2	Zfp382	ENSMUST00000098596;ENSMUST00000153792;MSTRG.28013.3;M
MSTRG.2	Gm10988	ENSMUST00000209150
MSTRG.2	Zfp146	MSTRG.28015.1;ENSMUST00000062181;MSTRG.28015.2;ENSMUS
MSTRG.2	Gm5113	ENSMUST00000209028;ENSMUST00000085668
MSTRG.2	Cox7a1	ENSMUST00000098594;ENSMUST00000208441
MSTRG.2	Capns1	ENSMUST00000207082;ENSMUST00000001845;ENSMUST0000012E

MSTRG.2	Gm26810	ENSMUST00000181153
MSTRG.2	Sobp	ENSMUST00000040275;ENSMUST00000189987
MSTRG.2	Tbcb	ENSMUST00000006254;ENSMUST00000169358;ENSMUST00000141
MSTRG.2	Polr2i	ENSMUST00000019882;ENSMUST00000149654;ENSMUST0000010[illegible]
MSTRG.2	Ovol3	ENSMUST00000189482
MSTRG.2	Wdr62	ENSMUST00000134570;ENSMUST00000145027;ENSMUST0000010[illegible]
MSTRG.2	Wdr62	MSTRG.28024.1
MSTRG.2	Rps12-p	ENSMUST00000118272
MSTRG.2	Thap8	ENSMUST00000108187;ENSMUST00000208866
MSTRG.2	Sdhaf1	ENSMUST00000098586
MSTRG.28029		MSTRG.28029.1
MSTRG.2	AC11226	ENSMUST00000213522;ENSMUST00000213469
MSTRG.2	Clip3	ENSMUST00000145253;ENSMUST00000150892;ENSMUST0000012[illegible]
MSTRG.2	Alkbh6;[illegible]	ENSMUST00000060834;ENSMUST00000136887;ENSMUST00000137
MSTRG.2	Syne4	ENSMUST00000176253;ENSMUST00000054594;ENSMUST0000017[illegible]
MSTRG.2	Tyrobp	ENSMUST00000207982;ENSMUST00000032800;ENSMUST0000020[illegible]
MSTRG.2	Hcst	ENSMUST00000208740;ENSMUST00000075062;ENSMUST00000207
MSTRG.2	Nfkbid	ENSMUST00000108176;ENSMUST00000108175;ENSMUST0000004[illegible]
MSTRG.2	Nphs1	ENSMUST00000126297;ENSMUST00000006825;ENSMUST00000131
MSTRG.2	Nphs1os	ENSMUST00000152625
MSTRG.2	Rps12-p	ENSMUST00000208689
MSTRG.2	Arhgap3	ENSMUST00000124276
MSTRG.2	Gm29245	ENSMUST00000187332
MSTRG.2	Arhgap3	ENSMUST00000044338;MSTRG.28040.1;ENSMUST00000207860;[illegible]
MSTRG.2	Proser3	ENSMUST00000215288;ENSMUST00000108165;ENSMUST00000062
MSTRG.2	1700019[illegible]	ENSMUST00000208842
MSTRG.2	Hspb6;G[illegible]	ENSMUST00000126989;ENSMUST00000123355;ENSMUST0000019[illegible]
MSTRG.2	Lin37	ENSMUST00000043975;ENSMUST00000146548;MSTRG.28044.2;[illegible]
MSTRG.2	Cox6b1	ENSMUST00000075738;ENSMUST00000208352;ENSMUST0000020[illegible]
MSTRG.28046		MSTRG.28046.1
MSTRG.28047		MSTRG.28047.1
MSTRG.2	Rbm42;G[illegible]	ENSMUST00000108141;ENSMUST00000042726;ENSMUST0000014[illegible]
MSTRG.2	Gm26610	ENSMUST00000181529
MSTRG.2	Atp4a	ENSMUST00000167761;ENSMUST00000005692;ENSMUST0000016[illegible]
MSTRG.2	Tmem147	ENSMUST00000207955;ENSMUST00000209065;ENSMUST00000207
MSTRG.2	Tmem147[illegible]	ENSMUST00000180820;MSTRG.28052.2;MSTRG.28052.3;MSTRG.
MSTRG.2	Gapdhs	ENSMUST00000182067;ENSMUST00000182634;ENSMUST00000074
MSTRG.2	Gapdhs	MSTRG.28054.1
MSTRG.2	Psenen	ENSMUST00000207747;ENSMUST00000043898;ENSMUST00000207
MSTRG.2	U2af1l4	ENSMUST00000166960;ENSMUST00000163330;ENSMUST0000016[illegible]
MSTRG.2	Kmt2b	ENSMUST00000006470;ENSMUST00000108154;ENSMUST00000131
MSTRG.2	Mir3569	ENSMUST00000185080
MSTRG.2	Zbtb32	ENSMUST00000108151;ENSMUST00000108150;ENSMUST00000144
MSTRG.2	9030612[illegible]	ENSMUST00000053792
MSTRG.2	Mag	ENSMUST00000187137;ENSMUST00000186422
MSTRG.2	Gm44662	ENSMUST00000206970
MSTRG.2	Hamp2	ENSMUST00000217812;ENSMUST00000074671;ENSMUST0000020[illegible]
MSTRG.2	Usf2-ps	MSTRG.28063.1;ENSMUST00000206657
MSTRG.2	Gm44662	MSTRG.28064.1
MSTRG.2	Hamp	ENSMUST00000062620
MSTRG.2	Usf2	ENSMUST00000058860;ENSMUST00000167285;ENSMUST0000017[illegible]
MSTRG.2	Gm17077	MSTRG.28067.1;ENSMUST00000133179;ENSMUST00000206345;[illegible]
MSTRG.2	Lsr	ENSMUST00000098553;ENSMUST00000001279;ENSMUST0000010[illegible]
MSTRG.2	Fxyd5;M	ENSMUST00000161805;ENSMUST00000161684;ENSMUST0000020[illegible]
MSTRG.2807		MSTRG.2807.1
MSTRG.2	Fxyd7	ENSMUST00000206341;ENSMUST00000073892
MSTRG.2	Fxyd1	ENSMUST00000206328;ENSMUST00000205807;ENSMUST0000020[illegible]

MSTRG.2	Hpn	ENSMUST00000039435;ENSMUST00000165124;ENSMUST0000010[illegible]
MSTRG.2	Scn1b	ENSMUST00000211945;ENSMUST00000098548;ENSMUST00000211
MSTRG.2	Gm37292	ENSMUST00000192062
MSTRG.2	Gramd1a	ENSMUST00000220635;ENSMUST00000185985;ENSMUST0000018[illegible]
MSTRG.2	Gm10640	MSTRG.28078.1;ENSMUST00000181958;ENSMUST00000187312;E
MSTRG.2	Uba2	ENSMUST00000102746;ENSMUST00000205711;ENSMUST00000152
MSTRG.2	Pdss2;A[illegible]	ENSMUST00000160823;ENSMUST00000095725;ENSMUST0000016[illegible]
MSTRG.2	Pdcd2l	ENSMUST00000002710;ENSMUST00000148381;MSTRG.28080.2;E
MSTRG.2	Lsm14a	MSTRG.28081.1;MSTRG.28081.2;ENSMUST00000085585;ENSMUS
MSTRG.2	Gm44567	ENSMUST00000205645
MSTRG.2	Gm12774	ENSMUST00000117881
MSTRG.2	Gm12764	MSTRG.28084.1;ENSMUST00000145961
MSTRG.2	2310043[illegible]	ENSMUST00000205403
MSTRG.2	Gpi1	ENSMUST00000038027;ENSMUST00000135243;ENSMUST00000205
MSTRG.2	Gm26762	ENSMUST00000180864
MSTRG.2	Gm12762	ENSMUST00000140881
MSTRG.2	Gm12758	ENSMUST00000127010;ENSMUST00000205940
MSTRG.2	Pdss2	MSTRG.2809.1
MSTRG.2	4931406[illegible]	ENSMUST00000108074;ENSMUST00000085592;MSTRG.28090.2;E
MSTRG.2	BC06029[illegible]	ENSMUST00000207005
MSTRG.2	Gm6096	ENSMUST00000105172
MSTRG.2	4931406[illegible]	MSTRG.28093.1
MSTRG.2	4931406[illegible]	MSTRG.28094.1
MSTRG.2	Kctd15	ENSMUST00000108069;ENSMUST00000108070;ENSMUST00000032
MSTRG.2	Gm12781	ENSMUST00000146269
MSTRG.2	Gm45091	ENSMUST00000205799
MSTRG.2	Cebpa	ENSMUST00000205391;ENSMUST00000042985
MSTRG.2	Pepd	ENSMUST00000075068;MSTRG.28099.2;ENSMUST00000161900;E
MSTRG.2	Lman2l	MSTRG.281.1;ENSMUST00000192969;ENSMUST00000125304;ENS
MSTRG.2	Pdss2	MSTRG.2810.1
MSTRG.2	Gm25922	ENSMUST00000104281
MSTRG.2	Cebpg;G[illegible]	ENSMUST00000130491;ENSMUST00000205752;ENSMUST0000007[illegible]
MSTRG.2	Gm45094	ENSMUST00000207013
MSTRG.2	Gm45095	ENSMUST00000206736
MSTRG.2	Gm45096	ENSMUST00000205833
MSTRG.28105		MSTRG.28105.1
MSTRG.2	Slc7a10	ENSMUST00000135452;ENSMUST00000001854;ENSMUST00000167
MSTRG.2	Lrp3	ENSMUST00000153163;ENSMUST00000155404;ENSMUST00000122
MSTRG.2	Gpatch1	ENSMUST00000079693;ENSMUST00000131213;ENSMUST00000131
MSTRG.2	Gpatch1	MSTRG.28109.1
MSTRG.2	Gm16546	ENSMUST00000159442
MSTRG.2	Gm44636	ENSMUST00000206130
MSTRG.2	Gpatch1	MSTRG.28111.1
MSTRG.2	Nudt19	ENSMUST00000040962;ENSMUST00000142930;ENSMUST0000012[illegible]
MSTRG.2	B230322[illegible]	ENSMUST00000181932
MSTRG.2	Rhpn2	ENSMUST00000032705;ENSMUST00000085556;ENSMUST0000015[illegible]
MSTRG.2	Faap24	ENSMUST00000032704;ENSMUST00000141001;ENSMUST00000154
MSTRG.2	Cep89	ENSMUST00000141704;ENSMUST00000079414;ENSMUST0000012[illegible]
MSTRG.2	Pdcd5	ENSMUST00000118501;ENSMUST00000120714;ENSMUST00000151
MSTRG.2	Ankrd27	ENSMUST00000040844;ENSMUST00000188906;ENSMUST0000018[illegible]
MSTRG.2	Dpy19l3	ENSMUST00000051377;ENSMUST00000143590;ENSMUST0000020[illegible]
MSTRG.2	AC15292[illegible]	ENSMUST00000213353
MSTRG.2	Dpy19l3	MSTRG.28120.1
MSTRG.2	Rpl17-p[illegible]	ENSMUST00000119541
MSTRG.2	Zfp507	ENSMUST00000061586;ENSMUST00000206615;MSTRG.28122.2;E
MSTRG.2	Gm26790	ENSMUST00000181078
MSTRG.2	Gm26790	MSTRG.28124.1

MSTRG.2	Zfp507	ENSMUST00000206761
MSTRG.2	E130304	ENSMUST00000187873
MSTRG.2	Ccne1	ENSMUST00000108023;ENSMUST00000128785;ENSMUST00000137
MSTRG.2	Ccne1	MSTRG.28128.1
MSTRG.2	Uri1	MSTRG.28129.1;MSTRG.28129.2
MSTRG.2	Rps19-p	ENSMUST00000160308
MSTRG.2	Uri1	MSTRG.28130.1;ENSMUST00000085513;ENSMUST00000205927;E
MSTRG.2	D530033	ENSMUST00000206282;ENSMUST00000205412
MSTRG.2	1600014	ENSMUST00000165308;ENSMUST00000178207;ENSMUST00000178
MSTRG.2	D530033	MSTRG.28133.1
MSTRG.2	D530033	MSTRG.28134.1
MSTRG.2	D530033	MSTRG.28135.1
MSTRG.28136		MSTRG.28136.1
MSTRG.2	Plekhf1	ENSMUST00000098513
MSTRG.2	Gm44573	ENSMUST00000191753
MSTRG.2	Gm37494	ENSMUST00000190789;ENSMUST00000191302;ENSMUST00000188
MSTRG.2	Pdss2	MSTRG.2814.1
MSTRG.2	Gm37494	MSTRG.28140.1
MSTRG.2	Gm37494	MSTRG.28141.1
MSTRG.2	Gm37494	MSTRG.28142.1
MSTRG.2	Gm26526	ENSMUST00000181075
MSTRG.2	Gm2058	ENSMUST00000178791
MSTRG.2	Gm6851	ENSMUST00000154478
MSTRG.2	AI98794	ENSMUST00000071804;ENSMUST00000206801;ENSMUST00000205
MSTRG.2	Gm20449	ENSMUST00000094532;ENSMUST00000165029;ENSMUST00000098
MSTRG.2	Gm44521	ENSMUST00000206469
MSTRG.2	Gm15470	ENSMUST00000119135
MSTRG.2	Gm3699	ENSMUST00000178135
MSTRG.2	Gm20449	MSTRG.28150.1
MSTRG.2	Ccnb1-p	ENSMUST00000155338
MSTRG.2	Hmgb1-p	ENSMUST00000173370
MSTRG.2	Gm7224	ENSMUST00000121359
MSTRG.2	2610021	ENSMUST00000205587;ENSMUST00000163475;ENSMUST00000205
MSTRG.2	2610021	MSTRG.28156.1
MSTRG.2	Zfp788	ENSMUST00000154942;ENSMUST00000100275;ENSMUST00000131
MSTRG.2	Zfp788	MSTRG.28158.1
MSTRG.2	Zfp141	ENSMUST00000174407;ENSMUST00000174489;ENSMUST00000173
MSTRG.2	Bend3	ENSMUST00000167488;ENSMUST00000216679;ENSMUST00000214
MSTRG.2	Gm19246	ENSMUST00000173599
MSTRG.2	Zfp141	MSTRG.28161.1
MSTRG.2	Zfp141	MSTRG.28162.1
MSTRG.28163		MSTRG.28163.1
MSTRG.2	Zfp976	ENSMUST00000107994;ENSMUST00000187616;ENSMUST00000098
MSTRG.2	Zfp975	ENSMUST00000107992;ENSMUST00000206356
MSTRG.2	Zfp975	MSTRG.28166.1
MSTRG.2	Gm17768	MSTRG.28169.1
MSTRG.2	Bend3	MSTRG.2817.1
MSTRG.2	Zfp715	ENSMUST00000012796;ENSMUST00000048015;ENSMUST00000107
MSTRG.2	Zfp715	MSTRG.28171.1
MSTRG.2	Cd33	ENSMUST00000205503;MSTRG.28172.2;ENSMUST00000004728;E
MSTRG.2	Zfp719	MSTRG.28173.1
MSTRG.2	Zfp658	ENSMUST00000107972;ENSMUST00000005597
MSTRG.2	Zfp719	ENSMUST00000058104;ENSMUST00000205769;ENSMUST00000147
MSTRG.2	Zfp719	MSTRG.28176.1
MSTRG.2	Nkg7	ENSMUST00000070518;ENSMUST00000206741;MSTRG.28177.2;E
MSTRG.2	Cldnd2	ENSMUST00000040227;ENSMUST00000206839
MSTRG.2	Etfb;Gm	ENSMUST00000004729;ENSMUST00000206286;ENSMUST00000203

MSTRG.2	AC16366	ENSMUST00000216546
MSTRG.2	Gm44816	ENSMUST00000206666
MSTRG.2	Gm36546	ENSMUST00000206372
MSTRG.2	Ctu1	ENSMUST00000038332
MSTRG.2	Klk14	ENSMUST00000056329
MSTRG.2	2310002	ENSMUST00000181454;ENSMUST00000181551;ENSMUST00000206
MSTRG.2	Klk13	ENSMUST00000066834;ENSMUST00000206554
MSTRG.2	Gm44814	ENSMUST00000207018
MSTRG.2	Klk12	ENSMUST00000107970;ENSMUST00000014063
MSTRG.2	Klk11	ENSMUST00000080211;ENSMUST00000171458
MSTRG.2	Gm9803	ENSMUST00000057649
MSTRG.2	Klk10	ENSMUST00000014058
MSTRG.2	Klk9	ENSMUST00000005891
MSTRG.2	2310002	MSTRG.28192.1
MSTRG.2	Klk9	MSTRG.28193.1
MSTRG.2	Klk8	ENSMUST00000085461;ENSMUST00000205537;ENSMUST00000206
MSTRG.2	Gm45122	ENSMUST00000206376
MSTRG.2	Gm45122	MSTRG.28196.1
MSTRG.2	Clec11a	ENSMUST00000004587
MSTRG.2	Shank1	ENSMUST00000107938;ENSMUST00000107935;ENSMUST00000134
MSTRG.2	Cnnm3	ENSMUST00000001166;ENSMUST00000097776;ENSMUST00000154
MSTRG.2	1700021	MSTRG.2820.1;ENSMUST00000147196;ENSMUST00000152926;EN
MSTRG.2	Gm44757	ENSMUST00000206373
MSTRG.2	Shank1	MSTRG.28201.1
MSTRG.2	1700008	ENSMUST00000107933;ENSMUST00000137946;ENSMUST00000150
MSTRG.2	Gm44780	ENSMUST00000205409
MSTRG.2	Syt3	ENSMUST00000118962;ENSMUST00000132399;ENSMUST00000118
MSTRG.2	Gm45124	ENSMUST00000206723
MSTRG.2	Gm10109	ENSMUST00000072123;ENSMUST00000206890
MSTRG.2	Klk1	ENSMUST00000075162;ENSMUST00000205329;ENSMUST00000206
MSTRG.2	1700028	ENSMUST00000206366;ENSMUST00000206686;ENSMUST00000037
MSTRG.2	Gm36864	ENSMUST00000223070
MSTRG.2	Cd24a	ENSMUST00000214532;ENSMUST00000058714;ENSMUST00000214
MSTRG.2	Gm25386	ENSMUST00000157814
MSTRG.2	2410002	ENSMUST00000055858;ENSMUST00000137742;ENSMUST00000137
MSTRG.2	Snord88	ENSMUST00000116853
MSTRG.2	Acpt	ENSMUST00000107945;ENSMUST00000118216
MSTRG.2	Spib	ENSMUST00000035323;ENSMUST00000205506;ENSMUST00000098
MSTRG.2	Pold1	ENSMUST00000049343;ENSMUST00000154783;ENSMUST00000138
MSTRG.2	Mir7053	ENSMUST00000184044
MSTRG.2	Gm44833	ENSMUST00000208368
MSTRG.2	Pold1	MSTRG.28218.1
MSTRG.2	Lrrc4b	ENSMUST00000127790;ENSMUST00000058667
MSTRG.2	AC15353	ENSMUST00000216740;MSTRG.2822.2;ENSMUST00000214651;EN
MSTRG.2	Aspdh	ENSMUST00000035929;ENSMUST00000207591
MSTRG.2	Josd2;M	ENSMUST00000035844;ENSMUST00000206210;MSTRG.28221.2;E
MSTRG.2	5430431	MSTRG.28222.1
MSTRG.2	5430431	ENSMUST00000206342
MSTRG.2	Emc10	MSTRG.28224.1;ENSMUST00000118808;MSTRG.28224.3;ENSMUS
MSTRG.2	Fam71e1	ENSMUST00000107927;ENSMUST00000205819;ENSMUST00000205
MSTRG.2	Gm44646	ENSMUST00000205847;ENSMUST00000207290
MSTRG.2	Mybpc2	ENSMUST00000165208
MSTRG.2	Napsa	ENSMUST00000002274;ENSMUST00000153124;ENSMUST00000207
MSTRG.2	Gm15396	ENSMUST00000209101
MSTRG.2	Qrsl1	ENSMUST00000020012;ENSMUST00000146937;ENSMUST00000154
MSTRG.2	Nr1h2	ENSMUST00000107912;ENSMUST00000073488;ENSMUST00000107
MSTRG.28231		MSTRG.28231.1

MSTRG.2	Zfp473	ENSMUST00000149011;ENSMUST00000060270;ENSMUST0000012
MSTRG.2	Vrk3	ENSMUST00000002275;ENSMUST00000134750;MSTRG.28233.2;
MSTRG.2	Vrk3	MSTRG.28234.1
MSTRG.2	Atf5	ENSMUST00000047356;ENSMUST00000107893;ENSMUST0000020
MSTRG.2	Nup62;I	ENSMUST00000208626;ENSMUST00000057195;ENSMUST0000020
MSTRG.2	Tbc1d17	ENSMUST00000047085;ENSMUST00000139947;ENSMUST0000014
MSTRG.2	Akt1s1;	ENSMUST00000107885;ENSMUST00000054343;ENSMUST00000142
MSTRG.2	Pnkp	ENSMUST00000107876;ENSMUST00000154968;MSTRG.28239.2;
MSTRG.2	Qrsl1	MSTRG.2824.1
MSTRG.2	Ptov1	ENSMUST00000207416;ENSMUST00000207303;ENSMUST0000004
MSTRG.2	Med25	ENSMUST00000208253;ENSMUST00000003049;ENSMUST0000020
MSTRG.2	D7Bwg08	ENSMUST00000208484
MSTRG.2	Fuz	ENSMUST00000071207;ENSMUST00000208600;ENSMUST0000020
MSTRG.2	Ap2a1	ENSMUST00000166972;ENSMUST00000085399;ENSMUST00000167
MSTRG.2	Rras	ENSMUST00000044111;ENSMUST00000210397;ENSMUST0000021
MSTRG.28246		MSTRG.28246.1
MSTRG.2	Prr12	ENSMUST00000057293;MSTRG.28247.1;ENSMUST00000210410
MSTRG.2	Prr12	MSTRG.28248.1
MSTRG.2	Prmt1;S	ENSMUST00000207370;ENSMUST00000207659;ENSMUST00000107
MSTRG.2	Rtn4ip1	ENSMUST00000054418;ENSMUST00000105492;ENSMUST00000214
MSTRG.2	Gm15545	ENSMUST00000141011;ENSMUST00000150613;ENSMUST00000123
MSTRG.2	Bcl2l12	ENSMUST00000003290;ENSMUST00000208128;MSTRG.28251.3;
MSTRG.2	Irf3	ENSMUST00000003284;ENSMUST00000209066;ENSMUST00000207
MSTRG.2	Prrg2	ENSMUST00000210690;ENSMUST00000007981;ENSMUST00000211
MSTRG.2	Nosip	ENSMUST00000107829;ENSMUST00000003513;ENSMUST00000211
MSTRG.2	Nosip	MSTRG.28255.1
MSTRG.2	Nosip	MSTRG.28256.1
MSTRG.2	Rcn3	ENSMUST00000019683;ENSMUST00000211352;ENSMUST0000020
MSTRG.2	Gm45669	ENSMUST00000209808
MSTRG.2	Fcgrt	ENSMUST00000003512;ENSMUST00000210642;ENSMUST00000211
MSTRG.2	Rtn4ip1	ENSMUST00000214453
MSTRG.2	Tead2	ENSMUST00000033060;ENSMUST00000210570;ENSMUST00000211
MSTRG.2	Cd37	ENSMUST00000098461;ENSMUST00000209779;ENSMUST0000003
MSTRG.2	Trpm4	ENSMUST00000211431;ENSMUST00000042194;ENSMUST00000211
MSTRG.2	Rpl14-p	ENSMUST00000026901
MSTRG.2	Mir150	ENSMUST00000083561
MSTRG.2	Gm45552	ENSMUST00000209340;MSTRG.28265.1;MSTRG.28265.2
MSTRG.2	Rps11;G	ENSMUST00000003521;MSTRG.28266.1;ENSMUST00000209838;
MSTRG.2	Snord35	ENSMUST00000083884
MSTRG.2	Snord34	ENSMUST00000083944
MSTRG.2	Snord32	ENSMUST00000083285
MSTRG.2	Crybg1	ENSMUST00000200401;ENSMUST00000020017
MSTRG.2	Aldh16a	ENSMUST00000209581;ENSMUST00000209963;ENSMUST00000211
MSTRG.2	Pih1d1	ENSMUST00000210139;ENSMUST00000211709;ENSMUST0000008
MSTRG.2	Ppfia3	ENSMUST00000210479;ENSMUST00000211067;ENSMUST00000211
MSTRG.2	Mtag2	ENSMUST00000058665
MSTRG.2	Lin7b	ENSMUST00000210865;ENSMUST00000003971;ENSMUST00000211
MSTRG.2	Gm45619	ENSMUST00000210360
MSTRG.28276		MSTRG.28276.1
MSTRG.2	Ntf5	ENSMUST00000058879;ENSMUST00000210347
MSTRG.2	Snrnp70	ENSMUST00000074575;ENSMUST00000210514;ENSMUST00000211
MSTRG.2	Gm22478	ENSMUST00000122570
MSTRG.2	Crybg1	MSTRG.2828.1
MSTRG.2	Ftl1;Gm	ENSMUST00000094434;ENSMUST00000211694;ENSMUST0000021
MSTRG.2	Gm10252	ENSMUST00000209541
MSTRG.2	Lhb	ENSMUST00000072453
MSTRG.2	Ruvbl2	ENSMUST00000211214;ENSMUST00000209426;ENSMUST0000021

MSTRG.2	Gys1	ENSMUST00000003964;ENSMUST00000211150;ENSMUST0000021
MSTRG.2	Gys1	MSTRG.28285.1
MSTRG.2	Bax;Gm4	ENSMUST00000211365;ENSMUST00000033093;ENSMUST0000021
MSTRG.2	Tulp2	ENSMUST00000211413;ENSMUST00000210813;ENSMUST00000107
MSTRG.2	Nucb1	ENSMUST00000211765;ENSMUST00000033096;ENSMUST0000021
MSTRG.2	Ppp1r15	ENSMUST00000211212;ENSMUST00000042105;ENSMUST00000167
MSTRG.2	Plekha4	ENSMUST00000211227;ENSMUST00000051810;ENSMUST00000211
MSTRG.2	Gm45564	ENSMUST00000210460
MSTRG.2	Hsd17b1	ENSMUST00000107752;ENSMUST00000210300;ENSMUST0000021
MSTRG.2	0610005	ENSMUST00000210383;ENSMUST00000210569;ENSMUST0000021
MSTRG.2	Bcat2	ENSMUST00000033098;ENSMUST00000209569;MSTRG.28295.2;
MSTRG.2	Dbp	ENSMUST00000080885;ENSMUST00000211513;ENSMUST00000211
MSTRG.2	Sphk2	ENSMUST00000107737;ENSMUST00000210060;ENSMUST00000072
MSTRG.2	Rpl18	ENSMUST00000210640;ENSMUST00000072503;ENSMUST00000211
MSTRG.2	Fam83e	ENSMUST00000129507;ENSMUST00000210434;ENSMUST00000211
MSTRG.2	Cnnm3	MSTRG.283.1
MSTRG.2	Atg5	ENSMUST00000039286;ENSMUST00000217412
MSTRG.2	Spaca4	ENSMUST00000094424
MSTRG.2	Sult2b1	ENSMUST00000075571;ENSMUST00000209739;ENSMUST0000021
MSTRG.2	Gm31597	ENSMUST00000211176
MSTRG.2	Lmtk3	ENSMUST00000209617;ENSMUST00000211309;ENSMUST0000020
MSTRG.2	Cyth2	ENSMUST00000107729;ENSMUST00000056820;ENSMUST0000021
MSTRG.2	Gm45444	ENSMUST00000211394
MSTRG.2	Kcnj14	ENSMUST00000071937;ENSMUST00000210232
MSTRG.2	Grwd1	ENSMUST00000131384;ENSMUST00000107723;ENSMUST0000020
MSTRG.2	Gm45441	ENSMUST00000209301
MSTRG.2	Grin2d	ENSMUST00000002848;ENSMUST00000211713;ENSMUST00000211
MSTRG.2	Atg5	MSTRG.2831.1
MSTRG.2	Kdelr1	ENSMUST00000002855;ENSMUST00000211716;ENSMUST00000211
MSTRG.2	Gm22098	ENSMUST00000157930
MSTRG.2	Tmem143	ENSMUST00000069772;ENSMUST00000210503;ENSMUST0000020
MSTRG.2	Tmem143	MSTRG.28313.1
MSTRG.2	Emp3	ENSMUST00000038876;ENSMUST00000164119;ENSMUST0000021
MSTRG.2	Gm45442	ENSMUST00000210038
MSTRG.2	Ccdc114	ENSMUST00000210602;ENSMUST00000211628;ENSMUST00000211
MSTRG.2	Gm9712	ENSMUST00000210842
MSTRG.2	Nomo1	ENSMUST00000033121;ENSMUST00000182564;ENSMUST00000182
MSTRG.2	Nomo1	MSTRG.28319.1
MSTRG.2	Atg5	MSTRG.2832.1
MSTRG.28320		MSTRG.28320.1
MSTRG.2	Sergef;	ENSMUST00000033127;ENSMUST00000216505;ENSMUST0000015
MSTRG.2	Gm22969	ENSMUST00000179018
MSTRG.2	Sergef	MSTRG.28323.1
MSTRG.2	Sergef	MSTRG.28324.1
MSTRG.2	Sergef	MSTRG.28325.1
MSTRG.2	Gm15700	ENSMUST00000139791
MSTRG.2	Gm45310	ENSMUST00000210436
MSTRG.2	Sergef	MSTRG.28328.1
MSTRG.2	Sergef	MSTRG.28329.1
MSTRG.2	Mir1929	ENSMUST00000158635
MSTRG.2	Sergef	MSTRG.28330.1
MSTRG.2	Gm45629	ENSMUST00000209629
MSTRG.2	Saal1	ENSMUST00000143082;ENSMUST00000210791;ENSMUST0000020
MSTRG.2	Saa2	ENSMUST00000210769;ENSMUST00000075982;ENSMUST00000211
MSTRG.2	Hps5;Gm	ENSMUST00000142663;ENSMUST00000107654;ENSMUST00000014
MSTRG.2	Gtf2h1	ENSMUST00000006774;ENSMUST00000165031;MSTRG.28335.2;
MSTRG.2	Gm9392	ENSMUST00000119337

MSTRG.2	Ldha	ENSMUST00000147535;ENSMUST00000048209;ENSMUST0000021C
MSTRG.2	C86187	ENSMUST00000209497
MSTRG.2	Prep	MSTRG.2834.1;ENSMUST00000099858;MSTRG.2834.3;MSTRG.28
MSTRG.2	Spty2d1	ENSMUST00000061639;ENSMUST00000208588
MSTRG.2	Gm32031	ENSMUST00000208993;ENSMUST00000208046;ENSMUST0000020E
MSTRG.2	Tmem86a	ENSMUST00000010451;ENSMUST00000211066
MSTRG.2	Ldhc	ENSMUST00000014545;ENSMUST00000126004
MSTRG.28344		MSTRG.28344.1
MSTRG.2	Tsg101	ENSMUST00000014546;ENSMUST00000146130;ENSMUST0000020S
MSTRG.2	Gm45628	ENSMUST00000210849
MSTRG.2	Uevld	ENSMUST00000094398;ENSMUST00000207986;ENSMUST00000207
MSTRG.2	Gm45737	ENSMUST00000208344
MSTRG.2	Uevld	MSTRG.28349.1
MSTRG.2	Prep	MSTRG.2835.1
MSTRG.2	Ptpn5	ENSMUST00000102626;ENSMUST00000209161;ENSMUST0000003З
MSTRG.2	Gm14377	ENSMUST00000207045
MSTRG.2	Mrgprx2	ENSMUST00000098433;ENSMUST00000186394
MSTRG.2	Mrgprx2	MSTRG.28354.1
MSTRG.2	Zdhhc13	ENSMUST00000118927;ENSMUST00000139125
MSTRG.2	Zdhhc13	MSTRG.28356.1
MSTRG.2	Gm45041	ENSMUST00000207366
MSTRG.2	Zdhhc13	MSTRG.28358.1
MSTRG.2	Zdhhc13	MSTRG.28359.1
MSTRG.2	Prep	MSTRG.2836.1
MSTRG.2	Htatip2	ENSMUST00000208048;ENSMUST00000085272;ENSMUST00000207
MSTRG.2	Htatip2	MSTRG.28361.1
MSTRG.2	Prmt3;G	ENSMUST00000140656;ENSMUST00000032715;ENSMUST00000152
MSTRG.2	Prmt3	MSTRG.28363.1
MSTRG.2	Prmt3	MSTRG.28364.1
MSTRG.2	Gm26856	ENSMUST00000181919
MSTRG.28366		MSTRG.28366.1
MSTRG.2	Nell1	ENSMUST00000081872
MSTRG.2	Nell1os	ENSMUST00000152166;ENSMUST00000136672
MSTRG.2	Nell1	MSTRG.28369.1
MSTRG.2	Prep	MSTRG.2837.1
MSTRG.2	4933405	ENSMUST00000119710
MSTRG.2	Nell1	MSTRG.28371.1
MSTRG.2	Gm7336	ENSMUST00000107041
MSTRG.2	E2f8	ENSMUST00000058745;ENSMUST00000119223;MSTRG.28373.3;M
MSTRG.2	Gm2788;	ENSMUST00000129551;ENSMUST00000210463;ENSMUST0000020S
MSTRG.2	Gm45282	ENSMUST00000209439
MSTRG.2	Gm18559	ENSMUST00000208249
MSTRG.2	Gm45207	ENSMUST00000207469
MSTRG.2	Gm32849	ENSMUST00000208696
MSTRG.2	9130015	ENSMUST00000208636;ENSMUST00000208621
MSTRG.2	Gm23766	ENSMUST00000103894
MSTRG.2	Gm37613	ENSMUST00000195677
MSTRG.2	Nav2	MSTRG.28381.1
MSTRG.2	Nav2	MSTRG.28382.1
MSTRG.2	Gm44913	ENSMUST00000208060
MSTRG.2	Nav2	MSTRG.28384.1
MSTRG.2	Gm45001	MSTRG.28385.1
MSTRG.2	Gm45235	ENSMUST00000208016
MSTRG.28387		MSTRG.28387.1
MSTRG.2	Fancf	ENSMUST00000169357
MSTRG.2	Gas2	ENSMUST00000129604;ENSMUST00000107589;ENSMUST00000132
MSTRG.2	Hace1	ENSMUST00000037044;ENSMUST00000131568;ENSMUST0000013€

MSTRG.28390		MSTRG.28390.1
MSTRG.28391		MSTRG.28391.1
MSTRG.2	Tubgcp5	ENSMUST00000032627;ENSMUST00000206789;ENSMUST0000020б
MSTRG.2	Cyfip1	ENSMUST00000168271;ENSMUST00000032629;ENSMUST00000173
MSTRG.2	Gm17907	ENSMUST00000173150
MSTRG.2	Gm44616	ENSMUST00000206417
MSTRG.2	Nipa2	ENSMUST00000117812;ENSMUST00000119201;ENSMUST00000032
MSTRG.2	Nipa2	MSTRG.28397.1
MSTRG.2	Nipa2	MSTRG.28398.1
MSTRG.2	A230056	ENSMUST00000149506;ENSMUST00000130189;ENSMUST00000154
MSTRG.2	AC15384	ENSMUST00000214524
MSTRG.2	Gm44509	ENSMUST00000205400
MSTRG.2	Nipa1	ENSMUST00000052204
MSTRG.2	Gm44508	ENSMUST00000206943
MSTRG.2	Gm44510	ENSMUST00000206288
MSTRG.2	Oca2	ENSMUST00000144739
MSTRG.2	Oca2	MSTRG.28407.1
MSTRG.2	Gm34121	ENSMUST00000206188;ENSMUST00000206544
MSTRG.2	Herc2	ENSMUST00000205653;MSTRG.28409.1;ENSMUST00000076226;E
MSTRG.2	Hace1	MSTRG.2841.1
MSTRG.2	Herc2	MSTRG.28410.1
MSTRG.2	Herc2	MSTRG.28411.1
MSTRG.2	Gm19187	ENSMUST00000206781
MSTRG.2	Gabrg3	ENSMUST00000068911;ENSMUST00000068394;ENSMUST00000171
MSTRG.2	Gm17984	ENSMUST00000205286
MSTRG.2	Gm44976	ENSMUST00000205543
MSTRG.2	Gm44977	ENSMUST00000205283
MSTRG.2	Gm18642	ENSMUST00000206642
MSTRG.2	Hace1	MSTRG.2842.1
MSTRG.2	Gabrg3	MSTRG.28420.1
MSTRG.2	Gm5596	ENSMUST00000207988
MSTRG.28422		MSTRG.28422.1
MSTRG.2	Atp10a	ENSMUST00000207668;MSTRG.28423.1;MSTRG.28423.4;ENSMUS
MSTRG.2	Atp10a	MSTRG.28424.1
MSTRG.2	Atp10a	MSTRG.28425.1
MSTRG.2	Gm44936	ENSMUST00000207324
MSTRG.2	Atp10a	MSTRG.28427.1
MSTRG.2	Atp10a	MSTRG.28428.1
MSTRG.2	Atp10a	MSTRG.28429.1
MSTRG.2	Hace1	MSTRG.2843.1
MSTRG.2	Gm44937	ENSMUST00000207705
MSTRG.2	Atp10a	MSTRG.28431.1
MSTRG.2	Gm6226	ENSMUST00000117765
MSTRG.2	Gm44742	ENSMUST00000207190
MSTRG.2	9330162	ENSMUST00000208498
MSTRG.2	Ube3a	ENSMUST00000202945;ENSMUST00000107537;ENSMUST00000201
MSTRG.2	Gm44562	ENSMUST00000207194
MSTRG.2	Gm44559	ENSMUST00000207177
MSTRG.2	Ube3a	MSTRG.28438.1
MSTRG.2	Ube3a	MSTRG.28439.1
MSTRG.2	Hace1	MSTRG.2844.1
MSTRG.2	Gm44560	ENSMUST00000207816
MSTRG.2	Gm44830	ENSMUST00000207667
MSTRG.2	Snhg14	ENSMUST00000190666;ENSMUST00000189653
MSTRG.2	Gm44829	ENSMUST00000208008
MSTRG.2	Gm7367	ENSMUST00000057611
MSTRG.2	Otud7a	ENSMUST00000058476

MSTRG.2	Gm5898	ENSMUST00000121016
MSTRG.2	Gm3307	ENSMUST00000176164
MSTRG.2	Klf13	ENSMUST00000063694;ENSMUST00000185175;ENSMUST00000183
MSTRG.2	Hace1	MSTRG.2845.1
MSTRG.2	Gm27252	MSTRG.28450.1;MSTRG.28450.2;MSTRG.28450.3;ENSMUST0000
MSTRG.28451		MSTRG.28451.1
MSTRG.2	Gm44627	ENSMUST00000206739
MSTRG.28453		MSTRG.28453.1
MSTRG.28454		MSTRG.28454.1
MSTRG.2	Gm32633	MSTRG.28455.1;ENSMUST00000205726;ENSMUST00000206557
MSTRG.2	Gm32633	MSTRG.28456.1
MSTRG.2	Gm32633	MSTRG.28457.1
MSTRG.2	Gm45212	ENSMUST00000205831
MSTRG.2	Mtmr10	ENSMUST00000206732;ENSMUST00000032736;ENSMUST0000020
MSTRG.2	AC13566	ENSMUST00000215636
MSTRG.2	Gm20457	ENSMUST00000173374
MSTRG.2	Fan1	ENSMUST00000163289;ENSMUST00000206778;ENSMUST0000020
MSTRG.2	Mphosph	ENSMUST00000032735;ENSMUST00000206047;ENSMUST0000020
MSTRG.2	Mcee	ENSMUST00000206138;ENSMUST00000037205;ENSMUST0000020
MSTRG.2	Mcee	MSTRG.28464.1
MSTRG.2	Mcee	MSTRG.28465.1
MSTRG.2	Mcee	MSTRG.28466.1
MSTRG.2	Apba2	ENSMUST00000032732;ENSMUST00000205613;ENSMUST0000020
MSTRG.2	Gm45082	ENSMUST00000206082
MSTRG.2	Gm44721	ENSMUST00000206978
MSTRG.2	Hace1	MSTRG.2847.1
MSTRG.2	Apba2	MSTRG.28470.1
MSTRG.2	Tjp1	ENSMUST00000102592;ENSMUST00000032729;ENSMUST0000020
MSTRG.2	Gm44794	ENSMUST00000206550
MSTRG.2	Gm44795	ENSMUST00000205496
MSTRG.2	Gm44792	ENSMUST00000207008
MSTRG.2	Gm7551	ENSMUST00000089593
MSTRG.2	Gm7546	ENSMUST00000211354
MSTRG.2	BC04625	ENSMUST00000154632
MSTRG.2	Tarsl2;'	ENSMUST00000126941;ENSMUST00000032728;ENSMUST0000020
MSTRG.2	Chsy1	ENSMUST00000036372
MSTRG.2	AC13566	ENSMUST00000213585;ENSMUST00000216742
MSTRG.2	Gm26827	ENSMUST00000181911
MSTRG.2	Gm26827	MSTRG.28481.1
MSTRG.2	Gm23042	ENSMUST00000083866
MSTRG.2	Gm18988	ENSMUST00000205601
MSTRG.2	Chsy1	MSTRG.28484.1
MSTRG.2	Gm20684	ENSMUST00000176744
MSTRG.2	Pcsk6;G	ENSMUST00000055576;ENSMUST00000098391;ENSMUST00000177
MSTRG.2	Gm45081	ENSMUST00000206281
MSTRG.2	Selenos	ENSMUST00000101801;ENSMUST00000205965;ENSMUST0000020
MSTRG.2	Lrrk1	ENSMUST00000015277;ENSMUST00000137181;ENSMUST00000145
MSTRG.2	AC15336	ENSMUST00000214811;ENSMUST00000217344
MSTRG.2	Gm44752	ENSMUST00000207140
MSTRG.2	Lrrk1	MSTRG.28491.1
MSTRG.2	Gm10974	ENSMUST00000107486
MSTRG.2	Gm23957	ENSMUST00000157209
MSTRG.2	Mir7057	ENSMUST00000184685
MSTRG.2	Aldh1a3	ENSMUST00000015278;ENSMUST00000173756;ENSMUST00000174
MSTRG.2	Asb7	ENSMUST00000131320;ENSMUST00000124899;ENSMUST00000207
MSTRG.2	Asb7	MSTRG.28497.1
MSTRG.2	Asb7	MSTRG.28498.1

MSTRG.2	Asb7	MSTRG.28499.1
MSTRG.2	Ankrd23	ENSMUST00000193083;MSTRG.285.1;ENSMUST00000191849;ENS
MSTRG.2850		MSTRG.2850.1
MSTRG.2	Adamts1	ENSMUST00000156396;ENSMUST00000148538;ENSMUST0000009
MSTRG.2	Gm44755	ENSMUST00000207830
MSTRG.2	Gm44609	ENSMUST00000207157
MSTRG.2	Adamts1	MSTRG.28503.1
MSTRG.2	Gm17909	ENSMUST00000207587
MSTRG.28505		MSTRG.28505.1
MSTRG.2	1700112	ENSMUST00000181631;ENSMUST00000208802
MSTRG.2	Lysmd4	ENSMUST00000208512;ENSMUST00000208213;ENSMUST0000005
MSTRG.2	Mef2a;G	ENSMUST00000156690;MSTRG.28508.2;ENSMUST00000107476;E
MSTRG.2	Mef2a	MSTRG.28509.1
MSTRG.2851		MSTRG.2851.1
MSTRG.2	Gm44668	ENSMUST00000207911
MSTRG.2	Lrrc28	ENSMUST00000053950;ENSMUST00000187953;ENSMUST0000019
MSTRG.2	Lrrc28	MSTRG.28512.1
MSTRG.2	Gm23233	ENSMUST00000157301
MSTRG.2	Gm32983	ENSMUST00000208500
MSTRG.2	Gm45100	ENSMUST00000209153
MSTRG.28516		MSTRG.28516.1
MSTRG.2	Ttc23	ENSMUST00000133710;ENSMUST00000032774;ENSMUST00000107
MSTRG.2	Gm44669	ENSMUST00000207774
MSTRG.2	Synm	ENSMUST00000074233;ENSMUST00000208231;ENSMUST00000051
MSTRG.2852		MSTRG.2852.1
MSTRG.2	Gm45102	ENSMUST00000207427
MSTRG.2	Arrdc4	ENSMUST00000048068;ENSMUST00000118110
MSTRG.28522		MSTRG.28522.1
MSTRG.2	Igf1r	ENSMUST00000005671;ENSMUST00000207621;ENSMUST00000207
MSTRG.2	Igf1r	MSTRG.28524.1
MSTRG.2	Igf1r	MSTRG.28525.1
MSTRG.2	Pgpep1l	ENSMUST00000032770;ENSMUST00000207874
MSTRG.2	Pgpep1l	MSTRG.28527.1
MSTRG.2	Pgpep1l	MSTRG.28528.1
MSTRG.2	Gm16157	ENSMUST00000135564;ENSMUST00000124813
MSTRG.2	AC15395	MSTRG.2853.1
MSTRG.2	Fam169b	ENSMUST00000125685;ENSMUST00000153805;ENSMUST0000021
MSTRG.2	Gm16157	MSTRG.28531.1
MSTRG.2	4930405	MSTRG.28532.1;ENSMUST00000207912
MSTRG.2	4930405	MSTRG.28533.1
MSTRG.2	Gm16158	ENSMUST00000123509;ENSMUST00000129965
MSTRG.28535		MSTRG.28535.1
MSTRG.2	Nr2f2	ENSMUST00000032768;ENSMUST00000207153;ENSMUST0000008
MSTRG.2	Nr2f2	MSTRG.28537.1
MSTRG.2	B130024	ENSMUST00000208041;ENSMUST00000189966;ENSMUST00000191
MSTRG.2	Gm28258	ENSMUST00000189033
MSTRG.2	AC15395	ENSMUST00000215196
MSTRG.2	Gm44532	ENSMUST00000207723
MSTRG.28541		MSTRG.28541.1
MSTRG.2	Rpl17-p	ENSMUST00000119383
MSTRG.2	Mctp2	ENSMUST00000079323;ENSMUST00000206466;ENSMUST0000020
MSTRG.2	Gm36696	ENSMUST00000205418
MSTRG.2	Mctp2	MSTRG.28545.1
MSTRG.2	Gm45715	ENSMUST00000206991
MSTRG.2	Kansl2-	ENSMUST00000181818
MSTRG.28548		MSTRG.28548.1
MSTRG.2	4930429	ENSMUST00000205260

MSTRG.2	AC15395	ENSMUST00000216959
MSTRG.2	A730056.	ENSMUST00000181299
MSTRG.2	Rgma	ENSMUST00000119206;ENSMUST00000094312;ENSMUST0000012
MSTRG.2	Gm44758	ENSMUST00000206096
MSTRG.2	Gm44645	ENSMUST00000205492
MSTRG.2	Chd2;Gm	ENSMUST00000169922;MSTRG.28554.2;MSTRG.28554.5;ENSMUS
MSTRG.2	Gm44686	ENSMUST00000206776
MSTRG.2	Slco3a1	ENSMUST00000098371;ENSMUST00000026897;MSTRG.28556.3;
MSTRG.2	Slco3a1	MSTRG.28557.1
MSTRG.2	Gm7580	ENSMUST00000132826
MSTRG.2	Slco3a1	MSTRG.28559.1;MSTRG.28559.2;MSTRG.28559.3
MSTRG.2	AC15395	ENSMUST00000213811
MSTRG.2	Slco3a1	MSTRG.28560.1
MSTRG.2	Slco3a1	MSTRG.28561.1;MSTRG.28561.2
MSTRG.2	Slco3a1	MSTRG.28562.1
MSTRG.2	Slco3a1	MSTRG.28563.1
MSTRG.2	Slco3a1	MSTRG.28564.1
MSTRG.28565		MSTRG.28565.1
MSTRG.2	Akap13	MSTRG.28566.1
MSTRG.28567		MSTRG.28567.1
MSTRG.28568		MSTRG.28568.1
MSTRG.2	AU02020	ENSMUST00000206358;MSTRG.28569.2;MSTRG.28569.1;ENSMUS
MSTRG.2	AC15395	MSTRG.2857.1
MSTRG.2	AU02020	MSTRG.28570.1
MSTRG.2	AU02020	MSTRG.28571.1;MSTRG.28571.2;MSTRG.28571.3;MSTRG.28571
MSTRG.2	Klhl25	ENSMUST00000171155;ENSMUST00000092073;ENSMUST0000020
MSTRG.2	Klhl25	MSTRG.28573.1
MSTRG.2	Klhl25	MSTRG.28574.1
MSTRG.2	Agbl1	ENSMUST00000156166;ENSMUST00000026854;ENSMUST0000010
MSTRG.2	Gm5597	ENSMUST00000182353
MSTRG.2	2310001	ENSMUST00000206600
MSTRG.2	Gm45210	ENSMUST00000206401
MSTRG.2	Akap13;	ENSMUST00000166315;ENSMUST00000207750;ENSMUST0000020
MSTRG.2858		MSTRG.2858.1
MSTRG.2	Gm44834	ENSMUST00000206671
MSTRG.2	Gm44695	ENSMUST00000205567
MSTRG.2	Akap13	MSTRG.28582.1
MSTRG.2	Gm25282	ENSMUST00000157271
MSTRG.2	Mrpl46	ENSMUST00000032841;ENSMUST00000133949
MSTRG.2	Gm45059	ENSMUST00000205558
MSTRG.2	Mrps11	ENSMUST00000032840;ENSMUST00000123706;ENSMUST0000013
MSTRG.2	5330411	ENSMUST00000205759
MSTRG.2	Det1	ENSMUST00000138062;ENSMUST00000107431;ENSMUST0000003
MSTRG.2	1700011	ENSMUST00000205762
MSTRG.2	Ascc3	ENSMUST00000035606;ENSMUST00000220355;ENSMUST0000021
MSTRG.2	Ntrk3	ENSMUST00000039431;ENSMUST00000195262;ENSMUST0000020
MSTRG.2	Gm44607	ENSMUST00000205611
MSTRG.2	4921513	ENSMUST00000206993
MSTRG.2	Ntrk3	MSTRG.28593.1
MSTRG.2	Ntrk3	MSTRG.28594.1
MSTRG.2	Gm39038	ENSMUST00000206774
MSTRG.2	Ntrk3	MSTRG.28596.1
MSTRG.2	Gm9885	ENSMUST00000064591;ENSMUST00000186234
MSTRG.2	Ntrk3	MSTRG.28598.1;MSTRG.28598.2;MSTRG.28598.3
MSTRG.2	Ntrk3	MSTRG.28599.1
MSTRG.2	Gm42417	MSTRG.286.1
MSTRG.2	Ascc3	MSTRG.2860.1

MSTRG.2	Ntrk3	MSTRG.28600.1
MSTRG.2	Aen	ENSMUST00000123824;ENSMUST00000107425;MSTRG.28601.3;E
MSTRG.2	Isg20	ENSMUST00000118867;ENSMUST00000121645;ENSMUST0000003
MSTRG.2	Gm26633	ENSMUST00000180546
MSTRG.2	Acan	ENSMUST00000032835;ENSMUST00000206779
MSTRG.2	Mfge8	ENSMUST00000206338;ENSMUST00000107409;ENSMUST00000032
MSTRG.2	Abhd2	MSTRG.28606.1
MSTRG.2	Gm31510	ENSMUST00000206795
MSTRG.2	Abhd2	ENSMUST00000037315;MSTRG.28608.2;ENSMUST00000135333;M
MSTRG.2	Gm44639	ENSMUST00000205859
MSTRG.2	AC15352	ENSMUST00000217818
MSTRG.2	Gm39041	ENSMUST00000205557
MSTRG.2	Gm24224	ENSMUST00000158828
MSTRG.2	Fanci	ENSMUST00000126456;ENSMUST00000118959;ENSMUST0000003
MSTRG.2	Gm16017	ENSMUST00000119834
MSTRG.2	Polg	ENSMUST00000073889;ENSMUST00000201662;ENSMUST0000013
MSTRG.2	Gm10616	ENSMUST00000205285
MSTRG.2	Ticrr	ENSMUST00000035977;ENSMUST00000205750;ENSMUST0000020
MSTRG.2	Kif7	ENSMUST00000183846;ENSMUST00000059836;ENSMUST0000017
MSTRG.2	9330171	ENSMUST00000035939;ENSMUST00000197011
MSTRG.2	Plin1	ENSMUST00000205915;MSTRG.28619.1;ENSMUST00000178257;E
MSTRG.2	Ascc3	MSTRG.2862.1
MSTRG.2	Anpep	ENSMUST00000107392;ENSMUST00000206682;ENSMUST0000004
MSTRG.2	Gm44974	ENSMUST00000206387
MSTRG.2	Arpin	ENSMUST00000048731
MSTRG.2	Ap3s2	ENSMUST00000075657;ENSMUST00000206725
MSTRG.2	Ap3s2	MSTRG.28624.1
MSTRG.2	Gm26646	ENSMUST00000180715;ENSMUST00000205705
MSTRG.2	5430400	ENSMUST00000181717
MSTRG.2	Gm44950	ENSMUST00000206403
MSTRG.2	Zfp710	ENSMUST00000164056;ENSMUST00000166250;ENSMUST0000004
MSTRG.2	Gm44951	MSTRG.28629.1;ENSMUST00000206449
MSTRG.2	AC15352	ENSMUST00000220079
MSTRG.2	Gm21057	ENSMUST00000206111;ENSMUST00000206022
MSTRG.2	Zfp710	MSTRG.28631.1
MSTRG.2	Gm44949	ENSMUST00000206423
MSTRG.28633		MSTRG.28633.1
MSTRG.2	Idh2	ENSMUST00000107384;ENSMUST00000134328;ENSMUST0000013
MSTRG.2	Gm45203	ENSMUST00000206384
MSTRG.2	Sema4b	ENSMUST00000032754;ENSMUST00000107383;ENSMUST0000020
MSTRG.2	Gm45206	ENSMUST00000206885
MSTRG.2	Cib1	ENSMUST00000065163;ENSMUST00000206084;ENSMUST00000071
MSTRG.2	Gdpgp1	ENSMUST00000062915
MSTRG.2	Ascc3	MSTRG.2864.1
MSTRG.2	Gm15504	ENSMUST00000119674
MSTRG.2	Gdpgp1	MSTRG.28641.1
MSTRG.2	Unc45a	ENSMUST00000107368;ENSMUST00000032748;ENSMUST00000154
MSTRG.2	Unc45a	MSTRG.28643.1
MSTRG.2	Ngrn	ENSMUST00000117989;ENSMUST00000138005
MSTRG.2	Vps33b	ENSMUST00000032749;ENSMUST00000150585;ENSMUST0000012
MSTRG.2	Vps33b	MSTRG.28646.1
MSTRG.2	Rccd1;6	ENSMUST00000130888;ENSMUST00000180467;MSTRG.28647.3;E
MSTRG.2	Prc1	ENSMUST00000163812;ENSMUST00000047558;ENSMUST00000172
MSTRG.2	Prc1	MSTRG.28649.1
MSTRG.2	AC15911	ENSMUST00000220349
MSTRG.2	Hddc3	ENSMUST00000032747;ENSMUST00000206480;ENSMUST0000020
MSTRG.2	Gm18310	MSTRG.28651.1;ENSMUST00000206494

MSTRG.2	Man2a2	ENSMUST00000205318;MSTRG.28652.1;MSTRG.28652.2;MSTRG.
MSTRG.2	Fes	ENSMUST00000205617;ENSMUST00000206002;ENSMUST0000008C
MSTRG.2	Furin	ENSMUST00000146771;ENSMUST00000122232;ENSMUST00000107
MSTRG.2	Gm44851	ENSMUST00000205735
MSTRG.2	Blm	ENSMUST00000206901;ENSMUST00000081314;MSTRG.28656.3;E
MSTRG.2	Blm	MSTRG.28657.1
MSTRG.2	Blm	MSTRG.28659.1
MSTRG.2	Lilrb4a	ENSMUST00000218123;ENSMUST00000102894;ENSMUST0000021S
MSTRG.2	Ndufab1	ENSMUST00000166096
MSTRG.2	Crtc3	ENSMUST00000122255;ENSMUST00000149176;ENSMUST00000127
MSTRG.2	Gm44931	ENSMUST00000206056
MSTRG.2	Crtc3	MSTRG.28663.1
MSTRG.2	Crtc3	MSTRG.28664.1
MSTRG.2	Gm15880	MSTRG.28665.1
MSTRG.2	Crtc3	MSTRG.28666.1
MSTRG.2	Zscan2	ENSMUST00000132163;ENSMUST00000205361;MSTRG.28667.4;E
MSTRG.2	Iqgap1	ENSMUST00000167377;ENSMUST00000205606;MSTRG.28668.2;E
MSTRG.2	Gm44649	ENSMUST00000206896;ENSMUST00000205608
MSTRG.2	AC15353	ENSMUST00000219828
MSTRG.2	Gm44649	MSTRG.28670.1
MSTRG.2	Iqgap1	MSTRG.28671.1
MSTRG.2	Iqgap1	MSTRG.28672.1
MSTRG.2	Iqgap1	MSTRG.28673.1
MSTRG.2	Wdr73	ENSMUST00000026816;ENSMUST00000139990;ENSMUST00000152
MSTRG.2	Nmb;Sec	ENSMUST00000146619;ENSMUST00000026817;ENSMUST0000014S
MSTRG.2	Gm16171	ENSMUST00000118004
MSTRG.2	Sec11a	MSTRG.28677.1
MSTRG.2	Gm45718	ENSMUST00000207577
MSTRG.2	Gm45718	MSTRG.28679.1
MSTRG.2	AC15297	ENSMUST00000218214
MSTRG.2	Gm18782	ENSMUST00000209023
MSTRG.2	Gm44899	ENSMUST00000209433;ENSMUST00000207451
MSTRG.2	Zfp592	ENSMUST00000107353;MSTRG.28682.2;ENSMUST00000176477;E
MSTRG.2	Gm44967	ENSMUST00000207047
MSTRG.2	Pde8a	ENSMUST00000026672;ENSMUST00000128154;ENSMUST0000013C
MSTRG.2	Gm7180	ENSMUST00000121753
MSTRG.2	Rps17	ENSMUST00000130487;ENSMUST00000080813;ENSMUST0000020E
MSTRG.2	2900076	ENSMUST00000181903;ENSMUST00000180385;ENSMUST00000181
MSTRG.2	Gm37829	ENSMUST00000193864
MSTRG.2	Whamm;G	ENSMUST00000207123;ENSMUST00000165460;ENSMUST0000020S
MSTRG.2	Ros1	ENSMUST00000219173;ENSMUST00000020045
MSTRG.2	Fam103a	ENSMUST00000042166;MSTRG.28690.2;ENSMUST00000133034;E
MSTRG.2	3110040	MSTRG.28691.1;ENSMUST00000026092;ENSMUST00000141075;E
MSTRG.2	Gm45016	ENSMUST00000208375
MSTRG.2	Gm45845	ENSMUST00000207326
MSTRG.2	Btbd1	ENSMUST00000026093;ENSMUST00000208114;ENSMUST0000020E
MSTRG.2	Gm45014	MSTRG.28695.1;ENSMUST00000207657
MSTRG.2	Gm10160	ENSMUST00000085094
MSTRG.2	Tm6sf1;	ENSMUST00000041890;ENSMUST00000124616;ENSMUST00000134
MSTRG.2	Hdgfl3	ENSMUST00000107305;ENSMUST00000026094;ENSMUST0000014S
MSTRG.2	Hdgfl3	MSTRG.28699.1
MSTRG.2	Ankrd39	MSTRG.287.1
MSTRG.2	Ros1	MSTRG.2870.1
MSTRG.2	Gm45088	ENSMUST00000207066
MSTRG.2	Mex3b	ENSMUST00000082237
MSTRG.2	Saxo2	ENSMUST00000056728;ENSMUST00000207693;ENSMUST00000151
MSTRG.2	Efl1;Gm	ENSMUST00000141726;ENSMUST00000179489;ENSMUST0000012E

MSTRG.2	Gm44917	ENSMUST00000208798
MSTRG.2	Efl1	MSTRG.28705.1
MSTRG.2	Gm44916	MSTRG.28706.1;ENSMUST00000208664
MSTRG.2	Efl1	MSTRG.28707.1
MSTRG.2	4933406	ENSMUST00000144828
MSTRG.2	n-R5s15	ENSMUST00000122513
MSTRG.2	Gm20630	ENSMUST00000177387
MSTRG.2	Gm44724	ENSMUST00000207119
MSTRG.2	Gm44724	MSTRG.28711.1
MSTRG.2	Gm7964	ENSMUST00000051522
MSTRG.2	Tlnrd1	ENSMUST00000094216
MSTRG.2	Mesd;AC	ENSMUST00000138803;ENSMUST00000094215;ENSMUST0000013
MSTRG.2	Tmc3	ENSMUST00000163297;ENSMUST00000165388;ENSMUST0000003
MSTRG.2	Gm16638	ENSMUST00000148687;ENSMUST00000208892
MSTRG.2	Gm16638	MSTRG.28717.1
MSTRG.2	Stard5;	ENSMUST00000075418;ENSMUST00000146469;MSTRG.28718.3;
MSTRG.2	Il16	ENSMUST00000001792;ENSMUST00000145610;MSTRG.28719.2;
MSTRG.2	Il16	MSTRG.28720.1
MSTRG.2	2310044	ENSMUST00000125131
MSTRG.2	Phgdh-p	ENSMUST00000207540
MSTRG.2	Abhd17c	ENSMUST00000117085;ENSMUST00000131505;ENSMUST0000020
MSTRG.2	Gm26708	ENSMUST00000208101;ENSMUST00000208236
MSTRG.2	Fah	ENSMUST00000032865;ENSMUST00000128460;ENSMUST0000020
MSTRG.28727		MSTRG.28727.1
MSTRG.2	Zfand6	ENSMUST00000209117;ENSMUST00000069537;MSTRG.28728.3;
MSTRG.2	Zfand6	MSTRG.28729.1
MSTRG.2	Gopc	ENSMUST00000217995;ENSMUST00000105475;ENSMUST0000002
MSTRG.2	2610206	ENSMUST00000129059;ENSMUST00000207313;ENSMUST0000016
MSTRG.28731		MSTRG.28731.1
MSTRG.28732		MSTRG.28732.1
MSTRG.2	Ctsc	ENSMUST00000032779;ENSMUST00000131108;ENSMUST0000012
MSTRG.2	Gm26522	ENSMUST00000181565
MSTRG.2	Rps13-p	ENSMUST00000182017
MSTRG.2	Tmem135	ENSMUST00000041968;ENSMUST00000156153;ENSMUST00000207
MSTRG.2	Gm44704	MSTRG.28737.1
MSTRG.2	Gm44704	ENSMUST00000208963
MSTRG.2	Tmem135	MSTRG.28739.1
MSTRG.2	AC15352	ENSMUST00000218299
MSTRG.2	Tmem135	MSTRG.28740.1
MSTRG.2	Gm24659	ENSMUST00000180212
MSTRG.2	Tmem135	MSTRG.28742.1
MSTRG.2	Gm44995	ENSMUST00000207056
MSTRG.2	Gm44996	ENSMUST00000208787
MSTRG.2	Fzd4	ENSMUST00000058755
MSTRG.2	Gm45064	MSTRG.28746.1
MSTRG.2	Me3	ENSMUST00000159504;ENSMUST00000162145;ENSMUST00000032
MSTRG.2	Me3	MSTRG.28750.1
MSTRG.2	Gm45793	ENSMUST00000207900
MSTRG.2	Hikeshi	ENSMUST00000153470;ENSMUST00000207309;ENSMUST0000007
MSTRG.2	Hikeshi	MSTRG.28753.1
MSTRG.2	Hikeshi	MSTRG.28754.1
MSTRG.2	Hikeshi	MSTRG.28755.1
MSTRG.2	Eed	ENSMUST00000107234;ENSMUST00000207980;ENSMUST00000207
MSTRG.2	Eed	MSTRG.28757.1
MSTRG.2	Gm45120	ENSMUST00000208880
MSTRG.2	Eed	MSTRG.28759.1
MSTRG.2	Gopc	MSTRG.2876.1

MSTRG.2	Eed	MSTRG.28760.1
MSTRG.2	E230029	ENSMUST00000207458;ENSMUST00000181784;ENSMUST0000020
MSTRG.2	Gm44860	ENSMUST00000208473
MSTRG.2	Gm44861	ENSMUST00000208325
MSTRG.2	2310010	ENSMUST00000181189;ENSMUST00000209166;ENSMUST0000020
MSTRG.2	Picalm;	ENSMUST00000207225;ENSMUST00000207484;ENSMUST0000020
MSTRG.2	Picalm	MSTRG.28766.1
MSTRG.2	Gm45220	ENSMUST00000207847
MSTRG.2	Tmem126	ENSMUST00000032843;ENSMUST00000123622
MSTRG.2	Dlg2	ENSMUST00000207095;ENSMUST00000209170
MSTRG.2	Gopc	MSTRG.2877.1
MSTRG.2	Sytl2	ENSMUST00000207578;ENSMUST00000208720;ENSMUST00000107
MSTRG.2	Tmem126	MSTRG.28771.1;ENSMUST00000136652;ENSMUST00000032844;E
MSTRG.28772		MSTRG.28772.1
MSTRG.28773		MSTRG.28773.1
MSTRG.2	Ccdc89	ENSMUST00000061391
MSTRG.2	Crebzf	ENSMUST00000061767;MSTRG.28775.1;MSTRG.28775.3;ENSMUS
MSTRG.2	Gm44907	ENSMUST00000207423
MSTRG.2	Dlg2;Gm	ENSMUST00000107196;ENSMUST00000146563;ENSMUST00000152
MSTRG.2	AC15352	ENSMUST00000218206
MSTRG.2	Gm45176	ENSMUST00000207696
MSTRG.2	Gm45177	ENSMUST00000209104
MSTRG.2	Gm45182	ENSMUST00000207579
MSTRG.2	Gm44679	ENSMUST00000207202
MSTRG.2	Gm44675	ENSMUST00000208188
MSTRG.2	Gm44676	ENSMUST00000208520
MSTRG.2	Nus1	ENSMUST00000023830;ENSMUST00000218983
MSTRG.2	Gm44677	ENSMUST00000209151
MSTRG.2	Gm44680	ENSMUST00000208414
MSTRG.2	Gm44681	ENSMUST00000208447
MSTRG.2	C030038	ENSMUST00000207840
MSTRG.2	Gm45130	ENSMUST00000207574
MSTRG.2	Gm45129	ENSMUST00000208444
MSTRG.2	4931412	ENSMUST00000207844
MSTRG.2	4930567	ENSMUST00000207593
MSTRG.2	B230206	ENSMUST00000207479
MSTRG.2	Cox5b	ENSMUST00000193210;ENSMUST00000195151;ENSMUST00000191
MSTRG.2880		MSTRG.2880.1
MSTRG.2	Gm45201	ENSMUST00000207187
MSTRG.2	Ccdc90b	ENSMUST00000153776;ENSMUST00000032842;ENSMUST0000008
MSTRG.2	Ankrd42	ENSMUST00000056106;ENSMUST00000118157;ENSMUST0000012
MSTRG.2	Gm37008	ENSMUST00000195712;MSTRG.28803.1;MSTRG.28803.3;ENSMUS
MSTRG.2	Pcf11	ENSMUST00000119954;ENSMUST00000151177;MSTRG.28804.2;
MSTRG.2	Pcf11	MSTRG.28805.1
MSTRG.2	Gm26981	ENSMUST00000182453;MSTRG.28806.1
MSTRG.2	4632427	MSTRG.28807.1;ENSMUST00000147198;ENSMUST00000182578;E
MSTRG.2	Rab30	ENSMUST00000032879;ENSMUST00000208945;ENSMUST00000107
MSTRG.2	Gm45200	ENSMUST00000207434
MSTRG.2	Cep85l	ENSMUST00000220443;ENSMUST00000220376;ENSMUST0000009
MSTRG.2	Gm15501	ENSMUST00000133910;ENSMUST00000140687
MSTRG.2	Ddias	ENSMUST00000032877;ENSMUST00000208356;ENSMUST00000207
MSTRG.2	Prcp	ENSMUST00000208686;ENSMUST00000076052;MSTRG.28812.2;E
MSTRG.2	9530078	ENSMUST00000208883
MSTRG.28814		MSTRG.28814.1
MSTRG.2	Gm31663	ENSMUST00000208481;ENSMUST00000207656
MSTRG.28816		MSTRG.28816.1
MSTRG.28817		MSTRG.28817.1

MSTRG.28818		MSTRG.28818.1
MSTRG.2	Usp35	ENSMUST00000168435;ENSMUST00000139582;ENSMUST00000181
MSTRG.2	AC10516	ENSMUST00000219618
MSTRG.2	Tenm4	ENSMUST00000107162;ENSMUST00000144263;ENSMUST00000133
MSTRG.2	Gm8319	ENSMUST00000205708
MSTRG.2	Mir708	ENSMUST00000102189
MSTRG.2	Mir6394	ENSMUST00000184738
MSTRG.2	Gm15412	ENSMUST00000137193
MSTRG.2	Rps11-p	ENSMUST00000117302
MSTRG.2	Gm15414	ENSMUST00000155217
MSTRG.2	Gm15413	ENSMUST00000155101
MSTRG.2	Gm15416	ENSMUST00000131080
MSTRG.2	Gm44633	ENSMUST00000205925
MSTRG.2	AC15352	ENSMUST00000217830
MSTRG.2	Gm25712	ENSMUST00000158634
MSTRG.2	C230038	ENSMUST00000136294;ENSMUST00000149463;ENSMUST00000129
MSTRG.2	Gm22226	ENSMUST00000157355
MSTRG.2	Nars2	MSTRG.28833.1;ENSMUST00000044466;ENSMUST00000149204;M
MSTRG.2	Nars2	MSTRG.28834.1
MSTRG.2	Nars2	MSTRG.28835.1
MSTRG.2	Nars2	MSTRG.28836.1
MSTRG.28837		MSTRG.28837.1
MSTRG.2	Gab2	ENSMUST00000004622;ENSMUST00000206791
MSTRG.2	Gab2	MSTRG.28839.1
MSTRG.2	Pln	ENSMUST00000219491;ENSMUST00000163319;ENSMUST00000046
MSTRG.2	Gab2	MSTRG.28840.1
MSTRG.2	Gab2	MSTRG.28841.1
MSTRG.2	Gab2	MSTRG.28842.1
MSTRG.2	Gab2	MSTRG.28843.1
MSTRG.2	Gab2	MSTRG.28844.1
MSTRG.2	Alg8	ENSMUST00000098300;ENSMUST00000147297;ENSMUST00000130
MSTRG.2	Ndufc2	ENSMUST00000032882;ENSMUST00000149122
MSTRG.2	Gm16053	ENSMUST00000120511
MSTRG.2	Ndufc2	MSTRG.28848.1
MSTRG.2	Thrsp	ENSMUST00000043077;ENSMUST00000206613
MSTRG.2	AC15352	ENSMUST00000218351;ENSMUST00000220401
MSTRG.2	Ints4	ENSMUST00000026126;ENSMUST00000206644;ENSMUST00000205
MSTRG.2	Gm24412	ENSMUST00000083426
MSTRG.2	Ints4	MSTRG.28852.1
MSTRG.2	Aamdc	ENSMUST00000042627;ENSMUST00000178078;ENSMUST00000136
MSTRG.2	Aamdc	MSTRG.28854.1
MSTRG.2	Aamdc	MSTRG.28855.1
MSTRG.2	Pak1	ENSMUST00000205757;ENSMUST00000206984;ENSMUST00000206
MSTRG.2	Rsf1	ENSMUST00000205536;ENSMUST00000156060;ENSMUST00000107
MSTRG.2	Rsf1	MSTRG.28858.1
MSTRG.2	Ywhaq-p	ENSMUST00000206623
MSTRG.2	AC15352	ENSMUST00000220436
MSTRG.2	Rpl15-p	ENSMUST00000205636
MSTRG.2	Rsf1os1	ENSMUST00000126902
MSTRG.2	Rsf1	MSTRG.28862.1
MSTRG.2	Rsf1os2	ENSMUST00000155074;ENSMUST00000147690
MSTRG.2	Gm45186	ENSMUST00000206874
MSTRG.2	Rsf1os2	MSTRG.28866.1
MSTRG.2	Clns1a	ENSMUST00000026506;ENSMUST00000148125;MSTRG.28867.3;M
MSTRG.2	Aqp11	ENSMUST00000206389;ENSMUST00000084986;ENSMUST00000205
MSTRG.2	Capn5	ENSMUST00000040971;ENSMUST00000107112;ENSMUST00000134
MSTRG.2	AC15594	ENSMUST00000220223

MSTRG.2	Omp	ENSMUST00000098281
MSTRG.2	Gm16938	ENSMUST00000205893;ENSMUST00000180517
MSTRG.2	B3gnt6	ENSMUST00000098278
MSTRG.2	Acer3	ENSMUST00000033020;ENSMUST00000120520;ENSMUST00000151
MSTRG.2	Acer3	MSTRG.28874.1
MSTRG.2	Acer3	MSTRG.28875.1
MSTRG.2	Acer3	MSTRG.28876.1
MSTRG.2	Lrrc32	ENSMUST00000205937;ENSMUST00000205956;ENSMUST00000165
MSTRG.2	Gm23040	ENSMUST00000083869
MSTRG.2	A630091	ENSMUST00000069035
MSTRG.2	Mcm9	MSTRG.2888.1;ENSMUST00000075540;ENSMUST00000219547;EN
MSTRG.2	A630091	MSTRG.28880.1
MSTRG.28881		MSTRG.28881.1
MSTRG.2	Emsy	ENSMUST00000205276;ENSMUST00000205911;MSTRG.28882.3;E
MSTRG.2	Emsy	MSTRG.28883.1
MSTRG.2	Emsy	MSTRG.28884.1
MSTRG.28885		MSTRG.28885.1
MSTRG.2	Gm15506	ENSMUST00000148776;ENSMUST00000208646
MSTRG.2	Gm15506	MSTRG.28887.1
MSTRG.2	Thap12	ENSMUST00000126356;ENSMUST00000033009;ENSMUST00000153
MSTRG.2	Thap12	MSTRG.28889.1
MSTRG.2	AC15594	ENSMUST00000218795
MSTRG.2	Gm19656	ENSMUST00000208543
MSTRG.2	Wnt11	ENSMUST00000165122;ENSMUST00000163913;ENSMUST00000067
MSTRG.2	Wnt11	MSTRG.28892.1
MSTRG.2	Gm8398	ENSMUST00000172081
MSTRG.2	Dgat2	ENSMUST00000033001;ENSMUST00000207491;ENSMUST00000207
MSTRG.2	Uvrag;G	ENSMUST00000037968;ENSMUST00000209123;ENSMUST00000207
MSTRG.2	Gm45188	MSTRG.28896.1;ENSMUST00000207592
MSTRG.2	Gm45187	ENSMUST00000209090;ENSMUST00000207697
MSTRG.2	Uvrag	MSTRG.28898.1
MSTRG.2	Gm23479	ENSMUST00000158876
MSTRG.2	Sema4c	ENSMUST00000191677;ENSMUST00000191642;MSTRG.289.2;ENS
MSTRG.2	AC15594	ENSMUST00000219731
MSTRG.2	Uvrag	MSTRG.28900.1
MSTRG.2	Uvrag	MSTRG.28901.1
MSTRG.2	Uvrag	MSTRG.28902.1
MSTRG.2	Gm33882	ENSMUST00000208990
MSTRG.2	Serpinh	ENSMUST00000169437;ENSMUST00000094154;ENSMUST00000208
MSTRG.2	Gdpd5	ENSMUST00000037528;ENSMUST00000207617;ENSMUST00000207
MSTRG.2	Gdpd5	MSTRG.28907.1
MSTRG.2	Klhl35	ENSMUST00000037359;ENSMUST00000153736;ENSMUST00000142
MSTRG.2	Klhl35	MSTRG.28909.1
MSTRG.2	Mcm9	MSTRG.2891.1
MSTRG.2	Rps3;Sn	ENSMUST00000032998;ENSMUST00000107096;ENSMUST00000208
MSTRG.2	Snord15	ENSMUST00000083888
MSTRG.2	Arrb1	MSTRG.28912.1
MSTRG.28914		MSTRG.28914.1
MSTRG.2	Arrb1	ENSMUST00000098266;ENSMUST00000179755;ENSMUST00000032
MSTRG.2	Mir326	ENSMUST00000083637
MSTRG.2	Gm44914	ENSMUST00000207191
MSTRG.2	Pold3	MSTRG.28918.1;ENSMUST00000032969;ENSMUST00000127128;E
MSTRG.2	AC15594	ENSMUST00000218476
MSTRG.2	Kcne3	ENSMUST00000178946;ENSMUST00000207358;ENSMUST00000049
MSTRG.2	Gm7067	ENSMUST00000207698
MSTRG.2	Pgm2l1	MSTRG.28922.1;ENSMUST00000084935;ENSMUST00000208158;E
MSTRG.2	Gpx2-ps	ENSMUST00000160622

MSTRG.2	Slco2b1	ENSMUST00000032985;MSTRG.28924.2;ENSMUST00000107086;E
MSTRG.2	Gm15635	ENSMUST00000131160;ENSMUST00000208024;ENSMUST0000014{
MSTRG.2	Xrra1	MSTRG.28926.1
MSTRG.2	Gm34280	ENSMUST00000208250;MSTRG.28927.2
MSTRG.2	Gm34280	MSTRG.28928.1
MSTRG.2	Gm44551	ENSMUST00000208804
MSTRG.2	Asf1a	ENSMUST00000219271;ENSMUST00000020004;MSTRG.2893.2;EN
MSTRG.2	Olfr520	ENSMUST00000220185;ENSMUST00000098264
MSTRG.2	Gm19182	ENSMUST00000207139
MSTRG.2	Olfr521	ENSMUST00000220001;ENSMUST00000098263
MSTRG.2	F730035	ENSMUST00000065741
MSTRG.28934		MSTRG.28934.1;MSTRG.28934.2
MSTRG.2	Neu3	MSTRG.28935.1;MSTRG.28935.2;MSTRG.28935.3;ENSMUST000C
MSTRG.2	Spcs2	ENSMUST00000036274;ENSMUST00000208477;ENSMUST00000207
MSTRG.2	Xrra1	MSTRG.28937.1
MSTRG.2	Xrra1	ENSMUST00000036155;MSTRG.28938.2;ENSMUST00000208354;E
MSTRG.2	Xrra1	MSTRG.28939.1
MSTRG.2	AC15394	ENSMUST00000219458
MSTRG.2	Gm38405	MSTRG.28940.1;MSTRG.28940.4;MSTRG.28940.2;ENSMUST000C
MSTRG.2	A930030	ENSMUST00000207625
MSTRG.2	Rnf169	ENSMUST00000080817;MSTRG.28942.2;ENSMUST00000174264;M
MSTRG.2	Gm18959	MSTRG.28943.1;MSTRG.28943.2;ENSMUST00000208954
MSTRG.2	Rnf169	MSTRG.28944.1
MSTRG.2	Rnf169	MSTRG.28945.1
MSTRG.2	Coa4	ENSMUST00000208772;ENSMUST00000054310
MSTRG.2	Gm39059	ENSMUST00000209160
MSTRG.2	Ucp2	ENSMUST00000126534;ENSMUST00000207890;ENSMUST0000012€
MSTRG.2	Ucp2	MSTRG.28949.1
MSTRG.2895		MSTRG.2895.1
MSTRG.2	Dnajb13	MSTRG.28950.1;ENSMUST00000207405;ENSMUST00000130534;E
MSTRG.2	Dnajb13	MSTRG.28951.1
MSTRG.2	P4ha3	ENSMUST00000057023;ENSMUST00000139790;ENSMUST0000014{
MSTRG.2	Ppme1	ENSMUST00000207634;MSTRG.28953.1;ENSMUST00000032963;M
MSTRG.2	C2cd3	ENSMUST00000098259;ENSMUST00000051777;ENSMUST0000013:
MSTRG.2	Gm44571	ENSMUST00000208092
MSTRG.2	Gm10603	ENSMUST00000183512;ENSMUST00000184420;ENSMUST00000184
MSTRG.2	Gm37716	ENSMUST00000193553
MSTRG.2	Ucp3	ENSMUST00000128044;ENSMUST00000032958;ENSMUST0000013:
MSTRG.2	Mrpl48	ENSMUST00000150042;ENSMUST00000064334;ENSMUST0000014€
MSTRG.2896		MSTRG.2896.1
MSTRG.2	Mrpl48	MSTRG.28960.1
MSTRG.2	Gm44648	ENSMUST00000208267
MSTRG.2	Cox20-p	ENSMUST00000119872
MSTRG.2	Rab6a	ENSMUST00000107048;ENSMUST00000032946;ENSMUST0000009{
MSTRG.2	Gm14382	ENSMUST00000117374
MSTRG.2	Plekhb1	ENSMUST00000107046;ENSMUST00000107045;ENSMUST0000007{
MSTRG.2	Gm45209	MSTRG.28966.1;ENSMUST00000207122
MSTRG.2	Fam168a	ENSMUST00000107042;ENSMUST00000208387;ENSMUST0000020{
MSTRG.2	Gm45209	MSTRG.28968.1
MSTRG.2	Gm3200	ENSMUST00000181493
MSTRG.2	Man1a	ENSMUST00000003843;ENSMUST00000105470;ENSMUST0000021{
MSTRG.2	Fam168a	MSTRG.28970.1
MSTRG.2	Gm44953	ENSMUST00000208745
MSTRG.2	Relt	ENSMUST00000008462;ENSMUST00000155413;ENSMUST0000015€
MSTRG.2	Arhgef1	ENSMUST00000107032;ENSMUST00000209041;ENSMUST0000020{
MSTRG.2	P2ry6	ENSMUST00000060174;ENSMUST00000209196;ENSMUST0000020{
MSTRG.2	Gm44545	ENSMUST00000208262

MSTRG.2	Clpb	ENSMUST00000137214;ENSMUST00000001884;ENSMUST00000106
MSTRG.2	Clpb	MSTRG.28977.1
MSTRG.2	Gm45380	ENSMUST00000210465
MSTRG.2	Phox2a	ENSMUST00000008090;ENSMUST00000209878
MSTRG.2	Man1a	MSTRG.2898.1
MSTRG.2	Inppl1	ENSMUST00000035836;ENSMUST00000165052;ENSMUST00000186
MSTRG.2	Gm10602	ENSMUST00000211428;ENSMUST00000098239;ENSMUST00000211
MSTRG.2	Folr2	ENSMUST00000094141;ENSMUST00000210598;ENSMUST00000211
MSTRG.2	Fchsd2	ENSMUST00000208439;MSTRG.28984.1;ENSMUST00000032931;E
MSTRG.2	Fchsd2	MSTRG.28985.1
MSTRG.2	Gm6341	ENSMUST00000119683
MSTRG.2	Fchsd2	MSTRG.28987.1
MSTRG.2	Fchsd2	MSTRG.28988.1
MSTRG.2	AC10763	ENSMUST00000213690
MSTRG.2	Man1a	MSTRG.2899.1;MSTRG.2899.2;MSTRG.2899.3
MSTRG.2	Fchsd2	MSTRG.28990.1
MSTRG.2	Atg16l2	ENSMUST00000207740;ENSMUST00000120267;ENSMUST00000143
MSTRG.2	Atg16l2	MSTRG.28992.1
MSTRG.2	Stard10	ENSMUST00000163799;ENSMUST00000032927;ENSMUST00000210
MSTRG.2	Gm20476	ENSMUST00000172644
MSTRG.2	Gm45344	ENSMUST00000211610
MSTRG.2	Gm45620	ENSMUST00000209980
MSTRG.2	Arap1;G	ENSMUST00000107010;ENSMUST00000137384;ENSMUST00000138
MSTRG.2	Gm45548	ENSMUST00000210076
MSTRG.2	Gm45837	MSTRG.28999.1
MSTRG.2	Rb1cc1	MSTRG.29.1
MSTRG.2	D430040	ENSMUST00000194426;ENSMUST00000149224
MSTRG.2	Man1a	MSTRG.2900.1
MSTRG.2	Gm45837	MSTRG.29000.1
MSTRG.2	Gm35363	ENSMUST00000209330
MSTRG.2	Art2a-p	ENSMUST00000173420
MSTRG.2	Gm7027	ENSMUST00000086958
MSTRG.2	Art2b	ENSMUST00000209526;ENSMUST00000063920
MSTRG.2	Rhog	ENSMUST00000106923;ENSMUST00000098230;ENSMUST00000209
MSTRG.2	Rrm1	MSTRG.29007.1
MSTRG.2	Trim21	ENSMUST00000033264;MSTRG.29008.2;ENSMUST00000106913;E
MSTRG.2	Stim1	MSTRG.29009.1;MSTRG.29009.2;ENSMUST00000033289;ENSMUS
MSTRG.2	AC16462	ENSMUST00000219148
MSTRG.2	Stim1	MSTRG.29010.1
MSTRG.2	Stim1	MSTRG.29011.1
MSTRG.2	Stim1	MSTRG.29012.1
MSTRG.2	Gm18255	MSTRG.29013.1
MSTRG.2	Gm18255	ENSMUST00000211316
MSTRG.2	Rrm1	ENSMUST00000033283;ENSMUST00000211720;MSTRG.29015.2;M
MSTRG.2	Gm45399	ENSMUST00000209955
MSTRG.2	Gm2996	MSTRG.29017.1
MSTRG.2	Trim68	ENSMUST00000082175;MSTRG.29018.2;ENSMUST00000210855;E
MSTRG.2	Gm45527	ENSMUST00000211052
MSTRG.2	Man1a	MSTRG.2902.1
MSTRG.2	Hbb-bt	ENSMUST00000098192
MSTRG.2	Hbb-bs	ENSMUST00000023934;ENSMUST00000131960;ENSMUST00000153
MSTRG.2	Trim34a	MSTRG.29022.1;ENSMUST00000060315;ENSMUST00000106848
MSTRG.2	AC12383	ENSMUST00000217156
MSTRG.2	Trim5	ENSMUST00000098179;ENSMUST00000051795
MSTRG.2	Trim5	MSTRG.29025.1
MSTRG.2	Trim5	MSTRG.29026.1
MSTRG.2	Trim12a	ENSMUST00000106837;ENSMUST00000106839;ENSMUST00000070

MSTRG.2	Trim34b	ENSMUST00000180136;ENSMUST00000106847;MSTRG.29029.3
MSTRG.2	Gm16998	ENSMUST00000181899
MSTRG.2	Trim34b	MSTRG.29030.1
MSTRG.2	Trim12c	ENSMUST00000059037;ENSMUST00000130139;ENSMUST00000184
MSTRG.2	Trim30a	MSTRG.29032.1;ENSMUST00000076922;ENSMUST00000142124;M
MSTRG.2	Gm25405	ENSMUST00000082973
MSTRG.2	Trim30a	MSTRG.29034.1
MSTRG.2	Trim30a	MSTRG.29035.1
MSTRG.29036		MSTRG.29036.1
MSTRG.2	Gm5900	MSTRG.29037.1
MSTRG.2	Folr1	ENSMUST00000106985;ENSMUST00000150184;ENSMUST00000124
MSTRG.2	Anapc15	ENSMUST00000209334;ENSMUST00000210984;ENSMUST0000010[illegible]
MSTRG.2904		MSTRG.2904.1
MSTRG.2	Gm22655	ENSMUST00000174949
MSTRG.2	Tomt	ENSMUST00000106969;ENSMUST00000106970
MSTRG.2	Lrrc51;[illegible]	MSTRG.29042.1;ENSMUST00000210566
MSTRG.2	Lamtor1	ENSMUST00000033131;ENSMUST00000193465;ENSMUST00000211
MSTRG.2	Lrrc51	ENSMUST00000106968;ENSMUST00000163903;ENSMUST0000010[illegible]
MSTRG.2	Lrrc51	MSTRG.29045.1
MSTRG.2	Numa1	MSTRG.29046.1;ENSMUST00000209639;ENSMUST00000210679;M
MSTRG.2	Numa1	MSTRG.29047.1
MSTRG.2	Gm8523	ENSMUST00000211386
MSTRG.2	Numa1	MSTRG.29049.1
MSTRG.2905		MSTRG.2905.1
MSTRG.2	Numa1	MSTRG.29050.1
MSTRG.2	Numa1	MSTRG.29051.1
MSTRG.2	Numa1	MSTRG.29052.1
MSTRG.2	Gm45313	MSTRG.29053.1;ENSMUST00000210584
MSTRG.2	Numa1	MSTRG.29054.1
MSTRG.2	Rnf121	MSTRG.29055.1
MSTRG.2	Il18bp	ENSMUST00000209844;ENSMUST00000094134;MSTRG.29056.3;E
MSTRG.2	Rnf121	ENSMUST00000096639;MSTRG.29057.2;ENSMUST00000089052;E
MSTRG.2	Xndc1;X[illegible]	ENSMUST00000106950;ENSMUST00000153176;ENSMUST00000141
MSTRG.2	Art5	ENSMUST00000094128;ENSMUST00000106937;ENSMUST0000010[illegible]
MSTRG.2906		MSTRG.2906.1
MSTRG.2	Art1	ENSMUST00000033300;ENSMUST00000210211;ENSMUST0000020[illegible]
MSTRG.2	Art5	MSTRG.29061.1
MSTRG.2	Chrna10	ENSMUST00000084830;ENSMUST00000211408
MSTRG.2	Nup98	ENSMUST00000210682;MSTRG.29063.1;MSTRG.29063.3;MSTRG.
MSTRG.2	Nup98	MSTRG.29064.1
MSTRG.2	Pgap2;A[illegible]	ENSMUST00000147536;ENSMUST00000140631;ENSMUST0000012[illegible]
MSTRG.29066		MSTRG.29066.1
MSTRG.2	Fam160a[illegible]	ENSMUST00000179474;ENSMUST00000211549;ENSMUST00000137
MSTRG.2	Gm5901	ENSMUST00000211523;ENSMUST00000106805;ENSMUST0000020[illegible]
MSTRG.2	Fam160a[illegible]	MSTRG.29069.1
MSTRG.2	Rps8-ps	ENSMUST00000172445
MSTRG.2	Gm5900	ENSMUST00000179862
MSTRG.2	Cavin3	ENSMUST00000047040
MSTRG.2	Gm45667	ENSMUST00000210380
MSTRG.2	Smpd1	ENSMUST00000046983;ENSMUST00000210934;ENSMUST00000211
MSTRG.2	Apbb1	ENSMUST00000191011;ENSMUST00000188368;ENSMUST0000019[illegible]
MSTRG.2	Tpp1	ENSMUST00000033184;ENSMUST00000210840;MSTRG.29075.3;E
MSTRG.2	Dchs1	ENSMUST00000078482;ENSMUST00000140959;ENSMUST00000154
MSTRG.2	Gm15645	ENSMUST00000131504
MSTRG.2	Mrpl17	ENSMUST00000124482;ENSMUST00000176158;ENSMUST00000033
MSTRG.2	Gm45912	ENSMUST00000210646
MSTRG.2	AC15294	ENSMUST00000219246

MSTRG.2	9130208	ENSMUST00000146911
MSTRG.29081		MSTRG.29081.1
MSTRG.2	Trim3	ENSMUST00000057525;ENSMUST00000106789;MSTRG.29082.4;E
MSTRG.2	Gm45632	ENSMUST00000211532
MSTRG.2	Arfip2	ENSMUST00000131446;ENSMUST00000142173;MSTRG.29084.2;E
MSTRG.2	Timm10b	ENSMUST00000106783;ENSMUST00000058333;ENSMUST0000021C
MSTRG.2	Mrps36-	ENSMUST00000118658
MSTRG.2	Rrp8	ENSMUST00000033179;ENSMUST00000098148;ENSMUST0000014[illegible]
MSTRG.2	Ilk	ENSMUST00000033182;ENSMUST00000149695;ENSMUST0000016[illegible]
MSTRG.2	Gja1	ENSMUST00000068581;ENSMUST00000218760;ENSMUST00000217
MSTRG.2	Taf10	MSTRG.29090.1;ENSMUST00000141116;ENSMUST00000131683;E
MSTRG.2	Gm4070;	ENSMUST00000106766;ENSMUST00000176467;ENSMUST0000017[illegible]
MSTRG.2	Gm20663	MSTRG.29093.1
MSTRG.2	Gm8982;	MSTRG.29094.1;MSTRG.29094.2
MSTRG.2	Gm4070;	MSTRG.29095.1;MSTRG.29095.2;MSTRG.29095.3
MSTRG.2	Gm27228	ENSMUST00000184500
MSTRG.2	Gm8979	ENSMUST00000183386
MSTRG.2	Gm27149	ENSMUST00000184716
MSTRG.2	Gm8982	ENSMUST00000184198
MSTRG.2	Fam178b	ENSMUST00000170295;ENSMUST00000194839;ENSMUST0000019[illegible]
MSTRG.2	Gvin1;G	ENSMUST00000006667;ENSMUST00000183409;MSTRG.29100.1;M
MSTRG.2	Gm22372	MSTRG.29101.1
MSTRG.2	Gm27150	ENSMUST00000183441
MSTRG.2	Gm22372	MSTRG.29103.1
MSTRG.2	Gm22372	ENSMUST00000104589
MSTRG.29105		MSTRG.29105.1
MSTRG.2	Gm1966	MSTRG.29106.1
MSTRG.2	Gm1966	MSTRG.29107.1
MSTRG.2	Gm10156	ENSMUST00000122364
MSTRG.29109		MSTRG.29109.1
MSTRG.2	AC16040	MSTRG.2911.1
MSTRG.2	Olfr482	ENSMUST00000217304;ENSMUST00000207291
MSTRG.2	Olfml1	ENSMUST00000120990
MSTRG.2	Gm44773	ENSMUST00000207374
MSTRG.2	Ppfibp2	ENSMUST00000207852;ENSMUST00000040056;ENSMUST0000020[illegible]
MSTRG.2	5330417	ENSMUST00000060348
MSTRG.2	Gm44772	ENSMUST00000207623
MSTRG.2	Cyb5r2	ENSMUST00000052438
MSTRG.2	Eif3f	ENSMUST00000033342;ENSMUST00000153596;ENSMUST00000147
MSTRG.2	Rpl27a;	ENSMUST00000143107;ENSMUST00000132877;ENSMUST0000014[illegible]
MSTRG.2	AC12445	ENSMUST00000198845;ENSMUST00000104387
MSTRG.2	Hsf2	ENSMUST00000220353;MSTRG.2912.2;ENSMUST00000079833;EN
MSTRG.2	St5	ENSMUST00000079282;ENSMUST00000168005;MSTRG.29120.1;E
MSTRG.2	St5	MSTRG.29121.1
MSTRG.2	St5	MSTRG.29122.1
MSTRG.2	Gm44864	ENSMUST00000207292
MSTRG.2	Akip1	ENSMUST00000033335;ENSMUST00000143581;ENSMUST00000147
MSTRG.2	BC05101	ENSMUST00000106735
MSTRG.2	Tmem9b	ENSMUST00000033333;ENSMUST00000118571;ENSMUST0000012[illegible]
MSTRG.2	4930431	ENSMUST00000207761;ENSMUST00000207570
MSTRG.2	Nrip3	ENSMUST00000041460;ENSMUST00000033331;ENSMUST00000207
MSTRG.2	Gm22822	ENSMUST00000177941
MSTRG.2	Serinc1	MSTRG.2913.1;ENSMUST00000020027;ENSMUST00000169122;EN
MSTRG.2	Dennd5a	ENSMUST00000130354;ENSMUST00000106722;ENSMUST0000008C
MSTRG.2	Dennd5a	MSTRG.29131.1
MSTRG.2	Tmem41b	ENSMUST00000094097;ENSMUST00000118429;ENSMUST0000012[illegible]
MSTRG.2	Tmem41b	MSTRG.29133.1

MSTRG.2	Tmem41b	MSTRG.29134.1
MSTRG.2	Gm24842	ENSMUST00000158681
MSTRG.2	Swap70	ENSMUST00000033325;ENSMUST00000209261;MSTRG.29136.2;E
MSTRG.2	Gm45515	ENSMUST00000210796
MSTRG.2	1600010I	ENSMUST00000191157;ENSMUST00000188662;ENSMUST0000018!
MSTRG.2	Gm23100	ENSMUST00000083455
MSTRG.2	Smpdl3a	ENSMUST00000151623;ENSMUST00000020022;ENSMUST0000012!
MSTRG.2	Ipo7	ENSMUST00000084731;ENSMUST00000208821;ENSMUST0000020
MSTRG.2	Gm25636	ENSMUST00000082666
MSTRG.2	Snora23	ENSMUST00000082517
MSTRG.2	AA47440	ENSMUST00000098110
MSTRG.2	Zfp143	ENSMUST00000209505;MSTRG.29144.2;ENSMUST00000211798;E
MSTRG.2	1600010I	MSTRG.29145.1
MSTRG.2	Gm45819	ENSMUST00000210940
MSTRG.2	Wee1	ENSMUST00000033326;MSTRG.29147.1
MSTRG.2	Adm	ENSMUST00000033054;ENSMUST00000185766
MSTRG.2	Gcc2	ENSMUST00000057659;ENSMUST00000162984;MSTRG.2915.3;EN
MSTRG.2	Sbf2	ENSMUST00000164759;ENSMUST00000033058;ENSMUST0000016(
MSTRG.2	Gm9064	ENSMUST00000163579
MSTRG.2	Gm17219	ENSMUST00000168439
MSTRG.2	Gm10087	ENSMUST00000167650
MSTRG.2	B430319	ENSMUST00000209731
MSTRG.2	Ampd3;A	ENSMUST00000170374;ENSMUST00000176487;ENSMUST0000021
MSTRG.2	Rnf141	ENSMUST00000148367;ENSMUST00000176210;MSTRG.29156.2;E
MSTRG.2	Rnf141	MSTRG.29157.1
MSTRG.2	Rnf141	MSTRG.29158.1
MSTRG.2	Ctr9	ENSMUST00000005749;ENSMUST00000152019;ENSMUST0000014(
MSTRG.2	Gcc2	MSTRG.2916.1
MSTRG.2	Galnt18	ENSMUST00000049430;ENSMUST00000106663
MSTRG.2	Galnt18	MSTRG.29161.1
MSTRG.2	Galnt18	MSTRG.29162.1
MSTRG.2	Galnt18	MSTRG.29163.1
MSTRG.2	Eif4g2;	ENSMUST00000162415;ENSMUST00000161051;ENSMUST0000016
MSTRG.2	1700012	ENSMUST00000209597;ENSMUST00000210788;ENSMUST0000020!
MSTRG.2	Gm45378	ENSMUST00000211063
MSTRG.2	1700012	MSTRG.29167.1
MSTRG.2	Dkk3	MSTRG.29168.1
MSTRG.2	Dkk3	ENSMUST00000033036
MSTRG.2	Gcc2	MSTRG.2917.1
MSTRG.2	Usp47	ENSMUST00000106653;ENSMUST00000215510;ENSMUST0000021
MSTRG.2	Usp47	MSTRG.29171.1
MSTRG.2	Parva	ENSMUST00000033030;ENSMUST00000139720;ENSMUST0000010(
MSTRG.2	Parva	MSTRG.29173.1
MSTRG.2	Parvaos	ENSMUST00000154848
MSTRG.2	2310014	ENSMUST00000146145;ENSMUST00000211302
MSTRG.2	Gm24154	ENSMUST00000175261
MSTRG.2	Gm45473	ENSMUST00000211452
MSTRG.2	Tead1	ENSMUST00000084705;ENSMUST00000059768;ENSMUST0000010(
MSTRG.2	Tead1	MSTRG.29179.1
MSTRG.2	Gcc2	MSTRG.2918.1
MSTRG.2	Gm44462	ENSMUST00000199696
MSTRG.2	Pth	ENSMUST00000079793
MSTRG.2	Btbd10	ENSMUST00000117577;ENSMUST00000047091;MSTRG.29182.3;E
MSTRG.2	Btbd10	MSTRG.29183.1
MSTRG.2	Gm15994	ENSMUST00000118029
MSTRG.2	Btbd10	MSTRG.29185.1
MSTRG.2	Btbd10	MSTRG.29186.1

MSTRG.2	Arntl	ENSMUST00000047321;MSTRG.29187.4;MSTRG.29187.2;MSTRG.
MSTRG.2	Arntl	MSTRG.29188.1
MSTRG.2	Gm45355	MSTRG.29189.1;ENSMUST00000211342
MSTRG.2	Lims1	ENSMUST00000020078;MSTRG.2919.2;MSTRG.2919.3;MSTRG.29
MSTRG.2	Far1	ENSMUST00000033018;ENSMUST00000129087;ENSMUST0000012З
MSTRG.2	Far1os	ENSMUST00000156875;ENSMUST00000138253
MSTRG.2	Spon1	ENSMUST00000046687;ENSMUST00000084696;MSTRG.29192.2
MSTRG.2	Spon1	MSTRG.29193.1
MSTRG.2	Rras2	MSTRG.29194.1;MSTRG.29194.2;ENSMUST00000069449;ENSMUS
MSTRG.2	Rras2	MSTRG.29195.1
MSTRG.2	Rras2	MSTRG.29196.1
MSTRG.2	Gm45615	MSTRG.29197.1
MSTRG.2	Gm45454	ENSMUST00000211484
MSTRG.2	Copb1	ENSMUST00000033012;ENSMUST00000209394;MSTRG.29199.3;M
MSTRG.2	Gm6428	ENSMUST00000161062
MSTRG.2	Gm23058	MSTRG.2920.1
MSTRG.29200		MSTRG.29200.1
MSTRG.2	Psma1	ENSMUST00000033008;ENSMUST00000135570;ENSMUST00000154
MSTRG.29202		MSTRG.29202.1
MSTRG.2	4933406	ENSMUST00000163996;ENSMUST00000124673
MSTRG.2	4933406	MSTRG.29204.1
MSTRG.2	Pde3b	ENSMUST00000032909;ENSMUST00000140007;ENSMUST0000021C
MSTRG.2	Pde3b	MSTRG.29206.1
MSTRG.2	Pde3b	MSTRG.29207.1
MSTRG.2	Cyp2r1	ENSMUST00000211506;ENSMUST00000032908;ENSMUST0000013E
MSTRG.29209		MSTRG.29209.1
MSTRG.2	Lims1	MSTRG.2921.1
MSTRG.2	Gm15500	ENSMUST00000071641;ENSMUST00000071523
MSTRG.2	Calcb	MSTRG.29211.1;MSTRG.29211.2;ENSMUST00000032902;ENSMUS
MSTRG.2	Insc	ENSMUST00000169913;ENSMUST00000139670;ENSMUST00000151
MSTRG.2	Insc	MSTRG.29213.1
MSTRG.2	Gm24982	ENSMUST00000104456
MSTRG.2	Sox6	ENSMUST00000206034;ENSMUST00000072804;ENSMUST0000016S
MSTRG.2	Sox6	MSTRG.29216.1
MSTRG.2	Gm45025	ENSMUST00000206043
MSTRG.2	Sox6os	ENSMUST00000032900
MSTRG.2	1110004	ENSMUST00000205450;ENSMUST00000032899;ENSMUST0000010E
MSTRG.2	Gm23058	ENSMUST00000093703
MSTRG.2	1700003	ENSMUST00000140689;ENSMUST00000206534
MSTRG.2	Gm4353	ENSMUST00000111755
MSTRG.2	1110004	MSTRG.29222.1
MSTRG.2	Plekha7	ENSMUST00000182834;ENSMUST00000214700;ENSMUST00000217
MSTRG.2	Plekha7	MSTRG.29224.1
MSTRG.2	Gm44867	ENSMUST00000206322;ENSMUST00000205675
MSTRG.2	Plekha7	MSTRG.29226.1
MSTRG.2	Plekha7	MSTRG.29227.1
MSTRG.2	Plekha7	MSTRG.29228.1
MSTRG.2	Nucb2	ENSMUST00000032895;ENSMUST00000182767;ENSMUST0000018З
MSTRG.2	Lims1	MSTRG.2923.1
MSTRG.2	Nucb2	MSTRG.29230.1
MSTRG.2	Gm45151	ENSMUST00000205445
MSTRG.2	Gm4366	ENSMUST00000201488
MSTRG.2	Rps13;S	ENSMUST00000170953;ENSMUST00000205490;ENSMUST0000020E
MSTRG.2	Gm44777	ENSMUST00000206930;ENSMUST00000205941;ENSMUST0000020E
MSTRG.2	Pik3c2a	ENSMUST00000170430;ENSMUST00000206805;MSTRG.29235.2;E
MSTRG.2	Pik3c2a	MSTRG.29236.1
MSTRG.2	Gm45033	ENSMUST00000206710

MSTRG.2	C430039	ENSMUST00000205973
MSTRG.2	Pik3c2a	MSTRG.29239.1
MSTRG.2	Lims1	MSTRG.2924.1
MSTRG.2	Pik3c2a	MSTRG.29240.1
MSTRG.2	AV35613	ENSMUST00000206201
MSTRG.29242		MSTRG.29242.1
MSTRG.2	Xylt1	ENSMUST00000160035;ENSMUST00000032892;ENSMUST00000161
MSTRG.2	Gm45153	ENSMUST00000205957
MSTRG.2	Xylt1	MSTRG.29245.1
MSTRG.2	Xylt1	MSTRG.29246.1
MSTRG.2	Xylt1	MSTRG.29247.1
MSTRG.2	Xylt1	MSTRG.29248.1
MSTRG.2	Xylt1	MSTRG.29249.1
MSTRG.2	Edar	ENSMUST00000003312
MSTRG.2	Xylt1	MSTRG.29250.1
MSTRG.2	Rps15a	ENSMUST00000131374;ENSMUST00000172457;ENSMUST00000106
MSTRG.2	Arl6ip1	ENSMUST00000032888;ENSMUST00000205182;ENSMUST00000203
MSTRG.2	Arl6ip1	MSTRG.29253.1
MSTRG.2	Itpripl	ENSMUST00000178344
MSTRG.2	Syt17	ENSMUST00000203796;ENSMUST00000203485;ENSMUST00000207
MSTRG.2	Syt17	MSTRG.29256.1
MSTRG.2	Coq7	ENSMUST00000207046;ENSMUST00000032887;ENSMUST00000209
MSTRG.2	Tmc5	ENSMUST00000098088;ENSMUST00000121715;ENSMUST00000121
MSTRG.2	Gde1	ENSMUST00000038791;ENSMUST00000132509;ENSMUST00000208
MSTRG.2	Ranbp2;	ENSMUST00000219332;MSTRG.2926.1;ENSMUST00000003310;EN
MSTRG.2	Ccp110	ENSMUST00000106557;MSTRG.29261.2;ENSMUST00000038650;E
MSTRG.2	Smg1	MSTRG.29262.7;MSTRG.29262.6;MSTRG.29262.5;MSTRG.29262
MSTRG.2	Gm25683	ENSMUST00000083940
MSTRG.2	Gm45084	MSTRG.29264.1;ENSMUST00000207096
MSTRG.2	Smg1	MSTRG.29265.1
MSTRG.2	4930583	ENSMUST00000138180
MSTRG.29267		MSTRG.29267.1
MSTRG.29268		MSTRG.29268.1
MSTRG.29269		MSTRG.29269.1
MSTRG.2	AC15859	ENSMUST00000219887
MSTRG.29270		MSTRG.29270.1
MSTRG.2	Gm45155	MSTRG.29271.1;MSTRG.29271.2;ENSMUST00000207365
MSTRG.2	Gp2	ENSMUST00000207887;ENSMUST00000033255
MSTRG.2	Acsm4	ENSMUST00000047045
MSTRG.2	Thumpd1	ENSMUST00000033236;ENSMUST00000209175
MSTRG.2	Gm44866	ENSMUST00000208774
MSTRG.2	Gm5601	ENSMUST00000207161;ENSMUST00000118909
MSTRG.2	9030624	ENSMUST00000059390;ENSMUST00000129334;ENSMUST00000175
MSTRG.2	1700016	ENSMUST00000209027
MSTRG.2	9030624	MSTRG.29279.1
MSTRG.2	Gm9987	ENSMUST00000069768
MSTRG.2	Gm44549	ENSMUST00000208351
MSTRG.2	9030624	MSTRG.29281.1
MSTRG.2	9030624	MSTRG.29282.1
MSTRG.2	9030624	MSTRG.29283.1
MSTRG.2	Gm44550	ENSMUST00000207311
MSTRG.2	9030624	MSTRG.29285.1
MSTRG.2	Gm26147	ENSMUST00000104479
MSTRG.2	Knop1	ENSMUST00000116280;ENSMUST00000063607;MSTRG.29287.4;E
MSTRG.2	Iqck	ENSMUST00000208658;ENSMUST00000153518;ENSMUST00000098
MSTRG.2	Iqck	MSTRG.29289.1
MSTRG.2	Sh3rf3	ENSMUST00000135526;ENSMUST00000153031;ENSMUST00000133

MSTRG.2	Gm44763	ENSMUST00000208287
MSTRG.2	Gprc5b	ENSMUST00000008878;ENSMUST00000207582;ENSMUST00000207
MSTRG.2	4930560(	ENSMUST00000207051
MSTRG.2	Tmem159	ENSMUST00000033210;ENSMUST00000155330;ENSMUST00000118
MSTRG.2	Acsm3	ENSMUST00000106528;ENSMUST00000106527;ENSMUST0000063
MSTRG.2	Eri2	ENSMUST00000106523;ENSMUST00000063902;ENSMUST0000125
MSTRG.2	Eri2	MSTRG.29296.1
MSTRG.2	Rexo5	ENSMUST00000033218;ENSMUST00000106520;ENSMUST0000133
MSTRG.2	Rexo5	MSTRG.29298.1
MSTRG.2	Rexo5	MSTRG.29299.1
MSTRG.2	Gm27672	ENSMUST00000183751
MSTRG.2	Rexo5	MSTRG.29300.1
MSTRG.2	Dcun1d3	ENSMUST00000059851;ENSMUST00000207233;ENSMUST0000098
MSTRG.2	Dcun1d3	MSTRG.29302.1
MSTRG.2	Dcun1d3	MSTRG.29303.1
MSTRG.2	Dcun1d3	MSTRG.29304.1
MSTRG.2	Dcun1d3	MSTRG.29305.1
MSTRG.2	Lyrm1	ENSMUST00000106518;ENSMUST00000207270;ENSMUST0000208
MSTRG.2	Lyrm1	MSTRG.29307.1
MSTRG.2	Dnah3	ENSMUST00000213149;ENSMUST00000209154;ENSMUST0000046
MSTRG.2	Gm25217	ENSMUST00000157547
MSTRG.2	Sept10	ENSMUST00000220156;ENSMUST00000165971;ENSMUST0000218
MSTRG.2	Uqcrc2	ENSMUST00000033176;ENSMUST00000150675;ENSMUST0000208
MSTRG.2	Pdzd9	ENSMUST00000033178
MSTRG.2	4930588(	ENSMUST00000208075
MSTRG.2	9030407	ENSMUST00000180933;ENSMUST00000180977;ENSMUST0000207
MSTRG.2	BC03033	ENSMUST00000207065;ENSMUST00000208454;ENSMUST000006C
MSTRG.2	BC03033	MSTRG.29315.1
MSTRG.2	Gm44661	MSTRG.29316.1
MSTRG.2	Gm44661	ENSMUST00000208830
MSTRG.2	Vwa3a	ENSMUST00000168600;ENSMUST00000033180;ENSMUST0000166
MSTRG.2	Eef2k	ENSMUST00000047875;ENSMUST00000143279;ENSMUST0000106
MSTRG.2	Gm23223	ENSMUST00000158119
MSTRG.2	Gm15774	ENSMUST00000141810;MSTRG.29320.2
MSTRG.2	Gm45047	ENSMUST00000207651
MSTRG.2	Polr3e	ENSMUST00000207481;ENSMUST00000106483;ENSMUST0000033
MSTRG.2	Gm9234	ENSMUST00000077747
MSTRG.2	Cdr2	ENSMUST00000033169;ENSMUST00000207722
MSTRG.2	Mfsd13b	ENSMUST00000033166
MSTRG.2	Mettl9	MSTRG.29326.1;ENSMUST00000033163;ENSMUST00000207332;E
MSTRG.2	Gm9905	ENSMUST00000065740
MSTRG.2	Gm45137	ENSMUST00000208652
MSTRG.2	Sowahc	ENSMUST00000182161
MSTRG.2	Gm44901	ENSMUST00000208157
MSTRG.2	2210406	ENSMUST00000207806
MSTRG.2	Igsf6	ENSMUST00000047194
MSTRG.2	Usp31	ENSMUST00000046929;ENSMUST00000205473
MSTRG.2	1700069	ENSMUST00000191155;ENSMUST00000065310
MSTRG.2	Scnn1b	ENSMUST00000033161;ENSMUST00000205520;ENSMUST0000206
MSTRG.2	Cog7	ENSMUST00000206817;ENSMUST00000205438;ENSMUST0000057
MSTRG.2	Gga2	ENSMUST00000124566;ENSMUST00000033160;ENSMUST0000144
MSTRG.2	Ears2	ENSMUST00000151530;ENSMUST00000147397;ENSMUST0000033
MSTRG.2934		MSTRG.2934.1
MSTRG.2	Ubfd1	ENSMUST00000033158;ENSMUST00000148060;MSTRG.29340.2;M
MSTRG.2	Ndufab1	ENSMUST00000033157;ENSMUST00000146964;ENSMUST0000123
MSTRG.2	Ndufab1	MSTRG.29342.1
MSTRG.2	Gm44986	ENSMUST00000206292

MSTRG.2	Palb2	ENSMUST00000106469;ENSMUST00000063587;ENSMUST0000009[illegible]
MSTRG.2	Dctn5	ENSMUST00000033156;ENSMUST00000123602;ENSMUST0000017[illegible]
MSTRG.2	Gm15489	MSTRG.29347.1;ENSMUST00000132811
MSTRG.2	Plk1	ENSMUST00000033154;ENSMUST00000206470;ENSMUST0000020[illegible]
MSTRG.2	Plk1	MSTRG.29349.1
MSTRG.2	P4ha1	ENSMUST00000009789;ENSMUST00000092512;ENSMUST0000010[illegible]
MSTRG.2	Gm44985	ENSMUST00000206202
MSTRG.2	Ern2	ENSMUST00000206198
MSTRG.2	Prkcb	MSTRG.29352.1
MSTRG.2	Prkcb;G[illegible]	ENSMUST00000143692;ENSMUST00000064989;MSTRG.29353.2;[illegible]
MSTRG.2	Prkcb	MSTRG.29354.1
MSTRG.2	Prkcb	MSTRG.29355.1
MSTRG.2	Gm44664	ENSMUST00000206841
MSTRG.2	Prkcb	MSTRG.29357.1;MSTRG.29357.2;MSTRG.29357.3;MSTRG.29357
MSTRG.2	Gm44663	ENSMUST00000206898
MSTRG.2	Gm14388	ENSMUST00000118119
MSTRG.2	P4ha1	MSTRG.2936.1
MSTRG.2	Gm26414	ENSMUST00000157674
MSTRG.2	Prkcb	MSTRG.29361.1
MSTRG.2	Gm14389	ENSMUST00000140453
MSTRG.2	Prkcb	MSTRG.29363.1
MSTRG.2	Rbbp6;G[illegible]	MSTRG.29364.1;MSTRG.29364.2;MSTRG.29364.4;MSTRG.29364
MSTRG.2	4930413[illegible]	ENSMUST00000205998
MSTRG.2	Gm45846	ENSMUST00000211170;ENSMUST00000211504;ENSMUST0000020[illegible]
MSTRG.2	Slc5a11	ENSMUST00000131933;ENSMUST00000140721;ENSMUST0000003[illegible]
MSTRG.2	Arhgap1	ENSMUST00000207010;ENSMUST00000098060;ENSMUST0000010[illegible]
MSTRG.2	Arhgap1	MSTRG.29369.1
MSTRG.2937		MSTRG.2937.1
MSTRG.2	Lcmt1	ENSMUST00000206721;ENSMUST00000033025;ENSMUST0000020[illegible]
MSTRG.2	Gm23559	ENSMUST00000157824
MSTRG.29372		MSTRG.29372.1
MSTRG.2	Kdm8	ENSMUST00000033010;ENSMUST00000135129
MSTRG.2	Kdm8	MSTRG.29374.1
MSTRG.29375		MSTRG.29375.1
MSTRG.2	Nsmce1	ENSMUST00000033006;ENSMUST00000138616;ENSMUST0000014[illegible]
MSTRG.2	Il4ra	ENSMUST00000033004;ENSMUST00000206217;ENSMUST0000020[illegible]
MSTRG.2	Gm44876	ENSMUST00000205394
MSTRG.2	Il4ra	MSTRG.29379.1
MSTRG.2	Gm10273	ENSMUST00000092511
MSTRG.2	Il21r	ENSMUST00000206234;MSTRG.29380.1;ENSMUST00000206103;[illegible]
MSTRG.2	Il21r	MSTRG.29381.1
MSTRG.29382		MSTRG.29382.1
MSTRG.2	Xpo6	MSTRG.29383.1
MSTRG.2	Gtf3c1	ENSMUST00000055506;ENSMUST00000206042;MSTRG.29384.3;[illegible]
MSTRG.2	Gtf3c1	MSTRG.29385.1;MSTRG.29385.2
MSTRG.2	D430042[illegible]	ENSMUST00000124223;ENSMUST00000069660;ENSMUST0000020[illegible]
MSTRG.2	Gsg1l	ENSMUST00000073935
MSTRG.2	Gm44874	ENSMUST00000206586
MSTRG.2	Sbk1	ENSMUST00000056028;MSTRG.29389.1;ENSMUST00000206424;[illegible]
MSTRG.2	Mcu	MSTRG.2939.1;ENSMUST00000020312;ENSMUST00000009791;M[illegible]
MSTRG.2	Gm10155	ENSMUST00000084598
MSTRG.2	Xpo6	ENSMUST00000168189;ENSMUST00000009344;ENSMUST0000016[illegible]
MSTRG.2	Xpo6	MSTRG.29392.1
MSTRG.2	Gm17137	ENSMUST00000164106;ENSMUST00000165729
MSTRG.2	Xpo6	MSTRG.29394.1
MSTRG.2	Gm17136	ENSMUST00000169993
MSTRG.2	Xpo6	MSTRG.29396.1

MSTRG.2	Lat;Mir	ENSMUST00000206793;ENSMUST00000032997;ENSMUST00000205
MSTRG.2	Nfatc2i	MSTRG.29398.1;ENSMUST00000075671;ENSMUST00000142947
MSTRG.2	Gm45141	ENSMUST00000206502
MSTRG.2	Actr1b	MSTRG.294.2;MSTRG.294.1;ENSMUST00000043951;ENSMUST000
MSTRG.2	Mcu	MSTRG.2940.1
MSTRG.2	Spns1	ENSMUST00000032994;ENSMUST00000119846;ENSMUST00000126
MSTRG.2	Tufm	ENSMUST00000098048;ENSMUST00000106392;ENSMUST00000205
MSTRG.2	Rabep2	ENSMUST00000151304;ENSMUST00000106407;MSTRG.29402.2;E
MSTRG.2	Atp2a1	ENSMUST00000032974;ENSMUST00000146973
MSTRG.2	Sh2b1	ENSMUST00000205440;ENSMUST00000032978;ENSMUST00000205
MSTRG.2	Atxn2l	ENSMUST00000040202;ENSMUST00000167759;ENSMUST00000206
MSTRG.2	Eif3c	ENSMUST00000032992;ENSMUST00000180459;ENSMUST00000205
MSTRG.2	Gm24596	ENSMUST00000122504
MSTRG.29408		MSTRG.29408.1
MSTRG.2	Cln3	ENSMUST00000084589;ENSMUST00000032962;ENSMUST00000116
MSTRG.2	Mcu	MSTRG.2941.1
MSTRG.2	Mir7059	ENSMUST00000184825
MSTRG.2	Apobr	ENSMUST00000039522;ENSMUST00000131860;ENSMUST00000137
MSTRG.2	Il27	ENSMUST00000058429
MSTRG.2	Mapk3	ENSMUST00000050201;ENSMUST00000057669;ENSMUST00000205
MSTRG.2	2510046	ENSMUST00000187458;ENSMUST00000084587
MSTRG.2	Sgf29	ENSMUST00000123571;ENSMUST00000032956;MSTRG.29415.1;E
MSTRG.2	Sult1a1	ENSMUST00000106373;ENSMUST00000106371;ENSMUST00000106
MSTRG.29417		MSTRG.29417.1
MSTRG.2	Gm6939;	ENSMUST00000122029;ENSMUST00000144897;ENSMUST00000084
MSTRG.2	Slx1b	MSTRG.29419.1
MSTRG.2	Mcu	MSTRG.2942.1
MSTRG.2	Bola2	ENSMUST00000144571;ENSMUST00000130498;ENSMUST00000052
MSTRG.2	Coro1a	ENSMUST00000106364;ENSMUST00000032949;MSTRG.29421.1;E
MSTRG.2	Gdpd3	ENSMUST00000032944;ENSMUST00000206858;ENSMUST00000205
MSTRG.2	Gm9967	ENSMUST00000205660;MSTRG.29423.2;ENSMUST00000206903;M
MSTRG.2	Gm9967	MSTRG.29424.1
MSTRG.2	Ypel3	ENSMUST00000038614;ENSMUST00000170882;ENSMUST00000106
MSTRG.2	Ppp4c	ENSMUST00000032936;ENSMUST00000206570;MSTRG.29426.3;M
MSTRG.2	Aldoa	ENSMUST00000032934;ENSMUST00000106348;ENSMUST00000087
MSTRG.2	Fam57b	ENSMUST00000205324;ENSMUST00000205888;ENSMUST00000206
MSTRG.2	Gm44856	ENSMUST00000206639
MSTRG.2	Mcu	MSTRG.2943.1
MSTRG.2	Doc2a	ENSMUST00000141805;ENSMUST00000064110;ENSMUST00000137
MSTRG.2	Ino80e	ENSMUST00000205619;ENSMUST00000050833;ENSMUST00000106
MSTRG.2	Hirip3	ENSMUST00000037248;ENSMUST00000205512;ENSMUST0000015C
MSTRG.2	Taok2	ENSMUST00000214525;ENSMUST00000071268;ENSMUST0000013C
MSTRG.2	Tmem219	ENSMUST00000154174;ENSMUST00000120007;ENSMUST00000137
MSTRG.2	Tmem219	MSTRG.29435.1
MSTRG.2	Gm20650	ENSMUST00000175690
MSTRG.2	Gm20650	MSTRG.29437.1
MSTRG.2	Kctd13;	ENSMUST00000032924;MSTRG.29439.2;MSTRG.29439.3;ENSMUS
MSTRG.2	Mcu	MSTRG.2944.1
MSTRG.2	Gm44735	ENSMUST00000206197
MSTRG.2	Asphd1	ENSMUST00000106340;ENSMUST00000052937
MSTRG.2	D830044	ENSMUST00000180704;ENSMUST00000205437;ENSMUST00000205
MSTRG.2	Cdipt	ENSMUST00000032920;ENSMUST00000206780;ENSMUST00000206
MSTRG.2	Maz	ENSMUST00000205568;ENSMUST00000032916;ENSMUST00000206
MSTRG.2	Mvp;Pag	ENSMUST00000165096;ENSMUST00000133172;ENSMUST00000172
MSTRG.2	Kif22	ENSMUST00000205754;ENSMUST00000032915;ENSMUST00000206
MSTRG.2	Gm31749	MSTRG.29447.1;ENSMUST00000206873
MSTRG.2	Zg16	ENSMUST00000051122;ENSMUST00000205424;ENSMUST00000205

MSTRG.2	Gm44939	ENSMUST00000206939
MSTRG.2	Gm10322	ENSMUST00000095646
MSTRG.2	Gm22747	ENSMUST00000082495
MSTRG.29451		MSTRG.29451.1
MSTRG.2	AI46760	MSTRG.29452.1;ENSMUST00000056288
MSTRG.2	Qprt;Sp	ENSMUST00000032912;MSTRG.29453.1;ENSMUST00000129332;M
MSTRG.2	Gm25735	ENSMUST00000175164
MSTRG.2	Cd2bp2	ENSMUST00000166791;ENSMUST00000206026;ENSMUST00000205
MSTRG.2	Tbc1d10	ENSMUST00000120705;ENSMUST00000205620;ENSMUST00000153
MSTRG.2	Zfp771	ENSMUST00000052509;ENSMUST00000151893;ENSMUST00000205
MSTRG.2	Dctpp1	ENSMUST00000035276
MSTRG.2	Mylpf	ENSMUST00000206204;MSTRG.29459.2;ENSMUST00000206772;E
MSTRG.2	Micu1	ENSMUST00000165563;ENSMUST00000167872;MSTRG.2946.2;EN
MSTRG.2	Sept1	ENSMUST00000131208;MSTRG.29460.1;ENSMUST00000106314;E
MSTRG.2	Zfp553	ENSMUST00000106312;ENSMUST00000056232;ENSMUST00000205
MSTRG.2	Sephs2	ENSMUST00000082428
MSTRG.2	Gm44729	ENSMUST00000206759
MSTRG.29464		MSTRG.29464.1
MSTRG.2	Gm17511	MSTRG.29465.1
MSTRG.2	Gm17511	ENSMUST00000172206
MSTRG.2	Gm31897	MSTRG.29467.1;MSTRG.29467.2;ENSMUST00000205924;ENSMUS
MSTRG.2	Zfp768	ENSMUST00000060783;ENSMUST00000205266
MSTRG.2	Itgal	ENSMUST00000117762;ENSMUST00000106306;ENSMUST0000012C
MSTRG.2	Gm17148	ENSMUST00000164702
MSTRG.2	Itgal	MSTRG.29470.1
MSTRG.2	Zfp747	ENSMUST00000067425;ENSMUST00000205832;ENSMUST00000206
MSTRG.2	9130019	MSTRG.29472.1;ENSMUST00000049052;ENSMUST00000164345
MSTRG.2	E430018	ENSMUST00000165495;ENSMUST00000074249;ENSMUST00000106
MSTRG.2	Smt3h2-	ENSMUST00000122370
MSTRG.2	Zfp764	ENSMUST00000059199;ENSMUST00000139118
MSTRG.2	Zfp689	ENSMUST00000053392;ENSMUST00000106299
MSTRG.2	B130055	ENSMUST00000147487;MSTRG.29477.1;ENSMUST00000144796;E
MSTRG.2	Gm45110	ENSMUST00000205704
MSTRG.2	B130055	MSTRG.29479.1
MSTRG.2	Micu1	MSTRG.2948.1
MSTRG.2	Prr14	MSTRG.29480.1
MSTRG.2	Fbrs	MSTRG.29481.1
MSTRG.29482		MSTRG.29482.1
MSTRG.2	Fbrs;AC	ENSMUST00000205432;ENSMUST00000206394;ENSMUST00000206
MSTRG.2	Mir7060	ENSMUST00000183486
MSTRG.2	Gm44760	ENSMUST00000206375
MSTRG.2	Bcl7c	ENSMUST00000106282;ENSMUST00000205977;ENSMUST00000206
MSTRG.2	Gm39090	ENSMUST00000205992;ENSMUST00000205284
MSTRG.2	Orai3	ENSMUST00000118865;ENSMUST00000061587;ENSMUST00000121
MSTRG.2	Gm17059	ENSMUST00000168284
MSTRG.2	Ctf1	ENSMUST00000047393;ENSMUST00000206997;ENSMUST00000206
MSTRG.2	Ctf2	ENSMUST00000206038
MSTRG.2	Fbxl19	ENSMUST00000205689;ENSMUST00000186207;ENSMUST00000189
MSTRG.2	Fbxl19	MSTRG.29493.1
MSTRG.2	1700008	ENSMUST00000185351
MSTRG.2	Srcap;G	ENSMUST00000185401;ENSMUST00000188124;MSTRG.29495.2;M
MSTRG.2	Gm28198	ENSMUST00000190938
MSTRG.2	Phkg2	ENSMUST00000129457;ENSMUST00000146383;ENSMUST00000205
MSTRG.2	Ccdc189	ENSMUST00000072155;ENSMUST00000205839;ENSMUST00000206
MSTRG.2	Rnf40	MSTRG.29499.1;ENSMUST00000205694;ENSMUST00000033088;E
MSTRG.2	4933424	ENSMUST00000192873;ENSMUST00000208994;ENSMUST00000195
MSTRG.2	Gm7399	ENSMUST00000032395

MSTRG.2	Gm44759	ENSMUST00000206057
MSTRG.2	Zfp629;	MSTRG.29501.1;ENSMUST00000191038;ENSMUST00000058038;E
MSTRG.29502		MSTRG.29502.1
MSTRG.29503		MSTRG.29503.1
MSTRG.2	Zfp668	MSTRG.29504.1
MSTRG.2	Setd1a;	ENSMUST00000047075;ENSMUST00000144406;ENSMUST0000012
MSTRG.2	9430064	ENSMUST00000206516
MSTRG.2	Stx1b	ENSMUST00000106267;ENSMUST00000156135
MSTRG.2	Gm45204	ENSMUST00000206297
MSTRG.2	Stx4a	ENSMUST00000152310;ENSMUST00000033075;ENSMUST00000121
MSTRG.2	Gm23803	ENSMUST00000104610
MSTRG.2	Zfp668	MSTRG.29510.1;ENSMUST00000054415;ENSMUST00000106263;M
MSTRG.2	Zfp646	ENSMUST00000205364;MSTRG.29511.2;MSTRG.29511.1;MSTRG.
MSTRG.2	Prss53;	ENSMUST00000121394;ENSMUST00000205300;ENSMUST0000003
MSTRG.2	Bckdk	ENSMUST00000071056;ENSMUST00000146574;ENSMUST00000151
MSTRG.2	Kat8	ENSMUST00000206071;MSTRG.29514.1;MSTRG.29514.2;ENSMUS
MSTRG.2	Kat8	MSTRG.29515.1
MSTRG.2	Prss8	ENSMUST00000032988;ENSMUST00000206568
MSTRG.2	Fus	ENSMUST00000106251;ENSMUST00000077609;ENSMUST00000137
MSTRG.2	Gm26690	ENSMUST00000181457;MSTRG.29518.2;ENSMUST00000033056;E
MSTRG.29519		MSTRG.29519.1
MSTRG.2	Micu1	MSTRG.2952.1
MSTRG.2	Gm45205	ENSMUST00000205745
MSTRG.2	9130023	ENSMUST00000078816
MSTRG.2	Armc5	ENSMUST00000206509;ENSMUST00000044660;MSTRG.29522.2
MSTRG.2	Tgfb1i1	ENSMUST00000164710;ENSMUST00000070656;ENSMUST0000016
MSTRG.2	Gm6916	ENSMUST00000205523
MSTRG.2	Gm6916	MSTRG.29525.1
MSTRG.2	Slc5a2	ENSMUST00000118169;ENSMUST00000171335
MSTRG.2	BC01715	ENSMUST00000147091;MSTRG.29528.1;ENSMUST00000033044;M
MSTRG.2	Rgs10;G	MSTRG.29529.1;MSTRG.29529.2;ENSMUST00000033133;MSTRG.
MSTRG.2	Dnajb12	ENSMUST00000147914;ENSMUST00000131810;ENSMUST0000014
MSTRG.2	Bag3	ENSMUST00000033136
MSTRG.2	2610306	ENSMUST00000206512
MSTRG.2	Tial1	ENSMUST00000106226;ENSMUST00000141079;MSTRG.29532.3;E
MSTRG.2	Gm32816	ENSMUST00000206497
MSTRG.2	Gm44672	ENSMUST00000206944
MSTRG.2	Sec23ip	ENSMUST00000042942;ENSMUST00000206504;MSTRG.29535.2;M
MSTRG.2	n-R5s15	ENSMUST00000104095
MSTRG.2	Gm45121	ENSMUST00000205396
MSTRG.2	Inpp5f;	ENSMUST00000098007;MSTRG.29539.2;ENSMUST00000043138;M
MSTRG.2	Dnajb12	MSTRG.2954.1
MSTRG.2	Inpp5f	MSTRG.29540.1
MSTRG.2	Mcmbp	ENSMUST00000057557;MSTRG.29541.2;MSTRG.29541.3;ENSMUS
MSTRG.2	Gm44673	ENSMUST00000205692
MSTRG.2	Gm44674	MSTRG.29543.1;ENSMUST00000157941
MSTRG.29544		MSTRG.29544.1
MSTRG.2	Wdr11	ENSMUST00000084519;ENSMUST00000143422;ENSMUST0000013
MSTRG.29546		MSTRG.29546.1
MSTRG.2	Edrf1	ENSMUST00000051169;ENSMUST00000128901;ENSMUST00000154
MSTRG.2	Edrf1	MSTRG.29548.1
MSTRG.2	Mmp21	ENSMUST00000131894;ENSMUST00000122136
MSTRG.2	Ddit4	MSTRG.2955.1;ENSMUST00000020308;MSTRG.2955.2
MSTRG.2	Uros	ENSMUST00000135760;ENSMUST00000106146;ENSMUST0000010
MSTRG.2	Bccip	ENSMUST00000033282;ENSMUST00000157077;ENSMUST00000132
MSTRG.2	Bccip	MSTRG.29552.1
MSTRG.2	Dhx32	ENSMUST00000033290;ENSMUST00000140593;ENSMUST0000010

MSTRG.2	Gm15483	ENSMUST00000120338
MSTRG.2	Gm45670	ENSMUST00000211382
MSTRG.2	Fank1	ENSMUST00000065359
MSTRG.2	Gm9333	ENSMUST00000205452
MSTRG.2	Gm9333	MSTRG.29558.1
MSTRG.2	Adam12;.	ENSMUST00000067680;MSTRG.29559.1;ENSMUST00000138363;E
MSTRG.2	Anapc16	MSTRG.2956.1;ENSMUST00000182898;MSTRG.2956.3;ENSMUST0
MSTRG.2	Adam12	MSTRG.29560.1
MSTRG.2	Gm23631	ENSMUST00000175291
MSTRG.2	Gm19514	ENSMUST00000206856
MSTRG.2	Adam12	MSTRG.29563.1
MSTRG.2	Adam12	MSTRG.29564.1
MSTRG.2	Adam12	MSTRG.29565.1
MSTRG.2	Adam12	MSTRG.29566.1
MSTRG.2	Adam12	MSTRG.29567.1
MSTRG.2	Adam12	MSTRG.29568.1
MSTRG.2	Dock1	ENSMUST00000211570;ENSMUST00000211593;ENSMUST00000084
MSTRG.2	Ascc1	ENSMUST00000219778;ENSMUST00000050516;ENSMUST00000164
MSTRG.2	Fam196a	ENSMUST00000210055;ENSMUST00000171394;ENSMUST0000021C
MSTRG.2	Gm45532	ENSMUST00000210342
MSTRG.2	Gm45597	ENSMUST00000211524
MSTRG.2	Gm24581	ENSMUST00000103962
MSTRG.2	Gm9347	ENSMUST00000211473
MSTRG.2	Ptpre	MSTRG.29575.1;ENSMUST00000211140;ENSMUST00000209268;E
MSTRG.2	Mir1962	ENSMUST00000157516
MSTRG.2	5830432	MSTRG.29577.1;MSTRG.29577.2;MSTRG.29577.3;MSTRG.29577
MSTRG.2	Mki67	MSTRG.29578.1;ENSMUST00000033310;MSTRG.29578.3;ENSMUS
MSTRG.2	Mki67	MSTRG.29579.1
MSTRG.2	Ascc1	MSTRG.2958.1
MSTRG.2	Gm45240	MSTRG.29580.1;ENSMUST00000211437
MSTRG.2	Mki67	MSTRG.29581.1
MSTRG.2	Mgmt	ENSMUST00000081510
MSTRG.2	Gm45280	ENSMUST00000210484
MSTRG.2	Mgmt	MSTRG.29584.1
MSTRG.2	Mgmt	MSTRG.29585.1
MSTRG.2	Mgmt	MSTRG.29586.1
MSTRG.2	Mgmt	MSTRG.29587.1
MSTRG.2	Ebf3	ENSMUST00000169486;ENSMUST00000033378;ENSMUST0000021C
MSTRG.2	Gm45278	ENSMUST00000209650
MSTRG.2	Spock2	ENSMUST00000138976;ENSMUST00000121820;ENSMUST00000165
MSTRG.2	Gm10578	ENSMUST00000209747;ENSMUST00000211070
MSTRG.2	Ebf3	MSTRG.29591.1
MSTRG.29592		MSTRG.29592.1
MSTRG.29593		MSTRG.29593.1
MSTRG.29594		MSTRG.29594.1
MSTRG.2	Glrx3	ENSMUST00000064404;ENSMUST00000211496;ENSMUST0000020S
MSTRG.2	Tcerg1l	ENSMUST00000160436;ENSMUST00000162222
MSTRG.2	Tcerg1l	MSTRG.29597.1
MSTRG.2	Mapk1ip	ENSMUST00000119664;ENSMUST00000075667;ENSMUST00000118
MSTRG.2	Ppp2r2d	ENSMUST00000128157;ENSMUST00000041097;ENSMUST00000148
MSTRG.2	Gm33533	ENSMUST00000193853
MSTRG.2	Mir6906	ENSMUST00000198998
MSTRG.2	Bnip3	ENSMUST00000106112;ENSMUST00000130500;ENSMUST00000141
MSTRG.2	Gm18258	ENSMUST00000210413
MSTRG.2	Pwwp2b	ENSMUST00000093993;ENSMUST00000172136;ENSMUST0000020S
MSTRG.2	Stk32c	ENSMUST00000016125
MSTRG.2	Stk32c	MSTRG.29605.1

MSTRG.2	Gm27975	ENSMUST00000184400
MSTRG.2	Gm45328	ENSMUST00000210942
MSTRG.2	Lrrc27	ENSMUST00000016124;ENSMUST00000106104;ENSMUST00000135
MSTRG.2	Inpp5a	ENSMUST00000106098;ENSMUST00000026550;ENSMUST00000097
MSTRG.2	Slc29a3	ENSMUST00000117513;ENSMUST00000119595;ENSMUST00000150
MSTRG.2	Inpp5a	MSTRG.29610.1
MSTRG.2	Inpp5a	MSTRG.29611.1
MSTRG.2	Inpp5a	MSTRG.29612.1
MSTRG.2	Inpp5a	MSTRG.29613.1
MSTRG.2	Inpp5a	MSTRG.29614.1
MSTRG.2	Nkx6-2	ENSMUST00000097974;ENSMUST00000106095
MSTRG.2	Fgfr2;C	ENSMUST00000120187;ENSMUST00000117872;ENSMUST00000153
MSTRG.2	Fgfr2	MSTRG.29617.1
MSTRG.2	Gm5903	ENSMUST00000121836
MSTRG.2	Ate1	ENSMUST00000035458;ENSMUST00000033139;ENSMUST00000208
MSTRG.2	Unc5b	ENSMUST00000077925;ENSMUST00000218316;ENSMUST00000218
MSTRG.2	Fgfr2	MSTRG.29620.1
MSTRG.2	Gm45719	MSTRG.29621.1;ENSMUST00000207954
MSTRG.2	Fgfr2	MSTRG.29622.1
MSTRG.2	Gm44647	ENSMUST00000207402
MSTRG.2	Gm4275	ENSMUST00000210214;ENSMUST00000209209;ENSMUST00000212
MSTRG.2	Gm27884	ENSMUST00000183865
MSTRG.2	Fgfr2	MSTRG.29626.1
MSTRG.2	Nsmce4a	ENSMUST00000160289;ENSMUST00000162510;ENSMUST00000159
MSTRG.2	Tacc2	ENSMUST00000207918;ENSMUST00000215492;ENSMUST00000207
MSTRG.2	4930513	ENSMUST00000207466
MSTRG.2	AC15351	ENSMUST00000218180
MSTRG.2	Gm6108	ENSMUST00000208365
MSTRG.2	Etos1	ENSMUST00000057134
MSTRG.2	Btbd16	ENSMUST00000207155;ENSMUST00000048453;ENSMUST00000208
MSTRG.2	Gm5602	ENSMUST00000146366
MSTRG.2	Plekha1	ENSMUST00000120441;ENSMUST00000148513;ENSMUST00000140
MSTRG.2	Mir7061	ENSMUST00000198082
MSTRG.2	Htra1	ENSMUST00000150717;ENSMUST00000006367;ENSMUST00000153
MSTRG.2	Dmbt1	ENSMUST00000213064;ENSMUST00000208860;ENSMUST00000084
MSTRG.2	Psap	ENSMUST00000105465;ENSMUST00000004316;ENSMUST00000177
MSTRG.2	5430419	ENSMUST00000050586;ENSMUST00000208921;ENSMUST00000128
MSTRG.2	Cuzd1	ENSMUST00000046611
MSTRG.2	1700007	ENSMUST00000188899;ENSMUST00000033146
MSTRG.2	Fam24a	ENSMUST00000207439;ENSMUST00000207784;ENSMUST00000084
MSTRG.2	2310057	ENSMUST00000059438;ENSMUST00000208526;ENSMUST00000207
MSTRG.2	Fgfr2	MSTRG.29645.1
MSTRG.2	Fgfr2	MSTRG.29646.1
MSTRG.2	Pstk	ENSMUST00000075610;ENSMUST00000145114;ENSMUST00000207
MSTRG.2	Gm44623	ENSMUST00000207934
MSTRG.2	Ikzf5	ENSMUST00000046306;ENSMUST00000121033;ENSMUST00000208
MSTRG.2	Cdh23	ENSMUST00000073242;ENSMUST00000105462;ENSMUST00000105
MSTRG.2	Gm44624	ENSMUST00000208700
MSTRG.2	Gm44625	ENSMUST00000207838
MSTRG.2	Acadsb	ENSMUST00000015829;ENSMUST00000117518;ENSMUST00000135
MSTRG.2	Gm44982	ENSMUST00000208795
MSTRG.2	Gm18600	ENSMUST00000207205
MSTRG.2	Gm44984	ENSMUST00000207802
MSTRG.2	Gm45105	ENSMUST00000207824
MSTRG.2	Hmx3	ENSMUST00000046093
MSTRG.2	Hmx2	ENSMUST00000051997
MSTRG.2	Bub3	ENSMUST00000084502;ENSMUST00000207736;ENSMUST00000208

MSTRG.2	Vsir	ENSMUST00000020301;ENSMUST00000105460;ENSMUST00000164
MSTRG.2	Fgfr2	MSTRG.29660.1
MSTRG.2	Gm16477	ENSMUST00000085365
MSTRG.2	Gm44546	ENSMUST00000208353
MSTRG.2	Gpr26	ENSMUST00000045840
MSTRG.2	Cpxm2	ENSMUST00000209155;ENSMUST00000033149;ENSMUST0000015C
MSTRG.2	n-R5s15	ENSMUST00000122681
MSTRG.2	Gm15677	ENSMUST00000152302
MSTRG.2	Gm17455	ENSMUST00000219863;ENSMUST00000218360
MSTRG.2	Chst15	ENSMUST00000077472;ENSMUST00000080215;ENSMUST00000132
MSTRG.2	Chst15	MSTRG.29671.1
MSTRG.2	Gm44891	ENSMUST00000208817
MSTRG.2	Chst15	ENSMUST00000207068
MSTRG.2	Gm10584	ENSMUST00000207036
MSTRG.2	Gm44892	ENSMUST00000208595
MSTRG.2	Gm34908	ENSMUST00000207222
MSTRG.2	Gm35147	ENSMUST00000208383
MSTRG.2	Gm4587	ENSMUST00000209135
MSTRG.2	Tbata	ENSMUST00000122261;ENSMUST00000121297;ENSMUST00000035
MSTRG.2	Oat	ENSMUST00000084500;ENSMUST00000211545
MSTRG.2	Nkx1-2	ENSMUST00000054562
MSTRG.2	Gm16764	ENSMUST00000148931
MSTRG.2	Lhpp	ENSMUST00000148669;ENSMUST00000033241;ENSMUST00000106
MSTRG.2	Gm15582	ENSMUST00000132849
MSTRG.2	Fam53b;[illegible]	MSTRG.29685.1;ENSMUST00000097999;ENSMUST00000065371;E
MSTRG.2	Fgfr2	MSTRG.29687.1
MSTRG.2	Gm45501	ENSMUST00000211468
MSTRG.2	Fgfr2	MSTRG.29689.1
MSTRG.2	Pcbd1	ENSMUST00000218005;ENSMUST00000020298
MSTRG.2	Abraxas[illegible]	ENSMUST00000084497;ENSMUST00000210077;MSTRG.29690.2;E
MSTRG.2	Fgfr2	MSTRG.29691.1
MSTRG.2	1500002[illegible]	ENSMUST00000209767;ENSMUST00000211184;ENSMUST0000018C
MSTRG.2	Gm45502	ENSMUST00000209561
MSTRG.2	Zranb1	MSTRG.29694.1;MSTRG.29694.2;ENSMUST00000106157;MSTRG.
MSTRG.2	Gm15718	ENSMUST00000131085
MSTRG.2	B930086[illegible]	ENSMUST00000209529
MSTRG.2	Cfap46	ENSMUST00000196540;ENSMUST00000129990;ENSMUST00000093
MSTRG.2	Gm4459	ENSMUST00000118750
MSTRG.2	Echs1	ENSMUST00000026538;ENSMUST00000155647;ENSMUST00000147
MSTRG.2	Zap70	MSTRG.297.1;ENSMUST00000027291;MSTRG.297.3;MSTRG.297.
MSTRG.2	Sgpl1	ENSMUST00000122259;ENSMUST00000092498;ENSMUST0000015C
MSTRG.2	Paox	ENSMUST00000211757;ENSMUST00000209908;ENSMUST0000021C
MSTRG.2	Adgra1	ENSMUST00000026548
MSTRG.2	Gm4974	ENSMUST00000209974
MSTRG.2	Kndc1	ENSMUST00000154782;ENSMUST00000121839;ENSMUST00000156
MSTRG.2	Utf1	ENSMUST00000168457
MSTRG.2	Gm16201	ENSMUST00000134312;ENSMUST00000139218;ENSMUST0000021C
MSTRG.2	Gm16201	MSTRG.29706.1
MSTRG.2	Gm2044;[illegible]	ENSMUST00000130771;ENSMUST00000200604;ENSMUST00000083
MSTRG.2	6430531[illegible]	ENSMUST00000146974;ENSMUST00000097970;ENSMUST0000021C
MSTRG.2	Adam8	ENSMUST00000156647;ENSMUST00000026546;ENSMUST00000106
MSTRG.2	Gm44308	ENSMUST00000197080
MSTRG.2	Mir7062	ENSMUST00000185038
MSTRG.2	Tubgcp2	ENSMUST00000210224;ENSMUST00000209930;ENSMUST00000026
MSTRG.2	Zfp511	ENSMUST00000210419;ENSMUST00000211789;ENSMUST00000168
MSTRG.2	Zfp511	MSTRG.29713.1
MSTRG.2	Cyp2e1	ENSMUST00000026552;ENSMUST00000209253;ENSMUST0000021C

MSTRG.2	Ifitm3	ENSMUST00000026565
MSTRG.2	Nlrp6	ENSMUST00000183845
MSTRG.2	Pgghg	ENSMUST00000164337;ENSMUST00000164580;ENSMUST0000016
MSTRG.2	Pgghg	MSTRG.29718.1
MSTRG.2	Ifitm5;	ENSMUST00000026562;ENSMUST00000211129;ENSMUST0000008
MSTRG.2	Prf1	ENSMUST00000219375;ENSMUST00000035419
MSTRG.2	Ifitm1	ENSMUST00000106042;ENSMUST00000106040;ENSMUST0000002
MSTRG.2	Zfp941;	ENSMUST00000080651;ENSMUST00000106052;ENSMUST0000020
MSTRG.2	Urah	ENSMUST00000106050;ENSMUST00000211372;ENSMUST0000002
MSTRG.2	C330022	ENSMUST00000190581
MSTRG.2	Scgb1c1	ENSMUST00000035300
MSTRG.2	Odf3	ENSMUST00000106049;ENSMUST00000135845
MSTRG.2	Ric8a	ENSMUST00000026558;ENSMUST00000151384;ENSMUST0000015
MSTRG.2	Sirt3	ENSMUST00000210296;ENSMUST00000106048;ENSMUST0000021
MSTRG.2	Psmd13	ENSMUST00000026560;ENSMUST00000151644;MSTRG.29728.2;
MSTRG.2	Gm15542	ENSMUST00000121585
MSTRG.2	Eif4ebp	MSTRG.2973.1;ENSMUST00000020288;ENSMUST00000167087;M
MSTRG.2	Cox8b	ENSMUST00000026561
MSTRG.2	Pkp3	ENSMUST00000106039;ENSMUST00000066873;ENSMUST0000016
MSTRG.2	Sigirr	ENSMUST00000210167;ENSMUST00000097958;MSTRG.29732.3;
MSTRG.2	Gm5905	ENSMUST00000211731
MSTRG.2	Ptdss2	ENSMUST00000172479;ENSMUST00000026568;ENSMUST0000017
MSTRG.2	Ptdss2	MSTRG.29735.1
MSTRG.2	Ptdss2	MSTRG.29736.1
MSTRG.2	Rnh1	ENSMUST00000210314;ENSMUST00000106033;ENSMUST0000016
MSTRG.2	Hras	ENSMUST00000026572;ENSMUST00000168550;ENSMUST0000012
MSTRG.2	Lrrc56	ENSMUST00000084446;ENSMUST00000070458;ENSMUST0000014
MSTRG.2	D830039	ENSMUST00000217740;ENSMUST00000051330
MSTRG.2	Lmntd2	ENSMUST00000026573;ENSMUST00000170841
MSTRG.2	Rassf7	ENSMUST00000046890;ENSMUST00000127613;ENSMUST0000020
MSTRG.2	Phrf1	ENSMUST00000155123;MSTRG.29742.4;MSTRG.29742.3;ENSMUS
MSTRG.2	Irf7	ENSMUST00000106023;ENSMUST00000026571;ENSMUST0000009
MSTRG.2	Taldo1	ENSMUST00000211654;ENSMUST00000026576;ENSMUST0000014
MSTRG.2	Gatd1	ENSMUST00000106008;ENSMUST00000156353;ENSMUST0000014
MSTRG.2	Drd4	ENSMUST00000026569;ENSMUST00000209649
MSTRG.2	Deaf1	ENSMUST00000080553;ENSMUST00000209600;ENSMUST0000021
MSTRG.2	Tmem80	ENSMUST00000155657;MSTRG.29748.1;ENSMUST00000126510;
MSTRG.2	Eps8l2	ENSMUST00000026577;ENSMUST00000140025;ENSMUST0000021
MSTRG.2	Lrrc20	ENSMUST00000049242
MSTRG.2	B230206	ENSMUST00000134845;ENSMUST00000129373
MSTRG.2	Rplp2;S	ENSMUST00000084434;ENSMUST00000140099;ENSMUST0000013
MSTRG.2	Cend1	ENSMUST00000084436;ENSMUST00000124444
MSTRG.2	Slc25a2	ENSMUST00000019226;ENSMUST00000106007;ENSMUST0000020
MSTRG.2	Pidd1	ENSMUST00000190068;ENSMUST00000128703;ENSMUST0000013
MSTRG.2	Pnpla2	ENSMUST00000164016;ENSMUST00000026583;ENSMUST0000016
MSTRG.2	Cracr2b	ENSMUST00000053670;ENSMUST00000167491;ENSMUST0000016
MSTRG.2	Gm10575	ENSMUST00000167900;ENSMUST00000097947;ENSMUST0000016
MSTRG.2	Cd151	ENSMUST00000211522;ENSMUST00000106000;ENSMUST0000020
MSTRG.2	Polr2l	ENSMUST00000043870
MSTRG.2	Lrrc20	MSTRG.2976.1
MSTRG.2	Tspan4	ENSMUST00000026585;ENSMUST00000138092;ENSMUST0000014
MSTRG.2	Chid1	ENSMUST00000153191;ENSMUST00000026586;ENSMUST0000016
MSTRG.2	Chid1	MSTRG.29762.1
MSTRG.2	Gm45416	ENSMUST00000209713
MSTRG.2	Ap2a2;M	ENSMUST00000003038;ENSMUST00000201320;MSTRG.29764.2;
MSTRG.2	Gm16982	MSTRG.29765.1
MSTRG.2	Muc6	MSTRG.29766.1;ENSMUST00000189314;ENSMUST00000190907

MSTRG.2	Muc5b	ENSMUST00000165147
MSTRG.2	Tollip	ENSMUST00000055819;ENSMUST00000001950;ENSMUST0000013C
MSTRG.2	Ifitm10	ENSMUST00000131791;ENSMUST00000059223;ENSMUST00000084
MSTRG.2	AC16516	ENSMUST00000220313
MSTRG.2	Ifitm10	MSTRG.29770.1
MSTRG.2	Brsk2;M	ENSMUST00000078200;ENSMUST00000018971;ENSMUST00000172
MSTRG.2	Mob2;Gm	ENSMUST00000211000;ENSMUST00000211206;ENSMUST00000084
MSTRG.2	Mrpl23	ENSMUST00000137345;ENSMUST00000038675;ENSMUST0000021C
MSTRG.2	Nctc1	ENSMUST00000132167;ENSMUST00000123668
MSTRG.2	Lsp1	ENSMUST00000105968;ENSMUST00000018963;MSTRG.29775.3;E
MSTRG.2	Prr33	MSTRG.29776.1;ENSMUST00000151580
MSTRG.2	Tnnt3	ENSMUST00000074187;ENSMUST00000078497;ENSMUST00000105
MSTRG.2	Tspan32	ENSMUST00000165444;ENSMUST00000131740
MSTRG.2	Tspan32	ENSMUST00000009396;ENSMUST00000141186;MSTRG.29779.2;E
MSTRG.2	AC16516	ENSMUST00000220094;ENSMUST00000219324;ENSMUST00000217
MSTRG.2	Tspan32	MSTRG.29780.1
MSTRG.2	R74862	ENSMUST00000133630;ENSMUST00000155952;ENSMUST00000131
MSTRG.2	Cd81	ENSMUST00000037941;ENSMUST00000128762;MSTRG.29782.2;E
MSTRG.2	Trpm5	ENSMUST00000148715;ENSMUST00000136602;ENSMUST00000126
MSTRG.2	Tssc4	ENSMUST00000060433;ENSMUST00000133410;ENSMUST00000207
MSTRG.2	Kcnq1	ENSMUST00000009689;ENSMUST00000185383;ENSMUST00000186
MSTRG.2	Gm38321	ENSMUST00000195053
MSTRG.2	Gm28821	ENSMUST00000185637
MSTRG.2	Kcnq1ot	ENSMUST00000185789;MSTRG.29789.1;MSTRG.29789.3
MSTRG.2	AC16516	ENSMUST00000219782
MSTRG.2	Gm27395	ENSMUST00000183938
MSTRG.2	Gm27389	ENSMUST00000183622
MSTRG.2	Gm27539	ENSMUST00000184971
MSTRG.2	Gm37364	ENSMUST00000192533
MSTRG.2	4933417	ENSMUST00000208893;ENSMUST00000188294
MSTRG.2	Gm38095	ENSMUST00000192896
MSTRG.2	Gm38065	ENSMUST00000193147
MSTRG.2	Nap1l4;	ENSMUST00000072727;ENSMUST00000208190;MSTRG.29798.5;M
MSTRG.2	Cars	ENSMUST00000010899;ENSMUST00000208575;ENSMUST00000105
MSTRG.2	Gm18828	ENSMUST00000181035;ENSMUST00000180475
MSTRG.2	AC12442	ENSMUST00000219114
MSTRG.2	Mir7064	ENSMUST00000184553
MSTRG.2	Cars	MSTRG.29801.1
MSTRG.2	Gm15580	ENSMUST00000121587
MSTRG.2	Tnfrsf2	ENSMUST00000208124;ENSMUST00000055723;MSTRG.29803.3
MSTRG.2	E230032	ENSMUST00000208797;ENSMUST00000207959;ENSMUST00000207
MSTRG.2	E230032	MSTRG.29805.1
MSTRG.2	Tnfrsf2	ENSMUST00000171066;ENSMUST00000075588;ENSMUST00000146
MSTRG.2	Tnfrsf2	ENSMUST00000035742;ENSMUST00000152703;ENSMUST00000208
MSTRG.2	Mrgpre	ENSMUST00000054048
MSTRG.2	Gm28447	ENSMUST00000186677
MSTRG.2	Osbpl5	ENSMUST00000137627;ENSMUST00000119499;ENSMUST0000002C
MSTRG.2	Nadsyn1	ENSMUST00000033415;ENSMUST00000156638;ENSMUST00000132
MSTRG.2	Dhcr7	ENSMUST00000139748;ENSMUST00000128454;ENSMUST00000073
MSTRG.2	Shank2	ENSMUST00000105902;ENSMUST00000213146;ENSMUST00000105
MSTRG.2	Mir3105	ENSMUST00000175480
MSTRG.2	Gm44999	ENSMUST00000207456
MSTRG.2	Gm14372	ENSMUST00000125355
MSTRG.2	Cttn	ENSMUST00000033407;ENSMUST00000103079;ENSMUST00000134
MSTRG.2	Gm14376	ENSMUST00000145297
MSTRG.2	Faddos	ENSMUST00000097928
MSTRG.2	Npffr1	ENSMUST00000020287

MSTRG.2	Fadd	ENSMUST00000033394
MSTRG.2	Ppfia1	ENSMUST00000168134;ENSMUST00000182982;ENSMUST00000182
MSTRG.2	Ano1	ENSMUST00000121758;ENSMUST00000118556;ENSMUST00000033
MSTRG.2	Gm44930	ENSMUST00000208733
MSTRG.2	Oraov1	ENSMUST00000128057;ENSMUST00000033388;ENSMUST00000093
MSTRG.2	Ccnd1	ENSMUST00000093962;ENSMUST00000208193;ENSMUST00000135
MSTRG.2	Mrgprf	ENSMUST00000033386;ENSMUST00000117718
MSTRG.2	Tpcn2	ENSMUST00000208328;ENSMUST00000209047;MSTRG.29827.3;E
MSTRG.2	1810010	ENSMUST00000208788;ENSMUST00000208264;ENSMUST00000207
MSTRG.2	Tpcn2	MSTRG.29829.1
MSTRG.2	Ppa1	ENSMUST00000020286;ENSMUST00000160470;ENSMUST0000016C
MSTRG.2	Tpcn2	MSTRG.29830.1
MSTRG.29831		MSTRG.29831.1
MSTRG.29832		MSTRG.29832.1
MSTRG.29833		MSTRG.29833.1
MSTRG.2	Insr	MSTRG.29834.2;MSTRG.29834.3;MSTRG.29834.4;ENSMUST0000C
MSTRG.2	Vmn2r-p	ENSMUST00000209168
MSTRG.2	Insr	MSTRG.29836.1
MSTRG.2	Gm16180	MSTRG.29838.1;ENSMUST00000128662
MSTRG.2	Zfp358	ENSMUST00000061508;ENSMUST00000207318;ENSMUST00000208
MSTRG.2	AC12442	ENSMUST00000219142
MSTRG.2	A430078	ENSMUST00000208363;MSTRG.29840.1;ENSMUST00000207059;M
MSTRG.2	Pex11g	ENSMUST00000004686;ENSMUST00000111081;ENSMUST00000118
MSTRG.2	Gm44775	ENSMUST00000207266
MSTRG.2	C330021	ENSMUST00000153341;ENSMUST00000136592;ENSMUST00000151
MSTRG.2	Rps23-p	ENSMUST00000146939
MSTRG.2	Mcoln1	ENSMUST00000004683;ENSMUST00000208739;ENSMUST0000016C
MSTRG.2	Pnpla6	ENSMUST00000111070;ENSMUST00000004681;MSTRG.29846.1;E
MSTRG.2	Pnpla6	MSTRG.29847.1
MSTRG.2	Camsap3	ENSMUST00000057028;ENSMUST00000171962;ENSMUST00000207
MSTRG.2	Xab2	ENSMUST00000163038;ENSMUST00000019614;ENSMUST00000159
MSTRG.2	Sar1a	ENSMUST00000220372;MSTRG.2985.1;ENSMUST00000020285;EN
MSTRG.2	Gm22204	ENSMUST00000122654
MSTRG.2	Pet100	ENSMUST00000208950;ENSMUST00000208185;ENSMUST00000156
MSTRG.2	Pcp2	ENSMUST00000145855;ENSMUST00000004749;ENSMUST00000128
MSTRG.2	Retn	ENSMUST00000169234;ENSMUST00000012849
MSTRG.2	Stxbp2	ENSMUST00000160708;ENSMUST00000162867;ENSMUST00000159
MSTRG.2	Rprl3	ENSMUST00000157400
MSTRG.2	Gm45231	ENSMUST00000208629
MSTRG.2	Trappc5	ENSMUST00000044857;ENSMUST00000207787
MSTRG.2	Fcer2a	ENSMUST00000005678;ENSMUST00000208145;ENSMUST00000208
MSTRG.2	Fcer2a	MSTRG.29859.1
MSTRG.2	Tysnd1	ENSMUST00000020284;MSTRG.2986.1;ENSMUST00000218135;MS
MSTRG.2	Clec4g	ENSMUST00000160527;ENSMUST00000062037
MSTRG.2	Gm16553	ENSMUST00000160490
MSTRG.2	Gm23886	ENSMUST00000122684
MSTRG.2	Gm16589	ENSMUST00000159522
MSTRG.2	Gm26750	ENSMUST00000181718
MSTRG.2	4932443	ENSMUST00000177130
MSTRG.2	Evi5l	ENSMUST00000177499;ENSMUST00000177053;ENSMUST00000176
MSTRG.2	Prr36	ENSMUST00000168386
MSTRG.2	Snapc2	ENSMUST00000011981;ENSMUST00000208316;ENSMUST00000208
MSTRG.2	Lrrc8e	ENSMUST00000053035;ENSMUST00000207770
MSTRG.2987		MSTRG.2987.1
MSTRG.2	Map2k7;	ENSMUST00000110998;ENSMUST00000062686;ENSMUST00000003
MSTRG.2	Ccl25	MSTRG.29871.1
MSTRG.2	Gm44869	ENSMUST00000208836

MSTRG.2	Gm45118	ENSMUST00000208928
MSTRG.2	Gm7434	ENSMUST00000208558
MSTRG.2	Gm45117	ENSMUST00000207250
MSTRG.2	Ctxn1	ENSMUST00000053252
MSTRG.2	Timm44	ENSMUST00000149827;ENSMUST00000003029;ENSMUST0000014[illegible]
MSTRG.2	Gm26102	ENSMUST00000157251
MSTRG.2	Elavl1	MSTRG.29879.1;ENSMUST00000098950;ENSMUST00000209010;[illegible]
MSTRG.2	Aifm2	ENSMUST00000105455;ENSMUST00000131361;ENSMUST00000067
MSTRG.2	Ccl25	ENSMUST00000155797;ENSMUST00000069762;MSTRG.29880.2;[illegible]
MSTRG.29881		MSTRG.29881.1
MSTRG.2	Zfp958	ENSMUST00000202692;ENSMUST00000073201
MSTRG.2	Zfp958	MSTRG.29883.1
MSTRG.2	Zfp958	MSTRG.29884.1
MSTRG.2	Gm2676	MSTRG.29885.1
MSTRG.2	Shcbp1	ENSMUST00000022945;ENSMUST00000208856;ENSMUST00000207
MSTRG.2	Gm6334	ENSMUST00000207083
MSTRG.29888		MSTRG.29888.1
MSTRG.2	Efnb2	ENSMUST00000001319;ENSMUST00000152698;ENSMUST0000020[illegible]
MSTRG.2	H2afy2	ENSMUST00000020283
MSTRG.2	E230020[illegible]	ENSMUST00000208097
MSTRG.2	Gm45074	ENSMUST00000209091
MSTRG.2	Gm45073	ENSMUST00000207688
MSTRG.2	4921522[illegible]	ENSMUST00000048606
MSTRG.2	Arglu1;[illegible]	ENSMUST00000048545;ENSMUST00000208252;ENSMUST00000207
MSTRG.2	Lig4	ENSMUST00000095476;ENSMUST00000170033
MSTRG.2	Abhd13	ENSMUST00000139793;ENSMUST00000048216;MSTRG.29896.3;[illegible]
MSTRG.2	Tnfsf13[illegible]	ENSMUST00000208683;ENSMUST00000207792;ENSMUST00000208
MSTRG.2	Gm44789	ENSMUST00000207195
MSTRG.2	Irs2	ENSMUST00000040514
MSTRG.299		MSTRG.299.1
MSTRG.2	Mir5108	ENSMUST00000175276
MSTRG.2	9530052[illegible]	ENSMUST00000180750;ENSMUST00000181315
MSTRG.2	9530052[illegible]	MSTRG.29901.1
MSTRG.2	Gm15418	ENSMUST00000151014
MSTRG.2	Col4a1	ENSMUST00000033898;ENSMUST00000209735;ENSMUST0000020[illegible]
MSTRG.2	Gm44716	ENSMUST00000209007
MSTRG.2	Col4a2	ENSMUST00000033899;ENSMUST00000145295;ENSMUST0000014[illegible]
MSTRG.2	Gm15419	ENSMUST00000148654
MSTRG.29907		MSTRG.29907.1
MSTRG.2	Ing1	ENSMUST00000211007;ENSMUST00000210740;ENSMUST0000021[illegible]
MSTRG.2	Rab20	ENSMUST00000033900
MSTRG.2	Gm5424	ENSMUST00000053865
MSTRG.2	Gm20100	ENSMUST00000209418
MSTRG.2	Rab20	MSTRG.29911.1
MSTRG.2	E230013[illegible]	ENSMUST00000180408
MSTRG.2	Naxd	ENSMUST00000177955;ENSMUST00000178817;ENSMUST0000003[illegible]
MSTRG.2	Cars2	ENSMUST00000049461;ENSMUST00000211395;ENSMUST00000211
MSTRG.2	Gm45592	ENSMUST00000209605
MSTRG.2	Gm45592	MSTRG.29916.1
MSTRG.2	Ankrd10	ENSMUST00000210530;ENSMUST00000169782;ENSMUST0000003[illegible]
MSTRG.2	1700016[illegible]	ENSMUST00000033906;ENSMUST00000190787
MSTRG.2	Gm7562	ENSMUST00000210502
MSTRG.2	Hk1	ENSMUST00000099691;MSTRG.2992.1;ENSMUST00000116238;E[illegible]
MSTRG.2	4930465	ENSMUST00000209697
MSTRG.2	Gm7600	ENSMUST00000209657
MSTRG.2	Gm6524	ENSMUST00000209673
MSTRG.2	Gm27854	ENSMUST00000175578

MSTRG.2	Arhgef7	MSTRG.29924.1;ENSMUST00000110909;ENSMUST00000033908;E
MSTRG.2	Gm18991	ENSMUST00000209225
MSTRG.2	Gm15875	ENSMUST00000152264
MSTRG.2	Tex29	ENSMUST00000033909;ENSMUST00000209704;ENSMUST00000144
MSTRG.2	Tubgcp3	ENSMUST00000164774;ENSMUST00000000776;ENSMUST00000168
MSTRG.2	Gm26483	ENSMUST00000158016
MSTRG.2	Hk1os	ENSMUST00000123457;ENSMUST00000143122;ENSMUST00000142
MSTRG.2	4931415	ENSMUST00000068194;ENSMUST00000131064
MSTRG.2	Proz	ENSMUST00000210050;ENSMUST00000211363;ENSMUST00000211
MSTRG.2	Pcid2	ENSMUST00000164416;ENSMUST00000168164;ENSMUST00000165
MSTRG.2	Pcid2	MSTRG.29933.1
MSTRG.2	Gm17022	ENSMUST00000166415
MSTRG.2	Cul4a	ENSMUST00000016680;ENSMUST00000135371;ENSMUST00000121
MSTRG.2	Mir7065	ENSMUST00000185110
MSTRG.2	Lamp1	ENSMUST00000033824;ENSMUST00000209709
MSTRG.2	Grtp1	ENSMUST00000209895;ENSMUST00000210317;ENSMUST00000165
MSTRG.2	Mir1968	ENSMUST00000157429
MSTRG.2	Hk1	MSTRG.2994.1
MSTRG.2	2810030	ENSMUST00000190673;ENSMUST00000190461
MSTRG.2	Atp11a	ENSMUST00000033818;ENSMUST00000091237;ENSMUST00000133
MSTRG.2	Gm15350	ENSMUST00000147398;ENSMUST00000135138
MSTRG.2	Gm15347	ENSMUST00000145372
MSTRG.2	Mcf2l;G	ENSMUST00000156560;ENSMUST00000095456;ENSMUST00000138
MSTRG.2	Gm15353	ENSMUST00000110860;ENSMUST00000149938
MSTRG.2	Gm15351	ENSMUST00000123920;ENSMUST00000153095;ENSMUST0000015C
MSTRG.2	Gm15349	ENSMUST00000121154
MSTRG.2	Dcun1d2	ENSMUST00000110840;ENSMUST00000110839;ENSMUST00000203
MSTRG.2	E330037	ENSMUST00000204295
MSTRG.2	Supv3l1	ENSMUST00000020273;ENSMUST00000162023;ENSMUST00000161
MSTRG.2	Gm25014	ENSMUST00000158401
MSTRG.2	Dcun1d2	MSTRG.29951.1
MSTRG.2	9530085	ENSMUST00000203963
MSTRG.2	Tmco3	ENSMUST00000210063;MSTRG.29953.1;ENSMUST00000211402;E
MSTRG.2	Tfdp1	ENSMUST00000209885;ENSMUST00000209396;ENSMUST0000021C
MSTRG.2	Tmem255	ENSMUST00000211356;ENSMUST00000167505;ENSMUST00000167
MSTRG.2	Gas6	ENSMUST00000033828
MSTRG.2	Rasa3	ENSMUST00000117551;ENSMUST00000137822;MSTRG.29957.2;E
MSTRG.2	Rasa3	MSTRG.29958.1
MSTRG.2	Rasa3	MSTRG.29959.1
MSTRG.2	4930507	ENSMUST00000181110
MSTRG.2	Cdc16	ENSMUST00000130173;ENSMUST00000043962;MSTRG.29960.1;E
MSTRG.2	Upf3a	ENSMUST00000043767;ENSMUST00000211724;ENSMUST00000211
MSTRG.2	Champ1	ENSMUST00000051870;ENSMUST00000128557
MSTRG.2	AC13918	ENSMUST00000197610;ENSMUST00000083883
MSTRG.2	Coprs	ENSMUST00000210249;ENSMUST00000033839;ENSMUST0000021C
MSTRG.2	Fbxo25	ENSMUST00000209913;ENSMUST00000043520;ENSMUST0000021C
MSTRG.29966		MSTRG.29966.1
MSTRG.2	Tdrp	ENSMUST00000062613;ENSMUST00000194703;MSTRG.29967.3;M
MSTRG.29968		MSTRG.29968.1
MSTRG.29969		MSTRG.29969.1
MSTRG.2	Srgn	MSTRG.2997.1
MSTRG.2	Erich1	ENSMUST00000110813;ENSMUST00000211242
MSTRG.2	Gm45520	ENSMUST00000210109
MSTRG.2	Gm10699	ENSMUST00000098916
MSTRG.2	Cln8	ENSMUST00000132001;ENSMUST00000123990;ENSMUST00000027
MSTRG.2	Kbtbd11	ENSMUST00000069399;ENSMUST00000184598;ENSMUST00000183
MSTRG.2	5830468	ENSMUST00000184288

MSTRG.2	Gm37844	ENSMUST00000192473
MSTRG.2	Arhgef1	ENSMUST00000084207;ENSMUST00000160619;ENSMUST00000161
MSTRG.2	Arhgef1	MSTRG.29978.1
MSTRG.2	Arhgef1	MSTRG.29979.1
MSTRG.2	Vps26a	ENSMUST00000092473;ENSMUST00000219574;MSTRG.2998.3;EN
MSTRG.2	Gm16350	ENSMUST00000162958
MSTRG.2	Gm45408	MSTRG.29982.1
MSTRG.2	Gm45675	ENSMUST00000211202
MSTRG.2	Gm45408	MSTRG.29984.1
MSTRG.2	Csmd1	ENSMUST00000082104;ENSMUST00000125551;ENSMUST00000131
MSTRG.2	Mir3106	ENSMUST00000175401
MSTRG.2	Gm16346	ENSMUST00000129158
MSTRG.2	Gm16347	ENSMUST00000149000;ENSMUST00000131707
MSTRG.2	Gm45393	ENSMUST00000211634
MSTRG.2	Ddx21	ENSMUST00000045866;ENSMUST00000218393;ENSMUST0000022
MSTRG.2	Csmd1	MSTRG.29990.1
MSTRG.2	Csmd1	MSTRG.29991.1
MSTRG.2	Gm45394	ENSMUST00000209240
MSTRG.2	Csmd1	MSTRG.29993.1
MSTRG.2	Gm25278	ENSMUST00000157267
MSTRG.2	Csmd1	MSTRG.29995.1
MSTRG.2	3110080	ENSMUST00000210736
MSTRG.2	Csmd1	MSTRG.29997.1
MSTRG.2	Csmd1	MSTRG.29998.1
MSTRG.2	Csmd1	MSTRG.29999.1
MSTRG.3	Gm25493	ENSMUST00000157375
MSTRG.3	Pcmtd1;	ENSMUST00000061280;ENSMUST00000182114;MSTRG.30.4;MSTR
MSTRG.3	Tmem131	ENSMUST00000027290;MSTRG.300.1;ENSMUST00000194563;MST
MSTRG.3	AC12196	ENSMUST00000218594
MSTRG.3	Csmd1	MSTRG.30000.1
MSTRG.3	Csmd1	MSTRG.30001.1
MSTRG.3	Csmd1	MSTRG.30002.1
MSTRG.3	Csmd1	MSTRG.30003.1
MSTRG.3	Csmd1	MSTRG.30004.1
MSTRG.3	Csmd1	MSTRG.30005.1
MSTRG.3	Csmd1	MSTRG.30006.1
MSTRG.3	Csmd1	MSTRG.30007.1
MSTRG.3	Csmd1	MSTRG.30008.1
MSTRG.3	Csmd1	MSTRG.30009.1
MSTRG.3	Srgn	ENSMUST00000160987;ENSMUST00000020271;MSTRG.3001.2;EN
MSTRG.3	Agpat5	ENSMUST00000149565;ENSMUST00000033847
MSTRG.3	Agpat5	MSTRG.30011.1
MSTRG.3	Gm45493	ENSMUST00000209494
MSTRG.3	Gm35934	ENSMUST00000213002
MSTRG.3	Mcph1	ENSMUST00000039412;MSTRG.30014.1;MSTRG.30014.3;ENSMUS
MSTRG.3	Gm25763	ENSMUST00000104099
MSTRG.3	Mcph1	MSTRG.30016.1
MSTRG.3	Gm16725	ENSMUST00000138744;MSTRG.30017.2
MSTRG.3	Gm17149	ENSMUST00000164712
MSTRG.3	Angpt2	ENSMUST00000033846;ENSMUST00000170200
MSTRG.3	Kif1bp	ENSMUST00000159704;ENSMUST00000162759;MSTRG.3002.3;EN
MSTRG.3	Rpl19-p	ENSMUST00000122422;ENSMUST00000184215
MSTRG.30021		MSTRG.30021.1
MSTRG.3	Gm35998	MSTRG.30023.1
MSTRG.30024		MSTRG.30024.1
MSTRG.3	Gm21119	MSTRG.30025.1
MSTRG.3	Gm21119	ENSMUST00000178438

MSTRG.30027		MSTRG.30027.1
MSTRG.30028		MSTRG.30028.1
MSTRG.3	Atp7b	ENSMUST00000006742;MSTRG.30029.2
MSTRG.3	4933428	ENSMUST00000199616
MSTRG.3	Alg11	ENSMUST00000072572;ENSMUST00000110737;ENSMUST00000134
MSTRG.3	Alg11	MSTRG.30031.1
MSTRG.3	Nek5	ENSMUST00000169834;ENSMUST00000213644
MSTRG.3	Nek3	ENSMUST00000033865;ENSMUST00000110730;ENSMUST0000017
MSTRG.3	Ckap2	ENSMUST00000046916;ENSMUST00000211629;ENSMUST00000211
MSTRG.3	Vps36	ENSMUST00000033866;ENSMUST00000153949
MSTRG.3	Vps36	MSTRG.30036.1
MSTRG.3	Vps36	MSTRG.30037.1
MSTRG.3	Thsd1	ENSMUST00000069828;ENSMUST00000160585
MSTRG.3	Rpl30-p	ENSMUST00000160070
MSTRG.3	Ddx50	MSTRG.3004.1
MSTRG.3	Gm45453	ENSMUST00000211758
MSTRG.3	Mrps31	ENSMUST00000033934;ENSMUST00000211083
MSTRG.3	Mrps31	MSTRG.30042.1
MSTRG.3	Vdac3	ENSMUST00000179233;MSTRG.30043.2;ENSMUST00000009036;E
MSTRG.3	Smim19	ENSMUST00000163774;ENSMUST00000033935;MSTRG.30044.3
MSTRG.3	Slc20a2	ENSMUST00000067786;ENSMUST00000209305;ENSMUST0000020
MSTRG.3	Gm17491	ENSMUST00000180411
MSTRG.3	Slc20a2	MSTRG.30047.1
MSTRG.3	Slc20a2	MSTRG.30048.1
MSTRG.3	Slc20a2	MSTRG.30049.1
MSTRG.3	Ddx50	ENSMUST00000218304;ENSMUST00000020270;MSTRG.3005.4;EN
MSTRG.3	Slc20a2	MSTRG.30050.1
MSTRG.3	Dkk4	ENSMUST00000033936
MSTRG.3	Polb	MSTRG.30052.1;ENSMUST00000033938;MSTRG.30052.3;MSTRG.
MSTRG.3	Gm26909	MSTRG.30053.1;ENSMUST00000181668
MSTRG.3	Ap3m2	ENSMUST00000163739;ENSMUST00000210656;ENSMUST0000021
MSTRG.3	1700041	ENSMUST00000186344;ENSMUST00000068068
MSTRG.3	Ikbkb	ENSMUST00000033939;ENSMUST00000135326;ENSMUST0000012
MSTRG.3	Gm15346	ENSMUST00000143638
MSTRG.30058		MSTRG.30058.1
MSTRG.3	Kat6a	ENSMUST00000110696;ENSMUST00000044331;MSTRG.30059.2;
MSTRG.3006		MSTRG.3006.1
MSTRG.3	Gm45555	ENSMUST00000210028
MSTRG.3	Gm7760	MSTRG.30061.1
MSTRG.3	4930467	MSTRG.30062.1
MSTRG.3	Gm6483;	MSTRG.30063.1;MSTRG.30063.2;MSTRG.30063.3;MSTRG.3006
MSTRG.3	Gm7760;	MSTRG.30064.1;MSTRG.30064.2;MSTRG.30064.3
MSTRG.3	Gm7760	MSTRG.30065.1;MSTRG.30065.2
MSTRG.3	4930467	MSTRG.30066.1
MSTRG.3	4930467	MSTRG.30067.1
MSTRG.3	Gm7760	ENSMUST00000212265
MSTRG.3	4930467	ENSMUST00000098909
MSTRG.3	Ccar1	MSTRG.3007.1;ENSMUST00000020268;ENSMUST00000219527;EN
MSTRG.3	Gm20786	MSTRG.30070.1
MSTRG.3	Gm31371	ENSMUST00000211848
MSTRG.3	Gm45754	ENSMUST00000212380
MSTRG.3	Gm20778	ENSMUST00000178524
MSTRG.3	Gm15319	MSTRG.30075.1;MSTRG.30075.2;ENSMUST00000178899
MSTRG.3	Gm15319	MSTRG.30076.1
MSTRG.3	Gm15319	MSTRG.30077.2;MSTRG.30077.1
MSTRG.3	Gm15319	MSTRG.30078.2;MSTRG.30078.1
MSTRG.3	Gm26804	MSTRG.30079.1

MSTRG.3	Ccar1	MSTRG.3008.1
MSTRG.3	6820431	ENSMUST00000150051;MSTRG.30080.2;MSTRG.30080.3;MSTRG.
MSTRG.3	Gm26804	MSTRG.30081.1
MSTRG.3	Gm26804	MSTRG.30082.1
MSTRG.3	Gm26804	MSTRG.30083.1
MSTRG.3	Gm26804	MSTRG.30084.1;ENSMUST00000181248;MSTRG.30084.2;MSTRG.
MSTRG.3	Gm15319	ENSMUST00000084046
MSTRG.3	Gm7807	ENSMUST00000212475
MSTRG.30087		MSTRG.30087.1
MSTRG.3	Gm21092	MSTRG.30088.1
MSTRG.30089		MSTRG.30089.1
MSTRG.3	Ccar1	MSTRG.3009.1
MSTRG.3	Gm21112	ENSMUST00000212224
MSTRG.3	Gpat4	MSTRG.30091.1;ENSMUST00000167004;ENSMUST00000210782;E
MSTRG.3	B230112	ENSMUST00000192979
MSTRG.3	Gins4	MSTRG.30093.1;ENSMUST00000033950
MSTRG.3	Golga7	ENSMUST00000051094;ENSMUST00000121783;ENSMUST0000013
MSTRG.3	Gm45412	ENSMUST00000210217
MSTRG.3	Sfrp1	ENSMUST00000033952
MSTRG.30097		MSTRG.30097.1
MSTRG.3	Gm26714	ENSMUST00000181338
MSTRG.3	1810011	ENSMUST00000052622
MSTRG.3	Gm37506	ENSMUST00000192008
MSTRG.30100		MSTRG.30100.1
MSTRG.3	Adam9	ENSMUST00000084035;ENSMUST00000208247;ENSMUST00000084
MSTRG.3	Tm2d2	ENSMUST00000033961;ENSMUST00000210758;ENSMUST0000021
MSTRG.3	Htra4	ENSMUST00000084031;ENSMUST00000176814
MSTRG.3	Gm16933	ENSMUST00000141741
MSTRG.3	Plekha2	ENSMUST00000064883;MSTRG.30105.2;MSTRG.30105.3;ENSMUS
MSTRG.3	Plekha2	MSTRG.30106.1
MSTRG.3	Plekha2	MSTRG.30107.1
MSTRG.3	Plekha2	MSTRG.30108.1
MSTRG.3	5830408	ENSMUST00000210072;ENSMUST00000211653;ENSMUST0000021
MSTRG.3	Snord98	ENSMUST00000116819
MSTRG.3	Gm45745	ENSMUST00000211447
MSTRG.3	5830408	MSTRG.30111.1
MSTRG.30112		MSTRG.30112.1
MSTRG.3	Tacc1;M	MSTRG.30113.1;MSTRG.30113.2;ENSMUST00000084512;ENSMUS
MSTRG.3	Tacc1	MSTRG.30114.1
MSTRG.3	Fgfr1	ENSMUST00000084027;ENSMUST00000178276;ENSMUST0000017
MSTRG.3	Gm16159	ENSMUST00000126122;ENSMUST00000140451
MSTRG.3	Letm2	ENSMUST00000079160;ENSMUST00000211133;ENSMUST0000021
MSTRG.3	Letm2	MSTRG.30118.1
MSTRG.3	Gm45570	ENSMUST00000211137
MSTRG.3	Ccar1	MSTRG.3012.1
MSTRG.3	D830025	ENSMUST00000210582
MSTRG.3	Plpp5	ENSMUST00000068916;ENSMUST00000124764;ENSMUST0000014
MSTRG.3	Nsd3;Gm	MSTRG.30122.1;ENSMUST00000155861;ENSMUST00000136107;E
MSTRG.3	Gm23184	ENSMUST00000122594
MSTRG.3	Nsd3	MSTRG.30124.1
MSTRG.3	Gm24992	ENSMUST00000157412
MSTRG.3	Kcnu1	ENSMUST00000098858
MSTRG.3	Kcnu1	MSTRG.30127.1
MSTRG.3	Hgsnat	MSTRG.30128.1
MSTRG.3	Hgsnat	ENSMUST00000037609;ENSMUST00000210894;ENSMUST00000211
MSTRG.3	AC12253	ENSMUST00000218355
MSTRG.3	Pomk	ENSMUST00000061850

MSTRG.3	Ddhd2	ENSMUST00000033975;ENSMUST00000210777;ENSMUST00000211
MSTRG.3	6430710I	ENSMUST00000210843
MSTRG.3	Gm17484	ENSMUST00000167899
MSTRG.3	Gm17484	MSTRG.30134.1
MSTRG.3	Bag4	ENSMUST00000038498;ENSMUST00000210103;ENSMUST0000020
MSTRG.3	Lsm1;St	ENSMUST00000038421;MSTRG.30136.1;ENSMUST00000210647;E
MSTRG.3	Ash2l	ENSMUST00000068892;ENSMUST00000139946;ENSMUST0000016
MSTRG.3	Gm32098	ENSMUST00000211305
MSTRG.3	Fnta	ENSMUST00000016138;ENSMUST00000209860;ENSMUST00000211
MSTRG.3	Tet1	MSTRG.3014.1;ENSMUST00000050826;ENSMUST00000174189;EN
MSTRG.3	Hook3	ENSMUST00000037182;ENSMUST00000211683;ENSMUST00000147
MSTRG.3	Gm45411	ENSMUST00000209647
MSTRG.3	Hook3	MSTRG.30142.1
MSTRG.3	Hook3	MSTRG.30143.1
MSTRG.3	Gm45413	ENSMUST00000210389
MSTRG.3	1700047	ENSMUST00000153552;ENSMUST00000144750
MSTRG.3	Rnf170;	ENSMUST00000014022;ENSMUST00000209300;ENSMUST00000124
MSTRG.3	Gm45251	MSTRG.30147.1;ENSMUST00000209486
MSTRG.3	Rnf170	MSTRG.30148.1
MSTRG.3	Gm45250	ENSMUST00000209414
MSTRG.3	AC16635	ENSMUST00000220243
MSTRG.3	Gm45572	ENSMUST00000211774
MSTRG.3	Zfp703	ENSMUST00000209610;ENSMUST00000127097;ENSMUST00000154
MSTRG.3	Gm45371	ENSMUST00000210723
MSTRG.3	Erlin2	ENSMUST00000209795;ENSMUST00000209976;ENSMUST0000003
MSTRG.3	Proscos	ENSMUST00000178990;ENSMUST00000163731
MSTRG.3	Prosc	ENSMUST00000033875;ENSMUST00000209525;ENSMUST0000020
MSTRG.3	Eif4ebp	ENSMUST00000033880;ENSMUST00000210959
MSTRG.3	5430430	ENSMUST00000209427
MSTRG.3	Adgra2	ENSMUST00000178514;ENSMUST00000033876;ENSMUST0000017
MSTRG.3	Brf2	ENSMUST00000033877;ENSMUST00000211236;ENSMUST00000211
MSTRG.3	Gm26479	ENSMUST00000122620
MSTRG.3	Rab11fi	ENSMUST00000054212;ENSMUST00000033878;ENSMUST0000020
MSTRG.3	4933416I	ENSMUST00000181424
MSTRG.3	Gm45267	ENSMUST00000211028
MSTRG.3	Gm45266	ENSMUST00000210111
MSTRG.30164		MSTRG.30164.1
MSTRG.30165		MSTRG.30165.1
MSTRG.30166		MSTRG.30166.1
MSTRG.3	Gm45861	ENSMUST00000209669;ENSMUST00000136749
MSTRG.3	Gm45861	MSTRG.30168.1
MSTRG.3	Gm22803	ENSMUST00000158049
MSTRG.3	Nudc-ps	ENSMUST00000209275
MSTRG.3	Unc5d	ENSMUST00000168630;ENSMUST00000211448
MSTRG.3	Unc5d	MSTRG.30172.1
MSTRG.3	Gm9911	ENSMUST00000211467
MSTRG.3	Gm45652	ENSMUST00000210926
MSTRG.3	Svet1	ENSMUST00000209370
MSTRG.30176		MSTRG.30176.1
MSTRG.30177		MSTRG.30177.1
MSTRG.3	Rnf122	ENSMUST00000046941;ENSMUST00000162640;ENSMUST00000217
MSTRG.3	Snord13	ENSMUST00000157627
MSTRG.3	Slc25a1	ENSMUST00000044977;ENSMUST00000138597;ENSMUST00000144
MSTRG.3	Tti2	ENSMUST00000209851;ENSMUST00000098842;ENSMUST0000021
MSTRG.3	Mak16	MSTRG.30181.1;ENSMUST00000033983;ENSMUST00000210278;E
MSTRG.3	Fut10	ENSMUST00000066173;ENSMUST00000161788;ENSMUST0000011
MSTRG.3	Fut10	MSTRG.30183.1

MSTRG.3	Gm8051	ENSMUST00000120464
MSTRG.3	Fut10	MSTRG.30185.1
MSTRG.3	Fut10	MSTRG.30186.1
MSTRG.3	Purg	MSTRG.30187.1;ENSMUST00000070340;ENSMUST00000078058
MSTRG.3	Purg	MSTRG.30188.1
MSTRG.3	Wrn	MSTRG.30189.1;ENSMUST00000033991;ENSMUST00000033990;E
MSTRG.3	Gm25862	ENSMUST00000158250
MSTRG.3	Wrn	MSTRG.30190.1
MSTRG.3	Wrn	MSTRG.30191.1
MSTRG.3	Wrn	MSTRG.30192.1
MSTRG.3	Wrn	MSTRG.30193.1
MSTRG.30194		MSTRG.30194.1
MSTRG.3	Ppp2cb	ENSMUST00000009774;MSTRG.30195.2;MSTRG.30195.3
MSTRG.3	Ubxn8	MSTRG.30196.1;MSTRG.30196.2;ENSMUST00000095349;ENSMUS
MSTRG.30197		MSTRG.30197.1
MSTRG.3	Gsr	ENSMUST00000154745;ENSMUST00000033992;ENSMUST00000149
MSTRG.3	Gm24727	ENSMUST00000158231
MSTRG.3	Gm38115	ENSMUST00000194317
MSTRG.3	Gm16128	ENSMUST00000117855
MSTRG.3	Gtf2e2	ENSMUST00000170705;MSTRG.30200.2;ENSMUST00000167264;E
MSTRG.3	Gtf2e2	MSTRG.30201.1
MSTRG.3	Smim18	ENSMUST00000179364
MSTRG.3	Gtf2e2	MSTRG.30203.1
MSTRG.3	Gtf2e2	MSTRG.30204.1
MSTRG.3	Rbpms	ENSMUST00000053251;ENSMUST00000191473;ENSMUST00000033
MSTRG.3	Gm26632	ENSMUST00000181120
MSTRG.3	Rbpms	MSTRG.30207.1
MSTRG.3	Rbpms	MSTRG.30208.1
MSTRG.3	Rbpms	MSTRG.30209.1
MSTRG.3	Dna2	ENSMUST00000131422;ENSMUST00000092462;ENSMUST00000131
MSTRG.3	Rbpms	MSTRG.30210.1
MSTRG.3	Gm26978	ENSMUST00000182843
MSTRG.3	Rbpms	MSTRG.30212.1
MSTRG.3	Rbpms	MSTRG.30213.1
MSTRG.3	Rbpms	MSTRG.30214.1
MSTRG.3	Gm10131	ENSMUST00000080152
MSTRG.3	Dctn6	ENSMUST00000155296;MSTRG.30216.1;ENSMUST00000033913;E
MSTRG.3	Gm26768	ENSMUST00000181097
MSTRG.3	Dctn6	MSTRG.30218.1
MSTRG.3	Leprotl	ENSMUST00000033910
MSTRG.3	Rufy2	ENSMUST00000148259;MSTRG.3022.1;ENSMUST00000131718;EN
MSTRG.3	Saraf	MSTRG.30220.1
MSTRG.3	Saraf	ENSMUST00000209470;ENSMUST00000033933;ENSMUST00000211
MSTRG.3	Saraf	MSTRG.30222.1
MSTRG.3	Mir6395	ENSMUST00000183885
MSTRG.30224		MSTRG.30224.1
MSTRG.3	Gm33831	MSTRG.30225.1
MSTRG.3	Gm33831	ENSMUST00000211696
MSTRG.30227		MSTRG.30227.1
MSTRG.30228		MSTRG.30228.1
MSTRG.30229		MSTRG.30229.1
MSTRG.3	Rufy2	MSTRG.3023.1
MSTRG.3	Gm45349	MSTRG.30230.1
MSTRG.3	Gm45349	ENSMUST00000210285
MSTRG.3	Gm45349	MSTRG.30232.1
MSTRG.3	Gm45349	MSTRG.30233.1
MSTRG.3	Dusp4	ENSMUST00000033930

MSTRG.3	Tnks	ENSMUST00000033929;ENSMUST00000209632;ENSMUST0000020[illegible]
MSTRG.3	Tnks	MSTRG.30236.1
MSTRG.3	Tnks	MSTRG.30237.1
MSTRG.3	Tnks	MSTRG.30238.1
MSTRG.3	Tnks	MSTRG.30239.1
MSTRG.3	Gm16135	ENSMUST00000129470
MSTRG.3	Tnks	MSTRG.30240.1
MSTRG.3	Gm9939	ENSMUST00000067161;ENSMUST00000210603;ENSMUST0000021[illegible]
MSTRG.3	Gm34368	ENSMUST00000209512
MSTRG.3	Gm45469	ENSMUST00000210468
MSTRG.3	Ppp1r3b	ENSMUST00000210337;ENSMUST00000070481;ENSMUST00000211
MSTRG.3	Ppp1r3b	MSTRG.30245.1
MSTRG.30246		MSTRG.30246.1
MSTRG.3	Eri1	ENSMUST00000033927;MSTRG.30247.2;MSTRG.30247.3;MSTRG.
MSTRG.3	Gm16793	ENSMUST00000181322
MSTRG.3	Mfhas1	ENSMUST00000037666;ENSMUST00000209953;MSTRG.30249.2;[illegible]
MSTRG.3	Hnrnph3	MSTRG.3025.1;ENSMUST00000020263;ENSMUST00000118898;E[illegible]
MSTRG.3	Mfhas1	MSTRG.30250.1
MSTRG.3	Rps12-p	ENSMUST00000182321
MSTRG.3	Prag1	ENSMUST00000110492;ENSMUST00000145386;ENSMUST0000015[illegible]
MSTRG.3	Prag1	MSTRG.30253.1
MSTRG.3	Lonrf1	ENSMUST00000065297
MSTRG.3	Lonrf1	MSTRG.30255.1
MSTRG.3	Lonrf1	MSTRG.30256.1
MSTRG.3	Lonrf1	MSTRG.30257.1
MSTRG.3	Dlc1	ENSMUST00000033923;ENSMUST00000098826;MSTRG.30259.1;[illegible]
MSTRG.3	Hnrnph3	MSTRG.3026.1
MSTRG.3	Dlc1	MSTRG.30260.1
MSTRG.3	Dlc1	MSTRG.30261.1
MSTRG.3	Dlc1	MSTRG.30262.1
MSTRG.30263		MSTRG.30263.1
MSTRG.3	Tusc3	ENSMUST00000209440;ENSMUST00000167992;ENSMUST00000211
MSTRG.3	Msr1	ENSMUST00000026021;ENSMUST00000170091;ENSMUST0000021[illegible]
MSTRG.3	Msr1	MSTRG.30266.1
MSTRG.3	Zdhhc2	ENSMUST00000049389;ENSMUST00000131672
MSTRG.3	Cnot7	ENSMUST00000034012;ENSMUST00000132032;ENSMUST00000144
MSTRG.3	Vps37a	ENSMUST00000098817;MSTRG.30269.1
MSTRG.3	Pbld2	ENSMUST00000124784;ENSMUST00000020262;ENSMUST0000013[illegible]
MSTRG.3	Vps37a	MSTRG.30270.1
MSTRG.3	Vps37a	MSTRG.30271.1
MSTRG.3	Mtmr7	ENSMUST00000048890;ENSMUST00000048898;ENSMUST0000017[illegible]
MSTRG.3	Pdgfrl	ENSMUST00000143216;ENSMUST00000034004
MSTRG.3	Mtus1	ENSMUST00000059115;ENSMUST00000093534;ENSMUST0000011[illegible]
MSTRG.3	Mtus1	MSTRG.30275.1
MSTRG.3	B430010	ENSMUST00000123703;ENSMUST00000152188
MSTRG.3	Gm16192	ENSMUST00000148357
MSTRG.3	Gm16193	ENSMUST00000125903
MSTRG.3	Smarce1	ENSMUST00000180067
MSTRG.3	Gm16143	ENSMUST00000155982
MSTRG.3	Frg1	ENSMUST00000033999;MSTRG.30280.1;ENSMUST00000210873
MSTRG.3	Frg1	MSTRG.30281.1
MSTRG.3	Frg1	MSTRG.30282.1
MSTRG.30283		MSTRG.30283.1
MSTRG.30284		MSTRG.30284.1
MSTRG.30285		MSTRG.30285.1
MSTRG.3	Adam34	MSTRG.30288.1
MSTRG.3	170012[illegible]	ENSMUST00000054837

MSTRG.30294		MSTRG.30294.1
MSTRG.3	Pcm1	ENSMUST00000211090;ENSMUST00000210862;ENSMUST0000004[illegible]
MSTRG.3	Asah1	ENSMUST00000034000;ENSMUST00000126561;ENSMUST00000131
MSTRG.3	Fat1	ENSMUST00000191428;ENSMUST00000189017;ENSMUST0000009[illegible]
MSTRG.3	Gm2366	ENSMUST00000180753
MSTRG.3	Klkb1;G[illegible]	ENSMUST00000026907;ENSMUST00000116473;ENSMUST0000009[illegible]
MSTRG.3	F830112[illegible]	ENSMUST00000194249
MSTRG.3	Herc4;A[illegible]	ENSMUST00000219856;ENSMUST00000219230;ENSMUST0000002[illegible]
MSTRG.3	Pdlim3	ENSMUST00000034053;ENSMUST00000211190;ENSMUST0000021[illegible]
MSTRG.3	Pdlim3	MSTRG.30301.1
MSTRG.30302		MSTRG.30302.1
MSTRG.3	Slc25a4	ENSMUST00000034049;ENSMUST00000155986
MSTRG.3	Actg-ps	ENSMUST00000099636;ENSMUST00000175156
MSTRG.3	Acsl1	ENSMUST00000034046;ENSMUST00000128746;MSTRG.30305.2;[illegible]
MSTRG.3	Gm45244	ENSMUST00000211328
MSTRG.3	Acsl1	MSTRG.30307.1
MSTRG.3	1700029[illegible]	ENSMUST00000095323;ENSMUST00000098786
MSTRG.3	Ufsp2	ENSMUST00000034051;ENSMUST00000210081;ENSMUST0000013[illegible]
MSTRG.3	AC15351	ENSMUST00000217955
MSTRG.3	Ufsp2	MSTRG.30310.1
MSTRG.3	Ankrd37	ENSMUST00000053558;ENSMUST00000153674;ENSMUST00000131
MSTRG.3	Lrp2bp	ENSMUST00000110380;ENSMUST00000066451;ENSMUST0000011[illegible]
MSTRG.3	Snx25	ENSMUST00000170416;ENSMUST00000110378;ENSMUST00000041
MSTRG.3	Snx25	MSTRG.30314.1
MSTRG.3	Snx25	MSTRG.30315.1
MSTRG.3	Gm23030	ENSMUST00000158004
MSTRG.3	Snx25	MSTRG.30317.1
MSTRG.3	Snx25	MSTRG.30318.1
MSTRG.3	Cfap97	ENSMUST00000154040;ENSMUST00000034048;MSTRG.30319.3;[illegible]
MSTRG.3	Gm26789	MSTRG.3032.1;ENSMUST00000181342
MSTRG.3	Gm15634	ENSMUST00000147206
MSTRG.3	Gm45693	ENSMUST00000211391
MSTRG.3	Cenpu	ENSMUST00000034045;ENSMUST00000211217;ENSMUST0000013[illegible]
MSTRG.3	Primpol	ENSMUST00000211400;ENSMUST00000040468;ENSMUST0000013[illegible]
MSTRG.3	Primpol	MSTRG.30324.1
MSTRG.3	Primpol	MSTRG.30325.1
MSTRG.3	Gm45607	ENSMUST00000211143
MSTRG.3	Casp3	ENSMUST00000211115;ENSMUST00000093517;ENSMUST0000020[illegible]
MSTRG.3	Gm16675	ENSMUST00000208508;ENSMUST00000181167
MSTRG.3	Gm16675	MSTRG.30329.1
MSTRG.3	Sirt1	ENSMUST00000120239;ENSMUST00000146028;ENSMUST0000002[illegible]
MSTRG.3	Gm16675	MSTRG.30330.1
MSTRG.3	Irf2	ENSMUST00000034041;ENSMUST00000208507;ENSMUST0000021[illegible]
MSTRG.3	Irf2	MSTRG.30332.1
MSTRG.3	Irf2	MSTRG.30333.1
MSTRG.3	Gm45479	ENSMUST00000211613
MSTRG.3	Gm31172	MSTRG.30336.1
MSTRG.3	Irf2	MSTRG.30338.1
MSTRG.3	Gm45481	ENSMUST00000209717
MSTRG.3	Gm16145	ENSMUST00000128807
MSTRG.3	Stox2	ENSMUST00000079195;ENSMUST00000211882;ENSMUST0000011[illegible]
MSTRG.3	Stox2	MSTRG.30341.1
MSTRG.3	Gm45537	ENSMUST00000211539
MSTRG.3	Stox2	MSTRG.30343.1
MSTRG.3	Stox2	MSTRG.30344.1
MSTRG.3	Stox2	MSTRG.30345.1
MSTRG.3	Stox2	MSTRG.30346.1

MSTRG.3	Stox2	MSTRG.30347.1
MSTRG.3	Stox2	MSTRG.30348.1
MSTRG.3	Stox2	MSTRG.30349.1
MSTRG.3	Dnajc12	ENSMUST00000129625;ENSMUST00000043317;ENSMUST0000013€
MSTRG.3	Stox2	MSTRG.30350.1
MSTRG.3	Stox2	MSTRG.30351.1
MSTRG.3	Stox2	MSTRG.30352.1
MSTRG.3	Gm8623	ENSMUST00000071573
MSTRG.30355		MSTRG.30355.1
MSTRG.3	Ing2	MSTRG.30356.1
MSTRG.3	Ing2	ENSMUST00000146625;ENSMUST00000080353;ENSMUST0000012£
MSTRG.3	AA38647	ENSMUST00000098781;ENSMUST00000211481
MSTRG.3	AA38647	MSTRG.30359.1
MSTRG.3036		MSTRG.3036.1
MSTRG.3	Trappc1	ENSMUST00000039061;ENSMUST00000123958;MSTRG.30360.3;E
MSTRG.3	Rwdd4a	ENSMUST00000175692;ENSMUST00000033973;ENSMUST0000017
MSTRG.3	Rwdd4a	MSTRG.30362.1
MSTRG.3	Cdkn2ai	ENSMUST00000038738;ENSMUST00000212175;ENSMUST0000021z
MSTRG.3	E030037	ENSMUST00000180928
MSTRG.3	Wwc2	ENSMUST00000057561;ENSMUST00000212282
MSTRG.3	Cldn22	ENSMUST00000038693
MSTRG.3	n-R5s97	ENSMUST00000082585
MSTRG.3	Wwc2	MSTRG.30369.1
MSTRG.3037		MSTRG.3037.1
MSTRG.3	Dctd	ENSMUST00000174278;ENSMUST00000170263;ENSMUST0000017
MSTRG.3	Dctd	MSTRG.30371.1
MSTRG.30372		MSTRG.30372.1
MSTRG.30373		MSTRG.30373.1
MSTRG.30374		MSTRG.30374.1
MSTRG.30375		MSTRG.30375.1
MSTRG.30376		MSTRG.30376.1
MSTRG.3	Gm9892	ENSMUST00000182415
MSTRG.3	Gm25813	ENSMUST00000082748
MSTRG.3	Tenm3	ENSMUST00000033965;ENSMUST00000190840;MSTRG.30379.3;E
MSTRG.3	AC15351	ENSMUST00000218961
MSTRG.3	Gm32842	ENSMUST00000212653
MSTRG.3	Gm45772	ENSMUST00000212961
MSTRG.3	Gm19744	ENSMUST00000190967;ENSMUST00000186135;ENSMUST0000018£
MSTRG.30383		MSTRG.30383.1
MSTRG.3	Aga	ENSMUST00000211424;ENSMUST00000033920;ENSMUST0000020£
MSTRG.3	Aga	MSTRG.30385.1
MSTRG.3	Neil3	MSTRG.30386.1;MSTRG.30386.2;ENSMUST00000047768;ENSMUS
MSTRG.3	Gm45553	ENSMUST00000209929
MSTRG.3	Spcs3	MSTRG.30388.1
MSTRG.3	Spcs3	ENSMUST00000067476
MSTRG.3039		MSTRG.3039.1
MSTRG.3	Asb5	ENSMUST00000209937;ENSMUST00000033918
MSTRG.3	5330439	ENSMUST00000162770
MSTRG.30393		MSTRG.30393.1
MSTRG.30394		MSTRG.30394.1
MSTRG.30395		MSTRG.30395.1
MSTRG.30396		MSTRG.30396.1
MSTRG.3	Hpgd	ENSMUST00000034026
MSTRG.3	Gm45540	ENSMUST00000209271
MSTRG.3	Wdr17	ENSMUST00000144711;ENSMUST00000176866;ENSMUST0000017€
MSTRG.3	Inpp4a	ENSMUST00000146676;ENSMUST00000027287;ENSMUST0000019£
MSTRG.3	AC15338	ENSMUST00000217953

MSTRG.3	Gm15758	ENSMUST00000161681;ENSMUST00000162822
MSTRG.3	Wdr17	MSTRG.30401.1
MSTRG.3	Gpm6a	MSTRG.30402.1;ENSMUST00000033915
MSTRG.3	n-R5s98	ENSMUST00000122549
MSTRG.3	Gm45263	ENSMUST00000210377
MSTRG.3	Gm45265	ENSMUST00000210852
MSTRG.3	Gm45264	ENSMUST00000210090
MSTRG.3	Gm45262	ENSMUST00000209917
MSTRG.3	A230085	ENSMUST00000209781
MSTRG.3	Fbxo8	MSTRG.30409.1
MSTRG.3	Egr2	ENSMUST00000145754;ENSMUST00000145936;ENSMUST00000048
MSTRG.30410		MSTRG.30410.1
MSTRG.3	Cep44	ENSMUST00000123493;ENSMUST00000040330;ENSMUST00000211
MSTRG.3	Cep44	MSTRG.30412.1
MSTRG.3	Fbxo8	ENSMUST00000156840;ENSMUST00000110322;MSTRG.30413.1;E
MSTRG.3	Gm2767;	ENSMUST00000209582;ENSMUST00000102437
MSTRG.3	Fbxo8	MSTRG.30415.1
MSTRG.3	Sap30	ENSMUST00000034022
MSTRG.3	2500002	ENSMUST00000180690;MSTRG.30417.1;ENSMUST00000210752;M
MSTRG.3	Galnt7	ENSMUST00000034021;ENSMUST00000110316;MSTRG.30418.2;E
MSTRG.3	Gm25379	ENSMUST00000104338
MSTRG.3	AC15338	ENSMUST00000217788;ENSMUST00000219739;ENSMUST00000218
MSTRG.3	Gm10284	ENSMUST00000145057
MSTRG.3	Galnt7	MSTRG.30421.1;MSTRG.30421.2
MSTRG.3	Galnt7	MSTRG.30422.1
MSTRG.3	Gm45534	ENSMUST00000211449
MSTRG.3	Gm45360	ENSMUST00000210698
MSTRG.3	Galnt7	MSTRG.30425.1
MSTRG.3	Gm19269	ENSMUST00000211401
MSTRG.3	Gm45358	ENSMUST00000209492
MSTRG.3	Galnt7	MSTRG.30428.1
MSTRG.3	4930412	ENSMUST00000211700
MSTRG.3	Reep3	ENSMUST00000020023;ENSMUST00000217841;ENSMUST00000218
MSTRG.3	AW04620	ENSMUST00000211284;ENSMUST00000210975;ENSMUST00000211
MSTRG.3	AW04620	MSTRG.30431.1
MSTRG.3	Gm45359	ENSMUST00000210635
MSTRG.3	Gm45644	ENSMUST00000210255
MSTRG.3	Hpf1	ENSMUST00000146863;ENSMUST00000149267;ENSMUST00000136
MSTRG.3	Clcn3	ENSMUST00000056508;ENSMUST00000093490;ENSMUST00000145
MSTRG.3	Clcn3	MSTRG.30437.1
MSTRG.3	Clcn3	MSTRG.30438.1
MSTRG.3	B230317	ENSMUST00000052189
MSTRG.3	Jmjd1c	ENSMUST00000174062;MSTRG.3044.1;ENSMUST00000173295;MS
MSTRG.3	Nek1;Gm	ENSMUST00000120689;ENSMUST00000034065;ENSMUST00000125
MSTRG.3	Nek1	MSTRG.30441.1
MSTRG.3	Gm45484	ENSMUST00000210310
MSTRG.3	Nek1	MSTRG.30443.1
MSTRG.3	Gm45483	ENSMUST00000209845
MSTRG.3	Gm16179	ENSMUST00000120685
MSTRG.3	Gm16178	ENSMUST00000129408
MSTRG.3	Sh3rf1	ENSMUST00000034060;ENSMUST00000209611;ENSMUST00000210
MSTRG.3	Sh3rf1	MSTRG.30448.1;MSTRG.30448.2
MSTRG.3	Sh3rf1	MSTRG.30449.1
MSTRG.3	Jmjd1c	MSTRG.3045.1
MSTRG.3	Sh3rf1	MSTRG.30450.1
MSTRG.3	Sh3rf1	MSTRG.30451.1
MSTRG.3	1700001	ENSMUST00000188654

MSTRG.3	Sh3rf1	MSTRG.30453.1
MSTRG.3	Gm10015	ENSMUST00000072570
MSTRG.3	Gm7432	ENSMUST00000210343
MSTRG.30456		MSTRG.30456.1
MSTRG.3	Palld	ENSMUST00000121200;ENSMUST00000121493;ENSMUST00000121
MSTRG.3	Gm18066	ENSMUST00000210904
MSTRG.3	4930512	ENSMUST00000133752;ENSMUST00000211790
MSTRG.3	Gm26359	ENSMUST00000158873
MSTRG.3	Gm45418	ENSMUST00000210693;ENSMUST00000210376
MSTRG.3	Ddx60	ENSMUST00000154398;ENSMUST00000156980;MSTRG.30461.2;M
MSTRG.3	Ddx60	MSTRG.30462.1
MSTRG.3	Ddx60	MSTRG.30463.1
MSTRG.3	Ddx60	MSTRG.30464.1
MSTRG.30465		MSTRG.30465.1
MSTRG.3	Cpe	ENSMUST00000048967;ENSMUST00000210680;ENSMUST00000211
MSTRG.3	Klhl2	ENSMUST00000034017;ENSMUST00000210166;ENSMUST0000020
MSTRG.3	Klhl2	MSTRG.30468.1
MSTRG.3	Gm10997	ENSMUST00000210294
MSTRG.3	Nrbf2	ENSMUST00000077839;ENSMUST00000159002
MSTRG.3	Klhl2	MSTRG.30470.1
MSTRG.3	Gm45345	ENSMUST00000211427
MSTRG.3	Gm45242	ENSMUST00000210666
MSTRG.3	Tmem192	ENSMUST00000026595;ENSMUST00000209852;ENSMUST0000020
MSTRG.3	Naf1	ENSMUST00000118009;ENSMUST00000212916
MSTRG.3	Nat2	ENSMUST00000093470;ENSMUST00000163856
MSTRG.3	Gm9755	ENSMUST00000032981
MSTRG.3	March1	ENSMUST00000110256;ENSMUST00000110255;ENSMUST0000017
MSTRG.3	March1	MSTRG.30479.1
MSTRG.3	Ado	ENSMUST00000075686
MSTRG.3	Gm24205	ENSMUST00000082953
MSTRG.3	Gm15354	ENSMUST00000132019
MSTRG.3	March1	MSTRG.30484.1
MSTRG.3	Gm15356	ENSMUST00000140669
MSTRG.3	Tma16	ENSMUST00000026681;ENSMUST00000143972;ENSMUST0000014
MSTRG.3	Sh2d4a	ENSMUST00000066594;ENSMUST00000212166
MSTRG.3	Gm45739	ENSMUST00000213094
MSTRG.3	Sh2d4a	MSTRG.30489.1
MSTRG.3	AC15337	ENSMUST00000218306
MSTRG.3	Gm19170	ENSMUST00000212440
MSTRG.3	Psd3	ENSMUST00000038959;ENSMUST00000098696;ENSMUST0000012
MSTRG.3	Psd3	MSTRG.30493.1
MSTRG.3	Psd3	MSTRG.30494.1
MSTRG.3	Psd3	MSTRG.30495.1
MSTRG.3	Psd3	MSTRG.30496.1
MSTRG.3	Psd3	MSTRG.30497.1
MSTRG.3	Gm15991	ENSMUST00000212123;ENSMUST00000127631
MSTRG.3	Gm22018	ENSMUST00000104357
MSTRG.3	Inpp4a	MSTRG.305.1
MSTRG.3	Gm10797	ENSMUST00000099661
MSTRG.3	Csgalna	ENSMUST00000078350;ENSMUST00000130214;ENSMUST0000013
MSTRG.3	1700125	ENSMUST00000124929;ENSMUST00000139265
MSTRG.3	Csgalna	MSTRG.30502.1
MSTRG.3	Gm15656	ENSMUST00000132076
MSTRG.3	Csgalna	MSTRG.30504.1
MSTRG.3	Csgalna	MSTRG.30505.1
MSTRG.3	Csgalna	MSTRG.30506.1
MSTRG.3	Csgalna	MSTRG.30507.1

MSTRG.3	Gm15654	ENSMUST00000152660
MSTRG.3	Csgalna	MSTRG.30509.1
MSTRG.3	AC15337	ENSMUST00000218174
MSTRG.3	Ints10	ENSMUST00000034328;ENSMUST00000110241;ENSMUST00000146
MSTRG.3	Gm15549	ENSMUST00000121595
MSTRG.3	Lpl	ENSMUST00000015712;ENSMUST00000213071;ENSMUST00000168
MSTRG.3	Lpl	MSTRG.30513.1
MSTRG.3	Slc18a1	ENSMUST00000037478;ENSMUST00000142548;ENSMUST0000014?
MSTRG.3	Gm15716	ENSMUST00000162457
MSTRG.3	Atp6v1b	ENSMUST00000006435;ENSMUST00000153079;ENSMUST0000015?
MSTRG.3	Atp6v1b	MSTRG.30517.1
MSTRG.3	Lzts1	MSTRG.30518.1
MSTRG.3	Lzts1	ENSMUST00000185176;ENSMUST00000037049;ENSMUST0000018l
MSTRG.3	AC15337	MSTRG.3052.1
MSTRG.3	Lzts1	MSTRG.30520.1
MSTRG.3	Lzts1	MSTRG.30521.1
MSTRG.3	Lzts1	MSTRG.30522.1
MSTRG.3	Zfp930	ENSMUST00000212681;ENSMUST00000212312;ENSMUST0000021?
MSTRG.3	Zfp930	MSTRG.30524.1
MSTRG.3	Zfp930	MSTRG.30525.1
MSTRG.3	Gm33178	ENSMUST00000212090
MSTRG.3	Zfp930	MSTRG.30527.1
MSTRG.3	D130040	ENSMUST00000212549;ENSMUST00000212443;ENSMUST0000021l
MSTRG.3	D130040	MSTRG.30529.1
MSTRG.3	Rtkn2	ENSMUST00000118160;ENSMUST00000105437;ENSMUST0000006?
MSTRG.3	D130040	MSTRG.30530.1
MSTRG.3	D130040	MSTRG.30531.1
MSTRG.3	Gm10033	ENSMUST00000213012;MSTRG.30532.2;ENSMUST00000211954;E
MSTRG.3	Gm10033	MSTRG.30533.1
MSTRG.3	Rps7-ps	MSTRG.30534.1
MSTRG.30535		MSTRG.30535.1
MSTRG.3	AC16675	ENSMUST00000216296;MSTRG.30536.2;MSTRG.30536.3
MSTRG.3	AC16675	MSTRG.30537.1
MSTRG.3	AC16675	MSTRG.30538.1
MSTRG.3	Zfp868;	ENSMUST00000121886;ENSMUST00000074982;MSTRG.30539.2;M
MSTRG.3	Rtkn2	MSTRG.3054.1
MSTRG.3	Zfp868	MSTRG.30540.1
MSTRG.3	Zfp964	ENSMUST00000204062;ENSMUST00000204285;ENSMUST0000016?
MSTRG.30542		MSTRG.30542.1
MSTRG.3	Gm7730	ENSMUST00000212486
MSTRG.3	Mau2	ENSMUST00000168013;ENSMUST00000050561;ENSMUST0000021?
MSTRG.3	Sugp1	ENSMUST00000011450;ENSMUST00000211808;ENSMUST0000021?
MSTRG.3	Tm6sf2	MSTRG.30546.1
MSTRG.3	Sugp1	MSTRG.30547.1
MSTRG.3	Tm6sf2	ENSMUST00000110160;ENSMUST00000049197;ENSMUST0000014?
MSTRG.3	Zfp963;	ENSMUST00000130458;ENSMUST00000149782;ENSMUST0000015?
MSTRG.3	Rtkn2	MSTRG.3055.1
MSTRG.3	Gm20422	MSTRG.30550.1
MSTRG.3	Gm20422	MSTRG.30551.1
MSTRG.3	Atp13a1	ENSMUST00000034326;MSTRG.30552.2;MSTRG.30552.3;ENSMUS
MSTRG.3	Lpar2	ENSMUST00000164890;ENSMUST00000034325
MSTRG.3	Pbx4	ENSMUST00000081503;ENSMUST00000151975;ENSMUST0000015?
MSTRG.3	Gatad2a	MSTRG.30555.1;MSTRG.30555.2
MSTRG.3	Cilp2	ENSMUST00000057831
MSTRG.3	Yjefn3;	MSTRG.30557.1;ENSMUST00000152938;ENSMUST00000180068;E
MSTRG.3	Yjefn3	MSTRG.30558.1
MSTRG.3	Tssk6	ENSMUST00000050373

MSTRG.3	Rtkn2	MSTRG.3056.1
MSTRG.3	Gatad2a	ENSMUST00000177851;ENSMUST00000065169;ENSMUST0000011[illegible]
MSTRG.3	Gatad2a	MSTRG.30561.1
MSTRG.3	Gatad2a	MSTRG.30562.1
MSTRG.3	Gatad2a	MSTRG.30563.1
MSTRG.3	Gatad2a	MSTRG.30564.1
MSTRG.3	Gatad2a	MSTRG.30565.1;MSTRG.30565.2
MSTRG.3	Rfxank	ENSMUST00000211970;ENSMUST00000075724;ENSMUST00000212
MSTRG.3	Nr2c2ap	ENSMUST00000211898;ENSMUST00000095273;ENSMUST00000212
MSTRG.3	Borcs8;[illegible]	ENSMUST00000002418;ENSMUST00000123976;MSTRG.30568.2;[illegible]
MSTRG.3	Tmem161[illegible]	ENSMUST00000125755;ENSMUST00000182715;ENSMUST00000002
MSTRG.3	Rtkn2	MSTRG.3057.1
MSTRG.3	Tmem161[illegible]	MSTRG.30570.1
MSTRG.3	Slc25a4[illegible]	ENSMUST00000110127;ENSMUST00000139333;MSTRG.30571.4;[illegible]
MSTRG.3	Slc25a4[illegible]	MSTRG.30572.1
MSTRG.3	Armc6	ENSMUST00000019679;ENSMUST00000139765;ENSMUST0000013[illegible]
MSTRG.3	Sugp2	ENSMUST00000164403;ENSMUST00000131489;ENSMUST0000009[illegible]
MSTRG.3	Homer3	ENSMUST00000087467;ENSMUST00000140212;ENSMUST0000011[illegible]
MSTRG.3	Homer3	MSTRG.30576.1
MSTRG.3	Ddx49	ENSMUST00000008004;MSTRG.30577.2;ENSMUST00000144890;[illegible]
MSTRG.3	Cope	ENSMUST00000066469;ENSMUST00000140363;MSTRG.30578.2;[illegible]
MSTRG.3	Cers1;G[illegible]	ENSMUST00000165819;ENSMUST00000136257;ENSMUST00000207
MSTRG.3	Gm16212	ENSMUST00000136190
MSTRG.3	Upf1	ENSMUST00000215817;ENSMUST00000075666;MSTRG.30580.3;[illegible]
MSTRG.3	Comp	ENSMUST00000003659;ENSMUST00000212439
MSTRG.3	Crtc1	ENSMUST00000076615;ENSMUST00000142769;ENSMUST0000012[illegible]
MSTRG.3	Crtc1	MSTRG.30583.1
MSTRG.3	Crtc1	MSTRG.30584.1
MSTRG.3	Klhl26	ENSMUST00000209415;ENSMUST00000210250;ENSMUST0000006[illegible]
MSTRG.3	Klhl26	MSTRG.30586.1
MSTRG.3	Crlf1	ENSMUST00000008032;ENSMUST00000127983
MSTRG.3	2810428	ENSMUST00000135773;ENSMUST00000143598;ENSMUST0000013[illegible]
MSTRG.3	Uba52;K[illegible]	ENSMUST00000140679;ENSMUST00000129909;ENSMUST0000012[illegible]
MSTRG.3	Arid5b	ENSMUST00000219238;ENSMUST00000020106;ENSMUST0000021[illegible]
MSTRG.3	Kxd1	MSTRG.30590.1
MSTRG.3	Fkbp8	ENSMUST00000119353;ENSMUST00000075491;ENSMUST00000144
MSTRG.3	Pgpep1	ENSMUST00000070173;ENSMUST00000211715;ENSMUST00000211
MSTRG.3	Lsm4	ENSMUST00000034311;MSTRG.30593.2;ENSMUST00000146972;[illegible]
MSTRG.3	Ell	ENSMUST00000210155;MSTRG.30594.1;MSTRG.30594.3;ENSMUS[illegible]
MSTRG.3	Ell	MSTRG.30595.1
MSTRG.3	Ell	MSTRG.30596.1
MSTRG.3	Isyna1	ENSMUST00000211117;ENSMUST00000210358;ENSMUST0000020[illegible]
MSTRG.3	Ssbp4	ENSMUST00000049908;ENSMUST00000210369;MSTRG.30598.3;[illegible]
MSTRG.3	Jund	ENSMUST00000095267
MSTRG.3	4930439[illegible]	ENSMUST00000137241
MSTRG.3	Arid5b	MSTRG.3060.1
MSTRG.3	Gm11175	ENSMUST00000211380;ENSMUST00000116172
MSTRG.3	Rab3a	ENSMUST00000110093;ENSMUST00000143118;ENSMUST00000034
MSTRG.3	Mpv17l2	ENSMUST00000038626;ENSMUST00000213010;ENSMUST00000212
MSTRG.3	Ifi30;P	ENSMUST00000034299;MSTRG.30603.1;ENSMUST00000034296;[illegible]
MSTRG.3	6330537[illegible]	ENSMUST00000212384
MSTRG.3	2010320[illegible]	ENSMUST00000191396
MSTRG.3	Ccdc124	ENSMUST00000007865;ENSMUST00000212680
MSTRG.3	Slc5a5	ENSMUST00000000809
MSTRG.3	Rpl18a	MSTRG.30608.1
MSTRG.3	Mast3	ENSMUST00000212140;MSTRG.30609.2;MSTRG.30609.3;ENSMUS[illegible]
MSTRG.3	Arid5b	MSTRG.3061.1

MSTRG.3	Mast3	MSTRG.30610.1
MSTRG.3	Mir7240	ENSMUST00000184074
MSTRG.3	Il12rb1	ENSMUST00000211936;ENSMUST00000000808;ENSMUST00000212
MSTRG.3	Il12rb1	MSTRG.30613.1
MSTRG.3	Rpl18a;I	ENSMUST00000054220;ENSMUST00000212796;ENSMUST00000212
MSTRG.3	Map1s	ENSMUST00000019405;MSTRG.30615.1;ENSMUST00000212697;E
MSTRG.3	Zfp709	ENSMUST00000188685;ENSMUST00000188374;ENSMUST00000203
MSTRG.3	Zfp882;	ENSMUST00000110002;ENSMUST00000131544;ENSMUST00000125
MSTRG.3	Zfp617	MSTRG.30618.1
MSTRG.3	Ccnb2-p	ENSMUST00000170898;ENSMUST00000118290
MSTRG.3	Arid5b	MSTRG.3062.1
MSTRG.3	Zfp617	MSTRG.30620.1
MSTRG.3	Zfp961	ENSMUST00000131237;ENSMUST00000138304;ENSMUST00000136
MSTRG.3	Zfp961	MSTRG.30622.1
MSTRG.3	Zfp961	MSTRG.30623.1
MSTRG.3	Zfp961	MSTRG.30624.1
MSTRG.3	Zfp961	MSTRG.30625.1
MSTRG.3	Gm45694	ENSMUST00000212022
MSTRG.3	Cyp4f18	ENSMUST00000003574;ENSMUST00000125448
MSTRG.3	Hsh2d	ENSMUST00000072097;ENSMUST00000165324
MSTRG.3	Hsh2d	MSTRG.30629.1
MSTRG.3	Arid5b	MSTRG.3063.1
MSTRG.3	Fam32a	ENSMUST00000003123;ENSMUST00000212117;MSTRG.30630.2
MSTRG.3	Gm45734	ENSMUST00000211916
MSTRG.3	Olfr372	ENSMUST00000215324;ENSMUST00000093434
MSTRG.3	Gm45544	ENSMUST00000211130
MSTRG.3	Gm45735	ENSMUST00000212779
MSTRG.3	Olfr373	ENSMUST00000213940;ENSMUST00000074540
MSTRG.3	Gm45543	ENSMUST00000209327
MSTRG.3	Olfr374	ENSMUST00000210435;ENSMUST00000209675;ENSMUST00000157
MSTRG.3	Arid5b	MSTRG.3064.1
MSTRG.30640		MSTRG.30640.1
MSTRG.3	Tpm4	ENSMUST00000123177;MSTRG.30641.1;ENSMUST00000003575;M
MSTRG.3	Gm26586	MSTRG.30642.1;ENSMUST00000181918
MSTRG.3	Gm16091	ENSMUST00000156664
MSTRG.3	Rab8a	ENSMUST00000003121;ENSMUST00000135613;ENSMUST00000128
MSTRG.3	Ap1m1	ENSMUST00000003117;ENSMUST00000212841;ENSMUST00000212
MSTRG.3	Ap1m1	MSTRG.30646.1
MSTRG.3	Gm39197	ENSMUST00000212069
MSTRG.3	Gm10282	ENSMUST00000075602
MSTRG.3	Klf2	ENSMUST00000067912
MSTRG.3	Arid5b	MSTRG.3065.1
MSTRG.3	Klf2	MSTRG.30650.1
MSTRG.30651		MSTRG.30651.1
MSTRG.3	Eps15l1	MSTRG.30652.1;ENSMUST00000163643;MSTRG.30652.4;MSTRG.
MSTRG.3	Smim7	ENSMUST00000167290;ENSMUST00000058733;ENSMUST00000163
MSTRG.3	Tmem38a	ENSMUST00000212763;ENSMUST00000034244;ENSMUST00000211
MSTRG.3	Calr3	ENSMUST00000019876;ENSMUST00000109974
MSTRG.3	1700030	MSTRG.30656.1
MSTRG.3	Gm3835	ENSMUST00000120752
MSTRG.3	1700030	ENSMUST00000064853;ENSMUST00000121902;ENSMUST00000139
MSTRG.3	Cherp;M	ENSMUST00000079510;MSTRG.30659.3;MSTRG.30659.2;ENSMUS
MSTRG.3	Arid5b	MSTRG.3066.1
MSTRG.3	Slc35e1	ENSMUST00000152080;ENSMUST00000141352
MSTRG.3	Gm17435	ENSMUST00000181452;ENSMUST00000212699
MSTRG.3	Med26	ENSMUST00000058534
MSTRG.3	Gm18646	MSTRG.30663.1;ENSMUST00000212240

MSTRG.3	Med26	MSTRG.30664.1
MSTRG.3	Gm35857	MSTRG.30665.2;MSTRG.30665.1;ENSMUST00000212739
MSTRG.3	Gm28019	ENSMUST00000178138
MSTRG.30667		MSTRG.30667.2;MSTRG.30667.1;MSTRG.30667.3
MSTRG.30668		MSTRG.30668.1
MSTRG.3	Nwd1	ENSMUST00000161386;ENSMUST00000093427;ENSMUST00000161
MSTRG.3	Arid5b	MSTRG.3067.1
MSTRG.3	Gm26237	ENSMUST00000175535
MSTRG.3	Haus8	ENSMUST00000123495;ENSMUST00000110071;ENSMUST0000003[illegible]
MSTRG.3	Myo9b	ENSMUST00000212935;ENSMUST00000170242;ENSMUST00000071
MSTRG.3	Myo9b	MSTRG.30673.1
MSTRG.3	Unc13a	MSTRG.30674.1
MSTRG.3	Use1	ENSMUST00000110053;ENSMUST00000110054;ENSMUST0000013[illegible]
MSTRG.3	Ocel1	ENSMUST00000110051;ENSMUST00000002469;ENSMUST0000011[illegible]
MSTRG.3	Nr2f6	ENSMUST00000132630;ENSMUST00000002466;ENSMUST00000137
MSTRG.3	Ushbp1	ENSMUST00000212626;ENSMUST00000049184;ENSMUST00000212
MSTRG.3	Babam1	ENSMUST00000002473;ENSMUST00000213093;ENSMUST00000212
MSTRG.3	Arid5b	MSTRG.3068.1
MSTRG.3	Babam1	MSTRG.30680.1
MSTRG.3	Ankle1	ENSMUST00000119976;ENSMUST00000125290;ENSMUST00000124
MSTRG.3	Abhd8	ENSMUST00000008094;ENSMUST00000212321;ENSMUST00000212
MSTRG.3	Mrpl34	ENSMUST00000048914
MSTRG.3	Dda1	ENSMUST00000147843;ENSMUST00000127885;ENSMUST00000124
MSTRG.3	Ano8	ENSMUST00000093450;ENSMUST00000213382;ENSMUST00000212
MSTRG.3	Gtpbp3	ENSMUST00000007754;ENSMUST00000168847;ENSMUST0000009[illegible]
MSTRG.3	Plvap	ENSMUST00000048452
MSTRG.3	C430049[illegible]	ENSMUST00000207913
MSTRG.3	Gm7850	ENSMUST00000212909
MSTRG.3	Arid5b	MSTRG.3069.1
MSTRG.3	Bst2	ENSMUST00000051672;MSTRG.30690.2;ENSMUST00000212386
MSTRG.3	Mvb12a	ENSMUST00000212954;ENSMUST00000212341;ENSMUST00000034
MSTRG.3	Tmem221	ENSMUST00000052072
MSTRG.3	Nxnl1	ENSMUST00000163659
MSTRG.3	Slc27a1	MSTRG.30694.1;ENSMUST00000212889;ENSMUST00000212989;[illegible]
MSTRG.3	Slc27a1	MSTRG.30695.1
MSTRG.3	Pgls	MSTRG.30696.1
MSTRG.3	Pgls;Fa[illegible]	ENSMUST00000034264;MSTRG.30697.1;ENSMUST00000127626;[illegible]
MSTRG.3	Colgalt	MSTRG.30698.1;ENSMUST00000047903;ENSMUST00000212216;[illegible]
MSTRG.3	Unc13a	ENSMUST00000030170;ENSMUST00000177517;ENSMUST00000177
MSTRG.3	Gm37135	ENSMUST00000192652
MSTRG.3	Rhobtb1	ENSMUST00000167286;ENSMUST00000164034;ENSMUST0000002[illegible]
MSTRG.3	Mir6769[illegible]	ENSMUST00000184793
MSTRG.3	Jak3	ENSMUST00000110013;ENSMUST00000051995;ENSMUST0000013[illegible]
MSTRG.3	Jak3	MSTRG.30702.1
MSTRG.3	B3gnt3	ENSMUST00000034260
MSTRG.3	Gm45756	ENSMUST00000212491
MSTRG.3	Gm18860	ENSMUST00000212029
MSTRG.3	Fcho1	ENSMUST00000093444;MSTRG.30706.1;ENSMUST00000146100;[illegible]
MSTRG.3	Gm35572	ENSMUST00000212427
MSTRG.3	Gm35572	MSTRG.30708.1
MSTRG.3	Gm35572	MSTRG.30709.1
MSTRG.3	A930033[illegible]	ENSMUST00000170048
MSTRG.3	Gm29705	ENSMUST00000212598
MSTRG.3	Crry-ps	ENSMUST00000079090;MSTRG.30711.2
MSTRG.3	Large1	ENSMUST00000004497;ENSMUST00000119826;ENSMUST00000212
MSTRG.3	Large1	MSTRG.30713.1
MSTRG.3	Large1	MSTRG.30714.1

MSTRG.3	Mir28b	ENSMUST00000175399
MSTRG.3	Large1	MSTRG.30716.1
MSTRG.3	Gm22532	ENSMUST00000158822
MSTRG.3	Large1	MSTRG.30718.1
MSTRG.3	Large1	MSTRG.30719.1
MSTRG.3	Rhobtb1	MSTRG.3072.1
MSTRG.3	Gm11033	ENSMUST00000127757;ENSMUST00000156185
MSTRG.3	Gm11033	MSTRG.30721.1
MSTRG.3	Sin3b	ENSMUST00000109950;ENSMUST00000004494;ENSMUST00000212
MSTRG.3	F2rl3	ENSMUST00000058099;ENSMUST00000212755
MSTRG.3	Gm45705	ENSMUST00000211831
MSTRG.3	Gm45706	ENSMUST00000213063
MSTRG.3	Gm45704	ENSMUST00000212582
MSTRG.3	Hmox1	ENSMUST00000005548;ENSMUST00000159631
MSTRG.3	Hmgxb4	ENSMUST00000041759;ENSMUST00000151062;ENSMUST00000145
MSTRG.3	Hmgxb4	MSTRG.30729.1
MSTRG.3	Rhobtb1	MSTRG.3073.1
MSTRG.3	Hmgxb4	MSTRG.30730.1
MSTRG.3	Gm45822	ENSMUST00000212231
MSTRG.3	Tom1	ENSMUST00000165630;ENSMUST00000212651;ENSMUST00000212
MSTRG.3	Gm45820	ENSMUST00000212068
MSTRG.3	Mcm5	ENSMUST00000164309;MSTRG.30734.2;MSTRG.30734.3;MSTRG.
MSTRG.3	Iqcm	ENSMUST00000212704
MSTRG.3	Iqcm	MSTRG.30736.1
MSTRG.3	Gm7901	ENSMUST00000120430
MSTRG.3	Gm10649	ENSMUST00000143324;ENSMUST00000136538;ENSMUST00000148
MSTRG.3	Cdk1	ENSMUST00000020099;ENSMUST00000119827;ENSMUST00000123
MSTRG.3	Gm5353	ENSMUST00000139800
MSTRG.3	Nr3c2	ENSMUST00000143284;ENSMUST00000109913;ENSMUST00000148
MSTRG.3	Gm24838	ENSMUST00000158684
MSTRG.3	Gm23260	ENSMUST00000158317
MSTRG.3	Arhgap1	ENSMUST00000076316;ENSMUST00000210519;ENSMUST0000021C
MSTRG.3	Gm45406	ENSMUST00000210161
MSTRG.3	Arhgap1	MSTRG.30747.1
MSTRG.3	4933421	ENSMUST00000209302
MSTRG.3	Gm45285	MSTRG.30749.1;ENSMUST00000210418
MSTRG.3	Ccdc6	ENSMUST00000147545;ENSMUST00000145990;ENSMUST00000156
MSTRG.3	Arhgap1	MSTRG.30750.1
MSTRG.3	Arhgap1	MSTRG.30751.1
MSTRG.3	Gm45407	ENSMUST00000209499
MSTRG.3	0610038	ENSMUST00000184762;ENSMUST00000181420
MSTRG.3	0610038	MSTRG.30754.1
MSTRG.3	Prmt9	ENSMUST00000056237;ENSMUST00000142824;MSTRG.30755.4;M
MSTRG.3	Prmt9	MSTRG.30756.1
MSTRG.3	Prmt9	MSTRG.30757.1
MSTRG.3	Gm23468	ENSMUST00000157125
MSTRG.3	Gm45286	ENSMUST00000209267
MSTRG.3	Ccdc6	MSTRG.3076.1
MSTRG.3	Tmem184	ENSMUST00000034030;MSTRG.30760.1;ENSMUST00000152168;E
MSTRG.3	Gm2225	ENSMUST00000209570
MSTRG.3	4933431	ENSMUST00000139140;ENSMUST00000153937;ENSMUST00000211
MSTRG.3	4933431	MSTRG.30764.1
MSTRG.3	Ednra	ENSMUST00000034029;ENSMUST00000146561
MSTRG.3	Lsm6	ENSMUST00000130325;ENSMUST00000051867;ENSMUST00000126
MSTRG.3	Rbmxl1	ENSMUST00000049245;ENSMUST00000211719
MSTRG.3	Slc10a7	ENSMUST00000211332;ENSMUST00000209490;ENSMUST00000034
MSTRG.3	Slc10a7	MSTRG.30769.1

MSTRG.3	Ccdc6	MSTRG.3077.1
MSTRG.30770		MSTRG.30770.1
MSTRG.3	Zfp827;(	ENSMUST00000087927;ENSMUST00000098614;ENSMUST0000011
MSTRG.3	Zfp827	MSTRG.30772.1
MSTRG.3	Zfp827	MSTRG.30773.1
MSTRG.3	Mmaa	ENSMUST00000048718;ENSMUST00000210231
MSTRG.3	Mmaa	MSTRG.30775.1
MSTRG.3	Mmaa	MSTRG.30776.1
MSTRG.3	Gm4890	ENSMUST00000181030
MSTRG.3	Smad1	ENSMUST00000066091;ENSMUST00000109885;ENSMUST0000021C
MSTRG.3	Smad1	MSTRG.30779.1
MSTRG.3	Ccdc6	MSTRG.3078.1
MSTRG.3	Smad1	MSTRG.30780.1
MSTRG.3	Otud4	ENSMUST00000173078;MSTRG.30781.2;MSTRG.30781.3;MSTRG.
MSTRG.3	Abce1	MSTRG.30782.1;ENSMUST00000080536;ENSMUST00000211509;E
MSTRG.3	Gm27006	ENSMUST00000182218
MSTRG.3	Anapc10	ENSMUST00000048147;ENSMUST00000210812;MSTRG.30784.3;E
MSTRG.3	Gm45435	ENSMUST00000211147
MSTRG.3	Gm45434	ENSMUST00000210550
MSTRG.30787		MSTRG.30787.1
MSTRG.3	Hhip	ENSMUST00000155850;ENSMUST00000079038
MSTRG.3079		MSTRG.3079.1
MSTRG.30790		MSTRG.30790.1
MSTRG.3	Frem3	ENSMUST00000039695
MSTRG.3	Frem3	MSTRG.30792.1
MSTRG.3	Smarca5	ENSMUST00000043359;ENSMUST00000122807;MSTRG.30793.2;M
MSTRG.3	Gab1	ENSMUST00000210676;ENSMUST00000034150;ENSMUST00000211
MSTRG.3	Gm7997	ENSMUST00000210546
MSTRG.3	Gab1	MSTRG.30796.1
MSTRG.3	Gab1	MSTRG.30797.1
MSTRG.3	Gm31105	ENSMUST00000211421
MSTRG.3	Usp38	MSTRG.30799.1;ENSMUST00000042724;MSTRG.30799.3;MSTRG.
MSTRG.3	Coa5	ENSMUST00000027286;ENSMUST00000192179
MSTRG.3	4930533	ENSMUST00000219192;ENSMUST00000058942;ENSMUST0000021
MSTRG.3	Gm9725	ENSMUST00000166229;ENSMUST00000209272
MSTRG.3	Inpp4b	ENSMUST00000172167;ENSMUST00000213285;ENSMUST0000016
MSTRG.3	Inpp4b	MSTRG.30802.1
MSTRG.3	Inpp4b	MSTRG.30803.1
MSTRG.3	Inpp4b	MSTRG.30804.1
MSTRG.3	Inpp4b	MSTRG.30805.1
MSTRG.3	Inpp4b	MSTRG.30806.1
MSTRG.3	Inpp4b	MSTRG.30807.1
MSTRG.3	Inpp4b	MSTRG.30808.1
MSTRG.3	Inpp4b	MSTRG.30809.1
MSTRG.3	AC08789	ENSMUST00000217867
MSTRG.3	Inpp4b	MSTRG.30810.1
MSTRG.3	4930579	ENSMUST00000168243
MSTRG.3	Inpp4b	MSTRG.30812.1
MSTRG.3	Gm17169	ENSMUST00000171382
MSTRG.3	Inpp4b	MSTRG.30814.1
MSTRG.3	Gm17072	ENSMUST00000166673
MSTRG.3	Inpp4b	MSTRG.30816.1
MSTRG.3	Inpp4b	MSTRG.30817.1
MSTRG.3	Inpp4b	MSTRG.30818.1
MSTRG.3	Inpp4b	MSTRG.30819.1
MSTRG.3	Inpp4b	MSTRG.30820.1
MSTRG.3	Inpp4b	MSTRG.30821.1

MSTRG.3	Inpp4b	MSTRG.30822.1
MSTRG.3	Inpp4b	MSTRG.30823.1
MSTRG.3	Inpp4b	MSTRG.30824.1
MSTRG.3	Il15	ENSMUST00000209363;ENSMUST00000209573;ENSMUST00000034
MSTRG.3	Il15	MSTRG.30826.1
MSTRG.30827		MSTRG.30827.1
MSTRG.3	Gm9655	ENSMUST00000211321
MSTRG.3	Gm9655	MSTRG.30829.1
MSTRG.3	Bicc1	ENSMUST00000143791;ENSMUST00000131445;ENSMUST00000014
MSTRG.3	Gm9655	MSTRG.30830.1
MSTRG.3	Zfp330	ENSMUST00000211451;ENSMUST00000209601;ENSMUST00000034
MSTRG.3	Rnf150	ENSMUST00000078525;ENSMUST00000211020;ENSMUST00000211
MSTRG.3	Gm18212	ENSMUST00000211678
MSTRG.3	Gm45449	ENSMUST00000211485
MSTRG.3	Gm10645	ENSMUST00000098605
MSTRG.3	Tbc1d9	ENSMUST00000034145;ENSMUST00000093393;ENSMUST00000142
MSTRG.3	Tbc1d9	MSTRG.30837.1
MSTRG.3	Gm17999	ENSMUST00000211568
MSTRG.3	Ucp1	ENSMUST00000034146
MSTRG.3	AC08789	ENSMUST00000219321
MSTRG.3	Elmod2	ENSMUST00000177594;ENSMUST00000053902;ENSMUST0000015C
MSTRG.3	Clgn	ENSMUST00000002259
MSTRG.3	Scoc	ENSMUST00000212473;ENSMUST00000212449;ENSMUST00000081
MSTRG.3	Scoc	MSTRG.30843.1
MSTRG.3	Ndufb7	ENSMUST00000036996;ENSMUST00000127162;MSTRG.30844.2
MSTRG.3	Tecr	ENSMUST00000212990;MSTRG.30845.1;ENSMUST00000212005;M
MSTRG.3	D830024I	ENSMUST00000212132
MSTRG.3	Tecr	MSTRG.30848.1
MSTRG.3	Gm18001	ENSMUST00000212023
MSTRG.3	Bicc1	MSTRG.3085.1
MSTRG.3	Tecr	MSTRG.30850.1
MSTRG.3	Dnajb1	ENSMUST00000005620;ENSMUST00000212300
MSTRG.3	Gipc1	ENSMUST00000019577;ENSMUST00000211985;ENSMUST00000212
MSTRG.3	Gipc1	MSTRG.30853.1
MSTRG.3	Pkn1	ENSMUST00000005616;ENSMUST00000144258;ENSMUST00000132
MSTRG.3	Ptger1	ENSMUST00000019608;ENSMUST00000159935;ENSMUST00000212
MSTRG.3	Ddx39	ENSMUST00000019576;ENSMUST00000127505;ENSMUST0000014C
MSTRG.3	Ddx39	MSTRG.30857.1
MSTRG.3	Adgre5;I	ENSMUST00000002964;ENSMUST00000166939;ENSMUST00000075
MSTRG.3	Samd1	ENSMUST00000095228;ENSMUST00000210523;ENSMUST0000020S
MSTRG.3	Gm15647	ENSMUST00000160178
MSTRG.3	Adgrl1	ENSMUST00000141158;ENSMUST00000124355;ENSMUST00000045
MSTRG.3	Adgrl1	MSTRG.30861.1
MSTRG.3	Gm44330	ENSMUST00000200106
MSTRG.3	Gm10644	ENSMUST00000098595
MSTRG.3	Asf1b	ENSMUST00000005607;MSTRG.30865.2;ENSMUST00000124913;E
MSTRG.3	Prkaca	ENSMUST00000005606;ENSMUST00000211558
MSTRG.3	Palm3	ENSMUST00000142367;ENSMUST00000055077;ENSMUST00000154
MSTRG.3	Palm3	MSTRG.30868.1
MSTRG.3	Il27ra	ENSMUST00000005601;MSTRG.30869.2;ENSMUST00000210245;E
MSTRG.3	AC07968	ENSMUST00000217960
MSTRG.3	Rln3	ENSMUST00000061923
MSTRG.3	Podnl1	ENSMUST00000093380;ENSMUST00000147175;MSTRG.30871.2
MSTRG.3	Gm10643	ENSMUST00000098592
MSTRG.3	C330011I	ENSMUST00000071067
MSTRG.3	Rfx1	ENSMUST00000211046;ENSMUST00000209362;ENSMUST0000021C
MSTRG.3	Mir709	ENSMUST00000102190

MSTRG.3	Dcaf15	MSTRG.30876.1;MSTRG.30876.2;MSTRG.30876.5;MSTRG.3087
MSTRG.3	Cc2d1a	ENSMUST00000117424;ENSMUST00000040383;ENSMUST00000154
MSTRG.3	4930432	ENSMUST00000143833;ENSMUST00000118856;ENSMUST0000009
MSTRG.3	Tfam	ENSMUST00000092430;ENSMUST00000145220;ENSMUST0000010
MSTRG.3	Nanos3	ENSMUST00000211482;ENSMUST00000070102;ENSMUST0000021
MSTRG.3	Mir181d	ENSMUST00000102383
MSTRG.3	Mir181c	ENSMUST00000083549
MSTRG.3	Gm26887	ENSMUST00000181282
MSTRG.3	Gm37352	ENSMUST00000193908;MSTRG.30884.1;MSTRG.30884.3
MSTRG.3	Mir23a	ENSMUST00000083677
MSTRG.3	Mir27a	ENSMUST00000083510
MSTRG.3	Mir24-2	ENSMUST00000083607;ENSMUST00000180630
MSTRG.3	Zswim4	ENSMUST00000039480;MSTRG.30889.2
MSTRG.3	Ank3	ENSMUST00000218194;MSTRG.3089.3;MSTRG.3089.5;MSTRG.3
MSTRG.3	D8Ertd7	MSTRG.30890.1;ENSMUST00000019506;MSTRG.30890.3
MSTRG.3	Mri1	ENSMUST00000126435;ENSMUST00000125498;MSTRG.30891.3;
MSTRG.3	Calr	MSTRG.30892.1;ENSMUST00000003912;ENSMUST00000154774;
MSTRG.3	Dand5	ENSMUST00000065539
MSTRG.30894		MSTRG.30894.1
MSTRG.3	Gadd45g	ENSMUST00000036734
MSTRG.3	Rad23a	ENSMUST00000144675;ENSMUST00000003911;MSTRG.30896.3;
MSTRG.3	Cacna1a	ENSMUST00000135382;ENSMUST00000121390;ENSMUST00000122
MSTRG.3	Cacna1a	MSTRG.30899.1
MSTRG.3	Unc50	ENSMUST00000114925;ENSMUST00000027285;ENSMUST00000144
MSTRG.3	AC13179	ENSMUST00000218846
MSTRG.3	Gm16183	ENSMUST00000127527
MSTRG.3	Ier2	ENSMUST00000060427
MSTRG.3	Gm26664	ENSMUST00000181140
MSTRG.3	Gm26664	MSTRG.30903.1
MSTRG.3	Nacc1	ENSMUST00000001975;MSTRG.30904.1;MSTRG.30904.2
MSTRG.3	Trmt1	ENSMUST00000175980;ENSMUST00000176914;ENSMUST00000177
MSTRG.3	Trmt1	MSTRG.30906.1
MSTRG.3	Nfix	ENSMUST00000148644;ENSMUST00000109764;MSTRG.30907.4;
MSTRG.3	Lyl1	ENSMUST00000037165
MSTRG.3	G430095	ENSMUST00000098571
MSTRG.3	AC12901	ENSMUST00000220152
MSTRG.3	Nfix	MSTRG.30910.1
MSTRG.3	Nfix	MSTRG.30911.1
MSTRG.3	Farsa;M	ENSMUST00000003906;ENSMUST00000129595;ENSMUST00000144
MSTRG.3	Mir7069	MSTRG.30913.1
MSTRG.3	Syce2	ENSMUST00000170296;ENSMUST00000136026;ENSMUST0000000
MSTRG.3	Gcdh	ENSMUST00000003907;ENSMUST00000109745;ENSMUST0000013
MSTRG.3	Junb	ENSMUST00000064922
MSTRG.3	Rtbdn	MSTRG.30917.1
MSTRG.3	Dnase2a	ENSMUST00000003910;ENSMUST00000109744;ENSMUST0000015
MSTRG.3	Mast1;G	ENSMUST00000109741;ENSMUST00000153000;MSTRG.30919.4;
MSTRG.3	Ank3	MSTRG.3092.1
MSTRG.3	Rtbdn	ENSMUST00000067472;ENSMUST00000152378
MSTRG.3	Rnaseh2	ENSMUST00000109738;ENSMUST00000109736;ENSMUST0000006
MSTRG.3	Rnaseh2	MSTRG.30922.1
MSTRG.3	Prdx2	ENSMUST00000109734;ENSMUST00000005292;ENSMUST0000013
MSTRG.3	Junb	ENSMUST00000209372
MSTRG.3	Gm42031	ENSMUST00000209823
MSTRG.3	Gm42031	MSTRG.30926.1
MSTRG.30927		MSTRG.30927.1
MSTRG.3	Man2b1	ENSMUST00000210991;ENSMUST00000209361;ENSMUST0000020
MSTRG.3	Man2b1	MSTRG.30929.1

MSTRG.3	Ank3	MSTRG.3093.1
MSTRG.3	Vps35	ENSMUST00000034131;ENSMUST00000209277;ENSMUST0000020[illegible]
MSTRG.3	Vps35	MSTRG.30931.1
MSTRG.3	Orc6	ENSMUST00000211597;ENSMUST00000211396;ENSMUST0000017[illegible]
MSTRG.3	4921524[illegible]	ENSMUST00000047749;ENSMUST00000209355;ENSMUST0000021[illegible]
MSTRG.3	4921524[illegible]	MSTRG.30934.1
MSTRG.3	Gm45594	ENSMUST00000211717
MSTRG.3	4921524[illegible]	MSTRG.30936.1
MSTRG.3	4921524[illegible]	MSTRG.30937.1
MSTRG.3	1700051[illegible]	ENSMUST00000194864;ENSMUST00000195451
MSTRG.3	Hook2	ENSMUST00000209764;ENSMUST00000064495;ENSMUST0000021[illegible]
MSTRG.3	Ank3	MSTRG.3094.1
MSTRG.3	Hook2	MSTRG.30940.1
MSTRG.3	Best2	ENSMUST00000209322;ENSMUST00000209421
MSTRG.3	Asna1	MSTRG.30942.1;ENSMUST00000064314;ENSMUST00000209834;[illegible]
MSTRG.3	Asna1	MSTRG.30943.1
MSTRG.3	Trir;Tn[illegible]	ENSMUST00000047281;MSTRG.30944.1;MSTRG.30944.3;MSTRG.[illegible]
MSTRG.3	Tnpo2	MSTRG.30946.1
MSTRG.3	A230103[illegible]	MSTRG.30947.1;ENSMUST00000125244;ENSMUST00000156084
MSTRG.3	Fbxw9	ENSMUST00000095220;ENSMUST00000139721;ENSMUST0000014[illegible]
MSTRG.3	Mir7070	ENSMUST00000183657
MSTRG.3	Ank3	MSTRG.3095.1
MSTRG.3	Gm5741	ENSMUST00000177563
MSTRG.30951		MSTRG.30951.1
MSTRG.3	Dhps	ENSMUST00000078665;ENSMUST00000152871;ENSMUST0000012[illegible]
MSTRG.3	Wdr83	ENSMUST00000131909;ENSMUST00000136696;ENSMUST0000009[illegible]
MSTRG.3	Wdr83os	ENSMUST00000079764;ENSMUST00000148592;ENSMUST0000014[illegible]
MSTRG.30955		MSTRG.30955.1
MSTRG.30956		MSTRG.30956.1
MSTRG.30957		MSTRG.30957.1
MSTRG.3	Gpt2	ENSMUST00000034136;ENSMUST00000132932;ENSMUST0000014[illegible]
MSTRG.3	Dnaja2	MSTRG.30959.1
MSTRG.3	Ank3	MSTRG.3096.1
MSTRG.3	Dnaja2	ENSMUST00000034138;MSTRG.30960.2;MSTRG.30960.3;MSTRG.[illegible]
MSTRG.3	Neto2	ENSMUST00000109686;ENSMUST00000209259;ENSMUST0000020[illegible]
MSTRG.3	Neto2	MSTRG.30962.1
MSTRG.3	Itfg1	ENSMUST00000034140;MSTRG.30964.1;ENSMUST00000210604;[illegible]
MSTRG.3	Gm39214	ENSMUST00000210912;MSTRG.30965.2
MSTRG.3	Phkb	ENSMUST00000159716;ENSMUST00000160611;ENSMUST0000005[illegible]
MSTRG.3	Phkb	MSTRG.30967.1
MSTRG.3	Gm16167	ENSMUST00000160428
MSTRG.3	Phkb	MSTRG.30969.1
MSTRG.3	Ank3	MSTRG.3097.1
MSTRG.3	Phkb	MSTRG.30970.1
MSTRG.3	Hm62979	ENSMUST00000183645
MSTRG.3	N4bp1	MSTRG.30972.1;ENSMUST00000034074;ENSMUST00000210029
MSTRG.3	Rps13-p	ENSMUST00000183478
MSTRG.3	Lonp2	ENSMUST00000034141;ENSMUST00000155433;ENSMUST0000015[illegible]
MSTRG.3	Gm16177	ENSMUST00000120728
MSTRG.3	Lonp2	MSTRG.30976.1
MSTRG.3	Gm22305	ENSMUST00000082690
MSTRG.3	Siah1a	MSTRG.30978.1;ENSMUST00000045296
MSTRG.3	Gm10638	ENSMUST00000098532
MSTRG.3	Ank3	MSTRG.3098.1
MSTRG.3	Cnep1r1	ENSMUST00000095214;ENSMUST00000131891;ENSMUST0000012[illegible]
MSTRG.3	Heatr3	ENSMUST00000034079;ENSMUST00000121949;ENSMUST0000013[illegible]
MSTRG.3	Gm9988	ENSMUST00000069786

MSTRG.3	Mir7071	ENSMUST00000184847
MSTRG.3	Papd5	ENSMUST00000066748;ENSMUST00000119033;ENSMUST0000011[illegible]
MSTRG.3	Papd5	MSTRG.30985.1
MSTRG.3	Papd5	MSTRG.30986.1
MSTRG.3	Papd5	MSTRG.30987.1
MSTRG.3	Papd5	MSTRG.30988.1
MSTRG.3	Papd5	MSTRG.30989.1
MSTRG.3	Ube2d1	MSTRG.3099.1;ENSMUST00000020085;ENSMUST00000144636;MS
MSTRG.3	Snx20	ENSMUST00000034087;ENSMUST00000209806
MSTRG.3	Adcy7	ENSMUST00000169037;ENSMUST00000168545;MSTRG.30991.3;M
MSTRG.3	Adcy7	MSTRG.30992.1
MSTRG.3	Brd7	ENSMUST00000131748;MSTRG.30993.2;ENSMUST00000034085;E
MSTRG.3	Cyld	MSTRG.30994.1;ENSMUST00000043526;ENSMUST00000211554;E
MSTRG.3	Cyld	MSTRG.30995.1
MSTRG.3	Gm17995	ENSMUST00000209266
MSTRG.3	Gm17910	ENSMUST00000211038
MSTRG.3	Chd9	MSTRG.30998.1
MSTRG.3	Gm45396	ENSMUST00000209208
MSTRG.3	Gm26983	MSTRG.31.1;ENSMUST00000182051
MSTRG.3	Mgat4a	ENSMUST00000042161;ENSMUST00000151952;ENSMUST00000154
MSTRG.3	AC15350	ENSMUST00000218529
MSTRG.3	Chd9	ENSMUST00000048665;ENSMUST00000209474;ENSMUST00000211
MSTRG.3	Gm45641	ENSMUST00000209775
MSTRG.3	Chd9	MSTRG.31002.1
MSTRG.3	Gm45640	ENSMUST00000210665
MSTRG.3	Chd9	MSTRG.31004.1
MSTRG.3	Chd9	MSTRG.31005.1
MSTRG.3	Chd9	MSTRG.31006.1
MSTRG.3	Gm45643	ENSMUST00000211364
MSTRG.3	Chd9	MSTRG.31008.1
MSTRG.3	Gm6658	ENSMUST00000209493;ENSMUST00000098517
MSTRG.3	Cisd1	ENSMUST00000045887
MSTRG.3	Chd9	MSTRG.31010.1
MSTRG.3	Chd9	MSTRG.31011.1
MSTRG.3	Chd9	MSTRG.31012.1
MSTRG.3	Rbl2	ENSMUST00000034091;ENSMUST00000210533;ENSMUST00000141
MSTRG.3	Rbl2	MSTRG.31014.1
MSTRG.3	Rbl2	MSTRG.31015.1
MSTRG.3	Aktip	ENSMUST00000120426;ENSMUST00000120213;ENSMUST0000012[illegible]
MSTRG.3	Gm45464	MSTRG.31017.1;ENSMUST00000210728
MSTRG.3	Gm8489	ENSMUST00000209876
MSTRG.3	Ipmk	ENSMUST00000079252;ENSMUST00000118381;ENSMUST00000121
MSTRG.3	Gm45666	ENSMUST00000211474
MSTRG.3	Gm3272	ENSMUST00000181181
MSTRG.3	Fto	ENSMUST00000069718;ENSMUST00000125471;ENSMUST0000012[illegible]
MSTRG.3	Fto	MSTRG.31024.1;MSTRG.31024.2
MSTRG.3	Fto	MSTRG.31025.1
MSTRG.3	Gm45427	ENSMUST00000209572
MSTRG.3	Fto	MSTRG.31027.1
MSTRG.3	Fto	MSTRG.31028.1
MSTRG.3	4831440	ENSMUST00000211287
MSTRG.3103		MSTRG.3103.1
MSTRG.3	Gm45292	ENSMUST00000211232
MSTRG.3	Gm45289	ENSMUST00000211048
MSTRG.3	Fto	MSTRG.31032.1
MSTRG.3	Gm45290	ENSMUST00000211177;MSTRG.31033.1
MSTRG.3	Fto	MSTRG.31034.3;MSTRG.31034.1;MSTRG.31034.2;MSTRG.31034

MSTRG.3	Gm45294	MSTRG.31035.2;MSTRG.31035.1;ENSMUST00000210685
MSTRG.3	Fto	MSTRG.31036.1
MSTRG.3	Fto	MSTRG.31037.1;MSTRG.31037.2
MSTRG.3	Fto	MSTRG.31038.1
MSTRG.3	Gm3235	ENSMUST00000168442
MSTRG.3	Gm9923	ENSMUST00000217785
MSTRG.3	Gm45293	ENSMUST00000209754
MSTRG.3	Gm45295	ENSMUST00000210393
MSTRG.3	Mmp2	ENSMUST00000034187;MSTRG.31042.2;MSTRG.31042.3;ENSMUS
MSTRG.3	Gm45472	ENSMUST00000211295
MSTRG.3	Lpcat2	ENSMUST00000046290;ENSMUST00000209265;ENSMUST0000021C
MSTRG.3	Capns2	ENSMUST00000104947
MSTRG.3	Gm22151	ENSMUST00000116963
MSTRG.3	Ces1d	ENSMUST00000034172;ENSMUST00000143256;ENSMUST0000014‹
MSTRG.3	Gm45909	ENSMUST00000212413
MSTRG.3	Ces1f	ENSMUST00000034178;ENSMUST00000156737;ENSMUST00000151
MSTRG.3	Gm9923	MSTRG.3105.1
MSTRG.3	Amfr	ENSMUST00000053766;ENSMUST00000139702;ENSMUST00000137
MSTRG.3	Gm45774	ENSMUST00000211992
MSTRG.3	Mt2	MSTRG.31052.1;ENSMUST00000034214;ENSMUST00000212806
MSTRG.3	Mt1	ENSMUST00000034215;ENSMUST00000212291;ENSMUST00000211
MSTRG.3	Nudt21	MSTRG.31054.1;ENSMUST00000034204;ENSMUST00000212622;E
MSTRG.3	Ogfod1	ENSMUST00000109556;ENSMUST00000093301;ENSMUST00000141
MSTRG.3	Ogfod1	MSTRG.31056.1
MSTRG.3	Gm45873	ENSMUST00000211865
MSTRG.3	Bbs2	ENSMUST00000034206;ENSMUST00000170208;ENSMUST00000172
MSTRG.3	Nup93	ENSMUST00000079961;ENSMUST00000212039;ENSMUST00000212
MSTRG.3	Zwint	ENSMUST00000020081;ENSMUST00000105431;ENSMUST0000016C
MSTRG.3	Rpl10-p	ENSMUST00000212707
MSTRG.3	Nup93	MSTRG.31061.1
MSTRG.3	Gm45804	ENSMUST00000212137
MSTRG.3	Ap3s1-p	ENSMUST00000178107
MSTRG.3	Gm15889	ENSMUST00000212669;ENSMUST00000159142
MSTRG.3	Gm15890	ENSMUST00000161024
MSTRG.3	Herpud1	ENSMUST00000161576;MSTRG.31066.2;ENSMUST00000034220;E
MSTRG.3	Nlrc5	MSTRG.31067.1;ENSMUST00000212382;ENSMUST00000211816;E
MSTRG.3	9330175	ENSMUST00000180612;ENSMUST00000180445
MSTRG.3	Nlrc5	MSTRG.31069.1
MSTRG.3	Bcr	ENSMUST00000164107;MSTRG.3107.2;MSTRG.3107.3;ENSMUSTC
MSTRG.3	Mir7072	ENSMUST00000184542
MSTRG.3	Ccl22	ENSMUST00000034231;ENSMUST00000156137
MSTRG.31072		MSTRG.31072.1
MSTRG.3	Arl2bp	ENSMUST00000034228;ENSMUST00000109527;ENSMUST00000212
MSTRG.3	Pllp	ENSMUST00000034227
MSTRG.3	Gm45767	ENSMUST00000212778
MSTRG.3	Cx3cl1	ENSMUST00000034230;ENSMUST00000211956;ENSMUST00000151
MSTRG.3	Ccl17	ENSMUST00000034232
MSTRG.3	Cpne2	ENSMUST00000048653;ENSMUST00000156894;ENSMUST0000013€
MSTRG.3	Fam192a	MSTRG.31079.1;ENSMUST00000034226;ENSMUST00000212262;E
MSTRG.3	Pcdh15	ENSMUST00000123398;ENSMUST00000148572;ENSMUST0000015€
MSTRG.3	Rspry1	ENSMUST00000060389;ENSMUST00000212729;ENSMUST00000212
MSTRG.3	Gm45797	ENSMUST00000212759
MSTRG.3	Rspry1	MSTRG.31082.1
MSTRG.3	Rspry1	MSTRG.31083.1
MSTRG.3	Ciapin1	ENSMUST00000162538;ENSMUST00000034233;ENSMUST00000161
MSTRG.3	Ciapin1	MSTRG.31085.1
MSTRG.3	Coq9	ENSMUST00000034234;ENSMUST00000159871;ENSMUST0000015‹

MSTRG.3	Ccdc102	ENSMUST00000077955;ENSMUST00000162020
MSTRG.3	Polr2c	ENSMUST00000109521;ENSMUST00000212744;MSTRG.31089.3;E
MSTRG.3	Gm37438	ENSMUST00000192813
MSTRG.3	Dok4	ENSMUST00000212810;ENSMUST00000046461;ENSMUST00000212
MSTRG.3	Adgrg5	ENSMUST00000153448;ENSMUST00000166802;ENSMUST00000074
MSTRG.3	Adgrg1	ENSMUST00000211850;ENSMUST00000212581;ENSMUST0000017
MSTRG.3	Adgrg1	MSTRG.31093.1
MSTRG.3	Adgrg3	ENSMUST00000051259;ENSMUST00000212558;ENSMUST00000211
MSTRG.3	Tepp	ENSMUST00000161029;ENSMUST00000212056;ENSMUST0000015
MSTRG.3	Zfp319	ENSMUST00000057717;MSTRG.31096.1
MSTRG.3	Katnb1	ENSMUST00000034239;ENSMUST00000212968;ENSMUST00000212
MSTRG.3	Gm45758	ENSMUST00000212448
MSTRG.3	Kifc3	ENSMUST00000169353;ENSMUST00000034240;ENSMUST0000016
MSTRG.3	4930594	ENSMUST00000192316;ENSMUST00000151866
MSTRG.3	Gm15398	ENSMUST00000147653;ENSMUST00000219998
MSTRG.3	Gm31224	ENSMUST00000212682
MSTRG.3	Gm31224	MSTRG.31101.1
MSTRG.3	Gm45812	ENSMUST00000212199
MSTRG.3	Gm31036	ENSMUST00000212925
MSTRG.3	Usb1	ENSMUST00000034245;ENSMUST00000213059;ENSMUST0000014
MSTRG.3	Mmp15	ENSMUST00000034243;ENSMUST00000212235
MSTRG.31106		MSTRG.31106.1
MSTRG.3	Cfap20	ENSMUST00000212731;ENSMUST00000034249;ENSMUST00000213
MSTRG.3	Csnk2a2	ENSMUST00000056919;ENSMUST00000212214;ENSMUST00000212
MSTRG.3	Gm45854	ENSMUST00000211857
MSTRG.3	Gm5778	ENSMUST00000171003
MSTRG.3	Csnk2a2	MSTRG.31110.1
MSTRG.3	Gm45853	ENSMUST00000212310
MSTRG.3	Gins3	ENSMUST00000034094;ENSMUST00000212434
MSTRG.3	Slc38a7	ENSMUST00000040481;ENSMUST00000212270;ENSMUST00000142
MSTRG.3	Got2	ENSMUST00000034097;ENSMUST00000211836;ENSMUST00000147
MSTRG.3	Gm45760	ENSMUST00000212190
MSTRG.3	Impdh2-	ENSMUST00000120826
MSTRG.3	Gm45422	MSTRG.31117.1
MSTRG.3	Gm8730	ENSMUST00000076373
MSTRG.3	Setd6	ENSMUST00000034096;MSTRG.31119.2;ENSMUST00000126888;M
MSTRG.3	AC11989	ENSMUST00000220399
MSTRG.3	Cnot1;G	ENSMUST00000098473;ENSMUST00000068452;ENSMUST00000211
MSTRG.3	Mir7073	ENSMUST00000197815
MSTRG.3	Gm45762	ENSMUST00000212863
MSTRG.3	Cnot1	MSTRG.31123.1
MSTRG.3	4930513	ENSMUST00000212647;ENSMUST00000145562;ENSMUST0000009
MSTRG.3	Gm31659	ENSMUST00000212289;ENSMUST00000211989;ENSMUST00000212
MSTRG.3	Gm29682	ENSMUST00000210896
MSTRG.3	Cdh5	ENSMUST00000034339;ENSMUST00000209911
MSTRG.3	Gm45887	ENSMUST00000212672
MSTRG.3	Gm45750	ENSMUST00000211966
MSTRG.3	AC11989	ENSMUST00000218804
MSTRG.3	Tk2	ENSMUST00000050211;ENSMUST00000212275;ENSMUST00000211
MSTRG.3	Cklf	ENSMUST00000034342;ENSMUST00000212433;MSTRG.31131.2;E
MSTRG.3	Cmtm2a;	ENSMUST00000034344;ENSMUST00000160596
MSTRG.3	Gm45711	MSTRG.31133.1
MSTRG.3	Cmtm4	ENSMUST00000179802
MSTRG.3	Gm45877	ENSMUST00000212110
MSTRG.3	Cmtm4	MSTRG.31136.1
MSTRG.3	Cmtm4	MSTRG.31137.1
MSTRG.3	Cmtm4	MSTRG.31138.1

MSTRG.3	Cmtm3	ENSMUST00000212139;ENSMUST00000212081;ENSMUST00000211
MSTRG.3	Gm15397	ENSMUST00000145790
MSTRG.31140		MSTRG.31140.1
MSTRG.3	Dync1li	MSTRG.31141.2;MSTRG.31141.1;ENSMUST00000041769;ENSMUS
MSTRG.3	Gm45731	ENSMUST00000211988
MSTRG.3	K230015	ENSMUST00000211969
MSTRG.3	Pdp2	ENSMUST00000059588
MSTRG.3	Rrad	ENSMUST00000034351;ENSMUST00000212163
MSTRG.3	Gm33023	ENSMUST00000212450;ENSMUST00000212328
MSTRG.3	Gm33023	MSTRG.31147.1
MSTRG.3	Fam96b	ENSMUST00000164884;ENSMUST00000163140;ENSMUST00000093
MSTRG.3	1700082	ENSMUST00000067136
MSTRG.3	Gm6407	ENSMUST00000176468
MSTRG.3	Terb1	ENSMUST00000064576;ENSMUST00000161520;ENSMUST00000162
MSTRG.3	Terb1	MSTRG.31151.1
MSTRG.3	Gm22148	ENSMUST00000104445
MSTRG.3	Nae1	ENSMUST00000161462;ENSMUST00000162466;ENSMUST00000034
MSTRG.3	Nae1	MSTRG.31154.1
MSTRG.3	Car7	ENSMUST00000212942;ENSMUST00000159416;ENSMUST00000056
MSTRG.3	Ces2f	ENSMUST00000076384;ENSMUST00000212820
MSTRG.3	Ces2g	ENSMUST00000043183;ENSMUST00000212330
MSTRG.3	Cbfb	ENSMUST00000052209;ENSMUST00000109392;ENSMUST0000010
MSTRG.3	Gm22063	ENSMUST00000082560
MSTRG.3	Pcdh15	MSTRG.3116.1
MSTRG.3	Cbfb	MSTRG.31160.1
MSTRG.3	Gm24629	ENSMUST00000122762
MSTRG.3	D230025	ENSMUST00000034361;ENSMUST00000156561;ENSMUST00000141
MSTRG.3	D230025	MSTRG.31163.1
MSTRG.3	D230025	MSTRG.31164.1
MSTRG.3	B3gnt9	ENSMUST00000136822;ENSMUST00000093217;ENSMUST00000161
MSTRG.3	Tradd	MSTRG.31166.1;ENSMUST00000034359;ENSMUST00000136239;M
MSTRG.3	Tradd	MSTRG.31167.1
MSTRG.3	Fbxl8	ENSMUST00000036221;ENSMUST00000126923
MSTRG.3	Hsf4	ENSMUST00000173640;ENSMUST00000036127;ENSMUST00000163
MSTRG.3	Specc1l	ENSMUST00000218766;ENSMUST00000218876;MSTRG.3117.3;MS
MSTRG.3	Nol3	ENSMUST00000014920
MSTRG.3	4931428	ENSMUST00000212922;ENSMUST00000014981;ENSMUST00000171
MSTRG.3	4931428	MSTRG.31172.1
MSTRG.3	Gm20163	ENSMUST00000212232;ENSMUST00000212288
MSTRG.3	Exoc3l	ENSMUST00000057855;ENSMUST00000212777;ENSMUST00000212
MSTRG.3	Tppp3	ENSMUST00000014990;ENSMUST00000177126;ENSMUST00000176
MSTRG.3	Zdhhc1	ENSMUST00000212303;ENSMUST00000212109;ENSMUST00000044
MSTRG.3	E2f4	ENSMUST00000015003;ENSMUST00000212037;MSTRG.31177.2;M
MSTRG.3	Elmo3	ENSMUST00000212046;ENSMUST00000109375;MSTRG.31178.2;E
MSTRG.3	AC13706	ENSMUST00000218702
MSTRG.3	Mir328	ENSMUST00000093622
MSTRG.3	Lrrc29	ENSMUST00000209964;ENSMUST00000070508;ENSMUST0000021C
MSTRG.3	Tmem208	ENSMUST00000015000;MSTRG.31182.1;ENSMUST00000153146;E
MSTRG.3	Fhod1	MSTRG.31183.1;MSTRG.31183.2;ENSMUST00000132777;ENSMUS
MSTRG.3	Slc9a5	ENSMUST00000073149;ENSMUST00000212772
MSTRG.3	Gfod2	ENSMUST00000013294;ENSMUST00000155038;ENSMUST00000135
MSTRG.3	Gfod2	MSTRG.31186.1
MSTRG.3	Gfod2	MSTRG.31187.1
MSTRG.3	Gfod2	MSTRG.31188.1
MSTRG.3	Ranbp10	MSTRG.31189.1;MSTRG.31189.2;ENSMUST00000041400;MSTRG.
MSTRG.3	Specc1l	MSTRG.3119.1
MSTRG.3	Ranbp10	MSTRG.31190.1

MSTRG.3	Tsnaxip	ENSMUST00000212566;ENSMUST00000034365;ENSMUST00000211
MSTRG.3	Tsnaxip	MSTRG.31192.1
MSTRG.3	Cenpt	ENSMUST00000212357;ENSMUST00000212431;ENSMUST0000004
MSTRG.3	Thap11	ENSMUST00000040445
MSTRG.3	4930578I	ENSMUST00000212850;ENSMUST00000212946
MSTRG.3	Nutf2	ENSMUST00000008594;ENSMUST00000212379;ENSMUST00000212
MSTRG.3	Hsd11b2	ENSMUST00000034363
MSTRG.3	Atp6v0d	ENSMUST00000013304;MSTRG.31198.1;ENSMUST00000144486;E
MSTRG.3	Atp6v0d	MSTRG.31199.1
MSTRG.3	Mgat4a	MSTRG.312.1
MSTRG.3	Adora2a	ENSMUST00000105420;MSTRG.3120.2;ENSMUST00000219044;EN
MSTRG.3	Gm38250	ENSMUST00000195108;ENSMUST00000192691
MSTRG.3	Gm45855	ENSMUST00000212546
MSTRG.3	Ripor1;I	MSTRG.31202.1;MSTRG.31202.2;MSTRG.31202.3;ENSMUST0000
MSTRG.3	Ctcf;Ca	MSTRG.31203.1;MSTRG.31203.2;ENSMUST00000195413;ENSMUS
MSTRG.3	Agrp	MSTRG.31204.1
MSTRG.3	Gm5915	ENSMUST00000116136
MSTRG.3	Acd	ENSMUST00000211870;ENSMUST00000042608;ENSMUST00000212
MSTRG.3	Pard6a	ENSMUST00000212634;ENSMUST00000212972;ENSMUST00000098
MSTRG.3	Enkd1	ENSMUST00000212162;ENSMUST00000212716;ENSMUST00000212
MSTRG.3	AC13706	ENSMUST00000219564;ENSMUST00000218467
MSTRG.3	4933405	ENSMUST00000013302
MSTRG.3	Edc4;Nr	ENSMUST00000040254;MSTRG.31211.3;MSTRG.31211.2;ENSMUS
MSTRG.3	Pskh1	ENSMUST00000049699
MSTRG.3	Ctrl	ENSMUST00000034368;ENSMUST00000212561
MSTRG.3	Gm16156	ENSMUST00000141168
MSTRG.3	Psmb10	ENSMUST00000212332;ENSMUST00000212686;ENSMUST00000034
MSTRG.3	Lcat	ENSMUST00000038896
MSTRG.3	Slc12a4	ENSMUST00000116429;MSTRG.31217.2;ENSMUST00000034370;E
MSTRG.3	Mir7074	ENSMUST00000183884
MSTRG.3	Pla2g15	ENSMUST00000212963;ENSMUST00000034377;ENSMUST00000212
MSTRG.3	Gucd1	ENSMUST00000145890;ENSMUST00000153125;ENSMUST0000003
MSTRG.31220		MSTRG.31220.1
MSTRG.3	Gm45752	ENSMUST00000212917
MSTRG.3	Dpep2;G	ENSMUST00000117555;ENSMUST00000081998;ENSMUST00000034
MSTRG.3	Dus2	ENSMUST00000142898;ENSMUST00000034375;ENSMUST0000011
MSTRG.3	Ddx28	ENSMUST00000058579
MSTRG.3	Dus2	MSTRG.31226.1
MSTRG.3	Dus2	MSTRG.31227.1
MSTRG.3	Dus2	MSTRG.31228.1
MSTRG.3	Nfatc3	ENSMUST00000212936;ENSMUST00000212742;ENSMUST00000211
MSTRG.3	Upb1	ENSMUST00000219052;ENSMUST00000039925;ENSMUST00000132
MSTRG.3	Nfatc3	MSTRG.31230.1
MSTRG.3	Gm27956	ENSMUST00000175604
MSTRG.3	Nfatc3	MSTRG.31232.1
MSTRG.3	Nfatc3	MSTRG.31233.1
MSTRG.3	Esrp2	MSTRG.31234.4;MSTRG.31234.1;MSTRG.31234.3;MSTRG.31234
MSTRG.3	1810019	ENSMUST00000137995;ENSMUST00000140509;ENSMUST00000145
MSTRG.3	Cdh3	ENSMUST00000080797
MSTRG.3	Cdh1	ENSMUST00000000312;ENSMUST00000167688;ENSMUST00000136
MSTRG.31238		MSTRG.31238.1
MSTRG.31239		MSTRG.31239.1
MSTRG.3	Gm25545	ENSMUST00000122673
MSTRG.3	Slc7a6	ENSMUST00000034378;ENSMUST00000213020;ENSMUST00000212
MSTRG.3	Slc7a6	MSTRG.31241.1
MSTRG.3	Slc7a6	MSTRG.31242.1
MSTRG.3	Slc7a6o	MSTRG.31243.1;ENSMUST00000035925;MSTRG.31243.4;MSTRG.

MSTRG.3	Prmt7	ENSMUST00000071592;ENSMUST00000109297;ENSMUST00000147
MSTRG.3	Prmt7	MSTRG.31245.1
MSTRG.3	Prmt7	MSTRG.31246.1
MSTRG.3	Gm26786	ENSMUST00000180410
MSTRG.3	Smpd3	ENSMUST00000067512;ENSMUST00000212896
MSTRG.3	Gm10629	ENSMUST00000137706
MSTRG.3	AC14446	ENSMUST00000218219
MSTRG.3	Zfp90	ENSMUST00000213045;ENSMUST00000211958;ENSMUST00000034
MSTRG.3	Tango6	ENSMUST00000048359;ENSMUST00000212764;ENSMUST00000211
MSTRG.3	Tango6	MSTRG.31252.1
MSTRG.3	Sntb2	ENSMUST00000212524;ENSMUST00000212298
MSTRG.3	Sntb2	MSTRG.31254.1
MSTRG.3	Sntb2	MSTRG.31255.1
MSTRG.3	Gm22085	ENSMUST00000158344
MSTRG.3	Has3	ENSMUST00000034385;ENSMUST00000176144
MSTRG.3	Chtf8	ENSMUST00000177068;ENSMUST00000169312;ENSMUST00000175
MSTRG.3	Utp4	ENSMUST00000212272;ENSMUST00000047629;MSTRG.31259.3;M
MSTRG.3	AC14446	ENSMUST00000220319
MSTRG.3	Gm16208	ENSMUST00000128476
MSTRG.3	Vps4a	ENSMUST00000034388;MSTRG.31261.2;ENSMUST00000144527;E
MSTRG.3	Gm16209	ENSMUST00000118457
MSTRG.3	Pdf;Cog	ENSMUST00000154271;ENSMUST00000095517;ENSMUST00000034
MSTRG.3	Nip7	ENSMUST00000034392;ENSMUST00000170962;ENSMUST00000212
MSTRG.3	Tmed6	ENSMUST00000034393
MSTRG.3	Terf2;M	ENSMUST00000133925;ENSMUST00000142616;MSTRG.31266.3;E
MSTRG.3	Terf2;R	MSTRG.31267.1;ENSMUST00000080443
MSTRG.3	C630050	ENSMUST00000193420
MSTRG.3	Cyb5b	ENSMUST00000034400;MSTRG.31269.2;ENSMUST00000212920
MSTRG.3	Snrpd3	ENSMUST00000020397;ENSMUST00000191097;ENSMUST0000022C
MSTRG.3	Nqo1	ENSMUST00000003947
MSTRG.3	Nfat5	ENSMUST00000151582;ENSMUST00000154474;MSTRG.31271.1;E
MSTRG.3	Nfat5	MSTRG.31272.1
MSTRG.3	Nob1	ENSMUST00000170796;ENSMUST00000169311;ENSMUST00000003
MSTRG.31274		MSTRG.31274.1
MSTRG.3	Psmd7	MSTRG.31275.1;ENSMUST00000044106;MSTRG.31275.3;ENSMUS
MSTRG.3	Wwp2;Mi	ENSMUST00000166615;ENSMUST00000213097;ENSMUST00000212
MSTRG.3	Rps26-p	ENSMUST00000077208;ENSMUST00000212102
MSTRG.3	Gm45900	ENSMUST00000211879
MSTRG.3	Wwp2	MSTRG.31279.1
MSTRG.3	Ggt5	ENSMUST00000219247;ENSMUST00000072217;ENSMUST00000189
MSTRG.3	Wwp2	MSTRG.31280.1
MSTRG.3	Wwp2	MSTRG.31281.1
MSTRG.3	Wwp2	MSTRG.31282.1
MSTRG.3	Gm1943	ENSMUST00000211890
MSTRG.3	Zfhx3	ENSMUST00000220518;ENSMUST00000221100;ENSMUST00000221
MSTRG.3	Gm39244	ENSMUST00000209663;ENSMUST00000211262
MSTRG.3	Lncbate	ENSMUST00000211953
MSTRG.3	Zfhx3	MSTRG.31287.1
MSTRG.3	Gm38042	ENSMUST00000191614
MSTRG.3	Gm9985	ENSMUST00000044101
MSTRG.3	Gm38318	ENSMUST00000195769
MSTRG.3	Zfhx3	MSTRG.31291.1
MSTRG.3	Zfhx3	MSTRG.31292.1
MSTRG.3	Gm20686	ENSMUST00000175846
MSTRG.3	Mir3108	ENSMUST00000175500
MSTRG.3	Dhx38	ENSMUST00000042601;MSTRG.31295.1;ENSMUST00000212667
MSTRG.3	Txnl4b	ENSMUST00000034159;ENSMUST00000178445

MSTRG.3	Txnl4b	MSTRG.31297.1
MSTRG.3	Hp	ENSMUST00000074898;ENSMUST00000212918;ENSMUST00000212
MSTRG.3	Dhodh	ENSMUST00000133353;ENSMUST00000125222;ENSMUST00000123
MSTRG.3	2010300	ENSMUST00000160023;ENSMUST00000162875;ENSMUST0000016C
MSTRG.3	Gm26402	ENSMUST00000158499
MSTRG.3	Pkd1l3	ENSMUST00000057344;ENSMUST00000109242;ENSMUST00000212
MSTRG.3	Ist1	ENSMUST00000034164;ENSMUST00000212562;ENSMUST00000212
MSTRG.3	Zfp821	ENSMUST00000212964;ENSMUST00000034163;ENSMUST00000212
MSTRG.3	Zfp821	MSTRG.31303.1
MSTRG.3	Atxn1l	ENSMUST00000093162;MSTRG.31304.3;MSTRG.31304.4;ENSMUS
MSTRG.3	Ap1g1	MSTRG.31305.1;MSTRG.31305.2;ENSMUST00000093157;ENSMUS
MSTRG.3	Snord71	ENSMUST00000104361
MSTRG.3	Gm25321	ENSMUST00000122558
MSTRG.3	Gm17344	ENSMUST00000173980;ENSMUST00000171173
MSTRG.3	Phlpp2	ENSMUST00000154262;ENSMUST00000179721;ENSMUST00000034
MSTRG.3	AC08754	ENSMUST00000218652
MSTRG.3	Gm16349	ENSMUST00000149990
MSTRG.3	Vac14	ENSMUST00000213003;ENSMUST00000034190;ENSMUST00000212
MSTRG.3	Vac14	MSTRG.31312.1
MSTRG.3	Mtss1l	ENSMUST00000052457;ENSMUST00000144041;ENSMUST00000141
MSTRG.3	Il34	ENSMUST00000154803;ENSMUST00000150680;ENSMUST0000007€
MSTRG.3	Gm15894	ENSMUST00000162828
MSTRG.3	Gm15895	ENSMUST00000157018
MSTRG.3	Pdpr	ENSMUST00000135302;MSTRG.31317.1;ENSMUST00000039333;E
MSTRG.3	Pdpr	MSTRG.31318.1
MSTRG.3	9430091	ENSMUST00000127301;ENSMUST00000143719
MSTRG.3	Gstt1	ENSMUST00000001713;ENSMUST00000120177;ENSMUST0000013S
MSTRG.3	Rfwd3	MSTRG.31320.1;ENSMUST00000038739;MSTRG.31320.3;ENSMUS
MSTRG.3	Glg1	MSTRG.31321.1;MSTRG.31321.2;MSTRG.31321.3;ENSMUST000C
MSTRG.3	Gm23193	ENSMUST00000102427
MSTRG.3	Glg1	MSTRG.31323.1;MSTRG.31323.2
MSTRG.3	Glg1	MSTRG.31324.1
MSTRG.3	Glg1	MSTRG.31325.1
MSTRG.3	Sf3b3;S	ENSMUST00000042012;MSTRG.31326.2;ENSMUST00000212515;E
MSTRG.3	Cog4	ENSMUST00000165867;ENSMUST00000174165;ENSMUST00000172
MSTRG.3	Gm26816	ENSMUST00000180679
MSTRG.3	Fuk	ENSMUST00000212971;ENSMUST00000041382;ENSMUST00000212
MSTRG.3	Ddt;Gst	ENSMUST00000172820;ENSMUST00000001716;ENSMUST00000001
MSTRG.3	St3gal2	ENSMUST00000117534;ENSMUST00000034197;ENSMUST0000012S
MSTRG.3	Gm26132	ENSMUST00000083166
MSTRG.3	Ddx19a	MSTRG.31332.1;ENSMUST00000040416;MSTRG.31332.3;ENSMUS
MSTRG.3	Ddx19b	MSTRG.31333.1
MSTRG.3	Ddx19b	ENSMUST00000040241;ENSMUST00000065784
MSTRG.3	Mir3473	ENSMUST00000175346
MSTRG.3	Aars;Ex	ENSMUST00000125268;ENSMUST00000154546;ENSMUST00000034
MSTRG.3	Clec18a	ENSMUST00000190778;ENSMUST00000190222
MSTRG.3	Zfp1	MSTRG.31339.1;MSTRG.31339.2;ENSMUST00000212072;ENSMUS
MSTRG.3	Susd2	ENSMUST00000095541;ENSMUST00000218896;ENSMUST0000021S
MSTRG.3	Zfp1	MSTRG.31340.1
MSTRG.3	Bcar1	ENSMUST00000166232;ENSMUST00000212349;ENSMUST00000212
MSTRG.3	Cfdp1	ENSMUST00000034432
MSTRG.3	Cfdp1	MSTRG.31343.1
MSTRG.3	Tmem170	ENSMUST00000034431
MSTRG.3	Gabarap	ENSMUST00000034428;ENSMUST00000130808;ENSMUST00000133
MSTRG.3	Gabarap	MSTRG.31346.1
MSTRG.3	Adat1	MSTRG.31347.1;ENSMUST00000034427;MSTRG.31347.3;ENSMUS
MSTRG.31348		MSTRG.31348.1

MSTRG.3	Kars	ENSMUST00000093120;ENSMUST00000164470;ENSMUST00000034
MSTRG.3	Cabin1	MSTRG.3135.1;MSTRG.3135.2;MSTRG.3135.3;ENSMUST0000000
MSTRG.3	Terf2ip	ENSMUST00000052138;ENSMUST00000212732
MSTRG.3	Gm6793	ENSMUST00000071732
MSTRG.3	Mon1b;S	ENSMUST00000179926;MSTRG.31352.2;MSTRG.31352.1;ENSMUS
MSTRG.3	Gm45789	ENSMUST00000213038
MSTRG.3	Fa2h	ENSMUST00000159336;ENSMUST00000038475
MSTRG.3	Wdr59	ENSMUST00000038193;ENSMUST00000034437;ENSMUST00000211
MSTRG.3	Wdr59	MSTRG.31358.1
MSTRG.3	Znrf1	MSTRG.31359.1;MSTRG.31359.2
MSTRG.3	Gstt2	ENSMUST00000220440;ENSMUST00000038257;MSTRG.3136.2;EN
MSTRG.3	Znrf1	ENSMUST00000172856;ENSMUST00000168428;ENSMUST00000171
MSTRG.3	Ldhd	ENSMUST00000173909;ENSMUST00000173521;ENSMUST0000007
MSTRG.3	Maf	ENSMUST00000069009;ENSMUST00000109104
MSTRG.3	Gm15655	ENSMUST00000134649
MSTRG.31364		MSTRG.31364.1
MSTRG.3	Wwox	ENSMUST00000004756;ENSMUST00000160862;ENSMUST0000010
MSTRG.3	Wwox	MSTRG.31366.1
MSTRG.3	Gm16116	ENSMUST00000159472
MSTRG.3	Wwox	MSTRG.31368.1
MSTRG.3	Gm16117	ENSMUST00000160544
MSTRG.3	Mif	ENSMUST00000038169;ENSMUST00000218267
MSTRG.3	Gm16118	ENSMUST00000161914
MSTRG.3	Wwox	MSTRG.31371.1
MSTRG.3	Wwox	MSTRG.31372.1
MSTRG.3	Wwox	MSTRG.31373.1
MSTRG.3	Wwox	MSTRG.31374.1
MSTRG.3	Wwox	MSTRG.31376.1
MSTRG.3	Wwox	MSTRG.31377.1
MSTRG.3	Wwox	MSTRG.31378.1
MSTRG.3	Wwox	MSTRG.31379.1
MSTRG.3	Derl3	ENSMUST00000009236;ENSMUST00000217811;ENSMUST0000021
MSTRG.3	Wwox	MSTRG.31380.1
MSTRG.3	Gm22556	ENSMUST00000157803
MSTRG.3	Wwox	MSTRG.31382.1
MSTRG.3	Cdyl2	MSTRG.31383.2;MSTRG.31383.1
MSTRG.3	Cdyl2	ENSMUST00000109102
MSTRG.3	Cdyl2	MSTRG.31385.1
MSTRG.3	Cdyl2	MSTRG.31386.1
MSTRG.3	Cdyl2	MSTRG.31387.1
MSTRG.3	Atmin	MSTRG.31388.1
MSTRG.3	Gcsh	ENSMUST00000040484;ENSMUST00000162548
MSTRG.3	Smarcb1	ENSMUST00000000925;ENSMUST00000133189;ENSMUST0000014
MSTRG.3	Cmc2	ENSMUST00000155260;ENSMUST00000148235;ENSMUST00000131
MSTRG.3	Cmc2	MSTRG.31391.1
MSTRG.3	Cenpn	ENSMUST00000212578;ENSMUST00000034205;ENSMUST00000212
MSTRG.3	Atmin	ENSMUST00000109099
MSTRG.3	Gan	ENSMUST00000064488;ENSMUST00000162997
MSTRG.3	Gan	MSTRG.31395.1
MSTRG.3	Mir7078	MSTRG.31396.1
MSTRG.31397		MSTRG.31397.1
MSTRG.31398		MSTRG.31398.1
MSTRG.3	Plcg2	ENSMUST00000081232
MSTRG.3	4930556	ENSMUST00000160659
MSTRG.3	Mmp11	ENSMUST00000132869;ENSMUST00000120281;ENSMUST0000000
MSTRG.3	Gm27361	ENSMUST00000175077
MSTRG.3	Cmip;Mi	ENSMUST00000166750;MSTRG.31401.2;ENSMUST00000211913;

MSTRG.3	Cmip	MSTRG.31402.1;MSTRG.31402.2;MSTRG.31402.3;MSTRG.31402
MSTRG.3	Mir7077	ENSMUST00000183991
MSTRG.31405		MSTRG.31405.1
MSTRG.3	Mphosph	ENSMUST00000213070;ENSMUST00000034303;ENSMUST00000211
MSTRG.3	Gm10617	ENSMUST00000098368
MSTRG.31408		MSTRG.31408.1
MSTRG.3	Cdh13	ENSMUST00000117160;ENSMUST00000145849;ENSMUST00000142
MSTRG.3	Chchd10	ENSMUST00000219839;ENSMUST00000058906;ENSMUST0000021[illegible]
MSTRG.3	Gm45722	ENSMUST00000213075
MSTRG.3	Gm24035	ENSMUST00000082867
MSTRG.3	Gm45721	ENSMUST00000212267
MSTRG.3	Gm45720	ENSMUST00000211881
MSTRG.3	Gm22999	ENSMUST00000157082
MSTRG.3	Cdh13	MSTRG.31415.1
MSTRG.3	Hsbp1	ENSMUST00000034300;ENSMUST00000213037;ENSMUST00000212
MSTRG.3	Mlycd	ENSMUST00000098367;ENSMUST00000145121
MSTRG.3	Mlycd	MSTRG.31418.1
MSTRG.3	Mlycd	MSTRG.31419.1
MSTRG.3	Gm867	ENSMUST00000059658;ENSMUST00000219979
MSTRG.3	Mbtps1	ENSMUST00000212601;MSTRG.31420.1;MSTRG.31420.2;ENSMUS
MSTRG.3	Cotl1	MSTRG.31421.1
MSTRG.3	Cotl1	ENSMUST00000034285;ENSMUST00000168698;ENSMUST00000211
MSTRG.3	Atp2c2	ENSMUST00000095171
MSTRG.3	Gm45747	ENSMUST00000212203
MSTRG.3	Tldc1	ENSMUST00000049156;ENSMUST00000212501;ENSMUST00000212
MSTRG.3	Hsdl1	MSTRG.31426.3;MSTRG.31426.4;MSTRG.31426.1;MSTRG.3142[illegible]
MSTRG.3	Dnaaf1	ENSMUST00000093100;MSTRG.31427.2
MSTRG.3	Taf1c;R[illegible]	ENSMUST00000212929;MSTRG.31428.2;ENSMUST00000093099;[illegible]
MSTRG.3	Klhl36	ENSMUST00000212636;ENSMUST00000212970;ENSMUST00000034
MSTRG.3	Zfp280b	ENSMUST00000218627;ENSMUST00000061617
MSTRG.3	Klhl36	MSTRG.31430.1
MSTRG.31431		MSTRG.31431.1;MSTRG.31431.2
MSTRG.31432		MSTRG.31432.1
MSTRG.31433		MSTRG.31433.1
MSTRG.31434		MSTRG.31434.1
MSTRG.31435		MSTRG.31435.1
MSTRG.31436		MSTRG.31436.1
MSTRG.31437		MSTRG.31437.1
MSTRG.3	Gm24459	MSTRG.31438.1
MSTRG.3	Gm24459	ENSMUST00000082730
MSTRG.3	Vpreb3	ENSMUST00000121151;ENSMUST00000000926
MSTRG.3	Gm24459	MSTRG.31440.1
MSTRG.3	Cog2	ENSMUST00000034460;ENSMUST00000176159;ENSMUST0000010[illegible]
MSTRG.3	Agt	ENSMUST00000063278
MSTRG.3	Capn9	ENSMUST00000093033;ENSMUST00000133086;ENSMUST0000013[illegible]
MSTRG.3	2310022[illegible]	ENSMUST00000034464
MSTRG.3	2310022[illegible]	MSTRG.31445.1
MSTRG.3	Arv1	MSTRG.31446.1
MSTRG.3	Fam89a	ENSMUST00000055257
MSTRG.3	Ttc13	ENSMUST00000214828;MSTRG.31448.2;ENSMUST00000117624;[illegible]
MSTRG.3	Ttc13	MSTRG.31449.1;MSTRG.31449.2;MSTRG.31449.3
MSTRG.3	Gm16221	ENSMUST00000117331
MSTRG.3	Ttc13	MSTRG.31450.1
MSTRG.3	Ttc13	MSTRG.31451.1
MSTRG.3	Arv1	ENSMUST00000034463;ENSMUST00000212036;ENSMUST00000212
MSTRG.3	Arv1	MSTRG.31453.1
MSTRG.3	Arv1	MSTRG.31454.1

MSTRG.3	Arv1	MSTRG.31455.1
MSTRG.3	Trim67;(	ENSMUST00000167588;ENSMUST00000161868;ENSMUST00000034
MSTRG.3	2810004	ENSMUST00000211971;ENSMUST00000034465;ENSMUST00000212
MSTRG.3	Exoc8	ENSMUST00000098312
MSTRG.3	Sprtn	ENSMUST00000034467;MSTRG.31459.2;ENSMUST00000212724
MSTRG.3	Gm5134;I	ENSMUST00000099577;ENSMUST00000134234;ENSMUST00000184
MSTRG.3	Gm45709	ENSMUST00000213052
MSTRG.3	Egln1	ENSMUST00000034469
MSTRG.3	Egln1	MSTRG.31462.1
MSTRG.3	Egln1	MSTRG.31464.1
MSTRG.31465		MSTRG.31465.1
MSTRG.3	Tsnax	ENSMUST00000075896;ENSMUST00000212603;MSTRG.31466.2
MSTRG.3	Tsnax	MSTRG.31467.1
MSTRG.3	Gm45874	ENSMUST00000212423
MSTRG.3	Tsnax	MSTRG.31469.1
MSTRG.3	Gm5134	MSTRG.3147.1
MSTRG.31470		MSTRG.31470.1
MSTRG.31471		MSTRG.31471.1
MSTRG.3	Sipa1l2	ENSMUST00000108775;ENSMUST00000212168;ENSMUST00000212
MSTRG.3	Ntpcr	ENSMUST00000146055;ENSMUST00000143504;ENSMUST00000151
MSTRG.3	Ntpcr	MSTRG.31474.1
MSTRG.3	Ntpcr	MSTRG.31475.1
MSTRG.3	Pcnx2	ENSMUST00000047239;ENSMUST00000119305;ENSMUST0000018(
MSTRG.31478		MSTRG.31478.1
MSTRG.3	Coa6	ENSMUST00000059093;ENSMUST00000211868
MSTRG.3	Gm16219	ENSMUST00000120096
MSTRG.3	Tarbp1	ENSMUST00000170518
MSTRG.3	Tarbp1	MSTRG.31481.1
MSTRG.31482		MSTRG.31482.1
MSTRG.3	Gm26759	ENSMUST00000181773
MSTRG.3	Gm26759	MSTRG.31484.1
MSTRG.3	Irf2bp2	ENSMUST00000054960
MSTRG.3	Gm31718	ENSMUST00000212406;ENSMUST00000212639
MSTRG.3	A630001(	ENSMUST00000212914
MSTRG.3	Gm31718	MSTRG.31488.1
MSTRG.3	Tomm20;(	ENSMUST00000179857;MSTRG.31489.2;MSTRG.31489.3;ENSMUS
MSTRG.3	Gm16220	ENSMUST00000131874
MSTRG.3	Rbm34	ENSMUST00000045994;ENSMUST00000212618;ENSMUST00000212
MSTRG.3	Rbm34	MSTRG.31491.1
MSTRG.3	Nrp1;Mi	ENSMUST00000212833;ENSMUST00000212746;ENSMUST00000122
MSTRG.3	Nrp1	MSTRG.31493.1
MSTRG.3	Nrp1	MSTRG.31494.1
MSTRG.31495		MSTRG.31495.1
MSTRG.3	Pard3	ENSMUST00000162309;ENSMUST00000079777;ENSMUST0000016C
MSTRG.3	Pard3	MSTRG.31497.1
MSTRG.3	Gm6921	ENSMUST00000161478
MSTRG.3	Gm22968	ENSMUST00000157955
MSTRG.3	4930470	ENSMUST00000192005
MSTRG.3	Prmt2;M	ENSMUST00000099571;ENSMUST00000020452;ENSMUST00000128
MSTRG.3	2610044(	ENSMUST00000190157;ENSMUST00000191043;ENSMUST00000188
MSTRG.3	2610044(	MSTRG.31501.1
MSTRG.31502		MSTRG.31502.1
MSTRG.3	Itgb1	ENSMUST00000090006;ENSMUST00000124826;ENSMUST00000135
MSTRG.3	Ccdc7a	ENSMUST00000217343;ENSMUST00000216104;ENSMUST00000213
MSTRG.3	Ccdc7a	MSTRG.31505.1
MSTRG.3	Gm22545	ENSMUST00000104350
MSTRG.3	Gm32856	MSTRG.31508.1;ENSMUST00000212180;ENSMUST00000212145

MSTRG.3	Gm32856	MSTRG.31509.1
MSTRG.3	Prmt2	MSTRG.3151.1
MSTRG.31510		MSTRG.31510.1
MSTRG.3	Gm26870	ENSMUST00000181242;MSTRG.31511.1;ENSMUST00000181572;E
MSTRG.3	Gm10722	ENSMUST00000151376
MSTRG.3	Gm11168	ENSMUST00000178348;ENSMUST00000179881
MSTRG.3	Gm10718	MSTRG.31514.1;ENSMUST00000099046
MSTRG.3	Gm10721	ENSMUST00000143083
MSTRG.3	Gm10720	ENSMUST00000099050
MSTRG.3	Gm10719	ENSMUST00000099049;ENSMUST00000179272;ENSMUST0000009
MSTRG.3	Gm10717	ENSMUST00000075573;ENSMUST00000179982;ENSMUST00000177
MSTRG.3	Gm17535	ENSMUST00000178641
MSTRG.3	S100b	ENSMUST00000036387
MSTRG.3	Alkbh8	ENSMUST00000212294;MSTRG.31521.2;ENSMUST00000211884;M
MSTRG.3	Gm24918	ENSMUST00000083022
MSTRG.3	Alkbh8	MSTRG.31523.1
MSTRG.3	Cwf19l2	ENSMUST00000027027;ENSMUST00000212644;MSTRG.31524.2;E
MSTRG.3	Cwf19l2	MSTRG.31525.1
MSTRG.3	Cwf19l2	MSTRG.31526.1
MSTRG.3	Gm5362	ENSMUST00000211968
MSTRG.3	Gucy1a2	ENSMUST00000115733;ENSMUST00000213060;ENSMUST00000212
MSTRG.3	Msantd4	ENSMUST00000212075;ENSMUST00000213011;ENSMUST00000047
MSTRG.3	Dip2a	ENSMUST00000162524;ENSMUST00000160048;ENSMUST0000015
MSTRG.31530		MSTRG.31530.1
MSTRG.3	Aasdhpp	ENSMUST00000051589;MSTRG.31531.1;ENSMUST00000212066;E
MSTRG.3	Kbtbd3	ENSMUST00000049648;ENSMUST00000212221
MSTRG.3	Kbtbd3	MSTRG.31533.1
MSTRG.3	Gm7390	ENSMUST00000212452
MSTRG.3	Gria4;G	ENSMUST00000027020;ENSMUST00000175155;ENSMUST00000163
MSTRG.3	Gria4	MSTRG.31536.1
MSTRG.3	Casp1	ENSMUST00000027015;MSTRG.31537.1
MSTRG.3	Casp4	ENSMUST00000162846;ENSMUST00000027012;ENSMUST0000016C
MSTRG.3	Casp12	ENSMUST00000027009;ENSMUST00000151788;ENSMUST0000014
MSTRG.3	Gm16240	ENSMUST00000161924
MSTRG.3	Casp12	MSTRG.31540.1
MSTRG.3	Pdgfd	ENSMUST00000058692;ENSMUST00000168039;ENSMUST00000214
MSTRG.3	Pdgfd	MSTRG.31542.1
MSTRG.3	Ddi1	ENSMUST00000051706
MSTRG.31544		MSTRG.31544.1
MSTRG.31545		MSTRG.31545.1
MSTRG.31546		MSTRG.31546.1
MSTRG.3	Dync2h1	ENSMUST00000048417;ENSMUST00000140466;ENSMUST00000147
MSTRG.3	Dync2h1	MSTRG.31548.1
MSTRG.3	Dcun1d5	ENSMUST00000215683;ENSMUST00000034499;MSTRG.31549.2;M
MSTRG.3	Pcnt	ENSMUST00000220395;ENSMUST00000217838;ENSMUST0000021
MSTRG.3	Tmem123	ENSMUST00000052865;ENSMUST00000136904;MSTRG.31550.5;M
MSTRG.31551		MSTRG.31551.1
MSTRG.3	Birc2	ENSMUST00000190341;ENSMUST00000074246;MSTRG.31552.2;M
MSTRG.3	Birc3	MSTRG.31553.1;ENSMUST00000013949;ENSMUST00000161642;E
MSTRG.31554		MSTRG.31554.1
MSTRG.3	Yap1	ENSMUST00000086580;ENSMUST00000065353;ENSMUST00000174
MSTRG.3	Gm20416	ENSMUST00000174760
MSTRG.3	Gm45494	ENSMUST00000211167
MSTRG.31558		MSTRG.31558.1
MSTRG.3	1700094	ENSMUST00000190491;ENSMUST00000187079
MSTRG.3	AC15590	ENSMUST00000215099
MSTRG.3	AC15590	MSTRG.31561.1

MSTRG.3	1700128	ENSMUST00000034483
MSTRG.3	Trpc6	ENSMUST00000050433;ENSMUST00000217462
MSTRG.3	Trpc6	MSTRG.31564.1
MSTRG.3	AC15679	ENSMUST00000215328
MSTRG.3	Pgr	ENSMUST00000189181;ENSMUST00000151080;ENSMUST00000098
MSTRG.3	Gm16485	ENSMUST00000104915
MSTRG.3	Arhgap4	MSTRG.31568.1;ENSMUST00000093893;ENSMUST00000182617;E
MSTRG.3	Arhgap4	MSTRG.31569.1
MSTRG.3	2610028	ENSMUST00000092406;ENSMUST00000105415
MSTRG.3	Arhgap4	MSTRG.31570.1
MSTRG.3	Arhgap4	MSTRG.31571.1
MSTRG.3	Gm16833	ENSMUST00000189204
MSTRG.3	AC15499	MSTRG.31575.1
MSTRG.3	Cntn5	ENSMUST00000160216
MSTRG.3	Mir6237	ENSMUST00000183808
MSTRG.3	Ybey	ENSMUST00000049185;ENSMUST00000218564
MSTRG.3	Cntn5	MSTRG.31580.1
MSTRG.3	AC10235	ENSMUST00000213753
MSTRG.3	Jrkl	ENSMUST00000110582
MSTRG.3	Mtmr2	ENSMUST00000034396;ENSMUST00000155679;ENSMUST00000136
MSTRG.3	Mtmr2	MSTRG.31588.1
MSTRG.3	Mtmr2	MSTRG.31589.1
MSTRG.3	Mcm3ap	MSTRG.3159.1;ENSMUST00000170795;ENSMUST00000218881;EN
MSTRG.3	Mtmr2	MSTRG.31590.1
MSTRG.3	Ccdc82;	ENSMUST00000169961;ENSMUST00000217444;ENSMUST0000011C
MSTRG.3	Gm26189	ENSMUST00000179879
MSTRG.3	Maml2	MSTRG.31593.1
MSTRG.3	Maml2	MSTRG.31594.1
MSTRG.3	Maml2	MSTRG.31595.1
MSTRG.3	Maml2	MSTRG.31596.1
MSTRG.3	Gm17571	MSTRG.31597.1
MSTRG.3	Maml2	MSTRG.31598.1
MSTRG.3	Maml2	MSTRG.31599.1
MSTRG.3	Gm19863	ENSMUST00000192480
MSTRG.3	Lss	ENSMUST00000159013;ENSMUST00000162475;ENSMUST00000048
MSTRG.3	Maml2	MSTRG.31600.1
MSTRG.3	Maml2	MSTRG.31601.1;MSTRG.31601.2
MSTRG.3	Maml2	MSTRG.31602.1
MSTRG.3	Maml2	MSTRG.31603.1
MSTRG.3	Cep57	ENSMUST00000142494;ENSMUST00000034398;ENSMUST00000148
MSTRG.3	Fam76b	ENSMUST00000059579;ENSMUST00000156680;ENSMUST00000129
MSTRG.3	Fam76b	MSTRG.31606.1
MSTRG.3	Sesn3	ENSMUST00000209187;ENSMUST00000034507;ENSMUST00000209
MSTRG.3	Sesn3	MSTRG.31608.1
MSTRG.3	Endod1	ENSMUST00000167549;ENSMUST00000213403;ENSMUST00000214
MSTRG.3	4930483	ENSMUST00000127576
MSTRG.3	Kdm4d	ENSMUST00000058796;ENSMUST00000115647
MSTRG.3	Cwc15	ENSMUST00000004200;ENSMUST00000213913;MSTRG.31611.2;E
MSTRG.3	Amotl1	ENSMUST00000013220;ENSMUST00000160770;ENSMUST00000223
MSTRG.3	CT03024	ENSMUST00000216646
MSTRG.3	CT03024	ENSMUST00000214010
MSTRG.3	CT03024	ENSMUST00000214920
MSTRG.3	Gpr83	ENSMUST00000034408;ENSMUST00000115624;ENSMUST00000133
MSTRG.3	Ankrd49	ENSMUST00000216372;ENSMUST00000214456;ENSMUST00000034
MSTRG.3	Mre11a	ENSMUST00000034405;ENSMUST00000115632;ENSMUST00000147
MSTRG.3	CT03024	ENSMUST00000216810
MSTRG.3	Col6a2	ENSMUST00000001181;ENSMUST00000105413;ENSMUST00000137

MSTRG.3	Mre11a	MSTRG.31620.1
MSTRG.3	Gm16379	ENSMUST00000161469
MSTRG.3	Gm16302	ENSMUST00000159268
MSTRG.3	Izumo1r	ENSMUST00000069408;ENSMUST00000117620;ENSMUST0000003
MSTRG.3	Izumo1r	MSTRG.31624.1
MSTRG.3	AC13674	ENSMUST00000216115
MSTRG.31626		MSTRG.31626.1
MSTRG.31627		MSTRG.31627.1
MSTRG.3	Panx1	MSTRG.31628.1;ENSMUST00000164273;ENSMUST00000056755;E
MSTRG.3	Panx1	MSTRG.31629.1
MSTRG.3	Col6a1	ENSMUST00000001147;ENSMUST00000137599
MSTRG.3	Panx1	MSTRG.31630.1
MSTRG.3	Vstm5	ENSMUST00000034413
MSTRG.3	Med17	ENSMUST00000034411;ENSMUST00000213788;ENSMUST0000021
MSTRG.3	Med17	MSTRG.31633.1
MSTRG.3	Smco4	ENSMUST00000215907;ENSMUST00000045620;ENSMUST0000017
MSTRG.3	Deup1	ENSMUST00000115592;ENSMUST00000115593;ENSMUST0000004
MSTRG.3	Deup1	MSTRG.31636.1
MSTRG.3	AC15524	ENSMUST00000216952
MSTRG.3	Slc36a4	ENSMUST00000115588;ENSMUST00000061568;ENSMUST0000021
MSTRG.3	Slc36a4	MSTRG.31639.1
MSTRG.3	Pcbp3	ENSMUST00000001148;ENSMUST00000168505;ENSMUST0000016
MSTRG.3	Slc36a4	MSTRG.31640.1
MSTRG.3	4931406	ENSMUST00000180339;ENSMUST00000034414;ENSMUST0000017
MSTRG.3	Taf1d;G	ENSMUST00000034415;ENSMUST00000214054;ENSMUST0000021
MSTRG.3	Taf1d	MSTRG.31643.1;MSTRG.31643.2
MSTRG.3	Cep295	ENSMUST00000160946;ENSMUST00000058041;ENSMUST0000005
MSTRG.3	Scarna9	ENSMUST00000104318
MSTRG.3	Gm24339	ENSMUST00000104206
MSTRG.3	Gm16568	ENSMUST00000066038
MSTRG.3	Pcbp3	MSTRG.3165.1
MSTRG.3	AC15469	ENSMUST00000213534
MSTRG.3	Chordc1	ENSMUST00000001825;ENSMUST00000216526;ENSMUST0000021
MSTRG.3	AC15469	ENSMUST00000217524
MSTRG.3	Naalad2	ENSMUST00000172171;ENSMUST00000166825;ENSMUST0000016
MSTRG.31655		MSTRG.31655.1
MSTRG.3	Zfp317	ENSMUST00000213725;MSTRG.31656.2;ENSMUST00000208694;E
MSTRG.3	Gm45699	MSTRG.31659.1
MSTRG.3	Pcbp3	MSTRG.3166.1
MSTRG.31660		MSTRG.31660.1
MSTRG.3	Zfp560	ENSMUST00000068079;ENSMUST00000214965;ENSMUST0000014
MSTRG.3	Zfp560	MSTRG.31662.1
MSTRG.3	AC16609	ENSMUST00000213976
MSTRG.3	Zfp26	ENSMUST00000159569;MSTRG.31664.1;ENSMUST00000098970;E
MSTRG.3	Gm26733	ENSMUST00000180846
MSTRG.3	AC17120	ENSMUST00000215252
MSTRG.3	Zfp426	ENSMUST00000168095;ENSMUST00000115562;ENSMUST0000016
MSTRG.3	Gm23316	ENSMUST00000157997
MSTRG.3	Gm26733	MSTRG.31669.1
MSTRG.3	Gm10941	ENSMUST00000105408
MSTRG.3	Zfp266	MSTRG.31670.1;ENSMUST00000174462;ENSMUST00000068296;E
MSTRG.3	AC17120	ENSMUST00000213332
MSTRG.31672		MSTRG.31672.1
MSTRG.3	Pin1	ENSMUST00000034689;MSTRG.31673.1
MSTRG.3	Olfm2	MSTRG.31674.1
MSTRG.3	Olfm2	ENSMUST00000034692;ENSMUST00000215039
MSTRG.3	Zfp846	ENSMUST00000214194;ENSMUST00000115557;ENSMUST0000014

MSTRG.3	Zfp846	MSTRG.31677.1
MSTRG.3	AC16456	ENSMUST00000217520
MSTRG.3	Fbxl12o	ENSMUST00000215582;ENSMUST00000136376;ENSMUST0000017C
MSTRG.3	Pofut2	ENSMUST00000020493;ENSMUST00000217993;ENSMUST0000021{
MSTRG.3	Gm26521	ENSMUST00000181576
MSTRG.3	Fbxl12	ENSMUST00000155301;MSTRG.31681.1;ENSMUST00000155280;E
MSTRG.3	Fbxl12	MSTRG.31682.1
MSTRG.3	Ubl5	MSTRG.31683.1;ENSMUST00000154429;ENSMUST00000129414;E
MSTRG.3	A230050	ENSMUST00000043911;ENSMUST00000175820;ENSMUST0000017€
MSTRG.3	Angptl6	ENSMUST00000043726
MSTRG.31686		MSTRG.31686.1
MSTRG.3	Ppan;Gm	ENSMUST00000004203;ENSMUST00000135608;ENSMUST0000014£
MSTRG.3	Eif3g	ENSMUST00000004206;ENSMUST00000213454;ENSMUST0000021З
MSTRG.3	Dnmt1	ENSMUST00000178110;ENSMUST00000004202;ENSMUST0000017
MSTRG.3	Slc19a1	ENSMUST00000136150;ENSMUST00000133059;ENSMUST00000144
MSTRG.3	Mir7081	ENSMUST00000184490
MSTRG.3	Dnmt1	MSTRG.31691.1
MSTRG.3	Mrpl4	ENSMUST00000003386;ENSMUST00000216824;ENSMUST0000021€
MSTRG.3	Icam1	ENSMUST00000215003;ENSMUST00000086399;MSTRG.31693.3;M
MSTRG.3	AC15931	ENSMUST00000216917;ENSMUST00000122714
MSTRG.3	Icam4	ENSMUST00000001040;ENSMUST00000215077
MSTRG.3	Icam5	ENSMUST00000019616
MSTRG.3	Gm26592	ENSMUST00000180870
MSTRG.3	Zglp1;A	ENSMUST00000115494;ENSMUST00000216967;ENSMUST0000021З
MSTRG.3	1700084	ENSMUST00000135674
MSTRG.3	2010300	MSTRG.317.1
MSTRG.3	Col18a1	ENSMUST00000081654;ENSMUST00000105409;ENSMUST00000072
MSTRG.3	1700084	MSTRG.31700.1
MSTRG.3	Tyk2;Cd	ENSMUST00000213717;ENSMUST00000001036;ENSMUST0000021€
MSTRG.3	Cdc37	MSTRG.31702.1
MSTRG.3	Gm16754	ENSMUST00000193875;ENSMUST00000181222;ENSMUST00000193
MSTRG.3	Keap1	ENSMUST00000194542;ENSMUST00000049567;ENSMUST00000164
MSTRG.3	S1pr5	ENSMUST00000122088
MSTRG.3	AC16363	ENSMUST00000215491
MSTRG.3	Atg4d	ENSMUST00000065005;ENSMUST00000216826;ENSMUST0000021£
MSTRG.3	Kri1	ENSMUST00000038671;ENSMUST00000184326;ENSMUST00000184
MSTRG.3	Cdkn2d	ENSMUST00000215619;ENSMUST00000086374;ENSMUST0000021З
MSTRG.3	AC15933	ENSMUST00000218871
MSTRG.3	Ap1m2	ENSMUST00000213762;ENSMUST00000115433;ENSMUST0000000З
MSTRG.3	Smarca4	MSTRG.31711.1
MSTRG.3	Gm26511	MSTRG.31712.1
MSTRG.3	Gm26511	ENSMUST00000181400
MSTRG.3	Gm26511	MSTRG.31714.1
MSTRG.3	Ldlr	ENSMUST00000034713;ENSMUST00000215917;ENSMUST0000021З
MSTRG.3	Spc24	ENSMUST00000098942;ENSMUST00000217382;ENSMUST0000021З
MSTRG.3	AC16699	ENSMUST00000216356
MSTRG.3	AC16699	MSTRG.31718.1
MSTRG.3	AC16699	MSTRG.31719.1
MSTRG.3	Fam207a	ENSMUST00000045454;MSTRG.3172.1;ENSMUST00000218414;EN
MSTRG.3	Rab3d	ENSMUST00000115351;ENSMUST00000122211;ENSMUST0000012{
MSTRG.3	Kank2	ENSMUST00000034717;ENSMUST00000213691
MSTRG.3	Dock6	ENSMUST00000217336;ENSMUST00000215729;ENSMUST00000034
MSTRG.3	Mir7083	ENSMUST00000184374
MSTRG.3	Angptl8	ENSMUST00000058777
MSTRG.3	Swsap1	ENSMUST00000053583
MSTRG.3	Epor	ENSMUST00000006397;ENSMUST00000213181
MSTRG.3	Rgl3	ENSMUST00000214026;ENSMUST00000214089;ENSMUST0000004£

MSTRG.3	Ccdc151	ENSMUST00000044926;ENSMUST00000214420
MSTRG.3	Prkcsh	ENSMUST00000216344;ENSMUST00000003493;ENSMUST00000115
MSTRG.3	2810425I	ENSMUST00000180790
MSTRG.3	AC12252	ENSMUST00000215184
MSTRG.3	Slc44a2	ENSMUST00000217461;ENSMUST00000215528;ENSMUST00000215
MSTRG.3	AC12252	ENSMUST00000217490;MSTRG.31733.2
MSTRG.3	Ilf3	ENSMUST00000067646;ENSMUST00000213518;ENSMUST00000214
MSTRG.3	Gm16853	MSTRG.31735.1;ENSMUST00000181812
MSTRG.3	Qtrt1	ENSMUST00000002902;ENSMUST00000213967;ENSMUST00000214
MSTRG.3	Dnm2	ENSMUST00000165766;ENSMUST00000173397;ENSMUST00000072
MSTRG.3	Dnm2	MSTRG.31738.1;MSTRG.31738.2
MSTRG.3	Dnm2	MSTRG.31739.1
MSTRG.3	Itgb2	ENSMUST00000000299;ENSMUST00000131023;MSTRG.3174.2;EN
MSTRG.3	Dnm2	MSTRG.31740.1
MSTRG.3	Mir199a	ENSMUST00000083613
MSTRG.3	Tmed1	ENSMUST00000213316;ENSMUST00000034698;MSTRG.31742.2;E
MSTRG.31743		MSTRG.31743.1
MSTRG.3	AB12461	ENSMUST00000086361;ENSMUST00000173769;ENSMUST00000076
MSTRG.3	Gm20519	ENSMUST00000173684
MSTRG.3	AB12461	MSTRG.31746.1
MSTRG.3	Carm1;T	ENSMUST00000034703;ENSMUST00000115395;ENSMUST00000115
MSTRG.3	Carm1	MSTRG.31748.1
MSTRG.3	Carm1	MSTRG.31749.1
MSTRG.3	Itgb2	MSTRG.3175.1
MSTRG.3	Carm1	MSTRG.31750.1
MSTRG.3	Yipf2	ENSMUST00000078572;ENSMUST00000034700;ENSMUST00000180
MSTRG.3	AC16137	ENSMUST00000217049
MSTRG.3	Mir1946	ENSMUST00000157853
MSTRG.31754		MSTRG.31754.1
MSTRG.3	Elavl3;	ENSMUST00000003501;ENSMUST00000215901;ENSMUST00000216
MSTRG.3	Gm16845	ENSMUST00000216270;ENSMUST00000180419
MSTRG.3	Ecsit	MSTRG.31757.1;ENSMUST00000179422;ENSMUST00000098937;E
MSTRG.3	Cnn1	ENSMUST00000214601;ENSMUST00000001384;ENSMUST00000216
MSTRG.3	Elof1;M	ENSMUST00000166335;ENSMUST00000013966;ENSMUST00000216
MSTRG.3	Pttg1ip	ENSMUST00000009435;ENSMUST00000218797;ENSMUST00000159
MSTRG.3	AC16362	ENSMUST00000215411;ENSMUST00000214165
MSTRG.3	Acp5	ENSMUST00000069330;ENSMUST00000165735;ENSMUST00000115
MSTRG.3	Pigyl	ENSMUST00000123680;ENSMUST00000148088
MSTRG.3	Rpl15-p	ENSMUST00000088480
MSTRG.3	Gm10182	ENSMUST00000178676
MSTRG.3	Zfp809	ENSMUST00000215902;ENSMUST00000213350;ENSMUST00000213
MSTRG.3	AC15930	ENSMUST00000213592
MSTRG.31767		MSTRG.31767.1
MSTRG.3	Zfp599	ENSMUST00000086281
MSTRG.3	AC15930	ENSMUST00000216780
MSTRG.3	Sumo3;U	ENSMUST00000020501;ENSMUST00000174113;ENSMUST00000137
MSTRG.31770		MSTRG.31770.1
MSTRG.3	Zfp810	ENSMUST00000086278;ENSMUST00000215202;ENSMUST00000214
MSTRG.3	Zfp810	MSTRG.31772.1
MSTRG.3	AC15930	ENSMUST00000216615
MSTRG.3	AC15930	ENSMUST00000216708
MSTRG.3	Zfp810	MSTRG.31775.1
MSTRG.3	Anln	ENSMUST00000040912;ENSMUST00000215006;ENSMUST00000217
MSTRG.3	Anln	MSTRG.31777.1
MSTRG.3	AC09392	ENSMUST00000214468
MSTRG.3	AC09392	ENSMUST00000213730
MSTRG.3	Trpm2;G	ENSMUST00000105401;ENSMUST00000105400;ENSMUST00000140

MSTRG.3	Rp9	ENSMUST00000215715;MSTRG.31780.2;ENSMUST00000034763;E
MSTRG.3	9530077	ENSMUST00000058868;ENSMUST00000213598;ENSMUST00000214
MSTRG.3	Rp9	MSTRG.31782.1
MSTRG.3	Gm17545	ENSMUST00000168332
MSTRG.3	Bbs9	ENSMUST00000039798;ENSMUST00000150395;ENSMUST00000142
MSTRG.3	Gm2976	ENSMUST00000160362
MSTRG.3	Gm16165	ENSMUST00000160140
MSTRG.3	Gm16164	ENSMUST00000160078
MSTRG.3	Bbs9	MSTRG.31788.1
MSTRG.3	Bbs9	MSTRG.31789.1
MSTRG.3	Trpm2	MSTRG.3179.1
MSTRG.3	Gm27639	ENSMUST00000184937
MSTRG.3	Gm10701	ENSMUST00000098926
MSTRG.3	Dpy19l1	ENSMUST00000115277;ENSMUST00000142064;ENSMUST0000017C
MSTRG.3	Dpy19l1	MSTRG.31794.1
MSTRG.3	Dpy19l1	MSTRG.31795.1
MSTRG.3	Herpud2	MSTRG.31796.1;ENSMUST00000008573;MSTRG.31796.3;ENSMUS
MSTRG.3	Gm29642	ENSMUST00000186170
MSTRG.3	Eepd1	ENSMUST00000040677;ENSMUST00000156237
MSTRG.3	Gm25861	ENSMUST00000158251
MSTRG.3	2010300	MSTRG.318.1
MSTRG.3	Trpm2	MSTRG.3180.1
MSTRG.3	Gm25346	ENSMUST00000104509
MSTRG.31801		MSTRG.31801.1
MSTRG.3	Sept7	ENSMUST00000115272;ENSMUST00000165594;ENSMUST00000215
MSTRG.3	CT02556	ENSMUST00000214972
MSTRG.3	Sept7	MSTRG.31804.1
MSTRG.3	AC14143	ENSMUST00000216055
MSTRG.31806		MSTRG.31806.1
MSTRG.3	Igsf9b	ENSMUST00000214357;ENSMUST00000115247;ENSMUST00000133
MSTRG.3	Acad8	ENSMUST00000129490;ENSMUST00000120367;ENSMUST0000006C
MSTRG.3	Acad8	MSTRG.31809.1
MSTRG.3	1810043	ENSMUST00000105397;MSTRG.3181.1;ENSMUST00000149074;EN
MSTRG.3	Acad8	MSTRG.31810.1
MSTRG.3	Acad8	MSTRG.31811.1
MSTRG.3	Thyn1	ENSMUST00000214910;ENSMUST00000213770;ENSMUST00000213
MSTRG.3	Vps26b	ENSMUST00000034470;ENSMUST00000213917
MSTRG.3	AC15616	ENSMUST00000215598
MSTRG.3	Ncapd3	ENSMUST00000073127;ENSMUST00000086198;ENSMUST00000217
MSTRG.3	Jam3	ENSMUST00000034472;ENSMUST00000215446;ENSMUST00000213
MSTRG.3	CT02566	ENSMUST00000217654;MSTRG.31817.1
MSTRG.3	Snx19	MSTRG.31819.1;ENSMUST00000164099;ENSMUST00000216552;M
MSTRG.3	Pfkl	ENSMUST00000020522;ENSMUST00000149271;ENSMUST0000022C
MSTRG.3	Adamts8	ENSMUST00000068135;ENSMUST00000163037
MSTRG.3	CT02565	ENSMUST00000214688;ENSMUST00000216803
MSTRG.3	Zbtb44	ENSMUST00000216649;ENSMUST00000115222;ENSMUST00000217
MSTRG.3	Zbtb44	MSTRG.31823.1
MSTRG.3	Gm17508	ENSMUST00000181215
MSTRG.3	St14	ENSMUST00000034478;ENSMUST00000148789;ENSMUST00000217
MSTRG.3	Gm16071	ENSMUST00000119589
MSTRG.3	Aplp2	ENSMUST00000217641;ENSMUST00000072634;ENSMUST0000007S
MSTRG.3	Aplp2	MSTRG.31828.1
MSTRG.3	CT02567	ENSMUST00000214795
MSTRG.3	Pfkl	MSTRG.3183.1
MSTRG.3	Prdm10	ENSMUST00000117389;MSTRG.31830.1;ENSMUST00000215499;E
MSTRG.3	Prdm10	MSTRG.31831.1
MSTRG.3	Prdm10	MSTRG.31832.1

MSTRG.3	Tmem45b	ENSMUST00000048050;ENSMUST00000217007;ENSMUST00000213
MSTRG.3	AC16724	ENSMUST00000213951
MSTRG.3	AC16724	ENSMUST00000213948
MSTRG.3	Nfrkb	ENSMUST00000086167;ENSMUST00000132329;ENSMUST00000131
MSTRG.3	Gm3756	ENSMUST00000169182
MSTRG.3	AC16063	ENSMUST00000216731
MSTRG.3	Aire	ENSMUST00000128241;ENSMUST00000130972;ENSMUST00000145
MSTRG.31840		MSTRG.31840.1
MSTRG.31841		MSTRG.31841.1
MSTRG.3	Gm44453	ENSMUST00000175161
MSTRG.3	Gm44453	MSTRG.31843.1
MSTRG.3	AC13460	ENSMUST00000214707
MSTRG.3	Arhgap3	ENSMUST00000174641;ENSMUST00000182310;ENSMUST00000174
MSTRG.3	AC13460	ENSMUST00000213983
MSTRG.3	Fli1;Gm	ENSMUST00000016231;MSTRG.31847.2;MSTRG.31847.3;ENSMUS
MSTRG.3	Gm38292	ENSMUST00000192646
MSTRG.3	Fli1	MSTRG.31849.1
MSTRG.3	Dnmt3l	ENSMUST00000135316;ENSMUST00000138785;ENSMUST0000000C
MSTRG.3	Gm27201	MSTRG.31850.1;ENSMUST00000183818;MSTRG.31850.2
MSTRG.3	Gm38346	ENSMUST00000192379
MSTRG.3	Ets1	ENSMUST00000183879;MSTRG.31853.1;ENSMUST00000184110;E
MSTRG.3	Gm27240	MSTRG.31854.1;MSTRG.31854.2;MSTRG.31854.3;ENSMUST0000
MSTRG.3	CT03064	ENSMUST00000217419
MSTRG.3	CT03064	MSTRG.31856.1
MSTRG.3	Gm37758	ENSMUST00000194275
MSTRG.3	CT03064	MSTRG.31858.1
MSTRG.3	Gm10698	ENSMUST00000182663
MSTRG.3186		MSTRG.3186.1
MSTRG.3	Gm10698	MSTRG.31860.1
MSTRG.3	Kirrel3	ENSMUST00000183580;ENSMUST00000115148;ENSMUST00000187
MSTRG.3	Gm38217	ENSMUST00000194358
MSTRG.3	Kirrel3	MSTRG.31863.1
MSTRG.3	Gm26662	ENSMUST00000181129
MSTRG.3	Kirrel3	MSTRG.31865.1
MSTRG.3	Kirrel3	ENSMUST00000066604
MSTRG.3	Gm23702	ENSMUST00000158840
MSTRG.3	St3gal4	MSTRG.31868.1
MSTRG.3	AC16011	ENSMUST00000214038
MSTRG.3	AC16457	ENSMUST00000218616
MSTRG.3	AC16011	MSTRG.31870.1
MSTRG.3	Cdon	ENSMUST00000151682;ENSMUST00000137200;ENSMUST0000011
MSTRG.3	Cdon	MSTRG.31872.1
MSTRG.3	Cdon	MSTRG.31873.1
MSTRG.3	Gm25630	ENSMUST00000084000
MSTRG.3	Gm24188	ENSMUST00000157980
MSTRG.3	Usp10;G	ENSMUST00000144458;ENSMUST00000108988;MSTRG.31876.3;E
MSTRG.3	Gm20388	MSTRG.31877.1;MSTRG.31877.2
MSTRG.3	Crispld	ENSMUST00000132583;ENSMUST00000034282;ENSMUST00000108
MSTRG.3	Gm15684	ENSMUST00000160605
MSTRG.3	D10Jhu8	ENSMUST00000001242;ENSMUST00000220359;ENSMUST0000021
MSTRG.3	Zdhhc7	ENSMUST00000034280;ENSMUST00000150444;ENSMUST00000124
MSTRG.3	Gm15898	MSTRG.31881.1
MSTRG.3	A130014	ENSMUST00000183021
MSTRG.3	Gm38379	ENSMUST00000194351
MSTRG.3	6430548	ENSMUST00000212474;ENSMUST00000034281;ENSMUST00000108
MSTRG.3	Fam92b	ENSMUST00000048786
MSTRG.3	A330074	ENSMUST00000183235

MSTRG.3	Gse1;Mi	ENSMUST00000180448;MSTRG.31887.1;MSTRG.31887.3;MSTRG.
MSTRG.3	Gse1	MSTRG.31889.1
MSTRG.3	Pwp2	ENSMUST00000042556
MSTRG.3	Gm26971	MSTRG.31890.1;ENSMUST00000182423
MSTRG.3	1700016	ENSMUST00000192004
MSTRG.3	Gins2	ENSMUST00000146526;ENSMUST00000034278;ENSMUST00000155
MSTRG.3	Gm10614	ENSMUST00000181414
MSTRG.3	Gm45834	ENSMUST00000212445
MSTRG.3	Gm26739	ENSMUST00000181041
MSTRG.3	1190005	ENSMUST00000123927;ENSMUST00000181333;ENSMUST00000180
MSTRG.3	Gm26537	ENSMUST00000181474;MSTRG.31897.2
MSTRG.3	Cox4i1	ENSMUST00000034276;ENSMUST00000181586;ENSMUST00000180
MSTRG.3	Gm33142	ENSMUST00000193913
MSTRG.3	Tsga10	ENSMUST00000041815;ENSMUST00000114902;ENSMUST00000088
MSTRG.3	Trappc1	MSTRG.3190.2;MSTRG.3190.1;ENSMUST00000000384;ENSMUST0
MSTRG.3	Gm24239	ENSMUST00000157791
MSTRG.3	Gm18709	ENSMUST00000180564
MSTRG.3	Irf8	ENSMUST00000047737;ENSMUST00000162775;MSTRG.31902.3;E
MSTRG.3	Gm26878	ENSMUST00000181778
MSTRG.3	Fendrr	ENSMUST00000181530;ENSMUST00000181231;ENSMUST00000182
MSTRG.3	Foxf1	ENSMUST00000181504
MSTRG.3	Mthfsd	ENSMUST00000047282;ENSMUST00000133058;ENSMUST00000116
MSTRG.3	Foxc2	ENSMUST00000054691
MSTRG.3	AC16040	ENSMUST00000218886;ENSMUST00000218143;ENSMUST00000218
MSTRG.3	Foxl1	ENSMUST00000181609
MSTRG.3	Gm37609	ENSMUST00000191778
MSTRG.3	5033426	ENSMUST00000180796;ENSMUST00000212635
MSTRG.3	Gm26815	ENSMUST00000181200
MSTRG.3	Gm26747	ENSMUST00000180597
MSTRG.3	1700018	ENSMUST00000034265;ENSMUST00000183280;ENSMUST00000182
MSTRG.3	Gm27045	ENSMUST00000182145
MSTRG.3	1700030	ENSMUST00000181133;ENSMUST00000181679
MSTRG.3	Fbxo31	ENSMUST00000059018;ENSMUST00000180979;ENSMUST00000180
MSTRG.3	AC16040	MSTRG.3192.1
MSTRG.3	Gm17786	ENSMUST00000182000
MSTRG.3	Map1lc3	ENSMUST00000034270;ENSMUST00000181826;MSTRG.31921.2;E
MSTRG.3	Zcchc14	ENSMUST00000046386;ENSMUST00000154725;ENSMUST00000139
MSTRG.3	Gm27030	ENSMUST00000182551
MSTRG.3	Gm45833	ENSMUST00000211931
MSTRG.3	Jph3	ENSMUST00000026357;ENSMUST00000167439;ENSMUST00000172
MSTRG.3	Klhdc4	ENSMUST00000045884;ENSMUST00000174709;ENSMUST00000174
MSTRG.3	Gm20406	ENSMUST00000173993
MSTRG.3	Gm20388	MSTRG.31929.1
MSTRG.3	Gm10146	ENSMUST00000072739
MSTRG.3	Gm20388	MSTRG.31930.1
MSTRG.3	Gm24283	ENSMUST00000104233
MSTRG.3	Gm42047	MSTRG.31932.1;ENSMUST00000212158;MSTRG.31932.2;ENSMUS
MSTRG.3	Gm20388	MSTRG.31933.1
MSTRG.3	Gm20388	MSTRG.31934.1
MSTRG.3	Gm26812	ENSMUST00000181776;ENSMUST00000181647
MSTRG.3	9530085	ENSMUST00000182121
MSTRG.3	Slc7a5	ENSMUST00000045557;ENSMUST00000182165;ENSMUST00000183
MSTRG.3	BC04864	ENSMUST00000181944;ENSMUST00000140928
MSTRG.3	Car5a	ENSMUST00000057653;ENSMUST00000151462
MSTRG.3	Agpat3;	ENSMUST00000001240;ENSMUST00000105389;ENSMUST00000166
MSTRG.3	Gm37497	ENSMUST00000195830
MSTRG.3	Gm27011	ENSMUST00000182352

MSTRG.3	Banp	ENSMUST00000172628;ENSMUST00000093078;ENSMUST0000017(
MSTRG.3	Gm20388	MSTRG.31943.1
MSTRG.3	Gm17709	ENSMUST00000174536
MSTRG.3	Mir7237	ENSMUST00000184699
MSTRG.3	Gm20388	MSTRG.31947.1
MSTRG.3	Gm20388	MSTRG.31948.1
MSTRG.3	Zfp469	ENSMUST00000187142
MSTRG.3	AC16040	ENSMUST00000219008
MSTRG.3	Zfpm1	ENSMUST00000054052;MSTRG.31950.2;MSTRG.31950.3;MSTRG.
MSTRG.3	Gm20388	MSTRG.31951.1
MSTRG.3	Gm20388	MSTRG.31952.1
MSTRG.3	Gm45353	ENSMUST00000210581
MSTRG.3	Trhr2	ENSMUST00000044123
MSTRG.3	Gm20681	ENSMUST00000176061
MSTRG.3	Zc3h18	ENSMUST00000017622;ENSMUST00000093073;ENSMUST0000017(
MSTRG.3	Cyba	MSTRG.31957.1;ENSMUST00000212600;ENSMUST00000017604;E
MSTRG.3	Gm20388	MSTRG.31958.1
MSTRG.3	Mvd;Rnf	MSTRG.31959.1;ENSMUST00000014614;MSTRG.31959.2;ENSMUS
MSTRG.3	Agpat3	MSTRG.3196.1
MSTRG.3	Il17c	ENSMUST00000050963
MSTRG.3	Mvd;Gm2	ENSMUST00000211883;ENSMUST00000006692;MSTRG.31961.1;E
MSTRG.3	Snai3	ENSMUST00000006762
MSTRG.3	Gm20388	MSTRG.31963.1
MSTRG.3	Rnf166	MSTRG.31964.1
MSTRG.3	Ctu2	ENSMUST00000116412;ENSMUST00000151855;ENSMUST0000014(
MSTRG.3	Piezo1	ENSMUST00000067252;ENSMUST00000156333;ENSMUST0000012(
MSTRG.3	Gm26497	ENSMUST00000083146
MSTRG.3	Piezo1	MSTRG.31968.1
MSTRG.3	Cdt1	ENSMUST00000006760;ENSMUST00000212201;MSTRG.31969.2;E
MSTRG.3	n-R5s77	ENSMUST00000083757
MSTRG.3	Aprt	ENSMUST00000213062;MSTRG.31970.1;ENSMUST00000006764;E
MSTRG.3	Galns	ENSMUST00000015171;ENSMUST00000212319;ENSMUST00000211
MSTRG.3	Gm45857	ENSMUST00000212207
MSTRG.3	Gm20388	MSTRG.31973.1
MSTRG.3	Trappc2	ENSMUST00000212477;ENSMUST00000211904;ENSMUST0000001E
MSTRG.3	Pabpn1l	ENSMUST00000093059
MSTRG.3	Mir6396	ENSMUST00000183603
MSTRG.3	Cbfa2t3	ENSMUST00000127984;ENSMUST00000006525;ENSMUST00000064
MSTRG.3	Cbfa2t3	MSTRG.31978.1;MSTRG.31978.2;MSTRG.31978.3
MSTRG.3	Cbfa2t3	MSTRG.31979.1
MSTRG.3	AC16041	ENSMUST00000219014;ENSMUST00000219695;ENSMUST0000021E
MSTRG.3	Gm20388	MSTRG.31980.1
MSTRG.3	Gm15899	ENSMUST00000128586
MSTRG.3	Cbfa2t3	MSTRG.31982.1
MSTRG.3	Gm10612	MSTRG.31983.1;ENSMUST00000162198
MSTRG.3	Gm20388	MSTRG.31984.1;MSTRG.31984.2
MSTRG.3	C230057	ENSMUST00000212444;ENSMUST00000212334
MSTRG.3	A530010	ENSMUST00000212007;ENSMUST00000212247
MSTRG.3	Gm20388	MSTRG.31987.1
MSTRG.3	Gm39271	ENSMUST00000212881
MSTRG.3	Acsf3	ENSMUST00000212903;ENSMUST00000015160;ENSMUST00000212
MSTRG.3	Cstb	ENSMUST00000005185
MSTRG.3	Gm45743	ENSMUST00000212701
MSTRG.3	Gm45893	ENSMUST00000211899
MSTRG.3	Gm16378	ENSMUST00000179168
MSTRG.3	Cdh15	ENSMUST00000034443
MSTRG.3	Gm45894	ENSMUST00000211940

MSTRG.3	Gm45894	ENSMUST00000212728
MSTRG.3	Ankrd11	MSTRG.31996.1;MSTRG.31996.3;MSTRG.31996.4;ENSMUST0000
MSTRG.3	Ankrd11	MSTRG.31997.2;MSTRG.31997.1;MSTRG.31997.3;MSTRG.31997
MSTRG.3	Gm24445	ENSMUST00000083981
MSTRG.3	Gm20388	MSTRG.31999.1;MSTRG.31999.2
MSTRG.3	Gm9826	ENSMUST00000029124
MSTRG.3	Tsga10	MSTRG.320.1
MSTRG.3	Rrp1	ENSMUST00000219374;MSTRG.3200.2;ENSMUST00000062678;EN
MSTRG.3	Ankrd11	MSTRG.32000.2;MSTRG.32000.1
MSTRG.3	Ankrd11	MSTRG.32001.1
MSTRG.3	Ankrd11	MSTRG.32002.1
MSTRG.3	2810013	ENSMUST00000186531;MSTRG.32003.1
MSTRG.3	Gm20388	MSTRG.32004.1
MSTRG.3	Spg7	ENSMUST00000142541;ENSMUST00000125975;ENSMUST0000014
MSTRG.3	Spg7	MSTRG.32006.1
MSTRG.3	Spg7	MSTRG.32007.1
MSTRG.3	Spg7	MSTRG.32008.1
MSTRG.3	Spg7	MSTRG.32009.1
MSTRG.3	Mir6907	ENSMUST00000183764
MSTRG.3	Spg7	MSTRG.32010.1
MSTRG.3	Rpl13;S	ENSMUST00000000756;ENSMUST00000152444;ENSMUST00000132
MSTRG.3	Cpne7;M	ENSMUST00000134235;ENSMUST00000037900;ENSMUST00000131
MSTRG.3	Sult5a1	ENSMUST00000000755;ENSMUST00000122819;ENSMUST00000137
MSTRG.3	Gm4316	ENSMUST00000212411;ENSMUST00000212490
MSTRG.3	Gm20388	MSTRG.32015.1
MSTRG.3	Dpep1	ENSMUST00000019422;ENSMUST00000212773;ENSMUST00000212
MSTRG.3	Chmp1a	ENSMUST00000000759;MSTRG.32017.1;ENSMUST00000167655;E
MSTRG.3	Chmp1a	MSTRG.32018.1
MSTRG.3	Rps12-p	ENSMUST00000139165
MSTRG.3	AC16041	ENSMUST00000218544;ENSMUST00000184204
MSTRG.3	Spata33	ENSMUST00000212760;ENSMUST00000212892;ENSMUST00000212
MSTRG.3	Cdk10;G	ENSMUST00000036880;ENSMUST00000212021;ENSMUST00000212
MSTRG.3	Spata2l	ENSMUST00000211845;ENSMUST00000166768;ENSMUST0000098
MSTRG.3	Vps9d1	ENSMUST00000149490;ENSMUST00000122363;MSTRG.32023.3;E
MSTRG.3	Zfp276	ENSMUST00000001092;ENSMUST00000150356;MSTRG.32024.2;E
MSTRG.3	Fanca	ENSMUST00000035495;ENSMUST00000213090;ENSMUST00000211
MSTRG.3	Fanca	MSTRG.32026.1
MSTRG.3	Gm20388	MSTRG.32027.1
MSTRG.3	Spire2	ENSMUST00000010298;ENSMUST00000212404
MSTRG.3	Tcf25	ENSMUST00000212571;ENSMUST00000212470;ENSMUST00000108
MSTRG.3	Pdxk	ENSMUST00000041616;ENSMUST00000218245
MSTRG.3	Mc1r	ENSMUST00000098324
MSTRG.3	Tubb3	ENSMUST00000071134;ENSMUST00000212743
MSTRG.3	Def8	ENSMUST00000212883;ENSMUST00000093049;ENSMUST00000001
MSTRG.3	Gm20388	MSTRG.32034.1
MSTRG.3	Gm25428	ENSMUST00000157608
MSTRG.3	Afg3l1	ENSMUST00000001520;ENSMUST00000098320;ENSMUST00000133
MSTRG.3	Dbndd1	ENSMUST00000176286;ENSMUST00000177240;ENSMUST00000176
MSTRG.3	Gas8	ENSMUST00000093043;ENSMUST00000212923;ENSMUST00000212
MSTRG.3	Gm22314	ENSMUST00000158007
MSTRG.3	Mir6908	ENSMUST00000184056
MSTRG.3	Gm20388	MSTRG.32040.1
MSTRG.3205		MSTRG.3205.1
MSTRG.3206		MSTRG.3206.1
MSTRG.3	Ilvbl	ENSMUST00000218061;ENSMUST00000218787;ENSMUST00000105
MSTRG.3	Syde1	ENSMUST00000040580
MSTRG.3	AC15388	ENSMUST00000218641

MSTRG.3 Rhou	ENSMUST00000136615;ENSMUST00000045487
MSTRG.3 Gm45781	ENSMUST00000212860
MSTRG.3 6030466	ENSMUST00000212339
MSTRG.3 4732419	ENSMUST00000181805
MSTRG.3 Rab4a	ENSMUST00000118535;ENSMUST00000117702;MSTRG.32097.3;E
MSTRG.3 2810455	ENSMUST00000138442;MSTRG.32098.1
MSTRG.3 Ccsap	ENSMUST00000034452;ENSMUST00000122421
MSTRG.3 Gm26805	ENSMUST00000181885
MSTRG.3 Olfr135	MSTRG.3210.1
MSTRG.3 Ccsap	MSTRG.32100.1
MSTRG.3 Gm4342	ENSMUST00000118982
MSTRG.3 Acta1	ENSMUST00000034453
MSTRG.3 Gm29773	ENSMUST00000212751
MSTRG.3 Nup133	ENSMUST00000044795;ENSMUST00000213089;ENSMUST00000212
MSTRG.3 Gm45890	ENSMUST00000212043;ENSMUST00000212188;ENSMUST00000212
MSTRG.3 Abcb10	ENSMUST00000075578
MSTRG.3 Taf5l	ENSMUST00000093039;ENSMUST00000165628;ENSMUST00000212
MSTRG.3 Gm17827	ENSMUST00000211825
MSTRG.3 Taf5l	MSTRG.32109.1
MSTRG.3 Gm37514	ENSMUST00000194512
MSTRG.3 Urb2	MSTRG.32110.1;ENSMUST00000173168;ENSMUST00000034457
MSTRG.3 Mir1967	ENSMUST00000157789
MSTRG.3 Gm45732	ENSMUST00000212460
MSTRG.3 Gm20388	MSTRG.32113.1
MSTRG.3 Gm3889	ENSMUST00000212467
MSTRG.3 Gm20388	MSTRG.32115.1;MSTRG.32115.2
MSTRG.3 Galnt2	MSTRG.32116.1
MSTRG.3 Galnt2	MSTRG.32117.1
MSTRG.3 Galnt2	MSTRG.32118.1;MSTRG.32118.2
MSTRG.3 Galnt2	MSTRG.32119.1
MSTRG.3212	MSTRG.3212.1
MSTRG.3 Galnt2	MSTRG.32120.1
MSTRG.3 Galnt2	MSTRG.32121.1
MSTRG.3 1810008	MSTRG.32122.1;ENSMUST00000162325
MSTRG.3 Gm20388	MSTRG.32123.1
MSTRG.3 Gm15775	MSTRG.32125.1;ENSMUST00000159796
MSTRG.3 St3gal4	MSTRG.32126.1;ENSMUST00000034537;MSTRG.32126.3;ENSMUS
MSTRG.3 St3gal4	MSTRG.32127.1
MSTRG.3 St3gal4	MSTRG.32128.1
MSTRG.3 Olfr135	ENSMUST00000203305;ENSMUST00000205085
MSTRG.3 4930581	ENSMUST00000093873;ENSMUST00000214597;ENSMUST0000015[illegible]
MSTRG.3 Dcps	ENSMUST00000034539;ENSMUST00000119847;ENSMUST0000015[illegible]
MSTRG.3 Gm24262	ENSMUST00000157431
MSTRG.3 Tirap	ENSMUST00000175765;ENSMUST00000176531;ENSMUST00000177
MSTRG.3 AC14044	ENSMUST00000214606
MSTRG.3 Tirap	MSTRG.32135.1
MSTRG.3 Tirap	MSTRG.32136.1
MSTRG.3 Srpr	ENSMUST00000138287;MSTRG.32137.1;ENSMUST00000034541;M
MSTRG.3 Foxred1	ENSMUST00000154691;ENSMUST00000130072;ENSMUST00000143
MSTRG.3 Fam118b	ENSMUST00000063782;ENSMUST00000121564;ENSMUST0000005[illegible]
MSTRG.3 Olfr135	MSTRG.3214.1
MSTRG.3 Fam118b	MSTRG.32140.1
MSTRG.3 Fam118b	MSTRG.32141.1
MSTRG.3 Fam118b	MSTRG.32142.1
MSTRG.3 Fam118b	MSTRG.32143.1
MSTRG.3 Rpusd4	ENSMUST00000034543;ENSMUST00000130552;ENSMUST00000146
MSTRG.32145	MSTRG.32145.1

MSTRG.3 Ddx25	ENSMUST00000034612
MSTRG.3 Pus3	ENSMUST00000135768;ENSMUST00000034615;ENSMUST00000121
MSTRG.3 Hyls1	ENSMUST00000115110;MSTRG.32148.2;ENSMUST00000132547
MSTRG.3 Pate2	ENSMUST00000098906;ENSMUST00000137309;ENSMUST0000011
MSTRG.3 Olfr135	MSTRG.3215.1
MSTRG.3 Gm6762	ENSMUST00000121769
MSTRG.3 Gm10105	ENSMUST00000184601
MSTRG.3 Pate4	ENSMUST00000034610
MSTRG.3 Pate3	ENSMUST00000178236
MSTRG.3 Pate1	ENSMUST00000183567
MSTRG.3 CT02561	ENSMUST00000216487;ENSMUST00000093868;ENSMUST00000184
MSTRG.3 CT02561	MSTRG.32156.1
MSTRG.3 AC15592	ENSMUST00000216568
MSTRG.3 Chek1	ENSMUST00000172702;ENSMUST00000034625;ENSMUST00000174
MSTRG.3 Stt3a	ENSMUST00000120381;ENSMUST00000135934;ENSMUST0000021
MSTRG.3 AC15388	ENSMUST00000217691
MSTRG.3 Stt3a	MSTRG.32160.1
MSTRG.3 Stt3a	MSTRG.32161.1
MSTRG.3 Gm26787	ENSMUST00000180474
MSTRG.3 Ei24	ENSMUST00000163192;ENSMUST00000115086;ENSMUST0000018
MSTRG.3 AC13828	ENSMUST00000216704
MSTRG.3 Pknox2	ENSMUST00000177218;ENSMUST00000039674;ENSMUST0000008
MSTRG.3 Gm3896	ENSMUST00000176020
MSTRG.3 n-R5s82	ENSMUST00000104213
MSTRG.3 AC16408	ENSMUST00000215174
MSTRG.3 AC16408	ENSMUST00000216206
MSTRG.3 AC15388	MSTRG.3217.1;ENSMUST00000219154;MSTRG.3217.2
MSTRG.3 Tmem218	ENSMUST00000034632
MSTRG.3 Slc37a2	MSTRG.32172.1;ENSMUST00000115068;ENSMUST00000161114;E
MSTRG.3 Gm10177	ENSMUST00000160052;ENSMUST00000161698
MSTRG.3 AC13828	ENSMUST00000214389
MSTRG.32175	MSTRG.32175.1
MSTRG.3 AC13828	ENSMUST00000215832
MSTRG.3 Ccdc15	MSTRG.32177.1;ENSMUST00000037275;ENSMUST00000213633;E
MSTRG.3 AC13828	ENSMUST00000214258
MSTRG.3 AC13828	ENSMUST00000217440
MSTRG.3218	MSTRG.3218.1
MSTRG.3 Ccdc15	MSTRG.32180.1
MSTRG.3 AC13828	ENSMUST00000217345
MSTRG.3 Msantd2	ENSMUST00000211060;ENSMUST00000048604;ENSMUST0000020
MSTRG.3 AC10595	ENSMUST00000214783
MSTRG.3 Esam	ENSMUST00000002011;ENSMUST00000146860;ENSMUST00000144
MSTRG.3 Vsig2	ENSMUST00000002008;ENSMUST00000215957;ENSMUST0000021
MSTRG.3 Nrgn	ENSMUST00000065668;ENSMUST00000182070
MSTRG.3 Siae	ENSMUST00000213126;ENSMUST00000215829;ENSMUST0000021
MSTRG.3 Spa17	ENSMUST00000002013;ENSMUST00000214786;ENSMUST0000021
MSTRG.3 Tbrg1	ENSMUST00000117654;ENSMUST00000142736;MSTRG.32189.3;E
MSTRG.3 2610008	ENSMUST00000039271;ENSMUST00000218854;ENSMUST0000022
MSTRG.32190	MSTRG.32190.1
MSTRG.32191	MSTRG.32191.1
MSTRG.32192	MSTRG.32192.1
MSTRG.32193	MSTRG.32193.1
MSTRG.3 Vwa5a	ENSMUST00000001544;ENSMUST00000118144;MSTRG.32194.3;E
MSTRG.3 Vwa5a	MSTRG.32195.1
MSTRG.3 Olfr920	ENSMUST00000074740
MSTRG.3 Scn3b	ENSMUST00000049941;ENSMUST00000175873;ENSMUST00000171
MSTRG.3 AC13535	ENSMUST00000215330

MSTRG.3	Gramd1b	ENSMUST00000137454;ENSMUST00000121357;ENSMUST0000004[illegible]
MSTRG.3	Lipt1	ENSMUST00000128085;ENSMUST00000041621;ENSMUST0000014[illegible]
MSTRG.3	2610008[illegible]	MSTRG.3220.1
MSTRG.3	1700110[illegible]	ENSMUST00000140807;ENSMUST00000156511
MSTRG.3	AC13247	ENSMUST00000213244
MSTRG.3	AC13247	ENSMUST00000217414
MSTRG.3	AC13247	ENSMUST00000214407
MSTRG.3	Gramd1b	MSTRG.32204.1
MSTRG.3	Gramd1b	MSTRG.32205.1
MSTRG.3	Gramd1b	MSTRG.32206.1
MSTRG.3	AC13247	ENSMUST00000215697
MSTRG.3	Gm17540	ENSMUST00000181617
MSTRG.3	Hspa8;S[illegible]	ENSMUST00000015800;ENSMUST00000149936;ENSMUST0000015[illegible]
MSTRG.3	2610008[illegible]	MSTRG.3221.1
MSTRG.3	Ubash3b	ENSMUST00000044155;ENSMUST00000151485;MSTRG.32210.2;[illegible]
MSTRG.3	Ubash3b	MSTRG.32211.1
MSTRG.3	Ubash3b	MSTRG.32212.1
MSTRG.3	Ubash3b	MSTRG.32213.1
MSTRG.3	Ubash3b	MSTRG.32214.1
MSTRG.3	AC15603	ENSMUST00000217288
MSTRG.3	Ubash3b	MSTRG.32216.1
MSTRG.3	Ubash3b	MSTRG.32217.1
MSTRG.3	Ubash3b	MSTRG.32218.1
MSTRG.3	Ubash3b	MSTRG.32219.1
MSTRG.3	AC16462	MSTRG.3222.1;ENSMUST00000217947
MSTRG.3	AC15603	ENSMUST00000215869
MSTRG.3	AC15603	ENSMUST00000217261
MSTRG.32222		MSTRG.32222.1
MSTRG.3	2610203[illegible]	MSTRG.32223.1;ENSMUST00000212373;ENSMUST00000188945;[illegible]
MSTRG.3	Gm25401	ENSMUST00000082975
MSTRG.3	CT00969	ENSMUST00000215342
MSTRG.3	2610203[illegible]	MSTRG.32226.1
MSTRG.3	CT00969	ENSMUST00000216668
MSTRG.3	2610203[illegible]	MSTRG.32228.1
MSTRG.3	CT00969	MSTRG.32229.1
MSTRG.3	Mier2	ENSMUST00000062855;ENSMUST00000165028;ENSMUST0000017[illegible]
MSTRG.3	2610203[illegible]	MSTRG.32230.1
MSTRG.3	CT00969	ENSMUST00000215745
MSTRG.3	CT00969	MSTRG.32232.1
MSTRG.3	2610203[illegible]	MSTRG.32234.1
MSTRG.3	Mirlet7[illegible]	ENSMUST00000175029
MSTRG.3	CT02561	MSTRG.32236.1;ENSMUST00000216299
MSTRG.3	2610203[illegible]	MSTRG.32237.1
MSTRG.3	CT00969	ENSMUST00000213487;ENSMUST00000213560
MSTRG.3	Gm28119	ENSMUST00000185538;ENSMUST00000175613
MSTRG.3	Tpgs1	ENSMUST00000020552;ENSMUST00000219585;ENSMUST0000022[illegible]
MSTRG.3	CT02561	ENSMUST00000216975
MSTRG.3	Sorl1	ENSMUST00000060989;ENSMUST00000134560;ENSMUST0000014[illegible]
MSTRG.3	Gm16214	ENSMUST00000143710
MSTRG.3	Sc5d	ENSMUST00000052725;ENSMUST00000169609;ENSMUST0000021[illegible]
MSTRG.3	Tbcel	ENSMUST00000125995;ENSMUST00000066148;ENSMUST0000006[illegible]
MSTRG.3	Gm16322	ENSMUST00000141275;ENSMUST00000125908
MSTRG.3	Oaf	ENSMUST00000034512;ENSMUST00000213286;ENSMUST0000021[illegible]
MSTRG.3	Arhgef1[illegible]	ENSMUST00000165665;MSTRG.32248.4;MSTRG.32248.3;ENSMUS[illegible]
MSTRG.3	Cdc34	ENSMUST00000020550;ENSMUST00000220133;ENSMUST0000016[illegible]
MSTRG.3	Trim29	ENSMUST00000034511;MSTRG.32250.1;ENSMUST00000217418;[illegible]
MSTRG.3	AC16293	ENSMUST00000215497;ENSMUST00000216604;ENSMUST0000021[illegible]

MSTRG.3	AC16293	ENSMUST00000213140
MSTRG.3	Nectin1	ENSMUST00000034510
MSTRG.3	AC12645	ENSMUST00000215774;MSTRG.32254.1;MSTRG.32254.3;MSTRG.
MSTRG.3	AC12645	MSTRG.32255.1
MSTRG.32256		MSTRG.32256.1
MSTRG.3	AC12645	ENSMUST00000217357;ENSMUST00000214440;ENSMUST0000021[illegible]
MSTRG.3	AC12645	ENSMUST00000214791
MSTRG.3	AC12645	MSTRG.32259.3;MSTRG.32259.2;MSTRG.32259.1;MSTRG.3225[illegible]
MSTRG.3	Bsg	ENSMUST00000179781;ENSMUST00000067036;ENSMUST0000017[illegible]
MSTRG.3	Thy1	MSTRG.32260.2;MSTRG.32260.1;MSTRG.32260.3;MSTRG.3226[illegible]
MSTRG.3	Ccdc153	ENSMUST00000216995;ENSMUST00000092426;ENSMUST00000217
MSTRG.3	Pdzd3	ENSMUST00000034618;ENSMUST00000213186
MSTRG.3	Nlrx1	ENSMUST00000034621;ENSMUST00000169651;ENSMUST0000016[illegible]
MSTRG.3	AC12457	ENSMUST00000215389
MSTRG.3	Nlrx1	MSTRG.32265.1
MSTRG.3	C2cd2l	ENSMUST00000065080;ENSMUST00000216222;ENSMUST0000021[illegible]
MSTRG.3	Rnf26;A[illegible]	ENSMUST00000185479;ENSMUST00000216448;ENSMUST0000006[illegible]
MSTRG.3	Usp2	ENSMUST00000034508;ENSMUST00000177054;ENSMUST00000114
MSTRG.3	Mfrp;C1[illegible]	MSTRG.32269.1;ENSMUST00000034654;ENSMUST00000114821;[illegible]
MSTRG.3	Hcn2	ENSMUST00000099513
MSTRG.3	Gm20444	ENSMUST00000174014
MSTRG.3	Rnf26	MSTRG.32271.1
MSTRG.3	AC14832	ENSMUST00000216960
MSTRG.3	Gm26737	ENSMUST00000181106;ENSMUST00000213646
MSTRG.3	Gm10687	ENSMUST00000180670
MSTRG.3	Mcam	ENSMUST00000149241;ENSMUST00000034650;ENSMUST0000009[illegible]
MSTRG.3	Cbl	ENSMUST00000206720;ENSMUST00000206147;ENSMUST0000020[illegible]
MSTRG.3	Cbl	MSTRG.32277.1
MSTRG.3	AC12457	ENSMUST00000214603
MSTRG.3	AC12457	ENSMUST00000214044
MSTRG.3	Polrmt	ENSMUST00000161765;ENSMUST00000161662;ENSMUST0000015[illegible]
MSTRG.3	Cbl	MSTRG.32280.1
MSTRG.3	Dpagt1	ENSMUST00000215248;ENSMUST00000216327;ENSMUST0000021[illegible]
MSTRG.3	AC12242	ENSMUST00000213709
MSTRG.3	H2afx	ENSMUST00000052686
MSTRG.3	Abcg4	ENSMUST00000159385;ENSMUST00000034648;ENSMUST00000161
MSTRG.3	AC12457	ENSMUST00000217413;MSTRG.32286.1;MSTRG.32286.2;ENSMUS
MSTRG.3	Hmbs	ENSMUST00000077353;ENSMUST00000097558;ENSMUST0000021[illegible]
MSTRG.3	Vps11	ENSMUST00000034644;ENSMUST00000214510;ENSMUST0000021[illegible]
MSTRG.3	Vps11	MSTRG.32289.1
MSTRG.3	Fgf22	ENSMUST00000219228;ENSMUST00000020577
MSTRG.3	AC12242	ENSMUST00000217460
MSTRG.3	Hyou1	ENSMUST00000066601;ENSMUST00000162560;ENSMUST00000161
MSTRG.3	Gm9830	ENSMUST00000178509
MSTRG.3	Gm9830	MSTRG.32293.1
MSTRG.3	Trappc4	ENSMUST00000213747;ENSMUST00000034623;ENSMUST00000217
MSTRG.3	Rps25	ENSMUST00000080300;ENSMUST00000213116;ENSMUST0000021[illegible]
MSTRG.3	Ccdc84	ENSMUST00000053286;ENSMUST00000215402;ENSMUST00000214
MSTRG.3	Ccdc84	MSTRG.32297.1
MSTRG.3	AC12242	ENSMUST00000213494
MSTRG.3	Foxr1	ENSMUST00000215375;ENSMUST00000098837
MSTRG.3	Mitd1	ENSMUST00000139725;ENSMUST00000027257;ENSMUST00000131
MSTRG.3	Rnf126	ENSMUST00000047203;ENSMUST00000218770;ENSMUST0000021[illegible]
MSTRG.3	C030014	ENSMUST00000213746;ENSMUST00000217463;ENSMUST0000008[illegible]
MSTRG.3	Gm22540	ENSMUST00000158168
MSTRG.3	AC12242	ENSMUST00000216321
MSTRG.3	AC12242	ENSMUST00000215551

MSTRG.3	Bcl9l	ENSMUST00000220303;ENSMUST00000217898;MSTRG.32304.2;E
MSTRG.3	Cxcr5	ENSMUST00000215293;ENSMUST00000062215;ENSMUST00000215
MSTRG.3	Ddx6;AC	MSTRG.32306.1;MSTRG.32306.2;MSTRG.32306.3;ENSMUST0000
MSTRG.3	AC15197	ENSMUST00000214422;ENSMUST00000215946
MSTRG.3	AC15197	ENSMUST00000217329
MSTRG.3	AC15197	ENSMUST00000216193
MSTRG.3231		MSTRG.3231.1
MSTRG.32310		MSTRG.32310.1
MSTRG.32311		MSTRG.32311.1
MSTRG.3	AC15197	ENSMUST00000214898
MSTRG.3	Ddx6	MSTRG.32313.1
MSTRG.3	AC15197	ENSMUST00000215131
MSTRG.3	Treh	ENSMUST00000034609;ENSMUST00000150822
MSTRG.3	Phldb1	ENSMUST00000147495;ENSMUST00000144251;ENSMUST00000156
MSTRG.3	AC15197	ENSMUST00000214717
MSTRG.3	Cd3g	ENSMUST00000002101;ENSMUST00000159019;MSTRG.32318.4;M
MSTRG.3	Cd3d	MSTRG.32319.3;MSTRG.32319.1;ENSMUST00000034602;MSTRG.
MSTRG.3	Fstl3	ENSMUST00000020575;ENSMUST00000170380;ENSMUST00000168
MSTRG.3	Cd3e	ENSMUST00000102832;MSTRG.32320.1;ENSMUST00000214582;E
MSTRG.3	AC12230	ENSMUST00000214762
MSTRG.3	Cd3e	MSTRG.32322.1
MSTRG.3	AC12230	ENSMUST00000216159
MSTRG.3	Arcn1	ENSMUST00000034607;MSTRG.32324.2;MSTRG.32324.3;MSTRG.
MSTRG.3	AC14211	ENSMUST00000217425
MSTRG.3	Ift46	ENSMUST00000154090;ENSMUST00000002099;ENSMUST00000128
MSTRG.3	Tmem25	ENSMUST00000213203;ENSMUST00000114705;ENSMUST00000132
MSTRG.3	AC14211	ENSMUST00000214931
MSTRG.3	Ttc36	ENSMUST00000044694;ENSMUST00000216449
MSTRG.3	Prss57	ENSMUST00000020573;ENSMUST00000169684;ENSMUST00000167
MSTRG.3	Kmt2a;A	MSTRG.32330.2;MSTRG.32330.1;MSTRG.32330.3;ENSMUST0000
MSTRG.3	AC06196	MSTRG.32331.1
MSTRG.3	AC06196	ENSMUST00000213180;MSTRG.32333.2;MSTRG.32333.3;ENSMUS
MSTRG.3	Atp5l	ENSMUST00000216815;MSTRG.32335.1;ENSMUST00000043675;E
MSTRG.3	AC06196	ENSMUST00000215803
MSTRG.3	Ube4a	MSTRG.32337.1;ENSMUST00000117506;MSTRG.32337.3;ENSMUS
MSTRG.3	AC06196	ENSMUST00000215720
MSTRG.32339		MSTRG.32339.1
MSTRG.3	Palm	ENSMUST00000046945;ENSMUST00000105379;ENSMUST00000218
MSTRG.3	Mpzl2	ENSMUST00000217097;ENSMUST00000215886;ENSMUST00000215
MSTRG.3	AC12230	ENSMUST00000214803
MSTRG.3	Mpzl3	ENSMUST00000114664;ENSMUST00000093856;ENSMUST00000114
MSTRG.3	Scn4b	ENSMUST00000060125
MSTRG.3	Jaml	ENSMUST00000050020;ENSMUST00000215880;ENSMUST00000216
MSTRG.3	Gm10684	ENSMUST00000098827;ENSMUST00000217151
MSTRG.3	AC12230	ENSMUST00000215148
MSTRG.3	Scn2b	ENSMUST00000170998
MSTRG.3	Tmprss4	ENSMUST00000034599;ENSMUST00000165263;ENSMUST00000163
MSTRG.3	AC15182	MSTRG.3235.1
MSTRG.3	BC04935	ENSMUST00000171277;ENSMUST00000164650;ENSMUST00000171
MSTRG.3	Il10ra	ENSMUST00000034594;ENSMUST00000176808;ENSMUST00000176
MSTRG.3	Gm20612	ENSMUST00000176318
MSTRG.3	Il10ra	MSTRG.32353.1;MSTRG.32353.2
MSTRG.3	Mir7086	ENSMUST00000183683
MSTRG.3	Fxyd2	ENSMUST00000214680;ENSMUST00000216289;ENSMUST00000213
MSTRG.3	AC16217	ENSMUST00000216490
MSTRG.3	Cep164	ENSMUST00000117194;ENSMUST00000216284;ENSMUST00000214
MSTRG.3	AC12680	ENSMUST00000215216

MSTRG.3	Misp	ENSMUST00000169041;ENSMUST00000219305;ENSMUST00000046
MSTRG.3	AC13458	MSTRG.32360.1
MSTRG.3	Zpr1	ENSMUST00000156440;ENSMUST00000114552;ENSMUST00000141
MSTRG.3	Bud13	ENSMUST00000138498;ENSMUST00000074957;MSTRG.32362.1;E
MSTRG.3	Bud13	MSTRG.32363.1
MSTRG.3	4931429	ENSMUST00000159565
MSTRG.3	AC16410	ENSMUST00000215849
MSTRG.3	4930448	ENSMUST00000215242;ENSMUST00000217385
MSTRG.3	Cadm1	ENSMUST00000114548;ENSMUST00000152459;ENSMUST00000143
MSTRG.3	AC12242	ENSMUST00000214736
MSTRG.3	AC12242	ENSMUST00000216522
MSTRG.3	Elane	ENSMUST00000046091
MSTRG.3	Cadm1	MSTRG.32370.1
MSTRG.3	Bace1	ENSMUST00000078111;ENSMUST00000034591;ENSMUST00000159
MSTRG.3	Gm16536	ENSMUST00000162209
MSTRG.3	AC12680	ENSMUST00000216609
MSTRG.3	Rnf214	ENSMUST00000161203;ENSMUST00000058720;ENSMUST00000161
MSTRG.3	Gm24646	ENSMUST00000157109
MSTRG.3	Rnf214	MSTRG.32376.1
MSTRG.3	Pcsk7	ENSMUST00000039059;ENSMUST00000215535;MSTRG.32377.2;M
MSTRG.3	Pcsk7	MSTRG.32378.1
MSTRG.3	Pcsk7	MSTRG.32379.1
MSTRG.3	Cfd	ENSMUST00000217837;ENSMUST00000061653
MSTRG.3	Tagln;S	ENSMUST00000034590;MSTRG.32380.1;ENSMUST00000216035;E
MSTRG.3	AC12227	ENSMUST00000213634;ENSMUST00000213884
MSTRG.3	AC12227	ENSMUST00000216147
MSTRG.3	Pafah1b	MSTRG.32383.1
MSTRG.3	Pafah1b	MSTRG.32384.1;ENSMUST00000172450;ENSMUST00000214179;E
MSTRG.3	Sik3	MSTRG.32385.1;MSTRG.32385.2
MSTRG.3	Sik3	ENSMUST00000126865;ENSMUST00000120463;ENSMUST00000120
MSTRG.3	Sik3	MSTRG.32387.1
MSTRG.3	Sik3	MSTRG.32388.1
MSTRG.3	Sik3	MSTRG.32389.1
MSTRG.3	Gm24019	ENSMUST00000178620
MSTRG.3	Sik3	MSTRG.32390.1
MSTRG.3	Sik3	MSTRG.32391.1
MSTRG.3	Gm5616	ENSMUST00000165252
MSTRG.3	Rbm7	ENSMUST00000213276;ENSMUST00000170000;ENSMUST00000214
MSTRG.3	Gm5617	ENSMUST00000048824
MSTRG.3	Rexo2	ENSMUST00000034524;ENSMUST00000216142;ENSMUST00000214
MSTRG.32396		MSTRG.32396.1
MSTRG.32397		MSTRG.32397.1
MSTRG.32398		MSTRG.32398.1
MSTRG.3	Zbtb16	MSTRG.32399.1
MSTRG.3	Mrpl30	ENSMUST00000027256;ENSMUST00000193673;ENSMUST00000161
MSTRG.3	Med16	ENSMUST00000105378;ENSMUST00000165684;ENSMUST00000170
MSTRG.3	Zbtb16	ENSMUST00000093852;ENSMUST00000216150
MSTRG.3	Zbtb16	MSTRG.32401.1
MSTRG.3	Zbtb16	MSTRG.32402.1
MSTRG.3	Zbtb16	MSTRG.32403.1
MSTRG.3	Zbtb16	MSTRG.32404.1
MSTRG.3	Zbtb16	MSTRG.32405.1
MSTRG.3	Zbtb16	MSTRG.32406.1
MSTRG.3	Zbtb16	MSTRG.32407.1
MSTRG.3	Zbtb16	MSTRG.32408.1
MSTRG.3	E130317	ENSMUST00000168521;ENSMUST00000217802
MSTRG.3	1700042	ENSMUST00000198232

MSTRG.3	Zw10	ENSMUST00000034803;ENSMUST00000215706;MSTRG.32411.3;M
MSTRG.3	Zw10	MSTRG.32412.1
MSTRG.3	Usp28	ENSMUST00000215788;ENSMUST00000216607;MSTRG.32413.2;E
MSTRG.3	Usp28	MSTRG.32414.1
MSTRG.3	Ttc12	ENSMUST00000055096;ENSMUST00000146666
MSTRG.3	Ttc12	MSTRG.32416.1
MSTRG.3	Ttc12	MSTRG.32417.1
MSTRG.3	Rpl10-p	ENSMUST00000076364
MSTRG.3	Plet1os	ENSMUST00000188703;ENSMUST00000190941
MSTRG.3	Ptbp1	ENSMUST00000172282;ENSMUST00000168988;MSTRG.3242.2;EN
MSTRG.3	Plet1	ENSMUST00000114474;ENSMUST00000188047
MSTRG.3	Pts	ENSMUST00000217146;ENSMUST00000034570;ENSMUST00000213
MSTRG.3	Sdhd	ENSMUST00000000175;MSTRG.32422.1
MSTRG.3	Timm8b	ENSMUST00000044051
MSTRG.3	AU01982	ENSMUST00000131351;ENSMUST00000171462;ENSMUST00000145
MSTRG.3	Pih1d2	ENSMUST00000000171;ENSMUST00000124029;ENSMUST00000151
MSTRG.3	Dlat	ENSMUST00000034567;ENSMUST00000126933;MSTRG.32426.2;E
MSTRG.3	Dixdc1	ENSMUST00000034566;ENSMUST00000141919;ENSMUST00000117
MSTRG.3	2310030	ENSMUST00000034564;ENSMUST00000215877
MSTRG.3	1110032	ENSMUST00000042468;ENSMUST00000177546;ENSMUST00000042
MSTRG.3	Plppr3	ENSMUST00000167250;ENSMUST00000113452;ENSMUST00000166
MSTRG.3	Fdxacb1	ENSMUST00000042391;ENSMUST00000176619;ENSMUST00000176
MSTRG.3	Alg9	ENSMUST00000034561;ENSMUST00000162073;ENSMUST00000159
MSTRG.3	Pou2af1	ENSMUST00000034554;ENSMUST00000214322
MSTRG.3	AC16005	ENSMUST00000213173
MSTRG.3	Colca2	ENSMUST00000114427;ENSMUST00000215038
MSTRG.3	2010007	ENSMUST00000186025;ENSMUST00000050829
MSTRG.3	Ppp2r1b	ENSMUST00000174555;ENSMUST00000174628;ENSMUST00000034
MSTRG.3	Gm25558	ENSMUST00000158083
MSTRG.3	Sik2	MSTRG.32438.1;ENSMUST00000176663;ENSMUST00000176824;E
MSTRG.3	Sik2	MSTRG.32439.1
MSTRG.3	Mir6910	ENSMUST00000184090
MSTRG.3	Sik2	MSTRG.32440.1
MSTRG.3	Sik2	MSTRG.32441.1
MSTRG.3	Sik2	MSTRG.32442.1
MSTRG.3	Arhgap2	ENSMUST00000124907;ENSMUST00000146509;ENSMUST00000065
MSTRG.3	Arhgap2	ENSMUST00000152203;ENSMUST00000142579;ENSMUST00000130
MSTRG.3	Fdx1	ENSMUST00000034552;ENSMUST00000214486;ENSMUST00000214
MSTRG.3	Rdx	ENSMUST00000163153;ENSMUST00000000590;ENSMUST00000061
MSTRG.3	Gm27686	ENSMUST00000185123
MSTRG.3	Zc3h12c	ENSMUST00000213645;ENSMUST00000165519
MSTRG.3	Gm17134	ENSMUST00000166593
MSTRG.3	Ddx10	ENSMUST00000065630;MSTRG.32450.2
MSTRG.3	AC11295	ENSMUST00000213563
MSTRG.3	4930550	ENSMUST00000215668;ENSMUST00000005262;ENSMUST00000217
MSTRG.3	Atm	MSTRG.32453.1;ENSMUST00000118282;ENSMUST00000126598;E
MSTRG.3	Npat	ENSMUST00000035850;MSTRG.32454.2;MSTRG.32454.1;ENSMUS
MSTRG.3	Acat1	ENSMUST00000034547;ENSMUST00000169317;ENSMUST00000167
MSTRG.3	Rab39	ENSMUST00000068449
MSTRG.3	Rps6-ps	ENSMUST00000118010
MSTRG.3	Cul5	ENSMUST00000166367;ENSMUST00000120122;ENSMUST00000141
MSTRG.3	Gm22304	ENSMUST00000082688
MSTRG.3	Prtn3	ENSMUST00000163188;ENSMUST00000006679;ENSMUST00000165
MSTRG.3	Cul5	MSTRG.32460.1
MSTRG.3	Gm16124	ENSMUST00000146938
MSTRG.3	Gm16124	MSTRG.32462.1
MSTRG.3	Slc35f2	ENSMUST00000048670;ENSMUST00000216515

MSTRG.3	Slc35f2	MSTRG.32464.1
MSTRG.3	Idh3a	ENSMUST00000217484;ENSMUST00000215273;ENSMUST0000021З
MSTRG.3	Acsbg1	ENSMUST00000034822;ENSMUST00000138315;ENSMUST0000012ß
MSTRG.3	AC15989	ENSMUST00000213253
MSTRG.3	AC15759	ENSMUST00000213307
MSTRG.3	Dnaja4	ENSMUST00000120452;ENSMUST00000070070
MSTRG.3	Wdr18	ENSMUST00000045247;ENSMUST00000220272;ENSMUST0000021ß
MSTRG.3	Mir5710	ENSMUST00000176884
MSTRG.3	Wdr61	ENSMUST00000118771;ENSMUST00000130368;ENSMUST00000051
MSTRG.3	Ireb2	MSTRG.32472.1;MSTRG.32472.2;MSTRG.32472.3;ENSMUST0000
MSTRG.3	AY07488	ENSMUST00000054018
MSTRG.3	Psma4	ENSMUST00000034848;ENSMUST00000171578;ENSMUST0000016
MSTRG.3	Ube2q2;	ENSMUST00000059555;MSTRG.32475.1;ENSMUST00000121677;E
MSTRG.3	Nrg4	ENSMUST00000139261;ENSMUST00000164721;ENSMUST00000144
MSTRG.3	Gm15625	ENSMUST00000117617
MSTRG.3	Etfa	ENSMUST00000034866;ENSMUST00000139342;ENSMUST0000015
MSTRG.3	Etfa	MSTRG.32479.1
MSTRG.3	R3hdm4	ENSMUST00000045628;ENSMUST00000219358;MSTRG.3248.3;EN
MSTRG.3	Etfa	MSTRG.32480.1
MSTRG.3	AC10781	MSTRG.32481.1
MSTRG.3	4930563	ENSMUST00000217105
MSTRG.3	Scaper	ENSMUST00000214747;ENSMUST00000037408;MSTRG.32483.2;E
MSTRG.3	Gm23407	ENSMUST00000082538
MSTRG.3	Scaper	MSTRG.32485.1
MSTRG.3	Scaper	MSTRG.32486.1
MSTRG.3	Scaper	MSTRG.32487.1
MSTRG.3	AC10781	ENSMUST00000214039;MSTRG.32488.2;ENSMUST00000216003
MSTRG.3	AC10781	MSTRG.32489.1
MSTRG.3	Rcn2	ENSMUST00000147842;ENSMUST00000114276;ENSMUST00000151
MSTRG.3	Tspan3	ENSMUST00000214970;ENSMUST00000034876;ENSMUST0000021
MSTRG.3	Pstpip1	ENSMUST00000059206;MSTRG.32492.3;MSTRG.32492.4;MSTRG.
MSTRG.3	Peak1	ENSMUST00000188142;ENSMUST00000061552;ENSMUST0000018
MSTRG.3	Gm29322	ENSMUST00000185378
MSTRG.3	Peak1os	ENSMUST00000181444
MSTRG.3	Gm37842	ENSMUST00000192330
MSTRG.3	Peak1	MSTRG.32498.1
MSTRG.3	Gm36940	ENSMUST00000194281
MSTRG.3	Txndc9	ENSMUST00000162031;ENSMUST00000193832;ENSMUST0000019
MSTRG.3	Kiss1r	ENSMUST00000219883;ENSMUST00000045529
MSTRG.3	Hmg20a	ENSMUST00000034879;ENSMUST00000215269;ENSMUST0000021
MSTRG.3	Hmg20a	MSTRG.32501.1
MSTRG.3	Hmg20a	MSTRG.32502.1
MSTRG.3	Snx33	MSTRG.32503.1
MSTRG.3	Snx33	ENSMUST00000050916;MSTRG.32504.1;MSTRG.32504.3
MSTRG.3	Imp3	ENSMUST00000034827
MSTRG.3	Snupn	ENSMUST00000068856
MSTRG.3	Snupn	MSTRG.32507.1
MSTRG.3	Commd4	ENSMUST00000214072;ENSMUST00000213161;ENSMUST0000006
MSTRG.3	Trcg1	ENSMUST00000093837
MSTRG.3	Arid3a	ENSMUST00000019708;ENSMUST00000105377;ENSMUST0000010
MSTRG.3	1700017	ENSMUST00000034846;ENSMUST00000215298;ENSMUST00000214
MSTRG.3	Gm6166	ENSMUST00000098689
MSTRG.3	Ppcdc	ENSMUST00000215961;ENSMUST00000214144;ENSMUST00000214
MSTRG.3	Ppcdc	MSTRG.32514.1
MSTRG.3	Cox5a	ENSMUST00000213678;ENSMUST00000000090;ENSMUST00000214
MSTRG.3	Gm26631	ENSMUST00000181462
MSTRG.3	Fam219b	ENSMUST00000093833;ENSMUST00000114200;ENSMUST0000021

MSTRG.3 Mpi	MSTRG.32518.1;ENSMUST00000034856;ENSMUST00000140671;E
MSTRG.3 Ptpn9	ENSMUST00000034832;ENSMUST00000213500;ENSMUST0000021€
MSTRG.3 Arid3a	MSTRG.3252.1
MSTRG.3 Gm10658	ENSMUST00000128125;ENSMUST00000135527
MSTRG.3 Sin3a	ENSMUST00000169879;MSTRG.32521.1;ENSMUST00000167715;E
MSTRG.3 2700012	MSTRG.32522.1;ENSMUST00000180525
MSTRG.3 Man2c1o	ENSMUST00000125333;ENSMUST00000162738;ENSMUST00000142
MSTRG.3 Man2c1	ENSMUST00000161393;ENSMUST00000160147;ENSMUST00000162
MSTRG.3 Neil1	MSTRG.32525.1;MSTRG.32525.2;MSTRG.32525.3;ENSMUST0000
MSTRG.3 Scamp2	ENSMUST00000045791;ENSMUST00000216986;ENSMUST0000021З
MSTRG.3 Scamp2	MSTRG.32527.1
MSTRG.3 Ulk3	ENSMUST00000053230;ENSMUST00000213212;ENSMUST0000021Е
MSTRG.3 Csk	ENSMUST00000034863;ENSMUST00000217314;ENSMUST0000021Е
MSTRG.3 Gm26243	ENSMUST00000179260
MSTRG.3 Csk	MSTRG.32530.1
MSTRG.3 Ubl7	ENSMUST00000216925;MSTRG.32531.2;ENSMUST00000163329;E
MSTRG.3 Sema7a	ENSMUST00000214314;ENSMUST00000043059;MSTRG.32532.3
MSTRG.3 Sema7a	MSTRG.32533.1
MSTRG.3 Edc3	ENSMUST00000043990;ENSMUST00000137245;ENSMUST00000142
MSTRG.3 Clk3	ENSMUST00000216095;ENSMUST00000065330;ENSMUST0000021Е
MSTRG.32536	MSTRG.32536.1
MSTRG.3 Gm17231	MSTRG.32537.1;ENSMUST00000165858;ENSMUST00000163186
MSTRG.3 Gm17231	MSTRG.32538.1
MSTRG.3 Arid3b	MSTRG.32539.1;MSTRG.32539.2;ENSMUST00000164010;ENSMUS
MSTRG.3 Grin3b	ENSMUST00000045085;ENSMUST00000149148
MSTRG.3 Gm17322	ENSMUST00000181289;ENSMUST00000188116;ENSMUST00000034
MSTRG.3 Cyp11a1	MSTRG.32541.1
MSTRG.3 Cyp11a1	MSTRG.32542.1
MSTRG.3 Pml	ENSMUST00000085673;ENSMUST00000114136;ENSMUST0000013Е
MSTRG.3 Pml	MSTRG.32545.1
MSTRG.3 Pml	MSTRG.32546.1
MSTRG.3 Stoml1	ENSMUST00000034883;ENSMUST00000215488;ENSMUST00000217
MSTRG.3 Nptn	ENSMUST00000177292;ENSMUST00000085651;MSTRG.32548.2;E
MSTRG.3 Nptn	MSTRG.32549.1
MSTRG.3 Tmem259	ENSMUST00000132080;ENSMUST00000126383;ENSMUST00000052
MSTRG.3 Nptn	MSTRG.32550.1
MSTRG.3 Nptn	MSTRG.32551.1
MSTRG.3 Rec114	ENSMUST00000175792;ENSMUST00000098674;ENSMUST0000018€
MSTRG.3 Ccdc33	ENSMUST00000042205;ENSMUST00000098682;ENSMUST0000021Е
MSTRG.3 Gm16130	ENSMUST00000153106
MSTRG.3 Stra6	ENSMUST00000145886;ENSMUST00000136154;ENSMUST0000012ξ
MSTRG.3 Gm16131	ENSMUST00000131007
MSTRG.3 Islr;Is	ENSMUST00000041477;ENSMUST00000168864;ENSMUST0000016З
MSTRG.3 Cnn2	ENSMUST00000004784;ENSMUST00000105374
MSTRG.3 1600029	ENSMUST00000179787;ENSMUST00000178259
MSTRG.3 Adpgk	ENSMUST00000217570;ENSMUST00000213413;ENSMUST0000002€
MSTRG.3 Bbs4	ENSMUST00000026265;ENSMUST00000217367;ENSMUST00000214
MSTRG.3 Neo1	ENSMUST00000068664;ENSMUST00000214547;ENSMUST0000021Е
MSTRG.3 CT03375	ENSMUST00000216941
MSTRG.3 Arih1;R	ENSMUST00000171975;ENSMUST00000026264;ENSMUST0000016Е
MSTRG.3 Arih1	MSTRG.32566.1
MSTRG.3 Tmem202	ENSMUST00000055345
MSTRG.3 Hexa	ENSMUST00000026262
MSTRG.3 Parp6	ENSMUST00000026267;ENSMUST00000050483;ENSMUST0000021€
MSTRG.3 Abca7	ENSMUST00000132517;ENSMUST00000171637;MSTRG.3257.3;MS
MSTRG.3 Pkm;AC1	ENSMUST00000034834;ENSMUST00000163694;ENSMUST00000214
MSTRG.3 Senp8	ENSMUST00000051039;ENSMUST00000177963;ENSMUST0000016З

MSTRG.3	Senp8	MSTRG.32572.1
MSTRG.3	Senp8	MSTRG.32573.1
MSTRG.3	Myo9a	ENSMUST00000128341;ENSMUST00000135298;ENSMUST0000013[illegible]
MSTRG.3	Gm7613	ENSMUST00000139070
MSTRG.3	Myo9a	MSTRG.32576.1
MSTRG.3	Myo9a	MSTRG.32577.1
MSTRG.3	AC15679	ENSMUST00000215963
MSTRG.3	AC15679	MSTRG.32579.1
MSTRG.3	Arhgap4	ENSMUST00000099501;MSTRG.3258.1;MSTRG.3258.3;ENSMUST[illegible]
MSTRG.3	AC15679	MSTRG.32580.1
MSTRG.3	Gm2735	ENSMUST00000136133
MSTRG.3	AC15679	MSTRG.32582.1
MSTRG.3	Myo9a	MSTRG.32583.1
MSTRG.3	AC15679	ENSMUST00000214655
MSTRG.3	AC15679	MSTRG.32585.1
MSTRG.3	Thsd4	ENSMUST00000171654;ENSMUST00000098660;ENSMUST00000034
MSTRG.3	Gm23668	ENSMUST00000157465
MSTRG.3	Thsd4	MSTRG.32588.1
MSTRG.3	AC15823	ENSMUST00000214235
MSTRG.3	Polr2e	ENSMUST00000147507;ENSMUST00000143438;ENSMUST00000004
MSTRG.3	AC15823	ENSMUST00000216899
MSTRG.3	Gm22099	ENSMUST00000157925
MSTRG.3	AC11268	ENSMUST00000216749;ENSMUST00000217430
MSTRG.3	AC11268	ENSMUST00000215297
MSTRG.3	AC11268	ENSMUST00000213119
MSTRG.3	AC16056	ENSMUST00000216170;MSTRG.32595.2
MSTRG.3	AC16039	ENSMUST00000217239;ENSMUST00000216861
MSTRG.3	AC16056	MSTRG.32597.1
MSTRG.3	Tle3	MSTRG.32598.1
MSTRG.3	AC14048	ENSMUST00000214104;ENSMUST00000213400
MSTRG.3	Eif5b	ENSMUST00000027252;ENSMUST00000192548;MSTRG.326.3;ENS
MSTRG.3	Gpx4	ENSMUST00000144698;ENSMUST00000105372;MSTRG.3260.3;EN
MSTRG.3	AC14048	MSTRG.32600.1
MSTRG.3	AC14048	MSTRG.32601.1
MSTRG.3	Gm10655	ENSMUST00000098658;ENSMUST00000187131
MSTRG.3	Tle3	ENSMUST00000178113;ENSMUST00000159386;MSTRG.32603.3;E
MSTRG.3	Glce;Mi	ENSMUST00000034785;ENSMUST00000185675;ENSMUST0000018[illegible]
MSTRG.3	Glce	MSTRG.32605.1
MSTRG.3	AC11329	ENSMUST00000216565
MSTRG.32607		MSTRG.32607.1
MSTRG.3	Anp32a	ENSMUST00000138226;ENSMUST00000085519;ENSMUST0000012[illegible]
MSTRG.32609		MSTRG.32609.1
MSTRG.3	Sbno2	MSTRG.3261.1;MSTRG.3261.2;ENSMUST00000219260;ENSMUST[illegible]
MSTRG.3	Rplp1	ENSMUST00000008036
MSTRG.3	Kif23	ENSMUST00000213159;MSTRG.32611.1;ENSMUST00000034815;E
MSTRG.3	Coro2b	ENSMUST00000048043;ENSMUST00000131981;ENSMUST00000164
MSTRG.3	Coro2b	MSTRG.32613.1
MSTRG.3	n-R5s84	ENSMUST00000122591
MSTRG.3	Itga11	ENSMUST00000034774
MSTRG.32616		MSTRG.32616.1
MSTRG.3	Fem1b	ENSMUST00000034775
MSTRG.3	Gm26609	ENSMUST00000181302
MSTRG.3	Map2k5	MSTRG.32619.1;ENSMUST00000034920;ENSMUST00000213604;E
MSTRG.3	Cirbp	ENSMUST00000154726;ENSMUST00000105365;MSTRG.3262.2;EN
MSTRG.3	Map2k5	MSTRG.32620.1
MSTRG.3	Map2k5	MSTRG.32621.1
MSTRG.3	Cln6	ENSMUST00000034776;ENSMUST00000141821;MSTRG.32622.2;E

MSTRG.3	Gm10653	ENSMUST00000132250;ENSMUST00000156423;ENSMUST0000013[illegible]
MSTRG.3	AC16461	ENSMUST00000216038
MSTRG.3	Calml4	ENSMUST00000213643;ENSMUST00000034777;ENSMUST0000021[illegible]
MSTRG.3	Pias1	ENSMUST00000098651;ENSMUST00000215501;MSTRG.32626.5;[illegible]
MSTRG.3	Pias1	MSTRG.32627.1;MSTRG.32627.2
MSTRG.3	Smad3	MSTRG.32629.3;ENSMUST00000034973;MSTRG.32629.1;MSTRG.
MSTRG.3	1600002	ENSMUST00000041882
MSTRG.3	Smad3	MSTRG.32630.1
MSTRG.3	Smad3	MSTRG.32631.1
MSTRG.3	2300009	ENSMUST00000186165;ENSMUST00000168665;ENSMUST00000187
MSTRG.3	2300009	MSTRG.32633.1
MSTRG.3	Gm16759	ENSMUST00000191455;ENSMUST00000126238;ENSMUST00000187
MSTRG.3	Iqch	ENSMUST00000042322;ENSMUST00000163624;ENSMUST0000017[illegible]
MSTRG.3	Gm25995	ENSMUST00000175396
MSTRG.3	Gm6257	ENSMUST00000163864
MSTRG.3	Aagab	ENSMUST00000041551;MSTRG.32638.1;MSTRG.32638.3;ENSMUS
MSTRG.3	Rpl4;Sn	ENSMUST00000034966;ENSMUST00000217055;ENSMUST0000008[illegible]
MSTRG.3	Efna2	ENSMUST00000003154
MSTRG.3	Gm23344	ENSMUST00000083818
MSTRG.3	Gm22571	ENSMUST00000082984
MSTRG.3	Gm23136	ENSMUST00000083425
MSTRG.3	Snapc5	ENSMUST00000034965
MSTRG.3	Lctl	ENSMUST00000034969;ENSMUST00000145011;ENSMUST0000011[illegible]
MSTRG.3	Zwilch	MSTRG.32645.1;ENSMUST00000122091;ENSMUST00000176848;[illegible]
MSTRG.3	Zwilch	MSTRG.32646.1
MSTRG.3	Tipin	ENSMUST00000215031;ENSMUST00000213165;MSTRG.32647.2;[illegible]
MSTRG.3	Gm24289	ENSMUST00000157629
MSTRG.3	Map2k1	MSTRG.32649.1;ENSMUST00000005066;ENSMUST00000147796;[illegible]
MSTRG.3	Stk11	ENSMUST00000144883;ENSMUST00000003152;ENSMUST0000010[illegible]
MSTRG.3	Gm24106	ENSMUST00000175498
MSTRG.3	AC16011	ENSMUST00000214497
MSTRG.3	Uchl4	ENSMUST00000039011
MSTRG.3	Map2k1	MSTRG.32653.1
MSTRG.3	Dis3l	ENSMUST00000168844;ENSMUST00000068367;ENSMUST0000011[illegible]
MSTRG.3	Rab11a;	ENSMUST00000172298;ENSMUST00000167569;ENSMUST00000172
MSTRG.3	Megf11	ENSMUST00000093829;ENSMUST00000118485;ENSMUST00000164
MSTRG.3	Megf11	MSTRG.32658.1
MSTRG.3	Dennd4a	ENSMUST00000038890;ENSMUST00000213926;ENSMUST0000021[illegible]
MSTRG.3	Cbarp	MSTRG.3266.1;ENSMUST00000169546;ENSMUST00000170219;E[illegible]
MSTRG.3	Dennd4a	MSTRG.32660.1
MSTRG.3	Gm24347	ENSMUST00000158711
MSTRG.3	Slc24a1	ENSMUST00000037798
MSTRG.3	Ints14	ENSMUST00000170517;ENSMUST00000037504;MSTRG.32663.3;[illegible]
MSTRG.3	Gm25640	ENSMUST00000175378
MSTRG.3	Hacd3	ENSMUST00000036615;MSTRG.32665.1
MSTRG.3	Igdcc4	ENSMUST00000035499;ENSMUST00000077696;ENSMUST0000021[illegible]
MSTRG.3	Igdcc4	MSTRG.32667.1
MSTRG.3	Parp16	ENSMUST00000216702;ENSMUST00000069000;ENSMUST0000021[illegible]
MSTRG.3	Gm25313	ENSMUST00000101817
MSTRG.3	Atp5d;M	MSTRG.3267.1;ENSMUST00000105367;ENSMUST00000003156;E[illegible]
MSTRG.3	Rnu5g	ENSMUST00000093721
MSTRG.3	Dpp8	MSTRG.32671.1;ENSMUST00000034960;MSTRG.32671.3;ENSMUS
MSTRG.3	AC12246	ENSMUST00000214559
MSTRG.3	Gm16218	MSTRG.32673.1
MSTRG.3	Cilp	ENSMUST00000048762;ENSMUST00000141382
MSTRG.3	Clpx	ENSMUST00000015501;ENSMUST00000113824;ENSMUST0000014[illegible]
MSTRG.3	Gm16218	ENSMUST00000155424

MSTRG.3 Clpx	MSTRG.32677.1
MSTRG.3 AC11464	ENSMUST00000214251
MSTRG.3 AC11023	ENSMUST00000215863
MSTRG.3 Mum1	ENSMUST00000020365;ENSMUST00000125784;ENSMUST0000013C
MSTRG.3 AC11464	ENSMUST00000215831
MSTRG.3 Pdcd7	ENSMUST00000048184;ENSMUST00000214433;ENSMUST00000215
MSTRG.3 Ubap1l	ENSMUST00000217103;ENSMUST00000147185;ENSMUST00000215
MSTRG.3 AC11464	ENSMUST00000216005
MSTRG.3 Mtfmt	ENSMUST00000074792;ENSMUST00000132966;ENSMUST0000014C
MSTRG.3 Mtfmt	MSTRG.32685.1
MSTRG.3 AC11464	ENSMUST00000214308
MSTRG.3 Spg21	ENSMUST00000034955;ENSMUST00000213957;ENSMUST00000215
MSTRG.3 Ankdd1a	ENSMUST00000217144;ENSMUST00000217646
MSTRG.3 Plekho2	ENSMUST00000214740;ENSMUST00000213554;ENSMUST0000006٤
MSTRG.3 Ndufs7	ENSMUST00000105364;ENSMUST00000152939;ENSMUST0000002C
MSTRG.3 Pif1	ENSMUST00000134538;ENSMUST00000136205;ENSMUST00000154
MSTRG.3 Snx1	MSTRG.32691.1
MSTRG.3 Ppib	ENSMUST00000213785;ENSMUST00000034947
MSTRG.3 Snx22	ENSMUST00000044711;ENSMUST00000214332
MSTRG.3 Snx1	ENSMUST00000034946;ENSMUST00000132841;ENSMUST00000143
MSTRG.3 Snx1	MSTRG.32695.1
MSTRG.3 Fam96a	ENSMUST00000034945
MSTRG.3 Dapk2	ENSMUST00000034944;ENSMUST00000129442;ENSMUST00000132
MSTRG.3 Dapk2	MSTRG.32698.1
MSTRG.3 Zfp609	MSTRG.32699.1;MSTRG.32699.2
MSTRG.3 Eif5b	MSTRG.327.1
MSTRG.3 Dazap1	ENSMUST00000156935;ENSMUST00000105362;ENSMUST00000105
MSTRG.3 Rbpms2	ENSMUST00000055844;ENSMUST00000216342;ENSMUST0000021٤
MSTRG.3 Oaz2	ENSMUST00000128928;ENSMUST00000154502;ENSMUST0000004٤
MSTRG.3 Zfp609;'	MSTRG.32702.2;ENSMUST00000159109;MSTRG.32702.1;ENSMUS
MSTRG.3 Zfp609	MSTRG.32703.1
MSTRG.3 Zfp609	MSTRG.32704.1
MSTRG.3 Zfp609	MSTRG.32705.1
MSTRG.3 n-R5s85	ENSMUST00000083453
MSTRG.3 Gm16073	ENSMUST00000116032
MSTRG.3 Trip4	MSTRG.32709.1
MSTRG.3 Mir6911	ENSMUST00000184241
MSTRG.3 Trip4	MSTRG.32710.1
MSTRG.3 Pclaf	ENSMUST00000045802;ENSMUST00000130308;ENSMUST00000153
MSTRG.3 Pclaf	MSTRG.32712.1
MSTRG.3 Gm23248	ENSMUST00000083367
MSTRG.3 Trip4	MSTRG.32714.1
MSTRG.3 Csnk1g1	ENSMUST00000205379;ENSMUST00000146069;ENSMUST0000020٤
MSTRG.3 Csnk1g1	MSTRG.32716.1
MSTRG.3 Csnk1g1	MSTRG.32717.1
MSTRG.3 Gm22657	ENSMUST00000104197
MSTRG.3 Csnk1g1	MSTRG.32719.1
MSTRG.3 Gm15122	MSTRG.3272.1;ENSMUST00000135434
MSTRG.3 Csnk1g1	MSTRG.32720.1
MSTRG.3 Csnk1g1	MSTRG.32721.1
MSTRG.3 Rps27l	ENSMUST00000040917;ENSMUST00000127896;ENSMUST00000147
MSTRG.3 Aph1c	ENSMUST00000169282
MSTRG.3 Gm17098	ENSMUST00000166507
MSTRG.3 Rab8b	ENSMUST00000041139;MSTRG.32725.2
MSTRG.3 Rab8b	MSTRG.32726.1
MSTRG.3 Lactb	ENSMUST00000215172;ENSMUST00000034929
MSTRG.3 Tpm1	ENSMUST00000113696;ENSMUST00000113705;ENSMUST00000113

MSTRG.3	Herc1	ENSMUST00000144280;MSTRG.32729.1;MSTRG.32729.3;MSTRG.
MSTRG.3	Rps15	ENSMUST00000068408;ENSMUST00000062674
MSTRG.3	Gm23428	ENSMUST00000122488
MSTRG.3	Herc1	MSTRG.32731.1
MSTRG.3	Gm28379	ENSMUST00000187676;ENSMUST00000186450
MSTRG.3	Fbxl22	ENSMUST00000056890;MSTRG.32733.1
MSTRG.3	Usp3	ENSMUST00000127569;ENSMUST00000139547;ENSMUST0000009[illegible]
MSTRG.3	Gm15563	ENSMUST00000124953
MSTRG.3	Usp3	MSTRG.32736.1
MSTRG.3	Vps13c	MSTRG.32737.1;ENSMUST00000077879;ENSMUST00000217386;[illegible]
MSTRG.3	Vps13c	MSTRG.32738.1
MSTRG.3	Rora	ENSMUST00000140351;ENSMUST00000034766;ENSMUST00000174
MSTRG.3	Apc2	ENSMUST00000105359;ENSMUST00000154212;ENSMUST0000014[illegible]
MSTRG.3	Rora	MSTRG.32740.1
MSTRG.3	Rora	MSTRG.32741.1
MSTRG.3	Rora	MSTRG.32742.1
MSTRG.3	Rora	MSTRG.32743.1
MSTRG.3	Rora	MSTRG.32744.1
MSTRG.3	Rora	MSTRG.32745.1
MSTRG.3	Rora	MSTRG.32746.1
MSTRG.3	Rora	MSTRG.32747.1
MSTRG.3	Rora	MSTRG.32748.1
MSTRG.3	Rora	MSTRG.32749.1
MSTRG.3	2310011	ENSMUST00000020341;MSTRG.3275.1
MSTRG.3	Rora	MSTRG.32750.1
MSTRG.3	Rora	MSTRG.32751.1
MSTRG.3	Rora	MSTRG.32752.1
MSTRG.3	Rora	MSTRG.32753.1
MSTRG.3	Rora	MSTRG.32754.1
MSTRG.3	Gm15511	ENSMUST00000152570
MSTRG.3	Gm15511	MSTRG.32756.1
MSTRG.3	Anxa2	MSTRG.32757.1;ENSMUST00000034756;ENSMUST00000123470;[illegible]
MSTRG.3	Mir3109	ENSMUST00000175301
MSTRG.3	Gm16144	ENSMUST00000131093
MSTRG.3	Mex3d	ENSMUST00000105350;ENSMUST00000123141
MSTRG.3	Ice2	ENSMUST00000034761;ENSMUST00000142743;ENSMUST0000012[illegible]
MSTRG.3	Foxb1	ENSMUST00000071281
MSTRG.3	B230323	ENSMUST00000180488;ENSMUST00000180914
MSTRG.3	B230323	MSTRG.32763.1
MSTRG.3	B230323	MSTRG.32764.1
MSTRG.3	AC15899	ENSMUST00000216018
MSTRG.3	Gtf2a2	MSTRG.32766.1
MSTRG.3	Mir5626	ENSMUST00000176002
MSTRG.3	Ccnb2	ENSMUST00000034742
MSTRG.3	Gm28731	ENSMUST00000185564
MSTRG.3	Bnip2	ENSMUST00000034754;ENSMUST00000085393;ENSMUST00000117
MSTRG.3	Bnip2	MSTRG.32771.1
MSTRG.3	Gtf2a2	ENSMUST00000118198;ENSMUST00000131078;ENSMUST0000011[illegible]
MSTRG.3	Myo1e	ENSMUST00000213863;ENSMUST00000214767;ENSMUST00000214
MSTRG.3	Myo1e	MSTRG.32774.1
MSTRG.3	Myo1e	MSTRG.32775.1
MSTRG.3	Rnf111	ENSMUST00000213208;MSTRG.32776.1;ENSMUST00000215848;[illegible]
MSTRG.3	Rnf111	MSTRG.32777.1
MSTRG.3	Rnf111	MSTRG.32778.1
MSTRG.3	Gm24615	ENSMUST00000083268
MSTRG.3	Mbd3	ENSMUST00000142997;ENSMUST00000092295;ENSMUST0000010[illegible]
MSTRG.3	Rnf111	MSTRG.32780.1

MSTRG.3	Gm10642	MSTRG.32781.1
MSTRG.3	Adam10	ENSMUST00000140205;ENSMUST00000067880;ENSMUST00000144
MSTRG.3	Sltm	ENSMUST00000216816;ENSMUST00000215714;MSTRG.32783.2;
MSTRG.3	Mindy2	MSTRG.32784.1;ENSMUST00000049031;MSTRG.32784.3;ENSMUS
MSTRG.3	Gm10642	ENSMUST00000098589
MSTRG.3	Polr2m;	ENSMUST00000034720;ENSMUST00000163972;ENSMUST0000016
MSTRG.3	Gm37660	ENSMUST00000192754
MSTRG.3	Cgnl1	ENSMUST00000072899;ENSMUST00000121322;ENSMUST00000152
MSTRG.3	Gm7866	ENSMUST00000183770
MSTRG.3	Uqcr11	ENSMUST00000138428;ENSMUST00000020372;ENSMUST00000141
MSTRG.3	Tcf12	ENSMUST00000183404;ENSMUST00000184770;ENSMUST00000034
MSTRG.3	Gm38057	ENSMUST00000195551
MSTRG.3	Gm38111	ENSMUST00000194817
MSTRG.3	Gm18821	ENSMUST00000184135
MSTRG.3	Tcf12	MSTRG.32794.1
MSTRG.3	Tcf12	MSTRG.32795.1
MSTRG.3	Gm27152	MSTRG.32796.1;ENSMUST00000183828
MSTRG.3	Gm37879	ENSMUST00000193112
MSTRG.3	Tcf12	MSTRG.32798.1
MSTRG.3	Gm37663	ENSMUST00000191887
MSTRG.3	Rev1	ENSMUST00000027251;ENSMUST00000194650;ENSMUST0000019
MSTRG.3	Tcf3	ENSMUST00000105344;ENSMUST00000105345;ENSMUST0000010
MSTRG.3	Tcf12	MSTRG.32800.1
MSTRG.3	Gm37023	ENSMUST00000192895
MSTRG.3	A430027	ENSMUST00000194599
MSTRG.3	Rpl15-p	MSTRG.32803.1;ENSMUST00000184247
MSTRG.3	Gm27450	MSTRG.32804.1;ENSMUST00000104632
MSTRG.3	Gm37137	ENSMUST00000195138
MSTRG.3	Tcf12	MSTRG.32806.1
MSTRG.3	Tcf12	MSTRG.32807.1
MSTRG.3	Tcf12	MSTRG.32808.1
MSTRG.32809		MSTRG.32809.1
MSTRG.3	Klf16	ENSMUST00000038558
MSTRG.3	Rab27a;	ENSMUST00000184146;ENSMUST00000034722;ENSMUST00000184
MSTRG.3	Rab27a	MSTRG.32811.1
MSTRG.3	Khdc3;R	ENSMUST00000172578;ENSMUST00000034737;ENSMUST0000016
MSTRG.3	Gm38215	ENSMUST00000192321
MSTRG.3	Dyx1c1;	ENSMUST00000034734;ENSMUST00000149692;ENSMUST00000037
MSTRG.3	Gm23567	ENSMUST00000178005
MSTRG.3	Gm37710	ENSMUST00000193501
MSTRG.3	Ccpg1os	ENSMUST00000124565;ENSMUST00000184123
MSTRG.3	Gm27353	ENSMUST00000184712
MSTRG.3	Pigb	ENSMUST00000183746;ENSMUST00000093819;MSTRG.32819.5;
MSTRG.3	Gm26710	ENSMUST00000181265
MSTRG.3	Gm38192	ENSMUST00000194364
MSTRG.3	Pigb	MSTRG.32821.1
MSTRG.3	Pigb	MSTRG.32822.1
MSTRG.3	2310009	ENSMUST00000184126;ENSMUST00000184319
MSTRG.3	Unc13c	ENSMUST00000184666
MSTRG.3	Gm37611	ENSMUST00000195250
MSTRG.3	Unc13c	MSTRG.32828.1
MSTRG.3	Gm37326	ENSMUST00000192828
MSTRG.3	Rexo1	ENSMUST00000182682;ENSMUST00000057910;ENSMUST00000182
MSTRG.3	CT03376	ENSMUST00000213947
MSTRG.3	Fam214a	ENSMUST00000216809;ENSMUST00000215370;MSTRG.32831.3;
MSTRG.3	Fam214a	MSTRG.32832.1
MSTRG.3	Fam214a	MSTRG.32833.1

MSTRG.3	Fam214a	MSTRG.32834.1
MSTRG.3	Fam214a	MSTRG.32835.1;MSTRG.32835.2
MSTRG.3	Arpp19	ENSMUST00000168166;ENSMUST00000169492;ENSMUST0000016[illegible]
MSTRG.3	Gm27188	ENSMUST00000184383
MSTRG.3	Gm7870	ENSMUST00000184467
MSTRG.3	Gm25163	ENSMUST00000158905
MSTRG.3	Rexo1	MSTRG.3284.1
MSTRG.3	Gm27253	ENSMUST00000183670
MSTRG.3	Zfp280d	ENSMUST00000184036;ENSMUST00000184517;ENSMUST0000009[illegible]
MSTRG.3	Zfp280d	MSTRG.32843.1
MSTRG.3	Gm37983	ENSMUST00000193207
MSTRG.3	BC06540	ENSMUST00000180997;ENSMUST00000184796
MSTRG.3	Gm37150	ENSMUST00000193900
MSTRG.3	Gm27230	MSTRG.32847.1
MSTRG.3	Gm27230	MSTRG.32848.1;ENSMUST00000184671;MSTRG.32848.2;ENSMUS
MSTRG.3	Abhd17a	ENSMUST00000003436;MSTRG.3285.2;ENSMUST00000191440;EN
MSTRG.3	Gm27254	ENSMUST00000183926
MSTRG.3	Mns1	ENSMUST00000183809;MSTRG.32851.1;ENSMUST00000184604;E
MSTRG.3	Gm36948	ENSMUST00000194708
MSTRG.3	Gm27231	ENSMUST00000185191
MSTRG.3	Tex9	MSTRG.32854.1;ENSMUST00000085358;ENSMUST00000184125;E
MSTRG.3	Tex9	MSTRG.32855.1
MSTRG.3	Gm28010	ENSMUST00000179485
MSTRG.3	4930509	ENSMUST00000181405
MSTRG.3	Rfx7;Gm	ENSMUST00000163401;ENSMUST00000093820;MSTRG.32858.5;E
MSTRG.3	Rfx7	MSTRG.32859.1
MSTRG.3	Scamp4;	ENSMUST00000220669;ENSMUST00000038411;ENSMUST0000007[illegible]
MSTRG.3	Gm37105	ENSMUST00000192251
MSTRG.3	Gm18014	ENSMUST00000183369
MSTRG.3	Gm27241	ENSMUST00000183517;ENSMUST00000185013
MSTRG.3	Gm27232	ENSMUST00000184373
MSTRG.3	Rfx7	MSTRG.32864.1
MSTRG.3	Nedd4	ENSMUST00000034740;ENSMUST00000184737;ENSMUST00000184
MSTRG.3	Nedd4	MSTRG.32867.1
MSTRG.3	A530064	ENSMUST00000195338
MSTRG.3	Gm7265	ENSMUST00000192735
MSTRG.3	Csnk1g2	MSTRG.3287.1;ENSMUST00000079773;ENSMUST00000085435;EN
MSTRG.3	Gm37955	ENSMUST00000195098
MSTRG.3	Gm36936	ENSMUST00000194508
MSTRG.3	Nedd4	MSTRG.32872.1
MSTRG.3	Myo5a	ENSMUST00000149823;ENSMUST00000123128;ENSMUST0000013[illegible]
MSTRG.3	Gm9719	ENSMUST00000162765
MSTRG.3	Gm16353	ENSMUST00000159458
MSTRG.3	Mapk6	ENSMUST00000173230;ENSMUST00000168937;ENSMUST0000004[illegible]
MSTRG.3	AC11588	ENSMUST00000214879
MSTRG.3	AC11588	ENSMUST00000215494
MSTRG.3	4933433	ENSMUST00000180574;ENSMUST00000180533
MSTRG.3	Btbd2	ENSMUST00000126980;ENSMUST00000003434;ENSMUST00000147
MSTRG.3	Leo1	ENSMUST00000213767;ENSMUST00000048937;ENSMUST00000213
MSTRG.3	Gm7972	ENSMUST00000208453;ENSMUST00000208434;ENSMUST0000020[illegible]
MSTRG.3	Tmod3	ENSMUST00000072232;ENSMUST00000214424;ENSMUST00000214
MSTRG.3	AC12383	ENSMUST00000215151
MSTRG.3	Lysmd2	ENSMUST00000034702;ENSMUST00000214961;ENSMUST00000213
MSTRG.32885		MSTRG.32885.1
MSTRG.3	Lrrc1	ENSMUST00000183873;ENSMUST00000113421;ENSMUST00000184
MSTRG.3	Lrrc1	MSTRG.32887.1
MSTRG.3	Lrrc1	MSTRG.32888.1

MSTRG.3	Gclc	ENSMUST00000034905;ENSMUST00000216315;ENSMUST0000021[illegible]
MSTRG.3	Btbd2	MSTRG.3289.1
MSTRG.3	Gclc	MSTRG.32890.1
MSTRG.3	Gclc	MSTRG.32891.1
MSTRG.3	Elovl5	ENSMUST00000034904;ENSMUST00000148398;MSTRG.32892.2;[illegible]
MSTRG.3	Elovl5	MSTRG.32893.1
MSTRG.3	AC16033	ENSMUST00000213816;ENSMUST00000216017
MSTRG.3	Slc17a5	ENSMUST00000052441;MSTRG.32895.2;ENSMUST00000117645;[illegible]
MSTRG.3	Slc17a5	MSTRG.32896.1
MSTRG.3	Gm8116	ENSMUST00000182917
MSTRG.3	Col12a1	ENSMUST00000135009;ENSMUST00000071750;MSTRG.32898.3;[illegible]
MSTRG.3	4930562	ENSMUST00000123894
MSTRG.3	Rev1	MSTRG.329.1
MSTRG.3	Mknk2	ENSMUST00000200082;ENSMUST00000003433;MSTRG.3290.4;MS[illegible]
MSTRG.3	Fbxo9	ENSMUST00000085311;ENSMUST00000001402;ENSMUST0000015[illegible]
MSTRG.3	Ick	ENSMUST00000118869;ENSMUST00000125615;ENSMUST0000014[illegible]
MSTRG.3	Ick;Rn7	MSTRG.32902.1;MSTRG.32902.2;MSTRG.32902.3
MSTRG.3	Ick	MSTRG.32903.1
MSTRG.3	Rn7sk	ENSMUST00000083103
MSTRG.3	C920006	ENSMUST00000180974
MSTRG.3	Gsta4	ENSMUST00000213215;ENSMUST00000034903
MSTRG.3	AC15931	ENSMUST00000216513
MSTRG.3	Gsta1	ENSMUST00000098537
MSTRG.3	Gm26541	ENSMUST00000181866
MSTRG.3	Gm3776	ENSMUST00000217203
MSTRG.3	Gm10639	ENSMUST00000119823
MSTRG.3	Gsta2	ENSMUST00000034902;ENSMUST00000125479;ENSMUST0000014[illegible]
MSTRG.3	Dppa5a	ENSMUST00000071991
MSTRG.3	Ooep	ENSMUST00000034900
MSTRG.3	Mob3a	ENSMUST00000003438;ENSMUST00000218090;ENSMUST0000022[illegible]
MSTRG.3	4930542	ENSMUST00000171096
MSTRG.3	Ddx43	ENSMUST00000113367
MSTRG.3	Gm8093	ENSMUST00000128935;ENSMUST00000129983;ENSMUST00000144
MSTRG.3	Mb21d1	ENSMUST00000070742;ENSMUST00000034898;ENSMUST00000127
MSTRG.3	Gm17324	ENSMUST00000165499
MSTRG.3	Mto1	ENSMUST00000034896;ENSMUST00000148238;ENSMUST0000015[illegible]
MSTRG.3	Eef1a1;[illegible]	ENSMUST00000042235;ENSMUST00000102184;ENSMUST0000012[illegible]
MSTRG.3	Cox7a2	ENSMUST00000034881;ENSMUST00000215933;ENSMUST0000021[illegible]
MSTRG.3	Tmem30a	ENSMUST00000034878;MSTRG.32928.1;ENSMUST00000120690;[illegible]
MSTRG.3	Tmem30a	MSTRG.32929.1
MSTRG.3	Izumo4	ENSMUST00000219987;ENSMUST00000219746;ENSMUST00000217
MSTRG.3	4930429	ENSMUST00000151676
MSTRG.3	Filip1	ENSMUST00000093811;ENSMUST00000172740;ENSMUST00000172
MSTRG.3	Gm20477	ENSMUST00000174028
MSTRG.3	Gm3211	ENSMUST00000172721
MSTRG.3	AC12731	ENSMUST00000213783
MSTRG.32935		MSTRG.32935.1
MSTRG.3	Gm9531	ENSMUST00000055820;ENSMUST00000183511
MSTRG.3	Senp6	ENSMUST00000175673;MSTRG.32937.1;ENSMUST00000037484;[illegible]
MSTRG.3	Gm20648	ENSMUST00000175815
MSTRG.3	Senp6	MSTRG.32939.1
MSTRG.3	Ap3d1	MSTRG.3294.1;MSTRG.3294.3;ENSMUST00000020420;MSTRG.32
MSTRG.3	Gm17477	MSTRG.32940.1;ENSMUST00000165458
MSTRG.3	Mei4	ENSMUST00000057067;ENSMUST00000189391
MSTRG.3	Mei4	MSTRG.32942.1
MSTRG.3	Gm37837	ENSMUST00000191743
MSTRG.3	Gm15896	ENSMUST00000118045

MSTRG.3 Phip	MSTRG.32946.1;ENSMUST00000034787;ENSMUST00000190774;E
MSTRG.3 Hmgn3	ENSMUST00000189777;ENSMUST00000187193;ENSMUST00000185
MSTRG.3 Gm2065	ENSMUST00000194697
MSTRG.3 Gm29054	ENSMUST00000194148;ENSMUST00000187589
MSTRG.3 Dot1l	ENSMUST00000105336;ENSMUST00000127740;ENSMUST00000147
MSTRG.3 Lca5	ENSMUST00000190514;ENSMUST00000034793;ENSMUST00000034
MSTRG.3 Rps27a-	ENSMUST00000078766
MSTRG.3 Gm2087	ENSMUST00000188217
MSTRG.3 Lca5	MSTRG.32953.1
MSTRG.3 Lca5	MSTRG.32954.1
MSTRG.3 Gm37307	ENSMUST00000193663
MSTRG.3 Sh3bgrl	ENSMUST00000188241;ENSMUST00000113215;ENSMUST00000188
MSTRG.3 AC12510	ENSMUST00000213703
MSTRG.3 Gm8226	ENSMUST00000190511
MSTRG.3 Bckdhb	ENSMUST00000190637;ENSMUST00000034801;ENSMUST0000019C
MSTRG.3 Gm17151	ENSMUST00000105760
MSTRG.3 Bckdhb	MSTRG.32961.1
MSTRG.3 Gm29164	ENSMUST00000185454
MSTRG.3 CT00976	ENSMUST00000217260;ENSMUST00000215223
MSTRG.3 Ttk	ENSMUST00000188445;ENSMUST00000185913;ENSMUST0000007C
MSTRG.3 Fam46a	ENSMUST00000034802;ENSMUST00000187711;MSTRG.32965.3
MSTRG.32966	MSTRG.32966.1
MSTRG.3 Gm5619	ENSMUST00000071172
MSTRG.3 Ibtk	ENSMUST00000039213;ENSMUST00000187521;ENSMUST00000186
MSTRG.3 4933431	ENSMUST00000195799
MSTRG.3 Plekhj1	ENSMUST00000218036;ENSMUST00000036805;ENSMUST00000219
MSTRG.3 Cyb5r4	ENSMUST00000168529;MSTRG.32970.2;ENSMUST00000174724;E
MSTRG.3 Me1	ENSMUST00000034989;ENSMUST00000185374;ENSMUST00000189
MSTRG.3 Gm38230	ENSMUST00000193572
MSTRG.3 A330041	ENSMUST00000150367
MSTRG.3 Gm25949	ENSMUST00000175333
MSTRG.3 Gm22138	ENSMUST00000158748
MSTRG.3 Gm37484	ENSMUST00000195378
MSTRG.3 Prss35	ENSMUST00000036426
MSTRG.3 Snap91	ENSMUST00000098495;ENSMUST00000190388;ENSMUST00000036
MSTRG.3 Gm37941	ENSMUST00000192170
MSTRG.3 Sf3a2	ENSMUST00000148665;ENSMUST00000151928;ENSMUST00000147
MSTRG.3 Dopey1	ENSMUST00000186290;ENSMUST00000190906;ENSMUST00000187
MSTRG.3 Dopey1	MSTRG.32981.1
MSTRG.3 Gm17530	ENSMUST00000194439;ENSMUST00000194336
MSTRG.3 Pgm3	ENSMUST00000070064;ENSMUST00000072585;ENSMUST0000019C
MSTRG.3 Rwdd2a	ENSMUST00000185566;ENSMUST00000034988
MSTRG.32985	MSTRG.32985.1
MSTRG.3 Gm37012	ENSMUST00000194991
MSTRG.3 Cep162	ENSMUST00000093802;ENSMUST00000139301;ENSMUST00000188
MSTRG.3 Gm24649	ENSMUST00000157106
MSTRG.3 Cep162	MSTRG.32989.1
MSTRG.3 Oaz1;Mi	ENSMUST00000060987;ENSMUST00000179894;ENSMUST00000177
MSTRG.3 Cep162	MSTRG.32990.1
MSTRG.3 Cep162	MSTRG.32991.1
MSTRG.3 Snhg5;S	ENSMUST00000182232;ENSMUST00000180563;ENSMUST00000183
MSTRG.3 Zfp949	ENSMUST00000162827;ENSMUST00000160652;ENSMUST00000162
MSTRG.3 Gm26461	ENSMUST00000175064
MSTRG.3 Zfp949	MSTRG.32995.1
MSTRG.3 Trim43a	ENSMUST00000164661;ENSMUST00000216686
MSTRG.3 9430037	ENSMUST00000180723;ENSMUST00000180982;ENSMUST00000177
MSTRG.3 Gm37226	ENSMUST00000191644

MSTRG.3	Gm30414	ENSMUST00000194886
MSTRG.3	Gm37062	ENSMUST00000195028
MSTRG.3	Sppl2b	MSTRG.3300.1
MSTRG.3	Nt5e	ENSMUST00000034992;ENSMUST00000186789;ENSMUST00000187
MSTRG.3	Nt5e	MSTRG.33001.1
MSTRG.3	Snx14;Gı	ENSMUST00000165315;ENSMUST00000140439;ENSMUST00000173
MSTRG.3	Gm10163	ENSMUST00000155008
MSTRG.3	Gm28229	ENSMUST00000188747
MSTRG.33005		MSTRG.33005.1
MSTRG.33006		MSTRG.33006.1
MSTRG.3	Mthfsl	ENSMUST00000113110;ENSMUST00000185519;ENSMUST00000186
MSTRG.3	Gm10634	ENSMUST00000188891
MSTRG.3	Bcl2a1d	ENSMUST00000098486
MSTRG.3	Lsm7	ENSMUST00000220225;ENSMUST00000035775;ENSMUST0000022C
MSTRG.3	Gm37560	ENSMUST00000194289
MSTRG.3	Gm26082	ENSMUST00000104070
MSTRG.3	Gm37614	ENSMUST00000195645
MSTRG.33013		MSTRG.33013.1
MSTRG.3	Gm29562	ENSMUST00000190113
MSTRG.3	Trim43c	ENSMUST00000163255
MSTRG.3	9330159I	ENSMUST00000188156;ENSMUST00000180712;ENSMUST0000018C
MSTRG.3	Gm26611	ENSMUST00000181449
MSTRG.3	Gm2396	ENSMUST00000188232;ENSMUST00000185501
MSTRG.3	Sppl2b	ENSMUST00000218007;ENSMUST00000035597;ENSMUST00000217
MSTRG.3	Bcl2a1a	ENSMUST00000098485
MSTRG.3	Gm37833	ENSMUST00000194342
MSTRG.3	Gm37993	ENSMUST00000195301
MSTRG.3	Gm24463	ENSMUST00000178417
MSTRG.3	Gm19498	ENSMUST00000191128
MSTRG.3	Trim43b	ENSMUST00000167113
MSTRG.3	4930579(	ENSMUST00000187015
MSTRG.3	Gm26423	ENSMUST00000104277
MSTRG.3	Bcl2a1b	ENSMUST00000068569;ENSMUST00000138109;ENSMUST00000127
MSTRG.3	Gadd45b	ENSMUST00000015456;ENSMUST00000220246;ENSMUST0000021S
MSTRG.3	Mthfs	MSTRG.33030.1
MSTRG.3	Mthfs	MSTRG.33031.1
MSTRG.3	Gm38091	ENSMUST00000192418
MSTRG.3	Mthfs	MSTRG.33033.1
MSTRG.3	Tmed3	ENSMUST00000058488;ENSMUST00000188808
MSTRG.33035		MSTRG.33035.1
MSTRG.3	Ctsh	ENSMUST00000132718;ENSMUST00000034915;ENSMUST00000127
MSTRG.3	Morf4l1	MSTRG.33037.1;ENSMUST00000085248;ENSMUST00000169860;E
MSTRG.3	Gm26882	ENSMUST00000181611
MSTRG.3	AC16294	MSTRG.33039.1
MSTRG.3	Tmprss9	ENSMUST00000219817
MSTRG.3	AC16294	ENSMUST00000214503
MSTRG.3	Gm22717	ENSMUST00000122703
MSTRG.33042		MSTRG.33042.1
MSTRG.3	Gm5620	ENSMUST00000077991
MSTRG.3	Adamts7	ENSMUST00000113059;ENSMUST00000167122;ENSMUST00000134
MSTRG.3	Tbc1d2b	MSTRG.33045.1;ENSMUST00000041767;MSTRG.33045.3;MSTRG.
MSTRG.3	Plscr1	ENSMUST00000093801;ENSMUST00000186364;ENSMUST00000187
MSTRG.3	Plscr1	MSTRG.33047.1
MSTRG.3	Mir6386	ENSMUST00000183558
MSTRG.3	Plscr2	ENSMUST00000034932;ENSMUST00000180154;ENSMUST00000126
MSTRG.3	Timm13	ENSMUST00000020440;ENSMUST00000219896;ENSMUST00000218
MSTRG.3	Gm29397	ENSMUST00000185205

MSTRG.3	Gm38391	ENSMUST00000191720;ENSMUST00000186512
MSTRG.3	Plod2	ENSMUST00000070522;ENSMUST00000160359;ENSMUST0000016C
MSTRG.3	Plod2	MSTRG.33053.1
MSTRG.3	1190002	ENSMUST00000113028
MSTRG.3	1190002	MSTRG.33055.1
MSTRG.3	Gm29395	ENSMUST00000188551
MSTRG.3	Slc9a9;	MSTRG.33057.1;ENSMUST00000033463;MSTRG.33057.11;MSTRG
MSTRG.3	Gm16262	MSTRG.33058.1;ENSMUST00000162306
MSTRG.3	Slc9a9	MSTRG.33059.1
MSTRG.3	Lmnb2	ENSMUST00000057623;ENSMUST00000179022;MSTRG.3306.2;EN
MSTRG.3	Slc9a9	MSTRG.33060.1
MSTRG.3	Slc9a9	MSTRG.33061.1
MSTRG.3	Slc9a9	MSTRG.33062.1
MSTRG.3	Slc9a9	MSTRG.33063.1
MSTRG.3	Slc9a9	MSTRG.33064.1;MSTRG.33064.2
MSTRG.3	Slc9a9	MSTRG.33065.1
MSTRG.3	AC12383	ENSMUST00000214362
MSTRG.3	Slc9a9	MSTRG.33067.1
MSTRG.3	Slc9a9	MSTRG.33068.1
MSTRG.3	Slc9a9	MSTRG.33069.1
MSTRG.3	Slc39a3	ENSMUST00000117276;ENSMUST00000059551;ENSMUST0000016E
MSTRG.3	Slc9a9	MSTRG.33070.1
MSTRG.3	Slc9a9	MSTRG.33071.1
MSTRG.3	Pls1	ENSMUST00000093800;MSTRG.33073.2;ENSMUST00000119760;E
MSTRG.3	U2surp	ENSMUST00000079659;ENSMUST00000078374;ENSMUST00000191
MSTRG.3	Gm28424	ENSMUST00000187393
MSTRG.3	Gk5	ENSMUST00000085217;ENSMUST00000122383;ENSMUST0000012S
MSTRG.3	Gk5	MSTRG.33077.1
MSTRG.3	BC04393	ENSMUST00000185694;ENSMUST00000070500
MSTRG.3	Rnf7	ENSMUST00000057500;ENSMUST00000071301;ENSMUST0000012E
MSTRG.3	Sgta	ENSMUST00000005067;ENSMUST00000218208;ENSMUST0000021S
MSTRG.3	Tfdp2;G	ENSMUST00000165120;ENSMUST00000034982;ENSMUST0000018E
MSTRG.3	Gm28924	ENSMUST00000190012
MSTRG.3	Tfdp2	MSTRG.33082.1
MSTRG.3	Tfdp2	MSTRG.33083.1
MSTRG.3	Tfdp2	MSTRG.33084.1
MSTRG.3	Atp1b3	ENSMUST00000034983;ENSMUST00000190045;MSTRG.33085.2;E
MSTRG.3	1700065	ENSMUST00000178038
MSTRG.3	Atr;Gm2	ENSMUST00000187356;ENSMUST00000034980;ENSMUST0000018E
MSTRG.3	Gm16794	ENSMUST00000180727;ENSMUST00000188854;ENSMUST0000018E
MSTRG.3	Gm16794	MSTRG.33089.1
MSTRG.3	Thop1	ENSMUST00000005057;ENSMUST00000167658;ENSMUST00000171
MSTRG.3	Xrn1;Gm	ENSMUST00000034981;ENSMUST00000185633;ENSMUST0000018S
MSTRG.3	C78334	ENSMUST00000190970;ENSMUST00000186632
MSTRG.3	Gm28085	ENSMUST00000189281
MSTRG.3	Zbtb38	ENSMUST00000152594;ENSMUST00000093798;MSTRG.33093.3;E
MSTRG.3	A930006	MSTRG.33094.1;ENSMUST00000186821
MSTRG.3	Rasa2	MSTRG.33095.1;ENSMUST00000034984;ENSMUST00000188853;E
MSTRG.3	n-R5s87	ENSMUST00000122752
MSTRG.3	Gm19280	ENSMUST00000190191
MSTRG.3	Rasa2	MSTRG.33098.1
MSTRG.3	Rasa2	MSTRG.33099.1
MSTRG.3	Pdcl3	ENSMUST00000027247;ENSMUST00000193489;ENSMUST00000194
MSTRG.3	Pias4	ENSMUST00000005064;ENSMUST00000134017;ENSMUST00000147
MSTRG.3	Gm10123	ENSMUST00000075723
MSTRG.3	Pxylp1	ENSMUST00000112951;ENSMUST00000078478;ENSMUST0000012C
MSTRG.3	Pxylp1	MSTRG.33102.1

MSTRG.3	Gm18291	ENSMUST00000190791
MSTRG.3	Gm19325	ENSMUST00000186238
MSTRG.3	Gm26767	MSTRG.33105.1;ENSMUST00000180936
MSTRG.3	Gm26767	MSTRG.33106.1
MSTRG.3	Slc25a3	MSTRG.33107.1;ENSMUST00000085206;ENSMUST00000035024;M
MSTRG.3	Gm16185	ENSMUST00000125071;MSTRG.33108.1
MSTRG.3	Nmnat3	ENSMUST00000134253;ENSMUST00000129771;ENSMUST00000112
MSTRG.3	Pias4	MSTRG.3311.1
MSTRG.3	Nmnat3	MSTRG.33110.1
MSTRG.3	Gm28530	ENSMUST00000190473
MSTRG.3	Nmnat3	MSTRG.33112.1
MSTRG.3	Nmnat3	MSTRG.33113.1
MSTRG.3	Rbp1	ENSMUST00000052068
MSTRG.3	4930579	ENSMUST00000058992
MSTRG.3	Copb2	ENSMUST00000188913;ENSMUST00000035033;ENSMUST00000186
MSTRG.3	AC14044	ENSMUST00000214600
MSTRG.3	Mrps22	ENSMUST00000035034;ENSMUST00000190150
MSTRG.3	Gm37637	ENSMUST00000195540
MSTRG.3	Pias4	MSTRG.3312.1
MSTRG.3	Faim	ENSMUST00000035038;ENSMUST00000112911;ENSMUST00000187
MSTRG.3	Gm1123	ENSMUST00000076730
MSTRG.3	Pik3cb	ENSMUST00000035037;ENSMUST00000136965;ENSMUST00000124
MSTRG.3	Pik3cb	MSTRG.33123.1
MSTRG.3	Pik3cb	MSTRG.33124.1
MSTRG.3	Pik3cb	MSTRG.33125.1
MSTRG.3	Gm37249	ENSMUST00000195185
MSTRG.3	Esyt3	ENSMUST00000042158;ENSMUST00000190002
MSTRG.3	Esyt3	MSTRG.33128.1
MSTRG.3	1700008	ENSMUST00000191694
MSTRG.3	Mras	ENSMUST00000035045;ENSMUST00000122384;ENSMUST00000119
MSTRG.3	Gm38327	ENSMUST00000195852
MSTRG.3	Cep70	ENSMUST00000185799;ENSMUST00000093795;ENSMUST00000189
MSTRG.3	Cep70	MSTRG.33133.1
MSTRG.3	AC15989	ENSMUST00000214024
MSTRG.3	AC15989	ENSMUST00000214625
MSTRG.3	4930422	ENSMUST00000191492
MSTRG.3	4930422	MSTRG.33137.1
MSTRG.3	Nme9	ENSMUST00000163199;ENSMUST00000056103
MSTRG.3	Armc8	ENSMUST00000035043;MSTRG.33139.2;MSTRG.33139.3;ENSMUS
MSTRG.3	Armc8	MSTRG.33140.1
MSTRG.3	Armc8	MSTRG.33141.1
MSTRG.3	Dbr1	ENSMUST00000066650;ENSMUST00000148987;ENSMUST00000156
MSTRG.3	Il20rb	ENSMUST00000098458;ENSMUST00000136606;ENSMUST00000136
MSTRG.3	Il20rb	MSTRG.33144.1
MSTRG.3	Il20rb	MSTRG.33145.1
MSTRG.3	Il20rb	MSTRG.33146.1
MSTRG.3	Nck1	ENSMUST00000116522;ENSMUST00000112874;ENSMUST00000186
MSTRG.3	Nck1	MSTRG.33148.1
MSTRG.3	Gm28586	ENSMUST00000189857
MSTRG.3	Map2k2	ENSMUST00000143517;ENSMUST00000105331;ENSMUST00000048
MSTRG.3	Nck1	MSTRG.33150.1
MSTRG.3	Slc35g2	MSTRG.33151.1
MSTRG.3	Slc35g2	ENSMUST00000093792
MSTRG.3	Pccb	ENSMUST00000149322;ENSMUST00000035116;ENSMUST00000149
MSTRG.33154		MSTRG.33154.1
MSTRG.3	Stag1	ENSMUST00000124487;ENSMUST00000146312;ENSMUST00000129
MSTRG.3	Gm8661	MSTRG.33157.1

MSTRG.3	Gm8661	ENSMUST00000118688
MSTRG.3	Stag1	MSTRG.33159.1
MSTRG.3	Zbtb7a	ENSMUST00000125261;ENSMUST00000048128;ENSMUST0000011
MSTRG.3	Stag1	MSTRG.33160.1
MSTRG.3	Gm37204	ENSMUST00000192389
MSTRG.3	Stag1	MSTRG.33162.1
MSTRG.3	Stag1	MSTRG.33163.1
MSTRG.3	Gm38297	ENSMUST00000195122
MSTRG.3	Stag1	MSTRG.33165.1
MSTRG.3	Stag1	MSTRG.33166.1
MSTRG.3	Stag1	MSTRG.33167.1
MSTRG.3	Stag1	MSTRG.33169.1
MSTRG.3	Eef2	ENSMUST00000047864;ENSMUST00000219943;ENSMUST0000021
MSTRG.3	Gm3081	ENSMUST00000193413
MSTRG.3	Stag1	MSTRG.33171.1
MSTRG.3	Msl2	ENSMUST00000189616;ENSMUST00000085177
MSTRG.3	Gm37553	ENSMUST00000194135
MSTRG.3	Ppp2r3a	MSTRG.33174.1;ENSMUST00000075941;ENSMUST00000066773;E
MSTRG.3	Gm37962	ENSMUST00000195535
MSTRG.3	Gm37621	ENSMUST00000194716
MSTRG.3	Gm37953	ENSMUST00000195293
MSTRG.3	Gm38344	ENSMUST00000195306
MSTRG.3	Gm37314	ENSMUST00000193545
MSTRG.3	Snord37	ENSMUST00000082507
MSTRG.3	Gm24338	ENSMUST00000104205
MSTRG.3	Gm29387	ENSMUST00000189074
MSTRG.3	Gm29387	MSTRG.33182.1
MSTRG.3	AL45031	ENSMUST00000214617
MSTRG.3	Cep63	ENSMUST00000162655;ENSMUST00000093791;ENSMUST00000191
MSTRG.3	Cep63	MSTRG.33185.1
MSTRG.3	Anapc13	ENSMUST00000190279;ENSMUST00000188398;ENSMUST0000003
MSTRG.3	Ryk	ENSMUST00000175883;ENSMUST00000035142;ENSMUST0000017
MSTRG.3	AC16416	ENSMUST00000214076
MSTRG.3	Ryk	MSTRG.33189.1
MSTRG.3	Eef2	MSTRG.3319.1
MSTRG.3	Slco2a1	ENSMUST00000185215;MSTRG.33190.2;MSTRG.33190.3;ENSMUS
MSTRG.3	Gm25842	ENSMUST00000122590
MSTRG.3	Gm22595	ENSMUST00000104250
MSTRG.3	Slco2a1	MSTRG.33194.1
MSTRG.3	AC15663	ENSMUST00000215971;ENSMUST00000216778
MSTRG.3	Srprb;G	ENSMUST00000035157;ENSMUST00000163567;ENSMUST0000016
MSTRG.3	Gm20425	MSTRG.33197.1
MSTRG.3	Cdv3	ENSMUST00000035484;ENSMUST00000116517;ENSMUST0000019
MSTRG.3	1300017	ENSMUST00000035163;ENSMUST00000151073;ENSMUST00000154
MSTRG.3	Pdcl3	MSTRG.332.1
MSTRG.3	4930442	ENSMUST00000056086
MSTRG.3	Topbp1	MSTRG.33200.2;MSTRG.33200.1;ENSMUST00000035164;ENSMUS
MSTRG.3	AC16525	ENSMUST00000216687
MSTRG.3	5830418	ENSMUST00000143564;ENSMUST00000145185;ENSMUST00000194
MSTRG.3	Bfsp2	ENSMUST00000136636;ENSMUST00000124310;MSTRG.33203.5;M
MSTRG.3	Bfsp2	MSTRG.33204.1
MSTRG.3	Bfsp2	MSTRG.33205.1
MSTRG.3	Gm16252	ENSMUST00000144420;ENSMUST00000216712;ENSMUST0000021
MSTRG.3	Tmem108	ENSMUST00000049452;ENSMUST00000189588;ENSMUST0000018
MSTRG.3	AC16525	ENSMUST00000214196
MSTRG.3	Dapk3	ENSMUST00000178422;ENSMUST00000217936;MSTRG.3321.2;EN
MSTRG.3	Gm37649	ENSMUST00000192079

MSTRG.3	4932413	ENSMUST00000190332
MSTRG.3	Gm38032	ENSMUST00000195052
MSTRG.3	Nphp3;A	ENSMUST00000193642;ENSMUST00000035167;ENSMUST00000194
MSTRG.3	Gm37200	ENSMUST00000193689
MSTRG.3	Uba5	MSTRG.33215.1;ENSMUST00000035166;MSTRG.33215.3;ENSMUS
MSTRG.3	Gm37750	ENSMUST00000193563
MSTRG.3	Ackr4	ENSMUST00000050139;ENSMUST00000076147;ENSMUST0000021
MSTRG.3	AC14573	ENSMUST00000214661
MSTRG.3	AC14573	ENSMUST00000216305
MSTRG.3	Dapk3	MSTRG.3322.1
MSTRG.3	Acpp	ENSMUST00000062723;ENSMUST00000131084;ENSMUST00000112
MSTRG.3	Dnajc13	ENSMUST00000035170;ENSMUST00000189813;ENSMUST0000018
MSTRG.3	Hmgb1-r	ENSMUST00000185927
MSTRG.3	Gm37563	ENSMUST00000192580
MSTRG.3	Mrpl3;G	ENSMUST00000149243;ENSMUST00000035177;ENSMUST0000019
MSTRG.3	AC16125	ENSMUST00000213426
MSTRG.3	AC16125	ENSMUST00000215114
MSTRG.33228		MSTRG.33228.1
MSTRG.3	Nudt16	ENSMUST00000185533;ENSMUST00000035179;ENSMUST0000019
MSTRG.3	Atcayos	ENSMUST00000129834;ENSMUST00000129950;ENSMUST0000015
MSTRG.3	Col6a5	ENSMUST00000190193;ENSMUST00000165165;ENSMUST0000018
MSTRG.3	Nek11	ENSMUST00000038648;ENSMUST00000177029;ENSMUST0000014
MSTRG.3	Gm37582	ENSMUST00000192953
MSTRG.3	Gm15619	ENSMUST00000128415
MSTRG.3	Aste1	ENSMUST00000035181;ENSMUST00000176597;ENSMUST00000123
MSTRG.3	Atp2c1	MSTRG.33236.1;ENSMUST00000176770;ENSMUST00000177074;E
MSTRG.3	Atp2c1	MSTRG.33237.1
MSTRG.3	Gm19667	MSTRG.33238.1;ENSMUST00000188984
MSTRG.3	Atp2c1	MSTRG.33239.1
MSTRG.3	Nmrk2	ENSMUST00000005069
MSTRG.3	Atp2c1	MSTRG.33241.1
MSTRG.3	Pik3r4	ENSMUST00000187573;ENSMUST00000190358;ENSMUST00000191
MSTRG.3	Gm22720	ENSMUST00000122710
MSTRG.3	Col6a6	ENSMUST00000098441;ENSMUST00000166431;ENSMUST0000006
MSTRG.3	Gm38314	ENSMUST00000195709
MSTRG.3	Wdr82	ENSMUST00000020490;ENSMUST00000191147;MSTRG.33246.2;E
MSTRG.3	Mirlet7	ENSMUST00000083506
MSTRG.3	Wdr82	MSTRG.33248.1
MSTRG.33249		MSTRG.33249.1
MSTRG.3	Atcay	ENSMUST00000146030;ENSMUST00000047408;ENSMUST00000133
MSTRG.3	Ppm1m	ENSMUST00000136431;ENSMUST00000076258;ENSMUST0000014
MSTRG.3	Tlr9	ENSMUST00000062241
MSTRG.3	Tlr9	MSTRG.33252.1
MSTRG.3	Twf2	ENSMUST00000024047;ENSMUST00000216348;ENSMUST00000187
MSTRG.3	Alas1	ENSMUST00000112524;ENSMUST00000141118;ENSMUST00000133
MSTRG.3	4930500	ENSMUST00000180701
MSTRG.3	Dusp7	ENSMUST00000172306;ENSMUST00000173051;ENSMUST00000173
MSTRG.3	Poc1a	ENSMUST00000072206;ENSMUST00000191434;ENSMUST00000216
MSTRG.3	Gm29123	ENSMUST00000185808
MSTRG.3	Poc1a	MSTRG.33259.1
MSTRG.3	Tjp3;Gm	MSTRG.3326.1;ENSMUST00000163009
MSTRG.3	Poc1a	MSTRG.33260.1
MSTRG.3	Abhd14a	ENSMUST00000048685;ENSMUST00000171678;ENSMUST00000171
MSTRG.3	Abhd14b	ENSMUST00000216130;ENSMUST00000187106;ENSMUST00000185
MSTRG.3	Pcbp4	ENSMUST00000185874;ENSMUST00000185854;ENSMUST00000024
MSTRG.3	Gpr62	ENSMUST00000164834
MSTRG.33266		MSTRG.33266.1

MSTRG.3	Parp3	ENSMUST00000067218;ENSMUST00000123555;ENSMUST0000014[illegible]
MSTRG.3	Rrp9	ENSMUST00000047721;ENSMUST00000125630;ENSMUST0000012[illegible]
MSTRG.3	Tex264	ENSMUST00000169068;ENSMUST00000163441;ENSMUST0000004[illegible]
MSTRG.3	Zfr2	ENSMUST00000144087;ENSMUST00000117798;ENSMUST0000012[illegible]
MSTRG.3	Rad54l2	MSTRG.33270.1
MSTRG.3	Rad54l2	ENSMUST00000046502;ENSMUST00000190363;ENSMUST00000191
MSTRG.3	Rad54l2	MSTRG.33272.1
MSTRG.3	Mapkapk	ENSMUST00000035194;ENSMUST00000143487;ENSMUST0000012[illegible]
MSTRG.3	Mapkapk	MSTRG.33274.1
MSTRG.3	Gm17040	ENSMUST00000170597;ENSMUST00000168672
MSTRG.3	Cish	ENSMUST00000085102;MSTRG.33276.2;ENSMUST00000168260;E
MSTRG.3	Gm17041	ENSMUST00000163196
MSTRG.3	Vprbp	ENSMUST00000159645;MSTRG.33278.2;ENSMUST00000055009;E
MSTRG.3	Vprbp	MSTRG.33279.1
MSTRG.3	Matk	ENSMUST00000119547;ENSMUST00000117488;ENSMUST0000010[illegible]
MSTRG.3	Vprbp	MSTRG.33280.1
MSTRG.3	Manf;Rb	ENSMUST00000159620;ENSMUST00000161272;ENSMUST0000005[illegible]
MSTRG.3	Vprbp	MSTRG.33282.1
MSTRG.3	Gm37612	ENSMUST00000193626
MSTRG.3	Manf	MSTRG.33284.1
MSTRG.3	4930524	ENSMUST00000193135;ENSMUST00000161822
MSTRG.3	Dock3	ENSMUST00000044532;ENSMUST00000166152;ENSMUST0000016[illegible]
MSTRG.3	Gm17205	ENSMUST00000168370
MSTRG.3	Mrpl54	ENSMUST00000046114;ENSMUST00000145452
MSTRG.3	AA54340	ENSMUST00000164248
MSTRG.3	Dock3	MSTRG.33292.1
MSTRG.33293		MSTRG.33293.1
MSTRG.3	A930036	ENSMUST00000194146
MSTRG.3	Sema3f	ENSMUST00000080560;ENSMUST00000192727;ENSMUST0000019[illegible]
MSTRG.3	Ifrd2;N	MSTRG.33296.1;MSTRG.33296.2;ENSMUST00000010201;ENSMUS
MSTRG.3	Tmem115	ENSMUST00000010189
MSTRG.3	Cyb561d	ENSMUST00000041459;ENSMUST00000195235;ENSMUST00000194
MSTRG.3	Zmynd10	ENSMUST00000010188;ENSMUST00000195627;ENSMUST0000019[illegible]
MSTRG.3	Gm37309	ENSMUST00000194994
MSTRG.3	Apba3;M	ENSMUST00000220297;ENSMUST00000217754;ENSMUST00000057
MSTRG.3	Gm34106	ENSMUST00000193782
MSTRG.3	Rassf1	MSTRG.33301.1;ENSMUST00000093786;ENSMUST00000125080;E
MSTRG.3	Gm9917	ENSMUST00000180865;ENSMUST00000194628;ENSMUST00000192
MSTRG.3	Tusc2	ENSMUST00000010198;ENSMUST00000193292;ENSMUST0000019[illegible]
MSTRG.3	Hyal2	ENSMUST00000192887;ENSMUST00000195752;ENSMUST0000001[illegible]
MSTRG.3	Gm16343	ENSMUST00000162027
MSTRG.3	Hyal1;N	ENSMUST00000112387;ENSMUST00000123005;ENSMUST0000001[illegible]
MSTRG.3	Ifrd2	ENSMUST00000010192;ENSMUST00000194932;MSTRG.33307.2;E
MSTRG.3	Lsmem2	ENSMUST00000191791
MSTRG.3	Gm38150	ENSMUST00000192996
MSTRG.3	Tjp3	ENSMUST00000045744;ENSMUST00000219479;ENSMUST0000021[illegible]
MSTRG.3	Sema3b	ENSMUST00000123926;ENSMUST00000102532;ENSMUST00000102
MSTRG.3	Gnai2;G	ENSMUST00000192615;MSTRG.33311.1;ENSMUST00000055704;E
MSTRG.3	Mon1a	ENSMUST00000191906;ENSMUST00000035202
MSTRG.3	Gm23856	ENSMUST00000158380
MSTRG.3	Mst1r	ENSMUST00000035203;ENSMUST00000195617;ENSMUST0000019[illegible]
MSTRG.3	Actl11	ENSMUST00000085073
MSTRG.3	Gm37974	ENSMUST00000193668
MSTRG.3	Camkv	ENSMUST00000035700;ENSMUST00000192318;ENSMUST0000019[illegible]
MSTRG.3	Traip	ENSMUST00000194538;ENSMUST00000049348;ENSMUST00000192
MSTRG.3	Rbm5	MSTRG.33319.1;ENSMUST00000182659;ENSMUST00000183307;E
MSTRG.3	Pip5k1c	ENSMUST00000163075;ENSMUST00000105327;ENSMUST0000016[illegible]

MSTRG.3	Rbm5	MSTRG.33320.1
MSTRG.3	Gm37850	MSTRG.33321.1
MSTRG.3	Gm37850	ENSMUST00000194573
MSTRG.3	Rbm6	ENSMUST00000035201;ENSMUST00000183032;ENSMUST00000181
MSTRG.3	Gm37436	ENSMUST00000193842
MSTRG.3	Rbm6	MSTRG.33325.1
MSTRG.3	Bsn;AC1	ENSMUST00000035208;ENSMUST00000124763;ENSMUST00000198
MSTRG.3	4930447	ENSMUST00000194283;ENSMUST00000194140;ENSMUST00000195
MSTRG.3	4930447	MSTRG.33328.1
MSTRG.3	Dohh;AC	ENSMUST00000072751;MSTRG.3333.1;ENSMUST00000142346;MS
MSTRG.3	Gm37247	ENSMUST00000192549
MSTRG.3	Gm37230	ENSMUST00000194491
MSTRG.3	Dag1	ENSMUST00000191899;ENSMUST00000080435;ENSMUST00000166
MSTRG.3	Nicn1	ENSMUST00000035227;ENSMUST00000194496;ENSMUST00000195
MSTRG.3	Gmppb	ENSMUST00000112295;ENSMUST00000150700;ENSMUST00000137
MSTRG.3	Rnf123	ENSMUST00000160249;ENSMUST00000162355;ENSMUST00000047
MSTRG.3	Amigo3	ENSMUST00000085060
MSTRG.3	Rnf123	MSTRG.33337.1
MSTRG.3	Mst1	ENSMUST00000035211;ENSMUST00000162886;ENSMUST00000161
MSTRG.3	Mir7088	ENSMUST00000184227
MSTRG.3	Mir6913	ENSMUST00000184244
MSTRG.3	Apeh	ENSMUST00000081309;ENSMUST00000193254;ENSMUST00000192
MSTRG.3	Gpx1	ENSMUST00000192401;ENSMUST00000082429;ENSMUST00000191
MSTRG.3	Amt	ENSMUST00000160025;ENSMUST00000035230;ENSMUST00000162
MSTRG.3	Tcta	ENSMUST00000044725;MSTRG.33343.1;ENSMUST00000192886;E
MSTRG.3	Rhoa	ENSMUST00000007959;ENSMUST00000193619;ENSMUST00000194
MSTRG.3	Gm37568	ENSMUST00000191755
MSTRG.3	Gm37401	ENSMUST00000192053
MSTRG.3	Usp4	ENSMUST00000035237;ENSMUST00000194224;ENSMUST00000193
MSTRG.3	Rps2-ps	ENSMUST00000195283
MSTRG.3	Uba7;Gm	ENSMUST00000176037;ENSMUST00000035216;ENSMUST00000176
MSTRG.3	Smim24	ENSMUST00000105322;ENSMUST00000118206;ENSMUST00000129
MSTRG.3	Fam212a	ENSMUST00000048568;ENSMUST00000175914
MSTRG.3	Gm24944	ENSMUST00000158330
MSTRG.3	4921523	ENSMUST00000175729
MSTRG.3	4930535	ENSMUST00000175891
MSTRG.3	Ip6k1	MSTRG.33354.1
MSTRG.3	Gm38134	MSTRG.33355.1;ENSMUST00000191858
MSTRG.3	Klhdc8b	ENSMUST00000193286;ENSMUST00000192245;ENSMUST00000035
MSTRG.3	Ccdc71	ENSMUST00000061209;ENSMUST00000193269;ENSMUST00000193
MSTRG.3	Qrich1;	ENSMUST00000195563;ENSMUST00000195513;ENSMUST00000192
MSTRG.3	Gm38366	ENSMUST00000193127
MSTRG.3	Cactin	ENSMUST00000050867;MSTRG.3336.1;MSTRG.3336.3;ENSMUST0
MSTRG.3	Impdh2	ENSMUST00000081111;ENSMUST00000193421;ENSMUST00000193
MSTRG.3	Ndufaf3	ENSMUST00000074208;ENSMUST00000194381;ENSMUST00000193
MSTRG.3	4833445	ENSMUST00000193193;ENSMUST00000194104;ENSMUST00000196
MSTRG.3	Lamb2	ENSMUST00000065014;ENSMUST00000195483;ENSMUST00000195
MSTRG.3	Usp19	ENSMUST00000006854;ENSMUST00000166103;ENSMUST00000085
MSTRG.3	Qars	ENSMUST00000006838;ENSMUST00000208069;ENSMUST00000207
MSTRG.3	Dalrd3	ENSMUST00000019183;ENSMUST00000195249;MSTRG.33367.2;E
MSTRG.3	Wdr6	MSTRG.33368.1;ENSMUST00000068700
MSTRG.3	P4htm	ENSMUST00000006853;ENSMUST00000191674;ENSMUST00000194
MSTRG.3	Tbxa2r	ENSMUST00000105325;ENSMUST00000220312;MSTRG.3337.1
MSTRG.3	Gm38163	ENSMUST00000194112
MSTRG.3	Slc25a2	ENSMUST00000194088;ENSMUST00000035222;ENSMUST00000195
MSTRG.3	Arih2	ENSMUST00000013338;ENSMUST00000193923;ENSMUST00000193
MSTRG.3	Arih2	MSTRG.33373.1

MSTRG.3	Prkar2a	ENSMUST00000192344;ENSMUST00000035220;ENSMUST00000195
MSTRG.3	Gm24259	ENSMUST00000083740
MSTRG.3	Ip6k2	ENSMUST00000192307;ENSMUST00000193560;ENSMUST00000193
MSTRG.3	Gm23034	ENSMUST00000158001
MSTRG.3	Nckipsd	ENSMUST00000035218;ENSMUST00000195323;ENSMUST00000194
MSTRG.3	Nckipsd	MSTRG.33379.1
MSTRG.3	Tbxa2r	MSTRG.3338.1
MSTRG.3	Uqcrc1	ENSMUST00000026743;ENSMUST00000194047;MSTRG.33380.2;E
MSTRG.3	Celsr3;(	ENSMUST00000024238;ENSMUST00000195255;ENSMUST00000195
MSTRG.3	Slc26a6	ENSMUST00000193825;ENSMUST00000192507;ENSMUST00000192
MSTRG.3	Tmem89	ENSMUST00000192852
MSTRG.33384		MSTRG.33384.1
MSTRG.3	Shisa5	ENSMUST00000112059;ENSMUST00000026737;ENSMUST00000198
MSTRG.3	Trex1;Gı	ENSMUST00000061973;ENSMUST00000160217;ENSMUST0000016C
MSTRG.3	Tma7	ENSMUST00000192801;ENSMUST00000167504;ENSMUST00000193
MSTRG.3	Ccdc51	ENSMUST00000026735
MSTRG.3	Ccdc51	MSTRG.33389.1
MSTRG.3	Gipc3	MSTRG.3339.1;ENSMUST00000045102
MSTRG.3	Gm7628	ENSMUST00000134690
MSTRG.3	Plxnb1	ENSMUST00000072093;ENSMUST00000131462;MSTRG.33391.2;E
MSTRG.3	3000002(	ENSMUST00000144161;ENSMUST00000125451;ENSMUST00000133
MSTRG.3	Nme6	ENSMUST00000200005;ENSMUST00000035053;ENSMUST00000199
MSTRG.3	Camp	ENSMUST00000112022
MSTRG.3	Cdc25a;I	ENSMUST00000094324;ENSMUST00000198848;ENSMUST00000198
MSTRG.3	Map4	ENSMUST00000035055;ENSMUST00000169851;MSTRG.33396.3;M
MSTRG.3	Map4	MSTRG.33397.1
MSTRG.3	Gm4734	ENSMUST00000200117
MSTRG.3	Map4	MSTRG.33399.1
MSTRG.3	Aff3	ENSMUST00000095027;MSTRG.334.2;ENSMUST00000039827;ENS
MSTRG.3	Hmg20b	ENSMUST00000105323;ENSMUST00000122993;ENSMUST00000132
MSTRG.3	Gm43732	ENSMUST00000200260
MSTRG.3	Map4	MSTRG.33401.1
MSTRG.3	Gm42433	ENSMUST00000196806
MSTRG.3	Gm42432	ENSMUST00000197902
MSTRG.3	Map4	MSTRG.33404.1
MSTRG.3	Elp6	ENSMUST00000199592;MSTRG.33405.2;ENSMUST00000068071;E
MSTRG.3	Elp6	MSTRG.33406.1
MSTRG.3	Elp6	MSTRG.33407.1
MSTRG.3	Elp6	MSTRG.33408.1
MSTRG.3	Dhx30;Gı	ENSMUST00000062368;ENSMUST00000198425;ENSMUST00000199
MSTRG.3	Mfsd12	ENSMUST00000044844;ENSMUST00000132368
MSTRG.3	Smarcc1	ENSMUST00000098355;ENSMUST00000088716;ENSMUST00000197
MSTRG.3	Gm23377	ENSMUST00000196828;ENSMUST00000082943
MSTRG.3	Smarcc1	MSTRG.33412.1
MSTRG.3	Smarcc1	MSTRG.33413.1
MSTRG.3	Smarcc1	MSTRG.33414.1
MSTRG.3	Ngp	ENSMUST00000035061
MSTRG.3	Scap	ENSMUST00000198976;ENSMUST00000198871;ENSMUST00000098
MSTRG.3	5830462	ENSMUST00000168137
MSTRG.3	Scap	MSTRG.33418.1
MSTRG.3	Ptpn23	ENSMUST00000040021;ENSMUST00000200531;MSTRG.33419.2;E
MSTRG.3	4930404	ENSMUST00000131736;ENSMUST00000124512;ENSMUST0000020
MSTRG.3	Klhl18	ENSMUST00000068025;ENSMUST00000198400;ENSMUST00000197
MSTRG.3	Klhl18	MSTRG.33421.1
MSTRG.3	Klhl18	MSTRG.33422.1
MSTRG.3	Kif9	ENSMUST00000198858;ENSMUST00000197248;ENSMUST00000061
MSTRG.3	Setd2;Gı	MSTRG.33424.3;MSTRG.33424.2;MSTRG.33424.1;ENSMUST0000

MSTRG.3	Nradd	ENSMUST00000035069;ENSMUST00000196735;ENSMUST0000019€
MSTRG.3	Nbeal2;l	ENSMUST00000133191;ENSMUST00000167320;ENSMUST0000019€
MSTRG.3	Lrrc2	ENSMUST00000035076;ENSMUST00000196834
MSTRG.3	Lrrc2	MSTRG.33428.1
MSTRG.3	Fzr1	ENSMUST00000140901;MSTRG.3343.2;MSTRG.3343.3;ENSMUSTC
MSTRG.3	Gm42470	ENSMUST00000198086
MSTRG.3	Ccdc12	ENSMUST00000019803;ENSMUST00000198077;ENSMUST0000019€
MSTRG.3	Ccdc12	MSTRG.33432.1
MSTRG.3	Ccdc12	MSTRG.33433.1
MSTRG.3	Gm35715	MSTRG.33434.1
MSTRG.3	Pth1r	ENSMUST00000198865;ENSMUST00000006005;ENSMUST0000019s
MSTRG.3	Myl3	ENSMUST00000079784;ENSMUST00000153142
MSTRG.3	Tmie	ENSMUST00000050958;ENSMUST00000198062;ENSMUST0000019s
MSTRG.3	Als2cl	ENSMUST00000143416;ENSMUST00000084926;ENSMUST0000013C
MSTRG.3	Gm43127	ENSMUST00000195890
MSTRG.3	Nfic	ENSMUST00000105321;ENSMUST00000078185;ENSMUST0000002C
MSTRG.33440		MSTRG.33440.1
MSTRG.3	Gm43814	ENSMUST00000197851
MSTRG.3	Ccrl2	MSTRG.33442.1
MSTRG.3	Ccrl2	ENSMUST00000111888;ENSMUST00000199839;ENSMUST0000019£
MSTRG.3	Ltf	ENSMUST00000198884;ENSMUST00000035077;ENSMUST0000019€
MSTRG.3	Lrrfip2	ENSMUST00000200094;ENSMUST00000035078;ENSMUST0000019€
MSTRG.3	Lrrfip2	MSTRG.33446.1
MSTRG.3	Lrrfip2	MSTRG.33447.1
MSTRG.3	Lrrfip2	MSTRG.33448.1
MSTRG.3	Mlh1	ENSMUST00000135218;ENSMUST00000035079;ENSMUST0000020C
MSTRG.3	Gm16104	ENSMUST00000156260
MSTRG.3	Mlh1	MSTRG.33450.1
MSTRG.3	Gm24860	ENSMUST00000158457
MSTRG.3	Epm2aip	ENSMUST00000135807;ENSMUST00000060711;ENSMUST0000020C
MSTRG.3	Pdcd6ip	MSTRG.33453.1;MSTRG.33453.2;ENSMUST00000035086;ENSMUS
MSTRG.3	Clasp2	MSTRG.33454.1;MSTRG.33454.2;ENSMUST00000216817;ENSMUS
MSTRG.3	Gm25059	ENSMUST00000157367
MSTRG.3	Clasp2	MSTRG.33456.1
MSTRG.3	Ubp1	ENSMUST00000009885;ENSMUST00000084885;ENSMUST00000214
MSTRG.3	Fbxl2	ENSMUST00000117537;ENSMUST00000035090;ENSMUST0000014?
MSTRG.3	CT02553	MSTRG.33459.1
MSTRG.3	Mir1191	ENSMUST00000199973
MSTRG.3	Susd5	ENSMUST00000135338
MSTRG.3	AC16611	ENSMUST00000214237
MSTRG.33462		MSTRG.33462.1
MSTRG.3	4921528	ENSMUST00000213446;ENSMUST00000214265
MSTRG.3	Bcl2a1c	ENSMUST00000066460
MSTRG.3	4930520	ENSMUST00000124664;ENSMUST00000147689;ENSMUST00000131
MSTRG.3	Crtap	ENSMUST00000084881;ENSMUST00000151377
MSTRG.3	Glb1;Tm	ENSMUST00000063042;ENSMUST00000111820;MSTRG.33467.2;M
MSTRG.3	Glb1	MSTRG.33468.1
MSTRG.3	Ccr4	MSTRG.33469.2;ENSMUST00000054414;MSTRG.33469.1;ENSMUS
MSTRG.3	Nfic	MSTRG.3347.1
MSTRG.3	Trim71	ENSMUST00000111816
MSTRG.3	C130032	ENSMUST00000180393
MSTRG.3	Cnot10	ENSMUST00000070117;MSTRG.33472.2;MSTRG.33472.1;ENSMUS
MSTRG.3	Gm23232	ENSMUST00000157300
MSTRG.3	Cnot10	MSTRG.33474.1
MSTRG.3	Dync1li	ENSMUST00000215345;MSTRG.33475.1;ENSMUST00000047404
MSTRG.3	Dync1li	MSTRG.33476.1;MSTRG.33476.2
MSTRG.3	Cmtm6	ENSMUST00000217637;ENSMUST00000035007

MSTRG.3	Cmtm6	MSTRG.33478.1
MSTRG.3	Cmtm6	MSTRG.33479.1
MSTRG.3	Gna11	ENSMUST00000043604;ENSMUST00000146984;ENSMUST00000134
MSTRG.3	Cmtm7	ENSMUST00000035009;MSTRG.33480.2;ENSMUST00000084867;M
MSTRG.3	Cmtm7	MSTRG.33481.1
MSTRG.3	Gm9888	ENSMUST00000150678;MSTRG.33482.2
MSTRG.3	Cmtm8	ENSMUST00000047013;ENSMUST00000213264;ENSMUST00000213
MSTRG.3	Gm23889	ENSMUST00000122689
MSTRG.3	Gpd1l	ENSMUST00000146623;MSTRG.33485.2;ENSMUST00000129305;E
MSTRG.33486		MSTRG.33486.1
MSTRG.33487		MSTRG.33487.1
MSTRG.33488		MSTRG.33488.1
MSTRG.3	Arpp21;I	ENSMUST00000070218;ENSMUST00000111872;ENSMUST00000164
MSTRG.3	Ncln;S1	ENSMUST00000020463;ENSMUST00000118498;ENSMUST00000151
MSTRG.3	Arpp21	MSTRG.33490.1;MSTRG.33490.2
MSTRG.3	Arpp21	MSTRG.33491.1
MSTRG.3	Mir128-	MSTRG.33492.1
MSTRG.3	Arpp21	MSTRG.33493.1
MSTRG.3	2310075	MSTRG.33494.1;ENSMUST00000161216
MSTRG.3	Arpp21	MSTRG.33495.1
MSTRG.3	2900079	ENSMUST00000149308;ENSMUST00000144424;ENSMUST0000013S
MSTRG.3	Gadl1	ENSMUST00000119291;ENSMUST00000069651
MSTRG.3	Gm16142	ENSMUST00000130584
MSTRG.3	Gm9385	ENSMUST00000117563
MSTRG.3	Gm16150	MSTRG.335.1;MSTRG.335.2;ENSMUST00000127620;MSTRG.335.
MSTRG.3	Gna15	ENSMUST00000147908;ENSMUST00000043709;ENSMUST0000015C
MSTRG.3	Tgfbr2	ENSMUST00000061101;MSTRG.33500.2;MSTRG.33500.4;MSTRG.
MSTRG.3	Osbpl10	ENSMUST00000183104;ENSMUST00000182384;ENSMUST00000183
MSTRG.3	Rps27rt	ENSMUST00000053150
MSTRG.3	Osbpl10	MSTRG.33503.1
MSTRG.3	Osbpl10	MSTRG.33504.1
MSTRG.3	Osbpl10	MSTRG.33505.1
MSTRG.3	Osbpl10	MSTRG.33506.1
MSTRG.3	Osbpl10	MSTRG.33507.1
MSTRG.3	Osbpl10	MSTRG.33508.1
MSTRG.3	Osbpl10	MSTRG.33509.1
MSTRG.3	Gm16106	ENSMUST00000119187;ENSMUST00000102273
MSTRG.3	Gm4665	ENSMUST00000183244
MSTRG.3	Osbpl10	MSTRG.33511.1
MSTRG.3	Gm26962	ENSMUST00000182545
MSTRG.3	Osbpl10	MSTRG.33513.1
MSTRG.3	Gm27002	ENSMUST00000182789
MSTRG.3	Osbpl10	MSTRG.33515.1
MSTRG.3	Stt3b	MSTRG.33516.2;MSTRG.33516.1;ENSMUST00000035010
MSTRG.3	Stt3b	MSTRG.33517.1
MSTRG.3	Stt3b	MSTRG.33518.1
MSTRG.3	AC16217	ENSMUST00000214793
MSTRG.3	AC15947	ENSMUST00000218454
MSTRG.3	Stt3b	MSTRG.33520.1;MSTRG.33520.2;MSTRG.33520.3
MSTRG.3	Rbms3	ENSMUST00000111773;ENSMUST00000068962;ENSMUST00000174
MSTRG.3	Rbms3	MSTRG.33522.1
MSTRG.3	Rbms3	MSTRG.33523.1
MSTRG.3	Rbms3	MSTRG.33524.1
MSTRG.3	Rbms3	MSTRG.33525.1
MSTRG.3	Rbms3	MSTRG.33526.1
MSTRG.3	Rbms3	MSTRG.33527.1
MSTRG.3	Gm18489	ENSMUST00000172565

MSTRG.3	Gm20397	ENSMUST00000174520
MSTRG.3	Aes	MSTRG.3353.1
MSTRG.3	Rbms3	MSTRG.33530.1
MSTRG.3	Rbms3	MSTRG.33531.1
MSTRG.3	Rbms3	MSTRG.33532.1
MSTRG.3	Rbms3	MSTRG.33533.1
MSTRG.3	Gm17396	ENSMUST00000172075
MSTRG.3	Gm22479	ENSMUST00000122566
MSTRG.3	Eomes	ENSMUST00000111763;ENSMUST00000035020;ENSMUST0000015C
MSTRG.3	Gm33460	ENSMUST00000212286
MSTRG.3	Gm26614	ENSMUST00000180386
MSTRG.3	Aes	ENSMUST00000220368;MSTRG.3354.3;ENSMUST00000002518;EN
MSTRG.3	Zcwpw2	ENSMUST00000111769;ENSMUST00000217232;ENSMUST00000191
MSTRG.3	Gm22008	ENSMUST00000104810
MSTRG.3	Azi2	ENSMUST00000044454;ENSMUST00000133580;ENSMUST00000134
MSTRG.3	Cmc1	MSTRG.33543.1;ENSMUST00000044220
MSTRG.3	Cmc1	MSTRG.33544.1
MSTRG.3	Cmc1	MSTRG.33545.1
MSTRG.3	Gm17399	ENSMUST00000181824
MSTRG.3	Golga4	ENSMUST00000084820;ENSMUST00000212593;MSTRG.33547.2;E
MSTRG.3	Gm18935	ENSMUST00000212692
MSTRG.3	Gm45897	ENSMUST00000212679
MSTRG.3	Gm15917	ENSMUST00000162771
MSTRG.3	Itga9	ENSMUST00000044165;ENSMUST00000125021;ENSMUST00000124
MSTRG.3	Itga9	MSTRG.33551.1
MSTRG.3	Gm10157	ENSMUST00000139408
MSTRG.3	Gm2415	ENSMUST00000134688;ENSMUST00000149952;ENSMUST0000012{
MSTRG.3	Gm16295	ENSMUST00000139512
MSTRG.3	Ctdspl	ENSMUST00000073109;ENSMUST00000172464;ENSMUST00000174
MSTRG.3	Ctdspl	MSTRG.33556.1
MSTRG.3	Mir26a-	ENSMUST00000083579
MSTRG.3	Vill	ENSMUST00000135872;ENSMUST00000051386;ENSMUST0000015{
MSTRG.3	Plcd1	ENSMUST00000214470;ENSMUST00000010804;ENSMUST0000021{
MSTRG.3	Tle2	ENSMUST00000140433;ENSMUST00000131794;ENSMUST0000013€
MSTRG.33560		MSTRG.33560.1
MSTRG.3	Scn11a	ENSMUST00000215718
MSTRG.3	Slc22a1	ENSMUST00000093775;ENSMUST00000170400
MSTRG.3	Slc22a1	ENSMUST00000084797
MSTRG.3	Slc22a1	ENSMUST00000173185
MSTRG.3	Oxsr1	ENSMUST00000040853;ENSMUST00000128880;ENSMUST0000013€
MSTRG.3	Gm22729	ENSMUST00000158088
MSTRG.3	Myd88	ENSMUST00000035092;ENSMUST00000150837;ENSMUST0000013{
MSTRG.3	Acaa1a	ENSMUST00000176351;ENSMUST00000177463;ENSMUST0000003{
MSTRG.3	Slc22a1	MSTRG.33569.1
MSTRG.3	Tle6	ENSMUST00000128278;ENSMUST00000142948;ENSMUST0000012{
MSTRG.3	Xylb	ENSMUST00000039610;ENSMUST00000215305;ENSMUST0000021{
MSTRG.3	Acvr2b	ENSMUST00000165044;ENSMUST00000035093;ENSMUST0000021{
MSTRG.3	AC16605	ENSMUST00000217028
MSTRG.3	Acvr2b	MSTRG.33573.1
MSTRG.3	4930516	ENSMUST00000181600
MSTRG.3	Exog	ENSMUST00000164213;ENSMUST00000035094;ENSMUST0000021{
MSTRG.3	Exog	MSTRG.33576.1
MSTRG.3	Exog	MSTRG.33577.1
MSTRG.3	Ccr8	ENSMUST00000048777
MSTRG.3	Slc25a3	ENSMUST00000035106;ENSMUST00000150093;ENSMUST0000013{
MSTRG.3	Gm10778	ENSMUST00000189672;ENSMUST00000105315
MSTRG.3	Gm2449	ENSMUST00000214307;ENSMUST00000180676

MSTRG.3	Wdr48	ENSMUST00000036561;ENSMUST00000217472;MSTRG.33581.2;E
MSTRG.3	Cx3cr1	ENSMUST00000177637;ENSMUST00000064165;ENSMUST0000021
MSTRG.3	Gorasp1	ENSMUST00000035099;ENSMUST00000214118;ENSMUST0000021
MSTRG.3	Ttc21a	ENSMUST00000035100;ENSMUST00000159128;ENSMUST0000016
MSTRG.3	Csrnp1	ENSMUST00000035101;ENSMUST00000215916;MSTRG.33585.3;M
MSTRG.3	AC11724	ENSMUST00000217388;ENSMUST00000214403
MSTRG.3	AC11724	ENSMUST00000215481
MSTRG.3	Xirp1	ENSMUST00000111635
MSTRG.3	Gm10778	MSTRG.3359.1
MSTRG.3	AC11724	ENSMUST00000216918
MSTRG.3	Rpsa;Gm	ENSMUST00000035105;ENSMUST00000217356;ENSMUST0000021
MSTRG.3	Gm26448	ENSMUST00000082446
MSTRG.3	Snora62	ENSMUST00000082991
MSTRG.3	AC11009	ENSMUST00000214963;ENSMUST00000216043
MSTRG.3	AC12370	ENSMUST00000213677
MSTRG.3	Eif1b	ENSMUST00000217553;MSTRG.33596.2;ENSMUST00000007139
MSTRG.3	Rpl14	ENSMUST00000165532;ENSMUST00000213310;ENSMUST0000021
MSTRG.3	Ctnnb1;	ENSMUST00000130466;ENSMUST00000007130;ENSMUST0000015
MSTRG.3	Aff3	MSTRG.336.1
MSTRG.3	Gm10778	MSTRG.3360.1
MSTRG.3	Ulk4	ENSMUST00000213408;MSTRG.33600.1;ENSMUST00000170406;E
MSTRG.3	Ulk4	MSTRG.33601.1
MSTRG.3	Ulk4	MSTRG.33602.1
MSTRG.3	Gm22987	ENSMUST00000122757
MSTRG.3	Gm17157	ENSMUST00000172397
MSTRG.3	AC16335	ENSMUST00000213695;ENSMUST00000215259
MSTRG.3	Lyzl4	ENSMUST00000077706;ENSMUST00000120918;ENSMUST0000012
MSTRG.3	Lyzl4os	ENSMUST00000156364
MSTRG.3	AC15981	ENSMUST00000214838
MSTRG.3	Vipr1	ENSMUST00000035115;ENSMUST00000213272;ENSMUST0000013
MSTRG.3	BC02592	ENSMUST00000126940;ENSMUST00000119492;ENSMUST0000011
MSTRG.3	Sec22c	ENSMUST00000078547;ENSMUST00000111560;ENSMUST0000013
MSTRG.3	Trak1;A	ENSMUST00000211301;ENSMUST00000211699;MSTRG.33611.2;E
MSTRG.3	Trak1	MSTRG.33612.1
MSTRG.3	Trak1	MSTRG.33613.1
MSTRG.3	Trak1	MSTRG.33614.1
MSTRG.3	Nktr	ENSMUST00000183070;ENSMUST00000035112;ENSMUST0000018
MSTRG.3	E530011	ENSMUST00000181325;ENSMUST00000216791
MSTRG.3	Zfp651	ENSMUST00000214732;ENSMUST00000093772;ENSMUST0000021
MSTRG.3	Higd1a	ENSMUST00000215007;ENSMUST00000060251;ENSMUST0000021
MSTRG.3	AC16508	ENSMUST00000217491
MSTRG.3	Gm26896	ENSMUST00000180482
MSTRG.3	AC16508	ENSMUST00000213591
MSTRG.33621		MSTRG.33621.1
MSTRG.3	Abhd5	ENSMUST00000156520;ENSMUST00000111497;ENSMUST0000017
MSTRG.3	A730085	ENSMUST00000177015
MSTRG.3	Snrk	ENSMUST00000119215;ENSMUST00000146832;MSTRG.33624.2;E
MSTRG.3	AC12039	ENSMUST00000217153
MSTRG.3	AC12039	ENSMUST00000217287
MSTRG.3	AC12039	ENSMUST00000213905
MSTRG.3	Ano10	ENSMUST00000042546;ENSMUST00000214409;ENSMUST0000021
MSTRG.3	Ano10	MSTRG.33629.1
MSTRG.3	Sirt6	ENSMUST00000119324;MSTRG.3363.2;ENSMUST00000042923;EN
MSTRG.3	Mir8106	ENSMUST00000184520
MSTRG.3	Gm26797	ENSMUST00000216452;ENSMUST00000213132;ENSMUST0000018
MSTRG.3	9530059	ENSMUST00000181107;ENSMUST00000215759;ENSMUST0000018
MSTRG.3	AC16461	ENSMUST00000216699

MSTRG.3	AC12477	MSTRG.33635.1
MSTRG.33636		MSTRG.33636.1
MSTRG.3	AC16461	ENSMUST00000215737;ENSMUST00000216314
MSTRG.3	AC12477	ENSMUST00000213492
MSTRG.3	AC12477	ENSMUST00000215837
MSTRG.3	Ankrd24	ENSMUST00000123896;ENSMUST00000153573;ENSMUST0000011
MSTRG.3	Tcaim	ENSMUST00000052740;ENSMUST00000149659;ENSMUST0000013
MSTRG.3	Gm25341	ENSMUST00000157461
MSTRG.3	Tcaim	MSTRG.33642.1
MSTRG.3	Gm35549	ENSMUST00000207349;ENSMUST00000203656
MSTRG.3	Gm35549	MSTRG.33644.1
MSTRG.3	Gm35549	MSTRG.33645.1
MSTRG.3	Zfp445	ENSMUST00000214626;MSTRG.33646.2;ENSMUST00000216063;E
MSTRG.3	AC12537	ENSMUST00000213573
MSTRG.3	A530083	ENSMUST00000180486;ENSMUST00000214426;ENSMUST0000021
MSTRG.3	Zkscan7	ENSMUST00000215872;ENSMUST00000063980;ENSMUST0000021
MSTRG.3	Zfp433	ENSMUST00000200889;ENSMUST00000201819;ENSMUST0000008
MSTRG.3	AC12537	ENSMUST00000214957;ENSMUST00000216462
MSTRG.3	AC12537	ENSMUST00000217053;ENSMUST00000214730;ENSMUST0000021
MSTRG.3	1110059	ENSMUST00000035154;ENSMUST00000118422;ENSMUST0000021
MSTRG.3	Kif15	ENSMUST00000040717;ENSMUST00000213745;ENSMUST0000021
MSTRG.3	AC13365	ENSMUST00000217243
MSTRG.3	Clec3b	ENSMUST00000026890
MSTRG.3	Cdcp1	ENSMUST00000148158;ENSMUST00000039229;ENSMUST0000014
MSTRG.3	Cdcp1	MSTRG.33657.1
MSTRG.3	AC13365	ENSMUST00000217077
MSTRG.3	Tmem42;	ENSMUST00000084733;ENSMUST00000213514;ENSMUST0000021
MSTRG.3	Zfp433	MSTRG.3366.1
MSTRG.3	AC13365	MSTRG.33660.1
MSTRG.3	Zdhhc3	ENSMUST00000140497;ENSMUST00000147563;ENSMUST0000002
MSTRG.3	Exosc7	ENSMUST00000026891;ENSMUST00000216412;ENSMUST0000021
MSTRG.3	Exosc7	MSTRG.33663.1
MSTRG.3	Scp2-ps	ENSMUST00000216438;ENSMUST00000213851;ENSMUST0000007
MSTRG.3	AC16412	ENSMUST00000213385
MSTRG.3	Lars2	ENSMUST00000038863;ENSMUST00000215674;ENSMUST0000021
MSTRG.3	Lars2	MSTRG.33667.1
MSTRG.3	Lars2	MSTRG.33668.1
MSTRG.3	Lars2	MSTRG.33669.1
MSTRG.3	AC16720	ENSMUST00000219225
MSTRG.3	Limd1	ENSMUST00000026269;MSTRG.33670.3;ENSMUST00000216352;M
MSTRG.3	Sacm1l	ENSMUST00000026270;ENSMUST00000217045;MSTRG.33671.2;E
MSTRG.3	Slc6a20	ENSMUST00000026273;ENSMUST00000166800;ENSMUST0000017
MSTRG.3	Ccr2	ENSMUST00000171719;ENSMUST00000168841;ENSMUST0000005
MSTRG.3	Slc6a20	ENSMUST00000171647;ENSMUST00000040960;ENSMUST0000017
MSTRG.3	Gm17120	ENSMUST00000164734
MSTRG.3	AC13285	ENSMUST00000214683
MSTRG.3	AC13285	ENSMUST00000216052
MSTRG.3	Gm17200	ENSMUST00000170214;ENSMUST00000163559;ENSMUST0000021
MSTRG.3	Gm10052	ENSMUST00000168019
MSTRG.3	Lztfl1	ENSMUST00000165754;ENSMUST00000098258;ENSMUST0000016
MSTRG.3	Ccr9	MSTRG.33681.1
MSTRG.3	Gm17020	ENSMUST00000172274
MSTRG.3	Gm17021	ENSMUST00000214413;ENSMUST00000172093;MSTRG.33683.3;E
MSTRG.3	Cxcr6	MSTRG.33684.1;ENSMUST00000049810;ENSMUST00000216072
MSTRG.3	Xcr1	ENSMUST00000182350
MSTRG.3	Ppp2r3d	ENSMUST00000180270;ENSMUST00000188509;MSTRG.33686.2
MSTRG.3	2010315	MSTRG.33687.1

MSTRG.3	CAAA011	MSTRG.33688.1;ENSMUST00000214845
MSTRG.3	2010315	MSTRG.33689.1;ENSMUST00000177714;ENSMUST00000071300;E
MSTRG.3	AC16720	ENSMUST00000219616
MSTRG.3	2010315	MSTRG.33690.1
MSTRG.33691		MSTRG.33691.1
MSTRG.33692		MSTRG.33692.1
MSTRG.3	Ppp2r3d	ENSMUST00000216876;MSTRG.33693.1;ENSMUST00000180233
MSTRG.3	Ppp2r3d	ENSMUST00000216394;ENSMUST00000189707
MSTRG.3	Ppp2r3d	ENSMUST00000217354
MSTRG.3	Gm14359	MSTRG.33697.1
MSTRG.33698		MSTRG.33698.1
MSTRG.33699		MSTRG.33699.1
MSTRG.3	Aff3	MSTRG.337.1
MSTRG.3	Gm3055	ENSMUST00000218939;ENSMUST00000105316;ENSMUST0000021
MSTRG.3	Clcn5	MSTRG.33700.1;ENSMUST00000004428;ENSMUST00000115746;E
MSTRG.3	Gm26401	ENSMUST00000158502
MSTRG.3	Mir500	ENSMUST00000093600
MSTRG.3	Mir501	ENSMUST00000102296
[truncated: 427,276 more chars]
